# Meta-Analysis of Differentiating Mouse Embryonic Stem Cell Gene Expression Kinetics Reveals Early Change of a Small Gene Set

**DOI:** 10.1371/journal.pcbi.0020158

**Published:** 2006-11-24

**Authors:** Clive H Glover, Michael Marin, Connie J Eaves, Cheryl D Helgason, James M Piret, Jennifer Bryan

**Affiliations:** 1 Michael Smith Laboratories, University of British Columbia, Vancouver, British Columbia, Canada; 2 Department of Statistics, University of British Columbia, Vancouver, British Columbia, Canada; 3 Department of Medical Genetics, University of British Columbia, Vancouver, British Columbia, Canada; 4 Terry Fox Laboratory, British Columbia Cancer Agency, Vancouver, British Columbia, Canada; 5 Department of Surgery, University of British Columbia, Vancouver, British Columbia, Canada; 6 Department of Cancer Endocrinology, British Columbia Cancer Agency, Vancouver, British Columbia, Canada; 7 Department of Chemical and Biological Engineering, University of British Columbia, Vancouver, British Columbia, Canada; University of Minnesota, United States of America

## Abstract

Stem cell differentiation involves critical changes in gene expression. Identification of these should provide endpoints useful for optimizing stem cell propagation as well as potential clues about mechanisms governing stem cell maintenance. Here we describe the results of a new meta-analysis methodology applied to multiple gene expression datasets from three mouse embryonic stem cell (ESC) lines obtained at specific time points during the course of their differentiation into various lineages. We developed methods to identify genes with expression changes that correlated with the altered frequency of functionally defined, undifferentiated ESC in culture. In each dataset, we computed a novel statistical confidence measure for every gene which captured the certainty that a particular gene exhibited an expression pattern of interest within that dataset. This permitted a joint analysis of the datasets, despite the different experimental designs. Using a ranking scheme that favored genes exhibiting patterns of interest, we focused on the top 88 genes whose expression was consistently changed when ESC were induced to differentiate. Seven of these (*103728_at, 8430410A17Rik, Klf2, Nr0b1, Sox2, Tcl1,* and *Zfp42*) showed a rapid decrease in expression concurrent with a decrease in frequency of undifferentiated cells and remained predictive when evaluated in additional maintenance and differentiating protocols. Through a novel meta-analysis, this study identifies a small set of genes whose expression is useful for identifying changes in stem cell frequencies in cultures of mouse ESC. The methods and findings have broader applicability to understanding the regulation of self-renewal of other stem cell types.

## Synopsis

Stem cells are able to develop into many specialized cell types and thus have the potential to be used to repair or replace damaged cells. One of the challenges that scientists face is learning how to multiply these cells in vitro without loss of their stem cell properties. The development of more rapid assays for stem cells in cultured populations would significantly aid the optimization of culture conditions for stem cells. The authors propose an assay for mouse embryonic stem cells based on the expression change of seven marker genes and show that it can detect both increases and decreases in the frequency of stem cells. The assay was developed by analyzing three independent microarray datasets that ask similar biological questions but use different experimental designs. Gene expression profiles were identified within each dataset that exhibited patterns consistent with loss of stem cell properties, and, using a novel statistical measure, these profiles were compared between datasets in an unbiased fashion. A similar experimental design could be used to develop other stem cell population assays, and the analytical methods are adaptable to unrelated biological questions where analysis of a diverse set of microarray experiments is useful.

## Introduction

Various types of stem cells are now recognized as responsible both for the generation of tissues and organs during embryonic development and also for the subsequent maintenance and repair of these tissues and organs throughout adult life. This has led to considerable interest in the potential of these stem cell populations to be exploited as cellular therapies for medical conditions where tissue damage or malfunction is severe and irreversible. The clinical realization of stem cell–based therapies will, however, rely on the development of robust, scalable methods for the ex vivo expansion and controlled manipulation of these cell populations. Development of such protocols requires extensive testing of multiple factors and culture conditions [1], but this is currently inhibited by the lack of rapid endpoints of stem cell frequency that can be used in high-throughput assays.

The specific identification of most stem cell types currently relies on the use of functional assays to detect their developmental potential, either in vitro or in vivo [2]. Such assays are thus, by their very nature, retrospective, protracted, cumbersome, and labor-intensive. These features make such assays impractical for large-scale studies and rapid screening methodologies. Monitoring critical changes in gene expression using either microarray or high-throughput quantitative reverse transcription PCR (Q–RT–PCR) platforms offers a potentially attractive solution but depends on the identification of a set of genes whose expression changes predict decreased stem cell frequency with adequate precision and specificity.

Recently, several groups have described differences in the gene expression profiles of several types of stem cells and their differentiating progeny [3–8]. Most of these investigations have resulted in lists of genes that are too large for comprehensive assessment of their functional significance or specificity. Moreover, many have focused on the detection of altered patterns of gene expression that are more likely to be indicative of emerging differentiated lineages than of altered transcription of genes responsible for sustaining the stem cell state. In many cases, the actual stem cell content of the population was insufficient to infer any changes in the stem cell subset. Mouse embryonic stem cells (ESC) are less problematic in this regard because of their ease of propagation in culture as a predominantly undifferentiated population [9,10] and the availability of well-defined protocols for inducing their rapid differentiation into particular lineages. A few genes that are important to the maintenance of the pluripotent status of mouse ESC, such as *Oct4* [11] and *Nanog* [12], have been identified. However, recent studies of the rate at which functional measures of stem cell frequency of mouse ESC are lost indicated that these occur well before changes in *Oct4* expression are initiated [13]. The goal of this study was to identify a robust set of early gene expression changes that would be reliable indicators of decreased pluripotent cell content in mouse ESC cultures, regardless of the differentiation stimulus applied. In the following, the ESC signature change is defined as a set of gene expression changes that are indicative of ESC loss from a population.

To identify candidates for inclusion in the ESC signature change, we sought genes that exhibited a pattern of expression consistent with functional assay output in three independently generated datasets from ESC-derived cell populations that had been treated for up to 96 h in several ways to induce differentiation. This strategy required innovation in statistical methodology since the ESC signature change is more complicated than simple differential expression. Here, we present a statistically rigorous approach where the probability that a gene exhibits a predetermined expression pattern is estimated using a semiparametric bootstrap. The definition of the ESC signature change was specific to each experimental context, and, therefore, we obtained from each dataset an objective summary of the evidence that a gene is part of the ESC signature change. Genes that exhibited the strongest evidence across all three datasets were then tested in other maintenance or differentiating conditions and shown to successfully predict functional assay readout, indicating their potential to be used as an assay in high-throughput screening experiments.

## Results

### Gene Expression Datasets

Gene expression data was obtained at several time points in three independent experiments in which various differentiation induction protocols were applied to three mouse ESC lines. Summaries of each of the experiments are shown in Table 1.

**Table 1:**
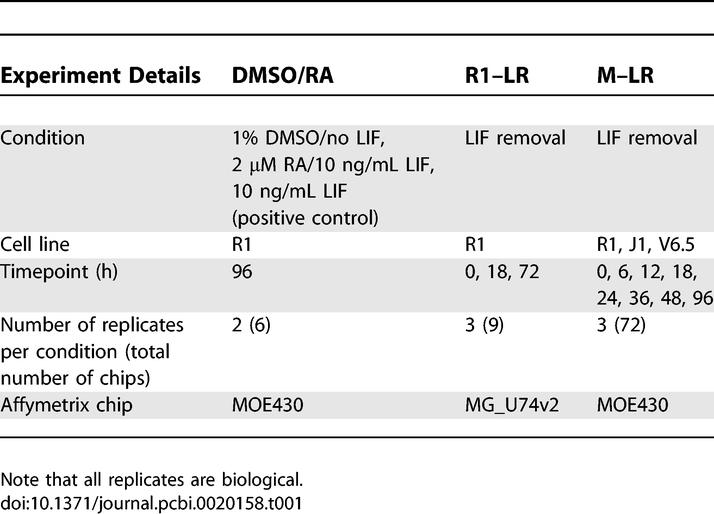
Summary of All Microarray Experiments Used in This Study

####  DMSO and retinoic acid-induced differentiation of R1 cells.

Independent, duplicate cultures of R1 cells [14] were cultured for 96 h with leukemia inhibitory factor (LIF) ± 2 μM retinoic acid (RA) or without LIF + 1% DMSO. The DMSO/RA dataset samples were hybridized to Affymetrix MOE430 A and B Genechips (Table 1).

#### Induction of R1 cell differentiation by LIF removal.

The data for R1 LIF removal (R1–LR dataset) have been described in detail previously [13]. Briefly, R1 cells were cultured in the absence of LIF for 0, 18, and 72 h. RNA for the 18-h sample was generated from cells cultured in suspension, while RNA for the 72-h sample was generated from cells cultured in methylcellulose-containing medium. Samples were generated independently in triplicate and hybridized to Affymetrix MGU74v2 A, B, and C Genechips (Table 1).

#### Induction of R1, J1, and V6.5 differentiation by LIF removal.

The multiple cell line LIF removal (M–LR) dataset is available from StemBase (http://www.scgp.ca:8080/StemBase/) [15]. R1, J1 [16], and V6.5 [17] cells were transferred onto 0.1% gelatin-coated dishes for 48 h with LIF prior to inoculation in petri dishes in the absence of LIF and RNA extracts obtained from 0 to 96 h later. RNA samples were generated independently in triplicate and hybridized to MOE430 A and B Affymetrix Genechips. (Table 1).

### Functional Assay Analysis

**Figure 1:**
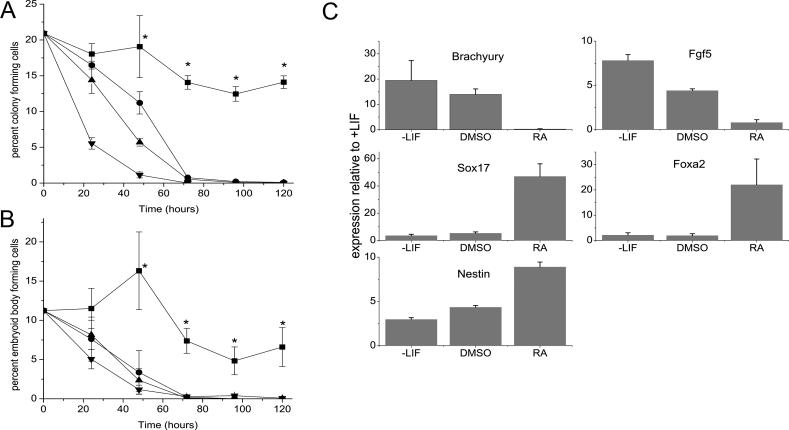
The Effect of LIF Removal with or without Addition of DMSO or RA on the Maintenance and Differentiation of ESC (A) CFC frequency and (B) EB-forming ability of cells from the ESC cultures assessed at varying times after initiation of the treatment (+LIF controls =▪ , –LIF = •, –LIF + DMSO = ▴, +LIF + RA = ▾). * denotes the data for the +LIF sample are significantly different from all other treatments. (C) Gene expression of differentiation markers was monitored by Q–RT–PCR after 96 h of treatment. Results shown are relative to the +LIF control cells.

To define the time course of changes in the biological properties of ESC subjected to the differentiation protocols used for gene expression analyses, R1 ESC were cultured on 0.1% gelatin-coated tissue-culture dishes with LIF ± RA or without LIF ± DMSO, and aliquots were sequentially tested in two-colony assays for undifferentiated cell activity The colony-forming cell (CFC) assay performed in liquid cultures containing LIF and the embryoid body (EB)-forming cell assay performed in a semisolid medium without LIF (Figure 1A and 1B). The loss of these activities more closely parallels the loss of stem cell activity measured by contribution to chimeric mice than the loss of expression of SSEA-1 or Oct4 [13]. In the starting population, 20.9 ± 0.2% of the R1 cells were detectable as CFC and 11.2 ± 0.4% gave rise to EB. Exposure to RA had the fastest effect, causing a reduction of both these values ∼20-fold within 24 h, whereas simply removing LIF (with or without DMSO addition) caused a corresponding reduction of these values ∼6-fold and 12-fold in the same time frame. After 96 h, CFC and EB-forming cell frequencies were less than 1% in all treated cultures. In control cultures the frequencies of both CFCs and EB-forming cells were sustained at half of the starting value, as noted previously when R1 cells were transferred from cultures containing feeders to gelatin-coated dishes [13].

To verify that each treatment induced cells to differentiate towards different lineages, we used Q–RT–PCR to monitor changes in transcript levels for five differentiation markers in samples obtained after 96 h of treatment with the three differentiation protocols (Figure 1C). All changes were statistically significant relative to the +LIF condition unless otherwise stated. As expected, removal of LIF, with or without DMSO treatment, induced the increased expression of genes associated with ectoderm, neural, and mesoderm differentiation (*Fgf5* [18], *Nestin* [19], and *brachyury* [20], respectively), but had little effect on the expression of genes associated with endodermal differentiation (*Foxa2* and *Sox17* [21]). In contrast, treatment with RA strongly induced the markers of neural and endodermal differentiation, but decreased the expression of *brachyury* (mesodermal differentiation) and had no significant effect on *Fgf5* expression (ectodermal differentiation). Overall, all treatments generated mixed populations of cells.

### Gene Expression Analysis

**Figure 2:**
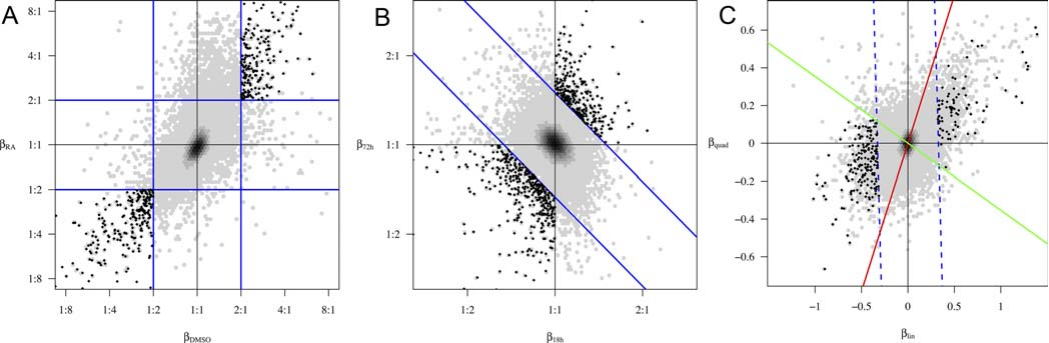
Transcriptome Plots of Estimated Expression Changes, Based on Fitting Models to Each Dataset All plots have density shading to demonstrate the number of points (genes) in different regions. Lines illustrate examples of some of the requirements that make up the definition of the ESC signature change and observed gene expression patterns that fulfilled all requirements are marked as ♦. Experiment-specific implementations of requirements are explained below. (A) DMSO/RA dataset. The requirement for large absolute changes is illustrated by the solid blue lines. Consistency across conditions implied that genes must exhibit a change in the same direction in both treatments (bottom left or top right quadrant). (B) R1–LR dataset. Note that the *y*-axis is the change seen at 72 h relative to that seen at 18 h. The requirement for large absolute changes is illustrated by the solid blue lines. The criterion for consistency was applied by requiring that the change 18 h after LIF removal be in the same direction as that after 72 h (i.e., in the lower left or upper right quadrants), regardless of its magnitude (C) M–LR dataset. The requirement for large absolute changes is illustrated by the dashed blue lines. To meet the consistency criterion, we required that a temporal gene expression trend either increase or decrease continuously over the duration of the experiment. This requirement was relaxed slightly to retain trends with a direction change occurring either very early (red line) or very late (green line).

For the data from each experiment we applied a gene-specific linear model to separate the observed expression into a level for that gene under a reference condition (e.g., +LIF or time 0) plus effects due to treatment and random fluctuation due to biological variability and measurement error. For example, to analyze the DMSO/RA dataset, the following model was used:

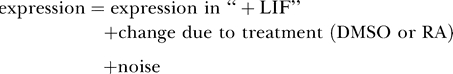
In this case, the three model parameters of primary interest were: (1) the change attributable to the effect of DMSO, (2) the change attributable to the effect of RA, and (3) the typical magnitude of the noise. These changes can be visualized easily by plotting parameter estimates on a “transcriptome plot” where each gene is represented by a single point (Figure 2A–2C). For the data from the DMSO/RA experiments, most of the genes in such a plot were found to lie close to the origin (Figure 2A), indicative of their unaltered expression following either treatment. However, it is interesting to note that for those genes whose observed expression change was greater than two in either treatment (463 genes), both treatments appeared to have similar effects, as indicated by the fact that 98% of these were either increased (209 genes, top right quadrant) or decreased (243 genes, bottom left quadrant) by both treatments. A similar model was fit to the R1–LR dataset to obtain estimates of expression changes 18 and 72 h after the removal of LIF, as well as a measure of the noise when this differentiation induction protocol was used (Figure 2B).

For the M–LR dataset, use of an ANOVA-type model, such as those described above, would have resulted in a large number of model parameters. Since interpretability of the parameters is so important in our context, we preferred a smaller, smoother quadratic model based on time. This model was able to capture the temporal trends of expression changes, and principal component analysis strongly suggested that a linear combination of constant, linear, and quadratic terms explained almost all of the data variability (Figure 2C).

### Identification of a Robust Set of Early Gene Expression Changes That Indicate Decreased Frequency of Undifferentiated ESC

We defined the ESC signature change as a set of gene expression changes that were associated with decreased frequencies of ESC as indicated by functional assay readouts during ESC culture. To identify genes that exhibit patterns consistent with the ESC signature change, we imposed three requirements on each dataset. When customized to a specific experimental context, these requirements constitute an expression-based definition of the ESC signature change, a prerequisite for developing an appropriate statistical procedure.

The three requirements used to select expression changes for inclusion were: (1) large change in absolute magnitude, (2) consistent change for all treatments and cell lines, and (3) large change relative to gene-specific variability. The first two requirements can be visualized as retaining expression changes falling in certain regions of the transcriptome plots shown in Figure 2 (namely, those regions containing black points). The third requirement cannot be visualized directly in a transcriptome plot, but its effect is revealed by the fact that some expression changes in the highlighted regions are not retained, due to large gene-specific variance. Applications of these requirements are shown in Figure 2A–2C, with full explanation detailed in Materials and Methods. Specific values of the thresholds used for each dataset are shown in Table 2.

**Table 2:**
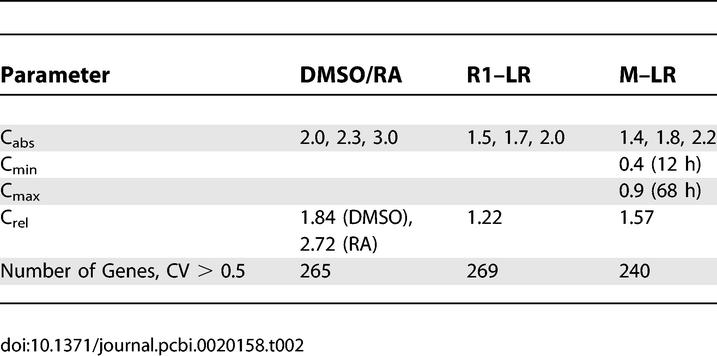
Thresholds Used in the Definition of the ESC Signature Change, in Terms of Gene Expression Changes

### Confidence Values

One way to detect ESC signature change genes is to identify those whose observed expression patterns fall in regions of interest in transcriptome plots (Figure 2). However, this approach ignores the biological and technical noise contained in the observed data. Furthermore, it fails to distinguish between genes whose observed expression changes barely fulfill our requirements from those that substantially exceed the specified thresholds. For genes of the latter type, we have more confidence that their true, long-run expression patterns are compatible with our definition of the ESC signature change. We therefore decided to define and exploit a probabilistic quantity that measured our confidence, given the observed data, that a gene exhibits an expression pattern consistent with the ESC signature change [22,23]. Within each experiment, we defined a quantity *p_g_* for each gene *g*: the probability that, in a hypothetical repeat of the experiment, the observed expression change of this gene would fulfill our requirements. Genes with true expression changes that substantially exceed all relevant thresholds have a *p_g_* greater than those that barely fulfill the requirements. If two genes shared common expression changes but differed with respect to their background variability, the *p_g_* of the gene with less variability would be greater. Also, as biological replication increases, the *p_g_* of true ESC signature change genes approach 1 and those of all other genes approach 0. Just as *p*-values are used to rank genes with respect to differential expression, we used *p_g_* to rank genes with respect to their consistency with the ESC signature change. Note that genes of primary interest have a *p_g_* value near 1, not near 0, as is the case with *p*-values.

By definition, knowing *p_g_* requires knowledge of the true change in expression following induction of differentiation, which is not available. Therefore, we estimated *p_g_* by calculating the proportion of bootstrap datasets in which gene *g* exhibited data that fulfilled our requirements [23] and referred to this quantity as the confidence value (CV) [24]. All CVs are given in Tables S1 and S2. The methods used to generate the bootstrap data are described in the Materials and Methods and a more detailed explanation is contained in Protocol S1.

### Meta-Analysis via Pareto Optimization

**Figure 3:**
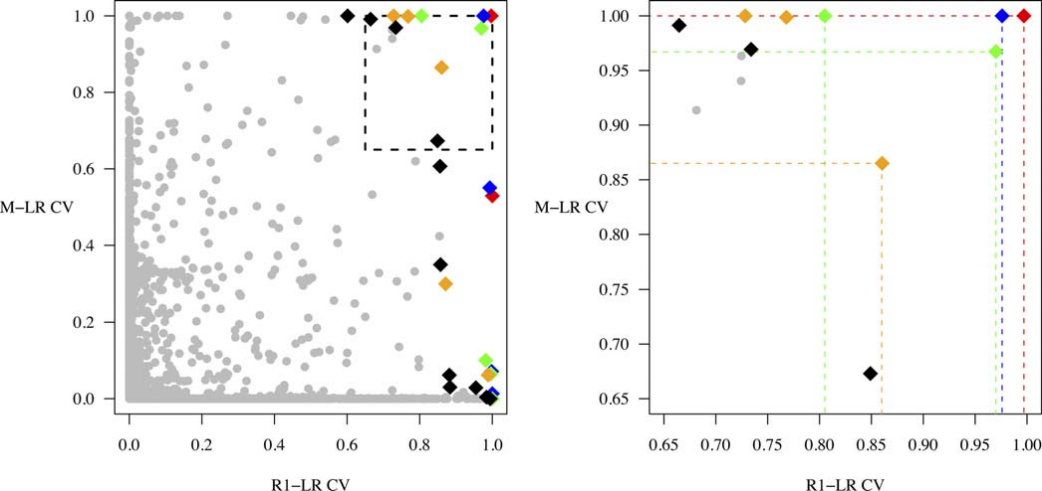
Two-way Pareto Front Analysis applied to CVs from the R1–LR Dataset and the M–LR Dataset (Left) Shows the CVs for all comparable genes with the first five Pareto fronts highlighted (red, first PF; blue, second PF; green, third PF; gold, fourth PF; black, fifth PF). (Right) Shows a magnification of the dashed box in the left panel. Here the red gene is said to dominate all other genes because, although it has an M–LR CV equal to that of several other genes, it has the highest R1–LR CV. Thus it lies on the first PF. In the same way, the blue gene dominates all genes except the red gene and thus lies on the second PF. It is not possible to choose between the two green genes because they each have larger CVs in one of the two experiments. They, therefore, lie on the same (third) PF. The highlighted yellow gene does have a larger CV in the R1–LR dataset than the green gene on the left but it falls on a lower PF because it is completely dominated by the green gene on the right.

After calculating CVs for all genes in the three experiments, we conducted a meta-analysis to identify the gene expression changes across all datasets most compatible with the ESC signature change. Genes with expression changes most correlated with decreased frequency of ESC pluripotency would have CVs near 1 in all three experiments. If we were working with only one dataset, we could rank the genes by CV in decreasing order. However, with CVs arising from two or more datasets, the task of ranking becomes considerably more difficult. In fact, it is only possible to partially order the genes, and we accomplished this with Pareto Front Analysis (PFA) [25]. Briefly, in PFA, a comparison is made between all pairs of genes and gene *g* is said to dominate gene *k* if, in all experiments, the CVs of gene *g* are greater than or equal to those of gene *k,* with strict inequality in at least one experiment. The set of genes not dominated by any others is called the first Pareto front (PF) and contains the most promising candidates for the ESC signature change. This set is removed from the analysis and the same principle of nondomination is then used to derive successive PFs (Figure 3). A more detailed explanation of PFA can be found in [25]. The first five PFs identified changes in expression of 89 probesets representing 88 genes (10, 7, 17, 27, and 28 probesets on PFs 1 to 5, respectively). The genes on PFs 1 and 2 are shown in Table 3 and the additional three are listed in Table S3.

**Table 3:**
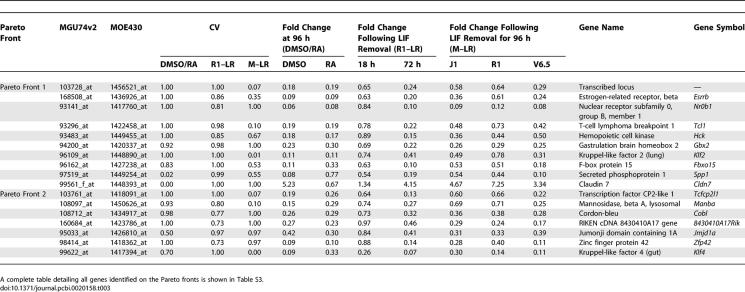
Genes Identified on the First Two Pareto Fronts

### Q–RT–PCR of Array Results

Experiments were undertaken to test, by an independent strategy, the consistency of the candidates identified from the analysis with the definition of the ESC signature change. Accordingly, RNA extracts were prepared from R1 and J1 cells cultured for 0, 24, 72, and 96 h with LIF ± RA or without LIF ± DMSO. Q–RT–PCR was used to measure the changes in levels of 22 selected transcripts (relative to the cells cultured in the presence of LIF). Nine of these were for genes in the first PF (*103728_at, Esrrb, Nr0b1, Tcl1, Hck, Gbx2, Klf2, Fbxo1,5* and *Spp1*), four for genes in the second PF (*Tcfcp2l1, 8430410A17Rik, Zfp42, Klf4*), five for genes in the third PF (*Sox2, Jam2, Morc, Podxl, Sod2*), two in the fourth PF (*Nr1d2, Kit*) and two in the fifth PF (*Mtf2, Nmyc1*). These genes were purposefully chosen to have both high and moderate confidence in their ESC signature change membership (i.e., from the first to the fifth PFs). This tested the breadth of the correlation between the Q–RT–PCR and array results across the complete set of genes contained in the first five PFs.

**Figure 4:**
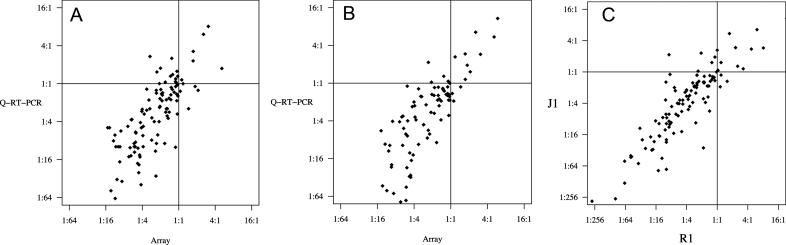
Comparison of Differently Measured Changes in Gene Expression within and between Two ESC Lines Grown for 0, 24, 72, and 96 h in +LIF ± RA or –LIF ± DMSO (A) Comparison of Q–RT–PCR and microarray results for the R1 line (r = 0.76). (B) Comparison of Q–RT–PCR and microarray results for the J1 line (r = 0.82). (C) Comparison of results between the R1 and J1 lines (r = 0.87).

Q–RT–PCR results were compared with their corresponding array data, except in the R1–LR dataset where the 24-hr Q–RT–PCR results were compared with the 18-h array data. All Q–RT–PCR data is shown in Table S4 and all comparisons of the datasets for matched treatments are plotted in Figure 4 (see Figure 4A and 4B). Results from the Q–RT–PCR measurements and the microarray analyses were strongly correlated in both cell lines (R1 cell line: Figure 4A, r = 0.76; J1 cell line: Figure 4B, r = 0.82), although the individual changes in gene expression measured by Q–RT–PCR were generally larger than those apparent from the microarray data. There was also a strong correlation between the data obtained for the two different cell lines tested (r = 0.86, Figure 4C). Overall, of the 22 genes tested, 18 demonstrated kinetics consistent with array results when assessed using Q–RT–PCR (Table S4).

**Figure 5:**
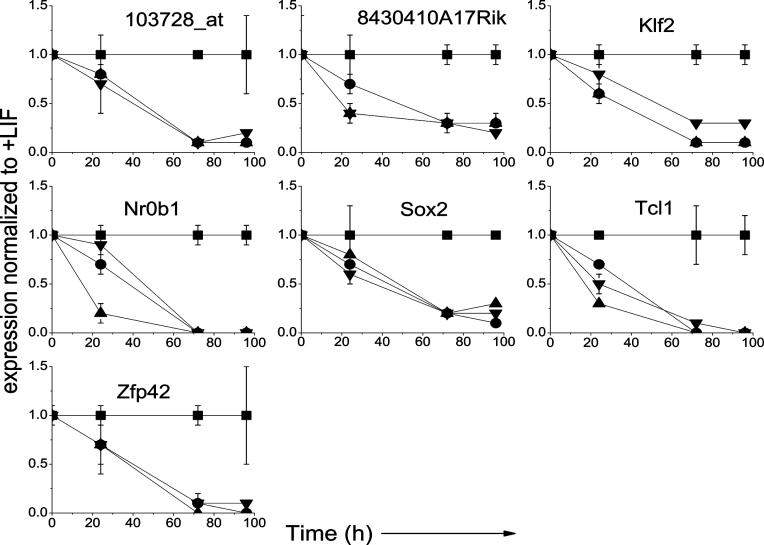
Q–RT–PCR Profiles of Transcript Levels for Seven Genes That Showed Rapid Decrease in ESC Populations When Subjected to a Variety of Differentiating Conditions All levels of expression are relative to the +LIF control. +LIF control = , –LIF = •, –LIF + DMSO = ▴, +LIF + RA = ▾.

In particular, seven genes evaluated (i.e., from the first PF: *103728_at, Klf2, Nr0b1,* and *Tcl1;* from the second PF: *8430410A17Rik* and *Zfp42;* and *Sox2* from the third PF) showed rapid (within 24 h) and sustained changes in expression in both ESC lines in all differentiation induction protocols (Figure 5). These seven genes were tested for their ability to predict the time course of functional changes in populations of ESC treated with another differentiation protocol, i.e., exposure to 50 μg/mL ascorbic acid (AA) in the absence of LIF, a treatment reported to promote the generation of cardiac myocytes from undifferentiated ESC [26]. Accordingly, R1 ESC were cultured for 5 d on 0.1% gelatin-coated tissue-culture dishes in standard maintenance conditions and with AA ± LIF and changes in gene expression compared with the loss of EB-forming potential.

**Figure 6:**
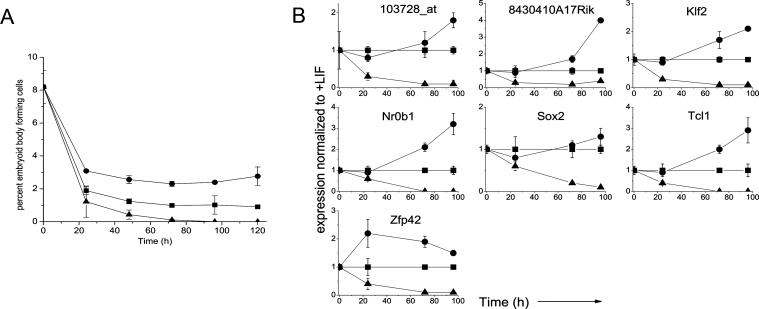
Comparison of Biological and Molecular Changes in ESC Stimulated to Differentiate by Exposure to AA (A) Changes in EB frequency. (B) Changes in the levels of transcripts for seven ESC signature change genes measured by Q–RT–PCR. +LIF (▪), +LIF + AA (•), and –LIF + AA (▴).

As expected, EB potential decreased to 40% of its starting value in the first 24 h after transferring the cells to the control (+LIF) conditions (without feeders) and then stayed constant over the remaining 5 d of the experiment (Figure 6, see Figure 6A). Cells cultured with AA in the absence of LIF showed a rapid decrease in EB potential to almost undetectable levels by day 3. Interestingly, in the presence of AA and LIF, there was an enhanced yield of EB-forming cells (with a doubling of the proportion of EB-forming cells when compared with the control +LIF conditions).

Figure 6B shows the time course of changes in transcript levels for the seven genes that had previously been identified as showing rapid changes in expression in all tested differentiation conditions. It can be seen that all were reduced in the cells exposed to AA in the absence of LIF, consistent with the concordant rapid loss in EB potential. Moreover, when AA was added in the presence of LIF, the level of expression of these genes increased relative to the +LIF control cells, consistent with their opposite biological response to the AA treated cells in the absence of LIF. *Zfp42* showed the most rapid increase in expression in the AA+LIF-treated cells, and the increase in expression of *103728_at* and *Sox2* were the most delayed. Nevertheless, significantly increased expression of all seven genes relative to the +LIF controls was seen by 96 h. Together, these results indicate that changes in expression of these seven genes can be used to infer concordant functional changes in populations of ESC in culture.

## Discussion

In this work, we have identified a small set of genes that exhibit the ESC signature change, i.e., whose altered expression is consistently and temporally correlated with an altered frequency of functionally defined, undifferentiated cells in ESC cultures. This result is important because we had previously found that significant changes of more established molecular markers of undifferentiated mouse ESC (Oct4 and SSEA-1) may not occur until well after the biological hallmarks of these cells have been lost [13]. By undertaking an integrated analysis of gene expression changes induced by exposure of ESC to multiple differentiation stimuli and the use of objective statistical methods, we identified seven genes whose altered expression correctly predicted concomitant functional changes induced by other treatments. Importantly, the expression changes of these genes reflected both decreased and increased frequency of ESC.

Four of these seven genes have been shown previously to be involved in the maintenance of mouse ESC or during early development. These include *Nr0b1* [27], *Sox2* [28], *Tcl1* [29], and *Zfp42* [30]. Among the remaining 81 genes on the first five PFs, an additional 11 have been reported to be involved in some aspect of development (see Table S5). Most notably *Hck* [31], *Fbx015* [32], *Dppa3/Stella* [33], and *Klf4* [34] have all been specifically implicated in maintenance or differentiation of ESC, while expression of *Eed* [35] is required for embryonic viability before implantation. Interestingly, *Oct4* [11] and *Nanog* [12] were not included in the first five PFs. While *Oct4* was differentially expressed in both the DMSO/RA and M–LR datasets, it was not changed in the R1–LR dataset as shown previously [13]. *Nanog* was differentially expressed in both the R1–LR and DMSO/RA datasets but did not show any change in the M–LR dataset.

Several previous microarray studies have been performed to uncover signalling pathways and regulatory factors required for the maintenance of human and mouse ESC [3–8,36,37]. These have each uncovered large numbers of genes whose expression was increased or absent in undifferentiated cells and, in some cases, little overlap has been found between the genes thus affected [38,39]. As a further step towards assessing the validity of our identified genes, we compared our data with two previously published datasets that sought to identify genes uniquely expressed in mouse ESC [4,5]. Of the 88 genes highlighted here, 75 were also changed in the other datasets (Table S6). This high degree of correspondence supported the validity of our very different approach to a similar biological question. Interestingly, a comparison of our results to genes whose expression has been reported to accompany the differentiation of human ESC [3,6,36,37], revealed far fewer similarities, as reported by others [40]. We found that only 26 of the 88 genes exhibited similar expression changes in at least one of the four human ESC studies. Moreover, in some cases, the gene expression level changed in the opposite direction. For example, in this work, *Podxl* was found to be strongly increased as mouse ESC differentiate, whereas the human homolog was found to be decreased in three studies of human ESC differentiation [3,6,37]. Overall, only six of the 88 genes were not identified as being altered in any other published datasets of differentiating ESC.

The goal of this work was to identify a small number of genes suitable for the development of an expression-based assay to estimate the frequency of ESC in culture. To achieve this, we have taken care to seek gene expression changes that fulfill several criteria beyond simple differential expression, including large, rapid, and consistent changes in more than one cell line following the induction of differentiation using multiple methods. In terms of statistical analysis, we required (1) a quantitative index that reflected each gene's compliance with the predefined ESC signature change (for use within each dataset); and (2) a meta-analytic procedure for ranking genes based on their compliance with the ESC signature change definition in multiple datasets.

As our quantitative index, we chose the probability that a gene would exhibit the ESC signature change in a hypothetical repeat of the experiment, instead of the more conventional *p*-value, the probability that a gene would exhibit data as, or more “extreme” than, the observed change if its true expression were unchanged in the study. This choice was necessitated because in our application the ESC signature change is defined by more than a requirement for differential expression. In the majority of microarray applications, the genes of interest are characterized by differential expression, where the complementarity of the null hypothesis (no differential expression) and the biologically interesting state (differential expression) permits the *p*-value to serve as an index of biological interest. Here we employed an index that originates with an explicit definition of the biologically interesting target profile. This is an application of methods previously described for identifying genes with biologically specific expression patterns [22,23]. The task of identifying ESC signature change genes can be viewed also as an instance of the so-called “problem of regions” [24], in which the term “confidence value” is first established. In certain settings, CVs can be shown to be approximations of Bayesian a posteriori probabilities. Although not formally established here, the CV can be interpreted heuristically as the posterior probability that a gene truly exhibits the ESC signature change.

As our method of meta-analysis, we used PFA [25] to (partially) rank genes based on three independent measures of ESC signature change compliance, as opposed to the more prevalent practice of integrating experiment-specific fold-changes [41,42], *p*-values[43,44], test statistics [45,46], or effect size estimates [47,48]. In these works, the common goal is a unified list of differentially expressed genes that is accompanied by an estimated error rate, usually the false discovery rate [49,50]. The methodological choices and innovations of this work are motivated by departures from this common goal, and our techniques may prove useful in other studies where biological interest is not synonymous with differential expression. PFA was first applied to gene expression data by Fleury et al. [25]. In that work, PFA was used to optimize multiple indices within one study as opposed to our use, which is the optimization of a comparable index, the CV, across distinct but related studies. Yang et al. present another compelling technique for the synthesis of competing measures of differential expression within a single experiment [51], and it may be possible to extend their methodology for use in meta-analysis.

Meta-analysis of microarray data is an increasingly common technique to capitalize on the combined power of biologically related but distinct datasets [41–48]. In addition to the usual advantage of increasing the effective sample size, the primary benefit of meta-analysis in our application is to insulate our biological findings from confounding experimental and biological effects [44,46]. For example, in the R1–LR dataset, changes induced by differentiation could have been confounded with changes caused by the removal of feeders from the culture. However, this effect was not present in the other datasets; therefore, any common gene expression changes cannot be attributed to the removal of feeders. An example of profound differences in gene expression caused by small changes in culture conditions was reported by Skottman et al., who demonstrated effects on 1,417 genes in human ESC cultured in serum containing versus serum-free conditions, despite comparable levels of expression of other markers of their undifferentiated state [52].

In summary, meta-analysis of multiple gene expression datasets from populations of mouse ESC induced to differentiate has revealed multiple genes whose altered expression provides a robust and timely indication of changes in pluripotency. These findings suggest the importance of the products of these genes in the molecular regulation of the undifferentiated state of ESC and provide a useful basis for developing high-throughput approaches for the bio-monitoring of ESC cultures.

## Materials and Methods

### ESC maintenance cultures.

J1 (passage 14) and R1 (passage 17) ESC lines were maintained on irradiated feeders at 37 °C in 5% CO_2_ in air with daily exchange of ESC maintenance medium consisting of high glucose Dulbecco's Modified Eagles Media supplemented with 15% pre-screened fetal bovine serum (FBS), 0.1 mM nonessential amino acids, 2 mM glutamine, 100 U/mL penicillin, 100 μg/mL streptomycin, 10 ng/mL LIF (all reagents from StemCell Technologies, http://www.stemcell.com) and 100 μM monothioglycerol (MTG, http://www.sigmaaldrich.com). Cells were passaged every second day. Primary mouse embryo feeders (StemCell Technologies) were maintained at 37 °C in 5% CO_2_ in air in DMEM supplemented with 10% FBS, 1 mM glutamine, 100 U/mL penicillin, 100 μg/mL streptomycin, and 100 μM MTG. Feeders were irradiated by exposure to 80 Gy 300 kvP X-rays.

### ESC experimental cultures.

Cells were thawed and maintained on irradiated feeders for two passages prior to initiation of differentiation cultures. To remove contaminating feeders, cells harvested from maintenance cultures were plated onto tissue culture dishes (Sarstedt, http://www.sarstedt.com/php/main.php) in maintenance medium for 1 h at 37 °C. All suspended and loosely adherent cells were harvested by gently pipetting medium onto the surface of the tissue-culture dish. Following this, feeder contamination was estimated at <1% based on cell size during counting.

Experimental cultures were performed on tissue culture dishes (Sarstedt), coated with 0.1% porcine gelatin (Sigma) with cells plated at a density of 80–1,500 cells per cm^2^ depending on the day of harvest. Differentiation media were based on maintenance medium with the following differences; (a) LIF removal—maintenance medium minus LIF, (b) DMSO—maintenance medium minus LIF plus 1% DMSO (Sigma), (c) RA—maintenance medium plus 2 μM RA (Sigma), (d) AA + LIF—maintenance medium plus 50 μg/mL AA (Sigma), and (e) AA-LIF—maintenance medium without LIF plus 50 μg/mL AA. Concentrated RA was prepared at a concentration of 10 mM by dissolving 30 mg powder in 10 mL 100% ethanol and stored at 4 °C in the dark. Media was prepared by adding 10 μL of RA stock solution to 50 mL maintenance media. Cells were harvested daily for functional assay analysis (CFC and EB assays, see below) or for RNA extraction.

### Colony forming cell assay.

Test cells were plated at a density of 1,000–2,500 cells on gelatinized 60-mm tissue-culture dishes (with grid) in maintenance medium at 37 °C in 5% CO_2_ in air. Five days later, colonies were stained for alkaline phosphatase (Kit 86-R; Sigma) and counted. Colonies were classified as differentiated (colourless), undifferentiated (pink), or mixed. Assay output was calculated as the percentage of undifferentiated colonies based on the assay seeding density.

### Embryoid body assay.

Test cells were plated at a density of 1,000–5,000 cells per 35-mm low-adherence petri dish in Iscove's Modified Dulbecco's Media supplemented with 15% prescreened FBS, 0.9% methylcellulose, 2 mM glutamine, and 150 μM MTG (all reagents from StemCell Technologies). EB were counted 5 d later and the frequency of EBs was calculated as the number of EBs generated per 100 ESC plated.

### RNA extraction and array hybridization.

Cytoplasmic RNA was extracted using the RNeasy mini kit (Qiagen, http://www1.qiagen.com). Standard Affymetrix protocols (Affymetrix, http://www.affymetrix.com/index.affx) were used to generate RNA probes from 5 μg of extracted RNA. Samples were hybridized to MOE430 A & B chips on a Genechip system (Affymetrix) at the Ottawa Genomics Innovation Centre (http://www.ottawagenomecenter.ca/) according to the manufacturer's instructions.

### Gene expression analysis.

In this analysis, as is common practice hybridization data for each probeset was considered independently, although we recognize that transcripts for many genes would be captured by multiple probesets. Furthermore, although the correspondence between probeset and gene is not, as a rule, one-to-one, we refer to the expression from each probeset as if it reflected the expression of one gene, unless otherwise stated.

All preprocessing, including background correction, normalization, probeset summarization, and log_2_ transformation, was carried out with the RMA algorithm [53] in the affy package [54] from Bioconductor [55] and processed data returned by RMA are referred to as expressions. The R code [56] for all the data analysis shown below is available from http://www.stat.ubc.ca/~jenny/webSupp/gloverSCmeta/index.html.

In the equations below we followed these conventions:

Observed intensities were denoted by *Y_i,cond_,* where *i* indexed the biological replicate, i.e., *i* ∈ {1, . . . , N}, and *cond* denoted the corresponding condition, i.e., treatment or time.

All models are gene-specific, although, for the sake of simplicity, an explicit index for gene was avoided.

Within the observations for one gene, the random errors *ɛ* were assumed to be independent and identically distributed and to have expectation zero and a finite, gene-specific variance 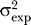 , where *exp* is DMSO/RA, R1–LR, or M–LR.

To summarize gene expression changes, we fit linear models to the RMA processed data. For the DMSO/RA data we used the following model:

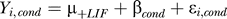
where *μ_+LIF_* was the expected intensity in the +LIF control condition, *β_+LIF_* = 0 by definition, and *β_DMSO_* (*β_RA_*) was the effect of DMSO (RA) treatment, relative to +LIF.

For the R1–LR data we used the following model:

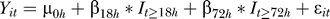
where *μ*_0*h*_ was the expected intensity at time 0, *β*_18*h*_ *(β*_72*h*_ *)* was the effect of 18 h versus 0 h (72 h versus 18 h), and *I_statement_* was 1 if the statement was true and *I_statement_* was 0 otherwise.

For the M–LR data we used the following model:

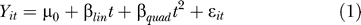
where *μ*_0_ was the expected intensity at time 0, *t* was log_2_ transformed and centered time where 0 h was changed to 3 h to avoid undefined values, and *β_lin_* and *β_quad_* gave the linear and quadratic effects of time, respectively.

### Defining the ESC signature change in terms of gene expression.

For the DMSO/RA data, a gene had to fulfill the following requirements to be included in the ESC signature change:

Absolute change: *β_cond_ > C_abs_,* or *β_cond_ <* –*C_abs_* for *cond = DMSO* and *cond = RA*

Change relative to variability: 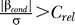 for *cond = DMSO* and *cond = RA*

Consistency: *β_DMSO_ >* 0 and *β_RA_ >* 0*,* or *β_DMSO_ <* 0 and *β_RA_ <* 0

For the R1–LR data, expression requirements were as follows:

Absolute change: *β*_18*h*_ *+ β*_72*h*_ *> C_abs,_* or *β*_18*h*_ *+ β*_72*h*_ *< C_abs_*

Change relative to variability: 
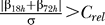


Consistency: *β*_18*h*_ *≥* 0 and *β*_72*h*_ *≥* 0*,* or *β*_18*h*_ *≤* 0 and *β*_72*h*_ *≤* 0

For the M–LR data, the definition of an interesting expression pattern was more complicated. We required a large absolute difference in the expected expression intensity between the start time, *t_min_*, of the study and the end, *t_max_*. Specifically, we required that

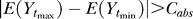



Given Equation 1, it can be shown that this is equivalent to the following requirement:

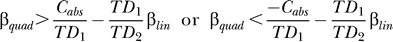
where 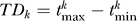 . The relative expression change requirement that

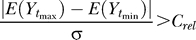
is equivalent to the following condition:

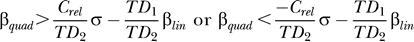



Consistency was built into the ESC signature change definition by noting the location in the time course of the vertex of the quadratic fit (Equation 1). The requirement for strictly increasing or decreasing expression patterns was relaxed by allowing genes whose vertex fell before *C_min_* or after *C_max_* to be retained, where *C_min_* and *C_max_* were specified relative to the standardized, transformed study time. This requirement is captured in the following condition:

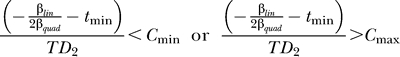



In all experiments, the specific values of the user-specified thresholds are given in Table 2. Note that the final results of this analysis are highly robust to modest differences in these thresholds.

### Confidence values.

One thousand simulated datasets were generated for each of the original datasets by adding the original fitted averages and a randomly sampled residual (with replacement) from the residuals associated with that gene within the dataset. Note that for the M–LR dataset, the bootstrap data was derived from time and cell line specific averages and residuals, not from quadratic fits. To maintain the covariance between genes, the same random selection of residuals were used for all genes in a simulated dataset. The simulated data was then assessed relative to the ESC signature change definitions given above. The proportion of times that a gene fulfilled the definition, i.e., the CV, was calculated. As a practical measure, within each experiment, we used several values for *C_abs_* and averaged the resulting CVs to obtain the CVs used for PFA. This was an expedient method for reducing the frequency of CVs equal to 0 or 1, which are highly undesirable for PFA. The use of multiple thresholds makes the final results of our analysis robust to modest changes. The most stringent values of *C_abs_* were chosen such that the number of genes retained in each dataset was approximately equal, and a range of less stringent thresholds was applied to create further distinction among the CVs.

### Comparison of MOE430 and MG_U74v2 chips.

To compare gene samples hybridized to the MOE430 and MG_U74 chips, six different comparisons were used. Two comparisons were generated from the Affymetrix-defined “good” or “best” comparisons (for more information see http://www.affymetrix.com). Resourcerer [57] was used to generate lists of comparable probesets based on EGO, Unigene, Locuslink, and Refseq comparisons. A list of two-way comparable probesets was generated by ordering evidence for comparison as follows: Affymetrix Best > Affymetrix Good > EGO > Unigene = Locuslink = Refseq. In this way 21,271 one-to-one mappings between the two chips were made (Table S7).

### Gene ontology.

Functional information about differentially expressed genes was obtained by loading Affymetrix identifiers into the Database for Annotation, Visualization, and Integrated Discovery 2.0 (DAVID2) [58]. Gene ontologies were determined and compiled at several different levels of the ontology.

### Quantitative reverse transcription-PCR.

Q–RT–PCR was performed as previously described [13]. Relative expression changes were determined with the 2^−ΔΔCT^ method [59] and the *Gapdh* transcript was used to normalize results between samples. Primers were manufactured by Invitrogen (http://www.invitrogen.com/) and sequences are shown in Table S8.

## Supporting Information

Protocol S1 Example of Confidence Value Calculation (89 KB DOC)

Table S1 Confidence Values for All Genes in the R1–LR Dataset (686 KB PDF)

Table S2 Confidence Values for All Genes in the DMSO/RA Dataset and M–LR Dataset (859 KB PDF)

Table S3 The First Five Pareto Fronts (18 Kb PDF)

Table S4 Raw Data from Q–RT–PCR (8 KB PDF)

Table S5 Biological Process and Molecular Function Classification of Genes in Pareto Fronts (8 KB PDF)

Table S6 Comparison of Table S3 with Other Publications (61 KB PDF)

Table S7 Probeset Translations between the MG_U74v2 and MOE430 Genechips (365 KB PDF)

Table S8 Primers Used for Q–RT–PCR (18 KB XLS)

### Accession Numbers

Accessions numbers from the Ensembl database (http://www.ensembl.org/index.html) for the genes mentioned in this paper are: *103728_at* (ENSMUST00000027649), *8430410A17Rik* (ENSMUST00000032141), *Klf2* (ENSMUST00000067912), *Nr0b1* (ENSMUST00000026036), *Sox2* (ENSMUST00000099151), *Tcl1* (ENSMUSG00000041359), *Zfp42* (ENSMUST00000082120), *Oct4* (ENSMUST00000025271), *Nanog* (ENSMUST00000012540), *Fgf5* (ENSMUST00000031280), *Nestin* (ENSMUST00000090973), *Brachyury* (ENSMUST00000074667), *Foxa2* (ENSMUST00000047315), *Sox17* (ENSMUST00000027035), *Esrrb* (ENSMUST00000021680), *Hck* (ENSMUST00000003370), *Gbx2* (ENSMUST00000036954), *Fbxo15* (ENSMUST00000037718), *Spp1* (ENSMUST00000031243), *Tcfcp2l1* (ENSMUST00000027629), *Jam2* (ENSMUST00000057513), *Morc* (ENSMUST00000023330), *Podxl* (ENSMUST00000026698), *Sod2* (ENSMUST00000007012), *Nr1d2* (ENSMUST00000090543), *Kit* (ENSMUST00000005815), *Mtf2* (ENSMUST00000081567), *Nmyc1* (ENSMUST00000043396), *Dppa3/Stella* (ENSMUST00000049644), *Klf4* (ENSMUST00000003116), *Eed* (ENSMUST00000032850), *Gdf3* (ENSMUST00000032211), *Krt1–18* (ENSMUST00000023803), *Krt1–19* (ENSMUST00000007317), *Cldn7* (ENSMUST00000018713), *Manba* (ENSMUST00000029814), *Cobl* (ENSMUSG00000020173), and *Jmjd1a* (ENSMUSG00000053470).

Accessions numbers from the ArrayExpress database (http://www.ebi.ac.uk/arrayexpress) for the microarrays mentioned in this paper are: for the DMSO/RA dataset (E-MEXP-412), and for the R1-LR dataset (E-MEXP-414).

Accession numbers from the Gene Expression Omnibus (http://www.ncbi.nlm.nih.gov/geo) for RNA samples mentioned in this paper are: R1 data (GSE2972), V6.5 data (GSE3231), and J1 data (GSE3749).

## Abbreviations

AA: ascorbic acid
CFC: colony forming cell
CV: confidence value
EB: embryoid body
ESC: embryonic stem cell(s)
LIF: leukemia inhibitory factor
M–LR: multiple cell line LIF removal
PF: Pareto front
PFA: Pareto front analysis
Q–RT–PCR: quantitative reverse transcription PCR
R1–LR: R1 LIF removal
RA: retinoic acid
