# Supplementary material for: Meta-Analysis of Differentiating Mouse Embryonic Stem Cell Gene Expression Kinetics Reveals Early Change of a Small Gene Set
Source: PLoS Comput Biol. 2006 Nov 24;2(11):e158. doi: 10.1371/journal.pcbi.0020158 (PMC1664699; doi:10.1371/journal.pcbi.0020158)
Supplement: Protocol S1 — (89 KB DOC) [file pcbi.0020158.sd001.doc]

Supplementary Methods – Example of Confidence Value Calculation


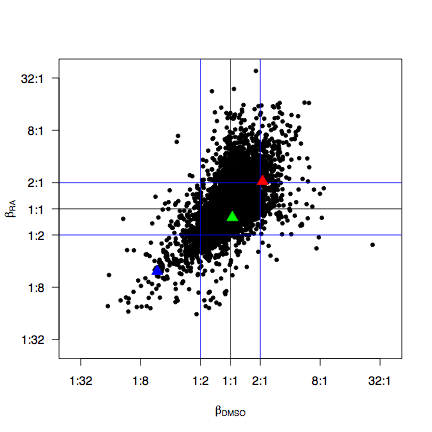
In this example, we will focus on 3 genes from the DMSO/RA data set: 1456521_at (blue triangle in Figure 1), 1416303_at (red triangle in Figure 1) and 1415894_at (green triangle in Figure 1).

Figure 1: Location of 3 examples genes in the complete data set transcriptome plot

# Generating Simulated Data

The post normalization data for each of the genes is shown in Table 1 below:

| **Probe set** | **LIF_1** | **LIF_2** | **DMSO_1** | **DMSO_2** | **RA_1** | **RA_2** |
| --- | --- | --- | --- | --- | --- | --- |
| **1456521_at** | 8.816387 | 8.01394 | 6.10392 | 5.839651 | 6.062873 | 5.981111 |
| **1416303_at** | 8.144175 | 8.314569 | 9.220502 | 9.391624 | 9.340817 | 9.208443 |
| **1415894_at** | 4.568746 | 4.68857 | 4.728089 | 4.660661 | 4.027839 | 4.562296 |

An ANOVA model is fit to this data generating the following fitted values (Table 2):

| **Probe set** | **LIF baseline** | **Adjustment due to DMSO treatment** | **Adjustment due to RA treatment** |
| --- | --- | --- | --- |
| **1456521_at** | 8.415164 | -2.443378 | -2.393172 |
| **1416303_at** | 8.229372 | 1.076691 | 1.045258 |
| **1415894_at** | 4.628657 | 0.065717 | -0.33359 |

Note that the LIF estimate is simply the average of the LIF_1 and LIF_2 data while the treatment effect estimates are differences between the treatment averages and the LIF baseline.

After fitting the model we end up with 6 residuals for each probe set, each one corresponding to the difference between the model fit and the original data. Since we only have two replicates in this data set, and the residuals must add to 0, the residuals are of the same magnitude but opposite sign (Table 3):

| **Probe set** | **LIF_1** | **LIF_2** | **DMSO_1** | **DMSO_2** | **RA_1** | **RA_2** |
| --- | --- | --- | --- | --- | --- | --- |
| **1456521_at** | 0.401 | -0.401 | 0.132 | -0.132 | 0.041 | -0.041 |
| **1416303_at** | -0.085 | 0.085 | -0.086 | 0.086 | 0.067 | -0.067 |
| **1415894_at** | -0.060 | 0.060 | 0.034 | -0.034 | -0.267 | 0.267 |

To simulate data, we add randomly sampled residuals (columns from Table 3) to the parameter estimates shown in Table 2. Note that replacement is allowed. To preserve covariance between genes, the same column of data is added to all genes.

For example, if the first simulated data set was generated by adding residuals from columns DMSO_1, RA_2, RA_2, LIF_2, LIF_1, DMSO_2 to the estimated parameters, then the following simulated data set would be generated:

| **Probe set** | **LIF_1_sim1** | **LIF_2_sim1** | **DMSO_1_sim1** | **DMSO_2_sim1** | **RA_1_sim1** | **RA_2_sim1** |
| --- | --- | --- | --- | --- | --- | --- |
| **1456521_at** | 8.54729837 | 8.37428281 | 5.93090481 | 5.57056262 | 6.42321538 | 5.88985763 |
| **1416303_at** | 8.14381115 | 8.16318531 | 9.23987631 | 9.39125994 | 9.18943306 | 9.36019085 |
| **1415894_at** | 4.66237058 | 4.89588553 | 4.96160253 | 4.75428588 | 4.23515512 | 4.26135342 |

A model is fit to this data in the same way as for the original data:

| **Probe set** | **LIF (baseline)** | **Adjustment due to DMSO treatment** | **Adjustment due to RA treatment** |
| --- | --- | --- | --- |
| **1456521_at** | 8.46079059 | -2.710056875 | -2.304254085 |
| **1416303_at** | 8.15349823 | 1.162069895 | 1.121313725 |
| **1415894_at** | 4.779128055 | 0.07881615 | -0.530873785 |

In our calculations, we generated 1000 data sets. In this example, we generate 10 simulated data sets. These are plotted in Figures 2 and 3.

## Applying Screening Criteria to Data sets

As described in the Results section, genes were considered to be part of the ESC signature change if they satisfied 3 criteria for inclusion. The particular thresholds used are shown in Table 2 of the paper. These screens were used on the simulated data sets. Figure 2 & 3 shows the results of applying a 2 fold cutoff to the probe sets and the resulting simulated data.


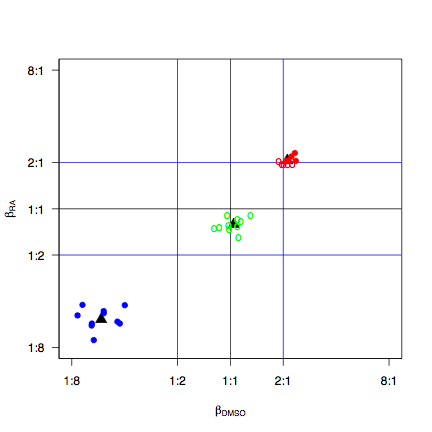


Figure 2: 10 simulated data sets for each of the 3 examples genes. Original data points are shown with triangles. Simulated data sets that pass the screening criteria are shown with filled circles, while those that failed are shown with open circles.

The confidence value is then the proportion of simulated data that pass all 3 criteria. In the case of 1456521_at, all simulated data passed the screen indicating a CV of 1 (Figure 2). For 1415894_at, none of the simulated data passed the screen leading to a CV of 0 (Figure 2). Half of the simulated data passed the criteria for 1416303_at, while half failed indicating a CV of 0.5 (Figures 2 & 3).


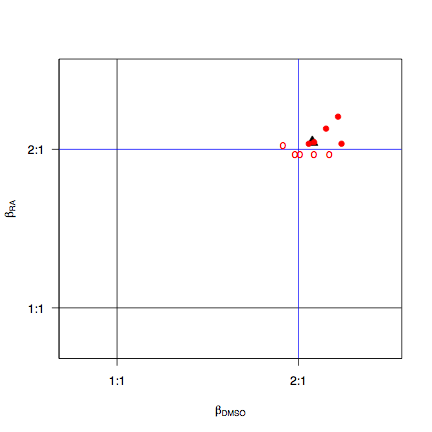


Figure 3: Magnification of the 10 simulated data sets in Figure 2 for gene 1416303_at.
