# Supplementary material for: Meta-Analysis of Differentiating Mouse Embryonic Stem Cell Gene Expression Kinetics Reveals Early Change of a Small Gene Set
Source: PLoS Comput Biol. 2006 Nov 24;2(11):e158. doi: 10.1371/journal.pcbi.0020158 (PMC1664699; doi:10.1371/journal.pcbi.0020158)
Supplement: Table S1 — (686 KB PDF) [file pcbi.0020158.st001.pdf]

| MG_U74v2 | Confidence Value |
|---|---|
| 100001_at | 0.00 |
| 100002_at | 0.00 |
| 100003_at | 0.00 |
| 100004_at | 0.00 |
| 100005_at | 0.03 |
| 100006_at | 0.00 |
| 100007_at | 0.00 |
| 100009_r_at | 0.67 |
| 100010_at | 0.00 |
| 100011_at | 0.07 |
| 100012_at | 0.03 |
| 100013_at | 0.00 |
| 100014_at | 0.00 |
| 100015_at | 0.32 |
| 100016_at | 0.00 |
| 100017_at | 0.00 |
| 100018_at | 0.00 |
| 100019_at | 0.00 |
| 100020_at | 0.00 |
| 100021_at | 0.00 |
| 100022_at | 0.00 |
| 100023_at | 0.05 |
| 100024_at | 0.39 |
| 100026_at | 0.00 |
| 100027_s_at | 0.00 |
| 100028_r_at | 0.00 |
| 100029_at | 0.00 |
| 100030_at | 0.91 |
| 100032_at | 0.32 |
| 100033_at | 0.00 |
| 100034_at | 0.00 |
| 100035_at | 0.00 |
| 100037_at | 0.01 |
| 100039_at | 0.05 |
| 100040_at | 0.00 |
| 100041_at | 0.00 |
| 100042_at | 0.00 |
| 100043_f_at | 0.00 |
| 100044_at | 0.00 |
| 100046_at | 0.13 |
| 100047_at | 0.00 |
| 100048_at | 0.00 |
| 100049_at | 0.00 |
| 100050_at | 0.28 |
| 100051_at | 0.00 |
| 100052_at | 0.00 |
| 100054_s_at | 0.01 |
| 100056_at | 0.00 |
| 100057_at | 0.00 |
| 100058_at | 0.00 |
| 100059_at | 0.04 |
| 100060_i_at | 0.00 |
| 100061_f_at | 0.00 |
| 100062_at | 0.00 |
| 100064_f_at | 0.00 |

| | |
|---|---|
| 100065_r_at | 0.00 |
| 100066_at | 0.00 |
| 100067_at | 0.00 |
| 100068_at | 0.00 |
| 100069_at | 0.00 |
| 100071_at | 0.00 |
| 100072_at | 0.00 |
| 100073_at | 0.41 |
| 100074_at | 0.02 |
| 100078_at | 0.00 |
| 100079_at | 0.00 |
| 100080_at | 0.00 |
| 100081_at | 0.01 |
| 100082_at | 0.00 |
| 100084_at | 0.00 |
| 100085_at | 0.00 |
| 100086_at | 0.00 |
| 100088_at | 0.03 |
| 100089_at | 0.01 |
| 100091_at | 0.00 |
| 100093_at | 0.00 |
| 100094_at | 0.01 |
| 100095_at | 0.00 |
| 100099_at | 0.00 |
| 100100_at | 0.00 |
| 100101_at | 0.00 |
| 100103_f_at | 0.00 |
| 100104_r_at | 0.00 |
| 100106_at | 0.00 |
| 100109_s_at | 0.00 |
| 100112_at | 0.05 |
| 100113_s_at | 0.00 |
| 100115_at | 0.00 |
| 100116_at | 0.02 |
| 100117_at | 0.00 |
| 100120_at | 0.19 |
| 100122_at | 0.00 |
| 100123_f_at | 0.00 |
| 100124_r_at | 0.00 |
| 100125_at | 0.18 |
| 100126_at | 0.00 |
| 100127_at | 0.00 |
| 100128_at | 0.00 |
| 100130_at | 0.00 |
| 100131_at | 0.00 |
| 100133_at | 0.14 |
| 100134_at | 0.00 |
| 100136_at | 0.01 |
| 100138_f_at | 0.00 |
| 100139_at | 0.00 |
| 100141_at | 0.00 |
| 100142_at | 0.00 |
| 100143_at | 0.00 |
| 100144_at | 0.00 |
| 100147_at | 0.00 |
| 100148_at | 0.00 |

| | |
|---|---|
| 100149_at | 0.00 |
| 100150_f_at | 0.00 |
| 100151_at | 0.00 |
| 100152_at | 0.00 |
| 100153_at | 0.00 |
| 100154_at | 0.27 |
| 100155_at | 0.50 |
| 100156_at | 0.00 |
| 100213_f_at | 0.00 |
| 100225_f_at | 0.00 |
| 100272_at | 0.00 |
| 100277_at | 0.03 |
| 100278_at | 0.00 |
| 100279_at | 0.00 |
| 100280_at | 0.00 |
| 100281_at | 0.00 |
| 100282_at | 0.00 |
| 100283_at | 0.00 |
| 100284_at | 0.00 |
| 100285_at | 0.00 |
| 100286_at | 0.00 |
| 100287_at | 0.00 |
| 100289_at | 0.00 |
| 100290_f_at | 0.03 |
| 100291_at | 0.00 |
| 100292_at | 0.00 |
| 100293_at | 0.00 |
| 100294_at | 0.00 |
| 100295_at | 0.00 |
| 100296_at | 0.00 |
| 100297_at | 0.00 |
| 100298_at | 0.00 |
| 100299_f_at | 0.00 |
| 100300_at | 0.00 |
| 100301_at | 0.57 |
| 100302_at | 0.00 |
| 100303_at | 0.00 |
| 100304_at | 0.00 |
| 100305_g_at | 0.00 |
| 100306_at | 0.00 |
| 100307_at | 0.00 |
| 100308_at | 0.05 |
| 100309_at | 0.00 |
| 100311_f_at | 0.00 |
| 100313_at | 0.00 |
| 100314_f_at | 0.00 |
| 100315_at | 0.00 |
| 100317_at | 0.00 |
| 100318_at | 0.00 |
| 100319_at | 0.00 |
| 100320_at | 0.02 |
| 100321_f_at | 0.00 |
| 100322_at | 0.00 |
| 100323_at | 0.03 |
| 100324_g_at | 0.03 |
| 100325_at | 0.31 |

| | |
|---|---|
| 100326_f_at | 0.00 |
| 100327_at | 0.00 |
| 100328_s_at | 0.00 |
| 100329_at | 0.00 |
| 100330_at | 0.00 |
| 100331_g_at | 0.00 |
| 100332_s_at | 0.00 |
| 100333_at | 0.00 |
| 100334_f_at | 0.00 |
| 100335_at | 0.00 |
| 100336_s_at | 0.00 |
| 100337_at | 0.00 |
| 100338_s_at | 0.00 |
| 100339_at | 0.00 |
| 100340_at | 0.00 |
| 100341_g_at | 0.00 |
| 100342_i_at | 0.08 |
| 100343_f_at | 0.00 |
| 100344_at | 0.00 |
| 100345_f_at | 0.25 |
| 100346_at | 0.00 |
| 100347_at | 0.00 |
| 100348_at | 0.17 |
| 100349_at | 0.06 |
| 100350_at | 0.00 |
| 100351_f_at | 0.00 |
| 100352_at | 0.00 |
| 100353_g_at | 0.00 |
| 100354_at | 0.00 |
| 100355_g_at | 0.00 |
| 100356_at | 0.00 |
| 100357_g_at | 0.00 |
| 100358_s_at | 0.00 |
| 100359_at | 0.00 |
| 100360_f_at | 0.00 |
| 100361_f_at | 0.00 |
| 100362_f_at | 0.00 |
| 100363_at | 0.00 |
| 100364_at | 0.00 |
| 100365_at | 0.00 |
| 100366_at | 0.00 |
| 100367_g_at | 0.00 |
| 100368_at | 0.00 |
| 100369_at | 0.00 |
| 100370_at | 0.00 |
| 100371_at | 0.10 |
| 100372_at | 0.00 |
| 100373_at | 0.00 |
| 100374_at | 0.21 |
| 100375_i_at | 0.04 |
| 100376_f_at | 0.00 |
| 100377_f_at | 0.00 |
| 100378_at | 0.00 |
| 100379_f_at | 0.00 |
| 100380_at | 0.00 |
| 100381_at | 0.00 |

| | |
|---|---|
| 100382_at | 0.00 |
| 100383_at | 0.00 |
| 100384_at | 0.00 |
| 100385_at | 0.00 |
| 100386_at | 0.00 |
| 100387_f_at | 0.00 |
| 100388_at | 0.00 |
| 100389_at | 0.00 |
| 100390_s_at | 0.00 |
| 100391_at | 0.00 |
| 100392_at | 0.00 |
| 100393_at | 0.00 |
| 100394_at | 0.00 |
| 100395_at | 0.17 |
| 100396_i_at | 0.00 |
| 100397_at | 0.00 |
| 100398_at | 0.00 |
| 100400_at | 0.04 |
| 100401_at | 0.00 |
| 100402_f_at | 0.00 |
| 100403_at | 0.00 |
| 100404_at | 0.00 |
| 100405_at | 0.09 |
| 100406_at | 0.00 |
| 100407_at | 0.00 |
| 100408_at | 0.00 |
| 100409_at | 0.00 |
| 100410_at | 0.00 |
| 100411_at | 0.00 |
| 100412_g_at | 0.00 |
| 100413_at | 0.00 |
| 100414_s_at | 0.00 |
| 100415_at | 0.00 |
| 100416_at | 0.00 |
| 100417_at | 0.12 |
| 100418_at | 0.41 |
| 100420_at | 0.00 |
| 100421_at | 0.00 |
| 100422_i_at | 0.00 |
| 100423_f_at | 0.00 |
| 100424_at | 0.00 |
| 100425_at | 0.00 |
| 100426_s_at | 0.00 |
| 100427_at | 0.00 |
| 100428_at | 0.00 |
| 100429_at | 0.00 |
| 100430_at | 0.00 |
| 100431_at | 0.00 |
| 100432_f_at | 0.18 |
| 100433_r_at | 0.00 |
| 100434_s_at | 0.00 |
| 100435_at | 0.00 |
| 100436_at | 0.00 |
| 100437_g_at | 0.00 |
| 100438_at | 0.00 |
| 100439_i_at | 0.00 |

| | |
|---|---|
| 100440_f_at | 0.00 |
| 100441_s_at | 0.00 |
| 100442_at | 0.00 |
| 100443_at | 0.00 |
| 100444_at | 0.00 |
| 100445_f_at | 0.00 |
| 100446_r_at | 0.00 |
| 100447_at | 0.00 |
| 100448_at | 0.00 |
| 100449_g_at | 0.00 |
| 100450_r_at | 0.00 |
| 100451_at | 0.00 |
| 100452_at | 0.00 |
| 100453_at | 0.00 |
| 100454_at | 0.00 |
| 100455_at | 0.00 |
| 100457_at | 0.05 |
| 100458_at | 0.00 |
| 100459_at | 0.00 |
| 100460_at | 0.00 |
| 100461_at | 0.00 |
| 100462_at | 0.00 |
| 100463_at | 0.00 |
| 100464_at | 0.00 |
| 100465_i_at | 0.00 |
| 100466_f_at | 0.00 |
| 100467_at | 0.00 |
| 100468_g_at | 0.00 |
| 100469_at | 0.00 |
| 100470_at | 0.00 |
| 100471_at | 0.05 |
| 100472_at | 0.61 |
| 100473_at | 0.01 |
| 100474_at | 0.00 |
| 100475_at | 0.14 |
| 100476_at | 0.00 |
| 100477_at | 0.04 |
| 100479_at | 0.00 |
| 100481_at | 0.00 |
| 100482_at | 0.00 |
| 100483_at | 0.00 |
| 100484_at | 0.49 |
| 100486_at | 0.00 |
| 100488_at | 0.00 |
| 100489_at | 0.01 |
| 100491_at | 0.00 |
| 100492_at | 0.15 |
| 100493_at | 0.00 |
| 100494_at | 0.00 |
| 100495_at | 0.00 |
| 100496_at | 0.00 |
| 100497_at | 0.00 |
| 100498_g_at | 0.13 |
| 100499_at | 0.02 |
| 100500_at | 0.00 |
| 100501_at | 0.00 |

| | |
|---|---|
| 100502_at | 0.00 |
| 100505_at | 0.00 |
| 100507_at | 0.00 |
| 100508_at | 0.00 |
| 100509_at | 0.00 |
| 100510_at | 0.00 |
| 100511_at | 0.00 |
| 100512_at | 0.00 |
| 100513_at | 0.00 |
| 100514_at | 0.24 |
| 100515_at | 0.00 |
| 100516_at | 0.00 |
| 100518_at | 0.00 |
| 100521_at | 0.00 |
| 100522_s_at | 0.00 |
| 100523_r_at | 0.17 |
| 100525_at | 0.00 |
| 100526_f_at | 0.00 |
| 100527_at | 0.00 |
| 100528_at | 0.00 |
| 100529_at | 0.00 |
| 100530_at | 0.00 |
| 100533_s_at | 0.00 |
| 100534_at | 0.00 |
| 100535_at | 0.00 |
| 100536_at | 0.00 |
| 100537_at | 0.00 |
| 100538_at | 0.21 |
| 100539_at | 0.00 |
| 100540_at | 0.00 |
| 100542_at | 0.00 |
| 100543_s_at | 0.00 |
| 100544_at | 0.00 |
| 100546_at | 0.00 |
| 100547_at | 0.00 |
| 100548_at | 0.00 |
| 100549_at | 0.00 |
| 100550_f_at | 0.03 |
| 100551_r_at | 0.00 |
| 100552_at | 0.00 |
| 100553_at | 0.00 |
| 100554_at | 0.00 |
| 100555_at | 0.00 |
| 100556_at | 0.00 |
| 100557_g_at | 0.00 |
| 100558_at | 0.00 |
| 100559_at | 0.33 |
| 100560_at | 0.01 |
| 100561_at | 0.03 |
| 100562_at | 0.00 |
| 100564_at | 0.00 |
| 100565_at | 0.00 |
| 100566_at | 0.00 |
| 100567_at | 0.00 |
| 100568_at | 0.00 |
| 100569_at | 0.34 |

| | |
|---|---|
| 100570_at | 0.00 |
| 100571_at | 0.10 |
| 100572_at | 0.00 |
| 100573_f_at | 0.00 |
| 100574_f_at | 0.00 |
| 100575_at | 0.00 |
| 100576_at | 0.00 |
| 100577_at | 0.00 |
| 100578_at | 0.00 |
| 100579_s_at | 0.00 |
| 100580_at | 0.00 |
| 100581_at | 0.13 |
| 100582_at | 0.00 |
| 100583_at | 0.00 |
| 100584_at | 0.05 |
| 100585_at | 0.00 |
| 100586_i_at | 0.00 |
| 100587_f_at | 0.00 |
| 100588_at | 0.14 |
| 100589_at | 0.00 |
| 100592_at | 0.00 |
| 100593_at | 0.00 |
| 100594_at | 0.00 |
| 100595_at | 0.00 |
| 100596_at | 0.00 |
| 100597_at | 0.00 |
| 100599_at | 0.00 |
| 100600_at | 0.00 |
| 100601_at | 0.01 |
| 100602_at | 0.00 |
| 100603_at | 0.00 |
| 100605_at | 0.00 |
| 100606_at | 0.00 |
| 100607_at | 0.00 |
| 100608_at | 0.00 |
| 100609_at | 0.00 |
| 100610_at | 0.01 |
| 100611_at | 0.41 |
| 100612_at | 0.00 |
| 100613_at | 0.00 |
| 100614_at | 0.00 |
| 100615_at | 0.00 |
| 100616_at | 0.00 |
| 100617_at | 0.00 |
| 100618_f_at | 0.00 |
| 100619_r_at | 0.00 |
| 100620_at | 0.00 |
| 100621_at | 0.00 |
| 100622_at | 0.00 |
| 100623_at | 0.00 |
| 100626_at | 0.08 |
| 100628_at | 0.00 |
| 100629_at | 0.02 |
| 100630_f_at | 0.00 |
| 100631_r_at | 0.00 |
| 100632_at | 0.00 |

| | |
|---|---|
| 100633_at | 0.00 |
| 100634_at | 0.00 |
| 100635_at | 0.00 |
| 100636_at | 0.01 |
| 100669_at | 0.00 |
| 100670_at | 0.00 |
| 100671_at | 0.00 |
| 100672_at | 0.00 |
| 100673_f_at | 0.01 |
| 100674_f_at | 0.00 |
| 100675_at | 0.00 |
| 100676_at | 0.00 |
| 100677_f_at | 0.00 |
| 100678_s_at | 0.00 |
| 100679_at | 0.00 |
| 100680_at | 0.38 |
| 100681_f_at | 0.00 |
| 100682_f_at | 0.01 |
| 100683_r_at | 0.00 |
| 100684_at | 0.00 |
| 100685_at | 0.00 |
| 100686_at | 0.00 |
| 100687_f_at | 0.00 |
| 100688_at | 0.00 |
| 100689_at | 0.00 |
| 100690_at | 0.00 |
| 100691_at | 0.00 |
| 100692_at | 0.00 |
| 100693_at | 0.00 |
| 100694_at | 0.00 |
| 100695_at | 0.00 |
| 100696_at | 0.00 |
| 100697_at | 0.00 |
| 100698_at | 0.00 |
| 100699_at | 0.00 |
| 100700_s_at | 0.00 |
| 100701_r_at | 0.01 |
| 100702_at | 0.00 |
| 100703_at | 0.00 |
| 100704_at | 0.00 |
| 100705_at | 0.00 |
| 100706_f_at | 0.13 |
| 100707_at | 0.00 |
| 100708_at | 0.00 |
| 100709_at | 0.00 |
| 100710_at | 0.00 |
| 100711_at | 0.00 |
| 100712_at | 0.00 |
| 100713_at | 0.01 |
| 100714_at | 0.00 |
| 100715_at | 0.00 |
| 100716_at | 0.00 |
| 100717_at | 0.00 |
| 100718_at | 0.04 |
| 100719_f_at | 0.00 |
| 100720_at | 0.00 |

| | |
|---|---|
| 100721_f_at | 0.00 |
| 100722_r_at | 0.00 |
| 100723_f_at | 0.00 |
| 100724_at | 0.00 |
| 100725_at | 0.00 |
| 100726_at | 0.00 |
| 100727_at | 0.00 |
| 100728_at | 0.00 |
| 100729_at | 0.00 |
| 100730_at | 0.00 |
| 100731_at | 0.00 |
| 100732_at | 0.00 |
| 100733_at | 0.00 |
| 100734_at | 0.00 |
| 100735_at | 0.00 |
| 100736_at | 0.00 |
| 100737_at | 0.00 |
| 100738_at | 0.00 |
| 100739_at | 0.00 |
| 100740_at | 0.00 |
| 100741_at | 0.00 |
| 100742_at | 0.02 |
| 100743_at | 0.00 |
| 100744_at | 0.00 |
| 100745_r_at | 0.00 |
| 100746_at | 0.00 |
| 100747_at | 0.00 |
| 100748_at | 0.00 |
| 100749_at | 0.00 |
| 100750_at | 0.00 |
| 100751_at | 0.00 |
| 100752_at | 0.00 |
| 100753_at | 0.00 |
| 100754_at | 0.00 |
| 100755_at | 0.00 |
| 100756_r_at | 0.00 |
| 100757_at | 0.00 |
| 100758_at | 0.00 |
| 100759_at | 0.00 |
| 100760_at | 0.00 |
| 100761_at | 0.00 |
| 100762_at | 0.00 |
| 100763_at | 0.00 |
| 100764_at | 0.00 |
| 100765_at | 0.00 |
| 100766_at | 0.00 |
| 100767_at | 0.00 |
| 100768_at | 0.00 |
| 100769_at | 0.00 |
| 100771_at | 0.00 |
| 100772_g_at | 0.02 |
| 100773_at | 0.00 |
| 100774_at | 0.00 |
| 100775_at | 0.00 |
| 100776_at | 0.00 |
| 100778_at | 0.00 |

| | |
|---|---|
| 100779_at | 0.00 |
| 100780_at | 0.00 |
| 100828_at | 0.00 |
| 100839_at | 0.00 |
| 100876_at | 0.33 |
| 100877_at | 0.00 |
| 100878_at | 0.04 |
| 100879_at | 0.00 |
| 100880_at | 0.00 |
| 100882_at | 0.00 |
| 100883_at | 0.00 |
| 100884_at | 0.04 |
| 100885_at | 0.00 |
| 100886_f_at | 0.00 |
| 100887_at | 0.02 |
| 100888_at | 0.00 |
| 100889_at | 0.00 |
| 100890_at | 0.00 |
| 100891_at | 0.00 |
| 100892_at | 0.00 |
| 100893_at | 0.00 |
| 100894_at | 0.00 |
| 100895_at | 0.00 |
| 100896_at | 0.00 |
| 100897_f_at | 0.00 |
| 100898_r_at | 0.01 |
| 100899_s_at | 0.00 |
| 100900_at | 0.00 |
| 100901_at | 0.04 |
| 100902_at | 0.00 |
| 100903_at | 0.00 |
| 100904_at | 0.00 |
| 100905_at | 0.00 |
| 100906_at | 0.00 |
| 100907_at | 0.00 |
| 100908_at | 0.00 |
| 100909_at | 0.00 |
| 100910_at | 0.00 |
| 100911_at | 0.00 |
| 100912_at | 0.00 |
| 100913_at | 0.00 |
| 100914_at | 0.00 |
| 100915_at | 0.05 |
| 100916_at | 0.00 |
| 100917_at | 0.00 |
| 100920_at | 0.09 |
| 100921_at | 0.00 |
| 100923_at | 0.02 |
| 100924_at | 0.02 |
| 100925_at | 0.00 |
| 100926_at | 0.00 |
| 100927_at | 0.00 |
| 100928_at | 0.60 |
| 100929_at | 0.14 |
| 100931_at | 0.05 |
| 100932_at | 0.00 |

| | |
|---|---|
| 100933_at | 0.00 |
| 100935_at | 0.00 |
| 100938_at | 0.00 |
| 100939_at | 0.00 |
| 100941_at | 0.13 |
| 100943_at | 0.00 |
| 100944_at | 0.00 |
| 100946_at | 0.00 |
| 100947_at | 0.01 |
| 100948_at | 0.00 |
| 100949_at | 0.00 |
| 100951_at | 0.00 |
| 100952_at | 0.00 |
| 100953_at | 0.00 |
| 100954_at | 0.19 |
| 100955_at | 0.04 |
| 100956_at | 0.00 |
| 100957_at | 0.00 |
| 100958_at | 0.02 |
| 100959_at | 0.70 |
| 100960_g_at | 0.00 |
| 100961_at | 0.00 |
| 100962_at | 0.00 |
| 100963_at | 0.00 |
| 100964_at | 0.00 |
| 100965_at | 0.00 |
| 100966_at | 0.00 |
| 100967_at | 0.03 |
| 100968_at | 0.00 |
| 100970_at | 0.00 |
| 100971_at | 0.00 |
| 100972_s_at | 0.00 |
| 100973_i_at | 0.00 |
| 100974_at | 0.13 |
| 100975_f_at | 0.00 |
| 100976_at | 0.18 |
| 100977_at | 0.02 |
| 100978_at | 0.00 |
| 100979_at | 0.00 |
| 100980_at | 0.00 |
| 100981_at | 0.01 |
| 100982_at | 0.00 |
| 100983_at | 0.00 |
| 100984_at | 0.00 |
| 100985_at | 0.00 |
| 100986_at | 0.00 |
| 100987_f_at | 0.00 |
| 100988_at | 0.00 |
| 100989_at | 0.00 |
| 100990_g_at | 0.01 |
| 100991_at | 0.00 |
| 100992_at | 0.00 |
| 100994_at | 0.00 |
| 100995_at | 0.00 |
| 100996_at | 0.00 |
| 100997_at | 0.00 |

| | |
|---|---|
| 100998_at | 0.00 |
| 101000_at | 0.05 |
| 101001_at | 0.00 |
| 101002_at | 0.00 |
| 101003_at | 0.00 |
| 101004_f_at | 0.01 |
| 101006_at | 0.00 |
| 101007_at | 0.03 |
| 101008_at | 0.00 |
| 101009_at | 0.69 |
| 101010_at | 0.00 |
| 101011_at | 0.00 |
| 101013_at | 0.00 |
| 101014_at | 0.00 |
| 101015_s_at | 0.00 |
| 101016_at | 0.00 |
| 101017_at | 0.00 |
| 101019_at | 0.04 |
| 101020_at | 0.00 |
| 101022_at | 0.00 |
| 101023_f_at | 0.00 |
| 101024_i_at | 0.02 |
| 101025_f_at | 0.00 |
| 101026_at | 0.00 |
| 101027_s_at | 0.00 |
| 101028_i_at | 0.24 |
| 101029_f_at | 0.00 |
| 101030_at | 0.02 |
| 101031_at | 0.00 |
| 101033_at | 0.00 |
| 101034_at | 0.00 |
| 101035_at | 0.00 |
| 101036_at | 0.00 |
| 101037_at | 0.00 |
| 101039_at | 0.00 |
| 101040_at | 0.06 |
| 101041_at | 0.00 |
| 101042_f_at | 0.00 |
| 101043_f_at | 0.00 |
| 101044_at | 0.05 |
| 101045_at | 0.00 |
| 101046_at | 0.00 |
| 101047_at | 0.05 |
| 101048_at | 0.00 |
| 101049_at | 0.00 |
| 101050_at | 0.00 |
| 101051_at | 0.00 |
| 101052_g_at | 0.00 |
| 101053_at | 0.00 |
| 101054_at | 0.00 |
| 101055_at | 0.00 |
| 101056_at | 0.00 |
| 101057_at | 0.00 |
| 101058_at | 0.00 |
| 101059_at | 0.01 |
| 101060_at | 0.04 |

| | |
|---|---|
| 101061_at | 0.04 |
| 101062_at | 0.00 |
| 101063_at | 0.00 |
| 101064_at | 0.00 |
| 101065_at | 0.00 |
| 101067_at | 0.02 |
| 101068_at | 0.00 |
| 101069_g_at | 0.00 |
| 101070_at | 0.00 |
| 101071_at | 0.00 |
| 101072_at | 0.00 |
| 101073_at | 0.00 |
| 101074_at | 0.00 |
| 101075_f_at | 0.00 |
| 101076_r_at | 0.00 |
| 101077_at | 0.00 |
| 101078_at | 0.03 |
| 101079_at | 0.00 |
| 101080_at | 0.05 |
| 101081_at | 0.00 |
| 101082_at | 0.01 |
| 101083_s_at | 0.00 |
| 101084_f_at | 0.00 |
| 101085_at | 0.03 |
| 101086_f_at | 0.00 |
| 101087_r_at | 0.00 |
| 101088_f_at | 0.00 |
| 101089_at | 0.55 |
| 101090_at | 0.00 |
| 101091_at | 0.00 |
| 101093_at | 0.00 |
| 101094_at | 0.00 |
| 101095_at | 0.00 |
| 101096_s_at | 0.00 |
| 101097_at | 0.00 |
| 101099_at | 0.00 |
| 101101_at | 0.13 |
| 101102_at | 0.00 |
| 101103_at | 0.00 |
| 101104_at | 0.00 |
| 101105_at | 0.00 |
| 101106_at | 0.00 |
| 101107_at | 0.00 |
| 101108_at | 0.00 |
| 101109_at | 0.00 |
| 101110_at | 0.18 |
| 101111_at | 0.00 |
| 101112_g_at | 0.00 |
| 101113_at | 0.10 |
| 101114_at | 0.00 |
| 101115_at | 0.00 |
| 101116_at | 0.00 |
| 101117_at | 0.00 |
| 101118_at | 0.00 |
| 101119_at | 0.00 |
| 101120_at | 0.00 |

| | |
|---|---|
| 101121_at | 0.00 |
| 101122_at | 0.00 |
| 101123_at | 0.01 |
| 101124_at | 0.00 |
| 101125_at | 0.00 |
| 101126_r_at | 0.00 |
| 101127_at | 0.00 |
| 101128_at | 0.00 |
| 101129_at | 0.00 |
| 101130_at | 0.29 |
| 101131_at | 0.00 |
| 101132_at | 0.00 |
| 101133_at | 0.00 |
| 101134_at | 0.00 |
| 101135_at | 0.88 |
| 101136_at | 0.00 |
| 101137_at | 0.00 |
| 101138_at | 0.00 |
| 101139_r_at | 0.03 |
| 101140_at | 0.00 |
| 101141_at | 0.00 |
| 101142_at | 0.00 |
| 101143_at | 0.00 |
| 101144_at | 0.00 |
| 101145_at | 0.00 |
| 101146_at | 0.00 |
| 101147_at | 0.00 |
| 101148_at | 0.02 |
| 101149_at | 0.00 |
| 101150_at | 0.18 |
| 101151_at | 0.00 |
| 101152_at | 0.00 |
| 101153_at | 0.00 |
| 101154_at | 0.00 |
| 101155_at | 0.00 |
| 101156_at | 0.00 |
| 101157_at | 0.00 |
| 101158_at | 0.00 |
| 101159_at | 0.00 |
| 101160_at | 0.54 |
| 101161_at | 0.00 |
| 101162_at | 0.00 |
| 101163_at | 0.00 |
| 101164_at | 0.00 |
| 101165_at | 0.00 |
| 101166_at | 0.00 |
| 101167_at | 0.00 |
| 101168_at | 0.00 |
| 101169_at | 0.00 |
| 101170_at | 0.00 |
| 101171_at | 0.00 |
| 101172_at | 0.00 |
| 101173_at | 0.00 |
| 101174_at | 0.00 |
| 101175_at | 0.00 |
| 101176_at | 0.00 |

| | |
|---|---|
| 101177_at | 0.00 |
| 101178_at | 0.00 |
| 101179_at | 0.22 |
| 101180_at | 0.02 |
| 101181_at | 0.00 |
| 101182_at | 0.04 |
| 101183_at | 0.00 |
| 101184_at | 0.00 |
| 101185_at | 0.00 |
| 101186_at | 0.00 |
| 101187_at | 0.00 |
| 101188_at | 0.06 |
| 101189_at | 0.00 |
| 101190_at | 0.00 |
| 101191_at | 0.00 |
| 101192_at | 0.00 |
| 101193_at | 0.00 |
| 101194_at | 0.00 |
| 101195_at | 0.00 |
| 101196_at | 0.00 |
| 101197_at | 0.00 |
| 101198_at | 0.00 |
| 101199_at | 0.00 |
| 101200_at | 0.00 |
| 101201_r_at | 0.00 |
| 101202_at | 0.00 |
| 101203_r_at | 0.00 |
| 101204_at | 0.00 |
| 101205_at | 0.00 |
| 101206_at | 0.00 |
| 101207_at | 0.00 |
| 101208_at | 0.00 |
| 101209_at | 0.00 |
| 101210_at | 0.00 |
| 101211_at | 0.00 |
| 101212_at | 0.00 |
| 101213_at | 0.00 |
| 101214_f_at | 0.00 |
| 101215_at | 0.00 |
| 101216_at | 0.00 |
| 101217_at | 0.01 |
| 101218_at | 0.00 |
| 101219_at | 0.00 |
| 101220_at | 0.00 |
| 101221_at | 0.00 |
| 101222_f_at | 0.00 |
| 101223_r_at | 0.00 |
| 101224_at | 0.00 |
| 101225_at | 0.00 |
| 101226_at | 0.00 |
| 101227_at | 0.00 |
| 101228_at | 0.00 |
| 101229_at | 0.00 |
| 101230_at | 0.00 |
| 101231_at | 0.00 |
| 101232_at | 0.00 |

| | |
|---|---|
| 101233_at | 0.00 |
| 101234_at | 0.00 |
| 101235_f_at | 0.00 |
| 101254_at | 0.00 |
| 101255_at | 0.00 |
| 101276_at | 0.00 |
| 101277_at | 0.00 |
| 101278_at | 0.00 |
| 101279_f_at | 0.00 |
| 101280_at | 0.00 |
| 101281_at | 0.00 |
| 101282_at | 0.00 |
| 101286_at | 0.00 |
| 101287_s_at | 0.05 |
| 101288_at | 0.00 |
| 101289_f_at | 0.00 |
| 101290_at | 0.00 |
| 101291_at | 0.00 |
| 101292_f_at | 0.00 |
| 101293_at | 0.00 |
| 101294_g_at | 0.11 |
| 101295_s_at | 0.00 |
| 101296_at | 0.00 |
| 101297_at | 0.00 |
| 101298_g_at | 0.00 |
| 101299_at | 0.00 |
| 101300_at | 0.00 |
| 101301_at | 0.00 |
| 101302_at | 0.00 |
| 101303_at | 0.00 |
| 101305_at | 0.00 |
| 101306_f_at | 0.01 |
| 101307_at | 0.00 |
| 101308_at | 0.00 |
| 101309_at | 0.00 |
| 101310_at | 0.00 |
| 101311_at | 0.00 |
| 101312_at | 0.00 |
| 101313_r_at | 0.00 |
| 101314_at | 0.00 |
| 101315_at | 0.00 |
| 101316_at | 0.00 |
| 101317_f_at | 0.00 |
| 101318_at | 0.00 |
| 101319_f_at | 0.00 |
| 101320_f_at | 0.00 |
| 101321_r_at | 0.00 |
| 101324_r_at | 0.00 |
| 101325_r_at | 0.00 |
| 101326_at | 0.00 |
| 101327_at | 0.00 |
| 101328_at | 0.00 |
| 101329_f_at | 0.00 |
| 101330_f_at | 0.00 |
| 101331_f_at | 0.00 |
| 101332_at | 0.00 |

| | |
|---|---|
| 101333_at | 0.00 |
| 101334_at | 0.00 |
| 101335_at | 0.00 |
| 101336_at | 0.00 |
| 101337_at | 0.00 |
| 101338_f_at | 0.00 |
| 101339_at | 0.00 |
| 101340_at | 0.00 |
| 101341_at | 0.00 |
| 101342_at | 0.00 |
| 101343_at | 0.00 |
| 101344_at | 0.00 |
| 101345_at | 0.00 |
| 101346_at | 0.00 |
| 101347_at | 0.00 |
| 101348_at | 0.00 |
| 101349_at | 0.00 |
| 101350_g_at | 0.00 |
| 101351_at | 0.00 |
| 101352_g_at | 0.00 |
| 101353_at | 0.00 |
| 101354_at | 0.00 |
| 101355_at | 0.00 |
| 101356_at | 0.00 |
| 101357_at | 0.01 |
| 101358_at | 0.00 |
| 101359_at | 0.00 |
| 101362_at | 0.00 |
| 101363_at | 0.00 |
| 101364_at | 0.00 |
| 101366_f_at | 0.00 |
| 101367_at | 0.00 |
| 101368_at | 0.76 |
| 101369_at | 0.00 |
| 101370_at | 0.00 |
| 101371_at | 0.00 |
| 101372_at | 0.00 |
| 101374_at | 0.00 |
| 101375_at | 0.00 |
| 101376_at | 0.00 |
| 101377_at | 0.00 |
| 101380_at | 0.00 |
| 101381_at | 0.00 |
| 101382_at | 0.00 |
| 101383_at | 0.00 |
| 101384_at | 0.00 |
| 101385_at | 0.00 |
| 101386_at | 0.00 |
| 101387_at | 0.00 |
| 101388_at | 0.00 |
| 101389_at | 0.13 |
| 101390_at | 0.00 |
| 101392_at | 0.00 |
| 101393_at | 0.03 |
| 101394_at | 0.00 |
| 101395_at | 0.00 |

| | |
|---|---|
| 101396_at | 0.00 |
| 101397_at | 0.00 |
| 101398_at | 0.00 |
| 101399_at | 0.00 |
| 101401_at | 0.00 |
| 101402_at | 0.00 |
| 101403_at | 0.00 |
| 101404_at | 0.06 |
| 101405_at | 0.00 |
| 101406_at | 0.00 |
| 101407_at | 0.00 |
| 101408_at | 0.00 |
| 101409_at | 0.00 |
| 101410_at | 0.03 |
| 101412_at | 0.00 |
| 101413_at | 0.00 |
| 101414_at | 0.00 |
| 101415_i_at | 0.00 |
| 101416_f_at | 0.00 |
| 101417_at | 0.00 |
| 101419_at | 0.00 |
| 101420_at | 0.00 |
| 101421_at | 0.00 |
| 101422_at | 0.00 |
| 101423_at | 0.03 |
| 101424_at | 0.00 |
| 101425_at | 0.00 |
| 101426_at | 0.00 |
| 101428_at | 0.00 |
| 101429_at | 0.00 |
| 101430_at | 0.03 |
| 101431_at | 0.00 |
| 101432_at | 0.00 |
| 101433_at | 0.00 |
| 101435_at | 0.00 |
| 101436_at | 0.00 |
| 101437_at | 0.00 |
| 101439_at | 0.00 |
| 101440_at | 0.00 |
| 101441_i_at | 0.00 |
| 101442_f_at | 0.00 |
| 101443_at | 0.00 |
| 101444_at | 0.00 |
| 101445_at | 0.00 |
| 101446_at | 0.01 |
| 101447_at | 0.00 |
| 101448_at | 0.00 |
| 101449_at | 0.01 |
| 101450_at | 0.00 |
| 101451_at | 0.03 |
| 101453_at | 0.00 |
| 101455_at | 0.07 |
| 101456_at | 0.03 |
| 101457_at | 0.00 |
| 101458_at | 0.32 |
| 101459_at | 0.00 |

| | |
|---|---|
| 101461_f_at | 0.00 |
| 101462_r_at | 0.00 |
| 101463_at | 0.00 |
| 101464_at | 0.39 |
| 101465_at | 0.01 |
| 101466_at | 0.00 |
| 101467_at | 0.00 |
| 101468_at | 0.00 |
| 101469_at | 0.00 |
| 101470_at | 0.00 |
| 101471_at | 0.00 |
| 101472_s_at | 0.00 |
| 101473_at | 0.00 |
| 101474_at | 0.00 |
| 101475_at | 0.00 |
| 101476_at | 0.00 |
| 101480_at | 0.00 |
| 101481_at | 0.00 |
| 101482_at | 0.00 |
| 101483_at | 0.00 |
| 101484_at | 0.00 |
| 101485_at | 0.00 |
| 101486_at | 0.42 |
| 101487_f_at | 0.01 |
| 101488_r_at | 0.00 |
| 101489_at | 0.00 |
| 101490_at | 0.00 |
| 101492_at | 0.00 |
| 101493_at | 0.00 |
| 101494_at | 0.00 |
| 101495_at | 0.00 |
| 101498_at | 0.00 |
| 101499_at | 0.13 |
| 101500_at | 0.00 |
| 101501_r_at | 0.00 |
| 101502_at | 0.00 |
| 101503_at | 0.03 |
| 101505_at | 0.00 |
| 101506_at | 0.01 |
| 101507_at | 0.00 |
| 101508_at | 0.02 |
| 101509_at | 0.00 |
| 101510_at | 0.12 |
| 101511_at | 0.00 |
| 101513_at | 0.00 |
| 101514_at | 0.00 |
| 101515_at | 0.00 |
| 101516_at | 0.00 |
| 101517_at | 0.00 |
| 101518_at | 0.00 |
| 101519_at | 0.00 |
| 101520_at | 0.00 |
| 101521_at | 0.00 |
| 101522_at | 0.00 |
| 101523_at | 0.05 |
| 101524_at | 0.01 |

| | |
|---|---|
| 101525_at | 0.00 |
| 101526_at | 0.03 |
| 101527_at | 0.00 |
| 101528_at | 0.00 |
| 101529_g_at | 0.03 |
| 101530_at | 0.00 |
| 101531_at | 0.00 |
| 101532_g_at | 0.00 |
| 101534_at | 0.00 |
| 101536_at | 0.00 |
| 101537_at | 0.00 |
| 101538_i_at | 0.00 |
| 101539_f_at | 0.00 |
| 101540_at | 0.03 |
| 101541_at | 0.00 |
| 101542_f_at | 0.00 |
| 101543_f_at | 0.00 |
| 101546_at | 0.00 |
| 101548_at | 0.00 |
| 101550_at | 0.00 |
| 101551_s_at | 0.00 |
| 101552_at | 0.00 |
| 101553_at | 0.00 |
| 101554_at | 0.42 |
| 101555_at | 0.00 |
| 101557_at | 0.00 |
| 101558_s_at | 0.00 |
| 101560_at | 0.60 |
| 101561_at | 0.00 |
| 101562_at | 0.00 |
| 101564_at | 0.00 |
| 101565_f_at | 0.00 |
| 101566_f_at | 0.00 |
| 101567_at | 0.00 |
| 101568_at | 0.17 |
| 101569_at | 0.00 |
| 101570_at | 0.00 |
| 101571_g_at | 0.00 |
| 101572_f_at | 0.00 |
| 101573_f_at | 0.00 |
| 101574_f_at | 0.00 |
| 101575_i_at | 0.00 |
| 101576_f_at | 0.00 |
| 101577_at | 0.00 |
| 101578_f_at | 0.00 |
| 101579_at | 0.00 |
| 101580_at | 0.00 |
| 101581_at | 0.00 |
| 101582_at | 0.00 |
| 101583_at | 0.00 |
| 101584_at | 0.00 |
| 101585_at | 0.02 |
| 101587_at | 0.01 |
| 101588_at | 0.00 |
| 101589_at | 0.00 |
| 101590_at | 0.00 |

| | |
|---|---|
| 101591_at | 0.01 |
| 101593_at | 0.30 |
| 101595_at | 0.00 |
| 101596_at | 0.00 |
| 101597_at | 0.00 |
| 101598_at | 0.00 |
| 101599_at | 0.00 |
| 101600_at | 0.00 |
| 101601_at | 0.00 |
| 101602_at | 0.00 |
| 101603_g_at | 0.00 |
| 101604_at | 0.00 |
| 101605_at | 0.00 |
| 101606_at | 0.00 |
| 101607_at | 0.00 |
| 101608_at | 0.00 |
| 101609_at | 0.00 |
| 101610_at | 0.00 |
| 101611_at | 0.00 |
| 101612_at | 0.00 |
| 101613_at | 0.00 |
| 101614_at | 0.00 |
| 101615_at | 0.00 |
| 101616_at | 0.00 |
| 101617_s_at | 0.06 |
| 101618_r_at | 0.00 |
| 101619_at | 0.00 |
| 101620_at | 0.00 |
| 101621_at | 0.00 |
| 101622_at | 0.00 |
| 101623_at | 0.00 |
| 101624_at | 0.00 |
| 101625_at | 0.00 |
| 101626_at | 0.00 |
| 101627_at | 0.00 |
| 101628_g_at | 0.00 |
| 101629_s_at | 0.00 |
| 101630_f_at | 0.00 |
| 101631_at | 0.57 |
| 101632_at | 0.00 |
| 101633_at | 0.00 |
| 101634_at | 0.00 |
| 101635_f_at | 0.02 |
| 101636_at | 0.00 |
| 101637_at | 0.00 |
| 101638_s_at | 0.00 |
| 101639_r_at | 0.00 |
| 101640_f_at | 0.00 |
| 101641_at | 0.00 |
| 101642_at | 0.00 |
| 101643_at | 0.00 |
| 101644_f_at | 0.00 |
| 101645_f_at | 0.00 |
| 101646_at | 0.00 |
| 101647_at | 0.00 |
| 101648_at | 0.00 |

| | |
|---|---|
| 101649_at | 0.00 |
| 101650_at | 0.00 |
| 101651_at | 0.00 |
| 101652_i_at | 0.00 |
| 101653_f_at | 0.00 |
| 101654_at | 0.00 |
| 101655_at | 0.00 |
| 101656_f_at | 0.00 |
| 101657_at | 0.00 |
| 101658_f_at | 0.00 |
| 101659_at | 0.00 |
| 101660_at | 0.00 |
| 101661_r_at | 0.00 |
| 101662_at | 0.00 |
| 101663_s_at | 0.00 |
| 101664_at | 0.00 |
| 101665_at | 0.00 |
| 101666_at | 0.00 |
| 101667_at | 0.00 |
| 101668_at | 0.00 |
| 101669_at | 0.00 |
| 101670_at | 0.00 |
| 101671_at | 0.00 |
| 101672_at | 0.00 |
| 101673_r_at | 0.00 |
| 101674_at | 0.00 |
| 101675_at | 0.00 |
| 101676_at | 0.21 |
| 101677_at | 0.00 |
| 101678_r_at | 0.00 |
| 101679_at | 0.00 |
| 101680_at | 0.00 |
| 101681_f_at | 0.00 |
| 101682_f_at | 0.02 |
| 101683_at | 0.00 |
| 101684_r_at | 0.02 |
| 101685_f_at | 0.00 |
| 101686_at | 0.00 |
| 101687_r_at | 0.00 |
| 101688_at | 0.00 |
| 101689_at | 0.00 |
| 101690_r_at | 0.00 |
| 101691_s_at | 0.00 |
| 101692_r_at | 0.00 |
| 101693_f_at | 0.00 |
| 101694_f_at | 0.00 |
| 101695_at | 0.00 |
| 101696_r_at | 0.00 |
| 101697_f_at | 0.00 |
| 101698_f_at | 0.00 |
| 101699_at | 0.00 |
| 101700_at | 0.00 |
| 101701_at | 0.00 |
| 101702_at | 0.00 |
| 101703_at | 0.00 |
| 101704_at | 0.00 |

| | |
|---|---|
| 101705_at | 0.00 |
| 101706_at | 0.00 |
| 101707_at | 0.00 |
| 101708_at | 0.47 |
| 101709_at | 0.00 |
| 101710_at | 0.00 |
| 101711_at | 0.00 |
| 101712_at | 0.00 |
| 101713_at | 0.00 |
| 101714_at | 0.00 |
| 101715_at | 0.00 |
| 101716_at | 0.00 |
| 101717_at | 0.00 |
| 101718_f_at | 0.00 |
| 101719_at | 0.00 |
| 101720_f_at | 0.00 |
| 101723_r_at | 0.00 |
| 101724_at | 0.00 |
| 101725_at | 0.00 |
| 101726_at | 0.00 |
| 101727_at | 0.00 |
| 101728_at | 0.00 |
| 101729_at | 0.00 |
| 101730_at | 0.00 |
| 101731_at | 0.00 |
| 101732_at | 0.03 |
| 101733_at | 0.00 |
| 101734_at | 0.00 |
| 101735_f_at | 0.00 |
| 101736_at | 0.00 |
| 101737_at | 0.00 |
| 101738_at | 0.00 |
| 101739_at | 0.00 |
| 101740_at | 0.00 |
| 101741_at | 0.00 |
| 101742_at | 0.00 |
| 101743_f_at | 0.00 |
| 101744_i_at | 0.00 |
| 101745_f_at | 0.00 |
| 101746_i_at | 0.00 |
| 101747_f_at | 0.00 |
| 101748_at | 0.00 |
| 101749_at | 0.00 |
| 101751_f_at | 0.00 |
| 101752_f_at | 0.00 |
| 101753_s_at | 0.42 |
| 101754_f_at | 0.00 |
| 101755_f_at | 0.00 |
| 101756_f_at | 0.00 |
| 101757_at | 0.02 |
| 101758_at | 0.00 |
| 101759_at | 0.00 |
| 101760_at | 0.00 |
| 101761_f_at | 0.00 |
| 101762_at | 0.00 |
| 101763_at | 0.00 |

| | |
|---|---|
| 101764_at | 0.00 |
| 101765_at | 0.00 |
| 101768_at | 0.00 |
| 101769_at | 0.00 |
| 101770_i_at | 0.00 |
| 101771_r_at | 0.00 |
| 101772_r_at | 0.00 |
| 101773_r_at | 0.00 |
| 101774_at | 0.00 |
| 101775_at | 0.00 |
| 101776_at | 0.00 |
| 101777_at | 0.00 |
| 101778_at | 0.00 |
| 101779_at | 0.00 |
| 101780_at | 0.00 |
| 101781_f_at | 0.00 |
| 101782_at | 0.00 |
| 101783_i_at | 0.00 |
| 101784_f_at | 0.00 |
| 101786_at | 0.00 |
| 101787_f_at | 0.02 |
| 101788_f_at | 0.00 |
| 101789_i_at | 0.00 |
| 101790_f_at | 0.00 |
| 101791_f_at | 0.00 |
| 101792_at | 0.00 |
| 101793_at | 0.00 |
| 101794_f_at | 0.00 |
| 101795_f_at | 0.00 |
| 101796_at | 0.00 |
| 101797_at | 0.00 |
| 101798_at | 0.00 |
| 101799_at | 0.00 |
| 101800_at | 0.00 |
| 101801_at | 0.00 |
| 101802_at | 0.00 |
| 101803_at | 0.00 |
| 101804_at | 0.00 |
| 101805_f_at | 0.00 |
| 101806_at | 0.00 |
| 101807_at | 0.00 |
| 101808_at | 0.00 |
| 101809_at | 0.00 |
| 101810_at | 0.00 |
| 101811_at | 0.00 |
| 101814_at | 0.00 |
| 101815_at | 0.00 |
| 101816_at | 0.00 |
| 101820_at | 0.00 |
| 101821_at | 0.00 |
| 101822_at | 0.00 |
| 101823_at | 0.00 |
| 101824_at | 0.00 |
| 101825_at | 0.00 |
| 101826_at | 0.00 |
| 101827_at | 0.00 |

| | |
|---|---|
| 101828_at | 0.00 |
| 101829_at | 0.00 |
| 101830_at | 0.00 |
| 101831_at | 0.00 |
| 101834_at | 0.00 |
| 101835_at | 0.00 |
| 101836_at | 0.13 |
| 101837_g_at | 0.13 |
| 101838_r_at | 0.00 |
| 101839_at | 0.00 |
| 101840_at | 0.00 |
| 101841_at | 0.00 |
| 101842_g_at | 0.00 |
| 101843_at | 0.00 |
| 101844_at | 0.00 |
| 101845_s_at | 0.00 |
| 101846_r_at | 0.00 |
| 101847_at | 0.00 |
| 101848_g_at | 0.00 |
| 101850_at | 0.00 |
| 101851_at | 0.00 |
| 101853_f_at | 0.00 |
| 101854_r_at | 0.00 |
| 101855_at | 0.00 |
| 101856_at | 0.00 |
| 101857_at | 0.00 |
| 101858_at | 0.00 |
| 101859_at | 0.00 |
| 101860_at | 0.00 |
| 101861_at | 0.00 |
| 101862_at | 0.00 |
| 101863_at | 0.08 |
| 101864_at | 0.00 |
| 101865_at | 0.00 |
| 101866_at | 0.00 |
| 101867_at | 0.00 |
| 101868_i_at | 0.00 |
| 101869_s_at | 0.00 |
| 101870_at | 0.00 |
| 101871_f_at | 0.00 |
| 101872_at | 0.00 |
| 101873_at | 0.00 |
| 101874_s_at | 0.00 |
| 101875_at | 0.00 |
| 101876_s_at | 0.00 |
| 101877_at | 0.00 |
| 101878_at | 0.00 |
| 101879_s_at | 0.00 |
| 101880_at | 0.00 |
| 101881_g_at | 0.00 |
| 101882_s_at | 0.00 |
| 101883_s_at | 0.00 |
| 101884_at | 0.00 |
| 101885_at | 0.00 |
| 101886_f_at | 0.00 |
| 101887_at | 0.00 |

| | |
|---|---|
| 101888_at | 0.00 |
| 101889_s_at | 0.00 |
| 101890_f_at | 0.00 |
| 101891_at | 0.00 |
| 101892_f_at | 0.01 |
| 101893_r_at | 0.00 |
| 101894_s_at | 0.02 |
| 101896_at | 0.00 |
| 101897_g_at | 0.00 |
| 101898_s_at | 0.00 |
| 101899_at | 0.00 |
| 101900_at | 0.02 |
| 101901_at | 0.00 |
| 101902_at | 0.36 |
| 101903_at | 0.00 |
| 101904_at | 0.00 |
| 101905_at | 0.00 |
| 101906_at | 0.00 |
| 101907_s_at | 0.00 |
| 101908_s_at | 0.00 |
| 101909_f_at | 0.01 |
| 101910_f_at | 0.00 |
| 101912_at | 0.00 |
| 101913_at | 0.10 |
| 101914_at | 0.03 |
| 101916_at | 0.00 |
| 101917_at | 0.00 |
| 101918_at | 0.00 |
| 101919_at | 0.04 |
| 101920_at | 0.00 |
| 101921_at | 0.00 |
| 101922_at | 0.03 |
| 101923_at | 0.00 |
| 101924_at | 0.00 |
| 101926_at | 0.81 |
| 101927_at | 0.00 |
| 101928_at | 0.00 |
| 101929_at | 0.01 |
| 101930_at | 0.00 |
| 101931_at | 0.00 |
| 101932_at | 0.00 |
| 101933_at | 0.00 |
| 101934_at | 0.00 |
| 101936_at | 0.01 |
| 101937_s_at | 0.01 |
| 101938_at | 0.00 |
| 101939_at | 0.00 |
| 101940_at | 0.00 |
| 101942_at | 0.00 |
| 101943_at | 0.00 |
| 101944_at | 0.00 |
| 101945_g_at | 0.00 |
| 101946_at | 0.00 |
| 101947_at | 0.00 |
| 101948_at | 0.00 |
| 101949_at | 0.00 |

| | |
|---|---|
| 101950_at | 0.00 |
| 101952_at | 0.00 |
| 101953_at | 0.00 |
| 101954_at | 0.00 |
| 101955_at | 0.00 |
| 101956_at | 0.00 |
| 101957_f_at | 0.01 |
| 101958_f_at | 0.00 |
| 101959_r_at | 0.00 |
| 101960_at | 0.00 |
| 101961_at | 0.00 |
| 101962_at | 0.00 |
| 101963_at | 0.00 |
| 101964_at | 0.00 |
| 101965_at | 0.00 |
| 101966_s_at | 0.01 |
| 101967_at | 0.00 |
| 101968_at | 0.00 |
| 101969_at | 0.00 |
| 101970_at | 0.00 |
| 101971_at | 0.00 |
| 101972_at | 0.00 |
| 101973_at | 0.00 |
| 101975_at | 0.00 |
| 101976_at | 0.00 |
| 101977_at | 0.00 |
| 101978_at | 0.00 |
| 101979_at | 0.10 |
| 101980_at | 0.01 |
| 101981_at | 0.01 |
| 101982_at | 0.01 |
| 101984_at | 0.00 |
| 101985_at | 0.00 |
| 101989_at | 0.00 |
| 101990_at | 0.05 |
| 101991_at | 0.00 |
| 101992_at | 0.00 |
| 101993_at | 0.71 |
| 101995_at | 0.02 |
| 101996_at | 0.00 |
| 101997_at | 0.00 |
| 101998_at | 0.00 |
| 102000_f_at | 0.00 |
| 102001_at | 0.00 |
| 102002_at | 0.03 |
| 102003_at | 0.00 |
| 102004_at | 0.00 |
| 102007_at | 0.04 |
| 102009_at | 0.21 |
| 102010_at | 0.00 |
| 102011_at | 0.00 |
| 102012_at | 0.87 |
| 102013_at | 0.00 |
| 102014_at | 0.00 |
| 102015_at | 0.00 |
| 102016_at | 0.00 |

| | |
|---|---|
| 102017_at | 0.00 |
| 102018_at | 0.06 |
| 102019_at | 0.01 |
| 102020_at | 0.00 |
| 102021_at | 0.00 |
| 102022_at | 0.00 |
| 102024_at | 0.01 |
| 102025_at | 0.00 |
| 102026_s_at | 0.00 |
| 102027_s_at | 0.00 |
| 102028_at | 0.00 |
| 102029_at | 0.00 |
| 102030_at | 0.14 |
| 102031_at | 0.00 |
| 102032_at | 0.00 |
| 102033_at | 0.00 |
| 102035_at | 0.00 |
| 102036_at | 0.00 |
| 102037_at | 0.01 |
| 102038_at | 0.00 |
| 102039_at | 0.00 |
| 102040_at | 0.00 |
| 102041_at | 0.01 |
| 102042_at | 0.00 |
| 102043_at | 0.00 |
| 102044_at | 0.13 |
| 102046_at | 0.00 |
| 102047_at | 0.00 |
| 102048_at | 0.75 |
| 102049_at | 0.01 |
| 102050_at | 0.00 |
| 102052_at | 0.11 |
| 102053_at | 0.00 |
| 102054_at | 0.00 |
| 102056_f_at | 0.10 |
| 102057_r_at | 0.00 |
| 102058_at | 0.00 |
| 102059_at | 0.00 |
| 102060_at | 0.02 |
| 102061_at | 0.00 |
| 102062_at | 0.00 |
| 102063_at | 0.03 |
| 102064_at | 0.00 |
| 102065_at | 0.00 |
| 102069_at | 0.65 |
| 102070_at | 0.00 |
| 102071_at | 0.00 |
| 102072_g_at | 0.00 |
| 102073_at | 0.00 |
| 102074_at | 0.00 |
| 102075_at | 0.00 |
| 102076_at | 0.00 |
| 102077_at | 0.00 |
| 102078_at | 0.00 |
| 102079_at | 0.00 |
| 102080_at | 0.00 |

| | |
|---|---|
| 102081_at | 0.00 |
| 102082_at | 0.00 |
| 102083_at | 0.00 |
| 102084_f_at | 0.00 |
| 102085_at | 0.00 |
| 102086_r_at | 0.00 |
| 102087_at | 0.00 |
| 102088_at | 0.00 |
| 102089_at | 0.00 |
| 102090_f_at | 0.00 |
| 102091_f_at | 0.01 |
| 102092_s_at | 0.04 |
| 102093_f_at | 0.00 |
| 102094_f_at | 0.00 |
| 102095_f_at | 0.00 |
| 102096_f_at | 0.00 |
| 102097_f_at | 0.00 |
| 102098_at | 0.00 |
| 102099_f_at | 0.00 |
| 102100_f_at | 0.05 |
| 102101_f_at | 0.00 |
| 102102_at | 0.00 |
| 102103_f_at | 0.01 |
| 102104_f_at | 0.00 |
| 102105_f_at | 0.00 |
| 102106_at | 0.00 |
| 102107_at | 0.00 |
| 102108_f_at | 0.10 |
| 102109_at | 0.00 |
| 102110_at | 0.00 |
| 102111_f_at | 0.02 |
| 102112_s_at | 0.00 |
| 102113_f_at | 0.00 |
| 102114_f_at | 0.00 |
| 102115_r_at | 0.00 |
| 102116_f_at | 0.00 |
| 102117_at | 0.00 |
| 102118_at | 0.00 |
| 102119_at | 0.00 |
| 102120_f_at | 0.00 |
| 102121_f_at | 0.00 |
| 102122_f_at | 0.00 |
| 102123_at | 0.00 |
| 102124_f_at | 0.00 |
| 102125_f_at | 0.00 |
| 102126_at | 0.00 |
| 102127_at | 0.00 |
| 102128_f_at | 0.01 |
| 102129_at | 0.00 |
| 102130_f_at | 0.00 |
| 102131_f_at | 0.00 |
| 102132_i_at | 0.00 |
| 102133_at | 0.01 |
| 102134_f_at | 0.00 |
| 102135_at | 0.00 |
| 102136_r_at | 0.00 |

| | |
|---|---|
| 102137_f_at | 0.00 |
| 102138_at | 0.00 |
| 102139_at | 0.00 |
| 102140_at | 0.00 |
| 102141_f_at | 0.00 |
| 102142_r_at | 0.03 |
| 102143_at | 0.00 |
| 102144_f_at | 0.00 |
| 102145_f_at | 0.00 |
| 102146_at | 0.00 |
| 102147_at | 0.00 |
| 102148_f_at | 0.00 |
| 102149_f_at | 0.00 |
| 102150_f_at | 0.00 |
| 102151_at | 0.00 |
| 102152_f_at | 0.00 |
| 102153_at | 0.00 |
| 102154_f_at | 0.00 |
| 102155_f_at | 0.00 |
| 102156_f_at | 0.00 |
| 102157_f_at | 0.00 |
| 102158_at | 0.00 |
| 102159_at | 0.00 |
| 102160_at | 0.00 |
| 102161_f_at | 0.00 |
| 102162_at | 0.00 |
| 102163_at | 0.00 |
| 102164_at | 0.00 |
| 102165_at | 0.00 |
| 102166_g_at | 0.00 |
| 102167_at | 0.00 |
| 102168_at | 0.00 |
| 102169_at | 0.00 |
| 102170_at | 0.00 |
| 102171_r_at | 0.00 |
| 102192_r_at | 0.00 |
| 102193_at | 0.00 |
| 102194_at | 0.00 |
| 102195_at | 0.00 |
| 102196_at | 0.00 |
| 102197_at | 0.00 |
| 102198_at | 0.00 |
| 102199_at | 0.00 |
| 102200_at | 0.00 |
| 102201_s_at | 0.00 |
| 102202_s_at | 0.00 |
| 102203_at | 0.00 |
| 102204_at | 0.00 |
| 102205_at | 0.00 |
| 102206_at | 0.00 |
| 102207_at | 0.00 |
| 102208_at | 0.47 |
| 102209_at | 0.00 |
| 102210_at | 0.00 |
| 102211_r_at | 0.00 |
| 102212_at | 0.00 |

| | |
|---|---|
| 102213_at | 0.00 |
| 102214_at | 0.00 |
| 102215_at | 0.00 |
| 102216_at | 0.00 |
| 102217_at | 0.00 |
| 102218_at | 0.00 |
| 102219_at | 0.01 |
| 102220_at | 0.00 |
| 102221_at | 0.00 |
| 102222_at | 0.03 |
| 102223_at | 0.00 |
| 102224_at | 0.00 |
| 102225_at | 0.00 |
| 102226_at | 0.00 |
| 102227_g_at | 0.00 |
| 102228_at | 0.00 |
| 102229_at | 0.00 |
| 102230_at | 0.00 |
| 102231_at | 0.00 |
| 102232_at | 0.00 |
| 102233_at | 0.00 |
| 102234_at | 0.00 |
| 102235_at | 0.33 |
| 102237_at | 0.00 |
| 102238_at | 0.00 |
| 102239_at | 0.64 |
| 102240_at | 0.00 |
| 102241_f_at | 0.00 |
| 102242_at | 0.00 |
| 102243_at | 0.00 |
| 102244_at | 0.00 |
| 102247_at | 0.01 |
| 102248_f_at | 0.00 |
| 102249_at | 0.00 |
| 102250_at | 0.00 |
| 102251_at | 0.00 |
| 102252_at | 0.11 |
| 102253_at | 0.00 |
| 102254_f_at | 0.00 |
| 102255_at | 0.34 |
| 102256_at | 0.00 |
| 102257_at | 0.00 |
| 102258_at | 0.00 |
| 102259_at | 0.19 |
| 102260_at | 0.00 |
| 102261_f_at | 0.00 |
| 102262_r_at | 0.00 |
| 102263_at | 0.00 |
| 102264_at | 0.00 |
| 102265_at | 0.00 |
| 102266_at | 0.00 |
| 102267_at | 0.00 |
| 102268_at | 0.00 |
| 102269_at | 0.00 |
| 102271_at | 0.00 |
| 102272_at | 0.00 |

| | |
|---|---|
| 102273_at | 0.00 |
| 102274_at | 0.00 |
| 102275_at | 0.04 |
| 102277_at | 0.02 |
| 102278_at | 0.01 |
| 102279_at | 0.00 |
| 102280_at | 0.04 |
| 102281_at | 0.00 |
| 102282_g_at | 0.00 |
| 102283_at | 0.06 |
| 102284_at | 0.00 |
| 102285_at | 0.00 |
| 102286_at | 0.00 |
| 102287_at | 0.00 |
| 102288_at | 0.00 |
| 102289_r_at | 0.00 |
| 102290_at | 0.00 |
| 102291_at | 0.00 |
| 102292_at | 0.71 |
| 102293_at | 0.00 |
| 102294_at | 0.00 |
| 102295_at | 0.00 |
| 102296_at | 0.00 |
| 102297_at | 0.00 |
| 102298_at | 0.00 |
| 102299_at | 0.00 |
| 102300_at | 0.00 |
| 102301_at | 0.00 |
| 102302_at | 0.00 |
| 102303_i_at | 0.00 |
| 102304_f_at | 0.00 |
| 102305_at | 0.00 |
| 102306_at | 0.00 |
| 102307_at | 0.00 |
| 102308_at | 0.13 |
| 102309_at | 0.00 |
| 102310_at | 0.00 |
| 102311_at | 0.00 |
| 102312_r_at | 0.00 |
| 102313_at | 0.00 |
| 102314_at | 0.00 |
| 102315_at | 0.00 |
| 102316_at | 0.00 |
| 102317_at | 0.00 |
| 102318_at | 0.00 |
| 102319_at | 0.00 |
| 102320_at | 0.00 |
| 102321_at | 0.00 |
| 102322_at | 0.00 |
| 102323_at | 0.00 |
| 102324_at | 0.00 |
| 102326_at | 0.00 |
| 102327_at | 0.00 |
| 102328_at | 0.00 |
| 102329_at | 0.00 |
| 102330_at | 0.00 |

| | |
|---|---|
| 102331_at | 0.00 |
| 102332_at | 0.99 |
| 102333_at | 0.00 |
| 102334_at | 0.00 |
| 102335_at | 0.81 |
| 102336_at | 0.00 |
| 102337_s_at | 0.02 |
| 102338_at | 0.00 |
| 102340_at | 0.00 |
| 102341_at | 0.00 |
| 102342_at | 0.00 |
| 102343_at | 0.00 |
| 102344_s_at | 0.11 |
| 102345_at | 0.20 |
| 102346_at | 0.00 |
| 102348_at | 0.08 |
| 102349_at | 0.01 |
| 102350_at | 0.00 |
| 102351_at | 0.00 |
| 102352_at | 0.00 |
| 102353_at | 0.00 |
| 102354_at | 0.00 |
| 102356_at | 0.00 |
| 102357_at | 0.00 |
| 102360_at | 0.00 |
| 102362_i_at | 0.09 |
| 102363_r_at | 0.06 |
| 102364_at | 0.02 |
| 102366_at | 0.00 |
| 102368_at | 0.00 |
| 102370_at | 0.08 |
| 102371_at | 0.00 |
| 102372_at | 0.00 |
| 102373_at | 0.00 |
| 102374_at | 0.00 |
| 102375_at | 0.00 |
| 102376_r_at | 0.00 |
| 102378_at | 0.00 |
| 102379_at | 0.01 |
| 102380_s_at | 0.00 |
| 102381_at | 0.00 |
| 102382_at | 0.00 |
| 102383_at | 0.00 |
| 102384_at | 0.00 |
| 102385_at | 0.00 |
| 102387_at | 0.00 |
| 102389_s_at | 0.00 |
| 102393_at | 0.00 |
| 102395_at | 0.00 |
| 102396_at | 0.00 |
| 102397_at | 0.00 |
| 102398_at | 0.00 |
| 102399_at | 0.12 |
| 102400_at | 0.03 |
| 102401_at | 0.47 |
| 102402_at | 0.00 |

| | |
|---|---|
| 102403_at | 0.00 |
| 102404_at | 0.00 |
| 102405_at | 0.00 |
| 102406_at | 0.00 |
| 102407_at | 0.00 |
| 102408_g_at | 0.00 |
| 102409_at | 0.00 |
| 102410_at | 0.00 |
| 102411_at | 0.00 |
| 102412_at | 0.00 |
| 102413_at | 0.00 |
| 102414_i_at | 0.00 |
| 102415_r_at | 0.00 |
| 102416_at | 0.00 |
| 102418_at | 0.07 |
| 102419_at | 0.04 |
| 102421_at | 0.00 |
| 102423_at | 0.00 |
| 102424_at | 0.01 |
| 102425_at | 0.34 |
| 102426_at | 0.00 |
| 102427_at | 0.00 |
| 102429_at | 0.00 |
| 102430_at | 0.00 |
| 102431_at | 0.38 |
| 102476_f_at | 0.00 |
| 102477_at | 0.00 |
| 102478_f_at | 0.00 |
| 102552_at | 0.00 |
| 102553_at | 0.00 |
| 102554_at | 0.00 |
| 102555_at | 0.00 |
| 102556_at | 0.00 |
| 102557_at | 0.00 |
| 102558_at | 0.00 |
| 102559_at | 0.00 |
| 102560_at | 0.00 |
| 102561_at | 0.00 |
| 102562_at | 0.00 |
| 102566_at | 0.00 |
| 102567_at | 0.00 |
| 102568_at | 0.00 |
| 102569_at | 0.00 |
| 102570_at | 0.00 |
| 102571_at | 0.00 |
| 102572_at | 0.00 |
| 102573_at | 0.00 |
| 102574_at | 0.00 |
| 102575_at | 0.00 |
| 102576_at | 0.00 |
| 102577_at | 0.00 |
| 102578_at | 0.00 |
| 102579_f_at | 0.00 |
| 102580_r_at | 0.00 |
| 102581_at | 0.00 |
| 102582_at | 0.00 |

| | |
|---|---|
| 102583_at | 0.00 |
| 102584_at | 0.00 |
| 102585_f_at | 0.00 |
| 102586_at | 0.00 |
| 102599_at | 0.00 |
| 102612_at | 0.00 |
| 102613_at | 0.00 |
| 102614_at | 0.00 |
| 102619_at | 0.00 |
| 102620_at | 0.00 |
| 102621_at | 0.00 |
| 102622_r_at | 0.00 |
| 102623_at | 0.00 |
| 102624_at | 0.00 |
| 102625_f_at | 0.00 |
| 102626_r_at | 0.05 |
| 102627_at | 0.08 |
| 102628_at | 0.00 |
| 102629_at | 0.00 |
| 102630_s_at | 0.00 |
| 102631_at | 0.00 |
| 102632_at | 0.02 |
| 102633_at | 0.00 |
| 102635_at | 0.00 |
| 102636_at | 0.00 |
| 102637_at | 0.00 |
| 102638_at | 0.00 |
| 102639_at | 0.00 |
| 102640_at | 0.00 |
| 102641_at | 0.00 |
| 102642_at | 0.00 |
| 102643_at | 0.00 |
| 102644_at | 0.00 |
| 102645_at | 0.00 |
| 102646_at | 0.02 |
| 102647_g_at | 0.00 |
| 102648_at | 0.00 |
| 102649_s_at | 0.82 |
| 102650_at | 0.00 |
| 102651_at | 0.00 |
| 102652_at | 0.15 |
| 102653_at | 0.00 |
| 102654_at | 0.00 |
| 102655_at | 0.00 |
| 102656_at | 0.00 |
| 102657_at | 0.00 |
| 102658_at | 0.00 |
| 102659_at | 0.00 |
| 102660_at | 0.00 |
| 102661_at | 0.00 |
| 102662_at | 0.00 |
| 102663_at | 0.00 |
| 102664_at | 0.00 |
| 102665_at | 0.30 |
| 102666_at | 0.00 |
| 102667_at | 0.00 |

| | |
|---|---|
| 102668_at | 0.00 |
| 102669_at | 0.00 |
| 102670_at | 0.03 |
| 102671_at | 0.00 |
| 102672_g_at | 0.00 |
| 102673_at | 0.00 |
| 102674_at | 0.00 |
| 102675_at | 0.03 |
| 102676_at | 0.00 |
| 102677_at | 0.01 |
| 102678_at | 0.00 |
| 102679_at | 0.00 |
| 102680_g_at | 0.00 |
| 102681_at | 0.00 |
| 102682_at | 0.00 |
| 102683_at | 0.00 |
| 102684_at | 0.00 |
| 102685_at | 0.00 |
| 102686_at | 0.00 |
| 102687_at | 0.00 |
| 102688_f_at | 0.00 |
| 102689_at | 0.03 |
| 102690_at | 0.00 |
| 102691_at | 0.00 |
| 102692_s_at | 0.00 |
| 102693_f_at | 0.00 |
| 102694_at | 0.00 |
| 102695_at | 0.00 |
| 102696_s_at | 0.06 |
| 102697_at | 0.00 |
| 102698_at | 0.00 |
| 102699_at | 0.00 |
| 102700_at | 0.00 |
| 102701_at | 0.00 |
| 102702_at | 0.00 |
| 102703_s_at | 0.00 |
| 102704_at | 0.00 |
| 102705_at | 0.00 |
| 102706_i_at | 0.00 |
| 102707_f_at | 0.00 |
| 102708_at | 0.00 |
| 102709_at | 0.00 |
| 102710_at | 0.24 |
| 102711_at | 0.00 |
| 102712_at | 0.33 |
| 102713_at | 0.00 |
| 102714_at | 0.00 |
| 102715_at | 0.00 |
| 102716_at | 0.00 |
| 102717_at | 0.00 |
| 102718_at | 0.00 |
| 102719_f_at | 0.00 |
| 102720_at | 0.00 |
| 102721_at | 0.00 |
| 102722_g_at | 0.00 |
| 102723_at | 0.00 |

| | |
|---|---|
| 102724_at | 0.00 |
| 102725_at | 0.00 |
| 102726_at | 0.00 |
| 102727_at | 0.01 |
| 102728_f_at | 0.00 |
| 102729_f_at | 0.00 |
| 102730_at | 0.00 |
| 102731_g_at | 0.00 |
| 102732_at | 0.00 |
| 102733_at | 0.00 |
| 102734_at | 0.00 |
| 102735_at | 0.00 |
| 102736_at | 0.65 |
| 102737_at | 0.00 |
| 102738_s_at | 0.00 |
| 102739_s_at | 0.00 |
| 102740_at | 0.00 |
| 102741_at | 0.00 |
| 102742_g_at | 0.01 |
| 102743_at | 0.00 |
| 102744_at | 0.00 |
| 102745_at | 0.00 |
| 102746_at | 0.00 |
| 102747_at | 0.00 |
| 102748_at | 0.00 |
| 102749_at | 0.00 |
| 102750_at | 0.12 |
| 102751_at | 0.00 |
| 102752_at | 0.00 |
| 102753_at | 0.00 |
| 102754_at | 0.00 |
| 102755_at | 0.00 |
| 102758_at | 0.00 |
| 102759_at | 0.00 |
| 102761_at | 0.00 |
| 102762_r_at | 0.00 |
| 102763_at | 0.00 |
| 102764_at | 0.35 |
| 102765_at | 0.00 |
| 102766_at | 0.00 |
| 102767_at | 0.00 |
| 102768_i_at | 0.00 |
| 102769_f_at | 0.00 |
| 102770_at | 0.00 |
| 102771_at | 0.00 |
| 102772_at | 0.00 |
| 102773_at | 0.00 |
| 102774_at | 0.00 |
| 102776_at | 0.00 |
| 102777_at | 0.00 |
| 102778_at | 0.00 |
| 102779_at | 0.00 |
| 102780_at | 0.00 |
| 102781_at | 0.00 |
| 102782_at | 0.00 |
| 102783_at | 0.00 |

| | |
|---|---|
| 102784_at | 0.00 |
| 102785_at | 0.00 |
| 102786_at | 0.00 |
| 102787_at | 0.00 |
| 102788_s_at | 0.21 |
| 102789_at | 0.00 |
| 102790_at | 0.00 |
| 102791_at | 0.01 |
| 102792_at | 0.46 |
| 102793_at | 0.00 |
| 102794_at | 0.00 |
| 102795_at | 0.12 |
| 102796_at | 0.23 |
| 102797_at | 0.00 |
| 102798_at | 0.01 |
| 102799_at | 0.00 |
| 102800_at | 0.00 |
| 102801_at | 0.00 |
| 102802_at | 0.00 |
| 102803_at | 0.00 |
| 102804_at | 0.00 |
| 102805_at | 0.17 |
| 102806_g_at | 0.17 |
| 102807_at | 0.02 |
| 102808_at | 0.00 |
| 102809_s_at | 0.42 |
| 102811_at | 0.00 |
| 102812_i_at | 0.00 |
| 102813_f_at | 0.01 |
| 102814_f_at | 0.00 |
| 102815_at | 0.01 |
| 102816_at | 0.00 |
| 102817_at | 0.00 |
| 102818_at | 0.01 |
| 102819_at | 0.05 |
| 102820_at | 0.00 |
| 102821_s_at | 0.00 |
| 102822_at | 0.00 |
| 102823_at | 0.00 |
| 102824_g_at | 0.00 |
| 102825_at | 0.00 |
| 102826_at | 0.00 |
| 102827_at | 0.00 |
| 102828_at | 0.00 |
| 102829_s_at | 0.00 |
| 102830_at | 0.00 |
| 102831_s_at | 0.00 |
| 102832_at | 0.00 |
| 102833_at | 0.06 |
| 102834_at | 0.02 |
| 102835_at | 0.00 |
| 102836_at | 0.00 |
| 102838_at | 0.00 |
| 102839_at | 0.00 |
| 102840_at | 0.00 |
| 102841_at | 0.00 |

| | |
|---|---|
| 102843_s_at | 0.00 |
| 102844_at | 0.00 |
| 102845_at | 0.06 |
| 102846_at | 0.00 |
| 102847_s_at | 0.00 |
| 102848_f_at | 0.01 |
| 102849_at | 0.00 |
| 102850_at | 0.02 |
| 102851_s_at | 0.00 |
| 102852_at | 0.01 |
| 102853_at | 0.01 |
| 102854_s_at | 0.15 |
| 102856_at | 0.00 |
| 102857_at | 0.00 |
| 102858_at | 0.00 |
| 102859_at | 0.06 |
| 102860_at | 0.00 |
| 102861_at | 0.00 |
| 102862_at | 0.00 |
| 102863_at | 0.00 |
| 102864_at | 0.00 |
| 102865_at | 0.00 |
| 102866_at | 0.00 |
| 102867_at | 0.00 |
| 102868_g_at | 0.00 |
| 102869_at | 0.00 |
| 102870_at | 0.00 |
| 102871_at | 0.00 |
| 102872_f_at | 0.00 |
| 102873_at | 0.00 |
| 102874_at | 0.00 |
| 102875_at | 0.00 |
| 102876_at | 0.00 |
| 102877_at | 0.00 |
| 102878_at | 0.00 |
| 102879_s_at | 0.00 |
| 102880_at | 0.00 |
| 102881_at | 0.00 |
| 102882_at | 0.00 |
| 102883_at | 0.01 |
| 102884_at | 0.12 |
| 102885_at | 0.00 |
| 102886_at | 0.00 |
| 102887_at | 0.00 |
| 102888_s_at | 0.00 |
| 102889_r_at | 0.00 |
| 102890_at | 0.00 |
| 102891_at | 0.00 |
| 102892_at | 0.00 |
| 102893_at | 0.33 |
| 102894_g_at | 0.25 |
| 102895_at | 0.00 |
| 102896_at | 0.00 |
| 102898_at | 0.00 |
| 102899_at | 0.00 |
| 102900_at | 0.00 |

| | |
|---|---|
| 102901_at | 0.00 |
| 102902_at | 0.00 |
| 102904_at | 0.00 |
| 102905_at | 0.00 |
| 102906_at | 0.01 |
| 102907_at | 0.00 |
| 102908_at | 0.00 |
| 102910_at | 0.29 |
| 102911_at | 0.00 |
| 102912_at | 0.00 |
| 102913_at | 0.00 |
| 102914_s_at | 0.01 |
| 102915_at | 0.00 |
| 102916_s_at | 0.00 |
| 102917_at | 0.00 |
| 102918_at | 0.00 |
| 102919_at | 0.00 |
| 102920_at | 0.41 |
| 102921_s_at | 0.00 |
| 102922_at | 0.13 |
| 102923_at | 0.00 |
| 102924_at | 0.00 |
| 102925_at | 0.04 |
| 102926_at | 0.00 |
| 102927_s_at | 0.00 |
| 102928_at | 0.00 |
| 102929_s_at | 0.00 |
| 102930_at | 0.00 |
| 102931_at | 0.00 |
| 102932_at | 0.52 |
| 102933_at | 0.00 |
| 102934_s_at | 0.00 |
| 102935_at | 0.00 |
| 102936_at | 0.22 |
| 102937_at | 0.00 |
| 102938_at | 0.00 |
| 102939_s_at | 0.00 |
| 102940_at | 0.00 |
| 102941_at | 0.00 |
| 102942_at | 0.00 |
| 102943_at | 0.00 |
| 102944_at | 0.00 |
| 102946_r_at | 0.00 |
| 102947_at | 0.00 |
| 102948_at | 0.00 |
| 102949_g_at | 0.00 |
| 102950_at | 0.00 |
| 102951_at | 0.00 |
| 102952_g_at | 0.05 |
| 102953_at | 0.00 |
| 102954_at | 0.00 |
| 102955_at | 0.00 |
| 102956_at | 0.01 |
| 102957_at | 0.00 |
| 102958_at | 0.00 |
| 102959_at | 0.00 |

| | |
|---|---|
| 102960_at | 0.00 |
| 102961_at | 0.00 |
| 102962_at | 0.08 |
| 102963_at | 0.00 |
| 102964_at | 0.06 |
| 102965_at | 0.00 |
| 102966_at | 0.00 |
| 102967_at | 0.00 |
| 102968_at | 0.00 |
| 102969_at | 0.00 |
| 102970_at | 0.00 |
| 102971_at | 0.00 |
| 102972_s_at | 0.00 |
| 102973_f_at | 0.00 |
| 102974_at | 0.01 |
| 102975_at | 0.00 |
| 102976_at | 0.00 |
| 102977_at | 0.00 |
| 102978_at | 0.00 |
| 102979_at | 0.00 |
| 102980_at | 0.00 |
| 102981_at | 0.12 |
| 102982_at | 0.00 |
| 102983_at | 0.61 |
| 102984_g_at | 0.72 |
| 102985_at | 0.00 |
| 102986_at | 0.00 |
| 102987_at | 0.00 |
| 102988_at | 0.00 |
| 102989_at | 0.00 |
| 102990_at | 0.00 |
| 102991_s_at | 0.03 |
| 102992_at | 0.00 |
| 102993_at | 0.00 |
| 102994_at | 0.01 |
| 102995_s_at | 0.00 |
| 102996_at | 0.00 |
| 102998_at | 0.00 |
| 103001_at | 0.00 |
| 103002_at | 0.00 |
| 103003_i_at | 0.00 |
| 103004_r_at | 0.00 |
| 103005_s_at | 0.12 |
| 103006_at | 0.00 |
| 103007_at | 0.00 |
| 103009_at | 0.00 |
| 103010_at | 0.00 |
| 103011_at | 0.00 |
| 103012_at | 0.00 |
| 103013_at | 0.00 |
| 103015_at | 0.00 |
| 103016_s_at | 0.00 |
| 103017_at | 0.00 |
| 103018_at | 0.00 |
| 103020_s_at | 0.04 |
| 103021_r_at | 0.01 |

| | |
|---|---|
| 103022_at | 0.00 |
| 103023_at | 0.00 |
| 103024_at | 0.01 |
| 103025_at | 0.05 |
| 103026_f_at | 0.00 |
| 103027_at | 0.05 |
| 103028_at | 0.00 |
| 103029_at | 0.00 |
| 103030_at | 0.00 |
| 103031_g_at | 0.00 |
| 103032_at | 0.00 |
| 103033_at | 0.00 |
| 103034_at | 0.00 |
| 103035_at | 0.77 |
| 103036_at | 0.00 |
| 103037_at | 0.00 |
| 103038_at | 0.01 |
| 103039_at | 0.01 |
| 103040_at | 0.00 |
| 103041_at | 0.00 |
| 103043_at | 0.01 |
| 103044_g_at | 0.00 |
| 103045_at | 0.00 |
| 103046_at | 0.05 |
| 103047_at | 0.57 |
| 103048_at | 0.39 |
| 103049_at | 0.00 |
| 103050_at | 0.00 |
| 103051_at | 0.00 |
| 103052_r_at | 0.00 |
| 103053_at | 0.00 |
| 103054_at | 0.00 |
| 103055_r_at | 0.00 |
| 103056_at | 0.13 |
| 103057_at | 0.00 |
| 103058_f_at | 0.00 |
| 103059_at | 0.00 |
| 103060_at | 0.00 |
| 103061_at | 0.95 |
| 103062_at | 0.00 |
| 103063_at | 0.00 |
| 103064_at | 0.00 |
| 103065_at | 0.00 |
| 103066_at | 0.00 |
| 103067_at | 0.01 |
| 103068_at | 0.00 |
| 103069_at | 0.00 |
| 103070_at | 0.00 |
| 103071_at | 0.00 |
| 103072_at | 0.00 |
| 103073_i_at | 0.00 |
| 103074_f_at | 0.00 |
| 103075_at | 0.00 |
| 103076_at | 0.09 |
| 103078_at | 0.01 |
| 103079_at | 0.22 |

| | |
|---|---|
| 103080_at | 0.01 |
| 103081_at | 0.00 |
| 103082_at | 0.04 |
| 103083_at | 0.00 |
| 103084_at | 0.00 |
| 103085_at | 0.00 |
| 103086_at | 0.00 |
| 103087_at | 0.00 |
| 103088_at | 0.00 |
| 103089_at | 0.00 |
| 103090_at | 0.00 |
| 103091_at | 0.00 |
| 103092_at | 0.00 |
| 103094_at | 0.05 |
| 103095_at | 0.00 |
| 103096_at | 0.00 |
| 103097_at | 0.00 |
| 103098_at | 0.00 |
| 103099_f_at | 0.00 |
| 103100_at | 0.00 |
| 103101_at | 0.00 |
| 103132_at | 0.00 |
| 103198_at | 0.00 |
| 103199_at | 0.00 |
| 103200_at | 0.00 |
| 103201_at | 0.00 |
| 103202_at | 0.02 |
| 103203_f_at | 0.32 |
| 103204_r_at | 0.01 |
| 103205_at | 0.00 |
| 103206_at | 0.00 |
| 103207_at | 0.01 |
| 103208_at | 0.00 |
| 103209_at | 0.00 |
| 103210_at | 0.00 |
| 103211_at | 0.00 |
| 103212_at | 0.09 |
| 103213_at | 0.00 |
| 103214_at | 0.00 |
| 103215_g_at | 0.00 |
| 103216_f_at | 0.00 |
| 103217_at | 0.00 |
| 103218_at | 0.00 |
| 103219_at | 0.00 |
| 103220_at | 0.00 |
| 103221_at | 0.00 |
| 103222_at | 0.03 |
| 103223_at | 0.00 |
| 103224_at | 0.00 |
| 103225_at | 0.00 |
| 103226_at | 0.00 |
| 103227_at | 0.00 |
| 103228_at | 0.00 |
| 103229_at | 0.00 |
| 103231_at | 0.00 |
| 103232_at | 0.00 |

| | |
|---|---|
| 103233_at | 0.00 |
| 103234_at | 0.01 |
| 103235_at | 0.00 |
| 103236_at | 0.00 |
| 103237_at | 0.00 |
| 103238_at | 0.00 |
| 103239_at | 0.00 |
| 103240_f_at | 0.00 |
| 103241_at | 0.00 |
| 103242_at | 0.01 |
| 103243_at | 0.00 |
| 103244_at | 0.00 |
| 103245_at | 0.00 |
| 103247_at | 0.00 |
| 103248_at | 0.00 |
| 103249_at | 0.00 |
| 103250_at | 0.00 |
| 103251_at | 0.00 |
| 103252_at | 0.00 |
| 103253_at | 0.00 |
| 103254_at | 0.00 |
| 103255_at | 0.00 |
| 103256_at | 0.00 |
| 103257_at | 0.00 |
| 103258_at | 0.63 |
| 103259_at | 0.00 |
| 103260_at | 0.01 |
| 103261_at | 0.00 |
| 103262_at | 0.00 |
| 103263_at | 0.00 |
| 103264_at | 0.00 |
| 103265_r_at | 0.00 |
| 103266_at | 0.00 |
| 103267_i_at | 0.00 |
| 103268_r_at | 0.00 |
| 103269_f_at | 0.02 |
| 103270_at | 0.00 |
| 103271_at | 0.00 |
| 103272_at | 0.00 |
| 103273_s_at | 0.00 |
| 103274_at | 0.00 |
| 103275_at | 0.00 |
| 103276_at | 0.00 |
| 103277_s_at | 0.35 |
| 103278_at | 0.00 |
| 103279_at | 0.00 |
| 103281_at | 0.07 |
| 103282_at | 0.00 |
| 103283_at | 0.00 |
| 103284_at | 0.00 |
| 103285_at | 0.02 |
| 103286_at | 0.00 |
| 103288_at | 0.10 |
| 103289_at | 0.00 |
| 103291_at | 0.00 |
| 103292_at | 0.00 |

| | |
|---|---|
| 103293_at | 0.00 |
| 103294_at | 0.00 |
| 103295_at | 0.00 |
| 103296_at | 0.00 |
| 103297_at | 0.00 |
| 103298_at | 0.01 |
| 103299_at | 0.00 |
| 103300_at | 0.00 |
| 103301_i_at | 0.13 |
| 103302_r_at | 0.00 |
| 103303_at | 0.01 |
| 103305_at | 0.00 |
| 103306_at | 0.41 |
| 103308_at | 0.23 |
| 103309_at | 0.00 |
| 103310_at | 0.01 |
| 103311_at | 0.00 |
| 103312_f_at | 0.12 |
| 103313_r_at | 0.00 |
| 103314_at | 0.00 |
| 103315_at | 0.00 |
| 103316_at | 0.00 |
| 103317_at | 0.11 |
| 103318_at | 0.00 |
| 103319_at | 0.00 |
| 103321_at | 0.00 |
| 103322_at | 0.00 |
| 103326_at | 0.00 |
| 103327_at | 0.00 |
| 103328_at | 0.00 |
| 103329_at | 0.00 |
| 103330_at | 0.01 |
| 103331_at | 0.00 |
| 103332_at | 0.00 |
| 103333_at | 0.00 |
| 103334_at | 0.00 |
| 103335_at | 0.04 |
| 103336_r_at | 0.00 |
| 103338_at | 0.00 |
| 103340_at | 0.00 |
| 103341_at | 0.00 |
| 103342_at | 0.86 |
| 103343_at | 0.00 |
| 103344_at | 0.00 |
| 103345_at | 0.00 |
| 103346_at | 0.00 |
| 103347_at | 0.00 |
| 103348_at | 0.00 |
| 103349_at | 0.00 |
| 103350_at | 0.00 |
| 103352_at | 0.00 |
| 103353_f_at | 0.00 |
| 103354_at | 0.00 |
| 103355_at | 0.00 |
| 103356_at | 0.00 |
| 103357_at | 0.00 |

| | |
|---|---|
| 103359_at | 0.00 |
| 103360_at | 0.00 |
| 103361_at | 0.00 |
| 103362_at | 0.00 |
| 103363_at | 0.00 |
| 103364_f_at | 0.00 |
| 103365_s_at | 0.00 |
| 103366_at | 0.00 |
| 103367_at | 0.00 |
| 103369_at | 0.00 |
| 103370_at | 0.00 |
| 103371_at | 0.00 |
| 103372_at | 0.00 |
| 103374_at | 0.00 |
| 103375_at | 0.03 |
| 103376_s_at | 0.00 |
| 103377_at | 0.46 |
| 103378_at | 0.00 |
| 103379_at | 0.01 |
| 103381_at | 0.00 |
| 103384_at | 0.00 |
| 103385_at | 0.02 |
| 103386_at | 0.01 |
| 103387_at | 0.00 |
| 103388_at | 0.00 |
| 103389_at | 0.45 |
| 103391_at | 0.00 |
| 103392_at | 0.00 |
| 103393_at | 0.00 |
| 103394_at | 0.01 |
| 103395_at | 0.00 |
| 103397_at | 0.00 |
| 103398_at | 0.00 |
| 103399_at | 0.23 |
| 103400_at | 0.01 |
| 103401_at | 0.00 |
| 103402_at | 0.02 |
| 103403_at | 0.00 |
| 103404_at | 0.01 |
| 103405_at | 0.00 |
| 103406_at | 0.00 |
| 103407_at | 0.00 |
| 103408_at | 0.00 |
| 103409_at | 0.00 |
| 103411_at | 0.00 |
| 103412_at | 0.00 |
| 103413_at | 0.00 |
| 103414_at | 0.00 |
| 103415_at | 0.00 |
| 103416_at | 0.00 |
| 103418_at | 0.00 |
| 103420_at | 0.00 |
| 103421_at | 0.03 |
| 103422_at | 0.00 |
| 103423_at | 0.00 |
| 103424_at | 0.00 |

| | |
|---|---|
| 103427_at | 0.00 |
| 103428_at | 0.00 |
| 103429_i_at | 0.00 |
| 103430_at | 0.01 |
| 103432_at | 0.00 |
| 103433_at | 0.00 |
| 103434_at | 0.00 |
| 103435_at | 0.00 |
| 103436_at | 0.00 |
| 103437_at | 0.24 |
| 103438_at | 0.00 |
| 103439_at | 0.00 |
| 103440_at | 0.51 |
| 103441_at | 0.00 |
| 103442_at | 0.00 |
| 103443_at | 0.03 |
| 103444_at | 0.00 |
| 103445_at | 0.00 |
| 103446_at | 0.00 |
| 103447_at | 0.00 |
| 103448_at | 0.00 |
| 103449_at | 0.00 |
| 103450_at | 0.00 |
| 103451_at | 0.00 |
| 103452_at | 0.00 |
| 103454_at | 0.00 |
| 103455_at | 0.00 |
| 103456_at | 0.00 |
| 103457_at | 0.00 |
| 103458_at | 0.00 |
| 103459_at | 0.01 |
| 103460_at | 0.00 |
| 103462_at | 0.00 |
| 103463_at | 0.00 |
| 103465_f_at | 0.00 |
| 103466_at | 0.00 |
| 103467_g_at | 0.00 |
| 103468_at | 0.01 |
| 103469_at | 0.00 |
| 103470_at | 0.00 |
| 103471_at | 0.02 |
| 103472_at | 0.00 |
| 103473_at | 0.00 |
| 103475_s_at | 0.02 |
| 103476_at | 0.00 |
| 103477_at | 0.00 |
| 103478_at | 0.00 |
| 103479_at | 0.00 |
| 103480_at | 0.00 |
| 103481_at | 0.02 |
| 103483_at | 0.07 |
| 103484_at | 0.00 |
| 103485_at | 0.00 |
| 103486_at | 0.00 |
| 103487_at | 0.00 |
| 103488_at | 0.00 |

| | |
|---|---|
| 103489_at | 0.00 |
| 103490_at | 0.00 |
| 103491_at | 0.08 |
| 103492_at | 0.00 |
| 103493_at | 0.25 |
| 103494_at | 0.00 |
| 103495_at | 0.00 |
| 103496_at | 0.06 |
| 103497_at | 0.00 |
| 103498_at | 0.00 |
| 103499_at | 0.00 |
| 103500_at | 0.00 |
| 103501_at | 0.11 |
| 103502_at | 0.00 |
| 103503_at | 0.46 |
| 103504_at | 0.70 |
| 103506_f_at | 0.41 |
| 103507_at | 0.03 |
| 103508_at | 0.02 |
| 103509_at | 0.00 |
| 103510_at | 0.00 |
| 103511_at | 0.00 |
| 103512_at | 0.00 |
| 103513_at | 0.00 |
| 103514_at | 0.00 |
| 103515_at | 0.00 |
| 103516_at | 0.00 |
| 103517_at | 0.00 |
| 103518_at | 0.00 |
| 103519_at | 0.00 |
| 103520_at | 0.20 |
| 103521_r_at | 0.00 |
| 103522_at | 0.00 |
| 103523_at | 0.00 |
| 103524_at | 0.00 |
| 103525_at | 0.00 |
| 103526_at | 0.00 |
| 103527_at | 0.00 |
| 103529_at | 0.00 |
| 103530_at | 0.00 |
| 103531_f_at | 0.08 |
| 103532_at | 0.25 |
| 103533_at | 0.00 |
| 103534_at | 0.00 |
| 103535_at | 0.00 |
| 103536_at | 0.00 |
| 103537_at | 0.00 |
| 103538_at | 0.99 |
| 103539_at | 0.00 |
| 103540_at | 0.00 |
| 103541_at | 0.00 |
| 103542_at | 0.00 |
| 103543_at | 0.00 |
| 103544_at | 0.00 |
| 103545_at | 0.00 |
| 103546_at | 0.00 |

| | |
|---|---|
| 103547_at | 0.00 |
| 103548_at | 0.00 |
| 103549_at | 0.17 |
| 103550_at | 0.00 |
| 103551_at | 0.02 |
| 103552_at | 0.00 |
| 103553_at | 0.00 |
| 103554_at | 0.03 |
| 103555_at | 0.00 |
| 103556_at | 0.01 |
| 103557_at | 0.00 |
| 103558_at | 0.00 |
| 103559_at | 0.02 |
| 103560_at | 0.03 |
| 103562_f_at | 0.00 |
| 103563_at | 0.00 |
| 103564_at | 0.00 |
| 103565_at | 0.00 |
| 103567_at | 0.00 |
| 103568_at | 0.00 |
| 103569_at | 0.00 |
| 103570_at | 0.00 |
| 103571_at | 0.00 |
| 103573_at | 0.00 |
| 103574_at | 0.01 |
| 103575_at | 0.34 |
| 103577_at | 0.00 |
| 103578_at | 0.00 |
| 103579_at | 0.00 |
| 103580_at | 0.00 |
| 103581_at | 0.00 |
| 103582_r_at | 0.00 |
| 103584_at | 0.00 |
| 103585_at | 0.00 |
| 103588_at | 0.00 |
| 103589_at | 0.00 |
| 103590_at | 0.00 |
| 103591_at | 0.00 |
| 103592_at | 0.00 |
| 103593_at | 0.00 |
| 103594_at | 0.00 |
| 103595_at | 0.00 |
| 103596_at | 0.00 |
| 103597_at | 0.00 |
| 103598_at | 0.00 |
| 103599_at | 0.00 |
| 103600_at | 0.38 |
| 103601_at | 0.00 |
| 103602_at | 0.00 |
| 103603_at | 0.00 |
| 103604_at | 0.00 |
| 103605_g_at | 0.00 |
| 103606_r_at | 0.01 |
| 103607_at | 0.00 |
| 103608_at | 0.00 |
| 103609_at | 0.00 |

| | |
|---|---|
| 103610_at | 0.00 |
| 103611_at | 0.00 |
| 103612_at | 0.00 |
| 103613_at | 0.00 |
| 103614_at | 0.01 |
| 103615_at | 0.00 |
| 103616_at | 0.00 |
| 103617_at | 0.00 |
| 103618_at | 0.00 |
| 103619_at | 0.01 |
| 103620_s_at | 0.00 |
| 103622_at | 0.00 |
| 103623_at | 0.00 |
| 103624_at | 0.00 |
| 103625_at | 0.00 |
| 103628_at | 0.00 |
| 103629_g_at | 0.00 |
| 103630_at | 0.00 |
| 103631_at | 0.00 |
| 103632_at | 0.00 |
| 103634_at | 0.00 |
| 103635_at | 0.00 |
| 103636_at | 0.00 |
| 103637_at | 0.00 |
| 103638_at | 0.03 |
| 103639_at | 0.00 |
| 103641_at | 0.00 |
| 103642_at | 0.00 |
| 103643_at | 0.00 |
| 103644_at | 0.00 |
| 103645_at | 0.00 |
| 103646_at | 0.00 |
| 103647_at | 0.17 |
| 103648_at | 0.02 |
| 103649_at | 0.00 |
| 103650_at | 0.00 |
| 103651_r_at | 0.00 |
| 103653_at | 0.95 |
| 103654_at | 0.00 |
| 103655_at | 0.00 |
| 103656_at | 0.00 |
| 103657_i_at | 0.64 |
| 103658_r_at | 0.40 |
| 103660_at | 0.00 |
| 103662_at | 0.00 |
| 103663_at | 0.00 |
| 103664_r_at | 0.00 |
| 103665_at | 0.00 |
| 103666_at | 0.00 |
| 103667_at | 0.00 |
| 103668_at | 0.00 |
| 103669_at | 0.00 |
| 103670_at | 0.00 |
| 103671_at | 0.00 |
| 103672_at | 0.00 |
| 103673_at | 0.00 |

| | |
|---|---|
| 103674_f_at | 0.00 |
| 103675_at | 0.00 |
| 103676_at | 0.00 |
| 103678_at | 0.00 |
| 103679_at | 0.00 |
| 103680_at | 0.05 |
| 103681_at | 0.00 |
| 103682_at | 0.00 |
| 103683_at | 0.00 |
| 103684_at | 0.00 |
| 103685_at | 0.00 |
| 103686_at | 0.00 |
| 103688_at | 0.00 |
| 103689_at | 0.00 |
| 103690_at | 0.00 |
| 103691_at | 0.00 |
| 103692_at | 0.00 |
| 103693_at | 0.00 |
| 103694_at | 0.00 |
| 103695_f_at | 0.00 |
| 103696_r_at | 0.00 |
| 103697_at | 0.61 |
| 103699_i_at | 0.12 |
| 103700_r_at | 0.00 |
| 103701_at | 0.00 |
| 103702_i_at | 0.00 |
| 103703_f_at | 0.00 |
| 103704_at | 0.00 |
| 103706_at | 0.00 |
| 103707_at | 0.00 |
| 103708_at | 0.02 |
| 103709_at | 0.05 |
| 103710_at | 0.00 |
| 103711_at | 0.00 |
| 103712_at | 0.02 |
| 103713_at | 0.07 |
| 103714_at | 0.00 |
| 103715_at | 0.00 |
| 103716_at | 0.00 |
| 103717_at | 0.00 |
| 103718_at | 0.00 |
| 103719_at | 0.00 |
| 103720_at | 0.38 |
| 103721_at | 0.02 |
| 103723_at | 0.00 |
| 103726_at | 0.00 |
| 103727_at | 0.00 |
| 103728_at | 1.00 |
| 103729_at | 0.41 |
| 103730_at | 0.00 |
| 103731_at | 0.00 |
| 103732_at | 0.00 |
| 103733_at | 0.00 |
| 103734_at | 0.00 |
| 103735_at | 0.00 |
| 103736_at | 0.01 |

| | |
|---|---|
| 103737_at | 0.69 |
| 103738_at | 0.00 |
| 103739_at | 0.00 |
| 103741_at | 0.00 |
| 103742_at | 0.00 |
| 103743_at | 0.00 |
| 103744_at | 0.00 |
| 103745_at | 0.00 |
| 103746_at | 0.17 |
| 103748_at | 0.00 |
| 103751_at | 0.37 |
| 103752_r_at | 0.00 |
| 103753_at | 0.00 |
| 103754_at | 0.00 |
| 103755_at | 0.00 |
| 103756_at | 0.05 |
| 103757_at | 0.00 |
| 103759_at | 0.00 |
| 103760_at | 0.00 |
| 103761_at | 1.00 |
| 103762_at | 0.00 |
| 103763_at | 0.00 |
| 103765_at | 0.00 |
| 103766_at | 0.00 |
| 103767_f_at | 0.00 |
| 103768_at | 0.01 |
| 103769_at | 0.00 |
| 103770_at | 0.00 |
| 103771_at | 0.00 |
| 103773_at | 0.00 |
| 103774_at | 0.08 |
| 103776_at | 0.00 |
| 103778_at | 0.00 |
| 103779_at | 0.00 |
| 103780_at | 0.11 |
| 103781_at | 0.01 |
| 103782_at | 0.00 |
| 103783_at | 0.00 |
| 103784_at | 0.00 |
| 103786_at | 0.00 |
| 103787_at | 0.00 |
| 103789_at | 0.31 |
| 103790_at | 0.00 |
| 103791_at | 0.02 |
| 103792_at | 0.00 |
| 103793_at | 0.00 |
| 103794_i_at | 0.00 |
| 103795_f_at | 0.00 |
| 103796_at | 0.31 |
| 103797_at | 0.00 |
| 103799_at | 0.07 |
| 103800_at | 0.00 |
| 103801_at | 0.00 |
| 103803_at | 0.00 |
| 103804_at | 0.00 |
| 103805_at | 0.00 |

| | |
|---|---|
| 103806_at | 0.02 |
| 103807_at | 0.00 |
| 103808_at | 0.00 |
| 103809_r_at | 0.00 |
| 103810_at | 0.00 |
| 103811_at | 0.00 |
| 103812_at | 0.09 |
| 103813_at | 0.00 |
| 103814_at | 0.00 |
| 103815_at | 0.00 |
| 103816_at | 0.02 |
| 103817_at | 0.00 |
| 103818_at | 0.00 |
| 103819_at | 0.00 |
| 103821_at | 0.00 |
| 103822_at | 0.00 |
| 103823_at | 0.00 |
| 103824_at | 0.00 |
| 103825_at | 0.00 |
| 103826_at | 0.00 |
| 103828_at | 0.00 |
| 103829_at | 0.00 |
| 103830_at | 0.00 |
| 103831_at | 0.00 |
| 103832_at | 0.00 |
| 103833_at | 0.00 |
| 103835_f_at | 0.00 |
| 103836_at | 0.00 |
| 103837_at | 0.00 |
| 103838_at | 0.00 |
| 103839_at | 0.00 |
| 103840_at | 0.00 |
| 103841_at | 0.00 |
| 103842_at | 0.01 |
| 103843_at | 0.00 |
| 103844_at | 0.00 |
| 103845_at | 0.00 |
| 103846_at | 0.00 |
| 103847_at | 0.02 |
| 103848_at | 0.00 |
| 103849_at | 0.00 |
| 103850_at | 0.00 |
| 103852_at | 0.00 |
| 103853_at | 0.00 |
| 103854_at | 0.00 |
| 103855_at | 0.00 |
| 103858_at | 0.00 |
| 103859_at | 0.00 |
| 103860_at | 0.00 |
| 103861_s_at | 0.00 |
| 103862_r_at | 0.00 |
| 103863_at | 0.00 |
| 103865_at | 0.00 |
| 103866_at | 0.00 |
| 103867_at | 0.00 |
| 103868_at | 0.00 |

| | |
|---|---|
| 103869_at | 0.26 |
| 103871_at | 0.00 |
| 103872_r_at | 0.00 |
| 103873_i_at | 0.00 |
| 103874_r_at | 0.00 |
| 103875_at | 0.00 |
| 103876_at | 0.00 |
| 103877_at | 0.00 |
| 103878_at | 0.00 |
| 103879_at | 0.00 |
| 103881_at | 0.03 |
| 103882_at | 0.00 |
| 103884_at | 0.00 |
| 103885_at | 0.00 |
| 103886_at | 0.00 |
| 103887_at | 0.00 |
| 103888_at | 0.01 |
| 103889_at | 0.01 |
| 103890_at | 0.00 |
| 103891_i_at | 0.00 |
| 103892_r_at | 0.00 |
| 103893_at | 0.00 |
| 103894_at | 0.00 |
| 103895_at | 0.00 |
| 103896_f_at | 0.00 |
| 103897_r_at | 0.00 |
| 103899_at | 0.54 |
| 103900_at | 0.00 |
| 103901_at | 0.00 |
| 103903_at | 0.00 |
| 103904_at | 0.00 |
| 103905_at | 0.00 |
| 103906_f_at | 0.04 |
| 103907_at | 0.00 |
| 103908_at | 0.00 |
| 103909_at | 0.00 |
| 103910_at | 0.00 |
| 103911_at | 0.00 |
| 103912_at | 0.00 |
| 103913_at | 0.14 |
| 103914_at | 0.00 |
| 103916_at | 0.00 |
| 103918_at | 0.00 |
| 103921_i_at | 0.00 |
| 103922_f_at | 0.00 |
| 103923_at | 0.00 |
| 103924_at | 0.00 |
| 103925_at | 0.00 |
| 103926_at | 0.22 |
| 103927_at | 0.00 |
| 103928_at | 0.00 |
| 103930_at | 0.31 |
| 103931_at | 0.00 |
| 103932_at | 0.00 |
| 103933_at | 0.00 |
| 103934_at | 0.00 |

| | |
|---|---|
| 103935_at | 0.00 |
| 103939_at | 0.00 |
| 103941_at | 0.00 |
| 103942_at | 0.00 |
| 103943_at | 0.00 |
| 103944_at | 0.00 |
| 103945_at | 0.00 |
| 103946_at | 0.00 |
| 103947_at | 0.00 |
| 103948_at | 0.00 |
| 103949_at | 0.00 |
| 103950_at | 0.04 |
| 103952_at | 0.00 |
| 103953_at | 0.00 |
| 103954_at | 0.00 |
| 103955_at | 0.00 |
| 103956_at | 0.00 |
| 103957_at | 0.10 |
| 103958_g_at | 0.03 |
| 103959_at | 0.00 |
| 103960_at | 0.00 |
| 103961_s_at | 0.00 |
| 103962_at | 0.00 |
| 103963_f_at | 0.00 |
| 103964_at | 0.00 |
| 103965_at | 0.00 |
| 103967_at | 0.00 |
| 103968_at | 0.00 |
| 103969_at | 0.01 |
| 103970_at | 0.16 |
| 103971_at | 0.00 |
| 103972_at | 0.00 |
| 103973_at | 0.00 |
| 103974_at | 0.00 |
| 103975_at | 0.00 |
| 103976_at | 0.00 |
| 103977_at | 0.00 |
| 103978_at | 0.07 |
| 103980_at | 0.03 |
| 103981_at | 0.00 |
| 103982_s_at | 0.00 |
| 103983_at | 0.00 |
| 103984_at | 0.00 |
| 103985_at | 0.00 |
| 103986_at | 0.00 |
| 103987_at | 0.00 |
| 103988_at | 0.00 |
| 103989_at | 0.39 |
| 103990_at | 0.46 |
| 103991_at | 0.00 |
| 103992_f_at | 0.00 |
| 103993_at | 0.00 |
| 103994_at | 0.11 |
| 103995_at | 0.00 |
| 103997_at | 0.00 |
| 103998_at | 0.00 |

| | |
|---|---|
| 103999_at | 0.00 |
| 104000_at | 0.00 |
| 104001_at | 0.00 |
| 104002_at | 0.00 |
| 104003_at | 0.00 |
| 104004_at | 0.00 |
| 104005_at | 0.01 |
| 104006_at | 0.00 |
| 104007_at | 0.00 |
| 104008_at | 0.00 |
| 104010_at | 0.00 |
| 104011_at | 0.00 |
| 104012_at | 0.00 |
| 104013_at | 0.00 |
| 104014_at | 0.00 |
| 104015_at | 0.00 |
| 104016_at | 0.09 |
| 104017_at | 0.00 |
| 104018_at | 0.00 |
| 104019_at | 0.00 |
| 104020_at | 0.00 |
| 104021_at | 0.00 |
| 104022_at | 0.00 |
| 104023_at | 0.00 |
| 104024_at | 0.00 |
| 104025_at | 0.02 |
| 104029_at | 0.00 |
| 104030_at | 0.36 |
| 104031_at | 0.03 |
| 104032_at | 0.00 |
| 104033_at | 0.00 |
| 104034_at | 0.00 |
| 104035_at | 0.00 |
| 104036_at | 0.00 |
| 104037_at | 0.00 |
| 104038_at | 0.00 |
| 104039_at | 0.00 |
| 104040_at | 0.00 |
| 104041_at | 0.01 |
| 104042_at | 0.00 |
| 104044_at | 0.02 |
| 104045_at | 0.00 |
| 104046_at | 0.00 |
| 104047_at | 0.00 |
| 104048_at | 0.01 |
| 104049_at | 0.00 |
| 104050_at | 0.00 |
| 104052_at | 0.00 |
| 104053_at | 0.00 |
| 104055_at | 0.00 |
| 104056_at | 0.14 |
| 104057_at | 0.00 |
| 104058_at | 0.00 |
| 104059_at | 0.00 |
| 104060_at | 0.05 |
| 104063_at | 0.06 |

| | |
|---|---|
| 104064_at | 0.00 |
| 104065_at | 0.00 |
| 104066_at | 0.00 |
| 104067_at | 0.00 |
| 104068_at | 0.00 |
| 104069_at | 0.00 |
| 104070_at | 0.15 |
| 104071_at | 0.00 |
| 104072_at | 0.00 |
| 104073_at | 0.00 |
| 104074_at | 0.00 |
| 104076_at | 0.08 |
| 104077_at | 0.01 |
| 104078_g_at | 0.03 |
| 104079_at | 0.00 |
| 104080_at | 0.00 |
| 104082_at | 0.00 |
| 104083_at | 0.00 |
| 104085_at | 0.00 |
| 104086_at | 0.00 |
| 104087_at | 0.00 |
| 104088_at | 0.00 |
| 104089_at | 0.00 |
| 104090_at | 0.00 |
| 104091_at | 0.00 |
| 104092_at | 0.10 |
| 104093_at | 0.00 |
| 104094_at | 0.00 |
| 104095_at | 0.00 |
| 104096_at | 0.00 |
| 104097_at | 0.00 |
| 104098_at | 0.00 |
| 104099_at | 0.00 |
| 104100_at | 0.08 |
| 104101_at | 0.00 |
| 104102_at | 0.00 |
| 104103_at | 0.00 |
| 104104_at | 0.00 |
| 104105_at | 0.00 |
| 104106_at | 0.38 |
| 104108_at | 0.00 |
| 104109_at | 0.00 |
| 104110_at | 0.00 |
| 104112_at | 0.00 |
| 104114_at | 0.00 |
| 104115_at | 0.00 |
| 104116_at | 0.16 |
| 104117_at | 0.02 |
| 104118_at | 0.00 |
| 104119_at | 0.00 |
| 104120_at | 0.00 |
| 104121_at | 0.06 |
| 104122_at | 0.00 |
| 104123_at | 0.00 |
| 104124_at | 0.00 |
| 104125_at | 0.00 |

| | |
|---|---|
| 104126_at | 0.00 |
| 104128_at | 0.00 |
| 104129_at | 0.00 |
| 104131_at | 0.00 |
| 104132_at | 0.00 |
| 104134_at | 0.00 |
| 104135_at | 0.23 |
| 104136_at | 0.00 |
| 104137_at | 0.00 |
| 104138_at | 0.00 |
| 104139_at | 0.45 |
| 104140_s_at | 0.00 |
| 104141_at | 0.00 |
| 104142_at | 0.00 |
| 104143_at | 0.00 |
| 104144_at | 0.00 |
| 104145_at | 0.00 |
| 104146_at | 0.00 |
| 104147_at | 0.01 |
| 104148_at | 0.00 |
| 104149_at | 0.47 |
| 104150_at | 0.02 |
| 104151_at | 0.00 |
| 104152_at | 0.00 |
| 104153_at | 0.00 |
| 104154_at | 0.02 |
| 104155_f_at | 0.00 |
| 104156_r_at | 0.00 |
| 104157_at | 0.00 |
| 104158_at | 0.43 |
| 104159_at | 0.00 |
| 104160_at | 0.00 |
| 104161_at | 0.00 |
| 104163_at | 0.00 |
| 104164_at | 0.00 |
| 104165_at | 0.00 |
| 104166_at | 0.00 |
| 104168_at | 0.08 |
| 104169_at | 0.00 |
| 104170_at | 0.00 |
| 104171_f_at | 0.00 |
| 104172_at | 0.00 |
| 104173_at | 0.00 |
| 104174_at | 0.00 |
| 104175_at | 0.00 |
| 104176_at | 0.00 |
| 104177_at | 0.00 |
| 104179_at | 0.07 |
| 104180_at | 0.00 |
| 104181_at | 0.00 |
| 104182_at | 0.00 |
| 104183_at | 0.00 |
| 104184_at | 0.00 |
| 104185_at | 0.00 |
| 104186_at | 0.00 |
| 104187_at | 0.00 |

| | |
|---|---|
| 104188_at | 0.00 |
| 104189_at | 0.00 |
| 104190_at | 0.00 |
| 104191_at | 0.00 |
| 104192_at | 0.16 |
| 104193_at | 0.00 |
| 104194_at | 0.00 |
| 104195_at | 0.01 |
| 104196_at | 0.00 |
| 104197_at | 0.00 |
| 104198_at | 0.03 |
| 104199_at | 0.00 |
| 104200_at | 0.00 |
| 104201_at | 0.00 |
| 104202_at | 0.00 |
| 104205_at | 0.00 |
| 104206_at | 0.00 |
| 104207_at | 0.00 |
| 104208_at | 0.00 |
| 104209_at | 0.00 |
| 104210_at | 0.00 |
| 104211_at | 0.00 |
| 104212_at | 0.00 |
| 104213_at | 0.00 |
| 104214_at | 0.00 |
| 104215_at | 0.02 |
| 104216_at | 0.07 |
| 104217_at | 0.00 |
| 104218_s_at | 0.08 |
| 104219_f_at | 0.00 |
| 104220_at | 0.00 |
| 104221_at | 0.00 |
| 104222_f_at | 0.01 |
| 104225_at | 0.63 |
| 104227_at | 0.00 |
| 104228_at | 0.02 |
| 104229_at | 0.00 |
| 104230_at | 0.00 |
| 104231_at | 0.00 |
| 104232_at | 0.79 |
| 104233_at | 0.00 |
| 104234_at | 0.00 |
| 104235_at | 0.00 |
| 104237_at | 0.00 |
| 104238_at | 0.00 |
| 104239_at | 0.00 |
| 104240_at | 0.00 |
| 104241_at | 0.00 |
| 104242_f_at | 0.00 |
| 104243_r_at | 0.00 |
| 104244_at | 0.00 |
| 104245_at | 0.00 |
| 104246_at | 0.00 |
| 104247_at | 0.00 |
| 104248_at | 0.02 |
| 104249_g_at | 0.18 |

| | |
|---|---|
| 104250_at | 0.13 |
| 104252_at | 0.02 |
| 104253_at | 0.00 |
| 104254_at | 0.00 |
| 104255_at | 0.09 |
| 104256_at | 0.00 |
| 104257_g_at | 0.00 |
| 104258_at | 0.04 |
| 104259_at | 0.00 |
| 104260_at | 0.00 |
| 104261_at | 0.01 |
| 104262_at | 0.00 |
| 104263_at | 0.00 |
| 104264_at | 0.00 |
| 104265_at | 0.00 |
| 104266_at | 0.01 |
| 104267_at | 0.00 |
| 104268_at | 0.00 |
| 104269_at | 0.00 |
| 104270_at | 0.00 |
| 104271_at | 0.00 |
| 104272_s_at | 0.00 |
| 104273_at | 0.00 |
| 104274_at | 0.00 |
| 104275_g_at | 0.01 |
| 104276_at | 0.00 |
| 104277_at | 0.00 |
| 104279_at | 0.00 |
| 104280_at | 0.00 |
| 104282_at | 0.00 |
| 104283_at | 0.02 |
| 104284_at | 0.00 |
| 104285_at | 0.01 |
| 104286_at | 0.09 |
| 104287_at | 0.00 |
| 104288_at | 0.16 |
| 104289_at | 0.00 |
| 104290_at | 0.00 |
| 104291_at | 0.00 |
| 104292_at | 0.00 |
| 104293_at | 0.00 |
| 104294_at | 0.00 |
| 104296_at | 0.00 |
| 104297_at | 0.00 |
| 104298_at | 0.00 |
| 104299_at | 0.00 |
| 104300_at | 0.00 |
| 104301_at | 0.00 |
| 104302_f_at | 0.25 |
| 104303_i_at | 0.00 |
| 104304_r_at | 0.00 |
| 104305_at | 0.00 |
| 104306_at | 0.00 |
| 104308_at | 0.00 |
| 104310_at | 0.00 |
| 104311_at | 0.00 |

| | |
|---|---|
| 104312_at | 0.00 |
| 104313_at | 0.00 |
| 104314_r_at | 0.28 |
| 104315_at | 0.08 |
| 104316_at | 0.00 |
| 104317_at | 0.00 |
| 104318_at | 0.00 |
| 104320_at | 0.00 |
| 104322_at | 0.00 |
| 104323_at | 0.00 |
| 104324_at | 0.00 |
| 104325_at | 0.00 |
| 104326_at | 0.00 |
| 104327_at | 0.13 |
| 104328_at | 0.00 |
| 104329_at | 0.00 |
| 104330_g_at | 0.01 |
| 104331_at | 0.00 |
| 104332_at | 0.00 |
| 104333_at | 0.00 |
| 104334_at | 0.00 |
| 104335_at | 0.00 |
| 104336_at | 0.00 |
| 104337_f_at | 0.23 |
| 104338_r_at | 0.04 |
| 104339_at | 0.00 |
| 104340_at | 0.00 |
| 104341_at | 0.00 |
| 104342_i_at | 0.25 |
| 104343_f_at | 0.00 |
| 104344_at | 0.00 |
| 104345_at | 0.00 |
| 104346_at | 0.00 |
| 104348_at | 0.05 |
| 104349_at | 0.00 |
| 104350_at | 0.03 |
| 104351_at | 0.00 |
| 104352_at | 0.00 |
| 104353_at | 0.00 |
| 104354_at | 0.00 |
| 104355_at | 0.00 |
| 104356_at | 0.00 |
| 104358_at | 0.00 |
| 104360_at | 0.00 |
| 104361_at | 0.00 |
| 104362_at | 0.00 |
| 104363_at | 0.00 |
| 104364_at | 0.00 |
| 104365_at | 0.00 |
| 104366_at | 0.00 |
| 104367_at | 0.15 |
| 104368_at | 0.00 |
| 104369_at | 0.00 |
| 104370_s_at | 0.00 |
| 104371_at | 0.00 |
| 104372_at | 0.00 |

| | |
|---|---|
| 104373_at | 0.02 |
| 104374_at | 0.00 |
| 104375_at | 0.00 |
| 104376_at | 0.16 |
| 104378_at | 0.12 |
| 104380_at | 0.01 |
| 104381_at | 0.00 |
| 104382_at | 0.00 |
| 104383_at | 0.00 |
| 104385_i_at | 0.00 |
| 104386_f_at | 0.00 |
| 104387_at | 0.00 |
| 104388_at | 0.53 |
| 104389_at | 0.17 |
| 104390_at | 0.00 |
| 104391_s_at | 0.00 |
| 104392_at | 0.00 |
| 104393_at | 0.00 |
| 104394_at | 0.00 |
| 104395_at | 0.01 |
| 104396_at | 0.00 |
| 104398_at | 0.00 |
| 104399_at | 0.00 |
| 104400_at | 0.15 |
| 104401_at | 0.00 |
| 104402_at | 0.00 |
| 104403_at | 0.00 |
| 104404_at | 0.00 |
| 104405_at | 0.00 |
| 104406_at | 0.02 |
| 104407_at | 0.00 |
| 104408_s_at | 0.00 |
| 104409_at | 0.01 |
| 104410_at | 0.00 |
| 104411_at | 0.00 |
| 104412_at | 0.00 |
| 104413_at | 0.06 |
| 104414_at | 0.00 |
| 104415_at | 0.00 |
| 104416_at | 0.00 |
| 104417_at | 0.01 |
| 104418_at | 0.00 |
| 104419_at | 0.00 |
| 104420_at | 0.00 |
| 104421_at | 0.00 |
| 104422_at | 0.00 |
| 104423_at | 0.02 |
| 104424_at | 0.00 |
| 104425_at | 0.00 |
| 104427_at | 0.00 |
| 104428_s_at | 0.00 |
| 104429_at | 0.00 |
| 104430_at | 0.00 |
| 104431_at | 0.00 |
| 104432_at | 0.00 |
| 104433_at | 0.00 |

| | |
|---|---|
| 104434_at | 0.00 |
| 104435_at | 0.00 |
| 104436_at | 0.00 |
| 104437_at | 0.00 |
| 104438_at | 0.00 |
| 104439_at | 0.00 |
| 104440_at | 0.00 |
| 104441_at | 0.00 |
| 104442_at | 0.03 |
| 104443_at | 0.00 |
| 104444_at | 0.00 |
| 104445_at | 0.00 |
| 104446_f_at | 0.00 |
| 104447_r_at | 0.00 |
| 104448_at | 0.00 |
| 104449_at | 0.00 |
| 104450_at | 0.00 |
| 104451_at | 0.00 |
| 104452_at | 0.00 |
| 104453_at | 0.00 |
| 104454_at | 0.00 |
| 104455_at | 0.00 |
| 104456_at | 0.00 |
| 104457_at | 0.00 |
| 104458_at | 0.00 |
| 104460_at | 0.00 |
| 104461_at | 0.03 |
| 104462_at | 0.00 |
| 104463_at | 0.00 |
| 104464_s_at | 0.32 |
| 104466_at | 0.00 |
| 104467_at | 0.02 |
| 104468_at | 0.03 |
| 104469_at | 0.01 |
| 104470_at | 0.00 |
| 104471_at | 0.02 |
| 104473_at | 0.00 |
| 104474_s_at | 0.00 |
| 104475_at | 0.00 |
| 104476_at | 0.00 |
| 104477_at | 0.00 |
| 104479_at | 0.00 |
| 104480_at | 0.13 |
| 104481_at | 0.00 |
| 104482_at | 0.00 |
| 104483_at | 0.00 |
| 104484_at | 0.00 |
| 104485_at | 0.00 |
| 104486_at | 0.01 |
| 104487_at | 0.00 |
| 104488_at | 0.00 |
| 104489_at | 0.00 |
| 104491_at | 0.00 |
| 104492_at | 0.00 |
| 104493_at | 0.00 |
| 104494_at | 0.00 |

| | |
|---|---|
| 104495_f_at | 0.00 |
| 104496_i_at | 0.00 |
| 104497_f_at | 0.00 |
| 104498_at | 0.00 |
| 104499_at | 0.00 |
| 104500_at | 0.00 |
| 104501_at | 0.00 |
| 104502_f_at | 0.11 |
| 104503_at | 0.00 |
| 104504_at | 0.00 |
| 104505_at | 0.00 |
| 104506_at | 0.00 |
| 104507_g_at | 0.00 |
| 104508_at | 0.00 |
| 104509_at | 0.00 |
| 104510_at | 0.00 |
| 104512_at | 0.00 |
| 104513_at | 0.01 |
| 104514_at | 0.00 |
| 104515_at | 0.00 |
| 104516_at | 0.00 |
| 104518_at | 0.00 |
| 104519_at | 0.00 |
| 104522_at | 0.00 |
| 104523_at | 0.09 |
| 104524_at | 0.00 |
| 104525_at | 0.00 |
| 104526_at | 0.16 |
| 104527_at | 0.00 |
| 104528_at | 0.00 |
| 104529_at | 0.00 |
| 104531_at | 0.00 |
| 104532_at | 0.00 |
| 104533_at | 0.07 |
| 104534_at | 0.15 |
| 104535_at | 0.00 |
| 104536_at | 0.00 |
| 104537_at | 0.00 |
| 104538_at | 0.00 |
| 104539_at | 0.00 |
| 104541_at | 0.00 |
| 104544_at | 0.90 |
| 104545_at | 0.21 |
| 104546_g_at | 0.00 |
| 104547_at | 0.00 |
| 104548_at | 0.00 |
| 104549_at | 0.00 |
| 104550_at | 0.00 |
| 104551_at | 0.00 |
| 104554_at | 0.00 |
| 104555_at | 0.00 |
| 104556_at | 0.00 |
| 104557_at | 0.00 |
| 104558_at | 0.00 |
| 104559_at | 0.00 |
| 104560_at | 0.00 |

| | |
|---|---|
| 104561_at | 0.00 |
| 104562_at | 0.00 |
| 104564_at | 0.00 |
| 104565_at | 0.00 |
| 104566_at | 0.00 |
| 104567_at | 0.00 |
| 104568_at | 0.01 |
| 104569_at | 0.00 |
| 104571_at | 0.00 |
| 104572_at | 0.00 |
| 104573_at | 0.00 |
| 104574_at | 0.00 |
| 104576_at | 0.00 |
| 104577_at | 0.00 |
| 104578_f_at | 0.00 |
| 104579_r_at | 0.02 |
| 104580_at | 0.10 |
| 104581_at | 0.00 |
| 104582_g_at | 0.00 |
| 104583_at | 0.00 |
| 104584_f_at | 0.48 |
| 104585_at | 0.02 |
| 104586_at | 0.00 |
| 104587_at | 0.01 |
| 104588_at | 0.00 |
| 104589_at | 0.10 |
| 104590_at | 0.00 |
| 104591_g_at | 0.00 |
| 104592_i_at | 0.00 |
| 104593_at | 0.00 |
| 104594_at | 0.00 |
| 104595_at | 0.04 |
| 104597_at | 0.23 |
| 104598_at | 0.30 |
| 104600_at | 0.03 |
| 104601_at | 0.40 |
| 104602_at | 0.00 |
| 104603_at | 0.04 |
| 104604_at | 0.02 |
| 104605_at | 0.00 |
| 104606_at | 0.00 |
| 104607_at | 0.00 |
| 104608_at | 0.00 |
| 104609_at | 0.00 |
| 104610_at | 0.24 |
| 104611_at | 0.00 |
| 104612_g_at | 0.03 |
| 104614_at | 0.00 |
| 104615_at | 0.00 |
| 104616_g_at | 0.00 |
| 104617_at | 0.00 |
| 104618_at | 0.00 |
| 104619_at | 0.00 |
| 104620_at | 0.00 |
| 104621_at | 0.00 |
| 104622_at | 0.00 |

| | |
|---|---|
| 104623_at | 0.00 |
| 104624_at | 0.00 |
| 104625_at | 0.00 |
| 104626_at | 0.00 |
| 104627_at | 0.00 |
| 104628_at | 0.00 |
| 104629_at | 0.00 |
| 104630_at | 0.00 |
| 104631_g_at | 0.00 |
| 104632_at | 0.00 |
| 104633_at | 0.00 |
| 104634_at | 0.00 |
| 104635_r_at | 0.11 |
| 104636_at | 0.00 |
| 104637_at | 0.00 |
| 104638_at | 0.00 |
| 104639_i_at | 0.09 |
| 104640_f_at | 0.10 |
| 104641_f_at | 0.00 |
| 104642_at | 0.00 |
| 104643_at | 0.01 |
| 104644_at | 0.00 |
| 104645_at | 0.08 |
| 104646_at | 0.00 |
| 104647_at | 0.28 |
| 104648_at | 0.00 |
| 104650_at | 0.00 |
| 104651_at | 0.00 |
| 104652_at | 0.00 |
| 104653_at | 0.00 |
| 104654_at | 0.00 |
| 104655_at | 0.00 |
| 104657_at | 0.00 |
| 104658_at | 0.00 |
| 104659_g_at | 0.00 |
| 104660_at | 0.00 |
| 104661_at | 0.01 |
| 104662_at | 0.00 |
| 104663_at | 0.00 |
| 104664_at | 0.00 |
| 104665_g_at | 0.00 |
| 104666_at | 0.00 |
| 104667_at | 0.00 |
| 104669_at | 0.00 |
| 104670_at | 0.00 |
| 104671_at | 0.07 |
| 104672_at | 0.01 |
| 104673_at | 0.04 |
| 104674_s_at | 0.00 |
| 104675_at | 0.00 |
| 104677_at | 0.00 |
| 104678_at | 0.00 |
| 104679_at | 0.04 |
| 104680_at | 0.00 |
| 104681_at | 0.05 |
| 104682_at | 0.00 |

| | |
|---|---|
| 104683_at | 0.00 |
| 104684_at | 0.00 |
| 104685_g_at | 0.00 |
| 104686_at | 0.00 |
| 104687_at | 0.00 |
| 104688_at | 0.00 |
| 104689_at | 0.00 |
| 104690_at | 0.00 |
| 104692_at | 0.00 |
| 104693_at | 0.03 |
| 104694_at | 0.00 |
| 104695_at | 0.00 |
| 104696_at | 0.00 |
| 104697_at | 0.04 |
| 104698_at | 0.01 |
| 104699_at | 0.00 |
| 104700_at | 0.00 |
| 104701_at | 0.40 |
| 104702_at | 0.00 |
| 104704_at | 0.03 |
| 104706_at | 0.00 |
| 104707_at | 0.00 |
| 104708_at | 0.00 |
| 104709_at | 0.00 |
| 104710_at | 0.00 |
| 104711_at | 0.00 |
| 104712_at | 0.00 |
| 104713_at | 0.00 |
| 104714_at | 0.00 |
| 104715_at | 0.00 |
| 104716_at | 0.00 |
| 104717_at | 0.00 |
| 104719_at | 0.00 |
| 104720_at | 0.00 |
| 104722_at | 0.00 |
| 104723_at | 0.00 |
| 104725_at | 0.00 |
| 104726_at | 0.00 |
| 104727_at | 0.00 |
| 104728_at | 0.02 |
| 104729_at | 0.00 |
| 104730_at | 0.00 |
| 104731_at | 0.00 |
| 104733_at | 0.00 |
| 104735_at | 0.00 |
| 104737_at | 0.00 |
| 104738_at | 0.00 |
| 104739_at | 0.00 |
| 104740_at | 0.07 |
| 104741_at | 0.00 |
| 104742_at | 0.00 |
| 104743_at | 0.00 |
| 104744_at | 0.52 |
| 104745_at | 0.32 |
| 104746_at | 0.00 |
| 104747_at | 0.00 |

| | |
|---|---|
| 104748_s_at | 0.00 |
| 104749_at | 0.00 |
| 104750_at | 0.00 |
| 104751_at | 0.81 |
| 104752_at | 0.00 |
| 104754_at | 0.00 |
| 104755_at | 0.00 |
| 104756_at | 0.00 |
| 104757_at | 0.00 |
| 104758_at | 0.00 |
| 104759_at | 0.00 |
| 104760_at | 0.00 |
| 104761_at | 0.00 |
| 104762_r_at | 0.00 |
| 104763_at | 0.00 |
| 104766_at | 0.00 |
| 104767_f_at | 0.00 |
| 160060_at | 0.00 |
| 160061_at | 0.00 |
| 160062_i_at | 0.00 |
| 160063_i_at | 0.00 |
| 160064_at | 0.00 |
| 160065_s_at | 0.00 |
| 160066_at | 0.00 |
| 160067_at | 0.00 |
| 160068_at | 0.53 |
| 160069_at | 0.00 |
| 160070_at | 0.00 |
| 160071_at | 0.00 |
| 160072_at | 0.00 |
| 160073_i_at | 0.00 |
| 160074_at | 0.01 |
| 160075_at | 0.00 |
| 160076_at | 0.00 |
| 160077_at | 0.00 |
| 160078_at | 0.00 |
| 160079_i_at | 0.00 |
| 160080_r_at | 0.00 |
| 160081_at | 0.00 |
| 160082_s_at | 0.00 |
| 160083_at | 0.00 |
| 160084_at | 0.00 |
| 160085_at | 0.03 |
| 160086_at | 0.00 |
| 160087_at | 0.00 |
| 160088_at | 0.00 |
| 160089_at | 0.07 |
| 160090_f_at | 0.02 |
| 160091_at | 0.00 |
| 160092_at | 0.00 |
| 160093_r_at | 0.00 |
| 160094_at | 0.18 |
| 160095_at | 0.35 |
| 160096_at | 0.00 |
| 160097_at | 0.00 |
| 160098_s_at | 0.00 |

| | |
|---|---|
| 160099_at | 0.00 |
| 160100_at | 0.00 |
| 160101_at | 0.00 |
| 160102_at | 0.00 |
| 160103_at | 0.01 |
| 160104_at | 0.00 |
| 160105_r_at | 0.00 |
| 160106_at | 0.01 |
| 160107_at | 0.00 |
| 160108_at | 0.54 |
| 160109_at | 0.00 |
| 160110_at | 0.00 |
| 160111_at | 0.10 |
| 160112_at | 0.04 |
| 160113_at | 0.00 |
| 160114_at | 0.00 |
| 160115_at | 0.00 |
| 160116_at | 0.00 |
| 160117_at | 0.00 |
| 160118_at | 0.00 |
| 160119_at | 0.00 |
| 160120_i_at | 0.00 |
| 160121_at | 0.00 |
| 160122_at | 0.00 |
| 160123_at | 0.01 |
| 160124_r_at | 0.00 |
| 160125_at | 0.00 |
| 160126_at | 0.00 |
| 160127_at | 0.00 |
| 160128_at | 0.00 |
| 160129_at | 0.00 |
| 160130_at | 0.03 |
| 160131_at | 0.02 |
| 160132_at | 0.00 |
| 160133_at | 0.00 |
| 160134_at | 0.04 |
| 160135_at | 0.00 |
| 160136_r_at | 0.00 |
| 160137_at | 0.03 |
| 160138_at | 0.61 |
| 160139_at | 0.00 |
| 160140_at | 0.00 |
| 160141_r_at | 0.17 |
| 160142_at | 0.00 |
| 160143_r_at | 0.00 |
| 160144_at | 0.00 |
| 160145_at | 0.00 |
| 160146_r_at | 0.00 |
| 160147_r_at | 0.00 |
| 160148_at | 0.00 |
| 160149_at | 0.00 |
| 160150_f_at | 0.02 |
| 160151_i_at | 0.00 |
| 160152_at | 0.00 |
| 160153_at | 0.00 |
| 160154_at | 0.00 |

| | |
|---|---|
| 160155_at | 0.00 |
| 160156_at | 0.00 |
| 160157_at | 0.00 |
| 160158_at | 0.03 |
| 160159_at | 0.01 |
| 160160_at | 0.00 |
| 160161_at | 0.00 |
| 160162_at | 0.10 |
| 160163_at | 0.00 |
| 160164_at | 0.00 |
| 160165_at | 0.01 |
| 160166_r_at | 0.00 |
| 160167_at | 0.00 |
| 160168_at | 0.00 |
| 160169_at | 0.01 |
| 160170_at | 0.00 |
| 160171_f_at | 0.00 |
| 160172_at | 0.04 |
| 160173_at | 0.21 |
| 160174_at | 0.00 |
| 160175_at | 0.00 |
| 160176_at | 0.00 |
| 160177_at | 0.00 |
| 160178_r_at | 0.00 |
| 160179_at | 0.00 |
| 160180_at | 0.00 |
| 160181_at | 0.00 |
| 160182_at | 0.00 |
| 160183_f_at | 0.02 |
| 160184_at | 0.00 |
| 160185_at | 0.00 |
| 160186_at | 0.00 |
| 160187_at | 0.00 |
| 160188_at | 0.08 |
| 160189_at | 0.06 |
| 160190_at | 0.00 |
| 160191_at | 0.24 |
| 160192_at | 0.00 |
| 160193_at | 0.00 |
| 160194_at | 0.00 |
| 160195_at | 0.03 |
| 160196_at | 0.00 |
| 160197_at | 0.02 |
| 160198_at | 0.00 |
| 160199_at | 0.00 |
| 160200_at | 0.00 |
| 160201_r_at | 0.00 |
| 160202_at | 0.01 |
| 160203_at | 0.04 |
| 160204_at | 0.26 |
| 160205_f_at | 0.00 |
| 160206_at | 0.09 |
| 160207_at | 0.00 |
| 160208_at | 0.02 |
| 160209_at | 0.00 |
| 160210_at | 0.00 |

| | |
|---|---|
| 160211_at | 0.00 |
| 160212_at | 0.00 |
| 160213_at | 0.00 |
| 160214_at | 0.00 |
| 160215_at | 0.09 |
| 160216_f_at | 0.00 |
| 160217_at | 0.00 |
| 160218_at | 0.00 |
| 160219_r_at | 0.00 |
| 160220_at | 0.06 |
| 160221_at | 0.00 |
| 160222_i_at | 0.00 |
| 160223_at | 0.00 |
| 160224_at | 0.00 |
| 160225_at | 0.00 |
| 160226_at | 0.00 |
| 160227_s_at | 0.00 |
| 160228_at | 0.00 |
| 160229_at | 0.00 |
| 160230_at | 0.00 |
| 160231_at | 0.00 |
| 160232_at | 0.00 |
| 160233_at | 0.00 |
| 160234_at | 0.06 |
| 160235_at | 0.01 |
| 160236_at | 0.04 |
| 160237_at | 0.01 |
| 160238_at | 0.00 |
| 160239_at | 0.00 |
| 160240_at | 0.24 |
| 160241_at | 0.00 |
| 160242_at | 0.01 |
| 160243_r_at | 0.00 |
| 160244_at | 0.00 |
| 160245_at | 0.00 |
| 160246_at | 0.01 |
| 160247_at | 0.00 |
| 160248_at | 0.04 |
| 160249_at | 0.00 |
| 160250_at | 0.00 |
| 160251_at | 0.00 |
| 160252_at | 0.00 |
| 160253_at | 0.74 |
| 160254_at | 0.00 |
| 160255_at | 0.00 |
| 160256_at | 0.00 |
| 160257_at | 0.00 |
| 160258_at | 0.00 |
| 160259_at | 0.01 |
| 160260_at | 0.14 |
| 160261_i_at | 0.02 |
| 160262_at | 0.00 |
| 160263_r_at | 0.00 |
| 160264_s_at | 0.00 |
| 160265_at | 0.00 |
| 160266_r_at | 0.00 |

| | |
|---|---|
| 160267_at | 0.00 |
| 160268_at | 0.00 |
| 160269_at | 0.00 |
| 160270_at | 0.00 |
| 160271_at | 0.00 |
| 160272_at | 0.01 |
| 160273_at | 0.48 |
| 160274_at | 0.00 |
| 160275_at | 0.00 |
| 160276_at | 0.06 |
| 160277_at | 0.00 |
| 160278_at | 0.27 |
| 160279_at | 0.00 |
| 160280_at | 0.45 |
| 160281_at | 0.00 |
| 160282_at | 0.00 |
| 160283_at | 0.00 |
| 160284_at | 0.00 |
| 160285_at | 0.07 |
| 160286_at | 0.00 |
| 160287_at | 0.06 |
| 160288_at | 0.05 |
| 160289_s_at | 0.02 |
| 160290_at | 0.02 |
| 160291_at | 0.00 |
| 160292_at | 0.10 |
| 160293_at | 0.00 |
| 160294_at | 0.00 |
| 160295_at | 0.00 |
| 160296_at | 0.20 |
| 160297_at | 0.00 |
| 160298_at | 0.03 |
| 160299_at | 0.00 |
| 160300_at | 0.00 |
| 160301_at | 0.00 |
| 160302_at | 0.00 |
| 160303_at | 0.00 |
| 160304_r_at | 0.00 |
| 160305_at | 0.00 |
| 160306_at | 0.00 |
| 160307_at | 0.00 |
| 160308_at | 0.00 |
| 160309_at | 0.00 |
| 160310_at | 0.00 |
| 160311_at | 0.00 |
| 160312_at | 0.00 |
| 160313_at | 0.05 |
| 160314_at | 0.02 |
| 160315_at | 0.00 |
| 160316_at | 0.79 |
| 160317_at | 0.00 |
| 160318_at | 0.00 |
| 160319_at | 0.00 |
| 160320_at | 0.07 |
| 160321_at | 0.00 |
| 160322_at | 0.00 |

| | |
|---|---|
| 160323_at | 0.00 |
| 160324_at | 0.01 |
| 160325_r_at | 0.00 |
| 160326_at | 0.01 |
| 160327_at | 0.00 |
| 160328_at | 0.00 |
| 160329_at | 0.00 |
| 160330_at | 0.00 |
| 160331_at | 0.00 |
| 160332_at | 0.00 |
| 160333_at | 0.00 |
| 160334_at | 0.00 |
| 160335_at | 0.51 |
| 160336_at | 0.00 |
| 160337_at | 0.37 |
| 160338_at | 0.00 |
| 160339_at | 0.00 |
| 160340_at | 0.00 |
| 160341_at | 0.00 |
| 160342_r_at | 0.00 |
| 160343_at | 0.00 |
| 160344_at | 0.00 |
| 160345_at | 0.00 |
| 160346_at | 0.00 |
| 160347_at | 0.00 |
| 160348_at | 0.00 |
| 160349_at | 0.00 |
| 160350_at | 0.00 |
| 160351_at | 0.00 |
| 160352_at | 0.01 |
| 160353_i_at | 0.03 |
| 160354_at | 0.00 |
| 160355_at | 0.00 |
| 160356_at | 0.00 |
| 160357_at | 0.00 |
| 160358_at | 0.00 |
| 160359_at | 0.00 |
| 160360_at | 0.00 |
| 160361_at | 0.01 |
| 160362_at | 0.00 |
| 160363_at | 0.00 |
| 160364_at | 0.00 |
| 160365_at | 0.42 |
| 160366_at | 0.00 |
| 160367_at | 0.75 |
| 160368_at | 0.00 |
| 160369_at | 0.01 |
| 160370_at | 0.70 |
| 160371_at | 0.00 |
| 160372_at | 0.00 |
| 160373_i_at | 0.31 |
| 160374_r_at | 0.00 |
| 160375_at | 0.30 |
| 160376_at | 0.00 |
| 160377_at | 0.00 |
| 160378_at | 0.00 |

| | |
|---|---|
| 160379_at | 0.00 |
| 160380_at | 0.00 |
| 160381_at | 0.00 |
| 160382_at | 0.00 |
| 160383_at | 0.00 |
| 160384_at | 0.00 |
| 160385_at | 0.00 |
| 160386_at | 0.00 |
| 160387_at | 0.00 |
| 160388_at | 0.00 |
| 160389_r_at | 0.00 |
| 160390_at | 0.00 |
| 160391_at | 0.00 |
| 160392_at | 0.00 |
| 160393_at | 0.00 |
| 160394_at | 0.00 |
| 160395_at | 0.00 |
| 160396_at | 0.00 |
| 160397_at | 0.02 |
| 160398_at | 0.00 |
| 160399_r_at | 0.52 |
| 160400_at | 0.00 |
| 160401_r_at | 0.00 |
| 160402_at | 0.00 |
| 160403_at | 0.00 |
| 160404_at | 0.00 |
| 160405_at | 0.00 |
| 160406_at | 0.00 |
| 160407_at | 0.00 |
| 160408_at | 0.00 |
| 160409_at | 0.00 |
| 160410_at | 0.00 |
| 160411_at | 0.00 |
| 160412_at | 0.00 |
| 160413_at | 0.00 |
| 160414_at | 0.00 |
| 160415_at | 0.00 |
| 160416_at | 0.00 |
| 160417_at | 0.00 |
| 160418_at | 0.00 |
| 160419_r_at | 0.00 |
| 160420_r_at | 0.51 |
| 160421_r_at | 0.00 |
| 160422_at | 0.00 |
| 160423_at | 0.00 |
| 160424_f_at | 0.01 |
| 160425_at | 0.00 |
| 160426_at | 0.00 |
| 160427_at | 0.00 |
| 160428_at | 0.00 |
| 160429_at | 0.00 |
| 160430_at | 0.00 |
| 160431_at | 0.04 |
| 160432_at | 0.00 |
| 160433_at | 0.00 |
| 160434_at | 0.01 |

| | |
|---|---|
| 160435_at | 0.00 |
| 160436_at | 0.00 |
| 160437_at | 0.00 |
| 160438_at | 0.00 |
| 160439_at | 0.01 |
| 160440_at | 0.00 |
| 160441_at | 0.00 |
| 160442_at | 0.00 |
| 160443_at | 0.00 |
| 160444_at | 0.00 |
| 160445_at | 0.00 |
| 160446_at | 0.00 |
| 160447_at | 0.03 |
| 160448_at | 0.00 |
| 160449_at | 0.00 |
| 160450_at | 0.00 |
| 160451_at | 0.00 |
| 160452_at | 0.00 |
| 160453_at | 0.01 |
| 160454_at | 0.00 |
| 160455_s_at | 0.00 |
| 160456_at | 0.01 |
| 160457_at | 0.00 |
| 160458_at | 0.00 |
| 160459_r_at | 0.00 |
| 160460_at | 0.00 |
| 160461_f_at | 0.03 |
| 160462_f_at | 0.00 |
| 160463_at | 0.00 |
| 160464_s_at | 0.00 |
| 160465_at | 0.00 |
| 160466_at | 0.00 |
| 160467_at | 0.00 |
| 160468_at | 0.00 |
| 160469_at | 0.35 |
| 160470_at | 0.06 |
| 160471_at | 0.00 |
| 160472_r_at | 0.04 |
| 160473_at | 0.04 |
| 160474_at | 0.00 |
| 160475_at | 0.00 |
| 160476_f_at | 0.00 |
| 160477_at | 0.00 |
| 160478_r_at | 0.00 |
| 160479_at | 0.00 |
| 160480_at | 0.15 |
| 160481_at | 0.00 |
| 160482_at | 0.03 |
| 160483_at | 0.03 |
| 160484_at | 0.00 |
| 160485_r_at | 0.00 |
| 160486_at | 0.00 |
| 160487_at | 0.00 |
| 160488_at | 0.00 |
| 160489_at | 0.00 |
| 160490_at | 0.00 |

| | |
|---|---|
| 160491_at | 0.00 |
| 160492_at | 0.00 |
| 160493_at | 0.00 |
| 160494_at | 0.00 |
| 160495_at | 0.00 |
| 160496_s_at | 0.00 |
| 160497_at | 0.00 |
| 160498_at | 0.01 |
| 160499_at | 0.00 |
| 160500_at | 0.00 |
| 160501_at | 0.00 |
| 160502_at | 0.00 |
| 160503_at | 0.00 |
| 160504_at | 0.00 |
| 160505_at | 0.00 |
| 160506_at | 0.00 |
| 160507_at | 0.43 |
| 160508_at | 0.00 |
| 160509_at | 0.00 |
| 160510_f_at | 0.00 |
| 160511_at | 0.00 |
| 160512_at | 0.00 |
| 160513_at | 0.00 |
| 160514_at | 0.00 |
| 160515_at | 0.00 |
| 160516_at | 0.00 |
| 160517_at | 0.05 |
| 160518_at | 0.00 |
| 160519_at | 0.13 |
| 160520_at | 0.00 |
| 160521_at | 0.00 |
| 160522_at | 0.00 |
| 160523_at | 0.00 |
| 160524_at | 0.00 |
| 160525_f_at | 0.00 |
| 160526_s_at | 0.00 |
| 160527_at | 0.00 |
| 160528_at | 0.00 |
| 160529_r_at | 0.02 |
| 160530_at | 0.00 |
| 160531_at | 0.00 |
| 160532_at | 0.00 |
| 160533_r_at | 0.00 |
| 160534_at | 0.00 |
| 160535_at | 0.02 |
| 160536_at | 0.00 |
| 160537_at | 0.00 |
| 160538_at | 0.00 |
| 160539_at | 0.06 |
| 160540_at | 0.00 |
| 160541_at | 0.00 |
| 160542_at | 0.00 |
| 160543_at | 0.00 |
| 160544_at | 0.02 |
| 160545_at | 0.85 |
| 160546_at | 0.03 |

| | |
|---|---|
| 160547_s_at | 0.00 |
| 160548_at | 0.00 |
| 160549_at | 0.01 |
| 160550_i_at | 0.00 |
| 160551_at | 0.00 |
| 160552_at | 0.00 |
| 160553_at | 0.00 |
| 160554_at | 0.00 |
| 160555_r_at | 0.00 |
| 160556_at | 0.00 |
| 160557_at | 0.00 |
| 160558_at | 0.00 |
| 160559_at | 0.00 |
| 160560_at | 0.00 |
| 160561_at | 0.60 |
| 160562_at | 0.00 |
| 160563_at | 0.00 |
| 160564_at | 0.00 |
| 160565_at | 0.00 |
| 160566_at | 0.00 |
| 160567_at | 0.00 |
| 160568_at | 0.00 |
| 160569_at | 0.00 |
| 160570_at | 0.00 |
| 160571_at | 0.24 |
| 160572_at | 0.00 |
| 160573_at | 0.00 |
| 160574_at | 0.00 |
| 160575_at | 0.41 |
| 160576_at | 0.00 |
| 160577_at | 0.00 |
| 160578_at | 0.02 |
| 160579_at | 0.00 |
| 160580_at | 0.00 |
| 160581_at | 0.00 |
| 160582_at | 0.00 |
| 160583_at | 0.00 |
| 160584_at | 0.00 |
| 160585_at | 0.00 |
| 160586_r_at | 0.00 |
| 160587_at | 0.00 |
| 160588_at | 0.01 |
| 160589_at | 0.00 |
| 160590_r_at | 0.00 |
| 160591_at | 0.01 |
| 160592_at | 0.00 |
| 160593_at | 0.00 |
| 160594_at | 0.00 |
| 160595_at | 0.00 |
| 160596_at | 0.00 |
| 160597_at | 0.00 |
| 160598_at | 0.00 |
| 160599_r_at | 0.00 |
| 160600_at | 0.13 |
| 160601_at | 0.00 |
| 160602_at | 0.00 |

| | |
|---|---|
| 160603_at | 0.04 |
| 160604_at | 0.00 |
| 160605_s_at | 0.00 |
| 160606_r_at | 0.00 |
| 160607_at | 0.00 |
| 160608_at | 0.00 |
| 160609_at | 0.06 |
| 160610_at | 0.00 |
| 160611_at | 0.00 |
| 160612_at | 0.00 |
| 160613_at | 0.00 |
| 160614_at | 0.38 |
| 160615_at | 0.00 |
| 160616_at | 0.00 |
| 160617_at | 0.03 |
| 160618_at | 0.00 |
| 160619_at | 0.00 |
| 160620_at | 0.25 |
| 160621_at | 0.00 |
| 160622_at | 0.00 |
| 160623_at | 0.00 |
| 160624_at | 0.00 |
| 160625_f_at | 0.00 |
| 160626_at | 0.02 |
| 160627_at | 0.00 |
| 160628_at | 0.00 |
| 160629_at | 0.00 |
| 160630_at | 0.00 |
| 160631_s_at | 0.00 |
| 160632_at | 0.01 |
| 160633_at | 0.05 |
| 160634_at | 0.00 |
| 160635_at | 0.00 |
| 160636_at | 0.00 |
| 160637_at | 0.00 |
| 160638_at | 0.00 |
| 160639_at | 0.00 |
| 160640_at | 0.00 |
| 160641_at | 0.00 |
| 160642_at | 0.01 |
| 160643_at | 0.00 |
| 160644_at | 0.00 |
| 160645_at | 0.00 |
| 160646_at | 0.03 |
| 160647_at | 0.00 |
| 160648_at | 0.00 |
| 160649_at | 0.00 |
| 160650_at | 0.00 |
| 160651_at | 0.32 |
| 160652_at | 0.24 |
| 160653_at | 0.00 |
| 160654_at | 0.00 |
| 160655_at | 0.00 |
| 160656_i_at | 0.00 |
| 160657_at | 0.00 |
| 160658_at | 0.00 |

| | |
|---|---|
| 160659_at | 0.00 |
| 160660_r_at | 0.00 |
| 160661_at | 0.00 |
| 160662_r_at | 0.00 |
| 160663_at | 0.00 |
| 160664_at | 0.00 |
| 160665_at | 0.00 |
| 160666_at | 0.00 |
| 160667_at | 0.01 |
| 160668_at | 0.04 |
| 160669_at | 0.00 |
| 160670_at | 0.01 |
| 160671_at | 0.00 |
| 160672_at | 0.00 |
| 160673_at | 0.00 |
| 160674_at | 0.00 |
| 160675_at | 0.00 |
| 160676_at | 0.00 |
| 160677_at | 0.00 |
| 160678_at | 0.06 |
| 160679_at | 0.10 |
| 160680_at | 0.00 |
| 160681_at | 0.05 |
| 160682_at | 0.00 |
| 160683_at | 0.70 |
| 160684_at | 0.73 |
| 160685_at | 0.00 |
| 160686_at | 0.00 |
| 160687_r_at | 0.00 |
| 160688_at | 0.00 |
| 160689_r_at | 0.00 |
| 160690_at | 0.00 |
| 160691_at | 0.00 |
| 160692_at | 0.00 |
| 160693_at | 0.00 |
| 160694_at | 0.00 |
| 160695_i_at | 0.04 |
| 160696_at | 0.09 |
| 160697_at | 0.00 |
| 160698_s_at | 0.00 |
| 160699_at | 0.00 |
| 160700_i_at | 0.00 |
| 160701_at | 0.00 |
| 160702_at | 0.25 |
| 160703_at | 0.00 |
| 160704_at | 0.00 |
| 160705_at | 0.00 |
| 160706_at | 0.00 |
| 160707_at | 0.00 |
| 160708_at | 0.22 |
| 160709_at | 0.02 |
| 160710_at | 0.00 |
| 160711_at | 0.01 |
| 160712_r_at | 0.36 |
| 160713_at | 0.00 |
| 160714_at | 0.04 |

| | |
|---|---|
| 160715_at | 0.00 |
| 160716_at | 0.00 |
| 160717_at | 0.00 |
| 160718_at | 0.00 |
| 160719_at | 0.00 |
| 160720_at | 0.00 |
| 160721_at | 0.00 |
| 160722_at | 0.00 |
| 160723_at | 0.00 |
| 160724_at | 0.00 |
| 160725_at | 0.00 |
| 160726_at | 0.64 |
| 160727_at | 0.00 |
| 160728_r_at | 0.00 |
| 160729_f_at | 0.00 |
| 160730_at | 0.00 |
| 160731_at | 0.00 |
| 160732_at | 0.00 |
| 160733_at | 0.00 |
| 160734_at | 0.00 |
| 160735_at | 0.00 |
| 160736_at | 0.00 |
| 160737_at | 0.00 |
| 160738_at | 0.00 |
| 160739_at | 0.26 |
| 160740_at | 0.01 |
| 160741_at | 0.00 |
| 160742_at | 0.00 |
| 160743_at | 0.00 |
| 160744_r_at | 0.00 |
| 160745_at | 0.00 |
| 160746_at | 0.02 |
| 160747_at | 0.00 |
| 160748_at | 0.00 |
| 160749_at | 0.03 |
| 160750_at | 0.00 |
| 160751_i_at | 0.00 |
| 160752_at | 0.00 |
| 160753_at | 0.00 |
| 160754_at | 0.00 |
| 160755_at | 0.01 |
| 160756_at | 0.00 |
| 160757_at | 0.00 |
| 160758_at | 0.00 |
| 160759_at | 0.00 |
| 160760_at | 0.00 |
| 160761_at | 0.00 |
| 160762_at | 0.00 |
| 160763_at | 0.00 |
| 160764_at | 0.00 |
| 160765_at | 0.01 |
| 160766_at | 0.00 |
| 160767_at | 0.05 |
| 160768_at | 0.04 |
| 160769_at | 0.00 |
| 160770_at | 0.00 |

| | |
|---|---|
| 160771_r_at | 0.00 |
| 160772_i_at | 0.00 |
| 160773_at | 0.00 |
| 160774_at | 0.00 |
| 160775_at | 0.00 |
| 160776_at | 0.00 |
| 160777_at | 0.00 |
| 160778_at | 0.17 |
| 160779_at | 0.00 |
| 160780_at | 0.19 |
| 160781_r_at | 0.01 |
| 160782_at | 0.00 |
| 160783_at | 0.08 |
| 160784_r_at | 0.00 |
| 160785_at | 0.00 |
| 160786_f_at | 0.00 |
| 160787_at | 0.00 |
| 160788_at | 0.00 |
| 160789_at | 0.00 |
| 160790_s_at | 0.01 |
| 160791_at | 0.00 |
| 160792_at | 0.00 |
| 160793_at | 0.00 |
| 160794_at | 0.00 |
| 160795_at | 0.00 |
| 160796_at | 0.00 |
| 160797_r_at | 0.00 |
| 160798_at | 0.00 |
| 160799_at | 0.00 |
| 160800_at | 0.00 |
| 160801_at | 0.00 |
| 160802_at | 0.01 |
| 160803_at | 0.00 |
| 160804_at | 0.00 |
| 160805_s_at | 0.00 |
| 160806_at | 0.26 |
| 160807_at | 0.00 |
| 160808_at | 0.01 |
| 160809_at | 0.00 |
| 160810_r_at | 0.00 |
| 160811_at | 0.00 |
| 160812_at | 0.00 |
| 160813_r_at | 0.01 |
| 160814_at | 0.00 |
| 160815_at | 0.00 |
| 160816_at | 0.00 |
| 160817_at | 0.00 |
| 160818_at | 0.00 |
| 160819_at | 0.00 |
| 160820_at | 0.00 |
| 160821_r_at | 0.00 |
| 160822_at | 0.00 |
| 160823_at | 0.00 |
| 160824_at | 0.00 |
| 160825_at | 0.00 |
| 160826_at | 0.00 |

| | |
|---|---|
| 160827_at | 0.00 |
| 160828_at | 0.62 |
| 160829_at | 0.07 |
| 160830_at | 0.00 |
| 160831_at | 0.00 |
| 160832_at | 0.53 |
| 160833_at | 0.00 |
| 160834_at | 0.00 |
| 160835_i_at | 0.00 |
| 160836_at | 0.00 |
| 160837_at | 0.00 |
| 160838_at | 0.00 |
| 160839_at | 0.00 |
| 160840_at | 0.00 |
| 160841_at | 0.00 |
| 160842_at | 0.00 |
| 160843_at | 0.04 |
| 160844_at | 0.00 |
| 160845_at | 0.00 |
| 160846_at | 0.00 |
| 160847_at | 0.00 |
| 160848_at | 0.02 |
| 160849_at | 0.00 |
| 160850_at | 0.01 |
| 160851_r_at | 0.00 |
| 160852_at | 0.00 |
| 160853_at | 0.00 |
| 160854_at | 0.00 |
| 160855_at | 0.00 |
| 160856_at | 0.00 |
| 160857_at | 0.02 |
| 160858_at | 0.00 |
| 160859_s_at | 0.25 |
| 160860_at | 0.00 |
| 160861_s_at | 0.00 |
| 160862_at | 0.00 |
| 160863_at | 0.00 |
| 160864_at | 0.00 |
| 160865_at | 0.00 |
| 160866_at | 0.00 |
| 160867_at | 0.00 |
| 160868_at | 0.00 |
| 160869_at | 0.00 |
| 160870_at | 0.00 |
| 160871_at | 0.00 |
| 160872_f_at | 0.00 |
| 160873_at | 0.00 |
| 160874_r_at | 0.00 |
| 160875_at | 0.00 |
| 160876_at | 0.01 |
| 160877_at | 0.00 |
| 160878_at | 0.00 |
| 160879_at | 0.01 |
| 160880_at | 0.00 |
| 160881_at | 0.00 |
| 160882_at | 0.00 |

| | |
|---|---|
| 160883_at | 0.00 |
| 160884_at | 0.00 |
| 160885_at | 0.00 |
| 160886_i_at | 0.00 |
| 160887_at | 0.05 |
| 160888_at | 0.00 |
| 160889_at | 0.00 |
| 160890_at | 0.00 |
| 160891_at | 0.00 |
| 160892_at | 0.00 |
| 160893_at | 0.00 |
| 160894_at | 0.00 |
| 160895_at | 0.00 |
| 160896_at | 0.00 |
| 160897_at | 0.11 |
| 160898_at | 0.00 |
| 160899_at | 0.02 |
| 160900_at | 0.00 |
| 160901_at | 0.10 |
| 160902_at | 0.00 |
| 160903_at | 0.00 |
| 160904_at | 0.00 |
| 160905_s_at | 0.00 |
| 160906_i_at | 0.09 |
| 160907_at | 0.00 |
| 160908_r_at | 0.00 |
| 160909_at | 0.00 |
| 160910_at | 0.00 |
| 160911_at | 0.00 |
| 160912_i_at | 0.07 |
| 160913_at | 0.00 |
| 160914_at | 0.00 |
| 160915_at | 0.00 |
| 160916_at | 0.00 |
| 160917_r_at | 0.00 |
| 160918_at | 0.00 |
| 160919_r_at | 0.00 |
| 160920_at | 0.00 |
| 160921_at | 0.00 |
| 160922_at | 0.00 |
| 160923_at | 0.00 |
| 160924_at | 0.00 |
| 160925_at | 0.07 |
| 160926_r_at | 0.00 |
| 160927_at | 0.00 |
| 160928_at | 0.00 |
| 160929_at | 0.00 |
| 160930_at | 0.00 |
| 160931_at | 0.00 |
| 160932_at | 0.00 |
| 160933_at | 0.00 |
| 160934_s_at | 0.33 |
| 160935_at | 0.01 |
| 160936_at | 0.03 |
| 160937_at | 0.05 |
| 160938_at | 0.00 |

| | |
|---|---|
| 160939_at | 0.00 |
| 160940_at | 0.00 |
| 160941_at | 0.00 |
| 160942_at | 0.00 |
| 160943_at | 0.06 |
| 160944_at | 0.00 |
| 160945_at | 0.00 |
| 160946_at | 0.00 |
| 160947_at | 0.00 |
| 160948_at | 0.00 |
| 160949_at | 0.39 |
| 160950_at | 0.00 |
| 160951_at | 0.00 |
| 160952_r_at | 0.00 |
| 160953_at | 0.00 |
| 160954_at | 0.00 |
| 160955_at | 0.01 |
| 160956_r_at | 0.00 |
| 160957_at | 0.00 |
| 160958_at | 0.00 |
| 160959_at | 0.00 |
| 160960_at | 0.00 |
| 160961_at | 0.00 |
| 160962_at | 0.01 |
| 160963_at | 0.05 |
| 160964_at | 0.00 |
| 160965_at | 0.07 |
| 160966_at | 0.00 |
| 160967_at | 0.00 |
| 160968_at | 0.16 |
| 160969_at | 0.00 |
| 160970_at | 0.04 |
| 160971_at | 0.00 |
| 160972_at | 0.00 |
| 160973_at | 0.00 |
| 160974_at | 0.00 |
| 160975_at | 0.00 |
| 160976_at | 0.36 |
| 160977_at | 0.07 |
| 160978_at | 0.00 |
| 160979_at | 0.42 |
| 160980_at | 0.00 |
| 160981_at | 0.00 |
| 160982_at | 0.00 |
| 160983_at | 0.00 |
| 160984_r_at | 0.00 |
| 160985_at | 0.00 |
| 160986_r_at | 0.00 |
| 160987_r_at | 0.00 |
| 160988_r_at | 0.02 |
| 160989_r_at | 0.00 |
| 160990_r_at | 0.00 |
| 160991_at | 0.00 |
| 160992_at | 0.00 |
| 160993_at | 0.00 |
| 160994_at | 0.00 |

| | |
|---|---|
| 160995_at | 0.00 |
| 160996_at | 0.00 |
| 160997_at | 0.00 |
| 160998_at | 0.00 |
| 160999_r_at | 0.00 |
| 161000_i_at | 0.00 |
| 161001_at | 0.00 |
| 161002_at | 0.00 |
| 161003_at | 0.00 |
| 161004_at | 0.31 |
| 161005_at | 0.00 |
| 161006_at | 0.00 |
| 161007_at | 0.00 |
| 161008_at | 0.00 |
| 161009_at | 0.00 |
| 161010_r_at | 0.00 |
| 161011_at | 0.00 |
| 161012_at | 0.00 |
| 161013_f_at | 0.30 |
| 161014_at | 0.00 |
| 161015_at | 0.00 |
| 161016_at | 0.00 |
| 161017_at | 0.00 |
| 161018_at | 0.00 |
| 161019_at | 0.00 |
| 161020_r_at | 0.00 |
| 161021_at | 0.00 |
| 161022_at | 0.00 |
| 161023_at | 0.00 |
| 161024_at | 0.00 |
| 161025_f_at | 0.00 |
| 161026_s_at | 0.00 |
| 161027_f_at | 0.00 |
| 161028_at | 0.00 |
| 161029_at | 0.00 |
| 161030_at | 0.00 |
| 161031_at | 0.00 |
| 161032_i_at | 0.00 |
| 161033_at | 0.00 |
| 161034_at | 0.00 |
| 161035_at | 0.00 |
| 161036_at | 0.05 |
| 161037_at | 0.00 |
| 161038_at | 0.00 |
| 161039_at | 0.00 |
| 161040_at | 0.00 |
| 161041_at | 0.00 |
| 161042_at | 0.75 |
| 161043_r_at | 0.00 |
| 161044_at | 0.00 |
| 161045_at | 0.00 |
| 161046_at | 0.21 |
| 161047_at | 0.00 |
| 161048_at | 0.00 |
| 161049_at | 0.00 |
| 161050_at | 0.00 |

| | |
|---|---|
| 161051_at | 0.00 |
| 161052_r_at | 0.00 |
| 161053_at | 0.00 |
| 161054_at | 0.00 |
| 161055_r_at | 0.00 |
| 161056_at | 0.00 |
| 161057_at | 0.00 |
| 161058_f_at | 0.00 |
| 161059_at | 0.00 |
| 161060_i_at | 0.00 |
| 161061_r_at | 0.00 |
| 161062_r_at | 0.00 |
| 161063_r_at | 0.00 |
| 161064_f_at | 0.00 |
| 161065_at | 0.00 |
| 161066_at | 0.00 |
| 161067_at | 0.00 |
| 161068_at | 0.00 |
| 161069_at | 0.00 |
| 161070_at | 0.12 |
| 161071_at | 0.00 |
| 161072_at | 0.61 |
| 161073_at | 0.00 |
| 161074_at | 0.00 |
| 161075_at | 0.00 |
| 161076_at | 0.00 |
| 161077_f_at | 0.00 |
| 161078_at | 0.00 |
| 161079_at | 0.00 |
| 161080_f_at | 0.00 |
| 161081_at | 0.00 |
| 161082_r_at | 0.00 |
| 161083_at | 0.00 |
| 161084_at | 0.00 |
| 161085_r_at | 0.00 |
| 161086_at | 0.03 |
| 161087_r_at | 0.00 |
| 161088_r_at | 0.01 |
| 161089_r_at | 0.00 |
| 161090_i_at | 0.00 |
| 161091_r_at | 0.00 |
| 161092_at | 0.00 |
| 161093_at | 0.00 |
| 161094_r_at | 0.00 |
| 161095_i_at | 0.00 |
| 161096_at | 0.15 |
| 161097_at | 0.00 |
| 161098_at | 0.00 |
| 161099_at | 0.00 |
| 161100_at | 0.00 |
| 161101_r_at | 0.00 |
| 161102_at | 0.00 |
| 161103_at | 0.00 |
| 161104_at | 0.05 |
| 161105_at | 0.00 |
| 161106_r_at | 0.00 |

| | |
|---|---|
| 161107_r_at | 0.02 |
| 161108_r_at | 0.00 |
| 161109_at | 0.00 |
| 161110_at | 0.00 |
| 161111_f_at | 0.00 |
| 161112_at | 0.00 |
| 161113_at | 0.00 |
| 161114_i_at | 0.00 |
| 161115_r_at | 0.00 |
| 161116_at | 0.00 |
| 161117_at | 0.00 |
| 161118_r_at | 0.00 |
| 161119_at | 0.00 |
| 161120_r_at | 0.00 |
| 161121_f_at | 0.36 |
| 161122_f_at | 0.01 |
| 161123_i_at | 0.00 |
| 161124_at | 0.00 |
| 161125_at | 0.00 |
| 161126_at | 0.00 |
| 161127_i_at | 0.00 |
| 161128_r_at | 0.00 |
| 161129_r_at | 0.00 |
| 161130_f_at | 0.00 |
| 161131_r_at | 0.00 |
| 161132_at | 0.00 |
| 161133_at | 0.01 |
| 161134_at | 0.00 |
| 161135_f_at | 0.00 |
| 161136_r_at | 0.00 |
| 161137_r_at | 0.00 |
| 161138_r_at | 0.00 |
| 161139_f_at | 0.00 |
| 161140_r_at | 0.00 |
| 161141_r_at | 0.00 |
| 161142_at | 0.00 |
| 161143_r_at | 0.00 |
| 161144_r_at | 0.00 |
| 161145_f_at | 0.00 |
| 161146_r_at | 0.00 |
| 161147_f_at | 0.00 |
| 161148_f_at | 0.00 |
| 161149_r_at | 0.02 |
| 161150_at | 0.00 |
| 161151_at | 0.00 |
| 161152_r_at | 0.00 |
| 161153_r_at | 0.00 |
| 161154_at | 0.00 |
| 161155_r_at | 0.00 |
| 161156_r_at | 0.00 |
| 161157_r_at | 0.01 |
| 161158_f_at | 0.00 |
| 161159_r_at | 0.00 |
| 161160_f_at | 0.00 |
| 161161_r_at | 0.11 |
| 161162_at | 0.00 |

| | |
|---|---|
| 161163_at | 0.00 |
| 161164_r_at | 0.00 |
| 161165_f_at | 0.00 |
| 161166_i_at | 0.01 |
| 161167_r_at | 0.00 |
| 161168_at | 0.00 |
| 161169_f_at | 0.00 |
| 161170_r_at | 0.00 |
| 161171_at | 0.00 |
| 161172_f_at | 0.00 |
| 161173_f_at | 0.00 |
| 161174_i_at | 0.00 |
| 161175_r_at | 0.00 |
| 161176_r_at | 0.00 |
| 161177_f_at | 0.00 |
| 161178_at | 0.00 |
| 161179_at | 0.00 |
| 161180_r_at | 0.00 |
| 161181_f_at | 0.00 |
| 161182_r_at | 0.00 |
| 161183_at | 0.04 |
| 161184_f_at | 0.00 |
| 161185_i_at | 0.00 |
| 161186_f_at | 0.00 |
| 161187_f_at | 0.00 |
| 161188_f_at | 0.00 |
| 161189_r_at | 0.00 |
| 161190_r_at | 0.00 |
| 161191_i_at | 0.00 |
| 161192_at | 0.00 |
| 161193_r_at | 0.00 |
| 161194_r_at | 0.00 |
| 161195_i_at | 0.00 |
| 161196_r_at | 0.00 |
| 161197_r_at | 0.00 |
| 161198_r_at | 0.00 |
| 161199_at | 0.00 |
| 161200_i_at | 0.00 |
| 161201_r_at | 0.00 |
| 161202_r_at | 0.00 |
| 161203_f_at | 0.00 |
| 161204_r_at | 0.00 |
| 161205_at | 0.00 |
| 161206_at | 0.00 |
| 161207_at | 0.00 |
| 161208_r_at | 0.00 |
| 161209_r_at | 0.00 |
| 161210_f_at | 0.00 |
| 161211_r_at | 0.00 |
| 161212_r_at | 0.00 |
| 161213_r_at | 0.00 |
| 161214_r_at | 0.00 |
| 161215_at | 0.00 |
| 161216_at | 0.00 |
| 161217_r_at | 0.00 |
| 161218_r_at | 0.00 |

| | |
|---|---|
| 161219_r_at | 0.00 |
| 161220_f_at | 0.00 |
| 161221_f_at | 0.04 |
| 161222_r_at | 0.00 |
| 161223_r_at | 0.00 |
| 161224_f_at | 0.00 |
| 161225_r_at | 0.00 |
| 161226_f_at | 0.00 |
| 161227_r_at | 0.00 |
| 161228_f_at | 0.41 |
| 161229_at | 0.00 |
| 161230_r_at | 0.00 |
| 161231_r_at | 0.00 |
| 161232_r_at | 0.00 |
| 161233_at | 0.03 |
| 161234_f_at | 0.00 |
| 161235_f_at | 0.00 |
| 161236_r_at | 0.00 |
| 161237_at | 0.01 |
| 161238_f_at | 0.00 |
| 161239_r_at | 0.00 |
| 161240_f_at | 0.00 |
| 161241_at | 0.00 |
| 161242_f_at | 0.00 |
| 161243_f_at | 0.00 |
| 161244_f_at | 0.00 |
| 161245_r_at | 0.00 |
| 161246_at | 0.00 |
| 161247_f_at | 0.00 |
| 161248_r_at | 0.00 |
| 161249_at | 0.00 |
| 161250_at | 0.00 |
| 161251_f_at | 0.00 |
| 161252_r_at | 0.00 |
| 161253_i_at | 0.00 |
| 161254_r_at | 0.00 |
| 161255_at | 0.00 |
| 161256_at | 0.00 |
| 161257_r_at | 0.00 |
| 161258_at | 0.00 |
| 161259_f_at | 0.00 |
| 161260_f_at | 0.00 |
| 161261_f_at | 0.00 |
| 161262_r_at | 0.05 |
| 161263_f_at | 0.00 |
| 161264_f_at | 0.00 |
| 161265_f_at | 0.00 |
| 161266_r_at | 0.00 |
| 161267_f_at | 0.00 |
| 161268_f_at | 0.00 |
| 161269_i_at | 0.00 |
| 161270_i_at | 0.23 |
| 161271_r_at | 0.00 |
| 161272_f_at | 0.00 |
| 161273_f_at | 0.00 |
| 161274_at | 0.00 |

| | |
|---|---|
| 161275_at | 0.00 |
| 161276_i_at | 0.00 |
| 161277_r_at | 0.00 |
| 161278_r_at | 0.00 |
| 161279_f_at | 0.00 |
| 161280_r_at | 0.00 |
| 161281_f_at | 0.00 |
| 161282_r_at | 0.00 |
| 161283_i_at | 0.00 |
| 161284_r_at | 0.00 |
| 161285_r_at | 0.00 |
| 161286_f_at | 0.00 |
| 161287_f_at | 0.00 |
| 161288_r_at | 0.00 |
| 161289_at | 0.00 |
| 161290_r_at | 0.01 |
| 161291_at | 0.00 |
| 161292_f_at | 0.00 |
| 161293_r_at | 0.00 |
| 161294_f_at | 0.06 |
| 161295_r_at | 0.00 |
| 161296_r_at | 0.00 |
| 161297_f_at | 0.00 |
| 161298_i_at | 0.00 |
| 161299_r_at | 0.00 |
| 161300_r_at | 0.00 |
| 161301_f_at | 0.00 |
| 161302_r_at | 0.14 |
| 161303_at | 0.00 |
| 161304_r_at | 0.00 |
| 161305_r_at | 0.00 |
| 161306_r_at | 0.00 |
| 161307_f_at | 0.00 |
| 161308_f_at | 0.00 |
| 161309_r_at | 0.00 |
| 161310_at | 0.00 |
| 161311_at | 0.00 |
| 161312_r_at | 0.00 |
| 161313_at | 0.00 |
| 161314_r_at | 0.09 |
| 161315_i_at | 0.00 |
| 161316_f_at | 0.00 |
| 161317_r_at | 0.00 |
| 161318_f_at | 0.00 |
| 161319_at | 0.00 |
| 161320_r_at | 0.00 |
| 161321_i_at | 0.00 |
| 161322_r_at | 0.00 |
| 161323_f_at | 0.00 |
| 161324_r_at | 0.00 |
| 161325_at | 0.00 |
| 161326_f_at | 0.00 |
| 161327_f_at | 0.00 |
| 161328_i_at | 0.00 |
| 161329_f_at | 0.00 |
| 161330_r_at | 0.00 |

| | |
|---|---|
| 161331_r_at | 0.00 |
| 161332_f_at | 0.00 |
| 161333_f_at | 0.00 |
| 161334_r_at | 0.00 |
| 161335_r_at | 0.00 |
| 161336_r_at | 0.00 |
| 161337_f_at | 0.00 |
| 161338_i_at | 0.00 |
| 161339_f_at | 0.00 |
| 161340_r_at | 0.00 |
| 161341_f_at | 0.00 |
| 161342_r_at | 0.02 |
| 161343_r_at | 0.00 |
| 161344_r_at | 0.01 |
| 161345_f_at | 0.02 |
| 161346_f_at | 0.00 |
| 161347_r_at | 0.00 |
| 161348_r_at | 0.00 |
| 161349_f_at | 0.00 |
| 161350_f_at | 0.00 |
| 161351_r_at | 0.00 |
| 161352_r_at | 0.00 |
| 161353_r_at | 0.00 |
| 161354_f_at | 0.00 |
| 161355_f_at | 0.00 |
| 161356_at | 0.00 |
| 161357_r_at | 0.00 |
| 161358_r_at | 0.00 |
| 161359_s_at | 0.00 |
| 161360_at | 0.00 |
| 161361_s_at | 0.00 |
| 161362_at | 0.00 |
| 161363_r_at | 0.00 |
| 161364_f_at | 0.00 |
| 161365_r_at | 0.00 |
| 161366_r_at | 0.01 |
| 161367_f_at | 0.00 |
| 161368_r_at | 0.01 |
| 161369_r_at | 0.01 |
| 161370_f_at | 0.00 |
| 161371_r_at | 0.00 |
| 161372_f_at | 0.00 |
| 161373_r_at | 0.00 |
| 161374_f_at | 0.00 |
| 161375_at | 0.00 |
| 161376_f_at | 0.00 |
| 161377_at | 0.00 |
| 161378_r_at | 0.00 |
| 161379_at | 0.00 |
| 161380_f_at | 0.00 |
| 161381_r_at | 0.00 |
| 161382_at | 0.00 |
| 161383_r_at | 0.00 |
| 161384_r_at | 0.00 |
| 161385_r_at | 0.01 |
| 161386_f_at | 0.00 |

| | |
|---|---|
| 161387_i_at | 0.00 |
| 161388_f_at | 0.00 |
| 161389_f_at | 0.00 |
| 161390_r_at | 0.00 |
| 161391_r_at | 0.01 |
| 161392_f_at | 0.00 |
| 161393_at | 0.00 |
| 161394_f_at | 0.00 |
| 161395_i_at | 0.01 |
| 161396_f_at | 0.00 |
| 161397_r_at | 0.00 |
| 161398_at | 0.00 |
| 161399_r_at | 0.00 |
| 161400_f_at | 0.00 |
| 161401_f_at | 0.00 |
| 161402_r_at | 0.00 |
| 161403_r_at | 0.00 |
| 161404_at | 0.00 |
| 161405_r_at | 0.00 |
| 161406_at | 0.00 |
| 161407_i_at | 0.00 |
| 161408_r_at | 0.00 |
| 161409_f_at | 0.00 |
| 161410_r_at | 0.00 |
| 161411_i_at | 0.00 |
| 161412_r_at | 0.00 |
| 161413_f_at | 0.00 |
| 161414_f_at | 0.00 |
| 161415_r_at | 0.00 |
| 161416_r_at | 0.00 |
| 161417_r_at | 0.00 |
| 161418_r_at | 0.00 |
| 161419_r_at | 0.00 |
| 161420_r_at | 0.00 |
| 161421_r_at | 0.01 |
| 161422_f_at | 0.00 |
| 161423_r_at | 0.00 |
| 161424_f_at | 0.00 |
| 161425_r_at | 0.00 |
| 161426_at | 0.00 |
| 161427_f_at | 0.00 |
| 161428_at | 0.00 |
| 161429_at | 0.00 |
| 161430_at | 0.00 |
| 161431_i_at | 0.02 |
| 161432_f_at | 0.00 |
| 161433_f_at | 0.00 |
| 161434_r_at | 0.00 |
| 161435_i_at | 0.00 |
| 161436_s_at | 0.00 |
| 161437_f_at | 0.00 |
| 161438_r_at | 0.00 |
| 161439_f_at | 0.00 |
| 161440_r_at | 0.00 |
| 161441_f_at | 0.00 |
| 161442_at | 0.00 |

| | |
|---|---|
| 161443_r_at | 0.00 |
| 161444_f_at | 0.00 |
| 161445_at | 0.00 |
| 161446_r_at | 0.00 |
| 161447_f_at | 0.00 |
| 161448_f_at | 0.00 |
| 161449_f_at | 0.00 |
| 161450_r_at | 0.00 |
| 161451_r_at | 0.00 |
| 161452_f_at | 0.00 |
| 161453_r_at | 0.00 |
| 161454_r_at | 0.00 |
| 161455_r_at | 0.00 |
| 161456_f_at | 0.00 |
| 161457_at | 0.00 |
| 161458_at | 0.00 |
| 161459_f_at | 0.00 |
| 161460_r_at | 0.00 |
| 161461_at | 0.00 |
| 161462_r_at | 0.01 |
| 161463_f_at | 0.00 |
| 161464_f_at | 0.00 |
| 161465_r_at | 0.00 |
| 161466_r_at | 0.00 |
| 161467_f_at | 0.00 |
| 161468_f_at | 0.00 |
| 161469_r_at | 0.00 |
| 161470_r_at | 0.00 |
| 161471_f_at | 0.00 |
| 161472_r_at | 0.00 |
| 161473_f_at | 0.00 |
| 161474_r_at | 0.00 |
| 161475_f_at | 0.00 |
| 161476_at | 0.00 |
| 161477_r_at | 0.00 |
| 161478_at | 0.00 |
| 161479_f_at | 0.00 |
| 161480_i_at | 0.00 |
| 161481_f_at | 0.00 |
| 161482_f_at | 0.01 |
| 161483_r_at | 0.00 |
| 161484_r_at | 0.00 |
| 161485_r_at | 0.00 |
| 161486_f_at | 0.00 |
| 161487_f_at | 0.00 |
| 161488_r_at | 0.00 |
| 161489_r_at | 0.00 |
| 161490_at | 0.00 |
| 161491_r_at | 0.00 |
| 161492_i_at | 0.00 |
| 161493_at | 0.00 |
| 161494_f_at | 0.00 |
| 161495_r_at | 0.00 |
| 161496_r_at | 0.00 |
| 161497_f_at | 0.00 |
| 161498_at | 0.00 |

| | |
|---|---|
| 161499_f_at | 0.00 |
| 161500_i_at | 0.00 |
| 161501_at | 0.00 |
| 161502_r_at | 0.00 |
| 161503_f_at | 0.00 |
| 161504_i_at | 0.03 |
| 161505_i_at | 0.00 |
| 161506_r_at | 0.00 |
| 161507_f_at | 0.00 |
| 161508_at | 0.00 |
| 161509_at | 0.00 |
| 161510_f_at | 0.00 |
| 161511_f_at | 0.15 |
| 161512_r_at | 0.00 |
| 161513_r_at | 0.00 |
| 161514_at | 0.00 |
| 161515_i_at | 0.00 |
| 161516_r_at | 0.00 |
| 161517_at | 0.00 |
| 161518_r_at | 0.00 |
| 161519_f_at | 0.00 |
| 161520_at | 0.00 |
| 161521_at | 0.00 |
| 161522_i_at | 0.01 |
| 161523_r_at | 0.00 |
| 161524_r_at | 0.04 |
| 161525_f_at | 0.00 |
| 161526_r_at | 0.00 |
| 161527_r_at | 0.00 |
| 161528_r_at | 0.01 |
| 161529_r_at | 0.00 |
| 161530_r_at | 0.00 |
| 161531_r_at | 0.01 |
| 161532_f_at | 0.00 |
| 161533_at | 0.00 |
| 161534_f_at | 0.00 |
| 161535_at | 0.00 |
| 161536_r_at | 0.00 |
| 161537_f_at | 0.00 |
| 161538_r_at | 0.00 |
| 161539_f_at | 0.00 |
| 161540_r_at | 0.00 |
| 161541_r_at | 0.00 |
| 161542_r_at | 0.00 |
| 161543_at | 0.00 |
| 161544_r_at | 0.00 |
| 161545_r_at | 0.00 |
| 161546_r_at | 0.00 |
| 161547_f_at | 0.00 |
| 161548_r_at | 0.00 |
| 161549_f_at | 0.00 |
| 161550_r_at | 0.00 |
| 161551_f_at | 0.00 |
| 161552_i_at | 0.00 |
| 161553_i_at | 0.00 |
| 161554_r_at | 0.00 |

| | |
|---|---|
| 161555_f_at | 0.00 |
| 161556_at | 0.00 |
| 161557_at | 0.00 |
| 161558_f_at | 0.00 |
| 161559_i_at | 0.00 |
| 161560_f_at | 0.00 |
| 161561_r_at | 0.00 |
| 161562_f_at | 0.00 |
| 161563_r_at | 0.00 |
| 161564_r_at | 0.02 |
| 161565_r_at | 0.00 |
| 161566_r_at | 0.02 |
| 161567_r_at | 0.00 |
| 161568_f_at | 0.00 |
| 161569_f_at | 0.00 |
| 161570_r_at | 0.00 |
| 161571_f_at | 0.00 |
| 161572_r_at | 0.00 |
| 161573_at | 0.00 |
| 161574_r_at | 0.00 |
| 161575_f_at | 0.00 |
| 161576_f_at | 0.00 |
| 161577_f_at | 0.00 |
| 161578_r_at | 0.00 |
| 161579_r_at | 0.00 |
| 161580_f_at | 0.00 |
| 161581_r_at | 0.00 |
| 161582_r_at | 0.00 |
| 161583_at | 0.00 |
| 161584_r_at | 0.00 |
| 161585_at | 0.00 |
| 161586_f_at | 0.00 |
| 161587_at | 0.00 |
| 161588_r_at | 0.00 |
| 161589_at | 0.00 |
| 161590_r_at | 0.00 |
| 161591_r_at | 0.05 |
| 161592_at | 0.00 |
| 161593_r_at | 0.00 |
| 161594_f_at | 0.00 |
| 161595_at | 0.02 |
| 161596_f_at | 0.01 |
| 161597_r_at | 0.00 |
| 161598_at | 0.00 |
| 161599_i_at | 0.00 |
| 161600_r_at | 0.00 |
| 161601_i_at | 0.00 |
| 161602_at | 0.00 |
| 161603_r_at | 0.42 |
| 161604_r_at | 0.00 |
| 161605_f_at | 0.00 |
| 161606_f_at | 0.00 |
| 161607_r_at | 0.00 |
| 161608_r_at | 0.00 |
| 161609_at | 0.17 |
| 161610_at | 0.00 |

| | |
|---|---|
| 161611_f_at | 0.00 |
| 161612_f_at | 0.00 |
| 161613_at | 0.00 |
| 161614_r_at | 0.00 |
| 161615_f_at | 0.00 |
| 161616_f_at | 0.00 |
| 161617_f_at | 0.00 |
| 161618_r_at | 0.00 |
| 161619_f_at | 0.00 |
| 161620_f_at | 0.00 |
| 161621_r_at | 0.00 |
| 161622_f_at | 0.00 |
| 161623_at | 0.00 |
| 161624_r_at | 0.00 |
| 161625_r_at | 0.00 |
| 161626_f_at | 0.00 |
| 161627_r_at | 0.00 |
| 161628_r_at | 0.00 |
| 161629_i_at | 0.00 |
| 161630_i_at | 0.00 |
| 161631_f_at | 0.00 |
| 161632_r_at | 0.00 |
| 161633_r_at | 0.00 |
| 161634_r_at | 0.00 |
| 161635_f_at | 0.00 |
| 161636_r_at | 0.00 |
| 161637_f_at | 0.00 |
| 161638_f_at | 0.00 |
| 161639_f_at | 0.00 |
| 161640_at | 0.00 |
| 161641_at | 0.00 |
| 161642_f_at | 0.00 |
| 161643_i_at | 0.00 |
| 161644_f_at | 0.00 |
| 161645_r_at | 0.00 |
| 161646_r_at | 0.00 |
| 161647_f_at | 0.00 |
| 161648_at | 0.00 |
| 161649_f_at | 0.00 |
| 161650_at | 0.00 |
| 161651_f_at | 0.00 |
| 161652_r_at | 0.00 |
| 161653_f_at | 0.27 |
| 161654_r_at | 0.00 |
| 161655_at | 0.00 |
| 161656_r_at | 0.00 |
| 161657_f_at | 0.00 |
| 161658_at | 0.00 |
| 161659_f_at | 0.00 |
| 161660_r_at | 0.00 |
| 161661_i_at | 0.00 |
| 161662_f_at | 0.00 |
| 161663_f_at | 0.00 |
| 161664_at | 0.00 |
| 161665_at | 0.00 |
| 161666_f_at | 0.00 |

| | |
|---|---|
| 161667_r_at | 0.00 |
| 161668_f_at | 0.00 |
| 161669_r_at | 0.00 |
| 161670_f_at | 0.00 |
| 161671_at | 0.00 |
| 161672_at | 0.00 |
| 161673_r_at | 0.00 |
| 161674_i_at | 0.00 |
| 161675_f_at | 0.00 |
| 161676_at | 0.00 |
| 161677_r_at | 0.00 |
| 161678_at | 0.00 |
| 161679_r_at | 0.00 |
| 161680_r_at | 0.00 |
| 161681_i_at | 0.00 |
| 161682_f_at | 0.00 |
| 161683_r_at | 0.00 |
| 161684_r_at | 0.00 |
| 161685_r_at | 0.00 |
| 161686_i_at | 0.00 |
| 161687_r_at | 0.00 |
| 161688_r_at | 0.00 |
| 161689_f_at | 0.00 |
| 161690_at | 0.00 |
| 161691_at | 0.00 |
| 161692_r_at | 0.00 |
| 161693_r_at | 0.00 |
| 161694_f_at | 0.00 |
| 161695_f_at | 0.00 |
| 161696_f_at | 0.00 |
| 161697_r_at | 0.00 |
| 161698_f_at | 0.02 |
| 161699_i_at | 0.00 |
| 161700_i_at | 0.00 |
| 161701_at | 0.00 |
| 161702_f_at | 0.00 |
| 161703_f_at | 0.65 |
| 161704_r_at | 0.02 |
| 161705_r_at | 0.00 |
| 161706_f_at | 0.01 |
| 161707_f_at | 0.00 |
| 161708_f_at | 0.00 |
| 161709_at | 0.00 |
| 161710_r_at | 0.00 |
| 161711_f_at | 0.00 |
| 161712_r_at | 0.00 |
| 161713_f_at | 0.00 |
| 161714_f_at | 0.00 |
| 161715_f_at | 0.00 |
| 161716_at | 0.00 |
| 161717_i_at | 0.00 |
| 161718_at | 0.00 |
| 161719_f_at | 0.00 |
| 161720_r_at | 0.00 |
| 161721_f_at | 0.00 |
| 161722_f_at | 0.00 |

| | |
|---|---|
| 161723_at | 0.00 |
| 161724_r_at | 0.01 |
| 161725_r_at | 0.00 |
| 161726_f_at | 0.00 |
| 161727_r_at | 0.00 |
| 161728_f_at | 0.00 |
| 161729_f_at | 0.00 |
| 161730_f_at | 0.00 |
| 161731_at | 0.00 |
| 161732_at | 0.00 |
| 161733_at | 0.00 |
| 161734_r_at | 0.00 |
| 161735_r_at | 0.00 |
| 161736_r_at | 0.00 |
| 161737_at | 0.00 |
| 161738_f_at | 0.00 |
| 161739_r_at | 0.00 |
| 161740_r_at | 0.00 |
| 161741_r_at | 0.00 |
| 161742_r_at | 0.00 |
| 161743_i_at | 0.00 |
| 161744_f_at | 0.00 |
| 161745_f_at | 0.00 |
| 161746_i_at | 0.00 |
| 161747_i_at | 0.00 |
| 161748_r_at | 0.00 |
| 161749_r_at | 0.00 |
| 161750_f_at | 0.00 |
| 161751_f_at | 0.00 |
| 161752_r_at | 0.00 |
| 161753_f_at | 0.00 |
| 161754_f_at | 0.01 |
| 161755_at | 0.00 |
| 161756_at | 0.04 |
| 161757_f_at | 0.00 |
| 161758_r_at | 0.00 |
| 161759_r_at | 0.00 |
| 161760_s_at | 0.00 |
| 161761_r_at | 0.00 |
| 161762_at | 0.00 |
| 161763_r_at | 0.03 |
| 161764_r_at | 0.00 |
| 161765_f_at | 0.00 |
| 161766_i_at | 0.00 |
| 161767_r_at | 0.00 |
| 161768_r_at | 0.00 |
| 161769_r_at | 0.00 |
| 161770_f_at | 0.00 |
| 161771_r_at | 0.00 |
| 161772_i_at | 0.00 |
| 161773_i_at | 0.00 |
| 161774_f_at | 0.00 |
| 161775_f_at | 0.00 |
| 161776_at | 0.00 |
| 161777_f_at | 0.00 |
| 161778_i_at | 0.00 |

| | |
|---|---|
| 161779_r_at | 0.00 |
| 161780_f_at | 0.00 |
| 161781_at | 0.00 |
| 161782_r_at | 0.00 |
| 161783_at | 0.00 |
| 161784_f_at | 0.00 |
| 161785_f_at | 0.00 |
| 161786_f_at | 0.00 |
| 161787_f_at | 0.03 |
| 161788_f_at | 0.00 |
| 161789_r_at | 0.00 |
| 161790_at | 0.00 |
| 161791_r_at | 0.00 |
| 161792_f_at | 0.00 |
| 161793_at | 0.00 |
| 161794_i_at | 0.00 |
| 161795_r_at | 0.00 |
| 161796_r_at | 0.00 |
| 161797_r_at | 0.08 |
| 161798_r_at | 0.00 |
| 161799_r_at | 0.05 |
| 161800_r_at | 0.00 |
| 161801_r_at | 0.00 |
| 161802_i_at | 0.01 |
| 161803_r_at | 0.00 |
| 161804_r_at | 0.00 |
| 161805_r_at | 0.00 |
| 161806_r_at | 0.04 |
| 161807_i_at | 0.00 |
| 161808_f_at | 0.00 |
| 161809_r_at | 0.00 |
| 161810_r_at | 0.00 |
| 161811_f_at | 0.00 |
| 161812_r_at | 0.00 |
| 161813_i_at | 0.00 |
| 161814_f_at | 0.00 |
| 161815_f_at | 0.00 |
| 161816_r_at | 0.00 |
| 161817_f_at | 0.72 |
| 161818_f_at | 0.00 |
| 161819_f_at | 0.01 |
| 161820_f_at | 0.00 |
| 161821_f_at | 0.00 |
| 161822_at | 0.00 |
| 161823_r_at | 0.00 |
| 161824_r_at | 0.00 |
| 161825_f_at | 0.00 |
| 161826_r_at | 0.00 |
| 161827_f_at | 0.00 |
| 161828_r_at | 0.00 |
| 161829_at | 0.00 |
| 161830_f_at | 0.00 |
| 161831_at | 0.00 |
| 161832_r_at | 0.00 |
| 161833_r_at | 0.00 |
| 161834_at | 0.00 |

| | |
|---|---|
| 161835_at | 0.00 |
| 161836_r_at | 0.00 |
| 161837_r_at | 0.01 |
| 161838_f_at | 0.00 |
| 161839_f_at | 0.00 |
| 161840_f_at | 0.00 |
| 161841_r_at | 0.00 |
| 161842_r_at | 0.00 |
| 161843_at | 0.00 |
| 161844_at | 0.00 |
| 161845_at | 0.00 |
| 161846_r_at | 0.00 |
| 161847_r_at | 0.00 |
| 161848_r_at | 0.00 |
| 161849_r_at | 0.00 |
| 161850_at | 0.00 |
| 161851_r_at | 0.01 |
| 161852_i_at | 0.00 |
| 161853_f_at | 0.00 |
| 161854_f_at | 0.00 |
| 161855_at | 0.00 |
| 161856_f_at | 0.00 |
| 161857_r_at | 0.00 |
| 161858_f_at | 0.00 |
| 161859_f_at | 0.00 |
| 161860_f_at | 0.00 |
| 161861_i_at | 0.00 |
| 161862_i_at | 0.10 |
| 161863_r_at | 0.00 |
| 161864_f_at | 0.00 |
| 161865_r_at | 0.01 |
| 161866_at | 0.00 |
| 161867_f_at | 0.00 |
| 161868_r_at | 0.00 |
| 161869_i_at | 0.00 |
| 161870_at | 0.00 |
| 161871_f_at | 0.00 |
| 161872_f_at | 0.00 |
| 161873_r_at | 0.00 |
| 161874_r_at | 0.00 |
| 161875_at | 0.00 |
| 161876_r_at | 0.00 |
| 161877_f_at | 0.00 |
| 161878_r_at | 0.00 |
| 161879_r_at | 0.00 |
| 161880_r_at | 0.00 |
| 161881_f_at | 0.00 |
| 161882_f_at | 0.00 |
| 161883_f_at | 0.00 |
| 161884_r_at | 0.00 |
| 161885_f_at | 0.00 |
| 161886_at | 0.00 |
| 161887_r_at | 0.00 |
| 161888_r_at | 0.00 |
| 161889_f_at | 0.02 |
| 161890_f_at | 0.00 |

| | |
|---|---|
| 161891_r_at | 0.00 |
| 161892_r_at | 0.00 |
| 161893_i_at | 0.00 |
| 161894_r_at | 0.00 |
| 161895_s_at | 0.00 |
| 161896_at | 0.00 |
| 161897_f_at | 0.07 |
| 161898_i_at | 0.00 |
| 161899_f_at | 0.00 |
| 161900_f_at | 0.10 |
| 161901_r_at | 0.08 |
| 161902_f_at | 0.00 |
| 161903_f_at | 0.00 |
| 161904_f_at | 0.00 |
| 161905_r_at | 0.00 |
| 161906_f_at | 0.00 |
| 161907_s_at | 0.00 |
| 161908_i_at | 0.03 |
| 161909_r_at | 0.00 |
| 161910_at | 0.00 |
| 161911_f_at | 0.00 |
| 161912_r_at | 0.00 |
| 161913_r_at | 0.00 |
| 161914_s_at | 0.00 |
| 161915_f_at | 0.00 |
| 161916_r_at | 0.00 |
| 161917_i_at | 0.00 |
| 161918_at | 0.00 |
| 161919_r_at | 0.00 |
| 161920_r_at | 0.00 |
| 161921_f_at | 0.00 |
| 161922_r_at | 0.00 |
| 161923_at | 0.00 |
| 161924_f_at | 0.00 |
| 161925_at | 0.00 |
| 161926_r_at | 0.00 |
| 161927_at | 0.00 |
| 161928_at | 0.00 |
| 161929_at | 0.00 |
| 161930_r_at | 0.00 |
| 161931_r_at | 0.00 |
| 161932_r_at | 0.00 |
| 161933_r_at | 0.00 |
| 161934_f_at | 0.00 |
| 161935_r_at | 0.00 |
| 161936_at | 0.00 |
| 161937_r_at | 0.01 |
| 161938_r_at | 0.00 |
| 161939_f_at | 0.00 |
| 161940_r_at | 0.00 |
| 161941_r_at | 0.00 |
| 161942_f_at | 0.00 |
| 161943_at | 0.00 |
| 161944_at | 0.01 |
| 161945_at | 0.00 |
| 161946_r_at | 0.01 |

| | |
|---|---|
| 161947_f_at | 0.00 |
| 161948_f_at | 0.00 |
| 161949_at | 0.00 |
| 161950_at | 0.00 |
| 161951_f_at | 0.00 |
| 161952_r_at | 0.01 |
| 161953_at | 0.00 |
| 161954_r_at | 0.00 |
| 161955_f_at | 0.00 |
| 161956_at | 0.00 |
| 161957_r_at | 0.00 |
| 161958_at | 0.00 |
| 161959_f_at | 0.00 |
| 161960_f_at | 0.00 |
| 161961_at | 0.00 |
| 161962_f_at | 0.00 |
| 161963_f_at | 0.00 |
| 161964_r_at | 0.00 |
| 161965_r_at | 0.00 |
| 161966_f_at | 0.00 |
| 161967_at | 0.00 |
| 161968_f_at | 0.00 |
| 161969_f_at | 0.00 |
| 161970_f_at | 0.00 |
| 161971_r_at | 0.00 |
| 161972_r_at | 0.00 |
| 161973_r_at | 0.00 |
| 161974_r_at | 0.00 |
| 161975_r_at | 0.00 |
| 161976_r_at | 0.00 |
| 161977_r_at | 0.00 |
| 161978_r_at | 0.00 |
| 161979_f_at | 0.00 |
| 161980_f_at | 0.13 |
| 161981_r_at | 0.00 |
| 161982_at | 0.00 |
| 161983_f_at | 0.00 |
| 161984_f_at | 0.41 |
| 161985_f_at | 0.00 |
| 161986_f_at | 0.00 |
| 161987_at | 0.00 |
| 161988_f_at | 0.00 |
| 161989_f_at | 0.00 |
| 161990_f_at | 0.00 |
| 161991_at | 0.00 |
| 161992_at | 0.00 |
| 161993_r_at | 0.00 |
| 161994_f_at | 0.00 |
| 161995_r_at | 0.00 |
| 161996_f_at | 0.02 |
| 161997_f_at | 0.01 |
| 161998_f_at | 0.00 |
| 161999_at | 0.00 |
| 162000_r_at | 0.00 |
| 162001_f_at | 0.00 |
| 162002_r_at | 0.00 |

| | |
|---|---|
| 162003_at | 0.00 |
| 162004_i_at | 0.00 |
| 162005_at | 0.00 |
| 162006_r_at | 0.00 |
| 162007_i_at | 0.00 |
| 162008_r_at | 0.00 |
| 162009_f_at | 0.00 |
| 162010_r_at | 0.00 |
| 162011_f_at | 0.00 |
| 162012_r_at | 0.00 |
| 162013_f_at | 0.00 |
| 162014_i_at | 0.00 |
| 162015_f_at | 0.00 |
| 162016_f_at | 0.00 |
| 162017_at | 0.00 |
| 162018_r_at | 0.00 |
| 162019_r_at | 0.00 |
| 162020_at | 0.00 |
| 162021_r_at | 0.00 |
| 162022_f_at | 0.00 |
| 162023_f_at | 0.00 |
| 162024_at | 0.00 |
| 162025_r_at | 0.00 |
| 162026_r_at | 0.00 |
| 162027_f_at | 0.00 |
| 162028_f_at | 0.00 |
| 162029_r_at | 0.00 |
| 162030_r_at | 0.01 |
| 162031_f_at | 0.00 |
| 162032_f_at | 0.00 |
| 162033_f_at | 0.00 |
| 162034_r_at | 0.00 |
| 162035_f_at | 0.00 |
| 162036_r_at | 0.00 |
| 162037_f_at | 0.00 |
| 162038_f_at | 0.00 |
| 162039_f_at | 0.00 |
| 162040_r_at | 0.00 |
| 162041_f_at | 0.00 |
| 162042_i_at | 0.00 |
| 162043_i_at | 0.00 |
| 162044_f_at | 0.02 |
| 162045_r_at | 0.00 |
| 162046_at | 0.00 |
| 162047_f_at | 0.00 |
| 162048_r_at | 0.00 |
| 162049_f_at | 0.00 |
| 162050_at | 0.00 |
| 162051_r_at | 0.00 |
| 162052_i_at | 0.00 |
| 162053_i_at | 0.06 |
| 162054_r_at | 0.00 |
| 162055_f_at | 0.00 |
| 162056_f_at | 0.00 |
| 162057_f_at | 0.00 |
| 162058_f_at | 0.00 |

| | |
|---|---|
| 162059_r_at | 0.00 |
| 162060_r_at | 0.00 |
| 162061_f_at | 0.00 |
| 162062_r_at | 0.00 |
| 162063_f_at | 0.00 |
| 162064_at | 0.00 |
| 162065_r_at | 0.00 |
| 162066_f_at | 0.00 |
| 162067_at | 0.00 |
| 162068_r_at | 0.00 |
| 162069_at | 0.00 |
| 162070_r_at | 0.00 |
| 162071_i_at | 0.00 |
| 162072_at | 0.00 |
| 162073_r_at | 0.00 |
| 162074_r_at | 0.00 |
| 162075_r_at | 0.00 |
| 162076_r_at | 0.00 |
| 162077_f_at | 0.05 |
| 162078_at | 0.00 |
| 162079_at | 0.00 |
| 162080_f_at | 0.00 |
| 162081_f_at | 0.00 |
| 162082_r_at | 0.00 |
| 162083_f_at | 0.00 |
| 162084_i_at | 0.00 |
| 162085_r_at | 0.00 |
| 162086_r_at | 0.00 |
| 162087_f_at | 0.00 |
| 162088_r_at | 0.00 |
| 162089_r_at | 0.00 |
| 162090_i_at | 0.00 |
| 162091_f_at | 0.00 |
| 162092_f_at | 0.00 |
| 162093_at | 0.00 |
| 162094_f_at | 0.00 |
| 162095_f_at | 0.00 |
| 162096_at | 0.01 |
| 162097_r_at | 0.00 |
| 162098_i_at | 0.00 |
| 162099_f_at | 0.00 |
| 162100_r_at | 0.00 |
| 162101_f_at | 0.41 |
| 162102_r_at | 0.00 |
| 162103_i_at | 0.00 |
| 162104_f_at | 0.00 |
| 162105_r_at | 0.00 |
| 162106_f_at | 0.00 |
| 162107_r_at | 0.00 |
| 162108_f_at | 0.00 |
| 162109_f_at | 0.00 |
| 162110_f_at | 0.00 |
| 162111_r_at | 0.00 |
| 162112_i_at | 0.00 |
| 162113_r_at | 0.01 |
| 162114_f_at | 0.00 |

| | |
|---|---|
| 162115_i_at | 0.00 |
| 162116_r_at | 0.00 |
| 162117_f_at | 0.00 |
| 162118_f_at | 0.00 |
| 162119_r_at | 0.05 |
| 162120_at | 0.00 |
| 162121_at | 0.00 |
| 162122_f_at | 0.00 |
| 162123_f_at | 0.00 |
| 162124_r_at | 0.01 |
| 162125_f_at | 0.00 |
| 162126_r_at | 0.00 |
| 162127_r_at | 0.00 |
| 162128_i_at | 0.17 |
| 162129_f_at | 0.00 |
| 162130_r_at | 0.00 |
| 162131_f_at | 0.00 |
| 162132_f_at | 0.00 |
| 162133_at | 0.00 |
| 162134_r_at | 0.00 |
| 162135_r_at | 0.00 |
| 162136_r_at | 0.00 |
| 162137_f_at | 0.00 |
| 162138_s_at | 0.15 |
| 162139_r_at | 0.00 |
| 162140_i_at | 0.00 |
| 162141_r_at | 0.00 |
| 162142_f_at | 0.00 |
| 162143_f_at | 0.00 |
| 162144_at | 0.00 |
| 162145_r_at | 0.00 |
| 162146_r_at | 0.00 |
| 162147_f_at | 0.00 |
| 162148_r_at | 0.00 |
| 162149_i_at | 0.00 |
| 162150_r_at | 0.00 |
| 162151_i_at | 0.00 |
| 162152_r_at | 0.00 |
| 162153_i_at | 0.00 |
| 162154_i_at | 0.00 |
| 162155_f_at | 0.00 |
| 162156_f_at | 0.00 |
| 162157_f_at | 0.00 |
| 162158_r_at | 0.00 |
| 162159_i_at | 0.00 |
| 162160_at | 0.02 |
| 162161_r_at | 0.00 |
| 162162_f_at | 0.00 |
| 162163_at | 0.00 |
| 162164_f_at | 0.00 |
| 162165_f_at | 0.00 |
| 162166_f_at | 0.00 |
| 162167_f_at | 0.00 |
| 162168_r_at | 0.00 |
| 162169_r_at | 0.00 |
| 162170_r_at | 0.00 |

| | |
|---|---|
| 162171_f_at | 0.00 |
| 162172_f_at | 0.00 |
| 162173_i_at | 0.00 |
| 162174_at | 0.00 |
| 162175_at | 0.00 |
| 162176_r_at | 0.00 |
| 162177_f_at | 0.00 |
| 162178_r_at | 0.00 |
| 162179_r_at | 0.01 |
| 162180_r_at | 0.07 |
| 162181_f_at | 0.00 |
| 162182_f_at | 0.00 |
| 162183_at | 0.00 |
| 162184_at | 0.00 |
| 162185_i_at | 0.00 |
| 162186_r_at | 0.00 |
| 162187_f_at | 0.00 |
| 162188_f_at | 0.00 |
| 162189_r_at | 0.00 |
| 162190_r_at | 0.00 |
| 162191_at | 0.00 |
| 162192_f_at | 0.00 |
| 162193_f_at | 0.00 |
| 162194_r_at | 0.00 |
| 162195_i_at | 0.00 |
| 162196_f_at | 0.00 |
| 162197_at | 0.00 |
| 162198_f_at | 0.00 |
| 162199_r_at | 0.01 |
| 162200_r_at | 0.00 |
| 162201_r_at | 0.00 |
| 162202_f_at | 0.00 |
| 162203_r_at | 0.00 |
| 162204_r_at | 0.00 |
| 162205_f_at | 0.00 |
| 162206_f_at | 0.87 |
| 162207_f_at | 0.00 |
| 162208_f_at | 0.00 |
| 162209_r_at | 0.00 |
| 162210_r_at | 0.00 |
| 162211_r_at | 0.00 |
| 162212_at | 0.00 |
| 162213_i_at | 0.00 |
| 162214_r_at | 0.00 |
| 162215_f_at | 0.00 |
| 162216_i_at | 0.00 |
| 162217_r_at | 0.00 |
| 162218_f_at | 0.00 |
| 162219_f_at | 0.04 |
| 162220_r_at | 0.03 |
| 162221_i_at | 0.00 |
| 162222_r_at | 0.00 |
| 162223_f_at | 0.00 |
| 162224_r_at | 0.00 |
| 162225_f_at | 0.00 |
| 162226_r_at | 0.00 |

| | |
|---|---|
| 162227_r_at | 0.00 |
| 162228_f_at | 0.03 |
| 162229_at | 0.00 |
| 162230_r_at | 0.00 |
| 162231_r_at | 0.03 |
| 162232_r_at | 0.00 |
| 162233_r_at | 0.00 |
| 162234_f_at | 0.27 |
| 162235_f_at | 0.00 |
| 162236_f_at | 0.00 |
| 162237_f_at | 0.00 |
| 162238_r_at | 0.00 |
| 162239_at | 0.00 |
| 162240_r_at | 0.00 |
| 162241_r_at | 0.00 |
| 162242_at | 0.00 |
| 162243_f_at | 0.00 |
| 162244_r_at | 0.00 |
| 162245_f_at | 0.00 |
| 162246_r_at | 0.00 |
| 162247_r_at | 0.00 |
| 162248_f_at | 0.00 |
| 162249_f_at | 0.00 |
| 162250_f_at | 0.00 |
| 162251_f_at | 0.00 |
| 162252_f_at | 0.00 |
| 162253_i_at | 0.00 |
| 162254_f_at | 0.00 |
| 162255_s_at | 0.00 |
| 162256_r_at | 0.00 |
| 162257_i_at | 0.00 |
| 162258_f_at | 0.00 |
| 162259_f_at | 0.00 |
| 162260_at | 0.00 |
| 162261_f_at | 0.00 |
| 162262_f_at | 0.00 |
| 162263_f_at | 0.00 |
| 162264_s_at | 0.00 |
| 162265_r_at | 0.00 |
| 162266_f_at | 0.00 |
| 162267_r_at | 0.00 |
| 162268_at | 0.00 |
| 162269_at | 0.00 |
| 162270_r_at | 0.00 |
| 162271_f_at | 0.00 |
| 162272_r_at | 0.00 |
| 162273_at | 0.00 |
| 162274_f_at | 0.00 |
| 162275_f_at | 0.00 |
| 162276_i_at | 0.00 |
| 162277_r_at | 0.00 |
| 162278_r_at | 0.00 |
| 162279_f_at | 0.00 |
| 162280_f_at | 0.00 |
| 162281_at | 0.00 |
| 162282_f_at | 0.00 |

| | |
|---|---|
| 162283_r_at | 0.00 |
| 162284_r_at | 0.00 |
| 162285_r_at | 0.00 |
| 162286_r_at | 0.00 |
| 162287_r_at | 0.05 |
| 162288_f_at | 0.00 |
| 162289_at | 0.00 |
| 162290_f_at | 0.00 |
| 162291_r_at | 0.01 |
| 162292_r_at | 0.00 |
| 162293_r_at | 0.00 |
| 162294_f_at | 0.00 |
| 162295_at | 0.00 |
| 162296_at | 0.00 |
| 162297_s_at | 0.00 |
| 162298_r_at | 0.00 |
| 162299_f_at | 0.00 |
| 162300_at | 0.00 |
| 162301_f_at | 0.00 |
| 162302_f_at | 0.00 |
| 162303_f_at | 0.00 |
| 162304_r_at | 0.00 |
| 162305_f_at | 0.00 |
| 162306_at | 0.00 |
| 162307_at | 0.00 |
| 162308_f_at | 0.00 |
| 162309_at | 0.00 |
| 162310_r_at | 0.00 |
| 162311_f_at | 0.00 |
| 162312_f_at | 0.00 |
| 162313_f_at | 0.06 |
| 162314_at | 0.00 |
| 162315_f_at | 0.00 |
| 162316_f_at | 0.00 |
| 162317_r_at | 0.00 |
| 162318_r_at | 0.01 |
| 162319_i_at | 0.00 |
| 162320_at | 0.00 |
| 162321_f_at | 0.00 |
| 162322_r_at | 0.01 |
| 162323_at | 0.00 |
| 162324_at | 0.00 |
| 162325_f_at | 0.00 |
| 162326_at | 0.00 |
| 162327_f_at | 0.00 |
| 162328_f_at | 0.00 |
| 162329_r_at | 0.00 |
| 162330_f_at | 0.00 |
| 162331_f_at | 0.00 |
| 162332_f_at | 0.00 |
| 162333_r_at | 0.00 |
| 162334_r_at | 0.01 |
| 162335_at | 0.00 |
| 162336_r_at | 0.00 |
| 162337_f_at | 0.00 |
| 162338_r_at | 0.00 |

| | |
|---|---|
| 162339_r_at | 0.00 |
| 162340_r_at | 0.00 |
| 162341_r_at | 0.00 |
| 162342_at | 0.00 |
| 162343_f_at | 0.00 |
| 162344_at | 0.00 |
| 162345_at | 0.00 |
| 162346_f_at | 0.00 |
| 162347_f_at | 0.00 |
| 162348_r_at | 0.00 |
| 162349_i_at | 0.02 |
| 162350_at | 0.00 |
| 162351_f_at | 0.00 |
| 162352_r_at | 0.00 |
| 162353_at | 0.00 |
| 162354_f_at | 0.00 |
| 162355_at | 0.00 |
| 162356_r_at | 0.00 |
| 162357_at | 0.00 |
| 162358_i_at | 0.00 |
| 162359_r_at | 0.00 |
| 162360_f_at | 0.00 |
| 162361_at | 0.00 |
| 162362_f_at | 0.48 |
| 162363_at | 0.00 |
| 162364_f_at | 0.00 |
| 162365_i_at | 0.00 |
| 162366_r_at | 0.00 |
| 162367_f_at | 0.00 |
| 162368_r_at | 0.03 |
| 162369_f_at | 0.00 |
| 162370_r_at | 0.00 |
| 162371_r_at | 0.00 |
| 162372_f_at | 0.00 |
| 162373_r_at | 0.01 |
| 162374_r_at | 0.00 |
| 162375_i_at | 0.00 |
| 162376_r_at | 0.00 |
| 162377_f_at | 0.00 |
| 162378_r_at | 0.00 |
| 162379_r_at | 0.03 |
| 162380_r_at | 0.00 |
| 162381_f_at | 0.00 |
| 162382_f_at | 0.00 |
| 162383_r_at | 0.00 |
| 162384_f_at | 0.18 |
| 162385_i_at | 0.02 |
| 162386_at | 0.00 |
| 162387_f_at | 0.00 |
| 162388_r_at | 0.00 |
| 162389_at | 0.00 |
| 162390_r_at | 0.00 |
| 162391_r_at | 0.00 |
| 162392_r_at | 0.00 |
| 162393_at | 0.00 |
| 162394_r_at | 0.00 |

| | |
|---|---|
| 162395_r_at | 0.00 |
| 162396_at | 0.00 |
| 162397_r_at | 0.02 |
| 162398_at | 0.00 |
| 162399_f_at | 0.00 |
| 162400_f_at | 0.00 |
| 162401_f_at | 0.09 |
| 162402_r_at | 0.01 |
| 162403_at | 0.00 |
| 162404_i_at | 0.00 |
| 162405_at | 0.00 |
| 162406_f_at | 0.00 |
| 162407_at | 0.00 |
| 162408_f_at | 0.00 |
| 162409_r_at | 0.01 |
| 162410_s_at | 0.00 |
| 162411_f_at | 0.00 |
| 162412_r_at | 0.00 |
| 162413_r_at | 0.00 |
| 162414_f_at | 0.00 |
| 162415_f_at | 0.00 |
| 162416_at | 0.00 |
| 162417_at | 0.00 |
| 162418_r_at | 0.00 |
| 162419_r_at | 0.00 |
| 162420_r_at | 0.00 |
| 162421_r_at | 0.00 |
| 162422_r_at | 0.00 |
| 162423_f_at | 0.03 |
| 162424_f_at | 0.00 |
| 162425_f_at | 0.00 |
| 162426_f_at | 0.00 |
| 162427_r_at | 0.00 |
| 162428_i_at | 0.01 |
| 162429_r_at | 0.00 |
| 162430_at | 0.00 |
| 162431_at | 0.00 |
| 162432_f_at | 0.00 |
| 162433_f_at | 0.00 |
| 162434_i_at | 0.00 |
| 162435_f_at | 0.00 |
| 162436_at | 0.00 |
| 162437_at | 0.00 |
| 162438_f_at | 0.00 |
| 162439_at | 0.00 |
| 162440_r_at | 0.00 |
| 162441_i_at | 0.00 |
| 162442_r_at | 0.00 |
| 162443_at | 0.00 |
| 162444_r_at | 0.02 |
| 162445_at | 0.00 |
| 162446_at | 0.00 |
| 162447_f_at | 0.00 |
| 162448_f_at | 0.00 |
| 162449_f_at | 0.00 |
| 162450_f_at | 0.00 |

| | |
|---|---|
| 162451_r_at | 0.00 |
| 162452_at | 0.00 |
| 162453_at | 0.00 |
| 162454_f_at | 0.00 |
| 162455_f_at | 0.00 |
| 162456_f_at | 0.00 |
| 162457_f_at | 0.00 |
| 162458_i_at | 0.00 |
| 162459_f_at | 0.07 |
| 162460_f_at | 0.00 |
| 162461_at | 0.01 |
| 162462_r_at | 0.00 |
| 162463_at | 0.00 |
| 162464_f_at | 0.00 |
| 162465_i_at | 0.00 |
| 162466_at | 0.00 |
| 162467_r_at | 0.00 |
| 162468_at | 0.00 |
| 162469_r_at | 0.00 |
| 162470_at | 0.00 |
| 162471_i_at | 0.00 |
| 162472_f_at | 0.00 |
| 162473_r_at | 0.00 |
| 162474_f_at | 0.00 |
| 162475_f_at | 0.00 |
| 162476_r_at | 0.00 |
| 162477_r_at | 0.00 |
| 162478_r_at | 0.00 |
| 162479_f_at | 0.00 |
| 162480_f_at | 0.00 |
| 162481_f_at | 0.00 |
| 162482_at | 0.00 |
| 162483_f_at | 0.00 |
| 162484_r_at | 0.00 |
| 162485_at | 0.00 |
| 162486_f_at | 0.00 |
| 162487_f_at | 0.00 |
| 162488_at | 0.00 |
| 162489_i_at | 0.00 |
| 162490_f_at | 0.00 |
| 162491_f_at | 0.00 |
| 162492_at | 0.00 |
| 162493_r_at | 0.00 |
| 162494_at | 0.00 |
| 162495_r_at | 0.00 |
| 162496_r_at | 0.00 |
| 162497_r_at | 0.00 |
| 162498_r_at | 0.00 |
| 162499_f_at | 0.00 |
| 162500_f_at | 0.00 |
| 162501_at | 0.00 |
| 162502_f_at | 0.00 |
| 162503_f_at | 0.00 |
| 162504_at | 0.00 |
| 92180_at | 0.00 |
| 92181_at | 0.00 |

| | |
|---|---|
| 92182_at | 0.00 |
| 92183_at | 0.00 |
| 92184_at | 0.00 |
| 92185_at | 0.12 |
| 92186_at | 0.00 |
| 92188_s_at | 0.01 |
| 92189_at | 0.00 |
| 92190_at | 0.40 |
| 92191_at | 0.68 |
| 92192_s_at | 0.00 |
| 92193_r_at | 0.07 |
| 92194_at | 0.00 |
| 92195_at | 0.00 |
| 92196_f_at | 0.00 |
| 92197_r_at | 0.00 |
| 92198_s_at | 0.01 |
| 92199_at | 0.00 |
| 92200_at | 0.00 |
| 92201_at | 0.00 |
| 92202_g_at | 0.00 |
| 92203_s_at | 0.00 |
| 92204_at | 0.00 |
| 92205_at | 0.00 |
| 92206_at | 0.00 |
| 92207_at | 0.00 |
| 92208_at | 0.00 |
| 92209_at | 0.37 |
| 92210_at | 0.00 |
| 92211_at | 0.01 |
| 92212_at | 0.00 |
| 92213_at | 0.00 |
| 92214_at | 0.00 |
| 92215_at | 0.00 |
| 92216_at | 0.00 |
| 92217_s_at | 0.00 |
| 92218_at | 0.00 |
| 92219_s_at | 0.00 |
| 92220_s_at | 0.27 |
| 92222_f_at | 0.10 |
| 92223_at | 0.00 |
| 92224_at | 0.00 |
| 92225_f_at | 0.00 |
| 92226_at | 0.04 |
| 92227_s_at | 0.00 |
| 92228_at | 0.00 |
| 92229_at | 0.00 |
| 92230_at | 0.00 |
| 92231_at | 0.48 |
| 92232_at | 0.81 |
| 92233_at | 0.00 |
| 92234_at | 0.00 |
| 92235_g_at | 0.00 |
| 92237_at | 0.00 |
| 92238_at | 0.00 |
| 92239_at | 0.00 |
| 92240_at | 0.00 |

| | |
|---|---|
| 92241_at | 0.00 |
| 92242_at | 0.00 |
| 92243_at | 0.05 |
| 92244_at | 0.00 |
| 92245_at | 0.00 |
| 92246_at | 0.00 |
| 92247_at | 0.03 |
| 92248_at | 0.00 |
| 92249_g_at | 0.00 |
| 92250_s_at | 0.05 |
| 92251_f_at | 0.00 |
| 92252_at | 0.00 |
| 92253_at | 0.00 |
| 92254_at | 0.00 |
| 92255_at | 0.00 |
| 92256_at | 0.00 |
| 92257_at | 0.09 |
| 92258_at | 0.00 |
| 92259_at | 0.00 |
| 92260_at | 0.00 |
| 92261_at | 0.00 |
| 92262_at | 0.00 |
| 92263_at | 0.00 |
| 92264_at | 0.00 |
| 92265_f_at | 0.00 |
| 92266_at | 0.00 |
| 92267_at | 0.00 |
| 92268_at | 0.01 |
| 92269_r_at | 0.00 |
| 92270_at | 0.07 |
| 92271_at | 0.00 |
| 92272_at | 0.00 |
| 92273_at | 0.00 |
| 92274_at | 0.00 |
| 92275_at | 0.95 |
| 92276_at | 0.00 |
| 92277_at | 0.00 |
| 92278_at | 0.00 |
| 92279_at | 0.00 |
| 92280_at | 0.07 |
| 92281_at | 0.00 |
| 92282_at | 0.00 |
| 92283_s_at | 0.00 |
| 92284_r_at | 0.01 |
| 92285_at | 0.00 |
| 92286_g_at | 0.00 |
| 92287_at | 0.00 |
| 92288_at | 0.10 |
| 92289_at | 0.00 |
| 92290_at | 0.00 |
| 92291_f_at | 0.00 |
| 92292_at | 0.56 |
| 92293_at | 0.00 |
| 92294_at | 0.00 |
| 92295_at | 0.00 |
| 92296_at | 0.00 |

| | |
|---|---|
| 92299_at | 0.00 |
| 92300_at | 0.00 |
| 92301_at | 0.00 |
| 92302_at | 0.02 |
| 92303_at | 0.00 |
| 92304_at | 0.02 |
| 92305_s_at | 0.00 |
| 92306_at | 0.00 |
| 92307_at | 0.00 |
| 92308_at | 0.00 |
| 92309_i_at | 0.00 |
| 92310_at | 0.00 |
| 92311_s_at | 0.00 |
| 92312_at | 0.00 |
| 92313_at | 0.00 |
| 92314_at | 0.00 |
| 92315_at | 0.00 |
| 92316_f_at | 0.00 |
| 92317_at | 0.07 |
| 92318_at | 0.00 |
| 92319_i_at | 0.00 |
| 92320_f_at | 0.00 |
| 92321_r_at | 0.00 |
| 92322_at | 0.00 |
| 92323_at | 0.41 |
| 92324_at | 0.00 |
| 92325_at | 0.21 |
| 92328_at | 0.00 |
| 92329_at | 0.00 |
| 92330_r_at | 0.00 |
| 92331_at | 0.02 |
| 92332_at | 0.00 |
| 92333_at | 0.00 |
| 92334_at | 0.00 |
| 92335_at | 0.00 |
| 92336_at | 0.00 |
| 92337_g_at | 0.00 |
| 92338_f_at | 0.29 |
| 92339_at | 0.00 |
| 92340_at | 0.00 |
| 92341_at | 0.00 |
| 92342_at | 0.00 |
| 92343_at | 0.00 |
| 92344_at | 0.00 |
| 92345_g_at | 0.01 |
| 92346_at | 0.24 |
| 92347_at | 0.00 |
| 92348_at | 0.00 |
| 92349_at | 0.00 |
| 92350_at | 0.00 |
| 92351_at | 0.00 |
| 92352_at | 0.00 |
| 92353_at | 0.00 |
| 92354_at | 0.00 |
| 92355_at | 0.00 |
| 92356_at | 0.00 |

| | |
|---|---|
| 92357_at | 0.00 |
| 92358_at | 0.00 |
| 92359_at | 0.00 |
| 92360_at | 0.00 |
| 92361_at | 0.00 |
| 92362_at | 0.00 |
| 92363_at | 0.00 |
| 92364_at | 0.00 |
| 92365_at | 0.00 |
| 92366_at | 0.00 |
| 92367_at | 0.00 |
| 92368_at | 0.00 |
| 92369_at | 0.00 |
| 92370_at | 0.00 |
| 92371_at | 0.00 |
| 92372_at | 0.00 |
| 92374_at | 0.00 |
| 92375_at | 0.00 |
| 92376_at | 0.00 |
| 92377_at | 0.00 |
| 92378_at | 0.00 |
| 92379_f_at | 0.00 |
| 92380_r_at | 0.00 |
| 92381_at | 0.01 |
| 92382_at | 0.00 |
| 92383_at | 0.03 |
| 92384_at | 0.00 |
| 92385_at | 0.00 |
| 92386_at | 0.00 |
| 92387_at | 0.00 |
| 92388_at | 0.00 |
| 92389_at | 0.00 |
| 92390_at | 0.00 |
| 92391_at | 0.00 |
| 92392_at | 0.00 |
| 92393_at | 0.00 |
| 92394_f_at | 0.00 |
| 92395_r_at | 0.00 |
| 92396_at | 0.00 |
| 92397_at | 0.00 |
| 92398_at | 0.00 |
| 92399_at | 0.00 |
| 92400_at | 0.00 |
| 92401_at | 0.00 |
| 92402_at | 0.00 |
| 92403_at | 0.00 |
| 92404_at | 0.00 |
| 92405_at | 0.00 |
| 92406_at | 0.00 |
| 92407_at | 0.00 |
| 92408_at | 0.00 |
| 92409_at | 0.00 |
| 92410_at | 0.01 |
| 92411_at | 0.00 |
| 92412_s_at | 0.00 |
| 92413_at | 0.00 |

| | |
|---|---|
| 92414_at | 0.00 |
| 92415_at | 0.00 |
| 92416_at | 0.00 |
| 92417_at | 0.00 |
| 92418_at | 0.00 |
| 92419_at | 0.00 |
| 92420_at | 0.00 |
| 92421_at | 0.00 |
| 92422_at | 0.00 |
| 92423_at | 0.00 |
| 92424_at | 0.03 |
| 92425_at | 0.00 |
| 92426_at | 0.00 |
| 92427_at | 0.00 |
| 92428_at | 0.00 |
| 92429_at | 0.00 |
| 92430_at | 0.00 |
| 92432_at | 0.00 |
| 92433_at | 0.00 |
| 92434_at | 0.00 |
| 92435_at | 0.00 |
| 92436_at | 0.00 |
| 92437_at | 0.00 |
| 92439_at | 0.00 |
| 92440_at | 0.07 |
| 92441_at | 0.00 |
| 92442_at | 0.00 |
| 92443_i_at | 0.00 |
| 92444_f_at | 0.00 |
| 92445_at | 0.00 |
| 92448_s_at | 0.00 |
| 92449_at | 0.00 |
| 92450_at | 0.00 |
| 92451_at | 0.00 |
| 92452_at | 0.00 |
| 92453_at | 0.00 |
| 92454_at | 0.00 |
| 92455_at | 0.00 |
| 92456_at | 0.00 |
| 92457_at | 0.00 |
| 92458_at | 0.00 |
| 92459_at | 0.00 |
| 92460_at | 0.00 |
| 92461_at | 0.00 |
| 92462_at | 0.00 |
| 92464_at | 0.00 |
| 92465_at | 0.00 |
| 92466_at | 0.00 |
| 92467_g_at | 0.00 |
| 92468_at | 0.00 |
| 92469_at | 0.00 |
| 92470_f_at | 0.00 |
| 92471_i_at | 0.00 |
| 92472_f_at | 0.00 |
| 92473_at | 0.00 |
| 92474_at | 0.00 |

| | |
|---|---|
| 92475_g_at | 0.00 |
| 92476_at | 0.33 |
| 92477_at | 0.05 |
| 92478_at | 0.00 |
| 92480_f_at | 0.00 |
| 92481_at | 0.00 |
| 92482_at | 0.00 |
| 92483_g_at | 0.00 |
| 92484_at | 0.28 |
| 92485_at | 0.00 |
| 92486_at | 0.00 |
| 92487_at | 0.00 |
| 92488_at | 0.00 |
| 92489_at | 0.00 |
| 92490_at | 0.00 |
| 92492_at | 0.00 |
| 92493_at | 0.00 |
| 92494_at | 0.00 |
| 92495_at | 0.04 |
| 92496_at | 0.00 |
| 92497_at | 0.00 |
| 92498_at | 0.00 |
| 92499_at | 0.00 |
| 92500_at | 0.01 |
| 92501_s_at | 0.00 |
| 92502_at | 0.00 |
| 92503_at | 0.05 |
| 92504_at | 0.00 |
| 92505_g_at | 0.01 |
| 92506_at | 0.00 |
| 92507_at | 0.14 |
| 92508_s_at | 0.02 |
| 92509_at | 0.00 |
| 92510_at | 0.05 |
| 92511_at | 0.00 |
| 92512_g_at | 0.00 |
| 92513_at | 0.58 |
| 92514_at | 0.00 |
| 92515_at | 0.00 |
| 92516_at | 0.00 |
| 92517_at | 0.00 |
| 92518_at | 0.00 |
| 92519_at | 0.00 |
| 92520_at | 0.00 |
| 92521_at | 0.02 |
| 92522_at | 0.00 |
| 92523_at | 0.00 |
| 92524_at | 0.00 |
| 92525_i_at | 0.00 |
| 92526_f_at | 0.00 |
| 92527_at | 0.00 |
| 92528_at | 0.00 |
| 92529_s_at | 0.05 |
| 92531_at | 0.00 |
| 92532_at | 0.00 |
| 92533_at | 0.00 |

| | |
|---|---|
| 92534_at | 0.00 |
| 92535_at | 0.00 |
| 92536_at | 0.00 |
| 92537_g_at | 0.00 |
| 92538_at | 0.00 |
| 92539_at | 0.12 |
| 92540_f_at | 0.00 |
| 92541_at | 0.00 |
| 92542_at | 0.00 |
| 92543_at | 0.00 |
| 92544_f_at | 0.00 |
| 92545_f_at | 0.00 |
| 92546_r_at | 0.00 |
| 92547_at | 0.00 |
| 92548_g_at | 0.16 |
| 92549_at | 0.00 |
| 92550_at | 0.63 |
| 92551_at | 0.00 |
| 92553_at | 0.00 |
| 92554_at | 0.22 |
| 92555_at | 0.73 |
| 92556_at | 0.00 |
| 92557_at | 0.00 |
| 92558_at | 0.28 |
| 92559_at | 0.64 |
| 92560_g_at | 0.02 |
| 92561_at | 0.00 |
| 92562_at | 0.00 |
| 92563_at | 0.00 |
| 92564_at | 0.03 |
| 92565_at | 0.05 |
| 92566_at | 0.00 |
| 92567_at | 0.24 |
| 92568_at | 0.06 |
| 92569_f_at | 0.01 |
| 92570_at | 0.00 |
| 92571_at | 0.00 |
| 92572_at | 0.00 |
| 92573_at | 0.00 |
| 92574_at | 0.00 |
| 92575_at | 0.00 |
| 92577_f_at | 0.00 |
| 92578_at | 0.00 |
| 92579_at | 0.00 |
| 92580_at | 0.00 |
| 92581_at | 0.41 |
| 92582_at | 0.00 |
| 92583_at | 0.00 |
| 92584_at | 0.00 |
| 92585_at | 0.00 |
| 92586_at | 0.00 |
| 92587_at | 0.00 |
| 92589_at | 0.10 |
| 92590_at | 0.00 |
| 92592_at | 0.00 |
| 92593_at | 0.41 |

| | |
|---|---|
| 92594_at | 0.00 |
| 92595_r_at | 0.00 |
| 92596_at | 0.25 |
| 92597_s_at | 0.00 |
| 92598_at | 0.00 |
| 92599_at | 0.00 |
| 92600_f_at | 0.00 |
| 92601_at | 0.00 |
| 92602_at | 0.00 |
| 92603_at | 0.02 |
| 92605_at | 0.00 |
| 92606_at | 0.00 |
| 92607_at | 0.22 |
| 92608_at | 0.03 |
| 92610_at | 0.00 |
| 92611_at | 0.00 |
| 92612_at | 0.00 |
| 92614_at | 0.00 |
| 92615_at | 0.00 |
| 92616_at | 0.00 |
| 92617_at | 0.00 |
| 92618_at | 0.02 |
| 92619_at | 0.00 |
| 92621_at | 0.00 |
| 92622_at | 0.07 |
| 92623_at | 0.00 |
| 92624_r_at | 0.00 |
| 92625_at | 0.00 |
| 92626_at | 0.00 |
| 92628_at | 0.00 |
| 92629_f_at | 0.00 |
| 92630_r_at | 0.00 |
| 92631_f_at | 0.01 |
| 92632_at | 0.00 |
| 92633_at | 0.06 |
| 92634_at | 0.00 |
| 92635_at | 0.00 |
| 92636_f_at | 0.00 |
| 92637_at | 0.00 |
| 92638_at | 0.00 |
| 92639_at | 0.00 |
| 92640_at | 0.00 |
| 92642_at | 0.30 |
| 92643_at | 0.03 |
| 92644_s_at | 0.04 |
| 92645_at | 0.00 |
| 92646_at | 0.00 |
| 92647_at | 0.00 |
| 92648_at | 0.00 |
| 92649_at | 0.01 |
| 92650_at | 0.00 |
| 92652_at | 0.01 |
| 92653_at | 0.00 |
| 92654_at | 0.00 |
| 92655_at | 0.00 |
| 92656_at | 0.00 |

| | |
|---|---|
| 92658_at | 0.00 |
| 92659_at | 0.00 |
| 92660_f_at | 0.02 |
| 92661_at | 0.00 |
| 92662_g_at | 0.00 |
| 92663_at | 0.00 |
| 92664_at | 0.01 |
| 92665_f_at | 0.00 |
| 92666_at | 0.00 |
| 92667_at | 0.00 |
| 92668_at | 0.00 |
| 92670_at | 0.00 |
| 92671_f_at | 0.00 |
| 92672_at | 0.00 |
| 92673_at | 0.00 |
| 92674_at | 0.00 |
| 92676_at | 0.00 |
| 92677_s_at | 0.00 |
| 92678_at | 0.00 |
| 92681_at | 0.16 |
| 92682_at | 0.00 |
| 92683_at | 0.00 |
| 92684_at | 0.00 |
| 92685_at | 0.00 |
| 92686_at | 0.00 |
| 92687_at | 0.00 |
| 92688_at | 0.00 |
| 92689_at | 0.00 |
| 92690_at | 0.00 |
| 92691_at | 0.00 |
| 92692_at | 0.00 |
| 92693_at | 0.00 |
| 92694_at | 0.00 |
| 92695_at | 0.00 |
| 92696_at | 0.03 |
| 92697_at | 0.00 |
| 92698_at | 0.00 |
| 92699_at | 0.00 |
| 92700_at | 0.00 |
| 92701_at | 0.00 |
| 92702_at | 0.00 |
| 92703_at | 0.08 |
| 92704_at | 0.00 |
| 92705_at | 0.00 |
| 92706_at | 0.00 |
| 92707_at | 0.00 |
| 92708_at | 0.00 |
| 92710_at | 0.00 |
| 92711_at | 0.00 |
| 92712_at | 0.00 |
| 92713_at | 0.00 |
| 92714_at | 0.00 |
| 92715_at | 0.00 |
| 92717_at | 0.00 |
| 92718_at | 0.00 |
| 92722_f_at | 0.00 |

| | |
|---|---|
| 92724_at | 0.00 |
| 92725_at | 0.00 |
| 92726_at | 0.00 |
| 92727_at | 0.01 |
| 92728_at | 0.00 |
| 92730_at | 0.27 |
| 92731_at | 0.00 |
| 92732_at | 0.00 |
| 92733_at | 0.00 |
| 92734_at | 0.00 |
| 92735_at | 0.00 |
| 92736_at | 0.00 |
| 92737_at | 0.00 |
| 92738_at | 0.01 |
| 92739_at | 0.00 |
| 92740_at | 0.00 |
| 92741_g_at | 0.00 |
| 92742_at | 0.00 |
| 92743_at | 0.00 |
| 92744_at | 0.00 |
| 92745_at | 0.00 |
| 92746_at | 0.00 |
| 92747_at | 0.00 |
| 92749_at | 0.00 |
| 92750_s_at | 0.00 |
| 92751_i_at | 0.00 |
| 92752_r_at | 0.00 |
| 92753_at | 0.00 |
| 92754_at | 0.00 |
| 92755_f_at | 0.00 |
| 92756_r_at | 0.00 |
| 92757_at | 0.00 |
| 92758_at | 0.00 |
| 92759_at | 0.00 |
| 92760_at | 0.00 |
| 92761_at | 0.00 |
| 92762_at | 0.00 |
| 92763_at | 0.00 |
| 92764_at | 0.00 |
| 92765_s_at | 0.00 |
| 92766_at | 0.00 |
| 92767_at | 0.30 |
| 92768_s_at | 0.00 |
| 92769_at | 0.00 |
| 92770_at | 0.98 |
| 92771_at | 0.00 |
| 92773_at | 0.06 |
| 92774_at | 0.00 |
| 92775_at | 0.02 |
| 92777_at | 0.00 |
| 92778_i_at | 0.00 |
| 92779_f_at | 0.00 |
| 92780_f_at | 0.00 |
| 92781_at | 0.00 |
| 92782_at | 0.00 |
| 92783_at | 0.00 |

| | |
|---|---|
| 92784_at | 0.00 |
| 92786_at | 0.00 |
| 92787_at | 0.00 |
| 92788_f_at | 0.00 |
| 92789_r_at | 0.00 |
| 92790_at | 0.01 |
| 92792_at | 0.00 |
| 92793_at | 0.00 |
| 92794_f_at | 0.01 |
| 92795_at | 0.00 |
| 92796_at | 0.14 |
| 92797_at | 0.00 |
| 92798_at | 0.00 |
| 92799_g_at | 0.00 |
| 92800_i_at | 0.00 |
| 92801_at | 0.00 |
| 92802_s_at | 0.00 |
| 92803_at | 0.00 |
| 92804_at | 0.00 |
| 92805_s_at | 0.00 |
| 92806_at | 0.00 |
| 92807_at | 0.00 |
| 92808_f_at | 0.00 |
| 92809_r_at | 0.00 |
| 92810_at | 0.00 |
| 92811_at | 0.00 |
| 92812_f_at | 0.00 |
| 92813_at | 0.00 |
| 92814_at | 0.00 |
| 92816_r_at | 0.00 |
| 92817_at | 0.00 |
| 92818_at | 0.00 |
| 92820_at | 0.00 |
| 92821_at | 0.00 |
| 92824_at | 0.00 |
| 92825_at | 0.00 |
| 92826_at | 0.00 |
| 92827_at | 0.05 |
| 92828_at | 0.00 |
| 92829_at | 0.00 |
| 92830_s_at | 0.00 |
| 92831_at | 0.00 |
| 92832_at | 0.00 |
| 92833_at | 0.00 |
| 92834_at | 0.00 |
| 92835_at | 0.00 |
| 92836_at | 0.00 |
| 92837_f_at | 0.00 |
| 92838_at | 0.01 |
| 92839_f_at | 0.00 |
| 92840_at | 0.00 |
| 92841_f_at | 0.00 |
| 92842_r_at | 0.00 |
| 92843_r_at | 0.01 |
| 92845_at | 0.78 |
| 92846_at | 0.00 |

| | |
|---|---|
| 92847_s_at | 0.00 |
| 92848_at | 0.09 |
| 92849_at | 0.00 |
| 92850_at | 0.08 |
| 92851_at | 0.00 |
| 92852_at | 0.06 |
| 92853_at | 0.00 |
| 92854_at | 0.00 |
| 92855_at | 0.00 |
| 92856_at | 0.00 |
| 92857_at | 0.00 |
| 92858_at | 0.00 |
| 92860_at | 0.00 |
| 92861_i_at | 0.02 |
| 92862_f_at | 0.00 |
| 92863_at | 0.00 |
| 92864_at | 0.00 |
| 92865_at | 0.00 |
| 92866_at | 0.00 |
| 92867_at | 0.00 |
| 92868_at | 0.00 |
| 92869_at | 0.00 |
| 92870_at | 0.00 |
| 92871_at | 0.00 |
| 92872_at | 0.03 |
| 92873_f_at | 0.00 |
| 92874_f_at | 0.00 |
| 92875_s_at | 0.00 |
| 92876_at | 0.00 |
| 92877_at | 0.00 |
| 92878_at | 0.00 |
| 92879_at | 0.00 |
| 92880_at | 0.04 |
| 92881_at | 0.14 |
| 92882_at | 0.00 |
| 92883_at | 0.00 |
| 92884_at | 0.00 |
| 92885_at | 0.00 |
| 92887_at | 0.00 |
| 92888_s_at | 0.01 |
| 92889_r_at | 0.00 |
| 92890_at | 0.00 |
| 92891_f_at | 0.00 |
| 92892_at | 0.00 |
| 92893_at | 0.00 |
| 92894_s_at | 0.00 |
| 92895_at | 0.00 |
| 92896_s_at | 0.00 |
| 92897_at | 0.00 |
| 92898_at | 0.00 |
| 92899_at | 0.00 |
| 92900_at | 0.00 |
| 92901_at | 0.06 |
| 92902_at | 0.00 |
| 92903_at | 0.00 |
| 92904_at | 0.01 |

| | |
|---|---|
| 92905_at | 0.00 |
| 92906_at | 0.00 |
| 92907_at | 0.08 |
| 92908_at | 0.05 |
| 92909_at | 0.00 |
| 92910_at | 0.00 |
| 92911_at | 0.00 |
| 92912_at | 0.00 |
| 92913_at | 0.00 |
| 92914_at | 0.00 |
| 92915_s_at | 0.00 |
| 92916_at | 0.00 |
| 92917_at | 0.00 |
| 92918_at | 0.00 |
| 92919_at | 0.00 |
| 92920_at | 0.00 |
| 92921_at | 0.00 |
| 92922_at | 0.00 |
| 92923_g_at | 0.00 |
| 92924_at | 0.00 |
| 92925_at | 0.04 |
| 92926_at | 0.00 |
| 92927_at | 0.00 |
| 92929_at | 0.00 |
| 92930_at | 0.00 |
| 92931_at | 0.34 |
| 92932_at | 0.00 |
| 92933_at | 0.00 |
| 92934_at | 0.00 |
| 92935_at | 0.00 |
| 92936_at | 0.00 |
| 92937_at | 0.00 |
| 92938_at | 0.00 |
| 92939_at | 0.00 |
| 92940_s_at | 0.00 |
| 92941_at | 0.00 |
| 92942_at | 0.00 |
| 92943_at | 0.00 |
| 92944_at | 0.00 |
| 92945_at | 0.00 |
| 92946_f_at | 0.00 |
| 92947_s_at | 0.00 |
| 92948_at | 0.00 |
| 92949_at | 0.00 |
| 92950_at | 0.00 |
| 92951_at | 0.00 |
| 92952_f_at | 0.00 |
| 92953_at | 0.00 |
| 92955_at | 0.00 |
| 92956_at | 0.18 |
| 92957_at | 0.00 |
| 92958_at | 0.26 |
| 92959_at | 0.00 |
| 92960_at | 0.00 |
| 92961_at | 0.00 |
| 92962_at | 0.00 |

| | |
|---|---|
| 92963_at | 0.00 |
| 92965_at | 0.00 |
| 92966_g_at | 0.00 |
| 92967_r_at | 0.00 |
| 92968_at | 0.00 |
| 92969_at | 0.00 |
| 92970_at | 0.00 |
| 92971_at | 0.00 |
| 92972_at | 0.00 |
| 92974_at | 0.00 |
| 92975_at | 0.00 |
| 92976_at | 0.00 |
| 92977_s_at | 0.00 |
| 92978_s_at | 0.50 |
| 92979_at | 0.00 |
| 92980_at | 0.00 |
| 92981_at | 0.00 |
| 92982_at | 0.00 |
| 92983_at | 0.00 |
| 92984_g_at | 0.00 |
| 92985_at | 0.00 |
| 92986_g_at | 0.00 |
| 92987_at | 0.00 |
| 92988_i_at | 0.01 |
| 92989_f_at | 0.00 |
| 92990_at | 0.00 |
| 92991_at | 0.00 |
| 92992_i_at | 0.00 |
| 92993_r_at | 0.00 |
| 92994_at | 0.00 |
| 92995_at | 0.00 |
| 92996_at | 0.00 |
| 92997_g_at | 0.00 |
| 92998_at | 0.00 |
| 92999_at | 0.00 |
| 93000_g_at | 0.00 |
| 93001_at | 0.00 |
| 93002_r_at | 0.01 |
| 93004_r_at | 0.00 |
| 93005_at | 0.00 |
| 93006_at | 0.00 |
| 93007_at | 0.00 |
| 93008_at | 0.00 |
| 93009_at | 0.00 |
| 93010_at | 0.00 |
| 93011_at | 0.00 |
| 93012_at | 0.00 |
| 93013_at | 0.00 |
| 93014_at | 0.00 |
| 93015_at | 0.00 |
| 93016_at | 0.02 |
| 93017_at | 0.00 |
| 93018_at | 0.00 |
| 93019_at | 0.01 |
| 93020_at | 0.01 |
| 93021_at | 0.05 |

| | |
|---|---|
| 93022_i_at | 0.00 |
| 93023_f_at | 0.01 |
| 93024_at | 0.00 |
| 93025_at | 0.00 |
| 93026_at | 0.04 |
| 93027_r_at | 0.00 |
| 93028_at | 0.54 |
| 93029_at | 0.00 |
| 93030_at | 0.00 |
| 93033_at | 0.00 |
| 93037_i_at | 0.73 |
| 93038_f_at | 0.62 |
| 93039_at | 0.00 |
| 93040_at | 0.00 |
| 93041_at | 0.00 |
| 93042_at | 0.00 |
| 93043_at | 0.01 |
| 93045_at | 0.00 |
| 93046_at | 0.00 |
| 93047_at | 0.00 |
| 93048_at | 0.00 |
| 93050_at | 0.00 |
| 93051_at | 0.02 |
| 93053_at | 0.00 |
| 93054_at | 0.00 |
| 93055_at | 0.00 |
| 93056_g_at | 0.00 |
| 93057_at | 0.00 |
| 93058_at | 0.22 |
| 93059_at | 0.00 |
| 93061_at | 0.00 |
| 93062_at | 0.00 |
| 93063_at | 0.00 |
| 93064_at | 0.02 |
| 93065_at | 0.00 |
| 93066_at | 0.00 |
| 93067_f_at | 0.00 |
| 93068_r_at | 0.00 |
| 93069_at | 0.17 |
| 93070_at | 0.00 |
| 93071_at | 0.00 |
| 93072_at | 0.00 |
| 93073_at | 0.00 |
| 93074_g_at | 0.00 |
| 93075_r_at | 0.00 |
| 93076_at | 0.05 |
| 93077_s_at | 0.00 |
| 93078_at | 0.21 |
| 93080_at | 0.32 |
| 93081_at | 0.00 |
| 93082_at | 0.00 |
| 93083_at | 0.21 |
| 93084_at | 0.00 |
| 93085_at | 0.00 |
| 93086_at | 0.00 |
| 93087_r_at | 0.00 |

| | |
|---|---|
| 93088_at | 0.12 |
| 93089_at | 0.41 |
| 93090_at | 0.07 |
| 93091_s_at | 0.00 |
| 93092_at | 0.00 |
| 93093_at | 0.21 |
| 93094_at | 0.00 |
| 93095_at | 0.08 |
| 93096_at | 0.00 |
| 93097_at | 0.00 |
| 93098_at | 0.00 |
| 93099_f_at | 0.00 |
| 93100_at | 0.36 |
| 93101_s_at | 0.00 |
| 93102_f_at | 0.05 |
| 93103_at | 0.00 |
| 93104_at | 0.79 |
| 93105_s_at | 0.00 |
| 93106_i_at | 0.00 |
| 93107_r_at | 0.00 |
| 93108_at | 0.00 |
| 93109_f_at | 0.00 |
| 93111_at | 0.01 |
| 93112_at | 0.00 |
| 93114_at | 0.00 |
| 93116_at | 0.01 |
| 93117_at | 0.00 |
| 93118_at | 0.00 |
| 93119_at | 0.01 |
| 93120_f_at | 0.00 |
| 93121_at | 0.00 |
| 93122_at | 0.00 |
| 93124_at | 0.00 |
| 93126_at | 0.07 |
| 93127_at | 0.00 |
| 93128_at | 0.00 |
| 93129_at | 0.00 |
| 93130_at | 0.00 |
| 93131_at | 0.00 |
| 93132_at | 0.00 |
| 93133_at | 0.00 |
| 93134_at | 0.00 |
| 93136_at | 0.00 |
| 93137_at | 0.00 |
| 93138_at | 0.00 |
| 93139_at | 0.00 |
| 93140_at | 0.00 |
| 93141_at | 0.81 |
| 93142_at | 0.01 |
| 93143_at | 0.10 |
| 93144_at | 0.00 |
| 93145_at | 0.00 |
| 93146_at | 0.00 |
| 93147_f_at | 0.00 |
| 93148_at | 0.00 |
| 93151_at | 0.00 |

| | |
|---|---|
| 93153_at | 0.00 |
| 93155_at | 0.00 |
| 93158_at | 0.00 |
| 93159_at | 0.00 |
| 93162_f_at | 0.00 |
| 93163_at | 0.00 |
| 93164_at | 0.01 |
| 93165_at | 0.00 |
| 93166_at | 0.00 |
| 93167_f_at | 0.00 |
| 93168_at | 0.00 |
| 93169_at | 0.00 |
| 93173_at | 0.00 |
| 93174_at | 0.05 |
| 93175_at | 0.00 |
| 93177_at | 0.00 |
| 93178_at | 0.00 |
| 93179_at | 0.00 |
| 93180_at | 0.00 |
| 93181_r_at | 0.00 |
| 93182_at | 0.00 |
| 93183_at | 0.00 |
| 93184_at | 0.00 |
| 93185_at | 0.00 |
| 93186_at | 0.00 |
| 93187_at | 0.00 |
| 93188_at | 0.00 |
| 93190_at | 0.00 |
| 93191_at | 0.00 |
| 93193_at | 0.00 |
| 93194_at | 0.00 |
| 93195_at | 0.00 |
| 93196_at | 0.00 |
| 93197_at | 0.00 |
| 93198_at | 0.00 |
| 93199_at | 0.00 |
| 93200_f_at | 0.12 |
| 93201_at | 0.00 |
| 93202_at | 0.01 |
| 93203_f_at | 0.05 |
| 93204_r_at | 0.00 |
| 93205_at | 0.00 |
| 93206_g_at | 0.00 |
| 93207_at | 0.00 |
| 93208_at | 0.00 |
| 93209_at | 0.00 |
| 93210_g_at | 0.00 |
| 93211_at | 0.00 |
| 93212_at | 0.03 |
| 93213_at | 0.00 |
| 93214_at | 0.02 |
| 93215_at | 0.00 |
| 93216_at | 0.00 |
| 93217_at | 0.00 |
| 93218_at | 0.01 |
| 93219_at | 0.03 |

| | |
|---|---|
| 93220_at | 0.00 |
| 93221_at | 0.00 |
| 93222_at | 0.00 |
| 93223_at | 0.00 |
| 93224_at | 0.00 |
| 93225_s_at | 0.00 |
| 93226_i_at | 0.00 |
| 93227_f_at | 0.00 |
| 93228_at | 0.02 |
| 93230_at | 0.00 |
| 93231_at | 0.00 |
| 93232_at | 0.00 |
| 93234_at | 0.26 |
| 93235_at | 0.00 |
| 93236_s_at | 0.01 |
| 93237_s_at | 0.00 |
| 93238_at | 0.00 |
| 93240_f_at | 0.00 |
| 93241_r_at | 0.00 |
| 93242_at | 0.00 |
| 93243_at | 0.00 |
| 93244_at | 0.00 |
| 93245_at | 0.00 |
| 93246_at | 0.30 |
| 93248_at | 0.00 |
| 93250_r_at | 0.01 |
| 93251_at | 0.00 |
| 93252_at | 0.00 |
| 93253_at | 0.29 |
| 93254_at | 0.00 |
| 93255_at | 0.00 |
| 93256_at | 0.00 |
| 93257_at | 0.00 |
| 93258_at | 0.00 |
| 93259_at | 0.00 |
| 93261_at | 0.00 |
| 93264_at | 0.00 |
| 93265_at | 0.00 |
| 93266_at | 0.00 |
| 93267_at | 0.00 |
| 93268_at | 0.05 |
| 93269_at | 0.00 |
| 93270_at | 0.00 |
| 93271_s_at | 0.01 |
| 93272_at | 0.00 |
| 93273_at | 0.00 |
| 93274_at | 0.08 |
| 93275_at | 0.00 |
| 93276_at | 0.00 |
| 93277_at | 0.00 |
| 93278_at | 0.00 |
| 93279_at | 0.00 |
| 93281_at | 0.10 |
| 93282_at | 0.00 |
| 93283_at | 0.00 |
| 93284_at | 0.00 |

| | |
|---|---|
| 93285_at | 0.11 |
| 93287_at | 0.00 |
| 93288_at | 0.00 |
| 93290_at | 0.00 |
| 93293_at | 0.00 |
| 93294_at | 0.01 |
| 93295_at | 0.00 |
| 93296_at | 0.98 |
| 93298_at | 0.00 |
| 93299_at | 0.00 |
| 93300_at | 0.00 |
| 93301_at | 0.02 |
| 93302_at | 0.00 |
| 93303_at | 0.00 |
| 93304_at | 0.00 |
| 93305_f_at | 0.18 |
| 93306_at | 0.00 |
| 93308_s_at | 0.00 |
| 93309_at | 0.02 |
| 93310_at | 0.00 |
| 93311_at | 0.00 |
| 93312_at | 0.00 |
| 93313_at | 0.00 |
| 93314_g_at | 0.00 |
| 93315_at | 0.00 |
| 93316_at | 0.10 |
| 93317_at | 0.00 |
| 93318_at | 0.00 |
| 93319_at | 0.05 |
| 93320_at | 0.00 |
| 93321_at | 0.00 |
| 93322_at | 0.00 |
| 93323_at | 0.12 |
| 93324_at | 0.61 |
| 93325_at | 0.08 |
| 93326_at | 0.56 |
| 93327_at | 0.19 |
| 93328_at | 0.00 |
| 93330_at | 0.00 |
| 93332_at | 0.00 |
| 93333_at | 0.01 |
| 93334_at | 0.00 |
| 93336_at | 0.00 |
| 93337_at | 0.00 |
| 93338_at | 0.00 |
| 93339_at | 0.26 |
| 93340_f_at | 0.03 |
| 93341_r_at | 0.00 |
| 93342_at | 0.00 |
| 93346_at | 0.00 |
| 93347_at | 0.00 |
| 93348_at | 0.00 |
| 93349_at | 0.48 |
| 93350_f_at | 0.01 |
| 93351_at | 0.00 |
| 93352_at | 0.00 |

| | |
|---|---|
| 93353_at | 0.13 |
| 93354_at | 0.14 |
| 93355_at | 0.00 |
| 93356_at | 0.00 |
| 93358_at | 0.03 |
| 93359_at | 0.00 |
| 93360_at | 0.00 |
| 93362_at | 0.00 |
| 93364_at | 0.00 |
| 93365_s_at | 0.00 |
| 93366_r_at | 0.00 |
| 93367_at | 0.00 |
| 93369_at | 0.00 |
| 93370_at | 0.00 |
| 93371_at | 0.00 |
| 93372_at | 0.29 |
| 93373_at | 0.00 |
| 93374_at | 0.00 |
| 93375_at | 0.00 |
| 93376_at | 0.00 |
| 93378_at | 0.00 |
| 93379_at | 0.00 |
| 93380_at | 0.00 |
| 93381_at | 0.00 |
| 93382_at | 0.00 |
| 93383_at | 0.00 |
| 93384_at | 0.00 |
| 93385_at | 0.00 |
| 93386_at | 0.00 |
| 93387_at | 0.00 |
| 93388_s_at | 0.00 |
| 93389_at | 0.40 |
| 93390_g_at | 0.13 |
| 93391_at | 0.00 |
| 93392_at | 0.00 |
| 93393_at | 0.00 |
| 93394_at | 0.00 |
| 93395_g_at | 0.00 |
| 93396_at | 0.00 |
| 93397_at | 0.00 |
| 93398_at | 0.00 |
| 93399_at | 0.00 |
| 93400_at | 0.00 |
| 93401_g_at | 0.01 |
| 93402_i_at | 0.00 |
| 93403_at | 0.00 |
| 93404_g_at | 0.00 |
| 93405_at | 0.00 |
| 93406_at | 0.00 |
| 93407_at | 0.00 |
| 93408_at | 0.00 |
| 93409_at | 0.00 |
| 93410_at | 0.00 |
| 93411_at | 0.00 |
| 93412_at | 0.00 |
| 93413_at | 0.00 |

| | |
|---|---|
| 93414_at | 0.02 |
| 93416_at | 0.03 |
| 93417_at | 0.00 |
| 93418_g_at | 0.00 |
| 93419_at | 0.00 |
| 93421_at | 0.11 |
| 93422_at | 0.00 |
| 93423_at | 0.00 |
| 93424_at | 0.01 |
| 93425_at | 0.00 |
| 93426_at | 0.00 |
| 93427_at | 0.00 |
| 93428_at | 0.00 |
| 93429_at | 0.00 |
| 93430_at | 0.39 |
| 93431_at | 0.00 |
| 93432_at | 0.00 |
| 93433_s_at | 0.00 |
| 93434_at | 0.00 |
| 93435_at | 0.01 |
| 93436_i_at | 0.00 |
| 93437_f_at | 0.00 |
| 93439_f_at | 0.00 |
| 93440_at | 0.00 |
| 93441_at | 0.00 |
| 93442_at | 0.00 |
| 93443_at | 0.00 |
| 93444_at | 0.00 |
| 93445_at | 0.00 |
| 93446_at | 0.00 |
| 93447_at | 0.00 |
| 93448_at | 0.00 |
| 93449_at | 0.00 |
| 93450_at | 0.00 |
| 93451_at | 0.01 |
| 93452_at | 0.00 |
| 93453_at | 0.00 |
| 93454_at | 0.00 |
| 93455_s_at | 0.42 |
| 93456_r_at | 0.83 |
| 93457_at | 0.00 |
| 93458_at | 0.59 |
| 93459_s_at | 0.00 |
| 93460_at | 0.00 |
| 93461_at | 0.00 |
| 93462_at | 0.00 |
| 93463_at | 0.02 |
| 93464_at | 0.08 |
| 93465_at | 0.00 |
| 93466_at | 0.01 |
| 93467_at | 0.73 |
| 93468_at | 0.00 |
| 93469_at | 0.00 |
| 93470_at | 0.00 |
| 93471_at | 0.00 |
| 93472_at | 0.00 |

| | |
|---|---|
| 93475_at | 0.00 |
| 93476_at | 0.00 |
| 93478_at | 0.48 |
| 93479_at | 0.00 |
| 93480_at | 0.00 |
| 93481_at | 0.00 |
| 93482_at | 0.00 |
| 93483_at | 0.85 |
| 93484_at | 0.00 |
| 93485_at | 0.00 |
| 93486_at | 0.00 |
| 93488_at | 0.00 |
| 93489_at | 0.00 |
| 93490_at | 0.00 |
| 93491_f_at | 0.00 |
| 93492_at | 0.00 |
| 93493_at | 0.01 |
| 93495_at | 0.00 |
| 93496_at | 0.00 |
| 93497_at | 0.00 |
| 93498_s_at | 0.00 |
| 93499_at | 0.00 |
| 93500_at | 0.00 |
| 93501_f_at | 0.00 |
| 93502_r_at | 0.00 |
| 93503_at | 0.21 |
| 93505_at | 0.00 |
| 93506_at | 0.00 |
| 93507_at | 0.17 |
| 93509_at | 0.00 |
| 93511_at | 0.26 |
| 93512_f_at | 0.00 |
| 93513_at | 0.00 |
| 93514_at | 0.00 |
| 93515_at | 0.00 |
| 93516_at | 0.00 |
| 93517_at | 0.00 |
| 93518_at | 0.00 |
| 93519_s_at | 0.00 |
| 93520_at | 0.00 |
| 93521_at | 0.00 |
| 93522_at | 0.00 |
| 93523_at | 0.00 |
| 93524_i_at | 0.00 |
| 93525_f_at | 0.00 |
| 93526_at | 0.00 |
| 93527_at | 0.04 |
| 93528_s_at | 0.15 |
| 93529_at | 0.00 |
| 93530_at | 0.03 |
| 93531_at | 0.00 |
| 93532_at | 0.00 |
| 93533_at | 0.00 |
| 93534_at | 0.55 |
| 93535_at | 0.01 |
| 93536_at | 0.00 |

| | |
|---|---|
| 93538_at | 0.36 |
| 93539_at | 0.01 |
| 93540_at | 0.08 |
| 93541_at | 0.00 |
| 93542_at | 0.00 |
| 93543_f_at | 0.00 |
| 93546_s_at | 0.25 |
| 93547_at | 0.22 |
| 93548_at | 0.00 |
| 93550_at | 0.07 |
| 93551_at | 0.00 |
| 93555_at | 0.00 |
| 93556_at | 0.00 |
| 93557_at | 0.11 |
| 93558_at | 0.00 |
| 93559_at | 0.00 |
| 93560_at | 0.00 |
| 93561_at | 0.00 |
| 93562_at | 0.00 |
| 93563_s_at | 0.00 |
| 93564_at | 0.00 |
| 93565_at | 0.00 |
| 93566_at | 0.00 |
| 93567_at | 0.00 |
| 93568_i_at | 0.00 |
| 93569_f_at | 0.00 |
| 93570_at | 0.00 |
| 93571_at | 0.00 |
| 93572_at | 0.00 |
| 93573_at | 0.00 |
| 93574_at | 0.35 |
| 93575_at | 0.00 |
| 93578_at | 0.00 |
| 93579_at | 0.00 |
| 93580_at | 0.00 |
| 93581_at | 0.00 |
| 93582_at | 0.01 |
| 93583_s_at | 0.00 |
| 93584_at | 0.00 |
| 93585_at | 0.00 |
| 93586_at | 0.00 |
| 93587_at | 0.00 |
| 93588_at | 0.00 |
| 93589_at | 0.00 |
| 93590_at | 0.00 |
| 93591_at | 0.00 |
| 93592_at | 0.00 |
| 93593_f_at | 0.09 |
| 93594_r_at | 0.00 |
| 93595_at | 0.01 |
| 93596_i_at | 0.02 |
| 93597_at | 0.00 |
| 93598_at | 0.01 |
| 93599_at | 0.00 |
| 93600_at | 0.00 |
| 93601_at | 0.00 |

| | |
|---|---|
| 93602_at | 0.00 |
| 93603_at | 0.00 |
| 93604_f_at | 0.27 |
| 93605_r_at | 0.00 |
| 93606_s_at | 0.03 |
| 93608_at | 0.00 |
| 93609_at | 0.00 |
| 93611_at | 0.00 |
| 93612_at | 0.00 |
| 93613_at | 0.00 |
| 93614_at | 0.03 |
| 93615_at | 0.63 |
| 93616_g_at | 0.40 |
| 93617_at | 0.00 |
| 93618_at | 0.00 |
| 93619_at | 0.00 |
| 93620_at | 0.00 |
| 93621_at | 0.00 |
| 93622_at | 0.00 |
| 93623_at | 0.00 |
| 93624_at | 0.00 |
| 93625_at | 0.00 |
| 93626_at | 0.00 |
| 93627_at | 0.00 |
| 93628_at | 0.00 |
| 93629_s_at | 0.00 |
| 93630_at | 0.10 |
| 93631_at | 0.00 |
| 93632_g_at | 0.00 |
| 93633_at | 0.00 |
| 93634_at | 0.01 |
| 93635_at | 0.00 |
| 93636_at | 0.00 |
| 93637_at | 0.00 |
| 93638_s_at | 0.00 |
| 93639_r_at | 0.00 |
| 93640_s_at | 0.00 |
| 93642_at | 0.10 |
| 93643_at | 0.00 |
| 93644_at | 0.00 |
| 93645_at | 0.00 |
| 93646_at | 0.06 |
| 93647_at | 0.00 |
| 93648_at | 0.00 |
| 93649_f_at | 0.00 |
| 93650_i_at | 0.00 |
| 93651_r_at | 0.00 |
| 93652_i_at | 0.00 |
| 93653_r_at | 0.00 |
| 93654_at | 0.00 |
| 93655_at | 0.00 |
| 93656_g_at | 0.04 |
| 93657_at | 0.00 |
| 93658_at | 0.00 |
| 93659_at | 0.00 |
| 93660_at | 0.00 |

| | |
|---|---|
| 93661_at | 0.00 |
| 93662_s_at | 0.00 |
| 93663_r_at | 0.00 |
| 93664_at | 0.00 |
| 93665_at | 0.00 |
| 93666_at | 0.01 |
| 93667_at | 0.00 |
| 93668_at | 0.00 |
| 93669_f_at | 0.27 |
| 93670_at | 0.05 |
| 93671_at | 0.00 |
| 93672_at | 0.00 |
| 93673_at | 0.00 |
| 93674_at | 0.06 |
| 93675_at | 0.00 |
| 93676_at | 0.00 |
| 93677_at | 0.00 |
| 93678_s_at | 0.00 |
| 93679_at | 0.00 |
| 93680_at | 0.00 |
| 93681_at | 0.16 |
| 93682_at | 0.00 |
| 93683_at | 0.00 |
| 93685_at | 0.00 |
| 93686_s_at | 0.00 |
| 93687_r_at | 0.00 |
| 93688_at | 0.00 |
| 93689_at | 0.00 |
| 93690_at | 0.06 |
| 93691_s_at | 0.10 |
| 93692_f_at | 0.00 |
| 93693_at | 0.00 |
| 93694_at | 0.00 |
| 93695_at | 0.00 |
| 93696_at | 0.00 |
| 93697_at | 0.00 |
| 93698_at | 0.00 |
| 93699_at | 0.00 |
| 93700_at | 0.00 |
| 93701_at | 0.00 |
| 93702_at | 0.00 |
| 93703_at | 0.99 |
| 93704_at | 0.00 |
| 93705_at | 0.00 |
| 93706_at | 0.00 |
| 93707_f_at | 0.00 |
| 93708_at | 0.00 |
| 93709_at | 0.00 |
| 93710_at | 0.00 |
| 93711_at | 0.01 |
| 93712_at | 0.00 |
| 93713_at | 0.04 |
| 93714_f_at | 0.00 |
| 93715_at | 0.00 |
| 93716_at | 0.00 |
| 93717_at | 0.00 |

| | |
|---|---|
| 93718_at | 0.00 |
| 93719_at | 0.00 |
| 93720_at | 0.00 |
| 93721_at | 0.06 |
| 93722_at | 0.00 |
| 93723_at | 0.00 |
| 93724_at | 0.00 |
| 93725_at | 0.00 |
| 93727_at | 0.00 |
| 93728_at | 0.01 |
| 93729_at | 0.00 |
| 93730_at | 0.00 |
| 93731_at | 0.01 |
| 93732_f_at | 0.01 |
| 93733_r_at | 0.00 |
| 93734_i_at | 0.00 |
| 93735_f_at | 0.00 |
| 93736_at | 0.00 |
| 93737_at | 0.00 |
| 93738_at | 0.56 |
| 93740_at | 0.00 |
| 93741_at | 0.00 |
| 93742_at | 0.02 |
| 93743_at | 0.00 |
| 93744_at | 0.00 |
| 93747_at | 0.00 |
| 93748_at | 0.00 |
| 93749_at | 0.00 |
| 93750_at | 0.24 |
| 93751_at | 0.00 |
| 93752_at | 0.03 |
| 93753_at | 0.01 |
| 93754_at | 0.13 |
| 93755_at | 0.00 |
| 93757_at | 0.00 |
| 93758_at | 0.00 |
| 93760_at | 0.00 |
| 93761_at | 0.00 |
| 93762_at | 0.00 |
| 93764_at | 0.00 |
| 93765_at | 0.00 |
| 93766_at | 0.00 |
| 93767_i_at | 0.00 |
| 93768_f_at | 0.17 |
| 93769_at | 0.00 |
| 93770_at | 0.01 |
| 93771_at | 0.00 |
| 93772_i_at | 0.00 |
| 93773_f_at | 0.06 |
| 93774_at | 0.00 |
| 93775_at | 0.31 |
| 93776_at | 0.00 |
| 93777_at | 0.00 |
| 93778_at | 0.00 |
| 93779_at | 0.00 |
| 93780_at | 0.23 |

| | |
|---|---|
| 93781_at | 0.00 |
| 93782_at | 0.10 |
| 93783_at | 0.00 |
| 93784_at | 0.00 |
| 93785_at | 0.20 |
| 93786_i_at | 0.01 |
| 93787_f_at | 0.00 |
| 93788_at | 0.00 |
| 93789_s_at | 0.01 |
| 93790_at | 0.00 |
| 93793_at | 0.00 |
| 93794_at | 0.01 |
| 93795_at | 0.05 |
| 93796_at | 0.00 |
| 93797_g_at | 0.52 |
| 93798_at | 0.44 |
| 93799_at | 0.00 |
| 93800_f_at | 0.00 |
| 93801_at | 0.00 |
| 93802_at | 0.00 |
| 93803_at | 0.01 |
| 93804_at | 0.53 |
| 93805_at | 0.02 |
| 93806_at | 0.00 |
| 93807_at | 0.00 |
| 93808_at | 0.00 |
| 93809_at | 0.00 |
| 93810_at | 0.00 |
| 93812_at | 0.00 |
| 93815_at | 0.00 |
| 93817_at | 0.00 |
| 93818_g_at | 0.20 |
| 93819_at | 0.06 |
| 93820_at | 0.00 |
| 93821_at | 0.12 |
| 93822_at | 0.00 |
| 93823_at | 0.00 |
| 93824_at | 0.00 |
| 93826_at | 0.00 |
| 93827_at | 0.00 |
| 93829_at | 0.00 |
| 93830_at | 0.00 |
| 93831_at | 0.01 |
| 93832_at | 0.00 |
| 93833_s_at | 0.19 |
| 93834_at | 0.00 |
| 93835_at | 0.04 |
| 93836_at | 0.08 |
| 93837_at | 0.00 |
| 93838_at | 0.03 |
| 93839_at | 0.00 |
| 93840_at | 0.00 |
| 93841_at | 0.00 |
| 93842_at | 0.00 |
| 93843_at | 0.00 |
| 93844_at | 0.00 |

| | |
|---|---|
| 93845_at | 0.00 |
| 93846_at | 0.00 |
| 93847_at | 0.00 |
| 93848_at | 0.00 |
| 93849_at | 0.00 |
| 93850_at | 0.06 |
| 93851_at | 0.00 |
| 93852_at | 0.00 |
| 93853_at | 0.00 |
| 93855_at | 0.00 |
| 93856_at | 0.01 |
| 93857_at | 0.12 |
| 93858_at | 0.00 |
| 93859_at | 0.00 |
| 93860_i_at | 0.00 |
| 93861_f_at | 0.00 |
| 93862_i_at | 0.00 |
| 93863_f_at | 0.00 |
| 93864_s_at | 0.48 |
| 93865_s_at | 0.00 |
| 93866_s_at | 0.45 |
| 93867_at | 0.06 |
| 93868_at | 0.01 |
| 93869_s_at | 0.00 |
| 93870_at | 0.00 |
| 93871_at | 0.04 |
| 93872_at | 0.00 |
| 93873_s_at | 0.00 |
| 93874_s_at | 0.01 |
| 93875_at | 0.00 |
| 93877_at | 0.00 |
| 93878_at | 0.00 |
| 93879_f_at | 0.00 |
| 93880_at | 0.26 |
| 93881_i_at | 0.01 |
| 93882_f_at | 0.18 |
| 93883_at | 0.02 |
| 93884_at | 0.00 |
| 93885_g_at | 0.00 |
| 93886_at | 0.00 |
| 93887_at | 0.02 |
| 93888_at | 0.00 |
| 93889_f_at | 0.01 |
| 93890_at | 0.00 |
| 93891_at | 0.00 |
| 93892_at | 0.00 |
| 93893_f_at | 0.00 |
| 93894_f_at | 0.00 |
| 93895_s_at | 0.00 |
| 93896_at | 0.00 |
| 93897_at | 0.00 |
| 93898_at | 0.00 |
| 93899_at | 0.00 |
| 93900_at | 0.01 |
| 93901_at | 0.00 |
| 93902_at | 0.03 |

| | |
|---|---|
| 93903_at | 0.01 |
| 93904_f_at | 0.00 |
| 93905_at | 0.00 |
| 93906_s_at | 0.00 |
| 93907_f_at | 0.00 |
| 93908_f_at | 0.01 |
| 93909_f_at | 0.03 |
| 93910_at | 0.00 |
| 93911_at | 0.00 |
| 93912_at | 0.07 |
| 93913_at | 0.00 |
| 93914_at | 0.00 |
| 93915_at | 0.00 |
| 93916_at | 0.06 |
| 93917_at | 0.00 |
| 93918_at | 0.11 |
| 93919_at | 0.00 |
| 93920_at | 0.00 |
| 93921_at | 0.01 |
| 93922_g_at | 0.00 |
| 93923_at | 0.00 |
| 93924_f_at | 0.00 |
| 93925_at | 0.00 |
| 93926_at | 0.00 |
| 93927_f_at | 0.00 |
| 93928_f_at | 0.00 |
| 93929_s_at | 0.00 |
| 93930_at | 0.08 |
| 93931_at | 0.00 |
| 93932_at | 0.00 |
| 93933_at | 0.00 |
| 93934_at | 0.00 |
| 93935_at | 0.00 |
| 93936_at | 0.00 |
| 93937_at | 0.02 |
| 93938_at | 0.00 |
| 93939_at | 0.00 |
| 93940_at | 0.00 |
| 93941_at | 0.01 |
| 93942_at | 0.00 |
| 93943_f_at | 0.03 |
| 93944_r_at | 0.00 |
| 93945_at | 0.00 |
| 93946_at | 0.00 |
| 93947_g_at | 0.00 |
| 93948_at | 0.00 |
| 93949_at | 0.00 |
| 93950_at | 0.00 |
| 93951_at | 0.00 |
| 93952_r_at | 0.00 |
| 93953_at | 0.00 |
| 93954_at | 0.00 |
| 93955_at | 0.00 |
| 93956_at | 0.00 |
| 93957_at | 0.00 |
| 93958_at | 0.02 |

| | |
|---|---|
| 93959_at | 0.00 |
| 93961_at | 0.00 |
| 93962_at | 0.00 |
| 93963_at | 0.00 |
| 93964_s_at | 0.40 |
| 93965_r_at | 0.35 |
| 93966_at | 0.05 |
| 93967_at | 0.02 |
| 93968_at | 0.00 |
| 93969_at | 0.00 |
| 93970_at | 0.22 |
| 93971_f_at | 0.00 |
| 93972_at | 0.00 |
| 93973_at | 0.00 |
| 93974_at | 0.00 |
| 93975_at | 0.00 |
| 93976_at | 0.00 |
| 93978_at | 0.00 |
| 93980_at | 0.00 |
| 93981_at | 0.00 |
| 93982_at | 0.00 |
| 93983_at | 0.00 |
| 93984_at | 0.00 |
| 93985_at | 0.01 |
| 93986_at | 0.00 |
| 93987_f_at | 0.00 |
| 93988_at | 0.03 |
| 93990_at | 0.00 |
| 93991_at | 0.00 |
| 93992_at | 0.05 |
| 93993_at | 0.01 |
| 93994_at | 0.00 |
| 93996_at | 0.00 |
| 93997_at | 0.00 |
| 93999_at | 0.02 |
| 94000_at | 0.00 |
| 94001_at | 0.00 |
| 94002_at | 0.02 |
| 94003_at | 0.00 |
| 94004_at | 0.01 |
| 94005_at | 0.01 |
| 94006_at | 0.00 |
| 94007_at | 0.00 |
| 94008_at | 0.01 |
| 94009_at | 0.00 |
| 94010_g_at | 0.00 |
| 94011_at | 0.00 |
| 94012_at | 0.00 |
| 94014_at | 0.00 |
| 94015_at | 0.00 |
| 94016_at | 0.00 |
| 94017_s_at | 0.00 |
| 94018_at | 0.04 |
| 94019_at | 0.32 |
| 94020_at | 0.00 |
| 94021_at | 0.00 |

| | |
|---|---|
| 94022_at | 0.00 |
| 94024_at | 0.01 |
| 94025_at | 0.00 |
| 94026_at | 0.00 |
| 94027_at | 0.07 |
| 94028_f_at | 0.01 |
| 94030_at | 0.00 |
| 94031_at | 0.00 |
| 94032_at | 0.00 |
| 94034_at | 0.00 |
| 94035_at | 0.00 |
| 94036_at | 0.00 |
| 94037_at | 0.00 |
| 94038_at | 0.00 |
| 94039_at | 0.00 |
| 94040_at | 0.00 |
| 94041_at | 0.00 |
| 94042_f_at | 0.00 |
| 94043_at | 0.01 |
| 94044_at | 0.33 |
| 94045_at | 0.00 |
| 94046_at | 0.00 |
| 94047_at | 0.01 |
| 94048_at | 0.00 |
| 94049_at | 0.00 |
| 94051_at | 0.00 |
| 94052_at | 0.00 |
| 94053_at | 0.00 |
| 94054_at | 0.00 |
| 94055_at | 0.09 |
| 94056_at | 0.26 |
| 94057_g_at | 0.32 |
| 94058_r_at | 0.03 |
| 94059_at | 0.00 |
| 94060_at | 0.00 |
| 94061_at | 0.07 |
| 94062_at | 0.00 |
| 94063_at | 0.00 |
| 94064_at | 0.00 |
| 94065_at | 0.00 |
| 94066_at | 0.01 |
| 94067_at | 0.00 |
| 94068_at | 0.00 |
| 94069_r_at | 0.00 |
| 94070_at | 0.00 |
| 94071_at | 0.09 |
| 94072_g_at | 0.01 |
| 94073_at | 0.00 |
| 94074_at | 0.00 |
| 94075_at | 0.00 |
| 94076_i_at | 0.01 |
| 94077_f_at | 0.00 |
| 94078_at | 0.00 |
| 94079_at | 0.00 |
| 94080_at | 0.00 |
| 94081_at | 0.00 |

| | |
|---|---|
| 94084_at | 0.00 |
| 94085_at | 0.00 |
| 94086_at | 0.00 |
| 94087_at | 0.00 |
| 94088_at | 0.24 |
| 94089_at | 0.00 |
| 94090_at | 0.00 |
| 94095_at | 0.00 |
| 94097_at | 0.21 |
| 94098_at | 0.00 |
| 94099_at | 0.00 |
| 94100_s_at | 0.00 |
| 94101_at | 0.00 |
| 94102_at | 0.00 |
| 94103_at | 0.00 |
| 94104_at | 0.00 |
| 94105_at | 0.00 |
| 94106_at | 0.00 |
| 94107_at | 0.00 |
| 94108_at | 0.00 |
| 94109_at | 0.00 |
| 94110_f_at | 0.00 |
| 94111_r_at | 0.05 |
| 94112_at | 0.00 |
| 94113_at | 0.00 |
| 94114_at | 0.00 |
| 94115_at | 0.00 |
| 94116_at | 0.00 |
| 94117_f_at | 0.00 |
| 94118_r_at | 0.00 |
| 94119_at | 0.00 |
| 94120_s_at | 0.00 |
| 94121_at | 0.00 |
| 94122_at | 0.00 |
| 94123_at | 0.00 |
| 94124_at | 0.00 |
| 94125_at | 0.00 |
| 94126_at | 0.00 |
| 94127_at | 0.00 |
| 94128_at | 0.00 |
| 94129_at | 0.00 |
| 94130_at | 0.00 |
| 94131_at | 0.00 |
| 94132_at | 0.00 |
| 94133_r_at | 0.00 |
| 94134_at | 0.00 |
| 94135_at | 0.00 |
| 94136_at | 0.00 |
| 94137_at | 0.00 |
| 94138_s_at | 0.00 |
| 94139_at | 0.00 |
| 94140_at | 0.00 |
| 94141_at | 0.00 |
| 94142_at | 0.00 |
| 94143_at | 0.00 |
| 94144_g_at | 0.00 |

| | |
|---|---|
| 94145_at | 0.00 |
| 94146_at | 0.00 |
| 94147_at | 0.65 |
| 94148_at | 0.00 |
| 94149_at | 0.00 |
| 94150_at | 0.00 |
| 94151_at | 0.00 |
| 94152_at | 0.00 |
| 94153_g_at | 0.00 |
| 94154_at | 0.00 |
| 94155_at | 0.00 |
| 94156_at | 0.00 |
| 94157_i_at | 0.00 |
| 94158_f_at | 0.00 |
| 94159_at | 0.00 |
| 94160_at | 0.00 |
| 94161_at | 0.00 |
| 94162_at | 0.00 |
| 94163_at | 0.00 |
| 94164_at | 0.00 |
| 94165_at | 0.00 |
| 94166_g_at | 0.00 |
| 94167_at | 0.00 |
| 94168_at | 0.00 |
| 94169_at | 0.00 |
| 94170_at | 0.00 |
| 94171_at | 0.00 |
| 94172_at | 0.00 |
| 94173_at | 0.00 |
| 94174_at | 0.32 |
| 94175_at | 0.00 |
| 94176_at | 0.00 |
| 94177_at | 0.00 |
| 94178_at | 0.00 |
| 94179_at | 0.00 |
| 94180_s_at | 0.00 |
| 94181_at | 0.00 |
| 94182_at | 0.00 |
| 94183_at | 0.00 |
| 94184_at | 0.00 |
| 94185_at | 0.00 |
| 94186_at | 0.00 |
| 94187_at | 0.00 |
| 94188_at | 0.00 |
| 94189_at | 0.01 |
| 94190_at | 0.00 |
| 94191_at | 0.00 |
| 94192_at | 0.82 |
| 94193_at | 0.00 |
| 94194_s_at | 0.00 |
| 94195_r_at | 0.00 |
| 94196_at | 0.00 |
| 94197_at | 0.00 |
| 94198_at | 0.00 |
| 94199_at | 0.00 |
| 94200_at | 0.98 |

| | |
|---|---|
| 94201_at | 0.00 |
| 94202_at | 0.00 |
| 94203_at | 0.00 |
| 94205_at | 0.00 |
| 94206_at | 0.00 |
| 94207_at | 0.00 |
| 94208_at | 0.05 |
| 94209_g_at | 0.00 |
| 94210_at | 0.00 |
| 94211_at | 0.00 |
| 94212_at | 0.00 |
| 94214_at | 0.32 |
| 94215_at | 0.00 |
| 94216_at | 0.00 |
| 94217_f_at | 0.00 |
| 94218_at | 0.00 |
| 94219_at | 0.00 |
| 94220_at | 0.00 |
| 94222_at | 0.00 |
| 94223_at | 0.00 |
| 94224_s_at | 0.22 |
| 94225_at | 0.00 |
| 94226_at | 0.00 |
| 94227_at | 0.00 |
| 94228_at | 0.00 |
| 94229_at | 0.00 |
| 94231_at | 0.00 |
| 94232_at | 0.00 |
| 94233_at | 0.00 |
| 94235_at | 0.05 |
| 94236_at | 0.00 |
| 94237_at | 0.00 |
| 94238_at | 0.16 |
| 94239_at | 0.00 |
| 94240_i_at | 0.00 |
| 94241_at | 0.00 |
| 94242_at | 0.00 |
| 94243_at | 0.00 |
| 94244_at | 0.00 |
| 94245_at | 0.00 |
| 94246_at | 0.00 |
| 94247_at | 0.29 |
| 94248_at | 0.00 |
| 94249_at | 0.00 |
| 94250_at | 0.00 |
| 94252_at | 0.00 |
| 94253_at | 0.00 |
| 94254_at | 0.00 |
| 94255_g_at | 0.00 |
| 94256_at | 0.00 |
| 94257_at | 0.00 |
| 94258_at | 0.34 |
| 94259_at | 0.03 |
| 94260_at | 0.00 |
| 94261_at | 0.00 |
| 94262_at | 0.00 |

| | |
|---|---|
| 94263_f_at | 0.00 |
| 94264_at | 0.00 |
| 94266_at | 0.00 |
| 94267_i_at | 0.00 |
| 94268_f_at | 0.00 |
| 94269_at | 0.00 |
| 94270_at | 0.86 |
| 94271_at | 0.00 |
| 94272_g_at | 0.00 |
| 94273_at | 0.08 |
| 94274_at | 0.00 |
| 94275_at | 0.00 |
| 94276_at | 0.00 |
| 94277_at | 0.00 |
| 94278_at | 0.00 |
| 94279_at | 0.00 |
| 94281_at | 0.00 |
| 94282_at | 0.00 |
| 94283_at | 0.00 |
| 94284_at | 0.00 |
| 94285_at | 0.00 |
| 94286_at | 0.00 |
| 94288_at | 0.00 |
| 94289_r_at | 0.00 |
| 94290_at | 0.00 |
| 94291_at | 0.00 |
| 94292_at | 0.00 |
| 94294_at | 0.00 |
| 94295_at | 0.04 |
| 94296_s_at | 0.02 |
| 94297_at | 0.01 |
| 94298_at | 0.00 |
| 94299_at | 0.01 |
| 94300_f_at | 0.00 |
| 94301_at | 0.14 |
| 94302_at | 0.00 |
| 94303_at | 0.00 |
| 94304_at | 0.00 |
| 94305_at | 0.22 |
| 94307_at | 0.00 |
| 94308_at | 0.00 |
| 94309_g_at | 0.05 |
| 94312_at | 0.01 |
| 94313_at | 0.00 |
| 94316_at | 0.00 |
| 94317_at | 0.00 |
| 94318_at | 0.00 |
| 94319_at | 0.01 |
| 94321_at | 0.00 |
| 94322_at | 0.00 |
| 94323_at | 0.00 |
| 94324_f_at | 0.00 |
| 94325_at | 0.00 |
| 94326_r_at | 0.00 |
| 94327_at | 0.00 |
| 94330_at | 0.06 |

| | |
|---|---|
| 94331_at | 0.00 |
| 94332_at | 0.00 |
| 94334_f_at | 0.14 |
| 94335_r_at | 0.42 |
| 94336_at | 0.00 |
| 94337_at | 0.00 |
| 94338_g_at | 0.46 |
| 94339_at | 0.00 |
| 94340_at | 0.00 |
| 94341_at | 0.27 |
| 94343_at | 0.00 |
| 94344_at | 0.00 |
| 94345_at | 0.69 |
| 94346_at | 0.03 |
| 94347_i_at | 0.00 |
| 94348_f_at | 0.00 |
| 94349_at | 0.00 |
| 94350_f_at | 0.04 |
| 94351_r_at | 0.23 |
| 94352_at | 0.00 |
| 94353_at | 0.00 |
| 94354_at | 0.98 |
| 94355_at | 0.00 |
| 94356_at | 0.00 |
| 94357_at | 0.00 |
| 94358_at | 0.00 |
| 94359_at | 0.43 |
| 94360_at | 0.00 |
| 94361_at | 0.00 |
| 94362_at | 0.00 |
| 94363_at | 0.00 |
| 94364_at | 0.00 |
| 94365_at | 0.00 |
| 94366_at | 0.00 |
| 94367_at | 0.00 |
| 94368_at | 0.00 |
| 94369_at | 0.10 |
| 94370_at | 0.00 |
| 94371_at | 0.00 |
| 94372_at | 0.00 |
| 94373_at | 0.00 |
| 94374_at | 0.00 |
| 94375_at | 0.01 |
| 94376_s_at | 0.00 |
| 94377_at | 0.00 |
| 94378_at | 0.37 |
| 94379_at | 0.00 |
| 94380_at | 0.00 |
| 94381_at | 0.00 |
| 94382_at | 0.00 |
| 94383_at | 0.00 |
| 94384_at | 0.62 |
| 94385_at | 0.00 |
| 94386_at | 0.00 |
| 94387_at | 0.00 |
| 94388_at | 0.00 |

| | |
|---|---|
| 94389_at | 0.00 |
| 94390_at | 0.00 |
| 94391_at | 0.00 |
| 94392_f_at | 0.00 |
| 94393_r_at | 0.00 |
| 94394_at | 0.00 |
| 94395_at | 0.32 |
| 94396_at | 0.03 |
| 94397_at | 0.00 |
| 94398_s_at | 0.00 |
| 94399_at | 0.00 |
| 94400_at | 0.00 |
| 94401_s_at | 0.00 |
| 94402_r_at | 0.00 |
| 94403_at | 0.00 |
| 94404_at | 0.00 |
| 94405_at | 0.01 |
| 94406_at | 0.00 |
| 94407_at | 0.00 |
| 94408_at | 0.00 |
| 94409_at | 0.00 |
| 94410_f_at | 0.00 |
| 94411_at | 0.00 |
| 94412_at | 0.00 |
| 94413_at | 0.00 |
| 94414_at | 0.00 |
| 94415_at | 0.00 |
| 94416_at | 0.00 |
| 94417_at | 0.00 |
| 94418_at | 0.04 |
| 94419_at | 0.00 |
| 94420_f_at | 0.00 |
| 94421_r_at | 0.00 |
| 94422_at | 0.00 |
| 94423_at | 0.00 |
| 94424_at | 0.00 |
| 94425_at | 0.00 |
| 94426_at | 0.00 |
| 94427_at | 0.04 |
| 94428_at | 0.00 |
| 94429_at | 0.00 |
| 94430_at | 0.02 |
| 94431_at | 0.00 |
| 94432_at | 0.58 |
| 94433_at | 0.26 |
| 94434_at | 0.00 |
| 94435_at | 0.00 |
| 94438_at | 0.00 |
| 94439_at | 0.00 |
| 94440_at | 0.00 |
| 94441_at | 0.00 |
| 94442_s_at | 0.00 |
| 94445_at | 0.00 |
| 94447_at | 0.00 |
| 94448_at | 0.00 |
| 94449_at | 0.00 |

| | |
|---|---|
| 94450_at | 0.00 |
| 94451_at | 0.00 |
| 94452_g_at | 0.00 |
| 94453_at | 0.03 |
| 94454_at | 0.00 |
| 94455_at | 0.00 |
| 94456_at | 0.08 |
| 94457_at | 0.00 |
| 94458_at | 0.02 |
| 94459_at | 0.00 |
| 94460_at | 0.31 |
| 94461_at | 0.00 |
| 94462_at | 0.00 |
| 94463_at | 0.00 |
| 94464_at | 0.00 |
| 94465_g_at | 0.01 |
| 94466_f_at | 0.00 |
| 94467_at | 0.01 |
| 94468_at | 0.00 |
| 94469_at | 0.00 |
| 94470_i_at | 0.00 |
| 94471_r_at | 0.00 |
| 94472_at | 0.00 |
| 94473_at | 0.00 |
| 94476_at | 0.04 |
| 94477_at | 0.03 |
| 94478_at | 0.00 |
| 94479_at | 0.00 |
| 94480_at | 0.00 |
| 94481_at | 0.00 |
| 94482_at | 0.00 |
| 94483_at | 0.01 |
| 94484_at | 0.00 |
| 94485_at | 0.00 |
| 94486_at | 0.00 |
| 94488_at | 0.00 |
| 94489_at | 0.00 |
| 94490_at | 0.01 |
| 94491_at | 0.00 |
| 94492_at | 0.00 |
| 94493_at | 0.00 |
| 94494_at | 0.00 |
| 94495_at | 0.00 |
| 94499_at | 0.00 |
| 94501_at | 0.00 |
| 94502_at | 0.00 |
| 94503_at | 0.00 |
| 94504_at | 0.00 |
| 94505_at | 0.01 |
| 94506_at | 0.00 |
| 94507_at | 0.00 |
| 94508_at | 0.00 |
| 94509_at | 0.00 |
| 94510_at | 0.00 |
| 94511_at | 0.00 |
| 94512_f_at | 0.00 |

| | |
|---|---|
| 94513_r_at | 0.00 |
| 94514_s_at | 0.00 |
| 94515_at | 0.00 |
| 94516_f_at | 0.06 |
| 94517_r_at | 0.00 |
| 94518_at | 0.00 |
| 94520_at | 0.00 |
| 94521_at | 0.00 |
| 94522_at | 0.00 |
| 94524_at | 0.00 |
| 94526_at | 0.00 |
| 94527_at | 0.00 |
| 94528_at | 0.00 |
| 94530_at | 0.00 |
| 94531_at | 0.00 |
| 94532_at | 0.00 |
| 94534_at | 0.00 |
| 94535_at | 0.00 |
| 94536_s_at | 0.00 |
| 94537_at | 0.00 |
| 94539_f_at | 0.00 |
| 94540_at | 0.00 |
| 94541_at | 0.00 |
| 94542_at | 0.02 |
| 94543_at | 0.00 |
| 94545_at | 0.00 |
| 94548_at | 0.26 |
| 94549_at | 0.12 |
| 94550_at | 0.00 |
| 94551_at | 0.00 |
| 94552_at | 0.00 |
| 94554_at | 0.00 |
| 94555_at | 0.00 |
| 94556_at | 0.00 |
| 94557_at | 0.00 |
| 94558_g_at | 0.00 |
| 94559_at | 0.00 |
| 94561_at | 0.11 |
| 94562_at | 0.00 |
| 94563_at | 0.00 |
| 94564_at | 0.00 |
| 94565_at | 0.00 |
| 94566_at | 0.00 |
| 94568_at | 0.00 |
| 94571_at | 0.00 |
| 94618_at | 0.00 |
| 94619_at | 0.00 |
| 94621_at | 0.00 |
| 94622_at | 0.00 |
| 94623_at | 0.00 |
| 94624_at | 0.00 |
| 94625_at | 0.00 |
| 94626_at | 0.00 |
| 94627_at | 0.00 |
| 94628_r_at | 0.00 |
| 94630_at | 0.00 |

| | |
|---|---|
| 94631_at | 0.00 |
| 94632_at | 0.00 |
| 94633_at | 0.00 |
| 94634_at | 0.00 |
| 94635_at | 0.00 |
| 94636_at | 0.00 |
| 94637_at | 0.00 |
| 94638_at | 0.00 |
| 94639_at | 0.00 |
| 94640_at | 0.00 |
| 94641_at | 0.00 |
| 94642_at | 0.00 |
| 94643_at | 0.03 |
| 94644_at | 0.00 |
| 94645_at | 0.00 |
| 94657_at | 0.00 |
| 94662_at | 0.00 |
| 94663_at | 0.18 |
| 94664_at | 0.00 |
| 94665_at | 0.00 |
| 94667_at | 0.00 |
| 94668_at | 0.00 |
| 94674_at | 0.00 |
| 94683_at | 0.00 |
| 94684_at | 0.00 |
| 94685_at | 0.00 |
| 94686_at | 0.00 |
| 94687_at | 0.00 |
| 94688_at | 0.00 |
| 94689_at | 0.00 |
| 94690_at | 0.00 |
| 94691_at | 0.00 |
| 94692_at | 0.00 |
| 94693_at | 0.00 |
| 94694_at | 0.00 |
| 94695_at | 0.00 |
| 94696_at | 0.00 |
| 94697_at | 0.00 |
| 94698_at | 0.00 |
| 94699_at | 0.00 |
| 94700_at | 0.00 |
| 94701_at | 0.00 |
| 94702_at | 0.00 |
| 94703_at | 0.00 |
| 94704_at | 0.00 |
| 94705_at | 0.00 |
| 94706_s_at | 0.00 |
| 94707_s_at | 0.00 |
| 94708_at | 0.00 |
| 94709_at | 0.00 |
| 94710_g_at | 0.00 |
| 94711_at | 0.00 |
| 94712_at | 0.48 |
| 94713_at | 0.00 |
| 94714_at | 0.00 |
| 94715_at | 0.35 |

| | |
|---|---|
| 94716_f_at | 0.00 |
| 94717_f_at | 0.00 |
| 94718_at | 0.00 |
| 94719_at | 0.00 |
| 94720_at | 0.00 |
| 94721_at | 0.00 |
| 94722_f_at | 0.00 |
| 94723_at | 0.01 |
| 94724_at | 0.13 |
| 94725_f_at | 0.00 |
| 94726_at | 0.00 |
| 94727_f_at | 0.91 |
| 94728_f_at | 0.00 |
| 94729_r_at | 0.00 |
| 94730_at | 0.00 |
| 94731_at | 0.00 |
| 94732_at | 0.00 |
| 94733_at | 0.00 |
| 94734_at | 0.00 |
| 94735_s_at | 0.00 |
| 94736_at | 0.00 |
| 94737_at | 0.00 |
| 94738_s_at | 0.01 |
| 94739_at | 0.00 |
| 94740_g_at | 0.00 |
| 94741_at | 0.00 |
| 94743_f_at | 0.00 |
| 94744_at | 0.00 |
| 94745_f_at | 1.00 |
| 94746_at | 0.00 |
| 94747_at | 0.00 |
| 94748_g_at | 0.00 |
| 94749_f_at | 0.62 |
| 94750_at | 0.10 |
| 94752_s_at | 0.00 |
| 94753_at | 0.00 |
| 94754_at | 0.00 |
| 94755_at | 0.00 |
| 94756_at | 0.00 |
| 94757_at | 0.09 |
| 94758_s_at | 0.00 |
| 94759_at | 0.00 |
| 94760_at | 0.00 |
| 94761_at | 0.31 |
| 94762_at | 0.00 |
| 94763_at | 0.00 |
| 94764_at | 0.00 |
| 94765_at | 0.00 |
| 94766_at | 0.00 |
| 94767_at | 0.00 |
| 94768_at | 0.03 |
| 94769_at | 0.00 |
| 94770_at | 0.00 |
| 94771_at | 0.00 |
| 94772_at | 0.00 |
| 94773_at | 0.00 |

| | |
|---|---|
| 94774_at | 0.00 |
| 94775_at | 0.00 |
| 94776_f_at | 0.00 |
| 94777_at | 0.00 |
| 94778_at | 0.00 |
| 94779_f_at | 0.00 |
| 94780_at | 0.00 |
| 94781_at | 0.00 |
| 94782_at | 0.00 |
| 94783_at | 0.00 |
| 94784_at | 0.00 |
| 94785_at | 0.00 |
| 94786_at | 0.00 |
| 94787_at | 0.00 |
| 94788_f_at | 0.00 |
| 94789_r_at | 0.00 |
| 94790_at | 0.00 |
| 94791_s_at | 0.00 |
| 94792_at | 0.00 |
| 94793_at | 0.00 |
| 94794_at | 0.02 |
| 94795_at | 0.00 |
| 94796_at | 0.01 |
| 94797_at | 0.00 |
| 94798_at | 0.00 |
| 94799_at | 0.00 |
| 94800_at | 0.00 |
| 94801_at | 0.00 |
| 94802_at | 0.38 |
| 94803_at | 0.00 |
| 94804_at | 0.00 |
| 94805_f_at | 0.00 |
| 94806_at | 0.00 |
| 94807_at | 0.04 |
| 94809_at | 0.00 |
| 94810_at | 0.00 |
| 94811_s_at | 0.02 |
| 94812_at | 0.00 |
| 94813_at | 0.00 |
| 94814_at | 0.01 |
| 94815_at | 0.02 |
| 94817_at | 0.06 |
| 94818_at | 0.03 |
| 94819_f_at | 0.00 |
| 94820_r_at | 0.08 |
| 94821_at | 0.85 |
| 94822_at | 0.00 |
| 94823_at | 0.00 |
| 94825_at | 0.00 |
| 94826_at | 0.00 |
| 94827_at | 0.00 |
| 94828_at | 0.00 |
| 94829_at | 0.00 |
| 94830_at | 0.00 |
| 94831_at | 0.00 |
| 94832_at | 0.00 |

| | |
|---|---|
| 94833_at | 0.05 |
| 94834_at | 0.00 |
| 94835_f_at | 0.02 |
| 94836_at | 0.00 |
| 94837_at | 0.00 |
| 94838_r_at | 0.00 |
| 94839_at | 0.00 |
| 94840_at | 0.01 |
| 94841_at | 0.00 |
| 94842_at | 0.06 |
| 94843_at | 0.00 |
| 94844_at | 0.00 |
| 94845_at | 0.01 |
| 94848_at | 0.00 |
| 94849_at | 0.00 |
| 94850_at | 0.01 |
| 94852_at | 0.00 |
| 94853_at | 0.06 |
| 94854_g_at | 0.01 |
| 94855_at | 0.00 |
| 94856_r_at | 0.00 |
| 94857_at | 0.00 |
| 94860_at | 0.00 |
| 94861_at | 0.00 |
| 94862_i_at | 0.00 |
| 94863_r_at | 0.00 |
| 94865_at | 0.01 |
| 94866_at | 0.00 |
| 94868_at | 0.00 |
| 94869_at | 0.00 |
| 94870_f_at | 0.00 |
| 94871_r_at | 0.00 |
| 94872_at | 0.00 |
| 94874_at | 0.00 |
| 94875_at | 0.01 |
| 94876_f_at | 0.00 |
| 94877_at | 0.00 |
| 94878_at | 0.00 |
| 94881_at | 0.00 |
| 94882_at | 0.00 |
| 94885_at | 0.00 |
| 94886_at | 0.00 |
| 94889_at | 0.00 |
| 94890_at | 0.00 |
| 94891_s_at | 0.00 |
| 94892_r_at | 0.00 |
| 94893_at | 0.00 |
| 94895_at | 0.01 |
| 94896_at | 0.00 |
| 94897_at | 0.69 |
| 94898_at | 0.00 |
| 94899_at | 0.00 |
| 94900_at | 0.00 |
| 94902_at | 0.00 |
| 94903_at | 0.00 |
| 94906_at | 0.00 |

| | |
|---|---|
| 94907_f_at | 0.00 |
| 94908_r_at | 0.00 |
| 94909_at | 0.00 |
| 94910_at | 0.04 |
| 94912_at | 0.19 |
| 94913_at | 0.00 |
| 94914_at | 0.00 |
| 94915_at | 0.05 |
| 94916_at | 0.00 |
| 94917_at | 0.00 |
| 94918_at | 0.00 |
| 94920_at | 0.00 |
| 94921_i_at | 0.00 |
| 94922_i_at | 0.00 |
| 94923_f_at | 0.00 |
| 94924_at | 0.00 |
| 94925_at | 0.00 |
| 94927_at | 0.00 |
| 94928_at | 0.00 |
| 94929_at | 0.00 |
| 94930_at | 0.15 |
| 94931_at | 0.00 |
| 94932_at | 0.00 |
| 94933_at | 0.00 |
| 94934_at | 0.08 |
| 94935_at | 0.00 |
| 94936_at | 0.02 |
| 94937_at | 0.00 |
| 94939_at | 0.39 |
| 94940_at | 0.00 |
| 94941_at | 0.00 |
| 94942_at | 0.00 |
| 94944_at | 0.00 |
| 94945_at | 0.00 |
| 94946_at | 0.00 |
| 94947_g_at | 0.06 |
| 94948_at | 0.44 |
| 94951_at | 0.00 |
| 94952_at | 0.48 |
| 94953_at | 0.00 |
| 94954_at | 0.00 |
| 94955_at | 0.00 |
| 94956_at | 0.00 |
| 94957_at | 0.00 |
| 94958_at | 0.00 |
| 94961_at | 0.00 |
| 94962_g_at | 0.00 |
| 94963_at | 0.00 |
| 94964_at | 0.04 |
| 94966_at | 0.02 |
| 94967_at | 0.00 |
| 94968_at | 0.00 |
| 94969_at | 0.00 |
| 94970_at | 0.00 |
| 94971_at | 0.00 |
| 94972_at | 0.00 |

| | |
|---|---|
| 94973_at | 0.12 |
| 94975_at | 0.01 |
| 94976_at | 0.00 |
| 94977_at | 0.00 |
| 94978_at | 0.00 |
| 94979_at | 0.00 |
| 94980_at | 0.00 |
| 94981_i_at | 0.35 |
| 94982_f_at | 0.87 |
| 94983_at | 0.00 |
| 94985_at | 0.00 |
| 94986_at | 0.61 |
| 94987_at | 0.00 |
| 94988_at | 0.00 |
| 94989_at | 0.10 |
| 94990_at | 0.00 |
| 94991_at | 0.00 |
| 94992_at | 0.00 |
| 94993_f_at | 0.00 |
| 94994_at | 0.00 |
| 94995_at | 0.00 |
| 94997_at | 0.00 |
| 94998_at | 0.05 |
| 94999_at | 0.00 |
| 95000_g_at | 0.00 |
| 95001_at | 0.00 |
| 95002_at | 0.00 |
| 95003_at | 0.00 |
| 95004_at | 0.00 |
| 95007_at | 0.00 |
| 95009_at | 0.00 |
| 95010_at | 0.00 |
| 95011_at | 0.00 |
| 95012_at | 0.00 |
| 95014_at | 0.00 |
| 95015_at | 0.00 |
| 95016_at | 0.21 |
| 95018_r_at | 0.00 |
| 95019_at | 0.00 |
| 95020_at | 0.01 |
| 95021_at | 0.21 |
| 95022_at | 0.00 |
| 95023_at | 0.00 |
| 95024_at | 0.02 |
| 95025_at | 0.00 |
| 95026_at | 0.00 |
| 95027_at | 0.00 |
| 95028_r_at | 0.00 |
| 95029_at | 0.03 |
| 95030_at | 0.00 |
| 95031_at | 0.00 |
| 95032_at | 0.00 |
| 95033_at | 0.97 |
| 95034_f_at | 0.00 |
| 95035_at | 0.00 |
| 95036_at | 0.01 |

| | |
|---|---|
| 95037_at | 0.00 |
| 95040_at | 0.01 |
| 95041_at | 0.00 |
| 95042_at | 0.00 |
| 95043_at | 0.00 |
| 95044_at | 0.00 |
| 95045_at | 0.01 |
| 95046_s_at | 0.00 |
| 95048_at | 0.00 |
| 95049_at | 0.00 |
| 95050_at | 0.00 |
| 95052_at | 0.00 |
| 95053_s_at | 0.00 |
| 95054_at | 0.00 |
| 95056_r_at | 0.00 |
| 95057_at | 0.00 |
| 95058_f_at | 0.03 |
| 95059_at | 0.00 |
| 95060_at | 0.02 |
| 95061_at | 0.05 |
| 95062_at | 0.00 |
| 95063_at | 0.00 |
| 95064_at | 0.00 |
| 95065_at | 0.00 |
| 95066_at | 0.00 |
| 95067_at | 0.00 |
| 95068_at | 0.02 |
| 95069_at | 0.01 |
| 95070_at | 0.00 |
| 95071_at | 0.00 |
| 95072_at | 0.00 |
| 95073_at | 0.00 |
| 95074_at | 0.00 |
| 95075_at | 0.00 |
| 95076_at | 0.00 |
| 95077_at | 0.12 |
| 95078_at | 0.00 |
| 95079_at | 0.00 |
| 95081_at | 0.00 |
| 95082_at | 0.29 |
| 95083_at | 0.29 |
| 95084_f_at | 0.00 |
| 95085_r_at | 0.00 |
| 95086_at | 0.00 |
| 95090_at | 0.00 |
| 95091_at | 0.00 |
| 95092_at | 0.00 |
| 95093_at | 0.09 |
| 95094_g_at | 0.09 |
| 95095_at | 0.00 |
| 95096_at | 0.02 |
| 95097_at | 0.00 |
| 95098_at | 0.00 |
| 95100_at | 0.00 |
| 95101_at | 0.00 |
| 95102_at | 0.00 |

| | |
|---|---|
| 95103_at | 0.00 |
| 95104_at | 0.00 |
| 95105_at | 0.00 |
| 95108_at | 0.00 |
| 95109_at | 0.00 |
| 95110_at | 0.00 |
| 95111_i_at | 0.02 |
| 95112_f_at | 0.01 |
| 95113_at | 0.00 |
| 95114_s_at | 0.00 |
| 95117_at | 0.00 |
| 95118_r_at | 0.00 |
| 95119_at | 0.00 |
| 95120_at | 0.12 |
| 95121_at | 0.02 |
| 95122_g_at | 0.03 |
| 95123_at | 0.00 |
| 95124_i_at | 0.00 |
| 95125_f_at | 0.00 |
| 95128_at | 0.00 |
| 95129_at | 0.00 |
| 95131_f_at | 0.00 |
| 95132_r_at | 0.00 |
| 95133_at | 0.19 |
| 95134_at | 0.00 |
| 95135_at | 0.00 |
| 95136_at | 0.01 |
| 95137_at | 0.00 |
| 95138_at | 0.01 |
| 95139_at | 0.00 |
| 95140_at | 0.00 |
| 95141_at | 0.00 |
| 95142_s_at | 0.01 |
| 95144_at | 0.01 |
| 95146_at | 0.00 |
| 95147_at | 0.00 |
| 95148_at | 0.00 |
| 95149_at | 0.00 |
| 95150_at | 0.00 |
| 95151_at | 0.00 |
| 95152_g_at | 0.00 |
| 95153_at | 0.00 |
| 95154_at | 0.00 |
| 95155_at | 0.00 |
| 95156_g_at | 0.00 |
| 95157_at | 0.00 |
| 95158_at | 0.00 |
| 95159_at | 0.00 |
| 95161_at | 0.00 |
| 95164_at | 0.00 |
| 95165_at | 0.00 |
| 95177_at | 0.00 |
| 95182_at | 0.00 |
| 95183_i_at | 0.03 |
| 95184_f_at | 0.16 |
| 95215_f_at | 0.00 |

| | |
|---|---|
| 95228_f_at | 0.00 |
| 95232_at | 0.05 |
| 95244_at | 0.00 |
| 95246_at | 0.00 |
| 95247_at | 0.00 |
| 95248_at | 0.00 |
| 95249_at | 0.00 |
| 95275_at | 0.00 |
| 95281_at | 0.00 |
| 95282_at | 0.02 |
| 95283_at | 0.00 |
| 95284_at | 0.00 |
| 95285_at | 0.00 |
| 95286_at | 0.67 |
| 95287_at | 0.00 |
| 95288_i_at | 0.00 |
| 95289_r_at | 0.00 |
| 95290_at | 0.00 |
| 95291_r_at | 0.00 |
| 95292_at | 0.00 |
| 95293_at | 0.00 |
| 95294_at | 0.00 |
| 95295_s_at | 0.00 |
| 95296_r_at | 0.00 |
| 95297_at | 0.00 |
| 95298_at | 0.00 |
| 95299_at | 0.00 |
| 95300_at | 0.00 |
| 95301_at | 0.00 |
| 95302_at | 0.00 |
| 95303_at | 0.00 |
| 95304_at | 0.00 |
| 95305_at | 0.00 |
| 95306_at | 0.00 |
| 95307_at | 0.00 |
| 95308_at | 0.00 |
| 95309_at | 0.00 |
| 95310_at | 0.00 |
| 95311_at | 0.00 |
| 95312_at | 0.00 |
| 95313_at | 0.00 |
| 95314_at | 0.00 |
| 95315_at | 0.00 |
| 95316_at | 0.01 |
| 95317_at | 0.00 |
| 95318_at | 0.00 |
| 95319_at | 0.00 |
| 95320_at | 0.00 |
| 95321_at | 0.00 |
| 95322_g_at | 0.00 |
| 95323_at | 0.00 |
| 95324_at | 0.00 |
| 95325_at | 0.00 |
| 95326_at | 0.00 |
| 95327_at | 0.00 |
| 95328_at | 0.00 |

| | |
|---|---|
| 95329_at | 0.00 |
| 95330_at | 0.00 |
| 95331_at | 0.00 |
| 95332_at | 0.00 |
| 95333_at | 0.00 |
| 95334_at | 0.00 |
| 95335_at | 0.00 |
| 95336_at | 0.00 |
| 95337_at | 0.00 |
| 95338_s_at | 0.03 |
| 95339_r_at | 0.01 |
| 95340_at | 0.00 |
| 95341_at | 0.03 |
| 95342_at | 0.00 |
| 95343_at | 0.00 |
| 95344_at | 0.00 |
| 95345_at | 0.05 |
| 95346_at | 0.49 |
| 95347_at | 0.00 |
| 95348_at | 0.49 |
| 95349_g_at | 0.00 |
| 95350_at | 0.00 |
| 95351_at | 0.02 |
| 95352_at | 0.00 |
| 95353_at | 0.00 |
| 95354_at | 0.00 |
| 95355_at | 0.05 |
| 95356_at | 0.00 |
| 95357_at | 0.07 |
| 95358_at | 0.00 |
| 95359_at | 0.00 |
| 95360_at | 0.00 |
| 95363_at | 0.00 |
| 95364_at | 0.00 |
| 95366_at | 0.01 |
| 95368_at | 0.00 |
| 95369_at | 0.00 |
| 95370_at | 0.00 |
| 95371_g_at | 0.00 |
| 95372_at | 0.00 |
| 95373_at | 0.00 |
| 95374_f_at | 0.00 |
| 95375_r_at | 0.00 |
| 95376_at | 0.00 |
| 95377_at | 0.00 |
| 95378_at | 0.00 |
| 95379_at | 0.03 |
| 95380_at | 0.00 |
| 95381_at | 0.00 |
| 95382_at | 0.00 |
| 95383_at | 0.00 |
| 95385_at | 0.00 |
| 95386_at | 0.00 |
| 95387_f_at | 0.05 |
| 95388_at | 0.00 |
| 95389_at | 0.00 |

| | |
|---|---|
| 95390_at | 0.00 |
| 95391_at | 0.00 |
| 95392_at | 0.00 |
| 95393_at | 0.00 |
| 95395_at | 0.00 |
| 95396_at | 0.00 |
| 95397_at | 0.00 |
| 95398_at | 0.00 |
| 95399_at | 0.00 |
| 95400_i_at | 0.17 |
| 95401_at | 0.00 |
| 95404_at | 0.00 |
| 95405_at | 0.00 |
| 95406_at | 0.00 |
| 95407_at | 0.00 |
| 95408_at | 0.00 |
| 95409_at | 0.00 |
| 95410_s_at | 0.00 |
| 95411_at | 0.00 |
| 95412_at | 0.00 |
| 95413_at | 0.00 |
| 95414_i_at | 0.00 |
| 95415_f_at | 0.00 |
| 95416_at | 0.00 |
| 95417_at | 0.00 |
| 95418_at | 0.00 |
| 95419_at | 0.00 |
| 95420_at | 0.00 |
| 95423_at | 0.08 |
| 95424_at | 0.00 |
| 95425_at | 0.29 |
| 95426_at | 0.01 |
| 95427_at | 0.00 |
| 95428_at | 0.00 |
| 95430_f_at | 0.06 |
| 95431_at | 0.00 |
| 95432_f_at | 0.00 |
| 95433_at | 0.00 |
| 95434_at | 0.00 |
| 95435_at | 0.00 |
| 95436_at | 0.00 |
| 95437_at | 0.00 |
| 95438_at | 0.00 |
| 95439_at | 0.02 |
| 95440_at | 0.34 |
| 95441_at | 0.00 |
| 95442_at | 0.00 |
| 95444_at | 0.00 |
| 95445_at | 0.00 |
| 95446_at | 0.00 |
| 95447_at | 0.00 |
| 95448_at | 0.00 |
| 95449_at | 0.00 |
| 95450_at | 0.00 |
| 95451_at | 0.00 |
| 95452_i_at | 0.00 |

| | |
|---|---|
| 95453_f_at | 0.00 |
| 95454_at | 0.00 |
| 95455_at | 0.00 |
| 95456_r_at | 0.02 |
| 95457_at | 0.03 |
| 95458_s_at | 0.01 |
| 95460_at | 0.00 |
| 95462_at | 0.20 |
| 95464_at | 0.00 |
| 95465_s_at | 0.00 |
| 95466_at | 0.01 |
| 95467_at | 0.00 |
| 95468_at | 0.00 |
| 95469_at | 0.00 |
| 95470_at | 0.00 |
| 95471_at | 0.24 |
| 95472_f_at | 0.00 |
| 95473_s_at | 0.00 |
| 95474_at | 0.00 |
| 95477_at | 0.00 |
| 95478_at | 0.01 |
| 95479_at | 0.01 |
| 95480_at | 0.00 |
| 95481_at | 0.00 |
| 95482_at | 0.02 |
| 95483_at | 0.00 |
| 95485_at | 0.00 |
| 95486_at | 0.01 |
| 95488_at | 0.01 |
| 95489_at | 0.06 |
| 95490_at | 0.00 |
| 95491_at | 0.00 |
| 95493_at | 0.00 |
| 95496_at | 0.00 |
| 95497_at | 0.00 |
| 95498_at | 0.00 |
| 95501_at | 0.00 |
| 95502_at | 0.00 |
| 95503_at | 0.00 |
| 95505_at | 0.00 |
| 95506_at | 0.00 |
| 95507_at | 0.56 |
| 95508_at | 0.00 |
| 95509_at | 0.00 |
| 95511_at | 0.06 |
| 95512_at | 0.00 |
| 95513_at | 0.00 |
| 95514_at | 0.00 |
| 95516_at | 0.00 |
| 95517_i_at | 0.00 |
| 95518_at | 0.13 |
| 95520_at | 0.01 |
| 95521_s_at | 0.00 |
| 95522_i_at | 0.00 |
| 95523_at | 0.01 |
| 95525_at | 0.00 |

| | |
|---|---|
| 95526_at | 0.00 |
| 95527_at | 0.00 |
| 95528_at | 0.00 |
| 95529_at | 0.00 |
| 95530_at | 0.00 |
| 95531_at | 0.08 |
| 95532_at | 0.00 |
| 95533_at | 0.00 |
| 95536_at | 0.00 |
| 95537_at | 0.00 |
| 95538_at | 0.00 |
| 95539_at | 0.00 |
| 95540_r_at | 0.00 |
| 95541_at | 0.04 |
| 95542_at | 0.01 |
| 95543_at | 0.09 |
| 95544_at | 0.00 |
| 95545_at | 0.00 |
| 95546_g_at | 0.00 |
| 95547_at | 0.03 |
| 95549_at | 0.00 |
| 95550_at | 0.01 |
| 95551_at | 0.04 |
| 95552_at | 0.00 |
| 95553_at | 0.00 |
| 95554_at | 0.00 |
| 95555_at | 0.00 |
| 95556_at | 0.00 |
| 95557_at | 0.00 |
| 95559_at | 0.00 |
| 95561_at | 0.00 |
| 95562_at | 0.75 |
| 95563_at | 0.00 |
| 95564_at | 0.44 |
| 95565_at | 0.00 |
| 95566_at | 0.00 |
| 95567_at | 0.00 |
| 95568_at | 0.11 |
| 95569_at | 0.00 |
| 95571_at | 0.00 |
| 95573_at | 0.00 |
| 95574_f_at | 0.00 |
| 95575_r_at | 0.00 |
| 95577_at | 0.05 |
| 95580_at | 0.04 |
| 95584_at | 0.08 |
| 95585_at | 0.00 |
| 95586_at | 0.00 |
| 95587_at | 0.00 |
| 95588_at | 0.00 |
| 95590_at | 0.02 |
| 95591_at | 0.00 |
| 95592_at | 0.01 |
| 95593_at | 0.00 |
| 95594_at | 0.00 |
| 95595_at | 0.00 |

| | |
|---|---|
| 95596_at | 0.00 |
| 95597_at | 0.00 |
| 95599_at | 0.00 |
| 95600_at | 0.00 |
| 95601_at | 0.00 |
| 95602_at | 0.00 |
| 95603_at | 0.00 |
| 95604_at | 0.00 |
| 95606_at | 0.00 |
| 95607_at | 0.00 |
| 95608_at | 0.01 |
| 95609_at | 0.00 |
| 95610_at | 0.26 |
| 95611_at | 0.00 |
| 95612_at | 0.00 |
| 95613_at | 0.00 |
| 95614_at | 0.01 |
| 95616_at | 0.00 |
| 95617_at | 0.01 |
| 95618_at | 0.00 |
| 95619_at | 0.04 |
| 95620_at | 0.00 |
| 95621_at | 0.00 |
| 95622_at | 0.24 |
| 95625_at | 0.00 |
| 95626_at | 0.00 |
| 95627_at | 0.04 |
| 95628_at | 0.00 |
| 95629_at | 0.00 |
| 95630_at | 0.00 |
| 95631_at | 0.05 |
| 95632_f_at | 0.00 |
| 95633_r_at | 0.00 |
| 95634_at | 0.00 |
| 95635_g_at | 0.00 |
| 95636_at | 0.00 |
| 95637_at | 0.00 |
| 95639_at | 0.00 |
| 95641_at | 0.00 |
| 95642_at | 0.00 |
| 95643_at | 0.07 |
| 95645_at | 0.00 |
| 95646_at | 0.00 |
| 95647_f_at | 0.00 |
| 95648_at | 0.00 |
| 95649_at | 0.00 |
| 95650_at | 0.00 |
| 95651_at | 0.00 |
| 95652_at | 0.00 |
| 95653_at | 0.00 |
| 95654_at | 0.18 |
| 95655_at | 0.24 |
| 95656_i_at | 0.00 |
| 95657_f_at | 0.00 |
| 95658_at | 0.00 |
| 95659_at | 0.04 |

| | |
|---|---|
| 95660_at | 0.00 |
| 95661_at | 0.63 |
| 95662_at | 0.00 |
| 95663_at | 0.00 |
| 95664_at | 0.08 |
| 95665_at | 0.10 |
| 95666_at | 0.00 |
| 95668_at | 0.00 |
| 95669_g_at | 0.06 |
| 95670_at | 0.37 |
| 95671_at | 0.00 |
| 95672_at | 0.00 |
| 95673_s_at | 0.00 |
| 95674_r_at | 0.00 |
| 95675_at | 0.01 |
| 95676_at | 0.00 |
| 95677_at | 0.00 |
| 95679_at | 0.01 |
| 95680_at | 0.00 |
| 95681_f_at | 0.00 |
| 95682_at | 0.00 |
| 95683_g_at | 0.00 |
| 95684_at | 0.00 |
| 95685_at | 0.00 |
| 95686_at | 0.00 |
| 95688_at | 0.00 |
| 95689_at | 0.00 |
| 95690_at | 0.00 |
| 95692_at | 0.00 |
| 95693_at | 0.01 |
| 95694_at | 0.00 |
| 95695_at | 0.00 |
| 95696_at | 0.00 |
| 95697_at | 0.00 |
| 95698_at | 0.07 |
| 95699_f_at | 0.00 |
| 95700_r_at | 0.00 |
| 95701_at | 0.00 |
| 95702_at | 0.00 |
| 95703_at | 0.00 |
| 95704_at | 0.00 |
| 95705_s_at | 0.05 |
| 95706_at | 0.52 |
| 95707_at | 0.01 |
| 95708_at | 0.00 |
| 95709_at | 0.00 |
| 95712_at | 0.00 |
| 95713_at | 0.00 |
| 95714_at | 0.00 |
| 95715_at | 0.00 |
| 95716_at | 0.00 |
| 95717_at | 0.00 |
| 95718_f_at | 0.00 |
| 95719_at | 0.00 |
| 95721_at | 0.02 |
| 95722_at | 0.00 |

| | |
|---|---|
| 95723_r_at | 0.00 |
| 95725_at | 0.00 |
| 95726_at | 0.00 |
| 95727_at | 0.00 |
| 95728_g_at | 0.00 |
| 95729_at | 0.00 |
| 95730_at | 0.00 |
| 95731_at | 0.00 |
| 95732_at | 0.00 |
| 95733_at | 0.22 |
| 95734_at | 0.00 |
| 95735_at | 0.00 |
| 95736_at | 0.00 |
| 95737_at | 0.00 |
| 95738_at | 0.00 |
| 95739_at | 0.00 |
| 95740_at | 0.00 |
| 95742_at | 0.00 |
| 95743_at | 0.01 |
| 95744_at | 0.00 |
| 95745_g_at | 0.00 |
| 95746_at | 0.00 |
| 95747_at | 0.00 |
| 95749_at | 0.00 |
| 95750_at | 0.00 |
| 95752_at | 0.01 |
| 95753_at | 0.00 |
| 95754_at | 0.00 |
| 95755_at | 0.00 |
| 95756_at | 0.00 |
| 95758_at | 0.40 |
| 95759_at | 0.00 |
| 95760_at | 0.00 |
| 95765_at | 0.00 |
| 95766_f_at | 0.02 |
| 95770_s_at | 0.00 |
| 95771_i_at | 0.00 |
| 95772_r_at | 0.00 |
| 95773_at | 0.00 |
| 95775_f_at | 0.00 |
| 95781_at | 0.00 |
| 95782_at | 0.00 |
| 95783_g_at | 0.00 |
| 95784_at | 0.00 |
| 95785_s_at | 0.00 |
| 95786_at | 0.00 |
| 95787_s_at | 0.00 |
| 95791_s_at | 0.00 |
| 95792_at | 0.00 |
| 95793_at | 0.00 |
| 95794_f_at | 0.00 |
| 95795_at | 0.00 |
| 95796_g_at | 0.00 |
| 95797_f_at | 0.00 |
| 95798_f_at | 0.00 |
| 95799_s_at | 0.00 |

| | |
|---|---|
| 95800_s_at | 0.05 |
| 95801_s_at | 0.01 |
| 95803_at | 0.00 |
| 95804_g_at | 0.00 |
| 95805_at | 0.00 |
| 95806_f_at | 0.00 |
| 95807_at | 0.00 |
| 95808_g_at | 0.00 |
| 95848_at | 0.00 |
| 95852_at | 0.00 |
| 95853_at | 0.00 |
| 95854_at | 0.00 |
| 95855_at | 0.00 |
| 95856_at | 0.00 |
| 95857_at | 0.00 |
| 95858_at | 0.00 |
| 95861_at | 0.00 |
| 95862_at | 0.00 |
| 95863_at | 0.00 |
| 95864_at | 0.00 |
| 95869_at | 0.00 |
| 95870_at | 0.01 |
| 95871_at | 0.00 |
| 95872_at | 0.00 |
| 95875_at | 0.00 |
| 95876_at | 0.00 |
| 95877_at | 0.00 |
| 95878_at | 0.00 |
| 95879_at | 0.00 |
| 95880_s_at | 0.00 |
| 95881_f_at | 0.00 |
| 95882_at | 0.00 |
| 95883_at | 0.78 |
| 95884_at | 0.00 |
| 95885_at | 0.00 |
| 95886_g_at | 0.00 |
| 95887_at | 0.24 |
| 95888_at | 0.21 |
| 95889_at | 0.00 |
| 95890_r_at | 0.00 |
| 95891_at | 0.00 |
| 95892_at | 0.00 |
| 95893_at | 0.00 |
| 95894_at | 0.00 |
| 95895_at | 0.00 |
| 95896_at | 0.00 |
| 95897_at | 0.00 |
| 95898_at | 0.00 |
| 95901_f_at | 0.00 |
| 95902_at | 0.00 |
| 95903_at | 0.00 |
| 95904_at | 0.00 |
| 95905_at | 0.00 |
| 95906_at | 0.00 |
| 95907_at | 0.00 |
| 95908_at | 0.00 |

| | |
|---|---|
| 95909_at | 0.00 |
| 95910_f_at | 0.00 |
| 95911_at | 0.01 |
| 95912_at | 0.00 |
| 95913_at | 0.01 |
| 95914_at | 0.05 |
| 95915_at | 0.00 |
| 95916_at | 0.00 |
| 95917_at | 0.00 |
| 95919_at | 0.00 |
| 95920_at | 0.00 |
| 95923_at | 0.00 |
| 95924_at | 0.00 |
| 95925_at | 0.00 |
| 95926_at | 0.00 |
| 95927_f_at | 0.16 |
| 95929_at | 0.00 |
| 95930_at | 0.00 |
| 95931_at | 0.00 |
| 95932_at | 0.00 |
| 95933_f_at | 0.00 |
| 95934_at | 0.00 |
| 95935_at | 0.00 |
| 95936_at | 0.01 |
| 95937_at | 0.00 |
| 95938_at | 0.00 |
| 95939_i_at | 0.00 |
| 95940_f_at | 0.00 |
| 95941_at | 0.00 |
| 95943_at | 0.00 |
| 95944_at | 0.40 |
| 95945_at | 0.00 |
| 95946_at | 0.00 |
| 95947_at | 0.00 |
| 95948_at | 0.00 |
| 95949_at | 0.00 |
| 95950_at | 0.00 |
| 95951_at | 0.07 |
| 95952_at | 0.00 |
| 95953_at | 0.00 |
| 95954_at | 0.51 |
| 95955_at | 0.00 |
| 95956_at | 0.00 |
| 95957_at | 0.00 |
| 95958_at | 0.00 |
| 95959_at | 0.00 |
| 95960_at | 0.00 |
| 95961_at | 0.01 |
| 95962_at | 0.00 |
| 95963_at | 0.01 |
| 95964_at | 0.54 |
| 95965_at | 0.00 |
| 95966_at | 0.00 |
| 95967_at | 0.00 |
| 95968_at | 0.00 |
| 95970_at | 0.00 |

| | |
|---|---|
| 95971_at | 0.00 |
| 95972_at | 0.00 |
| 95973_at | 0.00 |
| 95974_at | 0.25 |
| 95975_at | 0.00 |
| 95976_at | 0.00 |
| 95977_at | 0.00 |
| 95978_at | 0.03 |
| 95979_at | 0.00 |
| 95980_at | 0.00 |
| 95981_at | 0.00 |
| 95982_at | 0.00 |
| 95983_at | 0.00 |
| 95984_at | 0.00 |
| 95985_at | 0.00 |
| 95986_at | 0.00 |
| 95987_at | 0.00 |
| 95988_r_at | 0.00 |
| 95989_at | 0.00 |
| 95990_at | 0.00 |
| 95991_at | 0.00 |
| 95992_at | 0.00 |
| 95994_at | 0.00 |
| 95995_at | 0.00 |
| 95996_at | 0.00 |
| 95997_at | 0.00 |
| 95998_at | 0.00 |
| 95999_at | 0.00 |
| 96000_at | 0.00 |
| 96001_at | 0.00 |
| 96002_at | 0.00 |
| 96003_at | 0.00 |
| 96004_at | 0.00 |
| 96007_at | 0.00 |
| 96008_at | 0.00 |
| 96009_s_at | 0.00 |
| 96010_at | 0.00 |
| 96011_at | 0.07 |
| 96012_f_at | 0.02 |
| 96013_r_at | 0.08 |
| 96014_at | 0.00 |
| 96016_at | 0.93 |
| 96017_at | 0.00 |
| 96018_r_at | 0.00 |
| 96019_at | 0.00 |
| 96020_at | 0.12 |
| 96021_at | 0.00 |
| 96023_at | 0.00 |
| 96024_at | 0.00 |
| 96025_g_at | 0.00 |
| 96026_at | 0.00 |
| 96027_at | 0.00 |
| 96028_at | 0.00 |
| 96029_at | 0.00 |
| 96030_at | 0.00 |
| 96031_r_at | 0.00 |

| | |
|---|---|
| 96032_at | 0.00 |
| 96033_at | 0.00 |
| 96035_at | 0.02 |
| 96036_at | 0.00 |
| 96037_at | 0.01 |
| 96038_at | 0.24 |
| 96041_at | 0.00 |
| 96042_at | 0.72 |
| 96043_at | 0.00 |
| 96044_at | 0.00 |
| 96045_at | 0.00 |
| 96046_at | 0.04 |
| 96047_at | 0.00 |
| 96048_at | 0.03 |
| 96049_at | 0.44 |
| 96050_at | 0.00 |
| 96051_at | 0.00 |
| 96052_at | 0.00 |
| 96053_i_at | 0.08 |
| 96054_f_at | 0.01 |
| 96055_at | 0.00 |
| 96056_at | 0.01 |
| 96057_at | 0.00 |
| 96058_s_at | 0.02 |
| 96059_at | 0.00 |
| 96060_at | 0.02 |
| 96061_at | 0.00 |
| 96063_at | 0.04 |
| 96064_at | 0.00 |
| 96065_at | 0.00 |
| 96066_s_at | 0.00 |
| 96068_at | 0.03 |
| 96069_at | 0.00 |
| 96070_at | 0.00 |
| 96071_at | 0.00 |
| 96072_at | 0.00 |
| 96073_at | 0.00 |
| 96074_at | 0.00 |
| 96075_at | 0.00 |
| 96076_at | 0.00 |
| 96077_at | 0.00 |
| 96078_g_at | 0.00 |
| 96079_at | 0.00 |
| 96081_at | 0.00 |
| 96082_at | 0.00 |
| 96083_s_at | 0.00 |
| 96084_at | 0.00 |
| 96085_at | 0.04 |
| 96086_at | 0.00 |
| 96087_at | 0.00 |
| 96088_at | 0.00 |
| 96089_at | 0.00 |
| 96090_g_at | 0.00 |
| 96091_at | 0.00 |
| 96092_at | 0.00 |
| 96093_at | 0.00 |

| | |
|---|---|
| 96094_at | 0.00 |
| 96095_i_at | 0.00 |
| 96096_f_at | 0.00 |
| 96097_at | 0.00 |
| 96098_at | 0.00 |
| 96099_at | 0.00 |
| 96101_at | 0.00 |
| 96102_i_at | 0.00 |
| 96103_f_at | 0.00 |
| 96104_at | 0.00 |
| 96106_at | 0.01 |
| 96109_at | 1.00 |
| 96110_at | 0.01 |
| 96112_at | 0.00 |
| 96113_at | 0.00 |
| 96114_at | 0.00 |
| 96115_at | 0.09 |
| 96116_at | 0.00 |
| 96117_r_at | 0.01 |
| 96118_at | 0.00 |
| 96119_s_at | 0.00 |
| 96120_at | 0.25 |
| 96121_at | 0.00 |
| 96122_at | 0.00 |
| 96123_at | 0.00 |
| 96124_at | 0.00 |
| 96125_at | 0.00 |
| 96126_at | 0.01 |
| 96127_at | 0.02 |
| 96128_at | 0.00 |
| 96130_at | 0.00 |
| 96131_at | 0.00 |
| 96132_at | 0.00 |
| 96134_at | 0.12 |
| 96135_at | 0.00 |
| 96136_at | 0.00 |
| 96138_at | 0.00 |
| 96139_at | 0.04 |
| 96140_at | 0.01 |
| 96143_at | 0.00 |
| 96144_at | 0.00 |
| 96145_at | 0.00 |
| 96146_at | 0.00 |
| 96147_at | 0.00 |
| 96148_at | 0.00 |
| 96151_at | 0.00 |
| 96152_at | 0.10 |
| 96153_at | 0.00 |
| 96154_at | 0.00 |
| 96155_at | 0.00 |
| 96156_at | 0.61 |
| 96157_at | 0.00 |
| 96158_at | 0.05 |
| 96160_at | 0.00 |
| 96162_at | 1.00 |
| 96165_at | 0.00 |

| | |
|---|---|
| 96166_at | 0.00 |
| 96167_at | 0.37 |
| 96168_at | 0.00 |
| 96169_at | 0.00 |
| 96171_at | 0.00 |
| 96172_at | 0.00 |
| 96174_at | 0.13 |
| 96176_at | 0.00 |
| 96177_at | 0.00 |
| 96178_at | 0.02 |
| 96180_at | 0.00 |
| 96183_at | 0.01 |
| 96184_at | 0.05 |
| 96185_at | 0.00 |
| 96186_at | 0.23 |
| 96187_at | 0.00 |
| 96188_at | 0.11 |
| 96189_at | 0.35 |
| 96191_at | 0.05 |
| 96192_at | 0.03 |
| 96193_at | 0.00 |
| 96195_at | 0.00 |
| 96196_i_at | 0.00 |
| 96197_f_at | 0.00 |
| 96198_at | 0.00 |
| 96199_at | 0.02 |
| 96200_at | 0.00 |
| 96201_at | 0.00 |
| 96202_at | 0.00 |
| 96203_at | 0.27 |
| 96204_at | 0.00 |
| 96205_at | 0.00 |
| 96206_at | 0.00 |
| 96207_at | 0.00 |
| 96208_at | 0.01 |
| 96211_at | 0.00 |
| 96212_at | 0.00 |
| 96214_at | 0.00 |
| 96215_f_at | 0.01 |
| 96216_at | 0.00 |
| 96217_at | 0.00 |
| 96218_at | 0.00 |
| 96219_at | 0.00 |
| 96220_at | 0.00 |
| 96221_at | 0.00 |
| 96222_at | 0.01 |
| 96223_at | 0.00 |
| 96224_at | 0.00 |
| 96226_at | 0.00 |
| 96227_at | 0.00 |
| 96228_at | 0.00 |
| 96229_at | 0.00 |
| 96230_at | 0.00 |
| 96231_at | 0.00 |
| 96232_at | 0.00 |
| 96234_at | 0.00 |

| | |
|---|---|
| 96236_at | 0.00 |
| 96237_at | 0.00 |
| 96238_at | 0.05 |
| 96239_at | 0.00 |
| 96240_at | 0.00 |
| 96241_at | 0.00 |
| 96242_at | 0.00 |
| 96243_f_at | 0.00 |
| 96244_at | 0.00 |
| 96245_at | 0.00 |
| 96249_at | 0.00 |
| 96252_at | 0.00 |
| 96254_at | 0.00 |
| 96255_at | 0.00 |
| 96256_at | 0.00 |
| 96257_at | 0.00 |
| 96258_at | 0.08 |
| 96259_at | 0.00 |
| 96260_at | 0.06 |
| 96261_at | 0.00 |
| 96262_at | 0.00 |
| 96263_at | 0.00 |
| 96264_at | 0.00 |
| 96266_at | 0.00 |
| 96267_at | 0.00 |
| 96268_at | 0.00 |
| 96269_at | 0.00 |
| 96270_at | 0.00 |
| 96271_at | 0.00 |
| 96272_at | 0.33 |
| 96273_at | 0.00 |
| 96275_f_at | 0.02 |
| 96276_r_at | 0.00 |
| 96277_at | 0.00 |
| 96278_at | 0.07 |
| 96280_at | 0.00 |
| 96281_at | 0.00 |
| 96283_at | 0.00 |
| 96284_at | 0.01 |
| 96285_at | 0.00 |
| 96286_at | 0.00 |
| 96287_at | 0.00 |
| 96288_at | 0.34 |
| 96289_at | 0.00 |
| 96290_f_at | 0.00 |
| 96291_f_at | 0.00 |
| 96292_r_at | 0.00 |
| 96293_at | 0.00 |
| 96294_s_at | 0.00 |
| 96295_at | 0.27 |
| 96296_at | 0.11 |
| 96297_at | 0.00 |
| 96298_f_at | 0.00 |
| 96299_at | 0.03 |
| 96300_f_at | 0.00 |
| 96301_at | 0.00 |

| | |
|---|---|
| 96302_at | 0.02 |
| 96305_at | 0.00 |
| 96306_at | 0.00 |
| 96307_s_at | 0.00 |
| 96308_r_at | 0.00 |
| 96309_r_at | 0.03 |
| 96310_at | 0.00 |
| 96311_at | 0.00 |
| 96313_at | 0.00 |
| 96316_at | 0.01 |
| 96318_at | 0.00 |
| 96319_at | 0.00 |
| 96320_at | 0.00 |
| 96321_at | 0.00 |
| 96322_at | 0.04 |
| 96324_at | 0.00 |
| 96325_at | 0.01 |
| 96326_at | 0.00 |
| 96327_at | 0.00 |
| 96329_at | 0.00 |
| 96331_at | 0.00 |
| 96332_at | 0.00 |
| 96333_g_at | 0.00 |
| 96334_f_at | 0.00 |
| 96335_at | 0.00 |
| 96336_at | 0.77 |
| 96337_at | 0.00 |
| 96338_at | 0.00 |
| 96339_at | 0.00 |
| 96340_at | 0.04 |
| 96341_at | 0.00 |
| 96342_at | 0.52 |
| 96343_at | 0.00 |
| 96344_at | 0.00 |
| 96345_at | 0.01 |
| 96346_at | 0.00 |
| 96347_at | 0.00 |
| 96348_at | 0.00 |
| 96351_at | 0.01 |
| 96352_at | 0.02 |
| 96353_at | 0.01 |
| 96354_at | 0.00 |
| 96355_at | 0.00 |
| 96356_at | 0.00 |
| 96357_at | 0.00 |
| 96358_at | 0.00 |
| 96359_at | 0.00 |
| 96360_at | 0.02 |
| 96365_at | 0.00 |
| 96367_at | 0.00 |
| 96373_at | 0.00 |
| 96374_at | 0.00 |
| 96375_at | 0.06 |
| 96387_at | 0.00 |
| 96388_at | 0.00 |
| 96389_at | 0.00 |

| | |
|---|---|
| 96390_at | 0.00 |
| 96391_at | 0.00 |
| 96392_at | 0.00 |
| 96393_at | 0.00 |
| 96394_at | 0.00 |
| 96400_at | 0.00 |
| 96408_at | 0.00 |
| 96413_at | 0.00 |
| 96414_at | 0.00 |
| 96416_f_at | 0.00 |
| 96417_s_at | 0.00 |
| 96418_r_at | 0.00 |
| 96419_f_at | 0.00 |
| 96420_at | 0.00 |
| 96421_at | 0.00 |
| 96422_at | 0.00 |
| 96423_at | 0.00 |
| 96424_at | 0.00 |
| 96426_at | 0.60 |
| 96431_at | 0.00 |
| 96433_at | 0.00 |
| 96435_at | 0.00 |
| 96464_at | 0.00 |
| 96474_at | 0.00 |
| 96481_at | 0.00 |
| 96482_at | 0.00 |
| 96483_at | 0.00 |
| 96484_at | 0.00 |
| 96485_at | 0.00 |
| 96486_at | 0.00 |
| 96487_at | 0.00 |
| 96488_at | 0.00 |
| 96489_at | 0.16 |
| 96490_at | 0.00 |
| 96491_at | 0.02 |
| 96492_at | 0.00 |
| 96493_at | 0.00 |
| 96494_at | 0.27 |
| 96495_at | 0.00 |
| 96496_g_at | 0.00 |
| 96497_s_at | 0.00 |
| 96498_at | 0.00 |
| 96499_at | 0.00 |
| 96500_at | 0.00 |
| 96501_at | 0.00 |
| 96502_at | 0.00 |
| 96503_at | 0.00 |
| 96504_at | 0.00 |
| 96505_at | 0.00 |
| 96506_at | 0.00 |
| 96507_at | 0.00 |
| 96508_at | 0.00 |
| 96509_at | 0.00 |
| 96510_at | 0.00 |
| 96511_s_at | 0.00 |
| 96512_at | 0.00 |

| | |
|---|---|
| 96513_at | 0.00 |
| 96514_at | 0.00 |
| 96515_at | 0.00 |
| 96516_at | 0.00 |
| 96517_at | 0.00 |
| 96518_at | 0.01 |
| 96519_at | 0.00 |
| 96520_at | 0.00 |
| 96521_at | 0.00 |
| 96522_at | 0.00 |
| 96523_at | 0.00 |
| 96524_at | 0.00 |
| 96525_at | 0.00 |
| 96526_at | 0.00 |
| 96527_at | 0.00 |
| 96528_at | 0.00 |
| 96529_at | 0.00 |
| 96530_at | 0.04 |
| 96531_at | 0.00 |
| 96532_at | 0.09 |
| 96533_at | 0.00 |
| 96534_at | 0.00 |
| 96535_at | 0.00 |
| 96536_at | 0.00 |
| 96537_at | 0.00 |
| 96538_at | 0.00 |
| 96539_at | 0.02 |
| 96540_at | 0.00 |
| 96541_at | 0.00 |
| 96542_at | 0.00 |
| 96543_at | 0.00 |
| 96544_at | 0.00 |
| 96545_s_at | 0.00 |
| 96546_r_at | 0.00 |
| 96547_at | 0.00 |
| 96548_at | 0.00 |
| 96549_at | 0.00 |
| 96550_at | 0.00 |
| 96551_at | 0.00 |
| 96552_at | 0.00 |
| 96553_at | 0.00 |
| 96554_r_at | 0.00 |
| 96555_at | 0.00 |
| 96556_at | 0.00 |
| 96557_at | 0.08 |
| 96558_at | 0.00 |
| 96559_at | 0.00 |
| 96560_at | 0.00 |
| 96561_at | 0.54 |
| 96562_at | 0.00 |
| 96563_at | 0.00 |
| 96564_at | 0.00 |
| 96565_at | 0.00 |
| 96566_at | 0.00 |
| 96567_at | 0.00 |
| 96568_at | 0.00 |

| | |
|---|---|
| 96569_at | 0.00 |
| 96570_at | 0.00 |
| 96571_at | 0.00 |
| 96572_at | 0.00 |
| 96573_at | 0.00 |
| 96574_at | 0.00 |
| 96575_at | 0.00 |
| 96576_at | 0.00 |
| 96577_i_at | 0.06 |
| 96578_r_at | 0.00 |
| 96579_at | 0.00 |
| 96580_at | 0.60 |
| 96581_at | 0.00 |
| 96582_at | 0.00 |
| 96583_s_at | 0.00 |
| 96584_f_at | 0.44 |
| 96585_at | 0.00 |
| 96586_at | 0.00 |
| 96587_at | 0.02 |
| 96588_at | 0.00 |
| 96589_at | 0.00 |
| 96590_f_at | 0.00 |
| 96591_at | 0.06 |
| 96592_at | 0.00 |
| 96593_at | 0.00 |
| 96594_at | 0.09 |
| 96595_at | 0.00 |
| 96596_at | 0.00 |
| 96597_at | 0.00 |
| 96598_at | 0.02 |
| 96599_at | 0.01 |
| 96600_at | 0.00 |
| 96601_at | 0.00 |
| 96602_g_at | 0.00 |
| 96603_at | 0.05 |
| 96604_at | 0.00 |
| 96605_at | 0.01 |
| 96606_at | 0.00 |
| 96607_at | 0.00 |
| 96608_at | 0.00 |
| 96609_at | 0.00 |
| 96610_at | 0.00 |
| 96611_at | 0.00 |
| 96613_at | 0.00 |
| 96614_at | 0.00 |
| 96615_at | 0.00 |
| 96616_at | 0.00 |
| 96617_at | 0.00 |
| 96618_at | 0.00 |
| 96619_at | 0.00 |
| 96620_at | 0.00 |
| 96621_at | 0.00 |
| 96623_at | 0.00 |
| 96624_r_at | 0.00 |
| 96625_at | 0.00 |
| 96626_at | 0.03 |

| | |
|---|---|
| 96627_at | 0.01 |
| 96628_at | 0.01 |
| 96629_at | 0.00 |
| 96630_at | 0.01 |
| 96632_at | 0.00 |
| 96633_s_at | 0.01 |
| 96634_at | 0.00 |
| 96635_at | 0.00 |
| 96636_at | 0.02 |
| 96637_at | 0.00 |
| 96638_at | 0.00 |
| 96639_at | 0.00 |
| 96640_at | 0.00 |
| 96641_at | 0.16 |
| 96643_at | 0.00 |
| 96644_at | 0.00 |
| 96646_at | 0.00 |
| 96647_at | 0.00 |
| 96648_at | 0.02 |
| 96649_at | 0.00 |
| 96650_at | 0.00 |
| 96651_at | 0.26 |
| 96652_at | 0.00 |
| 96653_at | 0.00 |
| 96654_at | 0.00 |
| 96655_g_at | 0.00 |
| 96656_at | 0.00 |
| 96657_at | 0.00 |
| 96658_at | 0.00 |
| 96661_at | 0.00 |
| 96662_at | 0.11 |
| 96663_at | 0.00 |
| 96664_at | 0.00 |
| 96665_at | 0.00 |
| 96666_at | 0.01 |
| 96667_at | 0.00 |
| 96668_at | 0.00 |
| 96669_at | 0.00 |
| 96670_at | 0.00 |
| 96672_at | 0.03 |
| 96674_at | 0.00 |
| 96675_at | 0.00 |
| 96676_at | 0.00 |
| 96677_at | 0.25 |
| 96678_at | 0.10 |
| 96679_at | 0.52 |
| 96680_at | 0.80 |
| 96682_at | 0.00 |
| 96684_at | 0.01 |
| 96685_at | 0.00 |
| 96686_i_at | 0.32 |
| 96687_f_at | 0.29 |
| 96688_at | 0.00 |
| 96691_at | 0.00 |
| 96692_at | 0.00 |
| 96693_at | 0.00 |

| | |
|---|---|
| 96694_at | 0.00 |
| 96695_at | 0.06 |
| 96696_at | 0.00 |
| 96698_at | 0.00 |
| 96699_at | 0.00 |
| 96700_r_at | 0.00 |
| 96701_at | 0.00 |
| 96703_at | 0.00 |
| 96704_at | 0.57 |
| 96707_at | 0.06 |
| 96708_at | 0.00 |
| 96709_at | 0.00 |
| 96710_at | 0.00 |
| 96711_at | 0.00 |
| 96712_at | 0.00 |
| 96713_at | 0.00 |
| 96716_at | 0.03 |
| 96717_at | 0.00 |
| 96718_at | 0.00 |
| 96719_i_at | 0.00 |
| 96720_f_at | 0.00 |
| 96722_at | 0.00 |
| 96723_f_at | 0.00 |
| 96724_r_at | 0.00 |
| 96725_at | 0.15 |
| 96726_at | 0.00 |
| 96728_at | 0.00 |
| 96729_at | 0.00 |
| 96730_at | 0.00 |
| 96731_at | 0.00 |
| 96732_at | 0.00 |
| 96733_at | 0.17 |
| 96734_at | 0.00 |
| 96735_at | 0.12 |
| 96736_at | 0.00 |
| 96737_at | 0.00 |
| 96738_at | 0.06 |
| 96739_at | 0.00 |
| 96741_at | 0.00 |
| 96742_at | 0.00 |
| 96743_at | 0.00 |
| 96744_at | 0.10 |
| 96745_at | 0.00 |
| 96746_at | 0.00 |
| 96747_at | 0.31 |
| 96748_i_at | 0.50 |
| 96749_f_at | 0.00 |
| 96750_at | 0.00 |
| 96751_at | 0.00 |
| 96752_at | 0.99 |
| 96753_at | 0.02 |
| 96754_s_at | 0.00 |
| 96755_at | 0.00 |
| 96756_at | 0.00 |
| 96757_at | 0.00 |
| 96758_s_at | 0.00 |

| | |
|---|---|
| 96759_r_at | 0.03 |
| 96760_at | 0.00 |
| 96761_at | 0.00 |
| 96762_at | 0.00 |
| 96763_at | 0.00 |
| 96764_at | 0.00 |
| 96765_at | 0.00 |
| 96766_s_at | 0.01 |
| 96767_at | 0.00 |
| 96768_at | 0.00 |
| 96770_at | 0.00 |
| 96771_at | 0.66 |
| 96772_at | 0.00 |
| 96773_at | 0.00 |
| 96774_at | 0.01 |
| 96775_at | 0.00 |
| 96777_at | 0.01 |
| 96778_at | 0.00 |
| 96779_f_at | 0.00 |
| 96780_at | 0.01 |
| 96781_at | 0.01 |
| 96782_at | 0.00 |
| 96783_at | 0.00 |
| 96784_at | 0.00 |
| 96785_at | 0.00 |
| 96786_s_at | 0.00 |
| 96787_at | 0.00 |
| 96789_i_at | 0.00 |
| 96790_f_at | 0.00 |
| 96791_at | 0.00 |
| 96792_at | 0.00 |
| 96793_at | 0.00 |
| 96794_at | 0.00 |
| 96795_at | 0.00 |
| 96796_f_at | 0.00 |
| 96797_s_at | 0.00 |
| 96798_r_at | 0.00 |
| 96799_at | 0.00 |
| 96801_at | 0.05 |
| 96802_at | 0.00 |
| 96803_at | 0.23 |
| 96804_at | 0.00 |
| 96806_at | 0.00 |
| 96807_at | 0.00 |
| 96808_at | 0.00 |
| 96810_at | 0.00 |
| 96811_at | 0.21 |
| 96812_at | 0.40 |
| 96813_f_at | 0.00 |
| 96814_r_at | 0.00 |
| 96817_at | 0.00 |
| 96818_at | 0.00 |
| 96819_at | 0.00 |
| 96822_at | 0.00 |
| 96824_at | 0.03 |
| 96825_at | 0.00 |

| | |
|---|---|
| 96826_at | 0.00 |
| 96827_at | 0.01 |
| 96828_at | 0.00 |
| 96829_at | 0.00 |
| 96831_at | 0.00 |
| 96832_at | 0.13 |
| 96833_at | 0.01 |
| 96834_at | 0.00 |
| 96835_at | 0.00 |
| 96836_r_at | 0.00 |
| 96837_at | 0.00 |
| 96838_at | 0.00 |
| 96839_at | 0.00 |
| 96840_at | 0.52 |
| 96841_at | 1.00 |
| 96843_at | 0.00 |
| 96845_at | 0.00 |
| 96846_at | 0.00 |
| 96847_at | 0.00 |
| 96848_at | 0.00 |
| 96849_at | 0.00 |
| 96850_at | 0.05 |
| 96852_at | 0.00 |
| 96854_at | 0.00 |
| 96855_at | 0.00 |
| 96856_at | 0.00 |
| 96857_at | 0.00 |
| 96858_at | 0.09 |
| 96859_at | 0.00 |
| 96861_at | 0.00 |
| 96862_at | 0.01 |
| 96864_at | 0.00 |
| 96865_at | 0.00 |
| 96866_at | 0.01 |
| 96867_at | 0.00 |
| 96868_at | 0.00 |
| 96869_at | 0.00 |
| 96870_at | 0.00 |
| 96871_at | 0.00 |
| 96872_at | 0.00 |
| 96873_at | 0.00 |
| 96874_g_at | 0.00 |
| 96875_r_at | 0.02 |
| 96876_at | 0.02 |
| 96878_at | 0.00 |
| 96879_at | 0.00 |
| 96881_at | 0.00 |
| 96882_at | 0.00 |
| 96883_at | 0.00 |
| 96884_at | 0.00 |
| 96885_at | 0.03 |
| 96886_at | 0.00 |
| 96887_at | 0.00 |
| 96888_at | 0.01 |
| 96890_at | 0.00 |
| 96891_at | 0.00 |

| | |
|---|---|
| 96892_at | 0.00 |
| 96894_at | 0.00 |
| 96895_at | 0.00 |
| 96896_at | 0.06 |
| 96898_at | 0.00 |
| 96899_at | 0.00 |
| 96900_at | 0.62 |
| 96902_at | 0.05 |
| 96904_at | 0.00 |
| 96905_at | 0.00 |
| 96906_at | 0.00 |
| 96907_at | 0.00 |
| 96908_at | 0.00 |
| 96909_at | 0.00 |
| 96910_at | 0.00 |
| 96911_at | 0.01 |
| 96912_s_at | 0.01 |
| 96913_at | 0.00 |
| 96915_f_at | 0.01 |
| 96916_at | 0.00 |
| 96917_at | 0.00 |
| 96918_at | 0.00 |
| 96919_at | 0.00 |
| 96920_at | 0.27 |
| 96921_at | 0.00 |
| 96924_at | 0.02 |
| 96925_at | 0.00 |
| 96926_at | 0.00 |
| 96929_at | 0.00 |
| 96930_at | 0.00 |
| 96931_at | 0.00 |
| 96932_at | 0.00 |
| 96934_at | 0.00 |
| 96935_at | 0.00 |
| 96936_at | 0.00 |
| 96937_at | 0.00 |
| 96938_at | 0.00 |
| 96939_at | 0.00 |
| 96940_at | 0.78 |
| 96941_at | 0.00 |
| 96942_at | 0.00 |
| 96943_at | 0.00 |
| 96945_at | 0.00 |
| 96946_at | 0.00 |
| 96947_at | 0.03 |
| 96948_at | 0.07 |
| 96949_at | 0.05 |
| 96950_at | 0.00 |
| 96951_at | 0.00 |
| 96952_at | 0.00 |
| 96953_at | 0.00 |
| 96954_at | 0.00 |
| 96955_at | 0.00 |
| 96956_at | 0.00 |
| 96957_at | 0.00 |
| 96958_at | 0.00 |

| | |
|---|---|
| 96959_at | 0.00 |
| 96961_at | 0.01 |
| 96962_at | 0.00 |
| 96963_s_at | 0.00 |
| 96964_at | 0.00 |
| 96965_at | 0.00 |
| 96966_at | 0.00 |
| 96967_at | 0.00 |
| 96968_at | 0.00 |
| 96969_at | 0.00 |
| 96970_at | 0.00 |
| 96971_f_at | 0.00 |
| 96972_f_at | 0.00 |
| 96973_f_at | 0.00 |
| 96974_at | 0.00 |
| 96975_at | 0.00 |
| 96976_f_at | 0.00 |
| 96977_at | 0.00 |
| 96978_at | 0.00 |
| 96979_at | 0.00 |
| 96980_at | 0.01 |
| 96981_at | 0.00 |
| 96982_g_at | 0.00 |
| 96983_at | 0.00 |
| 96984_at | 0.00 |
| 96985_at | 0.00 |
| 96986_at | 0.00 |
| 96987_at | 0.00 |
| 96992_r_at | 0.00 |
| 96993_at | 0.00 |
| 96994_at | 0.00 |
| 96995_at | 0.00 |
| 96996_at | 0.00 |
| 96997_at | 0.00 |
| 96998_at | 0.00 |
| 96999_at | 0.00 |
| 97000_s_at | 0.00 |
| 97001_r_at | 0.00 |
| 97002_f_at | 0.00 |
| 97003_at | 0.00 |
| 97004_at | 0.00 |
| 97005_at | 0.00 |
| 97006_s_at | 0.00 |
| 97007_at | 0.00 |
| 97008_f_at | 0.00 |
| 97009_f_at | 0.00 |
| 97010_at | 0.00 |
| 97012_f_at | 0.00 |
| 97013_f_at | 0.01 |
| 97017_f_at | 0.00 |
| 97048_at | 0.00 |
| 97049_at | 0.00 |
| 97052_at | 0.00 |
| 97053_at | 0.00 |
| 97054_at | 0.00 |
| 97055_s_at | 0.00 |

| | |
|---|---|
| 97058_f_at | 0.00 |
| 97060_at | 0.00 |
| 97061_g_at | 0.00 |
| 97073_at | 0.00 |
| 97077_f_at | 0.00 |
| 97080_at | 0.00 |
| 97082_at | 0.00 |
| 97083_at | 0.99 |
| 97084_at | 0.00 |
| 97085_at | 0.00 |
| 97086_i_at | 0.00 |
| 97087_f_at | 0.00 |
| 97088_at | 0.00 |
| 97089_at | 0.00 |
| 97090_at | 0.00 |
| 97091_at | 0.00 |
| 97092_at | 0.00 |
| 97094_at | 0.00 |
| 97095_at | 0.00 |
| 97096_at | 0.00 |
| 97097_at | 0.00 |
| 97098_at | 0.00 |
| 97099_at | 0.00 |
| 97100_at | 0.00 |
| 97101_at | 0.00 |
| 97102_at | 0.00 |
| 97103_at | 0.00 |
| 97104_g_at | 0.00 |
| 97105_at | 0.00 |
| 97106_at | 0.00 |
| 97107_at | 0.00 |
| 97108_at | 0.00 |
| 97109_at | 0.00 |
| 97110_at | 0.00 |
| 97111_at | 0.15 |
| 97112_at | 0.00 |
| 97113_at | 0.00 |
| 97114_at | 0.00 |
| 97115_at | 0.00 |
| 97116_at | 0.01 |
| 97117_at | 0.00 |
| 97118_at | 0.00 |
| 97119_at | 0.00 |
| 97120_at | 0.00 |
| 97121_at | 0.00 |
| 97122_at | 0.00 |
| 97123_at | 0.00 |
| 97124_at | 0.01 |
| 97125_f_at | 0.00 |
| 97126_at | 0.00 |
| 97127_f_at | 0.02 |
| 97128_at | 0.00 |
| 97129_at | 0.05 |
| 97130_at | 0.01 |
| 97131_at | 0.00 |
| 97132_at | 0.00 |

| | |
|---|---|
| 97133_at | 0.00 |
| 97134_at | 0.00 |
| 97135_at | 0.00 |
| 97136_at | 0.00 |
| 97137_at | 0.00 |
| 97138_at | 0.00 |
| 97139_at | 0.00 |
| 97140_at | 0.00 |
| 97141_s_at | 0.00 |
| 97142_at | 0.00 |
| 97143_at | 0.00 |
| 97144_at | 0.00 |
| 97145_at | 0.00 |
| 97146_g_at | 0.00 |
| 97147_at | 0.00 |
| 97148_at | 0.01 |
| 97149_at | 0.00 |
| 97150_at | 0.00 |
| 97151_at | 0.00 |
| 97152_at | 0.00 |
| 97153_at | 0.00 |
| 97154_f_at | 0.29 |
| 97155_at | 0.00 |
| 97156_at | 0.00 |
| 97157_at | 0.00 |
| 97158_at | 0.00 |
| 97159_at | 0.00 |
| 97160_at | 0.01 |
| 97161_at | 0.22 |
| 97162_at | 0.00 |
| 97163_g_at | 0.00 |
| 97164_at | 0.00 |
| 97165_r_at | 0.13 |
| 97166_at | 0.00 |
| 97167_g_at | 0.00 |
| 97168_at | 0.00 |
| 97169_f_at | 0.68 |
| 97170_at | 0.00 |
| 97171_f_at | 0.00 |
| 97172_s_at | 0.00 |
| 97173_f_at | 0.00 |
| 97174_r_at | 0.00 |
| 97175_at | 0.00 |
| 97176_at | 0.00 |
| 97177_at | 0.00 |
| 97178_at | 0.00 |
| 97179_at | 0.00 |
| 97180_f_at | 0.00 |
| 97181_f_at | 0.00 |
| 97182_at | 0.00 |
| 97184_at | 0.00 |
| 97185_at | 0.00 |
| 97186_s_at | 0.00 |
| 97187_at | 0.00 |
| 97188_at | 0.00 |
| 97189_at | 0.00 |

| | |
|---|---|
| 97190_f_at | 0.00 |
| 97191_at | 0.00 |
| 97192_at | 0.00 |
| 97193_at | 0.48 |
| 97194_at | 0.00 |
| 97195_at | 0.01 |
| 97196_at | 0.00 |
| 97197_r_at | 0.61 |
| 97198_at | 0.01 |
| 97199_at | 0.00 |
| 97200_f_at | 0.00 |
| 97201_s_at | 0.00 |
| 97203_at | 0.01 |
| 97204_s_at | 0.00 |
| 97205_at | 0.00 |
| 97206_at | 0.53 |
| 97207_f_at | 0.00 |
| 97208_at | 0.00 |
| 97210_at | 0.00 |
| 97211_at | 0.12 |
| 97213_at | 0.00 |
| 97216_at | 0.00 |
| 97217_at | 0.00 |
| 97220_at | 0.00 |
| 97222_at | 0.00 |
| 97224_at | 0.00 |
| 97226_at | 0.00 |
| 97227_at | 0.08 |
| 97228_at | 0.00 |
| 97229_at | 0.00 |
| 97232_at | 0.00 |
| 97234_at | 0.00 |
| 97235_f_at | 0.00 |
| 97236_r_at | 0.01 |
| 97237_at | 0.00 |
| 97238_at | 0.01 |
| 97239_at | 0.00 |
| 97240_g_at | 0.00 |
| 97241_at | 0.00 |
| 97242_at | 0.00 |
| 97243_at | 0.02 |
| 97247_at | 0.00 |
| 97248_at | 0.00 |
| 97249_at | 0.00 |
| 97250_at | 0.00 |
| 97251_at | 0.00 |
| 97252_at | 0.00 |
| 97253_at | 0.00 |
| 97254_at | 0.00 |
| 97255_at | 0.11 |
| 97256_at | 0.00 |
| 97257_at | 0.00 |
| 97258_at | 0.06 |
| 97259_at | 0.12 |
| 97260_at | 0.33 |
| 97261_at | 0.00 |

| | |
|---|---|
| 97262_at | 0.00 |
| 97263_s_at | 0.01 |
| 97264_r_at | 0.00 |
| 97265_at | 0.00 |
| 97267_at | 0.00 |
| 97268_i_at | 0.00 |
| 97269_f_at | 0.02 |
| 97270_at | 0.00 |
| 97271_at | 0.00 |
| 97272_at | 0.00 |
| 97273_at | 0.00 |
| 97274_at | 0.00 |
| 97276_at | 0.00 |
| 97277_at | 0.00 |
| 97278_at | 0.00 |
| 97279_at | 0.17 |
| 97281_at | 0.00 |
| 97282_at | 0.00 |
| 97283_at | 0.83 |
| 97284_at | 0.00 |
| 97285_f_at | 0.00 |
| 97287_at | 0.00 |
| 97288_at | 0.00 |
| 97292_at | 0.00 |
| 97293_at | 0.00 |
| 97295_at | 0.00 |
| 97296_at | 0.00 |
| 97297_at | 0.00 |
| 97300_at | 0.00 |
| 97301_at | 0.00 |
| 97302_at | 0.09 |
| 97304_at | 0.01 |
| 97305_at | 0.00 |
| 97307_f_at | 0.00 |
| 97308_at | 0.00 |
| 97309_at | 0.00 |
| 97310_at | 0.00 |
| 97311_at | 0.00 |
| 97312_at | 0.00 |
| 97313_at | 0.00 |
| 97315_at | 0.00 |
| 97316_at | 0.00 |
| 97317_at | 1.00 |
| 97318_at | 0.00 |
| 97319_at | 0.09 |
| 97320_at | 0.00 |
| 97322_at | 0.00 |
| 97324_at | 0.01 |
| 97325_at | 0.00 |
| 97327_at | 0.00 |
| 97328_at | 0.00 |
| 97329_at | 0.00 |
| 97330_at | 0.00 |
| 97331_at | 0.00 |
| 97332_at | 0.00 |
| 97333_at | 0.00 |

| | |
|---|---|
| 97334_at | 0.78 |
| 97335_at | 0.00 |
| 97336_at | 0.00 |
| 97338_at | 0.00 |
| 97339_at | 0.00 |
| 97340_at | 0.00 |
| 97341_at | 0.00 |
| 97342_at | 0.00 |
| 97343_at | 0.00 |
| 97345_at | 0.03 |
| 97346_at | 0.00 |
| 97347_at | 0.05 |
| 97349_at | 0.00 |
| 97351_i_at | 0.07 |
| 97352_f_at | 0.03 |
| 97353_at | 0.00 |
| 97354_at | 0.00 |
| 97355_at | 0.00 |
| 97356_at | 0.00 |
| 97357_at | 0.00 |
| 97358_at | 0.05 |
| 97359_at | 0.00 |
| 97360_at | 0.00 |
| 97363_at | 0.00 |
| 97364_at | 0.00 |
| 97365_at | 0.00 |
| 97366_at | 0.00 |
| 97367_at | 0.00 |
| 97368_at | 0.00 |
| 97369_g_at | 0.01 |
| 97370_at | 0.00 |
| 97371_at | 0.00 |
| 97372_at | 0.00 |
| 97373_at | 0.00 |
| 97374_at | 0.00 |
| 97375_at | 0.00 |
| 97377_at | 0.00 |
| 97379_at | 0.00 |
| 97380_at | 0.00 |
| 97381_s_at | 0.00 |
| 97382_at | 0.00 |
| 97383_at | 0.07 |
| 97384_at | 0.00 |
| 97385_at | 0.00 |
| 97386_at | 0.00 |
| 97387_at | 0.00 |
| 97389_at | 0.00 |
| 97390_at | 0.00 |
| 97391_at | 0.00 |
| 97392_at | 0.00 |
| 97393_at | 0.00 |
| 97394_at | 0.06 |
| 97395_at | 0.00 |
| 97397_at | 0.00 |
| 97398_at | 0.00 |
| 97400_at | 0.00 |

| | |
|---|---|
| 97401_at | 0.01 |
| 97402_at | 0.00 |
| 97403_at | 0.00 |
| 97404_at | 0.00 |
| 97405_at | 0.00 |
| 97406_at | 0.22 |
| 97407_at | 0.00 |
| 97409_at | 0.09 |
| 97410_at | 0.00 |
| 97411_at | 0.18 |
| 97412_at | 0.00 |
| 97413_at | 0.00 |
| 97414_at | 0.00 |
| 97415_at | 0.00 |
| 97418_at | 0.00 |
| 97419_at | 0.00 |
| 97420_at | 0.00 |
| 97421_at | 0.00 |
| 97422_at | 0.00 |
| 97423_at | 0.00 |
| 97424_at | 0.01 |
| 97425_at | 0.00 |
| 97426_at | 0.96 |
| 97427_at | 0.00 |
| 97428_at | 0.00 |
| 97429_at | 0.02 |
| 97430_at | 0.00 |
| 97431_at | 0.00 |
| 97433_at | 0.00 |
| 97434_at | 0.00 |
| 97435_at | 0.00 |
| 97436_at | 0.00 |
| 97437_f_at | 0.00 |
| 97438_r_at | 0.00 |
| 97441_at | 0.00 |
| 97442_at | 0.14 |
| 97443_at | 0.00 |
| 97444_at | 0.77 |
| 97445_at | 0.00 |
| 97446_at | 0.00 |
| 97447_at | 0.00 |
| 97448_at | 0.00 |
| 97449_at | 0.00 |
| 97450_s_at | 0.00 |
| 97451_at | 0.00 |
| 97452_at | 0.73 |
| 97456_at | 0.63 |
| 97458_at | 0.12 |
| 97459_at | 0.00 |
| 97460_at | 0.07 |
| 97462_at | 0.00 |
| 97463_g_at | 0.00 |
| 97464_at | 0.00 |
| 97465_at | 0.00 |
| 97468_at | 0.00 |
| 97469_at | 0.00 |

| | |
|---|---|
| 97470_at | 0.00 |
| 97471_at | 0.00 |
| 97472_at | 0.00 |
| 97473_at | 0.00 |
| 97474_r_at | 0.00 |
| 97475_at | 0.00 |
| 97477_at | 0.02 |
| 97478_at | 0.00 |
| 97479_at | 0.00 |
| 97480_f_at | 0.00 |
| 97481_r_at | 0.00 |
| 97482_at | 0.00 |
| 97483_at | 0.00 |
| 97484_at | 0.22 |
| 97485_at | 0.00 |
| 97486_at | 0.02 |
| 97487_at | 0.00 |
| 97488_at | 0.00 |
| 97489_at | 0.00 |
| 97490_at | 0.01 |
| 97491_at | 0.13 |
| 97492_at | 0.04 |
| 97496_f_at | 0.00 |
| 97497_at | 0.00 |
| 97498_at | 0.04 |
| 97499_at | 0.00 |
| 97500_g_at | 0.00 |
| 97502_at | 0.00 |
| 97504_at | 0.00 |
| 97505_at | 0.00 |
| 97506_at | 0.00 |
| 97507_at | 0.21 |
| 97508_at | 0.00 |
| 97509_f_at | 0.08 |
| 97510_at | 0.00 |
| 97511_at | 0.00 |
| 97512_at | 0.00 |
| 97514_at | 0.00 |
| 97515_at | 0.00 |
| 97516_at | 0.00 |
| 97517_at | 0.00 |
| 97518_at | 0.00 |
| 97519_at | 0.99 |
| 97520_s_at | 0.97 |
| 97521_at | 0.00 |
| 97523_i_at | 0.00 |
| 97524_f_at | 0.00 |
| 97525_at | 0.00 |
| 97526_at | 0.00 |
| 97527_at | 0.00 |
| 97528_at | 0.00 |
| 97529_at | 0.02 |
| 97530_at | 0.00 |
| 97531_at | 0.00 |
| 97532_at | 0.00 |
| 97533_at | 0.00 |

| | |
|---|---|
| 97535_at | 0.00 |
| 97536_at | 0.01 |
| 97538_at | 0.00 |
| 97539_at | 0.00 |
| 97540_f_at | 0.00 |
| 97541_f_at | 0.00 |
| 97542_at | 0.00 |
| 97543_at | 0.00 |
| 97544_at | 0.00 |
| 97546_at | 0.00 |
| 97548_at | 0.00 |
| 97549_at | 0.00 |
| 97550_at | 0.00 |
| 97551_at | 0.00 |
| 97553_at | 0.00 |
| 97554_at | 0.00 |
| 97555_at | 0.00 |
| 97556_at | 0.01 |
| 97557_at | 0.16 |
| 97559_at | 0.00 |
| 97560_at | 0.01 |
| 97561_at | 0.00 |
| 97562_r_at | 0.00 |
| 97563_f_at | 0.00 |
| 97564_f_at | 0.00 |
| 97565_r_at | 0.00 |
| 97566_f_at | 0.00 |
| 97567_f_at | 0.00 |
| 97568_at | 0.00 |
| 97569_at | 0.00 |
| 97570_at | 0.00 |
| 97571_r_at | 0.00 |
| 97572_at | 0.00 |
| 97573_at | 0.00 |
| 97574_f_at | 0.00 |
| 97575_f_at | 0.00 |
| 97576_f_at | 0.00 |
| 97577_f_at | 0.00 |
| 97578_at | 0.00 |
| 97579_f_at | 0.00 |
| 97580_at | 0.00 |
| 97581_at | 0.00 |
| 97593_f_at | 0.00 |
| 97594_r_at | 0.00 |
| 97598_at | 0.00 |
| 97601_f_at | 0.00 |
| 97610_f_at | 0.00 |
| 97645_f_at | 0.56 |
| 97647_at | 0.00 |
| 97651_at | 0.00 |
| 97652_at | 0.00 |
| 97653_at | 0.00 |
| 97654_at | 0.00 |
| 97655_at | 0.00 |
| 97658_f_at | 0.00 |
| 97659_r_at | 0.00 |

| | |
|---|---|
| 97660_s_at | 0.00 |
| 97661_at | 0.00 |
| 97665_i_at | 0.00 |
| 97666_r_at | 0.00 |
| 97667_at | 0.00 |
| 97678_r_at | 0.00 |
| 97679_at | 0.00 |
| 97680_at | 0.00 |
| 97681_f_at | 0.00 |
| 97682_r_at | 0.00 |
| 97683_at | 0.00 |
| 97684_at | 0.00 |
| 97685_at | 0.00 |
| 97686_r_at | 0.00 |
| 97687_at | 0.00 |
| 97688_at | 0.00 |
| 97689_at | 0.37 |
| 97690_at | 0.01 |
| 97691_at | 0.00 |
| 97692_at | 0.00 |
| 97693_at | 0.00 |
| 97694_at | 0.00 |
| 97695_s_at | 0.00 |
| 97696_r_at | 0.00 |
| 97697_at | 0.00 |
| 97698_at | 0.00 |
| 97699_at | 0.00 |
| 97700_at | 0.00 |
| 97701_at | 0.00 |
| 97702_at | 0.00 |
| 97703_at | 0.00 |
| 97704_at | 0.00 |
| 97705_at | 0.01 |
| 97706_at | 0.00 |
| 97707_at | 0.00 |
| 97708_at | 0.00 |
| 97709_at | 0.00 |
| 97710_f_at | 0.00 |
| 97711_at | 0.00 |
| 97712_at | 0.00 |
| 97713_at | 0.00 |
| 97714_r_at | 0.00 |
| 97717_at | 0.01 |
| 97718_at | 0.00 |
| 97719_at | 0.00 |
| 97720_at | 0.00 |
| 97721_at | 0.00 |
| 97722_at | 0.02 |
| 97723_at | 0.00 |
| 97724_at | 0.00 |
| 97725_at | 0.00 |
| 97726_at | 0.00 |
| 97727_at | 0.00 |
| 97728_at | 0.00 |
| 97729_at | 0.00 |
| 97730_at | 0.00 |

| | |
|---|---|
| 97731_at | 0.00 |
| 97732_at | 0.00 |
| 97733_at | 0.00 |
| 97734_at | 0.00 |
| 97735_at | 0.00 |
| 97736_at | 0.28 |
| 97737_f_at | 0.00 |
| 97738_r_at | 0.00 |
| 97739_at | 0.00 |
| 97740_at | 0.49 |
| 97741_at | 0.00 |
| 97742_s_at | 0.04 |
| 97743_at | 0.00 |
| 97744_at | 0.00 |
| 97745_at | 0.00 |
| 97746_f_at | 0.00 |
| 97747_r_at | 0.00 |
| 97748_at | 0.00 |
| 97749_at | 0.00 |
| 97750_at | 0.00 |
| 97751_f_at | 0.00 |
| 97752_at | 0.00 |
| 97753_at | 0.00 |
| 97754_at | 0.00 |
| 97755_at | 0.00 |
| 97756_s_at | 0.00 |
| 97757_at | 0.00 |
| 97758_at | 0.00 |
| 97759_at | 0.00 |
| 97760_at | 0.00 |
| 97761_f_at | 0.00 |
| 97762_f_at | 0.00 |
| 97763_at | 0.00 |
| 97764_at | 0.00 |
| 97765_g_at | 0.00 |
| 97766_at | 0.00 |
| 97767_at | 0.00 |
| 97768_at | 0.00 |
| 97769_at | 0.00 |
| 97770_s_at | 0.09 |
| 97771_r_at | 0.00 |
| 97772_at | 0.00 |
| 97773_at | 0.00 |
| 97774_at | 0.00 |
| 97775_at | 0.00 |
| 97776_at | 0.00 |
| 97777_at | 0.00 |
| 97778_at | 0.00 |
| 97779_at | 0.29 |
| 97780_at | 0.00 |
| 97781_at | 0.00 |
| 97782_at | 0.00 |
| 97783_at | 0.00 |
| 97784_at | 0.00 |
| 97785_at | 0.00 |
| 97786_at | 0.00 |

| | |
|---|---|
| 97787_at | 0.00 |
| 97788_at | 0.00 |
| 97789_at | 0.74 |
| 97790_s_at | 0.00 |
| 97791_at | 0.00 |
| 97792_at | 0.00 |
| 97793_at | 0.00 |
| 97794_at | 0.00 |
| 97795_at | 0.00 |
| 97796_at | 0.00 |
| 97797_at | 0.00 |
| 97798_at | 0.11 |
| 97800_at | 0.00 |
| 97802_at | 0.00 |
| 97803_at | 0.05 |
| 97807_at | 0.00 |
| 97808_at | 0.08 |
| 97809_at | 0.00 |
| 97811_at | 0.00 |
| 97812_at | 0.00 |
| 97813_at | 0.00 |
| 97814_at | 0.00 |
| 97816_at | 0.04 |
| 97817_at | 0.02 |
| 97818_at | 0.00 |
| 97819_at | 0.01 |
| 97820_at | 0.14 |
| 97821_at | 0.00 |
| 97822_at | 0.00 |
| 97823_g_at | 0.00 |
| 97824_at | 0.00 |
| 97825_at | 0.09 |
| 97826_at | 0.00 |
| 97828_at | 0.00 |
| 97829_at | 0.00 |
| 97830_at | 0.00 |
| 97832_at | 0.00 |
| 97833_at | 0.14 |
| 97834_g_at | 0.21 |
| 97835_at | 0.00 |
| 97836_at | 0.00 |
| 97838_at | 0.00 |
| 97839_at | 0.00 |
| 97841_at | 0.00 |
| 97843_at | 0.00 |
| 97844_at | 0.00 |
| 97845_at | 0.00 |
| 97846_at | 0.49 |
| 97847_at | 0.00 |
| 97848_at | 0.03 |
| 97849_at | 0.00 |
| 97853_at | 0.00 |
| 97857_at | 0.04 |
| 97858_at | 0.00 |
| 97859_at | 0.00 |
| 97860_at | 0.00 |

| | |
|---|---|
| 97861_at | 0.00 |
| 97862_s_at | 0.00 |
| 97863_at | 0.00 |
| 97864_at | 0.02 |
| 97865_g_at | 0.03 |
| 97866_at | 0.03 |
| 97867_at | 0.00 |
| 97868_at | 0.00 |
| 97869_at | 0.00 |
| 97870_s_at | 0.00 |
| 97871_at | 0.00 |
| 97873_at | 0.00 |
| 97874_at | 0.16 |
| 97875_at | 0.00 |
| 97876_at | 0.00 |
| 97880_at | 0.01 |
| 97882_at | 0.00 |
| 97884_at | 0.00 |
| 97885_at | 0.00 |
| 97886_at | 0.00 |
| 97887_at | 0.00 |
| 97888_at | 0.00 |
| 97889_at | 0.00 |
| 97890_at | 0.88 |
| 97891_at | 0.00 |
| 97892_at | 0.03 |
| 97893_at | 0.00 |
| 97894_at | 0.01 |
| 97895_f_at | 0.02 |
| 97896_r_at | 0.13 |
| 97897_at | 0.00 |
| 97900_at | 0.00 |
| 97901_at | 0.00 |
| 97903_at | 0.00 |
| 97904_at | 0.00 |
| 97906_at | 0.00 |
| 97907_at | 0.00 |
| 97908_at | 0.00 |
| 97909_at | 0.03 |
| 97910_at | 0.00 |
| 97911_at | 0.01 |
| 97912_at | 0.00 |
| 97914_at | 0.00 |
| 97915_at | 0.00 |
| 97916_at | 0.00 |
| 97917_at | 0.00 |
| 97918_at | 0.32 |
| 97919_at | 0.01 |
| 97920_at | 0.00 |
| 97921_at | 0.00 |
| 97922_at | 0.00 |
| 97923_at | 0.00 |
| 97924_at | 0.00 |
| 97925_at | 0.01 |
| 97926_s_at | 0.00 |
| 97927_at | 0.00 |

| | |
|---|---|
| 97928_at | 0.00 |
| 97929_r_at | 0.00 |
| 97930_f_at | 0.08 |
| 97931_i_at | 0.00 |
| 97932_f_at | 0.01 |
| 97933_at | 0.00 |
| 97934_at | 0.00 |
| 97935_at | 0.00 |
| 97936_at | 0.00 |
| 97937_at | 0.87 |
| 97939_at | 0.00 |
| 97940_at | 0.00 |
| 97941_at | 0.00 |
| 97942_g_at | 0.00 |
| 97943_at | 0.00 |
| 97944_f_at | 0.00 |
| 97945_at | 0.00 |
| 97946_at | 0.00 |
| 97947_at | 0.00 |
| 97948_at | 0.01 |
| 97949_at | 0.00 |
| 97950_at | 0.00 |
| 97951_s_at | 0.00 |
| 97952_at | 0.00 |
| 97953_g_at | 0.00 |
| 97954_at | 0.00 |
| 97955_at | 0.00 |
| 97956_g_at | 0.00 |
| 97957_at | 0.00 |
| 97958_at | 0.60 |
| 97960_at | 0.07 |
| 97962_at | 0.00 |
| 97963_at | 0.00 |
| 97964_at | 0.00 |
| 97965_at | 0.00 |
| 97966_at | 0.00 |
| 97967_at | 0.00 |
| 97969_at | 0.00 |
| 97970_at | 0.00 |
| 97971_at | 0.00 |
| 97972_at | 0.01 |
| 97973_at | 0.00 |
| 97974_at | 0.00 |
| 97975_at | 0.06 |
| 97976_at | 0.00 |
| 97977_at | 0.39 |
| 97979_at | 0.00 |
| 97980_at | 0.00 |
| 97982_at | 0.00 |
| 97983_s_at | 0.00 |
| 97984_i_at | 0.00 |
| 97985_f_at | 0.00 |
| 97986_at | 0.00 |
| 97987_at | 0.00 |
| 97988_at | 0.00 |
| 97989_at | 0.00 |

| | |
|---|---|
| 97990_at | 0.00 |
| 97991_at | 0.55 |
| 97992_at | 0.00 |
| 97993_at | 0.00 |
| 97994_at | 0.74 |
| 97995_at | 0.22 |
| 97996_at | 0.03 |
| 97997_at | 0.00 |
| 97998_at | 0.05 |
| 98000_at | 0.00 |
| 98001_at | 0.27 |
| 98002_at | 0.00 |
| 98003_at | 0.00 |
| 98004_at | 0.00 |
| 98005_at | 0.00 |
| 98006_at | 0.00 |
| 98007_at | 0.01 |
| 98008_at | 0.03 |
| 98010_at | 0.00 |
| 98011_at | 0.00 |
| 98013_at | 0.00 |
| 98014_at | 0.00 |
| 98015_at | 0.00 |
| 98016_at | 0.03 |
| 98017_at | 0.00 |
| 98018_at | 0.04 |
| 98019_at | 0.00 |
| 98020_at | 0.00 |
| 98021_at | 0.00 |
| 98022_at | 0.00 |
| 98023_r_at | 0.00 |
| 98024_at | 0.00 |
| 98025_at | 0.00 |
| 98026_g_at | 0.00 |
| 98027_at | 0.00 |
| 98028_at | 0.00 |
| 98029_at | 0.00 |
| 98030_at | 0.00 |
| 98031_at | 0.02 |
| 98032_at | 0.00 |
| 98033_at | 0.06 |
| 98034_at | 0.00 |
| 98035_g_at | 0.00 |
| 98036_at | 0.00 |
| 98037_at | 0.00 |
| 98038_at | 0.03 |
| 98039_at | 0.00 |
| 98040_at | 0.00 |
| 98041_at | 0.00 |
| 98042_at | 0.37 |
| 98044_at | 0.00 |
| 98045_s_at | 0.00 |
| 98047_at | 0.01 |
| 98048_at | 0.00 |
| 98049_at | 0.00 |
| 98051_at | 0.00 |

| | |
|---|---|
| 98052_at | 0.00 |
| 98053_at | 0.00 |
| 98054_at | 0.00 |
| 98055_at | 0.00 |
| 98056_at | 0.00 |
| 98057_at | 0.00 |
| 98059_s_at | 0.33 |
| 98060_at | 0.00 |
| 98061_at | 0.00 |
| 98063_at | 0.00 |
| 98064_at | 0.00 |
| 98065_at | 0.00 |
| 98066_r_at | 0.00 |
| 98067_at | 0.00 |
| 98069_s_at | 0.00 |
| 98070_at | 0.00 |
| 98071_f_at | 0.00 |
| 98072_r_at | 0.04 |
| 98073_at | 0.00 |
| 98075_at | 0.00 |
| 98076_at | 0.00 |
| 98077_at | 0.09 |
| 98078_at | 0.00 |
| 98079_at | 0.03 |
| 98081_at | 0.00 |
| 98082_at | 0.00 |
| 98083_at | 0.00 |
| 98084_at | 0.09 |
| 98085_f_at | 0.00 |
| 98086_r_at | 0.00 |
| 98087_at | 0.00 |
| 98088_at | 0.00 |
| 98089_at | 0.00 |
| 98090_at | 0.00 |
| 98092_at | 0.00 |
| 98094_f_at | 0.01 |
| 98096_f_at | 0.00 |
| 98097_r_at | 0.00 |
| 98098_at | 0.00 |
| 98099_at | 0.00 |
| 98101_at | 0.00 |
| 98102_at | 0.00 |
| 98104_at | 0.19 |
| 98106_at | 0.00 |
| 98107_at | 0.00 |
| 98108_at | 0.28 |
| 98109_at | 0.00 |
| 98110_at | 0.00 |
| 98111_at | 0.00 |
| 98112_r_at | 0.33 |
| 98113_at | 0.00 |
| 98114_at | 0.00 |
| 98116_at | 0.00 |
| 98117_at | 0.00 |
| 98118_at | 0.00 |
| 98119_at | 0.00 |

| | |
|---|---|
| 98120_at | 0.11 |
| 98121_at | 0.01 |
| 98122_at | 0.18 |
| 98123_at | 0.00 |
| 98124_at | 0.00 |
| 98125_at | 0.00 |
| 98126_s_at | 0.00 |
| 98127_at | 0.04 |
| 98128_at | 0.00 |
| 98129_at | 0.00 |
| 98130_at | 0.00 |
| 98131_at | 0.00 |
| 98132_at | 0.00 |
| 98133_at | 0.01 |
| 98134_at | 0.07 |
| 98135_r_at | 0.00 |
| 98136_at | 0.01 |
| 98137_at | 0.00 |
| 98138_at | 0.06 |
| 98139_at | 0.00 |
| 98140_at | 0.00 |
| 98141_at | 0.01 |
| 98142_at | 0.00 |
| 98143_at | 0.00 |
| 98144_f_at | 0.00 |
| 98146_at | 0.00 |
| 98147_at | 0.00 |
| 98148_at | 0.00 |
| 98149_s_at | 0.00 |
| 98150_at | 0.00 |
| 98151_s_at | 0.03 |
| 98152_at | 0.00 |
| 98153_at | 0.00 |
| 98154_at | 0.00 |
| 98155_r_at | 0.00 |
| 98168_at | 0.00 |
| 98169_s_at | 0.00 |
| 98240_at | 0.00 |
| 98245_at | 0.02 |
| 98247_at | 0.00 |
| 98254_f_at | 0.00 |
| 98276_at | 0.00 |
| 98277_at | 0.00 |
| 98278_at | 0.00 |
| 98279_at | 0.00 |
| 98280_at | 0.00 |
| 98281_at | 0.00 |
| 98282_at | 0.00 |
| 98283_at | 0.06 |
| 98284_f_at | 0.00 |
| 98285_at | 0.00 |
| 98286_at | 0.00 |
| 98287_at | 0.00 |
| 98288_at | 0.00 |
| 98289_at | 0.00 |
| 98290_at | 0.00 |

| | |
|---|---|
| 98291_at | 0.00 |
| 98292_at | 0.00 |
| 98293_g_at | 0.00 |
| 98294_at | 0.00 |
| 98295_at | 0.00 |
| 98296_at | 0.00 |
| 98297_at | 0.00 |
| 98298_at | 0.00 |
| 98299_s_at | 0.00 |
| 98300_at | 0.00 |
| 98301_at | 0.00 |
| 98302_at | 0.00 |
| 98303_at | 0.00 |
| 98304_at | 0.00 |
| 98305_at | 0.01 |
| 98306_g_at | 0.00 |
| 98307_at | 0.00 |
| 98308_at | 0.00 |
| 98309_at | 0.00 |
| 98310_at | 0.00 |
| 98311_at | 0.00 |
| 98312_at | 0.00 |
| 98313_at | 0.02 |
| 98314_g_at | 0.00 |
| 98315_at | 0.00 |
| 98316_at | 0.00 |
| 98317_at | 0.00 |
| 98318_at | 0.00 |
| 98319_at | 0.00 |
| 98320_at | 0.03 |
| 98321_at | 0.00 |
| 98322_at | 0.00 |
| 98323_at | 0.00 |
| 98324_at | 0.00 |
| 98325_at | 0.00 |
| 98326_f_at | 0.00 |
| 98327_at | 0.00 |
| 98328_at | 0.00 |
| 98329_at | 0.00 |
| 98330_at | 0.08 |
| 98331_at | 0.07 |
| 98332_at | 0.00 |
| 98333_at | 0.00 |
| 98334_at | 0.00 |
| 98335_at | 0.00 |
| 98336_s_at | 0.04 |
| 98337_at | 0.00 |
| 98338_at | 0.00 |
| 98339_at | 0.00 |
| 98340_at | 0.00 |
| 98341_f_at | 0.00 |
| 98342_at | 0.00 |
| 98343_s_at | 0.00 |
| 98344_f_at | 0.00 |
| 98345_at | 0.00 |
| 98346_at | 0.02 |

| | |
|---|---|
| 98347_at | 0.00 |
| 98348_at | 0.00 |
| 98349_at | 0.16 |
| 98350_at | 0.00 |
| 98351_g_at | 0.00 |
| 98352_r_at | 0.00 |
| 98353_at | 0.00 |
| 98354_at | 0.00 |
| 98355_at | 0.00 |
| 98356_at | 0.00 |
| 98357_at | 0.00 |
| 98358_at | 0.00 |
| 98360_at | 0.00 |
| 98361_at | 0.00 |
| 98362_s_at | 0.00 |
| 98363_at | 0.00 |
| 98364_at | 0.00 |
| 98365_at | 0.00 |
| 98366_at | 0.00 |
| 98367_at | 0.00 |
| 98368_at | 0.00 |
| 98369_f_at | 0.00 |
| 98370_at | 0.00 |
| 98371_at | 0.00 |
| 98372_at | 0.00 |
| 98373_at | 0.00 |
| 98374_at | 0.00 |
| 98375_at | 0.00 |
| 98376_at | 0.00 |
| 98379_r_at | 0.00 |
| 98380_at | 0.00 |
| 98381_at | 0.00 |
| 98382_f_at | 0.00 |
| 98383_r_at | 0.00 |
| 98384_at | 0.00 |
| 98385_at | 0.00 |
| 98386_s_at | 0.00 |
| 98387_at | 0.00 |
| 98388_at | 0.00 |
| 98389_at | 0.00 |
| 98390_at | 0.00 |
| 98391_at | 0.00 |
| 98392_at | 0.00 |
| 98394_at | 0.00 |
| 98395_at | 0.00 |
| 98397_at | 0.00 |
| 98398_s_at | 0.00 |
| 98400_at | 0.00 |
| 98401_at | 0.00 |
| 98402_at | 0.00 |
| 98403_at | 0.00 |
| 98404_at | 0.00 |
| 98405_at | 0.00 |
| 98406_at | 0.00 |
| 98407_at | 0.00 |
| 98408_at | 0.00 |

| | |
|---|---|
| 98409_at | 0.00 |
| 98410_at | 0.00 |
| 98413_at | 0.00 |
| 98414_at | 0.73 |
| 98415_at | 0.01 |
| 98416_at | 0.00 |
| 98417_at | 0.00 |
| 98418_at | 0.00 |
| 98419_at | 0.00 |
| 98420_at | 0.00 |
| 98421_at | 0.02 |
| 98423_at | 0.00 |
| 98424_at | 0.40 |
| 98426_at | 0.00 |
| 98427_s_at | 0.00 |
| 98428_at | 0.00 |
| 98429_at | 0.00 |
| 98430_at | 0.00 |
| 98431_at | 0.00 |
| 98432_at | 0.00 |
| 98433_at | 0.00 |
| 98434_at | 0.00 |
| 98435_at | 0.00 |
| 98436_s_at | 0.01 |
| 98437_at | 0.00 |
| 98438_f_at | 0.00 |
| 98439_at | 0.00 |
| 98440_at | 0.51 |
| 98441_at | 0.00 |
| 98443_at | 0.00 |
| 98444_g_at | 0.00 |
| 98445_at | 0.00 |
| 98446_s_at | 0.00 |
| 98447_at | 0.00 |
| 98448_at | 0.00 |
| 98449_at | 0.00 |
| 98451_at | 0.00 |
| 98452_at | 0.00 |
| 98453_at | 0.00 |
| 98454_at | 0.00 |
| 98455_at | 0.00 |
| 98456_at | 0.00 |
| 98457_at | 0.01 |
| 98458_at | 0.00 |
| 98459_at | 0.00 |
| 98460_at | 0.00 |
| 98461_at | 0.00 |
| 98462_s_at | 0.00 |
| 98463_at | 0.00 |
| 98464_at | 0.00 |
| 98465_f_at | 0.26 |
| 98466_r_at | 0.00 |
| 98467_at | 0.00 |
| 98468_r_at | 0.00 |
| 98469_at | 0.00 |
| 98470_at | 0.00 |

| | |
|---|---|
| 98471_f_at | 0.01 |
| 98472_at | 0.01 |
| 98473_at | 0.00 |
| 98474_r_at | 0.00 |
| 98475_at | 0.00 |
| 98476_at | 0.00 |
| 98477_s_at | 0.00 |
| 98478_at | 0.20 |
| 98479_f_at | 0.00 |
| 98480_s_at | 0.00 |
| 98481_at | 0.00 |
| 98482_at | 0.00 |
| 98483_at | 0.00 |
| 98484_at | 0.00 |
| 98485_at | 0.03 |
| 98486_at | 0.00 |
| 98488_at | 0.00 |
| 98489_at | 0.00 |
| 98490_at | 0.08 |
| 98491_at | 0.00 |
| 98492_at | 0.00 |
| 98493_at | 0.00 |
| 98495_at | 0.00 |
| 98496_at | 0.00 |
| 98497_at | 0.00 |
| 98498_at | 0.00 |
| 98499_s_at | 0.00 |
| 98500_at | 0.68 |
| 98501_at | 0.50 |
| 98502_at | 0.00 |
| 98503_at | 0.01 |
| 98504_at | 0.01 |
| 98505_i_at | 0.00 |
| 98506_r_at | 0.00 |
| 98507_at | 0.00 |
| 98508_s_at | 0.26 |
| 98509_at | 0.00 |
| 98511_at | 0.37 |
| 98512_at | 0.00 |
| 98513_at | 0.00 |
| 98514_at | 0.71 |
| 98515_at | 0.00 |
| 98516_at | 0.00 |
| 98518_f_at | 0.08 |
| 98519_r_at | 0.00 |
| 98521_at | 0.16 |
| 98522_at | 0.00 |
| 98523_at | 0.00 |
| 98524_f_at | 0.00 |
| 98525_f_at | 0.00 |
| 98526_r_at | 0.00 |
| 98527_at | 0.00 |
| 98528_at | 0.01 |
| 98529_at | 0.00 |
| 98530_at | 0.00 |
| 98531_g_at | 0.17 |

| | |
|---|---|
| 98532_at | 0.01 |
| 98533_at | 0.43 |
| 98534_at | 0.00 |
| 98535_at | 0.12 |
| 98538_at | 0.00 |
| 98539_at | 0.00 |
| 98540_g_at | 0.00 |
| 98543_at | 0.15 |
| 98544_at | 0.00 |
| 98545_at | 0.00 |
| 98547_at | 0.01 |
| 98549_at | 0.00 |
| 98550_at | 0.16 |
| 98552_at | 0.00 |
| 98553_at | 0.00 |
| 98554_at | 0.00 |
| 98555_at | 0.00 |
| 98556_at | 0.00 |
| 98557_f_at | 0.00 |
| 98558_r_at | 0.00 |
| 98559_at | 0.00 |
| 98560_at | 0.00 |
| 98561_at | 0.00 |
| 98562_at | 0.01 |
| 98563_f_at | 0.00 |
| 98564_f_at | 0.00 |
| 98565_at | 0.00 |
| 98568_at | 0.00 |
| 98569_at | 0.00 |
| 98570_at | 0.00 |
| 98571_s_at | 0.00 |
| 98572_at | 0.01 |
| 98573_r_at | 0.00 |
| 98574_at | 0.00 |
| 98575_at | 0.00 |
| 98577_f_at | 0.66 |
| 98578_at | 0.00 |
| 98579_at | 0.96 |
| 98580_at | 0.00 |
| 98582_at | 0.00 |
| 98583_at | 0.00 |
| 98586_at | 0.00 |
| 98587_at | 0.00 |
| 98588_at | 0.00 |
| 98589_at | 0.00 |
| 98590_at | 0.01 |
| 98593_at | 0.00 |
| 98594_at | 0.00 |
| 98595_at | 0.00 |
| 98596_s_at | 0.00 |
| 98597_at | 0.00 |
| 98598_at | 0.00 |
| 98599_at | 0.00 |
| 98600_at | 0.01 |
| 98602_at | 0.00 |
| 98603_s_at | 0.07 |

| | |
|---|---|
| 98604_at | 0.00 |
| 98605_at | 0.00 |
| 98606_s_at | 0.00 |
| 98608_at | 0.00 |
| 98609_at | 0.00 |
| 98610_at | 0.00 |
| 98612_at | 0.00 |
| 98613_at | 0.00 |
| 98614_at | 0.00 |
| 98615_at | 0.00 |
| 98616_f_at | 0.00 |
| 98617_at | 0.00 |
| 98618_at | 0.00 |
| 98619_at | 0.00 |
| 98621_at | 0.00 |
| 98622_at | 0.00 |
| 98623_g_at | 0.16 |
| 98624_at | 0.00 |
| 98625_s_at | 0.00 |
| 98626_at | 0.00 |
| 98627_at | 0.00 |
| 98628_f_at | 0.00 |
| 98629_f_at | 0.00 |
| 98630_at | 0.00 |
| 98631_g_at | 0.01 |
| 98632_at | 0.00 |
| 98633_at | 0.00 |
| 98635_at | 0.00 |
| 98726_at | 0.00 |
| 98727_at | 0.00 |
| 98728_at | 0.00 |
| 98729_at | 0.00 |
| 98730_at | 0.65 |
| 98731_at | 0.00 |
| 98732_at | 0.00 |
| 98756_at | 0.02 |
| 98758_at | 0.39 |
| 98759_f_at | 0.00 |
| 98760_at | 0.00 |
| 98761_i_at | 0.02 |
| 98762_f_at | 0.07 |
| 98763_at | 0.00 |
| 98764_at | 0.00 |
| 98765_f_at | 0.00 |
| 98766_at | 0.00 |
| 98767_at | 0.00 |
| 98768_at | 0.00 |
| 98770_at | 0.00 |
| 98771_at | 0.00 |
| 98772_at | 0.02 |
| 98773_s_at | 0.00 |
| 98774_at | 0.00 |
| 98775_at | 0.00 |
| 98776_at | 0.00 |
| 98777_at | 0.00 |
| 98778_at | 0.00 |

| | |
|---|---|
| 98779_at | 0.00 |
| 98780_at | 0.00 |
| 98781_at | 0.00 |
| 98782_at | 0.00 |
| 98783_at | 0.00 |
| 98784_at | 0.00 |
| 98785_at | 0.00 |
| 98786_at | 0.00 |
| 98787_at | 0.00 |
| 98788_at | 0.00 |
| 98789_at | 0.00 |
| 98790_s_at | 0.00 |
| 98791_at | 0.00 |
| 98792_at | 0.00 |
| 98793_at | 0.00 |
| 98794_at | 0.00 |
| 98795_at | 0.00 |
| 98796_at | 0.00 |
| 98797_at | 0.00 |
| 98798_at | 0.00 |
| 98799_g_at | 0.00 |
| 98800_at | 0.00 |
| 98801_at | 0.00 |
| 98802_at | 0.00 |
| 98803_at | 0.00 |
| 98804_at | 0.00 |
| 98805_at | 0.00 |
| 98806_s_at | 0.00 |
| 98807_at | 0.00 |
| 98808_at | 0.00 |
| 98809_s_at | 0.00 |
| 98810_at | 0.00 |
| 98811_at | 0.00 |
| 98812_at | 0.00 |
| 98813_at | 0.00 |
| 98814_at | 0.00 |
| 98815_at | 0.50 |
| 98816_s_at | 0.00 |
| 98817_at | 0.08 |
| 98818_at | 0.02 |
| 98819_at | 0.00 |
| 98820_g_at | 0.00 |
| 98821_at | 0.00 |
| 98822_at | 0.03 |
| 98823_at | 0.00 |
| 98824_at | 0.00 |
| 98825_at | 0.00 |
| 98826_at | 0.01 |
| 98827_i_at | 0.00 |
| 98828_at | 0.00 |
| 98829_at | 0.00 |
| 98830_at | 0.00 |
| 98831_at | 0.00 |
| 98832_at | 0.00 |
| 98833_at | 0.44 |
| 98834_at | 0.00 |

| | |
|---|---|
| 98835_at | 0.00 |
| 98836_at | 0.00 |
| 98837_at | 0.00 |
| 98838_at | 0.00 |
| 98839_at | 0.00 |
| 98840_at | 0.00 |
| 98841_at | 0.00 |
| 98842_at | 0.00 |
| 98843_at | 0.04 |
| 98844_at | 0.00 |
| 98845_at | 0.47 |
| 98846_f_at | 0.00 |
| 98847_at | 0.00 |
| 98848_at | 0.00 |
| 98849_at | 0.00 |
| 98850_at | 0.00 |
| 98851_at | 0.00 |
| 98852_at | 0.01 |
| 98853_at | 0.00 |
| 98854_at | 0.00 |
| 98855_r_at | 0.58 |
| 98856_at | 0.00 |
| 98857_at | 0.00 |
| 98858_at | 0.00 |
| 98859_at | 0.00 |
| 98860_at | 0.00 |
| 98861_at | 0.00 |
| 98862_at | 0.00 |
| 98863_at | 0.00 |
| 98864_s_at | 0.00 |
| 98865_at | 0.00 |
| 98866_at | 0.00 |
| 98867_at | 0.00 |
| 98868_at | 0.01 |
| 98869_g_at | 0.00 |
| 98870_at | 0.00 |
| 98871_at | 0.00 |
| 98872_at | 0.00 |
| 98873_at | 0.00 |
| 98874_at | 0.01 |
| 98875_at | 0.00 |
| 98876_at | 0.00 |
| 98878_r_at | 0.00 |
| 98880_at | 0.11 |
| 98881_at | 0.00 |
| 98882_s_at | 0.00 |
| 98883_r_at | 0.00 |
| 98884_r_at | 0.00 |
| 98886_at | 0.00 |
| 98887_at | 0.00 |
| 98889_at | 0.00 |
| 98890_at | 0.00 |
| 98891_at | 0.00 |
| 98892_at | 0.00 |
| 98893_at | 0.00 |
| 98894_at | 0.00 |

| | |
|---|---|
| 98896_at | 0.26 |
| 98901_at | 0.00 |
| 98902_at | 0.00 |
| 98903_at | 0.00 |
| 98904_at | 0.00 |
| 98905_at | 0.00 |
| 98906_at | 0.00 |
| 98908_at | 0.00 |
| 98909_at | 0.00 |
| 98910_at | 0.00 |
| 98911_at | 0.00 |
| 98912_at | 0.00 |
| 98914_at | 0.00 |
| 98915_at | 0.05 |
| 98916_at | 0.00 |
| 98917_at | 0.00 |
| 98918_at | 0.00 |
| 98919_at | 0.00 |
| 98920_g_at | 0.00 |
| 98921_at | 0.00 |
| 98922_at | 0.00 |
| 98923_at | 0.00 |
| 98924_at | 0.00 |
| 98925_at | 0.00 |
| 98926_at | 0.00 |
| 98927_at | 0.04 |
| 98928_at | 0.00 |
| 98929_at | 0.00 |
| 98930_at | 0.00 |
| 98931_at | 0.00 |
| 98932_at | 0.00 |
| 98933_at | 0.00 |
| 98934_at | 0.00 |
| 98936_at | 0.04 |
| 98937_at | 0.00 |
| 98938_at | 0.00 |
| 98940_at | 0.00 |
| 98941_r_at | 0.03 |
| 98942_r_at | 0.00 |
| 98943_at | 0.00 |
| 98944_at | 0.00 |
| 98945_at | 0.00 |
| 98946_at | 0.12 |
| 98947_at | 0.00 |
| 98948_at | 0.00 |
| 98950_at | 0.00 |
| 98951_at | 0.00 |
| 98952_at | 0.02 |
| 98953_at | 0.00 |
| 98954_f_at | 0.00 |
| 98955_at | 0.00 |
| 98956_at | 0.12 |
| 98957_at | 0.00 |
| 98958_at | 0.05 |
| 98959_at | 0.06 |
| 98960_s_at | 0.00 |

| | |
|---|---|
| 98961_at | 0.00 |
| 98962_at | 0.00 |
| 98963_at | 0.00 |
| 98965_at | 0.00 |
| 98966_at | 0.03 |
| 98967_at | 0.00 |
| 98968_at | 0.00 |
| 98969_at | 0.00 |
| 98970_at | 0.00 |
| 98971_at | 0.00 |
| 98972_at | 0.00 |
| 98973_at | 0.00 |
| 98974_at | 0.00 |
| 98975_at | 0.02 |
| 98976_at | 0.00 |
| 98977_at | 0.00 |
| 98979_at | 0.00 |
| 98980_at | 0.00 |
| 98981_s_at | 0.00 |
| 98982_at | 0.01 |
| 98983_at | 0.00 |
| 98984_f_at | 0.09 |
| 98987_at | 0.00 |
| 98988_at | 0.06 |
| 98989_at | 0.02 |
| 98990_at | 0.01 |
| 98991_at | 0.05 |
| 98992_at | 0.00 |
| 98993_at | 0.00 |
| 98994_at | 0.00 |
| 98995_at | 0.00 |
| 98996_at | 0.00 |
| 98997_f_at | 0.00 |
| 98998_r_at | 0.00 |
| 98999_at | 0.00 |
| 99000_at | 0.00 |
| 99001_at | 0.29 |
| 99004_r_at | 0.00 |
| 99005_at | 0.02 |
| 99006_at | 0.01 |
| 99007_at | 0.00 |
| 99009_at | 0.11 |
| 99010_at | 0.00 |
| 99011_at | 0.81 |
| 99012_at | 0.00 |
| 99013_f_at | 0.00 |
| 99014_at | 0.00 |
| 99015_at | 0.00 |
| 99016_at | 0.00 |
| 99018_at | 0.00 |
| 99019_at | 0.00 |
| 99020_at | 0.00 |
| 99021_at | 0.00 |
| 99023_at | 0.00 |
| 99024_at | 0.00 |
| 99025_at | 0.00 |

| | |
|---|---|
| 99026_at | 0.00 |
| 99027_at | 0.00 |
| 99028_at | 0.00 |
| 99029_at | 0.00 |
| 99030_at | 0.00 |
| 99031_at | 0.00 |
| 99032_at | 0.01 |
| 99033_at | 0.32 |
| 99034_at | 0.00 |
| 99035_at | 0.00 |
| 99036_s_at | 0.00 |
| 99037_at | 0.00 |
| 99038_at | 0.00 |
| 99039_g_at | 0.02 |
| 99040_at | 0.00 |
| 99041_at | 0.01 |
| 99042_s_at | 0.00 |
| 99043_s_at | 0.00 |
| 99044_at | 0.00 |
| 99045_at | 0.00 |
| 99046_at | 0.00 |
| 99047_at | 0.00 |
| 99048_g_at | 0.00 |
| 99049_at | 0.00 |
| 99050_at | 0.00 |
| 99051_at | 0.34 |
| 99052_at | 0.54 |
| 99053_at | 0.00 |
| 99054_at | 0.00 |
| 99055_at | 0.00 |
| 99056_at | 0.01 |
| 99057_at | 0.20 |
| 99058_at | 0.05 |
| 99059_at | 0.00 |
| 99062_at | 0.00 |
| 99063_at | 0.00 |
| 99064_at | 0.00 |
| 99065_at | 0.00 |
| 99067_at | 0.00 |
| 99068_at | 0.00 |
| 99069_at | 0.00 |
| 99070_at | 0.00 |
| 99071_at | 0.01 |
| 99073_at | 0.00 |
| 99074_at | 0.00 |
| 99076_at | 0.93 |
| 99077_at | 0.00 |
| 99078_at | 0.00 |
| 99080_at | 0.25 |
| 99081_at | 0.00 |
| 99082_at | 0.00 |
| 99083_at | 0.00 |
| 99084_s_at | 0.00 |
| 99085_at | 0.00 |
| 99086_g_at | 0.00 |
| 99087_at | 0.00 |

| | |
|---|---|
| 99089_at | 0.00 |
| 99093_at | 0.00 |
| 99094_at | 0.00 |
| 99095_at | 0.01 |
| 99096_at | 0.00 |
| 99097_at | 0.00 |
| 99098_at | 0.00 |
| 99099_at | 0.00 |
| 99100_at | 0.12 |
| 99101_at | 0.00 |
| 99102_at | 0.01 |
| 99103_at | 0.00 |
| 99104_at | 0.00 |
| 99106_at | 0.00 |
| 99107_at | 0.00 |
| 99108_s_at | 0.00 |
| 99109_at | 0.79 |
| 99111_at | 0.00 |
| 99112_at | 0.02 |
| 99113_at | 0.00 |
| 99114_r_at | 0.00 |
| 99115_at | 0.00 |
| 99118_at | 0.00 |
| 99119_at | 0.01 |
| 99120_f_at | 0.00 |
| 99121_at | 0.40 |
| 99123_s_at | 0.00 |
| 99124_at | 0.00 |
| 99126_at | 0.00 |
| 99127_at | 0.00 |
| 99128_at | 0.00 |
| 99129_at | 0.00 |
| 99130_at | 0.00 |
| 99133_at | 0.00 |
| 99134_at | 0.00 |
| 99135_at | 0.00 |
| 99136_at | 0.00 |
| 99138_at | 0.00 |
| 99139_at | 0.00 |
| 99140_at | 0.00 |
| 99141_at | 0.00 |
| 99142_at | 0.00 |
| 99143_at | 0.02 |
| 99144_s_at | 0.00 |
| 99146_at | 0.00 |
| 99147_at | 0.00 |
| 99148_at | 0.00 |
| 99149_at | 0.05 |
| 99150_at | 0.00 |
| 99151_at | 0.00 |
| 99152_at | 0.00 |
| 99153_at | 0.00 |
| 99154_s_at | 0.00 |
| 99156_at | 0.00 |
| 99157_at | 0.00 |
| 99158_at | 0.00 |

| | |
|---|---|
| 99159_at | 0.01 |
| 99160_s_at | 0.01 |
| 99161_at | 0.00 |
| 99162_at | 0.00 |
| 99163_at | 0.00 |
| 99164_at | 0.00 |
| 99166_at | 0.00 |
| 99167_at | 0.00 |
| 99168_at | 0.00 |
| 99169_at | 0.01 |
| 99171_at | 0.00 |
| 99172_at | 0.00 |
| 99175_at | 0.01 |
| 99176_at | 0.00 |
| 99178_at | 0.00 |
| 99179_at | 0.00 |
| 99180_at | 0.00 |
| 99182_at | 0.00 |
| 99183_at | 0.00 |
| 99184_at | 0.00 |
| 99185_at | 0.06 |
| 99186_at | 0.00 |
| 99187_f_at | 0.00 |
| 99188_at | 0.00 |
| 99190_at | 0.00 |
| 99191_at | 0.01 |
| 99194_at | 0.00 |
| 99195_at | 0.00 |
| 99196_at | 0.00 |
| 99197_at | 0.00 |
| 99198_at | 0.00 |
| 99236_at | 0.00 |
| 99237_at | 0.00 |
| 99238_at | 0.00 |
| 99239_at | 0.00 |
| 99269_g_at | 0.00 |
| 99320_at | 0.00 |
| 99321_at | 0.00 |
| 99322_at | 0.00 |
| 99323_at | 0.00 |
| 99324_at | 0.00 |
| 99325_at | 0.25 |
| 99326_at | 0.00 |
| 99327_at | 0.00 |
| 99328_at | 0.00 |
| 99329_at | 0.01 |
| 99330_at | 0.00 |
| 99331_at | 0.00 |
| 99332_at | 0.00 |
| 99333_at | 0.00 |
| 99334_at | 0.00 |
| 99335_at | 0.00 |
| 99336_at | 0.00 |
| 99337_at | 0.00 |
| 99338_at | 0.00 |
| 99339_r_at | 0.00 |

| | |
|---|---|
| 99340_at | 0.00 |
| 99341_r_at | 0.00 |
| 99342_at | 0.00 |
| 99343_at | 0.02 |
| 99344_at | 0.00 |
| 99345_at | 0.00 |
| 99346_at | 0.00 |
| 99347_f_at | 0.00 |
| 99348_r_at | 0.00 |
| 99349_at | 0.00 |
| 99350_at | 0.00 |
| 99351_at | 0.00 |
| 99354_s_at | 0.00 |
| 99355_r_at | 0.00 |
| 99356_r_at | 0.00 |
| 99357_at | 0.00 |
| 99358_at | 0.00 |
| 99359_at | 0.00 |
| 99360_at | 0.00 |
| 99361_at | 0.01 |
| 99362_at | 0.00 |
| 99363_at | 0.00 |
| 99364_at | 0.00 |
| 99365_at | 0.00 |
| 99366_at | 0.19 |
| 99367_at | 0.00 |
| 99368_at | 0.00 |
| 99369_f_at | 0.00 |
| 99370_at | 0.00 |
| 99371_at | 0.00 |
| 99372_at | 0.00 |
| 99373_at | 0.00 |
| 99374_at | 0.00 |
| 99375_at | 0.00 |
| 99376_at | 0.00 |
| 99377_at | 0.00 |
| 99378_f_at | 0.00 |
| 99379_f_at | 0.00 |
| 99380_at | 0.00 |
| 99381_at | 0.00 |
| 99382_at | 0.00 |
| 99383_at | 0.00 |
| 99384_at | 0.46 |
| 99385_at | 0.00 |
| 99386_at | 0.00 |
| 99387_at | 0.00 |
| 99388_at | 0.00 |
| 99389_at | 0.00 |
| 99390_at | 0.00 |
| 99391_at | 0.00 |
| 99392_at | 0.00 |
| 99393_at | 0.00 |
| 99394_at | 0.00 |
| 99395_at | 0.00 |
| 99396_at | 0.00 |
| 99397_at | 0.02 |

| | |
|---|---|
| 99398_at | 0.00 |
| 99399_at | 0.00 |
| 99400_at | 0.00 |
| 99401_at | 0.00 |
| 99402_at | 0.00 |
| 99403_at | 0.00 |
| 99404_at | 0.00 |
| 99405_at | 0.00 |
| 99406_at | 0.00 |
| 99407_at | 0.00 |
| 99408_at | 0.00 |
| 99409_at | 0.00 |
| 99410_at | 0.00 |
| 99411_at | 0.00 |
| 99412_at | 0.00 |
| 99413_at | 0.00 |
| 99414_at | 0.00 |
| 99415_at | 0.00 |
| 99416_at | 0.00 |
| 99417_at | 0.00 |
| 99418_at | 0.00 |
| 99419_g_at | 0.00 |
| 99420_at | 0.00 |
| 99421_at | 0.00 |
| 99422_at | 0.00 |
| 99423_at | 0.00 |
| 99424_at | 0.00 |
| 99425_at | 0.06 |
| 99426_at | 0.00 |
| 99427_at | 0.00 |
| 99428_at | 0.00 |
| 99429_at | 0.00 |
| 99430_at | 0.00 |
| 99431_at | 0.00 |
| 99432_at | 0.00 |
| 99433_at | 0.00 |
| 99434_at | 0.00 |
| 99435_at | 0.00 |
| 99436_at | 0.00 |
| 99437_at | 0.00 |
| 99438_at | 0.00 |
| 99439_at | 0.00 |
| 99440_at | 0.75 |
| 99441_at | 0.01 |
| 99442_at | 0.00 |
| 99444_at | 0.00 |
| 99445_at | 0.02 |
| 99446_at | 0.00 |
| 99447_at | 0.00 |
| 99448_at | 0.00 |
| 99449_at | 0.00 |
| 99450_at | 0.00 |
| 99451_at | 0.01 |
| 99452_at | 0.43 |
| 99453_at | 0.00 |
| 99455_s_at | 0.00 |

| | |
|---|---|
| 99456_at | 0.00 |
| 99457_at | 0.02 |
| 99458_i_at | 0.06 |
| 99459_f_at | 0.05 |
| 99460_at | 0.00 |
| 99461_at | 0.00 |
| 99462_at | 0.00 |
| 99463_at | 0.00 |
| 99464_at | 0.00 |
| 99465_at | 0.00 |
| 99466_at | 0.00 |
| 99467_at | 0.00 |
| 99469_at | 0.00 |
| 99471_at | 0.00 |
| 99472_at | 0.00 |
| 99473_at | 0.00 |
| 99474_at | 0.00 |
| 99475_at | 0.11 |
| 99476_at | 0.00 |
| 99477_at | 0.00 |
| 99478_at | 0.00 |
| 99479_at | 0.00 |
| 99481_at | 0.00 |
| 99485_at | 0.01 |
| 99486_at | 0.00 |
| 99488_at | 0.00 |
| 99489_at | 0.00 |
| 99490_at | 0.08 |
| 99491_at | 0.00 |
| 99492_at | 0.00 |
| 99493_at | 0.00 |
| 99494_at | 0.00 |
| 99497_at | 0.00 |
| 99498_at | 0.00 |
| 99499_at | 0.00 |
| 99500_at | 0.00 |
| 99501_at | 0.00 |
| 99502_at | 0.01 |
| 99503_at | 0.00 |
| 99504_at | 0.09 |
| 99505_at | 0.05 |
| 99506_at | 0.00 |
| 99507_at | 0.00 |
| 99508_at | 0.00 |
| 99509_s_at | 0.00 |
| 99510_at | 0.00 |
| 99511_at | 0.00 |
| 99512_at | 0.00 |
| 99513_at | 0.01 |
| 99514_at | 0.07 |
| 99515_at | 0.00 |
| 99516_at | 0.00 |
| 99517_at | 0.00 |
| 99518_at | 0.01 |
| 99521_at | 0.05 |
| 99522_at | 0.00 |

| | |
|---|---|
| 99523_at | 0.00 |
| 99524_at | 0.00 |
| 99525_at | 0.00 |
| 99527_at | 0.00 |
| 99528_at | 0.00 |
| 99529_f_at | 0.47 |
| 99530_at | 0.00 |
| 99531_at | 0.00 |
| 99532_at | 0.34 |
| 99534_at | 0.00 |
| 99535_at | 0.68 |
| 99536_at | 0.00 |
| 99537_at | 0.00 |
| 99541_at | 0.00 |
| 99542_at | 0.00 |
| 99543_s_at | 0.00 |
| 99544_at | 0.00 |
| 99545_at | 0.00 |
| 99546_at | 0.00 |
| 99548_at | 0.00 |
| 99549_at | 0.00 |
| 99551_f_at | 0.00 |
| 99552_at | 0.00 |
| 99553_f_at | 0.00 |
| 99555_at | 0.00 |
| 99556_s_at | 0.00 |
| 99557_at | 0.00 |
| 99558_at | 0.00 |
| 99559_at | 0.00 |
| 99561_f_at | 1.00 |
| 99562_at | 0.00 |
| 99563_at | 0.00 |
| 99564_at | 0.01 |
| 99566_at | 0.00 |
| 99567_at | 0.00 |
| 99569_at | 0.00 |
| 99570_s_at | 0.00 |
| 99571_at | 0.00 |
| 99574_at | 0.01 |
| 99575_at | 0.00 |
| 99576_at | 0.00 |
| 99577_at | 0.00 |
| 99578_at | 0.00 |
| 99579_at | 0.00 |
| 99580_s_at | 0.01 |
| 99581_at | 0.00 |
| 99582_at | 0.48 |
| 99583_at | 0.00 |
| 99584_at | 0.00 |
| 99585_at | 0.00 |
| 99586_at | 0.01 |
| 99587_at | 0.00 |
| 99589_f_at | 0.00 |
| 99590_at | 0.00 |
| 99591_i_at | 0.00 |
| 99592_f_at | 0.00 |

| | |
|---|---|
| 99593_at | 0.00 |
| 99594_at | 0.00 |
| 99595_at | 0.00 |
| 99596_f_at | 0.00 |
| 99597_at | 0.00 |
| 99598_g_at | 0.05 |
| 99599_s_at | 0.01 |
| 99600_at | 0.20 |
| 99602_at | 0.06 |
| 99603_g_at | 0.03 |
| 99604_at | 0.01 |
| 99605_at | 0.00 |
| 99606_at | 0.00 |
| 99607_at | 0.17 |
| 99608_at | 0.00 |
| 99609_at | 0.00 |
| 99610_at | 0.00 |
| 99613_at | 0.00 |
| 99615_at | 0.00 |
| 99616_s_at | 0.00 |
| 99617_at | 0.00 |
| 99618_at | 0.00 |
| 99619_at | 0.00 |
| 99620_at | 0.00 |
| 99621_s_at | 0.06 |
| 99622_at | 1.00 |
| 99623_s_at | 0.00 |
| 99624_at | 0.17 |
| 99626_i_at | 0.00 |
| 99627_r_at | 0.00 |
| 99628_at | 0.02 |
| 99629_at | 0.00 |
| 99630_at | 0.02 |
| 99631_f_at | 0.00 |
| 99632_at | 0.00 |
| 99633_at | 0.09 |
| 99635_at | 0.00 |
| 99636_at | 0.00 |
| 99637_at | 0.00 |
| 99638_at | 0.00 |
| 99639_at | 0.00 |
| 99640_at | 0.01 |
| 99641_at | 0.00 |
| 99642_i_at | 0.01 |
| 99643_f_at | 0.01 |
| 99644_at | 0.00 |
| 99645_at | 0.00 |
| 99646_at | 0.00 |
| 99647_at | 0.00 |
| 99648_at | 0.00 |
| 99649_at | 0.03 |
| 99650_at | 0.02 |
| 99651_at | 0.00 |
| 99652_at | 0.00 |
| 99653_at | 0.00 |
| 99654_s_at | 0.00 |

| | |
|---|---|
| 99655_at | 0.00 |
| 99656_at | 0.00 |
| 99657_at | 0.00 |
| 99658_f_at | 0.00 |
| 99659_r_at | 0.03 |
| 99660_f_at | 0.00 |
| 99661_r_at | 0.00 |
| 99662_at | 0.00 |
| 99663_g_at | 0.00 |
| 99664_at | 0.00 |
| 99665_at | 0.15 |
| 99666_at | 0.00 |
| 99667_at | 0.00 |
| 99668_at | 0.03 |
| 99669_at | 0.30 |
| 99670_at | 0.00 |
| 99671_at | 0.00 |
| 99672_at | 0.00 |
| 99674_at | 0.00 |
| 99675_at | 0.00 |
| 99677_at | 0.00 |
| 99678_f_at | 0.00 |
| 99681_at | 0.00 |
| 99699_at | 0.00 |
| 99700_at | 0.00 |
| 99701_f_at | 0.01 |
| 99702_at | 0.00 |
| 99708_at | 0.00 |
| 99756_at | 0.00 |
| 99776_at | 0.00 |
| 99777_s_at | 0.00 |
| 99778_at | 0.00 |
| 99779_at | 0.00 |
| 99798_at | 0.00 |
| 99799_at | 0.00 |
| 99800_at | 0.00 |
| 99801_at | 0.06 |
| 99802_at | 0.00 |
| 99803_at | 0.00 |
| 99804_at | 0.00 |
| 99805_at | 0.00 |
| 99806_at | 0.00 |
| 99807_r_at | 0.01 |
| 99808_at | 0.00 |
| 99809_at | 0.00 |
| 99810_at | 0.00 |
| 99811_at | 0.00 |
| 99812_at | 0.00 |
| 99813_g_at | 0.00 |
| 99814_at | 0.00 |
| 99815_at | 0.00 |
| 99816_at | 0.00 |
| 99817_at | 0.00 |
| 99818_at | 0.00 |
| 99819_at | 0.00 |
| 99820_f_at | 0.01 |

| | |
|---|---|
| 99821_at | 0.00 |
| 99823_r_at | 0.03 |
| 99824_at | 0.00 |
| 99825_at | 0.00 |
| 99826_at | 0.00 |
| 99827_at | 0.00 |
| 99828_at | 0.00 |
| 99829_at | 0.00 |
| 99830_at | 0.00 |
| 99831_at | 0.00 |
| 99832_at | 0.00 |
| 99833_at | 0.00 |
| 99834_at | 0.00 |
| 99835_at | 0.00 |
| 99836_at | 0.00 |
| 99837_at | 0.00 |
| 99838_at | 0.09 |
| 99839_at | 0.00 |
| 99840_at | 0.00 |
| 99841_at | 0.00 |
| 99842_at | 0.00 |
| 99843_at | 0.00 |
| 99844_at | 0.00 |
| 99845_at | 0.00 |
| 99846_at | 0.00 |
| 99847_at | 0.00 |
| 99848_at | 0.00 |
| 99849_at | 0.74 |
| 99850_at | 0.00 |
| 99851_at | 0.00 |
| 99854_at | 0.00 |
| 99855_at | 0.02 |
| 99856_r_at | 0.05 |
| 99860_at | 0.00 |
| 99861_at | 0.00 |
| 99862_at | 0.00 |
| 99863_at | 0.00 |
| 99864_at | 0.00 |
| 99865_at | 0.00 |
| 99866_at | 0.00 |
| 99867_at | 0.00 |
| 99868_at | 0.00 |
| 99869_at | 0.00 |
| 99870_at | 0.00 |
| 99871_f_at | 0.00 |
| 99872_s_at | 0.00 |
| 99873_at | 0.00 |
| 99874_at | 0.00 |
| 99875_at | 0.00 |
| 99876_at | 0.00 |
| 99878_at | 0.24 |
| 99880_at | 0.00 |
| 99881_at | 0.00 |
| 99882_at | 0.00 |
| 99883_g_at | 0.00 |
| 99884_at | 0.00 |

| | |
|---|---|
| 99885_at | 0.00 |
| 99886_at | 0.00 |
| 99887_at | 0.00 |
| 99888_at | 0.00 |
| 99889_at | 0.00 |
| 99890_at | 0.00 |
| 99891_at | 0.00 |
| 99892_at | 0.00 |
| 99893_at | 0.00 |
| 99894_at | 0.00 |
| 99895_at | 0.00 |
| 99896_at | 0.00 |
| 99897_at | 0.04 |
| 99898_at | 0.00 |
| 99899_at | 0.00 |
| 99900_at | 0.00 |
| 99901_at | 0.00 |
| 99902_at | 0.00 |
| 99903_at | 0.00 |
| 99904_at | 0.00 |
| 99905_at | 0.00 |
| 99906_at | 0.00 |
| 99907_at | 0.00 |
| 99908_at | 0.00 |
| 99909_at | 0.00 |
| 99910_at | 0.00 |
| 99911_at | 0.00 |
| 99912_at | 0.00 |
| 99913_at | 0.00 |
| 99914_at | 0.00 |
| 99915_at | 0.00 |
| 99916_at | 0.00 |
| 99917_at | 0.00 |
| 99920_at | 0.26 |
| 99922_at | 0.00 |
| 99923_at | 0.00 |
| 99924_at | 0.00 |
| 99925_f_at | 0.00 |
| 99926_at | 0.00 |
| 99927_at | 0.00 |
| 99928_at | 0.00 |
| 99929_at | 0.00 |
| 99930_s_at | 0.14 |
| 99931_at | 0.00 |
| 99932_at | 0.00 |
| 99933_at | 0.00 |
| 99934_at | 0.00 |
| 99935_at | 0.00 |
| 99936_at | 0.00 |
| 99937_at | 0.00 |
| 99938_at | 0.00 |
| 99939_at | 0.00 |
| 99940_at | 0.00 |
| 99941_at | 0.00 |
| 99942_s_at | 0.04 |
| 99944_at | 0.00 |

| | |
|---|---|
| 99945_at | 0.00 |
| 99947_at | 0.00 |
| 99948_at | 0.02 |
| 99949_at | 0.00 |
| 99950_at | 0.00 |
| 99951_at | 0.00 |
| 99952_at | 0.00 |
| 99953_at | 0.00 |
| 99954_at | 0.00 |
| 99955_at | 0.03 |
| 99956_at | 1.00 |
| 99957_at | 0.00 |
| 99958_at | 0.00 |
| 99959_at | 0.81 |
| 99960_at | 0.03 |
| 99961_s_at | 0.00 |
| 99962_at | 0.00 |
| 99963_at | 0.00 |
| 99964_at | 0.00 |
| 99965_at | 0.00 |
| 99966_at | 0.00 |
| 99970_at | 0.01 |
| 99972_at | 0.00 |
| 99973_s_at | 0.00 |
| 99974_at | 0.00 |
| 99975_at | 0.00 |
| 99977_at | 0.00 |
| 99978_s_at | 0.00 |
| 99979_at | 0.00 |
| 99980_at | 0.01 |
| 99981_at | 0.00 |
| 99982_at | 0.00 |
| 99984_at | 0.00 |
| 99985_at | 0.19 |
| 99986_at | 0.00 |
| 99987_at | 0.00 |
| 99988_at | 0.00 |
| 99990_at | 0.00 |
| 99991_at | 0.00 |
| 99992_at | 0.00 |
| 99993_at | 0.00 |
| 99994_at | 0.00 |
| 99995_at | 0.00 |
| 99996_at | 0.00 |
| 99997_at | 0.00 |
| 99998_at | 0.00 |
| 99999_at | 0.00 |
| X-18SRNAMur/X00686_ | 0.00 |
| X-18SRNAMur/X00686_ | 0.28 |
| X-18SRNAMur/X00686_ | 0.03 |
| AFFX-BioB-3_at | 0.11 |
| AFFX-BioB-3_st_A | 0.00 |
| AFFX-BioB-5_at | 0.07 |
| AFFX-BioB-5_st_A | 0.00 |
| AFFX-BioB-M_at | 0.04 |
| AFFX-BioB-M_st_A | 0.00 |

| | |
|---|---|
| AFFX-BioC-3_at | 0.07 |
| AFFX-BioC-3_st_A | 0.00 |
| AFFX-BioC-5_at | 0.10 |
| AFFX-BioC-5_st_A | 0.00 |
| AFFX-BioDn-3_at | 0.06 |
| AFFX-BioDn-3_st_A | 0.02 |
| AFFX-BioDn-5_at | 0.08 |
| AFFX-BioDn-5_st_A | 0.03 |
| AFFX-CreX-3_at | 0.01 |
| AFFX-CreX-3_st_A | 0.00 |
| AFFX-CreX-5_at | 0.02 |
| AFFX-CreX-5_st_A | 0.00 |
| AFFX-DapX-3_at | 0.00 |
| AFFX-DapX-5_at | 0.00 |
| AFFX-DapX-M_at | 0.00 |
| FX-GapdhMur/M32599_ | 0.00 |
| -GapdhMur/M32599_3 | 0.02 |
| FX-GapdhMur/M32599_ | 0.13 |
| -GapdhMur/M32599_5 | 0.01 |
| X-GapdhMur/M32599_ | 0.00 |
| -GapdhMur/M32599_M | 0.17 |
| AFFX-LysX-3_at | 0.00 |
| AFFX-LysX-5_at | 0.00 |
| AFFX-LysX-M_at | 0.00 |
| AFFX-MURINE_B2_at | 0.00 |
| AFFX-MURINE_b1_at | 0.06 |
| AFFX-MUR_b2_at | 0.00 |
| AFFX-MurFAS_at | 0.00 |
| AFFX-MurIL10_at | 0.00 |
| AFFX-MurIL2_at | 0.00 |
| AFFX-MurIL4_at | 0.00 |
| AFFX-PheX-3_at | 0.00 |
| AFFX-PheX-5_at | 0.00 |
| AFFX-PheX-M_at | 0.00 |
| -PyruCarbMur/L09192_ | 0.00 |
| -PyruCarbMur/L09192_ | 0.00 |
| -PyruCarbMur/L09192_ | 0.00 |
| -PyruCarbMur/L09192_ | 0.00 |
| AFFX-ThrX-3_at | 0.00 |
| AFFX-ThrX-5_at | 0.00 |
| AFFX-ThrX-M_at | 0.00 |
| -TransRecMur/X57349_ | 0.12 |
| -TransRecMur/X57349_ | 0.33 |
| -TransRecMur/X57349_ | 0.18 |
| AFFX-TrpnX-3_at | 0.00 |
| AFFX-TrpnX-5_at | 0.00 |
| AFFX-TrpnX-M_at | 0.00 |
| FFX-YEL002c/WBP1_a | 0.00 |
| AFFX-YEL018w/_at | 0.00 |
| FFX-YEL021w/URA3_a | 0.00 |
| AFFX-YEL024w/RIP1_a | 0.00 |
| X-b-ActinMur/M12481_ | 0.01 |
| -b-ActinMur/M12481_3 | 0.15 |
| X-b-ActinMur/M12481_ | 0.04 |
| -b-ActinMur/M12481_5 | 0.04 |
| X-b-ActinMur/M12481_ | 0.05 |

| | |
|---|---|
| -b-ActinMur/M12481_M | 0.00 |
| 104769_at | 0.00 |
| 104770_at | 0.00 |
| 104771_at | 0.00 |
| 104772_at | 0.52 |
| 104773_at | 0.00 |
| 104774_at | 0.00 |
| 104775_at | 0.00 |
| 104776_at | 0.00 |
| 104777_at | 0.00 |
| 104778_at | 0.00 |
| 104779_at | 0.00 |
| 104780_at | 0.00 |
| 104781_at | 0.00 |
| 104782_at | 0.07 |
| 104783_at | 0.00 |
| 104784_at | 0.00 |
| 104785_at | 0.02 |
| 104786_at | 0.00 |
| 104787_at | 0.00 |
| 104788_at | 0.00 |
| 104789_at | 0.00 |
| 104790_at | 0.00 |
| 104791_at | 0.00 |
| 104792_at | 0.00 |
| 104793_at | 0.00 |
| 104794_at | 0.00 |
| 104795_at | 0.00 |
| 104796_at | 0.00 |
| 104798_at | 0.00 |
| 104799_at | 0.00 |
| 104800_at | 0.00 |
| 104801_at | 0.00 |
| 104802_at | 0.00 |
| 104803_at | 0.00 |
| 104804_at | 0.00 |
| 104805_at | 0.00 |
| 104806_at | 0.00 |
| 104807_at | 0.00 |
| 104809_at | 0.00 |
| 104810_at | 0.00 |
| 104811_at | 0.00 |
| 104812_at | 0.00 |
| 104813_at | 0.00 |
| 104814_at | 0.00 |
| 104815_at | 0.00 |
| 104816_at | 0.08 |
| 104817_at | 0.00 |
| 104818_at | 0.00 |
| 104819_at | 0.00 |
| 104820_at | 0.00 |
| 104821_at | 0.00 |
| 104823_at | 0.00 |
| 104824_at | 0.00 |
| 104825_g_at | 0.00 |
| 104826_at | 0.00 |

| | |
|---|---|
| 104827_f_at | 0.00 |
| 104828_r_at | 0.00 |
| 104829_at | 0.00 |
| 104830_at | 0.00 |
| 104831_at | 0.00 |
| 104832_at | 0.00 |
| 104833_at | 0.00 |
| 104834_at | 0.00 |
| 104835_at | 0.00 |
| 104836_at | 0.00 |
| 104837_at | 0.00 |
| 104838_at | 0.00 |
| 104839_at | 0.00 |
| 104840_at | 0.00 |
| 104841_i_at | 0.00 |
| 104842_f_at | 0.00 |
| 104843_at | 0.07 |
| 104844_at | 0.00 |
| 104845_at | 0.00 |
| 104846_at | 0.16 |
| 104847_at | 0.01 |
| 104848_at | 0.00 |
| 104849_at | 0.00 |
| 104850_at | 0.00 |
| 104851_at | 0.02 |
| 104852_at | 0.00 |
| 104853_at | 0.00 |
| 104854_at | 0.00 |
| 104855_at | 0.00 |
| 104856_at | 0.00 |
| 104857_at | 0.00 |
| 104858_at | 0.00 |
| 104859_at | 0.00 |
| 104860_at | 0.00 |
| 104861_at | 0.00 |
| 104862_at | 0.00 |
| 104863_at | 0.00 |
| 104864_at | 0.00 |
| 104865_at | 0.00 |
| 104866_at | 0.00 |
| 104867_at | 0.00 |
| 104868_at | 0.00 |
| 104869_at | 0.00 |
| 104870_at | 0.00 |
| 104871_at | 0.00 |
| 104872_at | 0.00 |
| 104873_at | 0.00 |
| 104874_at | 0.00 |
| 104875_at | 0.00 |
| 104876_at | 0.00 |
| 104877_at | 0.00 |
| 104878_at | 0.00 |
| 104880_at | 0.00 |
| 104882_at | 0.00 |
| 104885_at | 0.00 |
| 104886_at | 0.00 |

| | |
|---|---|
| 104887_at | 0.00 |
| 104888_at | 0.00 |
| 104889_at | 0.00 |
| 104890_at | 0.00 |
| 104891_at | 0.00 |
| 104892_at | 0.00 |
| 104893_at | 0.00 |
| 104894_at | 0.00 |
| 104895_at | 0.00 |
| 104896_at | 0.00 |
| 104897_at | 0.00 |
| 104898_at | 0.00 |
| 104899_at | 0.00 |
| 104900_at | 0.00 |
| 104901_at | 0.00 |
| 104902_at | 0.00 |
| 104903_at | 0.00 |
| 104904_at | 0.00 |
| 104905_at | 0.00 |
| 104906_at | 0.00 |
| 104907_at | 0.00 |
| 104908_at | 0.00 |
| 104909_at | 0.00 |
| 104910_at | 0.00 |
| 104911_at | 0.00 |
| 104912_at | 0.00 |
| 104913_at | 0.00 |
| 104914_at | 0.00 |
| 104915_at | 0.00 |
| 104916_at | 0.00 |
| 104917_at | 0.00 |
| 104918_at | 0.00 |
| 104919_at | 0.00 |
| 104920_at | 0.00 |
| 104921_at | 0.01 |
| 104922_at | 0.00 |
| 104923_at | 0.00 |
| 104924_at | 0.00 |
| 104925_at | 0.00 |
| 104926_at | 0.00 |
| 104927_at | 0.00 |
| 104928_at | 0.00 |
| 104929_at | 0.00 |
| 104930_at | 0.00 |
| 104931_at | 0.00 |
| 104932_at | 0.00 |
| 104933_at | 0.00 |
| 104934_at | 0.00 |
| 104935_at | 0.00 |
| 104936_at | 0.00 |
| 104937_at | 0.00 |
| 104938_at | 0.00 |
| 104939_at | 0.00 |
| 104940_at | 0.00 |
| 104941_at | 0.00 |
| 104942_at | 0.00 |

| | |
|---|---|
| 104943_at | 0.02 |
| 104944_at | 0.00 |
| 104945_at | 0.00 |
| 104946_at | 0.00 |
| 104947_at | 0.00 |
| 104948_at | 0.00 |
| 104949_at | 0.00 |
| 104950_at | 0.00 |
| 104951_f_at | 0.00 |
| 104952_r_at | 0.00 |
| 104953_at | 0.00 |
| 104954_at | 0.00 |
| 104955_at | 0.00 |
| 104956_at | 0.00 |
| 104957_at | 0.00 |
| 104958_at | 0.00 |
| 104959_at | 0.00 |
| 104960_at | 0.00 |
| 104961_at | 0.00 |
| 104962_at | 0.00 |
| 104963_at | 0.00 |
| 104964_at | 0.00 |
| 104965_at | 0.00 |
| 104966_at | 0.00 |
| 104967_at | 0.00 |
| 104968_at | 0.00 |
| 104969_at | 0.00 |
| 104970_at | 0.00 |
| 104971_at | 0.00 |
| 104972_at | 0.00 |
| 104973_at | 0.00 |
| 104974_at | 0.07 |
| 104975_at | 0.00 |
| 104976_at | 0.00 |
| 104977_at | 0.00 |
| 104978_at | 0.00 |
| 104979_at | 0.00 |
| 104980_at | 0.00 |
| 104981_at | 0.00 |
| 104982_at | 0.00 |
| 104983_at | 0.00 |
| 104984_at | 0.00 |
| 104985_f_at | 0.00 |
| 104986_at | 0.00 |
| 104987_at | 0.00 |
| 104988_at | 0.00 |
| 104989_at | 0.00 |
| 104990_at | 0.00 |
| 104991_at | 0.00 |
| 104992_at | 0.00 |
| 104993_at | 0.00 |
| 104994_at | 0.00 |
| 104995_g_at | 0.00 |
| 104996_at | 0.00 |
| 104997_at | 0.00 |
| 104998_at | 0.00 |

| | |
|---|---|
| 104999_at | 0.00 |
| 105000_at | 0.00 |
| 105001_at | 0.00 |
| 105002_at | 0.00 |
| 105003_at | 0.01 |
| 105004_at | 0.00 |
| 105005_at | 0.45 |
| 105006_at | 0.00 |
| 105007_at | 0.00 |
| 105008_at | 0.00 |
| 105009_at | 0.00 |
| 105010_at | 0.00 |
| 105011_at | 0.00 |
| 105012_at | 0.00 |
| 105013_at | 0.00 |
| 105014_at | 0.00 |
| 105015_at | 0.00 |
| 105016_at | 0.00 |
| 105017_at | 0.00 |
| 105018_at | 0.00 |
| 105019_r_at | 0.00 |
| 105020_i_at | 0.01 |
| 105021_f_at | 0.00 |
| 105022_at | 0.00 |
| 105023_at | 0.00 |
| 105024_at | 0.00 |
| 105025_at | 0.00 |
| 105026_at | 0.00 |
| 105027_at | 0.00 |
| 105028_at | 0.00 |
| 105029_at | 0.00 |
| 105030_at | 0.00 |
| 105031_at | 0.00 |
| 105032_at | 0.00 |
| 105033_at | 0.00 |
| 105035_at | 0.00 |
| 105036_at | 0.00 |
| 105037_at | 0.00 |
| 105038_at | 0.00 |
| 105039_at | 0.00 |
| 105040_at | 0.00 |
| 105041_at | 0.00 |
| 105042_at | 0.00 |
| 105043_at | 0.00 |
| 105044_at | 0.00 |
| 105045_at | 0.01 |
| 105046_at | 0.00 |
| 105047_at | 0.00 |
| 105048_at | 0.00 |
| 105049_at | 0.03 |
| 105050_at | 0.00 |
| 105051_at | 0.00 |
| 105052_at | 0.00 |
| 105053_at | 0.00 |
| 105054_f_at | 0.00 |
| 105055_at | 0.00 |

| | |
|---|---|
| 105056_at | 0.00 |
| 105057_at | 0.09 |
| 105058_at | 0.00 |
| 105059_at | 0.00 |
| 105060_at | 0.00 |
| 105061_at | 0.00 |
| 105063_at | 0.00 |
| 105064_at | 0.00 |
| 105065_at | 0.00 |
| 105066_at | 0.00 |
| 105067_at | 0.00 |
| 105068_at | 0.00 |
| 105069_at | 0.00 |
| 105070_at | 0.00 |
| 105071_at | 0.00 |
| 105072_at | 0.00 |
| 105073_at | 0.00 |
| 105074_at | 0.00 |
| 105075_at | 0.00 |
| 105076_at | 0.00 |
| 105077_at | 0.00 |
| 105078_at | 0.00 |
| 105079_at | 0.01 |
| 105080_at | 0.00 |
| 105081_at | 0.00 |
| 105082_at | 0.00 |
| 105083_at | 0.00 |
| 105084_at | 0.00 |
| 105086_at | 0.00 |
| 105087_at | 0.00 |
| 105088_at | 0.01 |
| 105089_at | 0.29 |
| 105090_at | 0.00 |
| 105091_at | 0.00 |
| 105092_at | 0.00 |
| 105093_at | 0.00 |
| 105094_at | 0.00 |
| 105095_at | 0.00 |
| 105096_s_at | 0.00 |
| 105097_at | 0.00 |
| 105098_at | 0.00 |
| 105099_at | 0.00 |
| 105100_at | 0.00 |
| 105101_at | 0.00 |
| 105102_at | 0.00 |
| 105103_at | 0.00 |
| 105104_at | 0.00 |
| 105105_at | 0.00 |
| 105106_at | 0.05 |
| 105107_at | 0.00 |
| 105108_at | 0.00 |
| 105109_at | 0.00 |
| 105110_at | 0.00 |
| 105111_at | 0.00 |
| 105112_at | 0.00 |
| 105113_at | 0.00 |

| | |
|---|---|
| 105114_at | 0.00 |
| 105115_at | 0.00 |
| 105116_at | 0.00 |
| 105117_at | 0.00 |
| 105119_i_at | 0.00 |
| 105120_f_at | 0.00 |
| 105121_at | 0.00 |
| 105122_at | 0.00 |
| 105123_at | 0.00 |
| 105124_at | 0.00 |
| 105125_at | 0.00 |
| 105126_at | 0.00 |
| 105127_at | 0.00 |
| 105128_at | 0.00 |
| 105129_at | 0.00 |
| 105131_at | 0.00 |
| 105132_at | 0.00 |
| 105133_at | 0.00 |
| 105134_at | 0.00 |
| 105135_at | 0.00 |
| 105137_at | 0.00 |
| 105138_at | 0.00 |
| 105139_at | 0.00 |
| 105140_at | 0.00 |
| 105141_at | 0.00 |
| 105142_at | 0.00 |
| 105143_at | 0.00 |
| 105144_at | 0.00 |
| 105145_at | 0.00 |
| 105146_at | 0.00 |
| 105147_at | 0.00 |
| 105148_at | 0.00 |
| 105149_at | 0.00 |
| 105150_at | 0.00 |
| 105151_at | 0.00 |
| 105152_at | 0.00 |
| 105153_at | 0.00 |
| 105154_at | 0.00 |
| 105155_at | 0.06 |
| 105156_at | 0.00 |
| 105157_at | 0.00 |
| 105158_at | 0.00 |
| 105159_at | 0.00 |
| 105160_at | 0.00 |
| 105161_at | 0.00 |
| 105162_at | 0.00 |
| 105163_at | 0.00 |
| 105164_at | 0.00 |
| 105165_at | 0.00 |
| 105166_at | 0.00 |
| 105167_at | 0.00 |
| 105168_at | 0.00 |
| 105169_at | 0.00 |
| 105170_at | 0.00 |
| 105171_at | 0.00 |
| 105172_at | 0.00 |

| | |
|---|---|
| 105173_at | 0.00 |
| 105174_at | 0.00 |
| 105175_at | 0.00 |
| 105176_at | 0.00 |
| 105177_at | 0.00 |
| 105178_at | 0.00 |
| 105179_at | 0.00 |
| 105180_at | 0.00 |
| 105181_at | 0.00 |
| 105182_at | 0.00 |
| 105183_at | 0.00 |
| 105184_at | 0.00 |
| 105186_at | 0.00 |
| 105187_at | 0.00 |
| 105188_at | 0.00 |
| 105189_at | 0.00 |
| 105190_at | 0.00 |
| 105191_at | 0.00 |
| 105192_at | 0.00 |
| 105193_at | 0.00 |
| 105194_at | 0.00 |
| 105195_at | 0.00 |
| 105196_at | 0.00 |
| 105197_at | 0.00 |
| 105198_at | 0.00 |
| 105200_at | 0.00 |
| 105201_at | 0.00 |
| 105202_at | 0.00 |
| 105203_at | 0.00 |
| 105204_at | 0.00 |
| 105205_at | 0.00 |
| 105206_at | 0.00 |
| 105207_at | 0.00 |
| 105208_at | 0.00 |
| 105209_at | 0.00 |
| 105210_at | 0.00 |
| 105211_at | 0.00 |
| 105212_at | 0.00 |
| 105213_at | 0.00 |
| 105214_at | 0.03 |
| 105215_at | 0.00 |
| 105216_at | 0.00 |
| 105217_at | 0.00 |
| 105218_at | 0.00 |
| 105219_at | 0.00 |
| 105220_at | 0.00 |
| 105221_at | 0.00 |
| 105222_at | 0.00 |
| 105223_at | 0.00 |
| 105224_at | 0.00 |
| 105225_at | 0.00 |
| 105226_at | 0.00 |
| 105227_r_at | 0.00 |
| 105228_at | 0.01 |
| 105229_at | 0.00 |
| 105230_at | 0.00 |

| | |
|---|---|
| 105231_at | 0.00 |
| 105232_at | 0.00 |
| 105233_at | 0.01 |
| 105234_at | 0.00 |
| 105235_at | 0.00 |
| 105236_at | 0.00 |
| 105237_at | 0.00 |
| 105238_at | 0.00 |
| 105239_at | 0.00 |
| 105240_at | 0.00 |
| 105241_at | 0.00 |
| 105242_at | 0.00 |
| 105243_at | 0.00 |
| 105244_at | 0.00 |
| 105245_at | 0.00 |
| 105246_at | 0.00 |
| 105247_at | 0.00 |
| 105248_at | 0.00 |
| 105249_at | 0.00 |
| 105250_at | 0.20 |
| 105251_at | 0.00 |
| 105252_at | 0.00 |
| 105253_at | 0.00 |
| 105254_at | 0.00 |
| 105255_at | 0.00 |
| 105256_at | 0.00 |
| 105257_at | 0.00 |
| 105258_at | 0.00 |
| 105259_at | 0.00 |
| 105260_at | 0.00 |
| 105261_at | 0.00 |
| 105262_at | 0.00 |
| 105263_at | 0.00 |
| 105264_at | 0.00 |
| 105265_at | 0.00 |
| 105266_at | 0.00 |
| 105267_at | 0.00 |
| 105268_at | 0.00 |
| 105269_at | 0.00 |
| 105270_at | 0.00 |
| 105271_at | 0.00 |
| 105272_at | 0.00 |
| 105273_at | 0.01 |
| 105274_at | 0.00 |
| 105275_at | 0.00 |
| 105276_f_at | 0.00 |
| 105277_at | 0.00 |
| 105278_f_at | 0.00 |
| 105279_at | 0.00 |
| 105280_at | 0.00 |
| 105281_at | 0.00 |
| 105282_at | 0.00 |
| 105283_at | 0.00 |
| 105284_at | 0.00 |
| 105285_at | 0.00 |
| 105286_at | 0.00 |

| | |
|---|---|
| 105287_at | 0.00 |
| 105288_at | 0.00 |
| 105289_at | 0.00 |
| 105290_at | 0.00 |
| 105291_at | 0.00 |
| 105292_at | 0.00 |
| 105293_at | 0.00 |
| 105294_at | 0.00 |
| 105295_at | 0.00 |
| 105296_at | 0.00 |
| 105297_at | 0.00 |
| 105298_at | 0.21 |
| 105299_at | 0.00 |
| 105300_r_at | 0.00 |
| 105301_at | 0.00 |
| 105302_at | 0.00 |
| 105303_at | 0.00 |
| 105304_at | 0.00 |
| 105305_at | 0.00 |
| 105306_at | 0.00 |
| 105307_at | 0.00 |
| 105308_at | 0.00 |
| 105309_at | 0.00 |
| 105310_at | 0.00 |
| 105311_at | 0.00 |
| 105312_at | 0.00 |
| 105313_at | 0.00 |
| 105314_at | 0.02 |
| 105315_at | 0.00 |
| 105316_at | 0.00 |
| 105317_at | 0.00 |
| 105318_at | 0.00 |
| 105319_at | 0.00 |
| 105320_at | 0.00 |
| 105321_at | 0.00 |
| 105322_at | 0.00 |
| 105323_at | 0.00 |
| 105324_at | 0.00 |
| 105325_at | 0.00 |
| 105326_at | 0.04 |
| 105327_at | 0.00 |
| 105328_at | 0.00 |
| 105329_at | 0.00 |
| 105330_at | 0.00 |
| 105331_at | 0.00 |
| 105332_at | 0.00 |
| 105333_at | 0.00 |
| 105334_at | 0.00 |
| 105335_at | 0.00 |
| 105336_at | 0.00 |
| 105337_at | 0.00 |
| 105338_at | 0.00 |
| 105339_at | 0.00 |
| 105340_at | 0.00 |
| 105341_f_at | 0.00 |
| 105342_at | 0.00 |

| | |
|---|---|
| 105343_at | 0.00 |
| 105344_at | 0.00 |
| 105345_at | 0.00 |
| 105346_at | 0.00 |
| 105347_at | 0.00 |
| 105348_at | 0.00 |
| 105349_at | 0.00 |
| 105350_at | 0.00 |
| 105351_at | 0.00 |
| 105352_at | 0.00 |
| 105353_at | 0.00 |
| 105354_at | 0.00 |
| 105355_at | 0.00 |
| 105356_at | 0.00 |
| 105357_at | 0.00 |
| 105358_at | 0.00 |
| 105359_at | 0.00 |
| 105360_at | 0.00 |
| 105361_at | 0.00 |
| 105362_at | 0.00 |
| 105363_at | 0.00 |
| 105364_at | 0.00 |
| 105365_at | 0.00 |
| 105366_at | 0.00 |
| 105367_at | 0.00 |
| 105368_at | 0.00 |
| 105369_at | 0.00 |
| 105370_at | 0.00 |
| 105371_at | 0.00 |
| 105372_at | 0.00 |
| 105373_at | 0.01 |
| 105374_at | 0.00 |
| 105375_at | 0.00 |
| 105376_at | 0.00 |
| 105377_at | 0.00 |
| 105378_at | 0.00 |
| 105379_at | 0.00 |
| 105380_at | 0.02 |
| 105381_at | 0.00 |
| 105382_at | 0.00 |
| 105384_at | 0.00 |
| 105385_at | 0.00 |
| 105386_at | 0.00 |
| 105387_at | 0.00 |
| 105388_at | 0.00 |
| 105389_at | 0.00 |
| 105390_at | 0.00 |
| 105391_at | 0.00 |
| 105392_at | 0.00 |
| 105393_at | 0.00 |
| 105394_at | 0.00 |
| 105395_at | 0.00 |
| 105396_at | 0.00 |
| 105397_at | 0.00 |
| 105398_at | 0.00 |
| 105399_at | 0.00 |

| | |
|---|---|
| 105400_at | 0.00 |
| 105401_at | 0.00 |
| 105402_at | 0.00 |
| 105403_at | 0.00 |
| 105404_at | 0.00 |
| 105405_at | 0.00 |
| 105406_at | 0.00 |
| 105407_at | 0.00 |
| 105409_at | 0.00 |
| 105410_at | 0.00 |
| 105411_at | 0.00 |
| 105412_at | 0.00 |
| 105413_at | 0.00 |
| 105414_at | 0.00 |
| 105415_at | 0.00 |
| 105416_at | 0.00 |
| 105417_s_at | 0.00 |
| 105418_r_at | 0.00 |
| 105419_at | 0.00 |
| 105420_at | 0.00 |
| 105421_at | 0.00 |
| 105422_at | 0.00 |
| 105423_at | 0.00 |
| 105424_at | 0.00 |
| 105425_at | 0.00 |
| 105427_at | 0.00 |
| 105428_at | 0.00 |
| 105429_at | 0.00 |
| 105430_at | 0.00 |
| 105431_at | 0.00 |
| 105432_at | 0.00 |
| 105434_at | 0.00 |
| 105435_at | 0.00 |
| 105436_at | 0.00 |
| 105437_at | 0.00 |
| 105438_at | 0.00 |
| 105439_at | 0.00 |
| 105440_at | 0.00 |
| 105441_at | 0.00 |
| 105442_at | 0.00 |
| 105443_at | 0.00 |
| 105444_at | 0.00 |
| 105445_at | 0.00 |
| 105446_at | 0.00 |
| 105447_at | 0.00 |
| 105448_at | 0.00 |
| 105449_at | 0.00 |
| 105450_at | 0.00 |
| 105451_at | 0.00 |
| 105453_at | 0.00 |
| 105454_at | 0.00 |
| 105455_at | 0.00 |
| 105456_at | 0.00 |
| 105457_at | 0.00 |
| 105458_at | 0.00 |
| 105459_at | 0.00 |

| | |
|---|---|
| 105460_at | 0.00 |
| 105461_at | 0.01 |
| 105464_at | 0.00 |
| 105465_at | 0.00 |
| 105466_at | 0.00 |
| 105467_at | 0.00 |
| 105468_at | 0.00 |
| 105469_at | 0.00 |
| 105470_at | 0.00 |
| 105471_at | 0.00 |
| 105472_at | 0.00 |
| 105473_at | 0.00 |
| 105474_at | 0.00 |
| 105475_at | 0.00 |
| 105476_at | 0.00 |
| 105477_at | 0.00 |
| 105478_at | 0.00 |
| 105479_at | 0.00 |
| 105480_at | 0.00 |
| 105481_at | 0.00 |
| 105483_at | 0.00 |
| 105484_at | 0.00 |
| 105485_f_at | 0.00 |
| 105486_r_at | 0.00 |
| 105487_at | 0.00 |
| 105488_at | 0.00 |
| 105489_at | 0.10 |
| 105490_at | 0.00 |
| 105491_at | 0.00 |
| 105492_at | 0.00 |
| 105493_at | 0.00 |
| 105494_at | 0.00 |
| 105495_at | 0.00 |
| 105496_at | 0.18 |
| 105497_at | 0.00 |
| 105498_at | 0.00 |
| 105499_at | 0.00 |
| 105500_at | 0.00 |
| 105501_at | 0.00 |
| 105502_at | 0.00 |
| 105503_at | 0.00 |
| 105504_at | 0.00 |
| 105505_at | 0.00 |
| 105506_at | 0.00 |
| 105507_at | 0.00 |
| 105508_at | 0.01 |
| 105509_at | 0.00 |
| 105510_at | 0.00 |
| 105511_at | 0.00 |
| 105512_at | 0.00 |
| 105513_at | 0.00 |
| 105514_at | 0.00 |
| 105515_at | 0.00 |
| 105516_f_at | 0.11 |
| 105517_at | 0.00 |
| 105518_at | 0.00 |

| | |
|---|---|
| 105519_s_at | 0.00 |
| 105520_at | 0.00 |
| 105521_at | 0.00 |
| 105522_at | 0.00 |
| 105523_at | 0.00 |
| 105524_at | 0.00 |
| 105525_at | 0.00 |
| 105526_at | 0.00 |
| 105527_at | 0.00 |
| 105528_at | 0.00 |
| 105529_at | 0.00 |
| 105530_at | 0.00 |
| 105531_at | 0.02 |
| 105532_at | 0.00 |
| 105533_at | 0.00 |
| 105534_at | 0.00 |
| 105535_at | 0.00 |
| 105536_at | 0.00 |
| 105537_at | 0.00 |
| 105538_at | 0.00 |
| 105539_at | 0.00 |
| 105540_at | 0.00 |
| 105541_at | 0.00 |
| 105542_at | 0.00 |
| 105543_at | 0.00 |
| 105544_at | 0.00 |
| 105545_at | 0.00 |
| 105546_at | 0.00 |
| 105547_at | 0.00 |
| 105548_at | 0.00 |
| 105549_at | 0.00 |
| 105550_at | 0.09 |
| 105551_at | 0.00 |
| 105552_at | 0.00 |
| 105554_at | 0.00 |
| 105555_at | 0.00 |
| 105556_at | 0.00 |
| 105557_at | 0.00 |
| 105558_at | 0.00 |
| 105559_at | 0.00 |
| 105560_at | 0.00 |
| 105561_at | 0.00 |
| 105562_at | 0.00 |
| 105563_at | 0.00 |
| 105564_at | 0.00 |
| 105565_at | 0.00 |
| 105566_at | 0.00 |
| 105567_at | 0.00 |
| 105569_at | 0.00 |
| 105570_at | 0.00 |
| 105571_at | 0.00 |
| 105572_at | 0.00 |
| 105573_at | 0.01 |
| 105574_i_at | 0.00 |
| 105575_f_at | 0.00 |
| 105576_at | 0.00 |

| | |
|---|---|
| 105577_g_at | 0.00 |
| 105578_at | 0.00 |
| 105579_at | 0.00 |
| 105580_at | 0.00 |
| 105581_at | 0.00 |
| 105582_at | 0.00 |
| 105583_at | 0.00 |
| 105584_at | 0.01 |
| 105585_at | 0.00 |
| 105586_at | 0.00 |
| 105587_at | 0.00 |
| 105588_at | 0.00 |
| 105589_at | 0.00 |
| 105590_at | 0.00 |
| 105591_at | 0.04 |
| 105592_at | 0.00 |
| 105593_at | 0.00 |
| 105594_at | 0.00 |
| 105595_at | 0.00 |
| 105596_at | 0.00 |
| 105597_at | 0.00 |
| 105598_at | 0.00 |
| 105599_at | 0.00 |
| 105600_at | 0.00 |
| 105601_at | 0.00 |
| 105602_at | 0.00 |
| 105603_at | 0.00 |
| 105604_at | 0.00 |
| 105605_at | 0.00 |
| 105606_at | 0.01 |
| 105607_at | 0.00 |
| 105608_at | 0.00 |
| 105609_at | 0.00 |
| 105610_at | 0.03 |
| 105611_at | 0.00 |
| 105612_at | 0.00 |
| 105613_at | 0.00 |
| 105614_at | 0.00 |
| 105615_at | 0.00 |
| 105616_at | 0.00 |
| 105617_at | 0.00 |
| 105618_at | 0.00 |
| 105619_at | 0.00 |
| 105620_at | 0.33 |
| 105621_at | 0.00 |
| 105623_at | 0.00 |
| 105624_at | 0.00 |
| 105625_at | 0.00 |
| 105626_at | 0.00 |
| 105627_at | 0.00 |
| 105629_at | 0.01 |
| 105630_at | 0.00 |
| 105631_at | 0.00 |
| 105632_at | 0.00 |
| 105633_at | 0.00 |
| 105634_at | 0.00 |

| | |
|---|---|
| 105635_at | 0.00 |
| 105636_at | 0.00 |
| 105637_g_at | 0.00 |
| 105638_at | 0.00 |
| 105639_at | 0.00 |
| 105640_at | 0.00 |
| 105641_at | 0.00 |
| 105642_at | 0.00 |
| 105643_at | 0.00 |
| 105644_at | 0.03 |
| 105645_at | 0.00 |
| 105646_at | 0.00 |
| 105647_at | 0.00 |
| 105648_at | 0.00 |
| 105649_at | 0.00 |
| 105650_at | 0.00 |
| 105651_g_at | 0.00 |
| 105652_at | 0.00 |
| 105653_at | 0.00 |
| 105655_at | 0.00 |
| 105656_at | 0.00 |
| 105657_at | 0.00 |
| 105658_at | 0.00 |
| 105659_f_at | 0.15 |
| 105660_at | 0.00 |
| 105661_at | 0.00 |
| 105662_at | 0.00 |
| 105663_at | 0.01 |
| 105664_at | 0.00 |
| 105665_at | 0.00 |
| 105666_at | 0.01 |
| 105667_at | 0.00 |
| 105668_at | 0.00 |
| 105669_at | 0.00 |
| 105670_at | 0.00 |
| 105673_at | 0.00 |
| 105674_at | 0.00 |
| 105675_at | 0.27 |
| 105676_at | 0.00 |
| 105677_at | 0.00 |
| 105678_at | 0.00 |
| 105679_at | 0.00 |
| 105680_at | 0.00 |
| 105683_at | 0.00 |
| 105684_at | 0.00 |
| 105685_at | 0.00 |
| 105686_at | 0.00 |
| 105687_at | 0.00 |
| 105688_f_at | 0.00 |
| 105689_r_at | 0.02 |
| 105691_at | 0.00 |
| 105692_at | 0.00 |
| 105693_at | 0.00 |
| 105694_at | 0.00 |
| 105695_r_at | 0.00 |
| 105696_at | 0.00 |

| | |
|---|---|
| 105697_at | 0.00 |
| 105698_at | 0.00 |
| 105699_at | 0.00 |
| 105700_at | 0.00 |
| 105701_at | 0.00 |
| 105702_at | 0.00 |
| 105703_at | 0.00 |
| 105704_at | 0.00 |
| 105705_at | 0.00 |
| 105706_at | 0.00 |
| 105707_at | 0.00 |
| 105708_at | 0.00 |
| 105709_at | 0.00 |
| 105710_at | 0.13 |
| 105711_at | 0.00 |
| 105712_at | 0.00 |
| 105713_at | 0.00 |
| 105714_at | 0.00 |
| 105715_at | 0.00 |
| 105716_at | 0.00 |
| 105717_at | 0.00 |
| 105718_at | 0.00 |
| 105719_at | 0.00 |
| 105720_at | 0.00 |
| 105721_at | 0.00 |
| 105722_at | 0.00 |
| 105723_at | 0.00 |
| 105724_at | 0.00 |
| 105725_at | 0.00 |
| 105726_at | 0.00 |
| 105727_at | 0.00 |
| 105728_at | 0.00 |
| 105729_at | 0.00 |
| 105730_at | 0.00 |
| 105732_at | 0.00 |
| 105733_at | 0.00 |
| 105734_at | 0.00 |
| 105735_at | 0.00 |
| 105736_at | 0.00 |
| 105737_at | 0.00 |
| 105738_at | 0.00 |
| 105739_at | 0.00 |
| 105740_at | 0.00 |
| 105741_at | 0.00 |
| 105742_at | 0.00 |
| 105743_at | 0.00 |
| 105744_at | 0.00 |
| 105745_at | 0.00 |
| 105746_at | 0.00 |
| 105747_at | 0.00 |
| 105748_at | 0.00 |
| 105749_at | 0.00 |
| 105750_at | 0.00 |
| 105751_at | 0.00 |
| 105752_f_at | 0.00 |
| 105753_r_at | 0.00 |

| | |
|---|---|
| 105754_at | 0.00 |
| 105755_at | 0.00 |
| 105756_at | 0.00 |
| 105757_at | 0.00 |
| 105758_at | 0.00 |
| 105759_at | 0.00 |
| 105760_at | 0.00 |
| 105761_at | 0.00 |
| 105762_at | 0.00 |
| 105764_at | 0.00 |
| 105765_at | 0.00 |
| 105766_at | 0.00 |
| 105767_i_at | 0.00 |
| 105768_r_at | 0.00 |
| 105769_at | 0.01 |
| 105770_at | 0.00 |
| 105771_at | 0.00 |
| 105772_at | 0.00 |
| 105774_at | 0.00 |
| 105775_at | 0.00 |
| 105776_at | 0.00 |
| 105777_at | 0.00 |
| 105778_at | 0.00 |
| 105779_at | 0.00 |
| 105780_at | 0.00 |
| 105781_at | 0.00 |
| 105782_at | 0.00 |
| 105783_at | 0.00 |
| 105785_at | 0.00 |
| 105786_at | 0.00 |
| 105787_at | 0.00 |
| 105788_at | 0.00 |
| 105789_at | 0.00 |
| 105790_at | 0.00 |
| 105791_at | 0.00 |
| 105792_at | 0.00 |
| 105793_at | 0.00 |
| 105794_at | 0.00 |
| 105795_at | 0.00 |
| 105796_at | 0.00 |
| 105797_at | 0.00 |
| 105798_at | 0.00 |
| 105799_at | 0.00 |
| 105800_at | 0.00 |
| 105801_at | 0.00 |
| 105802_at | 0.00 |
| 105803_at | 0.00 |
| 105804_at | 0.00 |
| 105805_at | 0.00 |
| 105806_at | 0.00 |
| 105807_at | 0.00 |
| 105808_at | 0.00 |
| 105809_at | 0.00 |
| 105811_at | 0.00 |
| 105812_at | 0.00 |
| 105813_at | 0.00 |

| | |
|---|---|
| 105814_at | 0.00 |
| 105815_at | 0.00 |
| 105816_at | 0.00 |
| 105817_at | 0.00 |
| 105818_g_at | 0.00 |
| 105819_at | 0.00 |
| 105820_at | 0.00 |
| 105821_at | 0.00 |
| 105822_at | 0.00 |
| 105823_at | 0.02 |
| 105824_at | 0.00 |
| 105825_at | 0.00 |
| 105826_at | 0.00 |
| 105828_at | 0.00 |
| 105829_at | 0.00 |
| 105830_at | 0.00 |
| 105831_at | 0.00 |
| 105832_at | 0.00 |
| 105833_at | 0.00 |
| 105834_at | 0.00 |
| 105835_at | 0.00 |
| 105836_at | 0.00 |
| 105837_at | 0.00 |
| 105838_at | 0.00 |
| 105839_at | 0.00 |
| 105840_at | 0.02 |
| 105841_at | 0.01 |
| 105842_at | 0.00 |
| 105843_at | 0.00 |
| 105844_at | 0.00 |
| 105845_at | 0.00 |
| 105846_at | 0.00 |
| 105847_at | 0.00 |
| 105848_at | 0.00 |
| 105849_at | 0.00 |
| 105850_at | 0.00 |
| 105851_at | 0.00 |
| 105852_at | 0.00 |
| 105853_at | 0.00 |
| 105854_at | 0.00 |
| 105855_at | 0.00 |
| 105856_at | 0.00 |
| 105857_at | 0.00 |
| 105858_at | 0.12 |
| 105859_at | 0.00 |
| 105860_at | 0.00 |
| 105861_at | 0.00 |
| 105862_at | 0.00 |
| 105863_at | 0.00 |
| 105864_at | 0.00 |
| 105865_at | 0.00 |
| 105866_at | 0.00 |
| 105867_at | 0.00 |
| 105868_at | 0.00 |
| 105869_at | 0.00 |
| 105870_at | 0.00 |

| | |
|---|---|
| 105871_at | 0.01 |
| 105872_at | 0.00 |
| 105874_at | 0.00 |
| 105875_at | 0.00 |
| 105876_at | 0.00 |
| 105877_at | 0.00 |
| 105878_at | 0.00 |
| 105879_at | 0.00 |
| 105880_at | 0.00 |
| 105881_at | 0.01 |
| 105882_at | 0.00 |
| 105883_at | 0.00 |
| 105884_at | 0.00 |
| 105885_at | 0.00 |
| 105886_r_at | 0.00 |
| 105887_at | 0.00 |
| 105888_at | 0.00 |
| 105889_at | 0.00 |
| 105890_at | 0.00 |
| 105892_at | 0.00 |
| 105893_at | 0.00 |
| 105895_at | 0.07 |
| 105896_at | 0.00 |
| 105897_at | 0.00 |
| 105898_at | 0.00 |
| 105899_at | 0.00 |
| 105900_at | 0.00 |
| 105901_at | 0.00 |
| 105902_at | 0.00 |
| 105903_at | 0.00 |
| 105904_at | 0.00 |
| 105905_at | 0.00 |
| 105906_at | 0.00 |
| 105907_at | 0.00 |
| 105908_at | 0.00 |
| 105909_at | 0.00 |
| 105910_at | 0.00 |
| 105911_at | 0.00 |
| 105912_at | 0.00 |
| 105913_at | 0.00 |
| 105914_at | 0.00 |
| 105915_at | 0.00 |
| 105916_at | 0.00 |
| 105917_at | 0.00 |
| 105918_at | 0.00 |
| 105919_at | 0.00 |
| 105921_at | 0.00 |
| 105922_at | 0.00 |
| 105923_at | 0.00 |
| 105924_at | 0.01 |
| 105925_at | 0.00 |
| 105926_at | 0.00 |
| 105927_at | 0.00 |
| 105928_at | 0.00 |
| 105929_at | 0.00 |
| 105930_at | 0.00 |

| | |
|---|---|
| 105931_at | 0.00 |
| 105932_at | 0.00 |
| 105933_at | 0.00 |
| 105934_at | 0.00 |
| 105935_at | 0.00 |
| 105936_at | 0.00 |
| 105937_at | 0.00 |
| 105939_at | 0.00 |
| 105940_at | 0.00 |
| 105941_at | 0.00 |
| 105942_at | 0.00 |
| 105943_at | 0.00 |
| 105944_at | 0.00 |
| 105945_at | 0.00 |
| 105946_at | 0.00 |
| 105947_at | 0.00 |
| 105948_at | 0.00 |
| 105949_i_at | 0.09 |
| 105950_at | 0.00 |
| 105951_at | 0.00 |
| 105952_at | 0.00 |
| 105953_at | 0.00 |
| 105954_at | 0.00 |
| 105955_at | 0.00 |
| 105956_at | 0.00 |
| 105957_at | 0.00 |
| 105958_at | 0.44 |
| 105959_at | 0.00 |
| 105960_at | 0.00 |
| 105961_at | 0.00 |
| 105962_at | 0.00 |
| 105963_at | 0.00 |
| 105964_at | 0.00 |
| 105965_at | 0.00 |
| 105967_at | 0.00 |
| 105972_r_at | 0.00 |
| 105975_at | 0.00 |
| 105977_f_at | 0.00 |
| 105978_at | 0.00 |
| 105981_at | 0.01 |
| 105991_at | 0.00 |
| 105993_at | 0.00 |
| 106004_at | 0.00 |
| 106005_at | 0.00 |
| 106006_at | 0.00 |
| 106007_at | 0.00 |
| 106008_at | 0.00 |
| 106009_at | 0.00 |
| 106010_at | 0.00 |
| 106011_at | 0.00 |
| 106012_at | 0.00 |
| 106015_at | 0.00 |
| 106016_at | 0.02 |
| 106017_at | 0.00 |
| 106018_at | 0.00 |
| 106019_f_at | 0.00 |

| | |
|---|---|
| 106020_r_at | 0.00 |
| 106023_at | 0.00 |
| 106025_at | 0.00 |
| 106026_at | 0.00 |
| 106029_at | 0.00 |
| 106031_at | 0.00 |
| 106033_at | 0.00 |
| 106039_at | 0.00 |
| 106041_at | 0.00 |
| 106042_at | 0.00 |
| 106043_g_at | 0.00 |
| 106044_at | 0.00 |
| 106046_at | 0.00 |
| 106049_at | 0.00 |
| 106050_at | 0.00 |
| 106051_at | 0.00 |
| 106053_s_at | 0.00 |
| 106054_at | 0.00 |
| 106056_at | 0.00 |
| 106057_at | 0.00 |
| 106058_at | 0.55 |
| 106059_at | 0.00 |
| 106060_at | 0.00 |
| 106061_at | 0.00 |
| 106062_at | 0.00 |
| 106063_at | 0.00 |
| 106064_at | 0.00 |
| 106065_at | 0.08 |
| 106066_at | 0.00 |
| 106070_at | 0.01 |
| 106071_at | 0.00 |
| 106072_at | 0.01 |
| 106073_at | 0.05 |
| 106074_at | 0.00 |
| 106075_at | 0.00 |
| 106076_at | 0.00 |
| 106077_at | 0.00 |
| 106078_at | 0.00 |
| 106079_g_at | 0.00 |
| 106080_at | 0.00 |
| 106081_at | 0.00 |
| 106082_at | 0.00 |
| 106083_at | 0.00 |
| 106084_at | 0.00 |
| 106085_at | 0.00 |
| 106086_at | 0.00 |
| 106087_at | 0.00 |
| 106088_at | 0.00 |
| 106089_at | 0.50 |
| 106090_at | 0.00 |
| 106091_g_at | 0.00 |
| 106092_at | 0.14 |
| 106093_at | 0.00 |
| 106094_at | 0.00 |
| 106095_at | 0.00 |
| 106096_at | 0.00 |

| | |
|---|---|
| 106097_at | 0.00 |
| 106098_at | 0.00 |
| 106099_at | 0.00 |
| 106100_at | 0.00 |
| 106101_at | 0.02 |
| 106102_at | 0.00 |
| 106103_at | 0.00 |
| 106104_at | 0.00 |
| 106105_at | 0.00 |
| 106106_at | 0.00 |
| 106107_at | 0.00 |
| 106108_at | 0.00 |
| 106109_at | 0.00 |
| 106111_at | 0.00 |
| 106112_at | 0.00 |
| 106113_at | 0.00 |
| 106114_at | 0.00 |
| 106115_at | 0.43 |
| 106117_at | 0.00 |
| 106118_at | 0.00 |
| 106119_at | 0.00 |
| 106120_at | 0.00 |
| 106121_at | 0.00 |
| 106123_at | 0.02 |
| 106124_at | 0.00 |
| 106125_at | 0.00 |
| 106126_at | 0.00 |
| 106128_at | 0.00 |
| 106129_at | 0.00 |
| 106130_at | 0.00 |
| 106131_at | 0.00 |
| 106132_at | 0.00 |
| 106133_at | 0.00 |
| 106134_at | 0.00 |
| 106135_at | 0.00 |
| 106136_at | 0.00 |
| 106137_at | 0.00 |
| 106138_at | 0.00 |
| 106140_at | 0.00 |
| 106141_at | 0.00 |
| 106142_at | 0.00 |
| 106143_at | 0.00 |
| 106144_at | 0.04 |
| 106145_at | 0.00 |
| 106146_at | 0.00 |
| 106147_at | 0.00 |
| 106148_g_at | 0.00 |
| 106149_at | 0.00 |
| 106150_at | 0.00 |
| 106151_at | 0.00 |
| 106152_at | 0.00 |
| 106153_g_at | 0.04 |
| 106154_at | 0.00 |
| 106155_at | 0.00 |
| 106156_at | 0.00 |
| 106157_at | 0.00 |

| | |
|---|---|
| 106158_at | 0.00 |
| 106159_at | 0.00 |
| 106160_at | 0.00 |
| 106161_at | 0.00 |
| 106162_at | 0.00 |
| 106163_at | 0.00 |
| 106164_at | 0.00 |
| 106165_at | 0.00 |
| 106166_at | 0.00 |
| 106167_at | 0.00 |
| 106168_at | 0.00 |
| 106169_at | 0.00 |
| 106170_at | 0.00 |
| 106171_at | 0.00 |
| 106172_at | 0.00 |
| 106173_at | 0.00 |
| 106175_at | 0.00 |
| 106176_at | 0.00 |
| 106177_at | 0.00 |
| 106178_at | 0.00 |
| 106179_at | 0.00 |
| 106181_at | 0.00 |
| 106182_at | 0.00 |
| 106183_at | 0.00 |
| 106184_at | 0.00 |
| 106185_at | 0.00 |
| 106186_at | 0.00 |
| 106187_at | 0.00 |
| 106188_at | 0.00 |
| 106189_at | 0.00 |
| 106190_at | 0.00 |
| 106191_at | 0.00 |
| 106192_at | 0.00 |
| 106194_at | 0.00 |
| 106195_at | 0.22 |
| 106196_at | 0.00 |
| 106198_at | 0.06 |
| 106199_at | 0.00 |
| 106200_at | 0.10 |
| 106203_at | 0.00 |
| 106204_at | 0.00 |
| 106205_at | 0.00 |
| 106207_at | 0.00 |
| 106208_at | 0.00 |
| 106209_at | 0.00 |
| 106210_at | 0.00 |
| 106213_at | 0.00 |
| 106214_at | 0.00 |
| 106215_at | 0.00 |
| 106218_at | 0.00 |
| 106219_at | 0.01 |
| 106222_at | 0.00 |
| 106225_at | 0.00 |
| 106228_at | 0.00 |
| 106237_at | 0.00 |
| 106238_at | 0.00 |

| | |
|---|---|
| 106242_at | 0.00 |
| 106243_s_at | 0.00 |
| 106244_at | 0.00 |
| 106245_at | 0.00 |
| 106248_at | 0.00 |
| 106249_at | 0.00 |
| 106250_at | 0.00 |
| 106252_at | 0.00 |
| 106253_at | 0.00 |
| 106254_at | 0.00 |
| 106255_at | 0.00 |
| 106256_at | 0.00 |
| 106259_at | 0.00 |
| 106260_at | 0.00 |
| 106262_at | 0.00 |
| 106263_at | 0.00 |
| 106264_at | 0.00 |
| 106265_at | 0.01 |
| 106267_s_at | 0.00 |
| 106268_at | 0.00 |
| 106269_at | 0.00 |
| 106270_at | 0.00 |
| 106272_at | 0.00 |
| 106274_at | 0.04 |
| 106275_at | 0.00 |
| 106276_at | 0.04 |
| 106277_at | 0.00 |
| 106279_at | 0.00 |
| 106280_at | 0.00 |
| 106281_f_at | 0.18 |
| 106282_r_at | 0.00 |
| 106283_at | 0.00 |
| 106284_at | 0.00 |
| 106285_at | 0.00 |
| 106286_at | 0.00 |
| 106287_at | 0.00 |
| 106288_at | 0.00 |
| 106289_at | 0.02 |
| 106290_at | 0.00 |
| 106291_at | 0.00 |
| 106292_at | 0.00 |
| 106294_at | 0.01 |
| 106295_at | 0.00 |
| 106297_at | 0.00 |
| 106298_r_at | 0.00 |
| 106299_at | 0.00 |
| 106300_at | 0.00 |
| 106301_at | 0.00 |
| 106302_at | 0.08 |
| 106303_at | 0.00 |
| 106304_at | 0.00 |
| 106306_at | 0.00 |
| 106309_at | 0.00 |
| 106310_at | 0.04 |
| 106311_at | 0.00 |
| 106312_at | 0.00 |

| | |
|---|---|
| 106313_at | 0.00 |
| 106434_at | 0.00 |
| 106435_at | 0.00 |
| 106436_at | 0.00 |
| 106437_at | 0.00 |
| 106438_at | 0.00 |
| 106439_at | 0.00 |
| 106440_at | 0.00 |
| 106441_at | 0.00 |
| 106442_at | 0.00 |
| 106443_at | 0.00 |
| 106444_at | 0.00 |
| 106447_at | 0.00 |
| 106448_at | 0.00 |
| 106449_at | 0.00 |
| 106452_s_at | 0.01 |
| 106453_i_at | 0.00 |
| 106454_at | 0.00 |
| 106455_at | 0.00 |
| 106456_at | 0.00 |
| 106457_at | 0.00 |
| 106458_at | 0.00 |
| 106459_at | 0.00 |
| 106460_at | 0.00 |
| 106461_at | 0.00 |
| 106462_g_at | 0.00 |
| 106463_at | 0.00 |
| 106464_at | 0.00 |
| 106465_at | 0.00 |
| 106466_at | 0.00 |
| 106467_at | 0.00 |
| 106468_at | 0.00 |
| 106469_at | 0.00 |
| 106470_at | 0.00 |
| 106471_at | 0.00 |
| 106472_at | 0.00 |
| 106473_at | 0.00 |
| 106474_at | 0.00 |
| 106475_at | 0.00 |
| 106476_at | 0.04 |
| 106477_at | 0.00 |
| 106478_at | 0.00 |
| 106479_at | 0.04 |
| 106480_at | 0.00 |
| 106481_at | 0.00 |
| 106482_at | 0.00 |
| 106483_at | 0.00 |
| 106484_at | 0.00 |
| 106485_at | 0.00 |
| 106486_at | 0.00 |
| 106487_at | 0.00 |
| 106488_at | 0.00 |
| 106489_at | 0.00 |
| 106490_at | 0.00 |
| 106491_at | 0.00 |
| 106492_at | 0.02 |

| | |
|---|---|
| 106493_at | 0.04 |
| 106494_at | 0.00 |
| 106495_at | 0.00 |
| 106496_at | 0.00 |
| 106497_at | 0.00 |
| 106498_i_at | 0.00 |
| 106499_at | 0.00 |
| 106500_f_at | 0.03 |
| 106501_at | 0.00 |
| 106502_at | 0.00 |
| 106503_at | 0.00 |
| 106504_at | 0.00 |
| 106505_at | 0.00 |
| 106506_at | 0.00 |
| 106507_at | 0.00 |
| 106508_at | 0.00 |
| 106509_at | 0.00 |
| 106510_f_at | 0.00 |
| 106511_at | 0.00 |
| 106512_at | 0.00 |
| 106513_at | 0.00 |
| 106515_at | 0.00 |
| 106516_at | 0.00 |
| 106517_at | 0.00 |
| 106518_at | 0.00 |
| 106519_at | 0.00 |
| 106520_at | 0.00 |
| 106521_at | 0.00 |
| 106522_at | 0.00 |
| 106524_at | 0.02 |
| 106525_at | 0.00 |
| 106526_at | 0.00 |
| 106527_at | 0.00 |
| 106528_at | 0.00 |
| 106529_at | 0.00 |
| 106530_at | 0.00 |
| 106531_at | 0.08 |
| 106532_at | 0.00 |
| 106533_at | 0.00 |
| 106534_at | 0.00 |
| 106535_at | 0.00 |
| 106536_f_at | 0.00 |
| 106537_at | 0.00 |
| 106538_at | 0.00 |
| 106539_at | 0.00 |
| 106540_at | 0.00 |
| 106541_at | 0.00 |
| 106542_at | 0.00 |
| 106543_at | 0.00 |
| 106544_at | 0.00 |
| 106545_at | 0.00 |
| 106546_at | 0.00 |
| 106547_at | 0.00 |
| 106548_at | 0.00 |
| 106549_at | 0.00 |
| 106550_at | 0.00 |

| | |
|---|---|
| 106551_at | 0.00 |
| 106552_at | 0.00 |
| 106553_at | 0.00 |
| 106554_at | 0.00 |
| 106555_at | 0.00 |
| 106556_at | 0.00 |
| 106557_at | 0.23 |
| 106558_at | 0.00 |
| 106561_at | 0.00 |
| 106562_at | 0.00 |
| 106564_at | 0.00 |
| 106565_at | 0.00 |
| 106567_at | 0.00 |
| 106568_at | 0.27 |
| 106569_at | 0.00 |
| 106570_at | 0.28 |
| 106571_at | 0.00 |
| 106572_at | 0.00 |
| 106573_at | 0.00 |
| 106574_at | 0.00 |
| 106575_at | 0.00 |
| 106576_at | 0.00 |
| 106577_at | 0.35 |
| 106578_at | 0.00 |
| 106580_at | 0.00 |
| 106581_at | 0.00 |
| 106582_at | 0.00 |
| 106583_at | 0.03 |
| 106584_at | 0.00 |
| 106586_at | 0.00 |
| 106587_at | 0.00 |
| 106588_at | 0.00 |
| 106590_at | 0.00 |
| 106592_at | 0.00 |
| 106593_g_at | 0.00 |
| 106594_at | 0.00 |
| 106596_at | 0.00 |
| 106597_at | 0.03 |
| 106598_at | 0.00 |
| 106600_at | 0.00 |
| 106602_at | 0.00 |
| 106603_at | 0.00 |
| 106604_at | 0.00 |
| 106605_at | 0.00 |
| 106606_at | 0.00 |
| 106607_at | 0.17 |
| 106608_at | 0.00 |
| 106609_at | 0.00 |
| 106612_at | 0.00 |
| 106614_at | 0.00 |
| 106615_at | 0.00 |
| 106616_at | 0.00 |
| 106617_at | 0.00 |
| 106618_at | 0.00 |
| 106619_at | 0.00 |
| 106620_at | 0.00 |

| | |
|---|---|
| 106623_at | 0.01 |
| 106624_at | 0.00 |
| 106625_at | 0.00 |
| 106627_at | 0.00 |
| 106629_at | 0.00 |
| 106630_at | 0.00 |
| 106632_at | 0.00 |
| 106633_at | 0.00 |
| 106634_at | 0.00 |
| 106635_at | 0.00 |
| 106636_s_at | 0.00 |
| 106637_r_at | 0.00 |
| 106638_at | 0.00 |
| 106640_at | 0.02 |
| 106642_at | 0.00 |
| 106643_at | 0.00 |
| 106644_at | 0.00 |
| 106647_at | 0.00 |
| 106648_at | 0.00 |
| 106649_at | 0.00 |
| 106650_at | 0.00 |
| 106651_at | 0.01 |
| 106652_at | 0.00 |
| 106653_at | 0.00 |
| 106654_at | 0.01 |
| 106656_at | 0.00 |
| 106657_at | 0.00 |
| 106659_at | 0.00 |
| 106661_at | 0.04 |
| 106663_at | 0.00 |
| 106664_at | 0.00 |
| 106666_at | 0.01 |
| 106667_at | 0.00 |
| 106670_at | 0.00 |
| 106672_at | 0.58 |
| 106673_at | 0.00 |
| 106745_f_at | 0.00 |
| 106757_f_at | 0.00 |
| 106794_at | 0.00 |
| 106795_at | 0.00 |
| 106796_at | 0.00 |
| 106797_at | 0.00 |
| 106798_at | 0.00 |
| 106799_at | 0.00 |
| 106800_at | 0.00 |
| 106801_at | 0.00 |
| 106802_at | 0.00 |
| 106803_at | 0.00 |
| 106804_at | 0.00 |
| 106805_at | 0.00 |
| 106806_at | 0.00 |
| 106807_i_at | 0.00 |
| 106808_r_at | 0.00 |
| 106809_at | 0.00 |
| 106810_at | 0.00 |
| 106811_at | 0.00 |

| | |
|---|---|
| 106812_at | 0.00 |
| 106813_at | 0.00 |
| 106814_at | 0.00 |
| 106815_at | 0.00 |
| 106816_at | 0.00 |
| 106817_at | 0.00 |
| 106819_at | 0.00 |
| 106820_at | 0.00 |
| 106821_at | 0.00 |
| 106822_at | 0.00 |
| 106823_at | 0.00 |
| 106824_at | 0.00 |
| 106825_at | 0.00 |
| 106826_at | 0.00 |
| 106827_at | 0.00 |
| 106828_at | 0.00 |
| 106829_at | 0.00 |
| 106830_at | 0.00 |
| 106831_at | 0.00 |
| 106832_at | 0.00 |
| 106833_at | 0.00 |
| 106834_at | 0.00 |
| 106835_at | 0.00 |
| 106836_at | 0.00 |
| 106837_at | 0.00 |
| 106838_at | 0.00 |
| 106839_at | 0.00 |
| 106840_at | 0.00 |
| 106841_at | 0.00 |
| 106842_at | 0.00 |
| 106843_at | 0.00 |
| 106844_at | 0.00 |
| 106845_at | 0.00 |
| 106846_at | 0.00 |
| 106847_at | 0.00 |
| 106848_at | 0.00 |
| 106849_at | 0.00 |
| 106850_at | 0.00 |
| 106851_at | 0.00 |
| 106852_at | 0.00 |
| 106853_at | 0.00 |
| 106854_at | 0.00 |
| 106855_at | 0.00 |
| 106856_at | 0.00 |
| 106857_at | 0.00 |
| 106858_at | 0.00 |
| 106859_at | 0.00 |
| 106860_at | 0.00 |
| 106861_at | 0.00 |
| 106862_at | 0.00 |
| 106863_at | 0.00 |
| 106864_at | 0.00 |
| 106865_at | 0.00 |
| 106866_at | 0.00 |
| 106867_at | 0.00 |
| 106868_at | 0.00 |

| | |
|---|---|
| 106869_at | 0.00 |
| 106870_at | 0.00 |
| 106871_at | 0.00 |
| 106872_at | 0.30 |
| 106873_at | 0.00 |
| 106874_at | 0.00 |
| 106875_at | 0.00 |
| 106876_at | 0.00 |
| 106877_at | 0.00 |
| 106878_at | 0.00 |
| 106879_at | 0.00 |
| 106880_at | 0.00 |
| 106881_at | 0.00 |
| 106882_at | 0.00 |
| 106883_at | 0.00 |
| 106884_at | 0.00 |
| 106885_at | 0.00 |
| 106886_at | 0.00 |
| 106888_at | 0.00 |
| 106889_at | 0.00 |
| 106890_at | 0.00 |
| 106891_at | 0.00 |
| 106892_at | 0.00 |
| 106893_at | 0.00 |
| 106894_at | 0.00 |
| 106895_at | 0.00 |
| 106896_at | 0.00 |
| 106897_at | 0.00 |
| 106898_at | 0.00 |
| 106899_at | 0.00 |
| 106900_at | 0.00 |
| 106901_at | 0.00 |
| 106902_at | 0.00 |
| 106903_at | 0.00 |
| 106905_at | 0.00 |
| 106906_at | 0.00 |
| 106907_at | 0.00 |
| 106908_at | 0.00 |
| 106909_at | 0.00 |
| 106910_at | 0.00 |
| 106911_at | 0.00 |
| 106913_at | 0.00 |
| 106914_at | 0.00 |
| 106915_at | 0.00 |
| 106916_at | 0.02 |
| 106917_at | 0.20 |
| 106918_at | 0.00 |
| 106919_at | 0.00 |
| 106920_at | 0.00 |
| 106921_at | 0.00 |
| 106922_at | 0.00 |
| 106923_at | 0.00 |
| 106925_at | 0.00 |
| 106927_at | 0.00 |
| 106928_i_at | 0.00 |
| 106929_r_at | 0.00 |

| | |
|---|---|
| 106930_at | 0.00 |
| 106931_at | 0.01 |
| 106933_at | 0.00 |
| 106934_at | 0.00 |
| 106935_at | 0.00 |
| 106936_at | 0.00 |
| 106938_at | 0.63 |
| 106939_at | 0.00 |
| 106940_at | 0.00 |
| 106941_at | 0.00 |
| 106942_at | 0.00 |
| 106943_at | 0.00 |
| 106944_g_at | 0.00 |
| 106945_at | 0.00 |
| 106947_at | 0.00 |
| 106948_at | 0.00 |
| 106949_at | 0.00 |
| 106951_at | 0.00 |
| 106952_at | 0.00 |
| 106953_i_at | 0.04 |
| 106954_f_at | 0.00 |
| 106955_at | 0.00 |
| 106956_at | 0.13 |
| 106957_f_at | 0.39 |
| 106958_r_at | 0.02 |
| 106959_at | 0.00 |
| 106961_at | 0.00 |
| 106962_at | 0.00 |
| 106963_at | 0.00 |
| 106964_at | 0.00 |
| 106965_at | 0.00 |
| 106966_at | 0.00 |
| 106967_at | 0.00 |
| 106968_at | 0.00 |
| 106970_at | 0.46 |
| 106971_at | 0.00 |
| 106972_at | 0.00 |
| 106973_at | 0.00 |
| 106975_at | 0.00 |
| 106976_at | 0.00 |
| 106977_at | 0.00 |
| 106978_at | 0.02 |
| 106979_at | 0.00 |
| 106980_at | 0.00 |
| 106981_at | 0.00 |
| 106983_at | 0.00 |
| 106984_at | 0.00 |
| 106985_at | 0.00 |
| 106986_at | 0.00 |
| 106987_at | 0.00 |
| 106988_at | 0.00 |
| 106989_at | 0.00 |
| 106990_at | 0.00 |
| 106991_at | 0.02 |
| 106992_at | 0.00 |
| 106993_at | 0.00 |

| | |
|---|---|
| 106997_at | 0.02 |
| 106998_at | 0.00 |
| 107001_at | 0.00 |
| 107002_at | 0.00 |
| 107003_at | 0.00 |
| 107005_at | 0.01 |
| 107007_at | 0.15 |
| 107008_at | 0.00 |
| 107009_at | 0.00 |
| 107012_at | 0.00 |
| 107013_at | 0.00 |
| 107014_g_at | 0.00 |
| 107015_at | 0.00 |
| 107016_at | 0.00 |
| 107017_at | 0.00 |
| 107018_at | 0.00 |
| 107019_at | 0.00 |
| 107020_at | 0.00 |
| 107021_at | 0.00 |
| 107022_at | 0.00 |
| 107023_at | 0.00 |
| 107024_at | 0.00 |
| 107025_at | 0.00 |
| 107026_at | 0.00 |
| 107027_at | 0.00 |
| 107028_s_at | 0.00 |
| 107029_at | 0.00 |
| 107030_at | 0.00 |
| 107031_at | 0.00 |
| 107032_at | 0.00 |
| 107033_at | 0.00 |
| 107037_f_at | 0.00 |
| 107038_r_at | 0.00 |
| 107041_at | 0.00 |
| 107042_at | 0.06 |
| 107043_at | 0.00 |
| 107044_at | 0.00 |
| 107045_at | 0.03 |
| 107046_at | 0.00 |
| 107047_at | 0.00 |
| 107049_at | 0.06 |
| 107050_at | 0.02 |
| 107051_at | 0.00 |
| 107053_at | 0.00 |
| 107055_at | 0.00 |
| 107056_at | 0.00 |
| 107057_at | 0.00 |
| 107058_at | 0.00 |
| 107059_at | 0.00 |
| 107060_at | 0.00 |
| 107062_at | 0.10 |
| 107063_at | 0.00 |
| 107064_at | 0.00 |
| 107065_at | 0.00 |
| 107066_s_at | 0.00 |
| 107067_at | 0.12 |

| | |
|---|---|
| 107068_at | 0.00 |
| 107071_at | 0.00 |
| 107072_at | 0.00 |
| 107074_at | 0.00 |
| 107075_at | 0.00 |
| 107077_at | 0.00 |
| 107078_at | 0.00 |
| 107080_at | 0.00 |
| 107081_at | 0.00 |
| 107082_at | 0.00 |
| 107083_at | 0.00 |
| 107085_at | 0.00 |
| 107086_at | 0.00 |
| 107087_at | 0.00 |
| 107088_at | 0.00 |
| 107089_at | 0.00 |
| 107090_at | 0.00 |
| 107091_at | 0.13 |
| 107092_at | 0.00 |
| 107094_at | 0.00 |
| 107095_at | 0.00 |
| 107096_at | 0.00 |
| 107099_at | 0.00 |
| 107100_at | 0.00 |
| 107101_at | 0.00 |
| 107102_at | 0.00 |
| 107103_at | 0.96 |
| 107105_at | 0.11 |
| 107109_at | 0.00 |
| 107110_at | 0.00 |
| 107111_at | 0.00 |
| 107112_at | 0.00 |
| 107113_at | 0.00 |
| 107115_s_at | 0.00 |
| 107116_at | 0.00 |
| 107117_at | 0.00 |
| 107119_at | 0.00 |
| 107120_at | 0.00 |
| 107121_at | 0.00 |
| 107122_at | 0.00 |
| 107123_at | 0.00 |
| 107124_at | 0.06 |
| 107127_at | 0.00 |
| 107128_at | 0.00 |
| 107130_at | 0.27 |
| 107131_at | 0.00 |
| 107132_at | 0.00 |
| 107133_at | 0.00 |
| 107135_at | 0.00 |
| 107136_at | 0.02 |
| 107137_at | 0.00 |
| 107139_at | 0.00 |
| 107142_at | 0.00 |
| 107143_at | 0.00 |
| 107144_at | 0.00 |
| 107145_at | 0.00 |

| | |
|---|---|
| 107146_at | 0.00 |
| 107147_at | 0.00 |
| 107149_at | 0.00 |
| 107150_at | 0.00 |
| 107151_at | 0.03 |
| 107152_at | 0.00 |
| 107154_f_at | 0.01 |
| 107162_f_at | 0.00 |
| 107173_f_at | 0.00 |
| 107180_at | 0.00 |
| 107201_f_at | 0.00 |
| 107220_i_at | 0.00 |
| 107249_at | 0.00 |
| 107273_at | 0.00 |
| 107274_at | 0.05 |
| 107275_at | 0.00 |
| 107276_at | 0.00 |
| 107277_at | 0.00 |
| 107278_at | 0.00 |
| 107279_at | 0.05 |
| 107281_at | 0.00 |
| 107282_at | 0.00 |
| 107283_at | 0.00 |
| 107284_at | 0.00 |
| 107285_at | 0.00 |
| 107286_at | 0.00 |
| 107287_at | 0.00 |
| 107288_at | 0.00 |
| 107289_at | 0.00 |
| 107290_at | 0.00 |
| 107291_at | 0.00 |
| 107292_at | 0.00 |
| 107293_at | 0.00 |
| 107294_at | 0.00 |
| 107295_at | 0.00 |
| 107296_at | 0.00 |
| 107297_at | 0.00 |
| 107298_at | 0.00 |
| 107299_at | 0.00 |
| 107300_at | 0.00 |
| 107301_at | 0.00 |
| 107302_at | 0.00 |
| 107303_at | 0.00 |
| 107304_at | 0.00 |
| 107305_at | 0.00 |
| 107306_at | 0.00 |
| 107307_at | 0.00 |
| 107308_at | 0.00 |
| 107309_at | 0.00 |
| 107310_at | 0.21 |
| 107311_at | 0.00 |
| 107312_at | 0.00 |
| 107313_at | 0.00 |
| 107314_at | 0.00 |
| 107315_at | 0.00 |
| 107316_at | 0.00 |

| | |
|---|---|
| 107317_at | 0.00 |
| 107318_at | 0.00 |
| 107319_at | 0.00 |
| 107320_at | 0.00 |
| 107321_at | 0.00 |
| 107322_at | 0.00 |
| 107323_at | 0.00 |
| 107324_at | 0.00 |
| 107325_at | 0.00 |
| 107326_at | 0.00 |
| 107327_at | 0.00 |
| 107328_at | 0.00 |
| 107329_at | 0.00 |
| 107330_at | 0.00 |
| 107331_at | 0.00 |
| 107332_at | 0.00 |
| 107333_at | 0.00 |
| 107334_r_at | 0.00 |
| 107335_at | 0.00 |
| 107336_i_at | 0.00 |
| 107337_r_at | 0.00 |
| 107338_at | 0.00 |
| 107339_at | 0.00 |
| 107340_at | 0.00 |
| 107341_at | 0.00 |
| 107342_at | 0.00 |
| 107343_at | 0.00 |
| 107344_at | 0.00 |
| 107345_at | 0.00 |
| 107346_at | 0.00 |
| 107347_at | 0.00 |
| 107348_at | 0.00 |
| 107349_at | 0.00 |
| 107350_at | 0.00 |
| 107351_at | 0.00 |
| 107352_at | 0.00 |
| 107353_at | 0.00 |
| 107354_at | 0.12 |
| 107355_r_at | 0.00 |
| 107356_at | 0.00 |
| 107357_at | 0.00 |
| 107358_at | 0.00 |
| 107359_at | 0.00 |
| 107360_at | 0.00 |
| 107361_at | 0.00 |
| 107362_at | 0.00 |
| 107363_at | 0.00 |
| 107364_at | 0.00 |
| 107365_at | 0.00 |
| 107366_at | 0.00 |
| 107367_at | 0.00 |
| 107368_at | 0.00 |
| 107369_at | 0.00 |
| 107370_at | 0.00 |
| 107371_at | 0.00 |
| 107372_at | 0.00 |

| | |
|---|---|
| 107373_at | 0.00 |
| 107374_at | 0.00 |
| 107375_at | 0.00 |
| 107376_at | 0.00 |
| 107377_at | 0.00 |
| 107378_at | 0.00 |
| 107379_at | 0.00 |
| 107380_at | 0.00 |
| 107381_at | 0.00 |
| 107382_at | 0.00 |
| 107383_at | 0.00 |
| 107385_at | 0.00 |
| 107386_at | 0.01 |
| 107387_at | 0.00 |
| 107388_at | 0.00 |
| 107389_at | 0.00 |
| 107390_at | 0.00 |
| 107391_at | 0.00 |
| 107392_at | 0.00 |
| 107393_at | 0.00 |
| 107394_at | 0.00 |
| 107395_at | 0.00 |
| 107396_at | 0.00 |
| 107397_at | 0.00 |
| 107398_at | 0.00 |
| 107399_at | 0.00 |
| 107400_at | 0.08 |
| 107401_at | 0.00 |
| 107402_at | 0.00 |
| 107403_at | 0.00 |
| 107404_at | 0.00 |
| 107406_at | 0.16 |
| 107407_at | 0.00 |
| 107408_at | 0.00 |
| 107410_at | 0.00 |
| 107411_at | 0.00 |
| 107412_at | 0.00 |
| 107415_at | 0.00 |
| 107416_at | 0.00 |
| 107417_at | 0.00 |
| 107418_at | 0.00 |
| 107419_at | 0.00 |
| 107420_at | 0.02 |
| 107421_at | 0.00 |
| 107422_at | 0.00 |
| 107423_at | 0.00 |
| 107424_at | 0.00 |
| 107425_at | 0.00 |
| 107426_at | 0.00 |
| 107427_at | 0.00 |
| 107428_at | 0.00 |
| 107429_at | 0.00 |
| 107431_at | 0.00 |
| 107433_at | 0.06 |
| 107434_at | 0.00 |
| 107435_at | 0.01 |

| | |
|---|---|
| 107436_at | 0.00 |
| 107437_at | 0.00 |
| 107438_at | 0.00 |
| 107439_at | 0.00 |
| 107440_at | 0.00 |
| 107441_at | 0.00 |
| 107442_at | 0.00 |
| 107443_at | 0.00 |
| 107444_at | 0.00 |
| 107445_at | 0.00 |
| 107446_at | 0.00 |
| 107447_at | 0.00 |
| 107448_at | 0.00 |
| 107449_at | 0.00 |
| 107450_at | 0.00 |
| 107453_at | 0.00 |
| 107454_at | 0.00 |
| 107455_at | 0.00 |
| 107456_at | 0.01 |
| 107457_at | 0.01 |
| 107459_at | 0.00 |
| 107460_at | 0.00 |
| 107461_at | 0.00 |
| 107462_at | 0.00 |
| 107463_at | 0.06 |
| 107464_at | 0.00 |
| 107465_at | 0.00 |
| 107466_at | 0.00 |
| 107467_at | 0.49 |
| 107468_at | 0.00 |
| 107469_at | 0.00 |
| 107470_at | 0.00 |
| 107471_at | 0.00 |
| 107472_at | 0.00 |
| 107473_at | 0.00 |
| 107474_at | 0.00 |
| 107475_at | 0.00 |
| 107476_at | 0.00 |
| 107477_at | 0.00 |
| 107478_at | 0.00 |
| 107479_at | 0.00 |
| 107480_at | 0.00 |
| 107481_at | 0.00 |
| 107482_at | 0.00 |
| 107483_at | 0.00 |
| 107485_at | 0.00 |
| 107486_g_at | 0.00 |
| 107487_at | 0.00 |
| 107488_at | 0.00 |
| 107489_at | 0.00 |
| 107490_at | 0.00 |
| 107491_at | 0.00 |
| 107492_at | 0.00 |
| 107493_at | 0.00 |
| 107494_at | 0.00 |
| 107495_at | 0.04 |

| | |
|---|---|
| 107497_at | 0.00 |
| 107498_at | 0.00 |
| 107501_at | 0.00 |
| 107502_at | 0.00 |
| 107503_at | 0.00 |
| 107505_at | 0.00 |
| 107506_at | 0.05 |
| 107507_at | 0.00 |
| 107508_at | 0.00 |
| 107509_at | 0.00 |
| 107510_at | 0.00 |
| 107511_at | 0.00 |
| 107512_at | 0.00 |
| 107513_at | 0.00 |
| 107515_at | 0.01 |
| 107516_at | 0.00 |
| 107517_at | 0.00 |
| 107518_at | 0.00 |
| 107519_at | 0.00 |
| 107520_at | 0.00 |
| 107521_at | 0.00 |
| 107523_at | 0.00 |
| 107525_at | 0.00 |
| 107526_at | 0.02 |
| 107527_at | 0.00 |
| 107528_at | 0.00 |
| 107529_at | 0.00 |
| 107530_at | 0.26 |
| 107531_at | 0.00 |
| 107532_at | 0.00 |
| 107533_at | 0.00 |
| 107534_at | 0.00 |
| 107536_at | 0.02 |
| 107537_at | 0.00 |
| 107538_at | 0.00 |
| 107539_at | 0.00 |
| 107541_at | 0.00 |
| 107542_at | 0.00 |
| 107543_s_at | 0.00 |
| 107544_r_at | 0.00 |
| 107545_at | 0.00 |
| 107546_at | 0.00 |
| 107547_at | 0.00 |
| 107548_at | 0.00 |
| 107549_f_at | 0.00 |
| 107552_at | 0.00 |
| 107553_at | 0.00 |
| 107554_at | 0.00 |
| 107555_at | 0.00 |
| 107556_at | 0.00 |
| 107557_at | 0.00 |
| 107558_at | 0.02 |
| 107560_at | 0.00 |
| 107561_at | 0.00 |
| 107562_g_at | 0.00 |
| 107563_at | 0.00 |

| | |
|---|---|
| 107564_at | 0.00 |
| 107565_at | 0.00 |
| 107566_at | 0.00 |
| 107567_at | 0.00 |
| 107568_at | 0.00 |
| 107569_at | 0.00 |
| 107571_at | 0.03 |
| 107572_at | 0.00 |
| 107574_at | 0.21 |
| 107575_at | 0.14 |
| 107577_at | 0.00 |
| 107579_at | 0.00 |
| 107580_g_at | 0.01 |
| 107581_at | 0.00 |
| 107582_at | 0.19 |
| 107583_at | 0.00 |
| 107585_at | 0.00 |
| 107587_at | 0.00 |
| 107588_at | 0.00 |
| 107589_at | 0.00 |
| 107590_at | 0.00 |
| 107591_at | 0.00 |
| 107592_at | 0.00 |
| 107593_at | 0.00 |
| 107594_at | 0.00 |
| 107596_at | 0.00 |
| 107597_f_at | 0.00 |
| 107598_at | 0.00 |
| 107599_at | 0.00 |
| 107600_at | 0.00 |
| 107601_at | 0.00 |
| 107602_at | 0.00 |
| 107605_at | 0.00 |
| 107608_at | 0.00 |
| 107609_at | 0.00 |
| 107612_at | 0.00 |
| 107613_at | 0.39 |
| 107614_at | 0.19 |
| 107615_f_at | 0.00 |
| 107616_at | 0.00 |
| 107617_at | 0.00 |
| 107618_at | 0.00 |
| 107619_s_at | 0.00 |
| 107620_at | 0.00 |
| 107621_at | 0.00 |
| 107622_at | 0.00 |
| 107623_at | 0.04 |
| 107624_at | 0.00 |
| 107626_at | 0.28 |
| 107629_at | 0.02 |
| 107630_at | 0.00 |
| 107631_at | 0.00 |
| 107632_at | 0.00 |
| 107664_at | 0.00 |
| 107677_at | 0.00 |
| 107752_at | 0.00 |

| | |
|---|---|
| 107753_at | 0.00 |
| 107754_f_at | 0.00 |
| 107755_f_at | 0.00 |
| 107756_at | 0.00 |
| 107758_at | 0.00 |
| 107759_at | 0.00 |
| 107763_at | 0.00 |
| 107764_at | 0.00 |
| 107766_at | 0.00 |
| 107767_at | 0.00 |
| 107769_r_at | 0.00 |
| 107772_at | 0.00 |
| 107774_at | 0.15 |
| 107775_at | 0.00 |
| 107776_at | 0.00 |
| 107777_at | 0.00 |
| 107779_at | 0.00 |
| 107780_at | 0.00 |
| 107781_at | 0.00 |
| 107782_g_at | 0.00 |
| 107784_at | 0.00 |
| 107786_at | 0.00 |
| 107787_at | 0.00 |
| 107788_at | 0.00 |
| 107789_at | 0.00 |
| 107790_at | 0.00 |
| 107791_at | 0.00 |
| 107792_at | 0.00 |
| 107793_at | 0.00 |
| 107795_at | 0.00 |
| 107796_at | 0.00 |
| 107797_at | 0.05 |
| 107802_at | 0.00 |
| 107804_at | 0.06 |
| 107806_at | 0.00 |
| 107807_at | 0.00 |
| 107808_at | 0.00 |
| 107809_at | 0.00 |
| 107810_at | 0.00 |
| 107811_at | 0.06 |
| 107812_at | 0.00 |
| 107813_at | 0.00 |
| 107815_at | 0.00 |
| 107817_at | 0.00 |
| 107818_at | 0.00 |
| 107821_at | 0.00 |
| 107825_at | 0.00 |
| 107826_at | 0.00 |
| 107827_at | 0.00 |
| 107828_at | 0.00 |
| 107829_at | 0.01 |
| 107830_at | 0.00 |
| 107832_at | 0.00 |
| 107833_at | 0.00 |
| 107835_at | 0.00 |
| 107836_at | 0.00 |

| | |
|---|---|
| 107843_at | 0.00 |
| 107844_at | 0.00 |
| 107845_at | 0.00 |
| 107846_at | 0.00 |
| 107847_at | 0.00 |
| 107848_at | 0.00 |
| 107849_at | 0.00 |
| 107850_at | 0.00 |
| 107851_at | 0.00 |
| 107852_at | 0.00 |
| 107853_at | 0.00 |
| 107854_at | 0.00 |
| 107855_at | 0.00 |
| 107856_at | 0.00 |
| 107857_at | 0.00 |
| 107859_at | 0.00 |
| 107860_at | 0.00 |
| 107861_at | 0.00 |
| 107862_at | 0.00 |
| 107863_at | 0.00 |
| 107864_at | 0.00 |
| 107865_at | 0.00 |
| 107866_at | 0.00 |
| 107867_at | 0.00 |
| 107868_at | 0.00 |
| 107869_at | 0.00 |
| 107870_at | 0.00 |
| 107871_at | 0.00 |
| 107872_r_at | 0.00 |
| 107874_at | 0.00 |
| 107875_at | 0.00 |
| 107876_at | 0.00 |
| 107877_at | 0.00 |
| 107878_at | 0.01 |
| 107879_at | 0.00 |
| 107880_at | 0.00 |
| 107881_at | 0.00 |
| 107882_at | 0.00 |
| 107883_at | 0.00 |
| 107884_at | 0.00 |
| 107885_at | 0.01 |
| 107886_at | 0.00 |
| 107887_at | 0.00 |
| 107888_at | 0.00 |
| 107889_at | 0.01 |
| 107890_at | 0.00 |
| 107893_at | 0.00 |
| 107894_at | 0.00 |
| 107895_at | 0.76 |
| 107897_at | 0.00 |
| 107898_at | 0.00 |
| 107900_at | 0.00 |
| 107904_at | 0.00 |
| 107906_at | 0.00 |
| 107907_at | 0.00 |
| 107910_at | 0.00 |

| | |
|---|---|
| 107911_at | 0.00 |
| 107912_at | 0.00 |
| 107914_at | 0.00 |
| 107915_at | 0.00 |
| 107916_at | 0.00 |
| 107917_at | 0.13 |
| 107918_at | 0.00 |
| 107920_at | 0.00 |
| 107921_at | 0.00 |
| 107922_at | 0.00 |
| 107923_at | 0.00 |
| 107924_at | 0.00 |
| 107925_at | 0.03 |
| 107926_at | 0.00 |
| 107927_at | 0.00 |
| 107928_at | 0.00 |
| 107930_at | 0.00 |
| 107932_at | 0.00 |
| 107934_at | 0.00 |
| 107935_at | 0.00 |
| 107936_at | 0.00 |
| 107937_at | 0.00 |
| 107938_at | 0.00 |
| 107939_at | 0.00 |
| 107942_at | 0.00 |
| 107946_at | 0.06 |
| 107947_at | 0.00 |
| 107948_at | 0.00 |
| 107949_at | 0.00 |
| 107950_at | 0.00 |
| 107951_at | 0.00 |
| 107952_i_at | 0.80 |
| 107953_r_at | 0.69 |
| 107954_at | 0.01 |
| 107956_at | 0.04 |
| 107957_at | 0.00 |
| 107958_at | 0.00 |
| 107959_at | 0.00 |
| 107960_at | 0.00 |
| 107961_at | 0.00 |
| 107964_at | 0.00 |
| 107968_at | 0.00 |
| 107969_at | 0.00 |
| 107970_at | 0.00 |
| 107971_at | 0.00 |
| 107976_at | 0.00 |
| 107983_at | 0.00 |
| 107984_at | 0.00 |
| 107985_at | 0.00 |
| 107986_at | 0.00 |
| 107988_at | 0.00 |
| 107989_at | 0.00 |
| 107991_at | 0.00 |
| 107992_g_at | 0.00 |
| 107993_at | 0.00 |
| 107994_at | 0.04 |

| | |
|---|---|
| 107995_at | 0.00 |
| 107996_at | 0.00 |
| 107997_at | 0.00 |
| 107999_at | 0.00 |
| 108001_at | 0.00 |
| 108003_at | 0.00 |
| 108004_at | 0.00 |
| 108005_at | 0.00 |
| 108006_at | 0.00 |
| 108007_at | 0.01 |
| 108008_at | 0.00 |
| 108009_at | 0.00 |
| 108010_at | 0.54 |
| 108011_at | 0.00 |
| 108012_at | 0.00 |
| 108013_at | 0.00 |
| 108014_at | 0.43 |
| 108016_at | 0.04 |
| 108017_at | 0.00 |
| 108018_at | 0.00 |
| 108019_f_at | 0.00 |
| 108020_r_at | 0.00 |
| 108021_f_at | 0.00 |
| 108022_at | 0.00 |
| 108023_at | 0.00 |
| 108024_at | 0.00 |
| 108025_at | 0.00 |
| 108027_at | 0.00 |
| 108028_at | 0.00 |
| 108029_at | 0.00 |
| 108030_at | 0.00 |
| 108032_at | 0.00 |
| 108034_at | 0.00 |
| 108036_at | 0.14 |
| 108037_at | 0.00 |
| 108038_at | 0.00 |
| 108039_at | 0.00 |
| 108040_at | 0.00 |
| 108041_at | 0.07 |
| 108042_at | 0.00 |
| 108044_at | 0.00 |
| 108045_at | 0.00 |
| 108046_at | 0.00 |
| 108047_at | 0.00 |
| 108048_at | 0.08 |
| 108049_at | 0.00 |
| 108051_at | 0.01 |
| 108053_at | 0.00 |
| 108054_at | 0.00 |
| 108055_at | 0.00 |
| 108056_at | 0.00 |
| 108057_at | 0.00 |
| 108058_at | 0.05 |
| 108059_at | 0.00 |
| 108060_at | 0.00 |
| 108061_at | 0.00 |

| | |
|---|---|
| 108062_at | 0.00 |
| 108064_at | 0.00 |
| 108066_at | 0.00 |
| 108068_at | 0.00 |
| 108069_at | 0.00 |
| 108070_s_at | 0.00 |
| 108071_at | 0.00 |
| 108072_at | 0.00 |
| 108073_at | 0.00 |
| 108075_at | 0.00 |
| 108076_at | 0.00 |
| 108077_at | 0.00 |
| 108078_at | 0.00 |
| 108079_at | 0.44 |
| 108080_at | 0.00 |
| 108081_at | 0.00 |
| 108082_at | 0.00 |
| 108084_at | 0.01 |
| 108085_at | 0.00 |
| 108087_at | 0.00 |
| 108088_at | 0.00 |
| 108090_at | 0.14 |
| 108091_at | 0.00 |
| 108093_at | 0.00 |
| 108094_at | 0.00 |
| 108095_at | 0.00 |
| 108096_at | 0.00 |
| 108097_at | 0.80 |
| 108098_f_at | 0.00 |
| 108099_at | 0.00 |
| 108100_at | 0.00 |
| 108101_at | 0.00 |
| 108104_at | 0.00 |
| 108105_at | 0.00 |
| 108106_at | 0.00 |
| 108108_at | 0.00 |
| 108109_at | 0.00 |
| 108230_at | 0.00 |
| 108231_at | 0.00 |
| 108232_r_at | 0.00 |
| 108233_at | 0.00 |
| 108236_at | 0.00 |
| 108238_at | 0.00 |
| 108240_at | 0.00 |
| 108241_at | 0.11 |
| 108242_at | 0.02 |
| 108243_at | 0.00 |
| 108244_at | 0.01 |
| 108245_at | 0.00 |
| 108246_at | 0.00 |
| 108247_at | 0.00 |
| 108248_at | 0.00 |
| 108249_at | 0.00 |
| 108250_at | 0.00 |
| 108251_at | 0.00 |
| 108252_at | 0.00 |

| | |
|---|---|
| 108253_at | 0.00 |
| 108254_at | 0.00 |
| 108255_at | 0.00 |
| 108256_at | 0.00 |
| 108257_at | 0.00 |
| 108260_at | 0.00 |
| 108261_at | 0.00 |
| 108262_at | 0.00 |
| 108263_at | 0.00 |
| 108264_at | 0.00 |
| 108265_at | 0.00 |
| 108266_at | 0.00 |
| 108267_at | 0.00 |
| 108268_at | 0.00 |
| 108269_at | 0.00 |
| 108270_at | 0.00 |
| 108271_at | 0.00 |
| 108272_at | 0.00 |
| 108273_at | 0.00 |
| 108274_at | 0.00 |
| 108275_at | 0.00 |
| 108276_at | 0.00 |
| 108277_at | 0.00 |
| 108278_at | 0.00 |
| 108279_at | 0.52 |
| 108280_at | 0.00 |
| 108281_at | 0.00 |
| 108282_at | 0.00 |
| 108283_at | 0.00 |
| 108284_at | 0.00 |
| 108286_at | 0.00 |
| 108287_r_at | 0.00 |
| 108288_at | 0.00 |
| 108289_at | 0.00 |
| 108290_at | 0.00 |
| 108291_r_at | 0.00 |
| 108293_at | 0.00 |
| 108294_at | 0.00 |
| 108295_at | 0.00 |
| 108296_at | 0.00 |
| 108298_at | 0.00 |
| 108300_at | 0.00 |
| 108302_at | 0.00 |
| 108303_at | 0.00 |
| 108304_at | 0.00 |
| 108305_at | 0.05 |
| 108306_at | 0.00 |
| 108307_at | 0.00 |
| 108308_at | 0.00 |
| 108309_at | 0.00 |
| 108310_at | 0.00 |
| 108311_at | 0.00 |
| 108312_at | 0.00 |
| 108313_at | 0.00 |
| 108314_at | 0.00 |
| 108315_at | 0.00 |

| | |
|---|---|
| 108316_at | 0.00 |
| 108317_at | 0.00 |
| 108318_at | 0.00 |
| 108319_at | 0.00 |
| 108320_at | 0.00 |
| 108321_at | 0.00 |
| 108322_at | 0.00 |
| 108323_f_at | 0.00 |
| 108324_r_at | 0.00 |
| 108325_at | 0.00 |
| 108326_at | 0.00 |
| 108327_at | 0.00 |
| 108328_at | 0.00 |
| 108329_at | 0.00 |
| 108330_at | 0.00 |
| 108331_at | 0.00 |
| 108332_at | 0.00 |
| 108333_at | 0.00 |
| 108334_at | 0.00 |
| 108335_r_at | 0.00 |
| 108336_at | 0.00 |
| 108337_at | 0.00 |
| 108339_at | 0.00 |
| 108340_at | 0.00 |
| 108341_at | 0.00 |
| 108344_at | 0.00 |
| 108345_at | 0.00 |
| 108346_at | 0.00 |
| 108347_at | 0.00 |
| 108348_at | 0.00 |
| 108349_at | 0.00 |
| 108350_at | 0.00 |
| 108352_at | 0.00 |
| 108353_at | 0.00 |
| 108354_at | 0.00 |
| 108355_at | 0.00 |
| 108356_at | 0.00 |
| 108357_at | 0.01 |
| 108358_at | 0.00 |
| 108359_at | 0.00 |
| 108360_at | 0.00 |
| 108361_at | 0.02 |
| 108362_g_at | 0.05 |
| 108365_at | 0.02 |
| 108366_at | 0.03 |
| 108367_at | 0.01 |
| 108368_at | 0.03 |
| 108369_at | 0.00 |
| 108370_at | 0.00 |
| 108371_at | 0.00 |
| 108373_at | 0.00 |
| 108375_at | 0.00 |
| 108376_at | 0.00 |
| 108377_at | 0.00 |
| 108379_at | 0.00 |
| 108380_at | 0.00 |

| | |
|---|---|
| 108381_at | 0.00 |
| 108382_at | 0.01 |
| 108383_at | 0.00 |
| 108384_g_at | 0.00 |
| 108385_at | 0.00 |
| 108389_at | 0.00 |
| 108397_r_at | 0.00 |
| 108399_at | 0.00 |
| 108408_at | 0.00 |
| 108415_at | 0.00 |
| 108416_at | 0.02 |
| 108417_at | 0.13 |
| 108418_at | 0.00 |
| 108420_at | 0.70 |
| 108421_at | 0.00 |
| 108423_at | 0.00 |
| 108425_at | 0.00 |
| 108426_f_at | 0.00 |
| 108427_at | 0.00 |
| 108433_at | 0.20 |
| 108440_at | 0.00 |
| 108458_at | 0.00 |
| 108460_at | 0.00 |
| 108461_at | 0.01 |
| 108464_at | 0.00 |
| 108465_at | 0.00 |
| 108466_at | 0.00 |
| 108467_at | 0.00 |
| 108468_at | 0.09 |
| 108470_at | 0.00 |
| 108471_at | 0.00 |
| 108473_at | 0.00 |
| 108474_at | 0.00 |
| 108475_at | 0.00 |
| 108476_at | 0.00 |
| 108477_at | 0.01 |
| 108478_f_at | 0.00 |
| 108479_at | 0.02 |
| 108481_at | 0.00 |
| 108484_at | 0.00 |
| 108486_at | 0.00 |
| 108487_at | 0.00 |
| 108488_at | 0.15 |
| 108489_at | 0.00 |
| 108490_at | 0.00 |
| 108491_at | 0.00 |
| 108492_at | 0.00 |
| 108493_at | 0.00 |
| 108494_at | 0.00 |
| 108495_at | 0.00 |
| 108496_at | 0.00 |
| 108497_at | 0.00 |
| 108499_at | 0.00 |
| 108500_at | 0.00 |
| 108502_at | 0.13 |
| 108503_at | 0.06 |

| | |
|---|---|
| 108504_at | 0.02 |
| 108505_at | 0.52 |
| 108506_at | 0.00 |
| 108507_at | 0.02 |
| 108511_at | 0.00 |
| 108512_at | 0.00 |
| 108513_at | 0.00 |
| 108515_at | 0.00 |
| 108516_at | 0.00 |
| 108519_at | 0.00 |
| 108520_at | 0.00 |
| 108521_at | 0.00 |
| 108523_at | 0.00 |
| 108524_at | 0.00 |
| 108526_at | 0.00 |
| 108530_at | 0.50 |
| 108531_at | 0.00 |
| 108532_at | 0.00 |
| 108533_at | 0.00 |
| 108534_at | 0.00 |
| 108535_at | 0.00 |
| 108536_at | 0.00 |
| 108537_at | 0.00 |
| 108538_at | 0.00 |
| 108539_at | 0.01 |
| 108540_at | 0.00 |
| 108541_at | 0.03 |
| 108542_at | 0.00 |
| 108543_at | 0.00 |
| 108545_at | 0.00 |
| 108546_at | 0.02 |
| 108547_at | 0.00 |
| 108548_at | 0.00 |
| 108551_at | 0.00 |
| 108552_at | 0.00 |
| 108553_at | 0.00 |
| 108554_at | 0.00 |
| 108555_at | 0.00 |
| 108556_at | 0.00 |
| 108558_at | 0.00 |
| 108559_at | 0.00 |
| 108560_at | 0.00 |
| 108561_at | 0.00 |
| 108562_at | 0.01 |
| 108564_at | 0.00 |
| 108565_at | 0.00 |
| 108566_at | 0.00 |
| 108567_g_at | 0.00 |
| 108568_at | 0.13 |
| 108569_at | 0.00 |
| 108570_at | 0.00 |
| 108571_at | 0.00 |
| 108574_at | 0.00 |
| 108575_at | 0.00 |
| 108576_at | 0.08 |
| 108577_at | 0.00 |

| | |
|---|---|
| 108579_at | 0.00 |
| 108580_at | 0.00 |
| 108581_at | 0.00 |
| 108582_at | 0.00 |
| 108585_at | 0.00 |
| 108586_at | 0.00 |
| 108587_at | 0.00 |
| 108614_f_at | 0.34 |
| 108709_at | 0.00 |
| 108710_at | 0.00 |
| 108711_at | 0.00 |
| 108712_at | 0.77 |
| 108713_at | 0.00 |
| 108714_f_at | 0.01 |
| 108717_at | 0.00 |
| 108718_at | 0.00 |
| 108719_at | 0.00 |
| 108721_at | 0.00 |
| 108722_at | 0.00 |
| 108723_at | 0.00 |
| 108726_at | 0.00 |
| 108727_g_at | 0.00 |
| 108728_at | 0.00 |
| 108729_at | 0.00 |
| 108730_at | 0.01 |
| 108732_at | 0.00 |
| 108733_at | 0.00 |
| 108734_at | 0.00 |
| 108735_at | 0.00 |
| 108736_at | 0.12 |
| 108737_at | 0.00 |
| 108739_at | 0.00 |
| 108741_at | 0.05 |
| 108742_at | 0.27 |
| 108743_at | 0.00 |
| 108744_at | 0.00 |
| 108745_at | 0.00 |
| 108747_at | 0.04 |
| 108748_g_at | 0.00 |
| 108749_at | 0.03 |
| 108750_at | 0.00 |
| 108751_at | 0.00 |
| 108752_at | 0.00 |
| 108753_at | 0.00 |
| 108754_at | 0.00 |
| 108755_at | 0.00 |
| 108756_at | 0.00 |
| 108758_at | 0.12 |
| 108759_at | 0.00 |
| 108760_at | 0.00 |
| 108762_at | 0.02 |
| 108763_at | 0.00 |
| 108764_at | 0.00 |
| 108765_at | 0.06 |
| 108766_at | 0.00 |
| 108767_at | 0.00 |

| | |
|---|---|
| 108770_at | 0.00 |
| 108773_at | 0.00 |
| 108777_at | 0.03 |
| 108780_at | 0.05 |
| 108781_at | 0.00 |
| 108782_at | 0.00 |
| 108783_at | 0.00 |
| 108784_at | 0.92 |
| 108785_at | 0.00 |
| 108786_at | 0.00 |
| 108787_at | 0.00 |
| 108788_at | 0.00 |
| 108789_at | 0.00 |
| 108790_at | 0.00 |
| 108791_i_at | 0.00 |
| 108792_r_at | 0.00 |
| 108794_at | 0.00 |
| 108795_at | 0.00 |
| 108796_at | 0.00 |
| 108797_at | 0.00 |
| 108798_at | 0.00 |
| 108802_at | 0.00 |
| 108803_f_at | 0.00 |
| 108804_r_at | 0.00 |
| 108805_f_at | 0.00 |
| 108806_r_at | 0.00 |
| 108807_at | 0.00 |
| 108808_at | 0.00 |
| 108809_at | 0.01 |
| 108811_at | 0.00 |
| 108812_at | 0.00 |
| 108813_at | 0.00 |
| 108814_at | 0.00 |
| 108816_at | 0.00 |
| 108817_at | 0.00 |
| 108818_at | 0.00 |
| 108820_at | 0.00 |
| 108822_at | 0.17 |
| 108823_at | 0.00 |
| 108825_at | 0.00 |
| 108826_i_at | 0.01 |
| 108827_f_at | 0.11 |
| 108828_at | 0.00 |
| 108829_at | 0.00 |
| 108830_at | 0.00 |
| 108831_at | 0.00 |
| 108832_at | 0.00 |
| 108833_at | 0.00 |
| 108835_at | 0.00 |
| 108836_at | 0.00 |
| 108837_at | 0.00 |
| 108838_at | 0.00 |
| 108839_at | 0.00 |
| 108840_at | 0.00 |
| 108841_at | 0.00 |
| 108842_at | 0.00 |

| | |
|---|---|
| 108843_at | 0.00 |
| 108844_at | 0.00 |
| 108845_at | 0.00 |
| 108846_at | 0.00 |
| 108847_at | 0.00 |
| 108848_g_at | 0.01 |
| 108849_at | 0.00 |
| 108850_at | 0.00 |
| 108851_at | 0.00 |
| 108852_at | 0.00 |
| 108853_at | 0.00 |
| 108855_at | 0.00 |
| 108856_at | 0.00 |
| 108859_at | 0.00 |
| 108860_at | 0.00 |
| 108864_at | 0.00 |
| 108865_at | 0.00 |
| 108866_at | 0.02 |
| 108867_at | 0.00 |
| 108868_at | 0.00 |
| 108869_at | 0.00 |
| 108870_at | 0.00 |
| 108871_at | 0.00 |
| 108872_at | 0.00 |
| 108873_at | 0.00 |
| 108874_at | 0.00 |
| 108875_at | 0.00 |
| 108876_at | 0.00 |
| 108877_at | 0.00 |
| 108878_at | 0.00 |
| 108879_at | 0.00 |
| 108880_at | 0.00 |
| 108882_at | 0.00 |
| 108883_at | 0.00 |
| 108884_at | 0.00 |
| 108885_at | 0.00 |
| 108886_at | 0.00 |
| 108887_at | 0.00 |
| 108888_at | 0.00 |
| 108889_at | 0.00 |
| 108890_at | 0.00 |
| 108891_at | 0.00 |
| 108893_at | 0.00 |
| 108894_at | 0.00 |
| 108895_at | 0.00 |
| 108896_at | 0.00 |
| 108897_at | 0.00 |
| 108898_at | 0.01 |
| 108899_at | 0.00 |
| 108900_at | 0.00 |
| 108901_at | 0.00 |
| 108902_at | 0.00 |
| 108903_at | 0.00 |
| 108904_at | 0.00 |
| 108905_at | 0.00 |
| 108906_at | 0.00 |

| | |
|---|---|
| 108907_at | 0.00 |
| 108908_at | 0.00 |
| 108910_at | 0.00 |
| 108911_at | 0.00 |
| 108912_at | 0.00 |
| 108913_at | 0.00 |
| 108915_at | 0.00 |
| 108916_at | 0.00 |
| 108917_at | 0.00 |
| 108918_at | 0.00 |
| 108919_at | 0.00 |
| 108920_at | 0.00 |
| 108921_at | 0.00 |
| 108922_at | 0.00 |
| 108923_at | 0.00 |
| 108924_at | 0.00 |
| 108925_at | 0.00 |
| 108926_at | 0.00 |
| 108927_at | 0.00 |
| 108928_at | 0.00 |
| 108929_at | 0.00 |
| 108930_at | 0.00 |
| 108931_at | 0.00 |
| 108932_at | 0.00 |
| 108933_at | 0.00 |
| 108934_at | 0.00 |
| 108935_at | 0.00 |
| 108936_at | 0.00 |
| 108937_at | 0.00 |
| 108938_at | 0.00 |
| 108939_g_at | 0.00 |
| 108940_at | 0.00 |
| 108941_at | 0.00 |
| 108942_at | 0.00 |
| 108943_at | 0.00 |
| 108944_at | 0.00 |
| 108945_g_at | 0.00 |
| 108946_at | 0.00 |
| 108947_at | 0.00 |
| 108953_at | 0.00 |
| 108954_at | 0.00 |
| 108956_at | 0.00 |
| 108957_at | 0.05 |
| 108958_at | 0.00 |
| 108961_at | 0.02 |
| 108962_at | 0.00 |
| 108967_at | 0.00 |
| 108969_at | 0.26 |
| 108973_at | 0.00 |
| 108979_at | 0.01 |
| 108981_at | 0.00 |
| 108983_at | 0.00 |
| 108985_at | 0.00 |
| 108986_at | 0.00 |
| 108994_at | 0.00 |
| 108995_at | 0.00 |

| | |
|---|---|
| 108996_at | 0.00 |
| 108997_at | 0.00 |
| 108998_at | 0.00 |
| 108999_at | 0.00 |
| 109000_at | 0.00 |
| 109001_at | 0.00 |
| 109002_at | 0.00 |
| 109003_at | 0.00 |
| 109004_at | 0.00 |
| 109005_at | 0.01 |
| 109006_at | 0.00 |
| 109007_at | 0.00 |
| 109008_at | 0.02 |
| 109009_at | 0.00 |
| 109010_at | 0.00 |
| 109011_at | 0.00 |
| 109012_at | 0.00 |
| 109013_at | 0.00 |
| 109015_at | 0.00 |
| 109016_at | 0.00 |
| 109017_at | 0.00 |
| 109020_at | 0.03 |
| 109021_at | 0.03 |
| 109028_at | 0.00 |
| 109029_at | 0.10 |
| 109030_at | 0.00 |
| 109031_at | 0.01 |
| 109033_at | 0.00 |
| 109039_at | 0.00 |
| 109040_at | 0.00 |
| 109042_at | 0.00 |
| 109045_at | 0.00 |
| 109047_at | 0.00 |
| 109049_at | 0.00 |
| 109051_at | 0.01 |
| 109055_at | 0.00 |
| 109056_at | 0.00 |
| 109057_at | 0.19 |
| 109058_at | 0.00 |
| 109059_at | 0.05 |
| 109060_at | 0.00 |
| 109061_g_at | 0.00 |
| 109062_f_at | 0.00 |
| 109063_r_at | 0.00 |
| 109064_at | 0.02 |
| 109065_r_at | 0.00 |
| 109066_at | 0.00 |
| 109067_at | 0.00 |
| 109069_at | 0.19 |
| 109070_i_at | 0.00 |
| 109071_f_at | 0.00 |
| 109073_f_at | 0.00 |
| 109074_r_at | 0.00 |
| 109075_at | 0.06 |
| 109076_at | 0.00 |
| 109077_at | 0.00 |

| | |
|---|---|
| 109078_at | 0.00 |
| 109079_f_at | 0.00 |
| 109080_r_at | 0.00 |
| 109081_at | 0.00 |
| 109083_at | 0.00 |
| 109084_at | 0.01 |
| 109085_at | 0.00 |
| 109086_at | 0.00 |
| 109088_at | 0.00 |
| 109089_at | 0.00 |
| 109090_at | 0.07 |
| 109091_at | 0.00 |
| 109092_at | 0.00 |
| 109093_at | 0.00 |
| 109094_at | 0.00 |
| 109095_at | 0.00 |
| 109097_at | 0.00 |
| 109098_at | 0.00 |
| 109099_at | 0.00 |
| 109102_r_at | 0.01 |
| 109103_f_at | 0.00 |
| 109104_at | 0.00 |
| 109105_i_at | 0.00 |
| 109106_f_at | 0.04 |
| 109107_at | 0.00 |
| 109108_at | 0.00 |
| 109109_at | 0.00 |
| 109110_at | 0.02 |
| 109111_at | 0.00 |
| 109114_at | 0.00 |
| 109115_at | 0.00 |
| 109117_at | 0.00 |
| 109118_at | 0.00 |
| 109119_at | 0.00 |
| 109120_at | 0.00 |
| 109121_at | 0.00 |
| 109122_at | 0.00 |
| 109123_at | 0.00 |
| 109124_at | 0.00 |
| 109125_at | 0.00 |
| 109126_at | 0.00 |
| 109128_at | 0.00 |
| 109130_at | 0.00 |
| 109131_at | 0.00 |
| 109132_at | 0.00 |
| 109133_at | 0.02 |
| 109134_r_at | 0.00 |
| 109135_at | 0.24 |
| 109136_at | 0.00 |
| 109137_at | 0.46 |
| 109138_at | 0.00 |
| 109139_at | 0.00 |
| 109140_at | 0.00 |
| 109141_at | 0.00 |
| 109142_at | 0.03 |
| 109143_at | 0.00 |

| | |
|---|---|
| 109144_at | 0.00 |
| 109145_at | 0.03 |
| 109147_at | 0.00 |
| 109148_at | 0.00 |
| 109149_at | 0.00 |
| 109150_at | 0.00 |
| 109151_at | 0.00 |
| 109152_g_at | 0.00 |
| 109153_at | 0.00 |
| 109154_at | 0.00 |
| 109155_at | 0.00 |
| 109156_at | 0.00 |
| 109157_at | 0.00 |
| 109158_at | 0.00 |
| 109159_f_at | 0.00 |
| 109160_at | 0.00 |
| 109161_at | 0.00 |
| 109163_f_at | 0.07 |
| 109164_r_at | 0.00 |
| 109165_at | 0.00 |
| 109167_at | 0.18 |
| 109168_at | 0.00 |
| 109169_at | 0.00 |
| 109170_at | 0.00 |
| 109171_at | 0.26 |
| 109172_at | 0.00 |
| 109173_at | 0.00 |
| 109175_at | 0.00 |
| 109176_at | 0.00 |
| 109177_at | 0.00 |
| 109178_g_at | 0.05 |
| 109179_at | 0.00 |
| 109183_at | 0.00 |
| 109184_at | 0.00 |
| 109185_at | 0.00 |
| 109187_at | 0.00 |
| 109296_at | 0.00 |
| 109297_at | 0.00 |
| 109298_f_at | 0.00 |
| 109299_f_at | 0.40 |
| 109300_at | 0.00 |
| 109301_i_at | 0.00 |
| 109302_at | 0.00 |
| 109303_at | 0.00 |
| 109304_r_at | 0.00 |
| 109305_at | 0.00 |
| 109306_at | 0.00 |
| 109307_at | 0.00 |
| 109308_at | 0.00 |
| 109309_at | 0.00 |
| 109311_at | 0.00 |
| 109317_at | 0.00 |
| 109318_at | 0.00 |
| 109320_at | 0.00 |
| 109322_at | 0.00 |
| 109323_at | 0.00 |

| | |
|---|---|
| 109325_at | 0.00 |
| 109326_at | 0.00 |
| 109327_at | 0.00 |
| 109328_at | 0.00 |
| 109329_at | 0.00 |
| 109330_at | 0.00 |
| 109331_at | 0.00 |
| 109332_at | 0.11 |
| 109333_at | 0.00 |
| 109334_at | 0.00 |
| 109335_at | 0.03 |
| 109336_at | 0.00 |
| 109337_at | 0.00 |
| 109338_at | 0.00 |
| 109339_at | 0.61 |
| 109341_at | 0.00 |
| 109342_at | 0.03 |
| 109343_at | 0.00 |
| 109344_at | 0.04 |
| 109345_at | 0.03 |
| 109346_at | 0.00 |
| 109347_at | 0.00 |
| 109348_at | 0.00 |
| 109350_f_at | 0.02 |
| 109351_r_at | 0.13 |
| 109352_s_at | 0.00 |
| 109353_at | 0.00 |
| 109355_at | 0.00 |
| 109356_at | 0.00 |
| 109357_at | 0.00 |
| 109358_f_at | 0.00 |
| 109359_r_at | 0.00 |
| 109360_at | 0.00 |
| 109361_at | 0.00 |
| 109362_at | 0.00 |
| 109363_at | 0.00 |
| 109364_at | 0.00 |
| 109365_at | 0.00 |
| 109366_at | 0.00 |
| 109367_at | 0.00 |
| 109368_at | 0.04 |
| 109369_at | 0.00 |
| 109370_at | 0.00 |
| 109371_at | 0.00 |
| 109372_at | 0.26 |
| 109373_g_at | 0.00 |
| 109374_at | 0.00 |
| 109375_at | 0.00 |
| 109377_at | 0.00 |
| 109378_at | 0.00 |
| 109379_at | 0.00 |
| 109380_at | 0.00 |
| 109381_s_at | 0.00 |
| 109382_r_at | 0.00 |
| 109383_at | 0.00 |
| 109384_at | 0.00 |

| | |
|---|---|
| 109385_at | 0.00 |
| 109386_at | 0.00 |
| 109387_f_at | 0.00 |
| 109388_r_at | 0.00 |
| 109389_at | 0.00 |
| 109390_at | 0.00 |
| 109391_at | 0.00 |
| 109392_at | 0.00 |
| 109393_at | 0.00 |
| 109394_at | 0.00 |
| 109395_at | 0.00 |
| 109397_at | 0.00 |
| 109398_at | 0.00 |
| 109399_at | 0.00 |
| 109401_at | 0.00 |
| 109402_at | 0.00 |
| 109403_at | 0.16 |
| 109404_at | 0.01 |
| 109405_at | 0.00 |
| 109407_at | 0.00 |
| 109408_at | 0.00 |
| 109410_at | 0.00 |
| 109411_at | 0.00 |
| 109414_at | 0.00 |
| 109415_at | 0.00 |
| 109416_at | 0.17 |
| 109418_at | 0.22 |
| 109419_at | 0.00 |
| 109421_at | 0.00 |
| 109422_at | 0.00 |
| 109423_at | 0.00 |
| 109424_at | 0.00 |
| 109425_at | 0.00 |
| 109426_at | 0.00 |
| 109427_at | 0.00 |
| 109428_at | 0.00 |
| 109429_at | 0.14 |
| 109430_at | 0.00 |
| 109431_at | 0.00 |
| 109432_at | 0.00 |
| 109433_at | 0.00 |
| 109434_at | 0.00 |
| 109435_at | 0.00 |
| 109436_at | 0.00 |
| 109437_at | 0.00 |
| 109438_at | 0.00 |
| 109439_at | 0.00 |
| 109440_at | 0.00 |
| 109441_at | 0.00 |
| 109442_at | 0.00 |
| 109443_at | 0.00 |
| 109444_at | 0.00 |
| 109445_at | 0.00 |
| 109446_at | 0.00 |
| 109447_at | 0.00 |
| 109448_at | 0.05 |

| | |
|---|---|
| 109450_at | 0.00 |
| 109451_at | 0.00 |
| 109453_at | 0.00 |
| 109454_at | 0.00 |
| 109455_at | 0.00 |
| 109456_at | 0.00 |
| 109457_at | 0.00 |
| 109458_f_at | 0.00 |
| 109460_at | 0.00 |
| 109462_at | 0.00 |
| 109463_at | 0.00 |
| 109464_at | 0.00 |
| 109465_at | 0.00 |
| 109466_at | 0.00 |
| 109467_at | 0.00 |
| 109468_at | 0.00 |
| 109469_at | 0.00 |
| 109470_at | 0.00 |
| 109471_at | 0.00 |
| 109472_at | 0.00 |
| 109473_at | 0.00 |
| 109474_r_at | 0.00 |
| 109475_at | 0.00 |
| 109476_at | 0.00 |
| 109477_at | 0.00 |
| 109478_at | 0.00 |
| 109479_at | 0.00 |
| 109480_at | 0.00 |
| 109481_at | 0.00 |
| 109482_at | 0.00 |
| 109483_at | 0.00 |
| 109484_at | 0.00 |
| 109485_at | 0.00 |
| 109486_at | 0.00 |
| 109487_at | 0.00 |
| 109488_at | 0.00 |
| 109489_at | 0.00 |
| 109490_at | 0.01 |
| 109491_at | 0.00 |
| 109492_at | 0.00 |
| 109493_at | 0.00 |
| 109494_at | 0.00 |
| 109495_at | 0.00 |
| 109496_at | 0.00 |
| 109498_at | 0.00 |
| 109499_at | 0.00 |
| 109500_at | 0.00 |
| 109501_at | 0.00 |
| 109502_at | 0.00 |
| 109503_at | 0.00 |
| 109505_at | 0.00 |
| 109506_at | 0.00 |
| 109507_at | 0.00 |
| 109508_at | 0.00 |
| 109509_at | 0.00 |
| 109511_at | 0.00 |

| | |
|---|---|
| 109512_at | 0.00 |
| 109513_r_at | 0.00 |
| 109515_at | 0.00 |
| 109517_at | 0.00 |
| 109518_at | 0.00 |
| 109519_at | 0.00 |
| 109520_at | 0.05 |
| 109521_at | 0.23 |
| 109522_at | 0.00 |
| 109523_at | 0.00 |
| 109524_at | 0.00 |
| 109525_at | 0.00 |
| 109526_at | 0.00 |
| 109527_at | 0.00 |
| 109528_at | 0.00 |
| 109529_at | 0.10 |
| 109530_at | 0.00 |
| 109531_at | 0.00 |
| 109532_at | 0.03 |
| 109533_at | 0.00 |
| 109535_at | 0.00 |
| 109536_at | 0.00 |
| 109537_at | 0.00 |
| 109538_at | 0.00 |
| 109539_at | 0.00 |
| 109540_at | 0.00 |
| 109541_at | 0.00 |
| 109542_at | 0.00 |
| 109543_at | 0.00 |
| 109544_at | 0.00 |
| 109545_at | 0.00 |
| 109546_at | 0.00 |
| 109547_at | 0.00 |
| 109550_at | 0.00 |
| 109551_at | 0.00 |
| 109553_at | 0.00 |
| 109554_at | 0.22 |
| 109556_at | 0.00 |
| 109560_at | 0.00 |
| 109561_at | 0.30 |
| 109562_at | 0.00 |
| 109563_at | 0.00 |
| 109564_at | 0.00 |
| 109565_at | 0.00 |
| 109569_at | 0.00 |
| 109570_at | 0.00 |
| 109574_at | 0.00 |
| 109575_at | 0.00 |
| 109578_at | 0.00 |
| 109579_at | 0.00 |
| 109580_at | 0.00 |
| 109581_at | 0.00 |
| 109582_at | 0.00 |
| 109585_at | 0.00 |
| 109587_at | 0.00 |
| 109588_r_at | 0.00 |

| | |
|---|---|
| 109589_at | 0.00 |
| 109593_at | 0.00 |
| 109594_g_at | 0.00 |
| 109595_at | 0.00 |
| 109598_at | 0.00 |
| 109601_at | 0.00 |
| 109602_at | 0.24 |
| 109603_at | 0.00 |
| 109607_at | 0.00 |
| 109608_at | 0.00 |
| 109615_i_at | 0.00 |
| 109616_f_at | 0.00 |
| 109617_at | 0.00 |
| 109618_at | 0.00 |
| 109620_at | 0.00 |
| 109622_at | 0.00 |
| 109623_at | 0.04 |
| 109628_at | 0.00 |
| 109630_at | 0.01 |
| 109632_at | 0.00 |
| 109635_at | 0.00 |
| 109636_at | 0.00 |
| 109637_at | 0.00 |
| 109638_at | 0.11 |
| 109639_at | 0.00 |
| 109640_at | 0.00 |
| 109641_at | 0.00 |
| 109642_at | 0.00 |
| 109643_at | 0.00 |
| 109644_at | 0.00 |
| 109645_at | 0.00 |
| 109646_at | 0.00 |
| 109647_at | 0.00 |
| 109648_at | 0.00 |
| 109649_at | 0.00 |
| 109650_at | 0.00 |
| 109651_at | 0.03 |
| 109652_at | 0.00 |
| 109653_at | 0.00 |
| 109655_at | 0.00 |
| 109656_at | 0.00 |
| 109657_at | 0.00 |
| 109658_at | 0.00 |
| 109661_at | 0.00 |
| 109662_at | 0.02 |
| 109663_at | 0.00 |
| 109664_at | 0.00 |
| 109665_at | 0.00 |
| 109667_at | 0.00 |
| 109668_at | 0.00 |
| 109669_at | 0.00 |
| 109670_r_at | 0.00 |
| 109672_at | 0.00 |
| 109673_at | 0.00 |
| 109674_at | 0.00 |
| 109678_at | 0.00 |

| | |
|---|---|
| 109679_at | 0.00 |
| 109680_at | 0.00 |
| 109681_at | 0.00 |
| 109682_at | 0.00 |
| 109683_at | 0.00 |
| 109684_at | 0.00 |
| 109685_at | 0.00 |
| 109686_at | 0.09 |
| 109687_at | 0.00 |
| 109689_at | 0.00 |
| 109690_at | 0.00 |
| 109691_at | 0.00 |
| 109692_at | 0.00 |
| 109693_at | 0.00 |
| 109694_at | 0.00 |
| 109695_at | 0.00 |
| 109696_at | 0.00 |
| 109697_at | 0.00 |
| 109698_at | 0.00 |
| 109700_at | 0.01 |
| 109701_at | 0.00 |
| 109702_at | 0.00 |
| 109703_at | 0.00 |
| 109705_s_at | 0.00 |
| 109706_at | 0.00 |
| 109708_at | 0.00 |
| 109709_at | 0.30 |
| 109710_at | 0.00 |
| 109711_at | 0.00 |
| 109712_at | 0.00 |
| 109713_at | 0.00 |
| 109714_at | 0.00 |
| 109715_at | 0.00 |
| 109716_at | 0.05 |
| 109718_at | 0.00 |
| 109719_at | 0.00 |
| 109721_at | 0.14 |
| 109722_at | 0.00 |
| 109723_at | 0.00 |
| 109724_at | 0.00 |
| 109726_at | 0.00 |
| 109727_at | 0.00 |
| 109728_at | 0.00 |
| 109729_at | 0.00 |
| 109730_at | 0.99 |
| 109733_at | 0.00 |
| 109734_at | 0.00 |
| 109735_at | 0.00 |
| 109737_at | 0.00 |
| 109738_at | 0.10 |
| 109739_at | 0.00 |
| 109740_at | 0.00 |
| 109742_at | 0.00 |
| 109744_at | 0.00 |
| 109745_at | 0.00 |
| 109746_at | 0.00 |

| | |
|---|---|
| 109747_at | 0.00 |
| 109748_at | 0.00 |
| 109749_at | 0.00 |
| 109750_at | 0.00 |
| 109751_at | 0.00 |
| 109752_at | 0.00 |
| 109753_at | 0.00 |
| 109754_at | 0.00 |
| 109755_at | 0.16 |
| 109756_at | 0.00 |
| 109757_at | 0.04 |
| 109758_at | 0.00 |
| 109759_at | 0.00 |
| 109760_at | 0.00 |
| 109761_g_at | 0.00 |
| 109762_at | 0.01 |
| 109763_at | 0.00 |
| 109764_at | 0.00 |
| 109765_f_at | 0.00 |
| 109766_r_at | 0.00 |
| 109767_at | 0.00 |
| 109768_at | 0.00 |
| 109769_f_at | 0.00 |
| 109770_r_at | 0.00 |
| 109771_at | 0.00 |
| 109772_at | 0.00 |
| 109773_at | 0.00 |
| 109774_at | 0.00 |
| 109775_at | 0.01 |
| 109776_at | 0.01 |
| 109777_at | 0.00 |
| 109778_g_at | 0.01 |
| 109780_at | 0.00 |
| 109781_at | 0.21 |
| 109785_at | 0.00 |
| 109786_at | 0.00 |
| 109787_at | 0.01 |
| 109788_f_at | 0.00 |
| 109789_s_at | 0.00 |
| 109790_at | 0.00 |
| 109791_at | 0.00 |
| 109792_at | 0.00 |
| 109793_s_at | 0.00 |
| 109794_f_at | 0.00 |
| 109795_at | 0.00 |
| 109796_f_at | 0.00 |
| 109797_at | 0.00 |
| 109798_at | 0.00 |
| 109799_at | 0.00 |
| 109800_at | 0.00 |
| 109801_at | 0.00 |
| 109802_f_at | 0.00 |
| 109803_at | 0.00 |
| 109804_at | 0.00 |
| 109805_f_at | 0.00 |
| 109806_at | 0.00 |

| | |
|---|---|
| 109807_f_at | 0.00 |
| 109808_at | 0.00 |
| 109809_at | 0.00 |
| 109810_f_at | 0.00 |
| 109811_f_at | 0.00 |
| 109812_f_at | 0.00 |
| 109813_f_at | 0.00 |
| 109814_r_at | 0.00 |
| 109815_f_at | 0.00 |
| 109816_s_at | 0.01 |
| 109817_f_at | 0.00 |
| 109818_f_at | 0.00 |
| 109819_f_at | 0.04 |
| 109820_f_at | 0.00 |
| 109821_f_at | 0.00 |
| 109822_f_at | 0.00 |
| 109823_f_at | 0.00 |
| 109824_f_at | 0.82 |
| 109825_at | 0.00 |
| 109826_f_at | 0.00 |
| 109827_at | 0.00 |
| 109828_f_at | 0.00 |
| 109829_r_at | 0.00 |
| 109830_at | 0.00 |
| 109831_s_at | 0.00 |
| 109832_f_at | 0.00 |
| 109870_at | 0.00 |
| 109907_at | 0.07 |
| 109909_at | 0.00 |
| 109910_at | 0.00 |
| 109911_at | 0.00 |
| 109912_at | 0.00 |
| 109913_i_at | 0.00 |
| 109914_f_at | 0.00 |
| 109915_at | 0.00 |
| 109916_at | 0.00 |
| 109917_at | 0.00 |
| 109918_at | 0.00 |
| 109920_at | 0.00 |
| 109921_at | 0.00 |
| 109922_at | 0.00 |
| 109923_at | 0.00 |
| 109925_at | 0.00 |
| 109926_at | 0.00 |
| 109927_at | 0.00 |
| 109928_at | 0.00 |
| 109929_at | 0.00 |
| 109930_at | 0.00 |
| 109931_at | 0.00 |
| 109932_at | 0.00 |
| 109933_at | 0.00 |
| 109934_at | 0.00 |
| 109935_at | 0.00 |
| 109936_i_at | 0.00 |
| 109937_f_at | 0.02 |
| 109938_at | 0.00 |

| | |
|---|---|
| 109940_at | 0.00 |
| 109941_at | 0.00 |
| 109942_at | 0.00 |
| 109943_at | 0.00 |
| 109944_at | 0.00 |
| 109945_at | 0.00 |
| 109946_at | 0.00 |
| 109947_at | 0.00 |
| 109948_at | 0.00 |
| 109950_at | 0.01 |
| 109951_at | 0.00 |
| 109952_at | 0.02 |
| 109953_at | 0.01 |
| 109954_at | 0.00 |
| 109955_at | 0.00 |
| 109956_r_at | 0.00 |
| 109957_at | 0.00 |
| 109958_at | 0.00 |
| 109959_at | 0.00 |
| 109961_at | 0.01 |
| 109962_at | 0.00 |
| 109963_f_at | 0.00 |
| 109965_s_at | 0.00 |
| 109967_at | 0.00 |
| 109968_at | 0.00 |
| 109969_at | 0.00 |
| 109970_at | 0.00 |
| 109971_at | 0.01 |
| 109972_at | 0.00 |
| 109973_at | 0.00 |
| 109975_at | 0.03 |
| 109976_at | 0.00 |
| 109977_at | 0.00 |
| 109978_s_at | 0.00 |
| 109979_i_at | 0.00 |
| 109980_at | 0.04 |
| 109982_at | 0.00 |
| 109984_at | 0.01 |
| 109985_at | 0.00 |
| 109986_at | 0.00 |
| 109987_at | 0.00 |
| 109988_at | 0.00 |
| 109989_at | 0.01 |
| 109990_at | 0.00 |
| 109991_at | 0.00 |
| 109992_at | 0.00 |
| 109993_at | 0.00 |
| 109994_at | 0.00 |
| 109995_at | 0.00 |
| 109996_at | 0.00 |
| 109997_at | 0.00 |
| 109998_at | 0.00 |
| 109999_at | 0.00 |
| 110000_at | 0.00 |
| 110001_at | 0.00 |
| 110002_at | 0.00 |

| | |
|---|---|
| 110003_at | 0.00 |
| 110004_at | 0.00 |
| 110005_at | 0.00 |
| 110006_at | 0.00 |
| 110007_at | 0.00 |
| 110008_at | 0.00 |
| 110009_at | 0.00 |
| 110011_at | 0.00 |
| 110012_f_at | 0.00 |
| 110013_r_at | 0.00 |
| 110014_at | 0.00 |
| 110015_at | 0.00 |
| 110016_at | 0.01 |
| 110017_at | 0.00 |
| 110018_at | 0.00 |
| 110020_at | 0.00 |
| 110022_at | 0.00 |
| 110023_at | 0.00 |
| 110024_at | 0.00 |
| 110026_at | 0.00 |
| 110027_at | 0.00 |
| 110028_at | 0.00 |
| 110029_at | 0.00 |
| 110030_at | 0.00 |
| 110031_at | 0.00 |
| 110032_at | 0.00 |
| 110033_at | 0.00 |
| 110034_at | 0.00 |
| 110035_at | 0.00 |
| 110036_at | 0.00 |
| 110038_at | 0.00 |
| 110040_at | 0.00 |
| 110041_s_at | 0.00 |
| 110042_at | 0.00 |
| 110043_at | 0.00 |
| 110044_at | 0.00 |
| 110045_at | 0.00 |
| 110046_at | 0.00 |
| 110047_at | 0.00 |
| 110048_at | 0.00 |
| 110049_at | 0.00 |
| 110050_at | 0.00 |
| 110051_at | 0.00 |
| 110052_at | 0.00 |
| 110053_at | 0.00 |
| 110054_at | 0.00 |
| 110055_at | 0.03 |
| 110056_at | 0.00 |
| 110057_at | 0.00 |
| 110058_at | 0.00 |
| 110059_at | 0.00 |
| 110060_at | 0.00 |
| 110061_at | 0.00 |
| 110062_at | 0.00 |
| 110063_at | 0.00 |
| 110064_at | 0.00 |

| | |
|---|---|
| 110065_at | 0.00 |
| 110066_at | 0.00 |
| 110067_at | 0.00 |
| 110068_at | 0.00 |
| 110069_at | 0.00 |
| 110070_at | 0.00 |
| 110071_at | 0.00 |
| 110072_at | 0.00 |
| 110074_at | 0.00 |
| 110075_at | 0.00 |
| 110076_at | 0.00 |
| 110077_at | 0.00 |
| 110078_at | 0.00 |
| 110079_g_at | 0.00 |
| 110080_at | 0.00 |
| 110081_at | 0.00 |
| 110082_at | 0.00 |
| 110083_at | 0.00 |
| 110084_at | 0.00 |
| 110085_at | 0.00 |
| 110086_at | 0.00 |
| 110087_at | 0.00 |
| 110088_at | 0.00 |
| 110089_at | 0.00 |
| 110090_at | 0.00 |
| 110091_at | 0.00 |
| 110092_at | 0.00 |
| 110093_at | 0.00 |
| 110094_at | 0.00 |
| 110095_at | 0.00 |
| 110096_at | 0.00 |
| 110097_at | 0.00 |
| 110098_at | 0.01 |
| 110099_at | 0.00 |
| 110100_at | 0.00 |
| 110101_at | 0.00 |
| 110102_at | 0.00 |
| 110103_at | 0.00 |
| 110104_at | 0.00 |
| 110105_at | 0.00 |
| 110106_at | 0.00 |
| 110107_at | 0.00 |
| 110108_at | 0.00 |
| 110109_at | 0.00 |
| 110110_at | 0.00 |
| 110111_at | 0.00 |
| 110112_at | 0.00 |
| 110113_at | 0.00 |
| 110114_at | 0.00 |
| 110115_at | 0.06 |
| 110116_at | 0.00 |
| 110117_at | 0.00 |
| 110118_at | 0.00 |
| 110119_at | 0.03 |
| 110120_at | 0.00 |
| 110121_at | 0.00 |

| | |
|---|---|
| 110122_at | 0.00 |
| 110123_at | 0.00 |
| 110124_at | 0.63 |
| 110125_at | 0.00 |
| 110126_at | 0.00 |
| 110128_at | 0.54 |
| 110129_at | 0.00 |
| 110131_at | 0.00 |
| 110132_at | 0.00 |
| 110133_at | 0.00 |
| 110134_s_at | 0.00 |
| 110135_at | 0.00 |
| 110136_at | 0.00 |
| 110137_at | 0.00 |
| 110138_at | 0.00 |
| 110139_at | 0.00 |
| 110140_f_at | 0.00 |
| 110141_r_at | 0.00 |
| 110142_at | 0.00 |
| 110143_at | 0.00 |
| 110144_at | 0.00 |
| 110145_at | 0.09 |
| 110147_at | 0.00 |
| 110150_at | 0.00 |
| 110151_at | 0.00 |
| 110152_at | 0.00 |
| 110153_at | 0.12 |
| 110156_at | 0.00 |
| 110157_at | 0.00 |
| 110159_at | 0.00 |
| 110160_at | 0.00 |
| 110163_at | 0.00 |
| 110164_at | 0.00 |
| 110165_at | 0.00 |
| 110167_at | 0.00 |
| 110168_at | 0.00 |
| 110169_at | 0.00 |
| 110170_at | 0.00 |
| 110171_at | 0.00 |
| 110172_at | 0.00 |
| 110173_at | 0.00 |
| 110174_at | 0.00 |
| 110176_at | 0.00 |
| 110177_at | 0.28 |
| 110178_at | 0.00 |
| 110179_at | 0.00 |
| 110180_at | 0.00 |
| 110181_at | 0.00 |
| 110182_at | 0.00 |
| 110183_at | 0.00 |
| 110184_at | 0.00 |
| 110185_at | 0.00 |
| 110186_at | 0.00 |
| 110187_at | 0.00 |
| 110188_at | 0.00 |
| 110189_at | 0.00 |

| | |
|---|---|
| 110190_at | 0.00 |
| 110191_at | 0.02 |
| 110192_at | 0.00 |
| 110193_at | 0.00 |
| 110194_at | 0.00 |
| 110196_at | 0.00 |
| 110197_at | 0.00 |
| 110198_at | 0.00 |
| 110201_at | 0.00 |
| 110202_at | 0.07 |
| 110203_at | 0.00 |
| 110204_at | 0.00 |
| 110205_at | 0.00 |
| 110206_at | 0.00 |
| 110207_at | 0.00 |
| 110208_at | 0.00 |
| 110210_at | 0.06 |
| 110214_at | 0.00 |
| 110217_at | 0.00 |
| 110218_at | 0.08 |
| 110221_at | 0.00 |
| 110222_at | 0.00 |
| 110223_at | 0.00 |
| 110224_at | 0.12 |
| 110225_at | 0.00 |
| 110226_at | 0.00 |
| 110228_at | 0.02 |
| 110229_at | 0.00 |
| 110232_at | 0.00 |
| 110233_at | 0.00 |
| 110234_at | 0.00 |
| 110236_at | 0.00 |
| 110239_at | 0.00 |
| 110240_g_at | 0.00 |
| 110241_at | 0.00 |
| 110246_at | 0.00 |
| 110248_at | 0.01 |
| 110253_at | 0.00 |
| 110258_at | 0.23 |
| 110259_at | 0.00 |
| 110260_at | 0.00 |
| 110261_at | 0.00 |
| 110262_at | 0.00 |
| 110263_at | 0.00 |
| 110264_at | 0.00 |
| 110266_at | 0.00 |
| 110267_g_at | 0.00 |
| 110268_at | 0.00 |
| 110269_at | 0.09 |
| 110270_at | 0.00 |
| 110272_at | 0.03 |
| 110273_at | 0.00 |
| 110274_at | 0.00 |
| 110275_at | 0.05 |
| 110276_at | 0.00 |
| 110277_at | 0.00 |

| | |
|---|---|
| 110278_at | 0.00 |
| 110279_at | 0.00 |
| 110280_at | 0.01 |
| 110281_g_at | 0.03 |
| 110284_at | 0.00 |
| 110286_at | 0.00 |
| 110287_at | 0.00 |
| 110288_at | 0.01 |
| 110289_at | 0.00 |
| 110290_at | 0.00 |
| 110291_at | 0.00 |
| 110292_at | 0.00 |
| 110293_at | 0.00 |
| 110294_at | 0.00 |
| 110295_at | 0.00 |
| 110296_at | 0.00 |
| 110297_at | 0.01 |
| 110298_at | 0.00 |
| 110299_at | 0.00 |
| 110300_at | 0.00 |
| 110301_at | 0.00 |
| 110303_at | 0.00 |
| 110305_at | 0.00 |
| 110306_at | 0.00 |
| 110307_at | 0.00 |
| 110308_at | 0.00 |
| 110309_at | 0.00 |
| 110310_at | 0.00 |
| 110311_at | 0.00 |
| 110312_at | 0.00 |
| 110314_at | 0.03 |
| 110315_at | 0.00 |
| 110316_at | 0.00 |
| 110318_at | 0.00 |
| 110319_at | 0.13 |
| 110320_at | 0.00 |
| 110322_at | 0.00 |
| 110323_at | 0.00 |
| 110324_at | 0.00 |
| 110326_at | 0.00 |
| 110327_at | 0.00 |
| 110328_at | 0.00 |
| 110329_at | 0.00 |
| 110330_at | 0.00 |
| 110331_at | 0.00 |
| 110333_at | 0.00 |
| 110334_at | 0.00 |
| 110335_at | 0.00 |
| 110336_at | 0.00 |
| 110337_at | 0.00 |
| 110338_at | 0.00 |
| 110339_at | 0.00 |
| 110341_at | 0.01 |
| 110343_f_at | 0.00 |
| 110344_at | 0.00 |
| 110345_at | 0.00 |

| | |
|---|---|
| 110346_at | 0.00 |
| 110347_i_at | 0.00 |
| 110348_f_at | 0.00 |
| 110351_at | 0.00 |
| 110352_f_at | 0.00 |
| 110353_at | 0.00 |
| 110354_at | 0.00 |
| 110355_at | 0.00 |
| 110356_at | 0.00 |
| 110357_at | 0.00 |
| 110358_at | 0.00 |
| 110360_at | 0.03 |
| 110361_at | 0.07 |
| 110362_at | 0.02 |
| 110363_at | 0.00 |
| 110365_at | 0.00 |
| 110367_at | 0.00 |
| 110368_at | 0.00 |
| 110370_at | 0.00 |
| 110371_at | 0.00 |
| 110372_at | 0.00 |
| 110373_at | 0.00 |
| 110374_at | 0.00 |
| 110375_at | 0.06 |
| 110376_at | 0.00 |
| 110377_at | 0.00 |
| 110378_at | 0.00 |
| 110379_at | 0.00 |
| 110380_at | 0.00 |
| 110381_at | 0.00 |
| 110382_at | 0.03 |
| 110384_at | 0.00 |
| 110385_at | 0.00 |
| 110387_at | 0.00 |
| 110388_at | 0.00 |
| 110389_at | 0.00 |
| 110390_g_at | 0.08 |
| 110392_at | 0.00 |
| 110393_at | 0.22 |
| 110395_at | 0.00 |
| 110396_at | 0.00 |
| 110397_at | 0.00 |
| 110398_f_at | 0.00 |
| 110399_at | 0.00 |
| 110400_f_at | 0.00 |
| 110406_at | 0.00 |
| 110407_at | 0.00 |
| 110408_at | 0.06 |
| 110410_at | 0.29 |
| 110411_at | 0.02 |
| 110414_at | 0.33 |
| 110415_f_at | 0.00 |
| 110416_r_at | 0.00 |
| 110417_at | 0.00 |
| 110419_at | 0.00 |
| 110420_at | 0.00 |

| | |
|---|---|
| 110421_at | 0.00 |
| 110422_at | 0.00 |
| 110423_at | 0.00 |
| 110424_at | 0.02 |
| 110425_at | 0.00 |
| 110428_at | 0.04 |
| 110429_at | 0.80 |
| 110431_at | 0.00 |
| 110432_at | 0.00 |
| 110433_at | 0.00 |
| 110434_at | 0.00 |
| 110435_at | 0.00 |
| 110437_at | 0.02 |
| 110439_at | 0.00 |
| 110440_at | 0.00 |
| 110441_at | 0.00 |
| 110442_at | 0.01 |
| 110443_at | 0.00 |
| 110444_at | 0.00 |
| 110445_at | 0.00 |
| 110446_at | 0.00 |
| 110447_at | 0.00 |
| 110448_i_at | 0.00 |
| 110449_f_at | 0.00 |
| 110450_at | 0.00 |
| 110451_at | 0.00 |
| 110452_i_at | 0.00 |
| 110453_r_at | 0.00 |
| 110454_at | 0.00 |
| 110455_at | 0.00 |
| 110456_at | 0.00 |
| 110457_at | 0.00 |
| 110458_at | 0.00 |
| 110459_at | 0.00 |
| 110460_at | 0.00 |
| 110461_at | 0.02 |
| 110462_at | 0.03 |
| 110463_at | 0.00 |
| 110464_at | 0.00 |
| 110465_at | 0.00 |
| 110466_at | 0.00 |
| 110467_at | 0.04 |
| 110468_at | 0.00 |
| 110469_at | 0.00 |
| 110471_i_at | 0.00 |
| 110472_f_at | 0.01 |
| 110473_at | 0.00 |
| 110474_at | 0.00 |
| 110476_at | 0.00 |
| 110478_at | 0.00 |
| 110479_i_at | 0.00 |
| 110480_f_at | 0.00 |
| 110482_at | 0.00 |
| 110483_at | 0.00 |
| 110486_at | 0.00 |
| 110491_at | 0.00 |

| | |
|---|---|
| 110492_at | 0.00 |
| 110493_at | 0.00 |
| 110494_at | 0.00 |
| 110495_at | 0.00 |
| 110499_f_at | 0.01 |
| 110502_at | 0.00 |
| 110503_at | 0.00 |
| 110504_at | 0.00 |
| 110505_at | 0.00 |
| 110506_at | 0.00 |
| 110507_at | 0.00 |
| 110508_at | 0.00 |
| 110509_at | 0.00 |
| 110510_at | 0.00 |
| 110511_at | 0.00 |
| 110512_at | 0.00 |
| 110513_at | 0.05 |
| 110514_at | 0.00 |
| 110515_at | 0.00 |
| 110516_at | 0.00 |
| 110517_at | 0.00 |
| 110518_at | 0.00 |
| 110519_at | 0.00 |
| 110520_at | 0.00 |
| 110521_at | 0.00 |
| 110522_at | 0.00 |
| 110523_at | 0.00 |
| 110524_at | 0.00 |
| 110526_at | 0.00 |
| 110527_at | 0.00 |
| 110528_at | 0.00 |
| 110529_at | 0.00 |
| 110530_at | 0.00 |
| 110531_at | 0.00 |
| 110532_at | 0.00 |
| 110533_at | 0.00 |
| 110535_at | 0.00 |
| 110536_at | 0.00 |
| 110537_at | 0.00 |
| 110538_at | 0.00 |
| 110539_at | 0.00 |
| 110540_at | 0.00 |
| 110541_at | 0.01 |
| 110542_at | 0.00 |
| 110543_at | 0.00 |
| 110544_at | 0.00 |
| 110545_at | 0.00 |
| 110546_at | 0.00 |
| 110547_at | 0.00 |
| 110548_at | 0.00 |
| 110549_at | 0.00 |
| 110554_at | 0.00 |
| 110558_at | 0.00 |
| 110560_at | 0.00 |
| 110562_at | 0.00 |
| 110564_at | 0.14 |

| | |
|---|---|
| 110565_r_at | 0.00 |
| 110566_at | 0.00 |
| 110567_at | 0.00 |
| 110569_at | 0.00 |
| 110570_at | 0.00 |
| 110571_at | 0.00 |
| 110573_at | 0.00 |
| 110574_at | 0.00 |
| 110575_at | 0.00 |
| 110576_at | 0.00 |
| 110577_at | 0.00 |
| 110578_at | 0.00 |
| 110579_at | 0.00 |
| 110580_at | 0.00 |
| 110581_at | 0.00 |
| 110582_at | 0.00 |
| 110583_at | 0.00 |
| 110584_at | 0.00 |
| 110585_at | 0.00 |
| 110586_at | 0.02 |
| 110587_at | 0.00 |
| 110588_g_at | 0.00 |
| 110589_at | 0.00 |
| 110590_at | 0.00 |
| 110591_at | 0.00 |
| 110592_at | 0.00 |
| 110593_at | 0.00 |
| 110594_at | 0.01 |
| 110596_at | 0.00 |
| 110597_f_at | 0.00 |
| 110598_r_at | 0.00 |
| 110599_at | 0.00 |
| 110600_at | 0.00 |
| 110601_at | 0.00 |
| 110602_at | 0.04 |
| 110603_at | 0.00 |
| 110604_at | 0.03 |
| 110605_at | 0.00 |
| 110606_at | 0.00 |
| 110607_at | 0.00 |
| 110608_at | 0.00 |
| 110609_s_at | 0.00 |
| 110610_at | 0.00 |
| 110611_at | 0.00 |
| 110612_at | 0.00 |
| 110613_at | 0.00 |
| 110614_at | 0.00 |
| 110615_at | 0.00 |
| 110616_at | 0.00 |
| 110617_at | 0.00 |
| 110618_at | 0.00 |
| 110619_at | 0.18 |
| 110620_at | 0.00 |
| 110621_at | 0.00 |
| 110622_at | 0.00 |
| 110623_at | 0.00 |

| | |
|---|---|
| 110624_at | 0.00 |
| 110625_at | 0.00 |
| 110626_at | 0.00 |
| 110627_at | 0.00 |
| 110628_i_at | 0.00 |
| 110629_at | 0.00 |
| 110630_at | 0.00 |
| 110631_at | 0.00 |
| 110632_at | 0.00 |
| 110634_at | 0.02 |
| 110635_at | 0.00 |
| 110636_at | 0.00 |
| 110637_at | 0.00 |
| 110638_at | 0.00 |
| 110639_at | 0.00 |
| 110640_at | 0.00 |
| 110641_at | 0.00 |
| 110642_at | 0.00 |
| 110643_at | 0.00 |
| 110644_s_at | 0.00 |
| 110645_at | 0.00 |
| 110646_at | 0.00 |
| 110647_at | 0.00 |
| 110648_at | 0.00 |
| 110649_at | 0.00 |
| 110650_at | 0.01 |
| 110651_at | 0.00 |
| 110652_at | 0.00 |
| 110653_at | 0.00 |
| 110654_at | 0.00 |
| 110655_at | 0.00 |
| 110656_at | 0.00 |
| 110657_at | 0.00 |
| 110658_at | 0.00 |
| 110659_at | 0.00 |
| 110660_at | 0.00 |
| 110661_at | 0.00 |
| 110662_at | 0.00 |
| 110663_at | 0.00 |
| 110664_g_at | 0.00 |
| 110665_i_at | 0.00 |
| 110666_r_at | 0.00 |
| 110667_at | 0.00 |
| 110669_at | 0.00 |
| 110670_at | 0.00 |
| 110672_at | 0.00 |
| 110673_at | 0.00 |
| 110674_at | 0.00 |
| 110675_at | 0.00 |
| 110676_at | 0.01 |
| 110677_at | 0.00 |
| 110678_at | 0.00 |
| 110679_at | 0.00 |
| 110680_at | 0.00 |
| 110681_at | 0.00 |
| 110682_at | 0.00 |

| | |
|---|---|
| 110683_at | 0.00 |
| 110684_at | 0.00 |
| 110685_at | 0.00 |
| 110686_at | 0.00 |
| 110687_at | 0.00 |
| 110688_at | 0.00 |
| 110689_at | 0.00 |
| 110690_at | 0.00 |
| 110691_at | 0.00 |
| 110692_at | 0.00 |
| 110693_at | 0.00 |
| 110694_at | 0.00 |
| 110696_at | 0.00 |
| 110699_at | 0.00 |
| 110701_at | 0.00 |
| 110702_at | 0.00 |
| 110703_at | 0.00 |
| 110704_at | 0.00 |
| 110705_at | 0.04 |
| 110706_at | 0.00 |
| 110707_at | 0.00 |
| 110708_at | 0.00 |
| 110709_at | 0.00 |
| 110710_at | 0.00 |
| 110711_at | 0.00 |
| 110712_at | 0.00 |
| 110713_at | 0.00 |
| 110715_at | 0.00 |
| 110716_at | 0.00 |
| 110719_at | 0.00 |
| 110720_at | 0.00 |
| 110721_at | 0.00 |
| 110722_at | 0.00 |
| 110727_at | 0.00 |
| 110728_at | 0.00 |
| 110730_at | 0.00 |
| 110731_at | 0.00 |
| 110732_at | 0.00 |
| 110736_at | 0.00 |
| 110737_at | 0.00 |
| 110738_at | 0.00 |
| 110739_at | 0.00 |
| 110740_at | 0.00 |
| 110741_at | 0.00 |
| 110743_at | 0.00 |
| 110744_at | 0.00 |
| 110745_i_at | 0.00 |
| 110746_f_at | 0.00 |
| 110748_at | 0.00 |
| 110750_at | 0.00 |
| 110751_at | 0.00 |
| 110752_at | 0.00 |
| 110753_at | 0.00 |
| 110754_at | 0.00 |
| 110755_at | 0.00 |
| 110756_at | 0.00 |

| | |
|---|---|
| 110757_at | 0.00 |
| 110758_at | 0.00 |
| 110760_at | 0.00 |
| 110761_at | 0.00 |
| 110763_at | 0.00 |
| 110764_r_at | 0.00 |
| 110765_at | 0.00 |
| 110766_at | 0.00 |
| 110767_r_at | 0.00 |
| 110768_at | 0.00 |
| 110769_at | 0.00 |
| 110770_at | 0.00 |
| 110772_at | 0.00 |
| 110774_at | 0.00 |
| 110777_at | 0.00 |
| 110778_at | 0.00 |
| 110783_at | 0.00 |
| 110785_f_at | 0.04 |
| 110786_at | 0.00 |
| 110788_at | 0.00 |
| 110789_at | 0.00 |
| 110790_at | 0.00 |
| 110792_at | 0.00 |
| 110793_at | 0.00 |
| 110795_at | 0.00 |
| 110796_at | 0.00 |
| 110805_at | 0.00 |
| 110806_at | 0.00 |
| 110807_at | 0.00 |
| 110808_at | 0.06 |
| 110809_at | 0.00 |
| 110810_at | 0.00 |
| 110812_at | 0.00 |
| 110816_at | 0.00 |
| 110817_at | 0.00 |
| 110819_at | 0.00 |
| 110822_at | 0.00 |
| 110823_s_at | 0.00 |
| 110824_at | 0.00 |
| 110826_at | 0.00 |
| 110828_at | 0.00 |
| 110829_at | 0.00 |
| 110830_at | 0.00 |
| 110833_at | 0.00 |
| 110834_at | 0.04 |
| 110835_at | 0.00 |
| 110836_at | 0.00 |
| 110839_at | 0.04 |
| 110840_at | 0.00 |
| 110841_at | 0.00 |
| 110842_at | 0.00 |
| 110844_at | 0.00 |
| 110845_at | 0.00 |
| 110846_at | 0.00 |
| 110847_at | 0.36 |
| 110848_at | 0.01 |

| | |
|---|---|
| 110850_f_at | 0.00 |
| 110851_r_at | 0.00 |
| 110852_at | 0.00 |
| 110853_at | 0.12 |
| 110854_at | 0.00 |
| 110855_at | 0.00 |
| 110856_at | 0.00 |
| 110857_at | 0.00 |
| 110858_at | 0.01 |
| 110859_at | 0.00 |
| 110860_at | 0.00 |
| 110861_g_at | 0.00 |
| 110862_at | 0.02 |
| 110863_at | 0.00 |
| 110957_at | 0.00 |
| 110958_at | 0.04 |
| 110960_at | 0.00 |
| 110961_at | 0.01 |
| 110962_at | 0.00 |
| 110963_at | 0.00 |
| 110964_at | 0.00 |
| 110966_at | 0.00 |
| 110967_at | 0.25 |
| 110968_at | 0.05 |
| 110969_at | 0.00 |
| 110972_at | 0.00 |
| 110974_at | 0.00 |
| 110980_at | 0.00 |
| 110982_at | 0.00 |
| 110983_at | 0.00 |
| 110985_at | 0.00 |
| 110986_at | 0.00 |
| 110987_at | 0.00 |
| 110988_at | 0.00 |
| 110989_at | 0.00 |
| 110990_at | 0.00 |
| 110994_at | 0.00 |
| 110995_at | 0.00 |
| 110998_at | 0.00 |
| 110999_at | 0.00 |
| 111000_at | 0.03 |
| 111002_at | 0.00 |
| 111005_at | 0.00 |
| 111006_at | 0.00 |
| 111007_at | 0.00 |
| 111008_at | 0.00 |
| 111009_f_at | 0.03 |
| 111010_r_at | 0.00 |
| 111011_at | 0.43 |
| 111012_at | 0.00 |
| 111013_at | 0.02 |
| 111014_at | 0.00 |
| 111015_at | 0.00 |
| 111016_at | 0.00 |
| 111018_at | 0.00 |
| 111020_at | 0.00 |

| | |
|---|---|
| 111022_at | 0.00 |
| 111023_at | 0.00 |
| 111024_f_at | 0.00 |
| 111025_r_at | 0.00 |
| 111026_at | 0.00 |
| 111027_at | 0.00 |
| 111029_at | 0.26 |
| 111030_at | 0.00 |
| 111031_at | 0.00 |
| 111032_s_at | 0.00 |
| 111033_at | 0.00 |
| 111035_at | 0.00 |
| 111036_f_at | 0.01 |
| 111037_at | 0.00 |
| 111038_at | 0.02 |
| 111039_at | 0.00 |
| 111041_at | 0.00 |
| 111042_at | 0.00 |
| 111043_i_at | 0.00 |
| 111044_f_at | 0.00 |
| 111045_at | 0.00 |
| 111046_r_at | 0.00 |
| 111048_at | 0.00 |
| 111053_at | 0.01 |
| 111054_at | 0.00 |
| 111055_at | 0.00 |
| 111056_at | 0.00 |
| 111057_at | 0.00 |
| 111058_at | 0.00 |
| 111059_at | 0.00 |
| 111060_at | 0.00 |
| 111061_at | 0.00 |
| 111062_at | 0.00 |
| 111064_at | 0.00 |
| 111065_at | 0.01 |
| 111066_at | 0.00 |
| 111067_at | 0.00 |
| 111068_at | 0.00 |
| 111070_at | 0.00 |
| 111071_at | 0.00 |
| 111072_at | 0.00 |
| 111073_at | 0.00 |
| 111075_at | 0.00 |
| 111076_at | 0.00 |
| 111078_at | 0.00 |
| 111079_at | 0.00 |
| 111080_at | 0.01 |
| 111081_at | 0.03 |
| 111082_at | 0.00 |
| 111083_at | 0.00 |
| 111084_at | 0.00 |
| 111085_at | 0.00 |
| 111086_at | 0.00 |
| 111087_at | 0.00 |
| 111088_at | 0.00 |
| 111089_at | 0.00 |

| | |
|---|---|
| 111090_at | 0.00 |
| 111092_at | 0.00 |
| 111093_at | 0.00 |
| 111094_at | 0.00 |
| 111095_at | 0.00 |
| 111096_at | 0.00 |
| 111097_at | 0.00 |
| 111098_at | 0.00 |
| 111099_at | 0.00 |
| 111101_at | 0.00 |
| 111102_at | 0.00 |
| 111103_at | 0.00 |
| 111104_at | 0.00 |
| 111105_at | 0.00 |
| 111107_at | 0.00 |
| 111108_at | 0.00 |
| 111109_r_at | 0.00 |
| 111110_at | 0.00 |
| 111111_at | 0.00 |
| 111112_at | 0.00 |
| 111113_at | 0.00 |
| 111114_g_at | 0.00 |
| 111115_at | 0.00 |
| 111116_at | 0.00 |
| 111117_at | 0.00 |
| 111118_at | 0.00 |
| 111119_at | 0.00 |
| 111120_at | 0.00 |
| 111121_at | 0.00 |
| 111122_at | 0.00 |
| 111123_s_at | 0.00 |
| 111124_at | 0.12 |
| 111125_at | 0.01 |
| 111126_at | 0.00 |
| 111127_at | 0.00 |
| 111128_at | 0.00 |
| 111129_at | 0.00 |
| 111130_at | 0.00 |
| 111131_g_at | 0.00 |
| 111132_at | 0.00 |
| 111133_at | 0.00 |
| 111135_at | 0.00 |
| 111136_at | 0.00 |
| 111137_at | 0.00 |
| 111138_at | 0.00 |
| 111139_at | 0.00 |
| 111140_at | 0.00 |
| 111141_at | 0.00 |
| 111142_at | 0.00 |
| 111143_at | 0.00 |
| 111144_at | 0.00 |
| 111145_at | 0.00 |
| 111146_at | 0.00 |
| 111147_at | 0.09 |
| 111148_at | 0.00 |
| 111149_at | 0.00 |

| | |
|---|---|
| 111150_at | 0.00 |
| 111151_f_at | 0.01 |
| 111152_at | 0.00 |
| 111153_at | 0.00 |
| 111154_at | 0.00 |
| 111155_at | 0.00 |
| 111156_at | 0.00 |
| 111157_at | 0.00 |
| 111158_at | 0.00 |
| 111159_at | 0.00 |
| 111160_at | 0.00 |
| 111161_at | 0.00 |
| 111162_f_at | 0.00 |
| 111163_at | 0.00 |
| 111164_at | 0.00 |
| 111165_at | 0.00 |
| 111166_at | 0.17 |
| 111168_at | 0.00 |
| 111170_at | 0.00 |
| 111171_at | 0.00 |
| 111172_at | 0.00 |
| 111173_at | 0.00 |
| 111174_at | 0.00 |
| 111176_at | 0.00 |
| 111177_at | 0.00 |
| 111178_at | 0.00 |
| 111181_at | 0.00 |
| 111182_at | 0.00 |
| 111183_at | 0.00 |
| 111184_at | 0.00 |
| 111185_at | 0.00 |
| 111186_at | 0.00 |
| 111187_at | 0.00 |
| 111188_at | 0.00 |
| 111189_at | 0.00 |
| 111190_at | 0.00 |
| 111191_at | 0.00 |
| 111193_at | 0.00 |
| 111194_at | 0.00 |
| 111195_at | 0.00 |
| 111197_at | 0.00 |
| 111198_at | 0.00 |
| 111199_at | 0.00 |
| 111200_at | 0.00 |
| 111201_at | 0.00 |
| 111202_at | 0.00 |
| 111203_at | 0.00 |
| 111204_at | 0.04 |
| 111205_at | 0.00 |
| 111206_at | 0.01 |
| 111207_at | 0.00 |
| 111208_at | 0.00 |
| 111209_at | 0.00 |
| 111210_at | 0.00 |
| 111211_at | 0.00 |
| 111212_at | 0.01 |

| | |
|---|---|
| 111213_at | 0.00 |
| 111214_at | 0.00 |
| 111215_at | 0.00 |
| 111216_at | 0.00 |
| 111217_at | 0.00 |
| 111218_at | 0.09 |
| 111220_at | 0.00 |
| 111221_at | 0.00 |
| 111222_at | 0.01 |
| 111224_at | 0.00 |
| 111225_at | 0.00 |
| 111226_at | 0.00 |
| 111227_at | 0.00 |
| 111228_at | 0.00 |
| 111229_at | 0.03 |
| 111230_at | 0.00 |
| 111231_at | 0.00 |
| 111232_at | 0.00 |
| 111233_at | 0.00 |
| 111234_at | 0.00 |
| 111235_at | 0.00 |
| 111236_at | 0.00 |
| 111237_at | 0.00 |
| 111238_at | 0.00 |
| 111239_at | 0.00 |
| 111240_at | 0.00 |
| 111241_at | 0.00 |
| 111242_at | 0.00 |
| 111243_at | 0.05 |
| 111244_at | 0.00 |
| 111245_at | 0.00 |
| 111246_at | 0.00 |
| 111247_at | 0.00 |
| 111249_at | 0.00 |
| 111250_at | 0.00 |
| 111252_at | 0.00 |
| 111254_at | 0.00 |
| 111255_r_at | 0.00 |
| 111256_at | 0.00 |
| 111258_at | 0.00 |
| 111259_at | 0.00 |
| 111260_at | 0.31 |
| 111261_at | 0.00 |
| 111262_at | 0.00 |
| 111263_at | 0.00 |
| 111264_at | 0.10 |
| 111265_f_at | 0.00 |
| 111266_i_at | 0.00 |
| 111267_f_at | 0.00 |
| 111268_at | 0.00 |
| 111271_at | 0.00 |
| 111272_at | 0.00 |
| 111273_at | 0.01 |
| 111275_at | 0.00 |
| 111276_r_at | 0.00 |
| 111277_at | 0.00 |

| | |
|---|---|
| 111278_at | 0.00 |
| 111279_at | 0.00 |
| 111280_at | 0.00 |
| 111281_at | 0.00 |
| 111283_at | 0.00 |
| 111287_at | 0.00 |
| 111288_at | 0.00 |
| 111289_at | 0.00 |
| 111291_at | 0.00 |
| 111292_at | 0.00 |
| 111293_at | 0.00 |
| 111294_at | 0.00 |
| 111295_at | 0.00 |
| 111298_at | 0.00 |
| 111299_at | 0.00 |
| 111305_at | 0.00 |
| 111306_at | 0.00 |
| 111307_at | 0.00 |
| 111308_at | 0.00 |
| 111309_at | 0.00 |
| 111310_at | 0.00 |
| 111311_at | 0.00 |
| 111312_at | 0.00 |
| 111313_at | 0.00 |
| 111314_at | 0.00 |
| 111315_at | 0.00 |
| 111316_at | 0.00 |
| 111317_at | 0.00 |
| 111318_at | 0.03 |
| 111319_at | 0.00 |
| 111322_at | 0.00 |
| 111325_at | 0.01 |
| 111327_at | 0.00 |
| 111328_at | 0.00 |
| 111329_at | 0.00 |
| 111331_at | 0.02 |
| 111332_at | 0.05 |
| 111334_at | 0.00 |
| 111335_at | 0.00 |
| 111336_at | 0.00 |
| 111337_at | 0.00 |
| 111339_at | 0.00 |
| 111340_at | 0.00 |
| 111341_at | 0.00 |
| 111342_at | 0.00 |
| 111343_i_at | 0.00 |
| 111344_f_at | 0.00 |
| 111345_at | 0.00 |
| 111347_at | 0.00 |
| 111349_at | 0.02 |
| 111350_at | 0.00 |
| 111352_at | 0.00 |
| 111353_at | 0.00 |
| 111354_at | 0.00 |
| 111355_at | 0.00 |
| 111356_at | 0.00 |

| | |
|---|---|
| 111358_at | 0.00 |
| 111359_at | 0.47 |
| 111360_at | 0.00 |
| 111361_at | 0.00 |
| 111363_at | 0.00 |
| 111364_at | 0.00 |
| 111365_at | 0.00 |
| 111366_at | 0.00 |
| 111367_at | 0.00 |
| 111371_at | 0.00 |
| 111372_at | 0.00 |
| 111375_at | 0.00 |
| 111377_at | 0.00 |
| 111378_at | 0.00 |
| 111379_at | 0.00 |
| 111380_at | 0.00 |
| 111381_r_at | 0.00 |
| 111382_at | 0.00 |
| 111383_at | 0.00 |
| 111384_at | 0.00 |
| 111385_at | 0.16 |
| 111386_at | 0.04 |
| 111389_at | 0.00 |
| 111390_at | 0.00 |
| 111391_at | 0.00 |
| 111392_f_at | 0.00 |
| 111393_r_at | 0.00 |
| 111394_at | 0.00 |
| 111395_at | 0.00 |
| 111396_r_at | 0.00 |
| 111397_at | 0.00 |
| 111398_at | 0.00 |
| 111399_at | 0.00 |
| 111402_at | 0.00 |
| 111405_at | 0.00 |
| 111406_at | 0.05 |
| 111407_at | 0.00 |
| 111408_at | 0.00 |
| 111409_at | 0.00 |
| 111410_at | 0.00 |
| 111411_at | 0.00 |
| 111412_at | 0.00 |
| 111413_at | 0.00 |
| 111414_at | 0.01 |
| 111416_at | 0.00 |
| 111417_at | 0.00 |
| 111419_at | 0.01 |
| 111420_at | 0.00 |
| 111421_at | 0.00 |
| 111423_at | 0.00 |
| 111424_at | 0.00 |
| 111425_at | 0.00 |
| 111426_at | 0.00 |
| 111427_at | 0.00 |
| 111428_at | 0.00 |
| 111429_f_at | 0.00 |

| | |
|---|---|
| 111430_r_at | 0.00 |
| 111431_at | 0.00 |
| 111432_at | 0.00 |
| 111433_at | 0.11 |
| 111434_at | 0.00 |
| 111435_at | 0.00 |
| 111437_at | 0.00 |
| 111438_at | 0.05 |
| 111439_at | 0.00 |
| 111440_at | 0.00 |
| 111441_at | 0.00 |
| 111442_s_at | 0.00 |
| 111443_at | 0.00 |
| 111444_at | 0.00 |
| 111445_at | 0.01 |
| 111446_at | 0.00 |
| 111447_at | 0.00 |
| 111448_f_at | 0.38 |
| 111449_r_at | 0.00 |
| 111451_at | 0.00 |
| 111452_at | 0.02 |
| 111453_at | 0.00 |
| 111454_at | 0.00 |
| 111455_at | 0.00 |
| 111457_at | 0.00 |
| 111458_at | 0.00 |
| 111459_at | 0.00 |
| 111464_at | 0.00 |
| 111465_at | 0.00 |
| 111466_at | 0.00 |
| 111468_at | 0.00 |
| 111469_at | 0.00 |
| 111471_at | 0.00 |
| 111472_at | 0.00 |
| 111473_at | 0.00 |
| 111474_g_at | 0.00 |
| 111475_at | 0.00 |
| 111476_at | 0.00 |
| 111477_at | 0.00 |
| 111478_at | 0.00 |
| 111479_at | 0.00 |
| 111480_at | 0.00 |
| 111481_at | 0.00 |
| 111482_at | 0.00 |
| 111483_at | 0.00 |
| 111485_at | 0.00 |
| 111486_at | 0.00 |
| 111487_at | 0.00 |
| 111488_at | 0.00 |
| 111489_at | 0.00 |
| 111491_at | 0.00 |
| 111492_at | 0.00 |
| 111493_at | 0.00 |
| 111494_at | 0.00 |
| 111495_at | 0.00 |
| 111496_at | 0.00 |

| | |
|---|---|
| 111497_at | 0.02 |
| 111498_at | 0.00 |
| 111499_at | 0.00 |
| 111500_at | 0.00 |
| 111501_at | 0.00 |
| 111502_s_at | 0.00 |
| 111503_r_at | 0.00 |
| 111504_at | 0.00 |
| 111506_at | 0.00 |
| 111507_at | 0.00 |
| 111508_at | 0.00 |
| 111509_at | 0.00 |
| 111510_at | 0.00 |
| 111511_at | 0.00 |
| 111512_at | 0.00 |
| 111514_at | 0.00 |
| 111515_at | 0.01 |
| 111516_r_at | 0.00 |
| 111517_at | 0.04 |
| 111518_at | 0.01 |
| 111519_at | 0.00 |
| 111521_f_at | 0.00 |
| 111522_r_at | 0.00 |
| 111523_at | 0.00 |
| 111524_at | 0.00 |
| 111525_at | 0.00 |
| 111526_at | 0.00 |
| 111527_at | 0.00 |
| 111528_at | 0.00 |
| 111529_at | 0.00 |
| 111531_at | 0.03 |
| 111532_at | 0.00 |
| 111533_at | 0.00 |
| 111535_at | 0.00 |
| 111538_at | 0.00 |
| 111539_at | 0.00 |
| 111540_at | 0.00 |
| 111541_at | 0.00 |
| 111542_at | 0.00 |
| 111543_at | 0.00 |
| 111544_at | 0.00 |
| 111545_at | 0.00 |
| 111546_at | 0.00 |
| 111547_at | 0.01 |
| 111548_g_at | 0.00 |
| 111549_at | 0.00 |
| 111550_at | 0.00 |
| 111551_at | 0.00 |
| 111552_at | 0.00 |
| 111553_at | 0.02 |
| 111554_i_at | 0.00 |
| 111555_f_at | 0.00 |
| 111558_at | 0.01 |
| 111559_at | 0.00 |
| 111560_at | 0.00 |
| 111561_at | 0.00 |

| | |
|---|---|
| 111562_at | 0.00 |
| 111564_at | 0.00 |
| 111565_at | 0.00 |
| 111566_at | 0.00 |
| 111567_at | 0.00 |
| 111568_at | 0.00 |
| 111569_at | 0.00 |
| 111570_at | 0.00 |
| 111572_at | 0.00 |
| 111573_at | 0.00 |
| 111575_at | 0.00 |
| 111576_at | 0.00 |
| 111578_at | 0.32 |
| 111579_at | 0.00 |
| 111581_at | 0.00 |
| 111582_at | 0.00 |
| 111583_at | 0.00 |
| 111584_at | 0.00 |
| 111586_at | 0.00 |
| 111587_g_at | 0.00 |
| 111588_at | 0.01 |
| 111589_at | 0.00 |
| 111590_at | 0.00 |
| 111591_at | 0.00 |
| 111593_at | 0.00 |
| 111594_at | 0.00 |
| 111595_at | 0.00 |
| 111596_at | 0.00 |
| 111597_at | 0.10 |
| 111599_at | 0.00 |
| 111600_at | 0.00 |
| 111601_at | 0.00 |
| 111602_at | 0.00 |
| 111604_at | 0.00 |
| 111605_at | 0.00 |
| 111606_at | 0.00 |
| 111607_at | 0.00 |
| 111608_s_at | 0.00 |
| 111609_r_at | 0.00 |
| 111610_at | 0.00 |
| 111611_at | 0.00 |
| 111612_at | 0.00 |
| 111613_at | 0.00 |
| 111614_at | 0.00 |
| 111615_at | 0.02 |
| 111616_at | 0.00 |
| 111617_at | 0.00 |
| 111618_at | 0.00 |
| 111619_at | 0.00 |
| 111620_at | 0.00 |
| 111621_at | 0.00 |
| 111622_at | 0.00 |
| 111623_at | 0.00 |
| 111624_at | 0.00 |
| 111625_at | 0.00 |
| 111626_at | 0.00 |

| | |
|---|---|
| 111627_at | 0.00 |
| 111628_at | 0.00 |
| 111629_at | 0.00 |
| 111630_at | 0.00 |
| 111631_at | 0.00 |
| 111632_at | 0.00 |
| 111633_at | 0.00 |
| 111634_at | 0.00 |
| 111635_at | 0.00 |
| 111636_at | 0.00 |
| 111637_at | 0.00 |
| 111638_at | 0.00 |
| 111639_at | 0.00 |
| 111640_at | 0.00 |
| 111641_at | 0.00 |
| 111642_at | 0.00 |
| 111643_at | 0.00 |
| 111644_at | 0.00 |
| 111645_at | 0.00 |
| 111646_at | 0.00 |
| 111647_at | 0.00 |
| 111648_at | 0.00 |
| 111649_at | 0.00 |
| 111650_at | 0.02 |
| 111651_at | 0.00 |
| 111653_at | 0.00 |
| 111654_at | 0.00 |
| 111656_at | 0.00 |
| 111657_at | 0.00 |
| 111658_at | 0.00 |
| 111659_at | 0.00 |
| 111660_at | 0.00 |
| 111661_at | 0.00 |
| 111662_at | 0.00 |
| 111663_at | 0.00 |
| 111665_f_at | 0.00 |
| 111666_at | 0.00 |
| 111667_at | 0.00 |
| 111668_at | 0.00 |
| 111670_at | 0.00 |
| 111671_at | 0.00 |
| 111672_at | 0.00 |
| 111676_at | 0.00 |
| 111677_at | 0.00 |
| 111678_at | 0.00 |
| 111679_at | 0.00 |
| 111680_at | 0.00 |
| 111681_f_at | 0.00 |
| 111682_at | 0.00 |
| 111683_at | 0.00 |
| 111684_at | 0.00 |
| 111685_at | 0.01 |
| 111686_r_at | 0.00 |
| 111687_at | 0.01 |
| 111688_at | 0.02 |
| 111689_at | 0.00 |

| | |
|---|---|
| 111690_at | 0.00 |
| 111691_at | 0.00 |
| 111692_at | 0.00 |
| 111694_at | 0.00 |
| 111695_at | 0.00 |
| 111697_at | 0.00 |
| 111698_at | 0.00 |
| 111699_at | 0.00 |
| 111700_at | 0.00 |
| 111701_at | 0.00 |
| 111703_at | 0.00 |
| 111704_at | 0.14 |
| 111706_at | 0.00 |
| 111707_at | 0.00 |
| 111708_at | 0.00 |
| 111709_at | 0.00 |
| 111711_at | 0.00 |
| 111712_at | 0.00 |
| 111713_at | 0.00 |
| 111714_at | 0.02 |
| 111715_at | 0.00 |
| 111716_at | 0.00 |
| 111717_at | 0.00 |
| 111718_at | 0.00 |
| 111719_at | 0.00 |
| 111720_at | 0.02 |
| 111721_at | 0.00 |
| 111722_at | 0.00 |
| 111723_at | 0.00 |
| 111724_at | 0.00 |
| 111726_at | 0.00 |
| 111727_at | 0.00 |
| 111728_at | 0.00 |
| 111729_at | 0.00 |
| 111730_at | 0.12 |
| 111731_at | 0.00 |
| 111732_at | 0.05 |
| 111733_at | 0.00 |
| 111734_at | 0.04 |
| 111735_at | 0.04 |
| 111736_at | 0.00 |
| 111738_at | 0.53 |
| 111740_at | 0.00 |
| 111741_at | 0.00 |
| 111742_at | 0.00 |
| 111743_at | 0.02 |
| 111744_at | 0.00 |
| 111745_at | 0.00 |
| 111746_at | 0.03 |
| 111747_at | 0.00 |
| 111748_at | 0.00 |
| 111749_at | 0.00 |
| 111750_at | 0.00 |
| 111751_at | 0.00 |
| 111753_at | 0.00 |
| 111754_at | 0.01 |

| | |
|---|---|
| 111756_f_at | 0.02 |
| 111757_at | 0.00 |
| 111758_at | 0.00 |
| 111759_at | 0.00 |
| 111760_at | 0.00 |
| 111761_at | 0.00 |
| 111762_at | 0.00 |
| 111763_at | 0.01 |
| 111764_at | 0.00 |
| 111765_at | 0.00 |
| 111766_at | 0.00 |
| 111767_at | 0.08 |
| 111768_at | 0.00 |
| 111769_at | 0.00 |
| 111770_at | 0.00 |
| 111772_at | 0.00 |
| 111774_at | 0.00 |
| 111775_at | 0.00 |
| 111777_at | 0.00 |
| 111778_at | 0.00 |
| 111781_at | 0.00 |
| 111782_at | 0.00 |
| 111783_at | 0.00 |
| 111784_at | 0.00 |
| 111786_at | 0.00 |
| 111787_at | 0.00 |
| 111789_at | 0.00 |
| 111790_at | 0.00 |
| 111793_at | 0.00 |
| 111794_at | 0.00 |
| 111795_at | 0.00 |
| 111796_at | 0.02 |
| 111800_at | 0.00 |
| 111801_g_at | 0.00 |
| 111802_at | 0.00 |
| 111804_at | 0.00 |
| 111805_at | 0.01 |
| 111806_at | 0.01 |
| 111808_f_at | 0.00 |
| 111809_r_at | 0.00 |
| 111810_at | 0.00 |
| 111812_at | 0.00 |
| 111814_at | 0.00 |
| 111815_s_at | 0.00 |
| 111816_at | 0.00 |
| 111818_at | 0.00 |
| 111819_at | 0.00 |
| 111820_at | 0.02 |
| 111821_at | 0.00 |
| 111822_at | 0.00 |
| 111823_at | 0.00 |
| 111825_at | 0.00 |
| 111826_at | 0.00 |
| 111827_f_at | 0.00 |
| 111829_at | 0.00 |
| 111830_at | 0.00 |

| | |
|---|---|
| 111831_at | 0.00 |
| 111832_at | 0.00 |
| 111833_at | 0.00 |
| 111834_at | 0.00 |
| 111835_at | 0.00 |
| 111836_at | 0.00 |
| 111837_at | 0.00 |
| 111840_at | 0.00 |
| 111841_at | 0.00 |
| 111842_at | 0.00 |
| 111843_at | 0.00 |
| 111845_at | 0.01 |
| 111846_at | 0.00 |
| 111847_at | 0.00 |
| 111849_at | 0.00 |
| 111850_at | 0.00 |
| 111852_at | 0.00 |
| 111853_at | 0.00 |
| 111855_at | 0.00 |
| 111859_at | 0.00 |
| 111860_at | 0.00 |
| 111861_at | 0.00 |
| 111863_at | 0.00 |
| 111864_at | 0.00 |
| 111865_at | 0.00 |
| 111867_at | 0.00 |
| 111869_at | 0.00 |
| 111872_at | 0.00 |
| 111873_at | 0.25 |
| 111875_at | 0.00 |
| 111876_at | 0.00 |
| 111877_at | 0.00 |
| 111880_at | 0.00 |
| 111882_r_at | 0.00 |
| 111883_at | 0.00 |
| 111884_at | 0.00 |
| 111886_at | 0.04 |
| 111887_at | 0.00 |
| 111889_at | 0.00 |
| 111890_at | 0.00 |
| 111891_f_at | 0.00 |
| 111892_r_at | 0.02 |
| 111893_at | 0.00 |
| 111894_at | 0.08 |
| 111895_at | 0.00 |
| 111896_at | 0.01 |
| 111897_at | 0.00 |
| 111898_at | 0.00 |
| 111899_at | 0.00 |
| 111900_at | 0.00 |
| 111902_f_at | 0.00 |
| 111903_at | 0.00 |
| 111904_at | 0.00 |
| 111906_at | 0.00 |
| 111907_at | 0.22 |
| 111909_f_at | 0.00 |

| | |
|---|---|
| 111910_r_at | 0.00 |
| 111911_at | 0.00 |
| 111912_at | 0.00 |
| 111915_at | 0.00 |
| 111916_at | 0.00 |
| 111917_at | 0.00 |
| 111918_at | 0.00 |
| 111919_at | 0.00 |
| 111920_at | 0.00 |
| 111921_at | 0.00 |
| 111924_at | 0.00 |
| 111925_at | 0.00 |
| 111926_at | 0.00 |
| 111927_at | 0.00 |
| 111928_at | 0.00 |
| 111930_at | 0.00 |
| 111931_at | 0.00 |
| 111932_at | 0.00 |
| 111933_at | 0.00 |
| 111934_at | 0.00 |
| 111935_at | 0.05 |
| 111936_at | 0.00 |
| 111937_at | 0.00 |
| 111938_at | 0.00 |
| 111939_at | 0.16 |
| 111940_at | 0.02 |
| 111941_at | 0.00 |
| 111942_f_at | 0.00 |
| 111943_r_at | 0.00 |
| 111944_at | 0.00 |
| 111945_f_at | 0.00 |
| 111946_r_at | 0.00 |
| 111947_at | 0.00 |
| 111948_at | 0.00 |
| 111949_at | 0.00 |
| 111950_at | 0.18 |
| 111951_at | 0.00 |
| 111952_at | 0.00 |
| 111954_r_at | 0.00 |
| 111955_at | 0.00 |
| 111956_at | 0.00 |
| 111957_at | 0.06 |
| 111958_at | 0.00 |
| 111960_at | 0.00 |
| 111962_at | 0.00 |
| 111963_at | 0.01 |
| 111964_at | 0.00 |
| 111965_at | 0.00 |
| 111966_at | 0.00 |
| 111967_at | 0.00 |
| 111968_at | 0.00 |
| 111969_at | 0.00 |
| 111970_at | 0.39 |
| 111971_at | 0.00 |
| 111972_at | 0.02 |
| 111974_at | 0.00 |

| | |
|---|---|
| 111975_at | 0.00 |
| 111976_at | 0.00 |
| 111977_s_at | 0.00 |
| 111978_at | 0.00 |
| 111980_at | 0.00 |
| 111981_at | 0.00 |
| 111982_at | 0.30 |
| 111983_at | 0.00 |
| 111984_at | 0.00 |
| 111985_at | 0.00 |
| 111986_at | 0.00 |
| 111987_at | 0.00 |
| 111988_g_at | 0.00 |
| 111989_at | 0.02 |
| 111990_at | 0.00 |
| 111991_at | 0.00 |
| 111992_at | 0.00 |
| 111993_at | 0.00 |
| 111994_at | 0.00 |
| 111995_at | 0.00 |
| 111996_at | 0.00 |
| 111997_s_at | 0.00 |
| 111998_at | 0.00 |
| 111999_at | 0.00 |
| 112000_at | 0.00 |
| 112001_at | 0.00 |
| 112002_at | 0.00 |
| 112003_at | 0.00 |
| 112004_at | 0.00 |
| 112005_at | 0.00 |
| 112007_at | 0.00 |
| 112009_at | 0.00 |
| 112010_at | 0.00 |
| 112011_at | 0.00 |
| 112012_at | 0.00 |
| 112013_at | 0.00 |
| 112014_at | 0.00 |
| 112015_at | 0.00 |
| 112016_at | 0.00 |
| 112017_at | 0.00 |
| 112018_at | 0.00 |
| 112019_at | 0.64 |
| 112020_g_at | 0.06 |
| 112021_at | 0.00 |
| 112022_at | 0.00 |
| 112023_at | 0.00 |
| 112024_at | 0.00 |
| 112025_at | 0.00 |
| 112026_at | 0.00 |
| 112027_at | 0.00 |
| 112028_at | 0.17 |
| 112029_at | 0.00 |
| 112030_at | 0.00 |
| 112031_g_at | 0.00 |
| 112032_at | 0.00 |
| 112033_at | 0.00 |

| | |
|---|---|
| 112034_f_at | 0.00 |
| 112035_r_at | 0.00 |
| 112036_at | 0.00 |
| 112037_at | 0.00 |
| 112038_at | 0.00 |
| 112039_at | 0.00 |
| 112040_at | 0.00 |
| 112041_at | 0.00 |
| 112042_at | 0.00 |
| 112043_at | 0.00 |
| 112044_at | 0.00 |
| 112045_at | 0.00 |
| 112048_at | 0.00 |
| 112049_at | 0.00 |
| 112050_at | 0.00 |
| 112051_at | 0.00 |
| 112052_at | 0.00 |
| 112053_at | 0.01 |
| 112054_at | 0.00 |
| 112055_at | 0.00 |
| 112056_at | 0.00 |
| 112057_at | 0.00 |
| 112058_at | 0.00 |
| 112059_at | 0.00 |
| 112060_at | 0.00 |
| 112061_at | 0.00 |
| 112062_at | 0.01 |
| 112063_at | 0.00 |
| 112064_at | 0.00 |
| 112065_at | 0.00 |
| 112066_at | 0.00 |
| 112067_at | 0.00 |
| 112068_at | 0.00 |
| 112070_at | 0.01 |
| 112072_at | 0.00 |
| 112073_at | 0.00 |
| 112074_at | 0.00 |
| 112076_at | 0.00 |
| 112077_at | 0.00 |
| 112078_at | 0.00 |
| 112079_at | 0.00 |
| 112080_at | 0.00 |
| 112081_at | 0.00 |
| 112082_at | 0.00 |
| 112083_at | 0.00 |
| 112084_at | 0.00 |
| 112085_at | 0.00 |
| 112086_at | 0.00 |
| 112087_at | 0.00 |
| 112088_at | 0.00 |
| 112089_at | 0.00 |
| 112090_at | 0.00 |
| 112091_at | 0.00 |
| 112092_at | 0.00 |
| 112093_at | 0.00 |
| 112094_at | 0.00 |

| | |
|---|---|
| 112095_at | 0.00 |
| 112096_at | 0.00 |
| 112097_at | 0.00 |
| 112098_at | 0.00 |
| 112100_at | 0.00 |
| 112101_at | 0.00 |
| 112102_at | 0.00 |
| 112103_at | 0.00 |
| 112104_at | 0.00 |
| 112105_at | 0.00 |
| 112107_at | 0.00 |
| 112108_at | 0.00 |
| 112109_at | 0.00 |
| 112110_at | 0.00 |
| 112114_at | 0.00 |
| 112155_at | 0.00 |
| 112157_at | 0.02 |
| 112159_at | 0.00 |
| 112160_at | 0.30 |
| 112162_at | 0.00 |
| 112163_at | 0.00 |
| 112164_at | 0.00 |
| 112165_at | 0.00 |
| 112166_at | 0.00 |
| 112167_f_at | 0.00 |
| 112168_r_at | 0.00 |
| 112169_at | 0.00 |
| 112170_at | 0.00 |
| 112172_at | 0.00 |
| 112174_at | 0.00 |
| 112175_at | 0.00 |
| 112176_at | 0.00 |
| 112179_at | 0.00 |
| 112180_at | 0.00 |
| 112181_at | 0.00 |
| 112182_at | 0.00 |
| 112183_g_at | 0.00 |
| 112185_at | 0.00 |
| 112186_at | 0.06 |
| 112191_at | 0.00 |
| 112192_at | 0.00 |
| 112193_at | 0.00 |
| 112194_f_at | 0.00 |
| 112195_r_at | 0.00 |
| 112196_at | 0.00 |
| 112197_at | 0.00 |
| 112198_at | 0.06 |
| 112199_f_at | 0.00 |
| 112200_at | 0.00 |
| 112203_at | 0.01 |
| 112204_at | 0.07 |
| 112205_at | 0.06 |
| 112206_at | 0.02 |
| 112209_at | 0.03 |
| 112210_at | 0.00 |
| 112211_at | 0.00 |

| | |
|---|---|
| 112212_at | 0.00 |
| 112213_at | 0.00 |
| 112217_at | 0.00 |
| 112220_at | 0.00 |
| 112221_at | 0.00 |
| 112223_at | 0.00 |
| 112226_at | 0.00 |
| 112236_at | 0.00 |
| 112237_at | 0.00 |
| 112238_at | 0.00 |
| 112239_r_at | 0.00 |
| 112244_at | 0.00 |
| 112245_at | 0.00 |
| 112247_at | 0.00 |
| 112248_at | 0.00 |
| 112249_g_at | 0.00 |
| 112251_at | 0.00 |
| 112253_at | 0.00 |
| 112254_at | 0.00 |
| 112255_at | 0.00 |
| 112256_at | 0.01 |
| 112257_at | 0.02 |
| 112258_at | 0.00 |
| 112260_at | 0.00 |
| 112262_at | 0.00 |
| 112263_at | 0.00 |
| 112264_i_at | 0.00 |
| 112265_r_at | 0.00 |
| 112266_at | 0.00 |
| 112268_at | 0.00 |
| 112269_at | 0.00 |
| 112271_at | 0.00 |
| 112276_at | 0.00 |
| 112277_at | 0.00 |
| 112278_at | 0.00 |
| 112279_r_at | 0.00 |
| 112280_i_at | 0.00 |
| 112281_f_at | 0.01 |
| 112282_s_at | 0.00 |
| 112283_at | 0.00 |
| 112284_at | 0.00 |
| 112286_at | 0.00 |
| 112287_at | 0.00 |
| 112288_at | 0.01 |
| 112289_g_at | 0.19 |
| 112290_at | 0.00 |
| 112291_r_at | 0.00 |
| 112292_at | 0.00 |
| 112293_at | 0.00 |
| 112294_at | 0.00 |
| 112295_at | 0.00 |
| 112296_at | 0.00 |
| 112300_at | 0.00 |
| 112302_at | 0.00 |
| 112303_at | 0.00 |
| 112304_at | 0.00 |

| | |
|---|---|
| 112305_at | 0.00 |
| 112306_at | 0.00 |
| 112307_at | 0.00 |
| 112308_at | 0.00 |
| 112309_at | 0.00 |
| 112310_r_at | 0.00 |
| 112311_at | 0.00 |
| 112312_at | 0.00 |
| 112313_at | 0.00 |
| 112314_at | 0.00 |
| 112315_at | 0.00 |
| 112316_at | 0.00 |
| 112317_at | 0.00 |
| 112318_at | 0.00 |
| 112320_at | 0.00 |
| 112321_at | 0.05 |
| 112322_at | 0.03 |
| 112323_at | 0.00 |
| 112324_at | 0.02 |
| 112325_at | 0.00 |
| 112330_at | 0.02 |
| 112331_at | 0.00 |
| 112333_at | 0.10 |
| 112335_at | 0.00 |
| 112336_at | 0.00 |
| 112337_at | 0.00 |
| 112338_at | 0.00 |
| 112340_at | 0.00 |
| 112343_at | 0.00 |
| 112344_at | 0.00 |
| 112345_at | 0.00 |
| 112346_at | 0.00 |
| 112348_at | 0.00 |
| 112349_at | 0.17 |
| 112350_at | 0.07 |
| 112351_at | 0.00 |
| 112352_at | 0.01 |
| 112353_at | 0.02 |
| 112355_at | 0.00 |
| 112356_at | 0.15 |
| 112357_at | 0.01 |
| 112358_at | 0.00 |
| 112361_at | 0.00 |
| 112362_at | 0.00 |
| 112363_at | 0.00 |
| 112364_at | 0.00 |
| 112365_at | 0.00 |
| 112366_at | 0.00 |
| 112367_at | 0.00 |
| 112368_at | 0.00 |
| 112369_g_at | 0.00 |
| 112370_at | 0.00 |
| 112372_at | 0.00 |
| 112373_at | 0.12 |
| 112374_at | 0.00 |
| 112375_at | 0.00 |

| | |
|---|---|
| 112376_at | 0.00 |
| 112378_at | 0.19 |
| 112379_at | 0.00 |
| 112381_at | 0.00 |
| 112383_at | 0.06 |
| 112384_at | 0.00 |
| 112385_at | 0.00 |
| 112386_at | 0.12 |
| 112387_at | 0.00 |
| 112388_at | 0.00 |
| 112389_at | 0.01 |
| 112390_at | 0.00 |
| 112391_at | 0.00 |
| 112392_at | 0.00 |
| 112393_at | 0.00 |
| 112394_at | 0.07 |
| 112395_at | 0.00 |
| 112396_at | 0.00 |
| 112397_at | 0.00 |
| 112398_at | 0.00 |
| 112399_at | 0.00 |
| 112400_at | 0.00 |
| 112401_at | 0.00 |
| 112402_at | 0.00 |
| 112403_at | 0.00 |
| 112404_at | 0.00 |
| 112405_at | 0.31 |
| 112406_at | 0.30 |
| 112407_at | 0.00 |
| 112408_at | 0.00 |
| 112409_g_at | 0.00 |
| 112410_at | 0.00 |
| 112411_at | 0.00 |
| 112412_at | 0.00 |
| 112413_at | 0.00 |
| 112414_at | 0.00 |
| 112415_at | 0.02 |
| 112416_at | 0.00 |
| 112420_at | 0.00 |
| 112421_at | 0.00 |
| 112422_at | 0.00 |
| 112423_at | 0.00 |
| 112425_r_at | 0.00 |
| 112426_r_at | 0.00 |
| 112427_at | 0.00 |
| 112429_at | 0.00 |
| 112431_at | 0.00 |
| 112432_at | 0.00 |
| 112433_at | 0.00 |
| 112434_at | 0.00 |
| 112435_at | 0.01 |
| 112437_at | 0.00 |
| 112438_at | 0.00 |
| 112440_at | 0.00 |
| 112441_at | 0.00 |
| 112442_at | 0.00 |

| | |
|---|---|
| 112443_at | 0.00 |
| 112444_r_at | 0.00 |
| 112445_at | 0.01 |
| 112446_at | 0.00 |
| 112447_at | 0.00 |
| 112448_at | 0.00 |
| 112449_at | 0.00 |
| 112451_at | 0.00 |
| 112452_at | 0.00 |
| 112453_at | 0.00 |
| 112454_at | 0.00 |
| 112455_at | 0.00 |
| 112456_at | 0.00 |
| 112457_at | 0.00 |
| 112458_at | 0.05 |
| 112459_at | 0.00 |
| 112460_at | 0.06 |
| 112461_at | 0.00 |
| 112462_at | 0.01 |
| 112464_at | 0.00 |
| 112465_at | 0.00 |
| 112466_at | 0.00 |
| 112467_at | 0.00 |
| 112468_at | 0.00 |
| 112471_at | 0.00 |
| 112472_at | 0.00 |
| 112473_at | 0.00 |
| 112474_f_at | 0.00 |
| 112475_r_at | 0.00 |
| 112476_at | 0.00 |
| 112477_at | 0.00 |
| 112478_at | 0.04 |
| 112479_at | 0.00 |
| 112480_at | 0.00 |
| 112481_at | 0.00 |
| 112482_at | 0.00 |
| 112484_at | 0.00 |
| 112485_at | 0.00 |
| 112486_at | 0.00 |
| 112487_at | 0.00 |
| 112488_at | 0.01 |
| 112489_at | 0.00 |
| 112490_at | 0.00 |
| 112491_at | 0.00 |
| 112492_at | 0.00 |
| 112493_at | 0.00 |
| 112495_at | 0.00 |
| 112496_at | 0.00 |
| 112497_at | 0.00 |
| 112498_at | 0.00 |
| 112499_at | 0.00 |
| 112500_at | 0.00 |
| 112501_at | 0.00 |
| 112502_at | 0.00 |
| 112503_at | 0.00 |
| 112504_at | 0.01 |

| | |
|---|---|
| 112505_at | 0.00 |
| 112506_at | 0.00 |
| 112508_at | 0.01 |
| 112509_at | 0.00 |
| 112510_at | 0.00 |
| 112512_at | 0.00 |
| 112513_at | 0.00 |
| 112514_at | 0.01 |
| 112515_at | 0.00 |
| 112516_at | 0.00 |
| 112531_at | 0.00 |
| 112638_at | 0.00 |
| 112644_at | 0.00 |
| 112645_at | 0.00 |
| 112646_at | 0.00 |
| 112647_at | 0.00 |
| 112648_f_at | 0.36 |
| 112650_at | 0.00 |
| 112651_at | 0.00 |
| 112652_at | 0.00 |
| 112653_at | 0.00 |
| 112654_at | 0.00 |
| 112655_at | 0.00 |
| 112656_at | 0.00 |
| 112657_at | 0.00 |
| 112658_at | 0.00 |
| 112659_at | 0.00 |
| 112660_at | 0.00 |
| 112661_at | 0.00 |
| 112662_at | 0.00 |
| 112663_at | 0.00 |
| 112664_at | 0.00 |
| 112665_at | 0.00 |
| 112668_at | 0.00 |
| 112669_at | 0.00 |
| 112671_at | 0.00 |
| 112672_at | 0.00 |
| 112673_at | 0.00 |
| 112674_g_at | 0.00 |
| 112675_at | 0.09 |
| 112676_at | 0.02 |
| 112677_at | 0.00 |
| 112678_at | 0.00 |
| 112679_at | 0.00 |
| 112680_at | 0.30 |
| 112681_at | 0.00 |
| 112682_at | 0.00 |
| 112683_at | 0.09 |
| 112684_at | 0.00 |
| 112685_at | 0.00 |
| 112686_at | 0.00 |
| 112687_at | 0.14 |
| 112688_at | 0.00 |
| 112689_at | 0.00 |
| 112690_at | 0.00 |
| 112691_at | 0.03 |

| | |
|---|---|
| 112692_at | 0.02 |
| 112693_at | 0.00 |
| 112694_at | 0.00 |
| 112695_at | 0.00 |
| 112696_at | 0.00 |
| 112697_at | 0.00 |
| 112698_at | 0.00 |
| 112699_at | 0.14 |
| 112700_at | 0.00 |
| 112701_at | 0.00 |
| 112702_at | 0.00 |
| 112703_at | 0.00 |
| 112704_at | 0.01 |
| 112705_at | 0.01 |
| 112706_at | 0.00 |
| 112707_at | 0.01 |
| 112708_f_at | 0.00 |
| 112709_r_at | 0.00 |
| 112710_at | 0.00 |
| 112711_at | 0.00 |
| 112712_at | 0.00 |
| 112713_g_at | 0.00 |
| 112714_at | 0.00 |
| 112715_at | 0.00 |
| 112716_at | 0.03 |
| 112718_at | 0.08 |
| 112719_at | 0.00 |
| 112720_at | 0.00 |
| 112721_at | 0.00 |
| 112722_at | 0.00 |
| 112723_at | 0.00 |
| 112724_at | 0.00 |
| 112725_at | 0.00 |
| 112726_at | 0.00 |
| 112727_at | 0.00 |
| 112728_at | 0.00 |
| 112729_at | 0.05 |
| 112730_at | 0.00 |
| 112731_at | 0.00 |
| 112732_g_at | 0.00 |
| 112733_at | 0.00 |
| 112734_at | 0.00 |
| 112735_at | 0.00 |
| 112736_at | 0.00 |
| 112737_at | 0.00 |
| 112738_at | 0.00 |
| 112739_at | 0.00 |
| 112740_at | 0.23 |
| 112741_at | 0.00 |
| 112742_at | 0.00 |
| 112743_at | 0.00 |
| 112744_at | 0.00 |
| 112745_at | 0.00 |
| 112746_at | 0.01 |
| 112747_at | 0.00 |
| 112748_at | 0.00 |

| | |
|---|---|
| 112750_at | 0.00 |
| 112751_s_at | 0.00 |
| 112752_f_at | 0.00 |
| 112753_at | 0.00 |
| 112754_at | 0.00 |
| 112755_at | 0.00 |
| 112756_at | 0.00 |
| 112757_at | 0.00 |
| 112758_at | 0.00 |
| 112759_g_at | 0.00 |
| 112760_at | 0.00 |
| 112761_at | 0.00 |
| 112763_at | 0.00 |
| 112764_at | 0.00 |
| 112766_at | 0.01 |
| 112767_s_at | 0.18 |
| 112768_at | 0.07 |
| 112769_at | 0.00 |
| 112770_at | 0.02 |
| 112771_f_at | 0.00 |
| 112772_r_at | 0.00 |
| 112773_at | 0.00 |
| 112774_i_at | 0.00 |
| 112775_r_at | 0.00 |
| 112778_at | 0.00 |
| 112779_at | 0.00 |
| 112780_at | 0.00 |
| 112781_at | 0.00 |
| 112783_at | 0.01 |
| 112784_at | 0.00 |
| 112786_i_at | 0.00 |
| 112787_f_at | 0.00 |
| 112788_at | 0.00 |
| 112790_at | 0.02 |
| 112791_at | 0.18 |
| 112792_at | 0.00 |
| 112793_at | 0.00 |
| 112794_at | 0.00 |
| 112795_at | 0.00 |
| 112797_at | 0.37 |
| 112799_at | 0.00 |
| 112802_at | 0.00 |
| 112804_at | 0.00 |
| 112807_at | 0.00 |
| 112808_at | 0.00 |
| 112809_at | 0.00 |
| 112813_at | 0.00 |
| 112814_at | 0.00 |
| 112816_at | 0.00 |
| 112817_at | 0.05 |
| 112819_at | 0.00 |
| 112820_at | 0.00 |
| 112822_at | 0.00 |
| 112823_at | 0.00 |
| 112824_at | 0.00 |
| 112825_at | 0.00 |

| | |
|---|---|
| 112826_at | 0.00 |
| 112827_at | 0.00 |
| 112828_at | 0.11 |
| 112829_at | 0.00 |
| 112830_at | 0.00 |
| 112831_at | 0.04 |
| 112832_at | 0.00 |
| 112833_at | 0.00 |
| 112835_at | 0.00 |
| 112837_at | 0.00 |
| 112838_at | 0.00 |
| 112840_at | 0.00 |
| 112842_at | 0.00 |
| 112843_at | 0.00 |
| 112844_at | 0.00 |
| 112845_at | 0.07 |
| 112846_at | 0.00 |
| 112847_at | 0.00 |
| 112848_at | 0.00 |
| 112850_at | 0.00 |
| 112851_at | 0.00 |
| 112852_at | 0.00 |
| 112853_at | 0.00 |
| 112854_s_at | 0.04 |
| 112855_at | 0.00 |
| 112856_at | 0.00 |
| 112857_g_at | 0.04 |
| 112858_at | 0.02 |
| 112859_at | 0.00 |
| 112860_at | 0.00 |
| 112861_at | 0.02 |
| 112862_at | 0.00 |
| 112864_at | 0.00 |
| 112865_at | 0.00 |
| 112867_at | 0.00 |
| 112868_at | 0.00 |
| 112869_at | 0.00 |
| 112870_at | 0.04 |
| 112874_at | 0.00 |
| 112875_at | 0.00 |
| 112876_at | 0.00 |
| 112877_at | 0.01 |
| 112878_at | 0.00 |
| 112880_at | 0.00 |
| 112881_at | 0.03 |
| 112883_at | 0.00 |
| 112885_i_at | 0.00 |
| 112886_f_at | 0.00 |
| 112887_at | 0.00 |
| 112888_at | 0.00 |
| 112889_at | 0.00 |
| 112890_at | 0.00 |
| 112891_at | 0.00 |
| 112892_at | 0.00 |
| 112893_at | 0.00 |
| 112894_at | 0.00 |

| | |
|---|---|
| 112895_at | 0.00 |
| 112896_at | 0.00 |
| 112898_at | 0.01 |
| 112900_at | 0.00 |
| 112901_at | 0.00 |
| 112902_at | 0.00 |
| 112903_at | 0.00 |
| 112904_at | 0.00 |
| 112905_at | 0.00 |
| 112906_at | 0.00 |
| 112908_at | 0.00 |
| 112910_f_at | 0.00 |
| 112911_r_at | 0.00 |
| 112912_at | 0.00 |
| 112913_at | 0.00 |
| 112914_at | 0.00 |
| 112915_at | 0.00 |
| 112916_at | 0.00 |
| 112917_at | 0.00 |
| 112918_at | 0.04 |
| 112919_at | 0.00 |
| 112920_at | 0.00 |
| 112921_at | 0.00 |
| 112922_i_at | 0.00 |
| 112923_at | 0.00 |
| 112924_at | 0.00 |
| 112925_at | 0.00 |
| 112927_at | 0.00 |
| 112928_at | 0.00 |
| 112929_at | 0.00 |
| 112930_at | 0.00 |
| 112932_at | 0.00 |
| 112933_at | 0.00 |
| 112934_at | 0.00 |
| 112935_at | 0.00 |
| 112936_at | 0.00 |
| 112937_at | 0.00 |
| 112938_at | 0.00 |
| 112939_at | 0.00 |
| 112940_i_at | 0.00 |
| 112941_f_at | 0.35 |
| 112942_at | 0.00 |
| 112943_at | 0.00 |
| 112944_at | 0.11 |
| 112945_at | 0.00 |
| 112946_at | 0.00 |
| 112947_at | 0.00 |
| 112948_at | 0.00 |
| 112949_at | 0.00 |
| 112950_at | 0.00 |
| 112951_at | 0.00 |
| 112953_at | 0.00 |
| 112955_at | 0.39 |
| 112956_at | 0.00 |
| 112957_at | 0.00 |
| 112958_at | 0.05 |

| | |
|---|---|
| 112959_at | 0.00 |
| 112961_at | 0.00 |
| 112962_at | 0.00 |
| 112963_at | 0.00 |
| 112964_at | 0.00 |
| 112965_at | 0.00 |
| 112966_at | 0.00 |
| 112967_at | 0.00 |
| 112968_at | 0.00 |
| 112969_at | 0.16 |
| 112970_at | 0.00 |
| 112971_at | 0.00 |
| 112972_at | 0.00 |
| 112973_at | 0.21 |
| 112974_at | 0.00 |
| 112975_at | 0.00 |
| 112976_at | 0.00 |
| 112977_at | 0.00 |
| 112978_at | 0.00 |
| 112979_at | 0.00 |
| 112980_at | 0.00 |
| 112981_at | 0.00 |
| 112983_at | 0.00 |
| 112984_at | 0.00 |
| 112985_at | 0.00 |
| 112986_at | 0.00 |
| 112987_at | 0.00 |
| 112988_at | 0.00 |
| 112989_at | 0.10 |
| 112990_at | 0.00 |
| 112992_at | 0.00 |
| 112993_at | 0.00 |
| 112994_at | 0.11 |
| 112996_at | 0.00 |
| 112997_at | 0.20 |
| 112998_at | 0.00 |
| 112999_at | 0.00 |
| 113000_at | 0.00 |
| 113001_at | 0.00 |
| 113003_at | 0.00 |
| 113005_at | 0.00 |
| 113006_r_at | 0.00 |
| 113007_at | 0.00 |
| 113008_at | 0.00 |
| 113011_at | 0.00 |
| 113012_at | 0.01 |
| 113013_s_at | 0.00 |
| 113014_at | 0.00 |
| 113015_at | 0.00 |
| 113016_at | 0.00 |
| 113017_at | 0.00 |
| 113018_at | 0.00 |
| 113019_at | 0.00 |
| 113020_at | 0.01 |
| 113021_at | 0.00 |
| 113022_at | 0.00 |

| | |
|---|---|
| 113023_at | 0.00 |
| 113024_at | 0.06 |
| 113025_at | 0.00 |
| 113026_at | 0.02 |
| 113027_at | 0.01 |
| 113028_at | 0.00 |
| 113029_at | 0.00 |
| 113030_at | 0.00 |
| 113031_at | 0.00 |
| 113032_at | 0.01 |
| 113033_at | 0.05 |
| 113034_at | 0.00 |
| 113035_at | 0.00 |
| 113036_at | 0.00 |
| 113037_at | 0.00 |
| 113038_at | 0.00 |
| 113039_at | 0.00 |
| 113040_at | 0.00 |
| 113041_at | 0.00 |
| 113042_at | 0.00 |
| 113043_at | 0.54 |
| 113044_at | 0.00 |
| 113045_at | 0.00 |
| 113046_at | 0.01 |
| 113047_at | 0.02 |
| 113048_at | 0.00 |
| 113049_at | 0.00 |
| 113050_at | 0.00 |
| 113051_at | 0.00 |
| 113052_at | 0.00 |
| 113053_at | 0.00 |
| 113054_at | 0.00 |
| 113055_at | 0.00 |
| 113057_at | 0.00 |
| 113058_at | 0.00 |
| 113059_at | 0.00 |
| 113061_at | 0.00 |
| 113062_at | 0.00 |
| 113064_at | 0.00 |
| 113065_at | 0.00 |
| 113066_at | 0.00 |
| 113067_at | 0.00 |
| 113068_at | 0.00 |
| 113069_at | 0.00 |
| 113070_at | 0.00 |
| 113071_at | 0.00 |
| 113072_at | 0.00 |
| 113073_at | 0.00 |
| 113074_at | 0.00 |
| 113075_at | 0.02 |
| 113076_at | 0.00 |
| 113077_at | 0.00 |
| 113078_at | 0.00 |
| 113079_at | 0.02 |
| 113080_at | 0.00 |
| 113081_at | 0.00 |

| | |
|---|---|
| 113082_at | 0.00 |
| 113083_at | 0.00 |
| 113084_at | 0.00 |
| 113085_at | 0.00 |
| 113086_at | 0.00 |
| 113088_at | 0.04 |
| 113089_at | 0.00 |
| 113090_at | 0.00 |
| 113092_at | 0.00 |
| 113093_at | 0.00 |
| 113094_at | 0.00 |
| 113098_at | 0.00 |
| 113099_at | 0.00 |
| 113100_at | 0.00 |
| 113101_f_at | 0.00 |
| 113105_at | 0.00 |
| 113106_at | 0.00 |
| 113107_at | 0.00 |
| 113108_at | 0.00 |
| 113110_at | 0.00 |
| 113112_at | 0.00 |
| 113113_at | 0.00 |
| 113114_at | 0.00 |
| 113115_at | 0.00 |
| 113116_at | 0.00 |
| 113117_at | 0.00 |
| 113118_at | 0.00 |
| 113119_at | 0.00 |
| 113121_at | 0.00 |
| 113122_i_at | 0.00 |
| 113123_r_at | 0.00 |
| 113124_at | 0.00 |
| 113125_at | 0.00 |
| 113126_at | 0.00 |
| 113128_at | 0.00 |
| 113129_at | 0.00 |
| 113130_at | 0.00 |
| 113131_at | 0.00 |
| 113132_at | 0.10 |
| 113133_at | 0.00 |
| 113135_at | 0.00 |
| 113136_at | 0.00 |
| 113139_at | 0.00 |
| 113140_at | 0.00 |
| 113141_at | 0.42 |
| 113142_at | 0.00 |
| 113143_at | 0.00 |
| 113144_at | 0.00 |
| 113145_at | 0.00 |
| 113146_at | 0.00 |
| 113147_at | 0.00 |
| 113148_at | 0.00 |
| 113149_at | 0.00 |
| 113151_at | 0.00 |
| 113152_at | 0.05 |
| 113153_at | 0.00 |

| | |
|---|---|
| 113154_at | 0.17 |
| 113155_at | 0.00 |
| 113156_at | 0.00 |
| 113160_at | 0.00 |
| 113161_at | 0.00 |
| 113162_at | 0.00 |
| 113164_at | 0.00 |
| 113165_at | 0.05 |
| 113166_at | 0.00 |
| 113167_at | 0.00 |
| 113168_at | 0.03 |
| 113171_at | 0.01 |
| 113172_at | 0.00 |
| 113173_at | 0.00 |
| 113174_at | 0.00 |
| 113176_at | 0.00 |
| 113177_at | 0.00 |
| 113178_at | 0.00 |
| 113179_at | 0.00 |
| 113180_at | 0.00 |
| 113181_r_at | 0.00 |
| 113182_at | 0.06 |
| 113183_at | 0.00 |
| 113186_at | 0.00 |
| 113187_at | 0.00 |
| 113188_at | 0.00 |
| 113189_at | 0.00 |
| 113190_at | 0.01 |
| 113191_at | 0.00 |
| 113193_at | 0.00 |
| 113194_g_at | 0.00 |
| 113195_at | 0.00 |
| 113196_at | 0.00 |
| 113198_at | 0.25 |
| 113199_at | 0.11 |
| 113200_at | 0.00 |
| 113201_at | 0.00 |
| 113203_at | 0.00 |
| 113204_at | 0.00 |
| 113205_g_at | 0.00 |
| 113206_at | 0.00 |
| 113207_at | 0.00 |
| 113208_at | 0.00 |
| 113210_at | 0.00 |
| 113211_at | 0.01 |
| 113212_at | 0.00 |
| 113213_at | 0.00 |
| 113214_at | 0.03 |
| 113215_i_at | 0.00 |
| 113216_f_at | 0.00 |
| 113219_at | 0.00 |
| 113222_at | 0.00 |
| 113223_at | 0.00 |
| 113225_at | 0.00 |
| 113226_at | 0.00 |
| 113227_at | 0.17 |

| | |
|---|---|
| 113228_at | 0.00 |
| 113229_at | 0.00 |
| 113230_at | 0.04 |
| 113231_at | 0.00 |
| 113232_at | 0.00 |
| 113234_at | 0.49 |
| 113235_at | 0.00 |
| 113239_at | 0.00 |
| 113240_at | 0.00 |
| 113241_at | 0.00 |
| 113242_at | 0.00 |
| 113243_at | 0.00 |
| 113244_at | 0.00 |
| 113246_at | 0.00 |
| 113247_at | 0.00 |
| 113248_at | 0.00 |
| 113249_at | 0.00 |
| 113250_at | 0.00 |
| 113251_at | 0.00 |
| 113252_at | 0.00 |
| 113253_r_at | 0.00 |
| 113255_at | 0.00 |
| 113256_at | 0.00 |
| 113257_at | 0.00 |
| 113258_at | 0.00 |
| 113259_at | 0.00 |
| 113260_at | 0.00 |
| 113261_at | 0.00 |
| 113262_at | 0.00 |
| 113264_at | 0.05 |
| 113265_at | 0.00 |
| 113266_at | 0.00 |
| 113267_at | 0.00 |
| 113268_s_at | 0.23 |
| 113269_r_at | 0.00 |
| 113270_at | 0.00 |
| 113271_at | 0.00 |
| 113274_at | 0.00 |
| 113275_at | 0.00 |
| 113276_at | 0.12 |
| 113277_at | 0.00 |
| 113278_at | 0.00 |
| 113279_at | 0.00 |
| 113280_at | 0.14 |
| 113282_at | 0.00 |
| 113283_at | 0.02 |
| 113284_at | 0.46 |
| 113285_at | 0.00 |
| 113286_at | 0.00 |
| 113287_at | 0.00 |
| 113288_at | 0.03 |
| 113290_at | 0.12 |
| 113291_at | 0.01 |
| 113292_at | 0.06 |
| 113293_at | 0.00 |
| 113294_at | 0.00 |

| | |
|---|---|
| 113295_at | 0.00 |
| 113296_at | 0.00 |
| 113297_at | 0.00 |
| 113298_at | 0.00 |
| 113299_at | 0.00 |
| 113300_at | 0.00 |
| 113301_at | 0.00 |
| 113302_at | 0.00 |
| 113303_at | 0.00 |
| 113304_at | 0.00 |
| 113305_at | 0.00 |
| 113306_at | 0.00 |
| 113307_at | 0.00 |
| 113308_at | 0.00 |
| 113309_at | 0.03 |
| 113310_f_at | 0.00 |
| 113311_r_at | 0.00 |
| 113312_at | 0.00 |
| 113314_at | 0.00 |
| 113316_at | 0.00 |
| 113317_at | 0.00 |
| 113318_at | 0.00 |
| 113319_at | 0.07 |
| 113320_at | 0.00 |
| 113321_at | 0.00 |
| 113322_at | 0.00 |
| 113323_at | 0.00 |
| 113324_at | 0.00 |
| 113325_r_at | 0.00 |
| 113326_at | 0.00 |
| 113327_at | 0.00 |
| 113328_at | 0.00 |
| 113329_at | 0.00 |
| 113330_at | 0.00 |
| 113331_at | 0.00 |
| 113332_at | 0.00 |
| 113333_at | 0.18 |
| 113334_at | 0.00 |
| 113335_at | 0.00 |
| 113336_at | 0.00 |
| 113337_at | 0.03 |
| 113338_at | 0.00 |
| 113339_at | 0.00 |
| 113341_at | 0.00 |
| 113342_g_at | 0.00 |
| 113343_at | 0.00 |
| 113344_at | 0.00 |
| 113345_at | 0.00 |
| 113346_at | 0.00 |
| 113347_at | 0.00 |
| 113348_at | 0.02 |
| 113349_at | 0.00 |
| 113352_at | 0.00 |
| 113353_at | 0.00 |
| 113396_f_at | 0.00 |
| 113397_f_at | 0.00 |

| | |
|---|---|
| 113421_at | 0.00 |
| 113422_at | 0.00 |
| 113423_at | 0.00 |
| 113424_at | 0.00 |
| 113425_at | 0.00 |
| 113426_at | 0.00 |
| 113427_at | 0.00 |
| 113428_at | 0.00 |
| 113429_at | 0.00 |
| 113430_at | 0.00 |
| 113431_at | 0.00 |
| 113432_at | 0.00 |
| 113433_at | 0.00 |
| 113434_at | 0.00 |
| 113435_at | 0.00 |
| 113436_at | 0.00 |
| 113437_at | 0.00 |
| 113438_at | 0.00 |
| 113439_at | 0.00 |
| 113441_at | 0.00 |
| 113442_at | 0.02 |
| 113443_r_at | 0.01 |
| 113444_at | 0.00 |
| 113445_at | 0.00 |
| 113446_at | 0.00 |
| 113447_at | 0.00 |
| 113448_at | 0.00 |
| 113449_at | 0.00 |
| 113450_at | 0.00 |
| 113451_at | 0.07 |
| 113452_at | 0.00 |
| 113453_at | 0.00 |
| 113454_at | 0.00 |
| 113455_at | 0.00 |
| 113456_at | 0.00 |
| 113457_at | 0.00 |
| 113458_at | 0.00 |
| 113459_at | 0.00 |
| 113460_at | 0.00 |
| 113461_at | 0.00 |
| 113462_at | 0.00 |
| 113463_at | 0.00 |
| 113464_at | 0.00 |
| 113465_at | 0.00 |
| 113466_at | 0.00 |
| 113467_at | 0.00 |
| 113468_at | 0.00 |
| 113469_at | 0.00 |
| 113470_at | 0.00 |
| 113472_at | 0.00 |
| 113473_at | 0.00 |
| 113474_at | 0.00 |
| 113475_at | 0.00 |
| 113476_at | 0.00 |
| 113478_at | 0.00 |
| 113479_at | 0.00 |

| | |
|---|---|
| 113480_at | 0.00 |
| 113481_at | 0.00 |
| 113482_at | 0.00 |
| 113483_at | 0.00 |
| 113484_at | 0.00 |
| 113485_at | 0.00 |
| 113486_at | 0.00 |
| 113487_at | 0.00 |
| 113488_at | 0.00 |
| 113489_at | 0.00 |
| 113490_at | 0.00 |
| 113491_at | 0.00 |
| 113492_at | 0.00 |
| 113493_at | 0.00 |
| 113494_at | 0.00 |
| 113495_at | 0.30 |
| 113496_at | 0.00 |
| 113497_at | 0.00 |
| 113498_at | 0.00 |
| 113499_at | 0.00 |
| 113500_at | 0.00 |
| 113501_at | 0.00 |
| 113502_at | 0.00 |
| 113503_at | 0.00 |
| 113504_at | 0.00 |
| 113505_at | 0.00 |
| 113506_at | 0.00 |
| 113507_at | 0.00 |
| 113508_at | 0.00 |
| 113509_at | 0.00 |
| 113510_s_at | 0.00 |
| 113511_at | 0.00 |
| 113512_at | 0.00 |
| 113515_at | 0.00 |
| 113516_at | 0.00 |
| 113517_at | 0.00 |
| 113518_f_at | 0.00 |
| 113519_r_at | 0.00 |
| 113520_at | 0.00 |
| 113521_at | 0.00 |
| 113522_at | 0.00 |
| 113523_at | 0.00 |
| 113524_at | 0.00 |
| 113525_at | 0.00 |
| 113526_i_at | 0.00 |
| 113527_f_at | 0.00 |
| 113528_at | 0.00 |
| 113529_at | 0.00 |
| 113530_at | 0.00 |
| 113531_at | 0.00 |
| 113532_at | 0.00 |
| 113533_at | 0.00 |
| 113534_at | 0.03 |
| 113535_at | 0.00 |
| 113537_at | 0.00 |
| 113538_at | 0.00 |

| | |
|---|---|
| 113539_at | 0.00 |
| 113541_at | 0.00 |
| 113542_at | 0.00 |
| 113543_at | 0.00 |
| 113544_at | 0.00 |
| 113545_at | 0.00 |
| 113546_at | 0.00 |
| 113548_at | 0.00 |
| 113549_at | 0.01 |
| 113550_at | 0.00 |
| 113551_at | 0.09 |
| 113552_at | 0.00 |
| 113553_at | 0.00 |
| 113554_at | 0.00 |
| 113555_at | 0.00 |
| 113556_at | 0.00 |
| 113561_at | 0.00 |
| 113562_at | 0.00 |
| 113563_at | 0.00 |
| 113564_at | 0.00 |
| 113565_at | 0.00 |
| 113567_at | 0.00 |
| 113568_at | 0.03 |
| 113569_at | 0.00 |
| 113570_at | 0.00 |
| 113574_at | 0.00 |
| 113575_at | 0.00 |
| 113577_at | 0.00 |
| 113578_at | 0.00 |
| 113579_at | 0.00 |
| 113580_at | 0.00 |
| 113581_at | 0.09 |
| 113584_i_at | 0.00 |
| 113585_f_at | 0.00 |
| 113587_at | 0.00 |
| 113588_f_at | 0.03 |
| 113589_r_at | 0.00 |
| 113590_at | 0.00 |
| 113591_at | 0.00 |
| 113592_at | 0.00 |
| 113593_at | 0.00 |
| 113595_at | 0.00 |
| 113596_at | 0.00 |
| 113597_g_at | 0.00 |
| 113598_at | 0.00 |
| 113599_at | 0.00 |
| 113600_at | 0.00 |
| 113601_at | 0.00 |
| 113602_at | 0.01 |
| 113604_at | 0.00 |
| 113606_at | 0.02 |
| 113608_at | 0.01 |
| 113610_at | 0.23 |
| 113611_at | 0.00 |
| 113612_at | 0.01 |
| 113613_at | 0.00 |

| | |
|---|---|
| 113615_r_at | 0.00 |
| 113617_f_at | 0.00 |
| 113618_r_at | 0.00 |
| 113620_at | 0.01 |
| 113621_at | 0.00 |
| 113622_at | 0.00 |
| 113624_at | 0.00 |
| 113626_at | 0.00 |
| 113627_at | 0.00 |
| 113629_at | 0.00 |
| 113630_at | 0.00 |
| 113631_at | 0.00 |
| 113632_at | 0.00 |
| 113633_at | 0.00 |
| 113636_at | 0.00 |
| 113638_at | 0.00 |
| 113639_r_at | 0.00 |
| 113640_at | 0.03 |
| 113641_at | 0.00 |
| 113642_at | 0.00 |
| 113643_at | 0.00 |
| 113645_s_at | 0.00 |
| 113646_i_at | 0.00 |
| 113647_at | 0.00 |
| 113648_at | 0.00 |
| 113649_at | 0.00 |
| 113650_at | 0.00 |
| 113651_at | 0.42 |
| 113652_at | 0.00 |
| 113653_at | 0.00 |
| 113654_at | 0.00 |
| 113656_at | 0.00 |
| 113657_at | 0.00 |
| 113658_at | 0.03 |
| 113662_at | 0.00 |
| 113664_at | 0.00 |
| 113665_at | 0.00 |
| 113668_at | 0.00 |
| 113671_at | 0.33 |
| 113672_at | 0.00 |
| 113673_at | 0.72 |
| 113674_at | 0.00 |
| 113676_at | 0.00 |
| 113680_at | 0.01 |
| 113681_at | 0.00 |
| 113682_at | 0.00 |
| 113683_at | 0.00 |
| 113684_at | 0.00 |
| 113685_at | 0.00 |
| 113686_at | 0.00 |
| 113688_at | 0.00 |
| 113689_at | 0.00 |
| 113691_at | 0.00 |
| 113692_at | 0.00 |
| 113693_at | 0.00 |
| 113694_at | 0.00 |

| | |
|---|---|
| 113695_at | 0.00 |
| 113696_at | 0.01 |
| 113698_at | 0.00 |
| 113699_at | 0.00 |
| 113701_at | 0.00 |
| 113702_at | 0.00 |
| 113703_i_at | 0.00 |
| 113704_r_at | 0.00 |
| 113705_at | 0.00 |
| 113706_at | 0.00 |
| 113707_at | 0.00 |
| 113708_at | 0.00 |
| 113709_at | 0.00 |
| 113712_at | 0.00 |
| 113713_at | 0.00 |
| 113714_at | 0.00 |
| 113715_at | 0.01 |
| 113716_at | 0.00 |
| 113717_at | 0.00 |
| 113720_at | 0.00 |
| 113721_at | 0.00 |
| 113722_at | 0.00 |
| 113724_at | 0.00 |
| 113725_at | 0.00 |
| 113726_at | 0.00 |
| 113727_at | 0.00 |
| 113728_at | 0.03 |
| 113729_at | 0.11 |
| 113730_at | 0.00 |
| 113731_at | 0.00 |
| 113732_at | 0.00 |
| 113733_at | 0.00 |
| 113734_at | 0.00 |
| 113735_at | 0.00 |
| 113736_at | 0.00 |
| 113737_at | 0.00 |
| 113738_at | 0.00 |
| 113739_at | 0.00 |
| 113740_at | 0.00 |
| 113741_at | 0.00 |
| 113742_at | 0.00 |
| 113743_at | 0.01 |
| 113744_at | 0.00 |
| 113746_at | 0.00 |
| 113747_at | 0.00 |
| 113748_at | 0.00 |
| 113749_at | 0.00 |
| 113750_at | 0.00 |
| 113751_at | 0.00 |
| 113752_at | 0.00 |
| 113753_at | 0.02 |
| 113756_at | 0.00 |
| 113757_at | 0.01 |
| 113758_at | 0.00 |
| 113759_at | 0.00 |
| 113760_at | 0.01 |

| | |
|---|---|
| 113761_at | 0.00 |
| 113762_at | 0.00 |
| 113763_r_at | 0.00 |
| 113764_at | 0.00 |
| 113765_at | 0.00 |
| 113766_at | 0.00 |
| 113767_at | 0.00 |
| 113768_at | 0.00 |
| 113769_at | 0.00 |
| 113770_at | 0.00 |
| 113771_g_at | 0.00 |
| 113772_at | 0.00 |
| 113775_at | 0.00 |
| 113776_at | 0.00 |
| 113777_at | 0.00 |
| 113778_at | 0.00 |
| 113779_i_at | 0.00 |
| 113780_f_at | 0.00 |
| 113781_at | 0.00 |
| 113783_at | 0.00 |
| 113784_at | 0.00 |
| 113785_at | 0.00 |
| 113786_at | 0.00 |
| 113787_at | 0.00 |
| 113788_at | 0.00 |
| 113789_at | 0.00 |
| 113790_at | 0.00 |
| 113792_at | 0.02 |
| 113793_at | 0.00 |
| 113794_at | 0.00 |
| 113795_at | 0.00 |
| 113796_at | 0.00 |
| 113797_at | 0.00 |
| 113798_at | 0.00 |
| 113799_at | 0.00 |
| 113800_at | 0.00 |
| 113801_at | 0.00 |
| 113802_at | 0.00 |
| 113803_at | 0.00 |
| 113804_at | 0.00 |
| 113805_at | 0.00 |
| 113806_at | 0.00 |
| 113807_at | 0.00 |
| 113808_at | 0.00 |
| 113809_at | 0.00 |
| 113810_at | 0.00 |
| 113811_at | 0.00 |
| 113812_at | 0.00 |
| 113813_at | 0.00 |
| 113814_at | 0.00 |
| 113815_at | 0.00 |
| 113816_at | 0.00 |
| 113817_at | 0.00 |
| 113820_i_at | 0.00 |
| 113821_at | 0.00 |
| 113822_at | 0.00 |

| | |
|---|---|
| 113823_at | 0.00 |
| 113824_at | 0.00 |
| 113825_at | 0.00 |
| 113826_at | 0.00 |
| 113827_f_at | 0.00 |
| 113828_at | 0.04 |
| 113829_at | 0.00 |
| 113830_at | 0.11 |
| 113831_at | 0.00 |
| 113832_at | 0.00 |
| 113833_at | 0.00 |
| 113834_at | 0.00 |
| 113835_s_at | 0.00 |
| 113836_at | 0.00 |
| 113837_f_at | 0.10 |
| 113838_at | 0.00 |
| 113839_at | 0.00 |
| 113840_at | 0.24 |
| 113841_at | 0.00 |
| 113842_at | 0.00 |
| 113843_at | 0.00 |
| 113844_at | 0.00 |
| 113845_at | 0.00 |
| 113846_at | 0.00 |
| 113847_at | 0.00 |
| 113848_at | 0.00 |
| 113849_at | 0.00 |
| 113850_at | 0.00 |
| 113851_at | 0.00 |
| 113852_at | 0.00 |
| 113853_at | 0.00 |
| 113854_at | 0.00 |
| 113855_at | 0.00 |
| 113856_at | 0.00 |
| 113857_at | 0.00 |
| 113858_at | 0.00 |
| 113859_at | 0.00 |
| 113860_at | 0.00 |
| 113861_at | 0.00 |
| 113862_at | 0.00 |
| 113863_at | 0.00 |
| 113864_at | 0.00 |
| 113865_at | 0.00 |
| 113866_at | 0.00 |
| 113867_at | 0.00 |
| 113868_at | 0.00 |
| 113869_at | 0.00 |
| 113870_at | 0.00 |
| 113871_at | 0.00 |
| 113872_at | 0.00 |
| 113873_at | 0.00 |
| 113874_at | 0.00 |
| 113875_at | 0.00 |
| 113876_at | 0.00 |
| 113877_at | 0.00 |
| 113878_at | 0.00 |

| | |
|---|---|
| 113879_at | 0.00 |
| 113880_at | 0.00 |
| 113881_at | 0.00 |
| 113883_at | 0.00 |
| 113884_at | 0.00 |
| 113885_at | 0.14 |
| 113886_at | 0.00 |
| 113887_at | 0.00 |
| 113888_at | 0.00 |
| 113889_at | 0.00 |
| 113890_at | 0.00 |
| 113891_at | 0.00 |
| 113892_at | 0.00 |
| 113893_at | 0.00 |
| 113894_at | 0.00 |
| 113895_at | 0.00 |
| 113896_at | 0.00 |
| 113897_at | 0.00 |
| 113898_at | 0.00 |
| 113899_at | 0.00 |
| 113900_at | 0.00 |
| 113901_at | 0.00 |
| 113902_at | 0.06 |
| 113903_at | 0.01 |
| 113904_at | 0.00 |
| 113907_at | 0.24 |
| 113908_at | 0.00 |
| 113910_at | 0.00 |
| 113911_at | 0.00 |
| 113912_at | 0.00 |
| 113913_at | 0.67 |
| 113914_at | 0.00 |
| 113915_at | 0.00 |
| 113916_at | 0.00 |
| 113918_at | 0.00 |
| 113919_at | 0.00 |
| 113920_at | 0.00 |
| 113921_at | 0.00 |
| 113926_at | 0.00 |
| 113930_at | 0.00 |
| 113931_at | 0.00 |
| 113932_g_at | 0.02 |
| 113933_at | 0.00 |
| 113935_at | 0.00 |
| 113936_at | 0.00 |
| 113937_at | 0.08 |
| 113938_at | 0.00 |
| 113939_at | 0.00 |
| 113940_at | 0.00 |
| 113941_at | 0.00 |
| 113946_at | 0.00 |
| 113953_at | 0.00 |
| 113957_at | 0.00 |
| 113959_at | 0.00 |
| 113964_at | 0.01 |
| 113966_at | 0.00 |

| | |
|---|---|
| 113968_at | 0.00 |
| 113969_at | 0.35 |
| 113970_at | 0.00 |
| 113974_at | 0.00 |
| 113975_at | 0.00 |
| 113976_at | 0.00 |
| 113978_at | 0.00 |
| 113981_at | 0.00 |
| 113982_at | 0.00 |
| 113984_f_at | 0.00 |
| 113985_at | 0.00 |
| 113986_at | 0.00 |
| 113989_at | 0.00 |
| 113990_at | 0.00 |
| 113992_at | 0.00 |
| 113994_at | 0.00 |
| 113995_at | 0.00 |
| 113997_at | 0.00 |
| 113998_at | 0.17 |
| 114001_at | 0.03 |
| 114002_at | 0.00 |
| 114004_at | 0.00 |
| 114005_at | 0.00 |
| 114008_at | 0.00 |
| 114011_at | 0.00 |
| 114014_at | 0.00 |
| 114015_at | 0.00 |
| 114016_at | 0.00 |
| 114018_at | 0.00 |
| 114020_at | 0.00 |
| 114021_at | 0.00 |
| 114022_r_at | 0.00 |
| 114023_at | 0.00 |
| 114024_at | 0.00 |
| 114025_at | 0.00 |
| 114026_at | 0.00 |
| 114027_at | 0.00 |
| 114028_at | 0.00 |
| 114029_at | 0.00 |
| 114030_at | 0.00 |
| 114031_at | 0.00 |
| 114032_f_at | 0.04 |
| 114034_at | 0.00 |
| 114036_at | 0.00 |
| 114037_at | 0.00 |
| 114038_at | 0.00 |
| 114040_at | 0.00 |
| 114042_at | 0.00 |
| 114043_at | 0.00 |
| 114044_r_at | 0.00 |
| 114045_at | 0.00 |
| 114048_at | 0.12 |
| 114049_at | 0.00 |
| 114050_at | 0.00 |
| 114051_at | 0.00 |
| 114052_at | 0.00 |

| | |
|---|---|
| 114054_at | 0.00 |
| 114055_at | 0.00 |
| 114056_at | 0.00 |
| 114057_at | 0.00 |
| 114058_at | 0.00 |
| 114059_at | 0.00 |
| 114060_at | 0.00 |
| 114061_at | 0.00 |
| 114062_at | 0.00 |
| 114064_at | 0.00 |
| 114065_at | 0.01 |
| 114066_at | 0.00 |
| 114068_at | 0.00 |
| 114069_at | 0.00 |
| 114073_at | 0.00 |
| 114075_at | 0.00 |
| 114076_at | 0.00 |
| 114082_at | 0.00 |
| 114083_at | 0.00 |
| 114084_at | 0.00 |
| 114085_at | 0.00 |
| 114086_at | 0.00 |
| 114087_at | 0.00 |
| 114088_at | 0.00 |
| 114089_at | 0.00 |
| 114091_at | 0.00 |
| 114092_at | 0.00 |
| 114093_at | 0.00 |
| 114094_at | 0.00 |
| 114095_at | 0.00 |
| 114096_at | 0.00 |
| 114097_at | 0.00 |
| 114098_at | 0.00 |
| 114102_at | 0.00 |
| 114103_f_at | 0.00 |
| 114104_r_at | 0.00 |
| 114105_at | 0.00 |
| 114106_at | 0.00 |
| 114107_at | 0.00 |
| 114108_at | 0.00 |
| 114109_at | 0.00 |
| 114110_at | 0.00 |
| 114112_at | 0.00 |
| 114115_at | 0.04 |
| 114116_at | 0.00 |
| 114117_f_at | 0.11 |
| 114119_at | 0.00 |
| 114120_at | 0.00 |
| 114121_at | 0.00 |
| 114122_at | 0.00 |
| 114123_at | 0.00 |
| 114126_at | 0.00 |
| 114127_at | 0.00 |
| 114129_at | 0.00 |
| 114130_at | 0.00 |
| 114131_at | 0.00 |

| | |
|---|---|
| 114133_at | 0.00 |
| 114136_at | 0.00 |
| 114138_at | 0.00 |
| 114139_at | 0.05 |
| 114140_at | 0.00 |
| 114142_at | 0.00 |
| 114143_at | 0.15 |
| 114144_at | 0.00 |
| 114145_at | 0.00 |
| 114146_at | 0.02 |
| 114147_at | 0.00 |
| 114148_at | 0.00 |
| 114149_at | 0.00 |
| 114150_at | 0.00 |
| 114151_at | 0.00 |
| 114152_at | 0.00 |
| 114153_at | 0.00 |
| 114154_at | 0.00 |
| 114155_at | 0.00 |
| 114156_at | 0.00 |
| 114157_at | 0.00 |
| 114158_at | 0.00 |
| 114159_at | 0.00 |
| 114160_at | 0.00 |
| 114161_at | 0.00 |
| 114162_at | 0.00 |
| 114163_at | 0.00 |
| 114164_at | 0.00 |
| 114165_at | 0.00 |
| 114166_at | 0.01 |
| 114167_at | 0.00 |
| 114168_at | 0.00 |
| 114169_at | 0.07 |
| 114170_at | 0.00 |
| 114171_at | 0.00 |
| 114172_at | 0.00 |
| 114173_f_at | 0.00 |
| 114174_at | 0.00 |
| 114175_at | 0.00 |
| 114176_at | 0.00 |
| 114177_at | 0.00 |
| 114178_at | 0.00 |
| 114179_at | 0.00 |
| 114180_g_at | 0.00 |
| 114181_at | 0.01 |
| 114182_at | 0.13 |
| 114184_at | 0.00 |
| 114185_at | 0.00 |
| 114186_at | 0.00 |
| 114187_at | 0.00 |
| 114188_at | 0.02 |
| 114189_at | 0.00 |
| 114190_at | 0.00 |
| 114191_at | 0.00 |
| 114192_at | 0.00 |
| 114193_at | 0.00 |

| | |
|---|---|
| 114194_at | 0.00 |
| 114196_at | 0.00 |
| 114197_at | 0.00 |
| 114198_at | 0.00 |
| 114199_at | 0.00 |
| 114200_at | 0.00 |
| 114201_at | 0.00 |
| 114203_at | 0.00 |
| 114204_at | 0.00 |
| 114205_g_at | 0.00 |
| 114206_at | 0.00 |
| 114207_at | 0.00 |
| 114208_at | 0.00 |
| 114209_at | 0.00 |
| 114210_at | 0.00 |
| 114211_at | 0.00 |
| 114212_at | 0.00 |
| 114213_at | 0.00 |
| 114214_at | 0.00 |
| 114215_at | 0.00 |
| 114216_at | 0.00 |
| 114217_at | 0.00 |
| 114218_at | 0.00 |
| 114219_at | 0.00 |
| 114220_at | 0.00 |
| 114221_at | 0.00 |
| 114222_at | 0.00 |
| 114223_at | 0.00 |
| 114224_at | 0.00 |
| 114225_at | 0.00 |
| 114226_at | 0.00 |
| 114228_at | 0.00 |
| 114229_at | 0.00 |
| 114230_at | 0.00 |
| 114231_at | 0.00 |
| 114232_at | 0.00 |
| 114233_at | 0.00 |
| 114234_f_at | 0.00 |
| 114235_at | 0.00 |
| 114236_at | 0.09 |
| 114237_at | 0.00 |
| 114238_at | 0.12 |
| 114239_at | 0.00 |
| 114240_at | 0.00 |
| 114241_at | 0.00 |
| 114242_at | 0.00 |
| 114243_at | 0.00 |
| 114244_at | 0.00 |
| 114245_at | 0.00 |
| 114246_at | 0.00 |
| 114247_at | 0.00 |
| 114248_at | 0.00 |
| 114249_at | 0.00 |
| 114250_at | 0.00 |
| 114251_at | 0.00 |
| 114252_at | 0.00 |

| | |
|---|---|
| 114253_at | 0.00 |
| 114254_at | 0.02 |
| 114255_at | 0.00 |
| 114256_at | 0.00 |
| 114257_r_at | 0.00 |
| 114258_at | 0.00 |
| 114259_at | 0.00 |
| 114260_at | 0.00 |
| 114263_at | 0.09 |
| 114264_at | 0.02 |
| 114266_at | 0.00 |
| 114268_at | 0.00 |
| 114270_at | 0.00 |
| 114271_at | 0.00 |
| 114274_at | 0.00 |
| 114275_at | 0.00 |
| 114277_f_at | 0.00 |
| 114278_r_at | 0.00 |
| 114280_at | 0.00 |
| 114281_at | 0.00 |
| 114285_at | 0.00 |
| 114287_at | 0.00 |
| 114288_at | 0.33 |
| 114289_f_at | 0.00 |
| 114290_r_at | 0.00 |
| 114291_at | 0.00 |
| 114293_at | 0.00 |
| 114294_i_at | 0.00 |
| 114295_f_at | 0.00 |
| 114296_at | 0.00 |
| 114297_f_at | 0.00 |
| 114298_r_at | 0.00 |
| 114299_at | 0.11 |
| 114300_at | 0.00 |
| 114301_at | 0.18 |
| 114302_at | 0.00 |
| 114303_at | 0.00 |
| 114304_at | 0.00 |
| 114305_at | 0.01 |
| 114306_at | 0.00 |
| 114308_at | 0.00 |
| 114309_at | 0.12 |
| 114311_at | 0.00 |
| 114312_g_at | 0.00 |
| 114313_at | 0.00 |
| 114314_at | 0.00 |
| 114315_at | 0.00 |
| 114316_at | 0.00 |
| 114319_at | 0.00 |
| 114321_at | 0.00 |
| 114322_at | 0.00 |
| 114323_at | 0.00 |
| 114324_at | 0.00 |
| 114326_at | 0.00 |
| 114327_i_at | 0.02 |
| 114328_f_at | 0.00 |

| | |
|---|---|
| 114329_at | 0.07 |
| 114330_at | 0.00 |
| 114331_at | 0.00 |
| 114332_at | 0.00 |
| 114333_s_at | 0.00 |
| 114334_at | 0.00 |
| 114337_at | 0.00 |
| 114338_at | 0.00 |
| 114340_at | 0.00 |
| 114342_at | 0.00 |
| 114344_at | 0.00 |
| 114345_at | 0.00 |
| 114346_at | 0.00 |
| 114347_at | 0.00 |
| 114349_at | 0.00 |
| 114350_r_at | 0.00 |
| 114351_at | 0.00 |
| 114352_at | 0.00 |
| 114353_at | 0.00 |
| 114355_at | 0.02 |
| 114356_at | 0.00 |
| 114357_at | 0.00 |
| 114358_at | 0.00 |
| 114359_at | 0.00 |
| 114360_at | 0.01 |
| 114361_at | 0.00 |
| 114362_at | 0.00 |
| 114363_at | 0.00 |
| 114364_at | 0.00 |
| 114365_at | 0.00 |
| 114366_r_at | 0.00 |
| 114372_at | 0.00 |
| 114373_at | 0.01 |
| 114374_i_at | 0.00 |
| 114375_f_at | 0.00 |
| 114376_at | 0.00 |
| 114377_at | 0.00 |
| 114378_at | 0.00 |
| 114379_at | 0.00 |
| 114380_at | 0.00 |
| 114381_at | 0.01 |
| 114382_at | 0.00 |
| 114383_at | 0.00 |
| 114384_at | 0.00 |
| 114388_at | 0.00 |
| 114389_at | 0.07 |
| 114390_at | 0.00 |
| 114392_at | 0.00 |
| 114393_at | 0.00 |
| 114394_at | 0.00 |
| 114395_at | 0.00 |
| 114396_at | 0.00 |
| 114397_at | 0.00 |
| 114398_at | 0.00 |
| 114399_at | 0.00 |
| 114401_at | 0.00 |

| | |
|---|---|
| 114402_at | 0.00 |
| 114403_at | 0.00 |
| 114404_at | 0.02 |
| 114406_at | 0.00 |
| 114407_at | 0.00 |
| 114408_at | 0.00 |
| 114409_at | 0.00 |
| 114411_at | 0.00 |
| 114412_at | 0.00 |
| 114414_at | 0.00 |
| 114415_at | 0.00 |
| 114416_at | 0.00 |
| 114417_at | 0.00 |
| 114418_at | 0.00 |
| 114419_at | 0.00 |
| 114420_at | 0.00 |
| 114421_at | 0.00 |
| 114422_at | 0.00 |
| 114423_at | 0.00 |
| 114424_at | 0.00 |
| 114425_at | 0.00 |
| 114426_at | 0.00 |
| 114427_at | 0.00 |
| 114429_at | 0.00 |
| 114430_at | 0.00 |
| 114432_at | 0.00 |
| 114433_at | 0.00 |
| 114434_at | 0.00 |
| 114436_at | 0.00 |
| 114438_at | 0.00 |
| 114440_at | 0.00 |
| 114441_at | 0.00 |
| 114442_at | 0.00 |
| 114443_at | 0.00 |
| 114444_at | 0.00 |
| 114445_at | 0.00 |
| 114449_at | 0.00 |
| 114450_at | 0.00 |
| 114451_at | 0.00 |
| 114452_at | 0.00 |
| 114453_at | 0.00 |
| 114454_at | 0.00 |
| 114456_i_at | 0.09 |
| 114457_f_at | 0.01 |
| 114458_at | 0.00 |
| 114459_at | 0.00 |
| 114461_at | 0.01 |
| 114462_at | 0.12 |
| 114463_at | 0.00 |
| 114464_at | 0.00 |
| 114466_at | 0.00 |
| 114467_at | 0.00 |
| 114468_at | 0.00 |
| 114469_at | 0.00 |
| 114470_at | 0.00 |
| 114471_at | 0.00 |

| | |
|---|---|
| 114472_at | 0.00 |
| 114473_at | 0.00 |
| 114474_at | 0.00 |
| 114475_at | 0.00 |
| 114476_at | 0.00 |
| 114478_at | 0.00 |
| 114481_at | 0.05 |
| 114482_at | 0.00 |
| 114483_at | 0.01 |
| 114484_at | 0.01 |
| 114485_at | 0.00 |
| 114486_at | 0.00 |
| 114487_at | 0.00 |
| 114488_at | 0.00 |
| 114489_at | 0.00 |
| 114490_at | 0.00 |
| 114491_f_at | 0.00 |
| 114493_at | 0.00 |
| 114494_at | 0.00 |
| 114495_at | 0.00 |
| 114496_at | 0.00 |
| 114497_at | 0.00 |
| 114498_at | 0.37 |
| 114499_at | 0.00 |
| 114500_at | 0.00 |
| 114501_at | 0.00 |
| 114502_at | 0.00 |
| 114505_at | 0.46 |
| 114506_at | 0.00 |
| 114507_at | 0.00 |
| 114508_at | 0.00 |
| 114509_at | 0.00 |
| 114510_at | 0.00 |
| 114511_at | 0.00 |
| 114512_at | 0.00 |
| 114513_at | 0.00 |
| 114514_at | 0.00 |
| 114515_at | 0.00 |
| 114516_at | 0.00 |
| 114517_at | 0.05 |
| 114518_at | 0.00 |
| 114520_at | 0.00 |
| 114521_at | 0.00 |
| 114522_at | 0.00 |
| 114523_at | 0.00 |
| 114524_at | 0.00 |
| 114525_at | 0.14 |
| 114526_at | 0.00 |
| 114527_at | 0.00 |
| 114528_at | 0.00 |
| 114530_at | 0.00 |
| 114531_f_at | 0.61 |
| 114532_at | 0.00 |
| 114533_at | 0.00 |
| 114534_at | 0.00 |
| 114535_f_at | 0.00 |

| | |
|---|---|
| 114536_at | 0.09 |
| 114537_at | 0.00 |
| 114538_at | 0.01 |
| 114539_at | 0.00 |
| 114540_at | 0.00 |
| 114541_at | 0.00 |
| 114542_at | 0.01 |
| 114543_at | 0.00 |
| 114544_at | 0.00 |
| 114545_at | 0.00 |
| 114546_at | 0.00 |
| 114547_at | 0.00 |
| 114548_at | 0.02 |
| 114549_at | 0.00 |
| 114550_at | 0.00 |
| 114551_at | 0.00 |
| 114552_at | 0.00 |
| 114553_at | 0.00 |
| 114554_at | 0.00 |
| 114555_at | 0.00 |
| 114556_at | 0.00 |
| 114557_at | 0.00 |
| 114558_at | 0.00 |
| 114559_at | 0.00 |
| 114560_at | 0.09 |
| 114561_at | 0.00 |
| 114562_at | 0.01 |
| 114563_at | 0.00 |
| 114564_at | 0.00 |
| 114565_at | 0.00 |
| 114566_at | 0.00 |
| 114567_at | 0.00 |
| 114568_at | 0.00 |
| 114569_at | 0.00 |
| 114570_at | 0.00 |
| 114571_at | 0.00 |
| 114572_at | 0.00 |
| 114573_at | 0.00 |
| 114574_at | 0.00 |
| 114575_at | 0.00 |
| 114576_at | 0.00 |
| 114578_at | 0.00 |
| 114579_at | 0.00 |
| 114580_at | 0.00 |
| 114581_at | 0.00 |
| 114582_at | 0.00 |
| 114583_at | 0.00 |
| 114584_at | 0.00 |
| 114585_at | 0.00 |
| 114586_at | 0.00 |
| 114587_at | 0.00 |
| 114588_at | 0.00 |
| 114589_at | 0.00 |
| 114590_at | 0.00 |
| 114591_at | 0.00 |
| 114592_at | 0.00 |

| | |
|---|---|
| 114593_at | 0.00 |
| 114594_at | 0.00 |
| 114595_at | 0.00 |
| 114596_at | 0.00 |
| 114597_at | 0.00 |
| 114598_at | 0.00 |
| 114599_at | 0.00 |
| 114600_at | 0.00 |
| 114601_at | 0.00 |
| 114602_at | 0.00 |
| 114603_at | 0.00 |
| 114604_at | 0.00 |
| 114605_at | 0.00 |
| 114606_at | 0.00 |
| 114607_at | 0.00 |
| 114608_at | 0.00 |
| 114609_at | 0.00 |
| 114610_at | 0.00 |
| 114611_at | 0.00 |
| 114612_at | 0.00 |
| 114613_at | 0.00 |
| 114614_at | 0.00 |
| 114615_at | 0.00 |
| 114616_at | 0.00 |
| 114617_at | 0.00 |
| 114618_at | 0.00 |
| 114619_at | 0.00 |
| 114621_at | 0.01 |
| 114622_at | 0.00 |
| 114624_at | 0.00 |
| 114625_at | 0.00 |
| 114626_at | 0.00 |
| 114628_at | 0.00 |
| 114629_at | 0.00 |
| 114630_at | 0.00 |
| 114631_at | 0.00 |
| 114632_at | 0.00 |
| 114633_at | 0.00 |
| 114634_at | 0.00 |
| 114635_at | 0.00 |
| 114636_at | 0.00 |
| 114638_at | 0.00 |
| 114639_at | 0.14 |
| 114641_at | 0.00 |
| 114642_at | 0.00 |
| 114644_at | 0.00 |
| 114651_at | 0.00 |
| 114652_at | 0.00 |
| 114653_at | 0.00 |
| 114654_at | 0.00 |
| 114655_at | 0.00 |
| 114656_at | 0.03 |
| 114657_at | 0.00 |
| 114658_at | 0.00 |
| 114659_at | 0.00 |
| 114663_at | 0.00 |

| | |
|---|---|
| 114665_at | 0.10 |
| 114666_at | 0.00 |
| 114667_at | 0.11 |
| 114668_at | 0.00 |
| 114669_g_at | 0.00 |
| 114670_at | 0.00 |
| 114671_at | 0.00 |
| 114672_at | 0.01 |
| 114674_at | 0.00 |
| 114675_at | 0.00 |
| 114676_at | 0.02 |
| 114677_at | 0.00 |
| 114678_g_at | 0.00 |
| 114679_at | 0.00 |
| 114680_at | 0.00 |
| 114681_at | 0.00 |
| 114682_at | 0.00 |
| 114683_at | 0.00 |
| 114684_at | 0.00 |
| 114685_at | 0.00 |
| 114686_at | 0.00 |
| 114687_at | 0.00 |
| 114688_at | 0.01 |
| 114689_at | 0.00 |
| 114690_at | 0.00 |
| 114692_at | 0.00 |
| 114694_at | 0.15 |
| 114695_at | 0.00 |
| 114696_at | 0.00 |
| 114697_at | 0.28 |
| 114698_at | 0.00 |
| 114699_at | 0.00 |
| 114701_at | 0.00 |
| 114702_at | 0.00 |
| 114704_at | 0.02 |
| 114705_at | 0.00 |
| 114706_at | 0.00 |
| 114707_at | 0.00 |
| 114708_at | 0.00 |
| 114709_at | 0.00 |
| 114710_at | 0.00 |
| 114711_at | 0.00 |
| 114712_at | 0.00 |
| 114713_at | 0.00 |
| 114714_at | 0.00 |
| 114715_at | 0.00 |
| 114716_at | 0.00 |
| 114717_at | 0.00 |
| 114718_at | 0.02 |
| 114719_at | 0.00 |
| 114721_at | 0.00 |
| 114722_at | 0.00 |
| 114723_at | 0.01 |
| 114724_at | 0.13 |
| 114725_at | 0.00 |
| 114726_at | 0.04 |

| | |
|---|---|
| 114727_at | 0.03 |
| 114728_at | 0.00 |
| 114730_at | 0.04 |
| 114732_at | 0.00 |
| 114733_at | 0.00 |
| 114734_at | 0.00 |
| 114736_at | 0.00 |
| 114737_at | 0.00 |
| 114738_at | 0.00 |
| 114739_at | 0.01 |
| 114741_at | 0.00 |
| 114743_at | 0.00 |
| 114746_at | 0.00 |
| 114747_at | 0.00 |
| 114748_at | 0.80 |
| 114749_at | 0.40 |
| 114750_at | 0.00 |
| 114751_at | 0.00 |
| 114752_at | 0.00 |
| 114753_at | 0.00 |
| 114755_at | 0.00 |
| 114756_at | 0.00 |
| 114757_at | 0.00 |
| 114758_at | 0.00 |
| 114759_at | 0.00 |
| 114760_at | 0.00 |
| 114762_at | 0.00 |
| 114763_at | 0.00 |
| 114764_at | 0.00 |
| 114765_at | 0.00 |
| 114766_at | 0.00 |
| 114767_at | 0.00 |
| 114768_at | 0.00 |
| 114770_at | 0.00 |
| 114771_s_at | 0.00 |
| 114772_r_at | 0.00 |
| 114774_at | 0.00 |
| 114775_at | 0.00 |
| 114776_at | 0.00 |
| 114777_i_at | 0.00 |
| 114778_f_at | 0.00 |
| 114779_at | 0.00 |
| 114780_at | 0.12 |
| 114781_at | 0.08 |
| 114782_at | 0.00 |
| 114783_at | 0.00 |
| 114784_at | 0.00 |
| 114785_at | 0.00 |
| 114786_at | 0.00 |
| 114787_at | 0.00 |
| 114788_at | 0.00 |
| 114789_at | 0.00 |
| 114790_at | 0.00 |
| 114791_at | 0.00 |
| 114792_at | 0.00 |
| 114793_at | 0.00 |

| | |
|---|---|
| 114794_at | 0.00 |
| 114795_at | 0.00 |
| 114796_at | 0.00 |
| 114797_at | 0.00 |
| 114799_at | 0.00 |
| 114800_at | 0.00 |
| 114801_at | 0.00 |
| 114802_at | 0.00 |
| 114803_at | 0.00 |
| 114804_at | 0.00 |
| 114805_at | 0.00 |
| 114806_at | 0.00 |
| 114807_at | 0.00 |
| 114808_at | 0.03 |
| 114809_at | 0.00 |
| 114810_at | 0.13 |
| 114811_at | 0.00 |
| 114812_at | 0.00 |
| 114814_at | 0.00 |
| 114815_at | 0.00 |
| 114816_at | 0.00 |
| 114818_at | 0.00 |
| 114819_at | 0.00 |
| 114820_at | 0.00 |
| 114821_at | 0.00 |
| 114822_f_at | 0.00 |
| 114823_r_at | 0.00 |
| 114825_at | 0.00 |
| 114826_at | 0.00 |
| 114827_at | 0.00 |
| 114829_at | 0.00 |
| 114830_at | 0.00 |
| 114831_at | 0.00 |
| 114832_at | 0.00 |
| 114833_at | 0.00 |
| 114834_at | 0.00 |
| 114835_s_at | 0.00 |
| 114836_at | 0.00 |
| 114837_at | 0.00 |
| 114838_at | 0.00 |
| 114840_at | 0.00 |
| 114841_at | 0.00 |
| 114842_g_at | 0.00 |
| 114843_at | 0.00 |
| 114844_at | 0.00 |
| 114845_s_at | 0.32 |
| 114847_at | 0.00 |
| 114848_at | 0.00 |
| 114849_at | 0.43 |
| 114850_at | 0.29 |
| 114851_at | 0.00 |
| 114853_at | 0.00 |
| 114854_at | 0.00 |
| 114855_at | 0.00 |
| 114856_at | 0.00 |
| 114857_at | 0.00 |

| | |
|---|---|
| 114858_at | 0.00 |
| 114859_at | 0.00 |
| 114860_at | 0.00 |
| 114861_at | 0.00 |
| 114862_at | 0.00 |
| 114863_at | 0.00 |
| 114864_at | 0.00 |
| 114865_at | 0.00 |
| 114866_at | 0.00 |
| 114867_at | 0.00 |
| 114868_at | 0.00 |
| 114870_at | 0.00 |
| 114871_at | 0.00 |
| 114872_at | 0.15 |
| 114873_at | 0.00 |
| 114874_at | 0.00 |
| 114875_at | 0.00 |
| 114876_at | 0.00 |
| 114877_at | 0.00 |
| 114879_at | 0.00 |
| 114880_at | 0.00 |
| 114881_at | 0.02 |
| 114882_at | 0.00 |
| 114883_at | 0.00 |
| 114884_at | 0.00 |
| 114885_at | 0.00 |
| 114886_at | 0.09 |
| 114887_at | 0.00 |
| 114888_at | 0.00 |
| 114889_at | 0.00 |
| 114890_at | 0.00 |
| 114891_at | 0.00 |
| 114892_at | 0.01 |
| 114893_at | 0.00 |
| 114894_at | 0.00 |
| 114895_at | 0.00 |
| 114896_at | 0.00 |
| 114897_f_at | 0.04 |
| 114898_at | 0.00 |
| 114899_at | 0.00 |
| 114900_at | 0.00 |
| 114901_at | 0.00 |
| 114902_at | 0.00 |
| 114903_at | 0.00 |
| 114904_at | 0.00 |
| 114905_at | 0.00 |
| 114906_at | 0.07 |
| 114907_at | 0.00 |
| 114908_at | 0.00 |
| 114909_at | 0.00 |
| 114910_at | 0.00 |
| 114911_at | 0.00 |
| 114913_at | 0.00 |
| 114914_at | 0.00 |
| 114915_at | 0.00 |
| 114916_at | 0.00 |

| | |
|---|---|
| 114917_at | 0.01 |
| 114918_at | 0.00 |
| 114919_at | 0.00 |
| 114920_at | 0.00 |
| 114921_at | 0.00 |
| 114922_at | 0.00 |
| 114924_at | 0.00 |
| 114925_at | 0.00 |
| 114926_at | 0.00 |
| 114927_at | 0.00 |
| 114928_at | 0.00 |
| 114929_at | 0.00 |
| 114930_at | 0.01 |
| 114931_at | 0.00 |
| 114932_at | 0.00 |
| 114933_at | 0.00 |
| 114934_at | 0.00 |
| 114935_at | 0.00 |
| 114936_at | 0.00 |
| 114937_at | 0.30 |
| 114938_at | 0.00 |
| 114939_at | 0.05 |
| 114940_at | 0.00 |
| 114941_at | 0.00 |
| 114942_at | 0.00 |
| 114943_at | 0.00 |
| 114944_at | 0.00 |
| 114945_at | 0.00 |
| 114946_at | 0.00 |
| 114947_at | 0.00 |
| 114948_at | 0.00 |
| 114949_at | 0.00 |
| 114950_at | 0.00 |
| 114951_at | 0.00 |
| 114952_at | 0.00 |
| 114953_at | 0.00 |
| 114954_at | 0.00 |
| 114955_at | 0.29 |
| 114956_at | 0.00 |
| 114957_at | 0.00 |
| 114958_at | 0.00 |
| 114959_at | 0.00 |
| 114960_at | 0.00 |
| 114961_at | 0.00 |
| 114963_at | 0.00 |
| 114964_at | 0.00 |
| 114965_at | 0.00 |
| 114966_at | 0.00 |
| 114967_at | 0.11 |
| 114968_at | 0.00 |
| 114969_at | 0.00 |
| 114970_at | 0.00 |
| 114971_at | 0.00 |
| 114972_at | 0.00 |
| 114973_at | 0.00 |
| 114974_at | 0.00 |

| | |
|---|---|
| 114975_at | 0.00 |
| 114976_at | 0.00 |
| 114977_at | 0.00 |
| 114978_at | 0.00 |
| 114979_at | 0.00 |
| 114980_at | 0.00 |
| 114981_at | 0.00 |
| 114982_at | 0.00 |
| 114983_at | 0.00 |
| 114984_at | 0.00 |
| 114985_at | 0.00 |
| 114986_at | 0.00 |
| 114988_at | 0.00 |
| 114989_at | 0.00 |
| 114990_at | 0.00 |
| 114991_at | 0.07 |
| 114992_at | 0.00 |
| 114993_at | 0.00 |
| 114994_at | 0.00 |
| 114996_at | 0.06 |
| 114997_at | 0.00 |
| 114998_at | 0.00 |
| 114999_at | 0.00 |
| 115000_at | 0.00 |
| 115001_at | 0.00 |
| 115002_at | 0.05 |
| 115003_at | 0.00 |
| 115004_at | 0.00 |
| 115005_at | 0.00 |
| 115009_at | 0.17 |
| 115010_at | 0.00 |
| 115011_at | 0.00 |
| 115012_at | 0.00 |
| 115013_at | 0.00 |
| 115014_at | 0.00 |
| 115015_at | 0.01 |
| 115016_at | 0.00 |
| 115017_at | 0.00 |
| 115018_at | 0.00 |
| 115020_at | 0.00 |
| 115021_at | 0.00 |
| 115022_at | 0.34 |
| 115024_at | 0.01 |
| 115025_at | 0.00 |
| 115026_at | 0.00 |
| 115027_at | 0.00 |
| 115028_at | 0.79 |
| 115029_at | 0.00 |
| 115030_at | 0.30 |
| 115031_at | 0.00 |
| 115032_i_at | 0.00 |
| 115033_f_at | 0.00 |
| 115035_at | 0.08 |
| 115037_at | 0.00 |
| 115038_i_at | 0.00 |
| 115039_f_at | 0.00 |

| | |
|---|---|
| 115041_at | 0.00 |
| 115042_at | 0.00 |
| 115043_at | 0.06 |
| 115044_at | 0.00 |
| 115045_at | 0.00 |
| 115046_at | 0.00 |
| 115047_at | 0.00 |
| 115048_at | 0.00 |
| 115049_at | 0.00 |
| 115050_at | 0.00 |
| 115051_at | 0.00 |
| 115053_at | 0.00 |
| 115055_at | 0.00 |
| 115056_at | 0.00 |
| 115057_at | 0.00 |
| 115058_at | 0.68 |
| 115059_at | 0.23 |
| 115061_at | 0.00 |
| 115062_at | 0.00 |
| 115064_at | 0.00 |
| 115065_at | 0.00 |
| 115066_at | 0.32 |
| 115067_at | 0.00 |
| 115068_at | 0.00 |
| 115069_at | 0.00 |
| 115070_at | 0.00 |
| 115071_at | 0.00 |
| 115072_at | 0.00 |
| 115073_g_at | 0.00 |
| 115074_at | 0.16 |
| 115076_at | 0.00 |
| 115077_f_at | 0.07 |
| 115078_r_at | 0.00 |
| 115079_at | 0.00 |
| 115080_f_at | 0.00 |
| 115081_r_at | 0.00 |
| 115082_at | 0.00 |
| 115084_at | 0.00 |
| 115085_at | 0.00 |
| 115086_at | 0.20 |
| 115087_at | 0.00 |
| 115088_at | 0.00 |
| 115089_at | 0.01 |
| 115090_at | 0.00 |
| 115091_at | 0.00 |
| 115093_at | 0.00 |
| 115094_at | 0.00 |
| 115096_at | 0.00 |
| 115097_at | 0.00 |
| 115098_at | 0.00 |
| 115099_at | 0.00 |
| 115100_at | 0.00 |
| 115101_g_at | 0.00 |
| 115102_at | 0.00 |
| 115103_g_at | 0.00 |
| 115104_at | 0.00 |

| | |
|---|---|
| 115105_at | 0.00 |
| 115106_at | 0.29 |
| 115107_at | 0.00 |
| 115109_at | 0.00 |
| 115110_at | 0.00 |
| 115111_at | 0.02 |
| 115112_at | 0.06 |
| 115113_at | 0.00 |
| 115114_at | 0.00 |
| 115115_at | 0.00 |
| 115116_at | 0.00 |
| 115117_at | 0.00 |
| 115118_at | 0.00 |
| 115119_at | 0.00 |
| 115120_at | 0.00 |
| 115121_at | 0.00 |
| 115123_at | 0.00 |
| 115124_at | 0.00 |
| 115126_at | 0.00 |
| 115127_at | 0.00 |
| 115128_at | 0.00 |
| 115129_at | 0.00 |
| 115131_at | 0.00 |
| 115132_at | 0.00 |
| 115133_at | 0.00 |
| 115134_at | 0.00 |
| 115135_at | 0.00 |
| 115136_at | 0.00 |
| 115137_at | 0.00 |
| 115138_at | 0.00 |
| 115139_at | 0.00 |
| 115140_at | 0.00 |
| 115141_at | 0.00 |
| 115142_at | 0.00 |
| 115143_at | 0.00 |
| 115144_f_at | 0.00 |
| 115145_at | 0.00 |
| 115146_g_at | 0.00 |
| 115147_at | 0.00 |
| 115148_at | 0.00 |
| 115149_at | 0.00 |
| 115150_at | 0.01 |
| 115151_at | 0.00 |
| 115152_at | 0.00 |
| 115153_at | 0.00 |
| 115155_at | 0.00 |
| 115156_at | 0.00 |
| 115157_at | 0.00 |
| 115158_at | 0.46 |
| 115159_at | 0.00 |
| 115160_at | 0.00 |
| 115161_at | 0.00 |
| 115162_at | 0.01 |
| 115164_at | 0.00 |
| 115165_at | 0.00 |
| 115166_at | 0.00 |

| | |
|---|---|
| 115167_at | 0.00 |
| 115168_at | 0.00 |
| 115169_at | 0.00 |
| 115170_at | 0.00 |
| 115171_at | 0.00 |
| 115172_at | 0.01 |
| 115173_at | 0.00 |
| 115175_at | 0.00 |
| 115176_at | 0.00 |
| 115177_at | 0.00 |
| 115178_at | 0.00 |
| 115179_at | 0.01 |
| 115180_at | 0.01 |
| 115181_at | 0.00 |
| 115182_at | 0.00 |
| 115183_at | 0.02 |
| 115184_at | 0.00 |
| 115185_at | 0.00 |
| 115187_at | 0.00 |
| 115188_at | 0.00 |
| 115189_r_at | 0.00 |
| 115190_at | 0.00 |
| 115191_at | 0.00 |
| 115193_at | 0.00 |
| 115194_at | 0.00 |
| 115195_at | 0.00 |
| 115196_at | 0.00 |
| 115197_at | 0.22 |
| 115198_at | 0.00 |
| 115199_at | 0.00 |
| 115200_at | 0.00 |
| 115201_at | 0.00 |
| 115202_at | 0.00 |
| 115203_at | 0.00 |
| 115205_at | 0.00 |
| 115206_at | 0.00 |
| 115207_at | 0.00 |
| 115208_at | 0.00 |
| 115209_at | 0.00 |
| 115210_at | 0.00 |
| 115211_at | 0.00 |
| 115212_at | 0.59 |
| 115213_at | 0.00 |
| 115214_at | 0.00 |
| 115215_at | 0.00 |
| 115216_at | 0.00 |
| 115217_at | 0.00 |
| 115218_at | 0.00 |
| 115219_at | 0.00 |
| 115220_at | 0.00 |
| 115221_at | 0.00 |
| 115222_at | 0.00 |
| 115223_at | 0.00 |
| 115224_at | 0.00 |
| 115225_at | 0.00 |
| 115226_at | 0.00 |

| | |
|---|---|
| 115228_at | 0.00 |
| 115229_at | 0.00 |
| 115230_at | 0.00 |
| 115231_at | 0.00 |
| 115233_at | 0.00 |
| 115234_at | 0.00 |
| 115235_at | 0.00 |
| 115236_at | 0.00 |
| 115237_at | 0.00 |
| 115238_at | 0.00 |
| 115239_at | 0.00 |
| 115240_at | 0.00 |
| 115241_at | 0.00 |
| 115242_at | 0.00 |
| 115243_at | 0.00 |
| 115244_at | 0.00 |
| 115245_at | 0.00 |
| 115246_at | 0.00 |
| 115247_at | 0.00 |
| 115248_at | 0.00 |
| 115249_at | 0.00 |
| 115250_at | 0.00 |
| 115251_at | 0.00 |
| 115252_at | 0.00 |
| 115253_at | 0.00 |
| 115254_at | 0.00 |
| 115255_at | 0.00 |
| 115256_at | 0.00 |
| 115258_at | 0.00 |
| 115259_at | 0.00 |
| 115260_at | 0.02 |
| 115261_at | 0.00 |
| 115262_at | 0.00 |
| 115263_at | 0.00 |
| 115264_at | 0.00 |
| 115265_at | 0.00 |
| 115266_at | 0.00 |
| 115267_at | 0.00 |
| 115268_at | 0.00 |
| 115269_at | 0.00 |
| 115270_at | 0.00 |
| 115271_at | 0.00 |
| 115272_at | 0.00 |
| 115273_at | 0.00 |
| 115274_at | 0.00 |
| 115275_at | 0.00 |
| 115276_at | 0.12 |
| 115277_at | 0.00 |
| 115278_at | 0.00 |
| 115279_at | 0.00 |
| 115280_at | 0.00 |
| 115281_at | 0.00 |
| 115282_at | 0.00 |
| 115283_at | 0.00 |
| 115284_at | 0.00 |
| 115285_at | 0.00 |

| | |
|---|---|
| 115286_at | 0.00 |
| 115287_at | 0.00 |
| 115288_at | 0.00 |
| 115289_at | 0.00 |
| 115290_at | 0.00 |
| 115291_at | 0.00 |
| 115292_at | 0.00 |
| 115293_at | 0.00 |
| 115294_at | 0.00 |
| 115295_at | 0.00 |
| 115296_at | 0.00 |
| 115297_at | 0.00 |
| 115298_at | 0.00 |
| 115299_at | 0.00 |
| 115300_at | 0.00 |
| 115301_r_at | 0.00 |
| 115302_at | 0.00 |
| 115303_at | 0.00 |
| 115304_at | 0.00 |
| 115305_at | 0.00 |
| 115306_at | 0.00 |
| 115307_at | 0.00 |
| 115308_at | 0.00 |
| 115309_at | 0.00 |
| 115310_at | 0.00 |
| 115311_at | 0.00 |
| 115312_at | 0.00 |
| 115313_at | 0.00 |
| 115314_at | 0.00 |
| 115315_at | 0.00 |
| 115316_at | 0.00 |
| 115317_at | 0.00 |
| 115318_at | 0.00 |
| 115319_at | 0.00 |
| 115320_at | 0.00 |
| 115321_at | 0.00 |
| 115322_at | 0.00 |
| 115323_at | 0.00 |
| 115324_at | 0.00 |
| 115325_at | 0.00 |
| 115326_at | 0.00 |
| 115327_at | 0.00 |
| 115328_at | 0.00 |
| 115329_at | 0.00 |
| 115330_at | 0.00 |
| 115331_at | 0.00 |
| 115332_at | 0.00 |
| 115333_at | 0.22 |
| 115334_at | 0.00 |
| 115335_at | 0.00 |
| 115336_at | 0.00 |
| 115337_at | 0.00 |
| 115338_at | 0.00 |
| 115339_at | 0.00 |
| 115340_at | 0.00 |
| 115341_at | 0.00 |

| | |
|---|---|
| 115342_at | 0.00 |
| 115344_at | 0.00 |
| 115346_at | 0.00 |
| 115347_at | 0.00 |
| 115348_i_at | 0.00 |
| 115349_r_at | 0.00 |
| 115350_at | 0.00 |
| 115351_at | 0.00 |
| 115352_at | 0.00 |
| 115353_at | 0.00 |
| 115354_at | 0.05 |
| 115355_at | 0.00 |
| 115356_s_at | 0.12 |
| 115357_at | 0.00 |
| 115358_at | 0.00 |
| 115360_at | 0.00 |
| 115361_at | 0.03 |
| 115362_at | 0.00 |
| 115363_at | 0.00 |
| 115364_at | 0.00 |
| 115365_at | 0.00 |
| 115367_at | 0.00 |
| 115369_at | 0.00 |
| 115370_at | 0.00 |
| 115371_at | 0.00 |
| 115372_at | 0.00 |
| 115373_at | 0.00 |
| 115374_r_at | 0.00 |
| 115376_at | 0.00 |
| 115378_at | 0.00 |
| 115379_at | 0.00 |
| 115380_at | 0.00 |
| 115381_at | 0.00 |
| 115382_at | 0.00 |
| 115385_at | 0.00 |
| 115386_at | 0.00 |
| 115387_at | 0.00 |
| 115389_at | 0.00 |
| 115390_at | 0.00 |
| 115391_at | 0.00 |
| 115392_at | 0.00 |
| 115393_at | 0.00 |
| 115394_at | 0.00 |
| 115395_at | 0.00 |
| 115396_at | 0.00 |
| 115397_at | 0.00 |
| 115398_at | 0.00 |
| 115400_at | 0.00 |
| 115401_at | 0.00 |
| 115402_at | 0.00 |
| 115403_at | 0.00 |
| 115405_at | 0.00 |
| 115406_at | 0.00 |
| 115407_at | 0.00 |
| 115408_at | 0.00 |
| 115409_at | 0.00 |

| | |
|---|---|
| 115410_at | 0.00 |
| 115411_at | 0.01 |
| 115412_at | 0.00 |
| 115414_at | 0.00 |
| 115415_at | 0.00 |
| 115416_at | 0.00 |
| 115417_at | 0.01 |
| 115418_at | 0.01 |
| 115419_f_at | 0.00 |
| 115420_r_at | 0.00 |
| 115421_at | 0.00 |
| 115422_at | 0.00 |
| 115423_at | 0.00 |
| 115424_at | 0.00 |
| 115425_at | 0.00 |
| 115426_at | 0.00 |
| 115427_at | 0.00 |
| 115428_at | 0.00 |
| 115429_at | 0.03 |
| 115431_at | 0.00 |
| 115432_at | 0.00 |
| 115433_at | 0.00 |
| 115434_at | 0.00 |
| 115435_at | 0.00 |
| 115436_at | 0.00 |
| 115437_at | 0.01 |
| 115438_at | 0.00 |
| 115439_at | 0.00 |
| 115440_at | 0.00 |
| 115441_at | 0.06 |
| 115442_at | 0.00 |
| 115443_g_at | 0.00 |
| 115444_at | 0.00 |
| 115445_at | 0.88 |
| 115446_at | 0.07 |
| 115447_g_at | 0.00 |
| 115449_at | 0.00 |
| 115450_at | 0.00 |
| 115451_at | 0.00 |
| 115452_at | 0.00 |
| 115453_at | 0.00 |
| 115455_at | 0.00 |
| 115456_at | 0.01 |
| 115459_at | 0.00 |
| 115460_f_at | 0.00 |
| 115461_at | 0.00 |
| 115463_at | 0.00 |
| 115464_at | 0.07 |
| 115465_f_at | 0.09 |
| 115466_at | 0.00 |
| 115467_at | 0.00 |
| 115469_at | 0.00 |
| 115470_at | 0.00 |
| 115473_at | 0.00 |
| 115474_g_at | 0.00 |
| 115475_at | 0.00 |

| | |
|---|---|
| 115476_at | 0.00 |
| 115477_at | 0.00 |
| 115478_at | 0.00 |
| 115479_at | 0.00 |
| 115480_at | 0.00 |
| 115481_at | 0.04 |
| 115482_at | 0.00 |
| 115483_at | 0.00 |
| 115485_at | 0.00 |
| 115486_at | 0.57 |
| 115487_at | 0.00 |
| 115488_at | 0.00 |
| 115489_at | 0.00 |
| 115490_at | 0.00 |
| 115491_at | 0.00 |
| 115492_at | 0.00 |
| 115493_at | 0.00 |
| 115494_s_at | 0.00 |
| 115495_r_at | 0.00 |
| 115496_at | 0.00 |
| 115497_at | 0.00 |
| 115498_at | 0.04 |
| 115499_g_at | 0.19 |
| 115500_at | 0.00 |
| 115501_at | 0.00 |
| 115502_at | 0.00 |
| 115503_at | 0.00 |
| 115504_g_at | 0.00 |
| 115505_at | 0.00 |
| 115506_at | 0.00 |
| 115507_at | 0.00 |
| 115508_at | 0.00 |
| 115509_at | 0.00 |
| 115510_at | 0.00 |
| 115511_at | 0.00 |
| 115512_at | 0.00 |
| 115513_at | 0.00 |
| 115514_at | 0.00 |
| 115515_at | 0.00 |
| 115516_at | 0.00 |
| 115517_at | 0.00 |
| 115518_at | 0.00 |
| 115519_at | 0.00 |
| 115520_at | 0.49 |
| 115521_at | 0.00 |
| 115522_at | 0.00 |
| 115523_at | 0.02 |
| 115525_at | 0.00 |
| 115526_at | 0.00 |
| 115527_at | 0.00 |
| 115528_at | 0.01 |
| 115529_at | 0.00 |
| 115530_at | 0.00 |
| 115533_at | 0.00 |
| 115535_at | 0.00 |
| 115536_at | 0.00 |

| | |
|---|---|
| 115537_at | 0.00 |
| 115538_at | 0.01 |
| 115539_at | 0.00 |
| 115540_at | 0.00 |
| 115541_at | 0.00 |
| 115543_at | 0.00 |
| 115544_at | 0.00 |
| 115545_at | 0.00 |
| 115546_at | 0.01 |
| 115547_at | 0.01 |
| 115548_at | 0.00 |
| 115549_at | 0.00 |
| 115550_at | 0.00 |
| 115551_at | 0.07 |
| 115553_at | 0.00 |
| 115554_at | 0.00 |
| 115555_at | 0.00 |
| 115556_s_at | 0.00 |
| 115557_r_at | 0.00 |
| 115558_at | 0.00 |
| 115559_at | 0.00 |
| 115560_at | 0.00 |
| 115561_at | 0.00 |
| 115562_at | 0.00 |
| 115564_at | 0.21 |
| 115565_at | 0.00 |
| 115566_at | 0.00 |
| 115567_r_at | 0.00 |
| 115569_i_at | 0.00 |
| 115570_f_at | 0.00 |
| 115571_at | 0.00 |
| 115572_at | 0.00 |
| 115573_at | 0.00 |
| 115575_at | 0.23 |
| 115576_at | 0.00 |
| 115577_at | 0.00 |
| 115578_at | 0.00 |
| 115579_at | 0.00 |
| 115580_at | 0.00 |
| 115581_at | 0.00 |
| 115582_at | 0.00 |
| 115583_at | 0.00 |
| 115584_at | 0.00 |
| 115585_at | 0.00 |
| 115586_at | 0.00 |
| 115587_at | 0.00 |
| 115588_at | 0.00 |
| 115589_at | 0.00 |
| 115590_at | 0.00 |
| 115591_at | 0.00 |
| 115592_at | 0.00 |
| 115593_at | 0.00 |
| 115594_at | 0.00 |
| 115595_at | 0.00 |
| 115596_at | 0.00 |
| 115597_at | 0.00 |

| | |
|---|---|
| 115598_at | 0.00 |
| 115599_at | 0.00 |
| 115600_at | 0.00 |
| 115601_at | 0.00 |
| 115602_at | 0.00 |
| 115603_at | 0.00 |
| 115604_at | 0.00 |
| 115605_at | 0.00 |
| 115606_at | 0.00 |
| 115607_at | 0.00 |
| 115608_at | 0.00 |
| 115609_at | 0.00 |
| 115610_at | 0.01 |
| 115611_f_at | 0.00 |
| 115612_at | 0.00 |
| 115613_at | 0.00 |
| 115614_at | 0.00 |
| 115615_at | 0.00 |
| 115616_at | 0.00 |
| 115617_at | 0.00 |
| 115618_at | 0.00 |
| 115619_at | 0.00 |
| 115620_at | 0.00 |
| 115621_i_at | 0.00 |
| 115622_f_at | 0.00 |
| 115623_at | 0.00 |
| 115624_at | 0.00 |
| 115625_at | 0.00 |
| 115626_at | 0.00 |
| 115627_at | 0.00 |
| 115628_at | 0.00 |
| 115629_at | 0.00 |
| 115630_at | 0.00 |
| 115631_at | 0.00 |
| 115632_at | 0.00 |
| 115633_at | 0.00 |
| 115634_at | 0.00 |
| 115635_at | 0.00 |
| 115636_at | 0.00 |
| 115637_at | 0.00 |
| 115638_at | 0.00 |
| 115639_at | 0.01 |
| 115640_at | 0.00 |
| 115641_at | 0.00 |
| 115642_at | 0.00 |
| 115643_at | 0.00 |
| 115644_at | 0.00 |
| 115645_at | 0.00 |
| 115646_at | 0.00 |
| 115647_at | 0.00 |
| 115648_at | 0.00 |
| 115649_at | 0.00 |
| 115650_at | 0.00 |
| 115651_at | 0.00 |
| 115652_at | 0.00 |
| 115653_at | 0.00 |

| | |
|---|---|
| 115654_at | 0.00 |
| 115655_at | 0.00 |
| 115657_at | 0.00 |
| 115658_at | 0.00 |
| 115659_at | 0.00 |
| 115660_at | 0.00 |
| 115661_at | 0.00 |
| 115662_at | 0.00 |
| 115663_at | 0.00 |
| 115664_at | 0.00 |
| 115665_at | 0.00 |
| 115666_at | 0.00 |
| 115667_at | 0.00 |
| 115668_at | 0.00 |
| 115669_at | 0.00 |
| 115670_at | 0.00 |
| 115672_at | 0.00 |
| 115673_r_at | 0.00 |
| 115674_at | 0.00 |
| 115675_at | 0.01 |
| 115676_at | 0.00 |
| 115677_at | 0.00 |
| 115678_at | 0.00 |
| 115679_at | 0.00 |
| 115680_at | 0.00 |
| 115681_at | 0.00 |
| 115682_at | 0.00 |
| 115683_at | 0.00 |
| 115684_at | 0.00 |
| 115685_at | 0.00 |
| 115686_at | 0.00 |
| 115687_at | 0.00 |
| 115688_at | 0.00 |
| 115689_at | 0.00 |
| 115690_at | 0.00 |
| 115691_at | 0.00 |
| 115692_r_at | 0.00 |
| 115693_at | 0.00 |
| 115694_at | 0.00 |
| 115695_r_at | 0.00 |
| 115696_at | 0.00 |
| 115697_at | 0.00 |
| 115699_at | 0.00 |
| 115700_at | 0.00 |
| 115701_at | 0.00 |
| 115702_at | 0.00 |
| 115703_at | 0.00 |
| 115704_at | 0.00 |
| 115705_at | 0.00 |
| 115706_at | 0.00 |
| 115707_at | 0.00 |
| 115708_at | 0.00 |
| 115709_at | 0.00 |
| 115710_at | 0.00 |
| 115711_at | 0.00 |
| 115712_at | 0.00 |

| | |
|---|---|
| 115713_at | 0.00 |
| 115714_at | 0.00 |
| 115715_at | 0.00 |
| 115716_at | 0.00 |
| 115717_at | 0.00 |
| 115718_at | 0.00 |
| 115719_at | 0.00 |
| 115727_at | 0.00 |
| 115728_at | 0.00 |
| 115729_at | 0.00 |
| 115731_at | 0.00 |
| 115732_at | 0.00 |
| 115733_at | 0.00 |
| 115734_f_at | 0.00 |
| 115735_r_at | 0.00 |
| 115736_at | 0.01 |
| 115740_at | 0.00 |
| 115741_at | 0.11 |
| 115742_at | 0.00 |
| 115743_at | 0.00 |
| 115744_at | 0.00 |
| 115745_at | 0.00 |
| 115746_at | 0.00 |
| 115747_at | 0.00 |
| 115748_at | 0.00 |
| 115749_at | 0.00 |
| 115750_at | 0.00 |
| 115751_at | 0.00 |
| 115752_at | 0.00 |
| 115753_at | 0.00 |
| 115754_at | 0.00 |
| 115755_g_at | 0.00 |
| 115756_at | 0.00 |
| 115757_at | 0.00 |
| 115759_at | 0.00 |
| 115760_at | 0.00 |
| 115761_at | 0.00 |
| 115762_at | 0.00 |
| 115764_at | 0.00 |
| 115765_at | 0.00 |
| 115766_at | 0.00 |
| 115768_f_at | 0.00 |
| 115769_at | 0.00 |
| 115770_at | 0.07 |
| 115771_at | 0.00 |
| 115772_at | 0.00 |
| 115774_at | 0.00 |
| 115775_at | 0.00 |
| 115776_at | 0.00 |
| 115779_at | 0.00 |
| 115780_at | 0.00 |
| 115781_at | 0.09 |
| 115782_at | 0.00 |
| 115783_at | 0.00 |
| 115784_at | 0.00 |
| 115786_at | 0.00 |

| | |
|---|---|
| 115787_at | 0.00 |
| 115788_at | 0.00 |
| 115789_at | 0.00 |
| 115790_at | 0.00 |
| 115791_at | 0.00 |
| 115792_at | 0.03 |
| 115793_at | 0.00 |
| 115794_at | 0.01 |
| 115795_at | 0.01 |
| 115796_at | 0.02 |
| 115797_at | 0.00 |
| 115798_at | 0.01 |
| 115800_at | 0.00 |
| 115801_at | 0.00 |
| 115802_at | 0.00 |
| 115803_at | 0.00 |
| 115804_at | 0.57 |
| 115805_at | 0.00 |
| 115806_at | 0.00 |
| 115807_f_at | 0.00 |
| 115808_at | 0.00 |
| 115809_i_at | 0.00 |
| 115810_f_at | 0.00 |
| 115811_at | 0.00 |
| 115812_at | 0.00 |
| 115813_at | 0.00 |
| 115814_at | 0.00 |
| 115815_at | 0.00 |
| 115816_at | 0.02 |
| 115817_at | 0.00 |
| 115818_at | 0.00 |
| 115819_at | 0.00 |
| 115820_at | 0.00 |
| 115821_at | 0.00 |
| 115823_at | 0.00 |
| 115824_at | 0.00 |
| 115825_at | 0.00 |
| 115827_at | 0.00 |
| 115828_at | 0.00 |
| 115829_at | 0.00 |
| 115830_at | 0.00 |
| 115831_at | 0.00 |
| 115832_at | 0.00 |
| 115833_at | 0.02 |
| 115835_at | 0.00 |
| 115837_at | 0.00 |
| 115838_at | 0.00 |
| 115840_at | 0.00 |
| 115841_at | 0.00 |
| 115842_at | 0.00 |
| 115843_at | 0.00 |
| 115844_at | 0.00 |
| 115845_at | 0.00 |
| 115846_g_at | 0.00 |
| 115847_i_at | 0.00 |
| 115848_at | 0.00 |

| | |
|---|---|
| 115849_at | 0.00 |
| 115850_at | 0.03 |
| 115851_at | 0.00 |
| 115852_at | 0.00 |
| 115853_at | 0.00 |
| 115854_at | 0.00 |
| 115855_at | 0.00 |
| 115856_at | 0.00 |
| 115857_at | 0.00 |
| 115858_at | 0.00 |
| 115859_at | 0.00 |
| 115860_at | 0.00 |
| 115861_at | 0.00 |
| 115862_at | 0.00 |
| 115863_at | 0.00 |
| 115864_at | 0.00 |
| 115865_at | 0.00 |
| 115869_at | 0.00 |
| 115870_at | 0.00 |
| 115872_at | 0.00 |
| 115873_at | 0.00 |
| 115874_at | 0.00 |
| 115875_at | 0.00 |
| 115876_at | 0.00 |
| 115877_at | 0.00 |
| 115878_at | 0.00 |
| 115879_at | 0.00 |
| 115880_at | 0.00 |
| 115881_g_at | 0.00 |
| 115882_at | 0.00 |
| 115883_g_at | 0.00 |
| 115884_at | 0.00 |
| 115885_at | 0.00 |
| 115887_at | 0.00 |
| 115888_at | 0.00 |
| 115891_at | 0.00 |
| 115892_at | 0.00 |
| 115893_at | 0.00 |
| 115894_at | 0.00 |
| 115895_at | 0.00 |
| 115896_at | 0.00 |
| 115897_at | 0.01 |
| 115898_at | 0.00 |
| 115899_at | 0.00 |
| 115901_at | 0.00 |
| 115902_at | 0.00 |
| 115903_at | 0.00 |
| 115904_at | 0.00 |
| 115906_at | 0.00 |
| 115907_at | 0.00 |
| 115908_at | 0.00 |
| 115909_at | 0.00 |
| 115910_at | 0.00 |
| 115911_at | 0.00 |
| 115912_at | 0.00 |
| 115913_at | 0.00 |

| | |
|---|---|
| 115915_at | 0.00 |
| 115916_at | 0.00 |
| 115917_at | 0.00 |
| 115919_at | 0.00 |
| 115920_at | 0.00 |
| 115921_at | 0.00 |
| 115922_i_at | 0.00 |
| 115923_f_at | 0.00 |
| 115924_at | 0.00 |
| 115925_at | 0.00 |
| 115926_at | 0.06 |
| 115927_at | 0.00 |
| 115928_at | 0.00 |
| 115929_at | 0.00 |
| 115930_at | 0.00 |
| 115931_at | 0.00 |
| 115932_at | 0.00 |
| 115933_at | 0.00 |
| 115934_at | 0.00 |
| 115935_at | 0.00 |
| 115936_at | 0.00 |
| 115937_at | 0.00 |
| 115938_at | 0.00 |
| 115939_at | 0.00 |
| 115940_at | 0.00 |
| 115941_at | 0.00 |
| 115942_at | 0.00 |
| 115943_at | 0.00 |
| 115944_at | 0.00 |
| 115945_at | 0.00 |
| 115946_at | 0.00 |
| 115947_at | 0.00 |
| 115948_at | 0.00 |
| 115949_at | 0.00 |
| 115950_at | 0.00 |
| 115951_at | 0.00 |
| 115952_at | 0.00 |
| 115953_at | 0.00 |
| 115954_at | 0.00 |
| 115955_at | 0.12 |
| 115956_at | 0.00 |
| 115957_at | 0.00 |
| 115958_at | 0.00 |
| 115959_at | 0.00 |
| 115960_at | 0.00 |
| 115961_at | 0.00 |
| 115962_at | 0.00 |
| 115963_at | 0.00 |
| 115964_at | 0.00 |
| 115965_at | 0.00 |
| 115966_at | 0.00 |
| 115967_at | 0.00 |
| 115968_at | 0.00 |
| 115969_at | 0.00 |
| 115970_at | 0.00 |
| 115971_at | 0.00 |

| | |
|---|---|
| 115972_at | 0.00 |
| 115973_at | 0.00 |
| 115974_at | 0.00 |
| 115975_r_at | 0.00 |
| 115976_at | 0.00 |
| 115977_at | 0.00 |
| 115978_at | 0.00 |
| 115979_at | 0.00 |
| 115980_at | 0.00 |
| 115981_at | 0.00 |
| 115982_at | 0.00 |
| 115983_at | 0.00 |
| 115985_r_at | 0.00 |
| 115986_at | 0.00 |
| 115987_at | 0.00 |
| 115988_at | 0.00 |
| 115989_at | 0.00 |
| 115990_at | 0.00 |
| 115991_at | 0.00 |
| 115992_at | 0.00 |
| 115993_at | 0.00 |
| 115994_at | 0.02 |
| 115995_at | 0.00 |
| 115996_at | 0.00 |
| 115997_at | 0.00 |
| 115998_at | 0.00 |
| 115999_at | 0.00 |
| 116000_at | 0.00 |
| 116001_at | 0.00 |
| 116002_at | 0.00 |
| 116003_at | 0.00 |
| 116004_at | 0.00 |
| 116005_at | 0.00 |
| 116006_at | 0.00 |
| 116007_at | 0.00 |
| 116008_at | 0.00 |
| 116009_at | 0.09 |
| 116010_at | 0.00 |
| 116011_at | 0.01 |
| 116012_at | 0.00 |
| 116013_at | 0.00 |
| 116014_at | 0.00 |
| 116015_at | 0.00 |
| 116016_at | 0.00 |
| 116017_at | 0.00 |
| 116018_at | 0.00 |
| 116019_at | 0.00 |
| 116020_at | 0.00 |
| 116021_at | 0.00 |
| 116022_at | 0.00 |
| 116023_at | 0.00 |
| 116024_at | 0.00 |
| 116025_at | 0.00 |
| 116026_at | 0.00 |
| 116027_at | 0.00 |
| 116028_at | 0.00 |

| | |
|---|---|
| 116029_at | 0.00 |
| 116030_at | 0.00 |
| 116031_at | 0.00 |
| 116032_at | 0.00 |
| 116033_at | 0.00 |
| 116034_at | 0.00 |
| 116035_at | 0.00 |
| 116036_at | 0.00 |
| 116037_at | 0.00 |
| 116039_at | 0.00 |
| 116040_at | 0.00 |
| 116041_at | 0.00 |
| 116042_at | 0.00 |
| 116043_at | 0.00 |
| 116044_at | 0.35 |
| 116046_at | 0.00 |
| 116047_at | 0.00 |
| 116048_at | 0.00 |
| 116049_at | 0.00 |
| 116050_at | 0.00 |
| 116051_at | 0.02 |
| 116052_at | 0.00 |
| 116053_at | 0.00 |
| 116054_at | 0.00 |
| 116055_at | 0.00 |
| 116056_at | 0.00 |
| 116057_at | 0.00 |
| 116058_at | 0.00 |
| 116059_at | 0.00 |
| 116060_at | 0.00 |
| 116062_at | 0.00 |
| 116063_at | 0.00 |
| 116065_at | 0.00 |
| 116066_at | 0.00 |
| 116067_at | 0.00 |
| 116068_at | 0.00 |
| 116069_at | 0.00 |
| 116070_at | 0.00 |
| 116071_at | 0.01 |
| 116072_at | 0.00 |
| 116073_at | 0.00 |
| 116074_at | 0.05 |
| 116075_at | 0.00 |
| 116076_f_at | 0.00 |
| 116077_r_at | 0.00 |
| 116080_at | 0.00 |
| 116081_at | 0.02 |
| 116083_at | 0.00 |
| 116084_at | 0.00 |
| 116085_at | 0.00 |
| 116087_at | 0.00 |
| 116088_at | 0.00 |
| 116089_at | 0.00 |
| 116090_at | 0.00 |
| 116091_at | 0.00 |
| 116092_at | 0.00 |

| | |
|---|---|
| 116093_at | 0.00 |
| 116094_at | 0.00 |
| 116095_at | 0.00 |
| 116096_at | 0.00 |
| 116098_at | 0.00 |
| 116099_at | 0.03 |
| 116100_f_at | 0.00 |
| 116102_at | 0.00 |
| 116103_at | 0.00 |
| 116104_at | 0.00 |
| 116105_at | 0.00 |
| 116106_at | 0.00 |
| 116107_at | 0.02 |
| 116108_at | 0.00 |
| 116109_at | 0.09 |
| 116111_at | 0.00 |
| 116112_at | 0.00 |
| 116113_at | 0.00 |
| 116114_at | 0.00 |
| 116115_at | 0.00 |
| 116116_at | 0.12 |
| 116117_at | 0.00 |
| 116119_at | 0.06 |
| 116120_at | 0.00 |
| 116121_at | 0.01 |
| 116122_at | 0.01 |
| 116123_at | 0.00 |
| 116124_at | 0.00 |
| 116125_at | 0.00 |
| 116126_at | 0.00 |
| 116127_at | 0.00 |
| 116128_at | 0.00 |
| 116129_at | 0.02 |
| 116130_at | 0.00 |
| 116131_at | 0.00 |
| 116132_at | 0.02 |
| 116133_at | 0.00 |
| 116134_at | 0.00 |
| 116135_at | 0.00 |
| 116136_at | 0.00 |
| 116137_at | 0.00 |
| 116139_at | 0.01 |
| 116140_at | 0.00 |
| 116143_at | 0.00 |
| 116144_at | 0.00 |
| 116145_at | 0.00 |
| 116146_at | 0.00 |
| 116147_at | 0.00 |
| 116148_at | 0.00 |
| 116149_at | 0.00 |
| 116150_at | 0.00 |
| 116151_at | 0.00 |
| 116152_at | 0.00 |
| 116154_at | 0.00 |
| 116155_at | 0.00 |
| 116156_at | 0.00 |

| | |
|---|---|
| 116157_g_at | 0.00 |
| 116158_at | 0.00 |
| 116159_at | 0.00 |
| 116160_at | 0.00 |
| 116161_at | 0.19 |
| 116162_g_at | 0.01 |
| 116163_at | 0.00 |
| 116164_at | 0.00 |
| 116165_at | 0.02 |
| 116166_at | 0.00 |
| 116170_at | 0.00 |
| 116173_at | 0.00 |
| 116174_at | 0.00 |
| 116175_at | 0.01 |
| 116176_at | 0.00 |
| 116177_at | 0.00 |
| 116178_at | 0.00 |
| 116179_at | 0.00 |
| 116180_at | 0.00 |
| 116181_at | 0.00 |
| 116182_at | 0.00 |
| 116183_at | 0.00 |
| 116184_at | 0.00 |
| 116185_at | 0.00 |
| 116186_at | 0.00 |
| 116187_at | 0.00 |
| 116188_at | 0.00 |
| 116189_at | 0.00 |
| 116190_at | 0.00 |
| 116191_at | 0.00 |
| 116192_at | 0.00 |
| 116193_at | 0.00 |
| 116194_at | 0.00 |
| 116195_at | 0.00 |
| 116196_at | 0.00 |
| 116197_at | 0.00 |
| 116198_at | 0.00 |
| 116199_at | 0.00 |
| 116200_at | 0.00 |
| 116201_at | 0.00 |
| 116202_at | 0.00 |
| 116203_at | 0.00 |
| 116204_at | 0.00 |
| 116205_at | 0.00 |
| 116206_at | 0.00 |
| 116207_at | 0.00 |
| 116208_at | 0.00 |
| 116209_at | 0.00 |
| 116210_at | 0.00 |
| 116211_at | 0.00 |
| 116212_at | 0.00 |
| 116213_at | 0.20 |
| 116214_at | 0.37 |
| 116215_at | 0.00 |
| 116216_at | 0.00 |
| 116217_at | 0.00 |

| | |
|---|---|
| 116218_at | 0.00 |
| 116219_g_at | 0.09 |
| 116220_at | 0.00 |
| 116221_at | 0.00 |
| 116222_at | 0.00 |
| 116223_at | 0.00 |
| 116224_at | 0.00 |
| 116225_at | 0.00 |
| 116226_at | 0.00 |
| 116227_at | 0.00 |
| 116228_at | 0.00 |
| 116229_at | 0.00 |
| 116230_at | 0.00 |
| 116231_at | 0.00 |
| 116232_at | 0.00 |
| 116233_at | 0.00 |
| 116234_at | 0.00 |
| 116235_at | 0.00 |
| 116236_at | 0.00 |
| 116237_at | 0.00 |
| 116238_at | 0.00 |
| 116239_at | 0.37 |
| 116240_at | 0.00 |
| 116241_at | 0.00 |
| 116242_at | 0.00 |
| 116243_at | 0.00 |
| 116244_at | 0.00 |
| 116245_at | 0.00 |
| 116246_at | 0.00 |
| 116247_at | 0.00 |
| 116248_at | 0.00 |
| 116249_at | 0.02 |
| 116250_at | 0.00 |
| 116251_at | 0.00 |
| 116252_at | 0.00 |
| 116253_at | 0.00 |
| 116254_at | 0.00 |
| 116255_at | 0.00 |
| 116256_at | 0.00 |
| 116257_at | 0.00 |
| 116258_at | 0.00 |
| 116259_at | 0.00 |
| 116260_at | 0.00 |
| 116261_at | 0.00 |
| 116262_at | 0.00 |
| 116263_at | 0.00 |
| 116264_at | 0.01 |
| 116265_at | 0.00 |
| 116266_at | 0.00 |
| 116267_at | 0.00 |
| 116268_at | 0.00 |
| 116269_at | 0.00 |
| 116270_at | 0.00 |
| 116271_at | 0.01 |
| 116272_at | 0.00 |
| 116273_at | 0.00 |

| | |
|---|---|
| 116274_at | 0.00 |
| 116275_at | 0.00 |
| 116276_at | 0.00 |
| 116277_at | 0.00 |
| 116278_at | 0.00 |
| 116279_at | 0.00 |
| 116280_at | 0.01 |
| 116281_at | 0.00 |
| 116282_at | 0.00 |
| 116283_at | 0.00 |
| 116284_at | 0.00 |
| 116285_at | 0.00 |
| 116286_at | 0.00 |
| 116287_at | 0.00 |
| 116288_at | 0.00 |
| 116289_at | 0.00 |
| 116290_at | 0.00 |
| 116291_at | 0.00 |
| 116293_at | 0.00 |
| 116294_at | 0.00 |
| 116295_at | 0.00 |
| 116296_at | 0.00 |
| 116297_at | 0.00 |
| 116299_at | 0.00 |
| 116300_r_at | 0.00 |
| 116301_at | 0.08 |
| 116302_at | 0.00 |
| 116303_at | 0.00 |
| 116304_at | 0.50 |
| 116305_at | 0.00 |
| 116306_at | 0.00 |
| 116307_g_at | 0.00 |
| 116308_at | 0.00 |
| 116309_at | 0.00 |
| 116310_at | 0.00 |
| 116311_at | 0.00 |
| 116312_at | 0.00 |
| 116313_at | 0.00 |
| 116314_at | 0.00 |
| 116315_at | 0.00 |
| 116316_at | 0.00 |
| 116317_at | 0.00 |
| 116318_at | 0.00 |
| 116319_at | 0.00 |
| 116320_at | 0.00 |
| 116321_at | 0.00 |
| 116322_at | 0.00 |
| 116323_at | 0.00 |
| 116324_g_at | 0.00 |
| 116325_at | 0.00 |
| 116327_at | 0.02 |
| 116329_at | 0.00 |
| 116330_at | 0.00 |
| 116332_at | 0.01 |
| 116333_at | 0.01 |
| 116334_at | 0.00 |

| | |
|---|---|
| 116335_at | 0.00 |
| 116336_at | 0.00 |
| 116337_at | 0.00 |
| 116338_at | 0.00 |
| 116339_at | 0.00 |
| 116340_at | 0.00 |
| 116341_at | 0.00 |
| 116342_at | 0.03 |
| 116344_at | 0.00 |
| 116345_at | 0.00 |
| 116346_at | 0.00 |
| 116347_at | 0.00 |
| 116348_at | 0.00 |
| 116349_at | 0.00 |
| 116350_at | 0.00 |
| 116354_r_at | 0.00 |
| 116355_at | 0.00 |
| 116357_at | 0.00 |
| 116358_at | 0.00 |
| 116359_at | 0.00 |
| 116360_at | 0.00 |
| 116361_at | 0.00 |
| 116362_at | 0.00 |
| 116363_at | 0.00 |
| 116366_at | 0.00 |
| 116368_at | 0.00 |
| 116369_at | 0.00 |
| 116370_at | 0.00 |
| 116371_at | 0.00 |
| 116372_at | 0.00 |
| 116373_at | 0.00 |
| 116374_at | 0.00 |
| 116375_at | 0.00 |
| 116376_at | 0.00 |
| 116377_at | 0.00 |
| 116378_at | 0.00 |
| 116379_at | 0.00 |
| 116380_at | 0.00 |
| 116381_at | 0.02 |
| 116382_at | 0.00 |
| 116383_at | 0.00 |
| 116386_at | 0.00 |
| 116387_at | 0.00 |
| 116388_at | 0.00 |
| 116389_at | 0.00 |
| 116390_at | 0.00 |
| 116391_at | 0.00 |
| 116392_at | 0.00 |
| 116393_at | 0.02 |
| 116395_at | 0.00 |
| 116396_at | 0.00 |
| 116397_at | 0.00 |
| 116400_at | 0.00 |
| 116403_at | 0.01 |
| 116405_at | 0.00 |
| 116406_at | 0.00 |

| | |
|---|---|
| 116407_at | 0.00 |
| 116408_at | 0.01 |
| 116409_at | 0.01 |
| 116410_at | 0.00 |
| 116411_f_at | 0.00 |
| 116412_r_at | 0.00 |
| 116414_at | 0.00 |
| 116415_at | 0.14 |
| 116416_at | 0.00 |
| 116417_at | 0.00 |
| 116418_at | 0.00 |
| 116420_at | 0.00 |
| 116421_at | 0.00 |
| 116422_at | 0.00 |
| 116423_at | 0.00 |
| 116424_at | 0.00 |
| 116425_at | 0.00 |
| 116426_at | 0.00 |
| 116427_at | 0.00 |
| 116428_at | 0.00 |
| 116429_at | 0.00 |
| 116431_at | 0.00 |
| 116432_at | 0.01 |
| 116433_at | 0.00 |
| 116434_at | 0.00 |
| 116435_at | 0.93 |
| 116436_at | 0.01 |
| 116437_at | 0.00 |
| 116438_at | 0.00 |
| 116439_at | 0.00 |
| 116440_at | 0.00 |
| 116443_at | 0.00 |
| 116444_at | 0.00 |
| 116445_at | 0.00 |
| 116448_at | 0.00 |
| 116449_at | 0.00 |
| 116450_at | 0.00 |
| 116451_at | 0.00 |
| 116452_at | 0.00 |
| 116453_f_at | 0.00 |
| 116454_r_at | 0.00 |
| 116455_at | 0.01 |
| 116456_at | 0.00 |
| 116457_at | 0.00 |
| 116458_s_at | 0.01 |
| 116459_r_at | 0.00 |
| 116460_at | 0.00 |
| 116461_at | 0.00 |
| 116463_at | 0.00 |
| 116464_at | 0.00 |
| 116465_at | 0.00 |
| 116466_at | 0.00 |
| 116468_at | 0.00 |
| 116469_at | 0.00 |
| 116470_at | 0.00 |
| 116471_at | 0.00 |

| | |
|---|---|
| 116472_at | 0.00 |
| 116473_at | 0.00 |
| 116474_at | 0.00 |
| 116475_at | 0.00 |
| 116476_at | 0.00 |
| 116477_at | 0.00 |
| 116478_at | 0.00 |
| 116479_at | 0.00 |
| 116480_at | 0.00 |
| 116481_at | 0.00 |
| 116482_at | 0.00 |
| 116483_at | 0.00 |
| 116484_at | 0.00 |
| 116485_f_at | 0.00 |
| 116486_r_at | 0.00 |
| 116487_at | 0.00 |
| 116488_f_at | 0.00 |
| 116489_r_at | 0.00 |
| 116490_at | 0.00 |
| 116491_at | 0.00 |
| 116492_at | 0.00 |
| 116493_at | 0.00 |
| 116494_at | 0.00 |
| 116495_at | 0.00 |
| 116496_at | 0.00 |
| 116497_at | 0.00 |
| 116498_at | 0.00 |
| 116499_at | 0.00 |
| 116500_at | 0.00 |
| 116501_at | 0.00 |
| 116502_at | 0.00 |
| 116503_at | 0.00 |
| 116504_at | 0.00 |
| 116505_at | 0.00 |
| 116506_at | 0.00 |
| 116507_at | 0.00 |
| 116508_at | 0.00 |
| 116509_at | 0.00 |
| 116510_at | 0.00 |
| 116511_at | 0.00 |
| 116512_at | 0.00 |
| 116513_at | 0.00 |
| 116514_at | 0.00 |
| 116515_at | 0.00 |
| 116516_at | 0.00 |
| 116517_at | 0.00 |
| 116518_at | 0.00 |
| 116519_at | 0.00 |
| 116520_at | 0.00 |
| 116521_at | 0.00 |
| 116522_at | 0.00 |
| 116523_at | 0.00 |
| 116524_at | 0.00 |
| 116525_at | 0.00 |
| 116526_at | 0.00 |
| 116527_f_at | 0.00 |

| | |
|---|---|
| 116528_at | 0.00 |
| 116529_at | 0.00 |
| 116530_at | 0.00 |
| 116531_at | 0.00 |
| 116532_at | 0.00 |
| 116533_at | 0.00 |
| 116534_at | 0.00 |
| 116535_at | 0.00 |
| 116536_at | 0.00 |
| 116537_at | 0.20 |
| 116538_at | 0.00 |
| 116539_at | 0.00 |
| 116540_at | 0.00 |
| 116541_at | 0.00 |
| 116542_at | 0.00 |
| 116543_at | 0.00 |
| 116544_at | 0.00 |
| 116545_at | 0.00 |
| 116546_at | 0.00 |
| 116547_at | 0.00 |
| 116548_at | 0.00 |
| 116549_at | 0.00 |
| 116550_at | 0.00 |
| 116552_at | 0.00 |
| 116553_at | 0.00 |
| 116554_at | 0.00 |
| 116555_at | 0.00 |
| 116556_at | 0.00 |
| 116557_at | 0.00 |
| 116558_at | 0.00 |
| 116560_at | 0.00 |
| 116561_at | 0.00 |
| 116562_at | 0.02 |
| 116563_at | 0.00 |
| 116564_at | 0.00 |
| 116565_at | 0.00 |
| 116566_at | 0.00 |
| 116567_at | 0.00 |
| 116568_at | 0.00 |
| 116569_at | 0.00 |
| 116570_at | 0.00 |
| 116571_at | 0.00 |
| 116572_at | 0.00 |
| 116573_at | 0.00 |
| 116574_at | 0.00 |
| 116575_at | 0.00 |
| 116576_at | 0.00 |
| 116577_at | 0.00 |
| 116578_at | 0.00 |
| 116579_at | 0.00 |
| 116580_at | 0.00 |
| 116581_at | 0.00 |
| 116582_at | 0.01 |
| 116583_at | 0.00 |
| 116584_at | 0.00 |
| 116585_at | 0.00 |

| | |
|---|---|
| 116586_at | 0.00 |
| 116587_at | 0.00 |
| 116588_at | 0.00 |
| 116589_at | 0.00 |
| 116590_at | 0.00 |
| 116591_at | 0.00 |
| 116592_at | 0.00 |
| 116593_at | 0.00 |
| 116594_at | 0.00 |
| 116595_at | 0.00 |
| 116596_at | 0.01 |
| 116597_at | 0.00 |
| 116598_at | 0.00 |
| 116599_at | 0.00 |
| 116600_at | 0.00 |
| 116601_at | 0.00 |
| 116602_at | 0.00 |
| 116603_at | 0.00 |
| 116604_at | 0.00 |
| 116605_at | 0.00 |
| 116606_at | 0.28 |
| 116607_at | 0.00 |
| 116608_at | 0.06 |
| 116609_at | 0.00 |
| 116610_at | 0.00 |
| 116611_at | 0.00 |
| 116612_at | 0.00 |
| 116613_at | 0.00 |
| 116614_at | 0.45 |
| 116615_at | 0.00 |
| 116616_at | 0.00 |
| 116617_at | 0.00 |
| 116619_at | 0.00 |
| 116620_at | 0.00 |
| 116621_at | 0.00 |
| 116623_at | 0.01 |
| 116624_at | 0.02 |
| 116626_at | 0.00 |
| 116627_at | 0.00 |
| 116628_at | 0.00 |
| 116629_at | 0.00 |
| 116630_at | 0.00 |
| 116631_at | 0.01 |
| 116632_at | 0.00 |
| 116633_at | 0.00 |
| 116634_at | 0.00 |
| 116635_at | 0.00 |
| 116636_at | 0.00 |
| 116637_at | 0.00 |
| 116638_at | 0.00 |
| 116640_at | 0.00 |
| 116641_f_at | 0.00 |
| 116642_f_at | 0.00 |
| 116643_at | 0.00 |
| 116644_at | 0.00 |
| 116645_at | 0.00 |

| | |
|---|---|
| 116646_at | 0.00 |
| 116647_at | 0.00 |
| 116648_at | 0.00 |
| 116649_at | 0.00 |
| 116650_at | 0.00 |
| 116651_at | 0.00 |
| 116652_at | 0.00 |
| 116653_at | 0.00 |
| 116654_at | 0.00 |
| 116655_at | 0.00 |
| 116656_at | 0.00 |
| 116657_at | 0.00 |
| 116658_at | 0.00 |
| 116659_at | 0.00 |
| 116660_at | 0.00 |
| 116661_at | 0.00 |
| 116662_at | 0.00 |
| 116663_at | 0.06 |
| 116664_at | 0.00 |
| 116665_at | 0.00 |
| 116666_at | 0.00 |
| 116668_at | 0.00 |
| 116670_at | 0.00 |
| 116671_at | 0.00 |
| 116672_at | 0.00 |
| 116673_at | 0.00 |
| 116674_at | 0.00 |
| 116676_at | 0.00 |
| 116678_at | 0.00 |
| 116679_at | 0.00 |
| 116680_at | 0.03 |
| 116681_at | 0.00 |
| 116682_g_at | 0.00 |
| 116683_at | 0.00 |
| 116684_at | 0.00 |
| 116685_at | 0.00 |
| 116686_at | 0.00 |
| 116688_at | 0.00 |
| 116689_at | 0.00 |
| 116690_at | 0.00 |
| 116691_at | 0.00 |
| 116692_at | 0.00 |
| 116694_at | 0.00 |
| 116695_at | 0.00 |
| 116696_at | 0.00 |
| 116697_at | 0.00 |
| 116698_at | 0.00 |
| 116699_at | 0.00 |
| 116701_at | 0.00 |
| 116702_at | 0.00 |
| 116703_at | 0.00 |
| 116705_at | 0.00 |
| 116706_at | 0.00 |
| 116707_at | 0.00 |
| 116708_at | 0.00 |
| 116709_at | 0.04 |

| | |
|---|---|
| 116710_at | 0.00 |
| 116711_at | 0.00 |
| 116714_at | 0.00 |
| 116715_at | 0.00 |
| 116716_at | 0.00 |
| 116717_at | 0.00 |
| 116718_at | 0.00 |
| 116719_at | 0.00 |
| 116720_at | 0.00 |
| 116721_at | 0.00 |
| 116722_at | 0.00 |
| 116725_at | 0.00 |
| 116726_at | 0.00 |
| 116727_at | 0.00 |
| 116728_at | 0.00 |
| 116729_at | 0.00 |
| 116730_r_at | 0.00 |
| 116731_at | 0.00 |
| 116733_at | 0.00 |
| 116734_at | 0.00 |
| 116735_at | 0.00 |
| 116736_at | 0.00 |
| 116737_at | 0.00 |
| 116738_at | 0.06 |
| 116739_at | 0.00 |
| 116740_at | 0.01 |
| 116741_at | 0.00 |
| 116742_at | 0.00 |
| 116743_at | 0.00 |
| 116744_at | 0.00 |
| 116745_at | 0.00 |
| 116746_at | 0.05 |
| 116747_at | 0.00 |
| 116748_at | 0.00 |
| 116749_at | 0.00 |
| 116750_at | 0.00 |
| 116751_at | 0.00 |
| 116752_at | 0.00 |
| 116753_at | 0.00 |
| 116754_at | 0.00 |
| 116755_at | 0.00 |
| 116756_at | 0.00 |
| 116757_at | 0.00 |
| 116758_at | 0.00 |
| 116760_at | 0.00 |
| 116761_at | 0.04 |
| 116762_at | 0.00 |
| 116763_at | 0.00 |
| 116764_at | 0.00 |
| 116765_at | 0.00 |
| 116766_at | 0.00 |
| 116767_at | 0.00 |
| 116768_at | 0.00 |
| 116769_at | 0.00 |
| 116771_at | 0.00 |
| 116773_at | 0.00 |

| | |
|---|---|
| 116774_at | 0.00 |
| 116775_at | 0.00 |
| 116776_at | 0.00 |
| 116777_at | 0.00 |
| 116778_at | 0.00 |
| 116779_at | 0.00 |
| 116780_at | 0.00 |
| 116781_at | 0.00 |
| 116782_at | 0.00 |
| 116783_at | 0.00 |
| 116784_at | 0.00 |
| 116785_at | 0.00 |
| 116786_at | 0.00 |
| 116787_at | 0.00 |
| 116788_at | 0.00 |
| 116789_at | 0.00 |
| 116790_at | 0.00 |
| 116791_at | 0.00 |
| 116792_at | 0.00 |
| 116793_at | 0.00 |
| 116794_at | 0.00 |
| 116795_at | 0.00 |
| 116796_at | 0.00 |
| 116797_at | 0.00 |
| 116798_at | 0.00 |
| 116799_at | 0.00 |
| 116800_at | 0.00 |
| 116803_at | 0.00 |
| 116804_at | 0.00 |
| 116805_at | 0.00 |
| 116806_at | 0.00 |
| 116807_at | 0.00 |
| 116808_at | 0.00 |
| 116809_at | 0.00 |
| 116810_at | 0.00 |
| 116811_at | 0.00 |
| 116812_at | 0.00 |
| 116813_at | 0.00 |
| 116814_at | 0.00 |
| 116815_at | 0.00 |
| 116816_at | 0.00 |
| 116817_at | 0.00 |
| 116818_at | 0.00 |
| 116819_at | 0.00 |
| 116820_at | 0.00 |
| 116821_at | 0.00 |
| 116822_at | 0.00 |
| 116823_at | 0.00 |
| 116826_at | 0.00 |
| 116827_at | 0.00 |
| 116828_at | 0.00 |
| 116829_at | 0.01 |
| 116830_at | 0.00 |
| 116831_at | 0.00 |
| 116832_at | 0.00 |
| 116833_at | 0.00 |

| | |
|---|---|
| 116834_at | 0.01 |
| 116835_at | 0.00 |
| 116836_at | 0.00 |
| 116837_at | 0.00 |
| 116838_at | 0.00 |
| 116839_at | 0.00 |
| 116841_at | 0.00 |
| 116842_at | 0.00 |
| 116843_at | 0.00 |
| 116844_at | 0.00 |
| 116846_at | 0.00 |
| 116849_at | 0.00 |
| 116850_at | 0.00 |
| 116852_at | 0.00 |
| 116853_at | 0.02 |
| 116855_at | 0.00 |
| 116856_at | 0.10 |
| 116857_at | 0.00 |
| 116858_at | 0.00 |
| 116859_at | 0.00 |
| 116860_at | 0.00 |
| 116865_at | 0.00 |
| 116868_at | 0.00 |
| 116869_at | 0.00 |
| 116870_at | 0.00 |
| 116871_at | 0.00 |
| 116872_at | 0.72 |
| 116873_at | 0.00 |
| 116874_at | 0.00 |
| 116875_at | 0.00 |
| 116877_at | 0.00 |
| 116878_at | 0.00 |
| 116879_at | 0.00 |
| 116880_at | 0.00 |
| 116881_at | 0.00 |
| 116882_at | 0.00 |
| 116883_at | 0.00 |
| 116884_at | 0.00 |
| 116885_i_at | 0.00 |
| 116886_f_at | 0.00 |
| 116887_at | 0.00 |
| 116889_at | 0.00 |
| 116890_at | 0.00 |
| 116891_at | 0.00 |
| 116895_at | 0.00 |
| 116896_at | 0.00 |
| 116898_at | 0.00 |
| 116900_at | 0.00 |
| 116901_g_at | 0.00 |
| 116902_at | 0.00 |
| 116903_at | 0.00 |
| 116904_at | 0.00 |
| 116905_at | 0.00 |
| 116906_at | 0.00 |
| 116907_at | 0.00 |
| 116908_at | 0.00 |

| | |
|---|---|
| 116909_at | 0.00 |
| 116910_at | 0.00 |
| 116911_at | 0.00 |
| 116912_at | 0.00 |
| 116913_at | 0.00 |
| 116914_at | 0.00 |
| 116916_at | 0.00 |
| 116917_at | 0.05 |
| 116919_f_at | 0.00 |
| 116920_r_at | 0.00 |
| 116921_at | 0.00 |
| 116925_at | 0.00 |
| 116927_at | 0.00 |
| 116929_at | 0.00 |
| 116930_at | 0.00 |
| 116931_at | 0.00 |
| 116934_at | 0.00 |
| 116935_i_at | 0.00 |
| 116936_f_at | 0.00 |
| 116937_at | 0.00 |
| 116938_at | 0.22 |
| 116939_at | 0.00 |
| 116940_at | 0.00 |
| 116942_at | 0.02 |
| 116943_at | 0.30 |
| 116944_at | 0.00 |
| 116945_at | 0.01 |
| 116946_at | 0.00 |
| 116947_at | 0.02 |
| 116948_at | 0.00 |
| 116949_at | 0.00 |
| 116952_at | 0.00 |
| 116953_at | 0.00 |
| 116954_at | 0.00 |
| 116955_at | 0.00 |
| 116956_at | 0.00 |
| 116958_at | 0.00 |
| 116959_at | 0.00 |
| 116961_at | 0.00 |
| 116962_at | 0.00 |
| 116963_at | 0.00 |
| 116964_at | 0.07 |
| 116965_at | 0.00 |
| 116966_at | 0.00 |
| 116967_at | 0.16 |
| 116968_at | 0.00 |
| 116969_at | 0.00 |
| 116970_at | 0.00 |
| 116971_at | 0.00 |
| 116972_at | 0.00 |
| 116973_at | 0.00 |
| 116974_at | 0.00 |
| 116975_at | 0.00 |
| 116976_at | 0.00 |
| 116977_at | 0.00 |
| 116978_at | 0.00 |

| | |
|---|---|
| 116979_at | 0.00 |
| 116980_at | 0.00 |
| 116982_at | 0.01 |
| 116983_at | 0.00 |
| 116984_at | 0.00 |
| 116985_at | 0.00 |
| 116986_at | 0.00 |
| 116987_at | 0.00 |
| 116988_at | 0.00 |
| 116989_at | 0.00 |
| 116990_at | 0.00 |
| 116991_g_at | 0.00 |
| 116992_at | 0.00 |
| 116993_at | 0.00 |
| 116994_at | 0.00 |
| 116995_at | 0.00 |
| 116996_at | 0.00 |
| 116997_at | 0.00 |
| 116999_at | 0.00 |
| 117000_at | 0.00 |
| 117002_at | 0.00 |
| 117003_at | 0.00 |
| 117004_at | 0.00 |
| 117005_at | 0.00 |
| 117006_at | 0.00 |
| 117007_at | 0.00 |
| 117008_at | 0.00 |
| 117009_at | 0.00 |
| 117010_at | 0.00 |
| 117011_at | 0.00 |
| 117012_g_at | 0.00 |
| 117013_at | 0.00 |
| 117014_at | 0.00 |
| 117015_at | 0.00 |
| 117017_at | 0.00 |
| 117018_at | 0.00 |
| 117019_at | 0.00 |
| 117020_at | 0.00 |
| 117021_at | 0.00 |
| 117022_at | 0.00 |
| 117023_at | 0.00 |
| 117024_g_at | 0.00 |
| 117025_at | 0.00 |
| 117026_at | 0.00 |
| 117027_at | 0.00 |
| 117028_at | 0.00 |
| 117029_at | 0.00 |
| 117030_at | 0.14 |
| 117031_at | 0.00 |
| 117032_g_at | 0.00 |
| 117035_at | 0.00 |
| 117036_at | 0.00 |
| 117037_at | 0.00 |
| 117038_at | 0.00 |
| 117039_at | 0.00 |
| 117041_at | 0.00 |

| | |
|---|---|
| 117042_at | 0.01 |
| 117043_at | 0.00 |
| 117044_at | 0.00 |
| 117046_at | 0.64 |
| 117047_at | 0.00 |
| 117048_at | 0.01 |
| 117049_at | 0.00 |
| 117050_at | 0.00 |
| 117051_at | 0.00 |
| 117052_at | 0.00 |
| 117053_at | 0.00 |
| 117054_at | 0.00 |
| 117055_at | 0.00 |
| 117056_at | 0.00 |
| 117057_at | 0.00 |
| 117058_at | 0.00 |
| 117059_at | 0.00 |
| 117060_at | 0.00 |
| 117061_at | 0.00 |
| 117062_at | 0.00 |
| 117063_at | 0.00 |
| 117064_f_at | 0.00 |
| 117065_at | 0.00 |
| 117066_at | 0.00 |
| 117067_at | 0.00 |
| 117068_at | 0.00 |
| 117069_at | 0.00 |
| 117070_at | 0.00 |
| 117071_at | 0.00 |
| 117072_at | 0.00 |
| 117073_at | 0.00 |
| 117074_at | 0.00 |
| 117075_at | 0.00 |
| 117076_at | 0.00 |
| 117077_at | 0.00 |
| 117078_at | 0.00 |
| 117079_at | 0.00 |
| 117080_at | 0.48 |
| 117081_at | 0.00 |
| 117082_at | 0.00 |
| 117084_at | 0.00 |
| 117085_at | 0.00 |
| 117087_at | 0.00 |
| 117088_at | 0.00 |
| 117089_at | 0.00 |
| 117090_at | 0.00 |
| 117093_at | 0.13 |
| 117094_at | 0.00 |
| 117095_at | 0.00 |
| 117096_at | 0.00 |
| 117097_at | 0.00 |
| 117098_at | 0.00 |
| 117099_at | 0.08 |
| 117101_at | 0.00 |
| 117102_at | 0.00 |
| 117103_at | 0.00 |

| | |
|---|---|
| 117106_at | 0.00 |
| 117107_at | 0.00 |
| 117108_at | 0.00 |
| 117110_at | 0.02 |
| 117111_at | 0.02 |
| 117112_at | 0.00 |
| 117113_at | 0.00 |
| 117116_at | 0.00 |
| 117118_at | 0.00 |
| 117120_at | 0.08 |
| 117121_at | 0.00 |
| 117122_at | 0.00 |
| 117123_at | 0.00 |
| 117125_at | 0.00 |
| 117126_at | 0.00 |
| 117127_at | 0.00 |
| 117128_at | 0.00 |
| 117129_at | 0.10 |
| 117131_at | 0.00 |
| 117132_at | 0.00 |
| 117133_at | 0.00 |
| 117134_at | 0.00 |
| 117136_at | 0.49 |
| 117137_at | 0.00 |
| 117138_at | 0.00 |
| 117139_at | 0.00 |
| 117140_at | 0.00 |
| 117141_at | 0.00 |
| 117142_at | 0.00 |
| 117143_s_at | 0.00 |
| 117144_r_at | 0.00 |
| 117145_at | 0.00 |
| 117146_at | 0.00 |
| 117147_at | 0.35 |
| 117148_at | 0.02 |
| 117149_at | 0.00 |
| 117151_at | 0.01 |
| 117153_at | 0.00 |
| 117154_at | 0.00 |
| 117155_at | 0.00 |
| 117156_at | 0.00 |
| 117157_at | 0.00 |
| 117158_at | 0.00 |
| 117159_at | 0.00 |
| 117160_at | 0.00 |
| 117161_at | 0.00 |
| 117163_at | 0.00 |
| 117164_g_at | 0.00 |
| 117166_at | 0.02 |
| 117168_at | 0.00 |
| 117170_at | 0.00 |
| 117171_at | 0.00 |
| 117172_at | 0.00 |
| 117173_s_at | 0.00 |
| 117174_r_at | 0.00 |
| 117175_at | 0.00 |

| | |
|---|---|
| 117176_at | 0.01 |
| 117177_at | 0.00 |
| 117178_at | 0.00 |
| 117179_at | 0.00 |
| 117181_at | 0.00 |
| 117182_at | 0.00 |
| 117183_at | 0.05 |
| 117184_at | 0.05 |
| 117186_at | 0.02 |
| 117188_at | 0.00 |
| 117189_at | 0.00 |
| 117190_at | 0.00 |
| 117191_at | 0.00 |
| 117192_at | 0.00 |
| 117193_at | 0.00 |
| 117194_at | 0.03 |
| 117195_at | 0.00 |
| 117196_at | 0.00 |
| 117197_at | 0.00 |
| 117198_at | 0.00 |
| 117199_at | 0.00 |
| 117200_at | 0.00 |
| 117201_at | 0.00 |
| 117204_at | 0.00 |
| 117206_at | 0.00 |
| 117207_at | 0.00 |
| 117208_at | 0.00 |
| 117210_at | 0.00 |
| 117211_at | 0.00 |
| 117212_at | 0.00 |
| 117213_at | 0.00 |
| 117215_at | 0.00 |
| 117216_at | 0.00 |
| 117217_at | 0.84 |
| 117218_at | 0.00 |
| 117219_f_at | 0.00 |
| 117220_r_at | 0.00 |
| 117221_at | 0.00 |
| 117222_at | 0.00 |
| 117223_at | 0.00 |
| 117224_g_at | 0.00 |
| 117225_at | 0.00 |
| 117226_at | 0.00 |
| 117227_at | 0.00 |
| 117228_at | 0.00 |
| 117229_at | 0.00 |
| 117230_at | 0.00 |
| 117231_at | 0.03 |
| 117232_at | 0.00 |
| 117233_at | 0.00 |
| 117234_at | 0.00 |
| 117235_at | 0.00 |
| 117236_at | 0.00 |
| 117238_at | 0.00 |
| 117239_at | 0.02 |
| 117240_at | 0.00 |

| | |
|---|---|
| 117241_at | 0.00 |
| 117242_at | 0.00 |
| 117243_at | 0.00 |
| 117244_at | 0.00 |
| 117245_s_at | 0.01 |
| 117246_at | 0.27 |
| 117248_at | 0.00 |
| 117250_at | 0.00 |
| 117251_at | 0.00 |
| 117252_at | 0.00 |
| 117253_at | 0.00 |
| 117257_at | 0.00 |
| 117258_at | 0.24 |
| 117259_at | 0.00 |
| 117260_at | 0.00 |
| 117262_at | 0.00 |
| 117263_at | 0.00 |
| 117265_at | 0.15 |
| 117266_at | 0.00 |
| 117267_at | 0.00 |
| 117269_at | 0.62 |
| 117270_at | 0.00 |
| 117271_at | 0.19 |
| 117272_at | 0.00 |
| 117273_at | 0.00 |
| 117276_at | 0.00 |
| 117277_at | 0.00 |
| 117279_at | 0.00 |
| 117280_at | 0.00 |
| 117281_at | 0.00 |
| 117284_at | 0.06 |
| 117285_g_at | 0.37 |
| 117286_at | 0.00 |
| 117287_at | 0.00 |
| 117289_at | 0.00 |
| 117290_at | 0.00 |
| 117291_at | 0.00 |
| 117292_at | 0.00 |
| 117293_at | 0.00 |
| 117294_at | 0.00 |
| 117295_at | 0.00 |
| 117297_at | 0.00 |
| 117298_at | 0.00 |
| 117299_at | 0.00 |
| 117300_at | 0.00 |
| 117301_at | 0.03 |
| 117302_at | 0.00 |
| 117306_at | 0.00 |
| 117307_at | 0.00 |
| 117308_at | 0.00 |
| 117309_at | 0.00 |
| 117310_at | 0.04 |
| 117316_at | 0.00 |
| 117317_at | 0.00 |
| 117319_at | 0.00 |
| 117320_at | 0.00 |

| | |
|---|---|
| 117321_at | 0.00 |
| 117324_at | 0.00 |
| 117327_at | 0.00 |
| 117331_at | 0.00 |
| 117332_at | 0.00 |
| 117333_at | 0.00 |
| 117334_f_at | 0.00 |
| 117335_r_at | 0.00 |
| 117336_at | 0.00 |
| 117338_at | 0.00 |
| 162520_at | 0.00 |
| 162521_i_at | 0.00 |
| 162522_f_at | 0.00 |
| 162523_at | 0.00 |
| 162524_at | 0.00 |
| 162525_at | 0.00 |
| 162526_at | 0.00 |
| 162527_s_at | 0.00 |
| 162528_at | 0.00 |
| 162529_at | 0.00 |
| 162530_at | 0.00 |
| 162531_at | 0.00 |
| 162532_r_at | 0.00 |
| 162533_at | 0.00 |
| 162534_at | 0.00 |
| 162535_at | 0.00 |
| 162536_r_at | 0.00 |
| 162537_at | 0.00 |
| 162538_at | 0.02 |
| 162539_at | 0.00 |
| 162540_at | 0.00 |
| 162541_i_at | 0.03 |
| 162542_r_at | 0.00 |
| 162543_r_at | 0.00 |
| 162544_i_at | 0.00 |
| 162545_at | 0.00 |
| 162546_at | 0.00 |
| 162547_at | 0.00 |
| 162548_at | 0.00 |
| 162549_at | 0.01 |
| 162550_f_at | 0.00 |
| 162551_at | 0.00 |
| 162552_at | 0.05 |
| 162553_at | 0.00 |
| 162554_at | 0.00 |
| 162555_i_at | 0.00 |
| 162556_at | 0.00 |
| 162557_at | 0.00 |
| 162558_i_at | 0.00 |
| 162559_at | 0.00 |
| 162560_at | 0.00 |
| 162561_at | 0.00 |
| 162562_at | 0.02 |
| 162563_r_at | 0.00 |
| 162564_at | 0.00 |
| 162565_r_at | 0.00 |

| | |
|---|---|
| 162566_at | 0.00 |
| 162567_at | 0.00 |
| 162568_at | 0.00 |
| 162569_at | 0.01 |
| 162570_at | 0.00 |
| 162571_at | 0.00 |
| 162572_i_at | 0.00 |
| 162573_at | 0.58 |
| 162574_at | 0.00 |
| 162575_at | 0.00 |
| 162576_at | 0.00 |
| 162577_at | 0.00 |
| 162578_at | 0.00 |
| 162579_i_at | 0.00 |
| 162580_at | 0.00 |
| 162581_at | 0.00 |
| 162582_at | 0.00 |
| 162583_at | 0.00 |
| 162584_at | 0.00 |
| 162585_at | 0.00 |
| 162586_at | 0.00 |
| 162587_at | 0.00 |
| 162588_i_at | 0.00 |
| 162589_at | 0.00 |
| 162590_at | 0.00 |
| 162591_at | 0.01 |
| 162592_at | 0.00 |
| 162593_at | 0.10 |
| 162594_at | 0.00 |
| 162595_at | 0.00 |
| 162596_at | 0.00 |
| 162597_at | 0.00 |
| 162598_at | 0.00 |
| 162599_i_at | 0.00 |
| 162600_at | 0.04 |
| 162601_i_at | 0.00 |
| 162602_at | 0.00 |
| 162603_at | 0.00 |
| 162604_at | 0.04 |
| 162605_at | 0.00 |
| 162606_at | 0.00 |
| 162607_i_at | 0.25 |
| 162608_at | 0.00 |
| 162609_at | 0.00 |
| 162610_at | 0.00 |
| 162611_i_at | 0.01 |
| 162612_at | 0.00 |
| 162613_at | 0.00 |
| 162614_at | 0.00 |
| 162615_at | 0.00 |
| 162616_at | 0.00 |
| 162617_at | 0.00 |
| 162618_at | 0.03 |
| 162619_at | 0.03 |
| 162620_at | 0.00 |
| 162621_at | 0.00 |

| | |
|---|---|
| 162622_at | 0.00 |
| 162623_at | 0.00 |
| 162624_at | 0.00 |
| 162625_at | 0.00 |
| 162626_at | 0.00 |
| 162627_at | 0.01 |
| 162628_at | 0.00 |
| 162629_at | 0.00 |
| 162630_at | 0.00 |
| 162631_at | 0.00 |
| 162632_at | 0.00 |
| 162633_at | 0.03 |
| 162634_at | 0.00 |
| 162635_at | 0.07 |
| 162636_at | 0.00 |
| 162637_at | 0.00 |
| 162638_at | 0.00 |
| 162639_at | 0.02 |
| 162640_at | 0.00 |
| 162641_r_at | 0.01 |
| 162642_at | 0.00 |
| 162643_at | 0.00 |
| 162644_at | 0.00 |
| 162645_at | 0.00 |
| 162646_at | 0.00 |
| 162647_at | 0.00 |
| 162648_at | 0.00 |
| 162649_at | 0.00 |
| 162650_at | 0.00 |
| 162651_at | 0.00 |
| 162652_f_at | 0.00 |
| 162653_at | 0.00 |
| 162654_at | 0.00 |
| 162655_at | 0.00 |
| 162656_i_at | 0.28 |
| 162657_at | 0.00 |
| 162658_at | 0.00 |
| 162659_at | 0.00 |
| 162660_at | 0.00 |
| 162661_at | 0.00 |
| 162662_at | 0.00 |
| 162663_at | 0.00 |
| 162664_at | 0.00 |
| 162665_at | 0.00 |
| 162666_at | 0.00 |
| 162667_at | 0.00 |
| 162668_at | 0.00 |
| 162669_at | 0.00 |
| 162670_at | 0.00 |
| 162671_f_at | 0.63 |
| 162672_at | 0.00 |
| 162673_at | 0.00 |
| 162674_at | 0.00 |
| 162675_f_at | 0.00 |
| 162676_at | 0.00 |
| 162677_at | 0.06 |

| | |
|---|---|
| 162678_at | 0.00 |
| 162679_at | 0.00 |
| 162680_at | 0.09 |
| 162681_at | 0.01 |
| 162682_at | 0.00 |
| 162683_at | 0.00 |
| 162684_at | 0.00 |
| 162685_r_at | 0.00 |
| 162686_at | 0.00 |
| 162687_at | 0.00 |
| 162688_at | 0.00 |
| 162689_at | 0.02 |
| 162690_at | 0.00 |
| 162691_at | 0.00 |
| 162692_at | 0.00 |
| 162693_at | 0.00 |
| 162694_at | 0.00 |
| 162695_at | 0.00 |
| 162696_at | 0.00 |
| 162697_at | 0.00 |
| 162698_at | 0.00 |
| 162699_at | 0.00 |
| 162700_at | 0.00 |
| 162701_at | 0.00 |
| 162702_at | 0.00 |
| 162703_at | 0.00 |
| 162704_i_at | 0.00 |
| 162705_at | 0.02 |
| 162706_at | 0.00 |
| 162707_i_at | 0.00 |
| 162708_at | 0.01 |
| 162709_at | 0.28 |
| 162710_at | 0.00 |
| 162711_at | 0.00 |
| 162712_at | 0.00 |
| 162713_at | 0.00 |
| 162714_at | 0.00 |
| 162715_at | 0.00 |
| 162716_at | 0.08 |
| 162717_at | 0.00 |
| 162718_f_at | 0.02 |
| 162719_at | 0.00 |
| 162720_at | 0.00 |
| 162721_at | 0.16 |
| 162722_at | 0.00 |
| 162723_at | 0.00 |
| 162724_at | 0.00 |
| 162725_at | 0.00 |
| 162726_i_at | 0.01 |
| 162727_at | 0.00 |
| 162728_at | 0.00 |
| 162729_at | 0.00 |
| 162730_at | 0.00 |
| 162731_at | 0.05 |
| 162732_i_at | 0.00 |
| 162733_at | 0.00 |

| | |
|---|---|
| 162734_at | 0.00 |
| 162735_at | 0.00 |
| 162736_at | 0.00 |
| 162737_i_at | 0.07 |
| 162738_at | 0.04 |
| 162739_at | 0.00 |
| 162740_at | 0.01 |
| 162741_at | 0.00 |
| 162742_at | 0.00 |
| 162743_at | 0.00 |
| 162744_at | 0.00 |
| 162745_at | 0.00 |
| 162746_at | 0.03 |
| 162747_at | 0.00 |
| 162748_at | 0.00 |
| 162749_at | 0.00 |
| 162750_at | 0.00 |
| 162751_at | 0.00 |
| 162752_i_at | 0.00 |
| 162753_at | 0.00 |
| 162754_at | 0.00 |
| 162755_at | 0.00 |
| 162756_at | 0.00 |
| 162757_at | 0.00 |
| 162758_at | 0.00 |
| 162759_at | 0.00 |
| 162760_at | 0.00 |
| 162761_at | 0.00 |
| 162762_at | 0.00 |
| 162763_at | 0.00 |
| 162764_at | 0.00 |
| 162765_at | 0.00 |
| 162766_at | 0.00 |
| 162767_at | 0.00 |
| 162768_at | 0.00 |
| 162769_at | 0.00 |
| 162770_at | 0.00 |
| 162771_at | 0.00 |
| 162772_f_at | 0.00 |
| 162773_at | 0.00 |
| 162774_at | 0.00 |
| 162775_at | 0.00 |
| 162776_f_at | 0.00 |
| 162777_at | 0.00 |
| 162778_at | 0.00 |
| 162779_i_at | 0.00 |
| 162780_at | 0.00 |
| 162781_r_at | 0.00 |
| 162782_at | 0.03 |
| 162783_r_at | 0.00 |
| 162784_at | 0.00 |
| 162785_at | 0.13 |
| 162786_r_at | 0.00 |
| 162787_at | 0.00 |
| 162788_at | 0.00 |
| 162789_at | 0.00 |

| | |
|---|---|
| 162790_i_at | 0.00 |
| 162791_at | 0.00 |
| 162792_at | 0.00 |
| 162793_at | 0.03 |
| 162794_at | 0.02 |
| 162795_at | 0.00 |
| 162796_at | 0.00 |
| 162797_at | 0.00 |
| 162798_at | 0.00 |
| 162799_at | 0.00 |
| 162800_at | 0.00 |
| 162801_at | 0.00 |
| 162802_at | 0.00 |
| 162803_at | 0.00 |
| 162804_at | 0.14 |
| 162805_f_at | 0.02 |
| 162806_at | 0.00 |
| 162807_at | 0.00 |
| 162808_at | 0.02 |
| 162809_at | 0.00 |
| 162810_at | 0.00 |
| 162811_at | 0.00 |
| 162812_i_at | 0.00 |
| 162813_at | 0.00 |
| 162814_at | 0.00 |
| 162815_at | 0.01 |
| 162816_at | 0.00 |
| 162817_at | 0.00 |
| 162818_at | 0.00 |
| 162819_at | 0.00 |
| 162820_i_at | 0.16 |
| 162821_at | 0.00 |
| 162822_at | 0.00 |
| 162823_at | 0.00 |
| 162824_at | 0.00 |
| 162825_at | 0.00 |
| 162826_r_at | 0.00 |
| 162827_at | 0.00 |
| 162828_at | 0.02 |
| 162829_i_at | 0.05 |
| 162830_at | 0.00 |
| 162831_at | 0.00 |
| 162832_at | 0.00 |
| 162833_at | 0.00 |
| 162834_at | 0.00 |
| 162835_at | 0.00 |
| 162836_at | 0.00 |
| 162837_at | 0.00 |
| 162838_at | 0.00 |
| 162839_i_at | 0.07 |
| 162840_at | 0.12 |
| 162841_i_at | 0.00 |
| 162842_at | 0.00 |
| 162843_at | 0.00 |
| 162844_at | 0.00 |
| 162845_at | 0.01 |

| | |
|---|---|
| 162846_at | 0.03 |
| 162847_at | 0.00 |
| 162848_at | 0.00 |
| 162849_at | 0.00 |
| 162850_at | 0.49 |
| 162851_at | 0.00 |
| 162852_s_at | 0.00 |
| 162853_r_at | 0.00 |
| 162854_at | 0.00 |
| 162855_at | 0.02 |
| 162856_at | 0.00 |
| 162857_at | 0.00 |
| 162858_at | 0.48 |
| 162859_at | 0.04 |
| 162860_r_at | 0.00 |
| 162861_at | 0.00 |
| 162862_at | 0.00 |
| 162863_at | 0.00 |
| 162864_i_at | 0.01 |
| 162865_r_at | 0.00 |
| 162866_at | 0.00 |
| 162867_r_at | 0.00 |
| 162868_at | 0.00 |
| 162869_at | 0.00 |
| 162870_at | 0.00 |
| 162871_at | 0.02 |
| 162872_at | 0.00 |
| 162873_at | 0.00 |
| 162874_at | 0.00 |
| 162875_at | 0.00 |
| 162876_at | 0.00 |
| 162877_at | 0.00 |
| 162878_at | 0.00 |
| 162879_at | 0.09 |
| 162880_at | 0.00 |
| 162881_at | 0.00 |
| 162882_at | 0.00 |
| 162883_at | 0.01 |
| 162884_at | 0.00 |
| 162885_at | 0.00 |
| 162886_r_at | 0.00 |
| 162887_at | 0.00 |
| 162888_at | 0.00 |
| 162889_at | 0.00 |
| 162890_at | 0.00 |
| 162891_at | 0.00 |
| 162892_at | 0.01 |
| 162893_at | 0.00 |
| 162894_r_at | 0.00 |
| 162895_at | 0.00 |
| 162896_at | 0.00 |
| 162897_at | 0.00 |
| 162898_at | 0.00 |
| 162899_at | 0.00 |
| 162900_at | 0.00 |
| 162901_i_at | 0.21 |

| | |
|---|---|
| 162902_at | 0.00 |
| 162903_at | 0.19 |
| 162904_at | 0.00 |
| 162905_i_at | 0.04 |
| 162906_at | 0.07 |
| 162907_at | 0.00 |
| 162908_at | 0.00 |
| 162909_at | 0.11 |
| 162910_at | 0.00 |
| 162911_at | 0.00 |
| 162912_at | 0.00 |
| 162913_at | 0.00 |
| 162914_i_at | 0.00 |
| 162915_at | 0.03 |
| 162916_at | 0.00 |
| 162917_at | 0.00 |
| 162918_at | 0.00 |
| 162919_at | 0.00 |
| 162920_at | 0.00 |
| 162921_i_at | 0.00 |
| 162922_at | 0.00 |
| 162923_at | 0.00 |
| 162924_at | 0.00 |
| 162925_at | 0.00 |
| 162926_at | 0.02 |
| 162927_at | 0.01 |
| 162928_f_at | 0.00 |
| 162929_at | 0.43 |
| 162930_at | 0.00 |
| 162931_at | 0.00 |
| 162932_at | 0.04 |
| 162933_at | 0.00 |
| 162934_at | 0.00 |
| 162935_at | 0.00 |
| 162936_at | 0.00 |
| 162937_at | 0.00 |
| 162938_at | 0.00 |
| 162939_at | 0.00 |
| 162940_at | 0.00 |
| 162941_at | 0.00 |
| 162942_at | 0.02 |
| 162943_r_at | 0.00 |
| 162944_at | 0.00 |
| 162945_at | 0.00 |
| 162946_at | 0.00 |
| 162947_at | 0.00 |
| 162948_s_at | 0.00 |
| 162949_at | 0.00 |
| 162950_i_at | 0.00 |
| 162951_at | 0.00 |
| 162952_at | 0.00 |
| 162953_at | 0.00 |
| 162954_at | 0.00 |
| 162955_at | 0.00 |
| 162956_at | 0.00 |
| 162957_at | 0.00 |

| | |
|---|---|
| 162958_at | 0.00 |
| 162959_i_at | 0.00 |
| 162960_at | 0.03 |
| 162961_at | 0.00 |
| 162962_at | 0.00 |
| 162963_at | 0.00 |
| 162964_at | 0.00 |
| 162965_at | 0.00 |
| 162966_i_at | 0.00 |
| 162967_r_at | 0.00 |
| 162968_at | 0.00 |
| 162969_at | 0.14 |
| 162970_at | 0.00 |
| 162971_r_at | 0.13 |
| 162972_at | 0.00 |
| 162973_at | 0.73 |
| 162974_at | 0.00 |
| 162975_at | 0.00 |
| 162976_at | 0.00 |
| 162977_at | 0.00 |
| 162978_at | 0.00 |
| 162979_at | 0.00 |
| 162980_at | 0.00 |
| 162981_at | 0.00 |
| 162982_at | 0.00 |
| 162983_at | 0.00 |
| 162984_at | 0.00 |
| 162985_at | 0.06 |
| 162986_at | 0.00 |
| 162987_at | 0.00 |
| 162988_at | 0.00 |
| 162989_at | 0.00 |
| 162990_at | 0.01 |
| 162991_at | 0.00 |
| 162992_at | 0.00 |
| 162993_at | 0.00 |
| 162994_at | 0.00 |
| 162995_at | 0.00 |
| 162996_at | 0.00 |
| 162997_at | 0.10 |
| 162998_at | 0.02 |
| 162999_at | 0.00 |
| 163000_at | 0.00 |
| 163001_at | 0.00 |
| 163002_at | 0.00 |
| 163003_at | 0.00 |
| 163004_i_at | 0.00 |
| 163005_s_at | 0.76 |
| 163006_at | 0.00 |
| 163007_at | 0.00 |
| 163008_at | 0.00 |
| 163009_at | 0.00 |
| 163010_at | 0.00 |
| 163011_at | 0.00 |
| 163012_at | 0.00 |
| 163013_at | 0.01 |

| | |
|---|---|
| 163014_at | 0.21 |
| 163015_at | 0.00 |
| 163016_at | 0.00 |
| 163017_i_at | 0.65 |
| 163018_at | 0.00 |
| 163019_i_at | 0.00 |
| 163020_at | 0.00 |
| 163021_at | 0.00 |
| 163022_at | 0.00 |
| 163023_at | 0.00 |
| 163024_at | 0.00 |
| 163025_at | 0.00 |
| 163026_at | 0.00 |
| 163027_at | 0.00 |
| 163028_at | 0.00 |
| 163029_at | 0.00 |
| 163030_at | 0.00 |
| 163031_at | 0.00 |
| 163032_at | 0.00 |
| 163033_at | 0.02 |
| 163034_at | 0.00 |
| 163035_at | 0.00 |
| 163036_at | 0.00 |
| 163037_at | 0.00 |
| 163038_at | 0.00 |
| 163039_at | 0.00 |
| 163040_i_at | 0.00 |
| 163041_at | 0.00 |
| 163042_at | 0.00 |
| 163043_r_at | 0.00 |
| 163044_at | 0.00 |
| 163045_at | 0.00 |
| 163046_at | 0.00 |
| 163047_at | 0.00 |
| 163048_at | 0.19 |
| 163049_at | 0.00 |
| 163050_r_at | 0.04 |
| 163051_at | 0.00 |
| 163052_at | 0.00 |
| 163053_at | 0.00 |
| 163054_at | 0.00 |
| 163055_at | 0.00 |
| 163056_at | 0.00 |
| 163057_at | 0.00 |
| 163058_at | 0.01 |
| 163059_at | 0.00 |
| 163060_at | 0.00 |
| 163061_at | 0.00 |
| 163062_at | 0.00 |
| 163063_i_at | 0.03 |
| 163064_at | 0.09 |
| 163065_r_at | 0.00 |
| 163066_at | 0.00 |
| 163067_at | 0.00 |
| 163068_at | 0.00 |
| 163069_i_at | 0.00 |

| | |
|---|---|
| 163070_at | 0.08 |
| 163071_at | 0.00 |
| 163072_at | 0.00 |
| 163073_at | 0.00 |
| 163074_at | 0.00 |
| 163075_at | 0.00 |
| 163076_at | 0.00 |
| 163077_at | 0.00 |
| 163078_at | 0.00 |
| 163079_at | 0.00 |
| 163080_at | 0.00 |
| 163081_at | 0.00 |
| 163082_i_at | 0.00 |
| 163083_at | 0.00 |
| 163084_at | 0.00 |
| 163085_i_at | 0.00 |
| 163086_at | 0.00 |
| 163087_at | 0.00 |
| 163088_r_at | 0.00 |
| 163089_at | 0.00 |
| 163090_at | 0.00 |
| 163091_at | 0.00 |
| 163092_at | 0.00 |
| 163093_at | 0.00 |
| 163094_at | 0.00 |
| 163095_at | 0.00 |
| 163096_at | 0.00 |
| 163097_at | 0.00 |
| 163098_at | 0.00 |
| 163099_at | 0.00 |
| 163100_at | 0.25 |
| 163101_r_at | 0.00 |
| 163102_r_at | 0.00 |
| 163103_at | 0.00 |
| 163104_at | 0.00 |
| 163105_at | 0.00 |
| 163106_at | 0.00 |
| 163107_at | 0.09 |
| 163108_at | 0.00 |
| 163109_at | 0.00 |
| 163110_at | 0.00 |
| 163111_at | 0.00 |
| 163112_at | 0.00 |
| 163113_at | 0.00 |
| 163114_at | 0.00 |
| 163115_at | 0.24 |
| 163116_at | 0.00 |
| 163117_at | 0.00 |
| 163118_r_at | 0.00 |
| 163119_i_at | 0.00 |
| 163120_at | 0.00 |
| 163121_at | 0.00 |
| 163122_at | 0.00 |
| 163123_at | 0.00 |
| 163124_s_at | 0.00 |
| 163125_at | 0.00 |

| | |
|---|---|
| 163126_at | 0.00 |
| 163127_at | 0.06 |
| 163128_at | 0.00 |
| 163129_at | 0.00 |
| 163130_at | 0.00 |
| 163131_at | 0.00 |
| 163132_at | 0.00 |
| 163133_at | 0.00 |
| 163134_at | 0.00 |
| 163135_at | 0.00 |
| 163136_at | 0.00 |
| 163137_at | 0.00 |
| 163138_at | 0.00 |
| 163139_at | 0.00 |
| 163140_at | 0.00 |
| 163141_at | 0.00 |
| 163142_at | 0.00 |
| 163143_at | 0.00 |
| 163144_at | 0.00 |
| 163145_at | 0.00 |
| 163146_at | 0.00 |
| 163147_at | 0.00 |
| 163148_at | 0.00 |
| 163149_at | 0.03 |
| 163150_at | 0.00 |
| 163151_at | 0.00 |
| 163152_at | 0.00 |
| 163153_at | 0.00 |
| 163154_at | 0.00 |
| 163155_at | 0.00 |
| 163156_at | 0.00 |
| 163157_at | 0.00 |
| 163158_at | 0.00 |
| 163159_at | 0.00 |
| 163160_at | 0.00 |
| 163161_at | 0.00 |
| 163162_at | 0.00 |
| 163163_at | 0.00 |
| 163164_at | 0.00 |
| 163165_at | 0.00 |
| 163166_at | 0.00 |
| 163167_at | 0.00 |
| 163168_r_at | 0.00 |
| 163169_at | 0.00 |
| 163170_at | 0.05 |
| 163171_at | 0.00 |
| 163172_at | 0.00 |
| 163173_at | 0.13 |
| 163174_at | 0.00 |
| 163175_at | 0.00 |
| 163176_at | 0.00 |
| 163177_at | 0.00 |
| 163178_at | 0.00 |
| 163179_at | 0.00 |
| 163180_at | 0.00 |
| 163181_at | 0.00 |

| | |
|---|---|
| 163182_at | 0.00 |
| 163183_at | 0.02 |
| 163184_at | 0.00 |
| 163185_at | 0.00 |
| 163186_at | 0.00 |
| 163187_at | 0.00 |
| 163188_at | 0.00 |
| 163189_i_at | 0.00 |
| 163190_at | 0.00 |
| 163191_at | 0.00 |
| 163192_at | 0.00 |
| 163193_at | 0.00 |
| 163194_at | 0.01 |
| 163195_at | 0.00 |
| 163196_i_at | 0.07 |
| 163197_i_at | 0.04 |
| 163198_at | 0.00 |
| 163199_at | 0.00 |
| 163200_at | 0.00 |
| 163201_at | 0.00 |
| 163202_at | 0.00 |
| 163203_at | 0.03 |
| 163204_at | 0.00 |
| 163205_at | 0.00 |
| 163206_at | 0.00 |
| 163207_at | 0.07 |
| 163208_at | 0.00 |
| 163209_at | 0.00 |
| 163210_at | 0.00 |
| 163211_at | 0.00 |
| 163212_at | 0.03 |
| 163213_at | 0.00 |
| 163214_at | 0.00 |
| 163215_at | 0.00 |
| 163216_at | 0.00 |
| 163217_at | 0.00 |
| 163218_at | 0.00 |
| 163219_at | 0.00 |
| 163220_at | 0.00 |
| 163221_at | 0.00 |
| 163222_at | 0.00 |
| 163223_at | 0.00 |
| 163224_at | 0.00 |
| 163225_at | 0.00 |
| 163226_at | 0.00 |
| 163227_at | 0.00 |
| 163228_at | 0.00 |
| 163229_at | 0.25 |
| 163230_at | 0.24 |
| 163231_at | 0.00 |
| 163232_at | 0.00 |
| 163233_at | 0.00 |
| 163234_at | 0.00 |
| 163235_at | 0.00 |
| 163236_at | 0.00 |
| 163237_at | 0.00 |

| | |
|---|---|
| 163238_f_at | 0.00 |
| 163239_r_at | 0.00 |
| 163240_i_at | 0.00 |
| 163241_at | 0.00 |
| 163242_at | 0.03 |
| 163243_at | 0.00 |
| 163244_at | 0.00 |
| 163245_at | 0.00 |
| 163246_at | 0.00 |
| 163247_at | 0.00 |
| 163248_at | 0.00 |
| 163249_at | 0.00 |
| 163250_at | 0.00 |
| 163251_at | 0.00 |
| 163252_at | 0.00 |
| 163253_at | 0.00 |
| 163254_at | 0.00 |
| 163255_at | 0.00 |
| 163256_i_at | 0.19 |
| 163257_at | 0.00 |
| 163258_at | 0.00 |
| 163259_at | 0.00 |
| 163260_at | 0.00 |
| 163261_at | 0.05 |
| 163262_at | 0.82 |
| 163263_at | 0.00 |
| 163264_at | 0.00 |
| 163265_at | 0.00 |
| 163266_at | 0.00 |
| 163267_at | 0.00 |
| 163268_at | 0.00 |
| 163269_at | 0.00 |
| 163270_i_at | 0.00 |
| 163271_at | 0.00 |
| 163272_at | 0.00 |
| 163273_at | 0.00 |
| 163274_at | 0.00 |
| 163275_at | 0.00 |
| 163276_at | 0.15 |
| 163277_at | 0.00 |
| 163278_at | 0.00 |
| 163279_at | 0.00 |
| 163280_i_at | 0.00 |
| 163281_at | 0.00 |
| 163282_at | 0.00 |
| 163283_at | 0.00 |
| 163284_at | 0.00 |
| 163285_at | 0.00 |
| 163286_at | 0.00 |
| 163287_at | 0.16 |
| 163288_at | 0.70 |
| 163289_at | 0.00 |
| 163290_at | 0.34 |
| 163291_at | 0.00 |
| 163292_at | 0.00 |
| 163293_at | 0.00 |

| | |
|---|---|
| 163294_at | 0.00 |
| 163295_at | 0.00 |
| 163296_at | 0.00 |
| 163297_at | 0.03 |
| 163298_at | 0.00 |
| 163299_at | 0.00 |
| 163300_at | 0.00 |
| 163301_at | 0.00 |
| 163302_at | 0.00 |
| 163303_at | 0.00 |
| 163304_at | 0.21 |
| 163305_at | 0.00 |
| 163306_at | 0.00 |
| 163307_at | 0.00 |
| 163308_at | 0.00 |
| 163309_s_at | 0.00 |
| 163310_at | 0.00 |
| 163311_at | 0.00 |
| 163312_i_at | 0.00 |
| 163313_at | 0.05 |
| 163314_at | 0.00 |
| 163315_at | 0.00 |
| 163316_at | 0.00 |
| 163317_at | 0.00 |
| 163318_at | 0.00 |
| 163319_at | 0.00 |
| 163320_at | 0.00 |
| 163321_i_at | 0.15 |
| 163322_at | 0.00 |
| 163323_at | 0.00 |
| 163324_at | 0.00 |
| 163325_s_at | 0.00 |
| 163326_i_at | 0.00 |
| 163327_at | 0.00 |
| 163328_at | 0.00 |
| 163329_i_at | 0.00 |
| 163330_at | 0.00 |
| 163331_at | 0.00 |
| 163332_at | 0.00 |
| 163333_at | 0.00 |
| 163334_at | 0.00 |
| 163335_at | 0.00 |
| 163336_at | 0.00 |
| 163337_at | 0.00 |
| 163338_at | 0.00 |
| 163339_at | 0.00 |
| 163340_at | 0.00 |
| 163341_at | 0.01 |
| 163342_at | 0.00 |
| 163343_at | 0.00 |
| 163344_at | 0.00 |
| 163345_at | 0.00 |
| 163346_at | 0.00 |
| 163347_at | 0.00 |
| 163348_at | 0.13 |
| 163349_at | 0.00 |

| | |
|---|---|
| 163350_at | 0.00 |
| 163351_at | 0.00 |
| 163352_at | 0.00 |
| 163353_at | 0.00 |
| 163354_at | 0.00 |
| 163355_at | 0.01 |
| 163356_i_at | 0.00 |
| 163357_at | 0.00 |
| 163358_at | 0.00 |
| 163359_at | 0.00 |
| 163360_at | 0.00 |
| 163361_i_at | 0.00 |
| 163362_at | 0.00 |
| 163363_at | 0.00 |
| 163364_at | 0.00 |
| 163365_r_at | 0.01 |
| 163366_at | 0.00 |
| 163367_at | 0.00 |
| 163368_at | 0.00 |
| 163369_at | 0.00 |
| 163370_at | 0.00 |
| 163371_at | 0.01 |
| 163372_at | 0.00 |
| 163373_at | 0.00 |
| 163374_at | 0.00 |
| 163375_at | 0.00 |
| 163376_at | 0.00 |
| 163377_at | 0.00 |
| 163378_at | 0.00 |
| 163379_at | 0.00 |
| 163380_f_at | 0.29 |
| 163381_at | 0.01 |
| 163382_at | 0.00 |
| 163383_at | 0.00 |
| 163384_i_at | 0.03 |
| 163385_at | 0.00 |
| 163386_at | 0.00 |
| 163387_at | 0.00 |
| 163388_at | 0.00 |
| 163389_at | 0.00 |
| 163390_r_at | 0.00 |
| 163391_at | 0.00 |
| 163392_at | 0.00 |
| 163393_at | 0.00 |
| 163394_at | 0.00 |
| 163395_at | 0.00 |
| 163396_at | 0.00 |
| 163397_r_at | 0.00 |
| 163398_at | 0.00 |
| 163399_at | 0.00 |
| 163400_at | 0.00 |
| 163401_at | 0.00 |
| 163402_at | 0.00 |
| 163403_r_at | 0.00 |
| 163404_at | 0.96 |
| 163405_at | 0.00 |

| | |
|---|---|
| 163406_at | 0.00 |
| 163407_at | 0.00 |
| 163408_at | 0.00 |
| 163409_at | 0.00 |
| 163410_at | 0.00 |
| 163411_at | 0.00 |
| 163412_at | 0.00 |
| 163413_at | 0.00 |
| 163414_f_at | 0.00 |
| 163415_at | 0.00 |
| 163416_r_at | 0.00 |
| 163417_at | 0.00 |
| 163418_at | 0.00 |
| 163419_at | 0.00 |
| 163420_at | 0.00 |
| 163421_at | 0.00 |
| 163422_i_at | 0.00 |
| 163423_at | 0.00 |
| 163424_at | 0.00 |
| 163425_at | 0.00 |
| 163426_at | 0.00 |
| 163427_s_at | 0.00 |
| 163428_at | 0.00 |
| 163429_at | 0.00 |
| 163430_at | 0.05 |
| 163431_at | 0.00 |
| 163432_at | 0.00 |
| 163433_r_at | 0.00 |
| 163434_at | 0.00 |
| 163435_at | 0.01 |
| 163436_at | 0.00 |
| 163437_at | 0.00 |
| 163438_at | 0.00 |
| 163439_at | 0.00 |
| 163440_at | 0.00 |
| 163441_at | 0.00 |
| 163442_at | 0.00 |
| 163443_at | 0.00 |
| 163444_at | 0.00 |
| 163445_at | 0.00 |
| 163446_at | 0.00 |
| 163447_at | 0.00 |
| 163448_at | 0.02 |
| 163449_at | 0.00 |
| 163450_at | 0.00 |
| 163451_at | 0.00 |
| 163452_at | 0.00 |
| 163453_at | 0.00 |
| 163454_at | 0.00 |
| 163455_at | 0.08 |
| 163456_at | 0.34 |
| 163457_f_at | 0.03 |
| 163458_at | 0.00 |
| 163459_at | 0.00 |
| 163460_at | 0.00 |
| 163461_at | 0.00 |

| | |
|---|---|
| 163462_at | 0.00 |
| 163463_at | 0.00 |
| 163464_at | 0.00 |
| 163465_at | 0.00 |
| 163466_at | 0.00 |
| 163467_at | 0.00 |
| 163468_at | 0.00 |
| 163469_at | 0.00 |
| 163470_at | 0.00 |
| 163471_at | 0.00 |
| 163472_at | 0.00 |
| 163473_at | 0.00 |
| 163474_at | 0.00 |
| 163475_at | 0.00 |
| 163476_at | 0.00 |
| 163477_at | 0.00 |
| 163478_at | 0.03 |
| 163479_at | 0.00 |
| 163480_at | 0.00 |
| 163481_at | 0.00 |
| 163482_at | 0.00 |
| 163483_r_at | 0.00 |
| 163484_at | 0.00 |
| 163485_at | 0.00 |
| 163486_at | 0.00 |
| 163487_r_at | 0.00 |
| 163488_at | 0.00 |
| 163489_at | 0.85 |
| 163490_at | 0.00 |
| 163491_at | 0.00 |
| 163492_at | 0.01 |
| 163493_at | 0.00 |
| 163494_at | 0.82 |
| 163495_at | 0.00 |
| 163496_i_at | 0.00 |
| 163497_at | 0.00 |
| 163498_i_at | 0.00 |
| 163499_at | 0.00 |
| 163500_at | 0.00 |
| 163501_at | 0.00 |
| 163502_at | 0.00 |
| 163503_at | 0.00 |
| 163504_at | 0.00 |
| 163505_at | 0.05 |
| 163506_at | 0.00 |
| 163507_at | 0.00 |
| 163508_at | 0.00 |
| 163509_at | 0.00 |
| 163510_at | 0.00 |
| 163511_at | 0.00 |
| 163512_at | 0.13 |
| 163513_f_at | 0.00 |
| 163514_at | 0.00 |
| 163515_r_at | 0.00 |
| 163516_at | 0.00 |
| 163517_at | 0.00 |

| | |
|---|---|
| 163518_at | 0.00 |
| 163519_at | 0.00 |
| 163520_at | 0.00 |
| 163521_at | 0.00 |
| 163522_at | 0.00 |
| 163523_i_at | 0.00 |
| 163524_at | 0.00 |
| 163525_at | 0.00 |
| 163526_at | 0.00 |
| 163527_i_at | 0.00 |
| 163528_at | 0.00 |
| 163529_at | 0.00 |
| 163530_at | 0.00 |
| 163531_at | 0.00 |
| 163532_at | 0.00 |
| 163533_at | 0.00 |
| 163534_f_at | 0.00 |
| 163535_at | 0.00 |
| 163536_at | 0.01 |
| 163537_at | 0.09 |
| 163538_at | 0.00 |
| 163539_at | 0.00 |
| 163540_i_at | 0.00 |
| 163541_at | 0.00 |
| 163542_at | 0.01 |
| 163543_r_at | 0.00 |
| 163544_at | 0.00 |
| 163545_at | 0.00 |
| 163546_at | 0.00 |
| 163547_at | 0.00 |
| 163548_at | 0.00 |
| 163549_at | 0.00 |
| 163550_at | 0.00 |
| 163551_at | 0.00 |
| 163552_at | 0.01 |
| 163553_at | 0.00 |
| 163554_at | 0.00 |
| 163555_at | 0.00 |
| 163556_at | 0.00 |
| 163557_at | 0.00 |
| 163558_at | 0.00 |
| 163559_at | 0.00 |
| 163560_at | 0.00 |
| 163561_at | 0.00 |
| 163562_at | 0.00 |
| 163563_at | 0.00 |
| 163564_at | 0.00 |
| 163565_at | 0.01 |
| 163566_at | 0.00 |
| 163567_at | 0.00 |
| 163568_at | 0.00 |
| 163569_at | 0.00 |
| 163570_at | 0.00 |
| 163571_at | 0.00 |
| 163572_at | 0.00 |
| 163573_at | 0.00 |

| | |
|---|---|
| 163574_at | 0.00 |
| 163575_at | 0.00 |
| 163576_at | 0.00 |
| 163577_at | 0.00 |
| 163578_at | 0.00 |
| 163579_at | 0.00 |
| 163580_r_at | 0.00 |
| 163581_at | 0.00 |
| 163582_at | 0.00 |
| 163583_at | 0.00 |
| 163584_at | 0.00 |
| 163585_at | 0.00 |
| 163586_at | 0.00 |
| 163587_at | 0.00 |
| 163588_at | 0.05 |
| 163589_at | 0.00 |
| 163590_at | 0.00 |
| 163591_at | 0.00 |
| 163592_at | 0.00 |
| 163593_i_at | 0.00 |
| 163594_at | 0.00 |
| 163595_at | 0.00 |
| 163596_at | 0.00 |
| 163597_at | 0.00 |
| 163598_at | 0.00 |
| 163599_at | 0.00 |
| 163600_at | 0.00 |
| 163601_at | 0.00 |
| 163602_at | 0.00 |
| 163603_at | 0.00 |
| 163604_at | 0.00 |
| 163605_at | 0.00 |
| 163606_at | 0.00 |
| 163607_at | 0.01 |
| 163608_at | 0.00 |
| 163609_at | 0.00 |
| 163610_at | 0.00 |
| 163611_f_at | 0.78 |
| 163612_at | 0.00 |
| 163613_i_at | 0.00 |
| 163614_at | 0.01 |
| 163615_at | 0.00 |
| 163616_at | 0.00 |
| 163617_at | 0.00 |
| 163618_i_at | 0.00 |
| 163619_at | 0.01 |
| 163620_at | 0.01 |
| 163621_at | 0.00 |
| 163622_at | 0.00 |
| 163623_at | 0.00 |
| 163624_i_at | 0.00 |
| 163625_at | 0.00 |
| 163626_at | 0.04 |
| 163627_f_at | 0.02 |
| 163628_at | 0.00 |
| 163629_at | 0.00 |

| | |
|---|---|
| 163630_at | 0.00 |
| 163631_at | 0.04 |
| 163632_at | 0.00 |
| 163633_at | 0.00 |
| 163634_at | 0.00 |
| 163635_at | 0.00 |
| 163636_at | 0.00 |
| 163637_at | 0.00 |
| 163638_i_at | 0.00 |
| 163639_at | 0.00 |
| 163640_at | 0.00 |
| 163641_at | 0.00 |
| 163642_at | 0.00 |
| 163643_i_at | 0.00 |
| 163644_at | 0.00 |
| 163645_at | 0.00 |
| 163646_at | 0.92 |
| 163647_at | 0.00 |
| 163648_at | 0.00 |
| 163649_at | 0.01 |
| 163650_at | 0.00 |
| 163651_at | 0.00 |
| 163652_at | 0.00 |
| 163653_at | 0.00 |
| 163654_r_at | 0.00 |
| 163655_s_at | 0.64 |
| 163656_at | 0.00 |
| 163657_at | 0.00 |
| 163658_at | 0.00 |
| 163659_at | 0.00 |
| 163660_at | 0.00 |
| 163661_at | 0.00 |
| 163662_at | 0.00 |
| 163663_at | 0.00 |
| 163664_at | 0.01 |
| 163665_at | 0.00 |
| 163666_at | 0.00 |
| 163667_at | 0.00 |
| 163668_at | 0.00 |
| 163669_at | 0.00 |
| 163670_f_at | 0.00 |
| 163671_at | 0.00 |
| 163672_at | 0.00 |
| 163673_at | 0.00 |
| 163674_at | 0.00 |
| 163675_r_at | 0.00 |
| 163676_at | 0.00 |
| 163677_at | 0.00 |
| 163678_at | 0.00 |
| 163679_at | 0.00 |
| 163680_at | 0.00 |
| 163681_at | 0.00 |
| 163682_at | 0.00 |
| 163683_at | 0.02 |
| 163684_at | 0.00 |
| 163685_at | 0.00 |

| | |
|---|---|
| 163686_at | 0.01 |
| 163687_at | 0.00 |
| 163688_at | 0.00 |
| 163689_at | 0.03 |
| 163690_at | 0.00 |
| 163691_at | 0.00 |
| 163692_at | 0.00 |
| 163693_at | 0.00 |
| 163694_at | 0.00 |
| 163695_at | 0.00 |
| 163696_at | 0.00 |
| 163697_at | 0.00 |
| 163698_at | 0.00 |
| 163699_at | 0.00 |
| 163700_at | 0.00 |
| 163701_at | 0.02 |
| 163702_i_at | 0.00 |
| 163703_r_at | 0.00 |
| 163704_at | 0.00 |
| 163705_at | 0.00 |
| 163706_at | 0.00 |
| 163707_at | 0.00 |
| 163708_at | 0.00 |
| 163709_i_at | 0.31 |
| 163710_at | 0.00 |
| 163711_at | 0.00 |
| 163712_at | 0.00 |
| 163713_at | 0.00 |
| 163714_at | 0.00 |
| 163715_at | 0.50 |
| 163716_at | 0.00 |
| 163717_r_at | 0.00 |
| 163718_at | 0.00 |
| 163719_at | 0.00 |
| 163720_at | 0.00 |
| 163721_at | 0.00 |
| 163722_at | 0.00 |
| 163723_at | 0.00 |
| 163724_at | 0.00 |
| 163725_at | 0.00 |
| 163726_at | 0.00 |
| 163727_at | 0.00 |
| 163728_at | 0.00 |
| 163729_at | 0.00 |
| 163730_i_at | 0.00 |
| 163731_at | 0.00 |
| 163732_at | 0.00 |
| 163733_at | 0.00 |
| 163734_at | 0.00 |
| 163735_at | 0.00 |
| 163736_at | 0.00 |
| 163737_at | 0.00 |
| 163738_at | 0.00 |
| 163739_at | 0.00 |
| 163740_at | 0.00 |
| 163741_at | 0.00 |

| | |
|---|---|
| 163742_i_at | 0.02 |
| 163743_at | 0.00 |
| 163744_at | 0.00 |
| 163745_at | 0.00 |
| 163746_at | 0.00 |
| 163747_at | 0.00 |
| 163748_at | 0.00 |
| 163749_at | 0.00 |
| 163750_at | 0.04 |
| 163751_i_at | 0.00 |
| 163752_at | 0.00 |
| 163753_at | 0.00 |
| 163754_at | 0.00 |
| 163755_at | 0.00 |
| 163756_at | 0.00 |
| 163757_at | 0.00 |
| 163758_at | 0.00 |
| 163759_at | 0.00 |
| 163760_at | 0.00 |
| 163761_at | 0.00 |
| 163762_at | 0.00 |
| 163763_at | 0.00 |
| 163764_at | 0.00 |
| 163765_at | 0.00 |
| 163766_at | 0.01 |
| 163767_at | 0.00 |
| 163768_at | 0.00 |
| 163769_at | 0.01 |
| 163770_at | 0.00 |
| 163771_at | 0.00 |
| 163772_at | 0.00 |
| 163773_at | 0.00 |
| 163774_at | 0.13 |
| 163775_at | 0.00 |
| 163776_at | 0.00 |
| 163777_at | 0.04 |
| 163778_at | 0.10 |
| 163779_at | 0.02 |
| 163780_at | 0.00 |
| 163781_at | 0.00 |
| 163782_at | 0.00 |
| 163783_at | 0.01 |
| 163784_at | 0.00 |
| 163785_at | 0.00 |
| 163786_at | 0.00 |
| 163787_at | 0.00 |
| 163788_at | 0.00 |
| 163789_at | 0.00 |
| 163790_at | 0.11 |
| 163791_f_at | 0.00 |
| 163792_at | 0.00 |
| 163793_at | 0.00 |
| 163794_r_at | 0.00 |
| 163795_at | 0.00 |
| 163796_at | 0.00 |
| 163797_i_at | 0.01 |

| | |
|---|---|
| 163798_at | 0.06 |
| 163799_f_at | 0.00 |
| 163800_at | 0.00 |
| 163801_at | 0.17 |
| 163802_at | 0.00 |
| 163803_r_at | 0.00 |
| 163804_i_at | 0.00 |
| 163805_at | 0.00 |
| 163806_at | 0.00 |
| 163807_i_at | 0.00 |
| 163808_at | 0.00 |
| 163809_r_at | 0.00 |
| 163810_at | 0.00 |
| 163811_at | 0.01 |
| 163812_at | 0.00 |
| 163813_at | 0.00 |
| 163814_r_at | 0.01 |
| 163815_at | 0.00 |
| 163816_at | 0.00 |
| 163817_at | 0.00 |
| 163818_at | 0.00 |
| 163819_at | 0.00 |
| 163820_at | 0.01 |
| 163821_at | 0.00 |
| 163822_at | 0.00 |
| 163823_at | 0.00 |
| 163824_i_at | 0.02 |
| 163825_at | 0.00 |
| 163826_at | 0.31 |
| 163827_at | 0.00 |
| 163828_at | 0.49 |
| 163829_r_at | 0.00 |
| 163830_at | 0.00 |
| 163831_at | 0.00 |
| 163832_at | 0.00 |
| 163833_at | 0.01 |
| 163834_at | 0.00 |
| 163835_r_at | 0.00 |
| 163836_at | 0.00 |
| 163837_at | 0.00 |
| 163838_at | 0.01 |
| 163839_at | 0.00 |
| 163840_at | 0.00 |
| 163841_f_at | 0.03 |
| 163842_at | 0.00 |
| 163843_i_at | 0.00 |
| 163844_at | 0.00 |
| 163845_i_at | 0.00 |
| 163846_at | 0.03 |
| 163847_at | 0.00 |
| 163848_i_at | 0.00 |
| 163849_r_at | 0.00 |
| 163850_at | 0.00 |
| 163851_at | 0.00 |
| 163852_at | 0.01 |
| 163853_at | 0.00 |

| | |
|---|---|
| 163854_at | 0.00 |
| 163855_at | 0.00 |
| 163856_at | 0.00 |
| 163857_at | 0.00 |
| 163858_at | 0.00 |
| 163859_f_at | 0.00 |
| 163860_at | 0.01 |
| 163861_at | 0.00 |
| 163862_at | 0.00 |
| 163863_at | 0.00 |
| 163864_i_at | 0.00 |
| 163865_at | 0.00 |
| 163866_at | 0.01 |
| 163867_at | 0.03 |
| 163868_at | 0.00 |
| 163869_at | 0.00 |
| 163870_at | 0.00 |
| 163871_at | 0.00 |
| 163872_i_at | 0.00 |
| 163873_at | 0.00 |
| 163874_at | 0.00 |
| 163875_at | 0.00 |
| 163876_at | 0.00 |
| 163877_at | 0.00 |
| 163878_at | 0.00 |
| 163879_r_at | 0.00 |
| 163880_at | 0.00 |
| 163881_at | 0.00 |
| 163882_at | 0.00 |
| 163883_at | 0.00 |
| 163884_f_at | 0.00 |
| 163885_at | 0.00 |
| 163886_at | 0.00 |
| 163887_at | 0.00 |
| 163888_at | 0.00 |
| 163889_at | 0.00 |
| 163890_at | 0.00 |
| 163891_at | 0.21 |
| 163892_at | 0.00 |
| 163893_at | 0.01 |
| 163894_at | 0.00 |
| 163895_at | 0.00 |
| 163896_at | 0.00 |
| 163897_i_at | 0.00 |
| 163898_at | 0.00 |
| 163899_at | 0.00 |
| 163900_at | 0.00 |
| 163901_at | 0.00 |
| 163902_at | 0.06 |
| 163903_at | 0.00 |
| 163904_at | 0.00 |
| 163905_at | 0.00 |
| 163906_at | 0.00 |
| 163907_at | 0.00 |
| 163908_at | 0.00 |
| 163909_at | 0.00 |

| | |
|---|---|
| 163910_i_at | 0.00 |
| 163911_at | 0.00 |
| 163912_at | 0.00 |
| 163913_at | 0.00 |
| 163914_at | 0.00 |
| 163915_at | 0.00 |
| 163916_i_at | 0.00 |
| 163917_at | 0.00 |
| 163918_at | 0.00 |
| 163919_at | 0.00 |
| 163920_at | 0.00 |
| 163921_at | 0.00 |
| 163922_at | 0.00 |
| 163923_i_at | 0.00 |
| 163924_at | 0.00 |
| 163925_at | 0.00 |
| 163926_at | 0.00 |
| 163927_at | 0.00 |
| 163928_at | 0.00 |
| 163929_at | 0.00 |
| 163930_at | 0.00 |
| 163931_at | 0.33 |
| 163932_i_at | 0.03 |
| 163933_at | 0.00 |
| 163934_at | 0.00 |
| 163935_at | 0.00 |
| 163936_at | 0.00 |
| 163937_at | 0.00 |
| 163938_at | 0.01 |
| 163939_at | 0.00 |
| 163940_at | 0.00 |
| 163941_at | 0.00 |
| 163942_at | 0.00 |
| 163943_at | 0.00 |
| 163944_at | 0.00 |
| 163945_r_at | 0.00 |
| 163946_at | 0.00 |
| 163947_f_at | 0.00 |
| 163948_at | 0.00 |
| 163949_r_at | 0.00 |
| 163950_r_at | 0.00 |
| 163951_at | 0.00 |
| 163952_at | 0.00 |
| 163953_at | 0.00 |
| 163954_at | 0.00 |
| 163955_at | 0.00 |
| 163956_at | 0.00 |
| 163957_at | 0.00 |
| 163958_at | 0.00 |
| 163959_at | 0.00 |
| 163960_at | 0.00 |
| 163961_at | 0.00 |
| 163962_at | 0.12 |
| 163963_at | 0.00 |
| 163964_at | 0.00 |
| 163965_at | 0.00 |

| | |
|---|---|
| 163966_at | 0.00 |
| 163967_at | 0.02 |
| 163968_at | 0.00 |
| 163969_at | 0.00 |
| 163970_s_at | 0.00 |
| 163971_at | 0.00 |
| 163972_at | 0.00 |
| 163973_at | 0.00 |
| 163974_at | 0.00 |
| 163975_at | 0.00 |
| 163976_at | 0.00 |
| 163977_at | 0.00 |
| 163978_at | 0.00 |
| 163979_at | 0.00 |
| 163980_at | 0.00 |
| 163981_at | 0.18 |
| 163982_at | 0.00 |
| 163983_at | 0.00 |
| 163984_at | 0.00 |
| 163985_at | 0.00 |
| 163986_at | 0.00 |
| 163987_at | 0.00 |
| 163988_at | 0.00 |
| 163989_at | 0.00 |
| 163990_at | 0.00 |
| 163991_at | 0.00 |
| 163992_at | 0.00 |
| 163993_at | 0.00 |
| 163994_at | 0.00 |
| 163995_at | 0.00 |
| 163996_at | 0.00 |
| 163997_at | 0.00 |
| 163998_at | 0.00 |
| 163999_at | 0.23 |
| 164000_at | 0.00 |
| 164001_at | 0.00 |
| 164002_f_at | 0.00 |
| 164003_at | 0.00 |
| 164004_at | 0.00 |
| 164005_at | 0.00 |
| 164006_at | 0.00 |
| 164007_at | 0.00 |
| 164008_at | 0.00 |
| 164009_at | 0.00 |
| 164010_at | 0.00 |
| 164011_at | 0.00 |
| 164012_at | 0.00 |
| 164013_i_at | 0.00 |
| 164014_at | 0.00 |
| 164015_at | 0.00 |
| 164016_at | 0.00 |
| 164017_at | 0.00 |
| 164018_i_at | 0.00 |
| 164019_at | 0.00 |
| 164020_at | 0.01 |
| 164021_at | 0.11 |

| | |
|---|---|
| 164022_i_at | 0.00 |
| 164023_at | 0.00 |
| 164024_at | 0.00 |
| 164025_at | 0.00 |
| 164026_at | 0.00 |
| 164027_at | 0.02 |
| 164028_at | 0.64 |
| 164029_at | 0.00 |
| 164030_at | 0.00 |
| 164031_at | 0.00 |
| 164032_at | 0.00 |
| 164033_at | 0.00 |
| 164034_at | 0.00 |
| 164035_at | 0.00 |
| 164036_at | 0.00 |
| 164037_at | 0.00 |
| 164038_at | 0.00 |
| 164039_at | 0.00 |
| 164040_at | 0.00 |
| 164041_at | 0.00 |
| 164042_at | 0.00 |
| 164043_at | 0.00 |
| 164044_r_at | 0.00 |
| 164045_at | 0.00 |
| 164046_f_at | 0.00 |
| 164047_at | 0.00 |
| 164048_at | 0.00 |
| 164049_at | 0.00 |
| 164050_at | 0.00 |
| 164051_at | 0.00 |
| 164052_at | 0.00 |
| 164053_at | 0.00 |
| 164054_at | 0.00 |
| 164055_at | 0.05 |
| 164056_at | 0.00 |
| 164057_at | 0.00 |
| 164058_i_at | 0.00 |
| 164059_f_at | 0.00 |
| 164060_at | 0.00 |
| 164061_at | 0.00 |
| 164062_at | 0.02 |
| 164063_at | 0.00 |
| 164064_at | 0.00 |
| 164065_at | 0.00 |
| 164066_at | 0.02 |
| 164067_at | 0.00 |
| 164068_at | 0.00 |
| 164069_at | 0.00 |
| 164070_at | 0.00 |
| 164071_at | 0.00 |
| 164072_at | 0.00 |
| 164073_at | 0.00 |
| 164074_at | 0.05 |
| 164075_at | 0.00 |
| 164076_at | 0.00 |
| 164077_at | 0.00 |

| | |
|---|---|
| 164078_at | 0.00 |
| 164079_at | 0.00 |
| 164080_at | 0.00 |
| 164081_at | 0.00 |
| 164082_at | 0.00 |
| 164083_at | 0.00 |
| 164084_at | 0.00 |
| 164085_at | 0.00 |
| 164086_at | 0.00 |
| 164087_at | 0.00 |
| 164088_at | 0.00 |
| 164089_at | 0.00 |
| 164090_i_at | 0.00 |
| 164091_at | 0.00 |
| 164092_at | 0.00 |
| 164093_at | 0.00 |
| 164094_at | 0.00 |
| 164095_i_at | 0.00 |
| 164096_at | 0.00 |
| 164097_at | 0.00 |
| 164098_at | 0.01 |
| 164099_at | 0.00 |
| 164100_at | 0.00 |
| 164101_i_at | 0.00 |
| 164102_at | 0.00 |
| 164103_at | 0.00 |
| 164104_at | 0.00 |
| 164105_at | 0.00 |
| 164106_at | 0.00 |
| 164107_at | 0.00 |
| 164108_at | 0.00 |
| 164109_at | 0.00 |
| 164110_at | 0.18 |
| 164111_at | 0.00 |
| 164112_at | 0.00 |
| 164113_at | 0.00 |
| 164114_at | 0.00 |
| 164115_at | 0.00 |
| 164116_at | 0.00 |
| 164117_at | 0.00 |
| 164118_at | 0.00 |
| 164119_at | 0.00 |
| 164120_at | 0.00 |
| 164121_at | 0.00 |
| 164122_at | 0.00 |
| 164123_at | 0.00 |
| 164124_at | 0.00 |
| 164125_at | 0.00 |
| 164126_at | 0.00 |
| 164127_at | 0.00 |
| 164128_at | 0.11 |
| 164129_at | 0.00 |
| 164130_at | 0.00 |
| 164131_at | 0.00 |
| 164132_at | 0.00 |
| 164133_at | 0.00 |

| | |
|---|---|
| 164134_at | 0.15 |
| 164135_at | 0.00 |
| 164136_at | 0.00 |
| 164137_at | 0.00 |
| 164138_at | 0.00 |
| 164139_at | 0.00 |
| 164140_at | 0.00 |
| 164141_at | 0.00 |
| 164142_at | 0.00 |
| 164143_at | 0.00 |
| 164144_at | 0.00 |
| 164145_at | 0.00 |
| 164146_i_at | 0.00 |
| 164147_at | 0.00 |
| 164148_at | 0.00 |
| 164149_at | 0.00 |
| 164150_at | 0.00 |
| 164151_at | 0.00 |
| 164152_at | 0.00 |
| 164153_at | 0.00 |
| 164154_at | 0.00 |
| 164155_at | 0.00 |
| 164156_at | 0.00 |
| 164157_at | 0.00 |
| 164158_at | 0.15 |
| 164159_at | 0.00 |
| 164160_at | 0.00 |
| 164161_s_at | 0.00 |
| 164162_at | 0.03 |
| 164163_at | 0.00 |
| 164164_at | 0.00 |
| 164165_at | 0.00 |
| 164166_at | 0.00 |
| 164167_at | 0.00 |
| 164168_at | 0.00 |
| 164169_at | 0.05 |
| 164170_at | 0.00 |
| 164171_at | 0.00 |
| 164172_at | 0.00 |
| 164173_at | 0.00 |
| 164174_f_at | 0.00 |
| 164175_at | 0.02 |
| 164176_at | 0.00 |
| 164177_at | 0.00 |
| 164178_i_at | 0.00 |
| 164179_at | 0.00 |
| 164180_s_at | 0.00 |
| 164181_f_at | 0.00 |
| 164182_at | 0.00 |
| 164183_at | 0.00 |
| 164184_at | 0.00 |
| 164185_at | 0.00 |
| 164186_at | 0.00 |
| 164187_at | 0.00 |
| 164188_f_at | 0.00 |
| 164189_at | 0.00 |

| | |
|---|---|
| 164190_at | 0.00 |
| 164191_at | 0.00 |
| 164192_at | 0.00 |
| 164193_at | 0.00 |
| 164194_at | 0.00 |
| 164195_at | 0.00 |
| 164196_at | 0.00 |
| 164197_at | 0.00 |
| 164198_at | 0.00 |
| 164199_at | 0.00 |
| 164200_i_at | 0.01 |
| 164201_at | 0.00 |
| 164202_at | 0.00 |
| 164203_at | 0.00 |
| 164204_at | 0.00 |
| 164205_at | 0.00 |
| 164206_at | 0.00 |
| 164207_at | 0.00 |
| 164208_at | 0.00 |
| 164209_at | 0.00 |
| 164210_r_at | 0.00 |
| 164211_at | 0.08 |
| 164212_at | 0.00 |
| 164213_at | 0.00 |
| 164214_at | 0.00 |
| 164215_at | 0.00 |
| 164216_at | 0.00 |
| 164217_at | 0.00 |
| 164218_i_at | 0.00 |
| 164219_at | 0.00 |
| 164220_at | 0.00 |
| 164221_at | 0.00 |
| 164222_at | 0.00 |
| 164223_at | 0.00 |
| 164224_at | 0.00 |
| 164225_at | 0.00 |
| 164226_at | 0.00 |
| 164227_at | 0.00 |
| 164228_at | 0.00 |
| 164229_at | 0.00 |
| 164230_at | 0.01 |
| 164231_at | 0.00 |
| 164232_at | 0.00 |
| 164233_at | 0.00 |
| 164234_at | 0.00 |
| 164235_at | 0.00 |
| 164236_at | 0.00 |
| 164237_at | 0.00 |
| 164238_at | 0.00 |
| 164239_at | 0.00 |
| 164240_at | 0.00 |
| 164241_at | 0.00 |
| 164242_r_at | 0.00 |
| 164243_at | 0.00 |
| 164244_at | 0.00 |
| 164245_at | 0.00 |

| | |
|---|---|
| 164246_at | 0.20 |
| 164247_at | 0.00 |
| 164248_at | 0.00 |
| 164249_at | 0.00 |
| 164250_at | 0.02 |
| 164251_at | 0.00 |
| 164252_at | 0.00 |
| 164253_at | 0.00 |
| 164254_at | 0.00 |
| 164255_at | 0.00 |
| 164256_at | 0.00 |
| 164257_at | 0.02 |
| 164258_at | 0.00 |
| 164259_at | 0.00 |
| 164260_at | 0.00 |
| 164261_at | 0.00 |
| 164262_at | 0.00 |
| 164263_at | 0.00 |
| 164264_s_at | 0.00 |
| 164265_at | 0.00 |
| 164266_at | 0.00 |
| 164267_at | 0.00 |
| 164268_at | 0.00 |
| 164269_at | 0.00 |
| 164270_r_at | 0.00 |
| 164271_r_at | 0.00 |
| 164272_at | 0.00 |
| 164273_at | 0.00 |
| 164274_f_at | 0.00 |
| 164275_at | 0.00 |
| 164276_at | 0.00 |
| 164277_at | 0.00 |
| 164278_at | 0.00 |
| 164279_f_at | 0.00 |
| 164280_f_at | 0.00 |
| 164281_at | 0.00 |
| 164282_at | 0.00 |
| 164283_r_at | 0.00 |
| 164284_r_at | 0.00 |
| 164285_f_at | 0.00 |
| 164286_f_at | 0.00 |
| 164287_i_at | 0.00 |
| 164288_r_at | 0.00 |
| 164289_f_at | 0.00 |
| 164290_i_at | 0.00 |
| 164291_r_at | 0.00 |
| 164292_r_at | 0.00 |
| 164293_at | 0.01 |
| 164294_r_at | 0.00 |
| 164295_i_at | 0.00 |
| 164296_r_at | 0.00 |
| 164297_r_at | 0.00 |
| 164298_r_at | 0.00 |
| 164299_f_at | 0.00 |
| 164300_i_at | 0.00 |
| 164301_f_at | 0.00 |

| | |
|---|---|
| 164302_at | 0.00 |
| 164303_at | 0.00 |
| 164304_r_at | 0.00 |
| 164305_at | 0.00 |
| 164306_f_at | 0.93 |
| 164307_f_at | 0.00 |
| 164308_f_at | 0.00 |
| 164309_at | 0.00 |
| 164310_f_at | 0.00 |
| 164311_f_at | 0.00 |
| 164312_i_at | 0.00 |
| 164313_f_at | 0.00 |
| 164314_f_at | 0.00 |
| 164315_f_at | 0.00 |
| 164316_f_at | 0.00 |
| 164317_f_at | 0.00 |
| 164318_f_at | 0.00 |
| 164319_r_at | 0.00 |
| 164320_f_at | 0.00 |
| 164321_at | 0.00 |
| 164322_f_at | 0.00 |
| 164323_at | 0.00 |
| 164324_f_at | 0.00 |
| 164325_f_at | 0.00 |
| 164326_r_at | 0.01 |
| 164327_at | 0.00 |
| 164328_f_at | 0.00 |
| 164329_f_at | 0.00 |
| 164330_f_at | 0.00 |
| 164331_f_at | 0.00 |
| 164332_r_at | 0.00 |
| 164333_r_at | 0.00 |
| 164334_r_at | 0.00 |
| 164335_f_at | 0.00 |
| 164336_i_at | 0.00 |
| 164337_at | 0.00 |
| 164338_f_at | 0.00 |
| 164339_f_at | 0.00 |
| 164340_at | 0.00 |
| 164341_f_at | 0.24 |
| 164342_f_at | 0.00 |
| 164343_at | 0.00 |
| 164344_f_at | 0.00 |
| 164345_r_at | 0.00 |
| 164346_f_at | 0.00 |
| 164347_i_at | 0.00 |
| 164348_r_at | 0.00 |
| 164349_r_at | 0.00 |
| 164350_i_at | 0.00 |
| 164351_r_at | 0.00 |
| 164352_f_at | 0.00 |
| 164353_r_at | 0.00 |
| 164354_r_at | 0.00 |
| 164355_f_at | 0.00 |
| 164356_at | 0.05 |
| 164357_i_at | 0.00 |

| | |
|---|---|
| 164358_f_at | 0.00 |
| 164359_i_at | 0.00 |
| 164360_r_at | 0.00 |
| 164361_r_at | 0.00 |
| 164362_at | 0.00 |
| 164363_at | 0.00 |
| 164364_at | 0.00 |
| 164365_r_at | 0.00 |
| 164366_f_at | 0.00 |
| 164367_f_at | 0.00 |
| 164368_i_at | 0.00 |
| 164369_at | 0.00 |
| 164370_f_at | 0.00 |
| 164371_i_at | 0.01 |
| 164372_at | 0.00 |
| 164373_r_at | 0.00 |
| 164374_f_at | 0.00 |
| 164375_f_at | 0.00 |
| 164376_r_at | 0.00 |
| 164377_f_at | 0.00 |
| 164378_f_at | 0.00 |
| 164379_i_at | 0.00 |
| 164380_i_at | 0.00 |
| 164381_i_at | 0.00 |
| 164382_at | 0.00 |
| 164383_f_at | 0.00 |
| 164384_i_at | 0.00 |
| 164385_r_at | 0.00 |
| 164386_i_at | 0.00 |
| 164387_r_at | 0.00 |
| 164388_r_at | 0.00 |
| 164389_at | 0.00 |
| 164390_f_at | 0.00 |
| 164391_at | 0.00 |
| 164392_at | 0.00 |
| 164393_f_at | 0.00 |
| 164394_at | 0.00 |
| 164395_r_at | 0.00 |
| 164396_at | 0.00 |
| 164397_f_at | 0.00 |
| 164398_at | 0.00 |
| 164399_r_at | 0.00 |
| 164400_s_at | 0.15 |
| 164401_at | 0.00 |
| 164402_r_at | 0.00 |
| 164403_i_at | 0.00 |
| 164404_r_at | 0.00 |
| 164405_f_at | 0.00 |
| 164406_r_at | 0.00 |
| 164407_f_at | 0.00 |
| 164408_at | 0.00 |
| 164409_i_at | 0.00 |
| 164410_at | 0.00 |
| 164411_f_at | 0.00 |
| 164412_at | 0.00 |
| 164413_i_at | 0.00 |

| | |
|---|---|
| 164414_r_at | 0.00 |
| 164415_r_at | 0.00 |
| 164416_at | 0.00 |
| 164417_f_at | 0.00 |
| 164418_f_at | 0.00 |
| 164419_i_at | 0.00 |
| 164420_i_at | 0.00 |
| 164421_i_at | 0.00 |
| 164422_at | 0.00 |
| 164423_at | 0.00 |
| 164424_at | 0.00 |
| 164425_f_at | 0.01 |
| 164426_f_at | 0.00 |
| 164427_f_at | 0.00 |
| 164428_i_at | 0.00 |
| 164429_f_at | 0.00 |
| 164430_at | 0.00 |
| 164431_at | 0.00 |
| 164432_at | 0.00 |
| 164433_at | 0.00 |
| 164434_f_at | 0.00 |
| 164435_i_at | 0.00 |
| 164436_at | 0.00 |
| 164437_i_at | 0.00 |
| 164438_i_at | 0.00 |
| 164439_f_at | 0.00 |
| 164440_f_at | 0.00 |
| 164441_at | 0.00 |
| 164442_f_at | 0.00 |
| 164443_at | 0.00 |
| 164444_r_at | 0.00 |
| 164445_i_at | 0.00 |
| 164446_r_at | 0.00 |
| 164447_r_at | 0.00 |
| 164448_r_at | 0.00 |
| 164449_at | 0.00 |
| 164450_r_at | 0.00 |
| 164451_i_at | 0.00 |
| 164452_f_at | 0.00 |
| 164453_i_at | 0.00 |
| 164454_i_at | 0.00 |
| 164455_at | 0.00 |
| 164456_f_at | 0.00 |
| 164457_r_at | 0.00 |
| 164458_f_at | 0.00 |
| 164459_r_at | 0.00 |
| 164460_at | 0.00 |
| 164461_f_at | 0.03 |
| 164462_f_at | 0.00 |
| 164463_r_at | 0.00 |
| 164464_i_at | 0.00 |
| 164465_f_at | 0.00 |
| 164466_i_at | 0.01 |
| 164467_f_at | 0.32 |
| 164468_f_at | 0.00 |
| 164469_i_at | 0.00 |

| | |
|---|---|
| 164470_r_at | 0.00 |
| 164471_f_at | 0.00 |
| 164472_f_at | 0.00 |
| 164473_i_at | 0.00 |
| 164474_r_at | 0.00 |
| 164475_i_at | 0.00 |
| 164476_r_at | 0.00 |
| 164477_f_at | 0.00 |
| 164478_r_at | 0.00 |
| 164479_f_at | 0.00 |
| 164480_f_at | 0.00 |
| 164481_i_at | 0.00 |
| 164482_i_at | 0.00 |
| 164483_r_at | 0.00 |
| 164484_f_at | 0.00 |
| 164485_at | 0.00 |
| 164486_f_at | 0.00 |
| 164487_at | 0.00 |
| 164488_i_at | 0.00 |
| 164489_i_at | 0.00 |
| 164490_r_at | 0.00 |
| 164491_at | 0.00 |
| 164492_f_at | 0.00 |
| 164493_i_at | 0.00 |
| 164494_at | 0.00 |
| 164495_i_at | 0.00 |
| 164496_r_at | 0.00 |
| 164497_r_at | 0.00 |
| 164498_r_at | 0.00 |
| 164499_r_at | 0.00 |
| 164500_i_at | 0.00 |
| 164501_f_at | 0.00 |
| 164502_r_at | 0.00 |
| 164503_at | 0.00 |
| 164504_r_at | 0.10 |
| 164505_r_at | 0.00 |
| 164506_r_at | 0.00 |
| 164507_f_at | 0.00 |
| 164508_r_at | 0.00 |
| 164509_i_at | 0.00 |
| 164510_i_at | 0.01 |
| 164511_r_at | 0.00 |
| 164512_i_at | 0.00 |
| 164513_f_at | 0.00 |
| 164514_r_at | 0.00 |
| 164515_i_at | 0.00 |
| 164516_i_at | 0.00 |
| 164517_r_at | 0.00 |
| 164518_at | 0.00 |
| 164519_i_at | 0.06 |
| 164520_f_at | 0.00 |
| 164521_r_at | 0.00 |
| 164522_r_at | 0.00 |
| 164523_f_at | 0.00 |
| 164524_at | 0.00 |
| 164525_f_at | 0.00 |

| | |
|---|---|
| 164526_f_at | 0.00 |
| 164527_f_at | 0.00 |
| 164528_at | 0.00 |
| 164529_f_at | 0.00 |
| 164530_f_at | 0.00 |
| 164531_f_at | 0.00 |
| 164532_r_at | 0.00 |
| 164533_f_at | 0.00 |
| 164534_at | 0.00 |
| 164535_r_at | 0.00 |
| 164536_f_at | 0.00 |
| 164537_r_at | 0.00 |
| 164538_f_at | 0.00 |
| 164539_f_at | 0.00 |
| 164540_i_at | 0.00 |
| 164541_f_at | 0.00 |
| 164542_f_at | 0.00 |
| 164543_f_at | 0.00 |
| 164544_f_at | 0.00 |
| 164545_f_at | 0.00 |
| 164546_i_at | 0.00 |
| 164547_r_at | 0.00 |
| 164548_i_at | 0.00 |
| 164549_r_at | 0.00 |
| 164550_f_at | 0.00 |
| 164551_r_at | 0.00 |
| 164552_i_at | 0.00 |
| 164553_r_at | 0.00 |
| 164554_i_at | 0.00 |
| 164555_i_at | 0.00 |
| 164556_r_at | 0.00 |
| 164557_f_at | 0.00 |
| 164558_at | 0.00 |
| 164559_at | 0.00 |
| 164560_at | 0.00 |
| 164561_f_at | 0.00 |
| 164562_r_at | 0.00 |
| 164563_r_at | 0.00 |
| 164564_r_at | 0.00 |
| 164565_at | 0.00 |
| 164566_f_at | 0.00 |
| 164567_r_at | 0.00 |
| 164568_i_at | 0.00 |
| 164569_i_at | 0.00 |
| 164570_r_at | 0.00 |
| 164571_i_at | 0.00 |
| 164572_s_at | 0.02 |
| 164573_f_at | 0.00 |
| 164574_at | 0.00 |
| 164575_f_at | 0.00 |
| 164576_r_at | 0.00 |
| 164577_f_at | 0.00 |
| 164578_i_at | 0.00 |
| 164579_r_at | 0.00 |
| 164580_at | 0.00 |
| 164581_at | 0.00 |

| | |
|---|---|
| 164582_f_at | 0.00 |
| 164583_i_at | 0.00 |
| 164584_f_at | 0.00 |
| 164585_f_at | 0.00 |
| 164586_at | 0.00 |
| 164587_f_at | 0.00 |
| 164588_at | 0.00 |
| 164589_f_at | 0.00 |
| 164590_r_at | 0.00 |
| 164591_at | 0.00 |
| 164592_at | 0.00 |
| 164593_r_at | 0.00 |
| 164594_f_at | 0.00 |
| 164595_at | 0.00 |
| 164596_i_at | 0.00 |
| 164597_r_at | 0.00 |
| 164598_i_at | 0.00 |
| 164599_at | 0.00 |
| 164600_i_at | 0.00 |
| 164601_f_at | 0.00 |
| 164602_at | 0.00 |
| 164603_f_at | 0.00 |
| 164604_f_at | 0.00 |
| 164605_at | 0.00 |
| 164606_f_at | 0.00 |
| 164607_i_at | 0.00 |
| 164608_at | 0.00 |
| 164609_at | 0.00 |
| 164610_f_at | 0.00 |
| 164611_r_at | 0.00 |
| 164612_at | 0.00 |
| 164613_f_at | 0.00 |
| 164614_i_at | 0.00 |
| 164615_f_at | 0.00 |
| 164616_at | 0.00 |
| 164617_i_at | 0.00 |
| 164618_f_at | 0.00 |
| 164619_at | 0.00 |
| 164620_r_at | 0.00 |
| 164621_i_at | 0.00 |
| 164622_f_at | 0.00 |
| 164623_at | 0.00 |
| 164624_f_at | 0.00 |
| 164625_at | 0.00 |
| 164626_i_at | 0.00 |
| 164627_r_at | 0.00 |
| 164628_i_at | 0.00 |
| 164629_at | 0.00 |
| 164630_r_at | 0.00 |
| 164631_f_at | 0.00 |
| 164632_i_at | 0.00 |
| 164633_f_at | 0.00 |
| 164634_f_at | 0.00 |
| 164635_i_at | 0.00 |
| 164636_f_at | 0.00 |
| 164637_f_at | 0.00 |

| | |
|---|---|
| 164638_f_at | 0.00 |
| 164639_i_at | 0.00 |
| 164640_r_at | 0.00 |
| 164641_r_at | 0.00 |
| 164642_i_at | 0.00 |
| 164643_i_at | 0.00 |
| 164644_r_at | 0.00 |
| 164645_f_at | 0.00 |
| 164646_at | 0.00 |
| 164647_r_at | 0.00 |
| 164648_r_at | 0.00 |
| 164649_i_at | 0.00 |
| 164650_i_at | 0.00 |
| 164651_i_at | 0.00 |
| 164652_r_at | 0.00 |
| 164653_at | 0.00 |
| 164654_f_at | 0.00 |
| 164655_f_at | 0.00 |
| 164656_i_at | 0.00 |
| 164657_r_at | 0.00 |
| 164658_at | 0.00 |
| 164659_f_at | 0.00 |
| 164660_r_at | 0.00 |
| 164661_r_at | 0.00 |
| 164662_i_at | 0.00 |
| 164663_r_at | 0.00 |
| 164664_i_at | 0.00 |
| 164665_at | 0.00 |
| 164666_f_at | 0.02 |
| 164667_f_at | 0.00 |
| 164668_at | 0.00 |
| 164669_at | 0.00 |
| 164670_at | 0.00 |
| 164671_i_at | 0.00 |
| 164672_f_at | 0.00 |
| 164673_r_at | 0.00 |
| 164674_i_at | 0.00 |
| 164675_i_at | 0.00 |
| 164676_f_at | 0.00 |
| 164677_r_at | 0.00 |
| 164678_i_at | 0.01 |
| 164679_at | 0.00 |
| 164680_s_at | 0.00 |
| 164681_i_at | 0.00 |
| 164682_at | 0.00 |
| 164683_r_at | 0.04 |
| 164684_r_at | 0.00 |
| 164685_r_at | 0.00 |
| 164686_f_at | 0.00 |
| 164687_i_at | 0.00 |
| 164688_r_at | 0.00 |
| 164689_i_at | 0.01 |
| 164690_at | 0.00 |
| 164691_r_at | 0.00 |
| 164692_i_at | 0.00 |
| 164693_r_at | 0.00 |

| | |
|---|---|
| 164694_r_at | 0.00 |
| 164695_f_at | 0.00 |
| 164696_r_at | 0.00 |
| 164697_i_at | 0.00 |
| 164698_at | 0.00 |
| 164699_f_at | 0.00 |
| 164700_r_at | 0.00 |
| 164701_f_at | 0.00 |
| 164702_r_at | 0.00 |
| 164703_i_at | 0.00 |
| 164704_s_at | 0.07 |
| 164705_at | 0.00 |
| 164706_at | 0.00 |
| 164707_r_at | 0.00 |
| 164708_i_at | 0.00 |
| 164709_at | 0.00 |
| 164710_f_at | 0.00 |
| 164711_r_at | 0.00 |
| 164712_at | 0.00 |
| 164713_r_at | 0.00 |
| 164714_f_at | 0.00 |
| 164715_i_at | 0.00 |
| 164716_r_at | 0.00 |
| 164717_f_at | 0.00 |
| 164718_r_at | 0.00 |
| 164719_at | 0.00 |
| 164720_i_at | 0.00 |
| 164721_i_at | 0.00 |
| 164722_f_at | 0.00 |
| 164723_f_at | 0.00 |
| 164724_r_at | 0.00 |
| 164725_f_at | 0.00 |
| 164726_i_at | 0.00 |
| 164727_r_at | 0.00 |
| 164728_i_at | 0.00 |
| 164729_f_at | 0.00 |
| 164730_i_at | 0.00 |
| 164731_f_at | 0.00 |
| 164732_i_at | 0.00 |
| 164733_at | 0.00 |
| 164734_f_at | 0.00 |
| 164735_at | 0.00 |
| 164736_r_at | 0.00 |
| 164737_r_at | 0.00 |
| 164738_i_at | 0.00 |
| 164739_at | 0.00 |
| 164740_at | 0.00 |
| 164741_r_at | 0.00 |
| 164742_i_at | 0.01 |
| 164743_i_at | 0.00 |
| 164744_at | 0.00 |
| 164745_r_at | 0.00 |
| 164746_f_at | 0.00 |
| 164747_i_at | 0.00 |
| 164748_at | 0.00 |
| 164749_f_at | 0.00 |

| | |
|---|---|
| 164750_f_at | 0.00 |
| 164751_f_at | 0.00 |
| 164752_i_at | 0.00 |
| 164753_f_at | 0.00 |
| 164754_at | 0.00 |
| 164755_i_at | 0.00 |
| 164756_r_at | 0.00 |
| 164757_at | 0.00 |
| 164758_i_at | 0.00 |
| 164759_i_at | 0.00 |
| 164760_f_at | 0.00 |
| 164761_f_at | 0.00 |
| 164762_f_at | 0.00 |
| 164763_f_at | 0.00 |
| 164764_i_at | 0.00 |
| 164765_at | 0.00 |
| 164766_f_at | 0.00 |
| 164767_at | 0.00 |
| 164768_f_at | 0.00 |
| 164769_f_at | 0.00 |
| 164770_i_at | 0.00 |
| 164771_at | 0.00 |
| 164772_f_at | 0.00 |
| 164773_r_at | 0.00 |
| 164774_f_at | 0.00 |
| 164775_f_at | 0.00 |
| 164776_f_at | 0.00 |
| 164777_i_at | 0.00 |
| 164778_r_at | 0.00 |
| 164779_r_at | 0.00 |
| 164780_at | 0.00 |
| 164781_at | 0.00 |
| 164782_i_at | 0.00 |
| 164783_f_at | 0.00 |
| 164784_at | 0.00 |
| 164785_f_at | 0.00 |
| 164786_at | 0.00 |
| 164787_at | 0.00 |
| 164788_f_at | 0.00 |
| 164789_at | 0.00 |
| 164790_at | 0.00 |
| 164791_i_at | 0.00 |
| 164792_at | 0.00 |
| 164793_at | 0.00 |
| 164794_i_at | 0.00 |
| 164795_f_at | 0.00 |
| 164796_s_at | 0.00 |
| 164797_f_at | 0.00 |
| 164798_i_at | 0.00 |
| 164799_at | 0.00 |
| 164800_at | 0.00 |
| 164801_at | 0.00 |
| 164802_i_at | 0.00 |
| 164803_r_at | 0.00 |
| 164804_f_at | 0.00 |
| 164805_at | 0.00 |

| | |
|---|---|
| 164806_r_at | 0.00 |
| 164807_at | 0.00 |
| 164808_i_at | 0.00 |
| 164809_at | 0.00 |
| 164810_r_at | 0.00 |
| 164811_at | 0.00 |
| 164812_i_at | 0.00 |
| 164813_f_at | 0.00 |
| 164814_i_at | 0.00 |
| 164815_i_at | 0.00 |
| 164816_f_at | 0.00 |
| 164817_at | 0.00 |
| 164818_at | 0.00 |
| 164819_f_at | 0.00 |
| 164820_i_at | 0.00 |
| 164821_f_at | 0.00 |
| 164822_r_at | 0.00 |
| 164823_r_at | 0.00 |
| 164824_i_at | 0.00 |
| 164825_at | 0.00 |
| 164826_at | 0.00 |
| 164827_r_at | 0.00 |
| 164828_r_at | 0.00 |
| 164829_f_at | 0.00 |
| 164830_r_at | 0.00 |
| 164831_f_at | 0.00 |
| 164832_f_at | 0.00 |
| 164833_f_at | 0.00 |
| 164834_r_at | 0.00 |
| 164835_at | 0.00 |
| 164836_i_at | 0.00 |
| 164837_f_at | 0.00 |
| 164838_f_at | 0.00 |
| 164839_r_at | 0.00 |
| 164840_r_at | 0.00 |
| 164841_at | 0.00 |
| 164842_i_at | 0.00 |
| 164843_i_at | 0.00 |
| 164844_at | 0.00 |
| 164845_f_at | 0.00 |
| 164846_f_at | 0.00 |
| 164847_f_at | 0.00 |
| 164848_f_at | 0.00 |
| 164849_f_at | 0.00 |
| 164850_f_at | 0.00 |
| 164851_f_at | 0.00 |
| 164852_at | 0.00 |
| 164853_r_at | 0.00 |
| 164854_r_at | 0.00 |
| 164855_at | 0.00 |
| 164856_at | 0.00 |
| 164857_f_at | 0.00 |
| 164858_f_at | 0.00 |
| 164859_f_at | 0.00 |
| 164860_at | 0.00 |
| 164861_r_at | 0.00 |

| | |
|---|---|
| 164862_f_at | 0.00 |
| 164863_r_at | 0.00 |
| 164864_i_at | 0.00 |
| 164865_i_at | 0.00 |
| 164866_f_at | 0.00 |
| 164867_i_at | 0.00 |
| 164868_f_at | 0.00 |
| 164869_r_at | 0.00 |
| 164870_f_at | 0.00 |
| 164871_i_at | 0.04 |
| 164872_i_at | 0.00 |
| 164873_r_at | 0.00 |
| 164874_f_at | 0.00 |
| 164875_r_at | 0.00 |
| 164876_i_at | 0.01 |
| 164877_r_at | 0.00 |
| 164878_r_at | 0.00 |
| 164879_i_at | 0.00 |
| 164880_at | 0.00 |
| 164881_f_at | 0.00 |
| 164882_r_at | 0.00 |
| 164883_at | 0.00 |
| 164884_i_at | 0.00 |
| 164885_f_at | 0.00 |
| 164886_f_at | 0.00 |
| 164887_i_at | 0.00 |
| 164888_f_at | 0.00 |
| 164889_f_at | 0.00 |
| 164890_r_at | 0.00 |
| 164891_i_at | 0.00 |
| 164892_at | 0.00 |
| 164893_f_at | 0.01 |
| 164894_at | 0.00 |
| 164895_f_at | 0.00 |
| 164896_f_at | 0.00 |
| 164897_f_at | 0.00 |
| 164898_i_at | 0.00 |
| 164899_r_at | 0.01 |
| 164900_at | 0.00 |
| 164901_r_at | 0.00 |
| 164902_r_at | 0.00 |
| 164903_i_at | 0.00 |
| 164904_i_at | 0.00 |
| 164905_r_at | 0.00 |
| 164906_f_at | 0.00 |
| 164907_f_at | 0.00 |
| 164908_f_at | 0.00 |
| 164909_r_at | 0.00 |
| 164910_s_at | 0.00 |
| 164911_i_at | 0.00 |
| 164912_r_at | 0.00 |
| 164913_r_at | 0.00 |
| 164914_at | 0.00 |
| 164915_i_at | 0.00 |
| 164916_r_at | 0.00 |
| 164917_at | 0.00 |

| | |
|---|---|
| 164918_i_at | 0.00 |
| 164919_at | 0.00 |
| 164920_r_at | 0.00 |
| 164921_r_at | 0.00 |
| 164922_at | 0.00 |
| 164923_f_at | 0.00 |
| 164924_at | 0.00 |
| 164925_i_at | 0.00 |
| 164926_r_at | 0.00 |
| 164927_i_at | 0.00 |
| 164928_f_at | 0.00 |
| 164929_f_at | 0.00 |
| 164930_at | 0.00 |
| 164931_r_at | 0.00 |
| 164932_f_at | 0.00 |
| 164933_f_at | 0.00 |
| 164934_r_at | 0.00 |
| 164935_r_at | 0.00 |
| 164936_at | 0.00 |
| 164937_r_at | 0.00 |
| 164938_f_at | 0.00 |
| 164939_f_at | 0.00 |
| 164940_r_at | 0.00 |
| 164941_f_at | 0.00 |
| 164942_r_at | 0.00 |
| 164943_at | 0.00 |
| 164944_f_at | 0.00 |
| 164945_at | 0.00 |
| 164946_f_at | 0.00 |
| 164947_f_at | 0.00 |
| 164948_i_at | 0.00 |
| 164949_at | 0.00 |
| 164950_f_at | 0.00 |
| 164951_f_at | 0.00 |
| 164952_i_at | 0.00 |
| 164953_i_at | 0.00 |
| 164954_r_at | 0.00 |
| 164955_f_at | 0.00 |
| 164956_f_at | 0.00 |
| 164957_i_at | 0.00 |
| 164958_f_at | 0.00 |
| 164959_r_at | 0.00 |
| 164960_i_at | 0.00 |
| 164961_at | 0.00 |
| 164962_r_at | 0.00 |
| 164963_r_at | 0.00 |
| 164964_f_at | 0.00 |
| 164965_f_at | 0.00 |
| 164966_r_at | 0.00 |
| 164967_f_at | 0.00 |
| 164968_i_at | 0.00 |
| 164969_r_at | 0.00 |
| 164970_at | 0.00 |
| 164971_r_at | 0.00 |
| 164972_r_at | 0.00 |
| 164973_r_at | 0.00 |

| | |
|---|---|
| 164974_f_at | 0.12 |
| 164975_i_at | 0.00 |
| 164976_r_at | 0.00 |
| 164977_f_at | 0.00 |
| 164978_f_at | 0.00 |
| 164979_i_at | 0.00 |
| 164980_at | 0.00 |
| 164981_f_at | 0.00 |
| 164982_at | 0.00 |
| 164983_r_at | 0.00 |
| 164984_i_at | 0.00 |
| 164985_i_at | 0.00 |
| 164986_i_at | 0.00 |
| 164987_s_at | 0.00 |
| 164988_f_at | 0.00 |
| 164989_i_at | 0.00 |
| 164990_f_at | 0.00 |
| 164991_f_at | 0.00 |
| 164992_f_at | 0.00 |
| 164993_r_at | 0.00 |
| 164994_i_at | 0.00 |
| 164995_at | 0.00 |
| 164996_f_at | 0.00 |
| 164997_i_at | 0.00 |
| 164998_f_at | 0.00 |
| 164999_i_at | 0.00 |
| 165000_r_at | 0.00 |
| 165001_r_at | 0.00 |
| 165002_f_at | 0.00 |
| 165003_f_at | 0.00 |
| 165004_i_at | 0.00 |
| 165005_r_at | 0.00 |
| 165006_f_at | 0.00 |
| 165007_i_at | 0.00 |
| 165008_r_at | 0.00 |
| 165009_f_at | 0.00 |
| 165010_f_at | 0.00 |
| 165011_f_at | 0.00 |
| 165012_at | 0.00 |
| 165013_i_at | 0.00 |
| 165014_r_at | 0.00 |
| 165015_f_at | 0.00 |
| 165016_f_at | 0.00 |
| 165017_i_at | 0.00 |
| 165018_f_at | 0.00 |
| 165019_f_at | 0.00 |
| 165020_f_at | 0.00 |
| 165021_at | 0.00 |
| 165022_f_at | 0.00 |
| 165023_r_at | 0.00 |
| 165024_f_at | 0.00 |
| 165025_f_at | 0.00 |
| 165026_i_at | 0.00 |
| 165027_at | 0.00 |
| 165028_r_at | 0.00 |
| 165029_f_at | 0.00 |

| | |
|---|---|
| 165030_r_at | 0.00 |
| 165031_at | 0.00 |
| 165032_i_at | 0.00 |
| 165033_f_at | 0.00 |
| 165034_i_at | 0.00 |
| 165035_i_at | 0.00 |
| 165036_at | 0.00 |
| 165037_r_at | 0.00 |
| 165038_i_at | 0.00 |
| 165039_r_at | 0.00 |
| 165040_r_at | 0.00 |
| 165041_f_at | 0.00 |
| 165042_f_at | 0.00 |
| 165043_i_at | 0.00 |
| 165044_i_at | 0.00 |
| 165045_r_at | 0.00 |
| 165046_i_at | 0.00 |
| 165047_r_at | 0.00 |
| 165048_f_at | 0.00 |
| 165049_r_at | 0.01 |
| 165050_at | 0.00 |
| 165051_at | 0.00 |
| 165052_f_at | 0.00 |
| 165053_at | 0.00 |
| 165054_i_at | 0.00 |
| 165055_i_at | 0.00 |
| 165056_r_at | 0.00 |
| 165057_i_at | 0.00 |
| 165058_f_at | 0.00 |
| 165059_at | 0.00 |
| 165060_r_at | 0.00 |
| 165061_i_at | 0.00 |
| 165062_f_at | 0.00 |
| 165063_r_at | 0.00 |
| 165064_at | 0.00 |
| 165065_r_at | 0.00 |
| 165066_at | 0.00 |
| 165067_f_at | 0.00 |
| 165068_at | 0.00 |
| 165069_at | 0.00 |
| 165070_i_at | 0.00 |
| 165071_r_at | 0.00 |
| 165072_f_at | 0.00 |
| 165073_f_at | 0.00 |
| 165074_i_at | 0.00 |
| 165075_r_at | 0.01 |
| 165076_f_at | 0.00 |
| 165077_i_at | 0.00 |
| 165078_i_at | 0.00 |
| 165079_i_at | 0.00 |
| 165080_r_at | 0.00 |
| 165081_i_at | 0.00 |
| 165082_r_at | 0.00 |
| 165083_r_at | 0.00 |
| 165084_r_at | 0.00 |
| 165085_f_at | 0.00 |

| | |
|---|---|
| 165086_f_at | 0.00 |
| 165087_r_at | 0.00 |
| 165088_r_at | 0.00 |
| 165089_f_at | 0.00 |
| 165090_r_at | 0.00 |
| 165091_r_at | 0.00 |
| 165092_at | 0.00 |
| 165093_i_at | 0.00 |
| 165094_i_at | 0.00 |
| 165095_i_at | 0.00 |
| 165096_f_at | 0.00 |
| 165097_r_at | 0.00 |
| 165098_f_at | 0.00 |
| 165099_f_at | 0.00 |
| 165100_i_at | 0.01 |
| 165101_r_at | 0.00 |
| 165102_at | 0.00 |
| 165103_f_at | 0.00 |
| 165104_f_at | 0.01 |
| 165105_f_at | 0.00 |
| 165106_i_at | 0.00 |
| 165107_f_at | 0.00 |
| 165108_r_at | 0.00 |
| 165109_r_at | 0.00 |
| 165110_f_at | 0.00 |
| 165111_f_at | 0.00 |
| 165112_f_at | 0.00 |
| 165113_f_at | 0.00 |
| 165114_i_at | 0.00 |
| 165115_i_at | 0.00 |
| 165116_f_at | 0.00 |
| 165117_r_at | 0.00 |
| 165118_i_at | 0.00 |
| 165119_f_at | 0.04 |
| 165120_at | 0.00 |
| 165121_r_at | 0.00 |
| 165122_i_at | 0.00 |
| 165123_at | 0.00 |
| 165124_r_at | 0.00 |
| 165125_f_at | 0.00 |
| 165126_i_at | 0.00 |
| 165127_i_at | 0.00 |
| 165128_r_at | 0.00 |
| 165129_i_at | 0.00 |
| 165130_r_at | 0.00 |
| 165131_f_at | 0.00 |
| 165132_r_at | 0.00 |
| 165133_f_at | 0.00 |
| 165134_f_at | 0.00 |
| 165135_i_at | 0.00 |
| 165136_i_at | 0.00 |
| 165137_r_at | 0.00 |
| 165138_f_at | 0.00 |
| 165139_f_at | 0.00 |
| 165140_f_at | 0.00 |
| 165141_at | 0.00 |

| | |
|---|---|
| 165142_f_at | 0.00 |
| 165143_at | 0.00 |
| 165144_r_at | 0.00 |
| 165145_s_at | 0.01 |
| 165146_f_at | 0.00 |
| 165147_r_at | 0.00 |
| 165148_i_at | 0.00 |
| 165149_i_at | 0.00 |
| 165150_i_at | 0.00 |
| 165151_r_at | 0.01 |
| 165152_f_at | 0.00 |
| 165153_f_at | 0.00 |
| 165154_f_at | 0.00 |
| 165155_f_at | 0.00 |
| 165156_i_at | 0.00 |
| 165157_r_at | 0.00 |
| 165158_i_at | 0.00 |
| 165159_r_at | 0.00 |
| 165160_at | 0.00 |
| 165161_at | 0.00 |
| 165162_f_at | 0.12 |
| 165163_i_at | 0.00 |
| 165164_i_at | 0.00 |
| 165165_r_at | 0.00 |
| 165166_f_at | 0.21 |
| 165167_r_at | 0.00 |
| 165168_i_at | 0.00 |
| 165169_at | 0.00 |
| 165170_i_at | 0.00 |
| 165171_r_at | 0.00 |
| 165172_r_at | 0.00 |
| 165173_at | 0.00 |
| 165174_i_at | 0.00 |
| 165175_r_at | 0.00 |
| 165176_f_at | 0.00 |
| 165177_f_at | 0.00 |
| 165178_f_at | 0.00 |
| 165179_f_at | 0.00 |
| 165180_f_at | 0.00 |
| 165181_i_at | 0.00 |
| 165182_i_at | 0.00 |
| 165183_s_at | 0.00 |
| 165184_at | 0.00 |
| 165185_r_at | 0.00 |
| 165186_at | 0.00 |
| 165187_i_at | 0.00 |
| 165188_r_at | 0.00 |
| 165189_i_at | 0.07 |
| 165190_f_at | 0.00 |
| 165191_at | 0.00 |
| 165192_r_at | 0.00 |
| 165193_i_at | 0.00 |
| 165194_at | 0.00 |
| 165195_r_at | 0.00 |
| 165196_i_at | 0.00 |
| 165197_f_at | 0.00 |

| | |
|---|---|
| 165198_f_at | 0.00 |
| 165199_f_at | 0.00 |
| 165200_f_at | 0.00 |
| 165201_i_at | 0.00 |
| 165202_r_at | 0.00 |
| 165203_i_at | 0.00 |
| 165204_i_at | 0.00 |
| 165205_r_at | 0.00 |
| 165206_r_at | 0.00 |
| 165207_f_at | 0.00 |
| 165208_f_at | 0.00 |
| 165209_f_at | 0.00 |
| 165210_at | 0.00 |
| 165211_f_at | 0.00 |
| 165212_i_at | 0.00 |
| 165213_r_at | 0.00 |
| 165214_i_at | 0.00 |
| 165215_f_at | 0.00 |
| 165216_r_at | 0.04 |
| 165217_at | 0.00 |
| 165218_f_at | 0.00 |
| 165219_s_at | 0.00 |
| 165220_r_at | 0.00 |
| 165221_at | 0.00 |
| 165222_s_at | 0.00 |
| 165223_f_at | 0.00 |
| 165224_i_at | 0.00 |
| 165225_f_at | 0.00 |
| 165226_r_at | 0.00 |
| 165227_r_at | 0.00 |
| 165228_r_at | 0.00 |
| 165229_r_at | 0.00 |
| 165230_r_at | 0.00 |
| 165231_r_at | 0.00 |
| 165232_at | 0.00 |
| 165233_f_at | 0.00 |
| 165234_f_at | 0.00 |
| 165235_f_at | 0.00 |
| 165236_f_at | 0.00 |
| 165237_f_at | 0.00 |
| 165238_r_at | 0.00 |
| 165239_f_at | 0.00 |
| 165240_i_at | 0.00 |
| 165241_f_at | 0.00 |
| 165242_r_at | 0.00 |
| 165243_i_at | 0.00 |
| 165244_f_at | 0.00 |
| 165245_i_at | 0.00 |
| 165246_r_at | 0.00 |
| 165247_f_at | 0.00 |
| 165248_f_at | 0.00 |
| 165249_f_at | 0.00 |
| 165250_at | 0.00 |
| 165251_i_at | 0.00 |
| 165252_r_at | 0.00 |
| 165253_f_at | 0.00 |

| | |
|---|---|
| 165254_i_at | 0.00 |
| 165255_at | 0.00 |
| 165256_f_at | 0.00 |
| 165257_r_at | 0.00 |
| 165258_r_at | 0.00 |
| 165259_r_at | 0.00 |
| 165260_f_at | 0.00 |
| 165261_i_at | 0.00 |
| 165262_i_at | 0.00 |
| 165263_at | 0.00 |
| 165264_f_at | 0.00 |
| 165265_f_at | 0.00 |
| 165266_i_at | 0.00 |
| 165267_i_at | 0.00 |
| 165268_i_at | 0.00 |
| 165269_i_at | 0.00 |
| 165270_i_at | 0.00 |
| 165271_at | 0.00 |
| 165272_i_at | 0.00 |
| 165273_f_at | 0.00 |
| 165274_f_at | 0.03 |
| 165275_r_at | 0.00 |
| 165276_r_at | 0.00 |
| 165277_at | 0.00 |
| 165278_r_at | 0.00 |
| 165279_r_at | 0.02 |
| 165280_f_at | 0.00 |
| 165281_at | 0.00 |
| 165282_r_at | 0.00 |
| 165283_r_at | 0.00 |
| 165284_at | 0.00 |
| 165285_i_at | 0.00 |
| 165286_r_at | 0.00 |
| 165287_i_at | 0.00 |
| 165288_f_at | 0.00 |
| 165289_r_at | 0.00 |
| 165290_f_at | 0.00 |
| 165291_i_at | 0.00 |
| 165292_i_at | 0.00 |
| 165293_i_at | 0.00 |
| 165294_at | 0.00 |
| 165295_at | 0.00 |
| 165296_f_at | 0.00 |
| 165297_at | 0.00 |
| 165298_f_at | 0.00 |
| 165299_f_at | 0.00 |
| 165300_i_at | 0.00 |
| 165301_f_at | 0.00 |
| 165302_at | 0.00 |
| 165303_f_at | 0.00 |
| 165304_at | 0.00 |
| 165305_i_at | 0.00 |
| 165306_f_at | 0.00 |
| 165307_r_at | 0.00 |
| 165308_f_at | 0.00 |
| 165309_r_at | 0.00 |

| | |
|---|---|
| 165310_r_at | 0.00 |
| 165311_i_at | 0.00 |
| 165312_f_at | 0.00 |
| 165313_at | 0.00 |
| 165314_f_at | 0.00 |
| 165315_at | 0.00 |
| 165316_at | 0.00 |
| 165317_r_at | 0.00 |
| 165318_f_at | 0.00 |
| 165319_at | 0.00 |
| 165320_f_at | 0.00 |
| 165321_r_at | 0.00 |
| 165322_i_at | 0.00 |
| 165323_at | 0.00 |
| 165324_at | 0.00 |
| 165325_r_at | 0.00 |
| 165326_at | 0.00 |
| 165327_r_at | 0.00 |
| 165328_r_at | 0.00 |
| 165329_r_at | 0.00 |
| 165330_r_at | 0.00 |
| 165331_at | 0.00 |
| 165332_r_at | 0.00 |
| 165333_f_at | 0.00 |
| 165334_s_at | 0.00 |
| 165335_i_at | 0.00 |
| 165336_f_at | 0.00 |
| 165337_i_at | 0.00 |
| 165338_r_at | 0.00 |
| 165339_f_at | 0.00 |
| 165340_f_at | 0.00 |
| 165341_i_at | 0.00 |
| 165342_i_at | 0.00 |
| 165343_i_at | 0.00 |
| 165344_r_at | 0.00 |
| 165345_at | 0.00 |
| 165346_r_at | 0.00 |
| 165347_f_at | 0.00 |
| 165348_r_at | 0.00 |
| 165349_f_at | 0.00 |
| 165350_f_at | 0.00 |
| 165351_f_at | 0.00 |
| 165352_r_at | 0.00 |
| 165353_f_at | 0.00 |
| 165354_r_at | 0.00 |
| 165355_r_at | 0.01 |
| 165356_r_at | 0.00 |
| 165357_f_at | 0.03 |
| 165358_r_at | 0.00 |
| 165359_f_at | 0.00 |
| 165360_i_at | 0.00 |
| 165361_f_at | 0.00 |
| 165362_f_at | 0.00 |
| 165363_i_at | 0.00 |
| 165364_f_at | 0.00 |
| 165365_i_at | 0.00 |

| | |
|---|---|
| 165366_i_at | 0.00 |
| 165367_f_at | 0.00 |
| 165368_at | 0.00 |
| 165369_f_at | 0.00 |
| 165370_at | 0.00 |
| 165371_i_at | 0.00 |
| 165372_at | 0.00 |
| 165373_at | 0.00 |
| 165374_i_at | 0.00 |
| 165375_at | 0.00 |
| 165376_f_at | 0.00 |
| 165377_r_at | 0.00 |
| 165378_i_at | 0.00 |
| 165379_f_at | 0.00 |
| 165380_f_at | 0.00 |
| 165381_r_at | 0.03 |
| 165382_r_at | 0.00 |
| 165383_r_at | 0.00 |
| 165384_f_at | 0.00 |
| 165385_at | 0.00 |
| 165386_r_at | 0.00 |
| 165387_i_at | 0.00 |
| 165388_at | 0.00 |
| 165389_i_at | 0.00 |
| 165390_at | 0.00 |
| 165391_r_at | 0.00 |
| 165392_i_at | 0.00 |
| 165393_f_at | 0.00 |
| 165394_f_at | 0.00 |
| 165395_i_at | 0.00 |
| 165396_at | 0.00 |
| 165397_f_at | 0.00 |
| 165398_i_at | 0.00 |
| 165399_i_at | 0.00 |
| 165400_f_at | 0.00 |
| 165401_r_at | 0.00 |
| X-18SRNAMur/X00686_ | 0.03 |
| X-18SRNAMur/X00686_ | 0.15 |
| X-18SRNAMur/X00686_ | 0.21 |
| AFFX-BioB-3_at | 0.03 |
| AFFX-BioB-3_st_B | 0.02 |
| AFFX-BioB-5_at | 0.04 |
| AFFX-BioB-5_st_B | 0.00 |
| AFFX-BioB-M_at | 0.07 |
| AFFX-BioB-M_st_B | 0.00 |
| AFFX-BioC-3_at | 0.06 |
| AFFX-BioC-3_st_B | 0.00 |
| AFFX-BioC-5_at | 0.02 |
| AFFX-BioC-5_st_B | 0.01 |
| AFFX-BioDn-3_at | 0.02 |
| AFFX-BioDn-3_st_B | 0.00 |
| AFFX-BioDn-5_at | 0.04 |
| AFFX-BioDn-5_st_B | 0.00 |
| AFFX-CreX-3_at | 0.01 |
| AFFX-CreX-3_st_B | 0.00 |
| AFFX-CreX-5_at | 0.02 |

| | |
|---|---|
| AFFX-CreX-5_st_B | 0.00 |
| AFFX-DapX-3_at | 0.00 |
| AFFX-DapX-5_at | 0.00 |
| AFFX-DapX-M_at | 0.00 |
| X-GapdhMur/M32599_ | 0.00 |
| -GapdhMur/M32599_3 | 0.01 |
| X-GapdhMur/M32599_ | 0.00 |
| -GapdhMur/M32599_5 | 0.00 |
| X-GapdhMur/M32599_ | 0.00 |
| -GapdhMur/M32599_M | 0.01 |
| AFFX-LysX-3_at | 0.00 |
| AFFX-LysX-5_at | 0.00 |
| AFFX-LysX-M_at | 0.00 |
| AFFX-MURINE_B2_at | 0.00 |
| AFFX-MURINE_b1_at | 0.07 |
| AFFX-MUR_b2_at | 0.00 |
| AFFX-MurFAS_at | 0.00 |
| AFFX-MurIL10_at | 0.00 |
| AFFX-MurIL2_at | 0.00 |
| AFFX-MurIL4_at | 0.00 |
| AFFX-PheX-3_at | 0.00 |
| AFFX-PheX-5_at | 0.00 |
| AFFX-PheX-M_at | 0.00 |
| -PyruCarbMur/L09192 | 0.00 |
| -PyruCarbMur/L09192 | 0.00 |
| -PyruCarbMur/L09192_ | 0.00 |
| -PyruCarbMur/L09192_ | 0.00 |
| AFFX-ThrX-3_at | 0.00 |
| AFFX-ThrX-5_at | 0.00 |
| AFFX-ThrX-M_at | 0.00 |
| -TransRecMur/X57349 | 0.05 |
| -TransRecMur/X57349 | 0.16 |
| -TransRecMur/X57349 | 0.14 |
| AFFX-TrpnX-3_at | 0.00 |
| AFFX-TrpnX-5_at | 0.00 |
| AFFX-TrpnX-M_at | 0.00 |
| FFX-YEL002c/WBP1_ | 0.00 |
| AFFX-YEL018w/_at | 0.00 |
| FFX-YEL021w/URA3_ | 0.00 |
| FFX-YEL024w/RIP1_a | 0.00 |
| X-b-ActinMur/M12481_ | 0.00 |
| -b-ActinMur/M12481_3 | 0.00 |
| X-b-ActinMur/M12481_ | 0.00 |
| -b-ActinMur/M12481_5 | 0.01 |
| X-b-ActinMur/M12481_ | 0.00 |
| -b-ActinMur/M12481_M | 0.00 |
| 128520_at | 0.00 |
| 128523_at | 0.00 |
| 128529_at | 0.00 |
| 128550_at | 0.00 |
| 128554_at | 0.00 |
| 128577_s_at | 0.00 |
| 128578_r_at | 0.00 |
| 128579_r_at | 0.00 |
| 128580_at | 0.00 |
| 128582_at | 0.00 |

| | |
|---|---|
| 128583_at | 0.18 |
| 128590_at | 0.00 |
| 128624_at | 0.05 |
| 128643_r_at | 0.00 |
| 128644_at | 0.00 |
| 128645_at | 0.01 |
| 128646_at | 0.00 |
| 128647_at | 0.00 |
| 128651_at | 0.00 |
| 128652_at | 0.00 |
| 128659_at | 0.00 |
| 128663_at | 0.00 |
| 128666_at | 0.00 |
| 128669_at | 0.00 |
| 128670_g_at | 0.00 |
| 128673_at | 0.00 |
| 128681_at | 0.00 |
| 128683_r_at | 0.00 |
| 128688_at | 0.00 |
| 128689_at | 0.00 |
| 128722_at | 0.00 |
| 128751_at | 0.00 |
| 128770_f_at | 0.00 |
| 128781_at | 0.00 |
| 128782_at | 0.00 |
| 128783_at | 0.00 |
| 128784_f_at | 0.00 |
| 128785_r_at | 0.00 |
| 128788_at | 0.00 |
| 128789_at | 0.00 |
| 128790_at | 0.00 |
| 128791_at | 0.00 |
| 128794_at | 0.00 |
| 128795_at | 0.00 |
| 128798_i_at | 0.00 |
| 128799_r_at | 0.00 |
| 128802_at | 0.00 |
| 128804_at | 0.00 |
| 128805_at | 0.00 |
| 128809_at | 0.00 |
| 128811_at | 0.00 |
| 128821_at | 0.03 |
| 128826_f_at | 0.00 |
| 128828_at | 0.00 |
| 128829_at | 0.00 |
| 128830_r_at | 0.00 |
| 128831_at | 0.00 |
| 128832_at | 0.00 |
| 128834_r_at | 0.00 |
| 128837_f_at | 0.00 |
| 128839_at | 0.00 |
| 128840_at | 0.00 |
| 128841_r_at | 0.00 |
| 128845_at | 0.00 |
| 128848_r_at | 0.00 |
| 128849_at | 0.00 |

| | |
|---|---|
| 128850_at | 0.00 |
| 128851_at | 0.00 |
| 128862_f_at | 0.00 |
| 128872_f_at | 0.00 |
| 128876_at | 0.00 |
| 128879_f_at | 0.00 |
| 128880_at | 0.00 |
| 128883_r_at | 0.00 |
| 128886_at | 0.00 |
| 128915_at | 0.00 |
| 128924_at | 0.00 |
| 128972_r_at | 0.00 |
| 128987_at | 0.00 |
| 129000_at | 0.00 |
| 129001_f_at | 0.00 |
| 129002_r_at | 0.00 |
| 129003_at | 0.00 |
| 129004_at | 0.00 |
| 129005_at | 0.00 |
| 129006_at | 0.00 |
| 129007_at | 0.00 |
| 129008_at | 0.00 |
| 129009_at | 0.00 |
| 129010_at | 0.00 |
| 129011_i_at | 0.00 |
| 129012_r_at | 0.00 |
| 129013_at | 0.00 |
| 129015_i_at | 0.00 |
| 129016_f_at | 0.00 |
| 129017_at | 0.00 |
| 129018_at | 0.00 |
| 129019_at | 0.00 |
| 129021_at | 0.00 |
| 129022_at | 0.00 |
| 129023_at | 0.00 |
| 129024_at | 0.00 |
| 129025_at | 0.00 |
| 129028_at | 0.00 |
| 129029_at | 0.00 |
| 129030_at | 0.00 |
| 129031_at | 0.00 |
| 129033_f_at | 0.00 |
| 129034_at | 0.00 |
| 129035_at | 0.00 |
| 129036_r_at | 0.00 |
| 129038_at | 0.00 |
| 129039_at | 0.00 |
| 129040_g_at | 0.00 |
| 129041_at | 0.00 |
| 129042_at | 0.01 |
| 129043_at | 0.00 |
| 129045_at | 0.00 |
| 129046_f_at | 0.03 |
| 129047_at | 0.00 |
| 129048_at | 0.00 |
| 129049_at | 0.00 |

| | |
|---|---|
| 129053_at | 0.00 |
| 129054_i_at | 0.00 |
| 129055_at | 0.00 |
| 129056_at | 0.00 |
| 129057_at | 0.00 |
| 129058_at | 0.00 |
| 129059_at | 0.00 |
| 129060_at | 0.00 |
| 129062_at | 0.00 |
| 129063_s_at | 0.00 |
| 129064_at | 0.00 |
| 129065_at | 0.00 |
| 129066_at | 0.00 |
| 129067_at | 0.00 |
| 129068_at | 0.00 |
| 129069_at | 0.00 |
| 129070_at | 0.00 |
| 129071_at | 0.06 |
| 129072_at | 0.00 |
| 129074_at | 0.00 |
| 129075_at | 0.00 |
| 129076_at | 0.00 |
| 129077_r_at | 0.00 |
| 129078_at | 0.00 |
| 129079_at | 0.00 |
| 129081_at | 0.00 |
| 129082_at | 0.00 |
| 129083_at | 0.00 |
| 129085_at | 0.00 |
| 129086_at | 0.00 |
| 129087_at | 0.00 |
| 129088_at | 0.00 |
| 129089_at | 0.00 |
| 129090_at | 0.00 |
| 129091_at | 0.01 |
| 129092_at | 0.00 |
| 129093_at | 0.00 |
| 129094_r_at | 0.00 |
| 129095_at | 0.00 |
| 129096_at | 0.00 |
| 129098_at | 0.00 |
| 129099_at | 0.00 |
| 129100_at | 0.00 |
| 129101_r_at | 0.00 |
| 129102_at | 0.00 |
| 129103_at | 0.00 |
| 129104_at | 0.00 |
| 129105_at | 0.00 |
| 129106_at | 0.00 |
| 129107_at | 0.00 |
| 129108_at | 0.00 |
| 129109_at | 0.00 |
| 129110_r_at | 0.00 |
| 129111_at | 0.00 |
| 129112_at | 0.00 |
| 129113_at | 0.00 |

| | |
|---|---|
| 129114_at | 0.00 |
| 129117_at | 0.00 |
| 129118_at | 0.00 |
| 129119_at | 0.00 |
| 129121_at | 0.00 |
| 129125_at | 0.00 |
| 129126_at | 0.00 |
| 129127_at | 0.00 |
| 129128_at | 0.00 |
| 129129_at | 0.00 |
| 129130_at | 0.00 |
| 129131_at | 0.00 |
| 129132_at | 0.00 |
| 129133_at | 0.00 |
| 129134_s_at | 0.05 |
| 129135_r_at | 0.00 |
| 129136_at | 0.00 |
| 129137_i_at | 0.00 |
| 129138_f_at | 0.00 |
| 129140_at | 0.00 |
| 129141_at | 0.00 |
| 129142_at | 0.00 |
| 129144_at | 0.00 |
| 129147_r_at | 0.29 |
| 129148_at | 0.00 |
| 129149_at | 0.00 |
| 129150_at | 0.00 |
| 129151_s_at | 0.00 |
| 129152_at | 0.00 |
| 129153_at | 0.00 |
| 129154_at | 0.00 |
| 129155_at | 0.00 |
| 129156_at | 0.00 |
| 129158_at | 0.00 |
| 129159_at | 0.00 |
| 129161_at | 0.00 |
| 129165_at | 0.00 |
| 129166_at | 0.00 |
| 129168_at | 0.14 |
| 129169_at | 0.00 |
| 129170_at | 0.00 |
| 129171_at | 0.00 |
| 129176_at | 0.00 |
| 129177_at | 0.00 |
| 129180_f_at | 0.21 |
| 129181_at | 0.00 |
| 129184_at | 0.00 |
| 129186_at | 0.00 |
| 129187_at | 0.00 |
| 129191_at | 0.00 |
| 129194_f_at | 0.00 |
| 129195_at | 0.02 |
| 129196_at | 0.00 |
| 129198_at | 0.00 |
| 129199_at | 0.00 |
| 129200_at | 0.00 |

| | |
|---|---|
| 129201_at | 0.00 |
| 129202_r_at | 0.00 |
| 129203_at | 0.00 |
| 129205_at | 0.00 |
| 129207_at | 0.00 |
| 129208_at | 0.00 |
| 129209_r_at | 0.00 |
| 129210_at | 0.00 |
| 129211_at | 0.00 |
| 129212_at | 0.00 |
| 129214_at | 0.00 |
| 129215_i_at | 0.00 |
| 129216_f_at | 0.00 |
| 129217_at | 0.00 |
| 129219_f_at | 0.00 |
| 129221_at | 0.00 |
| 129222_at | 0.00 |
| 129223_at | 0.00 |
| 129224_at | 0.00 |
| 129225_at | 0.34 |
| 129228_f_at | 0.26 |
| 129229_f_at | 0.00 |
| 129230_r_at | 0.00 |
| 129231_at | 0.00 |
| 129232_at | 0.00 |
| 129236_at | 0.00 |
| 129237_at | 0.00 |
| 129238_at | 0.00 |
| 129240_at | 0.00 |
| 129243_at | 0.00 |
| 129244_at | 0.00 |
| 129245_at | 0.00 |
| 129246_at | 0.00 |
| 129247_at | 0.00 |
| 129249_r_at | 0.00 |
| 129251_r_at | 0.00 |
| 129252_at | 0.00 |
| 129253_r_at | 0.00 |
| 129254_at | 0.00 |
| 129258_at | 0.00 |
| 129260_at | 0.00 |
| 129261_at | 0.00 |
| 129262_at | 0.00 |
| 129263_at | 0.00 |
| 129265_at | 0.00 |
| 129266_at | 0.00 |
| 129267_at | 0.02 |
| 129268_at | 0.00 |
| 129269_at | 0.00 |
| 129270_at | 0.00 |
| 129273_at | 0.00 |
| 129274_at | 0.00 |
| 129275_at | 0.00 |
| 129276_at | 0.00 |
| 129277_at | 0.00 |
| 129278_at | 0.00 |

| | |
|---|---|
| 129279_at | 0.00 |
| 129280_at | 0.00 |
| 129281_at | 0.00 |
| 129282_at | 0.01 |
| 129283_at | 0.00 |
| 129284_at | 0.00 |
| 129285_at | 0.00 |
| 129286_at | 0.04 |
| 129287_at | 0.00 |
| 129288_at | 0.00 |
| 129289_at | 0.00 |
| 129290_at | 0.00 |
| 129293_at | 0.00 |
| 129294_at | 0.00 |
| 129295_at | 0.00 |
| 129296_at | 0.00 |
| 129297_at | 0.04 |
| 129298_at | 0.00 |
| 129299_at | 0.00 |
| 129300_at | 0.00 |
| 129301_at | 0.00 |
| 129302_at | 0.02 |
| 129303_at | 0.00 |
| 129304_at | 0.19 |
| 129305_at | 0.00 |
| 129306_r_at | 0.00 |
| 129307_i_at | 0.00 |
| 129308_f_at | 0.00 |
| 129309_at | 0.00 |
| 129310_at | 0.08 |
| 129311_at | 0.00 |
| 129312_i_at | 0.00 |
| 129313_f_at | 0.00 |
| 129314_r_at | 0.00 |
| 129315_at | 0.07 |
| 129316_at | 0.00 |
| 129317_at | 0.00 |
| 129318_at | 0.00 |
| 129319_f_at | 0.00 |
| 129320_at | 0.00 |
| 129321_at | 0.15 |
| 129322_at | 0.01 |
| 129323_at | 0.00 |
| 129324_at | 0.00 |
| 129325_at | 0.00 |
| 129326_at | 0.00 |
| 129327_at | 0.00 |
| 129328_f_at | 0.00 |
| 129329_r_at | 0.00 |
| 129330_at | 0.00 |
| 129331_at | 0.00 |
| 129332_at | 0.00 |
| 129334_at | 0.00 |
| 129335_at | 0.00 |
| 129336_at | 0.00 |
| 129337_at | 0.00 |

| | |
|---|---|
| 129338_at | 0.00 |
| 129339_at | 0.00 |
| 129340_at | 0.00 |
| 129341_at | 0.00 |
| 129342_r_at | 0.00 |
| 129343_at | 0.00 |
| 129344_at | 0.00 |
| 129345_at | 0.00 |
| 129346_at | 0.00 |
| 129347_at | 0.00 |
| 129348_at | 0.00 |
| 129351_at | 0.00 |
| 129352_at | 0.00 |
| 129353_r_at | 0.00 |
| 129357_at | 0.00 |
| 129371_at | 0.00 |
| 129372_at | 0.00 |
| 129373_at | 0.00 |
| 129378_at | 0.00 |
| 129379_at | 0.00 |
| 129385_r_at | 0.00 |
| 129386_at | 0.00 |
| 129387_r_at | 0.00 |
| 129388_at | 0.00 |
| 129389_at | 0.00 |
| 129395_at | 0.00 |
| 129396_at | 0.00 |
| 129397_at | 0.00 |
| 129398_at | 0.00 |
| 129399_at | 0.00 |
| 129400_at | 0.00 |
| 129401_at | 0.00 |
| 129403_at | 0.00 |
| 129404_at | 0.00 |
| 129405_at | 0.00 |
| 129406_at | 0.00 |
| 129407_at | 0.00 |
| 129408_at | 0.00 |
| 129409_at | 0.00 |
| 129410_at | 0.00 |
| 129411_at | 0.00 |
| 129412_s_at | 0.00 |
| 129413_at | 0.00 |
| 129414_at | 0.00 |
| 129415_at | 0.00 |
| 129416_at | 0.00 |
| 129417_s_at | 0.00 |
| 129418_at | 0.00 |
| 129419_at | 0.00 |
| 129420_at | 0.00 |
| 129421_s_at | 0.00 |
| 129422_at | 0.00 |
| 129423_at | 0.00 |
| 129424_at | 0.00 |
| 129425_at | 0.00 |
| 129426_at | 0.00 |

| | |
|---|---|
| 129427_at | 0.00 |
| 129428_at | 0.00 |
| 129429_at | 0.00 |
| 129430_g_at | 0.00 |
| 129431_at | 0.00 |
| 129432_at | 0.00 |
| 129433_at | 0.00 |
| 129434_at | 0.00 |
| 129435_at | 0.00 |
| 129436_at | 0.00 |
| 129437_r_at | 0.00 |
| 129438_f_at | 0.00 |
| 129439_at | 0.00 |
| 129440_r_at | 0.00 |
| 129441_at | 0.00 |
| 129442_at | 0.00 |
| 129443_at | 0.00 |
| 129444_f_at | 0.00 |
| 129445_at | 0.00 |
| 129446_at | 0.00 |
| 129464_at | 0.00 |
| 129465_at | 0.00 |
| 129475_at | 0.00 |
| 129476_at | 0.00 |
| 129477_at | 0.00 |
| 129503_at | 0.00 |
| 129540_at | 0.00 |
| 129541_at | 0.00 |
| 129542_at | 0.00 |
| 129543_at | 0.00 |
| 129544_at | 0.00 |
| 129555_r_at | 0.00 |
| 129570_f_at | 0.00 |
| 129574_at | 0.00 |
| 129580_at | 0.00 |
| 129581_at | 0.00 |
| 129582_at | 0.00 |
| 129584_at | 0.00 |
| 129585_r_at | 0.00 |
| 129626_at | 0.00 |
| 129627_at | 0.00 |
| 129628_at | 0.00 |
| 129638_at | 0.00 |
| 129639_at | 0.00 |
| 129640_at | 0.00 |
| 129641_i_at | 0.00 |
| 129642_f_at | 0.00 |
| 129643_f_at | 0.00 |
| 129644_at | 0.00 |
| 129645_at | 0.00 |
| 129646_at | 0.00 |
| 129661_at | 0.00 |
| 129689_f_at | 0.00 |
| 129690_r_at | 0.00 |
| 129691_f_at | 0.00 |
| 129692_at | 0.00 |

| | |
|---|---|
| 129693_at | 0.00 |
| 129740_at | 0.00 |
| 129759_at | 0.00 |
| 129774_f_at | 0.00 |
| 129778_at | 0.00 |
| 129808_at | 0.00 |
| 129827_f_at | 0.00 |
| 129828_f_at | 0.00 |
| 129829_at | 0.00 |
| 129830_g_at | 0.00 |
| 129831_at | 0.00 |
| 129832_at | 0.00 |
| 129833_at | 0.00 |
| 129834_f_at | 0.00 |
| 129835_at | 0.00 |
| 129836_at | 0.00 |
| 129841_at | 0.00 |
| 129842_at | 0.09 |
| 129843_at | 0.00 |
| 129853_at | 0.00 |
| 129854_at | 0.00 |
| 129855_g_at | 0.00 |
| 129856_at | 0.00 |
| 129857_at | 0.00 |
| 129858_at | 0.00 |
| 129859_at | 0.00 |
| 129860_at | 0.00 |
| 129861_at | 0.00 |
| 129862_at | 0.00 |
| 129863_at | 0.00 |
| 129864_at | 0.00 |
| 129865_at | 0.00 |
| 129866_at | 0.00 |
| 129867_at | 0.00 |
| 129868_at | 0.00 |
| 129869_at | 0.00 |
| 129870_at | 0.00 |
| 129871_at | 0.00 |
| 129872_at | 0.00 |
| 129873_at | 0.00 |
| 129874_at | 0.00 |
| 129875_at | 0.00 |
| 129876_at | 0.00 |
| 129877_at | 0.00 |
| 129878_at | 0.00 |
| 129879_at | 0.00 |
| 129880_s_at | 0.64 |
| 129881_r_at | 0.17 |
| 129882_at | 0.00 |
| 129883_at | 0.00 |
| 129884_at | 0.00 |
| 129885_at | 0.00 |
| 129886_at | 0.00 |
| 129887_at | 0.00 |
| 129888_r_at | 0.00 |
| 129889_at | 0.00 |

| | |
|---|---|
| 129890_at | 0.00 |
| 129891_at | 0.00 |
| 129892_at | 0.00 |
| 129893_at | 0.00 |
| 129894_f_at | 0.00 |
| 129895_at | 0.00 |
| 129896_at | 0.00 |
| 129897_at | 0.00 |
| 129898_at | 0.00 |
| 129899_f_at | 0.00 |
| 129900_at | 0.00 |
| 129901_at | 0.00 |
| 129902_r_at | 0.00 |
| 129903_at | 0.10 |
| 129906_at | 0.00 |
| 129921_at | 0.00 |
| 129922_at | 0.00 |
| 129923_f_at | 0.00 |
| 129924_at | 0.00 |
| 129925_at | 0.00 |
| 129926_r_at | 0.00 |
| 129927_i_at | 0.07 |
| 129928_f_at | 0.34 |
| 129952_at | 0.00 |
| 129955_at | 0.00 |
| 129968_at | 0.00 |
| 129981_at | 0.00 |
| 129983_at | 0.00 |
| 130002_at | 0.00 |
| 130033_at | 0.00 |
| 130058_f_at | 0.00 |
| 130067_at | 0.00 |
| 130068_f_at | 0.00 |
| 130069_r_at | 0.00 |
| 130070_at | 0.00 |
| 130071_at | 0.00 |
| 130072_at | 0.00 |
| 130073_at | 0.00 |
| 130119_f_at | 0.00 |
| 130120_r_at | 0.00 |
| 130121_at | 0.01 |
| 130122_f_at | 0.00 |
| 130123_at | 0.00 |
| 130124_at | 0.00 |
| 130125_at | 0.00 |
| 130130_at | 0.00 |
| 130131_f_at | 0.00 |
| 130132_at | 0.00 |
| 130135_at | 0.00 |
| 130136_at | 0.00 |
| 130137_at | 0.00 |
| 130138_at | 0.00 |
| 130139_at | 0.00 |
| 130140_at | 0.00 |
| 130141_at | 0.00 |
| 130142_at | 0.00 |

| | |
|---|---|
| 130143_at | 0.00 |
| 130144_at | 0.00 |
| 130145_i_at | 0.00 |
| 130146_f_at | 0.00 |
| 130147_at | 0.00 |
| 130148_at | 0.00 |
| 130149_i_at | 0.00 |
| 130150_f_at | 0.00 |
| 130157_at | 0.00 |
| 130159_at | 0.00 |
| 130160_at | 0.00 |
| 130161_at | 0.00 |
| 130162_at | 0.00 |
| 130163_at | 0.00 |
| 130164_at | 0.00 |
| 130165_at | 0.00 |
| 130166_at | 0.00 |
| 130167_at | 0.00 |
| 130168_at | 0.00 |
| 130172_at | 0.00 |
| 130173_at | 0.00 |
| 130174_at | 0.00 |
| 130175_at | 0.00 |
| 130176_at | 0.00 |
| 130177_at | 0.00 |
| 130178_at | 0.00 |
| 130179_at | 0.00 |
| 130180_at | 0.00 |
| 130181_at | 0.00 |
| 130182_at | 0.00 |
| 130183_at | 0.01 |
| 130184_at | 0.00 |
| 130185_r_at | 0.00 |
| 130186_f_at | 0.00 |
| 130263_at | 0.00 |
| 130269_r_at | 0.00 |
| 130290_at | 0.00 |
| 130292_at | 0.00 |
| 130300_r_at | 0.00 |
| 130304_r_at | 0.00 |
| 130307_f_at | 0.00 |
| 130308_f_at | 0.00 |
| 130309_f_at | 0.00 |
| 130310_at | 0.00 |
| 130311_at | 0.00 |
| 130312_at | 0.00 |
| 130313_i_at | 0.00 |
| 130314_r_at | 0.00 |
| 130315_at | 0.00 |
| 130316_f_at | 0.00 |
| 130317_at | 0.00 |
| 130318_at | 0.00 |
| 130319_at | 0.00 |
| 130326_at | 0.00 |
| 130327_at | 0.00 |
| 130329_at | 0.00 |

| | |
|---|---|
| 130330_at | 0.00 |
| 130331_at | 0.00 |
| 130332_at | 0.00 |
| 130333_at | 0.00 |
| 130334_at | 0.00 |
| 130335_at | 0.00 |
| 130336_at | 0.00 |
| 130337_f_at | 0.00 |
| 130338_at | 0.00 |
| 130339_at | 0.00 |
| 130340_at | 0.00 |
| 130341_at | 0.00 |
| 130342_at | 0.00 |
| 130343_at | 0.00 |
| 130344_at | 0.00 |
| 130371_at | 0.00 |
| 130381_at | 0.00 |
| 130382_at | 0.00 |
| 130383_at | 0.00 |
| 130384_at | 0.00 |
| 130385_at | 0.00 |
| 130386_at | 0.00 |
| 130387_at | 0.00 |
| 130388_at | 0.00 |
| 130389_at | 0.00 |
| 130390_at | 0.00 |
| 130391_at | 0.00 |
| 130392_at | 0.00 |
| 130393_at | 0.00 |
| 130394_at | 0.01 |
| 130395_at | 0.00 |
| 130396_at | 0.00 |
| 130397_at | 0.00 |
| 130398_at | 0.00 |
| 130399_at | 0.00 |
| 130401_at | 0.00 |
| 130402_at | 0.00 |
| 130403_at | 0.00 |
| 130404_at | 0.00 |
| 130405_at | 0.00 |
| 130406_at | 0.00 |
| 130407_at | 0.00 |
| 130408_at | 0.00 |
| 130409_at | 0.00 |
| 130410_at | 0.00 |
| 130412_at | 0.00 |
| 130413_at | 0.00 |
| 130414_at | 0.00 |
| 130415_at | 0.00 |
| 130416_at | 0.00 |
| 130417_at | 0.00 |
| 130418_f_at | 0.00 |
| 130419_at | 0.00 |
| 130420_at | 0.00 |
| 130421_at | 0.00 |
| 130422_at | 0.00 |

| | |
|---|---|
| 130423_at | 0.00 |
| 130424_at | 0.00 |
| 130425_at | 0.00 |
| 130426_r_at | 0.00 |
| 130439_at | 0.00 |
| 130441_at | 0.00 |
| 130442_at | 0.62 |
| 130444_at | 0.13 |
| 130455_f_at | 0.00 |
| 130456_at | 0.00 |
| 130457_g_at | 0.00 |
| 130459_at | 0.00 |
| 130460_at | 0.00 |
| 130461_at | 0.05 |
| 130465_at | 0.00 |
| 130469_s_at | 0.00 |
| 130472_at | 0.00 |
| 130473_at | 0.00 |
| 130474_at | 0.00 |
| 130476_at | 0.00 |
| 130477_at | 0.00 |
| 130478_at | 0.00 |
| 130479_at | 0.00 |
| 130483_at | 0.00 |
| 130491_at | 0.00 |
| 130492_at | 0.00 |
| 130494_f_at | 0.00 |
| 130498_f_at | 0.01 |
| 130499_at | 0.03 |
| 130500_at | 0.00 |
| 130507_at | 0.00 |
| 130509_at | 0.00 |
| 130510_at | 0.00 |
| 130512_at | 0.00 |
| 130518_at | 0.00 |
| 130526_at | 0.00 |
| 130527_g_at | 0.00 |
| 130530_at | 0.00 |
| 130531_at | 0.01 |
| 130532_at | 0.11 |
| 130534_i_at | 0.01 |
| 130540_at | 0.00 |
| 130545_at | 0.00 |
| 130546_at | 0.00 |
| 130547_at | 0.00 |
| 130548_at | 0.00 |
| 130549_f_at | 0.00 |
| 130550_f_at | 0.00 |
| 130551_at | 0.00 |
| 130555_at | 0.06 |
| 130556_at | 0.00 |
| 130557_at | 0.00 |
| 130558_at | 0.00 |
| 130559_at | 0.00 |
| 130560_at | 0.00 |
| 130561_at | 0.00 |

| | |
|---|---|
| 130562_g_at | 0.00 |
| 130563_at | 0.00 |
| 130564_at | 0.00 |
| 130565_at | 0.00 |
| 130570_i_at | 0.00 |
| 130571_f_at | 0.00 |
| 130572_at | 0.00 |
| 130573_at | 0.00 |
| 130574_at | 0.00 |
| 130575_at | 0.00 |
| 130576_at | 0.00 |
| 130577_at | 0.00 |
| 130578_at | 0.00 |
| 130579_at | 0.00 |
| 130580_at | 0.00 |
| 130581_at | 0.00 |
| 130582_at | 0.00 |
| 130583_at | 0.00 |
| 130584_at | 0.00 |
| 130585_at | 0.00 |
| 130586_at | 0.02 |
| 130587_at | 0.00 |
| 130588_at | 0.00 |
| 130589_at | 0.00 |
| 130590_at | 0.00 |
| 130591_at | 0.00 |
| 130592_at | 0.00 |
| 130593_at | 0.00 |
| 130594_at | 0.00 |
| 130595_at | 0.00 |
| 130596_at | 0.00 |
| 130597_r_at | 0.00 |
| 130598_at | 0.00 |
| 130599_at | 0.00 |
| 130600_at | 0.00 |
| 130602_at | 0.00 |
| 130603_at | 0.00 |
| 130604_at | 0.00 |
| 130605_r_at | 0.00 |
| 130606_at | 0.00 |
| 130607_at | 0.00 |
| 130608_at | 0.01 |
| 130609_at | 0.00 |
| 130610_at | 0.00 |
| 130611_at | 0.00 |
| 130612_r_at | 0.00 |
| 130613_at | 0.00 |
| 130614_at | 0.00 |
| 130615_at | 0.00 |
| 130616_at | 0.00 |
| 130617_at | 0.00 |
| 130618_at | 0.00 |
| 130619_at | 0.00 |
| 130620_r_at | 0.00 |
| 130621_at | 0.00 |
| 130622_at | 0.00 |

| | |
|---|---|
| 130623_at | 0.00 |
| 130625_at | 0.00 |
| 130626_at | 0.00 |
| 130627_at | 0.00 |
| 130628_at | 0.00 |
| 130629_at | 0.00 |
| 130630_at | 0.00 |
| 130631_at | 0.00 |
| 130638_at | 0.00 |
| 130639_at | 0.00 |
| 130640_at | 0.00 |
| 130641_at | 0.00 |
| 130642_at | 0.00 |
| 130643_at | 0.00 |
| 130644_at | 0.00 |
| 130645_at | 0.00 |
| 130646_at | 0.00 |
| 130647_at | 0.00 |
| 130648_at | 0.00 |
| 130649_at | 0.00 |
| 130651_at | 0.00 |
| 130652_at | 0.00 |
| 130653_at | 0.00 |
| 130654_at | 0.00 |
| 130655_at | 0.00 |
| 130656_at | 0.00 |
| 130657_at | 0.00 |
| 130658_at | 0.00 |
| 130659_at | 0.00 |
| 130660_at | 0.00 |
| 130661_at | 0.00 |
| 130662_at | 0.00 |
| 130663_i_at | 0.00 |
| 130664_r_at | 0.00 |
| 130667_at | 0.00 |
| 130670_at | 0.00 |
| 130671_s_at | 0.00 |
| 130672_at | 0.00 |
| 130673_at | 0.00 |
| 130675_at | 0.00 |
| 130682_at | 0.00 |
| 130687_at | 0.00 |
| 130689_at | 0.00 |
| 130690_f_at | 0.06 |
| 130691_at | 0.00 |
| 130696_f_at | 0.00 |
| 130697_s_at | 0.00 |
| 130702_f_at | 0.00 |
| 130703_at | 0.00 |
| 130704_f_at | 0.00 |
| 130709_at | 0.00 |
| 130710_at | 0.00 |
| 130711_i_at | 0.00 |
| 130712_f_at | 0.00 |
| 130717_r_at | 0.00 |
| 130718_at | 0.00 |

| | |
|---|---|
| 130719_at | 0.00 |
| 130720_at | 0.00 |
| 130721_r_at | 0.00 |
| 130726_at | 0.00 |
| 130727_at | 0.00 |
| 130729_r_at | 0.00 |
| 130730_f_at | 0.01 |
| 130731_at | 0.00 |
| 130733_f_at | 0.00 |
| 130734_at | 0.00 |
| 130735_at | 0.00 |
| 130736_r_at | 0.00 |
| 130738_at | 0.68 |
| 130739_at | 0.00 |
| 130740_at | 0.00 |
| 130744_at | 0.00 |
| 130745_at | 0.00 |
| 130746_g_at | 0.00 |
| 130747_at | 0.00 |
| 130748_at | 0.00 |
| 130749_at | 0.00 |
| 130750_at | 0.00 |
| 130753_at | 0.00 |
| 130755_at | 0.00 |
| 130757_at | 0.00 |
| 130758_i_at | 0.00 |
| 130761_at | 0.00 |
| 130763_at | 0.00 |
| 130772_at | 0.00 |
| 130773_at | 0.00 |
| 130774_at | 0.09 |
| 130776_at | 0.00 |
| 130778_at | 0.00 |
| 130779_f_at | 0.00 |
| 130785_at | 0.00 |
| 130786_at | 0.00 |
| 130787_at | 0.00 |
| 130788_at | 0.00 |
| 130789_at | 0.00 |
| 130790_s_at | 0.00 |
| 130791_at | 0.00 |
| 130797_at | 0.00 |
| 130798_at | 0.00 |
| 130799_at | 0.00 |
| 130800_at | 0.00 |
| 130804_at | 0.00 |
| 130805_at | 0.00 |
| 130806_at | 0.00 |
| 130807_at | 0.00 |
| 130817_at | 0.00 |
| 130818_at | 0.00 |
| 130821_at | 0.00 |
| 130822_at | 0.00 |
| 130823_at | 0.00 |
| 130824_i_at | 0.52 |
| 130825_f_at | 0.62 |

| | |
|---|---|
| 130826_at | 0.00 |
| 130827_at | 0.00 |
| 130828_at | 0.00 |
| 130840_at | 0.00 |
| 130851_at | 0.00 |
| 130852_at | 0.00 |
| 130853_at | 0.00 |
| 130854_at | 0.00 |
| 130855_at | 0.00 |
| 130856_at | 0.00 |
| 130857_at | 0.00 |
| 130877_at | 0.00 |
| 130897_f_at | 0.00 |
| 130898_f_at | 0.00 |
| 130907_at | 0.00 |
| 130908_at | 0.00 |
| 130910_at | 0.00 |
| 130911_at | 0.07 |
| 130912_at | 0.00 |
| 130913_at | 0.00 |
| 130916_at | 0.04 |
| 130918_at | 0.00 |
| 130919_at | 0.00 |
| 130920_at | 0.00 |
| 130923_i_at | 0.00 |
| 130928_s_at | 0.00 |
| 130929_at | 0.00 |
| 130936_f_at | 0.00 |
| 130939_at | 0.00 |
| 130951_at | 0.00 |
| 130952_at | 0.00 |
| 130954_at | 0.00 |
| 130957_f_at | 0.22 |
| 130960_at | 0.00 |
| 130966_r_at | 0.00 |
| 130968_at | 0.00 |
| 130969_at | 0.02 |
| 130971_at | 0.00 |
| 130973_at | 0.00 |
| 130977_at | 0.00 |
| 130982_r_at | 0.00 |
| 130988_f_at | 0.03 |
| 130990_at | 0.00 |
| 130992_at | 0.00 |
| 130994_at | 0.00 |
| 130997_r_at | 0.00 |
| 130998_i_at | 0.00 |
| 130999_f_at | 0.00 |
| 131000_f_at | 0.00 |
| 131001_r_at | 0.01 |
| 131002_at | 0.00 |
| 131004_r_at | 0.00 |
| 131005_at | 0.00 |
| 131006_r_at | 0.00 |
| 131014_at | 0.00 |
| 131015_at | 0.00 |

| | |
|---|---|
| 131017_at | 0.00 |
| 131018_at | 0.00 |
| 131020_at | 0.00 |
| 131021_at | 0.26 |
| 131034_at | 0.00 |
| 131035_at | 0.00 |
| 131036_at | 0.00 |
| 131037_at | 0.00 |
| 131038_at | 0.00 |
| 131054_at | 0.00 |
| 131065_at | 0.00 |
| 131066_at | 0.00 |
| 131067_at | 0.00 |
| 131068_at | 0.00 |
| 131070_i_at | 0.00 |
| 131071_f_at | 0.00 |
| 131072_at | 0.00 |
| 131073_at | 0.00 |
| 131074_at | 0.00 |
| 131080_at | 0.00 |
| 131081_at | 0.00 |
| 131082_at | 0.00 |
| 131091_at | 0.00 |
| 131092_at | 0.00 |
| 131093_at | 0.00 |
| 131094_at | 0.00 |
| 131095_f_at | 0.00 |
| 131096_r_at | 0.00 |
| 131097_at | 0.00 |
| 131099_at | 0.00 |
| 131100_at | 0.00 |
| 131101_at | 0.00 |
| 131102_at | 0.00 |
| 131103_at | 0.00 |
| 131104_at | 0.00 |
| 131105_at | 0.00 |
| 131106_at | 0.00 |
| 131107_at | 0.00 |
| 131113_at | 0.00 |
| 131114_at | 0.00 |
| 131115_at | 0.00 |
| 131116_at | 0.00 |
| 131117_at | 0.00 |
| 131118_at | 0.00 |
| 131119_at | 0.00 |
| 131120_at | 0.00 |
| 131121_r_at | 0.00 |
| 131122_s_at | 0.00 |
| 131123_at | 0.00 |
| 131124_at | 0.00 |
| 131125_at | 0.00 |
| 131126_at | 0.00 |
| 131127_at | 0.00 |
| 131128_at | 0.13 |
| 131129_at | 0.00 |
| 131130_at | 0.00 |

| | |
|---|---|
| 131131_f_at | 0.00 |
| 131132_at | 0.00 |
| 131133_at | 0.00 |
| 131134_at | 0.00 |
| 131135_at | 0.00 |
| 131136_at | 0.00 |
| 131137_at | 0.13 |
| 131138_at | 0.00 |
| 131139_at | 0.00 |
| 131140_at | 0.00 |
| 131141_at | 0.00 |
| 131142_at | 0.00 |
| 131143_at | 0.00 |
| 131144_at | 0.00 |
| 131149_at | 0.00 |
| 131152_at | 0.00 |
| 131153_at | 0.00 |
| 131156_f_at | 0.00 |
| 131161_at | 0.00 |
| 131162_at | 0.00 |
| 131163_at | 0.00 |
| 131164_at | 0.00 |
| 131165_at | 0.00 |
| 131166_at | 0.00 |
| 131168_at | 0.00 |
| 131171_at | 0.00 |
| 131172_at | 0.00 |
| 131176_at | 0.00 |
| 131177_r_at | 0.00 |
| 131179_r_at | 0.00 |
| 131180_at | 0.00 |
| 131184_r_at | 0.00 |
| 131185_at | 0.00 |
| 131186_at | 0.00 |
| 131188_at | 0.07 |
| 131189_f_at | 0.00 |
| 131190_at | 0.00 |
| 131191_r_at | 0.00 |
| 131197_at | 0.00 |
| 131200_f_at | 0.00 |
| 131208_at | 0.11 |
| 131216_f_at | 0.56 |
| 131217_at | 0.00 |
| 131220_f_at | 0.00 |
| 131225_at | 0.00 |
| 131226_at | 0.00 |
| 131239_r_at | 0.00 |
| 131244_at | 0.00 |
| 131248_at | 0.00 |
| 131253_f_at | 0.00 |
| 131254_at | 0.00 |
| 131255_at | 0.00 |
| 131257_at | 0.00 |
| 131259_r_at | 0.00 |
| 131264_f_at | 0.00 |
| 131265_at | 0.00 |

| | |
|---|---|
| 131266_g_at | 0.00 |
| 131267_at | 0.00 |
| 131268_at | 0.00 |
| 131269_i_at | 0.00 |
| 131270_at | 0.00 |
| 131271_at | 0.00 |
| 131272_at | 0.00 |
| 131273_at | 0.00 |
| 131274_at | 0.00 |
| 131275_at | 0.00 |
| 131276_at | 0.00 |
| 131277_at | 0.00 |
| 131278_at | 0.00 |
| 131279_at | 0.00 |
| 131281_at | 0.00 |
| 131282_at | 0.00 |
| 131289_at | 0.00 |
| 131290_at | 0.00 |
| 131298_at | 0.00 |
| 131303_at | 0.00 |
| 131317_at | 0.00 |
| 131318_at | 0.00 |
| 131319_at | 0.00 |
| 131337_at | 0.00 |
| 131363_at | 0.00 |
| 131377_at | 0.00 |
| 131385_at | 0.00 |
| 131386_at | 0.02 |
| 131389_at | 0.00 |
| 131390_at | 0.00 |
| 131391_at | 0.00 |
| 131392_at | 0.00 |
| 131394_at | 0.00 |
| 131402_at | 0.00 |
| 131403_at | 0.00 |
| 131404_at | 0.00 |
| 131405_at | 0.00 |
| 131407_r_at | 0.01 |
| 131411_at | 0.35 |
| 131416_at | 0.00 |
| 131418_r_at | 0.00 |
| 131423_at | 0.00 |
| 131428_f_at | 0.00 |
| 131435_at | 0.00 |
| 131437_at | 0.00 |
| 131438_f_at | 0.00 |
| 131439_r_at | 0.00 |
| 131442_at | 0.00 |
| 131443_at | 0.00 |
| 131445_at | 0.00 |
| 131448_at | 0.00 |
| 131449_at | 0.00 |
| 131451_r_at | 0.00 |
| 131452_at | 0.00 |
| 131453_at | 0.00 |
| 131454_at | 0.00 |

| | |
|---|---|
| 131455_at | 0.03 |
| 131476_at | 0.00 |
| 131478_at | 0.00 |
| 131513_s_at | 0.00 |
| 131514_at | 0.00 |
| 131515_at | 0.00 |
| 131537_at | 0.00 |
| 131538_at | 0.00 |
| 131539_at | 0.00 |
| 131549_at | 0.00 |
| 131555_at | 0.00 |
| 131556_at | 0.00 |
| 131557_at | 0.00 |
| 131558_at | 0.00 |
| 131559_at | 0.00 |
| 131568_at | 0.00 |
| 131569_at | 0.00 |
| 131575_at | 0.00 |
| 131576_r_at | 0.00 |
| 131584_at | 0.00 |
| 131585_at | 0.00 |
| 131591_at | 0.00 |
| 131592_s_at | 0.00 |
| 131593_r_at | 0.00 |
| 131599_at | 0.00 |
| 131605_at | 0.00 |
| 131606_at | 0.00 |
| 131609_at | 0.00 |
| 131632_at | 0.00 |
| 131633_at | 0.00 |
| 131634_at | 0.00 |
| 131635_at | 0.00 |
| 131636_at | 0.00 |
| 131637_at | 0.01 |
| 131638_at | 0.00 |
| 131644_at | 0.00 |
| 131645_at | 0.00 |
| 131646_at | 0.00 |
| 131647_at | 0.00 |
| 131653_at | 0.00 |
| 131664_at | 0.00 |
| 131665_at | 0.00 |
| 131666_at | 0.00 |
| 131675_r_at | 0.00 |
| 131681_at | 0.00 |
| 131682_at | 0.00 |
| 131692_r_at | 0.00 |
| 131693_at | 0.00 |
| 131694_at | 0.00 |
| 131695_at | 0.00 |
| 131700_at | 0.00 |
| 131701_at | 0.00 |
| 131702_at | 0.00 |
| 131703_at | 0.00 |
| 131704_at | 0.00 |
| 131705_at | 0.00 |

| | |
|---|---|
| 131706_at | 0.00 |
| 131707_at | 0.00 |
| 131709_at | 0.00 |
| 131741_f_at | 0.00 |
| 131742_i_at | 0.00 |
| 131743_f_at | 0.00 |
| 131755_at | 0.00 |
| 131756_at | 0.00 |
| 131758_f_at | 0.00 |
| 131759_r_at | 0.00 |
| 131760_at | 0.00 |
| 131761_f_at | 0.00 |
| 131762_r_at | 0.00 |
| 131772_at | 0.00 |
| 131780_at | 0.00 |
| 131781_at | 0.00 |
| 131782_at | 0.00 |
| 131783_at | 0.00 |
| 131792_s_at | 0.00 |
| 131795_at | 0.00 |
| 131796_at | 0.00 |
| 131797_at | 0.00 |
| 131798_at | 0.00 |
| 131811_at | 0.00 |
| 131812_at | 0.00 |
| 131813_at | 0.00 |
| 131814_at | 0.00 |
| 131815_at | 0.00 |
| 131816_at | 0.00 |
| 131817_at | 0.00 |
| 131818_at | 0.00 |
| 131819_at | 0.00 |
| 131820_at | 0.00 |
| 131821_at | 0.00 |
| 131822_at | 0.00 |
| 131830_at | 0.00 |
| 131831_at | 0.00 |
| 131832_at | 0.00 |
| 131833_at | 0.00 |
| 131834_at | 0.00 |
| 131835_at | 0.00 |
| 131836_at | 0.00 |
| 131837_at | 0.00 |
| 131838_at | 0.00 |
| 131839_at | 0.00 |
| 131845_at | 0.00 |
| 131846_at | 0.00 |
| 131847_at | 0.00 |
| 131848_at | 0.00 |
| 131849_at | 0.00 |
| 131850_at | 0.00 |
| 131851_at | 0.00 |
| 131852_at | 0.00 |
| 131853_at | 0.00 |
| 131854_at | 0.00 |
| 131855_at | 0.00 |

| | |
|---|---|
| 131856_at | 0.00 |
| 131857_at | 0.00 |
| 131858_at | 0.00 |
| 131859_at | 0.00 |
| 131860_at | 0.00 |
| 131861_at | 0.00 |
| 131862_at | 0.00 |
| 131863_at | 0.00 |
| 131864_at | 0.00 |
| 131865_at | 0.00 |
| 131866_at | 0.00 |
| 131867_at | 0.00 |
| 131868_at | 0.00 |
| 131869_at | 0.00 |
| 131870_at | 0.00 |
| 131871_at | 0.00 |
| 131872_at | 0.00 |
| 131873_at | 0.00 |
| 131874_at | 0.00 |
| 131875_at | 0.00 |
| 131876_at | 0.00 |
| 131877_at | 0.00 |
| 131914_at | 0.72 |
| 131915_at | 0.01 |
| 131916_at | 0.00 |
| 131917_at | 0.00 |
| 131918_at | 0.00 |
| 131919_at | 0.00 |
| 131920_at | 0.00 |
| 131931_r_at | 0.00 |
| 131932_at | 0.00 |
| 131933_at | 0.00 |
| 131963_r_at | 0.00 |
| 131964_f_at | 0.02 |
| 131968_at | 0.00 |
| 131969_at | 0.00 |
| 131970_at | 0.00 |
| 131971_at | 0.00 |
| 131972_at | 0.00 |
| 131982_at | 0.00 |
| 131983_at | 0.23 |
| 131984_at | 0.00 |
| 131985_r_at | 0.00 |
| 131986_at | 0.00 |
| 131987_at | 0.00 |
| 131988_at | 0.00 |
| 132005_f_at | 0.00 |
| 132006_at | 0.00 |
| 132009_at | 0.00 |
| 132010_at | 0.00 |
| 132014_at | 0.00 |
| 132018_at | 0.00 |
| 132019_at | 0.00 |
| 132020_at | 0.00 |
| 132021_at | 0.00 |
| 132022_f_at | 0.00 |

| | |
|---|---|
| 132023_at | 0.00 |
| 132024_f_at | 0.00 |
| 132025_r_at | 0.00 |
| 132026_at | 0.00 |
| 132027_at | 0.00 |
| 132028_at | 0.00 |
| 132029_at | 0.00 |
| 132030_at | 0.00 |
| 132031_at | 0.00 |
| 132032_at | 0.00 |
| 132033_at | 0.02 |
| 132034_at | 0.00 |
| 132035_at | 0.00 |
| 132037_at | 0.00 |
| 132038_at | 0.00 |
| 132039_f_at | 0.15 |
| 132040_at | 0.00 |
| 132048_r_at | 0.00 |
| 132049_at | 0.00 |
| 132050_at | 0.00 |
| 132051_at | 0.00 |
| 132052_at | 0.00 |
| 132053_at | 0.00 |
| 132056_at | 0.00 |
| 132057_at | 0.00 |
| 132058_at | 0.00 |
| 132059_at | 0.00 |
| 132060_at | 0.00 |
| 132061_at | 0.00 |
| 132063_f_at | 0.00 |
| 132064_at | 0.00 |
| 132086_at | 0.00 |
| 132087_at | 0.00 |
| 132088_at | 0.04 |
| 132095_at | 0.00 |
| 132100_at | 0.00 |
| 132101_at | 0.00 |
| 132102_at | 0.00 |
| 132103_at | 0.00 |
| 132104_f_at | 0.00 |
| 132105_r_at | 0.00 |
| 132106_at | 0.00 |
| 132107_at | 0.00 |
| 132108_at | 0.00 |
| 132109_at | 0.00 |
| 132110_s_at | 0.00 |
| 132114_f_at | 0.00 |
| 132115_at | 0.00 |
| 132116_at | 0.00 |
| 132117_at | 0.00 |
| 132118_at | 0.00 |
| 132127_at | 0.00 |
| 132128_at | 0.00 |
| 132129_at | 0.00 |
| 132130_at | 0.00 |
| 132131_at | 0.00 |

| | |
|---|---|
| 132132_at | 0.00 |
| 132133_at | 0.00 |
| 132134_at | 0.00 |
| 132141_at | 0.00 |
| 132142_at | 0.00 |
| 132149_at | 0.00 |
| 132150_at | 0.00 |
| 132170_at | 0.00 |
| 132171_at | 0.00 |
| 132172_at | 0.00 |
| 132207_at | 0.00 |
| 132208_at | 0.00 |
| 132209_at | 0.00 |
| 132219_at | 0.00 |
| 132220_at | 0.00 |
| 132221_at | 0.00 |
| 132223_at | 0.00 |
| 132224_at | 0.01 |
| 132225_at | 0.00 |
| 132226_at | 0.00 |
| 132227_at | 0.26 |
| 132228_at | 0.00 |
| 132229_at | 0.00 |
| 132230_at | 0.00 |
| 132231_at | 0.00 |
| 132232_at | 0.00 |
| 132233_at | 0.00 |
| 132234_at | 0.00 |
| 132235_at | 0.00 |
| 132236_at | 0.00 |
| 132238_at | 0.00 |
| 132313_at | 0.00 |
| 132320_at | 0.00 |
| 132321_at | 0.00 |
| 132322_at | 0.00 |
| 132323_at | 0.00 |
| 132324_at | 0.00 |
| 132325_f_at | 0.00 |
| 132360_s_at | 0.00 |
| 132361_at | 0.00 |
| 132364_i_at | 0.00 |
| 132365_r_at | 0.00 |
| 132368_at | 0.00 |
| 132370_at | 0.00 |
| 132374_at | 0.05 |
| 132381_at | 0.00 |
| 132387_at | 0.00 |
| 132388_f_at | 0.00 |
| 132393_f_at | 0.00 |
| 132397_at | 0.00 |
| 132400_f_at | 0.00 |
| 132403_at | 0.00 |
| 132405_s_at | 0.00 |
| 132418_at | 0.00 |
| 132433_at | 0.00 |
| 132434_at | 0.00 |

| | |
|---|---|
| 132435_at | 0.00 |
| 132436_f_at | 0.00 |
| 132437_s_at | 0.00 |
| 132443_at | 0.00 |
| 132496_f_at | 0.00 |
| 132517_at | 0.00 |
| 132518_at | 0.00 |
| 132519_at | 0.00 |
| 132520_at | 0.00 |
| 132521_at | 0.00 |
| 132522_at | 0.00 |
| 132523_at | 0.00 |
| 132524_r_at | 0.00 |
| 132535_at | 0.00 |
| 132536_at | 0.00 |
| 132537_at | 0.00 |
| 132538_at | 0.00 |
| 132539_at | 0.00 |
| 132540_r_at | 0.00 |
| 132548_at | 0.00 |
| 132549_at | 0.00 |
| 132550_at | 0.00 |
| 132553_at | 0.00 |
| 132554_at | 0.00 |
| 132555_at | 0.00 |
| 132556_at | 0.00 |
| 132577_at | 0.00 |
| 132578_at | 0.00 |
| 132579_at | 0.00 |
| 132602_at | 0.00 |
| 132607_at | 0.00 |
| 132608_at | 0.00 |
| 132609_g_at | 0.02 |
| 132659_at | 0.00 |
| 132730_at | 0.01 |
| 132735_at | 0.00 |
| 132740_r_at | 0.00 |
| 132751_i_at | 0.00 |
| 132762_at | 0.00 |
| 132763_at | 0.00 |
| 132772_at | 0.00 |
| 132792_r_at | 0.00 |
| 132793_at | 0.00 |
| 132794_at | 0.00 |
| 132796_at | 0.00 |
| 132797_r_at | 0.00 |
| 132798_at | 0.00 |
| 132799_f_at | 0.00 |
| 132800_r_at | 0.00 |
| 132801_at | 0.00 |
| 132802_at | 0.00 |
| 132803_at | 0.00 |
| 132804_at | 0.00 |
| 132805_at | 0.00 |
| 132806_at | 0.00 |
| 132807_at | 0.00 |

| | |
|---|---|
| 132808_at | 0.00 |
| 132809_at | 0.00 |
| 132810_at | 0.00 |
| 132811_at | 0.00 |
| 132812_at | 0.00 |
| 132813_at | 0.00 |
| 132814_r_at | 0.00 |
| 132815_at | 0.11 |
| 132817_at | 0.09 |
| 132818_at | 0.00 |
| 132820_at | 0.00 |
| 132821_at | 0.00 |
| 132883_at | 0.00 |
| 132884_at | 0.00 |
| 132885_at | 0.00 |
| 132886_at | 0.00 |
| 132887_at | 0.00 |
| 132889_at | 0.00 |
| 132936_at | 0.00 |
| 133028_f_at | 0.00 |
| 133029_at | 0.00 |
| 133030_at | 0.00 |
| 133031_at | 0.00 |
| 133032_at | 0.00 |
| 133033_at | 0.00 |
| 133034_at | 0.00 |
| 133035_at | 0.00 |
| 133036_at | 0.00 |
| 133037_at | 0.00 |
| 133038_at | 0.00 |
| 133039_r_at | 0.00 |
| 133040_r_at | 0.00 |
| 133041_r_at | 0.00 |
| 133042_at | 0.00 |
| 133043_at | 0.00 |
| 133044_at | 0.00 |
| 133045_at | 0.00 |
| 133046_at | 0.00 |
| 133047_at | 0.00 |
| 133048_at | 0.00 |
| 133049_at | 0.00 |
| 133050_at | 0.00 |
| 133051_at | 0.00 |
| 133052_at | 0.46 |
| 133053_at | 0.00 |
| 133054_at | 0.00 |
| 133055_at | 0.00 |
| 133056_at | 0.00 |
| 133057_at | 0.00 |
| 133058_at | 0.00 |
| 133059_at | 0.00 |
| 133060_at | 0.00 |
| 133061_at | 0.00 |
| 133062_r_at | 0.00 |
| 133063_at | 0.00 |
| 133064_at | 0.00 |

| | |
|---|---|
| 133065_at | 0.00 |
| 133066_at | 0.00 |
| 133070_at | 0.00 |
| 133077_at | 0.00 |
| 133081_at | 0.01 |
| 133082_at | 0.00 |
| 133092_f_at | 0.00 |
| 133095_at | 0.00 |
| 133100_at | 0.00 |
| 133111_f_at | 0.00 |
| 133116_at | 0.00 |
| 133118_at | 0.00 |
| 133119_at | 0.00 |
| 133121_at | 0.00 |
| 133122_at | 0.00 |
| 133123_at | 0.00 |
| 133124_at | 0.00 |
| 133125_at | 0.00 |
| 133126_at | 0.01 |
| 133127_at | 0.00 |
| 133128_at | 0.00 |
| 133129_at | 0.00 |
| 133130_at | 0.00 |
| 133131_at | 0.00 |
| 133132_at | 0.00 |
| 133133_at | 0.00 |
| 133134_r_at | 0.00 |
| 133135_at | 0.00 |
| 133136_at | 0.00 |
| 133137_at | 0.00 |
| 133138_at | 0.00 |
| 133139_at | 0.00 |
| 133140_at | 0.00 |
| 133141_at | 0.00 |
| 133142_at | 0.00 |
| 133143_at | 0.00 |
| 133144_at | 0.00 |
| 133145_at | 0.00 |
| 133146_at | 0.00 |
| 133147_at | 0.00 |
| 133149_i_at | 0.00 |
| 133150_f_at | 0.00 |
| 133152_at | 0.00 |
| 133153_at | 0.00 |
| 133154_at | 0.00 |
| 133155_at | 0.00 |
| 133171_at | 0.02 |
| 133181_at | 0.00 |
| 133182_at | 0.00 |
| 133183_f_at | 0.06 |
| 133184_at | 0.00 |
| 133185_at | 0.00 |
| 133186_at | 0.00 |
| 133187_at | 0.00 |
| 133188_at | 0.00 |
| 133189_at | 0.00 |

| | |
|---|---|
| 133190_at | 0.00 |
| 133191_at | 0.00 |
| 133192_at | 0.00 |
| 133193_at | 0.00 |
| 133194_at | 0.00 |
| 133195_at | 0.00 |
| 133196_s_at | 0.00 |
| 133197_at | 0.00 |
| 133198_at | 0.00 |
| 133199_at | 0.00 |
| 133200_at | 0.01 |
| 133201_at | 0.00 |
| 133202_at | 0.00 |
| 133203_at | 0.00 |
| 133204_at | 0.47 |
| 133205_at | 0.00 |
| 133206_at | 0.00 |
| 133207_f_at | 0.02 |
| 133208_r_at | 0.00 |
| 133209_at | 0.00 |
| 133210_r_at | 0.00 |
| 133211_at | 0.00 |
| 133212_at | 0.00 |
| 133213_at | 0.00 |
| 133214_at | 0.00 |
| 133215_at | 0.00 |
| 133216_at | 0.00 |
| 133217_at | 0.00 |
| 133218_at | 0.00 |
| 133219_at | 0.00 |
| 133220_at | 0.00 |
| 133221_at | 0.00 |
| 133222_i_at | 0.00 |
| 133223_at | 0.00 |
| 133224_at | 0.00 |
| 133225_at | 0.00 |
| 133226_at | 0.00 |
| 133227_at | 0.00 |
| 133228_at | 0.00 |
| 133229_at | 0.00 |
| 133230_at | 0.00 |
| 133231_at | 0.00 |
| 133232_at | 0.00 |
| 133233_at | 0.00 |
| 133234_at | 0.00 |
| 133235_at | 0.00 |
| 133236_at | 0.00 |
| 133237_g_at | 0.00 |
| 133238_at | 0.00 |
| 133239_at | 0.00 |
| 133240_at | 0.00 |
| 133241_at | 0.00 |
| 133242_at | 0.00 |
| 133243_at | 0.00 |
| 133244_at | 0.00 |
| 133245_at | 0.00 |

| | |
|---|---|
| 133246_g_at | 0.00 |
| 133247_at | 0.00 |
| 133248_at | 0.00 |
| 133249_at | 0.00 |
| 133250_s_at | 0.00 |
| 133251_at | 0.00 |
| 133252_at | 0.00 |
| 133253_s_at | 0.00 |
| 133254_at | 0.00 |
| 133255_at | 0.00 |
| 133256_f_at | 0.00 |
| 133257_at | 0.00 |
| 133258_s_at | 0.00 |
| 133259_at | 0.00 |
| 133260_g_at | 0.00 |
| 133261_at | 0.00 |
| 133262_at | 0.04 |
| 133263_at | 0.00 |
| 133264_at | 0.00 |
| 133265_at | 0.00 |
| 133266_at | 0.00 |
| 133267_at | 0.00 |
| 133268_f_at | 0.00 |
| 133269_at | 0.00 |
| 133270_at | 0.00 |
| 133271_at | 0.00 |
| 133272_at | 0.00 |
| 133273_at | 0.00 |
| 133274_at | 0.00 |
| 133275_at | 0.00 |
| 133276_r_at | 0.00 |
| 133277_at | 0.00 |
| 133278_at | 0.00 |
| 133279_at | 0.00 |
| 133280_at | 0.00 |
| 133281_at | 0.00 |
| 133282_at | 0.00 |
| 133283_at | 0.00 |
| 133284_at | 0.00 |
| 133285_at | 0.00 |
| 133286_at | 0.00 |
| 133287_at | 0.00 |
| 133288_at | 0.00 |
| 133289_at | 0.00 |
| 133290_at | 0.00 |
| 133291_at | 0.00 |
| 133292_at | 0.00 |
| 133293_at | 0.00 |
| 133294_at | 0.00 |
| 133295_at | 0.00 |
| 133296_at | 0.00 |
| 133297_at | 0.00 |
| 133298_at | 0.00 |
| 133299_at | 0.00 |
| 133300_at | 0.00 |
| 133301_at | 0.00 |

| | |
|---|---|
| 133302_at | 0.00 |
| 133303_at | 0.00 |
| 133304_at | 0.00 |
| 133305_at | 0.00 |
| 133306_i_at | 0.00 |
| 133307_f_at | 0.00 |
| 133308_at | 0.00 |
| 133309_at | 0.00 |
| 133310_at | 0.00 |
| 133311_at | 0.00 |
| 133312_at | 0.00 |
| 133313_at | 0.00 |
| 133314_at | 0.17 |
| 133315_at | 0.00 |
| 133316_at | 0.00 |
| 133318_at | 0.00 |
| 133320_at | 0.00 |
| 133322_s_at | 0.00 |
| 133323_at | 0.00 |
| 133324_at | 0.00 |
| 133327_f_at | 0.00 |
| 133328_at | 0.00 |
| 133329_at | 0.00 |
| 133331_at | 0.00 |
| 133332_at | 0.00 |
| 133335_r_at | 0.00 |
| 133336_at | 0.00 |
| 133337_at | 0.00 |
| 133338_at | 0.00 |
| 133339_r_at | 0.00 |
| 133340_f_at | 0.00 |
| 133341_at | 0.00 |
| 133342_at | 0.00 |
| 133343_at | 0.00 |
| 133344_at | 0.00 |
| 133345_at | 0.00 |
| 133346_at | 0.00 |
| 133347_at | 0.00 |
| 133349_at | 0.00 |
| 133351_at | 0.00 |
| 133353_at | 0.00 |
| 133354_at | 0.00 |
| 133355_r_at | 0.00 |
| 133358_at | 0.00 |
| 133359_at | 0.00 |
| 133361_at | 0.00 |
| 133362_at | 0.00 |
| 133363_g_at | 0.00 |
| 133364_at | 0.01 |
| 133365_at | 0.86 |
| 133366_at | 0.00 |
| 133367_i_at | 0.00 |
| 133368_f_at | 0.00 |
| 133369_at | 0.00 |
| 133370_at | 0.00 |
| 133371_i_at | 0.00 |

| | |
|---|---|
| 133372_f_at | 0.11 |
| 133373_at | 0.29 |
| 133374_at | 0.00 |
| 133375_at | 0.00 |
| 133377_at | 0.00 |
| 133378_at | 0.00 |
| 133379_at | 0.00 |
| 133380_at | 0.01 |
| 133381_at | 0.02 |
| 133382_at | 0.00 |
| 133383_at | 0.00 |
| 133384_at | 0.00 |
| 133385_at | 0.73 |
| 133387_r_at | 0.00 |
| 133388_at | 0.00 |
| 133389_at | 0.08 |
| 133390_at | 0.00 |
| 133391_at | 0.00 |
| 133392_at | 0.00 |
| 133393_r_at | 0.00 |
| 133394_at | 0.00 |
| 133395_at | 0.00 |
| 133396_at | 0.00 |
| 133397_at | 0.00 |
| 133398_at | 0.00 |
| 133399_at | 0.00 |
| 133400_at | 0.00 |
| 133401_i_at | 0.00 |
| 133402_r_at | 0.00 |
| 133403_at | 0.00 |
| 133404_at | 0.00 |
| 133406_at | 0.00 |
| 133407_r_at | 0.00 |
| 133408_at | 0.00 |
| 133409_at | 0.00 |
| 133410_at | 0.00 |
| 133411_at | 0.00 |
| 133413_at | 0.00 |
| 133414_at | 0.00 |
| 133415_at | 0.00 |
| 133416_f_at | 0.00 |
| 133417_r_at | 0.00 |
| 133418_r_at | 0.00 |
| 133420_at | 0.00 |
| 133421_at | 0.00 |
| 133422_at | 0.00 |
| 133423_at | 0.00 |
| 133424_at | 0.00 |
| 133425_at | 0.00 |
| 133426_g_at | 0.00 |
| 133427_r_at | 0.00 |
| 133428_i_at | 0.00 |
| 133429_r_at | 0.00 |
| 133430_at | 0.00 |
| 133431_at | 0.00 |
| 133432_at | 0.00 |

| | |
|---|---|
| 133433_at | 0.00 |
| 133434_at | 0.00 |
| 133435_at | 0.00 |
| 133436_at | 0.00 |
| 133437_at | 0.00 |
| 133438_at | 0.00 |
| 133439_r_at | 0.00 |
| 133440_at | 0.00 |
| 133441_at | 0.00 |
| 133442_r_at | 0.00 |
| 133443_at | 0.00 |
| 133444_at | 0.00 |
| 133445_at | 0.00 |
| 133446_f_at | 0.00 |
| 133447_at | 0.00 |
| 133448_at | 0.00 |
| 133449_at | 0.00 |
| 133450_at | 0.00 |
| 133451_at | 0.00 |
| 133452_at | 0.00 |
| 133453_at | 0.00 |
| 133454_at | 0.00 |
| 133455_at | 0.00 |
| 133456_f_at | 0.00 |
| 133457_at | 0.01 |
| 133458_at | 0.00 |
| 133459_at | 0.00 |
| 133460_at | 0.00 |
| 133461_at | 0.00 |
| 133462_at | 0.00 |
| 133463_at | 0.00 |
| 133464_at | 0.00 |
| 133465_at | 0.00 |
| 133466_at | 0.00 |
| 133467_r_at | 0.00 |
| 133468_at | 0.00 |
| 133469_at | 0.00 |
| 133470_at | 0.00 |
| 133471_at | 0.00 |
| 133472_at | 0.00 |
| 133473_f_at | 0.00 |
| 133474_at | 0.00 |
| 133475_at | 0.00 |
| 133476_at | 0.00 |
| 133477_at | 0.00 |
| 133478_r_at | 0.00 |
| 133479_at | 0.00 |
| 133480_at | 0.00 |
| 133481_at | 0.00 |
| 133482_at | 0.00 |
| 133483_at | 0.01 |
| 133484_at | 0.00 |
| 133485_at | 0.00 |
| 133486_at | 0.00 |
| 133487_at | 0.00 |
| 133488_at | 0.00 |

| | |
|---|---|
| 133489_f_at | 0.00 |
| 133490_at | 0.00 |
| 133491_at | 0.00 |
| 133492_at | 0.00 |
| 133493_at | 0.00 |
| 133494_at | 0.00 |
| 133495_at | 0.00 |
| 133496_at | 0.00 |
| 133497_r_at | 0.00 |
| 133498_at | 0.00 |
| 133499_at | 0.00 |
| 133500_at | 0.00 |
| 133501_at | 0.00 |
| 133502_at | 0.00 |
| 133503_at | 0.00 |
| 133504_r_at | 0.00 |
| 133505_i_at | 0.00 |
| 133506_f_at | 0.00 |
| 133507_at | 0.00 |
| 133508_at | 0.00 |
| 133509_at | 0.00 |
| 133510_at | 0.00 |
| 133511_at | 0.00 |
| 133512_at | 0.00 |
| 133513_at | 0.00 |
| 133514_at | 0.00 |
| 133515_at | 0.00 |
| 133516_at | 0.00 |
| 133517_at | 0.00 |
| 133518_at | 0.00 |
| 133519_at | 0.00 |
| 133520_at | 0.00 |
| 133521_at | 0.00 |
| 133522_at | 0.00 |
| 133523_at | 0.00 |
| 133524_at | 0.00 |
| 133525_at | 0.00 |
| 133526_at | 0.00 |
| 133527_at | 0.00 |
| 133528_at | 0.00 |
| 133529_at | 0.00 |
| 133530_at | 0.00 |
| 133531_at | 0.00 |
| 133532_at | 0.00 |
| 133533_at | 0.00 |
| 133534_at | 0.00 |
| 133535_at | 0.00 |
| 133536_at | 0.04 |
| 133537_at | 0.00 |
| 133538_at | 0.00 |
| 133539_at | 0.00 |
| 133540_at | 0.00 |
| 133545_r_at | 0.00 |
| 133546_r_at | 0.00 |
| 133547_at | 0.00 |
| 133548_at | 0.27 |

| | |
|---|---|
| 133549_at | 0.00 |
| 133550_at | 0.00 |
| 133551_r_at | 0.00 |
| 133552_at | 0.05 |
| 133553_at | 0.01 |
| 133554_r_at | 0.00 |
| 133555_at | 0.00 |
| 133556_at | 0.00 |
| 133557_at | 0.00 |
| 133558_at | 0.00 |
| 133559_at | 0.00 |
| 133560_at | 0.00 |
| 133561_r_at | 0.00 |
| 133562_at | 0.00 |
| 133563_at | 0.00 |
| 133564_at | 0.00 |
| 133574_at | 0.01 |
| 133596_at | 0.00 |
| 133610_at | 0.00 |
| 133628_at | 0.00 |
| 133631_f_at | 0.02 |
| 133632_r_at | 0.00 |
| 133635_r_at | 0.00 |
| 133636_at | 0.04 |
| 133639_r_at | 0.00 |
| 133657_r_at | 0.00 |
| 133661_at | 0.00 |
| 133662_at | 0.00 |
| 133663_at | 0.00 |
| 133664_at | 0.00 |
| 133665_at | 0.00 |
| 133666_at | 0.00 |
| 133667_at | 0.00 |
| 133668_r_at | 0.00 |
| 133669_at | 0.00 |
| 133670_at | 0.00 |
| 133671_at | 0.00 |
| 133672_at | 0.00 |
| 133673_f_at | 0.00 |
| 133674_at | 0.00 |
| 133675_at | 0.00 |
| 133676_r_at | 0.00 |
| 133677_at | 0.00 |
| 133678_at | 0.00 |
| 133679_at | 0.00 |
| 133680_r_at | 0.00 |
| 133681_at | 0.00 |
| 133682_at | 0.00 |
| 133683_at | 0.00 |
| 133684_at | 0.00 |
| 133685_at | 0.00 |
| 133686_at | 0.00 |
| 133687_at | 0.00 |
| 133688_at | 0.00 |
| 133689_at | 0.00 |
| 133690_at | 0.00 |

| | |
|---|---|
| 133691_at | 0.00 |
| 133692_at | 0.00 |
| 133693_at | 0.00 |
| 133694_at | 0.00 |
| 133695_at | 0.00 |
| 133696_at | 0.00 |
| 133697_at | 0.00 |
| 133698_at | 0.00 |
| 133699_at | 0.00 |
| 133700_at | 0.00 |
| 133701_at | 0.00 |
| 133702_at | 0.00 |
| 133703_at | 0.00 |
| 133704_at | 0.00 |
| 133705_at | 0.00 |
| 133706_r_at | 0.00 |
| 133707_at | 0.00 |
| 133708_at | 0.00 |
| 133709_at | 0.00 |
| 133710_at | 0.00 |
| 133711_at | 0.01 |
| 133712_r_at | 0.00 |
| 133713_at | 0.00 |
| 133714_at | 0.00 |
| 133715_at | 0.00 |
| 133716_f_at | 0.00 |
| 133717_at | 0.00 |
| 133718_at | 0.00 |
| 133719_f_at | 0.00 |
| 133720_at | 0.00 |
| 133721_at | 0.00 |
| 133722_at | 0.00 |
| 133723_at | 0.00 |
| 133724_at | 0.00 |
| 133725_at | 0.00 |
| 133726_at | 0.24 |
| 133727_at | 0.00 |
| 133728_at | 0.00 |
| 133729_at | 0.00 |
| 133730_at | 0.00 |
| 133731_at | 0.00 |
| 133732_at | 0.74 |
| 133733_r_at | 0.00 |
| 133734_at | 0.07 |
| 133735_at | 0.00 |
| 133736_at | 0.00 |
| 133737_at | 0.00 |
| 133738_at | 0.00 |
| 133739_at | 0.00 |
| 133740_at | 0.00 |
| 133741_at | 0.00 |
| 133742_at | 0.00 |
| 133743_at | 0.00 |
| 133744_at | 0.00 |
| 133745_at | 0.00 |
| 133746_at | 0.00 |

| | |
|---|---|
| 133747_at | 0.00 |
| 133748_r_at | 0.00 |
| 133749_at | 0.00 |
| 133750_at | 0.00 |
| 133751_f_at | 0.00 |
| 133752_at | 0.00 |
| 133753_at | 0.00 |
| 133754_at | 0.00 |
| 133755_at | 0.00 |
| 133756_at | 0.00 |
| 133757_at | 0.00 |
| 133758_at | 0.00 |
| 133759_at | 0.00 |
| 133760_r_at | 0.00 |
| 133761_at | 0.00 |
| 133762_at | 0.00 |
| 133763_at | 0.00 |
| 133764_at | 0.00 |
| 133765_at | 0.00 |
| 133766_at | 0.00 |
| 133767_at | 0.00 |
| 133768_at | 0.00 |
| 133769_at | 0.00 |
| 133770_at | 0.00 |
| 133771_at | 0.00 |
| 133772_at | 0.00 |
| 133773_at | 0.00 |
| 133774_at | 0.00 |
| 133775_f_at | 0.00 |
| 133776_at | 0.00 |
| 133777_at | 0.00 |
| 133778_at | 0.00 |
| 133779_at | 0.00 |
| 133780_at | 0.00 |
| 133781_at | 0.08 |
| 133782_at | 0.00 |
| 133784_at | 0.00 |
| 133785_r_at | 0.00 |
| 133786_at | 0.00 |
| 133788_at | 0.00 |
| 133789_at | 0.00 |
| 133790_at | 0.00 |
| 133791_at | 0.00 |
| 133792_at | 0.00 |
| 133793_at | 0.00 |
| 133794_at | 0.00 |
| 133795_at | 0.00 |
| 133796_at | 0.00 |
| 133797_at | 0.00 |
| 133798_at | 0.00 |
| 133799_at | 0.00 |
| 133801_at | 0.00 |
| 133802_at | 0.00 |
| 133803_at | 0.00 |
| 133804_at | 0.00 |
| 133805_at | 0.00 |

| | |
|---|---|
| 133806_at | 0.00 |
| 133807_at | 0.00 |
| 133808_at | 0.00 |
| 133809_at | 0.00 |
| 133810_at | 0.12 |
| 133811_at | 0.00 |
| 133812_at | 0.02 |
| 133813_at | 0.03 |
| 133814_f_at | 0.00 |
| 133815_at | 0.00 |
| 133816_at | 0.00 |
| 133817_at | 0.00 |
| 133818_at | 0.00 |
| 133819_at | 0.66 |
| 133820_at | 0.00 |
| 133821_at | 0.00 |
| 133822_at | 0.00 |
| 133823_at | 0.00 |
| 133824_at | 0.00 |
| 133825_at | 0.00 |
| 133828_at | 0.00 |
| 133829_at | 0.00 |
| 133830_at | 0.10 |
| 133831_at | 0.00 |
| 133832_at | 0.00 |
| 133833_at | 0.00 |
| 133834_at | 0.00 |
| 133835_at | 0.00 |
| 133836_at | 0.00 |
| 133837_at | 0.00 |
| 133838_at | 0.00 |
| 133839_at | 0.00 |
| 133840_at | 0.00 |
| 133841_at | 0.00 |
| 133842_at | 0.00 |
| 133843_r_at | 0.00 |
| 133844_at | 0.02 |
| 133845_at | 0.00 |
| 133846_at | 0.00 |
| 133848_at | 0.00 |
| 133849_at | 0.00 |
| 133850_at | 0.00 |
| 133851_s_at | 0.00 |
| 133853_at | 0.00 |
| 133854_at | 0.18 |
| 133855_at | 0.00 |
| 133856_f_at | 0.00 |
| 133857_at | 0.00 |
| 133858_at | 0.00 |
| 133859_at | 0.00 |
| 133860_at | 0.00 |
| 133861_at | 0.00 |
| 133862_at | 0.00 |
| 133864_r_at | 0.00 |
| 133865_at | 0.00 |
| 133866_at | 0.00 |

| | |
|---|---|
| 133867_at | 0.00 |
| 133868_at | 0.00 |
| 133869_at | 0.00 |
| 133870_at | 0.00 |
| 133871_at | 0.00 |
| 133872_at | 0.00 |
| 133873_at | 0.00 |
| 133874_at | 0.00 |
| 133875_at | 0.00 |
| 133876_i_at | 0.00 |
| 133877_f_at | 0.00 |
| 133878_at | 0.00 |
| 133879_at | 0.00 |
| 133880_at | 0.00 |
| 133881_at | 0.00 |
| 133882_at | 0.00 |
| 133883_at | 0.00 |
| 133884_at | 0.00 |
| 133885_at | 0.00 |
| 133886_at | 0.00 |
| 133887_at | 0.00 |
| 133888_at | 0.00 |
| 133889_at | 0.00 |
| 133890_at | 0.00 |
| 133891_at | 0.00 |
| 133892_at | 0.00 |
| 133893_at | 0.00 |
| 133894_at | 0.00 |
| 133895_at | 0.00 |
| 133896_at | 0.00 |
| 133897_at | 0.00 |
| 133898_at | 0.00 |
| 133899_at | 0.00 |
| 133900_at | 0.00 |
| 133901_f_at | 0.00 |
| 133902_r_at | 0.00 |
| 133903_at | 0.00 |
| 133904_at | 0.00 |
| 133905_at | 0.00 |
| 133906_at | 0.00 |
| 133907_at | 0.00 |
| 133908_at | 0.00 |
| 133909_at | 0.08 |
| 133910_at | 0.00 |
| 133911_at | 0.00 |
| 133912_at | 0.00 |
| 133913_at | 0.03 |
| 133914_at | 0.00 |
| 133915_at | 0.00 |
| 133916_at | 0.00 |
| 133917_at | 0.00 |
| 133918_at | 0.00 |
| 133919_at | 0.00 |
| 133920_at | 0.00 |
| 133921_at | 0.00 |
| 133922_at | 0.00 |

| | |
|---|---|
| 133923_at | 0.00 |
| 133924_at | 0.00 |
| 133925_at | 0.00 |
| 133926_at | 0.01 |
| 133927_at | 0.00 |
| 133928_at | 0.00 |
| 133929_at | 0.00 |
| 133930_at | 0.00 |
| 133931_at | 0.00 |
| 133932_at | 0.00 |
| 133933_at | 0.00 |
| 133934_at | 0.00 |
| 133935_at | 0.00 |
| 133936_at | 0.00 |
| 133937_at | 0.00 |
| 133938_at | 0.00 |
| 133939_at | 0.00 |
| 133940_at | 0.00 |
| 133941_at | 0.00 |
| 133942_at | 0.00 |
| 133943_at | 0.00 |
| 133944_at | 0.00 |
| 133945_at | 0.00 |
| 133946_at | 0.00 |
| 133947_at | 0.00 |
| 133948_at | 0.00 |
| 133949_i_at | 0.00 |
| 133950_r_at | 0.00 |
| 133951_at | 0.00 |
| 133952_at | 0.00 |
| 133953_at | 0.00 |
| 133954_at | 0.00 |
| 133955_at | 0.00 |
| 133956_at | 0.00 |
| 133957_at | 0.00 |
| 133958_at | 0.00 |
| 133959_at | 0.00 |
| 133960_at | 0.00 |
| 133961_at | 0.00 |
| 133962_at | 0.00 |
| 133963_at | 0.00 |
| 133964_at | 0.00 |
| 133965_at | 0.00 |
| 133966_at | 0.00 |
| 133967_at | 0.00 |
| 133968_at | 0.00 |
| 133969_at | 0.00 |
| 133970_at | 0.00 |
| 133971_at | 0.00 |
| 133972_at | 0.00 |
| 133973_at | 0.00 |
| 133974_at | 0.00 |
| 133975_at | 0.00 |
| 133976_at | 0.00 |
| 133977_at | 0.00 |
| 133978_at | 0.00 |

| | |
|---|---|
| 133979_at | 0.00 |
| 133980_at | 0.00 |
| 133981_at | 0.00 |
| 133982_at | 0.00 |
| 133983_at | 0.00 |
| 133984_at | 0.00 |
| 133985_at | 0.00 |
| 133986_at | 0.00 |
| 133987_at | 0.00 |
| 133988_at | 0.00 |
| 133989_at | 0.00 |
| 133990_at | 0.00 |
| 133991_at | 0.00 |
| 133992_at | 0.00 |
| 133993_at | 0.00 |
| 133994_at | 0.00 |
| 133995_at | 0.00 |
| 133996_at | 0.00 |
| 133997_at | 0.00 |
| 133998_at | 0.00 |
| 133999_at | 0.00 |
| 134000_at | 0.00 |
| 134001_at | 0.00 |
| 134002_at | 0.00 |
| 134003_at | 0.00 |
| 134004_f_at | 0.00 |
| 134005_at | 0.00 |
| 134006_at | 0.00 |
| 134007_at | 0.00 |
| 134008_at | 0.00 |
| 134009_at | 0.00 |
| 134010_s_at | 0.00 |
| 134011_at | 0.00 |
| 134012_at | 0.00 |
| 134013_at | 0.00 |
| 134014_at | 0.00 |
| 134015_at | 0.00 |
| 134016_f_at | 0.04 |
| 134017_at | 0.00 |
| 134018_at | 0.00 |
| 134020_at | 0.00 |
| 134023_at | 0.00 |
| 134027_at | 0.00 |
| 134028_at | 0.00 |
| 134030_at | 0.00 |
| 134031_r_at | 0.00 |
| 134033_at | 0.00 |
| 134035_f_at | 0.02 |
| 134036_at | 0.00 |
| 134037_f_at | 0.00 |
| 134038_r_at | 0.00 |
| 134039_at | 0.00 |
| 134041_at | 0.00 |
| 134042_at | 0.00 |
| 134043_at | 0.00 |
| 134045_at | 0.13 |

| | |
|---|---|
| 134046_at | 0.00 |
| 134047_at | 0.47 |
| 134049_r_at | 0.00 |
| 134051_at | 0.00 |
| 134053_at | 0.00 |
| 134054_at | 0.00 |
| 134055_at | 0.00 |
| 134057_at | 0.00 |
| 134059_at | 0.00 |
| 134060_at | 0.00 |
| 134061_r_at | 0.00 |
| 134062_at | 0.00 |
| 134063_at | 0.00 |
| 134064_at | 0.00 |
| 134065_at | 0.00 |
| 134066_f_at | 0.00 |
| 134067_r_at | 0.00 |
| 134069_at | 0.00 |
| 134070_at | 0.00 |
| 134071_at | 0.00 |
| 134072_i_at | 0.00 |
| 134073_f_at | 0.00 |
| 134075_at | 0.00 |
| 134076_at | 0.00 |
| 134077_at | 0.00 |
| 134078_at | 0.00 |
| 134079_f_at | 0.00 |
| 134080_at | 0.00 |
| 134082_at | 0.00 |
| 134083_at | 0.00 |
| 134085_at | 0.00 |
| 134087_at | 0.00 |
| 134090_at | 0.00 |
| 134091_s_at | 0.00 |
| 134095_f_at | 0.00 |
| 134097_at | 0.00 |
| 134102_at | 0.00 |
| 134105_at | 0.00 |
| 134107_at | 0.09 |
| 134108_at | 0.00 |
| 134109_at | 0.00 |
| 134114_at | 0.00 |
| 134115_at | 0.00 |
| 134116_at | 0.00 |
| 134117_at | 0.00 |
| 134118_r_at | 0.00 |
| 134119_at | 0.00 |
| 134123_r_at | 0.00 |
| 134125_at | 0.00 |
| 134127_at | 0.00 |
| 134129_at | 0.00 |
| 134130_at | 0.00 |
| 134131_at | 0.00 |
| 134133_at | 0.00 |
| 134134_f_at | 0.00 |
| 134135_r_at | 0.00 |

| | |
|---|---|
| 134137_at | 0.00 |
| 134138_at | 0.00 |
| 134139_r_at | 0.00 |
| 134140_at | 0.00 |
| 134141_at | 0.01 |
| 134142_f_at | 0.00 |
| 134143_r_at | 0.00 |
| 134144_at | 0.00 |
| 134145_at | 0.00 |
| 134146_at | 0.00 |
| 134147_f_at | 0.00 |
| 134148_at | 0.00 |
| 134149_at | 0.00 |
| 134150_at | 0.00 |
| 134151_at | 0.00 |
| 134152_at | 0.05 |
| 134153_at | 0.00 |
| 134154_at | 0.00 |
| 134155_at | 0.00 |
| 134156_at | 0.00 |
| 134157_at | 0.00 |
| 134158_at | 0.00 |
| 134159_at | 0.00 |
| 134160_at | 0.00 |
| 134161_at | 0.00 |
| 134162_at | 0.00 |
| 134163_at | 0.00 |
| 134164_at | 0.00 |
| 134165_at | 0.00 |
| 134166_at | 0.00 |
| 134167_at | 0.00 |
| 134168_at | 0.00 |
| 134169_at | 0.00 |
| 134170_at | 0.00 |
| 134171_at | 0.00 |
| 134173_at | 0.00 |
| 134174_at | 0.00 |
| 134175_at | 0.00 |
| 134176_r_at | 0.00 |
| 134177_at | 0.00 |
| 134178_at | 0.00 |
| 134179_at | 0.00 |
| 134180_at | 0.00 |
| 134181_r_at | 0.03 |
| 134182_at | 0.00 |
| 134183_at | 0.00 |
| 134184_at | 0.00 |
| 134185_at | 0.00 |
| 134186_at | 0.00 |
| 134187_at | 0.01 |
| 134188_at | 0.00 |
| 134189_at | 0.00 |
| 134190_at | 0.00 |
| 134191_at | 0.00 |
| 134192_at | 0.00 |
| 134194_at | 0.00 |

| | |
|---|---|
| 134195_at | 0.00 |
| 134196_r_at | 0.00 |
| 134197_at | 0.00 |
| 134198_at | 0.00 |
| 134199_at | 0.00 |
| 134200_at | 0.00 |
| 134201_at | 0.00 |
| 134202_f_at | 0.00 |
| 134203_at | 0.00 |
| 134204_at | 0.00 |
| 134205_at | 0.08 |
| 134206_f_at | 0.00 |
| 134207_at | 0.00 |
| 134208_at | 0.00 |
| 134209_at | 0.00 |
| 134210_at | 0.00 |
| 134211_at | 0.00 |
| 134212_at | 0.00 |
| 134213_at | 0.00 |
| 134214_r_at | 0.00 |
| 134215_at | 0.00 |
| 134216_at | 0.00 |
| 134217_at | 0.00 |
| 134218_at | 0.00 |
| 134219_at | 0.00 |
| 134220_at | 0.00 |
| 134221_at | 0.07 |
| 134222_at | 0.00 |
| 134223_at | 0.00 |
| 134224_at | 0.00 |
| 134225_i_at | 0.00 |
| 134226_r_at | 0.00 |
| 134227_at | 0.00 |
| 134228_at | 0.00 |
| 134229_at | 0.00 |
| 134230_at | 0.49 |
| 134231_r_at | 0.00 |
| 134232_at | 0.00 |
| 134233_at | 0.00 |
| 134234_at | 0.01 |
| 134235_at | 0.00 |
| 134236_at | 0.00 |
| 134237_at | 0.00 |
| 134238_at | 0.00 |
| 134239_f_at | 0.00 |
| 134240_at | 0.00 |
| 134241_at | 0.00 |
| 134242_at | 0.00 |
| 134243_at | 0.00 |
| 134244_at | 0.00 |
| 134245_at | 0.00 |
| 134246_at | 0.00 |
| 134247_at | 0.00 |
| 134248_at | 0.00 |
| 134249_at | 0.66 |
| 134252_r_at | 0.00 |

| | |
|---|---|
| 134253_at | 0.00 |
| 134254_at | 0.00 |
| 134255_at | 0.00 |
| 134256_at | 0.00 |
| 134257_at | 0.00 |
| 134258_at | 0.00 |
| 134259_g_at | 0.00 |
| 134260_at | 0.00 |
| 134261_at | 0.00 |
| 134262_at | 0.00 |
| 134263_at | 0.00 |
| 134264_at | 0.00 |
| 134265_at | 0.00 |
| 134266_at | 0.00 |
| 134267_at | 0.00 |
| 134268_at | 0.00 |
| 134269_at | 0.00 |
| 134270_at | 0.00 |
| 134271_at | 0.00 |
| 134272_at | 0.00 |
| 134273_at | 0.00 |
| 134274_at | 0.00 |
| 134275_at | 0.00 |
| 134276_at | 0.00 |
| 134277_at | 0.00 |
| 134278_at | 0.00 |
| 134279_at | 0.00 |
| 134280_at | 0.00 |
| 134281_at | 0.00 |
| 134282_at | 0.00 |
| 134283_f_at | 0.00 |
| 134284_at | 0.00 |
| 134285_at | 0.00 |
| 134286_at | 0.00 |
| 134287_at | 0.00 |
| 134288_at | 0.09 |
| 134289_r_at | 0.00 |
| 134290_at | 0.00 |
| 134291_at | 0.00 |
| 134292_at | 0.00 |
| 134293_at | 0.00 |
| 134294_at | 0.00 |
| 134295_at | 0.00 |
| 134296_at | 0.00 |
| 134297_at | 0.00 |
| 134298_at | 0.00 |
| 134299_r_at | 0.00 |
| 134300_at | 0.00 |
| 134301_at | 0.00 |
| 134302_at | 0.00 |
| 134303_at | 0.00 |
| 134304_at | 0.00 |
| 134305_at | 0.00 |
| 134306_at | 0.00 |
| 134307_at | 0.00 |
| 134308_at | 0.00 |

| | |
|---|---|
| 134309_at | 0.00 |
| 134310_r_at | 0.00 |
| 134311_r_at | 0.00 |
| 134312_at | 0.00 |
| 134313_at | 0.00 |
| 134314_at | 0.00 |
| 134315_at | 0.00 |
| 134316_at | 0.00 |
| 134317_at | 0.00 |
| 134318_at | 0.00 |
| 134319_at | 0.00 |
| 134320_at | 0.00 |
| 134321_at | 0.00 |
| 134322_at | 0.00 |
| 134323_at | 0.04 |
| 134324_at | 0.00 |
| 134325_at | 0.00 |
| 134326_at | 0.00 |
| 134327_at | 0.00 |
| 134328_at | 0.00 |
| 134329_at | 0.00 |
| 134330_at | 0.00 |
| 134331_at | 0.00 |
| 134332_at | 0.00 |
| 134333_at | 0.00 |
| 134334_at | 0.00 |
| 134335_at | 0.00 |
| 134336_at | 0.00 |
| 134337_at | 0.00 |
| 134338_at | 0.00 |
| 134339_at | 0.00 |
| 134340_at | 0.00 |
| 134341_f_at | 0.00 |
| 134342_r_at | 0.00 |
| 134343_at | 0.00 |
| 134344_at | 0.00 |
| 134345_at | 0.00 |
| 134346_at | 0.00 |
| 134347_f_at | 0.00 |
| 134348_r_at | 0.00 |
| 134349_r_at | 0.00 |
| 134350_at | 0.00 |
| 134351_at | 0.00 |
| 134352_at | 0.00 |
| 134353_at | 0.00 |
| 134354_at | 0.00 |
| 134355_r_at | 0.00 |
| 134356_r_at | 0.00 |
| 134357_at | 0.00 |
| 134358_r_at | 0.00 |
| 134359_r_at | 0.00 |
| 134360_at | 0.00 |
| 134361_at | 0.00 |
| 134362_at | 0.00 |
| 134363_at | 0.00 |
| 134364_at | 0.00 |

| | |
|---|---|
| 134365_at | 0.02 |
| 134366_f_at | 0.00 |
| 134367_at | 0.00 |
| 134368_f_at | 0.00 |
| 134369_i_at | 0.00 |
| 134370_f_at | 0.00 |
| 134371_at | 0.00 |
| 134372_f_at | 0.00 |
| 134373_at | 0.00 |
| 134374_at | 0.00 |
| 134375_at | 0.00 |
| 134376_i_at | 0.00 |
| 134377_f_at | 0.00 |
| 134379_r_at | 0.00 |
| 134380_at | 0.00 |
| 134381_at | 0.01 |
| 134382_at | 0.00 |
| 134384_at | 0.00 |
| 134385_at | 0.00 |
| 134386_at | 0.00 |
| 134387_at | 0.13 |
| 134388_at | 0.00 |
| 134391_at | 0.00 |
| 134395_f_at | 0.00 |
| 134396_at | 0.00 |
| 134399_at | 0.00 |
| 134400_f_at | 0.00 |
| 134401_at | 0.00 |
| 134402_at | 0.00 |
| 134404_at | 0.00 |
| 134405_at | 0.00 |
| 134407_f_at | 0.00 |
| 134409_r_at | 0.00 |
| 134410_at | 0.00 |
| 134434_at | 0.00 |
| 134438_at | 0.00 |
| 134452_at | 0.00 |
| 134465_at | 0.00 |
| 134476_at | 0.00 |
| 134490_at | 0.00 |
| 134509_at | 0.00 |
| 134511_at | 0.00 |
| 134512_at | 0.00 |
| 134513_f_at | 0.00 |
| 134514_at | 0.00 |
| 134515_at | 0.03 |
| 134517_at | 0.00 |
| 134518_at | 0.00 |
| 134521_r_at | 0.00 |
| 134522_at | 0.00 |
| 134523_at | 0.00 |
| 134524_at | 0.00 |
| 134525_f_at | 0.00 |
| 134526_r_at | 0.00 |
| 134527_f_at | 0.00 |
| 134528_at | 0.00 |

| | |
|---|---|
| 134530_at | 0.00 |
| 134531_at | 0.00 |
| 134532_at | 0.00 |
| 134533_at | 0.00 |
| 134534_at | 0.00 |
| 134535_f_at | 0.00 |
| 134536_at | 0.00 |
| 134537_at | 0.00 |
| 134551_i_at | 0.00 |
| 134552_f_at | 0.00 |
| 134566_at | 0.23 |
| 134577_at | 0.00 |
| 134588_at | 0.00 |
| 134593_at | 0.00 |
| 134594_at | 0.00 |
| 134595_at | 0.00 |
| 134596_at | 0.00 |
| 134597_at | 0.00 |
| 134598_at | 0.00 |
| 134599_r_at | 0.00 |
| 134600_at | 0.00 |
| 134601_r_at | 0.00 |
| 134602_r_at | 0.00 |
| 134618_at | 0.00 |
| 134619_at | 0.00 |
| 134620_at | 0.00 |
| 134621_at | 0.00 |
| 134622_f_at | 0.02 |
| 134623_at | 0.00 |
| 134624_at | 0.00 |
| 134625_i_at | 0.00 |
| 134626_r_at | 0.00 |
| 134627_at | 0.00 |
| 134628_at | 0.00 |
| 134629_at | 0.00 |
| 134630_at | 0.01 |
| 134631_at | 0.00 |
| 134632_at | 0.00 |
| 134633_at | 0.00 |
| 134634_at | 0.00 |
| 134635_at | 0.00 |
| 134636_at | 0.00 |
| 134637_at | 0.00 |
| 134638_at | 0.01 |
| 134639_at | 0.00 |
| 134640_at | 0.00 |
| 134641_at | 0.00 |
| 134642_f_at | 0.00 |
| 134644_f_at | 0.00 |
| 134645_r_at | 0.00 |
| 134646_at | 0.00 |
| 134647_at | 0.00 |
| 134648_at | 0.00 |
| 134649_at | 0.00 |
| 134650_at | 0.00 |
| 134651_at | 0.00 |

| | |
|---|---|
| 134653_at | 0.00 |
| 134654_at | 0.00 |
| 134656_at | 0.00 |
| 134657_at | 0.00 |
| 134658_at | 0.00 |
| 134659_r_at | 0.00 |
| 134660_at | 0.03 |
| 134661_at | 0.00 |
| 134662_f_at | 0.05 |
| 134663_at | 0.00 |
| 134665_at | 0.00 |
| 134666_at | 0.00 |
| 134667_at | 0.00 |
| 134669_at | 0.00 |
| 134673_at | 0.00 |
| 134674_at | 0.00 |
| 134675_at | 0.00 |
| 134676_at | 0.00 |
| 134677_at | 0.02 |
| 134678_at | 0.00 |
| 134679_at | 0.00 |
| 134681_at | 0.01 |
| 134682_at | 0.00 |
| 134683_at | 0.00 |
| 134684_at | 0.00 |
| 134685_at | 0.02 |
| 134686_r_at | 0.00 |
| 134688_at | 0.00 |
| 134689_at | 0.00 |
| 134690_at | 0.00 |
| 134691_at | 0.00 |
| 134692_at | 0.00 |
| 134693_at | 0.00 |
| 134694_at | 0.00 |
| 134695_at | 0.00 |
| 134696_at | 0.00 |
| 134700_at | 0.00 |
| 134701_at | 0.00 |
| 134702_at | 0.00 |
| 134703_at | 0.00 |
| 134705_at | 0.00 |
| 134706_at | 0.00 |
| 134707_at | 0.00 |
| 134708_at | 0.00 |
| 134709_r_at | 0.00 |
| 134710_at | 0.00 |
| 134711_at | 0.00 |
| 134712_at | 0.00 |
| 134713_at | 0.00 |
| 134714_at | 0.00 |
| 134717_at | 0.01 |
| 134718_at | 0.00 |
| 134719_at | 0.00 |
| 134720_at | 0.00 |
| 134721_at | 0.00 |
| 134722_r_at | 0.00 |

| | |
|---|---|
| 134723_at | 0.00 |
| 134724_at | 0.00 |
| 134725_at | 0.00 |
| 134726_f_at | 0.00 |
| 134727_at | 0.00 |
| 134728_at | 0.00 |
| 134730_at | 0.00 |
| 134731_at | 0.00 |
| 134733_r_at | 0.00 |
| 134734_at | 0.00 |
| 134735_at | 0.00 |
| 134736_f_at | 0.00 |
| 134737_r_at | 0.00 |
| 134738_at | 0.00 |
| 134739_r_at | 0.00 |
| 134740_at | 0.00 |
| 134741_at | 0.00 |
| 134742_at | 0.00 |
| 134743_at | 0.00 |
| 134744_at | 0.00 |
| 134745_at | 0.00 |
| 134746_at | 0.00 |
| 134747_at | 0.00 |
| 134748_at | 0.00 |
| 134749_f_at | 0.00 |
| 134750_at | 0.00 |
| 134751_at | 0.01 |
| 134752_at | 0.00 |
| 134753_at | 0.00 |
| 134754_at | 0.00 |
| 134755_at | 0.00 |
| 134756_at | 0.00 |
| 134757_at | 0.00 |
| 134758_at | 0.00 |
| 134759_at | 0.00 |
| 134760_at | 0.00 |
| 134761_at | 0.00 |
| 134762_at | 0.00 |
| 134763_at | 0.00 |
| 134764_at | 0.00 |
| 134765_at | 0.00 |
| 134766_at | 0.00 |
| 134767_at | 0.00 |
| 134768_at | 0.00 |
| 134769_f_at | 0.00 |
| 134770_at | 0.00 |
| 134771_at | 0.00 |
| 134772_at | 0.00 |
| 134773_at | 0.00 |
| 134774_at | 0.00 |
| 134775_at | 0.01 |
| 134776_at | 0.00 |
| 134777_at | 0.00 |
| 134778_at | 0.00 |
| 134779_at | 0.00 |
| 134780_at | 0.00 |

| | |
|---|---|
| 134781_at | 0.00 |
| 134782_at | 0.00 |
| 134783_at | 0.00 |
| 134784_at | 0.00 |
| 134785_at | 0.00 |
| 134786_at | 0.00 |
| 134787_at | 0.00 |
| 134788_at | 0.00 |
| 134789_at | 0.00 |
| 134790_at | 0.00 |
| 134791_at | 0.00 |
| 134792_at | 0.00 |
| 134793_at | 0.00 |
| 134794_at | 0.00 |
| 134795_at | 0.00 |
| 134796_f_at | 0.00 |
| 134797_at | 0.09 |
| 134798_at | 0.01 |
| 134799_at | 0.00 |
| 134800_at | 0.00 |
| 134801_at | 0.00 |
| 134802_at | 0.00 |
| 134803_at | 0.00 |
| 134804_at | 0.00 |
| 134805_at | 0.00 |
| 134806_at | 0.00 |
| 134807_at | 0.00 |
| 134808_at | 0.00 |
| 134809_at | 0.00 |
| 134810_s_at | 0.00 |
| 134811_at | 0.00 |
| 134812_r_at | 0.00 |
| 134813_at | 0.00 |
| 134814_at | 0.00 |
| 134815_at | 0.00 |
| 134816_at | 0.00 |
| 134817_at | 0.00 |
| 134818_at | 0.00 |
| 134819_at | 0.00 |
| 134820_at | 0.00 |
| 134821_at | 0.00 |
| 134822_at | 0.00 |
| 134823_at | 0.00 |
| 134824_s_at | 0.00 |
| 134825_r_at | 0.00 |
| 134826_at | 0.00 |
| 134827_f_at | 0.00 |
| 134828_r_at | 0.00 |
| 134829_at | 0.00 |
| 134830_at | 0.00 |
| 134831_at | 0.00 |
| 134832_at | 0.00 |
| 134833_at | 0.00 |
| 134834_at | 0.00 |
| 134835_at | 0.00 |
| 134836_at | 0.00 |

| | |
|---|---|
| 134837_at | 0.00 |
| 134838_at | 0.00 |
| 134839_at | 0.00 |
| 134840_at | 0.00 |
| 134841_at | 0.00 |
| 134842_at | 0.00 |
| 134843_at | 0.00 |
| 134844_at | 0.00 |
| 134845_at | 0.00 |
| 134846_at | 0.00 |
| 134847_at | 0.00 |
| 134848_at | 0.00 |
| 134849_at | 0.00 |
| 134850_at | 0.00 |
| 134851_at | 0.00 |
| 134852_at | 0.00 |
| 134853_at | 0.01 |
| 134854_at | 0.00 |
| 134855_at | 0.00 |
| 134856_at | 0.00 |
| 134857_at | 0.00 |
| 134866_at | 0.00 |
| 134869_at | 0.00 |
| 134878_at | 0.00 |
| 134879_at | 0.00 |
| 134885_at | 0.00 |
| 134932_at | 0.00 |
| 134942_at | 0.00 |
| 134971_f_at | 0.00 |
| 135023_at | 0.00 |
| 135024_at | 0.00 |
| 135034_at | 0.00 |
| 135049_at | 0.00 |
| 135050_at | 0.00 |
| 135051_r_at | 0.00 |
| 135052_r_at | 0.00 |
| 135054_r_at | 0.00 |
| 135057_at | 0.00 |
| 135059_at | 0.00 |
| 135060_f_at | 0.01 |
| 135066_at | 0.00 |
| 135069_at | 0.02 |
| 135070_at | 0.00 |
| 135071_at | 0.00 |
| 135072_r_at | 0.00 |
| 135073_at | 0.00 |
| 135076_at | 0.00 |
| 135078_at | 0.00 |
| 135079_r_at | 0.00 |
| 135082_at | 0.00 |
| 135083_at | 0.00 |
| 135084_at | 0.00 |
| 135089_at | 0.00 |
| 135090_at | 0.00 |
| 135091_at | 0.00 |
| 135092_at | 0.00 |

| | |
|---|---|
| 135093_at | 0.00 |
| 135094_at | 0.00 |
| 135097_at | 0.00 |
| 135098_at | 0.00 |
| 135100_at | 0.00 |
| 135101_at | 0.00 |
| 135103_at | 0.00 |
| 135104_at | 0.00 |
| 135106_at | 0.00 |
| 135110_at | 0.00 |
| 135111_at | 0.00 |
| 135112_at | 0.00 |
| 135114_at | 0.00 |
| 135115_r_at | 0.00 |
| 135117_at | 0.00 |
| 135118_at | 0.00 |
| 135119_at | 0.00 |
| 135120_r_at | 0.00 |
| 135121_at | 0.00 |
| 135123_at | 0.00 |
| 135125_at | 0.00 |
| 135126_at | 0.00 |
| 135130_at | 0.00 |
| 135131_at | 0.00 |
| 135132_at | 0.00 |
| 135134_at | 0.00 |
| 135136_at | 0.00 |
| 135137_at | 0.00 |
| 135138_at | 0.00 |
| 135139_at | 0.00 |
| 135140_i_at | 0.00 |
| 135141_at | 0.00 |
| 135142_at | 0.00 |
| 135143_at | 0.00 |
| 135144_r_at | 0.00 |
| 135145_r_at | 0.00 |
| 135146_r_at | 0.00 |
| 135147_at | 0.00 |
| 135148_at | 0.00 |
| 135149_at | 0.00 |
| 135150_at | 0.00 |
| 135151_at | 0.00 |
| 135153_at | 0.00 |
| 135154_r_at | 0.00 |
| 135155_at | 0.00 |
| 135156_at | 0.00 |
| 135157_at | 0.00 |
| 135158_at | 0.00 |
| 135159_at | 0.00 |
| 135160_i_at | 0.00 |
| 135161_f_at | 0.00 |
| 135163_r_at | 0.00 |
| 135166_at | 0.00 |
| 135167_at | 0.00 |
| 135169_at | 0.00 |
| 135170_at | 0.00 |

| | |
|---|---|
| 135171_at | 0.12 |
| 135172_at | 0.00 |
| 135173_at | 0.00 |
| 135174_at | 0.00 |
| 135175_at | 0.00 |
| 135177_at | 0.00 |
| 135178_at | 0.03 |
| 135179_at | 0.00 |
| 135180_at | 0.00 |
| 135181_f_at | 0.00 |
| 135182_at | 0.00 |
| 135183_r_at | 0.00 |
| 135184_at | 0.00 |
| 135185_at | 0.00 |
| 135188_at | 0.00 |
| 135189_f_at | 0.33 |
| 135190_at | 0.00 |
| 135191_at | 0.00 |
| 135194_at | 0.00 |
| 135195_at | 0.00 |
| 135196_at | 0.00 |
| 135197_at | 0.00 |
| 135199_at | 0.00 |
| 135200_at | 0.00 |
| 135201_at | 0.00 |
| 135202_at | 0.00 |
| 135205_at | 0.00 |
| 135206_at | 0.00 |
| 135208_at | 0.00 |
| 135209_at | 0.00 |
| 135210_at | 0.00 |
| 135211_at | 0.00 |
| 135212_at | 0.00 |
| 135213_at | 0.00 |
| 135214_at | 0.00 |
| 135215_at | 0.00 |
| 135216_at | 0.00 |
| 135217_at | 0.00 |
| 135218_f_at | 0.00 |
| 135219_at | 0.00 |
| 135220_at | 0.00 |
| 135221_at | 0.00 |
| 135222_at | 0.00 |
| 135223_at | 0.00 |
| 135224_at | 0.00 |
| 135225_at | 0.00 |
| 135226_at | 0.00 |
| 135227_at | 0.00 |
| 135228_at | 0.00 |
| 135229_at | 0.00 |
| 135230_at | 0.00 |
| 135231_at | 0.00 |
| 135232_at | 0.00 |
| 135233_at | 0.00 |
| 135234_at | 0.00 |
| 135235_at | 0.00 |

| | |
|---|---|
| 135236_at | 0.00 |
| 135237_at | 0.00 |
| 135238_f_at | 0.00 |
| 135239_r_at | 0.00 |
| 135240_at | 0.00 |
| 135241_g_at | 0.00 |
| 135242_at | 0.00 |
| 135243_at | 0.00 |
| 135244_at | 0.00 |
| 135245_at | 0.00 |
| 135246_at | 0.00 |
| 135247_at | 0.00 |
| 135248_at | 0.00 |
| 135249_at | 0.00 |
| 135250_at | 0.00 |
| 135251_at | 0.00 |
| 135252_at | 0.00 |
| 135253_at | 0.00 |
| 135254_at | 0.00 |
| 135255_at | 0.00 |
| 135256_at | 0.00 |
| 135257_at | 0.00 |
| 135258_at | 0.00 |
| 135259_at | 0.00 |
| 135260_at | 0.00 |
| 135261_at | 0.00 |
| 135262_at | 0.00 |
| 135263_at | 0.00 |
| 135264_at | 0.00 |
| 135265_at | 0.00 |
| 135266_at | 0.00 |
| 135267_at | 0.00 |
| 135268_i_at | 0.00 |
| 135269_r_at | 0.00 |
| 135270_at | 0.00 |
| 135271_at | 0.00 |
| 135272_at | 0.00 |
| 135273_at | 0.00 |
| 135274_at | 0.00 |
| 135275_at | 0.00 |
| 135276_at | 0.00 |
| 135277_at | 0.00 |
| 135278_at | 0.00 |
| 135279_at | 0.00 |
| 135280_at | 0.00 |
| 135281_at | 0.00 |
| 135282_at | 0.00 |
| 135283_at | 0.00 |
| 135284_at | 0.00 |
| 135285_at | 0.00 |
| 135286_at | 0.00 |
| 135287_at | 0.00 |
| 135288_at | 0.00 |
| 135289_at | 0.00 |
| 135290_at | 0.00 |
| 135291_at | 0.00 |

| | |
|---|---|
| 135292_at | 0.00 |
| 135293_at | 0.00 |
| 135294_at | 0.00 |
| 135295_at | 0.00 |
| 135296_at | 0.00 |
| 135297_at | 0.00 |
| 135298_at | 0.00 |
| 135299_at | 0.00 |
| 135300_at | 0.00 |
| 135301_at | 0.00 |
| 135302_at | 0.00 |
| 135303_at | 0.00 |
| 135304_at | 0.00 |
| 135305_at | 0.00 |
| 135306_at | 0.00 |
| 135307_at | 0.00 |
| 135308_r_at | 0.00 |
| 135309_at | 0.00 |
| 135310_f_at | 0.00 |
| 135311_at | 0.00 |
| 135312_at | 0.00 |
| 135313_at | 0.00 |
| 135314_at | 0.15 |
| 135315_at | 0.00 |
| 135316_at | 0.01 |
| 135317_at | 0.00 |
| 135318_at | 0.00 |
| 135319_at | 0.00 |
| 135320_at | 0.00 |
| 135321_at | 0.00 |
| 135322_at | 0.00 |
| 135323_at | 0.00 |
| 135324_at | 0.00 |
| 135325_at | 0.00 |
| 135326_at | 0.00 |
| 135327_at | 0.00 |
| 135328_at | 0.00 |
| 135329_at | 0.00 |
| 135330_at | 0.00 |
| 135331_at | 0.00 |
| 135332_f_at | 0.00 |
| 135333_i_at | 0.00 |
| 135334_f_at | 0.00 |
| 135335_at | 0.00 |
| 135336_at | 0.00 |
| 135337_at | 0.00 |
| 135338_s_at | 0.20 |
| 135339_at | 0.00 |
| 135340_at | 0.00 |
| 135341_r_at | 0.00 |
| 135343_r_at | 0.02 |
| 135346_at | 0.00 |
| 135347_at | 0.02 |
| 135349_r_at | 0.00 |
| 135350_at | 0.00 |
| 135351_i_at | 0.00 |

| | |
|---|---|
| 135352_r_at | 0.00 |
| 135353_at | 0.00 |
| 135354_s_at | 0.33 |
| 135355_at | 0.27 |
| 135357_at | 0.00 |
| 135358_s_at | 0.00 |
| 135359_at | 0.00 |
| 135361_at | 0.00 |
| 135362_at | 0.00 |
| 135363_at | 0.00 |
| 135364_at | 0.00 |
| 135367_at | 0.00 |
| 135368_at | 0.30 |
| 135371_at | 0.00 |
| 135374_at | 0.00 |
| 135375_at | 0.00 |
| 135376_at | 0.00 |
| 135381_i_at | 0.00 |
| 135382_r_at | 0.04 |
| 135383_r_at | 0.00 |
| 135384_at | 0.00 |
| 135385_r_at | 0.00 |
| 135386_r_at | 0.00 |
| 135387_at | 0.00 |
| 135388_g_at | 0.00 |
| 135389_at | 0.00 |
| 135390_at | 0.00 |
| 135391_at | 0.00 |
| 135392_at | 0.00 |
| 135394_at | 0.00 |
| 135395_at | 0.00 |
| 135396_at | 0.00 |
| 135400_at | 0.00 |
| 135401_at | 0.00 |
| 135402_at | 0.00 |
| 135404_at | 0.00 |
| 135405_at | 0.00 |
| 135407_at | 0.00 |
| 135414_at | 0.00 |
| 135415_at | 0.00 |
| 135433_at | 0.00 |
| 135434_at | 0.00 |
| 135435_at | 0.00 |
| 135436_at | 0.00 |
| 135437_r_at | 0.00 |
| 135441_at | 0.12 |
| 135455_at | 0.00 |
| 135456_at | 0.00 |
| 135464_at | 0.00 |
| 135465_at | 0.00 |
| 135475_i_at | 0.00 |
| 135476_f_at | 0.00 |
| 135494_at | 0.00 |
| 135495_r_at | 0.00 |
| 135496_at | 0.00 |
| 135498_at | 0.00 |

| | |
|---|---|
| 135500_at | 0.00 |
| 135501_at | 0.00 |
| 135502_at | 0.00 |
| 135503_at | 0.05 |
| 135511_at | 0.00 |
| 135513_at | 0.00 |
| 135520_f_at | 0.00 |
| 135521_r_at | 0.00 |
| 135524_at | 0.00 |
| 135530_at | 0.00 |
| 135531_i_at | 0.01 |
| 135534_at | 0.00 |
| 135535_i_at | 0.00 |
| 135536_r_at | 0.00 |
| 135537_at | 0.00 |
| 135538_at | 0.00 |
| 135539_at | 0.00 |
| 135540_at | 0.00 |
| 135541_at | 0.00 |
| 135542_r_at | 0.00 |
| 135543_at | 0.00 |
| 135544_at | 0.00 |
| 135552_f_at | 0.00 |
| 135553_i_at | 0.00 |
| 135570_at | 0.00 |
| 135577_at | 0.00 |
| 135578_at | 0.00 |
| 135579_at | 0.00 |
| 135581_at | 0.00 |
| 135582_at | 0.00 |
| 135583_at | 0.01 |
| 135584_at | 0.00 |
| 135585_at | 0.00 |
| 135587_at | 0.00 |
| 135588_at | 0.00 |
| 135589_at | 0.00 |
| 135590_at | 0.00 |
| 135591_at | 0.00 |
| 135593_at | 0.00 |
| 135596_at | 0.00 |
| 135599_r_at | 0.00 |
| 135600_at | 0.00 |
| 135601_at | 0.00 |
| 135602_at | 0.00 |
| 135603_at | 0.00 |
| 135604_at | 0.02 |
| 135606_at | 0.00 |
| 135607_at | 0.00 |
| 135608_at | 0.00 |
| 135609_at | 0.04 |
| 135610_at | 0.00 |
| 135611_at | 0.00 |
| 135612_at | 0.00 |
| 135613_at | 0.11 |
| 135614_at | 0.44 |
| 135615_at | 0.00 |

| | |
|---|---|
| 135616_at | 0.00 |
| 135617_at | 0.00 |
| 135618_at | 0.02 |
| 135619_at | 0.01 |
| 135620_at | 0.00 |
| 135621_at | 0.00 |
| 135622_at | 0.00 |
| 135623_at | 0.00 |
| 135624_f_at | 0.00 |
| 135625_at | 0.00 |
| 135627_at | 0.00 |
| 135628_at | 0.00 |
| 135629_r_at | 0.00 |
| 135630_at | 0.00 |
| 135631_at | 0.00 |
| 135632_f_at | 0.00 |
| 135633_at | 0.00 |
| 135636_at | 0.00 |
| 135641_at | 0.00 |
| 135642_at | 0.00 |
| 135643_at | 0.00 |
| 135644_at | 0.00 |
| 135645_at | 0.00 |
| 135646_r_at | 0.00 |
| 135648_at | 0.00 |
| 135649_at | 0.01 |
| 135652_at | 0.07 |
| 135653_at | 0.00 |
| 135654_at | 0.00 |
| 135655_at | 0.00 |
| 135656_at | 0.00 |
| 135657_at | 0.00 |
| 135658_at | 0.00 |
| 135659_at | 0.00 |
| 135660_at | 0.00 |
| 135661_at | 0.00 |
| 135662_at | 0.00 |
| 135663_r_at | 0.00 |
| 135664_at | 0.00 |
| 135665_at | 0.00 |
| 135666_at | 0.00 |
| 135667_at | 0.00 |
| 135668_at | 0.00 |
| 135669_at | 0.00 |
| 135670_at | 0.00 |
| 135672_r_at | 0.00 |
| 135673_r_at | 0.00 |
| 135675_at | 0.09 |
| 135676_at | 0.00 |
| 135677_f_at | 0.00 |
| 135678_at | 0.00 |
| 135679_at | 0.00 |
| 135680_at | 0.00 |
| 135681_at | 0.00 |
| 135682_at | 0.00 |
| 135684_s_at | 0.10 |

| | |
|---|---|
| 135685_at | 0.00 |
| 135686_at | 0.00 |
| 135687_r_at | 0.00 |
| 135688_at | 0.00 |
| 135690_at | 0.00 |
| 135691_at | 0.02 |
| 135692_at | 0.00 |
| 135693_at | 0.00 |
| 135694_at | 0.00 |
| 135695_at | 0.00 |
| 135696_f_at | 0.00 |
| 135697_at | 0.00 |
| 135698_at | 0.01 |
| 135699_at | 0.00 |
| 135700_at | 0.00 |
| 135701_at | 0.00 |
| 135702_at | 0.00 |
| 135703_at | 0.00 |
| 135704_at | 0.00 |
| 135705_at | 0.00 |
| 135706_at | 0.00 |
| 135707_at | 0.00 |
| 135708_at | 0.00 |
| 135709_at | 0.00 |
| 135710_at | 0.00 |
| 135711_at | 0.00 |
| 135712_at | 0.00 |
| 135713_at | 0.00 |
| 135714_at | 0.00 |
| 135715_r_at | 0.00 |
| 135716_f_at | 0.05 |
| 135717_at | 0.00 |
| 135718_at | 0.00 |
| 135719_at | 0.00 |
| 135720_at | 0.00 |
| 135721_at | 0.00 |
| 135722_r_at | 0.00 |
| 135723_at | 0.00 |
| 135724_at | 0.00 |
| 135725_at | 0.00 |
| 135726_r_at | 0.00 |
| 135727_at | 0.00 |
| 135728_at | 0.00 |
| 135729_at | 0.00 |
| 135730_at | 0.00 |
| 135731_at | 0.00 |
| 135732_at | 0.00 |
| 135733_at | 0.00 |
| 135734_at | 0.00 |
| 135735_at | 0.00 |
| 135736_at | 0.00 |
| 135737_r_at | 0.00 |
| 135738_at | 0.00 |
| 135739_at | 0.00 |
| 135740_at | 0.00 |
| 135741_at | 0.00 |

| | |
|---|---|
| 135742_at | 0.00 |
| 135743_at | 0.00 |
| 135744_at | 0.00 |
| 135745_at | 0.00 |
| 135746_at | 0.00 |
| 135747_at | 0.00 |
| 135748_at | 0.00 |
| 135749_at | 0.00 |
| 135750_at | 0.00 |
| 135751_at | 0.00 |
| 135752_at | 0.00 |
| 135753_at | 0.00 |
| 135754_at | 0.00 |
| 135755_at | 0.00 |
| 135756_at | 0.00 |
| 135757_at | 0.00 |
| 135758_at | 0.00 |
| 135759_at | 0.00 |
| 135760_at | 0.00 |
| 135761_at | 0.00 |
| 135762_i_at | 0.00 |
| 135763_r_at | 0.00 |
| 135764_at | 0.00 |
| 135765_at | 0.00 |
| 135766_at | 0.00 |
| 135767_at | 0.00 |
| 135768_at | 0.00 |
| 135769_at | 0.00 |
| 135770_at | 0.00 |
| 135771_at | 0.00 |
| 135772_at | 0.00 |
| 135773_at | 0.00 |
| 135774_at | 0.00 |
| 135775_at | 0.00 |
| 135776_at | 0.00 |
| 135777_at | 0.00 |
| 135778_at | 0.00 |
| 135779_at | 0.00 |
| 135780_at | 0.00 |
| 135781_at | 0.00 |
| 135782_at | 0.00 |
| 135783_r_at | 0.00 |
| 135784_at | 0.00 |
| 135785_at | 0.00 |
| 135786_at | 0.00 |
| 135787_at | 0.00 |
| 135788_at | 0.00 |
| 135789_at | 0.00 |
| 135790_at | 0.00 |
| 135791_at | 0.00 |
| 135792_s_at | 0.00 |
| 135793_r_at | 0.00 |
| 135794_at | 0.00 |
| 135795_at | 0.00 |
| 135796_at | 0.00 |
| 135797_at | 0.00 |

| | |
|---|---|
| 135798_at | 0.00 |
| 135799_at | 0.00 |
| 135800_at | 0.00 |
| 135801_at | 0.00 |
| 135802_at | 0.00 |
| 135803_r_at | 0.00 |
| 135804_at | 0.00 |
| 135805_at | 0.00 |
| 135806_i_at | 0.00 |
| 135807_r_at | 0.00 |
| 135808_at | 0.00 |
| 135809_at | 0.00 |
| 135810_at | 0.00 |
| 135811_at | 0.00 |
| 135812_at | 0.00 |
| 135813_at | 0.00 |
| 135814_at | 0.00 |
| 135815_at | 0.00 |
| 135816_at | 0.00 |
| 135817_at | 0.00 |
| 135825_at | 0.00 |
| 135826_at | 0.00 |
| 135828_at | 0.00 |
| 135830_at | 0.00 |
| 135831_at | 0.00 |
| 135832_at | 0.00 |
| 135834_at | 0.00 |
| 135835_at | 0.00 |
| 135840_at | 0.00 |
| 135849_at | 0.00 |
| 135858_at | 0.00 |
| 135865_r_at | 0.00 |
| 135879_at | 0.00 |
| 135883_at | 0.00 |
| 135884_at | 0.00 |
| 135887_at | 0.00 |
| 135888_at | 0.46 |
| 135893_r_at | 0.00 |
| 135894_at | 0.00 |
| 135899_at | 0.00 |
| 135900_at | 0.00 |
| 135901_at | 0.00 |
| 135902_at | 0.00 |
| 135903_at | 0.00 |
| 135904_at | 0.00 |
| 135905_at | 0.00 |
| 135906_at | 0.00 |
| 135907_at | 0.00 |
| 135908_at | 0.00 |
| 135909_at | 0.00 |
| 135910_at | 0.00 |
| 135911_at | 0.00 |
| 135912_at | 0.00 |
| 135913_at | 0.00 |
| 135914_at | 0.00 |
| 135915_at | 0.00 |

| | |
|---|---|
| 135916_at | 0.00 |
| 135925_at | 0.00 |
| 135926_at | 0.00 |
| 135927_at | 0.00 |
| 135928_at | 0.00 |
| 135929_at | 0.00 |
| 135930_at | 0.00 |
| 135931_at | 0.00 |
| 135939_at | 0.00 |
| 135956_at | 0.00 |
| 135957_at | 0.00 |
| 135958_at | 0.00 |
| 135959_at | 0.00 |
| 135960_at | 0.00 |
| 135961_at | 0.00 |
| 135975_at | 0.00 |
| 135976_at | 0.00 |
| 135977_at | 0.00 |
| 135978_at | 0.00 |
| 135979_at | 0.00 |
| 135980_at | 0.00 |
| 135981_at | 0.00 |
| 135982_at | 0.00 |
| 135983_at | 0.00 |
| 135984_at | 0.00 |
| 136003_at | 0.00 |
| 136004_at | 0.00 |
| 136005_at | 0.00 |
| 136006_at | 0.00 |
| 136007_at | 0.00 |
| 136008_at | 0.00 |
| 136009_at | 0.00 |
| 136025_at | 0.00 |
| 136026_at | 0.00 |
| 136028_at | 0.00 |
| 136029_at | 0.00 |
| 136030_at | 0.00 |
| 136031_at | 0.00 |
| 136032_at | 0.00 |
| 136033_at | 0.00 |
| 136056_at | 0.00 |
| 136057_at | 0.00 |
| 136058_at | 0.00 |
| 136059_g_at | 0.00 |
| 136060_at | 0.00 |
| 136061_at | 0.01 |
| 136062_at | 0.00 |
| 136063_at | 0.00 |
| 136065_at | 0.00 |
| 136066_at | 0.00 |
| 136067_at | 0.00 |
| 136068_at | 0.00 |
| 136069_at | 0.00 |
| 136070_at | 0.00 |
| 136071_at | 0.00 |
| 136072_at | 0.00 |

| | |
|---|---|
| 136073_at | 0.00 |
| 136074_at | 0.02 |
| 136075_at | 0.00 |
| 136076_at | 0.00 |
| 136077_at | 0.00 |
| 136078_at | 0.00 |
| 136079_f_at | 0.00 |
| 136080_at | 0.00 |
| 136081_at | 0.00 |
| 136082_at | 0.00 |
| 136083_at | 0.00 |
| 136084_at | 0.00 |
| 136085_at | 0.00 |
| 136086_at | 0.00 |
| 136087_at | 0.00 |
| 136088_at | 0.00 |
| 136090_at | 0.00 |
| 136091_at | 0.00 |
| 136092_at | 0.00 |
| 136093_at | 0.00 |
| 136094_at | 0.00 |
| 136095_at | 0.00 |
| 136096_r_at | 0.00 |
| 136097_i_at | 0.00 |
| 136098_r_at | 0.00 |
| 136099_at | 0.00 |
| 136100_at | 0.00 |
| 136101_at | 0.00 |
| 136102_at | 0.00 |
| 136103_at | 0.00 |
| 136104_at | 0.00 |
| 136105_at | 0.04 |
| 136106_at | 0.00 |
| 136107_at | 0.00 |
| 136108_at | 0.00 |
| 136109_at | 0.00 |
| 136110_r_at | 0.02 |
| 136112_at | 0.00 |
| 136113_at | 0.00 |
| 136114_r_at | 0.00 |
| 136115_at | 0.00 |
| 136116_at | 0.00 |
| 136117_at | 0.00 |
| 136118_f_at | 0.00 |
| 136119_r_at | 0.00 |
| 136120_at | 0.00 |
| 136121_at | 0.00 |
| 136122_at | 0.00 |
| 136123_i_at | 0.00 |
| 136124_f_at | 0.00 |
| 136125_at | 0.00 |
| 136126_at | 0.00 |
| 136127_at | 0.00 |
| 136128_at | 0.00 |
| 136129_at | 0.00 |
| 136130_at | 0.00 |

| | |
|---|---|
| 136131_at | 0.00 |
| 136132_at | 0.00 |
| 136134_at | 0.00 |
| 136138_i_at | 0.00 |
| 136139_r_at | 0.00 |
| 136140_at | 0.00 |
| 136141_at | 0.00 |
| 136142_at | 0.00 |
| 136143_at | 0.00 |
| 136144_r_at | 0.00 |
| 136145_at | 0.00 |
| 136146_at | 0.00 |
| 136147_r_at | 0.00 |
| 136148_at | 0.00 |
| 136149_at | 0.00 |
| 136150_at | 0.00 |
| 136151_at | 0.00 |
| 136155_at | 0.00 |
| 136156_at | 0.00 |
| 136157_at | 0.00 |
| 136158_at | 0.00 |
| 136159_at | 0.00 |
| 136160_at | 0.00 |
| 136161_at | 0.00 |
| 136162_at | 0.00 |
| 136163_at | 0.00 |
| 136164_at | 0.15 |
| 136165_at | 0.00 |
| 136166_at | 0.00 |
| 136167_f_at | 0.00 |
| 136168_r_at | 0.00 |
| 136169_at | 0.00 |
| 136172_at | 0.03 |
| 136173_at | 0.00 |
| 136174_at | 0.00 |
| 136175_at | 0.00 |
| 136176_at | 0.00 |
| 136177_at | 0.00 |
| 136178_at | 0.00 |
| 136179_at | 0.00 |
| 136180_at | 0.00 |
| 136181_at | 0.00 |
| 136182_at | 0.00 |
| 136183_at | 0.00 |
| 136184_at | 0.00 |
| 136185_at | 0.00 |
| 136186_at | 0.00 |
| 136187_f_at | 0.00 |
| 136188_at | 0.00 |
| 136189_at | 0.00 |
| 136190_r_at | 0.00 |
| 136191_at | 0.00 |
| 136192_at | 0.00 |
| 136193_i_at | 0.05 |
| 136194_f_at | 0.00 |
| 136195_at | 0.00 |

| | |
|---|---|
| 136196_at | 0.00 |
| 136197_at | 0.00 |
| 136198_at | 0.00 |
| 136199_at | 0.00 |
| 136200_at | 0.00 |
| 136201_at | 0.00 |
| 136202_at | 0.00 |
| 136203_r_at | 0.00 |
| 136204_at | 0.00 |
| 136205_at | 0.00 |
| 136206_at | 0.00 |
| 136207_at | 0.00 |
| 136208_at | 0.00 |
| 136209_at | 0.00 |
| 136210_at | 0.00 |
| 136211_g_at | 0.00 |
| 136212_at | 0.00 |
| 136213_at | 0.00 |
| 136214_at | 0.00 |
| 136215_at | 0.00 |
| 136216_at | 0.00 |
| 136217_at | 0.00 |
| 136218_at | 0.00 |
| 136219_r_at | 0.00 |
| 136220_at | 0.00 |
| 136221_at | 0.00 |
| 136222_at | 0.00 |
| 136223_f_at | 0.00 |
| 136224_at | 0.00 |
| 136225_at | 0.00 |
| 136226_at | 0.00 |
| 136227_f_at | 0.00 |
| 136228_at | 0.00 |
| 136229_at | 0.00 |
| 136230_at | 0.00 |
| 136231_f_at | 0.01 |
| 136232_at | 0.00 |
| 136233_f_at | 0.00 |
| 136234_at | 0.00 |
| 136235_at | 0.00 |
| 136236_at | 0.00 |
| 136237_at | 0.00 |
| 136238_f_at | 0.00 |
| 136239_at | 0.00 |
| 136240_r_at | 0.00 |
| 136241_at | 0.00 |
| 136242_at | 0.00 |
| 136243_at | 0.00 |
| 136244_at | 0.00 |
| 136245_at | 0.01 |
| 136246_at | 0.00 |
| 136247_at | 0.00 |
| 136248_at | 0.00 |
| 136249_at | 0.00 |
| 136250_at | 0.00 |
| 136251_at | 0.00 |

| | |
|---|---|
| 136252_at | 0.00 |
| 136253_at | 0.00 |
| 136254_at | 0.00 |
| 136255_at | 0.00 |
| 136256_at | 0.00 |
| 136257_at | 0.00 |
| 136258_at | 0.00 |
| 136259_at | 0.00 |
| 136260_at | 0.00 |
| 136261_at | 0.00 |
| 136262_f_at | 0.00 |
| 136263_at | 0.00 |
| 136264_at | 0.00 |
| 136265_at | 0.00 |
| 136266_at | 0.00 |
| 136267_at | 0.00 |
| 136268_at | 0.00 |
| 136269_at | 0.00 |
| 136270_at | 0.00 |
| 136271_at | 0.00 |
| 136272_at | 0.00 |
| 136273_at | 0.00 |
| 136274_at | 0.00 |
| 136275_at | 0.00 |
| 136276_at | 0.00 |
| 136277_at | 0.00 |
| 136278_at | 0.00 |
| 136279_at | 0.00 |
| 136280_at | 0.00 |
| 136281_at | 0.00 |
| 136282_at | 0.00 |
| 136283_at | 0.00 |
| 136284_at | 0.00 |
| 136285_i_at | 0.00 |
| 136286_r_at | 0.00 |
| 136287_at | 0.00 |
| 136288_at | 0.00 |
| 136289_f_at | 0.00 |
| 136290_at | 0.00 |
| 136291_at | 0.00 |
| 136292_at | 0.00 |
| 136293_at | 0.00 |
| 136348_at | 0.00 |
| 136385_at | 0.00 |
| 136386_at | 0.00 |
| 136387_at | 0.00 |
| 136388_at | 0.00 |
| 136389_at | 0.00 |
| 136390_at | 0.00 |
| 136391_at | 0.00 |
| 136392_at | 0.00 |
| 136393_at | 0.00 |
| 136394_at | 0.00 |
| 136395_at | 0.00 |
| 136396_at | 0.00 |
| 136397_at | 0.00 |

| | |
|---|---|
| 136398_at | 0.00 |
| 136399_at | 0.00 |
| 136400_at | 0.00 |
| 136401_at | 0.00 |
| 136409_at | 0.00 |
| 136410_at | 0.00 |
| 136411_at | 0.00 |
| 136412_at | 0.00 |
| 136413_at | 0.00 |
| 136414_at | 0.00 |
| 136415_at | 0.00 |
| 136416_at | 0.00 |
| 136417_at | 0.00 |
| 136418_at | 0.00 |
| 136419_at | 0.00 |
| 136420_at | 0.00 |
| 136421_at | 0.00 |
| 136422_at | 0.00 |
| 136432_at | 0.00 |
| 136433_at | 0.00 |
| 136434_at | 0.00 |
| 136435_at | 0.00 |
| 136436_at | 0.00 |
| 136439_at | 0.00 |
| 136440_at | 0.00 |
| 136441_at | 0.00 |
| 136442_at | 0.00 |
| 136443_at | 0.00 |
| 136444_at | 0.00 |
| 136445_at | 0.00 |
| 136446_at | 0.00 |
| 136447_at | 0.00 |
| 136448_at | 0.00 |
| 136449_at | 0.00 |
| 136450_at | 0.00 |
| 136451_at | 0.00 |
| 136452_at | 0.00 |
| 136453_at | 0.00 |
| 136454_at | 0.00 |
| 136455_at | 0.00 |
| 136456_at | 0.00 |
| 136468_at | 0.00 |
| 136504_at | 0.00 |
| 136534_at | 0.00 |
| 136535_at | 0.09 |
| 136536_at | 0.00 |
| 136537_at | 0.00 |
| 136538_at | 0.00 |
| 136539_at | 0.00 |
| 136540_r_at | 0.00 |
| 136541_at | 0.00 |
| 136542_at | 0.00 |
| 136543_i_at | 0.00 |
| 136544_at | 0.00 |
| 136545_at | 0.00 |
| 136546_at | 0.01 |

| | |
|---|---|
| 136547_at | 0.00 |
| 136548_s_at | 0.00 |
| 136549_at | 0.00 |
| 136550_r_at | 0.00 |
| 136551_at | 0.00 |
| 136552_at | 0.01 |
| 136554_r_at | 0.00 |
| 136557_at | 0.00 |
| 136558_at | 0.00 |
| 136559_at | 0.00 |
| 136560_at | 0.00 |
| 136561_f_at | 0.06 |
| 136562_at | 0.00 |
| 136563_at | 0.00 |
| 136564_at | 0.00 |
| 136565_at | 0.00 |
| 136566_at | 0.00 |
| 136567_at | 0.00 |
| 136568_at | 0.00 |
| 136569_at | 0.00 |
| 136570_at | 0.00 |
| 136571_r_at | 0.00 |
| 136572_i_at | 0.00 |
| 136573_f_at | 0.00 |
| 136576_r_at | 0.00 |
| 136577_i_at | 0.00 |
| 136578_r_at | 0.00 |
| 136579_at | 0.00 |
| 136580_at | 0.00 |
| 136582_at | 0.00 |
| 136584_at | 0.00 |
| 136585_at | 0.00 |
| 136586_at | 0.22 |
| 136587_at | 0.00 |
| 136588_at | 0.00 |
| 136589_at | 0.00 |
| 136590_at | 0.00 |
| 136591_at | 0.00 |
| 136592_f_at | 0.09 |
| 136593_at | 0.00 |
| 136594_at | 0.00 |
| 136595_at | 0.00 |
| 136596_at | 0.00 |
| 136597_at | 0.00 |
| 136599_at | 0.00 |
| 136600_at | 0.00 |
| 136601_i_at | 0.00 |
| 136602_r_at | 0.00 |
| 136603_at | 0.00 |
| 136604_r_at | 0.00 |
| 136605_i_at | 0.00 |
| 136606_r_at | 0.00 |
| 136608_at | 0.00 |
| 136609_at | 0.01 |
| 136610_at | 0.00 |
| 136611_at | 0.00 |

| | |
|---|---|
| 136613_at | 0.00 |
| 136614_r_at | 0.00 |
| 136615_at | 0.00 |
| 136616_at | 0.00 |
| 136617_at | 0.00 |
| 136618_at | 0.00 |
| 136619_at | 0.00 |
| 136620_r_at | 0.00 |
| 136621_at | 0.00 |
| 136622_at | 0.00 |
| 136623_at | 0.00 |
| 136624_at | 0.00 |
| 136625_at | 0.00 |
| 136626_r_at | 0.00 |
| 136627_at | 0.00 |
| 136628_f_at | 0.00 |
| 136629_r_at | 0.00 |
| 136630_i_at | 0.00 |
| 136631_r_at | 0.00 |
| 136632_at | 0.00 |
| 136633_at | 0.00 |
| 136634_at | 0.00 |
| 136635_at | 0.00 |
| 136636_at | 0.00 |
| 136637_at | 0.00 |
| 136638_at | 0.00 |
| 136639_at | 0.00 |
| 136640_at | 0.00 |
| 136641_at | 0.00 |
| 136642_at | 0.00 |
| 136643_f_at | 0.04 |
| 136645_at | 0.00 |
| 136646_at | 0.00 |
| 136647_at | 0.00 |
| 136650_at | 0.00 |
| 136651_at | 0.00 |
| 136652_at | 0.00 |
| 136654_at | 0.00 |
| 136655_f_at | 0.00 |
| 136656_at | 0.00 |
| 136657_at | 0.00 |
| 136658_at | 0.00 |
| 136659_at | 0.00 |
| 136660_f_at | 0.00 |
| 136661_at | 0.01 |
| 136662_at | 0.00 |
| 136663_at | 0.00 |
| 136664_at | 0.00 |
| 136665_at | 0.00 |
| 136666_at | 0.00 |
| 136667_at | 0.00 |
| 136668_at | 0.00 |
| 136669_at | 0.00 |
| 136670_at | 0.00 |
| 136671_at | 0.00 |
| 136672_at | 0.00 |

| | |
|---|---|
| 136673_at | 0.00 |
| 136674_at | 0.00 |
| 136675_at | 0.00 |
| 136676_at | 0.00 |
| 136677_at | 0.00 |
| 136678_at | 0.00 |
| 136679_at | 0.00 |
| 136680_at | 0.00 |
| 136681_at | 0.00 |
| 136682_at | 0.00 |
| 136683_at | 0.00 |
| 136684_at | 0.00 |
| 136685_at | 0.00 |
| 136686_at | 0.00 |
| 136687_at | 0.00 |
| 136688_at | 0.00 |
| 136689_at | 0.01 |
| 136690_at | 0.00 |
| 136691_at | 0.00 |
| 136692_at | 0.00 |
| 136693_at | 0.00 |
| 136694_at | 0.00 |
| 136695_at | 0.00 |
| 136696_at | 0.00 |
| 136697_at | 0.00 |
| 136698_at | 0.00 |
| 136699_at | 0.00 |
| 136700_at | 0.00 |
| 136701_at | 0.00 |
| 136702_at | 0.00 |
| 136703_at | 0.00 |
| 136704_at | 0.00 |
| 136705_at | 0.00 |
| 136706_r_at | 0.00 |
| 136707_f_at | 0.00 |
| 136708_at | 0.00 |
| 136709_at | 0.00 |
| 136710_at | 0.00 |
| 136711_at | 0.00 |
| 136712_at | 0.00 |
| 136713_at | 0.00 |
| 136714_at | 0.00 |
| 136715_at | 0.00 |
| 136716_at | 0.00 |
| 136717_at | 0.00 |
| 136718_r_at | 0.00 |
| 136719_at | 0.00 |
| 136720_at | 0.00 |
| 136721_at | 0.00 |
| 136722_at | 0.00 |
| 136723_at | 0.00 |
| 136724_at | 0.00 |
| 136725_at | 0.00 |
| 136726_r_at | 0.00 |
| 136727_at | 0.00 |
| 136728_at | 0.00 |

| | |
|---|---|
| 136729_at | 0.00 |
| 136730_at | 0.00 |
| 136731_at | 0.00 |
| 136732_at | 0.00 |
| 136733_at | 0.46 |
| 136734_at | 0.00 |
| 136735_at | 0.00 |
| 136736_at | 0.00 |
| 136737_r_at | 0.00 |
| 136738_at | 0.00 |
| 136739_at | 0.00 |
| 136740_f_at | 0.00 |
| 136741_at | 0.00 |
| 136742_at | 0.00 |
| 136743_at | 0.00 |
| 136744_at | 0.00 |
| 136745_at | 0.00 |
| 136746_at | 0.00 |
| 136747_at | 0.00 |
| 136748_at | 0.00 |
| 136749_at | 0.00 |
| 136750_at | 0.00 |
| 136751_at | 0.00 |
| 136752_at | 0.00 |
| 136753_at | 0.00 |
| 136754_at | 0.00 |
| 136755_at | 0.00 |
| 136756_at | 0.00 |
| 136757_at | 0.00 |
| 136758_at | 0.00 |
| 136759_at | 0.00 |
| 136760_at | 0.00 |
| 136761_f_at | 0.00 |
| 136762_r_at | 0.00 |
| 136763_at | 0.00 |
| 136764_at | 0.00 |
| 136766_at | 0.00 |
| 136767_at | 0.00 |
| 136769_f_at | 0.00 |
| 136770_r_at | 0.00 |
| 136771_at | 0.00 |
| 136772_at | 0.00 |
| 136773_f_at | 0.00 |
| 136796_at | 0.00 |
| 136797_s_at | 0.00 |
| 136798_at | 0.04 |
| 136943_s_at | 0.00 |
| 136967_s_at | 0.00 |
| 136968_at | 0.00 |
| 137008_at | 0.00 |
| 137013_i_at | 0.00 |
| 137014_r_at | 0.00 |
| 137015_at | 0.00 |
| 137016_at | 0.00 |
| 137017_at | 0.00 |
| 137018_at | 0.00 |

| | |
|---|---|
| 137019_at | 0.00 |
| 137020_at | 0.00 |
| 137021_at | 0.00 |
| 137022_at | 0.27 |
| 137023_at | 0.00 |
| 137024_at | 0.00 |
| 137026_at | 0.00 |
| 137028_at | 0.03 |
| 137029_at | 0.00 |
| 137030_at | 0.00 |
| 137031_at | 0.00 |
| 137033_at | 0.02 |
| 137034_f_at | 0.55 |
| 137036_at | 0.00 |
| 137037_at | 0.00 |
| 137038_at | 0.00 |
| 137040_at | 0.00 |
| 137041_at | 0.13 |
| 137043_r_at | 0.00 |
| 137044_at | 0.00 |
| 137045_at | 0.00 |
| 137046_s_at | 0.00 |
| 137047_at | 0.00 |
| 137049_at | 0.00 |
| 137050_at | 0.00 |
| 137051_at | 0.00 |
| 137052_r_at | 0.00 |
| 137053_at | 0.00 |
| 137055_at | 0.00 |
| 137057_at | 0.00 |
| 137058_at | 0.00 |
| 137059_at | 0.00 |
| 137060_at | 0.00 |
| 137061_at | 0.00 |
| 137062_at | 0.00 |
| 137063_at | 0.00 |
| 137065_at | 0.00 |
| 137066_at | 0.00 |
| 137067_at | 0.00 |
| 137068_at | 0.50 |
| 137070_r_at | 0.00 |
| 137073_r_at | 0.00 |
| 137077_at | 0.00 |
| 137078_at | 0.00 |
| 137079_at | 0.00 |
| 137081_at | 0.00 |
| 137082_at | 0.00 |
| 137083_at | 0.00 |
| 137084_at | 0.00 |
| 137086_at | 0.00 |
| 137088_at | 0.00 |
| 137089_r_at | 0.00 |
| 137090_at | 0.03 |
| 137092_at | 0.00 |
| 137093_at | 0.00 |
| 137094_at | 0.00 |

| | |
|---|---|
| 137095_at | 0.00 |
| 137097_at | 0.00 |
| 137098_at | 0.00 |
| 137099_at | 0.00 |
| 137100_at | 0.00 |
| 137101_at | 0.00 |
| 137102_at | 0.00 |
| 137103_r_at | 0.00 |
| 137104_at | 0.00 |
| 137105_at | 0.00 |
| 137106_at | 0.02 |
| 137107_at | 0.00 |
| 137108_at | 0.00 |
| 137109_at | 0.00 |
| 137110_at | 0.00 |
| 137112_at | 0.00 |
| 137113_at | 0.00 |
| 137119_at | 0.00 |
| 137120_at | 0.00 |
| 137121_at | 0.00 |
| 137122_at | 0.00 |
| 137123_at | 0.00 |
| 137124_at | 0.00 |
| 137125_at | 0.00 |
| 137126_at | 0.01 |
| 137127_at | 0.00 |
| 137128_at | 0.00 |
| 137129_at | 0.00 |
| 137130_at | 0.00 |
| 137131_at | 0.00 |
| 137132_f_at | 0.00 |
| 137133_s_at | 0.00 |
| 137134_f_at | 0.02 |
| 137136_at | 0.00 |
| 137137_at | 0.00 |
| 137138_i_at | 0.00 |
| 137139_f_at | 0.00 |
| 137140_at | 0.00 |
| 137141_at | 0.00 |
| 137142_at | 0.00 |
| 137143_at | 0.00 |
| 137144_at | 0.00 |
| 137145_at | 0.00 |
| 137146_at | 0.00 |
| 137147_at | 0.00 |
| 137148_at | 0.00 |
| 137149_at | 0.00 |
| 137150_at | 0.00 |
| 137151_r_at | 0.00 |
| 137152_at | 0.01 |
| 137153_at | 0.00 |
| 137154_at | 0.00 |
| 137155_f_at | 0.00 |
| 137156_at | 0.00 |
| 137157_at | 0.00 |
| 137158_i_at | 0.00 |

| | |
|---|---|
| 137159_f_at | 0.00 |
| 137160_at | 0.00 |
| 137161_at | 0.00 |
| 137162_at | 0.00 |
| 137163_at | 0.00 |
| 137164_at | 0.00 |
| 137165_at | 0.00 |
| 137166_r_at | 0.00 |
| 137167_at | 0.00 |
| 137168_at | 0.00 |
| 137169_at | 0.00 |
| 137170_at | 0.00 |
| 137171_i_at | 0.00 |
| 137172_f_at | 0.00 |
| 137173_at | 0.00 |
| 137174_i_at | 0.00 |
| 137175_f_at | 0.00 |
| 137176_f_at | 0.00 |
| 137177_at | 0.00 |
| 137178_s_at | 0.00 |
| 137179_at | 0.00 |
| 137180_at | 0.00 |
| 137181_at | 0.00 |
| 137182_at | 0.00 |
| 137184_at | 0.00 |
| 137185_i_at | 0.00 |
| 137186_f_at | 0.00 |
| 137187_at | 0.00 |
| 137188_at | 0.00 |
| 137189_i_at | 0.00 |
| 137190_f_at | 0.00 |
| 137191_f_at | 0.00 |
| 137192_at | 0.00 |
| 137193_i_at | 0.00 |
| 137194_r_at | 0.00 |
| 137195_i_at | 0.00 |
| 137196_f_at | 0.00 |
| 137197_at | 0.00 |
| 137198_at | 0.00 |
| 137199_at | 0.00 |
| 137200_f_at | 0.00 |
| 137201_r_at | 0.00 |
| 137202_f_at | 0.00 |
| 137203_f_at | 0.00 |
| 137204_f_at | 0.00 |
| 137205_f_at | 0.00 |
| 137206_at | 0.00 |
| 137207_i_at | 0.00 |
| 137208_r_at | 0.00 |
| 137209_f_at | 0.00 |
| 137210_f_at | 0.00 |
| 137211_i_at | 0.00 |
| 137212_f_at | 0.00 |
| 137213_at | 0.00 |
| 137214_at | 0.00 |
| 137215_at | 0.00 |

| | |
|---|---|
| 137216_at | 0.00 |
| 137217_f_at | 0.00 |
| 137218_f_at | 0.00 |
| 137219_f_at | 0.00 |
| 137220_f_at | 0.00 |
| 137221_f_at | 0.00 |
| 137222_f_at | 0.00 |
| 137223_i_at | 0.00 |
| 137224_f_at | 0.01 |
| 137225_at | 0.00 |
| 137226_f_at | 0.00 |
| 137227_at | 0.00 |
| 137228_i_at | 0.00 |
| 137229_f_at | 0.00 |
| 137230_f_at | 0.00 |
| 137231_r_at | 0.00 |
| 137232_r_at | 0.00 |
| 137233_at | 0.00 |
| 137234_i_at | 0.00 |
| 137235_f_at | 0.00 |
| 137236_f_at | 0.00 |
| 137237_at | 0.00 |
| 137238_f_at | 0.00 |
| 137239_at | 0.00 |
| 137240_r_at | 0.00 |
| 137241_f_at | 0.00 |
| 137242_f_at | 0.00 |
| 137243_r_at | 0.00 |
| 137244_at | 0.00 |
| 137245_f_at | 0.00 |
| 137246_f_at | 0.00 |
| 137247_r_at | 0.00 |
| 137248_at | 0.00 |
| 137249_at | 0.00 |
| 137250_at | 0.00 |
| 137251_f_at | 0.00 |
| 137318_at | 0.00 |
| 137319_at | 0.00 |
| 137320_g_at | 0.00 |
| 137321_at | 0.00 |
| 137322_at | 0.00 |
| 137323_at | 0.00 |
| 137324_at | 0.00 |
| 137325_at | 0.00 |
| 137326_i_at | 0.00 |
| 137327_f_at | 0.00 |
| 137328_at | 0.00 |
| 137329_at | 0.00 |
| 137330_at | 0.00 |
| 137331_at | 0.00 |
| 137332_at | 0.00 |
| 137333_at | 0.00 |
| 137334_at | 0.00 |
| 137335_at | 0.00 |
| 137336_at | 0.00 |
| 137337_at | 0.00 |

| | |
|---|---|
| 137338_at | 0.00 |
| 137339_at | 0.00 |
| 137340_at | 0.00 |
| 137341_at | 0.00 |
| 137342_at | 0.00 |
| 137343_r_at | 0.00 |
| 137344_at | 0.00 |
| 137345_at | 0.00 |
| 137346_at | 0.00 |
| 137347_at | 0.00 |
| 137348_at | 0.00 |
| 137349_at | 0.00 |
| 137350_at | 0.00 |
| 137351_at | 0.00 |
| 137352_at | 0.00 |
| 137353_at | 0.00 |
| 137354_at | 0.00 |
| 137355_at | 0.00 |
| 137356_at | 0.00 |
| 137357_at | 0.00 |
| 137358_at | 0.00 |
| 137359_at | 0.14 |
| 137360_at | 0.00 |
| 137361_at | 0.00 |
| 137362_at | 0.00 |
| 137363_at | 0.00 |
| 137364_at | 0.00 |
| 137365_at | 0.00 |
| 137366_at | 0.00 |
| 137367_at | 0.00 |
| 137368_at | 0.00 |
| 137369_at | 0.00 |
| 137370_at | 0.00 |
| 137371_at | 0.00 |
| 137372_at | 0.00 |
| 137373_at | 0.00 |
| 137374_at | 0.00 |
| 137375_at | 0.00 |
| 137376_at | 0.00 |
| 137377_i_at | 0.00 |
| 137378_r_at | 0.00 |
| 137379_at | 0.00 |
| 137380_at | 0.00 |
| 137381_at | 0.00 |
| 137475_at | 0.00 |
| 137484_at | 0.00 |
| 137485_at | 0.00 |
| 137486_at | 0.00 |
| 137487_at | 0.00 |
| 137488_at | 0.00 |
| 137489_at | 0.00 |
| 137490_at | 0.00 |
| 137491_at | 0.00 |
| 137492_at | 0.00 |
| 137493_at | 0.00 |
| 137494_f_at | 0.00 |

| | |
|---|---|
| 137495_at | 0.00 |
| 137496_at | 0.00 |
| 137497_at | 0.00 |
| 137498_at | 0.00 |
| 137499_at | 0.00 |
| 137500_at | 0.00 |
| 137501_f_at | 0.00 |
| 137502_at | 0.00 |
| 137503_at | 0.04 |
| 137504_at | 0.00 |
| 137505_at | 0.00 |
| 137506_at | 0.00 |
| 137508_r_at | 0.00 |
| 137510_at | 0.00 |
| 137511_at | 0.00 |
| 137512_at | 0.00 |
| 137513_at | 0.00 |
| 137514_at | 0.00 |
| 137515_at | 0.00 |
| 137516_at | 0.00 |
| 137517_at | 0.00 |
| 137518_at | 0.00 |
| 137519_at | 0.00 |
| 137520_at | 0.00 |
| 137521_at | 0.00 |
| 137522_at | 0.00 |
| 137524_at | 0.00 |
| 137525_at | 0.00 |
| 137526_r_at | 0.00 |
| 137527_at | 0.00 |
| 137528_at | 0.00 |
| 137529_at | 0.00 |
| 137530_at | 0.00 |
| 137531_at | 0.00 |
| 137532_at | 0.00 |
| 137533_f_at | 0.00 |
| 137534_at | 0.00 |
| 137535_at | 0.00 |
| 137537_at | 0.00 |
| 137539_at | 0.00 |
| 137540_at | 0.00 |
| 137541_at | 0.00 |
| 137542_at | 0.00 |
| 137543_r_at | 0.00 |
| 137544_at | 0.00 |
| 137545_at | 0.00 |
| 137546_at | 0.00 |
| 137547_at | 0.00 |
| 137549_at | 0.00 |
| 137550_at | 0.00 |
| 137551_at | 0.00 |
| 137553_at | 0.00 |
| 137554_at | 0.00 |
| 137555_at | 0.01 |
| 137556_r_at | 0.00 |
| 137557_at | 0.00 |

| | |
|---|---|
| 137558_at | 0.00 |
| 137559_at | 0.00 |
| 137560_at | 0.00 |
| 137561_at | 0.33 |
| 137562_at | 0.00 |
| 137563_f_at | 0.00 |
| 137564_at | 0.00 |
| 137565_at | 0.00 |
| 137566_at | 0.00 |
| 137567_at | 0.00 |
| 137568_at | 0.00 |
| 137569_at | 0.00 |
| 137570_at | 0.00 |
| 137571_at | 0.00 |
| 137572_f_at | 0.01 |
| 137573_r_at | 0.00 |
| 137574_at | 0.00 |
| 137575_at | 0.00 |
| 137576_at | 0.00 |
| 137577_at | 0.00 |
| 137578_at | 0.00 |
| 137579_r_at | 0.00 |
| 137580_f_at | 0.00 |
| 137581_f_at | 0.00 |
| 137582_at | 0.00 |
| 137583_f_at | 0.00 |
| 137584_f_at | 0.00 |
| 137585_at | 0.00 |
| 137586_at | 0.00 |
| 137588_r_at | 0.00 |
| 137589_at | 0.00 |
| 137590_at | 0.00 |
| 137591_at | 0.00 |
| 137592_at | 0.00 |
| 137593_at | 0.00 |
| 137594_at | 0.00 |
| 137595_at | 0.00 |
| 137596_at | 0.00 |
| 137597_at | 0.00 |
| 137598_at | 0.00 |
| 137604_at | 0.00 |
| 137606_at | 0.00 |
| 137609_at | 0.00 |
| 137611_at | 0.00 |
| 137612_f_at | 0.00 |
| 137613_at | 0.00 |
| 137614_at | 0.00 |
| 137615_f_at | 0.00 |
| 137616_r_at | 0.00 |
| 137617_f_at | 0.00 |
| 137618_r_at | 0.00 |
| 137619_i_at | 0.00 |
| 137620_f_at | 0.00 |
| 137621_at | 0.00 |
| 137622_at | 0.00 |
| 137623_at | 0.00 |

| | |
|---|---|
| 137624_at | 0.00 |
| 137625_f_at | 0.00 |
| 137626_at | 0.00 |
| 137627_f_at | 0.05 |
| 137628_at | 0.00 |
| 137629_at | 0.00 |
| 137630_f_at | 0.00 |
| 137631_f_at | 0.00 |
| 137632_f_at | 0.00 |
| 137633_r_at | 0.00 |
| 137634_i_at | 0.00 |
| 137635_r_at | 0.00 |
| 137636_at | 0.00 |
| 137637_s_at | 0.00 |
| 137638_at | 0.00 |
| 137639_at | 0.00 |
| 137640_at | 0.00 |
| 137641_at | 0.00 |
| 137642_at | 0.31 |
| 137643_at | 0.03 |
| 137644_at | 0.00 |
| 137645_at | 0.00 |
| 137646_at | 0.00 |
| 137647_at | 0.00 |
| 137648_at | 0.00 |
| 137649_at | 0.00 |
| 137650_at | 0.01 |
| 137651_at | 0.00 |
| 137652_at | 0.00 |
| 137653_at | 0.00 |
| 137654_f_at | 0.00 |
| 137655_at | 0.00 |
| 137656_s_at | 0.00 |
| 137657_at | 0.00 |
| 137658_at | 0.00 |
| 137659_at | 0.00 |
| 137660_at | 0.00 |
| 137661_at | 0.00 |
| 137662_at | 0.00 |
| 137663_at | 0.00 |
| 137664_at | 0.00 |
| 137665_at | 0.01 |
| 137666_at | 0.00 |
| 137667_at | 0.00 |
| 137668_f_at | 0.00 |
| 137669_at | 0.00 |
| 137670_at | 0.00 |
| 137671_at | 0.00 |
| 137672_at | 0.00 |
| 137673_at | 0.00 |
| 137674_at | 0.00 |
| 137675_at | 0.00 |
| 137676_at | 0.00 |
| 137677_at | 0.00 |
| 137678_at | 0.00 |
| 137679_at | 0.00 |

| | |
|---|---|
| 137680_at | 0.01 |
| 137681_at | 0.00 |
| 137682_at | 0.00 |
| 137683_at | 0.00 |
| 137684_at | 0.00 |
| 137685_at | 0.02 |
| 137686_at | 0.00 |
| 137687_at | 0.00 |
| 137688_at | 0.00 |
| 137689_at | 0.00 |
| 137690_at | 0.00 |
| 137691_at | 0.00 |
| 137692_at | 0.00 |
| 137693_at | 0.00 |
| 137694_at | 0.00 |
| 137695_at | 0.00 |
| 137696_at | 0.00 |
| 137697_at | 0.00 |
| 137698_at | 0.00 |
| 137699_at | 0.00 |
| 137700_at | 0.00 |
| 137701_at | 0.00 |
| 137702_r_at | 0.00 |
| 137703_at | 0.00 |
| 137704_at | 0.00 |
| 137705_at | 0.00 |
| 137706_at | 0.00 |
| 137707_at | 0.00 |
| 137708_at | 0.00 |
| 137709_at | 0.00 |
| 137710_at | 0.00 |
| 137711_at | 0.00 |
| 137712_at | 0.00 |
| 137713_at | 0.00 |
| 137714_at | 0.00 |
| 137715_at | 0.00 |
| 137716_at | 0.00 |
| 137717_at | 0.00 |
| 137718_at | 0.00 |
| 137719_at | 0.00 |
| 137720_at | 0.00 |
| 137721_at | 0.00 |
| 137722_at | 0.00 |
| 137723_at | 0.00 |
| 137724_at | 0.00 |
| 137725_at | 0.00 |
| 137726_at | 0.00 |
| 137727_at | 0.00 |
| 137728_at | 0.00 |
| 137729_at | 0.00 |
| 137730_at | 0.00 |
| 137731_at | 0.00 |
| 137732_at | 0.00 |
| 137733_at | 0.00 |
| 137734_at | 0.00 |
| 137735_r_at | 0.00 |

| | |
|---|---|
| 137736_at | 0.00 |
| 137861_at | 0.00 |
| 137913_at | 0.00 |
| 137914_at | 0.00 |
| 137915_at | 0.00 |
| 137972_at | 0.00 |
| 137973_at | 0.09 |
| 137974_at | 0.00 |
| 137975_i_at | 0.00 |
| 137976_r_at | 0.00 |
| 137977_s_at | 0.00 |
| 137979_at | 0.00 |
| 137980_at | 0.00 |
| 137981_at | 0.00 |
| 137982_at | 0.00 |
| 137983_at | 0.00 |
| 137984_at | 0.00 |
| 137985_at | 0.00 |
| 137986_at | 0.00 |
| 137987_at | 0.00 |
| 137989_s_at | 0.00 |
| 137990_r_at | 0.00 |
| 137992_at | 0.00 |
| 137994_f_at | 0.00 |
| 137995_at | 0.00 |
| 137996_at | 0.00 |
| 137999_at | 0.00 |
| 138000_at | 0.00 |
| 138001_at | 0.00 |
| 138002_at | 0.00 |
| 138003_at | 0.00 |
| 138004_at | 0.00 |
| 138007_at | 0.00 |
| 138008_r_at | 0.00 |
| 138009_at | 0.00 |
| 138012_at | 0.00 |
| 138013_at | 0.00 |
| 138014_at | 0.00 |
| 138015_r_at | 0.00 |
| 138016_at | 0.00 |
| 138017_at | 0.00 |
| 138020_at | 0.00 |
| 138023_at | 0.00 |
| 138026_at | 0.00 |
| 138027_at | 0.00 |
| 138028_at | 0.00 |
| 138030_at | 0.00 |
| 138031_at | 0.00 |
| 138032_at | 0.00 |
| 138033_at | 0.00 |
| 138036_at | 0.00 |
| 138037_at | 0.02 |
| 138042_at | 0.01 |
| 138043_at | 0.00 |
| 138044_at | 0.00 |
| 138046_at | 0.00 |

| | |
|---|---|
| 138048_at | 0.00 |
| 138051_at | 0.00 |
| 138052_g_at | 0.03 |
| 138053_r_at | 0.00 |
| 138054_at | 0.00 |
| 138055_at | 0.00 |
| 138056_r_at | 0.00 |
| 138057_at | 0.00 |
| 138058_at | 0.00 |
| 138059_at | 0.00 |
| 138060_at | 0.30 |
| 138061_at | 0.00 |
| 138062_at | 0.00 |
| 138063_at | 0.00 |
| 138064_at | 0.00 |
| 138065_at | 0.07 |
| 138066_at | 0.00 |
| 138068_at | 0.00 |
| 138069_at | 0.15 |
| 138070_at | 0.00 |
| 138071_at | 0.00 |
| 138072_at | 0.00 |
| 138073_at | 0.02 |
| 138074_at | 0.00 |
| 138075_at | 0.00 |
| 138076_at | 0.00 |
| 138077_at | 0.00 |
| 138078_at | 0.00 |
| 138079_at | 0.00 |
| 138081_at | 0.01 |
| 138082_at | 0.00 |
| 138084_at | 0.00 |
| 138086_f_at | 0.00 |
| 138087_at | 0.00 |
| 138088_at | 0.00 |
| 138089_at | 0.06 |
| 138090_at | 0.00 |
| 138091_at | 0.00 |
| 138092_at | 0.00 |
| 138093_f_at | 0.00 |
| 138094_at | 0.00 |
| 138095_at | 0.00 |
| 138096_at | 0.00 |
| 138097_f_at | 0.00 |
| 138098_at | 0.00 |
| 138099_at | 0.00 |
| 138100_f_at | 0.00 |
| 138101_at | 0.00 |
| 138102_at | 0.00 |
| 138103_at | 0.00 |
| 138104_at | 0.00 |
| 138105_at | 0.00 |
| 138106_at | 0.00 |
| 138107_at | 0.00 |
| 138108_at | 0.00 |
| 138109_at | 0.00 |

| | |
|---|---|
| 138110_at | 0.00 |
| 138111_at | 0.00 |
| 138112_at | 0.00 |
| 138113_at | 0.00 |
| 138114_at | 0.00 |
| 138115_at | 0.00 |
| 138116_at | 0.00 |
| 138117_at | 0.00 |
| 138118_f_at | 0.00 |
| 138119_at | 0.00 |
| 138120_at | 0.00 |
| 138121_at | 0.00 |
| 138122_at | 0.00 |
| 138123_at | 0.00 |
| 138124_at | 0.00 |
| 138125_at | 0.00 |
| 138126_at | 0.05 |
| 138127_at | 0.00 |
| 138128_at | 0.00 |
| 138129_at | 0.00 |
| 138130_at | 0.00 |
| 138131_at | 0.00 |
| 138132_at | 0.00 |
| 138133_at | 0.00 |
| 138134_at | 0.00 |
| 138135_at | 0.00 |
| 138136_at | 0.00 |
| 138137_at | 0.00 |
| 138138_at | 0.00 |
| 138139_at | 0.00 |
| 138140_at | 0.00 |
| 138141_at | 0.00 |
| 138142_at | 0.00 |
| 138143_at | 0.00 |
| 138144_at | 0.00 |
| 138145_at | 0.00 |
| 138146_at | 0.00 |
| 138147_at | 0.00 |
| 138148_at | 0.00 |
| 138149_at | 0.00 |
| 138150_at | 0.00 |
| 138151_at | 0.00 |
| 138152_at | 0.00 |
| 138153_at | 0.00 |
| 138154_at | 0.00 |
| 138155_at | 0.00 |
| 138156_at | 0.00 |
| 138157_at | 0.00 |
| 138158_at | 0.00 |
| 138159_i_at | 0.00 |
| 138160_f_at | 0.00 |
| 138161_at | 0.00 |
| 138162_at | 0.00 |
| 138163_at | 0.00 |
| 138164_at | 0.00 |
| 138165_at | 0.00 |

| | |
|---|---|
| 138166_at | 0.00 |
| 138167_at | 0.00 |
| 138168_at | 0.00 |
| 138169_at | 0.00 |
| 138170_at | 0.00 |
| 138171_at | 0.00 |
| 138172_at | 0.00 |
| 138173_at | 0.00 |
| 138174_at | 0.00 |
| 138175_at | 0.00 |
| 138176_at | 0.00 |
| 138177_at | 0.00 |
| 138178_at | 0.00 |
| 138179_at | 0.00 |
| 138180_at | 0.00 |
| 138181_at | 0.00 |
| 138182_at | 0.00 |
| 138183_at | 0.00 |
| 138184_at | 0.00 |
| 138185_at | 0.00 |
| 138186_at | 0.00 |
| 138187_at | 0.00 |
| 138188_at | 0.00 |
| 138189_at | 0.00 |
| 138190_at | 0.00 |
| 138191_f_at | 0.00 |
| 138192_at | 0.00 |
| 138193_at | 0.00 |
| 138194_at | 0.00 |
| 138195_at | 0.00 |
| 138196_at | 0.00 |
| 138197_at | 0.00 |
| 138198_f_at | 0.00 |
| 138199_i_at | 0.00 |
| 138200_at | 0.01 |
| 138201_at | 0.00 |
| 138202_at | 0.00 |
| 138203_at | 0.00 |
| 138204_at | 0.00 |
| 138205_at | 0.00 |
| 138206_at | 0.00 |
| 138207_f_at | 0.00 |
| 138208_at | 0.00 |
| 138209_at | 0.00 |
| 138245_at | 0.00 |
| 138246_at | 0.00 |
| 138247_at | 0.00 |
| 138248_at | 0.00 |
| 138256_at | 0.00 |
| 138264_at | 0.00 |
| 138265_at | 0.01 |
| 138313_at | 0.00 |
| 138322_at | 0.00 |
| 138363_at | 0.01 |
| 138364_at | 0.00 |
| 138365_at | 0.00 |

| | |
|---|---|
| 138367_at | 0.00 |
| 138368_at | 0.00 |
| 138369_at | 0.00 |
| 138377_at | 0.00 |
| 138378_at | 0.00 |
| 138379_at | 0.00 |
| 138380_at | 0.00 |
| 138381_at | 0.00 |
| 138382_at | 0.00 |
| 138383_at | 0.00 |
| 138384_at | 0.00 |
| 138385_at | 0.00 |
| 138386_at | 0.00 |
| 138387_at | 0.00 |
| 138388_at | 0.00 |
| 138389_at | 0.00 |
| 138390_at | 0.00 |
| 138391_at | 0.00 |
| 138392_at | 0.00 |
| 138393_at | 0.00 |
| 138394_at | 0.00 |
| 138395_at | 0.00 |
| 138396_at | 0.00 |
| 138397_at | 0.00 |
| 138398_at | 0.00 |
| 138399_at | 0.00 |
| 138400_at | 0.00 |
| 138401_at | 0.00 |
| 138402_at | 0.00 |
| 138403_at | 0.00 |
| 138404_at | 0.00 |
| 138405_at | 0.00 |
| 138406_f_at | 0.00 |
| 138407_f_at | 0.00 |
| 138408_at | 0.00 |
| 138409_at | 0.00 |
| 138410_at | 0.00 |
| 138411_at | 0.00 |
| 138412_at | 0.00 |
| 138413_at | 0.00 |
| 138414_at | 0.00 |
| 138415_at | 0.00 |
| 138416_at | 0.00 |
| 138417_f_at | 0.00 |
| 138418_at | 0.00 |
| 138419_at | 0.00 |
| 138420_at | 0.00 |
| 138421_at | 0.00 |
| 138422_at | 0.00 |
| 138423_r_at | 0.00 |
| 138424_at | 0.00 |
| 138425_at | 0.00 |
| 138451_at | 0.00 |
| 138452_at | 0.00 |
| 138453_at | 0.00 |
| 138454_at | 0.00 |

| | |
|---|---|
| 138455_at | 0.00 |
| 138457_at | 0.00 |
| 138458_at | 0.00 |
| 138459_at | 0.00 |
| 138460_at | 0.00 |
| 138461_at | 0.00 |
| 138462_r_at | 0.00 |
| 138463_at | 0.00 |
| 138464_at | 0.00 |
| 138465_at | 0.00 |
| 138466_at | 0.00 |
| 138467_i_at | 0.00 |
| 138468_at | 0.00 |
| 138471_f_at | 0.00 |
| 138472_at | 0.08 |
| 138473_at | 0.18 |
| 138476_at | 0.00 |
| 138478_at | 0.00 |
| 138479_at | 0.00 |
| 138480_at | 0.00 |
| 138481_at | 0.00 |
| 138483_at | 0.00 |
| 138484_f_at | 0.00 |
| 138485_at | 0.00 |
| 138486_at | 0.00 |
| 138487_at | 0.00 |
| 138488_at | 0.00 |
| 138489_at | 0.00 |
| 138490_at | 0.00 |
| 138492_at | 0.00 |
| 138494_at | 0.00 |
| 138495_f_at | 0.00 |
| 138496_at | 0.00 |
| 138497_at | 0.00 |
| 138498_at | 0.00 |
| 138500_at | 0.00 |
| 138501_r_at | 0.00 |
| 138502_at | 0.00 |
| 138503_r_at | 0.00 |
| 138504_at | 0.00 |
| 138505_at | 0.00 |
| 138506_at | 0.00 |
| 138507_at | 0.00 |
| 138508_at | 0.00 |
| 138510_at | 0.00 |
| 138511_at | 0.00 |
| 138512_at | 0.00 |
| 138513_at | 0.00 |
| 138514_at | 0.00 |
| 138515_at | 0.00 |
| 138516_at | 0.00 |
| 138517_at | 0.00 |
| 138519_at | 0.00 |
| 138520_at | 0.00 |
| 138522_at | 0.00 |
| 138523_at | 0.00 |

| | |
|---|---|
| 138524_r_at | 0.00 |
| 138525_at | 0.00 |
| 138526_at | 0.00 |
| 138527_at | 0.00 |
| 138528_at | 0.00 |
| 138530_at | 0.00 |
| 138531_at | 0.00 |
| 138532_at | 0.00 |
| 138533_at | 0.00 |
| 138534_at | 0.00 |
| 138535_at | 0.00 |
| 138536_at | 0.00 |
| 138537_at | 0.00 |
| 138538_at | 0.00 |
| 138539_at | 0.00 |
| 138540_at | 0.00 |
| 138541_r_at | 0.00 |
| 138542_at | 0.00 |
| 138543_at | 0.00 |
| 138544_at | 0.01 |
| 138545_at | 0.00 |
| 138547_at | 0.00 |
| 138548_at | 0.00 |
| 138549_at | 0.00 |
| 138551_at | 0.00 |
| 138552_at | 0.00 |
| 138553_at | 0.08 |
| 138554_at | 0.00 |
| 138556_at | 0.00 |
| 138557_at | 0.01 |
| 138558_at | 0.00 |
| 138559_f_at | 0.00 |
| 138560_r_at | 0.00 |
| 138561_at | 0.00 |
| 138562_at | 0.00 |
| 138563_at | 0.00 |
| 138564_at | 0.00 |
| 138565_at | 0.00 |
| 138566_s_at | 0.00 |
| 138567_at | 0.00 |
| 138568_at | 0.00 |
| 138569_at | 0.00 |
| 138570_at | 0.00 |
| 138571_at | 0.00 |
| 138572_at | 0.00 |
| 138576_at | 0.00 |
| 138577_at | 0.03 |
| 138746_at | 0.00 |
| 138755_at | 0.00 |
| 138762_at | 0.00 |
| 138763_at | 0.00 |
| 138765_at | 0.00 |
| 138769_at | 0.00 |
| 138784_at | 0.00 |
| 138785_at | 0.00 |
| 138786_r_at | 0.00 |

| | |
|---|---|
| 138787_at | 0.00 |
| 138788_f_at | 0.00 |
| 138789_at | 0.00 |
| 138790_at | 0.00 |
| 138791_at | 0.00 |
| 138792_at | 0.16 |
| 138793_at | 0.00 |
| 138794_at | 0.00 |
| 138795_at | 0.00 |
| 138796_at | 0.00 |
| 138797_at | 0.00 |
| 138798_at | 0.00 |
| 138799_at | 0.00 |
| 138800_at | 0.00 |
| 138801_at | 0.00 |
| 138802_at | 0.00 |
| 138831_at | 0.00 |
| 138845_at | 0.00 |
| 138919_at | 0.00 |
| 138920_at | 0.00 |
| 138921_at | 0.00 |
| 138922_g_at | 0.00 |
| 138923_at | 0.00 |
| 138929_at | 0.00 |
| 138930_at | 0.00 |
| 138931_at | 0.00 |
| 138932_at | 0.00 |
| 138933_at | 0.00 |
| 138936_s_at | 0.00 |
| 138937_at | 0.00 |
| 138938_at | 0.00 |
| 138945_at | 0.19 |
| 138946_at | 0.00 |
| 138947_at | 0.00 |
| 138949_at | 0.00 |
| 138950_at | 0.00 |
| 138952_at | 0.00 |
| 138958_r_at | 0.00 |
| 138960_f_at | 0.00 |
| 138962_f_at | 0.00 |
| 138963_r_at | 0.00 |
| 138964_at | 0.01 |
| 138965_at | 0.00 |
| 138966_at | 0.00 |
| 138967_i_at | 0.00 |
| 138968_f_at | 0.00 |
| 138969_at | 0.00 |
| 138970_at | 0.00 |
| 138971_at | 0.00 |
| 138975_at | 0.00 |
| 138976_at | 0.00 |
| 138978_at | 0.00 |
| 138979_r_at | 0.00 |
| 138980_f_at | 0.01 |
| 138981_r_at | 0.00 |
| 138983_r_at | 0.00 |

| | |
|---|---|
| 138984_at | 0.00 |
| 138985_r_at | 0.00 |
| 138986_at | 0.14 |
| 138987_g_at | 0.00 |
| 138988_at | 0.24 |
| 138989_at | 0.00 |
| 138990_at | 0.00 |
| 138993_r_at | 0.00 |
| 138994_f_at | 0.00 |
| 138995_at | 0.00 |
| 138997_at | 0.00 |
| 138998_at | 0.00 |
| 138999_at | 0.00 |
| 139000_at | 0.00 |
| 139001_at | 0.00 |
| 139002_at | 0.00 |
| 139003_at | 0.00 |
| 139004_r_at | 0.00 |
| 139005_at | 0.00 |
| 139006_at | 0.00 |
| 139007_at | 0.00 |
| 139008_at | 0.00 |
| 139010_i_at | 0.01 |
| 139011_r_at | 0.00 |
| 139012_at | 0.00 |
| 139014_at | 0.00 |
| 139015_at | 0.00 |
| 139016_at | 0.00 |
| 139017_at | 0.00 |
| 139018_at | 0.00 |
| 139019_at | 0.05 |
| 139020_at | 0.00 |
| 139021_at | 0.00 |
| 139022_at | 0.00 |
| 139023_at | 0.00 |
| 139025_f_at | 0.00 |
| 139026_f_at | 0.00 |
| 139027_at | 0.00 |
| 139028_at | 0.00 |
| 139029_at | 0.04 |
| 139030_f_at | 0.00 |
| 139031_f_at | 0.00 |
| 139032_f_at | 0.00 |
| 139033_at | 0.00 |
| 139034_at | 0.00 |
| 139035_at | 0.05 |
| 139039_f_at | 0.00 |
| 139041_at | 0.00 |
| 139042_at | 0.00 |
| 139044_at | 0.00 |
| 139045_at | 0.00 |
| 139046_at | 0.00 |
| 139123_at | 0.00 |
| 139124_f_at | 0.00 |
| 139125_at | 0.00 |
| 139126_at | 0.00 |

| | |
|---|---|
| 139127_at | 0.00 |
| 139128_i_at | 0.00 |
| 139129_r_at | 0.00 |
| 139130_at | 0.31 |
| 139131_at | 0.00 |
| 139132_f_at | 0.00 |
| 139133_at | 0.00 |
| 139134_at | 0.00 |
| 139135_g_at | 0.00 |
| 139136_at | 0.00 |
| 139137_at | 0.00 |
| 139138_at | 0.00 |
| 139139_at | 0.00 |
| 139140_at | 0.00 |
| 139141_s_at | 0.00 |
| 139142_r_at | 0.00 |
| 139143_at | 0.00 |
| 139144_at | 0.00 |
| 139145_at | 0.00 |
| 139146_at | 0.00 |
| 139147_at | 0.00 |
| 139148_at | 0.00 |
| 139149_at | 0.00 |
| 139150_at | 0.00 |
| 139151_at | 0.00 |
| 139152_at | 0.00 |
| 139153_at | 0.00 |
| 139154_at | 0.00 |
| 139155_at | 0.00 |
| 139156_at | 0.00 |
| 139157_at | 0.00 |
| 139158_at | 0.00 |
| 139159_at | 0.00 |
| 139160_at | 0.00 |
| 139161_at | 0.00 |
| 139162_at | 0.00 |
| 139163_at | 0.00 |
| 139164_at | 0.00 |
| 139165_at | 0.00 |
| 139166_at | 0.00 |
| 139167_at | 0.00 |
| 139168_at | 0.00 |
| 139169_at | 0.00 |
| 139170_at | 0.00 |
| 139171_at | 0.00 |
| 139172_at | 0.00 |
| 139173_at | 0.00 |
| 139174_at | 0.00 |
| 139175_at | 0.00 |
| 139176_at | 0.00 |
| 139177_at | 0.00 |
| 139178_f_at | 0.00 |
| 139179_r_at | 0.00 |
| 139180_at | 0.00 |
| 139181_at | 0.00 |
| 139182_at | 0.00 |

| | |
|---|---|
| 139183_at | 0.00 |
| 139184_at | 0.00 |
| 139185_at | 0.00 |
| 139186_at | 0.00 |
| 139187_at | 0.00 |
| 139188_at | 0.00 |
| 139189_at | 0.00 |
| 139190_g_at | 0.00 |
| 139191_at | 0.00 |
| 139193_at | 0.00 |
| 139194_at | 0.00 |
| 139195_at | 0.00 |
| 139196_at | 0.00 |
| 139197_at | 0.00 |
| 139198_at | 0.00 |
| 139199_at | 0.00 |
| 139200_at | 0.00 |
| 139202_at | 0.00 |
| 139203_at | 0.00 |
| 139204_at | 0.00 |
| 139205_at | 0.00 |
| 139207_s_at | 0.29 |
| 139208_at | 0.00 |
| 139209_at | 0.00 |
| 139210_at | 0.00 |
| 139211_at | 0.00 |
| 139212_at | 0.00 |
| 139213_r_at | 0.00 |
| 139215_at | 0.00 |
| 139217_at | 0.00 |
| 139218_at | 0.00 |
| 139220_at | 0.00 |
| 139221_f_at | 0.02 |
| 139222_r_at | 0.00 |
| 139223_at | 0.00 |
| 139224_at | 0.00 |
| 139225_at | 0.00 |
| 139226_at | 0.00 |
| 139227_at | 0.00 |
| 139228_at | 0.08 |
| 139229_r_at | 0.00 |
| 139230_at | 0.00 |
| 139231_at | 0.00 |
| 139232_at | 0.00 |
| 139233_at | 0.00 |
| 139234_at | 0.00 |
| 139236_at | 0.00 |
| 139237_r_at | 0.00 |
| 139238_at | 0.00 |
| 139239_at | 0.00 |
| 139241_at | 0.00 |
| 139242_at | 0.00 |
| 139244_at | 0.00 |
| 139245_at | 0.00 |
| 139246_at | 0.00 |
| 139248_f_at | 0.00 |

| | |
|---|---|
| 139249_f_at | 0.00 |
| 139250_at | 0.00 |
| 139253_at | 0.08 |
| 139254_at | 0.00 |
| 139255_at | 0.00 |
| 139257_at | 0.00 |
| 139258_at | 0.00 |
| 139260_at | 0.00 |
| 139261_at | 0.00 |
| 139262_f_at | 0.00 |
| 139263_at | 0.00 |
| 139264_at | 0.00 |
| 139266_at | 0.00 |
| 139267_r_at | 0.00 |
| 139269_at | 0.02 |
| 139270_at | 0.00 |
| 139271_at | 0.00 |
| 139272_at | 0.00 |
| 139273_at | 0.00 |
| 139274_at | 0.00 |
| 139275_at | 0.00 |
| 139276_at | 0.00 |
| 139278_at | 0.00 |
| 139279_at | 0.00 |
| 139282_at | 0.00 |
| 139283_r_at | 0.00 |
| 139284_at | 0.00 |
| 139285_f_at | 0.00 |
| 139287_at | 0.00 |
| 139288_at | 0.00 |
| 139289_at | 0.00 |
| 139290_at | 0.00 |
| 139291_at | 0.00 |
| 139292_at | 0.00 |
| 139293_at | 0.00 |
| 139294_at | 0.00 |
| 139295_at | 0.00 |
| 139296_at | 0.00 |
| 139297_at | 0.00 |
| 139298_at | 0.00 |
| 139391_f_at | 0.00 |
| 139392_at | 0.00 |
| 139393_at | 0.46 |
| 139394_at | 0.00 |
| 139395_at | 0.00 |
| 139396_at | 0.00 |
| 139397_at | 0.00 |
| 139398_at | 0.00 |
| 139399_at | 0.00 |
| 139400_at | 0.00 |
| 139401_at | 0.00 |
| 139402_at | 0.00 |
| 139403_s_at | 0.00 |
| 139404_at | 0.00 |
| 139405_at | 0.00 |
| 139406_at | 0.00 |

| | |
|---|---|
| 139412_f_at | 0.00 |
| 139413_at | 0.06 |
| 139419_at | 0.00 |
| 139420_f_at | 0.00 |
| 139421_r_at | 0.00 |
| 139422_at | 0.00 |
| 139423_at | 0.00 |
| 139424_r_at | 0.00 |
| 139425_f_at | 0.00 |
| 139426_r_at | 0.00 |
| 139427_at | 0.00 |
| 139480_at | 0.00 |
| 139481_at | 0.00 |
| 139482_at | 0.00 |
| 139483_at | 0.00 |
| 139484_r_at | 0.00 |
| 139485_at | 0.00 |
| 139486_at | 0.00 |
| 139487_at | 0.00 |
| 139488_at | 0.00 |
| 139489_at | 0.00 |
| 139490_at | 0.00 |
| 139493_at | 0.00 |
| 139494_at | 0.00 |
| 139495_at | 0.00 |
| 139496_at | 0.00 |
| 139497_at | 0.00 |
| 139498_at | 0.00 |
| 139499_at | 0.00 |
| 139501_at | 0.00 |
| 139502_at | 0.00 |
| 139503_at | 0.00 |
| 139504_at | 0.00 |
| 139507_at | 0.00 |
| 139508_at | 0.00 |
| 139509_at | 0.00 |
| 139510_at | 0.00 |
| 139511_at | 0.00 |
| 139512_at | 0.00 |
| 139513_at | 0.00 |
| 139514_r_at | 0.00 |
| 139515_at | 0.00 |
| 139516_g_at | 0.00 |
| 139519_at | 0.00 |
| 139520_r_at | 0.00 |
| 139522_at | 0.00 |
| 139525_at | 0.00 |
| 139526_at | 0.00 |
| 139527_at | 0.00 |
| 139528_r_at | 0.00 |
| 139530_at | 0.00 |
| 139531_at | 0.00 |
| 139533_at | 0.04 |
| 139535_at | 0.00 |
| 139536_at | 0.00 |
| 139537_at | 0.03 |

| | |
|---|---|
| 139538_at | 0.00 |
| 139546_at | 0.00 |
| 139547_at | 0.00 |
| 139560_at | 0.00 |
| 139561_at | 0.00 |
| 139585_at | 0.00 |
| 139594_at | 0.33 |
| 139598_at | 0.00 |
| 139599_r_at | 0.00 |
| 139600_at | 0.00 |
| 139601_at | 0.00 |
| 139602_at | 0.00 |
| 139748_at | 0.00 |
| 139753_at | 0.00 |
| 139754_f_at | 0.00 |
| 139755_at | 0.00 |
| 139804_at | 0.00 |
| 139808_at | 0.00 |
| 139809_at | 0.00 |
| 139815_at | 0.02 |
| 139836_at | 0.00 |
| 139841_at | 0.00 |
| 139842_at | 0.00 |
| 139979_at | 0.00 |
| 139980_g_at | 0.02 |
| 139998_at | 0.36 |
| 140004_at | 0.00 |
| 140005_at | 0.01 |
| 140006_at | 0.00 |
| 140009_i_at | 0.00 |
| 140010_f_at | 0.02 |
| 140011_r_at | 0.00 |
| 140015_at | 0.00 |
| 140022_at | 0.00 |
| 140050_at | 0.00 |
| 140061_at | 0.00 |
| 140187_f_at | 0.00 |
| 140188_at | 0.00 |
| 140189_at | 0.00 |
| 140190_at | 0.00 |
| 140191_at | 0.00 |
| 140192_at | 0.00 |
| 140193_at | 0.00 |
| 140194_at | 0.00 |
| 140195_at | 0.00 |
| 140210_at | 0.00 |
| 140220_r_at | 0.00 |
| 140247_at | 0.07 |
| 140322_at | 0.00 |
| 140323_r_at | 0.00 |
| 140324_at | 0.05 |
| 140325_at | 0.00 |
| 140327_at | 0.00 |
| 140328_at | 0.00 |
| 140329_at | 0.00 |
| 140352_at | 0.00 |

| | |
|---|---|
| 140353_at | 0.00 |
| 140354_at | 0.00 |
| 140355_at | 0.00 |
| 140356_at | 0.00 |
| 140357_at | 0.00 |
| 140358_at | 0.00 |
| 140359_at | 0.00 |
| 140365_i_at | 0.00 |
| 140366_r_at | 0.00 |
| 140370_at | 0.00 |
| 140371_at | 0.00 |
| 140405_at | 0.00 |
| 140417_at | 0.00 |
| 140418_at | 0.00 |
| 140419_at | 0.00 |
| 140420_at | 0.00 |
| 140421_at | 0.00 |
| 140422_r_at | 0.00 |
| 140423_at | 0.00 |
| 140424_at | 0.00 |
| 140425_at | 0.00 |
| 140426_at | 0.00 |
| 140427_at | 0.00 |
| 140428_i_at | 0.00 |
| 140429_f_at | 0.00 |
| 140430_at | 0.00 |
| 140431_g_at | 0.11 |
| 140432_r_at | 0.00 |
| 140434_r_at | 0.01 |
| 140436_at | 0.16 |
| 140437_at | 0.00 |
| 140438_at | 0.00 |
| 140439_at | 0.00 |
| 140440_at | 0.00 |
| 140441_at | 0.00 |
| 140442_at | 0.00 |
| 140443_f_at | 0.00 |
| 140444_r_at | 0.00 |
| 140445_at | 0.00 |
| 140446_at | 0.00 |
| 140447_at | 0.00 |
| 140448_at | 0.00 |
| 140449_at | 0.00 |
| 140450_at | 0.00 |
| 140451_at | 0.00 |
| 140452_at | 0.00 |
| 140453_g_at | 0.00 |
| 140454_at | 0.00 |
| 140455_at | 0.00 |
| 140456_r_at | 0.00 |
| 140457_at | 0.00 |
| 140460_at | 0.00 |
| 140477_f_at | 0.00 |
| 140480_f_at | 0.00 |
| 140484_at | 0.02 |
| 140485_at | 0.00 |

| | |
|---|---|
| 140487_r_at | 0.00 |
| 140488_i_at | 0.00 |
| 140489_f_at | 0.00 |
| 140490_at | 0.00 |
| 140492_at | 0.00 |
| 140496_at | 0.00 |
| 140497_at | 0.00 |
| 140509_at | 0.00 |
| 140510_at | 0.00 |
| 140511_at | 0.00 |
| 140519_at | 0.00 |
| 140546_at | 0.00 |
| 140548_at | 0.00 |
| 140549_r_at | 0.00 |
| 140564_at | 0.00 |
| 140565_f_at | 0.06 |
| 140567_at | 0.00 |
| 140569_at | 0.00 |
| 140570_at | 0.00 |
| 140571_at | 0.00 |
| 140572_at | 0.00 |
| 140588_at | 0.00 |
| 140589_at | 0.00 |
| 140607_at | 0.00 |
| 140609_f_at | 0.00 |
| 140621_at | 0.00 |
| 140629_s_at | 0.00 |
| 140636_at | 0.00 |
| 140637_at | 0.00 |
| 140638_at | 0.00 |
| 140639_at | 0.00 |
| 140640_at | 0.00 |
| 140641_at | 0.00 |
| 140642_at | 0.00 |
| 140644_f_at | 0.00 |
| 140645_at | 0.02 |
| 140647_at | 0.00 |
| 140648_i_at | 0.00 |
| 140649_r_at | 0.00 |
| 140650_at | 0.00 |
| 140651_at | 0.00 |
| 140652_r_at | 0.00 |
| 140653_at | 0.00 |
| 140654_at | 0.06 |
| 140655_at | 0.00 |
| 140656_at | 0.00 |
| 140657_at | 0.00 |
| 140658_at | 0.00 |
| 140659_at | 0.00 |
| 140660_at | 0.00 |
| 140661_at | 0.00 |
| 140662_r_at | 0.00 |
| 140664_r_at | 0.00 |
| 140665_at | 0.00 |
| 140666_at | 0.00 |
| 140687_at | 0.00 |

| | |
|---|---|
| 140694_at | 0.00 |
| 140695_f_at | 0.00 |
| 140696_f_at | 0.00 |
| 140697_at | 0.00 |
| 140698_f_at | 0.00 |
| 140699_at | 0.00 |
| 140700_r_at | 0.00 |
| 140701_at | 0.00 |
| 140702_at | 0.00 |
| 140704_at | 0.00 |
| 140705_r_at | 0.00 |
| 140706_r_at | 0.00 |
| 140707_at | 0.00 |
| 140708_at | 0.00 |
| 140709_at | 0.00 |
| 140710_at | 0.00 |
| 140711_at | 0.00 |
| 140712_r_at | 0.00 |
| 140713_at | 0.00 |
| 140714_at | 0.00 |
| 140715_at | 0.00 |
| 140716_at | 0.00 |
| 140717_at | 0.00 |
| 140731_at | 0.00 |
| 140736_at | 0.00 |
| 140747_at | 0.00 |
| 140748_at | 0.00 |
| 140750_at | 0.00 |
| 140752_at | 0.00 |
| 140753_at | 0.00 |
| 140754_at | 0.00 |
| 140755_at | 0.00 |
| 140756_at | 0.02 |
| 140757_at | 0.00 |
| 140758_at | 0.00 |
| 140759_at | 0.00 |
| 140760_at | 0.00 |
| 140768_at | 0.00 |
| 140782_f_at | 0.00 |
| 140805_at | 0.00 |
| 140814_at | 0.00 |
| 140815_at | 0.00 |
| 140816_r_at | 0.00 |
| 140817_at | 0.00 |
| 140818_at | 0.00 |
| 140819_at | 0.00 |
| 140820_at | 0.16 |
| 140822_at | 0.00 |
| 140823_at | 0.00 |
| 140824_at | 0.00 |
| 140825_at | 0.00 |
| 140826_at | 0.00 |
| 140827_at | 0.00 |
| 140828_at | 0.00 |
| 140829_at | 0.00 |
| 140830_at | 0.00 |

| | |
|---|---|
| 140832_at | 0.00 |
| 140833_at | 0.00 |
| 140834_at | 0.00 |
| 140835_at | 0.00 |
| 140836_at | 0.00 |
| 140837_at | 0.00 |
| 140838_at | 0.00 |
| 140839_at | 0.00 |
| 140840_at | 0.00 |
| 140845_at | 0.00 |
| 140849_at | 0.01 |
| 140851_at | 0.00 |
| 140860_at | 0.11 |
| 140861_at | 0.00 |
| 140870_f_at | 0.00 |
| 140872_at | 0.00 |
| 140873_r_at | 0.00 |
| 140875_at | 0.00 |
| 140876_at | 0.00 |
| 140877_at | 0.00 |
| 140878_at | 0.00 |
| 140879_r_at | 0.00 |
| 140880_at | 0.00 |
| 140881_at | 0.00 |
| 140882_at | 0.00 |
| 140883_at | 0.00 |
| 140884_at | 0.01 |
| 140885_at | 0.00 |
| 140886_at | 0.00 |
| 140887_at | 0.00 |
| 140888_at | 0.00 |
| 140889_s_at | 0.00 |
| 140890_at | 0.00 |
| 140891_at | 0.00 |
| 140893_at | 0.00 |
| 140894_at | 0.00 |
| 140896_at | 0.00 |
| 140898_at | 0.00 |
| 140899_at | 0.00 |
| 140925_f_at | 0.00 |
| 140934_i_at | 0.00 |
| 140935_r_at | 0.00 |
| 140936_at | 0.00 |
| 140937_at | 0.00 |
| 140938_at | 0.00 |
| 140939_at | 0.00 |
| 140940_at | 0.00 |
| 140942_at | 0.16 |
| 140943_at | 0.00 |
| 140944_at | 0.00 |
| 140945_at | 0.00 |
| 140947_at | 0.00 |
| 140948_at | 0.00 |
| 140972_f_at | 0.00 |
| 140984_r_at | 0.00 |
| 140991_at | 0.00 |

| | |
|---|---|
| 140997_at | 0.00 |
| 140999_at | 0.00 |
| 141005_at | 0.04 |
| 141010_at | 0.00 |
| 141021_i_at | 0.00 |
| 141022_f_at | 0.00 |
| 141023_at | 0.00 |
| 141024_at | 0.00 |
| 141025_at | 0.07 |
| 141026_at | 0.00 |
| 141027_at | 0.01 |
| 141028_at | 0.00 |
| 141029_at | 0.00 |
| 141030_at | 0.00 |
| 141031_at | 0.00 |
| 141032_at | 0.00 |
| 141033_at | 0.00 |
| 141034_at | 0.01 |
| 141035_i_at | 0.00 |
| 141036_f_at | 0.00 |
| 141037_at | 0.00 |
| 141038_at | 0.00 |
| 141039_r_at | 0.00 |
| 141040_at | 0.00 |
| 141042_at | 0.00 |
| 141043_at | 0.00 |
| 141044_f_at | 0.02 |
| 141045_at | 0.00 |
| 141046_at | 0.00 |
| 141047_at | 0.00 |
| 141048_at | 0.00 |
| 141049_r_at | 0.00 |
| 141050_at | 0.00 |
| 141051_at | 0.00 |
| 141053_at | 0.00 |
| 141068_at | 0.00 |
| 141069_at | 0.00 |
| 141071_i_at | 0.00 |
| 141072_f_at | 0.00 |
| 141073_at | 0.00 |
| 141076_at | 0.00 |
| 141084_at | 0.00 |
| 141090_f_at | 0.24 |
| 141091_at | 0.00 |
| 141092_at | 0.00 |
| 141095_at | 0.00 |
| 141101_at | 0.00 |
| 141104_at | 0.00 |
| 141105_s_at | 0.00 |
| 141108_at | 0.00 |
| 141109_at | 0.00 |
| 141110_at | 0.00 |
| 141112_at | 0.00 |
| 141113_at | 0.00 |
| 141114_at | 0.00 |
| 141115_at | 0.00 |

| | |
|---|---|
| 141116_at | 0.00 |
| 141117_at | 0.00 |
| 141118_at | 0.00 |
| 141119_at | 0.10 |
| 141120_at | 0.00 |
| 141121_at | 0.00 |
| 141122_at | 0.00 |
| 141123_at | 0.00 |
| 141125_at | 0.00 |
| 141126_at | 0.00 |
| 141127_at | 0.00 |
| 141128_at | 0.00 |
| 141129_at | 0.00 |
| 141130_at | 0.00 |
| 141137_at | 0.00 |
| 141154_at | 0.00 |
| 141157_at | 0.00 |
| 141158_r_at | 0.00 |
| 141160_f_at | 0.00 |
| 141162_f_at | 0.02 |
| 141163_r_at | 0.00 |
| 141164_r_at | 0.00 |
| 141170_at | 0.00 |
| 141171_at | 0.00 |
| 141172_at | 0.00 |
| 141173_at | 0.00 |
| 141174_at | 0.00 |
| 141175_i_at | 0.49 |
| 141176_at | 0.00 |
| 141177_at | 0.01 |
| 141179_at | 0.00 |
| 141180_at | 0.00 |
| 141181_at | 0.00 |
| 165402_f_at | 0.00 |
| 165403_at | 0.00 |
| 165404_i_at | 0.00 |
| 165405_at | 0.00 |
| 165406_at | 0.00 |
| 165407_f_at | 0.00 |
| 165408_f_at | 0.00 |
| 165409_at | 0.00 |
| 165410_at | 0.00 |
| 165411_at | 0.00 |
| 165412_at | 0.00 |
| 165413_at | 0.00 |
| 165414_at | 0.00 |
| 165415_r_at | 0.00 |
| 165416_at | 0.00 |
| 165417_at | 0.00 |
| 165418_f_at | 0.00 |
| 165419_i_at | 0.00 |
| 165420_at | 0.00 |
| 165421_at | 0.00 |
| 165422_at | 0.00 |
| 165423_s_at | 0.00 |
| 165424_r_at | 0.00 |

| | |
|---|---|
| 165425_f_at | 0.02 |
| 165426_f_at | 0.00 |
| 165427_f_at | 0.00 |
| 165428_r_at | 0.00 |
| 165429_at | 0.00 |
| 165430_r_at | 0.00 |
| 165431_at | 0.00 |
| 165432_i_at | 0.00 |
| 165433_at | 0.00 |
| 165434_at | 0.00 |
| 165435_f_at | 0.00 |
| 165436_r_at | 0.00 |
| 165437_at | 0.00 |
| 165438_r_at | 0.00 |
| 165439_at | 0.00 |
| 165440_at | 0.00 |
| 165441_at | 0.01 |
| 165442_at | 0.00 |
| 165443_f_at | 0.00 |
| 165444_at | 0.00 |
| 165445_at | 0.00 |
| 165446_at | 0.00 |
| 165447_r_at | 0.00 |
| 165448_i_at | 0.10 |
| 165449_f_at | 0.20 |
| 165450_i_at | 0.00 |
| 165451_i_at | 0.01 |
| 165452_at | 0.15 |
| 165453_at | 0.00 |
| 165454_at | 0.00 |
| 165455_at | 0.00 |
| 165456_s_at | 0.00 |
| 165457_i_at | 0.00 |
| 165458_r_at | 0.00 |
| 165459_at | 0.00 |
| 165460_at | 0.52 |
| 165461_r_at | 0.00 |
| 165462_at | 0.00 |
| 165463_at | 0.00 |
| 165464_r_at | 0.00 |
| 165465_at | 0.00 |
| 165466_r_at | 0.00 |
| 165467_r_at | 0.25 |
| 165468_r_at | 0.00 |
| 165469_f_at | 0.00 |
| 165470_at | 0.00 |
| 165471_f_at | 0.00 |
| 165472_i_at | 0.00 |
| 165473_at | 0.00 |
| 165474_f_at | 0.00 |
| 165475_f_at | 0.00 |
| 165476_i_at | 0.00 |
| 165477_r_at | 0.00 |
| 165478_i_at | 0.00 |
| 165479_at | 0.00 |
| 165480_i_at | 0.00 |

| | |
|---|---|
| 165481_at | 0.00 |
| 165482_r_at | 0.00 |
| 165483_at | 0.00 |
| 165484_at | 0.00 |
| 165485_r_at | 0.00 |
| 165486_i_at | 0.00 |
| 165487_f_at | 0.00 |
| 165488_i_at | 0.00 |
| 165489_f_at | 0.00 |
| 165490_r_at | 0.00 |
| 165491_at | 0.00 |
| 165492_at | 0.00 |
| 165493_r_at | 0.00 |
| 165494_i_at | 0.00 |
| 165495_i_at | 0.00 |
| 165496_at | 0.00 |
| 165497_i_at | 0.00 |
| 165498_f_at | 0.00 |
| 165499_f_at | 0.00 |
| 165500_at | 0.00 |
| 165501_r_at | 0.00 |
| 165502_at | 0.00 |
| 165503_i_at | 0.00 |
| 165504_i_at | 0.00 |
| 165505_at | 0.00 |
| 165506_f_at | 0.05 |
| 165507_f_at | 0.00 |
| 165508_i_at | 0.00 |
| 165509_at | 0.00 |
| 165510_f_at | 0.00 |
| 165511_at | 0.00 |
| 165512_at | 0.00 |
| 165513_i_at | 0.00 |
| 165514_f_at | 0.00 |
| 165515_at | 0.00 |
| 165516_i_at | 0.00 |
| 165517_r_at | 0.00 |
| 165518_f_at | 0.00 |
| 165519_f_at | 0.00 |
| 165520_f_at | 0.00 |
| 165521_at | 0.00 |
| 165522_r_at | 0.00 |
| 165523_at | 0.00 |
| 165524_i_at | 0.00 |
| 165525_r_at | 0.00 |
| 165526_i_at | 0.00 |
| 165527_f_at | 0.00 |
| 165528_i_at | 0.10 |
| 165529_r_at | 0.00 |
| 165530_at | 0.00 |
| 165531_at | 0.00 |
| 165532_r_at | 0.00 |
| 165533_at | 0.00 |
| 165534_i_at | 0.00 |
| 165535_f_at | 0.00 |
| 165536_at | 0.00 |

| | |
|---|---|
| 165537_at | 0.01 |
| 165538_r_at | 0.00 |
| 165539_at | 0.00 |
| 165540_at | 0.00 |
| 165541_at | 0.00 |
| 165542_at | 0.00 |
| 165543_f_at | 0.00 |
| 165544_at | 0.00 |
| 165545_at | 0.00 |
| 165546_at | 0.00 |
| 165547_at | 0.00 |
| 165548_f_at | 0.00 |
| 165549_at | 0.00 |
| 165550_at | 0.00 |
| 165551_at | 0.00 |
| 165552_i_at | 0.00 |
| 165553_at | 0.00 |
| 165554_i_at | 0.00 |
| 165555_i_at | 0.00 |
| 165556_at | 0.00 |
| 165557_i_at | 0.04 |
| 165558_at | 0.00 |
| 165559_at | 0.00 |
| 165560_at | 0.00 |
| 165561_r_at | 0.00 |
| 165562_r_at | 0.00 |
| 165563_at | 0.00 |
| 165564_at | 0.00 |
| 165565_r_at | 0.11 |
| 165566_i_at | 0.00 |
| 165567_at | 0.00 |
| 165568_f_at | 0.00 |
| 165569_at | 0.01 |
| 165570_at | 0.00 |
| 165571_r_at | 0.00 |
| 165572_at | 0.00 |
| 165573_f_at | 0.00 |
| 165574_at | 0.00 |
| 165575_r_at | 0.00 |
| 165576_i_at | 0.00 |
| 165577_at | 0.00 |
| 165578_at | 0.00 |
| 165579_at | 0.00 |
| 165580_i_at | 0.00 |
| 165581_at | 0.00 |
| 165582_f_at | 0.00 |
| 165583_i_at | 0.02 |
| 165584_at | 0.00 |
| 165585_at | 0.00 |
| 165586_f_at | 0.00 |
| 165587_i_at | 0.00 |
| 165588_at | 0.00 |
| 165589_at | 0.00 |
| 165590_i_at | 0.00 |
| 165591_f_at | 0.00 |
| 165592_r_at | 0.00 |

| | |
|---|---|
| 165593_at | 0.00 |
| 165594_at | 0.00 |
| 165595_at | 0.00 |
| 165596_at | 0.00 |
| 165597_i_at | 0.23 |
| 165598_at | 0.00 |
| 165599_at | 0.00 |
| 165600_i_at | 0.00 |
| 165601_f_at | 0.00 |
| 165602_f_at | 0.00 |
| 165603_i_at | 0.00 |
| 165604_r_at | 0.00 |
| 165605_at | 0.00 |
| 165606_r_at | 0.01 |
| 165607_at | 0.00 |
| 165608_at | 0.00 |
| 165609_r_at | 0.02 |
| 165610_at | 0.00 |
| 165611_r_at | 0.00 |
| 165612_f_at | 0.00 |
| 165613_i_at | 0.03 |
| 165614_i_at | 0.00 |
| 165615_at | 0.11 |
| 165616_at | 0.00 |
| 165617_at | 0.15 |
| 165618_at | 0.06 |
| 165619_r_at | 0.05 |
| 165620_at | 0.00 |
| 165621_at | 0.00 |
| 165622_at | 0.00 |
| 165623_at | 0.00 |
| 165624_i_at | 0.01 |
| 165625_r_at | 0.00 |
| 165626_f_at | 0.00 |
| 165627_at | 0.00 |
| 165628_at | 0.00 |
| 165629_at | 0.00 |
| 165630_at | 0.00 |
| 165631_r_at | 0.00 |
| 165632_r_at | 0.00 |
| 165633_at | 0.00 |
| 165634_at | 0.00 |
| 165635_i_at | 0.00 |
| 165636_at | 0.00 |
| 165637_i_at | 0.00 |
| 165638_r_at | 0.00 |
| 165639_f_at | 0.00 |
| 165640_at | 0.03 |
| 165641_at | 0.00 |
| 165642_at | 0.00 |
| 165643_at | 0.00 |
| 165644_i_at | 0.00 |
| 165645_at | 0.00 |
| 165646_r_at | 0.00 |
| 165647_f_at | 0.00 |
| 165648_i_at | 0.00 |

| | |
|---|---|
| 165649_f_at | 0.00 |
| 165650_f_at | 0.00 |
| 165651_r_at | 0.01 |
| 165652_r_at | 0.00 |
| 165653_at | 0.00 |
| 165654_at | 0.00 |
| 165655_r_at | 0.00 |
| 165656_i_at | 0.01 |
| 165657_f_at | 0.00 |
| 165658_at | 0.00 |
| 165659_at | 0.05 |
| 165660_i_at | 0.05 |
| 165661_i_at | 0.00 |
| 165662_at | 0.00 |
| 165663_at | 0.00 |
| 165664_i_at | 0.01 |
| 165665_at | 0.00 |
| 165666_i_at | 0.00 |
| 165667_r_at | 0.00 |
| 165668_r_at | 0.01 |
| 165669_i_at | 0.04 |
| 165670_i_at | 0.00 |
| 165671_f_at | 0.00 |
| 165672_at | 0.00 |
| 165673_at | 0.00 |
| 165674_f_at | 0.00 |
| 165675_at | 0.00 |
| 165676_r_at | 0.00 |
| 165677_at | 0.00 |
| 165678_i_at | 0.15 |
| 165679_at | 0.00 |
| 165680_at | 0.00 |
| 165681_at | 0.00 |
| 165682_r_at | 0.00 |
| 165683_at | 0.00 |
| 165684_i_at | 0.00 |
| 165685_at | 0.00 |
| 165686_at | 0.00 |
| 165687_r_at | 0.00 |
| 165688_r_at | 0.00 |
| 165689_at | 0.00 |
| 165690_at | 0.00 |
| 165691_at | 0.27 |
| 165692_i_at | 0.00 |
| 165693_r_at | 0.00 |
| 165694_at | 0.49 |
| 165695_at | 0.00 |
| 165696_at | 0.00 |
| 165697_r_at | 0.00 |
| 165698_i_at | 0.02 |
| 165699_r_at | 0.00 |
| 165700_f_at | 0.00 |
| 165701_f_at | 0.00 |
| 165702_at | 0.00 |
| 165703_f_at | 0.00 |
| 165704_r_at | 0.00 |

| | |
|---|---|
| 165705_r_at | 0.00 |
| 165706_r_at | 0.09 |
| 165707_r_at | 0.00 |
| 165708_i_at | 0.00 |
| 165709_at | 0.00 |
| 165710_at | 0.00 |
| 165711_at | 0.01 |
| 165712_at | 0.00 |
| 165713_at | 0.43 |
| 165714_f_at | 0.00 |
| 165715_r_at | 0.00 |
| 165716_at | 0.00 |
| 165717_i_at | 0.00 |
| 165718_r_at | 0.00 |
| 165719_at | 0.00 |
| 165720_i_at | 0.00 |
| 165721_f_at | 0.00 |
| 165722_r_at | 0.00 |
| 165723_at | 0.00 |
| 165724_at | 0.00 |
| 165725_at | 0.00 |
| 165726_at | 0.00 |
| 165727_at | 0.00 |
| 165728_at | 0.00 |
| 165729_at | 0.00 |
| 165730_at | 0.00 |
| 165731_at | 0.00 |
| 165732_r_at | 0.00 |
| 165733_at | 0.00 |
| 165734_r_at | 0.00 |
| 165735_r_at | 0.00 |
| 165736_s_at | 0.00 |
| 165737_f_at | 0.00 |
| 165738_r_at | 0.00 |
| 165739_i_at | 0.00 |
| 165740_at | 0.00 |
| 165741_r_at | 0.00 |
| 165742_r_at | 0.00 |
| 165743_at | 0.00 |
| 165744_at | 0.00 |
| 165745_at | 0.00 |
| 165746_at | 0.00 |
| 165747_i_at | 0.01 |
| 165748_at | 0.00 |
| 165749_r_at | 0.00 |
| 165750_at | 0.00 |
| 165751_at | 0.00 |
| 165752_at | 0.00 |
| 165753_at | 0.00 |
| 165754_i_at | 0.00 |
| 165755_r_at | 0.22 |
| 165756_at | 0.00 |
| 165757_i_at | 0.00 |
| 165758_at | 0.00 |
| 165759_at | 0.00 |
| 165760_f_at | 0.00 |

| | |
|---|---|
| 165761_at | 0.00 |
| 165762_i_at | 0.00 |
| 165763_r_at | 0.00 |
| 165764_f_at | 0.00 |
| 165765_at | 0.00 |
| 165766_at | 0.00 |
| 165767_at | 0.00 |
| 165768_r_at | 0.00 |
| 165769_f_at | 0.00 |
| 165770_at | 0.00 |
| 165771_r_at | 0.00 |
| 165772_at | 0.00 |
| 165773_at | 0.00 |
| 165774_at | 0.00 |
| 165775_r_at | 0.00 |
| 165776_i_at | 0.06 |
| 165777_r_at | 0.00 |
| 165778_at | 0.00 |
| 165779_i_at | 0.00 |
| 165780_s_at | 0.00 |
| 165781_r_at | 0.00 |
| 165782_at | 0.00 |
| 165783_at | 0.00 |
| 165784_i_at | 0.00 |
| 165785_f_at | 0.51 |
| 165786_i_at | 0.00 |
| 165787_r_at | 0.00 |
| 165788_at | 0.00 |
| 165789_at | 0.00 |
| 165790_at | 0.02 |
| 165791_f_at | 0.00 |
| 165792_at | 0.00 |
| 165793_r_at | 0.00 |
| 165794_at | 0.01 |
| 165795_f_at | 0.01 |
| 165796_at | 0.00 |
| 165797_at | 0.00 |
| 165798_s_at | 0.00 |
| 165799_i_at | 0.00 |
| 165800_at | 0.00 |
| 165801_at | 0.01 |
| 165802_r_at | 0.00 |
| 165803_s_at | 0.00 |
| 165804_at | 0.00 |
| 165805_at | 0.00 |
| 165806_at | 0.00 |
| 165807_at | 0.00 |
| 165808_at | 0.00 |
| 165809_at | 0.00 |
| 165810_i_at | 0.00 |
| 165811_at | 0.00 |
| 165812_at | 0.00 |
| 165813_at | 0.00 |
| 165814_at | 0.00 |
| 165815_r_at | 0.00 |
| 165816_at | 0.00 |

| | |
|---|---|
| 165817_at | 0.00 |
| 165818_at | 0.00 |
| 165819_at | 0.01 |
| 165820_at | 0.00 |
| 165821_at | 0.04 |
| 165822_f_at | 0.00 |
| 165823_s_at | 0.00 |
| 165824_at | 0.00 |
| 165825_r_at | 0.00 |
| 165826_i_at | 0.12 |
| 165827_at | 0.00 |
| 165828_r_at | 0.00 |
| 165829_r_at | 0.00 |
| 165830_f_at | 0.00 |
| 165831_at | 0.00 |
| 165832_at | 0.00 |
| 165833_at | 0.00 |
| 165834_r_at | 0.00 |
| 165835_r_at | 0.00 |
| 165836_at | 0.00 |
| 165837_at | 0.00 |
| 165838_i_at | 0.02 |
| 165839_at | 0.00 |
| 165840_at | 0.00 |
| 165841_at | 0.00 |
| 165842_at | 0.00 |
| 165843_i_at | 0.00 |
| 165844_r_at | 0.00 |
| 165845_at | 0.00 |
| 165846_r_at | 0.00 |
| 165847_i_at | 0.00 |
| 165848_at | 0.00 |
| 165849_f_at | 0.00 |
| 165850_r_at | 0.00 |
| 165851_i_at | 0.00 |
| 165852_at | 0.00 |
| 165853_i_at | 0.00 |
| 165854_r_at | 0.00 |
| 165855_at | 0.00 |
| 165856_i_at | 0.01 |
| 165857_at | 0.00 |
| 165858_i_at | 0.00 |
| 165859_f_at | 0.00 |
| 165860_at | 0.00 |
| 165861_at | 0.00 |
| 165862_f_at | 0.03 |
| 165863_r_at | 0.00 |
| 165864_f_at | 0.00 |
| 165865_f_at | 0.00 |
| 165866_f_at | 0.10 |
| 165867_r_at | 0.00 |
| 165868_f_at | 0.10 |
| 165869_at | 0.00 |
| 165870_i_at | 0.00 |
| 165871_f_at | 0.08 |
| 165872_at | 0.00 |

| | |
|---|---|
| 165873_f_at | 0.05 |
| 165874_r_at | 0.00 |
| 165875_at | 0.00 |
| 165876_r_at | 0.00 |
| 165877_at | 0.00 |
| 165878_r_at | 0.00 |
| 165879_at | 0.00 |
| 165880_f_at | 0.00 |
| 165881_f_at | 0.00 |
| 165882_r_at | 0.00 |
| 165883_r_at | 0.00 |
| 165884_f_at | 0.00 |
| 165885_at | 0.00 |
| 165886_i_at | 0.00 |
| 165887_at | 0.00 |
| 165888_f_at | 0.00 |
| 165889_f_at | 0.00 |
| 165890_at | 0.00 |
| 165891_i_at | 0.00 |
| 165892_i_at | 0.03 |
| 165893_f_at | 0.00 |
| 165894_s_at | 0.00 |
| 165895_at | 0.00 |
| 165896_i_at | 0.00 |
| 165897_at | 0.55 |
| 165898_i_at | 0.00 |
| 165899_f_at | 0.00 |
| 165900_f_at | 0.00 |
| 165901_f_at | 0.00 |
| 165902_at | 0.00 |
| 165903_r_at | 0.00 |
| 165904_at | 0.00 |
| 165905_at | 0.00 |
| 165906_at | 0.00 |
| 165907_f_at | 0.00 |
| 165908_r_at | 0.00 |
| 165909_at | 0.00 |
| 165910_f_at | 0.00 |
| 165911_r_at | 0.00 |
| 165912_f_at | 0.00 |
| 165913_i_at | 0.01 |
| 165914_r_at | 0.00 |
| 165915_i_at | 0.05 |
| 165916_at | 0.00 |
| 165917_r_at | 0.01 |
| 165918_at | 0.00 |
| 165919_f_at | 0.00 |
| 165920_r_at | 0.00 |
| 165921_r_at | 0.00 |
| 165922_at | 0.00 |
| 165923_r_at | 0.00 |
| 165924_at | 0.00 |
| 165925_at | 0.00 |
| 165926_s_at | 0.00 |
| 165927_at | 0.00 |
| 165928_at | 0.00 |

| | |
|---|---|
| 165929_at | 0.00 |
| 165930_f_at | 0.00 |
| 165931_i_at | 0.00 |
| 165932_r_at | 0.00 |
| 165933_at | 0.00 |
| 165934_f_at | 0.00 |
| 165935_at | 0.00 |
| 165936_i_at | 0.00 |
| 165937_r_at | 0.00 |
| 165938_r_at | 0.00 |
| 165939_at | 0.00 |
| 165940_at | 0.00 |
| 165941_at | 0.00 |
| 165942_r_at | 0.00 |
| 165943_f_at | 0.00 |
| 165944_at | 0.00 |
| 165945_at | 0.00 |
| 165946_r_at | 0.00 |
| 165947_r_at | 0.00 |
| 165948_i_at | 0.00 |
| 165949_i_at | 0.00 |
| 165950_r_at | 0.00 |
| 165951_i_at | 0.00 |
| 165952_r_at | 0.00 |
| 165953_i_at | 0.00 |
| 165954_f_at | 0.00 |
| 165955_r_at | 0.00 |
| 165956_f_at | 0.00 |
| 165957_f_at | 0.00 |
| 165958_at | 0.00 |
| 165959_at | 0.00 |
| 165960_r_at | 0.00 |
| 165961_f_at | 0.00 |
| 165962_r_at | 0.00 |
| 165963_f_at | 0.00 |
| 165964_i_at | 0.00 |
| 165965_at | 0.06 |
| 165966_f_at | 0.20 |
| 165967_r_at | 0.00 |
| 165968_f_at | 0.01 |
| 165969_at | 0.00 |
| 165970_i_at | 0.00 |
| 165971_f_at | 0.00 |
| 165972_at | 0.00 |
| 165973_r_at | 0.00 |
| 165974_r_at | 0.01 |
| 165975_at | 0.00 |
| 165976_at | 0.01 |
| 165977_r_at | 0.00 |
| 165978_at | 0.08 |
| 165979_r_at | 0.00 |
| 165980_r_at | 0.00 |
| 165981_i_at | 0.00 |
| 165982_i_at | 0.00 |
| 165983_f_at | 0.00 |
| 165984_r_at | 0.00 |

| | |
|---|---|
| 165985_r_at | 0.00 |
| 165986_at | 0.00 |
| 165987_r_at | 0.00 |
| 165988_r_at | 0.00 |
| 165989_f_at | 0.00 |
| 165990_at | 0.00 |
| 165991_i_at | 0.00 |
| 165992_f_at | 0.00 |
| 165993_f_at | 0.00 |
| 165994_at | 0.01 |
| 165995_r_at | 0.00 |
| 165996_at | 0.00 |
| 165997_f_at | 0.00 |
| 165998_at | 0.06 |
| 165999_f_at | 0.14 |
| 166000_at | 0.00 |
| 166001_r_at | 0.00 |
| 166002_at | 0.00 |
| 166003_at | 0.00 |
| 166004_at | 0.00 |
| 166005_f_at | 0.02 |
| 166006_at | 0.00 |
| 166007_at | 0.06 |
| 166008_i_at | 0.00 |
| 166009_r_at | 0.00 |
| 166010_at | 0.00 |
| 166011_i_at | 0.01 |
| 166012_at | 0.34 |
| 166013_at | 0.00 |
| 166014_at | 0.00 |
| 166015_r_at | 0.00 |
| 166016_at | 0.00 |
| 166017_at | 0.00 |
| 166018_r_at | 0.00 |
| 166019_at | 0.00 |
| 166020_f_at | 0.02 |
| 166021_at | 0.00 |
| 166022_i_at | 0.00 |
| 166023_f_at | 0.00 |
| 166024_f_at | 0.00 |
| 166025_i_at | 0.00 |
| 166026_r_at | 0.00 |
| 166027_f_at | 0.00 |
| 166028_s_at | 0.00 |
| 166029_i_at | 0.01 |
| 166030_i_at | 0.00 |
| 166031_r_at | 0.00 |
| 166032_f_at | 0.00 |
| 166033_f_at | 0.00 |
| 166034_at | 0.00 |
| 166035_f_at | 0.00 |
| 166036_f_at | 0.00 |
| 166037_r_at | 0.00 |
| 166038_r_at | 0.00 |
| 166039_r_at | 0.00 |
| 166040_f_at | 0.00 |

| | |
|---|---|
| 166041_r_at | 0.00 |
| 166042_f_at | 0.00 |
| 166043_at | 0.00 |
| 166044_r_at | 0.00 |
| 166045_at | 0.00 |
| 166046_at | 0.00 |
| 166047_r_at | 0.00 |
| 166048_r_at | 0.00 |
| 166049_f_at | 0.00 |
| 166050_f_at | 0.00 |
| 166051_r_at | 0.00 |
| 166052_at | 0.00 |
| 166053_at | 0.00 |
| 166054_r_at | 0.00 |
| 166055_r_at | 0.00 |
| 166056_i_at | 0.00 |
| 166057_r_at | 0.00 |
| 166058_at | 0.00 |
| 166059_at | 0.00 |
| 166060_i_at | 0.00 |
| 166061_f_at | 0.00 |
| 166062_f_at | 0.00 |
| 166063_f_at | 0.00 |
| 166064_i_at | 0.00 |
| 166065_f_at | 0.06 |
| 166066_r_at | 0.00 |
| 166067_f_at | 0.00 |
| 166068_at | 0.00 |
| 166069_i_at | 0.00 |
| 166070_r_at | 0.00 |
| 166071_at | 0.00 |
| 166072_f_at | 0.00 |
| 166073_at | 0.00 |
| 166074_i_at | 0.00 |
| 166075_r_at | 0.00 |
| 166076_r_at | 0.00 |
| 166077_f_at | 0.55 |
| 166078_r_at | 0.00 |
| 166079_at | 0.00 |
| 166080_i_at | 0.00 |
| 166081_i_at | 0.00 |
| 166082_r_at | 0.00 |
| 166083_i_at | 0.00 |
| 166084_f_at | 0.00 |
| 166085_at | 0.00 |
| 166086_f_at | 0.00 |
| 166087_at | 0.00 |
| 166088_r_at | 0.00 |
| 166089_r_at | 0.00 |
| 166090_f_at | 0.00 |
| 166091_i_at | 0.00 |
| 166092_r_at | 0.00 |
| 166093_i_at | 0.22 |
| 166094_r_at | 0.00 |
| 166095_f_at | 0.13 |
| 166096_f_at | 0.00 |

| | |
|---|---|
| 166097_at | 0.00 |
| 166098_f_at | 0.00 |
| 166099_i_at | 0.00 |
| 166100_at | 0.00 |
| 166101_f_at | 0.00 |
| 166102_r_at | 0.00 |
| 166103_i_at | 0.00 |
| 166104_f_at | 0.00 |
| 166105_at | 0.00 |
| 166106_f_at | 0.00 |
| 166107_at | 0.00 |
| 166108_r_at | 0.00 |
| 166109_f_at | 0.00 |
| 166110_r_at | 0.00 |
| 166111_f_at | 0.00 |
| 166112_f_at | 0.13 |
| 166113_i_at | 0.00 |
| 166114_f_at | 0.00 |
| 166115_f_at | 0.00 |
| 166116_at | 0.00 |
| 166117_i_at | 0.00 |
| 166118_i_at | 0.00 |
| 166119_at | 0.00 |
| 166120_at | 0.00 |
| 166121_f_at | 0.00 |
| 166122_at | 0.04 |
| 166123_at | 0.00 |
| 166124_r_at | 0.00 |
| 166125_r_at | 0.00 |
| 166126_at | 0.00 |
| 166127_f_at | 0.00 |
| 166128_r_at | 0.00 |
| 166129_r_at | 0.00 |
| 166130_at | 0.00 |
| 166131_at | 0.76 |
| 166132_f_at | 0.00 |
| 166133_i_at | 0.00 |
| 166134_at | 0.00 |
| 166135_at | 0.00 |
| 166136_i_at | 0.00 |
| 166137_at | 0.03 |
| 166138_r_at | 0.00 |
| 166139_f_at | 0.00 |
| 166140_i_at | 0.00 |
| 166141_i_at | 0.00 |
| 166142_r_at | 0.00 |
| 166143_f_at | 0.00 |
| 166144_i_at | 0.00 |
| 166145_f_at | 0.00 |
| 166146_i_at | 0.00 |
| 166147_f_at | 0.00 |
| 166148_at | 0.00 |
| 166149_at | 0.00 |
| 166150_r_at | 0.00 |
| 166151_f_at | 0.00 |
| 166152_at | 0.00 |

| | |
|---|---|
| 166153_i_at | 0.00 |
| 166154_f_at | 0.00 |
| 166155_i_at | 0.00 |
| 166156_f_at | 0.00 |
| 166157_at | 0.00 |
| 166158_r_at | 0.00 |
| 166159_at | 0.00 |
| 166160_f_at | 0.03 |
| 166161_at | 0.74 |
| 166162_r_at | 0.00 |
| 166163_r_at | 0.00 |
| 166164_at | 0.00 |
| 166165_at | 0.00 |
| 166166_at | 0.00 |
| 166167_f_at | 0.00 |
| 166168_f_at | 0.00 |
| 166169_f_at | 0.00 |
| 166170_at | 0.00 |
| 166171_at | 0.00 |
| 166172_f_at | 0.00 |
| 166173_f_at | 0.33 |
| 166174_r_at | 0.00 |
| 166175_f_at | 0.00 |
| 166176_r_at | 0.00 |
| 166177_at | 0.00 |
| 166178_at | 0.00 |
| 166179_f_at | 0.00 |
| 166180_f_at | 0.00 |
| 166181_f_at | 0.00 |
| 166182_f_at | 0.00 |
| 166183_r_at | 0.00 |
| 166184_r_at | 0.00 |
| 166185_at | 0.00 |
| 166186_at | 0.00 |
| 166187_f_at | 0.00 |
| 166188_at | 0.00 |
| 166189_at | 0.00 |
| 166190_r_at | 0.00 |
| 166191_f_at | 0.00 |
| 166192_at | 0.00 |
| 166193_at | 0.00 |
| 166194_r_at | 0.00 |
| 166195_f_at | 0.00 |
| 166196_r_at | 0.00 |
| 166197_at | 0.00 |
| 166198_f_at | 0.06 |
| 166199_at | 0.02 |
| 166200_i_at | 0.00 |
| 166201_r_at | 0.00 |
| 166202_at | 0.00 |
| 166203_r_at | 0.00 |
| 166204_at | 0.00 |
| 166205_r_at | 0.00 |
| 166206_f_at | 0.00 |
| 166207_at | 0.00 |
| 166208_at | 0.00 |

| | |
|---|---|
| 166209_f_at | 0.00 |
| 166210_i_at | 0.03 |
| 166211_at | 0.00 |
| 166212_i_at | 0.00 |
| 166213_at | 0.00 |
| 166214_at | 0.00 |
| 166215_r_at | 0.00 |
| 166216_r_at | 0.00 |
| 166217_r_at | 0.00 |
| 166218_at | 0.00 |
| 166219_at | 0.00 |
| 166220_r_at | 0.00 |
| 166221_r_at | 0.00 |
| 166222_r_at | 0.00 |
| 166223_f_at | 0.00 |
| 166224_at | 0.00 |
| 166225_f_at | 0.00 |
| 166226_f_at | 0.00 |
| 166227_at | 0.00 |
| 166228_at | 0.00 |
| 166229_r_at | 0.00 |
| 166230_at | 0.00 |
| 166231_at | 0.00 |
| 166232_r_at | 0.00 |
| 166233_at | 0.00 |
| 166234_f_at | 0.00 |
| 166235_at | 0.00 |
| 166236_i_at | 0.00 |
| 166237_f_at | 0.14 |
| 166238_r_at | 0.00 |
| 166239_f_at | 0.00 |
| 166240_r_at | 0.00 |
| 166241_at | 0.00 |
| 166242_i_at | 0.00 |
| 166243_at | 0.00 |
| 166244_f_at | 0.00 |
| 166245_at | 0.00 |
| 166246_f_at | 0.00 |
| 166247_at | 0.02 |
| 166248_at | 0.00 |
| 166249_r_at | 0.00 |
| 166250_at | 0.00 |
| 166251_f_at | 0.00 |
| 166252_i_at | 0.00 |
| 166253_r_at | 0.00 |
| 166254_f_at | 0.00 |
| 166255_r_at | 0.00 |
| 166256_f_at | 0.00 |
| 166257_f_at | 0.00 |
| 166258_at | 0.00 |
| 166259_f_at | 0.00 |
| 166260_at | 0.00 |
| 166261_f_at | 0.00 |
| 166262_f_at | 0.00 |
| 166263_at | 0.00 |
| 166264_at | 0.00 |

| | |
|---|---|
| 166265_f_at | 0.12 |
| 166266_i_at | 0.00 |
| 166267_i_at | 0.00 |
| 166268_at | 0.00 |
| 166269_at | 0.00 |
| 166270_at | 0.00 |
| 166271_f_at | 0.00 |
| 166272_f_at | 0.00 |
| 166273_f_at | 0.00 |
| 166274_f_at | 0.00 |
| 166275_i_at | 0.00 |
| 166276_at | 0.00 |
| 166277_at | 0.00 |
| 166278_f_at | 0.00 |
| 166279_i_at | 0.00 |
| 166280_i_at | 0.00 |
| 166281_f_at | 0.11 |
| 166282_f_at | 0.14 |
| 166283_r_at | 0.00 |
| 166284_i_at | 0.00 |
| 166285_r_at | 0.00 |
| 166286_r_at | 0.01 |
| 166287_f_at | 0.00 |
| 166288_at | 0.00 |
| 166289_at | 0.00 |
| 166290_r_at | 0.00 |
| 166291_at | 0.00 |
| 166292_f_at | 0.00 |
| 166293_f_at | 0.00 |
| 166294_r_at | 0.00 |
| 166295_f_at | 0.00 |
| 166296_r_at | 0.00 |
| 166297_i_at | 0.00 |
| 166298_f_at | 0.00 |
| 166299_s_at | 0.00 |
| 166300_at | 0.00 |
| 166301_r_at | 0.00 |
| 166302_at | 0.00 |
| 166303_i_at | 0.00 |
| 166304_f_at | 0.03 |
| 166305_r_at | 0.00 |
| 166306_at | 0.00 |
| 166307_i_at | 0.00 |
| 166308_at | 0.00 |
| 166309_at | 0.00 |
| 166310_r_at | 0.00 |
| 166311_f_at | 0.02 |
| 166312_r_at | 0.00 |
| 166313_r_at | 0.00 |
| 166314_i_at | 0.00 |
| 166315_at | 0.00 |
| 166316_i_at | 0.00 |
| 166317_f_at | 0.00 |
| 166318_at | 0.00 |
| 166319_at | 0.01 |
| 166320_r_at | 0.00 |

| | |
|---|---|
| 166321_at | 0.00 |
| 166322_r_at | 0.00 |
| 166323_r_at | 0.00 |
| 166324_at | 0.00 |
| 166325_f_at | 0.00 |
| 166326_at | 0.00 |
| 166327_i_at | 0.00 |
| 166328_f_at | 0.02 |
| 166329_f_at | 0.00 |
| 166330_i_at | 0.00 |
| 166331_at | 0.00 |
| 166332_r_at | 0.00 |
| 166333_at | 0.00 |
| 166334_at | 0.31 |
| 166335_at | 0.00 |
| 166336_at | 0.00 |
| 166337_at | 0.00 |
| 166338_i_at | 0.00 |
| 166339_at | 0.00 |
| 166340_at | 0.06 |
| 166341_r_at | 0.00 |
| 166342_r_at | 0.00 |
| 166343_at | 0.00 |
| 166344_i_at | 0.00 |
| 166345_at | 0.00 |
| 166346_i_at | 0.00 |
| 166347_i_at | 0.00 |
| 166348_r_at | 0.00 |
| 166349_r_at | 0.00 |
| 166350_r_at | 0.00 |
| 166351_f_at | 0.04 |
| 166352_r_at | 0.00 |
| 166353_s_at | 0.06 |
| 166354_f_at | 0.04 |
| 166355_f_at | 0.00 |
| 166356_i_at | 0.00 |
| 166357_at | 0.00 |
| 166358_at | 0.00 |
| 166359_at | 0.00 |
| 166360_at | 0.01 |
| 166361_f_at | 0.00 |
| 166362_at | 0.00 |
| 166363_r_at | 0.00 |
| 166364_at | 0.00 |
| 166365_at | 0.00 |
| 166366_r_at | 0.00 |
| 166367_at | 0.00 |
| 166368_at | 0.00 |
| 166369_r_at | 0.00 |
| 166370_at | 0.00 |
| 166371_i_at | 0.00 |
| 166372_at | 0.00 |
| 166373_at | 0.21 |
| 166374_i_at | 0.00 |
| 166375_f_at | 0.00 |
| 166376_f_at | 0.00 |

| | |
|---|---|
| 166377_i_at | 0.00 |
| 166378_at | 0.00 |
| 166379_at | 0.00 |
| 166380_at | 0.00 |
| 166381_f_at | 0.00 |
| 166382_f_at | 0.00 |
| 166383_r_at | 0.00 |
| 166384_at | 0.00 |
| 166385_i_at | 0.00 |
| 166386_at | 0.00 |
| 166387_f_at | 0.00 |
| 166388_at | 0.00 |
| 166389_at | 0.00 |
| 166390_at | 0.00 |
| 166391_at | 0.00 |
| 166392_r_at | 0.00 |
| 166393_at | 0.00 |
| 166394_at | 0.00 |
| 166395_at | 0.00 |
| 166396_r_at | 0.00 |
| 166397_at | 0.00 |
| 166398_at | 0.12 |
| 166399_at | 0.00 |
| 166400_at | 0.00 |
| 166401_r_at | 0.02 |
| 166402_i_at | 0.01 |
| 166403_r_at | 0.00 |
| 166404_r_at | 0.00 |
| 166405_s_at | 0.00 |
| 166406_r_at | 0.00 |
| 166407_at | 0.00 |
| 166408_r_at | 0.00 |
| 166409_at | 0.00 |
| 166410_i_at | 0.00 |
| 166411_i_at | 0.05 |
| 166412_at | 0.00 |
| 166413_r_at | 0.00 |
| 166414_at | 0.00 |
| 166415_at | 0.12 |
| 166416_at | 0.00 |
| 166417_at | 0.00 |
| 166418_at | 0.00 |
| 166419_at | 0.00 |
| 166420_at | 0.00 |
| 166421_s_at | 0.14 |
| 166422_at | 0.02 |
| 166423_r_at | 0.11 |
| 166424_at | 0.00 |
| 166425_at | 0.00 |
| 166426_i_at | 0.00 |
| 166427_f_at | 0.72 |
| 166428_at | 0.00 |
| 166429_r_at | 0.00 |
| 166430_i_at | 0.29 |
| 166431_at | 0.00 |
| 166432_f_at | 0.00 |

| | |
|---|---|
| 166433_f_at | 0.00 |
| 166434_r_at | 0.00 |
| 166435_at | 0.00 |
| 166436_at | 0.00 |
| 166437_f_at | 0.01 |
| 166438_s_at | 0.01 |
| 166439_at | 0.00 |
| 166440_i_at | 0.00 |
| 166441_f_at | 0.00 |
| 166442_f_at | 0.08 |
| 166443_f_at | 0.00 |
| 166444_f_at | 0.00 |
| 166445_r_at | 0.00 |
| 166446_f_at | 0.00 |
| 166447_f_at | 0.00 |
| 166448_f_at | 0.00 |
| 166449_r_at | 0.00 |
| 166450_f_at | 0.00 |
| 166451_f_at | 0.00 |
| 166452_at | 0.00 |
| 166453_r_at | 0.00 |
| 166454_at | 0.00 |
| 166455_i_at | 0.00 |
| 166456_at | 0.01 |
| 166457_at | 0.00 |
| 166458_at | 0.00 |
| 166459_at | 0.00 |
| 166460_r_at | 0.00 |
| 166461_r_at | 0.00 |
| 166462_r_at | 0.00 |
| 166463_f_at | 0.00 |
| 166464_r_at | 0.00 |
| 166465_f_at | 0.00 |
| 166466_r_at | 0.00 |
| 166467_at | 0.00 |
| 166468_r_at | 0.00 |
| 166469_f_at | 0.00 |
| 166470_r_at | 0.00 |
| 166471_i_at | 0.00 |
| 166472_i_at | 0.01 |
| 166473_at | 0.00 |
| 166474_i_at | 0.00 |
| 166475_r_at | 0.00 |
| 166476_r_at | 0.00 |
| 166477_r_at | 0.00 |
| 166478_at | 0.00 |
| 166479_f_at | 0.00 |
| 166480_i_at | 0.00 |
| 166481_at | 0.00 |
| 166482_i_at | 0.00 |
| 166483_at | 0.00 |
| 166484_at | 0.00 |
| 166485_i_at | 0.00 |
| 166486_at | 0.00 |
| 166487_r_at | 0.03 |
| 166488_r_at | 0.00 |

| | |
|---|---|
| 166489_r_at | 0.00 |
| 166490_i_at | 0.00 |
| 166491_i_at | 0.00 |
| 166492_at | 0.00 |
| 166493_i_at | 0.00 |
| 166494_f_at | 0.00 |
| 166495_f_at | 0.01 |
| 166496_i_at | 0.00 |
| 166497_at | 0.00 |
| 166498_f_at | 0.00 |
| 166499_i_at | 0.00 |
| 166500_at | 0.00 |
| 166501_at | 0.00 |
| 166502_at | 0.00 |
| 166503_r_at | 0.00 |
| 166504_r_at | 0.01 |
| 166505_r_at | 0.00 |
| 166506_r_at | 0.00 |
| 166507_at | 0.00 |
| 166508_i_at | 0.01 |
| 166509_r_at | 0.00 |
| 166510_r_at | 0.02 |
| 166511_r_at | 0.00 |
| 166512_r_at | 0.00 |
| 166513_at | 0.00 |
| 166514_i_at | 0.00 |
| 166515_i_at | 0.00 |
| 166516_at | 0.00 |
| 166517_f_at | 0.00 |
| 166518_f_at | 0.00 |
| 166519_at | 0.02 |
| 166520_r_at | 0.00 |
| 166521_f_at | 0.01 |
| 166522_r_at | 0.00 |
| 166523_f_at | 0.00 |
| 166524_r_at | 0.00 |
| 166525_at | 0.00 |
| 166526_f_at | 0.00 |
| 166527_f_at | 0.00 |
| 166528_r_at | 0.00 |
| 166529_at | 0.00 |
| 166530_at | 0.00 |
| 166531_at | 0.04 |
| 166532_at | 0.00 |
| 166533_f_at | 0.00 |
| 166534_i_at | 0.01 |
| 166535_r_at | 0.00 |
| 166536_r_at | 0.00 |
| 166537_i_at | 0.00 |
| 166538_at | 0.00 |
| 166539_at | 0.00 |
| 166540_at | 0.00 |
| 166541_f_at | 0.00 |
| 166542_at | 0.00 |
| 166543_r_at | 0.00 |
| 166544_at | 0.00 |

| | |
|---|---|
| 166545_r_at | 0.00 |
| 166546_at | 0.00 |
| 166547_s_at | 0.00 |
| 166548_at | 0.00 |
| 166549_i_at | 0.00 |
| 166550_i_at | 0.00 |
| 166551_at | 0.00 |
| 166552_at | 0.00 |
| 166553_at | 0.00 |
| 166554_at | 0.00 |
| 166555_f_at | 0.01 |
| 166556_at | 0.00 |
| 166557_f_at | 0.00 |
| 166558_at | 0.00 |
| 166559_at | 0.00 |
| 166560_r_at | 0.00 |
| 166561_r_at | 0.00 |
| 166562_r_at | 0.00 |
| 166563_f_at | 0.00 |
| 166564_f_at | 0.00 |
| 166565_i_at | 0.00 |
| 166566_i_at | 0.00 |
| 166567_f_at | 0.40 |
| 166568_i_at | 0.00 |
| 166569_f_at | 0.00 |
| 166570_r_at | 0.00 |
| 166571_r_at | 0.00 |
| 166572_f_at | 0.00 |
| 166573_r_at | 0.00 |
| 166574_f_at | 0.00 |
| 166575_i_at | 0.00 |
| 166576_at | 0.00 |
| 166577_i_at | 0.00 |
| 166578_at | 0.00 |
| 166579_i_at | 0.00 |
| 166580_r_at | 0.00 |
| 166581_r_at | 0.00 |
| 166582_i_at | 0.00 |
| 166583_f_at | 0.00 |
| 166584_at | 0.00 |
| 166585_at | 0.00 |
| 166586_i_at | 0.00 |
| 166587_i_at | 0.00 |
| 166588_r_at | 0.00 |
| 166589_at | 0.08 |
| 166590_at | 0.00 |
| 166591_r_at | 0.00 |
| 166592_at | 0.00 |
| 166593_i_at | 0.00 |
| 166594_at | 0.00 |
| 166595_at | 0.00 |
| 166596_at | 0.00 |
| 166597_at | 0.00 |
| 166598_r_at | 0.00 |
| 166599_at | 0.00 |
| 166600_at | 0.24 |

| | |
|---|---|
| 166601_at | 0.00 |
| 166602_r_at | 0.00 |
| 166603_at | 0.00 |
| 166604_at | 0.00 |
| 166605_at | 0.00 |
| 166606_at | 0.00 |
| 166607_at | 0.00 |
| 166608_at | 0.00 |
| 166609_at | 0.00 |
| 166610_r_at | 0.00 |
| 166611_at | 0.00 |
| 166612_i_at | 0.00 |
| 166613_at | 0.00 |
| 166614_at | 0.00 |
| 166615_at | 0.00 |
| 166616_at | 0.00 |
| 166617_at | 0.00 |
| 166618_at | 0.00 |
| 166619_f_at | 0.00 |
| 166620_at | 0.06 |
| 166621_f_at | 0.00 |
| 166622_at | 0.00 |
| 166623_at | 0.00 |
| 166624_r_at | 0.00 |
| 166625_at | 0.00 |
| 166626_i_at | 0.00 |
| 166627_at | 0.00 |
| 166628_r_at | 0.00 |
| 166629_i_at | 0.01 |
| 166630_i_at | 0.00 |
| 166631_at | 0.00 |
| 166632_at | 0.00 |
| 166633_i_at | 0.00 |
| 166634_at | 0.00 |
| 166635_i_at | 0.01 |
| 166636_at | 0.00 |
| 166637_i_at | 0.00 |
| 166638_at | 0.00 |
| 166639_at | 0.00 |
| 166640_at | 0.00 |
| 166641_at | 0.00 |
| 166642_at | 0.00 |
| 166643_at | 0.00 |
| 166644_at | 0.00 |
| 166645_at | 0.00 |
| 166646_at | 0.00 |
| 166647_at | 0.00 |
| 166648_i_at | 0.00 |
| 166649_r_at | 0.00 |
| 166650_f_at | 0.00 |
| 166651_i_at | 0.00 |
| 166652_at | 0.00 |
| 166653_at | 0.00 |
| 166654_at | 0.00 |
| 166655_at | 0.00 |
| 166656_f_at | 0.00 |

| | |
|---|---|
| 166657_at | 0.00 |
| 166658_at | 0.00 |
| 166659_i_at | 0.00 |
| 166660_at | 0.00 |
| 166661_at | 0.00 |
| 166662_at | 0.00 |
| 166663_i_at | 0.00 |
| 166664_at | 0.01 |
| 166665_i_at | 0.00 |
| 166666_at | 0.05 |
| 166667_r_at | 0.08 |
| 166668_at | 0.00 |
| 166669_f_at | 0.00 |
| 166670_at | 0.00 |
| 166671_at | 0.00 |
| 166672_at | 0.00 |
| 166673_at | 0.05 |
| 166674_r_at | 0.00 |
| 166675_at | 0.00 |
| 166676_r_at | 0.00 |
| 166677_r_at | 0.00 |
| 166678_at | 0.00 |
| 166679_i_at | 0.08 |
| 166680_at | 0.70 |
| 166681_at | 0.01 |
| 166682_at | 0.00 |
| 166683_r_at | 0.42 |
| 166684_i_at | 0.00 |
| 166685_at | 0.00 |
| 166686_at | 0.00 |
| 166687_at | 0.03 |
| 166688_i_at | 0.00 |
| 166689_at | 0.00 |
| 166690_i_at | 0.00 |
| 166691_r_at | 0.00 |
| 166692_at | 0.00 |
| 166693_at | 0.00 |
| 166694_at | 0.00 |
| 166695_at | 0.00 |
| 166696_at | 0.07 |
| 166697_f_at | 0.00 |
| 166698_at | 0.00 |
| 166699_i_at | 0.00 |
| 166700_i_at | 0.04 |
| 166701_at | 0.00 |
| 166702_r_at | 0.00 |
| 166703_at | 0.00 |
| 166704_i_at | 0.00 |
| 166705_at | 0.00 |
| 166706_at | 0.00 |
| 166707_at | 0.00 |
| 166708_at | 0.00 |
| 166709_at | 0.00 |
| 166710_at | 0.00 |
| 166711_at | 0.00 |
| 166712_at | 0.00 |

| | |
|---|---|
| 166713_at | 0.00 |
| 166714_at | 0.00 |
| 166715_at | 0.00 |
| 166716_r_at | 0.00 |
| 166717_at | 0.00 |
| 166718_at | 0.00 |
| 166719_at | 0.00 |
| 166720_at | 0.00 |
| 166721_at | 0.00 |
| 166722_at | 0.00 |
| 166723_at | 0.01 |
| 166724_at | 0.00 |
| 166725_at | 0.00 |
| 166726_at | 0.12 |
| 166727_at | 0.00 |
| 166728_r_at | 0.00 |
| 166729_at | 0.00 |
| 166730_at | 0.00 |
| 166731_at | 0.00 |
| 166732_f_at | 0.32 |
| 166733_at | 0.00 |
| 166734_at | 0.00 |
| 166735_i_at | 0.00 |
| 166736_at | 0.00 |
| 166737_at | 0.00 |
| 166738_at | 0.00 |
| 166739_r_at | 0.00 |
| 166740_at | 0.08 |
| 166741_i_at | 0.01 |
| 166742_f_at | 0.00 |
| 166743_r_at | 0.02 |
| 166744_at | 0.00 |
| 166745_i_at | 0.00 |
| 166746_at | 0.00 |
| 166747_at | 0.00 |
| 166748_at | 0.59 |
| 166749_at | 0.00 |
| 166750_at | 0.00 |
| 166751_at | 0.00 |
| 166752_at | 0.00 |
| 166753_r_at | 0.00 |
| 166754_i_at | 0.00 |
| 166755_at | 0.00 |
| 166756_at | 0.00 |
| 166757_at | 0.00 |
| 166758_i_at | 0.00 |
| 166759_i_at | 0.00 |
| 166760_s_at | 0.00 |
| 166761_f_at | 0.00 |
| 166762_r_at | 0.00 |
| 166763_at | 0.00 |
| 166764_at | 0.00 |
| 166765_at | 0.00 |
| 166766_f_at | 0.00 |
| 166767_at | 0.01 |
| 166768_at | 0.00 |

| | |
|---|---|
| 166769_at | 0.01 |
| 166770_at | 0.00 |
| 166771_s_at | 0.00 |
| 166772_at | 0.00 |
| 166773_at | 0.00 |
| 166774_r_at | 0.00 |
| 166775_at | 0.00 |
| 166776_at | 0.00 |
| 166777_at | 0.00 |
| 166778_i_at | 0.00 |
| 166779_at | 0.00 |
| 166780_at | 0.00 |
| 166781_r_at | 0.00 |
| 166782_at | 0.01 |
| 166783_r_at | 0.00 |
| 166784_at | 0.00 |
| 166785_f_at | 0.00 |
| 166786_i_at | 0.00 |
| 166787_i_at | 0.00 |
| 166788_at | 0.00 |
| 166789_f_at | 0.00 |
| 166790_at | 0.00 |
| 166791_at | 0.00 |
| 166792_at | 0.00 |
| 166793_r_at | 0.00 |
| 166794_at | 0.01 |
| 166795_at | 0.01 |
| 166796_r_at | 0.00 |
| 166797_at | 0.00 |
| 166798_at | 0.00 |
| 166799_r_at | 0.00 |
| 166800_r_at | 0.00 |
| 166801_at | 0.06 |
| 166802_f_at | 0.12 |
| 166803_at | 0.00 |
| 166804_f_at | 0.00 |
| 166805_f_at | 0.00 |
| 166806_at | 0.00 |
| 166807_at | 0.00 |
| 166808_r_at | 0.00 |
| 166809_i_at | 0.00 |
| 166810_at | 0.00 |
| 166811_at | 0.00 |
| 166812_at | 0.00 |
| 166813_at | 0.00 |
| 166814_f_at | 0.00 |
| 166815_at | 0.00 |
| 166816_at | 0.01 |
| 166817_i_at | 0.00 |
| 166818_at | 0.00 |
| 166819_at | 0.00 |
| 166820_at | 0.46 |
| 166821_r_at | 0.00 |
| 166822_at | 0.00 |
| 166823_i_at | 0.00 |
| 166824_at | 0.00 |

| | |
|---|---|
| 166825_at | 0.00 |
| 166826_at | 0.00 |
| 166827_at | 0.00 |
| 166828_r_at | 0.00 |
| 166829_at | 0.00 |
| 166830_at | 0.00 |
| 166831_i_at | 0.00 |
| 166832_f_at | 0.00 |
| 166833_at | 0.00 |
| 166834_i_at | 0.00 |
| 166835_at | 0.01 |
| 166836_at | 0.00 |
| 166837_r_at | 0.00 |
| 166838_at | 0.00 |
| 166839_at | 0.00 |
| 166840_r_at | 0.00 |
| 166841_at | 0.00 |
| 166842_r_at | 0.00 |
| 166843_at | 0.00 |
| 166844_i_at | 0.00 |
| 166845_at | 0.00 |
| 166846_r_at | 0.00 |
| 166847_at | 0.00 |
| 166848_at | 0.00 |
| 166849_at | 0.95 |
| 166850_at | 0.00 |
| 166851_i_at | 0.02 |
| 166852_at | 0.00 |
| 166853_r_at | 0.00 |
| 166854_at | 0.00 |
| 166855_at | 0.00 |
| 166856_f_at | 0.00 |
| 166857_s_at | 0.00 |
| 166858_at | 0.00 |
| 166859_s_at | 0.00 |
| 166860_at | 0.00 |
| 166861_at | 0.00 |
| 166862_at | 0.00 |
| 166863_f_at | 0.00 |
| 166864_at | 0.00 |
| 166865_f_at | 0.00 |
| 166866_i_at | 0.08 |
| 166867_f_at | 0.00 |
| 166868_r_at | 0.00 |
| 166869_at | 0.00 |
| 166870_at | 0.00 |
| 166871_at | 0.00 |
| 166872_r_at | 0.00 |
| 166873_at | 0.00 |
| 166874_r_at | 0.00 |
| 166875_at | 0.03 |
| 166876_at | 0.00 |
| 166877_at | 0.00 |
| 166878_at | 0.00 |
| 166879_at | 0.00 |
| 166880_i_at | 0.00 |

| | |
|---|---|
| 166881_at | 0.00 |
| 166882_f_at | 0.22 |
| 166883_at | 0.00 |
| 166884_at | 0.00 |
| 166885_f_at | 0.00 |
| 166886_at | 0.00 |
| 166887_at | 0.00 |
| 166888_at | 0.00 |
| 166889_at | 0.00 |
| 166890_at | 0.00 |
| 166891_at | 0.00 |
| 166892_at | 0.00 |
| 166893_at | 0.00 |
| 166894_r_at | 0.00 |
| 166895_s_at | 0.00 |
| 166896_at | 0.00 |
| 166897_at | 0.00 |
| 166898_at | 0.00 |
| 166899_at | 0.00 |
| 166900_r_at | 0.00 |
| 166901_at | 0.00 |
| 166902_at | 0.00 |
| 166903_at | 0.00 |
| 166904_at | 0.00 |
| 166905_at | 0.00 |
| 166906_i_at | 0.31 |
| 166907_r_at | 0.00 |
| 166908_i_at | 0.00 |
| 166909_i_at | 0.00 |
| 166910_at | 0.00 |
| 166911_at | 0.00 |
| 166912_at | 0.00 |
| 166913_at | 0.00 |
| 166914_i_at | 0.00 |
| 166915_at | 0.00 |
| 166916_i_at | 0.00 |
| 166917_at | 0.00 |
| 166918_r_at | 0.00 |
| 166919_r_at | 0.00 |
| 166920_at | 0.00 |
| 166921_f_at | 0.00 |
| 166922_at | 0.00 |
| 166923_at | 0.00 |
| 166924_i_at | 0.00 |
| 166925_at | 0.00 |
| 166926_r_at | 0.00 |
| 166927_i_at | 0.00 |
| 166928_at | 0.00 |
| 166929_at | 0.00 |
| 166930_at | 0.00 |
| 166931_f_at | 0.00 |
| 166932_at | 0.00 |
| 166933_at | 0.00 |
| 166934_s_at | 0.00 |
| 166935_i_at | 0.00 |
| 166936_i_at | 0.00 |

| | |
|---|---|
| 166937_f_at | 0.00 |
| 166938_at | 0.00 |
| 166939_at | 0.00 |
| 166940_r_at | 0.00 |
| 166941_f_at | 0.00 |
| 166942_r_at | 0.00 |
| 166943_at | 0.00 |
| 166944_r_at | 0.00 |
| 166945_r_at | 0.00 |
| 166946_at | 0.00 |
| 166947_at | 0.00 |
| 166948_at | 0.00 |
| 166949_at | 0.00 |
| 166950_f_at | 0.00 |
| 166951_at | 0.00 |
| 166952_i_at | 0.00 |
| 166953_r_at | 0.00 |
| 166954_at | 0.00 |
| 166955_i_at | 0.02 |
| 166956_at | 0.00 |
| 166957_r_at | 0.00 |
| 166958_at | 0.00 |
| 166959_i_at | 0.00 |
| 166960_f_at | 0.00 |
| 166961_at | 0.00 |
| 166962_at | 0.00 |
| 166963_at | 0.00 |
| 166964_at | 0.00 |
| 166965_at | 0.00 |
| 166966_at | 0.00 |
| 166967_at | 0.00 |
| 166968_f_at | 0.00 |
| 166969_at | 0.00 |
| 166970_at | 0.00 |
| 166971_at | 0.00 |
| 166972_r_at | 0.00 |
| 166973_f_at | 0.00 |
| 166974_at | 0.00 |
| 166975_r_at | 0.00 |
| 166976_at | 0.12 |
| 166977_f_at | 0.00 |
| 166978_i_at | 0.00 |
| 166979_r_at | 0.00 |
| 166980_at | 0.00 |
| 166981_f_at | 0.00 |
| 166982_i_at | 0.00 |
| 166983_at | 0.00 |
| 166984_at | 0.00 |
| 166985_f_at | 0.01 |
| 166986_r_at | 0.00 |
| 166987_r_at | 0.00 |
| 166988_at | 0.00 |
| 166989_r_at | 0.02 |
| 166990_f_at | 0.00 |
| 166991_at | 0.00 |
| 166992_at | 0.00 |

| | |
|---|---|
| 166993_at | 0.00 |
| 166994_f_at | 0.00 |
| 166995_at | 0.00 |
| 166996_r_at | 0.00 |
| 166997_at | 0.00 |
| 166998_f_at | 0.00 |
| 166999_at | 0.17 |
| 167000_at | 0.00 |
| 167001_at | 0.00 |
| 167002_at | 0.00 |
| 167003_at | 0.00 |
| 167004_r_at | 0.00 |
| 167005_at | 0.01 |
| 167006_r_at | 0.00 |
| 167007_f_at | 0.00 |
| 167008_i_at | 0.00 |
| 167009_r_at | 0.00 |
| 167010_at | 0.00 |
| 167011_at | 0.00 |
| 167012_at | 0.00 |
| 167013_at | 0.00 |
| 167014_at | 0.00 |
| 167015_at | 0.00 |
| 167016_at | 0.00 |
| 167017_at | 0.00 |
| 167018_at | 0.00 |
| 167019_i_at | 0.00 |
| 167020_at | 0.00 |
| 167021_at | 0.00 |
| 167022_i_at | 0.00 |
| 167023_f_at | 0.41 |
| 167024_at | 0.00 |
| 167025_at | 0.00 |
| 167026_at | 0.00 |
| 167027_at | 0.00 |
| 167028_at | 0.13 |
| 167029_at | 0.00 |
| 167030_at | 0.00 |
| 167031_i_at | 0.00 |
| 167032_at | 0.00 |
| 167033_i_at | 0.00 |
| 167034_at | 0.00 |
| 167035_r_at | 0.00 |
| 167036_at | 0.00 |
| 167037_f_at | 0.00 |
| 167038_at | 0.00 |
| 167039_at | 0.00 |
| 167040_at | 0.00 |
| 167041_at | 0.00 |
| 167042_i_at | 0.00 |
| 167043_s_at | 0.27 |
| 167044_at | 0.00 |
| 167045_at | 0.00 |
| 167046_at | 0.00 |
| 167047_r_at | 0.00 |
| 167048_at | 0.00 |

| | |
|---|---|
| 167049_at | 0.00 |
| 167050_r_at | 0.00 |
| 167051_at | 0.00 |
| 167052_i_at | 0.00 |
| 167053_at | 0.00 |
| 167054_at | 0.00 |
| 167055_f_at | 0.13 |
| 167056_r_at | 0.00 |
| 167057_i_at | 0.00 |
| 167058_at | 0.00 |
| 167059_at | 0.00 |
| 167060_i_at | 0.00 |
| 167061_at | 0.00 |
| 167062_at | 0.00 |
| 167063_f_at | 0.00 |
| 167064_r_at | 0.00 |
| 167065_at | 0.00 |
| 167066_r_at | 0.00 |
| 167067_i_at | 0.00 |
| 167068_at | 0.00 |
| 167069_i_at | 0.00 |
| 167070_at | 0.00 |
| 167071_i_at | 0.00 |
| 167072_r_at | 0.00 |
| 167073_at | 0.00 |
| 167074_f_at | 0.00 |
| 167075_at | 0.00 |
| 167076_i_at | 0.00 |
| 167077_at | 0.00 |
| 167078_f_at | 0.00 |
| 167079_f_at | 0.00 |
| 167080_i_at | 0.00 |
| 167081_f_at | 0.00 |
| 167082_i_at | 0.00 |
| 167083_f_at | 0.01 |
| 167084_at | 0.00 |
| 167085_at | 0.00 |
| 167086_i_at | 0.00 |
| 167087_at | 0.00 |
| 167088_r_at | 0.47 |
| 167089_i_at | 0.00 |
| 167090_at | 0.00 |
| 167091_at | 0.00 |
| 167092_f_at | 0.00 |
| 167093_at | 0.00 |
| 167094_at | 0.01 |
| 167095_r_at | 0.00 |
| 167096_f_at | 0.00 |
| 167097_f_at | 0.00 |
| 167098_f_at | 0.00 |
| 167099_r_at | 0.00 |
| 167100_s_at | 0.00 |
| 167101_at | 0.08 |
| 167102_i_at | 0.00 |
| 167103_r_at | 0.00 |
| 167104_at | 0.00 |

| | |
|---|---|
| 167105_at | 0.00 |
| 167106_at | 0.00 |
| 167107_at | 0.00 |
| 167108_r_at | 0.00 |
| 167109_at | 0.00 |
| 167110_r_at | 0.00 |
| 167111_r_at | 0.00 |
| 167112_at | 0.00 |
| 167113_at | 0.00 |
| 167114_at | 0.00 |
| 167115_i_at | 0.00 |
| 167116_r_at | 0.00 |
| 167117_at | 0.00 |
| 167118_f_at | 0.19 |
| 167119_at | 0.00 |
| 167120_r_at | 0.00 |
| 167121_i_at | 0.00 |
| 167122_at | 0.01 |
| 167123_i_at | 0.00 |
| 167124_at | 0.00 |
| 167125_i_at | 0.00 |
| 167126_at | 0.00 |
| 167127_f_at | 0.00 |
| 167128_r_at | 0.00 |
| 167129_i_at | 0.00 |
| 167130_r_at | 0.00 |
| 167131_at | 0.00 |
| 167132_r_at | 0.00 |
| 167133_r_at | 0.00 |
| 167134_r_at | 0.00 |
| 167135_r_at | 0.00 |
| 167136_at | 0.00 |
| 167137_at | 0.00 |
| 167138_at | 0.00 |
| 167139_r_at | 0.00 |
| 167140_at | 0.00 |
| 167141_f_at | 0.00 |
| 167142_r_at | 0.00 |
| 167143_at | 0.00 |
| 167144_i_at | 0.00 |
| 167145_at | 0.00 |
| 167146_at | 0.00 |
| 167147_at | 0.00 |
| 167148_f_at | 0.00 |
| 167149_f_at | 0.00 |
| 167150_f_at | 0.00 |
| 167151_at | 0.00 |
| 167152_at | 0.00 |
| 167153_r_at | 0.00 |
| 167154_f_at | 0.00 |
| 167155_r_at | 0.00 |
| 167156_i_at | 0.00 |
| 167157_f_at | 0.00 |
| 167158_i_at | 0.00 |
| 167159_r_at | 0.00 |
| 167160_at | 0.00 |

| | |
|---|---|
| 167161_at | 0.00 |
| 167162_at | 0.00 |
| 167163_i_at | 0.00 |
| 167164_r_at | 0.00 |
| 167165_f_at | 0.01 |
| 167166_i_at | 0.00 |
| 167167_at | 0.00 |
| 167168_f_at | 0.00 |
| 167169_at | 0.00 |
| 167170_at | 0.00 |
| 167171_at | 0.00 |
| 167172_i_at | 0.02 |
| 167173_at | 0.00 |
| 167174_at | 0.12 |
| 167175_at | 0.00 |
| 167176_at | 0.00 |
| 167177_at | 0.00 |
| 167178_at | 0.00 |
| 167179_at | 0.00 |
| 167180_at | 0.00 |
| 167181_at | 0.00 |
| 167182_at | 0.00 |
| 167183_at | 0.00 |
| 167184_r_at | 0.00 |
| 167185_i_at | 0.06 |
| 167186_r_at | 0.00 |
| 167187_at | 0.00 |
| 167188_at | 0.09 |
| 167189_at | 0.00 |
| 167190_r_at | 0.00 |
| 167191_at | 0.00 |
| 167192_at | 0.00 |
| 167193_r_at | 0.00 |
| 167194_r_at | 0.00 |
| 167195_f_at | 0.00 |
| 167196_f_at | 0.00 |
| 167197_s_at | 0.00 |
| 167198_i_at | 0.00 |
| 167199_at | 0.00 |
| 167200_r_at | 0.00 |
| 167201_f_at | 0.29 |
| 167202_r_at | 0.00 |
| 167203_at | 0.00 |
| 167204_at | 0.00 |
| 167205_at | 0.00 |
| 167206_at | 0.00 |
| 167207_at | 0.00 |
| 167208_f_at | 0.00 |
| 167209_i_at | 0.00 |
| 167210_at | 0.00 |
| 167211_f_at | 0.00 |
| 167212_r_at | 0.00 |
| 167213_at | 0.00 |
| 167214_at | 0.00 |
| 167215_at | 0.00 |
| 167216_f_at | 0.05 |

| | |
|---|---|
| 167217_at | 0.00 |
| 167218_at | 0.00 |
| 167219_r_at | 0.00 |
| 167220_i_at | 0.00 |
| 167221_i_at | 0.01 |
| 167222_at | 0.00 |
| 167223_at | 0.00 |
| 167224_at | 0.00 |
| 167225_f_at | 0.00 |
| 167226_i_at | 0.00 |
| 167227_r_at | 0.00 |
| 167228_at | 0.00 |
| 167229_f_at | 0.00 |
| 167230_f_at | 0.01 |
| 167231_i_at | 0.00 |
| 167232_i_at | 0.00 |
| 167233_at | 0.00 |
| 167234_s_at | 0.31 |
| 167235_at | 0.00 |
| 167236_at | 0.00 |
| 167237_f_at | 0.00 |
| 167238_i_at | 0.03 |
| 167239_i_at | 0.02 |
| 167240_at | 0.00 |
| 167241_at | 0.00 |
| 167242_at | 0.19 |
| 167243_r_at | 0.00 |
| 167244_f_at | 0.00 |
| 167245_i_at | 0.00 |
| 167246_at | 0.00 |
| 167247_f_at | 0.04 |
| 167248_at | 0.00 |
| 167249_i_at | 0.00 |
| 167250_s_at | 0.02 |
| 167251_r_at | 0.00 |
| 167252_at | 0.00 |
| 167253_at | 0.00 |
| 167254_at | 0.00 |
| 167255_at | 0.00 |
| 167256_at | 0.00 |
| 167257_i_at | 0.00 |
| 167258_r_at | 0.00 |
| 167259_r_at | 0.00 |
| 167260_f_at | 0.00 |
| 167261_f_at | 0.38 |
| 167262_i_at | 0.04 |
| 167263_at | 0.00 |
| 167264_at | 0.00 |
| 167265_at | 0.23 |
| 167266_f_at | 0.00 |
| 167267_i_at | 0.00 |
| 167268_at | 0.00 |
| 167269_at | 0.00 |
| 167270_r_at | 0.00 |
| 167271_i_at | 0.00 |
| 167272_at | 0.00 |

| | |
|---|---|
| 167273_at | 0.00 |
| 167274_f_at | 0.00 |
| 167275_f_at | 0.00 |
| 167276_f_at | 0.00 |
| 167277_f_at | 0.00 |
| 167278_f_at | 0.04 |
| 167279_at | 0.00 |
| 167280_f_at | 0.00 |
| 167281_at | 0.00 |
| 167282_i_at | 0.00 |
| 167283_at | 0.00 |
| 167284_r_at | 0.00 |
| 167285_r_at | 0.00 |
| 167286_at | 0.08 |
| 167287_at | 0.00 |
| 167288_at | 0.00 |
| 167289_at | 0.00 |
| 167290_at | 0.00 |
| 167291_f_at | 0.00 |
| 167292_f_at | 0.01 |
| 167293_r_at | 0.00 |
| 167294_f_at | 0.00 |
| 167295_i_at | 0.00 |
| 167296_r_at | 0.00 |
| 167297_r_at | 0.00 |
| 167298_f_at | 0.00 |
| 167299_at | 0.00 |
| 167300_r_at | 0.00 |
| 167301_r_at | 0.00 |
| 167302_i_at | 0.00 |
| 167303_r_at | 0.00 |
| 167304_i_at | 0.00 |
| 167305_at | 0.00 |
| 167306_r_at | 0.00 |
| 167307_i_at | 0.00 |
| 167308_at | 0.00 |
| 167309_r_at | 0.00 |
| 167310_r_at | 0.00 |
| 167311_i_at | 0.00 |
| 167312_i_at | 0.00 |
| 167313_i_at | 0.00 |
| 167314_at | 0.00 |
| 167315_r_at | 0.00 |
| 167316_f_at | 0.00 |
| 167317_f_at | 0.00 |
| 167318_i_at | 0.00 |
| 167319_i_at | 0.00 |
| 167320_f_at | 0.00 |
| 167321_i_at | 0.00 |
| 167322_at | 0.00 |
| 167323_r_at | 0.00 |
| 167324_at | 0.00 |
| 167325_at | 0.00 |
| 167326_r_at | 0.00 |
| 167327_at | 0.00 |
| 167328_s_at | 0.00 |

| | |
|---|---|
| 167329_at | 0.00 |
| 167330_r_at | 0.00 |
| 167331_at | 0.00 |
| 167332_at | 0.00 |
| 167333_at | 0.00 |
| 167334_f_at | 0.00 |
| 167335_at | 0.00 |
| 167336_at | 0.00 |
| 167337_i_at | 0.00 |
| 167338_i_at | 0.00 |
| 167339_r_at | 0.01 |
| 167340_r_at | 0.02 |
| 167341_r_at | 0.00 |
| 167342_r_at | 0.00 |
| 167343_r_at | 0.00 |
| 167344_i_at | 0.00 |
| 167345_at | 0.00 |
| 167346_f_at | 0.00 |
| 167347_i_at | 0.00 |
| 167348_at | 0.00 |
| 167349_i_at | 0.00 |
| 167350_f_at | 0.00 |
| 167351_f_at | 0.00 |
| 167352_r_at | 0.00 |
| 167353_i_at | 0.00 |
| 167354_r_at | 0.00 |
| 167355_i_at | 0.00 |
| 167356_f_at | 0.00 |
| 167357_i_at | 0.00 |
| 167358_r_at | 0.00 |
| 167359_f_at | 0.00 |
| 167360_i_at | 0.00 |
| 167361_i_at | 0.00 |
| 167362_r_at | 0.00 |
| 167363_f_at | 0.00 |
| 167364_at | 0.00 |
| 167365_at | 0.00 |
| 167366_at | 0.00 |
| 167367_at | 0.00 |
| 167368_at | 0.00 |
| 167369_at | 0.00 |
| 167370_at | 0.00 |
| 167371_r_at | 0.00 |
| 167372_f_at | 0.00 |
| 167373_at | 0.00 |
| 167374_f_at | 0.00 |
| 167375_r_at | 0.00 |
| 167376_at | 0.00 |
| 167377_i_at | 0.00 |
| 167378_i_at | 0.00 |
| 167379_at | 0.00 |
| 167380_i_at | 0.00 |
| 167381_f_at | 0.00 |
| 167382_at | 0.00 |
| 167383_f_at | 0.07 |
| 167384_s_at | 0.31 |

| | |
|---|---|
| 167385_at | 0.00 |
| 167386_at | 0.00 |
| 167387_at | 0.00 |
| 167388_f_at | 0.00 |
| 167389_i_at | 0.00 |
| 167390_i_at | 0.00 |
| 167391_i_at | 0.00 |
| 167392_at | 0.00 |
| 167393_r_at | 0.00 |
| 167394_at | 0.00 |
| 167395_r_at | 0.00 |
| 167396_r_at | 0.00 |
| 167397_i_at | 0.00 |
| 167398_at | 0.00 |
| 167399_r_at | 0.00 |
| 167400_i_at | 0.01 |
| 167401_i_at | 0.00 |
| 167402_at | 0.00 |
| 167403_f_at | 0.00 |
| 167404_f_at | 0.00 |
| 167405_r_at | 0.00 |
| 167406_r_at | 0.00 |
| 167407_i_at | 0.00 |
| 167408_at | 0.00 |
| 167409_f_at | 0.00 |
| 167410_at | 0.00 |
| 167411_i_at | 0.00 |
| 167412_r_at | 0.00 |
| 167413_at | 0.00 |
| 167414_i_at | 0.00 |
| 167415_f_at | 0.00 |
| 167416_r_at | 0.00 |
| 167417_at | 0.00 |
| 167418_at | 0.00 |
| 167419_f_at | 0.00 |
| 167420_at | 0.00 |
| 167421_at | 0.00 |
| 167422_r_at | 0.00 |
| 167423_f_at | 0.00 |
| 167424_at | 0.00 |
| 167425_f_at | 0.00 |
| 167426_at | 0.00 |
| 167427_r_at | 0.00 |
| 167428_r_at | 0.00 |
| 167429_i_at | 0.01 |
| 167430_f_at | 0.44 |
| 167431_at | 0.00 |
| 167432_f_at | 0.00 |
| 167433_at | 0.00 |
| 167434_f_at | 0.02 |
| 167435_at | 0.00 |
| 167436_i_at | 0.00 |
| 167437_r_at | 0.00 |
| 167438_at | 0.00 |
| 167439_at | 0.00 |
| 167440_i_at | 0.00 |

| | |
|---|---|
| 167441_at | 0.00 |
| 167442_at | 0.00 |
| 167443_at | 0.00 |
| 167444_at | 0.00 |
| 167445_at | 0.00 |
| 167446_i_at | 0.00 |
| 167447_f_at | 0.00 |
| 167448_i_at | 0.00 |
| 167449_f_at | 0.00 |
| 167450_at | 0.00 |
| 167451_r_at | 0.00 |
| 167452_r_at | 0.00 |
| 167453_r_at | 0.00 |
| 167454_at | 0.00 |
| 167455_i_at | 0.00 |
| 167456_at | 0.00 |
| 167457_f_at | 0.02 |
| 167458_r_at | 0.00 |
| 167459_at | 0.00 |
| 167460_at | 0.00 |
| 167461_r_at | 0.00 |
| 167462_i_at | 0.00 |
| 167463_r_at | 0.00 |
| 167464_r_at | 0.00 |
| 167465_at | 0.00 |
| 167466_r_at | 0.00 |
| 167467_i_at | 0.00 |
| 167468_at | 0.01 |
| 167469_r_at | 0.00 |
| 167470_i_at | 0.00 |
| 167471_f_at | 0.00 |
| 167472_at | 0.00 |
| 167473_r_at | 0.00 |
| 167474_at | 0.01 |
| 167475_i_at | 0.00 |
| 167476_at | 0.00 |
| 167477_r_at | 0.00 |
| 167478_at | 0.00 |
| 167479_at | 0.00 |
| 167480_at | 0.00 |
| 167481_at | 0.00 |
| 167482_f_at | 0.00 |
| 167483_at | 0.00 |
| 167484_at | 0.00 |
| 167485_at | 0.00 |
| 167486_i_at | 0.00 |
| 167487_at | 0.00 |
| 167488_f_at | 0.00 |
| 167489_i_at | 0.00 |
| 167490_r_at | 0.00 |
| 167491_at | 0.00 |
| 167492_r_at | 0.00 |
| 167493_i_at | 0.00 |
| 167494_f_at | 0.00 |
| 167495_s_at | 0.01 |
| 167496_at | 0.00 |

| | |
|---|---|
| 167497_r_at | 0.00 |
| 167498_i_at | 0.00 |
| 167499_r_at | 0.00 |
| 167500_at | 0.00 |
| 167501_f_at | 0.00 |
| 167502_i_at | 0.01 |
| 167503_i_at | 0.00 |
| 167504_at | 0.00 |
| 167505_at | 0.00 |
| 167506_r_at | 0.00 |
| 167507_at | 0.00 |
| 167508_f_at | 0.00 |
| 167509_i_at | 0.00 |
| 167510_at | 0.00 |
| 167511_i_at | 0.00 |
| 167512_f_at | 0.03 |
| 167513_i_at | 0.00 |
| 167514_f_at | 0.00 |
| 167515_at | 0.00 |
| 167516_at | 0.00 |
| 167517_r_at | 0.00 |
| 167518_at | 0.00 |
| 167519_f_at | 0.00 |
| 167520_at | 0.00 |
| 167521_f_at | 0.21 |
| 167522_r_at | 0.00 |
| 167523_at | 0.00 |
| 167524_at | 0.00 |
| 167525_at | 0.00 |
| 167526_i_at | 0.00 |
| 167527_at | 0.00 |
| 167528_at | 0.00 |
| 167529_at | 0.00 |
| 167530_i_at | 0.00 |
| 167531_i_at | 0.00 |
| 167532_i_at | 0.00 |
| 167533_at | 0.00 |
| 167534_f_at | 0.00 |
| 167535_at | 0.00 |
| 167536_at | 0.00 |
| 167537_at | 0.00 |
| 167538_at | 0.00 |
| 167539_at | 0.00 |
| 167540_at | 0.00 |
| 167541_r_at | 0.00 |
| 167542_f_at | 0.00 |
| 167543_f_at | 0.00 |
| 167544_f_at | 0.00 |
| 167545_r_at | 0.00 |
| 167546_r_at | 0.00 |
| 167547_at | 0.00 |
| 167548_i_at | 0.00 |
| 167549_f_at | 0.00 |
| 167550_at | 0.00 |
| 167551_at | 0.00 |
| 167552_r_at | 0.00 |

| | |
|---|---|
| 167553_i_at | 0.00 |
| 167554_at | 0.00 |
| 167555_i_at | 0.00 |
| 167556_f_at | 0.00 |
| 167557_i_at | 0.00 |
| 167558_at | 0.00 |
| 167559_i_at | 0.00 |
| 167560_i_at | 0.00 |
| 167561_r_at | 0.00 |
| 167562_i_at | 0.00 |
| 167563_at | 0.00 |
| 167564_f_at | 0.00 |
| 167565_at | 0.00 |
| 167566_i_at | 0.00 |
| 167567_f_at | 0.00 |
| 167568_i_at | 0.00 |
| 167569_at | 0.00 |
| 167570_at | 0.00 |
| 167571_i_at | 0.00 |
| 167572_at | 0.00 |
| 167573_f_at | 0.00 |
| 167574_r_at | 0.00 |
| 167575_r_at | 0.00 |
| 167576_at | 0.00 |
| 167577_at | 0.00 |
| 167578_at | 0.00 |
| 167579_r_at | 0.00 |
| 167580_f_at | 0.00 |
| 167581_at | 0.00 |
| 167582_f_at | 0.00 |
| 167583_at | 0.00 |
| 167584_at | 0.00 |
| 167585_at | 0.00 |
| 167586_r_at | 0.00 |
| 167587_i_at | 0.00 |
| 167588_at | 0.00 |
| 167589_at | 0.00 |
| 167590_r_at | 0.00 |
| 167591_at | 0.00 |
| 167592_at | 0.00 |
| 167593_at | 0.00 |
| 167594_r_at | 0.00 |
| 167595_at | 0.00 |
| 167596_at | 0.01 |
| 167597_at | 0.00 |
| 167598_at | 0.00 |
| 167599_r_at | 0.00 |
| 167600_at | 0.00 |
| 167601_at | 0.00 |
| 167602_i_at | 0.01 |
| 167603_at | 0.00 |
| 167604_f_at | 0.00 |
| 167605_i_at | 0.00 |
| 167606_r_at | 0.00 |
| 167607_at | 0.00 |
| 167608_f_at | 0.00 |

| | |
|---|---|
| 167609_r_at | 0.00 |
| 167610_s_at | 0.00 |
| 167611_at | 0.04 |
| 167612_at | 0.00 |
| 167613_at | 0.00 |
| 167614_at | 0.00 |
| 167615_s_at | 0.00 |
| 167616_r_at | 0.00 |
| 167617_r_at | 0.00 |
| 167618_r_at | 0.00 |
| 167619_r_at | 0.00 |
| 167620_i_at | 0.00 |
| 167621_f_at | 0.00 |
| 167622_at | 0.00 |
| 167623_r_at | 0.00 |
| 167624_f_at | 0.00 |
| 167625_r_at | 0.00 |
| 167626_r_at | 0.00 |
| 167627_f_at | 0.40 |
| 167628_r_at | 0.00 |
| 167629_r_at | 0.00 |
| 167630_f_at | 0.00 |
| 167631_at | 0.00 |
| 167632_at | 0.00 |
| 167633_i_at | 0.00 |
| 167634_i_at | 0.00 |
| 167635_r_at | 0.00 |
| 167636_i_at | 0.00 |
| 167637_i_at | 0.00 |
| 167638_r_at | 0.00 |
| 167639_r_at | 0.00 |
| 167640_at | 0.00 |
| 167641_r_at | 0.00 |
| 167642_f_at | 0.00 |
| 167643_r_at | 0.00 |
| 167644_f_at | 0.00 |
| 167645_f_at | 0.00 |
| 167646_f_at | 0.00 |
| 167647_r_at | 0.01 |
| 167648_at | 0.00 |
| 167649_r_at | 0.00 |
| 167650_r_at | 0.00 |
| 167651_s_at | 0.00 |
| 167652_i_at | 0.00 |
| 167653_f_at | 0.00 |
| 167654_i_at | 0.00 |
| 167655_f_at | 0.00 |
| 167656_at | 0.00 |
| 167657_at | 0.00 |
| 167658_at | 0.00 |
| 167659_at | 0.00 |
| 167660_at | 0.00 |
| 167661_i_at | 0.00 |
| 167662_r_at | 0.00 |
| 167663_f_at | 0.00 |
| 167664_i_at | 0.00 |

| | |
|---|---|
| 167665_at | 0.03 |
| 167666_r_at | 0.00 |
| 167667_i_at | 0.00 |
| 167668_r_at | 0.00 |
| 167669_i_at | 0.00 |
| 167670_at | 0.00 |
| 167671_at | 0.00 |
| 167672_f_at | 0.00 |
| 167673_f_at | 0.00 |
| 167674_i_at | 0.03 |
| 167675_i_at | 0.00 |
| 167676_f_at | 0.02 |
| 167677_r_at | 0.00 |
| 167678_at | 0.00 |
| 167679_f_at | 0.00 |
| 167680_at | 0.00 |
| 167681_at | 0.00 |
| 167682_i_at | 0.00 |
| 167683_at | 0.03 |
| 167684_f_at | 0.00 |
| 167685_i_at | 0.00 |
| 167686_i_at | 0.00 |
| 167687_r_at | 0.00 |
| 167688_f_at | 0.00 |
| 167689_r_at | 0.00 |
| 167690_at | 0.02 |
| 167691_at | 0.00 |
| 167692_at | 0.02 |
| 167693_i_at | 0.03 |
| 167694_f_at | 0.00 |
| 167695_f_at | 0.00 |
| 167696_i_at | 0.00 |
| 167697_at | 0.00 |
| 167698_r_at | 0.00 |
| 167699_at | 0.00 |
| 167700_f_at | 0.00 |
| 167701_at | 0.00 |
| 167702_i_at | 0.00 |
| 167703_at | 0.00 |
| 167704_at | 0.00 |
| 167705_f_at | 0.00 |
| 167706_at | 0.00 |
| 167707_f_at | 0.00 |
| 167708_at | 0.00 |
| 167709_at | 0.00 |
| 167710_i_at | 0.00 |
| 167711_r_at | 0.00 |
| 167712_r_at | 0.00 |
| 167713_at | 0.00 |
| 167714_at | 0.00 |
| 167715_r_at | 0.01 |
| 167716_f_at | 0.00 |
| 167717_f_at | 0.00 |
| 167718_r_at | 0.00 |
| 167719_f_at | 0.00 |
| 167720_i_at | 0.00 |

| | |
|---|---|
| 167721_at | 0.00 |
| 167722_f_at | 0.00 |
| 167723_r_at | 0.00 |
| 167724_r_at | 0.00 |
| 167725_f_at | 0.46 |
| 167726_at | 0.00 |
| 167727_r_at | 0.00 |
| 167728_at | 0.00 |
| 167729_at | 0.00 |
| 167730_r_at | 0.00 |
| 167731_r_at | 0.00 |
| 167732_r_at | 0.00 |
| 167733_at | 0.00 |
| 167734_at | 0.08 |
| 167735_r_at | 0.00 |
| 167736_r_at | 0.00 |
| 167737_r_at | 0.00 |
| 167738_f_at | 0.00 |
| 167739_f_at | 0.00 |
| 167740_i_at | 0.00 |
| 167741_at | 0.00 |
| 167742_i_at | 0.00 |
| 167743_i_at | 0.00 |
| 167744_f_at | 0.00 |
| 167745_r_at | 0.00 |
| 167746_f_at | 0.05 |
| 167747_f_at | 0.00 |
| 167748_i_at | 0.00 |
| 167749_r_at | 0.00 |
| 167750_at | 0.00 |
| 167751_f_at | 0.00 |
| 167752_s_at | 0.00 |
| 167753_f_at | 0.00 |
| 167754_at | 0.00 |
| 167755_f_at | 0.29 |
| 167756_r_at | 0.00 |
| 167757_f_at | 0.00 |
| 167758_at | 0.02 |
| 167759_i_at | 0.00 |
| 167760_i_at | 0.00 |
| 167761_at | 0.00 |
| 167762_at | 0.00 |
| 167763_at | 0.00 |
| 167764_f_at | 0.41 |
| 167765_f_at | 0.00 |
| 167766_i_at | 0.01 |
| 167767_f_at | 0.00 |
| 167768_r_at | 0.00 |
| 167769_f_at | 0.00 |
| 167770_at | 0.00 |
| 167771_r_at | 0.00 |
| 167772_r_at | 0.00 |
| 167773_f_at | 0.00 |
| 167774_f_at | 0.00 |
| 167775_r_at | 0.00 |
| 167776_i_at | 0.00 |

| | |
|---|---|
| 167777_r_at | 0.00 |
| 167778_i_at | 0.00 |
| 167779_i_at | 0.00 |
| 167780_i_at | 0.00 |
| 167781_at | 0.00 |
| 167782_r_at | 0.00 |
| 167783_f_at | 0.01 |
| 167784_f_at | 0.00 |
| 167785_r_at | 0.00 |
| 167786_i_at | 0.00 |
| 167787_at | 0.00 |
| 167788_at | 0.00 |
| 167789_f_at | 0.00 |
| 167790_at | 0.00 |
| 167791_at | 0.00 |
| 167792_at | 0.00 |
| 167793_i_at | 0.00 |
| 167794_f_at | 0.31 |
| 167795_f_at | 0.00 |
| 167796_f_at | 0.00 |
| 167797_r_at | 0.00 |
| 167798_i_at | 0.00 |
| 167799_r_at | 0.01 |
| 167800_at | 0.00 |
| 167801_f_at | 0.00 |
| 167802_at | 0.00 |
| 167803_at | 0.00 |
| 167804_r_at | 0.00 |
| 167805_i_at | 0.00 |
| 167806_at | 0.00 |
| 167807_i_at | 0.00 |
| 167808_i_at | 0.00 |
| 167809_r_at | 0.00 |
| 167810_r_at | 0.00 |
| 167811_at | 0.00 |
| 167812_at | 0.08 |
| 167813_f_at | 0.00 |
| 167814_r_at | 0.00 |
| 167815_f_at | 0.00 |
| 167816_at | 0.00 |
| 167817_i_at | 0.00 |
| 167818_f_at | 0.00 |
| 167819_i_at | 0.00 |
| 167820_f_at | 0.00 |
| 167821_r_at | 0.00 |
| 167822_at | 0.00 |
| 167823_r_at | 0.00 |
| 167824_f_at | 0.00 |
| 167825_r_at | 0.00 |
| 167826_r_at | 0.00 |
| 167827_at | 0.00 |
| 167828_r_at | 0.00 |
| 167829_r_at | 0.00 |
| 167830_at | 0.00 |
| 167831_at | 0.00 |
| 167832_f_at | 0.00 |

| | |
|---|---|
| 167833_i_at | 0.00 |
| 167834_f_at | 0.00 |
| 167835_r_at | 0.00 |
| 167836_r_at | 0.00 |
| 167837_at | 0.00 |
| 167838_at | 0.00 |
| 167839_at | 0.00 |
| 167840_at | 0.08 |
| 167841_at | 0.00 |
| 167842_r_at | 0.00 |
| 167843_f_at | 0.01 |
| 167844_r_at | 0.00 |
| 167845_at | 0.00 |
| 167846_r_at | 0.00 |
| 167847_at | 0.00 |
| 167848_at | 0.00 |
| 167849_at | 0.00 |
| 167850_r_at | 0.00 |
| 167851_at | 0.00 |
| 167852_r_at | 0.00 |
| 167853_f_at | 0.00 |
| 167854_at | 0.00 |
| 167855_at | 0.00 |
| 167856_s_at | 0.00 |
| 167857_r_at | 0.01 |
| 167858_at | 0.00 |
| 167859_at | 0.00 |
| 167860_i_at | 0.07 |
| 167861_at | 0.00 |
| 167862_at | 0.00 |
| 167863_r_at | 0.00 |
| 167864_i_at | 0.00 |
| 167865_f_at | 0.00 |
| 167866_at | 0.00 |
| 167867_at | 0.00 |
| 167868_f_at | 0.00 |
| 167869_f_at | 0.00 |
| 167870_at | 0.00 |
| 167871_i_at | 0.00 |
| 167872_i_at | 0.00 |
| 167873_i_at | 0.00 |
| 167874_at | 0.01 |
| 167875_r_at | 0.00 |
| 167876_at | 0.00 |
| 167877_i_at | 0.00 |
| 167878_f_at | 0.00 |
| 167879_at | 0.00 |
| 167880_at | 0.02 |
| 167881_at | 0.00 |
| 167882_at | 0.00 |
| 167883_at | 0.00 |
| 167884_at | 0.00 |
| 167885_at | 0.00 |
| 167886_f_at | 0.04 |
| 167887_at | 0.00 |
| 167888_at | 0.00 |

| | |
|---|---|
| 167889_i_at | 0.00 |
| 167890_r_at | 0.00 |
| 167891_at | 0.00 |
| 167892_i_at | 0.01 |
| 167893_i_at | 0.00 |
| 167894_f_at | 0.00 |
| 167895_f_at | 0.00 |
| 167896_f_at | 0.00 |
| 167897_r_at | 0.00 |
| 167898_r_at | 0.00 |
| 167899_i_at | 0.00 |
| 167900_i_at | 0.00 |
| 167901_at | 0.00 |
| 167902_i_at | 0.00 |
| 167903_at | 0.00 |
| 167904_r_at | 0.00 |
| 167905_f_at | 0.00 |
| 167906_r_at | 0.00 |
| 167907_i_at | 0.00 |
| 167908_r_at | 0.00 |
| 167909_r_at | 0.00 |
| 167910_at | 0.00 |
| 167911_at | 0.00 |
| 167912_at | 0.04 |
| 167913_r_at | 0.00 |
| 167914_r_at | 0.00 |
| 167915_f_at | 0.00 |
| 167916_f_at | 0.00 |
| 167917_at | 0.00 |
| 167918_f_at | 0.00 |
| 167919_at | 0.00 |
| 167920_at | 0.00 |
| 167921_r_at | 0.00 |
| 167922_r_at | 0.00 |
| 167923_at | 0.00 |
| 167924_at | 0.00 |
| 167925_r_at | 0.00 |
| 167926_f_at | 0.00 |
| 167927_at | 0.00 |
| 167928_at | 0.00 |
| 167929_at | 0.00 |
| 167930_r_at | 0.00 |
| 167931_f_at | 0.00 |
| 167932_f_at | 0.00 |
| 167933_r_at | 0.00 |
| 167934_at | 0.00 |
| 167935_i_at | 0.00 |
| 167936_at | 0.00 |
| 167937_at | 0.00 |
| 167938_f_at | 0.00 |
| 167939_s_at | 0.00 |
| 167940_at | 0.00 |
| 167941_r_at | 0.00 |
| 167942_i_at | 0.00 |
| 167943_f_at | 0.00 |
| 167944_f_at | 0.00 |

| | |
|---|---|
| 167945_at | 0.00 |
| 167946_i_at | 0.00 |
| 167947_r_at | 0.00 |
| 167948_f_at | 0.00 |
| 167949_r_at | 0.00 |
| 167950_r_at | 0.00 |
| 167951_at | 0.00 |
| 167952_at | 0.00 |
| 167953_i_at | 0.00 |
| 167954_at | 0.00 |
| 167955_at | 0.00 |
| 167956_at | 0.00 |
| 167957_at | 0.00 |
| 167958_i_at | 0.00 |
| 167959_at | 0.00 |
| 167960_r_at | 0.00 |
| 167961_r_at | 0.00 |
| 167962_at | 0.00 |
| 167963_at | 0.00 |
| 167964_r_at | 0.00 |
| 167965_f_at | 0.47 |
| 167966_r_at | 0.00 |
| 167967_at | 0.00 |
| 167968_f_at | 0.00 |
| 167969_at | 0.00 |
| 167970_f_at | 0.00 |
| 167971_f_at | 0.02 |
| 167972_r_at | 0.00 |
| 167973_f_at | 0.00 |
| 167974_at | 0.00 |
| 167975_at | 0.00 |
| 167976_i_at | 0.00 |
| 167977_f_at | 0.00 |
| 167978_i_at | 0.00 |
| 167979_at | 0.00 |
| 167980_r_at | 0.00 |
| 167981_i_at | 0.00 |
| 167982_i_at | 0.00 |
| 167983_at | 0.00 |
| 167984_r_at | 0.00 |
| 167985_i_at | 0.00 |
| 167986_r_at | 0.00 |
| 167987_r_at | 0.00 |
| 167988_i_at | 0.00 |
| 167989_r_at | 0.00 |
| 167990_at | 0.00 |
| 167991_r_at | 0.00 |
| 167992_at | 0.00 |
| 167993_at | 0.00 |
| 167994_at | 0.00 |
| 167995_at | 0.00 |
| 167996_r_at | 0.00 |
| 167997_at | 0.00 |
| 167998_at | 0.00 |
| 167999_i_at | 0.01 |
| 168000_at | 0.00 |

| | |
|---|---|
| 168001_i_at | 0.01 |
| 168002_at | 0.00 |
| 168003_r_at | 0.00 |
| 168004_r_at | 0.00 |
| 168005_at | 0.00 |
| 168006_at | 0.00 |
| 168007_f_at | 0.00 |
| 168008_at | 0.00 |
| 168009_at | 0.00 |
| 168010_s_at | 0.00 |
| 168011_f_at | 0.00 |
| 168012_r_at | 0.00 |
| 168013_r_at | 0.01 |
| 168014_at | 0.00 |
| 168015_r_at | 0.00 |
| 168016_r_at | 0.02 |
| 168017_at | 0.00 |
| 168018_at | 0.00 |
| 168019_r_at | 0.00 |
| 168020_at | 0.00 |
| 168021_at | 0.00 |
| 168022_at | 0.00 |
| 168023_at | 0.00 |
| 168024_i_at | 0.00 |
| 168025_at | 0.00 |
| 168026_r_at | 0.00 |
| 168027_at | 0.00 |
| 168028_at | 0.00 |
| 168029_at | 0.00 |
| 168030_at | 0.00 |
| 168031_r_at | 0.00 |
| 168032_at | 0.00 |
| 168033_f_at | 0.00 |
| 168034_at | 0.00 |
| 168035_i_at | 0.00 |
| 168036_at | 0.00 |
| 168037_f_at | 0.00 |
| 168038_at | 0.00 |
| 168039_at | 0.00 |
| 168040_at | 0.00 |
| 168041_at | 0.00 |
| 168042_r_at | 0.00 |
| 168043_at | 0.00 |
| 168044_at | 0.00 |
| 168045_f_at | 0.00 |
| 168046_at | 0.00 |
| 168047_at | 0.00 |
| 168048_f_at | 0.00 |
| 168049_f_at | 0.00 |
| 168050_at | 0.00 |
| 168051_at | 0.00 |
| 168052_i_at | 0.00 |
| 168053_f_at | 0.00 |
| 168054_at | 0.00 |
| 168055_at | 0.00 |
| 168056_at | 0.00 |

| | |
|---|---|
| 168057_f_at | 0.03 |
| 168058_at | 0.00 |
| 168059_r_at | 0.00 |
| 168060_i_at | 0.00 |
| 168061_r_at | 0.00 |
| 168062_at | 0.00 |
| 168063_at | 0.00 |
| 168064_f_at | 0.00 |
| 168065_r_at | 0.00 |
| 168066_r_at | 0.00 |
| 168067_i_at | 0.00 |
| 168068_at | 0.00 |
| 168069_at | 0.00 |
| 168070_at | 0.00 |
| 168071_i_at | 0.00 |
| 168072_i_at | 0.00 |
| 168073_at | 0.00 |
| 168074_i_at | 0.00 |
| 168075_at | 0.00 |
| 168076_i_at | 0.00 |
| 168077_at | 0.02 |
| 168078_f_at | 0.04 |
| 168079_at | 0.00 |
| 168080_i_at | 0.00 |
| 168081_at | 0.00 |
| 168082_at | 0.00 |
| 168083_at | 0.00 |
| 168084_r_at | 0.00 |
| 168085_at | 0.00 |
| 168086_r_at | 0.00 |
| 168087_r_at | 0.01 |
| 168088_f_at | 0.00 |
| 168089_at | 0.00 |
| 168090_at | 0.00 |
| 168091_f_at | 0.00 |
| 168092_r_at | 0.00 |
| 168093_i_at | 0.00 |
| 168094_i_at | 0.00 |
| 168095_at | 0.00 |
| 168096_r_at | 0.00 |
| 168097_r_at | 0.00 |
| 168098_r_at | 0.00 |
| 168099_at | 0.30 |
| 168100_at | 0.00 |
| 168101_r_at | 0.01 |
| 168102_i_at | 0.00 |
| 168103_f_at | 0.00 |
| 168104_i_at | 0.01 |
| 168105_i_at | 0.00 |
| 168106_at | 0.03 |
| 168107_i_at | 0.00 |
| 168108_i_at | 0.00 |
| 168109_at | 0.00 |
| 168110_at | 0.00 |
| 168111_r_at | 0.00 |
| 168112_r_at | 0.00 |

| | |
|---|---|
| 168113_r_at | 0.00 |
| 168114_i_at | 0.00 |
| 168115_f_at | 0.00 |
| 168116_f_at | 0.01 |
| 168117_i_at | 0.00 |
| 168118_at | 0.00 |
| 168119_r_at | 0.00 |
| 168120_r_at | 0.00 |
| 168121_i_at | 0.00 |
| 168122_i_at | 0.00 |
| 168123_at | 0.01 |
| 168124_at | 0.00 |
| 168125_r_at | 0.06 |
| 168126_s_at | 0.02 |
| 168127_at | 0.00 |
| 168128_at | 0.00 |
| 168129_i_at | 0.00 |
| 168130_f_at | 0.02 |
| 168131_r_at | 0.00 |
| 168132_at | 0.00 |
| 168133_f_at | 0.00 |
| 168134_f_at | 0.00 |
| 168135_at | 0.00 |
| 168136_at | 0.00 |
| 168137_at | 0.00 |
| 168138_at | 0.00 |
| 168139_i_at | 0.00 |
| 168140_at | 0.00 |
| 168141_i_at | 0.00 |
| 168142_r_at | 0.00 |
| 168143_r_at | 0.00 |
| 168144_r_at | 0.00 |
| 168145_r_at | 0.00 |
| 168146_at | 0.00 |
| 168147_s_at | 0.00 |
| 168148_r_at | 0.00 |
| 168149_r_at | 0.00 |
| 168150_at | 0.00 |
| 168151_at | 0.00 |
| 168152_at | 0.00 |
| 168153_i_at | 0.89 |
| 168154_i_at | 0.00 |
| 168155_f_at | 0.16 |
| 168156_at | 0.00 |
| 168157_at | 0.00 |
| 168158_r_at | 0.00 |
| 168159_at | 0.00 |
| 168160_at | 0.00 |
| 168161_r_at | 0.00 |
| 168162_f_at | 0.00 |
| 168163_at | 0.00 |
| 168164_r_at | 0.00 |
| 168165_at | 0.00 |
| 168166_at | 0.00 |
| 168167_at | 0.00 |
| 168168_at | 0.00 |

| | |
|---|---|
| 168169_f_at | 0.00 |
| 168170_r_at | 0.00 |
| 168171_s_at | 0.00 |
| 168172_r_at | 0.00 |
| 168173_at | 0.00 |
| 168174_at | 0.00 |
| 168175_at | 0.00 |
| 168176_at | 0.02 |
| 168177_r_at | 0.00 |
| 168178_at | 0.00 |
| 168179_at | 0.00 |
| 168180_at | 0.00 |
| 168181_i_at | 0.00 |
| 168182_f_at | 0.00 |
| 168183_f_at | 0.00 |
| 168184_at | 0.00 |
| 168185_i_at | 0.00 |
| 168186_f_at | 0.00 |
| 168187_at | 0.00 |
| 168188_at | 0.00 |
| 168189_i_at | 0.00 |
| 168190_at | 0.00 |
| 168191_r_at | 0.00 |
| 168192_i_at | 0.00 |
| 168193_r_at | 0.00 |
| 168194_f_at | 0.00 |
| 168195_at | 0.00 |
| 168196_f_at | 0.00 |
| 168197_r_at | 0.00 |
| 168198_at | 0.00 |
| 168199_r_at | 0.00 |
| 168200_at | 0.00 |
| 168201_r_at | 0.00 |
| 168202_f_at | 0.08 |
| 168203_r_at | 0.00 |
| 168204_r_at | 0.00 |
| 168205_i_at | 0.00 |
| 168206_r_at | 0.00 |
| 168207_f_at | 0.00 |
| 168208_i_at | 0.00 |
| 168209_r_at | 0.00 |
| 168210_r_at | 0.00 |
| 168211_f_at | 0.00 |
| 168212_r_at | 0.00 |
| 168213_at | 0.00 |
| 168214_r_at | 0.00 |
| 168215_at | 0.00 |
| 168216_at | 0.00 |
| 168217_at | 0.00 |
| 168218_at | 0.00 |
| 168219_at | 0.00 |
| 168220_at | 0.00 |
| 168221_f_at | 0.00 |
| 168222_at | 0.00 |
| 168223_at | 0.01 |
| 168224_i_at | 0.00 |

| | |
|---|---|
| 168225_i_at | 0.00 |
| 168226_at | 0.00 |
| 168227_at | 0.00 |
| 168228_i_at | 0.00 |
| 168229_at | 0.00 |
| 168230_at | 0.00 |
| 168231_at | 0.00 |
| 168232_r_at | 0.00 |
| 168233_i_at | 0.00 |
| 168234_f_at | 0.00 |
| 168235_at | 0.00 |
| 168236_f_at | 0.00 |
| 168237_at | 0.00 |
| 168238_at | 0.00 |
| 168239_r_at | 0.00 |
| 168240_at | 0.00 |
| 168241_r_at | 0.00 |
| 168242_i_at | 0.00 |
| 168243_at | 0.00 |
| 168244_at | 0.00 |
| 168245_i_at | 0.00 |
| 168246_at | 0.00 |
| 168247_f_at | 0.00 |
| 168248_i_at | 0.00 |
| 168249_at | 0.00 |
| 168250_f_at | 0.01 |
| 168251_at | 0.00 |
| 168252_f_at | 0.02 |
| 168253_f_at | 0.00 |
| 168254_at | 0.00 |
| 168255_at | 0.01 |
| 168256_at | 0.00 |
| 168257_i_at | 0.01 |
| 168258_r_at | 0.00 |
| 168259_at | 0.00 |
| 168260_at | 0.00 |
| 168261_at | 0.00 |
| 168262_at | 0.00 |
| 168263_f_at | 0.00 |
| 168264_i_at | 0.00 |
| 168265_at | 0.00 |
| 168266_r_at | 0.00 |
| 168267_i_at | 0.00 |
| 168268_r_at | 0.00 |
| 168269_at | 0.00 |
| 168270_at | 0.00 |
| 168271_f_at | 0.00 |
| 168272_f_at | 0.02 |
| 168273_i_at | 0.00 |
| 168274_f_at | 0.17 |
| 168275_i_at | 0.00 |
| 168276_f_at | 0.00 |
| 168277_r_at | 0.00 |
| 168278_i_at | 0.09 |
| 168279_r_at | 0.00 |
| 168280_r_at | 0.00 |

| | |
|---|---|
| 168281_r_at | 0.00 |
| 168282_r_at | 0.01 |
| 168283_r_at | 0.00 |
| 168284_i_at | 0.00 |
| 168285_i_at | 0.01 |
| 168286_i_at | 0.00 |
| 168287_i_at | 0.00 |
| 168288_f_at | 0.00 |
| 168289_r_at | 0.00 |
| 168290_r_at | 0.00 |
| 168291_f_at | 0.00 |
| 168292_i_at | 0.00 |
| 168293_i_at | 0.00 |
| 168294_f_at | 0.00 |
| 168295_f_at | 0.00 |
| 168296_r_at | 0.00 |
| 168297_f_at | 0.00 |
| 168298_at | 0.00 |
| 168299_f_at | 0.00 |
| 168300_r_at | 0.00 |
| 168301_i_at | 0.00 |
| 168302_at | 0.00 |
| 168303_at | 0.00 |
| 168304_r_at | 0.00 |
| 168305_i_at | 0.00 |
| 168306_r_at | 0.00 |
| 168307_i_at | 0.00 |
| 168308_f_at | 0.00 |
| 168309_i_at | 0.00 |
| 168310_r_at | 0.00 |
| 168311_f_at | 0.00 |
| 168312_at | 0.00 |
| 168313_at | 0.00 |
| 168314_at | 0.00 |
| 168315_r_at | 0.00 |
| 168316_i_at | 0.00 |
| 168317_r_at | 0.00 |
| 168318_at | 0.00 |
| 168319_at | 0.05 |
| 168320_f_at | 0.00 |
| 168321_at | 0.00 |
| 168322_r_at | 0.00 |
| 168323_at | 0.00 |
| 168324_r_at | 0.00 |
| 168325_r_at | 0.00 |
| 168326_at | 0.00 |
| 168327_i_at | 0.00 |
| 168328_i_at | 0.00 |
| 168329_at | 0.00 |
| 168330_f_at | 0.00 |
| 168331_r_at | 0.00 |
| 168332_f_at | 0.00 |
| 168333_at | 0.00 |
| 168334_at | 0.00 |
| 168335_at | 0.00 |
| 168336_at | 0.00 |

| | |
|---|---|
| 168337_at | 0.00 |
| 168338_at | 0.02 |
| 168339_i_at | 0.00 |
| 168340_at | 0.00 |
| 168341_at | 0.00 |
| 168342_r_at | 0.00 |
| 168343_at | 0.02 |
| 168344_r_at | 0.00 |
| 168345_r_at | 0.00 |
| 168346_r_at | 0.48 |
| 168347_at | 0.00 |
| 168348_r_at | 0.00 |
| 168349_at | 0.00 |
| 168350_f_at | 0.00 |
| 168351_at | 0.00 |
| 168352_r_at | 0.00 |
| 168353_f_at | 0.00 |
| 168354_at | 0.00 |
| 168355_r_at | 0.00 |
| 168356_at | 0.00 |
| 168357_i_at | 0.00 |
| 168358_i_at | 0.01 |
| 168359_at | 0.00 |
| 168360_f_at | 0.03 |
| 168361_r_at | 0.00 |
| 168362_r_at | 0.00 |
| 168363_i_at | 0.06 |
| 168364_r_at | 0.00 |
| 168365_at | 0.00 |
| 168366_at | 0.00 |
| 168367_at | 0.00 |
| 168368_f_at | 0.00 |
| 168369_f_at | 0.27 |
| 168370_at | 0.00 |
| 168371_f_at | 0.00 |
| 168372_i_at | 0.00 |
| 168373_f_at | 0.00 |
| 168374_at | 0.00 |
| 168375_at | 0.00 |
| 168376_r_at | 0.00 |
| 168377_r_at | 0.11 |
| 168378_f_at | 0.00 |
| 168379_i_at | 0.20 |
| 168380_at | 0.00 |
| 168381_s_at | 0.00 |
| 168382_i_at | 0.08 |
| 168383_r_at | 0.00 |
| 168384_s_at | 0.40 |
| 168385_f_at | 0.00 |
| 168386_f_at | 0.00 |
| 168387_i_at | 0.00 |
| 168388_at | 0.00 |
| 168389_r_at | 0.00 |
| 168390_f_at | 0.00 |
| 168391_f_at | 0.00 |
| 168392_f_at | 0.00 |

| | |
|---|---|
| 168393_at | 0.00 |
| 168394_f_at | 0.02 |
| 168395_at | 0.00 |
| 168396_i_at | 0.00 |
| 168397_i_at | 0.00 |
| 168398_f_at | 0.00 |
| 168399_i_at | 0.00 |
| 168400_i_at | 0.00 |
| 168401_f_at | 0.00 |
| 168402_r_at | 0.00 |
| 168403_f_at | 0.00 |
| 168404_at | 0.00 |
| 168405_i_at | 0.00 |
| 168406_at | 0.00 |
| 168407_at | 0.00 |
| 168408_at | 0.00 |
| 168409_r_at | 0.00 |
| 168410_i_at | 0.00 |
| 168411_r_at | 0.00 |
| 168412_at | 0.00 |
| 168413_r_at | 0.00 |
| 168414_at | 0.00 |
| 168415_r_at | 0.00 |
| 168416_at | 0.00 |
| 168417_r_at | 0.00 |
| 168418_i_at | 0.00 |
| 168419_f_at | 0.00 |
| 168420_r_at | 0.00 |
| 168421_f_at | 0.00 |
| 168422_at | 0.00 |
| 168423_f_at | 0.00 |
| 168424_f_at | 0.01 |
| 168425_at | 0.00 |
| 168426_f_at | 0.06 |
| 168427_at | 0.00 |
| 168428_at | 0.00 |
| 168429_r_at | 0.01 |
| 168430_at | 0.00 |
| 168431_at | 0.00 |
| 168432_i_at | 0.00 |
| 168433_i_at | 0.00 |
| 168434_r_at | 0.00 |
| 168435_r_at | 0.00 |
| 168436_at | 0.00 |
| 168437_i_at | 0.02 |
| 168438_r_at | 0.00 |
| 168439_f_at | 0.00 |
| 168440_r_at | 0.00 |
| 168441_at | 0.00 |
| 168442_r_at | 0.00 |
| 168443_r_at | 0.00 |
| 168444_i_at | 0.00 |
| 168445_r_at | 0.00 |
| 168446_at | 0.00 |
| 168447_r_at | 0.00 |
| 168448_at | 0.00 |

| | |
|---|---|
| 168449_i_at | 0.00 |
| 168450_at | 0.00 |
| 168451_at | 0.00 |
| 168452_f_at | 0.00 |
| 168453_at | 0.00 |
| 168454_f_at | 0.00 |
| 168455_r_at | 0.00 |
| 168456_r_at | 0.00 |
| 168457_i_at | 0.00 |
| 168458_i_at | 0.00 |
| 168459_f_at | 0.00 |
| 168460_f_at | 0.00 |
| 168461_at | 0.00 |
| 168462_at | 0.00 |
| 168463_f_at | 0.00 |
| 168464_i_at | 0.00 |
| 168465_r_at | 0.00 |
| 168466_r_at | 0.00 |
| 168467_r_at | 0.01 |
| 168468_at | 0.00 |
| 168469_f_at | 0.00 |
| 168470_f_at | 0.00 |
| 168471_at | 0.00 |
| 168472_at | 0.00 |
| 168473_at | 0.00 |
| 168474_r_at | 0.00 |
| 168475_i_at | 0.00 |
| 168476_r_at | 0.00 |
| 168477_f_at | 0.00 |
| 168478_s_at | 0.00 |
| 168479_at | 0.00 |
| 168480_at | 0.00 |
| 168481_f_at | 0.00 |
| 168482_f_at | 0.00 |
| 168483_r_at | 0.00 |
| 168484_f_at | 0.00 |
| 168485_at | 0.00 |
| 168486_f_at | 0.00 |
| 168487_at | 0.00 |
| 168488_i_at | 0.00 |
| 168489_r_at | 0.00 |
| 168490_at | 0.00 |
| 168491_at | 0.00 |
| 168492_at | 0.00 |
| 168493_at | 0.04 |
| 168494_f_at | 0.00 |
| 168495_i_at | 0.01 |
| 168496_r_at | 0.00 |
| 168497_at | 0.00 |
| 168498_r_at | 0.00 |
| 168499_s_at | 0.48 |
| 168500_s_at | 0.00 |
| 168501_i_at | 0.00 |
| 168502_r_at | 0.00 |
| 168503_at | 0.00 |
| 168504_r_at | 0.00 |

| | |
|---|---|
| 168505_at | 0.00 |
| 168506_i_at | 0.00 |
| 168507_i_at | 0.01 |
| 168508_at | 0.86 |
| 168509_at | 0.00 |
| 168510_f_at | 0.00 |
| 168511_i_at | 0.00 |
| 168512_s_at | 0.00 |
| 168513_f_at | 0.01 |
| 168514_r_at | 0.00 |
| 168515_r_at | 0.00 |
| 168516_r_at | 0.00 |
| 168517_r_at | 0.00 |
| 168518_at | 0.00 |
| 168519_at | 0.00 |
| 168520_r_at | 0.00 |
| 168521_r_at | 0.00 |
| 168522_f_at | 0.00 |
| 168523_at | 0.00 |
| 168524_i_at | 0.00 |
| 168525_r_at | 0.00 |
| 168526_i_at | 0.00 |
| 168527_f_at | 0.00 |
| 168528_f_at | 0.14 |
| 168529_r_at | 0.05 |
| 168530_r_at | 0.00 |
| 168531_at | 0.00 |
| 168532_f_at | 0.00 |
| 168533_at | 0.00 |
| 168534_i_at | 0.00 |
| 168535_i_at | 0.00 |
| 168536_at | 0.00 |
| 168537_at | 0.00 |
| 168538_r_at | 0.00 |
| 168539_at | 0.00 |
| 168540_at | 0.00 |
| 168541_i_at | 0.00 |
| 168542_f_at | 0.02 |
| 168543_r_at | 0.00 |
| 168544_f_at | 0.00 |
| 168545_r_at | 0.00 |
| 168546_r_at | 0.00 |
| 168547_f_at | 0.00 |
| 168548_f_at | 0.05 |
| 168549_f_at | 0.00 |
| 168550_at | 0.12 |
| 168551_at | 0.00 |
| 168552_i_at | 0.00 |
| 168553_s_at | 0.00 |
| 168554_i_at | 0.00 |
| 168555_at | 0.00 |
| 168556_i_at | 0.00 |
| 168557_i_at | 0.00 |
| 168558_f_at | 0.00 |
| 168559_r_at | 0.00 |
| 168560_at | 0.01 |

| | |
|---|---|
| 168561_f_at | 0.00 |
| 168562_i_at | 0.00 |
| 168563_r_at | 0.00 |
| 168564_r_at | 0.00 |
| 168565_at | 0.00 |
| 168566_at | 0.00 |
| 168567_at | 0.00 |
| 168568_i_at | 0.00 |
| 168569_at | 0.00 |
| 168570_i_at | 0.00 |
| 168571_at | 0.00 |
| 168572_r_at | 0.00 |
| 168573_at | 0.00 |
| 168574_at | 0.00 |
| 168575_r_at | 0.00 |
| 168576_i_at | 0.00 |
| 168577_at | 0.00 |
| 168578_r_at | 0.00 |
| 168579_r_at | 0.00 |
| 168580_i_at | 0.00 |
| 168581_i_at | 0.00 |
| 168582_f_at | 0.00 |
| 168583_at | 0.00 |
| 168584_at | 0.00 |
| 168585_i_at | 0.00 |
| 168586_at | 0.00 |
| 168587_i_at | 0.00 |
| 168588_at | 0.00 |
| 168589_r_at | 0.00 |
| 168590_at | 0.00 |
| 168591_r_at | 0.00 |
| 168592_at | 0.00 |
| 168593_r_at | 0.00 |
| 168594_r_at | 0.00 |
| 168595_r_at | 0.00 |
| 168596_r_at | 0.00 |
| 168597_at | 0.00 |
| 168598_at | 0.00 |
| 168599_i_at | 0.00 |
| 168600_r_at | 0.00 |
| 168601_at | 0.00 |
| 168602_i_at | 0.00 |
| 168603_i_at | 0.01 |
| 168604_r_at | 0.00 |
| 168605_r_at | 0.00 |
| 168606_r_at | 0.00 |
| 168607_i_at | 0.00 |
| 168608_f_at | 0.03 |
| 168609_at | 0.00 |
| 168610_i_at | 0.00 |
| 168611_i_at | 0.00 |
| 168612_r_at | 0.00 |
| 168613_at | 0.04 |
| 168614_at | 0.00 |
| 168615_s_at | 0.00 |
| 168616_at | 0.00 |

| | |
|---|---|
| 168617_r_at | 0.00 |
| 168618_i_at | 0.00 |
| 168619_r_at | 0.00 |
| 168620_at | 0.00 |
| 168621_i_at | 0.00 |
| 168622_at | 0.00 |
| 168623_i_at | 0.00 |
| 168624_at | 0.00 |
| 168625_r_at | 0.00 |
| 168626_r_at | 0.00 |
| 168627_at | 0.00 |
| 168628_at | 0.00 |
| 168629_at | 0.00 |
| 168630_r_at | 0.00 |
| 168631_r_at | 0.00 |
| 168632_at | 0.00 |
| 168633_i_at | 0.00 |
| 168634_i_at | 0.00 |
| 168635_at | 0.00 |
| 168636_f_at | 0.00 |
| 168637_f_at | 0.00 |
| 168638_f_at | 0.00 |
| 168639_at | 0.00 |
| 168640_r_at | 0.00 |
| 168641_at | 0.00 |
| 168642_i_at | 0.00 |
| 168643_r_at | 0.00 |
| 168644_r_at | 0.00 |
| 168645_r_at | 0.00 |
| 168646_r_at | 0.00 |
| 168647_at | 0.00 |
| 168648_i_at | 0.00 |
| 168649_at | 0.00 |
| 168650_at | 0.00 |
| 168651_at | 0.00 |
| 168652_at | 0.00 |
| 168653_i_at | 0.00 |
| 168654_at | 0.00 |
| 168655_r_at | 0.00 |
| 168656_i_at | 0.00 |
| 168657_at | 0.00 |
| 168658_at | 0.00 |
| 168659_at | 0.00 |
| 168660_at | 0.00 |
| 168661_r_at | 0.00 |
| 168662_at | 0.00 |
| 168663_i_at | 0.00 |
| 168664_r_at | 0.00 |
| 168665_r_at | 0.00 |
| 168666_i_at | 0.00 |
| 168667_i_at | 0.00 |
| 168668_r_at | 0.00 |
| 168669_at | 0.00 |
| 168670_at | 0.00 |
| 168671_i_at | 0.00 |
| 168672_at | 0.00 |

| | |
|---|---|
| 168673_i_at | 0.00 |
| 168674_i_at | 0.00 |
| 168675_i_at | 0.02 |
| 168676_r_at | 0.00 |
| 168677_i_at | 0.00 |
| 168678_r_at | 0.00 |
| 168679_r_at | 0.00 |
| 168680_at | 0.00 |
| 168681_i_at | 0.00 |
| 168682_s_at | 0.02 |
| 168683_f_at | 0.00 |
| 168684_i_at | 0.00 |
| 168685_r_at | 0.00 |
| 168686_f_at | 0.00 |
| 168687_i_at | 0.00 |
| 168688_r_at | 0.00 |
| 168689_r_at | 0.00 |
| 168690_i_at | 0.02 |
| 168691_at | 0.00 |
| 168692_i_at | 0.00 |
| 168693_at | 0.00 |
| 168694_r_at | 0.00 |
| 168695_i_at | 0.00 |
| 168696_i_at | 0.00 |
| 168697_s_at | 0.00 |
| 168698_at | 0.00 |
| 168699_i_at | 0.00 |
| 168700_r_at | 0.00 |
| 168701_i_at | 0.00 |
| 168702_r_at | 0.00 |
| 168703_at | 0.00 |
| 168704_f_at | 0.00 |
| 168705_at | 0.00 |
| 168706_r_at | 0.00 |
| 168707_i_at | 0.00 |
| 168708_i_at | 0.00 |
| 168709_i_at | 0.00 |
| 168710_at | 0.00 |
| 168711_at | 0.00 |
| 168712_r_at | 0.00 |
| 168713_i_at | 0.00 |
| 168714_f_at | 0.00 |
| 168715_at | 0.00 |
| 168716_s_at | 0.00 |
| 168717_i_at | 0.00 |
| 168718_r_at | 0.00 |
| 168719_at | 0.00 |
| 168720_r_at | 0.00 |
| 168721_r_at | 0.00 |
| 168722_at | 0.00 |
| 168723_at | 0.00 |
| 168724_i_at | 0.00 |
| 168725_i_at | 0.00 |
| 168726_r_at | 0.00 |
| 168727_at | 0.00 |
| 168728_at | 0.00 |

| | |
|---|---|
| 168729_r_at | 0.00 |
| 168730_f_at | 0.00 |
| 168731_f_at | 0.00 |
| 168732_at | 0.00 |
| 168733_at | 0.00 |
| 168734_at | 0.00 |
| 168735_at | 0.00 |
| 168736_r_at | 0.00 |
| 168737_r_at | 0.00 |
| 168738_at | 0.00 |
| 168739_at | 0.00 |
| 168740_f_at | 0.00 |
| 168741_r_at | 0.00 |
| 168742_at | 0.00 |
| 168743_at | 0.00 |
| 168744_at | 0.00 |
| 168745_at | 0.00 |
| 168746_i_at | 0.00 |
| 168747_r_at | 0.00 |
| 168748_r_at | 0.00 |
| 168749_at | 0.00 |
| 168750_i_at | 0.00 |
| 168751_f_at | 0.00 |
| 168752_r_at | 0.00 |
| 168753_at | 0.00 |
| 168754_i_at | 0.00 |
| 168755_at | 0.00 |
| 168756_at | 0.00 |
| 168757_at | 0.00 |
| 168758_i_at | 0.00 |
| 168759_at | 0.00 |
| 168760_r_at | 0.00 |
| 168761_i_at | 0.02 |
| 168762_at | 0.00 |
| 168763_r_at | 0.00 |
| 168764_at | 0.00 |
| 168765_at | 0.00 |
| 168766_i_at | 0.00 |
| 168767_at | 0.00 |
| 168768_r_at | 0.00 |
| 168769_at | 0.00 |
| 168770_at | 0.00 |
| 168771_r_at | 0.01 |
| 168772_at | 0.00 |
| 168773_at | 0.00 |
| 168774_at | 0.00 |
| 168775_at | 0.00 |
| 168776_i_at | 0.00 |
| 168777_i_at | 0.00 |
| 168778_at | 0.00 |
| 168779_at | 0.00 |
| 168780_at | 0.00 |
| 168781_at | 0.00 |
| 168782_r_at | 0.00 |
| 168783_f_at | 0.00 |
| 168784_at | 0.00 |

| | |
|---|---|
| 168785_at | 0.00 |
| 168786_r_at | 0.00 |
| 168787_i_at | 0.00 |
| 168788_r_at | 0.00 |
| 168789_at | 0.00 |
| 168790_at | 0.00 |
| 168791_at | 0.00 |
| 168792_at | 0.00 |
| 168793_r_at | 0.00 |
| 168794_at | 0.00 |
| 168795_at | 0.00 |
| 168796_i_at | 0.00 |
| 168797_at | 0.00 |
| 168798_at | 0.00 |
| 168799_i_at | 0.00 |
| 168800_at | 0.00 |
| 168801_at | 0.00 |
| 168802_r_at | 0.00 |
| 168803_at | 0.05 |
| 168804_i_at | 0.00 |
| 168805_i_at | 0.00 |
| 168806_i_at | 0.00 |
| 168807_at | 0.00 |
| 168808_i_at | 0.00 |
| 168809_i_at | 0.00 |
| 168810_r_at | 0.00 |
| 168811_r_at | 0.00 |
| 168812_at | 0.00 |
| 168813_r_at | 0.00 |
| 168814_at | 0.00 |
| 168815_at | 0.00 |
| 168816_at | 0.00 |
| 168817_r_at | 0.00 |
| 168818_at | 0.00 |
| 168819_at | 0.00 |
| 168820_at | 0.00 |
| 168821_f_at | 0.00 |
| 168822_i_at | 0.00 |
| 168823_f_at | 0.00 |
| 168824_at | 0.00 |
| 168825_i_at | 0.00 |
| 168826_at | 0.00 |
| 168827_r_at | 0.00 |
| 168828_i_at | 0.00 |
| 168829_r_at | 0.00 |
| 168830_f_at | 0.00 |
| 168831_at | 0.00 |
| 168832_at | 0.00 |
| 168833_i_at | 0.00 |
| 168834_at | 0.00 |
| 168835_s_at | 0.00 |
| 168836_at | 0.00 |
| 168837_r_at | 0.00 |
| 168838_r_at | 0.00 |
| 168839_at | 0.00 |
| 168840_at | 0.00 |

| | |
|---|---|
| 168841_f_at | 0.00 |
| 168842_at | 0.00 |
| 168843_at | 0.00 |
| 168844_at | 0.00 |
| 168845_r_at | 0.00 |
| 168846_i_at | 0.05 |
| 168847_r_at | 0.00 |
| 168848_i_at | 0.00 |
| 168849_at | 0.00 |
| 168850_at | 0.00 |
| 168851_f_at | 0.00 |
| 168852_at | 0.00 |
| 168853_i_at | 0.00 |
| 168854_r_at | 0.00 |
| 168855_r_at | 0.00 |
| 168856_at | 0.00 |
| 168857_at | 0.00 |
| 168858_at | 0.00 |
| 168859_at | 0.00 |
| 168860_i_at | 0.00 |
| 168861_at | 0.00 |
| 168862_at | 0.00 |
| 168863_r_at | 0.00 |
| 168864_at | 0.00 |
| 168865_i_at | 0.00 |
| 168866_i_at | 0.00 |
| 168867_i_at | 0.00 |
| 168868_r_at | 0.00 |
| 168869_at | 0.00 |
| 168870_at | 0.00 |
| 168871_i_at | 0.00 |
| 168872_at | 0.00 |
| 168873_i_at | 0.00 |
| 168874_i_at | 0.00 |
| 168875_f_at | 0.00 |
| 168876_f_at | 0.00 |
| 168877_f_at | 0.00 |
| 168878_i_at | 0.00 |
| 168879_i_at | 0.01 |
| 168880_r_at | 0.00 |
| 168881_at | 0.00 |
| 168882_at | 0.00 |
| 168883_at | 0.00 |
| 168884_at | 0.00 |
| 168885_i_at | 0.00 |
| 168886_at | 0.00 |
| 168887_i_at | 0.00 |
| 168888_r_at | 0.00 |
| 168889_i_at | 0.00 |
| 168890_i_at | 0.00 |
| 168891_r_at | 0.00 |
| 168892_i_at | 0.00 |
| 168893_r_at | 0.00 |
| 168894_at | 0.00 |
| 168895_i_at | 0.00 |
| 168896_i_at | 0.00 |

| | |
|---|---|
| 168897_i_at | 0.00 |
| 168898_r_at | 0.00 |
| 168899_i_at | 0.00 |
| 168900_i_at | 0.00 |
| 168901_at | 0.00 |
| 168902_at | 0.00 |
| 168903_at | 0.00 |
| 168904_at | 0.02 |
| 168905_at | 0.00 |
| 168906_at | 0.00 |
| 168907_at | 0.00 |
| 168908_s_at | 0.00 |
| 168909_r_at | 0.00 |
| 168910_at | 0.00 |
| 168911_at | 0.00 |
| 168912_f_at | 0.05 |
| 168913_r_at | 0.00 |
| 168914_at | 0.00 |
| 168915_r_at | 0.00 |
| 168916_at | 0.00 |
| 168917_r_at | 0.00 |
| 168918_at | 0.00 |
| 168919_at | 0.00 |
| 168920_at | 0.00 |
| 168921_at | 0.00 |
| 168922_at | 0.00 |
| 168923_at | 0.00 |
| 168924_at | 0.00 |
| 168925_at | 0.00 |
| 168926_at | 0.00 |
| 168927_at | 0.00 |
| 168928_r_at | 0.00 |
| 168929_at | 0.00 |
| 168930_i_at | 0.00 |
| 168931_i_at | 0.00 |
| 168932_r_at | 0.00 |
| 168933_i_at | 0.00 |
| 168934_at | 0.00 |
| 168935_f_at | 0.00 |
| 168936_r_at | 0.00 |
| 168937_at | 0.00 |
| 168938_r_at | 0.00 |
| 168939_i_at | 0.00 |
| 168940_at | 0.00 |
| 168941_i_at | 0.00 |
| 168942_i_at | 0.00 |
| 168943_at | 0.00 |
| 168944_i_at | 0.00 |
| 168945_at | 0.00 |
| 168946_i_at | 0.00 |
| 168947_f_at | 0.00 |
| 168948_at | 0.00 |
| 168949_i_at | 0.00 |
| 168950_at | 0.00 |
| 168951_r_at | 0.00 |
| 168952_i_at | 0.00 |

| | |
|---|---|
| 168953_f_at | 0.00 |
| 168954_r_at | 0.00 |
| 168955_i_at | 0.00 |
| 168956_f_at | 0.00 |
| 168957_i_at | 0.00 |
| 168958_r_at | 0.00 |
| 168959_r_at | 0.00 |
| 168960_at | 0.00 |
| 168961_f_at | 0.00 |
| 168962_r_at | 0.00 |
| 168963_at | 0.00 |
| 168964_r_at | 0.00 |
| 168965_at | 0.00 |
| 168966_r_at | 0.00 |
| 168967_i_at | 0.00 |
| 168968_r_at | 0.00 |
| 168969_f_at | 0.07 |
| 168970_f_at | 0.00 |
| 168971_at | 0.00 |
| 168972_i_at | 0.05 |
| 168973_i_at | 0.00 |
| 168974_r_at | 0.00 |
| 168975_i_at | 0.00 |
| 168976_i_at | 0.02 |
| 168977_i_at | 0.00 |
| 168978_r_at | 0.00 |
| 168979_i_at | 0.00 |
| 168980_i_at | 0.00 |
| 168981_r_at | 0.00 |
| 168982_i_at | 0.00 |
| 168983_at | 0.00 |
| 168984_r_at | 0.00 |
| 168985_at | 0.00 |
| 168986_r_at | 0.00 |
| 168987_r_at | 0.00 |
| 168988_at | 0.00 |
| 168989_r_at | 0.00 |
| 168990_r_at | 0.00 |
| 168991_r_at | 0.00 |
| 168992_at | 0.00 |
| 168993_at | 0.00 |
| 168994_r_at | 0.00 |
| 168995_r_at | 0.00 |
| 168996_at | 0.00 |
| 168997_at | 0.00 |
| 168998_at | 0.00 |
| 168999_at | 0.00 |
| 169000_at | 0.00 |
| 169001_f_at | 0.00 |
| 169002_r_at | 0.00 |
| 169003_f_at | 0.00 |
| 169004_at | 0.00 |
| 169005_f_at | 0.00 |
| 169006_i_at | 0.00 |
| 169007_r_at | 0.00 |
| 169008_r_at | 0.00 |

| | |
|---|---|
| 169009_at | 0.00 |
| 169010_at | 0.00 |
| 169011_i_at | 0.00 |
| 169012_s_at | 0.05 |
| 169013_r_at | 0.00 |
| 169014_at | 0.00 |
| 169015_at | 0.00 |
| 169016_at | 0.00 |
| 169017_at | 0.00 |
| 169018_at | 0.00 |
| 169019_r_at | 0.00 |
| 169020_at | 0.00 |
| 169021_r_at | 0.00 |
| 169022_r_at | 0.00 |
| 169023_r_at | 0.00 |
| 169024_f_at | 0.00 |
| 169025_i_at | 0.00 |
| 169026_i_at | 0.00 |
| 169027_i_at | 0.00 |
| 169028_at | 0.00 |
| 169029_i_at | 0.00 |
| 169030_r_at | 0.00 |
| 169031_r_at | 0.00 |
| 169032_r_at | 0.00 |
| 169033_r_at | 0.00 |
| 169034_r_at | 0.00 |
| 169035_f_at | 0.00 |
| 169036_at | 0.00 |
| 169037_at | 0.00 |
| 169038_at | 0.00 |
| 169039_at | 0.00 |
| 169040_s_at | 0.00 |
| 169041_i_at | 0.00 |
| 169042_r_at | 0.00 |
| 169043_r_at | 0.00 |
| 169044_i_at | 0.00 |
| 169045_i_at | 0.00 |
| 169046_i_at | 0.00 |
| 169047_at | 0.00 |
| 169048_f_at | 0.00 |
| 169049_r_at | 0.00 |
| 169050_r_at | 0.00 |
| 169051_at | 0.00 |
| 169052_r_at | 0.00 |
| 169053_at | 0.00 |
| 169054_at | 0.00 |
| 169055_r_at | 0.00 |
| 169056_i_at | 0.00 |
| 169057_at | 0.00 |
| 169058_i_at | 0.00 |
| 169059_at | 0.00 |
| 169060_i_at | 0.00 |
| 169061_r_at | 0.00 |
| 169062_at | 0.00 |
| 169063_at | 0.00 |
| 169064_r_at | 0.00 |

| | |
|---|---|
| 169065_at | 0.00 |
| 169066_at | 0.00 |
| 169067_at | 0.00 |
| 169068_i_at | 0.00 |
| 169069_i_at | 0.00 |
| 169070_f_at | 0.00 |
| 169071_i_at | 0.00 |
| 169072_at | 0.00 |
| 169073_r_at | 0.00 |
| 169074_at | 0.00 |
| 169075_at | 0.00 |
| 169076_i_at | 0.00 |
| 169077_r_at | 0.00 |
| 169078_at | 0.00 |
| 169079_i_at | 0.00 |
| 169080_r_at | 0.00 |
| 169081_at | 0.00 |
| 169082_at | 0.00 |
| 169083_at | 0.00 |
| 169084_i_at | 0.00 |
| 169085_i_at | 0.00 |
| 169086_r_at | 0.00 |
| 169087_at | 0.00 |
| 169088_at | 0.00 |
| 169089_i_at | 0.00 |
| 169090_i_at | 0.01 |
| 169091_f_at | 0.00 |
| 169092_at | 0.00 |
| 169093_i_at | 0.00 |
| 169094_at | 0.00 |
| 169095_i_at | 0.00 |
| 169096_i_at | 0.00 |
| 169097_at | 0.00 |
| 169098_i_at | 0.00 |
| 169099_r_at | 0.00 |
| 169100_at | 0.00 |
| 169101_at | 0.00 |
| 169102_r_at | 0.00 |
| 169103_at | 0.00 |
| 169104_r_at | 0.00 |
| 169105_r_at | 0.00 |
| 169106_at | 0.00 |
| 169107_r_at | 0.00 |
| 169108_r_at | 0.00 |
| 169109_at | 0.00 |
| 169110_at | 0.00 |
| 169111_r_at | 0.00 |
| 169112_r_at | 0.00 |
| 169113_at | 0.00 |
| 169114_r_at | 0.00 |
| 169115_i_at | 0.00 |
| 169116_at | 0.00 |
| 169117_i_at | 0.00 |
| 169118_f_at | 0.00 |
| 169119_i_at | 0.00 |
| 169120_r_at | 0.00 |

| | |
|---|---|
| 169121_at | 0.00 |
| 169122_i_at | 0.00 |
| 169123_r_at | 0.00 |
| 169124_at | 0.00 |
| 169125_at | 0.00 |
| 169126_at | 0.00 |
| 169127_at | 0.00 |
| 169128_r_at | 0.00 |
| 169129_at | 0.00 |
| 169130_i_at | 0.00 |
| 169131_r_at | 0.00 |
| 169132_at | 0.00 |
| 169133_r_at | 0.00 |
| 169134_i_at | 0.00 |
| 169135_r_at | 0.00 |
| 169136_i_at | 0.00 |
| 169137_at | 0.00 |
| 169138_at | 0.00 |
| 169139_r_at | 0.00 |
| 169140_r_at | 0.00 |
| 169141_i_at | 0.00 |
| 169142_r_at | 0.00 |
| 169143_i_at | 0.00 |
| 169144_i_at | 0.00 |
| 169145_at | 0.00 |
| 169146_i_at | 0.00 |
| 169147_at | 0.00 |
| 169148_at | 0.00 |
| 169149_at | 0.00 |
| 169150_i_at | 0.00 |
| 169151_at | 0.00 |
| 169152_r_at | 0.00 |
| 169153_at | 0.00 |
| 169154_i_at | 0.00 |
| 169155_f_at | 0.00 |
| 169156_r_at | 0.00 |
| 169157_at | 0.00 |
| 169158_at | 0.00 |
| 169159_i_at | 0.00 |
| 169160_at | 0.00 |
| 169161_r_at | 0.00 |
| 169162_at | 0.00 |
| 169163_at | 0.00 |
| 169164_at | 0.00 |
| 169165_i_at | 0.03 |
| 169166_r_at | 0.00 |
| 169167_at | 0.00 |
| 169168_i_at | 0.00 |
| 169169_at | 0.00 |
| 169170_at | 0.00 |
| 169171_i_at | 0.00 |
| 169172_i_at | 0.02 |
| 169173_i_at | 0.00 |
| 169174_r_at | 0.00 |
| 169175_i_at | 0.00 |
| 169176_r_at | 0.00 |

| | |
|---|---|
| 169177_at | 0.00 |
| 169178_r_at | 0.00 |
| 169179_i_at | 0.00 |
| 169180_at | 0.00 |
| 169181_at | 0.00 |
| 169182_f_at | 0.00 |
| 169183_i_at | 0.00 |
| 169184_at | 0.00 |
| 169185_r_at | 0.00 |
| 169186_i_at | 0.00 |
| 169187_at | 0.00 |
| 169188_i_at | 0.00 |
| 169189_i_at | 0.00 |
| 169190_f_at | 0.00 |
| 169191_i_at | 0.00 |
| 169192_i_at | 0.00 |
| 169193_f_at | 0.00 |
| 169194_r_at | 0.00 |
| 169195_i_at | 0.00 |
| 169196_r_at | 0.00 |
| 169197_i_at | 0.00 |
| 169198_at | 0.00 |
| 169199_at | 0.00 |
| 169200_at | 0.00 |
| 169201_r_at | 0.00 |
| 169202_i_at | 0.00 |
| 169203_at | 0.00 |
| 169204_i_at | 0.00 |
| 169205_at | 0.00 |
| 169206_i_at | 0.00 |
| 169207_i_at | 0.00 |
| 169208_i_at | 0.00 |
| 169209_at | 0.00 |
| 169210_at | 0.00 |
| 169211_at | 0.00 |
| 169212_r_at | 0.00 |
| 169213_at | 0.00 |
| 169214_at | 0.00 |
| 169215_r_at | 0.00 |
| 169216_f_at | 0.00 |
| 169217_i_at | 0.00 |
| 169218_at | 0.00 |
| 169219_f_at | 0.00 |
| 169220_i_at | 0.00 |
| 169221_f_at | 0.00 |
| 169222_r_at | 0.00 |
| 169223_at | 0.00 |
| 169224_f_at | 0.00 |
| 169225_at | 0.00 |
| 169226_r_at | 0.00 |
| 169227_i_at | 0.00 |
| 169228_r_at | 0.00 |
| 169229_at | 0.00 |
| 169230_at | 0.00 |
| 169231_at | 0.00 |
| 169232_i_at | 0.00 |

| | |
|---|---|
| 169233_at | 0.00 |
| 169234_r_at | 0.00 |
| 169235_r_at | 0.00 |
| 169236_r_at | 0.00 |
| 169237_i_at | 0.00 |
| 169238_at | 0.00 |
| 169239_at | 0.00 |
| 169240_at | 0.00 |
| 169241_i_at | 0.00 |
| 169242_at | 0.00 |
| 169243_r_at | 0.00 |
| 169244_r_at | 0.00 |
| 169245_at | 0.00 |
| 169246_i_at | 0.00 |
| 169247_at | 0.00 |
| 169248_r_at | 0.00 |
| 169249_i_at | 0.00 |
| 169250_r_at | 0.00 |
| 169251_r_at | 0.00 |
| 169252_at | 0.00 |
| 169253_f_at | 0.01 |
| 169254_at | 0.00 |
| 169255_r_at | 0.00 |
| 169256_i_at | 0.00 |
| 169257_at | 0.00 |
| 169258_i_at | 0.00 |
| 169259_f_at | 0.14 |
| 169260_i_at | 0.00 |
| 169261_at | 0.00 |
| 169262_at | 0.00 |
| 169263_at | 0.00 |
| 169264_r_at | 0.00 |
| 169265_i_at | 0.00 |
| 169266_at | 0.00 |
| 169267_at | 0.00 |
| 169268_r_at | 0.00 |
| 169269_i_at | 0.00 |
| 169270_at | 0.00 |
| 169271_r_at | 0.00 |
| 169272_i_at | 0.00 |
| 169273_r_at | 0.00 |
| 169274_r_at | 0.00 |
| 169275_at | 0.00 |
| 169276_at | 0.00 |
| 169277_i_at | 0.00 |
| 169278_at | 0.00 |
| 169279_at | 0.00 |
| 169280_at | 0.00 |
| 169281_at | 0.00 |
| 169282_f_at | 0.01 |
| 169283_at | 0.00 |
| 169284_at | 0.00 |
| 169285_f_at | 0.00 |
| 169286_r_at | 0.00 |
| 169287_i_at | 0.00 |
| 169288_i_at | 0.00 |

| | |
|---|---|
| 169289_s_at | 0.00 |
| 169290_r_at | 0.00 |
| 169291_at | 0.00 |
| 169292_r_at | 0.00 |
| 169293_i_at | 0.00 |
| 169294_s_at | 0.00 |
| 169295_at | 0.00 |
| 169296_at | 0.00 |
| 169297_r_at | 0.00 |
| 169298_i_at | 0.00 |
| 169299_f_at | 0.00 |
| 169300_at | 0.00 |
| 169301_r_at | 0.00 |
| 169302_at | 0.00 |
| 169303_at | 0.00 |
| 169304_at | 0.00 |
| 169305_i_at | 0.00 |
| 169306_i_at | 0.00 |
| 169307_f_at | 0.00 |
| 169308_at | 0.00 |
| 169309_i_at | 0.00 |
| 169310_r_at | 0.00 |
| 169311_at | 0.01 |
| 169312_f_at | 0.00 |
| 169313_r_at | 0.00 |
| 169314_at | 0.00 |
| 169315_r_at | 0.00 |
| 169316_i_at | 0.00 |
| 169317_at | 0.00 |
| 169318_r_at | 0.00 |
| 169319_r_at | 0.00 |
| 169320_at | 0.00 |
| 169321_r_at | 0.00 |
| 169322_r_at | 0.00 |
| 169323_r_at | 0.00 |
| 169324_at | 0.00 |
| 169325_i_at | 0.00 |
| 169326_f_at | 0.00 |
| 169327_r_at | 0.00 |
| 169328_r_at | 0.04 |
| 169329_at | 0.00 |
| 169330_r_at | 0.00 |
| 169331_at | 0.00 |
| 169332_at | 0.00 |
| 169333_at | 0.00 |
| 169334_at | 0.00 |
| 169335_at | 0.00 |
| 169336_r_at | 0.00 |
| 169337_at | 0.00 |
| 169338_at | 0.00 |
| 169339_r_at | 0.00 |
| 169340_at | 0.00 |
| 169341_at | 0.00 |
| 169342_i_at | 0.00 |
| 169343_at | 0.00 |
| 169344_r_at | 0.00 |

| | |
|---|---|
| 169345_at | 0.00 |
| 169346_at | 0.00 |
| 169347_at | 0.00 |
| 169348_at | 0.00 |
| 169349_at | 0.00 |
| 169350_at | 0.00 |
| 169351_r_at | 0.00 |
| 169352_at | 0.00 |
| 169353_i_at | 0.00 |
| 169354_at | 0.00 |
| 169355_at | 0.00 |
| 169356_i_at | 0.00 |
| 169357_r_at | 0.00 |
| 169358_i_at | 0.00 |
| 169359_at | 0.00 |
| 169360_r_at | 0.00 |
| 169361_at | 0.00 |
| 169362_f_at | 0.00 |
| 169363_at | 0.00 |
| 169364_at | 0.00 |
| 169365_r_at | 0.00 |
| 169366_r_at | 0.00 |
| 169367_r_at | 0.00 |
| 169368_at | 0.00 |
| 169369_i_at | 0.00 |
| 169370_at | 0.00 |
| 169371_at | 0.00 |
| 169372_i_at | 0.00 |
| 169373_r_at | 0.00 |
| 169374_at | 0.00 |
| 169375_f_at | 0.00 |
| 169376_r_at | 0.00 |
| 169377_at | 0.00 |
| 169378_at | 0.00 |
| 169379_i_at | 0.00 |
| 169380_r_at | 0.00 |
| 169381_at | 0.00 |
| 169382_r_at | 0.00 |
| 169383_r_at | 0.00 |
| 169384_at | 0.00 |
| 169385_r_at | 0.00 |
| 169386_at | 0.00 |
| 169387_f_at | 0.00 |
| 169388_at | 0.00 |
| 169389_at | 0.00 |
| 169390_at | 0.00 |
| 169391_r_at | 0.00 |
| 169392_at | 0.00 |
| 169393_at | 0.00 |
| 169394_i_at | 0.00 |
| 169395_at | 0.00 |
| 169396_r_at | 0.00 |
| 169397_at | 0.00 |
| 169398_i_at | 0.04 |
| 169399_at | 0.00 |
| 169400_f_at | 0.00 |

| | |
|---|---|
| 169401_r_at | 0.00 |
| 169402_r_at | 0.01 |
| 169403_r_at | 0.00 |
| 169404_at | 0.00 |
| 169405_r_at | 0.00 |
| 169406_i_at | 0.00 |
| 169407_i_at | 0.00 |
| 169408_at | 0.00 |
| 169409_at | 0.00 |
| 169410_at | 0.00 |
| 169411_at | 0.00 |
| 169412_at | 0.00 |
| 169413_at | 0.00 |
| 169414_f_at | 0.00 |
| 169415_at | 0.00 |
| 169416_i_at | 0.00 |
| 169417_r_at | 0.00 |
| 169418_i_at | 0.00 |
| 169419_i_at | 0.00 |
| 169420_at | 0.00 |
| 169421_at | 0.00 |
| 169422_at | 0.00 |
| 169423_i_at | 0.00 |
| 169424_i_at | 0.00 |
| 169425_i_at | 0.00 |
| 169426_r_at | 0.00 |
| 169427_i_at | 0.00 |
| 169428_i_at | 0.00 |
| 169429_f_at | 0.00 |
| 169430_at | 0.00 |
| 169431_r_at | 0.00 |
| 169432_r_at | 0.00 |
| 169433_i_at | 0.00 |
| 169434_f_at | 0.00 |
| 169435_at | 0.00 |
| 169436_at | 0.00 |
| 169437_at | 0.00 |
| 169438_r_at | 0.00 |
| 169439_r_at | 0.00 |
| 169440_i_at | 0.00 |
| 169441_at | 0.00 |
| 169442_i_at | 0.00 |
| 169443_at | 0.00 |
| 169444_at | 0.00 |
| 169445_r_at | 0.00 |
| 169446_at | 0.00 |
| 169447_r_at | 0.00 |
| 169448_r_at | 0.00 |
| 169449_at | 0.00 |
| 169450_f_at | 0.00 |
| 169451_i_at | 0.00 |
| 169452_at | 0.00 |
| 169453_at | 0.00 |
| 169454_at | 0.00 |
| 169455_at | 0.00 |
| 169456_at | 0.00 |

| | |
|---|---|
| 169457_r_at | 0.00 |
| 169458_at | 0.00 |
| 169459_i_at | 0.00 |
| 169460_i_at | 0.00 |
| 169461_r_at | 0.00 |
| 169462_r_at | 0.00 |
| 169463_at | 0.00 |
| 169464_f_at | 0.01 |
| 169465_s_at | 0.02 |
| 169466_r_at | 0.00 |
| 169467_at | 0.00 |
| 169468_i_at | 0.00 |
| 169469_at | 0.00 |
| 169470_at | 0.00 |
| 169471_at | 0.00 |
| 169472_at | 0.00 |
| 169473_r_at | 0.00 |
| 169474_at | 0.00 |
| 169475_r_at | 0.00 |
| 169476_at | 0.00 |
| 169477_at | 0.00 |
| 169478_at | 0.00 |
| 169479_r_at | 0.00 |
| 169480_i_at | 0.00 |
| 169481_f_at | 0.00 |
| 169482_f_at | 0.00 |
| 169483_at | 0.00 |
| 169484_at | 0.00 |
| 169485_at | 0.00 |
| 169486_at | 0.00 |
| 169487_i_at | 0.00 |
| 169488_at | 0.00 |
| 169489_r_at | 0.00 |
| 169490_r_at | 0.00 |
| 169491_r_at | 0.00 |
| 169492_at | 0.00 |
| 169493_r_at | 0.00 |
| 169494_i_at | 0.00 |
| 169495_at | 0.00 |
| 169496_r_at | 0.00 |
| 169497_at | 0.00 |
| 169498_r_at | 0.00 |
| 169499_r_at | 0.00 |
| 169500_at | 0.00 |
| 169501_r_at | 0.00 |
| 169502_r_at | 0.00 |
| 169503_r_at | 0.00 |
| 169504_r_at | 0.00 |
| 169505_r_at | 0.00 |
| 169506_r_at | 0.00 |
| 169507_f_at | 0.00 |
| 169508_r_at | 0.00 |
| 169509_i_at | 0.00 |
| 169510_i_at | 0.00 |
| 169511_at | 0.00 |
| 169512_i_at | 0.00 |

| | |
|---|---|
| 169513_i_at | 0.00 |
| 169514_r_at | 0.00 |
| 169515_r_at | 0.00 |
| 169516_at | 0.00 |
| 169517_i_at | 0.00 |
| 169518_at | 0.00 |
| 169519_r_at | 0.00 |
| 169520_at | 0.00 |
| 169521_r_at | 0.00 |
| 169522_r_at | 0.00 |
| 169523_at | 0.00 |
| 169524_r_at | 0.00 |
| 169525_at | 0.00 |
| 169526_i_at | 0.00 |
| 169527_i_at | 0.01 |
| 169528_i_at | 0.00 |
| 169529_i_at | 0.00 |
| 169530_i_at | 0.00 |
| 169531_i_at | 0.00 |
| 169532_r_at | 0.00 |
| 169533_at | 0.00 |
| 169534_i_at | 0.00 |
| 169535_at | 0.00 |
| 169536_at | 0.04 |
| 169537_at | 0.00 |
| 169538_i_at | 0.00 |
| 169539_i_at | 0.00 |
| 169540_f_at | 0.00 |
| 169541_i_at | 0.00 |
| 169542_r_at | 0.00 |
| 169543_f_at | 0.00 |
| 169544_at | 0.00 |
| 169545_r_at | 0.00 |
| 169546_r_at | 0.00 |
| 169547_at | 0.00 |
| 169548_i_at | 0.00 |
| 169549_at | 0.00 |
| 169550_f_at | 0.00 |
| 169551_at | 0.00 |
| 169552_r_at | 0.00 |
| 169553_r_at | 0.00 |
| 169554_at | 0.00 |
| 169555_r_at | 0.00 |
| 169556_at | 0.00 |
| 169557_at | 0.00 |
| 169558_i_at | 0.00 |
| 169559_at | 0.00 |
| 169560_at | 0.00 |
| 169561_i_at | 0.00 |
| 169562_r_at | 0.00 |
| 169563_i_at | 0.00 |
| 169564_at | 0.00 |
| 169565_at | 0.00 |
| 169566_i_at | 0.00 |
| 169567_at | 0.00 |
| 169568_at | 0.00 |

| | |
|---|---|
| 169569_f_at | 0.00 |
| 169570_f_at | 0.00 |
| 169571_at | 0.00 |
| 169572_r_at | 0.00 |
| 169573_at | 0.00 |
| 169574_at | 0.00 |
| 169575_at | 0.00 |
| 169576_r_at | 0.00 |
| 169577_i_at | 0.00 |
| 169578_at | 0.00 |
| 169579_at | 0.00 |
| 169580_at | 0.00 |
| 169581_at | 0.00 |
| 169582_i_at | 0.00 |
| 169583_at | 0.00 |
| 169584_at | 0.00 |
| 169585_i_at | 0.00 |
| 169586_i_at | 0.00 |
| 169587_r_at | 0.00 |
| 169588_r_at | 0.00 |
| 169589_at | 0.00 |
| 169590_at | 0.00 |
| 169591_i_at | 0.00 |
| 169592_at | 0.00 |
| 169593_at | 0.00 |
| 169594_r_at | 0.00 |
| 169595_at | 0.00 |
| 169596_at | 0.00 |
| 169597_at | 0.00 |
| 169598_r_at | 0.00 |
| 169599_at | 0.00 |
| 169600_at | 0.00 |
| 169601_i_at | 0.00 |
| 169602_f_at | 0.00 |
| 169603_i_at | 0.00 |
| 169604_at | 0.00 |
| 169605_at | 0.00 |
| 169606_r_at | 0.00 |
| 169607_i_at | 0.00 |
| 169608_at | 0.00 |
| 169609_r_at | 0.00 |
| 169610_r_at | 0.00 |
| 169611_r_at | 0.00 |
| 169612_r_at | 0.00 |
| 169613_at | 0.00 |
| 169614_at | 0.00 |
| 169615_at | 0.00 |
| 169616_i_at | 0.00 |
| 169617_i_at | 0.00 |
| 169618_r_at | 0.00 |
| 169619_r_at | 0.00 |
| 169620_i_at | 0.00 |
| 169621_i_at | 0.00 |
| 169622_at | 0.00 |
| 169623_i_at | 0.00 |
| 169624_r_at | 0.00 |

| | |
|---|---|
| 169625_at | 0.00 |
| 169626_i_at | 0.00 |
| 169627_at | 0.00 |
| 169628_at | 0.00 |
| 169629_i_at | 0.00 |
| 169630_r_at | 0.00 |
| 169631_r_at | 0.00 |
| 169632_i_at | 0.01 |
| 169633_i_at | 0.00 |
| 169634_at | 0.00 |
| 169635_r_at | 0.00 |
| 169636_f_at | 0.00 |
| 169637_at | 0.00 |
| 169638_i_at | 0.02 |
| 169639_i_at | 0.00 |
| 169640_i_at | 0.00 |
| 169641_r_at | 0.00 |
| 169642_i_at | 0.00 |
| 169643_at | 0.00 |
| 169644_at | 0.00 |
| 169645_r_at | 0.00 |
| 169646_at | 0.00 |
| 169647_i_at | 0.00 |
| 169648_at | 0.00 |
| 169649_r_at | 0.00 |
| 169650_r_at | 0.00 |
| 169651_i_at | 0.00 |
| 169652_at | 0.00 |
| 169653_r_at | 0.00 |
| 169654_at | 0.00 |
| 169655_at | 0.00 |
| 169656_at | 0.00 |
| 169657_i_at | 0.00 |
| 169658_at | 0.00 |
| 169659_r_at | 0.00 |
| 169660_r_at | 0.00 |
| 169661_r_at | 0.00 |
| 169662_at | 0.00 |
| 169663_at | 0.00 |
| 169664_at | 0.00 |
| 169665_at | 0.00 |
| 169666_i_at | 0.00 |
| 169667_f_at | 0.02 |
| 169668_f_at | 0.00 |
| 169669_f_at | 0.00 |
| 169670_at | 0.00 |
| 169671_at | 0.00 |
| 169672_at | 0.00 |
| 169673_r_at | 0.00 |
| 169674_at | 0.00 |
| 169675_at | 0.00 |
| 169676_at | 0.00 |
| 169677_at | 0.00 |
| 169678_f_at | 0.00 |
| 169679_at | 0.00 |
| 169680_at | 0.00 |

| | |
|---|---|
| 169681_at | 0.00 |
| 169682_f_at | 0.00 |
| 169683_at | 0.00 |
| 169684_r_at | 0.00 |
| 169685_at | 0.00 |
| 169686_r_at | 0.00 |
| 169687_at | 0.00 |
| 169688_r_at | 0.00 |
| 169689_at | 0.00 |
| 169690_at | 0.00 |
| 169691_r_at | 0.00 |
| 169692_r_at | 0.00 |
| 169693_r_at | 0.00 |
| 169694_at | 0.00 |
| 169695_r_at | 0.00 |
| 169696_at | 0.00 |
| 169697_i_at | 0.00 |
| 169698_at | 0.00 |
| 169699_at | 0.00 |
| 169700_at | 0.00 |
| 169701_r_at | 0.00 |
| 169702_at | 0.00 |
| 169703_i_at | 0.00 |
| 169704_r_at | 0.00 |
| 169705_at | 0.00 |
| 169706_at | 0.00 |
| 169707_at | 0.00 |
| 169708_r_at | 0.00 |
| 169709_r_at | 0.00 |
| 169710_i_at | 0.01 |
| 169711_at | 0.00 |
| 169712_at | 0.00 |
| 169713_r_at | 0.00 |
| 169714_at | 0.00 |
| 169715_at | 0.00 |
| 169716_at | 0.00 |
| 169717_r_at | 0.00 |
| 169718_f_at | 0.20 |
| 169719_r_at | 0.00 |
| 169720_i_at | 0.00 |
| 169721_f_at | 0.00 |
| 169722_r_at | 0.00 |
| 169723_i_at | 0.00 |
| 169724_r_at | 0.00 |
| 169725_at | 0.00 |
| 169726_i_at | 0.00 |
| 169727_i_at | 0.00 |
| 169728_at | 0.00 |
| 169729_at | 0.00 |
| 169730_i_at | 0.00 |
| 169731_r_at | 0.00 |
| 169732_i_at | 0.00 |
| 169733_at | 0.00 |
| 169734_at | 0.00 |
| 169735_at | 0.00 |
| 169736_r_at | 0.00 |

| | |
|---|---|
| 169737_r_at | 0.00 |
| 169738_at | 0.00 |
| 169739_at | 0.00 |
| 169740_at | 0.00 |
| 169741_at | 0.00 |
| 169742_i_at | 0.00 |
| 169743_at | 0.00 |
| 169744_i_at | 0.00 |
| 169745_f_at | 0.00 |
| 169746_at | 0.00 |
| 169747_i_at | 0.00 |
| 169748_r_at | 0.00 |
| 169749_r_at | 0.00 |
| 169750_at | 0.00 |
| 169751_r_at | 0.00 |
| 169752_r_at | 0.00 |
| 169753_at | 0.00 |
| 169754_at | 0.00 |
| 169755_at | 0.00 |
| 169756_r_at | 0.00 |
| 169757_at | 0.00 |
| 169758_i_at | 0.00 |
| 169759_r_at | 0.00 |
| 169760_at | 0.00 |
| 169761_r_at | 0.00 |
| 169762_at | 0.00 |
| 169763_i_at | 0.00 |
| 169764_i_at | 0.00 |
| 169765_at | 0.00 |
| 169766_r_at | 0.00 |
| 169767_at | 0.00 |
| 169768_at | 0.00 |
| 169769_r_at | 0.00 |
| 169770_i_at | 0.00 |
| 169771_r_at | 0.01 |
| 169772_r_at | 0.00 |
| 169773_r_at | 0.00 |
| 169774_at | 0.00 |
| 169775_r_at | 0.00 |
| 169776_r_at | 0.00 |
| 169777_r_at | 0.00 |
| 169778_at | 0.00 |
| 169779_at | 0.00 |
| 169780_i_at | 0.00 |
| 169781_at | 0.00 |
| 169782_r_at | 0.00 |
| 169783_at | 0.00 |
| 169784_at | 0.00 |
| 169785_at | 0.00 |
| 169786_i_at | 0.00 |
| 169787_i_at | 0.00 |
| 169788_r_at | 0.00 |
| 169789_i_at | 0.00 |
| 169790_i_at | 0.00 |
| 169791_at | 0.00 |
| 169792_at | 0.00 |

| | |
|---|---|
| 169793_at | 0.00 |
| 169794_i_at | 0.00 |
| 169795_i_at | 0.00 |
| 169796_i_at | 0.00 |
| 169797_at | 0.00 |
| 169798_r_at | 0.00 |
| 169799_r_at | 0.00 |
| 169800_i_at | 0.00 |
| 169801_at | 0.00 |
| 169802_i_at | 0.00 |
| 169803_at | 0.00 |
| 169804_r_at | 0.00 |
| 169805_at | 0.00 |
| 169806_r_at | 0.00 |
| 169807_r_at | 0.00 |
| 169808_i_at | 0.00 |
| 169809_at | 0.00 |
| 169810_at | 0.00 |
| 169811_i_at | 0.00 |
| 169812_at | 0.00 |
| 169813_f_at | 0.00 |
| 169814_at | 0.00 |
| 169815_i_at | 0.00 |
| 169816_i_at | 0.00 |
| 169817_at | 0.00 |
| 169818_at | 0.00 |
| 169819_i_at | 0.00 |
| 169820_r_at | 0.00 |
| 169821_i_at | 0.00 |
| 169822_f_at | 0.00 |
| 169823_at | 0.00 |
| 169824_r_at | 0.00 |
| 169825_i_at | 0.00 |
| 169826_at | 0.00 |
| 169827_i_at | 0.00 |
| 169828_f_at | 0.00 |
| 169829_r_at | 0.00 |
| 169830_r_at | 0.00 |
| 169831_at | 0.00 |
| 169832_r_at | 0.00 |
| 169833_i_at | 0.00 |
| 169834_f_at | 0.00 |
| 169835_at | 0.00 |
| 169836_r_at | 0.00 |
| 169837_i_at | 0.00 |
| 169838_at | 0.00 |
| 169839_f_at | 0.00 |
| 169840_i_at | 0.00 |
| 169841_at | 0.00 |
| 169842_r_at | 0.00 |
| 169843_at | 0.00 |
| 169844_r_at | 0.00 |
| 169845_f_at | 0.00 |
| 169846_at | 0.00 |
| 169847_r_at | 0.00 |
| 169848_at | 0.00 |

| | |
|---|---|
| 169849_at | 0.00 |
| 169850_i_at | 0.00 |
| 169851_r_at | 0.00 |
| 169852_i_at | 0.00 |
| 169853_f_at | 0.00 |
| 169854_at | 0.00 |
| 169855_i_at | 0.00 |
| 169856_i_at | 0.00 |
| 169857_i_at | 0.00 |
| 169858_i_at | 0.00 |
| 169859_at | 0.00 |
| 169860_r_at | 0.00 |
| 169861_r_at | 0.00 |
| 169862_at | 0.00 |
| 169863_at | 0.00 |
| 169864_at | 0.00 |
| 169865_r_at | 0.00 |
| 169866_r_at | 0.00 |
| 169867_r_at | 0.00 |
| 169868_at | 0.00 |
| 169869_at | 0.00 |
| 169870_f_at | 0.00 |
| 169871_at | 0.00 |
| 169872_at | 0.00 |
| 169873_r_at | 0.00 |
| 169874_i_at | 0.00 |
| 169875_at | 0.00 |
| 169876_at | 0.00 |
| 169877_at | 0.01 |
| 169878_at | 0.00 |
| 169879_at | 0.00 |
| 169880_s_at | 0.01 |
| 169881_r_at | 0.00 |
| 169882_r_at | 0.00 |
| 169883_r_at | 0.00 |
| 169884_i_at | 0.00 |
| 169885_at | 0.00 |
| 169886_at | 0.00 |
| 169887_at | 0.00 |
| 169888_i_at | 0.00 |
| 169889_at | 0.00 |
| 169890_i_at | 0.00 |
| 169891_i_at | 0.00 |
| 169892_i_at | 0.01 |
| 169893_at | 0.00 |
| 169894_i_at | 0.00 |
| 169895_at | 0.00 |
| 169896_at | 0.00 |
| 169897_i_at | 0.00 |
| 169898_r_at | 0.00 |
| 169899_at | 0.00 |
| 169900_at | 0.00 |
| 169901_i_at | 0.00 |
| 169902_at | 0.00 |
| 169903_r_at | 0.00 |
| 169904_r_at | 0.00 |

| | |
|---|---|
| 169905_at | 0.00 |
| 169906_r_at | 0.00 |
| 169907_r_at | 0.00 |
| 169908_i_at | 0.00 |
| 169909_i_at | 0.00 |
| 169910_i_at | 0.00 |
| 169911_at | 0.00 |
| 169912_at | 0.00 |
| 169913_i_at | 0.00 |
| 169914_i_at | 0.00 |
| 169915_r_at | 0.00 |
| 169916_at | 0.00 |
| 169917_i_at | 0.00 |
| 169918_r_at | 0.00 |
| 169919_r_at | 0.00 |
| 169920_at | 0.00 |
| 169921_i_at | 0.00 |
| 169922_at | 0.00 |
| 169923_r_at | 0.00 |
| 169924_r_at | 0.00 |
| 169925_i_at | 0.00 |
| 169926_at | 0.00 |
| 169927_i_at | 0.00 |
| 169928_r_at | 0.00 |
| 169929_i_at | 0.00 |
| 169930_at | 0.00 |
| 169931_at | 0.00 |
| 169932_at | 0.00 |
| 169933_at | 0.00 |
| 169934_i_at | 0.00 |
| 169935_i_at | 0.00 |
| 169936_at | 0.00 |
| 169937_i_at | 0.00 |
| 169938_at | 0.00 |
| 169939_r_at | 0.00 |
| 169940_i_at | 0.00 |
| 169941_i_at | 0.00 |
| 169942_at | 0.00 |
| 169943_f_at | 0.00 |
| 169944_at | 0.00 |
| 169945_r_at | 0.00 |
| 169946_at | 0.00 |
| 169947_r_at | 0.00 |
| 169948_at | 0.00 |
| 169949_i_at | 0.00 |
| 169950_r_at | 0.00 |
| 169951_r_at | 0.00 |
| 169952_at | 0.00 |
| 169953_r_at | 0.00 |
| 169954_i_at | 0.00 |
| 169955_at | 0.00 |
| 169956_i_at | 0.00 |
| 169957_at | 0.00 |
| 169958_at | 0.00 |
| 169959_at | 0.00 |
| 169960_at | 0.00 |

| | |
|---|---|
| 169961_at | 0.00 |
| 169962_i_at | 0.00 |
| 169963_at | 0.00 |
| 169964_at | 0.00 |
| 169965_at | 0.00 |
| 169966_at | 0.00 |
| 169967_i_at | 0.00 |
| 169968_i_at | 0.00 |
| 169969_at | 0.00 |
| 169970_i_at | 0.00 |
| 169971_at | 0.00 |
| 169972_at | 0.00 |
| 169973_at | 0.00 |
| 169974_at | 0.00 |
| 169975_r_at | 0.00 |
| 169976_at | 0.00 |
| 169977_i_at | 0.01 |
| 169978_at | 0.00 |
| 169979_i_at | 0.00 |
| 169980_i_at | 0.00 |
| 169981_i_at | 0.00 |
| 169982_i_at | 0.00 |
| 169983_at | 0.00 |
| 169984_i_at | 0.00 |
| 169985_i_at | 0.00 |
| 169986_r_at | 0.00 |
| 169987_i_at | 0.00 |
| 169988_at | 0.00 |
| 169989_r_at | 0.00 |
| 169990_r_at | 0.00 |
| 169991_i_at | 0.00 |
| 169992_i_at | 0.00 |
| 169993_r_at | 0.00 |
| 169994_at | 0.00 |
| 169995_i_at | 0.00 |
| 169996_at | 0.00 |
| 169997_i_at | 0.00 |
| 169998_at | 0.00 |
| 169999_at | 0.00 |
| 170000_r_at | 0.00 |
| 170001_at | 0.00 |
| 170002_at | 0.00 |
| 170003_at | 0.00 |
| 170004_at | 0.00 |
| 170005_at | 0.00 |
| 170006_i_at | 0.00 |
| 170007_i_at | 0.00 |
| 170008_i_at | 0.00 |
| 170009_r_at | 0.00 |
| 170010_r_at | 0.00 |
| 170011_at | 0.00 |
| 170012_r_at | 0.00 |
| 170013_i_at | 0.00 |
| 170014_at | 0.00 |
| 170015_i_at | 0.00 |
| 170016_at | 0.00 |

| | |
|---|---|
| 170017_r_at | 0.00 |
| 170018_r_at | 0.00 |
| 170019_i_at | 0.01 |
| 170020_i_at | 0.00 |
| 170021_i_at | 0.00 |
| 170022_i_at | 0.00 |
| 170023_r_at | 0.00 |
| 170024_r_at | 0.00 |
| 170025_at | 0.00 |
| 170026_at | 0.00 |
| 170027_at | 0.00 |
| 170028_r_at | 0.00 |
| 170029_at | 0.00 |
| 170030_at | 0.00 |
| 170031_f_at | 0.00 |
| 170032_at | 0.00 |
| 170033_r_at | 0.00 |
| 170034_i_at | 0.00 |
| 170035_at | 0.00 |
| 170036_at | 0.00 |
| 170037_r_at | 0.00 |
| 170038_at | 0.00 |
| 170039_i_at | 0.01 |
| 170040_at | 0.00 |
| 170041_i_at | 0.00 |
| 170042_i_at | 0.00 |
| 170043_at | 0.00 |
| 170044_s_at | 0.01 |
| 170045_r_at | 0.00 |
| 170046_at | 0.00 |
| 170047_r_at | 0.00 |
| 170048_at | 0.00 |
| 170049_at | 0.00 |
| 170050_i_at | 0.00 |
| 170051_at | 0.00 |
| 170052_at | 0.00 |
| 170053_i_at | 0.00 |
| 170054_at | 0.00 |
| 170055_at | 0.00 |
| 170056_i_at | 0.00 |
| 170057_at | 0.00 |
| 170058_at | 0.00 |
| 170059_r_at | 0.00 |
| 170060_r_at | 0.00 |
| 170061_at | 0.00 |
| 170062_i_at | 0.00 |
| 170063_at | 0.00 |
| 170064_f_at | 0.22 |
| 170065_at | 0.00 |
| 170066_at | 0.01 |
| 170067_i_at | 0.00 |
| 170068_at | 0.00 |
| 170069_at | 0.00 |
| 170070_r_at | 0.00 |
| 170071_r_at | 0.00 |
| 170072_r_at | 0.00 |

| | |
|---|---|
| 170073_at | 0.00 |
| 170074_at | 0.00 |
| 170075_at | 0.00 |
| 170076_r_at | 0.00 |
| 170077_r_at | 0.00 |
| 170078_i_at | 0.00 |
| 170079_i_at | 0.00 |
| 170080_i_at | 0.00 |
| 170081_at | 0.00 |
| 170082_i_at | 0.00 |
| 170083_r_at | 0.00 |
| 170084_r_at | 0.00 |
| 170085_at | 0.00 |
| 170086_at | 0.00 |
| 170087_i_at | 0.00 |
| 170088_at | 0.00 |
| 170089_at | 0.00 |
| 170090_at | 0.00 |
| 170091_f_at | 0.00 |
| 170092_at | 0.00 |
| 170093_i_at | 0.00 |
| 170094_r_at | 0.00 |
| 170095_r_at | 0.00 |
| 170096_r_at | 0.00 |
| 170097_at | 0.00 |
| 170098_r_at | 0.00 |
| 170099_at | 0.00 |
| 170100_at | 0.00 |
| 170101_at | 0.00 |
| 170102_at | 0.00 |
| 170103_at | 0.00 |
| 170104_at | 0.00 |
| 170105_i_at | 0.00 |
| 170106_at | 0.00 |
| 170107_at | 0.00 |
| 170108_at | 0.00 |
| 170109_at | 0.00 |
| 170110_r_at | 0.00 |
| 170111_i_at | 0.00 |
| 170112_r_at | 0.00 |
| 170113_i_at | 0.00 |
| 170114_i_at | 0.00 |
| 170115_i_at | 0.00 |
| 170116_at | 0.00 |
| 170117_at | 0.00 |
| 170118_r_at | 0.00 |
| 170119_r_at | 0.00 |
| 170120_r_at | 0.00 |
| 170121_at | 0.00 |
| 170122_at | 0.00 |
| 170123_at | 0.00 |
| 170124_at | 0.00 |
| 170125_at | 0.00 |
| 170126_at | 0.00 |
| 170127_i_at | 0.00 |
| 170128_i_at | 0.00 |

| | |
|---|---|
| 170129_r_at | 0.00 |
| 170130_f_at | 0.00 |
| 170131_at | 0.00 |
| 170132_at | 0.00 |
| 170133_at | 0.00 |
| 170134_i_at | 0.00 |
| 170135_r_at | 0.00 |
| 170136_r_at | 0.00 |
| 170137_f_at | 0.14 |
| 170138_r_at | 0.00 |
| 170139_i_at | 0.00 |
| 170140_i_at | 0.00 |
| 170141_at | 0.00 |
| 170142_at | 0.00 |
| 170143_at | 0.00 |
| 170144_at | 0.00 |
| 170145_at | 0.00 |
| 170146_r_at | 0.00 |
| 170147_r_at | 0.03 |
| 170148_at | 0.00 |
| 170149_r_at | 0.00 |
| 170150_r_at | 0.00 |
| 170151_r_at | 0.00 |
| 170152_at | 0.00 |
| 170153_r_at | 0.00 |
| 170154_at | 0.00 |
| 170155_r_at | 0.00 |
| 170156_at | 0.00 |
| 170157_at | 0.00 |
| 170158_r_at | 0.00 |
| 170159_at | 0.00 |
| 170160_r_at | 0.00 |
| 170161_at | 0.00 |
| 170162_at | 0.00 |
| 170163_at | 0.00 |
| 170164_r_at | 0.00 |
| 170165_at | 0.00 |
| 170166_r_at | 0.00 |
| 170167_at | 0.00 |
| 170168_at | 0.00 |
| 170169_at | 0.00 |
| 170170_at | 0.00 |
| 170171_r_at | 0.00 |
| 170172_at | 0.00 |
| 170173_at | 0.00 |
| 170174_r_at | 0.00 |
| 170175_at | 0.00 |
| 170176_r_at | 0.00 |
| 170177_r_at | 0.00 |
| 170178_at | 0.00 |
| 170179_at | 0.00 |
| 170180_at | 0.00 |
| 170181_at | 0.00 |
| 170182_at | 0.00 |
| 170183_at | 0.00 |
| 170184_r_at | 0.00 |

| | |
|---|---|
| 170185_i_at | 0.00 |
| 170186_i_at | 0.00 |
| 170187_at | 0.00 |
| 170188_at | 0.00 |
| 170189_at | 0.00 |
| 170190_at | 0.00 |
| 170191_at | 0.00 |
| 170192_at | 0.00 |
| 170193_i_at | 0.00 |
| 170194_at | 0.00 |
| 170195_at | 0.00 |
| 170196_at | 0.00 |
| 170197_r_at | 0.00 |
| 170198_at | 0.00 |
| 170199_i_at | 0.00 |
| 170200_i_at | 0.00 |
| 170201_r_at | 0.00 |
| 170202_s_at | 0.00 |
| 170203_at | 0.00 |
| 170204_at | 0.00 |
| 170205_at | 0.00 |
| 170206_at | 0.00 |
| 170207_i_at | 0.00 |
| 170208_at | 0.00 |
| 170209_at | 0.00 |
| 170210_i_at | 0.00 |
| 170211_r_at | 0.00 |
| 170212_i_at | 0.00 |
| 170213_r_at | 0.00 |
| 170214_at | 0.00 |
| 170215_i_at | 0.00 |
| 170216_at | 0.00 |
| 170217_r_at | 0.00 |
| 170218_at | 0.00 |
| 170219_i_at | 0.00 |
| 170220_r_at | 0.00 |
| 170221_at | 0.00 |
| 170222_f_at | 0.00 |
| 170223_f_at | 0.00 |
| 170224_at | 0.00 |
| 170225_f_at | 0.00 |
| 170226_i_at | 0.00 |
| 170227_at | 0.00 |
| 170228_r_at | 0.00 |
| 170229_i_at | 0.00 |
| 170230_at | 0.00 |
| 170231_i_at | 0.00 |
| 170232_at | 0.00 |
| 170233_at | 0.03 |
| 170234_r_at | 0.00 |
| 170235_r_at | 0.00 |
| 170236_r_at | 0.00 |
| 170237_r_at | 0.00 |
| 170238_r_at | 0.00 |
| 170239_f_at | 0.00 |
| 170240_f_at | 0.00 |

| | |
|---|---|
| 170241_f_at | 0.00 |
| 170242_at | 0.00 |
| 170243_r_at | 0.00 |
| 170244_at | 0.00 |
| 170245_r_at | 0.00 |
| 170246_at | 0.00 |
| 170247_r_at | 0.00 |
| 170248_at | 0.00 |
| 170249_f_at | 0.00 |
| 170250_i_at | 0.00 |
| 170251_at | 0.00 |
| 170252_at | 0.00 |
| 170253_i_at | 0.00 |
| 170254_at | 0.00 |
| 170255_i_at | 0.00 |
| 170256_at | 0.00 |
| 170257_i_at | 0.00 |
| 170258_r_at | 0.00 |
| 170259_i_at | 0.00 |
| 170260_at | 0.00 |
| 170261_at | 0.00 |
| 170262_at | 0.01 |
| 170263_f_at | 0.00 |
| 170264_at | 0.00 |
| 170265_i_at | 0.00 |
| 170266_at | 0.00 |
| 170267_f_at | 0.00 |
| 170268_at | 0.00 |
| 170269_i_at | 0.00 |
| 170270_r_at | 0.00 |
| 170271_at | 0.00 |
| 170272_r_at | 0.00 |
| 170273_at | 0.00 |
| 170274_f_at | 0.02 |
| 170275_at | 0.00 |
| 170276_at | 0.00 |
| 170277_at | 0.00 |
| 170278_at | 0.00 |
| 170279_r_at | 0.00 |
| 170280_at | 0.00 |
| 170281_at | 0.00 |
| 170282_i_at | 0.00 |
| 170283_at | 0.00 |
| 170284_r_at | 0.00 |
| 170285_at | 0.00 |
| 170286_i_at | 0.00 |
| 170287_at | 0.00 |
| 170288_r_at | 0.00 |
| 170289_at | 0.00 |
| 170290_f_at | 0.00 |
| 170291_at | 0.00 |
| 170292_at | 0.00 |
| 170293_at | 0.00 |
| 170294_at | 0.00 |
| 170295_at | 0.00 |
| 170296_i_at | 0.00 |

| | |
|---|---|
| 170297_at | 0.00 |
| 170298_at | 0.00 |
| 170299_at | 0.00 |
| 170300_i_at | 0.00 |
| 170301_at | 0.00 |
| 170302_r_at | 0.00 |
| 170303_at | 0.00 |
| 170304_at | 0.00 |
| 170305_at | 0.00 |
| 170306_at | 0.00 |
| 170307_i_at | 0.00 |
| 170308_at | 0.00 |
| 170309_r_at | 0.00 |
| 170310_i_at | 0.00 |
| 170311_f_at | 0.00 |
| 170312_r_at | 0.00 |
| 170313_at | 0.00 |
| 170314_f_at | 0.00 |
| 170315_at | 0.00 |
| 170316_i_at | 0.00 |
| 170317_i_at | 0.00 |
| 170318_i_at | 0.01 |
| 170319_r_at | 0.00 |
| 170320_at | 0.00 |
| 170321_at | 0.00 |
| 170322_at | 0.00 |
| 170323_i_at | 0.00 |
| 170324_r_at | 0.00 |
| 170325_f_at | 0.00 |
| 170326_i_at | 0.00 |
| 170327_i_at | 0.00 |
| 170328_at | 0.00 |
| 170329_at | 0.00 |
| 170330_at | 0.00 |
| 170331_i_at | 0.00 |
| 170332_r_at | 0.00 |
| 170333_at | 0.00 |
| 170334_r_at | 0.00 |
| 170335_i_at | 0.00 |
| 170336_at | 0.00 |
| 170337_r_at | 0.00 |
| 170338_at | 0.00 |
| 170339_at | 0.00 |
| 170340_at | 0.00 |
| 170341_at | 0.00 |
| 170342_at | 0.00 |
| 170343_at | 0.00 |
| 170344_at | 0.00 |
| 170345_at | 0.00 |
| 170346_at | 0.00 |
| 170347_f_at | 0.09 |
| 170348_i_at | 0.00 |
| 170349_at | 0.00 |
| 170350_at | 0.00 |
| 170351_at | 0.00 |
| 170352_r_at | 0.00 |

| | |
|---|---|
| 170353_r_at | 0.00 |
| 170354_at | 0.00 |
| 170355_r_at | 0.00 |
| 170356_r_at | 0.00 |
| 170357_r_at | 0.00 |
| 170358_f_at | 0.00 |
| 170359_at | 0.00 |
| 170360_r_at | 0.00 |
| 170361_at | 0.00 |
| 170362_i_at | 0.00 |
| 170363_r_at | 0.00 |
| 170364_i_at | 0.00 |
| 170365_at | 0.00 |
| 170366_at | 0.00 |
| 170367_i_at | 0.00 |
| 170368_r_at | 0.00 |
| 170369_at | 0.00 |
| 170370_at | 0.00 |
| 170371_i_at | 0.00 |
| 170372_at | 0.00 |
| 170373_at | 0.00 |
| 170374_at | 0.00 |
| 170375_r_at | 0.00 |
| 170376_at | 0.00 |
| 170377_at | 0.00 |
| 170378_f_at | 0.00 |
| 170379_r_at | 0.00 |
| 170380_at | 0.00 |
| 170381_at | 0.00 |
| 170382_r_at | 0.00 |
| 170383_at | 0.00 |
| 170384_r_at | 0.00 |
| 170385_r_at | 0.00 |
| 170386_at | 0.00 |
| 170387_r_at | 0.00 |
| 170388_i_at | 0.00 |
| 170389_at | 0.00 |
| 170390_at | 0.00 |
| 170391_at | 0.00 |
| 170392_at | 0.00 |
| 170393_r_at | 0.00 |
| 170394_i_at | 0.00 |
| 170395_at | 0.00 |
| 170396_i_at | 0.00 |
| 170397_i_at | 0.00 |
| 170398_r_at | 0.00 |
| 170399_at | 0.00 |
| 170400_i_at | 0.00 |
| 170401_at | 0.00 |
| 170402_at | 0.00 |
| 170403_i_at | 0.00 |
| 170404_i_at | 0.00 |
| 170405_at | 0.00 |
| 170406_r_at | 0.00 |
| 170407_at | 0.00 |
| 170408_at | 0.00 |

| | |
|---|---|
| 170409_i_at | 0.00 |
| 170410_at | 0.00 |
| 170411_at | 0.00 |
| 170412_f_at | 0.00 |
| 170413_at | 0.00 |
| 170414_i_at | 0.00 |
| 170415_r_at | 0.00 |
| 170416_r_at | 0.00 |
| 170417_at | 0.00 |
| 170418_r_at | 0.00 |
| 170419_at | 0.00 |
| 170420_r_at | 0.00 |
| 170421_at | 0.00 |
| 170422_at | 0.00 |
| 170423_f_at | 0.00 |
| 170424_at | 0.00 |
| 170425_at | 0.00 |
| 170426_f_at | 0.00 |
| 170427_at | 0.00 |
| 170428_i_at | 0.00 |
| 170429_i_at | 0.00 |
| 170430_f_at | 0.00 |
| 170431_r_at | 0.00 |
| 170432_at | 0.00 |
| 170433_r_at | 0.00 |
| 170434_at | 0.00 |
| 170435_at | 0.00 |
| 170436_i_at | 0.00 |
| 170437_at | 0.00 |
| 170438_at | 0.00 |
| 170439_at | 0.00 |
| 170440_at | 0.00 |
| 170441_at | 0.00 |
| 170442_at | 0.00 |
| 170443_r_at | 0.00 |
| 170444_i_at | 0.00 |
| 170445_r_at | 0.00 |
| 170446_i_at | 0.00 |
| 170447_f_at | 0.00 |
| 170448_f_at | 0.00 |
| 170449_at | 0.00 |
| 170450_r_at | 0.00 |
| 170451_at | 0.00 |
| 170452_at | 0.00 |
| 170453_r_at | 0.00 |
| 170454_at | 0.00 |
| 170455_at | 0.00 |
| 170456_r_at | 0.00 |
| 170457_at | 0.00 |
| 170458_r_at | 0.00 |
| 170459_at | 0.00 |
| 170460_at | 0.00 |
| 170461_i_at | 0.00 |
| 170462_r_at | 0.00 |
| 170463_r_at | 0.00 |
| 170464_r_at | 0.00 |

| | |
|---|---|
| 170465_r_at | 0.00 |
| 170466_r_at | 0.00 |
| 170467_r_at | 0.00 |
| 170468_f_at | 0.26 |
| 170469_r_at | 0.00 |
| 170470_r_at | 0.00 |
| 170471_i_at | 0.00 |
| 170472_at | 0.00 |
| 170473_at | 0.00 |
| 170474_i_at | 0.00 |
| 170475_r_at | 0.00 |
| 170476_r_at | 0.00 |
| 170477_r_at | 0.00 |
| 170478_at | 0.00 |
| 170479_r_at | 0.00 |
| 170480_at | 0.00 |
| 170481_at | 0.00 |
| 170482_at | 0.00 |
| 170483_r_at | 0.00 |
| 170484_at | 0.00 |
| 170485_at | 0.00 |
| 170486_i_at | 0.00 |
| 170487_r_at | 0.01 |
| 170488_r_at | 0.00 |
| 170489_r_at | 0.00 |
| 170490_i_at | 0.00 |
| 170491_r_at | 0.00 |
| 170492_at | 0.00 |
| 170493_i_at | 0.00 |
| 170494_i_at | 0.00 |
| 170495_at | 0.00 |
| 170496_r_at | 0.00 |
| 170497_at | 0.00 |
| 170498_at | 0.00 |
| 170499_at | 0.00 |
| 170500_at | 0.00 |
| 170501_r_at | 0.00 |
| 170502_at | 0.00 |
| 170503_r_at | 0.00 |
| 170504_i_at | 0.00 |
| 170505_at | 0.00 |
| 170506_r_at | 0.00 |
| 170507_at | 0.00 |
| 170508_r_at | 0.00 |
| 170509_at | 0.00 |
| 170510_i_at | 0.00 |
| 170511_i_at | 0.00 |
| 170512_at | 0.00 |
| 170513_at | 0.00 |
| 170514_at | 0.00 |
| 170515_at | 0.00 |
| 170516_at | 0.00 |
| 170517_at | 0.00 |
| 170518_at | 0.00 |
| 170519_at | 0.00 |
| 170520_r_at | 0.00 |

| | |
|---|---|
| 170521_r_at | 0.00 |
| 170522_r_at | 0.00 |
| 170523_i_at | 0.00 |
| 170524_f_at | 0.00 |
| 170525_r_at | 0.00 |
| 170526_i_at | 0.00 |
| 170527_i_at | 0.00 |
| 170528_at | 0.00 |
| 170529_at | 0.00 |
| 170530_f_at | 0.03 |
| 170531_r_at | 0.00 |
| 170532_r_at | 0.00 |
| 170533_at | 0.00 |
| 170534_i_at | 0.00 |
| 170535_i_at | 0.00 |
| 170536_at | 0.00 |
| 170537_r_at | 0.01 |
| 170538_i_at | 0.00 |
| 170539_r_at | 0.00 |
| 170540_at | 0.00 |
| 170541_i_at | 0.00 |
| 170542_at | 0.00 |
| 170543_r_at | 0.00 |
| 170544_at | 0.00 |
| 170545_i_at | 0.00 |
| 170546_r_at | 0.00 |
| 170547_at | 0.00 |
| 170548_r_at | 0.00 |
| 170549_r_at | 0.00 |
| 170550_i_at | 0.00 |
| 170551_at | 0.00 |
| 170552_at | 0.00 |
| 170553_at | 0.00 |
| 170554_at | 0.00 |
| 170555_at | 0.00 |
| 170556_at | 0.00 |
| 170557_r_at | 0.00 |
| 170558_at | 0.00 |
| 170559_at | 0.00 |
| 170560_r_at | 0.00 |
| 170561_at | 0.00 |
| 170562_at | 0.00 |
| 170563_i_at | 0.00 |
| 170564_at | 0.00 |
| 170565_i_at | 0.00 |
| 170566_at | 0.00 |
| 170567_i_at | 0.00 |
| 170568_r_at | 0.00 |
| 170569_at | 0.00 |
| 170570_i_at | 0.00 |
| 170571_i_at | 0.00 |
| 170572_r_at | 0.00 |
| 170573_at | 0.00 |
| 170574_i_at | 0.00 |
| 170575_i_at | 0.00 |
| 170576_r_at | 0.01 |

| | |
|---|---|
| 170577_i_at | 0.00 |
| 170578_r_at | 0.00 |
| 170579_at | 0.00 |
| 170580_at | 0.00 |
| 170581_at | 0.00 |
| 170582_r_at | 0.00 |
| 170583_at | 0.00 |
| 170584_at | 0.00 |
| 170585_r_at | 0.00 |
| 170586_r_at | 0.00 |
| 170587_at | 0.00 |
| 170588_at | 0.00 |
| 170589_at | 0.00 |
| 170590_at | 0.00 |
| 170591_f_at | 0.00 |
| 170592_r_at | 0.00 |
| 170593_r_at | 0.00 |
| 170594_at | 0.00 |
| 170595_at | 0.00 |
| 170596_at | 0.00 |
| 170597_r_at | 0.00 |
| 170598_r_at | 0.00 |
| 170599_i_at | 0.00 |
| 170600_at | 0.00 |
| 170601_at | 0.00 |
| 170602_at | 0.00 |
| 170603_at | 0.00 |
| 170604_at | 0.00 |
| 170605_at | 0.00 |
| 170606_at | 0.00 |
| 170607_at | 0.00 |
| 170608_at | 0.00 |
| 170609_at | 0.00 |
| 170610_at | 0.00 |
| 170611_at | 0.00 |
| 170612_at | 0.00 |
| 170613_r_at | 0.00 |
| 170614_at | 0.00 |
| 170615_at | 0.00 |
| 170616_r_at | 0.00 |
| 170617_at | 0.00 |
| 170618_at | 0.00 |
| 170619_r_at | 0.00 |
| 170620_at | 0.00 |
| 170621_i_at | 0.00 |
| 170622_at | 0.00 |
| 170623_r_at | 0.00 |
| 170624_at | 0.00 |
| 170625_i_at | 0.00 |
| 170626_i_at | 0.00 |
| 170627_i_at | 0.00 |
| 170628_at | 0.00 |
| 170629_at | 0.00 |
| 170630_at | 0.00 |
| 170631_i_at | 0.00 |
| 170632_at | 0.00 |

| | |
|---|---|
| 170633_at | 0.00 |
| 170634_r_at | 0.00 |
| 170635_at | 0.00 |
| 170636_at | 0.00 |
| 170637_at | 0.00 |
| 170638_at | 0.00 |
| 170639_i_at | 0.00 |
| 170640_at | 0.00 |
| 170641_at | 0.00 |
| 170642_at | 0.00 |
| 170643_i_at | 0.00 |
| 170644_at | 0.00 |
| 170645_i_at | 0.00 |
| 170646_at | 0.00 |
| 170647_at | 0.00 |
| 170648_f_at | 0.00 |
| 170649_at | 0.00 |
| 170650_at | 0.00 |
| 170651_i_at | 0.00 |
| 170652_i_at | 0.00 |
| 170653_f_at | 0.00 |
| 170654_r_at | 0.00 |
| 170655_r_at | 0.00 |
| 170656_f_at | 0.00 |
| 170657_r_at | 0.00 |
| 170658_at | 0.00 |
| 170659_at | 0.00 |
| 170660_at | 0.00 |
| 170661_i_at | 0.00 |
| 170662_at | 0.00 |
| 170663_at | 0.00 |
| 170664_i_at | 0.00 |
| 170665_at | 0.00 |
| 170666_i_at | 0.00 |
| 170667_at | 0.00 |
| 170668_r_at | 0.00 |
| 170669_at | 0.03 |
| 170670_i_at | 0.00 |
| 170671_at | 0.00 |
| 170672_r_at | 0.00 |
| 170673_at | 0.00 |
| 170674_i_at | 0.00 |
| 170675_at | 0.00 |
| 170676_r_at | 0.00 |
| 170677_r_at | 0.00 |
| 170678_i_at | 0.00 |
| 170679_r_at | 0.00 |
| 170680_at | 0.00 |
| 170681_i_at | 0.00 |
| 170682_i_at | 0.00 |
| 170683_i_at | 0.00 |
| 170684_at | 0.00 |
| 170685_at | 0.00 |
| 170686_f_at | 0.00 |
| 170687_r_at | 0.00 |
| 170688_at | 0.00 |

| | |
|---|---|
| 170689_r_at | 0.00 |
| 170690_at | 0.00 |
| 170691_r_at | 0.00 |
| 170692_r_at | 0.00 |
| 170693_r_at | 0.00 |
| 170694_r_at | 0.00 |
| 170695_at | 0.00 |
| 170696_at | 0.00 |
| 170697_at | 0.00 |
| 170698_f_at | 0.00 |
| 170699_i_at | 0.00 |
| 170700_at | 0.00 |
| 170701_at | 0.00 |
| 170702_at | 0.00 |
| 170703_at | 0.00 |
| 170704_i_at | 0.00 |
| 170705_r_at | 0.00 |
| 170706_r_at | 0.00 |
| 170707_i_at | 0.00 |
| 170708_f_at | 0.00 |
| 170709_at | 0.00 |
| 170710_at | 0.00 |
| 170711_r_at | 0.00 |
| 170712_at | 0.00 |
| 170713_i_at | 0.00 |
| 170714_at | 0.00 |
| 170715_r_at | 0.00 |
| 170716_at | 0.00 |
| 170717_at | 0.00 |
| 170718_at | 0.00 |
| 170719_at | 0.02 |
| 170720_r_at | 0.00 |
| 170721_r_at | 0.00 |
| 170722_at | 0.00 |
| 170723_s_at | 0.00 |
| 170724_r_at | 0.00 |
| 170725_at | 0.00 |
| 170726_r_at | 0.00 |
| 170727_at | 0.00 |
| 170728_i_at | 0.00 |
| 170729_at | 0.00 |
| 170730_r_at | 0.03 |
| 170731_at | 0.00 |
| 170732_r_at | 0.00 |
| 170733_i_at | 0.00 |
| 170734_at | 0.00 |
| 170735_f_at | 0.00 |
| 170736_at | 0.00 |
| 170737_i_at | 0.00 |
| 170738_i_at | 0.00 |
| 170739_at | 0.00 |
| 170740_r_at | 0.00 |
| 170741_at | 0.00 |
| 170742_i_at | 0.00 |
| 170743_at | 0.00 |
| 170744_at | 0.00 |

| | |
|---|---|
| 170745_i_at | 0.00 |
| 170746_f_at | 0.00 |
| 170747_at | 0.00 |
| 170748_r_at | 0.00 |
| 170749_r_at | 0.00 |
| 170750_i_at | 0.00 |
| 170751_r_at | 0.00 |
| 170752_at | 0.00 |
| 170753_at | 0.00 |
| 170754_at | 0.00 |
| 170755_at | 0.00 |
| 170756_at | 0.00 |
| 170757_at | 0.00 |
| 170758_i_at | 0.00 |
| 170759_at | 0.00 |
| 170760_at | 0.00 |
| 170761_i_at | 0.00 |
| 170762_at | 0.00 |
| 170763_i_at | 0.00 |
| 170764_at | 0.00 |
| 170765_s_at | 0.00 |
| 170766_f_at | 0.00 |
| 170767_f_at | 0.00 |
| 170768_at | 0.00 |
| 170769_r_at | 0.00 |
| 170770_r_at | 0.00 |
| 170771_i_at | 0.00 |
| 170772_at | 0.00 |
| 170773_at | 0.00 |
| 170774_at | 0.00 |
| 170775_at | 0.00 |
| 170776_i_at | 0.00 |
| 170777_at | 0.00 |
| 170778_at | 0.00 |
| 170779_f_at | 0.00 |
| 170780_i_at | 0.00 |
| 170781_r_at | 0.00 |
| 170782_r_at | 0.00 |
| 170783_i_at | 0.00 |
| 170784_at | 0.00 |
| 170785_r_at | 0.00 |
| 170786_at | 0.00 |
| 170787_r_at | 0.00 |
| 170788_r_at | 0.00 |
| 170789_r_at | 0.00 |
| 170790_at | 0.00 |
| 170791_r_at | 0.00 |
| 170792_at | 0.00 |
| 170793_at | 0.00 |
| 170794_i_at | 0.00 |
| 170795_i_at | 0.00 |
| 170796_i_at | 0.00 |
| 170797_at | 0.00 |
| 170798_at | 0.00 |
| 170799_at | 0.00 |
| 170800_at | 0.00 |

| | |
|---|---|
| 170801_at | 0.00 |
| 170802_i_at | 0.00 |
| 170803_r_at | 0.00 |
| 170804_at | 0.00 |
| 170805_r_at | 0.00 |
| 170806_i_at | 0.00 |
| 170807_at | 0.00 |
| 170808_s_at | 0.03 |
| 170809_at | 0.00 |
| 170810_at | 0.00 |
| 170811_i_at | 0.00 |
| 170812_at | 0.02 |
| 170813_i_at | 0.00 |
| 170814_at | 0.00 |
| 170815_at | 0.00 |
| 170816_at | 0.00 |
| 170817_f_at | 0.00 |
| 170818_i_at | 0.00 |
| 170819_i_at | 0.00 |
| 170820_r_at | 0.00 |
| 170821_i_at | 0.00 |
| 170822_r_at | 0.00 |
| 170823_at | 0.00 |
| 170824_i_at | 0.00 |
| 170825_i_at | 0.00 |
| 170826_at | 0.00 |
| 170827_r_at | 0.00 |
| 170828_at | 0.00 |
| 170829_at | 0.00 |
| 170830_at | 0.00 |
| 170831_at | 0.00 |
| 170832_at | 0.00 |
| 170833_at | 0.00 |
| 170834_at | 0.00 |
| 170835_at | 0.00 |
| 170836_at | 0.00 |
| 170837_f_at | 0.00 |
| 170838_at | 0.00 |
| 170839_at | 0.00 |
| 170840_at | 0.00 |
| 170841_at | 0.00 |
| 170842_at | 0.00 |
| 170843_r_at | 0.00 |
| 170844_i_at | 0.01 |
| 170845_r_at | 0.00 |
| 170846_at | 0.00 |
| 170847_i_at | 0.00 |
| 170848_at | 0.00 |
| 170849_f_at | 0.00 |
| 170850_f_at | 0.00 |
| 170851_i_at | 0.00 |
| 170852_at | 0.00 |
| 170853_i_at | 0.00 |
| 170854_i_at | 0.00 |
| 170855_r_at | 0.00 |
| 170856_f_at | 0.00 |

| | |
|---|---|
| 170857_at | 0.00 |
| 170858_i_at | 0.00 |
| 170859_at | 0.00 |
| 170860_at | 0.00 |
| 170861_i_at | 0.00 |
| 170862_at | 0.00 |
| 170863_i_at | 0.00 |
| 170864_i_at | 0.00 |
| 170865_i_at | 0.00 |
| 170866_at | 0.00 |
| 170867_r_at | 0.00 |
| 170868_at | 0.00 |
| 170869_at | 0.00 |
| 170870_at | 0.00 |
| 170871_f_at | 0.00 |
| 170872_at | 0.00 |
| 170873_i_at | 0.00 |
| 170874_i_at | 0.00 |
| 170875_at | 0.00 |
| 170876_r_at | 0.00 |
| 170877_r_at | 0.00 |
| 170878_at | 0.00 |
| 170879_at | 0.00 |
| 170880_at | 0.00 |
| 170881_at | 0.00 |
| 170882_r_at | 0.00 |
| 170883_i_at | 0.00 |
| 170884_at | 0.00 |
| 170885_at | 0.00 |
| 170886_at | 0.00 |
| 170887_at | 0.00 |
| 170888_at | 0.00 |
| 170889_at | 0.00 |
| 170890_f_at | 0.00 |
| 170891_at | 0.00 |
| 170892_r_at | 0.00 |
| 170893_at | 0.00 |
| 170894_at | 0.00 |
| 170895_r_at | 0.00 |
| 170896_at | 0.00 |
| 170897_r_at | 0.00 |
| 170898_at | 0.00 |
| 170899_at | 0.00 |
| 170900_at | 0.00 |
| 170901_i_at | 0.00 |
| 170902_r_at | 0.01 |
| 170903_at | 0.00 |
| 170904_r_at | 0.00 |
| 170905_r_at | 0.00 |
| 170906_r_at | 0.00 |
| 170907_at | 0.00 |
| 170908_f_at | 0.00 |
| 170909_r_at | 0.00 |
| 170910_r_at | 0.00 |
| 170911_at | 0.00 |
| 170912_r_at | 0.00 |

| | |
|---|---|
| 170913_f_at | 0.00 |
| 170914_at | 0.00 |
| 170915_r_at | 0.00 |
| 170916_i_at | 0.00 |
| 170917_r_at | 0.00 |
| 170918_at | 0.00 |
| 170919_at | 0.00 |
| 170920_at | 0.00 |
| 170921_f_at | 0.01 |
| 170922_i_at | 0.00 |
| 170923_r_at | 0.00 |
| 170924_f_at | 0.00 |
| 170925_i_at | 0.00 |
| 170926_at | 0.00 |
| 170927_r_at | 0.00 |
| 170928_r_at | 0.00 |
| 170929_at | 0.00 |
| 170930_r_at | 0.00 |
| 170931_i_at | 0.00 |
| 170932_at | 0.00 |
| 170933_r_at | 0.00 |
| 170934_r_at | 0.00 |
| 170935_r_at | 0.00 |
| 170936_i_at | 0.00 |
| 170937_r_at | 0.00 |
| 170938_i_at | 0.00 |
| 170939_at | 0.00 |
| 170940_at | 0.00 |
| 170941_r_at | 0.00 |
| 170942_at | 0.00 |
| 170943_r_at | 0.00 |
| 170944_at | 0.00 |
| 170945_f_at | 0.00 |
| 170946_r_at | 0.00 |
| 170947_at | 0.00 |
| 170948_i_at | 0.00 |
| 170949_at | 0.00 |
| 170950_at | 0.00 |
| 170951_at | 0.00 |
| 170952_at | 0.00 |
| 170953_f_at | 0.23 |
| 170954_at | 0.00 |
| 170955_at | 0.00 |
| 170956_at | 0.00 |
| 170957_at | 0.00 |
| 170958_i_at | 0.00 |
| 170959_r_at | 0.00 |
| 170960_i_at | 0.00 |
| 170961_at | 0.00 |
| 170962_r_at | 0.00 |
| 170963_i_at | 0.00 |
| 170964_r_at | 0.00 |
| 170965_i_at | 0.00 |
| 170966_r_at | 0.00 |
| 170967_i_at | 0.00 |
| 170968_at | 0.00 |

| | |
|---|---|
| 170969_i_at | 0.05 |
| 170970_i_at | 0.04 |
| 170971_at | 0.00 |
| 170972_at | 0.00 |
| 170973_at | 0.00 |
| 170974_r_at | 0.00 |
| 170975_i_at | 0.00 |
| 170976_r_at | 0.03 |
| 170977_at | 0.00 |
| 170978_r_at | 0.00 |
| 170979_r_at | 0.00 |
| 170980_r_at | 0.00 |
| 170981_r_at | 0.00 |
| 170982_at | 0.00 |
| 170983_at | 0.00 |
| 170984_at | 0.00 |
| 170985_at | 0.00 |
| 170986_f_at | 0.00 |
| 170987_r_at | 0.00 |
| 170988_r_at | 0.00 |
| 170989_r_at | 0.00 |
| 170990_i_at | 0.00 |
| 170991_at | 0.00 |
| 170992_at | 0.00 |
| 170993_at | 0.00 |
| 170994_r_at | 0.00 |
| 170995_f_at | 0.00 |
| 170996_f_at | 0.00 |
| 170997_at | 0.00 |
| 170998_i_at | 0.00 |
| 170999_r_at | 0.00 |
| 171000_at | 0.00 |
| 171001_at | 0.00 |
| 171002_r_at | 0.00 |
| 171003_r_at | 0.00 |
| 171004_r_at | 0.00 |
| 171005_r_at | 0.00 |
| 171006_i_at | 0.00 |
| 171007_i_at | 0.00 |
| 171008_i_at | 0.00 |
| 171009_i_at | 0.00 |
| 171010_at | 0.00 |
| 171011_r_at | 0.00 |
| 171012_r_at | 0.00 |
| 171013_at | 0.00 |
| 171014_r_at | 0.00 |
| 171015_r_at | 0.00 |
| 171016_at | 0.00 |
| 171017_i_at | 0.00 |
| 171018_at | 0.00 |
| 171019_at | 0.00 |
| 171020_at | 0.00 |
| 171021_at | 0.00 |
| 171022_i_at | 0.00 |
| 171023_at | 0.00 |
| 171024_i_at | 0.00 |

| | |
|---|---|
| 171025_at | 0.00 |
| 171026_at | 0.00 |
| 171027_r_at | 0.00 |
| 171028_r_at | 0.00 |
| 171029_at | 0.00 |
| 171030_r_at | 0.00 |
| 171031_at | 0.00 |
| 171032_f_at | 0.00 |
| 171033_r_at | 0.00 |
| 171034_at | 0.00 |
| 171035_at | 0.00 |
| 171036_at | 0.00 |
| 171037_at | 0.00 |
| 171038_i_at | 0.00 |
| 171039_at | 0.01 |
| 171040_r_at | 0.00 |
| 171041_r_at | 0.00 |
| 171042_at | 0.00 |
| 171043_r_at | 0.00 |
| 171044_at | 0.00 |
| 171045_r_at | 0.00 |
| 171046_r_at | 0.00 |
| 171047_i_at | 0.00 |
| 171048_i_at | 0.00 |
| 171049_at | 0.00 |
| 171050_at | 0.00 |
| 171051_at | 0.00 |
| 171052_at | 0.00 |
| 171053_at | 0.00 |
| 171054_i_at | 0.00 |
| 171055_at | 0.00 |
| 171056_at | 0.00 |
| 171057_r_at | 0.00 |
| 171058_i_at | 0.00 |
| 171059_r_at | 0.00 |
| 171060_at | 0.00 |
| 171061_at | 0.00 |
| 171062_at | 0.00 |
| 171063_at | 0.00 |
| 171064_r_at | 0.00 |
| 171065_at | 0.00 |
| 171066_at | 0.00 |
| 171067_f_at | 0.00 |
| 171068_f_at | 0.00 |
| 171069_at | 0.00 |
| 171070_r_at | 0.00 |
| 171071_i_at | 0.00 |
| 171072_i_at | 0.00 |
| 171073_at | 0.00 |
| 171074_at | 0.00 |
| 171075_at | 0.00 |
| 171076_i_at | 0.00 |
| 171077_i_at | 0.00 |
| 171078_i_at | 0.00 |
| 171079_at | 0.00 |
| 171080_at | 0.00 |

| | |
|---|---|
| 171081_at | 0.00 |
| 171082_at | 0.00 |
| 171083_at | 0.00 |
| 171084_at | 0.00 |
| 171085_at | 0.00 |
| 171086_at | 0.00 |
| 171087_f_at | 0.00 |
| 171088_at | 0.00 |
| 171089_at | 0.00 |
| 171090_i_at | 0.00 |
| 171091_f_at | 0.00 |
| 171092_r_at | 0.00 |
| 171093_r_at | 0.00 |
| 171094_i_at | 0.00 |
| 171095_r_at | 0.00 |
| 171096_i_at | 0.00 |
| 171097_at | 0.00 |
| 171098_r_at | 0.00 |
| 171099_r_at | 0.00 |
| 171100_at | 0.00 |
| 171101_i_at | 0.00 |
| 171102_i_at | 0.00 |
| 171103_at | 0.00 |
| 171104_i_at | 0.00 |
| 171105_at | 0.00 |
| 171106_r_at | 0.00 |
| 171107_at | 0.00 |
| 171108_f_at | 0.00 |
| 171109_at | 0.00 |
| 171110_at | 0.00 |
| 171111_at | 0.00 |
| 171112_r_at | 0.00 |
| 171113_at | 0.00 |
| 171114_at | 0.00 |
| 171115_r_at | 0.00 |
| 171116_r_at | 0.01 |
| 171117_i_at | 0.00 |
| 171118_at | 0.00 |
| 171119_at | 0.00 |
| 171120_at | 0.00 |
| 171121_r_at | 0.00 |
| 171122_i_at | 0.00 |
| 171123_at | 0.00 |
| 171124_at | 0.00 |
| 171125_at | 0.00 |
| 171126_f_at | 0.00 |
| 171127_f_at | 0.00 |
| 171128_at | 0.00 |
| 171129_r_at | 0.00 |
| 171130_f_at | 0.00 |
| 171131_r_at | 0.00 |
| 171132_r_at | 0.00 |
| 171133_i_at | 0.00 |
| 171134_at | 0.00 |
| 171135_at | 0.00 |
| 171136_r_at | 0.00 |

| | |
|---|---|
| 171137_i_at | 0.00 |
| 171138_i_at | 0.00 |
| 171139_at | 0.00 |
| 171140_i_at | 0.00 |
| 171141_at | 0.00 |
| 171142_i_at | 0.00 |
| 171143_r_at | 0.00 |
| 171144_i_at | 0.00 |
| 171145_at | 0.00 |
| 171146_r_at | 0.00 |
| 171147_r_at | 0.00 |
| 171148_at | 0.00 |
| 171149_r_at | 0.00 |
| 171150_at | 0.00 |
| 171151_at | 0.00 |
| 171152_at | 0.00 |
| 171153_r_at | 0.00 |
| 171154_at | 0.00 |
| 171155_i_at | 0.00 |
| 171156_at | 0.00 |
| 171157_r_at | 0.00 |
| 171158_i_at | 0.00 |
| 171159_at | 0.00 |
| 171160_r_at | 0.00 |
| 171161_i_at | 0.00 |
| 171162_i_at | 0.00 |
| 171163_at | 0.00 |
| 171164_r_at | 0.00 |
| 171165_r_at | 0.00 |
| 171166_r_at | 0.00 |
| 171167_i_at | 0.00 |
| 171168_r_at | 0.00 |
| 171169_at | 0.00 |
| 171170_r_at | 0.00 |
| 171171_r_at | 0.00 |
| 171172_at | 0.00 |
| 171173_at | 0.00 |
| 171174_at | 0.00 |
| 171175_at | 0.00 |
| 171176_r_at | 0.00 |
| 171177_s_at | 0.00 |
| 171178_r_at | 0.00 |
| 171179_at | 0.00 |
| 171180_at | 0.00 |
| 171181_r_at | 0.00 |
| 171182_at | 0.00 |
| 171183_at | 0.00 |
| 171184_at | 0.00 |
| 171185_r_at | 0.00 |
| 171186_at | 0.00 |
| 171187_i_at | 0.00 |
| 171188_f_at | 0.00 |
| 171189_r_at | 0.00 |
| 171190_f_at | 0.24 |
| 171191_r_at | 0.00 |
| 171192_i_at | 0.00 |

| | |
|---|---|
| 171193_r_at | 0.00 |
| 171194_at | 0.00 |
| 171195_f_at | 0.00 |
| 171196_i_at | 0.00 |
| 171197_i_at | 0.00 |
| 171198_at | 0.00 |
| 171199_r_at | 0.00 |
| 171200_r_at | 0.00 |
| 171201_at | 0.00 |
| 171202_at | 0.00 |
| 171203_at | 0.00 |
| 171204_at | 0.00 |
| 171205_at | 0.00 |
| 171206_f_at | 0.00 |
| 171207_at | 0.00 |
| 171208_r_at | 0.00 |
| 171209_at | 0.00 |
| 171210_r_at | 0.00 |
| 171211_at | 0.00 |
| 171212_at | 0.00 |
| 171213_i_at | 0.00 |
| 171214_at | 0.00 |
| 171215_at | 0.00 |
| 171216_r_at | 0.05 |
| 171217_f_at | 0.00 |
| 171218_i_at | 0.00 |
| 171219_r_at | 0.00 |
| 171220_at | 0.00 |
| 171221_at | 0.00 |
| 171222_at | 0.00 |
| 171223_i_at | 0.00 |
| 171224_r_at | 0.00 |
| 171225_i_at | 0.00 |
| 171226_s_at | 0.00 |
| 171227_r_at | 0.00 |
| 171228_at | 0.00 |
| 171229_i_at | 0.00 |
| 171230_at | 0.00 |
| 171231_i_at | 0.00 |
| 171232_r_at | 0.00 |
| 171233_at | 0.00 |
| 171234_at | 0.00 |
| 171235_at | 0.00 |
| 171236_r_at | 0.00 |
| 171237_at | 0.00 |
| 171238_r_at | 0.00 |
| 171239_i_at | 0.00 |
| 171240_at | 0.00 |
| 171241_i_at | 0.00 |
| 171242_i_at | 0.00 |
| 171243_at | 0.00 |
| 171244_at | 0.00 |
| 171245_r_at | 0.00 |
| 171246_at | 0.00 |
| 171247_i_at | 0.00 |
| 171248_f_at | 0.00 |

| | |
|---|---|
| 171249_i_at | 0.00 |
| 171250_at | 0.00 |
| 171251_at | 0.00 |
| 171252_f_at | 0.00 |
| 171253_at | 0.00 |
| 171254_r_at | 0.00 |
| 171255_at | 0.00 |
| 171256_at | 0.00 |
| 171257_i_at | 0.00 |
| 171258_r_at | 0.00 |
| 171259_r_at | 0.00 |
| 171260_at | 0.00 |
| 171261_r_at | 0.00 |
| 171262_r_at | 0.00 |
| 171263_at | 0.00 |
| 171264_at | 0.00 |
| 171265_f_at | 0.00 |
| 171266_r_at | 0.00 |
| 171267_i_at | 0.00 |
| 171268_r_at | 0.00 |
| 171269_i_at | 0.00 |
| 171270_r_at | 0.00 |
| 171271_at | 0.00 |
| 171272_r_at | 0.00 |
| 171273_r_at | 0.00 |
| 171274_at | 0.00 |
| 171275_i_at | 0.00 |
| 171276_at | 0.00 |
| 171277_at | 0.00 |
| 171278_i_at | 0.00 |
| 171279_i_at | 0.00 |
| 171280_i_at | 0.00 |
| 171281_at | 0.00 |
| 171282_i_at | 0.00 |
| 171283_r_at | 0.00 |
| 171284_r_at | 0.00 |
| 171285_at | 0.00 |
| 171286_r_at | 0.00 |
| 171287_i_at | 0.00 |
| 171288_at | 0.00 |
| 171289_at | 0.00 |
| 171290_r_at | 0.00 |
| 171291_i_at | 0.00 |
| 171292_at | 0.00 |
| 171293_r_at | 0.00 |
| 171294_at | 0.00 |
| 171295_r_at | 0.00 |
| 171296_f_at | 0.00 |
| 171297_i_at | 0.00 |
| 171298_i_at | 0.00 |
| 171299_i_at | 0.00 |
| 171300_i_at | 0.00 |
| 171301_at | 0.00 |
| 171302_r_at | 0.00 |
| 171303_at | 0.00 |
| 171304_i_at | 0.00 |

| | |
|---|---|
| 171305_at | 0.00 |
| 171306_at | 0.00 |
| 171307_r_at | 0.00 |
| 171308_at | 0.00 |
| 171309_r_at | 0.00 |
| 171310_i_at | 0.00 |
| 171311_r_at | 0.00 |
| 171312_r_at | 0.00 |
| 171313_i_at | 0.00 |
| 171314_r_at | 0.00 |
| 171315_at | 0.00 |
| 171316_r_at | 0.00 |
| 171317_at | 0.00 |
| 171318_at | 0.00 |
| 171319_r_at | 0.00 |
| 171320_i_at | 0.00 |
| 171321_r_at | 0.00 |
| 171322_at | 0.00 |
| 171323_i_at | 0.00 |
| 171324_at | 0.00 |
| 171325_at | 0.00 |
| 171326_at | 0.00 |
| 171327_at | 0.00 |
| 171328_at | 0.00 |
| 171329_at | 0.00 |
| 171330_at | 0.00 |
| 171331_at | 0.00 |
| 171332_r_at | 0.00 |
| 171333_at | 0.00 |
| 171334_at | 0.00 |
| 171335_i_at | 0.00 |
| 171336_i_at | 0.00 |
| 171337_f_at | 0.00 |
| 171338_i_at | 0.00 |
| 171339_i_at | 0.00 |
| 171340_at | 0.00 |
| 171341_at | 0.00 |
| 171342_at | 0.00 |
| 171343_at | 0.00 |
| 171344_at | 0.00 |
| 171345_i_at | 0.00 |
| 171346_i_at | 0.00 |
| 171347_at | 0.00 |
| 171348_at | 0.00 |
| 171349_i_at | 0.00 |
| 171350_i_at | 0.00 |
| 171351_i_at | 0.00 |
| 171352_at | 0.00 |
| 171353_i_at | 0.00 |
| 171354_at | 0.00 |
| 171355_at | 0.00 |
| 171356_at | 0.00 |
| 171357_at | 0.00 |
| 171358_i_at | 0.00 |
| 171359_at | 0.00 |
| 171360_at | 0.00 |

| | |
|---|---|
| 171361_at | 0.00 |
| 171362_r_at | 0.00 |
| 171363_at | 0.00 |
| 171364_at | 0.00 |
| 171365_at | 0.00 |
| 171366_r_at | 0.00 |
| 171367_r_at | 0.00 |
| 171368_r_at | 0.00 |
| 171369_f_at | 0.00 |
| 171370_i_at | 0.00 |
| 171371_r_at | 0.00 |
| 171372_r_at | 0.00 |
| 171373_r_at | 0.00 |
| 171374_at | 0.00 |
| 171375_at | 0.00 |
| 171376_r_at | 0.00 |
| 171377_i_at | 0.00 |
| 171378_i_at | 0.00 |
| 171379_r_at | 0.00 |
| 171380_f_at | 0.00 |
| 171381_i_at | 0.00 |
| 171382_i_at | 0.00 |
| 171383_at | 0.00 |
| 171384_at | 0.00 |
| 171385_at | 0.00 |
| 171386_i_at | 0.00 |
| 171387_r_at | 0.00 |
| 171388_at | 0.00 |
| 171389_at | 0.00 |
| 171390_i_at | 0.00 |
| 171391_at | 0.00 |
| 171392_at | 0.00 |
| 171393_at | 0.00 |
| 171394_r_at | 0.00 |
| 171395_i_at | 0.00 |
| 171396_at | 0.00 |
| 171397_at | 0.00 |
| 171398_r_at | 0.00 |
| 171399_r_at | 0.00 |
| 171400_at | 0.00 |
| 171401_r_at | 0.00 |
| 171402_at | 0.00 |
| 171403_at | 0.00 |
| 171404_r_at | 0.00 |
| 171405_r_at | 0.00 |
| 171406_at | 0.00 |
| 171407_i_at | 0.00 |
| 171408_f_at | 0.00 |
| 171409_r_at | 0.00 |
| 171410_r_at | 0.00 |
| 171411_i_at | 0.00 |
| 171412_at | 0.00 |
| 171413_at | 0.00 |
| 171414_r_at | 0.00 |
| 171415_at | 0.00 |
| 171416_i_at | 0.00 |

| | |
|---|---|
| 171417_r_at | 0.00 |
| 171418_at | 0.00 |
| 171419_at | 0.00 |
| 171420_r_at | 0.00 |
| 171421_r_at | 0.00 |
| 171422_at | 0.00 |
| 171423_i_at | 0.00 |
| 171424_i_at | 0.00 |
| 171425_at | 0.00 |
| 171426_at | 0.00 |
| 171427_at | 0.00 |
| 171428_r_at | 0.00 |
| 171429_r_at | 0.00 |
| 171430_at | 0.00 |
| 171431_at | 0.00 |
| 171432_at | 0.00 |
| 171433_i_at | 0.00 |
| 171434_at | 0.00 |
| 171435_at | 0.00 |
| 171436_at | 0.00 |
| 171437_f_at | 0.00 |
| 171438_r_at | 0.00 |
| 171439_at | 0.00 |
| 171440_at | 0.00 |
| 171441_i_at | 0.00 |
| 171442_i_at | 0.00 |
| 171443_at | 0.00 |
| 171444_r_at | 0.00 |
| 171445_i_at | 0.00 |
| 171446_i_at | 0.00 |
| 171447_at | 0.00 |
| 171448_at | 0.00 |
| 171449_at | 0.00 |
| 171450_at | 0.00 |
| 171451_at | 0.00 |
| 171452_at | 0.00 |
| 171453_f_at | 0.00 |
| 171454_r_at | 0.00 |
| 171455_at | 0.00 |
| 171456_at | 0.00 |
| 171457_at | 0.00 |
| 171458_i_at | 0.00 |
| 171459_i_at | 0.00 |
| 171460_i_at | 0.00 |
| 171461_f_at | 0.00 |
| 171462_r_at | 0.00 |
| 171463_r_at | 0.00 |
| 171464_i_at | 0.00 |
| 171465_r_at | 0.00 |
| 171466_r_at | 0.00 |
| 171467_i_at | 0.00 |
| 171468_i_at | 0.00 |
| 171469_r_at | 0.00 |
| 171470_r_at | 0.00 |
| 171471_r_at | 0.00 |
| 171472_i_at | 0.00 |

| | |
|---|---|
| 171473_i_at | 0.00 |
| 171474_at | 0.00 |
| 171475_at | 0.00 |
| 171476_at | 0.00 |
| 171477_r_at | 0.00 |
| 171478_at | 0.00 |
| 171479_at | 0.00 |
| 171480_at | 0.00 |
| 171481_at | 0.00 |
| 171482_r_at | 0.00 |
| 171483_i_at | 0.00 |
| 171484_i_at | 0.00 |
| 171485_at | 0.00 |
| 171486_at | 0.00 |
| 171487_at | 0.00 |
| 171488_at | 0.00 |
| 171489_i_at | 0.00 |
| 171490_at | 0.01 |
| 171491_s_at | 0.00 |
| 171492_i_at | 0.00 |
| 171493_at | 0.00 |
| 171494_at | 0.00 |
| 171495_r_at | 0.00 |
| 171496_i_at | 0.00 |
| 171497_f_at | 0.00 |
| 171498_at | 0.00 |
| 171499_at | 0.00 |
| 171500_at | 0.00 |
| 171501_at | 0.00 |
| 171502_r_at | 0.00 |
| 171503_i_at | 0.00 |
| 171504_i_at | 0.00 |
| 171505_f_at | 0.00 |
| 171506_r_at | 0.00 |
| 171507_r_at | 0.00 |
| 171508_r_at | 0.00 |
| 171509_i_at | 0.00 |
| 171510_i_at | 0.00 |
| 171511_at | 0.00 |
| 171512_i_at | 0.00 |
| 171513_i_at | 0.00 |
| 171514_at | 0.00 |
| 171515_at | 0.00 |
| 171516_r_at | 0.00 |
| 171517_at | 0.00 |
| 171518_i_at | 0.00 |
| 171519_r_at | 0.00 |
| 171520_r_at | 0.00 |
| 171521_at | 0.00 |
| 171522_r_at | 0.00 |
| 171523_r_at | 0.00 |
| 171524_at | 0.00 |
| 171525_r_at | 0.00 |
| 171526_r_at | 0.00 |
| 171527_at | 0.00 |
| 171528_at | 0.00 |

| | |
|---|---|
| 171529_at | 0.00 |
| 171530_r_at | 0.00 |
| 171531_r_at | 0.00 |
| 171532_at | 0.00 |
| 171533_i_at | 0.00 |
| 171534_f_at | 0.00 |
| 171535_r_at | 0.00 |
| 171536_i_at | 0.00 |
| 171537_r_at | 0.00 |
| 171538_f_at | 0.00 |
| 171539_f_at | 0.14 |
| 171540_r_at | 0.00 |
| 171541_at | 0.00 |
| 171542_at | 0.00 |
| 171543_i_at | 0.00 |
| 171544_at | 0.00 |
| 171545_at | 0.00 |
| 171546_at | 0.00 |
| 171547_at | 0.00 |
| 171548_at | 0.00 |
| 171549_at | 0.00 |
| 171550_at | 0.00 |
| 171551_i_at | 0.00 |
| 171552_at | 0.00 |
| 171553_r_at | 0.00 |
| 171554_r_at | 0.00 |
| 171555_at | 0.00 |
| 171556_at | 0.00 |
| 171557_i_at | 0.00 |
| 171558_i_at | 0.00 |
| 171559_i_at | 0.00 |
| 171560_r_at | 0.00 |
| 171561_at | 0.00 |
| 171562_at | 0.00 |
| 171563_i_at | 0.00 |
| 171564_i_at | 0.00 |
| 171565_i_at | 0.00 |
| 171566_at | 0.00 |
| 171567_r_at | 0.00 |
| 171568_i_at | 0.00 |
| 171569_at | 0.00 |
| 171570_i_at | 0.00 |
| 171571_f_at | 0.00 |
| 171572_at | 0.00 |
| 171573_r_at | 0.00 |
| 171574_r_at | 0.00 |
| 171575_at | 0.00 |
| 171576_at | 0.00 |
| 171577_at | 0.00 |
| 171578_at | 0.00 |
| 171579_r_at | 0.00 |
| 171580_r_at | 0.00 |
| 171581_f_at | 0.00 |
| 171582_r_at | 0.00 |
| 171583_i_at | 0.00 |
| 171584_f_at | 0.00 |

| | |
|---|---|
| 171585_at | 0.00 |
| 171586_at | 0.00 |
| 171587_i_at | 0.00 |
| 171588_f_at | 0.00 |
| 171589_at | 0.00 |
| 171590_at | 0.00 |
| 171591_at | 0.00 |
| 171592_at | 0.00 |
| 171593_at | 0.00 |
| 171594_i_at | 0.00 |
| 171595_at | 0.00 |
| 171596_at | 0.00 |
| 171597_at | 0.00 |
| 171598_f_at | 0.00 |
| 171599_f_at | 0.01 |
| 171600_r_at | 0.00 |
| 171601_at | 0.00 |
| 171602_r_at | 0.01 |
| 171603_at | 0.00 |
| 171604_at | 0.00 |
| 171605_i_at | 0.00 |
| 171606_r_at | 0.00 |
| 171607_r_at | 0.00 |
| 171608_i_at | 0.00 |
| 171609_r_at | 0.00 |
| 171610_at | 0.00 |
| 171611_i_at | 0.00 |
| 171612_i_at | 0.00 |
| 171613_at | 0.00 |
| 171614_r_at | 0.00 |
| 171615_r_at | 0.00 |
| 171616_at | 0.00 |
| 171617_at | 0.00 |
| 171618_f_at | 0.00 |
| 171619_i_at | 0.00 |
| 171620_at | 0.00 |
| 171621_r_at | 0.00 |
| 171622_f_at | 0.00 |
| 171623_i_at | 0.00 |
| 171624_at | 0.00 |
| 171625_r_at | 0.00 |
| 171626_at | 0.00 |
| X-18SRNAMur/X00686_ | 0.01 |
| X-18SRNAMur/X00686_ | 0.24 |
| X-18SRNAMur/X00686_ | 0.07 |
| AFFX-BioB-3_at | 0.03 |
| AFFX-BioB-3_st_C | 0.00 |
| AFFX-BioB-5_at | 0.04 |
| AFFX-BioB-5_st_C | 0.00 |
| AFFX-BioB-M_at | 0.04 |
| AFFX-BioB-M_st_C | 0.00 |
| AFFX-BioC-3_at | 0.02 |
| AFFX-BioC-3_st_C | 0.00 |
| AFFX-BioC-5_at | 0.02 |
| AFFX-BioC-5_st_C | 0.00 |
| AFFX-BioDn-3_at | 0.01 |

| | |
|---|---|
| AFFX-BioDn-3_st_C | 0.00 |
| AFFX-BioDn-5_at | 0.02 |
| AFFX-BioDn-5_st_C | 0.00 |
| AFFX-CreX-3_at | 0.00 |
| AFFX-CreX-3_st_C | 0.00 |
| AFFX-CreX-5_at | 0.00 |
| AFFX-CreX-5_st_C | 0.00 |
| AFFX-DapX-3_at | 0.00 |
| AFFX-DapX-5_at | 0.00 |
| AFFX-DapX-M_at | 0.00 |
| FX-GapdhMur/M32599_ | 0.00 |
| -GapdhMur/M32599_3 | 0.01 |
| FX-GapdhMur/M32599_ | 0.00 |
| -GapdhMur/M32599_5 | 0.00 |
| X-GapdhMur/M32599_ | 0.00 |
| -GapdhMur/M32599_M | 0.01 |
| AFFX-LysX-3_at | 0.00 |
| AFFX-LysX-5_at | 0.00 |
| AFFX-LysX-M_at | 0.00 |
| AFFX-MURINE_B2_at | 0.00 |
| AFFX-MURINE_b1_at | 0.03 |
| AFFX-MUR_b2_at | 0.00 |
| AFFX-MurFAS_at | 0.00 |
| AFFX-MurIL10_at | 0.00 |
| AFFX-MurIL2_at | 0.00 |
| AFFX-MurIL4_at | 0.00 |
| AFFX-PheX-3_at | 0.00 |
| AFFX-PheX-5_at | 0.00 |
| AFFX-PheX-M_at | 0.00 |
| -PyruCarbMur/L09192 | 0.00 |
| -PyruCarbMur/L09192 | 0.00 |
| -PyruCarbMur/L09192_ | 0.00 |
| -PyruCarbMur/L09192_ | 0.00 |
| AFFX-ThrX-3_at | 0.00 |
| AFFX-ThrX-5_at | 0.00 |
| AFFX-ThrX-M_at | 0.00 |
| -TransRecMur/X57349 | 0.04 |
| -TransRecMur/X57349 | 0.13 |
| -TransRecMur/X57349 | 0.12 |
| AFFX-TrpnX-3_at | 0.00 |
| AFFX-TrpnX-5_at | 0.00 |
| AFFX-TrpnX-M_at | 0.00 |
| FFX-YEL002c/WBP1_a | 0.00 |
| AFFX-YEL018w/_at | 0.00 |
| FFX-YEL021w/URA3_a | 0.00 |
| FFX-YEL024w/RIP1_a | 0.00 |
| FX-b-ActinMur/M12481_ | 0.00 |
| -b-ActinMur/M12481_3 | 0.01 |
| FX-b-ActinMur/M12481_ | 0.00 |
| -b-ActinMur/M12481_5 | 0.01 |
| X-b-ActinMur/M12481_ | 0.00 |
| -b-ActinMur/M12481_M | 0.00 |
