# Supplementary material for: Meta-Analysis of Differentiating Mouse Embryonic Stem Cell Gene Expression Kinetics Reveals Early Change of a Small Gene Set
Source: PLoS Comput Biol. 2006 Nov 24;2(11):e158. doi: 10.1371/journal.pcbi.0020158 (PMC1664699; doi:10.1371/journal.pcbi.0020158)
Supplement: Table S2 — (859 KB PDF) [file pcbi.0020158.st002.pdf]

MOE430	Confidence Values	
	DMSO/RA	M-LR
1415670_at	0.00	0.00
1415671_at	0.00	0.00
1415672_at	0.00	0.00
1415673_at	0.01	0.11
1415674_a_at	0.00	0.00
1415675_at	0.00	0.00
1415676_a_at	0.00	0.00
1415677_at	0.00	0.00
1415678_at	0.00	0.00
1415679_at	0.00	0.00
1415680_at	0.00	0.00
1415681_at	0.00	0.00
1415682_at	0.00	0.00
1415683_at	0.00	0.00
1415684_at	0.00	0.00
1415685_at	0.00	0.00
1415686_at	0.00	0.00
1415687_a_at	0.00	0.01
1415688_at	0.00	0.00
1415689_s_at	0.00	0.00
1415690_at	0.00	0.00
1415691_at	0.00	0.00
1415692_s_at	0.00	0.00
1415693_at	0.00	0.00
1415694_at	0.00	0.00
1415695_at	0.00	0.00
1415696_at	0.00	0.00
1415697_at	0.00	0.93
1415698_at	0.00	0.00
1415699_a_at	0.00	0.26
1415700_a_at	0.00	0.00
1415701_x_at	0.00	0.00
1415702_a_at	0.00	0.00
1415703_at	0.00	0.00
1415704_a_at	0.00	0.00
1415705_at	0.00	0.00
1415706_at	0.00	0.00
1415707_at	0.00	0.00
1415708_at	0.00	0.11
1415709_s_at	0.00	0.00
1415710_at	0.00	0.00
1415711_at	0.00	0.00
1415712_at	0.00	0.00
1415713_a_at	0.00	0.01
1415714_a_at	0.00	0.00
1415715_at	0.00	0.00
1415716_a_at	0.00	0.00
1415717_at	0.00	0.00
1415718_at	0.00	0.00
1415719_s_at	0.00	0.00
1415720_s_at	0.00	0.00
1415721_a_at	0.00	0.00
1415722_a_at	0.00	0.00
1415723_at	0.00	0.00

1415724_a_at	0.00	0.00
1415725_at	0.03	0.35
1415726_at	0.00	0.00
1415727_at	0.00	0.18
1415728_at	0.00	0.00
1415729_at	0.00	0.00
1415730_at	0.00	0.00
1415731_at	0.00	0.00
1415732_at	0.00	0.00
1415733_a_at	0.00	0.32
1415734_at	0.00	0.00
1415735_at	0.00	0.00
1415736_at	0.00	0.00
1415737_at	0.00	0.00
1415738_at	0.00	0.00
1415739_at	0.00	0.00
1415740_at	0.00	0.36
1415741_at	0.00	0.00
1415742_at	0.00	0.00
1415743_at	0.00	0.00
1415744_at	0.00	0.29
1415745_a_at	0.00	0.00
1415746_at	0.00	0.00
1415747_s_at	0.00	0.00
1415748_a_at	0.00	0.00
1415749_a_at	0.00	0.00
1415750_at	0.00	0.00
1415751_at	0.00	0.00
1415752_at	0.00	0.00
1415753_at	0.00	0.00
1415754_at	0.00	0.07
1415755_a_at	0.00	0.00
1415756_a_at	0.00	0.00
1415757_at	0.00	0.00
1415758_at	0.00	0.00
1415759_a_at	0.00	0.00
1415760_s_at	0.00	0.02
1415761_at	0.00	0.32
1415762_x_at	0.00	0.21
1415763_a_at	0.00	0.00
1415764_at	0.00	0.00
1415765_at	0.00	0.00
1415766_at	0.00	0.00
1415767_at	0.00	0.00
1415768_a_at	0.00	0.00
1415769_at	0.00	0.00
1415770_at	0.00	0.00
1415771_at	0.00	0.06
1415772_at	0.00	0.04
1415773_at	0.00	0.35
1415774_at	0.00	0.00
1415775_at	0.00	0.00
1415776_at	0.00	0.00
1415777_at	0.00	0.00
1415778_at	0.00	0.00
1415779_s_at	0.00	0.00

1415780_a_at	0.00	0.00
1415781_a_at	0.00	0.00
1415782_at	0.00	0.00
1415783_at	0.00	0.00
1415784_at	0.00	0.00
1415785_a_at	0.00	0.08
1415786_at	0.00	0.00
1415787_at	0.00	0.00
1415788_at	0.00	0.00
1415789_a_at	0.00	0.00
1415790_at	0.00	0.00
1415791_at	0.00	0.00
1415792_at	0.00	0.00
1415793_at	0.00	0.00
1415794_a_at	0.00	0.00
1415795_at	0.00	0.00
1415796_at	0.00	0.00
1415797_at	0.00	0.00
1415798_at	0.00	0.00
1415799_at	0.00	0.01
1415800_at	0.01	0.00
1415801_at	0.00	0.00
1415802_at	0.00	0.59
1415803_at	0.00	0.00
1415804_at	0.00	0.00
1415805_at	0.00	0.00
1415806_at	0.00	0.00
1415807_s_at	0.00	0.35
1415808_at	0.00	0.00
1415809_at	0.00	0.00
1415810_at	0.00	0.24
1415811_at	0.00	0.24
1415812_at	0.01	0.00
1415813_at	0.00	0.00
1415814_at	0.00	0.00
1415815_at	0.00	0.00
1415816_at	0.00	0.00
1415817_s_at	0.00	0.00
1415818_at	0.00	0.00
1415819_a_at	0.00	0.00
1415820_x_at	0.00	0.00
1415821_at	0.00	0.00
1415822_at	0.53	0.00
1415823_at	0.24	0.00
1415824_at	0.54	0.00
1415825_s_at	0.00	0.00
1415826_at	0.00	0.00
1415827_a_at	0.00	0.00
1415828_a_at	0.00	0.00
1415829_at	0.00	0.00
1415830_at	0.01	0.20
1415831_at	0.00	0.00
1415832_at	0.00	0.00
1415833_x_at	0.00	0.00
1415834_at	0.21	0.00
1415835_at	0.00	0.00

1415836_at	0.00	0.00
1415837_at	0.00	0.00
1415838_at	0.00	0.00
1415839_a_at	0.00	0.00
1415840_at	0.00	0.00
1415841_at	0.00	0.00
1415842_at	0.00	0.00
1415843_at	0.00	0.00
1415844_at	0.00	0.00
1415845_at	0.00	0.00
1415846_a_at	0.00	0.00
1415847_at	0.00	0.00
1415848_at	0.00	0.00
1415849_s_at	0.20	0.01
1415850_at	0.00	0.00
1415851_a_at	0.00	0.00
1415852_at	0.00	0.00
1415853_at	0.00	0.00
1415854_at	0.00	0.00
1415855_at	0.00	0.00
1415856_at	0.00	1.00
1415857_at	0.00	1.00
1415858_at	0.00	0.02
1415859_at	0.00	0.00
1415860_at	0.00	0.00
1415861_at	0.00	0.00
1415862_at	0.00	0.00
1415863_at	0.00	0.48
1415864_at	0.00	0.00
1415865_s_at	0.00	0.00
1415866_at	0.00	0.00
1415867_at	0.00	0.00
1415868_at	0.00	0.00
1415869_a_at	0.00	0.20
1415870_at	0.00	0.00
1415871_at	0.00	0.00
1415872_at	0.00	0.00
1415873_a_at	0.00	0.00
1415874_at	0.00	0.33
1415875_at	0.00	0.00
1415876_a_at	0.00	0.00
1415877_at	0.00	0.00
1415878_at	0.00	0.00
1415879_a_at	0.00	0.00
1415880_a_at	0.00	0.00
1415881_at	0.00	0.00
1415882_at	0.00	0.00
1415883_a_at	0.00	0.00
1415884_at	0.00	0.00
1415885_at	0.00	0.00
1415886_at	0.00	0.00
1415887_at	0.00	0.00
1415888_at	0.00	0.00
1415889_a_at	0.00	0.00
1415890_at	0.00	0.11
1415891_at	0.00	0.00

1415892_at	0.00	0.07
1415893_at	0.00	0.00
1415894_at	0.00	0.00
1415895_at	0.00	0.00
1415896_x_at	0.00	0.00
1415897_a_at	0.00	0.00
1415898_at	0.00	0.00
1415899_at	0.00	0.00
1415900_a_at	0.00	0.00
1415901_at	0.00	0.00
1415902_at	0.00	0.00
1415903_at	0.00	0.00
1415904_at	0.00	0.00
1415905_at	0.00	0.00
1415906_at	0.00	0.09
1415907_at	0.00	0.00
1415908_at	0.00	0.00
1415909_at	0.00	0.00
1415910_s_at	0.00	0.00
1415911_at	0.00	0.00
1415912_a_at	0.00	0.00
1415913_at	0.00	0.00
1415914_at	0.00	0.00
1415915_at	0.00	0.00
1415916_a_at	0.00	0.00
1415917_at	0.00	0.00
1415918_a_at	0.00	0.00
1415919_at	0.00	0.00
1415920_at	0.00	0.16
1415921_a_at	0.00	0.00
1415922_s_at	0.00	0.00
1415923_at	0.05	0.00
1415924_at	0.00	0.00
1415925_a_at	0.00	0.23
1415926_at	0.00	0.00
1415927_at	0.00	0.00
1415928_a_at	0.00	0.00
1415929_at	0.00	0.00
1415930_a_at	0.00	0.00
1415931_at	0.00	0.00
1415932_x_at	0.00	0.00
1415933_a_at	0.00	0.00
1415934_at	0.00	0.00
1415935_at	0.00	0.00
1415936_at	0.00	0.00
1415937_s_at	0.00	0.00
1415938_at	1.00	0.00
1415939_at	0.00	0.00
1415940_at	0.00	0.00
1415941_s_at	0.00	0.00
1415942_at	0.00	0.00
1415943_at	0.00	0.00
1415944_at	0.00	0.00
1415945_at	0.00	0.27
1415946_at	0.00	0.00
1415947_at	0.00	0.11

1415948_at	0.00	0.02
1415949_at	0.00	0.00
1415950_a_at	0.00	0.00
1415951_at	0.00	0.00
1415952_at	0.00	0.00
1415953_s_at	0.00	0.00
1415954_at	0.00	0.00
1415955_x_at	0.00	0.00
1415956_a_at	0.00	0.00
1415957_a_at	0.00	0.00
1415958_at	0.00	0.00
1415959_at	0.00	0.00
1415960_at	0.00	0.00
1415961_at	0.00	0.44
1415962_at	0.00	0.00
1415963_at	0.00	0.11
1415964_at	0.00	0.00
1415965_at	0.00	0.00
1415966_a_at	0.00	0.00
1415967_at	0.00	0.00
1415968_a_at	0.00	0.00
1415969_s_at	0.00	0.00
1415970_at	0.00	0.00
1415971_at	0.02	0.00
1415972_at	0.38	0.00
1415973_at	0.00	0.00
1415974_at	0.00	0.00
1415975_at	0.01	0.36
1415976_a_at	0.00	0.00
1415977_at	0.00	0.00
1415978_at	0.00	0.60
1415979_x_at	0.00	0.00
1415980_at	0.00	0.00
1415981_at	0.00	0.00
1415982_at	0.00	0.00
1415983_at	0.00	0.00
1415984_at	0.01	0.01
1415985_at	0.00	0.00
1415986_at	0.00	0.00
1415987_at	0.00	0.00
1415988_at	0.00	0.00
1415989_at	0.00	0.00
1415990_at	0.00	0.00
1415991_a_at	0.00	0.00
1415992_at	0.00	0.00
1415993_at	0.00	0.00
1415994_at	0.00	0.00
1415995_at	0.00	0.04
1415996_at	0.00	0.00
1415997_at	0.00	0.00
1415998_at	0.00	0.00
1415999_at	0.00	0.01
1416000_a_at	0.00	0.00
1416001_a_at	0.00	0.33
1416002_x_at	0.00	0.33
1416003_at	0.00	0.00

1416004_at	0.00	0.00
1416005_at	0.00	0.34
1416006_at	0.00	0.00
1416007_at	0.00	0.00
1416008_at	0.00	0.00
1416009_at	0.00	0.00
1416010_a_at	0.00	0.00
1416011_x_at	0.00	0.00
1416012_at	0.00	0.00
1416013_at	0.00	0.00
1416014_at	0.00	0.03
1416015_s_at	0.00	0.08
1416016_at	0.00	0.00
1416017_at	0.00	0.00
1416018_at	0.00	0.01
1416019_at	0.00	0.00
1416020_a_at	0.00	0.20
1416021_a_at	0.00	0.00
1416022_at	0.00	0.00
1416023_at	0.00	0.00
1416024_x_at	0.00	0.00
1416025_at	0.00	0.00
1416026_a_at	0.00	0.00
1416027_at	0.00	0.00
1416028_a_at	0.00	0.00
1416029_at	0.00	0.00
1416030_a_at	0.00	0.00
1416031_s_at	0.00	0.00
1416032_at	0.00	0.00
1416033_at	0.00	0.00
1416034_at	0.00	0.48
1416035_at	0.00	0.00
1416036_at	0.00	0.00
1416037_a_at	0.00	0.00
1416038_at	0.00	0.00
1416039_x_at	0.99	0.00
1416040_at	0.00	0.00
1416041_at	0.83	0.06
1416042_s_at	0.00	0.15
1416043_at	0.00	0.15
1416044_at	0.00	0.00
1416045_a_at	0.00	0.00
1416046_a_at	0.00	0.00
1416047_at	0.00	0.00
1416048_at	0.00	0.00
1416049_at	0.24	0.00
1416050_a_at	0.00	0.33
1416051_at	0.00	0.00
1416052_at	0.49	0.00
1416053_at	0.00	0.00
1416054_at	0.00	0.00
1416055_at	0.00	0.00
1416056_a_at	0.00	0.00
1416057_at	0.00	0.00
1416058_s_at	0.00	0.00
1416059_at	0.00	0.00

1416060_at	0.00	0.00
1416061_at	0.00	0.00
1416062_at	0.00	0.00
1416063_x_at	0.00	0.00
1416064_a_at	0.00	0.00
1416065_a_at	0.01	0.03
1416066_at	0.00	0.00
1416067_at	0.00	0.19
1416068_at	0.00	0.00
1416069_at	0.91	0.06
1416070_a_at	0.00	0.11
1416071_at	0.00	0.19
1416072_at	0.00	0.00
1416073_a_at	0.00	0.01
1416074_a_at	0.00	0.00
1416075_at	0.00	0.00
1416076_at	0.00	0.22
1416077_at	0.00	0.00
1416078_s_at	0.00	0.10
1416079_a_at	0.00	0.00
1416080_at	0.00	0.00
1416081_at	0.00	0.08
1416082_at	0.00	0.00
1416083_at	0.00	0.00
1416084_at	0.00	0.00
1416085_s_at	0.00	0.00
1416086_at	0.00	0.00
1416087_at	0.00	0.00
1416088_a_at	0.00	0.00
1416089_at	0.00	0.00
1416090_at	0.00	0.04
1416091_at	0.00	0.01
1416092_a_at	0.00	0.00
1416093_a_at	0.00	0.34
1416094_at	0.28	0.00
1416095_x_at	0.00	0.02
1416096_at	0.00	0.00
1416097_at	0.00	0.00
1416098_at	0.00	0.00
1416099_at	0.00	0.00
1416100_at	0.00	0.00
1416101_a_at	0.00	0.00
1416102_at	0.00	0.00
1416103_at	0.00	0.00
1416104_at	0.00	0.00
1416105_at	0.00	0.00
1416106_at	0.00	0.08
1416107_at	0.00	0.00
1416108_a_at	0.00	0.12
1416109_at	0.00	0.00
1416110_at	0.00	0.00
1416111_at	0.00	0.00
1416112_at	0.00	0.00
1416113_at	0.00	0.00
1416114_at	0.00	0.00
1416115_at	0.00	0.00

1416116_at	0.00	0.00
1416117_at	0.00	0.00
1416118_at	0.00	0.01
1416119_at	0.00	0.00
1416120_at	0.00	0.30
1416121_at	0.00	0.00
1416122_at	0.00	0.00
1416123_at	0.00	0.00
1416124_at	0.00	0.00
1416125_at	0.03	0.00
1416126_at	0.00	0.15
1416127_a_at	0.00	0.00
1416128_at	0.00	0.00
1416129_at	0.00	0.00
1416130_at	0.00	0.11
1416131_s_at	0.00	0.00
1416132_at	0.00	0.00
1416133_at	0.00	0.01
1416134_at	0.41	0.00
1416135_at	0.00	0.45
1416136_at	0.00	0.26
1416137_at	0.00	0.00
1416138_at	0.00	0.00
1416139_at	0.00	0.00
1416140_a_at	0.00	0.00
1416141_a_at	0.00	0.00
1416142_at	0.00	0.00
1416143_at	0.00	0.03
1416144_a_at	0.00	0.11
1416145_at	0.00	0.00
1416146_at	0.00	0.42
1416147_at	0.00	0.00
1416148_at	0.00	0.33
1416149_at	0.00	0.00
1416150_a_at	0.00	0.19
1416151_at	0.00	0.00
1416152_a_at	0.00	0.02
1416153_at	0.00	0.00
1416154_at	0.00	0.00
1416155_at	0.27	0.10
1416156_at	0.31	0.00
1416157_at	0.30	0.00
1416158_at	0.00	0.00
1416159_at	0.00	0.00
1416160_at	0.00	0.00
1416161_at	0.00	0.00
1416162_at	0.00	0.00
1416163_at	0.00	0.10
1416164_at	0.00	0.00
1416165_at	0.00	0.00
1416166_a_at	0.00	0.00
1416167_at	0.00	0.00
1416168_at	0.00	0.00
1416169_at	0.00	0.00
1416170_at	0.00	0.00
1416171_at	0.00	0.34

1416172_at	0.00	0.08
1416173_at	0.00	0.00
1416174_at	0.00	0.00
1416175_a_at	0.00	0.00
1416176_at	0.00	0.00
1416177_at	0.00	0.31
1416178_a_at	0.00	0.00
1416179_a_at	0.00	0.00
1416180_a_at	0.00	0.00
1416181_at	0.00	0.00
1416182_at	0.00	0.00
1416183_a_at	0.00	0.02
1416184_s_at	0.00	0.00
1416185_a_at	0.00	0.01
1416186_at	0.00	0.00
1416187_s_at	0.00	0.00
1416188_at	0.00	0.00
1416189_a_at	0.00	0.00
1416190_a_at	0.00	0.00
1416191_at	0.00	0.00
1416192_at	0.00	0.00
1416193_at	0.00	0.00
1416194_at	0.00	0.00
1416195_at	0.00	0.00
1416196_at	0.00	0.00
1416197_at	0.00	0.00
1416198_at	0.00	0.00
1416199_at	0.00	0.00
1416200_at	0.00	0.00
1416201_at	0.00	0.00
1416202_at	0.00	0.04
1416203_at	0.00	0.00
1416204_at	0.00	0.00
1416205_at	0.00	0.00
1416206_at	0.00	0.00
1416207_at	0.00	0.00
1416208_at	0.00	0.00
1416209_at	0.00	0.00
1416210_at	0.00	0.00
1416211_a_at	0.00	0.00
1416212_at	0.00	0.32
1416213_x_at	0.00	0.00
1416214_at	0.00	0.00
1416215_at	0.00	0.00
1416216_at	0.00	0.00
1416217_a_at	0.00	0.00
1416218_x_at	0.00	0.00
1416219_at	0.00	0.00
1416220_at	0.01	0.00
1416221_at	0.10	0.00
1416222_at	0.00	0.00
1416223_at	0.00	0.00
1416224_at	0.00	0.00
1416225_at	0.00	0.00
1416226_at	0.00	0.00
1416227_at	0.00	0.00

1416228_at	0.00	0.02
1416229_at	0.00	0.00
1416230_at	0.00	0.00
1416231_at	0.00	0.00
1416232_at	0.00	0.00
1416233_at	0.00	0.00
1416234_at	0.00	0.00
1416235_at	0.00	0.00
1416236_a_at	0.00	0.00
1416237_at	0.00	0.00
1416238_at	0.00	0.00
1416239_at	0.00	0.00
1416240_at	0.00	0.00
1416241_at	0.00	0.00
1416242_at	0.03	0.00
1416243_a_at	0.00	0.00
1416244_a_at	0.00	0.00
1416245_at	0.00	0.09
1416246_a_at	0.00	0.00
1416247_at	0.00	0.00
1416248_at	0.00	0.00
1416249_at	0.00	0.00
1416250_at	0.00	0.26
1416251_at	0.00	0.00
1416252_at	0.00	0.00
1416253_at	0.00	0.00
1416254_a_at	0.00	0.00
1416255_at	0.00	0.00
1416256_a_at	0.00	0.00
1416257_at	0.69	0.00
1416258_at	0.00	0.00
1416259_at	0.00	0.00
1416260_a_at	0.00	0.00
1416261_at	0.00	0.00
1416262_at	0.00	0.00
1416263_at	0.00	0.00
1416264_at	0.00	0.00
1416265_at	0.00	0.00
1416266_at	0.00	0.00
1416267_at	0.00	0.00
1416268_at	0.00	0.00
1416269_at	0.00	0.12
1416270_at	0.00	0.00
1416271_at	0.00	0.00
1416272_at	0.00	0.00
1416273_at	0.00	0.00
1416274_at	0.00	0.00
1416275_at	0.00	0.00
1416276_a_at	0.00	0.00
1416277_a_at	0.00	0.00
1416278_a_at	0.00	0.00
1416279_at	0.00	0.00
1416280_at	0.00	0.43
1416281_at	0.00	0.00
1416282_at	0.00	0.32
1416283_at	0.00	0.12

1416284_at	0.00	0.00
1416285_at	0.00	0.00
1416286_at	0.00	0.00
1416287_at	0.00	0.00
1416288_at	0.00	0.05
1416289_at	0.00	0.00
1416290_a_at	0.00	0.31
1416291_at	0.00	0.33
1416292_at	0.00	0.03
1416293_at	0.00	0.00
1416294_at	0.00	0.00
1416295_a_at	0.00	0.00
1416296_at	0.00	0.00
1416297_s_at	0.00	0.00
1416298_at	0.00	0.00
1416299_at	0.00	0.09
1416300_a_at	0.00	0.00
1416301_a_at	0.00	0.00
1416302_at	0.00	0.00
1416303_at	0.22	0.26
1416304_at	0.19	0.00
1416305_at	0.00	0.00
1416306_at	0.00	0.00
1416307_at	0.00	0.00
1416308_at	0.00	0.00
1416309_at	0.00	0.00
1416310_at	0.00	0.00
1416311_s_at	0.00	0.00
1416312_at	0.00	0.00
1416313_at	0.00	0.00
1416314_at	0.00	0.00
1416315_at	0.00	0.27
1416316_at	0.73	0.09
1416317_a_at	0.00	0.00
1416318_at	0.00	0.00
1416319_at	0.00	0.00
1416320_at	0.00	0.00
1416321_s_at	0.00	0.00
1416322_at	0.00	0.00
1416323_at	0.00	0.00
1416324_s_at	0.00	0.00
1416325_at	0.00	0.00
1416326_at	0.00	0.00
1416327_at	0.00	0.00
1416328_a_at	0.00	0.00
1416329_at	0.00	0.00
1416330_at	0.00	0.00
1416331_a_at	0.00	0.00
1416332_at	0.00	0.00
1416333_at	0.00	0.00
1416334_at	0.00	0.00
1416335_at	0.00	0.00
1416336_s_at	0.00	0.00
1416337_at	0.00	0.00
1416338_at	0.00	0.00
1416339_a_at	0.00	0.00

1416340_a_at	0.00	0.00
1416341_at	0.00	0.00
1416342_at	0.00	0.00
1416343_a_at	0.00	0.00
1416344_at	0.00	0.00
1416345_at	0.00	0.31
1416346_at	0.00	0.30
1416347_at	0.00	0.00
1416348_at	0.00	0.00
1416349_at	0.00	0.00
1416350_at	0.00	0.00
1416351_at	0.00	0.00
1416352_s_at	0.00	0.00
1416353_at	0.00	0.00
1416354_at	0.00	0.00
1416355_at	0.00	0.00
1416356_at	0.00	0.00
1416357_a_at	0.00	0.00
1416358_at	0.00	0.00
1416359_at	0.00	0.00
1416360_at	0.00	0.00
1416361_a_at	0.00	0.00
1416362_a_at	0.00	0.00
1416363_at	0.00	0.00
1416364_at	0.00	0.00
1416365_at	0.00	0.00
1416366_at	0.00	0.00
1416367_at	0.00	0.00
1416368_at	1.00	0.12
1416369_at	0.00	0.00
1416370_at	0.00	0.00
1416371_at	0.00	0.00
1416372_at	0.00	0.00
1416373_at	0.00	0.00
1416374_at	0.00	0.00
1416375_at	0.00	0.00
1416376_at	0.00	0.00
1416377_at	0.00	0.00
1416378_at	0.00	0.00
1416379_at	0.00	0.00
1416380_at	0.00	0.00
1416381_a_at	0.00	0.00
1416382_at	0.00	0.40
1416383_a_at	0.00	0.00
1416384_a_at	0.00	0.00
1416385_a_at	0.00	0.00
1416386_a_at	0.00	0.00
1416387_at	0.00	0.00
1416388_at	0.00	0.00
1416389_a_at	0.01	0.72
1416390_at	0.00	0.00
1416391_at	0.00	0.33
1416392_a_at	0.00	0.00
1416393_at	0.00	0.29
1416394_at	0.00	0.00
1416395_at	0.00	0.04

1416396_at	0.00	0.00
1416397_at	0.00	0.00
1416398_at	0.00	0.00
1416399_a_at	0.00	0.00
1416400_at	0.00	0.00
1416401_at	0.00	0.00
1416402_at	0.00	0.00
1416403_at	0.00	0.00
1416404_s_at	0.00	0.00
1416405_at	0.00	0.00
1416406_at	0.00	0.00
1416407_at	0.00	0.33
1416408_at	0.00	0.00
1416409_at	0.00	0.00
1416410_at	0.00	0.00
1416411_at	0.00	0.00
1416412_at	0.00	0.00
1416413_at	0.00	0.00
1416414_at	0.00	0.00
1416415_a_at	0.00	0.33
1416416_x_at	0.00	0.00
1416417_a_at	0.00	0.00
1416418_at	0.00	0.58
1416419_s_at	0.00	0.69
1416420_a_at	0.00	0.00
1416421_a_at	0.00	0.10
1416422_a_at	0.00	0.01
1416423_x_at	0.00	0.00
1416424_at	0.00	0.00
1416425_at	0.00	0.00
1416426_at	0.00	0.00
1416427_at	0.00	0.26
1416428_at	0.00	0.00
1416429_a_at	0.00	0.00
1416430_at	0.00	0.00
1416431_at	0.61	0.00
1416432_at	0.03	0.02
1416433_at	0.00	0.09
1416434_at	0.00	0.00
1416435_at	0.00	0.00
1416436_a_at	0.00	0.00
1416437_a_at	0.00	0.00
1416438_at	0.00	0.00
1416439_at	0.00	0.02
1416440_at	0.00	0.00
1416441_at	0.00	0.00
1416442_at	0.00	0.33
1416443_a_at	0.00	0.00
1416444_at	0.00	0.00
1416445_at	0.00	0.00
1416446_at	0.00	0.00
1416447_at	0.00	0.04
1416448_at	0.00	0.31
1416449_x_at	0.00	0.00
1416450_at	0.00	0.00
1416451_s_at	0.00	0.00

1416452_at	0.47	0.31
1416453_x_at	0.00	0.00
1416454_s_at	0.05	0.00
1416455_a_at	0.00	0.00
1416456_a_at	0.00	0.00
1416457_at	0.00	0.26
1416458_at	0.00	0.00
1416459_at	0.00	0.00
1416460_at	0.00	0.00
1416461_at	0.00	0.34
1416462_at	0.00	0.29
1416463_at	0.00	0.00
1416464_at	0.00	0.00
1416465_a_at	0.00	0.00
1416466_at	0.00	0.00
1416467_at	0.00	0.00
1416468_at	0.00	0.00
1416469_at	0.00	0.00
1416470_a_at	0.00	0.00
1416471_at	0.00	0.00
1416472_at	0.00	0.00
1416473_a_at	0.00	0.00
1416474_at	0.00	0.00
1416475_at	0.00	0.00
1416476_a_at	0.00	0.00
1416477_at	0.00	0.00
1416478_a_at	0.00	0.00
1416479_a_at	0.00	0.00
1416480_a_at	0.00	0.00
1416481_s_at	0.00	0.00
1416482_at	0.00	0.00
1416483_at	0.00	0.00
1416484_at	0.00	0.00
1416485_at	0.00	0.00
1416486_at	0.00	0.00
1416487_a_at	0.00	0.00
1416488_at	0.00	0.00
1416489_at	0.00	0.00
1416490_at	0.00	0.00
1416491_at	0.00	0.00
1416492_at	0.60	0.57
1416493_at	0.00	0.00
1416494_at	0.00	0.00
1416495_s_at	0.00	0.01
1416496_at	0.00	0.00
1416497_at	0.00	0.00
1416498_at	0.08	0.02
1416499_a_at	0.00	0.00
1416500_at	0.00	0.00
1416501_at	0.00	0.00
1416502_a_at	0.00	0.00
1416503_at	0.00	0.00
1416504_at	0.03	0.00
1416505_at	0.00	0.00
1416506_at	0.00	0.06
1416507_at	0.00	0.00

1416508_at	0.00	0.00
1416509_at	0.00	0.00
1416510_at	0.00	0.02
1416511_a_at	0.00	0.00
1416512_at	0.01	1.00
1416513_at	0.00	0.00
1416514_a_at	0.00	0.07
1416515_at	0.01	0.00
1416516_at	0.01	0.00
1416517_at	0.00	0.00
1416518_at	0.00	0.00
1416519_at	0.00	0.00
1416520_x_at	0.00	0.00
1416521_at	0.00	0.00
1416522_a_at	0.00	0.00
1416523_at	0.00	0.00
1416524_at	0.00	0.00
1416525_at	0.00	0.00
1416526_a_at	0.00	0.00
1416527_at	0.00	0.00
1416528_at	0.00	0.00
1416529_at	0.00	0.00
1416530_a_at	0.34	1.00
1416531_at	0.00	0.00
1416532_at	0.00	0.00
1416533_at	0.00	0.00
1416534_at	0.00	0.00
1416535_at	0.00	0.00
1416536_at	0.00	0.00
1416537_at	0.00	0.00
1416538_at	0.00	0.00
1416539_at	0.00	0.00
1416540_at	0.00	0.00
1416541_at	0.00	0.00
1416542_at	0.00	0.00
1416543_at	0.00	0.00
1416544_at	0.00	0.13
1416545_at	0.00	0.00
1416546_a_at	0.00	0.00
1416547_at	0.00	0.00
1416548_at	0.00	0.00
1416549_at	0.00	0.00
1416550_at	0.00	0.00
1416551_at	0.00	0.00
1416552_at	0.99	0.00
1416553_at	0.00	0.00
1416554_at	0.00	0.00
1416555_at	0.00	0.00
1416556_at	0.00	0.00
1416557_a_at	0.00	0.01
1416558_at	0.00	0.00
1416559_at	0.00	0.00
1416560_at	0.00	0.00
1416561_at	0.00	0.00
1416562_at	0.00	0.00
1416563_at	0.00	0.00

1416564_at	0.00	0.00
1416565_at	0.00	0.00
1416566_at	0.00	0.09
1416567_s_at	0.00	0.00
1416568_a_at	0.00	0.32
1416569_at	0.00	0.29
1416570_s_at	0.00	0.32
1416571_at	0.00	0.00
1416572_at	0.00	0.00
1416573_at	0.00	0.00
1416574_at	0.00	0.00
1416575_at	0.00	0.00
1416576_at	0.00	0.00
1416577_a_at	0.00	0.00
1416578_at	0.00	0.00
1416579_a_at	0.00	0.00
1416580_a_at	0.00	0.00
1416581_at	0.00	0.00
1416582_a_at	0.00	0.00
1416583_at	0.00	0.00
1416584_at	0.00	0.01
1416585_at	0.00	0.26
1416586_at	0.00	0.00
1416587_a_at	0.00	0.00
1416588_at	0.00	0.00
1416589_at	0.52	0.00
1416590_a_at	0.00	0.00
1416591_at	0.00	0.00
1416592_at	0.00	0.00
1416593_at	0.00	0.00
1416594_at	0.00	0.00
1416595_at	0.00	0.10
1416596_at	0.00	0.00
1416597_at	0.00	0.00
1416598_at	0.00	0.00
1416599_at	0.00	0.00
1416600_a_at	0.00	0.00
1416601_a_at	0.00	0.00
1416602_a_at	0.00	0.00
1416603_at	0.00	0.00
1416604_at	0.00	0.00
1416605_at	0.02	0.51
1416606_s_at	0.00	0.38
1416607_at	0.00	0.00
1416608_a_at	0.00	0.00
1416609_at	0.00	0.00
1416610_a_at	0.00	0.00
1416611_at	0.00	0.00
1416612_at	0.00	0.00
1416613_at	0.00	0.00
1416614_at	0.00	0.00
1416615_at	0.00	0.33
1416616_s_at	0.00	0.63
1416617_at	0.00	0.00
1416618_at	0.00	0.00
1416619_at	0.00	0.00

1416620_at	0.00	0.00
1416621_at	0.00	0.00
1416622_at	0.00	0.00
1416623_at	0.00	0.00
1416624_a_at	0.00	0.00
1416625_at	0.00	0.00
1416626_at	0.00	0.34
1416627_at	0.00	0.00
1416628_at	0.00	0.00
1416629_at	0.00	0.00
1416630_at	0.00	0.00
1416631_at	0.00	0.00
1416632_at	0.00	0.33
1416633_a_at	0.00	0.00
1416634_at	0.00	0.00
1416635_at	0.00	0.00
1416636_at	0.00	0.00
1416637_at	0.00	0.00
1416638_at	0.00	0.04
1416639_at	0.00	0.00
1416640_at	0.00	0.00
1416641_at	0.00	0.00
1416642_a_at	0.00	0.00
1416643_at	0.00	0.00
1416644_a_at	0.00	0.00
1416645_a_at	0.00	0.00
1416646_at	0.00	0.00
1416647_at	0.00	0.00
1416648_at	0.00	0.00
1416649_at	0.00	0.00
1416650_at	0.00	0.00
1416651_at	0.00	0.00
1416652_at	0.00	0.00
1416653_at	0.00	0.00
1416654_at	0.00	0.00
1416655_at	0.00	0.00
1416656_at	0.42	0.29
1416657_at	0.00	0.00
1416658_at	0.00	0.00
1416659_at	0.00	0.00
1416660_at	0.00	0.00
1416661_at	0.00	0.00
1416662_at	0.00	0.00
1416663_at	0.00	0.00
1416664_at	0.00	0.00
1416665_at	0.00	0.00
1416666_at	0.00	0.00
1416667_at	0.00	0.00
1416668_at	0.00	0.00
1416669_s_at	0.00	0.00
1416670_at	0.00	0.00
1416671_a_at	0.00	0.00
1416672_s_at	0.00	0.00
1416673_at	0.00	0.00
1416674_at	0.00	0.00
1416675_s_at	0.00	0.02

1416676_at	0.00	0.00
1416677_at	0.00	0.00
1416678_at	0.00	0.01
1416679_at	0.00	0.00
1416680_at	0.00	0.11
1416681_at	0.00	0.00
1416682_at	0.00	0.00
1416683_at	0.00	0.05
1416684_at	0.00	0.00
1416685_s_at	0.00	0.00
1416686_at	0.00	0.01
1416687_at	0.00	0.10
1416688_at	0.00	0.00
1416689_at	0.00	0.03
1416690_at	0.00	0.00
1416691_at	0.00	0.00
1416692_at	0.00	0.02
1416693_at	0.00	0.00
1416694_at	0.00	0.00
1416695_at	0.00	0.00
1416696_at	0.00	0.00
1416697_at	0.00	0.00
1416698_a_at	0.00	0.00
1416699_at	0.00	0.00
1416700_at	0.00	0.00
1416701_at	0.00	0.01
1416702_at	0.00	0.00
1416703_at	0.00	0.00
1416704_at	0.00	0.00
1416705_at	0.00	0.00
1416706_at	0.00	0.00
1416707_a_at	0.00	0.00
1416708_a_at	0.00	0.00
1416709_a_at	0.00	0.25
1416710_at	0.00	0.00
1416711_at	0.00	0.00
1416712_at	0.00	0.00
1416713_at	0.00	0.00
1416714_at	0.00	0.00
1416715_at	0.49	0.00
1416716_at	0.00	0.00
1416717_at	0.00	0.00
1416718_at	0.00	0.00
1416719_a_at	0.00	0.00
1416720_at	0.00	0.00
1416721_s_at	0.00	0.19
1416722_at	0.00	0.03
1416723_at	0.00	0.00
1416724_x_at	0.00	0.00
1416725_at	0.00	0.00
1416726_s_at	0.00	0.00
1416727_a_at	0.00	0.03
1416728_at	0.00	0.00
1416729_at	0.00	0.00
1416730_at	0.00	0.00
1416731_at	0.17	0.00

1416732_at	0.01	0.00
1416733_at	0.00	0.00
1416734_at	0.00	0.00
1416735_at	0.00	0.00
1416736_at	0.00	0.00
1416737_at	0.00	0.00
1416738_at	0.00	0.00
1416739_a_at	0.00	0.00
1416740_at	0.41	0.00
1416741_at	0.00	0.00
1416742_at	0.00	0.00
1416743_at	0.00	0.00
1416744_at	0.00	0.00
1416745_x_at	0.00	0.00
1416746_at	0.00	0.00
1416747_at	0.00	0.00
1416748_a_at	0.00	0.01
1416749_at	0.00	0.00
1416750_at	0.00	0.00
1416751_a_at	0.00	0.35
1416752_at	0.00	0.00
1416753_at	0.00	0.00
1416754_at	0.00	0.00
1416755_at	0.00	0.00
1416756_at	0.00	0.00
1416757_at	0.00	0.00
1416758_at	0.00	0.00
1416759_at	0.07	0.00
1416760_at	0.00	0.00
1416761_at	0.00	0.00
1416762_at	0.06	0.00
1416763_at	0.00	0.01
1416764_at	0.00	0.00
1416765_s_at	0.00	0.00
1416766_at	0.00	0.00
1416767_a_at	0.00	0.00
1416768_at	0.00	0.22
1416769_s_at	0.00	0.00
1416770_at	0.00	0.00
1416771_at	0.00	0.00
1416772_at	0.00	0.51
1416773_at	0.00	0.00
1416774_at	0.00	0.00
1416775_at	0.00	0.00
1416776_at	0.00	0.00
1416777_at	0.00	0.00
1416778_at	0.00	0.00
1416779_at	0.00	0.00
1416780_at	0.00	0.33
1416781_at	0.00	0.00
1416782_s_at	0.00	0.00
1416783_at	0.00	0.00
1416784_at	0.00	0.00
1416785_at	0.00	0.00
1416786_at	0.00	0.01
1416787_at	0.00	0.00

1416788_a_at	0.00	0.00
1416789_at	0.00	0.00
1416790_a_at	0.00	0.00
1416791_a_at	0.00	0.19
1416792_at	0.00	0.02
1416793_at	0.00	0.00
1416794_at	0.00	0.00
1416795_at	0.00	0.00
1416796_at	0.00	0.00
1416797_at	0.00	0.00
1416798_a_at	0.00	0.00
1416799_at	0.00	0.00
1416800_at	0.00	0.00
1416801_at	0.00	0.00
1416802_a_at	0.00	0.00
1416803_at	0.00	0.00
1416804_at	0.00	0.00
1416805_at	0.00	0.00
1416806_at	0.00	0.00
1416807_at	0.00	0.00
1416808_at	0.00	0.00
1416809_at	0.00	0.00
1416810_at	0.00	0.00
1416811_s_at	0.00	0.00
1416812_at	0.00	0.00
1416813_at	0.00	0.00
1416814_at	0.00	0.00
1416815_s_at	0.00	0.00
1416816_at	0.00	0.00
1416817_at	0.00	0.00
1416818_at	0.58	0.00
1416819_at	0.00	0.00
1416820_at	0.00	0.00
1416821_at	0.00	0.00
1416822_at	0.00	0.00
1416823_a_at	0.00	0.00
1416824_at	0.00	0.00
1416825_at	0.00	0.00
1416826_a_at	0.00	0.00
1416827_at	0.00	0.00
1416828_at	0.00	0.00
1416829_at	0.00	0.00
1416830_at	0.00	0.00
1416831_at	0.00	0.92
1416832_at	0.00	1.00
1416833_at	0.00	0.00
1416834_x_at	0.00	0.00
1416835_s_at	0.00	0.00
1416836_at	0.02	0.01
1416837_at	0.00	0.01
1416838_at	0.00	0.00
1416839_at	0.00	0.00
1416840_at	0.00	0.00
1416841_at	0.00	0.00
1416842_at	0.00	0.32
1416843_at	0.00	0.00

1416844_at	0.00	0.00
1416845_at	0.00	0.00
1416846_a_at	0.00	0.00
1416847_s_at	0.00	0.00
1416848_at	0.00	0.00
1416849_at	0.00	0.24
1416850_s_at	0.00	0.00
1416851_at	0.00	0.00
1416852_a_at	0.00	0.02
1416853_at	0.00	0.00
1416854_at	0.00	0.00
1416855_at	0.00	0.00
1416856_at	0.00	0.00
1416857_at	0.00	0.00
1416858_a_at	0.00	0.00
1416859_at	0.00	0.00
1416860_s_at	0.00	0.02
1416861_at	0.00	0.00
1416862_at	0.00	0.00
1416863_at	0.00	0.00
1416864_at	0.00	0.00
1416865_at	0.17	0.00
1416866_at	0.00	0.00
1416867_at	0.00	0.00
1416868_at	0.00	0.00
1416869_x_at	0.00	0.00
1416870_at	0.00	0.00
1416871_at	0.00	0.00
1416872_at	0.00	0.00
1416873_a_at	0.00	0.00
1416874_a_at	0.00	0.00
1416875_at	0.00	0.00
1416876_at	0.00	0.00
1416877_a_at	0.00	0.31
1416878_at	0.00	0.00
1416879_at	0.00	0.00
1416880_at	0.00	0.00
1416881_at	0.00	0.22
1416882_at	0.00	0.00
1416883_at	0.00	0.00
1416884_at	0.00	0.07
1416885_at	0.00	0.00
1416886_at	0.00	0.00
1416887_at	0.00	0.00
1416888_at	0.00	0.00
1416889_at	0.00	0.00
1416890_at	0.00	0.36
1416891_at	0.00	0.00
1416892_s_at	0.10	0.02
1416893_at	0.02	0.00
1416894_at	0.00	0.00
1416895_at	0.00	0.00
1416896_at	0.00	0.00
1416897_at	0.00	0.00
1416898_a_at	0.00	0.00
1416899_at	0.88	0.00

1416900_s_at	0.00	0.00
1416901_at	0.00	0.54
1416902_a_at	0.00	0.00
1416903_at	0.00	0.00
1416904_at	0.00	0.00
1416905_at	0.00	0.00
1416906_at	0.00	0.00
1416907_at	0.00	0.00
1416908_s_at	0.00	0.00
1416909_at	0.00	0.00
1416910_at	0.00	0.00
1416911_a_at	0.00	0.00
1416912_at	0.00	0.00
1416913_at	0.00	0.00
1416914_s_at	0.00	0.00
1416915_at	0.71	0.14
1416916_at	0.00	0.00
1416917_at	0.00	0.01
1416918_at	0.00	0.00
1416919_a_at	0.00	0.00
1416920_at	0.00	0.00
1416921_x_at	0.00	0.00
1416922_a_at	0.00	0.00
1416923_a_at	0.03	0.00
1416924_at	0.00	0.33
1416925_at	0.00	0.12
1416926_at	0.00	0.00
1416927_at	0.00	0.00
1416928_at	0.00	0.14
1416929_at	0.00	0.00
1416930_at	0.00	0.00
1416931_at	0.00	0.00
1416932_at	0.00	0.00
1416933_at	0.00	0.00
1416934_at	0.00	0.00
1416935_at	0.00	0.00
1416936_at	0.00	0.00
1416937_at	0.00	0.15
1416938_at	0.00	0.00
1416939_at	0.00	0.56
1416940_at	0.00	0.00
1416941_s_at	0.00	0.00
1416942_at	0.00	0.00
1416943_at	0.05	0.00
1416944_a_at	0.00	0.00
1416945_at	0.00	0.00
1416946_a_at	0.00	0.00
1416947_s_at	0.00	0.00
1416948_at	0.00	0.15
1416949_s_at	0.00	0.00
1416950_at	0.13	0.01
1416951_a_at	0.00	0.00
1416952_at	0.00	0.00
1416953_at	0.00	0.00
1416954_at	0.00	0.00
1416955_at	0.00	0.00

1416956_at	0.00	0.00
1416957_at	0.00	0.00
1416958_at	0.00	0.00
1416959_at	0.00	0.00
1416960_at	0.00	0.00
1416961_at	0.00	0.00
1416962_at	0.00	0.00
1416963_at	0.00	0.00
1416964_at	0.00	0.00
1416965_at	0.00	0.00
1416966_at	0.00	0.00
1416967_at	1.00	0.00
1416968_a_at	0.00	0.00
1416969_at	0.00	0.00
1416970_a_at	0.00	0.00
1416971_at	0.00	0.00
1416972_at	0.00	0.11
1416973_at	0.00	0.29
1416974_at	0.00	0.00
1416975_at	0.00	0.00
1416976_at	0.00	0.00
1416977_at	0.00	0.00
1416978_at	0.00	0.00
1416979_at	0.00	0.00
1416980_at	0.00	0.00
1416981_at	0.00	0.00
1416982_at	0.00	0.00
1416983_s_at	0.00	0.00
1416984_at	0.00	0.00
1416985_at	0.00	0.00
1416986_a_at	0.00	0.00
1416987_at	0.00	0.00
1416988_at	0.00	0.00
1416989_at	0.00	0.00
1416990_at	0.00	0.00
1416991_at	0.00	0.23
1416992_at	0.00	0.00
1416993_at	0.00	0.00
1416994_at	0.00	0.00
1416995_at	0.00	0.00
1416996_at	0.00	0.00
1416997_a_at	0.00	0.00
1416998_at	0.00	0.06
1416999_at	0.00	0.00
1417000_at	0.00	0.00
1417001_a_at	0.00	0.00
1417002_at	0.00	0.00
1417003_at	0.00	0.00
1417004_at	0.00	0.00
1417005_at	0.00	0.00
1417006_at	0.00	0.01
1417007_a_at	0.00	0.00
1417008_at	0.00	0.00
1417009_at	0.00	0.00
1417010_at	0.00	0.00
1417011_at	0.00	0.00

1417012_at	0.00	0.00
1417013_at	0.00	0.00
1417014_at	0.00	0.00
1417015_at	0.00	0.00
1417016_at	0.00	0.00
1417017_at	0.00	0.00
1417018_at	0.00	0.00
1417019_a_at	0.00	0.00
1417020_at	0.00	0.00
1417021_a_at	0.00	0.00
1417022_at	0.04	0.41
1417023_a_at	0.00	0.00
1417024_at	0.00	0.02
1417025_at	0.00	0.00
1417026_at	0.00	0.00
1417027_at	1.00	0.24
1417028_a_at	1.00	0.34
1417029_a_at	0.99	0.30
1417030_at	0.00	0.00
1417031_at	0.00	0.00
1417032_at	0.00	0.00
1417033_at	0.00	0.00
1417034_at	0.00	0.00
1417035_at	0.00	0.00
1417036_at	0.00	0.00
1417037_at	0.00	0.27
1417038_at	0.00	0.00
1417039_a_at	0.00	0.00
1417040_a_at	0.00	0.00
1417041_at	0.00	0.02
1417042_at	0.00	0.00
1417043_at	0.00	0.00
1417044_at	0.00	0.00
1417045_at	0.00	0.00
1417046_at	0.00	0.00
1417047_at	0.00	0.00
1417048_at	0.00	0.00
1417049_at	0.00	0.00
1417050_at	0.00	0.00
1417051_at	0.00	0.00
1417052_at	0.00	0.33
1417053_at	0.00	0.38
1417054_a_at	0.00	0.22
1417055_at	0.00	0.26
1417056_at	0.00	0.33
1417057_a_at	0.00	0.28
1417058_a_at	0.00	0.00
1417059_at	0.00	0.00
1417060_at	0.00	0.00
1417061_at	0.00	0.20
1417062_at	0.00	0.00
1417063_at	0.00	0.00
1417064_at	0.00	0.00
1417065_at	0.00	0.00
1417066_at	0.00	0.00
1417067_s_at	0.00	0.00

1417068_a_at	0.00	0.00
1417069_a_at	0.00	0.00
1417070_at	0.00	0.00
1417071_s_at	0.00	0.00
1417072_at	0.00	0.00
1417073_a_at	0.00	0.00
1417074_at	0.00	0.00
1417075_at	0.00	0.30
1417076_at	0.00	0.00
1417077_at	0.00	0.00
1417078_at	0.00	0.00
1417079_s_at	0.00	0.00
1417080_a_at	0.00	0.33
1417081_a_at	0.00	0.00
1417082_at	0.00	0.01
1417083_at	0.00	0.00
1417084_at	0.00	0.00
1417085_at	0.00	0.00
1417086_at	0.00	0.00
1417087_at	0.00	0.00
1417088_at	0.00	0.00
1417089_a_at	0.00	0.00
1417090_at	0.36	0.17
1417091_at	0.00	0.00
1417092_at	0.00	0.05
1417093_a_at	0.00	0.09
1417094_at	0.00	0.00
1417095_a_at	0.00	0.00
1417096_at	0.00	0.05
1417097_at	0.00	0.00
1417098_s_at	0.00	0.00
1417099_at	0.00	0.00
1417100_at	0.00	0.00
1417101_at	0.00	0.00
1417102_a_at	0.00	0.00
1417103_at	0.00	0.00
1417104_at	0.00	0.00
1417105_at	0.00	0.00
1417106_at	0.00	0.00
1417107_at	0.00	0.00
1417108_at	0.00	0.00
1417109_at	0.06	0.00
1417110_at	0.03	0.00
1417111_at	0.00	0.00
1417112_at	0.00	0.00
1417113_at	0.00	0.00
1417114_at	0.00	0.00
1417115_at	0.00	0.00
1417116_at	0.00	0.00
1417117_at	0.00	0.00
1417118_a_at	0.00	0.03
1417119_at	0.00	0.00
1417120_at	0.00	0.00
1417121_at	0.00	0.00
1417122_at	0.00	0.00
1417123_at	0.00	0.00

1417124_at	0.00	0.00
1417125_at	0.00	0.00
1417126_a_at	0.00	0.00
1417127_at	0.00	0.00
1417128_at	0.00	0.00
1417129_a_at	0.00	0.00
1417130_s_at	0.01	0.02
1417131_at	0.00	0.00
1417132_at	0.00	0.00
1417133_at	0.24	0.00
1417134_at	0.00	0.00
1417135_at	0.00	0.00
1417136_s_at	0.00	0.00
1417137_at	0.00	0.00
1417138_s_at	0.00	0.00
1417139_at	0.00	0.00
1417140_a_at	0.00	0.00
1417141_at	0.00	0.00
1417142_at	0.00	0.00
1417143_at	0.00	0.00
1417144_at	0.00	0.00
1417145_at	0.00	0.04
1417146_at	0.00	0.00
1417147_at	0.00	0.00
1417148_at	0.00	0.00
1417149_at	0.00	0.18
1417150_at	0.00	0.00
1417151_a_at	0.00	0.00
1417152_at	0.00	0.00
1417153_at	0.00	0.00
1417154_at	0.00	0.00
1417155_at	0.98	0.64
1417156_at	0.98	0.00
1417157_at	0.00	0.00
1417158_at	0.00	0.00
1417159_at	0.00	0.00
1417160_s_at	0.00	0.00
1417161_at	0.00	0.00
1417162_at	0.08	0.00
1417163_at	0.00	0.00
1417164_at	0.00	0.00
1417165_at	0.00	0.00
1417166_at	0.00	0.00
1417167_at	0.00	0.00
1417168_a_at	0.00	0.00
1417169_at	0.00	0.00
1417170_at	0.00	0.00
1417171_at	0.00	0.00
1417172_at	0.00	0.00
1417173_at	0.00	0.00
1417174_at	0.00	0.00
1417175_at	0.00	0.29
1417176_at	0.00	0.53
1417177_at	0.00	0.33
1417178_at	0.00	0.00
1417179_at	0.00	0.01

1417180_at	0.00	0.00
1417181_a_at	0.00	0.00
1417182_at	0.00	0.00
1417183_at	0.00	0.03
1417184_s_at	0.00	0.00
1417185_at	0.00	0.00
1417186_at	0.00	0.00
1417187_at	0.00	0.00
1417188_s_at	0.00	0.00
1417189_at	0.15	0.12
1417190_at	0.00	0.00
1417191_at	0.00	0.00
1417192_at	0.00	0.59
1417193_at	0.38	0.85
1417194_at	0.00	0.00
1417195_at	0.02	0.00
1417196_s_at	0.00	0.00
1417197_at	0.00	0.07
1417198_at	0.00	0.00
1417199_at	0.00	0.00
1417200_at	0.00	0.00
1417201_at	0.00	0.00
1417202_s_at	0.00	0.00
1417203_at	0.00	0.00
1417204_at	0.00	0.00
1417205_at	0.00	0.00
1417206_at	0.00	0.00
1417207_at	0.00	0.00
1417208_at	0.00	0.00
1417209_at	0.00	0.00
1417210_at	0.00	0.00
1417211_a_at	0.00	0.00
1417212_at	0.00	0.00
1417213_a_at	0.00	0.00
1417214_at	0.00	0.00
1417215_at	0.00	0.00
1417216_at	0.00	0.00
1417217_at	0.00	0.00
1417218_at	0.00	0.00
1417219_s_at	0.00	0.02
1417220_at	0.00	0.00
1417221_at	0.00	0.00
1417222_a_at	0.00	0.00
1417223_at	0.00	0.00
1417224_a_at	0.00	0.04
1417225_at	0.00	0.00
1417226_at	0.00	0.19
1417227_at	0.00	0.00
1417228_at	0.00	0.00
1417229_at	0.00	0.00
1417230_at	0.00	0.00
1417231_at	0.00	0.00
1417232_at	0.00	0.00
1417233_at	0.00	0.04
1417234_at	0.00	0.00
1417235_at	0.00	0.00

1417236_at	0.00	0.00
1417237_at	0.00	0.00
1417238_at	0.00	0.00
1417239_at	0.00	0.00
1417240_at	0.62	0.17
1417241_at	0.00	0.00
1417242_at	0.00	0.23
1417243_at	0.00	0.00
1417244_a_at	0.00	0.00
1417245_at	0.00	0.00
1417246_at	0.00	0.00
1417247_at	0.00	0.00
1417248_at	0.00	0.00
1417249_at	0.00	0.00
1417250_at	0.00	0.00
1417251_at	0.00	0.00
1417252_at	0.00	0.00
1417253_at	0.00	0.00
1417254_at	0.00	0.00
1417255_at	0.00	0.00
1417256_at	0.00	0.00
1417257_at	0.00	0.00
1417258_at	0.00	0.00
1417259_a_at	0.00	0.00
1417260_at	0.00	0.00
1417261_at	0.00	0.00
1417262_at	0.00	0.00
1417263_at	0.00	0.00
1417264_at	0.00	0.00
1417265_s_at	0.00	0.00
1417266_at	0.00	0.00
1417267_s_at	0.00	0.00
1417268_at	0.00	0.00
1417269_at	0.00	0.00
1417270_at	0.00	0.00
1417271_a_at	0.00	0.00
1417272_at	0.00	0.00
1417273_at	0.00	0.00
1417274_at	0.00	0.00
1417275_at	0.00	0.00
1417276_at	0.00	0.00
1417277_at	0.00	0.00
1417278_a_at	0.00	0.00
1417279_at	0.00	0.00
1417280_at	0.00	0.00
1417281_a_at	0.00	0.00
1417282_at	0.00	0.00
1417283_at	0.00	0.00
1417284_at	0.00	0.00
1417285_a_at	0.00	0.29
1417286_at	0.00	0.09
1417287_at	0.00	0.00
1417288_at	0.08	0.05
1417289_at	0.14	0.00
1417290_at	0.00	0.00
1417291_at	0.00	0.00

1417292_at	0.00	0.00
1417293_at	0.00	0.00
1417294_at	0.00	0.10
1417295_at	0.00	0.00
1417296_at	0.00	0.15
1417297_at	0.00	0.00
1417298_at	0.00	0.00
1417299_at	0.00	0.00
1417300_at	0.20	0.00
1417301_at	0.00	0.00
1417302_at	0.33	0.00
1417303_at	0.00	0.00
1417304_at	0.00	0.00
1417305_at	0.00	0.00
1417306_at	0.00	0.00
1417307_at	0.00	0.00
1417308_at	0.00	0.00
1417309_at	0.00	0.00
1417310_at	0.00	0.00
1417311_at	0.00	0.00
1417312_at	0.00	0.00
1417313_at	0.00	0.00
1417314_at	0.00	0.00
1417315_at	0.00	0.00
1417316_at	0.00	0.01
1417317_s_at	0.00	0.00
1417318_at	0.00	0.00
1417319_at	0.00	0.00
1417320_at	0.00	0.00
1417321_at	0.00	0.26
1417322_at	0.00	0.00
1417323_at	0.00	0.00
1417324_at	0.00	0.00
1417325_at	0.00	0.00
1417326_a_at	0.00	0.00
1417327_at	0.00	0.00
1417328_at	0.00	0.00
1417329_at	0.00	0.00
1417330_at	0.00	0.00
1417331_a_at	0.00	0.28
1417332_at	0.01	0.00
1417333_at	0.00	0.00
1417334_at	0.00	0.00
1417335_at	0.00	0.00
1417336_a_at	0.00	0.00
1417337_at	0.00	0.00
1417338_at	0.00	0.00
1417339_a_at	0.00	0.00
1417340_at	0.00	0.00
1417341_a_at	0.00	0.00
1417342_at	0.00	0.00
1417343_at	0.00	0.01
1417344_at	0.00	0.00
1417345_at	0.00	0.00
1417346_at	0.75	0.00
1417347_at	0.00	0.00

1417348_at	0.00	0.00
1417349_at	0.00	0.00
1417350_at	0.00	0.00
1417351_a_at	0.00	0.18
1417352_s_at	0.00	0.06
1417353_x_at	0.00	0.17
1417354_at	0.00	0.09
1417355_at	0.98	0.13
1417356_at	1.00	0.00
1417357_at	0.00	0.00
1417358_s_at	0.00	0.00
1417359_at	0.00	0.00
1417360_at	0.00	0.00
1417361_at	0.00	0.00
1417362_at	0.00	0.00
1417363_at	0.00	0.00
1417364_at	0.00	0.00
1417365_a_at	0.00	0.00
1417366_s_at	0.00	0.00
1417367_at	0.00	0.00
1417368_s_at	0.00	0.00
1417369_at	0.00	0.00
1417370_at	0.00	0.00
1417371_at	0.00	0.00
1417372_a_at	0.00	0.00
1417373_a_at	0.46	0.81
1417374_at	0.33	0.43
1417375_at	0.00	0.00
1417376_a_at	0.00	0.38
1417377_at	0.00	0.03
1417378_at	0.00	0.33
1417379_at	0.00	0.00
1417380_at	0.00	0.00
1417381_at	0.00	0.00
1417382_at	0.00	0.00
1417383_at	0.00	0.00
1417384_at	0.00	0.00
1417385_at	0.00	0.00
1417386_at	0.00	0.00
1417387_at	0.00	0.00
1417388_at	0.00	0.03
1417389_at	0.00	0.02
1417390_at	0.00	0.30
1417391_a_at	0.00	0.00
1417392_a_at	0.28	0.00
1417393_a_at	0.00	0.03
1417394_at	0.70	0.00
1417395_at	0.34	0.00
1417396_at	0.03	0.64
1417397_at	0.00	0.00
1417398_at	0.42	0.00
1417399_at	0.00	0.00
1417400_at	0.00	0.00
1417401_at	0.00	0.00
1417402_at	0.00	0.00
1417403_at	0.00	0.00

1417404_at	0.00	0.00
1417405_at	0.00	0.00
1417406_at	0.00	0.00
1417407_at	0.00	0.00
1417408_at	0.00	0.00
1417409_at	0.67	0.26
1417410_s_at	0.00	0.00
1417411_at	0.00	0.00
1417412_at	0.00	0.00
1417413_at	0.00	0.00
1417414_at	0.00	0.00
1417415_at	0.00	0.00
1417416_at	0.00	0.00
1417417_a_at	0.00	0.00
1417418_s_at	0.00	0.00
1417419_at	0.05	0.21
1417420_at	0.00	0.56
1417421_at	0.00	0.00
1417422_at	0.00	0.00
1417423_at	0.00	0.70
1417424_at	0.00	0.00
1417425_at	0.00	0.00
1417426_at	0.00	0.01
1417427_at	0.00	0.00
1417428_at	0.00	0.00
1417429_at	0.00	0.00
1417430_at	0.00	0.00
1417431_a_at	0.00	0.00
1417432_a_at	0.00	0.00
1417433_at	0.00	0.00
1417434_at	0.00	0.00
1417435_at	0.00	0.00
1417436_at	0.00	0.00
1417437_at	0.00	0.00
1417438_at	0.00	0.00
1417439_at	0.00	0.00
1417440_at	0.04	0.00
1417441_at	0.00	0.00
1417442_a_at	0.00	0.00
1417443_at	0.00	0.00
1417444_at	0.00	0.02
1417445_at	0.00	0.00
1417446_at	0.00	0.00
1417447_at	0.00	0.00
1417448_at	0.00	0.00
1417449_at	0.00	0.00
1417450_a_at	0.00	0.00
1417451_a_at	0.00	0.00
1417452_a_at	0.00	0.00
1417453_at	0.00	0.00
1417454_at	0.00	0.00
1417455_at	0.00	0.00
1417456_at	0.00	0.00
1417457_at	0.00	0.00
1417458_s_at	0.00	0.00
1417459_at	0.00	0.00

1417460_at	0.10	0.00
1417461_at	0.00	0.00
1417462_at	0.00	0.00
1417463_a_at	0.00	0.00
1417464_at	0.00	0.00
1417465_at	0.00	0.00
1417466_at	0.00	0.00
1417467_a_at	0.00	0.00
1417468_at	0.00	0.00
1417469_at	0.00	0.00
1417470_at	0.36	0.00
1417471_s_at	0.00	0.00
1417472_at	0.11	0.15
1417473_a_at	0.00	0.00
1417474_at	0.00	0.00
1417475_at	0.00	0.00
1417476_at	0.00	0.00
1417477_at	0.00	0.00
1417478_a_at	0.00	0.14
1417479_at	0.00	0.00
1417480_at	0.00	0.05
1417481_at	0.00	0.00
1417482_at	0.00	0.00
1417483_at	0.00	0.00
1417484_at	0.00	0.00
1417485_at	0.00	0.00
1417486_at	0.00	0.00
1417487_at	0.00	0.00
1417488_at	0.00	0.00
1417489_at	0.00	0.00
1417490_at	0.00	0.00
1417491_at	0.00	0.00
1417492_at	0.00	0.00
1417493_at	0.00	0.00
1417494_a_at	0.00	0.00
1417495_x_at	0.00	0.00
1417496_at	0.00	0.00
1417497_at	0.00	0.00
1417498_at	0.00	0.00
1417499_at	0.00	0.36
1417500_a_at	0.00	0.10
1417501_at	0.00	0.00
1417502_at	0.00	0.00
1417503_at	0.00	0.00
1417504_at	0.00	0.00
1417505_s_at	0.00	0.00
1417506_at	0.00	0.01
1417507_at	0.00	0.00
1417508_at	0.08	0.00
1417509_at	0.07	0.00
1417510_at	0.00	0.00
1417511_at	0.00	0.00
1417512_at	0.00	0.00
1417513_at	0.00	0.00
1417514_at	0.00	0.00
1417515_at	0.00	0.00

1417516_at	0.00	0.01
1417517_at	0.00	0.00
1417518_at	0.00	0.00
1417519_at	0.00	0.00
1417520_at	0.00	0.00
1417521_at	0.00	0.00
1417522_at	0.00	0.00
1417523_at	0.00	0.00
1417524_at	0.00	0.00
1417525_at	0.00	0.00
1417526_at	0.00	0.00
1417527_at	0.00	0.00
1417528_at	0.00	0.00
1417529_at	0.00	0.00
1417530_a_at	0.00	0.00
1417531_at	0.00	0.00
1417532_at	0.00	0.00
1417533_a_at	0.00	0.00
1417534_at	0.00	0.00
1417535_at	0.00	0.00
1417536_at	0.00	0.00
1417537_at	0.00	0.00
1417538_at	0.00	0.00
1417539_at	0.00	0.00
1417540_at	0.00	0.00
1417541_at	0.08	0.38
1417542_at	0.00	0.01
1417543_at	0.00	0.00
1417544_a_at	0.00	0.04
1417545_at	0.00	0.00
1417546_at	0.00	0.00
1417547_at	0.00	0.00
1417548_at	0.00	0.00
1417549_at	0.00	0.00
1417550_a_at	0.00	0.00
1417551_at	0.00	0.00
1417552_at	0.00	0.00
1417553_at	0.00	0.00
1417554_at	0.00	0.00
1417555_at	0.00	0.00
1417556_at	0.00	0.00
1417557_at	0.00	0.00
1417558_at	0.00	0.00
1417559_at	0.00	0.00
1417560_at	0.00	0.00
1417561_at	0.00	0.00
1417562_at	0.00	0.00
1417563_at	0.00	0.00
1417564_at	0.00	0.00
1417565_at	0.00	0.00
1417566_at	0.00	0.00
1417567_at	0.00	0.00
1417568_at	0.00	0.00
1417569_at	0.00	0.00
1417570_at	0.00	0.00
1417571_at	0.00	0.00

1417572_at	0.00	0.00
1417573_at	0.00	0.00
1417574_at	0.00	0.00
1417575_at	0.00	0.00
1417576_a_at	0.00	0.00
1417577_at	0.00	0.00
1417578_a_at	0.00	0.00
1417579_x_at	0.00	0.00
1417580_s_at	0.00	0.00
1417581_at	0.00	0.00
1417582_s_at	0.13	0.08
1417583_a_at	0.00	0.00
1417584_at	0.00	0.00
1417585_at	0.00	0.00
1417586_at	0.00	0.00
1417587_at	0.00	0.00
1417588_at	0.00	0.00
1417589_at	0.00	0.00
1417590_at	0.00	0.00
1417591_at	0.00	0.00
1417592_at	0.00	0.00
1417593_at	0.00	0.00
1417594_at	0.00	0.00
1417595_at	0.00	0.00
1417596_at	0.00	0.00
1417597_at	0.00	0.00
1417598_a_at	0.00	0.00
1417599_at	0.00	0.00
1417600_at	0.00	0.00
1417601_at	0.00	0.00
1417602_at	0.00	0.00
1417603_at	0.00	0.00
1417604_at	0.00	0.00
1417605_s_at	0.00	0.00
1417606_a_at	0.00	0.00
1417607_at	0.00	0.00
1417608_a_at	0.00	0.00
1417609_at	0.00	0.00
1417610_at	0.00	0.00
1417611_at	0.00	0.00
1417612_at	0.65	0.33
1417613_at	0.00	0.00
1417614_at	0.00	0.00
1417615_a_at	0.00	0.00
1417616_at	0.00	0.00
1417617_at	0.00	0.00
1417618_at	0.00	0.00
1417619_at	0.00	0.24
1417620_at	0.00	0.00
1417621_at	0.00	0.00
1417622_at	0.00	0.00
1417623_at	0.00	0.00
1417624_at	0.03	0.00
1417625_s_at	0.00	0.00
1417626_at	0.00	0.00
1417627_a_at	0.00	0.00

1417628_at	0.00	0.00
1417629_at	0.00	0.00
1417630_at	0.00	0.00
1417631_at	0.00	0.00
1417632_at	0.00	0.00
1417633_at	0.00	0.00
1417634_at	0.00	0.00
1417635_at	0.00	0.00
1417636_at	0.00	0.00
1417637_a_at	0.00	0.00
1417638_at	1.00	0.00
1417639_at	0.00	0.00
1417640_at	0.00	0.00
1417641_at	0.00	0.00
1417642_at	0.00	0.00
1417643_at	0.00	0.00
1417644_at	0.00	0.00
1417645_at	0.00	0.00
1417646_a_at	0.00	0.31
1417647_at	0.00	0.10
1417648_s_at	0.00	0.16
1417649_at	0.00	0.00
1417650_at	0.00	0.00
1417651_at	0.00	0.00
1417652_a_at	0.00	0.00
1417653_at	0.00	0.00
1417654_at	0.00	0.00
1417655_a_at	0.00	0.33
1417656_at	0.94	0.00
1417657_s_at	0.00	0.10
1417658_at	0.05	0.18
1417659_at	0.00	0.00
1417660_s_at	0.00	0.00
1417661_at	0.00	0.00
1417662_at	0.00	0.00
1417663_a_at	0.00	0.00
1417664_a_at	0.00	0.00
1417665_a_at	0.00	0.00
1417666_at	0.00	0.00
1417667_a_at	0.00	0.00
1417668_at	0.00	0.00
1417669_at	0.00	0.00
1417670_at	0.00	0.00
1417671_at	0.00	0.00
1417672_at	0.00	0.00
1417673_at	0.00	0.00
1417674_s_at	0.00	0.00
1417675_a_at	0.00	0.14
1417676_a_at	0.00	0.00
1417677_at	0.00	0.00
1417678_at	0.00	0.00
1417679_at	0.00	0.00
1417680_at	0.00	0.00
1417681_at	0.00	0.00
1417682_a_at	0.00	0.00
1417683_at	0.00	0.00

1417684_at	0.00	0.01
1417685_at	0.00	0.00
1417686_at	0.00	0.00
1417687_at	0.00	0.00
1417688_at	0.00	0.00
1417689_a_at	0.00	0.00
1417690_at	0.00	0.00
1417691_at	0.00	0.00
1417692_at	0.00	0.05
1417693_a_at	0.00	0.00
1417694_at	0.00	0.00
1417695_a_at	0.62	0.41
1417696_at	0.34	0.04
1417697_at	0.75	0.47
1417698_at	0.00	0.24
1417699_at	0.00	0.33
1417700_at	0.00	0.00
1417701_at	0.00	0.00
1417702_a_at	0.00	0.00
1417703_at	0.00	0.00
1417704_a_at	0.00	0.00
1417705_at	0.00	0.00
1417706_at	0.00	0.01
1417707_at	0.00	0.00
1417708_at	0.00	0.00
1417709_at	0.00	0.00
1417710_at	0.00	0.00
1417711_at	0.00	0.00
1417712_at	0.00	0.00
1417713_at	0.00	0.00
1417714_x_at	0.00	0.00
1417715_a_at	0.00	0.00
1417716_at	0.00	0.00
1417717_a_at	0.00	0.00
1417718_at	0.00	0.00
1417719_at	0.11	0.01
1417720_at	0.00	0.26
1417721_s_at	0.00	0.00
1417722_at	0.00	0.00
1417723_at	0.00	0.00
1417724_at	0.00	0.23
1417725_a_at	0.00	0.33
1417726_at	0.00	0.28
1417727_at	0.00	0.00
1417728_at	0.00	0.07
1417729_at	0.00	0.00
1417730_at	0.00	0.00
1417731_at	0.00	0.00
1417732_at	0.00	0.00
1417733_at	0.00	0.00
1417734_at	0.00	0.00
1417735_at	0.00	0.00
1417736_at	0.00	0.00
1417737_at	0.00	0.12
1417738_at	0.00	0.00
1417739_at	0.00	0.00

1417740_at	0.00	0.00
1417741_at	0.00	0.04
1417742_a_at	0.00	0.00
1417743_at	0.00	0.00
1417744_a_at	0.00	0.30
1417745_at	0.00	0.00
1417746_at	0.00	0.00
1417747_at	0.00	0.00
1417748_x_at	0.00	0.00
1417749_a_at	0.00	0.00
1417750_a_at	0.00	0.00
1417751_at	0.00	0.00
1417752_at	0.00	0.03
1417753_at	0.00	0.00
1417754_at	0.00	0.26
1417755_at	0.00	0.00
1417756_a_at	0.00	0.00
1417757_at	0.00	0.00
1417758_at	0.00	0.00
1417759_at	0.00	0.00
1417760_at	1.00	1.00
1417761_at	0.00	0.00
1417762_a_at	0.00	0.00
1417763_at	0.00	0.00
1417764_at	0.00	0.00
1417765_a_at	0.00	0.00
1417766_at	0.00	0.00
1417767_at	0.00	0.00
1417768_at	0.00	0.00
1417769_at	0.00	0.00
1417770_s_at	0.00	0.00
1417771_a_at	0.00	0.00
1417772_at	0.00	0.00
1417773_at	0.00	0.00
1417774_at	0.00	0.00
1417775_at	0.00	0.30
1417776_at	0.00	0.00
1417777_at	0.00	0.00
1417778_at	0.00	0.00
1417779_at	0.00	0.00
1417780_at	0.00	0.24
1417781_at	0.00	0.00
1417782_at	0.00	0.00
1417783_at	0.00	0.00
1417784_at	0.00	0.00
1417785_at	0.00	0.03
1417786_a_at	0.00	0.00
1417787_at	0.00	0.00
1417788_at	0.00	0.00
1417789_at	0.00	0.00
1417790_at	0.00	0.00
1417791_a_at	0.00	0.10
1417792_at	0.00	0.00
1417793_at	0.00	0.00
1417794_at	0.00	0.00
1417795_at	0.00	0.00

1417796_at	0.00	0.00
1417797_a_at	0.00	0.00
1417798_at	0.00	0.00
1417799_at	0.00	0.00
1417800_at	0.00	0.00
1417801_a_at	0.00	0.00
1417802_at	0.00	0.00
1417803_at	0.00	0.00
1417804_at	0.47	0.73
1417805_at	0.00	0.00
1417806_at	0.00	0.00
1417807_at	0.00	0.00
1417808_at	0.00	0.00
1417809_at	0.00	0.00
1417810_a_at	0.00	0.00
1417811_at	0.00	0.00
1417812_a_at	0.00	0.00
1417813_at	0.00	0.00
1417814_at	0.00	0.00
1417815_a_at	0.02	0.00
1417816_s_at	0.00	0.00
1417817_a_at	0.14	0.00
1417818_at	0.83	0.18
1417819_at	0.00	0.00
1417820_at	0.00	0.00
1417821_at	0.00	0.30
1417822_at	0.00	0.00
1417823_at	0.00	0.00
1417824_at	0.00	0.00
1417825_at	0.00	0.00
1417826_at	0.00	0.00
1417827_at	0.00	0.00
1417828_at	0.00	0.00
1417829_a_at	0.00	0.22
1417830_at	0.00	0.00
1417831_at	0.00	0.00
1417832_at	0.00	0.00
1417833_at	0.00	0.00
1417834_at	0.00	0.00
1417835_at	0.00	0.00
1417836_at	0.00	0.00
1417837_at	0.00	0.34
1417838_at	0.00	0.00
1417839_at	0.00	0.00
1417840_at	0.00	0.00
1417841_at	0.00	0.00
1417842_at	0.00	0.00
1417843_s_at	0.00	0.00
1417844_at	0.00	0.00
1417845_at	0.00	0.69
1417846_at	0.00	0.00
1417847_at	0.03	0.00
1417848_at	0.00	0.00
1417849_at	0.00	0.00
1417850_at	0.00	0.00
1417851_at	0.00	0.00

1417852_x_at	0.00	0.00
1417853_at	0.00	0.00
1417854_at	0.00	0.00
1417855_at	0.00	0.00
1417856_at	0.00	0.00
1417857_at	0.00	0.00
1417858_at	0.00	0.00
1417859_at	0.00	0.00
1417860_a_at	0.00	0.00
1417861_at	0.00	0.00
1417862_at	0.00	0.00
1417863_at	0.00	0.00
1417864_at	0.00	0.00
1417865_at	0.00	0.00
1417866_at	0.00	0.00
1417867_at	0.00	0.00
1417868_a_at	0.00	0.00
1417869_s_at	0.00	0.00
1417870_x_at	0.00	0.01
1417871_at	0.00	0.00
1417872_at	0.00	0.00
1417873_at	0.00	0.14
1417874_at	0.07	0.00
1417875_at	0.00	0.00
1417876_at	0.00	0.00
1417877_at	0.00	0.00
1417878_at	0.00	0.00
1417879_at	0.00	0.00
1417880_at	0.00	0.00
1417881_at	0.00	0.00
1417882_at	0.00	0.00
1417883_at	0.00	0.00
1417884_at	0.00	0.00
1417885_at	0.00	0.00
1417886_at	0.00	0.01
1417887_at	0.00	0.05
1417888_at	0.04	0.00
1417889_at	0.00	0.00
1417890_at	0.00	0.00
1417891_at	0.00	0.00
1417892_a_at	0.00	0.00
1417893_at	0.00	0.00
1417894_at	0.00	0.00
1417895_a_at	0.00	0.00
1417896_at	0.00	0.00
1417897_at	0.00	0.00
1417898_a_at	0.00	0.00
1417899_at	0.00	0.00
1417900_a_at	0.00	0.00
1417901_a_at	0.00	0.00
1417902_at	0.00	0.00
1417903_at	0.00	0.00
1417904_at	0.00	0.00
1417905_at	0.00	0.00
1417906_at	0.00	0.00
1417907_at	0.00	0.00

1417908_s_at	0.00	0.00
1417909_at	0.00	0.00
1417910_at	0.00	0.00
1417911_at	0.00	0.00
1417912_at	0.00	0.00
1417913_at	0.00	0.00
1417914_at	0.00	0.00
1417915_at	0.00	0.00
1417916_a_at	0.00	0.00
1417917_at	0.78	0.00
1417918_at	0.00	0.00
1417919_at	0.00	0.00
1417920_at	0.00	0.00
1417921_at	0.00	0.33
1417922_at	0.00	0.00
1417923_at	0.00	0.00
1417924_at	0.00	0.00
1417925_at	0.00	0.00
1417926_at	0.00	0.00
1417927_at	0.00	0.00
1417928_at	0.00	0.11
1417929_at	0.00	0.00
1417930_at	0.00	0.00
1417931_at	0.00	0.00
1417932_at	0.00	0.00
1417933_at	0.00	0.00
1417934_at	0.00	0.00
1417935_at	0.00	0.00
1417936_at	0.00	0.00
1417937_at	0.00	0.00
1417938_at	0.00	0.00
1417939_at	0.00	0.00
1417940_s_at	0.00	0.00
1417941_at	0.00	0.00
1417942_at	0.00	0.00
1417943_at	0.00	0.00
1417944_at	0.00	0.00
1417945_at	0.38	0.57
1417946_at	0.00	0.00
1417947_at	0.00	0.01
1417948_s_at	0.00	0.11
1417949_at	0.00	0.01
1417950_a_at	0.00	0.00
1417951_at	0.00	0.00
1417952_at	0.00	0.00
1417953_at	0.00	0.00
1417954_at	0.00	0.00
1417955_at	0.00	0.00
1417956_at	0.00	0.00
1417957_a_at	0.00	0.00
1417958_at	0.00	0.00
1417959_at	0.57	0.02
1417960_at	0.00	0.00
1417961_a_at	0.00	0.00
1417962_s_at	0.00	0.00
1417963_at	0.00	0.00

1417964_at	0.00	0.00
1417965_at	0.00	0.00
1417966_at	0.00	0.03
1417967_at	0.00	0.00
1417968_a_at	0.00	0.00
1417969_at	0.00	0.00
1417970_at	0.00	0.00
1417971_at	0.00	0.04
1417972_s_at	0.00	0.00
1417973_at	0.00	0.00
1417974_at	0.00	0.02
1417975_at	0.00	0.00
1417976_at	0.00	0.34
1417977_at	0.00	0.00
1417978_at	0.00	0.00
1417979_at	0.00	0.00
1417980_a_at	0.00	0.00
1417981_at	0.00	0.00
1417982_at	0.00	0.00
1417983_a_at	0.00	0.01
1417984_at	0.00	0.00
1417985_at	0.00	0.00
1417986_at	0.00	0.00
1417987_at	0.00	0.00
1417988_at	0.00	0.00
1417989_at	0.00	0.00
1417990_at	0.00	0.00
1417991_at	0.00	0.00
1417992_a_at	0.00	0.00
1417993_at	0.00	0.00
1417994_a_at	0.00	0.00
1417995_at	0.00	0.00
1417996_at	0.00	0.00
1417997_at	0.00	0.00
1417998_at	0.00	0.00
1417999_at	0.00	0.05
1418000_a_at	0.00	0.33
1418001_at	0.00	0.00
1418002_at	0.00	0.00
1418003_at	0.00	0.00
1418004_a_at	0.00	0.00
1418005_at	0.00	0.00
1418006_at	0.00	0.00
1418007_at	0.00	0.00
1418008_at	0.00	0.00
1418009_at	0.00	0.00
1418010_a_at	0.00	0.00
1418011_a_at	0.00	0.05
1418012_at	0.01	0.05
1418013_at	0.00	0.00
1418014_a_at	0.00	0.00
1418015_at	0.00	0.00
1418016_at	0.00	0.00
1418017_at	0.00	0.00
1418018_at	0.00	0.28
1418019_at	0.00	0.00

1418020_s_at	0.02	0.00
1418021_at	0.00	0.00
1418022_at	0.00	0.22
1418023_at	0.00	0.06
1418024_at	0.00	0.00
1418025_at	0.02	0.00
1418026_at	0.00	0.00
1418027_at	0.00	0.00
1418028_at	0.00	0.00
1418029_at	0.00	0.00
1418030_at	0.00	0.00
1418031_at	0.00	0.00
1418032_at	0.00	0.00
1418033_s_at	0.00	0.00
1418034_at	0.00	0.00
1418035_a_at	0.00	0.00
1418036_at	0.00	0.00
1418037_at	0.00	0.00
1418038_s_at	0.00	0.00
1418039_at	0.00	0.00
1418040_at	0.00	0.00
1418041_at	0.00	0.00
1418042_a_at	0.00	0.00
1418043_at	0.00	0.00
1418044_at	0.00	0.00
1418045_at	0.00	0.00
1418046_at	0.00	0.00
1418047_at	0.00	0.00
1418048_at	0.00	0.00
1418049_at	0.00	0.00
1418050_at	0.00	0.00
1418051_at	0.00	0.00
1418052_at	0.00	0.00
1418053_at	0.00	0.00
1418054_at	0.00	0.00
1418055_at	0.00	0.00
1418056_at	0.00	0.00
1418057_at	0.04	0.00
1418058_at	0.00	0.00
1418059_at	0.00	0.00
1418060_a_at	0.00	0.00
1418061_at	0.00	0.00
1418062_at	0.00	0.00
1418063_at	0.00	0.00
1418064_at	0.00	0.00
1418065_at	0.00	0.00
1418066_at	0.00	0.00
1418067_at	0.00	0.00
1418068_at	0.00	0.00
1418069_at	0.00	0.00
1418070_at	0.19	0.14
1418071_s_at	0.00	0.00
1418072_at	0.00	0.00
1418073_at	0.00	0.00
1418074_at	0.00	0.00
1418075_at	0.00	0.00

1418076_at	0.00	0.01
1418077_at	0.00	0.00
1418078_at	0.00	0.03
1418079_at	0.00	0.00
1418080_at	0.00	0.00
1418081_at	0.00	0.00
1418082_at	0.00	0.00
1418083_at	0.00	0.00
1418084_at	0.00	0.00
1418085_at	0.00	0.13
1418086_at	0.00	0.00
1418087_at	0.00	0.00
1418088_a_at	0.00	0.00
1418089_at	0.00	0.00
1418090_at	0.00	0.00
1418091_at	1.00	0.07
1418092_s_at	0.00	0.00
1418093_a_at	0.00	0.00
1418094_s_at	0.00	1.00
1418095_at	0.00	0.00
1418096_at	0.00	0.00
1418097_a_at	0.00	0.00
1418098_at	0.00	0.00
1418099_at	0.00	0.00
1418100_at	0.00	0.00
1418101_a_at	0.00	0.03
1418102_at	0.00	0.00
1418103_at	0.00	0.00
1418104_at	0.00	0.00
1418105_at	0.00	0.00
1418106_at	0.00	0.00
1418107_at	0.00	0.00
1418108_at	0.00	0.00
1418109_at	0.00	0.00
1418110_a_at	0.00	0.00
1418111_at	0.00	0.00
1418112_at	0.00	0.00
1418113_at	0.00	0.00
1418114_at	0.01	0.00
1418115_s_at	0.00	0.00
1418116_at	0.00	0.00
1418117_at	0.00	0.00
1418118_at	0.00	0.00
1418119_at	0.00	0.01
1418120_at	0.00	0.00
1418121_at	0.00	0.00
1418122_at	0.00	0.00
1418123_at	0.00	0.00
1418124_at	0.00	0.00
1418125_at	0.00	0.18
1418126_at	0.00	0.00
1418127_a_at	0.00	0.03
1418128_at	0.00	0.11
1418129_at	0.00	0.00
1418130_at	0.00	0.00
1418131_at	0.00	0.00

1418132_a_at	0.00	0.00
1418133_at	0.00	0.00
1418134_at	0.00	0.00
1418135_at	0.00	0.00
1418136_at	0.03	0.00
1418137_at	0.00	0.00
1418138_at	0.00	0.00
1418139_at	0.00	0.00
1418140_at	0.00	0.00
1418141_at	0.00	0.00
1418142_at	0.00	0.00
1418143_at	0.00	0.00
1418144_a_at	0.00	0.00
1418145_at	0.00	0.00
1418146_a_at	0.00	0.00
1418147_at	0.00	0.00
1418148_at	0.00	0.00
1418149_at	0.00	0.00
1418150_at	0.00	0.00
1418151_at	0.00	0.00
1418152_at	0.00	0.00
1418153_at	0.00	0.00
1418154_at	0.00	0.00
1418155_at	0.00	0.00
1418156_at	0.00	0.00
1418157_at	0.00	0.00
1418158_at	0.00	0.00
1418159_at	0.00	0.00
1418160_at	0.00	0.00
1418161_at	0.00	0.00
1418162_at	0.00	0.00
1418163_at	0.00	0.00
1418164_at	0.00	0.00
1418165_at	0.00	0.00
1418166_at	0.00	0.00
1418167_at	0.00	0.00
1418168_at	0.00	0.00
1418169_at	0.00	0.00
1418170_a_at	0.00	0.00
1418171_at	0.00	0.00
1418172_at	0.00	0.00
1418173_at	0.00	0.00
1418174_at	0.00	0.00
1418175_at	0.00	0.00
1418176_at	0.00	0.00
1418177_at	0.00	0.00
1418178_at	0.55	0.03
1418179_at	0.00	0.00
1418180_at	0.00	0.00
1418181_at	0.00	0.00
1418182_at	0.00	0.00
1418183_a_at	0.00	0.00
1418184_at	0.00	0.00
1418185_at	0.00	0.00
1418186_at	0.00	0.00
1418187_at	0.00	0.50

1418188_a_at	0.01	0.00
1418189_s_at	0.00	0.00
1418190_at	0.00	0.00
1418191_at	0.00	0.00
1418192_at	0.00	0.00
1418193_at	0.00	0.00
1418194_at	0.00	0.00
1418195_at	0.00	0.00
1418196_at	0.00	0.00
1418197_at	0.00	0.00
1418198_a_at	0.00	0.00
1418199_at	0.00	0.00
1418200_at	0.00	0.00
1418201_at	0.09	0.32
1418202_a_at	0.00	0.00
1418203_at	0.00	0.01
1418204_s_at	0.00	0.00
1418205_at	0.00	0.00
1418206_at	0.00	0.00
1418207_at	0.00	0.00
1418208_at	0.00	0.00
1418209_a_at	0.00	0.07
1418210_at	0.00	0.04
1418211_at	0.00	0.00
1418212_at	0.00	0.00
1418213_at	0.00	0.00
1418214_at	0.00	0.00
1418215_at	0.00	0.00
1418216_at	0.00	0.00
1418217_at	0.00	0.00
1418218_at	0.00	0.00
1418219_at	0.00	0.00
1418220_at	0.00	0.00
1418221_at	0.00	0.00
1418222_at	0.00	0.00
1418223_at	0.00	0.00
1418224_at	0.00	0.00
1418225_at	0.00	0.29
1418226_at	0.00	0.33
1418227_at	0.00	0.12
1418228_at	0.00	0.62
1418229_s_at	0.00	0.32
1418230_a_at	0.00	0.00
1418231_at	0.00	0.00
1418232_s_at	0.00	0.00
1418233_a_at	0.00	0.00
1418234_s_at	0.00	0.22
1418235_at	0.00	0.00
1418236_s_at	0.00	0.00
1418237_s_at	0.00	0.00
1418238_at	0.00	0.00
1418239_at	0.00	0.00
1418240_at	0.12	0.00
1418241_at	0.00	0.00
1418242_at	0.00	0.00
1418243_at	0.00	0.00

1418244_at	0.00	0.00
1418245_a_at	0.00	0.00
1418246_at	0.00	0.00
1418247_s_at	0.00	0.00
1418248_at	0.00	0.00
1418249_at	0.00	0.00
1418250_at	0.00	0.00
1418251_at	0.00	0.00
1418252_at	0.00	0.00
1418253_a_at	0.00	0.00
1418254_at	0.00	0.00
1418255_s_at	0.00	0.00
1418256_at	0.00	0.00
1418257_at	0.00	0.00
1418258_s_at	0.00	0.00
1418259_a_at	0.00	0.00
1418260_at	0.00	0.00
1418261_at	0.00	0.00
1418262_at	0.00	0.00
1418263_at	0.00	0.00
1418264_at	0.00	0.00
1418265_s_at	0.00	0.00
1418266_at	0.00	0.00
1418267_at	0.00	0.00
1418268_at	0.00	0.00
1418269_at	0.00	0.00
1418270_at	0.00	0.00
1418271_at	0.00	0.00
1418272_at	0.00	0.00
1418273_a_at	0.00	0.00
1418274_at	0.00	0.01
1418275_a_at	0.00	0.00
1418276_at	0.00	0.00
1418277_at	0.00	0.00
1418278_at	0.00	0.00
1418279_a_at	0.00	0.01
1418280_at	0.00	0.33
1418281_at	0.00	0.00
1418282_x_at	0.00	0.00
1418283_at	0.00	0.00
1418284_at	0.00	0.00
1418285_at	0.00	0.00
1418286_a_at	0.00	0.00
1418287_a_at	0.00	0.00
1418288_at	0.00	0.01
1418289_at	0.00	0.00
1418290_a_at	0.00	0.00
1418291_at	0.00	0.00
1418292_at	0.00	0.26
1418293_at	0.00	0.00
1418294_at	0.00	0.00
1418295_s_at	0.00	0.00
1418296_at	0.00	0.00
1418297_at	0.00	0.00
1418298_s_at	0.00	0.00
1418299_at	0.00	0.00

1418300_a_at	0.00	0.00
1418301_at	0.00	0.09
1418302_at	0.00	0.00
1418303_at	0.00	0.00
1418304_at	0.00	0.00
1418305_s_at	0.00	0.00
1418306_at	0.00	0.00
1418307_a_at	0.00	0.00
1418308_at	0.00	0.00
1418309_at	0.00	0.00
1418310_a_at	0.00	0.00
1418311_at	0.00	0.00
1418312_at	0.00	0.00
1418313_at	0.00	0.00
1418314_a_at	0.00	0.00
1418315_at	0.00	0.00
1418316_a_at	0.00	0.00
1418317_at	0.00	0.00
1418318_at	0.87	0.99
1418319_at	0.00	0.00
1418320_at	0.00	0.49
1418321_at	0.00	0.00
1418322_at	0.00	0.00
1418323_at	0.00	0.00
1418324_at	0.00	0.24
1418325_at	0.00	0.00
1418326_at	0.00	0.00
1418327_at	0.00	0.00
1418328_at	0.00	0.00
1418329_at	0.00	0.00
1418330_at	0.00	0.02
1418331_at	0.00	0.00
1418332_a_at	0.00	0.00
1418333_at	0.00	0.00
1418334_at	0.01	0.01
1418335_a_at	0.00	0.00
1418336_at	0.00	0.00
1418337_at	0.00	0.00
1418338_at	0.00	0.00
1418339_at	0.00	0.00
1418340_at	0.00	0.00
1418341_at	0.00	0.00
1418342_at	0.00	0.00
1418343_at	0.00	0.00
1418344_at	0.00	0.00
1418345_at	0.00	0.00
1418346_at	0.00	0.00
1418347_at	0.00	0.00
1418348_a_at	0.00	0.00
1418349_at	0.00	0.00
1418350_at	0.00	0.00
1418351_a_at	0.00	0.00
1418352_at	0.00	0.00
1418353_at	0.00	0.00
1418354_at	0.00	0.00
1418355_at	0.00	0.00

1418356_at	0.00	0.00
1418357_at	0.00	0.00
1418358_at	0.00	0.00
1418359_at	0.00	0.17
1418360_at	0.00	0.00
1418361_at	0.00	0.00
1418362_at	1.00	0.97
1418363_at	0.00	0.00
1418364_a_at	0.00	0.00
1418365_at	0.00	0.00
1418366_at	0.00	0.00
1418367_x_at	0.00	0.00
1418368_at	0.00	0.00
1418369_at	0.00	0.00
1418370_at	0.01	0.04
1418371_at	0.00	0.00
1418372_at	0.00	0.09
1418373_at	0.00	0.00
1418374_at	0.00	0.00
1418375_at	0.00	0.00
1418376_at	0.53	0.00
1418377_a_at	0.00	0.00
1418378_at	0.00	0.00
1418379_s_at	0.00	0.00
1418380_at	0.00	0.00
1418381_at	0.00	0.00
1418382_at	0.00	0.00
1418383_at	0.00	0.00
1418384_at	0.00	0.00
1418385_at	0.00	0.00
1418386_at	0.00	0.45
1418387_at	0.00	0.00
1418388_s_at	0.00	0.00
1418389_at	0.00	0.00
1418390_at	0.00	0.00
1418391_at	0.00	0.00
1418392_a_at	0.00	0.00
1418393_a_at	0.00	0.00
1418394_a_at	0.00	0.00
1418395_at	0.55	0.00
1418396_at	0.00	0.00
1418397_at	0.00	0.00
1418398_a_at	0.00	0.00
1418399_at	0.00	0.00
1418400_at	0.00	0.00
1418401_a_at	0.00	0.32
1418402_at	0.00	0.00
1418403_at	0.00	0.00
1418404_at	0.00	0.00
1418405_at	0.00	0.00
1418406_at	0.00	0.00
1418407_at	0.00	0.00
1418408_at	0.00	0.00
1418409_at	0.00	0.00
1418410_at	0.00	0.00
1418411_at	0.00	0.00

1418412_at	0.00	0.00
1418413_at	0.00	0.00
1418414_at	0.00	0.00
1418415_at	0.00	0.00
1418416_x_at	0.00	0.00
1418417_at	0.00	0.00
1418418_a_at	0.00	0.00
1418419_at	0.00	0.00
1418420_at	0.00	0.00
1418421_at	0.00	0.00
1418422_at	0.00	0.00
1418423_s_at	0.00	0.00
1418424_at	0.00	0.00
1418425_at	0.00	0.00
1418426_at	0.00	0.00
1418427_at	0.00	0.00
1418428_at	0.00	0.00
1418429_at	0.00	0.00
1418430_at	0.00	0.00
1418431_at	0.00	0.00
1418432_at	0.00	0.00
1418433_at	0.00	0.00
1418434_at	0.63	0.00
1418435_at	0.61	0.00
1418436_at	0.00	0.92
1418437_a_at	0.00	0.00
1418438_at	0.00	0.00
1418439_at	0.00	0.00
1418440_at	0.00	0.00
1418441_at	0.00	0.00
1418442_at	0.00	0.00
1418443_at	0.00	0.03
1418444_a_at	0.00	0.00
1418445_at	0.00	0.00
1418446_at	0.00	0.00
1418447_at	0.00	0.00
1418448_at	0.31	0.72
1418449_at	0.00	0.00
1418450_at	0.00	0.00
1418451_at	0.00	0.00
1418452_at	0.00	0.00
1418453_a_at	0.00	0.00
1418454_at	0.00	0.00
1418455_at	0.00	0.00
1418456_a_at	0.00	0.00
1418457_at	0.00	0.00
1418458_at	0.00	0.00
1418459_at	0.00	0.00
1418460_at	0.00	0.01
1418461_at	0.00	0.00
1418462_at	0.00	0.00
1418463_at	0.00	0.00
1418464_at	0.00	0.00
1418465_at	0.00	0.00
1418466_at	0.00	0.00
1418467_at	0.00	0.00

1418468_at	0.01	0.00
1418469_at	0.00	0.00
1418470_at	0.00	0.00
1418471_at	0.00	0.00
1418472_at	0.00	0.00
1418473_at	0.00	0.00
1418474_at	0.00	0.00
1418475_at	0.00	0.00
1418476_at	0.00	0.00
1418477_at	0.00	0.00
1418478_at	0.00	0.00
1418479_at	0.00	0.00
1418480_at	0.00	0.00
1418481_at	0.00	0.00
1418482_at	0.00	0.00
1418483_a_at	0.00	0.00
1418484_at	0.00	0.00
1418485_at	0.00	0.00
1418486_at	0.00	0.00
1418487_at	0.00	0.01
1418488_s_at	0.00	0.42
1418489_a_at	0.00	0.00
1418490_at	0.00	0.00
1418491_a_at	0.00	0.00
1418492_at	0.00	0.00
1418493_a_at	0.00	0.00
1418494_at	0.00	0.00
1418495_at	0.00	0.00
1418496_at	0.00	0.00
1418497_at	0.16	0.00
1418498_at	0.00	0.00
1418499_a_at	0.00	0.00
1418500_at	0.00	0.00
1418501_a_at	0.00	0.00
1418502_a_at	0.00	0.00
1418503_at	0.05	0.04
1418504_at	0.00	0.31
1418505_at	0.00	0.00
1418506_a_at	0.00	0.00
1418507_s_at	0.99	0.28
1418508_a_at	0.00	0.00
1418509_at	0.00	0.00
1418510_s_at	0.00	0.25
1418511_at	0.00	0.00
1418512_at	0.00	0.00
1418513_at	0.00	0.00
1418514_at	0.17	0.31
1418515_at	0.10	0.00
1418516_at	0.05	0.00
1418517_at	0.00	0.00
1418518_at	0.00	0.28
1418519_at	0.00	0.00
1418520_at	0.00	0.00
1418521_a_at	0.00	0.00
1418522_at	0.00	0.00
1418523_at	0.00	0.00

1418524_at	0.00	0.00
1418525_at	0.00	0.00
1418526_at	0.00	0.01
1418527_a_at	0.00	0.55
1418528_a_at	0.00	0.00
1418529_at	0.00	0.00
1418530_at	0.00	0.25
1418531_at	0.00	0.00
1418532_at	0.02	0.32
1418533_s_at	0.18	0.38
1418534_at	0.00	0.00
1418535_at	0.00	0.00
1418536_at	0.00	0.00
1418537_at	0.00	0.00
1418538_at	0.00	0.00
1418539_a_at	0.00	0.00
1418540_a_at	0.00	0.00
1418541_at	0.00	0.00
1418542_s_at	0.00	0.00
1418543_s_at	0.00	0.00
1418544_at	0.00	0.00
1418545_at	0.00	0.00
1418546_a_at	0.00	0.00
1418547_at	0.00	0.00
1418548_at	0.00	0.00
1418549_at	0.00	0.00
1418550_x_at	0.00	0.00
1418551_at	0.00	0.00
1418552_at	0.00	0.00
1418553_at	0.00	0.00
1418554_at	0.00	0.00
1418555_x_at	0.00	0.00
1418556_at	0.00	0.00
1418557_s_at	0.00	0.00
1418558_at	0.00	0.00
1418559_at	0.00	0.00
1418560_at	0.00	0.00
1418561_at	0.00	0.00
1418562_at	0.00	0.00
1418563_at	0.00	0.00
1418564_s_at	0.00	0.00
1418565_at	0.00	0.00
1418566_s_at	0.00	0.00
1418567_a_at	0.00	0.00
1418568_x_at	0.00	0.00
1418569_at	0.59	0.00
1418570_at	0.00	0.00
1418571_at	0.53	0.00
1418572_x_at	0.64	0.00
1418573_a_at	0.00	0.00
1418574_a_at	0.00	0.00
1418575_at	0.00	0.00
1418576_at	0.00	0.00
1418577_at	0.00	0.00
1418578_at	0.00	0.00
1418579_at	0.00	0.00

1418580_at	0.00	0.00
1418581_a_at	0.00	0.00
1418582_at	0.00	0.00
1418583_at	0.00	0.00
1418584_at	0.00	0.00
1418585_at	0.00	0.00
1418586_at	0.00	0.00
1418587_at	0.00	0.00
1418588_at	0.00	0.00
1418589_a_at	0.00	0.00
1418590_at	0.00	0.00
1418591_at	0.00	0.00
1418592_at	0.00	0.00
1418593_at	0.00	0.00
1418594_a_at	0.00	0.00
1418595_at	0.00	0.00
1418596_at	0.00	0.00
1418597_at	0.00	0.00
1418598_at	0.00	0.00
1418599_at	0.00	0.00
1418600_at	0.00	0.00
1418601_at	0.00	0.00
1418602_at	0.00	0.00
1418603_at	0.00	0.00
1418604_at	0.00	0.00
1418605_at	0.00	0.00
1418606_at	0.00	0.00
1418607_at	0.00	0.00
1418608_at	0.00	0.00
1418609_at	0.00	0.00
1418610_at	0.00	0.00
1418611_at	0.00	0.00
1418612_at	0.00	0.00
1418613_at	0.00	0.00
1418614_at	0.00	0.00
1418615_at	0.00	0.00
1418616_at	0.00	0.00
1418617_x_at	0.00	0.00
1418618_at	0.00	0.00
1418619_at	0.00	0.00
1418620_at	0.00	0.00
1418621_at	0.00	0.00
1418622_at	0.00	0.02
1418623_at	0.00	0.00
1418624_at	0.00	0.00
1418625_s_at	0.00	0.00
1418626_a_at	0.04	0.00
1418627_at	0.00	0.00
1418628_at	0.00	0.00
1418629_a_at	0.00	0.00
1418630_at	0.00	0.00
1418631_at	0.00	0.33
1418632_at	0.00	0.30
1418633_at	0.00	0.00
1418634_at	0.00	0.00
1418635_at	0.00	0.00

1418636_at	0.00	0.00
1418637_at	0.00	0.00
1418638_at	0.00	0.00
1418639_at	0.00	0.00
1418640_at	0.28	0.52
1418641_at	0.00	0.00
1418642_at	0.00	0.00
1418643_at	0.00	0.62
1418644_a_at	0.00	0.00
1418645_at	0.00	0.00
1418646_at	0.00	0.00
1418647_at	0.00	0.00
1418648_at	0.00	0.00
1418649_at	0.00	0.00
1418650_at	0.00	0.00
1418651_at	0.00	0.00
1418652_at	0.00	0.00
1418653_at	0.00	0.00
1418654_at	0.00	0.00
1418655_at	0.00	0.00
1418656_at	0.00	0.04
1418657_at	0.00	0.00
1418658_at	0.00	0.00
1418659_at	0.00	0.00
1418660_at	0.00	0.00
1418661_at	0.00	0.00
1418662_at	0.00	0.00
1418663_at	0.00	0.00
1418664_at	0.50	0.00
1418665_at	0.00	0.00
1418666_at	0.00	0.00
1418667_at	0.00	0.33
1418668_at	0.00	0.00
1418669_at	0.00	0.00
1418670_s_at	0.00	0.00
1418671_at	0.00	0.00
1418672_at	0.00	0.00
1418673_at	0.00	0.00
1418674_at	0.00	0.00
1418675_at	0.00	0.00
1418676_at	0.00	0.00
1418677_at	0.00	0.00
1418678_at	0.00	0.00
1418679_at	0.00	0.00
1418680_at	0.00	0.00
1418681_at	0.00	0.00
1418682_at	0.00	0.00
1418683_at	0.00	0.00
1418684_at	0.00	0.00
1418685_at	0.00	0.00
1418686_at	0.00	0.00
1418687_at	0.00	0.00
1418688_at	0.00	0.00
1418689_at	0.00	0.00
1418690_at	0.00	0.00
1418691_at	0.00	0.00

1418692_at	0.00	0.00
1418693_at	0.00	0.00
1418694_at	0.00	0.00
1418695_a_at	0.00	0.00
1418696_at	0.00	0.00
1418697_at	0.00	0.00
1418698_a_at	0.00	0.00
1418699_s_at	0.00	0.00
1418700_at	0.00	0.00
1418701_at	0.00	0.00
1418702_a_at	0.00	0.00
1418703_at	0.36	0.11
1418704_at	0.00	0.00
1418705_at	0.00	0.00
1418706_at	0.00	0.00
1418707_at	0.00	0.00
1418708_at	0.00	0.00
1418709_at	0.00	0.00
1418710_at	0.00	0.00
1418711_at	0.00	0.00
1418712_at	0.00	0.00
1418713_at	0.00	0.22
1418714_at	0.00	0.00
1418715_at	0.00	0.00
1418716_at	0.00	0.00
1418717_at	0.00	0.00
1418718_at	0.00	0.00
1418719_at	0.00	0.00
1418720_at	0.00	0.00
1418721_at	0.00	0.00
1418722_at	0.00	0.00
1418723_at	0.00	0.00
1418724_at	0.00	0.00
1418725_at	0.00	0.00
1418726_a_at	0.00	0.00
1418727_at	0.00	0.31
1418728_at	0.00	0.00
1418729_at	0.00	0.00
1418730_at	0.00	0.00
1418731_at	0.00	0.00
1418732_s_at	0.00	0.00
1418733_at	0.00	0.00
1418734_at	0.00	0.00
1418735_at	0.00	0.00
1418736_at	0.00	0.00
1418737_at	0.00	0.00
1418738_at	0.00	0.00
1418739_at	0.00	0.00
1418740_at	0.00	0.00
1418741_at	0.00	0.00
1418742_at	0.00	0.00
1418743_a_at	0.00	0.00
1418744_s_at	0.00	0.00
1418745_at	0.00	0.00
1418746_at	0.00	0.00
1418747_at	0.00	0.00

1418748_at	0.00	0.00
1418749_at	0.00	0.00
1418750_at	0.00	0.00
1418751_at	0.00	0.00
1418752_at	0.00	0.00
1418753_at	0.53	0.33
1418754_at	0.00	0.00
1418755_at	0.00	0.00
1418756_at	1.00	0.00
1418757_at	0.00	0.00
1418758_a_at	0.00	0.00
1418759_at	0.00	0.00
1418760_at	0.00	0.00
1418761_at	0.00	0.00
1418762_at	0.00	0.00
1418763_at	0.00	0.22
1418764_a_at	0.00	0.00
1418765_at	0.00	0.00
1418766_s_at	0.00	0.00
1418767_at	0.00	0.00
1418768_at	0.00	0.00
1418769_at	0.00	0.00
1418770_at	0.00	0.00
1418771_a_at	0.00	0.00
1418772_at	0.00	0.00
1418773_at	0.00	0.00
1418774_a_at	0.00	0.00
1418775_at	0.00	0.00
1418776_at	0.00	0.00
1418777_at	0.00	0.00
1418778_at	0.00	0.00
1418779_at	0.00	0.00
1418780_at	0.00	0.00
1418781_at	0.00	0.00
1418782_at	0.00	0.00
1418783_at	0.00	0.00
1418784_at	0.00	0.00
1418785_at	0.00	0.00
1418786_at	0.00	0.00
1418787_at	0.00	0.00
1418788_at	0.00	0.00
1418789_at	0.00	0.00
1418790_at	0.00	0.00
1418791_at	0.00	0.00
1418792_at	0.07	0.00
1418793_at	0.00	0.00
1418794_at	0.00	0.00
1418795_at	0.00	0.00
1418796_at	0.00	0.00
1418797_at	0.00	0.00
1418798_s_at	0.00	0.00
1418799_a_at	0.00	0.00
1418800_at	0.00	0.00
1418801_at	0.00	0.00
1418802_at	0.00	0.00
1418803_a_at	0.00	0.00

1418804_at	0.00	0.00
1418805_at	0.00	0.00
1418806_at	0.00	0.00
1418807_at	0.00	0.00
1418808_at	0.00	0.00
1418809_at	0.00	0.00
1418810_at	0.00	0.00
1418811_at	0.00	0.00
1418812_a_at	0.00	0.00
1418813_at	0.00	0.00
1418814_s_at	0.00	0.00
1418815_at	0.80	0.00
1418816_at	0.00	0.00
1418817_at	0.00	0.00
1418818_at	0.00	0.00
1418819_at	0.00	0.00
1418820_s_at	0.02	0.50
1418821_at	0.00	0.00
1418822_a_at	0.00	0.00
1418823_at	0.00	0.00
1418824_at	0.00	0.00
1418825_at	0.00	0.00
1418826_at	0.00	0.00
1418827_at	0.00	0.00
1418828_at	0.00	0.00
1418829_a_at	0.00	0.00
1418830_at	0.00	0.00
1418831_at	0.00	0.00
1418832_at	0.00	0.00
1418833_at	0.00	0.00
1418834_at	0.00	0.00
1418835_at	0.38	0.00
1418836_at	0.00	0.00
1418837_at	0.00	0.00
1418838_at	0.00	0.00
1418839_at	0.00	0.00
1418840_at	0.00	0.29
1418841_s_at	0.00	0.00
1418842_at	0.00	0.00
1418843_at	0.00	0.00
1418844_at	0.00	0.00
1418845_at	0.00	0.00
1418846_at	0.00	0.00
1418847_at	0.00	0.00
1418848_at	0.00	0.00
1418849_x_at	0.00	0.00
1418850_at	0.00	0.00
1418851_at	0.00	0.00
1418852_at	0.00	0.00
1418853_at	0.00	0.00
1418854_at	0.00	0.00
1418855_at	0.00	0.00
1418856_a_at	0.00	0.00
1418857_at	0.00	0.00
1418858_at	0.00	0.00
1418859_at	0.00	0.00

1418860_a_at	0.00	0.00
1418861_at	0.00	0.00
1418862_at	0.00	0.00
1418863_at	0.00	0.26
1418864_at	0.00	0.17
1418865_at	0.00	0.00
1418866_at	0.00	0.00
1418867_at	0.00	0.00
1418868_at	0.00	0.00
1418869_a_at	0.00	0.00
1418870_at	0.00	0.00
1418871_a_at	0.00	0.00
1418872_at	0.00	0.00
1418873_at	0.00	0.00
1418874_a_at	0.00	0.00
1418875_at	0.00	0.00
1418876_at	0.00	0.00
1418877_at	0.00	0.00
1418878_at	0.00	0.00
1418879_at	0.00	0.00
1418880_at	0.00	0.00
1418881_at	0.00	0.00
1418882_at	0.00	0.00
1418883_a_at	0.00	0.00
1418884_x_at	0.00	0.00
1418885_a_at	0.00	0.00
1418886_s_at	0.00	0.00
1418887_a_at	0.00	0.00
1418888_a_at	0.00	0.00
1418889_a_at	0.00	0.00
1418890_a_at	0.00	0.00
1418891_a_at	0.00	0.00
1418892_at	0.00	0.00
1418893_at	0.00	0.10
1418894_s_at	0.00	0.11
1418895_at	0.00	0.30
1418896_a_at	0.00	0.00
1418897_at	0.00	0.00
1418898_at	0.00	0.00
1418899_at	0.00	0.08
1418900_at	0.00	0.00
1418901_at	0.00	0.02
1418902_at	0.00	0.00
1418903_at	0.00	0.00
1418904_at	0.00	0.00
1418905_at	0.00	0.00
1418906_at	0.00	0.00
1418907_at	0.00	0.00
1418908_at	0.00	0.02
1418909_at	0.00	0.00
1418910_at	0.00	0.31
1418911_s_at	0.00	0.00
1418912_at	0.00	0.00
1418913_at	0.00	0.00
1418914_s_at	0.00	0.00
1418915_at	0.01	0.34

1418916_a_at	0.00	0.00
1418917_at	0.00	0.00
1418918_at	0.00	0.00
1418919_at	0.00	0.00
1418920_at	0.00	0.00
1418921_at	0.00	0.00
1418922_at	0.00	0.00
1418923_at	0.00	0.00
1418924_at	0.00	0.00
1418925_at	0.00	0.00
1418926_at	0.00	0.00
1418927_a_at	0.00	0.00
1418928_a_at	0.00	0.00
1418929_at	0.00	0.00
1418930_at	0.00	0.00
1418931_at	0.00	0.00
1418932_at	0.00	0.00
1418933_at	0.00	0.00
1418934_at	0.00	0.00
1418935_at	0.00	0.00
1418936_at	0.00	0.00
1418937_at	0.00	0.00
1418938_at	0.00	0.00
1418939_at	0.00	0.00
1418940_at	0.00	0.00
1418941_at	0.00	0.00
1418942_at	0.00	0.00
1418943_at	0.00	0.00
1418944_at	0.00	0.00
1418945_at	0.00	0.00
1418946_at	0.00	0.00
1418947_at	0.00	0.00
1418948_at	0.00	0.00
1418949_at	0.00	0.00
1418950_at	0.00	0.00
1418951_at	0.00	0.00
1418952_at	0.00	0.00
1418953_at	0.00	0.00
1418954_at	0.00	0.00
1418955_at	0.00	0.00
1418956_at	0.00	0.00
1418957_at	0.00	0.00
1418958_at	0.00	0.00
1418959_at	0.00	0.00
1418960_at	0.00	0.00
1418961_at	0.00	0.00
1418962_at	0.00	0.00
1418963_at	0.00	0.00
1418964_at	0.00	0.00
1418965_at	0.00	0.27
1418966_a_at	0.00	0.00
1418967_a_at	0.00	0.00
1418968_at	0.00	0.00
1418969_at	0.00	0.00
1418970_a_at	0.00	0.00
1418971_x_at	0.00	0.00

1418972_at	0.00	0.00
1418973_at	0.00	0.00
1418974_at	0.00	0.00
1418975_at	0.00	0.00
1418976_s_at	0.00	0.00
1418977_at	0.00	0.00
1418978_at	0.00	0.00
1418979_at	0.00	0.00
1418980_a_at	0.00	0.00
1418981_at	0.00	0.00
1418982_at	0.00	0.00
1418983_at	0.00	0.00
1418984_at	0.00	0.31
1418985_at	0.00	0.00
1418986_a_at	0.00	0.03
1418987_at	0.00	0.00
1418988_at	0.00	0.00
1418989_at	0.00	0.00
1418990_at	0.00	0.00
1418991_at	0.00	0.00
1418992_at	0.00	0.00
1418993_s_at	0.00	0.00
1418994_at	0.88	0.16
1418995_at	0.00	0.00
1418996_a_at	0.00	0.00
1418997_at	0.00	0.00
1418998_at	0.00	0.00
1418999_at	0.00	0.00
1419000_at	0.00	0.00
1419001_at	0.00	0.00
1419002_s_at	0.00	0.00
1419003_at	0.00	0.00
1419004_s_at	0.00	0.00
1419005_at	0.00	0.00
1419006_s_at	0.00	0.00
1419007_at	0.00	0.07
1419008_at	0.00	0.00
1419009_at	0.00	0.00
1419010_x_at	0.00	0.00
1419011_at	0.00	0.00
1419012_at	0.00	0.00
1419013_at	0.00	0.00
1419014_at	0.00	0.00
1419015_at	0.00	0.00
1419016_at	0.00	0.00
1419017_at	0.00	0.00
1419018_at	0.00	0.00
1419019_a_at	0.00	0.00
1419020_at	0.00	0.00
1419021_at	0.53	0.00
1419022_a_at	0.00	0.00
1419023_x_at	0.00	0.00
1419024_at	0.00	0.00
1419025_at	0.00	0.00
1419026_at	0.00	0.00
1419027_s_at	0.00	0.00

1419028_at	0.00	0.00
1419029_at	0.00	0.00
1419030_at	0.00	0.02
1419031_at	0.00	0.00
1419032_at	0.00	0.00
1419033_at	0.00	0.00
1419034_at	0.00	0.01
1419035_s_at	0.00	0.00
1419036_at	0.00	0.00
1419037_at	0.00	0.00
1419038_a_at	0.00	0.00
1419039_at	0.00	0.00
1419040_at	0.00	0.00
1419041_at	0.00	0.00
1419042_at	0.00	0.00
1419043_a_at	0.00	0.00
1419044_at	0.00	0.00
1419045_at	0.00	0.29
1419046_at	0.00	0.00
1419047_at	0.00	0.00
1419048_at	0.00	0.00
1419049_at	0.00	0.00
1419050_at	0.00	0.00
1419051_at	0.00	0.00
1419052_at	0.00	0.00
1419053_at	0.00	0.00
1419054_a_at	0.03	0.12
1419055_a_at	0.00	0.00
1419056_at	0.00	0.00
1419057_at	0.00	0.00
1419058_at	0.00	0.00
1419059_at	0.00	0.00
1419060_at	0.00	0.00
1419061_at	0.00	0.00
1419062_at	0.00	0.38
1419063_at	0.00	0.00
1419064_a_at	0.00	0.00
1419065_at	0.00	0.00
1419066_at	0.00	0.00
1419067_a_at	0.00	0.00
1419068_at	0.00	0.00
1419069_at	0.00	0.00
1419070_at	0.00	0.00
1419071_at	0.00	0.00
1419072_at	0.00	0.00
1419073_at	0.00	0.00
1419074_at	0.00	0.00
1419075_s_at	0.00	0.00
1419076_a_at	0.00	0.00
1419077_at	0.00	0.00
1419078_at	0.00	0.00
1419079_at	0.00	0.00
1419080_at	0.00	0.00
1419081_at	0.00	0.00
1419082_at	0.00	0.00
1419083_at	0.00	0.00

1419084_a_at	0.00	0.00
1419085_at	0.00	0.00
1419086_at	0.00	0.00
1419087_s_at	0.00	0.00
1419088_at	0.00	0.00
1419089_at	0.00	0.00
1419090_x_at	0.00	0.00
1419091_a_at	0.69	0.00
1419092_a_at	0.00	0.00
1419093_at	0.00	0.00
1419094_at	0.00	0.00
1419095_a_at	0.00	0.00
1419096_at	0.00	0.00
1419097_a_at	0.00	0.00
1419098_at	0.00	0.00
1419099_x_at	0.00	0.01
1419100_at	0.00	0.00
1419101_at	0.00	0.00
1419102_at	0.00	0.00
1419103_a_at	0.00	0.00
1419104_at	0.00	0.00
1419105_at	0.00	0.00
1419106_at	0.00	0.00
1419107_at	0.00	0.00
1419108_at	0.00	0.00
1419109_at	0.00	0.00
1419110_at	0.00	0.00
1419111_at	0.00	0.00
1419112_at	0.00	0.01
1419113_at	0.00	0.00
1419114_at	0.00	0.00
1419115_at	0.00	0.00
1419116_at	0.00	0.00
1419117_at	0.00	0.00
1419118_at	0.00	0.00
1419119_at	0.00	0.00
1419120_at	0.00	0.00
1419121_at	0.00	0.00
1419122_at	0.00	0.00
1419123_a_at	0.00	1.00
1419124_at	0.00	0.00
1419125_at	0.00	0.00
1419126_at	0.00	0.00
1419127_at	0.00	0.00
1419128_at	0.00	0.00
1419129_at	0.00	0.00
1419130_at	0.00	0.00
1419131_at	0.00	0.00
1419132_at	0.00	0.00
1419133_at	0.00	0.00
1419134_at	0.00	0.00
1419135_at	0.00	0.00
1419136_at	0.00	0.00
1419137_at	0.00	0.00
1419138_at	0.00	0.00
1419139_at	0.00	0.00

1419140_at	0.00	0.00
1419141_at	0.00	0.00
1419142_at	0.00	0.00
1419143_at	0.00	0.00
1419144_at	0.00	0.00
1419145_at	0.00	0.00
1419146_a_at	0.00	0.00
1419147_at	0.00	0.00
1419148_at	0.00	0.00
1419149_at	0.00	0.00
1419150_at	0.00	0.00
1419151_at	0.00	0.00
1419152_at	0.00	0.00
1419153_at	0.00	0.00
1419154_at	0.00	0.31
1419155_a_at	0.00	0.00
1419156_at	0.00	0.00
1419157_at	0.00	0.00
1419158_a_at	0.00	0.00
1419159_at	0.00	0.00
1419160_at	0.00	0.00
1419161_a_at	0.00	0.00
1419162_s_at	0.00	0.00
1419163_s_at	0.00	0.00
1419164_at	0.00	0.00
1419165_at	0.00	0.00
1419166_at	0.00	0.00
1419167_at	0.00	0.00
1419168_at	0.00	0.00
1419169_at	0.00	0.00
1419170_at	0.00	0.00
1419171_at	0.00	0.00
1419172_at	0.00	0.00
1419173_at	0.00	0.00
1419174_at	0.00	0.17
1419175_a_at	0.00	0.00
1419176_at	0.00	0.00
1419177_at	0.00	0.00
1419178_at	0.00	0.00
1419179_at	0.00	0.00
1419180_at	0.00	0.00
1419181_at	0.00	0.00
1419182_at	0.00	0.00
1419183_at	0.00	0.00
1419184_a_at	0.00	0.00
1419185_a_at	0.00	0.00
1419186_a_at	0.00	0.00
1419187_at	0.00	0.00
1419188_s_at	0.00	0.00
1419189_at	0.00	0.00
1419190_at	0.00	0.00
1419191_at	0.00	0.00
1419192_at	0.00	0.00
1419193_a_at	0.00	0.00
1419194_s_at	0.00	0.00
1419195_at	0.00	0.00

1419196_at	0.00	0.00
1419197_x_at	0.00	0.00
1419198_at	0.00	0.00
1419199_at	0.00	0.00
1419200_at	0.00	0.00
1419201_at	0.00	0.00
1419202_at	0.00	0.00
1419203_at	0.00	0.00
1419204_at	0.00	0.00
1419205_x_at	0.00	0.05
1419206_at	0.00	0.00
1419207_at	0.00	0.00
1419208_at	0.00	0.00
1419209_at	0.00	0.00
1419210_at	0.00	0.00
1419211_s_at	0.00	0.00
1419212_at	0.00	0.00
1419213_at	0.00	0.00
1419214_at	0.00	0.00
1419215_at	0.00	0.00
1419216_at	0.00	0.00
1419217_at	0.00	0.00
1419218_at	0.00	0.00
1419219_at	0.00	0.00
1419220_at	0.00	0.00
1419221_a_at	0.00	0.00
1419222_at	0.00	0.00
1419223_a_at	0.00	0.00
1419224_at	0.00	0.00
1419225_at	0.00	0.00
1419226_at	0.00	0.00
1419227_at	0.00	0.00
1419228_at	0.00	0.00
1419229_at	0.00	0.00
1419230_at	0.00	0.00
1419231_s_at	0.00	0.00
1419232_a_at	0.00	0.18
1419233_x_at	0.00	0.06
1419234_at	0.44	0.11
1419235_s_at	0.57	0.00
1419236_at	0.00	0.00
1419237_at	0.00	0.00
1419238_at	0.00	0.00
1419239_at	0.00	0.00
1419240_at	0.00	0.00
1419241_a_at	0.00	0.00
1419242_at	0.00	0.00
1419243_at	0.00	0.00
1419244_a_at	0.00	0.00
1419245_at	0.00	0.00
1419246_s_at	0.00	0.00
1419247_at	0.00	0.00
1419248_at	0.00	0.00
1419249_at	0.00	0.00
1419250_a_at	0.00	0.00
1419251_at	0.00	0.00

1419252_at	0.00	0.00
1419253_at	0.00	0.00
1419254_at	0.00	0.00
1419255_at	0.00	0.00
1419256_at	0.00	0.00
1419257_at	0.00	0.02
1419258_at	0.00	0.00
1419259_at	0.00	0.00
1419260_a_at	0.00	0.00
1419261_at	0.00	0.00
1419262_at	0.00	0.00
1419263_a_at	0.00	0.00
1419264_at	0.00	0.00
1419265_at	0.00	0.00
1419266_at	0.00	0.00
1419267_at	0.00	0.00
1419268_at	0.00	0.00
1419269_at	0.00	0.00
1419270_a_at	0.00	0.27
1419271_at	0.00	0.00
1419272_at	0.00	0.00
1419273_at	0.12	0.00
1419274_at	0.00	0.00
1419275_at	0.00	0.00
1419276_at	0.00	0.00
1419277_at	0.00	0.00
1419278_at	0.00	0.00
1419279_at	0.00	0.00
1419280_at	0.00	0.00
1419281_a_at	0.00	0.33
1419282_at	0.00	0.00
1419283_s_at	0.00	0.00
1419284_at	0.00	0.00
1419285_s_at	0.00	0.00
1419286_s_at	0.00	0.03
1419287_at	0.00	0.00
1419288_at	0.00	0.00
1419289_a_at	0.00	0.00
1419290_at	0.00	0.00
1419291_x_at	0.00	0.00
1419292_at	0.00	0.00
1419293_at	0.00	0.00
1419294_at	0.00	0.00
1419295_at	0.00	0.00
1419296_at	0.00	0.00
1419297_at	0.00	0.00
1419298_at	0.00	0.00
1419299_at	0.00	0.00
1419300_at	0.00	0.00
1419301_at	0.00	0.00
1419302_at	0.00	0.00
1419303_at	0.00	0.00
1419304_at	0.00	0.00
1419305_a_at	0.00	0.00
1419306_at	0.00	0.00
1419307_at	0.00	0.00

1419308_at	0.00	0.00
1419309_at	0.00	0.00
1419310_s_at	0.00	0.00
1419311_at	0.00	0.00
1419312_at	0.00	0.00
1419313_at	0.00	0.00
1419314_at	0.00	0.00
1419315_at	0.00	0.00
1419316_s_at	0.00	0.00
1419317_x_at	0.00	0.00
1419318_at	0.00	0.00
1419319_at	0.00	0.00
1419320_at	0.00	0.00
1419321_at	0.00	0.00
1419322_at	0.00	0.00
1419323_at	0.00	0.00
1419324_at	0.00	0.00
1419325_at	0.00	0.00
1419326_at	0.00	0.00
1419327_at	0.00	0.00
1419328_at	0.00	0.00
1419329_at	0.00	0.00
1419330_a_at	0.37	0.00
1419331_at	0.00	0.00
1419332_at	0.00	0.00
1419333_at	0.00	0.00
1419334_at	0.00	0.00
1419335_at	0.00	0.00
1419336_at	0.00	0.00
1419337_at	0.00	0.00
1419338_at	0.00	0.00
1419339_at	0.00	0.00
1419340_at	0.00	0.00
1419341_at	0.00	0.00
1419342_at	0.00	0.00
1419343_at	0.00	0.00
1419344_at	0.00	0.00
1419345_at	0.00	0.00
1419346_a_at	0.00	0.00
1419347_x_at	0.00	0.00
1419348_at	0.00	0.00
1419349_a_at	0.00	0.00
1419350_at	0.08	0.00
1419351_a_at	0.00	0.10
1419352_at	0.00	0.15
1419353_at	0.00	0.00
1419354_at	0.00	0.00
1419355_at	0.00	0.00
1419356_at	0.00	0.00
1419357_at	0.00	0.00
1419358_at	0.00	0.00
1419359_at	0.00	0.00
1419360_a_at	0.00	0.00
1419361_at	0.00	0.00
1419362_at	0.00	0.00
1419363_a_at	0.00	0.34

1419364_a_at	0.00	0.00
1419365_at	0.00	0.00
1419366_at	0.00	0.00
1419367_at	0.00	0.00
1419368_a_at	0.02	0.06
1419369_at	0.00	0.00
1419370_a_at	0.00	0.00
1419371_s_at	0.00	0.00
1419372_at	0.00	0.00
1419373_at	0.00	0.00
1419374_at	0.00	0.00
1419375_at	0.00	0.00
1419376_at	0.00	0.00
1419377_at	0.00	0.00
1419378_a_at	0.00	0.00
1419379_x_at	0.00	0.00
1419380_at	0.01	0.07
1419381_at	0.00	0.00
1419382_a_at	0.00	0.00
1419383_at	0.00	0.00
1419384_at	0.00	0.00
1419385_a_at	0.00	0.00
1419386_at	0.00	0.00
1419387_s_at	0.00	0.01
1419388_at	0.00	0.00
1419389_at	0.00	0.00
1419390_at	0.00	0.00
1419391_at	0.00	0.00
1419392_at	0.00	0.00
1419393_at	0.00	0.00
1419394_s_at	0.00	0.00
1419395_at	0.00	0.00
1419396_at	0.00	0.00
1419397_at	0.00	0.21
1419398_a_at	0.89	0.70
1419399_at	0.00	0.00
1419400_at	0.00	0.00
1419401_at	0.00	0.00
1419402_at	0.00	0.00
1419403_at	0.00	0.00
1419404_s_at	0.00	0.01
1419405_at	0.00	0.00
1419406_a_at	0.00	0.00
1419407_at	0.00	0.00
1419408_at	0.00	0.00
1419409_at	0.00	0.00
1419410_at	0.00	0.00
1419411_at	0.00	0.00
1419412_at	0.00	0.00
1419413_at	0.00	0.00
1419414_at	0.00	0.00
1419415_a_at	0.07	0.34
1419416_a_at	0.38	0.65
1419417_at	0.00	0.30
1419418_a_at	1.00	0.99
1419419_at	0.00	0.00

1419420_at	0.00	0.00
1419421_at	0.00	0.00
1419422_at	0.00	0.00
1419423_at	0.00	0.00
1419424_at	0.00	0.00
1419425_at	0.00	0.00
1419426_s_at	0.00	0.00
1419427_at	0.00	0.00
1419428_a_at	0.00	0.00
1419429_at	0.00	0.00
1419430_at	0.22	0.73
1419431_at	0.00	0.00
1419432_at	0.00	0.00
1419433_at	0.00	0.00
1419434_at	0.00	0.00
1419435_at	0.00	0.00
1419436_at	0.00	0.00
1419437_at	0.00	0.00
1419438_at	0.00	0.00
1419439_at	0.00	0.00
1419440_at	0.00	0.00
1419441_at	0.00	0.00
1419442_at	0.00	0.00
1419443_at	0.00	0.00
1419444_at	0.00	0.00
1419445_s_at	0.00	0.00
1419446_at	0.00	0.00
1419447_s_at	0.00	0.00
1419448_at	0.00	0.00
1419449_a_at	0.00	0.00
1419450_at	0.00	0.00
1419451_at	0.00	0.00
1419452_at	0.00	0.53
1419453_at	0.00	0.00
1419454_x_at	0.08	0.18
1419455_at	0.03	0.00
1419456_at	0.00	0.05
1419457_at	0.00	0.03
1419458_at	0.00	0.00
1419459_a_at	0.00	0.00
1419460_at	0.00	0.00
1419461_at	0.00	0.00
1419462_s_at	0.00	0.00
1419463_at	0.00	0.00
1419464_at	0.00	0.01
1419465_at	0.00	0.00
1419466_at	0.00	0.00
1419467_at	0.00	0.00
1419468_at	0.00	0.00
1419469_at	0.00	0.00
1419470_at	0.00	0.00
1419471_a_at	0.00	0.00
1419472_s_at	0.00	0.00
1419473_a_at	0.00	0.00
1419474_a_at	0.00	0.00
1419475_a_at	0.00	0.00

1419476_at	0.00	0.00
1419477_at	0.00	0.00
1419478_at	0.00	0.00
1419479_at	0.00	0.00
1419480_at	0.00	0.00
1419481_at	0.00	0.00
1419482_at	0.00	0.00
1419483_at	0.00	0.00
1419484_a_at	0.00	0.00
1419485_at	0.00	0.00
1419486_at	0.00	0.00
1419487_at	0.00	0.00
1419488_at	0.00	0.00
1419489_at	0.00	0.00
1419490_at	0.00	0.00
1419491_at	0.00	0.00
1419492_s_at	0.00	0.00
1419493_a_at	0.00	0.00
1419494_a_at	0.00	0.00
1419495_at	0.00	0.00
1419496_at	0.00	0.00
1419497_at	0.00	0.00
1419498_at	0.00	0.00
1419499_at	0.00	0.00
1419500_at	0.00	0.00
1419501_at	0.00	0.00
1419502_at	0.00	0.00
1419503_at	0.00	0.00
1419504_at	0.00	0.00
1419505_a_at	0.00	0.00
1419506_at	0.00	0.00
1419507_at	0.00	0.00
1419508_at	0.00	0.00
1419509_a_at	0.00	0.00
1419510_at	0.00	0.00
1419511_at	0.00	0.00
1419512_at	0.00	0.00
1419513_a_at	0.00	0.00
1419514_at	0.00	0.00
1419515_at	0.00	0.00
1419516_at	0.00	0.00
1419517_at	0.00	0.00
1419518_at	0.00	0.00
1419519_at	0.00	0.00
1419520_at	0.00	0.00
1419521_at	0.00	0.00
1419522_at	0.00	0.00
1419523_at	0.00	0.00
1419524_at	0.00	0.00
1419525_at	0.00	0.00
1419526_at	0.00	0.00
1419527_at	0.00	0.00
1419528_at	0.00	0.00
1419529_at	0.00	0.00
1419530_at	0.00	0.00
1419531_at	0.00	0.00

1419532_at	0.00	0.00
1419533_at	0.00	0.00
1419534_at	0.00	0.00
1419535_at	0.00	0.00
1419536_a_at	0.00	0.00
1419537_at	0.00	0.00
1419538_at	0.00	0.00
1419539_at	0.00	0.00
1419540_at	0.00	0.00
1419541_at	0.00	0.00
1419542_at	0.00	0.00
1419543_a_at	0.00	0.17
1419544_at	0.00	0.00
1419545_a_at	0.00	0.00
1419546_at	0.00	0.00
1419547_at	0.00	0.00
1419548_at	0.00	0.00
1419549_at	0.00	0.00
1419550_a_at	0.00	0.00
1419551_s_at	0.00	0.00
1419552_at	0.00	0.00
1419553_a_at	0.00	0.00
1419554_at	0.00	0.00
1419555_at	0.00	0.00
1419556_at	0.00	0.00
1419557_a_at	0.00	0.27
1419558_at	0.00	0.00
1419559_at	0.00	0.00
1419560_at	0.00	0.00
1419561_at	0.00	0.00
1419562_at	0.00	0.01
1419563_at	0.00	0.00
1419564_at	0.00	0.00
1419565_a_at	0.00	0.00
1419566_at	0.00	0.00
1419567_at	0.00	0.00
1419568_at	0.00	0.00
1419569_a_at	0.00	0.00
1419570_at	0.00	0.00
1419571_at	0.00	0.00
1419572_a_at	0.00	0.05
1419573_a_at	0.29	0.00
1419574_at	0.00	0.00
1419575_s_at	0.00	0.00
1419576_at	0.00	0.00
1419577_at	0.00	0.00
1419578_at	0.00	0.00
1419579_at	0.00	0.00
1419580_at	0.00	0.00
1419581_at	0.00	0.00
1419582_at	0.00	0.00
1419583_at	0.00	0.00
1419584_at	0.00	0.00
1419585_at	0.00	0.00
1419586_at	0.00	0.00
1419587_s_at	0.00	0.00

1419588_at	0.00	0.00
1419589_at	0.00	0.00
1419590_at	0.00	0.00
1419591_at	0.00	0.00
1419592_at	0.00	0.00
1419593_at	0.00	0.00
1419594_at	0.00	0.00
1419595_a_at	0.00	0.00
1419596_at	0.00	0.00
1419597_at	0.00	0.00
1419598_at	0.00	0.00
1419599_s_at	0.00	0.00
1419600_at	0.00	0.00
1419601_at	0.00	0.00
1419602_at	0.00	0.00
1419603_at	0.00	0.00
1419604_at	0.00	0.00
1419605_at	0.00	0.00
1419606_a_at	0.00	0.28
1419607_at	0.00	0.00
1419608_a_at	0.00	0.00
1419609_at	0.00	0.00
1419610_at	0.00	0.00
1419611_at	0.00	0.00
1419612_at	0.00	0.00
1419613_at	0.00	0.00
1419614_at	0.00	0.00
1419615_at	0.00	0.00
1419616_at	0.00	0.00
1419617_at	0.00	0.00
1419618_at	0.00	0.00
1419619_at	0.00	0.00
1419620_at	0.00	0.00
1419621_at	0.00	0.00
1419622_at	0.00	0.00
1419623_at	0.00	0.00
1419624_a_at	0.00	0.00
1419625_at	0.00	0.00
1419626_at	0.00	0.00
1419627_s_at	0.00	0.00
1419628_at	0.00	0.00
1419629_at	0.00	0.00
1419630_a_at	0.00	0.00
1419631_at	0.00	0.00
1419632_at	0.00	0.00
1419633_at	0.00	0.00
1419634_a_at	0.00	0.00
1419635_at	0.00	0.00
1419636_at	0.00	0.00
1419637_s_at	0.00	0.00
1419638_at	0.33	0.00
1419639_at	0.00	0.00
1419640_at	0.00	0.00
1419641_at	0.00	0.00
1419642_at	0.00	0.00
1419643_s_at	0.00	0.00

1419644_at	0.00	0.00
1419645_at	0.00	0.00
1419646_a_at	0.00	0.00
1419647_a_at	0.47	0.00
1419648_at	0.00	0.00
1419649_s_at	0.00	0.00
1419650_at	0.00	0.00
1419651_at	0.00	0.00
1419652_s_at	0.00	0.00
1419653_a_at	0.00	0.17
1419654_at	0.00	0.00
1419655_at	0.00	0.00
1419656_at	0.00	0.00
1419657_a_at	0.12	0.00
1419658_at	0.00	0.00
1419659_s_at	0.00	0.00
1419660_at	0.00	0.00
1419661_at	0.00	0.00
1419662_at	0.00	0.00
1419663_at	0.00	0.00
1419664_at	0.00	0.00
1419665_a_at	0.00	0.00
1419666_x_at	0.00	0.00
1419667_at	0.00	0.00
1419668_at	0.00	0.00
1419669_at	0.00	0.00
1419670_at	0.00	0.00
1419671_a_at	0.00	0.00
1419672_at	0.00	0.00
1419673_at	0.00	0.00
1419674_a_at	0.00	0.00
1419675_at	0.00	0.00
1419676_at	0.00	0.00
1419677_at	0.00	0.00
1419678_at	0.00	0.00
1419679_at	0.00	0.00
1419680_a_at	0.00	0.33
1419681_a_at	0.00	0.00
1419682_a_at	0.00	0.00
1419683_at	0.00	0.00
1419684_at	0.00	0.00
1419685_at	0.00	0.01
1419686_at	0.00	0.00
1419687_at	0.00	0.00
1419688_at	0.00	0.00
1419689_at	0.00	0.00
1419690_at	0.00	0.00
1419691_at	0.00	0.00
1419692_a_at	0.00	0.00
1419693_at	0.00	0.00
1419694_at	0.00	0.00
1419695_at	0.00	0.00
1419696_at	0.00	0.00
1419697_at	0.00	0.00
1419698_at	0.00	0.00
1419699_at	0.00	0.00

1419700_a_at	0.00	0.90
1419701_s_at	0.00	0.00
1419702_at	0.00	0.00
1419703_at	0.00	0.00
1419704_at	0.00	0.00
1419705_at	0.00	0.00
1419706_a_at	0.00	0.00
1419707_at	0.00	0.00
1419708_at	0.00	0.00
1419709_at	0.00	0.00
1419710_at	0.00	0.00
1419711_at	0.00	0.00
1419712_at	0.00	0.00
1419713_at	0.00	0.00
1419714_at	0.00	0.00
1419715_at	0.00	0.00
1419716_a_at	0.00	0.00
1419717_at	0.00	0.00
1419718_at	0.00	0.00
1419719_at	0.00	0.00
1419720_at	0.00	0.00
1419721_at	0.00	0.00
1419722_at	0.00	0.00
1419723_at	0.00	0.00
1419724_at	0.00	0.00
1419725_at	0.00	0.00
1419726_at	0.00	0.00
1419727_at	0.00	0.00
1419728_at	0.00	0.00
1419729_at	0.00	0.00
1419730_at	0.00	0.00
1419731_at	0.00	0.00
1419732_at	0.00	0.00
1419733_at	0.00	0.00
1419734_at	0.00	0.00
1419735_at	0.00	0.00
1419736_a_at	0.00	0.00
1419737_a_at	0.00	0.00
1419738_a_at	0.05	0.00
1419739_at	0.00	0.00
1419740_at	0.00	0.00
1419741_at	0.00	0.03
1419742_at	0.00	0.00
1419743_s_at	0.00	0.00
1419744_at	0.00	0.00
1419745_at	0.00	0.00
1419746_at	0.00	0.00
1419747_at	0.00	0.00
1419748_at	0.00	0.00
1419749_at	0.00	0.00
1419750_at	0.00	0.00
1419751_x_at	0.00	0.00
1419752_at	0.00	0.00
1419753_at	0.00	0.00
1419754_at	0.00	0.00
1419755_at	0.00	0.00

1419756_at	0.00	0.00
1419757_at	0.00	0.00
1419758_at	0.12	0.01
1419759_at	0.06	0.18
1419760_a_at	0.00	0.00
1419761_a_at	0.00	0.00
1419762_at	0.00	0.00
1419763_at	0.00	0.00
1419764_at	0.00	0.00
1419765_at	0.00	0.00
1419766_at	0.00	0.00
1419767_at	0.00	0.02
1419768_at	0.00	0.00
1419769_at	0.00	0.00
1419770_at	0.00	0.00
1419771_at	0.00	0.00
1419772_at	0.00	0.00
1419773_at	0.00	0.00
1419774_at	0.00	0.00
1419775_at	0.00	0.00
1419776_at	0.00	0.00
1419777_at	0.00	0.00
1419778_at	0.00	0.00
1419779_at	0.00	0.00
1419780_at	0.00	0.00
1419782_at	0.00	0.00
1419785_at	0.00	0.00
1419789_at	0.00	0.00
1419790_at	0.00	0.00
1419795_at	0.00	0.00
1419796_at	0.00	0.00
1419797_at	0.00	0.00
1419798_at	0.00	0.00
1419799_at	0.00	0.00
1419800_at	0.00	0.00
1419801_x_at	0.00	0.00
1419802_at	0.00	0.00
1419803_s_at	0.00	0.00
1419805_s_at	0.00	0.00
1419806_at	0.00	0.00
1419807_at	0.00	0.00
1419808_at	0.00	0.00
1419809_s_at	0.00	0.00
1419810_x_at	0.00	0.00
1419811_at	0.00	0.00
1419812_s_at	0.00	0.00
1419814_s_at	0.00	0.05
1419815_at	0.00	0.00
1419816_s_at	0.00	0.00
1419817_s_at	0.00	0.00
1419818_x_at	0.00	0.00
1419819_s_at	0.00	0.00
1419820_at	0.00	0.00
1419821_s_at	0.00	0.00
1419822_at	0.00	0.00
1419823_s_at	0.00	0.00

1419826_at	0.00	0.00
1419827_s_at	0.00	0.00
1419830_at	0.00	0.00
1419831_at	0.00	0.00
1419832_s_at	0.00	0.00
1419833_s_at	0.01	0.00
1419834_x_at	0.00	0.00
1419835_s_at	0.00	0.00
1419836_at	0.00	0.00
1419838_s_at	0.00	0.00
1419839_x_at	0.00	0.19
1419840_at	0.00	0.00
1419842_at	0.00	0.00
1419843_at	0.00	0.00
1419845_at	0.00	0.00
1419846_at	0.00	0.00
1419847_at	0.00	0.00
1419848_x_at	0.00	0.00
1419849_at	0.00	0.00
1419851_at	0.00	0.00
1419857_at	0.00	0.00
1419858_at	0.00	0.00
1419859_at	0.00	0.00
1419861_at	0.00	0.00
1419863_at	0.00	0.00
1419864_x_at	0.00	0.00
1419866_s_at	0.00	0.00
1419867_a_at	0.00	0.00
1419869_s_at	0.00	0.00
1419872_at	0.00	0.00
1419873_s_at	0.00	0.00
1419874_x_at	0.00	0.00
1419875_at	0.00	0.00
1419876_at	0.00	0.00
1419877_x_at	0.00	0.00
1419879_s_at	0.23	0.00
1419880_x_at	0.00	0.00
1419881_x_at	0.00	0.00
1419882_at	0.00	0.00
1419883_s_at	0.00	0.00
1419884_at	0.00	0.00
1419885_at	0.00	0.00
1419886_at	0.00	0.00
1419887_at	0.00	0.00
1419888_at	0.00	0.00
1419889_at	0.00	0.00
1419890_at	0.00	0.00
1419891_s_at	0.00	0.00
1419893_at	0.00	0.00
1419894_at	0.00	0.00
1419895_at	0.00	0.00
1419896_at	0.72	0.06
1419897_at	0.00	0.00
1419898_s_at	0.00	0.00
1419899_at	0.00	0.00
1419900_at	0.00	0.00

1419905_s_at	0.00	0.00
1419906_at	0.00	0.00
1419907_s_at	0.00	0.00
1419908_at	0.00	0.00
1419909_at	0.00	0.00
1419910_at	0.00	0.00
1419911_at	0.00	0.00
1419912_s_at	0.00	0.00
1419913_at	0.00	0.00
1419914_s_at	0.00	0.00
1419915_at	0.00	0.00
1419916_at	0.00	0.00
1419917_s_at	0.00	0.00
1419918_at	0.00	0.00
1419919_at	0.00	0.00
1419920_s_at	0.00	0.33
1419921_s_at	0.02	0.34
1419927_s_at	0.00	0.04
1419928_at	0.00	0.00
1419929_at	0.00	0.00
1419930_at	0.00	0.00
1419931_at	0.00	0.00
1419932_s_at	0.00	0.00
1419933_at	0.00	0.00
1419934_at	0.00	0.00
1419935_s_at	0.00	0.00
1419936_at	0.00	0.00
1419937_at	0.00	0.00
1419940_at	0.00	0.00
1419941_at	0.00	0.00
1419942_at	0.00	0.00
1419943_s_at	0.00	0.21
1419944_at	0.00	0.00
1419945_s_at	0.00	0.00
1419946_s_at	0.00	0.00
1419947_at	0.00	0.00
1419948_at	0.00	0.00
1419949_at	0.00	0.00
1419950_s_at	0.00	0.00
1419951_at	0.00	0.00
1419952_at	0.00	0.00
1419953_at	0.00	0.00
1419954_s_at	0.00	0.01
1419955_at	0.00	0.00
1419956_at	0.00	0.00
1419957_at	0.00	0.00
1419958_at	0.00	0.00
1419959_s_at	0.00	0.00
1419960_at	0.00	0.00
1419961_s_at	0.00	0.00
1419962_at	0.00	0.00
1419963_at	0.00	0.00
1419964_s_at	0.00	0.00
1419965_at	0.00	0.00
1419966_at	0.00	0.00
1419967_at	0.00	0.00

1419968_at	0.00	0.00
1419969_at	0.00	0.00
1419970_at	0.00	0.00
1419971_s_at	0.00	0.00
1419972_at	0.00	0.00
1419973_at	0.00	0.00
1419974_at	0.00	0.00
1419975_at	0.00	0.00
1419976_s_at	0.00	0.00
1419977_s_at	0.00	0.00
1419978_s_at	0.00	0.00
1419979_s_at	0.00	0.00
1419980_at	0.00	0.00
1419981_at	0.00	0.00
1419982_s_at	0.00	0.00
1419983_at	0.00	0.00
1419984_s_at	0.00	0.00
1419987_at	0.00	0.00
1419988_at	0.00	0.00
1419989_at	0.00	0.00
1419991_at	0.00	0.00
1419992_x_at	0.00	0.00
1419993_at	0.00	0.00
1419994_s_at	0.00	0.00
1419995_at	0.00	0.00
1419997_at	0.00	0.00
1419998_at	0.00	0.00
1419999_at	0.00	0.00
1420000_s_at	0.00	0.00
1420001_at	0.00	0.00
1420002_at	0.00	0.00
1420003_at	0.00	0.00
1420004_s_at	0.00	0.00
1420007_at	0.00	0.00
1420008_s_at	0.00	0.00
1420009_at	0.00	0.00
1420010_at	0.00	0.00
1420011_s_at	0.00	0.00
1420012_at	0.00	0.00
1420013_s_at	0.00	0.00
1420014_at	0.00	0.00
1420017_at	0.00	0.00
1420018_s_at	0.00	0.00
1420019_at	0.00	0.00
1420020_at	0.00	0.00
1420021_s_at	0.00	0.00
1420022_s_at	0.00	0.00
1420023_at	0.00	0.00
1420024_s_at	0.00	0.25
1420025_s_at	0.00	0.00
1420026_at	0.00	0.00
1420027_at	0.00	0.00
1420028_s_at	0.00	0.00
1420029_at	0.00	0.00
1420030_at	0.00	0.00
1420031_at	0.00	0.00

1420032_at	0.00	0.00
1420033_s_at	0.00	0.33
1420034_at	0.00	0.00
1420037_at	0.00	0.00
1420038_at	0.00	0.00
1420039_s_at	0.00	0.00
1420040_at	0.00	0.00
1420041_at	0.00	0.00
1420042_at	0.00	0.00
1420043_s_at	0.00	0.00
1420044_at	0.00	0.00
1420045_at	0.00	0.00
1420046_s_at	0.00	0.00
1420047_at	0.00	0.00
1420048_at	0.00	0.00
1420049_at	0.00	0.00
1420050_at	0.00	0.00
1420051_at	0.00	0.00
1420052_x_at	0.00	0.00
1420053_at	0.00	0.00
1420054_s_at	0.00	0.00
1420055_at	0.00	0.00
1420056_s_at	0.00	0.00
1420057_at	0.00	0.00
1420058_s_at	0.00	0.00
1420059_at	0.00	0.00
1420060_s_at	0.00	0.00
1420061_s_at	0.00	0.00
1420062_at	0.00	0.00
1420063_at	0.00	0.00
1420064_s_at	0.00	0.00
1420065_at	0.00	0.00
1420066_s_at	0.00	0.00
1420067_at	0.00	0.00
1420068_at	0.00	0.00
1420069_at	0.00	0.00
1420072_s_at	0.00	0.00
1420073_s_at	0.00	0.00
1420074_at	0.00	0.00
1420075_at	0.00	0.00
1420076_at	0.00	0.00
1420078_at	0.00	0.00
1420079_at	0.00	0.00
1420083_at	0.00	0.00
1420084_at	0.00	0.00
1420085_at	1.00	0.31
1420086_x_at	1.00	0.32
1420087_at	0.00	0.00
1420088_at	0.00	0.00
1420089_at	0.00	0.00
1420090_at	0.00	0.00
1420091_s_at	0.00	0.03
1420092_at	0.00	0.00
1420093_s_at	0.00	0.11
1420094_at	0.00	0.00
1420095_s_at	0.00	0.00

1420096_at	0.00	0.00
1420101_at	0.00	0.00
1420102_at	0.00	0.00
1420103_at	0.00	0.00
1420104_at	0.00	0.00
1420105_at	0.00	0.00
1420106_at	0.00	0.00
1420108_at	0.00	0.00
1420109_at	0.00	0.00
1420110_s_at	0.00	0.00
1420111_at	0.00	0.00
1420113_s_at	0.00	0.00
1420114_s_at	0.00	0.00
1420115_at	0.00	0.00
1420116_s_at	0.00	0.00
1420117_at	0.00	0.00
1420118_s_at	0.00	0.00
1420121_at	0.00	0.00
1420122_at	0.00	0.00
1420123_at	0.00	0.00
1420124_s_at	0.00	0.00
1420125_at	0.00	0.00
1420126_at	0.00	0.00
1420129_s_at	0.00	0.00
1420130_s_at	0.00	0.00
1420131_s_at	0.00	0.00
1420132_s_at	0.00	0.00
1420138_at	0.00	0.00
1420140_at	0.00	0.00
1420141_at	0.00	0.00
1420142_s_at	0.00	0.26
1420146_at	0.00	0.00
1420147_at	0.00	0.00
1420148_at	0.00	0.00
1420149_at	0.00	0.00
1420150_at	0.00	0.00
1420155_at	0.00	0.00
1420156_at	0.00	0.00
1420157_s_at	0.00	0.00
1420158_s_at	0.00	0.00
1420159_at	0.00	0.00
1420160_s_at	0.00	0.00
1420161_at	0.00	0.00
1420162_at	0.00	0.00
1420163_at	0.00	0.00
1420164_at	0.00	0.00
1420165_s_at	0.00	0.00
1420166_at	0.00	0.00
1420168_at	0.00	0.00
1420169_at	0.00	0.00
1420170_at	0.00	0.00
1420171_s_at	0.26	0.00
1420172_at	0.51	0.00
1420173_at	0.00	0.00
1420174_s_at	0.00	0.00
1420175_at	0.00	0.00

1420176_x_at	0.00	0.00
1420177_at	0.00	0.00
1420179_at	0.00	0.00
1420180_at	0.00	0.00
1420183_at	0.00	0.00
1420184_at	0.00	0.00
1420185_at	0.00	0.00
1420186_at	0.00	0.00
1420187_at	0.00	0.00
1420189_at	0.00	0.00
1420191_s_at	0.00	0.00
1420192_at	0.00	0.00
1420193_at	0.00	0.00
1420194_at	0.00	0.00
1420195_at	0.00	0.00
1420196_s_at	0.00	0.00
1420197_at	0.00	0.00
1420198_at	0.00	0.00
1420200_at	0.00	0.00
1420201_at	0.00	0.00
1420202_at	0.00	0.00
1420203_at	0.00	0.00
1420204_at	0.00	0.00
1420210_at	0.00	0.00
1420211_at	0.00	0.00
1420217_x_at	0.00	0.00
1420218_at	0.00	0.00
1420222_at	0.00	0.00
1420223_at	0.00	0.00
1420224_at	0.00	0.00
1420225_at	0.00	0.00
1420226_x_at	0.00	0.00
1420227_at	0.00	0.00
1420228_at	0.00	0.00
1420229_at	0.00	0.00
1420230_at	0.00	0.00
1420231_at	0.00	0.00
1420232_at	0.00	0.00
1420234_at	0.00	0.00
1420235_at	0.00	0.00
1420236_at	0.00	0.00
1420237_at	0.00	0.00
1420240_at	0.00	0.00
1420241_at	0.00	0.00
1420243_at	0.00	0.00
1420244_at	0.00	0.00
1420246_at	0.00	0.00
1420247_at	0.00	0.00
1420248_at	0.00	0.00
1420249_s_at	0.00	0.00
1420250_at	0.00	0.00
1420251_at	0.00	0.00
1420252_at	0.00	0.00
1420253_at	0.00	0.00
1420254_at	0.00	0.00
1420255_at	0.00	0.00

1420256_x_at	0.00	0.00
1420257_at	0.00	0.00
1420258_at	0.00	0.00
1420259_at	0.00	0.00
1420260_at	0.00	0.00
1420261_at	0.00	0.00
1420262_at	0.00	0.00
1420263_at	0.00	0.00
1420264_at	0.00	0.00
1420267_at	0.00	0.00
1420268_x_at	0.00	0.00
1420269_at	0.00	0.00
1420270_at	0.00	0.00
1420272_at	0.00	0.00
1420273_x_at	0.00	0.00
1420274_at	0.00	0.00
1420275_at	0.00	0.00
1420278_at	0.00	0.00
1420279_at	0.00	0.00
1420280_x_at	0.00	0.00
1420281_at	0.00	0.00
1420282_s_at	0.00	0.00
1420284_at	0.00	0.00
1420285_at	0.00	0.00
1420286_at	0.00	0.00
1420287_at	0.00	0.00
1420288_at	0.00	0.00
1420289_at	0.00	0.00
1420290_at	0.00	0.00
1420291_at	0.00	0.00
1420292_x_at	0.00	0.00
1420295_x_at	0.00	0.00
1420296_at	0.00	0.00
1420297_at	0.00	0.00
1420298_at	0.00	0.00
1420301_at	0.00	0.00
1420302_at	0.00	0.00
1420303_x_at	0.00	0.00
1420304_x_at	0.00	0.00
1420305_at	0.00	0.00
1420307_a_at	0.00	0.00
1420318_at	0.00	0.00
1420325_at	0.00	0.00
1420326_s_at	0.00	0.00
1420328_at	0.00	0.00
1420329_at	0.00	0.00
1420330_at	0.00	0.00
1420331_at	0.00	0.00
1420332_x_at	0.00	0.00
1420333_at	0.00	0.00
1420334_at	0.00	0.00
1420335_at	0.00	0.00
1420336_at	0.00	0.00
1420337_at	0.92	1.00
1420338_at	0.00	0.00
1420339_at	0.00	0.00

1420340_at	0.00	0.00
1420341_at	0.00	0.00
1420342_at	0.00	0.00
1420343_at	0.00	0.00
1420344_x_at	0.00	0.00
1420345_at	0.00	0.00
1420346_at	0.00	0.00
1420347_at	0.00	0.00
1420348_at	0.00	0.00
1420349_at	0.00	0.00
1420350_at	0.00	0.00
1420351_at	0.00	0.00
1420352_at	0.00	0.00
1420353_at	0.00	0.00
1420354_at	0.00	0.00
1420355_at	0.00	0.00
1420356_at	0.00	0.00
1420357_s_at	0.00	0.00
1420358_at	0.00	0.00
1420359_at	0.00	0.00
1420360_at	0.04	0.00
1420361_at	0.00	0.00
1420362_a_at	0.00	0.00
1420363_at	0.00	0.00
1420364_at	0.00	0.00
1420365_a_at	0.00	0.00
1420366_at	0.00	0.00
1420367_at	0.00	0.00
1420368_at	0.00	0.23
1420369_a_at	0.00	0.00
1420370_s_at	0.00	0.00
1420371_at	0.00	0.00
1420372_at	0.00	0.00
1420373_at	0.00	0.00
1420374_at	0.00	0.00
1420375_at	0.00	0.00
1420376_a_at	0.08	0.00
1420377_at	0.00	0.00
1420378_at	0.00	0.00
1420379_at	0.00	0.00
1420380_at	0.00	0.00
1420381_a_at	0.00	0.00
1420382_at	0.00	0.00
1420383_a_at	0.00	0.00
1420384_at	0.00	0.00
1420385_at	0.00	0.00
1420386_at	0.00	0.00
1420387_at	0.00	0.00
1420388_at	0.00	0.04
1420389_at	0.00	0.00
1420390_s_at	0.00	0.00
1420391_at	0.00	0.00
1420392_at	0.00	0.00
1420393_at	0.00	0.00
1420394_s_at	0.00	0.00
1420395_a_at	0.00	0.00

1420396_at	0.00	0.00
1420397_a_at	0.00	0.00
1420398_at	0.00	0.00
1420399_at	0.00	0.00
1420400_at	0.00	0.00
1420401_a_at	0.00	0.00
1420402_at	0.00	0.00
1420403_at	0.00	0.00
1420404_at	0.00	0.00
1420405_at	0.00	0.00
1420406_at	0.00	0.00
1420407_at	0.00	0.00
1420408_a_at	0.00	0.00
1420409_at	0.00	0.00
1420410_at	1.00	0.13
1420411_a_at	0.00	0.00
1420412_at	0.00	0.00
1420413_at	0.00	0.00
1420414_at	0.00	0.00
1420415_at	0.00	0.00
1420416_at	0.00	0.00
1420417_at	0.00	0.00
1420418_at	0.00	0.00
1420419_a_at	0.00	0.00
1420420_at	0.00	0.00
1420421_s_at	0.00	0.00
1420422_at	0.00	0.00
1420423_at	0.00	0.00
1420424_at	0.00	0.00
1420425_at	0.05	0.00
1420426_at	0.00	0.00
1420427_a_at	0.00	0.00
1420428_at	0.00	0.00
1420429_at	0.00	0.00
1420430_a_at	0.00	0.00
1420431_at	0.00	0.00
1420432_at	0.00	0.00
1420433_at	0.00	0.00
1420434_at	0.00	0.00
1420435_at	0.00	0.00
1420436_x_at	0.00	0.00
1420437_at	0.00	0.00
1420438_at	0.00	0.00
1420439_at	0.00	0.00
1420440_at	0.00	0.00
1420441_at	0.00	0.00
1420442_at	0.00	0.00
1420443_at	0.00	0.00
1420444_at	0.00	0.00
1420445_at	0.00	0.00
1420446_at	0.00	0.00
1420447_at	0.00	0.00
1420448_at	0.00	0.00
1420449_at	0.00	0.00
1420450_at	0.00	0.00
1420451_at	0.00	0.00

1420452_at	0.00	0.00
1420453_at	0.00	0.00
1420454_at	0.00	0.00
1420455_at	0.00	0.00
1420456_at	0.00	0.00
1420457_at	0.00	0.00
1420458_at	0.00	0.00
1420459_at	0.00	0.00
1420460_a_at	0.00	0.00
1420461_at	0.00	0.00
1420462_at	0.00	0.00
1420463_at	0.00	0.00
1420464_s_at	0.00	0.00
1420465_s_at	0.00	0.00
1420466_at	0.00	0.00
1420467_at	0.00	0.00
1420468_at	0.00	0.00
1420469_at	0.00	0.00
1420470_at	0.00	0.00
1420471_at	0.00	0.00
1420472_at	0.00	0.00
1420473_at	0.00	0.00
1420474_at	0.00	0.00
1420475_at	0.00	0.00
1420476_a_at	0.00	0.00
1420477_at	0.00	0.00
1420478_at	0.00	0.07
1420479_a_at	0.00	0.00
1420480_at	0.00	0.00
1420481_at	0.00	0.00
1420482_at	0.00	0.00
1420483_at	0.00	0.00
1420484_a_at	0.00	0.00
1420485_at	0.00	0.00
1420486_at	0.00	0.00
1420487_at	0.00	0.00
1420488_at	0.00	0.00
1420489_at	0.00	0.21
1420490_at	0.00	0.00
1420491_at	0.00	0.00
1420492_s_at	0.00	0.00
1420493_a_at	0.00	0.00
1420494_x_at	0.00	0.00
1420495_a_at	0.00	0.00
1420496_at	0.00	0.00
1420497_a_at	0.00	0.13
1420498_a_at	0.00	0.98
1420499_at	0.00	0.32
1420500_at	0.00	0.00
1420501_at	0.00	0.00
1420502_at	0.03	0.00
1420503_at	0.00	0.00
1420504_at	0.00	0.00
1420505_a_at	0.03	0.29
1420506_a_at	0.00	0.00
1420507_a_at	0.00	0.00

1420508_at	0.00	0.00
1420509_at	0.00	0.00
1420510_at	0.00	0.00
1420511_at	0.00	0.00
1420512_at	0.00	0.00
1420513_at	0.00	0.00
1420514_at	0.00	0.00
1420515_a_at	0.00	0.00
1420516_at	0.00	0.00
1420517_at	0.00	0.00
1420518_a_at	0.00	0.00
1420519_a_at	0.00	0.00
1420520_x_at	0.00	0.00
1420521_at	0.00	0.00
1420522_at	0.00	0.00
1420523_at	0.00	0.00
1420524_a_at	0.00	0.00
1420525_a_at	0.00	0.00
1420526_at	0.00	0.00
1420527_s_at	0.00	0.00
1420528_at	0.00	0.00
1420529_at	0.00	0.00
1420530_at	0.00	0.00
1420531_at	0.00	0.00
1420532_at	0.00	0.00
1420533_at	0.00	0.00
1420534_at	0.00	0.00
1420535_a_at	0.00	0.00
1420536_at	0.00	0.00
1420537_at	0.00	0.00
1420538_at	0.00	0.00
1420539_a_at	0.00	0.00
1420540_a_at	0.00	0.00
1420541_at	0.00	0.00
1420542_at	0.00	0.00
1420543_at	0.00	0.00
1420544_at	0.00	0.00
1420545_a_at	0.00	0.00
1420546_at	0.00	0.00
1420547_at	0.00	0.00
1420548_a_at	0.00	0.00
1420549_at	0.10	0.00
1420550_at	0.00	0.00
1420551_at	0.00	0.00
1420552_s_at	0.00	0.00
1420553_x_at	0.00	0.00
1420554_a_at	0.00	0.00
1420555_at	0.00	0.00
1420556_at	0.00	0.00
1420557_at	0.00	0.00
1420558_at	0.00	0.00
1420559_a_at	0.00	0.00
1420560_at	0.00	0.00
1420561_at	0.00	0.00
1420562_at	0.00	0.00
1420563_at	0.00	0.00

1420564_at	0.00	0.00
1420565_at	0.67	0.00
1420566_at	0.00	0.00
1420567_at	0.00	0.00
1420568_at	0.00	0.00
1420569_at	0.00	0.00
1420570_x_at	0.00	0.00
1420571_at	0.00	0.00
1420572_at	0.00	0.00
1420573_at	0.00	0.00
1420574_at	0.00	0.00
1420575_at	0.00	0.00
1420576_at	0.00	0.00
1420577_at	0.00	0.00
1420578_at	0.00	0.00
1420579_s_at	0.00	0.00
1420580_at	0.00	0.00
1420581_at	0.00	0.00
1420582_at	0.00	0.00
1420583_a_at	0.00	0.00
1420584_at	0.00	0.00
1420585_a_at	0.00	0.00
1420586_at	0.00	0.00
1420587_at	0.00	0.00
1420588_at	0.00	0.00
1420589_at	0.00	0.00
1420590_at	0.00	0.00
1420591_at	0.00	0.00
1420592_a_at	0.00	0.05
1420593_a_at	0.00	0.00
1420594_at	0.00	0.00
1420595_at	0.00	0.00
1420596_at	0.00	0.00
1420597_a_at	0.00	0.00
1420598_x_at	0.00	0.00
1420599_at	0.00	0.00
1420600_at	0.00	0.00
1420601_at	0.00	0.00
1420602_a_at	0.00	0.00
1420603_s_at	0.00	0.00
1420604_at	0.00	0.00
1420605_at	0.00	0.00
1420606_at	0.00	0.00
1420607_at	0.00	0.00
1420608_at	0.00	0.00
1420609_at	0.00	0.00
1420610_at	0.00	0.00
1420611_at	0.00	0.00
1420612_s_at	0.02	0.00
1420613_at	0.00	0.00
1420614_at	0.00	0.02
1420615_at	0.00	0.00
1420616_at	0.00	0.00
1420617_at	0.00	0.00
1420618_at	0.00	0.00
1420619_a_at	0.00	0.00

1420620_a_at	0.00	0.00
1420621_a_at	0.17	0.43
1420622_a_at	0.00	0.00
1420623_x_at	0.00	0.00
1420624_a_at	0.01	0.42
1420625_at	0.00	0.00
1420626_at	0.00	0.22
1420627_a_at	0.00	0.00
1420628_at	0.00	0.01
1420629_a_at	0.00	0.00
1420630_at	0.00	0.00
1420631_a_at	0.00	0.00
1420632_a_at	0.00	0.00
1420633_a_at	0.00	0.00
1420634_a_at	0.00	0.00
1420635_a_at	0.00	0.00
1420636_a_at	0.00	0.00
1420637_at	0.00	0.00
1420638_at	0.00	0.00
1420639_at	0.00	0.00
1420640_at	0.00	0.00
1420641_a_at	0.00	0.00
1420642_a_at	0.00	0.00
1420643_at	0.00	0.00
1420644_a_at	0.00	0.00
1420645_at	0.00	0.00
1420646_at	0.00	0.00
1420647_a_at	0.87	0.00
1420648_at	0.00	0.00
1420649_at	0.07	0.00
1420650_at	0.00	0.00
1420651_at	0.00	0.00
1420652_at	0.00	0.00
1420653_at	0.00	0.00
1420654_a_at	0.00	0.00
1420655_at	0.00	0.00
1420656_at	0.00	0.00
1420657_at	0.00	0.00
1420658_at	0.00	0.00
1420659_at	0.00	0.00
1420660_at	0.00	0.00
1420661_a_at	0.00	0.00
1420662_at	0.00	0.00
1420663_at	0.00	0.00
1420664_s_at	0.02	0.00
1420665_at	0.00	0.00
1420666_at	0.00	0.00
1420667_at	0.00	0.00
1420668_a_at	0.00	0.00
1420669_at	0.00	0.00
1420670_at	0.00	0.00
1420671_x_at	0.00	0.00
1420672_at	0.00	0.00
1420673_a_at	0.00	0.00
1420674_at	0.00	0.00
1420675_at	0.00	0.00

1420676_at	0.00	0.00
1420677_x_at	0.00	0.00
1420678_a_at	0.00	0.00
1420679_a_at	0.00	0.00
1420680_at	0.00	0.00
1420681_at	0.00	0.00
1420682_at	0.06	0.00
1420683_at	0.00	0.05
1420684_at	0.00	0.00
1420685_at	0.00	0.00
1420686_at	0.00	0.00
1420687_at	0.00	0.00
1420688_a_at	0.00	0.28
1420689_at	0.00	0.00
1420690_at	0.00	0.00
1420691_at	0.00	0.00
1420692_at	0.00	0.00
1420693_at	0.00	0.00
1420694_a_at	0.00	0.00
1420695_at	0.00	0.00
1420696_at	0.00	0.00
1420697_at	0.00	0.00
1420698_at	0.00	0.00
1420699_at	0.00	0.00
1420700_s_at	0.00	0.00
1420701_at	0.00	0.00
1420702_at	0.00	0.00
1420703_at	0.00	0.00
1420704_at	0.00	0.00
1420705_at	0.00	0.00
1420706_at	0.00	0.00
1420707_a_at	0.00	0.00
1420708_at	0.00	0.00
1420709_s_at	0.00	0.00
1420710_at	0.00	0.00
1420711_a_at	0.08	0.00
1420712_a_at	0.00	0.10
1420713_a_at	0.00	0.00
1420714_at	0.00	0.00
1420715_a_at	0.00	0.00
1420716_at	0.00	0.00
1420717_at	0.00	0.00
1420718_at	0.00	0.00
1420719_at	0.00	0.00
1420720_at	0.00	0.00
1420721_at	0.00	0.00
1420722_at	0.00	0.00
1420723_at	0.00	0.00
1420724_at	0.00	0.00
1420725_at	0.00	0.00
1420726_x_at	0.00	0.00
1420727_a_at	0.00	0.00
1420728_at	0.00	0.00
1420729_at	0.00	0.00
1420730_a_at	0.00	0.00
1420731_a_at	0.00	0.00

1420732_at	0.00	0.00
1420733_at	0.00	0.00
1420734_at	0.00	0.00
1420735_at	0.00	0.00
1420736_at	0.00	0.00
1420737_at	0.00	0.00
1420738_at	0.00	0.00
1420739_at	0.00	0.00
1420740_at	0.00	0.00
1420741_x_at	0.00	0.00
1420742_at	0.00	0.00
1420743_a_at	0.00	0.00
1420744_at	0.00	0.00
1420745_a_at	0.00	0.00
1420746_at	0.00	0.00
1420747_at	0.00	0.00
1420748_a_at	0.00	0.00
1420749_a_at	0.00	0.00
1420750_at	0.00	0.00
1420751_at	0.00	0.00
1420752_at	0.00	0.00
1420753_at	0.00	0.00
1420754_at	0.00	0.00
1420755_a_at	0.00	0.00
1420756_at	0.00	0.00
1420757_at	0.00	0.00
1420758_at	0.00	0.00
1420759_s_at	0.00	0.00
1420760_s_at	0.00	0.00
1420761_at	0.00	0.00
1420762_a_at	0.00	0.00
1420763_at	0.00	0.00
1420764_at	0.00	0.00
1420765_a_at	0.00	0.00
1420766_at	0.00	0.00
1420767_at	0.00	0.00
1420768_a_at	0.00	0.00
1420769_at	0.00	0.00
1420770_at	0.00	0.00
1420771_at	0.00	0.00
1420772_a_at	0.00	0.00
1420773_at	0.00	0.00
1420774_a_at	0.00	0.00
1420775_at	0.00	0.00
1420776_a_at	0.00	0.00
1420777_a_at	0.00	0.00
1420778_at	0.00	0.00
1420779_at	0.00	0.00
1420780_at	0.00	0.00
1420781_at	0.00	0.00
1420782_at	0.00	0.00
1420783_at	0.00	0.00
1420784_at	0.00	0.00
1420785_at	0.00	0.00
1420786_a_at	0.00	0.00
1420787_at	0.00	0.00

1420788_at	0.00	0.00
1420789_at	0.00	0.00
1420790_x_at	0.00	0.00
1420791_x_at	0.00	0.00
1420792_at	0.00	0.00
1420793_at	0.00	0.00
1420794_at	0.00	0.00
1420795_at	0.00	0.00
1420796_at	0.00	0.00
1420797_at	0.00	0.00
1420798_s_at	0.00	0.00
1420799_at	0.00	0.00
1420800_a_at	0.00	0.00
1420801_at	0.00	0.00
1420802_at	0.00	0.00
1420803_at	0.00	0.00
1420804_s_at	0.00	0.00
1420805_at	0.00	0.00
1420806_at	0.00	0.00
1420807_a_at	0.00	0.00
1420808_at	0.00	0.00
1420809_a_at	0.00	0.00
1420810_at	0.00	0.00
1420811_a_at	0.00	0.00
1420812_at	0.00	0.00
1420813_at	0.00	0.00
1420814_at	0.00	0.00
1420815_at	0.00	0.00
1420816_at	0.00	0.11
1420817_at	0.00	0.00
1420818_at	0.00	0.00
1420819_at	0.00	0.00
1420820_at	0.00	0.00
1420821_at	0.00	0.00
1420822_s_at	0.00	0.00
1420823_at	0.00	0.00
1420824_at	0.00	0.00
1420825_at	0.00	0.00
1420826_at	0.00	0.00
1420827_a_at	0.00	0.00
1420828_s_at	0.00	0.00
1420829_a_at	0.00	0.00
1420830_x_at	0.00	0.00
1420831_at	0.00	0.00
1420832_at	0.00	0.00
1420833_at	0.00	0.00
1420834_at	0.00	0.00
1420835_at	0.00	0.36
1420836_at	0.00	0.51
1420837_at	0.00	0.00
1420838_at	0.00	0.00
1420839_at	0.00	0.00
1420840_at	0.00	0.00
1420841_at	0.00	0.00
1420842_at	0.00	0.00
1420843_at	0.00	0.00

1420844_at	0.00	0.00
1420845_at	0.00	0.00
1420846_at	0.00	0.00
1420847_a_at	0.00	0.00
1420848_at	0.00	0.00
1420849_at	0.00	0.00
1420850_at	0.00	0.53
1420851_at	0.00	0.00
1420852_a_at	0.00	0.00
1420853_at	0.00	0.00
1420854_at	0.00	0.00
1420855_at	0.00	0.00
1420856_a_at	0.00	0.00
1420857_at	0.00	0.00
1420858_at	0.00	0.00
1420859_at	0.00	0.00
1420860_at	0.00	0.00
1420861_at	0.00	0.00
1420862_at	0.00	0.00
1420863_at	0.00	0.00
1420864_at	0.00	0.00
1420865_at	0.00	0.00
1420866_at	0.00	0.00
1420867_at	0.00	0.00
1420868_s_at	0.00	0.00
1420869_at	0.00	0.00
1420870_at	0.00	0.00
1420871_at	0.00	0.00
1420872_at	0.00	0.00
1420873_at	0.00	0.00
1420874_at	0.00	0.00
1420875_at	0.00	0.00
1420876_a_at	0.00	0.00
1420877_at	0.00	0.00
1420878_a_at	0.00	0.03
1420879_a_at	0.00	0.00
1420880_a_at	0.00	0.00
1420881_at	0.00	0.00
1420882_a_at	0.00	0.00
1420883_at	0.00	0.00
1420884_at	0.00	0.00
1420885_a_at	0.00	0.00
1420886_a_at	0.00	0.00
1420887_a_at	0.00	0.00
1420888_at	0.00	0.00
1420889_at	0.00	0.00
1420890_at	0.00	0.00
1420891_at	0.00	0.00
1420892_at	0.00	0.00
1420893_a_at	0.00	0.00
1420894_at	0.00	0.00
1420895_at	0.00	0.00
1420896_at	0.00	0.00
1420897_at	0.00	0.00
1420898_at	0.00	0.00
1420899_at	0.00	0.00

1420900_a_at	0.00	0.00
1420901_a_at	0.00	0.00
1420902_at	0.00	0.00
1420903_at	0.00	0.00
1420904_at	0.00	0.00
1420905_at	0.00	0.00
1420906_at	0.00	0.00
1420907_at	0.00	0.00
1420908_at	0.00	0.00
1420909_at	0.00	0.02
1420910_at	0.00	0.00
1420911_a_at	0.00	0.00
1420912_at	0.00	0.00
1420913_at	0.00	0.00
1420914_at	0.00	0.00
1420915_at	0.00	0.00
1420916_at	0.00	0.02
1420917_at	0.00	0.00
1420918_at	0.00	0.00
1420919_at	0.00	0.00
1420920_a_at	0.00	0.00
1420921_at	0.00	0.00
1420922_at	0.00	0.00
1420923_at	0.00	0.00
1420924_at	0.00	0.00
1420925_at	0.00	0.00
1420926_at	0.00	0.00
1420927_at	0.00	0.00
1420928_at	0.09	0.00
1420929_at	0.00	0.00
1420930_s_at	0.40	0.00
1420931_at	0.00	0.00
1420932_at	0.00	0.00
1420933_a_at	0.00	0.00
1420934_a_at	0.00	0.00
1420935_a_at	0.00	0.00
1420936_s_at	0.00	0.00
1420937_at	0.00	0.00
1420938_at	0.00	0.00
1420939_at	0.00	0.00
1420940_x_at	0.00	0.00
1420941_at	0.00	0.00
1420942_s_at	0.00	0.00
1420943_at	0.00	0.00
1420944_at	0.00	0.00
1420945_at	0.00	0.00
1420946_at	0.00	0.00
1420947_at	0.00	0.00
1420948_s_at	0.00	0.00
1420949_at	0.00	0.00
1420950_at	0.00	0.00
1420951_a_at	0.00	0.00
1420952_at	0.00	0.17
1420953_at	0.00	0.00
1420954_a_at	0.00	0.00
1420955_at	0.00	0.00

1420956_at	0.00	0.00
1420957_at	0.00	0.00
1420958_at	0.00	0.00
1420959_at	0.00	0.00
1420960_at	0.00	0.00
1420961_a_at	0.00	0.00
1420962_at	0.00	0.00
1420963_at	0.00	0.00
1420964_at	0.00	0.00
1420965_a_at	0.00	0.00
1420966_at	0.00	0.00
1420967_at	0.00	0.00
1420968_at	0.00	0.00
1420969_at	0.00	0.00
1420970_at	0.00	0.00
1420971_at	0.00	0.00
1420972_at	0.00	0.00
1420973_at	0.00	0.00
1420974_at	0.00	0.00
1420975_at	0.00	0.00
1420976_at	0.00	0.00
1420977_at	0.00	0.00
1420978_at	0.00	0.00
1420979_at	0.00	0.00
1420980_at	0.00	0.00
1420981_a_at	0.00	0.00
1420982_at	0.00	0.00
1420983_at	0.00	0.00
1420984_at	0.00	0.00
1420985_at	0.00	0.00
1420986_s_at	0.00	0.00
1420987_at	0.00	0.00
1420988_at	0.00	0.00
1420989_at	0.00	0.00
1420990_at	0.00	0.00
1420991_at	0.19	0.00
1420992_at	0.00	0.00
1420993_at	0.00	0.00
1420994_at	0.53	0.00
1420995_at	0.00	0.00
1420996_at	0.00	0.00
1420997_a_at	0.00	0.00
1420998_at	0.00	0.27
1420999_at	0.00	0.00
1421000_at	0.00	0.00
1421001_a_at	0.00	0.00
1421002_at	0.00	0.00
1421003_at	0.00	0.00
1421004_at	0.00	0.00
1421005_at	0.00	0.00
1421006_at	0.00	0.00
1421007_at	0.00	0.00
1421008_at	0.00	0.00
1421009_at	0.00	0.00
1421010_at	0.00	0.00
1421011_at	0.00	0.00

1421012_at	0.00	0.00
1421013_at	0.00	0.00
1421014_a_at	0.00	0.01
1421015_s_at	0.00	0.00
1421016_at	0.00	0.00
1421017_at	0.00	0.00
1421018_at	0.00	0.00
1421019_at	0.00	0.00
1421020_at	0.00	0.00
1421021_at	0.00	0.00
1421022_x_at	0.00	0.00
1421023_at	0.00	0.00
1421024_at	0.00	0.00
1421025_at	0.00	0.00
1421026_at	0.00	0.00
1421027_a_at	0.00	0.00
1421028_a_at	0.00	0.00
1421029_a_at	0.00	0.00
1421030_at	0.00	0.00
1421031_a_at	0.00	0.00
1421032_a_at	0.00	0.00
1421033_a_at	0.00	0.03
1421034_a_at	0.00	0.00
1421035_a_at	0.00	0.00
1421036_at	0.00	0.00
1421037_at	0.00	0.00
1421038_a_at	0.00	0.00
1421039_at	0.00	0.00
1421040_a_at	0.00	0.00
1421041_s_at	0.00	0.00
1421042_at	0.00	0.00
1421043_s_at	0.00	0.00
1421044_at	0.00	0.00
1421045_at	0.00	0.00
1421046_a_at	0.00	0.00
1421047_at	0.00	0.00
1421048_a_at	0.00	0.00
1421049_at	0.00	0.00
1421050_at	0.00	0.00
1421051_s_at	0.00	0.00
1421052_a_at	0.15	0.00
1421053_at	0.00	0.05
1421054_at	0.00	0.00
1421055_at	0.00	0.00
1421056_at	0.00	0.00
1421057_at	0.00	0.00
1421058_at	0.00	0.00
1421059_a_at	0.00	0.00
1421060_at	0.00	0.00
1421061_at	0.00	0.00
1421062_s_at	0.00	0.00
1421063_s_at	0.00	0.00
1421064_at	0.00	0.00
1421065_at	0.00	0.00
1421066_at	0.00	0.01
1421067_a_at	0.00	0.00

1421068_at	0.00	0.00
1421069_at	0.00	0.00
1421070_at	0.00	0.00
1421071_at	0.00	0.00
1421072_at	0.00	0.00
1421073_a_at	0.00	0.00
1421074_at	0.00	0.00
1421075_s_at	0.00	0.00
1421076_at	0.00	0.00
1421077_at	0.00	0.00
1421078_at	0.00	0.00
1421079_at	0.00	0.00
1421080_at	0.00	0.00
1421081_a_at	0.00	0.00
1421082_s_at	0.00	0.00
1421083_x_at	0.00	0.00
1421084_at	0.00	0.00
1421085_at	0.00	0.00
1421086_at	0.00	0.00
1421087_at	0.00	0.00
1421088_at	0.00	0.00
1421089_a_at	0.00	0.00
1421090_at	0.00	0.00
1421091_at	0.00	0.00
1421092_at	0.00	0.00
1421093_at	0.00	0.00
1421094_at	0.00	0.00
1421095_a_at	0.00	0.00
1421096_at	0.00	0.00
1421097_at	0.00	0.00
1421098_at	0.00	0.00
1421099_at	0.00	0.00
1421100_a_at	0.00	0.00
1421101_a_at	0.00	0.00
1421102_a_at	0.00	0.00
1421103_at	0.00	0.00
1421104_at	0.00	0.00
1421105_at	0.00	0.00
1421106_at	0.02	0.00
1421107_at	0.00	0.00
1421108_at	0.00	0.00
1421109_at	0.00	0.00
1421110_at	0.00	0.00
1421111_at	0.00	0.00
1421112_at	0.00	0.00
1421113_at	0.00	0.00
1421114_a_at	0.00	0.00
1421115_a_at	0.00	0.00
1421116_a_at	0.00	0.00
1421117_at	0.00	0.00
1421118_a_at	0.00	0.00
1421119_at	0.00	0.00
1421120_at	0.00	0.00
1421121_at	0.00	0.00
1421122_at	0.00	0.00
1421123_at	0.00	0.00

1421124_at	0.00	0.00
1421125_at	0.00	0.00
1421126_at	0.00	0.00
1421127_at	0.00	0.00
1421128_at	0.00	0.00
1421129_a_at	0.00	0.00
1421130_at	0.00	0.00
1421131_a_at	0.00	0.00
1421132_at	0.00	0.00
1421133_at	0.00	0.00
1421134_at	0.00	0.00
1421135_a_at	0.00	0.00
1421136_at	0.00	0.00
1421137_a_at	0.00	0.00
1421138_a_at	0.00	0.00
1421139_a_at	0.00	0.00
1421140_a_at	0.02	0.01
1421141_a_at	0.11	0.39
1421142_s_at	0.04	0.22
1421143_at	0.00	0.00
1421144_at	0.00	0.01
1421145_at	0.00	0.00
1421146_at	0.00	0.00
1421147_at	0.00	0.00
1421148_a_at	0.00	0.00
1421149_a_at	0.01	0.00
1421150_at	0.00	0.00
1421151_a_at	0.00	0.26
1421152_a_at	0.00	0.00
1421153_at	0.00	0.00
1421154_at	0.00	0.00
1421155_at	0.00	0.00
1421156_a_at	0.01	0.00
1421157_at	0.00	0.00
1421158_at	0.00	0.00
1421159_at	0.00	0.00
1421160_a_at	0.00	0.00
1421161_at	0.00	0.00
1421162_a_at	0.00	0.00
1421163_a_at	0.00	0.00
1421164_a_at	0.00	0.00
1421165_at	0.00	0.00
1421166_at	0.00	0.00
1421167_at	0.00	0.00
1421168_at	0.00	0.00
1421169_at	0.00	0.00
1421170_a_at	0.00	0.00
1421171_at	0.00	0.00
1421172_at	0.00	0.00
1421173_at	0.00	0.00
1421174_at	0.00	0.00
1421175_at	0.00	0.00
1421176_at	0.00	0.00
1421177_at	0.00	0.00
1421178_at	0.00	0.00
1421179_at	0.00	0.00

1421180_at	0.00	0.00
1421181_at	0.00	0.00
1421182_at	0.00	0.00
1421183_at	0.00	0.00
1421184_a_at	0.00	0.00
1421185_at	0.00	0.00
1421186_at	0.00	0.00
1421187_at	0.00	0.00
1421188_at	0.00	0.00
1421189_at	0.00	0.00
1421190_at	0.00	0.00
1421191_s_at	0.00	0.00
1421192_a_at	0.00	0.00
1421193_a_at	0.00	0.00
1421194_at	0.00	0.00
1421195_at	0.00	0.00
1421196_at	0.00	0.00
1421197_a_at	0.00	0.00
1421198_at	0.00	0.00
1421199_at	0.00	0.00
1421200_at	0.00	0.00
1421201_a_at	0.00	0.00
1421202_at	0.00	0.00
1421203_at	0.00	0.00
1421204_a_at	0.00	0.00
1421205_at	0.00	0.00
1421206_at	0.00	0.00
1421207_at	0.00	0.00
1421208_at	0.00	0.00
1421209_s_at	0.00	0.00
1421210_at	0.00	0.00
1421211_a_at	0.00	0.00
1421212_at	0.00	0.00
1421213_at	0.00	0.00
1421214_at	0.00	0.00
1421215_a_at	0.00	0.00
1421216_a_at	0.00	0.00
1421217_a_at	0.00	0.00
1421218_at	0.00	0.00
1421219_at	0.00	0.00
1421220_at	0.00	0.00
1421221_at	0.00	0.00
1421222_at	0.00	0.00
1421223_a_at	0.00	0.22
1421224_a_at	0.00	0.00
1421225_a_at	0.00	0.00
1421226_at	0.00	0.00
1421227_at	0.00	0.00
1421228_at	0.00	0.00
1421229_at	0.00	0.00
1421230_a_at	0.00	0.00
1421231_at	0.00	0.00
1421232_at	0.00	0.00
1421233_at	0.00	0.00
1421234_at	0.00	0.00
1421235_s_at	0.00	0.00

1421236_at	0.00	0.00
1421237_at	0.00	0.00
1421238_a_at	0.00	0.00
1421239_at	0.00	0.00
1421240_at	0.00	0.00
1421241_at	0.00	0.00
1421242_at	0.00	0.00
1421243_at	0.00	0.00
1421244_at	0.00	0.00
1421245_at	0.00	0.00
1421246_at	0.00	0.00
1421247_at	0.00	0.00
1421248_at	0.00	0.00
1421249_at	0.00	0.00
1421250_at	0.00	0.00
1421251_at	0.00	0.00
1421252_a_at	0.00	0.00
1421253_at	0.00	0.00
1421254_a_at	0.00	0.00
1421255_a_at	0.00	0.00
1421256_at	0.00	0.00
1421257_at	0.00	0.00
1421258_a_at	0.00	0.00
1421259_at	0.00	0.00
1421260_a_at	0.02	0.40
1421261_at	0.00	0.00
1421262_at	0.00	0.00
1421263_at	0.00	0.00
1421264_at	0.00	0.00
1421265_a_at	0.00	0.00
1421266_s_at	0.00	0.00
1421267_a_at	0.01	0.00
1421268_at	0.00	0.00
1421269_at	0.00	0.01
1421270_at	0.00	0.00
1421271_at	0.00	0.00
1421272_at	0.00	0.00
1421273_at	0.00	0.00
1421274_at	0.00	0.00
1421275_s_at	0.00	0.00
1421276_a_at	0.00	0.00
1421277_at	0.00	0.00
1421278_s_at	0.00	0.00
1421279_at	0.00	0.00
1421280_at	0.00	0.00
1421281_at	0.00	0.00
1421282_at	0.00	0.00
1421283_at	0.00	0.00
1421284_at	0.00	0.00
1421285_at	0.00	0.00
1421286_a_at	0.00	0.00
1421287_a_at	0.66	0.00
1421288_at	0.00	0.00
1421289_at	0.00	0.00
1421290_at	0.00	0.00
1421291_at	0.00	0.00

1421292_a_at	0.00	0.00
1421293_at	0.00	0.00
1421294_at	0.00	0.00
1421295_at	0.00	0.00
1421296_at	0.00	0.00
1421297_a_at	0.00	0.00
1421298_a_at	0.00	0.00
1421299_a_at	0.00	0.00
1421300_at	0.00	0.00
1421301_at	0.00	0.00
1421302_a_at	0.00	0.00
1421303_at	0.00	0.00
1421304_at	0.00	0.00
1421305_x_at	0.00	0.00
1421306_a_at	0.00	0.00
1421307_at	0.00	0.00
1421308_at	0.00	0.00
1421309_at	0.00	0.00
1421310_at	0.00	0.00
1421311_at	0.00	0.00
1421312_a_at	0.00	0.00
1421313_s_at	0.00	0.00
1421314_at	0.00	0.00
1421315_s_at	0.00	0.00
1421316_at	0.00	0.00
1421317_x_at	0.00	0.00
1421318_at	0.00	0.00
1421319_at	0.00	0.00
1421320_a_at	0.00	0.00
1421321_a_at	0.00	0.00
1421322_a_at	0.00	0.00
1421323_a_at	0.00	0.00
1421324_a_at	0.00	0.00
1421325_at	0.00	0.00
1421326_at	0.00	0.00
1421327_at	0.00	0.00
1421328_at	0.00	0.00
1421329_a_at	0.00	0.00
1421330_at	0.00	0.00
1421331_at	0.00	0.00
1421332_at	0.00	0.00
1421333_a_at	0.00	0.00
1421334_x_at	0.00	0.00
1421335_a_at	0.00	0.00
1421336_at	0.00	0.00
1421337_at	0.00	0.00
1421338_at	0.00	0.00
1421339_at	0.00	0.00
1421340_at	0.00	0.00
1421341_at	0.00	0.00
1421342_at	0.00	0.00
1421343_at	0.00	0.00
1421344_a_at	0.00	0.00
1421345_at	0.00	0.00
1421346_a_at	0.00	0.00
1421347_at	0.00	0.00

1421348_a_at	0.00	0.00
1421349_x_at	0.00	0.00
1421350_a_at	0.00	0.00
1421351_at	0.00	0.00
1421352_at	0.00	0.00
1421353_at	0.00	0.00
1421354_at	0.00	0.00
1421355_at	0.00	0.00
1421356_at	0.00	0.00
1421357_at	0.00	0.00
1421358_at	0.00	0.00
1421359_at	0.00	0.00
1421360_at	0.00	0.00
1421361_at	0.00	0.00
1421362_a_at	0.00	0.00
1421363_at	0.00	0.00
1421364_at	0.00	0.00
1421365_at	0.30	0.00
1421366_at	0.00	0.00
1421367_at	0.00	0.00
1421368_at	0.00	0.00
1421369_a_at	0.00	0.00
1421370_a_at	0.00	0.00
1421371_at	0.00	0.00
1421372_at	0.00	0.00
1421373_at	0.00	0.00
1421374_a_at	0.00	0.00
1421375_a_at	0.38	0.00
1421376_at	0.00	0.00
1421377_at	0.00	0.00
1421378_s_at	0.00	0.00
1421379_at	0.00	0.00
1421380_at	0.00	0.00
1421381_a_at	0.00	0.00
1421382_at	0.00	0.00
1421383_at	0.00	0.00
1421384_at	0.00	0.00
1421385_a_at	0.00	0.00
1421386_at	0.00	0.00
1421387_at	0.00	0.00
1421388_at	0.00	0.00
1421389_a_at	0.00	0.00
1421390_at	0.00	0.00
1421391_at	0.00	0.00
1421392_a_at	0.00	0.00
1421393_at	0.00	0.00
1421394_a_at	0.00	0.00
1421395_at	0.00	0.00
1421396_at	0.00	0.00
1421397_a_at	0.00	0.00
1421398_at	0.00	0.00
1421399_at	0.00	0.00
1421400_at	0.00	0.00
1421401_at	0.00	0.00
1421402_at	0.00	0.00
1421403_at	0.00	0.00

1421404_at	0.00	0.00
1421405_at	0.00	0.00
1421406_at	0.00	0.00
1421407_at	0.00	0.00
1421408_at	0.00	0.00
1421409_at	0.00	0.00
1421410_a_at	0.00	0.00
1421411_at	0.00	0.00
1421412_at	0.00	0.00
1421413_a_at	0.00	0.00
1421414_a_at	0.00	0.00
1421415_s_at	0.00	0.00
1421416_at	0.00	0.00
1421417_s_at	0.00	0.00
1421418_a_at	0.00	0.00
1421419_at	0.00	0.00
1421420_at	0.00	0.00
1421421_at	0.00	0.00
1421422_at	0.00	0.00
1421423_at	0.00	0.00
1421424_a_at	0.00	0.00
1421425_a_at	0.00	0.00
1421426_at	0.00	0.00
1421427_at	0.00	0.00
1421428_at	0.00	0.00
1421429_a_at	0.00	0.00
1421430_at	0.00	0.00
1421431_at	0.00	0.00
1421432_at	0.00	0.00
1421433_at	0.00	0.00
1421434_at	0.00	0.00
1421435_at	0.00	0.00
1421436_at	0.00	0.00
1421437_x_at	0.00	0.00
1421438_at	0.00	0.00
1421439_at	0.00	0.00
1421440_at	0.00	0.00
1421441_at	0.00	0.00
1421442_at	0.00	0.00
1421443_at	0.00	0.00
1421444_at	0.00	0.00
1421445_at	0.00	0.00
1421446_at	0.00	0.00
1421447_at	0.00	0.00
1421448_at	0.00	0.00
1421449_at	0.00	0.00
1421450_a_at	0.00	0.07
1421451_at	0.00	0.00
1421452_at	0.00	0.00
1421453_at	0.00	0.00
1421454_at	0.00	0.00
1421455_at	0.00	0.00
1421456_at	0.00	0.00
1421457_a_at	0.00	0.00
1421458_at	0.00	0.00
1421459_a_at	0.00	0.00

1421460_at	0.00	0.00
1421461_at	0.00	0.00
1421462_a_at	0.00	0.00
1421463_at	0.00	0.00
1421464_at	0.00	0.00
1421465_at	0.00	0.00
1421466_at	0.00	0.00
1421467_at	0.00	0.00
1421468_at	0.00	0.00
1421469_a_at	0.00	0.00
1421470_at	0.00	0.00
1421471_at	0.00	0.00
1421472_at	0.00	0.00
1421473_at	0.00	0.00
1421474_a_at	0.00	0.00
1421475_at	0.00	0.00
1421476_a_at	0.00	0.00
1421477_at	0.00	0.00
1421478_a_at	0.00	0.00
1421479_at	0.00	0.00
1421480_a_at	0.00	0.00
1421481_at	0.00	0.00
1421482_at	0.00	0.00
1421483_at	0.00	0.00
1421484_at	0.00	0.00
1421485_at	0.00	0.00
1421486_at	0.00	0.00
1421487_a_at	0.00	0.00
1421488_at	0.00	0.00
1421489_a_at	0.00	0.00
1421490_at	0.00	0.00
1421491_a_at	0.00	0.00
1421492_at	0.00	0.00
1421493_a_at	0.00	0.00
1421494_at	0.00	0.00
1421495_a_at	0.00	0.00
1421496_at	0.14	0.00
1421497_at	0.00	0.00
1421498_a_at	0.01	0.15
1421499_a_at	0.00	0.00
1421500_at	0.00	0.00
1421501_a_at	0.00	0.00
1421502_at	0.00	0.00
1421503_at	0.00	0.00
1421504_at	0.00	0.00
1421505_at	0.00	0.00
1421506_at	0.00	0.00
1421507_at	0.00	0.00
1421508_at	0.00	0.00
1421509_at	0.00	0.00
1421510_at	0.00	0.00
1421511_at	0.00	0.00
1421512_at	0.00	0.00
1421513_at	0.00	0.00
1421514_a_at	0.00	0.00
1421515_at	0.00	0.01

1421516_at	0.00	0.00
1421517_at	0.00	0.00
1421518_at	0.00	0.00
1421519_a_at	0.00	0.00
1421520_at	0.00	0.00
1421521_at	0.00	0.00
1421522_at	0.00	0.00
1421523_at	0.00	0.21
1421524_at	0.00	0.00
1421525_a_at	0.00	0.00
1421526_at	0.00	0.00
1421527_at	0.00	0.00
1421528_a_at	0.00	0.00
1421529_a_at	0.00	0.00
1421530_a_at	0.00	0.00
1421531_at	0.00	0.00
1421532_at	0.00	0.00
1421533_at	0.00	0.00
1421534_at	0.00	0.00
1421535_a_at	0.00	0.00
1421536_at	0.00	0.00
1421537_at	0.00	0.00
1421538_at	0.00	0.00
1421539_at	0.00	0.00
1421540_at	0.00	0.00
1421541_a_at	0.00	0.00
1421542_at	0.00	0.00
1421543_at	0.00	0.00
1421544_at	0.00	0.00
1421545_a_at	0.00	0.00
1421546_a_at	0.00	0.01
1421547_at	0.00	0.00
1421548_at	0.00	0.00
1421549_at	0.00	0.00
1421550_a_at	0.00	0.00
1421551_s_at	0.00	0.00
1421552_at	0.00	0.00
1421553_at	0.00	0.00
1421554_at	0.00	0.00
1421555_at	0.00	0.00
1421556_at	0.00	0.00
1421557_x_at	0.00	0.00
1421558_at	0.00	0.00
1421559_at	0.00	0.00
1421560_at	0.00	0.00
1421561_at	0.00	0.00
1421562_at	0.00	0.00
1421563_at	0.00	0.00
1421564_at	0.00	0.00
1421565_at	0.00	0.00
1421566_at	0.00	0.00
1421567_at	0.00	0.00
1421568_at	0.00	0.00
1421569_at	0.00	0.00
1421570_at	0.00	0.00
1421571_a_at	0.00	0.00

1421572_at	0.00	0.00
1421573_at	0.00	0.00
1421574_at	0.00	0.00
1421575_at	0.00	0.00
1421576_at	0.00	0.00
1421577_at	0.00	0.00
1421578_at	0.00	0.00
1421579_at	0.00	0.00
1421580_at	0.00	0.00
1421581_at	0.00	0.00
1421582_a_at	0.00	0.00
1421583_at	0.00	0.00
1421584_at	0.00	0.00
1421585_at	0.00	0.00
1421586_a_at	0.00	0.00
1421587_at	0.00	0.00
1421588_at	0.00	0.00
1421589_at	0.00	0.00
1421590_at	0.00	0.00
1421591_at	0.00	0.00
1421592_at	0.00	0.00
1421593_at	0.00	0.00
1421594_a_at	0.00	0.00
1421595_at	0.00	0.00
1421596_s_at	0.00	0.00
1421597_a_at	0.00	0.00
1421598_at	0.00	0.00
1421599_at	0.00	0.00
1421600_a_at	0.00	0.00
1421601_at	0.00	0.00
1421602_at	0.00	0.00
1421603_a_at	0.00	0.00
1421604_a_at	0.00	0.00
1421605_a_at	0.00	0.00
1421606_a_at	0.00	0.12
1421607_at	0.00	0.00
1421608_at	0.00	0.00
1421609_a_at	0.00	0.00
1421610_at	0.00	0.00
1421611_at	0.00	0.00
1421612_a_at	0.00	0.00
1421613_at	0.00	0.00
1421614_at	0.00	0.00
1421615_at	0.00	0.00
1421616_at	0.00	0.00
1421617_at	0.00	0.00
1421618_at	0.00	0.00
1421619_at	0.00	0.00
1421620_at	0.00	0.00
1421621_at	0.00	0.00
1421622_a_at	0.00	0.00
1421623_at	0.00	0.00
1421624_a_at	0.04	1.00
1421625_a_at	0.00	0.00
1421626_at	0.00	0.00
1421627_at	0.00	0.00

1421628_at	0.00	0.00
1421629_at	0.00	0.00
1421630_at	0.00	0.00
1421631_at	0.00	0.00
1421632_at	0.00	0.00
1421633_a_at	0.00	0.00
1421634_at	0.00	0.00
1421635_at	0.00	0.00
1421636_at	0.00	0.00
1421637_at	0.00	0.00
1421638_at	0.00	0.00
1421639_at	0.00	0.00
1421640_a_at	0.00	0.00
1421641_at	0.00	0.00
1421642_a_at	0.00	0.00
1421643_at	0.00	0.00
1421644_at	0.00	0.00
1421645_at	0.00	0.00
1421646_a_at	0.00	0.00
1421647_at	0.00	0.00
1421648_at	0.00	0.00
1421649_at	0.00	0.00
1421650_at	0.00	0.00
1421651_at	0.00	0.00
1421652_at	0.00	0.00
1421653_a_at	0.00	0.00
1421654_a_at	0.00	0.00
1421655_a_at	0.00	0.00
1421656_at	0.00	0.00
1421657_a_at	0.00	0.33
1421658_x_at	0.00	0.00
1421659_at	0.00	0.00
1421660_at	0.00	0.00
1421661_at	0.00	0.00
1421662_a_at	0.00	0.00
1421663_at	0.00	0.00
1421664_a_at	0.00	0.00
1421665_a_at	0.00	0.00
1421666_a_at	0.00	0.00
1421667_at	0.00	0.00
1421668_x_at	0.00	0.00
1421669_at	0.00	0.00
1421670_a_at	0.00	0.00
1421671_at	0.00	0.00
1421672_at	0.00	0.00
1421673_s_at	0.00	0.00
1421674_at	0.00	0.00
1421675_at	0.00	0.00
1421676_at	0.00	0.00
1421677_at	0.00	0.00
1421678_at	0.00	0.00
1421679_a_at	0.00	0.00
1421680_at	0.00	0.00
1421681_at	0.00	0.00
1421682_a_at	0.00	0.00
1421683_at	0.00	0.00

1421684_at	0.00	0.00
1421685_at	0.00	0.00
1421686_at	0.00	0.00
1421687_at	0.00	0.00
1421688_a_at	0.00	0.00
1421689_at	0.00	0.00
1421690_s_at	0.00	0.00
1421691_at	0.00	0.00
1421692_at	0.00	0.00
1421693_a_at	0.00	0.00
1421694_a_at	0.00	0.00
1421695_at	0.00	0.00
1421696_at	0.00	0.00
1421697_at	0.00	0.00
1421698_a_at	0.00	0.00
1421699_at	0.00	0.00
1421700_at	0.00	0.00
1421701_at	0.00	0.00
1421702_at	0.00	0.00
1421703_at	0.00	0.00
1421704_a_at	0.00	0.00
1421705_at	0.00	0.00
1421706_at	0.00	0.00
1421707_at	0.00	0.00
1421708_a_at	0.00	0.00
1421709_a_at	0.00	0.00
1421710_at	0.00	0.00
1421711_at	0.00	0.00
1421712_at	0.00	0.00
1421713_at	0.00	0.00
1421714_at	0.00	0.00
1421715_at	0.00	0.00
1421716_at	0.00	0.00
1421717_at	0.00	0.00
1421718_at	0.00	0.00
1421719_at	0.00	0.00
1421720_a_at	0.00	0.00
1421721_a_at	0.00	0.00
1421722_at	0.00	0.00
1421723_at	0.00	0.00
1421724_at	0.00	0.00
1421725_at	0.00	0.00
1421726_at	0.00	0.00
1421727_at	0.00	0.00
1421728_at	0.00	0.00
1421729_a_at	0.00	0.00
1421730_at	0.00	0.00
1421731_a_at	0.00	0.27
1421732_at	0.00	0.00
1421733_a_at	0.00	0.00
1421734_at	0.00	0.00
1421735_a_at	0.00	0.00
1421736_at	0.00	0.00
1421737_at	0.00	0.00
1421738_at	0.00	0.00
1421739_a_at	0.00	0.00

1421740_at	0.00	0.00
1421741_at	0.00	0.00
1421742_at	0.00	0.00
1421743_a_at	0.00	0.00
1421744_at	0.00	0.00
1421745_at	0.00	0.00
1421746_a_at	0.00	0.00
1421747_at	0.00	0.00
1421748_a_at	0.00	0.00
1421749_at	0.00	0.00
1421750_a_at	0.00	0.00
1421751_a_at	0.00	0.01
1421752_a_at	0.00	0.00
1421753_a_at	0.00	0.00
1421754_at	0.00	0.00
1421755_at	0.00	0.00
1421756_a_at	0.00	0.00
1421757_at	0.00	0.00
1421758_at	0.00	0.00
1421759_a_at	0.00	0.00
1421760_at	0.00	0.00
1421761_a_at	0.00	0.00
1421762_at	0.00	0.00
1421763_at	0.00	0.00
1421764_at	0.00	0.00
1421765_at	0.00	0.00
1421766_at	0.00	0.00
1421767_at	0.00	0.00
1421768_a_at	0.00	0.00
1421769_at	0.00	0.00
1421770_a_at	0.00	0.00
1421771_a_at	0.00	0.00
1421772_a_at	0.00	0.00
1421773_at	0.00	0.00
1421774_at	0.00	0.00
1421775_at	0.00	0.00
1421776_at	0.00	0.00
1421777_at	0.00	0.00
1421778_at	0.00	0.00
1421779_at	0.00	0.00
1421780_a_at	0.00	0.00
1421781_at	0.00	0.00
1421782_a_at	0.00	0.00
1421783_a_at	0.00	0.00
1421784_a_at	0.00	0.00
1421785_at	0.00	0.00
1421786_at	0.00	0.00
1421787_at	0.00	0.00
1421788_x_at	0.00	0.00
1421789_s_at	0.00	0.00
1421790_a_at	0.00	0.00
1421791_at	0.00	0.00
1421792_s_at	0.00	0.00
1421793_at	0.00	0.00
1421794_at	0.00	0.00
1421795_s_at	0.00	0.00

1421796_a_at	0.00	0.00
1421797_a_at	0.00	0.00
1421798_at	0.00	0.00
1421799_at	0.00	0.00
1421800_at	0.00	0.00
1421801_at	0.00	0.00
1421802_at	0.00	0.00
1421803_at	0.00	0.00
1421804_at	0.00	0.00
1421805_at	0.00	0.00
1421806_at	0.00	0.00
1421807_at	0.00	0.00
1421808_at	0.00	0.00
1421809_at	0.00	0.00
1421810_at	0.00	0.00
1421811_at	0.84	0.00
1421812_at	0.06	0.66
1421813_a_at	0.00	0.00
1421814_at	0.01	0.00
1421815_at	0.00	0.00
1421816_at	0.00	0.00
1421817_at	0.00	0.00
1421818_at	0.00	0.00
1421819_a_at	0.00	0.00
1421820_a_at	0.00	0.00
1421821_at	0.00	0.00
1421822_at	0.00	0.00
1421823_a_at	0.00	0.00
1421824_at	0.00	0.00
1421825_at	0.00	0.00
1421826_at	0.00	0.00
1421827_at	0.00	0.00
1421828_at	0.00	0.00
1421829_at	0.00	0.00
1421830_at	0.00	0.00
1421831_at	0.00	0.00
1421832_at	0.00	0.01
1421833_at	0.00	0.00
1421834_at	0.00	0.00
1421835_at	0.00	0.00
1421836_at	0.00	0.00
1421837_at	0.00	0.00
1421838_at	0.00	0.00
1421839_at	0.00	0.00
1421840_at	0.00	0.00
1421841_at	0.00	0.00
1421842_a_at	0.00	0.00
1421843_at	0.00	0.00
1421844_at	0.00	0.00
1421845_at	0.00	0.00
1421846_at	0.00	0.00
1421847_at	0.00	0.00
1421848_at	0.00	0.00
1421849_at	0.00	0.06
1421850_at	0.00	0.00
1421851_at	0.00	0.00

1421852_at	0.48	0.00
1421853_at	0.00	0.00
1421854_at	0.00	0.00
1421855_at	0.00	0.00
1421856_at	0.00	0.00
1421857_at	0.00	0.00
1421858_at	0.00	0.00
1421859_at	0.00	0.00
1421860_at	0.00	0.00
1421861_at	0.00	0.00
1421862_a_at	0.00	0.00
1421863_at	0.00	0.00
1421864_at	0.00	0.00
1421865_at	0.00	0.00
1421866_at	0.00	0.00
1421867_at	0.00	0.00
1421868_a_at	0.00	0.00
1421869_at	0.00	0.00
1421870_at	0.00	0.00
1421871_at	0.28	0.02
1421872_at	0.00	0.00
1421873_s_at	0.00	0.00
1421874_a_at	0.00	0.06
1421875_a_at	0.00	0.00
1421876_at	0.00	0.00
1421877_at	0.00	0.00
1421878_at	0.00	0.00
1421879_at	0.00	0.00
1421880_at	0.00	0.00
1421881_a_at	0.11	0.00
1421882_a_at	0.95	0.00
1421883_at	0.72	0.00
1421884_at	0.00	0.00
1421885_at	0.00	0.00
1421886_at	0.00	0.00
1421887_a_at	0.00	0.00
1421888_x_at	0.00	0.00
1421889_a_at	0.00	0.00
1421890_at	0.00	0.00
1421891_at	0.00	0.00
1421892_at	0.00	0.00
1421893_a_at	0.00	0.00
1421894_a_at	0.00	0.00
1421895_at	0.00	0.27
1421896_at	0.00	0.00
1421897_at	0.00	0.00
1421898_a_at	0.00	0.00
1421899_a_at	0.00	0.00
1421900_at	0.00	0.00
1421901_at	0.00	0.00
1421902_at	0.00	0.00
1421903_at	0.00	0.16
1421904_at	0.00	0.00
1421905_at	0.00	0.00
1421906_at	0.00	0.00
1421907_at	0.00	0.00

1421908_a_at	0.04	0.00
1421909_at	0.00	0.00
1421910_at	0.00	0.00
1421911_at	0.00	0.00
1421912_at	0.00	0.00
1421913_at	0.00	0.00
1421914_s_at	0.00	0.42
1421915_a_at	0.00	0.00
1421916_at	0.00	0.00
1421917_at	0.00	0.03
1421918_at	0.00	0.00
1421919_a_at	0.00	0.00
1421920_a_at	0.00	0.00
1421921_at	0.00	0.00
1421922_at	0.00	0.01
1421923_at	0.00	0.00
1421924_at	0.00	0.67
1421925_at	0.00	0.00
1421926_at	0.00	0.00
1421927_at	0.00	0.00
1421928_at	0.00	0.00
1421929_at	0.00	0.00
1421930_at	0.00	0.00
1421931_at	0.00	0.00
1421932_at	0.00	0.00
1421933_at	0.00	0.00
1421934_at	0.00	0.00
1421935_at	0.00	0.00
1421936_at	0.22	0.33
1421937_at	0.00	0.00
1421938_at	0.00	0.00
1421939_a_at	0.00	0.00
1421940_at	0.00	0.00
1421941_at	0.00	0.00
1421942_s_at	0.00	0.00
1421943_at	0.00	0.00
1421944_a_at	0.00	0.00
1421945_a_at	0.00	0.34
1421946_at	0.00	0.00
1421947_at	0.08	0.01
1421948_a_at	0.00	0.00
1421949_a_at	0.00	0.00
1421950_at	0.00	0.00
1421951_at	0.00	0.00
1421952_at	0.00	0.00
1421953_at	0.00	0.00
1421954_at	0.00	0.00
1421955_a_at	0.00	0.00
1421956_at	0.00	0.00
1421957_a_at	0.00	0.00
1421958_at	0.00	0.00
1421959_s_at	0.00	0.00
1421960_at	0.00	0.00
1421961_a_at	0.00	0.00
1421962_at	0.00	0.00
1421963_a_at	0.00	0.00

1421964_at	0.00	0.03
1421965_s_at	0.00	0.34
1421966_at	0.00	0.00
1421967_at	0.00	0.00
1421968_a_at	0.07	0.00
1421969_a_at	0.00	0.00
1421970_a_at	0.00	0.00
1421971_a_at	0.00	0.00
1421972_s_at	0.00	0.00
1421973_at	0.00	0.00
1421974_at	0.00	0.00
1421975_a_at	0.00	0.00
1421976_at	0.00	0.00
1421977_at	0.00	0.00
1421978_at	0.00	0.00
1421979_at	0.00	0.00
1421980_at	0.00	0.00
1421981_at	0.00	0.00
1421982_a_at	0.00	0.00
1421983_s_at	0.00	0.01
1421984_at	0.00	0.00
1421985_a_at	0.00	0.00
1421986_at	0.00	0.00
1421987_at	0.00	0.00
1421988_at	0.00	0.00
1421989_s_at	0.00	0.00
1421990_at	0.00	0.00
1421991_a_at	0.00	0.00
1421992_a_at	0.01	0.04
1421993_a_at	0.00	0.00
1421994_a_at	0.00	0.00
1421995_at	0.00	0.00
1421996_at	0.00	0.00
1421997_s_at	0.16	0.00
1421998_at	0.00	0.00
1421999_at	0.00	0.00
1422000_at	0.00	0.00
1422001_at	0.00	0.00
1422002_at	0.00	0.00
1422003_at	0.00	0.00
1422004_at	0.00	0.00
1422005_at	0.00	0.00
1422006_at	0.00	0.00
1422007_at	0.00	0.00
1422008_a_at	0.00	0.00
1422009_at	0.00	0.00
1422010_at	0.00	0.00
1422011_s_at	0.00	0.00
1422012_at	0.00	0.00
1422013_at	0.00	0.00
1422014_at	0.00	0.00
1422015_a_at	0.00	0.00
1422016_a_at	0.00	0.00
1422017_s_at	0.00	0.00
1422018_at	0.03	0.00
1422019_at	0.00	0.00

1422020_at	0.00	0.00
1422021_at	0.00	0.02
1422022_at	0.00	0.00
1422023_at	0.00	0.00
1422024_at	0.00	0.00
1422025_at	0.00	0.00
1422026_at	0.00	0.00
1422027_a_at	0.00	0.00
1422028_a_at	0.00	0.00
1422029_at	0.00	0.00
1422030_at	0.00	0.00
1422031_a_at	0.00	0.00
1422032_a_at	0.00	0.00
1422033_a_at	0.00	0.00
1422034_a_at	0.00	0.00
1422035_at	0.00	0.00
1422036_at	0.00	0.00
1422037_at	0.00	0.00
1422038_a_at	0.00	0.00
1422039_at	0.00	0.00
1422040_at	0.00	0.00
1422041_at	0.00	0.00
1422042_at	0.00	0.00
1422043_at	0.00	0.00
1422044_at	0.00	0.00
1422045_a_at	0.00	0.00
1422046_at	0.00	0.00
1422047_at	0.00	0.00
1422048_at	0.00	0.00
1422049_at	0.00	0.00
1422050_at	0.00	0.00
1422051_a_at	0.00	0.00
1422052_at	0.00	0.00
1422053_at	0.00	0.00
1422054_a_at	0.00	0.00
1422055_at	0.00	0.00
1422056_at	0.00	0.00
1422057_at	0.00	0.00
1422058_at	0.00	0.00
1422059_at	0.00	0.00
1422060_at	0.00	0.00
1422061_at	0.00	0.00
1422062_at	0.00	0.00
1422063_a_at	0.00	0.00
1422064_a_at	0.00	0.00
1422065_at	0.00	0.00
1422066_x_at	0.00	0.00
1422067_at	0.00	0.00
1422068_at	0.00	0.00
1422069_at	0.00	0.00
1422070_at	0.00	0.00
1422071_at	0.00	0.00
1422072_a_at	0.00	0.00
1422073_a_at	0.00	0.00
1422074_at	0.00	0.00
1422075_at	0.00	0.00

1422076_at	0.00	0.00
1422077_at	0.00	0.00
1422078_at	0.00	0.00
1422079_at	0.00	0.00
1422080_at	0.00	0.00
1422081_at	0.00	0.00
1422082_a_at	0.00	0.00
1422083_at	0.00	0.00
1422084_at	0.00	0.00
1422085_at	0.00	0.00
1422086_at	0.00	0.00
1422087_at	0.00	0.00
1422088_at	0.00	0.00
1422089_at	0.00	0.00
1422090_a_at	0.00	0.00
1422091_at	0.00	0.00
1422092_at	0.00	0.00
1422093_at	0.00	0.00
1422094_a_at	0.00	0.00
1422095_a_at	0.00	0.00
1422096_at	0.00	0.00
1422097_at	0.00	0.00
1422098_at	0.00	0.00
1422099_a_at	0.00	0.00
1422100_at	0.00	0.00
1422101_at	0.00	0.00
1422102_a_at	0.00	0.00
1422103_a_at	0.00	0.00
1422104_at	0.00	0.00
1422105_at	0.00	0.00
1422106_a_at	0.00	0.00
1422107_at	0.00	0.00
1422108_at	0.00	0.00
1422109_at	0.00	0.00
1422110_at	0.00	0.00
1422111_at	0.00	0.00
1422112_at	0.00	0.00
1422113_at	0.00	0.00
1422114_at	0.00	0.00
1422115_a_at	0.00	0.00
1422116_at	0.00	0.00
1422117_s_at	0.00	0.00
1422118_at	0.00	0.00
1422119_at	0.00	0.00
1422120_at	0.00	0.00
1422121_at	0.00	0.00
1422122_at	0.00	0.00
1422123_s_at	0.00	0.00
1422124_a_at	0.00	0.00
1422125_at	0.00	0.00
1422126_a_at	0.00	0.00
1422127_at	0.00	0.00
1422128_at	0.00	0.00
1422129_at	0.00	0.00
1422130_at	0.00	0.00
1422131_at	0.00	0.00

1422132_at	0.00	0.00
1422133_at	0.00	0.00
1422134_at	0.00	0.00
1422135_at	0.00	0.00
1422136_at	0.00	0.00
1422137_at	0.00	0.00
1422138_at	0.00	0.00
1422139_at	0.00	0.00
1422140_at	0.00	0.00
1422141_s_at	0.00	0.00
1422142_at	0.02	0.11
1422143_at	0.00	0.00
1422144_at	0.00	0.00
1422145_at	0.00	0.00
1422146_at	0.00	0.00
1422147_a_at	0.00	0.00
1422148_at	0.00	0.00
1422149_at	0.00	0.00
1422150_at	0.00	0.00
1422151_at	0.00	0.00
1422152_at	0.00	0.00
1422153_a_at	0.00	0.00
1422154_at	0.00	0.00
1422155_at	0.00	0.00
1422156_a_at	0.00	0.00
1422157_a_at	0.00	0.00
1422158_at	0.00	0.00
1422159_at	0.00	0.00
1422160_at	0.00	0.00
1422161_at	0.00	0.00
1422162_at	0.00	0.00
1422163_at	0.00	0.00
1422164_at	0.00	0.00
1422165_at	0.00	0.00
1422166_at	0.00	0.00
1422167_at	0.00	0.00
1422168_a_at	0.00	0.00
1422169_a_at	0.00	0.00
1422170_at	0.00	0.00
1422171_at	0.00	0.00
1422172_x_at	0.00	0.00
1422173_at	0.00	0.00
1422174_at	0.00	0.00
1422175_at	0.00	0.00
1422176_at	0.00	0.00
1422177_at	0.00	0.00
1422178_a_at	0.00	0.00
1422179_at	0.00	0.00
1422180_a_at	0.00	0.00
1422181_at	0.00	0.00
1422182_at	0.00	0.00
1422183_a_at	0.00	0.00
1422184_a_at	0.00	0.00
1422185_a_at	0.00	0.00
1422186_s_at	0.00	0.00
1422187_at	0.00	0.00

1422188_s_at	0.00	0.00
1422189_x_at	0.00	0.00
1422190_at	0.00	0.00
1422191_at	0.00	0.00
1422192_at	0.00	0.00
1422193_at	0.00	0.00
1422194_at	0.00	0.00
1422195_s_at	0.00	0.00
1422196_at	0.00	0.00
1422197_at	0.00	0.00
1422198_a_at	0.67	0.40
1422199_at	0.00	0.00
1422200_at	0.00	0.00
1422201_at	0.00	0.00
1422202_at	0.00	0.00
1422203_at	0.00	0.00
1422204_at	0.00	0.00
1422205_at	0.00	0.00
1422206_at	0.00	0.00
1422207_at	0.00	0.00
1422208_a_at	0.00	0.00
1422209_s_at	0.00	0.00
1422210_at	0.00	0.00
1422211_a_at	0.00	0.00
1422212_at	0.00	0.00
1422213_s_at	0.00	0.00
1422214_at	0.00	0.00
1422215_at	0.00	0.00
1422216_at	0.00	0.00
1422217_a_at	0.00	0.00
1422218_at	0.00	0.00
1422219_a_at	0.00	0.00
1422220_at	0.00	0.00
1422221_at	0.00	0.00
1422222_at	0.00	0.00
1422223_at	0.00	0.00
1422224_at	0.00	0.00
1422225_s_at	0.00	0.00
1422226_at	0.00	0.00
1422227_at	0.00	0.00
1422228_at	0.00	0.00
1422229_at	0.00	0.00
1422230_s_at	0.00	0.00
1422231_a_at	0.00	0.00
1422232_at	0.00	0.00
1422233_at	0.00	0.00
1422234_at	0.00	0.00
1422235_at	0.00	0.00
1422236_at	0.00	0.00
1422237_at	0.00	0.00
1422238_at	0.00	0.00
1422239_at	0.00	0.00
1422240_s_at	0.00	0.00
1422241_a_at	0.00	0.00
1422242_at	0.00	0.00
1422243_at	0.00	0.00

1422244_at	0.00	0.00
1422245_a_at	0.00	0.00
1422246_at	0.00	0.00
1422247_a_at	0.00	0.00
1422248_at	0.00	0.00
1422249_s_at	0.00	0.00
1422250_at	0.00	0.00
1422251_at	0.00	0.00
1422252_a_at	0.00	0.00
1422253_at	0.00	0.00
1422254_a_at	0.00	0.00
1422255_at	0.00	0.00
1422256_at	0.00	0.00
1422257_s_at	0.00	0.00
1422258_at	0.00	0.00
1422259_a_at	0.00	0.00
1422260_x_at	0.00	0.00
1422261_a_at	0.00	0.00
1422262_a_at	0.00	0.00
1422263_at	0.00	0.00
1422264_s_at	0.23	0.00
1422265_at	0.00	0.00
1422266_at	0.00	0.00
1422267_at	0.00	0.00
1422268_a_at	0.00	0.00
1422269_at	0.00	0.00
1422270_a_at	0.00	0.00
1422271_at	0.00	0.00
1422272_at	0.00	0.00
1422273_at	0.00	0.00
1422274_at	0.00	0.00
1422275_at	0.00	0.00
1422276_at	0.00	0.00
1422277_at	0.00	0.00
1422278_at	0.00	0.00
1422279_at	0.00	0.00
1422280_at	0.00	0.00
1422281_at	0.00	0.00
1422282_at	0.00	0.00
1422283_at	0.00	0.00
1422284_at	0.00	0.00
1422285_at	0.00	0.00
1422286_a_at	0.00	0.00
1422287_at	0.00	0.00
1422288_at	0.00	0.00
1422289_a_at	0.00	0.00
1422290_at	0.00	0.00
1422291_at	0.00	0.00
1422292_at	0.00	0.00
1422293_a_at	0.00	0.00
1422294_at	0.00	0.00
1422295_at	0.00	0.00
1422296_at	0.00	0.00
1422297_at	0.00	0.00
1422298_at	0.00	0.00
1422299_a_at	0.00	0.00

1422300_at	0.00	0.00
1422301_at	0.00	0.00
1422302_s_at	0.00	0.00
1422303_a_at	0.00	0.00
1422304_at	0.00	0.00
1422305_at	0.00	0.00
1422306_at	0.00	0.00
1422307_at	0.00	0.00
1422308_a_at	0.00	0.00
1422309_a_at	0.00	0.00
1422310_at	0.00	0.00
1422311_a_at	0.00	0.00
1422312_a_at	0.00	0.00
1422313_a_at	0.00	0.00
1422314_at	0.00	0.00
1422315_x_at	0.00	0.00
1422316_at	0.00	0.00
1422317_a_at	0.00	0.00
1422318_at	0.00	0.00
1422319_at	0.00	0.00
1422320_x_at	0.00	0.00
1422321_a_at	0.01	0.00
1422322_at	0.00	0.00
1422323_a_at	0.00	0.00
1422324_a_at	0.00	0.00
1422325_at	0.00	0.00
1422326_at	0.00	0.00
1422327_s_at	0.00	0.00
1422328_at	0.00	0.00
1422329_a_at	0.00	0.00
1422330_at	0.00	0.00
1422331_at	0.00	0.00
1422332_at	0.00	0.00
1422333_at	0.00	0.00
1422334_a_at	0.00	0.00
1422335_at	0.00	0.00
1422336_at	0.00	0.00
1422337_at	0.00	0.00
1422338_at	0.00	0.00
1422339_at	0.00	0.00
1422340_a_at	0.00	0.00
1422341_s_at	0.00	0.00
1422342_at	0.00	0.00
1422343_at	0.00	0.00
1422344_s_at	0.00	0.00
1422345_s_at	0.00	0.00
1422346_at	0.00	0.00
1422347_at	0.00	0.00
1422348_at	0.00	0.00
1422349_at	0.00	0.00
1422350_at	0.00	0.00
1422351_at	0.00	0.00
1422352_at	0.00	0.00
1422353_at	0.00	0.00
1422354_at	0.00	0.00
1422355_at	0.00	0.00

1422356_at	0.00	0.00
1422357_at	0.00	0.00
1422358_at	0.00	0.00
1422359_at	0.00	0.00
1422360_at	0.00	0.00
1422361_at	0.00	0.00
1422362_s_at	0.00	0.00
1422363_at	0.00	0.00
1422364_at	0.00	0.00
1422365_at	0.00	0.00
1422366_at	0.00	0.00
1422367_at	0.00	0.00
1422368_at	0.00	0.00
1422369_at	0.00	0.00
1422370_at	0.00	0.00
1422371_at	0.00	0.00
1422372_at	0.00	0.00
1422373_at	0.00	0.00
1422374_s_at	0.00	0.00
1422375_a_at	0.00	0.00
1422376_at	0.00	0.00
1422377_at	0.00	0.00
1422378_at	0.00	0.00
1422379_x_at	0.00	0.00
1422380_at	0.00	0.00
1422381_at	0.00	0.00
1422382_at	0.00	0.00
1422383_at	0.00	0.00
1422384_at	0.00	0.00
1422385_at	0.00	0.00
1422386_at	0.00	0.00
1422387_at	0.00	0.00
1422388_at	0.00	0.00
1422389_at	0.00	0.00
1422390_at	0.00	0.00
1422391_at	0.00	0.00
1422392_at	0.00	0.00
1422393_at	0.00	0.00
1422394_at	0.00	0.00
1422395_at	0.00	0.00
1422396_s_at	0.00	0.00
1422397_a_at	0.00	0.00
1422398_at	0.00	0.00
1422399_a_at	0.00	0.01
1422400_a_at	0.00	0.00
1422401_at	0.00	0.00
1422402_a_at	0.00	0.00
1422403_at	0.00	0.00
1422404_x_at	0.00	0.00
1422405_at	0.00	0.00
1422406_at	0.00	0.00
1422407_s_at	0.00	0.00
1422408_at	0.00	0.00
1422409_at	0.00	0.00
1422410_at	0.00	0.00
1422411_s_at	0.00	0.00

1422412_x_at	0.00	0.00
1422413_at	0.00	0.00
1422414_a_at	0.00	0.00
1422415_at	0.00	0.00
1422416_s_at	0.00	0.00
1422417_at	0.00	0.00
1422418_s_at	0.00	0.32
1422419_s_at	0.00	0.00
1422420_at	0.00	0.00
1422421_at	0.00	0.00
1422422_at	0.00	0.00
1422423_at	0.00	0.00
1422424_at	0.00	0.00
1422425_at	0.00	0.00
1422426_at	0.00	0.00
1422427_a_at	0.00	0.00
1422428_at	0.00	0.00
1422429_at	0.00	0.00
1422430_at	0.00	0.00
1422431_at	0.00	0.00
1422432_at	0.00	0.00
1422433_s_at	0.00	0.00
1422434_a_at	0.00	0.00
1422435_at	0.00	0.00
1422436_at	0.00	0.00
1422437_at	0.00	0.00
1422438_at	0.00	0.00
1422439_a_at	0.00	0.00
1422440_at	0.00	0.00
1422441_x_at	0.00	0.00
1422442_at	0.00	0.01
1422443_at	0.00	0.00
1422444_at	0.00	0.00
1422445_at	0.00	0.00
1422446_x_at	0.00	0.00
1422447_at	0.00	0.00
1422448_at	0.00	0.00
1422449_s_at	0.36	0.00
1422450_at	0.00	0.00
1422451_at	0.00	0.00
1422452_at	0.00	0.00
1422453_at	0.00	0.03
1422454_at	0.00	0.00
1422455_s_at	0.00	0.00
1422456_at	0.00	0.01
1422457_s_at	0.00	0.00
1422458_at	1.00	0.10
1422459_a_at	0.00	0.00
1422460_at	0.00	0.00
1422461_at	0.00	0.00
1422462_at	0.00	0.00
1422463_a_at	0.00	0.00
1422464_at	0.00	0.06
1422465_a_at	0.00	0.00
1422466_at	0.00	0.00
1422467_at	0.00	0.00

1422468_at	0.00	0.00
1422469_at	0.00	0.00
1422470_at	0.00	0.00
1422471_at	0.00	0.00
1422472_at	0.00	0.00
1422473_at	0.00	0.00
1422474_at	0.00	0.00
1422475_a_at	0.00	0.00
1422476_at	0.00	0.27
1422477_at	0.00	0.00
1422478_a_at	0.00	0.00
1422479_at	0.00	0.05
1422480_at	0.00	0.00
1422481_at	0.00	0.00
1422482_at	0.00	0.00
1422483_a_at	0.00	0.00
1422484_at	0.00	0.10
1422485_at	0.00	0.00
1422486_a_at	0.00	0.27
1422487_at	0.00	0.00
1422488_at	0.00	0.00
1422489_at	0.00	0.00
1422490_at	0.10	0.00
1422491_a_at	0.35	0.00
1422492_at	0.00	0.00
1422493_at	0.00	0.00
1422494_s_at	0.00	0.00
1422495_a_at	0.00	0.00
1422496_at	0.00	0.00
1422497_at	0.00	0.00
1422498_at	0.00	0.00
1422499_at	0.00	0.00
1422500_at	0.00	0.00
1422501_s_at	0.00	0.00
1422502_at	0.00	0.00
1422503_s_at	0.00	0.00
1422504_at	0.00	0.00
1422505_at	0.00	0.00
1422506_a_at	0.02	0.01
1422507_at	0.00	0.07
1422508_at	0.00	0.00
1422509_at	0.00	0.05
1422510_at	0.00	0.09
1422511_a_at	0.00	0.00
1422512_a_at	0.00	0.00
1422513_at	0.00	0.00
1422514_at	0.00	0.00
1422515_at	0.00	0.00
1422516_a_at	0.00	0.00
1422517_a_at	0.00	0.40
1422518_at	0.00	0.00
1422519_at	0.00	0.00
1422520_at	0.00	0.00
1422521_at	0.00	0.00
1422522_at	0.00	0.00
1422523_at	0.00	0.00

1422524_at	0.00	0.00
1422525_at	0.00	0.00
1422526_at	0.00	0.00
1422527_at	0.00	0.00
1422528_a_at	0.00	0.00
1422529_s_at	0.00	0.00
1422530_at	0.00	0.00
1422531_at	0.00	0.00
1422532_at	0.00	0.00
1422533_at	0.00	0.00
1422534_at	0.00	0.00
1422535_at	0.00	0.00
1422536_at	0.00	0.00
1422537_a_at	0.00	0.00
1422538_at	0.00	0.00
1422539_at	0.00	0.00
1422540_at	0.00	0.22
1422541_at	0.00	0.00
1422542_at	0.00	0.00
1422543_at	0.00	0.00
1422544_at	0.00	0.01
1422545_at	0.00	0.00
1422546_at	0.00	0.00
1422547_at	0.00	0.00
1422548_at	0.00	0.00
1422549_at	0.00	0.00
1422550_a_at	0.00	0.00
1422551_at	0.00	0.00
1422552_at	0.00	0.00
1422553_at	0.00	0.00
1422554_at	0.00	0.00
1422555_s_at	0.00	0.01
1422556_at	0.00	0.00
1422557_s_at	0.00	0.00
1422558_at	0.00	0.00
1422559_at	0.00	0.01
1422560_at	0.00	0.00
1422561_at	0.00	0.00
1422562_at	0.00	0.00
1422563_at	0.00	0.00
1422564_at	0.00	0.00
1422565_s_at	0.00	0.00
1422566_at	0.00	0.00
1422567_at	0.00	0.24
1422568_at	0.00	0.00
1422569_at	0.00	0.00
1422570_at	0.00	0.00
1422571_at	0.00	0.00
1422572_at	0.00	0.00
1422573_at	0.00	0.00
1422574_at	0.00	0.00
1422575_at	0.00	0.00
1422576_at	0.00	0.00
1422577_at	0.00	0.00
1422578_at	0.00	0.00
1422579_at	0.00	0.16

1422580_at	0.00	0.00
1422581_at	0.00	0.00
1422582_at	0.00	0.00
1422583_at	0.00	0.00
1422584_at	0.00	0.00
1422585_at	0.00	0.00
1422586_at	0.00	0.00
1422587_at	0.00	0.00
1422588_at	0.00	0.00
1422589_at	0.00	0.00
1422590_at	0.00	0.00
1422591_at	0.00	0.00
1422592_at	0.00	0.00
1422593_at	0.00	0.00
1422594_at	0.00	0.00
1422595_s_at	0.00	0.00
1422596_at	0.00	0.00
1422597_at	0.00	0.05
1422598_at	0.00	0.00
1422599_s_at	0.00	0.00
1422600_at	0.00	0.00
1422601_at	0.07	0.00
1422602_a_at	0.00	0.00
1422603_at	0.00	0.00
1422604_at	0.00	0.00
1422605_at	0.00	0.00
1422606_at	0.00	0.00
1422607_at	0.00	0.00
1422608_at	0.00	0.05
1422609_at	0.00	0.00
1422610_s_at	0.01	0.32
1422611_s_at	0.00	0.33
1422612_at	0.00	0.10
1422613_a_at	0.00	0.00
1422614_s_at	0.00	0.00
1422615_at	0.00	0.00
1422616_s_at	0.00	0.00
1422617_at	0.00	0.00
1422618_x_at	0.00	0.00
1422619_at	0.00	0.00
1422620_s_at	0.00	0.00
1422621_at	0.00	0.17
1422622_at	0.00	0.00
1422623_x_at	0.00	0.00
1422624_at	0.00	0.00
1422625_at	0.00	0.00
1422626_at	0.00	0.00
1422627_a_at	0.00	0.00
1422628_at	0.00	0.00
1422629_s_at	0.00	0.00
1422630_at	0.00	0.00
1422631_at	0.00	0.09
1422632_at	0.00	0.00
1422633_at	0.00	0.00
1422634_a_at	0.00	0.00
1422635_at	0.00	0.00

1422636_at	0.00	0.00
1422637_at	0.00	0.00
1422638_s_at	0.00	0.00
1422639_at	0.00	0.00
1422640_at	0.00	0.00
1422641_at	0.00	0.00
1422642_at	0.00	0.00
1422643_at	0.00	0.00
1422644_at	0.00	0.00
1422645_at	0.00	0.00
1422646_at	0.00	0.00
1422647_at	0.00	0.00
1422648_at	0.00	0.00
1422649_at	0.00	0.00
1422650_a_at	0.00	0.11
1422651_at	0.00	0.00
1422652_at	0.00	0.00
1422653_at	0.00	0.00
1422654_at	0.00	0.00
1422655_at	0.77	0.22
1422656_at	0.00	0.00
1422657_at	0.00	0.00
1422658_at	0.00	0.00
1422659_at	0.00	0.00
1422660_at	0.00	0.52
1422661_at	0.00	0.00
1422662_at	0.00	0.00
1422663_at	0.00	0.00
1422664_at	0.00	0.00
1422665_a_at	0.00	0.00
1422666_at	0.00	0.00
1422667_at	0.00	0.00
1422668_at	0.00	0.00
1422669_at	0.00	0.00
1422670_at	0.00	0.00
1422671_s_at	0.00	0.00
1422672_at	0.00	0.00
1422673_at	0.00	0.00
1422674_s_at	0.00	0.00
1422675_at	0.00	0.00
1422676_at	0.00	0.00
1422677_at	0.00	0.00
1422678_at	0.00	0.00
1422679_s_at	0.00	0.00
1422680_at	0.00	0.00
1422681_at	0.00	0.00
1422682_s_at	0.00	0.00
1422683_at	0.00	0.00
1422684_a_at	0.00	0.02
1422685_at	0.00	0.00
1422686_s_at	0.00	0.00
1422687_at	0.00	0.00
1422688_a_at	0.00	0.29
1422689_at	0.00	0.00
1422690_at	0.00	0.00
1422691_at	0.00	0.00

1422692_at	0.00	0.00
1422693_a_at	0.00	0.16
1422694_at	0.00	0.00
1422695_at	0.00	0.00
1422696_at	0.00	0.00
1422697_s_at	1.00	0.10
1422698_s_at	1.00	0.10
1422699_at	0.00	0.00
1422700_at	0.00	0.00
1422701_at	0.00	0.00
1422702_at	0.00	0.00
1422703_at	0.00	0.00
1422704_at	0.00	0.00
1422705_at	0.00	0.00
1422706_at	0.00	0.00
1422707_at	0.00	0.00
1422708_at	0.00	0.00
1422709_a_at	0.00	0.00
1422710_a_at	0.00	0.00
1422711_a_at	0.00	0.00
1422712_a_at	0.00	0.00
1422713_a_at	0.00	0.00
1422714_at	0.00	0.00
1422715_s_at	0.00	0.00
1422716_a_at	0.00	0.00
1422717_at	0.00	0.00
1422718_at	0.00	0.00
1422719_s_at	0.00	0.00
1422720_at	0.00	0.00
1422721_at	0.00	0.00
1422722_at	0.00	0.00
1422723_at	0.00	0.00
1422724_at	0.00	0.00
1422725_at	0.00	0.00
1422726_x_at	0.00	0.00
1422727_at	0.00	0.00
1422728_at	0.00	0.00
1422729_at	0.00	0.00
1422730_at	0.00	0.00
1422731_at	0.00	0.00
1422732_at	0.00	0.01
1422733_at	0.00	0.00
1422734_a_at	0.00	0.00
1422735_at	0.00	0.00
1422736_at	0.00	0.14
1422737_at	0.01	0.00
1422738_at	0.00	0.00
1422739_at	0.00	0.00
1422740_at	0.00	0.00
1422741_a_at	0.00	0.00
1422742_at	0.00	0.00
1422743_at	0.00	0.00
1422744_at	0.00	0.00
1422745_at	0.00	0.00
1422746_s_at	0.00	0.00
1422747_at	0.00	0.00

1422748_at	0.00	0.00
1422749_at	0.00	0.00
1422750_a_at	0.00	0.00
1422751_at	0.00	0.00
1422752_at	0.00	0.00
1422753_a_at	0.00	0.00
1422754_at	0.00	0.00
1422755_at	0.00	0.00
1422756_at	0.00	0.00
1422757_at	0.00	0.00
1422758_at	0.00	0.00
1422759_a_at	0.00	0.00
1422760_at	0.00	0.00
1422761_at	0.00	0.00
1422762_at	0.00	0.00
1422763_at	0.00	0.00
1422764_at	0.00	0.00
1422765_at	0.00	0.00
1422766_at	0.00	0.00
1422767_at	0.00	0.28
1422768_at	0.00	0.98
1422769_at	0.00	0.98
1422770_at	0.00	0.00
1422771_at	0.00	0.00
1422772_at	0.00	0.00
1422773_at	0.00	0.00
1422774_at	0.00	0.00
1422775_at	0.00	0.00
1422776_at	0.00	0.00
1422777_at	0.00	0.00
1422778_at	0.00	0.00
1422779_at	0.00	0.00
1422780_at	0.00	0.00
1422781_at	0.00	0.00
1422782_s_at	0.00	0.00
1422783_a_at	0.00	0.00
1422784_at	0.00	0.00
1422785_at	0.00	0.00
1422786_at	0.00	0.00
1422787_at	0.00	0.00
1422788_at	0.00	0.00
1422789_at	0.00	0.00
1422790_at	0.00	0.00
1422791_at	0.00	0.00
1422792_at	0.00	0.00
1422793_at	0.00	0.00
1422794_at	0.00	0.00
1422795_at	0.00	0.00
1422796_at	0.00	0.00
1422797_at	0.00	0.00
1422798_at	0.00	0.00
1422799_at	0.00	0.00
1422800_at	0.00	0.00
1422801_at	0.00	0.00
1422802_at	0.00	0.00
1422803_at	0.00	0.00

1422804_at	0.00	0.00
1422805_a_at	0.00	0.23
1422806_x_at	0.00	0.00
1422807_at	0.00	0.00
1422808_s_at	0.00	0.00
1422809_at	0.00	0.00
1422810_at	0.00	0.00
1422811_at	0.00	0.00
1422812_at	0.00	0.00
1422813_at	0.00	0.00
1422814_at	0.00	0.00
1422815_at	0.00	0.00
1422816_a_at	0.00	0.00
1422817_at	0.00	0.00
1422818_at	0.29	0.00
1422819_at	0.00	0.09
1422820_at	0.00	0.00
1422821_s_at	0.00	0.00
1422822_at	0.00	0.00
1422823_at	0.00	0.00
1422824_s_at	0.00	0.00
1422825_at	0.00	0.00
1422826_at	0.00	0.00
1422827_x_at	0.00	0.00
1422828_at	0.00	0.00
1422829_at	0.00	0.00
1422830_s_at	0.00	0.00
1422831_at	0.00	0.00
1422832_at	0.00	0.00
1422833_at	0.00	0.15
1422834_at	0.00	0.00
1422835_at	0.00	0.00
1422836_at	0.00	0.00
1422837_at	0.00	0.00
1422838_at	0.00	0.00
1422839_at	0.00	0.00
1422840_at	0.00	0.00
1422841_at	0.00	0.00
1422842_at	0.00	0.00
1422843_at	0.00	0.00
1422844_a_at	0.00	0.31
1422845_at	0.00	0.00
1422846_at	0.00	0.00
1422847_a_at	0.00	0.09
1422848_a_at	0.00	0.00
1422849_a_at	0.00	0.00
1422850_at	0.00	0.00
1422851_at	0.23	0.00
1422852_at	0.00	0.03
1422853_at	0.00	0.00
1422854_at	0.00	0.00
1422855_at	0.00	0.00
1422856_at	0.00	0.00
1422857_at	0.00	0.00
1422858_at	0.00	0.00
1422859_a_at	0.00	0.00

1422860_at	0.00	0.00
1422861_s_at	0.00	0.00
1422862_at	0.00	0.00
1422863_s_at	0.00	0.00
1422864_at	0.00	0.00
1422865_at	0.00	0.00
1422866_at	0.00	0.00
1422867_at	0.00	0.00
1422868_s_at	0.00	0.00
1422869_at	0.00	0.00
1422870_at	0.00	0.00
1422871_at	0.00	0.00
1422872_at	0.00	0.00
1422873_at	0.00	0.00
1422874_at	0.00	0.00
1422875_at	0.00	0.00
1422876_at	0.00	0.00
1422877_at	0.00	0.00
1422878_at	0.00	0.00
1422879_at	0.00	0.20
1422880_at	0.00	0.06
1422881_s_at	0.00	0.00
1422882_at	0.00	0.00
1422883_at	0.00	0.00
1422884_at	0.00	0.13
1422885_at	0.00	0.00
1422886_a_at	0.00	0.00
1422887_a_at	0.00	0.40
1422888_at	0.00	0.00
1422889_at	0.00	0.00
1422890_at	0.00	0.00
1422891_at	0.00	0.00
1422892_s_at	0.00	0.00
1422893_at	0.00	0.00
1422894_at	0.00	0.00
1422895_at	0.00	0.00
1422896_at	0.00	0.00
1422897_at	0.00	0.00
1422898_s_at	0.00	0.00
1422899_at	0.00	0.00
1422900_at	0.00	0.00
1422901_at	0.00	0.00
1422902_s_at	0.00	0.00
1422903_at	0.00	0.00
1422904_at	0.00	0.00
1422905_s_at	0.00	0.00
1422906_at	0.00	0.00
1422907_at	0.00	0.00
1422908_at	0.00	0.00
1422909_at	0.00	0.00
1422910_s_at	0.00	0.00
1422911_at	0.00	0.00
1422912_at	0.00	0.03
1422913_at	0.00	0.00
1422914_at	0.00	0.00
1422915_at	0.00	0.00

1422916_at	0.00	0.00
1422917_at	0.00	0.00
1422918_at	0.00	0.00
1422919_at	0.00	0.00
1422920_at	0.00	0.00
1422921_at	0.00	0.00
1422922_at	0.00	0.00
1422923_at	0.00	0.00
1422924_at	0.00	0.00
1422925_s_at	0.00	0.00
1422926_at	0.00	0.00
1422927_at	0.00	0.00
1422928_at	0.00	0.00
1422929_s_at	0.00	0.00
1422930_at	0.00	0.00
1422931_at	0.00	0.00
1422932_a_at	0.00	0.00
1422933_at	0.00	0.00
1422934_x_at	0.00	0.00
1422935_x_at	0.00	0.00
1422936_at	0.00	0.00
1422937_at	0.00	0.00
1422938_at	0.00	0.00
1422939_at	0.00	0.00
1422940_x_at	0.00	0.00
1422941_at	0.00	0.00
1422942_at	0.00	0.00
1422943_a_at	0.00	0.00
1422944_a_at	0.00	0.00
1422945_a_at	0.00	0.00
1422946_a_at	0.00	0.00
1422947_at	0.00	0.00
1422948_s_at	0.00	0.36
1422949_at	0.00	0.00
1422950_at	0.00	0.00
1422951_at	0.00	0.00
1422952_at	0.00	0.00
1422953_at	0.00	0.00
1422954_at	0.00	0.00
1422955_at	0.00	0.00
1422956_at	0.00	0.03
1422957_at	0.00	0.00
1422958_at	0.00	0.00
1422959_s_at	0.00	0.00
1422960_at	0.00	0.00
1422961_at	0.00	0.00
1422962_a_at	0.00	0.07
1422963_at	0.00	0.00
1422964_at	0.00	0.00
1422965_at	0.01	0.00
1422966_a_at	0.00	0.00
1422967_a_at	0.00	0.00
1422968_at	0.00	0.00
1422969_s_at	0.00	0.00
1422970_at	0.00	0.00
1422971_at	0.00	0.00

1422972_s_at	0.00	0.00
1422973_a_at	0.00	0.00
1422974_at	0.00	0.00
1422975_at	0.00	0.00
1422976_x_at	0.00	0.00
1422977_at	0.00	0.00
1422978_at	0.00	0.00
1422979_at	0.00	0.00
1422980_a_at	0.00	0.00
1422981_at	0.00	0.00
1422982_at	0.00	0.00
1422983_at	0.00	0.00
1422984_at	0.00	0.00
1422985_at	0.00	0.00
1422986_at	1.00	0.44
1422987_at	0.00	0.00
1422988_at	0.00	0.00
1422989_a_at	0.00	0.00
1422990_at	0.00	0.00
1422991_at	0.00	0.00
1422992_s_at	0.00	0.00
1422993_s_at	0.00	0.59
1422994_at	0.00	0.00
1422995_at	0.00	0.00
1422996_at	0.00	0.00
1422997_s_at	0.00	0.05
1422998_a_at	0.00	0.00
1422999_at	0.00	0.00
1423000_a_at	0.00	0.00
1423001_at	0.00	0.00
1423002_at	0.00	0.00
1423003_at	0.00	0.00
1423004_at	0.00	0.00
1423005_a_at	0.00	0.00
1423006_at	0.00	0.00
1423007_a_at	0.00	0.00
1423008_at	0.00	0.00
1423009_at	0.00	0.00
1423010_at	0.00	0.00
1423011_at	0.00	0.00
1423012_at	0.00	0.00
1423013_at	0.00	0.00
1423014_at	0.00	0.00
1423015_at	0.00	0.00
1423016_a_at	0.00	0.00
1423017_a_at	0.00	0.00
1423018_at	0.00	0.00
1423019_at	0.00	0.00
1423020_at	0.00	0.00
1423021_s_at	0.00	0.00
1423022_at	0.00	0.00
1423023_at	0.00	0.00
1423024_at	0.00	0.00
1423025_a_at	0.00	0.06
1423026_at	0.00	0.00
1423027_at	0.00	0.00

1423028_at	0.00	0.00
1423029_at	0.00	0.00
1423030_at	0.00	0.00
1423031_at	0.00	0.00
1423032_at	0.00	0.00
1423033_at	0.00	0.00
1423034_at	0.00	0.00
1423035_s_at	0.00	0.00
1423036_at	0.00	0.00
1423037_at	0.00	0.00
1423038_at	0.00	0.00
1423039_a_at	0.00	0.00
1423040_at	0.00	0.31
1423041_a_at	0.00	0.32
1423042_at	0.00	0.00
1423043_s_at	0.00	0.00
1423044_at	0.00	0.00
1423045_at	0.00	0.00
1423046_s_at	0.00	0.01
1423047_at	0.00	0.09
1423048_a_at	0.00	0.00
1423049_a_at	0.96	0.00
1423050_s_at	0.00	0.00
1423051_at	0.00	0.23
1423052_at	0.00	0.00
1423053_at	0.00	0.00
1423054_at	0.00	0.00
1423055_at	0.00	0.00
1423056_at	0.00	0.00
1423057_at	0.00	0.00
1423058_at	0.00	0.00
1423059_at	0.00	0.00
1423060_at	0.00	0.00
1423061_at	0.00	0.00
1423062_at	0.00	0.00
1423063_at	0.00	0.00
1423064_at	0.37	0.00
1423065_at	0.00	0.00
1423066_at	0.57	0.00
1423067_at	0.00	0.00
1423068_at	0.00	0.00
1423069_at	0.00	0.00
1423070_at	0.00	0.00
1423071_x_at	0.00	0.00
1423072_at	0.00	0.00
1423073_at	0.00	0.00
1423074_at	0.00	0.00
1423075_at	0.00	0.00
1423076_at	0.00	0.00
1423077_at	0.00	0.00
1423078_a_at	0.02	0.00
1423079_a_at	0.00	0.00
1423080_at	0.00	0.00
1423081_a_at	0.00	0.00
1423082_at	0.00	0.00
1423083_at	0.00	0.00

1423084_at	0.00	0.00
1423085_at	0.00	0.00
1423086_at	0.00	0.52
1423087_a_at	0.00	0.04
1423088_at	0.00	0.00
1423089_at	0.00	0.00
1423090_x_at	0.00	0.00
1423091_a_at	0.00	0.00
1423092_at	0.00	0.00
1423093_at	0.00	0.00
1423094_at	0.00	0.00
1423095_s_at	0.00	0.00
1423096_at	0.00	0.00
1423097_s_at	0.00	0.00
1423098_at	0.00	0.00
1423099_a_at	0.00	0.09
1423100_at	0.00	0.00
1423101_at	0.00	0.00
1423102_a_at	0.00	0.00
1423103_at	0.00	0.00
1423104_at	0.69	0.44
1423105_a_at	0.00	0.00
1423106_at	0.00	0.00
1423107_at	0.00	0.00
1423108_at	0.00	0.00
1423109_s_at	0.00	0.00
1423110_at	0.00	0.00
1423111_at	0.00	0.00
1423112_at	0.00	0.00
1423113_a_at	0.00	0.05
1423114_at	0.00	0.00
1423115_at	0.00	0.00
1423116_at	0.00	0.00
1423117_at	0.00	0.00
1423118_at	0.00	0.00
1423119_at	0.00	0.21
1423120_at	0.00	0.00
1423121_at	0.00	0.00
1423122_at	0.00	0.00
1423123_at	0.00	0.00
1423124_x_at	0.00	0.00
1423125_at	0.00	0.00
1423126_at	0.00	0.00
1423127_at	0.00	0.00
1423128_at	0.00	0.00
1423129_at	0.00	0.00
1423130_a_at	0.00	0.00
1423131_at	0.00	0.00
1423132_a_at	0.00	0.00
1423133_at	0.00	0.00
1423134_at	0.00	0.00
1423135_at	0.00	0.00
1423136_at	0.00	0.00
1423137_at	0.00	0.00
1423138_at	0.00	0.00
1423139_at	0.00	0.00

1423140_at	0.00	0.00
1423141_at	0.00	0.00
1423142_a_at	0.00	0.06
1423143_at	0.00	0.11
1423144_at	0.00	0.00
1423145_a_at	0.00	0.00
1423146_at	0.00	0.00
1423147_at	0.00	0.00
1423148_at	0.00	0.00
1423149_at	0.00	0.00
1423150_at	0.00	0.00
1423151_at	0.00	0.00
1423152_at	0.00	0.00
1423153_x_at	0.00	0.00
1423154_at	0.00	0.00
1423155_at	0.00	0.00
1423156_at	0.00	0.02
1423157_at	0.00	0.00
1423158_at	0.06	0.29
1423159_at	0.00	0.00
1423160_at	0.00	0.00
1423161_s_at	0.00	0.35
1423162_s_at	0.00	0.00
1423163_at	0.00	0.00
1423164_at	0.00	0.00
1423165_a_at	0.00	0.00
1423166_at	0.00	0.00
1423167_at	0.00	0.05
1423168_at	0.00	0.00
1423169_at	0.00	0.00
1423170_at	0.00	0.00
1423171_at	0.00	0.00
1423172_at	0.00	0.00
1423173_at	0.00	0.00
1423174_a_at	0.01	0.27
1423175_s_at	0.00	0.00
1423176_at	0.00	0.00
1423177_a_at	0.00	0.00
1423178_at	0.00	0.00
1423179_at	0.00	0.00
1423180_at	0.00	0.00
1423181_s_at	0.00	0.51
1423182_at	0.00	0.00
1423183_at	0.00	0.00
1423184_at	0.00	0.00
1423185_a_at	0.00	0.00
1423186_at	0.00	0.00
1423187_at	0.00	0.00
1423188_a_at	0.00	0.00
1423189_at	0.00	0.00
1423190_at	0.00	0.00
1423191_at	0.00	0.00
1423192_at	0.00	0.00
1423193_at	0.00	0.00
1423194_at	0.00	0.00
1423195_at	0.00	0.00

1423196_at	0.00	0.00
1423197_a_at	0.00	0.00
1423198_a_at	0.00	0.00
1423199_at	0.00	0.00
1423200_at	0.00	0.01
1423201_at	0.03	0.00
1423202_a_at	0.01	0.00
1423203_a_at	0.00	0.00
1423204_at	0.00	0.00
1423205_at	0.00	0.00
1423206_s_at	0.00	0.32
1423207_at	0.00	0.00
1423208_at	0.00	0.00
1423209_at	0.00	0.00
1423210_a_at	0.00	0.01
1423211_at	0.01	0.33
1423212_at	0.29	0.00
1423213_at	0.00	0.00
1423214_at	0.00	0.00
1423215_at	0.00	0.00
1423216_a_at	0.00	0.05
1423217_a_at	0.00	0.17
1423218_a_at	0.00	0.00
1423219_a_at	0.00	0.07
1423220_at	0.00	0.00
1423221_at	0.00	0.00
1423222_at	0.00	0.00
1423223_a_at	0.00	0.00
1423224_at	0.00	0.00
1423225_at	0.00	0.00
1423226_at	0.00	0.00
1423227_at	0.00	0.08
1423228_at	0.00	0.00
1423229_at	0.00	0.00
1423230_at	0.00	0.00
1423231_at	0.00	0.00
1423232_at	0.00	0.32
1423233_at	0.00	0.00
1423234_at	0.00	0.00
1423235_at	0.00	0.00
1423236_at	0.00	0.00
1423237_at	0.00	0.14
1423238_at	0.00	0.00
1423239_at	0.00	0.00
1423240_at	0.00	0.00
1423241_a_at	0.00	0.32
1423242_at	0.00	0.00
1423243_at	0.00	0.00
1423244_at	0.00	0.00
1423245_at	0.00	0.00
1423246_at	0.00	0.00
1423247_at	0.00	0.00
1423248_at	0.00	0.00
1423249_at	0.00	0.00
1423250_a_at	0.42	0.00
1423251_at	0.00	0.00

1423252_at	0.00	0.00
1423253_at	0.00	0.00
1423254_x_at	0.00	0.00
1423255_at	0.00	0.31
1423256_a_at	0.00	0.00
1423257_at	0.00	0.00
1423258_at	0.00	0.00
1423259_at	0.00	0.00
1423260_at	0.00	0.00
1423261_at	0.00	0.00
1423262_a_at	0.00	0.00
1423263_at	0.00	0.00
1423264_at	0.00	0.38
1423265_at	0.00	0.00
1423266_at	0.00	0.00
1423267_s_at	0.00	0.00
1423268_at	0.00	0.00
1423269_a_at	0.00	0.00
1423270_at	0.00	0.00
1423271_at	0.00	0.01
1423272_at	0.00	0.00
1423273_at	0.00	0.00
1423274_at	0.00	0.00
1423275_at	0.00	0.11
1423276_at	0.00	0.00
1423277_at	0.00	0.00
1423278_at	0.00	0.00
1423279_at	0.00	0.00
1423280_at	0.00	1.00
1423281_at	0.00	1.00
1423282_at	0.00	0.00
1423283_at	0.00	0.00
1423284_at	0.00	0.00
1423285_at	0.00	0.00
1423286_at	0.00	0.00
1423287_at	0.00	0.00
1423288_s_at	0.00	0.00
1423289_a_at	0.00	0.01
1423290_at	0.00	0.00
1423291_s_at	0.00	0.00
1423292_a_at	0.00	0.00
1423293_at	0.00	0.18
1423294_at	0.00	0.00
1423295_at	0.00	0.00
1423296_at	0.00	0.00
1423297_at	0.00	0.00
1423298_at	0.00	0.00
1423299_at	0.00	0.00
1423300_at	0.00	0.00
1423301_at	0.00	0.00
1423302_a_at	0.00	0.00
1423303_at	0.00	0.00
1423304_a_at	0.00	0.00
1423305_at	0.00	0.00
1423306_at	0.00	0.00
1423307_s_at	0.00	0.00

1423308_at	0.00	0.00
1423309_at	0.00	0.00
1423310_at	0.00	0.00
1423311_s_at	0.00	0.00
1423312_at	0.00	0.00
1423313_at	0.00	0.00
1423314_s_at	0.00	0.00
1423315_at	0.00	0.00
1423316_at	0.00	0.00
1423317_at	0.00	0.10
1423318_at	0.00	0.00
1423319_at	0.00	0.00
1423320_at	0.00	0.00
1423321_at	0.04	0.00
1423322_at	0.00	0.00
1423323_at	0.00	0.00
1423324_at	0.00	0.00
1423325_at	0.00	0.07
1423326_at	0.00	0.00
1423327_at	0.53	0.00
1423328_at	0.00	0.00
1423329_at	0.00	0.00
1423330_at	0.00	0.00
1423331_a_at	0.00	0.00
1423332_at	0.00	0.01
1423333_at	0.00	0.00
1423334_at	0.00	0.00
1423335_at	0.00	0.06
1423336_at	0.00	0.00
1423337_at	0.00	0.00
1423338_at	0.00	0.00
1423339_s_at	0.00	0.00
1423340_at	0.00	0.00
1423341_at	0.00	0.00
1423342_at	0.00	0.00
1423343_at	0.00	0.00
1423344_at	0.00	0.00
1423345_at	0.00	0.00
1423346_at	0.00	0.00
1423347_at	0.00	0.00
1423348_at	0.00	0.00
1423349_at	0.00	0.00
1423350_at	0.00	0.00
1423351_at	0.00	0.00
1423352_at	0.00	0.00
1423353_at	0.00	0.00
1423354_at	0.00	0.00
1423355_at	0.00	0.00
1423356_at	0.00	0.00
1423357_at	0.00	0.00
1423358_at	0.00	0.00
1423359_at	0.00	0.00
1423360_at	0.00	0.00
1423361_at	0.00	0.44
1423362_at	0.00	0.00
1423363_at	0.00	0.00

1423364_a_at	0.00	0.00
1423365_at	0.00	0.00
1423366_at	0.00	0.00
1423367_at	0.00	0.00
1423368_at	0.00	0.03
1423369_at	0.00	0.00
1423370_a_at	0.00	0.00
1423371_at	0.00	0.00
1423372_at	0.00	0.31
1423373_at	0.00	0.00
1423374_at	0.00	0.00
1423375_at	0.00	0.00
1423376_a_at	0.00	0.00
1423377_at	0.00	0.00
1423378_at	0.26	0.00
1423379_at	0.00	0.00
1423380_s_at	0.00	0.00
1423381_at	0.00	0.00
1423382_a_at	0.00	0.03
1423383_a_at	0.00	0.00
1423384_s_at	0.00	0.00
1423385_at	0.00	0.00
1423386_at	0.00	0.00
1423387_at	0.00	0.00
1423388_at	0.00	0.00
1423389_at	0.00	0.00
1423390_at	0.00	0.00
1423391_at	0.00	0.00
1423392_at	0.11	0.27
1423393_at	0.17	0.55
1423394_at	0.00	0.00
1423395_at	0.00	0.00
1423396_at	0.00	0.00
1423397_at	0.00	0.00
1423398_at	0.00	0.00
1423399_a_at	0.81	0.40
1423400_at	0.00	0.00
1423401_at	0.00	0.00
1423402_at	0.00	0.00
1423403_at	0.00	0.00
1423404_at	0.00	0.00
1423405_at	0.00	0.00
1423406_at	0.00	0.00
1423407_a_at	0.00	0.00
1423408_a_at	0.00	0.00
1423409_a_at	0.00	0.00
1423410_at	0.00	0.00
1423411_at	0.00	0.00
1423412_at	0.00	0.00
1423413_at	0.00	0.00
1423414_at	0.00	0.00
1423415_at	0.00	0.00
1423416_at	0.00	0.00
1423417_at	0.00	0.00
1423418_at	0.00	0.00
1423419_at	0.00	0.00

1423420_at	0.00	0.00
1423421_at	0.00	0.00
1423422_at	0.00	0.00
1423423_at	0.00	0.00
1423424_at	0.08	0.28
1423425_at	0.00	0.00
1423426_at	0.00	0.00
1423427_at	0.00	0.00
1423428_at	0.00	0.00
1423429_at	0.00	0.00
1423430_at	0.00	0.34
1423431_a_at	0.00	0.33
1423432_at	0.00	0.00
1423433_at	0.00	0.00
1423434_at	0.00	0.00
1423435_at	0.00	0.00
1423436_at	0.00	0.00
1423437_at	0.00	0.00
1423438_at	0.00	0.00
1423439_at	0.00	0.00
1423440_at	0.00	0.00
1423441_at	0.00	0.00
1423442_a_at	0.00	0.00
1423443_at	0.00	0.00
1423444_at	0.00	0.00
1423445_at	0.00	0.00
1423446_at	0.00	0.00
1423447_at	0.00	0.00
1423448_at	0.00	0.00
1423449_a_at	0.00	0.00
1423450_a_at	0.00	0.00
1423451_at	0.00	0.00
1423452_at	0.00	0.08
1423453_at	0.00	0.00
1423454_a_at	0.00	0.00
1423455_at	0.00	0.00
1423456_at	0.00	0.00
1423457_at	0.00	0.00
1423458_at	0.00	0.00
1423459_at	0.00	0.17
1423460_at	0.00	0.00
1423461_a_at	0.00	0.08
1423462_at	0.00	0.00
1423463_a_at	0.00	0.00
1423464_at	0.00	0.00
1423465_at	0.12	0.00
1423466_at	0.00	0.00
1423467_at	0.00	0.00
1423468_at	0.00	0.00
1423469_at	0.00	0.00
1423470_at	0.00	0.00
1423471_at	0.00	0.00
1423472_at	0.00	0.00
1423473_at	0.00	0.00
1423474_at	0.00	0.00
1423475_at	0.00	0.00

1423476_at	0.00	0.00
1423477_at	0.00	0.00
1423478_at	0.00	0.00
1423479_at	0.00	0.40
1423480_at	0.00	0.24
1423481_at	0.00	0.35
1423482_at	0.00	0.00
1423483_s_at	0.00	0.03
1423484_at	0.00	0.00
1423485_at	0.00	0.00
1423486_at	0.00	0.00
1423487_at	0.00	0.00
1423488_at	0.00	0.00
1423489_at	0.00	0.00
1423490_at	0.00	0.00
1423491_at	0.00	0.00
1423492_at	0.00	0.11
1423493_a_at	0.00	0.00
1423494_at	0.00	0.00
1423495_at	0.00	0.00
1423496_a_at	0.00	0.00
1423497_at	0.00	0.00
1423498_at	0.00	0.00
1423499_at	0.00	0.00
1423500_a_at	0.00	0.00
1423501_at	0.00	0.00
1423502_at	0.00	0.00
1423503_at	0.00	0.00
1423504_at	0.00	0.00
1423505_at	0.97	0.00
1423506_a_at	0.08	0.00
1423507_a_at	0.00	0.00
1423508_at	0.56	0.02
1423509_a_at	0.00	0.00
1423510_at	0.00	0.00
1423511_at	0.00	0.28
1423512_at	0.00	0.00
1423513_at	0.00	0.00
1423514_at	0.00	0.00
1423515_at	0.00	0.00
1423516_a_at	0.00	0.00
1423517_at	0.00	0.01
1423518_at	0.00	0.00
1423519_at	0.00	0.00
1423520_at	0.00	0.14
1423521_at	0.00	0.00
1423522_at	0.00	0.00
1423523_at	0.94	0.00
1423524_at	0.00	0.00
1423525_at	0.00	0.00
1423526_at	0.00	0.28
1423527_at	0.00	0.00
1423528_at	0.00	0.00
1423529_at	0.00	0.00
1423530_at	0.00	0.00
1423531_a_at	0.00	0.00

1423532_at	0.00	0.00
1423533_a_at	0.00	0.00
1423534_at	0.00	0.00
1423535_at	0.00	0.00
1423536_at	0.00	0.00
1423537_at	0.00	0.00
1423538_at	0.00	0.00
1423539_at	0.00	0.00
1423540_at	0.00	0.00
1423541_at	0.00	0.00
1423542_at	0.00	0.00
1423543_at	0.00	0.00
1423544_at	0.00	0.00
1423545_a_at	0.00	0.41
1423546_at	0.00	0.00
1423547_at	0.00	0.00
1423548_s_at	0.00	0.00
1423549_at	0.00	0.00
1423550_at	0.00	0.00
1423551_at	0.00	0.00
1423552_at	0.00	0.00
1423553_at	0.00	0.00
1423554_at	0.00	0.00
1423555_a_at	0.00	0.00
1423556_at	0.00	0.00
1423557_at	0.00	0.00
1423558_at	0.00	0.00
1423559_at	0.00	0.00
1423560_at	0.00	0.00
1423561_at	0.00	0.00
1423562_at	0.00	0.00
1423563_at	0.00	0.00
1423564_a_at	0.00	0.00
1423565_at	0.00	0.00
1423566_a_at	0.00	0.28
1423567_a_at	0.00	0.00
1423568_at	0.00	0.00
1423569_at	0.00	0.00
1423570_at	0.00	0.00
1423571_at	0.00	0.00
1423572_at	0.00	0.00
1423573_at	0.00	0.00
1423574_s_at	0.00	0.00
1423575_a_at	0.00	0.00
1423576_a_at	0.00	0.00
1423577_at	0.00	0.00
1423578_at	0.00	0.00
1423579_a_at	0.00	0.00
1423580_at	0.00	0.00
1423581_at	0.00	0.00
1423582_at	0.00	0.00
1423583_at	0.00	0.00
1423584_at	0.00	0.00
1423585_at	0.00	0.00
1423586_at	0.00	0.00
1423587_a_at	0.00	0.00

1423588_at	0.00	0.06
1423589_at	0.00	0.00
1423590_at	0.03	0.00
1423591_at	0.00	0.00
1423592_at	0.00	0.00
1423593_a_at	0.00	0.00
1423594_a_at	0.00	0.00
1423595_at	0.00	0.00
1423596_at	0.00	0.00
1423597_at	0.00	0.24
1423598_at	0.00	0.00
1423599_a_at	0.00	0.00
1423600_a_at	0.14	0.00
1423601_s_at	0.02	0.12
1423602_at	0.00	0.00
1423603_at	0.06	0.09
1423604_at	0.00	0.00
1423605_a_at	0.00	0.00
1423606_at	0.00	0.00
1423607_at	0.00	0.00
1423608_at	0.01	0.00
1423609_a_at	0.00	0.00
1423610_at	0.00	0.07
1423611_at	0.00	0.00
1423612_at	0.00	0.00
1423613_at	0.00	0.00
1423614_at	0.00	0.01
1423615_at	0.00	0.00
1423616_at	0.00	0.33
1423617_at	0.00	0.00
1423618_at	0.00	0.00
1423619_at	0.00	0.00
1423620_at	0.00	0.00
1423621_a_at	0.00	0.00
1423622_a_at	0.00	0.00
1423623_at	0.00	0.00
1423624_at	0.00	0.00
1423625_a_at	0.00	0.00
1423626_at	0.00	0.00
1423627_at	0.00	0.00
1423628_s_at	0.00	0.00
1423629_at	0.00	0.00
1423630_at	0.00	0.00
1423631_at	0.00	0.00
1423632_at	0.00	0.00
1423633_at	0.00	0.00
1423634_at	0.00	0.00
1423635_at	0.00	0.00
1423636_at	0.00	0.00
1423637_at	0.00	0.00
1423638_at	0.00	0.00
1423639_at	0.00	0.00
1423640_at	0.00	0.00
1423641_s_at	0.00	0.00
1423642_at	0.00	0.02
1423643_at	0.00	0.21

1423644_at	0.00	0.00
1423645_a_at	0.00	0.00
1423646_at	0.00	0.00
1423647_a_at	0.00	0.00
1423648_at	0.00	0.02
1423649_at	0.00	0.00
1423650_at	0.00	0.00
1423651_at	0.00	0.00
1423652_at	0.00	0.00
1423653_at	0.00	0.01
1423654_a_at	0.00	0.00
1423655_a_at	0.00	0.00
1423656_x_at	0.00	0.00
1423657_at	0.00	0.00
1423658_at	0.00	0.00
1423659_a_at	0.00	0.00
1423660_at	0.00	0.00
1423661_s_at	0.00	0.00
1423662_at	0.00	0.00
1423663_at	0.00	0.00
1423664_at	0.01	0.00
1423665_a_at	0.00	0.00
1423666_s_at	0.00	0.00
1423667_at	0.00	0.00
1423668_at	0.00	0.00
1423669_at	0.00	0.00
1423670_a_at	0.00	0.00
1423671_at	0.00	0.00
1423672_at	0.00	0.00
1423673_at	0.00	0.00
1423674_at	0.00	0.00
1423675_at	0.00	0.16
1423676_at	0.00	0.00
1423677_at	0.00	0.00
1423678_at	0.00	0.00
1423679_at	0.00	0.00
1423680_at	0.00	0.00
1423681_at	0.00	0.00
1423682_a_at	0.00	0.00
1423683_at	0.00	0.00
1423684_at	0.00	0.04
1423685_at	0.00	0.17
1423686_a_at	0.00	0.00
1423687_a_at	0.00	0.00
1423688_at	0.00	0.00
1423689_a_at	0.00	0.00
1423690_s_at	0.00	0.00
1423691_x_at	0.94	0.00
1423692_at	0.00	0.00
1423693_at	0.00	0.00
1423694_at	0.09	0.00
1423695_at	0.00	0.00
1423696_a_at	0.00	0.01
1423697_at	0.00	0.28
1423698_at	0.00	0.00
1423699_at	0.00	0.01

1423700_at	0.00	0.00
1423701_at	0.00	0.00
1423702_at	0.00	0.08
1423703_at	0.00	0.33
1423704_at	0.00	0.00
1423705_at	0.00	0.02
1423706_a_at	0.00	0.01
1423707_at	0.00	0.00
1423708_a_at	0.00	0.06
1423709_s_at	0.00	0.20
1423710_at	0.00	0.00
1423711_at	0.00	0.00
1423712_a_at	0.00	0.00
1423713_at	0.00	0.00
1423714_at	0.00	0.00
1423715_a_at	0.00	0.01
1423716_s_at	0.00	0.00
1423717_at	0.00	0.01
1423718_at	0.00	0.00
1423719_at	0.00	0.00
1423720_a_at	0.00	0.00
1423721_at	0.97	0.00
1423722_at	0.01	0.00
1423723_s_at	0.00	0.16
1423724_at	0.00	0.00
1423725_at	0.00	0.41
1423726_at	0.00	0.00
1423727_at	0.00	0.00
1423728_at	0.00	0.32
1423729_a_at	0.00	0.00
1423730_at	0.00	0.00
1423731_at	0.00	0.00
1423732_at	0.07	0.00
1423733_a_at	0.04	0.33
1423734_at	0.00	0.00
1423735_a_at	0.00	0.17
1423736_a_at	0.00	0.00
1423737_at	0.00	0.00
1423738_at	0.00	0.00
1423739_x_at	0.00	0.00
1423740_a_at	0.00	0.10
1423741_at	0.00	0.00
1423742_at	0.00	0.00
1423743_at	0.00	0.00
1423744_x_at	0.00	0.13
1423745_at	0.00	0.00
1423746_at	0.00	0.33
1423747_a_at	0.61	0.00
1423748_at	0.00	0.00
1423749_s_at	0.00	0.00
1423750_a_at	0.00	0.00
1423751_at	0.00	0.00
1423752_at	0.00	0.00
1423753_at	0.00	0.00
1423754_at	0.06	0.31
1423755_at	0.00	0.00

1423756_s_at	0.66	0.13
1423757_x_at	0.00	0.00
1423758_at	0.00	0.49
1423759_a_at	0.00	0.00
1423760_at	0.07	0.00
1423761_at	0.00	0.00
1423762_at	0.00	0.00
1423763_x_at	0.00	0.00
1423764_s_at	0.00	0.01
1423765_at	0.00	0.00
1423766_at	0.00	0.15
1423767_at	0.13	0.60
1423768_at	0.00	0.00
1423769_at	0.00	0.00
1423770_at	0.00	0.00
1423771_at	0.00	0.00
1423772_x_at	0.00	0.24
1423773_at	0.00	0.00
1423774_a_at	0.00	0.00
1423775_s_at	0.00	0.00
1423776_s_at	0.00	0.00
1423777_at	0.00	0.00
1423778_at	0.00	0.00
1423779_at	0.00	0.00
1423780_at	0.00	0.00
1423781_at	0.00	0.00
1423782_at	0.00	0.00
1423783_at	0.00	0.00
1423784_at	0.00	0.00
1423785_at	0.00	0.00
1423786_at	1.00	1.00
1423787_at	0.00	0.19
1423788_at	0.00	0.00
1423789_at	0.00	0.00
1423790_at	0.00	0.07
1423791_at	0.00	0.00
1423792_a_at	0.00	0.00
1423793_at	0.00	0.00
1423794_at	0.00	0.00
1423795_at	0.00	0.01
1423796_at	0.00	0.00
1423797_at	0.00	0.00
1423798_a_at	0.00	0.00
1423799_at	0.00	0.00
1423800_at	0.00	0.00
1423801_a_at	0.00	0.00
1423802_at	0.00	0.00
1423803_s_at	0.00	0.00
1423804_a_at	0.00	0.00
1423805_at	0.00	0.00
1423806_at	0.00	0.02
1423807_a_at	0.00	0.00
1423808_at	0.00	0.00
1423809_at	0.00	0.00
1423810_at	0.00	0.00
1423811_at	0.00	0.34

1423812_s_at	0.00	0.00
1423813_at	0.00	0.00
1423814_at	0.00	0.00
1423815_at	0.00	0.32
1423816_at	0.00	0.00
1423817_s_at	0.00	0.00
1423818_a_at	0.00	0.00
1423819_s_at	0.00	0.00
1423820_at	0.00	0.02
1423821_at	0.00	0.00
1423822_a_at	0.00	0.00
1423823_at	0.00	0.00
1423824_at	1.00	0.00
1423825_at	0.00	0.00
1423826_at	0.00	0.00
1423827_s_at	0.00	0.33
1423828_at	0.00	0.00
1423829_at	0.00	0.00
1423830_a_at	0.00	0.00
1423831_at	0.00	0.00
1423832_at	0.00	0.00
1423833_a_at	0.00	0.00
1423834_s_at	0.00	0.00
1423835_at	0.00	0.00
1423836_at	0.00	0.00
1423837_at	0.00	0.00
1423838_s_at	0.00	0.02
1423839_a_at	0.00	0.01
1423840_at	0.00	0.00
1423841_at	0.00	0.18
1423842_a_at	0.00	0.00
1423843_at	0.00	0.00
1423844_s_at	0.00	0.00
1423845_at	0.00	0.00
1423846_x_at	0.00	0.00
1423847_at	0.00	0.00
1423848_at	0.00	0.00
1423849_a_at	0.00	0.00
1423850_at	0.00	0.15
1423851_a_at	0.00	0.00
1423852_at	0.00	0.00
1423853_at	0.00	0.00
1423854_a_at	0.00	0.00
1423855_x_at	0.00	0.00
1423856_at	0.00	0.00
1423857_at	0.00	0.00
1423858_a_at	0.00	0.00
1423859_a_at	0.00	0.00
1423860_at	0.00	0.00
1423861_at	0.00	0.00
1423862_at	0.00	0.00
1423863_at	0.00	0.30
1423864_at	0.00	0.00
1423865_at	0.00	0.00
1423866_at	0.00	0.00
1423867_at	0.00	0.00

1423868_at	0.00	0.00
1423869_s_at	0.00	0.00
1423870_at	0.00	0.00
1423871_at	0.00	0.00
1423872_a_at	0.00	0.00
1423873_at	0.00	0.27
1423874_at	0.00	0.00
1423875_at	0.00	0.00
1423876_at	0.00	0.00
1423877_at	0.00	0.00
1423878_at	0.00	0.00
1423879_at	0.00	0.00
1423880_at	0.00	0.00
1423881_at	0.00	0.00
1423882_at	0.00	0.00
1423883_at	0.02	0.00
1423884_at	0.00	0.19
1423885_at	0.00	0.00
1423886_at	0.00	0.00
1423887_a_at	0.00	0.00
1423888_at	0.00	0.00
1423889_at	0.00	0.00
1423890_x_at	0.00	0.00
1423891_at	0.00	0.00
1423892_at	0.00	0.01
1423893_x_at	0.00	0.01
1423894_a_at	0.00	0.00
1423895_a_at	0.00	0.00
1423896_a_at	0.00	0.00
1423897_at	0.00	0.00
1423898_a_at	0.00	0.01
1423899_at	0.00	0.00
1423900_at	0.00	0.00
1423901_at	0.00	0.00
1423902_s_at	0.00	0.00
1423903_at	0.00	0.00
1423904_a_at	0.00	0.00
1423905_at	0.00	0.00
1423906_at	0.00	0.00
1423907_a_at	0.00	0.00
1423908_at	0.00	0.00
1423909_at	0.00	0.00
1423910_at	0.00	0.00
1423911_at	0.00	0.00
1423912_at	0.00	0.29
1423913_at	0.00	0.00
1423914_at	0.00	0.00
1423915_at	0.00	0.00
1423916_s_at	0.00	0.00
1423917_a_at	0.00	0.00
1423918_at	0.00	0.00
1423919_at	0.00	0.21
1423920_at	0.00	0.00
1423921_at	0.00	0.00
1423922_s_at	0.00	0.00
1423923_a_at	0.00	0.00

1423924_s_at	0.00	0.00
1423925_at	0.00	0.17
1423926_at	0.00	0.00
1423927_at	0.00	0.00
1423928_at	0.00	0.00
1423929_at	0.00	0.00
1423930_at	0.00	0.00
1423931_s_at	0.00	0.00
1423932_at	0.00	0.00
1423933_a_at	0.00	0.00
1423934_at	0.00	0.00
1423935_x_at	0.00	0.00
1423936_at	0.00	0.00
1423937_at	0.00	0.00
1423938_at	0.00	0.00
1423939_a_at	0.00	0.00
1423940_at	0.00	0.00
1423941_at	0.00	0.00
1423942_a_at	0.00	0.00
1423943_at	0.00	0.00
1423944_at	0.00	0.00
1423945_a_at	0.00	0.00
1423946_at	0.05	0.33
1423947_at	0.00	0.00
1423948_at	0.33	0.04
1423949_at	0.00	0.00
1423950_at	0.00	0.00
1423951_at	0.00	0.00
1423952_a_at	0.00	0.05
1423953_at	0.00	0.00
1423954_at	0.00	0.00
1423955_a_at	0.00	0.00
1423956_at	0.00	0.00
1423957_at	0.00	0.00
1423958_a_at	0.00	0.14
1423959_at	0.00	0.00
1423960_at	0.00	0.00
1423961_at	0.00	0.00
1423962_at	0.00	0.00
1423963_at	0.00	0.00
1423964_at	0.00	0.00
1423965_at	0.00	0.00
1423966_at	0.00	0.00
1423967_at	0.00	0.00
1423968_at	0.00	0.00
1423969_at	0.00	0.04
1423970_at	0.00	0.00
1423971_at	0.00	0.00
1423972_at	0.00	0.00
1423973_a_at	0.00	0.00
1423974_at	0.00	0.00
1423975_s_at	0.00	0.00
1423976_at	0.00	0.59
1423977_at	0.00	0.00
1423978_at	0.00	0.00
1423979_a_at	0.00	0.00

1423980_at	0.00	0.00
1423981_x_at	0.00	0.00
1423982_at	0.00	0.44
1423983_at	0.00	0.00
1423984_a_at	0.00	0.00
1423985_at	0.00	0.00
1423986_a_at	0.24	0.00
1423987_at	0.00	0.16
1423988_at	0.00	0.00
1423989_at	0.00	0.00
1423990_at	0.00	0.00
1423991_at	0.00	0.00
1423992_at	0.00	0.00
1423993_at	0.00	0.00
1423994_at	0.09	0.00
1423995_at	0.00	0.00
1423996_a_at	0.00	0.00
1423997_at	0.00	0.00
1423998_at	0.00	0.00
1423999_at	0.00	0.00
1424000_a_at	0.00	0.00
1424001_at	0.00	0.87
1424002_at	0.00	0.00
1424003_at	0.00	0.00
1424004_x_at	0.00	0.00
1424005_at	0.00	0.00
1424006_at	0.00	0.00
1424007_at	0.00	0.00
1424008_a_at	0.00	0.00
1424009_at	0.00	0.00
1424010_at	0.00	0.00
1424011_at	0.00	0.00
1424012_at	0.00	0.15
1424013_at	0.00	0.17
1424014_at	0.00	0.00
1424015_at	0.00	0.00
1424016_at	0.00	0.00
1424017_a_at	0.00	0.00
1424018_at	0.00	0.00
1424019_at	0.00	0.34
1424020_at	0.00	0.00
1424021_at	0.00	0.00
1424022_at	0.00	0.00
1424023_at	0.00	0.00
1424024_at	0.00	0.00
1424025_at	0.00	0.00
1424026_s_at	0.00	0.00
1424027_at	0.00	0.00
1424028_at	0.00	0.00
1424029_at	0.00	0.00
1424030_at	0.00	0.00
1424031_at	0.00	0.00
1424032_at	0.00	0.00
1424033_at	0.00	0.00
1424034_at	0.00	0.00
1424035_at	0.00	0.00

1424036_at	0.00	0.00
1424037_at	0.00	0.00
1424038_a_at	0.00	0.00
1424039_at	0.02	0.20
1424040_at	0.28	0.04
1424041_s_at	0.00	0.00
1424042_at	0.00	0.00
1424043_at	0.00	0.21
1424044_at	0.00	0.00
1424045_at	0.00	0.00
1424046_at	0.00	0.00
1424047_at	0.00	0.00
1424048_a_at	0.00	0.00
1424049_at	0.00	0.00
1424050_s_at	0.00	0.00
1424051_at	0.15	0.00
1424052_at	0.00	0.00
1424053_a_at	0.00	0.00
1424054_at	0.00	0.00
1424055_at	0.00	0.00
1424056_at	0.03	0.00
1424057_at	0.00	0.00
1424058_at	0.00	0.00
1424059_at	0.00	0.00
1424060_at	0.00	0.00
1424061_at	0.00	0.00
1424062_at	0.00	0.00
1424063_at	0.00	0.00
1424064_at	0.00	0.00
1424065_at	0.00	0.00
1424066_at	0.00	0.04
1424067_at	0.00	0.06
1424068_at	0.00	0.00
1424069_at	0.00	0.00
1424070_at	0.00	0.00
1424071_s_at	0.00	0.00
1424072_at	0.00	0.00
1424073_at	0.00	0.00
1424074_at	0.00	0.00
1424075_at	0.00	0.00
1424076_at	0.00	0.00
1424077_at	0.00	0.00
1424078_s_at	0.00	0.00
1424079_x_at	0.00	0.00
1424080_at	0.00	0.00
1424081_at	0.00	0.11
1424082_at	0.00	0.00
1424083_at	0.00	0.00
1424084_at	0.00	0.00
1424085_at	0.00	0.00
1424086_at	0.00	0.00
1424087_at	0.00	0.00
1424088_at	0.00	0.00
1424089_a_at	0.00	0.00
1424090_at	0.00	0.00
1424091_at	0.00	0.00

1424092_at	0.00	0.00
1424093_x_at	0.00	0.00
1424094_at	0.00	0.07
1424095_at	0.00	0.00
1424096_at	0.00	0.00
1424097_at	0.00	0.00
1424098_at	0.00	0.00
1424099_at	0.09	0.00
1424100_s_at	0.00	0.00
1424101_at	0.00	0.00
1424102_at	0.00	0.00
1424103_at	0.00	0.00
1424104_at	0.00	0.15
1424105_a_at	0.00	0.00
1424106_at	0.00	0.00
1424107_at	0.00	0.00
1424108_at	0.00	0.00
1424109_a_at	0.00	0.00
1424110_a_at	0.00	0.28
1424111_at	0.00	0.00
1424112_at	0.00	0.50
1424113_at	0.00	0.00
1424114_s_at	0.00	0.00
1424115_at	0.00	0.00
1424116_x_at	0.00	0.00
1424117_at	0.00	0.00
1424118_a_at	0.00	0.00
1424119_at	0.00	0.00
1424120_at	0.00	0.00
1424121_at	0.00	0.00
1424122_s_at	0.00	0.00
1424123_at	0.00	0.00
1424124_at	0.00	0.00
1424125_at	0.00	0.00
1424126_at	0.00	0.00
1424127_at	0.00	0.00
1424128_x_at	0.00	0.00
1424129_at	0.00	0.00
1424130_a_at	0.68	0.00
1424131_at	0.00	0.00
1424132_at	0.00	0.00
1424133_at	0.00	0.04
1424134_at	0.00	0.00
1424135_at	0.00	0.00
1424136_a_at	0.00	0.22
1424137_at	0.00	0.00
1424138_at	0.00	0.00
1424139_at	0.00	0.00
1424140_at	0.00	0.00
1424141_at	0.00	0.00
1424142_at	0.00	0.03
1424143_a_at	0.11	0.00
1424144_at	0.03	0.01
1424145_at	0.00	0.00
1424146_at	0.00	0.00
1424147_at	0.00	0.06

1424148_a_at	0.00	0.00
1424149_at	0.00	0.00
1424150_at	0.00	0.00
1424151_at	0.00	0.04
1424152_at	0.01	0.00
1424153_s_at	0.01	0.00
1424154_a_at	0.00	0.00
1424155_at	0.00	0.00
1424156_at	0.00	0.00
1424157_at	0.00	0.00
1424158_at	0.00	0.00
1424159_at	0.00	0.00
1424160_at	0.00	0.00
1424161_at	0.00	0.33
1424162_at	0.00	0.00
1424163_at	0.41	0.87
1424164_at	0.00	0.00
1424165_a_at	0.00	0.00
1424166_at	0.00	0.00
1424167_a_at	0.39	0.00
1424168_a_at	0.00	0.00
1424169_at	0.64	0.00
1424170_at	0.00	0.35
1424171_a_at	0.00	0.00
1424172_at	0.00	0.00
1424173_at	0.00	0.00
1424174_at	0.00	0.00
1424175_at	0.00	0.00
1424176_a_at	0.00	0.11
1424177_at	0.00	0.00
1424178_at	0.00	0.00
1424179_at	0.00	0.00
1424180_a_at	0.00	0.00
1424181_at	0.00	0.00
1424182_at	0.00	0.00
1424183_at	0.00	0.00
1424184_at	0.00	0.00
1424185_a_at	0.00	0.00
1424186_at	0.00	0.00
1424187_at	0.00	0.00
1424188_at	0.00	0.00
1424189_at	0.00	0.00
1424190_at	0.00	0.00
1424191_a_at	0.00	0.00
1424192_at	0.04	0.62
1424193_at	0.00	0.01
1424194_at	0.00	0.00
1424195_a_at	0.00	0.00
1424196_at	0.00	0.00
1424197_s_at	0.00	0.00
1424198_at	0.00	0.00
1424199_at	0.00	0.00
1424200_s_at	0.00	0.00
1424201_a_at	0.00	0.00
1424202_at	0.00	0.00
1424203_at	0.00	0.00

1424204_at	0.00	0.00
1424205_at	0.00	0.00
1424206_at	0.00	0.21
1424207_at	0.00	0.20
1424208_at	0.00	0.00
1424209_at	0.00	0.00
1424210_at	0.00	0.00
1424211_at	0.00	0.00
1424212_at	0.00	0.00
1424213_at	0.00	0.00
1424214_at	0.00	0.09
1424215_at	0.00	0.00
1424216_a_at	0.00	0.01
1424217_at	0.00	0.00
1424218_a_at	0.00	0.00
1424219_at	0.00	0.00
1424220_a_at	0.00	0.00
1424221_at	0.00	0.07
1424222_s_at	0.00	0.00
1424223_at	0.00	0.00
1424224_at	0.00	0.00
1424225_at	0.00	0.00
1424226_at	0.00	0.00
1424227_at	0.00	0.00
1424228_at	0.00	0.00
1424229_at	0.00	0.03
1424230_at	0.00	0.00
1424231_s_at	0.00	0.00
1424232_a_at	0.00	0.00
1424233_at	0.00	0.00
1424234_s_at	0.00	0.00
1424235_at	0.00	0.00
1424236_at	0.00	0.00
1424237_at	0.00	0.00
1424238_at	0.00	0.00
1424239_at	0.10	0.27
1424240_at	0.00	0.00
1424241_at	0.00	0.00
1424242_at	0.00	0.00
1424243_at	0.00	0.00
1424244_at	0.00	0.00
1424245_at	0.00	0.00
1424246_a_at	0.27	0.01
1424247_at	0.00	0.00
1424248_at	0.00	0.00
1424249_a_at	0.00	0.00
1424250_a_at	0.00	0.00
1424251_a_at	0.00	0.01
1424252_at	0.00	0.00
1424253_at	0.00	0.00
1424254_at	1.00	0.00
1424255_at	0.00	0.00
1424256_at	0.00	0.00
1424257_at	0.00	0.00
1424258_at	0.00	0.34
1424259_at	0.00	0.00

1424260_at	0.00	0.00
1424261_at	0.00	0.00
1424262_at	0.00	0.00
1424263_at	0.00	0.13
1424264_at	0.00	0.00
1424265_at	0.00	0.00
1424266_s_at	0.00	0.00
1424267_at	0.00	0.00
1424268_at	0.00	0.00
1424269_a_at	0.02	0.00
1424270_at	0.00	0.00
1424271_at	0.00	0.00
1424272_at	0.00	0.00
1424273_at	0.00	0.00
1424274_at	0.00	0.00
1424275_s_at	0.00	0.00
1424276_at	0.00	0.00
1424277_at	0.00	0.00
1424278_a_at	0.00	0.36
1424279_at	0.00	0.00
1424280_at	0.00	0.32
1424281_at	0.00	0.00
1424282_at	0.00	0.00
1424283_at	0.00	0.00
1424284_at	0.00	0.00
1424285_s_at	0.00	0.00
1424286_at	0.00	0.00
1424287_at	0.00	0.00
1424288_at	0.00	0.00
1424289_at	0.00	0.00
1424290_at	0.00	0.00
1424291_at	0.00	0.22
1424292_at	0.00	0.00
1424293_s_at	0.00	0.18
1424294_at	0.00	0.17
1424295_at	0.68	0.00
1424296_at	0.00	0.00
1424297_at	0.00	0.00
1424298_at	0.00	0.00
1424299_at	0.00	0.00
1424300_at	0.00	0.27
1424301_at	0.00	0.00
1424302_at	0.00	0.00
1424303_at	0.00	0.00
1424304_at	0.00	0.00
1424305_at	0.00	0.00
1424306_at	0.00	0.00
1424307_at	0.00	0.00
1424308_at	0.00	0.00
1424309_a_at	0.00	0.31
1424310_at	0.00	0.00
1424311_at	0.00	0.00
1424312_at	0.00	0.00
1424313_a_at	0.00	0.03
1424314_at	0.00	0.32
1424315_at	0.00	0.00

1424316_at	0.00	0.00
1424317_at	0.00	0.00
1424318_at	0.00	0.00
1424319_at	0.00	0.00
1424320_a_at	0.00	0.00
1424321_at	0.00	0.00
1424322_at	0.00	0.00
1424323_at	0.00	0.00
1424324_at	0.00	0.00
1424325_at	0.00	0.00
1424326_at	0.00	0.00
1424327_at	0.00	0.11
1424328_s_at	0.00	0.12
1424329_a_at	0.00	0.00
1424330_at	0.00	0.00
1424331_at	0.00	0.00
1424332_at	0.00	0.00
1424333_at	0.00	0.00
1424334_at	0.00	0.00
1424335_at	0.00	0.00
1424336_at	0.00	0.00
1424337_at	0.00	0.00
1424338_at	0.00	0.00
1424339_at	0.00	0.00
1424340_at	0.00	0.00
1424341_s_at	0.00	0.00
1424342_at	0.00	0.00
1424343_a_at	0.00	0.00
1424344_s_at	0.00	0.00
1424345_s_at	0.00	0.00
1424346_at	0.00	0.00
1424347_at	0.00	0.00
1424348_at	0.00	0.00
1424349_a_at	0.05	0.01
1424350_s_at	0.00	0.00
1424351_at	0.00	0.00
1424352_at	0.00	0.00
1424353_at	0.00	0.00
1424354_at	0.00	0.00
1424355_a_at	0.00	0.09
1424356_a_at	0.00	0.00
1424357_at	0.00	0.00
1424358_at	0.00	0.00
1424359_at	0.00	0.00
1424360_at	0.00	0.00
1424361_at	0.00	0.00
1424362_at	0.00	0.00
1424363_at	0.00	0.00
1424364_a_at	0.00	0.00
1424365_at	0.00	0.00
1424366_at	0.00	0.00
1424367_a_at	0.00	0.00
1424368_s_at	0.00	0.00
1424369_at	0.00	0.00
1424370_s_at	0.00	0.00
1424371_at	0.00	0.00

1424372_at	0.00	0.00
1424373_at	0.00	0.00
1424374_at	0.00	0.00
1424375_s_at	0.00	0.00
1424376_at	0.00	0.00
1424377_at	0.00	0.47
1424378_at	0.00	0.00
1424379_at	0.00	0.00
1424380_at	0.00	0.00
1424381_at	0.00	0.00
1424382_at	0.00	0.02
1424383_at	0.00	0.00
1424384_a_at	0.00	0.00
1424385_at	0.00	0.00
1424386_at	0.00	0.00
1424387_at	0.00	0.00
1424388_at	0.00	0.00
1424389_at	0.00	0.00
1424390_at	0.00	0.00
1424391_at	0.00	0.00
1424392_at	0.00	0.00
1424393_s_at	0.00	0.00
1424394_at	0.00	0.00
1424395_at	0.00	0.00
1424396_a_at	0.00	0.00
1424397_at	0.00	0.01
1424398_at	0.00	0.00
1424399_at	0.00	0.00
1424400_a_at	0.00	0.00
1424401_at	0.00	0.00
1424402_at	0.00	0.00
1424403_a_at	0.00	0.00
1424404_at	0.00	0.00
1424405_at	0.00	0.00
1424406_at	0.00	0.00
1424407_s_at	0.00	0.00
1424408_at	0.00	0.00
1424409_at	0.00	0.00
1424410_at	0.00	0.00
1424411_at	0.00	0.00
1424412_at	0.00	0.00
1424413_at	0.00	0.00
1424414_at	0.00	0.00
1424415_s_at	0.00	0.00
1424416_at	0.00	0.00
1424417_at	0.00	0.00
1424418_at	0.00	0.00
1424419_at	0.00	0.00
1424420_at	0.00	0.00
1424421_at	0.00	0.00
1424422_s_at	0.00	0.00
1424423_at	0.00	0.00
1424424_at	0.00	0.00
1424425_a_at	0.00	0.00
1424426_at	0.00	0.00
1424427_at	0.00	0.00

1424428_at	0.00	0.15
1424429_s_at	0.00	0.00
1424430_at	0.00	0.00
1424431_at	0.00	0.00
1424432_at	0.00	0.00
1424433_at	0.04	0.03
1424434_at	0.00	0.00
1424435_a_at	0.00	0.01
1424436_at	0.00	0.00
1424437_s_at	0.00	0.00
1424438_a_at	0.00	0.00
1424439_at	0.00	0.00
1424440_at	0.00	0.00
1424441_at	0.00	0.00
1424442_a_at	0.00	0.07
1424443_at	0.00	0.00
1424444_a_at	0.00	0.00
1424445_at	0.00	0.00
1424446_at	0.00	0.00
1424447_at	0.00	0.00
1424448_at	0.00	0.05
1424449_at	0.00	0.00
1424450_at	0.01	0.25
1424451_at	0.00	0.00
1424452_at	0.00	0.00
1424453_at	0.00	0.00
1424454_at	0.00	0.00
1424455_at	0.00	0.00
1424456_at	0.00	0.03
1424457_at	0.00	0.00
1424458_at	0.00	0.04
1424459_at	0.00	0.00
1424460_s_at	0.00	0.00
1424461_at	0.00	0.00
1424462_at	0.00	0.00
1424463_at	0.00	0.00
1424464_s_at	0.00	0.00
1424465_at	0.00	0.00
1424466_at	0.00	0.00
1424467_at	0.00	0.00
1424468_s_at	0.00	0.00
1424469_a_at	0.00	0.00
1424470_a_at	0.00	0.00
1424471_at	0.00	0.00
1424472_at	0.00	0.00
1424473_at	0.00	0.13
1424474_a_at	0.00	0.00
1424475_at	0.00	0.00
1424476_at	0.00	0.00
1424477_at	0.00	0.00
1424478_at	0.00	0.13
1424479_at	0.00	0.00
1424480_s_at	0.00	0.00
1424481_s_at	0.00	0.00
1424482_at	0.00	0.00
1424483_at	0.00	0.00

1424484_at	0.00	0.00
1424485_at	0.00	0.00
1424486_a_at	0.00	0.00
1424487_x_at	0.00	0.00
1424488_a_at	0.00	0.00
1424489_a_at	0.00	0.00
1424490_at	0.00	0.86
1424491_at	0.00	0.00
1424492_at	0.00	0.00
1424493_s_at	0.00	0.00
1424494_s_at	0.00	0.00
1424495_a_at	0.00	0.00
1424496_at	0.00	0.00
1424497_at	0.00	0.00
1424498_at	0.00	0.00
1424499_s_at	0.00	0.00
1424500_at	0.00	0.11
1424501_at	0.00	0.00
1424502_at	0.00	0.00
1424503_at	0.00	0.00
1424504_at	0.00	0.00
1424505_at	0.00	0.00
1424506_at	0.00	0.00
1424507_at	0.00	0.00
1424508_at	0.00	0.00
1424509_at	0.00	0.00
1424510_at	0.00	0.00
1424511_at	0.00	0.00
1424512_a_at	0.00	0.00
1424513_at	0.00	0.00
1424514_at	0.00	0.00
1424515_at	0.01	0.00
1424516_at	0.00	0.13
1424517_at	0.00	0.00
1424518_at	0.00	0.00
1424519_at	0.00	0.00
1424520_at	0.00	0.00
1424521_at	0.00	0.00
1424522_at	0.00	0.47
1424523_at	0.00	0.00
1424524_at	0.00	0.00
1424525_at	0.00	0.00
1424526_a_at	0.00	0.00
1424527_at	0.00	0.00
1424528_at	0.00	0.01
1424529_s_at	0.00	0.00
1424530_at	0.00	0.00
1424531_a_at	0.04	0.61
1424532_at	0.00	0.00
1424533_a_at	0.00	0.00
1424534_at	0.00	0.00
1424535_at	0.00	0.00
1424536_at	0.00	0.00
1424537_at	0.00	0.00
1424538_at	0.00	0.00
1424539_at	0.00	0.00

1424540_at	0.00	0.00
1424541_at	0.00	0.00
1424542_at	0.00	0.00
1424543_at	0.00	0.00
1424544_at	0.00	0.00
1424545_at	0.00	0.00
1424546_at	0.00	0.00
1424547_at	0.00	0.00
1424548_at	0.00	0.00
1424549_at	0.00	0.00
1424550_at	0.00	0.00
1424551_at	0.00	0.00
1424552_at	0.00	0.00
1424553_at	0.00	0.00
1424554_at	0.00	0.24
1424555_at	0.00	0.00
1424556_at	0.02	0.00
1424557_at	0.00	0.00
1424558_a_at	0.00	0.00
1424559_at	0.00	0.00
1424560_at	0.00	0.00
1424561_at	0.00	0.00
1424562_a_at	0.00	0.00
1424563_at	0.00	0.00
1424564_at	0.00	0.00
1424565_at	0.00	0.00
1424566_s_at	0.00	0.00
1424567_at	0.98	0.00
1424568_at	0.39	0.00
1424569_at	0.00	0.07
1424570_at	0.00	0.38
1424571_at	0.00	0.00
1424572_a_at	0.00	0.00
1424573_at	0.00	0.00
1424574_at	0.00	0.00
1424575_at	0.00	0.00
1424576_s_at	0.00	0.00
1424577_at	0.00	0.00
1424578_at	0.00	0.00
1424579_at	0.00	0.00
1424580_at	0.00	0.00
1424581_at	0.00	0.00
1424582_at	0.00	0.00
1424583_at	0.00	0.00
1424584_a_at	0.00	0.00
1424585_at	0.00	0.00
1424586_at	0.00	0.00
1424587_at	0.00	0.00
1424588_at	0.00	0.00
1424589_s_at	0.00	0.15
1424590_at	0.00	0.00
1424591_at	0.00	0.24
1424592_a_at	0.00	0.00
1424593_at	0.00	0.00
1424594_at	0.65	0.00
1424595_at	0.00	0.00

1424596_s_at	0.00	0.00
1424597_at	0.00	0.00
1424598_at	0.00	0.00
1424599_at	0.00	0.00
1424600_at	0.00	0.00
1424601_at	0.00	0.00
1424602_s_at	0.00	0.00
1424603_at	0.00	0.00
1424604_s_at	0.00	0.00
1424605_at	0.00	0.00
1424606_at	0.00	0.00
1424607_a_at	0.00	0.00
1424608_a_at	0.00	0.00
1424609_a_at	0.00	0.00
1424610_at	0.00	0.23
1424611_x_at	0.00	0.26
1424612_at	0.00	0.00
1424613_at	0.00	0.00
1424614_at	0.00	0.00
1424615_at	0.00	0.00
1424616_s_at	0.00	0.00
1424617_at	0.00	0.00
1424618_at	0.00	0.00
1424619_at	0.00	0.00
1424620_at	0.00	0.49
1424621_at	0.00	0.00
1424622_at	0.00	0.00
1424623_at	0.00	0.00
1424624_at	0.00	0.00
1424625_a_at	0.00	0.00
1424626_at	0.00	0.00
1424627_at	0.00	0.00
1424628_a_at	0.00	0.00
1424629_at	0.08	0.00
1424630_a_at	0.16	0.00
1424631_a_at	0.00	0.00
1424632_a_at	0.00	0.00
1424633_at	0.00	0.00
1424634_at	0.00	0.00
1424635_at	0.00	0.00
1424636_at	0.00	0.00
1424637_s_at	0.00	0.00
1424638_at	0.11	0.00
1424639_a_at	0.00	0.00
1424640_at	0.00	0.00
1424641_a_at	0.00	0.06
1424642_at	0.00	0.11
1424643_at	0.00	0.34
1424644_at	0.00	0.00
1424645_at	0.00	0.00
1424646_at	0.00	0.03
1424647_at	0.00	0.00
1424648_at	0.00	0.00
1424649_a_at	0.00	0.00
1424650_at	0.00	0.00
1424651_at	0.00	0.00

1424652_at	0.00	0.00
1424653_at	0.00	0.00
1424654_at	0.00	0.00
1424655_at	0.00	0.00
1424656_s_at	0.00	0.00
1424657_at	0.00	0.00
1424658_at	0.00	0.00
1424659_at	0.00	0.00
1424660_s_at	0.00	0.00
1424661_at	0.00	0.00
1424662_at	0.00	0.00
1424663_at	0.00	0.00
1424664_at	0.00	0.00
1424665_at	0.00	0.00
1424666_at	0.00	0.00
1424667_a_at	0.00	0.00
1424668_a_at	0.00	0.00
1424669_at	0.00	0.00
1424670_s_at	0.00	0.00
1424671_at	0.00	0.00
1424672_at	0.00	0.00
1424673_at	0.00	0.00
1424674_at	0.00	0.00
1424675_at	0.00	0.00
1424676_s_at	0.00	0.00
1424677_at	0.00	0.00
1424678_at	0.00	0.00
1424679_at	0.00	0.00
1424680_at	0.00	0.00
1424681_a_at	0.00	0.17
1424682_at	0.00	0.00
1424683_at	0.00	1.00
1424684_at	0.00	0.00
1424685_at	0.00	0.29
1424686_at	0.00	0.00
1424687_at	0.00	0.00
1424688_at	0.00	0.00
1424689_at	0.00	0.00
1424690_at	0.00	0.00
1424691_at	0.00	0.00
1424692_at	0.00	0.00
1424693_at	0.00	0.00
1424694_at	0.00	0.00
1424695_at	0.00	0.00
1424696_at	0.00	0.00
1424697_at	0.00	0.00
1424698_s_at	0.00	0.00
1424699_at	0.00	0.00
1424700_at	0.00	0.00
1424701_at	0.00	0.00
1424702_a_at	0.00	0.00
1424703_at	0.00	0.00
1424704_at	0.00	0.00
1424705_at	0.00	0.00
1424706_at	0.00	0.00
1424707_at	0.00	0.00

1424708_at	0.00	0.00
1424709_at	0.00	0.00
1424710_a_at	0.00	0.00
1424711_at	0.00	0.00
1424712_at	0.00	0.02
1424713_at	0.06	0.92
1424714_at	0.00	0.00
1424715_at	0.00	0.00
1424716_at	0.00	0.00
1424717_at	0.00	0.00
1424718_at	0.00	0.00
1424719_a_at	0.00	0.00
1424720_at	0.00	0.00
1424721_at	0.00	0.00
1424722_at	0.00	0.00
1424723_s_at	0.00	0.00
1424724_a_at	0.43	0.30
1424725_at	0.00	0.00
1424726_at	0.00	0.00
1424727_at	0.00	0.00
1424728_at	0.00	0.00
1424729_at	0.00	0.00
1424730_a_at	0.00	0.00
1424731_at	0.00	0.00
1424732_s_at	0.00	0.00
1424733_at	0.00	0.00
1424734_at	0.00	0.00
1424735_at	0.00	0.00
1424736_at	0.00	0.13
1424737_at	0.00	0.00
1424738_at	0.00	0.00
1424739_at	0.00	0.00
1424740_at	0.00	0.00
1424741_s_at	0.20	0.00
1424742_at	0.00	0.00
1424743_at	0.00	0.00
1424744_at	0.00	0.00
1424745_at	0.00	0.00
1424746_at	0.01	0.55
1424747_at	0.00	0.00
1424748_at	0.00	0.00
1424749_at	0.00	0.00
1424750_at	0.00	0.00
1424751_at	0.00	0.00
1424752_x_at	0.00	0.10
1424753_at	0.00	0.04
1424754_at	0.00	0.00
1424755_at	0.00	0.00
1424756_at	0.00	0.00
1424757_at	0.00	0.00
1424758_s_at	0.00	0.00
1424759_at	0.00	0.00
1424760_a_at	0.00	0.00
1424761_at	0.00	0.00
1424762_at	0.00	0.00
1424763_at	0.00	0.00

1424764_at	0.00	0.00
1424765_at	0.00	0.00
1424766_at	0.00	0.31
1424767_at	0.00	0.00
1424768_at	0.91	0.00
1424769_s_at	0.82	0.00
1424770_at	0.45	0.33
1424771_at	0.00	0.27
1424772_at	0.00	0.00
1424773_at	0.00	0.00
1424774_s_at	0.00	0.00
1424775_at	0.00	0.00
1424776_a_at	0.00	0.00
1424777_at	0.00	0.00
1424778_at	0.00	0.00
1424779_at	0.00	0.00
1424780_a_at	0.00	0.02
1424781_at	0.06	0.00
1424782_at	0.00	0.00
1424783_a_at	0.00	0.00
1424784_at	0.00	0.02
1424785_at	0.00	0.00
1424786_s_at	0.00	0.00
1424787_a_at	0.00	0.00
1424788_at	0.00	0.00
1424789_at	0.00	0.00
1424790_at	0.00	0.00
1424791_a_at	0.00	0.00
1424792_at	0.00	0.00
1424793_a_at	0.00	0.00
1424794_at	0.00	0.00
1424795_a_at	0.00	0.00
1424796_at	0.00	0.00
1424797_a_at	0.72	0.53
1424798_a_at	0.00	0.00
1424799_a_at	0.00	0.00
1424800_at	0.00	0.18
1424801_at	0.00	0.16
1424802_a_at	0.00	0.01
1424803_at	0.00	0.00
1424804_at	0.00	0.00
1424805_a_at	0.00	0.00
1424806_s_at	0.00	0.00
1424807_at	0.00	0.00
1424808_at	0.00	0.00
1424809_at	0.00	0.29
1424810_at	0.00	0.00
1424811_at	0.00	0.00
1424812_at	0.00	0.00
1424813_at	0.00	0.00
1424814_a_at	0.00	0.00
1424815_at	0.00	0.00
1424816_at	0.00	0.00
1424817_at	0.00	0.00
1424818_at	0.00	0.00
1424819_a_at	0.00	0.00

1424820_a_at	0.00	0.00
1424821_at	0.00	0.02
1424822_at	0.00	0.00
1424823_s_at	0.00	0.00
1424824_at	0.00	0.00
1424825_a_at	0.00	0.00
1424826_s_at	0.01	0.00
1424827_a_at	0.00	0.00
1424828_a_at	0.00	0.00
1424829_at	0.00	0.00
1424830_at	0.00	0.00
1424831_at	0.00	0.00
1424832_at	0.00	0.00
1424833_at	0.00	0.00
1424834_s_at	0.00	0.00
1424835_at	0.00	0.00
1424836_a_at	0.00	0.00
1424837_at	0.00	0.00
1424838_at	0.00	0.00
1424839_a_at	0.00	0.01
1424840_at	0.00	0.00
1424841_s_at	0.00	0.00
1424842_a_at	0.00	0.00
1424843_a_at	0.00	0.00
1424844_at	0.00	0.00
1424845_a_at	0.00	0.00
1424846_at	0.00	0.00
1424847_at	0.92	0.78
1424848_at	0.00	0.00
1424849_at	0.00	0.00
1424850_at	0.00	0.00
1424851_at	0.00	0.00
1424852_at	0.00	0.00
1424853_s_at	0.00	0.00
1424854_at	0.00	0.00
1424855_at	0.00	0.00
1424856_at	0.00	0.00
1424857_a_at	0.00	0.00
1424858_at	0.00	0.00
1424859_at	0.00	0.00
1424860_at	0.00	0.00
1424861_at	0.00	0.00
1424862_s_at	0.00	0.00
1424863_a_at	0.00	0.00
1424864_at	0.00	0.00
1424865_at	0.00	0.00
1424866_at	0.00	0.00
1424867_a_at	0.00	0.00
1424868_at	0.00	0.00
1424869_at	0.00	0.00
1424870_at	0.00	0.00
1424871_s_at	0.00	0.00
1424872_at	0.00	0.16
1424873_at	0.00	0.00
1424874_a_at	0.00	0.00
1424875_at	0.00	0.00

1424876_s_at	0.00	0.00
1424877_a_at	0.00	0.00
1424878_at	0.00	0.00
1424879_at	0.00	0.00
1424880_at	0.00	0.00
1424881_at	0.00	0.00
1424882_a_at	0.00	0.00
1424883_s_at	0.00	0.29
1424884_at	0.00	0.00
1424885_at	0.00	0.00
1424886_at	0.00	0.00
1424887_at	0.00	0.24
1424888_at	0.00	0.00
1424889_at	0.00	0.00
1424890_at	0.00	0.00
1424891_a_at	0.00	0.00
1424892_at	0.00	0.00
1424893_at	0.00	0.00
1424894_at	0.00	0.00
1424895_at	0.00	0.00
1424896_at	0.00	0.00
1424897_at	0.00	0.00
1424898_at	0.00	0.00
1424899_at	0.00	0.00
1424900_at	0.00	0.00
1424901_at	0.00	0.00
1424902_at	0.00	0.00
1424903_at	0.00	0.00
1424904_at	0.00	0.00
1424905_a_at	0.00	0.00
1424906_at	0.00	0.00
1424907_a_at	0.00	0.23
1424908_at	0.00	0.00
1424909_at	0.00	0.00
1424910_at	0.00	0.00
1424911_a_at	0.00	0.00
1424912_at	0.00	0.00
1424913_at	0.00	0.00
1424914_at	0.00	0.00
1424915_s_at	0.00	0.00
1424916_x_at	0.00	0.00
1424917_a_at	0.00	0.01
1424918_at	0.00	0.00
1424919_at	0.00	0.00
1424920_at	0.00	0.00
1424921_at	0.00	0.39
1424922_a_at	0.00	0.00
1424923_at	0.00	0.00
1424924_at	0.00	0.00
1424925_at	0.00	0.00
1424926_at	0.00	0.00
1424927_at	0.00	0.00
1424928_at	0.00	0.00
1424929_a_at	0.00	0.00
1424930_s_at	0.00	0.00
1424931_s_at	0.00	0.00

1424932_at	0.10	0.00
1424933_at	0.00	0.00
1424934_at	0.00	0.00
1424935_at	0.00	0.00
1424936_a_at	0.00	0.00
1424937_at	0.00	0.00
1424938_at	0.00	0.00
1424939_at	0.00	0.00
1424940_s_at	0.00	0.00
1424941_at	0.00	0.00
1424942_a_at	0.00	0.72
1424943_at	0.00	0.00
1424944_at	0.00	0.00
1424945_at	0.00	0.00
1424946_a_at	0.00	0.00
1424947_at	0.00	0.00
1424948_x_at	0.00	0.00
1424949_at	0.00	0.00
1424950_at	0.00	0.00
1424951_at	0.00	0.37
1424952_at	0.00	0.00
1424953_at	0.00	0.00
1424954_a_at	0.00	0.00
1424955_at	0.00	0.00
1424956_at	0.00	0.00
1424957_at	0.00	0.00
1424958_at	0.00	0.00
1424959_at	0.00	0.00
1424960_at	0.00	0.00
1424961_at	0.00	0.00
1424962_at	0.00	0.00
1424963_at	0.00	0.00
1424964_at	0.00	0.00
1424965_at	0.00	0.00
1424966_at	0.00	0.00
1424967_x_at	0.00	0.00
1424968_at	0.00	0.00
1424969_s_at	0.00	0.00
1424970_at	0.00	0.28
1424971_at	0.00	0.00
1424972_at	0.00	0.00
1424973_at	0.00	0.00
1424974_at	0.00	0.00
1424975_at	0.00	0.00
1424976_at	0.00	0.00
1424977_at	0.00	0.00
1424978_at	0.00	0.00
1424979_at	0.00	0.00
1424980_s_at	0.00	0.00
1424981_at	0.00	0.00
1424982_a_at	0.00	0.00
1424983_a_at	0.00	0.00
1424984_at	0.00	0.00
1424985_a_at	0.00	0.00
1424986_s_at	0.00	0.00
1424987_at	0.00	0.00

1424988_at	0.00	0.03
1424989_at	0.00	0.00
1424990_at	0.00	0.00
1424991_s_at	0.00	0.01
1424992_at	0.00	0.00
1424993_at	0.00	0.00
1424994_at	0.00	0.00
1424995_at	0.00	0.00
1424996_at	0.00	0.00
1424997_at	0.00	0.00
1424998_at	0.00	0.00
1424999_at	0.00	0.00
1425000_s_at	0.00	0.00
1425001_at	0.00	0.00
1425002_at	0.00	0.00
1425003_at	0.00	0.00
1425004_s_at	0.00	0.00
1425005_at	0.00	0.00
1425006_a_at	0.00	0.00
1425007_at	0.02	0.19
1425008_a_at	0.00	0.00
1425009_at	0.00	0.00
1425010_at	0.00	0.00
1425011_x_at	0.00	0.00
1425012_at	0.00	0.00
1425013_at	0.00	0.00
1425014_at	0.00	0.00
1425015_at	0.00	0.00
1425016_at	0.00	0.00
1425017_at	0.00	0.00
1425018_at	0.00	0.00
1425019_at	0.00	0.00
1425020_at	0.00	0.00
1425021_a_at	0.00	0.00
1425022_at	0.00	0.20
1425023_at	0.00	0.01
1425024_at	0.00	0.00
1425025_at	0.00	0.00
1425026_at	0.00	0.00
1425027_s_at	0.00	0.00
1425028_a_at	0.04	0.00
1425029_a_at	0.00	0.00
1425030_at	0.00	0.00
1425031_at	0.00	0.00
1425032_at	0.00	0.00
1425033_at	0.00	0.00
1425034_at	0.00	0.00
1425035_s_at	1.00	0.00
1425036_a_at	0.00	0.00
1425037_at	0.00	0.00
1425038_at	0.00	0.00
1425039_at	0.00	0.00
1425040_at	0.00	0.00
1425041_at	0.00	0.00
1425042_s_at	0.00	0.00
1425043_s_at	0.00	0.00

1425044_at	0.00	0.00
1425045_at	0.00	0.00
1425046_at	0.00	0.00
1425047_a_at	0.00	0.00
1425048_a_at	0.00	0.00
1425049_at	0.00	0.00
1425050_at	0.00	0.00
1425051_at	0.00	0.00
1425052_at	0.00	0.01
1425053_at	0.00	0.00
1425054_a_at	0.00	0.00
1425055_at	0.00	0.00
1425056_s_at	0.00	0.00
1425057_at	0.00	0.00
1425058_at	0.00	0.00
1425059_at	0.00	0.00
1425060_s_at	0.00	0.00
1425061_at	0.00	0.00
1425062_at	0.00	0.00
1425063_at	0.00	0.00
1425064_at	0.00	0.00
1425065_at	0.00	0.00
1425066_a_at	0.00	0.00
1425067_at	0.00	0.00
1425068_a_at	0.00	0.00
1425069_at	0.00	0.00
1425070_at	0.00	0.00
1425071_s_at	0.00	0.00
1425072_at	0.00	0.00
1425073_at	0.00	0.00
1425074_at	0.00	0.00
1425075_at	0.00	0.00
1425076_at	0.00	0.00
1425077_at	0.00	0.00
1425078_x_at	0.00	0.00
1425079_at	0.00	0.00
1425080_at	0.00	0.00
1425081_at	0.00	0.00
1425082_s_at	0.00	0.00
1425083_at	0.00	0.00
1425084_at	0.00	0.00
1425085_at	0.00	0.00
1425086_a_at	0.00	0.00
1425087_at	0.00	0.00
1425088_at	0.00	0.00
1425089_at	0.00	0.00
1425090_s_at	0.00	0.00
1425091_at	0.00	0.00
1425092_at	0.00	0.00
1425093_at	0.00	0.00
1425094_a_at	0.00	0.00
1425095_at	0.00	0.00
1425096_a_at	0.00	0.00
1425097_a_at	0.00	0.00
1425098_at	0.00	0.00
1425099_a_at	0.00	0.00

1425100_a_at	0.00	0.00
1425101_a_at	0.00	0.00
1425102_a_at	0.00	0.00
1425103_at	0.00	0.00
1425104_at	0.00	0.00
1425105_at	0.00	0.00
1425106_a_at	0.00	0.00
1425107_a_at	0.00	0.00
1425108_a_at	0.00	0.00
1425109_at	0.00	0.00
1425110_at	0.00	0.00
1425111_at	0.00	0.00
1425112_at	0.00	0.00
1425113_x_at	0.00	0.00
1425114_at	0.00	0.00
1425115_at	0.00	0.00
1425116_a_at	0.00	0.00
1425117_at	0.00	0.00
1425118_at	0.00	0.00
1425119_at	0.00	0.00
1425120_x_at	0.00	0.00
1425121_a_at	0.00	0.00
1425122_at	0.00	0.00
1425123_at	0.00	0.00
1425124_at	0.00	0.00
1425125_at	0.00	0.00
1425126_at	0.00	0.00
1425127_at	0.00	0.00
1425128_at	0.00	0.00
1425129_a_at	0.00	0.00
1425130_a_at	0.00	0.00
1425131_at	0.00	0.00
1425132_at	0.00	0.00
1425133_s_at	0.00	0.00
1425134_a_at	0.00	0.00
1425135_a_at	0.00	0.00
1425136_x_at	0.00	0.00
1425137_a_at	0.00	0.00
1425138_at	0.00	0.00
1425139_at	0.00	0.00
1425140_at	0.00	0.00
1425141_at	0.00	0.00
1425142_a_at	0.00	0.33
1425143_a_at	0.00	0.28
1425144_at	0.00	0.00
1425145_at	0.00	0.00
1425146_at	0.00	0.00
1425147_at	0.00	0.00
1425148_a_at	0.00	0.00
1425149_a_at	0.00	0.00
1425150_at	0.00	0.00
1425151_a_at	0.00	0.00
1425152_s_at	0.00	0.00
1425153_at	0.00	0.00
1425154_a_at	0.00	0.00
1425155_x_at	0.00	0.00

1425156_at	0.00	0.00
1425157_x_at	0.00	0.00
1425158_at	0.00	0.00
1425159_at	0.00	0.00
1425160_at	0.00	0.00
1425161_a_at	0.00	0.00
1425162_at	0.00	0.00
1425163_at	0.00	0.00
1425164_a_at	0.00	0.00
1425165_at	0.00	0.00
1425166_at	0.00	0.00
1425167_a_at	0.00	0.00
1425168_at	0.00	0.00
1425169_at	0.00	0.00
1425170_a_at	0.00	0.00
1425171_at	0.00	0.00
1425172_at	0.00	0.00
1425173_s_at	0.00	0.00
1425174_at	0.00	0.00
1425175_at	0.00	0.00
1425176_at	0.00	0.00
1425177_at	0.00	0.00
1425178_s_at	0.00	0.00
1425179_at	0.41	0.31
1425180_at	0.00	0.00
1425181_at	0.00	0.00
1425182_x_at	0.00	0.00
1425183_a_at	0.00	0.00
1425184_at	0.00	0.00
1425185_at	0.00	0.00
1425186_at	0.00	0.00
1425187_at	0.00	0.00
1425188_s_at	0.00	0.00
1425189_a_at	0.12	0.02
1425190_a_at	0.00	0.00
1425191_at	0.00	0.00
1425192_at	0.00	0.00
1425193_at	0.00	0.00
1425194_a_at	0.00	0.06
1425195_a_at	0.00	0.00
1425196_a_at	0.00	0.00
1425197_at	0.00	0.00
1425198_at	0.00	0.00
1425199_a_at	0.00	0.00
1425200_at	0.00	0.00
1425201_a_at	0.00	0.00
1425202_a_at	0.00	0.00
1425203_at	0.00	0.00
1425204_s_at	0.00	0.00
1425205_at	0.00	0.00
1425206_a_at	0.00	0.00
1425207_at	0.00	0.00
1425208_at	0.00	0.00
1425209_at	0.00	0.00
1425210_s_at	0.00	0.00
1425211_at	0.00	0.00

1425212_a_at	0.00	0.00
1425213_at	0.00	0.00
1425214_at	0.00	0.00
1425215_at	0.00	0.00
1425216_at	0.00	0.00
1425217_a_at	0.00	0.00
1425218_a_at	0.00	0.00
1425219_x_at	0.00	0.00
1425220_x_at	0.00	0.00
1425221_at	0.00	0.00
1425222_x_at	0.00	0.00
1425223_at	0.00	0.00
1425224_at	0.00	0.00
1425225_at	0.00	0.00
1425226_x_at	0.00	0.00
1425227_a_at	0.00	0.00
1425228_a_at	0.00	0.00
1425229_a_at	0.00	0.00
1425230_at	0.00	0.00
1425231_a_at	0.00	0.00
1425232_x_at	0.00	0.00
1425233_at	0.00	0.00
1425234_at	0.00	0.00
1425235_s_at	0.00	0.00
1425236_at	0.00	0.00
1425237_at	0.00	0.00
1425238_at	0.00	0.00
1425239_at	0.00	0.00
1425240_at	0.00	0.00
1425241_a_at	0.00	0.00
1425242_at	0.00	0.00
1425243_at	0.00	0.00
1425244_a_at	0.00	0.00
1425245_a_at	0.00	0.00
1425246_at	0.00	0.00
1425247_a_at	0.00	0.00
1425248_a_at	0.00	0.08
1425249_a_at	0.00	0.00
1425250_a_at	0.00	0.00
1425251_at	0.00	0.00
1425252_a_at	0.00	0.00
1425253_a_at	0.00	0.00
1425254_at	0.00	0.00
1425255_s_at	0.00	0.00
1425256_a_at	0.00	0.00
1425257_at	0.00	0.00
1425258_at	0.00	0.00
1425259_x_at	0.00	0.00
1425260_at	0.00	0.00
1425261_at	0.00	0.00
1425262_at	0.00	0.00
1425263_a_at	0.00	0.00
1425264_s_at	0.00	0.10
1425265_a_at	0.00	0.00
1425266_a_at	0.00	0.00
1425267_a_at	0.00	0.00

1425268_a_at	0.00	0.00
1425269_at	0.00	0.00
1425270_at	0.00	0.00
1425271_at	0.00	0.00
1425272_at	0.00	0.00
1425273_s_at	0.00	0.00
1425274_at	0.00	0.00
1425275_at	0.00	0.00
1425276_at	0.00	0.00
1425277_at	0.00	0.00
1425278_at	0.00	0.00
1425279_at	0.00	0.00
1425280_at	0.00	0.00
1425281_a_at	0.00	0.00
1425282_at	0.00	0.00
1425283_a_at	0.00	0.00
1425284_a_at	0.00	0.00
1425285_a_at	0.00	0.00
1425286_at	0.00	0.00
1425287_at	0.00	0.00
1425288_at	0.00	0.00
1425289_a_at	0.00	0.00
1425290_at	0.00	0.00
1425291_at	0.00	0.00
1425292_at	0.00	0.00
1425293_a_at	0.00	0.00
1425294_at	0.00	0.00
1425295_at	0.00	0.00
1425296_a_at	0.00	0.00
1425297_at	0.00	0.00
1425298_a_at	0.00	0.00
1425299_s_at	0.00	0.00
1425300_at	0.00	0.00
1425301_at	0.00	0.00
1425302_at	0.00	0.00
1425303_at	0.00	0.00
1425304_s_at	0.00	0.00
1425305_at	0.00	0.00
1425306_at	0.00	0.00
1425307_at	0.00	0.00
1425308_at	0.00	0.00
1425309_at	0.00	0.00
1425310_a_at	0.00	0.00
1425311_at	0.00	0.00
1425312_s_at	0.60	0.00
1425313_at	0.00	0.00
1425314_at	0.00	0.00
1425315_at	0.00	0.00
1425316_at	0.00	0.00
1425317_x_at	0.00	0.00
1425318_a_at	0.00	0.00
1425319_s_at	0.00	0.00
1425320_at	0.00	0.00
1425321_a_at	0.00	0.00
1425322_at	0.00	0.00
1425323_a_at	0.00	0.00

1425324_x_at	0.00	0.00
1425325_at	0.00	0.00
1425326_at	0.00	0.00
1425327_at	0.00	0.00
1425328_at	0.00	0.00
1425329_a_at	0.00	0.00
1425330_a_at	0.00	0.00
1425331_at	0.00	0.03
1425332_at	0.00	0.00
1425333_at	0.00	0.00
1425334_at	0.00	0.00
1425335_at	0.00	0.00
1425336_x_at	0.00	0.00
1425337_at	0.00	0.00
1425338_at	0.00	0.00
1425339_at	0.00	0.00
1425340_a_at	0.00	0.31
1425341_at	0.00	0.00
1425342_a_at	0.00	0.00
1425343_at	0.00	0.00
1425344_at	0.00	0.00
1425345_at	0.00	0.00
1425346_at	0.00	0.00
1425347_a_at	0.00	0.00
1425348_a_at	0.00	0.00
1425349_a_at	0.00	0.22
1425350_a_at	0.00	0.00
1425351_at	0.00	0.00
1425352_at	0.00	0.00
1425353_at	0.00	0.00
1425354_a_at	0.00	0.24
1425355_at	0.00	0.00
1425356_at	0.00	0.00
1425357_a_at	0.00	0.00
1425358_at	0.00	0.00
1425359_at	0.00	0.00
1425360_at	0.00	0.00
1425361_at	0.00	0.00
1425362_at	0.00	0.00
1425363_at	0.00	0.00
1425364_a_at	0.00	0.00
1425365_a_at	0.00	0.00
1425366_a_at	0.00	0.00
1425367_at	0.00	0.00
1425368_a_at	0.00	0.00
1425369_a_at	0.00	0.00
1425370_a_at	0.00	0.00
1425371_at	0.00	0.00
1425372_at	0.00	0.00
1425373_a_at	0.00	0.00
1425374_at	0.00	0.00
1425375_at	0.00	0.00
1425376_at	0.00	0.00
1425377_at	0.00	0.00
1425378_at	0.00	0.00
1425379_at	0.00	0.00

1425380_at	0.00	0.00
1425381_a_at	0.00	0.00
1425382_a_at	0.00	0.00
1425383_a_at	0.00	0.00
1425384_a_at	0.00	0.00
1425385_a_at	0.00	0.00
1425386_at	0.00	0.00
1425387_at	0.00	0.00
1425388_a_at	0.00	0.00
1425389_a_at	0.00	0.00
1425390_at	0.00	0.00
1425391_a_at	0.00	0.00
1425392_a_at	0.00	0.00
1425393_a_at	0.00	0.00
1425394_at	0.00	0.00
1425395_at	0.00	0.00
1425396_a_at	0.00	0.83
1425397_at	0.00	0.00
1425398_at	0.00	0.00
1425399_at	0.00	0.00
1425400_a_at	0.00	0.00
1425401_at	0.00	0.00
1425402_at	0.00	0.00
1425403_at	0.00	0.00
1425404_a_at	0.00	0.00
1425405_a_at	0.00	0.00
1425406_at	0.00	0.00
1425407_s_at	0.00	0.00
1425408_a_at	0.00	0.00
1425409_at	0.00	0.00
1425410_at	0.00	0.00
1425411_at	0.00	0.00
1425412_at	0.00	0.00
1425413_at	0.00	0.00
1425414_at	0.00	0.00
1425415_a_at	0.00	0.00
1425416_s_at	0.00	0.00
1425417_x_at	0.00	0.00
1425418_at	0.00	0.00
1425419_a_at	0.00	0.30
1425420_s_at	0.00	0.00
1425421_at	0.00	0.00
1425422_a_at	0.00	0.00
1425423_at	0.00	0.00
1425424_at	0.00	0.00
1425425_a_at	0.00	0.00
1425426_a_at	0.00	0.00
1425427_at	0.00	0.00
1425428_at	0.00	0.00
1425429_s_at	0.00	0.00
1425430_at	0.00	0.00
1425431_at	0.00	0.00
1425432_at	0.00	0.00
1425433_a_at	0.00	0.00
1425434_a_at	0.00	0.00
1425435_at	0.00	0.00

1425436_x_at	0.00	0.00
1425437_a_at	0.00	0.00
1425438_at	0.00	0.00
1425439_a_at	0.00	0.00
1425440_x_at	0.00	0.00
1425441_at	0.00	0.00
1425442_at	0.00	0.00
1425443_at	0.00	0.00
1425444_a_at	0.00	0.00
1425445_a_at	0.00	0.00
1425446_at	0.00	0.00
1425447_at	0.00	0.00
1425448_x_at	0.00	0.00
1425449_at	0.00	0.00
1425450_at	0.00	0.00
1425451_s_at	0.00	0.00
1425452_s_at	0.00	0.00
1425453_x_at	0.00	0.00
1425454_a_at	0.00	0.00
1425455_a_at	0.00	0.00
1425456_a_at	0.00	0.00
1425457_a_at	0.00	0.00
1425458_a_at	0.00	0.00
1425459_at	0.00	0.00
1425460_at	0.00	0.09
1425461_at	0.00	0.00
1425462_at	0.00	0.00
1425463_at	0.00	0.00
1425464_at	0.00	0.00
1425465_a_at	0.00	0.00
1425466_at	0.00	0.00
1425467_a_at	0.00	0.00
1425468_at	0.00	0.00
1425469_a_at	0.00	0.00
1425470_at	0.00	0.00
1425471_x_at	0.00	0.00
1425472_a_at	0.00	0.00
1425473_at	0.00	0.12
1425474_a_at	0.00	0.00
1425475_at	0.00	0.00
1425476_at	0.00	0.00
1425477_x_at	0.00	0.00
1425478_x_at	0.00	0.00
1425479_at	0.00	0.01
1425480_at	0.00	0.00
1425481_at	0.00	0.00
1425482_s_at	0.00	0.00
1425483_at	0.00	0.00
1425484_at	0.00	0.00
1425485_at	0.00	0.00
1425486_s_at	0.00	0.00
1425487_at	0.00	0.00
1425488_at	0.00	0.00
1425489_at	0.00	0.00
1425490_a_at	0.00	0.00
1425491_at	0.00	0.00

1425492_at	0.00	0.00
1425493_at	0.00	0.00
1425494_s_at	0.00	0.00
1425495_at	0.00	0.00
1425496_at	0.01	0.00
1425497_a_at	0.00	0.00
1425498_at	0.00	0.61
1425499_at	0.00	0.00
1425500_x_at	0.00	0.00
1425501_at	0.00	0.00
1425502_x_at	0.00	0.00
1425503_at	0.00	0.00
1425504_at	0.00	0.00
1425505_at	0.00	0.00
1425506_at	0.00	0.00
1425507_at	0.00	0.00
1425508_s_at	0.00	0.00
1425509_at	0.00	0.00
1425510_at	0.00	0.00
1425511_at	0.00	0.00
1425512_at	0.00	0.00
1425513_at	0.00	0.00
1425514_at	0.00	0.00
1425515_at	0.00	0.00
1425516_at	0.00	0.00
1425517_s_at	0.00	0.00
1425518_at	0.00	0.00
1425519_a_at	0.00	0.00
1425520_a_at	0.00	0.00
1425521_at	0.00	0.00
1425522_at	0.00	0.00
1425523_at	0.00	0.03
1425524_at	0.00	0.00
1425525_a_at	0.00	0.18
1425526_a_at	0.00	0.00
1425527_at	0.00	0.00
1425528_at	0.00	0.00
1425529_s_at	0.00	0.00
1425530_a_at	0.00	0.00
1425531_at	0.00	0.66
1425532_a_at	0.03	0.00
1425533_a_at	0.00	0.00
1425534_at	0.00	0.00
1425535_at	0.00	0.00
1425536_at	0.00	0.37
1425537_at	0.00	0.01
1425538_x_at	0.00	0.00
1425539_a_at	0.00	0.00
1425540_at	0.00	0.00
1425541_at	0.00	0.00
1425542_a_at	0.00	0.00
1425543_s_at	0.00	0.00
1425544_at	0.00	0.00
1425545_x_at	0.00	0.20
1425546_a_at	0.00	0.00
1425547_a_at	0.00	0.00

1425548_a_at	0.00	0.00
1425549_at	0.00	0.00
1425550_a_at	0.00	0.00
1425551_at	0.00	0.00
1425552_at	0.00	0.00
1425553_s_at	0.00	0.00
1425554_a_at	0.00	0.00
1425555_at	0.00	0.00
1425556_at	0.00	0.00
1425557_x_at	0.00	0.00
1425558_at	0.00	0.00
1425559_a_at	0.00	0.00
1425560_a_at	0.00	0.00
1425561_at	0.00	0.00
1425562_s_at	0.00	0.24
1425563_s_at	0.00	0.00
1425564_at	0.31	0.15
1425565_at	0.68	0.00
1425566_at	0.00	0.00
1425567_a_at	0.23	0.00
1425568_a_at	0.00	0.00
1425569_a_at	0.00	0.00
1425570_at	0.00	0.00
1425571_at	0.00	0.00
1425572_a_at	0.00	0.00
1425573_a_at	0.00	0.00
1425574_at	0.00	0.00
1425575_at	0.00	0.00
1425576_at	0.00	0.11
1425577_at	0.00	0.00
1425578_a_at	0.00	0.00
1425579_at	0.00	0.00
1425580_a_at	0.00	0.01
1425581_s_at	0.00	0.65
1425582_a_at	0.00	0.00
1425583_at	0.00	0.00
1425584_x_at	0.00	0.00
1425585_at	0.00	0.13
1425586_a_at	0.00	0.00
1425587_a_at	0.00	0.00
1425588_at	0.00	0.00
1425589_at	0.00	0.00
1425590_s_at	0.00	0.00
1425591_a_at	0.00	0.00
1425592_at	0.00	0.00
1425593_at	0.00	0.00
1425594_at	0.00	0.00
1425595_at	0.00	0.00
1425596_at	0.00	0.00
1425597_a_at	0.00	0.00
1425598_a_at	0.00	0.00
1425599_a_at	0.00	0.00
1425600_a_at	0.00	0.00
1425601_a_at	0.00	0.00
1425602_a_at	0.00	0.00
1425603_at	0.00	0.00

1425604_at	0.00	0.00
1425605_a_at	0.00	0.00
1425606_at	0.00	0.00
1425607_at	0.00	0.00
1425608_at	0.00	0.00
1425609_at	0.00	0.00
1425610_s_at	0.00	0.18
1425611_a_at	0.00	0.00
1425612_at	0.00	0.00
1425613_at	0.00	0.00
1425614_x_at	0.00	0.00
1425615_a_at	0.00	0.00
1425616_a_at	0.00	0.00
1425617_at	0.00	0.50
1425618_at	0.00	0.00
1425619_s_at	0.00	0.00
1425620_at	0.00	0.00
1425621_at	0.00	0.00
1425622_at	0.00	0.00
1425623_a_at	0.00	0.00
1425624_at	0.00	0.00
1425625_at	0.00	0.00
1425626_at	0.00	0.00
1425627_x_at	0.00	0.02
1425628_a_at	0.00	0.00
1425629_a_at	0.00	0.00
1425630_at	0.00	0.00
1425631_at	0.00	0.00
1425632_a_at	0.00	0.00
1425633_at	0.00	0.00
1425634_a_at	0.00	0.00
1425635_at	0.00	0.00
1425636_at	0.00	0.00
1425637_at	0.00	0.00
1425638_at	0.00	0.00
1425639_at	0.00	0.00
1425640_at	0.00	0.00
1425641_at	0.00	0.00
1425642_at	0.00	0.00
1425643_at	0.00	0.00
1425644_at	0.00	0.00
1425645_s_at	0.00	0.00
1425646_at	0.00	0.00
1425647_at	0.00	0.00
1425648_at	0.00	0.00
1425649_at	0.00	0.00
1425650_at	0.20	0.61
1425651_at	0.00	0.00
1425652_s_at	0.00	0.00
1425653_at	0.00	0.00
1425654_a_at	0.00	0.00
1425655_at	0.00	0.00
1425656_a_at	0.00	0.00
1425657_at	0.00	0.00
1425658_at	0.00	0.00
1425659_at	0.00	0.00

1425660_at	0.00	0.00
1425661_at	0.00	0.00
1425662_at	0.00	0.00
1425663_at	0.00	0.00
1425664_at	0.00	0.00
1425665_a_at	0.00	0.00
1425666_at	0.00	0.00
1425667_at	0.00	0.00
1425668_a_at	0.00	0.00
1425669_at	0.00	0.00
1425670_at	0.00	0.00
1425671_at	0.00	0.00
1425672_a_at	0.00	0.00
1425673_at	0.00	0.00
1425674_a_at	0.00	0.00
1425675_s_at	0.10	0.00
1425676_a_at	0.00	0.04
1425677_a_at	0.00	0.00
1425678_a_at	0.00	0.00
1425679_a_at	0.00	0.00
1425680_a_at	0.00	0.00
1425681_a_at	0.00	0.00
1425682_a_at	0.00	0.00
1425683_at	0.00	0.00
1425684_at	0.00	0.00
1425685_at	0.00	0.00
1425686_at	0.00	0.00
1425687_at	0.00	0.00
1425688_a_at	0.00	0.00
1425689_at	0.00	0.00
1425690_at	0.00	0.00
1425691_at	0.00	0.00
1425692_a_at	0.00	0.00
1425693_at	0.00	0.00
1425694_at	0.00	0.00
1425695_at	0.00	0.00
1425696_at	0.00	0.00
1425697_at	0.00	0.00
1425698_a_at	0.00	0.00
1425699_a_at	0.00	0.00
1425700_at	0.00	0.00
1425701_a_at	0.00	0.00
1425702_a_at	0.00	0.07
1425703_at	0.00	0.00
1425704_at	0.00	0.00
1425705_a_at	0.00	0.00
1425706_a_at	0.00	0.00
1425707_a_at	0.00	0.00
1425708_at	0.00	0.00
1425709_at	0.00	0.00
1425710_a_at	0.00	0.00
1425711_a_at	0.00	0.00
1425712_at	0.00	0.00
1425713_a_at	0.00	0.00
1425714_a_at	0.00	0.00
1425715_at	0.00	0.00

1425716_s_at	0.00	0.00
1425717_at	0.00	0.00
1425718_a_at	0.00	0.00
1425719_a_at	0.00	0.00
1425720_at	0.00	0.00
1425721_at	0.00	0.00
1425722_at	0.00	0.00
1425723_at	0.00	0.00
1425724_at	0.00	0.00
1425725_s_at	0.00	0.00
1425726_x_at	0.00	0.00
1425727_at	0.00	0.00
1425728_at	0.00	0.00
1425729_at	0.00	0.00
1425730_at	0.00	0.00
1425731_at	0.00	0.00
1425732_a_at	0.00	0.00
1425733_a_at	0.00	0.00
1425734_a_at	0.00	0.00
1425735_at	0.00	0.00
1425736_at	0.00	0.00
1425737_at	0.00	0.00
1425738_at	0.00	0.00
1425739_at	0.00	0.00
1425740_at	0.00	0.00
1425741_at	0.00	0.00
1425742_a_at	0.00	0.00
1425743_at	0.00	0.00
1425744_a_at	0.00	0.00
1425745_a_at	0.00	0.00
1425746_at	0.00	0.00
1425747_at	0.00	0.00
1425748_at	0.00	0.00
1425749_at	0.00	0.00
1425750_a_at	0.00	0.00
1425751_at	0.00	0.00
1425752_at	0.00	0.00
1425753_a_at	0.07	0.00
1425754_a_at	0.00	0.00
1425755_at	0.00	0.00
1425756_at	0.00	0.00
1425757_a_at	0.00	0.00
1425758_a_at	0.00	0.00
1425759_at	0.00	0.00
1425760_a_at	0.00	0.00
1425761_a_at	0.00	0.00
1425762_a_at	0.00	0.00
1425763_x_at	0.00	0.00
1425764_a_at	0.15	0.24
1425765_at	0.00	0.00
1425766_x_at	0.00	0.00
1425767_a_at	0.00	0.00
1425768_at	0.00	0.00
1425769_x_at	0.00	0.00
1425770_at	0.00	0.00
1425771_at	0.00	0.00

1425772_at	0.00	0.00
1425773_s_at	0.00	0.00
1425774_at	0.00	0.00
1425775_at	0.00	0.00
1425776_a_at	0.00	0.00
1425777_at	0.00	0.00
1425778_at	0.00	0.00
1425779_a_at	0.00	0.00
1425780_a_at	0.00	0.00
1425781_a_at	0.00	0.00
1425782_at	0.00	0.00
1425783_at	0.00	0.00
1425784_a_at	0.00	0.25
1425785_a_at	0.00	0.00
1425786_a_at	0.00	0.00
1425787_a_at	0.00	0.00
1425788_a_at	0.00	0.00
1425789_s_at	0.00	0.00
1425790_a_at	0.00	0.00
1425791_at	0.00	0.00
1425792_a_at	0.00	0.00
1425793_a_at	0.00	0.00
1425794_at	0.00	0.00
1425795_a_at	0.00	0.00
1425796_a_at	0.00	0.00
1425797_a_at	0.00	0.00
1425798_a_at	0.00	0.00
1425799_at	0.00	0.00
1425800_at	0.00	0.00
1425801_x_at	0.00	0.27
1425802_a_at	0.00	0.00
1425803_a_at	0.00	0.00
1425804_at	0.00	0.00
1425805_a_at	0.00	0.00
1425806_a_at	0.00	0.21
1425807_at	0.00	0.00
1425808_a_at	0.00	0.00
1425809_at	0.00	0.00
1425810_a_at	0.87	0.00
1425811_a_at	0.76	0.00
1425812_a_at	0.00	0.00
1425813_at	0.00	0.00
1425814_a_at	0.00	0.00
1425815_a_at	0.00	0.00
1425816_at	0.00	0.00
1425817_a_at	0.00	0.00
1425818_at	0.00	0.00
1425819_at	0.00	0.00
1425820_x_at	0.00	0.00
1425821_at	0.00	0.00
1425822_a_at	0.00	0.00
1425823_at	0.00	0.00
1425824_a_at	0.00	0.00
1425825_at	0.00	0.00
1425826_a_at	0.00	0.00
1425827_at	0.00	0.00

1425828_at	0.00	0.00
1425829_a_at	0.00	0.00
1425830_a_at	0.00	0.00
1425831_at	0.00	0.00
1425832_a_at	0.00	0.00
1425833_a_at	0.00	0.00
1425834_a_at	0.00	0.00
1425835_a_at	0.00	0.00
1425836_a_at	0.00	0.00
1425837_a_at	0.00	0.00
1425838_at	0.00	0.00
1425839_at	0.00	0.00
1425840_a_at	0.00	0.00
1425841_at	0.00	0.00
1425842_at	0.00	0.00
1425843_at	0.00	0.00
1425844_a_at	0.00	0.00
1425845_a_at	0.00	0.00
1425846_a_at	0.00	0.00
1425847_a_at	0.00	0.00
1425848_a_at	0.00	0.00
1425849_at	0.00	0.00
1425850_a_at	0.00	0.00
1425851_a_at	0.00	0.00
1425852_at	0.00	0.00
1425853_s_at	0.00	0.00
1425854_x_at	0.00	0.00
1425855_a_at	0.00	0.00
1425856_at	0.00	0.00
1425857_at	0.00	0.00
1425858_at	0.00	0.00
1425859_a_at	0.00	0.00
1425860_x_at	0.00	0.00
1425861_x_at	0.00	0.00
1425862_a_at	0.01	0.00
1425863_a_at	0.00	0.00
1425864_a_at	0.00	0.00
1425865_a_at	0.00	0.00
1425866_a_at	0.00	0.00
1425867_at	0.00	0.00
1425868_at	0.00	0.00
1425869_a_at	0.00	0.00
1425870_a_at	0.00	0.00
1425871_a_at	0.00	0.00
1425872_at	0.00	0.00
1425873_a_at	0.00	0.00
1425874_at	0.00	0.00
1425875_a_at	0.00	0.00
1425876_a_at	0.00	0.00
1425877_at	0.00	0.00
1425878_at	0.00	0.00
1425879_at	0.00	0.00
1425880_x_at	0.00	0.00
1425881_at	0.00	0.00
1425882_at	0.00	0.00
1425883_at	0.00	0.00

1425884_at	0.00	0.00
1425885_a_at	0.00	0.00
1425886_at	0.00	0.00
1425887_at	0.00	0.00
1425888_at	0.00	0.00
1425889_at	0.00	0.00
1425890_at	0.00	0.00
1425891_a_at	0.00	0.00
1425892_a_at	0.00	0.00
1425893_a_at	0.00	0.00
1425894_at	0.00	0.00
1425895_a_at	0.00	0.00
1425896_a_at	0.00	0.00
1425897_at	0.00	0.00
1425898_x_at	0.00	0.00
1425899_a_at	0.00	0.00
1425900_at	0.00	0.00
1425901_at	0.00	0.00
1425902_a_at	0.00	0.00
1425903_at	0.00	0.00
1425904_at	0.00	0.00
1425905_at	0.00	0.00
1425906_a_at	0.00	0.00
1425907_s_at	0.00	0.00
1425908_at	0.00	0.00
1425909_at	0.00	0.00
1425910_at	0.00	0.00
1425911_a_at	0.01	0.00
1425912_at	0.00	0.00
1425913_a_at	0.00	0.00
1425914_a_at	0.00	0.00
1425915_at	0.00	0.00
1425916_at	0.00	0.00
1425917_at	0.00	0.00
1425918_at	0.00	0.00
1425919_at	0.00	0.00
1425920_at	0.00	0.00
1425921_a_at	0.00	0.00
1425922_a_at	0.65	0.38
1425923_at	0.00	0.00
1425924_at	0.00	0.00
1425925_at	0.00	0.00
1425926_a_at	0.58	0.04
1425927_a_at	0.00	0.00
1425928_at	0.00	0.00
1425929_a_at	0.00	0.00
1425930_a_at	0.00	0.10
1425931_a_at	0.00	0.00
1425932_a_at	0.00	0.00
1425933_a_at	0.00	0.00
1425934_a_at	0.00	0.00
1425935_at	0.00	0.00
1425936_a_at	0.00	0.00
1425937_a_at	0.00	0.07
1425938_a_at	0.00	0.00
1425939_at	0.00	0.00

1425940_a_at	0.00	0.34
1425941_a_at	0.00	0.00
1425942_a_at	0.00	0.00
1425943_at	0.00	0.00
1425944_a_at	0.00	0.00
1425945_at	0.00	0.00
1425946_at	0.00	0.00
1425947_at	0.00	0.00
1425948_a_at	0.00	0.27
1425949_at	0.00	0.00
1425950_at	0.00	0.00
1425951_a_at	0.00	0.00
1425952_a_at	0.00	0.00
1425953_at	0.00	0.00
1425954_a_at	0.00	0.00
1425955_at	0.00	0.00
1425956_a_at	0.00	0.01
1425957_x_at	0.00	0.00
1425958_at	0.00	0.00
1425959_x_at	0.00	0.00
1425960_s_at	0.00	0.00
1425961_at	0.00	0.00
1425962_at	0.00	0.00
1425963_at	0.00	0.00
1425964_x_at	0.00	0.00
1425965_at	0.00	0.00
1425966_x_at	0.00	0.00
1425967_a_at	0.00	0.00
1425968_s_at	0.00	0.00
1425969_a_at	0.00	0.00
1425970_a_at	0.00	0.00
1425971_at	0.00	0.00
1425972_a_at	0.00	0.00
1425973_at	0.00	0.00
1425974_a_at	0.41	0.00
1425975_a_at	0.00	0.00
1425976_x_at	0.00	0.00
1425977_a_at	0.00	0.00
1425978_at	0.00	0.00
1425979_a_at	0.00	0.00
1425980_at	0.00	0.00
1425981_a_at	0.00	0.00
1425982_a_at	0.00	0.00
1425983_x_at	0.00	0.00
1425984_at	0.00	0.00
1425985_s_at	0.00	0.00
1425986_a_at	0.00	0.00
1425987_a_at	0.00	0.00
1425988_a_at	0.00	0.00
1425989_a_at	0.00	0.00
1425990_a_at	0.00	0.00
1425991_a_at	0.00	0.00
1425992_at	0.00	0.00
1425993_a_at	0.00	0.23
1425994_a_at	0.00	0.00
1425995_s_at	0.00	0.00

1425996_a_at	0.00	0.00
1425997_a_at	0.00	0.00
1425998_at	0.00	0.00
1425999_at	0.00	0.00
1426000_at	0.00	0.00
1426001_at	0.00	0.32
1426002_a_at	0.00	0.00
1426003_at	0.00	0.00
1426004_a_at	0.00	0.00
1426005_at	0.00	0.00
1426006_at	0.00	0.00
1426007_a_at	0.00	0.00
1426008_a_at	0.00	0.00
1426009_a_at	0.00	0.00
1426010_a_at	0.00	0.29
1426011_a_at	0.00	0.00
1426012_a_at	0.00	0.00
1426013_s_at	0.00	0.09
1426014_a_at	0.00	0.00
1426015_s_at	0.00	0.00
1426016_a_at	0.00	0.00
1426017_a_at	0.00	0.00
1426018_a_at	0.00	0.00
1426019_at	0.00	0.00
1426020_at	0.00	0.00
1426021_a_at	0.00	0.00
1426022_a_at	0.00	0.00
1426023_a_at	0.00	0.00
1426024_a_at	0.26	0.00
1426025_s_at	0.00	0.00
1426026_at	0.00	0.00
1426027_a_at	0.00	0.00
1426028_a_at	0.00	0.00
1426029_a_at	0.00	0.00
1426030_a_at	0.00	0.02
1426031_a_at	0.00	0.00
1426032_at	0.00	0.00
1426033_at	0.00	0.00
1426034_a_at	0.00	0.00
1426035_at	0.00	0.00
1426036_a_at	0.00	0.00
1426037_a_at	0.00	0.00
1426038_at	0.00	0.00
1426039_a_at	0.00	0.00
1426040_a_at	0.05	0.00
1426041_a_at	0.00	0.00
1426042_at	0.00	0.00
1426043_a_at	0.00	0.00
1426044_a_at	0.00	0.00
1426045_at	0.00	0.00
1426046_a_at	0.00	0.00
1426047_a_at	0.00	0.00
1426048_s_at	0.00	0.00
1426049_a_at	0.00	0.00
1426050_at	0.00	0.00
1426051_a_at	0.00	0.00

1426052_at	0.00	0.00
1426053_a_at	0.00	0.00
1426054_at	0.00	0.00
1426055_a_at	0.00	0.00
1426056_at	0.00	0.00
1426057_a_at	0.00	0.00
1426058_a_at	0.00	0.00
1426059_at	0.00	0.00
1426060_at	0.00	0.00
1426061_x_at	0.00	0.00
1426062_a_at	0.00	0.00
1426063_a_at	0.00	0.00
1426064_at	0.00	0.00
1426065_a_at	0.00	0.00
1426066_a_at	0.00	0.00
1426067_x_at	0.00	0.00
1426068_at	0.00	0.00
1426069_s_at	0.00	0.00
1426070_a_at	0.00	0.00
1426071_at	0.00	0.00
1426072_at	0.00	0.00
1426073_at	0.00	0.00
1426074_at	0.00	0.00
1426075_at	0.00	0.00
1426076_at	0.00	0.00
1426077_at	0.00	0.00
1426078_a_at	0.00	0.00
1426079_at	0.00	0.00
1426080_a_at	0.00	0.00
1426081_a_at	0.00	0.00
1426082_a_at	0.00	0.00
1426083_a_at	0.00	0.62
1426084_a_at	0.00	0.00
1426085_a_at	0.00	0.00
1426086_a_at	0.00	0.00
1426087_at	0.00	0.00
1426088_at	0.00	0.00
1426089_a_at	0.00	0.00
1426090_a_at	0.00	0.00
1426091_a_at	0.00	0.00
1426092_a_at	0.00	0.00
1426093_at	0.00	0.00
1426094_at	0.00	0.00
1426095_a_at	0.00	0.00
1426096_at	0.00	0.00
1426097_a_at	0.00	0.00
1426098_a_at	0.00	0.00
1426099_at	0.00	0.00
1426100_a_at	0.00	0.00
1426101_at	0.00	0.00
1426102_at	0.00	0.00
1426103_a_at	0.00	0.00
1426104_at	0.00	0.00
1426105_a_at	0.00	0.00
1426106_a_at	0.00	0.00
1426107_at	0.00	0.00

1426108_s_at	0.00	0.00
1426109_a_at	0.00	0.00
1426110_a_at	0.00	0.00
1426111_x_at	0.00	0.00
1426112_a_at	0.00	0.00
1426113_x_at	0.00	0.00
1426114_at	0.00	0.00
1426115_a_at	0.00	0.00
1426116_at	0.00	0.00
1426117_a_at	0.00	0.00
1426118_a_at	0.00	0.28
1426119_at	0.00	0.00
1426120_a_at	0.00	0.00
1426121_at	0.00	0.00
1426122_a_at	0.00	0.00
1426123_a_at	0.00	0.00
1426124_a_at	0.00	0.00
1426125_a_at	0.00	0.00
1426126_a_at	0.00	0.00
1426127_x_at	0.00	0.00
1426128_a_at	0.00	0.00
1426129_at	0.00	0.00
1426130_at	0.00	0.00
1426131_at	0.00	0.00
1426132_at	0.00	0.00
1426133_a_at	0.00	0.00
1426134_at	0.00	0.00
1426135_a_at	0.00	0.00
1426136_x_at	0.00	0.00
1426137_at	0.40	0.00
1426138_a_at	0.00	0.00
1426139_a_at	0.00	0.00
1426140_x_at	0.00	0.00
1426141_at	0.00	0.00
1426142_a_at	0.00	0.00
1426143_at	0.00	0.00
1426144_x_at	0.00	0.00
1426145_at	0.00	0.00
1426146_a_at	0.00	0.00
1426147_s_at	0.00	0.00
1426148_at	0.00	0.00
1426149_at	0.00	0.00
1426150_at	0.00	0.00
1426151_a_at	0.00	0.18
1426152_a_at	0.00	0.00
1426153_a_at	0.00	0.00
1426154_s_at	0.00	0.00
1426155_a_at	0.00	0.00
1426156_at	0.00	0.00
1426157_a_at	0.00	0.00
1426158_at	0.00	0.00
1426159_x_at	0.00	0.00
1426160_a_at	0.00	0.00
1426161_at	0.00	0.00
1426162_a_at	0.00	0.00
1426163_x_at	0.00	0.00

1426164_a_at	0.00	0.06
1426165_a_at	0.00	0.00
1426166_at	0.00	0.00
1426167_a_at	0.00	0.00
1426168_a_at	0.00	0.00
1426169_a_at	0.00	0.00
1426170_a_at	0.00	0.00
1426171_x_at	0.00	0.00
1426172_a_at	0.00	0.00
1426173_at	0.00	0.00
1426174_s_at	0.00	0.00
1426175_a_at	0.00	0.00
1426176_a_at	0.00	0.00
1426177_a_at	0.00	0.00
1426178_at	0.00	0.00
1426179_a_at	0.00	0.00
1426180_a_at	0.00	0.00
1426181_a_at	0.00	0.00
1426182_a_at	0.00	0.00
1426183_a_at	0.00	0.00
1426184_a_at	0.00	0.00
1426185_at	0.00	0.00
1426186_a_at	0.00	0.31
1426187_a_at	0.00	0.00
1426188_s_at	0.00	0.00
1426189_at	0.00	0.00
1426190_at	0.00	0.00
1426191_a_at	0.00	0.00
1426192_at	0.00	0.00
1426193_at	0.00	0.00
1426194_x_at	0.00	0.00
1426195_a_at	0.00	0.01
1426196_at	0.00	0.00
1426197_at	0.00	0.00
1426198_at	0.00	0.00
1426199_x_at	0.00	0.00
1426200_at	0.00	0.00
1426201_at	0.00	0.00
1426202_at	0.00	0.00
1426203_at	0.00	0.00
1426204_a_at	0.00	0.00
1426205_at	0.00	0.00
1426206_at	0.00	0.00
1426207_at	0.00	0.00
1426208_x_at	0.20	0.00
1426209_at	0.00	0.00
1426210_x_at	0.00	0.00
1426211_at	0.00	0.00
1426212_s_at	0.00	0.00
1426213_at	0.00	0.46
1426214_at	0.00	0.00
1426215_at	0.00	0.63
1426216_at	0.00	0.00
1426217_at	0.00	0.00
1426218_at	0.00	0.00
1426219_at	0.00	0.00

1426220_at	0.00	0.00
1426221_at	0.87	0.00
1426222_s_at	0.00	0.00
1426223_at	0.00	0.00
1426224_x_at	0.00	0.00
1426225_at	0.00	0.00
1426226_at	0.00	0.00
1426227_s_at	0.00	0.00
1426228_at	0.00	0.00
1426229_s_at	0.00	0.00
1426230_at	0.00	0.00
1426231_at	0.00	0.00
1426232_at	0.00	0.31
1426233_at	0.00	0.00
1426234_s_at	0.00	0.00
1426235_a_at	0.00	0.00
1426236_a_at	0.00	0.00
1426237_at	0.10	0.00
1426238_at	0.00	0.00
1426239_s_at	0.00	0.00
1426240_at	0.00	0.00
1426241_a_at	0.00	0.00
1426242_at	0.00	0.00
1426243_at	0.00	0.08
1426244_at	0.00	0.00
1426245_s_at	0.00	0.00
1426246_at	0.00	0.00
1426247_at	0.00	0.00
1426248_at	0.00	0.00
1426249_at	0.00	0.00
1426250_s_at	0.00	0.00
1426251_at	0.00	0.00
1426252_a_at	0.00	0.00
1426253_at	0.00	0.00
1426254_at	0.00	0.00
1426255_at	0.00	0.00
1426256_at	0.00	0.07
1426257_a_at	0.00	0.00
1426258_at	0.00	0.00
1426259_at	0.00	0.00
1426260_a_at	0.00	0.00
1426261_s_at	0.00	0.00
1426262_at	0.00	0.00
1426263_at	0.00	0.00
1426264_at	0.00	0.00
1426265_x_at	0.00	0.00
1426266_s_at	0.00	0.29
1426267_at	0.00	0.00
1426268_at	0.00	0.00
1426269_at	0.00	0.00
1426270_at	0.00	0.00
1426271_at	0.00	0.00
1426272_at	0.00	0.00
1426273_at	0.00	0.00
1426274_at	0.00	0.00
1426275_a_at	0.00	0.00

1426276_at	0.00	0.00
1426277_at	0.00	0.00
1426278_at	0.00	0.00
1426279_at	0.00	0.00
1426280_at	0.00	0.00
1426281_at	0.00	0.00
1426282_at	0.00	0.00
1426283_at	0.00	0.00
1426284_at	0.00	0.00
1426285_at	0.00	0.00
1426286_at	0.00	0.33
1426287_at	0.00	0.00
1426288_at	0.00	0.00
1426289_at	0.00	0.00
1426290_at	0.00	0.00
1426291_at	0.00	0.00
1426292_at	0.00	0.00
1426293_at	0.00	0.00
1426294_at	0.00	0.00
1426295_at	0.00	0.00
1426296_at	0.00	0.00
1426297_at	0.00	0.00
1426298_at	0.00	0.00
1426299_at	0.00	0.00
1426300_at	0.00	0.00
1426301_at	0.00	0.00
1426302_at	0.00	0.00
1426303_at	0.00	0.00
1426304_x_at	0.00	0.00
1426305_at	0.00	0.00
1426306_a_at	0.02	0.00
1426307_at	0.00	0.00
1426308_at	0.00	0.00
1426309_at	0.00	0.00
1426310_at	0.00	0.00
1426311_s_at	0.00	0.00
1426312_at	0.00	0.00
1426313_at	0.00	0.00
1426314_at	0.00	0.00
1426315_a_at	0.00	0.00
1426316_at	0.00	0.00
1426317_at	0.00	0.00
1426318_at	0.00	0.00
1426319_at	0.00	0.00
1426320_at	0.00	0.00
1426321_at	0.00	0.00
1426322_a_at	0.00	0.00
1426323_x_at	0.00	0.00
1426324_at	0.00	0.00
1426325_at	0.00	0.00
1426326_at	0.00	0.00
1426327_s_at	0.00	0.00
1426328_a_at	0.00	0.00
1426329_s_at	0.00	0.00
1426330_at	0.00	0.00
1426331_a_at	0.00	0.00

1426332_a_at	0.00	0.15
1426333_a_at	0.00	0.00
1426334_a_at	0.00	0.00
1426335_at	0.00	0.00
1426336_at	0.00	0.00
1426337_a_at	0.00	0.00
1426338_a_at	0.00	0.00
1426339_at	0.00	0.00
1426340_at	0.00	0.00
1426341_at	0.00	0.00
1426342_at	0.00	0.00
1426343_at	0.00	0.00
1426344_at	0.00	0.00
1426345_at	0.00	0.00
1426346_at	0.00	0.00
1426347_at	0.00	0.00
1426348_at	0.04	0.16
1426349_s_at	0.00	0.00
1426350_at	0.00	0.00
1426351_at	0.00	0.31
1426352_s_at	0.00	0.00
1426353_at	0.00	0.00
1426354_at	0.00	0.00
1426355_a_at	0.00	0.00
1426356_at	0.00	0.00
1426357_at	0.00	0.00
1426358_at	0.00	0.00
1426359_at	0.00	0.00
1426360_at	0.00	0.00
1426361_at	0.00	0.00
1426362_at	0.00	0.00
1426363_x_at	0.00	0.00
1426364_at	0.00	0.00
1426365_at	0.00	0.00
1426366_at	0.00	0.00
1426367_at	0.00	0.00
1426368_at	0.00	0.00
1426369_at	0.00	0.00
1426370_at	0.00	0.00
1426371_at	0.00	0.00
1426372_a_at	0.00	0.00
1426373_at	0.00	0.00
1426374_at	0.00	0.00
1426375_s_at	0.00	0.00
1426376_at	0.00	0.00
1426377_at	0.00	0.01
1426378_at	0.00	0.00
1426379_at	0.00	0.00
1426380_at	0.00	0.00
1426381_at	0.00	0.00
1426382_at	0.00	0.00
1426383_at	0.00	0.00
1426384_a_at	0.00	0.00
1426385_x_at	0.00	0.00
1426386_at	0.00	0.13
1426387_x_at	0.00	0.00

1426388_s_at	0.00	0.00
1426389_at	0.00	0.00
1426390_a_at	0.00	0.00
1426391_at	0.00	0.00
1426392_a_at	0.00	0.00
1426393_a_at	0.00	0.00
1426394_at	0.00	0.25
1426395_s_at	0.00	0.01
1426396_at	0.00	0.00
1426397_at	0.00	0.00
1426398_at	0.00	0.00
1426399_at	0.00	0.00
1426400_a_at	0.00	0.00
1426401_at	0.03	0.06
1426402_at	0.00	0.61
1426403_at	0.00	0.00
1426404_a_at	0.21	0.01
1426405_at	0.00	0.00
1426406_at	0.00	0.00
1426407_at	0.00	0.00
1426408_at	0.00	0.00
1426409_at	0.00	0.33
1426410_at	0.00	0.00
1426411_a_at	0.00	0.00
1426412_at	0.99	0.70
1426413_at	0.00	0.00
1426414_a_at	0.00	0.00
1426415_a_at	0.00	0.00
1426416_a_at	0.00	0.00
1426417_at	0.00	0.00
1426418_at	0.00	0.00
1426419_at	0.00	0.00
1426420_at	0.00	0.00
1426421_s_at	0.00	0.00
1426422_at	0.00	0.00
1426423_at	0.00	0.03
1426424_at	0.00	0.00
1426425_at	0.00	0.00
1426426_at	0.00	0.33
1426427_at	0.00	0.00
1426428_at	0.00	0.00
1426429_at	0.00	0.00
1426430_at	0.00	0.00
1426431_at	0.00	0.00
1426432_a_at	0.00	0.00
1426433_at	0.00	0.00
1426434_at	0.00	0.00
1426435_at	0.00	0.00
1426436_at	0.00	0.00
1426437_s_at	0.00	0.00
1426438_at	0.00	0.61
1426439_at	0.00	0.00
1426440_at	0.00	0.00
1426441_at	0.00	0.00
1426442_at	0.00	0.00
1426443_at	0.00	0.00

1426444_at	0.00	0.00
1426445_at	0.00	0.00
1426446_at	0.00	0.00
1426447_at	0.00	0.47
1426448_at	0.00	0.00
1426449_a_at	0.00	0.00
1426450_at	0.00	0.00
1426451_at	0.00	0.00
1426452_a_at	0.00	0.00
1426453_at	0.00	0.00
1426454_at	0.00	0.00
1426455_at	0.00	0.00
1426456_a_at	0.17	0.34
1426457_at	0.00	0.00
1426458_at	0.00	0.00
1426459_s_at	0.00	0.00
1426460_a_at	0.00	0.00
1426461_at	0.00	0.00
1426462_at	0.00	0.00
1426463_at	0.00	0.00
1426464_at	0.00	0.00
1426465_at	0.00	0.00
1426466_s_at	0.00	0.00
1426467_s_at	0.00	0.00
1426468_at	0.00	0.00
1426469_a_at	0.00	0.00
1426470_at	0.00	0.00
1426471_at	0.00	0.00
1426472_at	0.00	0.00
1426473_at	0.00	0.00
1426474_at	0.00	0.00
1426475_at	0.00	0.00
1426476_at	0.00	0.00
1426477_at	0.00	0.00
1426478_at	0.00	0.00
1426479_a_at	0.00	0.00
1426480_at	0.00	0.01
1426481_at	0.00	0.00
1426482_at	0.00	0.00
1426483_at	0.00	0.00
1426484_at	0.00	0.00
1426485_at	0.00	0.00
1426486_at	0.00	0.00
1426487_a_at	0.00	0.00
1426488_at	0.00	0.00
1426489_s_at	0.00	0.00
1426490_at	0.00	0.00
1426491_at	0.00	0.00
1426492_at	0.00	0.00
1426493_a_at	0.00	0.00
1426494_at	0.00	0.01
1426495_at	0.00	0.14
1426496_at	0.00	0.00
1426497_at	0.00	0.00
1426498_at	0.00	0.00
1426499_at	0.00	0.00

1426500_at	0.00	0.00
1426501_a_at	0.00	0.00
1426502_s_at	0.00	0.00
1426503_a_at	0.00	0.00
1426504_a_at	0.00	0.00
1426505_at	0.00	0.00
1426506_at	0.00	0.00
1426507_at	0.00	0.00
1426508_at	0.00	0.00
1426509_s_at	0.00	0.00
1426510_at	0.36	0.51
1426511_at	0.00	0.00
1426512_at	0.00	0.00
1426513_at	0.00	0.00
1426514_at	0.00	0.00
1426515_a_at	0.00	0.00
1426516_a_at	0.00	0.00
1426517_at	0.00	0.00
1426518_at	0.00	0.00
1426519_at	0.00	0.89
1426520_at	0.00	0.00
1426521_at	0.00	0.00
1426522_at	0.00	0.06
1426523_a_at	0.00	0.00
1426524_at	0.00	0.00
1426525_at	0.00	0.00
1426526_s_at	0.00	0.00
1426527_at	0.00	0.34
1426528_at	0.00	0.00
1426529_a_at	0.52	0.00
1426530_a_at	0.00	0.00
1426531_at	0.00	0.00
1426532_at	0.00	0.00
1426533_at	0.00	0.13
1426534_a_at	0.00	0.00
1426535_at	0.00	0.02
1426536_at	0.00	0.00
1426537_at	0.00	0.00
1426538_a_at	0.00	0.46
1426539_at	0.00	0.00
1426540_at	0.00	0.00
1426541_a_at	0.00	0.00
1426542_at	0.00	0.00
1426543_x_at	0.00	0.00
1426544_a_at	0.00	0.00
1426545_at	0.00	0.00
1426546_at	0.03	0.00
1426547_at	0.00	0.00
1426548_a_at	0.00	0.00
1426549_at	0.00	0.00
1426550_at	0.00	0.00
1426551_at	0.00	0.00
1426552_a_at	0.00	0.00
1426553_at	0.00	0.00
1426554_a_at	0.00	0.00
1426555_at	0.00	0.00

1426556_at	0.00	0.00
1426557_at	0.00	0.00
1426558_x_at	0.00	0.00
1426559_at	0.00	0.00
1426560_a_at	0.00	0.00
1426561_a_at	0.00	0.00
1426562_a_at	0.00	0.00
1426563_at	0.00	0.00
1426564_at	0.00	0.00
1426565_at	0.00	0.24
1426566_s_at	0.00	0.00
1426567_a_at	0.00	0.00
1426568_at	0.00	0.00
1426569_a_at	0.00	0.00
1426570_a_at	0.00	0.00
1426571_at	0.00	0.00
1426572_at	0.00	0.00
1426573_at	0.00	0.00
1426574_a_at	0.00	0.00
1426575_at	0.00	0.00
1426576_at	0.00	0.00
1426577_a_at	0.00	0.00
1426578_s_at	0.00	0.00
1426579_at	0.00	0.00
1426580_at	0.00	0.00
1426581_at	0.00	0.00
1426582_at	0.00	0.00
1426583_at	0.00	0.00
1426584_a_at	0.00	0.00
1426585_s_at	0.00	0.00
1426586_at	0.00	0.00
1426587_a_at	0.00	0.00
1426588_at	0.00	0.00
1426589_at	0.00	0.00
1426590_at	0.00	0.33
1426591_at	0.00	0.00
1426592_a_at	0.00	0.00
1426593_a_at	0.07	0.00
1426594_at	0.00	0.00
1426595_at	0.00	0.00
1426596_a_at	0.00	0.21
1426597_s_at	0.00	0.00
1426598_at	0.00	0.00
1426599_a_at	0.00	0.00
1426600_at	0.00	0.00
1426601_at	0.00	0.00
1426602_at	0.00	0.00
1426603_at	0.00	0.00
1426604_at	0.00	0.00
1426605_at	0.00	0.00
1426606_at	0.00	0.00
1426607_at	0.00	0.00
1426608_at	0.00	0.00
1426609_at	0.00	0.59
1426610_a_at	0.00	0.00
1426611_at	0.00	0.08

1426612_at	0.03	0.48
1426613_a_at	0.00	0.09
1426614_at	0.00	0.12
1426615_s_at	0.00	0.00
1426616_at	0.00	0.00
1426617_a_at	0.00	0.00
1426618_a_at	0.00	0.00
1426619_at	0.00	0.00
1426620_at	0.00	0.00
1426621_a_at	0.00	0.00
1426622_a_at	0.00	0.00
1426623_a_at	0.00	0.00
1426624_a_at	0.00	0.28
1426625_at	0.00	0.00
1426626_at	0.00	0.00
1426627_at	0.00	0.00
1426628_at	0.00	0.00
1426629_at	0.00	0.01
1426630_at	0.00	0.00
1426631_at	0.00	0.00
1426632_at	0.00	0.00
1426633_s_at	0.00	0.00
1426634_at	0.00	0.00
1426635_at	0.00	0.00
1426636_a_at	0.00	0.00
1426637_a_at	0.00	0.00
1426638_at	0.00	0.00
1426639_a_at	0.00	0.00
1426640_s_at	0.00	0.00
1426641_at	0.00	0.00
1426642_at	0.00	0.00
1426643_at	0.00	0.00
1426644_at	0.00	0.00
1426645_at	0.00	0.00
1426646_at	0.00	0.00
1426647_at	0.00	0.00
1426648_at	0.13	0.00
1426649_at	0.00	0.00
1426650_at	0.00	0.00
1426651_at	0.00	0.00
1426652_at	0.00	0.00
1426653_at	0.00	0.00
1426654_at	0.00	0.00
1426655_a_at	0.00	0.09
1426656_at	0.00	0.22
1426657_s_at	0.00	0.00
1426658_x_at	0.00	0.00
1426659_a_at	0.00	0.00
1426660_x_at	0.00	0.00
1426661_at	0.00	0.00
1426662_at	0.00	0.00
1426663_s_at	0.00	0.00
1426664_x_at	0.00	0.00
1426665_at	0.00	0.00
1426666_a_at	0.00	0.00
1426667_a_at	0.00	0.00

1426668_at	0.00	0.00
1426669_at	0.00	0.00
1426670_at	0.00	0.00
1426671_a_at	0.00	0.06
1426672_at	0.00	0.00
1426673_at	0.00	0.01
1426674_at	0.00	0.33
1426675_at	0.00	0.00
1426676_s_at	0.00	0.33
1426677_at	0.19	0.00
1426678_at	0.00	0.00
1426679_at	0.00	0.00
1426680_at	0.33	0.33
1426681_at	0.00	0.00
1426682_at	0.00	0.00
1426683_at	0.00	0.00
1426684_at	0.00	0.00
1426685_a_at	0.00	0.00
1426686_s_at	0.00	0.00
1426687_at	0.00	0.00
1426688_at	0.00	0.00
1426689_s_at	0.00	0.00
1426690_a_at	0.00	0.00
1426691_at	0.00	0.00
1426692_at	0.00	0.00
1426693_x_at	0.00	0.00
1426694_at	0.00	0.00
1426695_at	0.00	0.00
1426696_at	0.00	0.00
1426697_a_at	0.00	0.00
1426698_a_at	0.00	0.41
1426699_at	0.00	0.00
1426700_a_at	0.00	0.00
1426701_at	0.00	0.00
1426702_at	0.00	0.00
1426703_at	0.00	0.00
1426704_at	0.00	0.00
1426705_s_at	0.00	0.00
1426706_s_at	0.00	0.00
1426707_at	0.00	0.00
1426708_at	0.17	0.00
1426709_a_at	0.00	0.00
1426710_at	0.00	0.00
1426711_at	0.00	0.00
1426712_at	0.00	0.00
1426713_s_at	0.00	0.00
1426714_at	0.00	0.00
1426715_s_at	0.00	0.00
1426716_at	0.00	0.00
1426717_at	0.00	0.00
1426718_at	0.00	0.13
1426719_at	0.00	0.00
1426720_at	0.00	0.00
1426721_s_at	0.00	0.00
1426722_at	0.00	0.05
1426723_at	0.00	0.00

1426724_at	0.00	0.00
1426725_s_at	0.02	0.00
1426726_at	0.00	0.01
1426727_s_at	0.00	0.01
1426728_x_at	0.00	0.00
1426729_at	0.00	0.00
1426730_a_at	0.00	0.00
1426731_at	0.00	0.00
1426732_at	0.00	0.00
1426733_at	0.00	0.02
1426734_at	0.00	0.00
1426735_at	0.00	0.00
1426736_at	0.00	0.30
1426737_at	0.00	0.00
1426738_at	0.00	0.00
1426739_at	0.00	0.00
1426740_s_at	0.00	0.00
1426741_a_at	0.01	0.42
1426742_at	0.00	0.01
1426743_at	0.00	0.08
1426744_at	0.00	0.00
1426745_at	0.00	0.00
1426746_at	0.00	0.00
1426747_at	0.00	0.00
1426748_s_at	0.00	0.00
1426749_at	0.00	0.27
1426750_at	0.70	0.00
1426751_s_at	0.00	0.00
1426752_at	0.00	0.00
1426753_at	0.00	0.00
1426754_x_at	0.00	0.00
1426755_at	0.00	0.00
1426756_at	0.00	0.13
1426757_at	0.00	0.00
1426758_s_at	0.00	0.00
1426759_at	0.00	0.00
1426760_at	0.00	0.00
1426761_at	0.00	0.00
1426762_s_at	0.00	0.00
1426763_at	0.00	0.00
1426764_at	0.00	0.00
1426765_at	0.00	0.00
1426766_at	0.00	0.00
1426767_at	0.00	0.00
1426768_at	0.00	0.00
1426769_s_at	0.00	0.00
1426770_at	0.00	0.00
1426771_at	0.00	0.00
1426772_x_at	0.00	0.00
1426773_at	0.00	0.00
1426774_at	0.00	0.00
1426775_s_at	0.00	0.63
1426776_at	0.00	0.00
1426777_a_at	0.00	0.00
1426778_at	0.00	0.00
1426779_x_at	0.00	0.00

1426780_at	0.00	0.00
1426781_at	0.00	0.00
1426782_at	0.00	0.00
1426783_at	0.00	0.00
1426784_at	0.00	0.00
1426785_s_at	0.00	0.00
1426786_s_at	0.00	0.00
1426787_at	0.00	0.00
1426788_a_at	0.00	0.00
1426789_s_at	0.00	0.00
1426790_at	0.00	0.00
1426791_at	0.00	0.00
1426792_s_at	0.00	0.00
1426793_a_at	0.00	0.00
1426794_at	0.00	0.00
1426795_at	0.00	0.00
1426796_at	0.00	0.00
1426797_at	0.00	0.00
1426798_a_at	0.00	0.00
1426799_at	0.01	0.00
1426800_at	0.00	0.00
1426801_at	0.00	0.67
1426802_at	0.00	0.14
1426803_at	0.00	0.00
1426804_at	0.00	0.00
1426805_at	0.00	0.00
1426806_at	0.00	0.00
1426807_at	0.00	0.00
1426808_at	0.00	0.70
1426809_at	0.00	0.00
1426810_at	0.50	0.97
1426811_at	0.00	0.00
1426812_a_at	0.00	0.00
1426813_at	0.00	0.27
1426814_at	0.00	0.00
1426815_s_at	0.00	0.00
1426816_at	0.00	0.00
1426817_at	0.00	0.00
1426818_at	0.00	0.00
1426819_at	0.00	0.00
1426820_at	0.00	0.00
1426821_at	0.00	0.00
1426822_at	0.00	0.00
1426823_s_at	0.00	0.00
1426824_at	0.00	0.00
1426825_at	0.00	0.00
1426826_at	0.00	0.00
1426827_at	0.00	0.01
1426828_at	0.00	0.00
1426829_at	0.00	0.00
1426830_a_at	0.00	0.34
1426831_at	0.03	0.22
1426832_at	0.00	0.00
1426833_at	0.00	0.00
1426834_s_at	0.00	0.00
1426835_at	0.00	0.00

1426836_s_at	0.00	0.00
1426837_at	0.00	0.00
1426838_at	0.00	0.00
1426839_at	0.00	0.00
1426840_at	0.00	0.00
1426841_at	0.00	0.00
1426842_at	0.00	0.00
1426843_at	0.00	0.00
1426844_a_at	0.00	0.02
1426845_at	0.00	0.00
1426846_at	0.00	0.17
1426847_at	0.00	0.00
1426848_at	0.00	0.00
1426849_at	0.00	0.00
1426850_a_at	0.00	0.00
1426851_a_at	0.00	0.00
1426852_x_at	0.00	0.00
1426853_at	0.00	0.81
1426854_a_at	0.00	0.00
1426855_at	0.00	0.00
1426856_at	0.00	0.00
1426857_a_at	0.01	0.00
1426858_at	0.76	0.25
1426859_at	0.00	0.00
1426860_at	0.00	0.00
1426861_at	0.00	0.00
1426862_at	0.00	0.00
1426863_at	0.00	0.00
1426864_a_at	0.00	0.00
1426865_a_at	0.00	0.00
1426866_at	0.00	0.00
1426867_at	0.00	0.00
1426868_x_at	0.00	0.00
1426869_at	0.00	0.00
1426870_at	0.00	0.00
1426871_at	0.00	0.00
1426872_at	0.00	0.00
1426873_s_at	0.00	0.00
1426874_at	0.00	0.00
1426875_s_at	0.00	0.00
1426876_at	0.00	0.00
1426877_a_at	0.00	0.00
1426878_at	0.00	0.00
1426879_at	0.00	0.04
1426880_at	0.00	0.00
1426881_at	0.00	0.02
1426882_at	0.00	0.00
1426883_at	0.00	0.00
1426884_at	0.00	0.00
1426885_a_at	0.00	0.00
1426886_at	0.00	0.00
1426887_at	0.00	0.00
1426888_at	0.00	0.00
1426889_at	0.00	0.00
1426890_a_at	0.00	0.00
1426891_at	0.00	0.00

1426892_at	0.00	0.00
1426893_at	0.00	0.00
1426894_s_at	0.00	0.00
1426895_at	0.00	0.01
1426896_at	0.00	0.00
1426897_at	0.00	0.01
1426898_at	0.00	0.00
1426899_at	0.00	0.00
1426900_at	0.00	0.37
1426901_s_at	0.00	0.00
1426902_at	0.00	0.00
1426903_at	0.00	0.00
1426904_s_at	0.00	0.00
1426905_a_at	0.00	0.00
1426906_at	0.00	0.00
1426907_s_at	0.00	0.00
1426908_at	0.00	0.33
1426909_at	0.00	0.00
1426910_at	0.02	0.00
1426911_at	0.48	0.00
1426912_at	0.00	0.00
1426913_at	0.00	0.00
1426914_at	0.00	0.06
1426915_at	0.00	0.00
1426916_at	0.00	0.00
1426917_s_at	0.00	0.00
1426918_at	0.40	0.00
1426919_at	0.00	0.00
1426920_x_at	0.00	0.00
1426921_at	0.00	0.00
1426922_s_at	0.00	0.00
1426923_at	0.00	0.00
1426924_at	0.00	0.00
1426925_at	0.00	0.00
1426926_at	0.00	0.00
1426927_at	0.00	0.00
1426928_at	0.00	0.00
1426929_at	0.00	0.00
1426930_at	0.00	0.00
1426931_s_at	0.00	0.34
1426932_at	0.00	0.00
1426933_at	0.00	0.00
1426934_at	0.00	0.00
1426935_at	0.00	0.00
1426936_at	0.00	0.00
1426937_at	0.00	0.00
1426938_at	0.00	0.00
1426939_at	0.00	0.18
1426940_at	0.02	0.00
1426941_at	0.00	0.00
1426942_at	0.00	0.00
1426943_at	0.00	0.00
1426944_at	0.00	0.00
1426945_at	0.00	0.00
1426946_at	0.00	0.00
1426947_x_at	0.00	0.00

1426948_at	0.00	0.00
1426949_s_at	0.00	0.25
1426950_at	0.00	0.00
1426951_at	0.02	0.00
1426952_at	0.00	0.00
1426953_at	0.00	0.33
1426954_at	0.00	0.00
1426955_at	0.00	0.00
1426956_a_at	0.00	0.00
1426957_at	0.00	0.00
1426958_at	0.00	0.00
1426959_at	0.00	0.00
1426960_a_at	0.00	0.00
1426961_at	0.00	0.00
1426962_at	0.01	0.00
1426963_at	0.00	0.01
1426964_at	0.23	0.00
1426965_at	0.00	0.00
1426966_at	0.00	0.00
1426967_at	0.00	0.00
1426968_a_at	0.00	0.00
1426969_at	0.00	0.00
1426970_a_at	0.00	0.00
1426971_at	0.00	0.00
1426972_at	0.00	0.00
1426973_at	0.00	0.00
1426974_at	0.00	0.00
1426975_at	0.00	0.00
1426976_at	0.00	0.12
1426977_at	0.00	0.02
1426978_at	0.00	0.00
1426979_at	0.00	0.00
1426980_s_at	0.81	0.01
1426981_at	0.00	0.00
1426982_at	0.00	0.00
1426983_at	0.00	0.00
1426984_at	0.00	0.00
1426985_s_at	0.00	0.00
1426986_at	0.00	0.00
1426987_at	0.00	0.00
1426988_at	0.00	0.00
1426989_at	0.00	0.00
1426990_at	0.00	0.00
1426991_at	0.00	0.00
1426992_at	0.00	0.01
1426993_at	0.00	0.00
1426994_at	0.00	0.00
1426995_a_at	0.00	0.00
1426996_at	0.00	0.00
1426997_at	0.00	0.00
1426998_at	0.03	0.49
1426999_at	0.00	0.00
1427000_at	0.00	0.00
1427001_s_at	0.00	0.00
1427002_s_at	0.00	0.00
1427003_at	0.00	0.00

1427004_at	0.00	0.00
1427005_at	0.00	0.00
1427006_at	0.00	0.00
1427007_at	0.00	0.00
1427008_at	0.00	0.00
1427009_at	0.00	0.00
1427010_s_at	0.00	0.00
1427011_a_at	0.00	0.00
1427012_at	0.00	0.00
1427013_at	0.00	0.00
1427014_at	0.00	0.00
1427015_at	0.00	0.00
1427016_at	0.00	0.00
1427017_at	0.00	0.00
1427018_at	0.00	0.00
1427019_at	0.00	0.00
1427020_at	0.00	0.00
1427021_s_at	0.00	0.00
1427022_at	0.00	0.00
1427023_at	0.00	0.00
1427024_at	0.00	0.00
1427025_at	0.00	0.00
1427026_at	0.00	0.00
1427027_a_at	0.00	0.00
1427028_at	0.00	0.00
1427029_at	0.00	0.00
1427030_at	0.00	0.00
1427031_s_at	0.00	0.00
1427032_at	0.00	0.00
1427033_at	0.00	0.00
1427034_at	0.00	0.00
1427035_at	0.03	0.00
1427036_a_at	0.00	0.00
1427037_at	0.00	0.01
1427038_at	0.00	0.00
1427039_at	0.00	0.00
1427040_at	0.00	0.00
1427041_at	0.00	0.00
1427042_at	0.00	0.00
1427043_s_at	0.00	0.00
1427044_a_at	0.00	0.00
1427045_at	0.00	0.00
1427046_at	0.00	0.00
1427047_at	0.00	0.00
1427048_at	0.00	0.00
1427049_s_at	0.00	0.00
1427050_at	0.00	0.00
1427051_at	0.00	0.00
1427052_at	0.00	0.00
1427053_at	0.00	0.00
1427054_s_at	0.00	0.00
1427055_at	0.00	0.00
1427056_at	0.00	0.00
1427057_at	0.00	0.00
1427058_at	0.00	0.00
1427059_at	0.00	0.00

1427060_at	0.00	0.00
1427061_at	0.00	0.00
1427062_at	0.00	0.00
1427063_at	0.00	0.00
1427064_a_at	0.00	0.00
1427065_at	0.00	0.00
1427066_a_at	0.00	0.00
1427067_at	0.00	0.00
1427068_x_at	0.00	0.00
1427069_at	0.00	0.00
1427070_at	0.00	0.15
1427071_at	0.00	0.00
1427072_at	0.00	0.00
1427073_at	0.00	0.00
1427074_at	0.00	0.13
1427075_s_at	0.00	0.00
1427076_at	0.00	0.00
1427077_a_at	0.00	0.00
1427078_at	0.00	0.00
1427079_at	0.00	0.00
1427080_at	0.00	0.00
1427081_at	0.00	0.00
1427082_at	0.00	0.00
1427083_a_at	0.00	0.00
1427084_a_at	0.00	0.01
1427085_at	0.00	0.00
1427086_at	0.00	0.00
1427087_at	0.00	0.37
1427088_at	0.00	0.00
1427089_at	0.10	0.10
1427090_at	0.00	0.00
1427091_at	0.00	0.00
1427092_at	0.00	0.00
1427093_at	0.00	0.00
1427094_at	0.00	0.00
1427095_at	0.00	0.03
1427096_s_at	0.00	0.00
1427097_at	0.00	0.00
1427098_at	0.00	0.00
1427099_at	0.00	0.00
1427100_at	0.00	0.00
1427101_at	0.00	0.00
1427102_at	0.00	0.00
1427103_at	0.00	0.00
1427104_at	0.00	0.00
1427105_at	0.00	0.00
1427106_at	0.00	0.00
1427107_at	0.00	0.00
1427108_at	0.00	0.00
1427109_at	0.00	0.00
1427110_at	0.00	0.00
1427111_s_at	0.00	0.00
1427112_at	0.00	0.00
1427113_s_at	0.00	0.00
1427114_at	0.00	0.00
1427115_at	0.00	0.00

1427116_at	0.00	0.00
1427117_at	0.00	0.00
1427118_at	0.00	0.00
1427119_at	0.00	0.00
1427120_at	0.00	0.00
1427121_at	0.00	0.00
1427122_at	0.00	0.00
1427123_s_at	0.00	0.00
1427124_at	0.00	0.00
1427125_s_at	0.00	0.00
1427126_at	0.00	0.00
1427127_x_at	0.00	0.00
1427128_at	0.00	0.00
1427129_a_at	0.00	0.30
1427130_x_at	0.00	0.00
1427131_s_at	0.00	0.00
1427132_at	0.00	0.00
1427133_s_at	0.00	0.01
1427134_at	0.00	0.00
1427135_at	0.00	0.00
1427136_s_at	0.00	0.00
1427137_at	0.00	0.00
1427138_at	0.00	0.00
1427139_at	0.00	0.00
1427140_at	0.00	0.00
1427141_at	0.00	0.00
1427142_s_at	0.07	0.00
1427143_at	0.21	0.00
1427144_at	0.00	0.00
1427145_at	0.00	0.00
1427146_at	0.00	0.00
1427147_at	0.00	0.00
1427148_at	0.00	0.00
1427149_at	0.00	0.00
1427150_at	0.00	0.00
1427151_at	0.00	0.00
1427152_at	0.00	0.07
1427153_at	0.00	0.00
1427154_at	0.00	0.00
1427155_at	0.00	0.00
1427156_s_at	0.00	0.00
1427157_at	0.00	0.00
1427158_at	0.00	0.00
1427159_at	0.00	0.00
1427160_at	0.00	0.00
1427161_at	0.00	0.00
1427162_a_at	0.00	0.00
1427163_at	0.00	0.00
1427164_at	0.00	0.00
1427165_at	0.00	0.00
1427166_a_at	0.00	0.00
1427167_at	0.00	0.00
1427168_a_at	0.00	0.00
1427169_at	0.00	0.00
1427170_at	0.33	0.02
1427171_at	0.00	0.00

1427172_at	0.00	0.00
1427173_a_at	0.00	0.00
1427174_at	0.10	0.27
1427175_at	0.00	0.00
1427176_s_at	0.00	0.00
1427177_at	0.00	0.00
1427178_at	0.00	0.10
1427179_at	0.00	0.00
1427180_at	0.00	0.00
1427181_at	0.00	0.00
1427182_s_at	0.00	0.00
1427183_at	0.05	0.00
1427184_at	0.00	0.00
1427185_at	0.00	0.00
1427186_a_at	0.00	0.00
1427187_at	0.00	0.00
1427188_at	0.00	0.00
1427189_at	0.00	0.00
1427190_at	0.00	0.00
1427191_at	0.00	0.00
1427192_a_at	0.00	0.00
1427193_at	0.00	0.00
1427194_a_at	0.00	0.00
1427195_at	0.00	0.00
1427196_at	0.00	0.00
1427197_at	0.00	0.00
1427198_at	0.00	0.00
1427199_at	0.00	0.00
1427200_at	0.00	0.00
1427201_at	0.00	0.00
1427202_at	0.00	0.00
1427203_at	0.00	0.00
1427204_at	0.00	0.00
1427205_x_at	0.00	0.00
1427206_at	0.00	0.00
1427207_s_at	0.00	0.00
1427208_at	0.02	0.00
1427209_at	0.00	0.00
1427210_at	0.00	0.00
1427211_at	0.00	0.00
1427212_at	0.00	0.00
1427213_at	0.00	0.00
1427214_at	0.00	0.00
1427215_at	0.00	0.00
1427216_at	0.00	0.00
1427217_at	0.00	0.00
1427218_at	0.00	0.00
1427219_at	0.00	0.00
1427220_a_at	0.00	0.00
1427221_at	0.00	0.00
1427222_a_at	0.00	0.00
1427223_a_at	0.00	0.00
1427224_at	0.00	0.00
1427225_at	0.00	0.00
1427226_at	0.00	0.00
1427227_at	0.00	0.00

1427228_at	0.00	0.00
1427229_at	0.00	0.00
1427230_at	0.00	0.00
1427231_at	0.00	0.00
1427232_at	0.00	0.00
1427233_at	0.00	0.00
1427234_at	0.00	0.00
1427235_at	0.00	0.00
1427236_a_at	0.00	0.00
1427237_at	0.00	0.00
1427238_at	0.83	0.53
1427239_at	0.00	0.00
1427240_at	0.00	0.00
1427241_at	0.00	0.00
1427242_at	0.00	0.00
1427243_at	0.00	0.00
1427244_at	0.00	0.00
1427245_at	0.00	0.00
1427246_at	0.00	0.00
1427247_at	0.00	0.00
1427248_at	0.00	0.00
1427249_x_at	0.00	0.00
1427250_at	0.00	0.00
1427251_at	0.00	0.00
1427252_at	0.00	0.00
1427253_s_at	0.00	0.00
1427254_at	0.00	0.00
1427255_s_at	0.00	0.00
1427256_at	0.00	0.00
1427257_at	0.00	0.00
1427258_at	0.00	0.16
1427259_at	0.00	0.00
1427260_a_at	0.00	0.00
1427261_at	0.00	0.00
1427262_at	0.00	0.00
1427263_at	0.00	0.00
1427264_at	0.00	0.00
1427265_at	0.00	0.00
1427266_at	0.00	0.00
1427267_at	0.00	0.00
1427268_at	0.00	0.00
1427269_at	0.00	0.00
1427270_a_at	0.00	0.00
1427271_at	0.00	0.00
1427272_at	0.00	0.00
1427273_at	0.00	0.00
1427274_at	0.00	0.00
1427275_at	0.00	0.00
1427276_at	0.00	0.00
1427277_at	0.00	0.00
1427278_at	0.00	0.00
1427279_at	0.00	0.00
1427280_at	0.00	0.00
1427281_at	0.00	0.00
1427282_a_at	0.00	0.00
1427283_at	0.00	0.00

1427284_a_at	0.02	0.00
1427285_s_at	0.00	0.00
1427286_at	0.00	0.00
1427287_s_at	0.00	0.00
1427288_at	0.00	0.00
1427289_at	0.00	0.00
1427290_at	0.00	0.00
1427291_at	0.00	0.00
1427292_at	0.00	0.00
1427293_a_at	0.00	0.00
1427294_a_at	0.00	0.00
1427295_at	0.00	0.00
1427296_at	0.00	0.00
1427297_at	0.00	0.00
1427298_at	0.00	0.00
1427299_at	0.00	0.00
1427300_at	0.00	0.00
1427301_at	0.00	0.00
1427302_at	1.00	0.00
1427303_at	0.00	0.00
1427304_at	0.00	0.00
1427305_at	0.00	0.00
1427306_at	0.00	0.00
1427307_a_at	0.00	0.00
1427308_at	0.00	0.00
1427309_at	0.00	0.00
1427310_at	0.00	0.00
1427311_at	0.00	0.00
1427312_at	0.00	0.00
1427313_at	0.00	0.00
1427314_at	0.00	0.00
1427315_at	0.00	0.00
1427316_s_at	0.00	0.00
1427317_at	0.00	0.00
1427318_s_at	0.00	0.00
1427319_at	0.00	0.00
1427320_at	0.00	0.00
1427321_s_at	0.00	0.00
1427322_at	0.00	0.00
1427323_s_at	0.00	0.00
1427324_at	0.00	0.00
1427325_s_at	0.00	0.00
1427326_at	0.00	0.00
1427327_at	0.00	0.00
1427328_a_at	0.00	0.00
1427329_a_at	0.00	0.00
1427330_at	0.00	0.00
1427331_at	0.00	0.00
1427332_at	0.00	0.00
1427333_s_at	0.00	0.00
1427334_s_at	0.00	0.00
1427335_at	0.00	0.00
1427336_at	0.00	0.00
1427337_at	0.00	0.00
1427338_at	0.00	0.00
1427339_at	0.00	0.00

1427340_at	0.00	0.00
1427341_at	0.00	0.00
1427342_at	0.00	0.00
1427343_at	0.00	0.00
1427344_s_at	0.00	0.00
1427345_a_at	0.00	0.00
1427346_at	0.00	0.00
1427347_s_at	0.00	0.00
1427348_at	0.00	0.00
1427349_x_at	0.00	0.00
1427350_a_at	0.00	0.00
1427351_s_at	0.00	0.00
1427352_at	0.00	0.00
1427353_at	0.00	0.00
1427354_at	0.00	0.00
1427355_at	0.00	0.00
1427356_at	0.00	0.00
1427357_at	0.00	0.02
1427358_a_at	0.00	0.00
1427359_at	0.00	0.00
1427360_at	0.00	0.00
1427361_at	0.00	0.00
1427362_x_at	0.00	0.00
1427363_at	0.00	0.00
1427364_a_at	0.00	0.03
1427365_at	0.00	0.00
1427366_at	0.00	0.00
1427367_at	0.00	0.00
1427368_x_at	0.00	0.00
1427369_at	0.00	0.00
1427370_at	0.00	0.00
1427371_at	0.00	0.00
1427372_at	0.00	0.00
1427373_at	0.00	0.00
1427374_at	0.00	0.00
1427375_at	0.00	0.01
1427376_a_at	0.00	0.00
1427377_x_at	0.00	0.00
1427378_at	0.00	0.00
1427379_at	0.00	0.00
1427380_at	0.00	0.00
1427381_at	0.00	0.00
1427382_a_at	0.00	0.00
1427383_at	0.00	0.00
1427384_at	0.00	0.00
1427385_s_at	0.09	0.00
1427386_at	0.00	0.00
1427387_a_at	0.00	0.00
1427388_at	0.00	0.00
1427389_at	0.00	0.00
1427390_at	0.00	0.00
1427391_a_at	0.00	0.00
1427392_at	0.00	0.00
1427393_at	0.00	0.00
1427394_at	0.00	0.00
1427395_a_at	0.00	0.00

1427396_a_at	0.00	0.00
1427397_at	0.00	0.00
1427398_at	0.00	0.00
1427399_a_at	0.00	0.00
1427400_at	0.00	0.00
1427401_at	0.00	0.00
1427402_at	0.00	0.00
1427403_at	0.00	0.00
1427404_x_at	0.00	0.00
1427405_s_at	0.00	0.00
1427406_at	0.00	0.00
1427407_s_at	0.00	0.00
1427408_a_at	0.00	0.00
1427409_at	0.00	0.00
1427410_at	0.00	0.00
1427411_s_at	0.00	0.00
1427412_s_at	0.00	0.00
1427413_a_at	0.00	0.00
1427414_at	0.00	0.00
1427415_at	0.00	0.00
1427416_x_at	0.00	0.00
1427417_at	0.00	0.00
1427418_a_at	0.00	0.00
1427419_x_at	0.00	0.00
1427420_at	0.00	0.00
1427421_at	0.00	0.00
1427422_at	0.00	0.00
1427423_at	0.00	0.00
1427424_at	0.00	0.00
1427425_at	0.00	0.00
1427426_at	0.00	0.00
1427427_at	0.00	0.00
1427428_at	0.00	0.00
1427429_at	0.00	0.00
1427430_at	0.00	0.00
1427431_at	0.00	0.00
1427432_a_at	0.00	0.22
1427433_s_at	0.00	0.00
1427434_at	0.00	0.00
1427435_at	0.00	0.00
1427436_at	0.00	0.00
1427437_at	0.00	0.00
1427438_at	0.00	0.00
1427439_s_at	0.00	0.11
1427440_a_at	0.00	0.00
1427441_a_at	0.00	0.00
1427442_a_at	0.71	1.00
1427443_at	0.00	0.00
1427444_at	0.00	0.00
1427445_a_at	0.00	0.00
1427446_s_at	0.00	0.00
1427447_a_at	0.00	0.00
1427448_at	0.00	0.00
1427449_a_at	0.00	0.00
1427450_x_at	0.00	0.00
1427451_a_at	0.00	0.00

1427452_at	0.00	0.00
1427453_at	0.00	0.00
1427454_at	0.00	0.00
1427455_x_at	0.00	0.00
1427456_at	0.00	0.00
1427457_a_at	0.00	0.00
1427458_at	0.00	0.00
1427459_at	0.00	0.00
1427460_at	0.00	0.00
1427461_at	0.00	0.00
1427462_at	0.00	0.00
1427463_at	0.00	0.00
1427464_s_at	0.00	0.00
1427465_at	0.00	0.00
1427466_at	0.00	0.00
1427467_a_at	0.00	0.00
1427468_at	0.00	0.00
1427469_at	0.00	0.00
1427470_s_at	0.00	0.00
1427471_at	0.00	0.00
1427472_a_at	0.00	0.00
1427473_at	0.00	0.00
1427474_s_at	0.00	0.31
1427475_a_at	0.00	0.00
1427476_a_at	0.00	0.01
1427477_at	0.00	0.00
1427478_at	0.00	0.00
1427479_at	0.01	0.00
1427480_at	0.00	0.00
1427481_a_at	0.05	0.00
1427482_a_at	0.00	0.00
1427483_at	0.03	0.00
1427484_at	0.00	0.00
1427485_at	0.00	0.00
1427486_at	0.00	0.00
1427487_at	0.00	0.00
1427488_a_at	0.00	0.00
1427489_at	0.00	0.00
1427490_at	0.00	0.00
1427491_at	0.00	0.00
1427492_at	0.00	0.00
1427493_at	0.00	0.00
1427494_at	0.00	0.00
1427495_at	0.00	0.00
1427496_at	0.00	0.00
1427497_at	0.00	0.00
1427498_a_at	0.00	0.00
1427499_at	0.00	0.00
1427500_at	0.00	0.00
1427501_at	0.00	0.00
1427502_at	0.00	0.00
1427503_at	0.00	0.00
1427504_s_at	0.00	0.03
1427505_a_at	0.00	0.00
1427506_at	0.00	0.00
1427507_at	0.00	0.00

1427508_at	0.00	0.00
1427509_at	0.00	0.00
1427510_at	0.00	0.00
1427511_at	0.00	0.00
1427512_a_at	0.00	0.00
1427513_at	0.00	0.00
1427514_at	0.00	0.00
1427515_at	0.00	0.00
1427516_a_at	0.00	0.00
1427517_at	0.00	0.00
1427518_at	0.00	0.00
1427519_at	0.00	0.00
1427520_a_at	0.00	0.00
1427521_a_at	0.00	0.00
1427522_at	0.00	0.00
1427523_at	0.00	0.00
1427524_a_at	0.00	0.00
1427525_at	0.00	0.00
1427526_at	0.00	0.00
1427527_a_at	0.00	0.00
1427528_a_at	0.00	0.00
1427529_at	0.00	0.00
1427530_at	0.00	0.00
1427531_a_at	0.00	0.00
1427532_at	0.00	0.00
1427533_at	0.00	0.00
1427534_at	0.00	0.00
1427535_s_at	0.00	0.00
1427536_at	0.00	0.00
1427537_at	0.00	0.00
1427538_at	0.00	0.00
1427539_a_at	0.00	0.00
1427540_at	0.00	0.00
1427541_x_at	0.00	0.00
1427542_at	0.00	0.00
1427543_s_at	0.00	0.00
1427544_a_at	0.00	0.00
1427545_at	0.00	0.00
1427546_at	0.00	0.00
1427547_a_at	0.00	0.00
1427548_a_at	0.00	0.30
1427549_s_at	0.00	0.00
1427550_at	0.06	0.00
1427551_at	0.00	0.00
1427552_a_at	0.00	0.00
1427553_at	0.00	0.00
1427554_at	0.00	0.00
1427555_at	0.00	0.00
1427556_at	0.00	0.00
1427557_at	0.00	0.00
1427558_s_at	0.00	0.00
1427559_a_at	0.00	0.00
1427560_at	0.00	0.00
1427561_a_at	0.00	0.00
1427562_a_at	0.00	0.00
1427563_at	0.00	0.00

1427564_at	0.00	0.00
1427565_a_at	0.00	0.00
1427566_at	0.00	0.00
1427567_a_at	0.00	0.00
1427568_a_at	0.00	0.00
1427569_a_at	0.00	0.00
1427570_at	0.00	0.00
1427571_at	0.00	0.00
1427572_at	0.00	0.00
1427573_at	0.00	0.00
1427574_s_at	0.00	0.00
1427575_at	0.00	0.00
1427576_at	0.00	0.00
1427577_x_at	0.00	0.00
1427578_a_at	0.00	0.52
1427579_at	0.00	0.00
1427580_a_at	0.00	0.00
1427581_at	0.00	0.00
1427582_at	0.00	0.00
1427583_at	0.00	0.00
1427584_at	0.00	0.07
1427585_at	0.00	0.00
1427586_at	0.00	0.00
1427587_at	0.00	0.00
1427588_a_at	0.00	0.00
1427589_at	0.00	0.00
1427590_at	0.00	0.00
1427591_at	0.00	0.00
1427592_at	0.00	0.00
1427593_at	0.00	0.00
1427594_at	0.00	0.00
1427595_at	0.00	0.00
1427596_at	0.00	0.00
1427597_at	0.00	0.00
1427598_at	0.00	0.00
1427599_at	0.00	0.00
1427600_at	0.00	0.00
1427601_at	0.00	0.00
1427602_at	0.00	0.00
1427603_at	0.00	0.00
1427604_a_at	0.00	0.00
1427605_at	0.00	0.00
1427606_at	0.00	0.00
1427607_at	0.00	0.00
1427608_a_at	0.00	0.00
1427609_at	0.00	0.00
1427610_at	0.08	0.00
1427611_at	0.00	0.00
1427612_at	0.00	0.00
1427613_at	0.00	0.00
1427614_at	0.00	0.00
1427615_at	0.00	0.00
1427616_at	0.00	0.00
1427617_at	0.00	0.00
1427618_at	0.00	0.00
1427619_a_at	0.00	0.00

1427620_at	0.00	0.00
1427621_at	0.00	0.00
1427622_at	0.00	0.00
1427623_at	0.00	0.00
1427624_s_at	0.00	0.00
1427625_a_at	0.00	0.00
1427626_at	0.00	0.00
1427627_at	0.00	0.00
1427628_at	0.00	0.00
1427629_at	0.00	0.00
1427630_x_at	0.00	0.00
1427631_x_at	0.00	0.00
1427632_x_at	0.00	0.00
1427633_a_at	0.00	0.00
1427634_at	0.00	0.00
1427635_at	0.00	0.00
1427636_at	0.00	0.00
1427637_a_at	0.00	0.00
1427638_at	0.00	0.00
1427639_a_at	0.00	0.00
1427640_a_at	0.00	0.00
1427641_at	0.00	0.00
1427642_at	0.00	0.00
1427643_at	0.00	0.00
1427644_at	0.00	0.00
1427645_a_at	0.00	0.00
1427646_a_at	0.00	0.00
1427647_at	0.00	0.00
1427648_at	0.00	0.00
1427649_at	0.00	0.00
1427650_a_at	0.00	0.00
1427651_x_at	0.00	0.00
1427652_x_at	0.00	0.00
1427653_at	0.00	0.00
1427654_a_at	0.00	0.00
1427655_a_at	0.00	0.00
1427656_at	0.00	0.00
1427657_at	0.00	0.00
1427658_at	0.00	0.00
1427659_at	0.00	0.00
1427660_x_at	0.00	0.00
1427661_a_at	0.00	0.00
1427662_at	0.00	0.00
1427663_a_at	0.00	0.00
1427664_at	0.00	0.00
1427665_a_at	0.00	0.00
1427666_a_at	0.00	0.00
1427667_s_at	0.00	0.00
1427668_at	0.00	0.00
1427669_a_at	0.00	0.00
1427670_a_at	0.07	0.10
1427671_a_at	0.00	0.00
1427672_a_at	0.00	0.00
1427673_a_at	0.00	0.00
1427674_a_at	0.00	0.00
1427675_at	0.00	0.00

1427676_a_at	0.00	0.00
1427677_a_at	0.00	0.00
1427678_at	0.00	0.00
1427679_at	0.00	0.00
1427680_a_at	0.00	0.00
1427681_s_at	0.00	0.00
1427682_a_at	0.00	0.00
1427683_at	0.00	0.00
1427684_at	0.00	0.00
1427685_a_at	0.00	0.00
1427686_at	0.00	0.00
1427687_at	0.00	0.00
1427688_a_at	0.00	0.00
1427689_a_at	0.00	0.00
1427690_a_at	0.00	0.00
1427691_a_at	0.00	0.00
1427692_a_at	0.00	0.00
1427693_at	0.00	0.00
1427694_at	0.00	0.00
1427695_a_at	0.00	0.00
1427696_at	0.00	0.00
1427697_a_at	0.00	0.00
1427698_at	0.00	0.00
1427699_a_at	0.00	0.00
1427700_x_at	0.00	0.00
1427701_a_at	0.00	0.00
1427702_at	0.00	0.00
1427703_at	0.00	0.00
1427704_a_at	0.00	0.00
1427705_a_at	0.00	0.00
1427706_a_at	0.00	0.00
1427707_a_at	0.00	0.00
1427708_a_at	0.20	0.33
1427709_at	0.00	0.00
1427710_at	0.00	0.00
1427711_a_at	0.00	0.00
1427712_at	0.00	0.00
1427713_x_at	0.00	0.00
1427714_at	0.00	0.00
1427715_a_at	0.00	0.00
1427716_at	0.00	0.00
1427717_at	0.00	0.00
1427718_a_at	0.00	0.00
1427719_s_at	0.00	0.00
1427720_a_at	0.00	0.00
1427721_at	0.00	0.00
1427722_at	0.00	0.00
1427723_at	0.00	0.00
1427724_at	0.00	0.00
1427725_a_at	0.00	0.00
1427726_at	0.00	0.00
1427727_x_at	0.00	0.00
1427728_at	0.00	0.00
1427729_at	0.00	0.00
1427730_a_at	0.00	0.00
1427731_at	0.00	0.00

1427732_s_at	0.00	0.00
1427733_a_at	0.00	0.00
1427734_a_at	0.00	0.00
1427735_a_at	0.81	0.00
1427736_a_at	0.00	0.00
1427737_a_at	0.00	0.00
1427738_at	0.00	0.00
1427739_a_at	0.00	0.39
1427740_a_at	0.00	0.00
1427741_x_at	0.00	0.00
1427742_a_at	0.00	0.34
1427743_at	0.00	0.00
1427744_at	0.00	0.00
1427745_x_at	0.00	0.00
1427746_x_at	0.00	0.00
1427747_a_at	0.00	0.00
1427748_at	0.00	0.00
1427749_at	0.00	0.00
1427750_at	0.00	0.00
1427751_a_at	0.00	0.00
1427752_a_at	0.00	0.00
1427753_at	0.00	0.00
1427754_a_at	0.00	0.00
1427755_at	0.00	0.00
1427756_x_at	0.00	0.00
1427757_at	0.00	0.00
1427758_x_at	0.00	0.00
1427759_a_at	0.00	0.00
1427760_s_at	0.00	0.00
1427761_at	0.00	0.00
1427762_x_at	0.00	0.23
1427763_a_at	0.00	0.00
1427764_a_at	0.00	0.00
1427765_a_at	0.00	0.00
1427766_at	0.00	0.00
1427767_a_at	0.00	0.00
1427768_s_at	0.00	0.00
1427769_x_at	0.00	0.00
1427770_a_at	0.00	0.61
1427771_x_at	0.00	0.00
1427772_at	0.00	0.00
1427773_a_at	0.00	0.00
1427774_at	0.00	0.00
1427775_at	0.00	0.00
1427776_a_at	0.00	0.00
1427777_x_at	0.00	0.00
1427778_at	0.00	0.00
1427779_a_at	0.00	0.00
1427780_at	0.00	0.00
1427781_at	0.00	0.00
1427782_a_at	0.00	0.00
1427783_at	0.00	0.00
1427784_at	0.00	0.00
1427785_x_at	0.00	0.00
1427786_at	0.00	0.00
1427787_at	0.00	0.00

1427788_at	0.00	0.00
1427789_s_at	0.00	0.00
1427790_at	0.00	0.00
1427791_a_at	0.00	0.00
1427792_at	0.00	0.00
1427793_at	0.00	0.00
1427794_at	0.00	0.00
1427795_s_at	0.00	0.00
1427796_at	0.00	0.00
1427797_s_at	0.00	0.00
1427798_x_at	0.00	0.00
1427799_x_at	0.00	0.00
1427800_at	0.00	0.00
1427801_at	0.00	0.00
1427802_a_at	0.00	0.00
1427803_at	0.00	0.00
1427804_at	0.00	0.00
1427805_at	0.00	0.00
1427806_at	0.00	0.00
1427807_at	0.00	0.00
1427808_at	0.00	0.00
1427809_at	0.00	0.00
1427810_at	0.00	0.00
1427811_at	0.00	0.00
1427812_at	0.00	0.00
1427813_at	0.00	0.00
1427814_at	0.00	0.00
1427815_at	0.00	0.00
1427816_at	0.00	0.00
1427817_at	0.00	0.00
1427818_at	0.00	0.00
1427819_at	0.00	0.00
1427820_at	0.00	0.00
1427821_at	0.00	0.00
1427822_a_at	0.00	0.00
1427823_at	0.00	0.00
1427824_at	0.00	0.00
1427825_at	0.00	0.00
1427826_a_at	0.00	0.00
1427827_at	0.00	0.00
1427828_at	0.00	0.00
1427829_at	0.00	0.00
1427830_at	0.00	0.00
1427831_s_at	0.00	0.00
1427832_at	0.00	0.00
1427833_at	0.00	0.00
1427834_at	0.00	0.00
1427835_at	0.00	0.00
1427836_at	0.00	0.00
1427837_at	0.00	0.00
1427838_at	0.00	0.00
1427839_at	0.00	0.00
1427840_at	0.00	0.00
1427841_at	0.00	0.00
1427842_at	0.00	0.00
1427843_at	0.00	0.00

1427844_a_at	0.00	0.00
1427845_at	0.00	0.00
1427846_x_at	0.00	0.00
1427847_at	0.00	0.00
1427848_at	0.00	0.00
1427849_a_at	0.00	0.00
1427850_x_at	0.00	0.00
1427851_x_at	0.00	0.00
1427852_x_at	0.00	0.00
1427853_a_at	0.00	0.00
1427854_x_at	0.00	0.00
1427855_at	0.00	0.00
1427856_a_at	0.00	0.00
1427857_x_at	0.00	0.00
1427858_at	0.00	0.00
1427859_at	0.00	0.00
1427860_at	0.00	0.00
1427861_at	0.00	0.00
1427862_at	0.00	0.00
1427863_at	0.00	0.00
1427864_at	0.00	0.00
1427865_at	0.00	0.00
1427866_x_at	0.00	0.00
1427867_at	0.00	0.00
1427868_x_at	0.00	0.00
1427869_at	0.00	0.00
1427870_x_at	0.00	0.00
1427871_at	0.00	0.00
1427872_at	0.00	0.00
1427873_at	0.00	0.00
1427874_at	0.00	0.01
1427875_a_at	0.00	0.00
1427876_at	0.00	0.00
1427877_at	0.00	0.00
1427878_at	0.00	0.00
1427879_at	0.00	0.00
1427880_at	0.00	0.00
1427881_at	0.00	0.19
1427882_at	0.00	0.21
1427883_a_at	0.00	0.00
1427884_at	0.00	0.00
1427885_at	0.00	0.00
1427886_at	0.00	0.00
1427887_at	0.00	0.00
1427888_a_at	0.00	0.00
1427889_at	0.00	0.00
1427890_a_at	0.00	0.00
1427891_at	0.00	0.00
1427892_at	0.00	0.00
1427893_a_at	0.00	0.00
1427894_at	0.00	0.00
1427895_at	0.00	0.00
1427896_at	0.00	0.00
1427897_s_at	0.00	0.00
1427898_at	0.00	0.00
1427899_at	0.00	0.00

1427900_at	0.00	0.00
1427901_at	0.00	0.31
1427902_at	0.00	0.00
1427903_at	0.00	0.00
1427904_s_at	0.00	0.00
1427905_at	0.00	0.00
1427906_at	0.00	0.00
1427907_at	0.00	0.00
1427908_at	0.00	0.00
1427909_at	0.00	0.00
1427910_at	0.00	0.00
1427911_at	0.00	0.00
1427912_at	0.75	0.00
1427913_at	0.00	0.00
1427914_a_at	0.00	0.00
1427915_s_at	0.00	0.00
1427916_at	0.00	0.00
1427917_s_at	0.00	0.00
1427918_a_at	0.00	0.34
1427919_at	0.00	0.00
1427920_at	0.00	0.00
1427921_s_at	0.00	0.00
1427922_at	0.00	0.00
1427923_at	0.00	0.00
1427924_at	0.00	0.00
1427925_at	0.00	0.00
1427926_at	0.00	0.00
1427927_at	0.00	0.00
1427928_s_at	0.00	0.00
1427929_a_at	0.00	0.00
1427930_at	0.00	0.00
1427931_s_at	0.00	0.00
1427932_s_at	0.00	0.00
1427933_at	0.00	0.00
1427934_at	0.00	0.00
1427935_at	0.00	0.00
1427936_at	0.00	0.00
1427937_at	0.00	0.00
1427938_at	0.00	0.00
1427939_s_at	0.00	0.00
1427940_s_at	0.00	0.00
1427941_at	0.00	0.04
1427942_at	0.00	0.00
1427943_at	0.00	0.00
1427944_at	0.00	0.00
1427945_at	0.00	0.00
1427946_s_at	0.00	0.00
1427947_at	0.00	0.00
1427948_a_at	0.00	0.00
1427949_at	0.00	0.00
1427950_at	0.00	0.00
1427951_s_at	0.00	0.00
1427952_at	0.00	0.00
1427953_at	0.00	0.00
1427954_at	0.00	0.00
1427955_a_at	0.00	0.00

1427956_at	0.00	0.00
1427957_at	0.00	0.00
1427958_at	0.00	0.00
1427959_at	0.00	0.00
1427960_at	0.00	0.00
1427961_s_at	0.00	0.00
1427962_at	0.00	0.00
1427963_s_at	0.00	0.00
1427964_at	0.03	0.23
1427965_at	0.00	0.18
1427966_at	0.00	0.00
1427967_at	0.00	0.00
1427968_at	0.00	0.00
1427969_s_at	0.00	0.00
1427970_at	0.00	0.00
1427971_at	0.00	0.01
1427972_at	0.00	0.00
1427973_s_at	0.00	0.00
1427974_s_at	0.00	0.00
1427975_at	0.00	0.00
1427976_at	0.00	0.00
1427977_x_at	0.00	0.00
1427978_at	0.00	0.00
1427979_at	0.00	0.00
1427980_at	0.00	0.00
1427981_a_at	0.00	0.00
1427982_s_at	0.00	0.00
1427983_at	0.00	0.00
1427984_at	0.00	0.00
1427985_at	0.00	0.00
1427986_a_at	0.00	0.00
1427987_at	0.00	0.00
1427988_s_at	0.00	0.00
1427989_at	0.00	0.00
1427990_at	0.00	0.00
1427991_s_at	0.00	0.00
1427992_a_at	0.00	0.00
1427993_at	0.00	0.00
1427994_at	0.00	0.00
1427995_at	0.00	0.00
1427996_at	0.00	0.00
1427997_at	0.00	0.22
1427998_at	0.00	0.00
1427999_at	0.00	0.00
1428000_at	0.00	0.00
1428001_at	0.00	0.00
1428002_at	0.00	0.00
1428003_s_at	0.00	0.00
1428004_at	0.00	0.00
1428005_at	0.00	0.00
1428006_at	0.00	0.00
1428007_at	0.00	0.00
1428008_at	0.00	0.00
1428009_a_at	0.00	0.00
1428010_at	0.00	0.00
1428011_a_at	0.00	0.00

1428012_at	0.00	0.00
1428013_at	0.00	0.00
1428014_at	0.00	0.00
1428015_at	0.00	0.00
1428016_a_at	0.17	0.14
1428017_at	0.00	0.00
1428018_a_at	0.00	0.00
1428019_at	0.00	0.00
1428020_at	0.00	0.00
1428021_at	0.00	0.00
1428022_at	0.00	0.00
1428023_at	0.00	0.00
1428024_at	0.00	0.00
1428025_s_at	0.00	0.01
1428026_at	0.00	0.00
1428027_at	0.00	0.00
1428028_at	0.00	0.00
1428029_a_at	0.00	0.00
1428030_at	0.00	0.00
1428031_at	0.00	0.00
1428032_at	0.00	0.00
1428033_at	0.00	0.00
1428034_a_at	0.00	0.00
1428035_at	0.00	0.00
1428036_at	0.00	0.00
1428037_at	0.00	0.00
1428038_at	0.00	0.00
1428039_at	0.00	0.00
1428040_at	0.00	0.00
1428041_at	0.00	0.00
1428042_at	0.00	0.00
1428043_a_at	0.00	0.00
1428044_at	0.00	0.00
1428045_a_at	0.00	0.00
1428046_a_at	0.00	0.00
1428047_s_at	0.00	0.00
1428048_at	0.00	0.00
1428049_a_at	0.00	0.00
1428050_a_at	0.00	0.00
1428051_a_at	0.00	0.00
1428052_a_at	0.16	0.52
1428053_at	0.00	0.00
1428054_at	0.00	0.00
1428055_at	0.00	0.00
1428056_at	0.00	0.00
1428057_a_at	0.00	0.00
1428058_at	0.00	0.00
1428059_at	0.00	0.00
1428060_at	0.00	0.00
1428061_at	0.00	0.00
1428062_at	0.00	0.00
1428063_at	0.00	0.00
1428064_at	0.00	0.00
1428065_at	0.00	0.00
1428066_at	0.00	0.00
1428067_at	0.00	0.00

1428068_at	0.00	0.02
1428069_at	0.00	0.00
1428070_at	0.00	0.00
1428071_at	0.00	0.00
1428072_a_at	0.00	0.00
1428073_a_at	0.00	0.00
1428074_at	0.00	0.00
1428075_at	0.00	0.00
1428076_s_at	0.00	0.00
1428077_at	0.00	0.00
1428078_at	0.01	0.00
1428079_at	0.00	0.00
1428080_at	0.00	0.00
1428081_at	0.00	0.00
1428082_at	0.00	0.00
1428083_at	0.00	0.00
1428084_at	0.00	0.33
1428085_at	0.00	0.00
1428086_at	0.00	0.00
1428087_at	0.00	0.00
1428088_at	0.00	0.00
1428089_at	0.00	0.00
1428090_at	0.00	0.83
1428091_at	0.00	0.00
1428092_at	0.00	0.07
1428093_at	0.00	0.00
1428094_at	0.02	0.27
1428095_a_at	0.00	0.00
1428096_at	0.00	0.00
1428097_at	0.00	0.00
1428098_a_at	0.00	0.00
1428099_a_at	0.00	0.32
1428100_at	0.00	0.02
1428101_at	0.00	0.00
1428102_at	0.00	0.00
1428103_at	0.06	0.00
1428104_at	0.00	0.00
1428105_at	0.00	0.00
1428106_at	0.00	0.00
1428107_at	0.12	0.01
1428108_x_at	0.00	0.00
1428109_at	0.00	0.00
1428110_x_at	0.00	0.00
1428111_at	0.00	0.00
1428112_at	0.00	0.00
1428113_at	0.00	0.00
1428114_at	0.00	0.00
1428115_a_at	0.00	0.00
1428116_a_at	0.00	0.00
1428117_x_at	0.00	0.00
1428118_at	0.00	0.00
1428119_a_at	0.00	0.00
1428120_at	0.00	0.00
1428121_at	0.00	0.00
1428122_s_at	0.00	0.00
1428123_at	0.00	0.00

1428125_at	0.00	0.00
1428126_a_at	0.00	0.00
1428127_at	0.00	0.00
1428128_at	0.00	0.00
1428129_at	0.00	0.00
1428130_at	0.00	0.00
1428131_a_at	0.00	0.00
1428132_at	0.00	0.01
1428133_at	0.00	0.00
1428134_at	0.00	0.00
1428135_a_at	0.00	0.00
1428137_at	0.00	0.00
1428138_s_at	0.00	0.00
1428140_at	0.00	0.00
1428141_at	0.00	0.00
1428143_a_at	0.00	0.00
1428144_at	0.00	0.00
1428145_at	0.00	0.00
1428146_s_at	0.00	0.00
1428155_at	0.00	0.00
1428159_s_at	0.00	0.00
1428160_at	0.00	0.00
1428161_a_at	0.00	0.02
1428163_at	0.00	0.00
1428164_at	0.00	0.00
1428165_at	0.00	0.00
1428169_at	0.00	0.00
1428171_at	0.00	0.00
1428172_at	0.00	0.00
1428179_at	0.00	0.00
1428181_at	0.00	0.00
1428182_at	0.00	0.00
1428187_at	0.00	0.00
1428188_at	0.00	0.00
1428189_at	0.00	0.00
1428191_s_at	0.00	0.00
1428193_at	0.00	0.00
1428194_at	0.00	0.00
1428195_at	0.00	0.00
1428196_a_at	0.00	0.00
1428197_at	0.00	0.00
1428200_a_at	0.00	0.00
1428201_at	0.00	0.00
1428209_at	0.00	0.00
1428212_x_at	0.00	0.00
1428213_at	0.00	0.00
1428214_at	0.00	0.00
1428215_x_at	0.00	0.00
1428216_s_at	0.00	0.00
1428217_at	0.00	0.00
1428218_a_at	0.00	0.00
1428224_at	0.00	0.05
1428225_s_at	0.00	0.00
1428226_at	0.00	0.19
1428229_at	0.00	0.00
1428230_at	0.00	0.00

1428235_at	0.00	0.00
1428237_at	0.00	0.00
1428238_at	0.00	0.00
1428241_at	0.00	0.00
1428242_at	0.00	0.00
1428244_at	0.00	0.00
1428245_at	0.00	0.01
1428246_at	0.00	0.00
1428247_at	0.00	0.00
1428248_at	0.01	0.00
1428249_at	0.00	0.00
1428255_at	0.00	0.33
1428257_s_at	0.00	0.00
1428258_at	0.00	0.00
1428262_s_at	0.00	0.00
1428263_a_at	0.00	0.00
1428264_at	0.00	0.20
1428265_at	0.00	0.00
1428266_at	0.00	0.00
1428267_at	0.00	0.00
1428272_at	0.00	0.00
1428277_at	0.00	0.51
1428279_a_at	0.00	0.00
1428280_at	0.00	0.02
1428282_at	0.00	0.00
1428283_at	0.00	0.00
1428286_at	0.00	0.00
1428288_at	0.31	0.13
1428289_at	0.12	0.05
1428294_at	0.00	0.00
1428296_at	0.00	0.26
1428297_at	0.00	0.00
1428299_at	0.00	0.00
1428301_at	0.00	0.57
1428302_at	0.00	0.00
1428303_at	0.00	0.00
1428306_at	0.00	0.00
1428308_at	0.00	0.00
1428309_s_at	0.00	0.00
1428310_at	0.00	0.00
1428311_at	0.00	0.00
1428312_at	0.00	0.00
1428314_at	0.00	0.00
1428315_at	0.00	0.32
1428316_a_at	0.00	0.00
1428317_at	0.00	0.00
1428319_at	0.57	0.00
1428320_at	0.00	0.00
1428322_a_at	0.00	0.33
1428326_s_at	0.00	0.00
1428327_at	0.00	0.00
1428328_at	0.00	0.00
1428329_a_at	0.00	0.00
1428330_at	0.00	0.00
1428331_at	0.00	0.00
1428333_at	0.00	0.00

1428335_a_at	0.00	0.00
1428337_at	0.00	0.00
1428340_s_at	0.00	0.00
1428346_at	0.00	0.00
1428349_s_at	0.00	0.00
1428351_at	0.00	0.00
1428353_at	0.00	0.01
1428354_at	0.00	0.00
1428357_at	0.00	0.00
1428358_at	0.00	0.00
1428359_s_at	0.00	0.00
1428360_x_at	0.00	0.00
1428361_x_at	0.00	0.00
1428362_at	0.00	0.14
1428363_at	0.00	0.00
1428364_at	0.00	0.00
1428365_a_at	0.00	0.00
1428367_at	0.00	0.00
1428368_at	0.00	0.00
1428369_s_at	0.00	0.00
1428372_at	0.00	0.00
1428374_at	0.00	0.00
1428379_at	0.00	0.00
1428380_at	0.00	0.00
1428381_a_at	0.00	0.00
1428388_at	0.00	0.01
1428389_s_at	0.00	0.23
1428390_at	0.00	0.00
1428392_at	0.00	0.00
1428394_at	0.00	0.00
1428401_at	0.00	0.00
1428402_at	0.00	0.00
1428405_at	0.00	0.00
1428406_s_at	0.00	0.04
1428421_a_at	0.00	0.00
1428439_at	0.00	0.00
1428442_at	0.00	0.00
1428443_a_at	0.00	0.00
1428448_a_at	0.00	0.01
1428449_at	0.00	0.00
1428452_at	0.00	0.00
1428453_at	0.00	0.00
1428454_at	0.00	0.00
1428455_at	0.00	0.00
1428456_at	0.00	0.00
1428464_at	0.00	0.00
1428465_at	0.00	0.00
1428467_at	0.00	0.00
1428468_at	0.00	0.00
1428469_a_at	0.00	0.00
1428472_at	0.00	0.00
1428475_at	0.00	0.04
1428476_a_at	0.00	0.00
1428477_at	0.01	0.26
1428483_a_at	0.00	0.00
1428485_at	0.00	0.00

1428488_at	0.00	0.00
1428492_at	0.78	0.00
1428494_a_at	0.00	0.34
1428500_at	0.00	0.00
1428501_at	0.00	0.00
1428502_at	0.00	0.00
1428503_a_at	0.00	0.00
1428505_at	0.00	0.00
1428507_at	0.00	0.00
1428510_at	0.00	0.00
1428511_at	0.00	0.00
1428515_at	0.00	0.00
1428526_at	0.00	0.00
1428528_at	0.00	0.00
1428529_at	0.00	0.29
1428530_x_at	0.00	0.00
1428531_at	0.00	0.00
1428532_at	0.00	0.00
1428534_at	0.00	0.00
1428537_at	0.00	0.00
1428538_s_at	0.00	0.00
1428543_at	0.00	0.62
1428552_at	0.00	0.00
1428554_a_at	0.00	0.44
1428563_at	0.00	0.00
1428570_at	0.00	0.00
1428571_at	0.00	0.00
1428572_at	0.00	0.00
1428573_at	0.00	0.00
1428574_a_at	0.00	0.00
1428575_at	0.00	0.00
1428577_at	0.00	0.00
1428578_s_at	0.00	0.00
1428580_at	0.00	0.00
1428585_at	0.00	0.00
1428586_at	0.00	0.00
1428587_at	0.00	0.00
1428588_a_at	0.00	0.00
1428589_at	0.00	0.00
1428590_at	0.00	0.00
1428591_at	0.00	0.00
1428592_s_at	0.00	0.00
1428608_at	0.00	0.00
1428609_at	0.00	0.00
1428610_at	0.00	0.00
1428611_at	0.00	0.00
1428612_at	0.00	0.00
1428616_at	0.00	0.00
1428619_at	0.00	0.01
1428620_at	0.00	0.00
1428621_a_at	0.00	0.00
1428625_a_at	0.00	0.00
1428626_at	0.00	0.00
1428631_a_at	0.00	0.00
1428635_at	0.00	0.00
1428639_at	0.00	0.00

1428645_at	0.00	0.00
1428648_at	0.00	0.00
1428649_at	0.00	0.00
1428655_at	0.00	0.00
1428657_at	0.00	0.00
1428662_a_at	0.00	0.00
1428664_at	0.00	0.00
1428666_at	0.00	0.26
1428667_at	0.00	0.00
1428669_at	0.00	0.00
1428674_at	0.00	0.00
1428675_at	0.00	0.00
1428679_s_at	0.00	0.00
1428684_at	0.00	0.00
1428689_at	0.00	0.15
1428690_at	0.00	0.00
1428697_at	0.00	0.00
1428698_at	0.00	0.00
1428699_at	0.00	0.00
1428701_at	0.00	0.00
1428706_at	0.41	0.00
1428707_at	0.00	0.00
1428708_x_at	0.00	0.00
1428709_a_at	0.00	0.00
1428710_at	0.00	0.00
1428714_at	0.00	0.00
1428722_at	0.00	0.00
1428728_at	0.00	0.00
1428736_at	0.00	0.00
1428737_s_at	0.00	0.00
1428740_a_at	0.00	0.00
1428741_at	0.00	0.00
1428742_at	0.00	0.00
1428745_a_at	0.00	0.00
1428751_at	0.00	0.00
1428752_at	0.00	0.00
1428753_a_at	0.00	0.00
1428760_at	0.00	0.00
1428761_a_at	0.00	0.00
1428762_at	0.00	0.00
1428766_at	0.00	0.00
1428772_at	0.00	0.24
1428776_at	0.00	0.00
1428780_at	0.00	0.00
1428781_at	0.00	0.00
1428782_a_at	0.00	0.00
1428784_at	0.00	0.00
1428786_at	0.00	0.00
1428787_at	0.00	0.00
1428788_at	0.00	0.00
1428789_at	0.00	0.00
1428790_at	0.00	0.00
1428794_at	0.01	0.00
1428797_at	0.00	0.00
1428798_s_at	0.00	0.00
1428803_at	0.00	0.00

1428810_at	0.00	0.00
1428816_a_at	0.00	0.00
1428819_at	0.00	0.00
1428820_at	0.00	0.00
1428823_at	0.00	0.00
1428835_at	0.00	0.00
1428838_a_at	0.00	0.00
1428842_a_at	0.00	0.00
1428843_at	0.00	0.00
1428844_a_at	0.00	0.62
1428845_at	0.00	0.00
1428847_a_at	0.00	0.00
1428848_a_at	0.00	0.00
1428849_at	0.00	0.00
1428850_x_at	0.00	0.00
1428853_at	1.00	0.00
1428855_at	0.00	0.00
1428856_at	0.00	0.00
1428862_at	0.00	0.00
1428868_a_at	0.00	0.00
1428869_at	0.01	0.59
1428870_at	0.00	0.08
1428871_at	0.00	0.00
1428872_at	0.00	0.00
1428873_a_at	0.00	0.00
1428874_at	0.00	0.00
1428875_at	0.00	0.00
1428878_a_at	0.00	0.00
1428879_at	0.00	0.00
1428880_at	0.00	0.00
1428881_at	0.00	0.00
1428882_at	0.00	0.21
1428888_at	0.00	0.00
1428891_at	0.00	0.13
1428895_at	0.00	0.00
1428904_at	0.00	0.00
1428905_at	0.00	0.00
1428907_at	0.00	0.15
1428908_at	0.06	0.56
1428917_at	0.00	0.00
1428920_at	0.00	0.00
1428922_at	0.00	0.00
1428929_s_at	0.00	0.00
1428931_a_at	0.00	0.00
1428935_at	0.00	0.00
1428942_at	0.00	0.00
1428943_at	0.00	0.00
1428954_at	0.00	0.00
1428955_x_at	0.00	0.00
1428961_a_at	0.00	0.00
1428966_at	0.00	0.00
1428982_at	0.00	0.00
1428983_at	0.00	0.00
1428988_at	0.00	0.00
1428995_at	0.00	0.00
1428997_at	0.00	0.00

1429002_at	0.00	0.00
1429003_at	0.00	0.00
1429005_at	0.00	0.00
1429014_at	0.00	0.00
1429015_at	0.00	0.00
1429016_at	0.00	0.00
1429033_at	0.00	0.00
1429035_at	0.00	0.00
1429038_at	0.00	0.00
1429039_s_at	0.00	0.00
1429040_at	0.00	0.00
1429041_at	0.00	0.00
1429043_at	0.00	0.05
1429052_at	0.00	0.00
1429054_at	0.00	0.00
1429061_at	0.00	0.00
1429062_at	0.00	0.00
1429063_s_at	0.00	0.00
1429076_a_at	0.00	0.00
1429077_x_at	0.00	0.00
1429078_a_at	0.00	0.00
1429080_at	0.00	0.00
1429086_at	0.00	0.00
1429090_at	0.00	0.00
1429103_at	0.00	0.01
1429104_at	0.00	0.00
1429108_at	0.00	0.00
1429109_at	0.00	0.00
1429110_a_at	0.00	0.16
1429115_at	0.00	0.00
1429117_at	0.00	0.00
1429122_a_at	0.00	0.20
1429124_s_at	0.00	0.00
1429126_at	0.00	0.00
1429128_x_at	0.00	0.00
1429137_at	0.00	0.00
1429139_at	0.10	0.00
1429144_at	0.00	0.00
1429150_at	0.00	0.00
1429159_at	0.00	0.00
1429168_at	0.00	0.00
1429170_a_at	0.00	0.00
1429171_a_at	0.00	0.00
1429172_a_at	0.00	0.00
1429173_at	0.00	0.00
1429177_x_at	0.00	0.39
1429183_at	0.02	0.00
1429186_a_at	0.00	0.00
1429193_at	0.00	0.00
1429198_at	0.00	0.00
1429199_s_at	0.00	0.00
1429200_at	0.00	0.00
1429207_at	0.00	0.00
1429208_at	0.00	0.00
1429212_a_at	0.00	0.00
1429219_at	0.00	0.00

1429220_at	0.00	0.00
1429221_at	0.00	0.00
1429222_at	0.00	0.00
1429227_x_at	0.00	0.00
1429238_a_at	0.00	0.00
1429239_a_at	0.00	0.00
1429240_at	0.00	0.14
1429244_at	0.00	0.00
1429246_a_at	0.00	0.00
1429247_at	0.00	0.00
1429252_at	0.00	0.00
1429253_at	0.00	0.00
1429265_a_at	0.00	0.87
1429270_a_at	0.00	0.00
1429280_at	0.00	0.00
1429287_a_at	0.00	0.00
1429288_x_at	0.00	0.00
1429291_at	0.00	0.00
1429292_a_at	0.00	0.00
1429294_at	0.00	0.12
1429295_s_at	0.00	0.42
1429296_at	0.00	0.00
1429301_at	0.00	0.00
1429302_at	0.00	0.00
1429303_at	0.00	0.00
1429317_at	0.00	0.00
1429318_a_at	0.00	0.00
1429319_at	0.00	0.00
1429321_at	0.00	0.00
1429328_at	0.00	0.00
1429339_a_at	0.00	0.00
1429347_at	0.00	0.00
1429349_at	0.00	0.00
1429352_at	0.00	0.00
1429356_s_at	0.00	0.00
1429359_s_at	0.00	0.00
1429360_at	0.00	0.00
1429367_at	0.00	0.00
1429369_at	0.00	0.00
1429370_a_at	0.00	0.00
1429379_at	0.00	0.00
1429381_x_at	0.00	0.00
1429382_at	0.00	0.00
1429388_at	0.73	0.00
1429400_at	0.02	0.33
1429410_at	0.00	0.00
1429411_a_at	0.02	0.00
1429412_at	0.00	0.00
1429427_s_at	0.00	0.00
1429428_at	0.00	0.00
1429439_at	0.00	0.00
1429451_at	0.00	0.00
1429453_a_at	0.00	0.00
1429456_a_at	0.00	0.00
1429457_at	0.00	0.00
1429473_at	0.00	0.00

1429474_at	0.00	0.00
1429483_at	0.00	0.00
1429485_a_at	0.00	0.00
1429490_at	0.00	0.00
1429491_s_at	0.18	0.12
1429492_x_at	0.00	0.00
1429497_s_at	0.00	0.00
1429514_at	0.00	0.00
1429527_a_at	0.00	0.00
1429528_at	0.00	0.00
1429530_a_at	0.15	0.34
1429531_at	0.00	0.00
1429533_at	0.00	0.20
1429534_a_at	0.00	0.04
1429536_at	0.00	0.00
1429541_at	0.00	0.00
1429553_at	0.00	0.00
1429554_at	0.00	0.00
1429555_at	0.00	0.04
1429558_a_at	0.00	0.00
1429560_at	0.00	0.00
1429562_at	0.00	0.00
1429563_x_at	0.00	0.00
1429566_a_at	0.00	0.00
1429568_x_at	0.00	0.00
1429569_a_at	0.00	0.00
1429574_at	0.00	0.00
1429581_at	0.00	0.00
1429582_at	0.00	0.00
1429583_at	0.00	0.00
1429584_at	0.00	0.00
1429585_s_at	0.00	0.00
1429597_at	0.65	0.00
1429615_at	0.00	0.00
1429616_at	0.00	0.00
1429619_a_at	0.00	0.00
1429620_at	0.00	0.00
1429623_at	0.00	0.00
1429626_at	0.00	0.00
1429632_at	0.00	0.00
1429638_at	0.00	0.00
1429643_a_at	0.00	0.00
1429650_at	0.00	0.00
1429654_at	0.05	0.91
1429655_at	0.00	0.00
1429681_a_at	0.00	0.09
1429692_s_at	0.00	0.00
1429707_at	0.00	0.00
1429708_at	0.00	0.00
1429709_at	0.00	0.00
1429710_at	0.00	0.00
1429711_at	0.00	0.00
1429715_at	0.00	0.00
1429721_s_at	0.00	0.00
1429723_at	0.00	0.00
1429730_at	0.00	0.00

1429739_a_at	0.00	0.00
1429745_at	0.00	0.00
1429748_at	0.00	0.00
1429752_x_at	0.00	0.00
1429758_at	0.00	0.09
1429761_at	0.00	0.00
1429763_at	0.00	0.00
1429768_at	0.00	0.00
1429775_a_at	0.00	0.15
1429776_a_at	0.00	0.26
1429777_at	0.00	0.00
1429782_at	0.00	0.00
1429783_at	0.00	0.00
1429786_a_at	0.00	0.00
1429787_x_at	0.00	0.00
1429794_a_at	0.00	0.00
1429800_at	0.00	0.00
1429806_at	0.00	0.00
1429819_at	0.00	0.00
1429830_a_at	0.00	0.21
1429832_at	0.00	0.00
1429835_at	0.00	0.00
1429839_a_at	0.45	0.63
1429845_at	0.00	0.00
1429848_at	0.00	0.00
1429850_x_at	0.00	0.00
1429859_a_at	0.00	0.00
1429866_at	0.00	0.00
1429878_a_at	0.00	0.00
1429884_at	0.00	0.00
1429888_a_at	0.00	0.04
1429894_a_at	0.00	0.00
1429897_a_at	0.22	0.00
1429907_at	0.00	0.00
1429908_at	0.00	0.00
1429921_at	0.00	0.00
1429939_at	0.00	0.00
1429947_a_at	0.00	0.00
1429948_x_at	0.00	0.00
1429956_at	0.00	0.00
1429962_at	0.00	0.00
1429976_at	0.00	0.00
1429979_a_at	0.00	0.00
1429980_x_at	0.00	0.00
1429981_a_at	0.00	0.00
1429982_at	0.00	0.00
1429994_s_at	0.00	0.00
1429999_at	0.00	0.00
1430005_a_at	0.00	0.00
1430007_a_at	0.00	0.00
1430018_at	0.00	0.00
1430019_a_at	0.00	0.00
1430020_x_at	0.00	0.00
1430021_a_at	0.00	0.00
1430022_at	0.00	0.00
1430025_at	0.00	0.00

1430029_a_at	0.00	0.00
1430032_at	0.00	0.00
1430045_at	0.00	0.00
1430053_a_at	0.00	0.00
1430078_a_at	0.00	0.00
1430092_at	0.00	0.00
1430111_a_at	0.00	0.00
1430117_a_at	0.00	0.00
1430123_a_at	0.00	0.00
1430124_x_at	0.00	0.00
1430125_s_at	0.00	0.09
1430127_a_at	0.00	0.00
1430128_a_at	0.11	0.00
1430147_a_at	0.00	0.00
1430153_at	0.00	0.00
1430164_a_at	0.00	0.00
1430167_a_at	0.00	0.00
1430171_at	0.00	0.00
1430172_a_at	0.00	0.00
1430173_x_at	0.00	0.00
1430195_at	0.00	0.00
1430197_a_at	0.00	0.00
1430205_a_at	0.00	0.00
1430219_at	0.00	0.00
1430231_a_at	0.00	0.00
1430233_a_at	0.14	0.00
1430240_a_at	0.00	0.00
1430259_at	0.00	0.00
1430265_at	0.00	0.00
1430271_x_at	0.00	0.00
1430274_a_at	0.00	0.00
1430275_a_at	0.00	0.00
1430278_a_at	0.00	0.00
1430283_s_at	0.00	0.00
1430289_a_at	0.00	0.50
1430290_at	0.00	0.00
1430291_at	0.00	0.00
1430292_a_at	0.00	0.00
1430293_a_at	0.00	0.00
1430295_at	0.00	0.00
1430297_a_at	0.00	0.00
1430306_a_at	0.00	0.00
1430307_a_at	0.00	0.35
1430320_at	0.00	0.00
1430326_s_at	0.00	0.00
1430332_a_at	0.00	0.00
1430335_a_at	0.00	0.00
1430354_x_at	0.00	0.00
1430355_a_at	0.00	0.00
1430371_x_at	0.00	0.00
1430375_a_at	0.00	0.00
1430385_a_at	0.00	0.00
1430388_a_at	0.00	0.00
1430391_a_at	0.00	0.00
1430394_a_at	0.00	0.00
1430406_at	0.00	0.00

1430417_s_at	0.00	0.00
1430419_at	0.00	0.00
1430421_a_at	0.00	0.00
1430427_a_at	0.00	0.00
1430437_a_at	0.00	0.00
1430443_at	0.00	0.00
1430447_a_at	0.00	0.00
1430453_a_at	0.00	0.00
1430454_x_at	0.00	0.00
1430457_at	0.00	0.00
1430463_a_at	0.00	0.00
1430474_a_at	0.00	0.00
1430483_a_at	0.00	0.00
1430486_at	0.00	0.00
1430487_at	0.00	0.00
1430492_at	0.00	0.00
1430500_s_at	0.00	0.00
1430509_at	0.00	0.00
1430512_a_at	0.00	0.00
1430514_a_at	0.00	0.00
1430519_a_at	0.00	0.22
1430522_a_at	0.00	0.00
1430523_s_at	0.00	0.00
1430526_a_at	0.00	0.00
1430527_a_at	0.00	0.00
1430533_a_at	0.00	0.00
1430536_a_at	0.00	0.34
1430542_a_at	0.00	0.29
1430545_at	0.00	0.00
1430549_at	0.00	0.00
1430559_at	0.00	0.00
1430562_at	0.00	0.00
1430571_s_at	0.00	0.26
1430573_s_at	0.00	0.00
1430575_a_at	0.00	0.00
1430576_at	0.00	0.00
1430586_at	0.00	0.00
1430598_at	0.00	0.00
1430599_at	0.00	0.00
1430604_a_at	0.00	0.00
1430610_at	0.00	0.00
1430619_a_at	0.00	0.00
1430634_a_at	0.25	0.14
1430643_at	0.00	0.00
1430656_a_at	0.00	0.00
1430661_at	0.00	0.00
1430668_a_at	0.00	0.00
1430671_a_at	0.00	0.00
1430676_at	0.00	0.00
1430681_at	0.00	0.00
1430692_a_at	0.00	0.00
1430694_at	0.00	0.00
1430698_a_at	0.00	0.00
1430700_a_at	0.00	0.00
1430701_a_at	0.00	0.00
1430708_a_at	0.00	0.00

1430713_s_at	0.00	0.00
1430718_s_at	0.00	0.17
1430727_at	0.00	0.00
1430734_at	0.00	0.00
1430749_at	0.00	0.00
1430769_s_at	0.00	0.00
1430771_a_at	0.00	0.00
1430772_at	0.00	0.00
1430773_a_at	0.00	0.00
1430776_s_at	0.00	0.00
1430777_a_at	0.00	0.00
1430778_a_at	0.00	0.02
1430780_a_at	0.02	0.00
1430798_x_at	0.00	0.31
1430805_s_at	0.00	0.00
1430811_a_at	0.00	0.00
1430818_at	0.00	0.00
1430820_a_at	0.00	0.29
1430824_at	0.00	0.00
1430826_s_at	0.00	0.00
1430827_a_at	0.00	0.00
1430829_s_at	0.00	0.00
1430837_a_at	0.00	0.00
1430838_x_at	0.00	0.00
1430845_at	0.00	0.00
1430848_a_at	0.00	0.00
1430853_a_at	0.00	0.00
1430869_a_at	0.00	0.00
1430875_a_at	0.00	0.18
1430889_a_at	0.00	0.00
1430891_at	0.00	0.00
1430899_at	0.00	0.00
1430902_at	0.00	0.00
1430912_a_at	0.00	0.00
1430956_at	0.00	0.00
1430962_at	0.00	0.00
1430970_a_at	0.00	0.00
1430971_a_at	0.00	0.07
1430972_x_at	0.00	0.00
1430976_a_at	0.00	0.00
1430978_at	0.00	0.28
1430979_a_at	0.00	0.00
1430980_a_at	0.00	0.00
1430982_at	0.00	0.02
1430983_at	0.00	0.00
1430986_at	0.00	0.00
1430989_a_at	0.00	0.00
1430993_at	0.00	0.00
1430994_at	0.00	0.00
1430998_at	0.00	0.00
1430999_a_at	0.00	0.00
1431003_a_at	0.00	0.00
1431006_at	0.00	0.00
1431008_at	0.00	0.00
1431010_a_at	0.00	0.00
1431011_at	0.00	0.00

1431012_a_at	0.00	0.00
1431013_at	0.00	0.00
1431017_at	0.00	0.00
1431020_a_at	0.00	0.00
1431022_at	0.00	0.00
1431024_a_at	0.00	0.00
1431028_a_at	0.00	0.00
1431030_a_at	0.00	0.00
1431031_at	0.00	0.00
1431032_at	0.00	0.00
1431033_x_at	0.00	0.00
1431036_a_at	0.00	0.00
1431037_a_at	0.00	0.00
1431040_at	0.00	0.00
1431048_at	0.00	0.00
1431055_a_at	0.00	0.00
1431056_a_at	0.00	0.00
1431057_a_at	0.00	0.00
1431058_at	0.00	0.00
1431059_x_at	0.00	0.00
1431062_a_at	0.00	0.00
1431070_a_at	0.00	0.00
1431072_a_at	0.00	0.00
1431074_a_at	0.00	0.00
1431075_a_at	0.00	0.00
1431078_at	0.00	0.00
1431081_a_at	0.00	0.00
1431082_a_at	0.00	0.00
1431085_a_at	0.00	0.00
1431086_s_at	0.00	0.00
1431092_at	0.00	0.00
1431099_at	0.00	0.00
1431105_a_at	0.00	0.00
1431107_at	0.00	0.00
1431117_x_at	0.00	0.00
1431119_at	0.00	0.00
1431124_at	0.00	0.00
1431125_a_at	0.00	0.14
1431132_x_at	0.00	0.00
1431135_at	0.00	0.00
1431145_a_at	0.00	0.00
1431162_a_at	0.07	0.27
1431164_at	0.00	0.00
1431166_at	0.00	0.00
1431170_at	0.00	0.00
1431177_a_at	0.00	0.00
1431181_a_at	0.00	0.00
1431182_at	0.00	0.00
1431188_a_at	0.00	0.00
1431191_a_at	0.00	0.00
1431203_at	0.00	0.00
1431208_a_at	0.00	0.00
1431210_at	0.00	0.00
1431213_a_at	0.00	0.00
1431214_at	0.00	0.00
1431219_at	0.00	0.00

1431226_a_at	0.00	0.00
1431227_at	0.00	0.00
1431231_at	0.00	0.00
1431232_a_at	0.00	0.00
1431238_at	0.00	0.00
1431239_at	0.00	0.00
1431240_at	0.00	0.00
1431241_at	0.00	0.00
1431262_at	0.00	0.00
1431270_a_at	0.00	0.00
1431274_a_at	0.00	0.22
1431280_at	0.00	0.00
1431282_at	0.00	0.00
1431284_a_at	0.00	0.00
1431287_at	0.00	0.00
1431292_a_at	0.29	0.00
1431293_a_at	0.00	0.33
1431295_a_at	0.00	0.00
1431299_a_at	0.00	0.00
1431301_at	0.00	0.00
1431302_a_at	0.00	0.00
1431304_a_at	0.00	0.00
1431314_a_at	0.00	0.00
1431320_a_at	0.00	0.00
1431326_a_at	0.00	0.00
1431328_at	0.00	0.00
1431330_at	0.00	0.00
1431331_at	0.00	0.00
1431332_a_at	0.00	0.00
1431335_a_at	0.00	0.00
1431336_at	0.00	0.00
1431337_a_at	0.00	0.00
1431339_a_at	0.00	0.31
1431345_a_at	0.00	0.00
1431349_at	0.00	0.00
1431350_at	0.00	0.00
1431354_a_at	0.00	0.00
1431357_a_at	0.00	0.00
1431359_a_at	0.00	0.14
1431362_a_at	0.00	0.00
1431363_at	0.00	0.00
1431373_at	0.00	0.00
1431375_s_at	0.06	0.00
1431379_a_at	0.00	0.00
1431382_a_at	0.00	0.00
1431385_a_at	0.00	0.00
1431386_s_at	0.00	0.00
1431388_at	0.00	0.00
1431390_a_at	0.00	0.00
1431394_a_at	0.00	0.00
1431395_a_at	0.00	0.00
1431396_at	0.00	0.00
1431400_a_at	0.00	0.00
1431405_a_at	0.00	0.00
1431411_a_at	0.00	0.00
1431415_a_at	0.00	0.00

1431416_a_at	0.14	0.44
1431417_at	0.01	0.01
1431419_at	0.00	0.00
1431420_s_at	0.00	0.00
1431421_x_at	0.00	0.00
1431422_a_at	0.67	0.00
1431423_a_at	0.00	0.22
1431428_a_at	0.00	0.00
1431429_a_at	0.65	0.00
1431431_a_at	0.00	0.00
1431434_at	0.00	0.00
1431435_at	0.00	0.00
1431442_at	0.00	0.00
1431464_a_at	0.00	0.00
1431469_a_at	0.00	0.00
1431475_a_at	0.00	0.00
1431487_at	0.00	0.00
1431505_at	0.00	0.00
1431506_s_at	0.00	0.13
1431507_a_at	0.00	0.00
1431527_at	0.00	0.00
1431530_a_at	0.00	0.00
1431539_at	0.00	0.00
1431541_at	0.00	0.00
1431542_at	0.00	0.00
1431549_at	0.00	0.00
1431553_at	0.00	0.00
1431554_a_at	0.00	0.00
1431569_a_at	0.00	0.00
1431575_at	0.00	0.00
1431591_s_at	0.00	0.00
1431592_a_at	0.00	0.00
1431593_a_at	0.00	0.00
1431594_at	0.00	0.00
1431597_a_at	0.00	0.00
1431598_a_at	0.00	0.00
1431602_a_at	0.00	0.00
1431604_a_at	0.00	0.00
1431606_a_at	0.00	0.00
1431609_a_at	0.00	0.00
1431611_a_at	0.00	0.00
1431613_a_at	0.00	0.00
1431619_a_at	0.00	0.00
1431624_a_at	0.00	0.00
1431630_a_at	0.00	0.00
1431644_a_at	0.00	0.00
1431645_a_at	0.00	0.00
1431646_a_at	0.00	0.00
1431653_at	0.00	0.00
1431655_a_at	0.00	0.00
1431663_a_at	0.00	0.00
1431665_a_at	0.00	0.00
1431668_at	0.00	0.00
1431675_a_at	0.00	0.00
1431676_x_at	0.00	0.00
1431680_a_at	0.00	0.00

1431686_a_at	0.00	0.00
1431687_at	0.00	0.00
1431691_a_at	0.00	0.00
1431692_a_at	0.00	0.00
1431693_a_at	0.00	0.00
1431694_a_at	0.00	0.00
1431697_at	0.00	0.00
1431700_at	0.00	0.00
1431701_a_at	0.00	0.31
1431702_at	0.00	0.00
1431704_a_at	0.00	0.00
1431705_a_at	0.00	0.00
1431706_at	0.00	0.00
1431707_a_at	0.00	0.00
1431711_a_at	0.00	0.00
1431712_a_at	0.01	0.00
1431715_a_at	0.00	0.00
1431716_at	0.00	0.00
1431719_a_at	0.00	0.00
1431721_a_at	0.00	0.00
1431722_a_at	0.00	0.00
1431724_a_at	0.00	0.00
1431725_at	0.00	0.00
1431726_a_at	0.00	0.00
1431731_at	0.00	0.00
1431734_a_at	0.00	0.00
1431737_at	0.00	0.00
1431739_at	0.00	0.00
1431740_at	0.00	0.00
1431741_a_at	0.00	0.00
1431743_a_at	0.00	0.00
1431744_a_at	0.00	0.00
1431745_a_at	0.00	0.00
1431746_a_at	0.00	0.00
1431748_a_at	0.00	0.00
1431749_a_at	0.00	0.00
1431750_at	0.00	0.00
1431752_a_at	0.00	0.00
1431753_x_at	0.00	0.00
1431760_a_at	0.00	0.00
1431761_at	0.00	0.00
1431762_at	0.00	0.00
1431763_a_at	0.00	0.00
1431765_a_at	0.00	0.00
1431766_x_at	0.00	0.00
1431768_a_at	0.01	0.46
1431771_a_at	0.00	0.00
1431772_a_at	0.00	0.00
1431773_at	0.00	0.00
1431774_a_at	0.00	0.00
1431777_a_at	0.01	0.00
1431781_at	0.00	0.00
1431782_s_at	0.00	0.00
1431784_a_at	0.00	0.25
1431791_a_at	0.00	0.21
1431792_a_at	0.00	0.00

1431795_a_at	0.00	0.00
1431800_at	0.00	0.00
1431803_at	0.00	0.00
1431804_a_at	0.00	0.00
1431805_a_at	0.00	0.01
1431808_a_at	0.00	0.00
1431810_a_at	0.00	0.00
1431812_a_at	0.00	0.00
1431815_a_at	0.00	0.00
1431816_at	0.00	0.00
1431821_a_at	0.00	0.00
1431822_a_at	0.00	0.00
1431824_at	0.00	0.00
1431827_a_at	0.00	0.00
1431828_a_at	0.00	0.00
1431829_a_at	0.00	0.32
1431830_at	0.00	0.00
1431832_x_at	0.00	0.00
1431833_a_at	0.00	0.00
1431834_a_at	0.00	0.00
1431843_a_at	0.00	0.00
1431844_at	0.00	0.00
1431848_at	0.00	0.00
1431856_a_at	0.00	0.00
1431857_at	0.00	0.00
1431861_a_at	0.00	0.00
1431862_x_at	0.00	0.00
1431868_at	0.00	0.00
1431875_a_at	0.00	0.00
1431877_a_at	0.00	0.00
1431878_at	0.00	0.00
1431885_a_at	0.00	0.00
1431886_at	0.00	0.00
1431890_a_at	0.00	0.00
1431893_a_at	0.00	0.71
1431900_a_at	0.00	0.00
1431901_a_at	0.00	0.00
1431913_a_at	0.00	0.00
1431914_at	0.00	0.00
1431917_at	0.00	0.00
1431920_a_at	0.00	0.00
1431921_a_at	0.00	0.00
1431926_a_at	0.00	0.00
1431929_a_at	0.00	0.00
1431933_a_at	0.00	0.00
1431936_a_at	0.00	0.00
1431938_a_at	0.00	0.00
1431939_a_at	0.00	0.22
1431942_at	0.00	0.00
1431946_a_at	0.00	0.00
1431947_at	0.00	0.00
1431951_a_at	0.00	0.00
1431960_at	0.00	0.00
1431962_a_at	0.00	0.00
1431973_at	0.00	0.00
1431980_a_at	0.00	0.00

1431981_at	0.00	0.00
1431995_at	0.00	0.00
1432000_a_at	0.00	0.00
1432003_a_at	0.00	0.00
1432004_a_at	0.00	0.00
1432005_at	0.00	0.00
1432006_at	0.00	0.00
1432007_s_at	0.00	0.00
1432012_a_at	0.00	0.00
1432013_a_at	0.00	0.00
1432016_a_at	0.00	0.00
1432018_at	0.00	0.00
1432022_at	0.00	0.00
1432027_a_at	0.00	0.00
1432029_a_at	0.00	0.00
1432032_a_at	0.00	0.00
1432034_at	0.00	0.00
1432042_a_at	0.00	0.00
1432043_at	0.00	0.00
1432050_at	0.00	0.00
1432052_at	0.00	0.00
1432057_a_at	0.00	0.00
1432066_at	0.00	0.00
1432068_a_at	0.00	0.00
1432072_at	0.00	0.00
1432075_a_at	0.00	0.00
1432081_at	0.00	0.00
1432083_a_at	0.00	0.00
1432086_a_at	0.00	0.00
1432091_a_at	0.00	0.00
1432094_a_at	0.00	0.01
1432096_at	0.00	0.00
1432097_a_at	0.00	0.00
1432099_a_at	0.00	0.00
1432103_a_at	0.00	0.00
1432104_a_at	0.00	0.00
1432108_at	0.00	0.00
1432115_a_at	0.00	0.00
1432125_at	0.00	0.00
1432129_a_at	0.00	0.00
1432130_a_at	0.00	0.00
1432136_s_at	0.00	0.00
1432139_at	0.00	0.00
1432143_a_at	0.00	0.00
1432144_a_at	0.00	0.00
1432151_at	0.00	0.00
1432155_at	0.00	0.00
1432156_a_at	0.00	0.00
1432158_a_at	0.00	0.00
1432159_a_at	0.00	0.00
1432160_at	0.00	0.00
1432164_a_at	0.00	0.00
1432167_at	0.00	0.00
1432174_a_at	0.00	0.00
1432176_a_at	0.00	0.00
1432177_a_at	0.00	0.00

1432179_x_at	0.00	0.00
1432181_s_at	0.00	0.00
1432187_at	0.00	0.01
1432188_s_at	0.00	0.00
1432189_a_at	0.00	0.00
1432190_at	0.00	0.00
1432195_s_at	0.00	0.00
1432207_a_at	0.00	0.02
1432211_a_at	0.00	0.00
1432227_at	0.00	0.00
1432235_at	0.00	0.00
1432236_a_at	0.00	0.00
1432249_a_at	0.00	0.00
1432255_at	0.00	0.00
1432263_a_at	0.00	0.00
1432264_x_at	0.00	0.02
1432269_a_at	0.00	0.00
1432270_a_at	0.00	0.00
1432271_a_at	0.00	0.17
1432272_a_at	0.00	0.00
1432273_a_at	0.00	0.00
1432275_at	0.00	0.00
1432281_a_at	0.00	0.00
1432282_a_at	0.00	0.00
1432287_a_at	0.00	0.00
1432295_a_at	0.00	0.00
1432296_a_at	0.00	0.00
1432301_a_at	0.00	0.00
1432312_a_at	0.00	0.00
1432322_at	0.00	0.00
1432329_a_at	0.00	0.00
1432331_a_at	0.00	0.00
1432332_a_at	0.00	0.01
1432344_a_at	0.00	0.00
1432346_a_at	0.00	0.00
1432349_a_at	0.00	0.00
1432350_at	0.00	0.00
1432360_a_at	0.00	0.00
1432367_a_at	0.00	0.02
1432372_a_at	0.00	0.18
1432377_x_at	0.00	0.00
1432381_a_at	0.00	0.00
1432384_a_at	0.00	0.00
1432385_a_at	0.00	0.00
1432392_at	0.00	0.00
1432394_a_at	0.00	0.03
1432399_a_at	0.00	0.00
1432400_at	0.00	0.00
1432405_a_at	0.00	0.00
1432408_a_at	0.00	0.00
1432410_a_at	0.00	0.00
1432411_a_at	0.00	0.00
1432414_at	0.00	0.00
1432415_at	0.00	0.00
1432416_a_at	0.00	0.00
1432417_a_at	0.00	0.00

1432418_a_at	0.00	0.00
1432419_a_at	0.00	0.00
1432426_a_at	0.00	0.00
1432430_a_at	0.00	0.00
1432431_s_at	0.00	0.00
1432432_a_at	0.00	0.00
1432435_s_at	0.00	0.00
1432436_a_at	0.00	0.00
1432444_a_at	0.00	0.00
1432453_a_at	0.00	0.00
1432459_a_at	0.00	0.00
1432462_a_at	0.00	0.00
1432466_a_at	0.09	0.03
1432474_a_at	0.00	0.00
1432478_a_at	0.00	0.00
1432479_at	0.00	0.00
1432480_a_at	0.00	0.00
1432486_a_at	0.00	0.00
1432488_a_at	0.00	0.49
1432490_a_at	0.00	0.00
1432491_at	0.00	0.00
1432492_a_at	0.00	0.00
1432499_a_at	0.00	0.00
1432503_a_at	0.00	0.00
1432517_a_at	0.00	0.00
1432526_a_at	0.00	0.00
1432533_a_at	0.00	0.00
1432538_a_at	0.00	0.00
1432539_a_at	0.00	0.01
1432543_a_at	0.00	0.00
1432558_a_at	0.00	0.00
1432622_a_at	0.00	0.00
1432623_at	0.00	0.00
1432628_at	0.00	0.00
1432634_at	0.00	0.00
1432635_a_at	0.00	0.00
1432646_a_at	0.00	0.00
1432647_at	0.00	0.00
1432657_at	0.00	0.00
1432675_at	0.00	0.00
1432763_at	0.00	0.00
1432820_at	0.00	0.00
1432821_at	0.00	0.00
1432826_a_at	0.00	0.00
1432827_x_at	0.00	0.00
1432828_at	0.00	0.00
1432829_at	0.00	0.00
1432834_at	0.00	0.00
1432835_at	0.00	0.00
1432845_at	0.00	0.00
1432848_a_at	0.00	0.00
1432849_at	0.00	0.00
1432862_at	0.00	0.00
1432863_a_at	0.00	0.00
1433029_at	0.00	0.00
1433052_at	0.00	0.00

1433139_at	0.00	0.00
1433140_a_at	0.01	0.00
1433144_at	0.00	0.00
1433156_at	0.00	0.00
1433210_at	0.00	0.00
1433211_at	0.00	0.00
1433407_at	0.00	0.15
1433408_a_at	0.00	0.14
1433428_x_at	0.00	0.00
1433429_at	0.00	0.00
1433430_s_at	0.00	0.00
1433431_at	0.00	0.00
1433432_x_at	0.00	0.00
1433433_at	0.00	0.00
1433436_s_at	0.00	0.00
1433437_at	0.00	0.00
1433438_x_at	0.00	0.00
1433439_at	0.00	0.00
1433440_x_at	0.00	0.40
1433442_at	0.01	0.00
1433443_a_at	0.00	0.00
1433444_at	0.00	0.00
1433445_x_at	0.00	0.00
1433446_at	0.00	0.00
1433447_x_at	0.00	0.00
1433448_at	0.00	0.00
1433450_at	0.00	0.00
1433451_at	0.00	0.00
1433457_s_at	0.00	0.00
1433459_x_at	0.00	0.00
1433460_at	0.00	0.00
1433461_at	0.00	0.00
1433462_a_at	0.00	0.00
1433463_at	0.00	0.00
1433467_at	0.00	0.00
1433470_a_at	0.00	0.25
1433471_at	0.00	0.14
1433472_x_at	0.00	0.00
1433473_x_at	0.00	0.00
1433474_at	0.00	0.00
1433475_a_at	0.00	0.00
1433476_at	0.00	0.00
1433477_at	0.00	0.00
1433478_at	0.00	0.36
1433479_at	0.00	0.00
1433480_at	0.00	0.00
1433482_a_at	0.00	0.01
1433483_s_at	0.00	0.00
1433485_x_at	0.00	0.00
1433486_at	0.00	0.00
1433487_at	0.00	0.00
1433488_x_at	0.01	0.32
1433489_s_at	0.00	0.01
1433490_s_at	0.00	0.00
1433491_at	0.00	0.00
1433492_at	0.00	0.00

1433493_at	0.00	0.00
1433494_at	0.00	0.00
1433495_at	0.00	0.00
1433496_at	0.00	0.00
1433497_at	0.00	0.04
1433502_s_at	0.00	0.23
1433504_at	0.00	0.25
1433507_a_at	0.02	0.00
1433508_at	0.00	0.26
1433509_s_at	0.07	0.00
1433510_x_at	0.00	0.00
1433511_at	0.00	0.00
1433512_at	0.00	0.00
1433513_x_at	0.00	0.00
1433514_at	0.00	0.00
1433515_s_at	0.00	0.00
1433516_a_at	0.00	0.00
1433517_at	0.00	0.00
1433518_at	0.00	0.00
1433519_at	0.00	0.00
1433520_at	0.00	0.00
1433521_at	0.00	0.00
1433527_at	0.00	0.00
1433530_at	0.00	0.00
1433531_at	0.06	0.00
1433532_a_at	0.00	0.00
1433533_x_at	0.00	0.00
1433534_a_at	0.00	0.00
1433535_x_at	0.00	0.00
1433538_at	0.00	0.00
1433540_x_at	0.00	0.00
1433541_a_at	0.00	0.00
1433543_at	0.00	0.00
1433545_s_at	0.00	0.00
1433546_at	0.00	0.33
1433548_at	0.00	0.00
1433549_x_at	0.00	0.00
1433550_at	0.00	0.00
1433552_a_at	0.00	0.53
1433554_at	0.00	0.00
1433558_at	0.00	0.00
1433562_s_at	0.00	0.00
1433563_s_at	0.00	0.00
1433565_at	0.00	0.00
1433568_at	0.00	0.00
1433569_x_at	0.00	0.01
1433570_s_at	0.00	0.00
1433572_a_at	0.00	0.00
1433573_x_at	0.00	0.00
1433574_at	0.00	0.00
1433575_at	0.00	0.00
1433576_at	0.00	0.01
1433579_at	0.00	0.00
1433580_at	0.00	0.12
1433581_at	0.00	0.00
1433582_at	0.00	0.00

1433584_at	0.00	0.00
1433585_at	0.00	0.00
1433588_at	0.00	0.00
1433589_at	0.00	0.00
1433591_at	0.00	0.00
1433593_at	0.00	0.00
1433594_at	0.00	0.00
1433595_at	0.00	0.00
1433597_at	0.00	0.87
1433598_at	0.00	0.00
1433603_at	0.00	0.00
1433604_x_at	0.00	0.01
1433605_at	0.00	0.00
1433606_at	0.00	0.00
1433609_s_at	0.00	0.00
1433611_s_at	0.00	0.00
1433612_at	0.00	0.00
1433613_at	0.00	0.00
1433616_a_at	0.00	0.00
1433618_at	0.00	0.00
1433627_at	0.00	0.00
1433628_at	0.00	0.00
1433629_s_at	0.00	0.00
1433631_at	0.00	0.00
1433636_at	0.00	0.00
1433639_at	0.00	0.00
1433640_at	0.00	0.01
1433645_at	0.00	0.00
1433654_at	0.00	0.00
1433655_at	0.00	0.00
1433656_a_at	0.00	0.18
1433658_x_at	0.00	0.00
1433659_at	0.00	0.00
1433660_at	0.00	0.00
1433661_at	0.00	0.00
1433662_s_at	0.00	0.00
1433663_s_at	0.00	0.00
1433665_at	0.00	0.00
1433666_s_at	0.00	0.00
1433668_at	0.00	0.07
1433669_at	0.00	0.00
1433670_at	0.00	0.00
1433671_at	0.00	0.00
1433674_a_at	0.00	0.00
1433675_at	0.00	0.00
1433676_at	0.00	0.00
1433678_at	0.00	0.00
1433680_x_at	0.00	0.00
1433683_at	0.00	0.00
1433684_at	0.00	0.00
1433685_a_at	0.00	0.00
1433688_x_at	0.00	0.00
1433689_s_at	0.00	0.00
1433690_at	0.00	0.00
1433691_at	0.00	0.00
1433693_x_at	0.00	0.00

1433698_a_at	0.00	0.00
1433702_at	0.00	0.00
1433704_s_at	0.00	0.00
1433705_at	0.00	0.00
1433706_a_at	0.00	0.00
1433708_at	0.00	0.00
1433709_at	0.00	0.00
1433711_s_at	0.00	0.00
1433717_at	0.00	0.00
1433718_a_at	0.11	0.04
1433720_s_at	1.00	0.00
1433721_x_at	0.00	0.00
1433723_s_at	0.00	0.00
1433724_at	0.00	0.00
1433725_at	0.00	0.00
1433729_x_at	0.00	0.00
1433733_a_at	0.00	0.00
1433736_at	0.00	0.00
1433737_at	0.00	0.00
1433741_at	0.00	0.00
1433745_at	0.00	0.02
1433750_at	0.00	0.32
1433753_x_at	0.00	0.00
1433756_at	0.00	0.00
1433757_a_at	0.00	0.00
1433758_at	0.00	0.00
1433760_a_at	0.00	0.00
1433765_at	0.00	0.00
1433773_at	0.00	0.00
1433774_x_at	0.00	0.00
1433775_at	0.00	0.00
1433780_at	0.00	0.00
1433783_at	0.00	0.00
1433784_at	0.00	0.00
1433785_at	0.00	0.00
1433786_x_at	0.00	0.00
1433797_at	0.00	0.00
1433798_a_at	0.00	0.00
1433799_at	0.00	0.00
1433800_a_at	0.00	0.00
1433803_at	0.00	0.00
1433804_at	0.04	0.00
1433805_at	0.00	0.00
1433806_x_at	0.00	0.00
1433808_at	0.00	0.23
1433809_at	0.00	0.00
1433810_x_at	0.00	0.00
1433816_at	0.00	0.00
1433822_x_at	0.00	0.00
1433824_x_at	0.00	0.00
1433829_a_at	0.00	0.83
1433830_at	0.00	0.00
1433832_at	0.00	0.00
1433834_at	0.00	0.00
1433835_at	0.00	0.00
1433842_at	0.01	0.00

1433843_at	0.00	0.01
1433844_a_at	0.00	0.00
1433845_x_at	0.00	0.00
1433850_at	0.00	0.00
1433851_at	0.00	0.00
1433855_at	0.00	0.00
1433859_at	0.00	0.00
1433860_at	0.00	0.00
1433863_at	0.00	0.00
1433866_x_at	0.00	0.00
1433867_at	0.00	0.00
1433869_at	0.00	0.00
1433871_at	0.00	0.00
1433878_at	0.00	0.22
1433880_at	0.00	0.22
1433881_at	0.00	0.00
1433883_at	0.95	0.06
1433886_at	0.00	0.00
1433887_at	0.00	0.00
1433888_at	0.00	0.00
1433890_a_at	0.00	0.00
1433892_at	0.00	0.00
1433893_s_at	0.00	0.00
1433899_x_at	0.00	0.00
1433901_at	0.00	0.00
1433903_at	0.00	0.07
1433904_at	0.00	0.00
1433906_at	0.00	0.00
1433908_a_at	0.00	0.00
1433909_at	0.00	0.00
1433910_at	0.00	0.00
1433912_at	0.00	0.00
1433913_at	0.00	0.00
1433916_at	0.00	0.05
1433918_at	0.00	0.00
1433919_at	0.00	0.00
1433923_at	0.00	0.00
1433924_at	1.00	0.00
1433928_a_at	0.00	0.00
1433935_at	0.00	0.12
1433936_at	0.00	0.00
1433940_at	0.00	0.00
1433941_at	0.00	0.00
1433942_at	0.00	0.26
1433946_at	0.00	0.00
1433948_at	0.00	0.00
1433949_x_at	0.00	0.00
1433951_at	0.00	0.00
1433952_at	0.00	0.14
1433953_at	0.00	0.00
1433954_at	0.00	0.00
1433956_at	0.00	0.00
1433958_at	0.00	0.00
1433961_at	0.00	0.00
1433963_a_at	0.00	0.00
1433964_s_at	0.00	0.00

1433966_x_at	0.00	0.00
1433984_a_at	0.00	0.00
1433991_x_at	0.00	0.00
1433995_s_at	0.00	0.00
1433999_at	0.00	0.00
1434000_at	0.00	0.00
1434003_a_at	0.00	0.00
1434004_at	0.00	0.00
1434005_at	0.27	0.26
1434009_at	0.00	0.00
1434010_at	0.00	0.00
1434011_a_at	0.00	0.16
1434012_at	0.00	0.00
1434016_at	0.00	0.00
1434017_at	0.00	0.00
1434019_at	0.00	0.01
1434020_at	0.00	0.00
1434025_at	0.00	0.12
1434027_at	0.00	0.00
1434032_at	0.00	0.00
1434033_at	0.00	0.00
1434034_at	0.00	0.00
1434035_at	0.00	0.00
1434036_at	0.00	0.05
1434037_s_at	0.07	0.32
1434038_at	0.00	0.06
1434043_a_at	0.00	0.00
1434044_at	0.00	0.00
1434045_at	0.00	0.00
1434047_x_at	0.00	0.55
1434048_at	0.00	0.00
1434053_x_at	0.00	0.27
1434054_at	0.00	0.00
1434056_a_at	0.00	0.00
1434057_at	0.00	0.00
1434059_at	0.00	0.00
1434060_at	0.00	0.00
1434062_at	0.00	0.00
1434066_at	0.00	0.00
1434071_a_at	0.00	0.00
1434074_x_at	0.00	0.00
1434078_at	0.00	0.00
1434079_s_at	0.00	0.00
1434080_at	0.00	0.00
1434087_at	0.00	0.00
1434089_at	0.00	0.00
1434092_at	0.00	0.00
1434099_at	0.00	0.00
1434100_x_at	0.00	0.00
1434103_at	0.00	0.00
1434104_at	0.00	0.00
1434108_at	0.00	0.00
1434109_at	0.00	0.00
1434113_a_at	0.00	0.15
1434114_at	0.00	0.00
1434116_at	0.00	0.00

1434117_at	0.00	0.00
1434118_at	0.00	0.00
1434119_at	0.00	0.00
1434120_a_at	0.00	0.15
1434121_at	0.00	0.00
1434124_x_at	0.00	0.00
1434127_a_at	0.00	0.00
1434128_a_at	0.00	0.00
1434131_at	0.00	0.00
1434133_s_at	0.00	0.00
1434134_at	0.00	0.00
1434135_at	0.00	0.00
1434137_x_at	0.00	0.00
1434138_at	0.00	0.00
1434143_at	0.00	0.00
1434145_s_at	0.00	0.00
1434147_at	0.00	0.00
1434148_at	0.00	0.00
1434149_at	0.00	0.00
1434150_a_at	0.00	0.00
1434151_at	0.00	0.00
1434155_a_at	0.00	0.00
1434162_at	0.00	0.00
1434163_at	0.00	0.00
1434164_s_at	0.00	0.00
1434167_at	0.00	0.00
1434175_s_at	0.00	0.00
1434176_x_at	0.00	0.00
1434177_at	0.00	0.00
1434180_at	0.00	0.00
1434181_at	0.00	0.00
1434184_s_at	0.00	0.00
1434190_at	0.00	0.00
1434195_at	0.00	0.00
1434196_at	0.00	0.00
1434198_at	0.00	0.00
1434199_at	0.00	0.00
1434204_x_at	0.00	0.05
1434205_at	0.00	0.00
1434206_s_at	0.00	0.10
1434210_s_at	0.00	0.00
1434212_at	0.00	0.00
1434213_x_at	0.00	0.00
1434214_at	0.00	0.00
1434215_at	0.00	0.00
1434216_a_at	0.00	0.01
1434224_at	0.00	0.00
1434227_at	0.00	0.00
1434229_a_at	0.00	0.00
1434230_at	0.00	0.00
1434231_x_at	0.00	0.00
1434235_at	0.00	0.00
1434237_at	0.00	0.00
1434239_at	0.00	0.29
1434243_s_at	0.00	0.00
1434244_x_at	0.00	0.00

1434250_at	0.00	0.00
1434251_at	0.00	0.00
1434254_at	0.00	0.00
1434256_s_at	0.00	0.00
1434261_at	0.00	0.21
1434262_at	0.00	0.00
1434271_at	0.00	0.00
1434272_at	0.00	0.00
1434273_at	0.00	0.00
1434276_x_at	0.00	0.00
1434278_at	0.00	0.00
1434279_at	0.00	0.00
1434280_at	0.00	0.00
1434281_at	0.00	0.00
1434282_at	0.00	0.00
1434287_at	0.00	0.00
1434291_a_at	0.00	0.00
1434292_at	0.00	0.00
1434299_x_at	0.00	0.00
1434300_at	0.00	0.00
1434305_at	0.00	0.00
1434306_at	0.00	0.00
1434309_at	0.00	0.06
1434311_at	0.00	0.00
1434312_at	0.00	0.00
1434317_s_at	0.00	0.37
1434319_at	0.00	0.00
1434320_at	0.00	0.00
1434323_at	0.00	0.00
1434324_x_at	0.00	0.00
1434325_x_at	0.00	0.00
1434326_x_at	0.00	0.00
1434328_at	0.00	0.01
1434329_s_at	0.00	0.00
1434330_at	0.00	0.00
1434332_at	0.00	0.07
1434333_a_at	0.00	0.00
1434334_at	0.00	0.00
1434335_at	0.00	0.00
1434336_s_at	0.00	0.00
1434340_at	0.00	0.00
1434341_x_at	0.00	0.00
1434342_at	0.00	0.00
1434343_at	0.00	0.00
1434345_at	0.00	0.00
1434348_at	0.00	0.00
1434353_at	0.00	0.00
1434356_a_at	0.00	0.31
1434357_a_at	0.00	0.06
1434358_x_at	0.00	0.00
1434363_x_at	0.00	0.00
1434364_at	0.00	0.00
1434366_x_at	0.00	0.00
1434367_s_at	0.00	0.00
1434369_a_at	0.00	0.00
1434370_s_at	0.00	0.00

1434372_at	0.00	0.00
1434377_x_at	0.00	0.00
1434378_a_at	0.00	0.00
1434379_at	0.00	0.00
1434380_at	0.00	0.00
1434386_at	0.00	0.00
1434390_at	0.00	0.00
1434391_at	0.00	0.00
1434392_at	0.00	0.00
1434393_at	0.00	0.00
1434396_a_at	0.00	0.00
1434400_at	0.00	0.00
1434402_at	0.00	0.00
1434403_at	0.00	0.00
1434410_at	0.00	0.00
1434412_x_at	0.00	0.00
1434416_a_at	0.00	0.00
1434417_at	0.00	0.00
1434419_s_at	0.00	0.00
1434420_x_at	0.00	0.00
1434425_at	0.00	0.00
1434427_a_at	0.00	0.00
1434432_at	0.00	0.00
1434433_x_at	0.00	0.00
1434434_s_at	0.00	0.12
1434435_s_at	0.00	0.00
1434436_at	0.09	0.00
1434437_x_at	0.00	0.26
1434438_at	0.00	0.00
1434440_at	0.00	0.00
1434442_at	0.15	0.00
1434445_at	0.00	0.00
1434449_at	0.00	0.00
1434453_at	0.00	0.00
1434465_x_at	0.00	0.00
1434466_at	0.00	0.00
1434467_at	0.00	0.00
1434468_at	0.00	0.00
1434469_at	0.00	0.00
1434471_at	0.00	0.00
1434472_at	0.00	0.01
1434479_at	0.02	0.00
1434481_at	0.00	0.00
1434483_at	0.00	0.00
1434484_at	0.00	0.00
1434485_a_at	0.00	0.00
1434486_x_at	0.00	0.00
1434487_at	0.00	0.24
1434489_at	0.00	0.00
1434491_a_at	0.00	0.00
1434496_at	0.00	0.00
1434499_a_at	0.00	0.00
1434500_at	0.00	0.00
1434502_x_at	0.00	0.00
1434503_s_at	0.00	0.19
1434507_at	0.00	0.00

1434510_at	0.00	0.00
1434511_at	0.00	0.00
1434512_x_at	0.00	0.00
1434513_at	0.00	0.00
1434514_at	0.00	0.00
1434515_at	0.00	0.00
1434516_at	0.00	0.00
1434517_at	0.00	0.00
1434518_at	0.00	0.00
1434523_x_at	0.00	0.00
1434528_at	0.00	0.00
1434529_x_at	0.00	0.00
1434533_x_at	0.00	0.00
1434537_at	0.00	0.00
1434538_x_at	0.00	0.05
1434540_a_at	0.00	0.04
1434541_x_at	0.00	0.00
1434542_at	0.00	0.00
1434543_a_at	0.00	0.04
1434544_at	0.00	0.04
1434545_x_at	0.00	0.01
1434546_at	0.00	0.00
1434547_at	0.00	0.01
1434549_at	0.00	0.02
1434551_at	0.00	0.00
1434552_at	0.00	0.01
1434553_at	0.00	0.00
1434554_at	0.00	0.00
1434555_at	0.00	0.00
1434557_at	0.08	0.00
1434560_at	0.00	0.00
1434561_at	0.00	0.00
1434563_at	0.00	0.00
1434569_at	0.00	0.00
1434575_at	0.00	0.00
1434578_x_at	0.00	0.05
1434579_x_at	0.00	0.00
1434585_at	0.00	0.00
1434586_a_at	0.00	0.00
1434587_x_at	0.00	0.00
1434588_x_at	0.00	0.00
1434589_x_at	0.00	0.00
1434596_at	0.00	0.00
1434597_at	0.00	0.00
1434598_at	0.00	0.00
1434599_a_at	0.00	0.00
1434600_at	0.00	0.00
1434604_at	0.00	0.00
1434605_at	0.00	0.00
1434606_at	0.00	0.00
1434607_at	0.00	0.00
1434608_at	0.00	0.00
1434609_at	0.00	0.00
1434610_at	0.00	0.00
1434611_at	0.00	0.00
1434612_s_at	0.00	0.00

1434614_at	0.00	0.00
1434615_x_at	0.00	0.00
1434616_at	0.00	0.00
1434617_x_at	0.00	0.00
1434618_at	0.00	0.00
1434620_s_at	0.00	0.00
1434623_at	0.00	0.00
1434624_x_at	0.00	0.00
1434625_at	0.00	0.00
1434627_at	0.00	0.00
1434628_a_at	0.00	0.02
1434633_at	0.00	0.00
1434637_x_at	0.00	0.00
1434641_x_at	0.00	0.00
1434642_at	0.00	0.00
1434643_at	0.00	0.00
1434644_at	0.00	0.00
1434646_s_at	0.00	0.00
1434648_a_at	0.00	0.00
1434649_at	0.00	0.00
1434651_a_at	0.00	0.17
1434653_at	0.00	0.00
1434658_at	0.00	0.00
1434659_at	0.00	0.00
1434661_at	0.00	0.00
1434674_at	0.00	0.00
1434676_at	0.00	0.00
1434679_at	0.00	0.00
1434681_at	0.63	0.00
1434688_x_at	0.00	0.00
1434690_at	0.00	0.00
1434691_at	0.00	0.00
1434692_at	0.00	0.00
1434694_at	0.00	0.00
1434695_at	0.00	0.00
1434697_at	0.00	0.00
1434698_at	0.00	0.00
1434702_at	0.00	0.00
1434703_at	0.00	0.00
1434704_at	0.00	0.00
1434705_at	0.03	0.50
1434709_at	0.00	0.00
1434714_at	0.00	0.00
1434719_at	0.00	0.00
1434731_x_at	0.00	0.00
1434732_x_at	0.00	0.00
1434735_at	0.00	0.00
1434736_at	0.00	0.00
1434739_at	0.00	0.00
1434743_x_at	0.00	0.00
1434744_at	0.00	0.01
1434745_at	0.00	0.00
1434747_at	0.00	0.00
1434748_at	0.00	0.00
1434751_at	0.00	0.00
1434752_at	0.00	0.00

1434755_at	0.00	0.00
1434757_at	0.07	0.01
1434767_at	0.00	0.00
1434768_at	0.00	0.00
1434772_at	0.00	0.00
1434773_a_at	0.00	0.00
1434775_at	0.00	0.00
1434776_at	0.00	0.00
1434777_at	0.00	0.00
1434783_at	0.00	0.03
1434784_s_at	0.00	0.30
1434785_at	0.00	0.00
1434787_at	0.00	0.00
1434790_a_at	0.00	0.00
1434791_at	0.00	0.00
1434793_at	0.00	0.00
1434796_at	0.00	0.00
1434799_x_at	0.00	0.01
1434801_x_at	0.00	0.08
1434803_a_at	0.00	0.00
1434805_at	0.00	0.00
1434813_x_at	0.00	0.00
1434814_x_at	0.00	0.00
1434820_s_at	0.00	0.00
1434821_at	0.00	0.00
1434823_x_at	0.00	0.00
1434824_at	0.00	0.00
1434827_at	0.00	0.00
1434831_a_at	0.00	0.00
1434832_at	0.00	0.00
1434836_at	0.00	0.00
1434839_s_at	0.00	0.00
1434840_at	0.00	0.00
1434842_s_at	0.01	0.03
1434844_at	0.00	0.00
1434846_at	0.00	0.00
1434850_at	0.00	0.00
1434851_s_at	0.00	0.61
1434852_at	0.00	0.00
1434853_x_at	0.88	0.00
1434854_a_at	0.00	0.00
1434856_at	0.00	0.00
1434859_at	0.00	0.00
1434865_a_at	0.00	0.00
1434866_x_at	0.00	0.00
1434868_at	0.00	0.00
1434871_at	0.00	0.00
1434872_x_at	0.00	0.00
1434875_a_at	0.00	0.00
1434877_at	0.00	0.00
1434879_at	0.00	0.00
1434881_s_at	0.00	0.00
1434882_at	0.00	0.06
1434883_at	0.00	0.00
1434884_at	0.00	0.00
1434886_at	0.00	0.00

1434888_a_at	0.00	0.00
1434889_at	0.00	0.00
1434892_x_at	0.00	0.00
1434893_at	0.00	0.00
1434896_at	0.00	0.00
1434897_a_at	0.00	0.00
1434898_at	0.00	0.00
1434899_s_at	0.00	0.00
1434900_at	0.00	0.00
1434901_at	0.00	0.00
1434904_at	0.00	0.00
1434906_at	0.00	0.00
1434914_at	0.00	0.00
1434920_a_at	0.00	0.00
1434922_at	0.00	0.00
1434923_at	0.00	0.00
1434924_at	0.00	0.00
1434927_at	0.00	0.00
1434930_at	0.00	0.00
1434931_at	0.00	0.06
1434932_at	0.00	0.00
1434935_at	0.00	0.00
1434937_at	0.00	0.00
1434938_at	0.00	0.00
1434940_x_at	0.00	0.00
1434941_s_at	0.00	0.10
1434942_at	0.00	0.00
1434943_at	0.00	0.00
1434944_at	0.01	0.00
1434946_at	0.00	0.00
1434953_at	0.00	0.00
1434954_at	0.00	0.16
1434958_at	0.00	0.00
1434959_at	0.00	0.00
1434962_x_at	0.00	0.00
1434964_at	0.00	0.00
1434968_a_at	0.00	0.00
1434970_a_at	0.00	0.00
1434971_x_at	0.03	0.00
1434972_x_at	0.00	0.56
1434975_x_at	0.00	0.00
1434976_x_at	0.00	0.03
1434981_at	0.00	0.00
1434985_a_at	0.00	0.01
1434986_a_at	0.00	0.00
1434987_at	0.00	0.00
1434988_x_at	0.00	0.00
1434992_at	0.00	0.00
1434994_at	0.00	0.00
1434995_s_at	0.00	0.00
1434998_at	0.00	0.00
1434999_at	0.00	0.00
1435003_at	0.00	0.00
1435006_s_at	0.00	0.00
1435007_s_at	0.00	0.00
1435011_x_at	0.00	0.00

1435012_x_at	0.00	0.00
1435013_at	0.00	0.00
1435015_at	0.00	0.00
1435018_at	0.00	0.00
1435019_at	0.00	0.00
1435023_at	0.00	0.00
1435026_at	0.00	0.00
1435030_at	0.00	0.00
1435035_at	0.00	0.14
1435039_a_at	0.00	0.00
1435041_at	0.00	0.00
1435042_at	0.00	0.00
1435053_s_at	0.00	0.00
1435054_at	0.00	0.03
1435055_a_at	0.00	0.00
1435056_x_at	0.00	0.00
1435057_x_at	0.00	0.04
1435058_x_at	0.00	0.00
1435064_a_at	0.00	0.00
1435066_at	0.00	0.00
1435067_at	0.00	0.00
1435077_at	0.00	0.00
1435086_s_at	0.32	0.00
1435087_at	0.00	0.00
1435089_at	0.00	0.00
1435091_at	0.00	0.00
1435097_at	0.00	0.00
1435101_at	0.00	0.00
1435102_a_at	0.00	0.00
1435103_x_at	0.01	0.14
1435105_at	0.00	0.00
1435106_at	0.00	0.00
1435110_at	0.53	0.32
1435112_a_at	0.00	0.00
1435113_x_at	0.00	0.00
1435114_at	0.00	0.00
1435117_a_at	0.00	0.00
1435122_x_at	0.00	0.00
1435128_at	0.00	0.00
1435129_at	0.02	0.00
1435130_at	0.00	0.00
1435133_at	0.00	0.23
1435137_s_at	0.00	0.00
1435139_at	0.00	0.06
1435140_at	0.00	0.00
1435143_at	0.00	0.00
1435148_at	0.00	0.00
1435151_a_at	0.00	0.00
1435152_at	0.00	0.00
1435156_at	0.00	0.00
1435160_at	0.00	0.00
1435162_at	0.00	0.00
1435164_s_at	0.00	0.00
1435169_at	0.00	0.00
1435170_at	0.00	0.00
1435172_at	0.00	0.06

1435174_at	0.00	0.00
1435176_a_at	0.00	0.00
1435177_a_at	0.00	0.13
1435178_x_at	0.00	0.00
1435184_at	0.00	0.00
1435191_at	0.00	0.00
1435192_at	0.00	0.00
1435193_at	0.00	0.00
1435194_at	0.00	0.00
1435199_at	0.00	0.00
1435202_at	0.00	0.00
1435214_at	0.00	0.00
1435216_a_at	0.00	0.00
1435219_x_at	0.00	0.00
1435220_s_at	0.00	0.00
1435221_at	0.00	0.00
1435222_at	0.00	0.00
1435226_at	0.00	0.00
1435228_at	0.00	0.00
1435231_at	0.00	0.00
1435232_x_at	0.04	0.00
1435235_at	0.00	0.00
1435239_at	0.00	0.00
1435240_at	0.00	0.00
1435243_at	0.00	0.00
1435250_at	0.00	0.00
1435252_at	0.00	0.00
1435258_at	0.00	0.00
1435259_s_at	0.00	0.00
1435269_at	0.00	0.00
1435270_x_at	0.00	0.10
1435271_at	0.00	0.00
1435272_at	0.00	0.00
1435275_at	0.00	0.37
1435276_a_at	0.00	0.00
1435277_x_at	0.00	0.15
1435288_at	0.00	0.00
1435290_x_at	0.00	0.00
1435300_at	0.00	0.00
1435301_at	0.00	0.00
1435304_at	0.00	0.00
1435306_a_at	0.00	0.00
1435309_at	0.00	0.00
1435315_s_at	0.00	0.00
1435316_at	0.00	0.00
1435317_x_at	0.00	0.00
1435321_at	0.00	0.00
1435322_at	0.00	0.00
1435323_a_at	0.00	0.00
1435324_x_at	0.00	0.00
1435326_at	0.00	0.02
1435327_at	0.00	0.00
1435330_at	0.00	0.00
1435331_at	0.00	0.00
1435333_at	0.00	0.06
1435335_a_at	0.00	0.00

1435336_at	0.00	0.00
1435341_at	0.00	0.08
1435347_at	0.00	0.00
1435350_at	0.00	0.00
1435352_at	0.00	0.00
1435353_a_at	0.00	0.00
1435357_at	0.00	0.00
1435358_at	0.00	0.00
1435360_at	0.00	0.00
1435362_at	0.00	0.00
1435364_at	0.00	0.00
1435368_a_at	0.00	0.00
1435369_at	0.00	0.03
1435370_a_at	0.00	0.00
1435371_x_at	0.00	0.00
1435372_a_at	0.00	0.00
1435375_at	0.00	0.00
1435376_at	0.00	0.00
1435382_at	0.09	0.00
1435383_x_at	0.01	0.00
1435386_at	0.00	0.00
1435390_at	0.00	0.00
1435394_s_at	0.01	0.00
1435395_s_at	0.00	0.21
1435405_at	0.00	0.00
1435413_x_at	0.00	0.00
1435414_s_at	0.00	0.00
1435415_x_at	0.00	0.00
1435416_x_at	0.00	0.00
1435417_at	0.00	0.00
1435418_at	0.00	0.00
1435420_at	0.00	0.00
1435429_x_at	0.00	0.00
1435430_at	0.00	0.00
1435431_at	0.00	0.00
1435442_at	0.00	0.09
1435444_at	0.00	0.00
1435446_a_at	0.00	0.00
1435447_at	0.00	0.00
1435448_at	0.00	0.00
1435449_at	0.00	0.00
1435450_at	0.00	0.00
1435454_a_at	0.00	0.00
1435458_at	0.00	0.00
1435463_s_at	0.00	0.00
1435468_at	0.00	0.00
1435476_a_at	0.00	0.00
1435477_s_at	0.00	0.00
1435479_at	0.00	0.00
1435493_at	0.54	0.00
1435494_s_at	0.84	0.00
1435495_at	0.00	0.00
1435496_at	0.00	0.00
1435502_a_at	0.00	0.00
1435504_at	0.00	0.00
1435507_x_at	0.00	0.00

1435508_x_at	0.00	0.00
1435509_x_at	0.00	0.10
1435517_x_at	0.00	0.00
1435518_at	0.01	0.00
1435519_at	0.00	0.00
1435522_a_at	0.00	0.00
1435524_at	0.00	0.00
1435525_at	0.00	0.00
1435526_at	0.00	0.00
1435527_at	0.00	0.00
1435528_at	0.00	0.00
1435530_at	0.00	0.00
1435532_at	0.00	0.00
1435533_s_at	0.00	0.00
1435534_a_at	0.00	0.00
1435537_at	0.00	0.00
1435541_at	0.00	0.00
1435551_at	0.00	0.00
1435559_at	0.00	0.00
1435561_at	0.00	0.00
1435565_at	0.00	0.00
1435567_at	0.00	0.00
1435572_at	0.00	0.00
1435573_at	0.00	0.00
1435577_at	0.00	0.00
1435578_s_at	0.00	0.00
1435586_at	0.00	0.00
1435587_at	0.00	0.00
1435592_at	0.00	0.00
1435593_x_at	0.00	0.00
1435602_at	0.00	0.23
1435611_x_at	0.00	0.00
1435613_x_at	0.00	0.00
1435614_s_at	0.00	0.00
1435622_at	0.00	0.00
1435625_at	0.00	0.00
1435626_a_at	0.00	0.00
1435627_x_at	0.00	0.00
1435628_x_at	0.00	0.00
1435630_s_at	0.00	0.00
1435631_x_at	0.00	0.00
1435634_at	0.00	0.00
1435635_at	0.00	0.00
1435637_at	0.00	0.00
1435638_at	0.00	0.00
1435639_at	0.00	0.00
1435643_x_at	0.00	0.00
1435651_a_at	0.00	0.02
1435652_a_at	0.00	0.00
1435655_at	0.00	0.00
1435659_a_at	0.00	0.00
1435660_at	0.00	0.00
1435666_at	0.00	0.00
1435669_at	0.63	0.27
1435680_a_at	0.00	0.00
1435681_s_at	0.00	0.00

1435683_a_at	0.00	0.00
1435684_at	0.00	0.00
1435685_x_at	0.00	0.00
1435690_at	0.00	0.00
1435695_a_at	0.13	0.02
1435697_a_at	0.00	0.00
1435702_s_at	0.00	0.00
1435709_at	0.00	0.00
1435712_a_at	0.00	0.00
1435714_x_at	0.00	0.00
1435715_x_at	0.00	0.00
1435716_x_at	0.00	0.00
1435725_x_at	0.00	0.00
1435729_at	0.00	0.00
1435731_x_at	0.00	0.00
1435732_x_at	0.00	0.00
1435733_x_at	0.00	0.00
1435734_x_at	0.00	0.00
1435735_x_at	0.00	0.00
1435736_x_at	0.00	0.00
1435737_a_at	0.00	0.00
1435738_x_at	0.00	0.00
1435742_at	0.00	0.30
1435747_at	0.00	0.00
1435754_at	0.00	0.07
1435755_at	0.00	0.00
1435756_at	0.00	0.00
1435757_a_at	0.00	0.00
1435758_at	0.00	0.00
1435759_at	0.00	0.00
1435762_at	0.00	0.05
1435764_a_at	0.00	0.00
1435767_at	0.00	0.00
1435768_at	0.00	0.00
1435780_at	0.00	0.00
1435782_at	0.00	0.00
1435783_at	0.01	0.35
1435788_at	0.00	0.00
1435789_x_at	0.00	0.00
1435791_x_at	0.00	0.00
1435792_at	0.00	0.00
1435798_a_at	0.00	0.00
1435799_at	0.00	0.00
1435800_a_at	0.00	0.05
1435802_at	0.00	0.33
1435803_a_at	0.00	0.01
1435804_at	0.00	0.00
1435807_at	0.00	0.27
1435811_a_at	0.00	0.00
1435812_at	0.00	0.00
1435816_at	0.00	0.00
1435817_x_at	0.00	0.00
1435820_x_at	0.00	0.00
1435821_s_at	0.00	0.30
1435823_x_at	0.00	0.00
1435824_at	0.00	0.00

1435830_a_at	0.00	0.00
1435833_at	0.00	0.00
1435834_at	0.00	0.00
1435837_at	0.00	0.00
1435840_x_at	0.00	0.00
1435843_x_at	0.00	0.00
1435847_at	0.00	0.00
1435853_at	0.00	0.00
1435855_x_at	0.00	0.00
1435856_x_at	0.00	0.00
1435857_s_at	0.58	0.00
1435859_x_at	0.00	0.03
1435860_at	0.00	0.00
1435862_at	0.00	0.00
1435863_at	0.00	0.00
1435864_a_at	0.00	0.01
1435865_at	0.00	0.00
1435866_s_at	0.00	0.00
1435869_s_at	0.00	0.00
1435870_at	0.00	0.00
1435871_at	0.00	0.00
1435872_at	0.00	0.00
1435873_a_at	0.00	0.00
1435881_at	0.00	0.00
1435882_at	0.00	0.00
1435884_at	0.00	0.00
1435885_s_at	0.00	0.00
1435886_at	0.00	0.00
1435889_at	0.00	0.00
1435896_at	0.00	0.00
1435898_x_at	0.00	0.00
1435903_at	0.00	0.00
1435906_x_at	0.05	0.00
1435919_at	0.00	0.00
1435920_x_at	0.00	0.00
1435924_at	0.00	0.00
1435934_at	0.00	0.00
1435939_s_at	0.00	0.00
1435941_at	0.00	0.00
1435943_at	0.00	0.00
1435944_s_at	0.00	0.00
1435945_a_at	0.00	0.00
1435946_at	0.00	0.00
1435949_at	0.00	0.00
1435950_at	0.00	0.00
1435951_at	0.00	0.00
1435958_at	0.00	0.00
1435962_at	0.00	0.00
1435964_a_at	0.00	0.00
1435965_at	0.00	0.00
1435966_x_at	0.00	0.00
1435967_s_at	0.00	0.00
1435970_at	0.00	0.00
1435977_at	0.00	0.00
1435978_at	0.00	0.00
1435979_a_at	0.00	0.00

1435981_at	0.00	0.00
1435986_x_at	0.00	0.00
1435988_x_at	0.00	0.00
1435989_x_at	0.84	0.00
1435995_at	0.00	0.00
1435998_at	0.44	0.56
1436000_a_at	0.00	0.00
1436005_at	0.00	0.00
1436007_a_at	0.00	0.00
1436012_s_at	0.00	0.00
1436014_a_at	0.00	0.00
1436016_x_at	0.00	0.00
1436019_a_at	0.00	0.00
1436021_at	0.00	0.00
1436026_at	0.00	0.00
1436027_at	0.00	0.00
1436030_at	0.00	0.00
1436031_at	0.00	0.00
1436032_at	0.00	0.00
1436034_at	0.00	0.00
1436038_a_at	0.00	0.00
1436042_at	0.00	0.00
1436046_x_at	0.00	0.00
1436048_at	0.00	0.00
1436049_at	0.00	0.00
1436050_x_at	0.00	0.00
1436057_at	0.00	0.00
1436058_at	0.00	0.00
1436064_x_at	0.00	0.00
1436066_at	0.00	0.00
1436073_at	0.00	0.00
1436077_a_at	0.00	0.00
1436078_at	0.00	0.00
1436079_s_at	0.00	0.00
1436092_at	0.00	0.00
1436097_x_at	0.00	0.00
1436112_at	0.00	0.00
1436121_a_at	0.00	0.00
1436137_at	0.00	0.00
1436144_at	0.00	0.00
1436149_at	0.00	0.00
1436152_a_at	0.00	0.00
1436153_a_at	0.00	0.00
1436158_at	0.00	0.00
1436162_at	0.00	0.00
1436165_at	0.00	0.00
1436167_at	0.00	0.00
1436170_a_at	0.00	0.00
1436172_at	0.00	0.00
1436176_at	0.00	0.00
1436179_a_at	0.00	0.00
1436180_at	0.00	0.00
1436182_at	0.00	0.00
1436186_at	0.00	0.32
1436187_at	0.00	0.00
1436188_a_at	0.00	0.00

1436198_at	0.00	0.00
1436201_x_at	0.00	0.00
1436207_at	0.00	0.00
1436212_at	0.00	0.00
1436213_a_at	0.00	0.00
1436214_at	0.00	0.00
1436222_at	0.00	0.00
1436226_at	0.00	0.00
1436228_at	0.00	0.00
1436232_a_at	0.00	0.00
1436234_at	0.00	0.00
1436235_x_at	0.00	0.00
1436236_x_at	0.00	0.03
1436241_s_at	0.00	0.00
1436242_a_at	0.00	0.00
1436243_at	0.00	0.00
1436244_a_at	0.00	0.00
1436253_at	0.00	0.00
1436255_at	0.00	0.00
1436261_at	0.00	0.00
1436262_x_at	0.00	0.00
1436266_x_at	0.00	0.01
1436267_a_at	0.00	0.00
1436269_s_at	0.00	0.00
1436270_at	0.00	0.00
1436272_at	0.00	0.00
1436277_at	0.00	0.00
1436289_x_at	0.00	0.00
1436291_a_at	0.69	0.00
1436292_a_at	0.00	0.00
1436297_a_at	0.00	0.31
1436298_x_at	0.01	0.00
1436300_at	0.00	0.00
1436301_at	0.00	0.00
1436307_at	0.00	0.00
1436308_at	0.00	0.00
1436315_at	0.00	0.00
1436316_at	0.00	0.00
1436320_at	0.00	0.00
1436327_a_at	0.00	0.00
1436336_at	0.00	0.00
1436337_at	0.00	0.00
1436339_at	0.00	0.00
1436341_at	0.00	0.00
1436342_a_at	0.00	0.00
1436343_at	0.00	0.00
1436344_at	0.00	0.00
1436348_at	0.00	0.00
1436349_at	0.00	0.00
1436351_at	0.00	0.00
1436357_at	0.00	0.00
1436362_x_at	0.00	0.00
1436363_a_at	0.00	0.00
1436364_x_at	0.00	0.00
1436366_at	0.00	0.00
1436370_at	0.00	0.00

1436372_a_at	0.00	0.00
1436374_x_at	0.00	0.00
1436382_at	0.00	0.00
1436384_at	0.00	0.00
1436385_at	0.00	0.00
1436388_a_at	0.00	0.00
1436390_a_at	0.00	0.00
1436391_s_at	0.00	0.00
1436392_s_at	0.55	0.00
1436393_a_at	0.00	0.00
1436394_at	0.00	0.00
1436399_s_at	0.00	0.00
1436402_at	0.00	0.00
1436404_at	0.00	0.00
1436409_at	0.00	0.00
1436416_x_at	0.00	0.00
1436419_a_at	0.35	0.00
1436420_a_at	0.00	0.00
1436421_s_at	0.00	0.00
1436427_at	0.00	0.00
1436442_at	0.00	0.02
1436443_a_at	0.00	0.00
1436448_a_at	0.00	0.00
1436451_a_at	0.00	0.00
1436452_x_at	0.00	0.00
1436454_x_at	0.00	0.00
1436462_at	0.00	0.00
1436477_x_at	0.00	0.00
1436479_a_at	0.00	0.00
1436480_at	0.00	0.00
1436482_a_at	0.00	0.00
1436487_x_at	0.00	0.00
1436490_x_at	0.00	0.00
1436494_x_at	0.00	0.02
1436495_s_at	0.00	0.00
1436498_at	0.00	0.04
1436500_at	0.00	0.00
1436504_x_at	0.00	0.00
1436505_at	0.00	0.00
1436506_a_at	0.00	0.00
1436510_a_at	0.00	0.00
1436512_at	0.87	0.70
1436517_at	0.00	0.00
1436519_a_at	0.00	0.00
1436522_at	0.00	0.00
1436525_at	0.00	0.00
1436528_at	0.00	0.00
1436542_at	0.00	0.00
1436547_at	0.00	0.00
1436549_a_at	0.00	0.00
1436558_at	0.00	0.03
1436559_a_at	0.00	0.00
1436561_at	0.00	0.00
1436563_at	0.00	0.00
1436565_at	0.00	0.00
1436566_at	0.00	0.00

1436567_a_at	0.00	0.00
1436570_at	0.00	0.00
1436573_at	0.00	0.00
1436580_at	0.00	0.00
1436584_at	0.00	0.76
1436586_x_at	0.00	0.00
1436588_at	0.00	0.00
1436589_x_at	0.00	0.00
1436609_a_at	0.00	0.00
1436613_at	0.00	0.00
1436615_a_at	0.00	0.00
1436616_at	0.00	0.00
1436620_at	0.00	0.00
1436623_at	0.00	0.00
1436639_at	0.00	0.00
1436643_x_at	0.00	0.00
1436645_a_at	0.00	0.36
1436653_at	0.00	0.00
1436655_at	0.00	0.00
1436658_at	0.00	0.00
1436664_a_at	0.00	0.00
1436665_a_at	0.54	0.56
1436668_at	0.00	0.00
1436669_at	0.00	0.00
1436670_x_at	0.00	0.00
1436674_at	0.00	0.00
1436677_at	0.00	0.00
1436680_s_at	0.00	0.00
1436681_x_at	0.00	0.00
1436682_at	0.00	0.00
1436684_a_at	0.00	0.02
1436686_at	0.00	0.00
1436687_x_at	0.00	0.00
1436688_x_at	0.00	0.00
1436689_a_at	0.00	0.00
1436691_x_at	0.00	0.00
1436693_x_at	0.00	0.00
1436696_x_at	0.00	0.00
1436699_x_at	0.00	0.00
1436703_x_at	0.00	0.00
1436704_x_at	0.00	0.00
1436707_x_at	0.00	0.00
1436708_x_at	0.00	0.02
1436713_s_at	0.00	0.00
1436714_at	0.55	0.00
1436715_s_at	0.00	0.00
1436716_at	0.00	0.00
1436717_x_at	0.00	0.00
1436722_a_at	0.00	0.00
1436724_a_at	0.00	0.13
1436726_s_at	0.00	0.00
1436727_x_at	0.00	0.00
1436730_at	0.00	0.00
1436736_x_at	0.41	0.00
1436737_a_at	0.00	0.00
1436746_at	0.02	0.00

1436747_at	0.11	0.00
1436748_at	0.00	0.00
1436750_a_at	0.00	0.00
1436756_x_at	0.00	0.00
1436757_a_at	0.00	0.00
1436759_x_at	0.00	0.00
1436760_a_at	0.00	0.00
1436761_s_at	0.00	0.00
1436762_x_at	0.00	0.00
1436763_a_at	0.00	0.00
1436766_at	0.00	0.03
1436767_at	0.00	0.00
1436768_x_at	0.00	0.00
1436769_at	0.00	0.00
1436770_x_at	0.00	0.00
1436771_x_at	0.00	0.25
1436773_x_at	0.00	0.00
1436775_a_at	0.00	0.00
1436776_x_at	0.00	0.00
1436780_at	0.00	0.00
1436781_at	0.00	0.00
1436782_at	0.00	0.00
1436783_x_at	0.00	0.00
1436784_x_at	0.00	0.00
1436788_at	0.00	0.00
1436790_a_at	0.01	0.06
1436791_at	0.00	0.00
1436797_a_at	0.00	0.00
1436798_at	0.00	0.00
1436801_x_at	0.00	0.00
1436802_at	0.00	0.00
1436803_a_at	0.00	0.00
1436804_s_at	0.00	0.00
1436806_at	0.00	0.00
1436807_x_at	0.00	0.00
1436809_a_at	0.00	0.00
1436810_x_at	0.00	0.00
1436816_at	0.00	0.00
1436819_at	0.00	0.00
1436820_at	0.00	0.00
1436822_x_at	0.00	0.00
1436823_x_at	0.00	0.00
1436824_x_at	0.00	0.00
1436828_a_at	0.00	0.00
1436833_x_at	0.00	0.00
1436834_x_at	0.00	0.00
1436835_at	0.00	0.00
1436836_x_at	0.00	0.00
1436838_x_at	0.00	0.01
1436839_at	0.00	0.00
1436840_x_at	0.00	0.00
1436845_at	0.00	0.00
1436846_x_at	0.00	0.00
1436848_x_at	0.00	0.00
1436849_x_at	0.00	0.00
1436851_at	0.00	0.00

1436853_a_at	0.00	0.00
1436854_at	0.00	0.00
1436856_x_at	0.00	0.00
1436859_at	0.00	0.00
1436861_at	0.00	0.00
1436862_at	0.00	0.00
1436863_at	0.00	0.00
1436871_at	0.00	0.03
1436872_at	0.00	0.00
1436874_x_at	0.00	0.29
1436879_x_at	0.00	0.00
1436880_at	0.00	0.00
1436881_x_at	0.00	0.00
1436882_at	0.00	0.00
1436884_x_at	0.00	0.00
1436885_a_at	0.00	0.13
1436886_x_at	0.00	0.06
1436887_x_at	0.00	0.24
1436889_at	0.00	0.00
1436890_at	0.00	0.15
1436893_a_at	0.00	0.00
1436896_at	0.00	0.00
1436898_at	0.01	0.00
1436899_at	0.00	0.00
1436900_x_at	0.00	0.00
1436902_x_at	0.00	0.00
1436905_x_at	0.04	0.37
1436906_at	0.00	0.00
1436908_at	0.00	0.00
1436909_at	0.00	0.00
1436910_at	0.00	0.00
1436912_at	0.00	0.00
1436915_x_at	0.00	0.41
1436917_s_at	0.00	0.00
1436921_at	0.00	0.00
1436922_at	0.00	0.00
1436923_at	0.00	0.00
1436924_x_at	0.00	0.00
1436926_at	1.00	0.35
1436928_s_at	0.00	0.00
1436929_x_at	0.00	0.00
1436930_x_at	0.00	0.00
1436934_s_at	0.00	0.00
1436935_x_at	0.00	0.33
1436936_s_at	0.00	0.00
1436940_at	0.00	0.00
1436944_x_at	0.00	0.00
1436945_x_at	0.00	0.00
1436946_s_at	0.00	0.00
1436947_a_at	0.00	0.00
1436949_a_at	0.00	0.00
1436950_at	0.00	0.00
1436951_x_at	0.00	0.00
1436952_at	0.00	0.00
1436953_at	0.00	0.00
1436954_at	0.00	0.00

1436955_at	0.00	0.00
1436956_at	0.00	0.00
1436958_x_at	0.00	0.00
1436959_x_at	0.65	0.43
1436963_x_at	0.00	0.00
1436965_at	0.00	0.00
1436970_a_at	0.00	0.00
1436971_x_at	0.00	0.00
1436973_at	0.00	0.00
1436979_x_at	0.00	0.06
1436980_x_at	0.00	0.00
1436981_a_at	0.02	0.00
1436986_at	0.00	0.00
1436989_s_at	0.00	0.00
1436990_s_at	1.00	0.00
1436991_x_at	0.00	0.00
1436992_x_at	0.00	0.00
1436993_x_at	0.00	0.00
1436994_a_at	0.00	0.00
1436995_a_at	0.00	0.00
1436996_x_at	0.00	0.00
1436997_x_at	0.05	0.03
1436998_at	0.00	0.00
1437000_at	0.00	0.00
1437004_at	0.00	0.00
1437005_a_at	0.00	0.00
1437006_x_at	0.00	0.00
1437007_x_at	0.00	0.02
1437008_x_at	0.00	0.00
1437009_a_at	0.00	0.00
1437012_x_at	0.00	0.00
1437013_x_at	0.00	0.00
1437014_x_at	0.00	0.00
1437015_x_at	0.17	0.98
1437016_x_at	0.00	0.00
1437024_at	0.00	0.20
1437025_at	0.00	0.00
1437026_at	0.00	0.00
1437027_x_at	0.00	0.00
1437028_at	0.00	0.00
1437029_at	0.00	0.00
1437032_x_at	0.00	0.07
1437033_a_at	0.00	0.00
1437034_x_at	0.00	0.00
1437035_x_at	0.00	0.00
1437036_at	0.00	0.00
1437037_x_at	0.00	0.00
1437039_at	0.00	0.03
1437043_a_at	0.00	0.00
1437044_a_at	0.00	0.00
1437045_at	0.00	0.00
1437046_x_at	0.00	0.00
1437048_x_at	0.00	0.00
1437050_s_at	0.00	0.00
1437051_at	0.00	0.00
1437052_s_at	0.00	0.34

1437053_x_at	0.00	0.00
1437054_x_at	0.00	0.00
1437055_x_at	0.00	0.00
1437061_at	0.00	0.00
1437062_s_at	0.00	0.00
1437067_at	0.00	0.00
1437074_at	0.00	0.00
1437078_at	0.00	0.00
1437080_s_at	0.00	0.00
1437081_at	0.00	0.00
1437082_at	0.00	0.00
1437097_at	0.00	0.00
1437099_x_at	0.00	0.02
1437100_x_at	0.00	0.00
1437101_at	0.13	0.00
1437102_at	0.00	0.00
1437103_at	0.00	0.15
1437110_at	0.00	0.00
1437111_at	0.00	0.00
1437112_at	0.00	0.00
1437113_s_at	0.00	0.00
1437120_at	0.00	0.00
1437131_x_at	0.00	0.01
1437132_x_at	0.01	0.00
1437133_x_at	0.12	1.00
1437134_at	0.00	0.00
1437138_at	0.00	0.00
1437141_x_at	0.00	0.00
1437142_a_at	0.00	0.00
1437143_a_at	0.17	0.02
1437144_x_at	0.00	0.18
1437145_s_at	0.00	0.00
1437148_at	0.00	0.00
1437155_a_at	0.15	0.00
1437157_at	0.00	0.00
1437159_at	0.00	0.00
1437161_x_at	0.00	0.02
1437163_x_at	0.00	0.26
1437164_x_at	0.00	0.00
1437165_a_at	0.00	1.00
1437169_at	0.00	0.00
1437170_x_at	0.00	0.00
1437171_x_at	0.00	0.00
1437172_x_at	0.00	0.23
1437175_at	0.00	0.00
1437180_at	0.00	0.00
1437185_s_at	0.00	0.00
1437188_at	0.00	0.00
1437189_x_at	0.00	0.00
1437192_x_at	0.00	0.00
1437194_x_at	0.00	0.00
1437195_x_at	0.00	0.00
1437198_at	0.00	0.00
1437203_at	0.00	0.00
1437204_a_at	0.00	0.00
1437205_at	0.00	0.00

1437208_at	0.00	0.00
1437210_a_at	0.00	0.00
1437211_x_at	0.00	0.00
1437216_at	0.00	0.00
1437218_at	0.00	0.00
1437220_x_at	0.00	0.00
1437223_s_at	0.00	0.00
1437225_x_at	0.00	0.00
1437226_x_at	0.00	0.00
1437233_x_at	0.00	0.00
1437234_x_at	0.08	0.00
1437235_x_at	0.00	0.00
1437236_a_at	0.01	0.04
1437237_x_at	0.00	0.07
1437238_x_at	0.00	0.23
1437239_x_at	0.08	0.00
1437243_at	0.00	0.00
1437246_x_at	0.00	0.00
1437253_at	0.00	0.00
1437256_at	0.00	0.00
1437262_x_at	0.00	0.00
1437267_x_at	0.00	0.00
1437270_a_at	0.00	0.00
1437271_at	0.00	0.00
1437275_at	0.00	0.00
1437277_x_at	0.00	0.00
1437278_a_at	0.00	0.60
1437279_x_at	0.00	0.00
1437280_s_at	0.00	0.00
1437281_x_at	0.00	0.00
1437288_at	0.00	0.14
1437289_at	0.00	0.34
1437290_at	0.00	0.37
1437294_at	0.00	0.00
1437297_at	0.00	0.00
1437301_a_at	0.00	0.00
1437302_at	0.00	0.00
1437308_s_at	0.00	0.00
1437309_a_at	0.00	0.32
1437313_x_at	0.00	0.00
1437314_a_at	0.00	0.02
1437317_at	0.00	0.00
1437320_s_at	0.00	0.00
1437323_a_at	0.00	0.00
1437324_x_at	0.00	0.00
1437325_x_at	0.00	0.00
1437326_x_at	0.00	0.00
1437327_x_at	0.00	0.20
1437328_x_at	0.00	0.00
1437330_at	0.00	0.00
1437331_a_at	0.00	0.00
1437333_x_at	0.00	0.00
1437334_x_at	0.00	0.00
1437335_x_at	0.00	0.00
1437336_x_at	0.00	0.00
1437338_x_at	0.00	0.00

1437340_x_at	0.00	0.00
1437341_x_at	0.00	0.00
1437342_x_at	0.00	0.00
1437343_x_at	0.00	0.02
1437344_x_at	0.00	0.00
1437345_a_at	0.00	0.09
1437349_at	0.00	0.00
1437350_at	0.00	0.00
1437354_at	0.00	0.00
1437358_at	0.00	0.35
1437364_at	0.00	0.00
1437367_at	0.00	0.00
1437368_at	0.00	0.00
1437369_at	0.00	0.00
1437377_a_at	0.00	0.07
1437378_x_at	0.00	0.03
1437379_x_at	0.00	0.00
1437380_x_at	0.00	0.24
1437381_x_at	0.00	0.00
1437382_at	0.00	0.00
1437389_x_at	0.00	0.00
1437390_x_at	0.00	0.00
1437391_x_at	0.00	0.00
1437394_at	0.00	0.00
1437396_at	0.00	0.00
1437398_a_at	0.00	0.00
1437399_at	0.00	0.00
1437401_at	0.00	0.00
1437402_x_at	0.00	0.00
1437405_a_at	0.15	0.00
1437406_x_at	0.00	0.00
1437413_x_at	0.00	0.00
1437419_at	0.00	0.00
1437420_at	0.00	0.00
1437423_a_at	0.00	0.00
1437425_at	0.00	0.00
1437428_x_at	0.00	0.00
1437430_at	0.00	0.00
1437432_a_at	0.00	0.00
1437434_a_at	1.00	0.07
1437437_x_at	0.00	0.00
1437438_x_at	0.00	0.00
1437450_x_at	0.00	0.00
1437452_x_at	0.00	0.00
1437454_a_at	0.00	0.00
1437455_a_at	0.00	0.12
1437456_x_at	0.00	0.00
1437457_a_at	0.00	0.00
1437458_x_at	0.12	0.00
1437459_x_at	0.00	0.00
1437461_s_at	0.00	0.15
1437463_x_at	0.00	0.00
1437465_a_at	0.00	0.00
1437468_x_at	0.00	0.00
1437472_at	0.00	0.00
1437477_at	0.00	0.00

1437480_at	0.00	0.00
1437489_x_at	0.00	0.00
1437490_x_at	0.00	0.00
1437491_at	0.00	0.00
1437497_a_at	0.00	0.00
1437498_at	0.00	0.00
1437502_x_at	0.00	0.24
1437503_a_at	0.11	0.00
1437508_at	0.00	0.00
1437509_x_at	0.00	0.00
1437510_x_at	0.00	0.00
1437511_x_at	0.00	0.00
1437512_x_at	0.00	0.00
1437513_a_at	0.00	0.00
1437515_at	0.00	0.00
1437519_x_at	0.00	0.00
1437520_a_at	0.00	0.49
1437521_s_at	0.00	0.00
1437522_x_at	0.00	0.00
1437523_s_at	0.00	0.00
1437525_a_at	0.04	0.49
1437526_x_at	0.00	0.00
1437527_x_at	0.00	0.00
1437531_at	0.00	0.00
1437533_at	0.00	0.00
1437534_at	0.00	0.00
1437537_at	0.00	0.00
1437540_at	0.00	0.00
1437541_at	0.00	0.00
1437543_at	0.00	0.00
1437544_at	0.00	0.00
1437545_at	0.00	0.07
1437546_at	0.00	0.00
1437547_s_at	0.00	0.00
1437548_at	0.00	0.00
1437553_at	0.00	0.00
1437563_at	0.00	0.00
1437564_at	0.00	0.00
1437565_a_at	0.00	0.00
1437566_at	0.00	0.00
1437568_at	0.00	0.00
1437582_at	0.00	0.00
1437583_x_at	0.00	0.00
1437585_x_at	0.00	0.00
1437586_at	0.00	0.00
1437589_x_at	0.00	0.00
1437591_a_at	0.00	0.00
1437592_x_at	0.00	0.00
1437593_x_at	0.00	0.00
1437607_at	0.00	0.00
1437608_x_at	0.00	0.00
1437610_x_at	0.00	0.00
1437611_x_at	0.00	0.00
1437612_at	0.00	0.00
1437614_x_at	0.00	0.00
1437615_s_at	0.00	0.00

1437616_x_at	0.00	0.00
1437618_x_at	0.00	0.00
1437619_x_at	0.00	0.00
1437620_x_at	0.00	0.00
1437621_x_at	0.00	0.00
1437622_x_at	0.00	0.00
1437624_x_at	0.00	0.00
1437626_at	0.00	0.08
1437628_s_at	0.00	0.00
1437630_at	0.00	0.33
1437634_at	0.00	0.00
1437642_at	0.00	0.00
1437644_at	0.00	0.00
1437645_at	0.00	0.00
1437649_x_at	0.00	0.00
1437651_a_at	0.00	0.00
1437652_at	0.00	0.00
1437658_a_at	0.00	0.00
1437661_at	0.00	0.00
1437666_x_at	0.00	0.00
1437667_a_at	0.00	0.00
1437670_x_at	0.00	0.00
1437671_x_at	0.00	0.00
1437672_at	0.00	0.00
1437674_at	0.00	0.00
1437679_a_at	0.00	0.00
1437680_x_at	0.00	0.00
1437682_x_at	0.00	0.00
1437683_x_at	0.00	0.00
1437684_at	0.00	0.00
1437685_x_at	0.00	0.00
1437686_x_at	0.00	0.00
1437687_x_at	0.00	0.00
1437688_x_at	0.00	0.00
1437689_x_at	0.38	0.00
1437690_x_at	0.00	0.00
1437691_at	0.00	0.00
1437692_x_at	0.00	0.00
1437693_at	0.00	0.26
1437696_at	0.00	0.00
1437697_at	0.00	0.00
1437706_x_at	0.00	0.00
1437708_x_at	0.00	0.00
1437709_x_at	0.00	0.00
1437711_x_at	0.00	0.41
1437712_x_at	0.00	0.00
1437713_x_at	0.00	0.00
1437714_x_at	0.00	0.00
1437715_x_at	0.00	0.08
1437716_x_at	0.00	0.00
1437718_x_at	0.00	0.00
1437719_x_at	0.00	0.00
1437721_at	0.00	0.00
1437722_x_at	0.00	0.00
1437723_s_at	0.00	0.00
1437724_x_at	0.00	0.00

1437725_x_at	0.00	0.00
1437726_x_at	0.00	0.00
1437728_at	0.00	0.00
1437729_at	0.00	0.00
1437730_at	0.00	0.00
1437732_at	0.00	0.00
1437733_at	0.00	0.00
1437738_at	0.00	0.00
1437741_at	0.00	0.00
1437742_at	0.00	0.00
1437746_at	0.00	0.00
1437747_at	0.00	0.00
1437750_at	0.00	0.00
1437751_at	0.00	0.00
1437752_at	0.00	0.00
1437758_a_at	0.00	0.00
1437765_at	0.00	0.00
1437767_s_at	0.00	0.00
1437772_s_at	0.00	0.00
1437773_x_at	0.00	0.00
1437779_at	0.00	0.00
1437782_at	0.00	0.00
1437783_x_at	0.00	0.00
1437786_at	0.00	0.00
1437790_at	0.00	0.00
1437791_s_at	0.00	0.00
1437801_at	0.00	0.00
1437802_x_at	0.00	0.00
1437803_at	0.00	0.00
1437804_at	0.00	0.00
1437805_at	0.00	0.00
1437806_x_at	0.00	0.00
1437807_x_at	0.00	0.00
1437808_x_at	0.00	0.00
1437810_a_at	0.00	0.00
1437812_x_at	0.00	0.00
1437816_at	0.00	0.00
1437830_x_at	0.00	0.00
1437832_x_at	0.00	0.00
1437835_a_at	0.00	0.03
1437836_x_at	0.00	0.00
1437837_x_at	0.00	0.00
1437838_x_at	0.00	0.00
1437839_x_at	0.00	0.00
1437841_x_at	0.00	0.00
1437843_s_at	0.00	0.00
1437844_x_at	0.00	0.00
1437845_x_at	0.01	0.00
1437846_x_at	0.00	0.00
1437847_x_at	0.00	0.00
1437848_x_at	0.00	0.00
1437849_x_at	0.00	0.00
1437850_a_at	0.00	0.00
1437851_x_at	0.00	0.00
1437852_x_at	0.00	0.00
1437853_x_at	0.00	0.00

1437855_at	0.00	0.00
1437859_x_at	0.00	0.00
1437863_at	0.00	0.00
1437867_at	0.00	0.00
1437874_s_at	0.00	0.00
1437881_at	0.00	0.00
1437882_s_at	0.00	0.00
1437885_at	0.00	0.00
1437889_x_at	0.00	0.00
1437890_at	0.00	0.00
1437901_a_at	0.00	0.00
1437902_s_at	0.00	0.00
1437903_at	0.00	0.00
1437905_at	0.00	0.00
1437906_x_at	0.00	0.00
1437907_a_at	0.03	0.00
1437908_a_at	0.00	0.00
1437909_at	0.00	0.00
1437913_at	0.00	0.00
1437915_at	0.00	0.00
1437917_at	0.00	0.00
1437920_at	0.00	0.00
1437922_at	0.00	0.00
1437924_at	0.00	0.00
1437925_at	0.00	0.00
1437932_a_at	0.00	0.00
1437938_x_at	0.00	0.00
1437943_s_at	0.00	0.00
1437945_x_at	0.00	0.00
1437946_x_at	0.00	0.00
1437947_x_at	0.00	0.00
1437948_x_at	0.00	0.00
1437949_x_at	0.00	0.00
1437952_at	0.00	0.00
1437958_at	0.00	0.00
1437968_at	0.00	0.00
1437969_s_at	0.00	0.00
1437970_at	0.00	0.00
1437971_at	0.00	0.00
1437974_a_at	0.00	0.00
1437975_a_at	0.00	0.00
1437976_x_at	0.00	0.00
1437981_x_at	0.00	0.00
1437982_x_at	0.00	0.00
1437984_x_at	0.00	0.18
1437985_a_at	0.00	0.00
1437990_x_at	0.00	0.00
1437991_x_at	0.00	0.00
1437992_x_at	0.00	0.00
1437993_x_at	0.05	0.00
1437994_x_at	0.00	0.00
1437995_x_at	0.00	0.00
1437997_x_at	0.00	0.00
1437998_at	0.00	0.00
1437999_x_at	0.00	0.00
1438000_x_at	0.00	0.00

1438001_x_at	0.82	0.69
1438006_at	0.00	0.01
1438009_at	0.00	0.00
1438011_at	0.00	0.00
1438013_x_at	0.00	0.00
1438014_at	0.00	0.00
1438017_at	0.00	0.00
1438019_at	0.00	0.00
1438023_at	0.00	0.00
1438033_at	0.00	0.00
1438034_at	0.00	0.00
1438040_a_at	0.00	0.00
1438045_at	0.00	0.00
1438050_x_at	0.00	0.00
1438056_x_at	0.00	0.00
1438058_s_at	0.00	0.03
1438064_at	0.00	0.00
1438067_at	0.00	0.00
1438069_a_at	0.00	0.00
1438070_at	0.00	0.00
1438076_at	0.02	0.00
1438080_at	0.00	0.00
1438082_at	0.00	0.00
1438083_at	0.00	0.00
1438090_x_at	0.00	0.00
1438091_a_at	0.00	0.29
1438092_x_at	0.01	0.64
1438093_x_at	0.00	0.00
1438094_x_at	0.00	0.00
1438095_x_at	0.00	0.73
1438096_a_at	0.00	0.50
1438097_at	0.00	0.17
1438114_x_at	0.00	0.00
1438115_a_at	0.00	0.11
1438116_x_at	0.00	0.00
1438117_x_at	0.00	0.00
1438118_x_at	0.43	0.00
1438119_at	0.00	0.00
1438120_x_at	0.00	0.00
1438133_a_at	1.00	0.00
1438138_a_at	0.00	0.00
1438140_a_at	0.00	0.00
1438143_s_at	0.00	0.00
1438144_x_at	0.00	0.00
1438150_at	0.00	0.00
1438151_x_at	0.00	0.00
1438152_at	0.00	0.00
1438153_x_at	0.00	0.00
1438154_x_at	0.00	0.00
1438155_x_at	0.00	0.00
1438156_x_at	0.00	0.00
1438159_x_at	0.00	0.00
1438161_s_at	0.00	0.04
1438163_x_at	0.00	0.00
1438164_x_at	0.02	0.17
1438165_x_at	0.00	0.00

1438166_x_at	0.00	0.00
1438167_x_at	0.00	0.00
1438168_x_at	0.00	0.47
1438169_a_at	0.00	0.01
1438170_x_at	0.00	0.00
1438171_x_at	0.00	0.00
1438172_x_at	0.00	0.00
1438173_x_at	0.00	0.00
1438174_x_at	0.00	0.00
1438176_x_at	0.00	0.00
1438177_x_at	0.00	0.00
1438178_x_at	0.00	0.18
1438179_s_at	0.00	0.00
1438180_x_at	0.00	0.00
1438181_x_at	0.00	0.00
1438182_x_at	0.00	0.00
1438183_x_at	0.00	0.00
1438184_a_at	0.00	0.00
1438187_at	0.00	0.00
1438188_x_at	0.00	0.00
1438190_x_at	0.00	0.00
1438192_s_at	0.00	0.00
1438199_at	0.00	0.00
1438202_at	0.00	0.00
1438204_at	0.00	0.00
1438206_a_at	0.00	0.00
1438211_s_at	0.00	0.00
1438215_at	0.00	0.00
1438216_at	0.00	0.00
1438219_at	0.00	0.00
1438220_at	0.00	0.00
1438221_at	0.00	0.00
1438223_at	0.00	0.00
1438225_x_at	0.00	0.00
1438233_at	0.00	0.00
1438234_at	0.00	0.00
1438243_at	0.00	0.00
1438244_at	0.00	0.00
1438245_at	0.00	0.00
1438246_at	0.00	0.00
1438248_at	0.00	0.00
1438250_s_at	0.00	0.00
1438251_x_at	0.00	0.00
1438252_at	0.00	0.00
1438260_at	0.00	0.00
1438261_at	0.00	0.00
1438264_a_at	0.00	0.00
1438267_x_at	0.00	0.00
1438274_at	0.00	0.00
1438278_a_at	0.00	0.09
1438286_at	0.00	0.00
1438287_x_at	0.00	0.00
1438289_a_at	0.00	0.19
1438290_x_at	0.00	0.00
1438291_x_at	0.00	0.00
1438292_x_at	0.00	0.00

1438297_at	0.00	0.00
1438302_at	0.00	0.00
1438312_s_at	0.00	0.00
1438314_at	0.00	0.00
1438315_x_at	0.00	0.00
1438316_a_at	0.00	0.00
1438317_a_at	0.00	0.29
1438318_x_at	0.00	0.30
1438319_x_at	0.00	0.04
1438320_s_at	0.00	0.00
1438321_x_at	0.00	0.37
1438322_x_at	0.00	0.00
1438332_at	0.00	0.00
1438343_at	0.00	0.00
1438354_x_at	0.00	0.00
1438357_at	0.00	0.00
1438358_x_at	0.00	0.00
1438360_x_at	0.02	0.16
1438365_x_at	0.00	0.37
1438366_x_at	0.00	0.00
1438367_x_at	0.00	0.00
1438368_a_at	0.00	0.00
1438369_x_at	0.00	0.00
1438370_x_at	0.00	0.00
1438371_x_at	0.00	0.23
1438373_at	0.00	0.00
1438374_x_at	0.00	0.00
1438376_s_at	0.00	0.00
1438377_x_at	0.00	0.00
1438379_x_at	0.00	0.00
1438380_at	0.00	0.00
1438381_x_at	0.00	0.00
1438383_x_at	0.00	0.00
1438385_s_at	0.00	0.00
1438386_x_at	0.00	0.03
1438387_x_at	0.00	0.00
1438389_x_at	0.00	0.00
1438390_s_at	0.00	0.00
1438391_x_at	0.00	0.00
1438397_a_at	0.00	0.02
1438398_at	0.00	0.00
1438403_s_at	0.00	0.01
1438415_s_at	0.00	0.00
1438416_at	0.00	0.00
1438418_at	0.00	0.00
1438422_at	0.00	0.00
1438427_at	0.00	0.00
1438430_at	0.00	0.00
1438438_at	0.00	0.00
1438441_at	0.00	0.00
1438443_at	0.00	0.00
1438446_x_at	0.00	0.00
1438453_at	0.00	0.00
1438458_a_at	0.00	0.00
1438459_x_at	0.00	0.00
1438461_at	0.00	0.00

1438462_x_at	0.00	0.00
1438463_x_at	0.00	0.00
1438465_at	0.00	0.00
1438467_at	0.00	0.00
1438468_at	0.00	0.00
1438476_a_at	0.00	0.00
1438477_a_at	0.00	0.00
1438478_a_at	0.00	0.00
1438480_a_at	0.00	0.03
1438483_at	0.00	0.00
1438485_at	0.00	0.00
1438492_at	0.00	0.00
1438494_at	0.00	0.00
1438501_at	0.00	0.00
1438502_x_at	0.00	0.00
1438503_x_at	0.00	0.00
1438504_x_at	0.00	0.00
1438506_s_at	0.00	0.00
1438507_x_at	0.00	0.00
1438509_at	0.00	0.00
1438510_a_at	0.00	0.44
1438511_a_at	0.00	0.00
1438524_x_at	0.00	0.00
1438527_at	0.00	0.00
1438534_x_at	0.00	0.00
1438535_at	0.00	0.00
1438537_at	0.00	0.00
1438545_at	0.00	0.01
1438546_x_at	0.00	0.22
1438547_x_at	0.27	0.00
1438548_x_at	0.00	0.00
1438549_a_at	0.00	0.00
1438550_x_at	0.00	0.00
1438551_at	0.00	0.00
1438552_x_at	0.00	0.00
1438554_x_at	0.00	0.00
1438556_a_at	0.00	0.00
1438557_x_at	0.00	0.00
1438559_x_at	0.00	0.00
1438560_x_at	0.00	0.00
1438562_a_at	0.00	0.00
1438563_s_at	0.00	0.00
1438564_at	0.00	0.00
1438571_at	0.00	0.00
1438578_a_at	0.00	0.00
1438597_x_at	0.00	0.00
1438601_at	0.00	0.00
1438602_s_at	0.00	0.00
1438603_x_at	0.00	0.00
1438606_a_at	0.26	0.04
1438608_at	0.00	0.00
1438609_x_at	0.00	0.00
1438610_a_at	0.00	0.00
1438611_at	0.00	0.00
1438612_a_at	0.00	0.00
1438616_x_at	0.00	0.00

1438619_x_at	0.00	0.00
1438620_x_at	0.00	0.00
1438621_x_at	0.00	0.00
1438622_x_at	0.00	0.00
1438623_x_at	0.00	0.00
1438625_s_at	0.00	0.00
1438626_x_at	0.00	0.00
1438627_x_at	0.00	0.15
1438629_x_at	0.00	0.00
1438630_x_at	0.00	0.03
1438631_x_at	0.00	0.00
1438632_x_at	0.00	0.00
1438633_x_at	0.00	0.00
1438634_x_at	0.00	0.00
1438637_x_at	0.00	0.02
1438640_x_at	0.00	0.00
1438642_at	0.00	0.00
1438644_x_at	0.00	0.00
1438645_x_at	0.00	0.00
1438646_x_at	0.00	0.00
1438647_x_at	0.00	0.01
1438649_x_at	0.00	0.00
1438650_x_at	0.00	0.00
1438651_a_at	0.01	0.00
1438652_x_at	0.00	0.00
1438653_x_at	0.00	0.00
1438654_x_at	0.00	0.00
1438655_a_at	0.00	0.00
1438656_x_at	0.00	0.00
1438657_x_at	0.00	0.00
1438659_x_at	0.00	0.00
1438661_a_at	0.00	0.00
1438669_at	0.00	0.00
1438670_at	0.00	0.00
1438673_at	0.00	0.05
1438674_a_at	0.00	0.00
1438675_at	0.00	0.00
1438676_at	0.00	0.00
1438677_at	0.00	0.00
1438679_at	0.00	0.00
1438680_at	0.00	0.00
1438685_at	0.00	0.00
1438686_at	0.00	0.02
1438690_at	0.00	0.00
1438694_at	0.00	0.00
1438695_at	0.00	0.00
1438696_at	0.00	0.00
1438700_at	0.00	0.00
1438705_at	0.00	0.00
1438708_x_at	0.00	0.00
1438710_at	0.00	0.00
1438711_at	0.00	0.00
1438712_at	0.00	0.00
1438714_at	0.00	0.00
1438716_at	0.00	0.00
1438717_a_at	0.00	0.00

1438721_a_at	0.00	0.00
1438723_a_at	0.00	0.00
1438731_at	0.00	0.00
1438736_at	0.00	0.00
1438741_at	0.00	0.00
1438743_at	0.00	0.00
1438746_at	0.00	0.00
1438748_at	0.00	0.00
1438755_at	0.00	0.00
1438758_at	0.00	0.00
1438759_x_at	0.00	0.00
1438760_x_at	0.00	0.00
1438761_a_at	0.00	0.15
1438764_at	0.00	0.00
1438769_a_at	0.00	0.00
1438776_x_at	0.00	0.00
1438777_a_at	0.00	0.00
1438782_at	0.00	0.00
1438789_s_at	0.00	0.00
1438790_x_at	0.00	0.00
1438792_at	0.00	0.00
1438793_x_at	0.00	0.00
1438794_x_at	0.00	0.00
1438795_x_at	0.00	0.00
1438797_at	0.00	0.00
1438802_at	0.00	0.00
1438808_at	0.00	0.00
1438809_at	0.00	0.00
1438812_x_at	0.00	0.00
1438817_at	0.00	0.00
1438824_at	0.00	0.00
1438825_at	0.00	0.00
1438826_x_at	0.00	0.00
1438832_x_at	0.00	0.01
1438835_a_at	0.00	0.24
1438836_at	0.00	0.00
1438839_a_at	0.00	0.00
1438840_x_at	0.00	0.00
1438841_s_at	0.00	0.00
1438842_at	0.00	0.00
1438843_x_at	0.00	0.00
1438844_x_at	0.00	0.00
1438845_at	0.00	0.00
1438846_x_at	0.00	0.00
1438847_at	0.00	0.00
1438850_at	0.00	0.00
1438851_x_at	0.00	0.00
1438852_x_at	0.00	0.01
1438853_x_at	0.00	0.00
1438854_x_at	0.00	0.00
1438855_x_at	0.00	0.00
1438856_x_at	0.00	0.00
1438857_x_at	0.00	0.00
1438859_x_at	0.00	0.00
1438860_a_at	0.00	0.00
1438865_at	0.00	0.00

1438886_at	0.00	0.00
1438887_a_at	0.00	0.00
1438888_at	0.00	0.00
1438902_a_at	0.00	0.00
1438903_at	0.00	0.00
1438908_at	0.00	0.00
1438909_at	0.00	0.00
1438910_a_at	0.00	0.00
1438912_at	0.00	0.00
1438913_x_at	0.00	0.00
1438914_at	0.00	0.00
1438917_x_at	0.00	0.22
1438918_at	0.00	0.00
1438919_x_at	0.00	0.00
1438922_x_at	0.00	0.10
1438923_at	0.00	0.00
1438924_x_at	0.00	0.00
1438925_x_at	0.00	0.00
1438926_at	0.00	0.00
1438927_x_at	0.00	0.00
1438928_x_at	0.00	0.00
1438931_s_at	0.00	0.00
1438932_at	0.99	0.88
1438933_x_at	0.70	0.87
1438934_x_at	0.00	0.00
1438938_x_at	0.00	0.01
1438939_x_at	0.00	0.00
1438940_x_at	0.00	0.02
1438941_x_at	0.00	0.00
1438942_x_at	0.00	0.00
1438943_x_at	0.00	0.00
1438945_x_at	0.00	0.00
1438948_x_at	0.00	0.00
1438949_at	0.00	0.00
1438950_x_at	0.00	0.00
1438951_x_at	0.01	0.03
1438952_x_at	0.00	0.00
1438953_at	0.00	0.00
1438954_x_at	0.00	0.00
1438955_x_at	0.00	0.00
1438956_x_at	0.00	0.00
1438957_x_at	0.00	0.28
1438958_x_at	0.00	0.00
1438961_s_at	0.00	0.00
1438963_s_at	0.00	0.00
1438964_x_at	0.00	0.00
1438966_x_at	0.00	0.00
1438968_x_at	0.00	0.00
1438969_x_at	0.00	0.31
1438973_x_at	0.00	0.00
1438974_x_at	0.00	0.00
1438975_x_at	0.00	0.00
1438976_x_at	0.00	0.05
1438977_x_at	0.00	0.05
1438978_x_at	0.00	0.00
1438980_x_at	0.00	0.00

1438982_s_at	0.00	0.00
1438983_x_at	0.00	0.00
1438984_x_at	0.00	0.08
1438986_x_at	0.00	0.00
1438988_x_at	0.00	0.00
1438990_x_at	0.00	0.00
1438991_x_at	0.00	0.00
1438992_x_at	0.00	0.00
1438993_a_at	0.00	0.00
1438999_a_at	0.00	0.00
1439002_s_at	0.00	0.00
1439005_x_at	0.00	0.00
1439008_at	0.00	0.00
1439012_a_at	0.00	0.00
1439013_x_at	0.00	0.00
1439016_x_at	0.00	0.00
1439017_x_at	0.00	0.00
1439018_at	0.00	0.00
1439022_at	0.00	0.00
1439028_at	0.00	0.00
1439030_at	0.00	0.00
1439032_at	0.00	0.00
1439036_a_at	0.00	0.00
1439037_at	0.00	0.00
1439040_at	0.00	0.00
1439042_at	0.00	0.00
1439045_x_at	0.00	0.00
1439047_s_at	0.00	0.00
1439049_at	0.00	0.00
1439051_a_at	0.00	0.00
1439054_at	0.00	0.00
1439060_s_at	0.00	0.06
1439061_at	0.00	0.00
1439064_at	0.00	0.00
1439065_x_at	0.63	0.49
1439069_a_at	0.00	0.00
1439070_x_at	0.00	0.00
1439074_a_at	0.00	0.00
1439075_at	0.00	0.00
1439079_a_at	0.00	0.00
1439080_at	0.00	0.00
1439081_at	0.00	0.00
1439083_at	0.00	0.00
1439102_at	0.00	0.00
1439103_at	0.00	0.00
1439110_at	0.00	0.00
1439111_at	0.00	0.00
1439113_at	0.00	0.00
1439116_at	0.00	0.00
1439119_a_at	0.00	0.00
1439120_at	0.00	0.00
1439121_at	0.00	0.00
1439122_at	0.00	0.00
1439142_at	0.00	0.00
1439144_at	0.00	0.00
1439148_a_at	0.00	0.00

1439150_x_at	0.00	0.00
1439154_at	0.00	0.00
1439164_at	0.00	0.00
1439167_at	0.00	0.00
1439182_at	0.00	0.00
1439184_s_at	0.00	0.00
1439185_x_at	0.00	0.00
1439191_at	0.00	0.00
1439199_at	0.00	0.00
1439200_x_at	0.00	0.00
1439206_at	0.00	0.00
1439210_at	0.00	0.00
1439214_a_at	0.00	0.18
1439234_a_at	0.00	0.00
1439235_x_at	0.00	0.00
1439239_at	0.00	0.00
1439240_x_at	0.00	0.00
1439241_x_at	0.00	0.00
1439243_x_at	0.00	0.00
1439244_a_at	0.00	0.00
1439245_at	0.00	0.00
1439246_x_at	0.00	0.00
1439251_at	0.00	0.00
1439253_x_at	0.00	0.00
1439255_s_at	0.00	0.00
1439256_x_at	0.00	0.00
1439257_x_at	0.00	0.00
1439258_at	0.00	0.00
1439259_x_at	0.00	0.10
1439260_a_at	0.26	0.00
1439261_x_at	0.00	0.00
1439262_x_at	0.00	0.00
1439263_at	0.00	0.00
1439264_x_at	0.00	0.00
1439266_a_at	0.00	0.26
1439267_x_at	0.00	0.00
1439268_x_at	0.00	0.01
1439269_x_at	0.00	0.00
1439270_x_at	0.00	0.00
1439271_x_at	0.00	0.00
1439272_at	0.00	0.00
1439283_at	0.00	0.00
1439287_at	0.00	0.00
1439295_x_at	0.00	0.00
1439296_at	0.00	0.00
1439323_a_at	0.00	0.00
1439332_at	0.00	0.00
1439350_s_at	0.00	0.00
1439356_at	0.00	0.00
1439360_x_at	0.00	0.00
1439364_a_at	0.00	0.33
1439366_at	0.00	0.00
1439367_x_at	0.00	0.00
1439368_a_at	0.00	0.00
1439369_x_at	0.00	0.00
1439370_x_at	0.00	0.00

1439371_x_at	0.00	0.00
1439372_at	0.00	0.00
1439373_x_at	0.00	0.00
1439374_x_at	0.00	0.00
1439375_x_at	0.00	0.00
1439376_x_at	0.00	0.00
1439377_x_at	0.00	0.00
1439379_x_at	0.00	0.00
1439380_x_at	0.00	0.00
1439381_x_at	0.00	0.00
1439382_x_at	0.00	0.00
1439383_x_at	0.00	0.00
1439384_at	0.00	0.00
1439385_x_at	0.00	0.00
1439386_x_at	0.00	0.01
1439388_s_at	0.00	0.00
1439389_s_at	0.39	0.00
1439390_at	0.00	0.00
1439392_x_at	0.00	0.00
1439393_x_at	0.00	0.00
1439394_x_at	0.00	0.00
1439396_x_at	0.00	0.00
1439398_x_at	0.00	0.00
1439399_a_at	0.00	0.00
1439403_x_at	0.00	0.00
1439405_x_at	0.00	0.00
1439406_x_at	0.00	0.00
1439407_x_at	0.02	0.00
1439408_a_at	0.00	0.00
1439409_x_at	0.00	0.00
1439410_x_at	0.00	0.00
1439411_a_at	0.00	0.24
1439413_x_at	0.00	0.00
1439415_x_at	0.00	0.00
1439416_x_at	0.00	0.00
1439417_at	0.00	0.00
1439418_x_at	0.00	0.00
1439421_x_at	0.00	0.00
1439422_a_at	0.00	0.00
1439423_x_at	0.00	0.00
1439424_x_at	0.00	0.00
1439426_x_at	0.00	0.00
1439429_x_at	0.00	0.00
1439432_x_at	0.00	0.00
1439433_a_at	0.00	0.00
1439435_x_at	0.00	0.00
1439436_x_at	0.00	0.13
1439437_x_at	0.00	0.00
1439438_a_at	0.00	0.04
1439439_x_at	0.00	0.03
1439440_x_at	0.01	0.00
1439441_x_at	0.05	0.00
1439443_x_at	0.00	0.00
1439444_x_at	0.00	0.00
1439445_x_at	0.00	0.00
1439447_x_at	0.00	0.00

1439448_x_at	0.00	0.00
1439450_x_at	0.00	0.00
1439451_x_at	0.00	0.00
1439452_x_at	0.00	0.00
1439453_x_at	0.00	0.00
1439454_x_at	0.00	0.00
1439455_x_at	0.00	0.00
1439456_x_at	0.00	0.00
1439457_x_at	0.00	0.00
1439458_x_at	0.00	0.00
1439459_x_at	0.00	0.00
1439460_a_at	0.00	0.00
1439461_x_at	0.00	0.00
1439462_x_at	0.00	0.00
1439463_x_at	0.00	0.01
1439464_s_at	0.00	0.19
1439465_x_at	0.00	0.00
1439466_s_at	0.00	0.11
1439476_at	0.03	0.00
1439479_at	0.00	0.00
1439482_at	0.00	0.00
1439483_at	0.00	0.00
1439506_at	0.00	0.00
1439516_at	0.00	0.00
1439548_at	0.00	0.00
1439567_at	0.00	0.00
1439625_at	0.00	0.00
1439749_at	0.00	0.00
1439771_s_at	0.00	0.00
1439803_at	0.00	0.00
1439809_at	0.00	0.00
1439810_s_at	0.00	0.00
1439959_at	0.00	0.00
1439962_at	0.00	0.00
1439964_at	0.00	0.00
1439973_at	0.00	0.00
1439995_at	0.00	0.00
1440192_at	0.00	0.00
1440195_at	0.00	0.00
1440201_at	0.00	0.00
1440213_a_at	0.00	0.00
1440221_at	0.00	0.00
1440230_at	0.00	0.00
1440252_at	0.00	0.00
1440253_at	0.00	0.00
1440255_at	0.00	0.00
1440323_at	0.00	0.00
1440499_at	0.00	0.00
1440591_at	0.00	0.00
1440831_at	0.00	0.28
1440865_at	0.00	0.00
1440874_at	0.00	0.00
1440936_at	0.00	0.00
1440959_s_at	0.00	0.00
1440963_at	0.00	0.00
1440964_s_at	0.00	0.00

1440971_x_at	0.00	0.00
1441013_at	0.00	0.00
1441023_at	0.00	0.00
1441115_at	0.00	0.00
1441134_at	0.00	0.00
1441146_at	0.00	0.00
1441147_at	0.00	0.00
1441182_at	0.00	0.00
1441342_at	0.00	0.00
1441344_at	0.00	0.00
1441350_at	0.00	0.00
1441394_at	0.00	0.00
1441401_at	0.00	0.00
1441421_at	0.00	0.00
1441433_at	0.00	0.00
1441583_at	0.00	0.00
1441618_at	0.00	0.00
1441659_at	0.00	0.00
1441866_s_at	0.00	0.00
1441986_at	0.00	0.00
1441992_at	0.00	0.00
1442006_at	0.00	0.00
1442025_a_at	0.00	0.00
1442026_at	0.00	0.00
1442028_at	0.00	0.00
1442145_at	0.00	0.00
1442169_at	0.00	0.00
1442211_at	0.00	0.00
1442296_at	0.00	0.00
1442344_at	0.00	0.00
1442374_at	0.00	0.00
1442378_x_at	0.00	0.00
1442384_at	0.00	0.00
1442484_at	0.00	0.00
1442492_at	0.00	0.00
1442494_at	0.00	0.00
1442526_at	0.00	0.00
1442529_at	0.00	0.00
1442531_at	0.00	0.00
1442540_at	0.00	0.00
1442554_s_at	0.00	0.00
1442566_at	0.00	0.00
1442597_at	0.00	0.00
1442661_at	0.00	0.00
1442665_at	0.00	0.00
1442669_at	0.00	0.00
1442694_at	0.00	0.00
1442744_at	0.00	0.00
1442745_x_at	0.00	0.00
1442778_at	0.00	0.00
1442833_at	0.00	0.00
1442900_at	0.00	0.00
1442932_at	0.00	0.00
1442941_at	0.00	0.00
1442948_at	0.00	0.00
1442974_at	0.00	0.00

1442975_at	0.00	0.00
1442976_at	0.00	0.00
1442984_at	0.00	0.00
1443001_at	0.00	0.00
1443312_at	0.00	0.00
1443317_at	0.00	0.00
1443326_at	0.00	0.00
1443352_at	0.00	0.00
1443385_at	0.00	0.00
1443414_at	0.00	0.00
1443416_at	0.00	0.00
1443427_at	0.00	0.00
1443428_at	0.00	0.00
1443431_at	0.00	0.00
1443461_at	0.00	0.00
1443464_at	0.00	0.00
1443472_at	0.00	0.00
1443473_at	0.00	0.00
1443482_at	0.00	0.00
1443589_at	0.00	0.00
1443605_at	0.00	0.00
1443695_at	0.00	0.00
1443696_s_at	0.00	0.00
1443706_at	0.00	0.00
1443711_at	0.00	0.00
1443762_s_at	0.00	0.00
1443856_at	0.00	0.00
1443874_at	0.00	0.00
1443892_at	1.00	0.97
1443949_at	0.00	0.00
1443969_at	0.00	0.12
1443996_at	0.00	0.00
1444028_s_at	0.00	0.00
1444052_at	0.00	0.00
1444160_at	0.00	0.00
1444186_at	0.00	0.00
1444274_at	0.00	0.00
1444292_at	0.00	0.00
1444508_s_at	0.00	0.00
1444829_at	0.00	0.00
1444866_at	0.00	0.00
1444887_at	0.00	0.00
1444943_at	0.00	0.00
1444952_a_at	0.00	0.04
1444953_at	0.00	0.00
1444959_at	0.00	0.00
1445047_at	0.00	0.00
1445116_at	0.00	0.00
1445125_at	0.00	0.00
1445172_at	0.00	0.00
1445367_at	0.00	0.00
1445459_at	0.00	0.00
1445485_at	0.00	0.00
1445535_at	0.00	0.00
1445689_at	0.00	0.00
1446008_at	0.00	0.00

1446086_s_at	0.17	0.00
1446147_at	0.00	0.00
1446148_x_at	0.00	0.00
1446165_at	0.00	0.00
1446244_at	0.00	0.00
1446378_at	0.00	0.00
1446379_at	0.00	0.00
1446572_at	0.00	0.00
1446695_at	0.00	0.00
1446790_at	0.00	0.00
1446828_at	0.00	0.00
1446829_at	0.00	0.00
1446856_at	0.00	0.00
1446914_at	0.00	0.00
1446966_at	0.00	0.00
1446971_at	0.00	0.00
1447130_at	0.00	0.00
1447146_s_at	0.00	0.00
1447182_at	0.00	0.00
1447276_at	0.00	0.00
1447287_at	0.00	0.00
1447350_at	0.00	0.00
1447351_x_at	0.00	0.00
1447377_at	0.00	0.00
1447427_at	0.00	0.00
1447456_x_at	0.00	0.00
1447462_at	0.00	0.00
1447494_at	0.00	0.00
1447604_at	0.00	0.00
1447631_at	0.00	0.00
1447632_at	0.00	0.00
1447653_x_at	0.00	0.00
1447820_x_at	0.00	0.00
1447858_x_at	0.00	0.00
1447888_x_at	0.00	0.00
1447919_x_at	0.00	0.00
1447924_at	0.00	0.08
1447926_at	0.00	0.00
1447935_at	0.00	0.00
1447942_x_at	0.00	0.00
1447945_at	0.00	0.00
1447947_at	0.00	0.00
1447952_at	0.00	0.00
1447953_at	0.00	0.00
1447956_at	0.00	0.00
1447957_at	0.00	0.00
1447958_at	0.00	0.00
1447959_at	0.00	0.00
1447961_s_at	0.00	0.00
1447962_at	0.00	0.00
1447963_at	0.00	0.00
1447964_at	0.00	0.00
1447965_at	0.00	0.00
1447968_at	0.00	0.00
1447969_at	0.00	0.00
1447970_at	0.00	0.00

1447976_at	0.00	0.00
1447977_x_at	0.23	0.31
1447978_at	0.00	0.00
1447981_at	0.00	0.00
1447982_at	0.00	0.00
1447983_at	0.00	0.00
1447984_at	0.00	0.00
1447985_s_at	0.00	0.00
1447986_at	0.00	0.00
1447989_at	0.00	0.00
1447990_at	0.00	0.00
1447997_s_at	0.00	0.00
1447998_at	0.00	0.00
1447999_x_at	0.00	0.00
1448000_at	0.00	0.00
1448001_x_at	0.00	0.00
1448002_x_at	0.00	0.00
1448005_at	0.00	0.00
1448008_at	0.00	0.00
1448009_at	0.00	0.00
1448010_at	0.00	0.00
1448011_at	0.00	0.00
1448012_at	0.00	0.00
1448013_at	0.00	0.00
1448014_s_at	0.00	0.00
1448016_at	0.00	0.00
1448017_at	0.00	0.00
1448018_at	0.00	0.00
1448019_at	0.00	0.00
1448020_at	0.00	0.00
1448021_at	0.00	0.00
1448022_at	0.00	0.00
1448024_at	0.00	0.00
1448026_at	0.00	0.00
1448028_at	0.00	0.00
1448029_at	0.00	0.00
1448030_at	0.00	0.00
1448031_at	0.00	0.00
1448032_at	0.00	0.00
1448034_at	0.00	0.00
1448037_at	0.00	0.00
1448038_at	0.00	0.00
1448040_at	0.00	0.00
1448041_at	0.00	0.00
1448042_s_at	0.00	0.00
1448043_x_at	0.00	0.00
1448046_at	0.00	0.00
1448047_at	0.00	0.00
1448048_at	0.00	0.00
1448050_s_at	0.00	0.00
1448051_at	0.00	0.00
1448053_at	0.00	0.00
1448054_at	0.00	0.00
1448055_at	0.00	0.00
1448056_at	0.00	0.00
1448058_s_at	0.00	0.00

1448059_at	0.00	0.00
1448060_at	0.00	0.00
1448061_at	0.00	0.00
1448062_at	0.00	0.00
1448064_at	0.00	0.00
1448065_at	0.00	0.00
1448067_at	0.00	0.00
1448068_at	0.00	0.00
1448069_at	0.00	0.00
1448070_at	0.00	0.00
1448071_at	0.00	0.00
1448072_at	0.00	0.00
1448073_at	0.00	0.00
1448074_at	0.00	0.00
1448075_at	0.00	0.00
1448076_at	0.00	0.00
1448078_at	0.00	0.00
1448081_at	0.00	0.00
1448082_at	0.00	0.00
1448085_at	0.00	0.00
1448086_at	0.00	0.00
1448088_at	0.00	0.00
1448089_at	0.00	0.00
1448090_at	0.00	0.00
1448091_at	0.00	0.00
1448093_s_at	0.00	0.00
1448095_at	0.00	0.00
1448097_at	0.00	0.00
1448098_at	0.00	0.00
1448100_at	0.00	0.00
1448101_s_at	0.00	0.00
1448102_a_at	0.00	0.00
1448103_s_at	0.00	0.00
1448104_at	0.00	0.00
1448105_at	0.00	0.00
1448106_at	0.00	0.00
1448107_x_at	0.00	0.00
1448108_at	0.00	0.00
1448109_a_at	0.00	0.00
1448110_at	0.00	0.00
1448111_at	0.00	0.00
1448112_at	0.00	0.00
1448113_at	0.01	0.00
1448114_a_at	0.00	0.00
1448115_at	0.00	0.00
1448116_at	0.00	0.00
1448117_at	0.00	0.00
1448118_a_at	0.00	0.00
1448119_at	0.00	0.00
1448120_at	0.00	0.00
1448121_at	0.01	0.26
1448122_at	0.00	0.00
1448123_s_at	0.00	0.02
1448124_at	0.00	0.00
1448125_at	0.00	0.00
1448126_at	0.00	0.00

1448127_at	0.00	0.00
1448128_at	0.00	0.00
1448129_at	0.00	0.00
1448130_at	0.00	0.00
1448131_at	0.00	0.00
1448132_at	0.00	0.00
1448133_at	0.00	0.11
1448134_at	0.00	0.00
1448135_at	0.00	0.00
1448136_at	0.00	0.00
1448137_at	0.00	0.00
1448138_at	0.00	0.00
1448139_at	0.00	0.00
1448140_at	0.00	0.15
1448141_at	0.00	0.00
1448142_x_at	0.00	0.00
1448143_at	0.00	0.00
1448144_at	0.00	0.31
1448145_at	0.00	0.00
1448146_at	0.00	0.00
1448147_at	0.97	0.00
1448148_at	0.00	0.00
1448149_at	0.00	0.00
1448150_at	0.00	0.00
1448151_at	0.00	0.00
1448152_at	0.00	0.00
1448153_at	0.00	0.00
1448154_at	0.00	0.00
1448155_at	0.00	0.31
1448156_at	0.00	0.00
1448157_s_at	0.00	0.00
1448158_at	0.00	0.00
1448159_at	0.00	0.00
1448160_at	0.00	0.00
1448161_a_at	0.00	0.00
1448162_at	0.00	0.00
1448163_at	0.07	0.00
1448164_at	0.00	0.00
1448165_at	0.00	0.00
1448166_a_at	0.00	0.00
1448167_at	0.00	0.00
1448168_a_at	0.00	0.00
1448169_at	0.85	0.00
1448170_at	0.00	0.00
1448171_at	0.00	0.00
1448172_at	0.00	0.00
1448173_a_at	0.00	0.00
1448174_at	0.00	0.00
1448175_at	0.00	0.00
1448176_a_at	0.00	0.00
1448177_at	0.00	0.00
1448178_a_at	0.00	0.00
1448179_at	0.00	0.00
1448180_a_at	0.00	0.00
1448181_at	0.00	0.00
1448182_a_at	0.00	0.67

1448183_a_at	0.00	0.00
1448184_at	0.00	0.00
1448185_at	0.00	0.00
1448186_at	0.00	0.00
1448187_at	0.00	0.00
1448188_at	0.00	0.00
1448189_a_at	0.00	0.00
1448190_at	0.00	0.00
1448191_at	0.00	0.00
1448192_s_at	0.11	0.46
1448193_at	0.00	0.00
1448194_a_at	0.00	0.00
1448195_at	0.03	0.00
1448196_at	0.00	0.00
1448197_at	0.00	0.00
1448198_a_at	0.00	0.00
1448199_at	0.00	0.26
1448200_at	0.00	0.29
1448201_at	0.00	0.00
1448202_x_at	0.00	0.00
1448203_at	0.00	0.40
1448204_at	0.00	0.00
1448205_at	0.00	0.00
1448206_at	0.00	0.07
1448207_at	0.00	0.00
1448208_at	0.00	0.07
1448209_a_at	0.00	0.00
1448210_at	0.00	0.00
1448211_at	0.00	0.00
1448212_at	0.00	0.00
1448213_at	0.00	0.00
1448214_at	0.00	0.00
1448215_a_at	0.00	0.00
1448216_at	0.00	0.00
1448217_a_at	0.00	0.00
1448218_s_at	0.00	0.00
1448219_a_at	0.00	0.00
1448220_at	0.00	0.00
1448221_at	0.00	0.09
1448222_x_at	0.00	0.00
1448223_at	0.00	0.00
1448224_at	0.00	0.00
1448225_at	0.00	0.00
1448226_at	0.00	0.36
1448227_at	0.00	0.00
1448228_at	0.00	0.00
1448229_s_at	0.00	0.00
1448230_at	0.00	0.00
1448231_at	0.00	0.00
1448232_x_at	0.00	0.00
1448233_at	0.45	0.00
1448234_at	0.00	0.12
1448235_s_at	0.00	0.00
1448236_at	0.00	0.00
1448237_x_at	0.00	0.00
1448238_at	0.00	0.00

1448239_at	0.00	0.00
1448240_at	0.00	0.00
1448241_at	0.00	0.23
1448242_at	0.00	0.00
1448243_at	0.00	0.00
1448244_at	0.08	0.00
1448245_at	0.00	0.00
1448246_at	0.00	0.23
1448247_at	0.00	0.00
1448248_at	0.00	0.00
1448249_at	0.00	0.00
1448250_at	0.00	0.00
1448251_at	0.00	0.00
1448252_a_at	0.00	0.00
1448253_at	0.00	0.00
1448254_at	0.00	0.00
1448255_a_at	0.00	0.00
1448256_at	0.00	0.00
1448257_at	0.00	0.00
1448258_a_at	0.02	0.00
1448259_at	0.12	0.00
1448260_at	0.00	0.00
1448261_at	0.00	0.00
1448262_at	0.00	0.00
1448263_a_at	0.00	0.00
1448264_a_at	0.00	0.00
1448265_x_at	0.00	0.00
1448266_at	0.00	0.00
1448267_at	0.00	0.00
1448268_at	0.00	0.00
1448269_a_at	0.04	0.00
1448270_at	0.00	0.39
1448271_a_at	0.00	0.75
1448272_at	0.00	0.17
1448273_at	0.00	0.00
1448274_at	0.00	0.00
1448275_at	0.00	0.00
1448276_at	0.00	0.00
1448277_at	0.00	0.00
1448278_at	0.00	0.02
1448279_at	0.00	0.00
1448280_at	0.00	0.00
1448281_a_at	0.00	0.00
1448282_at	0.00	0.00
1448283_a_at	0.03	0.42
1448284_a_at	0.00	0.00
1448285_at	0.00	0.00
1448286_at	0.00	0.00
1448287_at	0.00	0.00
1448288_at	0.00	0.00
1448289_at	0.31	0.02
1448290_at	0.00	0.00
1448291_at	0.00	0.00
1448292_at	0.00	0.01
1448293_at	0.00	0.00
1448294_at	0.00	0.00

1448295_at	0.00	0.00
1448296_x_at	0.00	0.00
1448297_a_at	0.00	0.00
1448298_at	0.00	0.00
1448299_at	0.00	0.00
1448300_at	0.00	0.00
1448301_s_at	0.00	0.00
1448302_at	0.00	0.00
1448303_at	0.00	0.00
1448304_a_at	0.00	0.01
1448305_at	0.00	0.00
1448306_at	0.00	0.01
1448307_at	0.00	0.00
1448308_at	0.00	0.00
1448309_at	0.00	0.00
1448310_at	0.00	0.00
1448311_at	0.00	0.00
1448312_at	0.00	0.00
1448313_at	0.00	0.00
1448314_at	0.00	0.00
1448315_a_at	0.00	0.00
1448316_at	0.01	0.00
1448317_at	0.23	0.00
1448318_at	0.00	0.00
1448319_at	0.12	1.00
1448320_at	0.00	0.00
1448321_at	0.00	0.00
1448322_a_at	0.00	0.00
1448323_a_at	0.00	0.00
1448324_at	0.00	0.00
1448325_at	0.00	0.00
1448326_a_at	0.00	0.00
1448327_at	0.00	0.00
1448328_at	0.00	0.00
1448329_at	0.00	0.00
1448330_at	0.00	0.00
1448331_at	0.00	0.00
1448332_at	0.00	0.00
1448333_at	0.00	0.00
1448334_a_at	0.00	0.00
1448335_s_at	0.00	0.12
1448336_at	0.00	0.00
1448337_at	0.00	0.00
1448338_at	0.00	0.00
1448339_at	0.00	0.06
1448340_at	0.00	0.02
1448341_a_at	0.00	0.00
1448342_at	0.00	0.00
1448343_a_at	0.00	0.00
1448344_at	0.00	0.00
1448345_at	0.00	0.00
1448346_at	0.00	0.00
1448347_a_at	0.00	0.48
1448348_at	0.00	0.08
1448349_at	0.00	0.00
1448350_at	0.00	0.00

1448351_at	0.00	0.00
1448352_at	0.00	0.00
1448353_x_at	0.00	0.00
1448354_at	0.00	0.00
1448355_at	0.00	0.00
1448356_at	0.00	0.00
1448357_at	0.00	0.29
1448358_s_at	0.00	0.24
1448359_a_at	0.00	0.00
1448360_s_at	0.00	0.00
1448361_at	0.01	0.00
1448362_at	0.00	0.57
1448363_at	0.00	0.00
1448364_at	0.00	0.00
1448365_at	0.00	0.00
1448366_at	0.00	0.00
1448367_at	0.00	0.00
1448368_at	0.00	0.00
1448369_at	0.00	0.00
1448370_at	0.38	0.00
1448371_at	0.08	0.00
1448372_a_at	0.00	0.00
1448373_at	0.02	0.59
1448374_at	0.00	0.00
1448375_at	0.00	0.00
1448376_at	0.00	0.18
1448377_at	0.00	0.00
1448378_at	0.00	0.00
1448379_at	0.00	0.00
1448380_at	0.00	0.00
1448381_at	0.00	0.00
1448382_at	0.00	0.00
1448383_at	0.00	0.00
1448384_at	0.00	0.00
1448385_at	0.00	0.00
1448386_a_at	0.00	0.00
1448387_at	0.00	0.00
1448388_a_at	0.00	0.00
1448389_at	0.00	0.00
1448390_a_at	0.00	0.00
1448391_at	0.00	0.00
1448392_at	0.38	0.00
1448393_at	0.00	1.00
1448394_at	0.00	0.00
1448395_at	0.00	0.00
1448396_at	0.00	0.01
1448397_at	0.00	0.00
1448398_s_at	0.00	0.71
1448399_at	0.00	0.00
1448400_a_at	0.00	0.00
1448401_at	0.00	0.00
1448402_at	0.00	0.00
1448403_at	0.00	0.48
1448404_at	0.00	0.00
1448405_a_at	0.00	0.00
1448406_at	0.00	0.00

1448407_at	0.00	0.00
1448408_at	0.00	0.00
1448409_at	0.00	0.00
1448410_at	0.00	0.00
1448411_at	0.00	0.00
1448412_a_at	0.00	0.00
1448413_at	0.00	0.00
1448414_at	0.00	0.08
1448415_a_at	0.00	0.00
1448416_at	0.00	0.00
1448417_at	0.00	0.00
1448418_s_at	0.00	0.00
1448419_at	0.00	0.00
1448420_a_at	0.00	0.13
1448421_s_at	0.00	0.00
1448422_at	0.00	0.00
1448423_at	0.00	0.00
1448424_at	0.00	0.00
1448425_at	0.00	0.00
1448426_at	0.00	0.00
1448427_at	0.00	0.04
1448428_at	0.00	0.00
1448429_at	0.00	0.01
1448430_a_at	0.00	0.00
1448431_at	0.00	0.00
1448432_at	0.00	0.02
1448433_a_at	0.00	0.35
1448434_at	0.14	0.00
1448435_at	0.00	0.00
1448436_a_at	0.00	0.00
1448437_a_at	0.00	0.00
1448438_at	0.00	0.00
1448439_at	0.00	0.00
1448440_x_at	0.00	0.00
1448441_at	0.00	0.00
1448442_a_at	0.00	0.00
1448443_at	0.00	0.00
1448444_at	0.00	0.00
1448445_at	0.00	0.06
1448446_at	0.00	0.00
1448447_at	0.00	0.00
1448448_a_at	0.00	0.01
1448449_at	0.00	0.02
1448450_at	0.00	0.00
1448451_at	0.00	0.00
1448452_at	0.00	0.00
1448453_at	0.00	0.00
1448454_at	0.00	0.43
1448455_at	0.00	0.00
1448456_at	0.00	0.00
1448457_at	0.00	0.00
1448458_at	0.00	0.00
1448459_at	0.00	0.00
1448460_at	0.00	0.00
1448461_a_at	0.00	0.00
1448462_at	0.00	0.00

1448463_at	0.00	0.00
1448464_at	0.00	0.00
1448465_at	0.00	0.00
1448466_at	0.00	0.01
1448467_a_at	0.00	0.00
1448468_a_at	0.00	0.00
1448469_at	0.00	0.00
1448470_at	0.00	0.00
1448471_a_at	0.00	0.00
1448472_at	0.00	0.00
1448473_at	0.00	0.00
1448474_at	0.00	0.02
1448475_at	0.00	0.00
1448476_at	0.00	0.00
1448477_at	0.00	0.00
1448478_at	0.00	0.00
1448479_at	0.00	0.00
1448480_at	0.00	0.77
1448481_at	0.00	0.00
1448482_at	0.00	0.89
1448483_a_at	0.00	0.00
1448484_at	0.00	0.01
1448485_at	0.00	0.00
1448486_at	0.00	0.00
1448487_at	0.00	0.00
1448488_at	0.00	0.00
1448489_at	0.00	0.00
1448490_at	0.00	0.00
1448491_at	0.00	0.33
1448492_a_at	0.00	0.44
1448493_at	0.00	0.00
1448494_at	0.01	0.00
1448495_at	0.00	0.00
1448496_a_at	0.00	0.00
1448497_at	0.00	0.14
1448498_at	0.00	0.00
1448499_a_at	0.00	0.00
1448500_a_at	0.00	0.00
1448501_at	0.07	0.00
1448502_at	0.00	0.00
1448503_at	0.00	0.10
1448504_a_at	0.00	0.00
1448505_at	0.00	0.27
1448506_at	0.00	0.00
1448507_at	0.00	0.00
1448508_at	0.00	0.00
1448509_at	0.00	0.00
1448510_at	0.00	0.00
1448511_at	0.00	0.00
1448512_at	0.00	0.00
1448513_a_at	0.00	0.35
1448514_at	0.00	0.00
1448515_at	0.00	0.22
1448516_at	0.00	0.00
1448517_at	0.00	0.00
1448518_at	0.00	0.00

1448519_at	0.00	0.00
1448520_at	0.00	0.00
1448521_at	0.00	0.00
1448522_at	0.00	0.00
1448523_at	0.00	0.00
1448524_s_at	0.00	0.00
1448525_a_at	0.00	0.00
1448526_at	0.00	0.00
1448527_at	0.00	0.10
1448528_at	0.00	0.00
1448529_at	0.00	0.00
1448530_at	0.00	0.00
1448531_at	0.00	0.00
1448532_at	0.00	0.00
1448533_at	0.00	0.00
1448534_at	0.00	0.00
1448535_at	0.00	0.00
1448536_at	0.00	0.00
1448537_at	0.00	0.00
1448538_a_at	0.00	0.00
1448539_a_at	0.00	0.00
1448540_a_at	0.00	0.00
1448541_at	0.00	0.00
1448542_at	0.00	0.14
1448543_at	0.00	0.28
1448544_at	0.00	0.00
1448545_at	0.00	0.00
1448546_at	0.00	0.03
1448547_at	0.00	0.00
1448548_at	0.00	0.00
1448549_a_at	0.00	0.00
1448550_at	0.00	0.00
1448551_a_at	0.99	0.57
1448552_s_at	0.00	0.00
1448553_at	0.00	0.00
1448554_s_at	0.00	0.00
1448555_at	0.00	0.00
1448556_at	0.00	0.00
1448557_at	0.00	0.00
1448558_a_at	0.00	0.00
1448559_at	0.00	0.20
1448560_at	0.00	0.00
1448561_at	0.00	0.00
1448562_at	0.34	0.01
1448563_at	0.00	0.93
1448564_at	0.00	0.00
1448565_at	0.00	0.00
1448566_at	0.00	0.27
1448567_at	0.00	0.00
1448568_a_at	0.00	0.00
1448569_at	0.00	0.00
1448570_at	0.00	0.00
1448571_a_at	0.00	0.00
1448572_at	0.00	0.00
1448573_a_at	0.00	0.00
1448574_at	0.00	0.00

1448575_at	0.00	0.00
1448576_at	0.00	0.00
1448577_x_at	0.00	0.00
1448578_at	0.00	0.00
1448579_at	0.00	0.00
1448580_at	0.00	0.00
1448581_at	0.00	0.00
1448582_at	0.00	0.00
1448583_at	0.00	0.00
1448584_at	0.00	0.00
1448585_at	0.00	0.22
1448586_at	0.00	0.06
1448587_at	0.00	0.00
1448588_at	0.00	0.00
1448589_at	0.00	0.00
1448590_at	0.00	0.00
1448591_at	0.00	0.00
1448592_at	0.00	0.00
1448593_at	0.00	0.00
1448594_at	0.02	0.00
1448595_a_at	0.00	0.00
1448596_at	0.00	0.00
1448597_at	0.00	0.00
1448598_at	0.00	0.00
1448599_s_at	0.00	0.00
1448600_s_at	0.00	0.00
1448601_s_at	0.00	0.00
1448602_at	0.00	0.00
1448603_at	0.00	0.00
1448604_at	0.00	0.00
1448605_at	0.00	0.00
1448606_at	0.00	0.00
1448607_at	0.00	0.00
1448608_at	0.00	0.00
1448609_at	0.09	0.00
1448610_a_at	0.53	0.94
1448611_at	0.00	0.16
1448612_at	0.00	0.00
1448613_at	0.00	0.00
1448614_at	0.00	0.00
1448615_at	0.00	0.00
1448616_at	0.00	0.00
1448617_at	0.00	0.00
1448618_at	0.00	0.00
1448619_at	0.00	0.00
1448620_at	0.00	0.00
1448621_a_at	0.00	0.07
1448622_at	0.00	0.00
1448623_at	0.49	0.00
1448624_at	0.00	0.07
1448625_at	0.00	0.00
1448626_at	0.00	0.01
1448627_s_at	0.00	0.00
1448628_at	0.00	0.00
1448629_at	0.00	0.00
1448630_a_at	0.00	0.00

1448631_a_at	0.00	0.00
1448632_at	0.00	0.00
1448633_at	0.00	0.24
1448634_at	0.00	0.00
1448635_at	0.00	0.04
1448636_at	0.00	0.00
1448637_at	0.00	0.00
1448638_at	0.00	0.00
1448639_a_at	0.00	0.00
1448640_at	0.00	0.00
1448641_at	0.00	0.00
1448642_at	0.00	0.00
1448643_at	0.00	0.00
1448644_at	0.00	0.00
1448645_at	0.00	0.00
1448646_at	0.00	0.09
1448647_at	0.00	0.00
1448648_at	0.00	0.00
1448649_at	0.00	0.00
1448650_a_at	0.00	0.00
1448651_at	0.00	0.19
1448652_at	0.00	0.00
1448653_at	0.11	0.61
1448654_at	0.00	0.00
1448655_at	0.00	0.00
1448656_at	0.00	0.00
1448657_a_at	0.00	0.01
1448658_at	0.00	0.30
1448659_at	0.00	0.00
1448660_at	0.00	0.00
1448661_at	0.00	0.00
1448662_at	0.00	0.00
1448663_s_at	0.00	0.00
1448664_a_at	0.00	0.00
1448665_at	0.00	0.00
1448666_s_at	0.00	0.00
1448667_x_at	0.00	0.00
1448668_a_at	0.00	0.00
1448669_at	0.00	0.00
1448670_at	0.00	0.00
1448671_at	0.00	0.00
1448672_a_at	0.00	0.00
1448673_at	0.00	0.00
1448674_at	0.00	0.00
1448675_at	0.00	0.00
1448676_at	0.00	0.00
1448677_at	0.00	0.03
1448678_at	0.00	0.00
1448679_at	0.00	0.00
1448680_at	0.00	0.00
1448681_at	0.00	0.00
1448682_at	0.00	0.00
1448683_at	0.00	0.00
1448684_at	0.00	0.00
1448685_at	0.00	0.00
1448686_at	0.00	0.00

1448687_at	0.00	0.00
1448688_at	0.87	1.00
1448689_at	0.13	0.00
1448690_at	0.00	0.00
1448691_at	0.00	0.00
1448692_at	0.02	0.00
1448693_at	0.00	0.00
1448694_at	0.00	0.00
1448695_at	0.00	0.00
1448696_at	0.00	0.00
1448697_s_at	0.00	0.00
1448698_at	0.00	0.33
1448699_at	0.00	0.00
1448700_at	0.00	0.00
1448701_a_at	0.00	0.00
1448702_at	0.00	0.00
1448703_at	0.00	0.00
1448704_s_at	0.00	0.00
1448705_at	0.00	0.00
1448706_at	0.00	0.00
1448707_at	0.00	0.60
1448708_at	0.00	0.00
1448709_at	0.00	0.00
1448710_at	0.00	0.00
1448711_at	0.00	0.00
1448712_at	0.00	0.00
1448713_at	0.00	0.00
1448714_at	0.00	0.00
1448715_x_at	0.00	0.22
1448716_at	0.00	0.00
1448717_at	0.00	0.00
1448718_at	0.00	0.00
1448719_at	0.00	0.00
1448720_at	0.00	0.00
1448721_at	0.00	0.00
1448722_s_at	0.00	0.42
1448723_at	0.00	0.00
1448724_at	0.00	0.00
1448725_at	0.00	0.01
1448726_at	0.00	0.00
1448727_at	0.00	0.00
1448728_a_at	0.00	0.00
1448729_a_at	0.00	0.12
1448730_at	0.00	0.00
1448731_at	0.00	0.00
1448732_at	0.00	0.00
1448733_at	0.00	0.00
1448734_at	0.00	0.00
1448735_at	0.00	0.00
1448736_a_at	0.00	0.00
1448737_at	0.00	0.00
1448738_at	0.00	0.00
1448739_x_at	0.00	0.00
1448740_at	0.00	0.00
1448741_at	0.00	0.00
1448742_at	0.01	0.00

1448743_at	0.00	0.00
1448744_at	0.00	0.00
1448745_s_at	0.00	0.00
1448746_at	0.00	0.00
1448747_at	0.00	0.00
1448748_at	0.00	0.00
1448749_at	0.00	0.00
1448750_at	0.00	0.00
1448751_at	0.00	0.00
1448752_at	0.00	0.00
1448753_at	0.00	0.00
1448754_at	0.29	0.00
1448755_at	0.00	0.00
1448756_at	0.00	0.00
1448757_at	0.55	0.00
1448758_at	0.00	0.00
1448759_at	0.00	0.00
1448760_at	0.00	0.00
1448761_a_at	0.00	0.00
1448762_at	0.00	0.07
1448763_at	0.00	0.00
1448764_a_at	0.00	0.00
1448765_at	0.00	0.00
1448766_at	0.00	0.00
1448767_s_at	0.00	0.00
1448768_at	0.00	0.00
1448769_at	0.00	0.00
1448770_a_at	0.00	0.00
1448771_a_at	0.00	0.00
1448772_at	0.00	0.14
1448773_at	0.00	0.00
1448774_at	0.00	0.00
1448775_at	0.00	0.00
1448776_at	0.00	0.00
1448777_at	0.01	0.00
1448778_at	0.00	0.00
1448779_at	0.00	0.02
1448780_at	0.00	0.00
1448781_at	0.00	0.00
1448782_at	0.00	0.00
1448783_at	0.00	0.00
1448784_at	0.00	0.00
1448785_at	0.00	0.00
1448786_at	0.00	0.00
1448787_at	0.00	0.00
1448788_at	0.00	0.00
1448789_at	0.00	0.00
1448790_at	0.00	0.00
1448791_at	0.00	0.30
1448792_a_at	0.00	0.00
1448793_a_at	0.00	0.03
1448794_s_at	0.00	0.00
1448795_a_at	0.00	0.01
1448796_s_at	0.00	0.23
1448797_at	0.00	0.00
1448798_at	0.00	0.00

1448799_s_at	0.00	0.17
1448800_at	0.00	0.00
1448801_a_at	0.00	0.00
1448802_at	0.00	0.01
1448803_at	0.00	0.00
1448804_at	0.00	0.00
1448805_at	0.00	0.00
1448806_at	0.00	0.00
1448807_at	0.00	0.00
1448808_a_at	0.00	0.00
1448809_at	0.00	0.00
1448810_at	0.00	0.00
1448811_at	0.00	0.00
1448812_at	0.00	0.33
1448813_at	0.00	0.00
1448814_at	0.00	0.00
1448815_at	0.00	0.00
1448816_at	0.00	0.00
1448817_at	0.00	0.00
1448818_at	0.00	0.00
1448819_at	0.00	0.00
1448820_a_at	0.00	0.00
1448821_at	0.00	0.00
1448822_at	0.00	0.00
1448823_at	0.00	0.00
1448824_at	0.01	0.00
1448825_at	0.00	0.00
1448826_at	0.00	0.00
1448827_s_at	0.00	0.00
1448828_at	0.00	0.00
1448829_at	0.00	0.00
1448830_at	0.20	0.00
1448831_at	0.00	0.00
1448832_a_at	0.00	0.00
1448833_at	0.00	0.00
1448834_at	0.00	0.00
1448835_at	0.00	0.00
1448836_s_at	0.00	0.00
1448837_at	0.00	0.00
1448838_at	0.00	0.02
1448839_at	0.23	0.03
1448840_at	0.00	0.00
1448841_at	0.00	0.00
1448842_at	0.00	0.00
1448843_at	0.00	0.00
1448844_at	0.00	0.36
1448845_at	0.69	1.00
1448846_a_at	0.00	0.00
1448847_at	0.00	0.00
1448848_at	0.00	0.00
1448849_at	0.00	0.32
1448850_a_at	0.00	0.00
1448851_a_at	0.00	0.00
1448852_at	0.00	0.00
1448853_at	0.00	0.00
1448854_s_at	0.00	0.00

1448855_at	0.00	0.00
1448856_a_at	0.00	0.00
1448857_a_at	0.00	0.00
1448858_at	0.00	0.10
1448859_at	0.00	0.00
1448860_at	0.00	0.00
1448861_at	0.00	0.00
1448862_at	0.00	0.00
1448863_a_at	0.00	0.00
1448864_at	0.00	0.00
1448865_at	0.00	0.00
1448866_at	0.00	0.10
1448867_at	0.00	0.00
1448868_at	0.00	0.14
1448869_a_at	0.00	0.00
1448870_at	0.01	0.00
1448871_at	0.00	0.00
1448872_at	0.00	0.00
1448873_at	0.00	0.33
1448874_a_at	0.00	0.00
1448875_at	0.00	0.00
1448876_at	0.02	0.00
1448877_at	0.00	0.00
1448878_at	0.00	0.00
1448879_at	0.00	0.00
1448880_at	0.00	0.00
1448881_at	0.00	0.00
1448882_at	0.00	0.00
1448883_at	0.00	0.01
1448884_at	0.00	0.00
1448885_at	0.00	0.00
1448886_at	0.03	0.00
1448887_x_at	0.00	0.00
1448888_at	0.00	0.12
1448889_at	0.00	0.00
1448890_at	1.00	0.01
1448891_at	0.00	0.00
1448892_at	0.00	0.00
1448893_at	0.00	0.00
1448894_at	0.00	0.00
1448895_a_at	0.00	0.00
1448896_at	0.00	0.00
1448897_at	0.00	0.00
1448898_at	0.00	0.00
1448899_s_at	0.00	0.00
1448900_at	0.00	0.00
1448901_at	0.00	0.00
1448902_at	0.00	0.00
1448903_at	0.00	0.00
1448904_at	0.00	0.00
1448905_at	0.00	0.00
1448906_at	0.00	0.00
1448907_at	0.00	0.03
1448908_at	0.00	0.00
1448909_a_at	0.00	0.00
1448910_at	0.00	0.00

1448911_at	0.00	0.00
1448912_at	0.00	0.00
1448913_at	0.00	0.00
1448914_a_at	0.02	0.00
1448915_at	0.00	0.00
1448916_at	0.00	0.00
1448917_at	0.00	0.00
1448918_at	0.00	0.00
1448919_at	0.00	0.00
1448920_at	0.00	0.00
1448921_a_at	0.00	0.27
1448922_at	0.00	0.00
1448923_at	0.00	0.21
1448924_at	0.00	0.00
1448925_at	0.00	0.00
1448926_at	0.00	0.00
1448927_at	0.00	0.00
1448928_at	0.00	0.00
1448929_at	0.00	0.00
1448930_at	0.00	0.00
1448931_at	0.00	0.00
1448932_at	0.00	0.00
1448933_at	0.00	0.00
1448934_at	0.00	0.00
1448935_at	0.00	0.00
1448936_at	0.00	0.00
1448937_at	0.00	0.00
1448938_at	0.00	0.00
1448939_at	0.00	0.00
1448940_at	0.00	0.00
1448941_at	0.00	0.00
1448942_at	0.00	0.00
1448943_at	0.00	0.00
1448944_at	0.00	0.00
1448945_at	0.00	0.00
1448946_at	0.00	0.00
1448947_at	0.00	0.24
1448948_at	0.00	0.00
1448949_at	0.00	1.00
1448950_at	0.00	0.00
1448951_at	0.00	0.00
1448952_at	0.00	0.00
1448953_at	0.00	0.00
1448954_at	0.00	0.00
1448955_s_at	0.00	0.00
1448956_at	0.00	0.33
1448957_at	0.00	0.00
1448958_at	0.00	0.00
1448959_at	0.00	0.00
1448960_at	0.00	0.00
1448961_at	0.00	0.00
1448962_at	0.00	0.00
1448963_at	0.00	0.34
1448964_at	0.00	0.00
1448965_at	0.00	0.00
1448966_a_at	0.00	0.00

1448967_at	0.00	0.00
1448968_at	0.00	0.14
1448969_at	0.00	0.00
1448970_at	0.00	0.00
1448971_at	0.00	0.00
1448972_at	0.00	0.00
1448973_at	0.00	0.00
1448974_at	0.00	0.00
1448975_s_at	0.00	0.00
1448976_at	0.00	0.00
1448977_at	0.58	0.00
1448978_at	0.00	0.00
1448979_at	0.00	0.00
1448980_at	0.00	0.00
1448981_x_at	0.00	0.00
1448982_at	0.00	0.00
1448983_at	0.00	0.00
1448984_at	0.00	0.00
1448985_at	0.00	0.00
1448986_x_at	0.00	0.00
1448987_at	0.00	0.62
1448988_at	0.00	0.00
1448989_a_at	0.00	0.00
1448990_a_at	0.00	0.08
1448991_a_at	0.96	0.04
1448992_at	0.97	0.00
1448993_at	0.00	0.00
1448994_at	0.00	0.00
1448995_at	0.00	0.00
1448996_at	0.00	0.00
1448997_at	0.00	0.00
1448998_at	0.00	0.00
1448999_at	0.00	0.00
1449000_at	0.00	0.00
1449001_at	0.00	0.00
1449002_at	0.00	0.00
1449003_a_at	0.00	0.00
1449004_at	0.00	0.00
1449005_at	0.00	0.00
1449006_at	0.00	0.00
1449007_at	0.00	0.00
1449008_at	0.00	0.00
1449009_at	0.00	0.00
1449010_at	0.00	0.00
1449011_at	0.00	0.00
1449012_s_at	0.00	0.00
1449013_at	0.00	0.00
1449014_at	0.00	0.00
1449015_at	0.00	0.00
1449016_at	0.00	0.00
1449017_at	0.00	0.00
1449018_at	0.00	0.10
1449019_at	0.00	0.00
1449020_at	0.00	0.00
1449021_at	0.00	0.00
1449022_at	0.06	0.00

1449023_a_at	0.00	0.01
1449024_a_at	0.00	0.99
1449025_at	0.00	0.00
1449026_at	0.00	0.00
1449027_at	0.00	0.71
1449028_at	0.00	0.87
1449029_at	0.00	0.00
1449030_at	0.00	0.00
1449031_at	0.00	0.01
1449032_at	0.00	0.00
1449033_at	0.00	0.00
1449034_at	0.00	0.00
1449035_at	0.00	0.00
1449036_at	0.83	0.33
1449037_at	0.00	0.00
1449038_at	0.00	0.00
1449039_a_at	0.00	0.02
1449040_a_at	0.02	0.08
1449041_a_at	0.00	0.00
1449042_at	0.00	0.00
1449043_at	0.00	0.05
1449044_at	0.00	0.00
1449045_at	0.00	0.00
1449046_a_at	0.00	0.00
1449047_at	0.00	0.00
1449048_s_at	0.00	0.00
1449049_at	0.00	0.00
1449050_at	0.00	0.00
1449051_at	0.00	0.00
1449052_a_at	0.00	0.00
1449053_s_at	0.00	0.00
1449054_a_at	0.00	0.00
1449055_x_at	0.00	0.00
1449056_at	0.00	0.00
1449057_at	0.00	0.00
1449058_at	1.00	0.68
1449059_a_at	0.00	0.00
1449060_at	0.00	0.00
1449061_a_at	0.00	0.00
1449062_at	0.00	0.00
1449063_at	0.00	0.00
1449064_at	1.00	0.65
1449065_at	0.00	0.08
1449066_a_at	0.00	0.00
1449067_at	0.00	0.00
1449068_at	0.00	0.00
1449069_at	0.00	0.00
1449070_x_at	0.00	0.00
1449071_at	0.00	0.00
1449072_a_at	0.00	0.67
1449073_at	0.56	0.00
1449074_at	0.00	0.00
1449075_at	0.00	0.00
1449076_x_at	0.00	0.00
1449077_at	0.00	0.00
1449078_at	0.00	0.00

1449079_s_at	0.00	0.00
1449080_at	0.00	0.00
1449081_at	0.00	0.00
1449082_at	0.00	0.00
1449083_at	0.00	0.00
1449084_s_at	0.00	0.41
1449085_at	0.00	0.00
1449086_at	0.00	0.00
1449087_at	0.00	0.00
1449088_at	0.00	0.00
1449089_at	0.00	0.00
1449090_a_at	0.00	0.05
1449091_at	0.00	0.00
1449092_at	0.00	0.00
1449093_at	0.00	0.00
1449094_at	0.00	0.00
1449095_at	0.00	0.00
1449096_at	0.00	0.00
1449097_at	0.00	0.00
1449098_a_at	0.00	0.00
1449099_at	0.00	0.00
1449100_at	0.00	0.00
1449101_at	0.00	0.00
1449102_at	0.00	0.00
1449103_at	0.00	0.00
1449104_at	0.00	0.00
1449105_at	0.00	0.00
1449106_at	0.00	0.34
1449107_at	0.00	0.00
1449108_at	0.00	0.00
1449109_at	0.95	0.00
1449110_at	1.00	0.02
1449111_a_at	0.00	0.00
1449112_at	0.00	0.00
1449113_at	0.00	0.00
1449114_at	0.00	0.00
1449115_at	0.19	0.22
1449116_a_at	0.01	0.63
1449117_at	0.00	0.02
1449118_at	0.00	0.00
1449119_at	0.00	0.00
1449120_a_at	0.00	0.00
1449121_at	0.00	0.00
1449122_at	0.00	0.00
1449123_at	0.00	0.00
1449124_at	0.00	0.00
1449125_at	0.00	0.00
1449126_at	0.00	0.00
1449127_at	0.00	0.00
1449128_at	0.00	0.00
1449129_a_at	0.00	0.00
1449130_at	0.00	0.00
1449131_s_at	0.00	0.00
1449132_at	0.00	0.00
1449133_at	0.00	0.00
1449134_s_at	0.00	0.00

1449135_at	0.00	0.00
1449136_at	0.00	0.00
1449137_at	0.00	0.00
1449138_at	0.00	0.00
1449139_at	0.00	0.00
1449140_at	0.00	0.00
1449141_at	0.42	0.06
1449142_a_at	0.00	0.00
1449143_at	0.00	0.00
1449144_at	0.00	0.00
1449145_a_at	0.00	0.00
1449146_at	0.00	0.00
1449147_at	0.00	0.00
1449148_a_at	0.00	0.00
1449149_at	0.00	0.00
1449150_at	0.00	0.00
1449151_at	0.00	0.00
1449152_at	0.00	0.00
1449153_at	0.00	0.00
1449154_at	0.00	0.00
1449155_at	0.00	0.08
1449156_at	0.00	0.00
1449157_at	0.00	0.00
1449158_at	0.00	0.00
1449159_at	0.00	0.00
1449160_at	0.00	0.00
1449161_at	0.00	0.00
1449162_at	0.00	0.02
1449163_at	0.00	0.00
1449164_at	0.00	0.00
1449165_at	0.00	0.00
1449166_at	0.00	0.00
1449167_at	0.00	0.01
1449168_a_at	0.00	0.00
1449169_at	0.00	0.00
1449170_at	0.00	0.00
1449171_at	0.00	0.00
1449172_a_at	0.00	0.00
1449173_at	0.00	0.00
1449174_at	0.00	0.00
1449175_at	0.00	0.00
1449176_a_at	0.00	0.00
1449177_at	0.00	0.00
1449178_at	0.00	0.13
1449179_at	0.00	0.00
1449180_at	0.00	0.00
1449181_at	0.00	0.00
1449182_at	0.00	0.00
1449183_at	0.00	0.00
1449184_at	0.00	0.00
1449185_at	0.00	0.00
1449186_at	0.00	0.05
1449187_at	0.00	0.00
1449188_at	0.00	0.00
1449189_at	0.00	0.00
1449190_a_at	0.00	0.00

1449191_at	0.00	0.00
1449192_at	0.16	0.00
1449193_at	0.00	0.00
1449194_at	0.00	0.00
1449195_s_at	0.00	0.00
1449196_a_at	0.00	0.00
1449197_at	0.00	0.00
1449198_a_at	0.00	0.00
1449199_at	0.00	0.00
1449200_at	0.00	0.00
1449201_at	0.00	0.00
1449202_at	0.00	0.00
1449203_at	0.00	0.00
1449204_at	0.00	0.00
1449205_at	0.00	0.00
1449206_at	0.00	0.00
1449207_a_at	0.00	0.22
1449208_at	0.00	0.00
1449209_a_at	0.00	0.00
1449210_at	0.00	0.00
1449211_at	0.00	0.00
1449212_at	0.00	0.00
1449213_at	0.00	0.00
1449214_a_at	0.00	0.00
1449215_at	0.00	0.00
1449216_at	0.00	0.00
1449217_at	0.00	0.00
1449218_at	0.00	0.00
1449219_at	0.00	0.00
1449220_at	0.00	0.00
1449221_a_at	0.00	0.00
1449222_at	0.00	0.00
1449223_at	0.00	0.00
1449224_at	0.00	0.00
1449225_a_at	0.00	0.00
1449226_at	0.00	0.00
1449227_at	0.00	0.00
1449228_at	0.00	0.00
1449229_a_at	0.00	0.00
1449230_at	0.00	0.00
1449231_at	0.48	0.16
1449232_at	0.00	0.00
1449233_at	0.00	0.00
1449234_at	0.00	0.00
1449235_at	0.00	0.00
1449236_at	0.00	0.00
1449237_at	0.00	0.00
1449238_at	0.00	0.00
1449239_at	0.00	0.00
1449240_at	0.00	0.00
1449241_at	0.00	0.00
1449242_s_at	0.00	0.00
1449243_a_at	0.00	0.00
1449244_at	0.84	0.00
1449245_at	0.00	0.00
1449246_at	0.00	0.00

1449247_at	0.00	0.00
1449248_at	0.00	0.00
1449249_at	0.00	0.00
1449250_at	0.00	0.00
1449251_at	0.00	0.00
1449252_at	0.00	0.00
1449253_at	0.00	0.05
1449254_at	0.02	0.55
1449255_a_at	0.00	0.00
1449256_a_at	0.01	0.01
1449257_at	0.00	0.00
1449258_at	0.00	0.00
1449259_at	0.00	0.00
1449260_at	0.00	0.00
1449261_at	0.00	0.00
1449262_s_at	0.00	0.00
1449263_at	0.00	0.00
1449264_at	0.00	0.00
1449265_at	0.00	0.00
1449266_at	0.00	0.00
1449267_at	0.00	0.00
1449268_at	0.00	0.00
1449269_at	0.00	0.00
1449270_at	0.00	0.00
1449271_a_at	0.00	0.00
1449272_at	0.00	0.00
1449273_at	0.00	0.34
1449274_at	0.00	0.00
1449275_at	0.00	0.00
1449276_at	0.00	0.00
1449277_at	0.00	0.00
1449278_at	0.00	0.00
1449279_at	0.00	0.00
1449280_at	0.00	0.00
1449281_at	0.00	0.00
1449282_at	0.00	0.00
1449283_a_at	0.00	0.02
1449284_at	0.00	0.00
1449285_at	0.00	0.00
1449286_at	0.00	0.00
1449287_at	0.00	0.00
1449288_at	1.00	0.75
1449289_a_at	0.90	0.08
1449290_at	0.00	0.00
1449291_a_at	0.00	0.00
1449292_at	0.00	0.00
1449293_a_at	0.00	0.00
1449294_at	0.00	0.00
1449295_at	0.00	0.00
1449296_a_at	0.00	0.00
1449297_at	0.00	0.00
1449298_a_at	0.00	0.00
1449299_at	0.00	0.03
1449300_at	0.00	0.00
1449301_at	0.00	0.00
1449302_at	0.00	0.00

1449303_at	0.00	0.06
1449304_at	0.00	0.00
1449305_at	0.00	0.00
1449306_at	0.00	0.00
1449307_at	0.00	0.00
1449308_at	0.00	0.00
1449309_at	0.00	0.00
1449310_at	0.00	0.00
1449311_at	0.00	0.00
1449312_at	0.00	0.00
1449313_at	0.00	0.00
1449314_at	0.00	0.00
1449315_at	0.00	0.00
1449316_at	0.00	0.00
1449317_at	0.00	0.00
1449318_at	0.00	0.00
1449319_at	0.00	0.00
1449320_at	0.00	0.00
1449321_x_at	0.00	0.00
1449322_at	0.00	0.00
1449323_a_at	0.00	0.00
1449324_at	0.00	0.00
1449325_at	0.00	0.00
1449326_x_at	0.00	0.00
1449327_at	0.00	0.00
1449328_at	0.00	0.00
1449329_at	0.00	0.00
1449330_at	0.00	0.00
1449331_a_at	0.00	0.00
1449332_at	0.00	0.00
1449333_at	0.00	0.00
1449334_at	0.00	0.00
1449335_at	0.00	0.00
1449336_a_at	0.00	0.00
1449337_at	0.00	0.00
1449338_at	0.00	0.01
1449339_at	0.00	0.00
1449340_at	0.00	0.00
1449341_a_at	0.00	0.00
1449342_at	0.00	0.00
1449343_s_at	0.00	0.00
1449344_s_at	0.00	0.00
1449345_at	0.00	0.00
1449346_s_at	0.00	0.00
1449347_a_at	0.00	0.00
1449348_at	0.00	0.00
1449349_at	0.00	0.06
1449350_at	0.00	0.00
1449351_s_at	0.00	0.33
1449352_at	0.00	0.00
1449353_at	0.00	0.00
1449354_at	0.00	0.00
1449355_a_at	0.00	0.00
1449356_at	0.00	0.00
1449357_at	0.00	0.30
1449358_at	0.00	0.00

1449359_at	0.00	0.00
1449360_at	0.00	0.00
1449361_at	0.00	0.00
1449362_a_at	0.00	0.00
1449363_at	0.07	0.00
1449364_at	0.00	0.00
1449365_at	0.00	0.00
1449366_at	0.00	0.00
1449367_at	0.00	0.00
1449368_at	0.00	0.00
1449369_at	0.00	0.02
1449370_at	0.00	0.00
1449371_at	0.00	0.00
1449372_at	0.00	0.00
1449373_at	0.00	0.00
1449374_at	0.62	0.00
1449375_at	0.00	0.00
1449376_at	0.00	0.00
1449377_at	0.00	0.00
1449378_at	0.00	0.00
1449379_at	0.00	0.00
1449380_at	0.00	0.00
1449381_a_at	0.00	0.00
1449382_at	0.00	0.00
1449383_at	0.00	0.01
1449384_at	0.00	0.00
1449385_at	0.00	0.00
1449386_at	0.00	0.00
1449387_at	0.00	0.00
1449388_at	0.00	0.00
1449389_at	0.00	0.00
1449390_at	0.00	0.05
1449391_at	0.00	0.00
1449392_at	0.00	0.00
1449393_at	0.00	0.00
1449394_at	0.00	0.00
1449395_at	0.00	0.00
1449396_at	0.00	0.00
1449397_at	0.00	0.00
1449398_at	0.00	0.00
1449399_a_at	0.00	0.00
1449400_at	0.00	0.00
1449401_at	0.00	0.00
1449402_at	0.00	0.00
1449403_at	0.00	0.00
1449404_at	0.00	0.00
1449405_at	0.00	0.00
1449406_at	0.00	0.00
1449407_at	0.00	0.00
1449408_at	0.67	1.00
1449409_at	0.00	0.00
1449410_a_at	0.00	0.00
1449411_at	0.00	0.00
1449412_at	0.00	0.00
1449413_at	0.00	0.00
1449414_at	0.00	0.00

1449415_at	0.00	0.00
1449416_at	0.00	0.00
1449417_at	0.00	0.00
1449418_s_at	0.00	0.00
1449419_at	0.00	0.00
1449420_at	0.00	0.00
1449421_a_at	0.00	0.00
1449422_at	0.00	0.00
1449423_at	0.00	0.00
1449424_at	0.00	0.00
1449425_at	0.00	0.00
1449426_a_at	0.00	0.00
1449427_at	0.00	0.00
1449428_at	0.00	0.00
1449429_at	0.00	0.00
1449430_a_at	0.00	0.00
1449431_at	0.00	0.00
1449432_a_at	0.00	0.00
1449433_at	0.00	0.00
1449434_at	0.00	0.00
1449435_at	0.00	0.00
1449436_s_at	0.00	0.00
1449437_at	0.00	0.02
1449438_at	0.00	0.00
1449439_at	0.00	0.00
1449440_at	0.00	0.00
1449441_a_at	0.00	0.00
1449442_at	0.00	0.00
1449443_at	0.00	0.00
1449444_a_at	0.00	0.12
1449445_x_at	0.00	0.00
1449446_at	0.00	0.00
1449447_at	0.00	0.00
1449448_at	0.00	0.01
1449449_at	0.00	0.00
1449450_at	0.00	0.00
1449451_at	0.00	0.00
1449452_a_at	0.00	0.00
1449453_at	0.00	0.00
1449454_at	0.00	0.00
1449455_at	1.00	0.67
1449456_a_at	0.00	0.00
1449457_at	0.00	0.00
1449458_at	0.00	0.00
1449459_s_at	0.00	0.00
1449460_at	0.00	0.00
1449461_at	0.00	0.00
1449462_at	0.00	0.00
1449463_at	0.00	0.00
1449464_at	0.00	0.00
1449465_at	0.00	0.00
1449466_at	0.00	0.00
1449467_at	0.00	0.00
1449468_at	0.00	0.00
1449469_at	0.00	0.00
1449470_at	0.00	0.00

1449471_at	0.00	0.00
1449472_at	0.00	0.00
1449473_s_at	0.00	0.00
1449474_a_at	0.00	0.00
1449475_at	0.00	0.00
1449476_at	0.00	0.00
1449477_s_at	0.00	0.00
1449478_at	0.00	0.00
1449479_at	0.00	0.00
1449480_at	0.00	0.00
1449481_at	0.00	0.00
1449482_at	0.00	0.00
1449483_at	0.00	0.00
1449484_at	0.00	0.00
1449485_at	0.00	0.00
1449486_at	0.00	0.00
1449487_at	0.00	0.00
1449488_at	0.00	0.00
1449489_at	0.00	0.00
1449490_at	0.00	0.00
1449491_at	0.54	0.00
1449492_a_at	0.00	0.00
1449493_at	0.00	0.00
1449494_at	0.00	0.00
1449495_at	0.00	0.00
1449496_at	0.00	0.00
1449497_at	0.00	0.00
1449498_at	0.00	0.00
1449499_at	0.00	0.00
1449500_at	0.00	0.00
1449501_a_at	0.00	0.00
1449502_at	0.00	0.00
1449503_at	0.00	0.00
1449504_at	0.00	0.02
1449505_at	0.00	0.00
1449506_a_at	0.00	0.00
1449507_a_at	0.03	0.00
1449508_at	0.00	0.00
1449509_at	0.00	0.00
1449510_at	0.00	0.00
1449511_a_at	0.00	0.00
1449512_a_at	0.00	0.00
1449513_at	0.00	0.00
1449514_at	0.00	0.00
1449515_at	0.00	0.00
1449516_a_at	0.00	0.00
1449517_at	0.00	0.00
1449518_at	0.00	0.00
1449519_at	0.00	0.00
1449520_at	0.00	0.00
1449521_at	0.00	0.00
1449522_at	0.00	0.00
1449523_at	0.00	0.00
1449524_at	0.00	0.00
1449525_at	0.00	0.00
1449526_a_at	0.00	0.00

1449527_at	0.00	0.00
1449528_at	0.00	0.00
1449529_s_at	0.00	0.00
1449530_at	0.00	0.00
1449531_at	0.00	0.00
1449532_at	0.00	0.00
1449533_at	0.00	0.00
1449534_at	0.13	0.09
1449535_at	0.00	0.00
1449536_at	0.00	0.00
1449537_at	0.00	0.00
1449538_a_at	0.00	0.00
1449539_at	0.00	0.00
1449540_at	0.00	0.00
1449541_x_at	0.00	0.00
1449542_at	0.00	0.00
1449543_at	0.00	0.00
1449544_a_at	0.00	0.00
1449545_at	0.00	0.00
1449546_a_at	0.00	0.00
1449547_at	0.00	0.00
1449548_at	0.00	0.00
1449549_at	0.00	0.00
1449550_at	0.00	0.00
1449551_at	0.01	0.00
1449552_at	0.00	0.00
1449553_at	0.00	0.00
1449554_at	0.00	0.00
1449555_a_at	0.00	0.00
1449556_at	0.00	0.00
1449557_at	0.00	0.00
1449558_at	0.00	0.00
1449559_at	0.02	0.00
1449560_at	0.00	0.00
1449561_at	0.00	0.00
1449562_at	0.00	0.00
1449563_at	0.00	0.00
1449564_at	0.00	0.00
1449565_at	0.00	0.00
1449566_at	0.00	0.00
1449567_at	0.00	0.00
1449568_at	0.00	0.00
1449569_at	0.00	0.00
1449570_at	0.00	0.00
1449571_at	0.00	0.00
1449572_at	0.00	0.00
1449573_at	0.00	0.00
1449574_a_at	0.00	0.00
1449575_a_at	0.00	0.00
1449576_at	0.00	0.00
1449577_x_at	0.00	0.00
1449578_at	0.00	0.25
1449579_at	0.00	0.00
1449580_s_at	0.00	0.00
1449581_at	0.00	0.00
1449582_at	0.00	0.00

1449583_at	0.00	0.00
1449584_at	0.00	0.00
1449585_at	0.00	0.00
1449586_at	0.00	0.00
1449587_a_at	0.00	0.00
1449588_at	0.00	0.00
1449589_x_at	0.00	0.00
1449590_a_at	0.00	0.03
1449591_at	0.00	0.00
1449592_at	0.57	0.34
1449593_at	0.00	0.00
1449594_at	0.00	0.00
1449595_at	0.00	0.00
1449596_at	0.00	0.00
1449597_at	0.00	0.00
1449598_at	0.00	0.00
1449599_at	0.00	0.00
1449600_at	0.00	0.00
1449603_at	0.00	0.00
1449604_at	0.00	0.00
1449605_at	0.00	0.00
1449609_at	0.00	0.00
1449611_at	0.00	0.00
1449614_s_at	0.00	0.00
1449615_s_at	0.00	0.00
1449617_at	0.00	0.00
1449618_s_at	0.00	0.00
1449619_s_at	0.00	0.00
1449620_s_at	0.00	0.00
1449621_s_at	0.00	0.00
1449622_s_at	0.00	0.00
1449623_at	0.00	0.00
1449624_at	0.00	0.00
1449625_at	0.00	0.00
1449628_s_at	0.00	0.00
1449629_s_at	0.00	0.00
1449630_s_at	0.00	0.00
1449631_at	0.00	0.00
1449632_s_at	0.00	0.00
1449633_s_at	0.00	0.00
1449635_at	0.00	0.24
1449637_at	0.00	0.00
1449638_at	0.00	0.00
1449639_at	0.00	0.00
1449640_at	0.00	0.00
1449641_at	0.00	0.00
1449642_at	0.00	0.00
1449643_s_at	0.00	0.00
1449644_at	0.00	0.00
1449645_s_at	0.00	0.02
1449646_s_at	0.00	0.00
1449647_at	0.00	0.00
1449648_s_at	0.00	0.06
1449649_at	0.00	0.00
1449650_at	0.00	0.00
1449651_x_at	0.00	0.00

1449652_at	0.00	0.00
1449653_at	0.00	0.00
1449654_s_at	0.00	0.00
1449656_at	0.00	0.00
1449657_at	0.00	0.00
1449658_at	0.00	0.00
1449659_s_at	0.00	0.00
1449660_s_at	0.00	0.00
1449661_at	0.00	0.00
1449662_at	0.00	0.00
1449663_at	0.00	0.00
1449664_s_at	0.00	0.00
1449665_at	0.00	0.00
1449669_at	0.00	0.00
1449670_x_at	0.00	0.00
1449671_at	0.00	0.00
1449672_s_at	0.00	0.00
1449673_s_at	0.00	0.00
1449674_s_at	0.00	0.00
1449675_at	0.00	0.03
1449676_at	0.00	0.00
1449677_s_at	0.00	0.00
1449678_at	0.00	0.00
1449679_s_at	0.00	0.00
1449680_at	0.00	0.00
1449681_at	0.00	0.00
1449682_s_at	0.00	0.35
1449683_x_at	0.00	0.00
1449684_at	0.00	0.00
1449685_s_at	0.00	0.00
1449686_s_at	0.00	0.00
1449687_at	0.00	0.00
1449688_at	0.00	0.00
1449689_at	0.00	0.00
1449690_x_at	0.00	0.00
1449691_at	0.00	0.00
1449692_at	0.00	0.00
1449693_at	0.00	0.00
1449694_s_at	0.00	0.00
1449697_s_at	0.00	0.00
1449698_at	0.00	0.00
1449699_s_at	0.00	0.00
1449700_at	0.00	0.00
1449701_at	0.00	0.00
1449702_at	0.00	0.00
1449703_at	0.00	0.00
1449704_at	0.00	0.00
1449705_x_at	0.00	0.00
1449706_s_at	0.00	0.00
1449707_at	0.00	0.00
1449708_s_at	0.00	0.00
1449710_s_at	0.00	0.00
1449711_at	0.00	0.00
1449712_s_at	0.00	0.00
1449713_at	0.00	0.00
1449714_at	0.00	0.00

1449715_at	0.00	0.00
1449716_s_at	0.00	0.00
1449717_at	0.00	0.00
1449718_s_at	0.00	0.16
1449719_at	0.00	0.00
1449720_at	0.00	0.00
1449721_at	0.00	0.00
1449724_s_at	0.00	0.00
1449726_at	0.00	0.00
1449727_x_at	0.00	0.00
1449728_at	0.00	0.00
1449729_at	0.44	0.02
1449730_s_at	0.00	0.00
1449731_s_at	0.00	0.00
1449732_at	0.00	0.00
1449733_s_at	0.00	0.00
1449735_at	0.00	0.00
1449736_at	0.00	0.00
1449738_s_at	0.00	0.00
1449739_at	0.00	0.00
1449740_s_at	0.00	0.00
1449741_at	0.00	0.00
1449742_at	0.00	0.00
1449744_at	0.00	0.00
1449745_at	0.00	0.00
1449747_at	0.00	0.00
1449748_at	0.00	0.00
1449749_s_at	0.00	0.00
1449750_at	0.00	0.00
1449751_at	0.00	0.00
1449752_at	0.00	0.00
1449753_at	0.00	0.00
1449754_at	0.00	0.00
1449755_at	0.00	0.00
1449756_at	0.00	0.00
1449757_x_at	0.00	0.00
1449759_at	0.00	0.00
1449760_at	0.00	0.00
1449761_at	0.00	0.00
1449764_x_at	0.00	0.00
1449765_at	0.00	0.00
1449768_at	0.00	0.00
1449770_x_at	0.00	0.00
1449771_at	0.00	0.00
1449772_at	0.00	0.00
1449773_s_at	0.51	0.00
1449774_at	0.00	0.00
1449776_at	0.00	0.00
1449779_at	0.00	0.00
1449780_at	0.00	0.00
1449781_at	0.00	0.00
1449782_at	0.00	0.00
1449783_at	0.00	0.00
1449784_at	0.00	0.00
1449785_at	0.00	0.00
1449787_at	0.00	0.00

1449788_at	0.00	0.00
1449793_at	0.00	0.00
1449795_at	0.00	0.00
1449796_at	0.00	0.00
1449797_x_at	0.00	0.00
1449799_s_at	0.00	0.00
1449800_x_at	0.00	0.00
1449801_at	0.00	0.00
1449803_x_at	0.00	0.00
1449804_at	0.00	0.00
1449805_at	0.00	0.00
1449806_at	0.00	0.00
1449808_at	0.00	0.00
1449813_at	0.00	0.00
1449814_at	0.00	0.00
1449815_a_at	0.00	0.00
1449816_at	0.00	0.00
1449817_at	0.00	0.00
1449818_at	0.00	0.00
1449819_at	0.00	0.00
1449820_at	0.00	0.00
1449821_a_at	0.00	0.00
1449822_at	0.00	0.00
1449823_at	0.00	0.00
1449824_at	0.00	0.00
1449825_at	0.00	0.00
1449826_a_at	0.00	0.00
1449827_at	0.00	0.00
1449828_at	0.00	0.00
1449829_at	0.00	0.00
1449830_at	0.00	0.00
1449831_at	0.00	0.00
1449832_at	0.00	0.00
1449833_at	0.00	0.00
1449834_at	0.00	0.00
1449835_at	0.00	0.00
1449836_x_at	0.00	0.00
1449837_at	0.00	0.00
1449838_at	0.00	0.00
1449839_at	0.00	0.00
1449840_at	0.00	0.00
1449841_at	0.00	0.00
1449842_at	0.00	0.00
1449843_at	0.00	0.00
1449844_at	0.00	0.00
1449845_a_at	0.00	0.00
1449846_at	0.00	0.00
1449847_a_at	0.00	0.00
1449848_at	0.00	0.00
1449849_a_at	0.00	0.00
1449850_at	0.00	0.00
1449851_at	0.00	0.00
1449852_a_at	0.00	0.00
1449853_at	0.00	0.00
1449854_at	0.00	0.00
1449855_s_at	0.00	0.11

1449856_at	0.00	0.00
1449857_at	0.00	0.00
1449858_at	0.00	0.00
1449859_at	0.00	0.00
1449860_at	0.00	0.00
1449861_at	0.00	0.00
1449862_a_at	0.00	0.00
1449863_a_at	0.00	0.00
1449864_at	0.00	0.00
1449865_at	0.00	0.00
1449866_at	0.00	0.00
1449867_at	0.00	0.00
1449868_at	0.00	0.00
1449869_at	0.00	0.00
1449870_a_at	0.00	0.00
1449871_at	0.00	0.00
1449872_at	0.00	0.00
1449873_at	0.00	0.00
1449874_at	0.00	0.00
1449875_s_at	0.00	0.17
1449876_at	0.00	0.00
1449877_s_at	0.00	0.00
1449878_a_at	0.00	0.00
1449879_at	0.00	0.00
1449880_s_at	0.00	0.00
1449881_a_at	0.00	0.00
1449882_a_at	0.00	0.00
1449883_at	0.00	0.00
1449884_at	0.00	0.00
1449885_at	0.00	0.00
1449886_a_at	0.00	0.00
1449887_at	0.00	0.00
1449888_at	0.24	0.00
1449889_a_at	0.00	0.00
1449890_at	0.00	0.00
1449891_a_at	0.00	0.00
1449892_at	0.00	0.00
1449893_a_at	0.00	0.00
1449894_at	0.00	0.00
1449895_at	0.00	0.00
1449896_at	0.00	0.00
1449897_a_at	0.00	0.00
1449898_at	0.16	0.05
1449899_at	0.00	0.00
1449900_at	0.00	0.00
1449901_a_at	0.00	0.00
1449902_at	0.00	0.00
1449903_at	0.00	0.00
1449904_at	0.00	0.00
1449905_at	0.00	0.00
1449906_at	0.00	0.00
1449907_at	0.00	0.00
1449908_at	0.00	0.00
1449909_at	0.00	0.00
1449910_at	0.00	0.00
1449911_at	0.00	0.00

1449912_at	0.00	0.00
1449913_at	0.00	0.00
1449914_at	0.00	0.00
1449915_at	0.00	0.00
1449916_at	0.00	0.00
1449917_at	0.00	0.00
1449918_at	0.00	0.00
1449919_at	0.00	0.00
1449920_at	0.00	0.00
1449921_s_at	0.00	0.00
1449922_at	0.00	0.00
1449923_at	0.00	0.00
1449924_at	0.00	0.00
1449925_at	0.00	0.00
1449926_at	0.00	0.00
1449927_at	0.00	0.00
1449928_at	0.00	0.00
1449929_at	0.00	0.00
1449930_a_at	0.00	0.00
1449931_at	0.00	0.00
1449932_at	0.00	0.00
1449933_a_at	0.00	0.00
1449934_at	0.00	0.00
1449935_a_at	0.00	0.08
1449936_at	0.00	0.00
1449937_at	0.00	0.00
1449938_at	0.00	0.00
1449939_s_at	0.00	0.01
1449940_a_at	0.00	0.02
1449941_at	0.00	0.00
1449942_a_at	0.28	0.24
1449943_at	0.00	0.00
1449944_a_at	0.00	0.00
1449945_at	0.00	0.00
1449946_a_at	0.00	0.00
1449947_s_at	0.00	0.00
1449948_at	0.00	0.00
1449949_a_at	0.00	0.00
1449950_at	0.00	0.00
1449951_at	0.00	0.00
1449952_s_at	0.00	0.00
1449953_at	0.00	0.00
1449954_at	0.00	0.00
1449955_at	0.00	0.00
1449956_at	0.00	0.00
1449957_at	0.00	0.00
1449958_a_at	0.00	0.00
1449959_x_at	0.00	0.00
1449960_at	0.00	0.00
1449961_at	0.00	0.00
1449962_at	0.00	0.00
1449963_at	0.00	0.00
1449964_a_at	0.00	0.00
1449965_at	0.00	0.00
1449966_s_at	0.00	0.00
1449967_at	0.00	0.00

1449968_s_at	0.00	0.04
1449969_at	0.00	0.00
1449970_at	0.00	0.00
1449971_a_at	0.00	0.00
1449972_s_at	0.07	0.23
1449973_a_at	0.00	0.00
1449974_at	0.00	0.00
1449975_a_at	0.00	0.00
1449976_a_at	0.00	0.00
1449977_at	0.00	0.00
1449978_at	0.00	0.00
1449979_a_at	0.00	0.00
1449980_a_at	0.00	0.00
1449981_a_at	0.00	0.00
1449982_at	0.00	0.00
1449983_a_at	0.00	0.00
1449984_at	0.00	0.00
1449985_at	0.00	0.00
1449986_at	0.00	0.00
1449987_at	0.00	0.00
1449988_at	0.00	0.00
1449989_at	0.00	0.00
1449990_at	0.00	0.00
1449991_at	0.00	0.00
1449992_at	0.00	0.00
1449993_at	0.00	0.00
1449994_at	0.00	0.00
1449995_at	0.00	0.00
1449996_a_at	0.00	0.00
1449997_at	0.00	0.00
1449998_at	0.00	0.00
1449999_a_at	0.00	0.00
1450000_at	0.00	0.00
1450001_a_at	0.00	0.00
1450002_at	0.00	0.00
1450003_at	0.00	0.00
1450004_at	0.00	0.00
1450005_x_at	0.00	0.00
1450006_at	0.00	0.00
1450007_at	0.00	0.00
1450008_a_at	0.00	0.00
1450009_at	0.00	0.00
1450010_at	0.00	0.00
1450011_at	0.00	0.00
1450012_x_at	0.00	0.00
1450013_at	0.00	0.00
1450014_at	0.00	0.00
1450015_x_at	0.00	0.00
1450016_at	0.00	0.00
1450017_at	0.00	0.00
1450018_s_at	0.00	0.31
1450019_at	0.00	0.00
1450020_at	0.00	0.00
1450021_at	0.00	0.00
1450022_at	0.00	0.00
1450023_at	0.00	0.00

1450024_at	0.00	0.00
1450025_at	0.00	0.00
1450026_a_at	0.00	0.00
1450027_at	0.00	0.00
1450028_a_at	0.00	0.00
1450029_s_at	0.00	0.00
1450030_at	0.00	0.00
1450031_at	0.00	0.00
1450032_at	0.00	0.00
1450033_a_at	0.00	0.00
1450034_at	0.00	0.00
1450035_a_at	0.00	0.00
1450036_at	0.00	0.00
1450037_at	0.00	0.00
1450038_s_at	0.00	0.00
1450039_at	0.00	0.00
1450040_at	0.00	0.00
1450041_a_at	0.00	0.00
1450042_at	0.00	0.00
1450043_at	0.00	0.00
1450044_at	0.00	0.19
1450045_at	0.00	0.00
1450046_at	0.00	0.00
1450047_at	0.01	0.00
1450048_a_at	0.00	0.35
1450049_a_at	0.00	0.00
1450050_at	0.00	0.00
1450051_at	0.00	0.00
1450052_at	0.00	0.00
1450053_at	0.00	0.00
1450054_at	0.00	0.00
1450055_at	0.00	0.00
1450056_at	0.00	0.00
1450057_at	0.00	0.00
1450058_at	0.00	0.06
1450059_at	0.00	0.00
1450060_at	0.00	0.00
1450061_at	0.00	0.00
1450062_a_at	0.00	0.00
1450063_at	0.00	0.00
1450064_at	0.00	0.00
1450065_at	0.00	0.00
1450066_at	0.00	0.00
1450067_a_at	0.00	0.00
1450068_at	0.00	0.00
1450069_a_at	0.00	0.00
1450070_s_at	0.00	0.00
1450071_at	0.00	0.00
1450072_at	0.00	0.00
1450073_at	0.00	0.00
1450074_at	0.00	0.00
1450075_at	0.00	0.00
1450076_at	0.00	0.00
1450077_at	0.00	0.00
1450078_at	0.00	0.00
1450079_at	0.00	0.33

1450080_at	0.00	0.00
1450081_x_at	0.00	0.00
1450082_s_at	0.00	0.01
1450083_at	0.00	0.00
1450084_s_at	0.00	0.00
1450085_at	0.00	0.00
1450086_at	0.00	0.00
1450087_a_at	0.00	0.35
1450088_a_at	0.00	0.00
1450089_a_at	0.00	0.00
1450090_at	0.00	0.00
1450091_at	0.00	0.00
1450092_at	0.00	0.00
1450093_s_at	0.00	0.00
1450094_at	0.00	0.00
1450095_a_at	0.00	0.00
1450096_at	0.00	0.00
1450097_s_at	0.00	0.00
1450098_at	0.00	0.00
1450099_a_at	0.00	0.00
1450100_a_at	0.00	0.00
1450101_a_at	0.00	0.00
1450102_a_at	0.46	0.01
1450103_a_at	0.00	0.00
1450104_at	0.00	0.00
1450105_at	0.00	0.00
1450106_a_at	0.00	0.00
1450107_a_at	0.00	0.00
1450108_at	0.00	0.00
1450109_s_at	0.00	0.00
1450110_at	0.00	0.00
1450111_a_at	0.00	0.00
1450112_a_at	0.00	0.00
1450113_at	0.00	0.00
1450114_at	0.00	0.00
1450115_at	0.00	0.00
1450116_at	0.00	0.00
1450117_at	0.02	0.00
1450118_a_at	0.00	0.00
1450119_at	0.00	0.00
1450120_at	0.00	0.00
1450121_at	0.00	0.00
1450122_at	0.00	0.00
1450123_at	0.00	0.00
1450124_a_at	0.00	0.00
1450125_at	0.00	0.00
1450126_at	0.00	0.00
1450127_a_at	0.00	0.00
1450128_at	0.00	0.00
1450129_a_at	0.00	0.00
1450130_at	0.00	0.00
1450131_a_at	0.00	0.00
1450132_at	0.00	0.00
1450133_at	0.00	0.00
1450134_at	0.00	0.00
1450135_at	0.00	0.00

1450136_at	0.00	0.00
1450137_at	0.00	0.00
1450138_a_at	0.00	0.00
1450139_at	0.00	0.00
1450140_a_at	0.00	0.00
1450141_at	0.00	0.00
1450142_a_at	0.00	0.00
1450143_at	0.00	0.00
1450144_at	0.00	0.00
1450145_at	0.00	0.00
1450146_at	0.00	0.00
1450147_at	0.00	0.00
1450148_at	0.00	0.00
1450149_a_at	0.00	0.00
1450150_a_at	0.00	0.00
1450151_at	0.00	0.00
1450152_at	0.00	0.00
1450153_at	0.00	0.00
1450154_at	0.00	0.00
1450155_at	0.00	0.00
1450156_a_at	0.00	0.00
1450157_a_at	0.00	0.00
1450158_at	0.00	0.00
1450159_at	0.00	0.00
1450160_at	0.00	0.00
1450161_at	0.00	0.00
1450162_at	0.00	0.00
1450163_a_at	0.00	0.00
1450164_at	0.00	0.00
1450165_at	0.00	0.00
1450166_at	0.00	0.00
1450167_at	0.00	0.00
1450168_at	0.00	0.00
1450169_at	0.00	0.00
1450170_x_at	0.00	0.00
1450171_x_at	0.00	0.00
1450172_at	0.00	0.00
1450173_at	0.00	0.00
1450174_at	0.00	0.00
1450175_a_at	0.00	0.00
1450176_at	0.00	0.00
1450177_at	0.00	0.00
1450178_at	0.00	0.00
1450179_at	0.00	0.00
1450180_a_at	0.00	0.01
1450181_at	0.00	0.00
1450182_at	0.00	0.00
1450183_a_at	0.00	0.00
1450184_s_at	0.00	0.00
1450185_a_at	0.00	0.00
1450186_s_at	0.58	0.88
1450187_a_at	0.00	0.00
1450188_s_at	0.00	0.00
1450189_at	0.00	0.00
1450190_at	0.00	0.00
1450191_a_at	0.00	0.00

1450192_at	0.00	0.00
1450193_at	0.00	0.00
1450194_a_at	0.00	0.00
1450195_at	0.00	0.00
1450196_s_at	0.00	0.00
1450197_at	0.00	0.00
1450198_at	0.00	0.00
1450199_a_at	0.00	0.00
1450200_s_at	0.00	0.00
1450201_at	0.00	0.00
1450202_at	0.00	0.00
1450203_at	0.00	0.00
1450204_a_at	0.00	0.00
1450205_at	0.00	0.00
1450206_at	0.04	0.00
1450207_at	0.00	0.00
1450208_a_at	0.00	0.00
1450209_at	0.00	0.00
1450210_at	0.00	0.00
1450211_at	0.00	0.00
1450212_at	0.00	0.00
1450213_at	0.00	0.00
1450214_at	0.00	0.00
1450215_at	0.00	0.00
1450216_at	0.00	0.00
1450217_at	0.00	0.00
1450218_at	0.00	0.00
1450219_at	0.00	0.00
1450220_a_at	0.00	0.00
1450221_at	0.00	0.00
1450222_x_at	0.00	0.00
1450223_at	0.00	0.00
1450224_at	0.00	0.00
1450225_at	0.00	0.00
1450226_at	0.00	0.00
1450227_at	0.00	0.00
1450228_a_at	0.00	0.00
1450229_at	0.00	0.00
1450230_at	0.00	0.00
1450231_a_at	0.00	0.00
1450232_at	0.00	0.00
1450233_a_at	0.00	0.00
1450234_at	0.00	0.00
1450235_at	0.00	0.00
1450236_at	0.00	0.00
1450237_at	0.00	0.00
1450238_at	0.00	0.00
1450239_at	0.00	0.00
1450240_a_at	0.00	0.00
1450241_a_at	0.00	0.00
1450242_at	0.00	0.00
1450243_a_at	0.00	0.00
1450244_a_at	0.00	0.00
1450245_at	0.00	0.00
1450246_at	0.00	0.00
1450247_a_at	0.00	0.00

1450248_at	0.00	0.00
1450249_s_at	0.00	0.00
1450250_at	0.00	0.00
1450251_a_at	0.00	0.00
1450252_at	0.00	0.00
1450253_a_at	0.00	0.27
1450254_at	0.00	0.00
1450255_at	0.00	0.00
1450256_at	0.00	0.00
1450257_at	0.00	0.00
1450258_a_at	0.00	0.00
1450259_a_at	0.00	0.00
1450260_at	0.00	0.00
1450261_a_at	0.00	0.00
1450262_at	0.00	0.00
1450263_at	0.00	0.00
1450264_a_at	0.00	0.00
1450265_at	0.00	0.00
1450266_at	0.00	0.00
1450267_at	0.00	0.00
1450268_at	0.00	0.00
1450269_a_at	0.00	0.00
1450270_at	0.00	0.00
1450271_at	0.00	0.00
1450272_at	0.00	0.00
1450273_at	0.00	0.00
1450274_at	0.00	0.00
1450275_x_at	0.00	0.00
1450276_a_at	0.00	0.00
1450277_at	0.00	0.00
1450278_at	0.00	0.00
1450279_at	0.00	0.00
1450280_a_at	0.00	0.00
1450281_a_at	0.00	0.00
1450282_at	0.10	0.02
1450283_at	0.00	0.00
1450284_at	0.00	0.00
1450285_at	0.00	0.00
1450286_at	0.00	0.00
1450287_at	0.00	0.00
1450288_at	0.00	0.00
1450289_at	0.00	0.00
1450290_at	0.00	0.00
1450291_s_at	0.00	0.00
1450292_a_at	0.00	0.00
1450293_at	0.00	0.00
1450294_a_at	0.00	0.00
1450295_s_at	0.00	0.01
1450296_at	0.00	0.00
1450297_at	0.00	0.00
1450298_at	0.00	0.00
1450299_at	0.00	0.00
1450300_at	0.00	0.00
1450301_at	0.00	0.00
1450302_at	0.00	0.00
1450303_at	0.00	0.00

1450304_at	0.00	0.00
1450305_at	0.00	0.00
1450306_at	0.00	0.00
1450307_x_at	0.00	0.00
1450308_a_at	0.00	0.00
1450309_at	0.00	0.00
1450310_at	0.00	0.00
1450311_at	0.00	0.00
1450312_at	0.00	0.00
1450313_at	0.00	0.00
1450314_at	0.00	0.00
1450315_at	0.00	0.00
1450316_at	0.00	0.00
1450317_at	0.00	0.00
1450318_a_at	0.00	0.00
1450319_at	0.00	0.00
1450320_at	0.00	0.00
1450321_at	0.00	0.00
1450322_s_at	0.00	0.00
1450323_at	0.00	0.00
1450324_a_at	0.00	0.00
1450325_at	0.00	0.00
1450326_at	0.00	0.00
1450327_at	0.00	0.00
1450328_at	0.00	0.00
1450329_a_at	0.00	0.00
1450330_at	0.00	0.00
1450331_s_at	0.00	0.00
1450332_s_at	0.00	0.00
1450333_a_at	0.00	0.00
1450334_at	0.00	0.00
1450335_at	0.00	0.00
1450336_at	0.00	0.00
1450337_a_at	0.00	0.00
1450338_x_at	0.00	0.00
1450339_a_at	0.00	0.00
1450340_a_at	0.00	0.00
1450341_at	0.00	0.00
1450342_at	0.00	0.00
1450343_at	0.00	0.00
1450344_a_at	0.00	0.00
1450345_at	0.00	0.00
1450346_at	0.00	0.00
1450347_at	0.00	0.00
1450348_at	0.00	0.00
1450349_at	0.00	0.00
1450350_a_at	0.00	0.00
1450351_a_at	0.00	0.00
1450352_at	0.00	0.00
1450353_at	0.00	0.00
1450354_a_at	0.00	0.00
1450355_a_at	0.00	0.00
1450356_at	0.00	0.00
1450357_a_at	0.00	0.00
1450358_at	0.00	0.00
1450359_at	0.00	0.00

1450360_at	0.00	0.00
1450361_at	0.00	0.00
1450362_at	0.00	0.00
1450363_at	0.00	0.00
1450364_a_at	0.00	0.00
1450365_at	0.00	0.00
1450366_at	0.00	0.00
1450367_at	0.00	0.00
1450368_a_at	0.00	0.00
1450369_at	0.00	0.00
1450370_a_at	0.00	0.00
1450371_at	0.00	0.00
1450372_a_at	0.00	0.00
1450373_at	0.00	0.00
1450374_at	0.00	0.00
1450375_at	0.00	0.00
1450376_at	0.00	0.02
1450377_at	0.46	0.00
1450378_at	0.01	0.24
1450379_at	0.22	0.00
1450380_at	0.00	0.00
1450381_a_at	0.00	0.00
1450382_at	0.00	0.00
1450383_at	0.00	0.00
1450384_at	0.00	0.00
1450385_at	0.00	0.01
1450386_at	0.00	0.00
1450387_s_at	0.00	0.00
1450388_s_at	0.00	0.23
1450389_s_at	0.00	0.00
1450390_x_at	0.00	0.00
1450391_a_at	0.00	0.00
1450392_at	0.00	0.00
1450393_a_at	0.00	0.29
1450394_at	0.00	0.00
1450395_at	0.00	0.00
1450396_at	0.00	0.00
1450397_at	0.00	0.00
1450398_at	0.00	0.00
1450399_at	0.00	0.00
1450400_at	0.00	0.00
1450401_at	0.00	0.00
1450402_at	0.00	0.00
1450403_at	0.00	0.00
1450404_at	0.00	0.00
1450405_at	0.00	0.38
1450406_a_at	0.00	0.00
1450407_a_at	0.00	0.00
1450408_at	0.00	0.00
1450409_a_at	0.00	0.00
1450410_a_at	0.00	0.00
1450411_at	0.00	0.00
1450412_at	0.00	0.00
1450413_at	0.00	0.00
1450414_at	0.00	0.00
1450415_at	0.00	0.00

1450416_at	0.00	0.00
1450417_a_at	0.00	0.00
1450418_a_at	0.00	0.00
1450419_at	0.00	0.00
1450420_at	0.00	0.00
1450421_at	0.00	0.00
1450422_a_at	0.00	0.00
1450423_s_at	0.00	0.21
1450424_a_at	0.00	0.00
1450425_a_at	0.00	0.00
1450426_at	0.00	0.00
1450427_at	0.00	0.00
1450428_at	0.00	0.00
1450429_at	0.00	0.08
1450430_at	0.00	0.00
1450431_a_at	0.00	0.00
1450432_s_at	0.00	0.00
1450433_at	0.00	0.00
1450434_s_at	0.00	0.00
1450435_at	0.00	0.00
1450436_s_at	0.00	0.00
1450437_a_at	0.00	0.00
1450438_at	0.00	0.00
1450439_at	0.00	0.00
1450440_at	0.00	0.00
1450441_at	0.00	0.00
1450442_at	0.00	0.00
1450443_at	0.00	0.00
1450444_a_at	0.00	0.00
1450445_at	0.00	0.00
1450446_a_at	0.00	0.00
1450447_at	0.00	0.00
1450448_at	0.00	0.00
1450449_a_at	0.00	0.00
1450450_at	0.00	0.00
1450451_at	0.00	0.00
1450452_a_at	0.00	0.00
1450453_a_at	0.00	0.00
1450454_at	0.00	0.00
1450455_s_at	0.00	0.00
1450456_at	0.00	0.00
1450457_at	0.00	0.00
1450458_at	0.00	0.00
1450459_at	0.00	0.00
1450460_at	0.00	0.00
1450461_at	0.00	0.01
1450462_at	0.00	0.00
1450463_at	0.00	0.00
1450464_at	0.00	0.00
1450465_at	0.00	0.00
1450466_at	0.00	0.00
1450467_at	0.00	0.00
1450468_at	0.00	0.00
1450469_at	0.00	0.00
1450470_at	0.00	0.00
1450471_at	0.00	0.00

1450472_s_at	0.00	0.00
1450473_at	0.00	0.00
1450474_at	0.00	0.00
1450475_at	0.00	0.00
1450476_at	0.00	0.00
1450477_at	0.00	0.00
1450478_a_at	0.00	0.00
1450479_x_at	0.00	0.00
1450480_a_at	0.00	0.13
1450481_at	0.00	0.00
1450482_a_at	0.41	0.31
1450483_at	0.00	0.00
1450484_a_at	0.00	0.00
1450485_at	0.00	0.00
1450486_a_at	0.00	0.00
1450487_at	0.00	0.00
1450488_at	0.00	0.00
1450489_at	0.56	0.00
1450490_at	0.00	0.00
1450491_at	0.00	0.00
1450492_at	0.00	0.00
1450493_at	0.00	0.00
1450494_x_at	0.00	0.00
1450495_a_at	0.00	0.00
1450496_a_at	0.00	0.00
1450497_at	0.00	0.00
1450498_at	0.00	0.00
1450499_at	0.00	0.00
1450500_at	0.00	0.00
1450501_at	0.00	0.00
1450502_at	0.00	0.00
1450503_at	0.00	0.00
1450504_a_at	0.00	0.33
1450505_a_at	0.00	0.00
1450506_a_at	0.00	0.00
1450507_at	0.00	0.00
1450508_at	0.00	0.00
1450509_at	0.00	0.00
1450510_a_at	0.00	0.00
1450511_at	0.00	0.00
1450512_at	0.00	0.00
1450513_at	0.00	0.00
1450514_at	0.00	0.00
1450515_at	0.00	0.00
1450516_a_at	0.00	0.00
1450517_at	0.00	0.00
1450518_at	0.00	0.00
1450519_a_at	0.00	0.00
1450520_at	0.00	0.00
1450521_a_at	0.00	0.00
1450522_a_at	0.00	0.10
1450523_at	0.00	0.00
1450524_at	0.00	0.00
1450525_at	0.00	0.00
1450526_at	0.00	0.00
1450527_at	0.00	0.00

1450528_at	0.00	0.00
1450529_at	0.00	0.00
1450530_at	0.00	0.00
1450531_at	0.00	0.00
1450532_at	0.00	0.00
1450533_a_at	0.00	0.00
1450534_x_at	0.00	0.00
1450535_at	0.00	0.00
1450536_s_at	0.00	0.00
1450537_at	0.00	0.00
1450538_s_at	0.00	0.00
1450539_at	0.00	0.00
1450540_x_at	0.00	0.00
1450541_at	0.00	0.00
1450542_s_at	0.00	0.00
1450543_at	0.00	0.00
1450544_at	0.00	0.00
1450545_a_at	0.00	0.00
1450546_at	0.00	0.00
1450547_x_at	0.00	0.00
1450548_at	0.00	0.00
1450549_s_at	0.00	0.00
1450550_at	0.00	0.00
1450551_x_at	0.00	0.00
1450552_at	0.00	0.00
1450553_at	0.00	0.00
1450554_at	0.00	0.00
1450555_at	0.00	0.00
1450556_at	0.00	0.00
1450557_at	0.00	0.00
1450558_at	0.00	0.00
1450559_at	0.00	0.00
1450560_a_at	0.00	0.00
1450561_a_at	0.00	0.00
1450562_at	0.00	0.00
1450563_at	0.00	0.00
1450564_x_at	0.00	0.00
1450565_at	0.00	0.00
1450566_at	0.00	0.00
1450567_a_at	0.00	0.06
1450568_at	0.00	0.00
1450569_a_at	0.00	0.00
1450570_a_at	0.00	0.00
1450571_a_at	0.00	0.00
1450572_at	0.00	0.00
1450573_at	0.00	0.00
1450574_at	0.00	0.00
1450575_at	0.00	0.00
1450576_a_at	0.02	0.00
1450577_at	0.00	0.00
1450578_at	0.00	0.00
1450579_x_at	0.00	0.00
1450580_at	0.00	0.00
1450581_at	0.00	0.00
1450582_at	0.00	0.00
1450583_s_at	0.00	0.00

1450584_at	0.00	0.00
1450585_at	0.00	0.00
1450586_at	0.00	0.00
1450587_at	0.00	0.00
1450588_at	0.00	0.00
1450589_at	0.00	0.00
1450590_at	0.00	0.00
1450591_at	0.00	0.00
1450592_at	0.00	0.00
1450593_at	0.00	0.00
1450594_at	0.00	0.00
1450595_at	0.00	0.00
1450596_at	0.00	0.00
1450597_at	0.00	0.00
1450598_at	0.00	0.00
1450599_at	0.00	0.00
1450600_at	0.00	0.00
1450601_at	0.00	0.00
1450602_at	0.00	0.00
1450603_s_at	0.00	0.00
1450604_at	0.00	0.00
1450605_at	0.00	0.00
1450606_at	0.00	0.00
1450607_s_at	0.00	0.00
1450608_at	0.00	0.00
1450609_at	0.00	0.00
1450610_at	0.00	0.00
1450611_at	0.00	0.00
1450612_a_at	0.00	0.00
1450613_x_at	0.00	0.00
1450614_x_at	0.00	0.00
1450615_at	0.00	0.00
1450616_at	0.00	0.00
1450617_at	0.00	0.00
1450618_a_at	0.00	0.00
1450619_x_at	0.00	0.00
1450620_at	0.00	0.00
1450621_a_at	0.00	0.00
1450622_at	0.00	0.00
1450623_at	0.00	0.00
1450624_at	0.00	0.00
1450625_at	0.00	0.00
1450626_at	0.93	0.10
1450627_at	0.00	0.00
1450628_at	0.00	0.00
1450629_at	0.00	0.00
1450630_at	0.00	0.02
1450631_x_at	0.00	0.00
1450632_at	0.00	0.00
1450633_at	0.00	0.00
1450634_at	0.00	0.00
1450635_at	0.00	0.00
1450636_s_at	0.00	0.00
1450637_a_at	0.00	0.00
1450638_at	0.00	0.00
1450639_at	0.00	0.00

1450640_x_at	0.00	0.00
1450641_at	0.47	0.00
1450642_at	0.00	0.00
1450643_s_at	0.00	0.00
1450644_at	0.00	0.00
1450645_at	0.00	0.00
1450646_at	0.00	0.00
1450647_at	0.00	0.00
1450648_s_at	0.00	0.00
1450649_at	0.04	0.00
1450650_at	0.00	0.00
1450651_at	0.00	0.01
1450652_at	0.00	0.00
1450653_at	0.00	0.00
1450654_a_at	0.00	0.12
1450655_at	0.00	0.00
1450656_at	0.00	0.00
1450657_at	0.00	0.00
1450658_at	0.00	0.00
1450659_at	0.00	0.00
1450660_at	0.00	0.00
1450661_x_at	0.00	0.00
1450662_at	0.00	0.00
1450663_at	0.00	0.00
1450664_at	0.02	0.12
1450665_at	0.00	0.40
1450666_s_at	0.00	0.00
1450667_a_at	0.00	0.00
1450668_s_at	0.00	0.05
1450669_at	0.00	0.00
1450670_at	0.00	0.00
1450671_at	0.00	0.00
1450672_a_at	0.00	0.00
1450673_at	0.00	0.00
1450674_at	0.00	0.00
1450675_at	0.00	0.00
1450676_at	0.00	0.00
1450677_at	0.00	0.00
1450678_at	0.00	0.00
1450679_at	0.00	0.00
1450680_at	0.00	0.00
1450681_at	0.00	0.00
1450682_at	0.00	0.00
1450683_at	0.00	0.00
1450684_at	0.00	0.00
1450685_at	0.00	0.00
1450686_at	0.00	0.00
1450687_at	0.00	0.06
1450688_at	0.00	0.00
1450689_at	0.00	0.00
1450690_at	0.00	0.00
1450691_at	0.00	0.00
1450692_at	0.00	0.00
1450693_at	0.00	0.00
1450694_at	0.00	0.00
1450695_at	0.00	0.00

1450696_at	0.00	0.00
1450697_at	0.00	0.00
1450698_at	0.00	0.00
1450699_at	0.00	0.00
1450700_at	0.00	0.00
1450701_a_at	0.00	0.00
1450702_at	0.00	0.00
1450703_at	0.00	0.00
1450704_at	0.00	0.00
1450705_at	0.00	0.00
1450706_a_at	0.00	0.00
1450707_at	0.00	0.00
1450708_at	0.00	0.00
1450709_at	0.00	0.00
1450710_at	1.00	0.75
1450711_at	0.00	0.00
1450712_at	0.00	0.00
1450713_at	0.00	0.00
1450714_at	0.00	0.00
1450715_at	0.00	0.00
1450716_at	0.00	0.00
1450717_at	0.00	0.00
1450718_at	0.00	0.00
1450719_at	0.00	0.00
1450720_at	0.00	0.00
1450721_at	0.00	0.00
1450722_at	0.00	0.23
1450723_at	0.00	0.00
1450724_at	0.00	0.00
1450725_s_at	0.00	0.11
1450726_at	0.00	0.00
1450727_a_at	0.00	0.00
1450728_at	0.00	0.00
1450729_at	0.00	0.00
1450730_at	0.00	0.00
1450731_s_at	0.00	0.00
1450732_a_at	0.00	0.00
1450733_at	0.00	0.00
1450734_at	0.00	0.00
1450735_at	0.00	0.70
1450736_a_at	0.00	0.00
1450737_at	0.00	0.00
1450738_at	0.00	0.37
1450739_at	0.00	0.00
1450740_a_at	0.00	0.00
1450741_at	0.00	0.00
1450742_at	0.00	0.07
1450743_s_at	0.00	0.64
1450744_at	0.00	0.00
1450745_at	0.00	0.00
1450746_at	0.00	0.00
1450747_at	0.00	0.00
1450748_at	0.00	0.00
1450749_a_at	0.00	0.00
1450750_a_at	0.00	0.00
1450751_at	0.00	0.00

1450752_at	0.00	0.00
1450753_at	0.00	0.00
1450754_at	0.00	0.00
1450755_at	0.00	0.00
1450756_s_at	0.00	0.00
1450757_at	0.01	0.00
1450758_at	0.00	0.00
1450759_at	0.00	0.00
1450760_a_at	0.00	0.00
1450761_s_at	0.00	0.00
1450762_s_at	0.00	0.00
1450763_x_at	0.00	0.00
1450764_at	0.09	0.46
1450765_a_at	0.00	0.00
1450766_at	0.00	0.00
1450767_at	0.00	0.00
1450768_at	0.00	0.00
1450769_s_at	0.00	0.00
1450770_at	0.00	0.00
1450771_at	0.00	0.00
1450772_at	0.00	0.00
1450773_at	0.00	0.00
1450774_at	0.00	0.00
1450775_at	0.00	0.00
1450776_at	0.00	0.00
1450777_at	0.00	0.00
1450778_a_at	0.00	0.09
1450779_at	0.00	0.00
1450780_s_at	0.33	0.01
1450781_at	0.29	0.00
1450782_at	0.00	0.00
1450783_at	0.00	0.00
1450784_at	0.00	0.06
1450785_at	0.00	0.00
1450786_x_at	0.10	0.00
1450787_at	0.00	0.00
1450788_at	0.00	0.00
1450789_at	0.00	0.00
1450790_at	0.00	0.00
1450791_at	0.00	0.00
1450792_at	0.00	0.00
1450793_at	0.00	0.00
1450794_at	0.00	0.00
1450795_at	0.00	0.00
1450796_at	0.00	0.00
1450797_a_at	0.00	0.00
1450798_at	0.00	0.00
1450799_at	0.00	0.00
1450800_at	0.00	0.00
1450801_at	0.00	0.00
1450802_at	0.00	0.00
1450803_at	0.00	0.00
1450804_at	0.00	0.00
1450805_at	0.00	0.00
1450806_at	0.00	0.00
1450807_at	0.00	0.00

1450808_at	0.00	0.00
1450809_at	0.00	0.00
1450810_at	0.00	0.00
1450811_at	0.00	0.00
1450812_at	0.00	0.00
1450813_a_at	0.00	0.00
1450814_a_at	0.00	0.00
1450815_s_at	0.00	0.00
1450816_at	0.00	0.00
1450817_at	0.00	0.00
1450818_a_at	0.00	0.00
1450819_at	0.00	0.00
1450820_a_at	0.00	0.00
1450821_at	0.01	0.00
1450822_at	0.00	0.00
1450823_at	0.00	0.00
1450824_at	0.00	0.00
1450825_at	0.00	0.00
1450826_a_at	0.00	0.00
1450827_at	0.00	0.00
1450828_at	0.00	0.00
1450829_at	0.00	0.00
1450830_a_at	0.00	0.00
1450831_at	0.00	0.00
1450832_at	0.00	0.00
1450833_at	0.00	0.00
1450834_at	0.00	0.00
1450835_a_at	0.00	0.00
1450836_at	0.00	0.00
1450837_at	0.00	0.00
1450838_x_at	0.00	0.00
1450839_at	0.10	0.00
1450840_a_at	0.00	0.00
1450841_at	0.00	0.00
1450842_a_at	0.00	0.00
1450843_a_at	0.86	0.00
1450844_at	0.00	0.00
1450845_a_at	0.00	0.02
1450846_at	0.00	0.28
1450847_at	0.00	0.31
1450848_at	0.00	0.00
1450849_at	0.00	0.00
1450850_at	0.00	0.00
1450851_at	0.00	0.00
1450852_s_at	0.00	0.00
1450853_at	0.33	0.44
1450854_at	0.00	0.00
1450855_at	0.00	0.00
1450856_at	0.00	0.00
1450857_a_at	0.00	0.00
1450858_a_at	0.00	0.00
1450859_s_at	0.00	0.00
1450860_at	0.06	0.37
1450861_at	0.00	0.00
1450862_at	0.00	0.00
1450863_a_at	0.00	0.00

1450864_at	0.00	0.00
1450865_s_at	0.00	0.00
1450866_a_at	0.00	0.00
1450867_at	0.00	0.00
1450868_at	0.00	0.00
1450869_at	0.00	0.00
1450870_at	0.00	0.00
1450871_a_at	0.00	0.00
1450872_s_at	0.00	0.00
1450873_at	0.00	0.27
1450874_at	0.00	0.00
1450875_at	0.00	0.00
1450876_at	0.00	0.00
1450877_at	0.00	0.05
1450878_at	0.00	0.00
1450879_at	0.00	0.00
1450880_at	0.00	0.00
1450881_s_at	0.00	0.00
1450882_s_at	0.00	0.00
1450883_a_at	0.00	0.00
1450884_at	0.00	0.00
1450885_at	0.00	0.00
1450886_at	0.00	0.00
1450887_at	0.00	0.00
1450888_at	0.00	0.00
1450889_at	0.07	0.00
1450890_a_at	0.00	0.00
1450891_at	0.00	0.31
1450892_a_at	0.00	0.00
1450893_a_at	0.00	0.00
1450894_a_at	0.00	0.00
1450895_a_at	0.00	0.00
1450896_at	0.00	0.00
1450897_at	0.00	0.00
1450898_at	0.00	0.00
1450899_at	0.00	0.00
1450900_at	0.00	0.00
1450901_a_at	0.00	0.00
1450902_at	0.00	0.00
1450903_at	0.00	0.00
1450904_at	0.00	0.00
1450905_at	0.00	0.00
1450906_at	0.00	0.00
1450907_at	0.00	0.00
1450908_at	0.00	0.10
1450909_at	0.00	0.00
1450910_at	0.00	0.00
1450911_at	0.00	0.00
1450912_at	0.00	0.00
1450913_at	0.00	0.00
1450914_at	0.00	0.00
1450915_at	0.00	0.00
1450916_at	0.00	0.00
1450917_at	0.00	0.00
1450918_s_at	0.00	0.00
1450919_at	0.00	0.00

1450920_at	0.00	0.00
1450921_at	0.00	0.00
1450922_a_at	0.37	0.00
1450923_at	0.02	0.00
1450924_at	0.00	0.00
1450925_a_at	0.00	0.00
1450926_at	0.00	0.00
1450927_at	0.00	0.00
1450928_at	0.00	0.00
1450929_at	0.86	0.29
1450930_at	0.00	0.00
1450931_at	0.00	0.00
1450932_s_at	0.00	0.00
1450933_at	0.00	0.00
1450934_at	0.00	0.00
1450935_at	0.00	0.00
1450936_a_at	0.00	0.00
1450937_at	0.00	0.00
1450938_at	0.00	0.29
1450939_at	0.00	0.00
1450940_at	0.00	0.00
1450941_at	0.00	0.00
1450942_at	0.00	0.00
1450943_at	0.00	0.00
1450944_at	0.00	0.00
1450945_at	0.00	0.00
1450946_at	0.00	0.00
1450947_at	0.00	0.11
1450948_a_at	0.00	0.00
1450949_at	0.00	0.00
1450950_at	0.00	0.00
1450951_at	0.00	0.00
1450952_at	0.00	0.00
1450953_at	0.00	0.00
1450954_at	0.00	0.21
1450955_s_at	0.00	0.00
1450956_at	0.00	0.00
1450957_a_at	0.00	0.00
1450958_at	0.00	0.00
1450959_at	0.00	0.00
1450960_at	0.00	0.00
1450961_a_at	0.00	0.00
1450962_at	0.00	0.00
1450963_at	0.00	0.00
1450964_a_at	0.00	0.00
1450965_at	0.00	0.00
1450966_at	0.00	0.00
1450967_at	0.00	0.00
1450968_at	0.00	0.00
1450969_at	0.00	0.00
1450970_at	0.03	0.00
1450971_at	0.06	0.01
1450972_at	0.00	0.00
1450973_s_at	0.00	0.00
1450974_at	0.00	0.00
1450975_at	0.00	0.00

1450976_at	0.00	0.00
1450977_s_at	0.00	0.00
1450978_at	0.00	0.00
1450979_at	0.00	0.00
1450980_at	0.00	0.00
1450981_at	0.35	0.00
1450982_at	0.00	0.06
1450983_at	0.00	0.20
1450984_at	0.00	0.00
1450985_a_at	0.00	0.00
1450986_at	0.00	0.04
1450987_a_at	0.00	0.00
1450988_at	0.00	0.00
1450989_at	0.78	0.05
1450990_at	0.04	0.70
1450991_at	0.00	0.00
1450992_a_at	0.00	0.00
1450993_at	0.00	0.00
1450994_at	0.00	0.00
1450995_at	0.00	0.00
1450996_at	0.00	0.00
1450997_at	0.00	0.07
1450998_at	0.00	0.00
1450999_a_at	0.00	0.00
1451000_at	0.00	0.24
1451001_at	0.00	0.00
1451002_at	0.00	0.00
1451003_at	0.00	0.00
1451004_at	0.00	0.00
1451005_at	0.00	0.05
1451006_at	0.00	0.00
1451007_at	0.00	0.00
1451008_at	0.00	0.00
1451009_at	0.00	0.00
1451010_at	0.00	0.00
1451011_at	0.00	0.00
1451012_a_at	0.00	0.20
1451013_at	0.00	0.00
1451014_at	0.00	0.00
1451015_at	0.00	0.00
1451016_at	0.00	0.06
1451017_at	0.00	0.00
1451018_at	0.00	0.00
1451019_at	0.00	0.00
1451020_at	0.00	0.00
1451021_a_at	0.00	0.00
1451022_at	0.00	0.00
1451023_at	0.00	0.00
1451024_at	0.00	0.00
1451025_at	0.00	0.00
1451026_at	0.00	0.51
1451027_at	0.00	0.00
1451028_at	0.00	0.00
1451029_at	0.00	0.00
1451030_at	0.00	0.00
1451031_at	0.00	0.00

1451032_at	0.00	0.00
1451033_a_at	0.00	0.00
1451034_at	0.00	0.00
1451035_a_at	0.00	0.00
1451036_at	0.00	0.00
1451037_at	0.25	0.00
1451038_at	0.00	0.00
1451039_at	0.00	0.00
1451040_at	0.00	0.00
1451041_at	0.00	0.00
1451042_a_at	0.00	0.00
1451043_at	0.00	0.00
1451044_at	0.00	0.01
1451045_at	0.00	0.00
1451046_at	0.01	0.31
1451047_at	0.00	0.00
1451048_at	0.00	0.00
1451049_at	0.00	0.00
1451050_at	0.00	0.00
1451051_a_at	0.00	0.00
1451052_at	0.00	0.00
1451053_a_at	0.00	0.00
1451054_at	0.00	0.00
1451055_at	0.00	0.00
1451056_at	0.00	0.25
1451057_x_at	0.00	0.00
1451058_at	0.00	0.00
1451059_at	0.00	0.00
1451060_at	0.00	0.00
1451061_at	0.00	0.00
1451062_a_at	0.00	0.00
1451063_at	0.00	0.00
1451064_a_at	0.00	0.67
1451065_a_at	0.00	0.49
1451066_at	0.00	0.00
1451067_at	0.00	0.00
1451068_s_at	0.00	0.00
1451069_at	0.00	0.00
1451070_at	0.00	0.28
1451071_a_at	0.00	0.00
1451072_a_at	0.00	0.00
1451073_at	0.00	0.00
1451074_at	0.00	0.00
1451075_s_at	0.00	0.22
1451076_s_at	0.00	0.00
1451077_at	0.00	0.00
1451078_at	0.00	0.00
1451079_at	0.00	0.00
1451080_at	0.00	0.25
1451081_a_at	0.00	0.08
1451082_at	0.00	0.00
1451083_s_at	0.00	0.31
1451084_at	0.00	0.00
1451085_at	0.00	0.00
1451086_s_at	0.00	0.00
1451087_at	0.00	0.00

1451088_a_at	0.00	0.00
1451089_a_at	0.00	0.00
1451090_a_at	0.00	0.10
1451091_at	0.00	0.23
1451092_a_at	0.00	0.00
1451093_at	0.00	0.00
1451094_at	0.00	0.00
1451095_at	0.00	0.00
1451096_at	0.00	0.00
1451097_at	0.00	0.00
1451098_at	0.00	0.00
1451099_at	0.00	0.00
1451100_a_at	0.00	0.00
1451101_a_at	0.00	0.00
1451102_at	0.00	0.00
1451103_at	0.00	0.00
1451104_a_at	0.00	0.00
1451105_at	0.00	0.00
1451106_at	0.00	0.00
1451107_at	0.00	0.00
1451108_at	0.00	0.00
1451109_a_at	0.00	0.00
1451110_at	0.00	0.00
1451111_at	0.00	0.03
1451112_s_at	0.00	0.00
1451113_a_at	0.00	0.00
1451114_at	0.00	0.00
1451115_at	0.00	0.00
1451116_at	0.00	0.00
1451117_a_at	0.00	0.00
1451118_a_at	0.00	0.00
1451119_a_at	0.00	0.66
1451120_at	0.00	0.01
1451121_a_at	0.00	0.00
1451122_at	0.00	0.00
1451123_at	0.02	0.23
1451124_at	0.00	0.05
1451125_at	0.00	0.00
1451126_at	0.00	0.00
1451127_at	0.00	0.00
1451128_s_at	0.00	0.03
1451129_at	0.00	0.00
1451130_at	0.00	0.00
1451131_at	0.00	0.00
1451132_at	0.00	0.00
1451133_s_at	0.00	0.00
1451134_a_at	0.02	0.00
1451135_at	0.00	0.00
1451136_a_at	0.00	0.00
1451137_a_at	0.00	0.00
1451138_x_at	0.00	0.00
1451139_at	0.01	0.00
1451140_s_at	0.00	0.00
1451141_at	0.00	0.00
1451142_at	0.00	0.00
1451143_at	0.00	0.00

1451144_at	0.00	0.57
1451145_s_at	0.00	0.00
1451146_at	0.00	0.00
1451147_x_at	0.00	0.00
1451148_at	0.00	0.00
1451149_at	0.00	0.00
1451150_at	0.00	0.00
1451151_s_at	0.00	0.00
1451152_a_at	0.00	0.00
1451153_a_at	0.00	0.00
1451154_a_at	0.00	0.00
1451155_at	0.00	0.00
1451156_s_at	0.00	0.00
1451157_at	0.00	0.00
1451158_at	0.00	0.00
1451159_at	0.00	0.00
1451160_s_at	0.00	0.00
1451161_a_at	0.00	0.00
1451162_at	0.00	0.00
1451163_at	0.00	0.00
1451164_a_at	0.00	0.49
1451165_at	0.00	0.00
1451166_a_at	0.00	0.00
1451167_at	0.00	0.00
1451168_a_at	0.00	0.00
1451169_at	0.00	0.00
1451170_s_at	0.00	0.00
1451171_at	0.00	0.00
1451172_at	0.00	0.00
1451173_at	0.00	0.00
1451174_at	0.00	0.00
1451175_at	0.00	0.00
1451176_at	0.00	0.00
1451177_at	0.00	0.00
1451178_at	0.00	0.00
1451179_a_at	0.00	0.00
1451180_a_at	0.00	0.00
1451181_at	0.00	0.00
1451182_s_at	0.00	0.00
1451183_at	0.00	0.00
1451184_at	0.00	0.00
1451185_at	0.00	0.00
1451186_at	0.00	0.00
1451187_at	0.00	0.00
1451188_at	0.00	0.00
1451189_at	0.53	0.00
1451190_a_at	0.00	0.00
1451191_at	0.00	0.00
1451192_a_at	0.00	0.00
1451193_x_at	0.00	0.00
1451194_at	0.00	0.00
1451195_a_at	0.13	0.01
1451196_at	0.00	0.00
1451197_s_at	0.00	0.00
1451198_at	0.00	0.00
1451199_at	0.00	0.18

1451200_at	0.00	0.01
1451201_s_at	0.00	0.00
1451202_at	0.00	0.00
1451203_at	0.00	0.00
1451204_at	0.00	0.00
1451205_at	0.00	0.01
1451206_s_at	0.00	0.00
1451207_at	0.00	0.00
1451208_at	0.00	0.61
1451209_at	0.00	0.00
1451210_at	0.00	0.00
1451211_a_at	0.00	0.07
1451212_at	0.00	0.00
1451213_at	0.00	0.00
1451214_at	0.00	0.17
1451215_at	0.00	0.00
1451216_at	0.00	0.00
1451217_a_at	0.00	0.00
1451218_at	0.00	0.00
1451219_at	0.00	0.00
1451220_at	0.00	0.00
1451221_at	0.00	0.00
1451222_at	0.00	0.00
1451223_a_at	0.00	0.01
1451224_at	0.00	0.00
1451225_at	0.01	0.00
1451226_at	0.00	0.00
1451227_a_at	0.00	0.00
1451228_a_at	0.00	0.00
1451229_at	0.00	0.00
1451230_a_at	0.04	0.37
1451231_a_at	0.00	0.01
1451232_at	0.00	0.00
1451233_at	0.00	0.00
1451234_at	0.00	0.00
1451235_at	0.00	0.00
1451236_at	0.00	0.00
1451237_s_at	0.00	0.00
1451238_at	0.00	0.00
1451239_a_at	0.00	0.00
1451240_a_at	0.00	0.05
1451241_at	0.00	0.00
1451242_a_at	0.00	0.00
1451243_at	0.00	0.00
1451244_a_at	0.00	0.00
1451245_at	0.00	0.00
1451246_s_at	0.00	0.00
1451247_at	0.00	0.00
1451248_at	0.00	0.00
1451249_at	0.00	0.09
1451250_at	0.00	0.00
1451251_at	0.00	0.00
1451252_at	0.00	0.00
1451253_at	0.00	0.00
1451254_at	0.00	0.36
1451255_at	0.00	0.00

1451256_at	0.00	0.00
1451257_at	0.00	0.00
1451258_at	0.00	0.00
1451259_at	0.00	0.37
1451260_at	0.00	0.01
1451261_s_at	0.00	0.00
1451262_a_at	0.00	0.01
1451263_a_at	0.00	0.00
1451264_at	0.00	0.00
1451265_at	0.00	0.33
1451266_at	0.00	0.02
1451267_at	0.00	0.00
1451268_at	0.00	0.00
1451269_at	0.00	0.00
1451270_at	0.00	0.00
1451271_a_at	0.00	0.00
1451272_a_at	0.00	0.00
1451273_x_at	0.00	0.00
1451274_at	0.00	0.00
1451275_at	0.00	0.00
1451276_at	0.00	0.00
1451277_at	0.00	0.00
1451278_a_at	0.00	0.00
1451279_at	0.00	0.00
1451280_at	0.00	0.00
1451281_at	0.00	0.00
1451282_at	0.00	0.00
1451283_at	0.00	0.00
1451284_at	0.00	0.00
1451285_at	0.00	0.01
1451286_s_at	0.00	0.30
1451287_s_at	0.00	0.00
1451288_s_at	0.00	0.00
1451289_at	0.00	0.00
1451290_at	0.00	0.00
1451291_at	0.00	0.00
1451292_at	0.00	0.00
1451293_at	0.00	0.18
1451294_s_at	0.00	0.01
1451295_a_at	0.00	0.00
1451296_x_at	0.00	0.00
1451297_at	0.00	0.00
1451298_at	0.00	0.00
1451299_at	0.00	0.00
1451300_a_at	0.00	0.00
1451301_at	0.00	0.00
1451302_at	0.00	0.00
1451303_at	0.00	0.02
1451304_at	0.00	0.00
1451305_at	0.00	0.00
1451306_at	0.00	0.00
1451307_at	0.00	0.00
1451308_at	0.00	0.00
1451309_at	0.00	0.00
1451310_a_at	0.00	0.30
1451311_a_at	0.00	0.00

1451312_at	0.00	0.00
1451313_a_at	0.00	0.00
1451314_a_at	0.00	0.00
1451315_at	0.00	0.00
1451316_a_at	0.00	0.00
1451317_at	0.00	0.00
1451318_a_at	0.00	0.00
1451319_at	0.00	0.00
1451320_at	0.00	0.00
1451321_a_at	0.00	0.00
1451322_at	0.00	0.00
1451323_at	0.00	0.00
1451324_s_at	0.00	0.00
1451325_at	0.00	0.00
1451326_at	0.00	0.00
1451327_a_at	0.00	0.00
1451328_at	0.00	0.00
1451329_at	0.00	0.00
1451330_a_at	0.00	0.00
1451331_at	0.00	0.00
1451332_at	0.00	0.00
1451333_a_at	0.00	0.00
1451334_at	0.00	0.00
1451335_at	0.00	0.00
1451336_at	0.00	0.00
1451337_at	0.00	0.00
1451338_at	0.00	0.00
1451339_at	0.00	0.01
1451340_at	0.00	0.00
1451341_s_at	0.00	0.01
1451342_at	0.00	0.00
1451343_at	0.00	0.00
1451344_at	0.00	0.00
1451345_at	0.00	0.00
1451346_at	0.00	0.00
1451347_at	0.00	0.02
1451348_at	0.00	0.00
1451349_at	0.00	0.00
1451350_a_at	0.00	0.00
1451351_at	0.00	0.00
1451352_s_at	0.00	0.00
1451353_at	0.00	0.00
1451354_at	0.00	0.00
1451355_at	0.00	0.00
1451356_at	0.00	0.00
1451357_at	0.00	0.00
1451358_a_at	0.00	0.00
1451359_at	0.00	0.05
1451360_at	0.00	0.00
1451361_a_at	0.00	0.00
1451362_at	0.00	0.00
1451363_a_at	0.00	0.00
1451364_at	0.00	0.00
1451365_at	0.00	0.38
1451366_at	0.00	0.30
1451367_at	0.00	0.00

1451368_at	0.00	0.00
1451369_at	0.00	0.00
1451370_at	0.00	0.00
1451371_at	0.00	0.00
1451372_a_at	0.00	0.00
1451373_at	0.00	0.00
1451374_x_at	0.00	0.00
1451375_at	0.00	0.00
1451376_at	0.00	0.00
1451377_a_at	0.00	0.00
1451378_at	0.00	0.03
1451379_at	0.00	0.00
1451380_at	0.00	0.00
1451381_at	0.00	0.00
1451382_at	0.00	0.00
1451383_a_at	0.00	0.00
1451384_at	0.00	0.00
1451385_at	0.00	0.00
1451386_at	0.00	0.26
1451387_s_at	0.00	0.00
1451388_a_at	0.00	0.00
1451389_at	0.00	0.00
1451390_s_at	0.00	0.00
1451391_at	0.00	0.00
1451392_at	0.00	0.00
1451393_at	0.00	0.00
1451394_at	0.00	0.00
1451395_at	0.00	0.00
1451396_at	0.00	0.00
1451397_at	0.00	0.00
1451398_at	0.00	0.00
1451399_at	0.00	0.00
1451400_at	0.00	0.00
1451401_a_at	0.00	0.00
1451402_at	0.00	0.00
1451403_at	0.00	0.00
1451404_at	0.00	0.00
1451405_at	0.00	0.00
1451406_a_at	0.00	0.00
1451407_at	0.00	0.00
1451408_at	0.00	0.00
1451409_at	0.00	0.00
1451410_a_at	0.00	0.00
1451411_at	0.00	0.00
1451412_a_at	0.00	0.00
1451413_at	0.00	0.00
1451414_at	0.00	0.00
1451415_at	0.00	0.00
1451416_a_at	0.00	0.99
1451417_at	0.01	0.00
1451418_a_at	0.00	0.00
1451419_at	0.00	0.00
1451420_at	0.00	0.00
1451421_a_at	0.00	0.00
1451422_at	0.00	0.00
1451423_at	0.00	0.00

1451424_at	0.00	0.00
1451425_a_at	0.57	0.00
1451426_at	0.00	0.00
1451427_a_at	0.00	0.00
1451428_x_at	0.00	0.00
1451429_at	0.00	0.00
1451430_at	0.00	0.00
1451431_a_at	0.00	0.01
1451432_x_at	0.00	0.00
1451433_at	0.00	0.00
1451434_s_at	0.00	0.00
1451435_at	0.00	0.00
1451436_at	0.00	0.04
1451437_at	0.01	0.00
1451438_s_at	0.00	0.00
1451439_at	0.00	0.06
1451440_at	0.00	0.00
1451441_at	0.00	0.00
1451442_at	0.00	0.00
1451443_at	0.00	0.00
1451444_s_at	0.00	0.00
1451445_at	0.00	0.00
1451446_at	0.00	0.00
1451447_at	0.00	0.00
1451448_a_at	0.00	0.00
1451449_at	0.00	0.00
1451450_at	0.00	0.00
1451451_at	0.00	0.00
1451452_a_at	0.00	0.00
1451453_at	0.00	0.00
1451454_at	0.00	0.00
1451455_at	0.00	0.00
1451456_at	0.00	0.00
1451457_at	0.16	0.00
1451458_at	0.00	0.00
1451459_at	0.00	0.00
1451460_a_at	0.00	0.00
1451461_a_at	0.02	0.00
1451462_a_at	0.00	0.00
1451463_at	0.00	0.00
1451464_at	0.00	0.00
1451465_at	0.00	0.00
1451466_at	0.00	0.00
1451467_s_at	0.00	0.00
1451468_s_at	0.00	0.00
1451469_at	0.00	0.00
1451470_s_at	0.00	0.00
1451471_at	0.00	0.00
1451472_at	0.00	0.00
1451473_a_at	0.00	0.00
1451474_a_at	0.00	0.00
1451475_at	0.00	0.00
1451476_at	0.00	0.00
1451477_at	0.00	0.00
1451478_at	0.00	0.00
1451479_a_at	0.00	0.00

1451480_at	0.00	0.00
1451481_s_at	0.00	0.00
1451482_at	0.00	0.00
1451483_s_at	0.00	0.00
1451484_a_at	0.00	0.00
1451485_at	0.00	0.00
1451486_at	0.00	0.00
1451487_at	0.00	0.00
1451488_at	0.00	0.00
1451489_at	0.00	0.00
1451490_at	0.00	0.00
1451491_at	0.00	0.00
1451492_at	0.00	0.00
1451493_at	0.00	0.02
1451494_at	0.00	0.00
1451495_at	0.00	0.00
1451496_at	0.00	0.00
1451497_at	0.00	0.00
1451498_at	0.00	0.00
1451499_at	0.00	0.00
1451500_at	0.00	0.00
1451501_a_at	0.00	0.00
1451502_at	0.00	0.00
1451503_at	0.00	0.00
1451504_at	0.00	0.00
1451505_at	0.00	0.00
1451506_at	0.00	0.00
1451507_at	0.00	0.00
1451508_at	0.00	0.00
1451509_at	0.00	0.00
1451510_s_at	0.00	0.00
1451511_at	0.00	0.00
1451512_s_at	0.00	0.00
1451513_x_at	0.00	0.00
1451514_at	0.00	0.00
1451515_s_at	0.00	0.00
1451516_at	0.00	0.00
1451517_at	0.00	0.00
1451518_at	0.00	0.00
1451519_at	0.00	0.00
1451520_at	0.00	0.15
1451521_x_at	0.00	0.00
1451522_s_at	0.00	0.00
1451523_a_at	0.00	0.00
1451524_at	0.00	0.00
1451525_at	0.00	0.00
1451526_at	0.00	0.00
1451527_at	0.37	0.67
1451528_at	0.00	0.00
1451529_at	0.00	0.00
1451530_at	0.00	0.00
1451531_at	0.00	0.00
1451532_s_at	0.00	0.00
1451533_at	0.00	0.00
1451534_at	0.00	0.00
1451535_at	0.00	0.00

1451536_at	0.00	0.00
1451537_at	0.00	0.00
1451538_at	0.00	0.00
1451539_at	0.00	0.00
1451540_at	0.00	0.00
1451541_at	0.00	0.00
1451542_at	0.00	0.00
1451543_at	0.00	0.00
1451544_at	0.00	0.00
1451545_at	0.00	0.00
1451546_s_at	0.00	0.00
1451547_at	0.00	0.00
1451548_at	0.00	0.00
1451549_at	0.00	0.00
1451550_at	0.00	0.00
1451551_at	0.00	0.00
1451552_at	0.00	0.00
1451553_at	0.00	0.00
1451554_a_at	0.00	0.00
1451555_at	0.00	0.00
1451556_a_at	0.00	0.00
1451557_at	0.00	0.00
1451558_at	0.00	0.00
1451559_a_at	0.00	0.00
1451560_at	0.00	0.00
1451561_at	0.00	0.00
1451562_at	0.00	0.00
1451563_at	0.00	0.00
1451564_at	0.00	0.00
1451565_s_at	0.00	0.00
1451566_at	0.00	0.00
1451567_a_at	0.00	0.00
1451568_at	0.00	0.00
1451569_at	0.00	0.00
1451570_a_at	0.00	0.00
1451571_s_at	0.00	0.00
1451572_a_at	0.00	0.00
1451573_a_at	0.00	0.00
1451574_at	0.00	0.00
1451575_a_at	0.00	0.00
1451576_at	0.00	0.00
1451577_at	0.00	0.00
1451578_at	0.00	0.00
1451579_at	0.00	0.00
1451580_a_at	0.00	0.00
1451581_at	0.00	0.00
1451582_at	0.00	0.00
1451583_a_at	0.00	0.00
1451584_at	0.00	0.00
1451585_x_at	0.00	0.00
1451586_at	0.00	0.00
1451587_a_at	0.00	0.00
1451588_at	0.00	0.00
1451589_at	0.00	0.07
1451590_at	0.00	0.00
1451591_a_at	0.00	0.00

1451592_at	0.00	0.00
1451593_at	0.00	0.00
1451594_s_at	0.00	0.00
1451595_a_at	0.00	0.00
1451596_a_at	0.00	0.00
1451597_at	0.00	0.00
1451598_at	0.00	0.00
1451599_at	0.00	0.00
1451600_s_at	0.00	0.00
1451601_a_at	0.00	0.00
1451602_at	0.00	0.00
1451603_at	0.00	0.00
1451604_a_at	0.00	0.00
1451605_at	0.00	0.00
1451606_at	0.00	0.00
1451607_at	0.00	0.00
1451608_a_at	0.00	0.00
1451609_at	0.00	0.00
1451610_at	0.00	0.00
1451611_at	0.00	0.00
1451612_at	0.00	0.00
1451613_at	0.00	0.00
1451614_a_at	0.00	0.00
1451615_at	0.00	0.00
1451616_at	0.00	0.00
1451617_at	0.00	0.00
1451618_at	0.00	0.00
1451619_at	0.00	0.00
1451620_at	0.00	0.00
1451621_at	0.00	0.00
1451622_at	0.01	0.00
1451623_at	0.00	0.00
1451624_a_at	0.00	0.00
1451625_a_at	0.00	0.00
1451626_x_at	0.00	0.00
1451627_a_at	0.00	0.00
1451628_a_at	0.00	0.00
1451629_at	0.00	0.00
1451630_at	0.00	0.00
1451631_at	0.00	0.00
1451632_a_at	0.00	0.00
1451633_a_at	0.00	0.00
1451634_at	0.00	0.00
1451635_at	0.00	0.00
1451636_at	0.00	0.00
1451637_a_at	0.00	0.00
1451638_s_at	0.00	0.00
1451639_at	0.00	0.00
1451640_a_at	0.00	0.00
1451641_at	0.00	0.47
1451642_at	0.00	0.00
1451643_a_at	0.00	0.00
1451644_a_at	0.00	0.00
1451645_at	0.00	0.00
1451646_at	0.00	0.00
1451647_at	0.00	0.00

1451648_a_at	0.00	0.00
1451649_a_at	0.00	0.37
1451650_at	0.00	0.00
1451651_at	0.00	0.00
1451652_a_at	0.03	0.00
1451653_a_at	0.00	0.00
1451654_x_at	0.00	0.00
1451655_at	0.00	0.00
1451656_at	0.00	0.00
1451657_a_at	0.00	0.00
1451658_a_at	0.00	0.00
1451659_at	0.00	0.00
1451660_a_at	0.00	0.00
1451661_at	0.00	0.00
1451662_x_at	0.00	0.00
1451663_a_at	0.00	0.00
1451664_x_at	0.00	0.00
1451665_a_at	0.00	0.13
1451666_at	0.00	0.00
1451667_at	0.00	0.00
1451668_at	0.00	0.00
1451669_at	0.00	0.00
1451670_at	0.00	0.00
1451671_at	0.00	0.00
1451672_at	0.00	0.18
1451673_at	0.00	0.00
1451674_at	0.00	0.00
1451675_a_at	0.00	0.00
1451676_at	0.00	0.00
1451677_at	0.00	0.00
1451678_at	0.00	0.00
1451679_at	0.00	0.04
1451680_at	0.00	0.00
1451681_at	0.00	0.00
1451682_at	0.00	0.00
1451683_x_at	0.00	0.00
1451684_a_at	0.00	0.00
1451685_at	0.00	0.00
1451686_x_at	0.00	0.00
1451687_a_at	0.00	0.00
1451688_s_at	0.00	0.00
1451689_a_at	0.00	0.00
1451690_a_at	0.00	0.00
1451691_at	0.00	0.00
1451692_at	0.00	0.00
1451693_a_at	0.00	0.00
1451694_at	0.00	0.00
1451695_a_at	0.00	0.00
1451696_at	0.00	0.00
1451697_a_at	0.00	0.00
1451698_at	0.00	0.00
1451699_at	0.00	0.00
1451700_a_at	0.00	0.00
1451701_x_at	0.00	0.34
1451702_at	0.00	0.00
1451703_s_at	0.00	0.00

1451704_at	0.00	0.00
1451705_a_at	0.00	0.00
1451706_a_at	0.00	0.00
1451707_s_at	0.00	0.00
1451708_at	0.00	0.00
1451709_at	0.00	0.00
1451710_at	0.00	0.00
1451711_at	0.00	0.00
1451712_at	0.00	0.00
1451713_a_at	0.00	0.00
1451714_a_at	0.00	0.00
1451715_at	0.00	0.00
1451716_at	0.00	0.00
1451717_s_at	0.00	0.00
1451718_at	0.00	0.00
1451719_at	0.00	0.00
1451720_at	0.00	0.00
1451721_a_at	0.00	0.00
1451722_s_at	0.00	0.24
1451723_at	0.00	0.00
1451724_at	0.00	0.00
1451725_a_at	0.00	0.00
1451726_at	0.00	0.00
1451727_at	0.00	0.00
1451728_at	0.00	0.00
1451729_at	0.00	0.00
1451730_at	0.00	0.00
1451731_at	0.00	0.00
1451732_at	0.00	0.00
1451733_at	0.00	0.00
1451734_a_at	0.02	0.00
1451735_at	0.00	0.00
1451736_a_at	0.00	0.00
1451737_at	0.00	0.00
1451738_at	0.00	0.00
1451739_at	0.00	0.00
1451740_at	0.00	0.00
1451741_a_at	0.00	0.35
1451742_a_at	0.00	0.00
1451743_at	0.00	0.00
1451744_a_at	0.00	0.00
1451745_a_at	0.00	0.71
1451746_a_at	0.00	0.00
1451747_a_at	0.00	0.00
1451748_a_at	0.00	0.00
1451749_at	0.00	0.00
1451750_at	0.00	0.00
1451751_at	0.00	0.00
1451752_at	0.00	0.00
1451753_at	0.00	0.00
1451754_a_at	0.00	0.00
1451755_a_at	0.00	0.00
1451756_at	0.00	0.00
1451757_at	0.00	0.00
1451758_at	0.00	0.00
1451759_at	0.00	0.00

1451760_s_at	0.00	0.00
1451761_at	0.00	0.00
1451762_a_at	0.00	0.00
1451763_at	0.00	0.00
1451764_at	0.00	0.00
1451765_a_at	0.00	0.00
1451766_at	0.00	0.00
1451767_at	0.00	0.00
1451768_a_at	0.00	0.00
1451769_s_at	0.00	0.00
1451770_s_at	0.00	0.53
1451771_at	0.00	0.00
1451772_at	0.00	0.00
1451773_s_at	0.00	0.00
1451774_at	0.00	0.00
1451775_s_at	0.00	0.00
1451776_s_at	0.00	0.00
1451777_at	0.00	0.00
1451778_at	0.00	0.00
1451779_at	0.00	0.00
1451780_at	0.00	0.00
1451781_at	0.00	0.00
1451782_a_at	0.69	0.49
1451783_a_at	0.00	0.00
1451784_x_at	0.04	0.10
1451785_at	0.00	0.00
1451786_at	0.00	0.00
1451787_at	0.00	0.00
1451788_at	0.00	0.00
1451789_a_at	0.00	0.00
1451790_a_at	0.00	0.00
1451791_at	0.00	0.00
1451792_a_at	0.00	0.00
1451793_at	0.00	0.00
1451794_at	0.00	0.00
1451795_at	0.00	0.00
1451796_s_at	0.00	0.00
1451797_at	0.00	0.00
1451798_at	0.00	0.00
1451799_at	0.00	0.00
1451800_at	0.00	0.00
1451801_at	0.00	0.00
1451802_at	0.00	0.00
1451803_a_at	0.00	0.01
1451804_a_at	0.00	0.00
1451805_at	0.00	0.00
1451806_at	0.00	0.00
1451807_at	0.00	0.00
1451808_at	0.00	0.00
1451809_s_at	0.00	0.00
1451810_at	0.00	0.00
1451811_at	0.00	0.00
1451812_at	0.00	0.00
1451813_at	0.00	0.00
1451814_a_at	0.00	0.00
1451815_at	0.00	0.00

1451816_at	0.00	0.00
1451817_at	0.00	0.00
1451818_at	0.00	0.00
1451819_at	0.00	0.00
1451820_at	0.00	0.00
1451821_a_at	0.00	0.00
1451822_a_at	0.00	0.00
1451823_at	0.00	0.00
1451824_at	0.00	0.00
1451825_a_at	0.00	0.01
1451826_at	0.00	0.00
1451827_a_at	0.00	0.00
1451828_a_at	0.00	0.00
1451829_a_at	0.00	0.00
1451830_a_at	0.00	0.00
1451831_at	0.00	0.00
1451832_at	0.00	0.00
1451833_a_at	0.00	0.00
1451834_at	0.00	0.00
1451835_at	0.00	0.00
1451836_at	0.00	0.00
1451837_at	0.00	0.00
1451838_a_at	0.00	0.00
1451839_a_at	0.00	0.00
1451840_at	0.00	0.00
1451841_a_at	0.00	0.00
1451842_a_at	0.00	0.00
1451843_a_at	0.00	0.00
1451844_at	0.00	0.00
1451845_a_at	0.00	0.09
1451846_at	0.00	0.00
1451847_s_at	0.00	0.00
1451848_a_at	0.00	0.00
1451849_a_at	0.00	0.00
1451850_at	0.00	0.00
1451851_a_at	0.00	0.00
1451852_at	0.00	0.00
1451853_at	0.00	0.00
1451854_a_at	0.00	0.15
1451855_at	0.00	0.00
1451856_at	0.00	0.00
1451857_a_at	0.15	0.36
1451858_at	0.00	0.00
1451859_at	0.00	0.00
1451860_a_at	0.00	0.00
1451861_at	0.00	0.00
1451862_a_at	0.00	0.00
1451863_at	0.00	0.00
1451864_at	0.00	0.00
1451865_at	0.00	0.00
1451866_a_at	0.00	0.00
1451867_x_at	0.00	0.00
1451868_at	0.00	0.00
1451869_at	0.00	0.00
1451870_a_at	0.00	0.00
1451871_a_at	0.00	0.00

1451872_a_at	0.00	0.00
1451873_a_at	0.00	0.00
1451874_at	0.00	0.00
1451875_at	0.00	0.00
1451876_a_at	0.00	0.00
1451877_at	0.00	0.00
1451878_a_at	0.00	0.00
1451879_a_at	0.00	0.00
1451880_at	0.00	0.00
1451881_at	0.00	0.00
1451882_a_at	0.00	0.00
1451883_at	0.00	0.00
1451884_a_at	0.00	0.00
1451885_at	0.00	0.00
1451886_at	0.00	0.00
1451887_at	0.00	0.00
1451888_a_at	0.00	0.00
1451889_at	0.00	0.00
1451890_at	0.00	0.00
1451891_a_at	0.00	0.00
1451892_at	0.00	0.00
1451893_s_at	0.00	0.00
1451894_a_at	0.00	0.00
1451895_a_at	0.00	0.00
1451896_a_at	0.00	0.00
1451897_a_at	0.00	0.00
1451898_a_at	0.00	0.00
1451899_a_at	0.00	0.14
1451900_at	0.00	0.00
1451901_at	0.00	0.00
1451902_at	0.00	0.00
1451903_at	0.00	0.00
1451904_a_at	0.00	0.00
1451905_a_at	0.00	0.00
1451906_at	0.00	0.00
1451907_a_at	0.00	0.00
1451908_a_at	0.00	0.00
1451909_a_at	0.00	0.00
1451910_a_at	0.00	0.00
1451911_a_at	0.00	0.00
1451912_a_at	0.00	0.00
1451913_a_at	0.00	0.00
1451914_a_at	0.00	0.00
1451915_at	0.00	0.00
1451916_s_at	0.00	0.00
1451917_a_at	0.00	0.00
1451918_a_at	0.00	0.00
1451919_a_at	0.00	0.00
1451920_a_at	0.00	0.00
1451921_a_at	0.00	0.00
1451922_at	0.00	0.00
1451923_at	0.00	0.00
1451924_a_at	0.01	0.00
1451925_at	0.00	0.00
1451926_at	0.00	0.00
1451927_a_at	0.00	0.00

1451928_a_at	0.00	0.00
1451929_a_at	0.00	0.00
1451930_at	0.00	0.00
1451931_x_at	0.02	0.07
1451932_a_at	0.00	0.00
1451933_a_at	0.00	0.00
1451934_at	0.00	0.00
1451935_a_at	0.00	0.00
1451936_a_at	0.00	0.00
1451937_at	0.00	0.00
1451938_a_at	0.00	0.00
1451939_a_at	0.00	0.00
1451940_x_at	0.00	0.00
1451941_a_at	0.00	0.00
1451942_x_at	0.00	0.00
1451943_a_at	0.00	0.00
1451944_a_at	0.00	0.00
1451945_at	0.00	0.00
1451946_a_at	0.00	0.00
1451947_at	0.00	0.00
1451948_at	0.00	0.00
1451949_at	0.00	0.00
1451950_a_at	0.00	0.00
1451951_at	0.00	0.00
1451952_at	0.00	0.00
1451953_at	0.00	0.00
1451954_at	0.00	0.00
1451955_a_at	0.00	0.00
1451956_a_at	0.00	0.00
1451957_at	0.00	0.00
1451958_at	0.00	0.00
1451959_a_at	0.00	0.00
1451960_a_at	0.00	0.00
1451961_a_at	0.00	0.00
1451962_at	0.00	0.00
1451963_at	0.00	0.00
1451964_at	0.00	0.00
1451965_at	0.00	0.00
1451966_at	0.00	0.00
1451967_x_at	0.00	0.01
1451968_at	0.49	0.48
1451969_s_at	0.00	0.00
1451970_at	0.00	0.00
1451971_at	0.00	0.02
1451972_at	0.00	0.00
1451973_at	0.00	0.00
1451974_at	0.00	0.00
1451975_at	0.00	0.00
1451976_s_at	0.00	0.00
1451977_at	0.00	0.00
1451978_at	0.00	0.00
1451979_at	0.00	0.00
1451980_at	0.00	0.00
1451981_at	0.00	0.00
1451982_at	0.00	0.00
1451983_at	0.00	0.00

1451984_at	0.00	0.00
1451985_at	0.00	0.00
1451986_s_at	0.00	0.00
1451987_at	0.00	0.00
1451988_s_at	0.00	0.00
1451989_a_at	0.00	0.00
1451990_at	0.00	0.00
1451991_at	0.00	0.00
1451992_at	0.00	0.00
1451993_at	0.00	0.00
1451994_s_at	0.00	0.00
1451995_at	0.00	0.00
1451996_at	0.00	0.00
1451997_at	0.00	0.00
1451998_at	0.00	0.00
1451999_at	0.00	0.00
1452000_s_at	0.00	0.00
1452001_at	0.00	0.00
1452002_at	0.00	0.00
1452003_at	0.00	0.00
1452004_at	0.00	0.00
1452005_at	0.00	0.00
1452006_x_at	0.00	0.00
1452007_at	0.01	0.00
1452008_at	0.00	0.00
1452009_at	0.00	0.00
1452010_at	0.00	0.00
1452011_a_at	0.00	0.00
1452012_a_at	0.00	0.27
1452013_at	0.00	0.00
1452014_a_at	0.00	0.00
1452015_at	0.00	0.00
1452016_at	0.00	0.00
1452017_at	0.00	0.00
1452018_at	0.00	0.00
1452019_at	0.00	0.00
1452020_a_at	0.00	0.00
1452021_a_at	0.00	0.00
1452022_at	0.00	0.00
1452023_at	0.00	0.00
1452024_a_at	0.01	0.00
1452025_a_at	0.00	0.00
1452026_a_at	0.00	0.00
1452027_a_at	0.00	0.00
1452028_a_at	0.00	0.00
1452029_a_at	0.00	0.00
1452030_a_at	0.00	0.25
1452031_at	0.00	0.00
1452032_at	0.00	0.01
1452033_at	0.00	0.00
1452034_at	0.00	0.00
1452035_at	0.12	0.00
1452036_a_at	0.00	0.01
1452037_at	0.00	0.00
1452038_at	0.00	0.00
1452039_a_at	0.00	0.00

1452040_a_at	0.00	0.00
1452041_at	0.00	0.00
1452042_a_at	0.00	0.00
1452043_at	0.00	0.00
1452044_at	0.00	0.00
1452045_at	0.00	0.00
1452046_a_at	0.00	0.00
1452047_at	0.01	0.35
1452048_at	0.00	0.07
1452049_at	0.00	0.48
1452050_at	0.00	0.00
1452051_at	0.00	0.00
1452052_s_at	0.00	0.05
1452053_a_at	0.00	0.00
1452054_at	0.00	0.00
1452055_at	0.00	0.00
1452056_s_at	0.00	0.00
1452057_at	0.00	0.00
1452058_a_at	0.00	0.33
1452059_at	0.00	0.00
1452060_a_at	0.00	0.00
1452061_s_at	0.00	0.00
1452062_at	0.00	0.00
1452063_at	0.95	0.72
1452064_at	0.00	0.00
1452065_at	0.00	0.00
1452066_a_at	0.00	0.00
1452067_at	0.00	0.00
1452068_at	0.00	0.00
1452069_a_at	0.00	0.00
1452070_at	0.00	0.00
1452071_at	0.00	0.00
1452072_at	0.00	0.00
1452073_at	0.00	0.00
1452074_at	0.00	0.00
1452075_at	0.00	0.00
1452076_at	0.00	0.00
1452077_at	0.00	0.00
1452078_a_at	0.00	0.00
1452079_s_at	0.00	0.00
1452080_a_at	0.00	0.00
1452081_a_at	0.00	0.00
1452082_at	0.00	0.00
1452083_a_at	0.00	0.00
1452084_at	0.00	0.00
1452085_at	0.00	0.00
1452086_at	0.00	0.00
1452087_at	0.00	0.00
1452088_at	0.00	0.00
1452089_at	0.00	0.00
1452090_a_at	0.00	0.00
1452091_a_at	0.00	0.00
1452092_at	0.00	0.10
1452093_at	0.00	0.00
1452094_at	0.00	1.00
1452095_a_at	0.00	0.00

1452096_s_at	0.00	0.00
1452097_a_at	0.00	0.00
1452098_at	0.00	0.01
1452099_at	0.00	0.00
1452100_at	0.00	0.00
1452101_at	0.00	0.00
1452102_at	0.00	0.00
1452103_at	0.00	0.00
1452104_at	0.00	0.00
1452105_a_at	0.00	0.00
1452106_at	0.00	0.00
1452107_s_at	0.00	0.00
1452108_at	0.00	0.00
1452109_at	0.00	0.00
1452110_at	0.00	0.00
1452111_at	0.00	0.00
1452112_a_at	0.00	0.00
1452113_a_at	0.00	0.00
1452114_s_at	0.00	0.00
1452115_a_at	0.00	0.00
1452116_s_at	0.00	0.00
1452117_a_at	0.00	0.00
1452118_at	0.00	0.00
1452119_at	0.00	0.00
1452120_at	0.00	0.00
1452121_at	0.00	0.00
1452122_at	0.00	0.00
1452123_s_at	0.00	0.00
1452124_at	0.00	0.09
1452125_at	0.00	0.00
1452126_at	0.00	0.00
1452127_a_at	0.00	0.34
1452128_a_at	0.00	0.00
1452129_at	0.00	0.00
1452130_at	0.00	0.00
1452131_at	0.00	0.00
1452132_at	0.00	0.00
1452133_at	0.00	0.00
1452134_at	0.00	0.00
1452135_at	0.00	0.00
1452136_at	0.00	0.00
1452137_at	0.00	0.00
1452138_a_at	0.00	0.00
1452139_at	0.00	0.00
1452140_at	0.00	0.00
1452141_a_at	0.00	0.00
1452142_at	0.00	0.00
1452143_at	0.00	0.00
1452144_a_at	0.00	0.00
1452145_at	0.00	0.00
1452146_a_at	0.00	0.00
1452147_at	0.00	0.00
1452148_at	0.00	0.00
1452149_at	0.00	0.00
1452150_at	0.00	0.02
1452151_at	0.00	0.02

1452152_at	0.00	0.00
1452153_at	0.00	0.00
1452154_at	0.00	0.00
1452155_a_at	0.00	0.00
1452156_a_at	0.00	0.00
1452157_at	0.00	0.00
1452158_at	0.00	0.00
1452159_at	0.00	0.00
1452160_at	0.00	0.00
1452161_at	0.00	0.00
1452162_at	0.00	0.00
1452163_at	0.73	0.00
1452164_at	0.00	0.00
1452165_at	0.00	0.00
1452166_a_at	0.00	0.00
1452167_at	0.00	0.00
1452168_x_at	0.00	0.33
1452169_a_at	0.00	0.00
1452170_at	0.00	0.00
1452171_at	0.00	0.33
1452172_at	0.00	0.30
1452173_at	0.00	0.00
1452174_at	0.00	0.00
1452175_at	0.00	0.00
1452176_at	0.00	0.01
1452177_at	0.00	0.00
1452178_at	0.00	0.33
1452179_at	0.02	0.01
1452180_at	0.00	0.00
1452181_at	0.00	0.00
1452182_at	0.00	0.00
1452183_a_at	0.00	0.00
1452184_at	0.00	0.00
1452185_at	0.00	0.00
1452186_at	0.00	0.00
1452187_at	0.00	0.00
1452188_at	0.00	0.00
1452189_at	0.00	0.00
1452190_at	0.00	0.00
1452191_at	0.00	0.00
1452192_at	0.00	0.00
1452193_a_at	0.00	0.00
1452194_at	0.00	0.00
1452195_s_at	0.00	0.00
1452196_a_at	0.00	0.00
1452197_at	0.00	0.00
1452198_at	0.00	0.00
1452199_at	0.00	0.00
1452200_at	0.00	0.00
1452201_at	0.00	0.00
1452202_at	0.00	0.00
1452203_at	0.00	0.00
1452204_at	0.00	0.00
1452205_x_at	0.00	0.00
1452206_at	0.00	0.00
1452207_at	0.04	0.00

1452208_at	0.00	0.00
1452209_at	0.00	0.00
1452210_at	0.01	0.00
1452211_at	0.00	0.00
1452212_at	0.00	0.00
1452213_at	0.00	0.00
1452214_at	0.00	0.00
1452215_at	0.00	0.01
1452216_at	0.00	0.00
1452217_at	0.75	0.00
1452218_at	0.00	0.00
1452219_at	0.00	0.00
1452220_at	0.00	0.00
1452221_a_at	0.00	0.00
1452222_at	0.00	0.00
1452223_s_at	0.00	0.00
1452224_at	0.00	0.06
1452225_at	0.00	0.00
1452226_at	0.00	0.32
1452227_at	0.00	0.07
1452228_at	0.00	0.00
1452229_at	0.00	0.00
1452230_at	0.00	0.00
1452231_x_at	0.00	0.00
1452232_at	0.00	0.06
1452233_at	0.00	0.00
1452234_s_at	0.00	0.00
1452235_at	0.00	0.00
1452236_at	0.00	0.00
1452237_at	0.00	0.00
1452238_at	0.00	0.00
1452239_at	0.00	0.00
1452240_at	0.00	0.00
1452241_at	0.00	0.00
1452242_at	0.00	0.02
1452243_at	0.00	0.00
1452244_at	0.00	0.00
1452245_at	0.00	0.00
1452246_at	0.00	0.00
1452247_at	0.00	0.00
1452248_at	0.00	0.00
1452249_at	0.35	0.14
1452250_a_at	0.00	0.00
1452251_at	0.00	0.00
1452252_at	0.00	0.39
1452253_at	0.00	0.00
1452254_at	0.00	0.00
1452255_at	0.00	0.00
1452256_at	0.00	0.01
1452257_at	0.00	0.00
1452258_at	0.00	0.00
1452259_at	0.00	0.00
1452260_at	0.00	0.00
1452261_at	0.00	0.00
1452262_at	0.00	0.00
1452263_at	0.00	0.00

1452264_at	0.00	0.00
1452265_at	0.00	0.00
1452266_at	0.00	0.19
1452267_at	0.00	0.00
1452268_at	0.00	0.00
1452269_at	0.00	0.00
1452270_s_at	0.00	0.00
1452271_at	0.00	0.00
1452272_a_at	0.00	0.00
1452273_at	0.00	0.00
1452274_at	0.00	0.25
1452275_at	0.00	0.00
1452276_at	0.12	0.60
1452277_at	0.00	0.00
1452278_a_at	0.00	0.00
1452279_at	0.00	0.00
1452280_at	0.16	0.33
1452281_at	0.00	0.00
1452282_at	0.00	0.00
1452283_at	0.00	0.00
1452284_at	0.00	0.00
1452285_a_at	0.00	0.00
1452286_at	0.00	0.00
1452287_at	0.00	0.06
1452288_at	0.00	0.00
1452289_a_at	0.00	0.00
1452290_at	0.00	0.00
1452291_at	0.00	0.00
1452292_at	0.00	0.00
1452293_at	0.00	0.00
1452294_at	0.01	0.54
1452295_at	0.00	0.00
1452296_at	0.00	0.00
1452297_at	0.00	0.00
1452298_a_at	0.00	0.00
1452299_at	0.00	0.00
1452300_at	0.00	0.00
1452301_at	0.00	0.00
1452302_at	0.00	0.00
1452303_at	0.00	0.00
1452304_a_at	0.00	0.00
1452305_s_at	0.00	0.00
1452306_at	0.00	0.00
1452307_at	0.00	0.05
1452308_a_at	0.00	0.00
1452309_at	0.00	0.00
1452310_at	0.00	0.00
1452311_at	0.00	0.00
1452312_at	0.00	0.00
1452313_at	0.00	0.00
1452314_at	0.00	0.00
1452315_at	0.00	0.04
1452316_at	0.00	0.00
1452317_at	0.00	0.00
1452318_a_at	0.00	0.00
1452319_at	0.00	0.00

1452320_at	0.00	0.40
1452321_at	0.00	0.00
1452322_a_at	0.00	0.00
1452323_at	0.00	0.00
1452324_at	0.00	0.00
1452325_at	0.00	0.00
1452326_at	0.00	0.00
1452327_at	0.00	0.00
1452328_s_at	0.00	0.00
1452329_at	0.00	0.00
1452330_a_at	0.00	0.00
1452331_s_at	0.00	0.00
1452332_at	0.00	0.00
1452333_at	0.00	0.00
1452334_at	0.00	0.00
1452335_at	0.00	0.00
1452336_at	0.00	0.00
1452337_at	0.00	0.00
1452338_s_at	0.00	0.00
1452339_at	0.00	0.00
1452340_at	0.00	0.13
1452341_at	0.00	0.00
1452342_at	0.00	0.00
1452343_at	0.00	0.00
1452344_at	0.00	0.00
1452345_at	0.00	0.00
1452346_at	0.00	0.00
1452347_at	0.00	0.00
1452348_s_at	0.00	0.00
1452349_x_at	0.00	0.00
1452350_at	0.00	0.00
1452351_at	0.00	0.00
1452352_at	0.00	0.00
1452353_at	0.00	0.00
1452354_at	0.00	0.00
1452355_at	0.00	0.00
1452356_at	0.00	0.00
1452357_at	0.00	0.00
1452358_at	0.00	0.00
1452359_at	0.00	0.00
1452360_a_at	0.00	0.00
1452361_at	0.00	0.00
1452362_at	0.00	0.00
1452363_a_at	0.00	0.00
1452364_at	0.00	0.00
1452365_at	0.00	0.00
1452366_at	0.00	0.00
1452367_at	0.00	0.01
1452368_at	0.00	0.03
1452369_at	0.00	0.00
1452370_s_at	0.00	0.00
1452371_at	0.00	0.00
1452372_at	0.00	0.00
1452373_at	0.00	0.00
1452374_at	0.00	0.00
1452375_at	0.00	0.00

1452376_at	0.00	0.00
1452377_at	0.00	0.00
1452378_at	0.00	0.00
1452379_at	0.00	0.00
1452380_at	0.00	0.00
1452381_at	0.00	0.00
1452382_at	0.00	0.00
1452383_at	0.00	0.00
1452384_at	0.28	0.00
1452385_at	0.00	0.00
1452386_at	0.00	0.00
1452387_a_at	0.34	0.13
1452388_at	0.00	0.00
1452389_at	0.00	0.00
1452390_at	0.00	0.00
1452391_at	0.00	0.00
1452392_a_at	0.00	0.00
1452393_at	0.00	0.00
1452394_at	0.00	0.00
1452395_at	0.00	0.00
1452396_at	0.00	0.00
1452397_at	0.00	0.18
1452398_at	0.00	0.00
1452399_at	0.00	0.00
1452400_a_at	0.00	0.00
1452401_at	0.00	0.00
1452402_at	0.00	0.00
1452403_a_at	0.00	0.00
1452404_at	0.00	0.00
1452405_x_at	0.00	0.00
1452406_x_at	0.00	0.08
1452407_at	0.00	0.00
1452408_at	0.00	0.00
1452409_at	0.00	0.00
1452410_a_at	0.00	0.00
1452411_at	0.00	0.00
1452412_at	0.00	0.00
1452413_at	0.00	0.00
1452414_s_at	0.00	0.07
1452415_at	0.00	0.00
1452416_at	0.00	0.00
1452417_x_at	0.00	0.00
1452418_at	0.00	0.00
1452419_at	0.00	0.14
1452420_at	0.00	0.00
1452421_at	0.00	0.00
1452422_a_at	0.00	0.17
1452423_at	0.00	0.00
1452424_at	0.00	0.00
1452425_at	0.00	0.00
1452426_x_at	0.00	0.00
1452427_s_at	0.00	0.00
1452428_a_at	0.87	0.51
1452429_s_at	0.00	0.00
1452430_s_at	0.00	0.31
1452431_s_at	0.00	0.00

1452432_at	0.00	0.00
1452433_at	0.00	0.00
1452434_s_at	0.00	0.00
1452435_at	0.00	0.00
1452436_at	0.01	0.08
1452437_at	0.00	0.00
1452438_s_at	0.05	0.03
1452439_s_at	0.00	0.40
1452440_at	0.00	0.00
1452441_at	0.00	0.00
1452442_at	0.00	0.00
1452443_s_at	0.00	0.00
1452444_at	0.00	0.00
1452445_at	0.00	0.00
1452446_a_at	0.00	0.00
1452447_at	0.00	0.00
1452448_at	0.00	0.00
1452449_at	0.00	0.00
1452450_at	0.00	0.00
1452451_at	0.00	0.00
1452452_at	0.00	0.00
1452453_a_at	0.00	0.00
1452454_at	0.00	0.35
1452455_at	0.00	0.00
1452456_at	0.00	0.00
1452457_a_at	0.00	0.00
1452458_s_at	0.00	0.13
1452459_at	0.00	0.00
1452460_at	0.00	0.00
1452461_a_at	0.00	0.00
1452462_a_at	0.00	0.00
1452463_x_at	0.00	0.00
1452464_a_at	0.00	0.00
1452465_at	0.00	0.00
1452466_a_at	0.00	0.00
1452467_at	0.00	0.00
1452468_s_at	0.00	0.00
1452469_a_at	0.00	0.00
1452470_at	0.00	0.00
1452471_at	0.00	0.00
1452472_at	0.00	0.00
1452473_at	0.00	0.00
1452474_a_at	0.00	0.00
1452475_at	0.00	0.00
1452476_at	0.00	0.00
1452477_at	0.00	0.00
1452478_at	0.00	0.00
1452479_at	0.00	0.00
1452480_at	0.00	0.00
1452481_at	0.00	0.00
1452482_at	0.00	0.00
1452483_a_at	0.00	0.00
1452484_at	0.00	0.00
1452485_at	0.00	0.00
1452486_a_at	0.00	0.00
1452487_x_at	0.00	0.00

1452488_at	0.00	0.00
1452489_at	0.00	0.00
1452490_a_at	0.00	0.00
1452491_at	0.00	0.00
1452492_a_at	0.00	0.00
1452493_s_at	0.00	0.00
1452494_s_at	0.00	0.00
1452495_at	0.00	0.00
1452496_at	0.00	0.00
1452497_a_at	0.00	0.00
1452498_at	0.00	0.00
1452499_a_at	0.00	0.00
1452500_at	0.00	0.00
1452501_at	0.00	0.00
1452502_at	0.00	0.00
1452503_a_at	0.00	0.00
1452504_s_at	0.00	0.01
1452505_at	0.00	0.00
1452506_a_at	0.00	0.00
1452507_at	0.00	0.00
1452508_x_at	0.00	0.00
1452509_at	0.00	0.00
1452510_at	0.00	0.00
1452511_at	0.00	0.00
1452512_a_at	0.00	0.00
1452513_a_at	0.00	0.00
1452514_a_at	0.00	0.00
1452515_a_at	0.00	0.00
1452516_at	0.00	0.00
1452517_at	0.00	0.01
1452518_a_at	0.00	0.00
1452519_a_at	0.00	0.00
1452520_a_at	0.00	0.00
1452521_a_at	0.00	0.00
1452522_at	0.00	0.00
1452523_a_at	0.00	0.00
1452524_a_at	0.00	0.00
1452525_a_at	0.00	0.00
1452526_a_at	0.00	0.00
1452527_a_at	0.00	0.16
1452528_a_at	0.00	0.00
1452529_a_at	0.00	0.00
1452530_a_at	0.00	0.00
1452531_at	0.00	0.00
1452532_x_at	0.00	0.00
1452533_at	0.00	0.00
1452534_a_at	0.00	0.00
1452535_at	0.00	0.00
1452536_s_at	0.00	0.00
1452537_at	0.00	0.00
1452538_at	0.00	0.00
1452539_a_at	0.00	0.00
1452540_a_at	0.01	0.51
1452541_at	0.00	0.00
1452542_x_at	0.00	0.00
1452543_a_at	0.00	0.00

1452544_x_at	0.00	0.00
1452545_a_at	0.00	0.00
1452546_x_at	0.00	0.00
1452547_s_at	0.00	0.00
1452548_x_at	0.00	0.00
1452549_at	0.00	0.00
1452550_a_at	0.00	0.00
1452551_at	0.00	0.00
1452552_at	0.00	0.00
1452553_at	0.00	0.00
1452554_at	0.00	0.00
1452555_at	0.00	0.00
1452556_at	0.00	0.00
1452557_a_at	0.00	0.00
1452558_at	0.00	0.00
1452559_at	0.00	0.00
1452560_a_at	0.00	0.00
1452561_at	0.00	0.00
1452562_at	0.00	0.00
1452563_a_at	0.00	0.00
1452564_at	0.00	0.00
1452565_x_at	0.00	0.00
1452566_at	0.00	0.00
1452567_at	0.00	0.00
1452568_at	0.00	0.00
1452569_at	0.00	0.00
1452570_at	0.00	0.00
1452571_at	0.00	0.00
1452572_at	0.00	0.00
1452573_a_at	0.00	0.00
1452574_x_at	0.00	0.00
1452575_at	0.00	0.00
1452576_at	0.00	0.00
1452577_at	0.00	0.00
1452578_at	0.00	0.00
1452579_at	0.00	0.00
1452580_a_at	0.00	0.00
1452581_at	0.00	0.00
1452582_at	0.00	0.00
1452583_s_at	0.00	0.00
1452584_at	0.00	0.00
1452585_at	0.00	0.00
1452586_at	0.00	0.00
1452587_at	0.00	0.00
1452588_at	0.00	0.00
1452589_at	0.00	0.00
1452590_a_at	0.00	0.00
1452591_a_at	0.00	0.00
1452592_at	0.00	0.00
1452593_a_at	0.00	0.00
1452594_at	0.00	0.00
1452595_at	0.00	0.00
1452596_at	0.00	0.00
1452597_at	0.00	0.00
1452598_at	0.01	0.24
1452599_s_at	0.00	0.00

1452600_at	0.00	0.00
1452601_a_at	0.00	0.00
1452602_a_at	0.00	0.00
1452603_at	0.00	0.00
1452604_at	0.00	0.00
1452605_at	0.00	0.00
1452606_at	0.00	0.00
1452607_at	0.00	0.00
1452608_at	0.00	0.00
1452609_at	0.00	0.00
1452610_at	0.00	0.00
1452611_at	0.00	0.00
1452612_at	0.00	0.07
1452613_at	0.00	0.00
1452614_at	0.00	0.00
1452615_s_at	0.00	0.00
1452616_s_at	0.00	0.13
1452617_at	0.00	0.00
1452618_at	0.00	0.00
1452619_a_at	0.00	0.00
1452620_at	0.00	0.00
1452621_at	0.00	0.00
1452622_a_at	0.00	0.00
1452623_at	0.00	0.00
1452624_at	0.00	0.00
1452625_at	0.00	0.00
1452626_a_at	0.00	0.00
1452627_at	0.00	0.00
1452628_at	0.00	0.00
1452629_at	0.00	0.00
1452630_at	0.00	0.00
1452631_at	0.00	0.00
1452632_at	0.00	0.00
1452633_s_at	0.00	0.00
1452634_at	0.00	0.00
1452635_x_at	0.00	0.00
1452636_x_at	0.00	0.00
1452637_a_at	0.00	0.00
1452638_s_at	0.00	0.00
1452639_at	0.00	0.00
1452640_at	0.00	0.00
1452641_at	0.00	0.00
1452642_at	0.00	0.00
1452643_at	0.00	0.00
1452644_at	0.00	0.00
1452645_x_at	0.00	0.00
1452646_at	0.00	0.00
1452647_a_at	0.00	0.00
1452648_at	0.00	0.00
1452649_at	0.00	0.00
1452650_at	0.00	0.00
1452651_a_at	0.00	0.00
1452652_at	0.00	0.00
1452653_at	0.00	0.00
1452654_at	0.00	0.00
1452655_at	0.00	0.00

1452656_at	0.04	0.00
1452657_at	0.00	0.00
1452658_at	0.00	0.21
1452659_at	0.00	0.24
1452660_s_at	0.00	0.00
1452661_at	0.00	0.00
1452662_a_at	0.00	0.23
1452663_at	0.00	0.00
1452664_a_at	0.00	0.00
1452665_at	0.00	0.24
1452666_a_at	0.01	0.58
1452667_at	0.00	0.00
1452668_x_at	0.00	0.00
1452669_at	0.00	0.00
1452670_at	0.00	0.00
1452671_s_at	0.00	0.00
1452673_at	0.00	0.00
1452674_a_at	0.00	0.00
1452675_at	0.00	0.00
1452676_a_at	0.00	0.00
1452677_at	0.00	0.00
1452679_at	0.00	0.22
1452680_at	0.00	0.01
1452681_at	0.01	0.66
1452683_at	0.00	0.00
1452685_at	0.00	0.00
1452686_s_at	0.00	0.00
1452688_at	0.00	0.00
1452691_at	0.00	0.00
1452692_a_at	0.00	0.00
1452694_at	0.00	0.00
1452697_at	0.00	0.00
1452699_at	0.00	0.00
1452701_x_at	0.00	0.00
1452703_at	0.00	0.00
1452704_at	0.00	0.00
1452705_at	0.00	0.00
1452708_a_at	0.00	0.33
1452709_at	0.00	0.00
1452710_at	0.00	0.00
1452712_at	0.00	0.00
1452713_a_at	0.00	0.04
1452714_at	0.00	0.00
1452715_at	0.00	0.00
1452716_at	0.00	0.61
1452718_at	0.06	0.34
1452720_a_at	0.00	0.00
1452721_a_at	0.00	0.00
1452723_at	0.00	0.00
1452726_a_at	0.00	0.00
1452730_at	0.00	0.32
1452731_x_at	0.00	0.45
1452732_at	0.00	0.00
1452734_at	0.00	0.00
1452735_at	0.00	0.00
1452736_at	0.00	0.00

1452742_at	0.00	0.00
1452743_at	0.00	0.00
1452744_at	0.00	0.00
1452746_at	0.00	0.00
1452747_at	0.00	0.00
1452751_at	0.00	0.00
1452752_at	0.00	0.00
1452753_at	0.00	0.00
1452754_at	0.00	0.00
1452757_s_at	0.00	0.00
1452758_s_at	0.00	0.32
1452764_at	0.00	0.00
1452765_at	0.00	0.00
1452767_at	0.00	0.00
1452768_at	0.00	0.00
1452769_at	0.00	0.00
1452770_at	0.00	0.00
1452772_at	0.00	0.00
1452773_at	0.00	0.00
1452774_at	0.00	0.27
1452776_a_at	0.00	0.00
1452777_a_at	0.00	0.00
1452778_x_at	0.00	0.00
1452780_at	0.00	0.00
1452781_a_at	0.00	0.00
1452782_a_at	0.00	0.00
1452784_at	0.00	0.00
1452787_a_at	0.00	0.40
1452790_x_at	0.00	0.00
1452792_at	0.00	0.00
1452793_at	0.00	0.00
1452799_at	0.00	0.00
1452801_at	0.00	0.00
1452803_at	0.23	0.00
1452812_at	0.00	0.00
1452813_a_at	0.00	0.00
1452822_at	0.00	0.00
1452823_at	0.00	0.19
1452828_at	0.00	0.32
1452829_at	0.00	0.00
1452830_s_at	0.00	0.00
1452831_s_at	0.00	0.19
1452835_a_at	0.00	0.00
1452836_at	0.00	0.03
1452837_at	0.00	0.00
1452838_at	0.00	0.35
1452839_at	0.00	0.00
1452843_at	0.00	0.00
1452844_at	0.00	0.00
1452846_at	0.00	0.00
1452862_at	0.00	0.00
1452864_at	0.00	0.00
1452866_at	0.00	0.00
1452869_at	0.00	0.00
1452870_at	0.00	0.00
1452877_at	0.00	0.00

1452878_at	0.00	0.00
1452880_at	0.00	0.17
1452882_at	0.00	0.00
1452884_at	0.00	0.00
1452885_at	0.00	0.00
1452889_at	0.00	0.00
1452894_at	0.00	0.00
1452895_at	0.00	0.00
1452896_at	0.00	0.00
1452899_at	0.00	0.00
1452900_at	0.00	0.00
1452907_at	0.00	0.00
1452913_at	0.00	0.00
1452917_at	0.01	0.00
1452919_a_at	0.00	0.00
1452920_a_at	0.00	0.00
1452925_a_at	0.00	0.00
1452927_x_at	0.00	0.00
1452929_at	0.00	0.00
1452931_at	0.00	0.00
1452939_a_at	0.00	0.00
1452940_x_at	0.00	0.00
1452941_at	0.00	0.00
1452946_a_at	0.00	0.00
1452954_at	0.00	0.00
1452957_at	0.00	0.00
1452959_a_at	0.00	0.00
1452961_at	0.00	0.00
1452976_a_at	0.00	0.00
1452981_at	0.00	0.00
1452986_at	0.00	0.00
1452987_at	0.00	0.00
1452997_at	0.00	0.00
1452999_at	0.00	0.10
1453004_at	0.00	0.52
1453005_at	0.00	0.00
1453013_at	0.00	0.46
1453014_a_at	0.00	0.00
1453015_at	0.00	0.00
1453016_at	0.00	0.06
1453018_at	0.00	0.02
1453019_at	0.00	0.21
1453025_at	0.00	0.00
1453026_at	0.00	0.00
1453030_at	0.00	0.00
1453033_at	0.00	0.00
1453037_at	0.00	0.00
1453047_at	0.00	0.00
1453060_at	0.00	0.00
1453063_at	0.28	0.61
1453064_at	0.06	0.14
1453076_at	0.00	0.00
1453077_a_at	0.00	0.02
1453084_s_at	0.00	0.00
1453086_at	0.00	0.00
1453089_at	0.00	0.00

1453090_x_at	0.00	0.00
1453092_at	0.00	0.00
1453095_at	0.00	0.00
1453096_x_at	0.00	0.00
1453097_a_at	0.00	0.00
1453098_at	0.00	0.00
1453099_at	0.00	0.00
1453100_at	0.00	0.00
1453106_a_at	0.00	0.17
1453107_s_at	0.00	0.00
1453111_a_at	0.00	0.00
1453112_a_at	0.00	0.00
1453113_at	0.00	0.00
1453117_at	0.00	0.00
1453124_at	0.00	0.00
1453128_at	0.00	0.00
1453132_a_at	0.00	0.00
1453147_at	0.00	0.00
1453156_s_at	0.00	0.00
1453162_at	0.00	0.00
1453164_a_at	0.00	0.00
1453169_a_at	0.00	0.06
1453175_at	0.00	0.00
1453181_x_at	0.00	0.00
1453182_a_at	0.00	0.03
1453184_at	0.00	0.00
1453195_at	0.00	0.00
1453196_a_at	0.00	0.09
1453198_at	0.00	0.00
1453199_at	0.00	0.00
1453200_at	0.00	0.00
1453206_at	0.00	0.00
1453207_at	0.00	0.00
1453208_at	0.00	0.00
1453223_s_at	0.03	1.00
1453237_at	0.00	0.00
1453238_s_at	0.00	0.00
1453239_a_at	0.00	0.00
1453249_a_at	0.00	0.00
1453251_at	0.00	0.00
1453253_a_at	0.00	0.00
1453256_at	0.00	0.00
1453258_at	0.00	0.00
1453260_a_at	0.00	0.00
1453265_at	0.00	0.00
1453278_a_at	0.00	0.00
1453281_at	0.00	0.00
1453283_at	0.00	0.00
1453287_at	0.00	0.00
1453298_at	0.00	0.00
1453299_a_at	0.56	1.00
1453301_a_at	0.00	0.00
1453307_a_at	0.00	0.00
1453314_x_at	0.00	0.00
1453317_a_at	0.00	0.00
1453321_at	0.00	0.00

1453324_at	0.00	0.00
1453338_at	0.00	0.00
1453343_s_at	0.00	0.00
1453355_at	0.00	0.05
1453360_a_at	0.00	0.00
1453362_x_at	0.00	0.00
1453367_a_at	0.00	0.00
1453369_a_at	0.00	0.00
1453389_a_at	0.00	0.00
1453392_at	0.00	0.01
1453393_a_at	0.00	0.00
1453406_a_at	0.00	0.00
1453410_at	0.00	0.00
1453412_a_at	0.00	0.00
1453413_at	0.00	0.00
1453426_a_at	0.00	0.00
1453427_at	0.00	0.00
1453450_at	0.00	0.00
1453461_at	0.00	0.00
1453466_at	0.00	0.00
1453467_s_at	0.00	0.00
1453468_at	0.00	0.00
1453470_a_at	0.00	0.00
1453472_a_at	0.00	0.00
1453473_a_at	0.00	0.00
1453474_at	0.00	0.00
1453486_a_at	0.00	0.00
1453497_a_at	0.00	0.00
1453498_x_at	0.00	0.00
1453501_at	0.00	0.00
1453505_a_at	0.00	0.00
1453515_at	0.00	0.00
1453517_at	0.00	0.00
1453527_a_at	0.00	0.00
1453528_at	0.00	0.00
1453550_a_at	0.00	0.00
1453553_at	0.00	0.00
1453554_a_at	0.00	0.00
1453556_x_at	0.00	0.00
1453559_a_at	0.00	0.00
1453560_at	0.00	0.00
1453564_a_at	0.00	0.00
1453567_s_at	0.00	0.00
1453569_s_at	0.00	0.00
1453570_x_at	0.00	0.00
1453571_at	0.00	0.00
1453572_a_at	0.00	0.00
1453573_at	0.00	0.00
1453574_at	0.00	0.00
1453576_at	0.00	0.00
1453578_at	0.00	0.00
1453589_a_at	0.00	0.00
1453596_at	0.00	0.00
1453604_a_at	0.00	0.00
1453609_s_at	0.00	0.00
1453612_at	0.00	0.00

1453613_at	0.00	0.00
1453614_a_at	0.00	0.00
1453621_at	0.00	0.00
1453623_a_at	0.00	0.00
1453631_at	0.00	0.00
1453633_a_at	0.00	0.00
1453634_a_at	0.00	0.00
1453644_at	0.00	0.00
1453647_at	0.00	0.00
1453651_a_at	0.00	0.01
1453659_at	0.00	0.00
1453666_at	0.00	0.00
1453674_at	0.00	0.00
1453677_a_at	0.00	0.00
1453678_at	0.00	0.00
1453683_a_at	0.00	0.00
1453686_x_at	0.00	0.00
1453710_at	0.00	0.00
1453712_a_at	0.00	0.00
1453714_a_at	0.00	0.00
1453721_a_at	0.00	0.00
1453722_s_at	0.00	0.33
1453723_x_at	0.00	0.00
1453724_a_at	0.00	0.00
1453725_a_at	0.00	0.01
1453728_a_at	0.00	0.30
1453729_a_at	0.00	0.00
1453731_a_at	0.00	0.00
1453733_a_at	0.00	0.00
1453740_a_at	0.00	0.00
1453741_x_at	0.00	0.00
1453744_a_at	0.00	0.00
1453748_a_at	0.00	0.00
1453750_x_at	0.00	0.00
1453752_at	0.00	0.00
1453753_at	0.00	0.00
1453758_at	0.00	0.00
1453767_a_at	0.00	0.00
1453768_a_at	0.00	0.00
1453772_at	0.00	0.00
1453777_a_at	0.00	0.00
1453780_at	0.00	0.00
1453784_at	0.00	0.00
1453788_at	0.00	0.00
1453796_a_at	0.00	0.00
1453804_a_at	0.00	0.00
1453806_at	0.00	0.00
1453811_at	0.00	0.00
1453815_at	0.00	0.00
1453816_at	0.00	0.00
1453819_x_at	0.00	0.00
1453820_at	0.00	0.00
1453827_at	0.00	0.00
1453830_at	0.00	0.00
1453833_a_at	0.00	0.00
1453836_a_at	0.00	0.00

1453839_a_at	0.00	0.00
1453840_at	0.00	0.00
1453848_s_at	0.00	0.00
1453849_s_at	0.00	0.06
1453851_a_at	0.00	0.00
1453853_a_at	0.00	0.00
1453856_at	0.00	0.00
1453864_at	0.00	0.00
1453865_a_at	0.00	0.00
1453866_a_at	0.00	0.00
1453867_at	0.00	0.00
1453876_at	0.00	0.00
1453881_x_at	0.00	0.00
1453886_a_at	0.00	0.00
1453887_a_at	0.00	0.00
1453891_at	0.00	0.00
1453901_at	0.00	0.00
1453902_at	0.00	0.00
1453911_at	0.00	0.00
1453913_a_at	0.00	0.00
1453914_at	0.00	0.00
1453915_a_at	0.00	0.00
1453920_a_at	0.00	0.00
1453924_a_at	0.00	0.00
1453928_a_at	0.00	0.04
1453939_x_at	0.00	0.00
1453943_a_at	0.00	0.00
1453946_a_at	0.00	0.00
1453950_a_at	0.00	0.00
1453954_a_at	0.00	0.00
1453956_a_at	0.00	0.00
1453957_a_at	0.00	0.00
1453960_a_at	0.00	0.00
1453962_at	0.00	0.00
1453972_x_at	0.00	0.00
1453977_at	0.00	0.00
1453981_at	0.00	0.00
1453985_at	0.00	0.00
1453986_at	0.00	0.00
1453988_a_at	0.00	0.00
1453989_at	0.00	0.00
1453991_at	0.00	0.00
1453993_a_at	0.08	0.00
1453995_a_at	0.00	0.00
1453996_a_at	0.00	0.00
1453997_a_at	0.00	0.00
1453998_at	0.00	0.00
1454005_at	0.00	0.00
1454006_a_at	0.00	0.00
1454007_a_at	0.00	0.00
1454008_at	0.00	0.00
1454011_a_at	0.00	0.31
1454014_a_at	0.00	0.00
1454015_a_at	0.00	0.00
1454016_at	0.00	0.00
1454018_at	0.00	0.00

1454021_a_at	0.00	0.01
1454022_at	0.00	0.00
1454023_a_at	0.00	0.00
1454026_a_at	0.00	0.00
1454030_at	0.00	0.00
1454034_a_at	0.00	0.00
1454036_a_at	0.00	0.00
1454037_a_at	0.00	0.00
1454041_at	0.00	0.00
1454042_a_at	0.00	0.00
1454043_a_at	0.00	0.00
1454044_a_at	0.00	0.00
1454045_a_at	0.00	0.00
1454046_x_at	0.00	0.00
1454047_a_at	0.00	0.00
1454048_a_at	0.00	0.00
1454060_a_at	0.00	0.00
1454061_at	0.00	0.00
1454064_a_at	0.21	0.05
1454066_at	0.00	0.00
1454067_a_at	0.00	0.00
1454070_a_at	0.00	0.00
1454074_a_at	0.00	0.00
1454078_a_at	0.00	0.00
1454082_a_at	0.00	0.00
1454086_a_at	0.00	0.00
1454090_at	0.00	0.00
1454092_a_at	0.00	0.00
1454106_a_at	0.00	0.00
1454107_a_at	0.00	0.00
1454109_a_at	0.00	0.00
1454114_a_at	0.08	0.00
1454116_a_at	0.00	0.00
1454119_at	0.00	0.00
1454120_a_at	0.00	0.19
1454136_a_at	0.00	0.00
1454138_a_at	0.00	0.00
1454142_a_at	0.00	0.15
1454144_a_at	0.00	0.00
1454145_at	0.00	0.00
1454149_a_at	0.00	0.00
1454152_a_at	0.00	0.00
1454157_a_at	0.00	0.00
1454159_a_at	0.00	0.33
1454161_s_at	0.00	0.00
1454167_at	0.00	0.00
1454168_a_at	0.00	0.00
1454169_a_at	0.00	0.00
1454174_a_at	0.00	0.00
1454176_at	0.00	0.00
1454183_at	0.00	0.00
1454184_a_at	0.00	0.00
1454197_a_at	0.00	0.16
1454206_a_at	0.00	0.00
1454211_a_at	0.00	0.08
1454219_at	0.00	0.00

1454221_a_at	0.00	0.00
1454228_a_at	0.00	0.00
1454229_a_at	0.00	0.00
1454231_a_at	0.00	0.00
1454236_a_at	0.00	0.00
1454240_at	0.00	0.00
1454247_a_at	0.10	0.00
1454260_at	0.00	0.00
1454265_a_at	0.00	0.00
1454267_a_at	0.00	0.00
1454268_a_at	0.10	0.26
1454305_at	0.00	0.00
1454312_at	0.00	0.00
1454313_at	0.00	0.00
1454317_at	0.00	0.00
1454368_at	0.00	0.00
1454369_a_at	0.00	0.00
1454372_at	0.00	0.00
1454373_x_at	0.00	0.00
1454438_at	0.00	0.00
1454454_at	0.00	0.00
1454504_at	0.00	0.00
1454602_s_at	0.00	0.00
1454603_a_at	0.00	0.00
1454604_s_at	0.00	0.00
1454605_a_at	0.00	0.00
1454606_at	0.00	0.00
1454607_s_at	0.00	0.48
1454608_x_at	0.00	0.00
1454610_at	0.00	0.00
1454611_a_at	0.02	0.00
1454613_at	0.00	0.00
1454615_x_at	0.00	0.00
1454616_at	0.00	0.00
1454618_at	0.00	0.00
1454620_x_at	0.00	0.00
1454621_s_at	0.00	0.00
1454622_at	0.00	0.00
1454623_at	0.00	0.00
1454626_at	0.00	0.00
1454627_a_at	0.00	0.00
1454628_at	0.00	0.00
1454629_at	0.00	0.00
1454631_at	0.00	0.00
1454632_at	0.00	0.00
1454633_at	0.00	0.00
1454635_at	0.00	0.00
1454636_at	0.00	0.00
1454638_a_at	0.00	0.00
1454639_x_at	0.00	0.00
1454640_at	0.00	0.00
1454641_at	0.00	0.00
1454643_at	0.00	0.00
1454647_at	0.00	0.00
1454648_s_at	0.00	0.00
1454651_x_at	0.00	0.00

1454652_at	0.00	0.00
1454656_at	0.00	0.00
1454657_s_at	0.00	0.00
1454661_at	0.00	0.00
1454663_at	0.00	0.13
1454664_a_at	0.00	0.00
1454668_at	0.00	0.00
1454669_at	0.00	0.00
1454670_at	0.00	0.12
1454673_at	0.00	0.00
1454674_at	0.00	0.00
1454675_at	0.00	0.00
1454677_at	0.00	0.00
1454678_s_at	0.00	0.00
1454681_at	0.00	0.00
1454682_at	0.00	0.00
1454686_at	0.00	0.00
1454688_x_at	0.00	0.00
1454689_at	0.00	0.00
1454690_at	0.00	0.00
1454692_x_at	0.00	0.00
1454694_a_at	0.00	0.00
1454696_at	0.00	0.00
1454697_at	0.00	0.02
1454698_at	0.00	0.00
1454699_at	0.00	0.00
1454703_x_at	0.00	0.00
1454704_at	0.00	0.00
1454705_at	0.00	0.00
1454706_at	0.00	0.00
1454708_at	0.00	0.00
1454711_at	0.00	0.22
1454712_at	0.00	0.00
1454713_s_at	0.00	0.00
1454714_x_at	0.00	0.00
1454716_x_at	0.00	0.00
1454719_at	0.00	0.00
1454722_at	0.00	0.00
1454725_at	0.00	0.00
1454732_at	0.00	0.00
1454735_at	0.05	0.00
1454736_at	0.00	0.00
1454737_at	0.00	0.00
1454738_x_at	0.00	0.00
1454747_a_at	0.00	0.00
1454750_a_at	0.00	0.00
1454753_at	0.00	0.17
1454754_a_at	0.00	0.00
1454758_a_at	0.00	0.00
1454759_at	0.00	0.00
1454760_at	0.00	0.00
1454763_at	0.00	0.00
1454770_at	0.00	0.00
1454771_at	0.00	0.00
1454773_at	0.00	0.00
1454778_x_at	0.00	0.00

1454779_s_at	0.00	0.00
1454781_x_at	0.00	0.00
1454783_at	0.00	0.00
1454785_at	0.00	0.00
1454786_at	0.00	0.00
1454787_at	0.00	0.00
1454789_x_at	0.00	0.00
1454791_a_at	0.00	0.00
1454793_x_at	0.00	0.17
1454794_at	0.00	0.00
1454796_at	0.00	0.00
1454798_at	0.00	0.00
1454801_at	0.00	0.00
1454802_x_at	0.00	0.00
1454803_a_at	0.00	0.00
1454804_at	0.00	0.00
1454805_at	0.00	0.91
1454807_a_at	0.00	0.00
1454811_a_at	0.00	0.00
1454813_at	0.00	0.00
1454814_s_at	0.00	0.34
1454815_at	0.00	0.00
1454817_at	0.00	0.00
1454820_at	0.00	0.00
1454822_x_at	0.00	0.00
1454831_at	0.00	0.00
1454833_at	0.00	0.00
1454837_at	0.00	0.02
1454838_s_at	0.86	0.02
1454839_a_at	0.00	0.00
1454842_a_at	0.00	0.00
1454849_x_at	0.03	0.00
1454856_x_at	0.00	0.00
1454858_x_at	0.00	0.00
1454859_a_at	0.00	0.00
1454860_x_at	0.00	0.00
1454862_at	0.77	0.00
1454865_at	0.00	0.00
1454870_x_at	0.00	0.00
1454872_at	0.00	0.00
1454875_a_at	0.00	0.00
1454879_s_at	0.00	0.00
1454881_s_at	0.00	0.00
1454884_at	0.00	0.00
1454887_at	0.00	0.00
1454890_at	0.35	1.00
1454891_at	0.00	0.00
1454893_at	0.00	0.00
1454897_at	0.00	0.00
1454898_s_at	0.00	0.00
1454899_at	0.23	0.00
1454903_at	0.00	0.00
1454904_at	0.00	0.00
1454905_at	0.00	0.00
1454906_at	0.00	0.00
1454907_at	0.00	0.00

1454908_x_at	0.00	0.00
1454912_at	0.00	0.00
1454914_at	0.00	0.09
1454921_at	0.00	0.00
1454925_x_at	0.00	0.00
1454928_at	0.00	0.00
1454929_s_at	0.00	0.00
1454930_at	0.00	0.00
1454932_at	0.00	0.00
1454940_at	0.00	0.00
1454941_at	0.00	0.00
1454943_a_at	0.00	0.00
1454946_at	0.83	0.15
1454947_a_at	0.00	0.00
1454953_at	0.00	0.00
1454954_at	0.00	0.00
1454955_at	0.00	0.00
1454959_s_at	0.00	0.00
1454963_at	0.00	0.14
1454964_at	0.00	0.35
1454967_at	0.00	0.00
1454970_at	0.00	0.00
1454971_x_at	0.00	0.00
1454972_at	0.00	0.00
1454974_at	0.22	0.00
1454977_at	0.00	0.00
1454979_at	0.00	0.00
1454984_at	0.00	0.00
1454985_at	0.00	0.00
1454987_a_at	0.00	0.00
1454993_a_at	0.00	0.07
1454998_at	0.00	0.00
1455001_x_at	0.00	0.00
1455002_at	0.00	0.00
1455005_s_at	0.00	0.00
1455006_at	0.00	0.00
1455007_s_at	0.00	0.00
1455008_at	0.00	0.00
1455009_at	0.02	0.00
1455012_s_at	0.00	0.00
1455013_at	0.00	0.00
1455017_a_at	0.00	0.00
1455019_x_at	0.00	0.00
1455021_at	0.00	0.00
1455026_at	0.00	0.00
1455030_at	0.00	0.00
1455035_s_at	0.00	0.45
1455036_s_at	0.00	0.00
1455039_a_at	0.00	0.00
1455040_s_at	0.00	0.00
1455041_at	0.00	0.00
1455042_at	0.00	0.00
1455045_at	0.00	0.00
1455047_at	0.00	0.00
1455053_a_at	0.00	0.00
1455054_a_at	0.00	0.00

1455056_at	0.00	0.00
1455058_at	0.00	0.00
1455060_at	0.00	0.00
1455061_a_at	0.00	0.00
1455065_x_at	0.25	0.00
1455066_s_at	0.00	0.00
1455069_x_at	0.00	0.00
1455071_at	0.00	0.00
1455072_at	0.00	0.00
1455073_at	0.00	0.00
1455076_a_at	0.00	0.00
1455077_a_at	0.00	0.00
1455084_x_at	0.00	0.38
1455089_at	0.00	0.00
1455090_at	0.00	0.00
1455092_at	0.00	0.00
1455093_a_at	0.00	0.00
1455098_a_at	0.00	0.00
1455099_at	0.00	0.33
1455100_at	0.00	0.00
1455105_at	0.00	0.01
1455106_a_at	0.02	0.00
1455109_at	0.00	0.00
1455115_a_at	0.00	0.01
1455126_x_at	0.00	0.00
1455128_x_at	0.00	0.00
1455129_at	0.00	0.00
1455131_at	0.00	0.00
1455134_at	0.00	0.00
1455136_at	0.00	0.00
1455138_x_at	0.00	0.27
1455141_at	0.00	0.00
1455143_at	0.00	0.00
1455149_at	0.00	0.01
1455151_at	0.00	0.00
1455152_at	0.00	0.00
1455155_at	0.00	0.00
1455156_at	0.00	0.00
1455158_at	0.31	0.00
1455167_at	0.00	0.00
1455168_a_at	0.00	0.00
1455171_at	0.00	0.00
1455175_at	0.00	0.00
1455176_a_at	0.00	0.00
1455177_at	0.00	0.00
1455179_at	0.00	0.00
1455180_at	0.00	0.00
1455182_at	0.00	0.00
1455184_at	0.00	0.00
1455191_x_at	0.00	0.00
1455195_at	0.00	0.00
1455198_a_at	0.00	0.00
1455201_x_at	0.00	0.05
1455202_at	0.00	0.00
1455204_at	0.02	0.00
1455205_a_at	0.00	0.00

1455206_at	0.00	0.00
1455207_at	0.00	0.00
1455209_at	0.00	0.00
1455211_a_at	0.00	0.00
1455214_at	0.00	0.00
1455220_at	0.00	0.00
1455222_a_at	0.00	0.00
1455229_x_at	0.00	0.00
1455231_s_at	0.00	0.00
1455232_at	0.00	0.00
1455233_at	0.00	0.00
1455235_x_at	0.00	0.00
1455236_x_at	0.00	0.00
1455237_at	0.00	0.00
1455239_at	0.00	0.00
1455242_at	0.00	0.00
1455245_x_at	0.00	0.00
1455252_at	0.00	0.02
1455253_at	0.00	0.00
1455254_at	0.00	0.00
1455255_at	0.00	0.00
1455264_at	0.00	0.00
1455265_a_at	0.00	0.00
1455269_a_at	0.00	0.00
1455271_at	0.00	0.00
1455276_x_at	0.00	0.00
1455279_at	0.00	0.00
1455281_at	0.00	0.00
1455282_x_at	0.00	0.00
1455283_x_at	0.00	0.00
1455284_x_at	0.00	0.00
1455285_at	0.00	0.00
1455286_at	0.00	0.02
1455288_at	0.00	0.00
1455290_at	0.00	0.00
1455291_s_at	0.00	0.00
1455298_at	0.00	0.00
1455302_at	0.00	0.00
1455305_x_at	0.00	0.00
1455310_at	0.00	0.00
1455315_at	0.00	0.00
1455316_x_at	0.00	0.31
1455319_x_at	0.00	0.00
1455326_at	0.00	0.00
1455332_x_at	0.00	0.00
1455335_at	0.00	0.00
1455338_at	0.00	0.00
1455339_at	0.00	0.00
1455343_at	0.00	0.00
1455346_at	0.00	0.00
1455348_x_at	0.00	0.00
1455349_at	0.00	0.00
1455350_at	0.00	0.00
1455356_at	0.00	0.00
1455357_x_at	0.00	0.00
1455363_at	0.00	0.00

1455364_a_at	0.00	0.00
1455366_at	0.00	0.00
1455367_at	0.00	0.00
1455371_at	0.00	0.00
1455372_at	0.00	0.00
1455382_at	0.00	0.00
1455384_x_at	0.00	0.00
1455385_at	0.00	0.00
1455388_at	0.00	0.00
1455393_at	0.00	0.00
1455405_at	0.00	0.00
1455408_at	0.00	0.00
1455414_at	0.00	0.00
1455421_x_at	0.00	0.00
1455422_x_at	0.00	0.00
1455423_at	0.00	0.00
1455424_at	0.00	0.00
1455431_at	0.00	0.00
1455433_at	0.00	0.00
1455434_a_at	0.00	0.00
1455435_s_at	0.00	0.00
1455439_a_at	0.21	0.00
1455441_at	0.00	0.00
1455446_x_at	0.00	0.00
1455447_at	0.00	0.00
1455456_a_at	0.00	0.00
1455457_at	0.00	0.00
1455458_x_at	0.00	0.00
1455462_at	0.00	0.00
1455466_at	0.00	0.00
1455470_x_at	0.00	0.00
1455474_at	0.00	0.00
1455475_at	0.00	0.00
1455477_s_at	0.00	0.00
1455479_a_at	0.00	0.00
1455480_s_at	0.00	0.05
1455482_at	0.00	0.00
1455484_at	0.00	0.00
1455485_x_at	0.00	0.00
1455486_at	0.00	0.00
1455488_at	0.00	0.21
1455490_at	0.00	0.00
1455491_at	0.00	0.10
1455493_at	0.00	0.00
1455494_at	0.00	0.00
1455504_a_at	0.66	0.31
1455505_at	0.00	0.00
1455511_at	0.00	0.00
1455517_at	0.00	0.00
1455526_at	0.00	0.00
1455530_at	0.00	0.00
1455531_at	0.00	0.00
1455534_s_at	0.00	0.00
1455539_at	0.00	0.00
1455540_at	0.00	0.00
1455546_s_at	0.00	0.00

1455550_x_at	0.00	0.00
1455551_at	0.00	0.00
1455556_at	0.00	0.00
1455562_at	0.00	0.00
1455563_at	0.00	0.07
1455570_x_at	0.00	0.00
1455571_x_at	0.00	0.00
1455572_x_at	0.00	0.00
1455573_at	0.00	0.00
1455575_at	0.00	0.00
1455578_x_at	0.00	0.00
1455579_at	0.00	0.00
1455581_x_at	0.00	0.00
1455582_at	0.00	0.00
1455587_at	0.00	0.00
1455593_at	0.00	0.01
1455601_at	0.00	0.00
1455602_x_at	0.00	0.00
1455605_at	0.00	0.07
1455611_at	0.00	0.00
1455615_at	0.00	0.00
1455618_x_at	0.00	0.00
1455625_at	0.00	0.00
1455626_at	0.00	0.00
1455628_at	0.00	0.00
1455631_at	0.00	0.00
1455632_at	0.00	0.00
1455639_at	0.00	0.00
1455640_a_at	0.00	0.00
1455641_at	0.00	0.00
1455642_a_at	0.00	0.00
1455644_at	0.00	0.00
1455650_at	0.00	0.00
1455653_at	0.00	0.00
1455655_a_at	0.00	0.01
1455662_x_at	0.00	0.00
1455675_a_at	0.00	0.00
1455676_x_at	0.00	0.00
1455677_s_at	0.00	0.00
1455678_at	0.04	0.01
1455683_a_at	0.00	0.00
1455685_at	0.00	0.00
1455691_at	0.00	0.00
1455692_x_at	0.00	0.00
1455693_x_at	0.00	0.00
1455694_at	0.00	0.00
1455696_a_at	0.00	0.00
1455698_at	0.00	0.04
1455700_at	0.00	0.00
1455710_x_at	0.00	0.00
1455713_x_at	0.00	0.00
1455715_at	0.00	0.00
1455719_at	0.00	0.00
1455725_a_at	0.00	0.00
1455727_at	0.00	0.00
1455730_at	0.00	0.00

1455731_at	0.00	0.00
1455733_at	0.00	0.00
1455734_at	0.00	0.00
1455740_at	0.00	0.00
1455741_a_at	0.00	0.00
1455742_x_at	0.00	0.00
1455747_at	0.00	0.00
1455748_at	0.00	0.00
1455749_x_at	0.00	0.00
1455752_a_at	0.00	0.00
1455758_at	0.00	0.00
1455764_at	0.00	0.00
1455765_a_at	0.00	0.00
1455767_x_at	0.00	0.00
1455776_x_at	0.00	0.00
1455777_x_at	0.00	0.00
1455781_at	0.00	0.00
1455787_x_at	0.00	0.00
1455788_x_at	0.00	0.00
1455789_x_at	0.00	0.00
1455791_at	0.00	0.00
1455792_x_at	0.00	0.00
1455796_x_at	0.00	0.00
1455797_x_at	0.00	0.00
1455798_at	0.00	0.01
1455800_x_at	0.00	0.33
1455801_x_at	0.00	0.00
1455802_x_at	0.00	0.00
1455804_x_at	0.00	0.00
1455805_x_at	0.00	0.00
1455806_x_at	0.00	0.00
1455808_at	0.00	0.00
1455809_x_at	0.00	0.00
1455810_a_at	0.00	0.00
1455811_at	0.00	0.00
1455812_x_at	0.00	0.00
1455813_at	0.00	0.00
1455814_x_at	0.00	0.61
1455815_a_at	0.00	0.00
1455819_at	0.00	0.40
1455820_x_at	0.00	0.07
1455821_x_at	0.00	0.00
1455822_x_at	0.00	0.00
1455824_x_at	0.00	0.00
1455825_s_at	0.00	0.00
1455826_a_at	0.00	0.00
1455828_at	0.00	0.00
1455829_at	0.00	0.00
1455831_at	0.00	0.00
1455832_a_at	0.00	0.00
1455834_x_at	0.00	0.00
1455836_at	0.00	0.00
1455841_s_at	0.00	0.66
1455844_at	0.00	0.00
1455853_x_at	0.00	0.00
1455855_x_at	0.00	0.08

1455857_a_at	0.00	0.00
1455858_x_at	0.00	0.00
1455860_at	0.00	0.00
1455866_x_at	0.00	0.00
1455867_at	0.00	0.00
1455868_a_at	0.00	0.00
1455869_at	0.00	0.00
1455871_s_at	0.90	0.00
1455873_a_at	0.00	0.00
1455874_at	0.00	0.00
1455875_x_at	0.00	0.00
1455877_a_at	0.00	0.00
1455880_s_at	0.00	0.00
1455883_a_at	0.00	0.00
1455886_at	0.00	0.00
1455890_x_at	0.00	0.00
1455892_x_at	0.00	0.00
1455893_at	0.00	0.00
1455894_at	0.00	0.00
1455895_x_at	0.00	0.00
1455896_a_at	0.00	0.20
1455897_x_at	0.00	0.20
1455898_x_at	0.00	0.53
1455899_x_at	0.00	0.00
1455900_x_at	0.00	0.00
1455901_at	0.00	0.00
1455904_at	0.00	0.09
1455905_at	0.00	0.00
1455908_a_at	0.00	0.00
1455910_at	0.00	0.00
1455911_x_at	0.00	0.00
1455912_x_at	0.00	0.00
1455913_x_at	0.00	0.00
1455915_at	0.00	0.00
1455918_at	0.00	0.00
1455927_x_at	0.00	0.00
1455928_x_at	0.00	0.00
1455929_x_at	0.00	0.00
1455930_at	0.08	0.00
1455931_at	0.00	0.00
1455932_at	0.00	0.00
1455934_at	0.00	0.00
1455936_a_at	0.00	0.00
1455938_x_at	0.00	0.00
1455939_x_at	0.00	0.00
1455940_x_at	0.00	0.27
1455941_s_at	0.00	0.00
1455946_x_at	0.00	0.00
1455949_at	0.00	0.00
1455950_x_at	0.00	0.00
1455953_x_at	0.00	0.00
1455954_x_at	0.00	0.00
1455955_s_at	0.00	0.00
1455956_x_at	0.00	0.00
1455957_x_at	0.00	0.00
1455958_s_at	0.00	0.00

1455959_s_at	0.00	0.00
1455961_at	0.00	0.00
1455965_at	0.00	0.00
1455968_x_at	0.00	0.00
1455972_x_at	0.00	0.00
1455974_at	0.00	0.00
1455975_x_at	0.00	0.00
1455976_x_at	0.00	0.00
1455977_x_at	0.00	0.00
1455978_a_at	0.00	0.00
1455981_at	0.00	0.00
1455984_at	0.00	0.00
1455985_x_at	0.00	0.00
1455987_at	0.00	0.00
1455988_a_at	0.00	0.12
1455989_at	0.00	0.00
1455990_at	0.00	0.00
1455991_at	0.00	0.00
1455992_at	0.00	0.00
1455994_x_at	0.00	0.00
1455996_x_at	0.00	0.00
1455997_a_at	0.00	0.00
1456000_at	0.00	0.00
1456001_at	0.00	0.00
1456002_at	0.00	0.00
1456003_a_at	0.00	0.00
1456004_x_at	0.00	0.00
1456005_a_at	0.00	0.00
1456006_at	0.00	0.00
1456007_at	0.00	0.00
1456009_x_at	0.00	0.00
1456010_x_at	0.00	0.00
1456011_x_at	0.00	0.00
1456012_x_at	0.00	0.00
1456013_x_at	0.00	0.00
1456014_s_at	0.00	0.00
1456015_x_at	0.00	0.00
1456016_x_at	0.00	0.00
1456017_x_at	0.00	0.00
1456028_x_at	0.03	0.00
1456029_a_at	0.00	0.00
1456030_at	0.00	0.00
1456031_at	0.00	0.00
1456032_x_at	0.00	0.30
1456036_x_at	0.00	0.00
1456037_x_at	0.00	0.00
1456039_at	0.00	0.00
1456040_at	0.00	0.00
1456042_s_at	0.00	0.00
1456043_at	0.00	0.00
1456048_at	0.00	0.00
1456049_at	0.00	0.00
1456054_a_at	0.00	0.00
1456055_x_at	0.00	0.00
1456056_a_at	0.00	0.00
1456057_x_at	0.00	0.00

1456059_at	0.00	0.00
1456062_at	0.00	0.00
1456064_at	0.00	0.00
1456066_a_at	0.00	0.00
1456069_at	0.00	0.00
1456071_a_at	0.00	0.00
1456075_at	0.00	0.00
1456078_x_at	0.00	0.00
1456079_x_at	0.00	0.60
1456080_a_at	0.01	0.01
1456081_a_at	0.00	0.00
1456082_x_at	0.00	0.00
1456083_x_at	0.00	0.03
1456084_x_at	0.00	0.00
1456085_x_at	0.00	0.00
1456086_x_at	0.00	0.00
1456088_at	0.00	0.00
1456094_at	0.00	0.00
1456095_at	0.00	0.00
1456097_a_at	0.00	0.00
1456098_a_at	0.00	0.00
1456100_at	0.00	0.00
1456101_at	0.00	0.00
1456104_at	0.00	0.00
1456106_x_at	0.00	0.00
1456107_x_at	0.00	0.00
1456108_x_at	0.00	0.00
1456109_a_at	0.00	0.00
1456112_at	0.00	0.00
1456115_at	0.00	0.00
1456117_at	0.00	0.28
1456120_at	0.00	0.00
1456124_x_at	0.00	0.00
1456125_a_at	0.00	0.00
1456128_at	0.00	0.00
1456131_x_at	0.00	0.00
1456132_x_at	0.00	0.00
1456133_x_at	0.00	0.00
1456134_x_at	0.00	0.00
1456135_s_at	0.00	0.00
1456136_at	0.00	0.00
1456140_at	0.00	0.00
1456142_x_at	0.00	0.00
1456148_a_at	0.00	0.00
1456151_at	0.00	0.00
1456154_at	0.00	0.00
1456155_x_at	0.00	0.00
1456169_at	0.00	0.00
1456170_x_at	0.00	0.00
1456173_at	0.00	0.00
1456174_x_at	0.00	0.00
1456175_a_at	0.00	0.00
1456176_x_at	0.00	0.00
1456177_x_at	0.00	0.00
1456182_x_at	0.00	0.00
1456188_at	0.00	0.00

1456190_a_at	0.00	0.00
1456193_x_at	0.00	0.00
1456194_a_at	0.00	0.00
1456195_x_at	0.00	0.00
1456196_x_at	0.00	0.00
1456199_x_at	0.00	0.00
1456205_x_at	0.00	0.00
1456206_at	0.00	0.00
1456212_x_at	0.00	0.00
1456213_x_at	0.00	0.00
1456218_at	0.00	0.00
1456222_at	0.00	0.00
1456225_x_at	0.00	0.00
1456226_x_at	0.00	0.00
1456227_x_at	0.00	0.00
1456228_x_at	0.00	0.00
1456237_x_at	0.00	0.00
1456239_at	0.00	0.15
1456240_x_at	0.00	0.00
1456241_a_at	0.00	0.00
1456243_x_at	0.00	0.00
1456244_x_at	0.01	0.00
1456245_x_at	0.00	0.00
1456246_x_at	0.00	0.00
1456247_x_at	0.00	0.00
1456249_x_at	0.00	0.00
1456250_x_at	0.00	0.00
1456251_x_at	0.00	0.00
1456252_x_at	0.00	0.00
1456262_at	0.00	0.00
1456263_at	0.00	0.00
1456266_at	0.00	0.00
1456269_at	0.00	0.00
1456270_s_at	0.00	0.00
1456275_at	0.00	0.00
1456278_x_at	0.00	0.00
1456279_a_at	0.00	0.00
1456283_at	0.00	0.00
1456289_at	0.00	0.00
1456290_x_at	0.00	0.00
1456292_a_at	0.57	0.00
1456293_s_at	0.00	0.00
1456302_at	0.00	0.00
1456305_x_at	0.00	0.00
1456306_a_at	0.00	0.00
1456307_s_at	0.00	0.00
1456309_x_at	0.00	0.00
1456310_a_at	0.00	0.00
1456312_x_at	0.02	0.00
1456313_x_at	0.00	0.00
1456314_x_at	0.00	0.00
1456315_a_at	0.00	0.00
1456316_a_at	0.00	0.00
1456319_at	0.00	0.00
1456322_at	0.00	0.00
1456323_at	0.00	0.00

1456325_at	0.00	0.00
1456330_at	0.00	0.00
1456333_a_at	0.00	0.00
1456340_at	0.00	0.00
1456341_a_at	0.63	0.02
1456342_at	0.00	0.00
1456349_x_at	0.00	0.03
1456352_a_at	0.00	0.00
1456355_s_at	0.00	0.00
1456360_at	0.00	0.00
1456365_at	0.00	0.00
1456370_s_at	0.00	0.00
1456371_a_at	0.00	0.00
1456373_x_at	0.00	0.00
1456374_x_at	0.00	0.01
1456375_x_at	0.00	0.00
1456377_x_at	0.30	0.32
1456379_x_at	0.00	0.00
1456380_x_at	0.00	0.00
1456381_x_at	0.00	0.00
1456383_at	0.00	0.10
1456385_x_at	0.00	0.00
1456386_at	0.00	0.00
1456388_at	0.00	0.00
1456390_at	0.00	0.00
1456393_at	0.00	0.78
1456395_at	0.00	0.00
1456398_at	0.00	0.00
1456399_at	0.00	0.00
1456405_at	0.00	0.35
1456411_at	0.00	0.00
1456412_a_at	0.00	0.00
1456417_at	0.00	0.00
1456424_s_at	0.00	0.00
1456427_at	0.00	0.00
1456430_at	0.00	0.00
1456431_at	0.00	0.00
1456433_at	0.00	0.00
1456434_x_at	0.00	0.00
1456436_x_at	0.00	0.00
1456437_x_at	0.00	0.00
1456438_x_at	0.00	0.00
1456439_x_at	0.03	0.00
1456442_at	0.00	0.00
1456447_at	0.00	0.00
1456455_at	0.00	0.00
1456456_x_at	0.00	0.00
1456457_at	0.00	0.00
1456458_at	0.00	0.00
1456459_x_at	0.00	0.00
1456461_at	0.00	0.00
1456462_x_at	0.00	0.00
1456464_x_at	0.00	0.00
1456465_at	0.00	0.00
1456466_x_at	0.00	0.00
1456469_x_at	0.00	0.00

1456470_x_at	0.00	0.00
1456471_x_at	0.00	0.00
1456473_x_at	0.00	0.00
1456474_at	0.00	0.00
1456481_at	0.00	0.00
1456486_at	0.00	0.00
1456488_at	0.00	0.00
1456492_at	0.00	0.00
1456493_at	0.00	0.00
1456494_a_at	0.00	0.00
1456495_s_at	0.00	0.00
1456496_at	0.00	0.00
1456497_x_at	0.00	0.00
1456510_x_at	0.00	0.00
1456511_x_at	1.00	0.59
1456515_s_at	0.00	0.00
1456516_x_at	0.00	0.00
1456521_at	1.00	0.07
1456525_at	0.00	0.00
1456528_x_at	0.00	0.00
1456529_at	0.00	0.00
1456530_x_at	0.00	0.03
1456534_at	0.00	0.00
1456540_s_at	0.00	0.00
1456541_x_at	0.00	0.26
1456542_s_at	0.00	0.00
1456543_at	0.00	0.00
1456546_at	0.00	0.00
1456565_s_at	0.00	0.00
1456566_x_at	0.00	0.00
1456567_x_at	0.00	0.00
1456568_at	0.00	0.00
1456569_x_at	0.00	0.00
1456571_at	0.00	0.00
1456572_x_at	0.00	0.00
1456573_x_at	0.00	0.00
1456575_at	0.00	0.00
1456576_x_at	0.00	0.00
1456577_x_at	0.00	0.00
1456578_x_at	0.00	0.00
1456579_x_at	0.00	0.00
1456580_s_at	0.00	0.00
1456581_x_at	0.00	0.00
1456582_x_at	0.00	0.00
1456583_x_at	0.00	0.00
1456584_x_at	0.00	0.00
1456585_x_at	0.00	0.00
1456588_x_at	0.00	0.00
1456590_x_at	0.20	1.00
1456595_x_at	0.00	0.00
1456598_at	0.00	0.00
1456600_a_at	0.00	0.00
1456601_x_at	0.00	0.00
1456603_at	0.00	0.00
1456604_a_at	0.00	0.00
1456605_at	0.00	0.00

1456606_a_at	0.00	0.00
1456608_at	0.00	0.00
1456612_at	0.00	0.00
1456613_at	0.00	0.00
1456615_a_at	0.00	0.00
1456616_a_at	0.00	0.06
1456617_a_at	0.00	0.00
1456618_at	0.00	0.00
1456620_at	0.00	0.00
1456622_at	0.00	0.00
1456623_at	0.76	0.04
1456624_at	0.00	0.00
1456626_a_at	0.00	0.23
1456627_at	0.00	0.00
1456628_x_at	0.00	0.00
1456629_at	0.00	0.00
1456630_x_at	0.00	0.00
1456635_at	0.00	0.00
1456636_at	0.00	0.00
1456637_at	0.00	0.00
1456642_x_at	0.05	0.00
1456644_at	0.00	0.00
1456647_a_at	0.00	0.00
1456651_a_at	0.00	0.03
1456658_at	0.00	0.00
1456663_x_at	0.00	0.00
1456664_x_at	0.00	0.07
1456672_at	0.00	0.00
1456676_a_at	0.00	0.00
1456691_s_at	0.00	0.00
1456694_x_at	0.00	0.00
1456695_x_at	0.00	0.00
1456697_x_at	0.00	0.00
1456699_s_at	0.00	0.00
1456700_x_at	0.01	0.00
1456701_at	0.00	0.00
1456702_x_at	0.00	0.00
1456706_at	0.00	0.00
1456707_at	0.00	0.00
1456714_at	0.00	0.00
1456716_s_at	0.00	0.00
1456724_x_at	0.00	0.00
1456725_x_at	0.00	0.00
1456726_x_at	0.00	0.00
1456727_a_at	0.00	0.00
1456728_x_at	0.00	0.00
1456730_x_at	0.00	0.05
1456731_x_at	0.00	0.00
1456732_at	0.00	0.00
1456733_x_at	0.45	0.26
1456734_at	0.00	0.00
1456736_x_at	0.00	0.00
1456737_x_at	0.00	0.00
1456739_x_at	0.11	0.00
1456740_x_at	0.00	0.00
1456741_s_at	0.00	0.00

1456743_x_at	0.00	0.00
1456744_x_at	0.00	0.00
1456745_x_at	0.00	0.00
1456746_a_at	0.00	0.00
1456747_x_at	0.00	0.00
1456748_a_at	0.01	0.00
1456757_at	0.00	0.00
1456770_at	0.00	0.00
1456869_at	0.00	0.00
1456870_at	0.00	0.00
1456972_at	0.00	0.00
1457094_at	0.00	0.00
1457126_at	0.00	0.00
1457147_at	0.00	0.00
1457265_at	0.00	0.00
1457272_at	0.00	0.00
1457285_at	0.00	0.00
1457303_at	0.00	0.00
1457308_at	0.00	0.00
1457372_at	0.00	0.00
1457379_at	0.00	0.00
1457455_at	0.00	0.00
1457475_at	0.00	0.00
1457476_at	0.00	0.00
1457520_at	0.00	0.00
1457521_at	0.00	0.00
1457588_at	0.00	0.00
1457594_at	0.00	0.00
1457675_at	0.00	0.00
1457676_at	0.00	0.00
1457695_at	0.00	0.00
1457738_at	0.00	0.00
1457776_at	0.00	0.00
1457810_at	0.00	0.00
1457833_at	0.00	0.00
1457834_at	0.00	0.00
1457841_at	0.00	0.00
1457849_at	0.00	0.00
1457860_at	0.00	0.00
1457872_at	0.00	0.00
1457887_at	0.00	0.00
1457899_at	0.00	0.00
1457905_at	0.00	0.00
1457954_at	0.00	0.00
1457964_at	0.00	0.00
1457970_at	0.00	0.00
1458011_s_at	0.00	0.00
1458041_at	0.00	0.00
1458173_at	0.00	0.00
1458196_at	0.00	0.00
1458215_at	0.00	0.00
1458219_at	0.00	0.00
1458414_at	0.00	0.00
1458613_at	0.00	0.00
1458795_at	0.00	0.00
1458846_at	0.00	0.00

1458854_at	0.00	0.00
1458862_at	0.00	0.00
1459021_at	0.00	0.00
1459085_at	0.00	0.00
1459092_at	0.00	0.00
1459211_at	0.04	0.00
1459220_at	0.00	0.00
1459410_at	0.00	0.00
1459478_at	0.00	0.00
1459546_s_at	0.00	0.00
1459701_x_at	0.00	0.00
1459725_s_at	0.00	0.00
1459814_at	0.00	0.00
1459816_x_at	0.00	0.00
1459817_at	0.00	0.00
1459880_at	0.00	0.00
1459882_at	0.00	0.00
1459884_at	0.00	0.00
1459885_s_at	0.00	0.00
1459889_at	0.00	0.00
1459890_s_at	0.00	0.00
1459891_at	0.00	0.00
1459892_at	0.00	0.00
1459893_at	0.00	0.00
1459894_at	0.00	0.00
1459900_at	0.00	0.00
1459902_at	0.00	0.00
1459903_at	0.00	0.00
1459908_at	0.00	0.00
1459909_at	0.00	0.00
1459911_at	0.00	0.00
1459912_at	0.00	0.00
1459914_at	0.00	0.00
1459916_at	0.00	0.00
1459917_at	0.00	0.00
1459918_at	0.00	0.00
1459920_at	0.00	0.00
1459921_at	0.00	0.00
1459922_at	0.00	0.00
1459923_at	0.00	0.00
1459924_at	0.00	0.00
1459925_at	0.00	0.00
1459926_at	0.00	0.00
1459927_at	0.00	0.00
1459928_at	0.00	0.00
1459929_at	0.00	0.00
1459930_at	0.00	0.00
1459931_a_at	0.00	0.00
1459932_at	0.00	0.00
1459933_at	0.00	0.00
1459934_at	0.00	0.00
1459935_at	0.00	0.00
1459952_at	0.00	0.00
1459983_at	0.00	0.00
1459985_at	0.00	0.00
1459986_a_at	0.00	0.00

1459987_s_at	0.00	0.00
1459990_at	0.00	0.00
1459992_x_at	0.00	0.00
1459994_x_at	0.00	0.00
1459996_at	0.00	0.00
1459999_at	0.00	0.00
1460004_x_at	0.04	0.00
1460007_at	0.00	0.00
1460008_x_at	0.00	0.00
1460009_at	0.00	0.00
1460010_a_at	0.00	0.00
1460017_at	0.00	0.04
1460034_at	0.00	0.00
1460038_at	0.00	0.00
1460042_at	0.00	0.00
1460164_at	0.00	0.00
1460165_at	0.00	0.00
1460166_at	0.00	0.00
1460167_at	0.00	0.00
1460168_at	0.00	0.09
1460169_a_at	0.00	0.00
1460170_at	0.00	0.00
1460171_at	0.00	0.18
1460172_at	0.00	0.00
1460173_at	0.00	0.00
1460174_at	0.00	0.00
1460175_at	0.00	0.00
1460176_at	0.00	0.00
1460177_at	0.00	0.00
1460178_at	0.00	0.00
1460179_at	0.00	0.00
1460180_at	0.00	0.00
1460181_at	0.00	0.00
1460182_at	0.00	0.00
1460183_at	0.00	0.00
1460184_at	0.00	0.00
1460185_at	0.00	0.00
1460186_at	0.02	0.00
1460187_at	0.01	0.00
1460188_at	0.00	0.00
1460189_at	0.00	0.00
1460190_at	0.00	0.00
1460191_at	0.00	0.05
1460192_at	0.02	0.00
1460193_at	0.00	0.00
1460194_at	0.00	0.00
1460195_at	0.00	0.08
1460196_at	0.00	0.00
1460197_a_at	0.00	0.00
1460198_a_at	0.00	0.17
1460199_a_at	0.00	0.00
1460200_s_at	0.00	0.00
1460201_a_at	0.00	0.00
1460202_at	0.00	0.00
1460203_at	0.00	0.00
1460204_at	0.00	0.00

1460205_at	0.00	0.00
1460206_at	0.00	0.00
1460207_s_at	0.00	0.00
1460208_at	0.00	0.00
1460209_at	0.00	0.00
1460210_at	0.00	0.00
1460211_a_at	0.00	0.34
1460212_at	0.00	0.00
1460213_at	0.00	0.00
1460214_at	0.00	0.00
1460215_at	0.00	0.00
1460216_at	0.00	0.00
1460217_at	0.00	0.00
1460218_at	0.00	0.00
1460219_at	0.00	0.00
1460220_a_at	0.23	0.00
1460221_at	0.00	0.00
1460222_at	0.00	0.00
1460223_a_at	0.16	0.00
1460224_at	0.00	0.00
1460225_at	0.00	0.00
1460226_at	0.00	0.00
1460227_at	0.00	0.10
1460228_at	0.00	0.00
1460229_at	0.00	0.00
1460230_at	0.00	0.00
1460231_at	0.00	0.00
1460232_s_at	0.00	0.00
1460233_at	0.00	0.00
1460234_at	0.00	0.00
1460235_at	0.00	0.00
1460236_at	0.00	0.00
1460237_at	0.00	0.00
1460238_at	0.00	0.00
1460239_at	0.00	0.31
1460240_a_at	0.00	0.00
1460241_a_at	0.00	0.00
1460242_at	0.00	0.00
1460243_at	0.00	0.00
1460244_at	0.00	0.00
1460245_at	0.00	0.00
1460246_at	0.00	0.00
1460247_a_at	0.00	0.00
1460248_at	0.00	0.00
1460249_at	0.00	0.00
1460250_at	0.00	0.00
1460251_at	0.00	0.00
1460252_s_at	0.00	0.00
1460253_at	0.00	0.00
1460254_at	0.00	0.00
1460255_at	0.00	0.00
1460256_at	0.00	0.00
1460257_a_at	0.00	0.00
1460258_at	0.00	0.00
1460259_s_at	0.00	0.00
1460260_s_at	0.00	0.00

1460261_at	0.00	0.00
1460262_a_at	0.00	0.00
1460263_at	0.00	0.00
1460264_at	0.00	0.00
1460265_at	0.00	0.00
1460266_at	0.00	0.00
1460268_at	0.00	0.00
1460269_at	0.00	0.00
1460271_at	0.00	0.00
1460272_at	0.00	0.00
1460273_a_at	0.00	0.00
1460274_at	0.00	0.00
1460275_at	0.00	0.00
1460276_a_at	0.00	0.00
1460277_at	0.00	0.00
1460278_a_at	0.00	0.00
1460279_a_at	0.00	0.00
1460280_at	0.00	0.00
1460281_at	0.00	0.00
1460282_at	0.00	0.00
1460283_at	0.00	0.00
1460284_at	0.00	0.00
1460285_at	0.00	0.00
1460286_at	0.00	0.00
1460287_at	0.00	0.00
1460288_a_at	0.00	0.28
1460289_at	0.00	0.00
1460290_at	0.00	0.00
1460291_at	0.00	0.00
1460292_a_at	0.00	0.00
1460293_at	0.00	0.00
1460294_at	0.00	0.00
1460295_s_at	0.00	0.00
1460296_a_at	0.00	0.00
1460297_at	0.00	0.00
1460298_at	0.00	0.00
1460299_at	0.00	0.00
1460300_a_at	0.00	0.00
1460301_at	0.00	0.00
1460302_at	0.57	0.00
1460303_at	0.00	0.00
1460304_a_at	0.00	0.24
1460305_at	0.20	0.00
1460306_at	0.00	0.00
1460307_at	0.00	0.00
1460308_a_at	0.00	0.00
1460309_at	0.00	0.00
1460310_a_at	0.00	0.00
1460311_at	0.00	0.00
1460312_at	0.00	0.00
1460313_at	0.00	0.00
1460314_s_at	0.01	0.12
1460315_s_at	0.00	0.00
1460316_at	0.00	0.00
1460317_s_at	0.00	0.00
1460318_at	0.00	0.00

1460319_at	0.00	0.00
1460320_at	0.00	0.00
1460321_at	0.00	0.00
1460322_at	0.00	0.00
1460323_at	0.00	0.30
1460324_at	0.00	0.00
1460325_at	0.00	0.05
1460326_at	0.00	0.00
1460327_at	0.00	0.00
1460328_at	0.00	0.00
1460329_at	0.00	0.00
1460330_at	0.86	0.00
1460331_at	0.00	0.00
1460332_at	0.00	0.00
1460333_at	0.00	0.00
1460334_at	0.00	0.00
1460335_at	0.03	0.00
1460336_at	0.00	0.00
1460337_at	0.00	0.00
1460338_a_at	0.00	0.01
1460339_at	0.00	0.00
1460340_at	0.00	0.00
1460341_at	0.00	0.00
1460342_s_at	0.00	0.00
1460343_at	0.00	0.00
1460344_at	0.00	0.00
1460345_at	0.00	0.00
1460346_at	0.00	0.11
1460347_at	0.00	0.00
1460348_at	0.00	0.00
1460349_at	0.00	0.00
1460350_at	0.00	0.00
1460351_at	0.92	0.00
1460352_s_at	0.00	0.03
1460353_at	0.00	0.00
1460354_a_at	0.00	0.00
1460355_at	0.00	0.00
1460356_at	0.00	0.00
1460357_at	0.00	0.10
1460358_s_at	0.00	0.00
1460359_at	0.00	0.00
1460360_at	0.00	0.00
1460361_at	0.05	0.00
1460362_at	0.00	0.00
1460363_at	0.00	0.00
1460364_at	0.00	0.00
1460365_a_at	0.00	0.00
1460366_at	0.01	0.00
1460367_at	0.00	0.00
1460368_at	0.00	0.00
1460369_at	0.00	0.00
1460370_at	0.00	0.00
1460371_at	0.00	0.00
1460372_at	0.00	0.00
1460373_a_at	0.00	0.00
1460374_at	0.00	0.00

1460375_at	0.00	0.00
1460376_a_at	0.00	0.00
1460377_a_at	0.00	0.00
1460378_a_at	0.15	0.00
1460379_at	0.00	0.00
1460380_at	0.00	0.00
1460381_at	0.00	0.00
1460382_at	0.00	0.00
1460383_at	0.00	0.00
1460384_a_at	0.00	0.00
1460385_a_at	0.00	0.00
1460386_a_at	0.00	0.00
1460387_a_at	0.00	0.00
1460388_at	0.00	0.00
1460389_at	0.00	0.00
1460390_at	0.00	0.00
1460391_at	0.00	0.00
1460392_a_at	0.01	0.00
1460393_a_at	0.00	0.00
1460394_a_at	0.00	0.32
1460395_at	0.00	0.00
1460396_at	0.00	0.00
1460397_at	0.00	0.29
1460398_at	0.00	0.00
1460399_at	0.00	0.00
1460400_at	0.00	0.00
1460401_at	0.00	0.00
1460402_at	0.00	0.00
1460403_at	0.00	0.05
1460404_at	0.00	0.00
1460405_at	0.00	0.00
1460406_at	0.00	0.00
1460407_at	0.00	0.00
1460408_at	0.00	0.00
1460409_at	0.00	0.00
1460410_at	0.00	0.00
1460411_s_at	0.18	0.42
1460412_at	0.00	0.00
1460413_s_at	0.00	0.00
1460414_at	0.00	0.00
1460415_a_at	0.00	0.00
1460416_s_at	0.00	0.00
1460417_at	0.00	0.00
1460418_x_at	0.00	0.00
1460419_a_at	0.00	0.00
1460420_a_at	0.00	0.00
1460421_at	0.00	0.00
1460422_at	0.00	0.00
1460423_x_at	0.00	0.00
1460424_at	0.00	0.00
1460425_at	0.00	0.00
1460426_at	0.00	0.00
1460427_a_at	0.00	0.00
1460428_at	0.00	0.00
1460429_at	0.08	0.03
1460430_at	0.02	0.17

1460432_a_at	0.00	0.00
1460433_at	0.00	0.00
1460434_at	0.00	0.00
1460436_at	0.00	0.00
1460438_at	0.00	0.00
1460442_at	0.00	0.00
1460444_at	0.00	0.00
1460445_at	0.00	0.00
1460450_at	0.00	0.00
1460455_at	0.00	0.00
1460460_a_at	0.00	0.00
1460464_at	0.00	0.00
1460469_at	0.00	0.00
1460480_at	0.00	0.00
1460481_at	0.00	0.00
1460486_at	0.00	0.00
1460490_at	0.00	0.00
1460495_s_at	0.00	0.00
1460498_a_at	0.00	0.00
1460504_at	0.00	0.00
1460508_at	0.00	0.00
1460511_at	0.00	0.00
1460512_a_at	0.00	0.00
1460513_a_at	0.00	0.00
1460514_s_at	0.00	0.00
1460519_a_at	0.00	0.00
1460521_a_at	0.00	0.00
1460541_at	0.00	0.00
1460542_s_at	0.00	0.00
1460543_x_at	0.00	0.00
1460544_at	0.00	0.00
1460545_at	0.00	0.00
1460547_a_at	0.00	0.08
1460548_a_at	0.00	0.00
1460549_a_at	0.00	0.00
1460551_at	0.00	0.00
1460555_at	0.00	0.00
1460557_at	0.00	0.23
1460561_x_at	0.00	0.00
1460562_at	0.00	0.00
1460565_at	0.00	0.00
1460568_at	0.00	0.00
1460569_x_at	0.00	0.64
1460571_at	0.00	0.01
1460579_at	0.00	0.00
1460581_a_at	0.00	0.00
1460583_at	0.00	0.00
1460590_s_at	0.00	0.00
1460592_at	0.00	0.00
1460594_a_at	0.00	0.00
1460596_at	0.00	0.00
1460605_at	0.00	0.00
1460609_at	0.00	0.00
1460610_at	0.00	0.00
1460613_x_at	0.00	0.00
1460621_x_at	0.00	0.00

1460626_at	0.00	0.00
1460629_at	0.00	0.00
1460631_at	0.00	0.00
1460633_at	0.00	0.01
1460634_at	0.00	0.01
1460635_at	0.00	0.00
1460636_at	0.00	0.00
1460637_s_at	0.00	0.00
1460638_at	0.00	0.00
1460639_a_at	0.00	0.00
1460640_at	0.00	0.00
1460641_a_at	0.00	0.00
1460642_at	0.00	0.00
1460643_at	0.00	0.00
1460644_at	0.00	0.00
1460645_at	0.00	0.08
1460646_at	0.00	0.00
1460647_a_at	0.00	0.00
1460648_at	0.00	0.00
1460649_at	0.00	0.00
1460650_at	0.00	0.00
1460651_at	0.00	0.00
1460652_at	0.00	0.00
1460653_at	0.00	0.00
1460654_at	0.00	0.00
1460655_a_at	0.00	0.00
1460656_a_at	0.00	0.00
1460657_at	0.00	0.00
1460658_at	0.00	0.00
1460659_at	0.00	0.00
1460660_x_at	0.00	0.00
1460661_at	0.00	0.00
1460662_at	0.00	0.00
1460663_at	0.00	0.00
1460664_at	0.00	0.00
1460665_a_at	0.00	0.00
1460666_a_at	0.00	0.00
1460667_at	0.00	0.00
1460668_at	0.00	0.00
1460669_at	0.00	0.00
1460670_at	0.00	0.00
1460671_at	0.00	0.00
1460672_at	0.00	0.07
1460673_at	0.00	0.00
1460674_at	0.00	0.00
1460675_at	0.00	0.00
1460676_at	0.00	0.00
1460677_at	0.00	0.00
1460678_at	0.31	0.00
1460679_at	0.00	0.00
1460680_a_at	0.00	0.00
1460681_at	0.00	0.00
1460682_s_at	0.10	0.00
1460683_at	0.00	0.00
1460684_at	0.00	0.00
1460685_at	0.00	0.00

1460686_at	0.00	0.01
1460687_at	0.00	0.00
1460688_s_at	0.00	0.00
1460689_at	0.00	0.00
1460690_at	0.00	0.00
1460691_at	0.00	0.00
1460692_at	0.00	0.00
1460693_a_at	0.00	0.00
1460694_s_at	0.00	0.00
1460695_a_at	0.00	0.00
1460696_at	0.00	0.00
1460697_s_at	0.00	0.00
1460698_a_at	0.00	0.00
1460699_at	0.00	0.00
1460700_at	0.00	0.00
1460701_a_at	0.00	0.00
1460702_at	0.00	0.00
1460703_at	0.00	0.00
1460704_at	0.00	0.02
1460705_at	0.00	0.00
1460706_s_at	0.00	0.00
1460707_at	0.00	0.00
1460708_s_at	0.00	0.00
1460709_a_at	0.00	0.00
1460710_at	0.00	0.00
1460711_at	0.76	0.46
1460712_s_at	0.00	0.00
1460713_at	0.00	0.00
1460714_at	0.00	0.00
1460715_x_at	0.00	0.00
1460716_a_at	0.00	0.00
1460717_at	0.00	0.00
1460718_s_at	0.00	0.00
1460719_a_at	0.00	0.00
1460720_at	0.00	0.00
1460722_at	0.00	0.00
1460723_at	0.00	0.00
1460724_at	0.00	0.00
1460725_at	0.00	0.00
1460726_at	0.00	0.00
1460727_at	0.00	0.00
1460728_s_at	0.00	0.00
1460729_at	0.00	0.00
1460730_at	0.00	0.00
1460731_at	0.00	0.00
1460732_a_at	0.00	0.00
1460733_at	0.00	0.00
1460734_at	0.00	0.00
1460735_at	0.00	0.00
1460736_at	0.00	0.00
1460737_at	0.00	0.00
1460738_at	0.00	0.00
1460739_at	0.00	0.00
1460740_at	0.13	0.24
1460741_x_at	0.00	0.00
1460742_at	0.00	0.00

1460743_at	0.00	0.00
1460744_at	0.00	0.00
1460745_at	0.00	0.00
1460746_at	0.00	0.00
8SRNAMur/X006	0.05	0.00
8SRNAMur/X006	0.09	0.00
8SRNAMur/X006	0.09	0.00
-ActinMur/M1248	0.00	0.00
-ActinMur/M1248	0.00	0.00
-ActinMur/M1248	0.00	0.00
AFFX-BioB-3_at	0.00	0.00
AFFX-BioB-5_at	0.00	0.00
AFFX-BioB-M_at	0.00	0.00
AFFX-BioC-3_at	0.00	0.00
AFFX-BioC-5_at	0.00	0.00
AFFX-BioDn-3_a	0.00	0.00
AFFX-BioDn-5_a	0.00	0.00
AFFX-CreX-3_at	0.00	0.00
AFFX-CreX-5_at	0.00	0.00
AFFX-DapX-3_at	0.00	0.00
AFFX-DapX-5_at	0.00	0.00
AFFX-DapX-M_a	0.00	0.00
GapdhMur/M3259	0.00	0.00
GapdhMur/M3259	0.00	0.00
GapdhMur/M3259	0.00	0.00
AFFX-LysX-3_at	0.00	0.00
AFFX-LysX-5_at	0.00	0.47
AFFX-LysX-M_at	0.00	0.00
AFFX-MUR_b2_a	0.00	0.00
FX-MURINE_b1_	0.00	0.00
FX-MURINE_B2_	0.00	0.00
AFFX-PheX-3_at	0.00	0.00
AFFX-PheX-5_at	0.00	0.00
AFFX-PheX-M_a	0.00	0.00
yruCarbMur/L091	0.00	0.00
yruCarbMur/L091	0.00	0.00
ruCarbMur/L0919	0.00	0.00
ruCarbMur/L0919	0.00	0.00
FFX-r2-Bs-dap-3_	0.00	0.00
FFX-r2-Bs-dap-5_	0.00	0.00
FFX-r2-Bs-dap-M_	0.00	0.00
FFX-r2-Bs-lys-3_	0.00	0.00
FFX-r2-Bs-lys-5_	0.00	0.00
FFX-r2-Bs-lys-M_	0.00	0.00
FFX-r2-Bs-phe-3_	0.00	0.00
FFX-r2-Bs-phe-5_	0.00	0.00
FFX-r2-Bs-phe-M_	0.00	0.00
FX-r2-Bs-thr-3_s	0.00	0.00
FX-r2-Bs-thr-5_s	0.00	0.00
FX-r2-Bs-thr-M_s	0.00	0.00
FX-r2-Ec-bioB-3_	0.00	0.00
FX-r2-Ec-bioB-5_	0.00	0.00
FX-r2-Ec-bioB-M_	0.00	0.00
FX-r2-Ec-bioC-3_	0.00	0.00
FX-r2-Ec-bioC-5_	0.00	0.00
FX-r2-Ec-bioD-3_	0.00	0.00

FX-r2-Ec-bioD-5_	0.00	0.00
FFX-r2-P1-cre-3_	0.00	0.00
FFX-r2-P1-cre-5_	0.00	0.00
AFFX-ThrX-3_at	0.00	0.00
AFFX-ThrX-5_at	0.00	0.00
AFFX-ThrX-M_at	0.00	0.00
ransRecMur/X573	0.00	0.00
ransRecMur/X573	0.00	0.00
ansRecMur/X573	0.00	0.00
AFFX-TrpnX-3_at	0.00	0.00
AFFX-TrpnX-5_at	0.00	0.00
AFFX-TrpnX-M_a	0.00	0.00
1415670_at	0.00	0.00
1415671_at	0.00	0.00
1415672_at	0.00	0.00
1415673_at	0.00	0.01
1415674_a_at	0.00	0.00
1415675_at	0.00	0.00
1415676_a_at	0.00	0.00
1415677_at	0.00	0.00
1415678_at	0.00	0.00
1415679_at	0.00	0.00
1415680_at	0.00	0.00
1415681_at	0.00	0.00
1415682_at	0.00	0.00
1415683_at	0.00	0.00
1415684_at	0.00	0.00
1415685_at	0.00	0.00
1415686_at	0.00	0.00
1415687_a_at	0.00	0.04
1415688_at	0.00	0.00
1415689_s_at	0.00	0.00
1415690_at	0.00	0.00
1415691_at	0.00	0.00
1415692_s_at	0.00	0.00
1415693_at	0.00	0.00
1415694_at	0.00	0.00
1415695_at	0.00	0.00
1415696_at	0.00	0.00
1415697_at	0.00	0.61
1415698_at	0.00	0.00
1415699_a_at	0.00	0.09
1415700_a_at	0.00	0.00
1415701_x_at	0.00	0.00
1415702_a_at	0.00	0.00
1415703_at	0.00	0.00
1415704_a_at	0.00	0.00
1415705_at	0.00	0.00
1415706_at	0.00	0.00
1415707_at	0.00	0.00
1415708_at	0.00	0.02
1415709_s_at	0.00	0.00
1415710_at	0.00	0.00
1415711_at	0.00	0.00
1415712_at	0.00	0.00
1415713_a_at	0.00	0.00

1415714_a_at	0.00	0.00
1415715_at	0.00	0.00
1415716_a_at	0.00	0.00
1415717_at	0.00	0.00
1415718_at	0.00	0.00
1415719_s_at	0.00	0.00
1415720_s_at	0.00	0.00
1415721_a_at	0.00	0.00
1415722_a_at	0.00	0.00
1415723_at	0.00	0.00
1415724_a_at	0.01	0.00
1415725_at	0.01	0.36
1415726_at	0.00	0.00
1415727_at	0.00	0.09
1415728_at	0.00	0.00
1415729_at	0.00	0.00
1415730_at	0.00	0.00
1415731_at	0.00	0.00
1415732_at	0.00	0.00
1415733_a_at	0.00	0.11
1415734_at	0.00	0.00
1415735_at	0.00	0.00
1415736_at	0.01	0.00
1415737_at	0.00	0.00
1415738_at	0.00	0.00
1415739_at	0.00	0.00
1415740_at	0.00	0.15
1415741_at	0.00	0.00
1415742_at	0.00	0.00
1415743_at	0.00	0.00
1415744_at	0.00	0.06
1415745_a_at	0.00	0.00
1415746_at	0.00	0.00
1415747_s_at	0.00	0.00
1415748_a_at	0.00	0.00
1415749_a_at	0.00	0.00
1415750_at	0.00	0.00
1415751_at	0.00	0.00
1415752_at	0.00	0.00
1415753_at	0.00	0.00
1415754_at	0.00	0.16
1415755_a_at	0.00	0.00
1415756_a_at	0.00	0.00
1415757_at	0.00	0.00
1415758_at	0.00	0.00
1415759_a_at	0.00	0.00
1415760_s_at	0.00	0.01
1415761_at	0.00	0.06
1415762_x_at	0.00	0.12
1415763_a_at	0.00	0.00
1415764_at	0.00	0.00
1415765_at	0.00	0.00
1415766_at	0.00	0.00
1415767_at	0.00	0.00
1415768_a_at	0.00	0.00
1415769_at	0.00	0.00

1419781_at	0.00	0.00
1419783_at	0.00	0.00
1419784_x_at	0.00	0.00
1419786_at	0.00	0.00
1419787_a_at	0.00	0.00
1419788_at	0.00	0.00
1419791_at	0.00	0.00
1419792_at	0.00	0.00
1419793_at	0.00	0.00
1419794_at	0.00	0.00
1419804_at	0.00	0.00
1419813_at	0.00	0.00
1419824_a_at	0.00	0.00
1419825_at	0.00	0.00
1419828_at	0.00	0.00
1419829_a_at	0.00	0.00
1419837_at	0.00	0.00
1419844_a_at	0.00	0.00
1419853_a_at	0.00	0.00
1419871_at	0.00	0.00
1419892_at	0.00	0.00
1419901_at	0.00	0.00
1419902_at	0.00	0.00
1419903_at	0.00	0.00
1419904_at	0.00	0.00
1419922_s_at	0.00	0.28
1419923_at	0.00	0.00
1419924_at	0.00	0.00
1419925_s_at	0.00	0.00
1419926_at	0.00	0.00
1419938_s_at	0.00	0.00
1419939_at	0.00	0.00
1419985_s_at	0.00	0.00
1419986_at	0.00	0.00
1419990_at	0.00	0.00
1419996_s_at	0.00	0.00
1420005_s_at	0.00	0.00
1420006_at	0.00	0.00
1420015_s_at	0.00	0.00
1420016_at	0.00	0.00
1420035_at	0.00	0.00
1420036_at	0.00	0.00
1420070_a_at	0.00	0.00
1420071_at	0.00	0.00
1420077_at	0.00	0.00
1420080_a_at	0.00	0.00
1420081_s_at	0.00	0.01
1420082_at	0.00	0.00
1420097_at	0.00	0.00
1420098_s_at	0.00	0.00
1420099_at	0.00	0.00
1420100_s_at	0.00	0.00
1420107_at	0.00	0.00
1420112_at	0.00	0.00
1420119_s_at	0.00	0.00
1420120_at	0.00	0.00

1420127_s_at	0.00	0.00
1420128_s_at	0.00	0.00
1420133_at	0.00	0.00
1420134_at	0.00	0.00
1420135_at	0.00	0.00
1420136_a_at	0.00	0.00
1420137_at	0.00	0.00
1420139_s_at	0.00	0.20
1420143_at	0.00	0.00
1420144_x_at	0.00	0.00
1420145_at	0.00	0.00
1420151_at	0.00	0.00
1420152_at	0.00	0.00
1420153_at	0.00	0.00
1420154_at	0.00	0.00
1420167_at	0.00	0.00
1420178_at	0.00	0.00
1420181_at	0.00	0.00
1420182_x_at	0.00	0.00
1420188_at	0.00	0.00
1420190_at	0.00	0.00
1420199_at	0.00	0.00
1420205_at	0.00	0.00
1420206_at	0.00	0.00
1420207_at	0.00	0.00
1420208_at	0.00	0.00
1420209_at	0.00	0.00
1420212_at	0.00	0.00
1420213_x_at	0.00	0.00
1420214_at	0.00	0.00
1420215_x_at	0.00	0.00
1420216_at	0.00	0.00
1420219_at	0.00	0.00
1420220_x_at	0.00	0.00
1420221_at	0.00	0.00
1420233_at	0.00	0.00
1420238_at	0.00	0.00
1420239_x_at	0.00	0.00
1420242_at	0.00	0.00
1420245_x_at	0.00	0.00
1420265_x_at	0.00	0.00
1420266_at	0.00	0.00
1420271_at	0.00	0.00
1420276_x_at	0.00	0.00
1420277_at	0.00	0.00
1420283_at	0.00	0.00
1420293_at	0.00	0.00
1420294_at	0.00	0.00
1420299_at	0.00	0.00
1420300_at	0.00	0.00
1420310_at	0.00	0.00
1420311_s_at	0.00	0.00
1420312_s_at	0.00	0.00
1420313_x_at	0.00	0.00
1420321_at	0.00	0.00
1428124_at	0.00	0.00

1428136_at	0.00	0.00
1428139_at	0.00	0.00
1428142_at	0.00	0.03
1428147_at	0.00	0.00
1428148_s_at	0.00	0.00
1428149_at	0.00	0.00
1428150_at	0.00	0.00
1428151_x_at	0.00	0.00
1428152_a_at	0.00	0.00
1428153_at	0.00	0.00
1428154_s_at	0.00	0.00
1428156_at	0.00	0.33
1428157_at	0.00	0.24
1428158_at	0.00	0.00
1428162_at	0.00	0.00
1428166_at	0.00	0.00
1428167_a_at	0.15	0.00
1428168_at	0.00	0.03
1428170_at	0.00	0.00
1428173_at	0.00	0.00
1428174_x_at	0.02	0.00
1428175_at	0.00	0.00
1428176_at	0.00	0.00
1428177_at	0.00	0.00
1428178_s_at	0.00	0.00
1428180_at	0.00	0.00
1428183_at	0.00	0.00
1428184_at	0.00	0.00
1428185_at	0.00	0.00
1428186_at	0.00	0.00
1428190_at	0.00	0.00
1428192_at	0.00	0.00
1428198_at	0.00	0.00
1428199_at	0.00	0.00
1428202_at	0.00	0.00
1428203_at	0.00	0.00
1428204_at	0.00	0.00
1428205_x_at	0.00	0.00
1428206_at	0.00	0.00
1428207_at	0.00	0.00
1428208_at	0.00	0.00
1428210_s_at	0.00	0.00
1428211_at	0.00	0.00
1428219_at	0.00	0.00
1428220_at	0.00	0.00
1428221_at	0.00	0.00
1428222_at	0.00	0.00
1428223_at	0.00	0.00
1428227_at	0.69	0.45
1428228_at	0.00	0.00
1428231_at	0.00	0.35
1428232_at	0.00	0.11
1428233_at	0.00	0.00
1428234_at	0.00	0.00
1428236_at	0.00	0.00
1428239_at	0.00	0.00

1428240_at	0.00	0.00
1428243_at	0.00	0.00
1428250_at	0.00	0.00
1428251_at	0.01	0.00
1428252_at	0.01	0.06
1428253_at	0.01	0.00
1428254_at	0.00	0.00
1428256_at	0.00	0.00
1428259_at	0.50	0.52
1428260_at	0.00	0.00
1428261_at	0.00	0.00
1428268_at	0.00	0.00
1428269_a_at	0.00	0.00
1428270_at	0.00	0.00
1428271_at	0.00	0.00
1428273_at	0.00	0.00
1428274_s_at	0.00	0.00
1428275_at	0.00	0.00
1428276_at	0.00	0.00
1428278_at	0.00	0.00
1428281_at	0.00	0.00
1428284_at	0.00	0.01
1428285_at	0.00	0.07
1428287_at	0.00	0.00
1428290_at	0.00	0.00
1428291_at	0.00	0.00
1428292_at	0.00	0.00
1428293_at	0.00	0.00
1428295_at	0.00	0.00
1428298_at	0.00	0.00
1428300_at	0.00	0.00
1428304_at	0.14	0.00
1428305_at	0.00	0.00
1428307_at	0.00	0.00
1428313_at	0.00	0.00
1428318_at	0.00	0.00
1428321_at	0.00	0.00
1428323_at	0.00	0.00
1428324_at	0.00	0.00
1428325_at	0.00	0.00
1428332_at	0.00	0.33
1428334_at	0.00	0.00
1428336_at	0.00	0.00
1428338_at	0.00	0.00
1428339_at	0.00	0.00
1428341_at	0.00	0.00
1428342_at	0.00	0.00
1428343_at	0.00	0.00
1428344_at	0.00	0.00
1428345_at	0.00	0.00
1428347_at	0.00	0.00
1428348_at	0.00	0.00
1428350_at	0.00	0.00
1428352_at	0.00	0.00
1428355_at	0.00	0.00
1428356_at	0.00	0.00

1428366_at	0.00	0.00
1428370_at	0.00	0.00
1428371_at	0.00	0.00
1428373_at	0.00	0.00
1428375_at	0.00	0.00
1428376_at	0.00	0.00
1428377_at	0.07	0.00
1428378_at	0.00	0.00
1428382_at	0.00	0.00
1428383_a_at	0.03	0.06
1428384_at	0.00	0.00
1428385_at	0.00	0.00
1428386_at	0.00	0.20
1428387_at	0.00	0.00
1428391_at	0.00	0.00
1428393_at	0.00	0.00
1428395_at	0.00	0.00
1428396_at	0.00	0.00
1428397_at	0.00	0.00
1428398_at	0.00	0.00
1428399_a_at	0.00	0.00
1428400_at	0.00	0.00
1428403_at	0.00	0.00
1428404_at	0.00	0.00
1428407_at	0.00	0.00
1428408_a_at	0.00	0.00
1428409_at	0.00	0.00
1428410_at	0.00	0.00
1428411_at	0.00	0.00
1428412_at	0.00	0.00
1428413_at	0.00	0.00
1428414_at	0.00	0.00
1428415_at	0.00	0.00
1428416_at	0.00	0.49
1428417_at	0.00	0.00
1428418_s_at	0.00	0.01
1428419_at	0.00	0.00
1428420_a_at	0.00	0.00
1428422_at	0.00	0.00
1428423_at	0.00	0.00
1428424_at	0.00	0.00
1428425_at	0.00	0.00
1428426_s_at	0.00	0.00
1428427_at	0.00	0.00
1428428_at	0.00	0.00
1428429_at	0.00	0.00
1428430_at	0.00	0.00
1428431_at	0.00	0.00
1428432_at	0.00	0.00
1428433_at	0.00	0.00
1428434_at	0.00	0.00
1428435_at	0.00	0.00
1428436_at	0.00	0.00
1428437_at	0.00	0.00
1428438_s_at	0.00	0.00
1428440_at	0.15	0.50

1428441_at	0.00	0.00
1428444_at	0.00	0.00
1428445_at	0.00	0.00
1428446_at	0.00	0.00
1428447_at	0.00	0.00
1428450_at	0.00	0.00
1428451_at	0.00	0.04
1428457_at	0.00	0.00
1428458_at	0.00	0.15
1428459_at	0.00	0.00
1428460_at	0.00	0.00
1428461_at	0.00	0.00
1428462_at	0.00	0.00
1428463_a_at	0.00	0.00
1428466_at	0.00	0.00
1428470_at	0.00	0.00
1428471_at	0.00	0.01
1428473_at	0.00	0.00
1428474_at	0.00	0.00
1428478_at	0.00	0.00
1428479_at	0.00	0.00
1428480_at	0.00	0.00
1428481_s_at	0.00	0.00
1428482_at	0.00	0.00
1428484_at	0.00	0.00
1428486_at	0.00	0.00
1428487_s_at	0.00	0.00
1428489_at	0.01	0.00
1428490_at	0.00	0.00
1428491_at	0.00	0.00
1428493_at	0.00	0.00
1428495_at	0.00	0.00
1428496_at	0.00	0.00
1428497_at	0.00	0.00
1428498_at	0.00	0.00
1428499_at	0.00	0.00
1428504_at	0.00	0.00
1428506_at	0.00	0.20
1428508_at	0.00	0.00
1428509_at	0.00	0.00
1428512_at	0.01	0.00
1428513_at	0.00	0.00
1428514_at	0.00	0.00
1428516_a_at	0.00	0.00
1428517_at	0.00	0.00
1428518_at	0.00	0.00
1428519_at	0.01	0.28
1428520_at	0.00	0.05
1428521_at	0.00	0.00
1428522_at	0.03	0.00
1428523_at	0.00	0.00
1428524_at	0.00	0.03
1428525_at	0.00	0.00
1428527_at	0.00	0.00
1428533_at	0.00	0.00
1428535_at	0.00	0.00

1428536_at	0.00	0.00
1428539_at	0.00	0.00
1428540_at	0.00	0.00
1428541_at	0.00	0.00
1428542_at	0.00	0.15
1428544_at	0.00	0.00
1428545_at	0.00	0.00
1428546_at	0.00	0.00
1428547_at	0.00	0.00
1428548_at	0.00	0.00
1428549_at	0.00	0.00
1428550_at	0.00	0.00
1428551_at	0.00	0.23
1428553_at	0.00	0.00
1428555_at	0.00	0.01
1428556_at	0.00	0.00
1428557_a_at	0.00	0.00
1428558_at	0.00	0.00
1428559_at	0.00	0.00
1428560_at	0.00	0.00
1428561_at	0.00	0.00
1428562_at	0.00	0.00
1428564_at	0.00	0.00
1428565_at	0.00	0.00
1428566_at	0.08	0.02
1428567_at	0.00	0.00
1428568_at	0.00	0.00
1428569_at	0.00	0.00
1428576_at	0.00	0.00
1428579_at	0.00	0.06
1428581_at	0.00	0.00
1428582_at	0.00	0.00
1428583_at	0.00	0.00
1428584_a_at	0.00	0.00
1428593_at	0.00	0.00
1428594_at	0.00	0.00
1428595_at	0.00	0.00
1428596_at	0.00	0.00
1428597_at	0.00	0.00
1428598_at	0.00	0.00
1428599_at	0.10	0.00
1428600_at	0.00	0.00
1428601_at	0.00	0.00
1428602_at	0.00	0.00
1428603_at	0.00	0.00
1428604_at	0.99	0.62
1428605_at	0.00	0.00
1428606_at	0.00	0.00
1428607_at	0.00	0.00
1428613_at	0.00	0.00
1428614_at	0.00	0.00
1428615_at	0.00	0.01
1428617_at	0.00	0.00
1428618_at	0.00	0.00
1428622_at	0.00	0.00
1428623_at	0.00	0.00

1428624_at	0.00	0.00
1428627_at	0.00	0.00
1428628_at	0.00	0.00
1428629_at	0.00	0.00
1428630_x_at	0.00	0.00
1428632_at	0.00	0.00
1428633_at	0.00	0.00
1428634_at	0.00	0.00
1428636_at	0.00	0.00
1428637_at	0.00	0.41
1428638_at	0.00	0.00
1428640_at	0.97	0.30
1428641_at	0.00	0.00
1428642_at	0.00	0.00
1428643_at	0.00	0.00
1428644_at	0.00	0.00
1428646_at	0.00	0.00
1428647_at	0.00	0.00
1428650_at	0.00	0.00
1428651_at	0.00	0.02
1428652_at	0.00	0.00
1428653_x_at	0.00	0.00
1428654_at	0.00	0.00
1428656_at	0.00	0.00
1428658_at	0.00	0.09
1428659_at	0.00	0.00
1428660_s_at	0.00	0.00
1428661_at	0.00	0.00
1428663_at	0.00	0.00
1428665_at	0.00	0.00
1428668_at	0.00	0.00
1428670_at	0.00	0.00
1428671_at	0.00	0.00
1428672_at	0.00	0.02
1428673_at	0.00	0.00
1428676_at	0.00	0.00
1428677_at	0.00	0.07
1428678_s_at	0.00	0.00
1428680_at	0.00	0.00
1428681_at	0.00	0.00
1428682_at	0.00	0.00
1428683_at	0.00	0.00
1428685_at	0.00	0.32
1428686_at	0.00	0.00
1428687_at	0.00	0.00
1428688_at	0.00	0.24
1428691_at	0.00	0.00
1428692_at	0.00	0.00
1428693_at	0.00	0.00
1428694_at	0.00	0.00
1428695_at	0.00	0.00
1428696_at	0.00	0.00
1428700_at	0.00	0.00
1428702_at	0.00	0.00
1428703_at	0.00	0.00
1428704_at	0.00	0.00

1428705_at	0.00	0.00
1428711_at	0.00	0.00
1428712_at	0.00	0.00
1428713_s_at	0.00	0.00
1428715_at	0.00	0.00
1428716_at	0.00	0.00
1428717_at	0.00	0.00
1428718_at	0.00	0.00
1428719_at	0.00	0.00
1428720_s_at	0.00	0.00
1428721_at	0.00	0.00
1428723_at	0.00	0.00
1428724_at	0.00	0.00
1428725_at	0.04	0.00
1428726_at	0.00	0.00
1428727_at	0.00	0.13
1428729_at	0.00	0.00
1428730_at	0.00	0.00
1428731_at	0.00	0.00
1428732_at	0.00	0.00
1428733_at	0.00	0.00
1428734_at	0.00	0.00
1428735_at	0.00	0.00
1428738_a_at	0.00	0.00
1428739_at	0.00	0.00
1428743_at	0.00	0.00
1428744_s_at	0.00	0.00
1428746_a_at	0.00	0.00
1428747_at	0.00	0.00
1428748_at	0.00	0.00
1428749_at	0.00	0.00
1428750_at	0.00	0.00
1428754_at	0.00	0.00
1428755_at	0.00	0.00
1428756_at	0.00	0.00
1428757_at	0.00	0.00
1428758_at	0.00	0.00
1428759_s_at	0.00	0.00
1428763_at	0.00	0.00
1428764_at	0.00	0.00
1428765_at	0.00	0.00
1428767_at	0.00	0.00
1428768_at	0.00	0.00
1428769_at	0.00	0.00
1428770_at	0.00	0.00
1428771_at	0.00	0.00
1428773_s_at	0.00	0.00
1428774_at	0.00	0.00
1428775_at	0.12	0.14
1428777_at	0.00	0.00
1428778_at	0.00	0.00
1428779_at	0.00	0.00
1428783_at	0.00	0.00
1428785_at	0.08	0.03
1428791_at	0.00	0.79
1428792_at	0.00	0.00

1428793_at	0.00	0.00
1428795_at	0.00	0.00
1428796_at	0.00	0.01
1428799_at	0.00	0.00
1428800_a_at	0.00	0.00
1428801_at	0.00	0.00
1428802_at	0.00	0.00
1428804_at	0.00	0.00
1428805_at	0.00	0.00
1428806_at	0.00	0.00
1428807_at	0.00	0.00
1428808_at	0.00	0.00
1428809_at	0.00	0.00
1428811_at	0.00	0.00
1428812_at	0.00	0.00
1428813_a_at	0.00	0.00
1428814_at	0.00	0.00
1428815_at	0.00	0.00
1428817_at	0.00	0.00
1428818_at	0.00	0.00
1428821_at	0.00	0.00
1428822_a_at	0.00	0.00
1428824_at	0.00	0.55
1428825_at	0.00	0.29
1428826_at	0.00	0.35
1428827_at	0.00	0.00
1428828_at	0.00	0.00
1428829_at	0.00	0.00
1428830_at	0.00	0.00
1428831_at	0.00	0.00
1428832_at	0.00	0.00
1428833_at	0.00	0.00
1428834_at	0.77	0.17
1428836_at	0.00	0.00
1428837_at	0.00	0.00
1428839_at	0.00	0.00
1428840_s_at	0.00	0.00
1428841_at	0.00	0.00
1428846_at	0.00	0.00
1428851_at	0.00	0.00
1428852_at	0.00	0.00
1428854_at	0.00	0.00
1428857_at	0.00	0.00
1428858_at	0.00	0.00
1428859_at	0.00	0.25
1428860_at	0.00	0.00
1428861_at	0.00	0.00
1428863_at	0.00	0.00
1428864_at	0.00	0.00
1428865_at	0.00	0.00
1428866_at	0.00	0.00
1428867_at	0.00	0.00
1428876_at	0.00	0.00
1428877_at	0.00	0.00
1428883_at	0.00	0.00
1428884_at	0.00	0.00

1428885_at	0.00	0.00
1428886_at	0.00	0.00
1428887_at	0.00	0.00
1428889_at	0.00	0.00
1428890_at	0.00	0.00
1428892_at	0.00	0.15
1428893_at	0.00	0.00
1428894_at	0.00	0.00
1428896_at	0.57	0.00
1428897_at	0.00	0.00
1428898_at	0.00	0.00
1428899_at	0.00	0.00
1428900_s_at	0.00	0.00
1428901_at	0.00	0.00
1428902_at	0.00	0.00
1428903_at	0.00	0.00
1428906_at	0.00	0.00
1428909_at	0.00	0.00
1428910_at	0.00	0.00
1428911_at	0.00	0.00
1428912_at	0.00	0.00
1428913_at	0.00	0.00
1428914_at	0.00	0.00
1428915_at	0.00	0.00
1428916_s_at	0.00	0.00
1428918_at	0.00	0.01
1428919_at	0.00	0.00
1428921_at	0.00	0.00
1428923_at	0.00	0.00
1428924_at	0.00	0.02
1428925_at	0.00	0.00
1428926_at	0.00	0.00
1428927_at	0.00	0.02
1428928_at	0.00	0.00
1428930_at	0.00	0.00
1428932_at	0.00	0.00
1428933_at	0.00	0.00
1428934_at	0.00	0.00
1428936_at	0.00	0.00
1428937_at	0.00	0.00
1428938_at	0.00	0.00
1428939_s_at	0.00	0.00
1428940_at	0.00	0.00
1428941_at	0.00	0.00
1428944_at	0.00	0.00
1428945_at	0.00	0.00
1428946_at	0.00	0.00
1428947_at	0.00	0.00
1428948_at	0.00	0.00
1428949_at	0.00	0.00
1428950_s_at	0.00	0.01
1428951_at	0.01	0.05
1428952_at	0.00	0.00
1428953_at	0.00	0.00
1428956_at	0.00	0.00
1428957_at	0.00	0.00

1428958_at	0.00	0.00
1428959_at	0.00	0.00
1428960_at	0.00	0.00
1428962_at	0.00	0.00
1428963_at	0.00	0.00
1428964_at	0.00	0.00
1428965_at	0.00	0.00
1428967_at	0.13	0.57
1428968_at	0.00	0.00
1428969_at	0.00	0.00
1428970_at	0.00	0.01
1428971_at	0.01	0.00
1428972_at	0.00	0.00
1428973_s_at	0.00	0.00
1428974_s_at	0.00	0.00
1428975_at	0.00	0.00
1428976_at	0.00	0.04
1428977_at	0.00	0.00
1428978_at	0.00	0.00
1428979_at	0.00	0.00
1428980_at	0.00	0.00
1428981_at	0.00	0.00
1428984_a_at	0.00	0.00
1428985_at	0.00	0.00
1428986_at	0.00	0.00
1428987_at	0.00	0.00
1428989_at	0.00	0.00
1428990_at	0.00	0.00
1428991_at	0.00	0.00
1428992_at	0.00	0.00
1428993_at	0.00	0.00
1428994_s_at	0.00	0.00
1428996_at	0.00	0.00
1428998_at	0.00	0.00
1428999_at	0.00	0.00
1429000_at	0.00	0.00
1429001_at	0.01	0.00
1429004_at	0.00	0.00
1429006_s_at	0.00	0.14
1429007_at	0.00	0.06
1429008_at	0.00	0.00
1429009_at	0.00	0.00
1429010_at	0.00	0.00
1429011_x_at	0.00	0.00
1429012_at	0.00	0.00
1429013_at	0.00	0.00
1429017_at	0.00	0.00
1429018_at	0.00	0.00
1429019_s_at	0.00	0.00
1429020_at	0.00	0.00
1429021_at	0.03	0.00
1429022_at	0.00	0.00
1429023_at	0.00	0.00
1429024_at	0.00	0.00
1429025_a_at	0.00	0.00
1429026_at	0.00	0.00

1429027_at	0.00	0.00
1429028_at	0.02	0.00
1429029_at	0.00	0.00
1429030_at	0.00	0.00
1429031_at	0.00	0.00
1429032_at	0.00	0.00
1429034_at	0.00	0.00
1429036_at	0.00	0.00
1429037_at	0.00	0.00
1429042_at	0.00	0.00
1429044_at	0.00	0.00
1429045_at	0.00	0.00
1429046_at	0.00	0.00
1429047_at	0.00	0.00
1429048_at	0.00	0.00
1429049_at	0.00	0.06
1429050_at	0.00	0.00
1429051_s_at	0.00	0.00
1429053_at	0.00	0.00
1429055_at	0.00	0.00
1429056_at	0.00	0.00
1429057_at	0.00	0.00
1429058_at	0.00	0.00
1429059_s_at	0.00	0.00
1429060_at	0.00	0.00
1429064_at	0.00	0.00
1429065_at	0.00	0.11
1429066_at	0.00	0.00
1429067_at	0.00	0.00
1429068_at	0.00	0.00
1429069_at	0.00	0.00
1429070_at	0.00	0.00
1429071_at	0.00	0.00
1429072_at	0.00	0.00
1429073_at	0.00	0.00
1429074_at	0.00	0.00
1429075_a_at	0.00	0.00
1429079_a_at	0.00	0.00
1429081_at	0.00	0.00
1429082_at	0.00	0.00
1429083_at	0.00	0.00
1429084_at	0.00	0.00
1429085_at	0.00	0.27
1429087_at	0.00	0.00
1429088_at	0.00	0.27
1429089_s_at	0.00	0.00
1429091_at	0.00	0.00
1429092_at	0.00	0.00
1429093_at	0.00	0.00
1429094_at	0.00	0.00
1429095_at	0.00	0.02
1429096_at	0.00	0.00
1429097_at	0.00	0.00
1429098_s_at	0.00	0.00
1429099_at	0.00	0.00
1429100_at	0.00	0.00

1429101_at	0.00	0.00
1429102_at	0.00	0.00
1429105_at	0.00	0.00
1429106_at	0.00	0.00
1429107_at	0.00	0.00
1429111_at	0.00	0.00
1429112_at	0.00	0.00
1429113_at	0.00	0.00
1429114_at	0.00	0.00
1429116_at	0.00	0.00
1429118_a_at	0.00	0.00
1429119_at	0.00	0.00
1429120_at	0.00	0.00
1429121_at	0.00	0.00
1429123_at	0.00	0.00
1429125_at	0.00	0.00
1429127_at	0.00	0.00
1429129_at	0.00	0.00
1429130_at	0.00	0.00
1429131_at	0.00	0.00
1429132_at	0.00	0.00
1429133_at	0.00	0.00
1429134_at	0.00	0.00
1429135_at	0.00	0.00
1429136_at	0.00	0.00
1429138_at	0.00	0.00
1429140_at	0.00	0.00
1429141_at	0.00	0.00
1429142_at	0.00	0.00
1429143_at	0.00	0.00
1429145_at	0.00	0.00
1429146_at	0.00	0.00
1429147_at	0.00	0.00
1429148_at	0.00	0.00
1429149_at	0.00	0.00
1429151_at	0.00	0.00
1429152_at	0.00	0.00
1429153_at	0.00	0.00
1429154_at	0.00	0.00
1429155_at	0.00	0.00
1429156_at	0.00	0.00
1429157_at	0.00	0.00
1429158_at	0.00	0.00
1429160_at	0.00	0.00
1429161_at	0.00	0.00
1429162_at	0.00	0.00
1429163_at	0.00	0.00
1429164_at	0.00	0.00
1429165_at	0.00	0.09
1429166_s_at	0.00	0.00
1429167_at	0.00	0.00
1429169_at	0.00	0.00
1429174_at	0.00	0.00
1429175_at	0.00	0.00
1429176_at	0.00	0.00
1429178_at	0.00	0.00

1429179_at	0.00	0.00
1429180_at	0.00	0.00
1429181_at	0.00	0.00
1429182_at	0.00	0.00
1429184_at	0.00	0.00
1429185_at	0.00	0.00
1429187_at	0.00	0.00
1429188_at	0.00	0.00
1429189_at	0.00	0.00
1429190_at	0.00	0.00
1429191_at	0.00	0.00
1429192_at	0.00	0.00
1429194_at	0.00	0.00
1429195_at	0.00	0.00
1429196_at	0.00	0.00
1429197_s_at	0.00	0.00
1429201_at	0.00	0.00
1429202_at	0.00	0.00
1429203_at	0.00	0.00
1429204_at	0.00	0.00
1429205_at	0.00	0.00
1429206_at	0.00	0.00
1429209_at	0.00	0.00
1429210_at	0.00	0.00
1429211_at	0.00	0.00
1429213_at	0.00	0.05
1429214_at	0.00	0.00
1429215_at	0.00	0.00
1429216_at	0.00	0.00
1429217_at	0.00	0.00
1429218_at	0.00	0.00
1429223_a_at	0.00	0.00
1429224_at	0.00	0.00
1429225_at	0.00	0.00
1429226_at	0.00	0.00
1429228_at	0.00	0.00
1429229_s_at	0.00	0.00
1429230_at	0.00	0.00
1429231_at	0.00	0.00
1429232_at	0.00	0.00
1429233_at	0.00	0.29
1429234_s_at	0.00	0.44
1429235_at	0.00	0.00
1429236_at	0.00	0.00
1429237_at	0.00	0.00
1429241_at	0.00	0.00
1429242_at	0.00	0.00
1429243_at	0.00	0.00
1429245_at	0.00	0.00
1429248_at	0.00	0.00
1429249_at	0.00	0.00
1429250_at	0.00	0.00
1429251_at	0.00	0.00
1429254_at	0.00	0.00
1429255_at	0.00	0.00
1429256_at	0.00	0.00

1429257_at	0.00	0.00
1429258_at	0.00	0.00
1429259_a_at	0.00	0.00
1429260_at	0.00	0.00
1429261_at	0.00	0.00
1429262_at	0.00	0.01
1429263_at	0.00	0.00
1429264_at	0.00	0.00
1429266_at	0.00	0.00
1429267_at	0.00	0.00
1429268_at	0.04	0.32
1429269_at	0.00	0.00
1429271_at	0.00	0.00
1429272_a_at	0.00	0.00
1429273_at	0.00	0.00
1429274_at	0.00	0.00
1429275_at	0.00	0.00
1429276_at	0.00	0.00
1429277_at	0.00	0.00
1429278_at	0.00	0.00
1429279_at	0.00	0.00
1429281_at	0.00	0.00
1429282_at	0.00	0.00
1429283_at	0.00	0.00
1429284_at	0.00	0.00
1429285_at	0.00	0.00
1429286_at	0.00	0.00
1429289_at	0.00	0.00
1429290_at	0.00	0.00
1429293_at	0.00	0.00
1429297_at	0.00	0.00
1429298_at	0.00	0.00
1429299_at	0.00	0.00
1429300_at	0.00	0.00
1429304_at	0.00	0.00
1429305_at	0.00	0.00
1429306_at	0.00	0.00
1429307_s_at	0.00	0.00
1429308_at	0.00	0.00
1429309_at	0.00	0.00
1429310_at	0.00	0.03
1429311_at	0.00	0.00
1429312_s_at	0.00	0.00
1429313_at	0.00	0.00
1429314_at	0.00	0.00
1429315_at	0.00	0.00
1429316_at	0.00	0.00
1429320_at	0.00	0.00
1429322_at	0.00	0.00
1429323_at	0.00	0.00
1429324_at	0.00	0.00
1429325_at	0.00	0.00
1429326_at	0.00	0.00
1429327_at	0.00	0.00
1429329_at	0.00	0.00
1429330_at	0.00	0.00

1429331_at	0.00	0.00
1429332_at	0.00	0.00
1429333_at	0.00	0.00
1429334_at	0.00	0.00
1429335_at	0.00	0.00
1429336_at	0.00	0.00
1429337_at	0.00	0.01
1429338_a_at	0.00	0.02
1429340_at	0.00	0.00
1429341_at	0.00	0.00
1429342_s_at	0.00	0.00
1429343_at	0.99	0.84
1429344_at	0.00	0.00
1429345_at	0.00	0.00
1429346_a_at	0.00	0.00
1429348_at	0.00	0.00
1429350_at	0.00	0.00
1429351_at	0.00	0.00
1429353_a_at	0.00	0.00
1429354_at	0.00	0.00
1429355_at	0.00	0.00
1429357_at	0.00	0.00
1429358_at	0.00	0.00
1429361_at	0.00	0.00
1429362_a_at	0.00	0.00
1429363_at	0.00	0.00
1429364_at	0.00	0.00
1429365_at	0.00	0.00
1429366_at	0.99	0.00
1429368_at	0.00	0.00
1429371_at	0.00	0.00
1429372_at	0.00	0.00
1429373_x_at	0.00	0.00
1429374_at	0.00	0.00
1429375_at	0.00	0.00
1429376_s_at	0.00	0.00
1429377_at	0.23	0.00
1429378_x_at	0.00	0.00
1429380_at	0.00	0.00
1429383_at	0.00	0.00
1429384_at	0.00	0.00
1429385_at	0.00	0.01
1429386_at	0.00	0.00
1429387_at	0.00	0.00
1429389_at	0.00	0.00
1429390_at	0.00	0.00
1429391_at	0.00	0.00
1429392_at	0.00	0.00
1429393_at	0.00	0.00
1429394_at	0.00	0.00
1429395_at	0.00	0.00
1429396_at	0.00	0.00
1429397_a_at	0.00	0.00
1429398_at	0.00	0.00
1429399_at	1.00	0.32
1429401_at	0.00	0.00

1429402_at	0.00	0.00
1429403_x_at	0.00	0.00
1429404_at	0.00	0.00
1429405_at	0.00	0.00
1429406_at	0.00	0.00
1429407_at	0.00	0.00
1429408_at	0.00	0.00
1429409_at	0.00	0.00
1429413_at	0.00	0.16
1429414_at	0.00	0.00
1429415_at	0.00	0.00
1429416_at	0.00	0.00
1429417_at	0.00	0.00
1429418_at	0.00	0.00
1429419_at	0.00	0.00
1429420_at	0.00	0.00
1429421_at	0.00	0.00
1429422_at	0.00	0.00
1429423_at	0.00	0.00
1429424_at	0.00	0.00
1429425_at	0.10	0.00
1429426_at	0.00	0.00
1429429_s_at	0.00	0.00
1429430_at	0.00	0.00
1429431_at	0.00	0.00
1429432_at	0.00	0.00
1429433_at	0.00	0.00
1429434_at	0.00	0.00
1429435_x_at	0.00	0.00
1429436_at	0.00	0.00
1429437_at	0.00	0.00
1429438_at	0.00	0.00
1429440_at	0.00	0.00
1429441_at	0.00	0.00
1429442_at	0.00	0.00
1429443_at	0.00	0.00
1429444_at	0.00	0.00
1429445_at	0.00	0.00
1429446_at	0.00	0.00
1429447_at	0.00	0.00
1429448_s_at	1.00	0.00
1429449_at	0.00	0.00
1429450_at	0.00	0.00
1429452_x_at	0.00	0.00
1429454_at	0.00	0.00
1429455_at	0.00	0.00
1429458_at	0.04	0.00
1429459_at	0.00	0.00
1429460_at	0.00	0.00
1429461_at	0.00	0.00
1429462_at	0.00	0.00
1429463_at	0.00	0.02
1429464_at	0.00	0.00
1429465_at	0.00	0.00
1429466_s_at	0.00	0.00
1429467_s_at	0.00	0.00

1429468_at	0.00	0.00
1429469_at	0.00	0.00
1429470_at	0.00	0.00
1429471_at	0.00	0.00
1429472_at	0.00	0.00
1429475_at	0.00	0.00
1429476_s_at	0.00	0.00
1429477_at	0.00	0.00
1429478_at	0.00	0.00
1429479_at	0.00	0.00
1429480_at	0.00	0.00
1429481_at	0.00	0.00
1429482_at	0.00	0.00
1429484_at	0.00	0.00
1429486_at	0.00	0.00
1429487_at	0.00	0.00
1429488_at	0.00	0.00
1429489_at	0.00	0.00
1429493_at	0.00	0.00
1429494_at	0.00	0.00
1429495_at	0.00	0.00
1429496_x_at	0.00	0.00
1429498_at	0.00	0.00
1429499_at	0.01	0.00
1429500_at	0.00	0.00
1429501_s_at	0.00	0.00
1429502_at	0.00	0.00
1429503_at	0.00	0.00
1429504_at	0.00	0.00
1429505_at	0.00	0.02
1429506_at	0.00	0.00
1429507_at	0.00	0.00
1429508_at	0.00	0.00
1429509_at	0.00	0.00
1429510_at	0.00	0.00
1429511_at	0.00	0.00
1429512_at	0.00	0.00
1429513_at	0.00	0.00
1429515_at	0.00	0.00
1429516_at	0.00	0.00
1429517_at	0.00	0.00
1429518_at	0.00	0.00
1429519_at	0.00	0.00
1429520_a_at	0.01	0.00
1429521_at	0.01	0.00
1429522_at	0.00	0.00
1429523_a_at	0.00	0.00
1429524_at	0.04	0.31
1429525_s_at	0.00	0.32
1429526_at	0.00	0.00
1429529_at	0.00	0.00
1429532_at	0.00	0.07
1429535_at	0.00	0.01
1429537_at	0.00	0.00
1429538_a_at	0.00	0.00
1429539_at	0.00	0.00

1429540_at	0.00	0.00
1429542_at	0.00	0.00
1429543_at	0.00	0.00
1429544_at	0.00	0.00
1429545_at	0.00	0.00
1429546_at	0.00	0.00
1429547_at	0.00	0.00
1429548_at	0.00	0.00
1429549_at	0.00	0.00
1429550_at	0.00	0.00
1429551_at	0.00	0.00
1429552_at	0.00	0.00
1429556_at	0.00	0.03
1429557_at	0.00	0.00
1429559_at	0.00	0.00
1429561_at	0.00	0.00
1429564_at	0.00	0.00
1429565_s_at	0.00	0.00
1429567_at	0.00	0.00
1429570_at	0.00	0.00
1429571_a_at	0.00	0.00
1429572_at	0.00	0.00
1429573_at	0.00	0.00
1429575_at	0.00	0.00
1429576_at	0.00	0.00
1429577_at	0.00	0.00
1429578_at	0.00	0.00
1429579_at	0.00	0.00
1429580_x_at	0.00	0.00
1429586_at	0.00	0.00
1429587_at	0.00	0.00
1429588_at	0.00	0.39
1429589_at	0.00	0.00
1429590_at	0.00	0.00
1429591_at	0.00	0.00
1429592_at	0.00	0.00
1429593_at	0.00	0.00
1429594_at	0.00	0.00
1429595_at	0.00	0.00
1429596_at	0.00	0.00
1429598_at	0.00	0.00
1429599_a_at	0.00	0.00
1429600_at	0.00	0.00
1429601_x_at	0.00	0.00
1429602_at	0.00	0.00
1429603_at	0.00	0.00
1429604_at	0.00	0.00
1429605_at	0.00	0.00
1429606_at	0.00	0.00
1429607_at	0.00	0.00
1429608_at	0.00	0.00
1429609_at	0.00	0.00
1429610_a_at	0.00	0.00
1429611_at	0.00	0.00
1429612_at	0.00	0.00
1429613_at	0.00	0.00

1429614_at	0.00	0.00
1429617_at	0.00	0.00
1429618_at	0.00	0.00
1429621_at	0.00	0.00
1429622_at	0.00	0.00
1429624_at	0.00	0.00
1429625_at	0.00	0.00
1429627_at	0.00	0.00
1429628_at	0.00	0.00
1429629_at	0.00	0.00
1429630_at	0.00	0.00
1429631_at	0.00	0.00
1429633_at	0.00	0.00
1429634_at	0.00	0.00
1429635_at	0.00	0.00
1429636_at	0.00	0.00
1429637_at	0.00	0.00
1429639_at	0.00	0.00
1429640_at	0.00	0.00
1429641_x_at	0.00	0.00
1429642_at	0.00	0.00
1429644_at	0.00	0.00
1429645_at	0.00	0.00
1429646_at	0.00	0.00
1429647_at	0.00	0.00
1429648_at	0.00	0.00
1429649_at	0.00	0.00
1429651_at	0.00	0.00
1429652_at	0.00	0.00
1429653_at	0.00	0.00
1429656_at	0.00	0.00
1429657_at	0.00	0.00
1429658_a_at	0.00	0.00
1429659_at	0.00	0.00
1429660_s_at	0.00	0.00
1429661_at	0.00	0.00
1429662_at	0.00	0.00
1429663_at	0.00	0.00
1429664_at	0.00	0.00
1429665_at	0.00	0.00
1429666_at	0.00	0.00
1429667_at	0.00	0.00
1429668_at	0.00	0.00
1429669_at	0.00	0.00
1429670_a_at	0.00	0.00
1429671_at	0.13	0.34
1429672_at	0.00	0.00
1429673_at	0.00	0.00
1429674_at	0.00	0.00
1429675_at	0.00	0.00
1429676_at	0.00	0.00
1429677_at	0.00	0.00
1429678_at	0.00	0.00
1429679_at	0.00	0.00
1429680_at	0.00	0.00
1429682_at	0.00	0.00

1429683_at	0.00	0.00
1429684_at	0.00	0.00
1429685_at	0.00	0.00
1429686_at	0.00	0.00
1429687_at	0.00	0.00
1429688_at	0.00	0.00
1429689_at	0.00	0.00
1429690_at	0.00	0.00
1429691_at	0.00	0.00
1429693_at	0.00	0.00
1429694_at	0.00	0.00
1429695_at	0.00	0.00
1429696_at	0.00	0.00
1429697_at	0.00	0.00
1429698_at	0.00	0.00
1429699_at	0.00	0.00
1429700_at	0.00	0.00
1429701_at	0.00	0.00
1429702_at	0.00	0.00
1429703_at	0.00	0.00
1429704_at	0.00	0.00
1429705_at	0.00	0.00
1429706_at	0.00	0.00
1429712_at	0.00	0.00
1429713_at	0.00	0.00
1429714_at	0.00	0.00
1429716_at	0.00	0.00
1429717_at	0.00	0.00
1429718_at	0.00	0.00
1429719_at	0.00	0.00
1429720_at	0.00	0.00
1429722_at	0.00	0.00
1429724_at	0.00	0.00
1429725_at	0.00	0.00
1429726_at	0.00	0.00
1429727_at	0.00	0.00
1429728_at	0.00	0.27
1429729_at	0.00	0.00
1429731_at	0.00	0.00
1429732_at	0.00	0.00
1429733_at	0.00	0.00
1429734_at	0.00	0.00
1429735_at	0.00	0.00
1429736_at	0.00	0.00
1429737_a_at	0.00	0.00
1429738_at	0.00	0.00
1429740_at	0.00	0.00
1429741_at	0.00	0.00
1429742_at	0.00	0.00
1429743_at	0.00	0.00
1429744_at	0.00	0.00
1429746_at	0.00	0.00
1429747_at	0.00	0.00
1429749_at	0.00	0.00
1429750_at	0.00	0.00
1429751_at	0.00	0.00

1429753_at	0.00	0.00
1429754_a_at	0.00	0.00
1429755_a_at	0.00	0.00
1429756_at	0.00	0.00
1429757_at	0.00	0.00
1429759_at	0.00	0.00
1429760_at	0.00	0.00
1429762_a_at	0.00	0.00
1429764_at	0.00	0.00
1429765_at	0.00	0.00
1429766_at	0.00	0.00
1429767_at	0.00	0.00
1429769_at	0.01	0.00
1429770_at	0.00	0.00
1429771_at	0.00	0.00
1429772_at	0.00	0.00
1429773_at	0.00	0.00
1429774_a_at	0.00	0.00
1429778_at	0.00	0.09
1429779_at	0.00	0.00
1429780_at	0.00	0.00
1429781_s_at	0.00	0.00
1429784_at	0.00	0.00
1429785_at	0.00	0.00
1429788_at	0.00	0.00
1429789_at	0.00	0.00
1429790_at	0.00	0.00
1429791_at	0.00	0.00
1429792_at	0.00	0.00
1429793_at	0.00	0.00
1429795_at	0.00	0.00
1429796_at	0.00	0.00
1429797_at	0.00	0.00
1429798_s_at	0.00	0.00
1429799_at	0.00	0.00
1429801_at	0.00	0.00
1429802_at	1.00	1.00
1429803_at	0.00	0.00
1429804_at	0.00	0.00
1429805_at	0.00	0.00
1429807_at	0.00	0.00
1429808_at	0.00	0.00
1429809_at	0.00	0.00
1429810_at	0.00	0.00
1429811_at	0.00	0.00
1429812_at	0.00	0.00
1429813_at	0.00	0.00
1429814_at	0.00	0.00
1429815_at	0.00	0.00
1429816_at	0.00	0.00
1429817_at	0.00	0.00
1429818_at	0.00	0.00
1429820_at	0.00	0.00
1429821_at	0.00	0.00
1429822_at	0.00	0.00
1429823_at	0.00	0.00

1429824_at	0.00	0.00
1429825_at	0.00	0.00
1429826_at	0.00	0.00
1429827_at	0.00	0.00
1429828_at	0.00	0.00
1429829_at	0.00	0.00
1429831_at	0.00	0.00
1429833_at	0.00	0.00
1429834_a_at	0.00	0.00
1429836_at	0.00	0.00
1429837_at	0.00	0.00
1429838_at	0.00	0.00
1429840_at	0.00	0.00
1429841_at	0.00	0.00
1429842_at	0.00	0.00
1429843_at	0.00	0.00
1429844_at	0.00	0.00
1429846_at	0.00	0.00
1429847_a_at	0.00	0.00
1429849_at	0.00	0.00
1429851_at	0.00	0.00
1429852_at	0.00	0.00
1429853_at	0.00	0.00
1429854_at	0.00	0.00
1429855_at	0.00	0.00
1429856_at	0.00	0.00
1429857_at	0.00	0.00
1429858_at	0.00	0.00
1429860_at	0.00	0.00
1429861_at	0.00	0.00
1429862_at	0.00	0.00
1429863_at	0.00	0.00
1429864_at	0.00	0.00
1429865_at	0.00	0.00
1429867_at	0.00	0.00
1429868_at	0.00	0.00
1429869_at	0.00	0.00
1429870_at	0.00	0.00
1429871_at	0.00	0.00
1429872_at	0.00	0.00
1429873_at	0.00	0.00
1429874_at	0.00	0.00
1429875_at	0.00	0.00
1429876_at	0.00	0.00
1429877_at	0.00	0.00
1429879_at	0.00	0.00
1429880_at	0.00	0.00
1429881_at	0.00	0.00
1429882_at	0.00	0.00
1429883_at	0.00	0.00
1429885_at	0.00	0.00
1429886_at	0.00	0.00
1429887_at	0.00	0.00
1429889_at	0.00	0.00
1429890_at	0.00	0.00
1429891_at	0.11	0.00

1429892_at	0.00	0.00
1429893_at	0.00	0.00
1429895_at	0.00	0.00
1429896_at	0.00	0.00
1429898_at	0.00	0.00
1429899_at	0.00	0.00
1429900_at	0.00	0.00
1429901_at	0.00	0.00
1429902_at	0.00	0.00
1429903_at	0.00	0.00
1429904_at	0.00	0.00
1429905_at	0.00	0.00
1429906_at	0.00	0.00
1429909_at	0.00	0.00
1429910_at	0.00	0.00
1429911_at	0.00	0.00
1429912_at	0.00	0.00
1429913_at	0.00	0.00
1429914_at	0.00	0.00
1429915_at	0.00	0.00
1429916_at	0.00	0.00
1429917_at	0.00	0.00
1429918_at	0.00	0.00
1429919_at	0.00	0.00
1429920_at	0.00	0.00
1429922_at	0.00	0.00
1429923_x_at	0.00	0.00
1429924_at	0.00	0.00
1429925_at	0.00	0.00
1429926_at	0.00	0.00
1429927_at	0.00	0.00
1429928_at	0.00	0.00
1429929_at	0.00	0.00
1429930_at	0.00	0.00
1429931_at	0.00	0.00
1429932_at	0.00	0.00
1429933_at	0.00	0.00
1429934_at	0.00	0.00
1429935_at	0.00	0.00
1429936_at	0.00	0.00
1429937_at	0.00	0.00
1429938_at	0.00	0.00
1429940_at	0.00	0.00
1429941_at	0.00	0.00
1429942_at	0.00	0.00
1429943_at	0.00	0.00
1429944_at	0.00	0.00
1429945_at	0.00	0.00
1429946_at	0.00	0.00
1429949_at	0.00	0.00
1429950_at	0.00	0.00
1429951_at	0.00	0.00
1429952_at	0.00	0.00
1429953_at	0.00	0.00
1429954_at	0.00	0.00
1429955_at	0.00	0.00

1429957_at	0.00	0.00
1429958_x_at	0.00	0.00
1429959_at	0.00	0.00
1429960_at	0.00	0.00
1429961_at	0.00	0.00
1429963_at	0.00	0.00
1429964_at	0.00	0.00
1429965_at	0.00	0.00
1429966_at	0.00	0.00
1429967_at	0.00	0.00
1429968_at	0.00	0.00
1429969_at	0.00	0.00
1429970_at	0.00	0.00
1429971_at	0.00	0.00
1429972_s_at	0.00	0.00
1429973_at	0.00	0.00
1429974_at	0.00	0.00
1429975_at	0.00	0.00
1429977_at	0.00	0.00
1429978_at	0.00	0.00
1429983_at	0.00	0.00
1429984_at	0.00	0.00
1429985_at	0.00	0.00
1429986_at	0.00	0.00
1429987_at	0.00	0.00
1429988_at	0.00	0.00
1429989_at	0.00	0.00
1429990_at	0.00	0.00
1429991_at	0.00	0.00
1429992_at	0.00	0.00
1429993_s_at	0.00	0.00
1429995_at	0.00	0.00
1429996_at	0.00	0.00
1429997_at	0.00	0.00
1429998_at	0.00	0.00
1430000_at	0.00	0.00
1430001_at	0.00	0.00
1430002_at	0.00	0.00
1430003_at	0.00	0.00
1430004_s_at	0.00	0.00
1430006_x_at	0.00	0.00
1430008_x_at	0.00	0.00
1430009_at	0.00	0.00
1430010_at	0.00	0.00
1430011_at	0.00	0.00
1430012_at	0.00	0.00
1430013_at	0.00	0.00
1430014_at	0.00	0.00
1430015_at	0.00	0.00
1430016_at	0.00	0.00
1430017_at	0.00	0.00
1430023_at	0.00	0.00
1430024_at	0.00	0.00
1430026_at	0.00	0.00
1430027_at	0.00	0.00
1430028_at	0.00	0.00

1430030_at	0.00	0.00
1430031_at	0.00	0.00
1430033_at	0.00	0.00
1430034_at	0.00	0.00
1430035_at	0.00	0.00
1430036_at	0.00	0.00
1430037_at	0.00	0.01
1430038_at	0.00	0.00
1430039_at	0.00	0.00
1430040_at	0.00	0.00
1430041_at	0.00	0.00
1430042_at	0.00	0.00
1430043_at	0.00	0.00
1430044_at	0.00	0.00
1430046_at	0.00	0.00
1430047_at	0.00	0.00
1430048_at	0.00	0.00
1430049_at	0.00	0.00
1430050_at	0.00	0.00
1430051_at	0.00	0.00
1430052_at	0.00	0.00
1430054_at	0.00	0.00
1430055_at	0.00	0.00
1430056_at	0.00	0.00
1430057_s_at	0.00	0.00
1430058_at	0.00	0.00
1430059_at	0.00	0.00
1430060_at	0.00	0.00
1430061_at	0.00	0.00
1430062_at	0.00	0.00
1430063_at	0.00	0.00
1430064_at	0.00	0.00
1430065_at	0.00	0.00
1430066_at	0.00	0.00
1430067_at	0.00	0.00
1430068_at	0.00	0.00
1430069_at	0.00	0.00
1430070_at	0.00	0.00
1430071_at	0.00	0.00
1430072_at	0.00	0.00
1430073_at	0.00	0.00
1430074_x_at	0.00	0.00
1430075_at	0.00	0.00
1430076_at	0.00	0.00
1430077_at	0.00	0.00
1430079_at	0.00	0.00
1430080_at	0.00	0.00
1430081_at	0.00	0.00
1430082_at	0.00	0.00
1430083_at	0.00	0.00
1430084_at	0.00	0.00
1430085_at	0.00	0.00
1430086_at	0.00	0.00
1430087_at	0.00	0.00
1430088_at	0.00	0.00
1430089_at	0.00	0.00

1430090_at	0.00	0.00
1430091_at	0.00	0.00
1430093_at	0.00	0.00
1430094_at	0.00	0.00
1430095_at	0.00	0.00
1430096_at	0.00	0.00
1430097_at	0.00	0.00
1430098_at	0.00	0.00
1430099_at	0.00	0.00
1430100_at	0.00	0.00
1430101_at	0.00	0.00
1430102_at	0.00	0.00
1430103_at	0.00	0.00
1430104_at	0.00	0.00
1430105_at	0.00	0.00
1430106_at	0.00	0.00
1430107_at	0.00	0.00
1430108_at	0.00	0.00
1430109_at	0.00	0.00
1430110_at	0.00	0.00
1430112_at	0.00	0.00
1430113_at	0.00	0.00
1430114_at	0.00	0.00
1430115_at	0.00	0.00
1430116_at	0.00	0.00
1430118_at	0.00	0.00
1430119_at	0.00	0.00
1430120_at	0.00	0.00
1430121_at	0.00	0.00
1430122_at	0.00	0.00
1430126_at	0.00	0.00
1430129_a_at	0.00	0.00
1430130_at	0.00	0.00
1430131_at	0.00	0.00
1430132_at	0.00	0.00
1430133_at	0.00	0.00
1430134_a_at	0.00	0.02
1430135_at	0.00	0.00
1430136_at	0.00	0.00
1430137_at	0.00	0.00
1430138_at	0.00	0.04
1430139_at	0.03	0.00
1430140_at	0.00	0.00
1430141_at	0.00	0.00
1430142_at	0.00	0.00
1430143_at	0.00	0.00
1430144_at	0.00	0.00
1430145_at	0.00	0.00
1430146_at	0.00	0.00
1430148_at	0.00	0.00
1430149_at	0.00	0.00
1430150_at	0.00	0.00
1430151_at	0.00	0.00
1430152_at	0.00	0.00
1430154_at	0.00	0.00
1430155_at	0.00	0.00

1430156_at	0.00	0.00
1430157_at	0.00	0.00
1430158_at	0.00	0.00
1430159_at	0.00	0.00
1430160_at	0.00	0.00
1430161_at	0.00	0.00
1430162_at	0.00	0.00
1430163_at	0.00	0.00
1430165_at	0.00	0.00
1430166_at	0.00	0.00
1430168_at	0.00	0.00
1430169_at	0.00	0.00
1430170_at	0.00	0.00
1430174_at	0.00	0.00
1430175_at	0.00	0.00
1430176_at	0.00	0.00
1430177_at	0.00	0.00
1430178_at	0.00	0.00
1430179_at	0.00	0.00
1430180_at	0.00	0.00
1430181_at	0.00	0.00
1430182_a_at	0.00	0.00
1430183_at	0.00	0.00
1430184_at	0.00	0.00
1430185_at	0.00	0.00
1430186_at	0.00	0.00
1430187_at	0.00	0.00
1430188_at	0.00	0.00
1430189_at	0.00	0.00
1430190_at	0.00	0.00
1430191_at	0.00	0.00
1430192_at	0.00	0.00
1430193_at	0.00	0.00
1430194_at	0.00	0.00
1430196_at	0.00	0.00
1430198_at	0.00	0.00
1430199_at	0.00	0.00
1430200_at	0.00	0.00
1430201_at	0.00	0.00
1430202_at	0.00	0.00
1430203_at	0.00	0.00
1430204_at	0.00	0.00
1430206_at	0.00	0.00
1430207_at	0.00	0.00
1430208_at	0.63	0.45
1430209_at	0.00	0.00
1430210_at	0.00	0.00
1430211_at	0.00	0.00
1430212_at	0.00	0.00
1430213_at	0.00	0.00
1430214_a_at	0.00	0.00
1430215_at	0.00	0.00
1430216_at	0.00	0.00
1430217_at	0.00	0.00
1430218_at	0.00	0.00
1430220_at	0.00	0.00

1430221_at	0.00	0.00
1430222_at	0.00	0.00
1430223_at	0.00	0.00
1430224_at	0.00	0.00
1430225_at	0.00	0.00
1430226_at	0.00	0.00
1430227_at	0.00	0.00
1430228_at	0.00	0.00
1430229_at	0.00	0.00
1430230_at	0.00	0.00
1430232_at	0.00	0.00
1430234_at	0.00	0.00
1430235_at	0.00	0.00
1430236_s_at	0.00	0.00
1430237_at	0.00	0.00
1430238_at	0.00	0.00
1430239_at	0.00	0.00
1430241_at	0.00	0.00
1430242_at	0.00	0.00
1430243_at	0.00	0.00
1430244_at	0.00	0.00
1430245_at	0.00	0.00
1430246_at	0.00	0.00
1430247_at	0.00	0.00
1430248_at	0.00	0.00
1430249_at	0.00	0.00
1430250_at	0.00	0.00
1430251_at	0.00	0.00
1430252_at	0.00	0.00
1430253_at	0.00	0.00
1430254_at	0.00	0.00
1430255_at	0.00	0.00
1430256_at	0.00	0.00
1430257_at	0.00	0.00
1430258_at	0.00	0.00
1430260_at	0.00	0.00
1430261_at	0.00	0.00
1430262_at	0.00	0.00
1430263_at	0.00	0.00
1430264_at	0.00	0.00
1430266_at	0.00	0.00
1430267_at	0.00	0.00
1430268_at	0.00	0.00
1430269_at	0.00	0.00
1430270_at	0.00	0.00
1430272_at	0.00	0.00
1430273_at	0.00	0.00
1430276_at	0.00	0.00
1430277_at	0.00	0.00
1430279_at	0.00	0.00
1430280_at	0.00	0.00
1430281_at	0.00	0.00
1430282_at	0.00	0.00
1430284_at	0.00	0.00
1430285_at	0.00	0.00
1430286_s_at	0.00	0.00

1430287_s_at	0.00	0.00
1430288_x_at	0.00	0.00
1430294_at	0.00	0.00
1430296_at	0.00	0.00
1430298_at	0.00	0.00
1430299_at	0.00	0.00
1430300_at	0.00	0.00
1430301_at	0.00	0.00
1430302_at	0.00	0.00
1430303_at	0.00	0.00
1430304_at	0.00	0.00
1430305_at	0.00	0.00
1430308_at	0.00	0.00
1430309_at	0.00	0.00
1430310_at	0.00	0.00
1430311_at	0.00	0.00
1430312_at	0.00	0.00
1430313_at	0.00	0.00
1430314_at	0.00	0.00
1430315_at	0.00	0.00
1430316_at	0.00	0.00
1430317_at	0.00	0.00
1430318_at	0.00	0.00
1430319_at	0.00	0.00
1430321_at	0.00	0.00
1430322_at	0.00	0.00
1430323_at	0.00	0.00
1430324_x_at	0.00	0.00
1430325_at	0.00	0.00
1430327_at	0.00	0.00
1430328_at	0.00	0.00
1430329_at	0.00	0.00
1430330_at	0.00	0.00
1430331_at	0.00	0.00
1430333_at	0.00	0.00
1430334_at	0.00	0.00
1430336_at	0.00	0.00
1430337_at	0.00	0.00
1430338_at	0.00	0.00
1430339_at	0.00	0.00
1430340_at	0.00	0.00
1430341_at	0.00	0.00
1430342_at	0.00	0.00
1430343_at	0.00	0.00
1430344_at	0.00	0.00
1430345_at	0.00	0.00
1430346_at	0.00	0.00
1430347_at	0.00	0.00
1430348_at	0.00	0.00
1430349_at	0.00	0.00
1430350_at	0.00	0.00
1430351_at	0.00	0.00
1430352_at	0.00	0.00
1430353_at	0.00	0.00
1430356_at	0.00	0.00
1430357_at	0.00	0.00

1430358_at	0.00	0.00
1430359_a_at	0.00	0.00
1430360_at	0.00	0.00
1430361_at	0.00	0.00
1430362_at	0.00	0.00
1430363_at	0.00	0.00
1430364_at	0.00	0.00
1430365_at	0.00	0.00
1430366_at	0.00	0.00
1430367_at	0.00	0.00
1430368_s_at	1.00	0.32
1430369_at	0.00	0.00
1430370_at	0.00	0.00
1430372_at	0.00	0.00
1430373_at	0.00	0.00
1430374_at	0.00	0.00
1430376_at	0.00	0.00
1430377_at	0.00	0.00
1430378_at	0.00	0.00
1430379_at	0.00	0.00
1430380_at	0.00	0.00
1430381_at	0.00	0.00
1430382_at	0.00	0.00
1430383_at	0.00	0.00
1430384_at	0.00	0.00
1430386_at	0.00	0.00
1430387_at	0.00	0.00
1430389_at	0.00	0.00
1430390_x_at	0.00	0.00
1430392_at	0.00	0.00
1430393_at	0.00	0.00
1430395_at	0.00	0.00
1430396_at	0.00	0.00
1430397_at	0.00	0.00
1430398_at	0.00	0.00
1430399_at	0.00	0.00
1430400_at	0.00	0.00
1430401_at	0.00	0.00
1430402_at	0.00	0.00
1430403_at	0.00	0.00
1430404_at	0.00	0.00
1430405_at	0.00	0.00
1430407_at	0.00	0.00
1430408_at	0.00	0.00
1430409_at	0.00	0.00
1430410_at	0.00	0.00
1430411_at	0.00	0.00
1430412_at	0.00	0.00
1430413_at	0.00	0.00
1430414_at	0.00	0.00
1430415_at	0.00	0.00
1430416_at	0.00	0.00
1430418_at	0.00	0.00
1430420_at	0.00	0.00
1430422_at	0.00	0.00
1430423_s_at	0.00	0.02

1430424_at	0.00	0.00
1430425_at	0.00	0.00
1430426_at	0.00	0.00
1430428_at	0.00	0.00
1430429_at	0.00	0.00
1430430_at	0.00	0.00
1430431_at	0.00	0.00
1430432_at	0.00	0.00
1430433_at	0.00	0.00
1430434_at	0.00	0.00
1430435_at	0.00	0.00
1430436_at	0.00	0.00
1430438_at	0.00	0.00
1430439_at	0.00	0.00
1430440_at	0.00	0.00
1430441_at	0.00	0.00
1430442_at	0.00	0.00
1430444_at	0.00	0.00
1430445_at	0.00	0.00
1430446_at	0.00	0.00
1430448_at	0.00	0.00
1430449_at	0.00	0.00
1430450_at	0.00	0.00
1430451_at	0.00	0.00
1430452_at	0.00	0.00
1430455_at	0.00	0.00
1430456_at	0.00	0.00
1430458_at	0.00	0.00
1430459_at	0.00	0.00
1430460_at	0.00	0.00
1430461_at	0.00	0.00
1430462_at	0.00	0.00
1430464_at	0.00	0.00
1430465_at	0.00	0.00
1430466_at	0.00	0.00
1430467_at	0.00	0.00
1430468_at	0.00	0.00
1430469_at	0.00	0.00
1430470_at	0.00	0.00
1430471_at	0.00	0.00
1430472_at	0.00	0.00
1430473_at	0.00	0.00
1430475_at	0.00	0.00
1430476_at	0.00	0.00
1430477_s_at	0.00	0.00
1430478_at	0.00	0.00
1430479_at	0.00	0.00
1430480_at	0.00	0.00
1430481_at	0.00	0.00
1430482_at	0.00	0.00
1430484_at	0.00	0.00
1430485_at	0.00	0.00
1430488_at	0.00	0.00
1430489_at	0.00	0.00
1430490_at	0.00	0.00
1430491_at	0.00	0.00

1430493_at	0.00	0.00
1430494_at	0.00	0.00
1430495_at	0.00	0.00
1430496_at	0.00	0.00
1430497_at	0.00	0.00
1430498_at	0.00	0.00
1430499_at	0.00	0.00
1430501_at	0.00	0.00
1430502_at	0.00	0.00
1430503_at	0.00	0.00
1430504_at	0.00	0.00
1430505_at	0.00	0.00
1430506_at	0.00	0.00
1430507_at	0.00	0.00
1430508_at	0.00	0.00
1430510_at	0.00	0.00
1430511_at	0.00	0.00
1430513_at	0.00	0.00
1430515_s_at	0.00	0.13
1430516_at	0.00	0.00
1430517_at	0.00	0.00
1430518_at	0.00	0.00
1430520_at	0.00	0.00
1430521_s_at	0.00	0.00
1430524_at	0.00	0.00
1430525_at	0.00	0.00
1430528_at	0.00	0.00
1430529_at	0.00	0.00
1430530_s_at	0.00	0.00
1430531_at	0.00	0.00
1430532_at	0.00	0.00
1430534_at	0.00	0.00
1430535_at	0.00	0.00
1430537_at	0.00	0.00
1430538_at	0.00	0.00
1430539_at	0.00	0.00
1430540_at	0.00	0.00
1430541_at	0.00	0.00
1430543_at	0.00	0.00
1430544_at	0.00	0.00
1430546_at	0.00	0.00
1430547_s_at	0.00	0.00
1430548_at	0.00	0.00
1430550_at	0.00	0.00
1430551_s_at	0.00	0.00
1430552_a_at	0.00	0.00
1430553_at	0.00	0.00
1430554_at	0.00	0.10
1430555_s_at	0.00	0.00
1430556_at	0.00	0.00
1430557_at	0.00	0.00
1430558_at	0.00	0.00
1430560_at	0.00	0.00
1430561_at	0.00	0.00
1430563_at	0.00	0.00
1430564_at	0.00	0.00

1430565_at	0.00	0.00
1430566_at	0.00	0.00
1430567_at	0.00	0.00
1430568_at	0.00	0.00
1430569_at	0.00	0.00
1430570_at	0.00	0.00
1430572_at	0.00	0.00
1430574_at	0.00	0.00
1430577_at	0.00	0.00
1430578_at	0.00	0.00
1430579_at	0.00	0.00
1430580_at	0.00	0.00
1430581_at	0.00	0.00
1430582_at	0.00	0.00
1430583_at	0.00	0.00
1430584_s_at	0.00	0.00
1430585_at	0.00	0.00
1430587_at	0.00	0.00
1430588_at	0.00	0.00
1430589_at	0.00	0.00
1430590_at	0.00	0.00
1430591_at	0.00	0.00
1430592_at	0.00	0.00
1430593_at	0.00	0.00
1430594_at	0.00	0.19
1430595_at	0.00	0.00
1430596_s_at	0.34	0.00
1430597_at	0.00	0.00
1430600_at	0.00	0.00
1430601_at	0.00	0.00
1430602_at	0.00	0.00
1430603_at	0.00	0.00
1430605_at	0.00	0.00
1430606_at	0.00	0.00
1430607_at	0.00	0.00
1430608_at	0.00	0.00
1430609_at	0.00	0.00
1430611_at	0.00	0.00
1430612_at	0.00	0.00
1430613_at	0.00	0.00
1430614_at	0.00	0.00
1430615_at	0.00	0.00
1430616_at	0.00	0.00
1430617_at	0.00	0.00
1430618_at	0.00	0.00
1430620_at	0.00	0.00
1430621_at	0.00	0.00
1430622_at	0.00	0.00
1430623_s_at	0.00	0.00
1430624_at	0.00	0.00
1430625_at	0.00	0.00
1430626_at	0.00	0.00
1430627_at	0.00	0.00
1430628_at	0.00	0.00
1430629_at	0.00	0.00
1430630_at	0.00	0.00

1430631_at	0.00	0.00
1430632_at	0.00	0.00
1430633_s_at	0.00	0.00
1430635_at	0.00	0.00
1430636_at	0.00	0.00
1430637_at	0.00	0.00
1430638_at	0.00	0.00
1430639_at	0.00	0.00
1430640_a_at	0.00	0.00
1430641_at	0.00	0.00
1430642_at	0.00	0.00
1430644_at	0.00	0.00
1430645_at	0.00	0.00
1430646_at	0.00	0.00
1430647_at	0.00	0.00
1430648_at	0.00	0.00
1430649_at	0.00	0.00
1430650_at	0.00	0.00
1430651_s_at	0.00	0.00
1430652_at	0.00	0.00
1430653_at	0.00	0.00
1430654_at	0.00	0.00
1430655_at	0.00	0.00
1430657_at	0.00	0.00
1430658_a_at	0.00	0.00
1430659_at	0.00	0.00
1430660_at	0.00	0.00
1430662_at	0.00	0.00
1430663_at	0.00	0.00
1430664_at	0.00	0.00
1430665_at	0.00	0.00
1430666_at	0.00	0.00
1430667_at	0.00	0.00
1430669_at	0.00	0.00
1430670_at	0.00	0.00
1430672_at	0.00	0.00
1430673_a_at	0.00	0.00
1430674_at	0.00	0.00
1430675_at	0.00	0.00
1430677_at	0.00	0.00
1430678_at	0.00	0.00
1430679_at	0.00	0.00
1430680_a_at	0.00	0.00
1430682_at	0.00	0.00
1430683_at	0.00	0.00
1430684_s_at	0.00	0.00
1430685_at	0.00	0.00
1430686_at	0.00	0.00
1430687_at	0.00	0.00
1430688_s_at	0.00	0.00
1430689_at	0.00	0.00
1430690_at	0.00	0.00
1430691_at	0.00	0.00
1430693_at	0.00	0.00
1430695_at	0.00	0.00
1430696_at	0.00	0.00

1430697_at	0.00	0.00
1430699_at	0.00	0.00
1430702_at	0.00	0.00
1430703_at	0.00	0.00
1430704_at	0.00	0.00
1430705_at	0.00	0.00
1430706_at	0.00	0.00
1430707_s_at	0.00	0.00
1430709_at	0.00	0.00
1430710_at	0.00	0.00
1430711_at	0.00	0.00
1430712_at	0.00	0.00
1430714_at	0.00	0.00
1430715_at	0.00	0.00
1430716_at	0.00	0.00
1430717_at	0.00	0.00
1430719_at	0.00	0.00
1430720_at	0.00	0.00
1430721_at	0.00	0.00
1430722_at	0.00	0.00
1430723_at	0.00	0.00
1430724_at	0.00	0.00
1430725_at	0.00	0.00
1430726_at	0.00	0.00
1430728_at	0.00	0.00
1430729_at	0.00	0.00
1430730_at	0.00	0.00
1430731_at	0.00	0.00
1430732_at	0.00	0.00
1430733_at	0.00	0.00
1430735_at	0.00	0.00
1430736_at	0.00	0.00
1430737_at	0.00	0.00
1430738_at	0.00	0.00
1430739_at	0.00	0.00
1430740_at	0.00	0.00
1430741_at	0.00	0.00
1430742_a_at	0.00	0.00
1430743_at	0.00	0.00
1430744_at	0.00	0.00
1430745_at	0.00	0.00
1430746_at	0.00	0.00
1430747_at	0.00	0.00
1430748_at	0.00	0.00
1430750_at	0.00	0.00
1430751_at	0.00	0.00
1430752_at	0.00	0.00
1430753_at	0.00	0.00
1430754_at	0.00	0.00
1430755_at	0.00	0.00
1430756_at	0.00	0.00
1430757_at	0.00	0.00
1430758_x_at	0.00	0.00
1430759_at	0.00	0.00
1430760_a_at	0.00	0.00
1430761_at	0.00	0.00

1430762_at	0.00	0.00
1430763_at	0.00	0.00
1430764_at	0.00	0.00
1430765_at	0.00	0.00
1430766_at	0.00	0.00
1430767_a_at	0.00	0.00
1430768_at	0.00	0.00
1430770_at	0.00	0.00
1430774_at	0.00	0.00
1430775_at	0.00	0.00
1430779_at	0.00	0.00
1430781_at	0.00	0.03
1430782_at	0.00	0.00
1430783_at	0.00	0.00
1430784_a_at	0.00	0.00
1430785_at	0.00	0.00
1430786_at	0.00	0.00
1430787_at	0.00	0.00
1430788_at	0.00	0.00
1430789_at	0.00	0.00
1430790_at	0.00	0.00
1430791_at	0.00	0.00
1430792_at	0.00	0.00
1430793_at	0.00	0.00
1430794_at	0.00	0.00
1430795_at	0.00	0.00
1430796_at	0.00	0.00
1430797_at	0.00	0.00
1430799_at	0.00	0.00
1430800_at	0.00	0.00
1430801_at	0.00	0.00
1430802_at	0.00	0.00
1430803_at	0.00	0.00
1430804_at	0.00	0.00
1430806_at	0.00	0.00
1430807_at	0.00	0.00
1430808_at	0.00	0.00
1430809_at	0.00	0.00
1430810_at	0.00	0.00
1430812_at	0.00	0.00
1430813_at	0.00	0.00
1430814_at	0.00	0.00
1430815_at	0.00	0.00
1430816_at	0.00	0.00
1430817_at	0.00	0.00
1430819_at	0.00	0.00
1430821_at	0.00	0.00
1430822_at	0.00	0.00
1430823_at	0.00	0.00
1430825_at	0.00	0.00
1430828_at	0.00	0.00
1430830_at	0.00	0.00
1430831_at	0.00	0.00
1430832_at	0.00	0.00
1430833_at	0.00	0.00
1430834_at	0.00	0.00

1430835_at	0.00	0.00
1430836_at	0.00	0.00
1430839_at	0.00	0.00
1430840_at	0.00	0.00
1430841_at	0.00	0.00
1430842_at	0.00	0.00
1430843_at	0.00	0.00
1430844_at	0.00	0.00
1430846_at	0.00	0.00
1430847_a_at	0.00	0.00
1430849_a_at	0.00	0.00
1430850_x_at	0.00	0.00
1430851_at	0.00	0.00
1430852_at	0.00	0.00
1430854_at	0.00	0.00
1430855_at	0.00	0.00
1430856_at	0.00	0.00
1430857_s_at	0.00	0.00
1430858_at	0.00	0.00
1430859_at	0.00	0.00
1430860_at	0.00	0.00
1430861_at	0.00	0.00
1430862_at	0.00	0.00
1430863_at	0.00	0.00
1430864_at	0.00	0.00
1430865_s_at	0.00	0.00
1430866_at	0.00	0.00
1430867_at	0.00	0.00
1430868_at	0.00	0.00
1430870_at	0.00	0.00
1430871_at	0.00	0.00
1430872_at	0.00	0.00
1430873_at	0.00	0.00
1430874_at	0.00	0.00
1430876_at	0.00	0.00
1430877_at	0.00	0.00
1430878_at	0.00	0.00
1430879_at	0.00	0.00
1430880_at	0.00	0.00
1430881_at	0.00	0.00
1430882_at	0.00	0.00
1430883_at	0.00	0.00
1430884_at	0.00	0.00
1430885_at	0.00	0.00
1430886_at	0.00	0.00
1430887_s_at	0.00	0.00
1430888_at	0.00	0.00
1430890_at	0.00	0.00
1430892_at	0.00	0.00
1430893_at	0.00	0.00
1430894_at	0.00	0.00
1430895_at	0.00	0.00
1430896_s_at	0.00	0.00
1430897_at	0.00	0.00
1430898_s_at	0.00	0.00
1430900_at	0.00	0.00

1430901_at	0.00	0.00
1430903_at	0.00	0.00
1430904_at	0.00	0.00
1430905_at	0.00	0.00
1430906_at	0.00	0.00
1430907_at	0.00	0.00
1430908_at	0.00	0.00
1430909_at	0.00	0.00
1430910_at	0.00	0.00
1430911_at	0.00	0.00
1430913_at	0.00	0.00
1430914_at	0.00	0.00
1430915_at	0.00	0.00
1430916_at	0.00	0.00
1430917_at	0.00	0.00
1430918_at	0.00	0.00
1430919_at	0.00	0.00
1430920_at	0.00	0.00
1430921_at	0.00	0.00
1430922_at	0.00	0.00
1430923_at	0.00	0.00
1430924_at	0.00	0.00
1430925_at	0.00	0.00
1430926_at	0.00	0.00
1430927_at	0.00	0.00
1430928_at	0.00	0.00
1430929_at	0.00	0.00
1430930_at	0.00	0.00
1430931_at	0.00	0.00
1430932_at	0.00	0.00
1430933_at	0.00	0.00
1430934_at	0.00	0.00
1430935_at	0.00	0.00
1430936_at	0.00	0.00
1430937_at	0.00	0.00
1430938_at	0.00	0.00
1430939_at	0.00	0.00
1430940_at	0.00	0.00
1430941_at	0.00	0.00
1430942_at	0.00	0.00
1430943_at	0.00	0.00
1430944_at	0.00	0.00
1430945_at	0.00	0.00
1430946_at	0.00	0.00
1430947_at	0.00	0.00
1430948_at	0.00	0.00
1430949_at	0.00	0.00
1430950_at	0.00	0.00
1430951_at	0.00	0.00
1430952_at	0.00	0.00
1430953_at	0.00	0.00
1430954_at	0.00	0.00
1430955_at	0.00	0.00
1430957_at	0.00	0.00
1430958_at	0.00	0.00
1430959_at	0.00	0.00

1430960_at	0.00	0.00
1430961_at	0.00	0.00
1430963_at	0.00	0.00
1430964_at	0.00	0.00
1430965_at	0.00	0.00
1430966_at	0.00	0.00
1430967_at	0.00	0.00
1430968_at	0.00	0.00
1430969_at	0.00	0.00
1430973_at	0.00	0.00
1430974_a_at	0.00	0.00
1430975_at	0.00	0.00
1430977_at	0.00	0.00
1430981_s_at	0.00	0.00
1430984_at	0.00	0.00
1430985_at	0.00	0.05
1430987_s_at	0.00	0.00
1430988_at	0.00	0.00
1430990_s_at	0.00	0.00
1430991_at	0.00	0.00
1430992_s_at	0.00	0.00
1430995_at	0.00	0.00
1430996_at	0.00	0.00
1430997_at	0.00	0.00
1431000_at	0.00	0.00
1431001_at	0.00	0.00
1431002_x_at	0.00	0.00
1431004_at	0.00	0.32
1431005_at	0.00	0.00
1431007_at	0.00	0.00
1431009_at	0.00	0.00
1431014_at	0.00	0.00
1431015_at	0.00	0.00
1431016_at	0.00	0.00
1431018_at	0.00	0.00
1431019_at	0.00	0.00
1431021_at	0.00	0.00
1431023_at	0.00	0.00
1431025_at	0.00	0.00
1431026_at	0.00	0.00
1431027_at	0.00	0.00
1431029_at	0.00	0.00
1431034_at	0.00	0.00
1431035_at	0.00	0.00
1431038_at	0.00	0.00
1431039_at	0.00	0.00
1431041_at	0.00	0.00
1431042_at	0.00	0.00
1431043_at	0.00	0.00
1431044_at	0.00	0.00
1431045_at	0.00	0.00
1431046_at	0.00	0.00
1431047_at	0.00	0.00
1431049_at	0.00	0.00
1431050_at	0.00	0.00
1431051_at	0.00	0.00

1431052_at	0.00	0.00
1431053_at	0.00	0.00
1431054_at	0.00	0.00
1431060_at	0.00	0.00
1431061_s_at	0.00	0.00
1431063_at	0.00	0.00
1431064_at	0.00	0.00
1431065_at	0.00	0.00
1431066_at	0.00	0.00
1431067_at	0.00	0.00
1431068_at	0.00	0.00
1431069_at	0.00	0.00
1431071_at	0.00	0.00
1431073_at	0.00	0.00
1431076_at	0.00	0.00
1431077_at	0.00	0.00
1431079_at	0.00	0.00
1431080_at	0.00	0.00
1431083_a_at	0.00	0.00
1431084_x_at	0.00	0.00
1431087_at	0.00	0.00
1431088_at	0.00	0.00
1431089_at	0.00	0.00
1431090_at	0.00	0.00
1431091_at	0.00	0.00
1431093_at	0.00	0.00
1431094_at	0.00	0.00
1431095_a_at	0.00	0.00
1431096_at	0.00	0.00
1431097_at	0.00	0.00
1431098_at	0.00	0.00
1431100_at	0.00	0.00
1431101_a_at	0.00	0.00
1431102_at	0.00	0.00
1431103_at	0.00	0.00
1431104_at	0.00	0.00
1431106_a_at	0.00	0.00
1431108_at	0.00	0.00
1431109_at	0.00	0.00
1431110_at	0.00	0.00
1431111_at	0.00	0.00
1431112_at	0.00	0.00
1431113_at	0.00	0.00
1431114_at	0.00	0.00
1431115_at	0.00	0.00
1431116_at	0.00	0.00
1431118_at	0.00	0.00
1431120_a_at	0.00	0.00
1431121_at	0.00	0.00
1431122_at	0.00	0.00
1431123_s_at	0.00	0.00
1431126_a_at	0.00	0.22
1431127_at	0.00	0.00
1431128_at	0.00	0.00
1431129_at	0.00	0.00
1431130_at	0.00	0.00

1431131_s_at	0.00	0.00
1431133_at	0.00	0.00
1431134_at	0.00	0.00
1431136_at	0.00	0.00
1431137_at	0.00	0.00
1431138_at	0.00	0.00
1431139_at	0.00	0.00
1431140_at	0.00	0.00
1431141_at	0.00	0.00
1431142_s_at	0.00	0.00
1431143_x_at	0.00	0.00
1431144_at	0.00	0.00
1431146_a_at	0.00	0.00
1431147_at	0.00	0.00
1431148_at	0.00	0.00
1431149_at	0.00	0.00
1431150_at	0.00	0.00
1431151_at	0.00	0.00
1431152_at	0.00	0.00
1431153_at	0.00	0.00
1431154_at	0.00	0.00
1431155_at	0.00	0.00
1431156_at	0.00	0.00
1431157_at	0.00	0.00
1431158_at	0.00	0.00
1431159_at	0.00	0.00
1431160_x_at	0.00	0.00
1431161_at	0.00	0.00
1431163_at	0.00	0.00
1431165_at	0.00	0.00
1431167_at	0.00	0.00
1431168_at	0.00	0.00
1431169_at	0.00	0.00
1431171_at	0.00	0.00
1431172_at	0.00	0.00
1431173_at	0.00	0.00
1431174_at	0.00	0.00
1431175_at	0.00	0.00
1431176_at	0.00	0.00
1431178_at	0.00	0.00
1431179_at	0.00	0.00
1431180_at	0.00	0.00
1431183_at	0.00	0.00
1431184_a_at	0.00	0.00
1431185_at	0.00	0.00
1431186_at	0.00	0.00
1431187_s_at	0.00	0.00
1431189_a_at	0.00	0.00
1431190_x_at	0.00	0.00
1431192_at	0.00	0.00
1431193_at	0.00	0.00
1431194_at	0.00	0.00
1431195_at	0.00	0.00
1431196_at	0.00	0.00
1431197_at	0.00	0.00
1431198_x_at	0.00	0.00

1431199_at	0.00	0.00
1431200_a_at	0.00	0.00
1431201_at	0.00	0.00
1431202_at	0.00	0.00
1431204_at	0.00	0.00
1431205_at	0.00	0.00
1431206_at	0.00	0.00
1431207_at	0.00	0.00
1431209_s_at	0.00	0.00
1431211_s_at	0.00	0.00
1431212_a_at	0.00	0.00
1431215_at	0.00	0.00
1431216_s_at	0.00	0.00
1431217_at	0.00	0.00
1431218_at	0.00	0.00
1431220_at	0.00	0.00
1431221_at	0.00	0.00
1431222_at	0.00	0.00
1431223_at	0.00	0.00
1431224_at	0.00	0.00
1431225_at	0.00	0.00
1431228_s_at	0.00	0.00
1431229_at	0.00	0.00
1431230_a_at	0.00	0.00
1431233_at	0.00	0.00
1431234_at	0.00	0.00
1431235_at	0.00	0.00
1431236_at	0.00	0.00
1431237_at	0.00	0.00
1431242_at	0.00	0.00
1431243_at	0.00	0.00
1431244_s_at	0.00	0.00
1431245_at	0.00	0.00
1431246_at	0.00	0.00
1431247_at	0.00	0.00
1431248_at	0.00	0.00
1431249_at	0.00	0.00
1431250_at	0.00	0.00
1431251_at	0.00	0.00
1431252_a_at	0.00	0.00
1431253_s_at	0.00	0.00
1431254_at	0.00	0.00
1431255_at	0.00	0.00
1431256_at	0.00	0.00
1431257_at	0.00	0.00
1431258_at	0.00	0.00
1431259_at	0.00	0.00
1431260_at	0.00	0.00
1431261_at	0.00	0.00
1431263_at	0.00	0.00
1431264_at	0.00	0.00
1431265_at	0.00	0.00
1431266_at	0.00	0.00
1431267_at	0.00	0.00
1431268_at	0.00	0.00
1431269_at	0.00	0.00

1431271_at	0.00	0.00
1431272_at	0.00	0.00
1431273_at	0.00	0.00
1431275_at	0.00	0.00
1431276_at	0.00	0.00
1431277_at	0.00	0.00
1431278_s_at	0.00	0.00
1431279_s_at	0.00	0.00
1431281_at	0.00	0.00
1431283_at	0.00	0.00
1431285_at	0.00	0.00
1431286_at	0.00	0.00
1431288_at	0.00	0.00
1431289_at	0.00	0.00
1431290_at	0.00	0.00
1431291_at	0.00	0.00
1431294_at	0.00	0.00
1431296_at	0.00	0.00
1431297_a_at	0.00	0.00
1431298_at	0.00	0.00
1431300_at	0.00	0.00
1431303_at	0.00	0.00
1431305_at	0.00	0.00
1431306_at	0.00	0.00
1431307_at	0.00	0.00
1431308_at	0.00	0.00
1431309_at	0.00	0.00
1431310_s_at	0.00	0.00
1431311_at	0.00	0.00
1431312_at	0.00	0.00
1431313_at	0.00	0.00
1431315_at	0.00	0.00
1431316_at	0.00	0.00
1431317_at	0.00	0.00
1431318_at	0.00	0.00
1431319_at	0.00	0.00
1431321_at	0.00	0.00
1431322_at	0.00	0.00
1431323_at	0.00	0.00
1431324_at	0.00	0.00
1431325_at	0.00	0.00
1431327_at	0.00	0.00
1431329_at	0.00	0.00
1431333_at	0.00	0.00
1431334_a_at	0.00	0.00
1431338_at	0.00	0.00
1431340_a_at	0.00	0.00
1431341_at	0.00	0.00
1431342_at	0.00	0.00
1431343_at	0.00	0.00
1431344_at	0.00	0.00
1431346_at	0.00	0.00
1431347_at	0.00	0.00
1431348_at	0.00	0.00
1431351_at	0.00	0.00
1431352_s_at	0.00	0.00

1431353_at	0.00	0.00
1431355_s_at	0.00	0.00
1431356_at	0.00	0.00
1431358_at	0.00	0.00
1431360_s_at	0.00	0.00
1431361_at	0.00	0.00
1431364_a_at	0.00	0.00
1431365_at	0.00	0.00
1431366_at	0.00	0.00
1431367_at	0.00	0.00
1431368_at	0.00	0.00
1431369_at	0.00	0.00
1431370_at	0.00	0.00
1431371_at	0.00	0.00
1431372_at	0.00	0.00
1431374_at	0.00	0.00
1431376_at	0.00	0.00
1431377_at	0.00	0.00
1431378_at	0.00	0.00
1431380_at	0.00	0.00
1431381_at	0.00	0.00
1431383_at	0.00	0.00
1431384_at	0.00	0.00
1431387_at	0.00	0.00
1431389_at	0.00	0.00
1431391_at	0.00	0.00
1431392_at	0.00	0.00
1431393_at	0.00	0.00
1431397_at	0.00	0.00
1431398_at	0.00	0.00
1431399_at	0.00	0.00
1431401_at	0.00	0.00
1431402_at	0.00	0.00
1431403_a_at	0.00	0.00
1431404_at	0.00	0.00
1431406_at	0.00	0.00
1431407_at	0.00	0.00
1431408_at	0.00	0.00
1431409_at	0.00	0.00
1431410_at	0.00	0.00
1431412_at	0.00	0.00
1431413_at	0.00	0.00
1431414_at	0.00	0.00
1431418_at	0.00	0.00
1431424_at	0.00	0.00
1431425_a_at	0.00	0.00
1431426_at	0.00	0.00
1431427_at	0.00	0.00
1431430_s_at	0.00	0.00
1431432_at	0.00	0.00
1431433_at	0.00	0.00
1431436_a_at	0.00	0.00
1431437_at	0.00	0.00
1431438_at	0.00	0.00
1431439_at	0.00	0.00
1431440_at	0.00	0.00

1431441_at	0.00	0.00
1431443_at	0.00	0.00
1431444_at	0.00	0.00
1431445_at	0.00	0.00
1431446_at	0.00	0.00
1431447_at	0.00	0.00
1431448_at	0.00	0.00
1431449_at	0.00	0.00
1431450_at	0.00	0.00
1431451_at	0.00	0.00
1431452_at	0.00	0.00
1431453_at	0.00	0.00
1431454_at	0.00	0.00
1431455_at	0.00	0.00
1431456_at	0.00	0.00
1431457_at	0.00	0.00
1431458_at	0.00	0.00
1431459_at	0.00	0.00
1431460_at	0.00	0.00
1431461_at	0.00	0.00
1431462_at	0.00	0.00
1431463_at	0.00	0.00
1431465_s_at	0.00	0.00
1431466_at	0.00	0.00
1431467_at	0.00	0.00
1431468_at	0.00	0.00
1431470_a_at	0.00	0.00
1431471_at	0.00	0.00
1431472_at	0.00	0.00
1431473_at	0.00	0.00
1431474_at	0.00	0.00
1431476_at	0.00	0.00
1431477_at	0.00	0.00
1431478_at	0.00	0.00
1431479_at	0.00	0.00
1431480_at	0.00	0.00
1431481_at	0.00	0.00
1431482_at	0.00	0.00
1431483_at	0.00	0.00
1431484_at	0.00	0.00
1431485_at	0.00	0.00
1431486_at	0.00	0.00
1431488_at	0.00	0.00
1431489_at	0.00	0.00
1431490_at	0.00	0.00
1431491_at	0.00	0.00
1431492_at	0.00	0.00
1431493_at	0.00	0.00
1431494_at	0.00	0.00
1431495_at	0.00	0.00
1431496_at	0.00	0.00
1431497_at	0.00	0.00
1431498_at	0.00	0.00
1431499_at	0.00	0.00
1431500_at	0.00	0.00
1431501_at	0.00	0.00

1431502_a_at	0.00	0.04
1431503_at	0.00	0.00
1431504_at	0.00	0.00
1431508_at	0.00	0.00
1431509_at	0.00	0.00
1431510_s_at	0.00	0.00
1431511_at	0.00	0.00
1431512_at	0.00	0.00
1431513_at	0.00	0.00
1431514_at	0.00	0.00
1431515_at	0.00	0.00
1431516_at	0.00	0.00
1431517_at	0.00	0.00
1431518_at	0.00	0.00
1431519_at	0.00	0.00
1431520_at	0.00	0.00
1431521_at	0.00	0.00
1431522_at	0.00	0.00
1431523_at	0.00	0.00
1431524_at	0.00	0.00
1431525_at	0.00	0.00
1431526_at	0.00	0.00
1431528_at	0.00	0.00
1431529_at	0.00	0.00
1431531_at	0.00	0.00
1431532_at	0.00	0.00
1431533_at	0.00	0.00
1431534_at	0.00	0.00
1431535_at	0.00	0.00
1431536_at	0.00	0.00
1431537_at	0.00	0.00
1431538_at	0.00	0.00
1431540_at	0.00	0.00
1431543_at	0.00	0.00
1431544_at	0.00	0.00
1431545_at	0.00	0.00
1431546_at	0.00	0.00
1431547_at	0.00	0.00
1431548_at	0.00	0.00
1431550_at	0.00	0.00
1431551_at	0.00	0.00
1431552_at	0.00	0.00
1431555_at	0.00	0.00
1431556_at	0.00	0.00
1431557_at	0.00	0.00
1431558_at	0.00	0.00
1431559_at	0.00	0.00
1431560_at	0.00	0.00
1431561_a_at	0.00	0.00
1431562_at	0.00	0.00
1431563_at	0.00	0.00
1431564_at	0.00	0.00
1431565_at	0.00	0.00
1431566_at	0.00	0.00
1431567_at	0.00	0.00
1431568_at	0.00	0.00

1431570_at	0.00	0.00
1431571_at	0.00	0.00
1431572_at	0.00	0.00
1431573_at	0.00	0.00
1431574_at	0.00	0.00
1431576_at	0.00	0.00
1431577_at	0.00	0.00
1431578_at	0.00	0.00
1431579_at	0.00	0.00
1431580_at	0.00	0.00
1431581_at	0.00	0.00
1431582_at	0.00	0.00
1431583_at	0.00	0.00
1431584_at	0.00	0.00
1431585_at	0.00	0.00
1431586_at	0.00	0.00
1431587_at	0.00	0.00
1431588_at	0.00	0.00
1431589_at	0.00	0.00
1431590_at	0.00	0.00
1431595_at	0.00	0.00
1431596_at	0.00	0.00
1431599_at	0.00	0.00
1431600_at	0.00	0.00
1431601_at	0.00	0.00
1431603_at	0.00	0.00
1431605_at	0.00	0.00
1431607_at	0.00	0.00
1431608_at	0.00	0.00
1431610_at	0.00	0.00
1431612_at	0.00	0.00
1431614_at	0.00	0.00
1431615_at	0.00	0.00
1431616_at	0.00	0.00
1431617_at	0.00	0.00
1431618_a_at	0.00	0.00
1431620_at	0.00	0.00
1431621_at	0.00	0.00
1431622_at	0.00	0.00
1431623_at	0.00	0.00
1431625_at	0.00	0.00
1431626_at	0.00	0.00
1431627_at	0.00	0.00
1431628_at	0.00	0.00
1431629_at	0.00	0.00
1431631_at	0.00	0.00
1431632_at	0.00	0.00
1431633_x_at	0.00	0.00
1431634_at	0.00	0.00
1431635_at	0.00	0.00
1431636_at	0.00	0.00
1431637_at	0.00	0.00
1431638_at	0.00	0.00
1431639_at	0.00	0.00
1431640_at	0.00	0.00
1431641_at	0.00	0.00

1431642_at	0.00	0.00
1431643_at	0.00	0.00
1431647_a_at	0.00	0.00
1431648_at	0.00	0.00
1431649_at	0.00	0.00
1431650_at	0.00	0.00
1431651_at	0.00	0.00
1431652_at	0.00	0.00
1431654_at	0.00	0.00
1431656_at	0.00	0.00
1431657_at	0.00	0.00
1431658_at	0.00	0.00
1431659_at	0.00	0.00
1431660_at	0.00	0.00
1431661_at	0.00	0.00
1431662_at	0.00	0.00
1431664_at	0.00	0.00
1431666_at	0.00	0.00
1431667_s_at	0.00	0.00
1431669_at	0.00	0.00
1431670_at	0.00	0.00
1431671_at	0.00	0.00
1431672_at	0.00	0.00
1431673_at	0.00	0.00
1431674_at	0.00	0.00
1431677_at	0.00	0.00
1431678_at	0.00	0.00
1431679_at	0.00	0.00
1431681_at	0.00	0.00
1431682_at	0.00	0.00
1431683_at	0.00	0.00
1431684_at	0.00	0.00
1431685_at	0.00	0.00
1431688_at	0.00	0.00
1431689_at	0.00	0.00
1431690_at	0.00	0.00
1431695_at	0.00	0.00
1431696_at	0.00	0.00
1431698_at	0.00	0.00
1431699_at	0.00	0.00
1431703_at	0.00	0.00
1431708_a_at	0.00	0.00
1431709_at	0.00	0.00
1431710_at	0.00	0.00
1431713_at	0.00	0.00
1431714_at	0.00	0.00
1431717_at	0.00	0.00
1431718_at	0.00	0.00
1431720_at	0.00	0.00
1431723_at	0.00	0.00
1431727_at	0.00	0.00
1431728_at	0.00	0.00
1431729_at	0.00	0.00
1431730_at	0.00	0.00
1431732_at	0.00	0.00
1431733_at	0.00	0.00

1431735_at	0.00	0.00
1431736_at	0.00	0.00
1431738_at	0.00	0.00
1431742_at	0.00	0.00
1431747_at	0.00	0.00
1431751_a_at	0.00	0.00
1431754_at	0.00	0.00
1431755_a_at	0.00	0.00
1431756_at	0.00	0.00
1431757_s_at	0.00	0.00
1431758_at	0.00	0.00
1431759_at	0.00	0.00
1431764_at	0.00	0.00
1431767_at	0.00	0.00
1431769_at	0.00	0.00
1431770_at	0.00	0.00
1431775_at	0.00	0.00
1431776_at	0.00	0.00
1431778_at	0.00	0.00
1431779_at	0.00	0.00
1431780_at	0.00	0.00
1431783_at	0.00	0.00
1431785_at	0.00	0.00
1431786_s_at	0.52	1.00
1431787_at	0.00	0.00
1431788_at	0.00	0.00
1431789_s_at	0.00	0.00
1431790_at	0.00	0.00
1431793_at	0.00	0.00
1431794_at	0.00	0.00
1431796_at	0.00	0.00
1431797_at	0.00	0.00
1431798_a_at	0.00	0.00
1431799_at	0.00	0.00
1431801_at	0.00	0.00
1431802_a_at	0.00	0.00
1431806_at	0.00	0.00
1431807_at	0.00	0.00
1431809_at	0.00	0.00
1431811_a_at	0.00	0.00
1431813_at	0.00	0.00
1431814_at	0.00	0.00
1431817_at	0.00	0.00
1431818_at	0.00	0.00
1431819_at	0.00	0.00
1431820_at	0.00	0.00
1431823_at	0.00	0.00
1431825_at	0.00	0.00
1431826_a_at	0.00	0.00
1431831_at	0.00	0.00
1431835_at	0.00	0.00
1431836_x_at	0.00	0.00
1431837_at	0.00	0.00
1431838_at	0.00	0.00
1431839_a_at	0.00	0.00
1431840_at	0.00	0.00

1431841_at	0.00	0.00
1431842_at	0.00	0.00
1431845_at	0.00	0.00
1431846_at	0.00	0.00
1431847_at	0.00	0.00
1431849_at	0.00	0.00
1431850_at	0.00	0.00
1431851_at	0.00	0.00
1431852_at	0.00	0.00
1431853_at	0.00	0.00
1431854_a_at	0.00	0.00
1431855_at	0.00	0.00
1431858_at	0.00	0.00
1431859_at	0.00	0.00
1431860_at	0.00	0.00
1431863_at	0.00	0.00
1431864_at	0.00	0.00
1431865_a_at	0.68	0.31
1431866_at	0.00	0.00
1431867_a_at	0.00	0.00
1431869_at	0.00	0.00
1431870_at	0.00	0.00
1431871_at	0.00	0.00
1431872_at	0.00	0.00
1431873_a_at	0.00	0.07
1431874_at	0.00	0.00
1431876_at	0.00	0.00
1431879_at	0.00	0.00
1431880_at	0.00	0.00
1431881_at	0.00	0.00
1431882_at	0.00	0.00
1431883_at	0.00	0.00
1431884_at	0.00	0.00
1431887_at	0.00	0.00
1431888_s_at	0.00	0.00
1431889_x_at	0.00	0.00
1431891_at	0.00	0.00
1431892_a_at	0.00	0.00
1431894_at	0.00	0.00
1431895_at	0.00	0.00
1431896_at	0.00	0.00
1431897_at	0.00	0.00
1431898_at	0.00	0.00
1431899_at	0.00	0.00
1431902_at	0.00	0.00
1431903_at	0.00	0.00
1431904_at	0.00	0.00
1431905_s_at	0.00	0.00
1431906_at	0.00	0.00
1431907_at	0.00	0.00
1431908_at	0.00	0.00
1431909_at	0.00	0.00
1431910_at	0.00	0.00
1431911_at	0.00	0.00
1431912_at	0.00	0.00
1431915_at	0.00	0.00

1431916_at	0.00	0.00
1431918_at	0.00	0.00
1431919_at	0.00	0.00
1431922_at	0.00	0.00
1431923_at	0.00	0.00
1431924_at	0.00	0.00
1431925_at	0.00	0.00
1431927_at	0.00	0.00
1431928_at	0.00	0.00
1431930_x_at	0.00	0.00
1431931_a_at	0.00	0.00
1431932_s_at	0.00	0.00
1431934_at	0.00	0.00
1431935_at	0.00	0.00
1431937_at	0.00	0.00
1431940_at	0.00	0.00
1431941_at	0.00	0.00
1431943_at	0.00	0.00
1431944_at	0.00	0.00
1431945_at	0.00	0.00
1431948_a_at	0.00	0.00
1431949_at	0.00	0.00
1431950_at	0.00	0.00
1431952_at	0.00	0.00
1431953_at	0.00	0.00
1431954_x_at	0.00	0.00
1431955_at	0.00	0.00
1431956_at	0.00	0.00
1431957_at	0.00	0.00
1431958_at	0.00	0.00
1431959_x_at	0.00	0.00
1431961_at	0.00	0.00
1431963_at	0.00	0.00
1431964_at	0.00	0.00
1431965_at	0.00	0.00
1431966_at	0.00	0.00
1431967_at	0.00	0.00
1431968_at	0.00	0.00
1431969_at	0.00	0.00
1431970_at	0.00	0.00
1431971_at	0.00	0.00
1431972_a_at	0.00	0.00
1431974_at	0.00	0.00
1431975_at	0.00	0.00
1431976_at	0.00	0.00
1431977_at	0.00	0.00
1431978_at	0.00	0.00
1431979_at	0.00	0.00
1431982_at	0.00	0.00
1431983_at	0.00	0.00
1431984_at	0.00	0.00
1431985_at	0.00	0.00
1431986_at	0.00	0.00
1431987_at	0.00	0.00
1431988_at	0.00	0.00
1431989_at	0.00	0.00

1431990_at	0.00	0.00
1431991_at	0.00	0.00
1431992_at	0.00	0.00
1431993_a_at	0.00	0.00
1431994_at	0.00	0.00
1431996_at	0.00	0.00
1431997_at	0.00	0.00
1431998_at	0.00	0.00
1431999_at	0.00	0.00
1432001_at	0.00	0.00
1432002_at	0.00	0.00
1432008_at	0.00	0.00
1432009_at	0.00	0.00
1432010_at	0.00	0.00
1432011_at	0.00	0.00
1432014_at	0.00	0.00
1432015_a_at	0.00	0.00
1432017_at	0.00	0.00
1432019_at	0.00	0.00
1432020_at	0.00	0.00
1432021_at	0.00	0.00
1432023_a_at	0.00	0.00
1432024_at	0.00	0.00
1432025_at	0.00	0.00
1432026_a_at	0.00	0.00
1432028_at	0.00	0.00
1432030_at	0.00	0.00
1432031_at	0.00	0.00
1432033_at	0.00	0.00
1432035_at	0.00	0.00
1432036_at	0.00	0.00
1432037_at	0.00	0.00
1432038_at	0.00	0.00
1432039_a_at	0.00	0.00
1432040_at	0.00	0.00
1432041_at	0.00	0.00
1432044_a_at	0.00	0.00
1432045_at	0.00	0.00
1432046_at	0.00	0.00
1432047_at	0.00	0.00
1432048_at	0.00	0.00
1432049_at	0.00	0.00
1432051_at	0.00	0.00
1432053_at	0.00	0.00
1432054_at	0.00	0.00
1432055_at	0.00	0.00
1432056_at	0.00	0.00
1432058_at	0.00	0.00
1432059_x_at	0.00	0.00
1432060_at	0.00	0.00
1432061_at	0.00	0.00
1432062_at	0.00	0.00
1432063_at	0.00	0.00
1432064_at	0.00	0.00
1432065_at	0.00	0.00
1432067_at	0.00	0.00

1432069_at	0.00	0.00
1432070_at	0.00	0.00
1432071_at	0.00	0.00
1432073_at	0.00	0.00
1432074_at	0.00	0.00
1432076_at	0.00	0.00
1432077_at	0.00	0.00
1432078_at	0.00	0.00
1432079_at	0.00	0.00
1432080_s_at	0.00	0.00
1432082_at	0.00	0.00
1432084_at	0.00	0.00
1432085_at	0.00	0.00
1432087_at	0.00	0.00
1432088_at	0.00	0.00
1432089_at	0.00	0.00
1432090_at	0.00	0.00
1432092_a_at	0.00	0.00
1432093_at	0.00	0.00
1432095_at	0.00	0.00
1432098_a_at	0.00	0.00
1432100_a_at	0.00	0.00
1432101_a_at	0.00	0.00
1432102_at	0.00	0.00
1432105_at	0.00	0.00
1432106_at	0.00	0.00
1432107_at	0.00	0.00
1432109_at	0.00	0.00
1432110_at	0.00	0.00
1432111_at	0.00	0.00
1432112_at	0.00	0.00
1432113_at	0.00	0.00
1432114_at	0.00	0.00
1432116_at	0.00	0.00
1432117_at	0.00	0.00
1432118_at	0.00	0.00
1432119_at	0.00	0.00
1432120_at	0.00	0.00
1432121_a_at	0.00	0.00
1432122_at	0.00	0.00
1432123_at	0.00	0.00
1432124_at	0.00	0.00
1432126_at	0.00	0.00
1432127_at	0.00	0.00
1432128_at	0.00	0.00
1432131_at	0.00	0.00
1432132_at	0.00	0.00
1432133_at	0.00	0.00
1432134_at	0.00	0.00
1432135_s_at	0.00	0.00
1432137_at	0.00	0.00
1432138_at	0.00	0.00
1432140_at	0.00	0.00
1432141_x_at	0.00	0.00
1432142_at	0.00	0.00
1432145_at	0.00	0.00

1432146_at	0.00	0.00
1432147_at	0.00	0.00
1432148_at	0.00	0.00
1432149_at	0.00	0.00
1432150_at	0.00	0.00
1432152_at	0.00	0.00
1432153_at	0.00	0.00
1432154_at	0.00	0.00
1432157_at	0.00	0.00
1432161_a_at	0.00	0.00
1432162_s_at	0.00	0.00
1432163_at	0.00	0.00
1432165_at	0.00	0.00
1432166_at	0.00	0.00
1432168_at	0.00	0.00
1432169_at	0.00	0.00
1432170_at	0.00	0.00
1432171_at	0.00	0.00
1432172_at	0.00	0.00
1432173_at	0.00	0.00
1432175_at	0.00	0.00
1432178_at	0.00	0.00
1432180_at	0.00	0.00
1432182_at	0.00	0.00
1432183_at	0.00	0.00
1432184_a_at	0.00	0.00
1432185_a_at	0.00	0.00
1432186_at	0.00	0.00
1432191_at	0.00	0.00
1432192_at	0.00	0.00
1432193_at	0.00	0.00
1432194_at	0.00	0.00
1432196_a_at	0.00	0.00
1432197_at	0.00	0.00
1432198_at	0.67	0.02
1432199_at	0.00	0.00
1432200_at	0.00	0.00
1432201_a_at	0.00	0.00
1432202_a_at	0.00	0.00
1432203_at	0.00	0.00
1432204_at	0.00	0.00
1432205_a_at	0.00	0.00
1432206_at	0.00	0.00
1432208_at	0.00	0.00
1432209_at	0.00	0.00
1432210_at	0.00	0.00
1432212_at	0.00	0.00
1432213_at	0.00	0.00
1432214_at	0.00	0.00
1432215_s_at	0.00	0.00
1432216_s_at	0.00	0.00
1432217_a_at	0.00	0.00
1432218_a_at	0.00	0.00
1432219_at	0.00	0.00
1432220_at	0.00	0.00
1432221_at	0.00	0.00

1432222_at	0.00	0.00
1432223_at	0.00	0.00
1432224_at	0.00	0.00
1432225_at	0.00	0.00
1432226_at	0.00	0.00
1432228_at	0.00	0.00
1432229_a_at	0.03	0.00
1432230_at	0.00	0.00
1432231_at	0.00	0.00
1432232_at	0.00	0.00
1432233_at	0.00	0.00
1432234_at	0.00	0.00
1432237_at	0.00	0.00
1432238_at	0.00	0.00
1432239_at	0.00	0.00
1432240_at	0.00	0.00
1432241_at	0.00	0.00
1432242_at	0.00	0.00
1432243_a_at	0.00	0.00
1432244_at	0.00	0.00
1432245_s_at	0.00	0.00
1432246_at	0.00	0.00
1432247_at	0.00	0.00
1432248_at	0.00	0.00
1432250_at	0.00	0.00
1432251_at	0.00	0.00
1432252_a_at	0.00	0.00
1432253_at	0.00	0.00
1432254_at	0.00	0.00
1432256_at	0.00	0.00
1432257_at	0.00	0.00
1432258_at	0.00	0.00
1432259_s_at	0.00	0.00
1432260_at	0.00	0.00
1432261_at	0.00	0.00
1432262_at	0.00	0.00
1432265_at	0.00	0.00
1432266_at	0.00	0.00
1432267_at	0.00	0.00
1432268_at	0.00	0.00
1432274_at	0.00	0.00
1432276_at	0.00	0.00
1432277_at	0.00	0.00
1432278_at	0.00	0.00
1432279_at	0.00	0.00
1432280_at	0.00	0.00
1432283_at	0.00	0.00
1432284_at	0.00	0.00
1432285_at	0.00	0.00
1432286_at	0.00	0.00
1432288_at	0.00	0.00
1432289_a_at	0.00	0.00
1432290_at	0.00	0.00
1432291_at	0.00	0.00
1432292_at	0.00	0.00
1432293_at	0.00	0.00

1432294_at	0.00	0.00
1432297_at	0.00	0.00
1432298_at	0.00	0.00
1432299_at	0.00	0.00
1432300_at	0.00	0.00
1432302_s_at	0.00	0.00
1432303_at	0.00	0.00
1432304_a_at	0.00	0.00
1432305_at	0.00	0.00
1432306_at	0.00	0.00
1432307_at	0.00	0.00
1432308_at	0.00	0.00
1432309_at	0.00	0.00
1432310_at	0.00	0.00
1432311_at	0.00	0.00
1432313_at	0.00	0.00
1432314_at	0.00	0.00
1432315_at	0.00	0.00
1432316_at	0.00	0.00
1432317_at	0.00	0.00
1432318_at	0.00	0.00
1432319_at	0.00	0.00
1432320_at	0.00	0.00
1432321_at	0.00	0.00
1432323_at	0.00	0.00
1432324_at	0.00	0.00
1432325_at	0.00	0.00
1432326_at	0.00	0.00
1432327_at	0.00	0.00
1432328_at	0.00	0.00
1432330_at	0.00	0.00
1432333_a_at	0.00	0.00
1432334_at	0.00	0.00
1432335_at	0.00	0.00
1432336_at	0.00	0.00
1432337_at	0.00	0.00
1432338_at	0.00	0.00
1432339_at	0.00	0.00
1432340_at	0.00	0.00
1432341_at	0.00	0.00
1432342_at	0.00	0.00
1432343_at	0.00	0.00
1432345_at	0.00	0.00
1432347_at	0.00	0.00
1432348_at	0.00	0.00
1432351_at	0.00	0.00
1432352_at	0.00	0.00
1432353_at	0.00	0.00
1432354_at	0.00	0.00
1432355_at	0.00	0.00
1432356_at	0.00	0.00
1432357_at	0.00	0.00
1432358_at	0.00	0.00
1432359_at	0.00	0.00
1432361_a_at	0.00	0.07
1432362_at	0.00	0.00

1432363_at	0.00	0.00
1432364_at	0.00	0.00
1432365_a_at	0.00	0.00
1432366_at	0.00	0.00
1432368_at	0.00	0.00
1432369_at	0.00	0.00
1432370_at	0.00	0.00
1432371_a_at	0.00	0.00
1432373_at	0.00	0.00
1432374_a_at	0.00	0.00
1432375_a_at	0.00	0.00
1432376_at	0.00	0.00
1432378_at	0.00	0.00
1432379_at	0.00	0.00
1432380_s_at	0.00	0.00
1432382_at	0.00	0.00
1432383_a_at	0.00	0.00
1432386_a_at	0.00	0.00
1432387_at	0.00	0.00
1432388_at	0.00	0.00
1432389_at	0.00	0.00
1432390_at	0.00	0.00
1432391_at	0.00	0.00
1432393_a_at	0.00	0.00
1432395_at	0.00	0.00
1432396_at	0.00	0.00
1432397_at	0.00	0.00
1432398_at	0.00	0.00
1432401_a_at	0.00	0.00
1432402_at	0.00	0.00
1432403_at	0.00	0.00
1432404_at	0.00	0.00
1432406_at	0.00	0.00
1432407_at	0.00	0.00
1432409_at	0.00	0.00
1432412_at	0.00	0.00
1432413_at	0.00	0.00
1432420_a_at	0.00	0.00
1432421_at	0.00	0.00
1432422_at	0.00	0.00
1432423_a_at	0.00	0.00
1432424_at	0.00	0.00
1432425_at	0.00	0.00
1432427_at	0.00	0.00
1432428_at	0.00	0.00
1432429_at	0.00	0.00
1432433_at	0.00	0.00
1432434_at	0.00	0.00
1432437_at	0.00	0.00
1432438_at	0.00	0.00
1432439_at	0.00	0.00
1432440_at	0.00	0.00
1432441_at	0.00	0.00
1432442_at	0.00	0.00
1432443_at	0.00	0.00
1432445_at	0.00	0.00

1432446_at	0.00	0.00
1432447_a_at	0.00	0.04
1432448_at	0.00	0.00
1432449_at	0.00	0.00
1432450_at	0.00	0.00
1432451_at	0.00	0.00
1432452_at	0.00	0.00
1432454_at	0.00	0.00
1432455_a_at	0.00	0.00
1432456_at	0.00	0.00
1432457_at	0.00	0.00
1432458_at	0.00	0.00
1432460_at	0.00	0.00
1432461_at	0.00	0.00
1432463_at	0.00	0.00
1432464_a_at	0.00	0.00
1432465_at	0.00	0.00
1432467_at	0.00	0.00
1432468_at	0.00	0.00
1432469_at	0.00	0.00
1432470_at	0.00	0.00
1432471_at	0.00	0.00
1432472_a_at	0.00	0.00
1432473_a_at	0.00	0.00
1432475_at	0.00	0.00
1432476_at	0.00	0.00
1432477_at	0.00	0.00
1432481_a_at	0.00	0.00
1432482_at	0.00	0.00
1432483_at	0.00	0.00
1432484_at	0.00	0.00
1432485_at	0.00	0.00
1432487_at	0.00	0.00
1432489_a_at	0.00	0.00
1432493_at	0.00	0.00
1432494_a_at	0.00	0.38
1432495_at	0.00	0.00
1432496_at	0.00	0.00
1432497_at	0.00	0.00
1432498_at	0.00	0.00
1432500_at	0.00	0.00
1432501_at	0.00	0.00
1432502_at	0.00	0.00
1432504_at	0.00	0.00
1432505_at	0.00	0.00
1432506_at	0.00	0.00
1432507_at	0.00	0.00
1432508_at	0.00	0.00
1432509_at	0.00	0.00
1432510_at	0.00	0.00
1432511_s_at	0.00	0.00
1432512_at	0.00	0.00
1432513_a_at	0.00	0.00
1432514_at	0.00	0.00
1432515_at	0.00	0.00
1432516_at	0.00	0.00

1432518_at	0.00	0.00
1432519_at	0.00	0.00
1432520_at	0.00	0.00
1432521_at	0.00	0.00
1432522_s_at	0.00	0.00
1432523_at	0.00	0.00
1432524_at	0.00	0.00
1432525_at	0.00	0.00
1432527_at	0.00	0.00
1432528_at	0.00	0.00
1432529_at	0.00	0.00
1432530_s_at	0.00	0.00
1432531_at	0.00	0.00
1432532_at	0.00	0.00
1432534_at	0.00	0.00
1432535_at	0.00	0.00
1432536_at	0.00	0.00
1432537_at	0.00	0.00
1432540_at	0.00	0.00
1432541_at	0.00	0.00
1432542_at	0.00	0.00
1432544_at	0.00	0.00
1432545_at	0.00	0.00
1432546_at	0.00	0.00
1432547_at	0.00	0.00
1432548_at	0.00	0.00
1432549_s_at	0.00	0.00
1432550_at	0.00	0.00
1432551_at	0.00	0.00
1432552_at	0.00	0.00
1432553_at	0.00	0.00
1432554_at	0.00	0.00
1432555_at	0.00	0.00
1432556_a_at	0.00	0.00
1432557_at	0.00	0.00
1432559_at	0.00	0.00
1432560_at	0.00	0.00
1432561_at	0.00	0.00
1432562_at	0.00	0.00
1432563_at	0.00	0.00
1432564_at	0.00	0.00
1432565_at	0.00	0.00
1432566_at	0.00	0.00
1432567_at	0.00	0.00
1432568_at	0.00	0.00
1432569_at	0.00	0.00
1432570_at	0.00	0.00
1432571_at	0.00	0.00
1432572_at	0.00	0.00
1432573_at	0.00	0.00
1432574_at	0.00	0.00
1432575_at	0.00	0.00
1432576_at	0.00	0.00
1432577_at	0.00	0.00
1432578_at	0.00	0.00
1432579_at	0.00	0.00

1432580_at	0.00	0.00
1432581_at	0.00	0.00
1432582_at	0.00	0.00
1432583_at	0.00	0.00
1432584_at	0.00	0.00
1432585_at	0.00	0.00
1432586_at	0.00	0.00
1432587_at	0.00	0.00
1432588_at	0.00	0.00
1432589_at	0.00	0.00
1432590_at	0.00	0.00
1432591_at	0.00	0.00
1432592_at	0.00	0.00
1432593_at	0.00	0.00
1432594_at	0.00	0.00
1432595_at	0.00	0.00
1432596_at	0.00	0.00
1432597_at	0.00	0.00
1432598_at	0.00	0.00
1432599_at	0.00	0.00
1432600_at	0.00	0.00
1432601_at	0.00	0.00
1432602_at	0.00	0.00
1432603_at	0.00	0.00
1432604_at	0.00	0.00
1432605_at	0.00	0.00
1432606_at	0.00	0.00
1432607_at	0.00	0.00
1432608_at	0.00	0.00
1432609_at	0.00	0.00
1432610_at	0.00	0.00
1432611_at	0.00	0.00
1432612_at	0.00	0.00
1432613_at	0.00	0.00
1432614_at	0.00	0.00
1432615_at	0.00	0.00
1432616_at	0.00	0.00
1432617_at	0.00	0.00
1432618_at	0.00	0.00
1432619_at	0.00	0.00
1432620_at	0.00	0.00
1432621_at	0.00	0.00
1432624_at	0.00	0.00
1432625_at	0.00	0.00
1432626_at	0.00	0.00
1432627_at	0.00	0.00
1432629_at	0.00	0.00
1432630_at	0.00	0.00
1432631_at	0.00	0.00
1432632_at	0.00	0.00
1432633_at	0.00	0.00
1432636_at	0.00	0.00
1432637_at	0.00	0.00
1432638_at	0.00	0.00
1432639_at	0.00	0.00
1432640_at	0.00	0.00

1432641_at	0.00	0.00
1432642_at	0.00	0.00
1432643_at	0.00	0.00
1432644_at	0.00	0.00
1432645_at	0.00	0.00
1432648_at	0.00	0.00
1432649_at	0.00	0.00
1432650_at	0.00	0.00
1432651_at	0.00	0.00
1432652_at	0.00	0.00
1432653_at	0.00	0.00
1432654_at	0.00	0.00
1432655_at	0.00	0.00
1432656_at	0.00	0.00
1432658_at	0.00	0.00
1432659_at	0.00	0.00
1432660_at	0.00	0.00
1432661_at	0.00	0.00
1432662_at	0.00	0.00
1432663_at	0.00	0.00
1432664_at	0.00	0.00
1432665_at	0.00	0.00
1432666_at	0.00	0.00
1432667_at	0.00	0.00
1432668_at	0.00	0.00
1432669_at	0.00	0.00
1432670_at	0.00	0.00
1432671_at	0.00	0.00
1432672_at	0.00	0.00
1432673_at	0.00	0.00
1432674_at	0.00	0.00
1432676_at	0.00	0.00
1432677_at	0.00	0.00
1432678_at	0.00	0.00
1432679_at	0.00	0.00
1432680_at	0.00	0.00
1432681_at	0.00	0.00
1432682_at	0.00	0.00
1432683_at	0.00	0.00
1432684_at	0.00	0.00
1432685_at	0.00	0.00
1432686_at	0.00	0.00
1432687_at	0.00	0.00
1432688_at	0.00	0.00
1432689_at	0.00	0.00
1432690_at	0.00	0.00
1432691_at	0.00	0.00
1432692_at	0.00	0.00
1432693_at	0.00	0.00
1432694_at	0.00	0.00
1432695_at	0.00	0.00
1432696_at	0.00	0.00
1432697_at	0.00	0.00
1432698_at	0.00	0.00
1432699_at	0.00	0.00
1432700_at	0.00	0.00

1432701_at	0.00	0.00
1432702_at	0.00	0.00
1432703_at	0.00	0.00
1432704_at	0.00	0.00
1432705_at	0.00	0.00
1432706_at	0.00	0.00
1432707_at	0.00	0.00
1432708_at	0.00	0.00
1432709_at	0.00	0.00
1432710_at	0.00	0.00
1432711_at	0.00	0.00
1432712_at	0.00	0.00
1432713_at	0.00	0.00
1432714_at	0.00	0.00
1432715_at	0.00	0.00
1432716_at	0.00	0.00
1432717_at	0.00	0.00
1432718_at	0.00	0.00
1432719_at	0.00	0.00
1432720_at	0.00	0.00
1432721_at	0.00	0.00
1432722_at	0.00	0.00
1432723_at	0.00	0.00
1432724_at	0.00	0.00
1432725_at	0.00	0.00
1432726_at	0.00	0.00
1432727_at	0.00	0.00
1432728_at	0.00	0.00
1432729_at	0.00	0.00
1432730_at	0.00	0.00
1432731_at	0.00	0.00
1432732_at	0.00	0.00
1432733_at	0.00	0.00
1432734_at	0.00	0.00
1432735_at	0.00	0.00
1432736_at	0.00	0.00
1432737_at	0.00	0.00
1432738_at	0.00	0.00
1432739_at	0.00	0.00
1432740_at	0.00	0.00
1432741_at	0.00	0.00
1432742_at	0.00	0.00
1432743_at	0.00	0.00
1432744_at	0.00	0.00
1432745_at	0.00	0.00
1432746_at	0.00	0.00
1432747_at	0.00	0.00
1432748_at	0.00	0.00
1432749_at	0.00	0.00
1432750_at	0.00	0.00
1432751_at	0.00	0.00
1432752_at	0.00	0.00
1432753_at	0.00	0.00
1432754_at	0.00	0.00
1432755_at	0.00	0.00
1432756_at	0.00	0.00

1432757_at	0.00	0.00
1432758_at	0.00	0.00
1432759_at	0.00	0.00
1432760_at	0.00	0.00
1432761_at	0.00	0.00
1432762_at	0.00	0.00
1432764_at	0.00	0.00
1432765_at	0.00	0.00
1432766_at	0.00	0.00
1432767_at	0.00	0.00
1432768_at	0.00	0.00
1432769_at	0.00	0.00
1432770_at	0.00	0.00
1432771_at	0.00	0.00
1432772_at	0.00	0.00
1432773_at	0.00	0.00
1432774_at	0.00	0.00
1432775_at	0.00	0.00
1432776_at	0.00	0.00
1432777_at	0.00	0.00
1432778_at	0.00	0.00
1432779_at	0.00	0.00
1432780_at	0.00	0.00
1432781_at	0.00	0.00
1432782_at	0.00	0.00
1432783_at	0.00	0.00
1432784_at	0.00	0.00
1432785_at	0.00	0.00
1432786_at	0.00	0.00
1432787_at	0.00	0.00
1432788_at	0.00	0.00
1432789_at	0.00	0.00
1432790_at	0.00	0.00
1432791_at	0.00	0.00
1432792_at	0.00	0.00
1432793_at	0.00	0.00
1432794_at	0.00	0.00
1432795_at	0.00	0.00
1432796_at	0.00	0.00
1432797_at	0.00	0.00
1432798_at	0.00	0.00
1432799_at	0.00	0.00
1432800_at	0.00	0.00
1432801_at	0.00	0.00
1432802_at	0.00	0.00
1432803_at	0.00	0.00
1432804_at	0.00	0.00
1432805_at	0.00	0.00
1432806_at	0.00	0.00
1432807_at	0.00	0.00
1432808_at	0.00	0.00
1432809_at	0.00	0.00
1432810_at	0.00	0.00
1432811_at	0.00	0.00
1432812_at	0.00	0.00
1432813_at	0.00	0.00

1432814_at	0.00	0.00
1432815_at	0.00	0.00
1432816_s_at	0.00	0.00
1432817_x_at	0.00	0.00
1432818_at	0.00	0.00
1432819_at	0.00	0.00
1432822_at	0.00	0.00
1432823_at	0.00	0.00
1432824_at	0.00	0.00
1432825_at	0.00	0.00
1432830_at	0.00	0.00
1432831_at	0.00	0.00
1432832_at	0.00	0.00
1432833_at	0.00	0.00
1432836_at	0.00	0.00
1432837_at	0.00	0.00
1432838_at	0.00	0.00
1432839_at	0.00	0.00
1432840_at	0.00	0.00
1432841_at	0.00	0.00
1432842_s_at	0.00	0.00
1432843_at	0.00	0.00
1432844_at	0.00	0.00
1432846_at	0.00	0.00
1432847_at	0.00	0.00
1432850_at	0.00	0.00
1432851_at	0.00	0.00
1432852_at	0.00	0.00
1432853_at	0.00	0.00
1432854_at	0.00	0.00
1432855_at	0.00	0.00
1432856_at	0.00	0.00
1432857_at	0.00	0.00
1432858_at	0.00	0.00
1432859_at	0.00	0.00
1432860_at	0.00	0.00
1432861_at	0.00	0.00
1432864_at	0.00	0.00
1432865_at	0.00	0.00
1432866_at	0.00	0.00
1432867_at	0.00	0.00
1432868_at	0.00	0.00
1432869_at	0.00	0.00
1432870_at	0.00	0.00
1432871_at	0.00	0.00
1432872_at	0.00	0.00
1432873_at	0.00	0.00
1432874_at	0.00	0.00
1432875_at	0.00	0.00
1432876_at	0.00	0.00
1432877_at	0.00	0.00
1432878_at	0.00	0.00
1432879_at	0.00	0.00
1432880_at	0.00	0.00
1432881_at	0.00	0.00
1432882_at	0.00	0.00

1432883_at	0.00	0.00
1432884_at	0.00	0.00
1432885_at	0.00	0.00
1432886_at	0.00	0.00
1432887_at	0.00	0.00
1432888_at	0.00	0.00
1432889_at	0.00	0.00
1432890_at	0.00	0.00
1432891_at	0.00	0.00
1432892_at	0.00	0.00
1432893_at	0.00	0.00
1432894_at	0.00	0.00
1432895_at	0.00	0.00
1432896_at	0.00	0.00
1432897_at	0.00	0.00
1432898_at	0.00	0.00
1432899_at	0.00	0.00
1432900_at	0.00	0.00
1432901_at	0.00	0.00
1432902_at	0.00	0.00
1432903_at	0.00	0.00
1432904_at	0.00	0.00
1432905_at	0.00	0.00
1432906_at	0.00	0.00
1432907_at	0.00	0.00
1432908_at	0.00	0.00
1432909_at	0.00	0.00
1432910_at	0.00	0.00
1432911_at	0.00	0.00
1432912_at	0.00	0.00
1432913_at	0.00	0.00
1432914_at	0.00	0.00
1432915_at	0.00	0.00
1432916_at	0.00	0.00
1432917_at	0.00	0.00
1432918_at	0.00	0.00
1432919_at	0.00	0.00
1432920_at	0.00	0.00
1432921_at	0.00	0.00
1432922_at	0.00	0.00
1432923_at	0.00	0.00
1432924_at	0.00	0.00
1432925_at	0.00	0.00
1432926_at	0.00	0.00
1432927_at	0.00	0.00
1432928_at	0.00	0.00
1432929_at	0.00	0.00
1432930_at	0.00	0.00
1432931_at	0.00	0.00
1432932_at	0.00	0.00
1432933_at	0.00	0.00
1432934_at	0.00	0.00
1432935_at	0.00	0.00
1432936_at	0.00	0.00
1432937_at	0.00	0.00
1432938_at	0.00	0.00

1432939_at	0.00	0.00
1432940_at	0.00	0.00
1432941_at	0.00	0.00
1432942_at	0.00	0.00
1432943_at	0.00	0.00
1432944_at	0.00	0.00
1432945_at	0.00	0.00
1432946_at	0.00	0.00
1432947_at	0.00	0.00
1432948_at	0.00	0.00
1432949_at	0.00	0.00
1432950_at	0.00	0.00
1432951_at	0.00	0.00
1432952_at	0.00	0.00
1432953_at	0.00	0.00
1432954_at	0.00	0.00
1432955_at	0.00	0.00
1432956_at	0.00	0.00
1432957_at	0.00	0.00
1432958_at	0.00	0.00
1432959_at	0.00	0.00
1432960_at	0.00	0.00
1432961_at	0.00	0.00
1432962_at	0.00	0.00
1432963_at	0.00	0.00
1432964_at	0.00	0.00
1432965_at	0.00	0.00
1432966_at	0.00	0.00
1432967_at	0.00	0.00
1432968_at	0.00	0.00
1432969_at	0.00	0.00
1432970_at	0.00	0.00
1432971_at	0.00	0.00
1432972_at	0.00	0.00
1432973_at	0.00	0.00
1432974_at	0.00	0.00
1432975_at	0.00	0.00
1432976_at	0.00	0.00
1432977_at	0.00	0.00
1432978_at	0.00	0.00
1432979_at	0.00	0.00
1432980_at	0.00	0.00
1432981_at	0.00	0.00
1432982_at	0.00	0.00
1432983_at	0.00	0.00
1432984_at	0.00	0.00
1432985_at	0.00	0.00
1432986_at	0.00	0.00
1432987_at	0.00	0.00
1432988_at	0.00	0.00
1432989_at	0.00	0.00
1432990_at	0.00	0.00
1432991_at	0.00	0.00
1432992_at	0.00	0.00
1432993_at	0.00	0.00
1432994_at	0.00	0.00

1432995_at	0.00	0.00
1432996_at	0.00	0.00
1432997_at	0.00	0.00
1432998_at	0.00	0.00
1432999_at	0.00	0.00
1433000_at	0.00	0.00
1433001_at	0.00	0.00
1433002_at	0.00	0.00
1433003_at	0.00	0.00
1433004_at	0.00	0.00
1433005_at	0.00	0.00
1433006_at	0.00	0.00
1433007_at	0.00	0.00
1433008_at	0.00	0.00
1433009_at	0.00	0.00
1433010_at	0.00	0.00
1433011_at	0.00	0.00
1433012_at	0.00	0.00
1433013_at	0.00	0.00
1433014_at	0.00	0.00
1433015_at	0.00	0.00
1433016_s_at	0.00	0.00
1433017_at	0.00	0.00
1433018_at	0.00	0.00
1433019_at	0.00	0.00
1433020_at	0.00	0.00
1433021_at	0.00	0.00
1433022_at	0.00	0.00
1433023_at	0.00	0.00
1433024_at	0.00	0.00
1433025_x_at	0.00	0.00
1433026_at	0.00	0.00
1433027_at	0.00	0.00
1433028_at	0.00	0.00
1433030_at	0.00	0.00
1433031_at	0.00	0.00
1433032_at	0.00	0.00
1433033_at	0.00	0.00
1433034_at	0.00	0.00
1433035_at	0.00	0.00
1433036_at	0.00	0.00
1433037_at	0.00	0.00
1433038_at	0.00	0.00
1433039_at	0.00	0.00
1433040_at	0.00	0.00
1433041_at	0.00	0.00
1433042_at	0.00	0.00
1433043_at	0.00	0.00
1433044_at	0.00	0.00
1433045_at	0.00	0.00
1433046_at	0.00	0.00
1433047_at	0.00	0.00
1433048_at	0.00	0.00
1433049_at	0.00	0.00
1433050_at	0.00	0.00
1433051_at	0.00	0.00

1433053_at	0.00	0.00
1433054_at	0.00	0.00
1433055_at	0.00	0.00
1433056_at	0.00	0.00
1433057_at	0.00	0.00
1433058_at	0.00	0.00
1433059_at	0.00	0.00
1433060_at	0.00	0.00
1433061_at	0.00	0.00
1433062_at	0.00	0.00
1433063_at	0.00	0.00
1433064_at	0.00	0.00
1433065_at	0.00	0.00
1433066_at	0.00	0.00
1433067_at	0.00	0.00
1433068_at	0.00	0.00
1433069_at	0.00	0.00
1433070_at	0.00	0.00
1433071_at	0.00	0.00
1433072_at	0.00	0.00
1433073_at	0.00	0.00
1433074_at	0.00	0.00
1433075_at	0.00	0.00
1433076_at	0.00	0.00
1433077_at	0.00	0.00
1433078_at	0.00	0.00
1433079_at	0.00	0.00
1433080_at	0.00	0.00
1433081_at	0.00	0.00
1433082_at	0.00	0.00
1433083_at	0.00	0.00
1433084_at	0.00	0.00
1433085_at	0.00	0.00
1433086_at	0.00	0.00
1433087_at	0.00	0.00
1433088_at	0.00	0.00
1433089_at	0.00	0.00
1433090_at	0.00	0.00
1433091_at	0.00	0.00
1433092_at	0.00	0.00
1433093_at	0.00	0.00
1433094_at	0.00	0.00
1433095_at	0.00	0.00
1433096_at	0.00	0.00
1433097_at	0.00	0.00
1433098_at	0.00	0.00
1433099_at	0.00	0.00
1433100_at	0.00	0.00
1433101_at	0.00	0.00
1433102_at	0.00	0.00
1433103_at	0.00	0.00
1433104_at	0.00	0.00
1433105_at	0.00	0.00
1433106_at	0.00	0.00
1433107_at	0.00	0.00
1433108_at	0.00	0.00

1433109_at	0.00	0.00
1433110_at	0.00	0.00
1433111_at	0.00	0.00
1433112_at	0.00	0.00
1433113_at	0.00	0.00
1433114_at	0.00	0.00
1433115_at	0.00	0.00
1433116_at	0.00	0.00
1433117_at	0.00	0.00
1433118_at	0.00	0.00
1433119_at	0.00	0.00
1433120_at	0.00	0.00
1433121_at	0.00	0.00
1433122_at	0.00	0.00
1433123_at	0.00	0.00
1433124_at	0.00	0.00
1433125_at	0.00	0.00
1433126_at	0.00	0.00
1433127_at	0.00	0.00
1433128_at	0.00	0.00
1433129_at	0.00	0.00
1433130_at	0.00	0.00
1433131_at	0.00	0.00
1433132_at	0.00	0.00
1433133_at	0.00	0.00
1433134_at	0.00	0.00
1433135_at	0.00	0.00
1433136_at	0.00	0.00
1433137_at	0.00	0.00
1433138_at	0.00	0.00
1433141_at	0.00	0.00
1433142_at	0.00	0.00
1433143_at	0.00	0.00
1433145_at	0.00	0.00
1433146_at	0.00	0.00
1433147_at	0.00	0.00
1433148_at	0.00	0.00
1433149_at	0.00	0.00
1433150_at	0.00	0.00
1433151_at	0.00	0.00
1433152_at	0.00	0.00
1433153_at	0.00	0.00
1433154_at	0.00	0.00
1433155_at	0.00	0.00
1433157_at	0.00	0.00
1433158_at	0.00	0.00
1433159_at	0.00	0.00
1433160_at	0.00	0.00
1433161_at	0.00	0.00
1433162_at	0.00	0.00
1433163_at	0.00	0.00
1433164_at	0.00	0.00
1433165_at	0.00	0.00
1433166_at	0.00	0.00
1433167_at	0.00	0.00
1433168_x_at	0.00	0.00

1433169_at	0.00	0.00
1433170_at	0.00	0.00
1433171_at	0.00	0.00
1433172_at	0.00	0.00
1433173_at	0.00	0.00
1433174_a_at	0.00	0.00
1433175_at	0.00	0.00
1433176_at	0.00	0.00
1433177_at	0.00	0.00
1433178_at	0.00	0.00
1433179_at	0.00	0.00
1433180_at	0.00	0.00
1433181_at	0.00	0.00
1433182_at	0.00	0.00
1433183_at	0.00	0.00
1433184_at	0.00	0.00
1433185_at	0.00	0.00
1433186_at	0.00	0.00
1433187_at	0.00	0.00
1433188_at	0.00	0.00
1433189_at	0.00	0.00
1433190_at	0.00	0.00
1433191_at	0.00	0.00
1433192_at	0.00	0.00
1433193_at	0.00	0.00
1433194_at	0.00	0.00
1433195_at	0.00	0.00
1433196_at	0.00	0.00
1433197_at	0.00	0.00
1433198_at	0.00	0.00
1433199_at	0.00	0.00
1433200_at	0.00	0.00
1433201_at	0.00	0.00
1433202_at	0.00	0.00
1433203_at	0.00	0.00
1433204_at	0.00	0.00
1433205_at	0.00	0.00
1433206_at	0.00	0.00
1433207_at	0.00	0.00
1433208_at	0.00	0.00
1433209_at	0.00	0.00
1433212_at	0.00	0.00
1433213_at	0.00	0.00
1433214_x_at	0.00	0.00
1433215_at	0.00	0.00
1433216_at	0.00	0.00
1433217_at	0.00	0.00
1433218_at	0.00	0.00
1433219_at	0.00	0.00
1433220_at	0.00	0.00
1433221_at	0.00	0.00
1433222_at	0.00	0.00
1433223_at	0.00	0.00
1433224_at	0.00	0.00
1433225_at	0.00	0.00
1433226_at	0.00	0.00

1433227_at	0.00	0.00
1433228_at	0.00	0.00
1433229_at	0.00	0.00
1433230_at	0.00	0.00
1433231_at	0.00	0.00
1433232_at	0.00	0.00
1433233_at	0.00	0.00
1433234_at	0.00	0.00
1433235_at	0.00	0.00
1433236_at	0.00	0.00
1433237_at	0.00	0.00
1433238_at	0.00	0.00
1433239_at	0.00	0.00
1433240_at	0.00	0.00
1433241_at	0.00	0.00
1433242_at	0.00	0.00
1433243_at	0.00	0.00
1433244_at	0.00	0.00
1433245_at	0.00	0.00
1433246_at	0.00	0.00
1433247_at	0.00	0.00
1433248_at	0.00	0.00
1433249_at	0.00	0.00
1433250_at	0.00	0.00
1433251_at	0.00	0.00
1433252_at	0.00	0.00
1433253_at	0.00	0.00
1433254_at	0.00	0.00
1433255_at	0.00	0.00
1433256_at	0.00	0.00
1433257_at	0.00	0.00
1433258_at	0.00	0.00
1433259_at	0.00	0.00
1433260_at	0.00	0.00
1433261_at	0.00	0.00
1433262_at	0.00	0.00
1433263_at	0.00	0.00
1433264_at	0.00	0.00
1433265_at	0.00	0.00
1433266_at	0.00	0.00
1433267_at	0.00	0.00
1433268_at	0.00	0.00
1433269_at	0.00	0.00
1433270_at	0.00	0.00
1433271_at	0.00	0.00
1433272_at	0.00	0.00
1433273_at	0.00	0.00
1433274_at	0.00	0.00
1433275_at	0.00	0.00
1433276_at	0.00	0.00
1433277_at	0.00	0.00
1433278_at	0.00	0.00
1433279_at	0.00	0.00
1433280_at	0.00	0.00
1433281_at	0.00	0.00
1433282_at	0.00	0.00

1433283_s_at	0.00	0.00
1433284_at	0.00	0.00
1433285_at	0.00	0.00
1433286_at	0.00	0.00
1433287_at	0.00	0.00
1433288_at	0.00	0.00
1433289_at	0.00	0.00
1433290_at	0.00	0.00
1433291_at	0.00	0.00
1433292_at	0.00	0.00
1433293_at	0.00	0.00
1433294_at	0.00	0.00
1433295_at	0.00	0.00
1433296_at	0.00	0.00
1433297_at	0.00	0.00
1433298_at	0.00	0.00
1433299_at	0.00	0.00
1433300_at	0.00	0.00
1433301_at	0.00	0.00
1433302_at	0.00	0.00
1433303_at	0.00	0.00
1433304_at	0.00	0.00
1433305_at	0.00	0.00
1433306_at	0.00	0.00
1433307_at	0.00	0.00
1433308_at	0.00	0.00
1433309_at	0.00	0.00
1433310_at	0.00	0.00
1433311_at	0.00	0.00
1433312_at	0.00	0.00
1433313_at	0.00	0.00
1433314_at	0.00	0.00
1433315_at	0.00	0.00
1433316_at	0.00	0.00
1433317_at	0.00	0.00
1433318_at	0.00	0.00
1433319_at	0.00	0.00
1433320_at	0.00	0.00
1433321_at	0.00	0.00
1433322_at	0.00	0.00
1433323_at	0.00	0.00
1433324_at	0.00	0.00
1433325_at	0.00	0.00
1433326_at	0.00	0.00
1433327_at	0.00	0.00
1433328_at	0.00	0.00
1433329_at	0.00	0.00
1433330_at	0.00	0.00
1433331_at	0.00	0.00
1433332_at	0.00	0.00
1433333_at	0.00	0.00
1433334_at	0.00	0.00
1433335_at	0.00	0.00
1433336_at	0.00	0.00
1433337_at	0.00	0.00
1433338_at	0.00	0.00

1433339_at	0.00	0.00
1433340_at	0.00	0.00
1433341_at	0.00	0.00
1433342_at	0.00	0.00
1433343_at	0.00	0.00
1433344_at	0.00	0.00
1433345_s_at	0.00	0.00
1433346_at	0.00	0.00
1433347_at	0.00	0.00
1433348_at	0.00	0.00
1433349_at	0.00	0.00
1433350_at	0.00	0.00
1433351_at	0.00	0.00
1433352_at	0.00	0.00
1433353_at	0.00	0.00
1433354_at	0.00	0.00
1433355_at	0.00	0.00
1433356_at	0.00	0.00
1433357_at	0.00	0.00
1433358_at	0.00	0.00
1433359_at	0.00	0.00
1433360_at	0.00	0.00
1433361_at	0.00	0.00
1433362_at	0.00	0.00
1433363_at	0.00	0.00
1433364_at	0.00	0.00
1433365_at	0.00	0.00
1433366_at	0.00	0.00
1433367_at	0.00	0.00
1433368_x_at	0.00	0.00
1433369_at	0.00	0.00
1433370_at	0.00	0.00
1433371_at	0.00	0.00
1433372_at	0.00	0.00
1433373_at	0.00	0.00
1433374_at	0.00	0.00
1433375_at	0.00	0.00
1433376_at	0.00	0.00
1433377_at	0.00	0.00
1433378_at	0.00	0.00
1433379_at	0.00	0.00
1433380_at	0.00	0.00
1433381_at	0.00	0.00
1433382_at	0.00	0.00
1433383_at	0.00	0.00
1433384_at	0.00	0.00
1433385_at	0.00	0.00
1433386_at	0.00	0.00
1433387_at	0.00	0.00
1433388_at	0.00	0.00
1433389_at	0.00	0.00
1433390_at	0.00	0.00
1433391_at	0.00	0.00
1433392_at	0.00	0.00
1433393_at	0.00	0.00
1433394_at	0.00	0.00

1433395_at	0.00	0.00
1433396_at	0.00	0.00
1433397_at	0.00	0.00
1433398_at	0.00	0.00
1433399_at	0.00	0.00
1433400_at	0.00	0.00
1433401_at	0.00	0.00
1433402_at	0.00	0.00
1433403_at	0.00	0.00
1433404_at	0.00	0.00
1433405_at	0.00	0.00
1433406_at	0.00	0.00
1433409_at	0.00	0.00
1433410_at	0.00	0.00
1433411_at	0.00	0.00
1433412_at	0.00	0.00
1433413_at	0.00	0.00
1433414_at	0.00	0.00
1433415_at	0.00	0.00
1433416_at	0.00	0.00
1433417_at	0.00	0.00
1433418_at	0.00	0.00
1433419_at	0.00	0.00
1433420_at	0.00	0.00
1433421_at	0.00	0.00
1433422_at	0.00	0.00
1433423_at	0.00	0.00
1433424_at	0.00	0.00
1433425_at	0.00	0.00
1433426_at	0.00	0.00
1433427_at	0.00	0.00
1433434_at	0.00	0.00
1433435_at	0.00	0.00
1433441_at	0.00	0.00
1433449_at	0.00	0.00
1433452_at	0.00	0.00
1433453_a_at	0.00	0.00
1433454_at	0.00	0.00
1433455_at	0.00	0.00
1433456_at	0.00	0.00
1433458_at	0.00	0.00
1433464_at	0.00	0.00
1433465_a_at	0.51	0.13
1433466_at	0.00	0.00
1433468_at	0.00	0.00
1433469_at	0.00	0.00
1433481_at	0.00	0.00
1433484_at	0.00	0.00
1433498_at	0.00	0.00
1433499_at	0.00	0.00
1433500_at	0.00	0.00
1433501_at	0.00	0.00
1433503_at	0.00	0.00
1433505_a_at	0.00	0.00
1433506_at	0.00	0.00
1433522_at	0.00	0.00

1433523_at	0.00	0.18
1433524_at	0.00	0.00
1433525_at	0.00	0.00
1433526_at	0.00	0.00
1433528_at	0.00	0.00
1433529_at	0.00	0.00
1433536_at	0.00	0.07
1433537_at	0.00	0.00
1433539_at	0.17	0.18
1433542_at	0.00	0.00
1433544_at	0.00	0.00
1433547_s_at	0.00	0.00
1433551_at	0.00	0.00
1433553_at	0.00	0.00
1433555_at	0.00	0.00
1433556_at	0.00	0.17
1433557_at	0.00	0.00
1433559_at	0.00	0.00
1433560_at	0.00	0.00
1433561_at	0.00	0.00
1433564_at	0.00	0.01
1433566_at	0.00	0.00
1433567_at	0.00	0.00
1433571_at	0.00	0.01
1433577_at	0.00	0.00
1433578_at	0.00	0.00
1433583_at	0.00	0.00
1433586_at	0.00	0.00
1433587_at	0.00	0.00
1433590_at	0.00	0.00
1433592_at	0.00	0.00
1433596_at	0.02	0.00
1433599_at	0.00	0.00
1433600_at	0.00	0.00
1433601_at	0.00	0.00
1433602_at	0.00	0.00
1433607_at	0.00	0.00
1433608_at	0.00	0.00
1433610_at	0.00	0.00
1433614_at	0.00	0.00
1433615_at	0.00	0.00
1433617_s_at	0.00	0.00
1433619_at	0.00	0.00
1433620_at	0.00	0.00
1433621_at	0.00	0.00
1433622_at	0.00	0.11
1433623_at	0.00	0.00
1433624_at	0.00	0.00
1433625_at	0.00	0.00
1433626_at	0.00	0.00
1433630_at	0.00	0.00
1433632_at	0.00	0.00
1433633_at	0.00	0.00
1433634_at	0.00	0.00
1433635_at	0.00	0.09
1433637_at	0.00	0.00

1433638_s_at	0.00	0.00
1433641_at	0.00	0.00
1433642_at	0.00	0.00
1433643_at	0.00	0.00
1433644_at	0.00	0.00
1433646_at	0.00	0.00
1433647_s_at	0.72	0.15
1433648_at	0.00	0.00
1433649_at	0.00	0.00
1433650_at	0.00	0.00
1433651_at	0.00	0.00
1433652_at	0.00	0.00
1433653_at	0.00	0.00
1433657_at	0.00	0.00
1433664_at	0.00	0.00
1433667_at	0.00	0.00
1433672_at	0.00	0.00
1433673_at	0.00	0.00
1433677_at	0.00	0.00
1433679_at	0.00	0.01
1433681_x_at	0.00	0.00
1433682_at	0.00	0.32
1433686_at	0.00	0.00
1433687_at	0.00	0.00
1433692_at	0.00	0.23
1433694_at	0.00	0.00
1433695_at	0.00	0.00
1433696_at	0.00	0.00
1433697_at	0.00	0.00
1433699_at	0.04	0.00
1433700_at	0.00	0.00
1433701_at	0.00	0.00
1433703_s_at	0.00	0.16
1433707_at	0.00	0.00
1433710_at	0.00	0.00
1433712_at	0.00	0.26
1433713_at	0.00	0.00
1433714_at	0.00	0.00
1433715_at	0.00	0.00
1433716_x_at	0.00	0.00
1433719_at	0.00	0.00
1433722_at	0.00	0.00
1433726_at	0.00	0.00
1433727_at	0.00	0.00
1433728_at	0.00	0.00
1433730_at	0.00	0.00
1433731_at	0.00	0.00
1433732_x_at	0.00	0.15
1433734_at	0.00	0.00
1433735_a_at	0.00	0.00
1433738_at	0.00	0.00
1433739_at	0.00	0.05
1433740_at	0.00	0.00
1433742_at	0.00	0.23
1433743_at	0.00	0.00
1433744_at	0.00	0.00

1433746_at	0.00	0.40
1433747_at	0.00	0.00
1433748_at	0.00	0.00
1433749_at	0.00	0.00
1433751_at	0.00	0.00
1433752_s_at	0.00	0.00
1433754_at	0.00	0.00
1433755_at	0.00	0.00
1433759_at	0.00	0.00
1433761_at	0.00	0.00
1433762_at	0.00	0.00
1433763_at	0.00	0.00
1433764_at	0.00	0.00
1433766_at	0.02	0.00
1433767_at	0.00	0.00
1433768_at	0.00	0.00
1433769_at	0.00	0.00
1433770_at	0.05	0.00
1433771_at	0.01	0.00
1433772_at	0.07	0.00
1433776_at	0.64	0.00
1433777_at	0.00	0.06
1433778_at	0.00	0.05
1433779_at	0.00	0.00
1433781_a_at	0.01	0.00
1433782_at	0.15	0.00
1433787_at	0.00	0.00
1433788_at	0.00	0.00
1433789_at	0.00	0.00
1433790_at	0.00	0.00
1433791_at	0.00	0.00
1433792_at	0.00	0.00
1433793_s_at	0.00	0.00
1433794_at	0.00	0.05
1433795_at	0.00	0.50
1433796_at	0.00	0.00
1433801_at	0.00	0.00
1433802_at	0.00	0.00
1433807_at	0.00	0.00
1433811_at	0.00	0.03
1433812_at	0.00	0.00
1433813_at	0.00	0.00
1433814_at	0.00	0.00
1433815_at	0.00	0.00
1433817_at	0.00	0.00
1433818_at	0.00	0.00
1433819_s_at	0.00	0.04
1433820_a_at	0.00	0.00
1433821_at	0.00	0.00
1433823_at	0.00	0.00
1433825_at	0.00	0.00
1433826_at	0.00	0.00
1433827_at	0.00	0.04
1433828_at	0.00	0.00
1433831_at	0.00	0.00
1433833_at	0.00	0.00

1433836_a_at	0.00	0.00
1433837_at	0.00	0.00
1433838_at	0.00	0.00
1433839_at	0.00	0.00
1433840_a_at	0.00	0.00
1433841_at	0.00	0.00
1433846_s_at	0.00	0.00
1433847_at	0.00	0.00
1433848_at	0.00	0.00
1433849_at	0.00	0.00
1433852_at	0.00	0.00
1433853_at	0.00	0.00
1433854_at	0.00	0.00
1433856_at	0.00	0.00
1433857_at	0.00	0.00
1433858_at	0.00	0.00
1433861_at	0.00	0.00
1433862_at	0.00	0.00
1433864_at	0.00	0.00
1433865_at	0.00	0.00
1433868_at	0.00	0.00
1433870_at	0.00	0.00
1433872_at	0.00	0.00
1433873_s_at	0.00	0.00
1433874_at	0.00	0.00
1433875_at	0.00	0.00
1433876_at	0.00	0.00
1433877_at	0.00	0.00
1433879_a_at	0.00	0.00
1433882_at	0.00	0.00
1433884_at	0.00	0.00
1433885_at	0.00	0.00
1433889_at	0.00	0.00
1433891_at	0.00	0.00
1433894_at	0.00	0.00
1433895_at	0.04	0.00
1433896_at	0.00	0.00
1433897_at	0.00	0.00
1433898_at	0.00	0.20
1433900_at	0.00	0.00
1433902_at	0.01	0.39
1433905_at	0.00	0.00
1433907_at	0.00	0.00
1433911_at	0.00	0.00
1433914_at	0.00	0.00
1433915_s_at	0.00	0.00
1433917_x_at	0.00	0.00
1433920_at	0.00	0.02
1433921_s_at	0.00	0.46
1433922_at	0.00	0.00
1433925_at	0.00	0.00
1433926_at	0.00	0.00
1433927_at	0.00	0.04
1433929_at	0.00	0.00
1433930_at	0.00	0.00
1433931_at	0.00	0.00

1433932_x_at	0.00	0.00
1433933_s_at	0.00	0.00
1433934_at	0.00	0.00
1433937_at	0.00	0.00
1433938_at	0.00	0.00
1433939_at	0.00	0.00
1433943_at	0.00	0.00
1433944_at	0.00	0.00
1433945_at	0.00	0.00
1433947_at	0.00	0.00
1433950_at	0.00	0.00
1433955_at	0.00	0.00
1433957_at	0.00	0.00
1433959_at	0.00	0.00
1433960_at	0.00	0.15
1433962_at	0.00	0.00
1433965_at	0.00	0.31
1433967_at	0.00	0.00
1433968_a_at	0.00	0.00
1433969_at	0.00	0.00
1433970_at	0.00	0.00
1433971_at	0.00	0.12
1433972_at	0.00	0.06
1433973_at	0.00	0.00
1433974_at	0.00	0.01
1433975_at	0.00	0.00
1433976_at	0.00	0.00
1433977_at	0.00	0.00
1433978_at	0.00	0.00
1433979_at	0.00	0.00
1433980_at	0.00	0.00
1433981_s_at	0.00	0.00
1433982_at	0.10	0.00
1433983_at	0.00	0.00
1433985_at	0.00	0.00
1433986_at	0.00	0.00
1433987_at	0.00	0.00
1433988_s_at	0.00	0.00
1433989_at	0.00	0.00
1433990_at	0.00	0.00
1433992_at	0.00	0.00
1433993_at	0.00	0.00
1433994_at	0.00	0.00
1433996_at	0.00	0.00
1433997_at	0.00	0.00
1433998_at	0.00	0.00
1434001_at	0.00	0.01
1434002_at	0.00	0.00
1434006_at	0.00	0.00
1434007_at	0.00	0.00
1434008_at	0.00	0.00
1434013_at	0.00	0.00
1434014_at	0.00	0.00
1434015_at	0.00	0.00
1434018_at	0.00	0.00
1434021_at	0.00	0.00

1434022_at	0.00	0.00
1434023_at	0.00	0.00
1434024_at	0.00	0.00
1434026_at	0.00	0.00
1434028_at	0.00	0.00
1434029_at	0.02	0.00
1434030_at	0.00	0.00
1434031_at	0.00	0.00
1434039_at	0.01	0.00
1434040_at	0.00	0.00
1434041_at	0.00	0.00
1434042_s_at	0.00	0.00
1434046_at	0.00	0.00
1434049_at	0.00	0.00
1434050_at	0.00	0.00
1434051_s_at	0.00	0.00
1434052_at	0.00	0.00
1434055_at	0.00	0.00
1434058_at	0.00	0.00
1434061_at	0.01	0.00
1434063_at	0.00	0.00
1434064_at	0.00	0.10
1434065_at	0.00	0.00
1434067_at	0.00	0.00
1434068_s_at	0.00	0.00
1434069_at	0.00	0.00
1434070_at	0.19	0.01
1434072_at	0.00	0.00
1434073_at	0.00	0.00
1434075_at	0.00	0.00
1434076_at	0.00	0.00
1434077_at	0.00	0.00
1434081_at	0.00	0.00
1434082_at	0.00	0.00
1434083_a_at	0.00	0.00
1434084_at	0.00	0.00
1434085_at	0.00	0.00
1434086_at	0.00	0.00
1434088_at	0.00	0.01
1434090_at	0.00	0.00
1434091_at	0.00	0.00
1434093_at	0.00	0.00
1434094_at	0.00	0.00
1434095_at	0.00	0.00
1434096_at	0.00	0.00
1434097_at	0.00	0.00
1434098_at	0.00	0.00
1434101_at	0.00	0.00
1434102_at	0.00	0.00
1434105_at	0.00	0.00
1434106_at	0.00	0.00
1434107_at	0.00	0.00
1434110_x_at	0.00	0.00
1434111_at	0.00	0.46
1434112_at	0.00	0.47
1434115_at	0.00	0.00

1434122_at	0.00	0.00
1434123_at	0.00	0.00
1434125_at	0.00	0.00
1434126_at	0.00	0.00
1434129_s_at	0.01	0.03
1434130_at	0.00	0.01
1434132_at	0.00	0.00
1434136_at	0.00	0.00
1434139_at	0.00	0.00
1434140_at	0.00	0.00
1434141_at	0.00	0.00
1434142_at	0.00	0.00
1434144_s_at	0.00	0.00
1434146_at	0.00	0.00
1434152_at	0.00	0.00
1434153_at	0.00	0.78
1434154_at	0.00	0.13
1434156_at	0.00	0.33
1434157_at	0.00	0.00
1434158_at	0.00	0.00
1434159_at	0.00	0.00
1434160_at	0.00	0.00
1434161_at	0.00	0.00
1434165_at	0.00	0.00
1434166_at	0.00	0.00
1434168_at	0.00	0.00
1434169_at	0.00	0.00
1434170_at	0.00	0.00
1434171_at	0.00	0.00
1434172_at	0.00	0.00
1434173_s_at	0.00	0.37
1434174_at	0.07	0.00
1434178_at	0.00	0.00
1434179_at	0.01	0.00
1434182_at	0.00	0.00
1434183_at	0.00	0.00
1434185_at	0.00	0.00
1434186_at	0.00	0.00
1434187_at	0.00	0.00
1434188_at	0.00	0.00
1434189_at	0.00	0.00
1434191_at	0.00	0.00
1434192_at	0.00	0.00
1434193_at	0.00	0.00
1434194_at	0.00	0.00
1434197_at	0.00	0.00
1434200_at	0.00	0.00
1434201_at	0.00	0.00
1434202_a_at	0.00	0.00
1434203_at	0.00	0.00
1434207_at	0.00	0.00
1434208_at	0.00	0.00
1434209_at	0.00	0.00
1434211_at	0.00	0.00
1434217_at	0.00	0.00
1434218_at	0.00	0.00

1434219_at	0.00	0.00
1434220_at	0.00	0.10
1434221_at	0.00	0.00
1434222_at	0.00	0.00
1434223_at	0.00	0.00
1434225_at	0.00	0.00
1434226_at	0.00	0.00
1434228_at	0.00	0.00
1434232_a_at	0.00	0.00
1434233_at	0.00	0.00
1434234_at	0.00	0.00
1434236_at	0.00	0.00
1434238_at	0.00	0.00
1434240_at	0.00	0.00
1434241_at	0.00	0.00
1434242_at	0.00	0.00
1434245_a_at	0.00	0.00
1434246_at	0.00	0.00
1434247_at	0.00	0.00
1434248_at	0.00	0.00
1434249_s_at	0.00	0.00
1434252_at	0.00	0.00
1434253_s_at	0.00	0.07
1434255_at	0.00	0.00
1434257_s_at	0.00	0.00
1434258_s_at	0.00	0.00
1434259_at	0.00	0.00
1434260_at	0.00	0.00
1434263_at	0.00	0.00
1434264_at	0.00	0.00
1434265_s_at	0.00	0.00
1434266_at	0.00	0.00
1434267_at	0.00	0.00
1434268_at	0.00	0.00
1434269_at	0.00	0.00
1434270_at	0.00	0.00
1434274_at	0.00	0.00
1434275_at	0.00	0.00
1434277_a_at	0.00	0.00
1434283_at	0.00	0.00
1434284_at	0.00	0.00
1434285_at	0.00	0.00
1434286_at	0.00	0.00
1434288_at	0.00	0.00
1434289_at	0.00	0.01
1434290_at	0.00	0.00
1434293_at	0.00	0.00
1434294_at	0.15	0.08
1434295_at	0.00	0.00
1434296_at	0.00	0.00
1434297_at	0.00	0.00
1434298_at	0.00	0.00
1434301_at	0.00	0.33
1434302_at	0.00	0.00
1434303_at	0.00	0.00
1434304_s_at	0.00	0.00

1434307_at	0.00	0.00
1434308_at	0.00	0.00
1434310_at	0.00	0.00
1434313_at	0.00	0.00
1434314_s_at	0.00	0.00
1434315_at	0.00	0.00
1434316_at	0.00	0.00
1434318_a_at	0.00	0.00
1434321_at	0.00	0.00
1434322_at	0.00	0.00
1434327_at	0.00	0.00
1434331_at	0.00	0.00
1434337_at	0.00	0.00
1434338_at	0.00	0.00
1434339_at	0.00	0.00
1434344_at	0.00	0.00
1434346_at	0.00	0.00
1434347_s_at	0.00	0.00
1434349_at	0.00	0.00
1434350_at	0.00	0.00
1434351_at	0.00	0.00
1434352_at	0.00	0.00
1434354_at	0.00	0.00
1434355_at	0.00	0.00
1434359_at	0.00	0.00
1434360_s_at	0.00	0.00
1434361_at	0.00	0.00
1434362_at	0.41	0.91
1434365_a_at	0.00	0.00
1434368_at	0.00	0.00
1434371_x_at	0.01	0.00
1434373_at	0.00	0.00
1434374_at	0.00	0.00
1434375_at	0.00	0.00
1434376_at	0.18	0.00
1434381_at	0.00	0.00
1434382_at	0.00	0.00
1434383_at	0.00	0.00
1434384_at	0.00	0.00
1434385_at	0.00	0.00
1434387_at	0.00	0.00
1434388_at	0.00	0.00
1434389_at	0.00	0.00
1434394_at	0.00	0.01
1434395_at	0.00	0.00
1434397_at	0.00	0.00
1434398_at	0.00	0.29
1434399_at	0.00	0.00
1434401_at	0.00	0.00
1434404_at	0.00	0.00
1434405_at	0.00	0.00
1434406_at	0.00	0.00
1434407_at	0.00	0.00
1434408_at	0.00	0.00
1434409_at	0.00	0.00
1434411_at	0.00	0.00

1434413_at	0.00	0.00
1434414_at	0.00	0.00
1434415_at	0.00	0.00
1434418_at	0.00	0.00
1434421_at	0.00	0.00
1434422_at	0.00	0.00
1434423_at	0.03	0.00
1434424_at	0.00	0.00
1434426_at	0.00	0.00
1434428_at	0.00	0.00
1434429_at	0.00	0.00
1434430_s_at	0.00	0.00
1434431_x_at	0.00	0.00
1434439_at	0.00	0.00
1434441_at	0.00	0.00
1434443_at	0.00	0.00
1434444_s_at	0.00	0.00
1434446_at	0.00	0.33
1434447_at	0.00	0.00
1434448_at	0.00	0.00
1434450_s_at	0.00	0.00
1434451_at	0.00	0.00
1434452_x_at	0.00	0.01
1434454_at	0.00	0.00
1434455_at	0.00	0.00
1434456_at	0.00	0.00
1434457_at	0.00	0.00
1434458_at	0.37	0.00
1434459_at	0.00	0.00
1434460_at	0.00	0.00
1434461_at	0.00	0.00
1434462_at	0.00	0.00
1434463_at	0.00	0.00
1434464_at	0.00	0.00
1434470_at	0.00	0.05
1434473_at	0.00	0.00
1434474_at	0.00	0.00
1434475_at	0.00	0.00
1434476_at	0.00	0.00
1434477_at	0.00	0.23
1434478_at	0.00	0.34
1434480_at	0.00	0.00
1434482_at	0.00	0.04
1434488_at	0.00	0.00
1434490_at	0.00	0.00
1434492_at	0.00	0.00
1434493_at	0.00	0.00
1434494_at	0.00	0.00
1434495_at	0.00	0.00
1434497_at	0.00	0.00
1434498_at	0.00	0.00
1434501_at	0.00	0.00
1434504_at	0.00	0.00
1434505_a_at	0.00	0.00
1434506_at	0.00	0.00
1434508_at	0.00	0.00

1434509_at	0.00	0.00
1434519_at	0.00	0.00
1434520_at	0.00	0.00
1434521_at	0.00	0.01
1434522_at	0.00	0.00
1434524_at	0.00	0.00
1434525_at	0.00	0.00
1434526_at	0.00	0.00
1434527_at	0.00	0.00
1434530_at	0.00	0.00
1434531_at	0.00	0.00
1434532_at	0.00	0.00
1434534_at	0.00	0.00
1434535_at	0.00	0.00
1434536_at	0.00	0.00
1434539_at	0.00	0.00
1434548_at	0.00	0.00
1434550_at	0.00	0.00
1434556_at	0.00	0.00
1434558_at	0.00	0.00
1434559_at	0.00	0.31
1434562_at	0.00	0.00
1434564_at	0.00	0.00
1434565_at	0.22	0.32
1434566_a_at	0.00	0.00
1434567_at	0.00	0.00
1434568_at	0.00	0.02
1434570_at	0.03	0.04
1434571_at	0.00	0.00
1434572_at	0.00	0.00
1434573_at	0.00	0.00
1434574_at	0.00	0.00
1434576_at	0.00	0.00
1434577_at	0.00	0.00
1434580_at	0.00	0.00
1434581_at	0.00	0.00
1434582_at	0.00	0.00
1434583_at	0.00	0.00
1434584_a_at	0.00	0.00
1434590_at	0.00	0.00
1434591_at	0.00	0.00
1434592_at	0.00	0.00
1434593_at	0.00	0.00
1434594_at	0.00	0.00
1434595_at	0.00	0.00
1434601_at	0.00	0.00
1434602_at	0.00	0.00
1434603_at	0.00	0.00
1434613_at	0.00	0.00
1434619_at	0.00	0.00
1434621_at	0.00	0.00
1434622_at	0.00	0.00
1434626_at	0.00	0.00
1434629_at	0.00	0.00
1434630_at	0.00	0.00
1434631_at	0.00	0.00

1434632_at	0.00	0.00
1434634_at	0.00	0.00
1434635_at	0.00	0.00
1434636_at	0.00	0.00
1434638_at	0.00	0.00
1434639_at	0.00	0.00
1434640_at	0.00	0.00
1434645_at	0.00	0.00
1434647_at	0.00	0.00
1434650_at	0.00	0.00
1434652_at	0.00	0.00
1434654_at	0.00	0.00
1434655_at	0.00	0.00
1434656_at	0.00	0.00
1434657_at	0.00	0.00
1434660_at	0.00	0.00
1434662_at	0.00	0.00
1434663_at	0.00	0.00
1434664_at	0.00	0.00
1434665_at	0.00	0.00
1434666_at	0.00	0.00
1434667_at	0.00	0.00
1434668_at	0.00	0.00
1434669_at	0.00	0.00
1434670_at	0.00	0.00
1434671_at	0.00	0.00
1434672_at	0.00	0.00
1434673_at	0.00	0.00
1434675_at	0.00	0.00
1434677_at	0.00	0.00
1434678_at	0.00	0.00
1434680_at	0.00	0.32
1434682_at	0.00	0.00
1434683_at	0.00	0.00
1434684_at	0.00	0.00
1434685_at	0.00	0.00
1434686_at	0.00	0.00
1434687_at	0.00	0.00
1434689_at	0.00	0.00
1434693_at	0.00	0.00
1434696_at	0.00	0.00
1434699_at	0.00	0.00
1434700_at	0.00	0.00
1434701_at	0.00	0.00
1434706_at	0.00	0.00
1434707_at	0.00	0.00
1434708_at	0.00	0.00
1434710_at	0.00	0.00
1434711_at	0.00	0.00
1434712_at	0.00	0.00
1434713_at	0.00	0.00
1434715_at	0.00	0.00
1434716_at	0.00	0.00
1434717_at	0.00	0.00
1434718_at	0.00	0.00
1434720_at	0.00	0.00

1434721_at	0.00	0.00
1434722_at	0.00	0.00
1434723_at	0.00	0.00
1434724_at	0.00	0.00
1434725_at	0.00	0.00
1434726_at	0.00	0.00
1434727_at	0.00	0.00
1434728_at	0.00	0.00
1434729_at	0.00	0.00
1434730_at	0.00	0.00
1434733_at	0.00	0.00
1434734_at	0.00	0.00
1434737_at	0.00	0.00
1434738_at	0.00	0.00
1434740_at	0.00	0.00
1434741_at	0.00	0.00
1434742_s_at	0.00	0.00
1434746_at	0.00	0.06
1434749_at	0.04	0.00
1434750_at	0.00	0.00
1434753_at	0.00	0.00
1434754_at	0.00	0.00
1434756_at	0.00	0.00
1434758_at	0.00	0.00
1434759_at	0.00	0.00
1434760_at	0.00	0.00
1434761_at	0.00	0.00
1434762_at	0.00	0.00
1434763_at	0.00	0.00
1434764_at	0.00	0.00
1434765_at	0.00	0.00
1434766_at	0.00	0.00
1434769_at	0.00	0.00
1434770_at	0.00	0.00
1434771_at	0.00	0.27
1434774_at	0.00	0.00
1434778_at	0.00	0.00
1434779_at	0.00	0.00
1434780_at	0.00	0.00
1434781_at	0.00	0.00
1434782_at	0.00	0.00
1434786_at	0.00	0.00
1434788_at	0.00	0.00
1434789_at	0.00	0.00
1434792_at	0.00	0.00
1434794_at	0.00	0.00
1434795_at	0.00	0.00
1434797_at	0.00	0.00
1434798_at	0.00	0.00
1434800_at	0.00	0.00
1434802_s_at	0.00	0.00
1434804_at	0.00	0.00
1434806_at	0.00	0.00
1434807_s_at	0.00	0.00
1434808_at	0.00	0.00
1434809_at	0.00	0.02

1434810_a_at	0.00	0.00
1434811_at	0.00	0.00
1434812_s_at	0.00	0.00
1434815_a_at	0.00	0.05
1434816_at	0.00	0.00
1434817_s_at	0.00	0.00
1434818_at	0.00	0.00
1434819_at	0.00	0.00
1434822_at	0.00	0.53
1434825_at	0.00	0.00
1434826_at	0.00	0.00
1434828_at	0.00	0.00
1434829_at	0.00	0.00
1434830_at	0.00	0.00
1434833_at	0.00	0.00
1434834_at	0.00	0.00
1434835_at	0.00	0.00
1434837_at	0.00	0.00
1434838_at	0.00	0.00
1434841_at	0.00	0.00
1434843_at	0.00	0.00
1434845_at	0.00	0.00
1434847_at	0.00	0.00
1434848_at	0.00	0.00
1434849_at	0.00	0.00
1434855_at	0.00	0.00
1434857_at	0.00	0.00
1434858_x_at	0.00	0.00
1434860_at	0.00	0.00
1434861_at	0.00	0.00
1434862_at	0.00	0.00
1434863_at	0.00	0.00
1434864_at	0.00	0.00
1434867_at	0.00	0.00
1434869_at	0.00	0.00
1434870_at	0.00	0.00
1434873_a_at	0.00	0.00
1434874_x_at	0.00	0.00
1434876_at	0.00	0.00
1434878_at	0.00	0.00
1434880_at	0.00	0.00
1434885_at	0.00	0.00
1434887_at	0.00	0.00
1434890_at	0.00	0.00
1434891_at	0.00	0.01
1434894_at	0.00	0.00
1434895_s_at	0.00	0.00
1434902_at	0.00	0.01
1434903_s_at	0.00	0.00
1434905_at	0.00	0.00
1434907_at	0.00	0.00
1434908_at	0.00	0.00
1434909_at	0.00	0.01
1434910_at	0.00	0.00
1434911_s_at	0.00	0.00
1434912_at	0.00	0.00

1434913_at	0.00	0.00
1434915_s_at	0.00	0.00
1434916_at	0.00	0.00
1434917_at	0.98	1.00
1434918_at	0.00	0.00
1434919_at	0.00	0.00
1434921_at	0.00	0.00
1434925_at	0.00	0.00
1434926_at	0.00	0.00
1434928_at	0.00	0.00
1434929_at	0.00	0.00
1434933_at	0.00	0.00
1434934_at	0.00	0.00
1434936_at	0.40	0.07
1434939_at	0.00	0.00
1434945_at	0.00	0.00
1434947_at	0.00	0.00
1434948_at	0.00	0.00
1434949_at	0.00	0.00
1434950_a_at	0.00	0.00
1434951_at	0.00	0.00
1434952_at	0.00	0.00
1434955_at	0.00	0.00
1434956_at	0.00	0.00
1434957_at	0.00	0.00
1434960_at	0.00	0.00
1434961_at	0.00	0.00
1434963_at	0.00	0.00
1434965_at	0.00	0.00
1434966_at	0.00	0.00
1434967_at	0.00	0.00
1434969_at	0.00	0.00
1434973_at	0.00	0.00
1434974_at	0.00	0.00
1434977_at	0.00	0.00
1434978_at	0.00	0.00
1434979_at	0.00	0.00
1434980_at	0.00	0.00
1434982_at	0.00	0.00
1434983_at	0.00	0.00
1434984_at	0.00	0.00
1434989_at	0.00	0.00
1434990_at	0.00	0.00
1434991_at	0.00	0.00
1434993_at	0.00	0.00
1434996_at	0.00	0.00
1434997_at	0.00	0.00
1435000_at	0.00	0.00
1435001_at	0.00	0.00
1435002_at	0.00	0.00
1435004_at	0.00	0.00
1435005_at	0.00	0.00
1435008_at	0.00	0.00
1435009_at	0.00	0.00
1435010_at	0.00	0.00
1435014_at	0.00	0.00

1435016_at	0.00	0.00
1435017_at	0.00	0.31
1435020_at	0.00	0.00
1435021_at	0.00	0.00
1435022_at	0.00	0.00
1435024_at	0.00	0.00
1435025_at	0.00	0.00
1435027_at	0.00	0.00
1435028_at	0.00	0.00
1435029_at	0.00	0.09
1435031_at	0.00	0.00
1435032_at	0.00	0.00
1435033_at	0.00	0.00
1435034_at	0.00	0.00
1435036_at	0.00	0.00
1435037_at	0.00	0.00
1435038_s_at	0.00	0.00
1435040_at	0.12	0.00
1435043_at	0.00	0.00
1435044_at	0.00	0.00
1435045_s_at	0.00	0.00
1435046_at	0.00	0.00
1435047_at	0.00	0.00
1435048_at	0.00	0.00
1435049_s_at	0.00	0.00
1435050_at	0.00	0.00
1435051_at	0.00	0.00
1435052_at	0.00	0.00
1435059_at	0.00	0.00
1435060_at	0.00	0.00
1435061_at	0.00	0.00
1435062_at	0.00	0.00
1435063_at	0.00	0.00
1435065_x_at	0.00	0.00
1435068_at	0.00	0.00
1435069_at	0.00	0.00
1435070_at	0.00	0.00
1435071_at	0.00	0.00
1435072_at	0.00	0.00
1435073_a_at	0.00	0.00
1435074_at	0.00	0.40
1435075_at	0.00	0.00
1435076_at	0.00	0.00
1435078_at	0.00	0.00
1435079_at	0.00	0.00
1435080_x_at	0.00	0.00
1435081_at	0.00	0.00
1435082_at	0.00	0.00
1435083_at	0.00	0.00
1435084_at	0.63	0.42
1435085_at	0.00	0.00
1435088_at	0.00	0.27
1435090_at	0.00	0.00
1435092_at	0.00	0.00
1435093_at	0.00	0.00
1435094_at	0.00	0.00

1435095_at	0.00	0.00
1435096_at	0.00	0.00
1435098_at	0.00	0.00
1435099_at	0.00	0.00
1435100_at	0.00	0.00
1435104_at	0.00	0.00
1435107_at	0.00	0.00
1435108_at	0.00	0.00
1435109_at	0.00	0.00
1435111_at	0.00	0.00
1435115_at	0.00	0.00
1435116_at	0.00	0.00
1435118_at	0.00	0.00
1435119_at	0.00	0.00
1435120_at	0.00	0.00
1435121_at	0.00	0.00
1435123_at	0.00	0.00
1435124_at	0.00	0.00
1435125_at	0.00	0.00
1435126_at	0.00	0.00
1435127_a_at	0.00	0.00
1435131_at	0.00	0.00
1435132_at	0.00	0.00
1435134_at	0.00	0.00
1435135_at	0.00	0.00
1435136_at	0.00	0.00
1435138_at	0.00	0.00
1435141_at	0.00	0.00
1435142_at	0.00	0.00
1435144_at	0.00	0.00
1435145_at	0.00	0.00
1435146_s_at	0.00	0.00
1435147_x_at	0.00	0.00
1435149_at	0.00	0.00
1435150_at	0.00	0.00
1435153_at	0.00	0.00
1435154_at	0.82	0.00
1435155_at	0.00	0.00
1435157_at	0.00	0.00
1435158_at	0.00	0.00
1435159_at	0.00	0.00
1435161_at	0.00	0.00
1435163_at	0.00	0.00
1435165_at	0.00	0.00
1435166_at	0.00	0.00
1435167_at	0.00	0.00
1435168_at	0.40	0.00
1435171_at	0.00	0.00
1435173_at	0.00	0.00
1435175_at	0.00	0.00
1435179_at	0.00	0.00
1435180_at	0.00	0.00
1435181_at	0.00	0.00
1435182_at	0.00	0.00
1435183_at	0.00	0.00
1435185_at	0.00	0.00

1435186_s_at	0.00	0.00
1435187_at	0.00	0.29
1435188_at	0.00	0.00
1435189_at	0.00	0.00
1435190_at	0.00	0.00
1435195_at	0.00	0.00
1435196_at	0.00	0.00
1435197_at	0.00	0.00
1435198_at	0.00	0.00
1435200_at	0.00	0.00
1435201_at	0.00	0.00
1435203_at	0.00	0.00
1435204_at	0.00	0.00
1435205_at	0.00	0.00
1435206_at	0.00	0.00
1435207_at	0.00	0.00
1435208_at	0.00	0.00
1435209_at	0.00	0.00
1435210_s_at	0.00	0.00
1435211_at	0.00	0.32
1435212_at	0.00	0.00
1435213_at	0.00	0.00
1435215_at	0.00	0.00
1435217_at	0.00	0.00
1435218_at	0.00	0.00
1435223_at	0.00	0.00
1435224_at	0.00	0.00
1435225_s_at	0.00	0.00
1435227_at	0.00	0.00
1435229_at	0.00	0.00
1435230_at	0.00	0.00
1435233_at	0.01	0.08
1435234_at	0.00	0.01
1435236_at	0.00	0.00
1435237_at	0.00	0.00
1435238_x_at	0.00	0.00
1435241_at	0.00	0.00
1435242_at	0.00	0.00
1435244_at	0.00	0.00
1435245_at	0.00	0.00
1435246_at	0.00	0.00
1435247_at	0.00	0.00
1435248_a_at	0.00	0.07
1435249_at	0.00	0.00
1435251_at	0.00	0.00
1435253_at	0.00	0.00
1435254_at	0.00	0.00
1435255_at	0.00	0.00
1435256_at	0.00	0.00
1435257_at	0.00	0.00
1435260_at	0.00	0.00
1435261_at	0.00	0.00
1435262_at	0.00	0.00
1435263_at	0.00	0.00
1435264_at	0.00	0.00
1435265_at	0.00	0.00

1435266_at	0.00	0.00
1435267_at	0.00	0.00
1435268_at	0.00	0.00
1435273_at	0.00	0.00
1435274_at	0.00	0.00
1435278_at	0.00	0.00
1435279_at	0.00	0.00
1435280_at	0.00	0.00
1435281_at	0.00	0.00
1435282_at	0.00	0.00
1435283_s_at	0.00	0.00
1435284_at	0.00	0.00
1435285_at	0.06	0.00
1435286_at	0.00	0.00
1435287_at	0.00	0.00
1435289_at	0.00	0.00
1435291_at	0.00	0.00
1435292_at	0.00	0.00
1435293_at	0.00	0.00
1435294_at	0.00	0.00
1435295_at	0.00	0.00
1435296_at	0.00	0.00
1435297_at	0.00	0.00
1435298_at	0.00	0.00
1435299_at	0.00	0.00
1435302_at	0.00	0.01
1435303_at	0.00	0.00
1435305_at	0.00	0.00
1435307_at	0.00	0.00
1435308_at	0.00	0.01
1435310_at	0.00	0.00
1435311_s_at	0.00	0.00
1435312_at	0.00	0.00
1435313_at	0.00	0.00
1435314_at	0.00	0.00
1435318_at	0.00	0.00
1435319_at	0.00	0.00
1435320_at	0.00	0.00
1435325_at	0.00	0.00
1435328_at	0.00	0.00
1435329_at	0.00	0.00
1435332_at	0.00	0.00
1435334_at	0.00	0.00
1435337_at	0.00	0.00
1435338_at	0.00	0.00
1435339_at	0.00	0.00
1435340_at	0.00	0.00
1435342_at	0.00	0.00
1435343_at	0.00	0.00
1435344_at	0.02	0.00
1435345_at	0.00	0.00
1435346_at	0.00	0.00
1435348_at	0.00	0.00
1435349_at	0.00	0.00
1435351_at	0.00	0.00
1435354_at	0.00	0.00

1435355_at	0.00	0.00
1435356_at	0.00	0.00
1435359_at	0.00	0.00
1435361_at	0.00	0.00
1435363_at	0.00	0.00
1435365_at	0.00	0.00
1435366_at	0.00	0.00
1435367_at	0.00	0.00
1435373_at	0.00	0.00
1435374_at	0.84	0.03
1435377_at	0.00	0.00
1435378_at	0.00	0.00
1435379_at	0.00	0.13
1435380_at	0.00	0.00
1435381_at	0.00	0.00
1435384_at	0.00	0.00
1435385_at	0.00	0.00
1435387_at	0.00	0.00
1435388_at	0.00	0.00
1435389_at	0.00	0.00
1435391_at	0.00	0.00
1435392_at	0.00	0.00
1435393_at	0.00	0.00
1435396_at	0.00	0.00
1435397_at	0.01	0.00
1435398_at	0.00	0.00
1435399_at	0.00	0.00
1435400_at	0.00	0.00
1435401_at	0.00	0.00
1435402_at	0.00	0.00
1435403_at	0.00	0.00
1435404_at	0.00	0.00
1435406_at	0.00	0.00
1435407_at	0.00	0.00
1435408_at	0.00	0.00
1435409_at	0.00	0.00
1435410_at	0.00	0.00
1435411_at	0.00	0.00
1435412_at	0.00	0.00
1435419_at	0.00	0.00
1435421_at	0.00	0.00
1435422_at	0.00	0.00
1435423_x_at	0.00	0.00
1435424_x_at	0.00	0.00
1435425_at	0.00	0.00
1435426_s_at	0.00	0.00
1435427_x_at	0.00	0.00
1435428_at	0.00	0.00
1435432_at	0.00	0.00
1435433_at	0.00	0.00
1435434_at	0.00	0.00
1435435_at	0.00	0.00
1435436_at	0.87	0.00
1435437_at	0.82	0.96
1435438_at	0.00	0.00
1435439_at	0.00	0.00

1435440_at	0.00	0.00
1435441_at	0.00	0.00
1435443_at	0.00	0.00
1435445_at	0.00	0.01
1435451_at	0.00	0.00
1435452_at	0.00	0.00
1435453_at	0.00	0.00
1435455_at	0.00	0.00
1435456_at	0.00	0.00
1435457_at	0.13	0.00
1435459_at	0.00	0.00
1435460_at	0.00	0.00
1435461_at	0.00	0.00
1435462_at	0.00	0.00
1435464_at	0.00	0.00
1435465_at	0.38	0.00
1435466_at	0.00	0.00
1435467_at	0.00	0.00
1435469_at	0.00	0.00
1435470_at	0.00	0.00
1435471_at	0.00	0.00
1435472_at	0.00	0.00
1435473_at	0.00	0.00
1435474_at	0.00	0.00
1435475_at	0.00	0.00
1435478_at	0.00	0.00
1435480_at	0.00	0.00
1435481_at	0.00	0.00
1435482_at	0.00	0.00
1435483_x_at	0.00	0.00
1435484_at	0.00	0.00
1435485_at	0.00	0.00
1435486_at	0.00	0.00
1435487_at	0.00	0.00
1435488_at	0.00	0.00
1435489_at	0.00	0.00
1435490_at	0.00	0.00
1435491_at	0.00	0.00
1435492_at	0.00	0.00
1435497_at	0.00	0.00
1435498_at	0.00	0.00
1435499_at	0.00	0.00
1435500_at	0.00	0.00
1435501_at	0.00	0.00
1435503_at	0.00	0.00
1435505_at	0.00	0.00
1435506_at	0.00	0.00
1435510_at	0.00	0.00
1435511_at	0.00	0.00
1435512_at	0.00	0.00
1435513_at	0.00	0.00
1435514_at	0.00	0.00
1435515_at	0.00	0.00
1435516_x_at	0.00	0.00
1435520_at	0.00	0.00
1435521_at	0.00	0.00

1435523_s_at	0.00	0.00
1435529_at	0.00	0.00
1435531_at	0.00	0.00
1435535_at	0.00	0.00
1435536_at	0.00	0.00
1435538_at	0.00	0.00
1435539_at	0.00	0.00
1435540_at	0.00	0.00
1435542_s_at	0.00	0.00
1435543_at	0.00	0.01
1435544_at	0.00	0.00
1435545_at	0.31	0.08
1435546_a_at	0.00	0.00
1435547_at	0.00	0.00
1435548_at	0.00	0.00
1435549_at	0.00	0.00
1435550_at	0.00	0.00
1435552_at	0.00	0.00
1435553_at	0.00	0.00
1435554_at	0.03	0.00
1435555_at	0.00	0.00
1435556_at	0.00	0.00
1435557_at	0.00	0.00
1435558_at	0.00	0.00
1435560_at	0.00	0.00
1435562_at	0.00	0.00
1435563_at	0.00	0.00
1435564_at	0.00	0.00
1435566_s_at	0.00	0.00
1435568_at	0.00	0.00
1435569_at	0.00	0.00
1435570_s_at	0.00	0.00
1435571_at	0.00	0.00
1435574_at	0.00	0.00
1435575_at	0.00	0.00
1435576_at	0.00	0.00
1435579_at	0.00	0.00
1435580_at	0.00	0.00
1435581_at	0.00	0.00
1435582_at	0.00	0.00
1435583_at	0.00	0.00
1435584_at	0.00	0.00
1435585_at	0.00	0.00
1435588_at	0.00	0.61
1435589_at	0.00	0.00
1435590_at	0.00	0.00
1435591_at	0.00	0.00
1435594_at	0.00	0.00
1435595_at	0.00	0.00
1435596_at	0.00	0.00
1435597_at	0.00	0.02
1435598_at	0.00	0.00
1435599_at	0.00	0.00
1435600_s_at	0.00	0.00
1435601_at	0.00	0.00
1435603_at	0.00	0.00

1435604_at	0.00	0.00
1435605_at	0.00	0.00
1435606_at	0.00	0.00
1435607_at	0.00	0.00
1435608_at	0.00	0.00
1435609_at	0.00	0.00
1435610_at	0.00	0.00
1435612_at	0.00	0.00
1435615_at	0.00	0.00
1435616_at	0.00	0.00
1435617_at	0.00	0.00
1435618_at	0.00	0.00
1435619_at	0.00	0.00
1435620_at	0.00	0.00
1435621_at	0.00	0.00
1435623_at	0.00	0.00
1435624_at	0.00	0.00
1435629_at	0.00	0.00
1435632_at	0.00	0.00
1435633_at	0.00	0.00
1435636_at	0.00	0.00
1435640_x_at	0.00	0.00
1435641_at	0.00	0.00
1435642_at	0.00	0.00
1435644_at	0.00	0.00
1435645_at	0.00	0.00
1435646_at	0.00	0.00
1435647_at	0.00	0.00
1435648_at	0.00	0.09
1435649_at	0.00	0.00
1435650_at	0.00	0.00
1435653_at	0.00	0.00
1435654_at	0.00	0.00
1435656_at	0.01	0.00
1435657_at	0.00	0.00
1435658_at	0.00	0.00
1435661_at	0.00	0.00
1435662_at	0.00	0.00
1435663_at	0.00	0.00
1435664_at	0.00	0.00
1435665_at	0.00	0.00
1435667_at	0.00	0.00
1435668_at	0.00	0.00
1435670_at	0.00	0.00
1435671_at	0.00	0.00
1435672_at	0.00	0.00
1435673_at	0.00	0.00
1435674_at	0.00	0.00
1435675_at	0.00	0.00
1435676_at	0.00	0.00
1435677_at	0.00	0.00
1435678_at	0.00	0.00
1435679_at	0.00	0.04
1435682_at	0.00	0.00
1435686_at	0.00	0.00
1435687_at	0.00	0.00

1435688_at	0.00	0.00
1435689_at	0.00	0.00
1435691_at	0.00	0.00
1435692_at	0.00	0.00
1435693_at	0.00	0.00
1435694_at	0.00	0.00
1435696_s_at	0.00	0.00
1435698_at	0.00	0.00
1435699_at	0.00	0.00
1435700_at	0.00	0.00
1435701_at	0.00	0.00
1435703_at	0.00	0.00
1435704_at	0.00	0.00
1435705_at	0.00	0.00
1435706_at	0.00	0.00
1435707_at	0.00	0.00
1435708_at	0.00	0.00
1435710_at	0.00	0.00
1435711_at	0.00	0.00
1435713_at	0.00	0.00
1435717_at	0.00	0.00
1435718_at	0.00	0.00
1435719_at	0.00	0.00
1435720_at	0.00	0.00
1435721_at	0.00	0.00
1435722_at	0.00	0.00
1435723_at	0.00	0.00
1435724_at	0.00	0.00
1435726_at	0.00	0.00
1435727_s_at	0.00	0.00
1435728_at	0.00	0.00
1435730_at	0.00	0.00
1435739_at	0.00	0.00
1435740_at	0.09	0.00
1435741_at	0.00	0.00
1435743_at	0.00	0.00
1435744_at	0.00	0.06
1435745_at	0.00	0.00
1435746_at	0.00	0.13
1435748_at	0.00	0.00
1435749_at	0.00	0.00
1435750_at	0.00	0.00
1435751_at	0.00	0.00
1435752_s_at	0.00	0.00
1435753_a_at	0.00	0.03
1435760_at	0.00	0.00
1435761_at	0.00	0.00
1435763_at	0.00	0.00
1435765_at	0.00	0.00
1435766_at	0.00	0.00
1435769_at	0.00	0.00
1435770_at	0.00	0.00
1435771_at	0.00	0.05
1435772_at	0.00	0.00
1435773_at	0.00	0.00
1435774_at	0.00	0.00

1435775_at	0.00	0.00
1435776_at	0.00	0.00
1435777_at	0.00	0.00
1435778_at	0.00	0.00
1435779_at	0.00	0.00
1435781_at	0.00	0.00
1435784_at	0.00	0.00
1435785_at	0.00	0.00
1435786_at	0.00	0.00
1435787_at	0.00	0.00
1435790_at	0.00	0.00
1435793_at	0.00	0.00
1435794_at	0.00	0.00
1435795_at	0.00	0.00
1435796_at	0.00	0.00
1435797_at	0.00	0.21
1435801_at	0.00	0.00
1435805_at	0.00	0.00
1435806_at	0.00	0.00
1435808_at	0.00	0.00
1435809_at	0.00	0.00
1435810_at	0.00	0.00
1435813_at	0.00	0.00
1435814_at	0.00	0.00
1435815_at	0.00	0.00
1435818_at	0.00	0.00
1435819_at	0.00	0.00
1435822_at	0.00	0.00
1435825_at	0.00	0.00
1435826_at	0.00	0.00
1435827_at	0.00	0.00
1435828_at	0.00	0.00
1435829_at	0.00	0.00
1435831_at	0.00	0.00
1435832_at	0.00	0.00
1435835_at	0.00	0.00
1435836_at	0.22	0.00
1435838_at	0.00	0.00
1435839_at	0.00	0.00
1435841_s_at	0.00	0.00
1435842_at	0.00	0.00
1435844_at	0.00	0.00
1435845_at	0.00	0.00
1435846_x_at	0.00	0.00
1435848_at	0.00	0.00
1435849_at	0.00	0.00
1435850_at	0.00	0.00
1435851_at	0.00	0.00
1435852_at	0.00	0.00
1435854_at	0.00	0.00
1435858_at	0.00	0.00
1435861_at	0.00	0.00
1435867_at	0.00	0.00
1435868_at	0.00	0.00
1435874_at	0.00	0.00
1435875_at	0.00	0.00

1435876_at	0.00	0.00
1435877_at	0.00	0.00
1435878_at	0.00	0.00
1435879_at	0.03	0.43
1435880_at	0.00	0.15
1435883_at	0.00	0.00
1435887_at	0.00	0.00
1435888_at	0.00	0.00
1435890_at	0.00	0.00
1435891_x_at	0.00	0.00
1435892_at	0.00	0.00
1435893_at	0.00	0.00
1435894_at	0.00	0.00
1435895_at	0.00	0.00
1435897_at	0.00	0.00
1435899_at	0.00	0.00
1435900_at	0.00	0.00
1435901_at	0.00	0.00
1435902_at	0.25	0.00
1435904_at	0.00	0.00
1435905_at	0.00	0.00
1435907_at	0.00	0.00
1435908_at	0.00	0.00
1435909_at	0.00	0.00
1435910_at	0.00	0.00
1435911_s_at	0.00	0.00
1435912_at	0.00	0.00
1435913_at	0.00	0.00
1435914_at	0.00	0.00
1435915_at	0.00	0.00
1435916_at	0.00	0.00
1435917_at	0.00	0.00
1435918_at	0.00	0.00
1435921_at	0.00	0.00
1435922_at	0.00	0.00
1435923_at	0.00	0.00
1435925_at	0.00	0.00
1435926_at	0.00	0.00
1435927_at	0.00	0.00
1435928_at	0.00	0.00
1435929_at	0.00	0.00
1435930_at	0.00	0.00
1435931_at	0.00	0.00
1435932_at	0.00	0.00
1435933_at	0.00	0.00
1435935_at	0.00	0.00
1435936_at	0.00	0.00
1435937_at	0.00	0.00
1435938_at	0.00	0.00
1435940_at	0.00	0.00
1435942_at	0.00	0.00
1435947_at	0.00	0.00
1435948_at	0.25	0.32
1435952_at	0.00	0.00
1435953_at	0.00	0.00
1435954_at	0.00	0.00

1435955_at	0.00	0.00
1435956_at	0.00	0.00
1435957_at	0.00	0.00
1435959_at	0.00	0.00
1435960_at	0.00	0.00
1435961_at	0.00	0.00
1435963_at	0.00	0.00
1435968_at	0.00	0.00
1435969_at	0.00	0.00
1435971_at	0.00	0.00
1435972_at	0.00	0.00
1435973_at	0.00	0.00
1435974_at	0.00	0.00
1435975_at	0.00	0.00
1435976_at	0.00	0.00
1435980_x_at	0.00	0.00
1435982_at	0.00	0.00
1435983_at	0.00	0.00
1435984_at	0.00	0.00
1435985_at	0.00	0.00
1435987_x_at	0.00	0.00
1435990_at	0.00	0.00
1435991_at	0.00	0.00
1435992_at	0.00	0.00
1435993_at	0.00	0.00
1435994_at	0.00	0.00
1435996_at	0.00	0.00
1435997_at	0.00	0.00
1435999_at	0.00	0.00
1436001_at	0.00	0.00
1436002_at	0.00	0.00
1436003_at	0.00	0.00
1436004_at	0.00	0.00
1436006_at	0.00	0.00
1436008_at	0.00	0.00
1436009_at	0.00	0.00
1436010_at	0.00	0.00
1436011_at	0.00	0.00
1436013_at	0.00	0.00
1436015_s_at	0.00	0.00
1436017_at	0.00	0.00
1436018_at	0.00	0.00
1436020_at	0.00	0.00
1436022_at	0.00	0.00
1436023_at	0.00	0.27
1436024_at	0.00	0.00
1436025_at	0.00	0.00
1436028_at	0.00	0.00
1436029_at	0.00	0.00
1436033_at	0.00	0.00
1436035_at	0.00	0.00
1436036_at	0.00	0.00
1436037_at	0.00	0.00
1436039_at	0.00	0.00
1436040_at	0.00	0.00
1436041_at	0.00	0.00

1436043_at	0.00	0.00
1436044_at	0.00	0.00
1436045_at	0.00	0.00
1436047_at	0.00	0.00
1436051_at	0.00	0.00
1436052_at	0.00	0.00
1436053_at	0.00	0.00
1436054_at	0.00	0.68
1436055_at	0.00	0.00
1436056_at	0.00	0.00
1436059_at	0.00	0.00
1436060_at	0.00	0.00
1436061_at	0.00	0.00
1436062_at	0.00	0.00
1436063_at	0.00	0.00
1436065_at	0.00	0.00
1436067_at	0.00	0.02
1436068_at	0.00	0.00
1436069_at	0.00	0.00
1436070_at	0.00	0.00
1436071_at	0.00	0.00
1436072_at	0.00	0.00
1436074_at	0.00	0.00
1436075_at	0.00	0.00
1436076_at	0.00	0.00
1436080_at	0.00	0.00
1436081_a_at	0.00	0.00
1436082_at	0.00	0.00
1436083_at	0.00	0.00
1436084_at	0.00	0.00
1436085_at	0.00	0.00
1436086_at	0.00	0.00
1436087_at	0.00	0.00
1436088_at	0.00	0.00
1436089_at	0.00	0.00
1436090_at	0.00	0.00
1436091_at	0.00	0.00
1436093_at	0.00	0.00
1436094_at	0.00	0.00
1436095_at	0.00	0.00
1436096_at	0.00	0.00
1436098_at	0.00	0.00
1436099_at	0.00	0.00
1436100_at	0.00	0.00
1436101_at	0.00	0.00
1436102_at	0.00	0.00
1436103_at	0.00	0.00
1436104_a_at	0.00	0.00
1436105_at	0.00	0.00
1436106_x_at	0.00	0.00
1436107_at	0.00	0.00
1436108_at	0.00	0.00
1436109_at	0.00	0.00
1436110_at	0.00	0.00
1436111_at	0.00	0.00
1436113_a_at	0.00	0.00

1436114_at	0.00	0.00
1436115_at	0.00	0.00
1436116_x_at	0.00	0.00
1436117_at	0.00	0.00
1436118_at	0.00	0.00
1436119_at	0.00	0.00
1436120_at	0.00	0.00
1436122_at	0.00	0.00
1436123_at	0.00	0.00
1436124_at	0.04	0.58
1436125_at	0.00	0.26
1436126_at	0.00	0.00
1436127_at	0.00	0.00
1436128_at	0.00	0.00
1436129_at	0.00	0.00
1436130_s_at	0.00	0.00
1436131_at	0.00	0.00
1436132_at	0.00	0.00
1436133_at	0.00	0.00
1436134_at	0.00	0.00
1436135_at	0.00	0.00
1436136_at	0.00	0.00
1436138_at	0.00	0.00
1436139_at	0.00	0.00
1436140_at	0.00	0.00
1436141_at	0.00	0.00
1436142_at	0.00	0.00
1436143_at	0.00	0.00
1436145_at	0.00	0.00
1436146_at	0.00	0.00
1436147_at	0.00	0.00
1436148_at	0.00	0.00
1436150_at	0.00	0.00
1436151_x_at	0.00	0.00
1436154_at	0.00	0.00
1436155_at	0.00	0.00
1436156_at	0.00	0.02
1436157_at	0.00	0.00
1436159_at	0.00	0.00
1436160_at	0.00	0.00
1436161_at	0.00	0.00
1436163_at	0.00	0.00
1436164_at	0.00	0.00
1436166_at	0.00	0.00
1436168_at	0.00	0.00
1436169_at	0.00	0.00
1436171_at	0.00	0.00
1436173_at	0.00	0.00
1436174_at	0.00	0.00
1436175_at	0.00	0.00
1436177_at	0.00	0.00
1436178_at	0.00	0.00
1436181_at	0.00	0.00
1436183_at	0.00	0.00
1436184_at	0.00	0.00
1436185_at	0.00	0.00

1436189_at	0.00	0.00
1436190_at	0.00	0.00
1436191_at	0.00	0.00
1436192_at	0.00	0.00
1436193_at	0.00	0.00
1436194_at	0.00	0.00
1436195_at	0.00	0.41
1436196_at	0.00	0.00
1436197_at	0.00	0.00
1436199_at	0.00	0.00
1436200_at	0.00	0.00
1436202_at	0.00	0.00
1436203_a_at	0.00	0.00
1436204_at	0.00	0.00
1436205_at	0.00	0.00
1436206_at	0.00	0.00
1436208_at	0.00	0.00
1436209_at	0.00	0.00
1436210_at	0.00	0.00
1436211_at	0.00	0.00
1436215_at	0.00	0.00
1436216_s_at	0.00	0.00
1436217_at	0.00	0.00
1436218_at	0.00	0.00
1436219_at	0.00	0.00
1436220_at	0.00	0.00
1436221_at	0.00	0.00
1436223_at	0.00	0.00
1436224_at	0.00	0.00
1436225_at	0.00	0.00
1436227_at	0.89	0.00
1436229_at	0.00	0.00
1436230_at	0.00	0.00
1436231_at	0.00	0.00
1436233_at	0.00	0.00
1436237_at	0.00	0.00
1436238_at	0.00	0.00
1436239_at	0.00	0.00
1436240_at	0.00	0.00
1436245_at	0.00	0.00
1436246_at	0.00	0.00
1436247_at	0.00	0.03
1436248_at	0.00	0.00
1436249_at	0.00	0.00
1436250_at	0.00	0.00
1436251_at	0.00	0.00
1436252_at	0.00	0.00
1436254_at	0.00	0.00
1436256_at	0.00	0.00
1436257_at	0.00	0.00
1436258_at	0.00	0.00
1436259_at	0.00	0.00
1436260_at	0.00	0.00
1436263_at	0.00	0.00
1436264_at	0.00	0.00
1436265_at	0.00	0.00

1436268_at	0.00	0.00
1436271_at	0.00	0.00
1436273_at	0.00	0.00
1436274_at	0.00	0.00
1436275_at	0.00	0.00
1436276_at	0.00	0.00
1436278_at	0.00	0.00
1436279_at	0.00	0.00
1436280_at	0.00	0.00
1436281_at	0.00	0.12
1436282_at	0.00	0.00
1436283_at	0.00	0.00
1436284_s_at	0.00	0.00
1436285_at	0.00	0.00
1436286_at	0.00	0.00
1436287_at	0.00	0.00
1436288_at	0.00	0.00
1436290_at	0.00	0.00
1436293_x_at	0.00	0.00
1436294_at	0.00	0.00
1436295_at	0.00	0.00
1436296_x_at	0.00	0.00
1436299_at	0.00	0.00
1436302_at	0.00	0.00
1436303_at	0.00	0.00
1436304_at	0.00	0.00
1436305_at	0.00	0.00
1436306_at	0.00	0.00
1436309_at	0.00	0.00
1436310_at	0.00	0.00
1436311_at	0.00	0.00
1436312_at	0.00	0.00
1436313_at	0.00	0.00
1436314_at	0.00	0.00
1436317_at	0.00	0.00
1436318_at	0.00	0.00
1436319_at	0.00	0.00
1436321_at	0.00	0.00
1436322_a_at	0.00	0.00
1436323_at	0.00	0.00
1436324_at	0.00	0.00
1436325_at	0.00	0.00
1436326_at	0.00	0.00
1436328_at	0.00	0.00
1436329_at	0.00	0.00
1436330_x_at	0.00	0.00
1436331_at	0.00	0.00
1436332_at	0.00	0.00
1436333_a_at	0.00	0.00
1436334_at	0.00	0.00
1436335_at	0.00	0.00
1436338_at	0.00	0.00
1436340_at	0.00	0.00
1436345_at	0.00	0.00
1436346_at	0.00	0.00
1436347_a_at	0.00	0.00

1436350_at	0.00	0.00
1436352_at	0.00	0.00
1436353_at	0.00	0.00
1436354_at	0.00	0.00
1436355_at	0.00	0.00
1436356_at	0.66	0.00
1436358_at	0.00	0.00
1436359_at	0.00	0.00
1436360_at	0.00	0.00
1436361_at	0.00	0.00
1436365_at	0.00	0.00
1436367_at	0.00	0.00
1436368_at	0.00	0.00
1436369_at	0.00	0.00
1436371_at	0.00	0.00
1436373_at	0.00	0.00
1436375_at	0.00	0.00
1436376_s_at	0.00	0.00
1436377_at	0.00	0.00
1436378_at	0.00	0.00
1436379_at	0.00	0.00
1436380_at	0.00	0.00
1436381_at	0.00	0.00
1436383_at	0.00	0.00
1436386_x_at	0.00	0.00
1436387_at	0.00	0.00
1436389_at	0.00	0.00
1436395_at	0.00	0.00
1436396_at	0.00	0.00
1436397_at	0.00	0.00
1436398_at	0.00	0.00
1436400_at	0.00	0.00
1436401_at	0.00	0.00
1436403_at	0.00	0.00
1436405_at	0.00	0.00
1436406_at	0.00	0.00
1436407_at	0.00	0.00
1436408_at	0.00	0.00
1436410_at	0.00	0.00
1436411_at	0.00	0.00
1436412_at	0.00	0.00
1436413_at	0.00	0.00
1436414_at	0.00	0.00
1436415_at	0.00	0.00
1436417_at	0.00	0.00
1436418_at	0.00	0.00
1436422_at	0.00	0.00
1436423_at	0.00	0.00
1436424_at	0.00	0.00
1436425_at	0.00	0.00
1436426_at	0.00	0.00
1436428_at	0.00	0.00
1436429_at	0.00	0.00
1436430_at	0.00	0.00
1436431_at	0.00	0.00
1436432_at	0.00	0.00

1436433_at	0.00	0.00
1436434_at	0.00	0.00
1436435_at	0.00	0.00
1436436_at	0.00	0.00
1436437_at	0.00	0.00
1436438_s_at	0.00	0.00
1436439_at	0.00	0.00
1436440_at	0.00	0.00
1436441_at	0.00	0.00
1436444_at	0.00	0.00
1436445_at	0.00	0.00
1436446_at	0.00	0.00
1436447_at	0.00	0.00
1436449_at	0.00	0.00
1436450_at	0.00	0.00
1436453_at	0.00	0.00
1436455_at	0.00	0.00
1436456_at	0.00	0.00
1436457_at	0.00	0.00
1436458_at	0.00	0.00
1436459_at	0.00	0.00
1436460_at	0.00	0.00
1436461_at	0.00	0.00
1436463_at	0.00	0.00
1436464_at	0.00	0.00
1436465_at	0.00	0.00
1436466_at	0.00	0.00
1436467_at	0.00	0.00
1436468_at	0.00	0.00
1436469_at	0.00	0.00
1436470_at	0.00	0.00
1436471_at	0.00	0.00
1436472_at	0.00	0.00
1436473_at	0.00	0.00
1436474_at	0.00	0.00
1436475_at	0.00	0.00
1436476_at	0.00	0.00
1436478_at	0.00	0.00
1436481_at	0.00	0.00
1436483_at	0.00	0.00
1436484_at	0.00	0.00
1436485_s_at	0.00	0.00
1436486_x_at	0.00	0.00
1436488_at	0.00	0.00
1436489_x_at	0.00	0.00
1436491_at	0.00	0.00
1436492_x_at	0.00	0.00
1436493_at	0.00	0.00
1436496_at	0.00	0.00
1436497_at	0.00	0.00
1436499_at	0.00	0.00
1436501_at	0.00	0.00
1436502_at	0.00	0.00
1436503_at	0.00	0.00
1436507_at	0.00	0.00
1436508_at	0.00	0.00

1436509_at	0.00	0.00
1436511_at	0.00	0.00
1436513_at	0.00	0.00
1436514_at	0.00	0.00
1436515_at	0.00	0.00
1436516_at	0.00	0.00
1436518_at	0.00	0.01
1436520_at	0.00	0.00
1436521_at	0.00	0.00
1436523_s_at	0.00	0.00
1436524_at	0.00	0.00
1436526_at	0.00	0.00
1436527_at	0.00	0.00
1436529_at	0.00	0.00
1436530_at	0.00	0.00
1436531_at	0.00	0.00
1436532_at	0.00	0.00
1436533_at	0.00	0.00
1436534_at	0.00	0.00
1436535_at	0.00	0.00
1436536_at	0.00	0.28
1436537_at	0.00	0.00
1436538_at	0.00	0.00
1436539_at	0.00	0.00
1436540_at	0.00	0.00
1436541_at	0.00	0.00
1436543_at	0.00	0.00
1436544_at	0.00	0.00
1436545_at	0.00	0.00
1436546_at	0.00	0.00
1436548_at	0.00	0.00
1436550_at	0.00	0.00
1436551_at	0.00	0.00
1436552_at	0.00	0.00
1436553_at	0.00	0.00
1436554_at	0.00	0.00
1436555_at	0.00	0.00
1436556_at	0.00	0.00
1436557_at	0.00	0.00
1436560_at	0.00	0.00
1436562_at	0.00	0.00
1436564_at	0.00	0.00
1436568_at	0.41	0.87
1436569_at	0.00	0.00
1436571_at	0.00	0.00
1436572_at	0.00	0.00
1436574_at	0.14	0.10
1436575_at	0.00	0.00
1436576_at	0.00	0.00
1436577_at	0.00	0.00
1436578_at	0.00	0.00
1436579_s_at	0.00	0.00
1436581_at	0.00	0.00
1436582_at	0.00	0.00
1436583_at	0.00	0.00
1436585_at	0.36	0.00

1436587_at	0.00	0.00
1436590_at	0.00	0.00
1436591_at	0.00	0.00
1436592_at	0.00	0.00
1436593_at	0.00	0.00
1436594_at	0.00	0.00
1436595_at	0.00	0.00
1436596_at	0.00	0.00
1436597_at	0.00	0.00
1436598_at	0.00	0.00
1436599_at	0.00	0.00
1436600_at	0.00	0.34
1436601_at	0.00	0.00
1436602_x_at	0.00	0.00
1436603_at	0.00	0.00
1436604_at	0.00	0.00
1436605_at	0.00	0.00
1436606_at	0.00	0.00
1436607_a_at	0.00	0.00
1436608_at	0.00	0.00
1436610_at	0.00	0.00
1436611_at	0.00	0.00
1436612_at	0.00	0.00
1436614_at	0.00	0.00
1436617_at	0.00	0.00
1436618_at	0.00	0.00
1436619_at	0.00	0.00
1436621_at	0.00	0.00
1436622_at	0.00	0.00
1436624_at	0.00	0.00
1436625_at	0.00	0.00
1436626_at	0.00	0.00
1436627_at	0.00	0.00
1436628_at	0.00	0.00
1436629_at	0.00	0.00
1436630_at	0.00	0.00
1436631_at	0.00	0.00
1436632_at	0.00	0.00
1436633_at	0.00	0.00
1436634_at	0.00	0.00
1436635_at	0.00	0.00
1436636_at	0.00	0.00
1436637_at	0.00	0.00
1436638_at	0.00	0.00
1436640_x_at	0.00	0.00
1436641_at	0.00	0.00
1436642_x_at	0.00	0.00
1436644_x_at	0.00	0.00
1436646_at	0.00	0.00
1436647_at	0.00	0.00
1436648_at	0.00	0.00
1436649_at	0.00	0.00
1436650_at	0.00	0.00
1436651_at	0.00	0.00
1436652_at	0.00	0.00
1436654_at	0.00	0.00

1436656_at	0.00	0.00
1436657_at	0.00	0.00
1436659_at	0.00	0.00
1436660_at	0.00	0.00
1436661_at	0.00	0.00
1436662_at	0.00	0.00
1436663_at	0.00	0.00
1436666_at	0.00	0.00
1436667_at	0.00	0.00
1436671_at	0.00	0.00
1436672_at	0.00	0.00
1436673_at	0.00	0.00
1436675_at	0.00	0.00
1436676_at	0.00	0.00
1436678_at	0.00	0.00
1436679_at	0.00	0.00
1436683_at	0.00	0.00
1436685_at	0.00	0.00
1436690_at	0.00	0.00
1436692_at	0.00	0.00
1436694_s_at	0.00	0.00
1436695_x_at	0.00	0.00
1436697_at	0.00	0.00
1436698_x_at	0.00	0.00
1436700_a_at	0.00	0.00
1436701_at	0.00	0.00
1436702_at	0.00	0.00
1436705_at	0.00	0.00
1436706_at	0.00	0.00
1436709_at	0.00	0.00
1436710_at	0.00	0.00
1436711_at	0.00	0.00
1436712_at	0.00	0.00
1436718_at	0.00	0.00
1436719_at	0.00	0.00
1436720_s_at	0.00	0.00
1436721_x_at	0.00	0.00
1436723_at	0.00	0.00
1436725_at	0.00	0.00
1436728_s_at	0.00	0.26
1436729_at	0.31	0.00
1436731_at	0.00	0.00
1436732_s_at	0.00	0.00
1436733_at	0.00	0.00
1436734_at	0.00	0.00
1436735_at	0.00	0.00
1436738_at	0.00	0.00
1436739_at	0.00	0.00
1436740_at	0.00	0.00
1436741_at	0.00	0.00
1436742_a_at	0.00	0.32
1436743_at	0.00	0.00
1436744_x_at	0.00	0.00
1436745_at	0.00	0.00
1436749_at	0.00	0.00
1436751_at	0.00	0.00

1436752_at	0.00	0.00
1436753_at	0.00	0.00
1436754_at	0.00	0.00
1436755_at	0.00	0.00
1436758_at	0.00	0.00
1436764_at	0.00	0.01
1436765_at	0.00	0.00
1436772_at	0.00	0.00
1436774_at	0.00	0.00
1436777_at	0.00	0.00
1436778_at	0.00	0.00
1436779_at	0.00	0.00
1436785_a_at	0.00	0.00
1436786_at	0.00	0.00
1436787_x_at	0.00	0.00
1436789_at	0.00	0.00
1436792_at	0.00	0.00
1436793_at	0.00	0.00
1436794_at	0.00	0.00
1436795_at	0.00	0.00
1436796_at	0.01	0.00
1436799_at	1.00	0.97
1436800_at	0.00	0.00
1436805_at	0.00	0.00
1436808_x_at	0.00	0.50
1436811_at	0.00	0.00
1436812_at	0.00	0.00
1436813_x_at	0.00	0.00
1436814_at	0.00	0.00
1436815_x_at	0.00	0.00
1436817_at	0.00	0.00
1436818_a_at	0.00	0.00
1436821_at	0.00	0.00
1436825_a_at	0.00	0.00
1436826_at	0.00	0.00
1436827_at	0.00	0.01
1436829_at	0.00	0.00
1436830_at	0.00	0.00
1436831_at	0.00	0.00
1436832_at	0.00	0.00
1436837_at	0.00	0.00
1436841_at	0.00	0.00
1436842_at	0.00	0.00
1436843_at	0.00	0.00
1436844_at	0.00	0.00
1436847_s_at	0.00	0.00
1436850_at	0.00	0.00
1436852_at	0.00	0.00
1436855_at	0.00	0.00
1436857_at	0.00	0.00
1436858_at	0.00	0.00
1436860_at	0.00	0.00
1436864_at	0.00	0.00
1436865_at	0.00	0.00
1436866_at	0.00	0.00
1436867_at	0.00	0.00

1436868_at	0.00	0.00
1436869_at	0.00	0.00
1436870_s_at	0.00	0.00
1436873_at	0.00	0.00
1436875_at	0.00	0.00
1436876_at	0.00	0.00
1436877_at	0.00	0.00
1436878_at	0.00	0.00
1436883_at	0.00	0.00
1436888_at	0.00	0.00
1436891_at	0.00	0.00
1436892_at	0.00	0.00
1436894_at	0.00	0.00
1436895_at	0.00	0.00
1436897_at	0.00	0.00
1436901_at	0.00	0.00
1436903_at	0.00	0.00
1436904_at	0.00	0.00
1436907_at	0.00	0.00
1436911_at	0.00	0.00
1436913_at	0.00	0.00
1436914_at	0.00	0.00
1436916_at	0.00	0.00
1436918_at	0.00	0.33
1436919_at	0.00	0.00
1436920_at	0.00	0.00
1436925_at	0.00	0.00
1436927_at	0.00	0.00
1436931_at	0.00	0.00
1436932_at	0.00	0.00
1436933_at	0.00	0.00
1436937_at	0.00	0.00
1436938_at	0.00	0.00
1436939_at	0.00	0.00
1436941_at	0.00	0.00
1436942_at	0.00	0.00
1436943_at	0.00	0.00
1436948_a_at	0.00	0.00
1436957_at	0.00	0.00
1436960_at	0.00	0.00
1436961_at	0.00	0.00
1436962_at	0.00	0.00
1436964_at	0.00	0.00
1436966_at	0.00	0.00
1436967_at	0.00	0.00
1436968_x_at	0.00	0.00
1436969_at	0.00	0.00
1436972_at	0.00	0.00
1436974_at	0.00	0.00
1436975_at	0.00	0.00
1436976_a_at	0.00	0.00
1436977_at	0.00	0.00
1436978_at	0.00	0.00
1436982_at	0.00	0.00
1436983_at	0.00	0.00
1436984_at	0.00	0.00

1436985_at	0.00	0.00
1436987_at	0.00	0.00
1436988_at	0.00	0.00
1436999_at	0.00	0.00
1437001_at	0.00	0.01
1437002_at	0.00	0.00
1437003_at	0.00	0.28
1437010_a_at	0.00	0.00
1437011_x_at	0.00	0.00
1437017_at	0.00	0.00
1437018_at	0.00	0.00
1437019_at	0.00	0.00
1437020_at	0.00	0.00
1437021_at	0.00	0.00
1437022_at	0.00	0.00
1437023_at	0.00	0.00
1437030_at	0.00	0.00
1437031_at	0.00	0.00
1437038_x_at	0.00	0.00
1437040_at	0.00	0.00
1437041_at	0.00	0.00
1437042_at	0.00	0.00
1437047_at	0.00	0.00
1437049_at	0.00	0.00
1437056_x_at	0.00	0.00
1437057_at	0.00	0.00
1437058_at	0.00	0.00
1437059_at	0.00	0.00
1437060_at	0.00	0.00
1437063_at	0.00	0.00
1437064_at	0.00	0.00
1437065_at	0.00	0.00
1437066_at	0.00	0.00
1437068_at	0.00	0.00
1437069_at	0.00	0.00
1437070_at	0.00	0.00
1437071_at	0.00	0.00
1437072_at	0.00	0.00
1437073_x_at	0.01	0.10
1437075_at	0.00	0.00
1437076_at	0.00	0.00
1437077_at	0.00	0.00
1437079_at	0.00	0.00
1437083_at	0.00	0.00
1437084_at	0.00	0.00
1437085_at	0.00	0.00
1437086_at	0.00	0.00
1437087_at	0.00	0.00
1437088_at	0.00	0.00
1437089_at	0.00	0.00
1437090_at	0.00	0.00
1437091_at	0.00	0.00
1437092_at	0.00	0.00
1437093_at	0.00	0.00
1437094_x_at	0.00	0.00
1437095_at	0.00	0.00

1437096_at	0.00	0.00
1437098_x_at	0.00	0.00
1437104_at	0.00	0.00
1437105_at	0.00	0.00
1437106_at	0.00	0.00
1437107_at	0.00	0.00
1437108_at	0.00	0.00
1437109_s_at	0.00	0.01
1437114_at	0.00	0.00
1437115_at	0.00	0.00
1437116_at	0.00	0.00
1437117_at	0.00	0.00
1437118_at	0.04	0.22
1437119_at	0.00	0.00
1437121_at	0.00	0.00
1437122_at	0.00	0.00
1437123_at	0.00	0.00
1437124_at	0.00	0.00
1437125_at	0.00	0.00
1437126_at	0.00	0.00
1437127_at	0.00	0.00
1437128_a_at	0.00	0.00
1437129_at	0.00	0.00
1437130_at	0.00	0.00
1437135_at	0.00	0.00
1437136_at	0.00	0.00
1437137_at	0.00	0.00
1437139_at	0.00	0.00
1437140_at	0.00	0.00
1437146_x_at	0.00	0.00
1437147_at	0.00	0.00
1437149_at	0.00	0.00
1437150_at	0.00	0.00
1437151_at	0.00	0.00
1437152_at	0.30	0.00
1437153_at	0.00	0.00
1437154_at	0.00	0.00
1437156_at	0.00	0.00
1437158_at	0.00	0.00
1437160_at	0.00	0.00
1437162_at	0.00	0.00
1437166_at	0.00	0.00
1437167_at	0.00	0.00
1437168_at	0.00	0.00
1437173_at	0.00	0.00
1437174_at	0.00	0.00
1437176_at	0.00	0.00
1437177_at	0.00	0.00
1437178_at	0.00	0.00
1437179_at	0.34	0.00
1437181_at	0.00	0.00
1437182_at	0.00	0.00
1437183_at	0.00	0.00
1437184_at	0.00	0.00
1437186_at	0.00	0.00
1437187_at	0.00	0.00

1437190_at	0.00	0.00
1437191_at	0.00	0.00
1437193_s_at	0.00	0.00
1437196_x_at	0.00	0.00
1437197_at	0.00	0.00
1437199_at	0.00	0.00
1437200_at	0.00	0.04
1437201_at	0.00	0.00
1437202_at	0.00	0.00
1437206_at	0.00	0.00
1437207_at	0.00	0.01
1437209_at	0.00	0.00
1437212_at	0.00	0.00
1437213_at	0.00	0.00
1437214_at	0.00	0.00
1437215_at	0.00	0.00
1437217_at	0.00	0.00
1437219_at	0.00	0.00
1437221_at	0.00	0.00
1437222_x_at	0.00	0.00
1437224_at	0.00	0.00
1437227_at	0.00	0.00
1437228_at	0.00	0.00
1437229_at	0.00	0.00
1437230_at	0.00	0.00
1437231_at	0.00	0.00
1437232_at	0.00	0.00
1437240_at	0.00	0.00
1437241_at	0.00	0.00
1437242_at	0.00	0.00
1437244_at	0.00	0.00
1437245_at	0.00	0.00
1437247_at	0.32	0.00
1437248_at	0.00	0.00
1437249_at	0.00	0.00
1437250_at	0.00	0.00
1437251_at	0.00	0.00
1437252_at	0.00	0.00
1437254_at	0.00	0.00
1437255_at	0.00	0.00
1437257_at	0.00	0.00
1437258_at	0.00	0.00
1437259_at	0.00	0.00
1437260_at	0.00	0.00
1437261_at	0.00	0.00
1437263_at	0.00	0.00
1437264_at	0.00	0.00
1437265_at	0.00	0.00
1437266_at	0.00	0.00
1437268_at	0.00	0.00
1437269_at	0.00	0.00
1437272_at	0.00	0.00
1437273_at	0.00	0.00
1437274_at	0.00	0.00
1437276_at	0.00	0.00
1437282_at	0.00	0.00

1437283_at	0.00	0.00
1437284_at	0.00	0.00
1437285_at	0.00	0.00
1437286_x_at	0.00	0.00
1437287_at	0.00	0.19
1437291_at	0.00	0.00
1437292_at	0.00	0.00
1437293_x_at	0.00	0.00
1437295_at	0.00	0.00
1437296_at	0.00	0.00
1437298_at	0.00	0.00
1437299_at	0.00	0.00
1437300_at	0.00	0.00
1437303_at	0.00	0.00
1437304_at	0.00	0.00
1437305_at	0.00	0.00
1437306_at	0.00	0.00
1437307_at	0.00	0.00
1437310_at	0.00	0.00
1437311_at	0.00	0.00
1437312_at	0.00	0.00
1437315_at	0.00	0.00
1437316_at	0.00	0.00
1437318_at	0.00	0.00
1437319_at	0.00	0.00
1437321_at	0.00	0.00
1437322_at	0.00	0.00
1437329_at	0.00	0.00
1437332_at	0.00	0.00
1437337_x_at	0.00	0.00
1437339_s_at	0.00	0.00
1437346_x_at	0.00	0.00
1437347_at	0.00	0.00
1437348_at	0.00	0.00
1437351_at	0.00	0.00
1437352_at	0.00	0.00
1437353_at	0.01	0.02
1437355_at	0.00	0.00
1437356_at	0.00	0.00
1437357_at	0.00	0.00
1437359_at	0.00	0.00
1437360_at	0.00	0.00
1437361_at	0.00	0.00
1437362_at	0.00	0.00
1437363_at	0.00	0.00
1437365_at	0.00	0.00
1437366_at	0.00	0.00
1437370_at	0.00	0.00
1437371_at	0.00	0.00
1437372_at	0.00	0.00
1437373_at	0.00	0.00
1437374_at	0.00	0.00
1437375_at	0.00	0.00
1437376_at	0.00	0.00
1437383_at	0.00	0.00
1437384_at	0.00	0.00

1437385_at	0.00	0.00
1437386_at	0.00	0.00
1437387_at	0.00	0.00
1437388_at	0.00	0.00
1437392_at	0.00	0.00
1437393_at	0.00	0.00
1437395_at	0.00	0.08
1437397_at	0.00	0.00
1437400_at	0.00	0.00
1437403_at	0.00	0.00
1437404_at	0.00	0.00
1437407_at	0.00	0.00
1437408_at	0.00	0.00
1437409_s_at	0.00	0.00
1437410_at	0.00	0.00
1437411_at	0.00	0.00
1437412_at	0.00	0.00
1437414_at	0.00	0.08
1437415_at	0.00	0.00
1437416_at	0.00	0.00
1437417_s_at	0.03	0.00
1437418_at	0.00	0.00
1437421_at	0.00	0.00
1437422_at	0.00	0.00
1437424_at	0.00	0.00
1437426_at	0.00	0.00
1437427_at	0.00	0.00
1437429_at	0.00	0.00
1437431_at	0.00	0.00
1437433_at	0.00	0.00
1437435_at	0.00	0.00
1437436_s_at	0.00	0.43
1437439_at	0.00	0.00
1437440_at	0.00	0.00
1437441_at	0.00	0.00
1437442_at	0.00	0.00
1437443_at	0.00	0.00
1437444_at	0.00	0.00
1437445_at	0.00	0.00
1437446_at	0.00	0.00
1437447_s_at	0.00	0.00
1437448_s_at	0.00	0.00
1437449_at	0.00	0.00
1437451_at	0.00	0.00
1437453_s_at	0.00	0.00
1437460_x_at	0.00	0.00
1437462_x_at	0.00	0.00
1437464_at	0.00	0.00
1437466_at	0.00	0.00
1437467_at	0.00	0.00
1437469_at	0.00	0.00
1437470_at	0.00	0.00
1437471_at	0.00	0.00
1437473_at	0.00	0.00
1437474_at	0.00	0.00
1437475_at	0.00	0.00

1437476_at	0.00	0.00
1437478_s_at	0.00	0.00
1437479_x_at	0.00	0.00
1437481_at	0.00	0.00
1437482_at	0.00	0.00
1437483_at	0.00	0.00
1437484_at	0.00	0.00
1437485_at	0.00	0.00
1437486_at	0.33	0.00
1437487_at	0.00	0.00
1437488_at	0.00	0.00
1437492_at	0.00	0.00
1437493_at	0.00	0.00
1437494_at	0.00	0.00
1437495_at	0.00	0.00
1437496_at	0.00	0.00
1437499_at	0.00	0.00
1437500_at	0.00	0.07
1437501_at	0.00	0.00
1437504_at	0.00	0.00
1437505_at	0.00	0.00
1437506_at	0.00	0.00
1437507_at	0.00	0.00
1437514_at	0.00	0.00
1437516_at	0.00	0.00
1437517_x_at	0.00	0.00
1437518_at	0.00	0.00
1437524_x_at	0.00	0.00
1437528_x_at	0.00	0.00
1437529_at	0.00	0.00
1437530_at	0.00	0.00
1437532_at	0.00	0.00
1437535_at	0.00	0.00
1437536_at	0.00	0.00
1437538_at	0.00	0.00
1437539_at	0.00	0.00
1437542_at	0.00	0.00
1437549_at	0.00	0.00
1437550_at	0.00	0.00
1437551_at	0.00	0.00
1437552_at	0.02	0.00
1437554_at	0.00	0.00
1437555_at	0.00	0.00
1437556_at	0.00	0.00
1437557_at	0.00	0.00
1437558_at	0.00	0.00
1437559_at	0.00	0.00
1437560_at	0.00	0.00
1437561_at	0.00	0.00
1437562_at	0.00	0.00
1437567_at	0.00	0.00
1437569_at	0.00	0.00
1437570_at	0.00	0.00
1437571_at	0.00	0.00
1437572_at	0.00	0.00
1437573_at	0.00	0.00

1437574_at	0.00	0.00
1437575_at	0.00	0.00
1437576_at	0.00	0.00
1437577_at	0.00	0.00
1437578_at	0.00	0.00
1437579_at	0.00	0.00
1437580_s_at	0.00	0.00
1437581_at	0.00	0.00
1437584_at	0.00	0.00
1437587_at	0.00	0.00
1437588_at	0.14	0.00
1437590_at	0.00	0.00
1437594_x_at	0.00	0.00
1437595_at	0.00	0.00
1437596_at	0.00	0.00
1437597_at	0.00	0.00
1437598_at	0.00	0.00
1437599_at	0.00	0.00
1437600_at	0.00	0.00
1437601_at	0.00	0.00
1437602_at	0.00	0.00
1437603_at	0.00	0.00
1437604_x_at	0.00	0.00
1437605_at	0.00	0.00
1437606_at	0.00	0.00
1437609_at	0.00	0.00
1437613_s_at	0.00	0.00
1437617_x_at	0.00	0.00
1437623_x_at	0.00	0.00
1437625_at	0.00	0.00
1437627_at	0.00	0.00
1437629_at	0.00	0.00
1437631_at	0.00	0.00
1437632_at	0.00	0.00
1437633_at	0.00	0.00
1437635_at	0.00	0.00
1437636_at	0.00	0.00
1437637_at	0.00	0.00
1437638_at	0.00	0.00
1437639_at	0.00	0.00
1437640_at	0.00	0.00
1437641_at	0.00	0.00
1437643_at	0.00	0.00
1437646_at	0.00	0.00
1437647_at	0.00	0.00
1437648_at	0.00	0.27
1437650_at	0.00	0.00
1437653_at	0.00	0.00
1437654_at	0.00	0.00
1437655_at	0.00	0.00
1437656_at	0.00	0.00
1437657_at	0.00	0.00
1437659_at	0.00	0.00
1437660_at	0.00	0.00
1437662_at	0.00	0.00
1437663_at	0.00	0.00

1437664_at	0.00	0.00
1437665_at	0.00	0.00
1437668_at	0.00	0.00
1437669_x_at	0.00	0.00
1437673_at	0.00	0.00
1437675_at	0.00	0.00
1437676_at	0.00	0.00
1437677_at	0.00	0.00
1437678_at	0.00	0.00
1437681_at	0.00	0.00
1437694_at	0.00	0.00
1437695_at	0.00	0.00
1437698_at	0.00	0.00
1437699_at	0.00	0.00
1437700_at	0.00	0.00
1437701_at	0.00	0.00
1437702_at	0.00	0.00
1437703_at	0.00	0.00
1437704_at	0.00	0.00
1437705_at	0.00	0.00
1437707_at	0.00	0.00
1437710_x_at	0.00	0.00
1437717_x_at	0.00	0.00
1437720_at	0.00	0.00
1437727_at	0.00	0.00
1437731_at	0.00	0.00
1437734_at	0.00	0.00
1437735_at	0.00	0.00
1437736_at	0.00	0.00
1437737_at	0.00	0.00
1437739_a_at	0.00	0.00
1437740_at	0.00	0.00
1437743_at	0.00	0.00
1437744_at	0.00	0.00
1437745_at	0.00	0.00
1437748_at	0.00	0.00
1437749_s_at	0.00	0.00
1437753_at	0.00	0.00
1437754_at	0.00	0.00
1437755_at	0.00	0.00
1437756_at	0.00	0.00
1437757_at	0.00	0.00
1437759_at	0.00	0.00
1437760_at	0.00	0.02
1437761_at	0.00	0.00
1437762_at	0.00	0.00
1437763_at	0.00	0.00
1437764_at	0.00	0.00
1437766_at	0.00	0.00
1437768_at	0.00	0.00
1437769_at	0.00	0.00
1437770_at	0.00	0.00
1437771_at	0.00	0.00
1437774_at	0.00	0.00
1437775_at	0.00	0.00
1437776_at	0.00	0.00

1437777_at	0.00	0.00
1437778_at	0.00	0.00
1437780_at	0.00	0.00
1437781_at	0.00	0.00
1437784_at	0.00	0.00
1437785_at	0.00	0.00
1437787_at	0.00	0.00
1437788_at	0.00	0.00
1437789_at	0.00	0.00
1437792_at	0.00	0.00
1437793_at	0.00	0.00
1437794_at	0.00	0.00
1437795_at	0.00	0.00
1437796_at	0.00	0.00
1437797_at	0.00	0.00
1437798_at	0.00	0.00
1437799_at	0.00	0.00
1437800_at	0.00	0.00
1437809_x_at	0.00	0.00
1437811_x_at	0.00	0.02
1437813_at	0.00	0.00
1437814_at	0.00	0.00
1437815_at	0.00	0.00
1437817_at	0.00	0.00
1437818_at	0.00	0.00
1437819_s_at	0.00	0.00
1437820_at	0.00	0.00
1437821_at	0.00	0.00
1437822_at	0.00	0.00
1437823_at	0.00	0.00
1437824_at	0.00	0.00
1437825_at	0.00	0.00
1437826_at	0.00	0.00
1437827_s_at	0.00	0.00
1437828_s_at	0.00	0.46
1437829_s_at	0.00	0.00
1437831_at	0.00	0.00
1437833_at	0.00	0.00
1437834_s_at	0.00	0.00
1437840_s_at	0.00	0.00
1437842_at	0.00	0.00
1437854_at	0.00	0.00
1437856_at	0.00	0.00
1437857_at	0.00	0.00
1437858_at	0.00	0.00
1437860_at	0.00	0.00
1437861_s_at	0.00	0.00
1437862_at	0.00	0.00
1437864_at	0.00	0.00
1437865_at	0.00	0.00
1437866_at	0.00	0.00
1437868_at	0.00	0.00
1437869_at	0.00	0.00
1437870_at	0.00	0.00
1437871_at	0.00	0.00
1437872_at	0.00	0.00

1437873_at	0.00	0.00
1437875_at	0.00	0.00
1437876_at	0.00	0.00
1437877_at	0.00	0.00
1437878_s_at	0.00	0.00
1437879_at	0.00	0.00
1437880_at	0.00	0.00
1437883_s_at	0.00	0.00
1437884_at	0.00	0.00
1437886_at	0.00	0.00
1437887_at	0.00	0.00
1437888_at	0.00	0.00
1437891_at	0.00	0.00
1437892_at	0.00	0.00
1437893_at	0.00	0.00
1437894_at	0.00	0.00
1437895_at	0.00	0.00
1437896_at	0.00	0.00
1437897_at	0.00	0.00
1437898_at	0.00	0.00
1437899_at	0.00	0.00
1437900_at	0.00	0.00
1437904_at	0.00	0.28
1437910_at	0.00	0.00
1437911_at	0.00	0.00
1437912_at	0.00	0.00
1437914_at	0.00	0.00
1437916_at	0.00	0.00
1437918_at	0.00	0.00
1437919_at	0.00	0.00
1437921_x_at	0.00	0.00
1437923_at	0.00	0.00
1437926_at	0.00	0.00
1437927_at	0.00	0.00
1437928_at	0.00	0.00
1437929_at	0.00	0.00
1437930_at	0.00	0.00
1437931_at	0.00	0.00
1437933_at	0.00	0.00
1437934_at	0.00	0.00
1437935_at	0.00	0.00
1437936_at	0.00	0.00
1437937_at	0.00	0.00
1437939_s_at	0.00	0.00
1437940_at	0.00	0.00
1437941_at	0.00	0.00
1437942_x_at	0.00	0.00
1437944_at	0.00	0.00
1437950_at	0.00	0.00
1437951_at	0.00	0.00
1437953_at	0.00	0.00
1437954_at	0.00	0.00
1437955_at	0.00	0.00
1437956_at	0.00	0.00
1437957_at	0.00	0.00
1437959_at	0.00	0.00

1437960_at	0.00	0.00
1437961_x_at	0.00	0.00
1437962_at	0.00	0.00
1437963_at	0.00	0.00
1437964_at	0.00	0.00
1437965_at	0.00	0.00
1437966_at	0.00	0.00
1437967_at	0.00	0.00
1437972_s_at	0.00	0.00
1437973_at	0.00	0.00
1437977_at	0.00	0.00
1437978_at	0.00	0.00
1437979_at	0.00	0.00
1437980_at	0.00	0.00
1437983_at	0.89	0.10
1437986_x_at	0.00	0.00
1437987_at	0.00	0.00
1437988_x_at	0.00	0.00
1437989_at	0.00	0.00
1437996_s_at	0.25	0.00
1438002_at	0.00	0.00
1438003_at	0.00	0.00
1438004_at	0.00	0.00
1438005_at	0.00	0.00
1438007_at	0.00	0.00
1438008_at	0.00	0.00
1438010_at	0.00	0.00
1438012_at	0.00	0.00
1438015_at	0.00	0.00
1438016_at	0.03	0.08
1438018_at	0.00	0.00
1438020_at	0.00	0.00
1438021_at	0.24	0.00
1438022_at	0.00	0.14
1438024_at	0.00	0.00
1438025_at	0.00	0.00
1438026_at	0.00	0.00
1438027_at	0.00	0.00
1438028_at	0.00	0.00
1438029_at	0.00	0.00
1438030_at	0.00	0.00
1438031_at	0.00	0.00
1438032_at	0.00	0.00
1438035_at	0.00	0.00
1438036_x_at	0.00	0.00
1438037_at	0.00	0.00
1438038_at	0.00	0.00
1438039_at	0.00	0.00
1438041_at	0.00	0.16
1438042_at	0.00	0.00
1438043_at	0.00	0.00
1438044_at	0.00	0.00
1438046_at	0.00	0.00
1438047_at	0.00	0.00
1438048_at	0.00	0.00
1438049_at	0.00	0.00

1438051_at	0.00	0.00
1438052_at	0.00	0.00
1438053_at	0.00	0.00
1438054_x_at	0.00	0.00
1438055_at	0.00	0.00
1438057_at	0.00	0.00
1438059_at	0.00	0.00
1438060_at	0.00	0.00
1438061_at	0.00	0.00
1438062_at	0.00	0.00
1438063_at	0.00	0.00
1438065_at	0.00	0.00
1438066_at	0.00	0.00
1438068_at	0.00	0.00
1438071_at	0.00	0.00
1438072_at	0.00	0.00
1438073_at	0.00	0.00
1438074_at	0.00	0.00
1438075_at	0.00	0.00
1438077_at	0.00	0.00
1438078_at	0.00	0.00
1438079_at	0.00	0.00
1438081_at	0.00	0.00
1438084_at	0.00	0.00
1438085_at	0.00	0.00
1438086_at	0.00	0.00
1438087_at	0.00	0.00
1438088_at	0.00	0.00
1438089_a_at	0.00	0.15
1438098_at	0.00	0.00
1438099_at	0.00	0.00
1438100_at	0.00	0.00
1438101_at	0.00	0.00
1438102_at	0.00	0.00
1438103_at	0.00	0.00
1438104_at	0.00	0.00
1438105_at	0.00	0.00
1438106_at	0.00	0.00
1438107_x_at	0.00	0.00
1438108_at	0.00	0.00
1438109_at	0.00	0.00
1438110_at	0.00	0.00
1438111_at	0.00	0.00
1438112_at	0.00	0.00
1438113_at	0.00	0.00
1438121_at	0.00	0.00
1438122_at	0.00	0.00
1438123_at	0.00	0.00
1438124_at	0.00	0.00
1438125_at	0.00	0.00
1438126_at	0.00	0.00
1438127_at	0.00	0.00
1438128_at	0.00	0.00
1438129_at	0.00	0.00
1438130_at	0.00	0.00
1438131_at	0.00	0.00

1438132_at	0.00	0.00
1438134_at	0.00	0.00
1438135_at	0.00	0.00
1438136_at	0.00	0.00
1438137_at	0.00	0.00
1438139_at	0.00	0.00
1438141_at	0.00	0.00
1438142_s_at	0.00	0.00
1438145_at	0.00	0.00
1438146_x_at	0.00	0.00
1438147_at	0.00	0.00
1438148_at	0.00	0.00
1438149_at	0.00	0.00
1438157_s_at	0.00	0.00
1438158_at	0.00	0.00
1438160_x_at	0.00	0.00
1438162_x_at	0.00	0.00
1438175_x_at	0.00	0.00
1438185_at	0.00	0.00
1438186_at	0.00	0.00
1438189_s_at	0.00	0.00
1438191_a_at	0.00	0.00
1438193_at	0.00	0.00
1438194_at	0.00	0.00
1438195_at	0.00	0.00
1438196_at	0.00	0.00
1438197_at	0.00	0.00
1438198_at	0.00	0.00
1438200_at	0.00	0.00
1438201_at	0.00	0.00
1438203_at	0.00	0.00
1438205_at	0.00	0.00
1438207_at	0.00	0.00
1438208_at	0.00	0.00
1438209_at	0.00	0.00
1438210_at	0.00	0.00
1438212_at	0.00	0.00
1438213_at	0.00	0.00
1438214_at	0.00	0.00
1438217_at	0.00	0.00
1438218_at	0.00	0.00
1438222_at	0.00	0.00
1438224_at	0.00	0.00
1438226_at	0.00	0.00
1438227_at	0.00	0.00
1438228_at	0.00	0.00
1438229_at	0.00	0.00
1438230_at	0.00	0.00
1438231_at	0.00	0.00
1438232_at	0.00	0.00
1438235_at	0.00	0.00
1438236_at	0.00	0.00
1438237_at	1.00	0.68
1438238_at	0.00	0.00
1438239_at	0.00	0.00
1438240_at	0.00	0.00

1438241_at	0.00	0.00
1438242_at	0.00	0.00
1438247_at	0.00	0.00
1438249_at	0.00	0.00
1438253_at	0.00	0.00
1438254_at	0.00	0.00
1438255_at	0.00	0.13
1438256_at	0.00	0.00
1438257_at	0.00	0.00
1438258_at	0.00	0.00
1438259_at	0.00	0.00
1438262_at	0.00	0.00
1438263_at	0.00	0.00
1438265_at	0.00	0.00
1438266_at	0.00	0.00
1438268_at	0.00	0.00
1438269_at	0.00	0.00
1438270_at	0.00	0.00
1438271_at	0.47	0.00
1438272_at	0.00	0.00
1438273_at	0.00	0.00
1438275_at	0.00	0.00
1438276_at	0.00	0.00
1438277_at	0.00	0.00
1438279_at	0.00	0.00
1438280_at	0.00	0.00
1438281_x_at	0.00	0.00
1438282_at	0.00	0.00
1438283_at	0.00	0.00
1438284_at	0.00	0.00
1438285_at	0.00	0.00
1438288_x_at	0.00	0.00
1438293_at	0.00	0.00
1438294_at	0.00	0.00
1438295_at	0.00	0.00
1438296_at	0.00	0.00
1438298_a_at	0.00	0.00
1438299_at	0.00	0.00
1438300_at	0.00	0.00
1438301_at	0.00	0.00
1438303_at	0.00	0.00
1438304_at	0.00	0.00
1438305_at	0.00	0.00
1438306_at	0.00	0.00
1438307_at	0.00	0.00
1438308_at	0.00	0.00
1438309_at	0.00	0.00
1438310_at	0.00	0.00
1438311_at	0.00	0.00
1438313_at	0.00	0.00
1438323_at	0.00	0.00
1438324_at	0.00	0.00
1438325_at	0.00	0.00
1438326_at	0.00	0.00
1438327_at	0.00	0.00
1438328_at	0.00	0.00

1438329_at	0.00	0.00
1438330_at	0.00	0.00
1438331_at	0.00	0.00
1438333_at	0.37	0.01
1438334_at	0.00	0.00
1438335_at	0.00	0.00
1438336_at	0.00	0.00
1438337_x_at	0.00	0.00
1438338_at	0.00	0.00
1438339_at	0.00	0.00
1438340_at	0.00	0.00
1438341_at	0.00	0.00
1438342_at	0.00	0.00
1438344_at	0.00	0.00
1438345_at	0.00	0.00
1438346_at	0.00	0.00
1438347_at	0.00	0.00
1438348_x_at	0.00	0.00
1438349_at	0.00	0.00
1438350_at	0.00	0.00
1438351_at	0.00	0.00
1438352_at	0.00	0.00
1438353_at	0.00	0.00
1438355_at	0.00	0.00
1438356_x_at	0.00	0.00
1438359_at	0.00	0.00
1438361_at	0.00	0.00
1438362_x_at	0.00	0.00
1438363_at	0.00	0.00
1438364_x_at	0.00	0.00
1438372_at	0.00	0.00
1438375_at	0.00	0.00
1438378_at	0.00	0.00
1438382_x_at	0.00	0.00
1438384_at	0.00	0.00
1438388_at	0.00	0.00
1438392_at	0.00	0.00
1438393_at	0.00	0.00
1438394_x_at	0.00	0.00
1438395_at	0.00	0.00
1438396_at	0.00	0.00
1438399_at	0.00	0.00
1438400_at	0.00	0.00
1438401_at	0.00	0.00
1438402_at	0.00	0.00
1438404_at	0.00	0.00
1438405_at	0.00	0.00
1438406_at	0.00	0.00
1438407_at	0.00	0.00
1438408_at	0.00	0.00
1438409_at	0.00	0.00
1438410_at	0.58	0.48
1438411_at	0.00	0.00
1438412_at	0.00	0.00
1438413_at	0.00	0.00
1438414_at	0.00	0.00

1438417_at	0.00	0.00
1438419_at	0.00	0.00
1438420_at	0.00	0.00
1438421_at	0.00	0.00
1438423_at	0.00	0.00
1438424_at	0.00	0.00
1438425_at	0.00	0.00
1438426_at	0.00	0.00
1438428_at	0.00	0.00
1438429_at	0.00	0.00
1438431_at	0.00	0.00
1438432_at	0.00	0.00
1438433_at	0.00	0.00
1438434_at	0.00	0.00
1438435_at	0.01	0.01
1438436_at	0.00	0.00
1438437_a_at	0.00	0.00
1438439_at	0.00	0.00
1438440_at	0.00	0.00
1438442_at	0.00	0.00
1438444_at	0.00	0.00
1438445_at	0.00	0.00
1438447_at	0.00	0.00
1438448_at	0.00	0.00
1438449_at	0.00	0.00
1438450_at	0.00	0.00
1438451_at	0.00	0.00
1438452_at	0.00	0.00
1438454_at	0.00	0.00
1438455_at	0.00	0.00
1438456_at	0.00	0.00
1438457_at	0.00	0.00
1438460_at	0.00	0.00
1438464_at	0.00	0.00
1438466_at	0.00	0.00
1438469_at	0.00	0.00
1438470_at	0.02	0.15
1438471_at	0.00	0.00
1438472_at	0.00	0.00
1438473_at	0.00	0.00
1438474_at	0.00	0.00
1438475_at	0.00	0.00
1438479_at	0.00	0.00
1438481_at	0.00	0.00
1438482_at	0.00	0.00
1438484_at	0.00	0.00
1438486_at	0.00	0.00
1438487_s_at	0.07	0.00
1438488_at	0.00	0.00
1438489_at	0.00	0.00
1438490_at	0.00	0.00
1438491_x_at	0.00	0.00
1438493_at	0.00	0.00
1438495_at	0.00	0.00
1438496_a_at	0.00	0.00
1438497_at	0.00	0.00

1438498_at	0.00	0.00
1438499_at	0.00	0.00
1438500_at	0.00	0.00
1438505_s_at	0.00	0.00
1438508_at	0.00	0.00
1438512_at	0.00	0.00
1438513_at	0.00	0.00
1438514_at	0.00	0.00
1438515_at	0.00	0.00
1438516_at	0.00	0.00
1438517_at	0.00	0.00
1438518_at	0.00	0.00
1438519_at	0.00	0.00
1438520_at	0.00	0.00
1438521_at	0.00	0.00
1438522_at	0.00	0.00
1438523_x_at	0.00	0.00
1438525_at	0.00	0.00
1438526_at	0.00	0.00
1438528_at	0.00	0.00
1438529_at	0.00	0.00
1438530_at	0.00	0.00
1438531_at	0.00	0.00
1438532_at	0.00	0.00
1438533_at	0.00	0.00
1438536_at	0.00	0.00
1438538_at	0.00	0.00
1438539_at	0.00	0.00
1438540_at	0.00	0.00
1438541_at	0.00	0.00
1438542_at	0.00	0.00
1438543_at	0.00	0.00
1438544_at	0.00	0.00
1438553_x_at	0.00	0.00
1438555_x_at	0.00	0.00
1438558_x_at	0.00	0.00
1438561_x_at	0.00	0.00
1438565_at	0.00	0.00
1438566_at	0.00	0.00
1438567_at	0.00	0.00
1438568_at	0.00	0.00
1438569_at	0.00	0.00
1438570_at	0.00	0.00
1438572_at	0.00	0.00
1438573_at	0.00	0.00
1438574_at	0.00	0.00
1438575_a_at	0.00	0.00
1438576_x_at	0.00	0.00
1438577_at	0.00	0.00
1438579_at	0.00	0.00
1438580_at	0.00	0.00
1438581_at	0.00	0.00
1438582_at	0.00	0.00
1438583_at	0.00	0.00
1438584_at	0.00	0.00
1438585_at	0.00	0.00

1438586_at	0.00	0.00
1438587_at	0.00	0.00
1438588_at	0.00	0.00
1438589_at	0.00	0.00
1438590_at	0.00	0.00
1438591_at	0.00	0.00
1438592_at	0.00	0.00
1438593_at	0.00	0.00
1438594_at	0.00	0.00
1438595_at	0.00	0.00
1438596_at	0.00	0.00
1438598_at	0.00	0.00
1438599_at	0.00	0.00
1438600_at	0.00	0.00
1438604_at	0.00	0.00
1438605_at	0.00	0.00
1438607_at	0.00	0.00
1438613_at	0.00	0.00
1438614_x_at	0.00	0.00
1438615_x_at	0.00	0.00
1438617_at	0.00	0.00
1438618_at	0.00	0.00
1438624_x_at	0.00	0.00
1438628_x_at	0.00	0.00
1438635_x_at	0.00	0.00
1438636_s_at	0.00	0.00
1438638_x_at	0.00	0.00
1438639_x_at	0.00	0.00
1438641_x_at	0.00	0.00
1438643_at	0.00	0.00
1438648_x_at	0.00	0.00
1438658_a_at	0.00	0.00
1438660_at	0.00	0.00
1438662_at	0.00	0.00
1438663_at	0.00	0.01
1438664_at	0.00	0.00
1438665_at	0.00	0.00
1438666_at	0.00	0.00
1438667_at	0.00	0.00
1438668_x_at	0.00	0.00
1438671_at	0.00	0.00
1438672_at	0.00	0.00
1438678_at	0.00	0.00
1438681_at	0.00	0.00
1438682_at	0.00	0.00
1438683_at	0.00	0.00
1438684_at	0.63	0.16
1438687_at	0.00	0.00
1438688_at	0.00	0.00
1438689_at	0.00	0.00
1438691_at	0.00	0.00
1438692_at	0.00	0.00
1438693_at	0.00	0.00
1438697_at	0.00	0.00
1438698_at	0.00	0.00
1438699_at	0.00	0.00

1438701_at	0.00	0.00
1438702_at	0.00	0.00
1438703_at	0.00	0.00
1438704_at	0.00	0.00
1438706_at	0.00	0.00
1438707_at	0.00	0.00
1438709_at	0.00	0.00
1438713_at	0.00	0.00
1438715_at	0.00	0.00
1438718_at	0.00	0.00
1438719_at	0.00	0.00
1438720_at	0.00	0.00
1438722_at	0.00	0.00
1438724_at	0.00	0.00
1438725_at	0.00	0.00
1438726_at	0.00	0.00
1438727_at	0.00	0.00
1438728_at	0.00	0.00
1438729_at	0.00	0.00
1438730_at	0.00	0.00
1438732_at	0.00	0.00
1438733_at	0.00	0.00
1438734_at	0.00	0.00
1438735_at	0.00	0.00
1438737_at	0.03	0.01
1438738_at	0.00	0.00
1438739_at	0.00	0.00
1438740_at	0.00	0.00
1438742_at	0.00	0.00
1438744_at	0.00	0.00
1438745_at	0.00	0.00
1438747_at	0.00	0.00
1438749_at	0.00	0.00
1438750_at	0.00	0.00
1438751_at	0.00	0.00
1438752_at	0.00	0.00
1438753_at	0.00	0.00
1438754_at	0.00	0.00
1438756_at	0.00	0.00
1438757_at	0.00	0.28
1438762_at	0.00	0.00
1438763_at	0.00	0.00
1438765_at	0.00	0.00
1438766_at	0.00	0.00
1438767_at	0.00	0.00
1438768_at	0.00	0.00
1438770_at	0.00	0.00
1438771_at	0.00	0.00
1438772_at	0.00	0.00
1438773_at	0.00	0.00
1438774_s_at	0.00	0.00
1438775_at	0.00	0.00
1438778_at	0.00	0.00
1438779_at	0.00	0.00
1438780_at	0.00	0.00
1438781_at	0.00	0.00

1438783_at	0.00	0.00
1438784_at	0.00	0.00
1438785_at	0.00	0.00
1438786_a_at	0.10	0.01
1438787_at	0.28	0.16
1438788_at	0.00	0.00
1438791_at	0.00	0.00
1438796_at	0.00	0.00
1438798_at	0.00	0.00
1438799_at	0.00	0.00
1438800_at	0.00	0.00
1438801_at	0.00	0.00
1438803_s_at	0.00	0.00
1438804_at	0.00	0.00
1438805_at	0.00	0.00
1438806_at	0.00	0.00
1438807_at	0.00	0.00
1438810_at	0.00	0.00
1438811_at	0.00	0.00
1438813_at	0.00	0.00
1438814_at	0.00	0.00
1438815_at	0.00	0.00
1438816_at	0.00	0.00
1438818_at	0.00	0.00
1438819_at	0.00	0.00
1438820_at	0.01	0.00
1438821_at	0.00	0.00
1438822_at	0.00	0.00
1438823_at	0.00	0.00
1438827_at	0.00	0.00
1438828_at	0.00	0.00
1438829_at	0.00	0.00
1438830_at	0.00	0.00
1438831_at	0.00	0.00
1438833_at	0.00	0.00
1438834_at	0.00	0.00
1438837_at	0.00	0.00
1438838_at	0.00	0.00
1438848_at	0.00	0.00
1438849_at	0.00	0.00
1438858_x_at	0.00	0.00
1438861_at	0.34	0.45
1438862_at	0.00	0.00
1438863_at	0.00	0.00
1438864_at	0.00	0.00
1438866_at	0.00	0.00
1438867_at	0.00	0.00
1438868_at	0.00	0.00
1438869_at	0.00	0.00
1438870_at	0.00	0.00
1438871_at	0.00	0.00
1438872_at	0.01	0.00
1438873_at	0.00	0.00
1438874_at	0.00	0.00
1438875_at	0.00	0.00
1438876_at	0.00	0.00

1438877_at	0.00	0.00
1438878_at	0.00	0.00
1438879_at	0.00	0.00
1438880_at	0.00	0.00
1438881_at	0.00	0.00
1438882_at	0.00	0.00
1438883_at	0.00	1.00
1438884_at	0.00	0.00
1438885_at	0.00	0.00
1438889_at	0.00	0.00
1438890_at	0.00	0.00
1438891_at	0.00	0.00
1438892_at	0.00	0.00
1438893_at	0.00	0.00
1438894_at	0.00	0.00
1438895_at	0.00	0.00
1438896_at	0.00	0.00
1438897_at	0.00	0.00
1438898_at	0.00	0.00
1438899_at	0.00	0.00
1438900_at	0.00	0.00
1438901_at	0.00	0.00
1438904_at	0.00	0.00
1438905_x_at	0.00	0.00
1438906_at	0.00	0.00
1438907_at	0.00	0.00
1438911_at	0.00	0.00
1438915_at	0.00	0.00
1438916_x_at	0.00	0.00
1438920_x_at	0.00	0.00
1438921_at	0.00	0.00
1438929_at	0.00	0.00
1438930_s_at	0.00	0.00
1438935_at	0.00	0.00
1438936_s_at	0.00	0.00
1438937_x_at	0.00	0.00
1438944_at	0.00	0.00
1438946_at	0.00	0.00
1438947_x_at	0.00	0.00
1438959_x_at	0.00	0.00
1438960_at	0.00	0.00
1438962_s_at	0.00	0.00
1438965_x_at	0.00	0.00
1438967_x_at	0.00	0.00
1438970_x_at	0.00	0.00
1438971_x_at	0.00	0.34
1438972_x_at	0.00	0.00
1438979_s_at	0.00	0.00
1438981_at	0.00	0.00
1438985_x_at	0.00	0.00
1438987_at	0.00	0.00
1438989_s_at	0.00	0.00
1438994_at	0.00	0.00
1438995_at	0.00	0.00
1438996_at	0.00	0.00
1438997_at	0.00	0.00

1438998_at	0.00	0.00
1439000_at	0.00	0.00
1439001_at	0.00	0.00
1439003_s_at	0.00	0.00
1439004_at	0.00	0.00
1439006_x_at	0.00	0.00
1439007_at	0.00	0.00
1439009_at	0.00	0.00
1439010_at	0.00	0.00
1439011_at	0.00	0.00
1439014_at	0.00	0.00
1439015_at	0.00	0.00
1439019_at	0.00	0.00
1439020_at	0.00	0.00
1439021_at	0.00	0.00
1439023_at	0.00	0.00
1439024_at	0.00	0.00
1439025_at	0.00	0.00
1439026_at	0.00	0.00
1439027_at	0.00	0.01
1439029_at	0.00	0.00
1439031_at	0.00	0.00
1439033_at	0.00	0.00
1439034_at	0.00	0.09
1439035_at	0.00	0.00
1439038_at	0.00	0.29
1439039_at	0.00	0.00
1439041_at	0.00	0.00
1439043_at	0.00	0.00
1439044_at	0.00	0.00
1439046_at	0.00	0.00
1439048_at	0.00	0.00
1439050_at	0.00	0.00
1439052_at	0.00	0.00
1439053_at	0.00	0.00
1439055_at	0.00	0.00
1439056_at	0.00	0.00
1439057_x_at	0.00	0.00
1439058_at	0.00	0.00
1439059_at	0.00	0.00
1439062_at	0.00	0.00
1439063_at	0.00	0.00
1439066_at	0.00	0.00
1439067_at	0.00	0.00
1439068_at	0.00	0.00
1439071_at	0.00	0.00
1439072_at	0.00	0.00
1439073_at	0.00	0.00
1439076_at	0.00	0.00
1439077_at	0.00	0.00
1439078_at	0.00	0.00
1439082_at	0.00	0.00
1439084_at	0.00	0.00
1439085_at	0.00	0.00
1439086_at	0.00	0.00
1439087_a_at	0.00	0.25

1439088_at	0.00	0.00
1439089_at	0.00	0.00
1439090_at	0.00	0.00
1439091_at	0.00	0.00
1439092_at	0.00	0.00
1439093_at	0.00	0.00
1439094_at	0.00	0.00
1439095_at	0.00	0.00
1439096_at	0.00	0.00
1439097_at	0.00	0.00
1439098_at	0.00	0.00
1439099_at	0.00	0.00
1439100_s_at	0.00	0.00
1439101_at	0.00	0.00
1439104_at	0.00	0.00
1439105_at	0.00	0.00
1439106_at	0.00	0.00
1439107_a_at	0.00	0.00
1439108_at	0.00	0.00
1439109_at	0.00	0.00
1439112_at	0.00	0.00
1439114_at	0.00	0.00
1439115_at	0.00	0.00
1439117_at	0.00	0.00
1439118_at	0.00	0.00
1439123_at	0.00	0.00
1439124_at	0.00	0.00
1439125_at	0.00	0.00
1439126_at	0.00	0.00
1439127_at	0.09	0.00
1439128_at	0.00	0.00
1439129_at	0.00	0.00
1439130_at	0.00	0.00
1439131_at	0.01	0.09
1439132_at	0.00	0.00
1439133_at	0.00	0.00
1439134_s_at	0.00	0.00
1439135_at	0.00	0.00
1439136_at	0.00	0.00
1439137_at	0.00	0.00
1439138_at	0.00	0.00
1439139_at	0.00	0.00
1439140_at	0.00	0.00
1439141_at	0.00	0.00
1439143_at	0.00	0.00
1439145_at	0.00	0.00
1439146_s_at	0.00	0.00
1439147_at	0.00	0.00
1439149_s_at	0.00	0.00
1439151_at	0.00	0.00
1439152_at	0.00	0.00
1439153_at	0.00	0.00
1439155_at	0.00	0.01
1439156_at	0.00	0.00
1439157_at	0.00	0.00
1439158_at	0.00	0.00

1439159_at	0.00	0.00
1439160_at	0.00	0.00
1439161_at	0.00	0.00
1439162_at	0.00	0.00
1439163_at	0.00	0.00
1439165_at	0.00	0.00
1439166_at	0.00	0.00
1439168_at	0.00	0.00
1439169_at	0.00	0.00
1439170_at	0.00	0.00
1439171_at	0.00	0.00
1439172_at	0.00	0.00
1439173_at	0.00	0.00
1439174_at	0.00	0.00
1439175_at	0.00	0.00
1439176_at	0.00	0.00
1439177_at	0.00	0.00
1439178_at	0.00	0.00
1439179_a_at	0.00	0.00
1439180_at	0.00	0.00
1439181_at	0.00	0.00
1439183_at	0.00	0.00
1439186_at	0.00	0.00
1439187_at	0.00	0.00
1439188_at	0.00	0.00
1439189_at	0.00	0.00
1439190_at	0.00	0.00
1439192_at	0.00	0.00
1439193_at	0.00	0.00
1439194_at	0.00	0.00
1439195_at	0.00	0.00
1439196_at	0.00	0.00
1439197_at	0.00	0.00
1439198_at	0.00	0.00
1439201_at	0.00	0.00
1439202_at	0.00	0.00
1439203_at	0.00	0.00
1439204_at	0.00	0.00
1439205_at	0.00	0.00
1439207_at	0.00	0.00
1439208_at	0.00	0.00
1439209_at	0.00	0.00
1439211_at	0.00	0.00
1439212_at	0.00	0.00
1439213_at	0.00	0.00
1439215_at	0.00	0.00
1439216_at	0.00	0.00
1439217_at	0.00	0.00
1439218_at	0.00	0.00
1439219_at	0.00	0.00
1439220_at	0.00	0.00
1439221_s_at	0.00	0.00
1439222_at	0.00	0.00
1439223_at	0.00	0.00
1439224_at	0.00	0.00
1439225_at	0.00	0.00

1439226_at	0.00	0.00
1439227_at	0.00	0.00
1439228_at	0.00	0.00
1439229_at	0.00	0.00
1439230_at	0.00	0.00
1439231_at	0.00	0.00
1439232_at	0.00	0.00
1439233_at	0.00	0.00
1439236_at	0.00	0.00
1439237_a_at	0.00	0.00
1439238_at	0.00	0.00
1439242_at	0.00	0.00
1439247_at	0.00	0.00
1439248_at	0.00	0.00
1439249_at	0.00	0.00
1439250_at	0.00	0.00
1439252_at	0.00	0.00
1439254_at	0.00	0.00
1439265_at	0.00	0.00
1439273_at	0.00	0.00
1439274_at	0.00	0.00
1439275_s_at	0.00	0.00
1439276_at	0.00	0.00
1439277_at	0.00	0.00
1439278_at	0.00	0.00
1439279_at	0.00	0.00
1439280_at	0.00	0.00
1439281_at	0.00	0.00
1439282_at	0.00	0.00
1439284_at	0.46	0.01
1439285_at	0.00	0.00
1439286_at	0.00	0.00
1439288_at	0.00	0.00
1439289_s_at	0.00	0.00
1439290_at	0.00	0.00
1439291_at	0.00	0.00
1439292_at	0.00	0.00
1439293_at	0.00	0.00
1439294_at	0.00	0.00
1439297_at	0.00	0.00
1439298_at	0.00	0.00
1439299_at	0.00	0.00
1439300_at	0.00	0.00
1439301_at	0.00	0.00
1439302_at	0.00	0.00
1439303_at	0.00	0.00
1439304_at	0.00	0.00
1439305_at	0.00	0.00
1439306_at	0.00	0.00
1439307_at	0.00	0.00
1439308_at	0.00	0.00
1439309_at	0.00	0.00
1439310_at	0.00	0.00
1439311_at	0.00	0.00
1439312_at	0.00	0.00
1439313_at	0.00	0.00

1439314_at	0.00	0.00
1439315_at	0.00	0.00
1439316_at	0.00	0.00
1439317_at	0.00	0.00
1439318_at	0.00	0.00
1439319_at	0.00	0.00
1439320_at	0.00	0.00
1439321_at	0.00	0.00
1439322_at	0.00	0.00
1439324_at	0.00	0.00
1439325_at	0.00	0.00
1439326_at	0.00	0.00
1439327_at	0.00	0.00
1439328_at	0.00	0.00
1439329_a_at	0.00	0.00
1439330_at	0.00	0.00
1439331_at	0.00	0.00
1439333_at	0.00	0.00
1439334_at	0.00	0.00
1439335_at	0.00	0.00
1439336_at	0.00	0.00
1439337_at	0.00	0.00
1439338_at	0.00	0.00
1439339_at	0.00	0.00
1439340_at	0.00	0.00
1439341_at	0.00	0.71
1439342_at	0.00	0.00
1439343_at	0.00	0.00
1439344_at	0.00	0.00
1439345_at	0.00	0.00
1439346_at	0.00	0.00
1439347_at	0.00	0.00
1439348_at	0.00	0.00
1439349_at	0.00	0.00
1439351_at	0.00	0.00
1439352_at	0.00	0.00
1439353_x_at	0.00	0.00
1439354_at	0.00	0.00
1439355_at	0.00	0.00
1439357_at	0.00	0.00
1439358_a_at	0.00	0.00
1439359_x_at	0.00	0.00
1439361_at	0.00	0.00
1439362_at	0.00	0.00
1439363_at	0.00	0.00
1439365_at	0.00	0.00
1439378_at	0.00	0.00
1439387_x_at	0.00	0.01
1439391_at	0.00	0.00
1439395_at	0.00	0.00
1439397_at	0.00	0.00
1439400_x_at	0.00	0.00
1439401_x_at	0.00	0.00
1439402_at	0.00	0.00
1439404_x_at	0.00	0.00
1439412_at	0.00	0.00

1439414_x_at	0.00	0.00
1439419_at	0.00	0.00
1439420_x_at	0.00	0.00
1439425_x_at	0.00	0.00
1439427_at	0.00	0.00
1439428_x_at	0.00	0.00
1439430_x_at	0.00	0.00
1439431_x_at	0.00	0.00
1439434_x_at	0.00	0.00
1439442_x_at	0.00	0.19
1439446_at	0.00	0.00
1439449_at	0.00	0.00
1439467_at	0.00	0.00
1439468_at	0.00	0.00
1439469_at	0.00	0.00
1439470_at	0.00	0.00
1439471_at	0.00	0.00
1439472_at	0.00	0.00
1439473_at	0.00	0.00
1439474_x_at	0.00	0.00
1439475_at	0.00	0.00
1439477_at	0.00	0.02
1439478_at	0.00	0.00
1439480_at	0.00	0.00
1439481_at	0.00	0.02
1439484_at	0.00	0.04
1439485_at	0.00	0.00
1439486_at	0.00	0.00
1439487_at	0.00	0.00
1439488_at	0.00	0.00
1439489_at	0.00	0.00
1439490_at	0.00	0.00
1439491_at	0.00	0.00
1439492_at	0.00	0.00
1439493_at	0.00	0.00
1439494_at	0.00	0.00
1439495_at	0.00	0.00
1439496_at	0.00	0.00
1439497_at	0.00	0.00
1439498_at	0.00	0.00
1439499_at	0.00	0.00
1439500_at	0.00	0.00
1439501_at	0.00	0.00
1439502_at	0.00	0.00
1439503_at	0.00	0.00
1439504_s_at	0.00	0.00
1439505_at	0.00	0.00
1439507_at	0.00	0.00
1439508_at	0.00	0.00
1439509_at	0.00	0.00
1439510_at	0.00	0.00
1439511_at	0.00	0.00
1439512_at	0.00	0.00
1439513_at	0.00	0.00
1439514_at	0.00	0.00
1439515_at	0.00	0.00

1439517_at	0.00	0.00
1439518_at	0.00	0.00
1439519_at	0.00	0.00
1439520_at	0.00	0.00
1439521_at	0.00	0.00
1439522_at	0.00	0.00
1439523_at	0.00	0.00
1439524_at	0.00	0.00
1439525_at	0.00	0.00
1439526_at	0.00	0.00
1439527_at	0.00	0.00
1439528_at	0.00	0.00
1439529_at	0.00	0.00
1439530_a_at	0.00	0.00
1439531_at	0.00	0.00
1439532_s_at	0.00	0.00
1439533_at	0.00	0.00
1439534_at	0.00	0.00
1439535_at	0.00	0.00
1439536_at	0.00	0.00
1439537_at	0.00	0.00
1439538_at	0.00	0.00
1439539_at	0.00	0.00
1439540_at	0.00	0.00
1439541_at	0.00	0.00
1439542_at	0.00	0.00
1439543_at	0.00	0.00
1439544_at	0.00	0.00
1439545_at	0.00	0.00
1439546_at	0.00	0.00
1439547_at	0.00	0.00
1439549_at	0.00	0.00
1439550_at	0.00	0.00
1439551_at	0.00	0.00
1439552_at	0.00	0.00
1439553_s_at	0.00	0.00
1439554_at	0.00	0.00
1439555_at	0.00	0.00
1439556_at	0.00	0.00
1439557_s_at	0.00	0.00
1439558_at	0.00	0.00
1439559_at	0.00	0.00
1439560_x_at	0.00	0.00
1439561_at	0.00	0.00
1439562_at	0.00	0.00
1439563_at	0.00	0.00
1439564_at	0.00	0.00
1439565_at	0.00	0.00
1439566_at	0.00	0.00
1439568_at	0.00	0.00
1439569_at	0.00	0.00
1439570_at	0.00	0.00
1439571_at	0.00	0.00
1439572_at	0.00	0.00
1439573_at	0.00	0.00
1439574_at	0.00	0.00

1439575_at	0.00	0.00
1439576_at	0.00	0.00
1439577_at	0.00	0.00
1439578_at	0.00	0.00
1439579_at	0.00	0.00
1439580_at	0.00	0.00
1439581_at	0.00	0.00
1439582_at	0.00	0.00
1439583_x_at	0.00	0.00
1439584_at	0.00	0.00
1439585_at	0.00	0.00
1439586_at	0.00	0.00
1439587_at	0.00	0.00
1439588_at	0.00	0.00
1439589_at	0.00	0.00
1439590_at	0.00	0.00
1439591_at	0.00	0.00
1439592_at	0.00	0.00
1439593_s_at	0.00	0.00
1439594_at	0.00	0.00
1439595_at	0.00	0.00
1439596_at	0.00	0.00
1439597_at	0.00	0.00
1439598_at	0.00	0.00
1439599_at	0.00	0.00
1439600_at	0.00	0.00
1439601_at	0.00	0.00
1439602_at	0.00	0.00
1439603_at	0.00	0.00
1439604_at	0.00	0.00
1439605_at	0.00	0.00
1439606_at	0.00	0.00
1439607_at	0.00	0.00
1439608_at	0.00	0.00
1439609_at	0.00	0.00
1439610_at	0.00	0.00
1439611_at	0.00	0.00
1439612_at	0.00	0.00
1439613_at	0.00	0.00
1439614_at	0.00	0.00
1439615_at	0.00	0.00
1439616_at	0.00	0.00
1439617_s_at	0.00	0.00
1439618_at	0.00	0.00
1439619_at	0.00	0.00
1439620_at	0.00	0.00
1439621_at	0.00	0.00
1439622_at	0.00	0.00
1439623_at	0.00	0.00
1439624_at	0.00	0.00
1439626_at	0.00	0.00
1439627_at	0.00	0.00
1439628_x_at	0.00	0.00
1439629_at	0.00	0.00
1439630_x_at	0.00	0.00
1439631_at	0.00	0.00

1439632_at	0.00	0.00
1439633_at	0.00	0.00
1439634_at	0.00	0.00
1439635_at	0.00	0.00
1439636_at	0.00	0.00
1439637_at	0.00	0.00
1439638_at	0.00	0.00
1439639_at	0.00	0.00
1439640_at	0.00	0.00
1439641_at	0.00	0.00
1439642_at	0.00	0.00
1439643_at	0.00	0.00
1439644_at	0.00	0.00
1439645_at	0.00	0.00
1439646_at	0.00	0.00
1439647_at	0.00	0.00
1439648_at	0.00	0.00
1439649_at	0.00	0.00
1439650_at	0.00	0.00
1439651_at	0.00	0.00
1439652_at	0.00	0.00
1439653_at	0.00	0.00
1439654_at	0.00	0.00
1439655_at	0.00	0.00
1439656_at	0.00	0.00
1439657_at	0.00	0.00
1439658_at	0.00	0.00
1439659_at	0.00	0.00
1439660_at	0.00	0.00
1439661_at	0.00	0.00
1439662_at	0.00	0.00
1439663_at	0.00	0.00
1439664_at	0.00	0.00
1439665_at	0.00	0.00
1439666_at	0.00	0.00
1439667_at	0.00	0.00
1439668_at	0.00	0.00
1439669_at	0.00	0.00
1439670_at	0.00	0.00
1439671_at	0.00	0.00
1439672_at	0.00	0.00
1439673_at	0.00	0.00
1439674_at	0.00	0.00
1439675_at	0.00	0.00
1439676_at	0.00	0.00
1439677_at	0.00	0.00
1439678_at	0.00	0.00
1439679_at	0.00	0.00
1439680_at	0.00	0.00
1439681_at	0.00	0.00
1439682_at	0.00	0.00
1439683_at	0.00	0.00
1439684_at	0.00	0.00
1439685_at	0.00	0.00
1439686_at	0.00	0.00
1439687_at	0.00	0.00

1439688_at	0.00	0.00
1439689_at	0.00	0.00
1439690_at	0.00	0.00
1439691_at	0.00	0.00
1439692_at	0.00	0.00
1439693_a_at	0.00	0.00
1439694_at	0.00	0.00
1439695_a_at	0.00	0.29
1439696_at	0.00	0.00
1439697_at	0.00	0.00
1439698_at	0.00	0.00
1439699_at	0.00	0.00
1439700_at	0.00	0.00
1439701_at	0.00	0.00
1439702_at	0.00	0.00
1439703_at	0.00	0.00
1439704_at	0.00	0.00
1439705_at	0.00	0.00
1439706_at	0.00	0.00
1439707_at	0.00	0.00
1439708_at	0.00	0.00
1439709_at	0.00	0.00
1439710_at	0.00	0.00
1439711_at	0.00	0.00
1439712_at	0.00	0.00
1439713_at	0.00	0.00
1439714_at	0.00	0.00
1439715_at	0.00	0.00
1439716_at	0.00	0.00
1439717_at	0.00	0.00
1439718_at	0.00	0.00
1439719_at	0.00	0.00
1439720_at	0.00	0.00
1439721_at	0.00	0.00
1439722_at	0.00	0.00
1439723_at	0.00	0.00
1439724_at	0.00	0.00
1439725_at	0.00	0.00
1439726_at	0.00	0.00
1439727_at	0.00	0.00
1439728_at	0.00	0.00
1439729_at	0.00	0.00
1439730_at	0.00	0.00
1439731_at	0.00	0.00
1439732_at	0.00	0.00
1439733_at	0.00	0.00
1439734_at	0.00	0.00
1439735_at	0.00	0.00
1439736_at	0.00	0.00
1439737_x_at	0.00	0.00
1439738_at	0.00	0.00
1439739_at	0.00	0.00
1439740_s_at	0.00	0.00
1439741_x_at	0.00	0.00
1439742_at	0.00	0.00
1439743_at	0.00	0.00

1439744_at	0.00	0.00
1439745_at	0.00	0.00
1439746_at	0.31	0.05
1439747_at	0.00	0.00
1439748_at	0.00	0.00
1439750_at	0.00	0.00
1439751_at	0.00	0.00
1439752_at	0.00	0.00
1439753_x_at	0.00	0.00
1439754_at	0.00	0.00
1439755_at	0.00	0.00
1439756_at	0.00	0.00
1439757_s_at	0.00	0.00
1439758_at	0.00	0.00
1439759_x_at	0.00	0.00
1439760_x_at	0.00	0.00
1439761_x_at	0.00	0.00
1439762_x_at	0.00	0.00
1439763_at	0.00	0.00
1439764_s_at	0.00	0.26
1439765_x_at	0.00	0.00
1439766_x_at	0.02	0.55
1439767_at	0.00	0.00
1439768_x_at	0.00	0.00
1439769_at	0.00	0.00
1439770_at	0.00	0.00
1439772_at	0.00	0.00
1439773_at	0.00	0.00
1439774_at	0.00	0.00
1439775_at	0.00	0.00
1439776_at	0.00	0.01
1439777_at	0.00	0.00
1439778_at	0.00	0.00
1439779_at	0.00	0.00
1439780_at	0.00	0.07
1439781_at	0.00	0.00
1439782_s_at	0.00	0.00
1439783_at	0.00	0.00
1439784_at	0.00	0.00
1439785_at	0.00	0.00
1439786_at	0.00	0.00
1439787_at	0.00	0.00
1439788_at	0.00	0.00
1439789_at	0.00	0.00
1439790_at	0.00	0.00
1439791_at	0.00	0.00
1439792_at	0.00	0.00
1439793_at	0.00	0.00
1439794_at	0.00	0.00
1439795_at	0.00	0.00
1439796_at	0.00	0.00
1439797_at	0.00	0.00
1439798_at	0.00	0.00
1439799_at	0.00	0.00
1439800_at	0.00	0.00
1439801_at	0.00	0.00

1439802_at	0.00	0.00
1439804_at	0.00	0.00
1439805_at	0.00	0.00
1439806_at	0.00	0.00
1439807_at	0.00	0.00
1439808_at	0.00	0.00
1439811_at	0.00	0.00
1439812_at	0.00	0.00
1439813_at	0.00	0.00
1439814_at	0.00	0.00
1439815_at	0.00	0.00
1439816_at	0.00	0.00
1439817_at	0.00	0.00
1439818_at	0.00	0.00
1439819_at	0.00	0.00
1439820_at	0.00	0.00
1439821_at	0.00	0.00
1439822_at	0.00	0.00
1439823_at	0.00	0.00
1439824_at	0.00	0.00
1439825_at	0.00	0.00
1439826_at	0.00	0.00
1439827_at	0.00	0.00
1439828_at	0.00	0.00
1439829_at	0.00	0.00
1439830_at	0.00	0.00
1439831_at	0.00	0.00
1439832_at	0.00	0.00
1439833_at	0.00	0.00
1439834_at	0.00	0.00
1439835_x_at	0.00	0.00
1439836_at	0.00	0.00
1439837_at	0.00	0.00
1439838_a_at	0.00	0.00
1439839_at	0.00	0.00
1439840_at	0.00	0.00
1439841_at	0.00	0.00
1439842_at	0.00	0.00
1439843_at	0.00	0.00
1439844_at	0.00	0.00
1439845_at	0.00	0.00
1439846_at	0.00	0.00
1439847_s_at	0.00	0.00
1439848_at	0.00	0.00
1439849_at	0.00	0.00
1439850_at	0.00	0.00
1439851_at	0.00	0.00
1439852_at	0.00	0.00
1439853_at	0.00	0.00
1439854_at	0.00	0.00
1439855_at	0.00	0.00
1439856_at	0.00	0.00
1439857_at	0.00	0.00
1439858_at	0.00	0.00
1439859_at	0.00	0.00
1439860_at	0.00	0.00

1439861_at	0.00	0.00
1439862_at	0.00	0.00
1439863_at	0.00	0.00
1439864_at	0.00	0.00
1439865_at	0.00	0.00
1439866_at	0.00	0.00
1439867_at	0.00	0.00
1439868_at	0.00	0.00
1439869_at	0.00	0.00
1439870_at	0.00	0.00
1439871_at	0.00	0.00
1439872_at	0.00	0.00
1439873_at	0.00	0.00
1439874_at	0.00	0.00
1439875_at	0.00	0.00
1439876_at	0.00	0.00
1439877_at	0.00	0.00
1439878_at	0.00	0.00
1439879_at	0.00	0.00
1439880_at	0.00	0.00
1439881_at	0.00	0.00
1439882_at	0.00	0.00
1439883_at	0.00	0.00
1439884_at	0.00	0.00
1439885_at	0.00	0.00
1439886_at	0.00	0.00
1439887_at	0.00	0.00
1439888_at	0.00	0.00
1439889_at	0.00	0.00
1439890_at	0.00	0.00
1439891_at	0.00	0.00
1439892_at	0.00	0.00
1439893_at	0.00	0.00
1439894_at	0.00	0.00
1439895_at	0.00	0.00
1439896_at	0.00	0.00
1439897_at	0.00	0.00
1439898_at	0.00	0.00
1439899_at	0.00	0.00
1439900_at	0.00	0.00
1439901_at	0.00	0.00
1439902_at	0.00	0.00
1439903_at	0.00	0.00
1439904_at	0.00	0.00
1439905_at	0.00	0.00
1439906_at	0.00	0.00
1439907_at	0.00	0.00
1439908_at	0.00	0.00
1439909_at	0.00	0.00
1439910_a_at	0.00	0.00
1439911_at	0.00	0.00
1439912_at	0.00	0.00
1439913_at	0.00	0.00
1439914_at	0.00	0.00
1439915_at	0.00	0.00
1439916_at	0.00	0.00

1439917_at	0.00	0.00
1439918_at	0.00	0.00
1439919_at	0.00	0.00
1439920_at	0.00	0.00
1439921_at	0.00	0.00
1439922_at	0.00	0.00
1439923_at	0.00	0.00
1439924_x_at	0.00	0.00
1439925_at	0.00	0.00
1439926_at	0.00	0.00
1439927_at	0.00	0.00
1439928_at	0.00	0.00
1439929_at	0.00	0.00
1439930_at	0.00	0.00
1439931_at	0.00	0.00
1439932_at	0.00	0.00
1439933_at	0.00	0.00
1439934_at	0.00	0.00
1439935_at	0.00	0.00
1439936_at	0.00	0.00
1439937_at	0.00	0.00
1439938_at	0.00	0.00
1439939_at	0.00	0.00
1439940_at	0.00	0.00
1439941_at	0.00	0.00
1439942_at	0.00	0.00
1439943_at	0.00	0.00
1439944_at	0.00	0.00
1439945_at	0.00	0.00
1439946_at	0.00	0.00
1439947_at	0.00	0.00
1439948_at	0.00	0.00
1439949_at	0.00	0.00
1439950_at	0.00	0.00
1439951_at	0.00	0.00
1439952_at	0.00	0.00
1439953_at	0.00	0.00
1439954_at	0.00	0.00
1439955_at	0.00	0.00
1439956_at	0.00	0.00
1439957_at	0.00	0.00
1439958_at	0.00	0.00
1439960_at	0.00	0.00
1439961_x_at	0.00	0.00
1439963_x_at	0.00	0.00
1439965_at	0.00	0.00
1439966_x_at	0.00	0.00
1439967_at	0.00	0.00
1439968_x_at	0.00	0.00
1439969_at	0.00	0.00
1439970_at	0.00	0.00
1439971_at	0.00	0.00
1439972_at	0.00	0.00
1439974_at	0.00	0.00
1439975_at	0.00	0.00
1439976_at	0.00	0.00

1439977_at	0.00	0.00
1439978_at	0.00	0.00
1439979_at	0.00	0.00
1439980_at	0.00	0.00
1439981_at	0.00	0.00
1439982_at	0.00	0.00
1439983_a_at	0.00	0.00
1439984_at	0.00	0.00
1439985_at	0.00	0.00
1439986_at	0.00	0.00
1439987_at	0.00	0.00
1439988_at	0.00	0.00
1439989_at	0.00	0.00
1439990_at	0.00	0.00
1439991_a_at	0.00	0.00
1439992_at	0.00	0.00
1439993_at	0.00	0.00
1439994_at	0.00	0.00
1439996_at	0.00	0.00
1439997_at	0.00	0.00
1439998_at	0.00	0.00
1439999_at	0.00	0.00
1440000_at	0.00	0.00
1440001_at	0.00	0.00
1440002_at	0.00	0.00
1440003_at	0.00	0.00
1440004_at	0.00	0.00
1440005_at	0.00	0.00
1440006_at	0.00	0.00
1440007_at	0.00	0.00
1440008_at	0.00	0.00
1440009_at	0.00	0.00
1440010_at	0.00	0.00
1440011_at	0.00	0.00
1440012_at	0.00	0.00
1440013_at	0.00	0.00
1440014_at	0.00	0.00
1440015_at	0.00	0.00
1440016_at	0.00	0.00
1440017_at	0.00	0.00
1440018_at	0.00	0.00
1440019_at	0.00	0.00
1440020_at	0.00	0.00
1440021_at	0.00	0.00
1440022_at	0.00	0.00
1440023_at	0.00	0.00
1440024_at	0.00	0.00
1440025_at	0.00	0.00
1440026_at	0.00	0.00
1440027_at	0.00	0.00
1440028_at	0.00	0.00
1440029_at	0.00	0.00
1440030_at	0.00	0.00
1440031_at	0.00	0.00
1440032_at	0.00	0.00
1440033_at	0.00	0.00

1440034_at	0.00	0.00
1440035_at	0.00	0.00
1440036_x_at	0.00	0.00
1440037_at	0.00	0.00
1440038_at	0.00	0.00
1440039_at	0.00	0.00
1440040_at	0.00	0.00
1440041_at	0.00	0.00
1440042_at	0.00	0.00
1440043_at	0.00	0.00
1440044_at	0.00	0.00
1440045_at	0.00	0.00
1440046_at	0.00	0.00
1440047_at	0.00	0.00
1440048_at	0.00	0.00
1440049_at	0.00	0.00
1440050_at	0.00	0.01
1440051_at	0.00	0.00
1440052_at	0.00	0.00
1440053_at	0.00	0.00
1440054_at	0.00	0.00
1440055_at	0.00	0.00
1440056_at	0.00	0.00
1440057_at	0.00	0.00
1440058_at	0.00	0.00
1440059_at	0.00	0.00
1440060_at	0.00	0.00
1440061_at	0.00	0.00
1440062_at	0.00	0.00
1440063_at	0.00	0.00
1440064_at	0.00	0.00
1440065_at	0.00	0.00
1440066_at	0.00	0.00
1440067_at	0.00	0.00
1440068_at	0.00	0.00
1440069_at	0.00	0.00
1440070_at	0.00	0.00
1440071_at	0.00	0.00
1440072_at	0.00	0.00
1440073_at	0.00	0.00
1440074_at	0.00	0.00
1440075_at	0.00	0.00
1440076_at	0.00	0.00
1440077_at	0.00	0.00
1440078_at	0.00	0.00
1440079_at	0.00	0.00
1440080_at	0.00	0.00
1440081_at	0.00	0.00
1440082_at	0.00	0.00
1440083_at	0.00	0.00
1440084_at	0.00	0.00
1440085_at	0.00	0.00
1440086_at	0.00	0.00
1440087_at	0.00	0.00
1440088_at	0.00	0.00
1440089_at	0.00	0.00

1440090_at	0.00	0.00
1440091_at	0.00	0.00
1440092_at	0.00	0.00
1440093_at	0.00	0.00
1440094_at	0.00	0.00
1440095_at	0.00	0.00
1440096_at	0.00	0.00
1440097_at	0.00	0.00
1440098_at	0.00	0.00
1440099_at	0.00	0.00
1440100_at	0.00	0.00
1440101_at	0.00	0.00
1440102_at	0.00	0.00
1440103_at	0.00	0.00
1440104_at	0.00	0.00
1440105_at	0.00	0.00
1440106_at	0.00	0.00
1440107_at	0.00	0.00
1440108_at	0.00	0.00
1440109_at	0.00	0.00
1440110_at	0.00	0.00
1440111_at	0.00	0.00
1440112_at	0.00	0.00
1440113_at	0.00	0.00
1440114_x_at	0.00	0.00
1440115_at	0.00	0.00
1440116_at	0.00	0.00
1440117_at	0.00	0.00
1440118_at	0.00	0.00
1440119_at	0.00	0.00
1440120_at	0.00	0.00
1440121_at	0.00	0.00
1440122_at	0.00	0.00
1440123_at	0.00	0.00
1440124_at	0.00	0.00
1440125_at	0.00	0.00
1440126_at	0.00	0.00
1440127_a_at	0.00	0.00
1440128_s_at	0.00	0.00
1440129_at	0.00	0.00
1440130_at	0.00	0.00
1440131_at	0.00	0.00
1440132_s_at	0.00	0.00
1440133_x_at	0.00	0.00
1440134_at	0.00	0.00
1440135_at	0.00	0.00
1440136_at	0.00	0.00
1440137_at	0.00	0.00
1440138_at	0.00	0.00
1440139_at	0.00	0.00
1440140_at	0.00	0.00
1440141_at	0.00	0.00
1440142_s_at	0.00	0.00
1440143_at	0.00	0.00
1440144_x_at	0.00	0.00
1440145_at	0.00	0.00

1440146_at	0.00	0.00
1440147_at	0.00	0.00
1440148_at	0.00	0.00
1440149_at	0.00	0.00
1440150_at	0.00	0.00
1440151_s_at	0.00	0.00
1440152_x_at	0.00	0.00
1440153_at	0.00	0.00
1440154_at	0.00	0.00
1440155_at	0.00	0.00
1440156_s_at	0.00	0.00
1440157_at	0.00	0.00
1440158_x_at	0.00	0.00
1440159_at	0.00	0.00
1440160_x_at	0.00	0.00
1440161_at	0.00	0.00
1440162_x_at	0.00	0.00
1440163_at	0.00	0.00
1440164_x_at	0.00	0.00
1440165_at	0.00	0.00
1440166_x_at	0.00	0.00
1440167_s_at	0.00	0.00
1440168_x_at	0.00	0.00
1440169_x_at	0.00	0.00
1440170_at	0.00	0.00
1440171_x_at	0.00	0.00
1440172_s_at	0.00	0.00
1440173_x_at	0.00	0.00
1440174_at	0.00	0.00
1440175_at	0.00	0.00
1440176_x_at	0.00	0.00
1440177_at	0.00	0.00
1440178_x_at	0.00	0.00
1440179_x_at	0.00	0.00
1440180_x_at	0.00	0.00
1440181_at	0.00	0.00
1440182_at	0.00	0.00
1440183_x_at	0.00	0.00
1440184_at	0.00	0.00
1440185_x_at	0.00	0.00
1440186_s_at	0.00	0.00
1440187_at	0.00	0.00
1440188_at	0.00	0.00
1440189_at	0.00	0.00
1440190_at	0.00	0.00
1440191_s_at	0.00	0.00
1440193_at	0.00	0.00
1440194_at	0.00	0.00
1440196_at	0.00	0.00
1440197_at	0.06	0.00
1440198_at	0.00	0.00
1440199_at	0.00	0.00
1440200_at	0.00	0.00
1440202_at	0.00	0.10
1440203_at	0.00	0.00
1440204_at	0.00	0.00

1440205_at	0.00	0.00
1440206_at	0.00	0.00
1440207_at	0.00	0.00
1440208_at	0.00	0.00
1440209_at	0.00	0.00
1440210_at	0.00	0.00
1440211_at	0.00	0.00
1440212_at	0.00	0.00
1440214_at	0.00	0.00
1440215_at	0.00	0.00
1440216_at	0.00	0.00
1440217_at	0.00	0.00
1440218_at	0.00	0.00
1440219_at	0.00	0.00
1440220_at	0.00	0.00
1440222_at	0.00	0.00
1440223_at	0.00	0.00
1440224_at	0.00	0.00
1440225_at	0.00	0.00
1440226_at	0.00	0.00
1440227_at	0.00	0.00
1440228_at	0.00	0.00
1440229_at	0.00	0.00
1440231_at	0.00	0.00
1440232_at	0.00	0.00
1440233_at	0.00	0.00
1440234_at	0.00	0.00
1440235_at	0.00	0.00
1440236_at	0.00	0.00
1440237_at	0.00	0.00
1440238_at	0.00	0.00
1440239_at	0.00	0.00
1440240_at	0.00	0.00
1440241_at	0.00	0.00
1440242_at	0.00	0.00
1440243_at	0.00	0.00
1440244_at	0.00	0.00
1440245_at	0.00	0.00
1440246_at	0.00	0.00
1440247_at	0.00	0.00
1440248_at	0.00	0.00
1440249_at	0.00	0.00
1440250_at	0.00	0.00
1440251_s_at	0.00	0.00
1440254_at	0.00	0.00
1440256_at	0.00	0.00
1440257_at	0.00	0.00
1440258_at	0.00	0.00
1440259_at	0.00	0.00
1440260_at	0.00	0.00
1440261_at	0.00	0.00
1440262_at	0.00	0.00
1440263_at	0.00	0.00
1440264_at	0.00	0.00
1440265_at	0.00	0.00
1440266_at	0.00	0.00

1440267_at	0.00	0.00
1440268_at	0.00	0.00
1440269_at	0.00	0.00
1440270_at	0.00	0.00
1440271_at	0.00	0.00
1440272_at	0.00	0.00
1440273_at	0.00	0.00
1440274_at	0.00	0.00
1440275_at	0.00	0.00
1440276_at	0.00	0.00
1440277_at	0.00	0.00
1440278_at	0.00	0.00
1440279_at	0.00	0.00
1440280_at	0.00	0.00
1440281_at	0.00	0.00
1440282_at	0.00	0.00
1440283_at	0.00	0.00
1440284_at	0.00	0.00
1440285_at	0.00	0.04
1440286_at	0.00	0.00
1440287_at	0.00	0.00
1440288_at	0.00	0.00
1440289_at	0.00	0.00
1440290_at	0.00	0.00
1440291_at	0.00	0.00
1440292_at	0.00	0.00
1440293_at	0.00	0.00
1440294_at	0.00	0.00
1440295_at	0.00	0.00
1440296_at	0.00	0.00
1440297_at	0.00	0.00
1440298_at	0.00	0.00
1440299_at	0.00	0.00
1440300_at	0.00	0.00
1440301_at	0.00	0.00
1440302_at	0.00	0.00
1440303_at	0.00	0.00
1440304_at	0.00	0.00
1440305_at	0.00	0.00
1440306_at	0.00	0.00
1440307_at	0.00	0.00
1440308_at	0.00	0.00
1440309_at	0.00	0.00
1440310_at	0.00	0.00
1440311_at	0.00	0.00
1440312_at	0.00	0.00
1440313_at	0.00	0.00
1440314_at	0.00	0.00
1440315_at	0.00	0.00
1440316_at	0.00	0.00
1440317_at	0.00	0.00
1440318_at	0.00	0.00
1440319_at	0.00	0.00
1440320_at	0.00	0.00
1440321_at	0.00	0.00
1440322_at	0.00	0.00

1440324_at	0.00	0.00
1440325_at	0.00	0.00
1440326_at	0.00	0.00
1440327_at	0.00	0.00
1440328_at	0.00	0.00
1440329_s_at	0.00	0.00
1440330_at	0.00	0.00
1440331_at	0.00	0.00
1440332_at	0.00	0.00
1440333_at	0.00	0.00
1440334_at	0.00	0.00
1440335_at	0.00	0.00
1440336_at	0.00	0.00
1440337_at	0.00	0.00
1440338_at	0.00	0.00
1440339_at	0.00	0.00
1440340_at	0.00	0.00
1440341_at	0.00	0.00
1440342_at	0.00	0.00
1440343_at	0.00	0.02
1440344_at	0.00	0.00
1440345_at	0.00	0.00
1440346_at	0.00	0.00
1440347_at	0.00	0.00
1440348_at	0.00	0.00
1440349_at	0.00	0.00
1440350_at	0.00	0.00
1440351_at	0.00	0.00
1440352_at	0.00	0.00
1440353_at	0.00	0.00
1440354_at	0.00	0.00
1440355_at	0.00	0.00
1440356_at	0.00	0.00
1440357_at	0.00	0.00
1440358_at	0.00	0.00
1440359_at	0.00	0.00
1440360_at	0.00	0.00
1440361_at	0.00	0.00
1440362_at	0.00	0.00
1440363_at	0.00	0.00
1440364_a_at	0.00	0.00
1440365_at	0.00	0.00
1440366_at	0.00	0.00
1440367_at	0.00	0.00
1440368_at	0.00	0.00
1440369_at	0.00	0.00
1440370_at	0.00	0.00
1440371_at	0.00	0.00
1440372_at	0.00	0.00
1440373_at	0.00	0.00
1440374_at	0.00	0.00
1440375_at	0.00	0.00
1440376_at	0.00	0.00
1440377_at	0.00	0.00
1440378_at	0.00	0.00
1440379_at	0.00	0.00

1440380_at	0.00	0.00
1440381_at	0.01	0.48
1440382_at	0.00	0.00
1440383_at	0.00	0.00
1440384_at	0.00	0.00
1440385_at	0.00	0.00
1440386_at	0.00	0.00
1440387_at	0.00	0.00
1440388_at	0.00	0.00
1440389_at	0.00	0.00
1440390_at	0.00	0.00
1440391_at	0.00	0.00
1440392_at	0.00	0.00
1440393_at	0.00	0.00
1440394_at	0.00	0.00
1440395_at	0.00	0.00
1440396_at	0.00	0.00
1440397_at	0.00	0.00
1440398_at	0.00	0.00
1440399_at	0.00	0.00
1440400_at	0.00	0.00
1440401_at	0.00	0.00
1440402_at	0.00	0.00
1440403_at	0.00	0.00
1440404_at	0.00	0.00
1440405_at	0.00	0.00
1440406_at	0.00	0.00
1440407_at	0.00	0.00
1440408_at	0.00	0.00
1440409_at	0.00	0.00
1440410_at	0.00	0.00
1440411_at	0.00	0.00
1440412_at	0.00	0.00
1440413_at	0.00	0.00
1440414_at	0.00	0.00
1440415_at	0.00	0.00
1440416_at	0.00	0.00
1440417_at	0.00	0.00
1440418_at	0.00	0.00
1440419_at	0.00	0.00
1440420_at	0.00	0.00
1440421_at	0.00	0.00
1440422_at	0.00	0.00
1440423_at	0.00	0.00
1440424_at	0.00	0.00
1440425_at	0.00	0.00
1440426_at	0.00	0.00
1440427_at	0.00	0.00
1440428_at	0.00	0.00
1440429_at	0.00	0.00
1440430_at	0.00	0.00
1440431_at	0.00	0.00
1440432_at	0.00	0.00
1440433_at	0.00	0.00
1440434_at	0.00	0.00
1440435_at	0.00	0.00

1440436_at	0.00	0.00
1440437_at	0.00	0.00
1440438_at	0.00	0.00
1440439_at	0.00	0.00
1440440_at	0.00	0.00
1440441_at	0.00	0.00
1440442_at	0.00	0.00
1440443_at	0.00	0.00
1440444_at	0.00	0.00
1440445_at	0.00	0.00
1440446_at	0.00	0.00
1440447_at	0.00	0.00
1440448_at	0.00	0.00
1440449_at	0.00	0.00
1440450_at	0.00	0.00
1440451_at	0.00	0.00
1440452_at	0.00	0.00
1440453_at	0.00	0.00
1440454_at	0.00	0.00
1440455_at	0.00	0.00
1440456_at	0.00	0.00
1440457_at	0.00	0.00
1440458_at	0.00	0.00
1440459_at	0.00	0.00
1440460_at	0.00	0.00
1440461_at	0.00	0.00
1440462_at	0.00	0.00
1440463_at	0.00	0.00
1440464_at	0.00	0.00
1440465_at	0.00	0.00
1440466_at	0.00	0.00
1440467_at	0.00	0.00
1440468_at	0.00	0.00
1440469_at	0.00	0.00
1440470_at	0.00	0.00
1440471_x_at	0.00	0.00
1440472_at	0.00	0.00
1440473_at	0.00	0.00
1440474_at	0.00	0.00
1440475_at	0.00	0.00
1440476_at	0.00	0.00
1440477_at	0.00	0.00
1440478_at	0.00	0.00
1440479_at	0.00	0.00
1440480_at	0.00	0.00
1440481_at	0.00	0.00
1440482_at	0.00	0.00
1440483_at	0.00	0.00
1440484_at	0.00	0.00
1440485_at	0.00	0.00
1440486_at	0.00	0.00
1440487_at	0.00	0.00
1440488_at	0.00	0.00
1440489_at	0.00	0.00
1440490_at	0.00	0.00
1440491_at	0.00	0.00

1440492_at	0.00	0.00
1440493_at	0.00	0.00
1440494_at	0.00	0.00
1440495_at	0.00	0.00
1440496_at	0.00	0.00
1440497_at	0.00	0.00
1440498_at	0.00	0.00
1440500_at	0.00	0.00
1440501_at	0.00	0.00
1440502_at	0.00	0.00
1440503_at	0.00	0.00
1440504_at	0.00	0.00
1440505_at	0.00	0.00
1440506_at	0.00	0.00
1440507_at	0.00	0.00
1440508_at	0.00	0.00
1440509_at	0.00	0.00
1440510_at	0.00	0.00
1440511_at	0.00	0.00
1440512_at	0.00	0.00
1440513_at	0.00	0.00
1440514_at	0.00	0.00
1440515_at	0.00	0.00
1440516_at	0.00	0.00
1440517_x_at	0.00	0.00
1440518_at	0.00	0.00
1440519_at	0.00	0.00
1440520_a_at	0.00	0.00
1440521_x_at	0.00	0.00
1440522_at	0.00	0.00
1440523_at	0.00	0.00
1440524_at	0.00	0.00
1440525_at	0.00	0.00
1440526_at	0.00	0.00
1440527_at	0.00	0.00
1440528_at	0.00	0.00
1440529_at	0.00	0.00
1440530_at	0.00	0.00
1440531_at	0.00	0.00
1440532_a_at	0.00	0.00
1440533_at	0.00	0.00
1440534_at	0.00	0.00
1440535_at	0.00	0.00
1440536_at	0.00	0.00
1440537_at	0.00	0.00
1440538_at	0.00	0.00
1440539_at	0.00	0.00
1440540_at	0.00	0.00
1440541_at	0.00	0.00
1440542_at	0.00	0.00
1440543_at	0.00	0.00
1440544_at	0.00	0.00
1440545_at	0.00	0.00
1440546_at	0.00	0.00
1440547_at	0.00	0.00
1440548_at	0.00	0.00

1440549_at	0.00	0.00
1440550_at	0.00	0.00
1440551_at	0.00	0.00
1440552_at	0.00	0.00
1440553_at	0.00	0.00
1440554_at	0.00	0.00
1440555_at	0.00	0.00
1440556_at	0.00	0.00
1440557_at	0.00	0.00
1440558_at	0.00	0.00
1440559_at	0.00	0.00
1440560_at	0.00	0.00
1440561_at	0.00	0.00
1440562_at	0.00	0.00
1440563_at	0.00	0.00
1440564_at	0.00	0.00
1440565_at	0.00	0.00
1440566_at	0.00	0.00
1440567_at	0.00	0.00
1440568_at	0.00	0.00
1440569_at	0.00	0.00
1440570_at	0.00	0.00
1440571_at	0.00	0.00
1440572_at	0.00	0.00
1440573_at	0.00	0.00
1440574_at	0.00	0.00
1440575_at	0.00	0.00
1440576_at	0.00	0.00
1440577_at	0.00	0.00
1440578_at	0.00	0.00
1440579_at	0.00	0.00
1440580_at	0.00	0.00
1440581_at	0.00	0.00
1440582_at	0.00	0.00
1440583_at	0.00	0.00
1440584_at	0.00	0.00
1440585_at	0.00	0.00
1440586_at	0.00	0.00
1440587_at	0.00	0.00
1440588_at	0.00	0.00
1440589_at	0.00	0.00
1440590_at	0.00	0.00
1440592_at	0.00	0.00
1440593_at	0.00	0.00
1440594_at	0.00	0.00
1440595_at	0.00	0.00
1440596_at	0.00	0.00
1440597_at	0.00	0.00
1440598_at	0.00	0.00
1440599_at	0.00	0.00
1440600_at	0.00	0.00
1440601_at	0.00	0.00
1440602_at	0.00	0.00
1440603_at	0.00	0.00
1440604_at	0.00	0.00
1440605_at	0.00	0.00

1440606_at	0.00	0.00
1440607_at	0.00	0.00
1440608_at	0.00	0.00
1440609_at	0.00	0.00
1440610_at	0.00	0.00
1440611_at	0.00	0.00
1440612_at	0.00	0.00
1440613_at	0.00	0.00
1440614_at	0.00	0.00
1440615_at	0.00	0.00
1440616_at	0.00	0.00
1440617_at	0.00	0.00
1440618_at	0.00	0.00
1440619_at	0.00	0.00
1440620_at	0.00	0.00
1440621_at	0.00	0.00
1440622_at	0.00	0.00
1440623_at	0.00	0.00
1440624_at	0.00	0.00
1440625_at	0.00	0.00
1440626_at	0.00	0.00
1440627_at	0.00	0.00
1440628_at	0.00	0.00
1440629_at	0.00	0.00
1440630_at	0.00	0.00
1440631_at	0.00	0.00
1440632_at	0.00	0.00
1440633_at	0.00	0.00
1440634_at	0.00	0.00
1440635_at	0.00	0.00
1440636_at	0.00	0.00
1440637_at	0.00	0.00
1440638_at	0.00	0.00
1440639_at	0.00	0.00
1440640_at	0.00	0.00
1440641_at	0.00	0.00
1440642_at	0.00	0.00
1440643_at	0.00	0.00
1440644_at	0.00	0.00
1440645_at	0.00	0.00
1440646_at	0.00	0.00
1440647_at	0.00	0.00
1440648_at	0.00	0.00
1440649_at	0.00	0.00
1440650_at	0.00	0.00
1440651_at	0.00	0.00
1440652_at	0.00	0.00
1440653_at	0.00	0.00
1440654_at	0.00	0.00
1440655_at	0.00	0.00
1440656_at	0.00	0.00
1440657_at	0.00	0.00
1440658_at	0.00	0.00
1440659_at	0.00	0.00
1440660_at	0.00	0.00
1440661_at	0.00	0.00

1440662_at	0.00	0.00
1440663_at	0.00	0.00
1440664_at	0.00	0.00
1440665_at	0.00	0.00
1440666_at	0.00	0.00
1440667_at	0.00	0.00
1440668_at	0.00	0.00
1440669_at	0.00	0.00
1440670_at	0.00	0.00
1440671_at	0.00	0.00
1440672_at	0.00	0.00
1440673_at	0.00	0.00
1440674_at	0.00	0.00
1440675_at	0.00	0.00
1440676_at	0.00	0.00
1440677_at	0.00	0.00
1440678_at	0.00	0.00
1440679_at	0.00	0.00
1440680_at	0.00	0.00
1440681_at	0.00	0.00
1440682_at	0.00	0.00
1440683_at	0.00	0.00
1440684_at	0.00	0.00
1440685_at	0.00	0.00
1440686_at	0.00	0.00
1440687_at	0.00	0.00
1440688_at	0.00	0.00
1440689_at	0.00	0.00
1440690_at	0.00	0.00
1440691_at	0.00	0.00
1440692_at	0.00	0.00
1440693_at	0.00	0.00
1440694_at	0.00	0.00
1440695_at	0.00	0.00
1440696_at	0.00	0.00
1440697_at	0.00	0.00
1440698_at	0.00	0.00
1440699_at	0.00	0.00
1440700_a_at	0.00	0.00
1440701_at	0.00	0.00
1440702_at	0.00	0.00
1440703_at	0.00	0.00
1440704_at	0.00	0.00
1440705_at	0.00	0.00
1440706_at	0.00	0.00
1440707_at	0.00	0.00
1440708_at	0.00	0.00
1440709_at	0.00	0.00
1440710_at	0.00	0.00
1440711_at	0.00	0.00
1440712_at	0.00	0.00
1440713_at	0.00	0.00
1440714_at	0.00	0.00
1440715_s_at	0.00	0.00
1440716_at	0.00	0.00
1440717_at	0.00	0.00

1440718_at	0.00	0.00
1440719_at	0.00	0.00
1440720_s_at	0.00	0.00
1440721_at	0.00	0.00
1440722_at	0.00	0.00
1440723_at	0.00	0.00
1440724_at	0.00	0.00
1440725_at	0.00	0.00
1440726_at	0.00	0.00
1440727_at	0.00	0.00
1440728_at	0.00	0.00
1440729_at	0.00	0.00
1440730_at	0.00	0.00
1440731_at	0.00	0.00
1440732_at	0.00	0.00
1440733_at	0.00	0.00
1440734_at	0.00	0.00
1440735_at	0.00	0.00
1440736_at	0.00	0.00
1440737_at	0.00	0.00
1440738_at	0.00	0.00
1440739_at	0.01	0.99
1440740_at	0.00	0.00
1440741_at	0.00	0.00
1440742_at	0.00	0.00
1440743_at	0.00	0.00
1440744_at	0.00	0.00
1440745_at	0.00	0.00
1440746_at	0.00	0.00
1440747_at	0.00	0.00
1440748_at	0.00	0.00
1440749_at	0.00	0.00
1440750_at	0.00	0.00
1440751_at	0.00	0.00
1440752_at	0.03	0.00
1440753_at	0.00	0.00
1440754_at	0.00	0.00
1440755_at	0.00	0.00
1440756_at	0.00	0.00
1440757_at	0.00	0.00
1440758_at	0.00	0.00
1440759_at	0.00	0.00
1440760_at	0.00	0.00
1440761_at	0.00	0.00
1440762_at	0.00	0.00
1440763_at	0.00	0.00
1440764_at	0.00	0.00
1440765_at	0.00	0.00
1440766_at	0.00	0.00
1440767_at	0.00	0.00
1440768_x_at	0.00	0.00
1440769_at	0.00	0.00
1440770_at	0.00	0.00
1440771_at	0.00	0.00
1440772_x_at	0.00	0.00
1440773_at	0.00	0.00

1440774_x_at	0.00	0.00
1440775_at	0.00	0.00
1440776_at	0.00	0.00
1440777_x_at	0.00	0.00
1440778_x_at	0.00	0.00
1440779_s_at	0.00	0.00
1440780_x_at	0.00	0.00
1440781_at	0.00	0.00
1440782_at	0.00	0.00
1440783_at	0.00	0.00
1440784_at	0.00	0.00
1440785_at	0.00	0.00
1440786_x_at	0.00	0.00
1440787_s_at	0.00	0.00
1440788_at	0.00	0.00
1440789_at	0.00	0.00
1440790_x_at	0.00	0.00
1440791_x_at	0.00	0.00
1440792_x_at	0.00	0.00
1440793_at	0.00	0.00
1440794_x_at	0.00	0.00
1440795_x_at	0.00	0.00
1440796_at	0.00	0.00
1440797_at	0.00	0.00
1440798_x_at	0.00	0.00
1440799_s_at	0.00	0.00
1440800_at	0.00	0.00
1440801_s_at	0.00	0.00
1440802_at	0.00	0.00
1440803_x_at	0.00	0.00
1440804_at	0.00	0.00
1440805_at	0.00	0.00
1440806_x_at	0.00	0.00
1440807_at	0.00	0.00
1440808_x_at	0.00	0.00
1440809_at	0.00	0.00
1440810_x_at	0.00	0.00
1440811_x_at	0.00	0.00
1440812_at	0.00	0.00
1440813_s_at	0.00	0.00
1440814_x_at	0.00	0.00
1440815_x_at	0.00	0.00
1440816_x_at	0.00	0.00
1440817_x_at	0.00	0.03
1440818_s_at	0.00	0.00
1440819_s_at	0.00	0.00
1440820_x_at	0.00	0.00
1440821_x_at	0.00	0.00
1440822_x_at	0.00	0.00
1440823_x_at	0.00	0.00
1440824_at	0.00	0.00
1440825_s_at	0.00	0.00
1440826_s_at	0.00	0.12
1440827_x_at	0.00	0.00
1440828_x_at	0.00	0.00
1440829_x_at	0.00	0.00

1440830_at	0.00	0.00
1440832_at	0.00	0.00
1440833_at	0.00	0.00
1440834_at	0.00	0.00
1440835_at	0.00	0.00
1440836_at	0.00	0.00
1440837_at	0.00	0.00
1440838_at	0.00	0.00
1440839_x_at	0.00	0.00
1440840_at	0.00	0.00
1440841_at	0.00	0.00
1440842_at	0.00	0.00
1440843_at	0.00	0.00
1440844_at	0.00	0.00
1440845_at	0.00	0.00
1440846_at	0.00	0.00
1440847_at	0.00	0.00
1440848_at	0.00	0.00
1440849_at	0.00	0.00
1440850_at	0.00	0.00
1440851_at	0.00	0.00
1440852_at	0.00	0.00
1440853_at	0.00	0.00
1440854_at	0.00	0.00
1440855_at	0.00	0.00
1440856_at	0.00	0.09
1440857_at	0.00	0.00
1440858_at	0.00	0.00
1440859_at	0.00	0.00
1440860_at	0.00	0.00
1440861_a_at	0.00	0.00
1440862_at	0.00	0.00
1440863_at	0.00	0.00
1440864_at	0.00	0.00
1440866_at	0.00	0.00
1440867_at	0.82	0.01
1440868_at	0.00	0.00
1440869_x_at	0.00	0.00
1440870_at	0.00	0.00
1440871_at	0.00	0.00
1440872_at	0.00	0.00
1440873_at	0.00	0.00
1440875_a_at	0.00	0.00
1440876_at	0.00	0.00
1440877_at	0.00	0.00
1440878_at	0.00	0.00
1440879_at	0.00	0.00
1440880_at	0.00	0.00
1440881_at	0.00	0.00
1440882_at	0.00	0.00
1440883_at	0.00	0.00
1440884_s_at	0.00	0.00
1440885_at	0.00	0.00
1440886_at	0.00	0.00
1440887_at	0.00	0.00
1440888_at	0.00	0.00

1440889_at	0.00	0.00
1440890_a_at	0.00	0.00
1440891_at	0.00	0.00
1440892_at	0.00	0.00
1440893_at	0.00	0.00
1440894_at	0.00	0.00
1440895_at	0.00	0.00
1440896_at	0.00	0.00
1440897_at	0.00	0.00
1440898_at	0.00	0.00
1440899_at	0.00	0.00
1440900_at	0.00	0.00
1440901_at	0.00	0.00
1440902_at	0.00	0.00
1440903_at	0.00	0.00
1440904_at	0.00	0.00
1440905_at	0.00	0.00
1440906_at	0.00	0.00
1440907_at	0.00	0.00
1440908_at	0.00	0.00
1440909_at	0.00	0.00
1440910_at	0.13	0.00
1440911_at	0.00	0.00
1440912_at	0.00	0.00
1440913_at	0.00	0.00
1440914_s_at	0.00	0.00
1440915_at	0.00	0.00
1440916_at	0.00	0.00
1440917_at	0.00	0.00
1440918_at	0.00	0.00
1440919_at	0.00	0.00
1440920_at	0.00	0.00
1440921_at	0.00	0.00
1440922_at	0.00	0.00
1440923_at	0.00	0.00
1440924_at	0.00	0.01
1440925_at	0.00	0.00
1440926_at	0.00	0.00
1440927_x_at	0.00	0.00
1440928_at	0.00	0.00
1440929_at	0.00	0.00
1440930_a_at	0.00	0.00
1440931_at	0.00	0.00
1440932_at	0.00	0.00
1440933_at	0.00	0.00
1440934_at	0.00	0.00
1440935_at	0.00	0.00
1440937_at	0.00	0.00
1440938_at	0.00	0.00
1440939_at	0.00	0.00
1440940_at	0.00	0.00
1440941_at	0.00	0.00
1440942_at	0.00	0.00
1440943_at	0.00	0.00
1440944_at	0.00	0.00
1440945_at	0.00	0.00

1440946_at	0.00	0.00
1440947_at	0.00	0.00
1440948_at	0.00	0.00
1440949_at	0.00	0.00
1440950_at	0.00	0.00
1440951_x_at	0.00	0.00
1440952_at	0.00	0.00
1440953_at	0.00	0.00
1440954_at	0.00	0.00
1440955_at	0.00	0.00
1440956_at	0.00	0.00
1440957_at	0.00	0.00
1440958_at	0.00	0.00
1440960_at	0.00	0.00
1440961_at	0.00	0.00
1440962_at	0.00	0.00
1440965_at	0.00	0.00
1440966_at	0.00	0.00
1440967_at	0.00	0.00
1440968_at	0.00	0.00
1440969_at	0.00	0.00
1440970_at	0.00	0.00
1440972_at	0.00	0.00
1440973_at	0.00	0.00
1440974_at	0.00	0.00
1440975_at	0.00	0.00
1440976_at	0.00	0.00
1440977_at	0.00	0.00
1440978_at	0.00	0.00
1440979_at	0.00	0.00
1440980_at	0.00	0.00
1440981_at	0.00	0.00
1440982_at	0.00	0.00
1440983_at	0.00	0.00
1440984_at	0.00	0.00
1440985_at	0.00	0.00
1440986_at	0.00	0.00
1440987_at	0.00	0.00
1440988_at	0.00	0.00
1440989_at	0.00	0.00
1440990_at	0.00	0.00
1440991_at	0.00	0.00
1440992_at	0.00	0.00
1440993_at	0.00	0.00
1440994_at	0.00	0.00
1440995_at	0.00	0.00
1440996_at	0.00	0.00
1440997_at	0.00	0.00
1440998_at	0.00	0.00
1440999_at	0.00	0.00
1441000_at	0.00	0.00
1441001_at	0.00	0.00
1441002_at	0.00	0.00
1441003_at	0.00	0.00
1441004_at	0.00	0.00
1441005_at	0.00	0.00

1441006_at	0.00	0.00
1441007_at	0.00	0.00
1441008_at	0.00	0.00
1441009_at	0.00	0.00
1441010_at	0.00	0.00
1441011_at	0.00	0.00
1441012_at	0.00	0.00
1441014_at	0.00	0.00
1441015_at	0.00	0.00
1441016_at	0.00	0.00
1441017_at	0.00	0.00
1441018_at	0.00	0.00
1441019_at	0.00	0.00
1441020_at	0.00	0.00
1441021_at	0.00	0.00
1441022_at	0.00	0.00
1441024_at	0.00	0.00
1441025_at	0.00	0.00
1441026_at	0.00	0.00
1441027_at	0.00	0.00
1441028_at	0.00	0.00
1441029_at	0.00	0.00
1441030_at	0.00	0.00
1441031_at	0.00	0.00
1441032_at	0.00	0.00
1441033_at	0.00	0.00
1441034_at	0.00	0.00
1441035_at	0.00	0.00
1441036_at	0.00	0.00
1441037_at	0.00	0.00
1441038_at	0.00	0.00
1441039_at	0.00	0.00
1441040_at	0.00	0.00
1441041_at	0.00	0.00
1441042_at	0.00	0.00
1441043_at	0.00	0.00
1441044_at	0.00	0.00
1441045_at	0.00	0.00
1441046_at	0.00	0.00
1441047_at	0.00	0.00
1441048_at	0.00	0.00
1441049_at	0.00	0.00
1441050_at	0.00	0.00
1441051_at	0.00	0.00
1441052_at	0.00	0.00
1441053_at	0.00	0.00
1441054_at	0.00	0.00
1441055_at	0.00	0.00
1441056_at	0.00	0.00
1441057_at	0.00	0.00
1441058_at	0.00	0.00
1441059_at	0.00	0.00
1441060_at	0.00	0.00
1441061_at	0.00	0.00
1441062_at	0.00	0.00
1441063_at	0.00	0.00

1441064_at	0.00	0.00
1441065_at	0.00	0.00
1441066_at	0.00	0.00
1441067_at	0.00	0.00
1441068_at	0.00	0.00
1441069_at	0.00	0.00
1441070_at	0.00	0.00
1441071_at	0.00	0.00
1441072_at	0.00	0.00
1441073_at	0.00	0.00
1441074_at	0.00	0.00
1441075_at	0.00	0.00
1441076_at	0.00	0.00
1441077_at	0.00	0.00
1441078_at	0.00	0.00
1441079_at	0.00	0.00
1441080_at	0.00	0.00
1441081_a_at	0.00	0.00
1441082_at	0.00	0.00
1441083_at	0.00	0.00
1441084_at	0.00	0.00
1441085_at	0.00	0.00
1441086_at	0.00	0.00
1441087_at	0.00	0.00
1441088_at	0.00	0.00
1441089_at	0.00	0.00
1441090_at	0.00	0.00
1441091_at	0.00	0.00
1441092_at	0.00	0.00
1441093_at	0.00	0.00
1441094_at	0.00	0.00
1441095_at	0.00	0.00
1441096_at	0.00	0.00
1441097_at	0.00	0.00
1441098_at	0.03	0.02
1441099_at	0.00	0.00
1441100_at	0.00	0.00
1441101_at	0.00	0.00
1441102_at	0.00	0.00
1441103_at	0.00	0.00
1441104_at	0.00	0.00
1441105_at	0.00	0.00
1441106_at	0.00	0.00
1441107_at	0.00	0.00
1441108_at	0.00	0.00
1441109_at	0.00	0.00
1441110_at	0.00	0.00
1441111_at	0.00	0.00
1441112_at	0.00	0.00
1441113_at	0.00	0.00
1441114_at	0.00	0.00
1441116_at	0.00	0.00
1441117_at	0.00	0.00
1441118_at	0.00	0.00
1441119_at	0.00	0.00
1441120_at	0.00	0.00

1441121_at	0.00	0.00
1441122_at	0.00	0.00
1441123_at	0.00	0.00
1441124_at	0.00	0.00
1441125_at	0.00	0.00
1441126_at	0.00	0.00
1441127_at	0.00	0.00
1441128_at	0.00	0.00
1441129_at	0.00	0.00
1441130_at	0.00	0.00
1441131_at	0.00	0.00
1441132_at	0.00	0.00
1441133_at	0.00	0.00
1441135_at	0.00	0.00
1441136_at	0.00	0.00
1441137_at	0.00	0.00
1441138_at	0.00	0.00
1441139_at	0.00	0.00
1441140_at	0.00	0.00
1441141_at	0.00	0.00
1441142_at	0.00	0.00
1441143_at	0.00	0.00
1441144_at	0.00	0.00
1441145_at	0.00	0.00
1441148_at	0.00	0.00
1441149_at	0.00	0.00
1441150_x_at	0.00	0.00
1441151_at	0.00	0.00
1441152_at	0.00	0.00
1441153_at	0.00	0.00
1441154_at	0.00	0.00
1441155_at	0.00	0.00
1441156_at	0.00	0.00
1441157_at	0.00	0.00
1441158_at	0.00	0.00
1441159_at	0.00	0.00
1441160_at	0.00	0.00
1441161_at	0.00	0.00
1441162_at	0.00	0.00
1441163_at	0.00	0.00
1441164_at	0.00	0.00
1441165_s_at	0.00	0.00
1441166_at	0.00	0.00
1441167_at	0.00	0.00
1441168_at	0.00	0.00
1441169_at	0.00	0.00
1441170_a_at	0.00	0.00
1441171_at	0.00	0.00
1441172_at	0.00	0.00
1441173_at	0.00	0.00
1441174_a_at	0.00	0.00
1441175_at	0.00	0.00
1441176_at	0.00	0.00
1441177_at	0.00	0.00
1441178_at	0.00	0.00
1441179_at	0.00	0.00

1441180_at	0.00	0.00
1441181_at	0.00	0.00
1441183_at	0.00	0.00
1441184_at	0.00	0.00
1441185_at	0.00	0.00
1441186_at	0.00	0.00
1441187_at	0.00	0.00
1441188_at	0.00	0.00
1441189_at	0.00	0.00
1441190_at	0.00	0.00
1441191_at	0.00	0.00
1441192_at	0.00	0.00
1441193_at	0.00	0.00
1441194_at	0.00	0.00
1441195_at	0.00	0.00
1441196_at	0.00	0.00
1441197_at	0.00	0.00
1441198_at	0.00	0.00
1441199_at	0.00	0.00
1441200_at	0.00	0.00
1441201_at	0.00	0.00
1441202_at	0.00	0.00
1441203_at	0.00	0.00
1441204_at	0.00	0.00
1441205_at	0.00	0.00
1441206_at	0.00	0.00
1441207_at	0.00	0.00
1441208_at	0.00	0.00
1441209_at	0.00	0.00
1441210_at	0.00	0.00
1441211_at	0.00	0.00
1441212_at	0.00	0.00
1441213_at	0.00	0.00
1441214_at	0.00	0.00
1441215_at	0.00	0.00
1441216_at	0.00	0.00
1441217_at	0.00	0.00
1441218_at	0.00	0.00
1441219_at	0.00	0.00
1441220_at	0.00	0.00
1441221_at	0.00	0.00
1441222_x_at	0.00	0.00
1441223_at	0.00	0.00
1441224_at	0.00	0.00
1441225_at	0.00	0.00
1441226_at	0.00	0.00
1441227_at	0.00	0.00
1441228_at	0.00	0.00
1441229_at	0.00	0.00
1441230_at	0.00	0.00
1441231_at	0.00	0.00
1441232_at	0.00	0.00
1441233_at	0.00	0.00
1441234_at	0.00	0.00
1441235_at	0.00	0.00
1441236_at	0.00	0.00

1441237_at	0.00	0.00
1441238_at	0.00	0.00
1441239_at	0.00	0.00
1441240_at	0.00	0.00
1441241_at	0.00	0.00
1441242_at	0.00	0.00
1441243_at	0.00	0.00
1441244_at	0.00	0.00
1441245_a_at	0.00	0.00
1441246_s_at	0.00	0.00
1441247_at	0.00	0.00
1441248_at	0.00	0.00
1441249_at	0.00	0.00
1441250_at	0.00	0.00
1441251_a_at	0.00	0.00
1441252_at	0.00	0.00
1441253_at	0.00	0.00
1441254_at	0.00	0.00
1441255_at	0.00	0.00
1441256_at	0.00	0.00
1441257_x_at	0.00	0.00
1441258_at	0.00	0.00
1441259_s_at	0.00	0.03
1441260_a_at	0.00	0.00
1441261_at	0.00	0.00
1441262_at	0.00	0.00
1441263_a_at	0.00	0.00
1441264_x_at	0.00	0.00
1441265_at	0.00	0.00
1441266_at	0.00	0.00
1441267_at	0.00	0.00
1441268_at	0.00	0.00
1441269_at	0.00	0.00
1441270_at	0.00	0.00
1441271_at	0.00	0.00
1441272_at	0.02	0.00
1441273_at	0.00	0.00
1441274_at	0.00	0.00
1441275_at	0.00	0.12
1441276_at	0.00	0.00
1441277_s_at	0.00	0.00
1441278_at	0.00	0.00
1441279_at	0.00	0.00
1441280_at	0.00	0.00
1441281_s_at	0.00	0.00
1441282_at	0.00	0.00
1441283_at	0.00	0.00
1441284_at	0.00	0.00
1441285_at	0.00	0.00
1441286_at	0.00	0.00
1441287_at	0.00	0.00
1441288_at	0.00	0.00
1441289_at	0.00	0.00
1441290_at	0.00	0.00
1441291_at	0.00	0.00
1441292_at	0.00	0.00

1441293_at	0.00	0.00
1441294_at	0.00	0.00
1441295_at	0.00	0.00
1441296_at	0.00	0.00
1441297_at	0.00	0.00
1441298_at	0.00	0.00
1441299_at	0.00	0.00
1441300_at	0.00	0.00
1441301_at	0.00	0.00
1441302_at	0.00	0.00
1441303_at	0.00	0.00
1441304_at	0.00	0.00
1441305_at	0.00	0.00
1441306_at	0.00	0.00
1441307_at	0.00	0.00
1441308_at	0.00	0.00
1441309_at	0.00	0.00
1441310_at	0.00	0.00
1441311_at	0.00	0.00
1441312_at	0.00	0.00
1441313_x_at	0.00	0.00
1441314_at	0.00	0.00
1441315_s_at	0.00	0.00
1441316_at	0.00	0.00
1441317_x_at	0.00	0.29
1441318_at	0.00	0.00
1441319_at	0.00	0.00
1441320_a_at	0.00	0.00
1441321_at	0.00	0.00
1441322_at	0.00	0.00
1441323_at	0.00	0.00
1441324_at	0.00	0.00
1441325_at	0.00	0.00
1441326_at	0.00	0.00
1441327_a_at	0.00	0.00
1441328_at	0.00	0.00
1441329_at	0.00	0.00
1441330_at	0.00	0.00
1441331_at	0.00	0.00
1441332_at	0.00	0.00
1441333_at	0.00	0.00
1441334_at	0.00	0.00
1441335_at	0.00	0.00
1441336_at	0.00	0.00
1441337_at	0.00	0.00
1441338_at	0.00	0.00
1441339_at	0.00	0.00
1441340_at	0.00	0.00
1441341_at	0.00	0.00
1441343_at	0.00	0.00
1441345_at	0.00	0.00
1441346_at	0.00	0.00
1441347_at	0.00	0.00
1441348_at	0.00	0.00
1441349_at	0.00	0.00
1441351_at	0.00	0.00

1441352_at	0.00	0.00
1441353_at	0.00	0.00
1441354_at	0.00	0.00
1441355_at	0.00	0.00
1441356_at	0.00	0.00
1441357_at	0.00	0.00
1441358_at	0.00	0.00
1441359_at	0.00	0.00
1441360_at	0.00	0.00
1441361_at	0.00	0.00
1441362_at	0.00	0.00
1441363_at	0.00	0.00
1441364_at	0.00	0.00
1441365_at	0.00	0.00
1441366_at	0.00	0.00
1441367_a_at	0.00	0.00
1441368_at	0.00	0.00
1441369_at	0.00	0.00
1441370_at	0.00	0.00
1441371_at	0.00	0.00
1441372_at	0.00	0.00
1441373_at	0.00	0.00
1441374_at	0.00	0.00
1441375_at	0.00	0.00
1441376_at	0.00	0.00
1441377_at	0.00	0.00
1441378_at	0.00	0.00
1441379_at	0.00	0.00
1441380_at	0.00	0.00
1441381_at	0.00	0.00
1441382_at	0.00	0.00
1441383_at	0.00	0.00
1441384_at	0.00	0.00
1441385_at	0.00	0.00
1441386_at	0.00	0.00
1441387_at	0.00	0.00
1441388_at	0.00	0.00
1441389_at	0.00	0.00
1441390_at	0.00	0.00
1441391_at	0.00	0.00
1441392_at	0.00	0.00
1441393_at	0.00	0.00
1441395_at	0.00	0.00
1441396_at	0.00	0.00
1441397_at	0.00	0.00
1441398_at	0.00	0.00
1441399_at	0.00	0.00
1441400_at	0.00	0.00
1441402_at	0.00	0.00
1441403_at	0.00	0.00
1441404_at	0.00	0.00
1441405_at	0.00	0.00
1441406_at	0.00	0.00
1441407_at	0.00	0.00
1441408_at	0.00	0.00
1441409_at	0.00	0.00

1441410_at	0.00	0.00
1441411_at	0.00	0.00
1441412_s_at	0.00	0.00
1441413_at	0.00	0.00
1441414_at	0.00	0.00
1441415_at	0.00	0.00
1441416_at	0.00	0.00
1441417_at	0.00	0.00
1441418_at	0.00	0.00
1441419_at	0.00	0.00
1441420_at	0.00	0.00
1441422_at	0.00	0.00
1441423_at	0.00	0.00
1441424_at	0.00	0.00
1441425_at	0.00	0.00
1441426_at	0.00	0.00
1441427_at	0.00	0.00
1441428_at	0.00	0.00
1441429_at	0.00	0.32
1441430_at	0.00	0.00
1441431_at	0.00	0.00
1441432_at	0.00	0.00
1441434_at	0.00	0.00
1441435_at	0.00	0.00
1441436_at	0.00	0.00
1441437_at	0.00	0.00
1441438_at	0.00	0.00
1441439_at	0.00	0.00
1441440_at	0.00	0.00
1441441_at	0.00	0.00
1441442_at	0.00	0.00
1441443_at	0.00	0.00
1441444_at	0.00	0.00
1441445_at	0.00	0.00
1441446_at	0.00	0.00
1441447_at	0.00	0.00
1441448_at	0.00	0.00
1441449_at	0.00	0.00
1441450_s_at	0.00	0.00
1441451_at	0.00	0.00
1441452_at	0.00	0.00
1441453_at	0.00	0.00
1441454_at	0.00	0.00
1441455_at	0.00	0.00
1441456_at	0.00	0.00
1441457_at	0.00	0.00
1441458_at	0.00	0.00
1441459_at	0.00	0.00
1441460_at	0.00	0.00
1441461_at	0.00	0.00
1441462_at	0.00	0.00
1441463_at	0.00	0.00
1441464_at	0.00	0.00
1441465_at	0.00	0.00
1441466_at	0.00	0.00
1441467_at	0.00	0.00

1441468_at	0.00	0.00
1441469_at	0.00	0.00
1441470_at	0.00	0.00
1441471_at	0.00	0.00
1441472_at	0.00	0.00
1441473_at	0.00	0.00
1441474_at	0.00	0.00
1441475_at	0.00	0.00
1441476_at	0.00	0.00
1441477_at	0.00	0.00
1441478_at	0.00	0.00
1441479_at	0.00	0.00
1441480_at	0.00	0.00
1441481_at	0.00	0.00
1441482_at	0.00	0.00
1441483_at	0.00	0.00
1441484_at	0.00	0.00
1441485_at	0.00	0.00
1441486_at	0.00	0.00
1441487_at	0.00	0.00
1441488_at	0.00	0.00
1441489_at	0.00	0.00
1441490_at	0.00	0.00
1441491_at	0.00	0.00
1441492_at	0.00	0.00
1441493_at	0.00	0.00
1441494_at	0.00	0.00
1441495_at	0.00	0.00
1441496_at	0.00	0.00
1441497_at	0.00	0.00
1441498_at	0.00	0.00
1441499_at	0.00	0.00
1441500_at	0.00	0.00
1441501_at	0.00	0.00
1441502_at	0.00	0.00
1441503_at	0.00	0.00
1441504_at	0.00	0.00
1441505_at	0.00	0.00
1441506_at	0.00	0.00
1441507_at	0.00	0.00
1441508_at	0.00	0.00
1441509_at	0.00	0.00
1441510_at	0.00	0.00
1441511_at	0.00	0.00
1441512_at	0.00	0.00
1441513_at	0.00	0.00
1441514_at	0.00	0.00
1441515_at	0.00	0.00
1441516_a_at	0.00	0.00
1441517_at	0.00	0.00
1441518_at	0.00	0.00
1441519_at	0.00	0.00
1441520_at	0.00	0.00
1441521_at	0.00	0.00
1441522_at	0.00	0.00
1441523_at	0.00	0.00

1441524_at	0.00	0.00
1441525_at	0.00	0.00
1441526_at	0.00	0.00
1441527_at	0.00	0.00
1441528_at	0.00	0.00
1441529_at	0.00	0.00
1441530_at	0.00	0.00
1441531_at	0.00	0.00
1441532_at	0.00	0.00
1441533_at	0.00	0.00
1441534_at	0.00	0.00
1441535_at	0.00	0.00
1441536_at	0.00	0.00
1441537_at	0.00	0.00
1441538_at	0.00	0.00
1441539_at	0.00	0.00
1441540_at	0.00	0.00
1441541_at	0.00	0.00
1441542_at	0.00	0.00
1441543_at	0.00	0.00
1441544_at	0.00	0.00
1441545_at	0.00	0.00
1441546_at	0.00	0.00
1441547_at	0.00	0.00
1441548_at	0.00	0.00
1441549_at	0.00	0.00
1441550_at	0.00	0.00
1441551_at	0.00	0.00
1441552_at	0.00	0.00
1441553_at	0.00	0.00
1441554_at	0.00	0.00
1441555_at	0.00	0.00
1441556_at	0.00	0.00
1441557_at	0.00	0.00
1441558_at	0.00	0.00
1441559_at	0.00	0.00
1441560_at	0.00	0.00
1441561_at	0.00	0.00
1441562_at	0.00	0.00
1441563_at	0.00	0.00
1441564_at	0.00	0.00
1441565_at	0.00	0.00
1441566_at	0.00	0.00
1441567_at	0.00	0.00
1441568_at	0.00	0.00
1441569_at	0.00	0.00
1441570_at	0.00	0.00
1441571_at	0.00	0.00
1441572_at	0.00	0.00
1441573_at	0.00	0.00
1441574_at	0.00	0.00
1441575_at	0.00	0.00
1441576_at	0.00	0.00
1441577_at	0.00	0.00
1441578_at	0.00	0.00
1441579_at	0.00	0.00

1441580_at	0.00	0.00
1441581_at	0.00	0.00
1441582_at	0.00	0.00
1441584_at	0.00	0.00
1441585_at	0.00	0.00
1441586_at	0.00	0.00
1441587_at	0.00	0.00
1441588_at	0.00	0.00
1441589_at	0.00	0.00
1441590_at	0.00	0.00
1441591_at	0.00	0.00
1441592_at	0.00	0.00
1441593_at	0.00	0.00
1441594_at	0.00	0.00
1441595_at	0.00	0.00
1441596_at	0.00	0.00
1441597_at	0.00	0.00
1441598_at	0.00	0.00
1441599_at	0.00	0.00
1441600_at	0.00	0.00
1441601_at	0.00	0.00
1441602_at	0.00	0.00
1441603_at	0.00	0.00
1441604_at	0.00	0.00
1441605_at	0.00	0.00
1441606_at	0.00	0.00
1441607_at	0.00	0.00
1441608_at	0.00	0.00
1441609_at	0.00	0.00
1441610_at	0.00	0.00
1441611_at	0.00	0.00
1441612_at	0.00	0.00
1441613_at	0.00	0.00
1441614_at	0.00	0.00
1441615_at	0.00	0.00
1441616_at	0.00	0.00
1441617_at	0.00	0.00
1441619_at	0.00	0.00
1441620_at	0.00	0.00
1441621_at	0.00	0.00
1441622_at	0.00	0.00
1441623_at	0.00	0.00
1441624_at	0.00	0.00
1441625_at	0.00	0.00
1441626_at	0.00	0.00
1441627_at	0.00	0.00
1441628_at	0.00	0.00
1441629_at	0.00	0.00
1441630_at	0.00	0.00
1441631_at	0.00	0.00
1441632_at	0.00	0.00
1441633_at	0.00	0.00
1441634_at	0.00	0.00
1441635_at	0.00	0.00
1441636_at	0.00	0.00
1441637_at	0.00	0.00

1441638_at	0.00	0.00
1441639_at	0.00	0.00
1441640_at	0.00	0.00
1441641_at	0.00	0.00
1441642_at	0.00	0.00
1441643_at	0.00	0.00
1441644_at	0.00	0.00
1441645_s_at	0.00	0.00
1441646_at	0.00	0.00
1441647_at	0.00	0.00
1441648_at	0.00	0.00
1441649_at	0.00	0.00
1441650_at	0.00	0.00
1441651_at	0.00	0.00
1441652_at	0.00	0.00
1441653_at	0.00	0.00
1441654_at	0.00	0.00
1441655_at	0.00	0.00
1441656_at	0.00	0.00
1441657_at	0.00	0.00
1441658_at	0.00	0.00
1441660_at	0.00	0.00
1441661_at	0.00	0.00
1441662_at	0.00	0.00
1441663_at	0.00	0.00
1441664_at	0.00	0.00
1441665_at	0.00	0.00
1441666_at	0.00	0.00
1441667_s_at	0.00	0.00
1441668_at	0.00	0.00
1441669_at	0.00	0.00
1441670_at	0.00	0.00
1441671_at	0.00	0.00
1441672_at	0.00	0.00
1441673_at	0.00	0.00
1441674_at	0.00	0.00
1441675_at	0.00	0.00
1441676_at	0.00	0.00
1441677_at	0.00	0.00
1441678_at	0.00	0.00
1441679_at	0.00	0.00
1441680_at	0.00	0.00
1441681_at	0.00	0.00
1441682_s_at	0.00	0.12
1441683_at	0.00	0.00
1441684_at	0.00	0.00
1441685_at	0.00	0.00
1441686_at	0.00	0.00
1441687_at	0.00	0.00
1441688_at	0.00	0.00
1441689_at	0.00	0.00
1441690_at	0.00	0.00
1441691_at	0.00	0.00
1441692_at	0.00	0.00
1441693_at	0.00	0.00
1441694_at	0.00	0.00

1441695_at	0.00	0.00
1441696_at	0.00	0.00
1441697_at	0.00	0.00
1441698_at	0.00	0.00
1441699_at	0.00	0.00
1441700_at	0.00	0.00
1441701_at	0.00	0.00
1441702_at	0.00	0.00
1441703_at	0.00	0.00
1441704_at	0.00	0.00
1441705_at	0.00	0.00
1441706_at	0.00	0.00
1441707_at	0.00	0.00
1441708_at	0.00	0.00
1441709_at	0.00	0.00
1441710_at	0.00	0.00
1441711_at	0.00	0.00
1441712_at	0.00	0.00
1441713_at	0.00	0.00
1441714_at	0.00	0.00
1441715_at	0.00	0.00
1441716_at	0.00	0.00
1441717_at	0.00	0.00
1441718_at	0.00	0.00
1441719_at	0.00	0.00
1441720_at	0.00	0.00
1441721_at	0.00	0.00
1441722_at	0.00	0.00
1441723_at	0.00	0.00
1441724_at	0.00	0.00
1441725_at	0.00	0.00
1441726_at	0.00	0.00
1441727_s_at	0.00	0.00
1441728_at	0.00	0.00
1441729_at	0.00	0.00
1441730_at	0.00	0.00
1441731_at	0.00	0.00
1441732_at	0.00	0.00
1441733_s_at	0.00	0.00
1441734_at	0.00	0.00
1441735_at	0.00	0.00
1441736_at	0.00	0.00
1441737_s_at	0.00	0.00
1441738_at	0.00	0.00
1441739_at	0.00	0.00
1441740_at	0.00	0.00
1441741_at	0.00	0.00
1441742_at	0.00	0.00
1441743_at	0.00	0.00
1441744_at	0.00	0.00
1441745_at	0.00	0.00
1441746_at	0.00	0.00
1441747_at	0.00	0.00
1441748_at	0.00	0.00
1441749_at	0.00	0.00
1441750_x_at	0.00	0.00

1441751_at	0.00	0.00
1441752_at	0.00	0.00
1441753_at	0.00	0.00
1441754_at	0.00	0.00
1441755_at	0.00	0.00
1441756_at	0.00	0.00
1441757_at	0.00	0.00
1441758_at	0.00	0.00
1441759_at	0.00	0.00
1441760_at	0.00	0.00
1441761_at	0.00	0.00
1441762_at	0.00	0.00
1441763_at	0.00	0.00
1441764_at	0.00	0.00
1441765_at	0.00	0.00
1441766_at	0.00	0.00
1441767_at	0.00	0.00
1441768_at	0.00	0.00
1441769_at	0.00	0.00
1441770_at	0.00	0.00
1441771_at	0.00	0.00
1441772_at	0.00	0.00
1441773_at	0.00	0.00
1441774_at	0.00	0.00
1441775_at	0.00	0.00
1441776_at	0.00	0.00
1441777_at	0.00	0.00
1441778_at	0.00	0.00
1441779_at	0.00	0.00
1441780_at	0.00	0.00
1441781_at	0.00	0.00
1441782_at	0.00	0.00
1441783_at	0.00	0.00
1441784_at	0.00	0.00
1441785_at	0.00	0.00
1441786_at	0.00	0.00
1441787_at	0.00	0.00
1441788_s_at	0.05	0.09
1441789_at	0.00	0.00
1441790_at	0.00	0.00
1441791_at	0.00	0.00
1441792_at	0.00	0.00
1441793_at	0.00	0.00
1441794_at	0.00	0.00
1441795_at	0.00	0.00
1441796_at	0.00	0.00
1441797_at	0.00	0.00
1441798_at	0.00	0.00
1441799_at	0.00	0.00
1441800_at	0.00	0.00
1441801_at	0.00	0.00
1441802_at	0.00	0.00
1441803_at	0.00	0.00
1441804_at	0.00	0.00
1441805_at	0.00	0.00
1441806_at	0.00	0.00

1441807_s_at	0.00	0.00
1441808_at	0.00	0.00
1441809_at	0.00	0.00
1441810_at	0.00	0.00
1441811_x_at	0.00	0.00
1441812_at	0.00	0.00
1441813_at	0.00	0.00
1441814_s_at	0.00	0.00
1441815_at	0.00	0.00
1441816_at	0.00	0.00
1441817_at	0.00	0.00
1441818_at	0.00	0.00
1441819_x_at	0.00	0.00
1441820_at	0.00	0.00
1441821_at	0.00	0.00
1441822_at	0.00	0.00
1441823_at	0.00	0.00
1441824_at	0.00	0.00
1441825_x_at	0.00	0.00
1441826_x_at	0.00	0.00
1441827_x_at	0.00	0.00
1441828_at	0.00	0.00
1441829_s_at	0.00	0.00
1441830_x_at	0.00	0.00
1441831_x_at	0.00	0.00
1441832_at	0.00	0.00
1441833_at	0.00	0.00
1441834_x_at	0.00	0.00
1441835_x_at	0.00	0.00
1441836_x_at	0.00	0.00
1441837_at	0.00	0.00
1441838_at	0.00	0.00
1441839_s_at	0.00	0.00
1441840_x_at	0.00	0.00
1441841_at	0.00	0.00
1441842_s_at	0.00	0.00
1441843_s_at	0.00	0.00
1441844_at	0.00	0.00
1441845_at	0.00	0.00
1441846_x_at	0.00	0.00
1441847_at	0.00	0.00
1441848_at	0.00	0.00
1441849_at	0.00	0.00
1441850_x_at	0.00	0.00
1441851_x_at	0.00	0.00
1441852_x_at	0.00	0.00
1441853_at	0.00	0.00
1441854_at	0.00	0.00
1441855_x_at	0.00	0.00
1441856_x_at	0.00	0.00
1441857_x_at	0.00	0.00
1441858_at	0.00	0.00
1441859_x_at	0.00	0.00
1441860_x_at	0.00	0.00
1441861_at	0.00	0.00
1441862_at	0.00	0.00

1441863_x_at	0.00	0.00
1441864_x_at	0.00	0.00
1441865_at	0.00	0.00
1441867_x_at	0.00	0.00
1441868_x_at	0.00	0.00
1441869_x_at	0.00	0.00
1441870_s_at	0.00	0.00
1441871_at	0.00	0.00
1441872_x_at	0.00	0.00
1441873_at	0.00	0.00
1441874_x_at	0.00	0.00
1441875_at	0.00	0.00
1441876_x_at	0.00	0.00
1441877_x_at	0.00	0.00
1441878_s_at	0.00	0.00
1441879_x_at	0.03	0.00
1441880_x_at	0.00	0.00
1441881_x_at	0.00	0.00
1441882_at	0.00	0.00
1441883_at	0.00	0.00
1441884_x_at	0.00	0.00
1441885_s_at	0.00	0.00
1441886_at	0.00	0.00
1441887_x_at	0.00	0.00
1441888_x_at	0.00	0.00
1441889_x_at	0.00	0.00
1441890_x_at	0.00	0.00
1441891_x_at	0.00	0.00
1441892_x_at	0.00	0.00
1441893_at	0.00	0.00
1441894_s_at	0.00	0.00
1441895_x_at	0.00	0.00
1441896_x_at	0.00	0.00
1441897_at	0.00	0.00
1441898_at	0.00	0.00
1441899_x_at	0.00	0.00
1441900_x_at	0.00	0.00
1441901_x_at	0.00	0.00
1441902_x_at	0.00	0.00
1441903_x_at	0.00	0.00
1441904_x_at	0.00	0.00
1441905_x_at	0.00	0.00
1441906_x_at	0.00	0.00
1441907_s_at	0.00	0.00
1441908_x_at	0.00	0.00
1441909_s_at	0.00	0.00
1441910_x_at	0.33	0.76
1441911_x_at	0.00	0.00
1441912_x_at	0.00	0.00
1441913_at	0.00	0.00
1441914_x_at	0.00	0.00
1441915_s_at	0.00	0.00
1441916_s_at	0.00	0.00
1441917_s_at	0.00	0.00
1441918_x_at	0.00	0.00
1441919_x_at	0.00	0.00

1441920_x_at	0.00	0.00
1441921_x_at	0.00	0.00
1441922_x_at	0.00	0.00
1441923_s_at	0.00	0.00
1441924_x_at	0.00	0.00
1441925_at	0.00	0.00
1441926_x_at	0.00	0.00
1441927_at	0.00	0.00
1441928_x_at	0.00	0.00
1441929_at	0.00	0.00
1441930_x_at	0.00	0.00
1441931_x_at	0.00	0.00
1441932_at	0.00	0.00
1441933_x_at	0.00	0.00
1441934_at	0.00	0.00
1441935_at	0.00	0.00
1441936_x_at	0.00	0.00
1441937_s_at	0.00	0.00
1441938_x_at	0.00	0.00
1441939_x_at	0.00	0.00
1441940_x_at	0.00	0.00
1441941_x_at	0.00	0.00
1441942_x_at	0.00	0.00
1441943_x_at	0.00	0.01
1441944_s_at	0.00	0.00
1441945_s_at	0.00	0.00
1441946_at	0.00	0.00
1441947_x_at	0.00	0.00
1441948_x_at	0.01	0.12
1441949_x_at	0.00	0.00
1441950_s_at	0.00	0.00
1441951_x_at	0.00	0.00
1441952_x_at	0.00	0.00
1441953_at	0.00	0.00
1441954_s_at	0.00	0.00
1441955_s_at	0.00	0.00
1441956_s_at	0.00	0.00
1441957_x_at	0.00	0.00
1441958_s_at	0.00	0.00
1441959_s_at	0.00	0.00
1441960_x_at	0.00	0.00
1441961_at	0.00	0.00
1441962_at	0.00	0.00
1441963_at	0.00	0.00
1441964_at	0.00	0.00
1441965_at	0.00	0.00
1441966_at	0.00	0.00
1441967_at	0.00	0.00
1441968_at	0.00	0.00
1441969_at	0.00	0.00
1441970_at	0.00	0.00
1441971_at	0.00	0.00
1441972_at	0.00	0.00
1441973_at	0.00	0.00
1441974_at	0.00	0.00
1441975_at	0.00	0.00

1441976_at	0.00	0.00
1441977_at	0.00	0.00
1441978_at	0.00	0.00
1441979_at	0.00	0.00
1441980_at	0.00	0.00
1441981_at	0.00	0.00
1441982_at	0.00	0.00
1441983_at	0.00	0.00
1441984_at	0.00	0.00
1441985_at	0.00	0.00
1441987_at	0.00	0.00
1441988_at	0.00	0.00
1441989_at	0.00	0.00
1441990_at	0.00	0.00
1441991_at	0.00	0.00
1441993_at	0.00	0.00
1441994_at	0.00	0.00
1441995_at	0.00	0.00
1441996_at	0.00	0.00
1441997_at	0.00	0.00
1441998_at	0.00	0.00
1441999_at	0.00	0.00
1442000_at	0.00	0.00
1442001_at	0.00	0.00
1442002_at	0.00	0.00
1442003_at	0.00	0.00
1442004_at	0.00	0.00
1442005_at	0.00	0.00
1442007_at	0.00	0.00
1442008_at	0.00	0.00
1442009_at	0.00	0.00
1442010_at	0.00	0.00
1442011_at	0.00	0.00
1442012_at	0.00	0.00
1442013_at	0.00	0.00
1442014_at	0.00	0.00
1442015_at	0.30	0.00
1442016_at	0.00	0.00
1442017_at	0.00	0.00
1442018_at	0.00	0.00
1442019_at	0.00	0.00
1442020_at	0.00	0.00
1442021_at	0.00	0.00
1442022_at	0.00	0.00
1442023_at	0.00	0.00
1442024_at	0.00	0.00
1442027_at	0.00	0.00
1442029_at	0.00	0.00
1442030_at	0.00	0.00
1442031_at	0.00	0.00
1442032_at	0.00	0.00
1442033_at	0.00	0.00
1442034_at	0.00	0.00
1442035_at	0.00	0.00
1442036_a_at	0.00	0.00
1442037_at	0.00	0.00

1442038_at	0.00	0.00
1442039_at	0.00	0.00
1442040_at	0.00	0.00
1442041_at	0.00	0.00
1442042_at	0.00	0.00
1442043_at	0.00	0.00
1442044_at	0.00	0.00
1442045_at	0.00	0.00
1442046_at	0.00	0.00
1442047_at	0.00	0.00
1442048_at	0.00	0.00
1442049_at	0.00	0.00
1442050_at	0.00	0.00
1442051_at	0.00	0.00
1442052_at	0.00	0.00
1442053_at	0.00	0.00
1442054_at	0.00	0.00
1442055_at	0.00	0.00
1442056_at	0.00	0.00
1442057_at	0.00	0.00
1442058_s_at	0.00	0.00
1442059_at	0.00	0.00
1442060_at	0.00	0.00
1442061_at	0.00	0.00
1442062_at	0.00	0.00
1442063_at	0.00	0.00
1442064_at	0.00	0.00
1442065_at	0.00	0.00
1442066_at	0.00	0.00
1442067_at	0.00	0.00
1442068_at	0.00	0.00
1442069_at	0.00	0.00
1442070_at	0.00	0.00
1442071_at	0.00	0.00
1442072_at	0.00	0.00
1442073_at	0.00	0.00
1442074_at	0.00	0.00
1442075_at	0.00	0.00
1442076_at	0.00	0.00
1442077_at	0.00	0.00
1442078_at	0.00	0.00
1442079_at	0.00	0.00
1442080_at	0.00	0.00
1442081_at	0.00	0.00
1442082_at	0.00	0.00
1442083_at	0.00	0.00
1442084_at	0.00	0.00
1442085_at	0.00	0.00
1442086_at	0.00	0.00
1442087_at	0.00	0.00
1442088_at	0.00	0.00
1442089_at	0.00	0.00
1442090_at	0.00	0.00
1442091_at	0.00	0.00
1442092_at	0.00	0.00
1442093_at	0.00	0.00

1442094_at	0.00	0.00
1442095_at	0.00	0.00
1442096_at	0.00	0.00
1442097_at	0.00	0.00
1442098_at	0.00	0.00
1442099_at	0.00	0.00
1442100_at	0.00	0.00
1442101_at	0.00	0.00
1442102_at	0.00	0.00
1442103_at	0.00	0.00
1442104_at	0.00	0.00
1442105_at	0.00	0.00
1442106_at	0.00	0.00
1442107_at	0.00	0.00
1442108_at	0.00	0.00
1442109_at	0.00	0.00
1442110_at	0.00	0.00
1442111_at	0.00	0.00
1442112_at	0.00	0.00
1442113_at	0.00	0.00
1442114_at	0.00	0.00
1442115_at	0.16	0.00
1442116_at	0.00	0.00
1442117_at	0.00	0.00
1442118_at	0.00	0.00
1442119_at	0.00	0.00
1442120_at	0.00	0.00
1442121_at	0.00	0.00
1442122_at	0.00	0.00
1442123_at	0.00	0.00
1442124_at	0.00	0.00
1442125_at	0.00	0.00
1442126_at	0.00	0.00
1442127_at	0.00	0.00
1442128_at	0.00	0.00
1442129_at	0.00	0.00
1442130_at	0.00	0.00
1442131_at	0.00	0.00
1442132_at	0.00	0.00
1442133_at	0.00	0.00
1442134_at	0.00	0.00
1442135_at	0.00	0.00
1442136_at	0.00	0.00
1442137_at	0.00	0.00
1442138_at	0.00	0.00
1442139_at	0.00	0.00
1442140_at	0.00	0.00
1442141_at	0.00	0.00
1442142_at	0.00	0.00
1442143_at	0.00	0.00
1442144_at	0.00	0.00
1442146_at	0.00	0.00
1442147_at	0.00	0.00
1442148_at	0.00	0.00
1442149_at	0.00	0.00
1442150_at	0.00	0.00

1442151_at	0.00	0.00
1442152_at	0.00	0.00
1442153_at	0.00	0.00
1442154_at	0.00	0.00
1442155_at	0.00	0.00
1442156_at	0.00	0.00
1442157_at	0.00	0.00
1442158_at	0.00	0.00
1442159_at	0.00	0.00
1442160_at	0.00	0.00
1442161_at	0.00	0.00
1442162_at	0.00	0.00
1442163_at	0.00	0.00
1442164_at	0.00	0.00
1442165_at	0.00	0.00
1442166_at	0.00	0.00
1442167_at	0.00	0.00
1442168_at	0.00	0.00
1442170_at	0.00	0.00
1442171_at	0.00	0.00
1442172_at	0.00	0.00
1442173_at	0.00	0.00
1442174_at	0.00	0.00
1442175_at	0.00	0.00
1442176_at	0.00	0.00
1442177_at	0.00	0.00
1442178_at	0.00	0.00
1442179_at	0.00	0.00
1442180_at	0.00	0.00
1442181_at	0.00	0.00
1442182_at	0.00	0.00
1442183_at	0.00	0.00
1442184_at	0.00	0.00
1442185_at	0.00	0.00
1442186_at	0.00	0.00
1442187_at	0.00	0.00
1442188_at	0.00	0.00
1442189_at	0.00	0.00
1442190_at	0.00	0.00
1442191_at	0.00	0.00
1442192_at	0.00	0.00
1442193_at	0.00	0.00
1442194_at	0.00	0.00
1442195_at	0.00	0.00
1442196_at	0.00	0.00
1442197_at	0.00	0.00
1442198_at	0.00	0.00
1442199_at	0.00	0.00
1442200_at	0.00	0.00
1442201_at	0.00	0.00
1442202_at	0.00	0.00
1442203_at	0.00	0.00
1442204_at	0.00	0.00
1442205_at	0.00	0.00
1442206_at	0.00	0.00
1442207_at	0.00	0.00

1442208_at	0.00	0.00
1442209_at	0.00	0.00
1442210_at	0.00	0.00
1442212_at	0.00	0.00
1442213_at	0.00	0.00
1442214_at	0.00	0.00
1442215_at	0.00	0.00
1442216_at	0.00	0.00
1442217_at	0.00	0.00
1442218_at	0.00	0.00
1442219_at	0.00	0.00
1442220_at	0.00	0.00
1442221_at	0.00	0.00
1442222_at	0.00	0.00
1442223_at	0.00	0.00
1442224_at	0.00	0.00
1442225_at	0.00	0.00
1442226_at	0.00	0.00
1442227_at	0.00	0.00
1442228_at	0.00	0.00
1442229_at	0.00	0.00
1442230_at	0.00	0.00
1442231_at	0.00	0.00
1442232_at	0.00	0.00
1442233_at	0.00	0.00
1442234_at	0.00	0.00
1442235_at	0.00	0.00
1442236_at	0.00	0.00
1442237_at	0.00	0.00
1442238_a_at	0.00	0.00
1442239_at	0.00	0.00
1442240_at	0.00	0.00
1442241_at	0.00	0.00
1442242_at	0.00	0.00
1442243_at	0.00	0.00
1442244_at	0.00	0.00
1442245_at	0.00	0.00
1442246_at	0.00	0.00
1442247_at	0.00	0.00
1442248_at	0.00	0.00
1442249_at	0.00	0.00
1442250_at	0.00	0.00
1442251_at	0.00	0.00
1442252_at	0.00	0.00
1442253_at	0.00	0.00
1442254_at	0.00	0.00
1442255_at	0.00	0.00
1442256_at	0.00	0.00
1442257_at	0.00	0.00
1442258_at	0.00	0.00
1442259_at	0.00	0.00
1442260_at	0.00	0.00
1442261_at	0.00	0.00
1442262_at	0.00	0.00
1442263_at	0.00	0.00
1442264_at	0.00	0.00

1442265_at	0.00	0.00
1442266_at	0.00	0.00
1442267_at	0.00	0.00
1442268_a_at	0.00	0.00
1442269_at	0.00	0.00
1442270_at	0.00	0.00
1442271_at	0.00	0.00
1442272_at	0.00	0.00
1442273_at	0.00	0.00
1442274_at	0.00	0.00
1442275_at	0.00	0.00
1442276_at	0.00	0.00
1442277_at	0.00	0.00
1442278_at	0.00	0.00
1442279_at	0.00	0.00
1442280_at	0.00	0.00
1442281_at	0.00	0.00
1442282_at	0.00	0.00
1442283_at	0.00	0.00
1442284_at	0.00	0.00
1442285_at	0.00	0.00
1442286_at	0.00	0.00
1442287_at	0.00	0.00
1442288_at	0.00	0.00
1442289_at	0.00	0.30
1442290_at	0.00	0.00
1442291_at	0.00	0.00
1442292_at	0.00	0.00
1442293_at	0.00	0.00
1442294_at	0.00	0.00
1442295_at	0.00	0.00
1442297_at	0.00	0.00
1442298_at	0.00	0.00
1442299_at	0.00	0.00
1442300_at	0.00	0.00
1442301_at	0.00	0.00
1442302_at	0.00	0.00
1442303_at	0.00	0.00
1442304_at	0.00	0.00
1442305_at	0.00	0.00
1442306_at	0.00	0.00
1442307_at	0.00	0.00
1442308_at	0.00	0.00
1442309_at	0.00	0.00
1442310_at	0.00	0.00
1442311_at	0.00	0.00
1442312_at	0.00	0.00
1442313_at	0.00	0.00
1442314_at	0.00	0.00
1442315_at	0.00	0.00
1442316_x_at	0.00	0.00
1442317_at	0.00	0.00
1442318_at	0.00	0.00
1442319_at	0.00	0.00
1442320_at	0.00	0.00
1442321_at	0.00	0.00

1442322_at	0.00	0.00
1442323_at	0.00	0.00
1442324_at	0.00	0.00
1442325_at	0.00	0.00
1442326_at	0.00	0.00
1442327_at	0.00	0.00
1442328_at	0.00	0.00
1442329_at	0.00	0.00
1442330_at	0.00	0.00
1442331_at	0.00	0.00
1442332_at	0.00	0.00
1442333_a_at	0.00	0.00
1442334_at	0.00	0.00
1442335_at	0.00	0.00
1442336_at	0.00	0.00
1442337_at	0.00	0.00
1442338_at	0.00	0.00
1442339_at	0.00	0.00
1442340_x_at	0.56	0.00
1442341_at	0.00	0.00
1442342_at	0.00	0.00
1442343_at	0.00	0.00
1442345_at	0.00	0.00
1442346_at	0.00	0.00
1442347_at	0.00	0.00
1442348_at	0.00	0.00
1442349_at	0.00	0.00
1442350_at	0.00	0.00
1442351_a_at	0.00	0.00
1442352_at	0.00	0.00
1442353_at	0.00	0.00
1442354_at	0.00	0.00
1442355_at	0.00	0.00
1442356_at	0.00	0.00
1442357_at	0.00	0.00
1442358_at	0.00	0.00
1442359_at	0.00	0.00
1442360_at	0.00	0.00
1442361_at	0.00	0.00
1442362_at	0.00	0.00
1442363_at	0.00	0.00
1442364_at	0.00	0.00
1442365_at	0.00	0.00
1442366_at	0.00	0.00
1442367_at	0.00	0.00
1442368_at	0.00	0.00
1442369_at	0.00	0.00
1442370_at	0.00	0.00
1442371_at	0.00	0.00
1442372_at	0.00	0.00
1442373_at	0.00	0.00
1442375_at	0.00	0.00
1442376_at	0.00	0.00
1442377_at	0.00	0.00
1442379_at	0.00	0.00
1442380_at	0.00	0.00

1442381_at	0.00	0.00
1442382_at	0.00	0.00
1442383_at	0.00	0.00
1442385_at	0.00	0.00
1442386_at	0.00	0.00
1442387_at	0.00	0.00
1442388_at	0.00	0.00
1442389_at	0.00	0.00
1442390_at	0.00	0.00
1442391_at	0.00	0.00
1442392_at	0.00	0.00
1442393_at	0.00	0.00
1442394_at	0.00	0.00
1442395_at	0.00	0.00
1442396_at	0.00	0.00
1442397_at	0.00	0.00
1442398_at	0.00	0.00
1442399_at	0.00	0.00
1442400_at	0.00	0.00
1442401_at	0.00	0.00
1442402_at	0.00	0.00
1442403_at	0.00	0.00
1442404_at	0.00	0.00
1442405_at	0.00	0.00
1442406_at	0.00	0.00
1442407_at	0.00	0.00
1442408_at	0.11	0.00
1442409_at	0.00	0.00
1442410_at	0.00	0.00
1442411_at	0.00	0.00
1442412_at	0.00	0.00
1442413_at	0.00	0.00
1442414_at	0.00	0.00
1442415_at	0.00	0.00
1442416_at	0.00	0.00
1442417_at	0.00	0.00
1442418_at	0.00	0.00
1442419_at	0.00	0.00
1442420_at	0.00	0.00
1442421_at	0.00	0.00
1442422_at	0.00	0.00
1442423_at	0.00	0.00
1442424_at	0.00	0.00
1442425_at	0.00	0.00
1442426_at	0.00	0.00
1442427_at	0.00	0.00
1442428_at	0.00	0.00
1442429_at	0.00	0.00
1442430_at	0.00	0.00
1442431_at	0.00	0.00
1442432_x_at	0.00	0.00
1442433_at	0.00	0.00
1442434_at	0.00	0.00
1442435_at	0.00	0.00
1442436_at	0.00	0.00
1442437_at	0.00	0.00

1442438_at	0.00	0.00
1442439_at	0.00	0.00
1442440_at	0.00	0.00
1442441_at	0.00	0.00
1442442_at	0.00	0.00
1442443_at	0.00	0.00
1442444_at	0.00	0.00
1442445_at	0.00	0.00
1442446_at	0.00	0.00
1442447_at	0.00	0.00
1442448_at	0.00	0.00
1442449_at	0.00	0.00
1442450_at	0.00	0.00
1442451_at	0.00	0.00
1442452_at	0.00	0.00
1442453_at	0.00	0.00
1442454_at	0.00	0.00
1442455_at	0.00	0.00
1442456_at	0.00	0.00
1442457_at	0.00	0.00
1442458_at	0.00	0.00
1442459_at	0.00	0.00
1442460_at	0.00	0.00
1442461_at	0.00	0.00
1442462_at	0.00	0.00
1442463_at	0.00	0.00
1442464_at	0.00	0.00
1442465_s_at	0.00	0.00
1442466_a_at	0.00	0.00
1442467_at	0.00	0.00
1442468_at	0.00	0.00
1442469_at	0.00	0.00
1442470_at	0.00	0.00
1442471_at	0.00	0.00
1442472_at	0.00	0.00
1442473_at	0.00	0.00
1442474_at	0.00	0.00
1442475_at	0.00	0.00
1442476_at	0.00	0.00
1442477_at	0.00	0.00
1442478_at	0.00	0.00
1442479_at	0.00	0.00
1442480_at	0.00	0.00
1442481_at	0.00	0.00
1442482_at	0.00	0.00
1442483_at	0.00	0.00
1442485_at	0.00	0.00
1442486_at	0.00	0.00
1442487_at	0.00	0.00
1442488_at	0.00	0.00
1442489_at	0.00	0.00
1442490_at	0.00	0.00
1442491_at	0.00	0.00
1442493_at	0.00	0.00
1442495_at	0.00	0.00
1442496_at	0.00	0.00

1442497_at	0.00	0.00
1442498_at	0.00	0.00
1442499_at	0.00	0.00
1442500_at	0.00	0.00
1442501_at	0.00	0.00
1442502_at	0.00	0.00
1442503_at	0.00	0.00
1442504_at	0.00	0.00
1442505_at	0.00	0.00
1442506_at	0.00	0.00
1442507_at	0.00	0.00
1442508_at	0.00	0.00
1442509_at	0.00	0.00
1442510_at	0.00	0.00
1442511_at	0.00	0.00
1442512_at	0.00	0.00
1442513_at	0.00	0.00
1442514_a_at	0.00	0.00
1442515_at	0.00	0.00
1442516_at	0.00	0.00
1442517_a_at	0.00	0.00
1442518_at	0.00	0.00
1442519_at	0.00	0.00
1442520_at	0.00	0.00
1442521_at	0.00	0.00
1442522_at	0.00	0.00
1442523_at	0.00	0.00
1442524_at	0.00	0.00
1442525_at	0.00	0.00
1442527_at	0.00	0.00
1442528_at	0.00	0.00
1442530_at	0.00	0.00
1442532_at	0.00	0.00
1442533_at	0.00	0.00
1442534_at	0.00	0.00
1442535_at	0.00	0.00
1442536_at	0.00	0.00
1442537_at	0.00	0.00
1442538_at	0.00	0.00
1442539_at	0.00	0.00
1442541_at	0.00	0.00
1442542_at	0.00	0.00
1442543_at	0.00	0.00
1442544_at	0.00	0.00
1442545_at	0.00	0.00
1442546_at	0.00	0.00
1442547_at	0.00	0.00
1442548_at	0.00	0.00
1442549_at	0.00	0.00
1442550_at	0.00	0.00
1442551_at	0.00	0.00
1442552_at	0.00	0.00
1442553_at	0.00	0.00
1442555_at	0.00	0.00
1442556_at	0.00	0.00
1442557_at	0.00	0.00

1442558_at	0.00	0.00
1442559_at	0.00	0.00
1442560_at	0.00	0.00
1442561_at	0.00	0.00
1442562_at	0.00	0.00
1442563_at	0.00	0.00
1442564_at	0.00	0.00
1442565_at	0.00	0.00
1442567_at	0.00	0.00
1442568_at	0.00	0.00
1442569_at	0.00	0.00
1442570_at	0.00	0.00
1442571_at	0.00	0.00
1442572_at	0.00	0.00
1442573_at	0.00	0.00
1442574_at	0.00	0.00
1442575_at	0.00	0.00
1442576_at	0.00	0.00
1442577_at	0.00	0.00
1442578_at	0.00	0.00
1442579_at	0.00	0.00
1442580_at	0.00	0.00
1442581_at	0.00	0.00
1442582_at	0.00	0.00
1442583_a_at	0.00	0.00
1442584_at	0.00	0.00
1442585_at	0.00	0.00
1442586_at	0.00	0.00
1442587_at	0.00	0.00
1442588_at	0.00	0.00
1442589_at	0.00	0.00
1442590_at	0.00	0.00
1442591_at	0.00	0.00
1442592_at	0.00	0.00
1442593_at	0.00	0.00
1442594_at	0.00	0.00
1442595_at	0.00	0.00
1442596_at	0.00	0.00
1442598_at	0.00	0.00
1442599_at	0.00	0.00
1442600_at	0.00	0.00
1442601_at	0.00	0.00
1442602_at	0.00	0.00
1442603_at	0.00	0.00
1442604_at	0.00	0.00
1442605_at	0.00	0.00
1442606_at	0.00	0.00
1442607_a_at	0.00	0.00
1442608_at	0.00	0.00
1442609_at	0.00	0.00
1442610_at	0.00	0.00
1442611_at	0.00	0.00
1442612_at	0.00	0.00
1442613_at	0.00	0.00
1442614_at	0.00	0.00
1442615_at	0.00	0.00

1442616_at	0.00	0.00
1442617_at	0.00	0.00
1442618_at	0.00	0.00
1442619_at	0.00	0.00
1442620_at	0.00	0.00
1442621_at	0.00	0.00
1442622_at	0.00	0.00
1442623_at	0.00	0.00
1442624_at	0.00	0.00
1442625_at	0.00	0.00
1442626_at	0.00	0.00
1442627_at	0.00	0.00
1442628_at	0.00	0.00
1442629_at	0.00	0.00
1442630_at	0.00	0.00
1442631_at	0.00	0.00
1442632_at	0.00	0.00
1442633_at	0.00	0.00
1442634_at	0.00	0.00
1442635_at	0.00	0.00
1442636_at	0.00	0.00
1442637_at	0.00	0.00
1442638_at	0.00	0.00
1442639_at	0.00	0.00
1442640_at	0.00	0.00
1442641_at	0.00	0.00
1442642_at	0.00	0.00
1442643_at	0.00	0.00
1442644_at	0.00	0.00
1442645_at	0.00	0.00
1442646_at	0.00	0.00
1442647_at	0.00	0.00
1442648_at	0.00	0.00
1442649_at	0.00	0.00
1442650_at	0.00	0.00
1442651_at	0.00	0.00
1442652_at	0.00	0.00
1442653_at	0.00	0.00
1442654_at	0.00	0.00
1442655_at	0.00	0.00
1442656_at	0.00	0.00
1442657_at	0.00	0.00
1442658_at	0.00	0.00
1442659_at	0.00	0.00
1442660_at	0.00	0.00
1442662_at	0.00	0.00
1442663_at	0.00	0.00
1442664_at	0.00	0.00
1442666_at	0.00	0.00
1442667_at	0.00	0.00
1442668_at	0.00	0.00
1442670_at	0.00	0.00
1442671_at	0.00	0.00
1442672_at	0.00	0.00
1442673_at	0.00	0.00
1442674_at	0.00	0.00

1442675_at	0.00	0.00
1442676_at	0.00	0.00
1442677_at	0.00	0.00
1442678_at	0.00	0.00
1442679_at	0.00	0.00
1442680_at	0.00	0.00
1442681_at	0.00	0.00
1442682_at	0.00	0.00
1442683_at	0.00	0.00
1442684_x_at	0.00	0.00
1442685_at	0.00	0.00
1442686_at	0.00	0.00
1442687_at	0.00	0.00
1442688_at	0.00	0.00
1442689_at	0.00	0.00
1442690_at	0.00	0.00
1442691_at	0.00	0.00
1442692_at	0.00	0.00
1442693_at	0.00	0.00
1442695_at	0.00	0.00
1442696_at	0.00	0.00
1442697_at	0.00	0.00
1442698_at	0.00	0.00
1442699_at	0.00	0.00
1442700_at	0.00	0.00
1442701_at	0.00	0.00
1442702_at	0.00	0.00
1442703_at	0.00	0.00
1442704_at	0.00	0.00
1442705_at	0.00	0.00
1442706_at	0.00	0.00
1442707_at	0.00	0.00
1442708_at	0.00	0.00
1442709_at	0.00	0.00
1442710_at	0.00	0.00
1442711_at	0.00	0.00
1442712_at	0.00	0.00
1442713_at	0.00	0.00
1442714_at	0.00	0.00
1442715_at	0.00	0.00
1442716_at	0.00	0.00
1442717_at	0.00	0.00
1442718_at	0.00	0.00
1442719_at	0.00	0.00
1442720_at	0.00	0.00
1442721_at	0.00	0.00
1442722_at	0.00	0.00
1442723_at	0.00	0.00
1442724_at	0.00	0.00
1442725_at	0.00	0.00
1442726_s_at	0.00	0.00
1442727_at	0.00	0.00
1442728_at	0.00	0.00
1442729_at	0.00	0.00
1442730_at	0.00	0.00
1442731_at	0.00	0.00

1442732_at	0.00	0.00
1442733_at	0.00	0.00
1442734_at	0.00	0.00
1442735_at	0.00	0.00
1442736_at	0.00	0.00
1442737_at	0.00	0.00
1442738_at	0.00	0.00
1442739_at	0.00	0.00
1442740_at	0.00	0.00
1442741_at	0.00	0.00
1442742_at	0.00	0.00
1442743_at	0.00	0.00
1442746_at	0.00	0.00
1442747_at	0.00	0.00
1442748_at	0.00	0.00
1442749_at	0.00	0.00
1442750_at	0.00	0.00
1442751_at	0.00	0.00
1442752_at	0.00	0.00
1442753_at	0.00	0.00
1442754_at	0.00	0.00
1442755_at	0.00	0.00
1442756_at	0.00	0.00
1442757_at	0.00	0.00
1442758_at	0.00	0.00
1442759_at	0.00	0.00
1442760_x_at	0.00	0.00
1442761_at	0.00	0.00
1442762_at	0.00	0.00
1442763_s_at	0.00	0.00
1442764_at	0.00	0.00
1442765_at	0.00	0.00
1442766_at	0.00	0.00
1442767_s_at	0.00	0.00
1442768_at	0.00	0.00
1442769_at	0.00	0.00
1442770_at	0.00	0.00
1442771_at	0.00	0.00
1442772_at	0.00	0.00
1442773_at	0.00	0.00
1442774_x_at	0.00	0.00
1442775_at	0.00	0.00
1442776_at	0.00	0.00
1442777_at	0.00	0.00
1442779_at	0.00	0.00
1442780_at	0.00	0.00
1442781_at	0.00	0.00
1442782_at	0.00	0.00
1442783_x_at	0.00	0.00
1442784_at	0.00	0.00
1442785_at	0.00	0.00
1442786_s_at	0.00	0.00
1442787_at	0.00	0.00
1442788_at	0.00	0.00
1442789_at	0.00	0.00
1442790_at	0.00	0.00

1442791_x_at	0.00	0.00
1442792_x_at	0.00	0.00
1442793_s_at	0.00	0.07
1442794_at	0.00	0.00
1442795_x_at	0.00	0.00
1442796_at	0.00	0.00
1442797_x_at	0.00	0.00
1442798_x_at	0.00	0.00
1442799_x_at	0.00	0.00
1442800_x_at	0.00	0.00
1442801_x_at	0.00	0.00
1442802_x_at	0.00	0.00
1442803_at	0.00	0.00
1442804_at	0.00	0.00
1442805_at	0.00	0.00
1442806_at	0.00	0.00
1442807_at	0.00	0.00
1442808_at	0.00	0.00
1442809_at	0.00	0.00
1442810_x_at	0.00	0.00
1442811_at	0.00	0.00
1442812_at	0.00	0.00
1442813_at	0.00	0.00
1442814_at	0.00	0.00
1442815_at	0.00	0.00
1442816_at	0.00	0.00
1442817_at	0.00	0.00
1442818_at	0.00	0.00
1442819_at	0.00	0.00
1442820_at	0.00	0.00
1442821_at	0.00	0.00
1442822_at	0.00	0.00
1442823_at	0.00	0.00
1442824_at	0.00	0.00
1442825_at	0.00	0.00
1442826_at	0.00	0.00
1442827_at	0.00	0.00
1442828_at	0.00	0.00
1442829_at	0.00	0.00
1442830_at	0.00	0.00
1442831_at	0.00	0.00
1442832_at	0.00	0.00
1442834_at	0.00	0.00
1442835_at	0.00	0.00
1442836_at	0.00	0.00
1442837_at	0.00	0.00
1442838_at	0.00	0.00
1442839_at	0.00	0.00
1442840_at	0.00	0.00
1442841_at	0.00	0.00
1442842_at	0.00	0.00
1442843_at	0.00	0.00
1442844_at	0.00	0.00
1442845_at	0.00	0.00
1442846_at	0.00	0.00
1442847_at	0.00	0.00

1442848_at	0.00	0.00
1442849_at	0.00	0.00
1442850_at	0.00	0.00
1442851_at	0.00	0.00
1442852_at	0.00	0.00
1442853_at	0.00	0.00
1442854_at	0.00	0.00
1442855_at	0.00	0.00
1442856_at	0.00	0.00
1442857_at	0.00	0.00
1442858_at	0.00	0.00
1442859_at	0.00	0.00
1442860_at	0.00	0.00
1442861_at	0.00	0.00
1442862_at	0.00	0.00
1442863_at	0.00	0.00
1442864_at	0.00	0.00
1442865_at	0.00	0.00
1442866_at	0.00	0.00
1442867_at	0.00	0.00
1442868_at	0.00	0.00
1442869_at	0.00	0.00
1442870_at	0.00	0.00
1442871_at	0.00	0.00
1442872_at	0.00	0.00
1442873_at	0.00	0.00
1442874_at	0.00	0.00
1442875_at	0.00	0.00
1442876_at	0.00	0.00
1442877_at	0.00	0.00
1442878_at	0.00	0.00
1442879_at	0.00	0.00
1442880_at	0.00	0.00
1442881_at	0.00	0.00
1442882_at	0.00	0.00
1442883_s_at	0.00	0.00
1442884_at	0.00	0.00
1442885_at	0.00	0.00
1442886_at	0.00	0.00
1442887_at	0.00	0.00
1442888_at	0.00	0.00
1442889_at	0.00	0.00
1442890_at	0.00	0.00
1442891_at	0.00	0.00
1442892_at	0.00	0.00
1442893_at	0.00	0.00
1442894_at	0.00	0.00
1442895_at	0.00	0.00
1442896_at	0.00	0.00
1442897_at	0.00	0.00
1442898_at	0.00	0.00
1442899_at	0.00	0.00
1442901_at	0.00	0.00
1442902_at	0.00	0.00
1442903_at	0.00	0.00
1442904_at	0.00	0.00

1442905_at	0.00	0.00
1442906_at	0.00	0.00
1442907_at	0.00	0.00
1442908_at	0.00	0.00
1442909_at	0.00	0.00
1442910_at	0.00	0.00
1442911_at	0.00	0.00
1442912_at	0.00	0.00
1442913_at	0.00	0.00
1442914_at	0.00	0.00
1442915_at	0.00	0.00
1442916_at	0.00	0.00
1442917_at	0.00	0.00
1442918_at	0.00	0.00
1442919_at	0.00	0.00
1442920_at	0.00	0.00
1442921_at	0.00	0.00
1442922_at	0.00	0.00
1442923_at	0.00	0.00
1442924_at	0.00	0.00
1442925_at	0.00	0.00
1442926_at	0.00	0.00
1442927_at	0.00	0.00
1442928_at	0.00	0.00
1442929_at	0.00	0.00
1442930_at	0.00	0.00
1442931_at	0.00	0.00
1442933_at	0.00	0.00
1442934_at	0.00	0.00
1442935_at	0.00	0.00
1442936_at	0.00	0.00
1442937_at	0.00	0.00
1442938_at	0.00	0.00
1442939_at	0.04	0.00
1442940_at	0.00	0.00
1442942_at	0.00	0.00
1442943_at	0.00	0.00
1442944_at	0.00	0.00
1442945_at	0.00	0.00
1442946_at	0.00	0.00
1442947_x_at	0.00	0.00
1442949_at	0.00	0.00
1442950_at	0.00	0.00
1442951_at	0.00	0.00
1442952_at	0.00	0.00
1442953_at	0.00	0.00
1442954_at	0.00	0.00
1442955_at	0.00	0.00
1442956_at	0.00	0.00
1442957_at	0.00	0.00
1442958_at	0.00	0.00
1442959_at	0.00	0.00
1442960_at	0.00	0.00
1442961_at	0.00	0.00
1442962_at	0.00	0.00
1442963_at	0.00	0.00

1442964_at	0.00	0.00
1442965_at	0.00	0.00
1442966_at	0.00	0.00
1442967_at	0.00	0.00
1442968_at	0.00	0.00
1442969_at	0.00	0.00
1442970_at	0.00	0.00
1442971_at	0.00	0.00
1442972_at	0.00	0.00
1442973_at	0.00	0.00
1442977_at	0.00	0.00
1442978_at	0.00	0.00
1442979_at	0.00	0.00
1442980_at	0.00	0.00
1442981_at	0.00	0.00
1442982_at	0.00	0.00
1442983_at	0.00	0.00
1442985_at	0.00	0.00
1442986_at	0.00	0.00
1442987_at	0.00	0.00
1442988_at	0.00	0.00
1442989_at	0.00	0.00
1442990_at	0.00	0.00
1442991_at	0.00	0.00
1442992_at	0.00	0.00
1442993_at	0.00	0.00
1442994_at	0.00	0.00
1442995_at	0.00	0.00
1442996_x_at	0.00	0.00
1442997_at	0.00	0.00
1442998_at	0.00	0.00
1442999_at	0.00	0.00
1443000_at	0.00	0.00
1443002_at	0.00	0.00
1443003_at	0.00	0.00
1443004_at	0.00	0.00
1443005_at	0.00	0.00
1443006_at	0.00	0.00
1443007_at	0.00	0.00
1443008_at	0.00	0.00
1443009_at	0.00	0.00
1443010_at	0.00	0.00
1443011_at	0.00	0.00
1443012_at	0.00	0.00
1443013_at	0.00	0.00
1443014_at	0.00	0.00
1443015_at	0.00	0.00
1443016_at	0.00	0.00
1443017_at	0.00	0.00
1443018_at	0.00	0.00
1443019_at	0.00	0.00
1443020_at	0.00	0.00
1443021_at	0.00	0.00
1443022_at	0.00	0.00
1443023_at	0.00	0.00
1443024_at	0.00	0.00

1443025_at	0.00	0.00
1443026_at	0.00	0.00
1443027_at	0.00	0.00
1443028_at	0.00	0.00
1443029_at	0.00	0.00
1443030_at	0.00	0.00
1443031_at	0.00	0.00
1443032_at	0.00	0.00
1443033_at	0.00	0.00
1443034_at	0.00	0.00
1443035_at	0.00	0.00
1443036_at	0.00	0.00
1443037_at	0.00	0.00
1443038_at	0.00	0.00
1443039_at	0.00	0.00
1443040_x_at	0.00	0.00
1443041_at	0.00	0.00
1443042_at	0.00	0.00
1443043_at	0.00	0.00
1443044_at	0.00	0.00
1443045_at	0.00	0.00
1443046_at	0.00	0.00
1443047_at	0.00	0.00
1443048_at	0.00	0.00
1443049_at	0.00	0.00
1443050_at	0.00	0.00
1443051_at	0.00	0.00
1443052_at	0.30	0.09
1443053_at	0.00	0.00
1443054_at	0.00	0.00
1443055_at	0.00	0.00
1443056_at	0.00	0.00
1443057_at	0.00	0.00
1443058_at	0.00	0.00
1443059_at	0.00	0.00
1443060_at	0.00	0.00
1443061_at	0.00	0.00
1443062_at	0.00	0.00
1443063_at	0.00	0.00
1443064_at	0.00	0.00
1443065_at	0.00	0.00
1443066_at	0.00	0.00
1443067_at	0.00	0.00
1443068_at	0.00	0.00
1443069_at	0.00	0.00
1443070_at	0.00	0.00
1443071_at	0.00	0.00
1443072_at	0.00	0.00
1443073_at	0.00	0.00
1443074_at	0.00	0.00
1443075_at	0.00	0.00
1443076_at	0.00	0.00
1443077_at	0.00	0.00
1443078_at	0.00	0.00
1443079_at	0.00	0.00
1443080_at	0.00	0.00

1443081_at	0.00	0.00
1443082_at	0.00	0.00
1443083_at	0.00	0.00
1443084_at	0.00	0.00
1443085_at	0.00	0.00
1443086_at	0.00	0.00
1443087_at	0.00	0.00
1443088_at	0.00	0.00
1443089_at	0.00	0.00
1443090_at	0.00	0.00
1443091_at	0.00	0.00
1443092_at	0.00	0.00
1443093_at	0.00	0.00
1443094_at	0.00	0.00
1443095_at	0.00	0.00
1443096_at	0.00	0.00
1443097_at	0.00	0.00
1443098_at	0.00	0.00
1443099_at	0.00	0.00
1443100_at	0.00	0.00
1443101_at	0.00	0.00
1443102_at	0.00	0.00
1443103_at	0.00	0.00
1443104_at	0.00	0.00
1443105_at	0.00	0.00
1443106_at	0.00	0.00
1443107_at	0.00	0.00
1443108_at	0.00	0.00
1443109_at	0.00	0.00
1443110_at	0.00	0.00
1443111_at	0.00	0.00
1443112_at	0.00	0.00
1443113_at	0.00	0.00
1443114_at	0.00	0.00
1443115_at	0.00	0.00
1443116_at	0.00	0.00
1443117_at	0.00	0.00
1443118_at	0.00	0.00
1443119_at	0.00	0.00
1443120_at	0.00	0.00
1443121_at	0.00	0.00
1443122_at	0.00	0.00
1443123_at	0.00	0.00
1443124_at	0.00	0.00
1443125_at	0.00	0.00
1443126_at	0.00	0.00
1443127_at	0.00	0.00
1443128_at	0.00	0.00
1443129_at	0.00	0.00
1443130_at	0.00	0.00
1443131_at	0.00	0.00
1443132_at	0.00	0.00
1443133_at	0.00	0.00
1443134_at	0.00	0.00
1443135_at	0.00	0.00
1443136_at	0.00	0.00

1443137_at	0.00	0.00
1443138_at	0.00	0.00
1443139_at	0.00	0.00
1443140_at	0.00	0.00
1443141_at	0.00	0.00
1443142_at	0.00	0.00
1443143_at	0.00	0.00
1443144_at	0.00	0.00
1443145_at	0.00	0.00
1443146_at	0.00	0.00
1443147_at	0.00	0.00
1443148_at	0.00	0.00
1443149_at	0.00	0.00
1443150_at	0.00	0.00
1443151_at	0.00	0.00
1443152_at	0.00	0.00
1443153_at	0.00	0.00
1443154_at	0.00	0.00
1443155_at	0.00	0.00
1443156_at	0.00	0.00
1443157_at	0.00	0.00
1443158_at	0.00	0.00
1443159_at	0.00	0.00
1443160_at	0.00	0.00
1443161_at	0.00	0.00
1443162_at	0.00	0.00
1443163_at	0.00	0.00
1443164_at	0.00	0.00
1443165_at	0.00	0.00
1443166_at	0.00	0.00
1443167_at	0.00	0.00
1443168_at	0.00	0.00
1443169_at	0.00	0.00
1443170_at	0.00	0.00
1443171_at	0.00	0.00
1443172_at	0.00	0.00
1443173_at	0.00	0.00
1443174_at	0.00	0.00
1443175_at	0.00	0.00
1443176_at	0.00	0.00
1443177_at	0.00	0.00
1443178_at	0.00	0.00
1443179_at	0.00	0.00
1443180_at	0.00	0.00
1443181_at	0.00	0.00
1443182_at	0.00	0.00
1443183_at	0.00	0.00
1443184_at	0.00	0.00
1443185_at	0.00	0.00
1443186_at	0.00	0.00
1443187_at	0.00	0.00
1443188_at	0.00	0.00
1443189_at	0.00	0.00
1443190_at	0.00	0.00
1443191_at	0.00	0.00
1443192_at	0.00	0.00

1443193_at	0.00	0.00
1443194_at	0.00	0.00
1443195_at	0.00	0.00
1443196_at	0.00	0.00
1443197_at	0.00	0.00
1443198_at	0.00	0.00
1443199_at	0.00	0.00
1443200_at	0.00	0.00
1443201_at	0.00	0.00
1443202_at	0.00	0.00
1443203_at	0.00	0.00
1443204_at	0.00	0.00
1443205_at	0.00	0.00
1443206_at	0.00	0.00
1443207_at	0.00	0.00
1443208_at	0.00	0.00
1443209_at	0.00	0.00
1443210_at	0.00	0.00
1443211_at	0.00	0.00
1443212_at	0.00	0.00
1443213_at	0.00	0.00
1443214_at	0.00	0.00
1443215_at	0.00	0.00
1443216_at	0.00	0.00
1443217_at	0.00	0.00
1443218_at	0.00	0.00
1443219_at	0.00	0.00
1443220_at	0.00	0.00
1443221_at	0.00	0.00
1443222_at	0.00	0.00
1443223_at	0.00	0.00
1443224_at	0.00	0.00
1443225_at	0.00	0.00
1443226_at	0.00	0.00
1443227_at	0.00	0.00
1443228_at	0.00	0.00
1443229_at	0.00	0.00
1443230_at	0.00	0.00
1443231_at	0.00	0.00
1443232_at	0.00	0.00
1443233_at	0.00	0.00
1443234_at	0.00	0.00
1443235_at	0.00	0.00
1443236_at	0.00	0.00
1443237_at	0.00	0.00
1443238_at	0.00	0.00
1443239_at	0.00	0.00
1443240_at	0.00	0.00
1443241_at	0.00	0.00
1443242_at	0.00	0.00
1443243_at	0.00	0.00
1443244_at	0.00	0.00
1443245_at	0.00	0.00
1443246_at	0.00	0.00
1443247_at	0.00	0.00
1443248_at	0.00	0.00

1443249_at	0.00	0.00
1443250_at	0.00	0.00
1443251_at	0.00	0.00
1443252_at	0.00	0.00
1443253_at	0.00	0.00
1443254_at	0.00	0.00
1443255_s_at	0.00	0.00
1443256_at	0.00	0.00
1443257_at	0.00	0.00
1443258_at	0.00	0.00
1443259_at	0.00	0.00
1443260_at	0.00	0.00
1443261_at	0.00	0.00
1443262_at	0.00	0.00
1443263_at	0.00	0.00
1443264_at	0.00	0.00
1443265_at	0.00	0.00
1443266_at	0.00	0.00
1443267_at	0.00	0.00
1443268_at	0.00	0.00
1443269_at	0.00	0.00
1443270_at	0.00	0.00
1443271_at	0.00	0.00
1443272_at	0.00	0.00
1443273_at	0.00	0.00
1443274_at	0.00	0.00
1443275_at	0.00	0.00
1443276_at	0.00	0.00
1443277_at	0.00	0.00
1443278_at	0.00	0.00
1443279_at	0.00	0.00
1443280_at	0.00	0.00
1443281_at	0.00	0.00
1443282_at	0.00	0.00
1443283_at	0.00	0.00
1443284_at	0.00	0.00
1443285_at	0.00	0.00
1443286_at	0.00	0.00
1443287_at	0.00	0.00
1443288_at	0.00	0.00
1443289_at	0.00	0.00
1443290_at	0.00	0.00
1443291_at	0.00	0.00
1443292_at	0.00	0.00
1443293_at	0.00	0.00
1443294_at	0.00	0.00
1443295_at	0.00	0.00
1443296_at	0.00	0.00
1443297_at	0.00	0.00
1443298_at	0.00	0.00
1443299_at	0.00	0.00
1443300_at	0.00	0.00
1443301_at	0.00	0.00
1443302_at	0.00	0.00
1443303_at	0.00	0.00
1443304_at	0.00	0.00

1443305_at	0.00	0.00
1443306_at	0.00	0.00
1443307_at	0.00	0.00
1443308_at	0.00	0.00
1443309_at	0.00	0.00
1443310_at	0.00	0.00
1443311_at	0.00	0.00
1443313_at	0.00	0.00
1443314_at	0.00	0.00
1443315_at	0.00	0.00
1443316_at	0.00	0.00
1443318_at	0.00	0.00
1443319_at	0.00	0.00
1443320_at	0.00	0.00
1443321_at	0.00	0.00
1443322_at	0.00	0.00
1443323_at	0.00	0.00
1443324_at	0.00	0.00
1443325_at	0.00	0.00
1443327_at	0.00	0.00
1443328_at	0.00	0.00
1443329_at	0.00	0.00
1443330_at	0.00	0.00
1443331_at	0.00	0.00
1443332_at	0.00	0.00
1443333_at	0.00	0.00
1443334_at	0.00	0.00
1443335_at	0.00	0.00
1443336_at	0.00	0.00
1443337_at	0.00	0.00
1443338_at	0.00	0.00
1443339_at	0.00	0.00
1443340_at	0.00	0.00
1443341_at	0.00	0.00
1443342_at	0.00	0.00
1443343_at	0.00	0.00
1443344_at	0.00	0.00
1443345_at	0.00	0.00
1443346_at	0.00	0.00
1443347_at	0.00	0.00
1443348_at	0.00	0.00
1443349_at	0.00	0.00
1443350_at	0.00	0.00
1443351_at	0.00	0.00
1443353_at	0.00	0.00
1443354_at	0.00	0.00
1443355_at	0.00	0.00
1443356_at	0.00	0.00
1443357_at	0.00	0.00
1443358_at	0.00	0.00
1443359_at	0.00	0.00
1443360_x_at	0.00	0.00
1443361_at	0.00	0.00
1443362_at	0.00	0.00
1443363_at	0.00	0.00
1443364_at	0.00	0.00

1443365_at	0.00	0.00
1443366_at	0.00	0.00
1443367_at	0.00	0.00
1443368_at	0.00	0.00
1443369_at	0.00	0.00
1443370_at	0.00	0.00
1443371_at	0.00	0.00
1443372_at	0.00	0.00
1443373_at	0.00	0.00
1443374_at	0.00	0.00
1443375_at	0.00	0.00
1443376_at	0.00	0.00
1443377_at	0.00	0.00
1443378_s_at	0.00	0.00
1443379_at	0.00	0.00
1443380_at	0.00	0.00
1443381_at	0.00	0.00
1443382_s_at	0.00	0.00
1443383_at	0.00	0.00
1443384_at	0.00	0.00
1443386_at	0.00	0.00
1443387_at	0.00	0.00
1443388_at	0.00	0.00
1443389_at	0.00	0.00
1443390_at	0.00	0.00
1443391_at	0.00	0.00
1443392_at	0.00	0.00
1443393_at	0.00	0.00
1443394_at	0.00	0.00
1443395_at	0.00	0.00
1443396_at	0.00	0.00
1443397_at	0.00	0.00
1443398_at	0.00	0.00
1443399_at	0.00	0.00
1443400_at	0.00	0.00
1443401_at	0.00	0.00
1443402_at	0.00	0.00
1443403_at	0.00	0.00
1443404_s_at	0.00	0.00
1443405_at	0.00	0.00
1443406_at	0.00	0.00
1443407_at	0.00	0.00
1443408_at	0.00	0.00
1443409_at	0.00	0.00
1443410_at	0.00	0.00
1443411_at	0.00	0.00
1443412_s_at	0.00	0.00
1443413_s_at	0.00	0.00
1443415_at	0.00	0.00
1443417_at	0.00	0.00
1443418_at	0.00	0.00
1443419_at	0.00	0.00
1443420_at	0.00	0.00
1443421_s_at	0.00	0.00
1443422_at	0.00	0.00
1443423_at	0.00	0.00

1443424_at	0.00	0.00
1443425_at	0.00	0.00
1443426_at	0.00	0.00
1443429_at	0.00	0.00
1443430_at	0.00	0.00
1443432_at	0.00	0.00
1443433_at	0.00	0.00
1443434_s_at	0.00	0.00
1443435_at	0.00	0.00
1443436_at	0.00	0.00
1443437_at	0.00	0.00
1443438_at	0.00	0.00
1443439_at	0.00	0.00
1443440_at	0.00	0.00
1443441_x_at	0.00	0.00
1443442_at	0.00	0.00
1443443_at	0.00	0.00
1443444_at	0.00	0.00
1443445_at	0.00	0.00
1443446_at	0.00	0.00
1443447_at	0.00	0.00
1443448_x_at	0.00	0.00
1443449_at	0.00	0.00
1443450_at	0.00	0.00
1443451_at	0.00	0.00
1443452_at	0.00	0.00
1443453_at	0.00	0.00
1443454_at	0.00	0.00
1443455_at	0.00	0.00
1443456_at	0.00	0.00
1443457_at	0.00	0.00
1443458_at	0.00	0.00
1443459_at	0.00	0.00
1443460_at	0.00	0.00
1443462_at	0.00	0.00
1443463_at	0.00	0.00
1443465_at	0.00	0.00
1443466_s_at	0.00	0.07
1443467_at	0.00	0.00
1443468_at	0.00	0.00
1443469_at	0.00	0.00
1443470_at	0.00	0.00
1443471_at	0.00	0.00
1443474_at	0.00	0.00
1443475_at	0.00	0.00
1443476_at	0.00	0.00
1443477_at	0.00	0.00
1443478_at	0.00	0.00
1443479_at	0.00	0.00
1443480_at	0.00	0.00
1443481_at	0.00	0.00
1443483_at	0.00	0.00
1443484_at	0.00	0.00
1443485_at	0.00	0.00
1443486_at	0.00	0.00
1443487_at	0.00	0.00

1443488_at	0.00	0.00
1443489_at	0.00	0.00
1443490_at	0.00	0.00
1443491_at	0.00	0.00
1443492_at	0.00	0.00
1443493_at	0.00	0.00
1443494_at	0.00	0.00
1443495_at	0.00	0.00
1443496_at	0.00	0.00
1443497_at	0.00	0.00
1443498_at	0.00	0.00
1443499_at	0.00	0.00
1443500_at	0.00	0.00
1443501_at	0.00	0.00
1443502_at	0.00	0.00
1443503_at	0.00	0.00
1443504_at	0.00	0.00
1443505_at	0.00	0.00
1443506_at	0.00	0.00
1443507_at	0.00	0.00
1443508_at	0.00	0.00
1443509_at	0.00	0.00
1443510_at	0.00	0.00
1443511_at	0.00	0.00
1443512_at	0.00	0.00
1443513_at	0.00	0.00
1443514_at	0.00	0.00
1443515_at	0.00	0.00
1443516_at	0.00	0.00
1443517_at	0.00	0.00
1443518_at	0.00	0.00
1443519_at	0.00	0.00
1443520_at	0.00	0.00
1443521_at	0.00	0.00
1443522_s_at	0.00	0.00
1443523_at	0.00	0.00
1443524_x_at	0.00	0.00
1443525_at	0.00	0.00
1443526_at	0.00	0.00
1443527_at	0.00	0.00
1443528_at	0.00	0.00
1443529_at	0.00	0.00
1443530_at	0.00	0.00
1443531_at	0.00	0.00
1443532_at	0.00	0.00
1443533_at	0.00	0.00
1443534_at	0.00	0.00
1443535_at	0.00	0.00
1443536_at	0.00	0.00
1443537_at	0.00	0.00
1443538_at	0.00	0.00
1443539_at	0.00	0.00
1443540_at	0.00	0.00
1443541_at	0.00	0.00
1443542_at	0.00	0.00
1443543_at	0.00	0.00

1443544_at	0.00	0.00
1443545_at	0.00	0.00
1443546_at	0.00	0.00
1443547_at	0.00	0.00
1443548_at	0.00	0.00
1443549_at	0.00	0.00
1443550_at	0.00	0.00
1443551_at	0.00	0.00
1443552_at	0.00	0.00
1443553_at	0.00	0.00
1443554_at	0.00	0.00
1443555_at	0.00	0.00
1443556_at	0.00	0.00
1443557_at	0.00	0.00
1443558_s_at	0.00	0.00
1443559_at	0.00	0.00
1443560_at	0.00	0.00
1443561_at	0.00	0.00
1443562_at	0.00	0.00
1443563_at	0.00	0.00
1443564_at	0.00	0.00
1443565_at	0.00	0.00
1443566_at	0.00	0.00
1443567_at	0.00	0.00
1443568_x_at	0.00	0.00
1443569_at	0.00	0.00
1443570_at	0.00	0.00
1443571_at	0.00	0.00
1443572_at	0.00	0.00
1443573_at	0.00	0.00
1443574_at	0.00	0.00
1443575_at	0.00	0.00
1443576_at	0.00	0.00
1443577_at	0.00	0.00
1443578_at	0.00	0.00
1443579_s_at	0.00	0.00
1443580_at	0.00	0.00
1443581_at	0.00	0.00
1443582_at	0.00	0.00
1443583_at	0.00	0.00
1443584_at	0.00	0.00
1443585_at	0.00	0.00
1443586_at	0.00	0.00
1443587_at	0.00	0.00
1443588_at	0.00	0.00
1443590_at	0.00	0.00
1443591_at	0.00	0.00
1443592_at	0.00	0.00
1443593_at	0.00	0.00
1443594_at	0.00	0.00
1443595_at	0.00	0.00
1443596_at	0.00	0.00
1443597_at	0.00	0.00
1443598_at	0.00	0.00
1443599_at	0.00	0.00
1443600_at	0.00	0.00

1443601_at	0.00	0.00
1443602_at	0.00	0.00
1443603_at	0.00	0.00
1443604_at	0.00	0.00
1443606_at	0.00	0.00
1443607_at	0.00	0.00
1443608_at	0.00	0.00
1443609_s_at	0.00	0.00
1443610_at	0.00	0.00
1443611_at	0.00	0.00
1443612_at	0.00	0.00
1443613_x_at	0.00	0.00
1443614_at	0.00	0.00
1443615_at	0.00	0.00
1443616_at	0.00	0.00
1443617_at	0.00	0.00
1443618_at	0.00	0.00
1443619_at	0.00	0.00
1443620_at	0.00	0.00
1443621_at	0.00	0.00
1443622_at	0.00	0.00
1443623_a_at	0.00	0.00
1443624_at	0.00	0.00
1443625_at	0.00	0.00
1443626_at	0.00	0.00
1443627_at	0.00	0.00
1443628_at	0.00	0.00
1443629_at	0.00	0.00
1443630_at	0.00	0.00
1443631_at	0.00	0.00
1443632_at	0.00	0.00
1443633_at	0.00	0.00
1443634_at	0.00	0.00
1443635_at	0.00	0.00
1443636_at	0.00	0.00
1443637_at	0.00	0.00
1443638_at	0.00	0.00
1443639_at	0.00	0.00
1443640_at	0.00	0.00
1443641_at	0.00	0.00
1443642_at	0.00	0.00
1443643_at	0.00	0.00
1443644_at	0.00	0.00
1443645_at	0.00	0.00
1443646_at	0.00	0.00
1443647_at	0.00	0.00
1443648_at	0.00	0.00
1443649_at	0.00	0.00
1443650_at	0.00	0.00
1443651_at	0.00	0.00
1443652_x_at	0.00	0.00
1443653_at	0.00	0.00
1443654_at	0.00	0.00
1443655_s_at	0.00	0.00
1443656_at	0.00	0.00
1443657_at	0.00	0.00

1443658_at	0.00	0.00
1443659_at	0.00	0.00
1443660_at	0.00	0.00
1443661_at	0.00	0.00
1443662_at	0.00	0.00
1443663_at	0.00	0.00
1443664_s_at	0.00	0.00
1443665_at	0.00	0.00
1443666_at	0.00	0.00
1443667_at	0.00	0.00
1443668_x_at	0.00	0.00
1443669_at	0.00	0.00
1443670_at	0.00	0.00
1443671_x_at	0.00	0.00
1443672_at	0.00	0.00
1443673_x_at	0.00	0.00
1443674_at	0.00	0.00
1443675_at	0.00	0.00
1443676_at	0.00	0.00
1443677_at	0.00	0.00
1443678_at	0.00	0.00
1443679_at	0.00	0.00
1443680_at	0.00	0.00
1443681_at	0.00	0.00
1443682_at	0.00	0.00
1443683_at	0.00	0.00
1443684_at	0.00	0.00
1443685_at	0.00	0.00
1443686_at	0.00	0.00
1443687_x_at	0.00	0.00
1443688_at	0.00	0.00
1443689_at	0.00	0.00
1443690_at	0.00	0.00
1443691_at	0.00	0.00
1443692_at	0.00	0.00
1443693_at	0.00	0.00
1443694_at	0.00	0.00
1443697_at	0.00	0.00
1443698_at	0.00	0.00
1443699_at	0.00	0.00
1443700_at	0.00	0.00
1443701_at	0.00	0.00
1443702_at	0.00	0.00
1443703_at	0.00	0.00
1443704_at	0.00	0.00
1443705_at	0.00	0.00
1443707_at	0.00	0.00
1443708_at	0.00	0.00
1443709_at	0.00	0.00
1443710_s_at	0.00	0.00
1443712_x_at	0.00	0.00
1443713_at	0.00	0.00
1443714_at	0.00	0.00
1443715_at	0.00	0.00
1443716_at	0.00	0.00
1443717_at	0.00	0.00

1443718_at	0.00	0.00
1443719_x_at	0.00	0.00
1443720_s_at	0.00	0.00
1443721_x_at	0.00	0.00
1443722_at	0.00	0.00
1443723_at	0.00	0.00
1443724_at	0.00	0.00
1443725_at	0.00	0.00
1443726_at	0.00	0.00
1443727_x_at	0.00	0.00
1443728_at	0.00	0.00
1443729_at	0.00	0.00
1443730_at	0.00	0.00
1443731_at	0.00	0.00
1443732_at	0.00	0.00
1443733_x_at	0.00	0.00
1443734_at	0.00	0.00
1443735_at	0.00	0.00
1443736_at	0.00	0.00
1443737_at	0.00	0.00
1443738_at	0.00	0.00
1443739_at	0.00	0.00
1443740_at	0.00	0.00
1443741_x_at	0.00	0.00
1443742_x_at	0.00	0.00
1443743_at	0.00	0.00
1443744_at	0.00	0.00
1443745_s_at	0.00	0.00
1443746_x_at	0.00	0.00
1443747_at	0.00	0.00
1443748_x_at	0.00	0.00
1443749_x_at	0.00	0.00
1443750_s_at	0.00	0.00
1443751_at	0.00	0.00
1443752_at	0.00	0.00
1443753_at	0.00	0.00
1443754_x_at	0.00	0.00
1443755_at	0.00	0.00
1443756_at	0.00	0.00
1443757_x_at	0.00	0.00
1443758_at	0.00	0.00
1443759_at	0.00	0.00
1443760_at	0.00	0.00
1443761_at	0.00	0.00
1443763_at	0.00	0.00
1443764_x_at	0.00	0.00
1443765_at	0.00	0.00
1443766_x_at	0.00	0.00
1443767_at	0.00	0.00
1443768_at	0.00	0.00
1443769_at	0.00	0.00
1443770_x_at	0.00	0.00
1443771_x_at	0.00	0.00
1443772_at	0.00	0.00
1443773_at	0.00	0.00
1443774_at	0.00	0.00

1443775_x_at	0.00	0.00
1443776_at	0.00	0.00
1443777_at	0.00	0.00
1443778_at	0.00	0.00
1443779_s_at	0.00	0.00
1443780_at	0.00	0.00
1443781_at	0.00	0.00
1443782_x_at	0.00	0.00
1443783_x_at	0.00	0.00
1443784_at	0.00	0.00
1443785_x_at	0.00	0.00
1443786_at	0.04	0.00
1443787_x_at	0.00	0.00
1443788_at	0.00	0.00
1443789_x_at	0.00	0.00
1443790_x_at	0.00	0.00
1443791_at	0.00	0.00
1443792_at	0.00	0.00
1443793_x_at	0.00	0.00
1443794_x_at	0.00	0.47
1443795_at	0.00	0.00
1443796_at	0.00	0.00
1443797_at	0.00	0.00
1443798_at	0.00	0.00
1443799_at	0.00	0.00
1443800_at	0.00	0.00
1443801_at	0.00	0.00
1443802_at	0.00	0.00
1443803_x_at	0.00	0.00
1443804_at	0.00	0.00
1443805_at	0.00	0.00
1443806_x_at	0.00	0.00
1443807_x_at	0.00	0.00
1443808_at	0.00	0.00
1443809_at	0.00	0.00
1443810_at	0.00	0.00
1443811_at	0.00	0.00
1443812_x_at	0.00	0.00
1443813_x_at	0.00	0.00
1443814_x_at	0.00	0.01
1443815_x_at	0.00	0.00
1443816_s_at	0.00	0.00
1443817_x_at	0.00	0.00
1443818_at	0.00	0.00
1443819_x_at	0.00	0.00
1443820_x_at	0.00	0.00
1443821_at	0.00	0.00
1443822_s_at	0.00	0.00
1443823_s_at	0.00	0.00
1443824_s_at	0.00	0.03
1443825_x_at	0.00	0.00
1443826_x_at	0.00	0.00
1443827_x_at	0.00	0.00
1443828_x_at	0.00	0.00
1443829_x_at	0.00	0.00
1443830_x_at	0.00	0.00

1443831_s_at	0.00	0.00
1443832_s_at	0.00	0.00
1443833_at	0.00	0.00
1443834_at	0.00	0.00
1443835_x_at	0.00	0.00
1443836_x_at	0.00	0.00
1443837_x_at	0.00	0.00
1443838_x_at	0.00	0.00
1443839_at	0.00	0.00
1443840_x_at	0.00	0.00
1443841_x_at	0.00	0.00
1443842_at	0.00	0.00
1443843_x_at	0.00	0.00
1443844_at	0.00	0.00
1443845_x_at	0.00	0.00
1443846_x_at	0.00	0.00
1443847_x_at	0.00	0.00
1443848_at	0.00	0.00
1443849_x_at	0.00	0.00
1443850_at	0.00	0.00
1443851_at	0.00	0.00
1443852_at	0.00	0.00
1443853_x_at	0.00	0.00
1443854_at	0.00	0.00
1443855_at	0.00	0.00
1443857_at	0.00	0.00
1443858_at	0.00	0.21
1443859_at	0.00	0.00
1443860_at	0.00	0.00
1443861_at	0.00	0.00
1443862_at	0.00	0.00
1443863_at	0.00	0.00
1443864_at	0.00	0.00
1443865_at	0.00	0.00
1443866_at	0.00	0.00
1443867_at	0.00	0.00
1443868_at	0.00	0.00
1443869_at	0.00	0.00
1443870_at	1.00	0.00
1443871_at	0.00	0.00
1443872_at	0.00	0.00
1443873_at	0.00	0.00
1443875_at	0.00	0.00
1443876_at	0.00	0.00
1443877_a_at	0.00	0.00
1443878_at	0.00	0.00
1443879_at	0.00	0.00
1443880_at	0.00	0.01
1443881_at	0.00	0.00
1443882_at	0.00	0.00
1443883_at	0.00	0.00
1443884_at	0.00	0.00
1443885_at	0.00	0.00
1443886_at	0.00	0.00
1443887_at	0.00	0.00
1443888_at	0.00	0.00

1443889_at	0.00	0.00
1443890_at	0.00	0.00
1443891_at	0.00	0.00
1443893_at	0.00	0.00
1443894_at	0.00	0.00
1443895_at	0.00	0.00
1443896_at	0.00	0.00
1443897_at	0.00	0.00
1443898_at	0.00	0.00
1443899_at	0.00	0.00
1443900_at	0.00	0.00
1443901_at	0.00	0.00
1443902_at	0.00	0.00
1443903_at	0.00	0.00
1443904_at	0.00	0.00
1443905_at	0.00	0.00
1443906_at	0.00	0.00
1443907_at	0.00	0.00
1443908_at	0.00	0.00
1443909_at	0.00	0.00
1443910_at	0.00	0.00
1443911_at	0.00	0.00
1443912_at	0.00	0.00
1443913_at	0.00	0.00
1443914_at	0.00	0.00
1443915_at	0.00	0.00
1443916_at	0.00	0.00
1443917_at	0.00	0.00
1443918_at	0.00	0.00
1443919_at	0.00	0.00
1443920_at	0.00	0.00
1443921_at	0.00	0.00
1443922_at	0.00	0.00
1443923_at	0.00	0.00
1443924_at	0.00	0.00
1443925_at	0.00	0.00
1443926_at	0.00	0.00
1443927_at	0.00	0.00
1443928_at	0.00	0.00
1443929_at	0.00	0.00
1443930_at	0.00	0.00
1443931_at	0.00	0.00
1443932_at	0.00	0.00
1443933_at	0.00	0.00
1443934_at	0.00	0.00
1443935_at	0.64	0.21
1443936_at	0.00	0.00
1443937_at	0.00	0.00
1443938_at	0.00	0.00
1443939_at	0.00	0.00
1443940_at	0.00	0.00
1443941_at	0.00	0.00
1443942_at	0.00	0.00
1443943_at	0.00	0.00
1443944_at	0.00	0.00
1443945_at	0.00	0.00

1443946_s_at	0.00	0.00
1443947_at	0.00	0.00
1443948_at	0.00	0.00
1443950_at	0.00	0.00
1443951_at	0.00	0.00
1443952_at	0.00	0.00
1443953_at	0.00	0.00
1443954_at	0.00	0.00
1443955_at	0.00	0.00
1443956_at	0.00	0.00
1443957_at	0.00	0.00
1443958_at	0.00	0.00
1443959_at	0.00	0.00
1443960_at	0.00	0.00
1443961_at	0.00	0.00
1443962_at	0.00	0.00
1443963_at	0.00	0.00
1443964_at	0.00	0.00
1443965_at	0.00	0.00
1443966_at	0.00	0.00
1443967_at	0.00	0.00
1443968_at	0.00	0.00
1443970_at	0.00	0.00
1443971_x_at	0.00	0.00
1443972_at	0.00	0.00
1443973_at	0.00	0.00
1443974_at	0.00	0.00
1443975_at	0.00	0.00
1443976_at	0.00	0.00
1443977_at	0.00	0.00
1443978_at	0.00	0.00
1443979_at	0.00	0.00
1443980_at	0.00	0.00
1443981_at	0.00	0.00
1443982_at	0.00	0.00
1443983_at	0.00	0.00
1443984_at	0.00	0.00
1443985_at	0.00	0.00
1443986_at	0.00	0.00
1443987_at	0.00	0.00
1443988_at	0.00	0.00
1443989_at	0.00	0.00
1443990_at	0.00	0.00
1443991_at	0.00	0.00
1443992_at	0.00	0.00
1443993_at	0.00	0.00
1443994_at	0.00	0.00
1443995_at	0.00	0.00
1443997_at	0.00	0.00
1443998_at	0.00	0.00
1443999_at	0.00	0.00
1444000_at	0.00	0.00
1444001_at	0.00	0.00
1444002_at	0.00	0.00
1444003_at	0.00	0.00
1444004_at	0.00	0.00

1444005_at	0.00	0.00
1444006_at	0.00	0.00
1444007_at	0.00	0.00
1444008_at	0.00	0.00
1444009_at	0.00	0.00
1444010_at	0.00	0.00
1444011_at	0.00	0.00
1444012_at	0.00	0.00
1444013_at	0.00	0.00
1444014_at	0.00	0.00
1444015_at	0.00	0.00
1444016_at	0.00	0.00
1444017_at	0.00	0.00
1444018_at	0.00	0.00
1444019_at	0.00	0.00
1444020_at	0.00	0.00
1444021_at	0.00	0.00
1444022_at	0.00	0.00
1444023_at	0.00	0.00
1444024_at	0.00	0.04
1444025_at	0.00	0.00
1444026_at	0.00	0.00
1444027_at	0.00	0.00
1444029_at	0.00	0.00
1444030_at	0.00	0.00
1444031_at	0.00	0.00
1444032_at	0.00	0.00
1444033_at	0.00	0.00
1444034_at	0.00	0.00
1444035_at	0.00	0.00
1444036_at	0.00	0.00
1444037_at	0.00	0.00
1444038_at	0.00	0.00
1444039_at	0.00	0.00
1444040_at	0.00	0.00
1444041_at	0.00	0.00
1444042_at	0.00	0.00
1444043_at	0.00	0.00
1444044_at	0.00	0.00
1444045_at	0.00	0.00
1444046_at	0.00	0.00
1444047_at	0.00	0.00
1444048_at	0.00	0.00
1444049_at	0.00	0.00
1444050_at	0.00	0.00
1444051_at	0.90	0.00
1444053_at	0.00	0.00
1444054_at	0.00	0.00
1444055_at	0.00	0.00
1444056_at	0.00	0.00
1444057_at	0.00	0.00
1444058_at	0.00	0.00
1444059_at	0.00	0.00
1444060_at	0.00	0.00
1444061_at	0.00	0.00
1444062_at	0.00	0.00

1444063_at	0.00	0.00
1444064_at	0.00	0.00
1444065_at	0.00	0.00
1444066_at	0.00	0.00
1444067_at	0.00	0.00
1444068_at	0.00	0.00
1444069_at	0.00	0.00
1444070_at	0.00	0.00
1444071_at	0.00	0.00
1444072_at	0.00	0.00
1444073_at	0.00	0.00
1444074_at	0.00	0.00
1444075_at	0.00	0.00
1444076_at	0.00	0.00
1444077_at	0.00	0.00
1444078_at	0.00	0.00
1444079_at	0.00	0.00
1444080_at	0.00	0.00
1444081_at	0.00	0.00
1444082_at	0.00	0.00
1444083_at	0.00	0.00
1444084_at	0.00	0.00
1444085_at	0.00	0.00
1444086_at	0.00	0.00
1444087_at	0.00	0.00
1444088_at	0.00	0.00
1444089_at	0.00	0.00
1444090_at	0.00	0.00
1444091_a_at	0.00	0.00
1444092_at	0.00	0.00
1444093_at	0.00	0.00
1444094_at	0.00	0.00
1444095_a_at	0.00	0.00
1444096_at	0.00	0.00
1444097_at	0.00	0.00
1444098_at	0.00	0.00
1444099_at	0.00	0.00
1444100_at	0.00	0.00
1444101_at	0.00	0.00
1444102_at	0.00	0.00
1444103_at	0.00	0.00
1444104_at	0.00	0.00
1444105_at	0.00	0.00
1444106_at	0.00	0.00
1444107_at	0.00	0.00
1444108_at	0.00	0.00
1444109_at	0.00	0.00
1444110_at	0.00	0.00
1444111_at	0.00	0.00
1444112_at	0.00	0.00
1444113_at	0.00	0.00
1444114_at	0.00	0.00
1444115_at	0.00	0.00
1444116_at	0.00	0.00
1444117_at	0.00	0.00
1444118_at	0.00	0.00

1444119_at	0.00	0.00
1444120_at	0.00	0.00
1444121_at	0.00	0.00
1444122_at	0.00	0.00
1444123_at	0.00	0.00
1444124_a_at	0.00	0.00
1444125_at	0.00	0.00
1444126_at	0.00	0.00
1444127_at	0.00	0.00
1444128_at	0.00	0.00
1444129_at	0.00	0.00
1444130_at	0.00	0.00
1444131_at	0.00	0.00
1444132_at	0.00	0.00
1444133_at	0.00	0.00
1444134_at	0.00	0.00
1444135_at	0.00	0.00
1444136_at	0.00	0.00
1444137_at	0.00	0.00
1444138_at	0.00	0.00
1444139_at	0.00	0.00
1444140_at	0.00	0.00
1444141_at	0.00	0.00
1444142_at	0.00	0.00
1444143_at	0.00	0.00
1444144_at	0.00	0.00
1444145_at	0.00	0.00
1444146_at	0.00	0.00
1444147_at	0.00	0.00
1444148_at	0.00	0.00
1444149_at	0.00	0.00
1444150_at	0.00	0.00
1444151_at	0.00	0.00
1444152_at	0.00	0.00
1444153_at	0.00	0.00
1444154_at	0.00	0.00
1444155_at	0.00	0.00
1444156_at	0.00	0.00
1444157_a_at	0.00	0.00
1444158_at	0.00	0.00
1444159_at	0.00	0.00
1444161_at	0.00	0.00
1444162_at	0.00	0.00
1444163_at	0.00	0.00
1444164_at	0.00	0.00
1444165_at	0.00	0.00
1444166_at	0.00	0.00
1444167_at	0.00	0.00
1444168_at	0.00	0.00
1444169_at	0.00	0.00
1444170_at	0.00	0.00
1444171_at	0.00	0.00
1444172_at	0.00	0.00
1444173_at	0.00	0.00
1444174_at	0.00	0.00
1444175_at	0.00	0.00

1444176_at	0.00	0.00
1444177_at	0.00	0.00
1444178_at	0.00	0.00
1444179_at	0.00	0.00
1444180_at	0.00	0.00
1444181_at	0.00	0.00
1444182_at	0.00	0.00
1444183_at	0.00	0.00
1444184_at	0.00	0.00
1444185_at	0.00	0.00
1444187_at	0.00	0.00
1444188_at	0.00	0.00
1444189_at	0.00	0.00
1444190_at	0.00	0.00
1444191_at	0.00	0.00
1444192_at	0.00	0.00
1444193_at	0.00	0.00
1444194_at	0.00	0.00
1444195_at	0.00	0.00
1444196_at	0.00	0.00
1444197_at	0.00	0.00
1444198_at	0.00	0.00
1444199_at	0.00	0.00
1444200_at	0.00	0.00
1444201_at	0.00	0.00
1444202_at	0.00	0.00
1444203_at	0.00	0.00
1444204_at	0.00	0.00
1444205_at	0.00	0.00
1444206_at	0.00	0.00
1444207_at	0.00	0.00
1444208_at	0.00	0.00
1444209_at	0.00	0.00
1444210_at	0.00	0.00
1444211_at	0.00	0.00
1444212_at	0.00	0.00
1444213_at	0.00	0.00
1444214_at	0.00	0.00
1444215_at	0.00	0.00
1444216_at	0.00	0.00
1444217_at	0.00	0.00
1444218_at	0.00	0.00
1444219_at	0.00	0.00
1444220_at	0.00	0.00
1444221_at	0.00	0.00
1444222_x_at	0.00	0.00
1444223_at	0.00	0.00
1444224_at	0.00	0.00
1444225_at	0.00	0.00
1444226_at	0.00	0.00
1444227_at	0.00	0.00
1444228_s_at	0.00	0.00
1444229_at	0.00	0.00
1444230_at	0.00	0.00
1444231_at	0.00	0.00
1444232_at	0.00	0.00

1444233_at	0.00	0.00
1444234_at	0.00	0.00
1444235_at	0.00	0.00
1444236_at	0.00	0.00
1444237_at	0.00	0.00
1444238_at	0.00	0.00
1444239_at	0.00	0.00
1444240_at	0.00	0.00
1444241_at	0.00	0.00
1444242_at	0.00	0.00
1444243_at	0.00	0.00
1444244_at	0.00	0.00
1444245_at	0.00	0.00
1444246_at	0.00	0.00
1444247_at	0.00	0.00
1444248_at	0.00	0.00
1444249_at	0.00	0.00
1444250_at	0.00	0.00
1444251_x_at	0.00	0.00
1444252_at	0.00	0.00
1444253_at	0.00	0.00
1444254_at	0.00	0.00
1444255_at	0.00	0.00
1444256_at	0.00	0.00
1444257_at	0.00	0.00
1444258_at	0.00	0.00
1444259_at	0.00	0.00
1444260_at	0.00	0.00
1444261_at	0.00	0.00
1444262_at	0.00	0.00
1444263_at	0.00	0.00
1444264_at	0.00	0.00
1444265_at	0.00	0.00
1444266_at	0.00	0.00
1444267_at	0.00	0.00
1444268_at	0.00	0.00
1444269_at	0.00	0.00
1444270_at	0.00	0.00
1444271_at	0.00	0.00
1444272_at	0.00	0.00
1444273_at	0.00	0.00
1444275_at	0.00	0.00
1444276_at	0.00	0.00
1444277_at	0.00	0.00
1444278_at	0.00	0.00
1444279_at	0.00	0.00
1444280_at	0.00	0.00
1444281_at	0.00	0.00
1444282_at	0.00	0.00
1444283_at	0.00	0.00
1444284_at	0.00	0.00
1444285_at	0.00	0.00
1444286_at	0.00	0.00
1444287_at	0.00	0.00
1444288_at	0.00	0.00
1444289_at	0.00	0.00

1444290_at	0.00	0.00
1444291_at	0.00	0.00
1444293_at	0.00	0.00
1444294_at	0.00	0.00
1444295_at	0.00	0.00
1444296_a_at	0.00	0.00
1444297_at	0.00	0.00
1444298_at	0.00	0.00
1444299_at	0.00	0.00
1444300_at	0.00	0.00
1444301_at	0.00	0.00
1444302_at	0.00	0.00
1444303_at	0.00	0.00
1444304_at	0.00	0.00
1444305_at	0.00	0.00
1444306_at	0.00	0.00
1444307_at	0.00	0.00
1444308_at	0.00	0.00
1444309_at	0.00	0.00
1444310_at	0.00	0.00
1444311_at	0.00	0.00
1444312_at	0.00	0.00
1444313_at	0.00	0.00
1444314_at	0.00	0.00
1444315_at	0.00	0.00
1444316_at	0.00	0.00
1444317_at	0.00	0.00
1444318_at	0.00	0.00
1444319_at	0.00	0.00
1444320_at	0.00	0.00
1444321_at	0.00	0.00
1444322_at	0.00	0.00
1444323_at	0.00	0.00
1444324_at	0.00	0.00
1444325_at	0.00	0.00
1444326_at	0.00	0.00
1444327_at	0.00	0.00
1444328_at	0.00	0.00
1444329_at	0.00	0.00
1444330_at	0.00	0.00
1444331_at	0.00	0.00
1444332_at	0.00	0.00
1444333_at	0.00	0.00
1444334_at	0.00	0.00
1444335_at	0.00	0.00
1444336_at	0.00	0.00
1444337_at	0.00	0.00
1444338_at	0.00	0.00
1444339_at	0.00	0.00
1444340_at	0.00	0.00
1444341_at	0.00	0.00
1444342_at	0.00	0.00
1444343_at	0.00	0.00
1444344_at	0.00	0.00
1444345_at	0.00	0.00
1444346_at	0.00	0.00

1444347_at	0.00	0.00
1444348_at	0.00	0.00
1444349_at	0.00	0.00
1444350_at	0.00	0.00
1444351_at	0.00	0.00
1444352_at	0.00	0.00
1444353_at	0.00	0.00
1444354_at	0.00	0.00
1444355_at	0.00	0.00
1444356_at	0.00	0.00
1444357_at	0.00	0.00
1444358_at	0.00	0.00
1444359_at	0.00	0.00
1444360_at	0.00	0.00
1444361_at	0.00	0.00
1444362_at	0.00	0.00
1444363_at	0.00	0.00
1444364_at	0.00	0.00
1444365_at	0.00	0.00
1444366_at	0.00	0.00
1444367_at	0.00	0.00
1444368_at	0.00	0.00
1444369_at	0.00	0.00
1444370_at	0.00	0.00
1444371_at	0.00	0.00
1444372_at	0.00	0.00
1444373_at	0.00	0.00
1444374_at	0.00	0.00
1444375_at	0.00	0.00
1444376_at	0.00	0.00
1444377_at	0.00	0.00
1444378_at	0.00	0.00
1444379_at	0.00	0.00
1444380_at	0.00	0.00
1444381_at	0.00	0.00
1444382_at	0.00	0.00
1444383_at	0.00	0.00
1444384_at	0.00	0.00
1444385_at	0.00	0.00
1444386_at	0.00	0.00
1444387_at	0.00	0.00
1444388_at	0.00	0.00
1444389_at	0.00	0.00
1444390_at	0.20	0.00
1444391_at	0.00	0.00
1444392_at	0.00	0.00
1444393_at	0.00	0.00
1444394_at	0.00	0.00
1444395_at	0.00	0.00
1444396_at	0.00	0.00
1444397_at	0.00	0.00
1444398_at	0.00	0.00
1444399_at	0.00	0.00
1444400_at	0.00	0.00
1444401_at	0.00	0.00
1444402_at	0.00	0.00

1444403_at	0.00	0.00
1444404_at	0.00	0.00
1444405_at	0.00	0.00
1444406_at	0.00	0.00
1444407_at	0.00	0.00
1444408_at	0.00	0.00
1444409_at	0.00	0.00
1444410_at	0.00	0.00
1444411_at	0.00	0.00
1444412_at	0.00	0.00
1444413_at	0.00	0.00
1444414_at	0.00	0.00
1444415_at	0.00	0.00
1444416_at	0.00	0.00
1444417_at	0.00	0.00
1444418_at	0.00	0.00
1444419_at	0.00	0.00
1444420_at	0.00	0.00
1444421_at	0.00	0.00
1444422_at	0.00	0.00
1444423_at	0.00	0.00
1444424_at	0.00	0.00
1444425_at	0.00	0.00
1444426_at	0.00	0.00
1444427_at	0.00	0.00
1444428_at	0.00	0.00
1444429_at	0.00	0.00
1444430_at	0.00	0.00
1444431_at	0.00	0.00
1444432_at	0.00	0.00
1444433_at	0.00	0.00
1444434_at	0.00	0.00
1444435_at	0.00	0.00
1444436_at	0.00	0.00
1444437_at	0.00	0.00
1444438_at	0.00	0.00
1444439_at	0.00	0.00
1444440_at	0.00	0.00
1444441_at	0.00	0.00
1444442_at	0.00	0.00
1444443_at	0.00	0.00
1444444_at	0.00	0.00
1444445_at	0.00	0.00
1444446_at	0.00	0.00
1444447_at	0.00	0.00
1444448_at	0.00	0.00
1444449_at	0.00	0.00
1444450_at	0.00	0.00
1444451_at	0.00	0.00
1444452_at	0.00	0.00
1444453_at	0.00	0.00
1444454_at	0.00	0.00
1444455_at	0.00	0.00
1444456_at	0.00	0.00
1444457_at	0.00	0.00
1444458_at	0.00	0.00

1444459_at	0.00	0.00
1444460_at	0.00	0.00
1444461_at	0.00	0.00
1444462_at	0.00	0.00
1444463_at	0.00	0.00
1444464_at	0.00	0.00
1444465_at	0.00	0.00
1444466_at	0.00	0.00
1444467_at	0.00	0.00
1444468_at	0.00	0.00
1444469_at	0.00	0.00
1444470_x_at	0.00	0.00
1444471_at	0.00	0.00
1444472_at	0.00	0.00
1444473_at	0.00	0.00
1444474_at	0.00	0.00
1444475_at	0.00	0.00
1444476_at	0.00	0.00
1444477_at	0.00	0.00
1444478_at	0.00	0.00
1444479_at	0.00	0.00
1444480_at	0.00	0.00
1444481_at	0.00	0.00
1444482_at	0.00	0.00
1444483_at	0.00	0.00
1444484_at	0.00	0.00
1444485_at	0.00	0.00
1444486_at	0.00	0.00
1444487_at	0.00	0.00
1444488_at	0.00	0.00
1444489_at	0.00	0.00
1444490_at	0.00	0.00
1444491_at	0.00	0.00
1444492_at	0.00	0.00
1444493_at	0.00	0.00
1444494_at	0.00	0.00
1444495_at	0.00	0.00
1444496_at	0.00	0.00
1444497_at	0.00	0.00
1444498_at	0.00	0.00
1444499_at	0.00	0.00
1444500_at	0.00	0.00
1444501_at	0.00	0.00
1444502_at	0.00	0.00
1444503_at	0.00	0.00
1444504_at	0.00	0.00
1444505_at	0.00	0.00
1444506_at	0.00	0.00
1444507_at	0.00	0.00
1444509_at	0.00	0.00
1444510_at	0.00	0.00
1444511_at	0.00	0.00
1444512_at	0.00	0.00
1444513_at	0.00	0.00
1444514_at	0.00	0.00
1444515_at	0.00	0.00

1444516_at	0.00	0.00
1444517_at	0.00	0.00
1444518_at	0.00	0.00
1444519_at	0.00	0.00
1444520_at	0.00	0.00
1444521_at	0.00	0.00
1444522_at	0.00	0.00
1444523_s_at	0.00	0.00
1444524_at	0.00	0.00
1444525_at	0.00	0.00
1444526_at	0.00	0.00
1444527_at	0.00	0.00
1444528_at	0.00	0.00
1444529_at	0.00	0.00
1444530_at	0.00	0.00
1444531_at	0.00	0.00
1444532_at	0.00	0.00
1444533_at	0.00	0.00
1444534_at	0.00	0.00
1444535_at	0.00	0.00
1444536_at	0.00	0.00
1444537_at	0.00	0.00
1444538_at	0.00	0.00
1444539_at	0.00	0.00
1444540_at	0.00	0.00
1444541_at	0.00	0.00
1444542_at	0.00	0.00
1444543_at	0.00	0.00
1444544_at	0.00	0.00
1444545_at	0.00	0.00
1444546_at	0.00	0.00
1444547_at	0.00	0.00
1444548_at	0.00	0.00
1444549_at	0.00	0.00
1444550_at	0.00	0.00
1444551_at	0.00	0.00
1444552_at	0.00	0.00
1444553_at	0.00	0.00
1444554_at	0.00	0.00
1444555_at	0.00	0.00
1444556_at	0.00	0.00
1444557_at	0.00	0.00
1444558_at	0.00	0.00
1444559_at	0.00	0.00
1444560_at	0.00	0.00
1444561_at	0.00	0.00
1444562_at	0.00	0.00
1444563_at	0.00	0.00
1444564_at	0.00	0.00
1444565_at	0.00	0.00
1444566_at	0.00	0.00
1444567_at	0.00	0.00
1444568_at	0.00	0.00
1444569_at	0.00	0.00
1444570_at	0.00	0.00
1444571_at	0.00	0.00

1444572_at	0.00	0.00
1444573_at	0.00	0.00
1444574_at	0.00	0.00
1444575_at	0.00	0.00
1444576_at	0.00	0.00
1444577_x_at	0.00	0.00
1444578_at	0.00	0.00
1444579_at	0.00	0.00
1444580_at	0.00	0.00
1444581_at	0.00	0.00
1444582_at	0.00	0.00
1444583_at	0.00	0.00
1444584_at	0.00	0.00
1444585_at	0.00	0.00
1444586_at	0.00	0.00
1444587_at	0.00	0.00
1444588_at	0.00	0.00
1444589_at	0.00	0.00
1444590_at	0.00	0.00
1444591_at	0.00	0.00
1444592_at	0.00	0.00
1444593_at	0.00	0.00
1444594_at	0.00	0.00
1444595_at	0.00	0.00
1444596_at	0.00	0.00
1444597_at	0.00	0.00
1444598_at	0.00	0.00
1444599_at	0.00	0.00
1444600_at	0.00	0.00
1444601_at	0.00	0.00
1444602_at	0.00	0.00
1444603_at	0.00	0.00
1444604_at	0.00	0.00
1444605_at	0.00	0.00
1444606_at	0.00	0.00
1444607_at	0.00	0.00
1444608_at	0.00	0.00
1444609_at	0.00	0.00
1444610_at	0.00	0.00
1444611_at	0.00	0.00
1444612_at	0.00	0.00
1444613_at	0.00	0.00
1444614_x_at	0.00	0.00
1444615_x_at	0.00	0.00
1444616_x_at	0.00	0.00
1444617_at	0.00	0.00
1444618_at	0.00	0.00
1444619_x_at	0.00	0.00
1444620_at	0.00	0.00
1444621_at	0.00	0.00
1444622_at	0.00	0.00
1444623_at	0.00	0.00
1444624_at	0.00	0.00
1444625_at	0.00	0.00
1444626_at	0.00	0.00
1444627_at	0.00	0.00

1444628_at	0.00	0.00
1444629_at	0.00	0.00
1444630_at	0.00	0.00
1444631_at	0.00	0.00
1444632_at	0.00	0.00
1444633_at	0.00	0.00
1444634_at	0.00	0.00
1444635_at	0.00	0.00
1444636_at	0.00	0.00
1444637_at	0.00	0.00
1444638_at	0.00	0.00
1444639_at	0.00	0.00
1444640_at	0.00	0.00
1444641_at	0.00	0.00
1444642_at	0.00	0.00
1444643_at	0.00	0.00
1444644_x_at	0.00	0.00
1444645_at	0.00	0.00
1444646_at	0.00	0.00
1444647_at	0.00	0.00
1444648_at	0.00	0.00
1444649_at	0.00	0.00
1444650_at	0.00	0.00
1444651_at	0.00	0.00
1444652_at	0.00	0.00
1444653_at	0.00	0.00
1444654_at	0.00	0.00
1444655_at	0.00	0.00
1444656_at	0.00	0.00
1444657_at	0.00	0.00
1444658_at	0.00	0.00
1444659_at	0.00	0.00
1444660_at	0.00	0.00
1444661_at	0.00	0.00
1444662_at	0.00	0.00
1444663_at	0.00	0.00
1444664_at	0.00	0.00
1444665_at	0.00	0.00
1444666_at	0.00	0.00
1444667_at	0.00	0.00
1444668_at	0.00	0.00
1444669_at	0.00	0.00
1444670_at	0.00	0.00
1444671_at	0.00	0.00
1444672_at	0.00	0.00
1444673_at	0.00	0.00
1444674_at	0.00	0.00
1444675_at	0.00	0.00
1444676_at	0.00	0.00
1444677_at	0.00	0.00
1444678_at	0.00	0.00
1444679_at	0.00	0.00
1444680_at	0.00	0.00
1444681_at	0.00	0.00
1444682_at	0.00	0.00
1444683_at	0.00	0.00

1444684_at	0.00	0.00
1444685_at	0.00	0.00
1444686_at	0.00	0.00
1444687_at	0.00	0.00
1444688_at	0.00	0.00
1444689_at	0.00	0.00
1444690_at	0.00	0.00
1444691_at	0.00	0.00
1444692_at	0.00	0.00
1444693_at	0.00	0.00
1444694_at	0.00	0.00
1444695_at	0.00	0.00
1444696_at	0.00	0.00
1444697_at	0.00	0.00
1444698_at	0.00	0.00
1444699_at	0.00	0.00
1444700_at	0.00	0.00
1444701_at	0.00	0.00
1444702_at	0.00	0.00
1444703_at	0.00	0.00
1444704_at	0.00	0.00
1444705_at	0.00	0.00
1444706_at	0.00	0.00
1444707_at	0.00	0.00
1444708_at	0.00	0.00
1444709_at	0.00	0.00
1444710_at	0.00	0.00
1444711_at	0.00	0.00
1444712_at	0.00	0.00
1444713_at	0.00	0.00
1444714_at	0.00	0.00
1444715_at	0.00	0.00
1444716_at	0.00	0.00
1444717_at	0.00	0.00
1444718_at	0.00	0.00
1444719_at	0.00	0.00
1444720_at	0.00	0.00
1444721_at	0.00	0.00
1444722_at	0.00	0.00
1444723_at	0.00	0.00
1444724_at	0.00	0.00
1444725_at	0.00	0.00
1444726_at	0.00	0.00
1444727_at	0.00	0.00
1444728_at	0.00	0.00
1444729_at	0.00	0.00
1444730_at	0.00	0.00
1444731_at	0.00	0.00
1444732_at	0.00	0.00
1444733_at	0.00	0.00
1444734_at	0.00	0.00
1444735_at	0.00	0.00
1444736_at	0.00	0.00
1444737_at	0.00	0.00
1444738_at	0.00	0.00
1444739_at	0.00	0.00

1444740_at	0.00	0.00
1444741_at	0.00	0.00
1444742_at	0.00	0.00
1444743_at	0.00	0.00
1444744_at	0.00	0.00
1444745_at	0.00	0.00
1444746_at	0.00	0.00
1444747_at	0.00	0.00
1444748_at	0.00	0.00
1444749_at	0.00	0.00
1444750_at	0.00	0.00
1444751_at	0.00	0.00
1444752_at	0.00	0.00
1444753_at	0.00	0.00
1444754_at	0.00	0.00
1444755_at	0.00	0.00
1444756_at	0.00	0.00
1444757_at	0.00	0.00
1444758_at	0.00	0.00
1444759_at	0.00	0.00
1444760_at	0.00	0.00
1444761_at	0.00	0.00
1444762_at	0.00	0.00
1444763_at	0.00	0.00
1444764_at	0.00	0.00
1444765_at	0.00	0.00
1444766_at	0.00	0.00
1444767_at	0.00	0.00
1444768_at	0.00	0.00
1444769_at	0.00	0.00
1444770_at	0.00	0.00
1444771_at	0.00	0.00
1444772_at	0.00	0.00
1444773_at	0.00	0.00
1444774_at	0.00	0.00
1444775_at	0.00	0.00
1444776_at	0.00	0.00
1444777_at	0.00	0.00
1444778_at	0.00	0.00
1444779_s_at	0.00	0.01
1444780_at	0.00	0.00
1444781_at	0.00	0.00
1444782_at	0.00	0.00
1444783_at	0.00	0.00
1444784_at	0.00	0.00
1444785_at	0.00	0.00
1444786_at	0.00	0.00
1444787_at	0.00	0.00
1444788_x_at	0.00	0.00
1444789_at	0.00	0.00
1444790_at	0.00	0.00
1444791_at	0.00	0.00
1444792_at	0.00	0.00
1444793_at	0.00	0.00
1444794_at	0.00	0.00
1444795_at	0.00	0.00

1444796_at	0.00	0.00
1444797_at	0.00	0.00
1444798_at	0.00	0.00
1444799_at	0.00	0.00
1444800_at	0.00	0.00
1444801_at	0.00	0.00
1444802_at	0.00	0.00
1444803_at	0.00	0.00
1444804_at	0.00	0.00
1444805_at	0.00	0.00
1444806_at	0.00	0.00
1444807_at	0.00	0.00
1444808_at	0.00	0.00
1444809_at	0.00	0.00
1444810_at	0.00	0.00
1444811_at	0.00	0.00
1444812_at	0.00	0.00
1444813_at	0.00	0.00
1444814_at	0.00	0.00
1444815_at	0.00	0.00
1444816_at	0.00	0.00
1444817_at	0.00	0.00
1444818_at	0.00	0.00
1444819_at	0.00	0.00
1444820_at	0.00	0.00
1444821_at	0.00	0.00
1444822_at	0.00	0.00
1444823_at	0.00	0.00
1444824_at	0.00	0.00
1444825_at	0.00	0.00
1444826_at	0.00	0.00
1444827_at	0.00	0.00
1444828_at	0.00	0.00
1444830_at	0.00	0.00
1444831_at	0.00	0.00
1444832_at	0.00	0.00
1444833_at	0.00	0.00
1444834_at	0.00	0.00
1444835_at	0.00	0.00
1444836_at	0.00	0.00
1444837_at	0.00	0.00
1444838_at	0.00	0.00
1444839_at	0.00	0.00
1444840_at	0.00	0.00
1444841_at	0.00	0.00
1444842_at	0.00	0.00
1444843_at	0.00	0.00
1444844_at	0.00	0.00
1444845_at	0.00	0.00
1444846_at	0.00	0.00
1444847_at	0.00	0.00
1444848_at	0.00	0.00
1444849_at	0.00	0.00
1444850_at	0.00	0.00
1444851_at	0.00	0.00
1444852_at	0.00	0.00

1444853_at	0.00	0.00
1444854_at	0.00	0.00
1444855_at	0.00	0.00
1444856_at	0.00	0.00
1444857_at	0.00	0.00
1444858_at	0.00	0.00
1444859_at	0.00	0.00
1444860_at	0.00	0.00
1444861_at	0.00	0.00
1444862_at	0.00	0.00
1444863_at	0.00	0.00
1444864_at	0.00	0.00
1444865_at	0.00	0.00
1444867_at	0.00	0.00
1444868_at	0.00	0.00
1444869_at	0.00	0.00
1444870_at	0.00	0.00
1444871_at	0.00	0.00
1444872_at	0.00	0.00
1444873_at	0.00	0.00
1444874_at	0.00	0.00
1444875_at	0.00	0.00
1444876_at	0.00	0.00
1444877_at	0.00	0.00
1444878_at	0.00	0.00
1444879_at	0.00	0.00
1444880_at	0.00	0.00
1444881_at	0.00	0.00
1444882_at	0.00	0.00
1444883_at	0.00	0.00
1444884_at	0.00	0.00
1444885_at	0.00	0.00
1444886_at	0.00	0.00
1444888_at	0.00	0.00
1444889_at	0.00	0.00
1444890_at	0.00	0.00
1444891_at	0.00	0.00
1444892_at	0.00	0.00
1444893_at	0.00	0.00
1444894_at	0.00	0.00
1444895_at	0.00	0.00
1444896_at	0.00	0.00
1444897_at	0.00	0.00
1444898_at	0.00	0.00
1444899_at	0.00	0.00
1444900_at	0.00	0.00
1444901_at	0.00	0.00
1444902_at	0.00	0.00
1444903_at	0.00	0.00
1444904_at	0.00	0.00
1444905_at	0.00	0.00
1444906_at	0.00	0.00
1444907_at	0.00	0.00
1444908_at	0.00	0.00
1444909_at	0.00	0.00
1444910_at	0.00	0.00

1444911_at	0.00	0.00
1444912_at	0.00	0.00
1444913_at	0.00	0.00
1444914_at	0.00	0.00
1444915_at	0.00	0.00
1444916_at	0.00	0.00
1444917_at	0.00	0.00
1444918_at	0.00	0.00
1444919_at	0.00	0.00
1444920_at	0.00	0.00
1444921_at	0.00	0.00
1444922_at	0.00	0.00
1444923_at	0.00	0.00
1444924_at	0.00	0.00
1444925_at	0.00	0.00
1444926_at	0.00	0.00
1444927_at	0.00	0.00
1444928_at	0.00	0.00
1444929_at	0.00	0.00
1444930_at	0.00	0.00
1444931_at	0.00	0.00
1444932_at	0.00	0.00
1444933_at	0.00	0.00
1444934_at	0.00	0.00
1444935_at	0.00	0.00
1444936_at	0.00	0.00
1444937_at	0.00	0.00
1444938_at	0.00	0.00
1444939_at	0.00	0.00
1444940_at	0.00	0.00
1444941_at	0.00	0.00
1444942_at	0.00	0.00
1444944_at	0.00	0.00
1444945_at	0.00	0.00
1444946_at	0.00	0.00
1444947_at	0.00	0.00
1444948_at	0.00	0.00
1444949_at	0.00	0.00
1444950_at	0.00	0.00
1444951_at	0.00	0.00
1444954_at	0.00	0.00
1444955_at	0.00	0.00
1444956_at	0.00	0.00
1444957_at	0.00	0.00
1444958_at	0.00	0.00
1444960_at	0.00	0.00
1444961_at	0.00	0.00
1444962_at	0.00	0.00
1444963_at	0.00	0.00
1444964_at	0.00	0.00
1444965_at	0.00	0.00
1444966_at	0.00	0.00
1444967_at	0.00	0.00
1444968_at	0.00	0.00
1444969_at	0.00	0.00
1444970_at	0.00	0.00

1444971_at	0.00	0.00
1444972_at	0.00	0.00
1444973_at	0.00	0.00
1444974_at	0.00	0.00
1444975_at	0.00	0.00
1444976_at	0.00	0.00
1444977_at	0.00	0.00
1444978_at	0.00	0.00
1444979_at	0.00	0.00
1444980_at	0.00	0.00
1444981_at	0.00	0.00
1444982_at	0.00	0.00
1444983_at	0.00	0.00
1444984_at	0.00	0.00
1444985_at	0.00	0.00
1444986_at	0.00	0.00
1444987_at	0.00	0.00
1444988_at	0.00	0.00
1444989_at	0.00	0.00
1444990_at	0.00	0.00
1444991_at	0.00	0.00
1444992_at	0.00	0.00
1444993_at	0.00	0.00
1444994_at	0.00	0.00
1444995_at	0.00	0.00
1444996_at	0.00	0.00
1444997_at	0.00	0.00
1444998_at	0.00	0.00
1444999_at	0.00	0.00
1445000_at	0.00	0.00
1445001_at	0.00	0.00
1445002_at	0.00	0.00
1445003_at	0.00	0.00
1445004_a_at	0.00	0.00
1445005_at	0.00	0.00
1445006_at	0.00	0.00
1445007_at	0.00	0.00
1445008_at	0.00	0.00
1445009_at	0.00	0.00
1445010_at	0.00	0.00
1445011_at	0.00	0.00
1445012_at	0.00	0.00
1445013_at	0.00	0.00
1445014_at	0.00	0.00
1445015_at	0.00	0.00
1445016_at	0.00	0.00
1445017_at	0.00	0.00
1445018_at	0.00	0.00
1445019_at	0.00	0.00
1445020_at	0.00	0.00
1445021_at	0.00	0.00
1445022_at	0.00	0.00
1445023_at	0.00	0.00
1445024_at	0.00	0.00
1445025_at	0.00	0.00
1445026_at	0.00	0.00

1445027_at	0.00	0.00
1445028_at	0.00	0.00
1445029_at	0.00	0.00
1445030_at	0.00	0.00
1445031_at	0.00	0.00
1445032_at	0.00	0.00
1445033_at	0.00	0.00
1445034_at	0.00	0.00
1445035_at	0.00	0.00
1445036_at	0.00	0.00
1445037_at	0.00	0.00
1445038_at	0.00	0.00
1445039_at	0.00	0.00
1445040_at	0.00	0.00
1445041_at	0.00	0.00
1445042_at	0.00	0.00
1445043_at	0.00	0.00
1445044_at	0.00	0.00
1445045_at	0.00	0.00
1445046_at	0.00	0.00
1445048_at	0.00	0.00
1445049_at	0.00	0.00
1445050_at	0.00	0.00
1445051_at	0.00	0.00
1445052_at	0.00	0.00
1445053_at	0.00	0.00
1445054_at	0.00	0.00
1445055_at	0.00	0.00
1445056_at	0.00	0.00
1445057_at	0.00	0.00
1445058_at	0.00	0.00
1445059_at	0.00	0.00
1445060_at	0.00	0.00
1445061_at	0.00	0.00
1445062_at	0.00	0.00
1445063_at	0.00	0.00
1445064_at	0.00	0.00
1445065_at	0.00	0.00
1445066_at	0.00	0.00
1445067_at	0.00	0.00
1445068_at	0.00	0.00
1445069_at	0.00	0.00
1445070_at	0.00	0.00
1445071_at	0.00	0.00
1445072_at	0.00	0.00
1445073_at	0.00	0.00
1445074_at	0.00	0.00
1445075_at	0.00	0.00
1445076_at	0.00	0.00
1445077_at	0.00	0.00
1445078_at	0.00	0.00
1445079_at	0.00	0.00
1445080_at	0.00	0.00
1445081_at	0.00	0.00
1445082_at	0.00	0.00
1445083_at	0.00	0.00

1445084_at	0.00	0.00
1445085_at	0.00	0.00
1445086_at	0.00	0.00
1445087_at	0.00	0.00
1445088_at	0.00	0.00
1445089_at	0.00	0.00
1445090_at	0.00	0.00
1445091_at	0.00	0.00
1445092_at	0.00	0.00
1445093_at	0.00	0.00
1445094_at	0.00	0.00
1445095_at	0.00	0.00
1445096_at	0.00	0.00
1445097_at	0.00	0.00
1445098_at	0.00	0.00
1445099_at	0.00	0.00
1445100_at	0.00	0.00
1445101_at	0.00	0.00
1445102_at	0.00	0.00
1445103_at	0.00	0.00
1445104_at	0.00	0.00
1445105_at	0.00	0.00
1445106_at	0.00	0.00
1445107_at	0.00	0.00
1445108_at	0.00	0.00
1445109_at	0.00	0.00
1445110_at	0.00	0.00
1445111_at	0.00	0.00
1445112_at	0.00	0.00
1445113_at	0.00	0.00
1445114_at	0.00	0.00
1445115_at	0.00	0.00
1445117_at	0.00	0.00
1445118_at	0.00	0.00
1445119_at	0.00	0.00
1445120_at	0.00	0.00
1445121_at	0.00	0.00
1445122_at	0.00	0.00
1445123_at	0.00	0.00
1445124_at	0.00	0.00
1445126_at	0.00	0.00
1445127_at	0.00	0.00
1445128_at	0.00	0.00
1445129_at	0.00	0.00
1445130_at	0.00	0.00
1445131_at	0.00	0.00
1445132_at	0.00	0.00
1445133_at	0.00	0.00
1445134_at	0.00	0.00
1445135_at	0.00	0.00
1445136_at	0.00	0.00
1445137_at	0.00	0.00
1445138_at	0.00	0.00
1445139_at	0.00	0.00
1445140_at	0.00	0.00
1445141_at	0.00	0.00

1445142_at	0.00	0.00
1445143_at	0.00	0.00
1445144_at	0.00	0.00
1445145_at	0.00	0.00
1445146_at	0.00	0.00
1445147_at	0.00	0.00
1445148_at	0.00	0.00
1445149_at	0.00	0.00
1445150_at	0.00	0.00
1445151_at	0.00	0.00
1445152_at	0.00	0.00
1445153_at	0.00	0.00
1445154_at	0.00	0.00
1445155_at	0.00	0.00
1445156_at	0.00	0.00
1445157_at	0.00	0.00
1445158_at	0.00	0.00
1445159_at	0.00	0.00
1445160_at	0.00	0.00
1445161_at	0.00	0.00
1445162_at	0.00	0.00
1445163_at	0.00	0.00
1445164_at	0.00	0.00
1445165_at	0.00	0.00
1445166_at	0.00	0.00
1445167_at	0.00	0.00
1445168_at	0.00	0.00
1445169_at	0.00	0.00
1445170_at	0.00	0.00
1445171_at	0.00	0.00
1445173_at	0.00	0.00
1445174_at	0.00	0.00
1445175_at	0.00	0.00
1445176_at	0.00	0.00
1445177_at	0.00	0.00
1445178_at	0.00	0.00
1445179_at	0.00	0.00
1445180_at	0.00	0.00
1445181_at	0.00	0.00
1445182_at	0.00	0.00
1445183_s_at	0.00	0.00
1445184_at	0.00	0.00
1445185_at	0.00	0.00
1445186_at	0.00	0.00
1445187_at	0.00	0.00
1445188_at	0.00	0.00
1445189_at	0.00	0.00
1445190_at	0.00	0.00
1445191_at	0.00	0.00
1445192_at	0.00	0.00
1445193_x_at	0.00	0.00
1445194_at	0.00	0.00
1445195_at	0.00	0.00
1445196_at	0.00	0.00
1445197_x_at	0.00	0.00
1445198_at	0.00	0.00

1445199_at	0.00	0.00
1445200_at	0.00	0.00
1445201_at	0.00	0.00
1445202_at	0.00	0.00
1445203_at	0.00	0.00
1445204_at	0.00	0.00
1445205_at	0.00	0.00
1445206_at	0.00	0.00
1445207_at	0.00	0.00
1445208_at	0.00	0.00
1445209_at	0.00	0.00
1445210_at	0.00	0.00
1445211_at	0.00	0.00
1445212_at	0.00	0.00
1445213_at	0.00	0.00
1445214_at	0.00	0.00
1445215_at	0.00	0.00
1445216_at	0.00	0.00
1445217_at	0.00	0.00
1445218_at	0.00	0.00
1445219_at	0.00	0.00
1445220_at	0.00	0.00
1445221_at	0.00	0.00
1445222_at	0.00	0.00
1445223_at	0.00	0.00
1445224_at	0.00	0.00
1445225_at	0.00	0.00
1445226_at	0.00	0.00
1445227_at	0.00	0.00
1445228_at	0.00	0.00
1445229_at	0.00	0.00
1445230_at	0.00	0.00
1445231_at	0.00	0.00
1445232_at	0.00	0.00
1445233_at	0.00	0.00
1445234_at	0.00	0.00
1445235_at	0.00	0.00
1445236_at	0.00	0.00
1445237_at	0.00	0.00
1445238_at	0.00	0.00
1445239_at	0.00	0.00
1445240_at	0.00	0.00
1445241_at	0.00	0.00
1445242_at	0.00	0.00
1445243_at	0.00	0.00
1445244_at	0.00	0.00
1445245_at	0.00	0.00
1445246_at	0.00	0.00
1445247_at	0.00	0.00
1445248_at	0.00	0.00
1445249_at	0.00	0.00
1445250_at	0.00	0.00
1445251_at	0.00	0.00
1445252_at	0.00	0.00
1445253_at	0.00	0.00
1445254_at	0.00	0.00

1445255_at	0.00	0.00
1445256_at	0.00	0.00
1445257_at	0.00	0.00
1445258_at	0.00	0.00
1445259_at	0.00	0.00
1445260_at	0.00	0.00
1445261_at	0.00	0.00
1445262_at	0.00	0.00
1445263_at	0.00	0.00
1445264_at	0.00	0.00
1445265_at	0.00	0.00
1445266_at	0.00	0.00
1445267_at	0.00	0.00
1445268_at	0.00	0.00
1445269_at	0.00	0.00
1445270_at	0.00	0.00
1445271_at	0.00	0.00
1445272_at	0.00	0.00
1445273_at	0.00	0.00
1445274_at	0.00	0.00
1445275_at	0.00	0.00
1445276_at	0.00	0.00
1445277_at	0.00	0.00
1445278_at	0.00	0.00
1445279_at	0.00	0.00
1445280_at	0.00	0.00
1445281_a_at	0.00	0.00
1445282_at	0.00	0.00
1445283_at	0.00	0.00
1445284_at	0.00	0.00
1445285_at	0.00	0.00
1445286_at	0.00	0.00
1445287_at	0.00	0.00
1445288_at	0.00	0.00
1445289_at	0.00	0.00
1445290_at	0.00	0.00
1445291_at	0.00	0.00
1445292_at	0.00	0.00
1445293_at	0.00	0.00
1445294_at	0.00	0.00
1445295_at	0.00	0.00
1445296_at	0.00	0.00
1445297_at	0.00	0.00
1445298_at	0.00	0.00
1445299_at	0.00	0.00
1445300_at	0.00	0.00
1445301_at	0.00	0.00
1445302_at	0.00	0.00
1445303_at	0.00	0.00
1445304_at	0.00	0.00
1445305_at	0.00	0.00
1445306_at	0.00	0.00
1445307_at	0.00	0.00
1445308_at	0.00	0.00
1445309_at	0.00	0.00
1445310_at	0.00	0.00

1445311_at	0.00	0.00
1445312_at	0.00	0.00
1445313_at	0.00	0.00
1445314_at	0.00	0.00
1445315_at	0.00	0.00
1445316_at	0.00	0.00
1445317_at	0.00	0.00
1445318_at	0.00	0.00
1445319_at	0.00	0.00
1445320_at	0.00	0.00
1445321_at	0.00	0.00
1445322_x_at	0.00	0.00
1445323_at	0.00	0.00
1445324_s_at	0.00	0.00
1445325_at	0.00	0.00
1445326_at	0.00	0.00
1445327_at	0.00	0.00
1445328_at	0.00	0.00
1445329_at	0.00	0.00
1445330_at	0.00	0.00
1445331_at	0.00	0.00
1445332_at	0.00	0.00
1445333_at	0.00	0.00
1445334_at	0.00	0.00
1445335_at	0.00	0.00
1445336_at	0.00	0.00
1445337_at	0.00	0.00
1445338_at	0.00	0.00
1445339_at	0.00	0.00
1445340_at	0.00	0.00
1445341_at	0.00	0.00
1445342_at	0.00	0.00
1445343_at	0.00	0.00
1445344_at	0.00	0.00
1445345_at	0.00	0.00
1445346_at	0.00	0.00
1445347_at	0.00	0.00
1445348_at	0.00	0.00
1445349_at	0.00	0.00
1445350_at	0.00	0.00
1445351_at	0.00	0.00
1445352_at	0.00	0.00
1445353_at	0.00	0.00
1445354_at	0.00	0.00
1445355_at	0.00	0.00
1445356_at	0.00	0.00
1445357_at	0.00	0.00
1445358_at	0.00	0.00
1445359_at	0.00	0.00
1445360_at	0.00	0.00
1445361_at	0.00	0.00
1445362_at	0.00	0.00
1445363_at	0.00	0.00
1445364_at	0.00	0.00
1445365_at	0.00	0.00
1445366_at	0.00	0.00

1445368_at	0.72	0.00
1445369_at	0.00	0.00
1445370_at	0.00	0.00
1445371_at	0.00	0.00
1445372_at	0.00	0.00
1445373_at	0.00	0.00
1445374_at	0.00	0.00
1445375_at	0.00	0.00
1445376_at	0.00	0.00
1445377_at	0.00	0.00
1445378_at	0.00	0.00
1445379_at	0.00	0.00
1445380_at	0.00	0.00
1445381_at	0.00	0.00
1445382_at	0.00	0.00
1445383_at	0.00	0.00
1445384_at	0.00	0.00
1445385_at	0.00	0.00
1445386_at	0.00	0.00
1445387_at	0.00	0.00
1445388_at	0.00	0.00
1445389_at	0.00	0.00
1445390_at	0.00	0.00
1445391_at	0.00	0.00
1445392_at	0.00	0.00
1445393_at	0.00	0.00
1445394_at	0.00	0.00
1445395_at	0.00	0.00
1445396_at	0.00	0.00
1445397_at	0.00	0.00
1445398_at	0.00	0.00
1445399_at	0.00	0.00
1445400_at	0.00	0.00
1445401_at	0.00	0.00
1445402_at	0.00	0.00
1445403_at	0.00	0.00
1445404_at	0.00	0.00
1445405_at	0.00	0.00
1445406_at	0.00	0.00
1445407_at	0.00	0.00
1445408_at	0.00	0.00
1445409_at	0.00	0.00
1445410_at	0.00	0.00
1445411_at	0.00	0.00
1445412_at	0.00	0.00
1445413_at	0.00	0.00
1445414_at	0.00	0.00
1445415_at	0.00	0.00
1445416_at	0.00	0.00
1445417_at	0.00	0.00
1445418_at	0.00	0.00
1445419_at	0.00	0.00
1445420_at	0.00	0.00
1445421_at	0.00	0.00
1445422_at	0.00	0.00
1445423_at	0.00	0.00

1445424_at	0.00	0.00
1445425_at	0.00	0.00
1445426_at	0.00	0.00
1445427_at	0.00	0.00
1445428_at	0.00	0.00
1445429_at	0.00	0.00
1445430_at	0.00	0.00
1445431_at	0.00	0.00
1445432_at	0.00	0.00
1445433_at	0.00	0.00
1445434_at	0.00	0.00
1445435_at	0.00	0.00
1445436_at	0.00	0.00
1445437_at	0.00	0.00
1445438_at	0.00	0.00
1445439_at	0.00	0.00
1445440_at	0.00	0.00
1445441_at	0.00	0.00
1445442_at	0.00	0.00
1445443_at	0.00	0.00
1445444_at	0.00	0.00
1445445_s_at	0.00	0.00
1445446_at	0.00	0.00
1445447_at	0.00	0.00
1445448_at	0.00	0.00
1445449_at	0.00	0.00
1445450_x_at	0.00	0.00
1445451_at	0.00	0.00
1445452_at	0.00	0.00
1445453_at	0.00	0.00
1445454_at	0.00	0.00
1445455_at	0.00	0.00
1445456_at	0.00	0.00
1445457_at	0.00	0.00
1445458_at	0.00	0.00
1445460_at	0.00	0.00
1445461_at	0.00	0.00
1445462_at	0.00	0.00
1445463_at	0.00	0.00
1445464_at	0.00	0.00
1445465_at	0.00	0.00
1445466_at	0.00	0.00
1445467_at	0.00	0.00
1445468_at	0.00	0.00
1445469_at	0.00	0.00
1445470_at	0.00	0.00
1445471_at	0.00	0.00
1445472_at	0.00	0.00
1445473_at	0.00	0.00
1445474_at	0.00	0.00
1445475_at	0.00	0.00
1445476_at	0.00	0.00
1445477_at	0.00	0.00
1445478_at	0.00	0.00
1445479_at	0.00	0.00
1445480_at	0.00	0.00

1445481_at	0.00	0.00
1445482_at	0.00	0.00
1445483_at	0.00	0.00
1445484_at	0.00	0.00
1445486_at	0.00	0.00
1445487_at	0.00	0.00
1445488_at	0.00	0.00
1445489_at	0.00	0.00
1445490_at	0.00	0.00
1445491_at	0.00	0.00
1445492_at	0.00	0.00
1445493_at	0.00	0.00
1445494_at	0.00	0.00
1445495_at	0.00	0.00
1445496_at	0.00	0.00
1445497_at	0.00	0.00
1445498_at	0.00	0.00
1445499_at	0.00	0.00
1445500_at	0.00	0.00
1445501_at	0.00	0.00
1445502_at	0.00	0.00
1445503_at	0.00	0.00
1445504_at	0.00	0.00
1445505_at	0.00	0.00
1445506_at	0.00	0.00
1445507_at	0.00	0.00
1445508_at	0.00	0.00
1445509_at	0.00	0.00
1445510_at	0.00	0.00
1445511_at	0.00	0.00
1445512_at	0.00	0.00
1445513_at	0.00	0.00
1445514_at	0.00	0.00
1445515_at	0.00	0.00
1445516_at	0.00	0.00
1445517_at	0.00	0.00
1445518_at	0.00	0.00
1445519_at	0.00	0.00
1445520_at	0.00	0.00
1445521_at	0.00	0.00
1445522_at	0.00	0.00
1445523_at	0.00	0.00
1445524_at	0.00	0.00
1445525_at	0.00	0.00
1445526_at	0.00	0.00
1445527_at	0.00	0.00
1445528_at	0.00	0.00
1445529_at	0.00	0.00
1445530_at	0.00	0.00
1445531_at	0.00	0.00
1445532_at	0.00	0.00
1445533_at	0.00	0.00
1445534_at	0.11	0.00
1445536_at	0.00	0.00
1445537_at	0.00	0.00
1445538_at	0.00	0.00

1445539_at	0.00	0.00
1445540_at	0.00	0.00
1445541_at	0.00	0.00
1445542_at	0.00	0.00
1445543_at	0.00	0.00
1445544_x_at	0.00	0.00
1445545_at	0.00	0.00
1445546_at	0.00	0.00
1445547_at	0.00	0.00
1445548_at	0.00	0.00
1445549_at	0.00	0.00
1445550_at	0.00	0.00
1445551_at	0.00	0.00
1445552_at	0.00	0.00
1445553_at	0.00	0.00
1445554_at	0.00	0.00
1445555_at	0.00	0.00
1445556_at	0.00	0.00
1445557_at	0.00	0.00
1445558_at	0.00	0.00
1445559_at	0.00	0.00
1445560_at	0.00	0.00
1445561_at	0.00	0.00
1445562_at	0.00	0.00
1445563_at	0.00	0.00
1445564_at	0.00	0.00
1445565_at	0.00	0.00
1445566_at	0.00	0.00
1445567_at	0.00	0.00
1445568_at	0.00	0.00
1445569_at	0.00	0.00
1445570_at	0.00	0.00
1445571_at	0.00	0.00
1445572_at	0.00	0.00
1445573_at	0.00	0.00
1445574_at	0.00	0.00
1445575_at	0.00	0.00
1445576_at	0.00	0.00
1445577_at	0.00	0.00
1445578_at	0.00	0.00
1445579_at	0.00	0.00
1445580_at	0.00	0.00
1445581_at	0.00	0.00
1445582_at	0.00	0.00
1445583_x_at	0.00	0.00
1445584_at	0.00	0.00
1445585_at	0.00	0.00
1445586_at	0.00	0.00
1445587_at	0.00	0.00
1445588_at	0.00	0.00
1445589_at	0.00	0.00
1445590_at	0.00	0.00
1445591_at	0.00	0.00
1445592_at	0.00	0.00
1445593_at	0.00	0.00
1445594_at	0.00	0.00

1445595_at	0.00	0.00
1445596_at	0.00	0.00
1445597_s_at	0.00	0.00
1445598_at	0.00	0.00
1445599_at	0.00	0.00
1445600_at	0.00	0.00
1445601_at	0.00	0.00
1445602_at	0.00	0.00
1445603_at	0.00	0.00
1445604_at	0.00	0.00
1445605_s_at	0.00	0.00
1445606_a_at	0.00	0.00
1445607_at	0.00	0.00
1445608_at	0.00	0.00
1445609_at	0.00	0.00
1445610_at	0.00	0.00
1445611_at	0.00	0.00
1445612_at	0.00	0.00
1445613_at	0.00	0.00
1445614_at	0.00	0.00
1445615_at	0.00	0.00
1445616_at	0.00	0.00
1445617_at	0.00	0.00
1445618_at	0.00	0.00
1445619_at	0.00	0.00
1445620_at	0.00	0.00
1445621_at	0.00	0.00
1445622_at	0.00	0.00
1445623_at	0.00	0.00
1445624_at	0.00	0.00
1445625_at	0.00	0.00
1445626_at	0.00	0.00
1445627_at	0.00	0.00
1445628_at	0.00	0.00
1445629_at	0.00	0.00
1445630_at	0.00	0.00
1445631_at	0.00	0.00
1445632_at	0.00	0.00
1445633_at	0.00	0.00
1445634_at	0.00	0.00
1445635_at	0.00	0.00
1445636_at	0.00	0.00
1445637_at	0.00	0.00
1445638_at	0.00	0.00
1445639_at	0.00	0.00
1445640_at	0.00	0.00
1445641_at	0.00	0.00
1445642_at	0.00	0.00
1445643_at	0.00	0.00
1445644_at	0.00	0.00
1445645_at	0.00	0.00
1445646_at	0.00	0.00
1445647_at	0.00	0.00
1445648_at	0.00	0.00
1445649_x_at	0.00	0.00
1445650_at	0.00	0.00

1445651_at	0.00	0.00
1445652_at	0.00	0.00
1445653_at	0.00	0.00
1445654_at	0.00	0.00
1445655_at	0.00	0.00
1445656_at	0.00	0.00
1445657_at	0.00	0.00
1445658_at	0.00	0.00
1445659_at	0.00	0.00
1445660_at	0.00	0.00
1445661_at	0.00	0.00
1445662_x_at	0.00	0.00
1445663_at	0.00	0.00
1445664_at	0.00	0.00
1445665_at	0.00	0.00
1445666_at	0.00	0.00
1445667_at	0.00	0.00
1445668_at	0.00	0.00
1445669_at	0.00	0.01
1445670_at	0.00	0.00
1445671_at	0.00	0.00
1445672_at	0.00	0.00
1445673_at	0.00	0.00
1445674_at	0.00	0.00
1445675_at	0.00	0.00
1445676_at	0.00	0.00
1445677_x_at	0.00	0.00
1445678_at	0.00	0.00
1445679_at	0.00	0.00
1445680_x_at	0.00	0.00
1445681_at	0.00	0.00
1445682_at	0.00	0.00
1445683_at	0.00	0.00
1445684_s_at	0.00	0.00
1445685_at	0.00	0.00
1445686_at	0.00	0.00
1445687_at	0.00	0.00
1445688_at	0.00	0.00
1445690_at	0.00	0.00
1445691_at	0.00	0.00
1445692_x_at	0.00	0.00
1445693_at	0.00	0.00
1445694_at	0.00	0.00
1445695_at	0.00	0.00
1445696_x_at	0.00	0.00
1445697_at	0.00	0.00
1445698_at	0.00	0.00
1445699_at	0.00	0.00
1445700_at	0.00	0.00
1445701_at	0.00	0.00
1445702_x_at	0.00	0.00
1445703_at	0.00	0.00
1445704_x_at	0.00	0.00
1445705_x_at	0.00	0.00
1445706_x_at	0.00	0.00
1445707_at	0.00	0.00

1445708_x_at	0.00	0.00
1445709_at	0.00	0.00
1445710_x_at	0.00	0.00
1445711_at	0.00	0.00
1445712_at	0.00	0.00
1445713_at	0.00	0.00
1445714_at	0.00	0.00
1445715_at	0.00	0.00
1445716_at	0.00	0.00
1445717_at	0.00	0.00
1445718_at	0.00	0.00
1445719_at	0.00	0.00
1445720_at	0.00	0.00
1445721_at	0.00	0.00
1445722_at	0.00	0.00
1445723_at	0.00	0.00
1445724_at	0.00	0.00
1445725_at	0.00	0.00
1445726_at	0.00	0.00
1445727_at	0.00	0.00
1445728_at	0.00	0.00
1445729_at	0.00	0.00
1445730_at	0.00	0.00
1445731_at	0.00	0.00
1445732_at	0.00	0.00
1445733_at	0.00	0.00
1445734_at	0.00	0.00
1445735_at	0.00	0.00
1445736_at	0.00	0.00
1445737_at	0.00	0.00
1445738_at	0.00	0.00
1445739_at	0.00	0.00
1445740_at	0.00	0.00
1445741_at	0.00	0.00
1445742_at	0.00	0.00
1445743_at	0.00	0.00
1445744_at	0.00	0.00
1445745_at	0.00	0.00
1445746_at	0.00	0.00
1445747_at	0.00	0.00
1445748_at	0.00	0.00
1445749_at	0.00	0.00
1445750_at	0.00	0.00
1445751_at	0.00	0.00
1445752_at	0.00	0.00
1445753_at	0.00	0.00
1445754_at	0.00	0.00
1445755_at	0.00	0.00
1445756_at	0.00	0.00
1445757_at	0.00	0.00
1445758_at	0.00	0.00
1445759_at	0.00	0.00
1445760_at	0.00	0.00
1445761_at	0.00	0.00
1445762_at	0.00	0.00
1445763_at	0.00	0.00

1445764_at	0.00	0.00
1445765_at	0.00	0.00
1445766_at	0.00	0.00
1445767_at	0.00	0.00
1445768_at	0.00	0.00
1445769_at	0.00	0.00
1445770_at	0.00	0.00
1445771_at	0.00	0.00
1445772_at	0.00	0.00
1445773_at	0.00	0.00
1445774_at	0.00	0.00
1445775_at	0.00	0.00
1445776_at	0.00	0.00
1445777_at	0.00	0.00
1445778_at	0.00	0.00
1445779_at	0.00	0.00
1445780_at	0.00	0.00
1445781_at	0.00	0.00
1445782_at	0.00	0.00
1445783_at	0.00	0.00
1445784_at	0.00	0.00
1445785_at	0.00	0.00
1445786_at	0.00	0.00
1445787_at	0.00	0.00
1445788_at	0.00	0.00
1445789_at	0.00	0.00
1445790_at	0.00	0.00
1445791_at	0.00	0.00
1445792_at	0.00	0.00
1445793_at	0.00	0.00
1445794_at	0.00	0.00
1445795_at	0.00	0.00
1445796_at	0.00	0.00
1445797_at	0.00	0.00
1445798_at	0.00	0.00
1445799_at	0.00	0.00
1445800_at	0.00	0.00
1445801_at	0.00	0.00
1445802_at	0.00	0.00
1445803_at	0.00	0.00
1445804_at	0.00	0.00
1445805_x_at	0.00	0.00
1445806_at	0.00	0.00
1445807_at	0.00	0.00
1445808_at	0.00	0.00
1445809_at	0.00	0.00
1445810_at	0.00	0.00
1445811_at	0.00	0.00
1445812_at	0.00	0.00
1445813_at	0.00	0.00
1445814_at	0.00	0.00
1445815_at	0.00	0.00
1445816_at	0.00	0.00
1445817_at	0.00	0.00
1445818_at	0.00	0.00
1445819_at	0.00	0.00

1445820_at	0.00	0.00
1445821_at	0.00	0.00
1445822_at	0.00	0.00
1445823_at	0.00	0.00
1445824_at	0.00	0.00
1445825_at	0.00	0.00
1445826_at	0.00	0.00
1445827_at	0.00	0.00
1445828_at	0.00	0.00
1445829_at	0.00	0.00
1445830_at	0.00	0.00
1445831_at	0.00	0.00
1445832_at	0.00	0.00
1445833_at	0.00	0.00
1445834_at	0.00	0.00
1445835_at	0.00	0.00
1445836_at	0.00	0.00
1445837_at	0.00	0.00
1445838_at	0.00	0.00
1445839_at	0.00	0.00
1445840_at	0.00	0.00
1445841_at	0.00	0.00
1445842_at	0.00	0.00
1445843_at	0.00	0.00
1445844_at	0.00	0.00
1445845_at	0.00	0.00
1445846_at	0.00	0.00
1445847_at	0.00	0.00
1445848_at	0.00	0.00
1445849_at	0.00	0.00
1445850_at	0.00	0.00
1445851_at	0.00	0.00
1445852_at	0.00	0.00
1445853_at	0.00	0.00
1445854_at	0.00	0.00
1445855_at	0.00	0.00
1445856_at	0.00	0.00
1445857_at	0.00	0.00
1445858_at	0.00	0.00
1445859_at	0.00	0.00
1445860_at	0.00	0.00
1445861_at	0.00	0.00
1445862_at	0.00	0.00
1445863_at	0.00	0.00
1445864_at	0.00	0.00
1445865_at	0.00	0.00
1445866_at	0.00	0.00
1445867_at	0.00	0.00
1445868_at	0.00	0.00
1445869_at	0.00	0.00
1445870_at	0.00	0.00
1445871_at	0.00	0.00
1445872_at	0.00	0.00
1445873_at	0.00	0.00
1445874_at	0.00	0.00
1445875_at	0.00	0.00

1445876_at	0.00	0.00
1445877_at	0.00	0.00
1445878_at	0.00	0.00
1445879_at	0.00	0.00
1445880_at	0.00	0.00
1445881_at	0.00	0.00
1445882_at	0.00	0.00
1445883_at	0.00	0.00
1445884_at	0.00	0.00
1445885_at	0.00	0.00
1445886_at	0.00	0.00
1445887_at	0.00	0.00
1445888_x_at	0.00	0.00
1445889_at	0.00	0.00
1445890_at	0.00	0.00
1445891_at	0.00	0.00
1445892_at	0.00	0.00
1445893_at	0.00	0.00
1445894_at	0.00	0.00
1445895_at	0.00	0.00
1445896_at	0.00	0.00
1445897_s_at	0.38	0.00
1445898_at	0.00	0.00
1445899_at	0.00	0.00
1445900_at	0.00	0.00
1445901_at	0.00	0.00
1445902_at	0.00	0.00
1445903_at	0.00	0.00
1445904_at	0.00	0.00
1445905_at	0.00	0.00
1445906_at	0.00	0.00
1445907_at	0.00	0.00
1445908_at	0.00	0.00
1445909_at	0.00	0.00
1445910_at	0.00	0.00
1445911_at	0.00	0.00
1445912_at	0.00	0.00
1445913_at	0.00	0.00
1445914_at	0.00	0.00
1445915_at	0.00	0.00
1445916_at	0.00	0.00
1445917_at	0.00	0.00
1445918_at	0.00	0.00
1445919_at	0.00	0.00
1445920_at	0.00	0.00
1445921_at	0.00	0.00
1445922_at	0.00	0.00
1445923_at	0.00	0.00
1445924_at	0.00	0.00
1445925_at	0.00	0.00
1445926_at	0.00	0.00
1445927_at	0.00	0.00
1445928_at	0.00	0.00
1445929_at	0.00	0.00
1445930_at	0.00	0.00
1445931_at	0.00	0.00

1445932_at	0.00	0.00
1445933_at	0.00	0.00
1445934_at	0.00	0.00
1445935_at	0.00	0.00
1445936_at	0.00	0.00
1445937_at	0.00	0.00
1445938_at	0.00	0.00
1445939_at	0.00	0.00
1445940_at	0.00	0.00
1445941_at	0.00	0.00
1445942_at	0.00	0.00
1445943_at	0.00	0.00
1445944_at	0.00	0.00
1445945_at	0.00	0.00
1445946_at	0.00	0.00
1445947_at	0.00	0.00
1445948_at	0.00	0.00
1445949_at	0.00	0.00
1445950_at	0.00	0.00
1445951_at	0.00	0.00
1445952_at	0.00	0.00
1445953_at	0.00	0.00
1445954_at	0.00	0.00
1445955_at	0.00	0.00
1445956_at	0.00	0.00
1445957_at	0.00	0.00
1445958_at	0.00	0.00
1445959_at	0.00	0.00
1445960_at	0.00	0.00
1445961_at	0.00	0.00
1445962_at	0.00	0.00
1445963_at	0.00	0.00
1445964_at	0.00	0.00
1445965_at	0.00	0.00
1445966_at	0.00	0.00
1445967_at	0.00	0.00
1445968_at	0.00	0.00
1445969_at	0.00	0.00
1445970_at	0.00	0.00
1445971_at	0.00	0.00
1445972_at	0.00	0.00
1445973_at	0.00	0.00
1445974_at	0.00	0.00
1445975_at	0.00	0.00
1445976_at	0.00	0.00
1445977_at	0.00	0.00
1445978_at	0.00	0.00
1445979_at	0.00	0.00
1445980_at	0.00	0.00
1445981_at	0.00	0.00
1445982_at	0.00	0.00
1445983_at	0.00	0.00
1445984_at	0.00	0.00
1445985_at	0.00	0.00
1445986_at	0.00	0.00
1445987_at	0.00	0.00

1445988_at	0.00	0.00
1445989_at	0.00	0.00
1445990_at	0.00	0.00
1445991_at	0.00	0.00
1445992_at	0.00	0.00
1445993_at	0.00	0.00
1445994_at	0.00	0.00
1445995_at	0.00	0.00
1445996_at	0.00	0.00
1445997_at	0.00	0.00
1445998_at	0.00	0.00
1445999_at	0.00	0.00
1446000_at	0.00	0.00
1446001_at	0.00	0.00
1446002_at	0.00	0.00
1446003_at	0.00	0.00
1446004_at	0.00	0.00
1446005_at	0.00	0.00
1446006_at	0.00	0.00
1446007_at	0.00	0.00
1446009_at	0.00	0.00
1446010_at	0.00	0.00
1446011_at	0.00	0.00
1446012_at	0.00	0.00
1446013_at	0.00	0.00
1446014_at	0.00	0.00
1446015_at	0.00	0.00
1446016_at	0.00	0.00
1446017_at	0.00	0.00
1446018_at	0.00	0.00
1446019_at	0.00	0.00
1446020_at	0.00	0.00
1446021_at	0.00	0.00
1446022_at	0.00	0.00
1446023_at	0.00	0.00
1446024_at	0.00	0.00
1446025_at	0.00	0.00
1446026_at	0.00	0.00
1446027_at	0.00	0.00
1446028_at	0.00	0.00
1446029_at	0.00	0.00
1446030_at	0.00	0.00
1446031_at	0.00	0.00
1446032_at	0.00	0.00
1446033_at	0.00	0.00
1446034_at	0.00	0.00
1446035_at	0.00	0.00
1446036_at	0.00	0.00
1446037_at	0.00	0.00
1446038_at	0.00	0.00
1446039_at	0.00	0.00
1446040_at	0.00	0.00
1446041_at	0.00	0.00
1446042_at	0.00	0.00
1446043_at	0.00	0.00
1446044_at	0.00	0.00

1446045_at	0.00	0.00
1446046_at	0.00	0.00
1446047_at	0.00	0.00
1446048_at	0.00	0.00
1446049_at	0.00	0.00
1446050_at	0.00	0.00
1446051_at	0.00	0.00
1446052_at	0.00	0.00
1446053_at	0.00	0.00
1446054_at	0.00	0.00
1446055_at	0.00	0.00
1446056_at	0.00	0.00
1446057_at	0.00	0.00
1446058_at	0.00	0.00
1446059_at	0.00	0.00
1446060_at	0.00	0.00
1446061_at	0.00	0.00
1446062_at	0.00	0.00
1446063_at	0.00	0.00
1446064_at	0.00	0.00
1446065_at	0.00	0.00
1446066_at	0.00	0.00
1446067_at	0.00	0.00
1446068_at	0.00	0.00
1446069_at	0.00	0.00
1446070_at	0.00	0.00
1446071_at	0.00	0.00
1446072_at	0.00	0.00
1446073_at	0.00	0.00
1446074_at	0.00	0.00
1446075_at	0.00	0.00
1446076_at	0.00	0.00
1446077_at	0.00	0.00
1446078_at	0.00	0.00
1446079_at	0.00	0.00
1446080_at	0.00	0.00
1446081_at	0.00	0.00
1446082_at	0.00	0.00
1446083_at	0.00	0.00
1446084_at	0.00	0.00
1446085_at	0.00	0.00
1446087_at	0.00	0.00
1446088_at	0.00	0.00
1446089_at	0.00	0.00
1446090_at	0.00	0.00
1446091_at	0.00	0.00
1446092_at	0.00	0.00
1446093_at	0.00	0.00
1446094_at	0.00	0.00
1446095_at	0.00	0.00
1446096_at	0.00	0.00
1446097_at	0.00	0.00
1446098_at	0.00	0.00
1446099_at	0.00	0.00
1446100_at	0.00	0.00
1446101_at	0.00	0.00

1446102_at	0.00	0.00
1446103_at	0.00	0.00
1446104_at	0.00	0.00
1446105_at	0.00	0.00
1446106_at	0.00	0.00
1446107_at	0.00	0.00
1446108_at	0.00	0.00
1446109_at	0.00	0.00
1446110_at	0.00	0.00
1446111_at	0.00	0.00
1446112_at	0.00	0.00
1446113_at	0.00	0.00
1446114_at	0.00	0.00
1446115_at	0.00	0.00
1446116_at	0.00	0.00
1446117_at	0.00	0.00
1446118_at	0.00	0.00
1446119_at	0.00	0.00
1446120_at	0.00	0.00
1446121_at	0.00	0.00
1446122_at	0.00	0.00
1446123_at	0.00	0.00
1446124_at	0.00	0.00
1446125_at	0.00	0.00
1446126_at	0.00	0.00
1446127_at	0.00	0.00
1446128_at	0.00	0.00
1446129_at	0.00	0.00
1446130_at	0.00	0.00
1446131_at	0.00	0.00
1446132_at	0.00	0.00
1446133_at	0.00	0.00
1446134_at	0.00	0.00
1446135_at	0.00	0.00
1446136_at	0.00	0.00
1446137_at	0.00	0.00
1446138_at	0.00	0.00
1446139_at	0.00	0.00
1446140_at	0.00	0.00
1446141_at	0.00	0.00
1446142_at	0.00	0.00
1446143_at	0.00	0.00
1446144_at	0.00	0.00
1446145_at	0.00	0.00
1446146_at	0.00	0.00
1446149_at	0.00	0.00
1446150_at	0.00	0.00
1446151_at	0.00	0.00
1446152_at	0.00	0.00
1446153_at	0.00	0.00
1446154_at	0.00	0.00
1446155_at	0.00	0.00
1446156_at	0.00	0.00
1446157_at	0.00	0.00
1446158_at	0.00	0.00
1446159_at	0.00	0.00

1446160_x_at	0.00	0.07
1446161_at	0.00	0.00
1446162_at	0.00	0.00
1446163_at	0.00	0.00
1446164_at	0.00	0.00
1446166_at	0.00	0.00
1446167_at	0.00	0.00
1446168_at	0.00	0.00
1446169_at	0.00	0.00
1446170_at	0.00	0.00
1446171_at	0.00	0.00
1446172_at	0.00	0.00
1446173_at	0.00	0.00
1446174_at	0.00	0.00
1446175_at	0.00	0.00
1446176_at	0.00	0.00
1446177_at	0.00	0.00
1446178_at	0.00	0.00
1446179_at	0.00	0.00
1446180_at	0.00	0.00
1446181_at	0.00	0.00
1446182_at	0.00	0.00
1446183_at	0.00	0.00
1446184_at	0.00	0.00
1446185_at	0.00	0.00
1446186_at	0.00	0.00
1446187_at	0.00	0.00
1446188_at	0.00	0.00
1446189_at	0.00	0.00
1446190_at	0.00	0.00
1446191_at	0.00	0.00
1446192_at	0.00	0.00
1446193_at	0.00	0.00
1446194_at	0.00	0.00
1446195_at	0.00	0.00
1446196_at	0.00	0.00
1446197_at	0.00	0.00
1446198_at	0.00	0.00
1446199_at	0.00	0.00
1446200_at	0.00	0.00
1446201_at	0.00	0.00
1446202_at	0.00	0.00
1446203_at	0.00	0.00
1446204_at	0.00	0.00
1446205_at	0.00	0.00
1446206_at	0.00	0.00
1446207_at	0.00	0.00
1446208_at	0.00	0.00
1446209_at	0.00	0.00
1446210_at	0.00	0.00
1446211_at	0.00	0.00
1446212_at	0.00	0.00
1446213_at	0.00	0.00
1446214_at	0.00	0.00
1446215_at	0.00	0.00
1446216_at	0.00	0.00

1446217_at	0.00	0.00
1446218_at	0.00	0.00
1446219_at	0.00	0.00
1446220_at	0.00	0.00
1446221_at	0.00	0.00
1446222_at	0.00	0.00
1446223_at	0.00	0.00
1446224_at	0.00	0.00
1446225_at	0.00	0.00
1446226_at	0.00	0.00
1446227_at	0.00	0.00
1446228_at	0.00	0.00
1446229_at	0.00	0.00
1446230_at	0.00	0.00
1446231_at	0.00	0.00
1446232_at	0.00	0.00
1446233_at	0.00	0.00
1446234_at	0.00	0.00
1446235_at	0.00	0.00
1446236_at	0.00	0.00
1446237_at	0.00	0.00
1446238_at	0.00	0.00
1446239_at	0.00	0.00
1446240_at	0.00	0.00
1446241_at	0.00	0.00
1446242_at	0.00	0.00
1446243_at	0.00	0.00
1446245_at	0.00	0.00
1446246_at	0.00	0.00
1446247_at	0.00	0.00
1446248_at	0.00	0.00
1446249_at	0.00	0.00
1446250_at	0.00	0.00
1446251_at	0.00	0.00
1446252_at	0.00	0.00
1446253_at	0.00	0.00
1446254_at	0.00	0.00
1446255_at	0.00	0.00
1446256_at	0.00	0.00
1446257_at	0.00	0.00
1446258_at	0.00	0.00
1446259_at	0.00	0.00
1446260_at	0.00	0.00
1446261_at	0.00	0.00
1446262_at	0.00	0.00
1446263_at	0.00	0.00
1446264_at	0.00	0.00
1446265_at	0.00	0.00
1446266_at	0.00	0.00
1446267_at	0.00	0.00
1446268_at	0.00	0.00
1446269_at	0.00	0.00
1446270_at	0.00	0.00
1446271_at	0.00	0.00
1446272_at	0.00	0.00
1446273_at	0.00	0.00

1446274_at	0.00	0.00
1446275_at	0.00	0.00
1446276_at	0.00	0.00
1446277_at	0.00	0.00
1446278_at	0.00	0.00
1446279_at	0.00	0.00
1446280_at	0.00	0.00
1446281_at	0.00	0.00
1446282_at	0.00	0.00
1446283_at	0.00	0.00
1446284_at	0.00	0.00
1446285_at	0.00	0.00
1446286_at	0.00	0.00
1446287_at	0.00	0.00
1446288_at	0.00	0.00
1446289_at	0.00	0.00
1446290_at	0.00	0.00
1446291_at	0.00	0.00
1446292_at	0.00	0.00
1446293_at	0.00	0.00
1446294_at	0.00	0.00
1446295_at	0.00	0.00
1446296_at	0.00	0.00
1446297_at	0.00	0.00
1446298_at	0.00	0.00
1446299_at	0.00	0.00
1446300_at	0.00	0.00
1446301_at	0.00	0.00
1446302_at	0.00	0.00
1446303_at	0.00	0.00
1446304_at	0.00	0.00
1446305_at	0.00	0.00
1446306_at	0.00	0.00
1446307_at	0.00	0.00
1446308_at	0.00	0.00
1446309_at	0.00	0.00
1446310_at	0.00	0.00
1446311_at	0.00	0.00
1446312_at	0.00	0.00
1446313_at	0.00	0.00
1446314_at	0.00	0.00
1446315_at	0.00	0.00
1446316_at	0.00	0.00
1446317_at	0.00	0.00
1446318_at	0.00	0.00
1446319_at	0.00	0.00
1446320_at	0.00	0.00
1446321_at	0.00	0.00
1446322_at	0.00	0.00
1446323_at	0.00	0.00
1446324_at	0.00	0.00
1446325_at	0.00	0.00
1446326_at	0.00	0.00
1446327_at	0.00	0.00
1446328_at	0.00	0.00
1446329_at	0.00	0.00

1446330_at	0.00	0.00
1446331_at	0.00	0.00
1446332_at	0.00	0.00
1446333_at	0.00	0.00
1446334_at	0.00	0.00
1446335_at	0.00	0.00
1446336_at	0.00	0.00
1446337_at	0.00	0.00
1446338_at	0.00	0.00
1446339_at	0.00	0.00
1446340_at	0.00	0.00
1446341_at	0.00	0.00
1446342_at	0.00	0.00
1446343_at	0.00	0.00
1446344_at	0.00	0.00
1446345_at	0.00	0.00
1446346_at	0.00	0.00
1446347_at	0.00	0.00
1446348_at	0.00	0.00
1446349_at	0.00	0.00
1446350_at	0.00	0.00
1446351_at	0.00	0.00
1446352_at	0.00	0.00
1446353_at	0.00	0.00
1446354_at	0.00	0.00
1446355_at	0.00	0.00
1446356_at	0.00	0.00
1446357_at	0.00	0.00
1446358_at	0.00	0.00
1446359_at	0.00	0.00
1446360_at	0.00	0.00
1446361_at	0.00	0.00
1446362_at	0.00	0.00
1446363_at	0.00	0.00
1446364_at	0.00	0.00
1446365_at	0.00	0.00
1446366_at	0.00	0.00
1446367_at	0.00	0.00
1446368_at	0.00	0.00
1446369_at	0.00	0.00
1446370_at	0.00	0.00
1446371_at	0.00	0.00
1446372_at	0.00	0.00
1446373_at	0.00	0.00
1446374_at	0.00	0.00
1446375_at	0.00	0.00
1446376_at	0.00	0.00
1446377_at	0.00	0.00
1446380_at	0.00	0.00
1446381_at	0.00	0.00
1446382_at	0.00	0.00
1446383_at	0.00	0.00
1446384_at	0.00	0.00
1446385_at	0.00	0.00
1446386_at	0.00	0.00
1446387_at	0.00	0.00

1446388_at	0.00	0.00
1446389_at	0.00	0.00
1446390_at	0.00	0.00
1446391_at	0.00	0.00
1446392_at	0.00	0.00
1446393_at	0.00	0.00
1446394_at	0.00	0.00
1446395_at	0.00	0.00
1446396_at	0.00	0.00
1446397_at	0.00	0.00
1446398_at	0.00	0.00
1446399_at	0.00	0.00
1446400_at	0.00	0.00
1446401_at	0.00	0.00
1446402_at	0.00	0.00
1446403_at	0.00	0.00
1446404_at	0.00	0.00
1446405_at	0.00	0.00
1446406_at	0.00	0.00
1446407_at	0.00	0.00
1446408_at	0.00	0.00
1446409_at	0.00	0.00
1446410_at	0.00	0.00
1446411_at	0.00	0.00
1446412_at	0.00	0.00
1446413_at	0.00	0.00
1446414_at	0.00	0.00
1446415_at	0.00	0.00
1446416_at	0.00	0.00
1446417_at	0.00	0.00
1446418_at	0.00	0.00
1446419_at	0.00	0.00
1446420_at	0.00	0.00
1446421_at	0.00	0.00
1446422_at	0.00	0.00
1446423_at	0.00	0.00
1446424_at	0.00	0.00
1446425_at	0.00	0.00
1446426_at	0.00	0.00
1446427_at	0.00	0.00
1446428_at	0.00	0.00
1446429_at	0.00	0.00
1446430_at	0.00	0.00
1446431_at	0.00	0.00
1446432_at	0.00	0.00
1446433_at	0.00	0.00
1446434_at	0.00	0.00
1446435_at	0.00	0.00
1446436_at	0.00	0.00
1446437_at	0.00	0.00
1446438_at	0.00	0.00
1446439_at	0.00	0.00
1446440_at	0.00	0.00
1446441_at	0.00	0.00
1446442_at	0.00	0.00
1446443_at	0.00	0.00

1446444_at	0.00	0.00
1446445_at	0.00	0.00
1446446_at	0.00	0.00
1446447_at	0.00	0.00
1446448_at	0.00	0.00
1446449_at	0.00	0.00
1446450_at	0.00	0.00
1446451_at	0.00	0.00
1446452_at	0.00	0.00
1446453_at	0.00	0.00
1446454_at	0.00	0.00
1446455_at	0.00	0.00
1446456_at	0.00	0.00
1446457_at	0.00	0.00
1446458_at	0.00	0.00
1446459_at	0.00	0.00
1446460_at	0.00	0.00
1446461_at	0.00	0.00
1446462_at	0.00	0.00
1446463_at	0.00	0.00
1446464_at	0.00	0.00
1446465_at	0.00	0.00
1446466_at	0.00	0.00
1446467_at	0.00	0.00
1446468_at	0.00	0.00
1446469_at	0.00	0.00
1446470_at	0.00	0.00
1446471_at	0.00	0.00
1446472_at	0.00	0.00
1446473_at	0.00	0.00
1446474_at	0.00	0.00
1446475_at	0.00	0.00
1446476_at	0.00	0.00
1446477_at	0.00	0.00
1446478_at	0.00	0.00
1446479_at	0.00	0.00
1446480_at	0.00	0.00
1446481_at	0.00	0.00
1446482_at	0.00	0.00
1446483_at	0.00	0.00
1446484_at	0.00	0.00
1446485_at	0.00	0.00
1446486_at	0.00	0.00
1446487_at	0.00	0.00
1446488_at	0.00	0.00
1446489_at	0.00	0.00
1446490_at	0.00	0.00
1446491_at	0.00	0.00
1446492_at	0.00	0.00
1446493_at	0.00	0.00
1446494_at	0.00	0.00
1446495_at	0.00	0.00
1446496_at	0.00	0.00
1446497_at	0.00	0.00
1446498_at	0.00	0.00
1446499_at	0.00	0.00

1446500_at	0.00	0.00
1446501_at	0.00	0.00
1446502_at	0.00	0.00
1446503_at	0.00	0.00
1446504_at	0.00	0.00
1446505_at	0.00	0.00
1446506_at	0.00	0.00
1446507_at	0.00	0.00
1446508_at	0.00	0.00
1446509_at	0.00	0.00
1446510_at	0.00	0.00
1446511_at	0.00	0.00
1446512_at	0.00	0.00
1446513_at	0.00	0.00
1446514_at	0.00	0.00
1446515_at	0.00	0.00
1446516_at	0.00	0.00
1446517_at	0.00	0.00
1446518_at	0.00	0.00
1446519_at	0.00	0.00
1446520_at	0.00	0.00
1446521_at	0.00	0.00
1446522_at	0.00	0.00
1446523_at	0.00	0.00
1446524_at	0.00	0.00
1446525_at	0.00	0.00
1446526_at	0.00	0.00
1446527_at	0.00	0.00
1446528_at	0.00	0.00
1446529_at	0.00	0.00
1446530_at	0.00	0.00
1446531_at	0.00	0.00
1446532_at	0.00	0.00
1446533_at	0.00	0.00
1446534_at	0.00	0.00
1446535_at	0.00	0.00
1446536_at	0.00	0.00
1446537_at	0.00	0.00
1446538_at	0.00	0.00
1446539_at	0.00	0.00
1446540_at	0.00	0.00
1446541_at	0.00	0.00
1446542_at	0.00	0.00
1446543_at	0.00	0.00
1446544_at	0.00	0.00
1446545_at	0.00	0.00
1446546_at	0.00	0.00
1446547_at	0.00	0.00
1446548_at	0.00	0.00
1446549_at	0.00	0.00
1446550_at	0.00	0.00
1446551_at	0.00	0.00
1446552_at	0.00	0.00
1446553_at	0.00	0.00
1446554_at	0.00	0.00
1446555_at	0.00	0.00

1446556_at	0.00	0.00
1446557_at	0.00	0.00
1446558_at	0.00	0.00
1446559_at	0.00	0.00
1446560_at	0.00	0.00
1446561_at	0.00	0.00
1446562_at	0.00	0.00
1446563_at	0.00	0.00
1446564_at	0.00	0.00
1446565_at	0.00	0.00
1446566_at	0.00	0.00
1446567_at	0.00	0.00
1446568_at	0.00	0.00
1446569_at	0.00	0.00
1446570_at	0.00	0.00
1446571_at	0.00	0.00
1446573_at	0.00	0.00
1446574_at	0.00	0.00
1446575_at	0.00	0.00
1446576_at	0.00	0.00
1446577_at	0.00	0.00
1446578_at	0.00	0.00
1446579_at	0.00	0.00
1446580_at	0.00	0.00
1446581_at	0.00	0.00
1446582_at	0.00	0.00
1446583_at	0.00	0.00
1446584_at	0.00	0.00
1446585_at	0.00	0.00
1446586_at	0.00	0.00
1446587_at	0.00	0.00
1446588_at	0.00	0.00
1446589_at	0.00	0.00
1446590_at	0.00	0.00
1446591_at	0.00	0.00
1446592_at	0.00	0.00
1446593_at	0.00	0.00
1446594_at	0.00	0.00
1446595_at	0.00	0.00
1446596_at	0.00	0.00
1446597_at	0.00	0.00
1446598_at	0.00	0.00
1446599_at	0.00	0.00
1446600_at	0.00	0.00
1446601_at	0.00	0.00
1446602_at	0.00	0.00
1446603_at	0.00	0.00
1446604_at	0.00	0.00
1446605_at	0.00	0.00
1446606_at	0.00	0.00
1446607_at	0.00	0.00
1446608_at	0.00	0.00
1446609_at	0.00	0.00
1446610_at	0.00	0.00
1446611_at	0.00	0.00
1446612_at	0.00	0.00

1446613_at	0.00	0.00
1446614_at	0.00	0.00
1446615_at	0.00	0.00
1446616_at	0.00	0.00
1446617_at	0.00	0.00
1446618_at	0.00	0.00
1446619_at	0.00	0.00
1446620_at	0.00	0.00
1446621_at	0.00	0.00
1446622_at	0.00	0.00
1446623_at	0.00	0.00
1446624_at	0.00	0.00
1446625_at	0.00	0.00
1446626_at	0.00	0.00
1446627_at	0.00	0.00
1446628_at	0.00	0.00
1446629_at	0.00	0.00
1446630_at	0.00	0.00
1446631_at	0.00	0.00
1446632_at	0.00	0.00
1446633_at	0.00	0.00
1446634_at	0.00	0.00
1446635_at	0.00	0.00
1446636_at	0.00	0.00
1446637_at	0.00	0.00
1446638_at	0.00	0.00
1446639_at	0.00	0.00
1446640_at	0.00	0.00
1446641_at	0.00	0.00
1446642_at	0.00	0.00
1446643_at	0.00	0.00
1446644_at	0.00	0.00
1446645_at	0.00	0.00
1446646_at	0.00	0.00
1446647_at	0.00	0.00
1446648_at	0.00	0.00
1446649_at	0.00	0.00
1446650_at	0.00	0.00
1446651_at	0.00	0.00
1446652_at	0.00	0.00
1446653_at	0.00	0.00
1446654_at	0.00	0.00
1446655_at	0.00	0.00
1446656_at	0.00	0.00
1446657_at	0.00	0.00
1446658_at	0.00	0.00
1446659_at	0.00	0.00
1446660_at	0.00	0.00
1446661_at	0.00	0.00
1446662_at	0.00	0.00
1446663_at	0.00	0.00
1446664_at	0.00	0.00
1446665_at	0.00	0.00
1446666_at	0.00	0.00
1446667_at	0.00	0.00
1446668_at	0.00	0.00

1446669_at	0.00	0.00
1446670_at	0.00	0.00
1446671_at	0.00	0.00
1446672_at	0.00	0.00
1446673_at	0.00	0.00
1446674_at	0.00	0.00
1446675_at	0.00	0.00
1446676_at	0.00	0.00
1446677_at	0.00	0.00
1446678_at	0.00	0.00
1446679_at	0.00	0.00
1446680_at	0.00	0.00
1446681_at	0.00	0.00
1446682_at	0.00	0.00
1446683_at	0.00	0.00
1446684_at	0.00	0.00
1446685_at	0.00	0.00
1446686_at	0.00	0.00
1446687_at	0.00	0.00
1446688_at	0.00	0.00
1446689_at	0.00	0.00
1446690_at	0.00	0.00
1446691_at	0.00	0.00
1446692_at	0.00	0.00
1446693_at	0.00	0.00
1446694_at	0.00	0.00
1446696_at	0.00	0.00
1446697_at	0.00	0.00
1446698_at	0.00	0.00
1446699_at	0.00	0.00
1446700_at	0.00	0.00
1446701_at	0.00	0.00
1446702_at	0.00	0.00
1446703_at	0.00	0.00
1446704_at	0.00	0.00
1446705_at	0.00	0.00
1446706_at	0.00	0.00
1446707_at	0.00	0.00
1446708_at	0.00	0.00
1446709_at	0.00	0.00
1446710_at	0.00	0.00
1446711_at	0.00	0.00
1446712_at	0.00	0.00
1446713_at	0.00	0.00
1446714_x_at	0.00	0.00
1446715_at	0.00	0.00
1446716_at	0.00	0.00
1446717_at	0.00	0.00
1446718_at	0.00	0.00
1446719_at	0.00	0.00
1446720_at	0.00	0.00
1446721_at	0.00	0.00
1446722_at	0.00	0.00
1446723_at	0.00	0.00
1446724_at	0.00	0.00
1446725_at	0.00	0.00

1446726_at	0.00	0.00
1446727_at	0.00	0.00
1446728_at	0.00	0.00
1446729_at	0.00	0.00
1446730_at	0.00	0.00
1446731_at	0.00	0.00
1446732_at	0.00	0.00
1446733_at	0.00	0.00
1446734_at	0.00	0.00
1446735_at	0.00	0.00
1446736_at	0.00	0.00
1446737_a_at	0.00	0.00
1446738_at	0.00	0.00
1446739_at	0.00	0.00
1446740_at	0.00	0.00
1446741_at	0.00	0.00
1446742_at	0.00	0.00
1446743_at	0.00	0.00
1446744_at	0.00	0.00
1446745_at	0.00	0.00
1446746_at	0.00	0.00
1446747_at	0.00	0.00
1446748_at	0.00	0.00
1446749_at	0.00	0.00
1446750_at	0.00	0.00
1446751_s_at	0.00	0.00
1446752_at	0.00	0.00
1446753_at	0.00	0.00
1446754_a_at	0.00	0.00
1446755_at	0.00	0.00
1446756_at	0.00	0.00
1446757_at	0.00	0.00
1446758_at	0.00	0.00
1446759_at	0.00	0.00
1446760_at	0.00	0.00
1446761_at	0.00	0.00
1446762_at	0.00	0.00
1446763_at	0.00	0.00
1446764_at	0.00	0.00
1446765_at	0.00	0.00
1446766_at	0.00	0.00
1446767_at	0.00	0.00
1446768_at	0.00	0.00
1446769_at	0.00	0.00
1446770_at	0.00	0.00
1446771_at	0.00	0.00
1446772_at	0.00	0.00
1446773_at	0.00	0.00
1446774_at	0.00	0.00
1446775_at	0.00	0.00
1446776_at	0.00	0.00
1446777_at	0.00	0.00
1446778_at	0.00	0.00
1446779_at	0.00	0.00
1446780_at	0.00	0.00
1446781_at	0.00	0.00

1446782_at	0.00	0.00
1446783_at	0.00	0.00
1446784_at	0.00	0.00
1446785_at	0.00	0.00
1446786_at	0.00	0.00
1446787_at	0.00	0.00
1446788_at	0.00	0.00
1446789_at	0.00	0.00
1446791_at	0.00	0.00
1446792_at	0.00	0.00
1446793_at	0.00	0.00
1446794_at	0.00	0.00
1446795_at	0.00	0.00
1446796_at	0.00	0.00
1446797_at	0.00	0.00
1446798_at	0.00	0.00
1446799_at	0.00	0.00
1446800_at	0.00	0.00
1446801_at	0.00	0.00
1446802_at	0.00	0.00
1446803_at	0.00	0.00
1446804_at	0.00	0.00
1446805_at	0.00	0.00
1446806_at	0.00	0.00
1446807_at	0.00	0.00
1446808_at	0.00	0.00
1446809_at	0.00	0.00
1446810_at	0.00	0.00
1446811_at	0.00	0.00
1446812_at	0.00	0.00
1446813_s_at	0.00	0.00
1446814_at	0.00	0.00
1446815_at	0.00	0.00
1446816_at	0.00	0.00
1446817_at	0.00	0.00
1446818_at	0.00	0.00
1446819_at	0.00	0.00
1446820_at	0.00	0.00
1446821_at	0.00	0.00
1446822_at	0.00	0.00
1446823_at	0.00	0.00
1446824_at	0.00	0.00
1446825_at	0.00	0.00
1446826_at	0.00	0.00
1446827_at	0.00	0.00
1446830_at	0.00	0.00
1446831_at	0.00	0.00
1446832_at	0.00	0.00
1446833_at	0.00	0.00
1446834_at	0.00	0.00
1446835_at	0.00	0.00
1446836_at	0.00	0.00
1446837_at	0.00	0.00
1446838_at	0.00	0.00
1446839_at	0.00	0.00
1446840_at	0.00	0.00

1446841_at	0.00	0.00
1446842_at	0.00	0.00
1446843_at	0.00	0.00
1446844_at	0.00	0.00
1446845_at	0.00	0.00
1446846_at	0.00	0.00
1446847_at	0.00	0.00
1446848_at	0.00	0.00
1446849_at	0.00	0.00
1446850_at	0.00	0.00
1446851_at	0.00	0.00
1446852_at	0.00	0.00
1446853_at	0.00	0.00
1446854_at	0.00	0.00
1446855_at	0.00	0.00
1446857_at	0.00	0.00
1446858_at	0.00	0.00
1446859_at	0.00	0.00
1446860_at	0.00	0.00
1446861_at	0.00	0.00
1446862_at	0.00	0.00
1446863_at	0.00	0.00
1446864_at	0.00	0.00
1446865_at	0.00	0.00
1446866_at	0.00	0.00
1446867_at	0.00	0.00
1446868_at	0.00	0.00
1446869_at	0.00	0.00
1446870_at	0.00	0.00
1446871_at	0.00	0.00
1446872_at	0.00	0.00
1446873_at	0.00	0.00
1446874_at	0.00	0.00
1446875_at	0.00	0.00
1446876_at	0.00	0.00
1446877_at	0.00	0.00
1446878_at	0.00	0.00
1446879_at	0.00	0.00
1446880_at	0.00	0.00
1446881_at	0.00	0.00
1446882_at	0.00	0.00
1446883_at	0.00	0.00
1446884_at	0.00	0.00
1446885_at	0.00	0.00
1446886_at	0.00	0.00
1446887_at	0.00	0.00
1446888_at	0.00	0.00
1446889_at	0.00	0.00
1446890_at	0.00	0.00
1446891_at	0.00	0.00
1446892_at	0.00	0.00
1446893_at	0.00	0.00
1446894_at	0.00	0.00
1446895_at	0.00	0.00
1446896_at	0.00	0.00
1446897_at	0.00	0.00

1446898_at	0.00	0.00
1446899_at	0.00	0.00
1446900_at	0.00	0.00
1446901_at	0.00	0.00
1446902_at	0.00	0.00
1446903_at	0.00	0.00
1446904_at	0.00	0.00
1446905_at	0.00	0.00
1446906_at	0.00	0.00
1446907_at	0.00	0.00
1446908_at	0.00	0.00
1446909_at	0.00	0.00
1446910_at	0.00	0.00
1446911_at	0.00	0.00
1446912_at	0.00	0.00
1446913_at	0.00	0.00
1446915_at	0.00	0.00
1446916_at	0.00	0.00
1446917_at	0.00	0.00
1446918_at	0.00	0.00
1446919_at	0.00	0.00
1446920_at	0.00	0.00
1446921_at	0.00	0.00
1446922_at	0.00	0.00
1446923_at	0.00	0.00
1446924_at	0.00	0.00
1446925_at	0.00	0.00
1446926_at	0.00	0.00
1446927_at	0.00	0.00
1446928_at	0.00	0.00
1446929_at	0.00	0.00
1446930_at	0.00	0.00
1446931_at	0.00	0.00
1446932_at	0.00	0.00
1446933_at	0.00	0.00
1446934_at	0.00	0.00
1446935_at	0.00	0.00
1446936_at	0.00	0.00
1446937_at	0.00	0.00
1446938_at	0.00	0.00
1446939_at	0.00	0.00
1446940_at	0.00	0.00
1446941_at	0.00	0.00
1446942_at	0.00	0.00
1446943_at	0.00	0.00
1446944_at	0.00	0.00
1446945_at	0.00	0.00
1446946_at	0.00	0.00
1446947_at	0.00	0.00
1446948_at	0.00	0.00
1446949_at	0.00	0.00
1446950_at	0.00	0.00
1446951_at	0.00	0.00
1446952_at	0.00	0.00
1446953_at	0.00	0.00
1446954_at	0.00	0.00

1446955_at	0.00	0.00
1446956_at	0.00	0.00
1446957_s_at	0.00	0.00
1446958_at	0.00	0.00
1446959_at	0.00	0.00
1446960_at	0.00	0.00
1446961_at	0.00	0.00
1446962_at	0.00	0.00
1446963_at	0.00	0.00
1446964_at	0.00	0.00
1446965_at	0.00	0.00
1446967_at	0.00	0.00
1446968_at	0.00	0.00
1446969_at	0.00	0.00
1446970_at	0.00	0.00
1446972_at	0.00	0.00
1446973_at	0.00	0.00
1446974_at	0.00	0.00
1446975_at	0.00	0.00
1446976_at	0.00	0.00
1446977_at	0.00	0.00
1446978_at	0.00	0.00
1446979_at	0.00	0.00
1446980_at	0.00	0.00
1446981_at	0.00	0.00
1446982_at	0.00	0.00
1446983_at	0.00	0.00
1446984_at	0.00	0.00
1446985_at	0.00	0.00
1446986_at	0.00	0.00
1446987_at	0.00	0.00
1446988_at	0.00	0.00
1446989_at	0.00	0.00
1446990_at	0.00	0.00
1446991_at	0.00	0.00
1446992_at	0.00	0.00
1446993_at	0.00	0.00
1446994_at	0.00	0.00
1446995_at	0.00	0.00
1446996_at	0.00	0.00
1446997_at	0.00	0.00
1446998_at	0.00	0.00
1446999_at	0.00	0.00
1447000_at	0.00	0.00
1447001_at	0.00	0.00
1447002_at	0.00	0.00
1447003_at	0.00	0.00
1447004_at	0.00	0.00
1447005_at	0.00	0.00
1447006_at	0.00	0.00
1447007_at	0.00	0.00
1447008_at	0.00	0.00
1447009_at	0.00	0.00
1447010_at	0.00	0.00
1447011_at	0.00	0.00
1447012_at	0.00	0.00

1447013_at	0.00	0.00
1447014_at	0.00	0.00
1447015_at	0.00	0.00
1447016_at	0.00	0.00
1447017_at	0.00	0.00
1447018_at	0.00	0.00
1447019_at	0.00	0.00
1447020_at	0.00	0.00
1447021_at	0.56	0.02
1447022_at	0.00	0.00
1447023_at	0.00	0.00
1447024_at	0.00	0.00
1447025_at	0.00	0.00
1447026_at	0.00	0.00
1447027_s_at	0.00	0.00
1447028_at	0.00	0.00
1447029_at	0.00	0.00
1447030_at	0.00	0.00
1447031_at	0.00	0.00
1447032_at	0.00	0.00
1447033_at	0.00	0.00
1447034_at	0.00	0.00
1447035_at	0.00	0.00
1447036_at	0.00	0.00
1447037_at	0.00	0.00
1447038_x_at	0.00	0.00
1447039_at	0.00	0.00
1447040_at	0.00	0.00
1447041_at	0.00	0.00
1447042_at	0.00	0.00
1447043_at	0.00	0.00
1447044_at	0.00	0.00
1447045_at	0.00	0.00
1447046_at	0.00	0.00
1447047_at	0.00	0.00
1447048_at	0.00	0.00
1447049_at	0.00	0.00
1447050_at	0.00	0.00
1447051_at	0.00	0.00
1447052_at	0.00	0.00
1447053_x_at	0.00	0.00
1447054_at	0.00	0.00
1447055_at	0.00	0.00
1447056_at	0.00	0.00
1447057_at	0.00	0.00
1447058_at	0.00	0.00
1447059_at	0.00	0.00
1447060_at	0.00	0.00
1447061_at	0.00	0.00
1447062_at	0.00	0.00
1447063_at	0.00	0.00
1447064_at	0.00	0.00
1447065_at	0.00	0.00
1447066_at	0.00	0.00
1447067_at	0.00	0.00
1447068_at	0.00	0.00

1447069_at	0.00	0.00
1447070_at	0.00	0.00
1447071_at	0.00	0.00
1447072_at	0.00	0.00
1447073_at	0.00	0.00
1447074_at	0.00	0.00
1447075_at	0.00	0.00
1447076_at	0.00	0.00
1447077_at	0.00	0.00
1447078_at	0.00	0.00
1447079_at	0.00	0.00
1447080_at	0.00	0.00
1447081_at	0.00	0.00
1447082_at	0.00	0.00
1447083_at	0.00	0.00
1447084_at	0.00	0.00
1447085_s_at	0.00	0.00
1447086_at	0.00	0.00
1447087_at	0.00	0.00
1447088_at	0.00	0.00
1447089_at	0.00	0.00
1447090_s_at	0.00	0.00
1447091_at	0.00	0.00
1447092_at	0.00	0.00
1447093_at	0.00	0.00
1447094_at	0.00	0.00
1447095_at	0.00	0.00
1447096_at	0.00	0.00
1447097_at	0.00	0.00
1447098_at	0.00	0.00
1447099_at	0.00	0.00
1447100_s_at	0.00	0.00
1447101_at	0.00	0.00
1447102_at	0.00	0.00
1447103_at	0.00	0.00
1447104_at	0.00	0.00
1447105_at	0.00	0.00
1447106_at	0.00	0.00
1447107_at	0.00	0.00
1447108_at	0.00	0.00
1447109_at	0.00	0.00
1447110_at	0.00	0.00
1447111_at	0.00	0.00
1447112_s_at	0.00	0.00
1447113_at	0.00	0.00
1447114_x_at	0.00	0.00
1447115_at	0.00	0.00
1447116_at	0.00	0.00
1447117_at	0.00	0.00
1447118_at	0.00	0.00
1447119_at	0.00	0.00
1447120_at	0.00	0.00
1447121_at	0.00	0.00
1447122_at	0.00	0.00
1447123_at	0.00	0.00
1447124_at	0.00	0.00

1447125_at	0.00	0.00
1447126_at	0.00	0.00
1447127_at	0.00	0.00
1447128_at	0.00	0.00
1447129_at	0.00	0.00
1447131_at	0.00	0.00
1447132_at	0.00	0.00
1447133_at	0.00	0.00
1447134_at	0.00	0.00
1447135_at	0.00	0.00
1447136_at	0.00	0.00
1447137_at	0.00	0.00
1447138_at	0.00	0.00
1447139_at	0.00	0.00
1447140_at	0.00	0.00
1447141_at	0.00	0.00
1447142_at	0.00	0.00
1447143_at	0.00	0.00
1447144_at	0.00	0.00
1447145_at	0.00	0.00
1447147_at	0.00	0.00
1447148_at	0.00	0.00
1447149_at	0.00	0.00
1447150_at	0.00	0.00
1447151_at	0.00	0.00
1447152_at	0.00	0.00
1447153_x_at	0.00	0.00
1447154_at	0.00	0.00
1447155_at	0.00	0.00
1447156_at	0.00	0.00
1447157_at	0.00	0.00
1447158_at	0.00	0.00
1447159_at	0.00	0.00
1447160_at	0.00	0.00
1447161_at	0.00	0.00
1447162_at	0.00	0.00
1447163_x_at	0.00	0.00
1447164_at	0.00	0.00
1447165_at	0.00	0.00
1447166_at	0.00	0.00
1447167_at	0.00	0.00
1447168_at	0.00	0.00
1447169_at	0.00	0.00
1447170_at	0.00	0.00
1447171_at	0.00	0.00
1447172_at	0.00	0.00
1447173_at	0.00	0.00
1447174_at	0.00	0.00
1447175_at	0.00	0.00
1447176_at	0.00	0.00
1447177_at	0.00	0.00
1447178_at	0.00	0.00
1447179_at	0.00	0.00
1447180_at	0.00	0.00
1447181_s_at	0.13	0.00
1447183_at	0.00	0.00

1447184_at	0.00	0.00
1447185_at	0.00	0.00
1447186_at	0.00	0.00
1447187_at	0.00	0.00
1447188_at	0.00	0.00
1447189_at	0.00	0.00
1447190_at	0.00	0.00
1447191_at	0.00	0.00
1447192_at	0.00	0.00
1447193_at	0.00	0.00
1447194_at	0.00	0.00
1447195_at	0.00	0.00
1447196_at	0.00	0.00
1447197_at	0.00	0.00
1447198_at	0.00	0.00
1447199_at	0.00	0.00
1447200_at	0.00	0.00
1447201_at	0.00	0.00
1447202_at	0.00	0.00
1447203_at	0.00	0.00
1447204_at	0.00	0.00
1447205_x_at	0.00	0.00
1447206_at	0.00	0.00
1447207_at	0.00	0.00
1447208_at	0.00	0.00
1447209_at	0.00	0.00
1447210_at	0.00	0.00
1447211_at	0.00	0.00
1447212_at	0.00	0.00
1447213_at	0.00	0.00
1447214_at	0.00	0.00
1447215_at	0.00	0.00
1447216_at	0.00	0.00
1447217_at	0.00	0.00
1447218_at	0.00	0.00
1447219_at	0.00	0.00
1447220_at	0.00	0.00
1447221_x_at	0.00	0.00
1447222_at	0.00	0.00
1447223_at	0.00	0.00
1447224_at	0.00	0.00
1447225_at	0.00	0.00
1447226_at	0.00	0.00
1447227_at	0.00	0.00
1447228_at	0.00	0.00
1447229_x_at	0.00	0.00
1447230_at	0.00	0.00
1447231_at	0.00	0.00
1447232_at	0.00	0.00
1447233_at	0.00	0.00
1447234_s_at	0.16	0.00
1447235_at	0.00	0.00
1447236_at	0.00	0.00
1447237_at	0.00	0.00
1447238_at	0.00	0.00
1447239_at	0.00	0.00

1447240_at	0.00	0.00
1447241_at	0.00	0.00
1447242_at	0.00	0.00
1447243_at	0.00	0.00
1447244_at	0.00	0.00
1447245_at	0.00	0.00
1447246_at	0.00	0.00
1447247_at	0.00	0.00
1447248_at	0.00	0.00
1447249_at	0.00	0.00
1447250_a_at	0.00	0.00
1447251_x_at	0.00	0.00
1447252_s_at	0.00	0.00
1447253_x_at	0.00	0.00
1447254_at	0.00	0.00
1447255_at	0.00	0.00
1447256_at	0.00	0.00
1447257_at	0.00	0.00
1447258_at	0.00	0.00
1447259_at	0.00	0.00
1447260_at	0.00	0.00
1447261_at	0.00	0.00
1447262_at	0.00	0.00
1447263_at	0.00	0.00
1447264_at	0.00	0.00
1447265_at	0.00	0.00
1447266_at	0.00	0.00
1447267_at	0.00	0.00
1447268_at	0.00	0.00
1447269_at	0.00	0.00
1447270_at	0.00	0.00
1447271_at	0.00	0.00
1447272_s_at	0.00	0.00
1447273_x_at	0.00	0.00
1447274_at	0.00	0.00
1447275_at	0.00	0.00
1447277_s_at	0.00	0.00
1447278_at	0.00	0.00
1447279_at	0.00	0.00
1447280_at	0.00	0.00
1447281_at	0.00	0.00
1447282_at	0.00	0.00
1447283_at	0.00	0.00
1447284_at	0.00	0.00
1447285_at	0.00	0.00
1447286_at	0.00	0.00
1447288_at	0.00	0.00
1447289_at	0.00	0.00
1447290_at	0.00	0.00
1447291_at	0.00	0.00
1447292_at	0.00	0.00
1447293_x_at	0.00	0.00
1447294_at	0.00	0.00
1447295_at	0.00	0.00
1447296_at	0.00	0.00
1447297_at	0.00	0.00

1447298_at	0.00	0.00
1447299_at	0.00	0.00
1447300_at	0.00	0.00
1447301_at	0.00	0.00
1447302_at	0.00	0.00
1447303_at	0.00	0.00
1447304_at	0.00	0.00
1447305_at	0.00	0.00
1447306_at	0.00	0.00
1447307_at	0.00	0.00
1447308_at	0.00	0.00
1447309_at	0.00	0.00
1447310_at	0.00	0.00
1447311_at	0.00	0.00
1447312_at	0.00	0.00
1447313_at	0.00	0.00
1447314_at	0.00	0.00
1447315_at	0.00	0.00
1447316_at	0.00	0.00
1447317_at	0.00	0.00
1447318_at	0.00	0.00
1447319_at	0.00	0.00
1447320_x_at	0.00	0.02
1447321_at	0.00	0.00
1447322_at	0.00	0.00
1447323_at	0.00	0.00
1447324_at	0.00	0.00
1447325_at	0.00	0.00
1447326_s_at	0.00	0.00
1447327_at	0.00	0.00
1447328_at	0.00	0.00
1447329_at	0.00	0.00
1447330_at	0.00	0.00
1447331_at	0.00	0.00
1447332_at	0.00	0.00
1447333_at	0.00	0.00
1447334_at	0.00	0.00
1447335_x_at	0.00	0.00
1447336_at	0.00	0.00
1447337_at	0.00	0.00
1447338_at	0.00	0.00
1447339_at	0.00	0.00
1447340_at	0.00	0.00
1447341_at	0.00	0.00
1447342_at	0.00	0.00
1447343_at	0.00	0.00
1447344_at	0.00	0.00
1447345_at	0.00	0.00
1447346_s_at	0.00	0.00
1447347_at	0.00	0.00
1447348_at	0.00	0.00
1447349_s_at	0.00	0.00
1447352_at	0.00	0.00
1447353_at	0.00	0.00
1447354_at	0.00	0.00
1447355_at	0.00	0.00

1447356_at	0.00	0.00
1447357_at	0.00	0.00
1447358_at	0.00	0.00
1447359_at	0.00	0.00
1447360_at	0.00	0.00
1447361_at	0.00	0.00
1447362_at	0.00	0.00
1447363_s_at	0.00	0.00
1447364_x_at	0.00	0.00
1447365_at	0.00	0.00
1447366_at	0.00	0.00
1447367_at	0.00	0.00
1447368_at	0.00	0.00
1447369_at	0.00	0.00
1447370_at	0.00	0.00
1447371_at	0.00	0.00
1447372_at	0.00	0.00
1447373_at	0.00	0.00
1447374_at	0.00	0.00
1447375_at	0.00	0.00
1447376_at	0.00	0.00
1447378_at	0.00	0.00
1447379_at	0.00	0.00
1447380_at	0.00	0.00
1447381_at	0.00	0.00
1447382_at	0.00	0.00
1447383_at	0.00	0.00
1447384_at	0.00	0.00
1447385_at	0.00	0.00
1447386_at	0.00	0.00
1447387_at	0.00	0.00
1447388_at	0.00	0.00
1447389_at	0.00	0.00
1447390_at	0.00	0.00
1447391_at	0.00	0.00
1447392_s_at	0.00	0.00
1447393_at	0.00	0.00
1447394_at	0.00	0.00
1447395_at	0.00	0.00
1447396_at	0.00	0.00
1447397_at	0.00	0.00
1447398_at	0.00	0.00
1447399_at	0.00	0.00
1447400_at	0.00	0.00
1447401_at	0.00	0.00
1447402_at	0.00	0.00
1447403_a_at	0.00	0.00
1447404_at	0.00	0.00
1447405_at	0.00	0.00
1447406_at	0.00	0.00
1447407_at	0.00	0.00
1447408_at	0.00	0.00
1447409_at	0.00	0.00
1447410_at	0.00	0.00
1447411_at	0.00	0.00
1447412_at	0.00	0.00

1447413_at	0.00	0.00
1447414_at	0.00	0.00
1447415_at	0.00	0.00
1447416_at	0.00	0.00
1447417_at	0.00	0.00
1447418_at	0.00	0.00
1447419_at	0.00	0.00
1447420_at	0.00	0.00
1447421_at	0.00	0.00
1447422_at	0.00	0.00
1447423_at	0.00	0.00
1447424_at	0.00	0.00
1447425_at	0.00	0.00
1447426_at	0.00	0.00
1447428_at	0.00	0.00
1447429_at	0.00	0.00
1447430_at	0.00	0.00
1447431_at	0.00	0.00
1447432_s_at	0.00	0.00
1447433_at	0.00	0.00
1447434_at	0.00	0.00
1447435_at	0.00	0.00
1447436_at	0.00	0.00
1447437_at	0.00	0.00
1447438_at	0.00	0.00
1447439_at	0.00	0.00
1447440_at	0.00	0.00
1447441_at	0.00	0.00
1447442_at	0.00	0.00
1447443_at	0.00	0.00
1447444_at	0.00	0.00
1447445_at	0.00	0.00
1447446_at	0.00	0.00
1447447_s_at	0.00	0.00
1447448_s_at	0.12	0.00
1447449_at	0.00	0.00
1447450_at	0.00	0.00
1447451_at	0.00	0.00
1447452_at	0.00	0.00
1447453_x_at	0.00	0.00
1447454_at	0.00	0.00
1447455_at	0.00	0.00
1447457_at	0.00	0.00
1447458_at	0.00	0.00
1447459_at	0.00	0.00
1447460_at	0.00	0.00
1447461_at	0.00	0.00
1447463_at	0.00	0.00
1447464_at	0.00	0.00
1447465_at	0.00	0.00
1447466_at	0.00	0.00
1447467_at	0.00	0.00
1447468_at	0.00	0.00
1447469_at	0.00	0.00
1447470_at	0.00	0.00
1447471_at	0.00	0.00

1447472_at	0.00	0.00
1447473_at	0.00	0.00
1447474_at	0.00	0.00
1447475_at	0.00	0.00
1447476_at	0.00	0.00
1447477_at	0.00	0.00
1447478_at	0.00	0.00
1447479_at	0.00	0.00
1447480_at	0.00	0.00
1447481_at	0.00	0.00
1447482_at	0.00	0.00
1447483_s_at	0.00	0.00
1447484_x_at	0.00	0.00
1447485_at	0.00	0.00
1447486_at	0.00	0.00
1447487_at	0.00	0.00
1447488_at	0.00	0.00
1447489_at	0.00	0.00
1447490_at	0.00	0.00
1447491_at	0.00	0.00
1447492_at	0.00	0.00
1447493_at	0.00	0.00
1447495_at	0.00	0.00
1447496_s_at	0.00	0.00
1447497_at	0.00	0.00
1447498_at	0.00	0.00
1447499_s_at	0.00	0.00
1447500_at	0.00	0.00
1447501_at	0.00	0.00
1447502_at	0.00	0.00
1447503_at	0.00	0.00
1447504_at	0.00	0.00
1447505_at	0.00	0.00
1447506_at	0.00	0.00
1447507_at	0.00	0.00
1447508_at	0.00	0.00
1447509_at	0.00	0.00
1447510_at	0.00	0.00
1447511_at	0.00	0.00
1447512_at	0.00	0.00
1447513_at	0.00	0.00
1447514_at	0.00	0.00
1447515_at	0.00	0.00
1447516_at	0.00	0.00
1447517_at	0.00	0.00
1447518_at	0.00	0.00
1447519_x_at	0.00	0.00
1447520_at	0.00	0.00
1447521_x_at	0.00	0.00
1447522_s_at	0.00	0.21
1447523_at	0.00	0.00
1447524_at	0.00	0.00
1447525_at	0.00	0.00
1447526_at	0.00	0.00
1447527_at	0.00	0.00
1447528_at	0.00	0.00

1447529_at	0.00	0.00
1447530_at	0.00	0.00
1447531_x_at	0.00	0.00
1447532_at	0.00	0.00
1447533_at	0.00	0.00
1447534_at	0.00	0.00
1447535_at	0.00	0.00
1447536_at	0.00	0.00
1447537_at	0.00	0.00
1447538_at	0.00	0.00
1447539_at	0.00	0.00
1447540_at	0.00	0.00
1447541_s_at	0.00	0.00
1447542_at	0.00	0.00
1447543_at	0.00	0.00
1447544_at	0.00	0.00
1447545_at	0.00	0.00
1447546_s_at	0.00	0.00
1447547_at	0.00	0.00
1447548_at	0.00	0.00
1447549_x_at	0.00	0.00
1447550_at	0.00	0.00
1447551_x_at	0.00	0.00
1447552_s_at	0.00	0.00
1447553_x_at	0.00	0.00
1447554_at	0.00	0.00
1447555_at	0.00	0.00
1447556_x_at	0.00	0.00
1447557_at	0.00	0.00
1447558_at	0.00	0.00
1447559_at	0.00	0.00
1447560_at	0.00	0.00
1447561_at	0.00	0.00
1447562_at	0.00	0.00
1447563_at	0.00	0.00
1447564_x_at	0.00	0.00
1447565_at	0.00	0.00
1447566_at	0.00	0.00
1447567_at	0.00	0.00
1447568_at	0.00	0.00
1447569_at	0.00	0.00
1447570_s_at	0.00	0.00
1447571_at	0.00	0.00
1447572_at	0.00	0.00
1447573_at	0.00	0.00
1447574_s_at	0.00	0.00
1447575_at	0.00	0.00
1447576_at	0.00	0.00
1447577_x_at	0.00	0.00
1447578_at	0.00	0.00
1447579_at	0.00	0.00
1447580_at	0.00	0.00
1447581_at	0.00	0.00
1447582_x_at	0.00	0.00
1447583_x_at	0.00	0.00
1447584_s_at	0.00	0.00

1447585_s_at	0.00	0.00
1447586_at	0.00	0.00
1447587_at	0.00	0.00
1447588_x_at	0.00	0.00
1447589_at	0.00	0.00
1447590_at	0.00	0.00
1447591_x_at	0.00	0.00
1447592_at	0.00	0.00
1447593_x_at	0.00	0.00
1447594_at	0.00	0.00
1447595_x_at	0.00	0.00
1447596_at	0.00	0.00
1447597_at	0.00	0.00
1447598_x_at	0.00	0.00
1447599_x_at	0.00	0.00
1447600_at	0.00	0.00
1447601_x_at	0.00	0.00
1447602_x_at	0.00	0.00
1447603_x_at	0.00	0.00
1447605_at	0.00	0.00
1447606_x_at	0.00	0.00
1447607_at	0.00	0.00
1447608_x_at	0.00	0.00
1447609_at	0.00	0.00
1447610_at	0.00	0.00
1447611_at	0.00	0.00
1447612_x_at	0.00	0.00
1447613_at	0.00	0.00
1447614_at	0.00	0.00
1447615_at	0.00	0.00
1447616_at	0.00	0.00
1447617_at	0.00	0.00
1447618_at	0.00	0.00
1447619_at	0.00	0.00
1447620_at	0.00	0.00
1447621_s_at	0.00	0.00
1447622_at	0.00	0.00
1447623_s_at	0.91	0.11
1447624_s_at	0.00	0.00
1447625_at	0.00	0.00
1447626_x_at	0.00	0.00
1447627_at	0.00	0.00
1447628_x_at	0.00	0.00
1447629_at	0.00	0.00
1447630_x_at	0.00	0.00
1447633_x_at	0.00	0.00
1447634_x_at	0.00	0.00
1447635_at	0.00	0.00
1447636_x_at	0.00	0.00
1447637_at	0.00	0.00
1447638_at	0.00	0.00
1447639_x_at	0.00	0.00
1447640_s_at	0.00	0.00
1447641_at	0.00	0.00
1447642_x_at	0.00	0.00
1447643_x_at	0.00	0.00

1447644_at	0.00	0.00
1447645_x_at	0.00	0.00
1447646_at	0.00	0.00
1447647_at	0.00	0.00
1447648_at	0.00	0.00
1447649_x_at	0.00	0.00
1447650_at	0.00	0.00
1447651_x_at	0.00	0.00
1447652_at	0.00	0.00
1447654_at	0.00	0.00
1447655_x_at	0.00	0.00
1447656_at	0.00	0.00
1447657_s_at	0.00	0.00
1447658_x_at	0.00	0.00
1447659_x_at	0.00	0.00
1447660_at	0.00	0.00
1447661_at	0.00	0.00
1447662_x_at	0.00	0.00
1447663_at	0.00	0.00
1447664_x_at	0.00	0.00
1447665_at	0.00	0.00
1447666_x_at	0.00	0.00
1447667_x_at	0.00	0.00
1447668_x_at	0.00	0.00
1447669_s_at	0.00	0.00
1447670_at	0.00	0.00
1447671_x_at	0.00	0.00
1447672_x_at	0.00	0.00
1447673_x_at	0.00	0.00
1447674_at	0.00	0.00
1447675_x_at	0.00	0.00
1447676_x_at	0.00	0.00
1447677_x_at	0.00	0.00
1447678_at	0.89	1.00
1447679_s_at	0.00	0.00
1447680_at	0.00	0.00
1447681_x_at	0.00	0.00
1447682_x_at	0.00	0.00
1447683_x_at	0.01	0.00
1447684_at	0.00	0.00
1447685_x_at	0.00	0.00
1447686_at	0.00	0.00
1447687_x_at	0.00	0.00
1447688_at	0.00	0.00
1447689_at	0.00	0.00
1447690_at	0.00	0.00
1447691_x_at	0.00	0.00
1447692_x_at	0.00	0.00
1447693_s_at	0.00	0.18
1447694_x_at	0.00	0.00
1447695_at	0.00	0.00
1447696_x_at	0.00	0.00
1447697_at	0.00	0.00
1447698_x_at	0.00	0.00
1447699_at	0.00	0.00
1447700_x_at	0.00	0.00

1447701_x_at	0.00	0.00
1447702_x_at	0.00	0.00
1447703_x_at	0.00	0.00
1447704_s_at	0.00	0.00
1447705_at	0.00	0.00
1447706_at	0.00	0.00
1447707_s_at	0.00	0.00
1447708_x_at	0.00	0.00
1447709_at	0.00	0.00
1447710_at	0.00	0.00
1447711_x_at	0.00	0.00
1447712_x_at	0.00	0.00
1447713_at	0.00	0.00
1447714_x_at	0.00	0.00
1447715_x_at	0.00	0.00
1447716_x_at	0.00	0.00
1447717_x_at	0.00	0.00
1447718_at	0.00	0.00
1447719_at	0.00	0.00
1447720_x_at	0.01	0.00
1447721_at	0.00	0.00
1447722_at	0.00	0.00
1447723_at	0.00	0.00
1447724_x_at	0.00	0.00
1447725_at	0.00	0.00
1447726_at	0.00	0.00
1447727_at	0.00	0.00
1447728_x_at	0.00	0.00
1447729_s_at	0.00	0.00
1447730_at	0.00	0.00
1447731_at	0.00	0.00
1447732_x_at	0.00	0.00
1447733_x_at	0.00	0.00
1447734_x_at	0.00	0.00
1447735_x_at	0.00	0.00
1447736_at	0.00	0.00
1447737_at	0.00	0.00
1447738_s_at	0.00	0.00
1447739_x_at	0.00	0.00
1447740_at	0.00	0.00
1447741_x_at	0.00	0.00
1447742_at	0.00	0.00
1447743_x_at	0.00	0.00
1447744_s_at	0.00	0.00
1447745_at	0.00	0.00
1447746_at	0.00	0.00
1447747_x_at	0.00	0.00
1447748_x_at	0.00	0.00
1447749_at	0.00	0.00
1447750_x_at	0.00	0.00
1447751_x_at	0.00	0.00
1447752_x_at	0.00	0.00
1447753_at	0.00	0.00
1447754_x_at	0.00	0.00
1447755_at	0.00	0.00
1447756_x_at	0.00	0.00

1447757_x_at	0.00	0.00
1447758_x_at	0.00	0.00
1447759_x_at	0.00	0.00
1447760_x_at	0.00	0.00
1447761_x_at	0.00	0.00
1447762_x_at	0.00	0.00
1447763_at	0.00	0.00
1447764_at	0.00	0.00
1447765_at	0.00	0.00
1447766_x_at	0.03	0.00
1447767_at	0.00	0.00
1447768_at	0.00	0.00
1447769_x_at	0.00	0.00
1447770_at	0.00	0.00
1447771_at	0.00	0.00
1447772_at	0.00	0.00
1447773_x_at	0.00	0.00
1447774_x_at	0.00	0.19
1447775_x_at	0.00	0.00
1447776_x_at	0.00	0.01
1447777_x_at	0.00	0.00
1447778_x_at	0.00	0.00
1447779_x_at	0.00	0.10
1447780_x_at	0.00	0.07
1447781_s_at	0.00	0.07
1447782_x_at	0.00	0.00
1447783_x_at	0.00	0.00
1447784_x_at	0.00	0.00
1447785_x_at	0.00	0.00
1447786_at	0.00	0.00
1447787_x_at	0.00	0.00
1447788_s_at	0.00	0.00
1447789_x_at	0.00	0.00
1447790_at	0.00	0.00
1447791_s_at	0.00	0.00
1447792_x_at	0.00	0.00
1447793_x_at	0.00	0.00
1447794_x_at	0.00	0.00
1447795_at	0.00	0.00
1447796_at	0.00	0.00
1447797_x_at	0.00	0.00
1447798_at	0.00	0.00
1447799_x_at	0.00	0.00
1447800_x_at	0.00	0.00
1447801_x_at	0.00	0.00
1447802_x_at	0.00	0.00
1447803_x_at	0.00	0.00
1447804_x_at	0.00	0.00
1447805_s_at	0.00	0.00
1447806_s_at	0.00	0.00
1447807_s_at	0.00	0.00
1447808_s_at	0.00	0.00
1447809_x_at	0.00	0.00
1447810_x_at	0.00	0.00
1447811_s_at	0.00	0.00
1447812_x_at	0.02	0.00

1447813_x_at	0.00	0.00
1447814_x_at	0.00	0.00
1447815_x_at	0.00	0.00
1447816_x_at	0.00	0.00
1447817_at	0.00	0.00
1447818_x_at	0.00	0.00
1447819_x_at	0.00	0.00
1447821_at	0.00	0.00
1447822_x_at	0.00	0.00
1447823_x_at	0.00	0.00
1447824_x_at	0.00	0.00
1447825_x_at	0.00	0.00
1447826_x_at	0.00	0.00
1447827_x_at	0.00	0.00
1447828_x_at	0.00	0.00
1447829_x_at	0.00	0.00
1447830_s_at	0.00	0.00
1447831_s_at	0.00	0.00
1447832_x_at	0.00	0.00
1447833_x_at	0.00	0.00
1447834_at	0.00	0.00
1447835_at	0.00	0.00
1447836_x_at	0.00	0.00
1447837_x_at	0.00	0.00
1447838_x_at	0.00	0.00
1447839_x_at	0.00	0.00
1447840_x_at	0.00	0.00
1447841_x_at	0.00	0.00
1447842_x_at	0.00	0.00
1447843_at	0.00	0.00
1447844_at	0.00	0.00
1447845_s_at	0.00	0.00
1447846_x_at	0.00	0.00
1447847_x_at	0.00	0.00
1447848_at	0.00	0.00
1447849_s_at	0.00	0.00
1447850_x_at	0.00	0.00
1447851_x_at	0.00	0.00
1447852_x_at	0.00	0.00
1447853_x_at	0.00	0.00
1447854_s_at	0.00	0.00
1447855_x_at	0.00	0.00
1447856_x_at	0.00	0.00
1447857_at	0.00	0.00
1447859_at	0.00	0.00
1447860_x_at	0.00	0.00
1447861_x_at	0.00	0.00
1447862_x_at	0.00	0.00
1447863_s_at	0.00	0.00
1447864_s_at	0.00	0.00
1447865_x_at	0.00	0.00
1447866_x_at	0.00	0.00
1447867_x_at	0.00	0.00
1447868_x_at	0.01	0.00
1447869_x_at	0.46	0.00
1447870_x_at	0.00	0.00

1447871_at	0.00	0.00
1447872_at	0.00	0.00
1447873_x_at	0.00	0.00
1447874_x_at	0.00	0.00
1447875_x_at	0.00	0.00
1447876_x_at	0.00	0.00
1447877_x_at	0.00	0.00
1447878_s_at	0.00	0.00
1447879_x_at	0.00	0.00
1447880_x_at	0.00	0.00
1447881_x_at	0.00	0.00
1447882_x_at	0.00	0.00
1447883_x_at	0.00	0.00
1447884_x_at	0.00	0.00
1447885_x_at	0.00	0.00
1447886_at	0.00	0.00
1447887_x_at	0.00	0.00
1447889_x_at	0.00	0.00
1447890_at	0.00	0.00
1447891_at	0.00	0.00
1447892_at	0.00	0.00
1447893_x_at	0.00	0.00
1447894_x_at	0.00	0.00
1447895_x_at	0.00	0.00
1447896_s_at	0.00	0.00
1447897_x_at	0.00	0.00
1447898_s_at	0.00	0.00
1447899_x_at	0.00	0.00
1447900_x_at	0.00	0.00
1447901_x_at	0.00	0.00
1447902_at	0.00	0.00
1447903_x_at	0.00	0.00
1447904_s_at	0.00	0.00
1447905_x_at	0.00	0.00
1447906_at	0.00	0.00
1447907_x_at	0.00	0.00
1447908_x_at	0.00	0.00
1447909_s_at	0.00	0.00
1447910_x_at	0.00	0.00
1447911_at	0.00	0.00
1447912_x_at	0.00	0.00
1447913_x_at	0.00	0.00
1447914_x_at	0.00	0.00
1447915_x_at	0.00	0.00
1447916_at	0.00	0.00
1447917_x_at	0.00	0.00
1447918_x_at	0.00	0.00
1447920_at	0.00	0.00
1447921_at	0.00	0.00
1447922_at	0.00	0.00
1447923_at	0.00	0.00
1447925_at	0.00	0.00
1447927_at	0.00	0.00
1447928_at	0.00	0.00
1447929_at	0.00	0.00
1447930_at	0.00	0.00

1447931_at	0.00	0.00
1447932_at	0.00	0.00
1447933_at	0.00	0.00
1447934_at	0.00	0.00
1447936_at	0.00	0.16
1447937_a_at	0.00	0.00
1447938_at	0.00	0.00
1447939_a_at	0.00	0.00
1447940_a_at	0.00	0.00
1447941_x_at	0.00	0.00
1447943_x_at	0.00	0.00
1447944_at	0.00	0.00
1447946_at	0.00	0.00
1447948_at	0.00	0.00
1447949_at	0.00	0.00
1447950_at	0.00	0.00
1447951_at	0.00	0.00
1447954_at	0.00	0.00
1447955_at	0.00	0.00
1447960_at	0.00	0.00
1447966_a_at	0.00	0.00
1447967_at	0.00	0.29
1447971_at	0.00	0.00
1447972_at	0.00	0.00
1447973_at	0.00	0.00
1447974_s_at	0.00	0.00
1447975_a_at	0.00	0.00
1447979_at	0.00	0.00
1447980_s_at	0.00	0.00
1447987_at	0.00	0.00
1447988_at	0.00	0.00
1447991_at	0.00	0.00
1447992_s_at	0.00	0.00
1447993_a_at	0.00	0.00
1447994_at	0.00	0.00
1447995_x_at	0.00	0.00
1447996_at	0.00	0.00
1448003_at	0.00	0.00
1448006_at	0.00	0.00
1448007_at	0.00	0.00
1448015_at	0.00	0.00
1448023_at	0.00	0.00
1448025_at	0.00	0.00
1448027_at	0.00	0.00
1448033_at	0.00	0.00
1448039_at	0.00	0.00
1448044_a_at	0.00	0.00
1448045_at	0.00	0.00
1448049_at	0.00	0.00
1448052_at	0.00	0.00
1448057_at	0.00	0.00
1448063_at	0.00	0.00
1448066_at	0.00	0.00
1448079_at	0.00	0.00
1448080_at	0.00	0.00
1448083_at	0.00	0.00

1448092_x_at	0.00	0.00
1448094_at	0.00	0.00
1448096_at	0.00	0.00
1448099_at	0.00	0.00
1449601_x_at	0.00	0.00
1449602_at	0.00	0.00
1449606_at	0.00	0.00
1449607_at	0.00	0.00
1449608_a_at	0.00	0.00
1449610_at	0.00	0.00
1449612_x_at	0.00	0.00
1449613_at	0.00	0.00
1449616_s_at	0.00	0.00
1449626_s_at	0.00	0.00
1449627_at	0.00	0.00
1449634_a_at	0.00	0.00
1449655_a_at	0.00	0.00
1449666_at	0.00	0.00
1449667_at	0.00	0.00
1449668_s_at	0.00	0.01
1449695_at	0.00	0.00
1449696_at	0.00	0.00
1449709_s_at	0.00	0.00
1449722_at	0.00	0.00
1449723_at	0.00	0.00
1449725_at	0.00	0.00
1449734_s_at	0.00	0.00
1449737_at	0.00	0.00
1449743_a_at	0.00	0.00
1449746_s_at	0.00	0.00
1449758_at	0.00	0.00
1449762_at	0.00	0.00
1449763_at	0.00	0.00
1449766_at	0.00	0.00
1449767_x_at	0.00	0.00
1449769_at	0.00	0.00
1449775_x_at	0.00	0.00
1449777_at	0.00	0.00
1449778_at	0.00	0.00
1449786_at	0.00	0.00
1449789_x_at	0.00	0.00
1449790_at	0.00	0.00
1449791_x_at	0.00	0.00
1449792_at	0.00	0.00
1449794_x_at	0.00	0.00
1449798_at	0.00	0.00
1449802_x_at	0.00	0.00
1449807_x_at	0.00	0.00
1452672_at	0.00	0.00
1452678_a_at	0.00	0.00
1452682_at	0.00	0.00
1452684_at	0.00	0.00
1452687_at	0.00	0.00
1452689_at	0.00	0.00
1452690_at	0.02	0.00
1452693_at	0.00	0.04

1452695_at	0.00	0.00
1452696_a_at	0.00	0.00
1452698_at	0.00	0.00
1452700_s_at	0.00	0.20
1452702_at	0.00	0.00
1452706_a_at	0.00	0.00
1452707_at	0.00	0.00
1452711_at	0.00	0.00
1452717_at	0.76	0.00
1452719_at	0.00	0.00
1452722_a_at	0.00	0.00
1452724_at	0.00	0.00
1452725_a_at	0.00	0.00
1452727_at	0.00	0.00
1452728_at	0.00	0.00
1452729_at	0.00	0.00
1452733_at	0.00	0.00
1452737_at	0.00	0.00
1452738_at	0.00	0.00
1452739_at	0.00	0.00
1452740_at	0.09	0.05
1452741_s_at	0.00	0.00
1452745_at	0.00	0.00
1452748_at	0.00	0.00
1452749_at	0.00	0.00
1452750_at	0.00	0.00
1452755_at	0.00	0.00
1452756_at	0.00	0.00
1452759_s_at	0.00	0.00
1452760_at	0.00	0.00
1452761_a_at	0.00	0.00
1452762_at	0.00	0.00
1452763_at	0.00	0.00
1452766_at	0.00	0.00
1452771_s_at	0.00	0.65
1452775_at	0.00	0.00
1452779_at	0.00	0.00
1452783_at	0.00	0.23
1452785_at	0.00	0.00
1452786_at	0.00	0.00
1452788_at	0.00	0.00
1452789_at	0.00	0.00
1452791_at	0.00	0.00
1452794_x_at	0.00	0.00
1452795_at	0.00	0.00
1452796_at	0.00	0.02
1452797_at	0.00	0.13
1452798_s_at	0.00	0.00
1452800_a_at	0.00	0.00
1452802_at	0.00	0.00
1452804_at	0.00	0.00
1452805_at	0.00	0.00
1452806_at	0.00	0.00
1452807_s_at	0.00	0.00
1452808_at	0.00	0.00
1452809_at	0.00	0.00

1452810_at	0.00	0.00
1452811_at	0.00	0.36
1452814_at	0.00	0.00
1452815_at	0.00	0.00
1452816_at	0.00	0.00
1452817_at	0.00	0.00
1452818_at	0.00	0.00
1452819_at	0.00	0.00
1452820_at	0.00	0.00
1452821_at	0.00	0.00
1452824_at	0.00	0.00
1452825_at	0.00	0.00
1452826_s_at	0.00	0.10
1452827_at	0.00	0.00
1452832_s_at	0.00	0.32
1452833_at	0.00	0.00
1452834_at	0.00	0.08
1452840_at	0.00	0.00
1452841_at	0.00	0.00
1452842_at	0.00	0.00
1452845_at	0.00	0.00
1452847_at	0.00	0.00
1452848_at	0.00	0.00
1452849_at	0.00	0.00
1452850_s_at	0.00	0.00
1452851_at	0.00	0.00
1452852_at	0.00	0.00
1452853_at	0.00	0.00
1452854_at	0.00	0.00
1452855_at	0.00	0.00
1452856_at	0.00	0.03
1452857_at	0.00	0.00
1452858_at	0.00	0.05
1452859_at	0.00	0.00
1452860_at	0.00	0.00
1452861_at	0.00	0.00
1452863_at	0.00	0.00
1452865_at	0.00	0.00
1452867_at	0.00	0.00
1452868_at	0.00	0.00
1452871_at	0.00	0.00
1452872_at	0.00	0.00
1452873_at	0.00	0.00
1452874_at	0.00	0.00
1452875_at	0.00	0.00
1452876_x_at	0.00	0.00
1452879_at	0.00	0.00
1452881_at	0.00	0.00
1452883_a_at	0.15	0.00
1452886_at	0.00	0.00
1452887_at	0.00	0.00
1452888_at	0.00	0.00
1452890_at	0.00	0.00
1452891_at	0.00	0.00
1452892_at	0.00	0.00
1452893_s_at	0.00	0.00

1452897_at	0.00	0.00
1452898_at	0.00	0.00
1452901_at	0.00	0.00
1452902_at	0.00	0.00
1452903_at	0.00	0.00
1452904_at	0.00	0.00
1452905_at	0.00	0.00
1452906_at	0.00	0.00
1452908_at	0.00	0.00
1452909_at	0.00	0.00
1452910_at	0.00	0.00
1452911_at	0.00	0.00
1452912_at	0.00	0.00
1452914_at	0.00	0.00
1452915_at	0.00	0.00
1452916_at	0.00	0.00
1452918_at	0.00	0.00
1452921_at	0.00	0.00
1452922_at	0.00	0.00
1452923_at	0.00	0.01
1452924_at	0.00	0.00
1452926_at	0.00	0.00
1452928_at	0.00	0.00
1452930_at	0.00	0.00
1452932_at	0.00	0.00
1452933_at	0.00	0.00
1452934_at	0.00	0.00
1452935_at	0.00	0.00
1452936_at	0.00	0.00
1452937_s_at	0.00	0.00
1452938_at	0.00	0.00
1452942_at	0.00	0.00
1452943_at	0.00	0.00
1452944_at	0.00	0.00
1452945_at	0.00	0.00
1452947_at	0.00	0.00
1452948_at	0.00	0.00
1452949_at	0.00	0.00
1452950_at	0.00	0.00
1452951_at	0.00	0.00
1452952_at	0.00	0.00
1452953_at	0.00	0.00
1452955_at	0.00	0.00
1452956_a_at	0.00	0.00
1452958_at	0.00	0.00
1452960_at	0.00	0.00
1452962_at	0.00	0.00
1452963_at	0.00	0.00
1452964_at	0.00	0.00
1452965_at	0.00	0.00
1452966_at	0.00	0.00
1452967_at	0.00	0.00
1452968_at	0.00	0.17
1452969_at	0.00	0.00
1452970_at	0.00	0.00
1452971_at	0.00	0.00

1452972_at	0.00	0.00
1452973_at	0.00	0.00
1452974_at	0.02	0.00
1452975_at	0.00	0.00
1452977_at	0.00	0.00
1452978_at	0.00	0.00
1452979_at	0.00	0.00
1452980_at	0.00	0.00
1452982_at	0.00	0.33
1452983_at	0.00	0.00
1452984_at	0.00	0.00
1452985_at	0.00	0.00
1452988_at	0.00	0.00
1452989_at	0.00	0.00
1452990_at	0.00	0.00
1452991_at	0.00	0.00
1452992_at	0.00	0.00
1452993_at	0.00	0.00
1452994_at	0.00	0.00
1452995_at	0.00	0.00
1452996_a_at	0.00	0.30
1452998_at	0.00	0.00
1453000_at	0.00	0.00
1453001_at	0.00	0.00
1453002_at	0.00	0.00
1453003_at	0.00	0.00
1453006_at	0.00	0.00
1453007_at	0.00	0.00
1453008_at	0.00	0.00
1453009_at	0.00	0.00
1453010_at	0.00	0.00
1453011_at	0.00	0.00
1453012_at	0.00	0.00
1453017_at	0.00	0.00
1453020_at	0.00	0.00
1453021_at	0.00	0.00
1453022_at	0.00	0.00
1453023_at	0.00	0.00
1453024_at	0.00	0.00
1453027_at	0.00	0.00
1453028_at	0.00	0.00
1453029_s_at	0.00	0.00
1453031_at	0.00	0.00
1453032_at	0.00	0.00
1453034_at	0.00	0.00
1453035_at	0.00	0.00
1453036_at	0.00	0.00
1453038_at	0.00	0.00
1453039_at	0.00	0.00
1453040_at	0.00	0.00
1453041_at	0.05	0.00
1453042_at	0.00	0.00
1453043_at	0.00	0.00
1453044_at	0.00	0.00
1453045_at	0.00	0.00
1453046_at	0.00	0.00

1453048_at	0.00	0.00
1453049_at	0.00	0.00
1453050_at	0.00	0.00
1453051_at	0.00	0.00
1453052_at	0.00	0.00
1453053_at	0.00	0.00
1453054_at	0.00	0.01
1453055_at	0.00	0.00
1453056_at	0.00	0.00
1453057_at	0.00	0.00
1453058_at	0.00	0.00
1453059_at	0.00	0.00
1453061_at	0.00	0.00
1453062_at	0.00	0.00
1453065_at	0.00	0.01
1453066_at	0.00	0.00
1453067_at	0.00	0.03
1453068_at	0.00	0.00
1453069_at	0.00	0.00
1453070_at	0.00	0.00
1453071_s_at	0.00	0.36
1453072_at	0.00	0.00
1453073_at	0.00	0.00
1453074_at	0.00	0.00
1453075_at	0.00	0.00
1453078_at	0.00	0.00
1453079_at	0.00	0.00
1453080_at	0.00	0.00
1453081_at	0.00	0.00
1453082_at	0.00	0.00
1453083_at	0.00	0.00
1453085_at	0.00	0.00
1453087_at	0.00	0.00
1453088_at	0.00	0.00
1453091_s_at	0.00	0.00
1453093_at	0.00	0.00
1453094_at	0.00	0.00
1453101_at	0.00	0.00
1453102_at	0.00	0.00
1453103_at	0.00	0.00
1453104_at	0.00	0.00
1453105_at	0.00	0.00
1453108_at	0.00	0.00
1453109_at	0.00	0.00
1453110_at	0.00	0.00
1453114_at	0.00	0.01
1453115_at	0.00	0.00
1453116_at	0.00	0.00
1453118_s_at	0.00	0.00
1453119_at	0.00	0.00
1453120_at	0.00	0.00
1453121_at	0.00	0.00
1453122_at	0.00	0.20
1453123_at	0.00	0.00
1453125_at	0.06	0.33
1453126_at	0.00	0.00

1453127_at	0.00	0.00
1453129_a_at	0.00	0.00
1453130_at	0.00	0.00
1453131_at	0.00	0.00
1453133_at	0.00	0.00
1453134_at	0.00	0.00
1453135_at	0.00	0.00
1453136_at	0.00	0.00
1453137_at	0.00	0.00
1453138_at	0.00	0.00
1453139_at	0.00	0.00
1453140_at	0.00	0.00
1453141_at	0.00	0.00
1453142_at	0.00	0.00
1453143_at	0.00	0.00
1453144_at	0.00	0.00
1453145_at	0.00	0.00
1453146_at	0.00	0.00
1453148_at	0.00	0.00
1453149_at	0.00	0.00
1453150_at	0.00	0.00
1453151_at	0.00	0.00
1453152_at	0.00	0.00
1453153_at	0.00	0.00
1453154_at	0.00	0.00
1453155_at	0.00	0.30
1453157_at	0.00	0.00
1453158_at	0.00	0.00
1453159_at	0.00	0.00
1453160_at	0.00	0.00
1453161_at	0.00	0.00
1453163_at	0.00	0.00
1453165_at	0.00	0.00
1453166_at	0.00	0.00
1453167_at	0.00	0.00
1453168_at	0.00	0.00
1453170_at	0.00	0.00
1453171_s_at	0.00	0.00
1453172_at	0.00	0.00
1453173_at	0.00	0.00
1453174_at	0.00	0.00
1453176_a_at	0.00	0.00
1453177_at	0.00	0.00
1453178_at	0.00	0.00
1453179_at	0.00	0.00
1453180_at	0.00	0.00
1453183_at	0.00	0.00
1453185_at	0.00	0.00
1453186_at	0.00	0.00
1453187_at	0.00	0.00
1453188_at	0.00	0.00
1453189_at	0.00	0.00
1453190_at	0.00	0.00
1453191_at	0.00	0.00
1453192_at	0.00	0.00
1453193_s_at	0.00	0.00

1453194_at	0.00	0.00
1453197_at	0.00	0.00
1453201_at	0.00	0.00
1453202_at	0.00	0.00
1453203_at	0.00	0.00
1453204_at	0.00	0.00
1453205_at	0.00	0.00
1453209_at	0.00	0.00
1453210_at	0.00	0.00
1453211_at	0.00	0.00
1453212_at	0.00	0.00
1453213_at	0.00	0.00
1453214_at	0.00	0.00
1453215_at	0.00	0.00
1453216_at	0.00	0.00
1453217_at	0.00	0.00
1453218_at	0.00	0.00
1453219_a_at	0.81	0.00
1453220_at	0.00	0.00
1453221_at	0.00	0.00
1453222_at	0.00	0.00
1453224_at	0.00	0.00
1453225_at	0.00	0.00
1453226_at	0.00	0.00
1453227_at	0.00	0.00
1453228_at	0.00	0.00
1453229_s_at	0.00	0.00
1453230_at	0.00	0.00
1453231_at	0.00	0.00
1453232_at	0.00	0.00
1453233_s_at	0.00	0.00
1453234_at	0.00	0.00
1453235_at	0.00	0.00
1453236_at	0.00	0.00
1453240_a_at	0.00	0.00
1453241_a_at	0.00	0.00
1453242_x_at	0.00	0.00
1453243_at	0.00	0.00
1453244_at	0.00	0.00
1453245_at	0.00	0.00
1453246_at	0.00	0.00
1453247_at	0.00	0.00
1453248_at	0.00	0.00
1453250_at	0.00	0.00
1453252_at	0.00	0.00
1453254_at	0.00	0.00
1453255_at	0.00	0.00
1453257_at	0.00	0.00
1453259_at	0.00	0.00
1453261_at	0.00	0.00
1453262_at	0.00	0.00
1453263_at	0.00	0.00
1453264_at	0.00	0.00
1453266_at	0.00	0.00
1453267_at	0.00	0.00
1453268_at	0.00	0.00

1453269_at	0.09	0.00
1453270_a_at	0.00	0.00
1453271_at	0.02	0.00
1453272_at	0.00	0.00
1453273_at	0.00	0.00
1453274_at	0.00	0.00
1453275_at	0.00	0.00
1453276_at	0.00	0.00
1453277_at	0.00	0.00
1453279_x_at	0.00	0.00
1453280_at	0.00	0.00
1453282_at	0.08	0.00
1453284_at	0.00	0.00
1453285_at	0.00	0.00
1453286_at	0.00	0.00
1453288_at	0.00	0.00
1453289_at	0.00	0.00
1453290_at	0.00	0.00
1453291_at	0.00	0.00
1453292_at	0.00	0.00
1453293_a_at	0.00	0.00
1453294_at	0.00	0.00
1453295_at	0.00	0.00
1453296_at	0.00	0.00
1453297_at	0.00	0.00
1453300_at	0.00	0.00
1453302_at	0.00	0.00
1453303_at	0.00	0.00
1453304_s_at	0.00	0.00
1453305_at	0.00	0.00
1453306_at	0.00	0.00
1453308_at	0.00	0.00
1453309_at	0.00	0.00
1453310_at	0.00	0.00
1453311_at	0.00	0.00
1453312_at	0.00	0.00
1453313_at	0.00	0.00
1453315_at	0.00	0.00
1453316_at	0.00	0.00
1453318_at	0.00	0.00
1453319_at	0.00	0.00
1453320_at	0.00	0.00
1453322_at	0.00	0.00
1453323_at	0.00	0.00
1453325_at	0.00	0.00
1453326_at	0.00	0.00
1453327_at	0.00	0.00
1453328_at	0.00	0.00
1453329_s_at	0.00	0.00
1453330_at	0.00	0.00
1453331_at	0.00	0.00
1453332_at	0.00	0.00
1453333_at	0.00	0.00
1453334_at	0.00	0.00
1453335_a_at	0.00	0.00
1453336_at	0.00	0.00

1453337_at	0.00	0.00
1453339_at	0.00	0.00
1453340_at	0.00	0.00
1453341_a_at	0.00	0.00
1453342_at	0.00	0.00
1453344_at	0.00	0.00
1453345_at	0.00	0.00
1453346_at	0.00	0.00
1453347_at	0.00	0.00
1453348_at	0.00	0.00
1453349_at	0.00	0.00
1453350_at	0.00	0.00
1453351_at	0.00	0.00
1453352_at	0.00	0.00
1453353_at	0.00	0.00
1453354_at	0.00	0.00
1453356_at	0.00	0.00
1453357_at	0.00	0.00
1453358_s_at	0.00	0.00
1453359_at	0.00	0.00
1453361_at	0.00	0.00
1453363_at	0.00	0.00
1453364_x_at	0.00	0.00
1453365_at	0.00	0.00
1453366_at	0.00	0.00
1453368_at	0.00	0.00
1453370_at	0.00	0.00
1453371_at	0.00	0.00
1453372_at	0.00	0.00
1453373_at	0.00	0.00
1453374_at	0.00	0.00
1453375_at	0.00	0.00
1453376_at	0.00	0.00
1453377_at	0.00	0.00
1453378_at	0.00	0.00
1453379_at	0.00	0.00
1453380_a_at	0.00	0.00
1453381_at	0.00	0.00
1453382_at	0.00	0.00
1453383_at	0.00	0.00
1453384_at	0.00	0.00
1453385_at	0.00	0.00
1453386_at	0.00	0.00
1453387_at	0.00	0.00
1453388_at	0.00	0.00
1453390_at	0.00	0.00
1453391_at	0.00	0.00
1453394_at	0.00	0.00
1453395_at	0.00	0.00
1453396_at	0.00	0.00
1453397_at	0.00	0.00
1453398_at	0.00	0.00
1453399_at	0.00	0.00
1453400_at	0.00	0.00
1453401_at	0.00	0.00
1453402_at	0.00	0.00

1453403_at	0.00	0.00
1453404_at	0.00	0.00
1453405_at	0.00	0.00
1453407_at	0.00	0.00
1453408_s_at	0.00	0.00
1453409_at	0.00	0.00
1453411_at	0.00	0.00
1453414_at	0.00	0.00
1453415_at	0.00	0.00
1453416_at	0.00	0.00
1453417_at	0.00	0.00
1453418_at	0.00	0.00
1453419_at	0.00	0.00
1453420_at	0.00	0.00
1453421_at	0.00	0.00
1453422_a_at	0.00	0.00
1453423_at	0.00	0.00
1453424_at	0.00	0.00
1453425_at	0.00	0.00
1453428_at	0.00	0.00
1453429_at	0.00	0.00
1453430_at	0.00	0.00
1453431_at	0.00	0.00
1453432_at	0.00	0.00
1453433_at	0.00	0.00
1453434_at	0.00	0.00
1453435_a_at	0.00	0.00
1453436_at	0.00	0.00
1453437_at	0.00	0.00
1453438_x_at	0.00	0.00
1453439_at	0.00	0.00
1453440_at	0.00	0.00
1453441_at	0.00	0.00
1453442_at	0.00	0.00
1453443_at	0.00	0.00
1453444_at	0.00	0.00
1453445_at	0.00	0.00
1453446_at	0.00	0.00
1453447_at	0.00	0.00
1453448_at	0.00	0.00
1453449_at	0.00	0.00
1453451_at	0.00	0.00
1453452_at	0.00	0.00
1453453_at	0.00	0.00
1453454_at	0.00	0.00
1453455_at	0.00	0.00
1453456_at	0.00	0.00
1453457_at	0.00	0.00
1453458_at	0.00	0.00
1453459_at	0.00	0.00
1453460_at	0.00	0.00
1453462_at	0.00	0.00
1453463_at	0.00	0.00
1453464_at	0.00	0.00
1453465_x_at	0.00	0.00
1453469_at	0.00	0.00

1453471_at	0.00	0.00
1453475_at	0.00	0.00
1453476_at	0.00	0.00
1453477_at	0.00	0.00
1453478_at	0.00	0.00
1453479_at	0.00	0.00
1453480_at	0.00	0.00
1453481_at	0.00	0.00
1453482_at	0.00	0.00
1453483_at	0.00	0.00
1453484_at	0.00	0.00
1453485_s_at	0.00	0.00
1453487_at	0.00	0.00
1453488_at	0.00	0.00
1453489_at	0.00	0.00
1453490_at	0.00	0.00
1453491_at	0.00	0.00
1453492_at	0.00	0.00
1453493_at	0.00	0.00
1453494_at	0.00	0.00
1453495_at	0.00	0.00
1453496_at	0.00	0.00
1453499_at	0.00	0.00
1453500_at	0.00	0.00
1453502_at	0.00	0.00
1453503_at	0.00	0.00
1453504_at	0.00	0.00
1453506_at	0.00	0.00
1453507_at	0.00	0.00
1453508_at	0.00	0.00
1453509_at	0.00	0.00
1453510_s_at	0.00	0.00
1453511_at	0.00	0.00
1453512_at	0.00	0.00
1453513_at	0.00	0.00
1453514_at	0.00	0.00
1453516_at	0.00	0.00
1453518_at	0.00	0.00
1453519_at	0.00	0.00
1453520_at	0.00	0.00
1453521_at	0.00	0.00
1453522_at	0.00	0.00
1453523_at	0.00	0.00
1453524_at	0.00	0.00
1453525_at	0.00	0.00
1453526_at	0.00	0.00
1453529_at	0.00	0.00
1453530_at	0.00	0.00
1453531_at	0.00	0.00
1453532_at	0.00	0.00
1453533_at	0.00	0.00
1453534_at	0.00	0.00
1453535_at	0.00	0.00
1453536_at	0.00	0.00
1453537_a_at	0.00	0.00
1453538_at	0.00	0.00

1453539_at	0.00	0.00
1453540_at	0.00	0.00
1453541_at	0.00	0.00
1453542_at	0.00	0.00
1453543_at	0.00	0.00
1453544_at	0.00	0.00
1453545_at	0.00	0.00
1453546_at	0.00	0.00
1453547_at	0.00	0.00
1453548_at	0.00	0.00
1453549_at	0.00	0.00
1453551_at	0.00	0.00
1453552_at	0.00	0.00
1453555_at	0.00	0.00
1453557_at	0.00	0.00
1453558_at	0.00	0.00
1453561_x_at	0.00	0.00
1453562_a_at	0.00	0.00
1453563_at	0.00	0.00
1453565_at	0.00	0.00
1453566_at	0.00	0.00
1453568_at	0.00	0.00
1453575_at	0.00	0.00
1453577_at	0.00	0.00
1453579_at	0.00	0.00
1453580_at	0.00	0.00
1453581_at	0.00	0.00
1453582_at	0.00	0.00
1453583_at	0.00	0.00
1453584_at	0.00	0.00
1453585_at	0.00	0.00
1453586_at	0.00	0.00
1453587_at	0.00	0.00
1453588_at	0.00	0.00
1453590_at	0.00	0.00
1453591_at	0.00	0.00
1453592_at	0.00	0.00
1453593_at	0.78	0.00
1453594_at	0.00	0.00
1453595_at	0.00	0.00
1453597_at	0.00	0.00
1453598_at	0.00	0.00
1453599_at	0.00	0.00
1453600_at	0.00	0.00
1453601_at	0.00	0.00
1453602_at	0.00	0.00
1453603_at	0.00	0.00
1453605_s_at	0.00	0.00
1453606_at	0.00	0.00
1453607_at	0.00	0.00
1453608_at	0.00	0.00
1453610_at	0.00	0.00
1453611_at	0.00	0.00
1453615_at	0.00	0.00
1453616_at	0.00	0.00
1453617_at	0.00	0.00

1453618_at	0.00	0.00
1453619_at	0.00	0.00
1453620_at	0.00	0.00
1453622_s_at	0.00	0.00
1453624_at	0.00	0.00
1453625_at	0.00	0.00
1453626_at	0.00	0.00
1453627_at	0.00	0.00
1453628_s_at	0.07	0.32
1453629_at	0.00	0.00
1453630_at	0.00	0.00
1453632_at	0.00	0.00
1453635_at	0.00	0.00
1453636_at	0.00	0.00
1453637_at	0.00	0.00
1453638_at	0.00	0.00
1453639_s_at	0.00	0.00
1453640_at	0.00	0.00
1453641_at	0.00	0.00
1453642_at	0.00	0.00
1453643_at	0.00	0.00
1453645_at	0.00	0.00
1453646_at	0.00	0.00
1453648_at	0.00	0.00
1453649_at	0.00	0.00
1453650_at	0.00	0.00
1453652_at	0.00	0.00
1453653_at	0.00	0.00
1453654_at	0.00	0.00
1453655_at	0.00	0.00
1453656_a_at	0.00	0.00
1453657_at	0.00	0.00
1453658_at	0.00	0.00
1453660_at	0.00	0.00
1453661_at	0.00	0.00
1453662_at	0.00	0.00
1453663_at	0.00	0.00
1453664_at	0.00	0.00
1453665_at	0.00	0.00
1453667_at	0.00	0.00
1453668_at	0.00	0.00
1453669_at	0.00	0.00
1453670_at	0.00	0.00
1453671_at	0.00	0.00
1453672_at	0.00	0.00
1453673_at	0.00	0.00
1453675_at	0.00	0.00
1453676_at	0.00	0.00
1453679_at	0.00	0.00
1453680_at	0.00	0.00
1453681_at	0.00	0.00
1453682_at	0.00	0.00
1453684_s_at	0.00	0.00
1453685_at	0.00	0.00
1453687_at	0.00	0.00
1453688_at	0.00	0.00

1453689_at	0.00	0.00
1453690_at	0.00	0.00
1453691_at	0.00	0.00
1453692_at	0.00	0.00
1453693_at	0.00	0.00
1453694_at	0.00	0.00
1453695_at	0.00	0.00
1453696_at	0.00	0.00
1453697_at	0.00	0.00
1453698_at	0.00	0.00
1453699_at	0.00	0.00
1453700_s_at	0.00	0.00
1453701_at	0.00	0.00
1453702_at	0.00	0.00
1453703_at	0.00	0.00
1453704_at	0.00	0.00
1453705_at	0.00	0.00
1453706_at	0.00	0.00
1453707_at	0.00	0.00
1453708_a_at	0.00	0.00
1453709_at	0.00	0.00
1453711_at	0.00	0.00
1453713_s_at	0.00	0.00
1453715_at	0.00	0.00
1453716_at	0.00	0.00
1453717_s_at	0.00	0.00
1453718_at	0.00	0.00
1453719_at	0.00	0.00
1453720_at	0.00	0.00
1453726_s_at	0.00	0.00
1453727_at	0.00	0.00
1453730_at	0.00	0.00
1453732_at	0.00	0.00
1453734_at	0.00	0.00
1453735_at	0.00	0.00
1453736_s_at	0.00	0.00
1453737_at	0.00	0.00
1453738_at	0.00	0.00
1453739_at	0.00	0.00
1453742_at	0.00	0.00
1453743_x_at	0.00	0.00
1453745_at	0.00	0.00
1453746_at	0.00	0.00
1453747_at	0.00	0.00
1453749_at	0.00	0.00
1453751_at	0.00	0.01
1453754_at	0.00	0.00
1453755_at	0.00	0.00
1453756_at	0.00	0.00
1453757_at	0.00	0.00
1453759_at	0.00	0.00
1453760_at	0.00	0.00
1453761_at	0.00	0.00
1453762_at	0.00	0.00
1453763_at	0.00	0.00
1453764_at	0.00	0.00

1453765_at	0.00	0.00
1453766_a_at	0.00	0.00
1453769_at	0.00	0.00
1453770_at	0.00	0.00
1453771_at	0.01	0.00
1453773_at	0.00	0.00
1453774_at	0.00	0.00
1453775_at	0.00	0.00
1453776_at	0.00	0.00
1453778_at	0.00	0.00
1453779_at	0.00	0.00
1453781_at	0.00	0.00
1453782_at	0.00	0.00
1453783_at	0.00	0.00
1453785_at	0.00	0.00
1453786_at	0.00	0.00
1453787_at	0.00	0.00
1453789_at	0.00	0.00
1453790_at	0.00	0.00
1453791_at	0.00	0.00
1453792_at	0.00	0.00
1453793_at	0.00	0.00
1453794_at	0.00	0.00
1453795_at	0.00	0.00
1453797_at	0.00	0.00
1453798_at	0.00	0.00
1453799_at	0.00	0.00
1453800_at	0.00	0.00
1453801_at	0.00	0.00
1453802_at	0.00	0.00
1453803_at	0.00	0.00
1453805_at	0.00	0.00
1453807_at	0.00	0.00
1453808_at	0.00	0.00
1453809_at	0.00	0.00
1453810_at	0.00	0.00
1453812_at	0.00	0.00
1453813_at	0.00	0.00
1453814_at	0.00	0.00
1453817_at	0.00	0.00
1453818_a_at	0.00	0.00
1453821_at	0.00	0.00
1453822_at	0.00	0.00
1453823_a_at	0.00	0.00
1453824_at	0.00	0.00
1453825_at	0.00	0.00
1453826_at	0.00	0.00
1453828_at	0.00	0.00
1453829_at	0.00	0.00
1453831_at	0.00	0.00
1453832_at	0.00	0.00
1453834_at	0.00	0.00
1453835_at	0.00	0.00
1453837_at	0.00	0.00
1453838_at	0.00	0.00
1453841_at	0.00	0.00

1453842_at	0.00	0.00
1453843_at	0.00	0.00
1453844_at	0.00	0.00
1453845_at	0.00	0.00
1453846_at	0.00	0.00
1453847_at	0.00	0.00
1453850_at	0.00	0.00
1453852_at	0.00	0.00
1453854_at	0.00	0.00
1453855_at	0.00	0.00
1453857_at	0.00	0.00
1453858_at	0.00	0.00
1453859_at	0.00	0.00
1453860_s_at	0.00	0.00
1453861_at	0.00	0.00
1453862_at	0.00	0.00
1453863_at	0.00	0.00
1453868_at	0.00	0.00
1453869_at	0.00	0.00
1453870_at	0.00	0.00
1453871_at	0.00	0.00
1453872_at	0.00	0.00
1453873_at	0.00	0.00
1453874_at	0.00	0.00
1453875_at	0.00	0.00
1453877_at	0.00	0.00
1453878_at	0.00	0.00
1453879_at	0.00	0.00
1453880_s_at	0.00	0.00
1453882_at	0.00	0.00
1453883_at	0.00	0.00
1453884_at	0.00	0.00
1453885_at	0.00	0.00
1453888_at	0.00	0.00
1453889_at	0.00	0.00
1453890_at	0.00	0.00
1453892_at	0.00	0.00
1453893_at	0.00	0.00
1453894_at	0.00	0.00
1453895_at	0.00	0.00
1453896_at	0.00	0.00
1453897_at	0.00	0.00
1453898_at	0.00	0.00
1453899_at	0.00	0.00
1453900_at	0.00	0.00
1453903_at	0.00	0.00
1453904_at	0.00	0.00
1453905_at	0.00	0.00
1453906_at	0.00	0.00
1453907_at	0.00	0.00
1453908_at	0.00	0.00
1453909_at	0.00	0.00
1453910_at	0.00	0.00
1453912_at	0.00	0.00
1453916_at	0.00	0.00
1453917_at	0.00	0.00

1453918_at	0.00	0.00
1453919_at	0.00	0.00
1453921_at	0.00	0.00
1453922_at	0.00	0.00
1453923_at	0.00	0.00
1453925_at	0.00	0.00
1453926_at	0.00	0.00
1453927_at	0.00	0.00
1453929_at	0.00	0.00
1453930_at	0.00	0.00
1453931_at	0.00	0.00
1453932_at	0.00	0.00
1453933_at	0.00	0.00
1453934_at	0.00	0.00
1453935_a_at	0.00	0.00
1453936_at	0.00	0.00
1453937_at	0.00	0.00
1453938_at	0.00	0.00
1453940_at	0.00	0.00
1453941_at	0.00	0.00
1453942_at	0.00	0.00
1453944_at	0.00	0.00
1453945_at	0.00	0.00
1453947_at	0.00	0.00
1453948_at	0.00	0.00
1453949_s_at	0.00	0.00
1453951_a_at	0.00	0.00
1453952_at	0.00	0.00
1453953_at	0.00	0.00
1453955_a_at	0.00	0.00
1453958_at	0.00	0.00
1453959_at	0.00	0.00
1453961_a_at	0.00	0.00
1453963_at	0.00	0.00
1453964_at	0.00	0.00
1453965_at	0.00	0.00
1453966_at	0.00	0.00
1453967_at	0.00	0.00
1453968_at	0.00	0.00
1453969_at	0.00	0.00
1453970_at	0.00	0.00
1453971_at	0.00	0.00
1453973_s_at	0.00	0.00
1453974_at	0.00	0.00
1453975_a_at	0.00	0.00
1453976_at	0.00	0.00
1453978_at	0.00	0.00
1453979_at	0.00	0.00
1453980_at	0.00	0.00
1453982_at	0.00	0.00
1453983_a_at	0.01	0.33
1453984_at	0.00	0.00
1453987_at	0.00	0.00
1453990_at	0.00	0.00
1453992_at	0.00	0.00
1453994_at	0.00	0.00

1453999_at	0.00	0.00
1454000_s_at	0.00	0.00
1454001_at	0.00	0.00
1454002_at	0.00	0.00
1454003_at	0.00	0.00
1454004_at	0.00	0.00
1454009_at	0.00	0.00
1454010_a_at	0.00	0.00
1454012_a_at	0.00	0.00
1454013_at	0.00	0.00
1454017_at	0.00	0.00
1454019_at	0.00	0.00
1454020_at	0.00	0.00
1454024_at	0.00	0.00
1454025_at	0.00	0.00
1454027_at	0.00	0.00
1454028_at	0.00	0.00
1454029_at	0.00	0.00
1454031_at	0.00	0.00
1454032_at	0.00	0.00
1454033_at	0.00	0.00
1454035_at	0.00	0.00
1454038_at	0.00	0.00
1454039_at	0.00	0.00
1454040_at	0.00	0.00
1454049_at	0.00	0.00
1454050_at	0.00	0.00
1454051_at	0.00	0.00
1454052_at	0.00	0.00
1454053_at	0.00	0.00
1454054_at	0.00	0.00
1454055_at	0.00	0.00
1454056_at	0.00	0.00
1454057_at	0.00	0.00
1454058_at	0.00	0.00
1454059_at	0.00	0.00
1454062_at	0.00	0.00
1454063_at	0.00	0.00
1454065_at	0.00	0.00
1454068_at	0.00	0.00
1454069_at	0.00	0.00
1454071_at	0.00	0.00
1454072_at	0.00	0.00
1454073_at	0.00	0.00
1454075_s_at	0.00	0.00
1454076_at	0.00	0.00
1454077_at	0.00	0.00
1454079_at	0.00	0.00
1454080_at	0.00	0.00
1454081_at	0.00	0.00
1454083_at	0.00	0.00
1454084_a_at	0.00	0.00
1454085_at	0.00	0.00
1454087_at	0.00	0.00
1454088_at	0.00	0.00
1454089_at	0.00	0.00

1454091_at	0.00	0.00
1454093_at	0.00	0.00
1454094_at	0.00	0.00
1454095_at	0.00	0.00
1454096_a_at	0.00	0.00
1454097_at	0.00	0.00
1454098_at	0.00	0.00
1454099_at	0.00	0.00
1454100_at	0.00	0.00
1454101_at	0.00	0.00
1454102_at	0.00	0.00
1454103_at	0.00	0.00
1454104_a_at	0.00	0.00
1454105_at	0.00	0.00
1454108_at	0.00	0.00
1454110_at	0.00	0.00
1454111_at	0.00	0.00
1454112_a_at	0.00	0.00
1454113_at	0.00	0.00
1454115_at	0.00	0.00
1454117_at	0.00	0.00
1454118_at	0.00	0.00
1454121_x_at	0.00	0.00
1454122_at	0.00	0.00
1454123_at	0.00	0.00
1454124_at	0.00	0.00
1454125_a_at	0.00	0.00
1454126_at	0.00	0.00
1454127_at	0.00	0.00
1454128_at	0.00	0.00
1454129_at	0.00	0.00
1454130_at	0.00	0.00
1454131_at	0.00	0.00
1454132_at	0.00	0.00
1454133_s_at	0.00	0.00
1454134_at	0.00	0.00
1454135_at	0.00	0.00
1454137_s_at	0.00	0.00
1454139_at	0.00	0.00
1454140_at	0.00	0.00
1454141_at	0.00	0.00
1454143_at	0.00	0.00
1454146_at	0.00	0.00
1454147_at	0.00	0.00
1454148_at	0.00	0.00
1454150_at	0.00	0.00
1454151_at	0.00	0.00
1454153_at	0.00	0.00
1454154_at	0.00	0.00
1454155_at	0.00	0.00
1454156_at	0.00	0.00
1454158_at	0.00	0.00
1454160_at	0.00	0.00
1454162_at	0.00	0.00
1454163_at	0.00	0.00
1454164_at	0.00	0.00

1454165_at	0.00	0.00
1454166_at	0.00	0.00
1454170_at	0.00	0.00
1454171_x_at	0.00	0.00
1454172_at	0.00	0.00
1454173_at	0.00	0.00
1454175_at	0.00	0.00
1454177_at	0.00	0.00
1454178_at	0.00	0.00
1454179_at	0.00	0.00
1454180_at	0.00	0.00
1454181_at	0.00	0.00
1454182_at	0.00	0.00
1454185_at	0.00	0.00
1454186_a_at	0.00	0.00
1454187_at	0.00	0.00
1454188_at	0.00	0.00
1454189_at	0.00	0.00
1454190_at	0.00	0.00
1454191_at	0.00	0.00
1454192_at	0.00	0.00
1454193_at	0.00	0.00
1454194_at	0.00	0.00
1454195_at	0.00	0.00
1454196_at	0.00	0.00
1454198_a_at	0.00	0.00
1454199_at	0.00	0.00
1454200_at	0.00	0.00
1454201_a_at	0.00	0.00
1454202_a_at	0.00	0.00
1454203_at	0.00	0.00
1454204_at	0.00	0.00
1454205_at	0.00	0.00
1454207_at	0.00	0.00
1454208_at	0.00	0.00
1454209_at	0.00	0.00
1454210_at	0.00	0.00
1454212_x_at	0.00	0.00
1454213_at	0.00	0.00
1454214_a_at	0.00	0.00
1454215_at	0.41	0.00
1454216_at	0.00	0.00
1454217_at	0.00	0.00
1454218_at	0.00	0.00
1454220_at	0.00	0.00
1454222_a_at	0.00	0.00
1454223_at	0.00	0.00
1454224_at	0.00	0.00
1454225_s_at	0.00	0.00
1454226_at	0.00	0.00
1454227_at	0.00	0.00
1454230_a_at	0.00	0.00
1454232_at	0.00	0.00
1454233_at	0.00	0.00
1454234_at	0.00	0.00
1454235_a_at	0.00	0.00

1454237_at	0.00	0.00
1454238_a_at	0.00	0.00
1454239_at	0.00	0.00
1454241_at	0.00	0.00
1454242_at	0.00	0.00
1454243_at	0.00	0.00
1454244_at	0.00	0.00
1454245_at	0.00	0.00
1454246_at	0.00	0.00
1454248_at	0.00	0.00
1454249_at	0.00	0.00
1454250_at	0.00	0.00
1454251_at	0.00	0.00
1454252_at	0.00	0.00
1454253_at	0.00	0.00
1454254_s_at	0.00	0.00
1454255_at	0.00	0.00
1454256_s_at	0.00	0.00
1454257_at	0.00	0.00
1454258_at	0.00	0.00
1454259_s_at	0.00	0.00
1454261_at	0.00	0.00
1454262_at	0.00	0.00
1454263_at	0.00	0.00
1454264_at	0.00	0.00
1454266_at	0.00	0.00
1454269_s_at	0.00	0.00
1454270_at	0.00	0.00
1454271_at	0.00	0.00
1454272_at	0.00	0.00
1454273_at	0.00	0.00
1454274_at	0.00	0.00
1454275_at	0.00	0.00
1454276_at	0.00	0.00
1454277_at	0.00	0.00
1454278_at	0.00	0.00
1454279_at	0.00	0.00
1454280_at	0.00	0.00
1454281_at	0.00	0.00
1454282_at	0.00	0.00
1454283_at	0.00	0.00
1454284_at	0.00	0.00
1454285_at	0.00	0.00
1454286_at	0.00	0.00
1454287_at	0.00	0.00
1454288_at	0.00	0.00
1454289_at	0.00	0.00
1454290_at	0.00	0.00
1454291_at	0.00	0.00
1454292_at	0.00	0.00
1454293_at	0.00	0.00
1454294_at	0.00	0.00
1454295_at	0.00	0.00
1454296_at	0.00	0.00
1454297_at	0.00	0.00
1454298_at	0.00	0.00

1454299_at	0.00	0.00
1454300_at	0.00	0.00
1454301_at	0.00	0.00
1454302_at	0.00	0.00
1454303_at	0.00	0.00
1454304_at	0.00	0.00
1454306_at	0.00	0.00
1454307_at	0.00	0.00
1454308_at	0.00	0.00
1454309_at	0.00	0.00
1454310_at	0.00	0.00
1454311_at	0.00	0.00
1454314_at	0.00	0.00
1454315_at	0.00	0.00
1454316_at	0.00	0.00
1454318_at	0.00	0.00
1454319_at	0.00	0.00
1454320_at	0.00	0.00
1454321_at	0.00	0.00
1454322_at	0.00	0.00
1454323_at	0.00	0.00
1454324_at	0.00	0.00
1454325_at	0.00	0.00
1454326_at	0.00	0.00
1454327_at	0.00	0.00
1454328_at	0.00	0.00
1454329_at	0.00	0.00
1454330_at	0.00	0.00
1454331_at	0.00	0.00
1454332_at	0.00	0.00
1454333_at	0.00	0.00
1454334_at	0.00	0.00
1454335_at	0.00	0.00
1454336_at	0.00	0.00
1454337_at	0.00	0.00
1454338_at	0.00	0.00
1454339_at	0.00	0.00
1454340_at	0.00	0.00
1454341_at	0.00	0.00
1454342_at	0.00	0.00
1454343_at	0.00	0.00
1454344_at	0.00	0.00
1454345_at	0.00	0.00
1454346_at	0.00	0.00
1454347_at	0.00	0.00
1454348_at	0.00	0.00
1454349_at	0.00	0.00
1454350_at	0.00	0.00
1454351_at	0.00	0.00
1454352_at	0.00	0.00
1454353_at	0.00	0.00
1454354_at	0.00	0.00
1454355_at	0.00	0.00
1454356_at	0.00	0.00
1454357_at	0.00	0.00
1454358_at	0.00	0.00

1454359_at	0.00	0.00
1454360_at	0.00	0.00
1454361_at	0.00	0.00
1454362_at	0.00	0.00
1454363_at	0.00	0.00
1454364_at	0.00	0.00
1454365_at	0.00	0.00
1454366_at	0.00	0.00
1454367_at	0.00	0.00
1454370_at	0.00	0.00
1454371_at	0.00	0.00
1454374_at	0.00	0.00
1454375_at	0.00	0.00
1454376_at	0.00	0.00
1454377_at	0.00	0.00
1454378_at	0.00	0.00
1454379_at	0.00	0.00
1454380_at	0.00	0.00
1454381_at	0.00	0.00
1454382_at	0.00	0.00
1454383_at	0.00	0.00
1454384_at	0.00	0.00
1454385_at	0.00	0.00
1454386_at	0.00	0.00
1454387_at	0.00	0.00
1454388_at	0.00	0.00
1454389_at	0.00	0.00
1454390_at	0.00	0.00
1454391_at	0.00	0.00
1454392_at	0.00	0.00
1454393_at	0.00	0.00
1454394_at	0.00	0.00
1454395_at	0.00	0.00
1454396_at	0.00	0.00
1454397_at	0.00	0.00
1454398_at	0.00	0.00
1454399_at	0.00	0.00
1454400_at	0.00	0.00
1454401_at	0.00	0.00
1454402_at	0.00	0.00
1454403_at	0.00	0.00
1454404_at	0.00	0.00
1454405_at	0.00	0.00
1454406_at	0.00	0.00
1454407_at	0.00	0.00
1454408_at	0.00	0.00
1454409_at	0.00	0.00
1454410_at	0.00	0.00
1454411_at	0.00	0.00
1454412_at	0.00	0.00
1454413_at	0.00	0.00
1454414_at	0.00	0.00
1454415_at	0.00	0.00
1454416_at	0.00	0.00
1454417_at	0.00	0.00
1454418_at	0.00	0.00

1454419_at	0.00	0.00
1454420_at	0.00	0.00
1454421_at	0.00	0.00
1454422_at	0.00	0.00
1454423_at	0.00	0.00
1454424_at	0.00	0.00
1454425_at	0.00	0.00
1454426_at	0.00	0.00
1454427_at	0.00	0.00
1454428_at	0.00	0.00
1454429_at	0.00	0.00
1454430_at	0.00	0.00
1454431_at	0.00	0.00
1454432_at	0.00	0.00
1454433_at	0.00	0.00
1454434_at	0.00	0.00
1454435_at	0.00	0.00
1454436_at	0.00	0.00
1454437_at	0.00	0.00
1454439_at	0.00	0.00
1454440_at	0.00	0.00
1454441_at	0.00	0.00
1454442_at	0.00	0.00
1454443_at	0.00	0.00
1454444_at	0.00	0.00
1454445_at	0.00	0.00
1454446_at	0.00	0.00
1454447_at	0.00	0.00
1454448_at	0.00	0.00
1454449_at	0.00	0.00
1454450_at	0.00	0.00
1454451_at	0.00	0.00
1454452_at	0.00	0.00
1454453_at	0.00	0.00
1454455_at	0.00	0.00
1454456_at	0.00	0.00
1454457_at	0.00	0.00
1454458_at	0.00	0.00
1454459_at	0.00	0.00
1454460_at	0.00	0.00
1454461_at	0.00	0.00
1454462_at	0.00	0.00
1454463_at	0.00	0.00
1454464_at	0.00	0.00
1454465_at	0.00	0.00
1454466_at	0.00	0.00
1454467_at	0.00	0.00
1454468_at	0.00	0.00
1454469_at	0.00	0.00
1454470_at	0.00	0.00
1454471_at	0.00	0.00
1454472_at	0.00	0.00
1454473_at	0.00	0.00
1454474_at	0.00	0.00
1454475_at	0.00	0.00
1454476_at	0.00	0.00

1454477_at	0.00	0.00
1454478_at	0.00	0.00
1454479_at	0.00	0.00
1454480_at	0.00	0.00
1454481_at	0.00	0.00
1454482_at	0.00	0.00
1454483_at	0.00	0.00
1454484_at	0.00	0.00
1454485_at	0.00	0.00
1454486_at	0.00	0.00
1454487_at	0.00	0.00
1454488_at	0.00	0.00
1454489_at	0.00	0.00
1454490_at	0.00	0.00
1454491_at	0.00	0.00
1454492_at	0.00	0.00
1454493_at	0.00	0.00
1454494_at	0.00	0.00
1454495_at	0.00	0.00
1454496_at	0.00	0.00
1454497_at	0.00	0.00
1454498_at	0.00	0.00
1454499_at	0.00	0.00
1454500_at	0.00	0.00
1454501_at	0.00	0.00
1454502_at	0.00	0.00
1454503_at	0.00	0.00
1454505_at	0.00	0.00
1454506_at	0.00	0.00
1454507_at	0.00	0.00
1454508_at	0.00	0.00
1454509_at	0.00	0.00
1454510_at	0.00	0.00
1454511_at	0.00	0.00
1454512_at	0.00	0.00
1454513_at	0.00	0.00
1454514_at	0.00	0.00
1454515_at	0.00	0.00
1454516_at	0.00	0.00
1454517_at	0.00	0.00
1454518_at	0.00	0.00
1454519_at	0.00	0.00
1454520_at	0.00	0.00
1454521_at	0.00	0.00
1454522_at	0.00	0.00
1454523_at	0.00	0.00
1454524_at	0.00	0.00
1454525_at	0.00	0.00
1454526_at	0.00	0.00
1454527_at	0.00	0.00
1454528_at	0.00	0.00
1454529_at	0.00	0.00
1454530_at	0.00	0.00
1454531_at	0.00	0.00
1454532_at	0.00	0.00
1454533_at	0.00	0.00

1454534_at	0.00	0.00
1454535_at	0.00	0.00
1454536_at	0.00	0.00
1454537_at	0.00	0.00
1454538_at	0.00	0.00
1454539_at	0.00	0.00
1454540_at	0.00	0.00
1454541_at	0.00	0.00
1454542_at	0.00	0.00
1454543_at	0.00	0.00
1454544_at	0.00	0.00
1454545_at	0.00	0.00
1454546_at	0.00	0.00
1454547_at	0.00	0.00
1454548_at	0.00	0.00
1454549_at	0.00	0.00
1454550_at	0.00	0.00
1454551_at	0.00	0.00
1454552_at	0.00	0.00
1454553_at	0.00	0.00
1454554_at	0.00	0.00
1454555_at	0.00	0.00
1454556_at	0.00	0.00
1454557_at	0.00	0.00
1454558_at	0.00	0.00
1454559_at	0.00	0.00
1454560_at	0.00	0.00
1454561_at	0.00	0.00
1454562_at	0.00	0.00
1454563_at	0.00	0.00
1454564_at	0.00	0.00
1454565_at	0.00	0.00
1454566_at	0.00	0.00
1454567_at	0.00	0.00
1454568_at	0.00	0.00
1454569_at	0.00	0.00
1454570_at	0.00	0.00
1454571_at	0.00	0.00
1454572_at	0.00	0.00
1454573_at	0.00	0.00
1454574_at	0.00	0.00
1454575_at	0.00	0.00
1454576_at	0.00	0.00
1454577_at	0.00	0.00
1454578_at	0.00	0.00
1454579_at	0.00	0.00
1454580_at	0.00	0.00
1454581_at	0.00	0.00
1454582_at	0.00	0.00
1454583_at	0.00	0.00
1454584_at	0.00	0.00
1454585_at	0.00	0.00
1454586_at	0.00	0.00
1454587_at	0.00	0.00
1454588_at	0.00	0.00
1454589_at	0.00	0.00

1454590_at	0.00	0.00
1454591_at	0.00	0.00
1454592_at	0.00	0.00
1454593_at	0.00	0.00
1454594_at	0.00	0.00
1454595_at	0.00	0.00
1454596_at	0.00	0.00
1454597_at	0.00	0.00
1454598_at	0.00	0.00
1454599_at	0.00	0.00
1454600_at	0.00	0.00
1454601_at	0.00	0.00
1454609_x_at	0.00	0.00
1454612_at	0.01	0.00
1454614_at	0.00	0.00
1454617_at	0.00	0.00
1454619_at	0.00	0.00
1454624_at	0.00	0.00
1454625_at	0.00	0.00
1454630_at	0.00	0.00
1454634_at	0.00	0.00
1454637_at	0.00	0.00
1454642_a_at	0.01	0.00
1454644_at	0.00	0.00
1454645_at	0.00	0.00
1454646_at	0.00	0.00
1454649_at	0.00	0.00
1454650_at	0.00	0.00
1454653_at	0.00	0.00
1454654_at	0.07	0.00
1454655_at	0.00	0.05
1454658_at	0.00	0.00
1454659_at	0.00	0.00
1454660_at	0.00	0.00
1454662_at	0.00	0.00
1454665_at	0.00	0.00
1454666_at	0.00	0.37
1454667_at	0.00	0.00
1454671_at	0.00	0.00
1454672_at	0.00	0.00
1454676_s_at	0.00	0.00
1454679_at	0.00	0.00
1454680_at	0.00	0.21
1454683_at	0.00	0.00
1454684_at	0.00	0.00
1454685_at	0.00	0.00
1454687_at	0.00	0.00
1454691_at	0.00	0.00
1454693_at	0.00	0.00
1454695_at	0.00	0.00
1454700_at	0.00	0.00
1454701_at	0.00	0.00
1454702_at	0.00	0.00
1454707_at	0.00	0.00
1454709_at	0.00	0.00
1454710_at	0.00	0.00

1454715_at	0.00	0.00
1454717_at	0.00	0.00
1454718_at	0.00	0.00
1454720_at	0.00	0.00
1454721_at	0.00	0.00
1454723_at	0.00	0.00
1454724_x_at	0.00	0.02
1454726_s_at	0.00	0.00
1454727_at	0.00	0.00
1454728_s_at	0.00	0.01
1454729_at	0.00	0.00
1454730_at	0.00	0.00
1454731_at	0.00	0.00
1454733_at	0.00	0.00
1454734_at	0.00	0.00
1454739_at	0.00	0.00
1454740_at	0.23	0.29
1454741_s_at	0.30	0.19
1454742_at	0.00	0.00
1454743_at	0.00	0.00
1454744_at	0.00	0.01
1454745_at	0.00	0.01
1454746_at	0.00	0.00
1454748_at	0.00	0.26
1454749_at	0.00	0.00
1454751_at	0.00	0.00
1454752_at	0.00	0.00
1454755_at	0.00	0.00
1454756_at	0.00	0.00
1454757_s_at	0.00	0.00
1454761_at	0.00	0.00
1454762_at	0.00	0.00
1454764_s_at	0.00	0.00
1454765_at	0.00	0.00
1454766_at	0.02	0.24
1454767_at	0.00	0.00
1454768_at	0.00	0.00
1454769_at	0.00	0.00
1454772_at	0.00	0.00
1454774_at	0.00	0.00
1454775_at	0.00	0.00
1454776_at	0.00	0.00
1454777_at	0.00	0.00
1454780_at	0.00	0.00
1454782_at	0.00	0.00
1454784_at	0.00	0.00
1454788_at	0.67	1.00
1454790_at	0.00	0.00
1454792_s_at	0.00	0.00
1454795_at	0.00	0.00
1454797_at	0.00	0.00
1454799_at	0.00	0.00
1454800_at	0.00	0.00
1454806_at	0.00	0.00
1454808_at	0.00	0.07
1454809_at	0.00	0.00

1454810_s_at	0.00	0.00
1454812_at	0.00	0.00
1454816_at	0.00	0.00
1454818_at	0.00	0.00
1454819_at	0.00	0.00
1454821_at	0.00	0.00
1454823_at	0.00	0.00
1454824_s_at	0.00	0.00
1454825_at	0.00	0.00
1454826_at	0.00	0.00
1454827_at	0.00	0.00
1454828_at	0.00	0.00
1454829_at	0.00	0.00
1454830_at	0.00	0.00
1454832_at	0.00	0.00
1454834_at	0.00	0.00
1454835_at	0.00	0.00
1454836_at	0.00	0.00
1454840_at	0.00	0.00
1454841_at	0.00	0.07
1454843_at	0.00	0.00
1454844_at	0.00	0.00
1454845_x_at	0.00	0.00
1454846_at	0.00	0.00
1454847_at	0.00	0.00
1454848_at	0.00	0.00
1454850_at	0.00	0.00
1454851_at	0.00	0.00
1454852_at	0.00	0.00
1454853_s_at	0.00	0.00
1454854_at	0.00	0.00
1454855_at	0.00	0.00
1454857_at	0.00	0.00
1454861_at	0.00	0.00
1454863_at	0.00	0.00
1454864_at	0.00	0.00
1454866_s_at	0.00	0.08
1454867_at	0.00	0.00
1454868_at	0.00	0.00
1454869_at	0.00	0.00
1454871_at	0.00	0.00
1454873_at	0.00	0.00
1454874_at	0.00	0.02
1454876_at	0.00	0.00
1454877_at	0.48	0.30
1454878_at	0.00	0.00
1454880_s_at	0.00	0.00
1454882_at	0.00	0.00
1454883_at	0.00	0.00
1454885_at	0.10	0.00
1454886_x_at	0.00	0.00
1454888_at	0.00	0.00
1454889_x_at	0.00	0.00
1454892_at	0.00	0.00
1454894_at	0.00	0.05
1454895_at	0.00	0.00

1454896_at	0.00	0.00
1454900_s_at	0.00	0.00
1454901_at	0.00	0.00
1454902_at	0.00	0.00
1454909_at	0.00	0.00
1454910_at	0.00	0.00
1454911_at	0.00	0.00
1454913_at	0.00	0.00
1454915_at	0.00	0.00
1454916_s_at	0.00	0.00
1454917_at	0.00	0.02
1454918_at	0.00	0.00
1454919_at	0.00	0.00
1454920_at	0.00	0.08
1454922_at	0.00	0.00
1454923_at	0.00	0.00
1454924_at	0.00	0.00
1454926_at	0.00	0.00
1454927_at	0.00	0.00
1454931_at	0.00	0.00
1454933_at	0.00	0.00
1454934_at	0.00	0.00
1454935_at	0.00	0.00
1454936_at	0.00	0.00
1454937_at	0.00	0.07
1454938_at	0.00	0.00
1454939_at	0.00	0.00
1454942_at	0.00	0.00
1454944_at	0.00	0.00
1454945_at	0.00	0.00
1454948_at	0.02	0.02
1454949_at	0.00	0.00
1454950_at	0.00	0.00
1454951_at	0.00	0.00
1454952_s_at	0.00	0.00
1454956_at	0.00	0.00
1454957_at	0.00	0.33
1454958_at	0.00	0.00
1454960_at	0.00	0.22
1454961_at	0.00	0.00
1454962_at	0.00	0.01
1454965_at	0.00	0.00
1454966_at	0.00	0.00
1454968_at	0.00	0.00
1454969_at	0.01	0.00
1454973_at	0.11	0.00
1454975_at	0.00	0.00
1454976_at	0.07	0.00
1454978_at	0.00	0.00
1454980_at	0.00	0.00
1454981_at	0.00	0.00
1454982_at	0.00	0.00
1454983_at	0.00	0.00
1454986_at	0.00	0.00
1454988_s_at	0.00	0.00
1454989_at	0.00	0.00

1454990_at	0.00	0.00
1454991_at	0.00	0.00
1454992_at	0.00	0.00
1454994_at	0.00	0.00
1454995_at	0.00	0.00
1454996_at	0.00	0.21
1454997_at	0.04	0.00
1454999_at	0.00	0.00
1455000_at	0.00	0.00
1455003_at	0.00	0.00
1455004_at	0.00	0.00
1455010_at	0.00	0.00
1455011_at	0.00	0.13
1455014_at	0.00	0.00
1455015_at	0.00	0.00
1455016_at	0.00	0.00
1455018_at	0.00	0.00
1455020_at	0.00	0.00
1455022_at	0.00	0.00
1455023_at	0.00	0.00
1455024_at	0.00	0.00
1455025_at	0.00	0.00
1455027_at	0.00	0.00
1455028_at	0.00	0.00
1455029_at	0.00	0.41
1455031_at	0.00	0.00
1455032_at	0.00	0.00
1455033_at	0.00	0.00
1455034_at	0.00	0.00
1455037_at	0.00	0.00
1455038_at	0.00	0.00
1455043_at	0.00	0.00
1455044_at	0.00	0.00
1455046_a_at	0.00	0.00
1455048_at	0.00	0.00
1455049_at	0.00	0.00
1455050_at	0.00	0.00
1455051_at	0.00	0.00
1455052_a_at	0.00	0.00
1455055_at	0.00	0.00
1455057_at	0.00	0.00
1455059_at	0.00	0.00
1455062_at	0.00	0.00
1455063_at	0.00	0.00
1455064_at	0.00	0.00
1455067_at	0.00	0.00
1455068_at	0.00	0.00
1455070_at	0.00	0.00
1455074_at	0.00	0.00
1455075_at	0.00	0.00
1455078_at	0.00	0.00
1455079_at	0.00	0.00
1455080_at	0.00	0.00
1455081_at	0.00	0.00
1455082_at	0.00	0.00
1455083_at	0.00	0.00

1455085_at	0.00	0.00
1455086_at	0.00	0.00
1455087_at	0.00	0.00
1455088_at	0.00	0.00
1455091_at	0.00	0.00
1455094_s_at	0.00	0.00
1455095_at	0.00	0.00
1455096_at	0.00	0.00
1455097_at	0.00	0.00
1455101_at	0.00	0.00
1455102_at	0.00	0.00
1455103_at	0.00	0.00
1455104_at	0.00	0.00
1455107_at	0.00	0.00
1455108_at	0.00	0.00
1455110_at	0.00	0.00
1455111_at	0.00	0.00
1455112_at	0.00	0.00
1455113_at	0.00	0.00
1455114_at	0.00	0.00
1455116_at	0.00	0.00
1455117_at	0.00	0.00
1455118_at	0.00	0.00
1455119_at	0.00	0.00
1455120_at	0.00	0.00
1455121_at	0.00	0.00
1455122_at	0.00	0.00
1455123_at	0.00	0.00
1455124_at	0.00	0.00
1455125_at	0.00	0.00
1455127_at	0.00	0.00
1455130_at	0.15	0.00
1455132_at	0.00	0.00
1455133_s_at	0.00	0.00
1455135_at	0.00	0.00
1455137_at	0.00	0.02
1455139_at	0.00	0.00
1455140_at	0.00	0.00
1455142_at	0.01	0.00
1455144_s_at	0.00	0.00
1455145_at	0.00	0.00
1455146_at	0.00	0.00
1455147_at	0.00	0.00
1455148_at	0.00	0.00
1455150_at	0.00	0.00
1455153_at	0.00	0.00
1455154_at	0.81	0.00
1455157_a_at	0.00	0.00
1455159_at	0.00	0.00
1455160_at	0.00	0.00
1455161_at	0.00	0.00
1455162_at	0.00	0.00
1455163_at	0.00	0.00
1455164_at	0.00	0.00
1455165_at	0.00	0.00
1455166_at	0.00	0.00

1455169_at	0.00	0.00
1455170_at	0.00	0.00
1455172_at	0.00	0.15
1455173_at	0.00	0.00
1455174_at	0.00	0.00
1455178_at	0.00	0.00
1455181_at	0.00	0.00
1455183_at	0.00	0.00
1455185_s_at	0.00	0.00
1455186_a_at	0.00	0.11
1455187_at	0.00	0.00
1455188_at	0.00	0.00
1455189_at	0.00	0.16
1455190_at	0.00	0.00
1455192_at	0.00	0.00
1455193_at	0.00	0.00
1455194_at	0.00	0.00
1455196_s_at	0.00	0.00
1455197_at	0.00	0.00
1455199_at	0.00	0.00
1455200_at	0.00	0.00
1455203_at	0.00	0.00
1455208_at	0.00	0.00
1455210_at	0.00	0.00
1455212_at	0.00	0.00
1455213_at	0.00	0.00
1455215_at	0.00	0.00
1455216_at	0.00	0.00
1455217_at	0.00	0.00
1455218_at	0.00	0.00
1455219_at	0.00	0.00
1455221_at	0.00	0.00
1455223_at	0.00	0.00
1455224_at	0.00	0.00
1455225_at	0.00	0.00
1455226_at	0.00	0.00
1455227_at	0.00	0.00
1455228_at	0.00	0.00
1455230_at	0.00	0.00
1455234_at	0.00	0.00
1455238_at	0.98	0.38
1455240_x_at	0.00	0.00
1455241_at	0.00	0.00
1455243_at	0.00	0.00
1455244_at	0.29	0.00
1455246_at	0.00	0.00
1455247_at	0.01	0.00
1455248_at	0.00	0.00
1455249_at	0.00	0.00
1455250_at	0.00	0.00
1455251_at	0.00	0.00
1455256_at	0.00	0.00
1455257_at	0.00	0.00
1455258_at	0.00	0.00
1455259_a_at	0.00	0.00
1455260_at	0.00	0.00

1455261_at	0.00	0.00
1455262_at	0.00	0.00
1455263_at	0.00	0.00
1455266_at	0.00	0.17
1455267_at	0.00	0.00
1455268_at	0.00	0.00
1455270_at	0.00	0.00
1455272_at	0.00	0.00
1455273_at	0.00	0.00
1455274_at	0.00	0.00
1455275_at	0.00	0.00
1455277_at	0.00	0.00
1455278_at	0.00	0.00
1455280_at	0.00	0.00
1455287_at	0.00	0.00
1455289_at	0.00	0.00
1455292_x_at	0.00	0.00
1455293_at	0.00	0.07
1455294_at	0.00	0.00
1455295_at	0.00	0.00
1455296_at	0.00	0.00
1455297_at	0.00	0.00
1455299_at	0.32	0.00
1455300_at	0.50	0.63
1455301_at	0.00	0.00
1455303_at	0.00	0.00
1455304_at	0.00	0.00
1455306_at	0.00	0.00
1455307_at	0.00	0.00
1455308_at	0.00	0.00
1455309_at	0.00	0.00
1455311_at	0.00	0.00
1455312_at	0.00	0.00
1455313_at	0.00	0.00
1455314_at	0.38	0.00
1455317_at	0.00	0.00
1455318_at	0.00	0.00
1455320_at	0.00	0.02
1455321_at	0.00	0.00
1455322_at	0.00	0.02
1455323_at	0.00	0.00
1455324_at	0.00	0.00
1455325_at	0.00	0.00
1455327_at	0.00	0.00
1455328_at	0.00	0.00
1455329_at	0.00	0.00
1455330_at	0.00	0.04
1455331_at	0.00	0.00
1455333_at	0.01	0.08
1455334_at	0.00	0.00
1455336_at	0.00	0.00
1455337_at	0.00	0.00
1455340_at	0.00	0.00
1455341_at	0.00	0.00
1455342_at	0.00	0.07
1455344_at	0.00	0.00

1455345_at	0.00	0.00
1455347_at	0.00	0.00
1455351_at	0.09	0.00
1455352_at	0.00	0.00
1455353_at	0.00	0.00
1455354_at	0.00	0.00
1455355_at	0.00	0.00
1455358_at	0.00	0.00
1455359_at	0.00	0.00
1455360_at	0.00	0.00
1455361_at	0.00	0.00
1455362_at	0.00	0.00
1455365_at	0.00	0.00
1455368_at	0.00	0.00
1455369_at	0.00	0.00
1455370_at	0.00	0.00
1455373_at	0.00	0.00
1455374_at	0.00	0.00
1455375_at	0.00	0.00
1455376_at	0.00	0.00
1455377_at	0.00	0.00
1455378_at	0.00	0.00
1455379_at	0.00	0.00
1455380_at	0.00	0.00
1455381_at	0.00	0.00
1455383_at	0.00	0.00
1455386_at	0.00	0.00
1455387_at	0.00	0.01
1455389_s_at	0.00	0.02
1455390_at	0.00	0.00
1455391_at	0.00	0.00
1455392_at	0.00	0.00
1455394_at	0.00	0.00
1455395_at	0.00	0.00
1455396_at	0.00	0.00
1455397_at	0.00	0.00
1455398_at	0.00	0.00
1455399_at	0.00	0.00
1455400_at	0.00	0.00
1455401_at	0.00	0.00
1455402_at	0.00	0.00
1455403_at	0.09	0.00
1455404_at	0.00	0.00
1455406_at	0.00	0.00
1455407_at	0.00	0.00
1455409_at	0.00	0.23
1455410_at	0.00	0.00
1455411_at	0.00	0.00
1455412_at	0.00	0.00
1455413_at	0.00	0.00
1455415_at	0.00	0.00
1455416_at	0.00	0.00
1455417_at	0.00	0.00
1455418_at	0.00	0.00
1455419_at	0.00	0.00
1455420_at	0.00	0.00

1455425_at	1.00	0.99
1455426_at	0.00	0.00
1455427_at	0.00	0.00
1455428_at	0.00	0.00
1455429_at	0.00	0.00
1455430_at	0.00	0.00
1455432_at	0.00	0.00
1455436_at	0.00	0.00
1455437_at	0.00	0.00
1455438_at	0.00	0.00
1455440_at	0.00	0.00
1455442_at	0.00	0.00
1455443_at	0.00	0.00
1455444_at	0.00	0.00
1455445_at	0.00	0.00
1455448_at	0.00	0.00
1455449_at	0.00	0.00
1455450_at	0.00	0.00
1455451_at	0.00	0.00
1455452_x_at	0.00	0.00
1455453_at	0.00	0.00
1455454_at	0.00	0.00
1455455_at	0.00	0.00
1455459_at	0.00	0.00
1455460_at	0.00	0.00
1455461_at	0.00	0.00
1455463_at	0.00	0.00
1455464_x_at	0.00	0.00
1455465_at	0.00	0.00
1455467_at	0.00	0.00
1455468_at	0.00	0.00
1455469_at	0.00	0.00
1455471_at	0.08	0.00
1455472_at	0.00	0.00
1455473_at	0.00	0.00
1455476_a_at	0.00	0.00
1455478_at	0.00	0.00
1455481_at	0.00	0.00
1455483_at	0.00	0.00
1455487_at	0.00	0.00
1455489_at	0.00	0.00
1455492_at	0.00	0.00
1455495_at	0.00	0.00
1455496_at	0.00	0.09
1455497_at	0.00	0.00
1455498_at	0.00	0.00
1455499_at	0.00	0.00
1455500_at	0.00	0.00
1455501_at	0.00	0.00
1455502_at	0.00	0.00
1455503_at	0.00	0.00
1455506_at	0.00	0.00
1455507_s_at	0.00	0.00
1455508_at	0.00	0.00
1455509_at	0.00	0.00
1455510_at	0.00	0.00

1455512_at	0.00	0.00
1455513_at	0.00	0.00
1455514_at	0.00	0.00
1455515_at	0.00	0.00
1455516_at	0.00	0.00
1455518_at	0.00	0.00
1455519_at	0.00	0.00
1455520_at	0.00	0.00
1455521_at	0.00	0.00
1455522_at	0.00	0.00
1455523_at	0.00	0.00
1455524_at	0.00	0.00
1455525_at	0.00	0.00
1455527_at	0.00	0.00
1455528_at	0.00	0.00
1455529_at	0.00	0.00
1455532_at	0.00	0.00
1455533_at	0.00	0.00
1455535_at	0.00	0.00
1455536_at	0.00	0.00
1455537_at	0.00	0.00
1455538_at	0.00	0.00
1455541_a_at	0.00	0.00
1455542_at	0.00	0.00
1455543_at	0.00	0.00
1455544_at	0.00	0.00
1455545_at	0.00	0.00
1455547_at	0.00	0.00
1455548_at	0.00	0.00
1455549_at	0.00	0.00
1455552_at	0.00	0.00
1455553_at	0.00	0.00
1455554_at	0.00	0.00
1455555_at	0.00	0.00
1455557_at	0.00	0.00
1455558_at	0.00	0.00
1455559_at	0.00	0.00
1455560_at	0.00	0.00
1455561_at	0.00	0.00
1455564_at	0.00	0.00
1455565_at	0.00	0.00
1455566_s_at	0.00	0.00
1455567_at	0.00	0.00
1455568_at	0.00	0.00
1455569_at	0.00	0.00
1455574_at	0.00	0.00
1455576_at	0.00	0.00
1455577_at	0.00	0.00
1455580_at	0.00	0.00
1455583_at	0.00	0.00
1455584_at	0.00	0.00
1455585_at	0.00	0.04
1455586_at	0.00	0.00
1455588_at	0.00	0.00
1455589_at	0.00	0.00
1455590_at	0.00	0.00

1455591_at	0.00	0.00
1455592_at	0.00	0.00
1455594_at	0.00	0.00
1455595_at	0.00	0.00
1455596_a_at	0.00	0.00
1455597_at	0.00	0.00
1455598_at	0.00	0.00
1455599_at	0.00	0.00
1455600_at	0.00	0.00
1455603_at	0.00	0.00
1455604_at	0.91	0.02
1455606_at	0.00	0.00
1455607_at	0.00	0.00
1455608_at	0.00	0.00
1455609_at	0.00	0.00
1455610_at	0.00	0.00
1455612_at	0.00	0.00
1455613_at	0.00	0.00
1455614_at	0.00	0.00
1455616_at	0.00	0.00
1455617_at	0.00	0.00
1455619_at	0.00	0.00
1455620_at	0.00	0.00
1455621_at	0.00	0.00
1455622_at	0.00	0.00
1455623_at	0.00	0.00
1455624_at	0.00	0.00
1455627_at	0.00	0.00
1455629_at	0.00	0.00
1455630_at	0.00	0.00
1455633_at	0.00	0.00
1455634_at	0.00	0.00
1455635_at	0.00	0.00
1455636_at	0.00	0.00
1455637_x_at	0.00	0.00
1455638_at	0.00	0.00
1455643_s_at	0.00	0.26
1455645_at	0.00	0.00
1455646_at	0.00	0.00
1455647_at	0.00	0.00
1455648_at	0.00	0.00
1455649_at	0.00	0.00
1455651_at	0.00	0.00
1455652_at	0.00	0.00
1455654_at	0.00	0.00
1455656_at	0.00	0.00
1455657_at	0.00	0.00
1455658_at	0.00	0.00
1455659_at	0.00	0.00
1455660_at	0.00	0.00
1455661_at	0.00	0.00
1455663_at	0.00	0.00
1455664_at	0.00	0.00
1455665_at	0.00	0.00
1455666_at	0.00	0.00
1455667_at	0.00	0.00

1455668_at	0.00	0.00
1455669_at	0.00	0.00
1455670_at	0.00	0.00
1455671_at	0.00	0.00
1455672_s_at	0.00	0.00
1455673_at	0.00	0.00
1455674_at	0.00	0.00
1455679_at	0.00	0.00
1455680_at	0.00	0.01
1455681_at	0.00	0.00
1455682_at	0.00	0.00
1455684_at	0.00	0.00
1455686_at	0.00	0.00
1455687_at	0.00	0.00
1455688_at	0.00	0.00
1455689_at	0.00	0.00
1455690_at	0.00	0.00
1455695_at	0.00	0.00
1455697_at	0.00	0.00
1455699_at	0.00	0.00
1455701_at	0.00	0.00
1455702_at	0.00	0.00
1455703_at	0.00	0.00
1455704_at	0.00	0.00
1455705_at	0.00	0.00
1455706_at	0.00	0.00
1455707_at	0.00	0.00
1455708_at	0.00	0.00
1455709_at	0.00	0.00
1455711_at	0.00	0.00
1455712_at	0.00	0.00
1455714_at	0.00	0.00
1455716_at	0.00	0.00
1455717_s_at	0.00	0.00
1455718_at	0.00	0.00
1455720_at	0.00	0.00
1455721_at	0.00	0.00
1455722_at	0.01	0.00
1455723_at	0.00	0.00
1455724_at	0.00	0.00
1455726_at	0.00	0.00
1455728_at	0.00	0.00
1455729_at	0.00	0.00
1455732_at	0.00	0.00
1455735_at	0.00	0.13
1455736_at	0.00	0.00
1455737_at	0.00	0.00
1455738_at	0.00	0.00
1455739_at	0.00	0.00
1455743_at	0.00	0.00
1455744_at	0.00	0.00
1455745_at	0.00	0.00
1455746_at	0.00	0.00
1455750_at	0.00	0.03
1455751_at	0.00	0.00
1455753_at	0.00	0.00

1455754_at	0.00	0.00
1455755_at	0.00	0.00
1455756_at	0.00	0.00
1455757_at	0.00	0.00
1455759_a_at	0.00	0.00
1455760_at	0.00	0.01
1455761_at	0.00	0.00
1455762_at	0.00	0.00
1455763_at	0.00	0.00
1455766_at	0.00	0.00
1455768_at	0.00	0.00
1455769_at	0.00	0.00
1455770_at	0.00	0.00
1455771_at	0.00	0.00
1455772_at	0.00	0.00
1455773_at	0.00	0.00
1455774_at	0.00	0.00
1455775_at	0.00	0.00
1455778_at	0.00	0.01
1455779_at	0.00	0.00
1455780_at	0.00	0.00
1455782_at	0.00	0.00
1455783_at	0.00	0.00
1455784_at	0.00	0.00
1455785_at	0.00	0.00
1455786_at	0.00	0.00
1455790_at	0.00	0.00
1455793_at	0.00	0.00
1455794_at	0.00	0.00
1455795_at	0.00	0.00
1455799_at	0.00	0.00
1455803_at	0.00	0.00
1455807_at	0.00	0.00
1455816_a_at	0.00	0.00
1455817_x_at	0.00	0.00
1455818_at	0.00	0.00
1455823_at	0.00	0.00
1455827_at	0.00	0.05
1455830_s_at	0.00	0.00
1455833_at	0.00	0.00
1455835_at	0.00	0.00
1455837_at	0.00	0.00
1455838_at	0.00	0.00
1455839_at	0.00	0.00
1455840_at	0.00	0.15
1455842_x_at	0.00	0.00
1455843_at	0.00	0.00
1455845_at	0.00	0.00
1455846_at	0.00	0.00
1455847_at	0.00	0.00
1455848_at	0.00	0.00
1455849_at	0.00	0.00
1455850_at	0.00	0.00
1455851_at	0.00	0.00
1455852_at	0.00	0.00
1455854_a_at	0.00	0.00

1455856_at	0.00	0.00
1455859_at	0.00	0.00
1455861_at	0.00	0.00
1455862_at	0.10	0.00
1455863_at	0.00	0.00
1455864_at	0.00	0.00
1455865_at	0.00	0.00
1455870_at	0.00	0.00
1455872_at	0.00	0.00
1455876_at	0.00	0.00
1455878_at	0.01	0.00
1455879_at	0.00	0.00
1455881_at	0.00	0.00
1455882_x_at	0.00	0.00
1455884_at	0.00	0.00
1455885_at	0.00	0.00
1455887_at	0.00	0.00
1455888_at	0.00	0.00
1455889_at	0.00	0.00
1455891_at	0.00	0.00
1455902_x_at	0.00	0.00
1455903_at	0.00	0.00
1455906_at	0.00	0.00
1455907_x_at	0.00	0.00
1455909_at	0.00	0.00
1455914_at	0.00	0.00
1455916_at	0.00	0.00
1455917_at	0.00	0.00
1455919_at	0.00	0.00
1455920_x_at	0.00	0.00
1455921_at	0.00	0.00
1455922_at	0.00	0.00
1455923_at	0.00	0.00
1455924_at	0.00	0.00
1455925_at	0.00	0.00
1455926_at	0.00	0.00
1455933_at	0.00	0.00
1455935_at	0.00	0.00
1455937_at	0.00	0.00
1455942_at	0.00	0.00
1455943_at	0.00	0.00
1455944_at	0.00	0.03
1455945_at	0.00	0.00
1455947_at	0.00	0.00
1455948_x_at	0.00	0.01
1455951_at	0.00	0.02
1455952_at	0.00	0.00
1455960_at	0.00	0.00
1455962_at	0.00	0.00
1455963_at	0.00	0.00
1455964_at	0.00	0.17
1455966_s_at	0.00	0.00
1455967_at	0.00	0.00
1455969_at	0.00	0.00
1455970_at	0.00	0.00
1455971_at	0.00	0.00

1455973_at	0.00	0.00
1455979_at	0.00	0.00
1455980_a_at	0.00	0.00
1455982_at	0.00	0.00
1455983_at	0.00	0.00
1455986_at	0.00	0.00
1455993_at	0.00	0.00
1455995_at	0.00	0.00
1455998_at	0.00	0.00
1455999_at	0.00	0.00
1456008_at	0.00	0.00
1456018_at	0.00	0.00
1456019_at	0.02	0.00
1456020_at	0.00	0.00
1456021_at	0.00	0.00
1456022_at	0.00	0.00
1456023_at	0.00	0.00
1456024_at	0.00	0.00
1456025_at	0.00	0.00
1456026_at	0.00	0.13
1456027_at	0.00	0.00
1456033_at	0.00	0.00
1456034_at	0.00	0.00
1456035_at	0.00	0.00
1456038_at	0.00	0.03
1456041_at	0.00	0.00
1456044_at	0.00	0.00
1456045_at	0.00	0.00
1456046_at	0.00	0.00
1456047_at	0.00	0.00
1456050_at	0.00	0.00
1456051_at	0.00	0.00
1456052_at	0.00	0.00
1456053_at	0.00	0.00
1456058_at	0.00	0.00
1456060_at	0.00	0.00
1456061_at	0.00	0.00
1456063_at	0.00	0.00
1456065_at	0.00	0.00
1456067_at	0.05	0.00
1456068_at	0.00	0.00
1456070_at	0.00	0.00
1456072_at	0.00	0.14
1456073_s_at	0.00	0.00
1456074_at	0.00	0.00
1456076_at	0.00	0.00
1456077_x_at	0.00	0.00
1456087_at	0.00	0.00
1456089_at	0.00	0.00
1456090_at	0.00	0.01
1456091_at	0.00	0.00
1456092_at	0.00	0.00
1456093_at	0.00	0.00
1456096_at	0.00	0.00
1456099_at	0.00	0.00
1456102_a_at	0.00	0.00

1456103_at	0.00	0.00
1456105_at	0.00	0.00
1456110_at	0.00	0.00
1456111_at	0.00	0.00
1456113_at	0.00	0.00
1456114_at	0.00	0.00
1456116_at	0.00	0.00
1456118_at	0.00	0.00
1456119_at	0.00	0.00
1456121_at	0.00	0.00
1456122_at	0.00	0.00
1456123_at	0.00	0.00
1456126_at	0.00	0.00
1456127_at	0.04	0.00
1456129_at	0.00	0.00
1456130_at	0.00	0.00
1456137_at	0.00	0.00
1456138_at	0.00	0.00
1456139_at	0.00	0.00
1456141_x_at	0.00	0.00
1456143_at	0.31	0.00
1456144_at	0.00	0.00
1456145_at	0.00	0.00
1456146_at	0.00	0.00
1456147_at	0.00	0.00
1456149_at	0.00	0.00
1456150_at	0.00	0.00
1456152_at	0.00	0.00
1456153_at	0.00	0.00
1456156_at	0.00	0.00
1456157_at	0.00	0.00
1456158_at	0.00	0.00
1456159_at	0.00	0.00
1456160_at	0.00	0.00
1456161_at	0.00	0.00
1456162_x_at	0.00	0.00
1456163_at	0.00	0.04
1456164_at	0.00	0.00
1456165_at	0.00	0.00
1456166_at	0.00	0.00
1456167_at	0.00	0.00
1456168_at	0.00	0.00
1456171_at	0.00	0.00
1456172_at	0.00	0.00
1456178_at	0.00	0.00
1456179_at	0.51	0.00
1456180_at	0.00	0.00
1456181_at	0.00	0.00
1456183_at	0.00	0.00
1456184_at	0.00	0.00
1456185_at	0.00	0.00
1456186_at	0.00	0.00
1456187_at	0.00	0.00
1456189_x_at	0.00	0.00
1456191_x_at	0.00	0.00
1456192_x_at	0.00	0.00

1456197_x_at	0.00	0.00
1456198_at	0.00	0.00
1456200_at	0.00	0.00
1456201_at	0.00	0.00
1456202_at	0.00	0.00
1456203_at	0.00	0.00
1456204_at	0.00	0.03
1456207_at	0.00	0.00
1456208_at	0.00	0.00
1456209_x_at	0.00	0.00
1456210_at	0.00	0.00
1456211_at	0.00	0.00
1456214_at	0.00	0.00
1456215_at	0.00	0.00
1456216_at	0.00	0.00
1456217_at	0.00	0.00
1456219_at	0.00	0.00
1456220_at	0.00	0.00
1456221_at	0.00	0.00
1456223_at	0.00	0.00
1456224_x_at	0.00	0.00
1456229_at	0.00	0.00
1456230_at	0.00	0.00
1456231_at	0.00	0.00
1456232_at	0.00	0.00
1456233_at	0.00	0.00
1456234_at	0.00	0.00
1456235_at	0.00	0.00
1456236_s_at	0.00	0.00
1456238_at	0.00	0.00
1456242_at	0.41	0.78
1456248_at	0.00	0.00
1456253_s_at	0.00	0.00
1456254_at	0.00	0.00
1456255_at	0.00	0.00
1456256_at	0.00	0.00
1456257_at	0.00	0.00
1456258_at	0.00	0.00
1456259_at	0.00	0.00
1456260_at	0.00	0.00
1456261_at	0.00	0.00
1456264_at	0.00	0.00
1456265_at	0.00	0.00
1456267_at	0.00	0.00
1456268_at	0.00	0.00
1456271_at	0.00	0.00
1456272_at	0.00	0.00
1456273_x_at	0.00	0.00
1456274_at	0.00	0.00
1456276_at	0.00	0.00
1456277_at	0.00	0.00
1456280_at	0.00	0.00
1456281_at	0.00	0.00
1456282_at	0.00	0.00
1456284_at	0.00	0.00
1456285_at	0.00	0.00

1456286_at	0.00	0.00
1456287_at	0.00	0.00
1456288_at	0.00	0.00
1456291_x_at	0.00	0.00
1456294_at	0.00	0.00
1456295_at	0.00	0.00
1456296_at	0.00	0.00
1456297_at	0.00	0.00
1456298_at	0.00	0.00
1456299_at	0.00	0.00
1456300_at	0.00	0.00
1456301_at	0.00	0.00
1456303_at	0.00	0.00
1456304_at	0.00	0.00
1456308_x_at	0.00	0.03
1456311_x_at	0.00	0.00
1456317_at	0.00	0.00
1456318_at	0.00	0.00
1456320_at	0.00	0.00
1456321_at	0.00	0.00
1456324_at	0.00	0.00
1456326_at	0.00	0.01
1456327_at	0.00	0.00
1456328_at	0.00	0.00
1456329_at	0.58	0.72
1456331_at	0.00	0.00
1456332_at	0.00	0.00
1456334_s_at	0.00	0.00
1456335_at	0.00	0.00
1456336_at	0.00	0.00
1456337_at	0.00	0.00
1456338_at	0.00	0.00
1456339_at	0.00	0.00
1456343_at	0.00	0.00
1456344_at	0.00	0.00
1456345_at	0.00	0.00
1456346_at	0.00	0.00
1456347_at	0.00	0.00
1456348_x_at	0.00	0.00
1456350_at	0.00	0.00
1456351_at	0.00	0.00
1456353_at	0.00	0.00
1456354_at	0.00	0.00
1456356_at	0.00	0.00
1456357_at	0.00	0.00
1456358_at	0.00	0.00
1456359_at	0.00	0.00
1456361_at	0.00	0.00
1456362_at	0.00	0.00
1456363_at	0.00	0.00
1456364_at	0.00	0.00
1456366_at	0.00	0.00
1456367_at	0.00	0.00
1456368_at	0.00	0.00
1456369_at	0.00	0.00
1456372_at	0.00	0.00

1456376_at	0.00	0.00
1456378_s_at	0.00	0.01
1456382_at	0.00	0.00
1456384_at	0.00	0.00
1456387_at	0.00	0.00
1456389_at	0.00	0.00
1456391_at	0.00	0.00
1456392_at	0.00	0.00
1456394_at	0.00	0.00
1456396_at	0.00	0.00
1456397_at	0.00	0.00
1456400_at	0.00	0.00
1456401_at	0.00	0.00
1456402_at	0.00	0.00
1456403_at	0.00	0.00
1456404_at	0.00	0.00
1456406_at	0.00	0.00
1456407_a_at	0.00	0.00
1456408_x_at	0.00	0.00
1456409_at	0.00	0.00
1456410_at	0.00	0.00
1456413_at	0.00	0.00
1456414_at	0.00	0.00
1456415_at	0.00	0.00
1456416_at	0.00	0.00
1456418_at	0.00	0.00
1456419_at	0.00	0.00
1456420_at	0.00	0.00
1456421_at	0.00	0.00
1456422_at	0.00	0.00
1456423_at	0.00	0.00
1456425_at	0.00	0.00
1456426_at	0.00	0.00
1456428_at	0.00	0.00
1456429_at	0.00	0.00
1456432_at	0.00	0.00
1456435_at	0.00	0.00
1456440_s_at	0.00	0.00
1456441_at	0.00	0.00
1456443_at	0.00	0.00
1456444_at	0.00	0.00
1456445_at	0.00	0.00
1456446_at	0.00	0.00
1456448_at	0.00	0.00
1456449_at	0.00	0.00
1456450_at	0.00	0.00
1456451_at	0.00	0.00
1456452_at	0.00	0.00
1456453_at	0.00	0.00
1456454_at	0.00	0.00
1456460_at	0.00	0.00
1456463_at	0.00	0.00
1456467_s_at	0.00	0.00
1456468_x_at	0.00	0.00
1456472_at	0.00	0.00
1456475_s_at	0.00	0.12

1456476_at	0.00	0.00
1456477_at	0.00	0.00
1456478_at	0.00	0.00
1456479_at	0.00	0.00
1456480_at	0.00	0.00
1456482_at	0.00	0.00
1456483_at	0.00	0.00
1456484_at	0.00	0.00
1456485_at	0.00	0.00
1456487_at	0.00	0.00
1456489_at	0.00	0.00
1456490_at	0.00	0.00
1456491_at	0.00	0.00
1456498_at	0.00	0.00
1456499_at	0.00	0.00
1456500_at	0.00	0.00
1456501_at	0.00	0.00
1456502_at	0.00	0.00
1456503_at	0.00	0.00
1456504_at	0.00	0.00
1456505_at	0.00	0.00
1456506_at	0.00	0.00
1456507_at	0.00	0.00
1456508_at	0.00	0.00
1456509_at	0.00	0.00
1456512_at	0.00	0.00
1456513_at	0.00	0.00
1456514_at	0.00	0.00
1456517_at	0.00	0.00
1456518_at	0.00	0.00
1456519_at	0.00	0.00
1456520_at	0.00	0.01
1456522_at	0.00	0.00
1456523_at	0.00	0.00
1456524_at	0.00	0.00
1456526_at	0.00	0.00
1456527_at	0.00	0.00
1456531_x_at	0.00	0.01
1456532_at	0.00	0.00
1456533_at	0.00	0.00
1456535_at	0.00	0.00
1456536_at	0.00	0.00
1456537_at	0.00	0.00
1456538_at	0.00	0.00
1456539_at	0.00	0.00
1456544_at	0.00	0.00
1456545_at	0.00	0.00
1456547_at	0.00	0.00
1456548_at	0.00	0.00
1456549_at	0.00	0.00
1456550_at	0.00	0.00
1456551_at	0.00	0.00
1456552_at	0.00	0.00
1456553_at	0.00	0.00
1456554_at	0.00	0.00
1456555_at	0.00	0.00

1456556_at	0.00	0.00
1456557_at	0.00	0.00
1456558_s_at	0.00	0.00
1456559_at	0.00	0.00
1456560_at	0.00	0.00
1456561_s_at	0.00	0.00
1456562_x_at	0.00	0.00
1456563_at	0.00	0.00
1456564_at	0.00	0.00
1456570_at	0.00	0.00
1456574_at	0.00	0.00
1456586_x_at	0.00	0.00
1456587_x_at	0.00	0.00
1456589_x_at	0.00	0.00
1456591_x_at	0.00	0.00
1456592_at	0.00	0.00
1456593_at	0.00	0.00
1456594_at	0.00	0.00
1456596_at	0.00	0.00
1456597_at	0.00	0.31
1456599_at	0.00	0.00
1456602_at	0.00	0.00
1456607_at	0.00	0.00
1456609_at	0.00	0.00
1456610_at	0.00	0.08
1456611_at	0.00	0.00
1456614_at	0.00	0.00
1456619_at	0.00	0.00
1456621_at	0.00	0.00
1456625_at	0.00	0.00
1456631_at	0.00	0.00
1456632_at	0.00	0.00
1456633_at	0.00	0.00
1456634_at	0.00	0.00
1456638_at	0.00	0.00
1456639_at	0.00	0.00
1456640_at	0.00	0.00
1456641_at	0.00	0.00
1456643_at	0.00	0.00
1456645_at	0.00	0.00
1456646_at	0.00	0.00
1456648_at	0.00	0.00
1456649_at	0.00	0.00
1456650_at	0.00	0.00
1456652_at	0.00	0.00
1456653_a_at	0.00	0.00
1456654_at	0.00	0.00
1456655_at	0.00	0.00
1456656_at	0.00	0.00
1456657_at	0.00	0.00
1456659_at	0.00	0.00
1456660_a_at	0.00	0.00
1456661_at	0.00	0.00
1456662_at	0.00	0.00
1456665_at	0.00	0.00
1456666_at	0.00	0.00

1456667_at	0.00	0.00
1456668_at	0.00	0.00
1456669_at	0.00	0.00
1456670_at	0.00	0.00
1456671_at	0.00	0.00
1456673_at	0.00	0.00
1456674_at	0.00	0.00
1456675_at	0.00	0.00
1456677_at	0.00	0.00
1456678_at	0.00	0.00
1456679_at	0.00	0.00
1456680_at	0.00	0.00
1456681_at	0.00	0.00
1456682_at	0.00	0.00
1456683_at	0.00	0.00
1456684_at	0.00	0.00
1456685_at	0.00	0.00
1456686_at	0.00	0.00
1456687_at	0.00	0.00
1456688_at	0.00	0.00
1456689_at	0.00	0.00
1456690_at	0.00	0.00
1456692_at	0.00	0.00
1456693_at	0.00	0.00
1456696_x_at	0.00	0.00
1456698_s_at	0.00	0.06
1456703_at	0.00	0.00
1456704_at	0.00	0.00
1456705_at	0.00	0.00
1456708_at	0.00	0.00
1456709_at	0.00	0.00
1456710_at	0.00	0.00
1456711_at	0.00	0.00
1456712_at	0.00	0.00
1456713_at	0.00	0.00
1456715_at	0.00	0.00
1456717_at	0.00	0.00
1456718_at	0.00	0.00
1456719_at	0.00	0.00
1456720_at	0.00	0.00
1456721_at	0.00	0.00
1456722_at	0.00	0.00
1456723_at	0.00	0.00
1456729_x_at	0.00	0.00
1456735_x_at	0.00	0.00
1456738_s_at	0.00	0.00
1456742_x_at	0.00	0.00
1456749_at	0.00	0.00
1456750_at	0.00	0.00
1456751_x_at	0.00	0.00
1456752_at	0.00	0.00
1456753_at	0.00	0.02
1456754_at	0.00	0.00
1456755_at	0.00	0.00
1456756_at	0.01	0.00
1456758_at	0.00	0.00

1456759_at	0.00	0.00
1456760_at	0.00	0.00
1456761_at	0.00	0.00
1456762_at	0.00	0.00
1456763_at	0.00	0.00
1456764_at	0.00	0.00
1456765_at	0.00	0.00
1456766_at	0.00	0.00
1456767_at	0.00	0.00
1456768_a_at	0.00	0.00
1456769_at	0.00	0.00
1456771_at	0.00	0.00
1456772_at	0.00	0.00
1456773_at	0.00	0.00
1456774_at	0.00	0.00
1456775_at	0.00	0.00
1456776_at	0.00	0.00
1456777_at	0.00	0.00
1456778_at	0.00	0.00
1456779_a_at	0.00	0.00
1456780_at	0.00	0.00
1456781_at	0.00	0.00
1456782_at	0.00	0.00
1456783_at	0.00	0.00
1456784_at	0.00	0.00
1456785_at	0.00	0.00
1456786_at	0.00	0.00
1456787_at	0.00	0.00
1456788_at	0.00	0.00
1456789_at	0.00	0.00
1456790_at	0.00	0.00
1456791_at	0.00	0.00
1456792_at	0.00	0.00
1456793_at	0.00	0.00
1456794_at	0.00	0.00
1456795_at	0.04	0.29
1456796_at	0.00	0.00
1456797_at	0.00	0.00
1456798_at	0.00	0.00
1456799_at	0.00	0.00
1456800_a_at	0.00	0.00
1456801_at	0.00	0.00
1456802_at	0.00	0.00
1456803_at	0.00	0.00
1456804_at	0.00	0.00
1456805_a_at	0.00	0.00
1456806_at	0.00	0.00
1456807_at	0.00	0.00
1456808_at	0.00	0.00
1456809_at	0.00	0.00
1456810_at	0.00	0.00
1456811_at	0.00	0.00
1456812_at	0.00	0.00
1456813_at	0.00	0.00
1456814_at	0.00	0.00
1456815_at	0.00	0.00

1456816_at	0.00	0.00
1456817_at	0.00	0.00
1456818_at	0.00	0.00
1456819_at	0.00	0.00
1456820_at	0.00	0.00
1456821_at	0.00	0.00
1456822_at	0.00	0.00
1456823_at	0.00	0.00
1456824_at	0.00	0.00
1456825_at	0.00	0.00
1456826_at	0.00	0.00
1456827_at	0.00	0.00
1456828_at	0.00	0.00
1456829_at	0.00	0.00
1456830_at	0.00	0.00
1456831_at	0.00	0.00
1456832_at	0.00	0.00
1456833_at	0.00	0.00
1456834_at	0.00	0.00
1456835_at	0.00	0.00
1456836_at	0.00	0.00
1456837_at	0.00	0.00
1456838_at	0.00	0.00
1456839_at	0.00	0.00
1456840_at	0.00	0.00
1456841_at	0.00	0.00
1456842_at	0.00	0.00
1456843_at	0.00	0.00
1456844_at	0.00	0.00
1456845_at	0.00	0.00
1456846_at	0.00	0.00
1456847_at	0.00	0.00
1456848_at	0.00	0.00
1456849_at	0.00	0.00
1456850_at	0.00	0.00
1456851_at	0.00	0.00
1456852_at	0.00	0.00
1456853_at	0.00	0.00
1456854_at	0.00	0.00
1456855_at	0.00	0.00
1456856_at	0.00	0.00
1456857_at	0.00	0.00
1456858_at	0.00	0.00
1456859_at	0.00	0.00
1456860_at	0.00	0.00
1456861_at	0.00	0.00
1456862_at	0.00	0.00
1456863_at	0.00	0.00
1456864_at	0.00	0.00
1456865_x_at	0.00	0.08
1456866_x_at	0.00	0.00
1456867_x_at	0.00	0.00
1456868_at	0.00	0.00
1456871_a_at	0.00	0.00
1456872_a_at	0.00	0.00
1456873_at	0.00	0.00

1456874_at	0.00	0.00
1456875_at	0.00	0.00
1456876_at	0.00	0.00
1456877_at	0.00	0.00
1456878_at	0.00	0.00
1456879_at	0.00	0.00
1456880_at	0.00	0.00
1456881_at	0.00	0.00
1456882_at	0.00	0.00
1456883_at	0.00	0.00
1456884_at	0.00	0.00
1456885_at	0.00	0.00
1456886_at	0.00	0.00
1456887_at	0.00	0.00
1456888_at	0.00	0.00
1456889_at	0.00	0.00
1456890_at	0.00	0.00
1456891_at	0.00	0.00
1456892_at	0.00	0.00
1456893_at	0.00	0.00
1456894_at	0.00	0.00
1456895_at	0.00	0.00
1456896_at	0.00	0.00
1456897_at	0.00	0.00
1456898_at	0.00	0.00
1456899_at	0.00	0.00
1456900_at	0.00	0.00
1456901_at	0.00	0.00
1456902_at	0.00	0.00
1456903_at	0.00	0.00
1456904_at	0.00	0.00
1456905_at	0.00	0.00
1456906_at	0.00	0.00
1456907_at	0.00	0.00
1456908_at	0.00	0.00
1456909_at	0.00	0.00
1456910_at	0.00	0.00
1456911_at	0.00	0.00
1456912_at	0.00	0.00
1456913_at	0.00	0.00
1456914_at	0.00	0.00
1456915_at	0.00	0.00
1456916_at	0.00	0.00
1456917_at	0.00	0.00
1456918_at	0.00	0.00
1456919_at	0.00	0.00
1456920_at	0.00	0.00
1456921_at	0.00	0.00
1456922_at	0.00	0.00
1456923_at	0.00	0.00
1456924_at	0.00	0.00
1456925_at	0.00	0.00
1456926_at	0.00	0.00
1456927_at	0.00	0.00
1456928_at	0.00	0.00
1456929_at	0.00	0.00

1456930_at	0.00	0.00
1456931_at	0.00	0.00
1456932_at	0.00	0.00
1456933_at	0.00	0.00
1456934_at	0.00	0.00
1456935_at	0.00	0.00
1456936_at	0.00	0.00
1456937_at	0.00	0.00
1456938_at	0.00	0.00
1456939_at	0.00	0.00
1456940_at	0.00	0.00
1456941_at	0.00	0.00
1456942_x_at	0.00	0.00
1456943_a_at	0.00	0.00
1456944_at	0.00	0.00
1456945_at	0.00	0.00
1456946_at	0.00	0.00
1456947_at	0.00	0.00
1456948_at	0.00	0.00
1456949_at	0.00	0.00
1456950_at	0.00	0.00
1456951_at	0.00	0.00
1456952_at	0.00	0.00
1456953_at	0.00	0.00
1456954_at	0.00	0.00
1456955_at	0.00	0.00
1456956_at	0.00	0.00
1456957_at	0.00	0.00
1456958_at	0.00	0.00
1456959_at	0.00	0.00
1456960_at	0.00	0.00
1456961_at	0.00	0.00
1456962_at	0.00	0.00
1456963_at	0.00	0.00
1456964_at	0.00	0.00
1456965_at	0.00	0.00
1456966_at	0.00	0.00
1456967_at	0.00	0.00
1456968_at	0.00	0.00
1456969_at	0.00	0.00
1456970_at	0.00	0.00
1456971_at	0.00	0.00
1456973_at	0.00	0.00
1456974_at	0.00	0.00
1456975_at	0.00	0.00
1456976_at	0.00	0.00
1456977_at	0.00	0.00
1456978_s_at	0.00	0.00
1456979_at	0.00	0.00
1456980_at	0.00	0.00
1456981_at	0.00	0.02
1456982_at	0.00	0.00
1456983_at	0.00	0.00
1456984_at	0.00	0.00
1456985_at	0.00	0.00
1456986_at	0.00	0.00

1456987_at	0.00	0.00
1456988_at	0.00	0.00
1456989_at	0.00	0.00
1456990_at	0.00	0.00
1456991_at	0.00	0.00
1456992_at	0.00	0.00
1456993_at	0.00	0.00
1456994_at	0.00	0.00
1456995_at	0.00	0.00
1456996_at	0.00	0.00
1456997_at	0.00	0.00
1456998_at	0.00	0.00
1456999_at	0.00	0.00
1457000_at	0.00	0.00
1457001_at	0.00	0.00
1457002_at	0.00	0.00
1457003_at	0.00	0.00
1457004_at	0.00	0.00
1457005_at	0.00	0.00
1457006_at	0.00	0.00
1457007_at	0.00	0.00
1457008_at	0.00	0.00
1457009_at	0.00	0.00
1457010_at	0.00	0.00
1457011_at	0.00	0.00
1457012_at	0.00	0.00
1457013_at	0.00	0.00
1457014_x_at	0.00	0.00
1457015_at	0.00	0.00
1457016_at	0.00	0.00
1457017_at	0.00	0.00
1457018_at	0.00	0.00
1457019_s_at	0.00	0.00
1457020_at	0.00	0.00
1457021_x_at	0.00	0.00
1457022_at	0.00	0.00
1457023_at	0.00	0.00
1457024_x_at	0.00	0.00
1457025_at	0.00	0.00
1457026_at	0.26	0.00
1457027_at	0.00	0.00
1457028_at	0.00	0.00
1457029_at	0.00	0.00
1457030_at	0.00	0.00
1457031_at	0.00	0.00
1457032_at	0.00	0.00
1457033_at	0.00	0.00
1457034_at	0.11	0.01
1457035_at	0.00	0.00
1457036_at	0.00	0.00
1457037_at	0.00	0.00
1457038_at	0.00	0.00
1457039_at	0.00	0.00
1457040_at	0.00	0.00
1457041_at	0.00	0.00
1457042_at	0.00	0.00

1457043_at	0.00	0.00
1457044_at	0.00	0.00
1457045_at	0.00	0.00
1457046_s_at	0.00	0.00
1457047_at	0.00	0.00
1457048_at	0.00	0.00
1457049_at	0.00	0.00
1457050_at	0.00	0.00
1457051_at	0.00	0.00
1457052_at	0.00	0.00
1457053_at	0.00	0.00
1457054_a_at	0.00	0.00
1457055_at	0.00	0.00
1457056_at	0.00	0.00
1457057_at	0.00	0.00
1457058_at	0.00	0.00
1457059_at	0.00	0.00
1457060_at	0.00	0.00
1457061_at	0.00	0.00
1457062_at	0.00	0.00
1457063_at	0.00	0.00
1457064_at	0.00	0.00
1457065_at	0.00	0.00
1457066_at	0.00	0.00
1457067_at	0.00	0.00
1457068_at	0.00	0.00
1457069_at	0.00	0.00
1457070_at	0.00	0.00
1457071_x_at	0.00	0.00
1457072_at	0.00	0.00
1457073_at	0.00	0.00
1457074_at	0.00	0.00
1457075_at	0.00	0.00
1457076_at	0.00	0.00
1457077_at	0.00	0.00
1457078_at	0.00	0.17
1457079_at	0.00	0.00
1457080_at	0.00	0.00
1457081_at	0.00	0.00
1457082_at	0.00	0.00
1457083_at	0.00	0.21
1457084_at	0.00	0.00
1457085_at	0.00	0.00
1457086_at	0.00	0.00
1457087_at	0.00	0.00
1457088_at	0.00	0.00
1457089_at	0.00	0.00
1457090_at	0.00	0.00
1457091_at	0.00	0.00
1457092_at	0.00	0.00
1457093_at	0.00	0.00
1457095_at	0.00	0.00
1457096_at	0.00	0.00
1457097_at	0.00	0.00
1457098_at	0.00	0.00
1457099_at	0.00	0.00

1457100_at	0.00	0.00
1457101_at	0.00	0.00
1457102_at	0.00	0.00
1457103_at	0.00	0.00
1457104_at	0.00	0.00
1457105_at	0.00	0.00
1457106_at	0.00	0.00
1457107_at	0.00	0.00
1457108_at	0.00	0.00
1457109_x_at	0.00	0.00
1457110_at	0.00	0.00
1457111_at	0.00	0.00
1457112_at	0.00	0.00
1457113_at	0.00	0.00
1457114_at	0.00	0.00
1457115_at	0.00	0.00
1457116_at	0.00	0.00
1457117_at	0.00	0.00
1457118_at	0.00	0.00
1457119_at	0.00	0.00
1457120_at	0.00	0.00
1457121_at	0.00	0.00
1457122_at	0.00	0.00
1457123_at	0.00	0.00
1457124_at	0.00	0.00
1457125_at	0.00	0.00
1457127_at	0.00	0.00
1457128_at	0.00	0.00
1457129_at	0.00	0.00
1457130_at	0.00	0.00
1457131_at	0.00	0.00
1457132_at	0.00	0.00
1457133_at	0.00	0.00
1457134_at	0.00	0.00
1457135_at	0.00	0.00
1457136_at	0.00	0.00
1457137_at	0.00	0.00
1457138_x_at	0.00	0.00
1457139_at	0.00	0.00
1457140_s_at	0.00	0.00
1457141_at	0.00	0.00
1457142_at	0.00	0.00
1457143_at	0.00	0.00
1457144_at	0.00	0.00
1457145_at	0.00	0.00
1457146_at	0.00	0.00
1457148_at	0.00	0.00
1457149_at	0.00	0.00
1457150_at	0.00	0.00
1457151_at	0.00	0.00
1457152_at	0.00	0.00
1457153_at	0.00	0.00
1457154_at	0.00	0.00
1457155_at	0.00	0.00
1457156_at	0.00	0.00
1457157_at	0.00	0.00

1457158_at	0.00	0.00
1457159_at	0.00	0.00
1457160_at	0.00	0.00
1457161_at	0.00	0.00
1457162_at	0.00	0.00
1457163_at	0.00	0.00
1457164_at	0.00	0.00
1457165_at	0.00	0.00
1457166_at	0.00	0.00
1457167_at	0.00	0.00
1457168_at	0.00	0.00
1457169_at	0.00	0.00
1457170_at	0.00	0.00
1457171_at	0.00	0.00
1457172_at	0.00	0.00
1457173_at	0.00	0.00
1457174_at	0.00	0.00
1457175_at	0.00	0.00
1457176_at	0.00	0.00
1457177_at	0.00	0.00
1457178_at	0.00	0.00
1457179_at	0.00	0.00
1457180_at	0.00	0.00
1457181_at	0.00	0.00
1457182_at	0.00	0.00
1457183_at	0.00	0.00
1457184_at	0.00	0.00
1457185_at	0.00	0.00
1457186_at	0.00	0.00
1457187_at	0.00	0.00
1457188_at	0.00	0.00
1457189_at	0.00	0.00
1457190_at	0.00	0.00
1457191_at	0.00	0.00
1457192_at	0.00	0.00
1457193_at	0.00	0.00
1457194_at	0.00	0.00
1457195_at	0.00	0.00
1457196_at	0.00	0.00
1457197_at	0.00	0.00
1457198_at	0.00	0.00
1457199_at	0.00	0.00
1457200_at	0.00	0.00
1457201_at	0.00	0.00
1457202_at	0.00	0.00
1457203_at	0.00	0.00
1457204_at	0.00	0.00
1457205_at	0.00	0.00
1457206_at	0.00	0.00
1457207_at	0.00	0.00
1457208_at	0.00	0.00
1457209_at	0.00	0.00
1457210_at	0.00	0.00
1457211_at	0.00	0.00
1457212_at	0.00	0.00
1457213_a_at	0.00	0.00

1457214_at	0.00	0.00
1457215_at	0.00	0.00
1457216_at	0.00	0.00
1457217_at	0.00	0.00
1457218_at	0.00	0.00
1457219_at	0.00	0.00
1457220_at	0.00	0.00
1457221_at	0.00	0.00
1457222_at	0.00	0.00
1457223_at	0.00	0.00
1457224_at	0.00	0.00
1457225_at	0.00	0.00
1457226_at	0.00	0.00
1457227_at	0.00	0.00
1457228_x_at	0.00	0.00
1457229_at	0.00	0.00
1457230_at	0.00	0.00
1457231_at	0.00	0.00
1457232_at	0.00	0.00
1457233_at	0.00	0.00
1457234_at	0.00	0.00
1457235_at	0.00	0.00
1457236_at	0.00	0.00
1457237_at	0.00	0.00
1457238_at	0.00	0.00
1457239_at	0.00	0.00
1457240_at	0.00	0.00
1457241_at	0.00	0.00
1457242_at	0.00	0.00
1457243_at	0.00	0.00
1457244_at	0.00	0.00
1457245_at	0.00	0.00
1457246_at	0.00	0.00
1457247_at	0.00	0.00
1457248_x_at	0.00	0.00
1457249_at	0.00	0.00
1457250_x_at	0.00	0.00
1457251_x_at	0.00	0.00
1457252_x_at	0.00	0.00
1457253_at	0.00	0.00
1457254_x_at	0.00	0.00
1457255_x_at	0.00	0.00
1457256_x_at	0.00	0.00
1457257_x_at	0.00	0.00
1457258_at	0.00	0.00
1457259_at	0.00	0.00
1457260_at	0.00	0.00
1457261_at	0.00	0.00
1457262_at	0.00	0.00
1457263_at	0.00	0.00
1457264_at	0.00	0.00
1457266_at	0.00	0.00
1457267_at	0.00	0.00
1457268_at	0.00	0.00
1457269_at	0.00	0.00
1457270_at	0.00	0.00

1457271_at	0.00	0.00
1457273_at	0.00	0.00
1457274_at	0.00	0.00
1457275_at	0.00	0.00
1457276_at	0.00	0.00
1457277_at	0.00	0.00
1457278_at	0.00	0.00
1457279_at	0.00	0.00
1457280_at	0.00	0.00
1457281_at	0.74	0.17
1457282_x_at	0.00	0.00
1457283_at	0.00	0.00
1457284_at	0.00	0.00
1457286_at	0.00	0.00
1457287_at	0.00	0.00
1457288_at	0.00	0.00
1457289_at	0.00	0.00
1457290_at	0.00	0.00
1457291_at	0.00	0.00
1457292_at	0.00	0.26
1457293_at	0.00	0.00
1457294_at	0.00	0.00
1457295_at	0.00	0.00
1457296_at	0.00	0.00
1457297_at	0.00	0.00
1457298_at	0.00	0.00
1457299_at	0.00	0.00
1457300_at	0.00	0.00
1457301_at	0.00	0.00
1457302_at	0.00	0.00
1457304_at	0.00	0.00
1457305_at	0.00	0.00
1457306_at	0.00	0.00
1457307_at	0.00	0.00
1457309_at	0.00	0.00
1457310_x_at	0.00	0.00
1457311_at	0.00	0.00
1457312_at	0.00	0.00
1457313_at	0.00	0.00
1457314_at	0.03	0.97
1457315_at	0.00	0.00
1457316_at	0.00	0.00
1457317_at	0.00	0.00
1457318_at	0.00	0.00
1457319_at	0.00	0.00
1457320_at	0.00	0.00
1457321_at	0.00	0.00
1457322_at	0.00	0.00
1457323_at	0.00	0.00
1457324_at	0.00	0.00
1457325_at	0.00	0.00
1457326_at	0.00	0.00
1457327_at	0.00	0.00
1457328_at	0.00	0.00
1457329_at	0.00	0.00
1457330_at	0.00	0.00

1457331_at	0.00	0.00
1457332_at	0.00	0.00
1457333_at	0.00	0.00
1457334_at	0.00	0.00
1457335_at	0.00	0.00
1457336_at	0.00	0.00
1457337_at	0.00	0.00
1457338_at	0.00	0.00
1457339_at	0.00	0.00
1457340_at	0.00	0.00
1457341_at	0.00	0.00
1457342_at	0.00	0.00
1457343_at	0.00	0.00
1457344_at	0.00	0.00
1457345_at	0.00	0.00
1457346_at	0.00	0.00
1457347_at	0.00	0.00
1457348_at	0.00	0.00
1457349_at	0.00	0.00
1457350_at	0.00	0.00
1457351_at	0.00	0.00
1457352_x_at	0.00	0.00
1457353_at	0.00	0.00
1457354_at	0.00	0.00
1457355_at	0.00	0.00
1457356_at	0.00	0.00
1457357_at	0.00	0.00
1457358_at	0.00	0.00
1457359_at	0.00	0.00
1457360_at	0.00	0.00
1457361_at	0.00	0.00
1457362_at	0.00	0.00
1457363_at	0.00	0.00
1457364_at	0.00	0.00
1457365_at	0.00	0.00
1457366_at	0.00	0.00
1457367_at	0.00	0.00
1457368_at	0.00	0.00
1457369_at	0.00	0.00
1457370_at	0.00	0.00
1457371_at	0.00	0.00
1457373_at	0.00	0.00
1457374_at	0.00	0.00
1457375_at	0.00	0.00
1457376_at	0.00	0.00
1457377_at	0.00	0.00
1457378_at	0.00	0.00
1457380_at	0.00	0.00
1457381_at	0.00	0.00
1457382_at	0.00	0.00
1457383_at	0.00	0.00
1457384_at	0.00	0.00
1457385_at	0.00	0.00
1457386_at	0.00	0.00
1457387_at	0.00	0.00
1457388_at	0.00	0.00

1457389_at	0.00	0.00
1457390_at	0.00	0.00
1457391_at	0.00	0.00
1457392_at	0.00	0.00
1457393_at	0.00	0.00
1457394_at	0.00	0.00
1457395_at	0.00	0.00
1457396_at	0.00	0.00
1457397_at	0.00	0.00
1457398_at	0.00	0.00
1457399_at	0.00	0.00
1457400_at	0.00	0.00
1457401_at	0.00	0.00
1457402_at	0.00	0.00
1457403_at	0.00	0.00
1457404_at	0.00	0.00
1457405_at	0.00	0.00
1457406_at	0.00	0.00
1457407_at	0.00	0.00
1457408_at	0.00	0.00
1457409_at	0.00	0.00
1457410_at	0.00	0.00
1457411_at	0.00	0.00
1457412_at	0.00	0.00
1457413_at	0.00	0.00
1457414_at	0.00	0.00
1457415_a_at	0.00	0.00
1457416_at	0.00	0.00
1457417_at	0.00	0.00
1457418_at	0.00	0.00
1457419_s_at	0.00	0.00
1457420_at	0.00	0.00
1457421_at	0.00	0.00
1457422_at	0.00	0.00
1457423_at	0.00	0.00
1457424_at	0.00	0.00
1457425_at	0.00	0.00
1457426_at	0.00	0.00
1457427_at	0.00	0.00
1457428_at	0.00	0.00
1457429_s_at	0.00	0.00
1457430_at	0.00	0.00
1457431_at	0.00	0.00
1457432_at	0.00	0.00
1457433_x_at	0.00	0.00
1457434_s_at	0.00	0.00
1457435_x_at	0.00	0.00
1457436_at	0.00	0.00
1457437_at	0.00	0.00
1457438_at	0.00	0.00
1457439_at	0.00	0.00
1457440_at	0.00	0.00
1457441_at	0.00	0.00
1457442_at	0.00	0.00
1457443_at	0.00	0.00
1457444_at	0.00	0.00

1457445_at	0.00	0.00
1457446_at	0.00	0.00
1457447_at	0.00	0.00
1457448_at	0.00	0.00
1457449_at	0.00	0.00
1457450_at	0.00	0.00
1457451_at	0.00	0.00
1457452_at	0.00	0.00
1457453_at	0.00	0.00
1457454_at	0.00	0.00
1457456_at	0.00	0.00
1457457_at	0.00	0.00
1457458_at	0.00	0.00
1457459_at	0.00	0.00
1457460_at	0.00	0.00
1457461_at	0.00	0.00
1457462_at	0.00	0.00
1457463_at	0.00	0.00
1457464_at	0.00	0.00
1457465_at	0.00	0.00
1457466_at	0.00	0.00
1457467_at	0.00	0.00
1457468_at	0.00	0.00
1457469_at	0.00	0.00
1457470_at	0.00	0.00
1457471_at	0.00	0.00
1457472_at	0.00	0.00
1457473_at	0.00	0.00
1457474_at	0.00	0.00
1457477_at	0.00	0.00
1457478_at	0.00	0.00
1457479_at	0.00	0.00
1457480_at	0.00	0.00
1457481_at	0.00	0.00
1457482_at	0.00	0.00
1457483_at	0.00	0.00
1457484_at	0.00	0.00
1457485_at	0.00	0.00
1457486_at	0.00	0.00
1457487_at	0.00	0.00
1457488_at	0.00	0.00
1457489_at	0.00	0.00
1457490_at	0.00	0.00
1457491_at	0.00	0.00
1457492_at	0.00	0.00
1457493_at	0.00	0.00
1457494_at	0.00	0.00
1457495_at	0.00	0.00
1457496_at	0.00	0.00
1457497_at	0.00	0.00
1457498_at	0.00	0.00
1457499_at	0.00	0.00
1457500_at	0.00	0.00
1457501_at	0.00	0.00
1457502_at	0.00	0.00
1457503_at	0.00	0.00

1457504_at	0.00	0.00
1457505_at	0.00	0.00
1457506_at	0.00	0.00
1457507_at	0.00	0.00
1457508_at	0.00	0.00
1457509_at	0.00	0.00
1457510_at	0.00	0.00
1457511_at	0.00	0.00
1457512_at	0.00	0.00
1457513_at	0.00	0.00
1457514_at	0.00	0.00
1457515_at	0.00	0.00
1457516_at	0.00	0.00
1457517_at	0.00	0.00
1457518_at	0.00	0.00
1457519_at	0.00	0.00
1457522_at	0.00	0.00
1457523_at	0.00	0.00
1457524_at	0.00	0.00
1457525_at	0.00	0.00
1457526_at	0.00	0.00
1457527_at	0.00	0.00
1457528_at	0.00	0.00
1457529_x_at	0.00	0.00
1457530_at	0.00	0.00
1457531_a_at	0.00	0.00
1457532_at	0.00	0.00
1457533_at	0.00	0.00
1457534_at	0.00	0.00
1457535_at	0.00	0.00
1457536_at	0.00	0.00
1457537_at	0.00	0.00
1457538_at	0.00	0.00
1457539_at	0.00	0.00
1457540_at	0.00	0.00
1457541_at	0.00	0.00
1457542_at	0.00	0.00
1457543_at	0.00	0.00
1457544_at	0.00	0.00
1457545_at	0.00	0.00
1457546_at	0.00	0.00
1457547_at	0.00	0.00
1457548_at	0.00	0.00
1457549_at	0.00	0.00
1457550_at	0.00	0.00
1457551_at	0.00	0.00
1457552_at	0.00	0.00
1457553_at	0.00	0.00
1457554_at	0.00	0.00
1457555_at	0.00	0.00
1457556_at	0.00	0.00
1457557_at	0.00	0.00
1457558_at	0.00	0.00
1457559_at	0.00	0.00
1457560_at	0.00	0.00
1457561_at	0.00	0.00

1457562_at	0.00	0.00
1457563_at	0.00	0.00
1457564_at	0.00	0.00
1457565_at	0.00	0.00
1457566_at	0.00	0.00
1457567_at	0.00	0.00
1457568_at	0.00	0.00
1457569_at	0.00	0.00
1457570_at	0.00	0.00
1457571_at	0.00	0.00
1457572_at	0.00	0.00
1457573_at	0.00	0.00
1457574_at	0.00	0.00
1457575_at	0.00	0.00
1457576_at	0.00	0.00
1457577_at	0.00	0.00
1457578_at	0.00	0.00
1457579_at	0.00	0.00
1457580_at	0.00	0.00
1457581_at	0.00	0.00
1457582_at	0.00	0.00
1457583_at	0.00	0.00
1457584_at	0.00	0.00
1457585_at	0.00	0.00
1457586_at	0.00	0.00
1457587_at	0.00	0.00
1457589_at	0.00	0.00
1457590_at	0.00	0.00
1457591_at	0.00	0.00
1457592_x_at	0.00	0.00
1457593_at	0.00	0.00
1457595_at	0.00	0.00
1457596_at	0.00	0.00
1457597_at	0.00	0.00
1457598_at	0.00	0.00
1457599_at	0.00	0.00
1457600_x_at	0.00	0.00
1457601_at	0.00	0.00
1457602_at	0.00	0.00
1457603_at	0.00	0.00
1457604_x_at	0.00	0.00
1457605_at	0.00	0.00
1457606_x_at	0.00	0.00
1457607_at	0.00	0.00
1457608_at	0.00	0.00
1457609_at	0.00	0.00
1457610_at	0.00	0.00
1457611_x_at	0.00	0.00
1457612_at	0.00	0.00
1457613_at	0.00	0.00
1457614_at	0.00	0.00
1457615_at	0.00	0.00
1457616_at	0.00	0.00
1457617_at	0.00	0.00
1457618_at	0.00	0.00
1457619_at	0.00	0.00

1457620_at	0.00	0.00
1457621_at	0.00	0.00
1457622_at	0.00	0.00
1457623_x_at	0.00	0.00
1457624_at	0.00	0.00
1457625_s_at	0.00	0.00
1457626_at	0.00	0.00
1457627_x_at	0.00	0.00
1457628_at	0.00	0.00
1457629_at	0.00	0.00
1457630_at	0.00	0.00
1457631_at	0.00	0.00
1457632_s_at	0.00	0.00
1457633_x_at	0.00	0.00
1457634_at	0.00	0.00
1457635_s_at	0.00	0.00
1457636_x_at	0.00	0.00
1457637_at	0.00	0.00
1457638_x_at	0.00	0.00
1457639_at	0.00	0.00
1457640_x_at	0.00	0.00
1457641_at	0.00	0.00
1457642_at	0.00	0.00
1457643_x_at	0.00	0.00
1457644_s_at	0.00	0.00
1457645_at	0.00	0.00
1457646_at	0.00	0.00
1457647_x_at	0.00	0.00
1457648_x_at	0.00	0.00
1457649_x_at	0.00	0.00
1457650_x_at	0.00	0.00
1457651_x_at	0.00	0.00
1457652_x_at	0.00	0.00
1457653_at	0.00	0.00
1457654_at	0.00	0.00
1457655_x_at	0.00	0.00
1457656_s_at	0.00	0.00
1457657_at	0.00	0.00
1457658_x_at	0.00	0.01
1457659_x_at	0.00	0.00
1457660_x_at	0.00	0.00
1457661_at	0.00	0.00
1457662_x_at	0.00	0.00
1457663_at	0.00	0.00
1457664_x_at	0.00	0.00
1457665_x_at	0.00	0.00
1457666_s_at	0.00	0.00
1457667_x_at	0.00	0.00
1457668_x_at	0.00	0.00
1457669_x_at	0.00	0.00
1457670_s_at	0.00	0.00
1457671_at	0.00	0.00
1457672_at	0.00	0.00
1457673_at	0.00	0.00
1457674_at	0.00	0.00
1457677_at	0.00	0.00

1457678_at	0.00	0.00
1457679_at	0.00	0.00
1457680_a_at	0.00	0.00
1457681_at	0.00	0.00
1457682_at	0.00	0.00
1457683_at	0.00	0.00
1457684_at	0.00	0.00
1457685_at	0.00	0.00
1457686_at	0.00	0.00
1457687_at	0.00	0.00
1457688_at	0.00	0.00
1457689_at	0.00	0.00
1457690_at	0.00	0.00
1457691_at	0.00	0.00
1457692_at	0.00	0.00
1457693_a_at	0.00	0.00
1457694_at	0.00	0.00
1457696_at	0.00	0.00
1457697_at	0.00	0.00
1457698_at	0.00	0.00
1457699_at	0.00	0.00
1457700_at	0.00	0.00
1457701_at	0.00	0.00
1457702_at	0.00	0.00
1457703_at	0.00	0.00
1457704_at	0.00	0.00
1457705_at	0.00	0.00
1457706_at	0.00	0.00
1457707_at	0.00	0.00
1457708_at	0.00	0.00
1457709_a_at	0.00	0.00
1457710_at	0.00	0.00
1457711_at	0.00	0.00
1457712_at	0.00	0.00
1457713_at	0.00	0.00
1457714_at	0.00	0.00
1457715_at	0.00	0.00
1457716_at	0.03	0.00
1457717_at	0.00	0.00
1457718_at	0.00	0.00
1457719_at	0.00	0.00
1457720_at	0.00	0.00
1457721_at	0.00	0.00
1457722_at	0.00	0.00
1457723_at	0.00	0.00
1457724_at	0.00	0.00
1457725_at	0.00	0.00
1457726_at	0.00	0.00
1457727_at	0.00	0.00
1457728_at	0.00	0.00
1457729_at	0.00	0.00
1457730_at	0.00	0.00
1457731_at	0.00	0.00
1457732_at	0.00	0.00
1457733_at	0.00	0.00
1457734_at	0.00	0.00

1457735_at	0.00	0.00
1457736_at	0.00	0.00
1457737_at	0.00	0.00
1457739_at	0.00	0.00
1457740_at	0.00	0.00
1457741_at	0.00	0.00
1457742_at	0.00	0.00
1457743_at	0.00	0.00
1457744_at	0.00	0.00
1457745_at	0.00	0.00
1457746_at	0.00	0.00
1457747_at	0.00	0.00
1457748_at	0.00	0.00
1457749_at	0.00	0.00
1457750_at	0.00	0.00
1457751_at	0.00	0.00
1457752_at	0.00	0.00
1457753_at	0.00	0.00
1457754_at	0.00	0.00
1457755_at	0.00	0.00
1457756_at	0.00	0.00
1457757_at	0.00	0.00
1457758_at	0.00	0.00
1457759_at	0.00	0.00
1457760_at	0.00	0.00
1457761_at	0.00	0.00
1457762_at	0.00	0.00
1457763_at	0.00	0.00
1457764_at	0.00	0.00
1457765_at	0.00	0.00
1457766_at	0.00	0.00
1457767_at	0.00	0.00
1457768_at	0.00	0.00
1457769_at	0.00	0.00
1457770_at	0.00	0.00
1457771_at	0.00	0.00
1457772_at	0.00	0.00
1457773_at	0.00	0.00
1457774_at	0.00	0.00
1457775_at	0.00	0.00
1457777_at	0.00	0.00
1457778_at	0.00	0.00
1457779_at	0.00	0.00
1457780_at	0.00	0.00
1457781_at	0.00	0.00
1457782_at	0.00	0.00
1457783_at	0.00	0.00
1457784_at	0.00	0.00
1457785_at	0.00	0.00
1457786_at	0.00	0.00
1457787_at	0.00	0.00
1457788_at	0.00	0.00
1457789_at	0.00	0.00
1457790_at	0.00	0.00
1457791_at	0.00	0.00
1457792_at	0.00	0.00

1457793_a_at	0.00	0.00
1457794_at	0.00	0.00
1457795_at	0.00	0.00
1457796_at	0.00	0.00
1457797_at	0.00	0.00
1457798_at	0.00	0.00
1457799_at	0.00	0.00
1457800_at	0.00	0.00
1457801_at	0.00	0.00
1457802_at	0.00	0.00
1457803_at	0.00	0.00
1457804_at	0.00	0.00
1457805_at	0.00	0.00
1457806_at	0.00	0.00
1457807_at	0.00	0.00
1457808_at	0.00	0.00
1457809_at	0.00	0.00
1457811_at	0.00	0.00
1457812_at	0.00	0.00
1457813_at	0.00	0.00
1457814_at	0.00	0.00
1457815_at	0.00	0.00
1457816_at	0.00	0.00
1457817_at	0.00	0.00
1457818_at	0.00	0.00
1457819_at	0.00	0.00
1457820_at	0.00	0.00
1457821_at	0.00	0.00
1457822_at	0.00	0.00
1457823_at	0.34	0.01
1457824_at	0.00	0.00
1457825_x_at	0.00	0.08
1457826_a_at	0.00	0.00
1457827_at	0.00	0.00
1457828_at	0.00	0.00
1457829_at	0.00	0.00
1457830_at	0.00	0.00
1457831_at	0.00	0.00
1457832_at	0.00	0.00
1457835_at	0.00	0.00
1457836_at	0.00	0.00
1457837_at	0.00	0.00
1457838_at	0.00	0.00
1457839_at	0.00	0.00
1457840_at	0.00	0.00
1457842_at	0.00	0.00
1457843_at	0.00	0.00
1457844_a_at	0.00	0.00
1457845_at	0.00	0.00
1457846_at	0.00	0.00
1457847_at	0.00	0.00
1457848_at	0.00	0.00
1457850_at	0.00	0.00
1457851_at	0.00	0.00
1457852_at	0.00	0.00
1457853_at	0.00	0.00

1457854_at	0.00	0.00
1457855_at	0.00	0.00
1457856_at	0.00	0.00
1457857_at	0.00	0.00
1457858_at	0.00	0.00
1457859_at	0.00	0.00
1457861_at	0.00	0.00
1457862_at	0.00	0.00
1457863_at	0.00	0.00
1457864_at	0.00	0.00
1457865_at	0.00	0.00
1457866_at	0.00	0.00
1457867_at	0.00	0.00
1457868_at	0.00	0.00
1457869_at	0.00	0.01
1457870_at	0.00	0.00
1457871_at	0.00	0.00
1457873_at	0.00	0.00
1457874_at	0.00	0.00
1457875_at	0.00	0.00
1457876_at	0.00	0.00
1457877_at	0.00	0.00
1457878_at	0.00	0.00
1457879_at	0.00	0.00
1457880_at	0.00	0.00
1457881_at	0.00	0.00
1457882_at	0.00	0.00
1457883_at	0.00	0.00
1457884_at	0.00	0.00
1457885_at	0.00	0.00
1457886_at	0.00	0.00
1457888_at	0.00	0.00
1457889_at	0.00	0.00
1457890_at	0.00	0.00
1457891_at	0.00	0.00
1457892_at	0.00	0.00
1457893_at	0.00	0.00
1457894_at	0.00	0.00
1457895_at	0.00	0.00
1457896_at	0.00	0.00
1457897_at	0.00	0.00
1457898_at	0.00	0.00
1457900_at	0.00	0.00
1457901_at	0.00	0.00
1457902_at	0.00	0.00
1457903_at	0.00	0.00
1457904_at	0.00	0.00
1457906_at	0.00	0.00
1457907_at	0.00	0.00
1457908_at	0.00	0.00
1457909_at	0.00	0.00
1457910_at	0.00	0.00
1457911_at	0.00	0.00
1457912_at	0.00	0.00
1457913_at	0.00	0.00
1457914_at	0.00	0.00

1457915_at	0.00	0.00
1457916_at	0.00	0.00
1457917_at	0.00	0.00
1457918_at	0.00	0.00
1457919_at	0.00	0.00
1457920_at	0.00	0.00
1457921_at	0.00	0.00
1457922_at	0.00	0.00
1457923_at	0.00	0.00
1457924_at	0.00	0.00
1457925_at	0.00	0.00
1457926_at	0.00	0.00
1457927_at	0.00	0.00
1457928_at	0.00	0.00
1457929_at	0.00	0.00
1457930_at	0.00	0.00
1457931_at	0.00	0.00
1457932_at	0.00	0.00
1457933_at	0.00	0.00
1457934_at	0.00	0.00
1457935_at	0.00	0.00
1457936_at	0.00	0.06
1457937_at	0.00	0.00
1457938_at	0.00	0.00
1457939_at	0.00	0.00
1457940_at	0.00	0.00
1457941_at	0.00	0.00
1457942_at	0.00	0.00
1457943_at	0.00	0.00
1457944_at	0.00	0.00
1457945_at	0.00	0.00
1457946_at	0.00	0.00
1457947_at	0.00	0.00
1457948_at	0.00	0.00
1457949_at	0.00	0.00
1457950_at	0.00	0.00
1457951_at	0.00	0.00
1457952_at	0.00	0.00
1457953_at	0.00	0.00
1457955_at	0.00	0.00
1457956_at	0.00	0.00
1457957_at	0.00	0.00
1457958_at	0.00	0.00
1457959_at	0.00	0.00
1457960_at	0.00	0.00
1457961_at	0.00	0.00
1457962_at	0.00	0.00
1457963_at	0.00	0.00
1457965_at	0.00	0.00
1457966_at	0.00	0.00
1457967_at	0.00	0.00
1457968_at	0.00	0.00
1457969_at	0.00	0.00
1457971_at	0.00	0.00
1457972_at	0.00	0.00
1457973_at	0.00	0.00

1457974_at	0.00	0.00
1457975_at	0.00	0.00
1457976_at	0.00	0.00
1457977_at	0.00	0.00
1457978_at	0.00	0.00
1457979_at	0.00	0.00
1457980_x_at	0.00	0.00
1457981_x_at	0.00	0.00
1457982_at	0.00	0.00
1457983_s_at	0.00	0.00
1457984_at	0.00	0.00
1457985_at	0.00	0.00
1457986_at	0.00	0.00
1457987_at	0.00	0.00
1457988_at	0.00	0.00
1457989_at	0.00	0.00
1457990_at	0.00	0.00
1457991_at	0.00	0.00
1457992_at	0.00	0.00
1457993_at	0.00	0.00
1457994_at	0.00	0.00
1457995_at	0.00	0.00
1457996_at	0.00	0.00
1457997_at	0.00	0.00
1457998_at	0.00	0.00
1457999_at	0.00	0.00
1458000_at	0.00	0.00
1458001_at	0.00	0.00
1458002_at	0.00	0.00
1458003_at	0.00	0.00
1458004_at	0.00	0.00
1458005_at	0.00	0.00
1458006_at	0.00	0.00
1458007_at	0.00	0.00
1458008_at	0.00	0.00
1458009_at	0.00	0.00
1458010_at	0.00	0.00
1458012_at	0.00	0.00
1458013_at	0.00	0.00
1458014_at	0.00	0.00
1458015_at	0.00	0.00
1458016_at	0.00	0.00
1458017_at	0.00	0.00
1458018_at	0.00	0.00
1458019_at	0.00	0.00
1458020_at	0.00	0.00
1458021_at	0.00	0.00
1458022_at	0.00	0.00
1458023_at	0.00	0.00
1458024_at	0.00	0.00
1458025_at	0.00	0.00
1458026_at	0.00	0.00
1458027_at	0.00	0.00
1458028_at	0.00	0.00
1458029_at	0.00	0.00
1458030_at	0.00	0.00

1458031_at	0.00	0.00
1458032_at	0.00	0.00
1458033_at	0.00	0.00
1458034_at	0.00	0.00
1458035_at	0.00	0.00
1458036_at	0.00	0.00
1458037_at	0.00	0.00
1458038_at	0.00	0.00
1458039_at	0.00	0.00
1458040_at	0.00	0.00
1458042_at	0.00	0.00
1458043_at	0.00	0.00
1458044_at	0.00	0.00
1458045_at	0.00	0.00
1458046_at	0.00	0.00
1458047_at	0.00	0.00
1458048_at	0.00	0.00
1458049_at	0.00	0.00
1458050_at	0.00	0.00
1458051_at	0.00	0.00
1458052_at	0.00	0.00
1458053_at	0.00	0.00
1458054_at	0.00	0.00
1458055_at	0.00	0.00
1458056_at	0.00	0.00
1458057_at	0.00	0.00
1458058_at	0.00	0.00
1458059_at	0.00	0.00
1458060_at	0.00	0.00
1458061_at	0.00	0.00
1458062_at	0.00	0.00
1458063_at	0.00	0.00
1458064_at	0.00	0.00
1458065_at	0.00	0.00
1458066_at	0.00	0.00
1458067_at	0.00	0.00
1458068_at	0.00	0.00
1458069_at	0.00	0.00
1458070_at	0.00	0.00
1458071_at	0.00	0.00
1458072_at	0.00	0.00
1458073_at	0.00	0.00
1458074_at	0.00	0.00
1458075_at	0.00	0.00
1458076_at	0.00	0.00
1458077_at	0.00	0.00
1458078_at	0.00	0.00
1458079_at	0.00	0.00
1458080_at	0.00	0.00
1458081_at	0.00	0.00
1458082_at	0.00	0.00
1458083_at	0.00	0.00
1458084_at	0.00	0.00
1458085_at	0.00	0.00
1458086_at	0.00	0.00
1458087_at	0.00	0.00

1458088_at	0.00	0.00
1458089_at	0.00	0.00
1458090_at	0.00	0.00
1458091_at	0.00	0.00
1458092_at	0.00	0.00
1458093_at	0.00	0.00
1458094_at	0.00	0.00
1458095_at	0.00	0.00
1458096_at	0.00	0.00
1458097_at	0.00	0.00
1458098_at	0.00	0.00
1458099_at	0.00	0.00
1458100_at	0.00	0.00
1458101_at	0.00	0.00
1458102_at	0.00	0.00
1458103_at	0.00	0.00
1458104_a_at	0.00	0.00
1458105_at	0.00	0.00
1458106_at	0.00	0.00
1458107_at	0.00	0.00
1458108_at	0.00	0.00
1458109_at	0.00	0.00
1458110_at	0.00	0.00
1458111_at	0.00	0.00
1458112_at	0.00	0.00
1458113_at	0.00	0.00
1458114_at	0.00	0.00
1458115_at	0.00	0.00
1458116_at	0.00	0.00
1458117_at	0.00	0.00
1458118_at	0.00	0.00
1458119_at	0.00	0.00
1458120_at	0.00	0.00
1458121_at	0.00	0.00
1458122_at	0.00	0.00
1458123_at	0.00	0.00
1458124_at	0.00	0.00
1458125_at	0.00	0.00
1458126_at	0.00	0.00
1458127_at	0.00	0.00
1458128_at	0.00	0.00
1458129_at	0.00	0.00
1458130_at	0.00	0.00
1458131_at	0.00	0.00
1458132_at	0.00	0.00
1458133_at	0.00	0.00
1458134_at	0.00	0.00
1458135_at	0.00	0.00
1458136_at	0.00	0.00
1458137_at	0.00	0.00
1458138_at	0.00	0.00
1458139_at	0.00	0.00
1458140_at	0.00	0.00
1458141_at	0.00	0.00
1458142_at	0.00	0.00
1458143_at	0.00	0.00

1458144_at	0.00	0.00
1458145_at	0.00	0.00
1458146_at	0.00	0.00
1458147_at	0.00	0.00
1458148_at	0.00	0.00
1458149_at	0.00	0.00
1458150_at	0.00	0.00
1458151_at	0.00	0.00
1458152_at	0.00	0.00
1458153_at	0.00	0.00
1458154_at	0.00	0.00
1458155_at	0.00	0.00
1458156_at	0.00	0.00
1458157_at	0.00	0.00
1458158_at	0.00	0.00
1458159_at	0.00	0.00
1458160_at	0.00	0.00
1458161_at	0.00	0.00
1458162_at	0.00	0.00
1458163_at	0.00	0.00
1458164_at	0.00	0.00
1458165_at	0.00	0.00
1458166_at	0.00	0.00
1458167_at	0.00	0.00
1458168_at	0.00	0.00
1458169_at	0.00	0.00
1458170_at	0.00	0.00
1458171_at	0.00	0.00
1458172_at	0.00	0.00
1458174_at	0.00	0.00
1458175_at	0.00	0.00
1458176_at	0.00	0.00
1458177_at	0.00	0.00
1458178_at	0.00	0.00
1458179_at	0.00	0.00
1458180_at	0.00	0.00
1458181_at	0.00	0.00
1458182_at	0.00	0.00
1458183_at	0.00	0.00
1458184_at	0.00	0.00
1458185_at	0.00	0.00
1458186_at	0.00	0.00
1458187_at	0.00	0.00
1458188_at	0.00	0.00
1458189_at	0.00	0.00
1458190_at	0.00	0.00
1458191_at	0.00	0.00
1458192_at	0.00	0.00
1458193_at	0.00	0.00
1458194_at	0.00	0.00
1458195_at	0.00	0.00
1458197_x_at	0.00	0.00
1458198_at	0.00	0.00
1458199_at	0.00	0.00
1458200_at	0.00	0.00
1458201_at	0.00	0.00

1458202_at	0.00	0.00
1458203_at	0.00	0.00
1458204_at	0.00	0.00
1458205_at	0.00	0.00
1458206_at	0.00	0.00
1458207_at	0.00	0.00
1458208_s_at	0.00	0.00
1458209_at	0.00	0.00
1458210_at	0.00	0.00
1458211_at	0.00	0.00
1458212_at	0.00	0.00
1458213_at	0.00	0.00
1458214_at	0.00	0.00
1458216_at	0.00	0.00
1458217_s_at	0.00	0.00
1458218_s_at	0.00	0.00
1458220_at	0.00	0.00
1458221_at	0.00	0.00
1458222_at	0.00	0.00
1458223_at	0.00	0.00
1458224_at	0.00	0.00
1458225_at	0.00	0.00
1458226_at	0.07	0.00
1458227_at	0.00	0.00
1458228_at	0.00	0.00
1458229_at	0.00	0.00
1458230_at	0.00	0.00
1458231_at	0.00	0.00
1458232_at	0.00	0.00
1458233_at	0.00	0.00
1458234_at	0.00	0.00
1458235_at	0.00	0.00
1458236_at	0.00	0.00
1458237_at	0.00	0.00
1458238_at	0.00	0.00
1458239_at	0.00	0.00
1458240_at	0.00	0.00
1458241_at	0.00	0.00
1458242_at	0.00	0.00
1458243_at	0.00	0.00
1458244_at	0.00	0.00
1458245_at	0.00	0.00
1458246_at	0.00	0.00
1458247_s_at	0.00	0.00
1458248_at	0.00	0.00
1458249_at	0.00	0.00
1458250_at	0.00	0.00
1458251_at	0.00	0.00
1458252_at	0.00	0.00
1458253_at	0.00	0.00
1458254_at	0.00	0.00
1458255_at	0.00	0.00
1458256_at	0.00	0.00
1458257_at	0.00	0.00
1458258_at	0.00	0.00
1458259_x_at	0.00	0.00

1458260_at	0.00	0.00
1458261_at	0.00	0.00
1458262_at	0.00	0.00
1458263_at	0.00	0.00
1458264_at	0.00	0.00
1458265_at	0.00	0.00
1458266_at	0.00	0.00
1458267_at	0.00	0.00
1458268_s_at	0.00	0.00
1458269_at	0.00	0.00
1458270_at	0.00	0.00
1458271_at	0.00	0.00
1458272_at	0.00	0.00
1458273_at	0.00	0.00
1458274_at	0.00	0.00
1458275_at	0.00	0.00
1458276_x_at	0.00	0.00
1458277_at	0.00	0.00
1458278_at	0.00	0.00
1458279_at	0.00	0.00
1458280_at	0.00	0.00
1458281_at	0.00	0.00
1458282_at	0.00	0.00
1458283_at	0.00	0.00
1458284_at	0.00	0.00
1458285_at	0.00	0.00
1458286_at	0.00	0.00
1458287_at	0.00	0.00
1458288_at	0.00	0.00
1458289_at	0.00	0.00
1458290_at	0.00	0.00
1458291_at	0.00	0.00
1458292_at	0.00	0.00
1458293_at	0.00	0.00
1458294_at	0.00	0.00
1458295_at	0.00	0.00
1458296_at	0.00	0.00
1458297_s_at	0.00	0.00
1458298_at	0.00	0.00
1458299_s_at	0.02	0.00
1458300_at	0.00	0.00
1458301_x_at	0.00	0.00
1458302_at	0.00	0.00
1458303_at	0.00	0.00
1458304_at	0.00	0.00
1458305_at	0.00	0.00
1458306_at	0.00	0.00
1458307_at	0.00	0.00
1458308_at	0.00	0.00
1458309_at	0.00	0.00
1458310_at	0.00	0.00
1458311_at	0.00	0.00
1458312_at	0.00	0.00
1458313_at	0.00	0.00
1458314_at	0.00	0.00
1458315_at	0.00	0.00

1458316_at	0.00	0.00
1458317_at	0.00	0.00
1458318_at	0.00	0.00
1458319_at	0.00	0.00
1458320_at	0.00	0.00
1458321_at	0.00	0.00
1458322_x_at	0.00	0.00
1458323_at	0.00	0.00
1458324_x_at	0.00	0.00
1458325_x_at	0.00	0.00
1458326_at	0.00	0.00
1458327_x_at	0.00	0.00
1458328_x_at	0.00	0.00
1458329_x_at	0.00	0.00
1458330_x_at	0.00	0.00
1458331_x_at	0.00	0.00
1458332_x_at	0.00	0.00
1458333_x_at	0.00	0.00
1458334_at	0.00	0.00
1458335_x_at	0.00	0.00
1458336_at	0.00	0.00
1458337_at	0.00	0.00
1458338_x_at	0.00	0.00
1458339_at	0.00	0.00
1458340_at	0.00	0.00
1458341_x_at	0.00	0.00
1458342_at	0.00	0.00
1458343_x_at	0.00	0.00
1458344_at	0.00	0.00
1458345_s_at	0.00	0.00
1458346_x_at	0.00	0.00
1458347_s_at	0.02	0.16
1458348_at	0.00	0.00
1458349_s_at	0.00	0.00
1458350_at	0.00	0.00
1458351_s_at	0.00	0.00
1458352_at	0.00	0.00
1458353_at	0.00	0.00
1458354_x_at	0.00	0.00
1458355_x_at	0.00	0.00
1458356_at	0.00	0.00
1458357_x_at	0.00	0.00
1458358_at	0.00	0.00
1458359_at	0.00	0.00
1458360_at	0.00	0.00
1458361_at	0.00	0.00
1458362_at	0.00	0.00
1458363_at	0.00	0.00
1458364_s_at	0.00	0.00
1458365_at	0.00	0.00
1458366_at	0.00	0.00
1458367_at	0.00	0.00
1458368_at	0.00	0.00
1458369_at	0.00	0.00
1458370_at	0.00	0.00
1458371_at	0.00	0.00

1458372_at	0.00	0.00
1458373_at	0.00	0.00
1458374_at	0.00	0.00
1458375_at	0.00	0.00
1458376_at	0.00	0.00
1458377_at	0.00	0.00
1458378_at	0.00	0.00
1458379_at	0.00	0.00
1458380_at	0.00	0.00
1458381_at	0.00	0.00
1458382_a_at	0.00	0.00
1458383_at	0.00	0.00
1458384_at	0.00	0.00
1458385_at	0.00	0.00
1458386_at	0.00	0.00
1458387_at	0.00	0.00
1458388_at	0.00	0.00
1458389_at	0.00	0.00
1458390_at	0.00	0.00
1458391_at	0.00	0.00
1458392_at	0.00	0.00
1458393_at	0.00	0.00
1458394_at	0.00	0.00
1458395_at	0.00	0.00
1458396_at	0.00	0.00
1458397_at	0.00	0.00
1458398_at	0.00	0.00
1458399_at	0.00	0.00
1458400_at	0.00	0.00
1458401_at	0.00	0.00
1458402_at	0.00	0.00
1458403_at	0.00	0.00
1458404_at	0.00	0.00
1458405_at	0.00	0.00
1458406_at	0.00	0.00
1458407_s_at	0.00	0.00
1458408_at	0.00	0.00
1458409_at	0.00	0.00
1458410_at	0.00	0.00
1458411_at	0.00	0.00
1458412_at	0.00	0.00
1458413_at	0.00	0.00
1458415_at	0.00	0.00
1458416_at	0.00	0.00
1458417_at	0.00	0.00
1458418_at	0.00	0.00
1458419_at	0.00	0.00
1458420_at	0.00	0.00
1458421_at	0.00	0.00
1458422_at	0.00	0.00
1458423_at	0.00	0.00
1458424_at	0.00	0.00
1458425_at	0.00	0.00
1458426_at	0.00	0.00
1458427_at	0.00	0.00
1458428_at	0.00	0.00

1458429_at	0.00	0.00
1458430_at	0.00	0.00
1458431_at	0.00	0.00
1458432_at	0.00	0.00
1458433_at	0.00	0.00
1458434_at	0.00	0.00
1458435_at	0.00	0.00
1458436_at	0.00	0.00
1458437_at	0.00	0.00
1458438_at	0.00	0.00
1458439_a_at	0.00	0.00
1458440_at	0.00	0.00
1458441_at	0.00	0.00
1458442_at	0.00	0.00
1458443_at	0.00	0.00
1458444_at	0.00	0.00
1458445_at	0.00	0.00
1458446_at	0.00	0.00
1458447_at	0.00	0.00
1458448_at	0.00	0.00
1458449_at	0.00	0.00
1458450_at	0.00	0.00
1458451_at	0.00	0.00
1458452_at	0.00	0.00
1458453_at	0.00	0.00
1458454_at	0.00	0.00
1458455_at	0.00	0.00
1458456_x_at	0.00	0.00
1458457_at	0.00	0.00
1458458_at	0.00	0.00
1458459_a_at	0.00	0.00
1458460_at	0.00	0.00
1458461_at	0.00	0.00
1458462_at	0.00	0.00
1458463_at	0.00	0.00
1458464_at	0.00	0.00
1458465_at	0.00	0.00
1458466_at	0.00	0.00
1458467_at	0.00	0.00
1458468_at	0.00	0.00
1458469_at	0.00	0.00
1458470_at	0.00	0.00
1458471_at	0.00	0.00
1458472_at	0.00	0.00
1458473_at	0.00	0.00
1458474_at	0.00	0.00
1458475_at	0.00	0.00
1458476_at	0.00	0.00
1458477_at	0.00	0.00
1458478_at	0.00	0.00
1458479_at	0.00	0.00
1458480_at	0.00	0.00
1458481_at	0.00	0.00
1458482_at	0.00	0.00
1458483_at	0.00	0.00
1458484_at	0.00	0.00

1458485_at	0.00	0.00
1458486_at	0.00	0.00
1458487_at	0.00	0.00
1458488_at	0.00	0.00
1458489_at	0.00	0.00
1458490_at	0.00	0.00
1458491_at	0.00	0.00
1458492_x_at	0.00	0.00
1458493_a_at	0.00	0.00
1458494_at	0.00	0.00
1458495_at	0.00	0.00
1458496_at	0.00	0.00
1458497_at	0.00	0.00
1458498_at	0.00	0.00
1458499_at	0.00	0.00
1458500_at	0.00	0.00
1458501_at	0.00	0.00
1458502_at	0.00	0.00
1458503_at	0.00	0.00
1458504_at	0.00	0.00
1458505_at	0.00	0.00
1458506_at	0.00	0.00
1458507_at	0.00	0.00
1458508_at	0.00	0.00
1458509_at	0.00	0.00
1458510_at	0.00	0.00
1458511_at	0.00	0.00
1458512_at	0.00	0.00
1458513_at	0.00	0.00
1458514_at	0.00	0.00
1458515_at	0.00	0.00
1458516_at	0.00	0.00
1458517_at	0.00	0.00
1458518_at	0.00	0.00
1458519_at	0.00	0.00
1458520_at	0.00	0.00
1458521_at	0.00	0.00
1458522_at	0.00	0.00
1458523_at	0.00	0.00
1458524_at	0.00	0.00
1458525_at	0.00	0.00
1458526_at	0.00	0.00
1458527_at	0.00	0.00
1458528_at	0.00	0.00
1458529_at	0.00	0.00
1458530_at	0.00	0.00
1458531_at	0.00	0.00
1458532_at	0.00	0.00
1458533_at	0.00	0.00
1458534_at	0.00	0.00
1458535_at	0.00	0.00
1458536_at	0.00	0.00
1458537_at	0.00	0.00
1458538_at	0.00	0.00
1458539_at	0.00	0.00
1458540_at	0.00	0.00

1458541_at	0.00	0.00
1458542_at	0.00	0.00
1458543_at	0.00	0.00
1458544_at	0.00	0.00
1458545_at	0.00	0.00
1458546_at	0.00	0.00
1458547_at	0.00	0.00
1458548_at	0.00	0.00
1458549_at	0.00	0.00
1458550_at	0.00	0.00
1458551_at	0.00	0.00
1458552_at	0.00	0.00
1458553_at	0.00	0.00
1458554_at	0.00	0.00
1458555_at	0.00	0.00
1458556_at	0.00	0.00
1458557_at	0.00	0.00
1458558_at	0.00	0.00
1458559_at	0.00	0.00
1458560_at	0.00	0.00
1458561_at	0.00	0.00
1458562_at	0.00	0.00
1458563_at	0.00	0.00
1458564_at	0.00	0.00
1458565_at	0.00	0.00
1458566_at	0.00	0.00
1458567_at	0.00	0.00
1458568_at	0.00	0.00
1458569_at	0.00	0.00
1458570_at	0.00	0.00
1458571_at	0.00	0.00
1458572_at	0.00	0.00
1458573_at	0.00	0.00
1458574_at	0.00	0.00
1458575_at	0.00	0.00
1458576_at	0.00	0.00
1458577_at	0.00	0.00
1458578_at	0.00	0.00
1458579_at	0.00	0.00
1458580_at	0.00	0.00
1458581_at	0.00	0.00
1458582_at	0.00	0.00
1458583_at	0.00	0.00
1458584_at	0.00	0.00
1458585_at	0.00	0.00
1458586_at	0.00	0.00
1458587_at	0.00	0.00
1458588_at	0.00	0.00
1458589_at	0.00	0.00
1458590_at	0.00	0.00
1458591_at	0.00	0.00
1458592_at	0.00	0.00
1458593_at	0.00	0.00
1458594_at	0.00	0.00
1458595_at	0.00	0.00
1458596_at	0.00	0.00

1458597_at	0.00	0.00
1458598_at	0.00	0.00
1458599_at	0.00	0.00
1458600_at	0.00	0.00
1458601_at	0.00	0.00
1458602_at	0.00	0.00
1458603_at	0.00	0.00
1458604_at	0.00	0.00
1458605_at	0.00	0.00
1458606_at	0.00	0.00
1458607_at	0.00	0.00
1458608_at	0.00	0.00
1458609_at	0.00	0.00
1458610_at	0.00	0.00
1458611_at	0.00	0.00
1458612_at	0.00	0.00
1458614_at	0.00	0.00
1458615_at	0.00	0.00
1458616_at	0.00	0.00
1458617_at	0.00	0.00
1458618_at	0.00	0.00
1458619_at	0.00	0.00
1458620_at	0.00	0.00
1458621_at	0.00	0.00
1458622_at	0.00	0.00
1458623_at	0.00	0.00
1458624_at	0.00	0.00
1458625_at	0.00	0.00
1458626_at	0.00	0.00
1458627_at	0.00	0.00
1458628_at	0.00	0.00
1458629_at	0.00	0.00
1458630_at	0.00	0.00
1458631_at	0.00	0.00
1458632_at	0.00	0.00
1458633_at	0.00	0.00
1458634_at	0.00	0.00
1458635_at	0.00	0.00
1458636_at	0.00	0.00
1458637_x_at	0.00	0.00
1458638_at	0.00	0.00
1458639_at	0.00	0.00
1458640_at	0.00	0.00
1458641_at	0.00	0.00
1458642_at	0.00	0.00
1458643_at	0.00	0.00
1458644_at	0.00	0.00
1458645_at	0.00	0.00
1458646_at	0.00	0.00
1458647_at	0.00	0.00
1458648_at	0.00	0.00
1458649_at	0.00	0.00
1458650_at	0.00	0.00
1458651_at	0.00	0.00
1458652_at	0.00	0.00
1458653_at	0.00	0.00

1458654_at	0.00	0.00
1458655_at	0.00	0.00
1458656_at	0.00	0.00
1458657_at	0.00	0.00
1458658_at	0.00	0.00
1458659_at	0.00	0.00
1458660_at	0.00	0.00
1458661_at	0.00	0.00
1458662_at	0.00	0.00
1458663_at	0.00	0.00
1458664_at	0.00	0.00
1458665_at	0.00	0.00
1458666_at	0.00	0.00
1458667_at	0.00	0.00
1458668_at	0.00	0.00
1458669_at	0.00	0.00
1458670_at	0.00	0.00
1458671_at	0.00	0.00
1458672_at	0.00	0.00
1458673_at	0.00	0.00
1458674_at	0.00	0.00
1458675_at	0.00	0.00
1458676_at	0.00	0.00
1458677_at	0.00	0.00
1458678_at	0.00	0.00
1458679_a_at	0.00	0.00
1458680_at	0.00	0.00
1458681_at	0.00	0.00
1458682_at	0.00	0.00
1458683_at	0.00	0.00
1458684_at	0.00	0.00
1458685_at	0.00	0.00
1458686_at	0.00	0.00
1458687_at	0.00	0.00
1458688_at	0.00	0.00
1458689_at	0.00	0.00
1458690_at	0.00	0.00
1458691_at	0.00	0.00
1458692_at	0.00	0.00
1458693_at	0.00	0.00
1458694_at	0.00	0.00
1458695_at	0.00	0.00
1458696_at	0.00	0.00
1458697_at	0.00	0.00
1458698_at	0.00	0.00
1458699_at	0.00	0.00
1458700_at	0.00	0.00
1458701_at	0.00	0.00
1458702_at	0.00	0.00
1458703_at	0.00	0.00
1458704_at	0.00	0.00
1458705_at	0.00	0.00
1458706_at	0.00	0.00
1458707_at	0.00	0.00
1458708_at	0.00	0.00
1458709_a_at	0.00	0.00

1458710_at	0.00	0.00
1458711_at	0.00	0.00
1458712_at	0.00	0.00
1458713_at	0.00	0.00
1458714_at	0.00	0.00
1458715_at	0.00	0.00
1458716_at	0.21	0.01
1458717_at	0.00	0.00
1458718_at	0.00	0.00
1458719_at	0.00	0.00
1458720_at	0.00	0.00
1458721_at	0.00	0.00
1458722_at	0.00	0.00
1458723_at	0.00	0.00
1458724_at	0.00	0.00
1458725_at	0.00	0.00
1458726_at	0.00	0.00
1458727_at	0.00	0.00
1458728_at	0.00	0.00
1458729_at	0.00	0.00
1458730_at	0.00	0.00
1458731_at	0.00	0.00
1458732_at	0.00	0.00
1458733_at	0.00	0.00
1458734_at	0.00	0.00
1458735_at	0.00	0.00
1458736_at	0.00	0.00
1458737_at	0.00	0.00
1458738_at	0.00	0.00
1458739_at	0.00	0.00
1458740_at	0.00	0.00
1458741_at	0.00	0.00
1458742_at	0.00	0.00
1458743_at	0.00	0.00
1458744_at	0.00	0.00
1458745_at	0.00	0.00
1458746_at	0.00	0.00
1458747_at	0.00	0.00
1458748_at	0.00	0.00
1458749_at	0.00	0.00
1458750_at	0.00	0.00
1458751_at	0.00	0.00
1458752_at	0.00	0.00
1458753_at	0.00	0.00
1458754_at	0.00	0.00
1458755_at	0.00	0.00
1458756_at	0.00	0.00
1458757_at	0.00	0.00
1458758_at	0.00	0.00
1458759_at	0.00	0.00
1458760_at	0.00	0.00
1458761_at	0.00	0.00
1458762_at	0.00	0.00
1458763_at	0.00	0.00
1458764_at	0.00	0.00
1458765_at	0.00	0.00

1458766_at	0.00	0.00
1458767_at	0.00	0.00
1458768_at	0.00	0.00
1458769_at	0.00	0.00
1458770_at	0.00	0.00
1458771_at	0.00	0.00
1458772_at	0.00	0.00
1458773_at	0.00	0.00
1458774_at	0.00	0.00
1458775_at	0.00	0.00
1458776_at	0.00	0.00
1458777_at	0.00	0.00
1458778_at	0.00	0.00
1458779_at	0.00	0.00
1458780_at	0.00	0.00
1458781_at	0.00	0.00
1458782_at	0.00	0.00
1458783_at	0.00	0.00
1458784_at	0.00	0.00
1458785_at	0.00	0.00
1458786_at	0.00	0.00
1458787_at	0.00	0.00
1458788_at	0.00	0.00
1458789_at	0.00	0.00
1458790_at	0.00	0.00
1458791_at	0.00	0.00
1458792_at	0.00	0.00
1458793_at	0.00	0.00
1458794_at	0.00	0.00
1458796_at	0.00	0.00
1458797_at	0.00	0.00
1458798_at	0.00	0.00
1458799_at	0.00	0.00
1458800_at	0.00	0.00
1458801_at	0.00	0.00
1458802_at	0.00	0.00
1458803_at	0.00	0.00
1458804_at	0.00	0.00
1458805_at	0.00	0.00
1458806_at	0.00	0.00
1458807_at	0.00	0.00
1458808_at	0.00	0.00
1458809_at	0.00	0.00
1458810_at	0.00	0.00
1458811_at	0.00	0.00
1458812_at	0.00	0.00
1458813_at	0.00	0.00
1458814_at	0.00	0.00
1458815_at	0.00	0.00
1458816_at	0.00	0.00
1458817_at	0.00	0.00
1458818_at	0.00	0.00
1458819_at	0.00	0.00
1458820_at	0.00	0.00
1458821_at	0.00	0.00
1458822_at	0.00	0.00

1458823_at	0.00	0.00
1458824_at	0.00	0.00
1458825_at	0.00	0.00
1458826_at	0.00	0.00
1458827_at	0.00	0.00
1458828_at	0.00	0.00
1458829_at	0.00	0.00
1458830_at	0.00	0.00
1458831_at	0.00	0.00
1458832_at	0.00	0.00
1458833_at	0.00	0.00
1458834_at	0.00	0.00
1458835_at	0.00	0.00
1458836_at	0.00	0.00
1458837_at	0.00	0.00
1458838_at	0.00	0.00
1458839_at	0.00	0.00
1458840_at	0.00	0.00
1458841_at	0.00	0.00
1458842_at	0.00	0.00
1458843_at	0.00	0.00
1458844_at	0.00	0.00
1458845_at	0.00	0.00
1458847_at	0.00	0.00
1458848_at	0.00	0.00
1458849_at	0.00	0.00
1458850_at	0.00	0.00
1458851_at	0.00	0.00
1458852_at	0.00	0.00
1458853_at	0.00	0.00
1458855_at	0.00	0.00
1458856_at	0.00	0.00
1458857_at	0.00	0.00
1458858_at	0.00	0.00
1458859_at	0.00	0.00
1458860_at	0.00	0.00
1458861_at	0.00	0.00
1458863_at	0.00	0.00
1458864_at	0.00	0.00
1458865_at	0.00	0.00
1458866_at	0.00	0.00
1458867_at	0.00	0.00
1458868_at	0.00	0.00
1458869_at	0.00	0.00
1458870_x_at	0.00	0.00
1458871_at	0.00	0.00
1458872_at	0.00	0.00
1458873_at	0.00	0.00
1458874_at	0.00	0.00
1458875_at	0.00	0.00
1458876_at	0.00	0.00
1458877_at	0.00	0.00
1458878_at	0.00	0.00
1458879_at	0.00	0.00
1458880_at	0.00	0.00
1458881_at	0.00	0.00

1458882_at	0.00	0.00
1458883_at	0.00	0.00
1458884_at	0.00	0.00
1458885_at	0.00	0.00
1458886_at	0.00	0.00
1458887_at	0.00	0.00
1458888_at	0.00	0.00
1458889_at	0.00	0.00
1458890_at	0.00	0.00
1458891_at	0.00	0.00
1458892_at	0.00	0.00
1458893_at	0.00	0.00
1458894_at	0.00	0.00
1458895_at	0.00	0.00
1458896_at	0.00	0.00
1458897_at	0.00	0.00
1458898_at	0.00	0.00
1458899_at	0.00	0.00
1458900_at	0.00	0.00
1458901_at	0.00	0.00
1458902_at	0.00	0.00
1458903_at	0.00	0.00
1458904_at	0.00	0.00
1458905_x_at	0.00	0.00
1458906_at	0.00	0.00
1458907_at	0.00	0.00
1458908_at	0.00	0.00
1458909_at	0.00	0.00
1458910_at	0.00	0.00
1458911_at	0.00	0.00
1458912_at	0.00	0.00
1458913_at	0.00	0.00
1458914_at	0.00	0.00
1458915_at	0.00	0.00
1458916_at	0.00	0.00
1458917_at	0.00	0.00
1458918_at	0.00	0.00
1458919_at	0.00	0.00
1458920_at	0.00	0.00
1458921_at	0.00	0.00
1458922_at	0.00	0.00
1458923_at	0.00	0.00
1458924_at	0.00	0.00
1458925_at	0.00	0.00
1458926_at	0.00	0.00
1458927_at	0.00	0.00
1458928_at	0.00	0.00
1458929_at	0.00	0.00
1458930_at	0.00	0.00
1458931_at	0.00	0.00
1458932_at	0.00	0.00
1458933_at	0.00	0.00
1458934_at	0.00	0.00
1458935_at	0.00	0.00
1458936_at	0.00	0.00
1458937_at	0.00	0.00

1458938_at	0.00	0.00
1458939_at	0.00	0.00
1458940_at	0.00	0.00
1458941_at	0.00	0.00
1458942_at	0.00	0.00
1458943_at	0.00	0.00
1458944_at	0.00	0.00
1458945_at	0.00	0.00
1458946_at	0.00	0.00
1458947_at	0.00	0.00
1458948_at	0.00	0.00
1458949_at	0.00	0.00
1458950_at	0.00	0.00
1458951_at	0.00	0.00
1458952_at	0.00	0.00
1458953_at	0.00	0.00
1458954_at	0.00	0.00
1458955_at	0.00	0.00
1458956_at	0.00	0.00
1458957_at	0.00	0.00
1458958_at	0.00	0.00
1458959_at	0.00	0.00
1458960_at	0.00	0.00
1458961_at	0.00	0.00
1458962_at	0.00	0.00
1458963_at	0.00	0.04
1458964_at	0.00	0.00
1458965_at	0.00	0.00
1458966_at	0.00	0.00
1458967_at	0.00	0.00
1458968_at	0.00	0.00
1458969_at	0.00	0.00
1458970_at	0.00	0.00
1458971_at	0.00	0.00
1458972_at	0.00	0.00
1458973_at	0.00	0.00
1458974_at	0.00	0.00
1458975_at	0.00	0.00
1458976_at	0.00	0.00
1458977_at	0.00	0.00
1458978_at	0.00	0.00
1458979_at	0.00	0.00
1458980_at	0.00	0.00
1458981_at	0.00	0.00
1458982_at	0.00	0.00
1458983_at	0.00	0.00
1458984_at	0.00	0.00
1458985_at	0.00	0.00
1458986_at	0.00	0.00
1458987_at	0.00	0.00
1458988_at	0.00	0.00
1458989_at	0.00	0.00
1458990_at	0.00	0.00
1458991_at	0.00	0.00
1458992_at	0.00	0.00
1458993_at	0.00	0.00

1458994_at	0.00	0.00
1458995_at	0.00	0.00
1458996_at	0.00	0.00
1458997_at	0.00	0.00
1458998_at	0.00	0.00
1458999_at	0.00	0.00
1459000_at	0.00	0.00
1459001_at	0.00	0.00
1459002_at	0.00	0.00
1459003_at	0.00	0.00
1459004_at	0.00	0.00
1459005_at	0.00	0.00
1459006_a_at	0.00	0.00
1459007_at	0.00	0.00
1459008_at	0.00	0.00
1459009_at	0.00	0.00
1459010_at	0.00	0.00
1459011_at	0.00	0.00
1459012_at	0.00	0.00
1459013_at	0.00	0.00
1459014_at	0.00	0.00
1459015_at	0.00	0.00
1459016_at	0.00	0.00
1459017_at	0.00	0.00
1459018_at	0.00	0.00
1459019_at	0.00	0.00
1459020_at	0.00	0.00
1459022_at	0.00	0.00
1459023_at	0.00	0.00
1459024_at	0.00	0.00
1459025_at	0.00	0.00
1459026_at	0.00	0.00
1459027_at	0.00	0.00
1459028_at	0.00	0.00
1459029_at	0.00	0.00
1459030_at	0.00	0.00
1459031_at	0.00	0.00
1459032_at	0.00	0.00
1459033_at	0.00	0.00
1459034_at	0.00	0.00
1459035_at	0.00	0.00
1459036_at	0.00	0.00
1459037_at	0.00	0.00
1459038_at	0.00	0.00
1459039_at	0.00	0.00
1459040_at	0.00	0.00
1459041_at	0.00	0.00
1459042_at	0.00	0.00
1459043_at	0.00	0.00
1459044_at	0.00	0.00
1459045_at	0.00	0.00
1459046_at	0.00	0.00
1459047_x_at	0.00	0.00
1459048_s_at	0.00	0.00
1459049_at	0.00	0.00
1459050_at	0.00	0.00

1459051_at	0.00	0.00
1459052_at	0.00	0.00
1459053_at	0.00	0.00
1459054_at	0.00	0.00
1459055_at	0.00	0.00
1459056_at	0.00	0.00
1459057_at	0.00	0.00
1459058_at	0.00	0.00
1459059_at	0.00	0.00
1459060_at	0.00	0.00
1459061_at	0.00	0.00
1459062_x_at	0.00	0.00
1459063_at	0.00	0.00
1459064_at	0.00	0.00
1459065_at	0.00	0.00
1459066_at	0.00	0.00
1459067_at	0.00	0.00
1459068_at	0.00	0.00
1459069_at	0.00	0.00
1459070_at	0.00	0.00
1459071_at	0.00	0.00
1459072_at	0.00	0.00
1459073_x_at	0.00	0.00
1459074_at	0.00	0.00
1459075_at	0.00	0.00
1459076_at	0.00	0.00
1459077_at	0.00	0.00
1459078_at	0.00	0.00
1459079_at	0.00	0.00
1459080_at	0.00	0.00
1459081_at	0.00	0.00
1459082_at	0.00	0.00
1459083_at	0.00	0.00
1459084_at	0.00	0.00
1459086_at	0.00	0.00
1459087_at	0.00	0.00
1459088_at	0.00	0.00
1459089_at	0.00	0.00
1459090_at	0.00	0.00
1459091_at	0.00	0.00
1459093_at	0.00	0.00
1459094_at	0.00	0.00
1459095_at	0.00	0.00
1459096_at	0.00	0.00
1459097_at	0.00	0.00
1459098_at	0.00	0.00
1459099_at	0.00	0.00
1459100_at	0.00	0.00
1459101_at	0.00	0.00
1459102_at	0.00	0.00
1459103_at	0.00	0.00
1459104_at	0.00	0.00
1459105_at	0.00	0.00
1459106_at	0.00	0.00
1459107_at	0.00	0.00
1459108_a_at	0.00	0.00

1459109_at	0.00	0.00
1459110_at	0.00	0.00
1459111_at	0.00	0.00
1459112_at	0.00	0.00
1459113_at	0.00	0.00
1459114_at	0.00	0.00
1459115_at	0.00	0.00
1459116_at	0.00	0.00
1459117_at	0.00	0.00
1459118_at	0.00	0.00
1459119_at	0.00	0.00
1459120_at	0.00	0.00
1459121_at	0.00	0.00
1459122_at	0.00	0.00
1459123_at	0.00	0.00
1459124_at	0.00	0.00
1459125_at	0.00	0.00
1459126_at	0.00	0.00
1459127_at	0.00	0.00
1459128_at	0.00	0.00
1459129_at	0.00	0.00
1459130_at	0.00	0.00
1459131_at	0.00	0.00
1459132_at	0.00	0.00
1459133_at	0.00	0.00
1459134_at	0.00	0.00
1459135_at	0.00	0.00
1459136_at	0.00	0.00
1459137_at	0.00	0.00
1459138_at	0.00	0.00
1459139_at	0.00	0.00
1459140_at	0.00	0.00
1459141_at	0.00	0.00
1459142_at	0.00	0.00
1459143_at	0.00	0.00
1459144_at	0.00	0.00
1459145_at	0.00	0.00
1459146_at	0.00	0.00
1459147_at	0.00	0.00
1459148_at	0.00	0.00
1459149_at	0.00	0.00
1459150_at	0.00	0.00
1459151_x_at	0.00	0.00
1459152_at	0.00	0.00
1459153_at	0.00	0.00
1459154_at	0.00	0.00
1459155_at	0.00	0.00
1459156_at	0.00	0.00
1459157_at	0.00	0.00
1459158_at	0.00	0.00
1459159_a_at	0.00	0.00
1459160_at	0.00	0.00
1459161_at	0.00	0.00
1459162_at	0.00	0.00
1459163_at	0.00	0.00
1459164_at	0.00	0.00

1459165_at	0.00	0.00
1459166_at	0.00	0.00
1459167_at	0.00	0.00
1459168_at	0.00	0.00
1459169_at	0.00	0.00
1459170_at	0.00	0.00
1459171_at	0.00	0.00
1459172_at	0.00	0.00
1459173_at	0.00	0.00
1459174_at	0.00	0.00
1459175_at	0.00	0.00
1459176_at	0.00	0.00
1459177_at	0.00	0.00
1459178_at	0.00	0.00
1459179_at	0.00	0.00
1459180_at	0.00	0.00
1459181_at	0.00	0.00
1459182_at	0.00	0.00
1459183_at	0.00	0.00
1459184_at	0.00	0.00
1459185_at	0.00	0.00
1459186_at	0.00	0.00
1459187_at	0.00	0.00
1459188_at	0.00	0.00
1459189_at	0.00	0.00
1459190_at	0.00	0.00
1459191_at	0.00	0.00
1459192_at	0.00	0.00
1459193_at	0.00	0.00
1459194_at	0.00	0.00
1459195_at	0.00	0.00
1459196_at	0.00	0.00
1459197_at	0.00	0.00
1459198_at	0.00	0.00
1459199_at	0.00	0.00
1459200_at	0.00	0.00
1459201_at	0.00	0.00
1459202_at	0.00	0.00
1459203_at	0.00	0.00
1459204_at	0.00	0.00
1459205_at	0.00	0.00
1459206_at	0.00	0.00
1459207_at	0.00	0.00
1459208_at	0.00	0.00
1459209_at	0.00	0.00
1459210_at	0.00	0.00
1459212_at	0.00	0.00
1459213_at	0.00	0.00
1459214_at	0.00	0.00
1459215_at	0.00	0.00
1459216_at	0.00	0.00
1459217_at	0.00	0.00
1459218_at	0.00	0.00
1459219_at	0.00	0.00
1459221_at	0.00	0.00
1459222_x_at	0.00	0.00

1459223_at	0.00	0.00
1459224_at	0.00	0.00
1459225_at	0.00	0.00
1459226_at	0.00	0.00
1459227_at	0.00	0.00
1459228_at	0.00	0.00
1459229_at	0.00	0.00
1459230_at	0.00	0.00
1459231_at	0.00	0.00
1459232_at	0.00	0.00
1459233_at	0.00	0.00
1459234_at	0.00	0.00
1459235_at	0.00	0.00
1459236_at	0.00	0.00
1459237_at	0.00	0.00
1459238_at	0.00	0.00
1459239_at	0.00	0.00
1459240_at	0.00	0.00
1459241_at	0.00	0.00
1459242_at	0.00	0.00
1459243_at	0.00	0.00
1459244_at	0.00	0.00
1459245_s_at	0.00	0.00
1459246_at	0.00	0.00
1459247_at	0.00	0.00
1459248_at	0.00	0.00
1459249_at	0.00	0.00
1459250_at	0.00	0.00
1459251_at	0.00	0.00
1459252_at	0.00	0.00
1459253_at	0.00	0.00
1459254_at	0.00	0.00
1459255_at	0.00	0.00
1459256_at	0.00	0.00
1459257_at	0.00	0.00
1459258_at	0.00	0.00
1459259_at	0.00	0.00
1459260_at	0.00	0.00
1459261_at	0.00	0.00
1459262_at	0.00	0.00
1459263_at	0.00	0.00
1459264_at	0.00	0.00
1459265_at	0.00	0.00
1459266_at	0.00	0.00
1459267_at	0.00	0.00
1459268_at	0.00	0.00
1459269_at	0.00	0.00
1459270_at	0.00	0.00
1459271_at	0.00	0.00
1459272_at	0.00	0.00
1459273_at	0.00	0.00
1459274_at	0.00	0.00
1459275_at	0.00	0.00
1459276_at	0.00	0.00
1459277_at	0.00	0.00
1459278_at	0.00	0.00

1459279_at	0.00	0.00
1459280_at	0.00	0.00
1459281_at	0.00	0.00
1459282_at	0.00	0.00
1459283_at	0.00	0.00
1459284_at	0.00	0.00
1459285_at	0.00	0.00
1459286_at	0.00	0.00
1459287_at	0.00	0.00
1459288_at	0.00	0.00
1459289_at	0.00	0.00
1459290_at	0.00	0.00
1459291_at	0.00	0.00
1459292_at	0.00	0.00
1459293_at	0.00	0.00
1459294_at	0.00	0.00
1459295_at	0.00	0.00
1459296_at	0.00	0.00
1459297_at	0.00	0.00
1459298_at	0.00	0.00
1459299_at	0.00	0.00
1459300_at	0.00	0.00
1459301_at	0.00	0.00
1459302_at	0.00	0.00
1459303_at	0.00	0.00
1459304_at	0.00	0.00
1459305_at	0.00	0.00
1459306_at	0.00	0.00
1459307_at	0.00	0.00
1459308_at	0.00	0.00
1459309_at	0.00	0.00
1459310_at	0.00	0.00
1459311_at	0.00	0.00
1459312_at	0.00	0.00
1459313_at	0.00	0.00
1459314_at	0.00	0.00
1459315_at	0.00	0.00
1459316_at	0.00	0.00
1459317_at	0.00	0.00
1459318_at	0.00	0.00
1459319_at	0.00	0.00
1459320_at	0.00	0.00
1459321_at	0.00	0.00
1459322_at	0.00	0.00
1459323_at	0.00	0.00
1459324_at	0.00	0.00
1459325_at	0.00	0.00
1459326_at	0.00	0.00
1459327_at	0.00	0.00
1459328_at	0.00	0.00
1459329_at	0.00	0.00
1459330_at	0.00	0.00
1459331_at	0.00	0.00
1459332_at	0.00	0.00
1459333_at	0.00	0.00
1459334_at	0.00	0.00

1459335_at	0.00	0.00
1459336_at	0.00	0.00
1459337_at	0.00	0.00
1459338_at	0.00	0.00
1459339_at	0.00	0.00
1459340_at	0.00	0.00
1459341_at	0.00	0.00
1459342_at	0.00	0.00
1459343_at	0.00	0.00
1459344_at	0.00	0.00
1459345_at	0.00	0.00
1459346_at	0.00	0.00
1459347_at	0.00	0.00
1459348_at	0.00	0.00
1459349_at	0.00	0.00
1459350_at	0.00	0.00
1459351_at	0.00	0.00
1459352_at	0.00	0.00
1459353_at	0.00	0.00
1459354_at	0.00	0.00
1459355_at	0.00	0.00
1459356_at	0.00	0.00
1459357_at	0.00	0.00
1459358_at	0.00	0.00
1459359_at	0.00	0.00
1459360_at	0.00	0.00
1459361_at	0.00	0.00
1459362_at	0.00	0.00
1459363_at	0.00	0.00
1459364_at	0.00	0.00
1459365_at	0.00	0.00
1459366_at	0.00	0.00
1459367_at	0.00	0.00
1459368_at	0.00	0.00
1459369_at	0.00	0.00
1459370_at	0.00	0.00
1459371_at	0.00	0.00
1459372_at	0.00	0.00
1459373_at	0.00	0.00
1459374_at	0.00	0.00
1459375_at	0.00	0.00
1459376_at	0.00	0.00
1459377_at	0.00	0.00
1459378_at	0.00	0.00
1459379_at	0.00	0.00
1459380_at	0.00	0.00
1459381_at	0.00	0.00
1459382_at	0.00	0.00
1459383_at	0.00	0.00
1459384_at	0.00	0.00
1459385_at	0.00	0.00
1459386_at	0.00	0.00
1459387_at	0.00	0.00
1459388_at	0.00	0.00
1459389_at	0.00	0.00
1459390_at	0.00	0.00

1459391_at	0.00	0.00
1459392_at	0.00	0.00
1459393_at	0.00	0.00
1459394_at	0.00	0.00
1459395_at	0.00	0.00
1459396_at	0.00	0.00
1459397_at	0.00	0.00
1459398_at	0.00	0.00
1459399_at	0.00	0.00
1459400_at	0.00	0.00
1459401_at	0.00	0.00
1459402_at	0.00	0.00
1459403_at	0.00	0.00
1459404_at	0.00	0.00
1459405_at	0.00	0.00
1459406_at	0.00	0.00
1459407_at	0.00	0.00
1459408_at	0.00	0.00
1459409_at	0.00	0.00
1459411_at	0.00	0.00
1459412_at	0.00	0.00
1459413_at	0.00	0.00
1459414_at	0.00	0.00
1459415_at	0.00	0.00
1459416_at	0.00	0.00
1459417_at	0.00	0.00
1459418_at	0.00	0.00
1459419_at	0.00	0.00
1459420_at	0.00	0.00
1459421_at	0.00	0.00
1459422_at	0.00	0.00
1459423_at	0.00	0.00
1459424_at	0.00	0.00
1459425_at	0.00	0.00
1459426_at	0.00	0.00
1459427_at	0.00	0.00
1459428_at	0.00	0.00
1459429_at	0.00	0.00
1459430_at	0.00	0.00
1459431_at	0.00	0.00
1459432_at	0.00	0.00
1459433_at	0.00	0.00
1459434_at	0.00	0.00
1459435_at	0.00	0.00
1459436_at	0.00	0.00
1459437_at	0.00	0.00
1459438_at	0.00	0.00
1459439_at	0.00	0.00
1459440_at	0.00	0.00
1459441_at	0.00	0.00
1459442_at	0.00	0.00
1459443_at	0.00	0.00
1459444_at	0.00	0.00
1459445_at	0.00	0.00
1459446_at	0.00	0.00
1459447_at	0.00	0.00

1459448_at	0.00	0.00
1459449_at	0.00	0.00
1459450_at	0.00	0.00
1459451_at	0.00	0.00
1459452_at	0.00	0.00
1459453_at	0.00	0.00
1459454_at	0.00	0.00
1459455_at	0.00	0.00
1459456_at	0.00	0.00
1459457_at	0.00	0.00
1459458_at	0.00	0.00
1459459_at	0.00	0.00
1459460_at	0.00	0.00
1459461_at	0.00	0.00
1459462_at	0.00	0.00
1459463_at	0.00	0.00
1459464_at	0.00	0.00
1459465_at	0.00	0.00
1459466_at	0.00	0.00
1459467_at	0.00	0.00
1459468_at	0.00	0.00
1459469_at	0.00	0.00
1459470_at	0.00	0.00
1459471_at	0.00	0.00
1459472_at	0.00	0.00
1459473_at	0.00	0.00
1459474_at	0.00	0.00
1459475_at	0.00	0.00
1459476_s_at	0.00	0.00
1459477_at	0.00	0.00
1459479_at	0.00	0.00
1459480_at	0.00	0.00
1459481_at	0.00	0.00
1459482_at	0.00	0.00
1459483_at	0.00	0.00
1459484_at	0.00	0.00
1459485_at	0.00	0.00
1459486_at	0.00	0.00
1459487_at	0.00	0.00
1459488_at	0.00	0.00
1459489_at	0.00	0.00
1459490_at	0.00	0.00
1459491_at	0.00	0.00
1459492_at	0.00	0.00
1459493_at	0.00	0.00
1459494_at	0.00	0.00
1459495_at	0.00	0.00
1459496_at	0.00	0.00
1459497_at	0.00	0.00
1459498_at	0.00	0.00
1459499_at	0.00	0.00
1459500_at	0.00	0.00
1459501_at	0.00	0.00
1459502_at	0.00	0.00
1459503_at	0.00	0.00
1459504_at	0.00	0.00

1459505_at	0.00	0.00
1459506_at	0.00	0.00
1459507_at	0.00	0.00
1459508_at	0.00	0.00
1459509_at	0.00	0.00
1459510_at	0.00	0.00
1459511_at	0.00	0.00
1459512_at	0.00	0.00
1459513_at	0.00	0.00
1459514_at	0.00	0.00
1459515_at	0.00	0.00
1459516_at	0.00	0.00
1459517_at	0.00	0.00
1459518_at	0.00	0.00
1459519_at	0.00	0.00
1459520_at	0.00	0.00
1459521_at	0.00	0.00
1459522_s_at	0.03	0.18
1459523_at	0.00	0.00
1459524_at	0.00	0.00
1459525_at	0.00	0.00
1459526_at	0.00	0.00
1459527_at	0.00	0.00
1459528_at	0.00	0.00
1459529_at	0.00	0.00
1459530_at	0.00	0.00
1459531_at	0.00	0.00
1459532_at	0.00	0.00
1459533_at	0.00	0.00
1459534_at	0.00	0.00
1459535_at	0.00	0.00
1459536_at	0.00	0.00
1459537_at	0.00	0.00
1459538_at	0.00	0.00
1459539_at	0.00	0.00
1459540_at	0.00	0.00
1459541_at	0.00	0.00
1459542_at	0.00	0.00
1459543_at	0.00	0.00
1459544_at	0.00	0.00
1459545_at	0.00	0.00
1459547_at	0.00	0.00
1459548_at	0.00	0.00
1459549_at	0.00	0.00
1459550_at	0.00	0.00
1459551_at	0.00	0.00
1459552_at	0.00	0.00
1459553_at	0.00	0.00
1459554_at	0.00	0.00
1459555_at	0.00	0.00
1459556_at	0.00	0.00
1459557_at	0.00	0.00
1459558_at	0.00	0.00
1459559_at	0.00	0.00
1459560_at	0.00	0.00
1459561_at	0.00	0.00

1459562_at	0.00	0.00
1459563_x_at	0.00	0.00
1459564_at	0.00	0.00
1459565_at	0.00	0.00
1459566_at	0.00	0.00
1459567_at	0.00	0.00
1459568_at	0.00	0.00
1459569_at	0.00	0.00
1459570_at	0.00	0.00
1459571_at	0.00	0.00
1459572_at	0.00	0.00
1459573_at	0.00	0.00
1459574_at	0.00	0.00
1459575_at	0.00	0.00
1459576_at	0.00	0.00
1459577_at	0.00	0.00
1459578_at	0.00	0.00
1459579_at	0.00	0.00
1459580_at	0.00	0.00
1459581_at	0.00	0.00
1459582_at	0.00	0.00
1459583_at	0.00	0.00
1459584_at	0.00	0.00
1459585_at	0.00	0.00
1459586_at	0.00	0.00
1459587_at	0.00	0.00
1459588_at	0.00	0.00
1459589_at	0.00	0.00
1459590_at	0.00	0.00
1459591_at	0.00	0.00
1459592_a_at	0.00	0.00
1459593_x_at	0.00	0.00
1459594_at	0.00	0.00
1459595_at	0.00	0.00
1459596_at	0.00	0.00
1459597_at	0.00	0.00
1459598_at	0.00	0.00
1459599_at	0.00	0.00
1459600_at	0.00	0.00
1459601_at	0.00	0.00
1459602_at	0.00	0.00
1459603_at	0.00	0.00
1459604_at	0.00	0.00
1459605_at	0.00	0.00
1459606_at	0.00	0.00
1459607_at	0.00	0.00
1459608_at	0.00	0.00
1459609_at	0.00	0.00
1459610_at	0.00	0.00
1459611_at	0.00	0.00
1459612_at	0.00	0.00
1459613_at	0.00	0.00
1459614_at	0.00	0.00
1459615_at	0.00	0.00
1459616_at	0.00	0.00
1459617_at	0.00	0.00

1459618_at	0.00	0.00
1459619_at	0.00	0.00
1459620_at	0.00	0.00
1459621_at	0.00	0.00
1459622_at	0.00	0.00
1459623_at	0.00	0.00
1459624_at	0.00	0.00
1459625_at	0.00	0.00
1459626_at	0.00	0.00
1459627_at	0.00	0.00
1459628_at	0.00	0.00
1459629_at	0.00	0.00
1459630_at	0.00	0.00
1459631_at	0.00	0.00
1459632_at	0.00	0.00
1459633_at	0.00	0.00
1459634_at	0.00	0.00
1459635_at	0.00	0.00
1459636_at	0.00	0.00
1459637_at	0.00	0.00
1459638_at	0.00	0.00
1459639_at	0.00	0.00
1459640_at	0.00	0.00
1459641_at	0.00	0.00
1459642_at	0.00	0.00
1459643_at	0.00	0.00
1459644_at	0.00	0.00
1459645_at	0.00	0.00
1459646_at	0.00	0.00
1459647_at	0.00	0.00
1459648_at	0.00	0.00
1459649_at	0.00	0.00
1459650_at	0.00	0.00
1459651_s_at	0.00	0.00
1459652_at	0.00	0.00
1459653_at	0.00	0.00
1459654_at	0.00	0.00
1459655_at	0.00	0.00
1459656_at	0.00	0.00
1459657_s_at	0.00	0.02
1459658_at	0.00	0.00
1459659_at	0.00	0.00
1459660_at	0.00	0.00
1459661_at	0.00	0.00
1459662_at	0.00	0.00
1459663_at	0.00	0.00
1459664_at	0.00	0.00
1459665_s_at	0.00	0.00
1459666_at	0.00	0.00
1459667_at	0.00	0.00
1459668_at	0.00	0.00
1459669_at	0.00	0.00
1459670_at	0.00	0.00
1459671_at	0.00	0.00
1459672_at	0.00	0.00
1459673_at	0.00	0.00

1459674_at	0.00	0.00
1459675_at	0.00	0.00
1459676_at	0.00	0.00
1459677_at	0.00	0.00
1459678_at	0.00	0.00
1459679_s_at	0.00	0.15
1459680_at	0.00	0.00
1459681_at	0.00	0.00
1459682_at	0.00	0.00
1459683_at	0.00	0.00
1459684_at	0.00	0.00
1459685_at	0.00	0.00
1459686_at	0.00	0.00
1459687_x_at	0.00	0.00
1459688_at	0.00	0.00
1459689_at	0.00	0.00
1459690_at	0.00	0.00
1459691_at	0.00	0.00
1459692_at	0.00	0.00
1459693_x_at	0.00	0.00
1459694_at	0.00	0.00
1459695_at	0.00	0.00
1459696_at	0.00	0.00
1459697_at	0.00	0.00
1459698_at	0.00	0.00
1459699_at	0.00	0.00
1459700_at	0.00	0.00
1459702_at	0.00	0.00
1459703_at	0.00	0.00
1459704_at	0.00	0.00
1459705_at	0.00	0.00
1459706_at	0.00	0.00
1459707_at	0.00	0.00
1459708_at	0.00	0.00
1459709_at	0.00	0.00
1459710_at	0.00	0.00
1459711_at	0.00	0.00
1459712_at	0.00	0.00
1459713_s_at	0.00	0.00
1459714_at	0.00	0.00
1459715_at	0.00	0.00
1459716_at	0.00	0.00
1459717_at	0.00	0.00
1459718_x_at	0.00	0.00
1459719_at	0.00	0.00
1459720_x_at	0.00	0.00
1459721_at	0.00	0.00
1459722_at	0.00	0.00
1459723_at	0.00	0.00
1459724_at	0.00	0.00
1459726_at	0.00	0.00
1459727_at	0.00	0.00
1459728_at	0.00	0.00
1459729_at	0.00	0.00
1459730_at	0.00	0.00
1459731_at	0.00	0.00

1459732_at	0.00	0.00
1459733_at	0.00	0.00
1459734_at	0.00	0.00
1459735_at	0.00	0.00
1459736_at	0.00	0.00
1459737_s_at	0.00	0.00
1459738_x_at	0.00	0.00
1459739_at	0.00	0.00
1459740_s_at	0.00	0.00
1459741_x_at	0.00	0.00
1459742_at	0.00	0.00
1459743_at	0.00	0.00
1459744_at	0.00	0.00
1459745_at	0.00	0.00
1459746_at	0.00	0.00
1459747_at	0.00	0.00
1459748_at	0.00	0.00
1459749_s_at	0.00	0.00
1459750_s_at	0.00	0.00
1459751_s_at	0.00	0.00
1459752_at	0.00	0.00
1459753_x_at	0.00	0.00
1459754_x_at	0.00	0.00
1459755_x_at	0.00	0.00
1459756_at	0.00	0.00
1459757_x_at	0.00	0.00
1459758_at	0.00	0.00
1459759_s_at	0.00	0.00
1459760_at	0.00	0.00
1459761_x_at	0.00	0.00
1459762_x_at	0.00	0.00
1459763_at	0.00	0.00
1459764_x_at	0.00	0.00
1459765_s_at	0.00	0.00
1459766_x_at	0.00	0.00
1459767_x_at	0.00	0.00
1459768_x_at	0.00	0.00
1459769_at	0.00	0.00
1459770_at	0.00	0.00
1459771_x_at	0.00	0.00
1459772_at	0.00	0.00
1459773_x_at	0.00	0.00
1459774_at	0.00	0.00
1459775_at	0.00	0.00
1459776_x_at	0.00	0.00
1459777_at	0.00	0.00
1459778_at	0.00	0.00
1459779_s_at	0.00	0.00
1459780_at	0.00	0.00
1459781_x_at	0.00	0.00
1459782_x_at	0.00	0.00
1459783_s_at	0.00	0.00
1459784_x_at	0.00	0.00
1459785_at	0.00	0.00
1459786_at	0.00	0.00
1459787_at	0.00	0.00

1459788_at	0.00	0.00
1459789_at	0.00	0.00
1459790_x_at	0.00	0.00
1459791_at	0.00	0.00
1459792_at	0.00	0.00
1459793_s_at	0.00	0.00
1459794_at	0.00	0.00
1459795_at	0.00	0.00
1459796_at	0.00	0.00
1459797_at	0.00	0.00
1459798_x_at	0.00	0.00
1459799_at	0.00	0.00
1459800_s_at	0.00	0.36
1459801_at	0.00	0.00
1459802_at	0.00	0.00
1459803_x_at	0.00	0.00
1459804_at	0.00	0.00
1459805_x_at	0.03	0.30
1459806_x_at	0.00	0.00
1459807_x_at	0.00	0.00
1459808_at	0.00	0.00
1459809_x_at	0.00	0.00
1459810_at	0.00	0.00
1459811_at	0.00	0.00
1459812_x_at	0.00	0.00
1459813_at	0.00	0.00
1459815_at	0.00	0.00
1459818_x_at	0.00	0.00
1459819_at	0.00	0.00
1459820_at	0.00	0.00
1459821_x_at	0.00	0.00
1459822_at	0.00	0.00
1459823_at	0.00	0.00
1459824_at	0.00	0.00
1459825_x_at	0.00	0.00
1459826_at	0.00	0.00
1459827_x_at	0.00	0.00
1459828_at	0.00	0.00
1459829_x_at	0.00	0.00
1459830_at	0.00	0.00
1459831_s_at	0.00	0.00
1459832_s_at	0.00	0.00
1459833_x_at	0.00	0.00
1459834_x_at	0.00	0.00
1459835_s_at	0.00	0.00
1459836_x_at	0.00	0.00
1459837_at	0.00	0.00
1459838_s_at	0.97	0.00
1459839_x_at	0.00	0.00
1459840_s_at	0.00	0.00
1459841_x_at	0.00	0.00
1459842_x_at	0.11	0.51
1459843_s_at	0.35	0.13
1459844_at	0.00	0.00
1459845_at	0.00	0.00
1459846_x_at	0.00	0.00

1459847_x_at	0.00	0.00
1459848_x_at	0.00	0.00
1459849_x_at	0.00	0.00
1459850_x_at	0.00	0.00
1459851_x_at	0.00	0.00
1459852_x_at	0.00	0.00
1459853_x_at	0.00	0.00
1459854_s_at	0.05	0.00
1459855_x_at	0.00	0.00
1459856_at	0.00	0.00
1459857_at	0.00	0.00
1459858_x_at	0.00	0.00
1459859_x_at	0.00	0.00
1459860_x_at	1.00	0.43
1459861_s_at	0.00	0.00
1459862_at	0.00	0.00
1459863_x_at	0.00	0.00
1459864_at	0.00	0.00
1459865_x_at	0.00	0.00
1459866_x_at	0.00	0.00
1459867_x_at	0.00	0.00
1459868_x_at	0.00	0.00
1459869_x_at	0.00	0.00
1459870_x_at	0.00	0.00
1459871_x_at	0.00	0.00
1459872_x_at	0.00	0.00
1459873_x_at	0.00	0.00
1459874_s_at	0.00	0.00
1459875_x_at	0.00	0.00
1459876_at	0.00	0.00
1459877_x_at	0.00	0.00
1459878_a_at	0.00	0.00
1459879_at	0.00	0.00
1459881_at	0.00	0.00
1459883_at	0.00	0.00
1459886_at	0.00	0.00
1459887_at	0.00	0.00
1459888_at	0.00	0.00
1459895_at	0.00	0.00
1459896_at	0.00	0.00
1459897_a_at	0.00	0.00
1459898_at	0.00	0.00
1459901_at	0.00	0.00
1459904_at	0.00	0.00
1459905_at	0.00	0.00
1459906_at	0.00	0.00
1459907_a_at	0.00	0.00
1459910_at	0.00	0.00
1459913_at	0.00	0.00
1459919_a_at	0.00	0.00
1459936_at	0.00	0.00
1459937_at	0.00	0.00
1459938_at	0.00	0.00
1459939_at	0.00	0.00
1459940_at	0.00	0.00
1459941_at	0.00	0.00

1459942_at	0.00	0.00
1459943_at	0.00	0.00
1459944_at	0.00	0.00
1459945_at	0.00	0.00
1459946_at	0.00	0.00
1459947_at	0.00	0.00
1459948_at	0.00	0.00
1459949_at	0.00	0.00
1459950_at	0.00	0.00
1459951_at	0.00	0.00
1459953_at	0.00	0.00
1459954_at	0.00	0.00
1459955_at	0.00	0.00
1459956_at	0.00	0.00
1459957_at	0.00	0.00
1459958_at	0.00	0.00
1459959_at	0.00	0.00
1459960_at	0.00	0.00
1459961_a_at	0.00	0.00
1459962_at	0.00	0.00
1459963_at	0.00	0.00
1459964_at	0.00	0.00
1459965_at	0.00	0.00
1459966_at	0.00	0.00
1459967_at	0.00	0.00
1459968_at	0.00	0.00
1459969_x_at	0.00	0.00
1459970_at	0.00	0.00
1459971_at	0.00	0.00
1459972_x_at	0.00	0.00
1459973_x_at	0.00	0.00
1459974_x_at	0.00	0.00
1459975_at	0.00	0.00
1459976_s_at	0.00	0.06
1459977_x_at	0.00	0.00
1459978_x_at	0.00	0.00
1459979_x_at	0.00	0.00
1459980_x_at	0.00	0.00
1459981_s_at	0.00	0.00
1459982_a_at	0.00	0.00
1459984_at	0.00	0.00
1459988_at	0.00	0.00
1459989_at	0.00	0.00
1459991_at	0.00	0.00
1459993_at	0.00	0.00
1459995_at	0.00	0.00
1459997_s_at	0.00	0.00
1459998_at	0.00	0.00
1460000_at	0.00	0.00
1460001_at	0.00	0.00
1460002_at	0.00	0.00
1460003_at	0.00	0.00
1460005_at	0.00	0.00
1460006_at	0.07	0.00
1460011_at	0.00	0.00
1460012_at	0.00	0.00

1460013_at	0.00	0.00
1460014_at	0.00	0.00
1460015_at	0.00	0.00
1460016_at	0.00	0.00
1460018_at	0.00	0.00
1460019_at	0.00	0.00
1460020_at	0.00	0.00
1460021_at	0.00	0.00
1460022_at	0.00	0.00
1460023_at	0.00	0.00
1460024_at	0.00	0.00
1460025_at	0.00	0.00
1460026_s_at	0.00	0.00
1460027_at	0.00	0.00
1460028_at	0.00	0.00
1460029_at	0.00	0.00
1460030_at	0.00	0.00
1460031_at	0.00	0.00
1460032_at	0.00	0.00
1460033_at	0.00	0.00
1460035_at	0.00	0.00
1460036_at	0.00	0.00
1460037_at	0.00	0.03
1460039_at	0.00	0.00
1460040_at	0.00	0.00
1460041_at	0.00	0.00
1460043_at	0.00	0.00
1460044_at	0.00	0.00
1460045_at	0.00	0.00
1460046_at	0.00	0.00
1460047_at	0.00	0.00
1460048_at	0.00	0.00
1460049_s_at	0.00	0.00
1460050_x_at	0.00	0.00
1460051_at	0.00	0.00
1460052_at	0.00	0.00
1460053_at	0.00	0.00
1460054_at	0.00	0.00
1460055_at	0.00	0.00
1460056_at	0.00	0.00
1460057_at	0.11	0.22
1460058_at	0.00	0.00
1460059_at	0.00	0.00
1460060_at	0.00	0.00
1460061_at	0.00	0.00
1460062_at	0.00	0.00
1460063_at	0.00	0.00
1460064_at	0.00	0.00
1460065_at	0.00	0.00
1460066_at	0.00	0.00
1460067_at	0.00	0.00
1460068_at	0.00	0.00
1460069_at	0.00	0.00
1460070_at	0.00	0.00
1460071_at	0.00	0.00
1460072_at	0.00	0.00

1460073_at	0.00	0.00
1460074_x_at	0.00	0.00
1460075_x_at	0.00	0.00
1460076_x_at	0.00	0.00
1460077_at	0.00	0.00
1460078_at	0.00	0.00
1460079_at	0.00	0.00
1460080_at	0.00	0.00
1460081_at	0.00	0.00
1460082_at	0.00	0.00
1460083_at	0.00	0.00
1460084_at	0.00	0.00
1460085_at	0.00	0.01
1460086_at	0.00	0.00
1460087_at	0.00	0.00
1460088_at	0.00	0.00
1460089_at	0.00	0.00
1460090_at	0.00	0.00
1460091_at	0.00	0.00
1460092_at	0.00	0.00
1460093_at	0.00	0.00
1460094_at	0.00	0.00
1460095_at	0.00	0.00
1460096_at	0.00	0.00
1460097_at	0.00	0.00
1460098_at	0.00	0.00
1460099_at	0.00	0.00
1460100_at	0.00	0.00
1460101_at	0.00	0.00
1460102_at	0.00	0.00
1460103_at	0.00	0.00
1460104_at	0.00	0.00
1460105_at	0.00	0.00
1460106_at	0.00	0.00
1460107_at	0.00	0.00
1460108_at	0.00	0.00
1460109_at	0.00	0.00
1460110_at	0.00	0.00
1460111_at	0.00	0.00
1460112_at	0.00	0.00
1460113_at	0.00	0.00
1460114_at	0.00	0.00
1460115_at	0.00	0.00
1460116_s_at	0.00	0.00
1460117_at	0.00	0.00
1460118_at	0.00	0.00
1460119_at	0.00	0.00
1460120_at	0.00	0.00
1460121_at	0.00	0.00
1460122_at	0.00	0.00
1460123_at	0.00	0.00
1460124_at	0.00	0.00
1460125_at	0.00	0.00
1460126_at	0.00	0.00
1460127_at	0.00	0.00
1460128_at	0.00	0.00

1460129_at	0.00	0.00
1460130_at	0.00	0.00
1460131_at	0.00	0.00
1460132_at	0.00	0.00
1460133_at	0.00	0.00
1460134_at	0.00	0.00
1460135_at	0.00	0.00
1460136_at	0.00	0.00
1460137_at	0.00	0.00
1460138_at	0.00	0.00
1460139_at	0.00	0.00
1460140_at	0.00	0.00
1460141_at	0.00	0.00
1460142_at	0.00	0.00
1460143_at	0.00	0.00
1460144_at	0.00	0.00
1460145_at	0.00	0.00
1460146_at	0.00	0.00
1460147_at	0.00	0.00
1460148_at	0.00	0.00
1460149_at	0.00	0.00
1460150_at	0.00	0.00
1460151_at	0.00	0.00
1460152_at	0.00	0.00
1460153_at	0.00	0.00
1460154_at	0.00	0.00
1460155_at	0.00	0.00
1460156_at	0.00	0.00
1460157_at	0.00	0.00
1460158_at	0.00	0.00
1460159_at	0.00	0.00
1460160_at	0.00	0.00
1460161_at	0.00	0.00
1460162_at	0.00	0.00
1460163_at	0.00	0.00
1460267_at	0.00	0.00
1460431_at	0.11	0.16
1460435_at	0.00	0.00
1460437_at	0.00	0.00
1460439_at	0.00	0.00
1460440_at	0.00	0.00
1460441_at	0.00	0.00
1460443_at	0.00	0.00
1460446_at	0.00	0.26
1460447_at	0.00	0.00
1460448_s_at	0.00	0.00
1460449_at	0.00	0.00
1460451_at	0.00	0.00
1460452_at	0.00	0.00
1460453_at	0.00	0.00
1460454_at	0.00	0.09
1460456_at	0.00	0.00
1460457_at	0.00	0.00
1460458_at	0.00	0.00
1460459_at	0.00	0.00
1460461_at	0.00	0.00

1460462_at	0.00	0.00
1460463_at	0.00	0.00
1460465_at	0.00	0.00
1460466_at	0.00	0.00
1460467_at	0.00	0.00
1460468_s_at	0.00	0.00
1460470_at	0.00	0.00
1460471_at	0.98	0.00
1460472_at	0.00	0.00
1460473_at	0.00	0.00
1460474_at	0.00	0.00
1460475_at	0.00	0.00
1460476_s_at	0.00	0.00
1460477_at	0.00	0.00
1460478_at	0.00	0.00
1460479_at	0.00	0.00
1460482_at	0.00	0.00
1460483_at	0.00	0.00
1460484_at	0.00	0.00
1460485_at	0.00	0.00
1460487_at	0.00	0.00
1460488_at	0.00	0.00
1460489_at	0.00	0.00
1460491_at	0.00	0.00
1460492_at	0.00	0.00
1460493_at	0.00	0.00
1460494_at	0.00	0.00
1460496_at	0.00	0.00
1460497_a_at	0.00	0.00
1460499_at	0.00	0.00
1460500_at	0.00	0.00
1460501_at	0.00	0.00
1460502_at	0.00	0.00
1460503_at	0.00	0.00
1460505_at	0.00	0.00
1460506_s_at	0.00	0.00
1460507_at	0.00	0.00
1460509_at	0.00	0.00
1460510_a_at	0.00	0.00
1460515_at	0.00	0.00
1460516_at	0.00	0.00
1460517_at	0.00	0.00
1460518_at	0.00	0.00
1460520_at	0.00	0.00
1460522_at	0.00	0.00
1460523_at	0.00	0.00
1460524_at	0.00	0.00
1460525_at	0.00	0.00
1460526_at	0.00	0.00
1460527_at	0.00	0.00
1460528_at	0.00	0.00
1460529_at	0.00	0.00
1460530_at	0.00	0.00
1460531_at	0.00	0.00
1460532_at	0.00	0.00
1460533_at	0.00	0.00

1460534_at	0.00	0.00
1460535_at	0.00	0.00
1460536_at	0.00	0.00
1460537_at	0.00	0.00
1460538_at	0.00	0.00
1460539_at	0.00	0.00
1460540_at	0.00	0.00
1460546_at	0.00	0.00
1460550_at	0.00	0.00
1460552_at	0.00	0.19
1460553_at	0.00	0.00
1460554_s_at	0.00	0.01
1460556_at	0.00	0.00
1460558_at	0.00	0.00
1460559_at	0.00	0.00
1460560_at	0.00	0.00
1460563_at	0.00	0.00
1460564_at	0.00	0.00
1460566_at	0.00	0.00
1460567_at	0.00	0.00
1460570_at	0.00	0.00
1460572_a_at	0.00	0.00
1460573_at	0.00	0.00
1460574_at	0.00	0.00
1460575_at	0.00	0.17
1460576_at	0.00	0.00
1460577_at	0.00	0.00
1460578_at	0.00	0.00
1460580_at	0.00	0.00
1460582_x_at	0.00	0.00
1460584_at	0.00	0.00
1460585_x_at	0.00	0.00
1460586_at	0.00	0.00
1460587_at	0.00	0.00
1460588_at	0.00	0.00
1460589_at	0.00	0.00
1460591_at	0.00	0.00
1460593_at	0.00	0.00
1460595_at	0.00	0.00
1460597_at	0.00	0.00
1460598_at	0.00	0.00
1460599_at	0.00	0.00
1460600_at	0.00	0.00
1460601_at	0.00	0.00
1460602_at	0.00	0.00
1460603_at	0.00	0.00
1460604_at	0.00	0.00
1460606_at	0.00	0.00
1460607_at	0.00	0.00
1460608_at	0.00	0.00
1460611_at	0.00	0.00
1460612_at	0.00	0.00
1460614_at	0.00	0.00
1460615_at	0.00	0.00
1460616_at	0.00	0.00
1460617_s_at	0.00	0.00

1460618_x_at	0.00	0.00
1460619_at	0.00	0.00
1460620_at	0.00	0.00
1460622_x_at	0.00	0.00
1460623_at	0.00	0.00
1460624_at	0.00	0.00
1460625_at	0.00	0.00
1460627_at	0.00	0.00
1460628_at	0.00	0.00
1460630_at	0.00	0.00
1460632_at	0.00	0.00
8SRNAMur/X006	0.04	0.00
8SRNAMur/X006	0.12	0.00
8SRNAMur/X006	0.06	0.00
-ActinMur/M1248	0.00	0.00
-ActinMur/M1248	0.00	0.00
-ActinMur/M1248	0.00	0.00
AFFX-BioB-3_at	0.00	0.00
AFFX-BioB-5_at	0.00	0.00
AFFX-BioB-M_at	0.00	0.00
AFFX-BioC-3_at	0.00	0.00
AFFX-BioC-5_at	0.00	0.00
AFFX-BioDn-3_a	0.00	0.00
AFFX-BioDn-5_a	0.00	0.00
AFFX-CreX-3_at	0.00	0.00
AFFX-CreX-5_at	0.00	0.00
AFFX-DapX-3_at	0.00	0.00
AFFX-DapX-5_at	0.00	0.00
AFFX-DapX-M_a	0.00	0.00
GapdhMur/M3259	0.00	0.00
GapdhMur/M3259	0.00	0.00
GapdhMur/M3259	0.00	0.00
AFFX-LysX-3_at	0.00	0.00
AFFX-LysX-5_at	0.00	0.05
AFFX-LysX-M_at	0.00	0.00
AFFX-MUR_b2_a	0.00	0.00
FFX-MURINE_b1_	0.00	0.00
FFX-MURINE_B2_	0.00	0.00
AFFX-PheX-3_at	0.00	0.00
AFFX-PheX-5_at	0.00	0.00
AFFX-PheX-M_a	0.00	0.00
yruCarbMur/L091	0.00	0.00
yruCarbMur/L091	0.00	0.00
ruCarbMur/L0919	0.00	0.00
ruCarbMur/L0919	0.00	0.00
FFX-r2-Bs-dap-3_	0.00	0.00
FFX-r2-Bs-dap-5_	0.00	0.00
FFX-r2-Bs-dap-M_	0.00	0.00
FFX-r2-Bs-lys-3_	0.00	0.00
FFX-r2-Bs-lys-5_	0.00	0.00
FFX-r2-Bs-lys-M_	0.00	0.00
FFX-r2-Bs-phe-3_	0.00	0.00
FFX-r2-Bs-phe-5_	0.00	0.00
FFX-r2-Bs-phe-M_	0.00	0.00
FX-r2-Bs-thr-3_s	0.00	0.00
FX-r2-Bs-thr-5_s	0.00	0.00

FX-r2-Bs-thr-M_s	0.00	0.00
FFX-r2-Ec-bioB-3_	0.00	0.00
FFX-r2-Ec-bioB-5_	0.00	0.00
FX-r2-Ec-bioB-M_	0.00	0.00
FFX-r2-Ec-bioC-3_	0.00	0.00
FFX-r2-Ec-bioC-5_	0.00	0.00
FFX-r2-Ec-bioD-3_	0.00	0.00
FFX-r2-Ec-bioD-5_	0.00	0.00
FFX-r2-P1-cre-3_	0.00	0.00
FFX-r2-P1-cre-5_	0.00	0.00
AFFX-ThrX-3_at	0.00	0.00
AFFX-ThrX-5_at	0.00	0.00
AFFX-ThrX-M_at	0.00	0.00
ransRecMur/X573	0.00	0.00
ransRecMur/X573	0.00	0.00
ansRecMur/X573	0.00	0.00
AFFX-TrpnX-3_at	0.00	0.00
AFFX-TrpnX-5_at	0.00	0.00
AFFX-TrpnX-M_a	0.00	0.00
