# Supplementary material for: Meta-Analysis of Differentiating Mouse Embryonic Stem Cell Gene Expression Kinetics Reveals Early Change of a Small Gene Set
Source: PLoS Comput Biol. 2006 Nov 24;2(11):e158. doi: 10.1371/journal.pcbi.0020158 (PMC1664699; doi:10.1371/journal.pcbi.0020158)
Supplement: Table S3 — (18 Kb PDF) [file pcbi.0020158.st003.pdf]

		Confidence Values			Fold change at 96 h		Fold change following LIF removal (R1LR)		Fold change following LIF removal for 96 h (MLR)				
MGU74v2	MOE430	R1LR	DMSO/RA	MLR	DMSO	RA	18 h	72 h	J1	R1	V6.5	Gene Name	Gene Symbol
**Pareto Front 1**													
103728_at	1456521_at	1.00	1.00	0.07	0.18	0.19	0.65	0.24	0.58	0.64	0.29	Transcribed locus	---
168508_at	1436926_at	0.86	1.00	0.35	0.09	0.09	0.63	0.20	0.36	0.61	0.24	estrogen related receptor, beta	Esrrb
93141_at	1417760_at	0.81	1.00	1.00	0.06	0.08	0.84	0.10	0.09	0.12	0.08	nuclear receptor subfamily 0, gro	Nr0b1
93296_at	1422458_at	0.98	1.00	0.10	0.19	0.19	0.78	0.22	0.48	0.73	0.42	T-cell lymphoma breakpoint 1	Tcl1
93483_at	1449455_at	0.85	1.00	0.67	0.18	0.17	0.89	0.15	0.36	0.44	0.50	hemopoietic cell kinase	Hck
94200_at	1420337_at	0.98	0.92	1.00	0.23	0.30	0.69	0.22	0.26	0.29	0.25	gastrulation brain homeobox 2	Gbx2
96109_at	1448890_at	1.00	1.00	0.01	0.11	0.11	0.74	0.41	0.49	0.78	0.31	Kruppel-like factor 2 (lung)	Klf2
96162_at	1427238_at	1.00	0.83	0.53	0.11	0.33	0.63	0.10	0.53	0.51	0.18	F-box protein 15	Fbxo15
97519_at	1449254_at	0.99	0.02	0.55	0.08	0.77	0.54	0.19	0.54	0.44	0.10	secreted phosphoprotein 1	Spp1
99561_f_at	1448393_at	1.00	0.00	1.00	5.23	0.67	1.34	4.15	4.67	7.25	3.34	claudin 7	Cldn7
**Pareto Front 2**													
103761_at	1418091_at	1.00	1.00	0.07	0.19	0.26	0.64	0.13	0.60	0.66	0.22	transcription factor CP2-like 1	Tcfcp2l1
108097_at	1450626_at	0.80	0.93	0.10	0.15	0.29	0.74	0.27	0.69	0.71	0.25	mannosidase, beta A, lysosomal	Manba
108712_at	1434917_at	0.77	0.98	1.00	0.26	0.29	0.73	0.32	0.36	0.38	0.28	cordon-bleu	Cobl
160684_at	1423786_at	0.73	1.00	1.00	0.27	0.23	0.97	0.46	0.29	0.24	0.17	RIKEN cDNA 8430410A17 gene	8430410A17Rik
95033_at	1426810_at	0.97	0.50	0.97	0.42	0.30	0.84	0.41	0.31	0.33	0.39	jumonji domain containing 1A	Jmjd1a
98414_at	1418362_at	0.73	1.00	0.97	0.09	0.10	0.88	0.14	0.28	0.40	0.11	zinc finger protein 42	Zfp42
99622_at	1417394_at	1.00	0.70	0.00	0.09	0.33	0.26	0.07	0.30	0.14	0.11	Kruppel-like factor 4 (gut)	Klf4
**Pareto Front 3**													
100009_r_a	1416967_at	0.67	1.00	0.00	0.16	0.07	0.89	0.36	0.59	1.11	0.39	SRY-box containing gene 2	Sox2
101560_at	1415856_at	0.60	0.00	1.00	1.22	0.43	1.09	1.85	3.37	4.71	3.22	embigin	Emb
102012_at	1418895_at	0.87	0.00	0.30	0.61	2.18	0.74	0.48	0.54	0.66	0.52	src family associated phosphopro	Scap2
102332_at	1448370_at	0.99	0.38	0.00	0.45	0.42	0.79	0.38	1.02	0.95	0.78	Unc-51 like kinase 1 (C. elegans)	Ulk1
108784_at	1455604_at	0.92	0.91	0.02	0.32	0.25	0.51	0.36	0.46	0.63	0.58	expressed sequence AI427138	AI427138
112828_at	1448688_at	0.11	0.87	1.00	3.07	5.89	1.18	1.45	10.45	4.96	4.45	podocalyxin-like	Podxl
115445_at	1435374_at	0.88	0.84	0.03	0.22	0.32	0.64	0.34	0.75	0.56	0.38	Transcribed locus	---
116872_at	1435437_at	0.72	0.82	0.96	3.08	3.01	1.27	2.07	2.78	2.49	2.84	SET domain-containing protein 7	MGI:1920501
117246_at	1448845_at	0.27	0.69	1.00	0.23	0.31	0.77	0.65	0.29	0.26	0.32	ribonuclease P 25 subunit (huma	Rpp25
133365_at	1436568_at	0.86	0.41	0.87	0.23	0.44	0.74	0.42	0.41	0.31	0.44	junction adhesion molecule 2	Jam2
133819_at	1419418_a_	0.66	1.00	0.99	0.08	0.09	0.79	0.24	0.38	0.33	0.09	microrchidia	Morc
163005_s_a	1429366_at	0.76	0.99	0.00	0.19	0.22	0.65	0.28	0.49	0.91	0.34	leucine rich repeat containing 34	Lrrc34
92770_at	1421375_a_	0.98	0.38	0.00	2.11	5.38	0.33	0.16	0.36	0.35	0.05	S100 calcium binding protein A6	S100a6
93864_s_at	1421624_a_	0.48	0.04	1.00	0.30	0.65	1.03	0.36	0.38	0.35	0.34	enabled homolog (Drosophila)	Enah
94745_f_at	1427479_at	1.00	0.01	0.00	0.73	5.61	0.49	0.26	0.89	0.35	1.00	eukaryotic translation initiation fa	Eif1a
96752_at	1424067_at	0.99	0.00	0.06	0.63	0.80	0.69	0.41	0.67	0.75	0.61	intercellular adhesion molecule	Icam1
97890_at	1416041_at	0.88	0.83	0.06	0.21	0.32	0.87	0.38	0.57	0.73	0.23	serum/glucocorticoid regulated ki	Sgk
**Pareto Front 4**													
100030_at	1448562_at	0.91	0.34	0.01	0.47	0.17	0.83	0.36	0.25	0.39	0.85	uridine phosphorylase 1	Upp1

100301_at	1422986_at	0.57	1.00	0.44	0.21	0.21	0.92	0.54	0.40	0.57	0.37	estrogen related receptor, beta	Esrrb
103342_at	1448653_at	0.86	0.11	0.61	0.45	0.55	0.72	0.49	0.41	0.33	0.25	embryonic ectoderm developmen	Eed
103653_at	1449590_a_	0.95	0.00	0.03	0.38	1.16	0.73	0.47	0.39	0.39	0.41	muscle and microspikes RAS	Mras
104139_at	1452094_at	0.45	0.00	1.00	1.50	1.88	0.91	0.57	5.73	3.69	2.91	procollagen-proline, 2-oxoglutara	P4ha1
104544_at	1423327_at	0.90	0.53	0.00	0.16	0.41	0.75	0.43	0.88	0.36	0.42	RIKEN cDNA 4930517K11 gene	4930517K11Rik
107103_at	1416958_at	0.96	0.00	0.00	0.51	0.73	0.60	0.43	0.80	0.91	0.72	nuclear receptor subfamily 1, gro	Nr1d2
108010_at	1418318_at	0.54	0.87	0.99	3.13	16.53	1.17	1.87	4.61	3.73	2.54	ring finger protein 128	Rnf128
108048_at	1454788_at	0.08	0.67	1.00	2.62	5.18	1.00	1.37	3.02	3.47	3.13	ADP-ribosylation factor-like 7	Arl7
113673_at	1423508_at	0.72	0.56	0.02	0.28	0.42	0.69	0.38	0.57	0.59	0.36	MYST histone acetyltransferase r	Myst4
115804_at	1438237_at	0.57	1.00	0.68	0.16	0.14	0.88	0.50	0.49	0.22	0.12	RIKEN cDNA C230088H06 gene	---
133204_at	1455425_at	0.47	1.00	0.99	0.14	0.08	0.96	0.43	0.28	0.33	0.26	Expressed sequence BB001228	BB001228
160370_at	1416552_at	0.70	0.99	0.00	0.17	0.25	0.91	0.44	0.93	1.08	0.44	developmental pluripotency asso	Dppa5
160828_at	1426858_at	0.62	0.76	0.25	0.31	0.33	0.64	0.53	0.38	0.38	0.31	inhibin beta-B	Inhbb
93063_at	1427442_a_	0.00	0.71	1.00	2.70	9.60	0.50	0.85	6.52	3.84	3.02	amyloid beta (A4) precursor prote	App
93104_at	1426083_a_	0.79	0.00	0.62	0.92	1.83	0.65	0.48	2.74	3.04	1.93	B-cell translocation gene 1, anti-p	Btg1
94270_at	1448169_at	0.86	0.85	0.00	4.12	12.01	1.27	2.57	12.20	18.17	1.76	keratin complex 1, acidic, gene 1	Krt1-18
94354_at	1421840_at	0.98	0.00	0.00	0.37	0.83	0.53	0.36	1.12	0.67	0.75	ATP-binding cassette, sub-family	Abca1
95518_at	1424683_at	0.13	0.00	1.00	1.74	3.14	1.22	1.43	7.05	5.20	3.40	RIKEN cDNA 1810015C04 gene	1810015C04Rik
95531_at	1454890_at	0.08	0.35	1.00	3.90	2.22	0.79	3.31	20.76	7.86	5.62	angiomotin	Amot
96042_at	1448610_a_	0.72	0.53	0.94	0.43	0.32	0.75	0.52	0.30	0.42	0.28	superoxide dismutase 2, mitocho	Sod2
96841_at	1451069_at	1.00	0.00	0.00	0.71	0.46	0.73	0.41	1.39	1.26	1.09	proviral integration site 3	Pim3
96900_at	1433720_s_	0.62	1.00	0.00	0.18	0.12	0.92	0.54	0.45	0.85	0.48	Nur77 downstream gene 2	MGI:2143558
97317_at	1415894_at	1.00	0.00	0.00	1.05	0.79	2.00	5.20	1.05	1.00	1.21	Ectonucleotide pyrophosphatase/	Enpp2
97426_at	1416529_at	0.96	0.00	0.00	0.67	6.50	0.28	0.15	0.75	0.63	0.57	epithelial membrane protein 1	Emp1
97520_s_at	1423506_a_	0.97	0.08	0.00	1.73	3.84	1.21	2.33	0.66	1.13	1.39	neuronatin	Nnat
99956_at	1452514_a_	1.00	0.00	0.00	0.67	0.96	0.49	0.25	2.31	1.57	1.04	kit oncogene	Kit
**Pareto Front 5**													
103048_at	1417155_at	0.39	0.98	0.64	0.30	0.25	0.96	0.58	0.35	0.50	0.35	neuroblastoma myc-related onco	Nmyc1
103234_at	1424847_at	0.01	0.92	0.78	0.29	0.25	1.02	0.94	0.43	0.35	0.45	neurofilament, heavy polypeptide	Nefh
103737_at	1418753_at	0.69	0.53	0.33	0.39	0.43	0.95	0.51	0.40	0.63	0.65	---	---
108279_at	1455300_at	0.52	0.50	0.63	0.35	0.42	0.93	0.35	0.37	0.51	0.29	RIKEN cDNA E130014J05 gene	E130014J05Rik
110429_at	1455333_at	0.80	0.01	0.08	0.28	0.66	0.72	0.36	0.39	0.32	0.25	cDNA sequence BC023928	BC023928
111970_at	1460711_at	0.39	0.76	0.46	0.36	0.24	0.92	0.57	0.54	0.54	0.40	RIKEN cDNA 4930461P20 gene	4930461P20Rik
115058_at	1434362_at	0.68	0.41	0.91	2.65	2.23	1.25	2.04	2.47	2.28	2.83	expressed sequence AW550831	AW550831
116214_at	1456329_at	0.37	0.58	0.72	2.44	3.11	1.19	1.91	2.29	2.11	2.45	RIKEN cDNA A230098A12 gene	A230098A12Rik
116435_at	1418076_at	0.93	0.00	0.01	1.90	0.78	1.65	2.22	1.70	2.03	1.24	suppression of tumorigenicity 14	St14
160253_at	1423754_at	0.74	0.06	0.31	0.34	0.56	0.82	0.51	0.53	0.66	0.34	interferon induced transmembran	Ifitm3
161042_at	1427912_at	0.75	0.75	0.00	0.35	0.29	1.27	1.92	0.80	0.75	0.77	carbonyl reductase 3	Cbr3
161106_r_a	1443892_at	0.00	1.00	0.97	0.21	0.16	1.09	0.78	0.36	0.21	0.20	---	---
162522_f_a	1437015_x_	0.00	0.17	0.98	0.51	0.22	0.95	0.78	0.29	0.42	0.39	phospholipase A2, group IB, panc	Pla2g1b
163288_at	1460471_at	0.70	0.98	0.00	0.23	0.26	0.82	0.36	0.78	0.93	0.40	RIKEN cDNA 2410146L05 gene	2410146L05Rik
163489_at	1418488_s_	0.85	0.00	0.42	1.52	1.06	1.35	2.24	3.03	2.75	1.72	receptor-interacting serine-threon	Ripk4
163715_at	1429399_at	0.50	1.00	0.32	0.19	0.17	0.92	0.47	0.34	0.49	0.59	ring finger protein 125	Rnf125
165699_r_a	1453299_a_	0.00	0.56	1.00	0.42	0.37	0.98	1.02	0.30	0.23	0.29	purine-nucleoside phosphorylase	Pnp

166142_r_a	1436799_at	0.00	1.00	0.97	0.13	0.13	1.16	0.96	0.39	0.27	0.17	RIKEN cDNA D230005D02 gene	D230005D02Rik
167088_r_a	1456242_at	0.47	0.41	0.78	0.34	0.45	0.98	0.13	0.27	0.47	0.24	LOC433110	LOC433110
92275_at	1418147_at	0.95	0.00	0.00	0.56	0.45	0.77	0.42	1.25	1.48	0.92	transcription factor AP-2, gamma	Tcfap2c
92476_at	1449288_at	0.33	1.00	0.75	0.23	0.16	0.85	0.62	0.43	0.47	0.29	growth differentiation factor 3	Gdf3
92550_at	1417156_at	0.63	0.98	0.00	5.12	5.63	1.22	2.04	1.90	1.70	1.22	keratin complex 1, acidic, gene 1	Krt1-19
93271_s_at	1450186_s_	0.01	0.58	0.88	2.44	3.17	0.82	1.38	2.24	2.90	2.98	GNAS (guanine nucleotide bindin	Gnas
95584_at	1453223_s_	0.08	0.03	1.00	0.25	0.64	1.08	0.63	0.34	0.23	0.11	developmental pluripotency asso	Dppa2
96203_at	1424713_at	0.27	0.06	0.92	0.50	0.59	1.06	0.54	0.23	0.43	0.11	calmodulin-like 4	Calml4
97083_at	1441023_at	0.99	0.00	0.00	1.00	0.92	0.51	0.35	1.10	0.93	1.07	eukaryotic translation initiation fa	Eif2s2
97283_at	1424295_at	0.83	0.68	0.00	0.37	0.32	0.84	0.36	1.14	2.48	0.44	developmental pluripotency-asso	Dppa3
97442_at	1416832_at	0.14	0.00	1.00	1.20	1.05	1.08	1.47	7.27	3.55	4.90	solute carrier family 39 (metal ion	Slc39a8
