# Supplementary material for: Meta-Analysis of Differentiating Mouse Embryonic Stem Cell Gene Expression Kinetics Reveals Early Change of a Small Gene Set
Source: PLoS Comput Biol. 2006 Nov 24;2(11):e158. doi: 10.1371/journal.pcbi.0020158 (PMC1664699; doi:10.1371/journal.pcbi.0020158)
Supplement: Table S4 — (8 KB PDF) [file pcbi.0020158.st004.pdf]

Sample	Time	103728_a	Esrbb	Nr0b1	Tcl1	Hck	Gbx2	Klf2	Fbxo15	Spp1	Tcfcp2l1	3430410A17Ri
R1												
+LIF	0	1.0	1.0	1.0	1.0	1.0	1.0	1.0	1.0	1.0	1.0	1.0
+LIF	24	1.0	1.0	1.0	1.0	1.0	1.0	1.0	1.0	1.0	1.0	1.0
+LIF	72	1.0	1.0	1.0	1.0	1.0	1.0	1.0	1.0	1.0	1.0	1.0
+LIF	96	1.0	1.0	1.0	1.0	1.0	1.0	1.0	1.0	1.0	1.0	1.0
-LIF	0	1.0	1.0	1.0	1.0	1.0	1.0	1.0	1.0	1.0	1.0	1.0
-LIF	24	0.7	0.7	0.7	0.8	1.2	0.6	0.6	0.5	0.7	0.3	0.6
-LIF	72	0.1	0.1	0.0	0.0	0.3	0.1	0.1	0.1	0.1	0.2	0.3
-LIF	96	0.1	0.0	0.0	0.0	0.2	0.0	0.1	0.1	0.1	0.1	0.3
DMSO	0	1.0	1.0	1.0	1.0	1.0	1.0	1.0	1.0	1.0	1.0	1.0
DMSO	24	0.8	0.4	0.2	0.5	0.4	0.2	0.6	0.6	0.4	0.3	0.4
DMSO	72	0.1	0.0	0.0	0.0	0.0	0.1	0.1	0.0	0.2	0.2	0.3
DMSO	96	0.1	0.1	0.0	0.0	0.1	0.1	0.1	0.1	0.1	0.2	0.3
RA	0	1.0	1.0	1.0	1.0	1.0	1.0	1.0	1.0	1.0	1.0	1.0
RA	24	0.7	1.0	0.9	0.8	0.8	1.7	0.8	0.8	0.8	1.1	0.4
RA	72	0.1	0.2	0.0	0.0	0.3	0.8	0.6	0.4	2.5	0.6	0.3
RA	96	0.2	0.1	0.0	0.1	0.3	0.8	0.2	0.2	2.5	0.2	0.2
J1												
+LIF	0	1.0	1.0	1.0	1.0	1.0	1.0	1.0	1.0	1.0	1.0	1.0
+LIF	24	1.0	1.0	1.0	1.0	1.0	1.0	1.0	1.0	1.0	1.0	1.0
+LIF	72	1.0	1.0	1.0	1.0	1.0	1.0	1.0	1.0	1.0	1.0	1.0
+LIF	96	1.0	1.0	1.00	1.0	1.0	1.0	1.0	1.0	1.0	1.0	1.0
-LIF	0	1.0	1.0	1.0	1.0	1.0	1.0	1.0	1.0	1.0	1.0	1.0
-LIF	24	0.6	0.5	0.7	0.7	0.9	0.3	0.8	0.9	0.8	0.4	0.5
-LIF	72	0.1	0.1	0.0	0.0	0.3	0.0	0.1	0.1	0.9	0.3	0.3
-LIF	96	0.1	0.1	0.01	0.0	0.3	0.0	0.1	0.1	0.1	0.2	0.3
DMSO	0	1.0	1.0	1.0	1.0	1.0	1.0	1.0	1.0	1.0	1.0	1.0
DMSO	24	0.4	0.1	0.4	0.3	0.2	0.3	0.5	0.6	0.5	0.4	0.3
DMSO	72	0.0	0.1	0.0	0.0	0.1	0.0	0.0	0.0	ND	0.1	0.2
DMSO	96	0.0	0.0	0.00	0.0	0.2	0.1	0.0	0.0	0.1	0.1	0.3
RA	0	1.0	1.0	1.0	1.0	1.0	1.0	1.0	1.0	1.0	1.0	1.0
RA	24	0.4	0.3	0.4	0.5	0.5	2.5	0.4	1.0	0.9	0.7	0.3
RA	72	0.1	0.6	0.14	0.1	0.6	1.8	0.6	1.1	ND	0.6	0.6
RA	96	0.0	0.1	0.02	0.0	0.2	0.8	0.2	0.6	1.3	0.3	0.4

Sample	Time	Zfp42	Klf4	Sox2	Podxl	Jam2	Morc	Sod2	Nr1d2	Kit	Nmyc1	Mtf2
**R1**												
+LIF	0	1.0	1.0	1.0	1.0	1.0	1.0	1.0	1.0	1.0	1.0	1.0
+LIF	24	1.0	1.0	1.0	1.0	1.0	1.0	1.0	1.0	1.0	1.0	1.0
+LIF	72	1.0	1.0	1.0	1.0	1.0	1.0	1.0	1.0	1.0	1.0	1.0
+LIF	96	1.0	1.0	1.0	1.0	1.0	1.0	1.0	1.0	1.0	1.0	1.0
-LIF	0	1.0	1.0	1.0	1.0	1.0	1.0	1.0	1.0	1.0	1.0	1.0
-LIF	24	0.1	0.0	0.9	1.0	0.7	1.4	0.9	1.1	0.6	1.0	1.1
-LIF	72	0.1	0.2	0.1	0.4	0.0	0.0	0.4	0.7	0.4	0.4	0.8
-LIF	96	0.0	0.2	0.1	4.7	0.1	0.0	0.4	0.7	0.3	0.3	0.6
DMSO	0	1.0	1.0	1.0	1.0	1.0	1.0	1.0	1.0	1.0	1.0	1.0
DMSO	24	0.6	0.0	0.8	0.4	0.5	0.9	1.1	1.3	0.4	0.7	0.8
DMSO	72	0.1	0.2	0.2	3.0	0.0	0.0	0.6	1.0	0.7	0.2	0.4
DMSO	96	0.0	0.1	0.1	8.1	0.1	0.0	0.7	1.0	0.3	0.4	0.8
RA	0	1.0	1.0	1.0	1.0	1.0	1.0	1.0	1.0	1.0	1.0	1.0
RA	24	0.7	0.1	0.6	0.6	0.8	0.8	1.1	1.0	ND	0.3	1.1
RA	72	0.1	2.0	0.2	10.4	0.6	0.1	0.6	1.1	1.2	0.1	1.6
RA	96	0.0	2.7	0.2	23.3	1.5	0.2	0.6	1.8	0.6	0.4	1.3
**J1**												
+LIF	0	1.0	ND	1.0	1.0	ND	ND	1.0	ND	ND	1.0	ND
+LIF	24	1.0	ND	1.0	1.0	ND	ND	1.0	ND	ND	1.0	ND
+LIF	72	1.0	ND	1.0	1.0	ND	ND	1.0	ND	ND	1.0	ND
+LIF	96	1.0	ND	1.0	1.0	ND	ND	1.0	ND	ND	1.0	ND
-LIF	0	1.0	ND	1.0	1.0	ND	ND	1.0	ND	ND	1.0	ND
-LIF	24	0.6	ND	0.7	2.6	ND	ND	0.8	ND	ND	1.0	ND
-LIF	72	0.1	ND	0.2	0.9	ND	ND	0.5	ND	ND	0.5	ND
-LIF	96	0.0	ND	0.1	2.8	ND	ND	0.4	ND	ND	0.4	ND
DMSO	0	1.0	ND	1.0	1.0	ND	ND	1.0	ND	ND	1.0	ND
DMSO	24	0.7	ND	0.7	3.2	ND	ND	0.7	ND	ND	1.0	ND
DMSO	72	0.0	ND	0.2	ND	ND	ND	0.6	ND	ND	0.3	ND
DMSO	96	0.0	ND	0.3	2.9	ND	ND	0.2	ND	ND	0.1	ND
RA	0	1.0	ND	1.0	1.0	ND	ND	1.0	ND	ND	1.0	ND
RA	24	0.7	ND	0.6	1.0	ND	ND	0.7	ND	ND	0.2	ND
RA	72	0.1	ND	0.2	ND	ND	ND	0.9	ND	ND	0.4	ND
RA	96	0.1	ND	0.2	10.8	ND	ND	0.6	ND	ND	0.5	ND
