# Supplementary material for: Meta-Analysis of Differentiating Mouse Embryonic Stem Cell Gene Expression Kinetics Reveals Early Change of a Small Gene Set
Source: PLoS Comput Biol. 2006 Nov 24;2(11):e158. doi: 10.1371/journal.pcbi.0020158 (PMC1664699; doi:10.1371/journal.pcbi.0020158)
Supplement: Table S5 — (8 KB PDF) [file pcbi.0020158.st005.pdf]

Biological Process

TERM	COUNT	PERCENTAGE
REGULATION OF BIOLOGICAL PROCESS	23	25.6
PHYSIOLOGICAL PROCESS	56	62.2
NEGATIVE REGULATION OF METABOLISM	5	5.6
METABOLISM	40	44.4
AXONOGENESIS	4	4.4
PROTEIN METABOLISM	20	22.2
DEVELOPMENT	15	16.7
CELLULAR PHYSIOLOGICAL PROCESS	28	31.1
MACROMOLECULE METABOLISM	22	24.4
PROTEIN AMINO ACID PHOSPHORYLATION	8	8.9
PROTEIN MODIFICATION	11	12.2
CELL MIGRATION	4	4.4
REGULATION OF METABOLISM	16	17.8
PHOSPHATE METABOLISM	9	10
PHOSPHORUS METABOLISM	9	10
REGULATION OF CELL PROLIFERATION	4	4.4
REGULATION OF PHYSIOLOGICAL PROCESS	16	17.8
CELL GROWTH AND/OR MAINTENANCE	24	26.7
PHOSPHORYLATION	8	8.9
MORPHOGENESIS	10	11.1
NEGATIVE REGULATION OF CYTOKINE BIOSYNTHESIS	2	2.2
NEGATIVE REGULATION OF CYTOKINE PRODUCTION	2	2.2
REGULATION OF TRANSCRIPTION	14	15.6
REGULATION OF NUCLEOBASE, NUCLEOSIDE, NUCLEOTIDE AND NUCLEIC ACID MET	14	15.6
TRANSCRIPTION	14	15.6
CHEMOTAXIS	3	3.3
TAXIS	3	3.3
CELL MOTILITY	4	4.4
REGULATION OF TRANSCRIPTION, DNA-DEPENDENT	13	14.4
NEGATIVE REGULATION OF PROTEIN BIOSYNTHESIS	2	2.2
NUCLEOBASE, NUCLEOSIDE, NUCLEOTIDE AND NUCLEIC ACID METABOLISM	18	20
NEGATIVE REGULATION OF BIOSYNTHESIS	2	2.2
TRANSCRIPTION, DNA-DEPENDENT	13	14.4
NEGATIVE REGULATION OF TRANSCRIPTION	3	3.3
ORGANOGENESIS	8	8.9
NEGATIVE REGULATION OF NUCLEOBASE, NUCLEOSIDE, NUCLEOTIDE AND NUCLEI	3	3.3
GROWTH	3	3.3
RESPONSE TO CHEMICAL SUBSTANCE	4	4.4

Molecular Function

TERM	COUNT	PERCENTAG
TRANSCRIPTION REGULATOR ACTIVITY	13	14.4
PROTEIN SERINE/THREONINE KINASE ACTIVITY	8	8.9
TRANSCRIPTION FACTOR ACTIVITY	11	12.2
PROTEIN KINASE ACTIVITY	8	8.9
BINDING	46	51.1
PURINE NUCLEOTIDE BINDING	13	14.4
TRANSFERASE ACTIVITY	13	14.4
NUCLEOTIDE BINDING	13	14.4
PROTEIN-TYROSINE KINASE ACTIVITY	6	6.7
PHOSPHOTRANSFERASE ACTIVITY, ALCOHOL GROUP AS ACCEPTOR	8	8.9
STEROID HORMONE RECEPTOR ACTIVITY	3	3.3
LIGAND-DEPENDENT NUCLEAR RECEPTOR ACTIVITY	3	3.3
GROWTH FACTOR ACTIVITY	4	4.4
ATP BINDING	10	11.1
ADENYL NUCLEOTIDE BINDING	10	11.1
NUCLEIC ACID BINDING	19	21.1
KINASE ACTIVITY	8	8.9
TRANSFERASE ACTIVITY, TRANSFERRING PHOSPHORUS-CONTAINING G	8	8.9
DNA BINDING	13	14.4
CATALYTIC ACTIVITY	25	27.8
PROTEIN BINDING	14	15.6
TRANSFERASE ACTIVITY, TRANSFERRING PENTOSYL GROUPS	2	2.2
