# Supplementary material for: Meta-Analysis of Differentiating Mouse Embryonic Stem Cell Gene Expression Kinetics Reveals Early Change of a Small Gene Set
Source: PLoS Comput Biol. 2006 Nov 24;2(11):e158. doi: 10.1371/journal.pcbi.0020158 (PMC1664699; doi:10.1371/journal.pcbi.0020158)
Supplement: Table S6 — (61 KB PDF) [file pcbi.0020158.st006.pdf]

| MGU74v2 | MOE430 | Gene Name | Gene Symbo | Bhattacharya | Brandenburger | Sato | Sperger | Overlap with human data sets | Ivanova | Ramalho-Santos | Overlap with mouse data sets | Overlap with all publications |
|---|---|---|---|---|---|---|---|---|---|---|---|---|
| 100009_r_a | 1416967_a | SRY-box containing gen | Sox2 | 1 | 1 | 0 | 1 | **3** | 1 | 0 | **1** | **4** |
| 100030_at | 1448562_a | uridine phosphorylase 1 | Upp1 | 0 | 0 | 0 | 0 | **0** | 1 | 1 | **2** | **2** |
| 101560_at | 1415856_a | embigin | Emb | 0 | 0 | 0 | 0 | **0** | 0 | 0 | **0** | **0** |
| 102012_at | 1418895_a | src family associated ph | Scap2 | 0 | 0 | 0 | 0 | **0** | 1 | 0 | **1** | **1** |
| 102332_at | 1448370_a | Unc-51 like kinase 1 (C. | Ulk1 | 0 | 0 | 0 | 0 | **0** | 0 | 0 | **0** | **0** |
| 103048_at | 1417155_a | neuroblastoma myc-rela | Nmyc1 | 0 | 0 | 0 | 0 | **0** | 1 | 1 | **2** | **2** |
| 103234_at | 1424847_a | neurofilament, heavy po | Nefh | 0 | 0 | 1 | 1 | **2** | 0 | 1 | **1** | **3** |
| 103342_at | 1448653_a | embryonic ectoderm dev | Eed | 0 | 0 | 0 | 0 | **0** | 1 | 0 | **1** | **1** |
| 103653_at | 449590_a_ | muscle and microspikes | Mras | 0 | 0 | 0 | 0 | **0** | 1 | 0 | **1** | **1** |
| 103728_at | 1456521_a | Transcribed locus | --- | 0 | 0 | 0 | 0 | **0** | 1 | 1 | **2** | **2** |
| 103737_at | 1418753_a | --- | --- | 0 | 0 | 0 | 1 | **1** | 1 | 0 | **1** | **2** |
| 103761_at | 1418091_a | transcription factor CP2- | Tcfcp2l1 | 0 | 0 | 0 | 0 | **0** | 1 | 1 | **2** | **2** |
| 104139_at | 1452094_a | procollagen-proline, 2-o | P4ha1 | 0 | 0 | 0 | 0 | **0** | 0 | 0 | **0** | **0** |
| 104544_at | 1423327_a | RIKEN cDNA 4930517K | 930517K11Ri | 0 | 0 | 0 | 0 | **0** | 1 | 1 | **2** | **2** |
| 107103_at | 1416958_a | nuclear receptor subfam | Nr1d2 | 0 | 0 | 0 | 0 | **0** | 1 | 0 | **1** | **1** |
| 108010_at | 1418318_a | ring finger protein 128 | Rnf128 | 0 | 0 | 0 | 0 | **0** | 0 | 0 | **0** | **0** |
| 108048_at | 1454788_a | ADP-ribosylation factor-l | Arl7 | 0 | 0 | 0 | 1 | **1** | 0 | 0 | **0** | **1** |
| 108097_at | 1450626_a | mannosidase, beta A, ly | Manba | 0 | 0 | 0 | 0 | **0** | 1 | 0 | **1** | **1** |
| 108279_at | 1455300_a | RIKEN cDNA E130014J | 130014J05Ri | 0 | 0 | 0 | 0 | **0** | 1 | 0 | **1** | **1** |
| 108712_at | 1434917_a | cordon-bleu | Cobl | 0 | 0 | 0 | 1 | **1** | 1 | 0 | **1** | **2** |
| 108784_at | 1455604_a | expressed sequence AI4 | AI427138 | 0 | 0 | 0 | 0 | **0** | 1 | 0 | **1** | **1** |
| 110429_at | 1455333_a | tensin 3 | Tns3 | 0 | 0 | 0 | 1 | **1** | 1 | 0 | **1** | **2** |
| 111970_at | 1460711_a | RIKEN cDNA 4930461P | 930461P20Ri | 0 | 0 | 0 | 0 | **0** | 1 | 0 | **1** | **1** |
| 112828_at | 1448688_a | podocalyxin-like | Podxl | 1 | 1 | 1 | 0 | **3** | 0 | 0 | **0** | **3** |
| 113673_at | 1423508_a | MYST histone acetyltran | Myst4 | 0 | 0 | 0 | 1 | **1** | 1 | 0 | **1** | **2** |
| 115058_at | 1434362_a | expressed sequence AW | AW550831 | 0 | 0 | 0 | 0 | **0** | 1 | 0 | **1** | **1** |
| 115445_at | 1435374_a | Transcribed locus | --- | 0 | 0 | 0 | 0 | **0** | 1 | 0 | **1** | **1** |
| 115804_at | 1438237_a | RIKEN cDNA C230088H | --- | 0 | 0 | 0 | 0 | **0** | 1 | 0 | **1** | **1** |
| 116214_at | 1456329_a | RIKEN cDNA A230098A | 230098A12R | 0 | 0 | 0 | 0 | **0** | 1 | 0 | **1** | **1** |
| 116435_at | 1418076_a | suppression of tumorige | St14 | 0 | 0 | 0 | 0 | **0** | 1 | 0 | **1** | **1** |
| 116872_at | 1435437_a | SET domain-containing | MGI:1920501 | 0 | 0 | 0 | 0 | **0** | 1 | 0 | **1** | **1** |
| 117246_at | 1448845_a | ribonuclease P 25 subur | Rpp25 | 0 | 0 | 0 | 0 | **0** | 1 | 0 | **1** | **1** |
| 133204_at | 1455425_a | Expressed sequence BE | BB001228 | 0 | 0 | 0 | 0 | **0** | 1 | 1 | **2** | **2** |
| 133365_at | 1436568_a | junction adhesion molec | Jam2 | 0 | 1 | 0 | 0 | **1** | 1 | 0 | **1** | **2** |

| | | | | | | | | | | | | |
|---|---|---|---|---|---|---|---|---|---|---|---|---|
| 133819_at | 419418_a_ | microrchidia | Morc | 0 | 0 | 0 | 0 | **0** | 1 | 0 | **1** | **1** |
| 160253_at | 1423754_a | interferon induced transn | Ifitm3 | 0 | 0 | 0 | 0 | **0** | 0 | 0 | **0** | **0** |
| 160370_at | 1416552_a | developmental pluripoter | Dppa5 | 0 | 0 | 0 | 0 | **0** | 1 | 1 | **2** | **2** |
| 160684_at | 1423786_a | RIKEN cDNA 8430410A | 430410A17Ri | 0 | 0 | 0 | 0 | **0** | 1 | 1 | **2** | **2** |
| 160828_at | 1426858_a | inhibin beta-B | Inhbb | 0 | 0 | 0 | 0 | **0** | 1 | 1 | **2** | **2** |
| 161042_at | 1427912_a | carbonyl reductase 3 | Cbr3 | 0 | 0 | 0 | 0 | **0** | 0 | 1 | **1** | **1** |
| 161106_r_a | 1443892_a | --- | --- | 0 | 0 | 0 | 0 | **0** | 1 | 1 | **2** | **2** |
| 162522_f_a | 437015_x_ | phospholipase A2, grour | Pla2g1b | 0 | 1 | 0 | 1 | **2** | 1 | 0 | **1** | **3** |
| 163005_s_a | 1429366_a | leucine rich repeat conta | Lrrc34 | 0 | 0 | 0 | 0 | **0** | 1 | 0 | **1** | **1** |
| 163288_at | 1460471_a | RIKEN cDNA 2410146L | 410146L05Ri | 0 | 0 | 0 | 0 | **0** | 1 | 0 | **1** | **1** |
| 163489_at | 418488_s_ | receptor-interacting serir | Ripk4 | 0 | 0 | 0 | 0 | **0** | 1 | 0 | **1** | **1** |
| 163715_at | 1429399_a | ring finger protein 125 | Rnf125 | 0 | 0 | 0 | 0 | **0** | 1 | 0 | **1** | **1** |
| 165699_r_a | 453299_a_ | purine-nucleoside phosp | Pnp | 0 | 0 | 0 | 0 | **0** | 1 | 0 | **1** | **1** |
| 166142_r_a | 1436799_a | RIKEN cDNA D230005D | 230005D02R | 0 | 0 | 0 | 0 | **0** | 1 | 0 | **1** | **1** |
| 167088_r_a | 1456242_a | LOC433110 | LOC433110 | 0 | 0 | 0 | 0 | **0** | 1 | 0 | **1** | **1** |
| 168508_at | 1436926_a | estrogen related recepto | Esrrb | 0 | 0 | 0 | 0 | **0** | 1 | 0 | **1** | **1** |
| 92275_at | 1418147_a | transcription factor AP-2 | Tcfap2c | 0 | 0 | 0 | 1 | **1** | 1 | 1 | **2** | **3** |
| 92476_at | 1449288_a | growth differentiation fac | Gdf3 | 1 | 0 | 1 | 0 | **2** | 1 | 1 | **2** | **4** |
| 92550_at | 1417156_a | keratin complex 1, acidic | Krt1-19 | 0 | 1 | 1 | 1 | **3** | 0 | 0 | **0** | **3** |
| 92770_at | 421375_a_ | S100 calcium binding pro | S100a6 | 0 | 0 | 0 | 0 | **0** | 1 | 0 | **1** | **1** |
| 93063_at | 427442_a_ | amyloid beta (A4) precur | App | 0 | 0 | 1 | 1 | **2** | 1 | 0 | **1** | **3** |
| 93104_at | 426083_a_ | B-cell translocation gene | Btg1 | 0 | 0 | 0 | 0 | **0** | 1 | 0 | **1** | **1** |
| 93141_at | 1417760_a | nuclear receptor subfam | Nr0b1 | 0 | 0 | 0 | 0 | **0** | 1 | 1 | **2** | **2** |
| 93271_s_at | 450186_s_ | GNAS (guanine nucleoti | Gnas | 0 | 1 | 0 | 1 | **2** | 0 | 0 | **0** | **2** |
| 93296_at | 1422458_a | T-cell lymphoma breakpo | Tcl1 | 0 | 0 | 0 | 0 | **0** | 1 | 1 | **2** | **2** |
| 93483_at | 1449455_a | hemopoietic cell kinase | Hck | 0 | 0 | 0 | 0 | **0** | 1 | 0 | **1** | **1** |
| 93864_s_at | 421624_a_ | enabled homolog (Drosc | Enah | 0 | 0 | 0 | 1 | **1** | 0 | 0 | **0** | **1** |
| 94200_at | 1420337_a | gastrulation brain homec | Gbx2 | 0 | 0 | 0 | 0 | **0** | 1 | 1 | **2** | **2** |
| 94270_at | 1448169_a | keratin complex 1, acidic | Krt1-18 | 1 | 1 | 1 | 0 | **3** | 0 | 0 | **0** | **3** |
| 94354_at | 1421840_a | ATP-binding cassette, su | Abca1 | 0 | 0 | 0 | 0 | **0** | 1 | 0 | **1** | **1** |
| 94745_f_at | 1427479_a | similar to Eukaryotic tran | MGC107533 | 0 | 0 | 0 | 1 | **1** | 1 | 1 | **2** | **3** |
| 95033_at | 1426810_a | jumonji domain containin | Jmjd1a | 0 | 0 | 1 | 1 | **2** | 1 | 1 | **2** | **4** |
| 95518_at | 1424683_a | RIKEN cDNA 1810015C | 810015C04R | 0 | 0 | 0 | 0 | **0** | 1 | 0 | **1** | **1** |
| 95531_at | 1454890_a | angiomotin | Amot | 0 | 0 | 0 | 1 | **1** | 1 | 1 | **2** | **3** |
| 95584_at | 453223_s_ | developmental pluripoter | Dppa2 | 0 | 0 | 0 | 0 | **0** | 1 | 1 | **2** | **2** |
| 96042_at | 448610_a_ | superoxide dismutase 2, | Sod2 | 0 | 0 | 1 | 0 | **1** | 1 | 1 | **2** | **3** |
| 96109_at | 1448890_a | Kruppel-like factor 2 (lun | Klf2 | 0 | 0 | 0 | 0 | **0** | 0 | 1 | **1** | **1** |
| 96162_at | 1427238_a | F-box protein 15 | Fbxo15 | 0 | 0 | 0 | 0 | **0** | 1 | 1 | **2** | **2** |
| 96203_at | 1424713_a | calmodulin-like 4 | Calml4 | 0 | 0 | 0 | 0 | **0** | 1 | 0 | **1** | **1** |

| | | | | | | | | | | | | |
|---|---|---|---|---|---|---|---|---|---|---|---|---|
| 96752_at | 1424067_a | intercellular adhesion mo | Icam1 | 0 | 0 | 0 | 0 | **0** | 1 | 0 | **1** | **1** |
| 96841_at | 1451069_a | proviral integration site 3 | Pim3 | 0 | 0 | 0 | 0 | **0** | 1 | 0 | **1** | **1** |
| 96900_at | 433720_s_ | Nur77 downstream gene | MGI:2143558 | 0 | 0 | 0 | 0 | **0** | 1 | 0 | **1** | **1** |
| 97083_at | 1441023_a | eukaryotic translation ini | Eif2s2 | 0 | 0 | 0 | 1 | **1** | 1 | 0 | **1** | **2** |
| 97283_at | 1424295_a | developmental pluripoten | Dppa3 | 0 | 0 | 0 | 0 | **0** | 0 | 1 | **1** | **1** |
| 97317_at | 1415894_a | ectonucleotide pyrophos | Enpp2 | 0 | 0 | 0 | 0 | **0** | 0 | 0 | **0** | **0** |
| 97426_at | 1416529_a | epithelial membrane pro | Emp1 | 0 | 0 | 0 | 0 | **0** | 1 | 1 | **2** | **2** |
| 97442_at | 1416832_a | solute carrier family 39 ( | Slc39a8 | 0 | 0 | 0 | 0 | **0** | 1 | 0 | **1** | **1** |
| 97519_at | 1449254_a | secreted phosphoprotein | Spp1 | 0 | 0 | 0 | 0 | **0** | 1 | 1 | **2** | **2** |
| 97520_s_at | 423506_a_ | neuronatin | Nnat | 0 | 1 | 0 | 1 | **2** | 0 | 0 | **0** | **2** |
| 97890_at | 1416041_a | serum/glucocorticoid reg | Sgk | 0 | 0 | 0 | 0 | **0** | 1 | 1 | **2** | **2** |
| 98414_at | 1418362_a | zinc finger protein 42 | Zfp42 | 1 | 0 | 0 | 0 | **1** | 1 | 1 | **2** | **3** |
| 99561_f_at | 1448393_a | claudin 7 | Cldn7 | 0 | 0 | 0 | 1 | **1** | 1 | 0 | **1** | **2** |
| 99622_at | 1417394_a | Kruppel-like factor 4 (gut | Klf4 | 0 | 0 | 0 | 0 | **0** | 1 | 0 | **1** | **1** |
| 99956_at | 452514_a_ | kit oncogene | Kit | 0 | 0 | 0 | 1 | **1** | 1 | 0 | **1** | **2** |
