# Supplementary material for: Meta-Analysis of Differentiating Mouse Embryonic Stem Cell Gene Expression Kinetics Reveals Early Change of a Small Gene Set
Source: PLoS Comput Biol. 2006 Nov 24;2(11):e158. doi: 10.1371/journal.pcbi.0020158 (PMC1664699; doi:10.1371/journal.pcbi.0020158)
Supplement: Table S7 — (365 KB PDF) [file pcbi.0020158.st007.pdf]

| MG_U74v2 | MOE430 |
|---|---|
| 160684_at | 1423786_at |
| 93141_at | 1417760_at |
| 94200_at | 1420337_at |
| 99622_at | 1417394_at |
| 115804_at | 1438237_at |
| 133819_at | 1419418_a_at |
| 98414_at | 1418362_at |
| 108712_at | 1434917_at |
| 93483_at | 1449455_at |
| 95033_at | 1426810_at |
| 133204_at | 1455425_at |
| 163715_at | 1429399_at |
| 133365_at | 1436568_at |
| 160712_r_at | 1426980_s_at |
| 104544_at | 1423327_at |
| 97283_at | 1424295_at |
| 117246_at | 1448845_at |
| 96162_at | 1427238_at |
| 94817_at | 1450843_a_at |
| 108010_at | 1418318_at |
| 160828_at | 1426858_at |
| 160469_at | 1421811_at |
| 100030_at | 1448562_at |
| 103342_at | 1448653_at |
| 101009_at | 1423691_x_at |
| 94270_at | 1448169_at |
| 93088_at | 1449289_a_at |
| 165755_r_at | 1428604_at |
| 103437_at | 1450929_at |
| 108505_at | 1434025_at |
| 103048_at | 1417155_at |
| 94341_at | 1422698_s_at |
| 96042_at | 1448610_a_at |
| 112386_at | 1424769_s_at |
| 94097_at | 1449058_at |
| 92476_at | 1449288_at |
| 102665_at | 1418756_at |
| 116872_at | 1435437_at |
| 114665_at | 1435436_at |
| 163115_at | 1448551_a_at |
| 92770_at | 1421375_a_at |
| 95346_at | 1426137_at |
| 97154_f_at | 1439065_x_at |
| 108279_at | 1455300_at |
| 109059_at | 1424768_at |
| 111970_at | 1460711_at |
| 114667_at | 1452063_at |
| 111011_at | 1436574_at |
| 93864_s_at | 1421624_a_at |
| 167088_r_at | 1456242_at |
| 165826_i_at | 1456511_x_at |
| 115564_at | 1421882_a_at |
| 95584_at | 1453223_s_at |
| 106957_f_at | 1456179_at |
| 161004_at | 1436419_a_at |

| | |
|---|---|
| 162721_at | 1424163_at |
| 92881_at | 1448371_at |
| 97977_at | 1454974_at |
| 92550_at | 1417156_at |
| 112160_at | 1420930_s_at |
| 167764_f_at | 1436905_x_at |
| 111218_at | 1435084_at |
| 102809_s_at | 1425396_a_at |
| 115058_at | 1434362_at |
| 96203_at | 1424713_at |
| 97846_at | 1460429_at |
| 96189_at | 1418820_s_at |
| 110847_at | 1455186_a_at |
| 115059_at | 1418640_at |
| 110153_at | 1450764_at |
| 160979_at | 1434705_at |
| 102344_s_at | 1424531_a_at |
| 108736_at | 1417302_at |
| 102910_at | 1419759_at |
| 113998_at | 1453628_s_at |
| 92554_at | 1422887_a_at |
| 95610_at | 1428092_at |
| 93296_at | 1422458_at |
| 96109_at | 1448890_at |
| 103728_at | 1456521_at |
| 103761_at | 1418091_at |
| 102332_at | 1448370_at |
| 97890_at | 1416041_at |
| 108784_at | 1455604_at |
| 92275_at | 1418147_at |
| 115445_at | 1435374_at |
| 168508_at | 1436926_at |
| 108097_at | 1450626_at |
| 160370_at | 1416552_at |
| 163005_s_at | 1429366_at |
| 103389_at | 1423523_at |
| 160901_at | 1423100_at |
| 163494_at | 1435040_at |
| 100009_r_at | 1416967_at |
| 163288_at | 1460471_at |
| 113673_at | 1423508_at |
| 114697_at | 1434376_at |
| 109403_at | 1423760_at |
| 103737_at | 1418753_at |
| 130738_at | 1425312_s_at |
| 160253_at | 1423754_at |
| 133732_at | 1437588_at |
| 104030_at | 1428853_at |
| 161042_at | 1427912_at |
| 160683_at | 1419657_a_at |
| 102069_at | 1418514_at |
| 94384_at | 1419647_a_at |
| 96900_at | 1433720_s_at |
| 100301_at | 1422986_at |
| 161072_at | 1429388_at |
| 160068_at | 1417719_at |

| | |
|---|---|
| 116304_at | 1434070_at |
| 95507_at | 1416052_at |
| 97520_s_at | 1423506_a_at |
| 96561_at | 1434836_at |
| 110128_at | 1429897_a_at |
| 103440_at | 1450664_at |
| 102792_at | 1425753_a_at |
| 109137_at | 1418070_at |
| 102345_at | 1417638_at |
| 103600_at | 1460223_a_at |
| 109709_at | 1433776_at |
| 99669_at | 1419573_a_at |
| 100569_at | 1419091_a_at |
| 95733_at | 1451782_a_at |
| 100023_at | 1417656_at |
| 103720_at | 1425565_at |
| 112648_f_at | 1449115_at |
| 93083_at | 1425567_a_at |
| 166732_f_at | 1418994_at |
| 92581_at | 1415984_at |
| 92209_at | 1416504_at |
| 95758_at | 1415822_at |
| 107613_at | 1429491_s_at |
| 106577_at | 1428288_at |
| 113141_at | 1453285_at |
| 131411_at | 1437179_at |
| 100475_at | 1425974_a_at |
| 114937_at | 1435545_at |
| 160204_at | 1428706_at |
| 98112_r_at | 1450860_at |
| 95286_at | 1418626_a_at |
| 110177_at | 1421852_at |
| 103308_at | 1434767_at |
| 162656_i_at | 1448269_a_at |
| 104070_at | 1434037_s_at |
| 113671_at | 1436291_a_at |
| 104598_at | 1448830_at |
| 99033_at | 1426733_at |
| 100395_at | 1459211_at |
| 160507_at | 1428078_at |
| 112406_at | 1454976_at |
| 134566_at | 1436227_at |
| 97406_at | 1434757_at |
| 115197_at | 1427240_at |
| 106917_at | 1435669_at |
| 163304_at | 1422697_s_at |
| 165691_at | 1424556_at |
| 109135_at | 1427143_at |
| 92596_at | 1452047_at |
| 165467_r_at | 1451123_at |
| 102044_at | 1448594_at |
| 107310_at | 1417027_at |
| 96557_at | 1422655_at |
| 112955_at | 1435110_at |
| 163999_at | 1428951_at |
| 129304_at | 1450489_at |

| | |
|---|---|
| 110619_at | 1454946_at |
| 108433_at | 1419330_a_at |
| 163048_at | 1428304_at |
| 104589_at | 1419273_at |
| 162858_at | 1452654_at |
| 163981_at | 1417028_a_at |
| 107614_at | 1442939_at |
| 93528_s_at | 1428289_at |
| 133854_at | 1442015_at |
| 131216_f_at | 1433712_at |
| 93143_at | 1452609_at |
| 115086_at | 1455507_s_at |
| 113969_at | 1433647_s_at |
| 160367_at | 1428319_at |
| 98330_at | 1423424_at |
| 111730_at | 1434681_at |
| 92317_at | 1421881_a_at |
| 108417_at | 1422965_at |
| 103506_f_at | 1426911_at |
| 100974_at | 1449511_a_at |
| 96134_at | 1430128_a_at |
| 166430_i_at | 1457434_s_at |
| 103047_at | 1420711_a_at |
| 160206_at | 1452226_at |
| 93316_at | 1460192_at |
| 96738_at | 1416094_at |
| 99475_at | 1449109_at |
| 163778_at | 1437552_at |
| 104092_at | 1424056_at |
| 102962_at | 1449898_at |
| 109738_at | 1429458_at |
| 112831_at | 1424770_at |
| 113132_at | 1417661_at |
| 106302_at | 1434179_at |
| 109686_at | 1417619_at |
| 93028_at | 1448194_a_at |
| 99561_f_at | 1448393_at |
| 95706_at | 1426808_at |
| 94214_at | 1416023_at |
| 101560_at | 1415856_at |
| 109069_at | 1417061_at |
| 97444_at | 1422476_at |
| 104139_at | 1452094_at |
| 97937_at | 1451021_a_at |
| 110429_at | 1455333_at |
| 93104_at | 1426083_a_at |
| 93089_at | 1450934_at |
| 114845_s_at | 1421113_at |
| 94745_f_at | 1427479_at |
| 114048_at | 1426438_at |
| 99535_at | 1425837_a_at |
| 163489_at | 1418488_s_at |
| 96156_at | 1436506_a_at |
| 103653_at | 1449590_a_at |
| 100073_at | 1418386_at |
| 92292_at | 1421924_at |

| | |
|---|---|
| 167965_f_at | 1438157_s_at |
| 96426_at | 1415906_at |
| 94749_f_at | 1425220_x_at |
| 97452_at | 1424572_a_at |
| 104580_at | 1416675_s_at |
| 102335_at | 1448690_at |
| 93750_at | 1415812_at |
| 97994_at | 1433471_at |
| 97169_f_at | 1425427_at |
| 97519_at | 1449254_at |
| 108969_at | 1452287_at |
| 101554_at | 1448306_at |
| 93354_at | 1417561_at |
| 104149_at | 1449731_s_at |
| 96771_at | 1434606_at |
| 100024_at | 1422629_s_at |
| 160968_at | 1434528_at |
| 113913_at | 1434822_at |
| 93200_f_at | 1422956_at |
| 96120_at | 1448234_at |
| 116938_at | 1436584_at |
| 99624_at | 1427108_at |
| 100680_at | 1420773_at |
| 96840_at | 1423187_at |
| 98730_at | 1421523_at |
| 133385_at | 1436619_at |
| 112680_at | 1425940_a_at |
| 98579_at | 1417065_at |
| 138792_at | 1438750_at |
| 96295_at | 1451064_a_at |
| 100706_f_at | 1434353_at |
| 163646_at | 1419288_at |
| 106607_at | 1433754_at |
| 116435_at | 1418076_at |
| 93738_at | 1426599_a_at |
| 102239_at | 1418133_at |
| 94712_at | 1419417_at |
| 107574_at | 1429234_s_at |
| 115520_at | 1443858_at |
| 93326_at | 1448737_at |
| 94433_at | 1426722_at |
| 97820_at | 1417177_at |
| 96744_at | 1448445_at |
| 100559_at | 1423925_at |
| 103377_at | 1452320_at |
| 94897_at | 1451695_a_at |
| 115486_at | 1455029_at |
| 102259_at | 1420816_at |
| 93058_at | 1424343_a_at |
| 94986_at | 1417428_at |
| 93246_at | 1418024_at |
| 97491_at | 1427129_a_at |
| 93754_at | 1448491_at |
| 113284_at | 1434111_at |
| 97995_at | 1450461_at |
| 113280_at | 1436194_at |

| | |
|---|---|
| 97918_at | 1427243_at |
| 107062_at | 1426382_at |
| 94359_at | 1417965_at |
| 94952_at | 1437103_at |
| 160138_at | 1450376_at |
| 92796_at | 1423611_at |
| 115770_at | 1455172_at |
| 93836_at | 1422470_at |
| 113551_at | 1433794_at |
| 103780_at | 1421019_at |
| 104744_at | 1424293_s_at |
| 95655_at | 1452203_at |
| 162785_at | 1454671_at |
| 95883_at | 1452180_at |
| 111359_at | 1425020_at |
| 163017_i_at | 1454136_a_at |
| 92589_at | 1415673_at |
| 163229_at | 1429556_at |
| 166820_at | 1433583_at |
| 107626_at | 1418762_at |
| 93349_at | 1448433_a_at |
| 164028_at | 1449052_a_at |
| 99109_at | 1416442_at |
| 117046_at | 1454896_at |
| 113199_at | 1417829_a_at |
| 141175_i_at | 1454011_a_at |
| 96152_at | 1418022_at |
| 98042_at | 1426555_at |
| 97279_at | 1423780_at |
| 100120_at | 1416808_at |
| 163774_at | 1416568_a_at |
| 111767_at | 1420628_at |
| 164110_at | 1436883_at |
| 98424_at | 1452127_a_at |
| 93455_s_at | 1422912_at |
| 93324_at | 1450644_at |
| 117271_at | 1429003_at |
| 108090_at | 1426409_at |
| 104135_at | 1450706_a_at |
| 102796_at | 1423522_at |
| 106672_at | 1434000_at |
| 111982_at | 1435497_at |
| 160273_at | 1437626_at |
| 102401_at | 1448436_a_at |
| 95661_at | 1416066_at |
| 138060_at | 1435656_at |
| 100472_at | 1424800_at |
| 97426_at | 1416529_at |
| 99956_at | 1452514_a_at |
| 94174_at | 1420929_at |
| 94561_at | 1422499_at |
| 97740_at | 1418401_a_at |
| 95944_at | 1424398_at |
| 98478_at | 1416488_at |
| 94192_at | 1420342_at |
| 103930_at | 1446957_s_at |

| | |
|---|---|
| 113495_at | 1437003_at |
| 100374_at | 1423582_at |
| 96812_at | 1427049_s_at |
| 117265_at | 1436202_at |
| 163173_at | 1428142_at |
| 162206_f_at | 1456212_x_at |
| 163828_at | 1436925_at |
| 92338_f_at | 1424872_at |
| 113333_at | 1428727_at |
| 160614_at | 1422553_at |
| 102012_at | 1418895_at |
| 112405_at | 1449005_at |
| 98059_s_at | 1421654_a_at |
| 98077_at | 1422884_at |
| 104635_r_at | 1449094_at |
| 102048_at | 1420992_at |
| 109730_at | 1434283_at |
| 109339_at | 1427133_s_at |
| 106058_at | 1460190_at |
| 92607_at | 1423294_at |
| 115276_at | 1442514_a_at |
| 99959_at | 1450387_s_at |
| 108420_at | 1419033_at |
| 109299_f_at | 1459089_at |
| 93078_at | 1417185_at |
| 103277_s_at | 1425585_at |
| 109372_at | 1434613_at |
| 101926_at | 1417216_at |
| 160702_at | 1454681_at |
| 93540_at | 1448333_at |
| 96662_at | 1429514_at |
| 93234_at | 1418417_at |
| 165713_at | 1436932_at |
| 113651_at | 1429001_at |
| 110564_at | 1456661_at |
| 104701_at | 1418025_at |
| 165640_at | 1452834_at |
| 162600_at | 1426397_at |
| 109167_at | 1424008_a_at |
| 160137_at | 1450026_a_at |
| 111907_at | 1449044_at |
| 109561_at | 1434058_at |
| 114309_at | 1452857_at |
| 93372_at | 1450407_a_at |
| 112767_s_at | 1452222_at |
| 105710_at | 1427271_at |
| 108957_at | 1455878_at |
| 102922_at | 1435066_at |
| 115551_at | 1428694_at |
| 102920_at | 1452009_at |
| 100012_at | 1426025_s_at |
| 134047_at | 1435397_at |
| 106089_at | 1419656_at |
| 106568_at | 1452224_at |
| 112699_at | 1436173_at |
| 100032_at | 1418180_at |

| | |
|---|---|
| 99121_at | 1452247_at |
| 95954_at | 1444292_at |
| 110410_at | 1451740_at |
| 114967_at | 1456898_at |
| 107103_at | 1416958_at |
| 101902_at | 1418114_at |
| 140860_at | 1419277_at |
| 92901_at | 1450180_a_at |
| 96296_at | 1434970_a_at |
| 93100_at | 1416454_s_at |
| 115030_at | 1435363_at |
| 100015_at | 1449090_a_at |
| 92191_at | 1460519_a_at |
| 107467_at | 1424801_at |
| 94238_at | 1431057_a_at |
| 97411_at | 1419513_a_at |
| 96841_at | 1451069_at |
| 163321_i_at | 1427235_at |
| 112378_at | 1460337_at |
| 161070_at | 1434403_at |
| 98946_at | 1425241_a_at |
| 113043_at | 1452242_at |
| 139393_at | 1438516_at |
| 160739_at | 1436746_at |
| 103402_at | 1452664_a_at |
| 117147_at | 1427917_s_at |
| 105620_at | 1438781_at |
| 163100_at | 1448935_at |
| 103035_at | 1416016_at |
| 99607_at | 1423149_at |
| 112740_at | 1434602_at |
| 96049_at | 1416405_at |
| 160335_at | 1418627_at |
| 95077_at | 1419553_a_at |
| 99001_at | 1449515_at |
| 107406_at | 1455246_at |
| 113198_at | 1436040_at |
| 162671_f_at | 1437019_at |
| 104610_at | 1426381_at |
| 94354_at | 1421840_at |
| 113885_at | 1439106_at |
| 103989_at | 1432057_a_at |
| 96940_at | 1448519_at |
| 104106_at | 1426559_at |
| 94147_at | 1419149_at |
| 96342_at | 1419287_at |
| 102425_at | 1422751_at |
| 105516_f_at | 1458667_at |
| 112356_at | 1451370_at |
| 103025_at | 1416380_at |
| 97960_at | 1456043_at |
| 101089_at | 1449178_at |
| 93507_at | 1420924_at |
| 132227_at | 1439665_at |
| 101389_at | 1460235_at |
| 113290_at | 1429431_at |

| | |
|---|---|
| 114143_at | 1435657_at |
| 92555_at | 1416872_at |
| 103005_s_at | 1452483_a_at |
| 94395_at | 1433640_at |
| 162901_i_at | 1454609_x_at |
| 99532_at | 1423176_at |
| 97798_at | 1426655_a_at |
| 160173_at | 1436713_s_at |
| 165868_f_at | 1435655_at |
| 160316_at | 1434390_at |
| 160976_at | 1454979_at |
| 95462_at | 1423456_at |
| 102252_at | 1421950_at |
| 96752_at | 1424067_at |
| 108530_at | 1426752_at |
| 92231_at | 1449167_at |
| 160365_at | 1448819_at |
| 110414_at | 1449530_at |
| 163611_f_at | 1426887_at |
| 109755_at | 1435017_at |
| 163790_at | 1437108_at |
| 101130_at | 1450857_a_at |
| 98988_at | 1417483_at |
| 163348_at | 1429298_at |
| 98331_at | 1427883_a_at |
| 93467_at | 1436030_at |
| 93026_at | 1415897_a_at |
| 98001_at | 1421164_a_at |
| 114724_at | 1434414_at |
| 103061_at | 1416561_at |
| 92232_at | 1416576_at |
| 97083_at | 1441023_at |
| 103538_at | 1448029_at |
| 101993_at | 1416342_at |
| 96016_at | 1436349_at |
| 100928_at | 1423407_a_at |
| 163404_at | 1424410_at |
| 103990_at | 1422134_at |
| 97317_at | 1415894_at |
| 96680_at | 1417191_at |
| 161703_f_at | 1448213_at |
| 99076_at | 1416959_at |
| 103697_at | 1454984_at |
| 160095_at | 1416121_at |
| 102649_s_at | 1420603_s_at |
| 98500_at | 1425145_at |
| 94727_f_at | 1420729_at |
| 101368_at | 1423429_at |
| 98514_at | 1451791_at |
| 94305_at | 1423669_at |
| 102292_at | 1449519_at |
| 160545_at | 1415907_at |
| 94821_at | 1420886_a_at |
| 98501_at | 1422317_a_at |
| 104647_at | 1417262_at |
| 93574_at | 1416168_at |

| | |
|---|---|
| 98577_f_at | 1453629_at |
| 99051_at | 1424542_at |
| 98833_at | 1418945_at |
| 95562_at | 1429483_at |
| 93534_at | 1449368_at |
| 100484_at | 1417256_at |
| 92567_at | 1422437_at |
| 160280_at | 1449145_a_at |
| 161817_f_at | 1421474_a_at |
| 92593_at | 1423606_at |
| 103258_at | 1449328_at |
| 107895_at | 1439050_at |
| 93866_s_at | 1448416_at |
| 102764_at | 1460226_at |
| 94761_at | 1421228_at |
| 94345_at | 1452843_at |
| 101160_at | 1449984_at |
| 104225_at | 1437074_at |
| 97789_at | 1422212_at |
| 104388_at | 1417936_at |
| 160108_at | 1419665_a_at |
| 110124_at | 1436562_at |
| 117217_at | 1418545_at |
| 100611_at | 1423547_at |
| 160373_i_at | 1416778_at |
| 160726_at | 1417073_a_at |
| 97456_at | 1428082_at |
| 93833_s_at | 1418072_at |
| 97645_f_at | 1447377_at |
| 106557_at | 1424086_at |
| 93458_at | 1419959_s_at |
| 98855_r_at | 1435169_at |
| 165897_at | 1441836_x_at |
| 95964_at | 1448017_at |
| 102710_at | 1425269_at |
| 97779_at | 1449965_at |
| 101708_at | 1421618_at |
| 103899_at | 1456388_at |
| 102712_at | 1450826_a_at |
| 99052_at | 1418926_at |
| 117136_at | 1429883_at |
| 160832_at | 1421821_at |
| 102932_at | 1449706_s_at |
| 96038_at | 1422603_at |
| 137068_at | 1438245_at |
| 104158_at | 1429002_at |
| 93430_at | 1417625_s_at |
| 165694_at | 1426430_at |
| 166849_at | 1435061_at |
| 104601_at | 1448529_at |
| 160949_at | 1448725_at |
| 167384_s_at | 1443832_s_at |
| 133052_at | 1456832_at |
| 135888_at | 1437303_at |
| 97991_at | 1451979_at |
| 99384_at | 1423006_at |

| | |
|---|---|
| 114748_at | 1426915_at |
| 95564_at | 1460399_at |
| 99057_at | 1423135_at |
| 94939_at | 1448617_at |
| 96584_f_at | 1451852_at |
| 94258_at | 1426454_at |
| 98108_at | 1448326_a_at |
| 92558_at | 1448162_at |
| 96920_at | 1416749_at |
| 103729_at | 1418153_at |
| 163290_at | 1452050_at |
| 99582_at | 1416579_a_at |
| 101464_at | 1460227_at |
| 160575_at | 1454641_at |
| 94224_s_at | 1452348_s_at |
| 103306_at | 1451594_s_at |
| 111939_at | 1435284_at |
| 96288_at | 1424058_at |
| 112994_at | 1425357_a_at |
| 102255_at | 1418674_at |
| 137561_at | 1429169_at |
| 94378_at | 1426037_a_at |
| 114505_at | 1427358_a_at |
| 94460_at | 1416252_at |
| 112797_at | 1450760_a_at |
| 93538_at | 1448706_at |
| 139207_s_at | 1425213_at |
| 116239_at | 1439535_at |
| 108079_at | 1452785_at |
| 103789_at | 1424922_a_at |
| 115022_at | 1437870_at |
| 163456_at | 1437862_at |
| 92931_at | 1419204_at |
| 93253_at | 1419568_at |
| 163709_i_at | 1421000_at |
| 103503_at | 1426926_at |
| 93785_at | 1450995_at |
| 114288_at | 1451177_at |
| 100155_at | 1415797_at |
| 101458_at | 1416773_at |
| 94044_at | 1420021_s_at |
| 114955_at | 1437923_at |
| 100876_at | 1454674_at |
| 160620_at | 1452239_at |
| 106872_at | 1447946_at |
| 96167_at | 1422452_at |
| 105958_at | 1433992_at |
| 105005_at | 1455345_at |
| 162573_at | 1452825_at |
| 93775_at | 1452956_a_at |
| 95016_at | 1418084_at |
| 104751_at | 1422530_at |
| 93339_at | 1460542_s_at |
| 99920_at | 1418380_at |
| 135368_at | 1416173_at |
| 129147_r_at | 1420518_a_at |

| | |
|---|---|
| 93080_at | 1420632_a_at |
| 98508_s_at | 1422620_s_at |
| 160708_at | 1423025_a_at |
| 92845_at | 1428140_at |
| 98543_at | 1448591_at |
| 105089_at | 1441354_at |
| 92190_at | 1418605_at |
| 105298_at | 1455616_at |
| 111029_at | 1451458_at |
| 107530_at | 1417293_at |
| 92730_at | 1418350_at |
| 131021_at | 1448727_at |
| 105675_at | 1457409_at |
| 97736_at | 1442384_at |
| 110967_at | 1420098_s_at |
| 107130_at | 1454625_at |
| 96494_at | 1451793_at |
| 102418_at | 1417482_at |
| 166683_r_at | 1427167_at |
| 100514_at | 1422555_s_at |
| 160191_at | 1452120_at |
| 116537_at | 1445717_at |
| 160337_at | 1415977_at |
| 98440_at | 1417777_at |
| 162820_i_at | 1428446_at |
| 94948_at | 1449041_a_at |
| 167028_at | 1418773_at |
| 99011_at | 1417588_at |
| 113840_at | 1460564_at |
| 167043_s_at | 1420329_at |
| 100348_at | 1456319_at |
| 103554_at | 1418402_at |
| 97507_at | 1448380_at |
| 166726_at | 1453012_at |
| 160571_at | 1422433_s_at |
| 111873_at | 1426963_at |
| 103647_at | 1416205_at |
| 162969_at | 1433474_at |
| 105250_at | 1453017_at |
| 115333_at | 1438214_at |
| 139998_at | 1456662_at |
| 103399_at | 1426241_a_at |
| 101135_at | 1418688_at |
| 104337_f_at | 1449799_s_at |
| 109418_at | 1419577_at |
| 130442_at | 1424306_at |
| 108822_at | 1448303_at |
| 110393_at | 1429418_at |
| 101110_at | 1424131_at |
| 92513_at | 1450396_at |
| 129321_at | 1435831_at |
| 160296_at | 1421847_at |
| 136164_at | 1431227_at |
| 96020_at | 1417063_at |
| 110258_at | 1435763_at |
| 112289_g_at | 1426573_at |

| | |
|---|---|
| 94930_at | 1450663_at |
| 93093_at | 1416880_at |
| 169259_f_at | 1430308_at |
| 164246_at | 1419403_at |
| 93798_at | 1423653_at |
| 100954_at | 1426923_at |
| 107582_at | 1455070_at |
| 164158_at | 1442109_at |
| 135314_at | 1456398_at |
| 113154_at | 1418480_at |
| 111578_at | 1420514_at |
| 111950_at | 1435217_at |
| 162607_i_at | 1452219_at |
| 98349_at | 1421239_at |
| 95133_at | 1451095_at |
| 163962_at | 1431007_at |
| 162384_f_at | 1458699_at |
| 166237_f_at | 1429037_at |
| 109554_at | 1435603_at |
| 163801_at | 1435774_at |
| 104846_at | 1438222_at |
| 109416_at | 1449187_at |
| 97874_at | 1424628_a_at |
| 104526_at | 1422566_at |
| 114810_at | 1419599_s_at |
| 162709_at | 1427879_at |
| 96725_at | 1415746_at |
| 114238_at | 1442018_at |
| 165452_at | 1425891_a_at |
| 162128_i_at | 1430332_a_at |
| 111166_at | 1439948_at |
| 112349_at | 1448967_at |
| 164134_at | 1436155_at |
| 160887_at | 1418102_at |
| 108488_at | 1418467_at |
| 114694_at | 1429680_at |
| 94724_at | 1420450_at |
| 93353_at | 1423607_at |
| 163014_at | 1415936_at |
| 102030_at | 1420948_s_at |
| 132039_f_at | 1420406_at |
| 111704_at | 1446956_at |
| 96272_at | 1420842_at |
| 97334_at | 1452021_a_at |
| 99452_at | 1451255_at |
| 114872_at | 1427969_s_at |
| 99985_at | 1421529_a_at |
| 103501_at | 1449934_at |
| 166095_f_at | 1438967_x_at |
| 104534_at | 1453283_at |
| 102652_at | 1422068_at |
| 161511_f_at | 1431591_s_at |
| 166680_at | 1454966_at |
| 129168_at | 1459666_at |
| 167055_f_at | 1456243_x_at |
| 104367_at | 1419252_at |

| | |
|---|---|
| 133726_at | 1438455_at |
| 102884_at | 1418110_a_at |
| 165617_at | 1437329_at |
| 100418_at | 1418451_at |
| 94476_at | 1430271_x_at |
| 108036_at | 1419278_at |
| 165460_at | 1455176_a_at |
| 96704_at | 1448612_at |
| 96188_at | 1425405_a_at |
| 102750_at | 1416182_at |
| 167812_at | 1443229_at |
| 96336_at | 1423569_at |
| 103994_at | 1426366_at |
| 104327_at | 1433784_at |
| 103056_at | 1454817_at |
| 92644_s_at | 1450194_a_at |
| 140436_at | 1435367_at |
| 166112_f_at | 1437602_at |
| 95665_at | 1453412_a_at |
| 94973_at | 1428320_at |
| 97165_r_at | 1458414_at |
| 135613_at | 1440268_at |
| 93691_s_at | 1425457_a_at |
| 97206_at | 1416627_at |
| 94715_at | 1422217_a_at |
| 117099_at | 1425528_at |
| 166976_at | 1422668_at |
| 137041_at | 1441011_at |
| 162973_at | 1454672_at |
| 135171_at | 1438530_at |
| 115741_at | 1453069_at |
| 103869_at | 1416623_at |
| 160696_at | 1416812_at |
| 101486_at | 1448632_at |
| 108014_at | 1452123_s_at |
| 99100_at | 1460700_at |
| 100046_at | 1419253_at |
| 168278_i_at | 1443495_at |
| 102308_at | 1449008_at |
| 93557_at | 1418325_at |
| 162909_at | 1434285_at |
| 97260_at | 1450685_at |
| 100920_at | 1450317_at |
| 111124_at | 1456145_at |
| 166423_r_at | 1455208_at |
| 100308_at | 1418440_at |
| 163230_at | 1418260_at |
| 93550_at | 1420731_a_at |
| 104376_at | 1415743_at |
| 104314_r_at | 1417211_a_at |
| 95568_at | 1454074_a_at |
| 114299_at | 1435524_at |
| 104286_at | 1448889_at |
| 114780_at | 1437494_at |
| 93630_at | 1425932_a_at |
| 93930_at | 1448207_at |

| | |
|---|---|
| 114749_at | 1434186_at |
| 104745_at | 1416793_at |
| 167188_at | 1447483_s_at |
| 165866_f_at | 1458326_at |
| 162593_at | 1437150_at |
| 100471_at | 1455288_at |
| 110269_at | 1452257_at |
| 108241_at | 1454967_at |
| 100929_at | 1455058_at |
| 114536_at | 1431018_at |
| 103970_at | 1423526_at |
| 103504_at | 1449815_a_at |
| 104348_at | 1426312_at |
| 98896_at | 1424028_at |
| 101979_at | 1453851_a_at |
| 103812_at | 1417852_x_at |
| 112373_at | 1451341_s_at |
| 93782_at | 1423654_a_at |
| 94055_at | 1421313_s_at |
| 162929_at | 1416638_at |
| 115112_at | 1454995_at |
| 160651_at | 1423323_at |
| 111597_at | 1440847_at |
| 103774_at | 1419867_a_at |
| 141119_at | 1439104_at |
| 97302_at | 1450084_s_at |
| 161106_r_at | 1443892_at |
| 112828_at | 1448688_at |
| 101046_at | 1450641_at |
| 100059_at | 1454268_a_at |
| 95518_at | 1424683_at |
| 95531_at | 1454890_at |
| 160158_at | 1450990_at |
| 98045_s_at | 1420498_a_at |
| 160705_at | 1449031_at |
| 101019_at | 1416382_at |
| 101990_at | 1416183_a_at |
| 103046_at | 1418094_s_at |
| 160547_s_at | 1415996_at |
| 111413_at | 1450947_at |
| 114302_at | 1417175_at |
| 97887_at | 1418069_at |
| 94834_at | 1418365_at |
| 95668_at | 1423281_at |
| 96047_at | 1426225_at |
| 163671_at | 1454866_s_at |
| 160548_at | 1415997_at |
| 165699_r_at | 1453299_a_at |
| 96094_at | 1419232_a_at |
| 95369_at | 1452141_a_at |
| 95000_g_at | 1452270_s_at |
| 93261_at | 1448883_at |
| 140322_at | 1434503_s_at |
| 95606_at | 1422768_at |
| 94999_at | 1426990_at |
| 163800_at | 1450079_at |

| | |
|---|---|
| 160918_at | 1448964_at |
| 160064_at | 1418436_at |
| 96747_at | 1449027_at |
| 93063_at | 1427442_a_at |
| 97288_at | 1431701_a_at |
| 163015_at | 1417920_at |
| 113649_at | 1447936_at |
| 104990_at | 1457314_at |
| 167827_at | 1417420_at |
| 101590_at | 1428094_at |
| 93002_r_at | 1450989_at |
| 169407_i_at | 1456590_x_at |
| 166142_r_at | 1436799_at |
| 98590_at | 1417654_at |
| 101963_at | 1451310_a_at |
| 97442_at | 1416832_at |
| 93290_at | 1416530_a_at |
| 94394_at | 1418448_at |
| 94840_at | 1449024_a_at |
| 94913_at | 1415697_at |
| 108048_at | 1454788_at |
| 97681_f_at | 1427474_s_at |
| 167673_f_at | 1448793_a_at |
| 96634_at | 1452716_at |
| 163020_at | 1433898_at |
| 101676_at | 1449106_at |
| 92857_at | 1448398_s_at |
| 100909_at | 1418320_at |
| 92440_at | 1418301_at |
| 101588_at | 1415802_at |
| 101383_at | 1419606_a_at |
| 106640_at | 1448271_a_at |
| 116890_at | 1436729_at |
| 164520_f_at | 1458347_s_at |
| 108051_at | 1417896_at |
| 162840_at | 1416389_a_at |
| 162011_f_at | 1449028_at |
| 162989_at | 1420626_at |
| 93118_at | 1433829_a_at |
| 168319_at | 1437110_at |
| 97925_at | 1417176_at |
| 166425_at | 1458351_s_at |
| 101093_at | 1452035_at |
| 100528_at | 1428791_at |
| 101106_at | 1423758_at |
| 102017_at | 1425498_at |
| 94330_at | 1424265_at |
| 103029_at | 1418840_at |
| 103034_at | 1416492_at |
| 96115_at | 1438001_x_at |
| 96643_at | 1424351_at |
| 160451_at | 1451208_at |
| 162611_i_at | 1424490_at |
| 95356_at | 1432466_a_at |
| 93271_s_at | 1450186_s_at |
| 93133_at | 1417022_at |

| | |
|---|---|
| 94085_at | 1417426_at |
| 104712_at | 1424942_a_at |
| 94855_at | 1448563_at |
| 103234_at | 1424847_at |
| 102959_at | 1425650_at |
| 113036_at | 1424921_at |
| 104448_at | 1419399_at |
| 97352_f_at | 1435275_at |
| 94831_at | 1417490_at |
| 93840_at | 1419095_a_at |
| 101039_at | 1424051_at |
| 110361_at | 1428440_at |
| 165116_f_at | 1436915_x_at |
| 108809_at | 1419380_at |
| 94985_at | 1426402_at |
| 160362_at | 1433576_at |
| 98589_at | 1448318_at |
| 93009_at | 1416411_at |
| 165058_f_at | 1449072_a_at |
| 93780_at | 1417316_at |
| 98550_at | 1426853_at |
| 100629_at | 1416842_at |
| 162604_at | 1451386_at |
| 112677_at | 1429413_at |
| 114356_at | 1436023_at |
| 160400_at | 1428090_at |
| 96183_at | 1421141_a_at |
| 104231_at | 1455491_at |
| 103075_at | 1417945_at |
| 115794_at | 1434398_at |
| 105871_at | 1428834_at |
| 93953_at | 1420388_at |
| 104463_at | 1452252_at |
| 100597_at | 1448429_at |
| 98124_at | 1431126_a_at |
| 96630_at | 1415938_at |
| 92633_at | 1417869_s_at |
| 92887_at | 1416457_at |
| 93011_at | 1416418_at |
| 113059_at | 1455189_at |
| 94231_at | 1417419_at |
| 160084_at | 1427364_a_at |
| 95021_at | 1433597_at |
| 113622_at | 1426801_at |
| 103835_f_at | 1448812_at |
| 94415_at | 1440831_at |
| 110233_at | 1419400_at |
| 99141_at | 1448241_at |
| 95693_at | 1450048_a_at |
| 98135_r_at | 1428277_at |
| 95428_at | 1426213_at |
| 113451_at | 1425035_s_at |
| 94493_at | 1451701_x_at |
| 107113_at | 1416751_a_at |
| 94346_at | 1454805_at |
| 95120_at | 1418643_at |

| | |
|---|---|
| 116136_at | 1454996_at |
| 108041_at | 1452771_s_at |
| 92608_at | 1425811_a_at |
| 92794_f_at | 1424110_a_at |
| 115547_at | 1428231_at |
| 104469_at | 1419309_at |
| 108749_at | 1435554_at |
| 135753_at | 1426519_at |
| 98627_at | 1454159_a_at |
| 92854_at | 1449256_a_at |
| 101410_at | 1418283_at |
| 104548_at | 1417837_at |
| 139228_at | 1436124_at |
| 165790_at | 1451416_a_at |
| 97305_at | 1448480_at |
| 160532_at | 1423721_at |
| 160344_at | 1416901_at |
| 97237_at | 1450735_at |
| 103845_at | 1433750_at |
| 92853_at | 1422943_a_at |
| 99948_at | 1451347_at |
| 160961_at | 1434261_at |
| 165215_f_at | 1437342_x_at |
| 164140_at | 1451365_at |
| 98937_at | 1452648_at |
| 92323_at | 1449283_a_at |
| 92185_at | 1436512_at |
| 112317_at | 1424294_at |
| 104702_at | 1437828_s_at |
| 94032_at | 1415727_at |
| 163852_at | 1428052_a_at |
| 113565_at | 1452666_a_at |
| 101861_at | 1420688_a_at |
| 93559_at | 1416135_at |
| 99147_at | 1425531_at |
| 97871_at | 1449324_at |
| 162522_f_at | 1437015_x_at |
| 160763_at | 1448871_at |
| 160921_at | 1416617_at |
| 99583_at | 1449575_a_at |
| 111917_at | 1434153_at |
| 97160_at | 1448392_at |
| 104816_at | 1427334_s_at |
| 100606_at | 1448233_at |
| 98111_at | 1425993_a_at |
| 160249_at | 1419493_a_at |
| 163448_at | 1433523_at |
| 102912_at | 1447522_s_at |
| 112870_at | 1430368_s_at |
| 103100_at | 1417818_at |
| 104633_at | 1423805_at |
| 104094_at | 1417928_at |
| 116139_at | 1450087_a_at |
| 93806_at | 1428107_at |
| 110375_at | 1429377_at |
| 115392_at | 1428458_at |

| | |
|---|---|
| 94473_at | 1420131_s_at |
| 104143_at | 1418455_at |
| 160060_at | 1452880_at |
| 101510_at | 1417056_at |
| 94931_at | 1418899_at |
| 95497_at | 1426612_at |
| 160139_at | 1417013_at |
| 93352_at | 1433428_x_at |
| 171290_r_at | 1441910_x_at |
| 93014_at | 1448203_at |
| 98435_at | 1449383_at |
| 108004_at | 1435074_at |
| 107043_at | 1450997_at |
| 116945_at | 1451679_at |
| 163254_at | 1428551_at |
| 103974_at | 1419154_at |
| 96735_at | 1448956_at |
| 95453_f_at | 1419814_s_at |
| 116214_at | 1456329_at |
| 109171_at | 1419456_at |
| 167429_i_at | 1455605_at |
| 112237_at | 1448482_at |
| 92979_at | 1423232_at |
| 102419_at | 1419415_a_at |
| 110751_at | 1428715_at |
| 113309_at | 1427072_at |
| 98618_at | 1449116_a_at |
| 100443_at | 1425764_a_at |
| 94012_at | 1417499_at |
| 113227_at | 1424263_at |
| 162558_i_at | 1428658_at |
| 98560_at | 1460740_at |
| 107997_at | 1429265_a_at |
| 103282_at | 1417804_at |
| 113937_at | 1425523_at |
| 165915_i_at | 1435222_at |
| 94875_at | 1416093_a_at |
| 108093_at | 1424522_at |
| 97920_at | 1423952_a_at |
| 102212_at | 1419018_at |
| 108365_at | 1451527_at |
| 160749_at | 1424192_at |
| 163217_at | 1452996_a_at |
| 109089_at | 1454745_at |
| 97313_at | 1451070_at |
| 95159_at | 1451164_a_at |
| 162562_at | 1449036_at |
| 99586_at | 1426195_a_at |
| 163528_at | 1437143_a_at |
| 92771_at | 1423545_a_at |
| 114757_at | 1420850_at |
| 115480_at | 1421014_a_at |
| 97347_at | 1436665_a_at |
| 102675_at | 1419007_at |
| 97983_s_at | 1420505_a_at |
| 112438_at | 1428416_at |

| | |
|---|---|
| 92646_at | 1416948_at |
| 99018_at | 1428844_a_at |
| 161666_f_at | 1449773_s_at |
| 160502_at | 1415948_at |
| 117142_at | 1429085_at |
| 98619_at | 1452681_at |
| 93294_at | 1416953_at |
| 163806_at | 1428519_at |
| 107407_at | 1453313_at |
| 113353_at | 1436156_at |
| 94256_at | 1438606_a_at |
| 106609_at | 1433746_at |
| 96222_at | 1438278_a_at |
| 160065_s_at | 1425810_a_at |
| 94266_at | 1428405_at |
| 100967_at | 1416316_at |
| 98914_at | 1423511_at |
| 113595_at | 1427074_at |
| 100600_at | 1448182_a_at |
| 103923_at | 1429775_a_at |
| 110382_at | 1433634_at |
| 162703_at | 1427087_at |
| 113549_at | 1434220_at |
| 102009_at | 1449273_at |
| 129228_f_at | 1438410_at |
| 94343_at | 1433887_at |
| 167741_at | 1429203_at |
| 110274_at | 1432494_a_at |
| 109641_at | 1420836_at |
| 169366_r_at | 1424951_at |
| 160393_at | 1433514_at |
| 94450_at | 1423850_at |
| 167937_at | 1436054_at |
| 97250_at | 1423210_a_at |
| 94508_at | 1451000_at |
| 103776_at | 1442028_at |
| 114429_at | 1449303_at |
| 98539_at | 1423459_at |
| 104085_at | 1453016_at |
| 164052_at | 1418395_at |
| 95075_at | 1426447_at |
| 100588_at | 1417189_at |
| 167680_at | 1452999_at |
| 93471_at | 1438673_at |
| 160384_at | 1448221_at |
| 98485_at | 1431805_a_at |
| 100089_at | 1416498_at |
| 96708_at | 1416108_a_at |
| 160090_f_at | 1416921_x_at |
| 94269_at | 1427773_a_at |
| 160266_r_at | 1451144_at |
| 160706_at | 1453004_at |
| 165833_at | 1435412_at |
| 100581_at | 1422506_a_at |
| 101439_at | 1417331_a_at |
| 113720_at | 1451641_at |

| | |
|---|---|
| 165986_at | 1429213_at |
| 108011_at | 1416106_at |
| 112706_at | 1455048_at |
| 165775_r_at | 1424759_at |
| 98934_at | 1419351_a_at |
| 163956_at | 1415874_at |
| 102788_s_at | 1424797_a_at |
| 98320_at | 1419430_at |
| 109051_at | 1429588_at |
| 161967_at | 1437171_x_at |
| 102322_at | 1416308_at |
| 116601_at | 1429165_at |
| 160829_at | 1418835_at |
| 96849_at | 1416345_at |
| 98038_at | 1416155_at |
| 106638_at | 1434891_at |
| 97934_at | 1423236_at |
| 102925_at | 1433845_x_at |
| 160795_at | 1426775_s_at |
| 93488_at | 1433502_s_at |
| 162645_at | 1456475_s_at |
| 97928_at | 1454837_at |
| 160613_at | 1417109_at |
| 92805_s_at | 1431429_a_at |
| 100095_at | 1416050_a_at |
| 160100_at | 1431339_a_at |
| 166741_i_at | 1451439_at |
| 94968_at | 1448963_at |
| 164098_at | 1450044_at |
| 95974_at | 1420549_at |
| 93812_at | 1423181_s_at |
| 96743_at | 1428554_a_at |
| 109701_at | 1417541_at |
| 160479_at | 1416429_a_at |
| 160314_at | 1416939_at |
| 103817_at | 1448592_at |
| 160462_f_at | 1415978_at |
| 163624_i_at | 1426412_at |
| 113995_at | 1428167_a_at |
| 93789_s_at | 1424355_a_at |
| 100394_at | 1417396_at |
| 96041_at | 1422660_at |
| 96305_at | 1433611_s_at |
| 163334_at | 1428113_at |
| 101295_s_at | 1427548_a_at |
| 94445_at | 1423725_at |
| 110291_at | 1417741_at |
| 111053_at | 1434570_at |
| 97974_at | 1451046_at |
| 93440_at | 1415766_at |
| 100977_at | 1423748_at |
| 116635_at | 1451731_at |
| 107490_at | 1434154_at |
| 95914_at | 1456405_at |
| 103598_at | 1425617_at |
| 113740_at | 1425581_s_at |

| | |
|---|---|
| 93298_at | 1415890_at |
| 107462_at | 1430701_a_at |
| 103406_at | 1417390_at |
| 163684_at | 1429535_at |
| 160878_at | 1423264_at |
| 132393_f_at | 1460456_at |
| 97498_at | 1417872_at |
| 160777_at | 1433460_at |
| 110862_at | 1434383_at |
| 100571_at | 1416148_at |
| 95553_at | 1428543_at |
| 94881_at | 1424638_at |
| 93277_at | 1426351_at |
| 92540_f_at | 1421260_a_at |
| 160943_at | 1460229_at |
| 160468_at | 1420473_at |
| 95685_at | 1416890_at |
| 110365_at | 1416931_at |
| 106931_at | 1434500_at |
| 101082_at | 1416632_at |
| 106263_at | 1426900_at |
| 101123_at | 1417999_at |
| 93071_at | 1415869_a_at |
| 116837_at | 1433942_at |
| 101929_at | 1418258_s_at |
| 163798_at | 1452811_at |
| 93551_at | 1428296_at |
| 94034_at | 1451259_at |
| 162957_at | 1428824_at |
| 98154_at | 1452781_a_at |
| 111753_at | 1452612_at |
| 100915_at | 1417472_at |
| 104252_at | 1433935_at |
| 168498_r_at | 1455387_at |
| 96283_at | 1415961_at |
| 94826_at | 1427578_a_at |
| 110469_at | 1438684_at |
| 97489_at | 1433504_at |
| 100154_at | 1421812_at |
| 100499_at | 1425536_at |
| 94438_at | 1416780_at |
| 160731_at | 1417738_at |
| 98632_at | 1417976_at |
| 164017_at | 1426609_at |
| 95646_at | 1416772_at |
| 106594_at | 1451260_at |
| 160756_at | 1417744_a_at |
| 160456_at | 1424136_a_at |
| 162465_i_at | 1424887_at |
| 160182_at | 1448454_at |
| 160310_at | 1426931_s_at |
| 160892_at | 1416918_at |
| 134016_f_at | 1437693_at |
| 113344_at | 1428471_at |
| 113729_at | 1435744_at |
| 100529_at | 1418631_at |

| | |
|---|---|
| 133431_at | 1424766_at |
| 104725_at | 1427918_a_at |
| 117113_at | 1434478_at |
| 97312_at | 1416440_at |
| 163631_at | 1434665_at |
| 93048_at | 1416615_at |
| 109360_at | 1418794_at |
| 92880_at | 1420911_a_at |
| 95649_at | 1424170_at |
| 103885_at | 1448633_at |
| 96868_at | 1428079_at |
| 99934_at | 1424456_at |
| 160495_at | 1422631_at |
| 114360_at | 1434205_at |
| 100461_at | 1417720_at |
| 162747_at | 1423924_s_at |
| 105742_at | 1435879_at |
| 113920_at | 1433795_at |
| 95556_at | 1423492_at |
| 103783_at | 1426992_at |
| 105045_at | 1427152_at |
| 114301_at | 1429839_a_at |
| 164190_at | 1450665_at |
| 96056_at | 1448605_at |
| 166308_at | 1456026_at |
| 97273_at | 1417655_a_at |
| 112384_at | 1433730_at |
| 97114_at | 1415687_a_at |
| 97809_at | 1460692_at |
| 113171_at | 1436890_at |
| 96281_at | 1423256_a_at |
| 97909_at | 1415849_s_at |
| 100156_at | 1415945_at |
| 95146_at | 1424039_at |
| 112847_at | 1428688_at |
| 160074_at | 1426215_at |
| 160679_at | 1424746_at |
| 103502_at | 1436399_s_at |
| 105102_at | 1440381_at |
| 96347_at | 1424382_at |
| 93084_at | 1434897_a_at |
| 160176_at | 1418228_at |
| 161007_at | 1450253_a_at |
| 108007_at | 1424033_at |
| 96701_at | 1448283_a_at |
| 107557_at | 1433648_at |
| 96711_at | 1422517_a_at |
| 96075_at | 1450851_at |
| 114997_at | 1417780_at |
| 98845_at | 1426186_a_at |
| 114983_at | 1422993_s_at |
| 117293_at | 1428477_at |
| 111989_at | 1426629_at |
| 96037_at | 1416924_at |
| 98608_at | 1420024_s_at |
| 103400_at | 1415708_at |

| | |
|---|---|
| 94303_at | 1425142_a_at |
| 98886_at | 1423767_at |
| 160245_at | 1453013_at |
| 114240_at | 1455409_at |
| 96310_at | 1425264_s_at |
| 108790_at | 1449231_at |
| 97921_at | 1426670_at |
| 99158_at | 1452246_at |
| 96029_at | 1423811_at |
| 105648_at | 1438096_a_at |
| 129315_at | 1437353_at |
| 160955_at | 1417075_at |
| 111600_at | 1448965_at |
| 163783_at | 1434020_at |
| 98482_at | 1417092_at |
| 97242_at | 1427878_at |
| 116112_at | 1428967_at |
| 104126_at | 1415920_at |
| 115489_at | 1433770_at |
| 166281_f_at | 1441948_x_at |
| 104109_at | 1452828_at |
| 101047_at | 1453556_x_at |
| 160494_at | 1438317_a_at |
| 101010_at | 1423430_at |
| 109684_at | 1451422_at |
| 94839_at | 1416903_at |
| 106583_at | 1417288_at |
| 160279_at | 1427881_at |
| 103065_at | 1448568_a_at |
| 160202_at | 1423662_at |
| 163525_at | 1453071_s_at |
| 160153_at | 1448362_at |
| 95408_at | 1423206_s_at |
| 169053_at | 1429671_at |
| 162614_at | 1448627_s_at |
| 92569_f_at | 1450986_at |
| 135182_at | 1452858_at |
| 102001_at | 1448226_at |
| 99171_at | 1423275_at |
| 165812_at | 1451058_at |
| 109716_at | 1452856_at |
| 104040_at | 1434702_at |
| 114704_at | 1452953_at |
| 101521_at | 1424278_a_at |
| 92777_at | 1438133_a_at |
| 94564_at | 1421606_a_at |
| 164062_at | 1421936_at |
| 92517_at | 1423540_at |
| 103708_at | 1424344_s_at |
| 103101_at | 1423616_at |
| 160782_at | 1454964_at |
| 163111_at | 1416928_at |
| 96340_at | 1423707_at |
| 110482_at | 1424349_a_at |
| 97395_at | 1460633_at |
| 92611_at | 1448347_a_at |

| | |
|---|---|
| 111823_at | 1426998_at |
| 92956_at | 1421965_s_at |
| 98138_at | 1452049_at |
| 94004_at | 1450981_at |
| 104145_at | 1424643_at |
| 110974_at | 1423228_at |
| 161646_r_at | 1435086_s_at |
| 96866_at | 1423122_at |
| 104039_at | 1454814_s_at |
| 135530_at | 1422021_at |
| 113762_at | 1455320_at |
| 100955_at | 1422462_at |
| 112773_at | 1432094_a_at |
| 108047_at | 1428542_at |
| 101536_at | 1423200_at |
| 98007_at | 1417542_at |
| 117035_at | 1433965_at |
| 102313_at | 1420499_at |
| 95756_at | 1451026_at |
| 93736_at | 1448200_at |
| 93279_at | 1416455_a_at |
| 160835_i_at | 1431359_a_at |
| 96700_r_at | 1455349_at |
| 101108_at | 1416042_s_at |
| 96653_at | 1452734_at |
| 113706_at | 1454841_at |
| 93889_f_at | 1452540_a_at |
| 104700_at | 1422198_a_at |
| 163055_at | 1454766_at |
| 93727_at | 1426736_at |
| 162854_at | 1434559_at |
| 92863_at | 1448121_at |
| 111421_at | 1451357_at |
| 97947_at | 1428103_at |
| 95613_at | 1448339_at |
| 101485_at | 1417242_at |
| 103804_at | 1450784_at |
| 93023_f_at | 1460314_s_at |
| 95542_at | 1433883_at |
| 104241_at | 1434487_at |
| 94232_at | 1448698_at |
| 96266_at | 1426698_a_at |
| 113288_at | 1454666_at |
| 108049_at | 1423946_at |
| 103054_at | 1426242_at |
| 163867_at | 1451199_at |
| 93701_at | 1424207_at |
| 165682_r_at | 1423841_at |
| 96696_at | 1452787_a_at |
| 112989_at | 1455322_at |
| 133930_at | 1437904_at |
| 109942_at | 1452151_at |
| 115460_f_at | 1435325_at |
| 161126_at | 1435998_at |
| 106558_at | 1417785_at |
| 112388_at | 1423489_at |

| | |
|---|---|
| 99662_at | 1437520_a_at |
| 98089_at | 1428475_at |
| 103038_at | 1421061_at |
| 165536_at | 1452708_a_at |
| 160492_at | 1416071_at |
| 92571_at | 1416146_at |
| 163498_i_at | 1418097_a_at |
| 102749_at | 1418709_at |
| 117148_at | 1455665_at |
| 166540_at | 1422486_a_at |
| 134809_at | 1433407_at |
| 160343_at | 1450891_at |
| 106072_at | 1428892_at |
| 93971_f_at | 1448492_a_at |
| 104627_at | 1434256_s_at |
| 106250_at | 1419045_at |
| 93997_at | 1418116_at |
| 111096_at | 1452454_at |
| 97863_at | 1450868_at |
| 113652_at | 1452012_a_at |
| 109130_at | 1427997_at |
| 160221_at | 1417079_s_at |
| 98409_at | 1459679_s_at |
| 109930_at | 1433835_at |
| 102381_at | 1433531_at |
| 104716_at | 1448754_at |
| 110360_at | 1451485_at |
| 116109_at | 1452294_at |
| 161289_at | 1455871_s_at |
| 104089_at | 1428529_at |
| 160809_at | 1423047_at |
| 109006_at | 1425199_a_at |
| 95109_at | 1455035_s_at |
| 160345_at | 1416349_at |
| 92848_at | 1416452_at |
| 109692_at | 1426726_at |
| 160639_at | 1420712_a_at |
| 97274_at | 1421751_a_at |
| 169297_r_at | 1423368_at |
| 104843_at | 1456795_at |
| 101995_at | 1450957_a_at |
| 162894_r_at | 1456597_at |
| 102049_at | 1417273_at |
| 94282_at | 1416735_at |
| 111735_at | 1436183_at |
| 109780_at | 1417080_a_at |
| 115376_at | 1438006_at |
| 98048_at | 1423982_at |
| 103581_at | 1449065_at |
| 114996_at | 1429461_at |
| 165830_f_at | 1433679_at |
| 102940_at | 1419135_at |
| 95001_at | 1450983_at |
| 100535_at | 1428362_at |
| 95479_at | 1448505_at |
| 162795_at | 1426716_at |

| | |
|---|---|
| 97315_at | 1424258_at |
| 160246_at | 1416950_at |
| 95490_at | 1460211_a_at |
| 163718_at | 1419205_x_at |
| 93529_at | 1419041_at |
| 116263_at | 1455680_at |
| 112675_at | 1452178_at |
| 93975_at | 1416129_at |
| 109090_at | 1428227_at |
| 100901_at | 1450439_at |
| 107575_at | 1428259_at |
| 103799_at | 1452254_at |
| 163627_f_at | 1452968_at |
| 96781_at | 1415725_at |
| 167815_f_at | 1452760_at |
| 103630_at | 1448403_at |
| 109184_at | 1429648_at |
| 117110_at | 1452233_at |
| 98114_at | 1423086_at |
| 117134_at | 1419495_at |
| 112013_at | 1435478_at |
| 111829_at | 1455010_at |
| 97824_at | 1416606_s_at |
| 116613_at | 1436436_at |
| 160534_at | 1415740_at |
| 94917_at | 1418510_s_at |
| 104782_at | 1438663_at |
| 93323_at | 1453572_a_at |
| 160773_at | 1417321_at |
| 101954_at | 1416415_a_at |
| 113997_at | 1417879_at |
| 94978_at | 1416171_at |
| 95004_at | 1428255_at |
| 112657_at | 1434317_s_at |
| 94812_at | 1453169_a_at |
| 95457_at | 1437290_at |
| 92553_at | 1417825_at |
| 96240_at | 1434783_at |
| 99444_at | 1418187_at |
| 115066_at | 1418351_a_at |
| 104730_at | 1417675_a_at |
| 114701_at | 1429390_at |
| 112807_at | 1460431_at |
| 95028_r_at | 1447977_x_at |
| 109759_at | 1454197_a_at |
| 113448_at | 1435233_at |
| 95618_at | 1434314_s_at |
| 112959_at | 1428924_at |
| 97886_at | 1432372_a_at |
| 96069_at | 1417294_at |
| 93681_at | 1418532_at |
| 97958_at | 1426614_at |
| 98918_at | 1451091_at |
| 95409_at | 1415733_a_at |
| 162797_at | 1426741_a_at |
| 108354_at | 1417699_at |

| | |
|---|---|
| 97939_at | 1451195_a_at |
| 163324_at | 1426232_at |
| 138245_at | 1455777_x_at |
| 116080_at | 1435341_at |
| 116249_at | 1440343_at |
| 100151_at | 1417815_a_at |
| 134220_at | 1436728_s_at |
| 104066_at | 1460411_s_at |
| 96778_at | 1416998_at |
| 115357_at | 1435008_at |
| 98587_at | 1420478_at |
| 163389_at | 1434001_at |
| 94132_at | 1460671_at |
| 104383_at | 1448289_at |
| 96306_at | 1428494_a_at |
| 93821_at | 1448611_at |
| 96063_at | 1451968_at |
| 109341_at | 1434156_at |
| 114407_at | 1451857_a_at |
| 98931_at | 1433546_at |
| 108478_f_at | 1428615_at |
| 103980_at | 1421151_a_at |
| 100612_at | 1415878_at |
| 110719_at | 1424669_at |
| 111886_at | 1436499_at |
| 102387_at | 1424377_at |
| 93560_at | 1450095_a_at |
| 96804_at | 1424019_at |
| 99648_at | 1417950_a_at |
| 101079_at | 1416791_a_at |
| 93272_at | 1426160_a_at |
| 109946_at | 1452832_s_at |
| 96756_at | 1435333_at |
| 162704_i_at | 1452132_at |
| 104154_at | 1426538_a_at |
| 94361_at | 1448270_at |
| 100884_at | 1448894_at |
| 94040_at | 1430536_a_at |
| 103001_at | 1451803_a_at |
| 160377_at | 1423723_s_at |
| 165373_at | 1422948_s_at |
| 93120_f_at | 1425336_x_at |
| 114702_at | 1430985_at |
| 163764_at | 1426495_at |
| 97976_at | 1455434_a_at |
| 97487_at | 1416666_at |
| 95014_at | 1417501_at |
| 160896_at | 1417090_at |
| 101017_at | 1422439_a_at |
| 95523_at | 1428333_at |
| 96641_at | 1433478_at |
| 163057_at | 1451254_at |
| 165707_r_at | 1424591_at |
| 129405_at | 1438861_at |
| 162711_at | 1428347_at |
| 110419_at | 1437370_at |

| | |
|---|---|
| 98503_at | 1417512_at |
| 100902_at | 1428357_at |
| 97492_at | 1415738_at |
| 110376_at | 1423104_at |
| 94507_at | 1423883_at |
| 109304_r_at | 1454885_at |
| 93959_at | 1456204_at |
| 160237_at | 1448427_at |
| 105758_at | 1448761_a_at |
| 100908_at | 1425340_a_at |
| 107058_at | 1424397_at |
| 163355_at | 1429433_at |
| 96954_at | 1426248_at |
| 160608_at | 1438097_at |
| 162641_r_at | 1433901_at |
| 167331_at | 1444051_at |
| 95117_at | 1424111_at |
| 93285_at | 1415834_at |
| 104331_at | 1448709_at |
| 93987_f_at | 1417126_a_at |
| 160847_at | 1425562_s_at |
| 106578_at | 1423317_at |
| 116879_at | 1435543_at |
| 104614_at | 1417389_at |
| 138042_at | 1440856_at |
| 98278_at | 1422020_at |
| 108300_at | 1428284_at |
| 165540_at | 1439038_at |
| 105079_at | 1441275_at |
| 109355_at | 1437200_at |
| 134085_at | 1429660_s_at |
| 106295_at | 1428388_at |
| 164376_r_at | 1452780_at |
| 98952_at | 1451081_a_at |
| 160550_i_at | 1416212_at |
| 92564_at | 1433842_at |
| 104699_at | 1425580_a_at |
| 105830_at | 1416447_at |
| 100607_at | 1416013_at |
| 163619_at | 1452838_at |
| 96138_at | 1455951_at |
| 101078_at | 1456616_a_at |
| 106195_at | 1454877_at |
| 163605_at | 1450018_s_at |
| 103016_s_at | 1449164_at |
| 115369_at | 1419062_at |
| 137642_at | 1419270_a_at |
| 111491_at | 1426978_at |
| 94861_at | 1449718_s_at |
| 168548_f_at | 1459976_s_at |
| 96046_at | 1448246_at |
| 93267_at | 1426671_a_at |
| 100923_at | 1450650_at |
| 96871_at | 1448543_at |
| 160637_at | 1424309_a_at |
| 99031_at | 1454763_at |

| | |
|---|---|
| 98459_at | 1425179_at |
| 104595_at | 1421849_at |
| 115798_at | 1455102_at |
| 163371_at | 1438021_at |
| 93669_f_at | 1436790_a_at |
| 162655_at | 1454680_at |
| 100409_at | 1426673_at |
| 160900_at | 1417594_at |
| 97217_at | 1426830_a_at |
| 93803_at | 1418078_at |
| 104549_at | 1433497_at |
| 163696_at | 1433960_at |
| 99162_at | 1429043_at |
| 96127_at | 1415892_at |
| 94084_at | 1423987_at |
| 109758_at | 1426743_at |
| 95470_at | 1423766_at |
| 109978_s_at | 1423380_s_at |
| 163205_at | 1452797_at |
| 161444_f_at | 1417887_at |
| 94006_at | 1431822_a_at |
| 96263_at | 1423119_at |
| 114991_at | 1434296_at |
| 104254_at | 1428789_at |
| 111806_at | 1460325_at |
| 141025_at | 1436585_at |
| 161161_r_at | 1435277_x_at |
| 103959_at | 1455175_at |
| 94510_at | 1422844_a_at |
| 98104_at | 1416769_s_at |
| 94752_s_at | 1422054_a_at |
| 115134_at | 1435902_at |
| 163104_at | 1428269_a_at |
| 162548_at | 1423787_at |
| 98574_at | 1422453_at |
| 160152_at | 1416005_at |
| 105326_at | 1436125_at |
| 112333_at | 1428506_at |
| 160653_at | 1426118_a_at |
| 95742_at | 1422459_a_at |
| 94239_at | 1450938_at |
| 117131_at | 1427971_at |
| 98473_at | 1418847_at |
| 112394_at | 1433833_at |
| 102698_at | 1449888_at |
| 110004_at | 1434568_at |
| 109528_at | 1425706_a_at |
| 165870_i_at | 1429177_x_at |
| 100900_at | 1433736_at |
| 93373_at | 1417706_at |
| 101525_at | 1428322_a_at |
| 165425_f_at | 1433622_at |
| 94850_at | 1449968_s_at |
| 103852_at | 1423958_a_at |
| 103207_at | 1419397_at |
| 101008_at | 1434434_s_at |

| | |
|---|---|
| 116590_at | 1455159_at |
| 93243_at | 1418910_at |
| 160652_at | 1448111_at |
| 168398_f_at | 1447800_x_at |
| 163642_at | 1428156_at |
| 104275_g_at | 1427739_a_at |
| 162925_at | 1451649_a_at |
| 108515_at | 1434930_at |
| 114454_at | 1453727_at |
| 163207_at | 1419454_x_at |
| 102385_at | 1428389_s_at |
| 93980_at | 1426426_at |
| 94896_at | 1448144_at |
| 169510_i_at | 1436935_x_at |
| 94344_at | 1419174_at |
| 104122_at | 1433808_at |
| 93527_at | 1456341_a_at |
| 103905_at | 1428485_at |
| 108024_at | 1417145_at |
| 100895_at | 1437630_at |
| 160957_at | 1460571_at |
| 114849_at | 1426221_at |
| 161009_at | 1434436_at |
| 98826_at | 1420086_x_at |
| 92904_at | 1420425_at |
| 97327_at | 1421731_a_at |
| 97901_at | 1453097_a_at |
| 113523_at | 1438041_at |
| 99113_at | 1416678_at |
| 95419_at | 1423702_at |
| 97201_s_at | 1417285_a_at |
| 163129_at | 1418667_at |
| 103791_at | 1418023_at |
| 97166_at | 1419680_a_at |
| 94462_at | 1460730_at |
| 93841_at | 1460575_at |
| 101492_at | 1416228_at |
| 92797_at | 1422794_at |
| 104091_at | 1428197_at |
| 97205_at | 1426395_s_at |
| 160757_at | 1428101_at |
| 94458_at | 1415995_at |
| 160171_f_at | 1418073_at |
| 112496_at | 1436181_at |
| 94041_at | 1423684_at |
| 95485_at | 1460184_at |
| 102936_at | 1460329_at |
| 114723_at | 1428826_at |
| 107149_at | 1449186_at |
| 129316_at | 1437424_at |
| 99801_at | 1422058_at |
| 115142_at | 1434477_at |
| 93112_at | 1448777_at |
| 94456_at | 1426854_a_at |
| 101934_at | 1434348_at |
| 114930_at | 1427310_at |

| | |
|---|---|
| 115784_at | 1420916_at |
| 112860_at | 1424239_at |
| 95760_at | 1423820_at |
| 112492_at | 1433777_at |
| 94971_at | 1430574_at |
| 101499_at | 1449942_a_at |
| 97682_r_at | 1427473_at |
| 96245_at | 1423815_at |
| 105054_f_at | 1435753_a_at |
| 162637_at | 1428520_at |
| 163083_at | 1419170_at |
| 104709_at | 1423347_at |
| 111478_at | 1428976_at |
| 163604_at | 1434749_at |
| 115800_at | 1434339_at |
| 102047_at | 1415683_at |
| 108046_at | 1417010_at |
| 134045_at | 1437500_at |
| 109029_at | 1420984_at |
| 99106_at | 1451366_at |
| 163384_i_at | 1434565_at |
| 162910_at | 1428949_at |
| 160223_at | 1452665_at |
| 167320_f_at | 1433905_at |
| 96119_s_at | 1417130_s_at |
| 107631_at | 1424161_at |
| 96831_at | 1424650_at |
| 104585_at | 1424809_at |
| 94247_at | 1435493_at |
| 116676_at | 1435807_at |
| 116964_at | 1449351_s_at |
| 110621_at | 1435899_at |
| 113601_at | 1428859_at |
| 98083_at | 1433508_at |
| 162690_at | 1434936_at |
| 94079_at | 1448729_a_at |
| 162269_at | 1429560_at |
| 160192_at | 1416177_at |
| 100927_at | 1417963_at |
| 94368_at | 1460557_at |
| 160538_at | 1422440_at |
| 113145_at | 1428560_at |
| 160263_r_at | 1452066_a_at |
| 114444_at | 1434949_at |
| 102783_at | 1452799_at |
| 96884_at | 1415975_at |
| 109926_at | 1427284_a_at |
| 168444_i_at | 1459861_s_at |
| 101583_at | 1448272_at |
| 104132_at | 1448677_at |
| 162838_at | 1424589_s_at |
| 101894_s_at | 1423364_a_at |
| 92580_at | 1417024_at |
| 107520_at | 1433561_at |
| 102315_at | 1423884_at |
| 166666_at | 1434566_a_at |

| | |
|---|---|
| 114468_at | 1452908_at |
| 112321_at | 1433555_at |
| 111499_at | 1454731_at |
| 162741_at | 1429624_at |
| 107925_at | 1427089_at |
| 110017_at | 1437395_at |
| 109756_at | 1451190_a_at |
| 95482_at | 1419920_s_at |
| 104586_at | 1428100_at |
| 160311_at | 1416683_at |
| 96869_at | 1416937_at |
| 165618_at | 1426013_s_at |
| 107889_at | 1429268_at |
| 116146_at | 1429484_at |
| 96112_at | 1423972_at |
| 96207_at | 1434005_at |
| 96792_at | 1455593_at |
| 95631_at | 1460288_a_at |
| 162463_at | 1419494_a_at |
| 100408_at | 1421015_s_at |
| 163294_at | 1454774_at |
| 108582_at | 1424054_at |
| 168493_at | 1435967_s_at |
| 97951_s_at | 1452105_a_at |
| 160912_i_at | 1426456_a_at |
| 92925_at | 1418901_at |
| 93598_at | 1417648_s_at |
| 160471_at | 1460168_at |
| 98606_s_at | 1425106_a_at |
| 160820_at | 1460675_at |
| 93541_at | 1423505_at |
| 111381_r_at | 1424303_at |
| 160427_at | 1428084_at |
| 116427_at | 1439837_at |
| 100479_at | 1423066_at |
| 110014_at | 1418530_at |
| 160767_at | 1417696_at |
| 109344_at | 1434294_at |
| 100065_r_at | 1415800_at |
| 102383_at | 1433639_at |
| 100048_at | 1424139_at |
| 96098_at | 1422819_at |
| 109031_at | 1435035_at |
| 115000_at | 1455496_at |
| 99872_s_at | 1418364_a_at |
| 134685_at | 1434521_at |
| 101372_at | 1429294_at |
| 129317_at | 1426744_at |
| 129054_i_at | 1449364_at |
| 102717_at | 1424775_at |
| 97414_at | 1423827_s_at |
| 163621_at | 1440826_s_at |
| 113301_at | 1418507_s_at |
| 130988_f_at | 1459874_s_at |
| 110844_at | 1449446_at |
| 92879_at | 1416792_at |

| | |
|---|---|
| 106975_at | 1427481_a_at |
| 106073_at | 1434301_at |
| 163625_at | 1455293_at |
| 114059_at | 1438487_s_at |
| 160164_at | 1415755_a_at |
| 102089_at | 1422148_at |
| 97948_at | 1417850_at |
| 111372_at | 1428754_at |
| 111256_at | 1429891_at |
| 96019_at | 1422880_at |
| 103460_at | 1428306_at |
| 106245_at | 1433811_at |
| 96852_at | 1452032_at |
| 102689_at | 1450378_at |
| 94422_at | 1434038_at |
| 109391_at | 1418324_at |
| 114886_at | 1420119_s_at |
| 115497_at | 1460446_at |
| 102993_at | 1418483_a_at |
| 104741_at | 1454787_at |
| 95095_at | 1448559_at |
| 163552_at | 1423733_a_at |
| 112446_at | 1428796_at |
| 101355_at | 1422396_s_at |
| 160734_at | 1422593_at |
| 100136_at | 1416344_at |
| 97703_at | 1421945_a_at |
| 97394_at | 1424206_at |
| 93973_at | 1426674_at |
| 94890_at | 1437943_s_at |
| 116187_at | 1418727_at |
| 106643_at | 1428415_at |
| 110253_at | 1452303_at |
| 93895_s_at | 1417279_at |
| 160228_at | 1429555_at |
| 96647_at | 1432271_a_at |
| 95460_at | 1460171_at |
| 99537_at | 1416585_at |
| 113656_at | 1437043_a_at |
| 95135_at | 1416840_at |
| 104287_at | 1448866_at |
| 100538_at | 1451124_at |
| 95007_at | 1426949_s_at |
| 108368_at | 1435742_at |
| 106567_at | 1434113_a_at |
| 108587_at | 1460403_at |
| 95637_at | 1426750_at |
| 112784_at | 1454704_at |
| 160330_at | 1460645_at |
| 100992_at | 1423212_at |
| 95158_at | 1415744_at |
| 112103_at | 1435139_at |
| 92531_at | 1455698_at |
| 162634_at | 1454748_at |
| 134014_at | 1439648_at |
| 99594_at | 1416877_a_at |

| | |
|---|---|
| 111137_at | 1443870_at |
| 130739_at | 1451303_at |
| 97445_at | 1417057_a_at |
| 92282_at | 1427887_at |
| 95427_at | 1423293_at |
| 96535_at | 1435802_at |
| 101506_at | 1417351_a_at |
| 106653_at | 1460552_at |
| 115416_at | 1424554_at |
| 96907_at | 1451896_a_at |
| 95161_at | 1451075_s_at |
| 160286_at | 1452659_at |
| 100066_at | 1416283_at |
| 100589_at | 1433470_a_at |
| 101498_at | 1436848_x_at |
| 114085_at | 1426802_at |
| 114858_at | 1449668_s_at |
| 95714_at | 1417055_at |
| 160542_at | 1420937_at |
| 96135_at | 1426964_at |
| 95489_at | 1416044_at |
| 111352_at | 1428672_at |
| 163881_at | 1452030_a_at |
| 114557_at | 1455185_s_at |
| 92838_at | 1416514_a_at |
| 164642_i_at | 1437309_a_at |
| 107447_at | 1435445_at |
| 106615_at | 1436775_a_at |
| 101065_at | 1417947_at |
| 101414_at | 1448531_at |
| 101429_at | 1417516_at |
| 101063_at | 1418370_at |
| 93880_at | 1426001_at |
| 101404_at | 1451265_at |
| 94011_at | 1415739_at |
| 114521_at | 1460567_at |
| 117260_at | 1418335_a_at |
| 163969_at | 1421968_a_at |
| 96260_at | 1416038_at |
| 96801_at | 1422184_a_at |
| 163951_at | 1424314_at |
| 96623_at | 1435133_at |
| 94489_at | 1438657_x_at |
| 95054_at | 1460323_at |
| 95431_at | 1426676_s_at |
| 163871_at | 1429658_a_at |
| 131868_at | 1435728_at |
| 113850_at | 1429724_at |
| 109925_at | 1456058_at |
| 115099_at | 1423138_at |
| 163531_at | 1416299_at |
| 160589_at | 1436505_at |
| 160791_at | 1424802_a_at |
| 107047_at | 1427901_at |
| 98558_r_at | 1438984_x_at |
| 99119_at | 1448346_at |

| | |
|---|---|
| 160364_at | 1427432_a_at |
| 116301_at | 1429278_at |
| 101971_at | 1423729_a_at |
| 108973_at | 1435247_at |
| 112108_at | 1437253_at |
| 164233_at | 1429310_at |
| 111958_at | 1436983_at |
| 103350_at | 1451056_at |
| 160078_at | 1450914_at |
| 160546_at | 1451461_a_at |
| 99917_at | 1416544_at |
| 97342_at | 1420489_at |
| 111650_at | 1424296_at |
| 103096_at | 1419281_a_at |
| 115096_at | 1434088_at |
| 160381_at | 1451266_at |
| 160121_at | 1455798_at |
| 95659_at | 1416569_at |
| 169171_i_at | 1444319_at |
| 101584_at | 1419259_at |
| 160623_at | 1449229_a_at |
| 164036_at | 1434860_at |
| 106046_at | 1428228_at |
| 104440_at | 1420762_a_at |
| 112239_r_at | 1428970_at |
| 160322_at | 1423759_a_at |
| 92388_at | 1415710_at |
| 160769_at | 1448438_at |
| 113020_at | 1419922_s_at |
| 95723_r_at | 1428707_at |
| 114169_at | 1455266_at |
| 100401_at | 1420952_at |
| 92428_at | 1418292_at |
| 97897_at | 1433475_a_at |
| 93503_at | 1448201_at |
| 162314_at | 1430519_a_at |
| 93810_at | 1448118_a_at |
| 95061_at | 1418234_s_at |
| 99078_at | 1417322_at |
| 103617_at | 1460242_at |
| 115319_at | 1429886_at |
| 92637_at | 1450269_a_at |
| 99949_at | 1424428_at |
| 97717_at | 1449592_at |
| 162710_at | 1429587_at |
| 103359_at | 1451141_at |
| 110988_at | 1436020_at |
| 104288_at | 1451971_at |
| 93043_at | 1415821_at |
| 92827_at | 1420881_at |
| 112186_at | 1438255_at |
| 108541_at | 1427059_at |
| 105712_at | 1435948_at |
| 164107_at | 1439024_at |
| 112495_at | 1460237_at |
| 98804_at | 1420604_at |

| | |
|---|---|
| 92889_r_at | 1422210_at |
| 99099_at | 1426587_a_at |
| 95395_at | 1426840_at |
| 96027_at | 1449333_at |
| 94360_at | 1452877_at |
| 111014_at | 1429728_at |
| 92997_g_at | 1421657_a_at |
| 160440_at | 1422487_at |
| 111964_at | 1424478_at |
| 99564_at | 1415810_at |
| 163537_at | 1433983_at |
| 115206_at | 1454930_at |
| 94945_at | 1449694_s_at |
| 97553_at | 1452182_at |
| 99777_s_at | 1455488_at |
| 100561_at | 1417380_at |
| 162938_at | 1456038_at |
| 102791_at | 1422962_a_at |
| 104488_at | 1427070_at |
| 107984_at | 1435249_at |
| 108106_at | 1418916_a_at |
| 160807_at | 1450504_a_at |
| 137094_at | 1434721_at |
| 166681_at | 1433779_at |
| 108511_at | 1452733_at |
| 101581_at | 1416680_at |
| 167862_at | 1434577_at |
| 135749_at | 1457832_at |
| 129269_at | 1455330_at |
| 96904_at | 1453725_a_at |
| 105003_at | 1456623_at |
| 130662_at | 1434861_at |
| 162967_r_at | 1454697_at |
| 104648_at | 1435762_at |
| 114105_at | 1455735_at |
| 160561_at | 1416006_at |
| 112721_at | 1456396_at |
| 165885_at | 1428685_at |
| 101558_s_at | 1415676_a_at |
| 102040_at | 1451672_at |
| 104567_at | 1449004_at |
| 160170_at | 1460181_at |
| 95732_at | 1437972_s_at |
| 164232_at | 1455402_at |
| 110763_at | 1451229_at |
| 94832_at | 1415963_at |
| 160924_at | 1436896_at |
| 110608_at | 1425007_at |
| 113182_at | 1415988_at |
| 110329_at | 1426527_at |
| 92562_at | 1416543_at |
| 160531_at | 1416393_at |
| 100112_at | 1417574_at |
| 104097_at | 1424046_at |
| 133830_at | 1429893_at |
| 93999_at | 1448357_at |

| | |
|---|---|
| 100579_s_at | 1434540_a_at |
| 111275_at | 1437414_at |
| 96854_at | 1415706_at |
| 160383_at | 1432264_x_at |
| 93013_at | 1435176_a_at |
| 101393_at | 1460330_at |
| 116953_at | 1422594_at |
| 104261_at | 1435077_at |
| 95018_r_at | 1433903_at |
| 160234_at | 1451080_at |
| 130586_at | 1445849_at |
| 107016_at | 1428825_at |
| 114770_at | 1454799_at |
| 165544_at | 1418942_at |
| 97884_at | 1460195_at |
| 162576_at | 1448689_at |
| 115780_at | 1454952_s_at |
| 99522_at | 1450886_at |
| 165549_at | 1428449_at |
| 96949_at | 1415767_at |
| 101055_at | 1448128_at |
| 111464_at | 1460397_at |
| 99084_s_at | 1425023_at |
| 162851_at | 1428293_at |
| 162620_at | 1433536_at |
| 114332_at | 1454874_at |
| 160131_at | 1452387_a_at |
| 171582_r_at | 1423431_a_at |
| 101004_f_at | 1416150_a_at |
| 106654_at | 1426953_at |
| 109017_at | 1428652_at |
| 111957_at | 1434835_at |
| 97433_at | 1448711_at |
| 117049_at | 1435758_at |
| 129202_r_at | 1452899_at |
| 162912_at | 1424066_at |
| 97559_at | 1424736_at |
| 162752_i_at | 1452176_at |
| 92927_at | 1422607_at |
| 112476_at | 1455317_at |
| 112977_at | 1435029_at |
| 103888_at | 1437161_x_at |
| 104125_at | 1417250_at |
| 107619_s_at | 1428877_at |
| 107996_at | 1428555_at |
| 109004_at | 1448584_at |
| 96942_at | 1423728_at |
| 113337_at | 1428725_at |
| 100496_at | 1418908_at |
| 160123_at | 1427903_at |
| 108898_at | 1423685_at |
| 93728_at | 1425742_a_at |
| 94257_at | 1428905_at |
| 160379_at | 1424092_at |
| 96084_at | 1428224_at |
| 101056_at | 1448236_at |

| | |
|---|---|
| 109681_at | 1452727_at |
| 106570_at | 1417196_s_at |
| 93346_at | 1417864_at |
| 101107_at | 1415870_at |
| 112985_at | 1419614_at |
| 115347_at | 1451556_a_at |
| 99645_at | 1428125_at |
| 109079_f_at | 1454724_x_at |
| 113767_at | 1452099_at |
| 160313_at | 1426827_at |
| 92907_at | 1448873_at |
| 95473_s_at | 1417343_at |
| 95139_at | 1428882_at |
| 108581_at | 1416926_at |
| 107088_at | 1425806_a_at |
| 100054_s_at | 1434119_at |
| 116887_at | 1435400_at |
| 170812_at | 1457139_at |
| 107617_at | 1423847_at |
| 95707_at | 1448685_at |
| 109990_at | 1434290_at |
| 114974_at | 1434235_at |
| 104626_at | 1427964_at |
| 107045_at | 1428251_at |
| 93794_at | 1423781_at |
| 108040_at | 1454957_at |
| 112861_at | 1448546_at |
| 106252_at | 1416991_at |
| 113282_at | 1428890_at |
| 104279_at | 1415754_at |
| 101027_s_at | 1424105_a_at |
| 103078_at | 1424726_at |
| 111744_at | 1431802_a_at |
| 114581_at | 1455114_at |
| 98084_at | 1417112_at |
| 97485_at | 1447277_s_at |
| 111900_at | 1426266_s_at |
| 97539_at | 1426749_at |
| 163681_at | 1452693_at |
| 160431_at | 1452048_at |
| 97839_at | 1429497_s_at |
| 103674_f_at | 1417210_at |
| 104404_at | 1431026_at |
| 106044_at | 1434446_at |
| 114658_at | 1435155_at |
| 166246_f_at | 1447679_s_at |
| 101030_at | 1449110_at |
| 97816_at | 1428907_at |
| 93603_at | 1448849_at |
| 111937_at | 1433856_at |
| 99906_at | 1420602_a_at |
| 100931_at | 1460346_at |
| 162979_at | 1456599_at |
| 167635_r_at | 1455948_x_at |
| 171441_i_at | 1438737_at |
| 98149_s_at | 1452730_at |

| | |
|---|---|
| 93020_at | 1448595_a_at |
| 115262_at | 1438349_at |
| 103875_at | 1416709_a_at |
| 160676_at | 1427131_s_at |
| 105209_at | 1426842_at |
| 100515_at | 1418518_at |
| 97345_at | 1420592_a_at |
| 97866_at | 1423217_a_at |
| 116455_at | 1435771_at |
| 101980_at | 1422693_a_at |
| 100576_at | 1416410_at |
| 106458_at | 1450778_a_at |
| 113425_at | 1456072_at |
| 114686_at | 1451202_at |
| 115901_at | 1424611_x_at |
| 116031_at | 1452833_at |
| 96198_at | 1418085_at |
| 162654_at | 1436837_at |
| 109721_at | 1452740_at |
| 113039_at | 1436742_a_at |
| 160788_at | 1416172_at |
| 162756_at | 1417768_at |
| 106475_at | 1435187_at |
| 102200_at | 1417828_at |
| 104706_at | 1418988_at |
| 114800_at | 1433934_at |
| 95596_at | 1428353_at |
| 97404_at | 1429038_at |
| 98956_at | 1423732_at |
| 113581_at | 1460430_at |
| 115249_at | 1429769_at |
| 163281_at | 1428870_at |
| 102364_at | 1449117_at |
| 166654_at | 1440050_at |
| 95688_at | 1423346_at |
| 94286_at | 1415705_at |
| 160092_at | 1416067_at |
| 112950_at | 1436827_at |
| 103415_at | 1448376_at |
| 110354_at | 1428236_at |
| 111292_at | 1426628_at |
| 112404_at | 1454962_at |
| 95526_at | 1415682_at |
| 96861_at | 1424164_at |
| 160091_at | 1426554_a_at |
| 98629_f_at | 1427418_a_at |
| 98483_at | 1448656_at |
| 162963_at | 1456609_at |
| 161731_at | 1448127_at |
| 102062_at | 1423416_at |
| 115089_at | 1455840_at |
| 97434_at | 1424839_a_at |
| 101984_at | 1415760_s_at |
| 100733_at | 1448206_at |
| 104260_at | 1435326_at |
| 106306_at | 1448947_at |

| | |
|---|---|
| 108108_at | 1452213_at |
| 110691_at | 1436573_at |
| 112788_at | 1455174_at |
| 114670_at | 1455585_at |
| 116557_at | 1435797_at |
| 163078_at | 1454863_at |
| 98981_s_at | 1427670_a_at |
| 115812_at | 1435379_at |
| 160098_s_at | 1434369_a_at |
| 94895_at | 1460357_at |
| 101509_at | 1421750_a_at |
| 110424_at | 1439477_at |
| 160709_at | 1435755_at |
| 109961_at | 1424657_at |
| 94830_at | 1450877_at |
| 102811_at | 1417730_at |
| 97293_at | 1423740_a_at |
| 93676_at | 1448899_s_at |
| 102389_s_at | 1423537_at |
| 104259_at | 1450416_at |
| 113275_at | 1453104_at |
| 114815_at | 1434394_at |
| 116072_at | 1429240_at |
| 93318_at | 1448417_at |
| 110822_at | 1423174_a_at |
| 99477_at | 1421947_at |
| 162606_at | 1454612_at |
| 160521_at | 1426813_at |
| 97528_at | 1423727_at |
| 93802_at | 1434882_at |
| 168550_at | 1438408_at |
| 160248_at | 1433482_a_at |
| 103404_at | 1454670_at |
| 114739_at | 1436595_at |
| 103330_at | 1452061_s_at |
| 167433_at | 1434489_at |
| 98893_at | 1426877_a_at |
| 102043_at | 1416689_at |
| 103853_at | 1452171_at |
| 108579_at | 1448651_at |
| 109056_at | 1423597_at |
| 116904_at | 1455584_at |
| 98906_at | 1417480_at |
| 163491_at | 1434666_at |
| 161077_f_at | 1448400_a_at |
| 95149_at | 1451825_a_at |
| 93299_at | 1416915_at |
| 101069_g_at | 1455504_a_at |
| 99180_at | 1423142_a_at |
| 109907_at | 1436766_at |
| 114612_at | 1458417_at |
| 109342_at | 1436165_at |
| 100584_at | 1421223_a_at |
| 167324_at | 1454860_x_at |
| 101091_at | 1451005_at |
| 114576_at | 1435746_at |

| | |
|---|---|
| 92795_at | 1416091_at |
| 95662_at | 1417241_at |
| 95468_at | 1423785_at |
| 92839_f_at | 1426613_a_at |
| 116333_at | 1418201_at |
| 96126_at | 1415893_at |
| 98943_at | 1416433_at |
| 111347_at | 1448570_at |
| 165423_s_at | 1437227_at |
| 93130_at | 1456117_at |
| 160544_at | 1416021_a_at |
| 107105_at | 1416046_a_at |
| 112790_at | 1436038_a_at |
| 99366_at | 1424906_at |
| 162737_i_at | 1433575_at |
| 92703_at | 1427266_at |
| 95614_at | 1454636_at |
| 93025_at | 1424820_a_at |
| 102915_at | 1448931_at |
| 112295_at | 1434064_at |
| 101967_at | 1416857_at |
| 102026_s_at | 1448448_a_at |
| 102924_at | 1425822_a_at |
| 106291_at | 1454785_at |
| 111846_at | 1434132_at |
| 93620_at | 1417775_at |
| 94829_at | 1424541_at |
| 168204_r_at | 1430515_s_at |
| 169469_at | 1439755_at |
| 160636_at | 1452599_s_at |
| 106956_at | 1452982_at |
| 161320_r_at | 1425028_a_at |
| 108101_at | 1424280_at |
| 100010_at | 1421604_a_at |
| 116252_at | 1432052_at |
| 97377_at | 1416692_at |
| 114349_at | 1451459_at |
| 162790_i_at | 1425149_a_at |
| 111385_at | 1418712_at |
| 98532_at | 1426885_a_at |
| 111419_at | 1434063_at |
| 96717_at | 1423752_at |
| 104108_at | 1424015_at |
| 93082_at | 1422032_a_at |
| 104313_at | 1451149_at |
| 106301_at | 1422456_at |
| 107445_at | 1429532_at |
| 109718_at | 1434934_at |
| 110416_r_at | 1429081_at |
| 110757_at | 1434890_at |
| 111152_at | 1424720_at |
| 113128_at | 1433931_at |
| 114363_at | 1433725_at |
| 116335_at | 1460353_at |
| 138798_at | 1435781_at |
| 94016_at | 1423642_at |

| | |
|---|---|
| 99988_at | 1423736_a_at |
| 93572_at | 1425143_a_at |
| 163113_at | 1429337_at |
| 96070_at | 1449018_at |
| 160719_at | 1451845_a_at |
| 96785_at | 1448839_at |
| 102024_at | 1422737_at |
| 109364_at | 1455196_s_at |
| 116319_at | 1428680_at |
| 116587_at | 1434462_at |
| 99574_at | 1434016_at |
| 160102_at | 1415785_a_at |
| 114313_at | 1428624_at |
| 106981_at | 1434075_at |
| 99490_at | 1423970_at |
| 163358_at | 1435867_at |
| 113285_at | 1454990_at |
| 105960_at | 1456783_at |
| 100144_at | 1415771_at |
| 101975_at | 1449939_s_at |
| 109411_at | 1449214_a_at |
| 111228_at | 1451148_at |
| 111377_at | 1455024_at |
| 114014_at | 1418086_at |
| 116841_at | 1449047_at |
| 116986_at | 1435281_at |
| 95722_at | 1416592_at |
| 163093_at | 1416672_s_at |
| 95650_at | 1416972_at |
| 162099_f_at | 1417224_a_at |
| 99067_at | 1417399_at |
| 162536_r_at | 1418319_at |
| 97888_at | 1424616_s_at |
| 95539_at | 1434306_at |
| 93114_at | 1435395_s_at |
| 160962_at | 1423948_at |
| 94972_at | 1418703_at |
| 100979_at | 1454064_a_at |
| 165569_at | 1434174_at |
| 111820_at | 1424373_at |
| 94466_f_at | 1420497_a_at |
| 162140_i_at | 1438832_x_at |
| 165841_at | 1422910_s_at |
| 108008_at | 1428336_at |
| 168123_at | 1429628_at |
| 116320_at | 1437533_at |
| 104237_at | 1452621_at |
| 95677_at | 1452713_a_at |
| 106479_at | 1424988_at |
| 109075_at | 1416315_at |
| 105573_at | 1452340_at |
| 162754_at | 1428755_at |
| 160501_at | 1449207_a_at |
| 104157_at | 1435369_at |
| 98423_at | 1423271_at |
| 103665_at | 1417404_at |

| | |
|---|---|
| 108538_at | 1450690_at |
| 114864_at | 1454765_at |
| 116644_at | 1419687_at |
| 129059_at | 1453748_a_at |
| 131197_at | 1453256_at |
| 96943_at | 1415699_a_at |
| 94454_at | 1415796_at |
| 98545_at | 1416202_at |
| 160416_at | 1416858_a_at |
| 95625_at | 1417116_at |
| 99999_at | 1417791_a_at |
| 98573_r_at | 1422547_at |
| 93844_at | 1430326_s_at |
| 97425_at | 1433645_at |
| 162667_at | 1450904_at |
| 163064_at | 1421883_at |
| 113620_at | 1452280_at |
| 171626_at | 1417289_at |
| 110226_at | 1456019_at |
| 163339_at | 1452737_at |
| 112205_at | 1426556_at |
| 94340_at | 1428772_at |
| 109799_at | 1435291_at |
| 163097_at | 1448646_at |
| 95458_s_at | 1437288_at |
| 103950_at | 1452448_at |
| 94253_at | 1452662_a_at |
| 110268_at | 1428451_at |
| 100957_at | 1427965_at |
| 98999_at | 1418372_at |
| 94643_at | 1450295_s_at |
| 113828_at | 1418328_at |
| 107994_at | 1424133_at |
| 94491_at | 1422471_at |
| 160688_at | 1420116_s_at |
| 113052_at | 1439775_at |
| 104719_at | 1418257_at |
| 107525_at | 1453196_a_at |
| 111911_at | 1428445_at |
| 166158_r_at | 1419644_at |
| 97061_g_at | 1460590_s_at |
| 101948_at | 1424114_s_at |
| 104208_at | 1435003_at |
| 104760_at | 1451016_at |
| 106018_at | 1433740_at |
| 106255_at | 1428648_at |
| 111213_at | 1455099_at |
| 112391_at | 1426011_a_at |
| 112401_at | 1454960_at |
| 113044_at | 1434809_at |
| 94307_at | 1422540_at |
| 98575_at | 1423828_at |
| 162991_at | 1424214_at |
| 95448_at | 1426611_at |
| 168289_r_at | 1433844_a_at |
| 92834_at | 1433928_a_at |

| | |
|---|---|
| 162627_at | 1438016_at |
| 100984_at | 1417296_at |
| 115877_at | 1439027_at |
| 102767_at | 1455089_at |
| 109667_at | 1429521_at |
| 162904_at | 1448796_s_at |
| 113946_at | 1452274_at |
| 104212_at | 1424353_at |
| 103057_at | 1448187_at |
| 110968_at | 1434817_s_at |
| 168437_i_at | 1447776_x_at |
| 130507_at | 1428902_at |
| 98894_at | 1426878_at |
| 93819_at | 1431036_a_at |
| 110629_at | 1429505_at |
| 133841_at | 1434608_at |
| 108088_at | 1426821_at |
| 93090_at | 1433489_s_at |
| 95232_at | 1424101_at |
| 111517_at | 1449017_at |
| 115106_at | 1434909_at |
| 104643_at | 1427261_at |
| 167230_f_at | 1457658_x_at |
| 92971_at | 1428891_at |
| 103840_at | 1448762_at |
| 106527_at | 1436247_at |
| 111319_at | 1418965_at |
| 97812_at | 1422736_at |
| 100064_f_at | 1438650_x_at |
| 102210_at | 1437651_a_at |
| 104573_at | 1434544_at |
| 105835_at | 1436518_at |
| 107442_at | 1435235_at |
| 110286_at | 1434354_at |
| 111088_at | 1438075_at |
| 112290_at | 1434482_at |
| 116481_at | 1434404_at |
| 116832_at | 1455558_at |
| 116965_at | 1439481_at |
| 160941_at | 1418406_at |
| 165468_r_at | 1419557_a_at |
| 160127_at | 1420827_a_at |
| 94841_at | 1424681_a_at |
| 170148_at | 1424784_at |
| 94852_at | 1426236_a_at |
| 95652_at | 1428360_x_at |
| 164012_at | 1428559_at |
| 96121_at | 1434012_at |
| 163849_r_at | 1437627_at |
| 170002_at | 1439387_x_at |
| 167069_i_at | 1439515_at |
| 92586_at | 1448253_at |
| 95154_at | 1448868_at |
| 94294_at | 1450920_at |
| 162448_f_at | 1456434_x_at |
| 94020_at | 1431292_a_at |

| | |
|---|---|
| 103421_at | 1423465_at |
| 111445_at | 1424433_at |
| 162832_at | 1423994_at |
| 107051_at | 1454815_at |
| 160655_at | 1418018_at |
| 98521_at | 1433916_at |
| 160802_at | 1423703_at |
| 110145_at | 1424970_at |
| 114630_at | 1452598_at |
| 160388_at | 1423078_a_at |
| 96124_at | 1428327_at |
| 114115_at | 1452045_at |
| 115061_at | 1428950_s_at |
| 162549_at | 1415981_at |
| 160231_at | 1424907_a_at |
| 163289_at | 1417454_at |
| 103374_at | 1428402_at |
| 107885_at | 1433872_at |
| 162713_at | 1433544_at |
| 101385_at | 1417728_at |
| 101511_at | 1448524_s_at |
| 107956_at | 1430981_s_at |
| 114305_at | 1433724_at |
| 115386_at | 1453067_at |
| 92568_at | 1423441_at |
| 99633_at | 1416852_a_at |
| 95471_at | 1417649_at |
| 98927_at | 1448304_a_at |
| 98079_at | 1450725_s_at |
| 97243_at | 1450982_at |
| 116942_at | 1451316_a_at |
| 96833_at | 1447924_at |
| 100473_at | 1460444_at |
| 97843_at | 1420808_at |
| 168469_f_at | 1441937_s_at |
| 96911_at | 1450623_at |
| 92708_at | 1424590_at |
| 167744_f_at | 1459783_s_at |
| 94977_at | 1460203_at |
| 110316_at | 1450393_a_at |
| 110854_at | 1423298_at |
| 112953_at | 1423432_at |
| 97797_at | 1430656_a_at |
| 92782_at | 1452036_a_at |
| 101916_at | 1423938_at |
| 102215_at | 1419286_s_at |
| 102321_at | 1418128_at |
| 103248_at | 1449429_at |
| 103832_at | 1448976_at |
| 104565_at | 1451665_a_at |
| 106168_at | 1436383_at |
| 106260_at | 1417272_at |
| 111336_at | 1424394_at |
| 112913_at | 1434459_at |
| 114625_at | 1424905_a_at |
| 115342_at | 1435118_at |

| | |
|---|---|
| 115577_at | 1452367_at |
| 117211_at | 1418561_at |
| 117229_at | 1434816_at |
| 136056_at | 1457936_at |
| 137709_at | 1435618_at |
| 138476_at | 1434418_at |
| 160572_at | 1416144_a_at |
| 92628_at | 1416519_at |
| 99167_at | 1417006_at |
| 95712_at | 1417037_at |
| 93823_at | 1417948_s_at |
| 98400_at | 1418264_at |
| 96072_at | 1419737_a_at |
| 96959_at | 1422559_at |
| 160612_at | 1423570_at |
| 95653_at | 1423764_s_at |
| 93311_at | 1423849_a_at |
| 95620_at | 1426440_at |
| 162818_at | 1428281_at |
| 162701_at | 1433692_at |
| 160627_at | 1434607_at |
| 164054_at | 1435224_at |
| 160107_at | 1448736_a_at |
| 95716_at | 1450012_x_at |
| 99546_at | 1450694_at |
| 92254_at | 1452298_a_at |
| 160303_at | 1453206_at |
| 161573_at | 1455876_at |
| 168384_s_at | 1447623_s_at |
| 160461_f_at | 1416431_at |
| 97721_at | 1418376_at |
| 109775_at | 1426680_at |
| 113326_at | 1455244_at |
| 116943_at | 1454741_s_at |
| 95622_at | 1460678_at |
| 160255_at | 1452217_at |
| 99462_at | 1416731_at |
| 115354_at | 1417300_at |
| 168680_at | 1431417_at |
| 160797_r_at | 1433718_a_at |
| 135069_at | 1436515_at |
| 109651_at | 1438470_at |
| 108280_at | 1443962_at |
| 110650_at | 1434384_at |
| 100894_at | 1415830_at |
| 114917_at | 1424458_at |
| 113168_at | 1452883_a_at |
| 93019_at | 1416746_at |
| 102002_at | 1450021_at |
| 162699_at | 1437001_at |
| 115463_at | 1455795_at |
| 163536_at | 1429384_at |
| 160112_at | 1428252_at |
| 97825_at | 1416271_at |
| 102199_at | 1431125_a_at |
| 94105_at | 1415724_a_at |

| | |
|---|---|
| 129267_at | 1449855_s_at |
| 163804_i_at | 1429156_at |
| 114715_at | 1456200_at |
| 98493_at | 1424328_s_at |
| 100962_at | 1417930_at |
| 102336_at | 1448396_at |
| 163841_f_at | 1459853_x_at |
| 113177_at | 1417233_at |
| 95592_at | 1428874_at |
| 96862_at | 1448388_a_at |
| 116641_f_at | 1424752_x_at |
| 96894_at | 1448422_at |
| 113231_at | 1416917_at |
| 115365_at | 1453149_at |
| 110277_at | 1433682_at |
| 141026_at | 1442434_at |
| 134152_at | 1437873_at |
| 101476_at | 1415728_at |
| 108476_at | 1424300_at |
| 107085_at | 1433873_s_at |
| 100492_at | 1435869_s_at |
| 103370_at | 1450937_at |
| 160426_at | 1417041_at |
| 94909_at | 1453728_a_at |
| 116381_at | 1429759_at |
| 108479_at | 1428233_at |
| 99007_at | 1417544_a_at |
| 162784_at | 1426623_a_at |
| 163002_at | 1429307_s_at |
| 160704_at | 1451313_a_at |
| 130444_at | 1429007_at |
| 97227_at | 1421026_at |
| 129842_at | 1441971_at |
| 106065_at | 1434002_at |
| 94235_at | 1423901_at |
| 166007_at | 1429327_at |
| 115833_at | 1455757_at |
| 100477_at | 1422587_at |
| 94019_at | 1423039_a_at |
| 107274_at | 1427408_a_at |
| 101061_at | 1449930_a_at |
| 107495_at | 1438026_at |
| 114656_at | 1425937_a_at |
| 97458_at | 1454696_at |
| 103712_at | 1433871_at |
| 93795_at | 1416448_at |
| 162926_at | 1448838_at |
| 104285_at | 1427229_at |
| 140434_r_at | 1436767_at |
| 160094_at | 1423589_at |
| 92381_at | 1420975_at |
| 96766_s_at | 1425248_a_at |
| 163811_at | 1428546_at |
| 93333_at | 1417652_a_at |
| 92585_at | 1416351_at |
| 160643_at | 1449080_at |

| | |
|---|---|
| 116563_at | 1436541_at |
| 99528_at | 1415795_at |
| 110303_at | 1431502_a_at |
| 161076_at | 1423919_at |
| 93936_at | 1434360_s_at |
| 113692_at | 1427983_at |
| 94252_at | 1423744_x_at |
| 93627_at | 1454801_at |
| 160929_at | 1423099_a_at |
| 100951_at | 1417753_at |
| 100970_at | 1416657_at |
| 101901_at | 1416104_at |
| 103085_at | 1418172_at |
| 103393_at | 1423192_at |
| 103654_at | 1418152_at |
| 103753_at | 1434332_at |
| 104272_s_at | 1421450_a_at |
| 105504_at | 1456651_a_at |
| 105611_at | 1438695_at |
| 106648_at | 1434337_at |
| 107019_at | 1455430_at |
| 107086_at | 1437009_a_at |
| 107947_at | 1429659_at |
| 108545_at | 1420368_at |
| 109471_at | 1447966_a_at |
| 109948_at | 1428533_at |
| 110486_at | 1455133_s_at |
| 110796_at | 1416814_at |
| 111018_at | 1428654_at |
| 111135_at | 1429436_at |
| 111523_at | 1424383_at |
| 111723_at | 1429248_at |
| 111872_at | 1433464_at |
| 111897_at | 1434065_at |
| 112477_at | 1424731_at |
| 112948_at | 1434914_at |
| 113124_at | 1448181_at |
| 113542_at | 1428678_s_at |
| 113688_at | 1434007_at |
| 115004_at | 1455137_at |
| 115187_at | 1449202_at |
| 115544_at | 1434956_at |
| 116077_r_at | 1436330_x_at |
| 116205_at | 1435778_at |
| 116346_at | 1429055_at |
| 116598_at | 1428419_at |
| 116817_at | 1419238_at |
| 116977_at | 1452302_at |
| 117317_at | 1433897_at |
| 128830_r_at | 1453065_at |
| 132740_r_at | 1433849_at |
| 94249_at | 1415679_at |
| 96892_at | 1415695_at |
| 96300_f_at | 1415716_a_at |
| 160499_at | 1415889_a_at |
| 93495_at | 1416166_a_at |

| | |
|---|---|
| 93041_at | 1416214_at |
| 160888_at | 1416216_at |
| 94246_at | 1416268_at |
| 167020_at | 1416584_at |
| 93536_at | 1416837_at |
| 167285_r_at | 1419440_at |
| 94953_at | 1421546_a_at |
| 93737_at | 1421901_at |
| 98132_at | 1422484_at |
| 95626_at | 1422532_at |
| 160953_at | 1422710_a_at |
| 99536_at | 1422852_at |
| 95420_at | 1423706_a_at |
| 160233_at | 1423717_at |
| 99675_at | 1423940_at |
| 97966_at | 1424055_at |
| 96341_at | 1424104_at |
| 96083_s_at | 1424251_a_at |
| 166715_at | 1425109_at |
| 92780_f_at | 1425469_a_at |
| 97422_at | 1426735_at |
| 164018_i_at | 1428453_at |
| 163059_at | 1428463_a_at |
| 163370_at | 1428484_at |
| 166212_i_at | 1428633_at |
| 93426_at | 1428982_at |
| 160305_at | 1429370_a_at |
| 164638_f_at | 1429690_at |
| 163372_at | 1429833_at |
| 162717_at | 1433567_at |
| 161010_r_at | 1434045_at |
| 98524_f_at | 1434278_at |
| 165708_i_at | 1434428_at |
| 160485_r_at | 1435702_s_at |
| 166889_at | 1438412_at |
| 167904_r_at | 1440816_x_at |
| 160101_at | 1448239_at |
| 94068_at | 1449243_a_at |
| 162993_at | 1449437_at |
| 95046_s_at | 1449940_a_at |
| 162783_r_at | 1452215_at |
| 96670_at | 1452823_at |
| 92856_at | 1453118_s_at |
| 163356_i_at | 1453230_at |
| 96614_at | 1454606_at |
| 94428_at | 1454658_at |
| 94025_at | 1460198_a_at |
| 168329_at | 1426737_at |
| 96085_at | 1416368_at |
| 160254_at | 1424254_at |
| 96765_at | 1433924_at |
| 170095_r_at | 1437434_a_at |
| 101001_at | 1423824_at |
| 101451_at | 1417355_at |
| 160670_at | 1448147_at |
| 102220_at | 1416899_at |

| | |
|---|---|
| 98600_at | 1460351_at |
| 106173_at | 1455154_at |
| 106310_at | 1417346_at |
| 99942_s_at | 1417917_at |
| 95297_at | 1420565_at |
| 102907_at | 1454862_at |
| 94332_at | 1452163_at |
| 92258_at | 1429597_at |
| 110203_at | 1424594_at |
| 93327_at | 1424169_at |
| 101844_at | 1449374_at |
| 162702_at | 1431422_a_at |
| 117021_at | 1436356_at |
| 97830_at | 1421287_a_at |
| 102852_at | 1418815_at |
| 101040_at | 1416257_at |
| 99015_at | 1448757_at |
| 100143_at | 1417240_at |
| 107072_at | 1416818_at |
| 94430_at | 1451189_at |
| 110228_at | 1428896_at |
| 113484_at | 1449491_at |
| 101070_at | 1418434_at |
| 160162_at | 1426529_a_at |
| 104930_at | 1420994_at |
| 93887_at | 1418664_at |
| 100381_at | 1427735_a_at |
| 108539_at | 1418571_at |
| 114270_at | 1435465_at |
| 167657_at | 1447869_x_at |
| 94874_at | 1416134_at |
| 112504_at | 1435168_at |
| 93360_at | 1424167_a_at |
| 107592_at | 1424568_at |
| 115111_at | 1417470_at |
| 93281_at | 1422449_s_at |
| 162903_at | 1452249_at |
| 166354_f_at | 1427170_at |
| 164092_at | 1430596_s_at |
| 110437_at | 1455299_at |
| 94963_at | 1416156_at |
| 164097_at | 1452384_at |
| 103818_at | 1417392_a_at |
| 166319_at | 1436714_at |
| 110639_at | 1457026_at |
| 92357_at | 1423378_at |
| 95603_at | 1416049_at |
| 95102_at | 1423986_a_at |
| 107124_at | 1448317_at |
| 165598_at | 1437486_at |
| 104276_at | 1452803_at |
| 94964_at | 1416157_at |
| 93753_at | 1416303_at |
| 100976_at | 1451037_at |
| 95721_at | 1426648_at |
| 113760_at | 1449192_at |

| | |
|---|---|
| 104116_at | 1434442_at |
| 101551_s_at | 1460378_a_at |
| 166589_at | 1446086_s_at |
| 110411_at | 1428775_at |
| 166084_f_at | 1426208_x_at |
| 107587_at | 1455862_at |
| 116860_at | 1455314_at |
| 98910_at | 1436747_at |
| 160085_at | 1448609_at |
| 166904_at | 1428599_at |
| 100509_at | 1417508_at |
| 93064_at | 1422490_at |
| 162557_at | 1419350_at |
| 93674_at | 1416865_at |
| 94833_at | 1448259_at |
| 93018_at | 1417460_at |
| 94432_at | 1420928_at |
| 165819_at | 1428566_at |
| 95520_at | 1417162_at |
| 94024_at | 1424143_a_at |
| 98136_at | 1421052_a_at |
| 102859_at | 1433509_s_at |
| 101469_at | 1422818_at |
| 115792_at | 1433982_at |
| 92673_at | 1418792_at |
| 101886_f_at | 1451784_x_at |
| 102976_at | 1424629_at |
| 101080_at | 1416740_at |
| 100565_at | 1448163_at |
| 103683_at | 1417582_s_at |
| 162879_at | 1454973_at |
| 137022_at | 1456067_at |
| 138078_at | 1432417_a_at |
| 104100_at | 1424130_a_at |
| 100595_at | 1460707_at |
| 162552_at | 1424099_at |
| 163596_at | 1437152_at |
| 103385_at | 1448126_at |
| 106757_f_at | 1447812_x_at |
| 94995_at | 1435695_a_at |
| 96169_at | 1424152_at |
| 97972_at | 1448434_at |
| 106642_at | 1450786_x_at |
| 113213_at | 1423306_at |
| 107491_at | 1421498_a_at |
| 104761_at | 1426708_at |
| 136166_at | 1429281_at |
| 109033_at | 1434557_at |
| 162913_at | 1451622_at |
| 112445_at | 1427035_at |
| 116106_at | 1428377_at |
| 160580_at | 1417110_at |
| 106039_at | 1451352_s_at |
| 163216_at | 1435285_at |
| 95387_f_at | 1455678_at |
| 92867_at | 1416048_at |

| | |
|---|---|
| 111408_at | 1448195_at |
| 114790_at | 1455403_at |
| 98547_at | 1448799_s_at |
| 109176_at | 1435740_at |
| 107400_at | 1455204_at |
| 103310_at | 1427142_s_at |
| 101973_at | 1452207_at |
| 94408_at | 1417624_at |
| 98432_at | 1448134_at |
| 102219_at | 1417332_at |
| 94297_at | 1416125_at |
| 102283_at | 1418057_at |
| 115731_at | 1450889_at |
| 168148_r_at | 1454114_a_at |
| 101972_at | 1423590_at |
| 96736_at | 1454899_at |
| 95627_at | 1417817_a_at |
| 94060_at | 1423694_at |
| 110408_at | 1454997_at |
| 103691_at | 1417888_at |
| 114136_at | 1426237_at |
| 162599_i_at | 1434458_at |
| 105373_at | 1453041_at |
| 95023_at | 1426940_at |
| 105629_at | 1424153_s_at |
| 160970_at | 1454735_at |
| 104480_at | 1449740_s_at |
| 100626_at | 1426040_a_at |
| 108825_at | 1448876_at |
| 137579_r_at | 1427388_at |
| 94289_r_at | 1426306_a_at |
| 97207_f_at | 1448244_at |
| 106222_at | 1434129_s_at |
| 161034_at | 1451502_at |
| 99058_at | 1450780_s_at |
| 102956_at | 1449559_at |
| 98405_at | 1422601_at |
| 112052_at | 1452436_at |
| 116624_at | 1441098_at |
| 114636_at | 1451225_at |
| 138472_at | 1455471_at |
| 167141_f_at | 1439407_x_at |
| 97300_at | 1426677_at |
| 101450_at | 1460220_a_at |
| 108315_at | 1427208_at |
| 92878_at | 1454642_a_at |
| 112956_at | 1453269_at |
| 92220_s_at | 1425532_a_at |
| 101502_at | 1422286_a_at |
| 98817_at | 1421365_at |
| 93126_at | 1455106_a_at |
| 97914_at | 1418503_at |
| 96590_f_at | 1429139_at |
| 114263_at | 1428334_at |
| 100708_at | 1420376_a_at |
| 100139_at | 1416965_at |

| | |
|---|---|
| 101957_f_at | 1435368_a_at |
| 162731_at | 1429425_at |
| 104603_at | 1417883_at |
| 160641_at | 1416432_at |
| 104031_at | 1450824_at |
| 112226_at | 1421106_at |
| 106997_at | 1455009_at |
| 96186_at | 1416836_at |
| 114442_at | 1427185_at |
| 94477_at | 1455930_at |
| 107136_at | 1417297_at |
| 161116_at | 1450821_at |
| 97768_at | 1421156_a_at |
| 98921_at | 1451134_a_at |
| 168984_r_at | 1426592_a_at |
| 115402_at | 1428512_at |
| 163889_at | 1428522_at |
| 98555_at | 1448361_at |
| 137341_at | 1455375_at |
| 160308_at | 1421814_at |
| 104282_at | 1435327_at |
| 115370_at | 1435344_at |
| 101913_at | 1429400_at |
| 96948_at | 1423664_at |
| 165433_at | 1416844_at |
| 92521_at | 1420649_at |
| 96035_at | 1416647_at |
| 113640_at | 1424529_s_at |
| 101059_at | 1415923_at |
| 114188_at | 1448027_at |
| 160470_at | 1448488_at |
| 164053_at | 1455120_at |
| 160339_at | 1448315_a_at |
| 97320_at | 1451139_at |
| 108357_at | 1429621_at |
| 100041_at | 1453111_a_at |
| 106140_at | 1435675_at |
| 95316_at | 1449545_at |
| 97249_at | 1417167_at |
| 108085_at | 1455247_at |
| 93729_at | 1421836_at |
| 104210_at | 1455158_at |
| 97360_at | 1448316_at |
| 111386_at | 1429499_at |
| 167216_f_at | 1447720_x_at |
| 115996_at | 1438872_at |
| 111990_at | 1435475_at |
| 162733_at | 1456315_a_at |
| 98495_at | 1460361_at |
| 102395_at | 1417133_at |
| 96285_at | 1423321_at |
| 113548_at | 1435076_at |
| 98433_at | 1417045_at |
| 168468_at | 1429021_at |
| 168070_at | 1432034_at |
| 162948_s_at | 1424780_a_at |

| | |
|---|---|
| 113297_at | 1433772_at |
| 93705_at | 1420682_at |
| 92484_at | 1422018_at |
| 108084_at | 1428785_at |
| 131386_at | 1452738_at |
| 94331_at | 1426353_at |
| 96657_at | 1420502_at |
| 104006_at | 1419251_at |
| 92481_at | 1422747_at |
| 94936_at | 1418215_at |
| 117125_at | 1434096_at |
| 102952_g_at | 1427505_a_at |
| 160780_at | 1450117_at |
| 109345_at | 1447930_at |
| 167843_f_at | 1445710_x_at |
| 95612_at | 1452917_at |
| 99491_at | 1419455_at |
| 104195_at | 1429207_at |
| 113189_at | 1450679_at |
| 114517_at | 1435951_at |
| 111831_at | 1421023_at |
| 95444_at | 1423722_at |
| 163222_at | 1429056_at |
| 162126_r_at | 1455206_at |
| 93251_at | 1448465_at |
| 140655_at | 1426951_at |
| 165441_at | 1428550_at |
| 103444_at | 1452210_at |
| 160498_at | 1452024_a_at |
| 93051_at | 1448499_a_at |
| 111476_at | 1435888_at |
| 95514_at | 1426897_at |
| 110604_at | 1426962_at |
| 160687_r_at | 1418334_at |
| 165747_i_at | 1433781_a_at |
| 116594_at | 1435880_at |
| 104467_at | 1434547_at |
| 102737_at | 1451924_a_at |
| 114426_at | 1428774_at |
| 98415_at | 1460338_a_at |
| 113837_f_at | 1449459_s_at |
| 93472_at | 1434479_at |
| 111935_at | 1424515_at |
| 104211_at | 1460305_at |
| 115444_at | 1424082_at |
| 111080_at | 1428904_at |
| 138014_at | 1441733_s_at |
| 110853_at | 1431777_a_at |
| 93195_at | 1429005_at |
| 165638_r_at | 1449098_a_at |
| 162889_at | 1428106_at |
| 111963_at | 1441340_at |
| 98990_at | 1450622_at |
| 100013_at | 1424617_at |
| 160067_at | 1423705_at |
| 97182_at | 1422535_at |

| | |
|---|---|
| 111896_at | 1436796_at |
| 116843_at | 1449325_at |
| 112947_at | 1431712_a_at |
| 135609_at | 1418648_at |
| 103499_at | 1435386_at |
| 134115_at | 1424900_at |
| 93147_f_at | 1452240_at |
| 163381_at | 1417301_at |
| 99881_at | 1420360_at |
| 93111_at | 1448526_at |
| 109950_at | 1455606_at |
| 96277_at | 1448129_at |
| 107811_at | 1448692_at |
| 114462_at | 1455359_at |
| 107278_at | 1452258_at |
| 116137_at | 1429089_s_at |
| 94637_at | 1427586_at |
| 139533_at | 1452259_at |
| 116013_at | 1437570_at |
| 165449_f_at | 1448824_at |
| 115015_at | 1451388_a_at |
| 96117_r_at | 1417287_at |
| 99935_at | 1417749_a_at |
| 167076_i_at | 1447683_x_at |
| 166003_at | 1438451_at |
| 116410_at | 1437635_at |
| 134638_at | 1455861_at |
| 108305_at | 1435743_at |
| 139234_at | 1423222_at |
| 104651_at | 1456039_at |
| 92197_r_at | 1450576_a_at |
| 99933_at | 1417703_at |
| 114750_at | 1434699_at |
| 93511_at | 1423608_at |
| 168280_r_at | 1455854_a_at |
| 160174_at | 1448278_at |
| 102302_at | 1427153_at |
| 165118_i_at | 1448994_at |
| 162687_at | 1453106_a_at |
| 163130_at | 1434310_at |
| 93623_at | 1448665_at |
| 111318_at | 1428634_at |
| 160396_at | 1423955_a_at |
| 101527_at | 1419258_at |
| 92311_s_at | 1425862_a_at |
| 160522_at | 1450839_at |
| 108543_at | 1417344_at |
| 92539_at | 1416762_at |
| 96865_at | 1415973_at |
| 96191_at | 1415711_at |
| 111076_at | 1455352_at |
| 106171_at | 1455342_at |
| 111493_at | 1419584_at |
| 105519_s_at | 1429619_a_at |
| 108302_at | 1422673_at |
| 103553_at | 1433408_a_at |

| | |
|---|---|
| 163296_at | 1458364_s_at |
| 95506_at | 1419649_s_at |
| 102815_at | 1418468_at |
| 165741_r_at | 1451323_at |
| 160285_at | 1428267_at |
| 108502_at | 1433745_at |
| 95573_at | 1438192_s_at |
| 117183_at | 1419927_s_at |
| 110848_at | 1428527_at |
| 103276_at | 1417435_at |
| 98625_s_at | 1416185_a_at |
| 110352_f_at | 1451146_at |
| 97386_at | 1436026_at |
| 107064_at | 1433501_at |
| 93902_at | 1448814_at |
| 92787_at | 1416065_a_at |
| 115805_at | 1460559_at |
| 92302_at | 1452281_at |
| 98019_at | 1418136_at |
| 94794_at | 1448771_a_at |
| 101440_at | 1432431_s_at |
| 114112_at | 1453271_at |
| 100516_at | 1450264_a_at |
| 108762_at | 1454903_at |
| 160690_at | 1419037_at |
| 96255_at | 1416923_a_at |
| 160857_at | 1419639_at |
| 160448_at | 1426844_a_at |
| 106991_at | 1427183_at |
| 94003_at | 1433676_at |
| 103760_at | 1428797_at |
| 163053_at | 1416700_at |
| 104704_at | 1415986_at |
| 106215_at | 1452214_at |
| 93642_at | 1416900_s_at |
| 114467_at | 1428535_at |
| 94385_at | 1429763_at |
| 101589_at | 1433507_a_at |
| 108076_at | 1417585_at |
| 108741_at | 1450916_at |
| 160130_at | 1451188_at |
| 104625_at | 1434035_at |
| 95927_f_at | 1439516_at |
| 96741_at | 1434922_at |
| 96606_at | 1450007_at |
| 102885_at | 1452007_at |
| 103549_at | 1449022_at |
| 112687_at | 1433599_at |
| 162648_at | 1416655_at |
| 101727_at | 1458299_s_at |
| 109695_at | 1418012_at |
| 111884_at | 1433453_a_at |
| 165637_i_at | 1452606_at |
| 113674_at | 1460366_at |
| 101073_at | 1448655_at |
| 111414_at | 1448691_at |

| | |
|---|---|
| 160549_at | 1415741_at |
| 112007_at | 1435745_at |
| 104389_at | 1429758_at |
| 103830_at | 1448742_at |
| 163742_i_at | 1429348_at |
| 115897_at | 1424792_at |
| 114248_at | 1455142_at |
| 162560_at | 1452256_at |
| 92458_at | 1422663_at |
| 108780_at | 1424711_at |
| 99970_at | 1419054_a_at |
| 163163_at | 1437217_at |
| 165848_at | 1429520_a_at |
| 93300_at | 1450923_at |
| 112464_at | 1451437_at |
| 114687_at | 1424301_at |
| 110720_at | 1426503_a_at |
| 92660_f_at | 1416943_at |
| 94356_at | 1426956_a_at |
| 93187_at | 1426784_at |
| 166387_f_at | 1429449_at |
| 108568_at | 1452059_at |
| 166786_i_at | 1456103_at |
| 97904_at | 1426392_a_at |
| 104315_at | 1451309_at |
| 108877_at | 1455033_at |
| 92908_at | 1422742_at |
| 160198_at | 1433442_at |
| 99448_at | 1448402_at |
| 111000_at | 1418791_at |
| 93496_at | 1415840_at |
| 97847_at | 1416354_at |
| 99955_at | 1416891_at |
| 106297_at | 1416775_at |
| 163588_at | 1433891_at |
| 113126_at | 1428383_a_at |
| 102942_at | 1428794_at |
| 98770_at | 1420441_at |
| 104597_at | 1418240_at |
| 95561_at | 1429270_a_at |
| 92456_at | 1423483_s_at |
| 160276_at | 1418394_a_at |
| 100924_at | 1448886_at |
| 94624_at | 1419439_at |
| 99005_at | 1448414_at |
| 116011_at | 1425521_at |
| 104007_at | 1420967_at |
| 108077_at | 1434472_at |
| 109726_at | 1451652_a_at |
| 114031_at | 1424956_at |
| 97255_at | 1451154_a_at |
| 110792_at | 1460466_at |
| 104441_at | 1451663_a_at |
| 97930_f_at | 1424093_x_at |
| 97359_at | 1418198_a_at |
| 102807_at | 1434138_at |

| | |
|---|---|
| 104095_at | 1452150_at |
| 160628_at | 1417823_at |
| 97367_at | 1449019_at |
| 103636_at | 1418746_at |
| 162663_at | 1428011_a_at |
| 104491_at | 1437067_at |
| 163337_at | 1436897_at |
| 160150_f_at | 1436836_x_at |
| 110288_at | 1424333_at |
| 103229_at | 1418594_a_at |
| 162715_at | 1434778_at |
| 103718_at | 1420645_at |
| 111127_at | 1454969_at |
| 168961_f_at | 1429701_at |
| 160871_at | 1451762_a_at |
| 160116_at | 1454677_at |
| 111360_at | 1429764_at |
| 92767_at | 1425493_at |
| 104673_at | 1421928_at |
| 93858_at | 1418930_at |
| 104324_at | 1428467_at |
| 92508_s_at | 1427569_a_at |
| 162624_at | 1428540_at |
| 163569_at | 1438474_at |
| 105219_at | 1427121_at |
| 160215_at | 1420619_a_at |
| 94036_at | 1416511_a_at |
| 97868_at | 1449935_a_at |
| 113870_at | 1441320_a_at |
| 99471_at | 1449628_s_at |
| 160714_at | 1417694_at |
| 165776_i_at | 1416350_at |
| 106529_at | 1440222_at |
| 96216_at | 1451219_at |
| 95446_at | 1425194_a_at |
| 94426_at | 1436007_a_at |
| 96703_at | 1450062_a_at |
| 115859_at | 1438666_at |
| 104053_at | 1454711_at |
| 116746_at | 1436381_at |
| 162619_at | 1425934_a_at |
| 135199_at | 1415921_a_at |
| 108057_at | 1422662_at |
| 117270_at | 1429095_at |
| 163256_i_at | 1437678_at |
| 102890_at | 1416825_at |
| 166339_at | 1452004_at |
| 92205_at | 1443969_at |
| 92269_r_at | 1427550_at |
| 97211_at | 1415780_a_at |
| 102317_at | 1422895_at |
| 103449_at | 1455155_at |
| 166200_i_at | 1434424_at |
| 101561_at | 1428942_at |
| 96007_at | 1415700_a_at |
| 168417_r_at | 1429661_at |

| | |
|---|---|
| 112049_at | 1435234_at |
| 163223_at | 1421891_at |
| 166915_at | 1429178_at |
| 163711_at | 1450172_at |
| 101457_at | 1421066_at |
| 99489_at | 1418253_a_at |
| 104017_at | 1451828_a_at |
| 113901_at | 1450910_at |
| 138073_at | 1442166_at |
| 113685_at | 1434808_at |
| 96271_at | 1417222_a_at |
| 108765_at | 1424928_at |
| 100426_s_at | 1425797_a_at |
| 109764_at | 1434648_a_at |
| 99499_at | 1419122_at |
| 95577_at | 1452122_at |
| 104482_at | 1418164_at |
| 114838_at | 1452401_at |
| 163701_at | 1460510_a_at |
| 110442_at | 1436185_at |
| 112203_at | 1417069_a_at |
| 140878_at | 1421020_at |
| 163582_at | 1417134_at |
| 93220_at | 1425475_at |
| 98498_at | 1448659_at |
| 102930_at | 1422523_at |
| 105453_at | 1458440_at |
| 114639_at | 1423825_at |
| 98968_at | 1419754_at |
| 116421_at | 1436514_at |
| 94942_at | 1448597_at |
| 98018_at | 1420664_s_at |
| 160110_at | 1448145_at |
| 95689_at | 1460718_s_at |
| 114792_at | 1434352_at |
| 116063_at | 1452248_at |
| 95557_at | 1426238_at |
| 95413_at | 1449889_a_at |
| 101067_at | 1428709_a_at |
| 110425_at | 1426458_at |
| 116595_at | 1453174_at |
| 162811_at | 1428162_at |
| 102033_at | 1450662_at |
| 92280_at | 1427385_s_at |
| 107059_at | 1436705_at |
| 98492_at | 1460253_at |
| 165170_i_at | 1460614_at |
| 103064_at | 1449708_s_at |
| 106031_at | 1433862_at |
| 98385_at | 1421499_a_at |
| 163461_at | 1430483_a_at |
| 109080_r_at | 1433771_at |
| 109323_at | 1451591_a_at |
| 116362_at | 1456094_at |
| 108054_at | 1451348_at |
| 103569_at | 1418011_a_at |

| | |
|---|---|
| 107920_at | 1423624_at |
| 160585_at | 1450423_s_at |
| 97536_at | 1434560_at |
| 97369_g_at | 1418279_a_at |
| 102886_at | 1421088_at |
| 92817_at | 1416210_at |
| 96068_at | 1451217_a_at |
| 99175_at | 1450649_at |
| 101178_at | 1420696_at |
| 97451_at | 1424024_at |
| 163016_at | 1439009_at |
| 104059_at | 1437297_at |
| 104558_at | 1434336_s_at |
| 98067_at | 1421679_a_at |
| 107797_at | 1456351_at |
| 108415_at | 1450351_a_at |
| 96218_at | 1434309_at |
| 163562_at | 1424229_at |
| 93670_at | 1435561_at |
| 112400_at | 1429653_at |
| 116312_at | 1452192_at |
| 115441_at | 1429185_at |
| 103545_at | 1419798_at |
| 113568_at | 1438672_at |
| 111849_at | 1455928_x_at |
| 129277_at | 1436035_at |
| 103298_at | 1448664_a_at |
| 97421_at | 1448635_at |
| 101405_at | 1448913_at |
| 94099_at | 1449960_at |
| 101585_at | 1423451_at |
| 94390_at | 1433669_at |
| 112014_at | 1435913_at |
| 110407_at | 1436300_at |
| 164707_r_at | 1451987_at |
| 96651_at | 1422676_at |
| 104418_at | 1455291_s_at |
| 107095_at | 1433700_at |
| 163247_at | 1438024_at |
| 115431_at | 1454922_at |
| 103735_at | 1419708_at |
| 109123_at | 1454978_at |
| 112254_at | 1422028_a_at |
| 95129_at | 1448893_at |
| 112682_at | 1451200_at |
| 161636_r_at | 1434496_at |
| 101060_at | 1423423_at |
| 160772_i_at | 1451727_at |
| 98129_at | 1417219_s_at |
| 104531_at | 1422847_a_at |
| 94550_at | 1416260_a_at |
| 105881_at | 1450350_a_at |
| 93250_r_at | 1437313_x_at |
| 108095_at | 1451110_at |
| 100130_at | 1448694_at |
| 102839_at | 1453181_x_at |

| | |
|---|---|
| 111975_at | 1416598_at |
| 99954_at | 1451293_at |
| 109169_at | 1454725_at |
| 116200_at | 1427088_at |
| 99188_at | 1451130_at |
| 165862_f_at | 1447896_s_at |
| 100953_at | 1417586_at |
| 103710_at | 1455433_at |
| 104326_at | 1451079_at |
| 92492_at | 1423718_at |
| 113805_at | 1426497_at |
| 93839_at | 1418101_a_at |
| 103477_at | 1449582_at |
| 115772_at | 1425042_s_at |
| 133328_at | 1443842_at |
| 112300_at | 1451744_a_at |
| 138091_at | 1454082_a_at |
| 163509_at | 1444478_at |
| 94814_at | 1428645_at |
| 103270_at | 1416969_at |
| 95345_at | 1423311_s_at |
| 133155_at | 1440999_at |
| 109084_at | 1439010_at |
| 109393_at | 1436318_at |
| 103796_at | 1452870_at |
| 113129_at | 1424236_at |
| 104141_at | 1460689_at |
| 111698_at | 1434039_at |
| 102348_at | 1424609_a_at |
| 103418_at | 1424321_at |
| 136594_at | 1428806_at |
| 161130_f_at | 1447739_x_at |
| 162892_at | 1452759_s_at |
| 105978_at | 1450966_at |
| 96607_at | 1420809_a_at |
| 167487_at | 1447713_at |
| 116119_at | 1454826_at |
| 97557_at | 1423783_at |
| 114867_at | 1455353_at |
| 94536_s_at | 1420820_at |
| 163532_at | 1428919_at |
| 100635_at | 1415696_at |
| 97965_at | 1422147_a_at |
| 162807_at | 1428154_s_at |
| 163428_at | 1428342_at |
| 100020_at | 1416637_at |
| 94813_at | 1416855_at |
| 103039_at | 1423267_s_at |
| 109928_at | 1452852_at |
| 116337_at | 1451526_at |
| 94461_at | 1417190_at |
| 163041_at | 1429787_x_at |
| 100636_at | 1417562_at |
| 166782_at | 1458602_at |
| 103412_at | 1435321_at |
| 107598_at | 1424378_at |

| | |
|---|---|
| 129294_at | 1433816_at |
| 162825_at | 1424395_at |
| 163313_at | 1436740_at |
| 160355_at | 1427099_at |
| 92935_at | 1448785_at |
| 96824_at | 1452017_at |
| 102328_at | 1424552_at |
| 110323_at | 1455394_at |
| 94367_at | 1448604_at |
| 104628_at | 1448647_at |
| 111684_at | 1436036_at |
| 115873_at | 1451044_at |
| 99655_at | 1418223_at |
| 99901_at | 1421431_at |
| 96206_at | 1424468_s_at |
| 94069_r_at | 1451420_at |
| 98905_at | 1454610_at |
| 160298_at | 1424186_at |
| 115456_at | 1435308_at |
| 104372_at | 1416863_at |
| 106812_at | 1428930_at |
| 109917_at | 1424356_a_at |
| 112293_at | 1416860_s_at |
| 167407_i_at | 1418167_at |
| 162793_at | 1451536_at |
| 170773_at | 1429984_at |
| 94899_at | 1460342_s_at |
| 103209_at | 1448870_at |
| 107142_at | 1428385_at |
| 96649_at | 1452130_at |
| 163033_at | 1432503_a_at |
| 97919_at | 1452869_at |
| 100879_at | 1418677_at |
| 160376_at | 1452646_at |
| 103288_at | 1449089_at |
| 160872_f_at | 1420548_a_at |
| 162529_at | 1424313_a_at |
| 170772_at | 1436856_x_at |
| 96259_at | 1417386_at |
| 92208_at | 1427944_at |
| 162855_at | 1420969_at |
| 135384_at | 1438868_at |
| 106270_at | 1434183_at |
| 163650_at | 1424211_at |
| 163925_at | 1430314_at |
| 97474_r_at | 1448254_at |
| 98011_at | 1455021_at |
| 101094_at | 1416480_a_at |
| 97438_r_at | 1429193_at |
| 92243_at | 1434609_at |
| 103565_at | 1417886_at |
| 104188_at | 1455556_at |
| 114345_at | 1428335_a_at |
| 98053_at | 1455815_a_at |
| 93615_at | 1447640_s_at |
| 107132_at | 1428124_at |

| | |
|---|---|
| 110225_at | 1436534_at |
| 101104_at | 1415745_a_at |
| 106644_at | 1420895_at |
| 96254_at | 1416755_at |
| 166517_f_at | 1426300_at |
| 160697_at | 1433775_at |
| 96640_at | 1448509_at |
| 162636_at | 1452208_at |
| 98880_at | 1433588_at |
| 103238_at | 1450782_at |
| 94916_at | 1450646_at |
| 117030_at | 1436866_at |
| 163246_at | 1437151_at |
| 160203_at | 1426473_at |
| 162759_at | 1424791_a_at |
| 101466_at | 1448896_at |
| 160385_at | 1432164_a_at |
| 104740_at | 1424427_at |
| 162644_at | 1424030_at |
| 160428_at | 1427441_a_at |
| 160824_at | 1428657_at |
| 96004_at | 1450878_at |
| 92565_at | 1416095_x_at |
| 107623_at | 1433468_at |
| 106966_at | 1436354_at |
| 111893_at | 1450948_a_at |
| 103716_at | 1455072_at |
| 160602_at | 1416843_at |
| 104681_at | 1421143_at |
| 98101_at | 1433528_at |
| 112352_at | 1455129_at |
| 101651_at | 1419429_at |
| 104293_at | 1428081_at |
| 109645_at | 1449128_at |
| 93431_at | 1434944_at |
| 99541_at | 1435306_a_at |
| 162941_at | 1448939_at |
| 130532_at | 1453002_at |
| 163686_at | 1431993_a_at |
| 116032_at | 1439663_at |
| 96244_at | 1448260_at |
| 98535_at | 1449183_at |
| 103855_at | 1419835_s_at |
| 94804_at | 1425383_a_at |
| 112852_at | 1455548_at |
| 99975_at | 1426483_at |
| 94009_at | 1427915_s_at |
| 167497_r_at | 1429096_at |
| 98789_at | 1450140_a_at |
| 109602_at | 1420650_at |
| 166340_at | 1447934_at |
| 161104_at | 1437208_at |
| 160260_at | 1448461_a_at |
| 107050_at | 1428428_at |
| 117236_at | 1428562_at |
| 160149_at | 1453095_at |

| | |
|---|---|
| 104176_at | 1434935_at |
| 112503_at | 1428629_at |
| 97484_at | 1426531_at |
| 160220_at | 1450998_at |
| 104523_at | 1423614_at |
| 166778_i_at | 1453005_at |
| 103202_at | 1418392_a_at |
| 164188_f_at | 1443849_x_at |
| 165998_at | 1456515_s_at |
| 103709_at | 1455494_at |
| 160768_at | 1423879_at |
| 97998_at | 1421149_a_at |
| 160977_at | 1452304_a_at |
| 117111_at | 1426849_at |
| 130969_at | 1435653_at |
| 97199_at | 1433439_at |
| 107075_at | 1422669_at |
| 107588_at | 1417974_at |
| 139261_at | 1427405_s_at |
| 95593_at | 1415698_at |
| 115418_at | 1426961_at |
| 101877_at | 1455285_at |
| 103233_at | 1419191_at |
| 109011_at | 1418469_at |
| 163108_at | 1423362_at |
| 94935_at | 1434644_at |
| 99596_f_at | 1435652_a_at |
| 96939_at | 1452670_at |
| 96353_at | 1416479_a_at |
| 93493_at | 1423645_a_at |
| 116417_at | 1433738_at |
| 109415_at | 1438018_at |
| 111299_at | 1429710_at |
| 98407_at | 1418285_at |
| 168095_at | 1422793_at |
| 160412_at | 1423534_at |
| 93102_f_at | 1422340_a_at |
| 112808_at | 1434401_at |
| 110651_at | 1424020_at |
| 101657_at | 1450342_at |
| 103941_at | 1421278_s_at |
| 108556_at | 1435223_at |
| 109397_at | 1451480_at |
| 94925_at | 1423625_a_at |
| 163000_at | 1424189_at |
| 160880_at | 1425975_a_at |
| 167922_r_at | 1438079_at |
| 164082_at | 1452841_at |
| 92196_f_at | 1455546_s_at |
| 112357_at | 1434603_at |
| 108064_at | 1433838_at |
| 111371_at | 1453120_at |
| 114490_at | 1423129_at |
| 116105_at | 1433657_at |
| 96327_at | 1415689_s_at |
| 163012_at | 1424241_at |

| | |
|---|---|
| 162628_at | 1448720_at |
| 95118_r_at | 1451128_s_at |
| 160207_at | 1451666_at |
| 116596_at | 1423528_at |
| 114688_at | 1427382_a_at |
| 166384_at | 1431035_at |
| 103356_at | 1448892_at |
| 110786_at | 1433979_at |
| 98486_at | 1418105_at |
| 164632_i_at | 1437021_at |
| 92445_at | 1450510_a_at |
| 166370_at | 1453054_at |
| 103303_at | 1448026_at |
| 97509_f_at | 1424050_s_at |
| 166356_i_at | 1415982_at |
| 115382_at | 1428656_at |
| 96564_at | 1420622_a_at |
| 163592_at | 1433985_at |
| 103756_at | 1435228_at |
| 117277_at | 1454933_at |
| 99494_at | 1448443_at |
| 101444_at | 1417684_at |
| 106056_at | 1428518_at |
| 107461_at | 1429723_at |
| 115821_at | 1437647_at |
| 96236_at | 1425554_a_at |
| 92310_at | 1427005_at |
| 97346_at | 1428552_at |
| 98596_s_at | 1449198_a_at |
| 165609_r_at | 1419522_at |
| 115355_at | 1423276_at |
| 105861_at | 1435605_at |
| 111379_at | 1449852_a_at |
| 114104_r_at | 1439007_at |
| 114716_at | 1433755_at |
| 115198_at | 1455255_at |
| 115565_at | 1435568_at |
| 160930_at | 1416500_at |
| 162520_at | 1417866_at |
| 93776_at | 1428226_at |
| 163342_at | 1433920_at |
| 166831_i_at | 1435204_at |
| 163208_at | 1452199_at |
| 163127_at | 1450724_at |
| 98936_at | 1426257_a_at |
| 97319_at | 1422562_at |
| 103471_at | 1416061_at |
| 94301_at | 1416328_a_at |
| 103455_at | 1436048_at |
| 111996_at | 1432464_a_at |
| 129476_at | 1440896_at |
| 96663_at | 1416864_at |
| 98848_at | 1419329_at |
| 93097_at | 1419549_at |
| 164903_i_at | 1435766_at |
| 160595_at | 1448555_at |

| | |
|---|---|
| 163760_at | 1449195_s_at |
| 95541_at | 1417953_at |
| 161046_at | 1418476_at |
| 108997_at | 1437251_at |
| 95002_at | 1460741_x_at |
| 111804_at | 1435536_at |
| 116437_at | 1433875_at |
| 116852_at | 1438413_at |
| 162678_at | 1423806_at |
| 163640_at | 1424350_s_at |
| 98633_at | 1426434_at |
| 168029_at | 1426455_at |
| 96882_at | 1451076_s_at |
| 162848_at | 1452805_at |
| 165978_at | 1420973_at |
| 133844_at | 1455673_at |
| 105584_at | 1436508_at |
| 96106_at | 1452725_a_at |
| 92652_at | 1449146_at |
| 168356_at | 1429174_at |
| 106667_at | 1460352_s_at |
| 113255_at | 1452128_a_at |
| 132659_at | 1452949_at |
| 160449_at | 1416018_at |
| 93609_at | 1424876_s_at |
| 163825_at | 1437199_at |
| 93516_at | 1453473_a_at |
| 103222_at | 1422823_at |
| 106916_at | 1439833_at |
| 99665_at | 1416007_at |
| 109690_at | 1438688_at |
| 98152_at | 1422450_at |
| 105138_at | 1456023_at |
| 105481_at | 1436446_at |
| 105686_at | 1442175_at |
| 112978_at | 1428472_at |
| 116831_at | 1431334_a_at |
| 116868_at | 1452328_s_at |
| 160990_r_at | 1424243_at |
| 168532_f_at | 1447864_s_at |
| 165331_at | 1417033_at |
| 131208_at | 1424358_at |
| 103559_at | 1450519_a_at |
| 109776_at | 1428914_at |
| 163664_at | 1419031_at |
| 113757_at | 1427941_at |
| 116970_at | 1435900_at |
| 160199_at | 1418693_at |
| 168001_i_at | 1458341_x_at |
| 95456_r_at | 1418574_a_at |
| 103275_at | 1460650_at |
| 162561_at | 1456293_s_at |
| 98948_at | 1433656_a_at |
| 103241_at | 1455462_at |
| 107066_s_at | 1426944_at |
| 107096_at | 1451212_at |

| | |
|---|---|
| 107753_at | 1438440_at |
| 110301_at | 1452080_a_at |
| 112045_at | 1421991_a_at |
| 97727_at | 1422300_at |
| 163422_i_at | 1425537_at |
| 161050_at | 1435981_at |
| 160434_at | 1448867_at |
| 107526_at | 1437735_at |
| 104004_at | 1428875_at |
| 162708_at | 1427902_at |
| 160668_at | 1422511_a_at |
| 94262_at | 1452125_at |
| 98455_at | 1452116_s_at |
| 93859_at | 1415685_at |
| 115776_at | 1450422_a_at |
| 162869_at | 1420965_a_at |
| 93573_at | 1422557_s_at |
| 93017_at | 1450941_at |
| 95433_at | 1460396_at |
| 103095_at | 1448227_at |
| 103668_at | 1417628_at |
| 110322_at | 1426369_at |
| 113187_at | 1433625_at |
| 160401_r_at | 1423641_s_at |
| 163821_at | 1428261_at |
| 96934_at | 1433516_a_at |
| 164255_at | 1437748_at |
| 96526_at | 1437885_at |
| 99656_at | 1451249_at |
| 163060_at | 1452791_at |
| 164275_at | 1453601_at |
| 167802_at | 1451849_a_at |
| 108468_at | 1425492_at |
| 100050_at | 1425895_a_at |
| 93596_i_at | 1416567_s_at |
| 103468_at | 1419402_at |
| 160755_at | 1437611_x_at |
| 97210_at | 1419742_at |
| 110370_at | 1453102_at |
| 111329_at | 1435016_at |
| 113746_at | 1435552_at |
| 115188_at | 1434627_at |
| 115559_at | 1429503_at |
| 115765_at | 1452261_at |
| 116126_at | 1426561_a_at |
| 134790_at | 1427933_at |
| 98945_at | 1418010_a_at |
| 95424_at | 1422457_s_at |
| 98911_at | 1433803_at |
| 97220_at | 1448307_at |
| 97447_at | 1451290_at |
| 163495_at | 1460247_a_at |
| 104283_at | 1416060_at |
| 117210_at | 1418255_s_at |
| 95547_at | 1419061_at |
| 113777_at | 1435338_at |

| | |
|---|---|
| 107456_at | 1433819_s_at |
| 166330_i_at | 1447752_x_at |
| 160351_at | 1451243_at |
| 97997_at | 1448395_at |
| 110628_i_at | 1455576_at |
| 110795_at | 1417441_at |
| 116042_at | 1424858_at |
| 116440_at | 1453035_at |
| 116846_at | 1434161_at |
| 116963_at | 1434887_at |
| 117149_at | 1439052_at |
| 137687_at | 1436594_at |
| 162876_at | 1424261_at |
| 160785_at | 1428369_s_at |
| 164799_at | 1429712_at |
| 96898_at | 1433562_s_at |
| 98594_at | 1433581_at |
| 96762_at | 1433585_at |
| 94535_at | 1450054_at |
| 98143_at | 1460319_at |
| 95608_at | 1417492_at |
| 100133_at | 1448765_at |
| 100947_at | 1421910_at |
| 116774_at | 1443924_at |
| 131186_at | 1435954_at |
| 160484_at | 1452649_at |
| 100026_at | 1450871_a_at |
| 105975_at | 1426717_at |
| 106071_at | 1433617_s_at |
| 109935_at | 1426504_a_at |
| 111327_at | 1415976_a_at |
| 114376_at | 1416686_at |
| 114994_at | 1436059_at |
| 115827_at | 1428339_at |
| 130731_at | 1452119_at |
| 135538_at | 1455418_at |
| 97390_at | 1417132_at |
| 161829_at | 1418289_at |
| 99144_s_at | 1418520_at |
| 163630_at | 1420488_at |
| 99073_at | 1422513_at |
| 160309_at | 1423462_at |
| 95690_at | 1428068_at |
| 162613_at | 1429618_at |
| 167207_at | 1459664_at |
| 98966_at | 1449118_at |
| 111212_at | 1435632_at |
| 97912_at | 1416009_at |
| 109710_at | 1418371_at |
| 92717_at | 1426413_at |
| 97838_at | 1433675_at |
| 100372_at | 1460408_at |
| 104901_at | 1443305_at |
| 113201_at | 1418040_at |
| 114602_at | 1435890_at |
| 129055_at | 1435474_at |

| | |
|---|---|
| 133146_at | 1435615_at |
| 93898_at | 1419667_at |
| 96779_f_at | 1420113_s_at |
| 99014_at | 1423892_at |
| 165434_at | 1428496_at |
| 167136_at | 1429792_at |
| 163144_at | 1434219_at |
| 168349_at | 1435557_at |
| 95215_f_at | 1437666_x_at |
| 164102_at | 1437872_at |
| 168544_f_at | 1447845_s_at |
| 96166_at | 1452768_at |
| 162917_at | 1452814_at |
| 94018_at | 1423461_a_at |
| 94768_at | 1416161_at |
| 111732_at | 1454958_at |
| 111438_at | 1436309_at |
| 113941_at | 1418391_at |
| 94932_at | 1418711_at |
| 102019_at | 1460354_a_at |
| 166303_i_at | 1436791_at |
| 103295_at | 1455066_s_at |
| 100311_f_at | 1421802_at |
| 103928_at | 1439510_at |
| 105029_at | 1440226_at |
| 106007_at | 1426530_a_at |
| 107828_at | 1427349_x_at |
| 111328_at | 1429187_at |
| 113914_at | 1438200_at |
| 114028_at | 1451827_a_at |
| 114303_at | 1428136_at |
| 115026_at | 1437203_at |
| 134597_at | 1439945_at |
| 95010_at | 1418587_at |
| 169471_at | 1419369_at |
| 160911_at | 1421884_at |
| 99619_at | 1428586_at |
| 163298_at | 1436191_at |
| 99129_at | 1448212_at |
| 92915_s_at | 1452493_s_at |
| 95696_at | 1456244_x_at |
| 160657_at | 1460669_at |
| 92437_at | 1460684_at |
| 160375_at | 1449434_at |
| 160746_at | 1450994_at |
| 163833_at | 1429320_at |
| 92790_at | 1415860_at |
| 112435_at | 1433598_at |
| 103721_at | 1452106_at |
| 101955_at | 1416064_a_at |
| 92579_at | 1416422_a_at |
| 111864_at | 1418083_at |
| 116591_at | 1429048_at |
| 163909_at | 1436826_at |
| 104458_at | 1436207_at |
| 170400_i_at | 1432539_a_at |

| | |
|---|---|
| 104340_at | 1417968_a_at |
| 107872_r_at | 1455607_at |
| 112010_at | 1450414_at |
| 112343_at | 1424081_at |
| 113222_at | 1418327_at |
| 115062_at | 1422624_at |
| 116719_at | 1435315_s_at |
| 116877_at | 1436094_at |
| 131695_at | 1454951_at |
| 135662_at | 1437312_at |
| 96674_at | 1419950_s_at |
| 97927_at | 1422572_at |
| 163644_at | 1422692_at |
| 162966_i_at | 1422752_at |
| 168028_at | 1424492_at |
| 99623_s_at | 1426562_a_at |
| 163862_at | 1434884_at |
| 92828_at | 1449438_at |
| 98559_at | 1452469_a_at |
| 162834_at | 1455400_at |
| 111894_at | 1424372_at |
| 98511_at | 1418573_a_at |
| 109142_at | 1434910_at |
| 96587_at | 1421789_s_at |
| 94835_f_at | 1427347_s_at |
| 99599_s_at | 1438058_s_at |
| 95020_at | 1428187_at |
| 113167_at | 1435225_s_at |
| 114894_at | 1429167_at |
| 101090_at | 1460208_at |
| 107531_at | 1428593_at |
| 92294_at | 1447926_at |
| 100138_f_at | 1436979_x_at |
| 100605_at | 1419739_at |
| 101142_at | 1448662_at |
| 101905_at | 1415769_at |
| 102840_at | 1450479_x_at |
| 103391_at | 1455991_at |
| 104723_at | 1423912_at |
| 109088_at | 1451216_at |
| 109693_at | 1433927_at |
| 110647_at | 1449014_at |
| 111355_at | 1436684_a_at |
| 111365_at | 1454795_at |
| 111903_at | 1433559_at |
| 113257_at | 1421064_at |
| 114463_at | 1434659_at |
| 115913_at | 1423089_at |
| 116962_at | 1424830_at |
| 134338_at | 1443939_at |
| 95708_at | 1415827_a_at |
| 97446_at | 1416140_a_at |
| 161040_at | 1422985_at |
| 160635_at | 1435102_a_at |
| 163427_s_at | 1448701_a_at |
| 160511_at | 1448823_at |

| | |
|---|---|
| 97505_at | 1451025_at |
| 97308_at | 1454669_at |
| 163432_at | 1454878_at |
| 114781_at | 1437123_at |
| 97258_at | 1425140_at |
| 111746_at | 1449415_at |
| 116327_at | 1417941_at |
| 99856_r_at | 1422592_at |
| 111877_at | 1436303_at |
| 100557_g_at | 1426378_at |
| 163861_at | 1424135_at |
| 102000_f_at | 1435757_a_at |
| 113968_at | 1418583_at |
| 115093_at | 1428883_at |
| 171533_i_at | 1429818_at |
| 99467_at | 1426476_at |
| 93747_at | 1433706_a_at |
| 160823_at | 1450899_at |
| 111341_at | 1428845_at |
| 135257_at | 1437216_at |
| 100425_at | 1418261_at |
| 103577_at | 1456676_a_at |
| 111384_at | 1426895_at |
| 112001_at | 1434908_at |
| 114138_at | 1428323_at |
| 114322_at | 1435117_a_at |
| 115673_r_at | 1455817_x_at |
| 115936_at | 1429633_at |
| 116983_at | 1433988_s_at |
| 130425_at | 1438084_at |
| 160113_at | 1416738_at |
| 94526_at | 1416850_s_at |
| 163209_at | 1417517_at |
| 98865_at | 1418678_at |
| 163434_at | 1418943_at |
| 94164_at | 1419694_at |
| 98586_at | 1420477_at |
| 93612_at | 1422597_at |
| 162897_at | 1424871_s_at |
| 162821_at | 1428185_at |
| 160076_at | 1430500_s_at |
| 96658_at | 1433480_at |
| 170835_at | 1440371_at |
| 99866_at | 1445689_at |
| 94469_at | 1448196_at |
| 92544_f_at | 1448442_a_at |
| 93062_at | 1448909_a_at |
| 160881_at | 1456565_s_at |
| 167335_at | 1441842_s_at |
| 102018_at | 1435965_at |
| 107042_at | 1428219_at |
| 99521_at | 1421830_at |
| 109623_at | 1434660_at |
| 163183_at | 1420618_at |
| 163149_at | 1417565_at |
| 115009_at | 1436305_at |

| | |
|---|---|
| 109762_at | 1424049_at |
| 160159_at | 1448205_at |
| 112929_at | 1418379_s_at |
| 97699_at | 1421232_at |
| 162010_r_at | 1426582_at |
| 101194_at | 1420568_at |
| 116116_at | 1438039_at |
| 96522_at | 1417365_a_at |
| 99191_at | 1448405_a_at |
| 92473_at | 1449029_at |
| 109407_at | 1426909_at |
| 96065_at | 1416503_at |
| 100628_at | 1448284_a_at |
| 111683_at | 1424778_at |
| 112910_f_at | 1454830_at |
| 98005_at | 1420859_at |
| 100527_at | 1419812_s_at |
| 101003_at | 1416151_at |
| 101072_at | 1437733_at |
| 101171_at | 1420457_at |
| 101387_at | 1419173_at |
| 101922_at | 1417204_at |
| 102231_at | 1419295_at |
| 102427_at | 1451324_s_at |
| 102797_at | 1448390_a_at |
| 102944_at | 1455060_at |
| 103328_at | 1421640_a_at |
| 103823_at | 1448173_a_at |
| 103868_at | 1448802_at |
| 103952_at | 1452317_at |
| 104184_at | 1450791_at |
| 105588_at | 1420510_at |
| 106186_at | 1434969_at |
| 106618_at | 1426223_at |
| 107020_at | 1419355_at |
| 107392_at | 1423258_at |
| 109098_at | 1437875_at |
| 109389_at | 1434238_at |
| 109511_at | 1429617_at |
| 109719_at | 1439051_a_at |
| 110378_at | 1426511_at |
| 111339_at | 1419267_at |
| 111420_at | 1417632_at |
| 111624_at | 1434797_at |
| 111680_at | 1434828_at |
| 113716_at | 1453068_at |
| 114052_at | 1429171_a_at |
| 114057_at | 1455440_at |
| 114629_at | 1421043_s_at |
| 115844_at | 1451382_at |
| 117216_at | 1421191_s_at |
| 129759_at | 1430849_a_at |
| 136558_at | 1455323_at |
| 160069_at | 1417506_at |
| 160423_at | 1420846_at |
| 167396_r_at | 1421350_a_at |

| | |
|---|---|
| 93724_at | 1423428_at |
| 160391_at | 1423680_at |
| 162697_at | 1424916_x_at |
| 162833_at | 1426791_at |
| 160144_at | 1426905_a_at |
| 163140_at | 1427003_at |
| 163890_at | 1427433_s_at |
| 160281_at | 1428437_at |
| 96713_at | 1434510_at |
| 96881_at | 1435863_at |
| 160945_at | 1437786_at |
| 167246_at | 1442802_x_at |
| 99817_at | 1450303_at |
| 165602_f_at | 1451173_at |
| 94797_at | 1451239_a_at |
| 96337_at | 1452357_at |
| 98901_at | 1452795_at |
| 169991_i_at | 1448580_at |
| 97808_at | 1418562_at |
| 93274_at | 1426124_a_at |
| 112718_at | 1438429_at |
| 104016_at | 1449204_at |
| 97383_at | 1437149_at |
| 92503_at | 1418308_at |
| 115356_s_at | 1425494_s_at |
| 101593_at | 1417311_at |
| 165448_i_at | 1424062_at |
| 92643_at | 1421820_a_at |
| 109335_at | 1455574_at |
| 93069_at | 1448356_at |
| 113753_at | 1429239_a_at |
| 112791_at | 1437536_at |
| 115183_at | 1453127_at |
| 160290_at | 1423120_at |
| 102853_at | 1450950_at |
| 110191_at | 1431068_at |
| 112330_at | 1417535_at |
| 99960_at | 1426233_at |
| 161036_at | 1426712_at |
| 133364_at | 1442630_at |
| 109971_at | 1428886_at |
| 160287_at | 1415930_a_at |
| 100713_at | 1449546_a_at |
| 100321_f_at | 1449910_at |
| 93805_at | 1448643_at |
| 114065_at | 1455547_at |
| 98120_at | 1415690_at |
| 103067_at | 1423137_at |
| 101182_at | 1450525_at |
| 109989_at | 1435542_s_at |
| 111423_at | 1434036_at |
| 161000_i_at | 1416309_at |
| 102686_at | 1424059_at |
| 116311_at | 1425718_a_at |
| 97251_at | 1428153_at |
| 92763_at | 1427490_at |

| | |
|---|---|
| 103807_at | 1418202_a_at |
| 101484_at | 1448343_a_at |
| 111350_at | 1424083_at |
| 129306_r_at | 1430038_at |
| 95730_at | 1421971_a_at |
| 165606_r_at | 1435661_at |
| 96146_at | 1449007_at |
| 104244_at | 1435889_at |
| 104569_at | 1452377_at |
| 99133_at | 1425364_a_at |
| 100148_at | 1418330_at |
| 100403_at | 1449071_at |
| 101034_at | 1418508_a_at |
| 101125_at | 1451913_a_at |
| 101516_at | 1429830_a_at |
| 103201_at | 1449171_at |
| 103256_at | 1418990_at |
| 103314_at | 1451474_a_at |
| 103592_at | 1417854_at |
| 103744_at | 1434109_at |
| 103745_at | 1454938_at |
| 103821_at | 1417019_a_at |
| 104470_at | 1435099_at |
| 105260_at | 1424472_at |
| 105637_g_at | 1439960_at |
| 106025_at | 1416471_at |
| 106546_at | 1457236_at |
| 106586_at | 1454679_at |
| 108028_at | 1454717_at |
| 108795_at | 1456485_at |
| 109760_at | 1448850_a_at |
| 109766_r_at | 1419275_at |
| 110399_at | 1436375_at |
| 110583_at | 1425059_at |
| 110675_at | 1434355_at |
| 110764_r_at | 1448666_s_at |
| 111361_at | 1454879_s_at |
| 111774_at | 1427937_at |
| 111790_at | 1434022_at |
| 112497_at | 1452812_at |
| 112645_at | 1426672_at |
| 112833_at | 1427259_at |
| 112844_at | 1423626_at |
| 113193_at | 1427006_at |
| 113842_at | 1439091_at |
| 113976_at | 1448985_at |
| 114060_at | 1451516_at |
| 114837_at | 1416545_at |
| 115218_at | 1437891_at |
| 115783_at | 1435451_at |
| 116659_at | 1428643_at |
| 117198_at | 1436189_at |
| 129017_at | 1437879_at |
| 133268_f_at | 1425178_s_at |
| 133851_s_at | 1437069_at |
| 135812_at | 1455273_at |

| | |
|---|---|
| 99674_at | 1415681_at |
| 160800_at | 1415712_at |
| 160167_at | 1415926_at |
| 160904_at | 1417147_at |
| 96768_at | 1417226_at |
| 96177_at | 1417967_at |
| 92710_at | 1419457_at |
| 94292_at | 1419912_s_at |
| 97550_at | 1420813_at |
| 164094_at | 1421122_at |
| 99982_at | 1421266_s_at |
| 95404_at | 1422792_at |
| 97229_at | 1423132_a_at |
| 163265_at | 1423524_at |
| 162934_at | 1423991_at |
| 92804_at | 1424473_at |
| 117014_at | 1425899_a_at |
| 160271_at | 1428380_at |
| 169555_r_at | 1428985_at |
| 160736_at | 1430805_s_at |
| 92840_at | 1433580_at |
| 94967_at | 1433702_at |
| 169616_i_at | 1435287_at |
| 95755_at | 1435800_a_at |
| 92883_at | 1436034_at |
| 162890_at | 1436867_at |
| 164034_at | 1436870_s_at |
| 171604_at | 1437328_x_at |
| 171499_at | 1438955_x_at |
| 166072_f_at | 1439003_s_at |
| 166751_at | 1441315_s_at |
| 170944_at | 1443735_at |
| 98155_r_at | 1448277_at |
| 92934_at | 1449126_at |
| 166302_at | 1449170_at |
| 94803_at | 1449542_at |
| 95364_at | 1449848_at |
| 164024_at | 1455046_a_at |
| 163868_at | 1455843_at |
| 164081_at | 1456163_at |
| 166784_at | 1456518_at |
| 134288_at | 1428848_a_at |
| 165871_f_at | 1436242_a_at |
| 94750_at | 1449502_at |
| 103493_at | 1419405_at |
| 163107_at | 1428111_at |
| 100277_at | 1422053_at |
| 112944_at | 1434506_at |
| 136535_at | 1437404_at |
| 166673_at | 1455500_at |
| 114329_at | 1426359_at |
| 162680_at | 1433768_at |
| 116009_at | 1424860_at |
| 131983_at | 1442274_at |
| 138553_at | 1436892_at |
| 166421_s_at | 1452608_at |

| | |
|---|---|
| 166740_at | 1434243_s_at |
| 113319_at | 1438069_a_at |
| 103076_at | 1432270_a_at |
| 104218_s_at | 1442603_at |
| 115158_at | 1427042_at |
| 95511_at | 1422445_at |
| 106200_at | 1426982_at |
| 94934_at | 1415832_at |
| 96858_at | 1418127_a_at |
| 110218_at | 1436986_at |
| 94757_at | 1420579_s_at |
| 95951_at | 1420804_s_at |
| 93838_at | 1428381_a_at |
| 167101_at | 1442038_at |
| 115926_at | 1428663_at |
| 163070_at | 1455794_at |
| 134107_at | 1454229_a_at |
| 95423_at | 1416497_at |
| 92257_at | 1418659_at |
| 103713_at | 1428193_at |
| 115035_at | 1426421_s_at |
| 96678_at | 1451559_a_at |
| 98822_at | 1453939_x_at |
| 130957_f_at | 1434645_at |
| 104255_at | 1435551_at |
| 98533_at | 1416727_a_at |
| 104121_at | 1426873_s_at |
| 93464_at | 1455151_at |
| 160188_at | 1418505_at |
| 94027_at | 1434615_x_at |
| 96258_at | 1448300_at |
| 104772_at | 1436304_at |
| 113230_at | 1417018_at |
| 92850_at | 1452767_at |
| 160783_at | 1426780_at |
| 133389_at | 1443286_at |
| 103281_at | 1420906_at |
| 102370_at | 1434642_at |
| 163455_at | 1422008_a_at |
| 106115_at | 1416474_at |
| 136733_at | 1440177_at |
| 104179_at | 1434312_at |
| 130911_at | 1455059_at |
| 96594_at | 1435194_at |
| 99425_at | 1451766_at |
| 134221_at | 1458910_at |
| 92773_at | 1448943_at |
| 103531_f_at | 1434714_at |
| 160876_at | 1417077_at |
| 166442_f_at | 1437876_at |
| 110513_at | 1455682_at |
| 160609_at | 1418774_a_at |
| 96278_at | 1423686_a_at |
| 111264_at | 1420980_at |
| 113610_at | 1451301_at |
| 100011_at | 1429360_at |

| | |
|---|---|
| 102727_at | 1422169_a_at |
| 104533_at | 1435458_at |
| 115464_at | 1429719_at |
| 104671_at | 1422573_at |
| 110202_at | 1449068_at |
| 137034_f_at | 1423310_at |
| 103079_at | 1426525_at |
| 162635_at | 1417895_a_at |
| 98915_at | 1429321_at |
| 103978_at | 1426361_at |
| 160320_at | 1425826_a_at |
| 163891_at | 1436845_at |
| 167860_i_at | 1429963_at |
| 162839_i_at | 1429504_at |
| 113830_at | 1449388_at |
| 96011_at | 1450874_at |
| 98815_at | 1421627_at |
| 166065_f_at | 1459823_at |
| 104302_f_at | 1428155_at |
| 115002_at | 1426601_at |
| 139019_at | 1438816_at |
| 115575_at | 1417701_at |
| 160480_at | 1426794_at |
| 98283_at | 1450308_a_at |
| 109448_at | 1427197_at |
| 92529_s_at | 1421721_a_at |
| 160517_at | 1423521_at |
| 94853_at | 1417432_a_at |
| 110115_at | 1440857_at |
| 117269_at | 1422520_at |
| 105155_at | 1438495_at |
| 93646_at | 1420873_at |
| 165659_at | 1436738_at |
| 97975_at | 1452497_a_at |
| 112460_at | 1433664_at |
| 165528_i_at | 1450980_at |
| 95430_f_at | 1451036_at |
| 137973_at | 1418117_at |
| 93268_at | 1424109_a_at |
| 92852_at | 1426642_at |
| 108576_at | 1431054_at |
| 96896_at | 1452587_at |
| 107091_at | 1428872_at |
| 130555_at | 1444872_at |
| 101000_at | 1426763_at |
| 96603_at | 1420832_at |
| 133734_at | 1443649_at |
| 93713_at | 1422853_at |
| 114808_at | 1427136_s_at |
| 165965_at | 1437088_at |
| 98336_s_at | 1451920_a_at |
| 101900_at | 1449152_at |
| 94548_at | 1451247_at |
| 109332_at | 1419685_at |
| 129225_at | 1435577_at |
| 160897_at | 1451359_at |

| | |
|---|---|
| 162997_at | 1453183_at |
| 138089_at | 1455079_at |
| 102235_at | 1434777_at |
| 109781_at | 1416131_s_at |
| 138945_at | 1419490_at |
| 168099_at | 1444759_at |
| 103335_at | 1421217_a_at |
| 116917_at | 1437360_at |
| 165660_i_at | 1438105_at |
| 132815_at | 1420644_a_at |
| 133262_at | 1440841_at |
| 113292_at | 1429739_a_at |
| 101150_at | 1421773_at |
| 110428_at | 1424621_at |
| 135503_at | 1438700_at |
| 96695_at | 1417609_at |
| 112458_at | 1418996_a_at |
| 116738_at | 1434073_at |
| 117129_at | 1452821_at |
| 96375_at | 1442484_at |
| 163430_at | 1433623_at |
| 95800_s_at | 1422249_s_at |
| 94061_at | 1416326_at |
| 99505_at | 1451643_a_at |
| 103507_at | 1451161_a_at |
| 103913_at | 1449944_a_at |
| 93319_at | 1415850_at |
| 110275_at | 1427228_at |
| 92270_at | 1426016_a_at |
| 102100_f_at | 1449633_s_at |
| 107506_at | 1455831_at |
| 111332_at | 1434696_at |
| 160472_r_at | 1426649_at |
| 109520_at | 1456610_at |
| 107774_at | 1449885_at |
| 165678_i_at | 1434572_at |
| 96158_at | 1451125_at |
| 100039_at | 1448372_a_at |
| 94912_at | 1422451_at |
| 160603_at | 1421907_at |
| 112958_at | 1437571_at |
| 113033_at | 1435526_at |
| 116614_at | 1451751_at |
| 162677_at | 1433857_at |
| 110834_at | 1433466_at |
| 93867_at | 1419572_a_at |
| 96803_at | 1420654_a_at |
| 129286_at | 1459879_at |
| 109368_at | 1424523_at |
| 96850_at | 1426833_at |
| 93076_at | 1428537_at |
| 101523_at | 1428262_s_at |
| 99080_at | 1428311_at |
| 162859_at | 1433537_at |
| 97803_at | 1450919_at |
| 102833_at | 1434116_at |

| | |
|---|---|
| 106493_at | 1443862_at |
| 103027_at | 1433867_at |
| 108058_at | 1428409_at |
| 113902_at | 1428558_at |
| 93095_at | 1416176_at |
| 98127_at | 1423057_at |
| 106276_at | 1436033_at |
| 95619_at | 1429122_a_at |
| 94989_at | 1454722_at |
| 109145_at | 1451361_a_at |
| 99600_at | 1416945_at |
| 166122_at | 1420774_a_at |
| 100878_at | 1423535_at |
| 135178_at | 1441229_at |
| 100125_at | 1450854_at |
| 103496_at | 1419396_at |
| 108546_at | 1452682_at |
| 109020_at | 1453224_at |
| 109757_at | 1437173_at |
| 114032_f_at | 1428603_at |
| 167174_at | 1420638_at |
| 102399_at | 1429359_s_at |
| 103751_at | 1452273_at |
| 110839_at | 1436164_at |
| 115781_at | 1453100_at |
| 131128_at | 1444969_at |
| 132088_at | 1435555_at |
| 134660_at | 1435162_at |
| 167912_at | 1429637_at |
| 96599_at | 1435420_at |
| 106274_at | 1423519_at |
| 93871_at | 1451798_at |
| 162738_at | 1452661_at |
| 104471_at | 1448928_at |
| 107463_at | 1436764_at |
| 92495_at | 1420999_at |
| 101919_at | 1425972_a_at |
| 94807_at | 1428190_at |
| 136643_f_at | 1455038_at |
| 104056_at | 1433995_s_at |
| 97259_at | 1422608_at |
| 160843_at | 1460164_at |
| 100400_at | 1416668_at |
| 162985_at | 1428646_at |
| 166667_r_at | 1435594_at |
| 160278_at | 1418702_a_at |
| 110958_at | 1445967_at |
| 102063_at | 1416501_at |
| 136798_at | 1440072_at |
| 99009_at | 1416105_at |
| 160482_at | 1416947_s_at |
| 101456_at | 1425331_at |
| 110785_f_at | 1451477_at |
| 114897_f_at | 1453028_at |
| 162808_at | 1417327_at |
| 93563_s_at | 1423516_a_at |

| | |
|---|---|
| 113276_at | 1455161_at |
| 99149_at | 1416118_at |
| 107151_at | 1437177_at |
| 94418_at | 1417403_at |
| 166679_i_at | 1438256_at |
| 93721_at | 1417462_at |
| 98843_at | 1421301_at |
| 113658_at | 1423063_at |
| 163242_at | 1418245_a_at |
| 166687_at | 1423633_at |
| 92383_at | 1451977_at |
| 104468_at | 1423093_at |
| 104728_at | 1426246_at |
| 108758_at | 1454700_at |
| 94549_at | 1424129_at |
| 92510_at | 1426431_at |
| 99514_at | 1428669_at |
| 166137_at | 1442344_at |
| 100088_at | 1426205_at |
| 93752_at | 1426705_s_at |
| 99185_at | 1428588_a_at |
| 112257_at | 1433812_at |
| 112383_at | 1455719_at |
| 112478_at | 1417559_at |
| 160633_at | 1417724_at |
| 116099_at | 1421333_a_at |
| 95440_at | 1423679_at |
| 103638_at | 1426983_at |
| 117194_at | 1453622_s_at |
| 103212_at | 1451306_at |
| 103285_at | 1449490_at |
| 92226_at | 1417875_at |
| 96299_at | 1428286_at |
| 164128_at | 1434172_at |
| 97848_at | 1426863_at |
| 163846_at | 1433741_at |
| 161787_f_at | 1455791_at |
| 96192_at | 1431804_a_at |
| 92424_at | 1434031_at |
| 164162_at | 1441753_at |
| 102222_at | 1427672_a_at |
| 105214_at | 1435973_at |
| 110314_at | 1418206_at |
| 115429_at | 1432394_a_at |
| 139537_at | 1436631_at |
| 163826_at | 1457096_at |
| 162633_at | 1448678_at |
| 117093_at | 1427308_at |
| 133636_at | 1459746_at |
| 93992_at | 1424073_at |
| 92642_at | 1448752_at |
| 162639_at | 1416164_at |
| 95551_at | 1452736_at |
| 102670_at | 1449095_at |
| 111688_at | 1452198_at |
| 160236_at | 1424824_at |

| | |
|---|---|
| 162541_i_at | 1448532_at |
| 96626_at | 1449672_s_at |
| 107571_at | 1416708_a_at |
| 139413_at | 1459670_at |
| 162915_at | 1420875_at |
| 164169_at | 1450756_s_at |
| 94842_at | 1452101_at |
| 110319_at | 1426936_at |
| 133081_at | 1428373_at |
| 95058_f_at | 1423833_a_at |
| 160172_at | 1426758_s_at |
| 160937_at | 1416776_at |
| 160646_at | 1421817_at |
| 94910_at | 1435737_a_at |
| 166415_at | 1455565_at |
| 101085_at | 1450865_s_at |
| 104350_at | 1422854_at |
| 104600_at | 1452720_a_at |
| 105049_at | 1436332_at |
| 105591_at | 1457038_at |
| 163297_at | 1428491_at |
| 102142_r_at | 1449597_at |
| 102819_at | 1418046_at |
| 104442_at | 1441992_at |
| 160483_at | 1416723_at |
| 165615_at | 1439014_at |
| 93340_f_at | 1452102_at |
| 102964_at | 1424237_at |
| 162942_at | 1422706_at |
| 96947_at | 1428181_at |
| 166620_at | 1444451_at |
| 97892_at | 1451137_a_at |
| 102883_at | 1426890_a_at |
| 140010_f_at | 1426272_at |
| 164027_at | 1427046_at |
| 92872_at | 1448106_at |
| 116856_at | 1437064_at |
| 93416_at | 1419083_at |
| 102632_at | 1422814_at |
| 103459_at | 1424674_at |
| 114389_at | 1435021_at |
| 160447_at | 1423195_at |
| 163932_i_at | 1430616_at |
| 166906_i_at | 1449402_at |
| 94818_at | 1460631_at |
| 100550_f_at | 1434491_a_at |
| 102208_at | 1449078_at |
| 102991_s_at | 1454987_a_at |
| 115043_at | 1435148_at |
| 92696_at | 1421515_at |
| 98989_at | 1448619_at |
| 168057_f_at | 1455947_at |
| 168176_at | 1432023_a_at |
| 96060_at | 1450138_a_at |
| 168904_at | 1450896_at |
| 101148_at | 1448695_at |

| | |
|---|---|
| 109008_at | 1441442_at |
| 112322_at | 1434067_at |
| 112845_at | 1434481_at |
| 112858_at | 1456443_at |
| 113442_at | 1455980_a_at |
| 96707_at | 1416370_at |
| 167400_i_at | 1425169_at |
| 97358_at | 1428510_at |
| 116680_at | 1418785_at |
| 162746_at | 1416133_at |
| 92681_at | 1417217_at |
| 161086_at | 1418898_at |
| 114727_at | 1451640_a_at |
| 163197_i_at | 1428799_at |
| 162846_at | 1436982_at |
| 165669_i_at | 1454772_at |
| 101007_at | 1418300_a_at |
| 95978_at | 1442145_at |
| 166851_i_at | 1457639_at |
| 106597_at | 1435764_a_at |
| 111497_at | 1435163_at |
| 114718_at | 1437543_at |
| 96139_at | 1422489_at |
| 94088_at | 1423470_at |
| 167690_at | 1424997_at |
| 104406_at | 1449450_at |
| 117120_at | 1434246_at |
| 140645_at | 1441045_at |
| 160848_at | 1448875_at |
| 111349_at | 1451505_at |
| 112881_at | 1437158_at |
| 114146_at | 1429689_at |
| 100074_at | 1439448_x_at |
| 103491_at | 1446497_at |
| 96912_s_at | 1416811_s_at |
| 163505_at | 1437865_at |
| 95887_at | 1448068_at |
| 94273_at | 1449142_a_at |
| 103881_at | 1424488_a_at |
| 110462_at | 1440055_at |
| 115260_at | 1434951_at |
| 96238_at | 1434549_at |
| 101503_at | 1415877_at |
| 108416_at | 1452395_at |
| 97996_at | 1424217_at |
| 93463_at | 1424656_s_at |
| 96672_at | 1428662_a_at |
| 169464_f_at | 1459269_at |
| 104215_at | 1435444_at |
| 113606_at | 1416472_at |
| 168252_f_at | 1430395_at |
| 92622_at | 1431284_a_at |
| 105606_at | 1416701_at |
| 162782_at | 1424299_at |
| 103481_at | 1420083_at |
| 104117_at | 1451220_at |

| | |
|---|---|
| 113283_at | 1460603_at |
| 97460_at | 1454031_at |
| 104943_at | 1427991_s_at |
| 108366_at | 1431823_at |
| 115523_at | 1449481_at |
| 116409_at | 1418390_at |
| 94396_at | 1448496_a_at |
| 100742_at | 1420161_at |
| 111756_f_at | 1426052_at |
| 160588_at | 1428616_at |
| 101180_at | 1421205_at |
| 103556_at | 1455090_at |
| 109975_at | 1452227_at |
| 112415_at | 1444218_at |
| 138069_at | 1419063_at |
| 95766_f_at | 1427873_at |
| 99668_at | 1444120_at |
| 100081_at | 1415909_at |
| 95936_at | 1448022_at |
| 104502_f_at | 1436050_x_at |
| 114498_at | 1436999_at |
| 166519_at | 1427485_at |
| 167172_i_at | 1429772_at |
| 164074_at | 1435292_at |
| 93016_at | 1460621_x_at |
| 97529_at | 1417732_at |
| 92304_at | 1427305_at |
| 96530_at | 1442006_at |
| 96902_at | 1452721_a_at |
| 102060_at | 1417674_s_at |
| 107629_at | 1424514_at |
| 163824_i_at | 1429087_at |
| 95096_at | 1451179_a_at |
| 102275_at | 1420943_at |
| 112676_at | 1460597_at |
| 113728_at | 1434700_at |
| 113792_at | 1439847_s_at |
| 114404_at | 1435546_a_at |
| 136592_f_at | 1435336_at |
| 95024_at | 1418191_at |
| 106289_at | 1426210_x_at |
| 116051_at | 1445605_s_at |
| 160535_at | 1416331_a_at |
| 93966_at | 1448410_at |
| 111940_at | 1434668_at |
| 93021_at | 1428209_at |
| 93967_at | 1417326_a_at |
| 165706_r_at | 1451545_at |
| 104266_at | 1439506_at |
| 116853_at | 1435634_at |
| 138065_at | 1442347_at |
| 160197_at | 1416400_at |
| 99457_at | 1426817_at |
| 166020_f_at | 1439765_x_at |
| 100457_at | 1448579_at |
| 100887_at | 1448828_at |

| | |
|---|---|
| 101101_at | 1421823_a_at |
| 101188_at | 1421468_at |
| 106661_at | 1426914_at |
| 111406_at | 1435645_at |
| 128624_at | 1456652_at |
| 136074_at | 1450119_at |
| 98772_at | 1419728_at |
| 95282_at | 1438902_a_at |
| 162705_at | 1450949_at |
| 92618_at | 1455236_x_at |
| 102974_at | 1449498_at |
| 107954_at | 1451629_at |
| 114264_at | 1452236_at |
| 116608_at | 1455656_at |
| 96811_at | 1416165_at |
| 99445_at | 1428065_at |
| 102337_s_at | 1435477_s_at |
| 113075_at | 1430343_at |
| 140756_at | 1437322_at |
| 96875_r_at | 1420174_s_at |
| 97486_at | 1422509_at |
| 101914_at | 1415731_at |
| 110272_at | 1426774_at |
| 111013_at | 1452002_at |
| 99628_at | 1423130_a_at |
| 103483_at | 1450935_at |
| 166510_r_at | 1440248_at |
| 94663_at | 1440874_at |
| 92775_at | 1451296_x_at |
| 104025_at | 1448907_at |
| 111734_at | 1423574_s_at |
| 114254_at | 1435676_at |
| 114906_at | 1449439_at |
| 95643_at | 1415770_at |
| 93421_at | 1419249_at |
| 108504_at | 1428861_at |
| 112770_at | 1434663_at |
| 166247_at | 1427090_at |
| 160617_at | 1432543_a_at |
| 164257_at | 1452772_at |
| 103816_at | 1424595_at |
| 108503_at | 1425480_at |
| 112256_at | 1418409_at |
| 112766_at | 1428130_at |
| 117301_at | 1426474_at |
| 131915_at | 1437936_at |
| 137090_at | 1435473_at |
| 138037_at | 1435319_at |
| 139815_at | 1429526_at |
| 160529_r_at | 1415990_at |
| 96174_at | 1427886_at |
| 96178_at | 1433433_at |
| 100958_at | 1454656_at |
| 101936_at | 1422886_a_at |
| 111892_r_at | 1451251_at |
| 116107_at | 1420951_a_at |

| | |
|---|---|
| 167683_at | 1424577_at |
| 99580_s_at | 1424783_a_at |
| 96491_at | 1447947_at |
| 99325_at | 1450763_x_at |
| 100610_at | 1426400_a_at |
| 102277_at | 1427120_at |
| 102850_at | 1448298_at |
| 104423_at | 1434695_at |
| 107005_at | 1419806_at |
| 107558_at | 1423882_at |
| 110808_at | 1437752_at |
| 160578_at | 1415729_at |
| 97722_at | 1417764_at |
| 163967_at | 1427375_at |
| 166875_at | 1439108_at |
| 96924_at | 1452189_at |
| 100116_at | 1419153_at |
| 101044_at | 1424877_a_at |
| 112053_at | 1429109_at |
| 112206_at | 1428897_at |
| 116129_at | 1418449_at |
| 96199_at | 1416934_at |
| 160397_at | 1451113_a_at |
| 102052_at | 1417566_at |
| 103375_at | 1428401_at |
| 103889_at | 1434839_s_at |
| 104250_at | 1434694_at |
| 116132_at | 1427234_at |
| 116165_at | 1424695_at |
| 160169_at | 1429707_at |
| 101357_at | 1460724_at |
| 104735_at | 1434881_s_at |
| 110461_at | 1435402_at |
| 113264_at | 1424745_at |
| 94845_at | 1415764_at |
| 160765_at | 1417591_at |
| 93988_at | 1423567_a_at |
| 164250_at | 1437759_at |
| 94998_at | 1450870_at |
| 112783_at | 1428177_at |
| 114461_at | 1437696_at |
| 134515_at | 1424894_at |
| 99650_at | 1424827_a_at |
| 99513_at | 1448558_a_at |
| 166723_at | 1451867_x_at |
| 166629_i_at | 1456673_at |
| 163607_at | 1428238_at |
| 101892_f_at | 1437767_s_at |
| 102111_f_at | 1447970_at |
| 112028_at | 1429415_at |
| 115417_at | 1422653_at |
| 133171_at | 1427138_at |
| 141044_f_at | 1456942_x_at |
| 160326_at | 1415704_a_at |
| 96677_at | 1460687_at |
| 103260_at | 1430291_at |

| | |
|---|---|
| 104661_at | 1455134_at |
| 111553_at | 1449290_at |
| 94505_at | 1417371_at |
| 166360_at | 1424396_a_at |
| 93958_at | 1425929_a_at |
| 167758_at | 1436967_at |
| 165994_at | 1437180_at |
| 102400_at | 1433663_s_at |
| 102906_at | 1449009_at |
| 106016_at | 1423169_at |
| 107067_at | 1436732_s_at |
| 109102_r_at | 1452067_at |
| 111972_at | 1427144_at |
| 116175_at | 1429478_at |
| 133553_at | 1443584_at |
| 160473_at | 1416798_a_at |
| 98975_at | 1424291_at |
| 162932_at | 1425913_a_at |
| 99102_at | 1428194_at |
| 115180_at | 1428849_at |
| 94005_at | 1429709_at |
| 96309_r_at | 1438655_a_at |
| 113443_r_at | 1437154_at |
| 97857_at | 1423646_at |
| 94295_at | 1425628_a_at |
| 93530_at | 1435637_at |
| 160850_at | 1460673_at |
| 104587_at | 1424807_at |
| 106666_at | 1427927_at |
| 108777_at | 1428804_at |
| 109953_at | 1456614_at |
| 110634_at | 1416019_at |
| 111147_at | 1436829_at |
| 97819_at | 1416531_at |
| 97424_at | 1417225_at |
| 96733_at | 1425266_a_at |
| 166816_at | 1428287_at |
| 98959_at | 1449821_a_at |
| 101568_at | 1423044_at |
| 101981_at | 1416353_at |
| 102994_at | 1448713_at |
| 108730_at | 1427959_at |
| 110248_at | 1433574_at |
| 160195_at | 1415721_a_at |
| 166438_s_at | 1424933_at |
| 163938_at | 1436443_a_at |
| 166769_at | 1456951_at |
| 163814_r_at | 1438588_at |
| 100320_at | 1417975_at |
| 106101_at | 1454809_at |
| 109133_at | 1417783_at |
| 113165_at | 1451302_at |
| 115736_at | 1417306_at |
| 98758_at | 1420338_at |
| 103094_at | 1449509_at |
| 160925_at | 1422687_at |

| | |
|---|---|
| 95617_at | 1425981_a_at |
| 95870_at | 1442974_at |
| 95439_at | 1448813_at |
| 96598_at | 1455653_at |
| 93212_at | 1452427_s_at |
| 101937_s_at | 1427663_a_at |
| 103842_at | 1426439_at |
| 107515_at | 1423316_at |
| 108367_at | 1428517_at |
| 134751_at | 1436010_at |
| 166199_at | 1436941_at |
| 167874_at | 1439913_at |
| 166985_f_at | 1447897_x_at |
| 97894_at | 1448472_at |
| 100914_at | 1437867_at |
| 104076_at | 1417402_at |
| 107878_at | 1438070_at |
| 112288_at | 1426572_at |
| 115150_at | 1418359_at |
| 117048_at | 1451280_at |
| 95698_at | 1416417_a_at |
| 95963_at | 1419968_at |
| 96885_at | 1424882_a_at |
| 97817_at | 1428131_a_at |
| 160352_at | 1449055_x_at |
| 98047_at | 1454616_at |
| 101591_at | 1422443_at |
| 103394_at | 1418296_at |
| 105273_at | 1435698_at |
| 108507_at | 1424141_at |
| 112508_at | 1424698_s_at |
| 136172_at | 1440292_at |
| 137665_at | 1456223_at |
| 138200_at | 1439026_at |
| 99659_r_at | 1419547_at |
| 168379_i_at | 1419693_at |
| 114181_at | 1421912_at |
| 168013_r_at | 1428846_at |
| 162538_at | 1429290_at |
| 168972_i_at | 1430789_at |
| 94296_s_at | 1460279_a_at |
| 168272_f_at | 1437420_at |
| 100981_at | 1450783_at |
| 101732_at | 1422320_x_at |
| 104198_at | 1427095_at |
| 109700_at | 1460345_at |
| 110467_at | 1431262_at |
| 113152_at | 1419551_s_at |
| 113680_at | 1450642_at |
| 116408_at | 1455082_at |
| 94071_at | 1419371_s_at |
| 96048_at | 1428326_s_at |
| 96777_at | 1449138_at |
| 162883_at | 1452789_at |
| 167495_s_at | 1453406_a_at |
| 104921_at | 1445212_at |

| | |
|---|---|
| 160089_at | 1415880_a_at |
| 99112_at | 1416954_at |
| 167647_r_at | 1429875_at |
| 167502_i_at | 1434919_at |
| 165795_f_at | 1437618_x_at |
| 94854_g_at | 1439600_at |
| 168513_f_at | 1441942_x_at |
| 167122_at | 1449540_at |
| 103024_at | 1416871_at |
| 104005_at | 1448941_at |
| 104645_at | 1419354_at |
| 106198_at | 1451105_at |
| 106265_at | 1427233_at |
| 112704_at | 1458362_at |
| 94427_at | 1416017_at |
| 95679_at | 1417161_at |
| 93842_at | 1423790_at |
| 99041_at | 1431345_a_at |
| 96684_at | 1433457_s_at |
| 94490_at | 1451102_at |
| 97556_at | 1451356_at |
| 92325_at | 1452589_at |
| 97705_at | 1453077_a_at |
| 103551_at | 1425543_s_at |
| 107354_at | 1429718_at |
| 108244_at | 1440586_at |
| 114939_at | 1425778_at |
| 97203_at | 1415922_s_at |
| 99006_at | 1430749_at |
| 160678_at | 1454604_s_at |
| 101449_at | 1424275_s_at |
| 102041_at | 1450917_at |
| 110341_at | 1454991_at |
| 112692_at | 1419032_at |
| 129297_at | 1439136_at |
| 132730_at | 1422979_at |
| 93835_at | 1416109_at |
| 162591_at | 1418184_at |
| 97409_at | 1418825_at |
| 92198_s_at | 1419727_at |
| 162160_at | 1423121_at |
| 93218_at | 1423543_at |
| 97490_at | 1448247_at |
| 93164_at | 1451519_at |
| 104380_at | 1417538_at |
| 110961_at | 1453036_at |
| 111687_at | 1426544_a_at |
| 116122_at | 1426803_at |
| 141027_at | 1432007_s_at |
| 99820_f_at | 1424816_at |
| 165651_r_at | 1430297_a_at |
| 94975_at | 1433527_at |
| 165557_i_at | 1439528_at |
| 101540_at | 1416790_a_at |
| 112389_at | 1423152_at |
| 116947_at | 1449012_s_at |

| | |
|---|---|
| 134775_at | 1451358_a_at |
| 94405_at | 1420148_at |
| 97198_at | 1421839_at |
| 163435_at | 1434558_at |
| 94966_at | 1448354_at |
| 99390_at | 1456976_at |
| 103317_at | 1423285_at |
| 103386_at | 1417449_at |
| 103532_at | 1435172_at |
| 106070_at | 1422479_at |
| 111398_at | 1454734_at |
| 111754_at | 1444318_at |
| 112877_at | 1451804_a_at |
| 113027_at | 1437634_at |
| 133813_at | 1440263_at |
| 136061_at | 1440850_at |
| 136245_at | 1452655_at |
| 93903_at | 1419140_at |
| 98874_at | 1421376_at |
| 96605_at | 1423909_at |
| 95801_s_at | 1427831_s_at |
| 162804_at | 1428547_at |
| 166029_i_at | 1428721_at |
| 116074_at | 1438111_at |
| 168495_i_at | 1442915_at |
| 96591_at | 1449465_at |
| 168104_i_at | 1453603_at |
| 102247_at | 1420971_at |
| 102798_at | 1416077_at |
| 108562_at | 1428582_at |
| 115528_at | 1438270_at |
| 117042_at | 1451486_at |
| 93301_at | 1417199_at |
| 94189_at | 1418421_at |
| 163797_i_at | 1424292_at |
| 99071_at | 1427076_at |
| 98033_at | 1448786_at |
| 98421_at | 1456612_at |
| 100094_at | 1424255_at |
| 102133_at | 1459929_at |
| 109404_at | 1455107_at |
| 110016_at | 1429220_at |
| 111204_at | 1452267_at |
| 116631_at | 1431052_at |
| 117184_at | 1426403_at |
| 130183_at | 1439273_at |
| 94865_at | 1415788_at |
| 96648_at | 1416246_a_at |
| 165668_r_at | 1416390_at |
| 98457_at | 1421225_a_at |
| 92410_at | 1422964_at |
| 165664_i_at | 1425681_a_at |
| 92958_at | 1434831_a_at |
| 167292_f_at | 1450036_at |
| 101587_at | 1422438_at |
| 102278_at | 1450657_at |

| | |
|---|---|
| 102677_at | 1448660_at |
| 111325_at | 1428756_at |
| 115179_at | 1460377_a_at |
| 115411_at | 1429376_s_at |
| 115816_at | 1460632_at |
| 136689_at | 1440034_at |
| 98528_at | 1415763_a_at |
| 94815_at | 1415865_s_at |
| 99159_at | 1416940_at |
| 98562_at | 1417381_at |
| 97885_at | 1418004_a_at |
| 160711_at | 1419367_at |
| 95082_at | 1423062_at |
| 99980_at | 1427454_at |
| 166801_at | 1437051_at |
| 133812_at | 1440588_at |
| 160324_at | 1448938_at |
| 96325_at | 1451078_at |
| 103379_at | 1455047_at |
| 104395_at | 1435272_at |
| 104486_at | 1434719_at |
| 110210_at | 1455588_at |
| 114373_at | 1417740_at |
| 114381_at | 1428417_at |
| 116264_at | 1439180_at |
| 98031_at | 1417040_a_at |
| 97124_at | 1421534_at |
| 94375_at | 1422612_at |
| 160165_at | 1424345_s_at |
| 97706_at | 1446474_at |
| 98472_at | 1449556_at |
| 98471_f_at | 1449877_s_at |
| 96628_at | 1452157_at |
| 163261_at | 1454882_at |
| 164020_at | 1416132_at |
| 171490_at | 1453740_a_at |
| 102424_at | 1419561_at |
| 109787_at | 1435589_at |
| 109980_at | 1423379_at |
| 110586_at | 1454823_at |
| 112716_at | 1450913_at |
| 93874_s_at | 1417505_s_at |
| 92888_s_at | 1421664_a_at |
| 163013_at | 1423851_a_at |
| 97429_at | 1425678_a_at |
| 168250_f_at | 1437130_at |
| 167602_i_at | 1447892_at |
| 99504_at | 1451008_at |
| 95036_at | 1451129_at |
| 166795_at | 1456948_at |
| 103242_at | 1460712_s_at |
| 104461_at | 1460326_at |
| 106294_at | 1452152_at |
| 110297_at | 1431087_at |
| 111273_at | 1416942_at |
| 114621_at | 1428473_at |

| | |
|---|---|
| 129296_at | 1443992_at |
| 134234_at | 1431115_at |
| 135583_at | 1429890_at |
| 164066_at | 1417915_at |
| 99897_at | 1421189_at |
| 94066_at | 1422429_at |
| 161704_r_at | 1423166_at |
| 98941_r_at | 1429144_at |
| 163614_at | 1449348_at |
| 96888_at | 1451035_a_at |
| 163194_at | 1455162_at |
| 106623_at | 1420851_at |
| 107457_at | 1435550_at |
| 108382_at | 1423484_at |
| 111558_at | 1452350_at |
| 111588_at | 1428647_at |
| 111685_at | 1434218_at |
| 112728_at | 1433848_at |
| 112898_at | 1419356_at |
| 112918_at | 1452291_at |
| 113717_at | 1428459_at |
| 115024_at | 1427342_at |
| 116403_at | 1456140_at |
| 141177_at | 1430428_at |
| 163838_at | 1418151_at |
| 99441_at | 1426462_at |
| 164055_at | 1426514_at |
| 92722_f_at | 1427277_at |
| 98305_at | 1437138_at |
| 95743_at | 1448493_at |
| 99502_at | 1449069_at |
| 166011_i_at | 1449494_at |
| 166767_at | 1451072_a_at |
| 96360_at | 1451168_a_at |
| 96827_at | 1452830_s_at |
| 166521_f_at | 1456335_at |
| 101465_at | 1450034_at |
| 104041_at | 1454668_at |
| 105508_at | 1421525_a_at |
| 106123_at | 1439035_at |
| 106219_at | 1423207_at |
| 109630_at | 1424672_at |
| 113046_at | 1423308_at |
| 115437_at | 1426822_at |
| 134853_at | 1438807_at |
| 136661_at | 1435827_at |
| 138363_at | 1459906_at |
| 138964_at | 1426606_at |
| 160208_at | 1415985_at |
| 95029_at | 1417475_at |
| 93720_at | 1421024_at |
| 93309_at | 1423042_at |
| 93731_at | 1423677_at |
| 95675_at | 1426759_at |
| 163542_at | 1448860_at |
| 95961_at | 1455694_at |

| | |
|---|---|
| 162096_at | 1459816_x_at |
| 162564_at | 1425942_a_at |
| 100291_at | 1450457_at |
| 101982_at | 1451097_at |
| 104147_at | 1417773_at |
| 104417_at | 1444052_at |
| 104679_at | 1425785_a_at |
| 113608_at | 1429537_at |
| 113964_at | 1448565_at |
| 114542_at | 1429740_at |
| 115546_at | 1424813_at |
| 133574_at | 1429847_a_at |
| 134717_at | 1442125_at |
| 160103_at | 1420609_at |
| 92331_at | 1422975_at |
| 97880_at | 1423710_at |
| 98436_s_at | 1426165_a_at |
| 92727_at | 1427288_at |
| 95550_at | 1427467_a_at |
| 160350_at | 1427552_a_at |
| 165605_at | 1428352_at |
| 96322_at | 1448266_at |
| 163457_f_at | 1452296_at |
| 169880_s_at | 1452324_at |
| 100673_f_at | 1420789_at |
| 103043_at | 1449897_a_at |
| 103806_at | 1449299_at |
| 104568_at | 1427283_at |
| 106651_at | 1423069_at |
| 113088_at | 1429488_at |
| 113696_at | 1458618_at |
| 92603_at | 1415671_at |
| 95097_at | 1417157_at |
| 167766_i_at | 1426262_at |
| 98402_at | 1428847_a_at |
| 165856_i_at | 1428855_at |
| 160292_at | 1432181_s_at |
| 162864_i_at | 1433978_at |
| 92268_at | 1436859_at |
| 93142_at | 1449311_at |
| 96184_at | 1454753_at |
| 95913_at | 1457265_at |
| 163492_at | 1460005_at |
| 97304_at | 1460655_a_at |
| 103736_at | 1448005_at |
| 107829_at | 1431405_a_at |
| 108979_at | 1434181_at |
| 110541_at | 1460599_at |
| 110648_at | 1434894_at |
| 115610_at | 1436582_at |
| 130531_at | 1453733_a_at |
| 130992_at | 1434086_at |
| 134323_at | 1438765_at |
| 134630_at | 1443884_at |
| 134798_at | 1440961_at |
| 140849_at | 1439878_at |

| | |
|---|---|
| 95142_s_at | 1417259_a_at |
| 161946_r_at | 1417358_s_at |
| 95590_at | 1424160_at |
| 162730_at | 1428171_at |
| 96915_f_at | 1428464_at |
| 113903_at | 1428557_a_at |
| 165794_at | 1437070_at |
| 162179_r_at | 1438961_s_at |
| 95060_at | 1448502_at |
| 94453_at | 1449412_at |
| 93350_f_at | 1451244_a_at |
| 167715_r_at | 1453332_at |
| 160740_at | 1454921_at |
| 165537_at | 1460206_at |
| 102963_at | 1417878_at |
| 105949_i_at | 1423560_at |
| 109165_at | 1416961_at |
| 110676_at | 1436414_at |
| 113612_at | 1430129_a_at |
| 115538_at | 1439772_at |
| 128645_at | 1429040_at |
| 136549_at | 1457173_at |
| 160361_at | 1415674_a_at |
| 93770_at | 1416809_at |
| 94259_at | 1417998_at |
| 99169_at | 1419743_s_at |
| 167857_r_at | 1421324_a_at |
| 99329_at | 1421378_s_at |
| 95138_at | 1429367_at |
| 162990_at | 1429382_at |
| 93614_at | 1431164_at |
| 96774_at | 1434180_at |
| 163213_at | 1434302_at |
| 166446_f_at | 1438853_x_at |
| 96780_at | 1448971_at |
| 163782_at | 1451531_at |
| 166598_r_at | 1452994_at |
| 101684_r_at | 1460311_at |
| 103397_at | 1426922_s_at |
| 103441_at | 1419036_at |
| 103486_at | 1449399_a_at |
| 109021_at | 1416897_at |
| 113291_at | 1435492_at |
| 114166_at | 1435578_s_at |
| 114483_at | 1428854_at |
| 115487_at | 1431113_at |
| 115679_at | 1436544_at |
| 116582_at | 1436178_at |
| 116834_at | 1453140_at |
| 136504_at | 1452412_at |
| 95467_at | 1416267_at |
| 96627_at | 1416667_at |
| 163269_at | 1417831_at |
| 96344_at | 1417951_at |
| 95686_at | 1419244_a_at |
| 160808_at | 1424119_at |

| | |
|---|---|
| 97324_at | 1424496_at |
| 93667_at | 1424986_s_at |
| 167114_at | 1429928_at |
| 162815_at | 1436623_at |
| 162960_at | 1436812_at |
| 162322_r_at | 1441863_x_at |
| 171612_i_at | 1446155_at |
| 98006_at | 1448369_at |
| 95600_at | 1449119_at |
| 162692_at | 1449849_a_at |
| 160106_at | 1450355_a_at |
| 92830_s_at | 1452519_a_at |
| 99649_at | 1455959_s_at |
| 165635_i_at | 1456284_at |
| 99987_at | 1456486_at |
| 100005_at | 1460642_at |
| 102037_at | 1451989_a_at |
| 103020_s_at | 1424850_at |
| 103345_at | 1427888_a_at |
| 104044_at | 1430293_a_at |
| 104228_at | 1437534_at |
| 105458_at | 1439089_at |
| 107380_at | 1444199_at |
| 108427_at | 1431465_s_at |
| 111827_f_at | 1438771_at |
| 113715_at | 1455182_at |
| 114005_at | 1428176_at |
| 116073_at | 1435520_at |
| 116432_at | 1433492_at |
| 129223_at | 1431111_at |
| 136608_at | 1455699_at |
| 137134_f_at | 1422641_at |
| 138791_at | 1458920_at |
| 140654_at | 1450025_at |
| 141105_s_at | 1434868_at |
| 162598_at | 1417410_s_at |
| 99549_at | 1419662_at |
| 99855_at | 1421340_at |
| 96054_f_at | 1422715_s_at |
| 96284_at | 1423370_a_at |
| 94299_at | 1424461_at |
| 166402_i_at | 1424579_at |
| 160626_at | 1425350_a_at |
| 95752_at | 1426480_at |
| 162852_s_at | 1429538_a_at |
| 160667_at | 1434920_a_at |
| 94243_at | 1437349_at |
| 168166_at | 1440002_at |
| 166437_f_at | 1445815_at |
| 167571_i_at | 1447744_s_at |
| 97401_at | 1460676_at |
| 104258_at | 1427943_at |
| 105823_at | 1425092_at |
| 109161_at | 1448908_at |
| 110499_f_at | 1454965_at |
| 111230_at | 1456683_at |

| | |
|---|---|
| 111465_at | 1456820_at |
| 112027_at | 1452411_at |
| 113331_at | 1435028_at |
| 113725_at | 1426597_s_at |
| 114398_at | 1423208_at |
| 115153_at | 1455483_at |
| 115751_at | 1453119_at |
| 131637_at | 1420580_at |
| 95040_at | 1415937_s_at |
| 93214_at | 1427763_a_at |
| 92724_at | 1436549_a_at |
| 162751_at | 1436680_s_at |
| 163479_at | 1436723_at |
| 167646_f_at | 1438990_x_at |
| 167932_f_at | 1441935_at |
| 160111_at | 1449576_at |
| 162569_at | 1453255_at |
| 93451_at | 1455056_at |
| 165514_f_at | 1456055_x_at |
| 102848_f_at | 1429244_at |
| 103574_at | 1454708_at |
| 104002_at | 1418397_at |
| 104693_at | 1429126_at |
| 105484_at | 1452140_at |
| 105554_at | 1421107_at |
| 107003_at | 1453739_at |
| 108489_at | 1455456_a_at |
| 109005_at | 1450420_at |
| 109490_at | 1453245_at |
| 110206_at | 1428857_at |
| 110964_at | 1452202_at |
| 111065_at | 1435937_at |
| 111206_at | 1419585_at |
| 111518_at | 1454806_at |
| 114355_at | 1416379_at |
| 116332_at | 1422643_at |
| 128829_at | 1434135_at |
| 133315_at | 1456659_at |
| 133474_at | 1436457_at |
| 134046_at | 1448818_at |
| 135894_at | 1454030_at |
| 140488_i_at | 1455857_a_at |
| 140884_at | 1457598_at |
| 95068_at | 1417182_at |
| 98406_at | 1418126_at |
| 161595_at | 1420243_at |
| 168707_i_at | 1422657_at |
| 95580_at | 1424385_at |
| 99509_s_at | 1425750_a_at |
| 93856_at | 1425995_s_at |
| 160801_at | 1426567_a_at |
| 95069_at | 1426788_a_at |
| 162653_at | 1428220_at |
| 97374_at | 1428452_at |
| 163406_at | 1428570_at |
| 165590_i_at | 1429142_at |

| | |
|---|---|
| 163763_at | 1429596_at |
| 162732_i_at | 1434471_at |
| 165520_f_at | 1437140_at |
| 96836_r_at | 1437585_x_at |
| 163795_at | 1438417_at |
| 97690_at | 1449641_at |
| 98169_s_at | 1449730_s_at |
| 163913_at | 1449862_a_at |
| 97532_at | 1451242_a_at |
| 160369_at | 1451895_a_at |
| 94211_at | 1452073_at |
| 95426_at | 1452341_at |
| 100489_at | 1423313_at |
| 104866_at | 1457552_at |
| 105550_at | 1449169_at |
| 105769_at | 1434593_at |
| 109330_at | 1425921_a_at |
| 111405_at | 1454617_at |
| 111789_at | 1439852_at |
| 112338_at | 1425914_a_at |
| 113211_at | 1417252_at |
| 114624_at | 1436854_at |
| 115353_at | 1416682_at |
| 115414_at | 1418458_at |
| 116096_at | 1455171_at |
| 116253_at | 1439774_at |
| 116280_at | 1453264_at |
| 117176_at | 1434265_s_at |
| 141034_at | 1445440_at |
| 167488_f_at | 1416528_at |
| 98961_at | 1418736_at |
| 105803_at | 1418911_s_at |
| 98980_at | 1419206_at |
| 171483_i_at | 1419766_at |
| 95136_at | 1424285_s_at |
| 163620_at | 1424732_s_at |
| 163975_at | 1424738_at |
| 96728_at | 1424786_s_at |
| 99544_at | 1425228_a_at |
| 162828_at | 1428759_s_at |
| 99545_at | 1432075_a_at |
| 167855_at | 1438833_at |
| 163689_at | 1439478_at |
| 168500_s_at | 1447621_s_at |
| 167865_f_at | 1447750_x_at |
| 166208_at | 1448626_at |
| 93591_at | 1448644_at |
| 99905_at | 1450547_x_at |
| 92537_g_at | 1455918_at |
| 100380_at | 1423262_a_at |
| 100486_at | 1449023_a_at |
| 101445_at | 1422946_a_at |
| 101691_s_at | 1448093_s_at |
| 103037_at | 1449093_at |
| 103318_at | 1436232_a_at |
| 103422_at | 1449130_at |

| | |
|---|---|
| 103434_at | 1431707_a_at |
| 103457_at | 1424632_a_at |
| 104316_at | 1433749_at |
| 104334_at | 1450987_a_at |
| 104413_at | 1433595_at |
| 105088_at | 1457089_at |
| 105924_at | 1417862_at |
| 107435_at | 1456153_at |
| 108560_at | 1452800_a_at |
| 109595_at | 1455781_at |
| 111845_at | 1453207_at |
| 113561_at | 1428431_at |
| 113683_at | 1435480_at |
| 114672_at | 1425682_a_at |
| 115098_at | 1429125_at |
| 116829_at | 1426886_at |
| 134042_at | 1429896_at |
| 137367_at | 1445009_at |
| 138960_f_at | 1453040_at |
| 139200_at | 1431110_at |
| 98008_at | 1415803_at |
| 160288_at | 1415929_at |
| 94806_at | 1416090_at |
| 96319_at | 1416664_at |
| 98075_at | 1416763_at |
| 98609_at | 1417038_at |
| 165480_i_at | 1418056_at |
| 97497_at | 1418634_at |
| 99056_at | 1418713_at |
| 166432_f_at | 1418734_at |
| 163373_at | 1418778_at |
| 165922_at | 1419203_at |
| 98245_at | 1419935_s_at |
| 94197_at | 1421268_at |
| 163576_at | 1422609_at |
| 99640_at | 1423265_at |
| 166745_i_at | 1424975_at |
| 93870_at | 1425693_at |
| 94008_at | 1427914_a_at |
| 163250_at | 1429335_at |
| 166662_at | 1429824_at |
| 97477_at | 1431665_a_at |
| 96629_at | 1434216_a_at |
| 160230_at | 1434435_s_at |
| 93424_at | 1434937_at |
| 166422_at | 1436719_at |
| 167431_at | 1439788_at |
| 161836_r_at | 1440907_at |
| 97518_at | 1448130_at |
| 160272_at | 1448504_a_at |
| 96252_at | 1449674_s_at |
| 99387_at | 1450808_at |
| 94915_at | 1450911_at |
| 98398_s_at | 1451755_a_at |
| 168077_at | 1453386_at |
| 94958_at | 1454893_at |

| | |
|---|---|
| 166785_f_at | 1456215_at |
| 97175_at | 1460520_at |
| 165268_i_at | 1450727_a_at |
| 167409_f_at | 1451779_at |
| 100153_at | 1426864_a_at |
| 100412_g_at | 1450637_a_at |
| 101035_at | 1415813_at |
| 101635_f_at | 1426166_at |
| 101997_at | 1451747_a_at |
| 102195_at | 1448050_s_at |
| 103220_at | 1422554_at |
| 103901_at | 1426899_at |
| 104375_at | 1435026_at |
| 104487_at | 1440252_at |
| 104746_at | 1416803_at |
| 106993_at | 1429713_at |
| 108016_at | 1436709_at |
| 111793_at | 1424389_at |
| 113930_at | 1436538_at |
| 115104_at | 1450400_at |
| 115202_at | 1436545_at |
| 115591_at | 1441989_at |
| 115639_at | 1435892_at |
| 129319_f_at | 1437126_at |
| 135244_at | 1436492_x_at |
| 137055_at | 1436537_at |
| 139248_f_at | 1436100_at |
| 140497_at | 1456908_at |
| 93937_at | 1418361_at |
| 95486_at | 1420056_s_at |
| 96818_at | 1421720_a_at |
| 94723_at | 1421758_at |
| 160082_s_at | 1423052_at |
| 160184_at | 1423334_at |
| 160672_at | 1423612_at |
| 160242_at | 1423663_at |
| 162927_at | 1423671_at |
| 165556_at | 1424003_at |
| 96832_at | 1424424_at |
| 99036_s_at | 1425393_a_at |
| 160453_at | 1426388_s_at |
| 163170_at | 1427476_a_at |
| 165696_at | 1428087_at |
| 98016_at | 1428310_at |
| 162688_at | 1428487_s_at |
| 94363_at | 1433636_at |
| 98468_r_at | 1434821_at |
| 167130_r_at | 1435595_at |
| 97514_at | 1435864_a_at |
| 167005_at | 1440765_at |
| 168255_at | 1441481_at |
| 167328_s_at | 1443838_x_at |
| 96753_at | 1449523_at |
| 93539_at | 1452586_at |
| 95567_at | 1452884_at |
| 166945_r_at | 1453407_at |

| | |
|---|---|
| 97226_at | 1455008_at |
| 96729_at | 1455575_at |
| 100905_at | 1427032_at |
| 101366_f_at | 1453018_at |
| 101965_at | 1451074_at |
| 102103_f_at | 1449726_at |
| 102196_at | 1449144_at |
| 102726_at | 1416783_at |
| 103080_at | 1418131_at |
| 103443_at | 1426942_at |
| 103614_at | 1425902_a_at |
| 103619_at | 1417766_at |
| 106051_at | 1448669_at |
| 106925_at | 1439859_at |
| 108369_at | 1449461_at |
| 110008_at | 1420397_a_at |
| 110503_at | 1429154_at |
| 110667_at | 1457294_at |
| 111383_at | 1433868_at |
| 111895_at | 1452085_at |
| 112827_at | 1426220_at |
| 112850_at | 1450449_a_at |
| 112992_at | 1434126_at |
| 113328_at | 1455481_at |
| 114162_at | 1420576_at |
| 115970_at | 1420985_at |
| 115993_at | 1438788_at |
| 116740_at | 1431569_a_at |
| 117302_at | 1431799_at |
| 128529_at | 1445607_at |
| 129195_at | 1455406_at |
| 129412_s_at | 1426305_at |
| 131162_at | 1429631_at |
| 131405_at | 1427344_s_at |
| 135666_at | 1444787_at |
| 137130_at | 1436025_at |
| 95144_at | 1416079_a_at |
| 160540_at | 1416394_at |
| 98133_at | 1417504_at |
| 95045_at | 1417710_at |
| 94366_at | 1417779_at |
| 93976_at | 1418433_at |
| 99327_at | 1419722_at |
| 98818_at | 1421866_at |
| 162394_r_at | 1423021_s_at |
| 160439_at | 1423273_at |
| 96666_at | 1423538_at |
| 98454_at | 1423967_at |
| 160695_i_at | 1424367_a_at |
| 93893_f_at | 1425436_x_at |
| 160666_at | 1428502_at |
| 166409_at | 1429456_a_at |
| 167390_i_at | 1429904_at |
| 164175_at | 1430177_at |
| 167044_at | 1431239_at |
| 166868_r_at | 1439498_at |

| | |
|---|---|
| 162900_at | 1440254_at |
| 170537_r_at | 1445173_at |
| 167148_f_at | 1447676_x_at |
| 161192_at | 1447858_x_at |
| 96847_at | 1448447_at |
| 166577_i_at | 1456231_at |
| 168285_i_at | 1457654_at |
| 160899_at | 1460214_at |
| 161334_r_at | 1443362_at |
| 100042_at | 1424172_at |
| 100460_at | 1422679_s_at |
| 100964_at | 1449003_a_at |
| 101139_r_at | 1449587_a_at |
| 101158_at | 1449913_at |
| 101437_at | 1449336_a_at |
| 101447_at | 1450056_at |
| 102022_at | 1438477_a_at |
| 103568_at | 1451939_a_at |
| 103611_at | 1419554_at |
| 103781_at | 1451573_a_at |
| 103969_at | 1434704_at |
| 104301_at | 1452591_a_at |
| 104415_at | 1435221_at |
| 104604_at | 1451281_at |
| 104947_at | 1457302_at |
| 105666_at | 1458694_at |
| 105721_at | 1447996_at |
| 106103_at | 1447439_at |
| 106469_at | 1437224_at |
| 107594_at | 1436424_at |
| 108535_at | 1424425_a_at |
| 109120_at | 1428851_at |
| 110263_at | 1424571_at |
| 110373_at | 1433831_at |
| 110464_at | 1449091_at |
| 111239_at | 1423331_a_at |
| 111272_at | 1447931_at |
| 112365_at | 1436113_a_at |
| 114083_at | 1456324_at |
| 114346_at | 1431693_a_at |
| 114409_at | 1437660_at |
| 114562_at | 1426288_at |
| 115717_at | 1427161_at |
| 131455_at | 1453840_at |
| 131964_f_at | 1455689_at |
| 133927_at | 1437979_at |
| 133963_at | 1457184_at |
| 134878_at | 1456927_at |
| 135694_at | 1453419_at |
| 136174_at | 1442999_at |
| 137680_at | 1427353_at |
| 138467_i_at | 1428860_at |
| 139205_at | 1435179_at |
| 140832_at | 1439276_at |
| 95513_at | 1415774_at |
| 96613_at | 1416633_a_at |

| | |
|---|---|
| 94376_s_at | 1416748_a_at |
| 94047_at | 1416830_at |
| 160200_at | 1416856_at |
| 97238_at | 1417450_a_at |
| 98121_at | 1417465_at |
| 168125_r_at | 1418912_at |
| 160591_at | 1418967_a_at |
| 98802_at | 1419431_at |
| 166637_i_at | 1420386_at |
| 93718_at | 1422399_a_at |
| 98929_at | 1423242_at |
| 92216_at | 1423389_at |
| 99032_at | 1423619_at |
| 97496_f_at | 1423771_at |
| 94379_at | 1425270_at |
| 167203_at | 1425881_at |
| 98982_at | 1426349_s_at |
| 160235_at | 1428465_at |
| 95477_at | 1432150_at |
| 96212_at | 1434155_a_at |
| 165405_at | 1434579_x_at |
| 166203_r_at | 1434636_at |
| 163560_at | 1437475_at |
| 166258_at | 1437929_at |
| 167704_at | 1438558_x_at |
| 96539_at | 1440195_at |
| 137685_at | 1442061_at |
| 165997_f_at | 1446850_at |
| 166701_at | 1447569_at |
| 93962_at | 1448020_at |
| 169553_r_at | 1449500_at |
| 160129_at | 1449506_a_at |
| 160844_at | 1450660_at |
| 160615_at | 1451115_at |
| 99466_at | 1452376_at |
| 163320_at | 1455104_at |
| 164404_r_at | 1457715_at |
| 162219_f_at | 1418446_at |
| 100397_at | 1450792_at |
| 100433_r_at | 1425924_at |
| 101682_f_at | 1420793_at |
| 101966_s_at | 1420620_a_at |
| 102221_at | 1419289_a_at |
| 102863_at | 1418801_at |
| 103225_at | 1429173_at |
| 103525_at | 1425255_s_at |
| 103631_at | 1452588_at |
| 103704_at | 1435376_at |
| 104663_at | 1418144_a_at |
| 104672_at | 1416658_at |
| 104776_at | 1442443_at |
| 105384_at | 1454047_a_at |
| 106292_at | 1419406_a_at |
| 106588_at | 1454851_at |
| 108298_at | 1416927_at |
| 109337_at | 1455043_at |

| | |
|---|---|
| 109533_at | 1460595_at |
| 110179_at | 1427949_at |
| 110345_at | 1454646_at |
| 110632_at | 1427996_at |
| 111786_at | 1448049_at |
| 111795_at | 1420482_at |
| 111850_at | 1454848_at |
| 112210_at | 1438005_at |
| 112813_at | 1455390_at |
| 113912_at | 1428488_at |
| 113974_at | 1452808_at |
| 113986_at | 1418649_at |
| 114784_at | 1417673_at |
| 114931_at | 1442087_at |
| 115064_at | 1428884_at |
| 115191_at | 1426370_at |
| 115461_at | 1430560_at |
| 115995_at | 1447601_x_at |
| 116360_at | 1418585_at |
| 116913_at | 1424885_at |
| 117324_at | 1434837_at |
| 130584_at | 1446919_at |
| 131020_at | 1426927_at |
| 133680_r_at | 1457884_at |
| 133926_at | 1444761_at |
| 135316_at | 1423950_at |
| 135665_at | 1449471_at |
| 137152_at | 1437705_at |
| 137542_at | 1452390_at |
| 138569_at | 1453087_at |
| 97468_at | 1416698_a_at |
| 93303_at | 1418087_at |
| 93374_at | 1418161_at |
| 99552_at | 1418673_at |
| 99961_s_at | 1418841_s_at |
| 93435_at | 1418866_at |
| 160109_at | 1419156_at |
| 92649_at | 1419516_at |
| 168973_i_at | 1419540_at |
| 93498_s_at | 1421888_x_at |
| 93664_at | 1422009_at |
| 97530_at | 1422714_at |
| 96052_at | 1422716_a_at |
| 95717_at | 1426643_at |
| 160903_at | 1426719_at |
| 93138_at | 1428515_at |
| 163775_at | 1428760_at |
| 163377_at | 1431014_at |
| 167393_r_at | 1431455_at |
| 98912_at | 1434597_at |
| 94810_at | 1436884_x_at |
| 166665_i_at | 1437365_at |
| 166238_r_at | 1447949_at |
| 95912_at | 1448041_at |
| 94002_at | 1448174_at |
| 95701_at | 1448960_at |

| | |
|---|---|
| 165801_at | 1450693_at |
| 98092_at | 1451335_at |
| 166335_at | 1452228_at |
| 93199_at | 1452488_at |
| 163880_at | 1452985_at |
| 167094_at | 1453517_at |
| 96110_at | 1460196_at |
| 163049_at | 1460454_at |
| 100539_at | 1417094_at |
| 100560_at | 1448578_at |
| 101909_f_at | 1427249_x_at |
| 102202_s_at | 1420387_at |
| 102319_at | 1421797_a_at |
| 102795_at | 1426557_at |
| 103011_at | 1449343_s_at |
| 103420_at | 1417357_at |
| 103858_at | 1420520_x_at |
| 104057_at | 1417320_at |
| 104222_f_at | 1419805_s_at |
| 104263_at | 1438685_at |
| 104980_at | 1442439_at |
| 106576_at | 1434701_at |
| 107596_at | 1433651_at |
| 108531_at | 1426365_at |
| 109148_at | 1451398_at |
| 109309_at | 1451171_at |
| 109390_at | 1449079_s_at |
| 109722_at | 1433976_at |
| 110372_at | 1428254_at |
| 110810_at | 1455642_a_at |
| 110812_at | 1428745_a_at |
| 111315_at | 1433955_at |
| 111998_at | 1434585_at |
| 112017_at | 1436068_at |
| 112663_at | 1449011_at |
| 112703_at | 1454931_at |
| 112908_at | 1434350_at |
| 113758_at | 1429189_at |
| 114016_at | 1425404_a_at |
| 114185_at | 1437002_at |
| 114954_at | 1457562_at |
| 115195_at | 1436055_at |
| 115482_at | 1434524_at |
| 115530_at | 1436302_at |
| 115569_i_at | 1452278_a_at |
| 115795_at | 1421144_at |
| 116160_at | 1430032_at |
| 116547_at | 1443041_at |
| 116577_at | 1426988_at |
| 116597_at | 1440842_at |
| 116984_at | 1452443_s_at |
| 117116_at | 1433556_at |
| 117138_at | 1436159_at |
| 117160_at | 1429158_at |
| 133711_at | 1451169_at |
| 135258_at | 1452989_at |

| | |
|---|---|
| 137178_s_at | 1441151_at |
| 140329_at | 1432232_at |
| 140836_at | 1445629_at |
| 160552_at | 1416087_at |
| 92535_at | 1416301_a_at |
| 95660_at | 1417203_at |
| 96867_at | 1417776_at |
| 92759_at | 1417812_a_at |
| 164093_at | 1417978_at |
| 98857_at | 1418047_at |
| 92277_at | 1420160_s_at |
| 99807_r_at | 1422279_at |
| 165751_at | 1422596_at |
| 161491_r_at | 1423491_at |
| 166170_at | 1423862_at |
| 160477_at | 1424085_at |
| 160444_at | 1425134_a_at |
| 97257_at | 1425141_at |
| 99451_at | 1426893_at |
| 99126_at | 1427262_at |
| 97548_at | 1428172_at |
| 165679_at | 1428313_at |
| 93877_at | 1428500_at |
| 163305_at | 1431948_a_at |
| 160811_at | 1433554_at |
| 97353_at | 1433558_at |
| 95386_at | 1434690_at |
| 166736_at | 1435001_at |
| 166377_i_at | 1437918_at |
| 167606_r_at | 1443743_at |
| 95917_at | 1445535_at |
| 170034_i_at | 1446274_at |
| 167889_i_at | 1447620_at |
| 167769_f_at | 1447757_x_at |
| 93066_at | 1448148_at |
| 92223_at | 1449401_at |
| 93742_at | 1449933_a_at |
| 163581_at | 1450863_a_at |
| 160134_at | 1451311_a_at |
| 93235_at | 1452288_at |
| 99194_at | 1452676_a_at |
| 98094_f_at | 1453911_at |
| 161001_at | 1455180_at |
| 166254_f_at | 1455805_x_at |
| 103274_at | 1457066_at |
| 95896_at | 1459885_s_at |
| 100127_at | 1451191_at |
| 100540_at | 1434790_a_at |
| 100699_at | 1451781_at |
| 101109_at | 1426778_at |
| 101208_at | 1421533_at |
| 101564_at | 1460665_a_at |
| 101670_at | 1431242_at |
| 102254_f_at | 1435840_x_at |
| 102691_at | 1418865_at |
| 102828_at | 1426850_a_at |

| | |
|---|---|
| 102875_at | 1434563_at |
| 103560_at | 1428626_at |
| 103624_at | 1424079_x_at |
| 103625_at | 1449045_at |
| 103770_at | 1435572_at |
| 104229_at | 1449553_at |
| 104290_at | 1449217_at |
| 104356_at | 1451509_at |
| 105233_at | 1436141_at |
| 105274_at | 1423033_at |
| 105292_at | 1457534_at |
| 106304_at | 1424041_s_at |
| 106967_at | 1417453_at |
| 107053_at | 1424253_at |
| 107137_at | 1428620_at |
| 107388_at | 1439612_at |
| 107615_f_at | 1419290_at |
| 107877_at | 1438900_at |
| 107923_at | 1434409_at |
| 108018_at | 1448475_at |
| 108053_at | 1424412_at |
| 108070_s_at | 1436333_a_at |
| 108962_at | 1427998_at |
| 109099_at | 1424443_at |
| 109984_at | 1433551_at |
| 110003_at | 1432374_a_at |
| 110858_at | 1434375_at |
| 111023_at | 1424999_at |
| 111283_at | 1428768_at |
| 111378_at | 1429473_at |
| 111472_at | 1427447_a_at |
| 111837_at | 1452139_at |
| 113076_at | 1425510_at |
| 113190_at | 1431619_a_at |
| 113507_at | 1454944_at |
| 113797_at | 1456625_at |
| 114408_at | 1424582_at |
| 114573_at | 1443592_at |
| 114834_at | 1425346_at |
| 114901_at | 1452441_at |
| 114943_at | 1435912_at |
| 115793_at | 1434911_s_at |
| 115829_at | 1424879_at |
| 116140_at | 1452336_at |
| 117151_at | 1434447_at |
| 129283_at | 1431266_at |
| 129542_at | 1458891_at |
| 129543_at | 1448096_at |
| 130121_at | 1424761_at |
| 130562_g_at | 1457858_at |
| 130755_at | 1439508_at |
| 131838_at | 1447532_at |
| 135434_at | 1453056_at |
| 135603_at | 1440207_at |
| 135664_at | 1438235_at |
| 136033_at | 1441022_at |

| | |
|---|---|
| 137983_at | 1435421_at |
| 138969_at | 1436393_a_at |
| 139220_at | 1436426_at |
| 139488_at | 1458360_at |
| 141164_r_at | 1443984_at |
| 99068_at | 1415680_at |
| 93809_at | 1415742_at |
| 96636_at | 1415748_a_at |
| 160621_at | 1416595_at |
| 94274_at | 1416726_s_at |
| 97341_at | 1416781_at |
| 98626_at | 1417105_at |
| 98470_at | 1417154_at |
| 96793_at | 1417742_a_at |
| 93680_at | 1417751_at |
| 160862_at | 1418181_at |
| 160692_at | 1418340_at |
| 94383_at | 1418547_at |
| 162714_at | 1419265_at |
| 166787_i_at | 1419332_at |
| 167186_r_at | 1419388_at |
| 96055_at | 1419473_a_at |
| 166586_i_at | 1421037_at |
| 99239_at | 1421341_at |
| 96581_at | 1421593_at |
| 96730_at | 1421893_a_at |
| 94534_at | 1422500_at |
| 99176_at | 1422516_a_at |
| 98441_at | 1423369_at |
| 98150_at | 1423448_at |
| 163776_at | 1423923_a_at |
| 160419_r_at | 1423928_at |
| 160642_at | 1424042_at |
| 114622_at | 1424342_at |
| 166417_at | 1424474_a_at |
| 99148_at | 1424828_a_at |
| 163280_i_at | 1426269_at |
| 92632_at | 1426710_at |
| 161084_at | 1427104_at |
| 97989_at | 1427468_at |
| 166256_f_at | 1429420_at |
| 165517_r_at | 1429551_at |
| 161296_r_at | 1429748_at |
| 98756_at | 1430195_at |
| 96208_at | 1434009_at |
| 96541_at | 1434964_at |
| 169078_at | 1435071_at |
| 166451_f_at | 1435999_at |
| 99004_r_at | 1437004_at |
| 166418_at | 1437228_at |
| 95517_i_at | 1438534_x_at |
| 96570_at | 1439103_at |
| 166714_at | 1440272_at |
| 167570_at | 1440953_at |
| 165629_at | 1442031_at |
| 166217_r_at | 1443823_s_at |

| | |
|---|---|
| 166447_f_at | 1447905_x_at |
| 95894_at | 1447935_at |
| 160257_at | 1448184_at |
| 94038_at | 1448238_at |
| 93595_at | 1448313_at |
| 95132_r_at | 1448483_a_at |
| 95505_at | 1448848_at |
| 92263_at | 1449531_at |
| 98107_at | 1449660_s_at |
| 95872_at | 1449755_at |
| 99677_at | 1450057_at |
| 170212_i_at | 1450270_at |
| 162588_i_at | 1450653_at |
| 99953_at | 1450688_at |
| 95328_at | 1450771_at |
| 96767_at | 1451099_at |
| 165600_i_at | 1451599_at |
| 97742_s_at | 1451882_a_at |
| 163477_at | 1452191_at |
| 98852_at | 1452386_at |
| 94956_at | 1452434_s_at |
| 162801_at | 1452865_at |
| 160342_r_at | 1453806_at |
| 163341_at | 1454857_at |
| 163344_at | 1456501_at |
| 167464_r_at | 1457796_at |
| 95890_r_at | 1459916_at |
| 95318_at | 1460252_s_at |
| 167906_r_at | 1441448_at |
| 168443_r_at | 1451723_at |
| 100476_at | 1417484_at |
| 100633_at | 1416766_at |
| 100968_at | 1424723_s_at |
| 101358_at | 1448661_at |
| 101468_at | 1452279_at |
| 101579_at | 1448753_at |
| 101918_at | 1420653_at |
| 101921_at | 1418341_at |
| 101939_at | 1433655_at |
| 102136_r_at | 1445047_at |
| 102280_at | 1449249_at |
| 102402_at | 1419484_a_at |
| 102826_at | 1423556_at |
| 102872_f_at | 1424706_at |
| 102908_at | 1417337_at |
| 103018_at | 1424274_at |
| 103036_at | 1417437_at |
| 103052_r_at | 1416158_at |
| 103239_at | 1422074_at |
| 103336_r_at | 1418764_a_at |
| 103346_at | 1417743_at |
| 103530_at | 1450059_at |
| 103664_r_at | 1425830_a_at |
| 103871_at | 1433627_at |
| 103890_at | 1415866_at |
| 104087_at | 1419994_s_at |

| | |
|---|---|
| 104161_at | 1420936_s_at |
| 104361_at | 1434689_at |
| 104366_at | 1435087_at |
| 104378_at | 1450686_at |
| 104444_at | 1425191_at |
| 104503_at | 1437296_at |
| 104557_at | 1420307_a_at |
| 104698_at | 1425463_at |
| 104979_at | 1430651_s_at |
| 105509_at | 1437375_at |
| 105625_at | 1451542_at |
| 105642_at | 1441046_at |
| 106029_at | 1428186_at |
| 106057_at | 1451196_at |
| 106617_at | 1452283_at |
| 107322_at | 1449584_at |
| 107383_at | 1424728_at |
| 107408_at | 1434928_at |
| 107547_at | 1417473_a_at |
| 108039_at | 1429393_at |
| 108243_at | 1416938_at |
| 109140_at | 1417865_at |
| 109607_at | 1423599_a_at |
| 109652_at | 1433481_at |
| 109965_s_at | 1449369_at |
| 110433_at | 1437022_at |
| 110585_at | 1416360_at |
| 111308_at | 1433769_at |
| 112449_at | 1434389_at |
| 112906_at | 1456049_at |
| 112980_at | 1429262_at |
| 113166_at | 1419366_at |
| 113265_at | 1435810_at |
| 113430_at | 1449614_s_at |
| 113893_at | 1436641_at |
| 114331_at | 1433859_at |
| 114344_at | 1441967_at |
| 114372_at | 1452847_at |
| 114475_at | 1433826_at |
| 114511_at | 1420978_at |
| 114575_at | 1448918_at |
| 115137_at | 1423577_at |
| 115138_at | 1420467_at |
| 115147_at | 1455103_at |
| 115409_at | 1435575_at |
| 115601_at | 1455016_at |
| 115632_at | 1441079_at |
| 115831_at | 1455778_at |
| 115852_at | 1439076_at |
| 115874_at | 1450070_s_at |
| 115888_at | 1427379_at |
| 116065_at | 1434177_at |
| 116108_at | 1455210_at |
| 116143_at | 1458686_at |
| 116382_at | 1435349_at |
| 116830_at | 1436780_at |

| | |
|---|---|
| 117095_at | 1438254_at |
| 117272_at | 1428279_a_at |
| 129075_at | 1457246_at |
| 129107_at | 1458005_at |
| 130977_at | 1437174_at |
| 131653_at | 1456773_at |
| 133562_at | 1456126_at |
| 133703_at | 1429430_at |
| 135179_at | 1441273_at |
| 135212_at | 1457569_at |
| 135435_at | 1441707_at |
| 135531_i_at | 1453810_at |
| 135644_at | 1455551_at |
| 136244_at | 1435832_at |
| 138466_at | 1434982_at |
| 138565_at | 1450063_at |
| 139018_at | 1437926_at |
| 139127_at | 1452332_at |
| 140323_r_at | 1422565_s_at |
| 140480_f_at | 1442302_at |
| 140653_at | 1445278_at |
| 140830_at | 1440308_at |
| 96610_at | 1415826_at |
| 97449_at | 1415902_at |
| 93330_at | 1416203_at |
| 93592_at | 1416371_at |
| 94327_at | 1416984_at |
| 92818_at | 1417202_s_at |
| 97515_at | 1417369_at |
| 98429_at | 1417433_at |
| 96352_at | 1417463_a_at |
| 93010_at | 1417731_at |
| 94177_at | 1417871_at |
| 94323_at | 1418274_at |
| 160417_at | 1418429_at |
| 166171_at | 1418500_at |
| 95379_at | 1418934_at |
| 96764_at | 1419043_a_at |
| 98872_at | 1419064_a_at |
| 92738_at | 1419080_at |
| 101076_r_at | 1419268_at |
| 98996_at | 1419838_s_at |
| 93535_at | 1419866_s_at |
| 96518_at | 1420007_at |
| 92737_at | 1421173_at |
| 97709_at | 1422080_at |
| 98118_at | 1422241_a_at |
| 92429_at | 1422926_at |
| 98365_at | 1422949_at |
| 92200_at | 1423427_at |
| 96946_at | 1423873_at |
| 95406_at | 1424365_at |
| 160686_at | 1424452_at |
| 97116_at | 1424930_s_at |
| 96913_at | 1426522_at |
| 93985_at | 1426721_s_at |

| | |
|---|---|
| 96270_at | 1426745_at |
| 95609_at | 1426798_a_at |
| 92706_at | 1427140_at |
| 93405_at | 1427462_at |
| 97195_at | 1427510_at |
| 96975_at | 1427858_at |
| 95478_at | 1427955_a_at |
| 94531_at | 1428134_at |
| 160632_at | 1428229_at |
| 162686_at | 1428600_at |
| 160329_at | 1428675_at |
| 160982_at | 1428762_at |
| 167056_r_at | 1429705_at |
| 168249_at | 1429756_at |
| 170301_at | 1430529_at |
| 99179_at | 1431145_a_at |
| 169575_at | 1431517_at |
| 140861_at | 1432485_at |
| 99183_at | 1433591_at |
| 167518_at | 1434862_at |
| 163638_i_at | 1435116_at |
| 166865_f_at | 1435347_at |
| 164163_at | 1435671_at |
| 163464_at | 1435769_at |
| 165898_i_at | 1437792_at |
| 92408_at | 1438221_at |
| 167029_at | 1441840_x_at |
| 94786_at | 1443605_at |
| 95955_at | 1443949_at |
| 161127_i_at | 1447653_x_at |
| 165972_at | 1447698_x_at |
| 161789_r_at | 1447874_x_at |
| 166508_i_at | 1447943_x_at |
| 96563_at | 1448014_s_at |
| 93774_at | 1448202_x_at |
| 95434_at | 1448279_at |
| 165634_at | 1448674_at |
| 98306_g_at | 1448834_at |
| 96346_at | 1448842_at |
| 92876_at | 1448959_at |
| 98339_at | 1449264_at |
| 92500_at | 1449315_at |
| 167835_r_at | 1449791_x_at |
| 99802_at | 1450003_at |
| 160981_at | 1450666_s_at |
| 92610_at | 1450705_at |
| 162968_at | 1451143_at |
| 163236_at | 1451570_a_at |
| 160786_f_at | 1452057_at |
| 163707_at | 1452898_at |
| 94483_at | 1453099_at |
| 93521_at | 1454689_at |
| 163900_at | 1454769_at |
| 162720_at | 1454980_at |
| 94007_at | 1455077_a_at |
| 166456_at | 1457746_at |

| | |
|---|---|
| 165993_f_at | 1458343_x_at |
| 97450_s_at | 1460167_at |
| 97001_r_at | 1460312_at |
| 100142_at | 1421810_at |
| 100281_at | 1417114_at |
| 100297_at | 1438234_at |
| 100309_at | 1422990_at |
| 100370_at | 1425337_at |
| 100410_at | 1429558_a_at |
| 100469_at | 1452560_a_at |
| 100534_at | 1423395_at |
| 100599_at | 1448135_at |
| 100601_at | 1417533_a_at |
| 100621_at | 1451201_s_at |
| 100747_at | 1420155_at |
| 100767_at | 1458196_at |
| 100778_at | 1450136_at |
| 100986_at | 1419184_a_at |
| 101088_f_at | 1437850_a_at |
| 101359_at | 1416513_at |
| 101419_at | 1423221_at |
| 101473_at | 1432517_a_at |
| 101542_f_at | 1423043_s_at |
| 101557_at | 1460644_at |
| 101595_at | 1448064_at |
| 101696_r_at | 1419881_x_at |
| 101883_s_at | 1420357_s_at |
| 101898_s_at | 1425137_a_at |
| 101933_at | 1422664_at |
| 102014_at | 1424859_at |
| 102118_at | 1459952_at |
| 102201_s_at | 1421853_at |
| 102205_at | 1452461_a_at |
| 102207_at | 1424544_at |
| 102233_at | 1427202_at |
| 102307_at | 1418139_at |
| 102320_at | 1454807_a_at |
| 102342_at | 1422455_s_at |
| 102371_at | 1416505_at |
| 102645_at | 1438045_at |
| 102700_at | 1416711_at |
| 102745_at | 1452558_at |
| 102780_at | 1426875_s_at |
| 102970_at | 1425271_at |
| 102972_s_at | 1427307_a_at |
| 103069_at | 1428639_at |
| 103309_at | 1417592_at |
| 103362_at | 1424208_at |
| 103416_at | 1419169_at |
| 103438_at | 1418937_at |
| 103470_at | 1456270_s_at |
| 103511_at | 1451463_at |
| 103584_at | 1453015_at |
| 103602_at | 1420709_s_at |
| 103642_at | 1422801_at |
| 103754_at | 1423112_at |

| | |
|---|---|
| 103766_at | 1422167_at |
| 103849_at | 1421953_at |
| 103981_at | 1455727_at |
| 103982_s_at | 1422070_at |
| 103992_f_at | 1455423_at |
| 104093_at | 1417756_a_at |
| 104096_at | 1453804_a_at |
| 104296_at | 1434551_at |
| 104308_at | 1419128_at |
| 104317_at | 1448809_at |
| 104371_at | 1418295_s_at |
| 104409_at | 1418784_at |
| 104411_at | 1419893_at |
| 104538_at | 1448816_at |
| 104547_at | 1419172_at |
| 104766_at | 1418305_s_at |
| 104778_at | 1438224_at |
| 105068_at | 1444517_at |
| 105397_at | 1456968_at |
| 105709_at | 1434904_at |
| 105849_at | 1418944_at |
| 105952_at | 1459700_at |
| 106120_at | 1457027_at |
| 106287_at | 1436779_at |
| 106497_at | 1419610_at |
| 106505_at | 1455377_at |
| 106620_at | 1417972_s_at |
| 106664_at | 1451050_at |
| 106831_at | 1446150_at |
| 106920_at | 1434515_at |
| 107089_at | 1428093_at |
| 107534_at | 1419283_s_at |
| 107541_at | 1420014_at |
| 107543_s_at | 1417537_at |
| 107581_at | 1434652_at |
| 107622_at | 1434115_at |
| 107795_at | 1435428_at |
| 108371_at | 1451449_at |
| 108473_at | 1451142_at |
| 108497_at | 1454915_at |
| 108820_at | 1454920_at |
| 108850_at | 1437267_x_at |
| 109085_at | 1417247_at |
| 109408_at | 1434277_a_at |
| 109425_at | 1439834_at |
| 109639_at | 1417600_at |
| 109647_at | 1454834_at |
| 109657_at | 1427117_at |
| 109728_at | 1437291_at |
| 110005_at | 1435342_at |
| 110053_at | 1442027_at |
| 110072_at | 1453291_at |
| 110178_at | 1424555_at |
| 110181_at | 1456046_at |
| 110276_at | 1456477_at |
| 110318_at | 1420907_at |

| | |
|---|---|
| 110351_at | 1426133_a_at |
| 110355_at | 1424671_at |
| 110460_at | 1419459_a_at |
| 110681_at | 1455674_at |
| 110699_at | 1437407_at |
| 110767_r_at | 1420909_at |
| 110819_at | 1431755_a_at |
| 110833_at | 1423831_at |
| 110852_at | 1416532_at |
| 111056_at | 1422030_at |
| 111345_at | 1428739_at |
| 111814_at | 1429811_at |
| 111853_at | 1449271_a_at |
| 111915_at | 1416559_at |
| 111991_at | 1454846_at |
| 111995_at | 1434534_at |
| 112021_at | 1458769_at |
| 112212_at | 1436939_at |
| 112363_at | 1453003_at |
| 112393_at | 1451087_at |
| 112472_at | 1419488_at |
| 112488_at | 1453008_at |
| 112733_at | 1419066_at |
| 112795_at | 1434148_at |
| 112840_at | 1452607_at |
| 112889_at | 1417890_at |
| 112937_at | 1457030_at |
| 112963_at | 1421519_a_at |
| 113121_at | 1452169_a_at |
| 113556_at | 1434883_at |
| 113933_at | 1416235_at |
| 114020_at | 1451291_at |
| 114268_at | 1452111_at |
| 114319_at | 1443881_at |
| 114458_at | 1436858_at |
| 114696_at | 1437241_at |
| 114698_at | 1460348_at |
| 114789_at | 1455983_at |
| 114807_at | 1435665_at |
| 114812_at | 1418203_at |
| 115014_at | 1460303_at |
| 115053_at | 1435434_at |
| 115084_at | 1436555_at |
| 115152_at | 1443945_at |
| 115269_at | 1426012_a_at |
| 115285_at | 1458399_at |
| 115410_at | 1439015_at |
| 115479_at | 1450085_at |
| 115527_at | 1436663_at |
| 115677_at | 1440206_at |
| 115943_at | 1457287_at |
| 116071_at | 1438416_at |
| 116147_at | 1423322_at |
| 116340_at | 1429092_at |
| 116349_at | 1418092_s_at |
| 116366_at | 1452216_at |

| | |
|---|---|
| 116400_at | 1424431_at |
| 116616_at | 1428969_at |
| 116815_at | 1418553_at |
| 116940_at | 1424597_at |
| 116979_at | 1433751_at |
| 117064_f_at | 1435616_at |
| 117098_at | 1429093_at |
| 117225_at | 1455324_at |
| 117267_at | 1424913_at |
| 117321_at | 1451465_at |
| 129191_at | 1443531_at |
| 129300_at | 1456889_at |
| 129503_at | 1453173_at |
| 130696_f_at | 1424404_at |
| 130778_at | 1451467_s_at |
| 130920_at | 1442086_at |
| 130936_f_at | 1451403_at |
| 130997_r_at | 1428731_at |
| 131190_at | 1455334_at |
| 131423_at | 1418495_at |
| 132437_s_at | 1433822_x_at |
| 133211_at | 1445237_at |
| 133340_f_at | 1424405_at |
| 133701_at | 1447034_at |
| 133882_at | 1444611_at |
| 133941_at | 1440748_at |
| 134533_at | 1422741_a_at |
| 134646_at | 1451605_at |
| 134823_at | 1442562_at |
| 135163_r_at | 1443934_at |
| 135201_at | 1425039_at |
| 135591_at | 1440294_at |
| 136026_at | 1449510_at |
| 136134_at | 1440854_at |
| 136587_at | 1457113_at |
| 136591_at | 1455634_at |
| 136634_at | 1442653_at |
| 137659_at | 1445032_at |
| 137713_at | 1456918_at |
| 138387_at | 1430313_at |
| 138557_at | 1445867_at |
| 138572_at | 1453643_at |
| 139509_at | 1457215_at |
| 140650_at | 1450004_at |
| 160265_at | 1415723_at |
| 98950_at | 1415749_a_at |
| 94457_at | 1415792_at |
| 92598_at | 1415814_at |
| 160533_r_at | 1415924_at |
| 95666_at | 1415934_at |
| 94073_at | 1416270_at |
| 162719_at | 1416274_at |
| 160693_at | 1416387_at |
| 165428_r_at | 1416473_a_at |
| 160147_r_at | 1416518_at |
| 99938_at | 1416587_a_at |

| | |
|---|---|
| 93460_at | 1416786_at |
| 93119_at | 1416902_a_at |
| 162780_at | 1416981_at |
| 94929_at | 1417068_a_at |
| 165459_at | 1417096_at |
| 98088_at | 1417268_at |
| 163858_at | 1417407_at |
| 97527_at | 1417458_s_at |
| 97423_at | 1417474_at |
| 92498_at | 1417757_at |
| 92301_at | 1417813_at |
| 96734_at | 1417834_at |
| 160869_at | 1417892_a_at |
| 97254_at | 1418119_at |
| 162959_i_at | 1418155_at |
| 93163_at | 1418177_at |
| 160363_at | 1418284_at |
| 164137_at | 1418304_at |
| 165677_at | 1418348_a_at |
| 93755_at | 1418368_at |
| 160972_at | 1418423_s_at |
| 163963_at | 1418781_at |
| 104848_at | 1418863_at |
| 98338_at | 1418868_at |
| 92290_at | 1419404_s_at |
| 92295_at | 1419717_at |
| 93470_at | 1419749_at |
| 93974_at | 1419816_s_at |
| 160737_at | 1420013_s_at |
| 167142_r_at | 1420245_x_at |
| 94795_at | 1420531_at |
| 93116_at | 1420611_at |
| 98055_at | 1420631_a_at |
| 161038_at | 1420637_at |
| 163087_at | 1420919_at |
| 98348_at | 1421157_at |
| 94753_at | 1421302_a_at |
| 166136_i_at | 1421404_at |
| 93648_at | 1421446_at |
| 93007_at | 1421471_at |
| 160919_r_at | 1421841_at |
| 93626_at | 1422906_at |
| 97712_at | 1422994_at |
| 98572_at | 1423151_at |
| 96273_at | 1423231_at |
| 160894_at | 1423233_at |
| 92468_at | 1423421_at |
| 160347_at | 1423698_at |
| 93270_at | 1423784_at |
| 98875_at | 1423793_at |
| 99335_at | 1423855_x_at |
| 162947_at | 1423865_at |
| 94054_at | 1423917_a_at |
| 95753_at | 1423920_at |
| 94042_f_at | 1423985_at |
| 95658_at | 1424121_at |

| | |
|---|---|
| 163926_at | 1424205_at |
| 160658_at | 1424257_at |
| 94078_at | 1424364_a_at |
| 165477_r_at | 1424387_at |
| 97284_at | 1424406_at |
| 95501_at | 1424564_at |
| 165583_i_at | 1424831_at |
| 92870_at | 1425187_at |
| 94777_at | 1425260_at |
| 98993_at | 1425542_a_at |
| 93533_at | 1425674_a_at |
| 168568_i_at | 1426308_at |
| 94885_at | 1426666_a_at |
| 163688_at | 1426797_at |
| 98463_at | 1426804_at |
| 99638_at | 1426955_at |
| 163634_at | 1426968_a_at |
| 163829_r_at | 1427419_x_at |
| 92757_at | 1427674_a_at |
| 98604_at | 1427720_a_at |
| 96215_f_at | 1427820_at |
| 167585_at | 1427948_a_at |
| 98099_at | 1428164_at |
| 95086_at | 1428201_at |
| 162621_at | 1428325_at |
| 98973_at | 1428390_at |
| 97835_at | 1428538_s_at |
| 166067_f_at | 1428665_at |
| 95636_at | 1428679_s_at |
| 92350_at | 1428819_at |
| 99604_at | 1428843_at |
| 164063_at | 1428901_at |
| 163941_at | 1429024_at |
| 165559_at | 1429074_at |
| 163527_i_at | 1429277_at |
| 165896_i_at | 1429482_at |
| 160466_at | 1429528_at |
| 163449_at | 1429547_at |
| 166287_f_at | 1429746_at |
| 167382_at | 1429991_at |
| 166884_at | 1430329_at |
| 167232_i_at | 1431192_at |
| 166218_at | 1431561_a_at |
| 162129_f_at | 1432221_at |
| 160885_at | 1433519_at |
| 94424_at | 1433521_at |
| 162798_at | 1433670_at |
| 97354_at | 1434066_at |
| 165432_i_at | 1434195_at |
| 97911_at | 1434214_at |
| 95897_at | 1434386_at |
| 94784_at | 1434850_at |
| 161092_at | 1434859_at |
| 92647_at | 1434892_x_at |
| 93923_at | 1435019_at |
| 94869_at | 1435070_at |

| | |
|---|---|
| 92656_at | 1436382_at |
| 92180_at | 1436517_at |
| 166043_at | 1437040_at |
| 98951_at | 1437205_at |
| 163636_at | 1437392_at |
| 167366_at | 1437422_at |
| 165426_f_at | 1437464_at |
| 94838_r_at | 1437515_at |
| 166375_f_at | 1437609_at |
| 167873_i_at | 1437673_at |
| 160883_at | 1437840_s_at |
| 166082_r_at | 1438059_at |
| 161530_r_at | 1438934_x_at |
| 168450_at | 1439126_at |
| 163761_at | 1439620_at |
| 166879_at | 1439727_at |
| 95532_at | 1439771_s_at |
| 167757_f_at | 1441946_at |
| 169241_i_at | 1442051_at |
| 95949_at | 1442492_at |
| 167113_at | 1442824_at |
| 99974_at | 1443054_at |
| 167038_at | 1443774_at |
| 164283_r_at | 1444595_at |
| 167277_f_at | 1445871_at |
| 160879_at | 1446899_at |
| 167165_f_at | 1447633_x_at |
| 166998_f_at | 1447707_s_at |
| 167021_at | 1447719_at |
| 161122_f_at | 1447919_x_at |
| 96389_at | 1447978_at |
| 97751_f_at | 1447999_x_at |
| 97538_at | 1448124_at |
| 93762_at | 1448138_at |
| 93099_f_at | 1448191_at |
| 97882_at | 1448242_at |
| 161023_at | 1448681_at |
| 99630_at | 1448699_at |
| 98434_at | 1449066_a_at |
| 96950_at | 1449277_at |
| 165907_f_at | 1449398_at |
| 92403_at | 1449468_at |
| 96760_at | 1449886_a_at |
| 99962_at | 1450053_at |
| 163844_at | 1450761_s_at |
| 98967_at | 1450779_at |
| 93491_f_at | 1450858_a_at |
| 164132_at | 1450924_at |
| 99156_at | 1451269_at |
| 160218_at | 1451385_at |
| 167694_f_at | 1451608_a_at |
| 96691_at | 1451995_at |
| 96654_at | 1452162_at |
| 95401_at | 1452164_at |
| 163609_at | 1452220_at |
| 92541_at | 1452651_a_at |

| | |
|---|---|
| 160270_at | 1452671_s_at |
| 94062_at | 1452692_a_at |
| 163035_at | 1452863_at |
| 99566_at | 1452927_x_at |
| 163934_at | 1453055_at |
| 163018_at | 1453130_at |
| 93320_at | 1453303_at |
| 161114_i_at | 1453324_at |
| 160445_at | 1453848_s_at |
| 162659_at | 1454727_at |
| 98064_at | 1454754_a_at |
| 163147_at | 1454919_at |
| 166771_s_at | 1455003_at |
| 166613_at | 1455340_at |
| 99926_at | 1455490_at |
| 166209_f_at | 1455891_at |
| 168336_at | 1456069_at |
| 163698_at | 1456089_at |
| 97148_at | 1456136_at |
| 165923_r_at | 1456198_at |
| 164043_at | 1456272_at |
| 92638_at | 1456390_at |
| 167875_r_at | 1459790_x_at |
| 161828_r_at | 1459817_at |
| 97780_at | 1459934_at |
| 97261_at | 1460179_at |
| 163855_at | 1460467_at |
| 92279_at | 1460583_at |
| 161800_r_at | 1453852_at |
| 162070_r_at | 1418867_at |
| 166143_f_at | 1449440_at |
| 167596_at | 1454037_a_at |
| 168256_at | 1425835_a_at |
| 100001_at | 1419178_at |
| 100002_at | 1449123_at |
| 100003_at | 1427306_at |
| 100004_at | 1428531_at |
| 100007_at | 1451252_at |
| 100014_at | 1416944_a_at |
| 100016_at | 1417234_at |
| 100017_at | 1419487_at |
| 100018_at | 1418333_at |
| 100019_at | 1427256_at |
| 100021_at | 1418852_at |
| 100022_at | 1448724_at |
| 100028_r_at | 1458153_at |
| 100029_at | 1419053_at |
| 100033_at | 1416988_at |
| 100034_at | 1421752_a_at |
| 100035_at | 1449160_at |
| 100040_at | 1450866_a_at |
| 100043_f_at | 1440186_s_at |
| 100044_at | 1416003_at |
| 100047_at | 1416828_at |
| 100049_at | 1416184_s_at |
| 100051_at | 1419097_a_at |

| | |
|---|---|
| 100052_at | 1452522_at |
| 100056_at | 1423442_a_at |
| 100057_at | 1436121_a_at |
| 100058_at | 1451975_at |
| 100068_at | 1416468_at |
| 100069_at | 1448792_a_at |
| 100071_at | 1430893_at |
| 100072_at | 1450953_at |
| 100078_at | 1417761_at |
| 100079_at | 1452184_at |
| 100080_at | 1419348_at |
| 100082_at | 1421234_at |
| 100084_at | 1450850_at |
| 100085_at | 1448485_at |
| 100086_at | 1452148_at |
| 100091_at | 1448769_at |
| 100093_at | 1460169_a_at |
| 100099_at | 1448621_a_at |
| 100101_at | 1417274_at |
| 100106_at | 1417370_at |
| 100113_s_at | 1451783_a_at |
| 100115_at | 1426289_at |
| 100122_at | 1422208_a_at |
| 100126_at | 1422505_at |
| 100128_at | 1448314_at |
| 100131_at | 1423150_at |
| 100134_at | 1417271_a_at |
| 100141_at | 1417452_a_at |
| 100147_at | 1423644_at |
| 100152_at | 1421966_at |
| 100213_f_at | 1455578_x_at |
| 100272_at | 1452563_a_at |
| 100278_at | 1419497_at |
| 100279_at | 1421890_at |
| 100280_at | 1450608_at |
| 100282_at | 1422235_at |
| 100283_at | 1451689_a_at |
| 100284_at | 1421539_at |
| 100286_at | 1418267_at |
| 100287_at | 1430512_a_at |
| 100292_at | 1449105_at |
| 100294_at | 1421229_at |
| 100298_at | 1450375_at |
| 100300_at | 1422978_at |
| 100303_at | 1450587_at |
| 100306_at | 1459902_at |
| 100307_at | 1459909_at |
| 100313_at | 1427489_at |
| 100317_at | 1422159_at |
| 100318_at | 1431119_at |
| 100319_at | 1450330_at |
| 100322_at | 1451948_at |
| 100326_f_at | 1425417_x_at |
| 100328_s_at | 1420464_s_at |
| 100331_g_at | 1418506_a_at |
| 100332_s_at | 1423223_a_at |

| | |
|---|---|
| 100333_at | 1450788_at |
| 100334_f_at | 1421788_x_at |
| 100335_at | 1450283_at |
| 100336_s_at | 1449880_s_at |
| 100337_at | 1421692_at |
| 100339_at | 1424898_at |
| 100344_at | 1460696_at |
| 100346_at | 1420795_at |
| 100347_at | 1447986_at |
| 100350_at | 1441583_at |
| 100358_s_at | 1422225_s_at |
| 100359_at | 1427421_at |
| 100364_at | 1426106_a_at |
| 100365_at | 1423012_at |
| 100378_at | 1459701_x_at |
| 100382_at | 1421517_at |
| 100383_at | 1460385_a_at |
| 100384_at | 1425786_a_at |
| 100385_at | 1422277_at |
| 100390_s_at | 1450571_a_at |
| 100391_at | 1420931_at |
| 100392_at | 1451038_at |
| 100398_at | 1449841_at |
| 100402_f_at | 1417899_at |
| 100404_at | 1448652_at |
| 100406_at | 1423544_at |
| 100407_at | 1460668_at |
| 100411_at | 1459298_at |
| 100413_at | 1424532_at |
| 100414_s_at | 1415960_at |
| 100416_at | 1416558_at |
| 100420_at | 1427268_at |
| 100421_at | 1418735_at |
| 100424_at | 1437447_s_at |
| 100427_at | 1417676_a_at |
| 100428_at | 1421279_at |
| 100429_at | 1416618_at |
| 100430_at | 1425873_a_at |
| 100431_at | 1425644_at |
| 100434_s_at | 1420713_a_at |
| 100435_at | 1417143_at |
| 100438_at | 1421756_a_at |
| 100444_at | 1450674_at |
| 100447_at | 1448679_at |
| 100451_at | 1424622_at |
| 100452_at | 1418600_at |
| 100453_at | 1448676_at |
| 100454_at | 1416674_at |
| 100455_at | 1416688_at |
| 100458_at | 1423173_at |
| 100459_at | 1422630_at |
| 100463_at | 1420633_a_at |
| 100464_at | 1428468_at |
| 100466_f_at | 1434498_at |
| 100470_at | 1448342_at |
| 100474_at | 1420377_at |

| | |
|---|---|
| 100481_at | 1449154_at |
| 100482_at | 1460691_at |
| 100483_at | 1449026_at |
| 100488_at | 1450099_a_at |
| 100491_at | 1418445_at |
| 100493_at | 1416761_at |
| 100494_at | 1450869_at |
| 100495_at | 1417207_at |
| 100500_at | 1416888_at |
| 100501_at | 1448911_at |
| 100502_at | 1426036_a_at |
| 100505_at | 1448125_at |
| 100507_at | 1426851_a_at |
| 100508_at | 1416992_at |
| 100510_at | 1418053_at |
| 100511_at | 1428242_at |
| 100513_at | 1425572_a_at |
| 100518_at | 1416458_at |
| 100521_at | 1426526_s_at |
| 100525_at | 1448115_at |
| 100526_f_at | 1448329_at |
| 100530_at | 1460634_at |
| 100536_at | 1433785_at |
| 100537_at | 1455006_at |
| 100543_s_at | 1448521_at |
| 100546_at | 1416920_at |
| 100547_at | 1452112_a_at |
| 100548_at | 1416406_at |
| 100549_at | 1448716_at |
| 100552_at | 1448167_at |
| 100553_at | 1448101_s_at |
| 100554_at | 1416554_at |
| 100555_at | 1416600_a_at |
| 100562_at | 1448740_at |
| 100564_at | 1417103_at |
| 100566_at | 1452114_s_at |
| 100567_at | 1417023_a_at |
| 100568_at | 1416015_s_at |
| 100570_at | 1420535_a_at |
| 100575_at | 1417118_a_at |
| 100577_at | 1416336_s_at |
| 100578_at | 1415852_at |
| 100580_at | 1421062_s_at |
| 100583_at | 1421653_a_at |
| 100592_at | 1415882_at |
| 100593_at | 1418726_a_at |
| 100594_at | 1415946_at |
| 100596_at | 1450699_at |
| 100603_at | 1415841_at |
| 100608_at | 1422690_at |
| 100613_at | 1416113_at |
| 100614_at | 1451203_at |
| 100615_at | 1422420_at |
| 100617_at | 1423772_x_at |
| 100620_at | 1424618_at |
| 100623_at | 1416306_at |

| | |
|---|---|
| 100632_at | 1417690_at |
| 100634_at | 1448723_at |
| 100669_at | 1426145_at |
| 100670_at | 1421660_at |
| 100671_at | 1422332_at |
| 100674_f_at | 1426140_x_at |
| 100675_at | 1450300_at |
| 100678_s_at | 1427681_s_at |
| 100679_at | 1421701_at |
| 100684_at | 1416339_a_at |
| 100685_at | 1422481_at |
| 100686_at | 1422156_a_at |
| 100688_at | 1420536_at |
| 100690_at | 1420546_at |
| 100691_at | 1421705_at |
| 100692_at | 1427824_at |
| 100693_at | 1422187_at |
| 100694_at | 1416277_a_at |
| 100695_at | 1449161_at |
| 100696_at | 1450415_at |
| 100697_at | 1420389_at |
| 100698_at | 1450361_at |
| 100702_at | 1421602_at |
| 100703_at | 1417489_at |
| 100705_at | 1450192_at |
| 100707_at | 1421270_at |
| 100709_at | 1415808_at |
| 100710_at | 1423030_at |
| 100711_at | 1431177_a_at |
| 100712_at | 1420801_at |
| 100714_at | 1421464_at |
| 100715_at | 1421687_at |
| 100716_at | 1420590_at |
| 100717_at | 1460667_at |
| 100719_f_at | 1420490_at |
| 100720_at | 1418883_a_at |
| 100724_at | 1427587_at |
| 100726_at | 1421616_at |
| 100727_at | 1416074_a_at |
| 100728_at | 1418663_at |
| 100729_at | 1448109_a_at |
| 100730_at | 1421547_at |
| 100731_at | 1450373_at |
| 100732_at | 1436760_a_at |
| 100734_at | 1449323_a_at |
| 100735_at | 1427842_at |
| 100736_at | 1421761_a_at |
| 100737_at | 1450252_at |
| 100738_at | 1426149_at |
| 100739_at | 1427834_at |
| 100740_at | 1422817_at |
| 100741_at | 1421661_at |
| 100744_at | 1441134_at |
| 100745_r_at | 1459892_at |
| 100746_at | 1457594_at |
| 100748_at | 1446971_at |

| | |
|---|---|
| 100749_at | 1457810_at |
| 100750_at | 1420301_at |
| 100751_at | 1450104_at |
| 100752_at | 1450280_a_at |
| 100753_at | 1449710_s_at |
| 100755_at | 1422928_at |
| 100757_at | 1452476_at |
| 100758_at | 1423763_x_at |
| 100759_at | 1421044_at |
| 100760_at | 1421460_at |
| 100761_at | 1422036_at |
| 100762_at | 1421414_a_at |
| 100763_at | 1442374_at |
| 100764_at | 1417546_at |
| 100765_at | 1448055_at |
| 100766_at | 1421136_at |
| 100773_at | 1425454_a_at |
| 100774_at | 1428015_at |
| 100779_at | 1419530_at |
| 100780_at | 1416276_a_at |
| 100828_at | 1457126_at |
| 100877_at | 1436339_at |
| 100880_at | 1434380_at |
| 100882_at | 1419492_s_at |
| 100883_at | 1418250_at |
| 100885_at | 1417299_at |
| 100888_at | 1460390_at |
| 100889_at | 1460034_at |
| 100890_at | 1423877_at |
| 100891_at | 1418358_at |
| 100892_at | 1423711_at |
| 100893_at | 1460243_at |
| 100896_at | 1422813_at |
| 100899_s_at | 1448705_at |
| 100903_at | 1417800_at |
| 100904_at | 1452699_at |
| 100906_at | 1418741_at |
| 100907_at | 1416640_at |
| 100910_at | 1433609_s_at |
| 100911_at | 1448225_at |
| 100912_at | 1452839_at |
| 100913_at | 1417443_at |
| 100916_at | 1418118_at |
| 100917_at | 1426496_at |
| 100921_at | 1422536_at |
| 100925_at | 1453208_at |
| 100932_at | 1417228_at |
| 100933_at | 1448366_at |
| 100935_at | 1418437_a_at |
| 100939_at | 1424298_at |
| 100944_at | 1434372_at |
| 100946_at | 1427126_at |
| 100948_at | 1450627_at |
| 100949_at | 1417688_at |
| 100961_at | 1449544_a_at |
| 100963_at | 1451399_at |

| | |
|---|---|
| 100965_at | 1455167_at |
| 100966_at | 1418680_at |
| 100972_s_at | 1430375_a_at |
| 100973_i_at | 1434962_x_at |
| 100978_at | 1417253_at |
| 100982_at | 1418399_at |
| 100983_at | 1422796_at |
| 100985_at | 1449733_s_at |
| 100988_at | 1435448_at |
| 100994_at | 1416936_at |
| 100997_at | 1426731_at |
| 100998_at | 1451721_a_at |
| 101002_at | 1450714_at |
| 101011_at | 1415867_at |
| 101013_at | 1428868_a_at |
| 101014_at | 1427691_a_at |
| 101015_s_at | 1451462_a_at |
| 101016_at | 1426390_a_at |
| 101023_f_at | 1433936_at |
| 101026_at | 1419620_at |
| 101031_at | 1450561_a_at |
| 101036_at | 1423079_a_at |
| 101042_f_at | 1416712_at |
| 101043_f_at | 1415954_at |
| 101045_at | 1448286_at |
| 101048_at | 1422124_a_at |
| 101049_at | 1416026_a_at |
| 101053_at | 1424036_at |
| 101054_at | 1425519_a_at |
| 101057_at | 1454682_at |
| 101058_at | 1417765_a_at |
| 101062_at | 1416399_a_at |
| 101064_at | 1448282_at |
| 101071_at | 1448826_at |
| 101074_at | 1416493_at |
| 101075_f_at | 1455802_x_at |
| 101077_at | 1416464_at |
| 101081_at | 1415702_a_at |
| 101084_f_at | 1452729_at |
| 101086_f_at | 1416244_a_at |
| 101095_at | 1417359_at |
| 101096_s_at | 1426187_a_at |
| 101097_at | 1452580_a_at |
| 101099_at | 1423056_at |
| 101102_at | 1460737_at |
| 101103_at | 1423257_at |
| 101105_at | 1421082_s_at |
| 101112_g_at | 1437628_s_at |
| 101115_at | 1450009_at |
| 101116_at | 1420109_at |
| 101117_at | 1420163_at |
| 101118_at | 1447341_at |
| 101119_at | 1449781_at |
| 101121_at | 1420236_at |
| 101122_at | 1421527_at |
| 101126_r_at | 1420069_at |

| | |
|---|---|
| 101127_at | 1419628_at |
| 101128_at | 1420442_at |
| 101129_at | 1423665_a_at |
| 101131_at | 1450299_at |
| 101133_at | 1422306_at |
| 101134_at | 1452568_at |
| 101136_at | 1450272_at |
| 101137_at | 1435151_a_at |
| 101138_at | 1450319_at |
| 101140_at | 1450219_at |
| 101141_at | 1427806_at |
| 101143_at | 1450043_at |
| 101144_at | 1421628_at |
| 101145_at | 1421512_at |
| 101146_at | 1450002_at |
| 101147_at | 1420544_at |
| 101149_at | 1450326_at |
| 101151_at | 1450215_at |
| 101152_at | 1422207_at |
| 101153_at | 1452564_at |
| 101154_at | 1420141_at |
| 101155_at | 1449306_at |
| 101156_at | 1452552_at |
| 101159_at | 1422025_at |
| 101161_at | 1422069_at |
| 101162_at | 1420757_at |
| 101163_at | 1418065_at |
| 101164_at | 1421744_at |
| 101166_at | 1450514_at |
| 101167_at | 1421598_at |
| 101168_at | 1452382_at |
| 101169_at | 1420511_at |
| 101170_at | 1450548_at |
| 101172_at | 1421710_at |
| 101173_at | 1420983_at |
| 101175_at | 1422250_at |
| 101176_at | 1422304_at |
| 101177_at | 1422098_at |
| 101181_at | 1422181_at |
| 101183_at | 1449866_at |
| 101184_at | 1421671_at |
| 101185_at | 1423027_at |
| 101186_at | 1420747_at |
| 101187_at | 1418813_at |
| 101189_at | 1450366_at |
| 101190_at | 1450352_at |
| 101191_at | 1420666_at |
| 101192_at | 1420348_at |
| 101193_at | 1421395_at |
| 101195_at | 1420805_at |
| 101197_at | 1421732_at |
| 101198_at | 1417747_at |
| 101199_at | 1426072_at |
| 101200_at | 1449920_at |
| 101202_at | 1420297_at |
| 101205_at | 1449808_at |

| | |
|---|---|
| 101206_at | 1420289_at |
| 101207_at | 1417451_a_at |
| 101209_at | 1421775_at |
| 101211_at | 1447494_at |
| 101212_at | 1419364_a_at |
| 101213_at | 1419441_at |
| 101214_f_at | 1418625_s_at |
| 101215_at | 1421738_at |
| 101216_at | 1420287_at |
| 101220_at | 1449765_at |
| 101221_at | 1419992_x_at |
| 101223_r_at | 1449690_x_at |
| 101224_at | 1445125_at |
| 101225_at | 1448085_at |
| 101226_at | 1444943_at |
| 101227_at | 1448090_at |
| 101228_at | 1459918_at |
| 101229_at | 1448088_at |
| 101231_at | 1446695_at |
| 101232_at | 1444829_at |
| 101233_at | 1448082_at |
| 101234_at | 1458862_at |
| 101254_at | 1433569_x_at |
| 101255_at | 1449436_s_at |
| 101278_at | 1432032_a_at |
| 101280_at | 1460313_at |
| 101281_at | 1422370_at |
| 101282_at | 1422275_at |
| 101286_at | 1422274_at |
| 101288_at | 1422319_at |
| 101289_f_at | 1425182_x_at |
| 101302_at | 1421538_at |
| 101303_at | 1422372_at |
| 101305_at | 1422331_at |
| 101307_at | 1424352_at |
| 101309_at | 1450577_at |
| 101310_at | 1460309_at |
| 101311_at | 1427765_a_at |
| 101312_at | 1422223_at |
| 101315_at | 1427832_at |
| 101317_f_at | 1450613_x_at |
| 101318_at | 1450527_at |
| 101325_r_at | 1425276_at |
| 101327_at | 1448160_at |
| 101329_f_at | 1427837_at |
| 101331_f_at | 1427860_at |
| 101332_at | 1426178_at |
| 101333_at | 1423637_at |
| 101334_at | 1452528_a_at |
| 101335_at | 1450465_at |
| 101337_at | 1426077_at |
| 101341_at | 1450529_at |
| 101342_at | 1451889_at |
| 101343_at | 1423024_at |
| 101344_at | 1460663_at |
| 101345_at | 1447953_at |

| | |
|---|---|
| 101346_at | 1452535_at |
| 101347_at | 1427750_at |
| 101348_at | 1427753_at |
| 101354_at | 1422203_at |
| 101356_at | 1426100_a_at |
| 101363_at | 1460710_at |
| 101364_at | 1427519_at |
| 101367_at | 1422521_at |
| 101369_at | 1433705_at |
| 101370_at | 1419548_at |
| 101371_at | 1424469_a_at |
| 101374_at | 1451859_at |
| 101375_at | 1420751_at |
| 101376_at | 1425872_at |
| 101380_at | 1424753_at |
| 101381_at | 1421305_x_at |
| 101382_at | 1418894_s_at |
| 101384_at | 1424549_at |
| 101386_at | 1417735_at |
| 101388_at | 1416784_at |
| 101390_at | 1427374_at |
| 101392_at | 1451300_a_at |
| 101395_at | 1436940_at |
| 101396_at | 1451687_a_at |
| 101397_at | 1450628_at |
| 101398_at | 1448341_a_at |
| 101399_at | 1418670_s_at |
| 101401_at | 1416867_at |
| 101402_at | 1424180_a_at |
| 101403_at | 1418777_at |
| 101406_at | 1417335_at |
| 101407_at | 1427282_a_at |
| 101408_at | 1422558_at |
| 101409_at | 1451211_a_at |
| 101412_at | 1432103_a_at |
| 101417_at | 1427252_at |
| 101420_at | 1422756_at |
| 101421_at | 1422888_at |
| 101424_at | 1425719_a_at |
| 101425_at | 1448273_at |
| 101426_at | 1434034_at |
| 101428_at | 1451911_a_at |
| 101431_at | 1418808_at |
| 101432_at | 1455315_at |
| 101435_at | 1449168_a_at |
| 101436_at | 1418652_at |
| 101446_at | 1418412_at |
| 101448_at | 1416540_at |
| 101453_at | 1419608_a_at |
| 101459_at | 1450077_at |
| 101463_at | 1418708_at |
| 101467_at | 1434342_at |
| 101470_at | 1434793_at |
| 101471_at | 1438711_at |
| 101472_s_at | 1421259_at |
| 101474_at | 1419578_at |

| | |
|---|---|
| 101475_at | 1448733_at |
| 101480_at | 1416870_at |
| 101481_at | 1415791_at |
| 101482_at | 1450149_a_at |
| 101483_at | 1420745_a_at |
| 101489_at | 1448484_at |
| 101490_at | 1423651_at |
| 101495_at | 1416330_at |
| 101500_at | 1433490_s_at |
| 101501_r_at | 1415911_at |
| 101505_at | 1426644_at |
| 101507_at | 1428364_at |
| 101513_at | 1438358_x_at |
| 101514_at | 1416771_at |
| 101515_at | 1416408_at |
| 101517_at | 1450965_at |
| 101518_at | 1451412_a_at |
| 101519_at | 1418567_a_at |
| 101520_at | 1418651_at |
| 101522_at | 1448069_at |
| 101526_at | 1448601_s_at |
| 101530_at | 1416557_a_at |
| 101534_at | 1422419_s_at |
| 101537_at | 1416913_at |
| 101538_i_at | 1449081_at |
| 101539_f_at | 1435370_a_at |
| 101541_at | 1456474_at |
| 101552_at | 1423279_at |
| 101553_at | 1424279_at |
| 101555_at | 1451086_s_at |
| 101562_at | 1417095_a_at |
| 101566_f_at | 1420465_s_at |
| 101569_at | 1448457_at |
| 101572_f_at | 1420553_x_at |
| 101577_at | 1416141_a_at |
| 101582_at | 1426579_at |
| 101596_at | 1420048_at |
| 101597_at | 1446829_at |
| 101598_at | 1458795_at |
| 101599_at | 1419940_at |
| 101600_at | 1444887_at |
| 101601_at | 1419885_at |
| 101603_g_at | 1457849_at |
| 101604_at | 1446379_at |
| 101605_at | 1446008_at |
| 101606_at | 1459085_at |
| 101607_at | 1438755_at |
| 101608_at | 1458041_at |
| 101609_at | 1428752_at |
| 101610_at | 1449684_at |
| 101611_at | 1420173_at |
| 101612_at | 1460264_at |
| 101614_at | 1419890_at |
| 101615_at | 1420149_at |
| 101619_at | 1450593_at |
| 101620_at | 1422316_at |

| | |
|---|---|
| 101621_at | 1421803_at |
| 101622_at | 1421737_at |
| 101623_at | 1420445_at |
| 101624_at | 1421518_at |
| 101625_at | 1421342_at |
| 101626_at | 1420742_at |
| 101629_s_at | 1421673_s_at |
| 101632_at | 1419214_at |
| 101634_at | 1415839_a_at |
| 101636_at | 1420466_at |
| 101641_at | 1427455_x_at |
| 101642_at | 1427841_at |
| 101643_at | 1450349_at |
| 101646_at | 1426055_a_at |
| 101647_at | 1421767_at |
| 101648_at | 1422318_at |
| 101649_at | 1421833_at |
| 101650_at | 1420798_s_at |
| 101655_at | 1419521_at |
| 101659_at | 1425127_at |
| 101660_at | 1458532_at |
| 101661_r_at | 1420290_at |
| 101664_at | 1422475_a_at |
| 101666_at | 1421730_at |
| 101668_at | 1449761_at |
| 101671_at | 1420084_at |
| 101674_at | 1453584_at |
| 101677_at | 1453827_at |
| 101679_at | 1419723_at |
| 101681_f_at | 1450531_at |
| 101687_r_at | 1442569_at |
| 101688_at | 1448089_at |
| 101689_at | 1419831_at |
| 101693_f_at | 1447997_s_at |
| 101694_f_at | 1447631_at |
| 101695_at | 1419822_at |
| 101699_at | 1422287_at |
| 101700_at | 1422272_at |
| 101701_at | 1422052_at |
| 101702_at | 1421085_at |
| 101704_at | 1450518_at |
| 101705_at | 1421800_at |
| 101709_at | 1420796_at |
| 101710_at | 1421351_at |
| 101711_at | 1422295_at |
| 101712_at | 1422218_at |
| 101713_at | 1420413_at |
| 101714_at | 1421508_at |
| 101715_at | 1450615_at |
| 101716_at | 1450616_at |
| 101717_at | 1422281_at |
| 101719_at | 1450606_at |
| 101724_at | 1423029_at |
| 101725_at | 1450530_at |
| 101726_at | 1422342_at |
| 101728_at | 1422190_at |

| | |
|---|---|
| 101729_at | 1423019_at |
| 101730_at | 1450288_at |
| 101733_at | 1422349_at |
| 101734_at | 1421435_at |
| 101735_f_at | 1422415_at |
| 101736_at | 1422271_at |
| 101737_at | 1450996_at |
| 101738_at | 1450795_at |
| 101739_at | 1450619_x_at |
| 101740_at | 1421659_at |
| 101742_at | 1421715_at |
| 101748_at | 1450586_at |
| 101755_f_at | 1450811_at |
| 101756_f_at | 1422425_at |
| 101760_at | 1450246_at |
| 101762_at | 1422401_at |
| 101763_at | 1450346_at |
| 101764_at | 1422290_at |
| 101765_at | 1450573_at |
| 101769_at | 1427687_at |
| 101772_r_at | 1425613_at |
| 101774_at | 1425772_at |
| 101775_at | 1421769_at |
| 101776_at | 1452482_at |
| 101777_at | 1427783_at |
| 101778_at | 1422192_at |
| 101779_at | 1422278_at |
| 101786_at | 1422255_at |
| 101788_f_at | 1450564_x_at |
| 101793_at | 1417876_at |
| 101794_f_at | 1422802_at |
| 101795_f_at | 1422421_at |
| 101796_at | 1427592_at |
| 101797_at | 1419408_at |
| 101800_at | 1422953_at |
| 101801_at | 1422350_at |
| 101802_at | 1422358_at |
| 101803_at | 1430899_at |
| 101806_at | 1450581_at |
| 101807_at | 1450594_at |
| 101808_at | 1422423_at |
| 101809_at | 1422777_at |
| 101810_at | 1450810_at |
| 101811_at | 1450796_at |
| 101814_at | 1427723_at |
| 101815_at | 1421763_at |
| 101821_at | 1450575_at |
| 101822_at | 1422237_at |
| 101825_at | 1422961_at |
| 101827_at | 1422424_at |
| 101828_at | 1421359_at |
| 101829_at | 1450833_at |
| 101830_at | 1452533_at |
| 101831_at | 1427571_at |
| 101834_at | 1427060_at |
| 101835_at | 1448409_at |

| | |
|---|---|
| 101839_at | 1432301_a_at |
| 101842_g_at | 1460420_a_at |
| 101843_at | 1448874_a_at |
| 101850_at | 1417635_at |
| 101851_at | 1448788_at |
| 101853_f_at | 1450876_at |
| 101855_at | 1422550_a_at |
| 101856_at | 1435525_at |
| 101857_at | 1448603_at |
| 101858_at | 1460704_at |
| 101859_at | 1418848_at |
| 101860_at | 1448571_a_at |
| 101862_at | 1419590_at |
| 101864_at | 1424678_at |
| 101865_at | 1419279_at |
| 101866_at | 1425507_at |
| 101867_at | 1419499_at |
| 101868_i_at | 1419744_at |
| 101870_at | 1425247_a_at |
| 101871_f_at | 1452577_at |
| 101872_at | 1421040_a_at |
| 101873_at | 1427092_at |
| 101874_s_at | 1449529_s_at |
| 101875_at | 1450560_a_at |
| 101878_at | 1426112_a_at |
| 101879_s_at | 1422563_at |
| 101884_at | 1449347_a_at |
| 101887_at | 1423396_at |
| 101888_at | 1424034_at |
| 101889_s_at | 1420583_a_at |
| 101891_at | 1418352_at |
| 101899_at | 1418897_at |
| 101903_at | 1441667_s_at |
| 101904_at | 1427714_at |
| 101906_at | 1452197_at |
| 101910_f_at | 1427631_x_at |
| 101912_at | 1434484_at |
| 101917_at | 1420692_at |
| 101920_at | 1427094_at |
| 101927_at | 1416754_at |
| 101928_at | 1417498_at |
| 101930_at | 1423493_a_at |
| 101931_at | 1416372_at |
| 101932_at | 1418540_a_at |
| 101938_at | 1419500_at |
| 101940_at | 1416999_at |
| 101942_at | 1435799_at |
| 101943_at | 1434117_at |
| 101947_at | 1417734_at |
| 101949_at | 1431938_a_at |
| 101950_at | 1417178_at |
| 101952_at | 1460373_a_at |
| 101953_at | 1423571_at |
| 101956_at | 1452181_at |
| 101960_at | 1423880_at |
| 101961_at | 1416815_s_at |

| | |
|---|---|
| 101962_at | 1452155_a_at |
| 101964_at | 1451015_at |
| 101968_at | 1422585_at |
| 101969_at | 1448428_at |
| 101970_at | 1419928_at |
| 101976_at | 1416163_at |
| 101977_at | 1451575_a_at |
| 101978_at | 1451624_a_at |
| 101985_at | 1416729_at |
| 101989_at | 1428782_a_at |
| 101991_at | 1417429_at |
| 101992_at | 1448822_at |
| 101996_at | 1417140_a_at |
| 101998_at | 1419636_at |
| 102003_at | 1415709_s_at |
| 102010_at | 1427487_at |
| 102011_at | 1421948_a_at |
| 102013_at | 1420541_at |
| 102015_at | 1435681_s_at |
| 102016_at | 1452260_at |
| 102020_at | 1426058_a_at |
| 102021_at | 1421034_a_at |
| 102025_at | 1417851_at |
| 102028_at | 1422638_s_at |
| 102029_at | 1448686_at |
| 102031_at | 1453833_a_at |
| 102032_at | 1450388_s_at |
| 102035_at | 1430889_a_at |
| 102036_at | 1452069_a_at |
| 102038_at | 1450251_a_at |
| 102039_at | 1417093_a_at |
| 102042_at | 1428202_at |
| 102046_at | 1449121_at |
| 102053_at | 1448961_at |
| 102054_at | 1417685_at |
| 102058_at | 1427297_at |
| 102059_at | 1449377_at |
| 102061_at | 1418589_a_at |
| 102064_at | 1449265_at |
| 102065_at | 1418243_at |
| 102073_at | 1420755_a_at |
| 102074_at | 1422285_at |
| 102075_at | 1427755_at |
| 102077_at | 1426173_at |
| 102078_at | 1419345_at |
| 102079_at | 1426190_at |
| 102080_at | 1426038_at |
| 102081_at | 1421565_at |
| 102082_at | 1421740_at |
| 102083_at | 1421779_at |
| 102084_f_at | 1452501_at |
| 102085_at | 1421399_at |
| 102086_r_at | 1448091_at |
| 102087_at | 1452421_at |
| 102088_at | 1422227_at |
| 102102_at | 1447989_at |

| | |
|---|---|
| 102106_at | 1459893_at |
| 102109_at | 1450150_a_at |
| 102110_at | 1449745_at |
| 102112_s_at | 1419836_at |
| 102123_at | 1423141_at |
| 102126_at | 1448344_at |
| 102127_at | 1445172_at |
| 102130_f_at | 1457841_at |
| 102135_at | 1442211_at |
| 102138_at | 1447276_at |
| 102139_at | 1420105_at |
| 102140_at | 1447287_at |
| 102143_at | 1449631_at |
| 102146_at | 1450225_at |
| 102147_at | 1450572_at |
| 102148_f_at | 1422406_at |
| 102149_f_at | 1422404_x_at |
| 102151_at | 1423420_at |
| 102157_f_at | 1427799_x_at |
| 102161_f_at | 1422348_at |
| 102164_at | 1422328_at |
| 102167_at | 1422383_at |
| 102192_r_at | 1425559_a_at |
| 102193_at | 1435390_at |
| 102194_at | 1427085_at |
| 102197_at | 1418355_at |
| 102198_at | 1421038_a_at |
| 102203_at | 1449937_at |
| 102204_at | 1451715_at |
| 102206_at | 1422787_at |
| 102209_at | 1417621_at |
| 102211_r_at | 1426848_at |
| 102213_at | 1419220_at |
| 102214_at | 1423292_a_at |
| 102216_at | 1418266_at |
| 102217_at | 1449514_at |
| 102218_at | 1450297_at |
| 102223_at | 1460732_a_at |
| 102225_at | 1434062_at |
| 102228_at | 1460651_at |
| 102229_at | 1423581_at |
| 102230_at | 1449954_at |
| 102232_at | 1452765_at |
| 102237_at | 1417597_at |
| 102238_at | 1450164_at |
| 102240_at | 1460336_at |
| 102241_f_at | 1425229_a_at |
| 102242_at | 1460662_at |
| 102243_at | 1419474_a_at |
| 102244_at | 1418559_at |
| 102248_f_at | 1422518_at |
| 102249_at | 1419148_at |
| 102250_at | 1449508_at |
| 102251_at | 1418935_at |
| 102253_at | 1425160_at |
| 102256_at | 1418755_at |

| | |
|---|---|
| 102257_at | 1421233_at |
| 102258_at | 1422723_at |
| 102260_at | 1420399_at |
| 102263_at | 1450681_at |
| 102264_at | 1418612_at |
| 102265_at | 1419150_at |
| 102266_at | 1422728_at |
| 102267_at | 1419138_at |
| 102268_at | 1429124_s_at |
| 102271_at | 1460739_at |
| 102272_at | 1420066_s_at |
| 102273_at | 1449870_a_at |
| 102274_at | 1419297_at |
| 102279_at | 1426970_a_at |
| 102284_at | 1419677_at |
| 102285_at | 1421309_at |
| 102286_at | 1426602_at |
| 102287_at | 1426396_at |
| 102288_at | 1426079_at |
| 102289_r_at | 1425289_a_at |
| 102290_at | 1422699_at |
| 102291_at | 1427393_at |
| 102293_at | 1421303_at |
| 102295_at | 1417680_at |
| 102296_at | 1448312_at |
| 102297_at | 1418033_s_at |
| 102298_at | 1419675_at |
| 102300_at | 1449331_a_at |
| 102301_at | 1418556_at |
| 102305_at | 1424146_at |
| 102306_at | 1450729_at |
| 102309_at | 1419181_at |
| 102310_at | 1417925_at |
| 102311_at | 1419720_at |
| 102314_at | 1415958_at |
| 102316_at | 1418671_at |
| 102318_at | 1419186_a_at |
| 102323_at | 1416997_a_at |
| 102324_at | 1417934_at |
| 102326_at | 1448561_at |
| 102327_at | 1449396_at |
| 102329_at | 1418976_s_at |
| 102330_at | 1418204_s_at |
| 102331_at | 1428372_at |
| 102333_at | 1427226_at |
| 102334_at | 1416333_at |
| 102338_at | 1449403_at |
| 102340_at | 1423595_at |
| 102341_at | 1449006_at |
| 102343_at | 1417100_at |
| 102346_at | 1448497_at |
| 102350_at | 1427449_a_at |
| 102351_at | 1448730_at |
| 102352_at | 1418195_at |
| 102353_at | 1450678_at |
| 102354_at | 1423809_at |

| | |
|---|---|
| 102356_at | 1448418_s_at |
| 102357_at | 1454977_at |
| 102360_at | 1434087_at |
| 102366_at | 1449182_at |
| 102368_at | 1427934_at |
| 102372_at | 1424305_at |
| 102373_at | 1448649_at |
| 102374_at | 1434027_at |
| 102375_at | 1451722_s_at |
| 102376_r_at | 1419084_a_at |
| 102378_at | 1417644_at |
| 102379_at | 1448855_at |
| 102380_s_at | 1450209_at |
| 102382_at | 1425099_a_at |
| 102384_at | 1430526_a_at |
| 102396_at | 1423068_at |
| 102397_at | 1418582_at |
| 102398_at | 1416990_at |
| 102403_at | 1416575_at |
| 102405_at | 1460219_at |
| 102409_at | 1448703_at |
| 102410_at | 1423450_a_at |
| 102411_at | 1416586_at |
| 102412_at | 1426751_s_at |
| 102413_at | 1418478_at |
| 102416_at | 1417017_at |
| 102421_at | 1424259_at |
| 102423_at | 1452011_a_at |
| 102426_at | 1422598_at |
| 102429_at | 1422898_s_at |
| 102430_at | 1419272_at |
| 102552_at | 1422339_at |
| 102554_at | 1422382_at |
| 102555_at | 1422417_at |
| 102556_at | 1423022_at |
| 102560_at | 1460321_at |
| 102567_at | 1422239_at |
| 102572_at | 1421621_at |
| 102574_at | 1421793_at |
| 102575_at | 1450611_at |
| 102576_at | 1419210_at |
| 102577_at | 1421790_a_at |
| 102578_at | 1421765_at |
| 102581_at | 1422266_at |
| 102582_at | 1450497_at |
| 102584_at | 1422254_a_at |
| 102586_at | 1451014_at |
| 102599_at | 1416642_a_at |
| 102612_at | 1450946_at |
| 102613_at | 1426071_at |
| 102614_at | 1421336_at |
| 102619_at | 1450235_at |
| 102620_at | 1421961_a_at |
| 102621_at | 1421452_at |
| 102623_at | 1425840_a_at |
| 102624_at | 1449484_at |

| | |
|---|---|
| 102628_at | 1425893_a_at |
| 102629_at | 1419607_at |
| 102630_s_at | 1420353_at |
| 102631_at | 1448953_at |
| 102633_at | 1460280_at |
| 102635_at | 1419190_at |
| 102636_at | 1418214_at |
| 102637_at | 1425620_at |
| 102638_at | 1419202_at |
| 102639_at | 1422758_at |
| 102640_at | 1450959_at |
| 102641_at | 1418747_at |
| 102642_at | 1417584_at |
| 102643_at | 1419602_at |
| 102644_at | 1427040_at |
| 102648_at | 1420378_at |
| 102650_at | 1425245_a_at |
| 102651_at | 1425303_at |
| 102653_at | 1450123_at |
| 102654_at | 1449232_at |
| 102655_at | 1450155_at |
| 102656_at | 1427615_at |
| 102657_at | 1418939_at |
| 102658_at | 1419532_at |
| 102659_at | 1417551_at |
| 102660_at | 1422870_at |
| 102661_at | 1427682_a_at |
| 102662_at | 1419747_at |
| 102663_at | 1452521_a_at |
| 102664_at | 1421123_at |
| 102666_at | 1448821_at |
| 102667_at | 1422093_at |
| 102668_at | 1449051_at |
| 102669_at | 1422161_at |
| 102674_at | 1450476_at |
| 102676_at | 1421071_at |
| 102678_at | 1418077_at |
| 102680_g_at | 1425253_a_at |
| 102681_at | 1420351_at |
| 102682_at | 1419341_at |
| 102683_at | 1460654_at |
| 102684_at | 1421782_a_at |
| 102685_at | 1451953_at |
| 102687_at | 1436561_at |
| 102688_f_at | 1420343_at |
| 102690_at | 1419731_at |
| 102692_s_at | 1427726_at |
| 102693_f_at | 1419090_x_at |
| 102694_at | 1449238_at |
| 102695_at | 1427608_a_at |
| 102699_at | 1419676_at |
| 102701_at | 1422257_s_at |
| 102702_at | 1435358_at |
| 102703_s_at | 1425382_a_at |
| 102705_at | 1449990_at |
| 102708_at | 1419175_a_at |

| | |
|---|---|
| 102709_at | 1436241_s_at |
| 102711_at | 1419221_a_at |
| 102713_at | 1418864_at |
| 102714_at | 1419625_at |
| 102715_at | 1418157_at |
| 102716_at | 1425989_a_at |
| 102718_at | 1424727_at |
| 102719_f_at | 1422260_x_at |
| 102720_at | 1418788_at |
| 102723_at | 1447998_at |
| 102724_at | 1427448_at |
| 102725_at | 1448468_a_at |
| 102728_f_at | 1450171_x_at |
| 102729_f_at | 1460232_s_at |
| 102731_g_at | 1421358_at |
| 102732_at | 1436042_at |
| 102733_at | 1421256_at |
| 102734_at | 1418854_at |
| 102735_at | 1421356_at |
| 102739_s_at | 1420492_s_at |
| 102740_at | 1418160_at |
| 102741_at | 1445596_at |
| 102743_at | 1424718_at |
| 102744_at | 1422188_s_at |
| 102746_at | 1456100_at |
| 102748_at | 1418907_at |
| 102751_at | 1417455_at |
| 102752_at | 1416329_at |
| 102753_at | 1416347_at |
| 102754_at | 1419562_at |
| 102755_at | 1418165_at |
| 102758_at | 1452466_a_at |
| 102759_at | 1418463_at |
| 102761_at | 1452262_at |
| 102762_r_at | 1419014_at |
| 102763_at | 1418595_at |
| 102765_at | 1418720_at |
| 102766_at | 1421165_at |
| 102770_at | 1425643_at |
| 102771_at | 1451833_a_at |
| 102772_at | 1423999_at |
| 102773_at | 1427482_a_at |
| 102774_at | 1418093_a_at |
| 102776_at | 1428592_s_at |
| 102777_at | 1422226_at |
| 102778_at | 1418830_at |
| 102781_at | 1454149_a_at |
| 102782_at | 1449346_s_at |
| 102784_at | 1426974_at |
| 102785_at | 1418477_at |
| 102786_at | 1433486_at |
| 102787_at | 1433485_x_at |
| 102789_at | 1450333_a_at |
| 102790_at | 1424283_at |
| 102793_at | 1427211_at |
| 102794_at | 1448710_at |

| | |
|---|---|
| 102799_at | 1418037_at |
| 102800_at | 1416693_at |
| 102802_at | 1417932_at |
| 102804_at | 1460681_at |
| 102808_at | 1418738_at |
| 102816_at | 1421921_at |
| 102817_at | 1449354_at |
| 102818_at | 1422617_at |
| 102820_at | 1449479_at |
| 102822_at | 1418679_at |
| 102827_at | 1416816_at |
| 102831_s_at | 1449858_at |
| 102832_at | 1427790_at |
| 102836_at | 1416195_at |
| 102838_at | 1419480_at |
| 102841_at | 1435782_at |
| 102843_s_at | 1451632_a_at |
| 102846_at | 1449489_at |
| 102847_s_at | 1422230_s_at |
| 102849_at | 1418142_at |
| 102851_s_at | 1460188_at |
| 102856_at | 1425369_a_at |
| 102857_at | 1419019_a_at |
| 102858_at | 1421137_a_at |
| 102860_at | 1424923_at |
| 102861_at | 1417809_at |
| 102862_at | 1436344_at |
| 102864_at | 1449499_at |
| 102865_at | 1421047_at |
| 102866_at | 1460683_at |
| 102869_at | 1417521_at |
| 102871_at | 1418051_at |
| 102874_at | 1427699_a_at |
| 102876_at | 1422867_at |
| 102877_at | 1419060_at |
| 102878_at | 1416602_a_at |
| 102880_at | 1427341_at |
| 102882_at | 1421169_at |
| 102887_at | 1449033_at |
| 102888_s_at | 1417627_a_at |
| 102889_r_at | 1425836_a_at |
| 102891_at | 1450163_a_at |
| 102892_at | 1416956_at |
| 102895_at | 1453576_at |
| 102896_at | 1417790_at |
| 102898_at | 1425379_at |
| 102899_at | 1420902_at |
| 102900_at | 1427523_at |
| 102901_at | 1426638_at |
| 102902_at | 1425041_at |
| 102904_at | 1422891_at |
| 102905_at | 1449591_at |
| 102911_at | 1419076_a_at |
| 102913_at | 1437913_at |
| 102916_s_at | 1450798_at |
| 102917_at | 1421210_at |

| | |
|---|---|
| 102918_at | 1449199_at |
| 102919_at | 1418616_at |
| 102921_s_at | 1460251_at |
| 102923_at | 1425892_a_at |
| 102926_at | 1418880_at |
| 102927_s_at | 1425969_a_at |
| 102929_s_at | 1422115_a_at |
| 102931_at | 1448386_a_at |
| 102933_at | 1420995_at |
| 102938_at | 1449492_a_at |
| 102939_s_at | 1419768_at |
| 102941_at | 1448009_at |
| 102943_at | 1428612_at |
| 102946_r_at | 1449974_at |
| 102947_at | 1419117_at |
| 102950_at | 1422400_a_at |
| 102953_at | 1460238_at |
| 102954_at | 1452511_at |
| 102955_at | 1418932_at |
| 102957_at | 1418641_at |
| 102958_at | 1418494_at |
| 102960_at | 1448948_at |
| 102961_at | 1449242_s_at |
| 102965_at | 1427091_at |
| 102966_at | 1434311_at |
| 102967_at | 1423328_at |
| 102968_at | 1418216_at |
| 102969_at | 1430417_s_at |
| 102971_at | 1422105_at |
| 102975_at | 1425335_at |
| 102977_at | 1420516_at |
| 102978_at | 1448010_at |
| 102979_at | 1436167_at |
| 102980_at | 1454941_at |
| 102982_at | 1454872_at |
| 102985_at | 1454042_a_at |
| 102986_at | 1418420_at |
| 102987_at | 1429100_at |
| 102988_at | 1460394_a_at |
| 102989_at | 1450645_at |
| 102990_at | 1427884_at |
| 102995_s_at | 1417898_a_at |
| 102996_at | 1460643_at |
| 102998_at | 1450715_at |
| 103002_at | 1418014_a_at |
| 103006_at | 1425927_a_at |
| 103007_at | 1416895_at |
| 103009_at | 1450050_at |
| 103012_at | 1419426_s_at |
| 103013_at | 1460228_at |
| 103015_at | 1421818_at |
| 103017_at | 1449670_x_at |
| 103023_at | 1417043_at |
| 103028_at | 1417171_at |
| 103032_at | 1421733_a_at |
| 103033_at | 1418021_at |

| | |
|---|---|
| 103040_at | 1416111_at |
| 103041_at | 1421784_a_at |
| 103044_g_at | 1455710_x_at |
| 103045_at | 1425755_at |
| 103050_at | 1417447_at |
| 103051_at | 1417160_s_at |
| 103053_at | 1419391_at |
| 103055_r_at | 1422311_a_at |
| 103058_f_at | 1420527_s_at |
| 103059_at | 1418374_at |
| 103060_at | 1434121_at |
| 103063_at | 1425495_at |
| 103066_at | 1450484_a_at |
| 103068_at | 1417826_at |
| 103070_at | 1416985_at |
| 103071_at | 1452241_at |
| 103072_at | 1448453_at |
| 103081_at | 1434824_at |
| 103084_at | 1460318_at |
| 103087_at | 1427345_a_at |
| 103088_at | 1417795_at |
| 103089_at | 1427301_at |
| 103090_at | 1448337_at |
| 103091_at | 1417856_at |
| 103092_at | 1455012_s_at |
| 103098_at | 1425656_a_at |
| 103099_f_at | 1452221_a_at |
| 103132_at | 1446790_at |
| 103198_at | 1455239_at |
| 103199_at | 1419412_at |
| 103200_at | 1460555_at |
| 103205_at | 1420635_a_at |
| 103208_at | 1423559_at |
| 103210_at | 1449360_at |
| 103211_at | 1434517_at |
| 103213_at | 1418602_at |
| 103216_f_at | 1454690_at |
| 103217_at | 1425687_at |
| 103218_at | 1451227_a_at |
| 103219_at | 1419832_s_at |
| 103223_at | 1452878_at |
| 103224_at | 1417529_at |
| 103226_at | 1450430_at |
| 103227_at | 1427961_s_at |
| 103228_at | 1427025_at |
| 103231_at | 1429319_at |
| 103232_at | 1454732_at |
| 103235_at | 1419127_at |
| 103236_at | 1422647_at |
| 103237_at | 1422970_at |
| 103240_f_at | 1422411_s_at |
| 103243_at | 1425272_at |
| 103244_at | 1418796_at |
| 103245_at | 1424479_at |
| 103247_at | 1419077_at |
| 103249_at | 1417304_at |

| | |
|---|---|
| 103250_at | 1417903_at |
| 103251_at | 1455073_at |
| 103253_at | 1449172_a_at |
| 103254_at | 1428346_at |
| 103255_at | 1448861_at |
| 103257_at | 1434553_at |
| 103259_at | 1417679_at |
| 103261_at | 1418109_at |
| 103262_at | 1421298_a_at |
| 103263_at | 1418850_at |
| 103264_at | 1421879_at |
| 103266_at | 1420407_at |
| 103271_at | 1451022_at |
| 103272_at | 1433681_x_at |
| 103273_s_at | 1455765_a_at |
| 103278_at | 1422760_at |
| 103279_at | 1449393_at |
| 103283_at | 1419555_at |
| 103284_at | 1449309_at |
| 103289_at | 1419017_at |
| 103291_at | 1422325_at |
| 103292_at | 1421984_at |
| 103293_at | 1448256_at |
| 103294_at | 1420940_x_at |
| 103296_at | 1449285_at |
| 103297_at | 1427213_at |
| 103299_at | 1433678_at |
| 103300_at | 1435006_s_at |
| 103305_at | 1427387_a_at |
| 103311_at | 1425391_a_at |
| 103315_at | 1434898_at |
| 103316_at | 1435530_at |
| 103319_at | 1436559_a_at |
| 103321_at | 1455184_at |
| 103322_at | 1418337_at |
| 103326_at | 1434330_at |
| 103327_at | 1432331_a_at |
| 103329_at | 1421125_at |
| 103331_at | 1451085_at |
| 103332_at | 1426782_at |
| 103333_at | 1417880_at |
| 103334_at | 1418249_at |
| 103338_at | 1428931_a_at |
| 103340_at | 1417049_at |
| 103341_at | 1416563_at |
| 103343_at | 1453768_a_at |
| 103344_at | 1420501_at |
| 103347_at | 1420326_s_at |
| 103348_at | 1460350_at |
| 103352_at | 1448549_a_at |
| 103353_f_at | 1416194_at |
| 103354_at | 1417737_at |
| 103355_at | 1440213_a_at |
| 103357_at | 1449067_at |
| 103360_at | 1417989_at |
| 103363_at | 1416451_s_at |

| | |
|---|---|
| 103364_f_at | 1428189_at |
| 103365_s_at | 1449619_s_at |
| 103367_at | 1418655_at |
| 103369_at | 1436316_at |
| 103371_at | 1416949_s_at |
| 103372_at | 1427509_at |
| 103376_s_at | 1425760_a_at |
| 103378_at | 1448572_at |
| 103381_at | 1453014_a_at |
| 103387_at | 1416542_at |
| 103388_at | 1451592_at |
| 103392_at | 1450065_at |
| 103398_at | 1424027_at |
| 103401_at | 1460216_at |
| 103403_at | 1452889_at |
| 103405_at | 1428188_at |
| 103407_at | 1424722_at |
| 103408_at | 1434333_a_at |
| 103409_at | 1451539_at |
| 103411_at | 1434254_at |
| 103413_at | 1417873_at |
| 103414_at | 1422584_at |
| 103423_at | 1417507_at |
| 103424_at | 1454632_at |
| 103427_at | 1454635_at |
| 103429_i_at | 1449076_x_at |
| 103432_at | 1419569_a_at |
| 103433_at | 1418758_a_at |
| 103435_at | 1434184_s_at |
| 103436_at | 1450023_at |
| 103439_at | 1435780_at |
| 103442_at | 1424869_at |
| 103445_at | 1451660_a_at |
| 103446_at | 1426276_at |
| 103447_at | 1434445_at |
| 103448_at | 1419394_s_at |
| 103450_at | 1428454_at |
| 103451_at | 1434653_at |
| 103452_at | 1432099_a_at |
| 103456_at | 1455741_a_at |
| 103458_at | 1419407_at |
| 103462_at | 1422808_s_at |
| 103463_at | 1438468_at |
| 103469_at | 1420722_at |
| 103472_at | 1447604_at |
| 103473_at | 1428548_at |
| 103476_at | 1454796_at |
| 103478_at | 1456869_at |
| 103479_at | 1435756_at |
| 103480_at | 1419696_at |
| 103484_at | 1423856_at |
| 103485_at | 1439064_at |
| 103487_at | 1422625_at |
| 103488_at | 1449127_at |
| 103489_at | 1426105_a_at |
| 103490_at | 1450772_at |

| | |
|---|---|
| 103492_at | 1448901_at |
| 103494_at | 1420018_s_at |
| 103495_at | 1449650_at |
| 103498_at | 1450127_a_at |
| 103500_at | 1437382_at |
| 103509_at | 1460469_at |
| 103510_at | 1449382_at |
| 103512_at | 1453047_at |
| 103513_at | 1422602_a_at |
| 103514_at | 1450731_s_at |
| 103515_at | 1433709_at |
| 103516_at | 1418925_at |
| 103518_at | 1452352_at |
| 103519_at | 1449486_at |
| 103521_r_at | 1455179_at |
| 103522_at | 1450805_at |
| 103523_at | 1435152_at |
| 103524_at | 1428166_at |
| 103526_at | 1418252_at |
| 103527_at | 1436693_x_at |
| 103529_at | 1449125_at |
| 103533_at | 1450736_a_at |
| 103535_at | 1450621_a_at |
| 103536_at | 1419073_at |
| 103537_at | 1449278_at |
| 103539_at | 1460204_at |
| 103540_at | 1448038_at |
| 103541_at | 1425009_at |
| 103542_at | 1427051_at |
| 103543_at | 1424764_at |
| 103544_at | 1418869_a_at |
| 103546_at | 1422931_at |
| 103547_at | 1460565_at |
| 103548_at | 1419411_at |
| 103552_at | 1443856_at |
| 103555_at | 1451988_s_at |
| 103557_at | 1426227_s_at |
| 103558_at | 1421589_at |
| 103563_at | 1435226_at |
| 103564_at | 1454706_at |
| 103567_at | 1415758_at |
| 103570_at | 1422606_at |
| 103571_at | 1425548_a_at |
| 103573_at | 1421834_at |
| 103578_at | 1431188_a_at |
| 103579_at | 1417620_at |
| 103580_at | 1424025_at |
| 103582_r_at | 1452054_at |
| 103585_at | 1454794_at |
| 103588_at | 1416080_at |
| 103589_at | 1448932_at |
| 103591_at | 1418593_at |
| 103593_at | 1456062_at |
| 103594_at | 1417855_at |
| 103595_at | 1448915_at |
| 103596_at | 1418578_at |

| | |
|---|---|
| 103597_at | 1418028_at |
| 103599_at | 1451053_a_at |
| 103601_at | 1436336_at |
| 103603_at | 1460562_at |
| 103607_at | 1417836_at |
| 103608_at | 1431354_a_at |
| 103612_at | 1418903_at |
| 103613_at | 1423498_at |
| 103615_at | 1435331_at |
| 103618_at | 1428722_at |
| 103622_at | 1449964_a_at |
| 103623_at | 1422831_at |
| 103632_at | 1429349_at |
| 103634_at | 1421322_a_at |
| 103635_at | 1424526_a_at |
| 103637_at | 1449043_at |
| 103639_at | 1418293_at |
| 103641_at | 1455587_at |
| 103643_at | 1421115_a_at |
| 103644_at | 1435943_at |
| 103645_at | 1449223_at |
| 103646_at | 1417008_at |
| 103649_at | 1418654_at |
| 103650_at | 1416989_at |
| 103655_at | 1426437_s_at |
| 103656_at | 1427011_a_at |
| 103660_at | 1449442_at |
| 103662_at | 1418465_at |
| 103663_at | 1426618_a_at |
| 103666_at | 1418415_at |
| 103667_at | 1434513_at |
| 103669_at | 1433798_a_at |
| 103670_at | 1418821_at |
| 103671_at | 1451814_a_at |
| 103672_at | 1428997_at |
| 103673_at | 1416051_at |
| 103675_at | 1427231_at |
| 103676_at | 1448912_at |
| 103678_at | 1434612_s_at |
| 103681_at | 1434791_at |
| 103684_at | 1418484_at |
| 103685_at | 1436570_at |
| 103686_at | 1431705_a_at |
| 103688_at | 1436998_at |
| 103689_at | 1428988_at |
| 103692_at | 1423085_at |
| 103693_at | 1457676_at |
| 103694_at | 1427047_at |
| 103701_at | 1427030_at |
| 103706_at | 1435896_at |
| 103707_at | 1419483_at |
| 103711_at | 1437537_at |
| 103714_at | 1451547_at |
| 103715_at | 1450276_a_at |
| 103717_at | 1448146_at |
| 103719_at | 1449537_at |

| | |
|---|---|
| 103723_at | 1454783_at |
| 103727_at | 1434840_at |
| 103731_at | 1455252_at |
| 103732_at | 1433941_at |
| 103733_at | 1448019_at |
| 103738_at | 1454820_at |
| 103739_at | 1428374_at |
| 103742_at | 1449522_at |
| 103743_at | 1435106_at |
| 103748_at | 1427980_at |
| 103752_r_at | 1455156_at |
| 103755_at | 1418460_at |
| 103757_at | 1440255_at |
| 103759_at | 1449454_at |
| 103762_at | 1417698_at |
| 103763_at | 1450072_at |
| 103765_at | 1418200_at |
| 103767_f_at | 1433773_at |
| 103769_at | 1421843_at |
| 103771_at | 1435105_at |
| 103773_at | 1439482_at |
| 103778_at | 1448012_at |
| 103779_at | 1434900_at |
| 103782_at | 1455677_s_at |
| 103786_at | 1435946_at |
| 103787_at | 1417416_at |
| 103790_at | 1460742_at |
| 103793_at | 1448618_at |
| 103797_at | 1426002_a_at |
| 103800_at | 1438056_x_at |
| 103801_at | 1419310_s_at |
| 103803_at | 1419767_at |
| 103805_at | 1448746_at |
| 103808_at | 1418447_at |
| 103809_r_at | 1416361_a_at |
| 103810_at | 1418158_at |
| 103811_at | 1419308_at |
| 103813_at | 1449579_at |
| 103814_at | 1420460_a_at |
| 103819_at | 1417486_at |
| 103822_at | 1428961_a_at |
| 103824_at | 1448411_at |
| 103825_at | 1422268_a_at |
| 103826_at | 1450093_s_at |
| 103828_at | 1447894_x_at |
| 103829_at | 1426491_at |
| 103831_at | 1416734_at |
| 103833_at | 1425983_x_at |
| 103836_at | 1419375_at |
| 103837_at | 1418748_at |
| 103838_at | 1449206_at |
| 103839_at | 1451596_a_at |
| 103841_at | 1421030_at |
| 103843_at | 1421152_a_at |
| 103844_at | 1460383_at |
| 103846_at | 1422308_a_at |

| | |
|---|---|
| 103848_at | 1436950_at |
| 103850_at | 1451978_at |
| 103854_at | 1422549_at |
| 103863_at | 1460656_a_at |
| 103865_at | 1456598_at |
| 103866_at | 1453281_at |
| 103872_r_at | 1450741_at |
| 103876_at | 1417583_a_at |
| 103877_at | 1460731_at |
| 103878_at | 1423235_at |
| 103879_at | 1451354_at |
| 103882_at | 1417745_at |
| 103884_at | 1424682_at |
| 103887_at | 1448756_at |
| 103893_at | 1452255_at |
| 103894_at | 1424174_at |
| 103895_at | 1426459_s_at |
| 103896_f_at | 1418918_at |
| 103900_at | 1423910_at |
| 103903_at | 1433881_at |
| 103904_at | 1417933_at |
| 103908_at | 1453195_at |
| 103909_at | 1448615_at |
| 103910_at | 1448784_at |
| 103911_at | 1424604_s_at |
| 103912_at | 1428265_at |
| 103914_at | 1420493_a_at |
| 103916_at | 1426436_at |
| 103918_at | 1424730_a_at |
| 103924_at | 1434287_at |
| 103927_at | 1435091_at |
| 103931_at | 1418611_at |
| 103932_at | 1435442_at |
| 103934_at | 1424338_at |
| 103935_at | 1421129_a_at |
| 103939_at | 1451511_at |
| 103942_at | 1436370_at |
| 103943_at | 1428578_s_at |
| 103944_at | 1421430_at |
| 103945_at | 1421524_at |
| 103946_at | 1424560_at |
| 103947_at | 1424347_at |
| 103948_at | 1450806_at |
| 103949_at | 1450704_at |
| 103954_at | 1449495_at |
| 103955_at | 1416795_at |
| 103956_at | 1419216_at |
| 103960_at | 1449246_at |
| 103963_f_at | 1419042_at |
| 103964_at | 1460652_at |
| 103965_at | 1460433_at |
| 103968_at | 1422216_at |
| 103971_at | 1460719_a_at |
| 103972_at | 1418614_at |
| 103973_at | 1418613_at |
| 103975_at | 1418492_at |

| | |
|---|---|
| 103976_at | 1449605_at |
| 103977_at | 1449305_at |
| 103984_at | 1419820_at |
| 103985_at | 1450244_a_at |
| 103986_at | 1422001_at |
| 103987_at | 1448768_at |
| 103988_at | 1426843_at |
| 103991_at | 1450636_s_at |
| 103993_at | 1422106_a_at |
| 103997_at | 1423344_at |
| 103998_at | 1429063_s_at |
| 103999_at | 1455326_at |
| 104000_at | 1424968_at |
| 104001_at | 1446862_at |
| 104003_at | 1434518_at |
| 104008_at | 1450220_a_at |
| 104010_at | 1418607_at |
| 104011_at | 1419435_at |
| 104012_at | 1427694_at |
| 104013_at | 1427731_at |
| 104014_at | 1422645_at |
| 104015_at | 1426836_s_at |
| 104018_at | 1439479_at |
| 104019_at | 1424539_at |
| 104020_at | 1448885_at |
| 104021_at | 1420414_at |
| 104022_at | 1429080_at |
| 104023_at | 1428018_a_at |
| 104024_at | 1424973_at |
| 104029_at | 1430240_a_at |
| 104032_at | 1435666_at |
| 104034_at | 1435417_at |
| 104035_at | 1456492_at |
| 104036_at | 1435680_a_at |
| 104038_at | 1448463_at |
| 104042_at | 1423927_at |
| 104045_at | 1424029_at |
| 104046_at | 1436498_at |
| 104047_at | 1420932_at |
| 104049_at | 1427898_at |
| 104050_at | 1419650_at |
| 104052_at | 1433912_at |
| 104058_at | 1428655_at |
| 104064_at | 1428954_at |
| 104065_at | 1424065_at |
| 104067_at | 1426707_at |
| 104069_at | 1460677_at |
| 104072_at | 1419059_at |
| 104074_at | 1436522_at |
| 104080_at | 1434019_at |
| 104082_at | 1427992_a_at |
| 104083_at | 1433956_at |
| 104086_at | 1452311_at |
| 104088_at | 1427908_at |
| 104090_at | 1433430_s_at |
| 104098_at | 1417841_at |

| | |
|---|---|
| 104099_at | 1449184_at |
| 104101_at | 1454865_at |
| 104102_at | 1460495_s_at |
| 104104_at | 1460605_at |
| 104105_at | 1422759_a_at |
| 104110_at | 1423992_at |
| 104112_at | 1437742_at |
| 104114_at | 1460401_at |
| 104115_at | 1426823_s_at |
| 104118_at | 1417062_at |
| 104119_at | 1434620_s_at |
| 104120_at | 1452603_at |
| 104123_at | 1436078_at |
| 104124_at | 1428580_at |
| 104128_at | 1416747_at |
| 104129_at | 1418767_at |
| 104131_at | 1460276_a_at |
| 104134_at | 1424057_at |
| 104136_at | 1426465_at |
| 104137_at | 1449302_at |
| 104138_at | 1448489_at |
| 104140_s_at | 1451170_s_at |
| 104144_at | 1448437_a_at |
| 104148_at | 1452145_at |
| 104151_at | 1451522_s_at |
| 104152_at | 1429650_at |
| 104153_at | 1418238_at |
| 104159_at | 1418218_at |
| 104160_at | 1422717_at |
| 104163_at | 1426760_at |
| 104164_at | 1428689_at |
| 104165_at | 1418486_at |
| 104166_at | 1449476_at |
| 104169_at | 1423477_at |
| 104171_f_at | 1419141_at |
| 104172_at | 1451648_a_at |
| 104173_at | 1450912_at |
| 104174_at | 1459546_s_at |
| 104175_at | 1419580_at |
| 104177_at | 1436058_at |
| 104180_at | 1420554_a_at |
| 104181_at | 1420723_at |
| 104182_at | 1418405_at |
| 104185_at | 1424689_at |
| 104186_at | 1429108_at |
| 104187_at | 1418375_at |
| 104189_at | 1421377_at |
| 104190_at | 1435350_at |
| 104193_at | 1452268_at |
| 104194_at | 1448696_at |
| 104197_at | 1434938_at |
| 104199_at | 1460743_at |
| 104200_at | 1435860_at |
| 104201_at | 1417553_at |
| 104202_at | 1435430_at |
| 104205_at | 1449827_at |

| | |
|---|---|
| 104206_at | 1453287_at |
| 104207_at | 1439018_at |
| 104209_at | 1449406_at |
| 104213_at | 1424969_s_at |
| 104214_at | 1417929_at |
| 104217_at | 1449526_a_at |
| 104220_at | 1422771_at |
| 104221_at | 1418326_at |
| 104227_at | 1417894_at |
| 104230_at | 1417904_at |
| 104235_at | 1433832_at |
| 104238_at | 1451553_at |
| 104239_at | 1418398_a_at |
| 104240_at | 1425611_a_at |
| 104245_at | 1452929_at |
| 104246_at | 1434561_at |
| 104247_at | 1451380_at |
| 104253_at | 1434805_at |
| 104262_at | 1422324_a_at |
| 104264_at | 1449099_at |
| 104265_at | 1449379_at |
| 104267_at | 1417329_at |
| 104268_at | 1452416_at |
| 104269_at | 1422531_at |
| 104270_at | 1451992_at |
| 104271_at | 1418554_at |
| 104273_at | 1419001_at |
| 104274_at | 1435450_at |
| 104277_at | 1421059_a_at |
| 104280_at | 1417788_at |
| 104284_at | 1454786_at |
| 104289_at | 1423713_at |
| 104292_at | 1424127_at |
| 104294_at | 1454905_at |
| 104297_at | 1428096_at |
| 104298_at | 1424915_s_at |
| 104299_at | 1423668_at |
| 104300_at | 1434998_at |
| 104305_at | 1424209_at |
| 104306_at | 1448215_a_at |
| 104310_at | 1428245_at |
| 104311_at | 1426486_at |
| 104312_at | 1425699_a_at |
| 104318_at | 1426770_at |
| 104320_at | 1427929_a_at |
| 104322_at | 1434748_at |
| 104323_at | 1449062_at |
| 104325_at | 1451371_at |
| 104328_at | 1424011_at |
| 104332_at | 1423769_at |
| 104333_at | 1417822_at |
| 104335_at | 1416199_at |
| 104336_at | 1424841_s_at |
| 104339_at | 1428249_at |
| 104341_at | 1431299_a_at |
| 104344_at | 1449034_at |

| | |
|---|---|
| 104345_at | 1434865_a_at |
| 104349_at | 1428133_at |
| 104351_at | 1439030_at |
| 104352_at | 1450711_at |
| 104354_at | 1419873_s_at |
| 104358_at | 1426855_at |
| 104360_at | 1460694_s_at |
| 104362_at | 1433765_at |
| 104363_at | 1435191_at |
| 104364_at | 1417016_at |
| 104365_at | 1416611_at |
| 104368_at | 1427079_at |
| 104369_at | 1422784_at |
| 104370_s_at | 1427700_x_at |
| 104374_at | 1419100_at |
| 104381_at | 1450444_a_at |
| 104387_at | 1450404_at |
| 104390_at | 1434555_at |
| 104392_at | 1452702_at |
| 104393_at | 1418389_at |
| 104394_at | 1416402_at |
| 104396_at | 1434017_at |
| 104398_at | 1455618_x_at |
| 104399_at | 1419645_at |
| 104401_at | 1439803_at |
| 104402_at | 1434320_at |
| 104403_at | 1434162_at |
| 104405_at | 1434953_at |
| 104407_at | 1426301_at |
| 104408_s_at | 1449135_at |
| 104410_at | 1449188_at |
| 104412_at | 1454959_s_at |
| 104414_at | 1448031_at |
| 104416_at | 1421442_at |
| 104419_at | 1426903_at |
| 104420_at | 1420393_at |
| 104421_at | 1449525_at |
| 104422_at | 1416588_at |
| 104424_at | 1422815_at |
| 104425_at | 1418983_at |
| 104427_at | 1443762_s_at |
| 104428_s_at | 1421739_a_at |
| 104430_at | 1419226_at |
| 104431_at | 1426044_a_at |
| 104434_at | 1460733_at |
| 104436_at | 1421370_a_at |
| 104437_at | 1449813_at |
| 104438_at | 1446313_at |
| 104439_at | 1419213_at |
| 104443_at | 1423466_at |
| 104445_at | 1429159_at |
| 104449_at | 1422504_at |
| 104450_at | 1425971_at |
| 104451_at | 1426441_at |
| 104453_at | 1415718_at |
| 104454_at | 1423539_at |

| | |
|---|---|
| 104455_at | 1421941_at |
| 104456_at | 1436049_at |
| 104457_at | 1448011_at |
| 104460_at | 1423365_at |
| 104462_at | 1449226_at |
| 104466_at | 1417120_at |
| 104474_s_at | 1426204_a_at |
| 104475_at | 1434104_at |
| 104476_at | 1424156_at |
| 104477_at | 1448021_at |
| 104479_at | 1450318_a_at |
| 104483_at | 1421381_a_at |
| 104484_at | 1419528_at |
| 104485_at | 1452523_a_at |
| 104489_at | 1420371_at |
| 104492_at | 1460666_a_at |
| 104493_at | 1425433_a_at |
| 104494_at | 1454736_at |
| 104495_f_at | 1449313_at |
| 104498_at | 1425671_at |
| 104499_at | 1425710_a_at |
| 104500_at | 1418793_at |
| 104501_at | 1436079_s_at |
| 104504_at | 1451894_a_at |
| 104508_at | 1428787_at |
| 104509_at | 1449227_at |
| 104510_at | 1449999_a_at |
| 104512_at | 1454881_s_at |
| 104514_at | 1427039_at |
| 104515_at | 1429678_at |
| 104516_at | 1417839_at |
| 104518_at | 1450671_at |
| 104519_at | 1417618_at |
| 104522_at | 1435885_s_at |
| 104525_at | 1428351_at |
| 104527_at | 1418281_at |
| 104528_at | 1416651_at |
| 104529_at | 1417842_at |
| 104532_at | 1448345_at |
| 104535_at | 1423360_at |
| 104536_at | 1420634_a_at |
| 104537_at | 1424505_at |
| 104539_at | 1418940_at |
| 104541_at | 1419669_at |
| 104550_at | 1428283_at |
| 104554_at | 1460647_a_at |
| 104555_at | 1460202_at |
| 104559_at | 1460690_at |
| 104560_at | 1424776_a_at |
| 104561_at | 1423998_at |
| 104562_at | 1434343_at |
| 104564_at | 1448628_at |
| 104566_at | 1448350_at |
| 104571_at | 1448995_at |
| 104572_at | 1433910_at |
| 104574_at | 1415730_at |

| | |
|---|---|
| 104576_at | 1426373_at |
| 104577_at | 1417360_at |
| 104583_at | 1423300_at |
| 104588_at | 1419197_x_at |
| 104593_at | 1451166_a_at |
| 104605_at | 1434329_s_at |
| 104606_at | 1460218_at |
| 104607_at | 1419056_at |
| 104608_at | 1451996_at |
| 104609_at | 1415765_at |
| 104617_at | 1434906_at |
| 104618_at | 1416174_at |
| 104619_at | 1417431_a_at |
| 104620_at | 1423649_at |
| 104621_at | 1433834_at |
| 104622_at | 1418107_at |
| 104623_at | 1419655_at |
| 104624_at | 1426815_s_at |
| 104629_at | 1417361_at |
| 104634_at | 1418232_s_at |
| 104641_f_at | 1439113_at |
| 104642_at | 1449905_at |
| 104644_at | 1450692_at |
| 104646_at | 1418949_at |
| 104650_at | 1422635_at |
| 104652_at | 1449158_at |
| 104654_at | 1422564_at |
| 104655_at | 1422545_at |
| 104657_at | 1450207_at |
| 104660_at | 1418751_at |
| 104662_at | 1422303_a_at |
| 104666_at | 1435481_at |
| 104667_at | 1432083_a_at |
| 104669_at | 1417244_a_at |
| 104670_at | 1434846_at |
| 104675_at | 1425824_a_at |
| 104677_at | 1452235_at |
| 104678_at | 1417859_at |
| 104680_at | 1417481_at |
| 104682_at | 1419518_at |
| 104684_at | 1437968_at |
| 104686_at | 1450202_at |
| 104687_at | 1451024_at |
| 104688_at | 1439323_a_at |
| 104689_at | 1450464_at |
| 104690_at | 1417249_at |
| 104692_at | 1420558_at |
| 104694_at | 1451902_at |
| 104695_at | 1453184_at |
| 104696_at | 1418989_at |
| 104707_at | 1424445_at |
| 104708_at | 1455042_at |
| 104710_at | 1418991_at |
| 104711_at | 1417510_at |
| 104713_at | 1433565_at |
| 104714_at | 1450932_s_at |

| | |
|---|---|
| 104715_at | 1424281_at |
| 104717_at | 1419984_s_at |
| 104720_at | 1433780_at |
| 104722_at | 1451165_at |
| 104726_at | 1451625_a_at |
| 104727_at | 1437077_at |
| 104729_at | 1416255_at |
| 104731_at | 1419564_at |
| 104733_at | 1418579_at |
| 104737_at | 1435639_at |
| 104739_at | 1424068_at |
| 104742_at | 1452592_at |
| 104743_at | 1423551_at |
| 104747_at | 1448299_at |
| 104749_at | 1449664_s_at |
| 104750_at | 1417292_at |
| 104752_at | 1418254_at |
| 104754_at | 1448778_at |
| 104755_at | 1427689_a_at |
| 104756_at | 1435431_at |
| 104757_at | 1424891_a_at |
| 104758_at | 1452710_at |
| 104759_at | 1454998_at |
| 104762_r_at | 1432462_a_at |
| 104770_at | 1444153_at |
| 104771_at | 1440208_at |
| 104775_at | 1452331_s_at |
| 104780_at | 1451711_at |
| 104781_at | 1439900_at |
| 104783_at | 1428644_at |
| 104786_at | 1440220_at |
| 104789_at | 1430050_at |
| 104790_at | 1451788_at |
| 104794_at | 1440276_at |
| 104795_at | 1457717_at |
| 104800_at | 1439957_at |
| 104802_at | 1418789_at |
| 104803_at | 1430681_at |
| 104804_at | 1437363_at |
| 104805_at | 1436621_at |
| 104806_at | 1443642_at |
| 104807_at | 1437166_at |
| 104809_at | 1456777_at |
| 104810_at | 1450406_a_at |
| 104811_at | 1444097_at |
| 104812_at | 1457566_at |
| 104819_at | 1445592_at |
| 104820_at | 1452295_at |
| 104823_at | 1458264_at |
| 104833_at | 1427967_at |
| 104836_at | 1442178_at |
| 104837_at | 1435142_at |
| 104839_at | 1458585_at |
| 104850_at | 1439614_at |
| 104852_at | 1457376_at |
| 104859_at | 1435700_at |

| | |
|---|---|
| 104860_at | 1445329_at |
| 104861_at | 1436218_at |
| 104863_at | 1436211_at |
| 104867_at | 1422700_at |
| 104869_at | 1440384_at |
| 104871_at | 1441792_at |
| 104874_at | 1429731_at |
| 104875_at | 1456411_at |
| 104878_at | 1450237_at |
| 104882_at | 1441102_at |
| 104886_at | 1424796_at |
| 104888_at | 1455492_at |
| 104890_at | 1451656_at |
| 104892_at | 1438870_at |
| 104894_at | 1458621_at |
| 104895_at | 1440262_at |
| 104897_at | 1447151_at |
| 104900_at | 1436154_at |
| 104902_at | 1430382_at |
| 104904_at | 1447344_at |
| 104906_at | 1444175_at |
| 104909_at | 1422782_s_at |
| 104910_at | 1438744_at |
| 104912_at | 1442074_at |
| 104916_at | 1434939_at |
| 104917_at | 1444509_at |
| 104919_at | 1440209_at |
| 104922_at | 1421345_at |
| 104926_at | 1436052_at |
| 104932_at | 1435387_at |
| 104933_at | 1457282_x_at |
| 104937_at | 1455193_at |
| 104939_at | 1435958_at |
| 104940_at | 1439204_at |
| 104941_at | 1442084_at |
| 104946_at | 1457032_at |
| 104948_at | 1435337_at |
| 104949_at | 1444181_at |
| 104950_at | 1435373_at |
| 104957_at | 1436245_at |
| 104958_at | 1457999_at |
| 104960_at | 1440345_at |
| 104962_at | 1449477_s_at |
| 104965_at | 1432013_a_at |
| 104966_at | 1437756_at |
| 104970_at | 1439537_at |
| 104972_at | 1439657_at |
| 104973_at | 1457606_x_at |
| 104977_at | 1444325_at |
| 104982_at | 1459273_at |
| 104985_f_at | 1437841_x_at |
| 104986_at | 1429423_at |
| 104987_at | 1424499_s_at |
| 104994_at | 1425062_at |
| 104995_g_at | 1425063_at |
| 104996_at | 1429153_at |

| | |
|---|---|
| 104997_at | 1433587_at |
| 105000_at | 1422707_at |
| 105001_at | 1416310_at |
| 105007_at | 1444289_at |
| 105009_at | 1442417_at |
| 105010_at | 1445546_at |
| 105011_at | 1433174_a_at |
| 105012_at | 1436921_at |
| 105013_at | 1453302_at |
| 105018_at | 1447980_s_at |
| 105023_at | 1440271_at |
| 105024_at | 1444587_at |
| 105025_at | 1456498_at |
| 105026_at | 1445647_at |
| 105036_at | 1440485_at |
| 105038_at | 1422774_at |
| 105040_at | 1455562_at |
| 105041_at | 1431141_at |
| 105047_at | 1421467_at |
| 105048_at | 1443375_at |
| 105050_at | 1442425_at |
| 105051_at | 1459896_at |
| 105053_at | 1456924_at |
| 105055_at | 1441028_at |
| 105058_at | 1451611_at |
| 105059_at | 1437557_at |
| 105060_at | 1449993_at |
| 105061_at | 1427423_at |
| 105066_at | 1455993_at |
| 105067_at | 1458918_at |
| 105069_at | 1440974_at |
| 105070_at | 1436610_at |
| 105072_at | 1427359_at |
| 105073_at | 1443443_at |
| 105075_at | 1428038_at |
| 105077_at | 1444440_at |
| 105078_at | 1428036_at |
| 105080_at | 1456784_at |
| 105081_at | 1435109_at |
| 105083_at | 1460055_at |
| 105086_at | 1455966_s_at |
| 105087_at | 1442012_at |
| 105090_at | 1436605_at |
| 105091_at | 1458042_at |
| 105096_s_at | 1437115_at |
| 105097_at | 1425445_a_at |
| 105099_at | 1444554_at |
| 105101_at | 1438914_at |
| 105103_at | 1441039_at |
| 105105_at | 1420112_at |
| 105107_at | 1434531_at |
| 105109_at | 1447971_at |
| 105110_at | 1442190_at |
| 105111_at | 1426167_a_at |
| 105112_at | 1419844_a_at |
| 105114_at | 1453431_at |

| | |
|---|---|
| 105119_i_at | 1452562_at |
| 105120_f_at | 1424903_at |
| 105121_at | 1420687_at |
| 105127_at | 1444154_at |
| 105129_at | 1436593_at |
| 105132_at | 1440003_at |
| 105133_at | 1440973_at |
| 105135_at | 1458596_at |
| 105137_at | 1428978_at |
| 105140_at | 1443937_at |
| 105143_at | 1442757_at |
| 105147_at | 1455313_at |
| 105148_at | 1442135_at |
| 105150_at | 1444247_at |
| 105152_at | 1430377_at |
| 105158_at | 1455461_at |
| 105159_at | 1454880_s_at |
| 105161_at | 1440037_at |
| 105163_at | 1445379_at |
| 105165_at | 1436910_at |
| 105167_at | 1434669_at |
| 105172_at | 1445944_at |
| 105177_at | 1438108_at |
| 105180_at | 1417781_at |
| 105181_at | 1457765_at |
| 105182_at | 1453906_at |
| 105183_at | 1458653_at |
| 105184_at | 1451566_at |
| 105187_at | 1441580_at |
| 105190_at | 1450835_a_at |
| 105192_at | 1451466_at |
| 105193_at | 1457731_at |
| 105194_at | 1439502_at |
| 105196_at | 1457187_at |
| 105197_at | 1442132_at |
| 105201_at | 1437274_at |
| 105207_at | 1457876_at |
| 105210_at | 1419477_at |
| 105211_at | 1425215_at |
| 105212_at | 1425883_at |
| 105216_at | 1444123_at |
| 105217_at | 1435975_at |
| 105221_at | 1452481_at |
| 105222_at | 1439879_at |
| 105223_at | 1456622_at |
| 105224_at | 1439060_s_at |
| 105226_at | 1445301_at |
| 105227_r_at | 1447450_at |
| 105229_at | 1455459_at |
| 105230_at | 1437364_at |
| 105237_at | 1434209_at |
| 105240_at | 1440374_at |
| 105241_at | 1453319_at |
| 105246_at | 1419670_at |
| 105247_at | 1435471_at |
| 105251_at | 1435501_at |

| | |
|---|---|
| 105252_at | 1440993_at |
| 105257_at | 1457743_at |
| 105258_at | 1455502_at |
| 105262_at | 1434863_at |
| 105265_at | 1453863_at |
| 105270_at | 1447466_at |
| 105271_at | 1442253_at |
| 105283_at | 1457028_at |
| 105286_at | 1443407_at |
| 105288_at | 1447254_at |
| 105289_at | 1445591_at |
| 105293_at | 1437910_at |
| 105295_at | 1455371_at |
| 105296_at | 1416822_at |
| 105299_at | 1444209_at |
| 105300_r_at | 1436345_at |
| 105301_at | 1437774_at |
| 105302_at | 1440886_at |
| 105303_at | 1427263_at |
| 105304_at | 1450340_a_at |
| 105307_at | 1420617_at |
| 105309_at | 1435388_at |
| 105310_at | 1457732_at |
| 105312_at | 1444785_at |
| 105313_at | 1458034_at |
| 105315_at | 1419006_s_at |
| 105316_at | 1442011_at |
| 105317_at | 1419690_at |
| 105318_at | 1460589_at |
| 105321_at | 1439942_at |
| 105323_at | 1435713_at |
| 105324_at | 1444048_at |
| 105327_at | 1421398_at |
| 105328_at | 1442017_at |
| 105329_at | 1436011_at |
| 105331_at | 1444405_at |
| 105334_at | 1438453_at |
| 105338_at | 1441307_at |
| 105341_f_at | 1456558_s_at |
| 105344_at | 1444045_at |
| 105348_at | 1439141_at |
| 105353_at | 1441761_at |
| 105359_at | 1456888_at |
| 105361_at | 1416463_at |
| 105365_at | 1438355_at |
| 105366_at | 1455472_at |
| 105368_at | 1453295_at |
| 105369_at | 1439191_at |
| 105376_at | 1432548_at |
| 105381_at | 1455967_at |
| 105382_at | 1442161_at |
| 105385_at | 1438212_at |
| 105389_at | 1436071_at |
| 105391_at | 1440200_at |
| 105394_at | 1442389_at |
| 105395_at | 1439098_at |

| | |
|---|---|
| 105396_at | 1434349_at |
| 105401_at | 1458640_at |
| 105402_at | 1449657_at |
| 105406_at | 1458628_at |
| 105407_at | 1457671_at |
| 105411_at | 1436051_at |
| 105414_at | 1429692_s_at |
| 105415_at | 1444453_at |
| 105420_at | 1439687_at |
| 105421_at | 1442266_at |
| 105422_at | 1435485_at |
| 105425_at | 1458649_at |
| 105429_at | 1457753_at |
| 105434_at | 1438921_at |
| 105435_at | 1441058_at |
| 105436_at | 1455619_at |
| 105439_at | 1421934_at |
| 105443_at | 1443910_at |
| 105448_at | 1459887_at |
| 105451_at | 1439827_at |
| 105454_at | 1457797_at |
| 105455_at | 1437584_at |
| 105460_at | 1438680_at |
| 105465_at | 1459733_at |
| 105466_at | 1433653_at |
| 105467_at | 1436080_at |
| 105469_at | 1437366_at |
| 105470_at | 1447369_at |
| 105472_at | 1444090_at |
| 105474_at | 1441110_at |
| 105475_at | 1438123_at |
| 105476_at | 1447686_at |
| 105477_at | 1444195_at |
| 105478_at | 1458494_at |
| 105480_at | 1447207_at |
| 105483_at | 1424415_s_at |
| 105490_at | 1440306_at |
| 105492_at | 1416820_at |
| 105493_at | 1457315_at |
| 105494_at | 1425079_at |
| 105498_at | 1424868_at |
| 105500_at | 1435584_at |
| 105501_at | 1425238_at |
| 105502_at | 1442133_at |
| 105503_at | 1456211_at |
| 105505_at | 1436075_at |
| 105506_at | 1439205_at |
| 105507_at | 1441300_at |
| 105510_at | 1424446_at |
| 105513_at | 1457770_at |
| 105517_at | 1442827_at |
| 105518_at | 1444516_at |
| 105520_at | 1442317_at |
| 105522_at | 1440452_at |
| 105523_at | 1456935_at |
| 105524_at | 1457334_at |

| | |
|---|---|
| 105526_at | 1438819_at |
| 105528_at | 1428474_at |
| 105529_at | 1460600_at |
| 105532_at | 1432474_a_at |
| 105534_at | 1419792_at |
| 105536_at | 1438464_at |
| 105539_at | 1448927_at |
| 105541_at | 1442233_at |
| 105544_at | 1459133_at |
| 105547_at | 1430154_at |
| 105548_at | 1431514_at |
| 105549_at | 1444011_at |
| 105555_at | 1452926_at |
| 105556_at | 1435806_at |
| 105557_at | 1457064_at |
| 105559_at | 1441957_x_at |
| 105561_at | 1455303_at |
| 105562_at | 1435108_at |
| 105563_at | 1459695_at |
| 105564_at | 1449059_a_at |
| 105565_at | 1444429_at |
| 105566_at | 1440876_at |
| 105570_at | 1436557_at |
| 105571_at | 1442263_at |
| 105572_at | 1438796_at |
| 105578_at | 1441286_at |
| 105581_at | 1428888_at |
| 105582_at | 1439164_at |
| 105583_at | 1424790_at |
| 105585_at | 1435826_at |
| 105589_at | 1425163_at |
| 105590_at | 1427546_at |
| 105592_at | 1439869_at |
| 105594_at | 1456914_at |
| 105595_at | 1451671_at |
| 105596_at | 1422107_at |
| 105597_at | 1449606_at |
| 105599_at | 1455523_at |
| 105600_at | 1430152_at |
| 105604_at | 1435305_at |
| 105607_at | 1448079_at |
| 105612_at | 1435407_at |
| 105613_at | 1421255_a_at |
| 105614_at | 1458524_at |
| 105615_at | 1435848_at |
| 105616_at | 1443205_at |
| 105618_at | 1455559_at |
| 105619_at | 1429887_at |
| 105623_at | 1428866_at |
| 105626_at | 1438878_at |
| 105627_at | 1442467_at |
| 105630_at | 1423239_at |
| 105632_at | 1454906_at |
| 105633_at | 1440376_at |
| 105636_at | 1453138_at |
| 105638_at | 1452005_at |

| | |
|---|---|
| 105640_at | 1455419_at |
| 105641_at | 1437544_at |
| 105643_at | 1454109_a_at |
| 105645_at | 1445651_at |
| 105646_at | 1438258_at |
| 105647_at | 1435985_at |
| 105650_at | 1444119_at |
| 105651_g_at | 1434374_at |
| 105652_at | 1442403_at |
| 105653_at | 1444743_at |
| 105655_at | 1429849_at |
| 105662_at | 1438409_at |
| 105668_at | 1448052_at |
| 105669_at | 1436658_at |
| 105670_at | 1427203_at |
| 105676_at | 1436209_at |
| 105678_at | 1455258_at |
| 105679_at | 1436093_at |
| 105680_at | 1439743_at |
| 105683_at | 1435991_at |
| 105684_at | 1440370_at |
| 105685_at | 1436226_at |
| 105687_at | 1418921_at |
| 105691_at | 1435210_s_at |
| 105694_at | 1429106_at |
| 105695_r_at | 1436076_at |
| 105697_at | 1458254_at |
| 105698_at | 1441143_at |
| 105699_at | 1457354_at |
| 105700_at | 1431191_a_at |
| 105701_at | 1447138_at |
| 105702_at | 1418917_at |
| 105704_at | 1439084_at |
| 105705_at | 1417519_at |
| 105706_at | 1443960_at |
| 105707_at | 1455598_at |
| 105708_at | 1444560_at |
| 105713_at | 1440535_at |
| 105714_at | 1440853_at |
| 105715_at | 1455554_at |
| 105717_at | 1459723_at |
| 105719_at | 1439581_at |
| 105720_at | 1436013_at |
| 105722_at | 1440749_at |
| 105723_at | 1447236_at |
| 105724_at | 1459699_at |
| 105725_at | 1457446_at |
| 105727_at | 1440148_at |
| 105728_at | 1430750_at |
| 105729_at | 1439882_at |
| 105730_at | 1455557_at |
| 105732_at | 1438432_at |
| 105733_at | 1443855_at |
| 105734_at | 1433449_at |
| 105738_at | 1436259_at |
| 105740_at | 1427409_at |

| | |
|---|---|
| 105741_at | 1455219_at |
| 105744_at | 1439854_at |
| 105745_at | 1435642_at |
| 105748_at | 1455023_at |
| 105749_at | 1456759_at |
| 105750_at | 1457004_at |
| 105754_at | 1436657_at |
| 105755_at | 1429696_at |
| 105756_at | 1442129_at |
| 105757_at | 1436096_at |
| 105761_at | 1434052_at |
| 105762_at | 1455963_at |
| 105765_at | 1427594_at |
| 105770_at | 1444196_at |
| 105771_at | 1434675_at |
| 105774_at | 1442348_at |
| 105776_at | 1439777_at |
| 105778_at | 1436733_at |
| 105780_at | 1444501_at |
| 105782_at | 1428292_at |
| 105785_at | 1435293_at |
| 105786_at | 1422200_at |
| 105788_at | 1456444_at |
| 105789_at | 1455708_at |
| 105792_at | 1457077_at |
| 105793_at | 1418681_at |
| 105797_at | 1457136_at |
| 105798_at | 1444236_at |
| 105799_at | 1455717_s_at |
| 105800_at | 1457819_at |
| 105801_at | 1447322_at |
| 105802_at | 1441998_at |
| 105804_at | 1456812_at |
| 105808_at | 1456137_at |
| 105812_at | 1450707_at |
| 105813_at | 1421035_a_at |
| 105815_at | 1456838_at |
| 105816_at | 1455537_at |
| 105820_at | 1419137_at |
| 105821_at | 1419750_at |
| 105824_at | 1435963_at |
| 105825_at | 1439889_at |
| 105828_at | 1457333_at |
| 105829_at | 1434187_at |
| 105831_at | 1433839_at |
| 105832_at | 1438257_at |
| 105833_at | 1439552_at |
| 105834_at | 1419423_at |
| 105836_at | 1440838_at |
| 105839_at | 1444287_at |
| 105842_at | 1451985_at |
| 105844_at | 1425277_at |
| 105845_at | 1449329_at |
| 105852_at | 1419791_at |
| 105854_at | 1437765_at |
| 105855_at | 1454687_at |

| | |
|---|---|
| 105857_at | 1460035_at |
| 105859_at | 1436535_at |
| 105860_at | 1442040_at |
| 105862_at | 1436579_s_at |
| 105863_at | 1430168_at |
| 105864_at | 1439546_at |
| 105865_at | 1437230_at |
| 105869_at | 1455499_at |
| 105870_at | 1435918_at |
| 105872_at | 1444380_at |
| 105877_at | 1457052_at |
| 105879_at | 1441324_at |
| 105880_at | 1434147_at |
| 105882_at | 1439750_at |
| 105883_at | 1455652_at |
| 105885_at | 1443565_at |
| 105886_r_at | 1451250_at |
| 105887_at | 1442228_at |
| 105888_at | 1439892_at |
| 105889_at | 1458411_at |
| 105890_at | 1436445_at |
| 105892_at | 1456996_at |
| 105893_at | 1439500_at |
| 105896_at | 1425061_at |
| 105899_at | 1440086_at |
| 105901_at | 1435411_at |
| 105902_at | 1442043_at |
| 105903_at | 1443895_at |
| 105904_at | 1442328_at |
| 105905_at | 1441963_at |
| 105906_at | 1418851_at |
| 105907_at | 1442039_at |
| 105909_at | 1444266_at |
| 105910_at | 1436340_at |
| 105911_at | 1436358_at |
| 105913_at | 1444168_at |
| 105914_at | 1428958_at |
| 105915_at | 1451411_at |
| 105918_at | 1437188_at |
| 105919_at | 1427224_at |
| 105921_at | 1457778_at |
| 105922_at | 1428118_at |
| 105925_at | 1443868_at |
| 105928_at | 1457821_at |
| 105930_at | 1456656_at |
| 105937_at | 1420405_at |
| 105941_at | 1440210_at |
| 105942_at | 1455703_at |
| 105943_at | 1431076_at |
| 105944_at | 1438671_at |
| 105945_at | 1434430_s_at |
| 105946_at | 1455392_at |
| 105947_at | 1441080_at |
| 105950_at | 1436060_at |
| 105951_at | 1442506_at |
| 105953_at | 1428895_at |

| | |
|---|---|
| 105957_at | 1435842_at |
| 105959_at | 1438063_at |
| 105961_at | 1455984_at |
| 105962_at | 1435200_at |
| 105964_at | 1450483_at |
| 105965_at | 1424714_at |
| 105967_at | 1424390_at |
| 105972_r_at | 1428607_at |
| 105977_f_at | 1424245_at |
| 105991_at | 1416222_at |
| 106005_at | 1422642_at |
| 106006_at | 1423115_at |
| 106008_at | 1424489_a_at |
| 106009_at | 1427236_a_at |
| 106010_at | 1417362_at |
| 106011_at | 1422905_s_at |
| 106012_at | 1428104_at |
| 106015_at | 1425328_at |
| 106017_at | 1452657_at |
| 106023_at | 1452647_a_at |
| 106026_at | 1426481_at |
| 106033_at | 1435717_at |
| 106041_at | 1431379_a_at |
| 106049_at | 1455936_a_at |
| 106054_at | 1426030_a_at |
| 106059_at | 1417099_at |
| 106061_at | 1424534_at |
| 106062_at | 1451151_s_at |
| 106064_at | 1448857_a_at |
| 106066_at | 1449557_at |
| 106074_at | 1417298_at |
| 106075_at | 1448832_a_at |
| 106076_at | 1429225_at |
| 106077_at | 1438599_at |
| 106081_at | 1443528_at |
| 106082_at | 1455166_at |
| 106083_at | 1440205_at |
| 106086_at | 1457745_at |
| 106088_at | 1446228_at |
| 106095_at | 1436656_at |
| 106096_at | 1439020_at |
| 106098_at | 1449365_at |
| 106099_at | 1456782_at |
| 106100_at | 1438494_at |
| 106104_at | 1451429_at |
| 106108_at | 1457949_at |
| 106109_at | 1439563_at |
| 106111_at | 1431443_at |
| 106112_at | 1451319_at |
| 106117_at | 1455986_at |
| 106118_at | 1440834_at |
| 106119_at | 1457305_at |
| 106121_at | 1439884_at |
| 106124_at | 1442154_at |
| 106126_at | 1424457_at |
| 106130_at | 1442446_at |

| | |
|---|---|
| 106133_at | 1446442_at |
| 106134_at | 1438007_at |
| 106135_at | 1455610_at |
| 106137_at | 1440025_at |
| 106141_at | 1425341_at |
| 106142_at | 1439829_at |
| 106143_at | 1441751_at |
| 106146_at | 1459984_at |
| 106149_at | 1435389_at |
| 106150_at | 1458897_at |
| 106154_at | 1453828_at |
| 106155_at | 1426937_at |
| 106157_at | 1434015_at |
| 106159_at | 1440004_at |
| 106160_at | 1425210_s_at |
| 106161_at | 1435130_at |
| 106162_at | 1428596_at |
| 106164_at | 1438246_at |
| 106166_at | 1440229_at |
| 106169_at | 1440859_at |
| 106170_at | 1447489_at |
| 106172_at | 1444221_at |
| 106176_at | 1436678_at |
| 106178_at | 1435793_at |
| 106183_at | 1457394_at |
| 106184_at | 1460593_at |
| 106185_at | 1435581_at |
| 106189_at | 1425027_s_at |
| 106190_at | 1427093_at |
| 106192_at | 1435214_at |
| 106194_at | 1453380_a_at |
| 106196_at | 1455311_at |
| 106199_at | 1417707_at |
| 106203_at | 1449916_at |
| 106204_at | 1429523_a_at |
| 106205_at | 1417840_at |
| 106207_at | 1433686_at |
| 106208_at | 1454798_at |
| 106213_at | 1416875_at |
| 106214_at | 1452265_at |
| 106218_at | 1427737_a_at |
| 106225_at | 1451339_at |
| 106228_at | 1454630_at |
| 106237_at | 1417669_at |
| 106238_at | 1433937_at |
| 106242_at | 1417438_at |
| 106243_s_at | 1425293_a_at |
| 106244_at | 1428088_at |
| 106248_at | 1428889_at |
| 106253_at | 1436752_at |
| 106254_at | 1435365_at |
| 106256_at | 1430811_a_at |
| 106259_at | 1419898_s_at |
| 106264_at | 1452853_at |
| 106267_s_at | 1451405_at |
| 106269_at | 1428832_at |

| | |
|---|---|
| 106272_at | 1417806_at |
| 106275_at | 1426492_at |
| 106277_at | 1417830_at |
| 106279_at | 1426581_at |
| 106280_at | 1416720_at |
| 106283_at | 1425018_at |
| 106284_at | 1434273_at |
| 106285_at | 1417578_a_at |
| 106286_at | 1423248_at |
| 106288_at | 1433846_s_at |
| 106290_at | 1434601_at |
| 106298_r_at | 1449394_at |
| 106300_at | 1452153_at |
| 106303_at | 1452867_at |
| 106309_at | 1418081_at |
| 106311_at | 1417348_at |
| 106312_at | 1450630_at |
| 106313_at | 1417691_at |
| 106434_at | 1429164_at |
| 106435_at | 1450450_at |
| 106436_at | 1455613_at |
| 106437_at | 1429583_at |
| 106439_at | 1428991_at |
| 106440_at | 1429766_at |
| 106441_at | 1426097_a_at |
| 106442_at | 1455190_at |
| 106447_at | 1453286_at |
| 106448_at | 1433900_at |
| 106449_at | 1460570_at |
| 106455_at | 1428986_at |
| 106456_at | 1429134_at |
| 106457_at | 1457227_at |
| 106459_at | 1457070_at |
| 106460_at | 1433744_at |
| 106463_at | 1436552_at |
| 106464_at | 1455543_at |
| 106465_at | 1419763_at |
| 106466_at | 1434925_at |
| 106467_at | 1437653_at |
| 106470_at | 1443970_at |
| 106471_at | 1437940_at |
| 106472_at | 1439971_at |
| 106474_at | 1428696_at |
| 106481_at | 1423802_at |
| 106482_at | 1453145_at |
| 106484_at | 1443872_at |
| 106485_at | 1428636_at |
| 106486_at | 1441236_at |
| 106488_at | 1443732_at |
| 106489_at | 1445338_at |
| 106491_at | 1455508_at |
| 106494_at | 1460003_at |
| 106499_at | 1439540_at |
| 106502_at | 1436674_at |
| 106503_at | 1440926_at |
| 106504_at | 1435512_at |

| | |
|---|---|
| 106506_at | 1445141_at |
| 106508_at | 1442590_at |
| 106512_at | 1435489_at |
| 106516_at | 1459857_at |
| 106517_at | 1456807_at |
| 106519_at | 1442442_at |
| 106521_at | 1442725_at |
| 106522_at | 1457751_at |
| 106525_at | 1455879_at |
| 106526_at | 1416160_at |
| 106528_at | 1435911_s_at |
| 106530_at | 1458409_at |
| 106535_at | 1440897_at |
| 106537_at | 1443162_at |
| 106538_at | 1425649_at |
| 106540_at | 1425535_at |
| 106542_at | 1438598_at |
| 106544_at | 1456304_at |
| 106545_at | 1426517_at |
| 106547_at | 1447198_at |
| 106549_at | 1454832_at |
| 106553_at | 1446652_at |
| 106554_at | 1417424_at |
| 106555_at | 1450452_a_at |
| 106562_at | 1418771_a_at |
| 106564_at | 1428412_at |
| 106565_at | 1451182_s_at |
| 106569_at | 1425460_at |
| 106571_at | 1428974_s_at |
| 106572_at | 1425486_s_at |
| 106573_at | 1448864_at |
| 106574_at | 1428876_at |
| 106580_at | 1422431_at |
| 106581_at | 1448464_at |
| 106582_at | 1428736_at |
| 106584_at | 1460550_at |
| 106590_at | 1428382_at |
| 106596_at | 1455164_at |
| 106598_at | 1428316_a_at |
| 106600_at | 1424408_at |
| 106602_at | 1428391_at |
| 106603_at | 1423941_at |
| 106606_at | 1448131_at |
| 106608_at | 1454214_a_at |
| 106612_at | 1453253_a_at |
| 106614_at | 1448420_a_at |
| 106616_at | 1433455_at |
| 106625_at | 1451289_at |
| 106627_at | 1428726_at |
| 106629_at | 1438400_at |
| 106630_at | 1424157_at |
| 106632_at | 1418531_at |
| 106633_at | 1460443_at |
| 106634_at | 1434081_at |
| 106635_at | 1427891_at |
| 106647_at | 1432144_a_at |

| | |
|---|---|
| 106649_at | 1454934_at |
| 106650_at | 1455478_at |
| 106652_at | 1419498_at |
| 106656_at | 1454764_s_at |
| 106657_at | 1427957_at |
| 106659_at | 1454750_a_at |
| 106663_at | 1423891_at |
| 106670_at | 1455942_at |
| 106673_at | 1423704_at |
| 106794_at | 1447370_at |
| 106795_at | 1439428_x_at |
| 106797_at | 1440189_at |
| 106798_at | 1440143_at |
| 106799_at | 1447190_at |
| 106802_at | 1438724_at |
| 106804_at | 1458542_at |
| 106805_at | 1456338_at |
| 106809_at | 1456891_at |
| 106816_at | 1430344_at |
| 106817_at | 1442301_at |
| 106823_at | 1441664_at |
| 106828_at | 1449860_at |
| 106829_at | 1433701_at |
| 106830_at | 1424482_at |
| 106832_at | 1438209_at |
| 106834_at | 1435045_s_at |
| 106835_at | 1416562_at |
| 106837_at | 1435849_at |
| 106838_at | 1441742_at |
| 106839_at | 1435433_at |
| 106840_at | 1442607_a_at |
| 106841_at | 1444359_at |
| 106845_at | 1456025_at |
| 106846_at | 1430537_at |
| 106849_at | 1442431_at |
| 106850_at | 1439049_at |
| 106851_at | 1442101_at |
| 106852_at | 1440362_at |
| 106853_at | 1424972_at |
| 106856_at | 1435720_at |
| 106857_at | 1458269_at |
| 106859_at | 1428216_s_at |
| 106861_at | 1436525_at |
| 106863_at | 1458587_at |
| 106865_at | 1441025_at |
| 106866_at | 1439199_at |
| 106867_at | 1458400_at |
| 106869_at | 1426622_a_at |
| 106873_at | 1439874_at |
| 106874_at | 1438594_at |
| 106875_at | 1436850_at |
| 106876_at | 1443515_at |
| 106877_at | 1428022_at |
| 106878_at | 1445395_at |
| 106880_at | 1442359_at |
| 106881_at | 1436844_at |

| | |
|---|---|
| 106884_at | 1456505_at |
| 106888_at | 1452988_at |
| 106890_at | 1418312_at |
| 106893_at | 1460546_at |
| 106895_at | 1439042_at |
| 106897_at | 1438399_at |
| 106899_at | 1421103_at |
| 106900_at | 1435914_at |
| 106901_at | 1441047_at |
| 106906_at | 1453414_at |
| 106907_at | 1422770_at |
| 106908_at | 1442172_at |
| 106910_at | 1450442_at |
| 106911_at | 1456346_at |
| 106915_at | 1455828_at |
| 106918_at | 1437912_at |
| 106919_at | 1439242_at |
| 106921_at | 1428479_at |
| 106922_at | 1456884_at |
| 106927_at | 1449397_at |
| 106930_at | 1438406_at |
| 106933_at | 1456606_a_at |
| 106934_at | 1453074_at |
| 106935_at | 1437864_at |
| 106936_at | 1428541_at |
| 106939_at | 1455916_at |
| 106940_at | 1425577_at |
| 106941_at | 1424842_a_at |
| 106945_at | 1439171_at |
| 106947_at | 1435558_at |
| 106948_at | 1435186_s_at |
| 106949_at | 1449056_at |
| 106951_at | 1435441_at |
| 106952_at | 1436198_at |
| 106955_at | 1418686_at |
| 106959_at | 1434979_at |
| 106961_at | 1449457_at |
| 106962_at | 1432030_at |
| 106963_at | 1427045_at |
| 106964_at | 1452747_at |
| 106965_at | 1418731_at |
| 106971_at | 1439187_at |
| 106972_at | 1435871_at |
| 106973_at | 1440265_at |
| 106977_at | 1435960_at |
| 106979_at | 1456151_at |
| 106980_at | 1438421_at |
| 106983_at | 1425473_at |
| 106985_at | 1448006_at |
| 106988_at | 1429543_at |
| 106989_at | 1456529_at |
| 106990_at | 1437183_at |
| 106992_at | 1434182_at |
| 106998_at | 1439529_at |
| 107001_at | 1424553_at |
| 107002_at | 1429329_at |

| | |
|---|---|
| 107008_at | 1437484_at |
| 107009_at | 1434415_at |
| 107012_at | 1452022_at |
| 107015_at | 1436081_a_at |
| 107018_at | 1436177_at |
| 107023_at | 1455192_at |
| 107024_at | 1439039_at |
| 107026_at | 1419470_at |
| 107029_at | 1424419_at |
| 107031_at | 1429651_at |
| 107032_at | 1451529_at |
| 107033_at | 1433624_at |
| 107041_at | 1448693_at |
| 107044_at | 1433996_at |
| 107046_at | 1427064_a_at |
| 107055_at | 1456478_at |
| 107056_at | 1438774_s_at |
| 107057_at | 1451389_at |
| 107060_at | 1418408_at |
| 107063_at | 1436029_at |
| 107065_at | 1433710_at |
| 107068_at | 1426383_at |
| 107071_at | 1434318_a_at |
| 107074_at | 1448530_at |
| 107078_at | 1435641_at |
| 107081_at | 1424118_a_at |
| 107082_at | 1450900_at |
| 107083_at | 1431337_a_at |
| 107087_at | 1451297_at |
| 107090_at | 1417797_a_at |
| 107092_at | 1424576_s_at |
| 107094_at | 1424031_at |
| 107099_at | 1419050_at |
| 107100_at | 1454840_at |
| 107101_at | 1423094_at |
| 107102_at | 1452749_at |
| 107109_at | 1416178_a_at |
| 107110_at | 1424277_at |
| 107111_at | 1427899_at |
| 107112_at | 1459713_s_at |
| 107117_at | 1448856_a_at |
| 107120_at | 1424421_at |
| 107122_at | 1425043_s_at |
| 107127_at | 1454808_at |
| 107128_at | 1448473_at |
| 107131_at | 1435159_at |
| 107133_at | 1440211_at |
| 107135_at | 1418837_at |
| 107139_at | 1452915_at |
| 107143_at | 1424348_at |
| 107144_at | 1422552_at |
| 107145_at | 1438008_at |
| 107152_at | 1455596_a_at |
| 107162_f_at | 1437415_at |
| 107249_at | 1459194_at |
| 107276_at | 1449527_at |

| | |
|---|---|
| 107281_at | 1442632_at |
| 107282_at | 1434918_at |
| 107284_at | 1437555_at |
| 107287_at | 1439202_at |
| 107289_at | 1440512_at |
| 107292_at | 1438207_at |
| 107293_at | 1436961_at |
| 107295_at | 1435257_at |
| 107296_at | 1456535_at |
| 107299_at | 1459905_at |
| 107300_at | 1459886_at |
| 107301_at | 1454986_at |
| 107302_at | 1442049_at |
| 107303_at | 1442238_a_at |
| 107305_at | 1436624_at |
| 107306_at | 1439802_at |
| 107309_at | 1429211_at |
| 107314_at | 1435587_at |
| 107316_at | 1422083_at |
| 107317_at | 1430347_at |
| 107320_at | 1443781_at |
| 107321_at | 1449971_a_at |
| 107323_at | 1437031_at |
| 107324_at | 1457843_at |
| 107325_at | 1439486_at |
| 107326_at | 1455721_at |
| 107327_at | 1453859_at |
| 107328_at | 1457420_at |
| 107329_at | 1455044_at |
| 107330_at | 1460625_at |
| 107331_at | 1457933_at |
| 107332_at | 1455690_at |
| 107333_at | 1438073_at |
| 107338_at | 1440849_at |
| 107339_at | 1443692_at |
| 107340_at | 1457075_at |
| 107341_at | 1437472_at |
| 107342_at | 1456499_at |
| 107344_at | 1451847_s_at |
| 107345_at | 1431323_at |
| 107348_at | 1424508_at |
| 107352_at | 1442397_at |
| 107353_at | 1421240_at |
| 107355_r_at | 1443887_at |
| 107361_at | 1429191_at |
| 107362_at | 1438479_at |
| 107363_at | 1424787_a_at |
| 107366_at | 1434504_at |
| 107367_at | 1430144_at |
| 107368_at | 1424940_s_at |
| 107369_at | 1442351_a_at |
| 107370_at | 1444323_at |
| 107373_at | 1430046_at |
| 107375_at | 1453594_at |
| 107376_at | 1456811_at |
| 107378_at | 1419829_a_at |

| | |
|---|---|
| 107381_at | 1422878_at |
| 107382_at | 1440930_a_at |
| 107387_at | 1439088_at |
| 107389_at | 1435165_at |
| 107390_at | 1416275_at |
| 107391_at | 1450758_at |
| 107394_at | 1436131_at |
| 107395_at | 1438286_at |
| 107396_at | 1435149_at |
| 107397_at | 1443863_at |
| 107398_at | 1420481_at |
| 107402_at | 1456761_at |
| 107404_at | 1424987_at |
| 107410_at | 1434845_at |
| 107411_at | 1453134_at |
| 107415_at | 1435549_at |
| 107416_at | 1453081_at |
| 107417_at | 1450887_at |
| 107418_at | 1428536_at |
| 107421_at | 1438715_at |
| 107422_at | 1425459_at |
| 107424_at | 1428285_at |
| 107425_at | 1429084_at |
| 107426_at | 1436758_at |
| 107427_at | 1451811_at |
| 107428_at | 1424312_at |
| 107429_at | 1419180_at |
| 107431_at | 1428710_at |
| 107436_at | 1438500_at |
| 107437_at | 1422731_at |
| 107438_at | 1424411_at |
| 107439_at | 1427132_at |
| 107440_at | 1427925_at |
| 107441_at | 1436138_at |
| 107443_at | 1434952_at |
| 107444_at | 1436161_at |
| 107446_at | 1455960_at |
| 107450_at | 1444144_at |
| 107453_at | 1435384_at |
| 107455_at | 1451904_a_at |
| 107459_at | 1428457_at |
| 107460_at | 1428808_at |
| 107464_at | 1437483_at |
| 107465_at | 1426389_at |
| 107466_at | 1456502_at |
| 107468_at | 1438198_at |
| 107469_at | 1418256_at |
| 107470_at | 1424044_at |
| 107472_at | 1449350_at |
| 107473_at | 1439843_at |
| 107474_at | 1457043_at |
| 107475_at | 1456878_at |
| 107477_at | 1452600_at |
| 107478_at | 1455339_at |
| 107479_at | 1440191_s_at |
| 107480_at | 1433722_at |

| | |
|---|---|
| 107487_at | 1442097_at |
| 107489_at | 1450955_s_at |
| 107492_at | 1451561_at |
| 107493_at | 1448288_at |
| 107497_at | 1426551_at |
| 107498_at | 1425632_a_at |
| 107502_at | 1457320_at |
| 107505_at | 1423661_s_at |
| 107507_at | 1452310_at |
| 107508_at | 1428989_at |
| 107509_at | 1451471_at |
| 107510_at | 1448891_at |
| 107511_at | 1448952_at |
| 107512_at | 1429256_at |
| 107513_at | 1454916_s_at |
| 107516_at | 1417981_at |
| 107517_at | 1435570_s_at |
| 107518_at | 1424533_a_at |
| 107519_at | 1435343_at |
| 107521_at | 1428508_at |
| 107523_at | 1448833_at |
| 107527_at | 1436818_a_at |
| 107528_at | 1451717_s_at |
| 107529_at | 1433933_s_at |
| 107533_at | 1451207_at |
| 107538_at | 1455170_at |
| 107539_at | 1454818_at |
| 107542_at | 1455914_at |
| 107544_r_at | 1417536_at |
| 107545_at | 1418058_at |
| 107546_at | 1418696_at |
| 107548_at | 1423453_at |
| 107552_at | 1419659_s_at |
| 107553_at | 1449342_at |
| 107554_at | 1417787_at |
| 107555_at | 1424224_at |
| 107556_at | 1460606_at |
| 107563_at | 1457621_at |
| 107564_at | 1450425_a_at |
| 107565_at | 1424676_s_at |
| 107566_at | 1428758_at |
| 107567_at | 1454797_at |
| 107568_at | 1418719_at |
| 107569_at | 1438678_at |
| 107572_at | 1416207_at |
| 107577_at | 1452756_at |
| 107583_at | 1423289_a_at |
| 107585_at | 1428597_at |
| 107589_at | 1422858_at |
| 107590_at | 1427000_at |
| 107593_at | 1416560_at |
| 107597_f_at | 1422016_a_at |
| 107600_at | 1428408_a_at |
| 107601_at | 1433850_at |
| 107602_at | 1455181_at |
| 107608_at | 1433922_at |

| | |
|---|---|
| 107612_at | 1423659_a_at |
| 107616_at | 1428614_at |
| 107618_at | 1424519_at |
| 107620_at | 1424540_at |
| 107621_at | 1451160_s_at |
| 107624_at | 1452223_s_at |
| 107632_at | 1451108_at |
| 107752_at | 1445281_a_at |
| 107758_at | 1444776_at |
| 107764_at | 1455763_at |
| 107769_r_at | 1453024_at |
| 107775_at | 1425040_at |
| 107776_at | 1454876_at |
| 107786_at | 1440957_at |
| 107787_at | 1429064_at |
| 107791_at | 1445499_at |
| 107793_at | 1453586_at |
| 107796_at | 1444754_at |
| 107802_at | 1426932_at |
| 107807_at | 1436088_at |
| 107808_at | 1452631_at |
| 107809_at | 1456509_at |
| 107810_at | 1455663_at |
| 107812_at | 1430301_at |
| 107817_at | 1418952_at |
| 107821_at | 1445895_at |
| 107825_at | 1442700_at |
| 107832_at | 1452873_at |
| 107835_at | 1440764_at |
| 107836_at | 1430994_at |
| 107843_at | 1429916_at |
| 107848_at | 1440301_at |
| 107854_at | 1444072_at |
| 107855_at | 1427247_at |
| 107856_at | 1446290_at |
| 107860_at | 1451503_at |
| 107862_at | 1438285_at |
| 107864_at | 1442202_at |
| 107865_at | 1429463_at |
| 107868_at | 1441699_at |
| 107870_at | 1435406_at |
| 107871_at | 1455030_at |
| 107874_at | 1436841_at |
| 107875_at | 1424370_s_at |
| 107876_at | 1435678_at |
| 107879_at | 1442504_at |
| 107880_at | 1437252_at |
| 107882_at | 1419111_at |
| 107883_at | 1427110_at |
| 107884_at | 1422694_at |
| 107886_at | 1448177_at |
| 107887_at | 1435229_at |
| 107888_at | 1429258_at |
| 107890_at | 1451932_a_at |
| 107897_at | 1453189_at |
| 107898_at | 1423426_at |

| | |
|---|---|
| 107904_at | 1437654_at |
| 107906_at | 1449859_at |
| 107907_at | 1452978_at |
| 107910_at | 1429186_a_at |
| 107911_at | 1419179_at |
| 107912_at | 1416886_at |
| 107915_at | 1435938_at |
| 107916_at | 1419466_at |
| 107918_at | 1419683_at |
| 107921_at | 1436509_at |
| 107922_at | 1449086_at |
| 107924_at | 1445632_at |
| 107926_at | 1434880_at |
| 107927_at | 1430054_at |
| 107928_at | 1425977_a_at |
| 107932_at | 1455509_at |
| 107934_at | 1431043_at |
| 107935_at | 1427919_at |
| 107936_at | 1439053_at |
| 107948_at | 1453474_at |
| 107949_at | 1438434_at |
| 107957_at | 1429150_at |
| 107958_at | 1455529_at |
| 107959_at | 1434413_at |
| 107960_at | 1436120_at |
| 107964_at | 1426494_at |
| 107968_at | 1442089_at |
| 107969_at | 1437467_at |
| 107970_at | 1429046_at |
| 107976_at | 1452299_at |
| 107983_at | 1431179_at |
| 107985_at | 1431843_a_at |
| 107986_at | 1439773_at |
| 107988_at | 1434931_at |
| 107989_at | 1422551_at |
| 107993_at | 1458727_at |
| 107999_at | 1428300_at |
| 108003_at | 1428395_at |
| 108005_at | 1419686_at |
| 108006_at | 1434576_at |
| 108009_at | 1424149_at |
| 108012_at | 1426206_at |
| 108013_at | 1428513_at |
| 108017_at | 1453689_at |
| 108022_at | 1424213_at |
| 108027_at | 1425482_s_at |
| 108029_at | 1451442_at |
| 108030_at | 1450031_at |
| 108032_at | 1428899_at |
| 108034_at | 1433727_at |
| 108037_at | 1431075_a_at |
| 108038_at | 1424430_at |
| 108042_at | 1417913_at |
| 108044_at | 1426939_at |
| 108045_at | 1452752_at |
| 108055_at | 1428650_at |

| | |
|---|---|
| 108059_at | 1438004_at |
| 108060_at | 1430292_a_at |
| 108061_at | 1424069_at |
| 108062_at | 1426355_a_at |
| 108066_at | 1426861_at |
| 108068_at | 1417310_at |
| 108069_at | 1449505_at |
| 108071_at | 1454961_at |
| 108073_at | 1416758_at |
| 108075_at | 1433614_at |
| 108078_at | 1425608_at |
| 108080_at | 1423937_at |
| 108081_at | 1423936_at |
| 108082_at | 1423918_at |
| 108087_at | 1423197_a_at |
| 108091_at | 1433726_at |
| 108094_at | 1456316_a_at |
| 108098_f_at | 1424717_at |
| 108099_at | 1437594_x_at |
| 108104_at | 1426734_at |
| 108105_at | 1437636_at |
| 108109_at | 1428490_at |
| 108233_at | 1453061_at |
| 108245_at | 1420582_at |
| 108246_at | 1444495_at |
| 108247_at | 1429852_at |
| 108249_at | 1455969_at |
| 108252_at | 1436406_at |
| 108253_at | 1443143_at |
| 108255_at | 1450198_at |
| 108257_at | 1447147_at |
| 108260_at | 1430389_at |
| 108265_at | 1442724_at |
| 108266_at | 1438863_at |
| 108267_at | 1441710_at |
| 108268_at | 1457092_at |
| 108270_at | 1439919_at |
| 108271_at | 1458535_at |
| 108272_at | 1435894_at |
| 108275_at | 1457422_at |
| 108277_at | 1444604_at |
| 108284_at | 1417849_at |
| 108286_at | 1418857_at |
| 108289_at | 1427352_at |
| 108290_at | 1458175_at |
| 108296_at | 1434718_at |
| 108304_at | 1439046_at |
| 108310_at | 1452319_at |
| 108311_at | 1425286_at |
| 108312_at | 1441972_at |
| 108313_at | 1453421_at |
| 108314_at | 1447119_at |
| 108316_at | 1430876_at |
| 108318_at | 1423303_at |
| 108328_at | 1427012_at |
| 108330_at | 1458947_at |

| | |
|---|---|
| 108333_at | 1445481_at |
| 108335_r_at | 1439618_at |
| 108336_at | 1442243_at |
| 108337_at | 1444368_at |
| 108345_at | 1439865_at |
| 108346_at | 1451483_s_at |
| 108348_at | 1437485_at |
| 108350_at | 1429831_at |
| 108352_at | 1427911_at |
| 108355_at | 1434032_at |
| 108359_at | 1434826_at |
| 108360_at | 1450737_at |
| 108370_at | 1435664_at |
| 108373_at | 1454950_at |
| 108376_at | 1453608_at |
| 108385_at | 1453342_at |
| 108389_at | 1450175_a_at |
| 108397_r_at | 1460332_at |
| 108399_at | 1426279_at |
| 108418_at | 1424865_at |
| 108423_at | 1452959_a_at |
| 108426_f_at | 1440832_at |
| 108440_at | 1436327_a_at |
| 108458_at | 1452086_at |
| 108460_at | 1452070_at |
| 108465_at | 1419651_at |
| 108467_at | 1439107_a_at |
| 108470_at | 1428222_at |
| 108471_at | 1434215_at |
| 108474_at | 1426357_at |
| 108475_at | 1434786_at |
| 108477_at | 1434316_at |
| 108484_at | 1424518_at |
| 108486_at | 1451321_a_at |
| 108487_at | 1428239_at |
| 108491_at | 1426846_at |
| 108492_at | 1454746_at |
| 108493_at | 1417066_at |
| 108494_at | 1451328_at |
| 108495_at | 1424599_at |
| 108496_at | 1424485_at |
| 108499_at | 1424520_at |
| 108500_at | 1428980_at |
| 108512_at | 1416869_x_at |
| 108513_at | 1424162_at |
| 108516_at | 1426443_at |
| 108519_at | 1422510_at |
| 108520_at | 1424005_at |
| 108521_at | 1453562_a_at |
| 108523_at | 1429212_a_at |
| 108524_at | 1449555_a_at |
| 108526_at | 1433813_at |
| 108532_at | 1450697_at |
| 108533_at | 1451331_at |
| 108534_at | 1454733_at |
| 108536_at | 1448385_at |

| | |
|---|---|
| 108537_at | 1424393_s_at |
| 108540_at | 1424444_a_at |
| 108542_at | 1448404_at |
| 108547_at | 1417511_at |
| 108548_at | 1455053_a_at |
| 108551_at | 1418737_at |
| 108555_at | 1448702_at |
| 108558_at | 1450701_a_at |
| 108561_at | 1455922_at |
| 108564_at | 1428516_a_at |
| 108565_at | 1433529_at |
| 108570_at | 1422524_at |
| 108571_at | 1453023_at |
| 108574_at | 1424744_at |
| 108575_at | 1433968_a_at |
| 108577_at | 1420673_a_at |
| 108580_at | 1428207_at |
| 108585_at | 1436720_s_at |
| 108710_at | 1435925_at |
| 108711_at | 1450918_s_at |
| 108713_at | 1427947_at |
| 108717_at | 1438241_at |
| 108718_at | 1427122_at |
| 108719_at | 1421878_at |
| 108721_at | 1428660_s_at |
| 108722_at | 1429440_at |
| 108723_at | 1427015_at |
| 108728_at | 1455560_at |
| 108732_at | 1449304_at |
| 108733_at | 1423164_at |
| 108734_at | 1434399_at |
| 108735_at | 1435466_at |
| 108737_at | 1452289_a_at |
| 108743_at | 1428122_s_at |
| 108745_at | 1438402_at |
| 108750_at | 1432202_a_at |
| 108751_at | 1437409_s_at |
| 108752_at | 1437453_s_at |
| 108753_at | 1435917_at |
| 108754_at | 1454780_at |
| 108755_at | 1454844_at |
| 108756_at | 1417568_at |
| 108760_at | 1433672_at |
| 108763_at | 1435024_at |
| 108764_at | 1440955_at |
| 108766_at | 1453042_at |
| 108767_at | 1426984_at |
| 108770_at | 1460584_at |
| 108773_at | 1420640_at |
| 108781_at | 1426270_at |
| 108782_at | 1435467_at |
| 108785_at | 1455441_at |
| 108786_at | 1427004_at |
| 108789_at | 1437914_at |
| 108796_at | 1455202_at |
| 108797_at | 1439569_at |

| | |
|---|---|
| 108798_at | 1441078_at |
| 108807_at | 1452807_s_at |
| 108808_at | 1434581_at |
| 108811_at | 1435264_at |
| 108812_at | 1440225_at |
| 108813_at | 1435939_s_at |
| 108814_at | 1435601_at |
| 108816_at | 1429136_at |
| 108818_at | 1424814_a_at |
| 108823_at | 1434095_at |
| 108831_at | 1439668_at |
| 108832_at | 1440529_at |
| 108833_at | 1439292_at |
| 108835_at | 1453653_at |
| 108836_at | 1437601_at |
| 108838_at | 1443646_at |
| 108839_at | 1443595_at |
| 108840_at | 1456928_at |
| 108841_at | 1457056_at |
| 108843_at | 1456781_at |
| 108844_at | 1441077_at |
| 108845_at | 1440399_at |
| 108846_at | 1426471_at |
| 108847_at | 1425037_at |
| 108859_at | 1430349_at |
| 108864_at | 1434672_at |
| 108865_at | 1438575_a_at |
| 108868_at | 1416154_at |
| 108869_at | 1452845_at |
| 108872_at | 1430037_at |
| 108873_at | 1431170_at |
| 108874_at | 1444151_at |
| 108876_at | 1436239_at |
| 108878_at | 1459434_at |
| 108879_at | 1441199_at |
| 108882_at | 1447526_at |
| 108884_at | 1425852_at |
| 108886_at | 1456771_at |
| 108887_at | 1440311_at |
| 108888_at | 1457713_at |
| 108891_at | 1436260_at |
| 108895_at | 1437901_a_at |
| 108897_at | 1426287_at |
| 108901_at | 1439607_at |
| 108902_at | 1439348_at |
| 108904_at | 1457813_at |
| 108905_at | 1430765_at |
| 108906_at | 1442865_at |
| 108908_at | 1437042_at |
| 108915_at | 1418610_at |
| 108918_at | 1429394_at |
| 108919_at | 1457941_at |
| 108920_at | 1459029_at |
| 108921_at | 1439985_at |
| 108922_at | 1435583_at |
| 108923_at | 1436337_at |

| | |
|---|---|
| 108924_at | 1447929_at |
| 108925_at | 1425110_at |
| 108926_at | 1459656_at |
| 108927_at | 1438492_at |
| 108933_at | 1455651_at |
| 108935_at | 1455517_at |
| 108936_at | 1460070_at |
| 108937_at | 1442918_at |
| 108940_at | 1443614_at |
| 108943_at | 1441166_at |
| 108945_g_at | 1443623_a_at |
| 108946_at | 1441211_at |
| 108947_at | 1458298_at |
| 108953_at | 1439043_at |
| 108954_at | 1417985_at |
| 108967_at | 1424267_at |
| 108981_at | 1460001_at |
| 108983_at | 1440911_at |
| 108985_at | 1424696_at |
| 108998_at | 1437242_at |
| 109000_at | 1453072_at |
| 109001_at | 1425541_at |
| 109009_at | 1430669_at |
| 109012_at | 1425850_a_at |
| 109013_at | 1429116_at |
| 109015_at | 1456895_at |
| 109028_at | 1435571_at |
| 109039_at | 1455532_at |
| 109040_at | 1455793_at |
| 109042_at | 1438733_at |
| 109045_at | 1417736_at |
| 109047_at | 1458384_at |
| 109055_at | 1451496_at |
| 109067_at | 1455685_at |
| 109076_at | 1439102_at |
| 109077_at | 1452149_at |
| 109081_at | 1424032_at |
| 109083_at | 1452225_at |
| 109086_at | 1434511_at |
| 109091_at | 1424238_at |
| 109092_at | 1434387_at |
| 109094_at | 1418968_at |
| 109095_at | 1451210_at |
| 109103_f_at | 1441081_a_at |
| 109104_at | 1416469_at |
| 109111_at | 1424332_at |
| 109114_at | 1427201_at |
| 109115_at | 1417733_at |
| 109117_at | 1424654_at |
| 109118_at | 1448477_at |
| 109121_at | 1428441_at |
| 109124_at | 1436501_at |
| 109126_at | 1429446_at |
| 109128_at | 1452672_at |
| 109131_at | 1453795_at |
| 109132_at | 1424335_at |

| | |
|---|---|
| 109136_at | 1424646_at |
| 109138_at | 1450855_at |
| 109139_at | 1424697_at |
| 109143_at | 1433997_at |
| 109144_at | 1426825_at |
| 109147_at | 1425788_a_at |
| 109149_at | 1451275_at |
| 109150_at | 1448583_at |
| 109154_at | 1453105_at |
| 109155_at | 1426364_at |
| 109157_at | 1427158_at |
| 109160_at | 1423575_a_at |
| 109168_at | 1426577_a_at |
| 109170_at | 1433520_at |
| 109172_at | 1424047_at |
| 109173_at | 1452625_at |
| 109175_at | 1417573_at |
| 109177_at | 1452381_at |
| 109183_at | 1434255_at |
| 109185_at | 1435000_at |
| 109187_at | 1454645_at |
| 109297_at | 1420133_at |
| 109298_f_at | 1425820_x_at |
| 109300_at | 1440344_at |
| 109305_at | 1439532_s_at |
| 109306_at | 1447859_at |
| 109307_at | 1441701_at |
| 109308_at | 1424507_at |
| 109311_at | 1455609_at |
| 109317_at | 1452965_at |
| 109318_at | 1431810_a_at |
| 109320_at | 1453180_at |
| 109322_at | 1424535_at |
| 109325_at | 1435479_at |
| 109326_at | 1434575_at |
| 109327_at | 1455051_at |
| 109328_at | 1452282_at |
| 109331_at | 1433764_at |
| 109333_at | 1455998_at |
| 109336_at | 1455582_at |
| 109338_at | 1426876_at |
| 109343_at | 1452618_at |
| 109346_at | 1424666_at |
| 109347_at | 1435209_at |
| 109348_at | 1435156_at |
| 109352_s_at | 1459991_at |
| 109353_at | 1425848_a_at |
| 109356_at | 1438046_at |
| 109357_at | 1437295_at |
| 109361_at | 1435261_at |
| 109362_at | 1452779_at |
| 109363_at | 1434268_at |
| 109365_at | 1436166_at |
| 109366_at | 1417810_a_at |
| 109367_at | 1454942_at |
| 109369_at | 1418776_at |

| | |
|---|---|
| 109371_at | 1420913_at |
| 109377_at | 1424471_at |
| 109378_at | 1425034_at |
| 109379_at | 1455015_at |
| 109380_at | 1424210_at |
| 109383_at | 1418909_at |
| 109384_at | 1421824_at |
| 109385_at | 1421009_at |
| 109386_at | 1429115_at |
| 109392_at | 1449300_at |
| 109398_at | 1440212_at |
| 109399_at | 1426994_at |
| 109401_at | 1449189_at |
| 109402_at | 1448552_s_at |
| 109405_at | 1434684_at |
| 109410_at | 1454776_at |
| 109414_at | 1429113_at |
| 109419_at | 1448645_at |
| 109421_at | 1433893_s_at |
| 109422_at | 1438566_at |
| 109423_at | 1424060_at |
| 109424_at | 1429339_a_at |
| 109426_at | 1427480_at |
| 109427_at | 1436168_at |
| 109428_at | 1443704_at |
| 109430_at | 1440449_at |
| 109432_at | 1458674_at |
| 109436_at | 1442072_at |
| 109438_at | 1435707_at |
| 109441_at | 1421497_at |
| 109443_at | 1457801_at |
| 109445_at | 1455287_at |
| 109447_at | 1455416_at |
| 109450_at | 1456794_at |
| 109451_at | 1459910_at |
| 109455_at | 1453766_a_at |
| 109456_at | 1431917_at |
| 109457_at | 1456766_at |
| 109462_at | 1442325_at |
| 109464_at | 1456021_at |
| 109465_at | 1431053_at |
| 109466_at | 1420683_at |
| 109469_at | 1419714_at |
| 109470_at | 1458605_at |
| 109472_at | 1442016_at |
| 109473_at | 1435097_at |
| 109474_r_at | 1452815_at |
| 109478_at | 1442304_at |
| 109479_at | 1442164_at |
| 109481_at | 1439890_at |
| 109485_at | 1421379_at |
| 109488_at | 1427146_at |
| 109494_at | 1457806_at |
| 109496_at | 1438247_at |
| 109498_at | 1429105_at |
| 109499_at | 1453021_at |

| | |
|---|---|
| 109501_at | 1429665_at |
| 109506_at | 1457370_at |
| 109508_at | 1444603_at |
| 109509_at | 1436044_at |
| 109512_at | 1424339_at |
| 109513_r_at | 1425279_at |
| 109517_at | 1438784_at |
| 109519_at | 1419517_at |
| 109522_at | 1438539_at |
| 109523_at | 1440023_at |
| 109524_at | 1421124_at |
| 109526_at | 1460023_at |
| 109531_at | 1443606_at |
| 109537_at | 1421967_at |
| 109538_at | 1439667_at |
| 109539_at | 1459969_x_at |
| 109540_at | 1437559_at |
| 109541_at | 1441562_at |
| 109543_at | 1460059_at |
| 109544_at | 1451816_at |
| 109547_at | 1442428_at |
| 109550_at | 1417803_at |
| 109551_at | 1451899_a_at |
| 109553_at | 1428455_at |
| 109560_at | 1447187_at |
| 109562_at | 1435025_at |
| 109563_at | 1437023_at |
| 109564_at | 1433626_at |
| 109574_at | 1434160_at |
| 109575_at | 1418591_at |
| 109578_at | 1426427_at |
| 109585_at | 1438341_at |
| 109587_at | 1443938_at |
| 109589_at | 1419365_at |
| 109598_at | 1431077_at |
| 109603_at | 1417284_at |
| 109608_at | 1422533_at |
| 109617_at | 1437586_at |
| 109618_at | 1434072_at |
| 109622_at | 1416305_at |
| 109632_at | 1424219_at |
| 109636_at | 1423778_at |
| 109640_at | 1455849_at |
| 109642_at | 1418598_at |
| 109643_at | 1455110_at |
| 109646_at | 1451621_at |
| 109649_at | 1435279_at |
| 109650_at | 1431109_at |
| 109653_at | 1422860_at |
| 109655_at | 1430036_at |
| 109656_at | 1424414_at |
| 109663_at | 1436306_at |
| 109664_at | 1426537_at |
| 109668_at | 1454839_a_at |
| 109669_at | 1426972_at |
| 109672_at | 1429539_at |

| | |
|---|---|
| 109673_at | 1450024_at |
| 109674_at | 1434530_at |
| 109678_at | 1426017_a_at |
| 109679_at | 1416760_at |
| 109680_at | 1436919_at |
| 109682_at | 1416356_at |
| 109685_at | 1417032_at |
| 109687_at | 1424418_at |
| 109689_at | 1423037_at |
| 109691_at | 1417922_at |
| 109697_at | 1456484_at |
| 109698_at | 1452669_at |
| 109702_at | 1438205_at |
| 109708_at | 1448917_at |
| 109711_at | 1418185_at |
| 109713_at | 1432526_a_at |
| 109714_at | 1418306_at |
| 109715_at | 1426407_at |
| 109723_at | 1435093_at |
| 109724_at | 1416215_at |
| 109727_at | 1434924_at |
| 109729_at | 1433613_at |
| 109733_at | 1426479_a_at |
| 109734_at | 1424947_at |
| 109735_at | 1415917_at |
| 109737_at | 1450027_at |
| 109739_at | 1425933_a_at |
| 109740_at | 1418832_at |
| 109742_at | 1416223_at |
| 109744_at | 1434159_at |
| 109745_at | 1451984_at |
| 109746_at | 1452346_at |
| 109747_at | 1451720_at |
| 109748_at | 1424852_at |
| 109749_at | 1451446_at |
| 109750_at | 1460205_at |
| 109751_at | 1418529_at |
| 109752_at | 1421089_a_at |
| 109753_at | 1448275_at |
| 109754_at | 1416261_at |
| 109761_g_at | 1436179_a_at |
| 109767_at | 1426249_at |
| 109768_at | 1436322_a_at |
| 109771_at | 1418570_at |
| 109772_at | 1429254_at |
| 109773_at | 1424268_at |
| 109774_at | 1448825_at |
| 109785_at | 1424954_a_at |
| 109786_at | 1433450_at |
| 109794_f_at | 1454171_x_at |
| 109797_at | 1429031_at |
| 109801_at | 1419316_s_at |
| 109870_at | 1443679_at |
| 109909_at | 1455354_at |
| 109910_at | 1426987_at |
| 109911_at | 1436389_at |

| | |
|---|---|
| 109912_at | 1450395_at |
| 109915_at | 1434438_at |
| 109916_at | 1453578_at |
| 109920_at | 1436192_at |
| 109921_at | 1452820_at |
| 109922_at | 1426239_s_at |
| 109927_at | 1426989_at |
| 109931_at | 1418156_at |
| 109932_at | 1455545_at |
| 109933_at | 1440848_at |
| 109934_at | 1455442_at |
| 109938_at | 1427177_at |
| 109940_at | 1439792_at |
| 109941_at | 1438693_at |
| 109943_at | 1435955_at |
| 109944_at | 1433767_at |
| 109945_at | 1424588_at |
| 109947_at | 1456331_at |
| 109951_at | 1437476_at |
| 109954_at | 1427043_s_at |
| 109955_at | 1435784_at |
| 109957_at | 1435170_at |
| 109958_at | 1429223_a_at |
| 109959_at | 1434780_at |
| 109963_f_at | 1424317_at |
| 109967_at | 1435198_at |
| 109968_at | 1429598_at |
| 109969_at | 1435808_at |
| 109970_at | 1434762_at |
| 109972_at | 1452271_at |
| 109973_at | 1424226_at |
| 109976_at | 1436590_at |
| 109979_i_at | 1432821_at |
| 109985_at | 1434023_at |
| 109986_at | 1434106_at |
| 109988_at | 1454900_s_at |
| 109991_at | 1455321_at |
| 109992_at | 1435189_at |
| 109994_at | 1427376_a_at |
| 109995_at | 1433926_at |
| 109997_at | 1451487_at |
| 109998_at | 1424386_at |
| 109999_at | 1417969_at |
| 110000_at | 1418482_at |
| 110001_at | 1424262_at |
| 110006_at | 1431314_a_at |
| 110007_at | 1420111_at |
| 110009_at | 1452837_at |
| 110011_at | 1454718_at |
| 110018_at | 1455577_at |
| 110020_at | 1435861_at |
| 110022_at | 1430535_at |
| 110023_at | 1417668_at |
| 110024_at | 1417848_at |
| 110026_at | 1428378_at |
| 110029_at | 1439584_at |

| | |
|---|---|
| 110030_at | 1443631_at |
| 110033_at | 1441371_at |
| 110034_at | 1458263_at |
| 110035_at | 1435795_at |
| 110041_s_at | 1438867_at |
| 110042_at | 1442751_at |
| 110046_at | 1449831_at |
| 110054_at | 1459036_at |
| 110056_at | 1438309_at |
| 110057_at | 1444418_at |
| 110060_at | 1440492_at |
| 110061_at | 1443327_at |
| 110063_at | 1419531_at |
| 110064_at | 1425086_a_at |
| 110066_at | 1437989_at |
| 110067_at | 1418148_at |
| 110068_at | 1423463_a_at |
| 110070_at | 1439509_at |
| 110071_at | 1456648_at |
| 110076_at | 1457356_at |
| 110077_at | 1457124_at |
| 110079_g_at | 1424985_a_at |
| 110080_at | 1429606_at |
| 110084_at | 1430655_at |
| 110085_at | 1460472_at |
| 110086_at | 1435452_at |
| 110088_at | 1453116_at |
| 110089_at | 1439779_at |
| 110093_at | 1430622_at |
| 110096_at | 1456929_at |
| 110099_at | 1442570_at |
| 110104_at | 1423050_s_at |
| 110107_at | 1457682_at |
| 110110_at | 1450071_at |
| 110111_at | 1452013_at |
| 110113_at | 1459722_at |
| 110116_at | 1443990_at |
| 110117_at | 1447105_at |
| 110118_at | 1444534_at |
| 110121_at | 1420416_at |
| 110122_at | 1426142_a_at |
| 110125_at | 1442717_at |
| 110126_at | 1437766_at |
| 110129_at | 1435313_at |
| 110132_at | 1457076_at |
| 110134_s_at | 1450152_at |
| 110135_at | 1441785_at |
| 110142_at | 1442195_at |
| 110143_at | 1449850_at |
| 110144_at | 1455412_at |
| 110147_at | 1455250_at |
| 110156_at | 1435302_at |
| 110157_at | 1424434_at |
| 110159_at | 1455327_at |
| 110160_at | 1452087_at |
| 110164_at | 1452707_at |

| | |
|---|---|
| 110165_at | 1455877_a_at |
| 110167_at | 1436819_at |
| 110173_at | 1447403_a_at |
| 110174_at | 1438253_at |
| 110180_at | 1455318_at |
| 110182_at | 1450435_at |
| 110184_at | 1439849_at |
| 110185_at | 1458467_at |
| 110187_at | 1428786_at |
| 110190_at | 1437549_at |
| 110192_at | 1436136_at |
| 110194_at | 1435829_at |
| 110198_at | 1416490_at |
| 110201_at | 1447993_a_at |
| 110204_at | 1455737_at |
| 110207_at | 1436612_at |
| 110214_at | 1421221_at |
| 110217_at | 1417251_at |
| 110221_at | 1455225_at |
| 110222_at | 1441987_at |
| 110223_at | 1451704_at |
| 110229_at | 1424965_at |
| 110232_at | 1438466_at |
| 110234_at | 1456721_at |
| 110236_at | 1437304_at |
| 110241_at | 1444461_at |
| 110246_at | 1460371_at |
| 110259_at | 1418168_at |
| 110261_at | 1437888_at |
| 110262_at | 1434123_at |
| 110270_at | 1417844_at |
| 110273_at | 1460435_at |
| 110278_at | 1432091_a_at |
| 110279_at | 1451066_at |
| 110284_at | 1429454_at |
| 110287_at | 1427214_at |
| 110289_at | 1424400_a_at |
| 110290_at | 1435004_at |
| 110292_at | 1418706_at |
| 110293_at | 1417666_at |
| 110294_at | 1428981_at |
| 110295_at | 1424376_at |
| 110296_at | 1428151_x_at |
| 110298_at | 1432492_a_at |
| 110299_at | 1452860_at |
| 110300_at | 1418853_at |
| 110305_at | 1420977_at |
| 110306_at | 1436163_at |
| 110307_at | 1429322_at |
| 110308_at | 1452944_at |
| 110309_at | 1433646_at |
| 110310_at | 1434476_at |
| 110311_at | 1417278_a_at |
| 110312_at | 1422930_at |
| 110315_at | 1451039_at |
| 110320_at | 1427162_a_at |

| | |
|---|---|
| 110324_at | 1424106_at |
| 110327_at | 1428403_at |
| 110328_at | 1417277_at |
| 110330_at | 1427014_at |
| 110331_at | 1426832_at |
| 110333_at | 1416259_at |
| 110334_at | 1426812_a_at |
| 110335_at | 1424188_at |
| 110336_at | 1433914_at |
| 110338_at | 1454730_at |
| 110339_at | 1453103_at |
| 110343_f_at | 1426518_at |
| 110344_at | 1428422_at |
| 110346_at | 1455025_at |
| 110353_at | 1424545_at |
| 110357_at | 1456206_at |
| 110358_at | 1428273_at |
| 110363_at | 1428256_at |
| 110367_at | 1425345_at |
| 110368_at | 1454755_at |
| 110371_at | 1446198_at |
| 110374_at | 1426954_at |
| 110377_at | 1433975_at |
| 110379_at | 1451668_at |
| 110380_at | 1425400_a_at |
| 110381_at | 1420819_at |
| 110384_at | 1424707_at |
| 110392_at | 1435752_s_at |
| 110396_at | 1435036_at |
| 110397_at | 1435283_s_at |
| 110398_f_at | 1437153_at |
| 110400_f_at | 1436794_at |
| 110406_at | 1434267_at |
| 110415_f_at | 1433865_at |
| 110417_at | 1423403_at |
| 110420_at | 1460244_at |
| 110422_at | 1451376_at |
| 110431_at | 1454902_at |
| 110432_at | 1419320_at |
| 110434_at | 1443908_at |
| 110435_at | 1426771_at |
| 110439_at | 1427411_s_at |
| 110440_at | 1450411_at |
| 110441_at | 1435265_at |
| 110443_at | 1455249_at |
| 110444_at | 1422822_at |
| 110445_at | 1455648_at |
| 110446_at | 1434843_at |
| 110447_at | 1434635_at |
| 110450_at | 1433610_at |
| 110451_at | 1426505_at |
| 110454_at | 1434473_at |
| 110455_at | 1451415_at |
| 110456_at | 1424815_at |
| 110457_at | 1434736_at |
| 110458_at | 1442057_at |

| | |
|---|---|
| 110463_at | 1439784_at |
| 110465_at | 1434136_at |
| 110466_at | 1429194_at |
| 110473_at | 1439055_at |
| 110474_at | 1454911_at |
| 110476_at | 1440914_s_at |
| 110478_at | 1454827_at |
| 110491_at | 1427322_at |
| 110494_at | 1429686_at |
| 110495_at | 1436606_at |
| 110502_at | 1437084_at |
| 110504_at | 1452947_at |
| 110506_at | 1438472_at |
| 110507_at | 1441782_at |
| 110508_at | 1425180_at |
| 110510_at | 1447954_at |
| 110512_at | 1440246_at |
| 110515_at | 1455731_at |
| 110516_at | 1441625_at |
| 110517_at | 1430652_at |
| 110519_at | 1442241_at |
| 110520_at | 1436868_at |
| 110521_at | 1456880_at |
| 110522_at | 1441189_at |
| 110523_at | 1442780_at |
| 110524_at | 1450265_at |
| 110527_at | 1440729_at |
| 110529_at | 1457275_at |
| 110530_at | 1441136_at |
| 110532_at | 1444480_at |
| 110533_at | 1444756_at |
| 110542_at | 1441027_at |
| 110543_at | 1436548_at |
| 110544_at | 1440935_at |
| 110547_at | 1447973_at |
| 110548_at | 1428996_at |
| 110549_at | 1444077_at |
| 110554_at | 1437866_at |
| 110562_at | 1439844_at |
| 110566_at | 1458256_at |
| 110567_at | 1429959_at |
| 110573_at | 1456027_at |
| 110574_at | 1429956_at |
| 110575_at | 1457967_at |
| 110576_at | 1418839_at |
| 110579_at | 1438238_at |
| 110580_at | 1455222_a_at |
| 110584_at | 1426513_at |
| 110590_at | 1435750_at |
| 110591_at | 1418461_at |
| 110592_at | 1456632_at |
| 110593_at | 1437595_at |
| 110599_at | 1436217_at |
| 110600_at | 1447786_at |
| 110601_at | 1452325_at |
| 110603_at | 1421330_at |

| | |
|---|---|
| 110611_at | 1440875_a_at |
| 110613_at | 1425123_at |
| 110616_at | 1452960_at |
| 110622_at | 1440883_at |
| 110623_at | 1458619_at |
| 110624_at | 1444548_at |
| 110625_at | 1442251_at |
| 110630_at | 1434188_at |
| 110631_at | 1456871_a_at |
| 110635_at | 1455552_at |
| 110637_at | 1455542_at |
| 110641_at | 1451543_at |
| 110649_at | 1435180_at |
| 110652_at | 1434200_at |
| 110653_at | 1434841_at |
| 110654_at | 1450726_at |
| 110657_at | 1456354_at |
| 110661_at | 1459907_a_at |
| 110662_at | 1457793_a_at |
| 110670_at | 1425814_a_at |
| 110672_at | 1419605_at |
| 110674_at | 1455026_at |
| 110677_at | 1459989_at |
| 110678_at | 1428564_at |
| 110679_at | 1425193_at |
| 110680_at | 1428009_a_at |
| 110682_at | 1423989_at |
| 110686_at | 1439153_at |
| 110688_at | 1417811_at |
| 110690_at | 1452888_at |
| 110692_at | 1418475_at |
| 110694_at | 1437178_at |
| 110696_at | 1437754_at |
| 110701_at | 1436507_at |
| 110702_at | 1455671_at |
| 110703_at | 1456120_at |
| 110704_at | 1431128_at |
| 110706_at | 1419709_at |
| 110707_at | 1440331_at |
| 110713_at | 1424692_at |
| 110715_at | 1437113_s_at |
| 110716_at | 1460611_at |
| 110721_at | 1420862_at |
| 110722_at | 1437319_at |
| 110727_at | 1460598_at |
| 110728_at | 1427892_at |
| 110732_at | 1429652_at |
| 110737_at | 1438010_at |
| 110738_at | 1435144_at |
| 110740_at | 1417545_at |
| 110741_at | 1451688_s_at |
| 110743_at | 1455050_at |
| 110748_at | 1426217_at |
| 110750_at | 1448413_at |
| 110752_at | 1456087_at |
| 110753_at | 1424982_a_at |

| | |
|---|---|
| 110754_at | 1424463_at |
| 110755_at | 1434185_at |
| 110756_at | 1454628_at |
| 110758_at | 1460197_a_at |
| 110760_at | 1452970_at |
| 110761_at | 1455031_at |
| 110765_at | 1451277_at |
| 110766_at | 1429498_at |
| 110768_at | 1448979_at |
| 110770_at | 1452798_s_at |
| 110772_at | 1417425_at |
| 110774_at | 1454867_at |
| 110777_at | 1424148_a_at |
| 110789_at | 1437539_at |
| 110790_at | 1434141_at |
| 110793_at | 1423874_at |
| 110806_at | 1419183_at |
| 110807_at | 1435111_at |
| 110809_at | 1424334_at |
| 110817_at | 1424061_at |
| 110823_s_at | 1426499_at |
| 110824_at | 1426340_at |
| 110826_at | 1435127_a_at |
| 110828_at | 1455729_at |
| 110829_at | 1434470_at |
| 110830_at | 1451174_at |
| 110835_at | 1416359_at |
| 110836_at | 1452653_at |
| 110840_at | 1438203_at |
| 110841_at | 1424644_at |
| 110842_at | 1451423_at |
| 110845_at | 1423647_a_at |
| 110846_at | 1428673_at |
| 110851_r_at | 1428114_at |
| 110855_at | 1428095_a_at |
| 110857_at | 1451279_at |
| 110859_at | 1424159_at |
| 110863_at | 1452868_at |
| 110957_at | 1437041_at |
| 110960_at | 1434885_at |
| 110962_at | 1424581_at |
| 110966_at | 1434584_a_at |
| 110969_at | 1435696_s_at |
| 110972_at | 1426424_at |
| 110980_at | 1435050_at |
| 110982_at | 1418739_at |
| 110983_at | 1454956_at |
| 110985_at | 1434105_at |
| 110986_at | 1435085_at |
| 110987_at | 1455591_at |
| 110995_at | 1417857_at |
| 110999_at | 1434710_at |
| 111002_at | 1419126_at |
| 111005_at | 1425207_at |
| 111006_at | 1435043_at |
| 111007_at | 1425712_at |

| | |
|---|---|
| 111008_at | 1429292_a_at |
| 111012_at | 1433797_at |
| 111015_at | 1425185_at |
| 111016_at | 1424962_at |
| 111020_at | 1427982_s_at |
| 111022_at | 1444111_at |
| 111026_at | 1424366_at |
| 111027_at | 1417650_at |
| 111030_at | 1449385_at |
| 111031_at | 1426488_at |
| 111033_at | 1449817_at |
| 111035_at | 1458442_at |
| 111037_at | 1434915_s_at |
| 111039_at | 1434655_at |
| 111041_at | 1417590_at |
| 111042_at | 1450133_at |
| 111048_at | 1436503_at |
| 111054_at | 1424493_s_at |
| 111055_at | 1439880_at |
| 111057_at | 1426452_a_at |
| 111058_at | 1426706_s_at |
| 111060_at | 1433911_at |
| 111061_at | 1418923_at |
| 111062_at | 1424652_at |
| 111066_at | 1452464_a_at |
| 111067_at | 1436021_at |
| 111068_at | 1436132_at |
| 111070_at | 1437538_at |
| 111071_at | 1428426_s_at |
| 111072_at | 1448937_at |
| 111073_at | 1455549_at |
| 111078_at | 1439551_at |
| 111079_at | 1444494_at |
| 111082_at | 1435037_at |
| 111085_at | 1430673_a_at |
| 111086_at | 1445932_at |
| 111087_at | 1458582_at |
| 111090_at | 1430353_at |
| 111092_at | 1436006_at |
| 111094_at | 1441380_at |
| 111097_at | 1434847_at |
| 111103_at | 1435592_at |
| 111105_at | 1428329_a_at |
| 111109_r_at | 1446434_at |
| 111110_at | 1443669_at |
| 111112_at | 1447222_at |
| 111117_at | 1435119_at |
| 111118_at | 1435878_at |
| 111120_at | 1436725_at |
| 111122_at | 1422920_at |
| 111128_at | 1440008_at |
| 111133_at | 1456113_at |
| 111136_at | 1444703_at |
| 111138_at | 1424645_at |
| 111139_at | 1458541_at |
| 111140_at | 1453378_at |

| | |
|---|---|
| 111144_at | 1444305_at |
| 111148_at | 1444234_at |
| 111153_at | 1441002_at |
| 111155_at | 1421274_at |
| 111156_at | 1420639_at |
| 111157_at | 1430671_a_at |
| 111162_f_at | 1416075_at |
| 111163_at | 1419040_at |
| 111164_at | 1438806_at |
| 111165_at | 1431364_a_at |
| 111168_at | 1453837_at |
| 111170_at | 1455611_at |
| 111174_at | 1430365_at |
| 111182_at | 1455294_at |
| 111184_at | 1454883_at |
| 111185_at | 1460413_s_at |
| 111186_at | 1458499_at |
| 111190_at | 1435253_at |
| 111195_at | 1442775_at |
| 111197_at | 1453494_at |
| 111201_at | 1459067_at |
| 111203_at | 1437477_at |
| 111205_at | 1436912_at |
| 111207_at | 1453886_a_at |
| 111208_at | 1436385_at |
| 111211_at | 1456063_at |
| 111214_at | 1455586_at |
| 111215_at | 1416539_at |
| 111224_at | 1439487_at |
| 111225_at | 1424271_at |
| 111226_at | 1435002_at |
| 111227_at | 1424286_at |
| 111233_at | 1434248_at |
| 111235_at | 1434525_at |
| 111237_at | 1435078_at |
| 111240_at | 1440224_at |
| 111241_at | 1427338_at |
| 111242_at | 1438043_at |
| 111244_at | 1443983_at |
| 111246_at | 1455221_at |
| 111247_at | 1416227_at |
| 111249_at | 1420551_at |
| 111250_at | 1443967_at |
| 111252_at | 1426841_at |
| 111254_at | 1427052_at |
| 111255_r_at | 1438770_at |
| 111258_at | 1453007_at |
| 111259_at | 1440198_at |
| 111271_at | 1426979_at |
| 111277_at | 1426317_at |
| 111279_at | 1439151_at |
| 111280_at | 1458567_at |
| 111281_at | 1434691_at |
| 111287_at | 1441793_at |
| 111289_at | 1457415_a_at |
| 111294_at | 1433512_at |

| | |
|---|---|
| 111295_at | 1418163_at |
| 111298_at | 1425431_at |
| 111305_at | 1455014_at |
| 111307_at | 1434564_at |
| 111310_at | 1436053_at |
| 111312_at | 1429383_at |
| 111313_at | 1433851_at |
| 111314_at | 1440483_at |
| 111316_at | 1436074_at |
| 111317_at | 1435303_at |
| 111322_at | 1433641_at |
| 111334_at | 1424095_at |
| 111335_at | 1429148_at |
| 111342_at | 1429516_at |
| 111354_at | 1448302_at |
| 111358_at | 1418090_at |
| 111363_at | 1423359_at |
| 111366_at | 1452706_a_at |
| 111367_at | 1417047_at |
| 111375_at | 1417827_at |
| 111380_at | 1427893_a_at |
| 111382_at | 1433592_at |
| 111389_at | 1454792_s_at |
| 111390_at | 1424016_at |
| 111391_at | 1435910_at |
| 111394_at | 1423861_at |
| 111395_at | 1455018_at |
| 111396_r_at | 1455901_at |
| 111397_at | 1459898_at |
| 111402_at | 1434621_at |
| 111407_at | 1427923_at |
| 111409_at | 1424702_a_at |
| 111412_at | 1434069_at |
| 111416_at | 1418797_at |
| 111424_at | 1428271_at |
| 111425_at | 1418637_at |
| 111426_at | 1454619_at |
| 111427_at | 1452034_at |
| 111431_at | 1417212_at |
| 111432_at | 1447923_at |
| 111434_at | 1455992_at |
| 111435_at | 1426746_at |
| 111437_at | 1451368_at |
| 111439_at | 1455083_at |
| 111440_at | 1434258_s_at |
| 111441_at | 1429163_at |
| 111444_at | 1434197_at |
| 111446_at | 1436062_at |
| 111447_at | 1426860_at |
| 111451_at | 1453369_a_at |
| 111454_at | 1434417_at |
| 111457_at | 1418998_at |
| 111458_at | 1418662_at |
| 111459_at | 1438452_at |
| 111466_at | 1447933_at |
| 111468_at | 1425252_a_at |

| | |
|---|---|
| 111469_at | 1428947_at |
| 111471_at | 1424624_at |
| 111475_at | 1428632_at |
| 111477_at | 1451379_at |
| 111480_at | 1451224_at |
| 111481_at | 1455595_at |
| 111482_at | 1419618_at |
| 111483_at | 1458369_at |
| 111485_at | 1436098_at |
| 111486_at | 1434782_at |
| 111487_at | 1436233_at |
| 111488_at | 1417549_at |
| 111489_at | 1428767_at |
| 111492_at | 1421025_at |
| 111494_at | 1424908_at |
| 111495_at | 1423913_at |
| 111498_at | 1428858_at |
| 111500_at | 1435916_at |
| 111504_at | 1429047_at |
| 111506_at | 1434175_s_at |
| 111507_at | 1451982_at |
| 111508_at | 1424466_at |
| 111510_at | 1454850_at |
| 111512_at | 1457263_at |
| 111514_at | 1439096_at |
| 111516_r_at | 1435876_at |
| 111519_at | 1460294_at |
| 111529_at | 1451589_at |
| 111532_at | 1418044_at |
| 111533_at | 1429591_at |
| 111538_at | 1417977_at |
| 111539_at | 1436313_at |
| 111540_at | 1428243_at |
| 111541_at | 1424276_at |
| 111542_at | 1434050_at |
| 111543_at | 1455566_s_at |
| 111544_at | 1450432_s_at |
| 111545_at | 1455428_at |
| 111549_at | 1455113_at |
| 111550_at | 1435682_at |
| 111551_at | 1448782_at |
| 111560_at | 1429779_at |
| 111562_at | 1440699_at |
| 111564_at | 1454939_at |
| 111565_at | 1456514_at |
| 111566_at | 1428979_at |
| 111569_at | 1418336_at |
| 111570_at | 1457264_at |
| 111573_at | 1430339_at |
| 111575_at | 1437072_at |
| 111576_at | 1431726_a_at |
| 111582_at | 1441265_at |
| 111583_at | 1441062_at |
| 111584_at | 1429147_at |
| 111591_at | 1436560_at |
| 111593_at | 1442092_at |

| | |
|---|---|
| 111594_at | 1460291_at |
| 111595_at | 1456754_at |
| 111596_at | 1441723_at |
| 111599_at | 1428761_a_at |
| 111601_at | 1444145_at |
| 111602_at | 1419373_at |
| 111611_at | 1435432_at |
| 111614_at | 1434764_at |
| 111617_at | 1445598_at |
| 111619_at | 1447409_at |
| 111620_at | 1456969_at |
| 111622_at | 1440899_at |
| 111623_at | 1454949_at |
| 111627_at | 1424888_at |
| 111630_at | 1447988_at |
| 111632_at | 1456207_at |
| 111636_at | 1436667_at |
| 111638_at | 1444506_at |
| 111639_at | 1456281_at |
| 111640_at | 1440997_at |
| 111641_at | 1453350_at |
| 111644_at | 1444413_at |
| 111645_at | 1447546_s_at |
| 111647_at | 1442118_at |
| 111648_at | 1433615_at |
| 111649_at | 1442091_at |
| 111651_at | 1428922_at |
| 111656_at | 1445505_at |
| 111659_at | 1435556_at |
| 111660_at | 1453953_at |
| 111662_at | 1451541_at |
| 111670_at | 1453322_at |
| 111672_at | 1418150_at |
| 111676_at | 1439896_at |
| 111678_at | 1437871_at |
| 111679_at | 1457687_at |
| 111681_f_at | 1434152_at |
| 111682_at | 1435141_at |
| 111689_at | 1437637_at |
| 111690_at | 1448214_at |
| 111691_at | 1428348_at |
| 111692_at | 1419507_at |
| 111694_at | 1424198_at |
| 111695_at | 1431798_a_at |
| 111697_at | 1441980_at |
| 111699_at | 1443975_at |
| 111701_at | 1419264_at |
| 111703_at | 1458695_at |
| 111706_at | 1449371_at |
| 111711_at | 1456061_at |
| 111724_at | 1457037_at |
| 111726_at | 1419048_at |
| 111727_at | 1456372_at |
| 111728_at | 1429066_at |
| 111729_at | 1455503_at |
| 111731_at | 1439085_at |

| | |
|---|---|
| 111740_at | 1426218_at |
| 111745_at | 1418381_at |
| 111747_at | 1434967_at |
| 111748_at | 1419011_at |
| 111749_at | 1449607_at |
| 111750_at | 1458439_a_at |
| 111751_at | 1439008_at |
| 111760_at | 1451447_at |
| 111762_at | 1457817_at |
| 111764_at | 1420515_a_at |
| 111765_at | 1434756_at |
| 111766_at | 1435535_at |
| 111768_at | 1439140_at |
| 111769_at | 1453479_at |
| 111770_at | 1424308_at |
| 111777_at | 1435378_at |
| 111781_at | 1426630_at |
| 111782_at | 1452918_at |
| 111783_at | 1429202_at |
| 111794_at | 1420507_a_at |
| 111802_at | 1423630_at |
| 111809_r_at | 1428574_a_at |
| 111810_at | 1423777_at |
| 111815_s_at | 1419211_s_at |
| 111816_at | 1426757_at |
| 111821_at | 1451305_at |
| 111822_at | 1431428_a_at |
| 111825_at | 1454643_at |
| 111826_at | 1418975_at |
| 111830_at | 1428691_at |
| 111833_at | 1456098_a_at |
| 111834_at | 1427078_at |
| 111835_at | 1452293_at |
| 111836_at | 1451402_at |
| 111840_at | 1427279_at |
| 111841_at | 1424524_at |
| 111842_at | 1460554_s_at |
| 111847_at | 1434707_at |
| 111852_at | 1439100_s_at |
| 111855_at | 1428594_at |
| 111859_at | 1424103_at |
| 111860_at | 1451234_at |
| 111861_at | 1433939_at |
| 111863_at | 1451912_a_at |
| 111865_at | 1436299_at |
| 111869_at | 1421018_at |
| 111875_at | 1428295_at |
| 111876_at | 1428539_at |
| 111882_r_at | 1415908_at |
| 111883_at | 1433981_s_at |
| 111887_at | 1417723_at |
| 111889_at | 1434483_at |
| 111898_at | 1431016_at |
| 111899_at | 1434886_at |
| 111902_f_at | 1418855_at |
| 111904_at | 1417799_at |

| | |
|---|---|
| 111906_at | 1429742_at |
| 111912_at | 1423810_at |
| 111916_at | 1416722_at |
| 111919_at | 1453108_at |
| 111921_at | 1424992_at |
| 111925_at | 1435361_at |
| 111928_at | 1427951_s_at |
| 111930_at | 1435311_s_at |
| 111931_at | 1434448_at |
| 111932_at | 1421117_at |
| 111933_at | 1438716_at |
| 111934_at | 1417970_at |
| 111944_at | 1435183_at |
| 111947_at | 1431302_a_at |
| 111948_at | 1417982_at |
| 111949_at | 1427946_s_at |
| 111951_at | 1441073_at |
| 111952_at | 1423291_s_at |
| 111955_at | 1434289_at |
| 111956_at | 1429356_s_at |
| 111962_at | 1440903_at |
| 111965_at | 1429679_at |
| 111966_at | 1449482_at |
| 111967_at | 1434351_at |
| 111968_at | 1436861_at |
| 111969_at | 1426450_at |
| 111971_at | 1436458_at |
| 111974_at | 1438273_at |
| 111976_at | 1435346_at |
| 111980_at | 1424653_at |
| 111981_at | 1416673_at |
| 111983_at | 1452933_at |
| 111984_at | 1429304_at |
| 111985_at | 1436556_at |
| 111997_s_at | 1416847_s_at |
| 111999_at | 1434580_at |
| 112000_at | 1433524_at |
| 112002_at | 1424688_at |
| 112003_at | 1436909_at |
| 112004_at | 1455067_at |
| 112005_at | 1455669_at |
| 112009_at | 1420655_at |
| 112012_at | 1437869_at |
| 112015_at | 1438670_at |
| 112016_at | 1441251_a_at |
| 112018_at | 1435005_at |
| 112022_at | 1427086_at |
| 112023_at | 1458115_at |
| 112024_at | 1429192_at |
| 112025_at | 1452306_at |
| 112026_at | 1454821_at |
| 112029_at | 1454935_at |
| 112032_at | 1452309_at |
| 112033_at | 1429054_at |
| 112036_at | 1440217_at |
| 112037_at | 1438486_at |

| | |
|---|---|
| 112039_at | 1438475_at |
| 112041_at | 1458722_at |
| 112044_at | 1458077_at |
| 112051_at | 1419333_at |
| 112057_at | 1448092_x_at |
| 112058_at | 1429856_at |
| 112063_at | 1443866_at |
| 112065_at | 1428700_at |
| 112068_at | 1435487_at |
| 112072_at | 1436229_at |
| 112076_at | 1432143_a_at |
| 112078_at | 1452630_at |
| 112079_at | 1455292_x_at |
| 112081_at | 1432073_at |
| 112083_at | 1448748_at |
| 112084_at | 1439831_at |
| 112087_at | 1435439_at |
| 112088_at | 1448481_at |
| 112089_at | 1441226_at |
| 112104_at | 1440247_at |
| 112105_at | 1421892_at |
| 112110_at | 1418954_at |
| 112155_at | 1434326_x_at |
| 112162_at | 1430705_at |
| 112163_at | 1451351_at |
| 112164_at | 1425697_at |
| 112166_at | 1421072_at |
| 112169_at | 1438436_at |
| 112172_at | 1435095_at |
| 112174_at | 1449891_a_at |
| 112176_at | 1435499_at |
| 112179_at | 1427472_a_at |
| 112180_at | 1429840_at |
| 112181_at | 1457054_a_at |
| 112191_at | 1437396_at |
| 112192_at | 1420374_at |
| 112193_at | 1433728_at |
| 112196_at | 1455257_at |
| 112199_f_at | 1439714_at |
| 112213_at | 1424677_at |
| 112217_at | 1430632_at |
| 112221_at | 1437264_at |
| 112223_at | 1439574_at |
| 112238_at | 1421159_at |
| 112245_at | 1434303_at |
| 112247_at | 1425642_at |
| 112251_at | 1426603_at |
| 112253_at | 1416801_at |
| 112260_at | 1432212_at |
| 112262_at | 1448759_at |
| 112263_at | 1419635_at |
| 112266_at | 1443915_at |
| 112268_at | 1437470_at |
| 112269_at | 1434157_at |
| 112271_at | 1438674_a_at |
| 112276_at | 1424503_at |

| | |
|---|---|
| 112277_at | 1418871_a_at |
| 112278_at | 1434046_at |
| 112284_at | 1436521_at |
| 112286_at | 1423942_a_at |
| 112287_at | 1427910_at |
| 112291_r_at | 1434744_at |
| 112292_at | 1429580_x_at |
| 112294_at | 1418891_a_at |
| 112302_at | 1428350_at |
| 112303_at | 1452862_at |
| 112304_at | 1416289_at |
| 112305_at | 1425333_at |
| 112306_at | 1419971_s_at |
| 112307_at | 1433703_s_at |
| 112308_at | 1436741_at |
| 112309_at | 1426367_at |
| 112311_at | 1422739_at |
| 112312_at | 1450730_at |
| 112313_at | 1418048_at |
| 112314_at | 1451444_s_at |
| 112315_at | 1451455_at |
| 112318_at | 1428695_at |
| 112320_at | 1433500_at |
| 112323_at | 1425151_a_at |
| 112325_at | 1452039_a_at |
| 112331_at | 1451558_at |
| 112335_at | 1452174_at |
| 112336_at | 1440830_at |
| 112337_at | 1455308_at |
| 112340_at | 1435529_at |
| 112344_at | 1438195_at |
| 112345_at | 1447486_at |
| 112346_at | 1433837_at |
| 112348_at | 1434327_at |
| 112351_at | 1434509_at |
| 112355_at | 1435241_at |
| 112358_at | 1454975_at |
| 112361_at | 1426902_at |
| 112364_at | 1421963_a_at |
| 112366_at | 1426781_at |
| 112367_at | 1417637_a_at |
| 112372_at | 1434013_at |
| 112374_at | 1427245_at |
| 112375_at | 1454909_at |
| 112376_at | 1417569_at |
| 112379_at | 1433759_at |
| 112385_at | 1423878_at |
| 112387_at | 1418769_at |
| 112390_at | 1418015_at |
| 112392_at | 1453187_at |
| 112395_at | 1434192_at |
| 112396_at | 1426694_at |
| 112398_at | 1452615_s_at |
| 112402_at | 1455306_at |
| 112403_at | 1419853_a_at |
| 112407_at | 1424779_at |

| | |
|---|---|
| 112410_at | 1435174_at |
| 112412_at | 1434385_at |
| 112413_at | 1426882_at |
| 112414_at | 1426259_at |
| 112420_at | 1424420_at |
| 112421_at | 1455118_at |
| 112422_at | 1435201_at |
| 112423_at | 1455262_at |
| 112429_at | 1456611_at |
| 112431_at | 1426684_at |
| 112432_at | 1436182_at |
| 112433_at | 1460255_at |
| 112437_at | 1422211_a_at |
| 112440_at | 1435356_at |
| 112441_at | 1429004_at |
| 112442_at | 1422721_at |
| 112447_at | 1455376_at |
| 112448_at | 1441999_at |
| 112452_at | 1426874_at |
| 112453_at | 1451218_at |
| 112454_at | 1421748_a_at |
| 112455_at | 1429157_at |
| 112459_at | 1427212_at |
| 112461_at | 1417315_at |
| 112465_at | 1436778_at |
| 112466_at | 1427080_at |
| 112467_at | 1427230_at |
| 112468_at | 1418230_a_at |
| 112471_at | 1433998_at |
| 112473_at | 1439219_at |
| 112479_at | 1455071_at |
| 112481_at | 1452323_at |
| 112482_at | 1456483_at |
| 112484_at | 1418535_at |
| 112485_at | 1450174_at |
| 112486_at | 1424794_at |
| 112487_at | 1427156_s_at |
| 112489_at | 1430606_at |
| 112490_at | 1436965_at |
| 112491_at | 1435032_at |
| 112493_at | 1436221_at |
| 112499_at | 1437057_at |
| 112500_at | 1435175_at |
| 112501_at | 1455515_at |
| 112505_at | 1452663_at |
| 112506_at | 1427139_at |
| 112509_at | 1434667_at |
| 112510_at | 1427103_at |
| 112512_at | 1452921_at |
| 112513_at | 1460405_at |
| 112515_at | 1434622_at |
| 112516_at | 1427033_at |
| 112531_at | 1427508_at |
| 112638_at | 1438327_at |
| 112644_at | 1460448_s_at |
| 112646_at | 1437350_at |

| | |
|---|---|
| 112647_at | 1429034_at |
| 112652_at | 1418827_at |
| 112655_at | 1444578_at |
| 112659_at | 1448614_at |
| 112660_at | 1458470_at |
| 112661_at | 1436546_at |
| 112662_at | 1438300_at |
| 112664_at | 1430330_at |
| 112668_at | 1456395_at |
| 112669_at | 1449375_at |
| 112671_at | 1451564_at |
| 112678_at | 1454773_at |
| 112679_at | 1435454_a_at |
| 112688_at | 1459913_at |
| 112689_at | 1422925_s_at |
| 112690_at | 1438310_at |
| 112695_at | 1434933_at |
| 112696_at | 1434774_at |
| 112702_at | 1452071_at |
| 112711_at | 1421243_at |
| 112723_at | 1429683_at |
| 112724_at | 1425031_at |
| 112730_at | 1424574_at |
| 112731_at | 1438677_at |
| 112734_at | 1417958_at |
| 112735_at | 1417060_at |
| 112736_at | 1421099_at |
| 112737_at | 1429656_at |
| 112743_at | 1434706_at |
| 112744_at | 1435391_at |
| 112745_at | 1423468_at |
| 112747_at | 1425025_at |
| 112750_at | 1434903_s_at |
| 112751_s_at | 1451681_at |
| 112753_at | 1427215_at |
| 112754_at | 1419510_at |
| 112758_at | 1419166_at |
| 112759_g_at | 1455005_s_at |
| 112763_at | 1426685_a_at |
| 112769_at | 1434223_at |
| 112778_at | 1452292_at |
| 112780_at | 1429879_at |
| 112792_at | 1436312_at |
| 112793_at | 1427207_s_at |
| 112794_at | 1452190_at |
| 112802_at | 1460363_at |
| 112804_at | 1451235_at |
| 112809_at | 1426515_a_at |
| 112814_at | 1434203_at |
| 112816_at | 1455868_a_at |
| 112822_at | 1437399_at |
| 112823_at | 1440194_at |
| 112824_at | 1426943_at |
| 112825_at | 1428674_at |
| 112826_at | 1456506_at |
| 112829_at | 1429237_at |

| | |
|---|---|
| 112830_at | 1436805_at |
| 112835_at | 1416993_at |
| 112837_at | 1441962_at |
| 112838_at | 1426960_a_at |
| 112842_at | 1434221_at |
| 112843_at | 1420963_at |
| 112846_at | 1428817_at |
| 112848_at | 1456030_at |
| 112859_at | 1434270_at |
| 112864_at | 1454647_at |
| 112865_at | 1417411_at |
| 112868_at | 1454044_a_at |
| 112869_at | 1429711_at |
| 112874_at | 1452056_s_at |
| 112876_at | 1455436_at |
| 112880_at | 1417282_at |
| 112883_at | 1454982_at |
| 112887_at | 1433961_at |
| 112890_at | 1426277_at |
| 112892_at | 1428910_at |
| 112894_at | 1457704_at |
| 112895_at | 1443886_at |
| 112901_at | 1456627_at |
| 112902_at | 1426811_at |
| 112903_at | 1450639_at |
| 112904_at | 1435273_at |
| 112905_at | 1454864_at |
| 112912_at | 1419046_at |
| 112914_at | 1417671_at |
| 112915_at | 1417439_at |
| 112916_at | 1435067_at |
| 112917_at | 1436592_at |
| 112920_at | 1435115_at |
| 112921_at | 1419072_at |
| 112923_at | 1418544_at |
| 112924_at | 1455022_at |
| 112925_at | 1423134_at |
| 112927_at | 1456394_at |
| 112930_at | 1454761_at |
| 112933_at | 1450470_at |
| 112934_at | 1435256_at |
| 112935_at | 1427267_at |
| 112938_at | 1425802_a_at |
| 112939_at | 1424960_at |
| 112942_at | 1434723_at |
| 112943_at | 1423304_a_at |
| 112945_at | 1420552_s_at |
| 112946_at | 1429508_at |
| 112949_at | 1434260_at |
| 112951_at | 1437394_at |
| 112957_at | 1452379_at |
| 112962_at | 1434963_at |
| 112964_at | 1434059_at |
| 112966_at | 1455112_at |
| 112967_at | 1419028_at |
| 112968_at | 1437071_at |

| | |
|---|---|
| 112971_at | 1451450_at |
| 112972_at | 1428005_at |
| 112974_at | 1423447_at |
| 112975_at | 1435245_at |
| 112976_at | 1421087_at |
| 112979_at | 1455770_at |
| 112981_at | 1452890_at |
| 112983_at | 1429313_at |
| 112984_at | 1450281_a_at |
| 112986_at | 1439542_at |
| 112987_at | 1450441_at |
| 112988_at | 1434026_at |
| 112990_at | 1460560_at |
| 112993_at | 1435455_at |
| 112996_at | 1427041_at |
| 112998_at | 1452337_at |
| 112999_at | 1424862_s_at |
| 113000_at | 1457269_at |
| 113001_at | 1455141_at |
| 113003_at | 1434844_at |
| 113006_r_at | 1455388_at |
| 113007_at | 1415886_at |
| 113008_at | 1434048_at |
| 113011_at | 1435354_at |
| 113013_s_at | 1426432_a_at |
| 113014_at | 1422893_at |
| 113015_at | 1418542_s_at |
| 113016_at | 1455522_at |
| 113017_at | 1455435_s_at |
| 113019_at | 1438682_at |
| 113021_at | 1433761_at |
| 113022_at | 1460568_at |
| 113029_at | 1439808_at |
| 113030_at | 1455626_at |
| 113034_at | 1424867_a_at |
| 113035_at | 1456123_at |
| 113037_at | 1417448_at |
| 113038_at | 1434965_at |
| 113040_at | 1438208_at |
| 113041_at | 1434677_at |
| 113047_at | 1416846_a_at |
| 113048_at | 1419467_at |
| 113050_at | 1448435_at |
| 113051_at | 1434849_at |
| 113053_at | 1417159_at |
| 113054_at | 1440327_at |
| 113055_at | 1454855_at |
| 113057_at | 1424866_at |
| 113058_at | 1450556_at |
| 113061_at | 1434715_at |
| 113062_at | 1438712_at |
| 113068_at | 1435741_at |
| 113070_at | 1432834_at |
| 113071_at | 1438719_at |
| 113072_at | 1426634_at |
| 113077_at | 1439570_at |

| | |
|---|---|
| 113078_at | 1423375_at |
| 113080_at | 1436633_at |
| 113089_at | 1450754_at |
| 113090_at | 1427337_at |
| 113098_at | 1424961_at |
| 113101_f_at | 1416549_at |
| 113106_at | 1422740_at |
| 113107_at | 1448067_at |
| 113108_at | 1442739_at |
| 113110_at | 1425074_at |
| 113112_at | 1422309_a_at |
| 113113_at | 1421681_at |
| 113115_at | 1424884_at |
| 113116_at | 1454804_at |
| 113117_at | 1451473_a_at |
| 113118_at | 1423926_at |
| 113119_at | 1427022_at |
| 113125_at | 1422103_a_at |
| 113130_at | 1434508_at |
| 113131_at | 1428064_at |
| 113133_at | 1423512_at |
| 113135_at | 1451343_at |
| 113136_at | 1430769_s_at |
| 113139_at | 1426640_s_at |
| 113142_at | 1428561_at |
| 113143_at | 1425534_at |
| 113146_at | 1449162_at |
| 113147_at | 1436811_at |
| 113148_at | 1427112_at |
| 113151_at | 1423105_a_at |
| 113153_at | 1428598_at |
| 113155_at | 1450654_a_at |
| 113156_at | 1418999_at |
| 113160_at | 1415750_at |
| 113161_at | 1434750_at |
| 113162_at | 1426625_at |
| 113164_at | 1423797_at |
| 113172_at | 1416537_at |
| 113173_at | 1452874_at |
| 113174_at | 1451098_at |
| 113176_at | 1460358_s_at |
| 113178_at | 1417050_at |
| 113179_at | 1448945_at |
| 113180_at | 1417245_at |
| 113181_r_at | 1420840_at |
| 113183_at | 1417466_at |
| 113191_at | 1453197_at |
| 113195_at | 1452100_at |
| 113196_at | 1451344_at |
| 113200_at | 1438578_a_at |
| 113203_at | 1428413_at |
| 113207_at | 1454644_at |
| 113208_at | 1449113_at |
| 113210_at | 1424223_at |
| 113212_at | 1417264_at |
| 113223_at | 1455135_at |

| | |
|---|---|
| 113226_at | 1449267_at |
| 113228_at | 1448993_at |
| 113229_at | 1424575_at |
| 113232_at | 1430989_a_at |
| 113235_at | 1419703_at |
| 113239_at | 1424810_at |
| 113240_at | 1424721_at |
| 113241_at | 1456763_at |
| 113242_at | 1436877_at |
| 113243_at | 1455661_at |
| 113244_at | 1451363_a_at |
| 113246_at | 1436520_at |
| 113247_at | 1452126_at |
| 113248_at | 1426501_a_at |
| 113249_at | 1458849_at |
| 113250_at | 1426965_at |
| 113251_at | 1429773_at |
| 113256_at | 1424550_at |
| 113258_at | 1435181_at |
| 113260_at | 1429118_a_at |
| 113261_at | 1436039_at |
| 113262_at | 1424664_at |
| 113266_at | 1429733_at |
| 113267_at | 1423330_at |
| 113270_at | 1438229_at |
| 113277_at | 1436208_at |
| 113278_at | 1420996_at |
| 113279_at | 1434770_at |
| 113287_at | 1437900_at |
| 113295_at | 1423761_at |
| 113296_at | 1427069_at |
| 113298_at | 1434929_at |
| 113300_at | 1439538_at |
| 113302_at | 1423555_a_at |
| 113303_at | 1435538_at |
| 113304_at | 1437357_at |
| 113306_at | 1434315_at |
| 113311_r_at | 1434408_at |
| 113312_at | 1418183_a_at |
| 113314_at | 1460434_at |
| 113316_at | 1434331_at |
| 113318_at | 1434480_at |
| 113320_at | 1436196_at |
| 113323_at | 1434054_at |
| 113324_at | 1428622_at |
| 113325_r_at | 1460455_at |
| 113327_at | 1456328_at |
| 113329_at | 1436407_at |
| 113330_at | 1436735_at |
| 113332_at | 1435090_at |
| 113334_at | 1458415_at |
| 113335_at | 1437868_at |
| 113336_at | 1424834_s_at |
| 113338_at | 1429584_at |
| 113339_at | 1440879_at |
| 113345_at | 1460619_at |

| | |
|---|---|
| 113346_at | 1425596_at |
| 113347_at | 1436328_at |
| 113349_at | 1425607_at |
| 113352_at | 1440239_at |
| 113421_at | 1435692_at |
| 113422_at | 1439279_at |
| 113423_at | 1441093_at |
| 113424_at | 1441099_at |
| 113426_at | 1460078_at |
| 113427_at | 1417527_at |
| 113428_at | 1431067_at |
| 113429_at | 1421275_s_at |
| 113431_at | 1431030_a_at |
| 113432_at | 1442895_at |
| 113433_at | 1440389_at |
| 113434_at | 1440539_at |
| 113435_at | 1418882_at |
| 113436_at | 1437952_at |
| 113437_at | 1441398_at |
| 113439_at | 1440702_at |
| 113441_at | 1434494_at |
| 113444_at | 1439681_at |
| 113445_at | 1441661_at |
| 113446_at | 1429743_at |
| 113447_at | 1442231_at |
| 113449_at | 1441414_at |
| 113453_at | 1442602_at |
| 113454_at | 1442677_at |
| 113455_at | 1451574_at |
| 113460_at | 1427987_at |
| 113462_at | 1437745_at |
| 113464_at | 1460126_at |
| 113465_at | 1439895_at |
| 113467_at | 1455457_at |
| 113468_at | 1426568_at |
| 113469_at | 1418941_at |
| 113472_at | 1418356_at |
| 113474_at | 1419489_at |
| 113475_at | 1441976_at |
| 113478_at | 1443946_s_at |
| 113479_at | 1439550_at |
| 113480_at | 1444041_at |
| 113482_at | 1440501_at |
| 113485_at | 1458104_a_at |
| 113486_at | 1440277_at |
| 113487_at | 1447141_at |
| 113489_at | 1435188_at |
| 113490_at | 1440199_at |
| 113491_at | 1430096_at |
| 113492_at | 1442477_at |
| 113493_at | 1443463_at |
| 113494_at | 1442405_at |
| 113496_at | 1435498_at |
| 113497_at | 1442515_at |
| 113498_at | 1437059_at |
| 113499_at | 1437770_at |

| | |
|---|---|
| 113500_at | 1456920_at |
| 113501_at | 1442460_at |
| 113502_at | 1442668_at |
| 113503_at | 1440420_at |
| 113504_at | 1428105_at |
| 113505_at | 1442313_at |
| 113506_at | 1442990_at |
| 113508_at | 1441408_at |
| 113509_at | 1441379_at |
| 113510_s_at | 1441222_x_at |
| 113511_at | 1442688_at |
| 113512_at | 1440401_at |
| 113515_at | 1441154_at |
| 113516_at | 1440538_at |
| 113517_at | 1456410_at |
| 113521_at | 1441168_at |
| 113525_at | 1435838_at |
| 113528_at | 1439819_at |
| 113529_at | 1446320_at |
| 113531_at | 1453780_at |
| 113533_at | 1419571_at |
| 113537_at | 1442019_at |
| 113538_at | 1422922_at |
| 113543_at | 1417692_at |
| 113544_at | 1429079_a_at |
| 113545_at | 1434893_at |
| 113550_at | 1417515_at |
| 113552_at | 1423266_at |
| 113555_at | 1451918_a_at |
| 113562_at | 1421875_a_at |
| 113563_at | 1449020_at |
| 113564_at | 1452626_a_at |
| 113567_at | 1427100_at |
| 113569_at | 1417891_at |
| 113570_at | 1426453_at |
| 113574_at | 1419292_at |
| 113575_at | 1426371_at |
| 113577_at | 1421316_at |
| 113578_at | 1452775_at |
| 113579_at | 1434737_at |
| 113580_at | 1425262_at |
| 113587_at | 1433884_at |
| 113590_at | 1436388_a_at |
| 113591_at | 1434550_at |
| 113592_at | 1450111_a_at |
| 113599_at | 1427324_at |
| 113600_at | 1460349_at |
| 113604_at | 1428556_at |
| 113611_at | 1450921_at |
| 113613_at | 1428605_at |
| 113621_at | 1437532_at |
| 113624_at | 1452971_at |
| 113627_at | 1433713_at |
| 113629_at | 1416976_at |
| 113632_at | 1417230_at |
| 113633_at | 1424203_at |

| | |
|---|---|
| 113636_at | 1417467_a_at |
| 113638_at | 1416824_at |
| 113639_r_at | 1424946_a_at |
| 113641_at | 1434382_at |
| 113642_at | 1430540_at |
| 113643_at | 1426828_at |
| 113648_at | 1434923_at |
| 113650_at | 1421031_a_at |
| 113654_at | 1453344_at |
| 113657_at | 1448582_at |
| 113662_at | 1428203_at |
| 113664_at | 1436635_at |
| 113665_at | 1422026_at |
| 113668_at | 1429957_at |
| 113672_at | 1421860_at |
| 113676_at | 1431759_at |
| 113681_at | 1442298_at |
| 113682_at | 1442053_at |
| 113684_at | 1428831_at |
| 113686_at | 1454994_at |
| 113691_at | 1435874_at |
| 113693_at | 1424284_at |
| 113694_at | 1455597_at |
| 113695_at | 1429204_at |
| 113699_at | 1419641_at |
| 113701_at | 1451476_at |
| 113702_at | 1434867_at |
| 113705_at | 1454981_at |
| 113707_at | 1460343_at |
| 113708_at | 1426542_at |
| 113712_at | 1457798_at |
| 113713_at | 1433649_at |
| 113714_at | 1440922_at |
| 113721_at | 1435072_at |
| 113722_at | 1436630_at |
| 113724_at | 1455660_at |
| 113727_at | 1418032_at |
| 113731_at | 1450879_at |
| 113732_at | 1430657_at |
| 113733_at | 1435161_at |
| 113734_at | 1427007_at |
| 113735_at | 1449150_at |
| 113741_at | 1436396_at |
| 113747_at | 1435968_at |
| 113748_at | 1418730_at |
| 113750_at | 1416527_at |
| 113752_at | 1436524_at |
| 113756_at | 1418291_at |
| 113759_at | 1422474_at |
| 113761_at | 1429012_at |
| 113763_r_at | 1425599_a_at |
| 113764_at | 1435262_at |
| 113765_at | 1454983_at |
| 113766_at | 1436984_at |
| 113768_at | 1424357_at |
| 113769_at | 1434395_at |

| | |
|---|---|
| 113772_at | 1453220_at |
| 113775_at | 1428920_at |
| 113778_at | 1425090_s_at |
| 113783_at | 1448150_at |
| 113784_at | 1440576_at |
| 113785_at | 1452872_at |
| 113788_at | 1452253_at |
| 113793_at | 1452470_at |
| 113794_at | 1444580_at |
| 113798_at | 1438033_at |
| 113799_at | 1435401_at |
| 113802_at | 1458195_at |
| 113803_at | 1457277_at |
| 113804_at | 1445588_at |
| 113807_at | 1430051_at |
| 113808_at | 1452016_at |
| 113811_at | 1422639_at |
| 113813_at | 1439178_at |
| 113814_at | 1436647_at |
| 113815_at | 1434747_at |
| 113816_at | 1447359_at |
| 113820_i_at | 1455213_at |
| 113821_at | 1438147_at |
| 113822_at | 1453495_at |
| 113823_at | 1438323_at |
| 113827_f_at | 1442120_at |
| 113829_at | 1420714_at |
| 113831_at | 1428893_at |
| 113832_at | 1455087_at |
| 113833_at | 1442281_at |
| 113834_at | 1437698_at |
| 113835_s_at | 1451635_at |
| 113839_at | 1456217_at |
| 113841_at | 1427182_s_at |
| 113843_at | 1460736_at |
| 113844_at | 1419788_at |
| 113845_at | 1457808_at |
| 113847_at | 1456397_at |
| 113848_at | 1435488_at |
| 113849_at | 1426545_at |
| 113851_at | 1429459_at |
| 113853_at | 1445576_at |
| 113855_at | 1439192_at |
| 113857_at | 1455369_at |
| 113858_at | 1455188_at |
| 113860_at | 1436539_at |
| 113861_at | 1419003_at |
| 113862_at | 1435953_at |
| 113865_at | 1439965_at |
| 113866_at | 1440455_at |
| 113868_at | 1419710_at |
| 113869_at | 1434522_at |
| 113874_at | 1435500_at |
| 113875_at | 1441638_at |
| 113880_at | 1444932_at |
| 113883_at | 1460080_at |

| | |
|---|---|
| 113888_at | 1451397_at |
| 113891_at | 1419224_at |
| 113892_at | 1443672_at |
| 113896_at | 1439983_a_at |
| 113898_at | 1420734_at |
| 113899_at | 1436620_at |
| 113904_at | 1434461_at |
| 113908_at | 1437466_at |
| 113910_at | 1435351_at |
| 113916_at | 1418818_at |
| 113918_at | 1449092_at |
| 113919_at | 1424439_at |
| 113921_at | 1455839_at |
| 113926_at | 1438404_at |
| 113936_at | 1417957_a_at |
| 113938_at | 1452973_at |
| 113939_at | 1454211_a_at |
| 113940_at | 1418860_a_at |
| 113953_at | 1449074_at |
| 113957_at | 1418346_at |
| 113959_at | 1423576_a_at |
| 113966_at | 1434927_at |
| 113970_at | 1416414_at |
| 113978_at | 1423765_at |
| 113981_at | 1428671_at |
| 113982_at | 1426155_a_at |
| 113985_at | 1424354_at |
| 113989_at | 1429311_at |
| 113990_at | 1418095_at |
| 113992_at | 1419317_x_at |
| 113994_at | 1450532_at |
| 114002_at | 1424191_a_at |
| 114018_at | 1448449_at |
| 114021_at | 1423887_a_at |
| 114022_r_at | 1440844_at |
| 114023_at | 1439152_at |
| 114024_at | 1418948_at |
| 114025_at | 1440878_at |
| 114026_at | 1436070_at |
| 114034_at | 1438747_at |
| 114036_at | 1444099_at |
| 114037_at | 1449174_at |
| 114038_at | 1428470_at |
| 114040_at | 1436865_at |
| 114042_at | 1450066_at |
| 114043_at | 1435670_at |
| 114044_r_at | 1434958_at |
| 114045_at | 1438731_at |
| 114050_at | 1454936_at |
| 114055_at | 1435104_at |
| 114056_at | 1438419_at |
| 114058_at | 1453226_at |
| 114061_at | 1428462_at |
| 114064_at | 1435443_at |
| 114066_at | 1423932_at |
| 114068_at | 1424319_at |

| | |
|---|---|
| 114069_at | 1434008_at |
| 114073_at | 1435624_at |
| 114076_at | 1415992_at |
| 114084_at | 1456885_at |
| 114086_at | 1454816_at |
| 114087_at | 1434789_at |
| 114088_at | 1421863_at |
| 114089_at | 1428959_at |
| 114091_at | 1435190_at |
| 114092_at | 1455738_at |
| 114093_at | 1434228_at |
| 114095_at | 1434656_at |
| 114096_at | 1451426_at |
| 114097_at | 1433630_at |
| 114106_at | 1434313_at |
| 114107_at | 1435775_at |
| 114108_at | 1435927_at |
| 114109_at | 1438418_at |
| 114116_at | 1455002_at |
| 114119_at | 1434194_at |
| 114120_at | 1435847_at |
| 114121_at | 1434804_at |
| 114122_at | 1452204_at |
| 114123_at | 1426941_at |
| 114126_at | 1438699_at |
| 114127_at | 1451270_at |
| 114129_at | 1435993_at |
| 114130_at | 1435718_at |
| 114131_at | 1433716_x_at |
| 114133_at | 1449197_at |
| 114140_at | 1434492_at |
| 114144_at | 1445504_at |
| 114145_at | 1429175_at |
| 114148_at | 1430187_at |
| 114150_at | 1452595_at |
| 114152_at | 1427327_at |
| 114155_at | 1425985_s_at |
| 114156_at | 1460028_at |
| 114159_at | 1440314_at |
| 114161_at | 1459497_at |
| 114164_at | 1436223_at |
| 114167_at | 1455561_at |
| 114170_at | 1435419_at |
| 114172_at | 1456785_at |
| 114174_at | 1453587_at |
| 114175_at | 1458421_at |
| 114177_at | 1440382_at |
| 114191_at | 1456146_at |
| 114194_at | 1419938_s_at |
| 114196_at | 1457679_at |
| 114197_at | 1440840_at |
| 114198_at | 1429099_at |
| 114199_at | 1440339_at |
| 114200_at | 1447306_at |
| 114201_at | 1458620_at |
| 114207_at | 1439887_at |

| | |
|---|---|
| 114209_at | 1444605_at |
| 114210_at | 1426566_s_at |
| 114211_at | 1447393_at |
| 114212_at | 1421045_at |
| 114213_at | 1436056_at |
| 114215_at | 1420807_a_at |
| 114217_at | 1449518_at |
| 114218_at | 1428992_at |
| 114219_at | 1437697_at |
| 114220_at | 1442777_at |
| 114224_at | 1441516_a_at |
| 114226_at | 1449423_at |
| 114228_at | 1455706_at |
| 114229_at | 1439519_at |
| 114230_at | 1439561_at |
| 114232_at | 1442290_at |
| 114233_at | 1437930_at |
| 114234_f_at | 1449959_x_at |
| 114235_at | 1450337_a_at |
| 114237_at | 1431158_at |
| 114239_at | 1434093_at |
| 114241_at | 1457331_at |
| 114244_at | 1455413_at |
| 114245_at | 1458639_at |
| 114250_at | 1435299_at |
| 114251_at | 1430167_a_at |
| 114253_at | 1426292_at |
| 114255_at | 1430305_at |
| 114256_at | 1443928_at |
| 114258_at | 1460026_s_at |
| 114259_at | 1444537_at |
| 114260_at | 1437361_at |
| 114271_at | 1416320_at |
| 114274_at | 1457826_a_at |
| 114275_at | 1452186_at |
| 114280_at | 1421048_a_at |
| 114281_at | 1434193_at |
| 114285_at | 1452903_at |
| 114287_at | 1428478_at |
| 114293_at | 1423357_at |
| 114296_at | 1451508_at |
| 114300_at | 1453721_a_at |
| 114306_at | 1437176_at |
| 114308_at | 1452188_at |
| 114314_at | 1426933_at |
| 114316_at | 1438422_at |
| 114321_at | 1451214_at |
| 114323_at | 1452796_at |
| 114324_at | 1428080_at |
| 114326_at | 1448638_at |
| 114330_at | 1449291_a_at |
| 114337_at | 1448204_at |
| 114340_at | 1429944_at |
| 114350_r_at | 1418817_at |
| 114351_at | 1417113_at |
| 114352_at | 1418029_at |

| | |
|---|---|
| 114353_at | 1451153_a_at |
| 114357_at | 1434140_at |
| 114359_at | 1449413_at |
| 114361_at | 1451448_a_at |
| 114362_at | 1425065_at |
| 114364_at | 1424077_at |
| 114365_at | 1433748_at |
| 114366_r_at | 1455830_s_at |
| 114377_at | 1438027_at |
| 114378_at | 1418563_at |
| 114379_at | 1423182_at |
| 114380_at | 1424475_at |
| 114382_at | 1436231_at |
| 114383_at | 1434520_at |
| 114384_at | 1439105_at |
| 114388_at | 1425038_at |
| 114390_at | 1437568_at |
| 114392_at | 1455622_at |
| 114393_at | 1424248_at |
| 114394_at | 1435893_at |
| 114395_at | 1423438_at |
| 114396_at | 1417255_at |
| 114397_at | 1434169_at |
| 114399_at | 1438725_at |
| 114401_at | 1434999_at |
| 114402_at | 1448251_at |
| 114403_at | 1451491_at |
| 114406_at | 1436180_at |
| 114412_at | 1435996_at |
| 114416_at | 1455226_at |
| 114417_at | 1434977_at |
| 114418_at | 1434978_at |
| 114419_at | 1456121_at |
| 114420_at | 1426981_at |
| 114422_at | 1460437_at |
| 114424_at | 1423101_at |
| 114425_at | 1435928_at |
| 114427_at | 1435582_at |
| 114432_at | 1439317_at |
| 114434_at | 1434693_at |
| 114436_at | 1436089_at |
| 114438_at | 1454829_at |
| 114440_at | 1433548_at |
| 114441_at | 1418347_at |
| 114443_at | 1423914_at |
| 114445_at | 1433827_at |
| 114449_at | 1434674_at |
| 114451_at | 1455140_at |
| 114452_at | 1417702_a_at |
| 114453_at | 1455473_at |
| 114459_at | 1431027_at |
| 114464_at | 1457727_at |
| 114466_at | 1418951_at |
| 114469_at | 1455884_at |
| 114470_at | 1437120_at |
| 114471_at | 1456619_at |

| | |
|---|---|
| 114472_at | 1428732_at |
| 114474_at | 1455237_at |
| 114476_at | 1429910_at |
| 114478_at | 1452362_at |
| 114482_at | 1453266_at |
| 114487_at | 1435254_at |
| 114488_at | 1427016_at |
| 114489_at | 1429152_at |
| 114491_f_at | 1427055_at |
| 114493_at | 1419619_at |
| 114495_at | 1419331_at |
| 114497_at | 1429053_at |
| 114501_at | 1432131_at |
| 114506_at | 1443883_at |
| 114507_at | 1442305_at |
| 114510_at | 1435440_at |
| 114512_at | 1460440_at |
| 114514_at | 1440335_at |
| 114516_at | 1428012_at |
| 114518_at | 1429501_s_at |
| 114520_at | 1457728_at |
| 114524_at | 1436377_at |
| 114528_at | 1421109_at |
| 114530_at | 1436566_at |
| 114532_at | 1442858_at |
| 114534_at | 1442047_at |
| 114535_f_at | 1437316_at |
| 114537_at | 1440234_at |
| 114540_at | 1424548_at |
| 114545_at | 1458520_at |
| 114547_at | 1442197_at |
| 114549_at | 1457309_at |
| 114553_at | 1458455_at |
| 114555_at | 1431079_at |
| 114556_at | 1449573_at |
| 114559_at | 1442315_at |
| 114563_at | 1439170_at |
| 114566_at | 1425847_a_at |
| 114568_at | 1455341_at |
| 114570_at | 1439135_at |
| 114572_at | 1436197_at |
| 114579_at | 1443386_at |
| 114582_at | 1451563_at |
| 114583_at | 1456933_at |
| 114584_at | 1458100_at |
| 114585_at | 1436617_at |
| 114586_at | 1429817_at |
| 114587_at | 1457909_at |
| 114589_at | 1418170_a_at |
| 114590_at | 1435213_at |
| 114591_at | 1457975_at |
| 114595_at | 1458385_at |
| 114597_at | 1456327_at |
| 114598_at | 1444777_at |
| 114599_at | 1456903_at |
| 114601_at | 1435779_at |

| | |
|---|---|
| 114603_at | 1419318_at |
| 114604_at | 1445606_a_at |
| 114605_at | 1442293_at |
| 114606_at | 1423204_at |
| 114608_at | 1431044_at |
| 114611_at | 1434751_at |
| 114614_at | 1440889_at |
| 114616_at | 1460128_at |
| 114617_at | 1446552_at |
| 114619_at | 1460402_at |
| 114628_at | 1437750_at |
| 114631_at | 1460344_at |
| 114632_at | 1426669_at |
| 114633_at | 1451175_at |
| 114635_at | 1420915_at |
| 114644_at | 1434855_at |
| 114651_at | 1454889_x_at |
| 114652_at | 1436860_at |
| 114653_at | 1433637_at |
| 114654_at | 1426084_a_at |
| 114655_at | 1455818_at |
| 114659_at | 1460367_at |
| 114666_at | 1438431_at |
| 114671_at | 1429485_a_at |
| 114679_at | 1423376_a_at |
| 114680_at | 1434598_at |
| 114681_at | 1450069_a_at |
| 114682_at | 1435064_a_at |
| 114683_at | 1433852_at |
| 114684_at | 1452053_a_at |
| 114685_at | 1426959_at |
| 114689_at | 1423205_at |
| 114690_at | 1460257_a_at |
| 114692_at | 1437755_at |
| 114699_at | 1429151_at |
| 114705_at | 1416932_at |
| 114706_at | 1449083_at |
| 114707_at | 1423620_at |
| 114708_at | 1460558_at |
| 114709_at | 1433796_at |
| 114710_at | 1431510_s_at |
| 114711_at | 1423253_at |
| 114712_at | 1421027_a_at |
| 114713_at | 1449493_at |
| 114719_at | 1431028_a_at |
| 114721_at | 1422548_at |
| 114722_at | 1432360_a_at |
| 114728_at | 1419161_a_at |
| 114732_at | 1418647_at |
| 114733_at | 1435166_at |
| 114736_at | 1455790_at |
| 114737_at | 1456321_at |
| 114738_at | 1429749_at |
| 114741_at | 1459974_x_at |
| 114743_at | 1436613_at |
| 114746_at | 1433825_at |

| | |
|---|---|
| 114747_at | 1456389_at |
| 114751_at | 1455395_at |
| 114752_at | 1427054_s_at |
| 114753_at | 1451375_at |
| 114755_at | 1455037_at |
| 114758_at | 1425223_at |
| 114759_at | 1440320_at |
| 114760_at | 1429912_at |
| 114762_at | 1438522_at |
| 114764_at | 1451409_at |
| 114765_at | 1430328_at |
| 114766_at | 1454691_at |
| 114767_at | 1418668_at |
| 114771_s_at | 1458613_at |
| 114772_r_at | 1444508_s_at |
| 114774_at | 1429428_at |
| 114775_at | 1435591_at |
| 114776_at | 1418031_at |
| 114777_i_at | 1460436_at |
| 114779_at | 1433590_at |
| 114782_at | 1422670_at |
| 114783_at | 1428613_at |
| 114785_at | 1440909_at |
| 114786_at | 1435027_at |
| 114787_at | 1416799_at |
| 114791_at | 1435195_at |
| 114793_at | 1433620_at |
| 114794_at | 1427312_at |
| 114795_at | 1455429_at |
| 114796_at | 1435905_at |
| 114797_at | 1429695_at |
| 114799_at | 1455122_at |
| 114801_at | 1434671_at |
| 114802_at | 1435553_at |
| 114804_at | 1419165_at |
| 114805_at | 1420659_at |
| 114806_at | 1420404_at |
| 114809_at | 1418096_at |
| 114811_at | 1451653_a_at |
| 114814_at | 1448407_at |
| 114816_at | 1438442_at |
| 114818_at | 1433925_at |
| 114820_at | 1455331_at |
| 114821_at | 1421119_at |
| 114825_at | 1454231_a_at |
| 114826_at | 1435160_at |
| 114827_at | 1421937_at |
| 114829_at | 1429111_at |
| 114830_at | 1424925_at |
| 114831_at | 1419393_at |
| 114832_at | 1437471_at |
| 114833_at | 1435157_at |
| 114835_s_at | 1424785_at |
| 114836_at | 1435193_at |
| 114840_at | 1427978_at |
| 114844_at | 1452398_at |

| | |
|---|---|
| 114847_at | 1424022_at |
| 114848_at | 1428676_at |
| 114851_at | 1423351_at |
| 114853_at | 1439092_at |
| 114854_at | 1439789_at |
| 114855_at | 1436379_at |
| 114857_at | 1435739_at |
| 114859_at | 1440090_at |
| 114865_at | 1440257_at |
| 114866_at | 1434456_at |
| 114870_at | 1439211_at |
| 114873_at | 1440544_at |
| 114874_at | 1439347_at |
| 114875_at | 1437957_at |
| 114876_at | 1439596_at |
| 114879_at | 1442629_at |
| 114880_at | 1453001_at |
| 114882_at | 1460065_at |
| 114883_at | 1424422_s_at |
| 114884_at | 1457539_at |
| 114887_at | 1429517_at |
| 114889_at | 1456425_at |
| 114890_at | 1455864_at |
| 114891_at | 1460046_at |
| 114893_at | 1441240_at |
| 114895_at | 1436425_at |
| 114898_at | 1456257_at |
| 114900_at | 1457916_at |
| 114902_at | 1420110_s_at |
| 114903_at | 1439568_at |
| 114907_at | 1458227_at |
| 114909_at | 1421167_at |
| 114910_at | 1458062_at |
| 114911_at | 1441116_at |
| 114915_at | 1458592_at |
| 114916_at | 1456825_at |
| 114918_at | 1436109_at |
| 114920_at | 1449316_at |
| 114922_at | 1434754_at |
| 114924_at | 1429946_at |
| 114925_at | 1455256_at |
| 114928_at | 1420420_at |
| 114933_at | 1435048_at |
| 114936_at | 1424934_at |
| 114938_at | 1425540_at |
| 114940_at | 1443991_at |
| 114946_at | 1457132_at |
| 114947_at | 1442951_at |
| 114949_at | 1421401_at |
| 114951_at | 1443403_at |
| 114952_at | 1439935_at |
| 114956_at | 1442310_at |
| 114957_at | 1457853_at |
| 114958_at | 1442240_at |
| 114959_at | 1436569_at |
| 114961_at | 1429970_at |

| | |
|---|---|
| 114963_at | 1424953_at |
| 114970_at | 1455780_at |
| 114971_at | 1442048_at |
| 114972_at | 1444551_at |
| 114975_at | 1425000_s_at |
| 114977_at | 1428867_at |
| 114978_at | 1435935_at |
| 114980_at | 1419114_at |
| 114981_at | 1437049_at |
| 114982_at | 1434766_at |
| 114984_at | 1455630_at |
| 114985_at | 1429455_at |
| 114986_at | 1453262_at |
| 114988_at | 1434490_at |
| 114990_at | 1451610_at |
| 114992_at | 1429275_at |
| 114993_at | 1426901_s_at |
| 114998_at | 1419195_at |
| 114999_at | 1433743_at |
| 115001_at | 1426740_s_at |
| 115003_at | 1419559_at |
| 115005_at | 1458679_a_at |
| 115010_at | 1434308_at |
| 115011_at | 1453020_at |
| 115012_at | 1454676_s_at |
| 115013_at | 1427116_at |
| 115016_at | 1426126_a_at |
| 115017_at | 1455487_at |
| 115018_at | 1422494_s_at |
| 115020_at | 1451974_at |
| 115021_at | 1455432_at |
| 115025_at | 1442034_at |
| 115027_at | 1435031_at |
| 115029_at | 1437187_at |
| 115031_at | 1424996_at |
| 115037_at | 1460406_at |
| 115042_at | 1456561_s_at |
| 115044_at | 1438074_at |
| 115045_at | 1433801_at |
| 115046_at | 1427200_at |
| 115047_at | 1419996_s_at |
| 115048_at | 1453039_at |
| 115049_at | 1429595_at |
| 115050_at | 1427956_at |
| 115055_at | 1426907_s_at |
| 115056_at | 1451391_at |
| 115065_at | 1419296_at |
| 115068_at | 1434457_at |
| 115069_at | 1455094_s_at |
| 115070_at | 1434449_at |
| 115071_at | 1425103_at |
| 115072_at | 1450732_a_at |
| 115073_g_at | 1422746_s_at |
| 115076_at | 1451510_s_at |
| 115079_at | 1420005_s_at |
| 115082_at | 1423902_s_at |

| | |
|---|---|
| 115085_at | 1437300_at |
| 115087_at | 1460398_at |
| 115088_at | 1428833_at |
| 115090_at | 1434168_at |
| 115097_at | 1452687_at |
| 115105_at | 1442025_a_at |
| 115107_at | 1419358_at |
| 115109_at | 1456755_at |
| 115110_at | 1448293_at |
| 115113_at | 1455732_at |
| 115114_at | 1434406_at |
| 115115_at | 1456317_at |
| 115116_at | 1453288_at |
| 115118_at | 1455594_at |
| 115119_at | 1424749_at |
| 115124_at | 1424795_a_at |
| 115126_at | 1429195_at |
| 115127_at | 1440884_s_at |
| 115129_at | 1439488_at |
| 115131_at | 1420630_at |
| 115132_at | 1437899_at |
| 115133_at | 1419479_at |
| 115135_at | 1458981_at |
| 115136_at | 1452859_at |
| 115139_at | 1453487_at |
| 115140_at | 1435083_at |
| 115141_at | 1419185_a_at |
| 115143_at | 1450364_a_at |
| 115149_at | 1449220_at |
| 115151_at | 1423333_at |
| 115155_at | 1450436_s_at |
| 115157_at | 1419760_a_at |
| 115159_at | 1455450_at |
| 115160_at | 1444222_x_at |
| 115161_at | 1427719_s_at |
| 115165_at | 1436598_at |
| 115166_at | 1451340_at |
| 115167_at | 1424694_at |
| 115169_at | 1434834_at |
| 115170_at | 1448708_at |
| 115171_at | 1434107_at |
| 115175_at | 1435903_at |
| 115176_at | 1435399_at |
| 115178_at | 1452948_at |
| 115181_at | 1455664_at |
| 115182_at | 1453038_at |
| 115185_at | 1449345_at |
| 115189_r_at | 1419071_at |
| 115190_at | 1418861_at |
| 115194_at | 1455153_at |
| 115196_at | 1456409_at |
| 115199_at | 1455404_at |
| 115200_at | 1455396_at |
| 115201_at | 1436596_at |
| 115203_at | 1428343_at |
| 115205_at | 1444299_at |

| | |
|---|---|
| 115207_at | 1436301_at |
| 115208_at | 1420986_s_at |
| 115209_at | 1455823_at |
| 115210_at | 1429549_at |
| 115213_at | 1459996_at |
| 115214_at | 1424227_at |
| 115215_at | 1447084_at |
| 115217_at | 1438999_a_at |
| 115219_at | 1427298_at |
| 115220_at | 1453315_at |
| 115221_at | 1451565_s_at |
| 115222_at | 1457358_at |
| 115223_at | 1434812_s_at |
| 115224_at | 1434806_at |
| 115226_at | 1425606_at |
| 115230_at | 1455568_at |
| 115235_at | 1434727_at |
| 115237_at | 1431046_at |
| 115239_at | 1444322_at |
| 115240_at | 1418906_at |
| 115241_at | 1420678_a_at |
| 115242_at | 1422094_a_at |
| 115246_at | 1444504_at |
| 115250_at | 1418108_at |
| 115251_at | 1426665_at |
| 115252_at | 1425101_a_at |
| 115255_at | 1435182_at |
| 115261_at | 1443600_at |
| 115263_at | 1435385_at |
| 115264_at | 1457576_at |
| 115265_at | 1458406_at |
| 115266_at | 1428420_a_at |
| 115267_at | 1439860_at |
| 115268_at | 1419377_at |
| 115271_at | 1442143_at |
| 115272_at | 1440007_at |
| 115273_at | 1439587_at |
| 115274_at | 1458580_at |
| 115277_at | 1453159_at |
| 115278_at | 1441384_at |
| 115279_at | 1460129_at |
| 115281_at | 1420432_at |
| 115286_at | 1455295_at |
| 115288_at | 1436252_at |
| 115289_at | 1435721_at |
| 115291_at | 1444616_x_at |
| 115292_at | 1440958_at |
| 115293_at | 1441341_at |
| 115295_at | 1456815_at |
| 115297_at | 1451360_at |
| 115299_at | 1450467_at |
| 115300_at | 1428977_at |
| 115301_r_at | 1430357_at |
| 115302_at | 1440240_at |
| 115303_at | 1439073_at |
| 115304_at | 1445246_at |

| | |
|---|---|
| 115305_at | 1459888_at |
| 115306_at | 1460137_at |
| 115307_at | 1431360_s_at |
| 115311_at | 1458547_at |
| 115317_at | 1458208_s_at |
| 115322_at | 1442429_at |
| 115323_at | 1442001_at |
| 115336_at | 1442207_at |
| 115338_at | 1453010_at |
| 115341_at | 1448359_a_at |
| 115344_at | 1449516_a_at |
| 115346_at | 1450672_a_at |
| 115350_at | 1424381_at |
| 115351_at | 1431217_at |
| 115352_at | 1438274_at |
| 115358_at | 1419212_at |
| 115360_at | 1434717_at |
| 115362_at | 1454917_at |
| 115363_at | 1429302_at |
| 115364_at | 1436652_at |
| 115367_at | 1454779_s_at |
| 115371_at | 1417700_at |
| 115372_at | 1418780_at |
| 115373_at | 1424763_at |
| 115374_r_at | 1435891_x_at |
| 115378_at | 1459997_s_at |
| 115380_at | 1435255_at |
| 115381_at | 1428120_at |
| 115385_at | 1460020_at |
| 115387_at | 1435153_at |
| 115389_at | 1454695_at |
| 115390_at | 1456060_at |
| 115391_at | 1455144_s_at |
| 115393_at | 1454634_at |
| 115394_at | 1417971_at |
| 115395_at | 1434980_at |
| 115396_at | 1417235_at |
| 115397_at | 1435208_at |
| 115398_at | 1452342_at |
| 115401_at | 1454892_at |
| 115405_at | 1434991_at |
| 115407_at | 1456165_at |
| 115408_at | 1418499_a_at |
| 115412_at | 1429018_at |
| 115415_at | 1449317_at |
| 115422_at | 1436193_at |
| 115423_at | 1426682_at |
| 115425_at | 1457721_at |
| 115426_at | 1435096_at |
| 115427_at | 1426015_s_at |
| 115428_at | 1460591_at |
| 115432_at | 1456435_at |
| 115433_at | 1452043_at |
| 115434_at | 1439031_at |
| 115435_at | 1455564_at |
| 115436_at | 1429271_at |

| | |
|---|---|
| 115438_at | 1452748_at |
| 115439_at | 1433877_at |
| 115449_at | 1442075_at |
| 115450_at | 1457698_at |
| 115452_at | 1435022_at |
| 115453_at | 1434798_at |
| 115466_at | 1429955_at |
| 115470_at | 1428724_at |
| 115474_g_at | 1421600_a_at |
| 115475_at | 1424929_a_at |
| 115476_at | 1455078_at |
| 115477_at | 1452449_at |
| 115478_at | 1425021_a_at |
| 115483_at | 1428769_at |
| 115490_at | 1442076_at |
| 115493_at | 1434926_at |
| 115500_at | 1435251_at |
| 115502_at | 1428742_at |
| 115505_at | 1429616_at |
| 115506_at | 1438430_at |
| 115507_at | 1429250_at |
| 115509_at | 1453571_at |
| 115510_at | 1429229_s_at |
| 115511_at | 1457595_at |
| 115513_at | 1435886_at |
| 115516_at | 1434269_at |
| 115517_at | 1448744_at |
| 115518_at | 1435230_at |
| 115519_at | 1455744_at |
| 115521_at | 1429966_at |
| 115525_at | 1435547_at |
| 115526_at | 1436143_at |
| 115529_at | 1431856_a_at |
| 115533_at | 1435010_at |
| 115535_at | 1434753_at |
| 115536_at | 1451276_at |
| 115537_at | 1434769_at |
| 115539_at | 1435280_at |
| 115540_at | 1435339_at |
| 115541_at | 1434713_at |
| 115543_at | 1426451_at |
| 115545_at | 1435459_at |
| 115548_at | 1456267_at |
| 115549_at | 1427286_at |
| 115550_at | 1437393_at |
| 115553_at | 1419642_at |
| 115554_at | 1439804_at |
| 115555_at | 1451500_at |
| 115562_at | 1454828_at |
| 115566_at | 1423074_at |
| 115567_r_at | 1434765_at |
| 115571_at | 1451433_at |
| 115572_at | 1444409_at |
| 115573_at | 1435748_at |
| 115576_at | 1423443_at |
| 115578_at | 1444484_at |

| | |
|---|---|
| 115586_at | 1436230_at |
| 115587_at | 1430092_at |
| 115594_at | 1436572_at |
| 115595_at | 1439857_at |
| 115597_at | 1418660_at |
| 115600_at | 1440297_at |
| 115602_at | 1440996_at |
| 115603_at | 1423473_at |
| 115611_f_at | 1441292_at |
| 115613_at | 1419786_at |
| 115615_at | 1441141_at |
| 115620_at | 1453496_at |
| 115631_at | 1445899_at |
| 115634_at | 1427434_at |
| 115635_at | 1448374_at |
| 115636_at | 1442199_at |
| 115641_at | 1429563_x_at |
| 115642_at | 1441090_at |
| 115647_at | 1434629_at |
| 115648_at | 1458611_at |
| 115649_at | 1442114_at |
| 115651_at | 1445422_at |
| 115652_at | 1419144_at |
| 115654_at | 1455411_at |
| 115655_at | 1443879_at |
| 115657_at | 1425861_x_at |
| 115659_at | 1444149_at |
| 115660_at | 1445370_at |
| 115661_at | 1424765_at |
| 115662_at | 1441010_at |
| 115664_at | 1460412_at |
| 115665_at | 1444780_at |
| 115666_at | 1429179_at |
| 115668_at | 1440227_at |
| 115669_at | 1436200_at |
| 115672_at | 1435710_at |
| 115680_at | 1440278_at |
| 115681_at | 1438053_at |
| 115684_at | 1457133_at |
| 115686_at | 1431149_at |
| 115690_at | 1459436_at |
| 115694_at | 1456791_at |
| 115695_r_at | 1423558_at |
| 115699_at | 1428661_at |
| 115700_at | 1429726_at |
| 115701_at | 1447100_s_at |
| 115702_at | 1447829_x_at |
| 115703_at | 1434960_at |
| 115704_at | 1434657_at |
| 115705_at | 1456765_at |
| 115708_at | 1427209_at |
| 115709_at | 1456048_at |
| 115710_at | 1428827_at |
| 115711_at | 1429218_at |
| 115712_at | 1431681_at |
| 115713_at | 1422825_at |

| | |
|---|---|
| 115714_at | 1449057_at |
| 115715_at | 1455863_at |
| 115716_at | 1453316_at |
| 115719_at | 1429230_at |
| 115727_at | 1430318_at |
| 115728_at | 1419311_at |
| 115729_at | 1457271_at |
| 115733_at | 1419187_at |
| 115740_at | 1428887_at |
| 115742_at | 1429627_at |
| 115744_at | 1457677_at |
| 115745_at | 1418472_at |
| 115746_at | 1455431_at |
| 115748_at | 1441094_at |
| 115749_at | 1436438_s_at |
| 115752_at | 1438233_at |
| 115753_at | 1416966_at |
| 115756_at | 1419515_at |
| 115757_at | 1430538_at |
| 115759_at | 1435348_at |
| 115760_at | 1423871_at |
| 115761_at | 1428692_at |
| 115762_at | 1424324_at |
| 115764_at | 1428681_at |
| 115766_at | 1418924_at |
| 115768_f_at | 1435377_at |
| 115769_at | 1459988_at |
| 115774_at | 1425029_a_at |
| 115775_at | 1452316_at |
| 115779_at | 1456833_at |
| 115782_at | 1434945_at |
| 115786_at | 1417926_at |
| 115787_at | 1437641_at |
| 115788_at | 1455495_at |
| 115789_at | 1452897_at |
| 115790_at | 1423251_at |
| 115791_at | 1452964_at |
| 115797_at | 1439480_at |
| 115801_at | 1436128_at |
| 115802_at | 1435205_at |
| 115803_at | 1427065_at |
| 115807_f_at | 1436355_at |
| 115808_at | 1453171_s_at |
| 115811_at | 1455452_x_at |
| 115813_at | 1454739_at |
| 115815_at | 1453585_at |
| 115817_at | 1428729_at |
| 115818_at | 1455405_at |
| 115819_at | 1423542_at |
| 115820_at | 1436907_at |
| 115823_at | 1452891_at |
| 115824_at | 1449443_at |
| 115825_at | 1450691_at |
| 115828_at | 1453059_at |
| 115830_at | 1439128_at |
| 115832_at | 1427461_at |

| | |
|---|---|
| 115835_at | 1442576_at |
| 115837_at | 1448650_a_at |
| 115838_at | 1436739_at |
| 115840_at | 1455157_a_at |
| 115841_at | 1425150_at |
| 115842_at | 1453480_at |
| 115843_at | 1438827_at |
| 115847_i_at | 1442769_at |
| 115848_at | 1436476_at |
| 115849_at | 1436428_at |
| 115854_at | 1437642_at |
| 115857_at | 1456287_at |
| 115858_at | 1430359_a_at |
| 115860_at | 1436175_at |
| 115861_at | 1419519_at |
| 115862_at | 1429050_at |
| 115869_at | 1455646_at |
| 115870_at | 1431920_a_at |
| 115872_at | 1428916_s_at |
| 115876_at | 1454819_at |
| 115878_at | 1438226_at |
| 115879_at | 1428788_at |
| 115885_at | 1435947_at |
| 115887_at | 1437540_at |
| 115891_at | 1438038_at |
| 115892_at | 1451432_x_at |
| 115893_at | 1438325_at |
| 115894_at | 1444590_at |
| 115895_at | 1423995_at |
| 115896_at | 1439796_at |
| 115899_at | 1424838_at |
| 115904_at | 1436516_at |
| 115907_at | 1443988_at |
| 115909_at | 1436338_at |
| 115910_at | 1460012_at |
| 115911_at | 1429373_x_at |
| 115915_at | 1436502_at |
| 115916_at | 1422785_at |
| 115920_at | 1422685_at |
| 115921_at | 1448490_at |
| 115925_at | 1434295_at |
| 115928_at | 1438694_at |
| 115930_at | 1452614_at |
| 115932_at | 1428793_at |
| 115933_at | 1429432_at |
| 115935_at | 1452871_at |
| 115937_at | 1425481_at |
| 115941_at | 1431222_at |
| 115945_at | 1442300_at |
| 115946_at | 1429942_at |
| 115948_at | 1442496_at |
| 115949_at | 1458882_at |
| 115951_at | 1441988_at |
| 115954_at | 1439793_at |
| 115956_at | 1451469_at |
| 115958_at | 1459299_at |

| | |
|---|---|
| 115962_at | 1435936_at |
| 115963_at | 1429975_at |
| 115964_at | 1458642_at |
| 115966_at | 1458707_at |
| 115967_at | 1444258_at |
| 115969_at | 1423305_at |
| 115976_at | 1455329_at |
| 115982_at | 1458650_at |
| 115985_r_at | 1440284_at |
| 115987_at | 1453336_at |
| 115988_at | 1430034_at |
| 115989_at | 1444570_at |
| 115991_at | 1440287_at |
| 115998_at | 1427369_at |
| 116001_at | 1455187_at |
| 116006_at | 1452629_at |
| 116010_at | 1442327_at |
| 116012_at | 1453742_at |
| 116014_at | 1442248_at |
| 116016_at | 1425950_at |
| 116018_at | 1441003_at |
| 116019_at | 1436319_at |
| 116021_at | 1457061_at |
| 116026_at | 1447972_at |
| 116036_at | 1436395_at |
| 116037_at | 1457069_at |
| 116039_at | 1444245_at |
| 116040_at | 1421691_at |
| 116046_at | 1438294_at |
| 116048_at | 1444089_at |
| 116049_at | 1456347_at |
| 116052_at | 1453523_at |
| 116056_at | 1438764_at |
| 116057_at | 1418400_at |
| 116058_at | 1434207_at |
| 116060_at | 1458366_at |
| 116062_at | 1451489_at |
| 116066_at | 1452984_at |
| 116067_at | 1452809_at |
| 116068_at | 1429663_at |
| 116075_at | 1430728_at |
| 116083_at | 1421422_at |
| 116084_at | 1436118_at |
| 116085_at | 1417893_at |
| 116087_at | 1449173_at |
| 116091_at | 1426214_at |
| 116092_at | 1455520_at |
| 116093_at | 1428198_at |
| 116094_at | 1455437_at |
| 116095_at | 1444321_at |
| 116098_at | 1439063_at |
| 116102_at | 1422804_at |
| 116103_at | 1454767_at |
| 116104_at | 1438687_at |
| 116111_at | 1417897_at |
| 116113_at | 1434191_at |

| | |
|---|---|
| 116114_at | 1438709_at |
| 116115_at | 1430145_at |
| 116117_at | 1426490_at |
| 116120_at | 1456154_at |
| 116123_at | 1421204_a_at |
| 116124_at | 1426975_at |
| 116127_at | 1437959_at |
| 116128_at | 1440858_at |
| 116131_at | 1439790_at |
| 116133_at | 1436047_at |
| 116134_at | 1421927_at |
| 116135_at | 1436788_at |
| 116144_at | 1438762_at |
| 116148_at | 1423899_at |
| 116149_at | 1421338_at |
| 116150_at | 1428748_at |
| 116151_at | 1428750_at |
| 116152_at | 1435380_at |
| 116155_at | 1455356_at |
| 116163_at | 1418546_a_at |
| 116166_at | 1452719_at |
| 116170_at | 1432176_a_at |
| 116173_at | 1433687_at |
| 116174_at | 1421055_at |
| 116176_at | 1457044_at |
| 116178_at | 1440559_at |
| 116179_at | 1453290_at |
| 116181_at | 1453191_at |
| 116182_at | 1436447_at |
| 116183_at | 1430181_at |
| 116184_at | 1444184_at |
| 116186_at | 1431150_at |
| 116188_at | 1436102_at |
| 116189_at | 1430053_a_at |
| 116190_at | 1451159_at |
| 116191_at | 1431023_at |
| 116192_at | 1450488_at |
| 116193_at | 1450718_at |
| 116194_at | 1442823_at |
| 116195_at | 1423209_at |
| 116196_at | 1455486_at |
| 116197_at | 1439795_at |
| 116198_at | 1441713_at |
| 116199_at | 1437763_at |
| 116202_at | 1444177_at |
| 116203_at | 1421880_at |
| 116206_at | 1444189_at |
| 116207_at | 1448738_at |
| 116209_at | 1443578_at |
| 116210_at | 1439787_at |
| 116211_at | 1439798_at |
| 116212_at | 1448063_at |
| 116215_at | 1457150_at |
| 116221_at | 1458402_at |
| 116224_at | 1437373_at |
| 116227_at | 1448807_at |

| | |
|---|---|
| 116228_at | 1458459_a_at |
| 116230_at | 1458454_at |
| 116232_at | 1429546_at |
| 116233_at | 1429068_at |
| 116235_at | 1440915_at |
| 116236_at | 1440522_at |
| 116237_at | 1436331_at |
| 116238_at | 1448712_at |
| 116240_at | 1435623_at |
| 116241_at | 1429468_at |
| 116243_at | 1421213_at |
| 116244_at | 1444589_at |
| 116245_at | 1442088_at |
| 116246_at | 1452329_at |
| 116251_at | 1457442_at |
| 116255_at | 1446116_at |
| 116256_at | 1449608_a_at |
| 116257_at | 1435703_at |
| 116259_at | 1423162_s_at |
| 116260_at | 1424310_at |
| 116262_at | 1451492_at |
| 116268_at | 1454897_at |
| 116269_at | 1457772_at |
| 116272_at | 1435673_at |
| 116273_at | 1455720_at |
| 116275_at | 1442314_at |
| 116278_at | 1434591_at |
| 116281_at | 1453150_at |
| 116282_at | 1452041_at |
| 116284_at | 1441385_at |
| 116285_at | 1437605_at |
| 116288_at | 1444520_at |
| 116289_at | 1434463_at |
| 116291_at | 1442136_at |
| 116295_at | 1451292_at |
| 116296_at | 1425004_s_at |
| 116297_at | 1433673_at |
| 116299_at | 1438077_at |
| 116302_at | 1429114_at |
| 116303_at | 1434286_at |
| 116305_at | 1455536_at |
| 116309_at | 1439119_a_at |
| 116310_at | 1420961_a_at |
| 116313_at | 1436513_at |
| 116314_at | 1433807_at |
| 116316_at | 1451147_x_at |
| 116317_at | 1421264_at |
| 116321_at | 1437175_at |
| 116322_at | 1429161_at |
| 116325_at | 1459883_at |
| 116329_at | 1417088_at |
| 116330_at | 1419699_at |
| 116334_at | 1434876_at |
| 116336_at | 1455454_at |
| 116338_at | 1431618_a_at |
| 116339_at | 1455370_at |

| | |
|---|---|
| 116341_at | 1429486_at |
| 116344_at | 1440965_at |
| 116345_at | 1433719_at |
| 116347_at | 1452368_at |
| 116348_at | 1435038_s_at |
| 116350_at | 1451678_at |
| 116355_at | 1456753_at |
| 116357_at | 1444006_at |
| 116363_at | 1435123_at |
| 116368_at | 1452972_at |
| 116369_at | 1434226_at |
| 116370_at | 1453372_at |
| 116373_at | 1429771_at |
| 116374_at | 1425387_at |
| 116375_at | 1456068_at |
| 116377_at | 1444040_at |
| 116378_at | 1428830_at |
| 116379_at | 1454707_at |
| 116383_at | 1434662_at |
| 116389_at | 1430143_at |
| 116390_at | 1456873_at |
| 116391_at | 1455081_at |
| 116392_at | 1455745_at |
| 116395_at | 1436205_at |
| 116405_at | 1436754_at |
| 116406_at | 1455028_at |
| 116407_at | 1437645_at |
| 116416_at | 1455027_at |
| 116418_at | 1457266_at |
| 116420_at | 1452118_at |
| 116422_at | 1442041_at |
| 116424_at | 1448544_at |
| 116425_at | 1435196_at |
| 116426_at | 1435460_at |
| 116428_at | 1430126_at |
| 116429_at | 1444047_at |
| 116431_at | 1435266_at |
| 116433_at | 1436553_at |
| 116434_at | 1436404_at |
| 116438_at | 1455659_at |
| 116443_at | 1418694_at |
| 116444_at | 1425104_at |
| 116445_at | 1454988_s_at |
| 116449_at | 1438518_at |
| 116450_at | 1444242_at |
| 116451_at | 1438055_at |
| 116452_at | 1425190_a_at |
| 116453_f_at | 1455813_at |
| 116456_at | 1417884_at |
| 116457_at | 1456917_at |
| 116461_at | 1449621_s_at |
| 116463_at | 1449411_at |
| 116464_at | 1435482_at |
| 116465_at | 1434729_at |
| 116466_at | 1458087_at |
| 116470_at | 1455743_at |

| | |
|---|---|
| 116471_at | 1441059_at |
| 116473_at | 1427134_at |
| 116475_at | 1442042_at |
| 116478_at | 1441245_a_at |
| 116483_at | 1417523_at |
| 116492_at | 1422708_at |
| 116493_at | 1440697_at |
| 116494_at | 1419794_at |
| 116495_at | 1453776_at |
| 116496_at | 1444346_at |
| 116499_at | 1437677_at |
| 116501_at | 1442926_at |
| 116502_at | 1439564_at |
| 116505_at | 1457151_at |
| 116509_at | 1425978_at |
| 116510_at | 1438775_at |
| 116513_at | 1444022_at |
| 116514_at | 1458393_at |
| 116517_at | 1428943_at |
| 116518_at | 1440325_at |
| 116519_at | 1442124_at |
| 116520_at | 1427496_at |
| 116521_at | 1428903_at |
| 116522_at | 1435218_at |
| 116523_at | 1453073_at |
| 116526_at | 1443652_x_at |
| 116531_at | 1457378_at |
| 116533_at | 1447951_at |
| 116535_at | 1460470_at |
| 116536_at | 1444388_at |
| 116539_at | 1452887_at |
| 116540_at | 1440983_at |
| 116543_at | 1439671_at |
| 116544_at | 1436271_at |
| 116545_at | 1425084_at |
| 116546_at | 1442548_at |
| 116549_at | 1430028_at |
| 116554_at | 1455270_at |
| 116555_at | 1440298_at |
| 116560_at | 1458076_at |
| 116567_at | 1442122_at |
| 116568_at | 1452966_at |
| 116581_at | 1449147_at |
| 116584_at | 1436367_at |
| 116585_at | 1456406_at |
| 116586_at | 1428898_at |
| 116588_at | 1434297_at |
| 116589_at | 1431220_at |
| 116593_at | 1420384_at |
| 116599_at | 1435658_at |
| 116600_at | 1455160_at |
| 116602_at | 1438031_at |
| 116603_at | 1438030_at |
| 116604_at | 1455448_at |
| 116605_at | 1435644_at |
| 116607_at | 1453034_at |

| | |
|---|---|
| 116610_at | 1420382_at |
| 116611_at | 1418685_at |
| 116612_at | 1429044_at |
| 116617_at | 1433977_at |
| 116619_at | 1422064_a_at |
| 116620_at | 1428921_at |
| 116621_at | 1456294_at |
| 116626_at | 1435082_at |
| 116627_at | 1434678_at |
| 116628_at | 1436601_at |
| 116630_at | 1433987_at |
| 116632_at | 1426816_at |
| 116633_at | 1449560_at |
| 116634_at | 1429323_at |
| 116637_at | 1457276_at |
| 116638_at | 1434916_at |
| 116640_at | 1436380_at |
| 116647_at | 1458678_at |
| 116649_at | 1455567_at |
| 116650_at | 1418859_at |
| 116653_at | 1440329_s_at |
| 116656_at | 1437572_at |
| 116657_at | 1442064_at |
| 116661_at | 1428290_at |
| 116666_at | 1455136_at |
| 116668_at | 1434838_at |
| 116670_at | 1422821_s_at |
| 116671_at | 1434562_at |
| 116674_at | 1436755_at |
| 116678_at | 1418844_at |
| 116679_at | 1439513_at |
| 116683_at | 1450713_at |
| 116686_at | 1429267_at |
| 116688_at | 1460586_at |
| 116689_at | 1434807_s_at |
| 116690_at | 1424437_s_at |
| 116691_at | 1456487_at |
| 116692_at | 1433986_at |
| 116694_at | 1424757_at |
| 116695_at | 1435805_at |
| 116696_at | 1434028_at |
| 116701_at | 1445186_at |
| 116706_at | 1430570_at |
| 116708_at | 1430363_at |
| 116714_at | 1444536_at |
| 116716_at | 1425570_at |
| 116718_at | 1436587_at |
| 116720_at | 1444471_at |
| 116721_at | 1460136_at |
| 116722_at | 1440947_at |
| 116725_at | 1440231_at |
| 116726_at | 1435711_at |
| 116728_at | 1434582_at |
| 116730_r_at | 1425378_at |
| 116731_at | 1438532_at |
| 116734_at | 1452619_a_at |

| | |
|---|---|
| 116742_at | 1447288_at |
| 116743_at | 1442203_at |
| 116744_at | 1443963_at |
| 116747_at | 1439848_at |
| 116749_at | 1419901_at |
| 116750_at | 1429832_at |
| 116751_at | 1441732_at |
| 116752_at | 1456760_at |
| 116753_at | 1425695_at |
| 116754_at | 1444348_at |
| 116756_at | 1457773_at |
| 116757_at | 1416388_at |
| 116758_at | 1441107_at |
| 116764_at | 1455337_at |
| 116765_at | 1453374_at |
| 116768_at | 1430689_at |
| 116769_at | 1431756_at |
| 116771_at | 1452639_at |
| 116773_at | 1458233_at |
| 116778_at | 1422006_at |
| 116779_at | 1458607_at |
| 116780_at | 1457355_at |
| 116783_at | 1430183_at |
| 116786_at | 1436346_at |
| 116787_at | 1460054_at |
| 116791_at | 1457752_at |
| 116792_at | 1425439_a_at |
| 116793_at | 1440913_at |
| 116794_at | 1430246_at |
| 116796_at | 1455165_at |
| 116797_at | 1444278_at |
| 116798_at | 1449918_at |
| 116803_at | 1436622_at |
| 116804_at | 1444588_at |
| 116807_at | 1424762_at |
| 116809_at | 1436248_at |
| 116810_at | 1444784_at |
| 116811_at | 1418411_at |
| 116812_at | 1452993_at |
| 116813_at | 1458717_at |
| 116816_at | 1443485_at |
| 116819_at | 1444752_at |
| 116820_at | 1454873_at |
| 116823_at | 1431324_at |
| 116826_at | 1457031_at |
| 116827_at | 1419129_at |
| 116828_at | 1425639_at |
| 116833_at | 1438196_at |
| 116836_at | 1427459_at |
| 116838_at | 1428837_at |
| 116842_at | 1427922_at |
| 116844_at | 1443889_at |
| 116849_at | 1434118_at |
| 116850_at | 1419496_at |
| 116855_at | 1421861_at |
| 116869_at | 1439872_at |

| | |
|---|---|
| 116871_at | 1460624_at |
| 116873_at | 1455924_at |
| 116874_at | 1455289_at |
| 116875_at | 1423246_at |
| 116878_at | 1452969_at |
| 116880_at | 1433752_s_at |
| 116882_at | 1458380_at |
| 116883_at | 1419601_at |
| 116884_at | 1456115_at |
| 116891_at | 1419124_at |
| 116895_at | 1429197_s_at |
| 116898_at | 1435068_at |
| 116902_at | 1452175_at |
| 116903_at | 1455032_at |
| 116905_at | 1455538_at |
| 116908_at | 1419819_s_at |
| 116910_at | 1436702_at |
| 116911_at | 1456491_at |
| 116912_at | 1455215_at |
| 116916_at | 1419668_at |
| 116921_at | 1435772_at |
| 116925_at | 1435514_at |
| 116927_at | 1453200_at |
| 116929_at | 1457260_at |
| 116930_at | 1455612_at |
| 116931_at | 1424625_a_at |
| 116934_at | 1427320_at |
| 116939_at | 1428864_at |
| 116944_at | 1456894_at |
| 116948_at | 1455989_at |
| 116949_at | 1438217_at |
| 116952_at | 1435486_at |
| 116954_at | 1434661_at |
| 116958_at | 1455336_at |
| 116959_at | 1455620_at |
| 116968_at | 1437855_at |
| 116969_at | 1434829_at |
| 116973_at | 1435822_at |
| 116978_at | 1428341_at |
| 116987_at | 1428714_at |
| 116988_at | 1420686_at |
| 116989_at | 1451332_at |
| 116990_at | 1420035_at |
| 116991_g_at | 1459982_a_at |
| 116992_at | 1441978_at |
| 116993_at | 1434943_at |
| 116994_at | 1439811_at |
| 116995_at | 1455116_at |
| 116996_at | 1438780_at |
| 116999_at | 1436263_at |
| 117000_at | 1428801_at |
| 117002_at | 1440923_at |
| 117003_at | 1434781_at |
| 117004_at | 1455484_at |
| 117005_at | 1435667_at |
| 117007_at | 1428525_at |

| | |
|---|---|
| 117008_at | 1424194_at |
| 117009_at | 1456490_at |
| 117013_at | 1437421_at |
| 117015_at | 1428577_at |
| 117017_at | 1452327_at |
| 117019_at | 1433861_at |
| 117025_at | 1437650_at |
| 117028_at | 1419005_at |
| 117038_at | 1435668_at |
| 117039_at | 1433869_at |
| 117041_at | 1435785_at |
| 117043_at | 1459895_at |
| 117044_at | 1455080_at |
| 117047_at | 1447471_at |
| 117050_at | 1436334_at |
| 117051_at | 1434266_at |
| 117052_at | 1428392_at |
| 117053_at | 1428139_at |
| 117055_at | 1418474_at |
| 117056_at | 1455580_at |
| 117060_at | 1445476_at |
| 117065_at | 1440357_at |
| 117067_at | 1427690_a_at |
| 117074_at | 1429214_at |
| 117075_at | 1440359_at |
| 117076_at | 1435562_at |
| 117077_at | 1420725_at |
| 117078_at | 1453353_at |
| 117079_at | 1455672_s_at |
| 117082_at | 1436360_at |
| 117085_at | 1422477_at |
| 117087_at | 1428211_at |
| 117088_at | 1437890_at |
| 117090_at | 1439475_at |
| 117096_at | 1436920_at |
| 117097_at | 1455463_at |
| 117101_at | 1436629_at |
| 117102_at | 1429444_at |
| 117103_at | 1418775_at |
| 117106_at | 1434830_at |
| 117107_at | 1435787_at |
| 117108_at | 1447927_at |
| 117118_at | 1436618_at |
| 117123_at | 1443952_at |
| 117126_at | 1459045_at |
| 117128_at | 1419823_s_at |
| 117133_at | 1449449_at |
| 117139_at | 1439021_at |
| 117140_at | 1418419_at |
| 117141_at | 1452652_at |
| 117146_at | 1435540_at |
| 117154_at | 1439646_at |
| 117155_at | 1436084_at |
| 117156_at | 1436400_at |
| 117157_at | 1455493_at |
| 117159_at | 1434288_at |

| | |
|---|---|
| 117161_at | 1437397_at |
| 117168_at | 1435227_at |
| 117170_at | 1435801_at |
| 117177_at | 1452961_at |
| 117178_at | 1438022_at |
| 117179_at | 1442140_at |
| 117181_at | 1454972_at |
| 117189_at | 1439797_at |
| 117190_at | 1436238_at |
| 117191_at | 1448998_at |
| 117195_at | 1435295_at |
| 117196_at | 1434225_at |
| 117197_at | 1451338_at |
| 117199_at | 1423356_at |
| 117200_at | 1439232_at |
| 117201_at | 1428321_at |
| 117204_at | 1428963_at |
| 117207_at | 1423341_at |
| 117208_at | 1428926_at |
| 117213_at | 1429083_at |
| 117215_at | 1429155_at |
| 117218_at | 1460452_at |
| 117227_at | 1435491_at |
| 117228_at | 1427002_s_at |
| 117232_at | 1429274_at |
| 117234_at | 1418537_at |
| 117235_at | 1455401_at |
| 117238_at | 1428400_at |
| 117240_at | 1438426_at |
| 117243_at | 1426422_at |
| 117244_at | 1428524_at |
| 117250_at | 1434373_at |
| 117251_at | 1428338_at |
| 117253_at | 1426739_at |
| 117257_at | 1452946_a_at |
| 117259_at | 1419087_s_at |
| 117262_at | 1425343_at |
| 117263_at | 1435240_at |
| 117266_at | 1424818_at |
| 117273_at | 1456399_at |
| 117276_at | 1426869_at |
| 117279_at | 1456339_at |
| 117280_at | 1433907_at |
| 117281_at | 1428723_at |
| 117286_at | 1429022_at |
| 117287_at | 1450961_a_at |
| 117289_at | 1451571_s_at |
| 117290_at | 1455639_at |
| 117291_at | 1435239_at |
| 117292_at | 1430600_at |
| 117294_at | 1418360_at |
| 117295_at | 1435809_at |
| 117297_at | 1435207_at |
| 117298_at | 1455850_at |
| 117299_at | 1436415_at |
| 117300_at | 1422766_at |

| | |
|---|---|
| 117307_at | 1448803_at |
| 117308_at | 1421425_a_at |
| 117316_at | 1452741_s_at |
| 117327_at | 1427274_at |
| 117332_at | 1453767_a_at |
| 117333_at | 1424494_s_at |
| 117336_at | 1434641_x_at |
| 117338_at | 1428148_s_at |
| 128550_at | 1429209_at |
| 128554_at | 1429365_at |
| 128577_s_at | 1434779_at |
| 128579_r_at | 1442054_at |
| 128580_at | 1435073_a_at |
| 128590_at | 1437768_at |
| 128651_at | 1442112_at |
| 128663_at | 1429421_at |
| 128666_at | 1454629_at |
| 128681_at | 1436220_at |
| 128688_at | 1435883_at |
| 128689_at | 1419299_at |
| 128722_at | 1453425_at |
| 128751_at | 1437294_at |
| 128783_at | 1437212_at |
| 128784_f_at | 1437020_at |
| 128785_r_at | 1439521_at |
| 128791_at | 1453214_at |
| 128794_at | 1430683_at |
| 128795_at | 1447227_at |
| 128802_at | 1420400_at |
| 128804_at | 1458801_at |
| 128809_at | 1454686_at |
| 128828_at | 1437383_at |
| 128832_at | 1426299_at |
| 128834_r_at | 1444193_at |
| 128841_r_at | 1438805_at |
| 128845_at | 1444206_at |
| 128849_at | 1420356_at |
| 128862_f_at | 1417347_at |
| 128880_at | 1430098_at |
| 128886_at | 1439603_at |
| 128915_at | 1436876_at |
| 128972_r_at | 1439167_at |
| 128987_at | 1429846_at |
| 129005_at | 1443621_at |
| 129006_at | 1458648_at |
| 129008_at | 1442840_at |
| 129009_at | 1441609_at |
| 129013_at | 1439194_at |
| 129018_at | 1456846_at |
| 129019_at | 1460122_at |
| 129021_at | 1439275_s_at |
| 129023_at | 1424138_at |
| 129024_at | 1442953_at |
| 129029_at | 1441395_at |
| 129030_at | 1442962_at |
| 129031_at | 1442098_at |

| | |
|---|---|
| 129034_at | 1441695_at |
| 129036_r_at | 1443888_at |
| 129038_at | 1456130_at |
| 129041_at | 1442857_at |
| 129043_at | 1458224_at |
| 129045_at | 1443446_at |
| 129049_at | 1442167_at |
| 129064_at | 1440721_at |
| 129065_at | 1457454_at |
| 129066_at | 1457401_at |
| 129069_at | 1441672_at |
| 129072_at | 1441376_at |
| 129074_at | 1458169_at |
| 129079_at | 1443557_at |
| 129081_at | 1441409_at |
| 129082_at | 1434984_at |
| 129083_at | 1457167_at |
| 129086_at | 1442816_at |
| 129088_at | 1436832_at |
| 129090_at | 1442696_at |
| 129092_at | 1441060_at |
| 129093_at | 1425586_a_at |
| 129094_r_at | 1427149_at |
| 129096_at | 1445093_at |
| 129098_at | 1442216_at |
| 129099_at | 1443618_at |
| 129103_at | 1429995_at |
| 129104_at | 1441294_at |
| 129106_at | 1441149_at |
| 129109_at | 1441163_at |
| 129111_at | 1442961_at |
| 129112_at | 1442534_at |
| 129113_at | 1431049_at |
| 129117_at | 1457865_at |
| 129125_at | 1455768_at |
| 129127_at | 1442791_x_at |
| 129128_at | 1430637_at |
| 129131_at | 1434869_at |
| 129144_at | 1440532_a_at |
| 129148_at | 1456980_at |
| 129149_at | 1443684_at |
| 129152_at | 1436122_at |
| 129154_at | 1442954_at |
| 129155_at | 1439236_at |
| 129161_at | 1454835_at |
| 129166_at | 1447493_at |
| 129170_at | 1442153_at |
| 129176_at | 1455399_at |
| 129177_at | 1456796_at |
| 129181_at | 1432499_a_at |
| 129187_at | 1442510_at |
| 129194_f_at | 1436650_at |
| 129196_at | 1455697_at |
| 129203_at | 1455298_at |
| 129205_at | 1457012_at |
| 129209_r_at | 1458548_at |

| | |
|---|---|
| 129210_at | 1442194_at |
| 129211_at | 1440027_at |
| 129212_at | 1419314_at |
| 129217_at | 1458544_at |
| 129231_at | 1460451_at |
| 129232_at | 1439680_at |
| 129238_at | 1457247_at |
| 129240_at | 1443071_at |
| 129243_at | 1458911_at |
| 129249_r_at | 1444709_at |
| 129251_r_at | 1428493_at |
| 129253_r_at | 1459694_at |
| 129260_at | 1449610_at |
| 129262_at | 1425112_at |
| 129265_at | 1456978_s_at |
| 129266_at | 1450454_at |
| 129268_at | 1433874_at |
| 129273_at | 1451320_at |
| 129275_at | 1436642_x_at |
| 129278_at | 1444056_at |
| 129280_at | 1457897_at |
| 129285_at | 1439599_at |
| 129295_at | 1441602_at |
| 129303_at | 1435835_at |
| 129311_at | 1449262_s_at |
| 129318_at | 1448008_at |
| 129326_at | 1438239_at |
| 129331_at | 1444884_at |
| 129338_at | 1421502_at |
| 129339_at | 1451410_a_at |
| 129341_at | 1447318_at |
| 129342_r_at | 1442919_at |
| 129346_at | 1441033_at |
| 129347_at | 1426925_at |
| 129352_at | 1459117_at |
| 129353_r_at | 1440453_at |
| 129357_at | 1439339_at |
| 129371_at | 1460161_at |
| 129373_at | 1445069_at |
| 129386_at | 1459703_at |
| 129387_r_at | 1459572_at |
| 129388_at | 1459018_at |
| 129389_at | 1442958_at |
| 129395_at | 1455541_a_at |
| 129396_at | 1444989_at |
| 129398_at | 1445035_at |
| 129399_at | 1458061_at |
| 129400_at | 1443478_at |
| 129403_at | 1445060_at |
| 129404_at | 1447332_at |
| 129409_at | 1444823_at |
| 129411_at | 1446867_at |
| 129413_at | 1459594_at |
| 129415_at | 1457550_at |
| 129416_at | 1447944_at |
| 129417_s_at | 1457084_at |

| | |
|---|---|
| 129418_at | 1445822_at |
| 129419_at | 1444840_at |
| 129420_at | 1459610_at |
| 129423_at | 1446907_at |
| 129424_at | 1456711_at |
| 129425_at | 1446033_at |
| 129426_at | 1445950_at |
| 129428_at | 1458782_at |
| 129433_at | 1429949_at |
| 129434_at | 1442859_at |
| 129442_at | 1441547_at |
| 129541_at | 1459309_at |
| 129544_at | 1439505_at |
| 129555_r_at | 1437259_at |
| 129574_at | 1434878_at |
| 129661_at | 1437273_at |
| 129740_at | 1430565_at |
| 129774_f_at | 1418120_at |
| 129808_at | 1425408_a_at |
| 129833_at | 1452399_at |
| 129864_at | 1459682_at |
| 129866_at | 1436283_at |
| 129867_at | 1455397_at |
| 129870_at | 1436440_at |
| 129871_at | 1459063_at |
| 129872_at | 1458272_at |
| 129873_at | 1447302_at |
| 129875_at | 1442627_at |
| 129877_at | 1443032_at |
| 129879_at | 1445297_at |
| 129882_at | 1454067_a_at |
| 129885_at | 1454075_s_at |
| 129887_at | 1459031_at |
| 129890_at | 1457892_at |
| 129893_at | 1447468_at |
| 129895_at | 1430206_at |
| 129899_f_at | 1442637_at |
| 129901_at | 1447183_at |
| 129902_r_at | 1452131_at |
| 129906_at | 1429464_at |
| 129924_at | 1441391_at |
| 129955_at | 1456550_at |
| 129981_at | 1456157_at |
| 130058_f_at | 1444112_at |
| 130067_at | 1447404_at |
| 130071_at | 1436472_at |
| 130072_at | 1458090_at |
| 130124_at | 1420447_at |
| 130125_at | 1444201_at |
| 130132_at | 1451998_at |
| 130138_at | 1453817_at |
| 130140_at | 1459467_at |
| 130146_f_at | 1421979_at |
| 130175_at | 1453423_at |
| 130178_at | 1436008_at |
| 130182_at | 1442582_at |

| | |
|---|---|
| 130269_r_at | 1430303_at |
| 130300_r_at | 1460404_at |
| 130311_at | 1437574_at |
| 130315_at | 1426098_a_at |
| 130319_at | 1452875_at |
| 130326_at | 1436627_at |
| 130327_at | 1459204_at |
| 130329_at | 1444891_at |
| 130331_at | 1442448_at |
| 130332_at | 1445302_at |
| 130336_at | 1439978_at |
| 130337_f_at | 1443469_at |
| 130340_at | 1447303_at |
| 130342_at | 1459936_at |
| 130395_at | 1444897_at |
| 130396_at | 1458171_at |
| 130398_at | 1442362_at |
| 130399_at | 1458573_at |
| 130401_at | 1453429_at |
| 130402_at | 1460148_at |
| 130404_at | 1445740_at |
| 130405_at | 1445792_at |
| 130407_at | 1459227_at |
| 130408_at | 1440000_at |
| 130409_at | 1445753_at |
| 130410_at | 1459244_at |
| 130412_at | 1459115_at |
| 130415_at | 1459093_at |
| 130416_at | 1455696_a_at |
| 130417_at | 1446267_at |
| 130419_at | 1445119_at |
| 130420_at | 1458851_at |
| 130421_at | 1459044_at |
| 130459_at | 1450020_at |
| 130460_at | 1453135_at |
| 130472_at | 1446213_at |
| 130477_at | 1449362_a_at |
| 130478_at | 1430764_at |
| 130483_at | 1416192_at |
| 130492_at | 1417709_at |
| 130500_at | 1434640_at |
| 130509_at | 1450234_at |
| 130510_at | 1460459_at |
| 130512_at | 1452879_at |
| 130546_at | 1444795_at |
| 130550_f_at | 1441569_at |
| 130559_at | 1446740_at |
| 130563_at | 1444331_at |
| 130564_at | 1445839_at |
| 130565_at | 1440833_at |
| 130573_at | 1459960_at |
| 130575_at | 1458840_at |
| 130577_at | 1445763_at |
| 130578_at | 1442971_at |
| 130579_at | 1458950_at |
| 130580_at | 1447188_at |

| | |
|---|---|
| 130583_at | 1443496_at |
| 130587_at | 1453391_at |
| 130597_r_at | 1439670_at |
| 130600_at | 1459106_at |
| 130602_at | 1444678_at |
| 130603_at | 1445803_at |
| 130605_r_at | 1442029_at |
| 130607_at | 1458564_at |
| 130609_at | 1431780_at |
| 130610_at | 1439562_at |
| 130612_r_at | 1444640_at |
| 130620_r_at | 1444859_at |
| 130622_at | 1456958_at |
| 130626_at | 1459575_at |
| 130627_at | 1437314_a_at |
| 130631_at | 1446110_at |
| 130638_at | 1458555_at |
| 130642_at | 1445819_at |
| 130643_at | 1437215_at |
| 130645_at | 1422245_a_at |
| 130647_at | 1440409_at |
| 130649_at | 1453078_at |
| 130651_at | 1447605_at |
| 130656_at | 1446036_at |
| 130670_at | 1437269_at |
| 130672_at | 1453345_at |
| 130673_at | 1426633_s_at |
| 130675_at | 1430401_at |
| 130682_at | 1455755_at |
| 130691_at | 1418814_s_at |
| 130703_at | 1442436_at |
| 130704_f_at | 1435846_x_at |
| 130709_at | 1460596_at |
| 130710_at | 1436244_a_at |
| 130718_at | 1423405_at |
| 130719_at | 1434794_at |
| 130720_at | 1440041_at |
| 130721_r_at | 1455714_at |
| 130733_f_at | 1433715_at |
| 130735_at | 1447170_at |
| 130740_at | 1451408_at |
| 130744_at | 1428057_a_at |
| 130747_at | 1442956_at |
| 130750_at | 1422725_at |
| 130761_at | 1421108_at |
| 130763_at | 1430115_at |
| 130772_at | 1417283_at |
| 130779_f_at | 1429727_at |
| 130785_at | 1444249_at |
| 130786_at | 1459180_at |
| 130787_at | 1444818_at |
| 130788_at | 1445284_at |
| 130789_at | 1445949_at |
| 130791_at | 1445094_at |
| 130797_at | 1445023_at |
| 130804_at | 1440215_at |

| | |
|---|---|
| 130821_at | 1459252_at |
| 130822_at | 1447137_at |
| 130826_at | 1446924_at |
| 130827_at | 1446976_at |
| 130828_at | 1441113_at |
| 130851_at | 1457327_at |
| 130852_at | 1446925_at |
| 130877_at | 1442412_at |
| 130907_at | 1455589_at |
| 130908_at | 1451630_at |
| 130912_at | 1444055_at |
| 130913_at | 1460447_at |
| 130919_at | 1417860_a_at |
| 130923_i_at | 1439183_at |
| 130928_s_at | 1451986_s_at |
| 130929_at | 1452263_at |
| 130939_at | 1428763_at |
| 130951_at | 1456768_a_at |
| 130960_at | 1429774_a_at |
| 130968_at | 1435203_at |
| 130971_at | 1443664_s_at |
| 130973_at | 1429130_at |
| 130994_at | 1434275_at |
| 131015_at | 1459878_a_at |
| 131017_at | 1448039_at |
| 131018_at | 1451498_at |
| 131035_at | 1432019_at |
| 131037_at | 1440731_at |
| 131054_at | 1456091_at |
| 131067_at | 1445935_at |
| 131068_at | 1459577_at |
| 131081_at | 1459516_at |
| 131082_at | 1458815_at |
| 131091_at | 1459178_at |
| 131093_at | 1446831_at |
| 131094_at | 1446887_at |
| 131097_at | 1445243_at |
| 131100_at | 1444312_at |
| 131101_at | 1447356_at |
| 131102_at | 1459236_at |
| 131103_at | 1431515_at |
| 131113_at | 1456523_at |
| 131114_at | 1446343_at |
| 131117_at | 1456998_at |
| 131118_at | 1446032_at |
| 131119_at | 1445470_at |
| 131120_at | 1439355_at |
| 131124_at | 1436405_at |
| 131129_at | 1444830_at |
| 131130_at | 1446275_at |
| 131131_f_at | 1443918_at |
| 131133_at | 1458804_at |
| 131134_at | 1445992_at |
| 131135_at | 1459207_at |
| 131138_at | 1444975_at |
| 131139_at | 1446045_at |

| | |
|---|---|
| 131140_at | 1445756_at |
| 131142_at | 1444945_at |
| 131143_at | 1459515_at |
| 131144_at | 1442801_x_at |
| 131149_at | 1454092_a_at |
| 131152_at | 1424846_at |
| 131153_at | 1424023_at |
| 131156_f_at | 1438142_s_at |
| 131161_at | 1455261_at |
| 131163_at | 1440478_at |
| 131164_at | 1440094_at |
| 131165_at | 1455583_at |
| 131171_at | 1440594_at |
| 131177_r_at | 1456875_at |
| 131184_r_at | 1422599_s_at |
| 131189_f_at | 1433600_at |
| 131217_at | 1449327_at |
| 131220_f_at | 1434222_at |
| 131225_at | 1428987_at |
| 131226_at | 1456767_at |
| 131239_r_at | 1431071_at |
| 131244_at | 1447326_s_at |
| 131248_at | 1449082_at |
| 131253_f_at | 1440353_at |
| 131255_at | 1434252_at |
| 131271_at | 1444066_at |
| 131272_at | 1432104_a_at |
| 131275_at | 1446344_at |
| 131282_at | 1444265_at |
| 131289_at | 1431148_at |
| 131337_at | 1445918_at |
| 131377_at | 1456643_at |
| 131389_at | 1435660_at |
| 131390_at | 1443901_at |
| 131391_at | 1433484_at |
| 131392_at | 1445857_at |
| 131394_at | 1421397_a_at |
| 131402_at | 1434716_at |
| 131403_at | 1457103_at |
| 131416_at | 1448924_at |
| 131443_at | 1445054_at |
| 131449_at | 1446613_at |
| 131453_at | 1444892_at |
| 131454_at | 1459159_a_at |
| 131478_at | 1423495_at |
| 131513_s_at | 1435990_at |
| 131549_at | 1431236_at |
| 131555_at | 1428318_at |
| 131556_at | 1459678_at |
| 131569_at | 1456905_at |
| 131584_at | 1446154_at |
| 131591_at | 1453153_at |
| 131599_at | 1419147_at |
| 131606_at | 1457543_at |
| 131634_at | 1442366_at |
| 131646_at | 1447310_at |

| | |
|---|---|
| 131665_at | 1436931_at |
| 131682_at | 1438742_at |
| 131692_r_at | 1459615_at |
| 131693_at | 1451759_at |
| 131702_at | 1456962_at |
| 131703_at | 1441623_at |
| 131705_at | 1447279_at |
| 131706_at | 1436430_at |
| 131707_at | 1452354_at |
| 131741_f_at | 1430525_at |
| 131772_at | 1459325_at |
| 131792_s_at | 1434049_at |
| 131796_at | 1444043_at |
| 131797_at | 1441061_at |
| 131813_at | 1444960_at |
| 131816_at | 1427722_at |
| 131821_at | 1441499_at |
| 131822_at | 1446894_at |
| 131831_at | 1420135_at |
| 131832_at | 1445691_at |
| 131834_at | 1436274_at |
| 131835_at | 1445622_at |
| 131845_at | 1417758_at |
| 131846_at | 1445835_at |
| 131847_at | 1457965_at |
| 131852_at | 1447338_at |
| 131853_at | 1457195_at |
| 131854_at | 1455383_at |
| 131855_at | 1443505_at |
| 131856_at | 1445259_at |
| 131858_at | 1445038_at |
| 131859_at | 1445636_at |
| 131860_at | 1458893_at |
| 131861_at | 1422989_a_at |
| 131862_at | 1447525_at |
| 131863_at | 1425403_at |
| 131864_at | 1444736_at |
| 131866_at | 1437482_at |
| 131873_at | 1443865_at |
| 131874_at | 1431459_at |
| 131918_at | 1443521_at |
| 131919_at | 1427106_at |
| 131933_at | 1457880_at |
| 131968_at | 1443615_at |
| 131971_at | 1440312_at |
| 131984_at | 1441639_at |
| 131987_at | 1457364_at |
| 132009_at | 1444740_at |
| 132014_at | 1431852_at |
| 132019_at | 1455784_at |
| 132020_at | 1419340_at |
| 132023_at | 1430082_at |
| 132025_r_at | 1439785_at |
| 132028_at | 1451364_at |
| 132030_at | 1448080_at |
| 132035_at | 1443147_at |

| | |
|---|---|
| 132048_r_at | 1436531_at |
| 132049_at | 1441640_at |
| 132051_at | 1444029_at |
| 132053_at | 1419903_at |
| 132063_f_at | 1449358_at |
| 132064_at | 1435845_at |
| 132086_at | 1446619_at |
| 132087_at | 1435121_at |
| 132102_at | 1435359_at |
| 132103_at | 1441802_at |
| 132106_at | 1437495_at |
| 132107_at | 1442901_at |
| 132108_at | 1456987_at |
| 132110_s_at | 1455704_at |
| 132114_f_at | 1427196_at |
| 132117_at | 1443288_at |
| 132118_at | 1441818_at |
| 132132_at | 1453399_at |
| 132134_at | 1425010_at |
| 132150_at | 1427325_s_at |
| 132172_at | 1451651_at |
| 132208_at | 1444993_at |
| 132220_at | 1444935_at |
| 132223_at | 1430059_at |
| 132231_at | 1436463_at |
| 132238_at | 1437124_at |
| 132313_at | 1420648_at |
| 132361_at | 1429261_at |
| 132370_at | 1435381_at |
| 132388_f_at | 1426502_s_at |
| 132397_at | 1435069_at |
| 132403_at | 1439989_at |
| 132496_f_at | 1460379_at |
| 132523_at | 1455346_at |
| 132535_at | 1420740_at |
| 132549_at | 1441541_at |
| 132553_at | 1458307_at |
| 132577_at | 1442891_at |
| 132579_at | 1444672_at |
| 132602_at | 1435049_s_at |
| 132735_at | 1421486_at |
| 132751_i_at | 1435423_x_at |
| 132762_at | 1456634_at |
| 132772_at | 1427299_at |
| 132794_at | 1444458_at |
| 132806_at | 1442654_at |
| 132807_at | 1457342_at |
| 132808_at | 1441212_at |
| 132809_at | 1424901_at |
| 132811_at | 1457815_at |
| 132812_at | 1437493_at |
| 132813_at | 1439873_at |
| 132883_at | 1457116_at |
| 132884_at | 1437664_at |
| 132889_at | 1432847_at |
| 133028_f_at | 1443295_at |

| | |
|---|---|
| 133029_at | 1445972_at |
| 133032_at | 1447312_at |
| 133035_at | 1458361_at |
| 133036_at | 1437662_at |
| 133037_at | 1459551_at |
| 133043_at | 1448094_at |
| 133045_at | 1424976_at |
| 133046_at | 1445401_at |
| 133049_at | 1427497_at |
| 133050_at | 1452644_at |
| 133053_at | 1427339_at |
| 133055_at | 1459520_at |
| 133056_at | 1447396_at |
| 133057_at | 1440451_at |
| 133058_at | 1445086_at |
| 133059_at | 1444946_at |
| 133061_at | 1443306_at |
| 133065_at | 1440347_at |
| 133070_at | 1430070_at |
| 133092_f_at | 1435952_at |
| 133095_at | 1425198_at |
| 133111_f_at | 1427217_at |
| 133116_at | 1425622_at |
| 133122_at | 1443291_at |
| 133125_at | 1437562_at |
| 133127_at | 1451778_at |
| 133128_at | 1449936_at |
| 133129_at | 1439117_at |
| 133130_at | 1426328_a_at |
| 133131_at | 1440407_at |
| 133132_at | 1441232_at |
| 133136_at | 1445670_at |
| 133137_at | 1456837_at |
| 133139_at | 1416287_at |
| 133142_at | 1421080_at |
| 133143_at | 1453329_s_at |
| 133145_at | 1457328_at |
| 133147_at | 1458651_at |
| 133153_at | 1421458_at |
| 133154_at | 1457273_at |
| 133181_at | 1436206_at |
| 133184_at | 1440616_at |
| 133185_at | 1459333_at |
| 133186_at | 1432649_at |
| 133187_at | 1460277_at |
| 133188_at | 1447383_at |
| 133190_at | 1453445_at |
| 133192_at | 1436675_at |
| 133193_at | 1425254_at |
| 133194_at | 1425759_at |
| 133196_s_at | 1460089_at |
| 133197_at | 1446905_at |
| 133199_at | 1456848_at |
| 133200_at | 1446017_at |
| 133202_at | 1455109_at |
| 133203_at | 1444079_at |

| | |
|---|---|
| 133209_at | 1457274_at |
| 133212_at | 1444885_at |
| 133213_at | 1445331_at |
| 133214_at | 1445997_at |
| 133215_at | 1459084_at |
| 133216_at | 1445645_at |
| 133217_at | 1431716_at |
| 133218_at | 1445733_at |
| 133219_at | 1444938_at |
| 133224_at | 1458731_at |
| 133227_at | 1444921_at |
| 133235_at | 1446728_at |
| 133242_at | 1455527_at |
| 133244_at | 1452471_at |
| 133247_at | 1452297_at |
| 133248_at | 1449330_at |
| 133252_at | 1438136_at |
| 133256_f_at | 1457179_at |
| 133258_s_at | 1450135_at |
| 133261_at | 1443714_at |
| 133267_at | 1458634_at |
| 133271_at | 1436257_at |
| 133275_at | 1456679_at |
| 133280_at | 1457602_at |
| 133281_at | 1441121_at |
| 133286_at | 1457911_at |
| 133294_at | 1425925_at |
| 133303_at | 1428345_at |
| 133316_at | 1441654_at |
| 133320_at | 1440929_at |
| 133322_s_at | 1439991_a_at |
| 133324_at | 1443417_at |
| 133327_f_at | 1456974_at |
| 133329_at | 1442527_at |
| 133331_at | 1442462_at |
| 133336_at | 1430993_at |
| 133337_at | 1439549_at |
| 133338_at | 1420483_at |
| 133347_at | 1436256_at |
| 133351_at | 1453793_at |
| 133353_at | 1419793_at |
| 133355_r_at | 1443370_at |
| 133374_at | 1435694_at |
| 133375_at | 1458231_at |
| 133382_at | 1442854_at |
| 133384_at | 1426470_at |
| 133387_r_at | 1458009_at |
| 133388_at | 1440530_at |
| 133390_at | 1457575_at |
| 133391_at | 1442967_at |
| 133392_at | 1441399_at |
| 133393_r_at | 1458013_at |
| 133394_at | 1442493_at |
| 133395_at | 1443607_at |
| 133397_at | 1442862_at |
| 133398_at | 1442162_at |

| | |
|---|---|
| 133399_at | 1442163_at |
| 133403_at | 1458262_at |
| 133406_at | 1427323_s_at |
| 133407_r_at | 1441628_at |
| 133408_at | 1458251_at |
| 133409_at | 1443419_at |
| 133411_at | 1443759_at |
| 133413_at | 1446813_s_at |
| 133414_at | 1442861_at |
| 133420_at | 1458154_at |
| 133422_at | 1443506_at |
| 133430_at | 1446252_at |
| 133436_at | 1445898_at |
| 133440_at | 1446657_at |
| 133443_at | 1458277_at |
| 133452_at | 1443084_at |
| 133453_at | 1459462_at |
| 133456_f_at | 1424733_at |
| 133458_at | 1459111_at |
| 133460_at | 1446737_a_at |
| 133461_at | 1446006_at |
| 133465_at | 1453328_at |
| 133466_at | 1440099_at |
| 133467_r_at | 1459721_at |
| 133471_at | 1458317_at |
| 133472_at | 1440960_at |
| 133479_at | 1427428_at |
| 133485_at | 1442354_at |
| 133487_at | 1456910_at |
| 133489_f_at | 1438061_at |
| 133493_at | 1447275_at |
| 133494_at | 1418642_at |
| 133496_at | 1446609_at |
| 133499_at | 1455228_at |
| 133503_at | 1442193_at |
| 133510_at | 1453390_at |
| 133515_at | 1456557_at |
| 133537_at | 1443556_at |
| 133540_at | 1442595_at |
| 133546_r_at | 1440338_at |
| 133547_at | 1427531_a_at |
| 133549_at | 1444328_at |
| 133557_at | 1441037_at |
| 133558_at | 1442342_at |
| 133563_at | 1438802_at |
| 133610_at | 1452385_at |
| 133628_at | 1439298_at |
| 133666_at | 1443122_at |
| 133667_at | 1455475_at |
| 133672_at | 1442544_at |
| 133674_at | 1420577_at |
| 133676_r_at | 1445409_at |
| 133681_at | 1435332_at |
| 133684_at | 1459284_at |
| 133686_at | 1444042_at |
| 133687_at | 1444635_at |

| | |
|---|---|
| 133692_at | 1445864_at |
| 133694_at | 1459681_at |
| 133696_at | 1459336_at |
| 133699_at | 1459262_at |
| 133704_at | 1440527_at |
| 133705_at | 1446229_at |
| 133709_at | 1428039_at |
| 133714_at | 1430446_at |
| 133718_at | 1440516_at |
| 133720_at | 1445565_at |
| 133727_at | 1443907_at |
| 133729_at | 1423070_at |
| 133730_at | 1431291_at |
| 133738_at | 1422108_at |
| 133743_at | 1456532_at |
| 133750_at | 1441553_at |
| 133755_at | 1438467_at |
| 133757_at | 1436690_at |
| 133759_at | 1456981_at |
| 133763_at | 1440355_at |
| 133766_at | 1441522_at |
| 133768_at | 1458962_at |
| 133778_at | 1458252_at |
| 133782_at | 1443035_at |
| 133784_at | 1443014_at |
| 133785_r_at | 1458035_at |
| 133788_at | 1441717_at |
| 133790_at | 1443376_at |
| 133792_at | 1441104_at |
| 133793_at | 1424994_at |
| 133798_at | 1440736_at |
| 133799_at | 1446095_at |
| 133801_at | 1439494_at |
| 133802_at | 1455621_at |
| 133805_at | 1449585_at |
| 133806_at | 1449602_at |
| 133808_at | 1442352_at |
| 133816_at | 1442474_at |
| 133818_at | 1442644_at |
| 133820_at | 1458480_at |
| 133822_at | 1442841_at |
| 133823_at | 1430249_at |
| 133831_at | 1442137_at |
| 133834_at | 1444060_at |
| 133836_at | 1439898_at |
| 133837_at | 1458660_at |
| 133838_at | 1435663_at |
| 133840_at | 1442522_at |
| 133848_at | 1444565_at |
| 133849_at | 1421434_at |
| 133850_at | 1422933_at |
| 133853_at | 1421609_a_at |
| 133859_at | 1420529_at |
| 133861_at | 1435969_at |
| 133864_r_at | 1457881_at |
| 133867_at | 1420419_a_at |

| | |
|---|---|
| 133871_at | 1458661_at |
| 133881_at | 1421219_at |
| 133889_at | 1447969_at |
| 133908_at | 1429264_at |
| 133919_at | 1424195_a_at |
| 133931_at | 1425399_at |
| 133932_at | 1443632_at |
| 133935_at | 1457067_at |
| 133936_at | 1425504_at |
| 133937_at | 1438411_at |
| 133938_at | 1446331_at |
| 133939_at | 1458677_at |
| 133943_at | 1460130_at |
| 133951_at | 1440435_at |
| 133952_at | 1442612_at |
| 133954_at | 1442085_at |
| 133966_at | 1453482_at |
| 133970_at | 1430048_at |
| 133972_at | 1444399_at |
| 133975_at | 1449723_at |
| 133981_at | 1447411_at |
| 133985_at | 1430223_at |
| 133987_at | 1432428_at |
| 133992_at | 1460052_at |
| 133997_at | 1441975_at |
| 134006_at | 1436475_at |
| 134009_at | 1444575_at |
| 134011_at | 1444179_at |
| 134013_at | 1430256_at |
| 134028_at | 1428149_at |
| 134030_at | 1421168_at |
| 134031_r_at | 1443239_at |
| 134033_at | 1457795_at |
| 134039_at | 1418452_at |
| 134041_at | 1453228_at |
| 134043_at | 1415913_at |
| 134051_at | 1455147_at |
| 134054_at | 1439573_at |
| 134055_at | 1417819_at |
| 134057_at | 1419338_at |
| 134060_at | 1428587_at |
| 134061_r_at | 1436321_at |
| 134062_at | 1443941_at |
| 134067_r_at | 1460082_at |
| 134070_at | 1449543_at |
| 134071_at | 1431831_at |
| 134077_at | 1441227_at |
| 134078_at | 1450733_at |
| 134079_f_at | 1419582_at |
| 134080_at | 1438071_at |
| 134097_at | 1457714_at |
| 134116_at | 1425274_at |
| 134117_at | 1427477_at |
| 134119_at | 1419623_at |
| 134131_at | 1431929_a_at |
| 134133_at | 1453617_at |

| | |
|---|---|
| 134147_f_at | 1459998_at |
| 134148_at | 1457748_at |
| 134149_at | 1444542_at |
| 134150_at | 1440300_at |
| 134151_at | 1435100_at |
| 134154_at | 1453472_a_at |
| 134163_at | 1439578_at |
| 134165_at | 1429684_at |
| 134171_at | 1444601_at |
| 134174_at | 1444727_at |
| 134175_at | 1436604_at |
| 134177_at | 1440337_at |
| 134182_at | 1439817_at |
| 134183_at | 1438266_at |
| 134186_at | 1456657_at |
| 134194_at | 1447392_s_at |
| 134200_at | 1444525_at |
| 134206_f_at | 1434569_at |
| 134209_at | 1424325_at |
| 134218_at | 1459844_at |
| 134222_at | 1442341_at |
| 134223_at | 1442320_at |
| 134237_at | 1429112_at |
| 134241_at | 1438220_at |
| 134245_at | 1457824_at |
| 134248_at | 1431066_at |
| 134253_at | 1430792_at |
| 134255_at | 1446758_at |
| 134256_at | 1455617_at |
| 134257_at | 1438713_at |
| 134263_at | 1457956_at |
| 134265_at | 1442803_at |
| 134269_at | 1419047_at |
| 134273_at | 1444129_at |
| 134277_at | 1446966_at |
| 134280_at | 1448044_a_at |
| 134281_at | 1445539_at |
| 134286_at | 1447460_at |
| 134289_r_at | 1457780_at |
| 134291_at | 1420334_at |
| 134296_at | 1444585_at |
| 134297_at | 1428026_at |
| 134298_at | 1446694_at |
| 134300_at | 1459685_at |
| 134305_at | 1457440_at |
| 134316_at | 1439094_at |
| 134329_at | 1442871_at |
| 134334_at | 1431171_at |
| 134341_f_at | 1434639_at |
| 134342_r_at | 1457718_at |
| 134354_at | 1444927_at |
| 134360_at | 1420144_x_at |
| 134377_f_at | 1439693_a_at |
| 134380_at | 1437886_at |
| 134384_at | 1430883_at |
| 134386_at | 1416548_at |

| | |
|---|---|
| 134388_at | 1419160_at |
| 134391_at | 1442356_at |
| 134399_at | 1429493_at |
| 134401_at | 1421501_a_at |
| 134405_at | 1436329_at |
| 134410_at | 1442083_at |
| 134434_at | 1444790_at |
| 134438_at | 1436119_at |
| 134465_at | 1453082_at |
| 134509_at | 1447570_s_at |
| 134511_at | 1449931_at |
| 134513_f_at | 1429027_at |
| 134514_at | 1429867_at |
| 134524_at | 1442768_at |
| 134531_at | 1437629_at |
| 134532_at | 1445134_at |
| 134534_at | 1436371_at |
| 134595_at | 1448439_at |
| 134600_at | 1444406_at |
| 134620_at | 1434733_at |
| 134621_at | 1442013_at |
| 134623_at | 1443635_at |
| 134627_at | 1460063_at |
| 134628_at | 1425556_at |
| 134633_at | 1458680_at |
| 134634_at | 1457161_at |
| 134635_at | 1435314_at |
| 134637_at | 1425230_at |
| 134639_at | 1459704_at |
| 134640_at | 1438242_at |
| 134641_at | 1458704_at |
| 134642_f_at | 1429880_at |
| 134647_at | 1460620_at |
| 134651_at | 1443235_at |
| 134658_at | 1458929_at |
| 134661_at | 1453738_at |
| 134663_at | 1432235_at |
| 134665_at | 1445711_at |
| 134666_at | 1453300_at |
| 134669_at | 1457805_at |
| 134675_at | 1456020_at |
| 134676_at | 1427975_at |
| 134679_at | 1457145_at |
| 134682_at | 1445164_at |
| 134686_r_at | 1457706_at |
| 134688_at | 1438231_at |
| 134689_at | 1443145_at |
| 134692_at | 1442119_at |
| 134693_at | 1459294_at |
| 134696_at | 1444547_at |
| 134700_at | 1430066_at |
| 134703_at | 1429682_at |
| 134707_at | 1442991_at |
| 134718_at | 1418750_at |
| 134720_at | 1458020_at |
| 134724_at | 1447436_at |

| | |
|---|---|
| 134727_at | 1444620_at |
| 134730_at | 1459690_at |
| 134731_at | 1457051_at |
| 134733_r_at | 1426172_a_at |
| 134734_at | 1439799_at |
| 134736_f_at | 1460509_at |
| 134737_r_at | 1455234_at |
| 134747_at | 1429809_at |
| 134748_at | 1447340_at |
| 134752_at | 1457641_at |
| 134756_at | 1430252_at |
| 134758_at | 1452238_at |
| 134759_at | 1459187_at |
| 134766_at | 1457988_at |
| 134768_at | 1450513_at |
| 134774_at | 1440967_at |
| 134783_at | 1447389_at |
| 134786_at | 1438886_at |
| 134795_at | 1442804_at |
| 134801_at | 1428803_at |
| 134804_at | 1443027_at |
| 134807_at | 1442422_at |
| 134808_at | 1459259_at |
| 134811_at | 1449424_at |
| 134812_r_at | 1436043_at |
| 134813_at | 1457502_at |
| 134816_at | 1436599_at |
| 134818_at | 1439033_at |
| 134822_at | 1439565_at |
| 134826_at | 1420503_at |
| 134830_at | 1418691_at |
| 134833_at | 1460417_at |
| 134834_at | 1442419_at |
| 134838_at | 1441353_at |
| 134855_at | 1443066_at |
| 134857_at | 1431864_at |
| 134866_at | 1455443_at |
| 134869_at | 1434467_at |
| 134971_f_at | 1439606_at |
| 135023_at | 1430150_at |
| 135024_at | 1437676_at |
| 135034_at | 1453044_at |
| 135051_r_at | 1440470_at |
| 135070_at | 1457193_at |
| 135083_at | 1425117_at |
| 135089_at | 1442985_at |
| 135091_at | 1436199_at |
| 135098_at | 1444105_at |
| 135101_at | 1459511_at |
| 135103_at | 1444200_at |
| 135112_at | 1460025_at |
| 135114_at | 1455453_at |
| 135123_at | 1457008_at |
| 135125_at | 1458453_at |
| 135126_at | 1435790_at |
| 135131_at | 1440235_at |

| | |
|---|---|
| 135132_at | 1437937_at |
| 135137_at | 1453247_at |
| 135138_at | 1445371_at |
| 135142_at | 1427572_at |
| 135144_r_at | 1421292_a_at |
| 135145_r_at | 1435765_at |
| 135148_at | 1458718_at |
| 135151_at | 1440658_at |
| 135156_at | 1457720_at |
| 135158_at | 1441653_at |
| 135166_at | 1460027_at |
| 135167_at | 1449458_at |
| 135169_at | 1425214_at |
| 135174_at | 1453306_at |
| 135175_at | 1429284_at |
| 135177_at | 1427164_at |
| 135183_r_at | 1424021_at |
| 135184_at | 1452980_at |
| 135185_at | 1447314_at |
| 135188_at | 1439198_at |
| 135190_at | 1457123_at |
| 135194_at | 1453466_at |
| 135196_at | 1458395_at |
| 135197_at | 1430418_at |
| 135200_at | 1447082_at |
| 135205_at | 1431180_at |
| 135208_at | 1440881_at |
| 135210_at | 1437564_at |
| 135211_at | 1457977_at |
| 135216_at | 1451418_a_at |
| 135223_at | 1443571_at |
| 135225_at | 1430402_at |
| 135226_at | 1440533_at |
| 135228_at | 1442686_at |
| 135230_at | 1435854_at |
| 135234_at | 1455762_at |
| 135243_at | 1435629_at |
| 135245_at | 1440901_at |
| 135249_at | 1436251_at |
| 135251_at | 1418122_at |
| 135253_at | 1444669_at |
| 135255_at | 1445308_at |
| 135256_at | 1441225_at |
| 135264_at | 1439941_at |
| 135265_at | 1449627_at |
| 135271_at | 1446311_at |
| 135272_at | 1457990_at |
| 135278_at | 1453734_at |
| 135287_at | 1430451_at |
| 135288_at | 1445274_at |
| 135293_at | 1458443_at |
| 135296_at | 1434146_at |
| 135302_at | 1438834_at |
| 135304_at | 1451623_at |
| 135305_at | 1441323_at |
| 135307_at | 1457412_at |

| | |
|---|---|
| 135310_f_at | 1439531_at |
| 135312_at | 1435650_at |
| 135317_at | 1441801_at |
| 135327_at | 1434673_at |
| 135331_at | 1441382_at |
| 135336_at | 1440962_at |
| 135359_at | 1427443_at |
| 135361_at | 1442361_at |
| 135362_at | 1441156_at |
| 135367_at | 1439830_at |
| 135374_at | 1424919_at |
| 135375_at | 1443264_at |
| 135376_at | 1459054_at |
| 135386_r_at | 1439823_at |
| 135391_at | 1421183_at |
| 135392_at | 1457574_at |
| 135394_at | 1459735_at |
| 135400_at | 1458358_at |
| 135401_at | 1435438_at |
| 135402_at | 1425374_at |
| 135404_at | 1443211_at |
| 135407_at | 1430594_at |
| 135414_at | 1451772_at |
| 135455_at | 1448023_at |
| 135456_at | 1452360_a_at |
| 135494_at | 1435979_a_at |
| 135495_r_at | 1446069_at |
| 135513_at | 1430694_at |
| 135524_at | 1457856_at |
| 135537_at | 1418584_at |
| 135539_at | 1459138_at |
| 135541_at | 1427514_at |
| 135543_at | 1445482_at |
| 135570_at | 1438706_at |
| 135581_at | 1457607_at |
| 135585_at | 1439081_at |
| 135589_at | 1429098_s_at |
| 135596_at | 1440746_at |
| 135600_at | 1447455_at |
| 135611_at | 1453813_at |
| 135617_at | 1457750_at |
| 135623_at | 1425455_a_at |
| 135632_f_at | 1437567_at |
| 135633_at | 1431748_a_at |
| 135641_at | 1436638_at |
| 135643_at | 1444053_at |
| 135654_at | 1420781_at |
| 135655_at | 1439979_at |
| 135656_at | 1452611_at |
| 135663_r_at | 1443972_at |
| 135667_at | 1452437_at |
| 135677_f_at | 1428450_at |
| 135682_at | 1459617_at |
| 135686_at | 1455533_at |
| 135690_at | 1457939_at |
| 135692_at | 1439786_at |

| | |
|---|---|
| 135693_at | 1436576_at |
| 135696_f_at | 1434429_at |
| 135699_at | 1436295_at |
| 135704_at | 1423278_at |
| 135707_at | 1437181_at |
| 135710_at | 1430503_at |
| 135719_at | 1454048_a_at |
| 135720_at | 1444001_at |
| 135727_at | 1435206_at |
| 135728_at | 1440147_at |
| 135730_at | 1447245_at |
| 135734_at | 1441106_at |
| 135739_at | 1441327_a_at |
| 135747_at | 1447987_at |
| 135748_at | 1455636_at |
| 135750_at | 1443453_at |
| 135752_at | 1441161_at |
| 135755_at | 1442209_at |
| 135758_at | 1441610_at |
| 135766_at | 1456829_at |
| 135767_at | 1443287_at |
| 135770_at | 1443350_at |
| 135771_at | 1456856_at |
| 135773_at | 1440282_at |
| 135776_at | 1436449_at |
| 135779_at | 1442662_at |
| 135780_at | 1446071_at |
| 135793_r_at | 1442116_at |
| 135796_at | 1450905_at |
| 135797_at | 1439845_at |
| 135800_at | 1441729_at |
| 135803_r_at | 1445801_at |
| 135804_at | 1435830_a_at |
| 135805_at | 1447696_x_at |
| 135808_at | 1446676_at |
| 135813_at | 1451268_at |
| 135816_at | 1430709_at |
| 135828_at | 1441075_at |
| 135887_at | 1430097_at |
| 135893_r_at | 1430498_at |
| 135902_at | 1428434_at |
| 135903_at | 1457095_at |
| 135905_at | 1445851_at |
| 135910_at | 1445382_at |
| 135911_at | 1445492_at |
| 135913_at | 1444304_at |
| 135915_at | 1447470_at |
| 135927_at | 1447513_at |
| 135928_at | 1431991_at |
| 135939_at | 1430546_at |
| 135959_at | 1445165_at |
| 135960_at | 1456682_at |
| 135976_at | 1459255_at |
| 135979_at | 1457414_at |
| 135982_at | 1442127_at |
| 135983_at | 1459732_at |

| | |
|---|---|
| 135984_at | 1444914_at |
| 136005_at | 1441066_at |
| 136006_at | 1424221_at |
| 136007_at | 1455711_at |
| 136009_at | 1441089_at |
| 136025_at | 1423343_at |
| 136030_at | 1436258_at |
| 136032_at | 1435215_at |
| 136057_at | 1426302_at |
| 136063_at | 1456872_a_at |
| 136067_at | 1451304_at |
| 136068_at | 1439163_at |
| 136077_at | 1458643_at |
| 136080_at | 1460094_at |
| 136082_at | 1440273_at |
| 136091_at | 1441183_at |
| 136092_at | 1442486_at |
| 136093_at | 1457875_at |
| 136096_r_at | 1457862_at |
| 136099_at | 1432185_a_at |
| 136100_at | 1457390_at |
| 136101_at | 1442949_at |
| 136103_at | 1442776_at |
| 136104_at | 1458029_at |
| 136106_at | 1457535_at |
| 136112_at | 1420136_a_at |
| 136115_at | 1447485_at |
| 136116_at | 1438800_at |
| 136117_at | 1442404_at |
| 136122_at | 1430773_a_at |
| 136125_at | 1437256_at |
| 136132_at | 1455203_at |
| 136140_at | 1424606_at |
| 136143_at | 1458655_at |
| 136146_at | 1443859_at |
| 136148_at | 1451606_at |
| 136149_at | 1455398_at |
| 136156_at | 1457657_at |
| 136157_at | 1447461_at |
| 136178_at | 1429052_at |
| 136187_f_at | 1428734_at |
| 136188_at | 1457437_at |
| 136189_at | 1459454_at |
| 136196_at | 1445840_at |
| 136198_at | 1438042_at |
| 136199_at | 1441338_at |
| 136201_at | 1442695_at |
| 136203_r_at | 1456828_at |
| 136204_at | 1456892_at |
| 136207_at | 1425067_at |
| 136215_at | 1458276_x_at |
| 136220_at | 1446980_at |
| 136224_at | 1425081_at |
| 136225_at | 1432293_at |
| 136229_at | 1423320_at |
| 136236_at | 1447382_at |

| | |
|---|---|
| 136238_f_at | 1434679_at |
| 136239_at | 1459006_a_at |
| 136247_at | 1447265_at |
| 136250_at | 1443724_at |
| 136252_at | 1441461_at |
| 136259_at | 1445854_at |
| 136260_at | 1441363_at |
| 136261_at | 1460030_at |
| 136267_at | 1444707_at |
| 136268_at | 1444273_at |
| 136270_at | 1436127_at |
| 136275_at | 1438491_x_at |
| 136277_at | 1445328_at |
| 136283_at | 1458536_at |
| 136284_at | 1446041_at |
| 136288_at | 1457867_at |
| 136293_at | 1442206_at |
| 136348_at | 1439669_at |
| 136388_at | 1436103_at |
| 136389_at | 1430793_at |
| 136390_at | 1446939_at |
| 136409_at | 1420078_at |
| 136414_at | 1459139_at |
| 136434_at | 1447243_at |
| 136436_at | 1448301_s_at |
| 136440_at | 1446519_at |
| 136442_at | 1438443_at |
| 136443_at | 1451396_at |
| 136444_at | 1445874_at |
| 136446_at | 1443454_at |
| 136448_at | 1444636_at |
| 136451_at | 1455640_a_at |
| 136453_at | 1439724_at |
| 136534_at | 1439910_a_at |
| 136540_r_at | 1440928_at |
| 136542_at | 1429903_at |
| 136543_i_at | 1456150_at |
| 136544_at | 1440919_at |
| 136547_at | 1440705_at |
| 136548_s_at | 1441678_at |
| 136557_at | 1447388_at |
| 136562_at | 1442338_at |
| 136565_at | 1425300_at |
| 136567_at | 1442850_at |
| 136571_r_at | 1440187_at |
| 136580_at | 1451655_at |
| 136596_at | 1456985_at |
| 136610_at | 1441422_at |
| 136611_at | 1457470_at |
| 136618_at | 1430062_at |
| 136620_r_at | 1440302_at |
| 136622_at | 1445512_at |
| 136624_at | 1439633_at |
| 136626_r_at | 1453139_at |
| 136633_at | 1444598_at |
| 136637_at | 1447133_at |

| | |
|---|---|
| 136639_at | 1445674_at |
| 136641_at | 1428764_at |
| 136646_at | 1439622_at |
| 136647_at | 1429967_at |
| 136657_at | 1455344_at |
| 136658_at | 1445099_at |
| 136659_at | 1454784_at |
| 136662_at | 1457726_at |
| 136665_at | 1458015_at |
| 136666_at | 1419828_at |
| 136667_at | 1439954_at |
| 136669_at | 1445013_at |
| 136673_at | 1443979_at |
| 136674_at | 1425988_a_at |
| 136675_at | 1435124_at |
| 136677_at | 1425982_a_at |
| 136678_at | 1445549_at |
| 136679_at | 1451684_a_at |
| 136680_at | 1455373_at |
| 136682_at | 1440365_at |
| 136685_at | 1453309_at |
| 136688_at | 1435699_at |
| 136690_at | 1438481_at |
| 136691_at | 1457523_at |
| 136692_at | 1442369_at |
| 136702_at | 1447725_at |
| 136703_at | 1419237_at |
| 136705_at | 1443268_at |
| 136710_at | 1434055_at |
| 136711_at | 1459967_at |
| 136712_at | 1445856_at |
| 136715_at | 1445524_at |
| 136716_at | 1452505_at |
| 136718_r_at | 1457904_at |
| 136721_at | 1456988_at |
| 136724_at | 1451534_at |
| 136725_at | 1436246_at |
| 136726_r_at | 1441369_at |
| 136727_at | 1446000_at |
| 136728_at | 1443073_at |
| 136731_at | 1439904_at |
| 136732_at | 1453366_at |
| 136734_at | 1422649_at |
| 136744_at | 1457501_at |
| 136748_at | 1426647_at |
| 136750_at | 1425667_at |
| 136755_at | 1439196_at |
| 136758_at | 1439851_at |
| 136759_at | 1446360_at |
| 136763_at | 1419588_at |
| 136764_at | 1435982_at |
| 136766_at | 1417996_at |
| 136772_at | 1433792_at |
| 136796_at | 1447289_at |
| 136943_s_at | 1457225_at |
| 136968_at | 1441142_at |

| | |
|---|---|
| 137008_at | 1457863_at |
| 137015_at | 1448781_at |
| 137018_at | 1445206_at |
| 137019_at | 1460117_at |
| 137023_at | 1441366_at |
| 137038_at | 1425119_at |
| 137040_at | 1440477_at |
| 137044_at | 1440223_at |
| 137046_s_at | 1460592_at |
| 137047_at | 1445327_at |
| 137050_at | 1457791_at |
| 137060_at | 1443682_at |
| 137078_at | 1457326_at |
| 137081_at | 1445536_at |
| 137082_at | 1460628_at |
| 137088_at | 1440251_s_at |
| 137089_r_at | 1444597_at |
| 137095_at | 1444592_at |
| 137098_at | 1437924_at |
| 137100_at | 1448025_at |
| 137104_at | 1443931_at |
| 137105_at | 1447575_at |
| 137120_at | 1455277_at |
| 137122_at | 1442358_at |
| 137123_at | 1456450_at |
| 137124_at | 1449945_at |
| 137128_at | 1436571_at |
| 137129_at | 1441191_at |
| 137131_at | 1436575_at |
| 137132_f_at | 1455512_at |
| 137133_s_at | 1435730_at |
| 137137_at | 1452451_at |
| 137143_at | 1423742_at |
| 137144_at | 1446075_at |
| 137153_at | 1440468_at |
| 137154_at | 1460425_at |
| 137161_at | 1448104_at |
| 137165_at | 1447301_at |
| 137170_at | 1427561_a_at |
| 137177_at | 1459994_x_at |
| 137180_at | 1452827_at |
| 137190_f_at | 1436751_at |
| 137208_r_at | 1447191_at |
| 137213_at | 1428200_a_at |
| 137214_at | 1456183_at |
| 137215_at | 1425304_s_at |
| 137225_at | 1420367_at |
| 137227_at | 1442555_at |
| 137236_f_at | 1429973_at |
| 137323_at | 1459579_at |
| 137325_at | 1445656_at |
| 137330_at | 1419044_at |
| 137332_at | 1446253_at |
| 137336_at | 1440558_at |
| 137337_at | 1459669_at |
| 137340_at | 1441244_at |

| | |
|---|---|
| 137344_at | 1442188_at |
| 137346_at | 1443009_at |
| 137347_at | 1460150_at |
| 137350_at | 1444332_at |
| 137351_at | 1441511_at |
| 137353_at | 1444208_at |
| 137356_at | 1441203_at |
| 137357_at | 1444345_at |
| 137358_at | 1457086_at |
| 137361_at | 1442889_at |
| 137362_at | 1441725_at |
| 137363_at | 1444633_at |
| 137364_at | 1459683_at |
| 137365_at | 1435289_at |
| 137366_at | 1442741_at |
| 137369_at | 1445221_at |
| 137371_at | 1436111_at |
| 137373_at | 1458861_at |
| 137374_at | 1442383_at |
| 137375_at | 1441453_at |
| 137376_at | 1444124_a_at |
| 137380_at | 1458246_at |
| 137381_at | 1460306_at |
| 137484_at | 1458025_at |
| 137486_at | 1444023_at |
| 137492_at | 1456088_at |
| 137493_at | 1421494_at |
| 137494_f_at | 1457098_at |
| 137496_at | 1442981_at |
| 137497_at | 1441283_at |
| 137499_at | 1459692_at |
| 137506_at | 1440925_at |
| 137513_at | 1452894_at |
| 137515_at | 1430373_at |
| 137517_at | 1445694_at |
| 137525_at | 1441799_at |
| 137526_r_at | 1427451_a_at |
| 137528_at | 1458498_at |
| 137529_at | 1430361_at |
| 137533_f_at | 1438488_at |
| 137535_at | 1447571_at |
| 137540_at | 1442256_at |
| 137550_at | 1436422_at |
| 137551_at | 1435887_at |
| 137557_at | 1455075_at |
| 137559_at | 1444212_at |
| 137562_at | 1437385_at |
| 137567_at | 1440283_at |
| 137569_at | 1421057_at |
| 137570_at | 1442295_at |
| 137574_at | 1458546_at |
| 137577_at | 1421098_at |
| 137578_at | 1419813_at |
| 137581_f_at | 1444457_at |
| 137584_f_at | 1444577_x_at |
| 137589_at | 1445679_at |

| | |
|---|---|
| 137594_at | 1457784_at |
| 137595_at | 1442495_at |
| 137606_at | 1453770_at |
| 137609_at | 1457473_at |
| 137614_at | 1455681_at |
| 137626_at | 1457486_at |
| 137629_at | 1446890_at |
| 137638_at | 1442750_at |
| 137639_at | 1431582_at |
| 137641_at | 1453177_at |
| 137652_at | 1455415_at |
| 137653_at | 1439137_at |
| 137657_at | 1445229_at |
| 137669_at | 1426109_a_at |
| 137670_at | 1458936_at |
| 137671_at | 1446715_at |
| 137672_at | 1442102_at |
| 137676_at | 1447566_at |
| 137677_at | 1425846_a_at |
| 137678_at | 1445459_at |
| 137684_at | 1439632_at |
| 137688_at | 1458024_at |
| 137692_at | 1444357_at |
| 137693_at | 1436532_at |
| 137696_at | 1430112_at |
| 137697_at | 1457155_at |
| 137699_at | 1438531_at |
| 137701_at | 1458060_at |
| 137712_at | 1457135_at |
| 137717_at | 1416117_at |
| 137718_at | 1440057_at |
| 137719_at | 1438572_at |
| 137722_at | 1456818_at |
| 137723_at | 1446206_at |
| 137724_at | 1425424_at |
| 137727_at | 1439237_a_at |
| 137728_at | 1440116_at |
| 137731_at | 1436577_at |
| 137733_at | 1460051_at |
| 137913_at | 1429909_at |
| 137914_at | 1444825_at |
| 137972_at | 1455064_at |
| 137974_at | 1455119_at |
| 137977_s_at | 1435759_at |
| 137979_at | 1456944_at |
| 137980_at | 1445403_at |
| 137981_at | 1436150_at |
| 137982_at | 1447223_at |
| 137984_at | 1442032_at |
| 137985_at | 1419825_at |
| 137987_at | 1459056_at |
| 137994_f_at | 1434590_at |
| 137996_at | 1459727_at |
| 138001_at | 1420354_at |
| 138004_at | 1439976_at |
| 138008_r_at | 1437944_at |

| | |
|---|---|
| 138009_at | 1458638_at |
| 138012_at | 1444046_at |
| 138020_at | 1445479_at |
| 138026_at | 1443525_at |
| 138027_at | 1457531_a_at |
| 138028_at | 1445375_at |
| 138030_at | 1449613_at |
| 138031_at | 1424902_at |
| 138032_at | 1447402_at |
| 138033_at | 1438662_at |
| 138036_at | 1441815_at |
| 138046_at | 1459728_at |
| 138048_at | 1440673_at |
| 138054_at | 1455410_at |
| 138055_at | 1442899_at |
| 138056_r_at | 1456965_at |
| 138058_at | 1435404_at |
| 138059_at | 1436137_at |
| 138062_at | 1439358_a_at |
| 138063_at | 1434536_at |
| 138066_at | 1445357_at |
| 138068_at | 1457423_at |
| 138070_at | 1434800_at |
| 138071_at | 1437787_at |
| 138074_at | 1445634_at |
| 138079_at | 1424812_at |
| 138084_at | 1440258_at |
| 138086_f_at | 1434596_at |
| 138087_at | 1440270_at |
| 138092_at | 1444000_at |
| 138093_f_at | 1440797_at |
| 138096_at | 1456524_at |
| 138101_at | 1440426_at |
| 138104_at | 1447186_at |
| 138107_at | 1459127_at |
| 138108_at | 1459210_at |
| 138110_at | 1440479_at |
| 138114_at | 1441666_at |
| 138118_f_at | 1455632_at |
| 138120_at | 1451029_at |
| 138121_at | 1439590_at |
| 138122_at | 1442706_at |
| 138123_at | 1424734_at |
| 138127_at | 1441487_at |
| 138137_at | 1443479_at |
| 138138_at | 1435994_at |
| 138142_at | 1425146_at |
| 138148_at | 1453400_at |
| 138149_at | 1445415_at |
| 138151_at | 1445521_at |
| 138152_at | 1445394_at |
| 138153_at | 1436148_at |
| 138155_at | 1458956_at |
| 138158_at | 1446207_at |
| 138165_at | 1440534_at |
| 138166_at | 1447445_at |

| | |
|---|---|
| 138168_at | 1441786_at |
| 138169_at | 1457463_at |
| 138175_at | 1439717_at |
| 138177_at | 1450427_at |
| 138178_at | 1443628_at |
| 138182_at | 1435974_at |
| 138185_at | 1446506_at |
| 138187_at | 1459717_at |
| 138193_at | 1446663_at |
| 138194_at | 1431897_at |
| 138197_at | 1438962_s_at |
| 138204_at | 1424561_at |
| 138206_at | 1455526_at |
| 138209_at | 1449655_a_at |
| 138247_at | 1456931_at |
| 138248_at | 1459523_at |
| 138256_at | 1459629_at |
| 138313_at | 1447095_at |
| 138365_at | 1459564_at |
| 138367_at | 1457850_at |
| 138368_at | 1444906_at |
| 138369_at | 1442940_at |
| 138377_at | 1440668_at |
| 138383_at | 1458881_at |
| 138384_at | 1423530_at |
| 138385_at | 1447125_at |
| 138386_at | 1451657_a_at |
| 138388_at | 1447319_at |
| 138389_at | 1459730_at |
| 138390_at | 1447017_at |
| 138391_at | 1457597_at |
| 138392_at | 1437129_at |
| 138393_at | 1423183_at |
| 138395_at | 1439725_at |
| 138396_at | 1459634_at |
| 138398_at | 1445477_at |
| 138399_at | 1445590_at |
| 138401_at | 1444998_at |
| 138402_at | 1444026_at |
| 138403_at | 1457346_at |
| 138404_at | 1441155_at |
| 138408_at | 1441388_at |
| 138409_at | 1436411_at |
| 138411_at | 1444363_at |
| 138413_at | 1444381_at |
| 138414_at | 1420578_at |
| 138416_at | 1453352_at |
| 138421_at | 1448352_at |
| 138422_at | 1458598_at |
| 138423_r_at | 1456402_at |
| 138425_at | 1454079_at |
| 138452_at | 1437560_at |
| 138453_at | 1434989_at |
| 138454_at | 1455753_at |
| 138455_at | 1419745_at |
| 138457_at | 1439738_at |

| | |
|---|---|
| 138458_at | 1455785_at |
| 138459_at | 1440406_at |
| 138461_at | 1458273_at |
| 138462_r_at | 1419732_at |
| 138464_at | 1436268_at |
| 138465_at | 1439754_at |
| 138468_at | 1436095_at |
| 138479_at | 1424633_at |
| 138480_at | 1445685_at |
| 138481_at | 1453566_at |
| 138483_at | 1455365_at |
| 138484_f_at | 1436123_at |
| 138485_at | 1447559_at |
| 138486_at | 1421629_at |
| 138488_at | 1440864_at |
| 138494_at | 1453486_a_at |
| 138495_f_at | 1435511_at |
| 138497_at | 1447467_at |
| 138498_at | 1419781_at |
| 138500_at | 1429836_at |
| 138502_at | 1444531_at |
| 138504_at | 1445863_at |
| 138505_at | 1443773_at |
| 138506_at | 1455623_at |
| 138507_at | 1455146_at |
| 138510_at | 1457183_at |
| 138514_at | 1459798_x_at |
| 138515_at | 1460013_at |
| 138517_at | 1444468_at |
| 138519_at | 1453170_at |
| 138523_at | 1459716_at |
| 138524_r_at | 1434720_at |
| 138525_at | 1447540_at |
| 138526_at | 1456519_at |
| 138527_at | 1435503_at |
| 138528_at | 1433802_at |
| 138531_at | 1457087_at |
| 138532_at | 1435246_at |
| 138533_at | 1455544_at |
| 138538_at | 1442726_s_at |
| 138539_at | 1433888_at |
| 138541_r_at | 1457323_at |
| 138542_at | 1455756_at |
| 138543_at | 1444095_a_at |
| 138545_at | 1439912_at |
| 138548_at | 1426094_at |
| 138551_at | 1457785_at |
| 138552_at | 1440504_at |
| 138554_at | 1448083_at |
| 138556_at | 1428317_at |
| 138558_at | 1418606_at |
| 138763_at | 1445735_at |
| 138769_at | 1446944_at |
| 138787_at | 1428033_at |
| 138788_f_at | 1426268_at |
| 138793_at | 1424122_s_at |

| | |
|---|---|
| 138795_at | 1459602_at |
| 138796_at | 1418205_at |
| 138799_at | 1420435_at |
| 138800_at | 1441708_at |
| 138801_at | 1440126_at |
| 138802_at | 1420727_a_at |
| 138929_at | 1442309_at |
| 138930_at | 1436378_at |
| 138936_s_at | 1442583_a_at |
| 138946_at | 1460081_at |
| 138947_at | 1436275_at |
| 138949_at | 1449872_at |
| 138950_at | 1418947_at |
| 138965_at | 1431167_at |
| 138966_at | 1416322_at |
| 138970_at | 1460482_at |
| 138971_at | 1435300_at |
| 138975_at | 1455701_at |
| 138976_at | 1451499_at |
| 138978_at | 1424993_at |
| 138989_at | 1442887_at |
| 138990_at | 1458483_at |
| 138993_r_at | 1458095_at |
| 138994_f_at | 1434347_s_at |
| 138997_at | 1457149_at |
| 138998_at | 1453359_at |
| 138999_at | 1442762_at |
| 139001_at | 1447232_at |
| 139008_at | 1456288_at |
| 139012_at | 1457969_at |
| 139016_at | 1441741_at |
| 139021_at | 1441295_at |
| 139022_at | 1444789_at |
| 139023_at | 1429215_at |
| 139025_f_at | 1445662_x_at |
| 139027_at | 1458407_s_at |
| 139041_at | 1456621_at |
| 139044_at | 1440203_at |
| 139045_at | 1447442_at |
| 139046_at | 1442732_at |
| 139126_at | 1439867_at |
| 139132_f_at | 1447124_at |
| 139136_at | 1459626_at |
| 139140_at | 1447501_at |
| 139144_at | 1456187_at |
| 139146_at | 1456883_at |
| 139147_at | 1435120_at |
| 139148_at | 1440562_at |
| 139149_at | 1447373_at |
| 139150_at | 1453709_at |
| 139152_at | 1438098_at |
| 139153_at | 1458255_at |
| 139162_at | 1440636_at |
| 139164_at | 1447452_at |
| 139169_at | 1441109_at |
| 139170_at | 1447464_at |

| | |
|---|---|
| 139171_at | 1460327_at |
| 139174_at | 1437925_at |
| 139180_at | 1455925_at |
| 139181_at | 1457979_at |
| 139182_at | 1443716_at |
| 139184_at | 1427020_at |
| 139194_at | 1419757_at |
| 139199_at | 1449878_a_at |
| 139202_at | 1441367_a_at |
| 139211_at | 1419104_at |
| 139223_at | 1433947_at |
| 139225_at | 1455518_at |
| 139231_at | 1442370_at |
| 139232_at | 1447412_at |
| 139237_r_at | 1450243_a_at |
| 139238_at | 1443028_at |
| 139239_at | 1438505_s_at |
| 139242_at | 1442645_at |
| 139244_at | 1441777_at |
| 139246_at | 1452356_at |
| 139249_f_at | 1452979_at |
| 139250_at | 1436408_at |
| 139254_at | 1440031_at |
| 139255_at | 1457295_at |
| 139257_at | 1440968_at |
| 139262_f_at | 1452640_at |
| 139264_at | 1439940_at |
| 139267_r_at | 1444185_at |
| 139270_at | 1439099_at |
| 139272_at | 1457040_at |
| 139276_at | 1428923_at |
| 139278_at | 1440286_at |
| 139279_at | 1447508_at |
| 139284_at | 1442930_at |
| 139285_f_at | 1455754_at |
| 139288_at | 1442225_at |
| 139289_at | 1442212_at |
| 139290_at | 1439493_at |
| 139291_at | 1455514_at |
| 139292_at | 1443683_at |
| 139293_at | 1447237_at |
| 139294_at | 1457888_at |
| 139295_at | 1441019_at |
| 139297_at | 1457957_at |
| 139397_at | 1440058_at |
| 139398_at | 1423015_at |
| 139401_at | 1459618_at |
| 139403_s_at | 1435276_a_at |
| 139406_at | 1457460_at |
| 139421_r_at | 1445660_at |
| 139422_at | 1424754_at |
| 139423_at | 1438701_at |
| 139481_at | 1443685_at |
| 139482_at | 1459792_at |
| 139483_at | 1460102_at |
| 139487_at | 1427107_at |

| | |
|---|---|
| 139489_at | 1444117_at |
| 139490_at | 1457951_at |
| 139493_at | 1449634_a_at |
| 139494_at | 1459409_at |
| 139495_at | 1442204_at |
| 139496_at | 1435852_at |
| 139497_at | 1432509_at |
| 139498_at | 1435621_at |
| 139499_at | 1441820_at |
| 139504_at | 1457010_at |
| 139519_at | 1455444_at |
| 139522_at | 1449629_s_at |
| 139526_at | 1458656_at |
| 139527_at | 1437386_at |
| 139528_r_at | 1441309_at |
| 139530_at | 1436335_at |
| 139531_at | 1420188_at |
| 139538_at | 1449669_at |
| 139547_at | 1444741_at |
| 139748_at | 1437496_at |
| 139753_at | 1445358_at |
| 139755_at | 1420593_a_at |
| 139804_at | 1431316_at |
| 139808_at | 1422929_s_at |
| 139836_at | 1442738_at |
| 140006_at | 1443146_at |
| 140015_at | 1436431_at |
| 140022_at | 1419395_at |
| 140189_at | 1453434_at |
| 140190_at | 1445498_at |
| 140192_at | 1456086_x_at |
| 140194_at | 1437449_at |
| 140195_at | 1440762_at |
| 140210_at | 1453582_at |
| 140220_r_at | 1442174_at |
| 140353_at | 1443465_at |
| 140354_at | 1453765_at |
| 140358_at | 1443738_at |
| 140359_at | 1444572_at |
| 140371_at | 1451807_at |
| 140405_at | 1430345_at |
| 140427_at | 1449963_at |
| 140432_r_at | 1420178_at |
| 140438_at | 1438730_at |
| 140439_at | 1420070_a_at |
| 140440_at | 1447121_at |
| 140451_at | 1425412_at |
| 140453_g_at | 1426135_a_at |
| 140457_at | 1431063_at |
| 140485_at | 1443111_at |
| 140489_f_at | 1436923_at |
| 140492_at | 1447457_at |
| 140510_at | 1445643_at |
| 140519_at | 1451801_at |
| 140546_at | 1426509_s_at |
| 140548_at | 1437307_at |

| | |
|---|---|
| 140549_r_at | 1439530_a_at |
| 140564_at | 1434364_at |
| 140569_at | 1442881_at |
| 140570_at | 1425138_at |
| 140571_at | 1443821_at |
| 140572_at | 1442033_at |
| 140588_at | 1447520_at |
| 140607_at | 1431296_at |
| 140609_f_at | 1434397_at |
| 140641_at | 1431251_at |
| 140642_at | 1457626_at |
| 140652_r_at | 1442311_at |
| 140656_at | 1453789_at |
| 140659_at | 1419697_at |
| 140660_at | 1440304_at |
| 140661_at | 1419871_at |
| 140665_at | 1459774_at |
| 140707_at | 1458425_at |
| 140708_at | 1441582_at |
| 140709_at | 1429222_at |
| 140710_at | 1440763_at |
| 140711_at | 1428396_at |
| 140712_r_at | 1459072_at |
| 140713_at | 1443659_at |
| 140715_at | 1445416_at |
| 140716_at | 1459726_at |
| 140750_at | 1425808_a_at |
| 140759_at | 1436324_at |
| 140768_at | 1445879_at |
| 140805_at | 1436135_at |
| 140817_at | 1425057_at |
| 140819_at | 1420430_a_at |
| 140828_at | 1431147_at |
| 140833_at | 1456655_at |
| 140837_at | 1459745_at |
| 140839_at | 1440824_at |
| 140872_at | 1447580_at |
| 140876_at | 1442898_at |
| 140877_at | 1435579_at |
| 140879_r_at | 1440942_at |
| 140880_at | 1455497_at |
| 140881_at | 1429232_at |
| 140883_at | 1457907_at |
| 140885_at | 1458622_at |
| 140888_at | 1457217_at |
| 140889_s_at | 1439862_at |
| 140891_at | 1442346_at |
| 140893_at | 1444091_a_at |
| 140894_at | 1431025_at |
| 140896_at | 1439608_at |
| 140972_f_at | 1430762_at |
| 140984_r_at | 1456876_at |
| 140991_at | 1416804_at |
| 141010_at | 1422050_at |
| 141032_at | 1443950_at |
| 141033_at | 1438752_at |

| | |
|---|---|
| 141037_at | 1419359_at |
| 141040_at | 1440001_at |
| 141048_at | 1439770_at |
| 141049_r_at | 1457249_at |
| 141051_at | 1444344_at |
| 141069_at | 1430746_at |
| 141084_at | 1454112_a_at |
| 141101_at | 1442220_at |
| 141108_at | 1447535_at |
| 141110_at | 1457578_at |
| 141114_at | 1455982_at |
| 141115_at | 1446755_at |
| 141117_at | 1440415_at |
| 141118_at | 1440497_at |
| 141123_at | 1442480_at |
| 141125_at | 1438304_at |
| 141154_at | 1440709_at |
| 141170_at | 1458474_at |
| 141172_at | 1430630_at |
| 96936_at | 1415670_at |
| 93256_at | 1415672_at |
| 94052_at | 1415675_at |
| 93843_at | 1415677_at |
| 98580_at | 1415678_at |
| 94225_at | 1415684_at |
| 93312_at | 1415688_at |
| 160849_at | 1415691_at |
| 94886_at | 1415692_s_at |
| 93982_at | 1415693_at |
| 98605_at | 1415694_at |
| 95128_at | 1415707_at |
| 99096_at | 1415713_a_at |
| 99651_at | 1415714_a_at |
| 97392_at | 1415715_at |
| 160282_at | 1415717_at |
| 97471_at | 1415719_s_at |
| 95565_at | 1415720_s_at |
| 94488_at | 1415722_a_at |
| 99652_at | 1415732_at |
| 99587_at | 1415734_at |
| 96257_at | 1415751_at |
| 160366_at | 1415752_at |
| 160792_at | 1415756_a_at |
| 98037_at | 1415759_a_at |
| 93081_at | 1415775_at |
| 99559_at | 1415776_at |
| 92601_at | 1415777_at |
| 96573_at | 1415779_s_at |
| 92640_at | 1415783_at |
| 97516_at | 1415787_at |
| 94920_at | 1415793_at |
| 99563_at | 1415794_a_at |
| 98617_at | 1415799_at |
| 93981_at | 1415806_at |
| 166975_r_at | 1415809_at |
| 160562_at | 1415816_at |

| | |
|---|---|
| 94304_at | 1415818_at |
| 92573_at | 1415819_a_at |
| 97262_at | 1415825_s_at |
| 160890_at | 1415829_at |
| 98523_at | 1415833_x_at |
| 160443_at | 1415835_at |
| 95738_at | 1415836_at |
| 98513_at | 1415842_at |
| 170392_at | 1415843_at |
| 160190_at | 1415844_at |
| 168114_i_at | 1415845_at |
| 93103_at | 1415847_at |
| 97940_at | 1415848_at |
| 160490_at | 1415853_at |
| 99577_at | 1415855_at |
| 94837_at | 1415858_at |
| 97954_at | 1415861_at |
| 93990_at | 1415872_at |
| 98564_f_at | 1415876_a_at |
| 160530_at | 1415881_at |
| 162530_at | 1415884_at |
| 96268_at | 1415891_at |
| 160742_at | 1415901_at |
| 95611_at | 1415904_at |
| 160213_at | 1415905_at |
| 93257_at | 1415915_at |
| 92626_at | 1415919_at |
| 160577_at | 1415932_x_at |
| 96926_at | 1415935_at |
| 99152_at | 1415939_at |
| 160713_at | 1415940_at |
| 98342_at | 1415942_at |
| 96033_at | 1415944_at |
| 93124_at | 1415950_a_at |
| 99082_at | 1415951_at |
| 96267_at | 1415967_at |
| 94199_at | 1415969_s_at |
| 100551_r_at | 1415970_at |
| 97695_s_at | 1415979_x_at |
| 94278_at | 1415983_at |
| 94322_at | 1415993_at |
| 93996_at | 1415994_at |
| 95671_at | 1415999_at |
| 97535_at | 1416004_at |
| 96930_at | 1416012_at |
| 98153_at | 1416024_x_at |
| 93096_at | 1416025_at |
| 95412_at | 1416027_at |
| 93356_at | 1416030_a_at |
| 160378_at | 1416032_at |
| 98628_f_at | 1416035_at |
| 162544_i_at | 1416040_at |
| 161045_at | 1416053_at |
| 99336_at | 1416054_at |
| 96887_at | 1416056_a_at |
| 98944_at | 1416059_at |

| | |
|---|---|
| 160441_at | 1416063_x_at |
| 96324_at | 1416068_at |
| 160358_at | 1416072_at |
| 94264_at | 1416078_s_at |
| 160321_at | 1416083_at |
| 92868_at | 1416086_at |
| 94527_at | 1416096_at |
| 163255_at | 1416098_at |
| 99101_at | 1416100_at |
| 160413_at | 1416107_at |
| 160319_at | 1416114_at |
| 162766_at | 1416115_at |
| 167515_at | 1416116_at |
| 92807_at | 1416119_at |
| 97504_at | 1416123_at |
| 92225_f_at | 1416126_at |
| 93587_at | 1416137_at |
| 98128_at | 1416143_at |
| 165742_r_at | 1416149_at |
| 161792_f_at | 1416159_at |
| 160551_at | 1416175_a_at |
| 95405_at | 1416181_at |
| 95059_at | 1416186_at |
| 160291_at | 1416189_a_at |
| 98098_at | 1416193_at |
| 94313_at | 1416197_at |
| 94015_at | 1416198_at |
| 97963_at | 1416206_at |
| 162364_f_at | 1416209_at |
| 93822_at | 1416217_a_at |
| 97483_at | 1416219_at |
| 99932_at | 1416224_at |
| 94906_at | 1416225_at |
| 96023_at | 1416231_at |
| 94486_at | 1416234_at |
| 94125_at | 1416237_at |
| 99936_at | 1416238_at |
| 94263_f_at | 1416240_at |
| 95091_at | 1416241_at |
| 94518_at | 1416245_at |
| 94522_at | 1416247_at |
| 94521_at | 1416253_at |
| 160410_at | 1416254_a_at |
| 96081_at | 1416258_at |
| 92418_at | 1416263_at |
| 97331_at | 1416265_at |
| 99840_at | 1416266_at |
| 160126_at | 1416272_at |
| 160489_at | 1416273_at |
| 99128_at | 1416278_a_at |
| 95704_at | 1416279_at |
| 96264_at | 1416281_at |
| 96652_at | 1416284_at |
| 162964_at | 1416286_at |
| 96256_at | 1416292_at |
| 98015_at | 1416294_at |

| | |
|---|---|
| 99957_at | 1416298_at |
| 93506_at | 1416300_a_at |
| 94248_at | 1416307_at |
| 93924_f_at | 1416311_s_at |
| 96693_at | 1416312_at |
| 95503_at | 1416313_at |
| 93512_f_at | 1416319_at |
| 166317_f_at | 1416321_s_at |
| 99940_at | 1416323_at |
| 93122_at | 1416325_at |
| 97807_at | 1416327_at |
| 93284_at | 1416332_at |
| 166724_at | 1416334_at |
| 94849_at | 1416335_at |
| 95472_f_at | 1416337_at |
| 93275_at | 1416338_at |
| 99562_at | 1416340_a_at |
| 160146_r_at | 1416341_at |
| 160458_at | 1416357_a_at |
| 162866_at | 1416358_at |
| 95359_at | 1416364_at |
| 93359_at | 1416366_at |
| 98917_at | 1416369_at |
| 95065_at | 1416373_at |
| 95137_at | 1416376_at |
| 96802_at | 1416377_at |
| 97332_at | 1416378_at |
| 96956_at | 1416381_a_at |
| 93308_s_at | 1416383_a_at |
| 98930_at | 1416384_a_at |
| 92847_s_at | 1416385_a_at |
| 96919_at | 1416392_a_at |
| 98544_at | 1416395_at |
| 99584_at | 1416401_at |
| 97647_at | 1416404_s_at |
| 160826_at | 1416412_at |
| 162532_r_at | 1416413_at |
| 95074_at | 1416425_at |
| 94478_at | 1416426_at |
| 162769_at | 1416428_at |
| 96044_at | 1416434_at |
| 97980_at | 1416435_at |
| 96329_at | 1416438_at |
| 93039_at | 1416441_at |
| 95703_at | 1416443_a_at |
| 94393_r_at | 1416444_at |
| 95451_at | 1416445_at |
| 94882_at | 1416446_at |
| 111121_at | 1416450_at |
| 160509_at | 1416456_a_at |
| 93781_at | 1416460_at |
| 94889_at | 1416465_a_at |
| 93991_at | 1416478_a_at |
| 95441_at | 1416485_at |
| 92578_at | 1416486_at |
| 163853_at | 1416489_at |

| | |
|---|---|
| 163810_at | 1416491_at |
| 99593_at | 1416495_s_at |
| 160457_at | 1416496_at |
| 160327_at | 1416499_a_at |
| 96952_at | 1416506_at |
| 95044_at | 1416508_at |
| 95736_at | 1416510_at |
| 99595_at | 1416521_at |
| 94206_at | 1416522_a_at |
| 160096_at | 1416524_at |
| 95491_at | 1416526_a_at |
| 96338_at | 1416533_at |
| 96073_at | 1416534_at |
| 98563_f_at | 1416535_at |
| 99111_at | 1416541_at |
| 96962_at | 1416546_a_at |
| 93562_at | 1416547_at |
| 99570_s_at | 1416551_at |
| 93526_at | 1416553_at |
| 99629_at | 1416555_at |
| 92487_at | 1416564_at |
| 160226_at | 1416570_s_at |
| 160318_at | 1416580_a_at |
| 97845_at | 1416581_at |
| 99670_at | 1416582_a_at |
| 160317_at | 1416591_at |
| 96761_at | 1416596_at |
| 98599_at | 1416597_at |
| 95072_at | 1416604_at |
| 99979_at | 1416612_at |
| 162603_at | 1416619_at |
| 162896_at | 1416620_at |
| 99054_at | 1416621_at |
| 162770_at | 1416622_at |
| 99081_at | 1416625_at |
| 96017_at | 1416628_at |
| 92582_at | 1416629_at |
| 92614_at | 1416630_at |
| 162758_at | 1416631_at |
| 94872_at | 1416635_at |
| 160839_at | 1416639_at |
| 92551_at | 1416641_at |
| 97333_at | 1416648_at |
| 94045_at | 1416649_at |
| 93449_at | 1416650_at |
| 92648_at | 1416653_at |
| 162556_at | 1416654_at |
| 94250_at | 1416659_at |
| 96321_at | 1416663_at |
| 93582_at | 1416665_at |
| 98571_s_at | 1416669_s_at |
| 93837_at | 1416676_at |
| 94318_at | 1416677_at |
| 93045_at | 1416679_at |
| 160503_at | 1416684_at |
| 165413_at | 1416694_at |

| | |
|---|---|
| 93042_at | 1416695_at |
| 96318_at | 1416696_at |
| 160333_at | 1416699_at |
| 99978_s_at | 1416703_at |
| 99647_at | 1416705_at |
| 164120_at | 1416706_at |
| 98552_at | 1416707_a_at |
| 165464_r_at | 1416710_at |
| 95108_at | 1416713_at |
| 98002_at | 1416714_at |
| 96195_at | 1416716_at |
| 92825_at | 1416717_at |
| 92700_at | 1416718_at |
| 96099_at | 1416728_at |
| 98923_at | 1416730_at |
| 98496_at | 1416737_at |
| 93784_at | 1416742_at |
| 97281_at | 1416744_at |
| 94828_at | 1416750_at |
| 160354_at | 1416752_at |
| 94944_at | 1416753_at |
| 160258_at | 1416765_s_at |
| 98955_at | 1416770_at |
| 160504_at | 1416777_at |
| 163570_at | 1416785_at |
| 93029_at | 1416788_a_at |
| 93948_at | 1416796_at |
| 167691_at | 1416800_at |
| 160699_at | 1416802_a_at |
| 92754_at | 1416806_at |
| 160081_at | 1416807_at |
| 99135_at | 1416819_at |
| 160574_at | 1416821_at |
| 92387_at | 1416827_at |
| 96938_at | 1416833_at |
| 160717_at | 1416841_at |
| 97310_at | 1416851_at |
| 98994_at | 1416854_at |
| 92360_at | 1416861_at |
| 166573_r_at | 1416862_at |
| 162590_at | 1416866_at |
| 160638_at | 1416868_at |
| 94412_at | 1416873_a_at |
| 160629_at | 1416882_at |
| 95713_at | 1416883_at |
| 94233_at | 1416885_at |
| 93532_at | 1416889_at |
| 97405_at | 1416896_at |
| 96354_at | 1416904_at |
| 96051_at | 1416905_at |
| 95100_at | 1416906_at |
| 94495_at | 1416909_at |
| 97204_s_at | 1416910_at |
| 97267_at | 1416911_a_at |
| 96718_at | 1416914_s_at |
| 99059_at | 1416916_at |

| | |
|---|---|
| 98614_at | 1416919_a_at |
| 160553_at | 1416930_at |
| 99019_at | 1416933_at |
| 98963_at | 1416935_at |
| 96951_at | 1416951_a_at |
| 93915_at | 1416957_at |
| 94407_at | 1416960_at |
| 162775_at | 1416962_at |
| 97407_at | 1416963_at |
| 163551_at | 1416964_at |
| 160104_at | 1416968_a_at |
| 93820_at | 1416970_a_at |
| 97533_at | 1416978_at |
| 94014_at | 1416979_at |
| 163982_at | 1416987_at |
| 162580_at | 1416995_at |
| 99166_at | 1417002_at |
| 94809_at | 1417004_at |
| 93565_at | 1417005_at |
| 93337_at | 1417007_a_at |
| 95104_at | 1417011_at |
| 165588_at | 1417021_a_at |
| 94285_at | 1417025_at |
| 96937_at | 1417026_at |
| 165839_at | 1417031_at |
| 162992_at | 1417034_at |
| 97891_at | 1417035_at |
| 97430_at | 1417042_at |
| 162550_f_at | 1417046_at |
| 93579_at | 1417064_at |
| 97431_at | 1417072_at |
| 160729_f_at | 1417076_at |
| 96891_at | 1417082_at |
| 93548_at | 1417083_at |
| 94353_at | 1417084_at |
| 92556_at | 1417085_at |
| 94364_at | 1417087_at |
| 160565_at | 1417089_a_at |
| 99070_at | 1417091_at |
| 160798_at | 1417098_s_at |
| 163077_at | 1417101_at |
| 97307_f_at | 1417102_a_at |
| 97253_at | 1417106_at |
| 98461_at | 1417108_at |
| 160579_at | 1417111_at |
| 161631_f_at | 1417117_at |
| 162767_at | 1417119_at |
| 92939_at | 1417121_at |
| 163116_at | 1417122_at |
| 94492_at | 1417124_at |
| 95150_at | 1417128_at |
| 97389_at | 1417131_at |
| 163240_i_at | 1417139_at |
| 160933_at | 1417141_at |
| 99924_at | 1417144_at |
| 160867_at | 1417148_at |

| | |
|---|---|
| 98983_at | 1417149_at |
| 99040_at | 1417150_at |
| 93137_at | 1417151_a_at |
| 168302_at | 1417158_at |
| 163480_at | 1417163_at |
| 163992_at | 1417164_at |
| 160833_at | 1417165_at |
| 97853_at | 1417166_at |
| 92821_at | 1417168_a_at |
| 92820_at | 1417169_at |
| 98597_at | 1417172_at |
| 163391_at | 1417179_at |
| 98022_at | 1417180_at |
| 92547_at | 1417186_at |
| 166746_at | 1417187_at |
| 94275_at | 1417206_at |
| 95588_at | 1417208_at |
| 163979_at | 1417214_at |
| 98588_at | 1417220_at |
| 94940_at | 1417227_at |
| 93934_at | 1417232_at |
| 94371_at | 1417237_at |
| 95103_at | 1417243_at |
| 97216_at | 1417246_at |
| 99939_at | 1417257_at |
| 93295_at | 1417258_at |
| 98404_at | 1417260_at |
| 97397_at | 1417265_s_at |
| 92849_at | 1417266_at |
| 97964_at | 1417267_s_at |
| 95037_at | 1417269_at |
| 163787_at | 1417270_at |
| 99089_at | 1417275_at |
| 160863_at | 1417276_at |
| 97420_at | 1417290_at |
| 92793_at | 1417291_at |
| 160770_at | 1417303_at |
| 96066_s_at | 1417308_at |
| 93188_at | 1417312_at |
| 97907_at | 1417313_at |
| 93513_at | 1417317_s_at |
| 166806_at | 1417318_at |
| 99062_at | 1417319_at |
| 162596_at | 1417323_at |
| 160938_at | 1417324_at |
| 98456_at | 1417334_at |
| 96978_at | 1417336_a_at |
| 96298_f_at | 1417339_a_at |
| 95681_f_at | 1417341_a_at |
| 95680_at | 1417342_at |
| 135772_at | 1417345_at |
| 162657_at | 1417349_at |
| 96280_at | 1417368_s_at |
| 92561_at | 1417382_at |
| 162622_at | 1417387_at |
| 99922_at | 1417397_at |

| | |
|---|---|
| 163285_at | 1417400_at |
| 95607_at | 1417405_at |
| 160834_at | 1417406_at |
| 160902_at | 1417412_at |
| 165507_f_at | 1417413_at |
| 97787_at | 1417415_at |
| 96828_at | 1417422_at |
| 98938_at | 1417427_at |
| 93094_at | 1417430_at |
| 161017_at | 1417445_at |
| 92450_at | 1417446_at |
| 94562_at | 1417456_at |
| 93765_at | 1417459_at |
| 160075_at | 1417468_at |
| 160597_at | 1417471_s_at |
| 96799_at | 1417476_at |
| 160691_at | 1417479_at |
| 99835_at | 1417487_at |
| 92256_at | 1417491_at |
| 160759_at | 1417503_at |
| 99527_at | 1417520_at |
| 162935_at | 1417522_at |
| 160830_at | 1417524_at |
| 92766_at | 1417525_at |
| 92814_at | 1417532_at |
| 160721_at | 1417540_at |
| 97340_at | 1417547_at |
| 96620_at | 1417550_a_at |
| 92441_at | 1417552_at |
| 92869_at | 1417554_at |
| 94075_at | 1417556_at |
| 168944_i_at | 1417557_at |
| 92831_at | 1417560_at |
| 162608_at | 1417564_at |
| 94857_at | 1417571_at |
| 163200_at | 1417575_at |
| 163029_at | 1417576_a_at |
| 163908_at | 1417577_at |
| 171571_f_at | 1417581_at |
| 160142_at | 1417593_at |
| 98419_at | 1417595_at |
| 98992_at | 1417596_at |
| 93694_at | 1417602_at |
| 99667_at | 1417607_at |
| 160175_at | 1417614_at |
| 99055_at | 1417616_at |
| 99500_at | 1417622_at |
| 160761_at | 1417626_at |
| 162728_at | 1417629_at |
| 162735_at | 1417630_at |
| 94902_at | 1417633_at |
| 96160_at | 1417636_at |
| 92497_at | 1417639_at |
| 161012_at | 1417640_at |
| 98372_at | 1417642_at |
| 93585_at | 1417651_at |

| | |
|---|---|
| 96719_i_at | 1417653_at |
| 97876_at | 1417659_at |
| 99984_at | 1417662_at |
| 98554_at | 1417663_a_at |
| 93542_at | 1417667_a_at |
| 98106_at | 1417670_at |
| 163574_at | 1417672_at |
| 166707_at | 1417677_at |
| 94506_at | 1417681_at |
| 92873_f_at | 1417682_a_at |
| 166408_r_at | 1417686_at |
| 96935_at | 1417689_a_at |
| 92916_at | 1417708_at |
| 94781_at | 1417714_x_at |
| 93355_at | 1417716_at |
| 96883_at | 1417718_at |
| 95147_at | 1417722_at |
| 96834_at | 1417727_at |
| 160596_at | 1417739_at |
| 163354_at | 1417750_a_at |
| 96575_at | 1417762_a_at |
| 163228_at | 1417769_at |
| 93310_at | 1417771_a_at |
| 98032_at | 1417778_at |
| 92742_at | 1417789_at |
| 98410_at | 1417793_at |
| 163139_at | 1417796_at |
| 160771_r_at | 1417801_a_at |
| 163007_at | 1417802_at |
| 163168_r_at | 1417807_at |
| 164099_at | 1417824_at |
| 162668_at | 1417833_at |
| 162682_at | 1417843_s_at |
| 95537_at | 1417846_at |
| 92417_at | 1417858_at |
| 99558_at | 1417861_at |
| 165474_f_at | 1417863_at |
| 99671_at | 1417867_at |
| 160916_at | 1417877_at |
| 93173_at | 1417881_at |
| 165796_at | 1417882_at |
| 170081_at | 1417885_at |
| 98484_at | 1417901_a_at |
| 97946_at | 1417905_at |
| 170146_r_at | 1417906_at |
| 97479_at | 1417908_s_at |
| 96846_at | 1417909_at |
| 99186_at | 1417910_at |
| 140455_at | 1417914_at |
| 98876_at | 1417918_at |
| 97979_at | 1417919_at |
| 161021_at | 1417923_at |
| 99025_at | 1417927_at |
| 92400_at | 1417931_at |
| 163796_at | 1417937_at |
| 163822_at | 1417942_at |

| | |
|---|---|
| 163572_at | 1417943_at |
| 99898_at | 1417944_at |
| 92813_at | 1417952_at |
| 95436_at | 1417954_at |
| 99994_at | 1417956_at |
| 160680_at | 1417960_at |
| 162086_r_at | 1417961_a_at |
| 96185_at | 1417964_at |
| 98014_at | 1417973_at |
| 165912_f_at | 1417979_at |
| 160247_at | 1417983_a_at |
| 95469_at | 1417987_at |
| 99442_at | 1417988_at |
| 165491_at | 1417990_at |
| 95552_at | 1417991_at |
| 99910_at | 1417994_a_at |
| 92356_at | 1417995_at |
| 95105_at | 1418002_at |
| 160359_at | 1418003_at |
| 95053_s_at | 1418005_at |
| 92233_at | 1418007_at |
| 92835_at | 1418013_at |
| 93580_at | 1418016_at |
| 92244_at | 1418026_at |
| 162920_at | 1418034_at |
| 95549_at | 1418036_at |
| 162707_i_at | 1418039_at |
| 163708_at | 1418041_at |
| 93942_at | 1418045_at |
| 160133_at | 1418049_at |
| 95566_at | 1418050_at |
| 94123_at | 1418054_at |
| 99000_at | 1418060_a_at |
| 92335_at | 1418061_at |
| 94429_at | 1418062_at |
| 93387_at | 1418063_at |
| 163086_at | 1418064_at |
| 97549_at | 1418066_at |
| 160284_at | 1418068_at |
| 160232_at | 1418074_at |
| 96682_at | 1418075_at |
| 163663_at | 1418080_at |
| 99063_at | 1418088_a_at |
| 95308_at | 1418098_at |
| 94928_at | 1418099_at |
| 160905_s_at | 1418100_at |
| 165490_r_at | 1418111_at |
| 98612_at | 1418113_at |
| 96239_at | 1418121_at |
| 95555_at | 1418123_at |
| 162602_at | 1418134_at |
| 160537_at | 1418138_at |
| 94404_at | 1418143_at |
| 92422_at | 1418149_at |
| 94135_at | 1418159_at |
| 169994_at | 1418162_at |

| | |
|---|---|
| 98240_at | 1418166_at |
| 93801_at | 1418173_at |
| 160841_at | 1418174_at |
| 99965_at | 1418175_at |
| 99964_at | 1418176_at |
| 95019_at | 1418186_at |
| 96895_at | 1418190_at |
| 92300_at | 1418192_at |
| 93367_at | 1418196_at |
| 99507_at | 1418197_at |
| 94402_r_at | 1418199_at |
| 162643_at | 1418207_at |
| 96504_at | 1418208_at |
| 93567_at | 1418210_at |
| 97082_at | 1418211_at |
| 163157_at | 1418213_at |
| 161037_at | 1418219_at |
| 99846_at | 1418220_at |
| 98814_at | 1418221_at |
| 96074_at | 1418239_at |
| 98987_at | 1418242_at |
| 162982_at | 1418244_at |
| 160080_r_at | 1418248_at |
| 97986_at | 1418259_a_at |
| 161041_at | 1418263_at |
| 92919_at | 1418268_at |
| 98119_at | 1418273_a_at |
| 160514_at | 1418277_at |
| 99479_at | 1418287_a_at |
| 98892_at | 1418288_at |
| 93379_at | 1418297_at |
| 98426_at | 1418302_at |
| 162601_i_at | 1418303_at |
| 98830_at | 1418307_a_at |
| 92435_at | 1418310_a_at |
| 165671_f_at | 1418311_at |
| 163632_at | 1418314_a_at |
| 98933_at | 1418316_a_at |
| 98527_at | 1418321_at |
| 160656_i_at | 1418323_at |
| 163661_at | 1418331_at |
| 163716_at | 1418332_a_at |
| 98335_at | 1418342_at |
| 161080_f_at | 1418343_at |
| 163915_at | 1418344_at |
| 93637_at | 1418353_at |
| 161049_at | 1418357_at |
| 92994_at | 1418363_at |
| 92599_at | 1418373_at |
| 97828_at | 1418377_a_at |
| 96132_at | 1418382_at |
| 93061_at | 1418393_a_at |
| 94441_at | 1418396_at |
| 99050_at | 1418413_at |
| 98380_at | 1418414_at |
| 99530_at | 1418418_a_at |

| | |
|---|---|
| 161115_r_at | 1418422_at |
| 98474_r_at | 1418424_at |
| 166111_f_at | 1418426_at |
| 97889_at | 1418438_at |
| 94228_at | 1418442_at |
| 96905_at | 1418444_a_at |
| 99010_at | 1418450_at |
| 96953_at | 1418456_a_at |
| 96835_at | 1418464_at |
| 92909_at | 1418471_at |
| 163160_at | 1418473_at |
| 112502_at | 1418479_at |
| 92987_at | 1418485_at |
| 97795_at | 1418489_a_at |
| 93139_at | 1418490_at |
| 92697_at | 1418496_at |
| 99893_at | 1418498_at |
| 93808_at | 1418509_at |
| 96742_at | 1418511_at |
| 98775_at | 1418513_at |
| 99034_at | 1418517_at |
| 98123_at | 1418519_at |
| 94277_at | 1418521_a_at |
| 96008_at | 1418528_a_at |
| 98438_f_at | 1418536_at |
| 97698_at | 1418548_at |
| 94700_at | 1418549_at |
| 160582_at | 1418551_at |
| 97149_at | 1418558_at |
| 98102_at | 1418560_at |
| 160395_at | 1418566_s_at |
| 92527_at | 1418586_at |
| 92892_at | 1418588_at |
| 167344_i_at | 1418592_at |
| 92937_at | 1418596_at |
| 94124_at | 1418597_at |
| 94778_at | 1418601_at |
| 92532_at | 1418603_at |
| 163044_at | 1418609_at |
| 92702_at | 1418615_at |
| 96523_at | 1418618_at |
| 92714_at | 1418619_at |
| 92980_at | 1418620_at |
| 94031_at | 1418622_at |
| 93740_at | 1418624_at |
| 98034_at | 1418638_at |
| 94900_at | 1418639_at |
| 160372_at | 1418644_a_at |
| 92833_at | 1418645_at |
| 98403_at | 1418646_at |
| 94389_at | 1418656_at |
| 163052_at | 1418658_at |
| 92731_at | 1418666_at |
| 97661_at | 1418682_at |
| 166648_i_at | 1418687_at |
| 94503_at | 1418692_at |

| | |
|---|---|
| 97402_at | 1418697_at |
| 98909_at | 1418700_at |
| 92362_at | 1418714_at |
| 163978_at | 1418716_at |
| 96153_at | 1418722_at |
| 99927_at | 1418724_at |
| 171107_at | 1418725_at |
| 161784_f_at | 1418728_at |
| 163993_at | 1418729_at |
| 160405_at | 1418732_s_at |
| 98028_at | 1418733_at |
| 162582_at | 1418740_at |
| 160274_at | 1418742_at |
| 165623_at | 1418744_s_at |
| 93369_at | 1418745_at |
| 99548_at | 1418752_at |
| 94737_at | 1418754_at |
| 166054_r_at | 1418757_at |
| 93658_at | 1418759_at |
| 160135_at | 1418763_at |
| 97335_at | 1418765_at |
| 95373_at | 1418770_at |
| 96838_at | 1418779_at |
| 92237_at | 1418782_at |
| 97427_at | 1418787_at |
| 92229_at | 1418790_at |
| 92436_at | 1418798_s_at |
| 160594_at | 1418799_a_at |
| 161058_f_at | 1418802_at |
| 93198_at | 1418806_at |
| 166002_at | 1418807_at |
| 95784_at | 1418809_at |
| 95290_at | 1418810_at |
| 163131_at | 1418816_at |
| 97322_at | 1418826_at |
| 163521_at | 1418828_at |
| 99045_at | 1418829_a_at |
| 160944_at | 1418831_at |
| 93625_at | 1418833_at |
| 164624_f_at | 1418836_at |
| 98969_at | 1418838_at |
| 99461_at | 1418842_at |
| 95571_at | 1418843_at |
| 95585_at | 1418845_at |
| 163154_at | 1418858_at |
| 163251_at | 1418870_at |
| 93414_at | 1418872_at |
| 167294_f_at | 1418873_at |
| 94302_at | 1418874_a_at |
| 99531_at | 1418875_at |
| 160996_at | 1418876_at |
| 92897_at | 1418878_at |
| 163331_at | 1418879_at |
| 160275_at | 1418888_a_at |
| 97415_at | 1418890_a_at |
| 163694_at | 1418900_at |

| | |
|---|---|
| 94528_at | 1418905_at |
| 97418_at | 1418914_s_at |
| 163985_at | 1418920_at |
| 165409_at | 1418928_a_at |
| 165933_at | 1418931_at |
| 99845_at | 1418933_at |
| 163984_at | 1418936_at |
| 99847_at | 1418946_at |
| 97776_at | 1418950_at |
| 92990_at | 1418955_at |
| 166167_f_at | 1418956_at |
| 94731_at | 1418957_at |
| 93386_at | 1418958_at |
| 93769_at | 1418962_at |
| 94448_at | 1418970_a_at |
| 98397_at | 1418973_at |
| 98447_at | 1418982_at |
| 115972_at | 1418985_at |
| 96501_at | 1418993_s_at |
| 98808_at | 1418995_at |
| 94115_at | 1419008_at |
| 165642_at | 1419009_at |
| 92355_at | 1419012_at |
| 94704_at | 1419015_at |
| 166024_f_at | 1419016_at |
| 92690_at | 1419020_at |
| 160568_at | 1419023_x_at |
| 94150_at | 1419025_at |
| 96125_at | 1419026_at |
| 164216_at | 1419029_at |
| 163541_at | 1419051_at |
| 94357_at | 1419057_at |
| 98021_at | 1419058_at |
| 163345_at | 1419070_at |
| 163416_r_at | 1419074_at |
| 103465_f_at | 1419075_s_at |
| 92693_at | 1419078_at |
| 163054_at | 1419103_a_at |
| 97969_at | 1419105_at |
| 92371_at | 1419109_at |
| 92594_at | 1419113_at |
| 168428_at | 1419118_at |
| 163210_at | 1419119_at |
| 163706_at | 1419121_at |
| 92273_at | 1419125_at |
| 97435_at | 1419131_at |
| 166412_at | 1419132_at |
| 163295_at | 1419133_at |
| 163617_at | 1419136_at |
| 98793_at | 1419139_at |
| 165585_at | 1419142_at |
| 165822_f_at | 1419143_at |
| 165685_at | 1419145_at |
| 165853_i_at | 1419152_at |
| 163072_at | 1419158_a_at |
| 93951_at | 1419159_at |

| | |
|---|---|
| 92409_at | 1419164_at |
| 163123_at | 1419176_at |
| 164056_at | 1419182_at |
| 96515_at | 1419192_at |
| 99508_at | 1419200_at |
| 165962_r_at | 1419201_at |
| 92974_at | 1419207_at |
| 97106_at | 1419208_at |
| 163174_at | 1419215_at |
| 93633_at | 1419217_at |
| 92685_at | 1419222_at |
| 98300_at | 1419225_at |
| 92419_at | 1419227_at |
| 97788_at | 1419230_at |
| 97159_at | 1419241_a_at |
| 108294_at | 1419243_at |
| 97301_at | 1419246_s_at |
| 97844_at | 1419248_at |
| 93422_at | 1419250_a_at |
| 163813_at | 1419261_at |
| 163010_at | 1419262_at |
| 97875_at | 1419263_a_at |
| 98024_at | 1419266_at |
| 96287_at | 1419269_at |
| 92271_at | 1419271_at |
| 93717_at | 1419282_at |
| 166391_at | 1419294_at |
| 93940_at | 1419298_at |
| 98452_at | 1419300_at |
| 93941_at | 1419304_at |
| 98326_f_at | 1419306_at |
| 170894_at | 1419307_at |
| 98126_s_at | 1419312_at |
| 93712_at | 1419313_at |
| 164089_at | 1419315_at |
| 92242_at | 1419319_at |
| 92918_at | 1419321_at |
| 131254_at | 1419322_at |
| 95343_at | 1419323_at |
| 93643_at | 1419324_at |
| 161032_i_at | 1419325_at |
| 97713_at | 1419327_at |
| 92460_at | 1419328_at |
| 97718_at | 1419334_at |
| 99236_at | 1419336_at |
| 165591_f_at | 1419337_at |
| 162174_at | 1419349_a_at |
| 164263_at | 1419353_at |
| 99610_at | 1419360_a_at |
| 99986_at | 1419372_at |
| 94827_at | 1419378_a_at |
| 161679_r_at | 1419381_at |
| 168530_r_at | 1419383_at |
| 97684_at | 1419384_at |
| 95601_at | 1419385_a_at |
| 98000_at | 1419387_s_at |

| | |
|---|---|
| 97753_at | 1419392_at |
| 93444_at | 1419410_at |
| 97783_at | 1419413_at |
| 166930_at | 1419414_at |
| 97785_at | 1419419_at |
| 99892_at | 1419425_at |
| 94142_at | 1419427_at |
| 160516_at | 1419428_a_at |
| 92743_at | 1419432_at |
| 93385_at | 1419433_at |
| 92291_f_at | 1419436_at |
| 92896_s_at | 1419437_at |
| 171370_i_at | 1419438_at |
| 98475_at | 1419442_at |
| 96637_at | 1419447_s_at |
| 98065_at | 1419450_at |
| 170930_r_at | 1419451_at |
| 160474_at | 1419460_at |
| 99182_at | 1419461_at |
| 93588_at | 1419462_s_at |
| 163333_at | 1419464_at |
| 98976_at | 1419476_at |
| 160604_at | 1419486_at |
| 97651_at | 1419501_at |
| 164144_at | 1419504_at |
| 98970_at | 1419505_a_at |
| 97091_at | 1419508_at |
| 97385_at | 1419509_a_at |
| 165550_at | 1419511_at |
| 167026_at | 1419520_at |
| 99463_at | 1419523_at |
| 99972_at | 1419524_at |
| 98137_at | 1419525_at |
| 94697_at | 1419526_at |
| 165807_at | 1419527_at |
| 95929_at | 1419533_at |
| 93167_f_at | 1419534_at |
| 97813_at | 1419536_a_at |
| 95295_s_at | 1419538_at |
| 160357_at | 1419542_at |
| 160124_r_at | 1419545_a_at |
| 93338_at | 1419558_at |
| 98962_at | 1419560_at |
| 93697_at | 1419583_at |
| 93454_at | 1419589_at |
| 92924_at | 1419594_at |
| 93575_at | 1419595_a_at |
| 165820_at | 1419600_at |
| 164112_at | 1419611_at |
| 93383_at | 1419613_at |
| 99865_at | 1419616_at |
| 97786_at | 1419621_at |
| 96796_f_at | 1419622_at |
| 97092_at | 1419629_at |
| 94021_at | 1419630_a_at |
| 92760_at | 1419631_at |

| | |
|---|---|
| 98315_at | 1419632_at |
| 92499_at | 1419633_at |
| 160910_at | 1419634_a_at |
| 162528_at | 1419643_s_at |
| 96311_at | 1419646_a_at |
| 168861_at | 1419660_at |
| 160877_at | 1419663_at |
| 161054_at | 1419672_at |
| 92459_at | 1419684_at |
| 92322_at | 1419691_at |
| 92401_at | 1419692_a_at |
| 92339_at | 1419702_at |
| 95022_at | 1419706_a_at |
| 92246_at | 1419707_at |
| 92406_at | 1419711_at |
| 92955_at | 1419712_at |
| 167533_at | 1419713_at |
| 168874_i_at | 1419715_at |
| 167699_at | 1419718_at |
| 92749_at | 1419719_at |
| 170152_at | 1419726_at |
| 166231_at | 1419733_at |
| 99065_at | 1419735_at |
| 94701_at | 1419740_at |
| 92913_at | 1419748_at |
| 97970_at | 1419755_at |
| 92715_at | 1419762_at |
| 92694_at | 1419764_at |
| 94760_at | 1419770_at |
| 95399_at | 1419771_at |
| 94800_at | 1419772_at |
| 92318_at | 1419773_at |
| 95977_at | 1419774_at |
| 94685_at | 1419775_at |
| 95991_at | 1419776_at |
| 97186_s_at | 1419777_at |
| 95926_at | 1419778_at |
| 97192_at | 1419779_at |
| 95990_at | 1419780_at |
| 97134_at | 1419782_at |
| 166227_at | 1419784_x_at |
| 94683_at | 1419785_at |
| 95294_at | 1419789_at |
| 98294_at | 1419790_at |
| 95925_at | 1419795_at |
| 95930_at | 1419796_at |
| 96544_at | 1419797_at |
| 97691_at | 1419799_at |
| 99867_at | 1419800_at |
| 99347_f_at | 1419801_x_at |
| 96392_at | 1419807_at |
| 98356_at | 1419811_at |
| 166818_at | 1419827_s_at |
| 99870_at | 1419834_x_at |
| 167269_at | 1419837_at |
| 95528_at | 1419840_at |

| | |
|---|---|
| 94129_at | 1419842_at |
| 95293_at | 1419843_at |
| 97117_at | 1419845_at |
| 95291_r_at | 1419846_at |
| 95372_at | 1419847_at |
| 95398_at | 1419849_at |
| 97099_at | 1419851_at |
| 95901_f_at | 1419857_at |
| 97162_at | 1419858_at |
| 95909_at | 1419859_at |
| 99826_at | 1419861_at |
| 96547_at | 1419863_at |
| 99356_r_at | 1419864_x_at |
| 96359_at | 1419869_s_at |
| 99868_at | 1419875_at |
| 96527_at | 1419877_x_at |
| 92597_s_at | 1419883_s_at |
| 95965_at | 1419891_s_at |
| 98345_at | 1419895_at |
| 93351_at | 1419905_s_at |
| 95284_at | 1419907_s_at |
| 99345_at | 1419909_at |
| 94435_at | 1419914_s_at |
| 95861_at | 1419929_at |
| 96388_at | 1419953_at |
| 96393_at | 1419962_at |
| 94836_at | 1419965_at |
| 92784_at | 1419980_at |
| 98247_at | 1420025_s_at |
| 96408_at | 1420041_at |
| 160229_at | 1420044_at |
| 96917_at | 1420058_s_at |
| 95941_at | 1420060_s_at |
| 161110_at | 1420064_s_at |
| 94095_at | 1420068_at |
| 95869_at | 1420072_s_at |
| 97176_at | 1420075_at |
| 165421_at | 1420080_a_at |
| 95877_at | 1420101_at |
| 99016_at | 1420103_at |
| 95081_at | 1420115_at |
| 95249_at | 1420121_at |
| 166439_at | 1420127_s_at |
| 168635_at | 1420151_at |
| 171049_at | 1420153_at |
| 97330_at | 1420157_s_at |
| 168644_r_at | 1420167_at |
| 95248_at | 1420168_at |
| 168555_at | 1420182_x_at |
| 162453_at | 1420189_at |
| 161496_r_at | 1420193_at |
| 94403_at | 1420196_s_at |
| 161705_r_at | 1420202_at |
| 164588_at | 1420207_at |
| 168039_at | 1420209_at |
| 167206_at | 1420215_x_at |

| | |
|---|---|
| 99681_at | 1420223_at |
| 161493_at | 1420224_at |
| 161362_at | 1420229_at |
| 161769_r_at | 1420235_at |
| 162084_i_at | 1420240_at |
| 161693_r_at | 1420246_at |
| 162151_i_at | 1420248_at |
| 162198_f_at | 1420249_s_at |
| 161351_r_at | 1420251_at |
| 95862_at | 1420253_at |
| 161450_r_at | 1420261_at |
| 168120_r_at | 1420268_x_at |
| 162330_f_at | 1420270_at |
| 162161_r_at | 1420272_at |
| 171323_i_at | 1420276_x_at |
| 162103_i_at | 1420280_x_at |
| 162212_at | 1420292_x_at |
| 162229_at | 1420303_x_at |
| 162244_r_at | 1420304_x_at |
| 167153_r_at | 1420328_at |
| 96551_at | 1420331_at |
| 166368_at | 1420333_at |
| 96498_at | 1420335_at |
| 163990_at | 1420341_at |
| 165620_at | 1420345_at |
| 167806_at | 1420346_at |
| 97769_at | 1420349_at |
| 164264_s_at | 1420350_at |
| 164259_at | 1420355_at |
| 94183_at | 1420358_at |
| 97757_at | 1420359_at |
| 96562_at | 1420361_at |
| 93287_at | 1420363_at |
| 93117_at | 1420365_a_at |
| 99130_at | 1420369_a_at |
| 115929_at | 1420375_at |
| 98803_at | 1420390_s_at |
| 92490_at | 1420395_a_at |
| 92368_at | 1420401_a_at |
| 95324_at | 1420402_at |
| 97172_s_at | 1420408_a_at |
| 94112_at | 1420412_at |
| 163729_at | 1420424_at |
| 169544_at | 1420426_at |
| 95719_at | 1420427_a_at |
| 92181_at | 1420428_at |
| 94732_at | 1420431_at |
| 97731_at | 1420437_at |
| 94734_at | 1420438_at |
| 93140_at | 1420439_at |
| 94148_at | 1420440_at |
| 169300_at | 1420444_at |
| 96997_at | 1420451_at |
| 93391_at | 1420453_at |
| 98791_at | 1420454_at |
| 97719_at | 1420461_at |

| | |
|---|---|
| 96421_at | 1420470_at |
| 94098_at | 1420471_at |
| 98549_at | 1420484_a_at |
| 160398_at | 1420486_at |
| 166055_r_at | 1420491_at |
| 96665_at | 1420495_a_at |
| 92509_at | 1420496_at |
| 161117_at | 1420512_at |
| 164157_at | 1420513_at |
| 163831_at | 1420517_at |
| 103859_at | 1420519_a_at |
| 163699_at | 1420522_at |
| 166279_i_at | 1420534_at |
| 170900_at | 1420537_at |
| 92376_at | 1420540_a_at |
| 170786_at | 1420543_at |
| 170474_i_at | 1420545_a_at |
| 93131_at | 1420547_at |
| 164918_i_at | 1420550_at |
| 96423_at | 1420555_at |
| 94775_at | 1420556_at |
| 98376_at | 1420557_at |
| 99043_s_at | 1420559_a_at |
| 96537_at | 1420562_at |
| 97793_at | 1420563_at |
| 98279_at | 1420564_at |
| 164777_i_at | 1420566_at |
| 99913_at | 1420569_at |
| 93628_at | 1420571_at |
| 95340_at | 1420575_at |
| 98871_at | 1420581_at |
| 99326_at | 1420584_at |
| 99394_at | 1420589_at |
| 94128_at | 1420594_at |
| 98873_at | 1420595_at |
| 94182_at | 1420596_at |
| 166983_at | 1420597_a_at |
| 94738_s_at | 1420598_x_at |
| 98832_at | 1420600_at |
| 94711_at | 1420601_at |
| 96505_at | 1420605_at |
| 94898_at | 1420608_at |
| 96030_at | 1420627_a_at |
| 98431_at | 1420636_a_at |
| 94515_at | 1420641_a_at |
| 160601_at | 1420643_at |
| 165625_r_at | 1420656_at |
| 93392_at | 1420657_at |
| 160952_r_at | 1420660_at |
| 160331_at | 1420663_at |
| 92910_at | 1420669_at |
| 171425_at | 1420670_at |
| 98373_at | 1420671_x_at |
| 98825_at | 1420672_at |
| 168141_i_at | 1420681_at |
| 167058_at | 1420684_at |

| | |
|---|---|
| 165895_at | 1420689_at |
| 95976_at | 1420690_at |
| 92407_at | 1420693_at |
| 94180_s_at | 1420694_a_at |
| 162679_at | 1420697_at |
| 94692_at | 1420698_at |
| 95775_f_at | 1420701_at |
| 168242_i_at | 1420702_at |
| 99330_at | 1420703_at |
| 94687_at | 1420705_at |
| 94130_at | 1420706_at |
| 92434_at | 1420707_a_at |
| 98813_at | 1420710_at |
| 97926_s_at | 1420715_a_at |
| 92389_at | 1420718_at |
| 167010_at | 1420721_at |
| 97767_at | 1420728_at |
| 97381_s_at | 1420730_a_at |
| 99839_at | 1420735_at |
| 98311_at | 1420737_at |
| 94101_at | 1420738_at |
| 96568_at | 1420739_at |
| 169741_at | 1420741_x_at |
| 160948_at | 1420743_a_at |
| 99891_at | 1420749_a_at |
| 98850_at | 1420753_at |
| 96435_at | 1420754_at |
| 94138_s_at | 1420756_at |
| 96549_at | 1420761_at |
| 98288_at | 1420763_at |
| 94181_at | 1420764_at |
| 169088_at | 1420770_at |
| 95793_at | 1420771_at |
| 96650_at | 1420776_a_at |
| 167960_r_at | 1420777_a_at |
| 94190_at | 1420782_at |
| 98276_at | 1420785_at |
| 98783_at | 1420786_a_at |
| 95320_at | 1420788_at |
| 95908_at | 1420790_x_at |
| 99402_at | 1420794_at |
| 94625_at | 1420797_at |
| 95319_at | 1420799_at |
| 99450_at | 1420800_a_at |
| 94168_at | 1420802_at |
| 160430_at | 1420811_a_at |
| 160114_at | 1420815_at |
| 99876_at | 1420818_at |
| 94501_at | 1420821_at |
| 160836_at | 1420824_at |
| 98926_at | 1420834_at |
| 99069_at | 1420837_at |
| 93091_s_at | 1420847_a_at |
| 168842_at | 1420849_at |
| 98847_at | 1420853_at |
| 92836_at | 1420855_at |

| | |
|---|---|
| 98004_at | 1420858_at |
| 163501_at | 1420861_at |
| 96474_at | 1420864_at |
| 93878_at | 1420869_at |
| 163315_at | 1420870_at |
| 93954_at | 1420871_at |
| 163907_at | 1420872_at |
| 92462_at | 1420876_a_at |
| 99027_at | 1420888_at |
| 160573_at | 1420890_at |
| 92404_at | 1420891_at |
| 163340_at | 1420892_at |
| 92427_at | 1420894_at |
| 96945_at | 1420898_at |
| 99991_at | 1420904_at |
| 99992_at | 1420905_at |
| 92186_at | 1420926_at |
| 94431_at | 1420927_at |
| 96180_at | 1420941_at |
| 163980_at | 1420944_at |
| 95157_at | 1420950_at |
| 163408_at | 1420956_at |
| 97729_at | 1420964_at |
| 133279_at | 1420970_at |
| 170419_at | 1420974_at |
| 92650_at | 1420976_at |
| 164249_at | 1420979_at |
| 160647_at | 1421001_a_at |
| 98303_at | 1421004_at |
| 163892_at | 1421007_at |
| 99046_at | 1421010_at |
| 166838_at | 1421017_at |
| 94484_at | 1421029_a_at |
| 95390_at | 1421036_at |
| 98796_at | 1421039_at |
| 92774_at | 1421046_a_at |
| 94107_at | 1421056_at |
| 93695_at | 1421058_at |
| 92898_at | 1421075_s_at |
| 166019_at | 1421077_at |
| 166085_at | 1421078_at |
| 160951_at | 1421092_at |
| 94740_g_at | 1421095_a_at |
| 168643_r_at | 1421096_at |
| 161291_at | 1421100_a_at |
| 93682_at | 1421101_a_at |
| 160523_at | 1421104_at |
| 92747_at | 1421112_at |
| 93136_at | 1421114_a_at |
| 92382_at | 1421120_at |
| 163162_at | 1421121_at |
| 163591_at | 1421127_at |
| 93398_at | 1421128_at |
| 99915_at | 1421134_at |
| 95935_at | 1421145_at |
| 93413_at | 1421147_at |

| | |
|---|---|
| 99947_at | 1421148_a_at |
| 98291_at | 1421155_at |
| 95310_at | 1421161_at |
| 92894_s_at | 1421162_a_at |
| 92465_at | 1421170_a_at |
| 92414_at | 1421172_at |
| 96496_g_at | 1421175_at |
| 98307_at | 1421176_at |
| 134709_r_at | 1421177_at |
| 165639_f_at | 1421182_at |
| 93397_at | 1421188_at |
| 95292_at | 1421194_at |
| 92252_at | 1421195_at |
| 97441_at | 1421197_a_at |
| 98366_at | 1421198_at |
| 92960_at | 1421206_at |
| 93407_at | 1421212_at |
| 94644_at | 1421218_at |
| 162159_i_at | 1421224_a_at |
| 97108_at | 1421235_s_at |
| 93945_at | 1421244_at |
| 98838_at | 1421246_at |
| 95244_at | 1421251_at |
| 160913_at | 1421253_at |
| 94645_at | 1421263_at |
| 98624_at | 1421265_a_at |
| 92998_at | 1421272_at |
| 92938_at | 1421281_at |
| 99393_at | 1421282_at |
| 92296_at | 1421284_at |
| 94163_at | 1421286_a_at |
| 94191_at | 1421288_at |
| 95333_at | 1421291_at |
| 99869_at | 1421293_at |
| 98386_s_at | 1421297_a_at |
| 98801_at | 1421304_at |
| 93891_at | 1421312_a_at |
| 97760_at | 1421327_at |
| 98289_at | 1421337_at |
| 167011_at | 1421347_at |
| 98304_at | 1421352_at |
| 98368_at | 1421354_at |
| 96566_at | 1421355_at |
| 98295_at | 1421363_at |
| 93040_at | 1421374_a_at |
| 93926_at | 1421382_at |
| 168951_r_at | 1421383_at |
| 94736_at | 1421384_at |
| 94713_at | 1421385_a_at |
| 94941_at | 1421389_a_at |
| 98375_at | 1421391_at |
| 160645_at | 1421392_a_at |
| 99382_at | 1421393_at |
| 94694_at | 1421396_at |
| 99832_at | 1421400_at |
| 94762_at | 1421407_at |

| | |
|---|---|
| 98851_at | 1421409_at |
| 99433_at | 1421411_at |
| 94187_at | 1421412_at |
| 99037_at | 1421416_at |
| 94185_at | 1421419_at |
| 99993_at | 1421424_a_at |
| 92370_at | 1421432_at |
| 96510_at | 1421441_at |
| 98726_at | 1421444_at |
| 164100_at | 1421454_at |
| 93384_at | 1421455_at |
| 97168_at | 1421456_at |
| 164784_at | 1421457_a_at |
| 92926_at | 1421461_at |
| 94126_at | 1421465_at |
| 98781_at | 1421470_at |
| 94755_at | 1421473_at |
| 96509_at | 1421475_at |
| 98782_at | 1421477_at |
| 97683_at | 1421481_at |
| 167745_r_at | 1421484_at |
| 166957_r_at | 1421489_a_at |
| 98383_r_at | 1421490_at |
| 98860_at | 1421500_at |
| 92991_at | 1421504_at |
| 99324_at | 1421510_at |
| 99904_at | 1421511_at |
| 168015_r_at | 1421516_at |
| 96548_at | 1421522_at |
| 98430_at | 1421528_a_at |
| 94622_at | 1421530_a_at |
| 98367_at | 1421537_at |
| 169570_f_at | 1421540_at |
| 94774_at | 1421551_s_at |
| 98347_at | 1421552_at |
| 94175_at | 1421558_at |
| 96597_at | 1421566_at |
| 94719_at | 1421568_at |
| 94619_at | 1421569_at |
| 95275_at | 1421570_at |
| 93077_s_at | 1421571_a_at |
| 95336_at | 1421572_at |
| 167791_at | 1421576_at |
| 96507_at | 1421577_at |
| 94146_at | 1421578_at |
| 92745_at | 1421579_at |
| 94626_at | 1421580_at |
| 92524_at | 1421581_at |
| 94178_at | 1421586_a_at |
| 163284_at | 1421594_a_at |
| 92912_at | 1421597_a_at |
| 99888_at | 1421614_at |
| 97128_at | 1421620_at |
| 92659_at | 1421622_a_at |
| 99323_at | 1421623_at |
| 98846_f_at | 1421638_at |

| | |
|---|---|
| 99408_at | 1421641_at |
| 94630_at | 1421643_at |
| 98299_s_at | 1421644_at |
| 93708_at | 1421646_a_at |
| 98728_at | 1421649_at |
| 94696_at | 1421655_a_at |
| 92512_g_at | 1421666_a_at |
| 99417_at | 1421667_at |
| 99854_at | 1421669_at |
| 99349_at | 1421672_at |
| 167662_r_at | 1421675_at |
| 94565_at | 1421678_at |
| 99134_at | 1421682_a_at |
| 94166_g_at | 1421688_a_at |
| 97152_at | 1421689_at |
| 99399_at | 1421690_s_at |
| 99842_at | 1421698_a_at |
| 94623_at | 1421699_at |
| 94108_at | 1421704_a_at |
| 99333_at | 1421712_at |
| 99700_at | 1421719_at |
| 96400_at | 1421722_at |
| 94705_at | 1421727_at |
| 99321_at | 1421729_a_at |
| 94137_at | 1421734_at |
| 99320_at | 1421735_a_at |
| 94642_at | 1421742_at |
| 92621_at | 1421743_a_at |
| 94566_at | 1421755_at |
| 98354_at | 1421760_at |
| 99376_at | 1421762_at |
| 97679_at | 1421770_a_at |
| 94699_at | 1421771_a_at |
| 95391_at | 1421774_at |
| 98370_at | 1421781_at |
| 95304_at | 1421785_at |
| 95228_f_at | 1421791_at |
| 99887_at | 1421796_a_at |
| 94927_at | 1421804_at |
| 162553_at | 1421825_at |
| 163429_at | 1421827_at |
| 134007_at | 1421844_at |
| 98322_at | 1421848_at |
| 162289_at | 1421854_at |
| 97949_at | 1421855_at |
| 160241_at | 1421858_at |
| 163030_at | 1421865_at |
| 168002_at | 1421868_a_at |
| 93347_at | 1421873_s_at |
| 96593_at | 1421896_at |
| 93911_at | 1421904_at |
| 97090_at | 1421909_at |
| 97778_at | 1421915_a_at |
| 95079_at | 1421917_at |
| 98323_at | 1421919_a_at |
| 98766_at | 1421922_at |

| | |
|---|---|
| 164342_f_at | 1421925_at |
| 98282_at | 1421931_at |
| 92478_at | 1421939_a_at |
| 93433_s_at | 1421944_a_at |
| 96229_at | 1421946_at |
| 92961_at | 1421951_at |
| 99800_at | 1421958_at |
| 160870_at | 1421960_at |
| 92947_s_at | 1421970_a_at |
| 164320_f_at | 1421977_at |
| 92899_at | 1421978_at |
| 92347_at | 1421980_at |
| 96675_at | 1421982_a_at |
| 93005_at | 1421990_at |
| 160334_at | 1421993_a_at |
| 96533_at | 1421998_at |
| 98328_at | 1421999_at |
| 94688_at | 1422002_at |
| 99841_at | 1422003_at |
| 93672_at | 1422005_at |
| 92665_f_at | 1422011_s_at |
| 98395_at | 1422012_at |
| 92762_at | 1422013_at |
| 94702_at | 1422019_at |
| 95317_at | 1422023_at |
| 94698_at | 1422024_at |
| 94720_at | 1422027_a_at |
| 97794_at | 1422040_at |
| 92516_at | 1422044_at |
| 98828_at | 1422046_at |
| 97580_at | 1422048_at |
| 98337_at | 1422049_at |
| 92219_s_at | 1422055_at |
| 97723_at | 1422059_at |
| 94140_at | 1422062_at |
| 94744_at | 1422067_at |
| 92364_at | 1422073_a_at |
| 99916_at | 1422079_at |
| 98840_at | 1422084_at |
| 97781_at | 1422089_at |
| 98329_at | 1422090_a_at |
| 99404_at | 1422100_at |
| 92199_at | 1422102_a_at |
| 99880_at | 1422109_at |
| 99875_at | 1422110_at |
| 98309_at | 1422112_at |
| 93671_at | 1422114_at |
| 99912_at | 1422116_at |
| 98731_at | 1422119_at |
| 166910_at | 1422120_at |
| 99332_at | 1422121_at |
| 94141_at | 1422122_at |
| 95332_at | 1422125_at |
| 162984_at | 1422126_a_at |
| 99861_at | 1422127_at |
| 93134_at | 1422130_at |

| | |
|---|---|
| 98285_at | 1422135_at |
| 99401_at | 1422136_at |
| 167708_at | 1422137_at |
| 97772_at | 1422139_at |
| 96413_at | 1422143_at |
| 97153_at | 1422144_at |
| 99423_at | 1422145_at |
| 97791_at | 1422146_at |
| 96987_at | 1422149_at |
| 92977_s_at | 1422150_at |
| 94102_at | 1422152_at |
| 163438_at | 1422153_a_at |
| 97151_at | 1422154_at |
| 94746_at | 1422160_at |
| 95327_at | 1422163_at |
| 99386_at | 1422165_at |
| 110045_at | 1422168_a_at |
| 98856_at | 1422171_at |
| 98821_at | 1422173_at |
| 95344_at | 1422177_at |
| 163757_at | 1422178_a_at |
| 99373_at | 1422179_at |
| 93622_at | 1422180_a_at |
| 92340_at | 1422183_a_at |
| 94284_at | 1422186_s_at |
| 98727_at | 1422193_at |
| 98810_at | 1422196_at |
| 99380_at | 1422197_at |
| 99407_at | 1422199_at |
| 98795_at | 1422202_at |
| 92342_at | 1422205_at |
| 94227_at | 1422209_s_at |
| 98788_at | 1422220_at |
| 99914_at | 1422221_at |
| 92739_at | 1422222_at |
| 99361_at | 1422228_at |
| 98317_at | 1422232_at |
| 98732_at | 1422233_at |
| 94634_at | 1422234_at |
| 94121_at | 1422240_s_at |
| 99435_at | 1422243_at |
| 94721_at | 1422246_at |
| 92921_at | 1422253_at |
| 161968_f_at | 1422259_a_at |
| 98387_at | 1422263_at |
| 94116_at | 1422265_at |
| 94618_at | 1422267_at |
| 99398_at | 1422280_at |
| 94635_at | 1422282_at |
| 98812_at | 1422283_at |
| 96422_at | 1422284_at |
| 99411_at | 1422288_at |
| 163022_at | 1422289_a_at |
| 99895_at | 1422291_at |
| 97053_at | 1422294_at |
| 94145_at | 1422305_at |

| | |
|---|---|
| 99814_at | 1422312_a_at |
| 95334_at | 1422329_a_at |
| 93225_s_at | 1422333_at |
| 99804_at | 1422335_at |
| 94636_at | 1422336_at |
| 97000_s_at | 1422337_at |
| 96999_at | 1422343_at |
| 99811_at | 1422347_at |
| 97005_at | 1422367_at |
| 97004_at | 1422373_at |
| 96984_at | 1422384_at |
| 96985_at | 1422385_at |
| 94718_at | 1422398_at |
| 97156_at | 1422409_at |
| 94639_at | 1422427_a_at |
| 162524_at | 1422428_at |
| 160648_at | 1422430_at |
| 97248_at | 1422432_at |
| 167151_at | 1422436_at |
| 160177_at | 1422442_at |
| 161849_r_at | 1422446_x_at |
| 165489_f_at | 1422447_at |
| 93302_at | 1422448_at |
| 99632_at | 1422460_at |
| 95734_at | 1422464_at |
| 97270_at | 1422466_at |
| 98087_at | 1422469_at |
| 160543_at | 1422480_at |
| 160422_at | 1422482_at |
| 160429_at | 1422488_at |
| 96699_at | 1422495_a_at |
| 160917_r_at | 1422498_at |
| 95746_at | 1422508_at |
| 99516_at | 1422522_at |
| 93092_at | 1422527_at |
| 93053_at | 1422529_s_at |
| 162970_at | 1422538_at |
| 92308_at | 1422541_at |
| 162587_at | 1422543_at |
| 163137_at | 1422560_at |
| 99127_at | 1422576_at |
| 160487_at | 1422580_at |
| 99029_at | 1422581_at |
| 160868_at | 1422583_at |
| 163937_at | 1422586_at |
| 94316_at | 1422588_at |
| 105126_at | 1422590_at |
| 95536_at | 1422591_at |
| 96313_at | 1422600_at |
| 92606_at | 1422604_at |
| 98168_at | 1422613_a_at |
| 97917_at | 1422614_s_at |
| 167048_at | 1422616_s_at |
| 94167_at | 1422622_at |
| 98280_at | 1422626_at |
| 92214_at | 1422632_at |

| | |
|---|---|
| 165564_at | 1422633_at |
| 162683_at | 1422634_a_at |
| 96205_at | 1422644_at |
| 92736_at | 1422648_at |
| 99104_at | 1422651_at |
| 160631_s_at | 1422654_at |
| 166035_f_at | 1422658_at |
| 162860_r_at | 1422659_at |
| 160618_at | 1422661_at |
| 161079_at | 1422671_s_at |
| 96586_at | 1422674_s_at |
| 93208_at | 1422682_s_at |
| 162954_at | 1422683_at |
| 93661_at | 1422701_at |
| 97525_at | 1422704_at |
| 169442_i_at | 1422712_a_at |
| 94388_at | 1422718_at |
| 92515_at | 1422720_at |
| 162827_at | 1422722_at |
| 93675_at | 1422724_at |
| 165750_at | 1422727_at |
| 160066_at | 1422730_at |
| 99636_at | 1422732_at |
| 92658_at | 1422735_at |
| 92969_at | 1422738_at |
| 92519_at | 1422744_at |
| 163319_at | 1422750_a_at |
| 92668_at | 1422755_at |
| 99367_at | 1422764_at |
| 94156_at | 1422790_at |
| 99023_at | 1422791_at |
| 99164_at | 1422797_at |
| 92968_at | 1422807_at |
| 93486_at | 1422811_at |
| 96558_at | 1422816_a_at |
| 97987_at | 1422826_at |
| 92683_at | 1422828_at |
| 97755_at | 1422829_at |
| 95331_at | 1422832_at |
| 93950_at | 1422833_at |
| 99339_r_at | 1422835_at |
| 161132_at | 1422837_at |
| 99410_at | 1422838_at |
| 97792_at | 1422839_at |
| 97475_at | 1422843_at |
| 169621_i_at | 1422845_at |
| 92811_at | 1422846_at |
| 96234_at | 1422855_at |
| 93570_at | 1422856_at |
| 92399_at | 1422864_at |
| 164069_at | 1422868_s_at |
| 92698_at | 1422869_at |
| 98829_at | 1422871_at |
| 97725_at | 1422872_at |
| 92728_at | 1422873_at |
| 99456_at | 1422874_at |

| | |
|---|---|
| 99833_at | 1422876_at |
| 98972_at | 1422883_at |
| 92454_at | 1422899_at |
| 94425_at | 1422903_at |
| 98807_at | 1422907_at |
| 93242_at | 1422913_at |
| 171232_r_at | 1422914_at |
| 98797_at | 1422915_at |
| 165847_i_at | 1422916_at |
| 92712_at | 1422918_at |
| 93957_at | 1422921_at |
| 92415_at | 1422924_at |
| 171228_at | 1422935_x_at |
| 99430_at | 1422942_at |
| 95628_at | 1422944_a_at |
| 93446_at | 1422954_at |
| 93370_at | 1422955_at |
| 92240_at | 1422958_at |
| 93202_at | 1422974_at |
| 160649_at | 1422977_at |
| 92667_at | 1422982_at |
| 99432_at | 1422984_at |
| 97728_at | 1422987_at |
| 92390_at | 1422992_s_at |
| 98777_at | 1423004_at |
| 92449_at | 1423007_a_at |
| 99403_at | 1423008_at |
| 99375_at | 1423009_at |
| 93704_at | 1423013_at |
| 92393_at | 1423018_at |
| 94717_f_at | 1423028_at |
| 160450_at | 1423031_at |
| 94242_at | 1423035_s_at |
| 99146_at | 1423038_at |
| 94509_at | 1423045_at |
| 161463_f_at | 1423055_at |
| 97363_at | 1423059_at |
| 96155_at | 1423067_at |
| 160356_at | 1423073_at |
| 160593_at | 1423076_at |
| 99013_f_at | 1423088_at |
| 92636_f_at | 1423090_x_at |
| 99178_at | 1423091_a_at |
| 93758_at | 1423092_at |
| 160718_at | 1423096_at |
| 96859_at | 1423102_a_at |
| 163219_at | 1423103_at |
| 93509_at | 1423106_at |
| 95695_at | 1423109_s_at |
| 163885_at | 1423114_at |
| 97428_at | 1423116_at |
| 162980_at | 1423118_at |
| 99579_at | 1423126_at |
| 92575_at | 1423128_at |
| 99616_s_at | 1423133_at |
| 92602_at | 1423145_a_at |

| | |
|---|---|
| 161051_at | 1423146_at |
| 92864_at | 1423147_at |
| 168448_at | 1423154_at |
| 97502_at | 1423159_at |
| 96003_at | 1423165_a_at |
| 163579_at | 1423171_at |
| 167365_at | 1423179_at |
| 167841_at | 1423184_at |
| 96286_at | 1423185_a_at |
| 160796_at | 1423186_at |
| 97311_at | 1423188_a_at |
| 169717_r_at | 1423190_at |
| 99995_at | 1423203_a_at |
| 96855_at | 1423215_at |
| 166298_f_at | 1423224_at |
| 160403_at | 1423225_at |
| 99446_at | 1423226_at |
| 96848_at | 1423229_at |
| 96698_at | 1423234_at |
| 163038_at | 1423238_at |
| 96334_f_at | 1423244_at |
| 92874_f_at | 1423245_at |
| 96773_at | 1423247_at |
| 160409_at | 1423282_at |
| 163845_i_at | 1423284_at |
| 92932_at | 1423287_at |
| 98522_at | 1423296_at |
| 162765_at | 1423297_at |
| 94992_at | 1423301_at |
| 166222_r_at | 1423315_at |
| 167587_i_at | 1423318_at |
| 98408_at | 1423319_at |
| 95411_at | 1423335_at |
| 167861_at | 1423337_at |
| 163747_at | 1423339_s_at |
| 92903_at | 1423340_at |
| 92514_at | 1423342_at |
| 163474_at | 1423349_at |
| 167678_at | 1423352_at |
| 167914_r_at | 1423353_at |
| 163786_at | 1423358_at |
| 160071_at | 1423373_at |
| 95525_at | 1423374_at |
| 163091_at | 1423385_at |
| 162930_at | 1423406_at |
| 93599_at | 1423408_a_at |
| 160219_r_at | 1423410_at |
| 92734_at | 1423415_at |
| 99098_at | 1423418_at |
| 163555_at | 1423422_at |
| 113546_at | 1423425_at |
| 93015_at | 1423437_at |
| 160481_at | 1423439_at |
| 160987_r_at | 1423440_at |
| 92566_at | 1423446_at |
| 167509_i_at | 1423469_at |

| | |
|---|---|
| 97543_at | 1423472_at |
| 95694_at | 1423474_at |
| 97993_at | 1423482_at |
| 93760_at | 1423486_at |
| 163741_at | 1423494_at |
| 163459_at | 1423497_at |
| 98066_r_at | 1423502_at |
| 98957_at | 1423503_at |
| 95502_at | 1423507_a_at |
| 99488_at | 1423510_at |
| 165663_at | 1423514_at |
| 94046_at | 1423518_at |
| 166108_r_at | 1423527_at |
| 95330_at | 1423529_at |
| 97272_at | 1423531_a_at |
| 160209_at | 1423532_at |
| 95438_at | 1423533_a_at |
| 162577_at | 1423536_at |
| 167439_at | 1423541_at |
| 96345_at | 1423548_s_at |
| 160694_at | 1423557_at |
| 92358_at | 1423561_at |
| 96808_at | 1423563_at |
| 160920_at | 1423572_at |
| 166148_at | 1423580_at |
| 97213_at | 1423583_at |
| 96136_at | 1423587_a_at |
| 96692_at | 1423591_at |
| 160846_at | 1423596_at |
| 94186_at | 1423602_at |
| 96087_at | 1423609_a_at |
| 162943_r_at | 1423613_at |
| 160201_r_at | 1423621_a_at |
| 94449_at | 1423628_s_at |
| 163914_at | 1423635_at |
| 99815_at | 1423639_at |
| 95098_at | 1423650_at |
| 160787_at | 1423655_a_at |
| 97829_at | 1423657_at |
| 93757_at | 1423670_a_at |
| 160908_r_at | 1423672_at |
| 93423_at | 1423673_at |
| 160512_at | 1423678_at |
| 98049_at | 1423681_at |
| 96200_at | 1423683_at |
| 97436_at | 1423687_a_at |
| 93531_at | 1423692_at |
| 93783_at | 1423693_at |
| 160789_at | 1423695_at |
| 160239_at | 1423696_a_at |
| 94241_at | 1423701_at |
| 94868_at | 1423712_a_at |
| 97364_at | 1423714_at |
| 93519_s_at | 1423715_a_at |
| 94279_at | 1423716_s_at |
| 160446_at | 1423719_at |

| | |
|---|---|
| 160386_at | 1423730_at |
| 162806_at | 1423731_at |
| 96899_at | 1423737_at |
| 96086_at | 1423745_at |
| 98602_at | 1423749_s_at |
| 162761_at | 1423755_at |
| 164965_f_at | 1423762_at |
| 160592_at | 1423770_at |
| 95032_at | 1423775_s_at |
| 93561_at | 1423779_at |
| 97554_at | 1423789_at |
| 99620_at | 1423796_at |
| 92855_at | 1423799_at |
| 98940_at | 1423800_at |
| 93283_at | 1423801_a_at |
| 93293_at | 1423807_a_at |
| 160663_at | 1423814_at |
| 99609_at | 1423816_at |
| 160371_at | 1423818_a_at |
| 160889_at | 1423821_at |
| 97252_at | 1423823_at |
| 160156_at | 1423829_at |
| 98445_at | 1423834_s_at |
| 160660_r_at | 1423835_at |
| 96669_at | 1423837_at |
| 98759_f_at | 1423846_x_at |
| 163633_at | 1423853_at |
| 95418_at | 1423854_a_at |
| 96082_at | 1423857_at |
| 93845_at | 1423863_at |
| 94059_at | 1423864_at |
| 92583_at | 1423867_at |
| 162642_at | 1423868_at |
| 160779_at | 1423881_at |
| 165281_at | 1423889_at |
| 160346_at | 1423894_a_at |
| 93743_at | 1423906_at |
| 93826_at | 1423911_at |
| 163245_at | 1423915_at |
| 95726_at | 1423916_s_at |
| 97329_at | 1423922_s_at |
| 94954_at | 1423931_s_at |
| 97413_at | 1423933_a_at |
| 96604_at | 1423943_at |
| 98116_at | 1423944_at |
| 92549_at | 1423945_a_at |
| 98635_at | 1423949_at |
| 98125_at | 1423951_at |
| 93497_at | 1423954_at |
| 160196_at | 1423956_at |
| 160506_at | 1423959_at |
| 98965_at | 1423960_at |
| 94053_at | 1423964_at |
| 160435_at | 1423965_at |
| 94976_at | 1423974_at |
| 136222_at | 1423983_at |

| | |
|---|---|
| 92753_at | 1423984_a_at |
| 99493_at | 1423990_at |
| 94767_at | 1424000_a_at |
| 160268_at | 1424002_at |
| 160860_at | 1424007_at |
| 162533_at | 1424009_at |
| 96826_at | 1424010_at |
| 95759_at | 1424014_at |
| 99581_at | 1424018_at |
| 96320_at | 1424038_a_at |
| 160814_at | 1424052_at |
| 94628_r_at | 1424063_at |
| 163773_at | 1424070_at |
| 94979_at | 1424071_s_at |
| 160958_at | 1424075_at |
| 163544_at | 1424076_at |
| 94067_at | 1424080_at |
| 94543_at | 1424087_at |
| 95651_at | 1424088_at |
| 162810_at | 1424090_at |
| 162605_at | 1424091_at |
| 94317_at | 1424096_at |
| 166862_at | 1424097_at |
| 94551_at | 1424102_at |
| 163270_i_at | 1424107_at |
| 93269_at | 1424108_at |
| 98516_at | 1424117_at |
| 99028_at | 1424120_at |
| 160622_at | 1424123_at |
| 93500_at | 1424126_at |
| 160536_at | 1424132_at |
| 113864_at | 1424137_at |
| 93566_at | 1424140_at |
| 162931_at | 1424145_at |
| 162049_f_at | 1424150_at |
| 96021_at | 1424154_a_at |
| 162956_at | 1424165_a_at |
| 160488_at | 1424166_at |
| 95141_at | 1424168_a_at |
| 160117_at | 1424175_at |
| 95042_at | 1424196_at |
| 160758_at | 1424200_s_at |
| 163231_at | 1424202_at |
| 162961_at | 1424215_at |
| 162581_at | 1424218_a_at |
| 162632_at | 1424231_s_at |
| 99937_at | 1424234_s_at |
| 160193_at | 1424235_at |
| 99497_at | 1424240_at |
| 96231_at | 1424242_at |
| 160840_at | 1424250_a_at |
| 163935_at | 1424256_at |
| 99094_at | 1424260_at |
| 95043_at | 1424273_at |
| 97343_at | 1424288_at |
| 163765_at | 1424289_at |

| | |
|---|---|
| 98003_at | 1424302_at |
| 97373_at | 1424316_at |
| 97366_at | 1424326_at |
| 94924_at | 1424329_a_at |
| 162750_at | 1424331_at |
| 160610_at | 1424341_s_at |
| 160821_r_at | 1424346_at |
| 162951_at | 1424359_at |
| 169646_at | 1424361_at |
| 167463_r_at | 1424368_s_at |
| 96172_at | 1424375_s_at |
| 163310_at | 1424379_at |
| 92398_at | 1424380_at |
| 165684_i_at | 1424388_at |
| 96661_at | 1424391_at |
| 94381_at | 1424399_at |
| 162908_at | 1424409_at |
| 92584_at | 1424416_at |
| 170644_at | 1424417_at |
| 165572_at | 1424432_at |
| 93600_at | 1424438_a_at |
| 162664_at | 1424440_at |
| 97957_at | 1424441_at |
| 163575_at | 1424447_at |
| 99571_at | 1424451_at |
| 163291_at | 1424455_at |
| 160151_i_at | 1424462_at |
| 160297_at | 1424465_at |
| 160558_at | 1424480_s_at |
| 162571_at | 1424497_at |
| 94370_at | 1424502_at |
| 113872_at | 1424504_at |
| 164058_i_at | 1424506_at |
| 165417_at | 1424509_at |
| 165641_at | 1424510_at |
| 92639_at | 1424511_at |
| 160960_at | 1424513_at |
| 165567_at | 1424525_at |
| 166032_f_at | 1424537_at |
| 160856_at | 1424538_at |
| 168471_at | 1424546_at |
| 166211_at | 1424547_at |
| 163767_at | 1424557_at |
| 163601_at | 1424559_at |
| 163264_at | 1424586_at |
| 92865_at | 1424592_a_at |
| 160923_at | 1424600_at |
| 165730_at | 1424623_at |
| 165563_at | 1424627_at |
| 163590_at | 1424634_at |
| 94766_at | 1424635_at |
| 94324_f_at | 1424639_a_at |
| 169112_r_at | 1424647_at |
| 96941_at | 1424648_at |
| 92688_at | 1424655_at |
| 163470_at | 1424659_at |

| | |
|---|---|
| 165815_r_at | 1424679_at |
| 96262_at | 1424684_at |
| 169267_at | 1424693_at |
| 165560_at | 1424699_at |
| 163677_at | 1424703_at |
| 92676_at | 1424704_at |
| 93336_at | 1424708_at |
| 94876_f_at | 1424710_a_at |
| 95026_at | 1424715_at |
| 96237_at | 1424729_at |
| 160306_at | 1424737_at |
| 94520_at | 1424743_at |
| 160640_at | 1424748_at |
| 163433_r_at | 1424750_at |
| 160898_at | 1424751_at |
| 96787_at | 1424758_s_at |
| 160794_at | 1424760_a_at |
| 96688_at | 1424782_at |
| 163155_at | 1424793_a_at |
| 99474_at | 1424798_a_at |
| 160673_at | 1424803_at |
| 163748_at | 1424811_at |
| 93475_at | 1424817_at |
| 98051_at | 1424819_a_at |
| 98063_at | 1424825_a_at |
| 163376_at | 1424835_at |
| 169345_at | 1424844_at |
| 92430_at | 1424851_at |
| 92600_f_at | 1424853_s_at |
| 167990_at | 1424870_at |
| 97506_at | 1424873_at |
| 93896_at | 1424886_at |
| 165835_r_at | 1424889_at |
| 93371_at | 1424890_at |
| 94434_at | 1424892_at |
| 163869_at | 1424893_at |
| 164096_at | 1424911_a_at |
| 97472_at | 1424912_at |
| 163325_s_at | 1424918_at |
| 99350_at | 1424926_at |
| 93638_s_at | 1424931_s_at |
| 165570_at | 1424937_at |
| 162452_at | 1424944_at |
| 163458_at | 1424950_at |
| 163368_at | 1424952_at |
| 163966_at | 1424955_at |
| 166848_at | 1424958_at |
| 163746_at | 1424959_at |
| 160845_at | 1424971_at |
| 98078_at | 1424979_at |
| 92230_at | 1424981_at |
| 97393_at | 1425006_a_at |
| 98479_f_at | 1425014_at |
| 98771_at | 1425015_at |
| 101773_r_at | 1425017_at |
| 96794_at | 1425030_at |

| | |
|---|---|
| 166864_at | 1425045_at |
| 97247_at | 1425052_at |
| 167539_at | 1425055_at |
| 95285_at | 1425058_at |
| 93419_at | 1425066_a_at |
| 94226_at | 1425068_a_at |
| 164170_at | 1425073_at |
| 93778_at | 1425078_x_at |
| 164253_at | 1425083_at |
| 167199_at | 1425088_at |
| 94176_at | 1425094_a_at |
| 97732_at | 1425100_a_at |
| 94423_at | 1425108_a_at |
| 163666_at | 1425111_at |
| 99364_at | 1425114_at |
| 165944_at | 1425120_x_at |
| 93162_f_at | 1425122_at |
| 95066_at | 1425129_a_at |
| 98391_at | 1425144_at |
| 166595_at | 1425147_at |
| 163040_i_at | 1425152_s_at |
| 92218_at | 1425153_at |
| 93244_at | 1425161_a_at |
| 97094_at | 1425164_a_at |
| 96567_at | 1425171_at |
| 93777_at | 1425173_s_at |
| 94365_at | 1425196_a_at |
| 166677_r_at | 1425197_at |
| 163751_i_at | 1425201_a_at |
| 163695_at | 1425211_at |
| 165031_at | 1425218_a_at |
| 164743_i_at | 1425224_at |
| 93897_at | 1425244_a_at |
| 167215_at | 1425246_at |
| 96589_at | 1425251_at |
| 167399_r_at | 1425283_a_at |
| 98831_at | 1425291_at |
| 160747_at | 1425296_a_at |
| 93461_at | 1425298_a_at |
| 96582_at | 1425301_at |
| 167660_at | 1425309_at |
| 97982_at | 1425310_a_at |
| 167583_at | 1425314_at |
| 111629_at | 1425321_a_at |
| 96968_at | 1425322_at |
| 94933_at | 1425323_a_at |
| 95533_at | 1425332_at |
| 94764_at | 1425367_at |
| 99848_at | 1425376_at |
| 94134_at | 1425377_at |
| 160674_at | 1425381_a_at |
| 93849_at | 1425388_a_at |
| 160515_at | 1425422_a_at |
| 163423_at | 1425425_a_at |
| 97774_at | 1425432_at |
| 95381_at | 1425437_a_at |

| | |
|---|---|
| 96043_at | 1425449_at |
| 95450_at | 1425458_a_at |
| 160764_at | 1425462_at |
| 92802_s_at | 1425467_a_at |
| 92801_at | 1425468_at |
| 98060_at | 1425472_a_at |
| 93482_at | 1425506_at |
| 163548_at | 1425514_at |
| 163318_at | 1425518_at |
| 95586_at | 1425525_a_at |
| 99021_at | 1425527_at |
| 163440_at | 1425530_a_at |
| 98462_s_at | 1425547_a_at |
| 97551_at | 1425553_s_at |
| 94212_at | 1425557_x_at |
| 95747_at | 1425560_a_at |
| 95298_at | 1425574_at |
| 97841_at | 1425591_a_at |
| 160864_at | 1425601_a_at |
| 97763_at | 1425609_at |
| 97398_at | 1425615_a_at |
| 162610_at | 1425623_a_at |
| 93790_at | 1425630_at |
| 93933_at | 1425631_at |
| 94994_at | 1425633_at |
| 93468_at | 1425640_at |
| 95393_at | 1425660_at |
| 170250_i_at | 1425663_at |
| 134805_at | 1425664_at |
| 96224_at | 1425680_a_at |
| 95299_at | 1425692_a_at |
| 168011_f_at | 1425700_at |
| 94198_at | 1425703_at |
| 167159_r_at | 1425715_at |
| 93696_at | 1425723_at |
| 94708_at | 1425724_at |
| 166660_at | 1425740_at |
| 170047_r_at | 1425756_at |
| 92235_g_at | 1425762_a_at |
| 130139_at | 1425771_at |
| 164222_at | 1425773_s_at |
| 98615_at | 1425780_a_at |
| 98864_s_at | 1425790_a_at |
| 99951_at | 1425793_a_at |
| 162253_i_at | 1425796_a_at |
| 160946_at | 1425805_a_at |
| 98360_at | 1425812_a_at |
| 99525_at | 1425817_a_at |
| 94993_f_at | 1425823_at |
| 94051_at | 1425842_at |
| 170745_i_at | 1425857_at |
| 93213_at | 1425871_a_at |
| 167367_at | 1425898_x_at |
| 170523_i_at | 1425916_at |
| 164609_at | 1425946_at |
| 99334_at | 1425947_at |

| | |
|---|---|
| 170254_at | 1425956_a_at |
| 104382_at | 1425967_a_at |
| 99331_at | 1425968_s_at |
| 163487_r_at | 1425979_a_at |
| 93075_r_at | 1425990_a_at |
| 94743_f_at | 1425999_at |
| 99903_at | 1426005_at |
| 165511_at | 1426007_a_at |
| 96783_at | 1426014_a_at |
| 94825_at | 1426019_at |
| 92781_at | 1426020_at |
| 99907_at | 1426033_at |
| 92677_s_at | 1426034_a_at |
| 93851_at | 1426046_a_at |
| 92303_at | 1426047_a_at |
| 161014_at | 1426049_a_at |
| 99026_at | 1426050_at |
| 93128_at | 1426054_at |
| 98499_s_at | 1426062_a_at |
| 92534_at | 1426063_a_at |
| 161067_at | 1426065_a_at |
| 99371_at | 1426070_a_at |
| 166935_i_at | 1426075_at |
| 162729_at | 1426078_a_at |
| 115451_at | 1426082_a_at |
| 92750_s_at | 1426091_a_at |
| 97177_at | 1426101_at |
| 96514_at | 1426103_a_at |
| 99103_at | 1426111_x_at |
| 98332_at | 1426115_a_at |
| 94776_f_at | 1426127_x_at |
| 133779_at | 1426134_at |
| 93719_at | 1426139_a_at |
| 93105_s_at | 1426159_x_at |
| 162410_s_at | 1426170_a_at |
| 97762_f_at | 1426171_x_at |
| 97109_at | 1426175_a_at |
| 99370_at | 1426182_a_at |
| 160675_at | 1426192_at |
| 165587_i_at | 1426212_s_at |
| 162540_at | 1426216_at |
| 95787_s_at | 1426219_at |
| 165778_at | 1426231_at |
| 98509_at | 1426234_s_at |
| 163955_at | 1426250_s_at |
| 92605_at | 1426252_a_at |
| 94860_at | 1426256_at |
| 112362_at | 1426258_at |
| 96746_at | 1426265_x_at |
| 163629_at | 1426273_at |
| 92718_at | 1426278_at |
| 166749_at | 1426280_at |
| 166358_at | 1426281_at |
| 163670_f_at | 1426282_at |
| 95621_at | 1426284_at |
| 92366_at | 1426285_at |

| | |
|---|---|
| 162958_at | 1426290_at |
| 164032_at | 1426291_at |
| 92506_at | 1426294_at |
| 98040_at | 1426297_at |
| 162749_at | 1426298_at |
| 97922_at | 1426307_at |
| 166248_at | 1426309_at |
| 161029_at | 1426316_at |
| 170804_at | 1426318_at |
| 94064_at | 1426326_at |
| 93706_at | 1426333_a_at |
| 99419_g_at | 1426334_a_at |
| 95702_at | 1426342_at |
| 96635_at | 1426344_at |
| 94957_at | 1426345_at |
| 96045_at | 1426347_at |
| 95417_at | 1426350_at |
| 163061_at | 1426362_at |
| 163275_at | 1426399_at |
| 160205_f_at | 1426405_at |
| 166394_at | 1426418_at |
| 98553_at | 1426457_at |
| 94481_at | 1426461_at |
| 98507_at | 1426464_at |
| 166907_r_at | 1426466_s_at |
| 99950_at | 1426469_a_at |
| 93258_at | 1426475_at |
| 99990_at | 1426487_a_at |
| 98583_at | 1426520_at |
| 162837_at | 1426524_at |
| 97811_at | 1426534_a_at |
| 163096_at | 1426535_at |
| 165774_at | 1426541_a_at |
| 99197_at | 1426547_at |
| 165505_at | 1426549_at |
| 116247_at | 1426550_at |
| 163463_at | 1426553_at |
| 92959_at | 1426570_a_at |
| 93254_at | 1426585_s_at |
| 160295_at | 1426586_at |
| 170201_r_at | 1426607_at |
| 160819_at | 1426615_s_at |
| 92691_at | 1426621_a_at |
| 96615_at | 1426624_a_at |
| 93402_i_at | 1426636_a_at |
| 160243_r_at | 1426650_at |
| 95750_at | 1426654_at |
| 93127_at | 1426658_x_at |
| 94823_at | 1426660_x_at |
| 98593_at | 1426662_at |
| 96845_at | 1426668_at |
| 95432_f_at | 1426675_at |
| 97464_at | 1426678_at |
| 165705_r_at | 1426681_at |
| 94946_at | 1426686_s_at |
| 93264_at | 1426690_a_at |

| | |
|---|---|
| 162638_at | 1426691_at |
| 163095_at | 1426692_at |
| 94397_at | 1426700_a_at |
| 162787_at | 1426701_at |
| 162700_at | 1426703_at |
| 164278_at | 1426704_at |
| 160710_at | 1426709_a_at |
| 162693_at | 1426711_at |
| 94339_at | 1426714_at |
| 165462_at | 1426729_at |
| 160525_f_at | 1426730_a_at |
| 97419_at | 1426765_at |
| 95559_at | 1426766_at |
| 162878_at | 1426767_at |
| 171245_r_at | 1426768_at |
| 95594_at | 1426773_at |
| 160745_at | 1426783_at |
| 97510_at | 1426785_s_at |
| 160624_at | 1426796_at |
| 168809_i_at | 1426807_at |
| 162774_at | 1426809_at |
| 98538_at | 1426820_at |
| 163126_at | 1426826_at |
| 99464_at | 1426829_at |
| 163771_at | 1426866_at |
| 165740_at | 1426867_at |
| 163583_at | 1426870_at |
| 97826_at | 1426872_at |
| 163011_at | 1426879_at |
| 164135_at | 1426881_at |
| 161640_at | 1426883_at |
| 97908_at | 1426884_at |
| 95123_at | 1426889_at |
| 163662_at | 1426898_at |
| 95437_at | 1426912_at |
| 161020_r_at | 1426924_at |
| 160992_at | 1426928_at |
| 93070_at | 1426946_at |
| 93517_at | 1426947_x_at |
| 160701_at | 1426966_at |
| 163187_at | 1426973_at |
| 163571_at | 1426991_at |
| 160269_at | 1426995_a_at |
| 99077_at | 1426997_at |
| 163987_at | 1427008_at |
| 99931_at | 1427009_at |
| 167049_at | 1427013_at |
| 163903_at | 1427017_at |
| 163025_at | 1427018_at |
| 92378_at | 1427019_at |
| 98488_at | 1427026_at |
| 94558_g_at | 1427027_a_at |
| 93480_at | 1427029_at |
| 160927_at | 1427034_at |
| 161005_at | 1427050_at |
| 161073_at | 1427057_at |

| | |
|---|---|
| 163332_at | 1427061_at |
| 163132_at | 1427063_at |
| 164637_f_at | 1427071_at |
| 162763_at | 1427075_s_at |
| 169708_r_at | 1427081_at |
| 163199_at | 1427082_at |
| 163090_at | 1427097_at |
| 92315_at | 1427102_at |
| 96837_at | 1427109_at |
| 165686_at | 1427114_at |
| 160753_at | 1427115_at |
| 93555_at | 1427118_at |
| 94358_at | 1427119_at |
| 93702_at | 1427128_at |
| 93441_at | 1427141_at |
| 94703_at | 1427154_at |
| 167814_r_at | 1427159_at |
| 99923_at | 1427160_at |
| 111993_at | 1427163_at |
| 162776_f_at | 1427166_a_at |
| 99476_at | 1427168_a_at |
| 164109_at | 1427169_at |
| 92826_at | 1427173_a_at |
| 97814_at | 1427179_at |
| 92686_at | 1427180_at |
| 93852_at | 1427186_a_at |
| 95563_at | 1427188_at |
| 166461_r_at | 1427190_at |
| 169378_at | 1427199_at |
| 166708_at | 1427205_x_at |
| 98842_at | 1427220_a_at |
| 166100_at | 1427241_at |
| 160394_at | 1427244_at |
| 160616_at | 1427248_at |
| 93265_at | 1427260_a_at |
| 93457_at | 1427264_at |
| 160619_at | 1427270_a_at |
| 96782_at | 1427290_at |
| 92692_at | 1427291_at |
| 160414_at | 1427295_at |
| 94754_at | 1427300_at |
| 98811_at | 1427313_at |
| 160842_at | 1427317_at |
| 93583_s_at | 1427329_a_at |
| 163668_at | 1427331_at |
| 92306_at | 1427346_at |
| 97745_at | 1427354_at |
| 92533_at | 1427355_at |
| 163731_at | 1427357_at |
| 93556_at | 1427365_at |
| 97470_at | 1427366_at |
| 99836_at | 1427372_at |
| 98401_at | 1427377_x_at |
| 94714_at | 1427380_at |
| 92314_at | 1427391_a_at |
| 166668_at | 1427415_at |

| | |
|---|---|
| 93182_at | 1427420_at |
| 96998_at | 1427424_at |
| 166812_at | 1427426_at |
| 97126_at | 1427427_at |
| 92948_at | 1427429_at |
| 134821_at | 1427430_at |
| 98839_at | 1427436_at |
| 94563_at | 1427440_a_at |
| 97697_at | 1427452_at |
| 92701_at | 1427458_at |
| 99481_at | 1427465_at |
| 92952_f_at | 1427470_s_at |
| 92289_at | 1427486_at |
| 164103_at | 1427492_at |
| 92914_at | 1427494_at |
| 92412_s_at | 1427498_a_at |
| 92733_at | 1427502_at |
| 164160_at | 1427507_at |
| 97790_s_at | 1427512_a_at |
| 98308_at | 1427520_a_at |
| 99844_at | 1427529_at |
| 92617_at | 1427543_s_at |
| 97122_at | 1427560_at |
| 97654_at | 1427563_at |
| 94127_at | 1427564_at |
| 163499_at | 1427568_a_at |
| 98316_at | 1427573_at |
| 99405_at | 1427577_x_at |
| 97121_at | 1427582_at |
| 95396_at | 1427585_at |
| 92184_at | 1427588_a_at |
| 95376_at | 1427591_at |
| 98780_at | 1427605_at |
| 97652_at | 1427611_at |
| 94640_at | 1427618_at |
| 97653_at | 1427622_at |
| 165954_f_at | 1427626_at |
| 94202_at | 1427628_at |
| 98827_i_at | 1427635_at |
| 94103_at | 1427636_at |
| 97655_at | 1427637_a_at |
| 97541_f_at | 1427651_x_at |
| 92328_at | 1427655_a_at |
| 166252_i_at | 1427657_at |
| 93086_at | 1427660_x_at |
| 160557_at | 1427661_a_at |
| 92953_at | 1427671_a_at |
| 95342_at | 1427675_at |
| 93132_at | 1427676_a_at |
| 92726_at | 1427677_a_at |
| 96420_at | 1427693_at |
| 98427_s_at | 1427705_a_at |
| 92367_at | 1427707_a_at |
| 163074_at | 1427715_a_at |
| 93905_at | 1427717_at |
| 92305_s_at | 1427725_a_at |

| | |
|---|---|
| 97196_at | 1427727_x_at |
| 93617_at | 1427736_a_at |
| 98764_at | 1427738_at |
| 98382_f_at | 1427745_x_at |
| 160564_at | 1427747_a_at |
| 161052_r_at | 1427751_a_at |
| 133140_at | 1427754_a_at |
| 161649_f_at | 1427759_a_at |
| 93224_at | 1427766_at |
| 94758_s_at | 1427767_a_at |
| 160097_at | 1427788_at |
| 98281_at | 1427794_at |
| 92260_at | 1427804_at |
| 99884_at | 1427813_at |
| 99699_at | 1427828_at |
| 96964_at | 1427859_at |
| 96416_f_at | 1427864_at |
| 94215_at | 1427874_at |
| 96307_s_at | 1427875_a_at |
| 160251_at | 1427876_at |
| 94843_at | 1427885_at |
| 162999_at | 1427890_a_at |
| 162574_at | 1427894_at |
| 99157_at | 1427895_at |
| 98070_at | 1427896_at |
| 98069_s_at | 1427897_s_at |
| 166245_at | 1427900_at |
| 95011_at | 1427905_at |
| 163188_at | 1427906_at |
| 163712_at | 1427907_at |
| 98039_at | 1427909_at |
| 160299_at | 1427913_at |
| 163071_at | 1427916_at |
| 163669_at | 1427924_at |
| 93183_at | 1427928_s_at |
| 96519_at | 1427931_s_at |
| 166159_at | 1427962_at |
| 164045_at | 1427966_at |
| 166107_at | 1427968_at |
| 164047_at | 1427974_s_at |
| 164902_r_at | 1427976_at |
| 98954_f_at | 1427977_x_at |
| 99184_at | 1427981_a_at |
| 168898_r_at | 1427985_at |
| 162976_at | 1427986_a_at |
| 97412_at | 1428004_at |
| 96710_at | 1428029_a_at |
| 93688_at | 1428043_a_at |
| 98061_at | 1428049_a_at |
| 165431_at | 1428062_at |
| 93054_at | 1428063_at |
| 163851_at | 1428066_at |
| 163439_at | 1428067_at |
| 95063_at | 1428069_at |
| 95119_at | 1428071_at |
| 94373_at | 1428072_a_at |

| | |
|---|---|
| 98146_at | 1428073_a_at |
| 166316_i_at | 1428077_at |
| 94382_at | 1428085_at |
| 160931_at | 1428086_at |
| 163178_at | 1428089_at |
| 162535_at | 1428102_at |
| 162873_at | 1428109_at |
| 95749_at | 1428112_at |
| 163425_at | 1428115_a_at |
| 160812_at | 1428141_at |
| 96348_at | 1428143_a_at |
| 97488_at | 1428144_at |
| 95064_at | 1428146_s_at |
| 94290_at | 1428158_at |
| 96909_at | 1428159_s_at |
| 96906_at | 1428161_a_at |
| 96002_at | 1428165_at |
| 160581_at | 1428169_at |
| 162812_i_at | 1428170_at |
| 163009_at | 1428173_at |
| 162831_at | 1428175_at |
| 95496_at | 1428182_at |
| 165773_at | 1428183_at |
| 163514_at | 1428184_at |
| 169258_i_at | 1428195_at |
| 95078_at | 1428196_a_at |
| 93986_at | 1428213_at |
| 160256_at | 1428215_x_at |
| 98891_at | 1428217_at |
| 163503_at | 1428221_at |
| 162916_at | 1428223_at |
| 92574_at | 1428235_at |
| 96819_at | 1428241_at |
| 94260_at | 1428244_at |
| 99139_at | 1428246_at |
| 97512_at | 1428258_at |
| 160402_at | 1428263_a_at |
| 93514_at | 1428266_at |
| 163843_i_at | 1428268_at |
| 98953_at | 1428272_at |
| 160140_at | 1428282_at |
| 162779_i_at | 1428298_at |
| 96059_at | 1428302_at |
| 161082_r_at | 1428303_at |
| 163266_at | 1428307_at |
| 92806_at | 1428308_at |
| 160752_at | 1428312_at |
| 99196_at | 1428314_at |
| 93046_at | 1428328_at |
| 163186_at | 1428330_at |
| 160513_at | 1428331_at |
| 162526_at | 1428337_at |
| 163411_at | 1428344_at |
| 166099_i_at | 1428355_at |
| 160145_at | 1428358_at |
| 160328_at | 1428365_a_at |

| | |
|---|---|
| 163850_at | 1428366_at |
| 93590_at | 1428367_at |
| 163175_at | 1428370_at |
| 163443_at | 1428371_at |
| 165717_i_at | 1428384_at |
| 162937_at | 1428393_at |
| 162877_at | 1428411_at |
| 165213_r_at | 1428414_at |
| 98613_at | 1428421_a_at |
| 166482_i_at | 1428427_at |
| 163507_at | 1428430_at |
| 165232_at | 1428435_at |
| 160822_at | 1428443_a_at |
| 162672_at | 1428444_at |
| 163166_at | 1428447_at |
| 165743_at | 1428460_at |
| 162885_at | 1428461_at |
| 97295_at | 1428480_at |
| 166419_at | 1428482_at |
| 160784_r_at | 1428483_a_at |
| 165522_r_at | 1428486_at |
| 163788_at | 1428497_at |
| 163300_at | 1428498_at |
| 163375_at | 1428499_at |
| 94400_at | 1428501_at |
| 160991_at | 1428503_a_at |
| 98881_at | 1428507_at |
| 169974_at | 1428509_at |
| 97971_at | 1428511_at |
| 163676_at | 1428521_at |
| 166345_at | 1428523_at |
| 97271_at | 1428526_at |
| 99447_at | 1428528_at |
| 160654_at | 1428534_at |
| 163146_at | 1428549_at |
| 163036_at | 1428571_at |
| 163407_at | 1428573_at |
| 165419_i_at | 1428601_at |
| 163566_at | 1428602_at |
| 94537_at | 1428608_at |
| 162673_at | 1428611_at |
| 163328_at | 1428617_at |
| 95480_at | 1428621_a_at |
| 166916_i_at | 1428623_at |
| 166893_at | 1428627_at |
| 165435_f_at | 1428628_at |
| 160590_r_at | 1428635_at |
| 166144_i_at | 1428638_at |
| 170096_r_at | 1428642_at |
| 168615_s_at | 1428653_x_at |
| 166448_f_at | 1428664_at |
| 93749_at | 1428667_at |
| 163267_at | 1428682_at |
| 163302_at | 1428683_at |
| 160723_at | 1428684_at |
| 163259_at | 1428686_at |

| | |
|---|---|
| 167949_r_at | 1428687_at |
| 163387_at | 1428697_at |
| 96211_at | 1428698_at |
| 163172_at | 1428701_at |
| 163399_at | 1428702_at |
| 165650_f_at | 1428703_at |
| 163920_at | 1428716_at |
| 163628_at | 1428717_at |
| 92316_f_at | 1428720_s_at |
| 161060_i_at | 1428728_at |
| 160893_at | 1428733_at |
| 164220_at | 1428735_at |
| 93484_at | 1428737_s_at |
| 96145_at | 1428740_a_at |
| 163986_at | 1428741_at |
| 162981_at | 1428744_s_at |
| 162863_at | 1428746_a_at |
| 160634_at | 1428751_at |
| 164148_at | 1428765_at |
| 160722_at | 1428766_at |
| 163988_at | 1428771_at |
| 93624_at | 1428776_at |
| 162623_at | 1428778_at |
| 166800_r_at | 1428779_at |
| 97098_at | 1428780_at |
| 93647_at | 1428784_at |
| 163412_at | 1428790_at |
| 163286_at | 1428792_at |
| 165436_r_at | 1428807_at |
| 164136_at | 1428809_at |
| 160980_at | 1428810_at |
| 166110_r_at | 1428812_at |
| 162995_at | 1428813_a_at |
| 165689_at | 1428815_at |
| 162612_at | 1428821_at |
| 162813_at | 1428822_a_at |
| 160659_at | 1428823_at |
| 93481_at | 1428835_at |
| 163045_at | 1428836_at |
| 165465_at | 1428840_s_at |
| 165647_f_at | 1428841_at |
| 93148_at | 1428862_at |
| 163936_at | 1428865_at |
| 97935_at | 1428871_at |
| 163382_at | 1428873_a_at |
| 163237_at | 1428900_s_at |
| 163398_at | 1428917_at |
| 165558_at | 1428932_at |
| 160180_at | 1428935_at |
| 162843_at | 1428945_at |
| 168117_i_at | 1428948_at |
| 163414_f_at | 1428952_at |
| 165633_at | 1428960_at |
| 163584_at | 1428964_at |
| 163819_at | 1428965_at |
| 93059_at | 1428966_at |

| | |
|---|---|
| 162857_at | 1428975_at |
| 161030_at | 1428983_at |
| 163726_at | 1428984_a_at |
| 165777_r_at | 1428990_at |
| 163312_i_at | 1428994_s_at |
| 94480_at | 1429014_at |
| 163559_at | 1429023_at |
| 166813_at | 1429025_a_at |
| 163533_at | 1429029_at |
| 166725_at | 1429030_at |
| 163518_at | 1429032_at |
| 161065_at | 1429035_at |
| 162816_at | 1429042_at |
| 165731_at | 1429067_at |
| 165813_at | 1429069_at |
| 165808_at | 1429071_at |
| 166712_at | 1429073_at |
| 165797_at | 1429076_a_at |
| 92875_s_at | 1429078_a_at |
| 106827_at | 1429082_at |
| 162724_at | 1429086_at |
| 163921_at | 1429102_at |
| 160689_r_at | 1429117_at |
| 167275_f_at | 1429127_at |
| 163534_f_at | 1429129_at |
| 163195_at | 1429135_at |
| 160999_r_at | 1429137_at |
| 163732_at | 1429140_at |
| 163736_at | 1429141_at |
| 165593_at | 1429143_at |
| 162949_at | 1429146_at |
| 164121_at | 1429166_s_at |
| 167422_r_at | 1429172_a_at |
| 161169_f_at | 1429198_at |
| 163282_at | 1429206_at |
| 164038_at | 1429216_at |
| 167728_at | 1429231_at |
| 160922_at | 1429238_a_at |
| 167108_r_at | 1429260_at |
| 165733_at | 1429263_at |
| 167302_i_at | 1429282_at |
| 166929_at | 1429283_at |
| 164156_at | 1429285_at |
| 165811_at | 1429286_at |
| 92803_at | 1429287_a_at |
| 95483_at | 1429291_at |
| 162893_at | 1429300_at |
| 92324_at | 1429303_at |
| 164022_i_at | 1429316_at |
| 161071_at | 1429317_at |
| 93428_at | 1429326_at |
| 166530_at | 1429330_at |
| 165827_at | 1429331_at |
| 165496_at | 1429334_at |
| 165484_at | 1429340_at |
| 171517_at | 1429342_s_at |

| | |
|---|---|
| 163898_at | 1429346_a_at |
| 165594_at | 1429347_at |
| 170454_at | 1429350_at |
| 96151_at | 1429352_at |
| 165523_at | 1429353_a_at |
| 165681_at | 1429364_at |
| 162555_i_at | 1429371_at |
| 165439_at | 1429374_at |
| 160583_at | 1429379_at |
| 164015_at | 1429380_at |
| 163616_at | 1429385_at |
| 170641_at | 1429392_at |
| 163134_at | 1429395_at |
| 163081_at | 1429397_a_at |
| 165975_at | 1429401_at |
| 169193_f_at | 1429405_at |
| 163682_at | 1429407_at |
| 166388_at | 1429408_at |
| 163496_i_at | 1429409_at |
| 166395_at | 1429414_at |
| 163656_at | 1429416_at |
| 163943_at | 1429417_at |
| 165495_i_at | 1429419_at |
| 165487_f_at | 1429424_at |
| 162781_r_at | 1429429_s_at |
| 167668_r_at | 1429438_at |
| 165526_i_at | 1429445_at |
| 166653_at | 1429447_at |
| 167773_f_at | 1429450_at |
| 94893_at | 1429451_at |
| 98109_at | 1429453_a_at |
| 163875_at | 1429460_at |
| 163006_at | 1429465_at |
| 163759_at | 1429466_s_at |
| 165473_at | 1429471_at |
| 166191_f_at | 1429480_at |
| 164182_at | 1429494_at |
| 168012_r_at | 1429500_at |
| 165889_f_at | 1429513_at |
| 163558_at | 1429518_at |
| 165709_at | 1429519_at |
| 165323_at | 1429522_at |
| 163451_at | 1429529_at |
| 166492_at | 1429536_at |
| 162861_at | 1429540_at |
| 167540_at | 1429542_at |
| 165899_f_at | 1429544_at |
| 165683_at | 1429550_at |
| 163252_at | 1429552_at |
| 163976_at | 1429561_at |
| 162675_f_at | 1429565_s_at |
| 166030_i_at | 1429572_at |
| 160884_at | 1429574_at |
| 167899_i_at | 1429575_at |
| 167370_at | 1429577_at |
| 166242_i_at | 1429578_at |

| | |
|---|---|
| 92525_i_at | 1429582_at |
| 163403_r_at | 1429599_a_at |
| 169600_at | 1429602_at |
| 168156_at | 1429603_at |
| 170598_r_at | 1429610_a_at |
| 165860_at | 1429611_at |
| 162921_i_at | 1429614_at |
| 96157_at | 1429615_at |
| 160478_r_at | 1429622_at |
| 163019_i_at | 1429626_at |
| 163623_at | 1429634_at |
| 166386_at | 1429635_at |
| 165886_i_at | 1429636_at |
| 163724_at | 1429639_at |
| 167301_r_at | 1429640_at |
| 165963_f_at | 1429641_x_at |
| 166355_f_at | 1429642_at |
| 166367_at | 1429645_at |
| 168262_at | 1429646_at |
| 163837_at | 1429662_at |
| 163808_at | 1429670_a_at |
| 166294_r_at | 1429675_at |
| 171342_at | 1429677_at |
| 165792_at | 1429693_at |
| 166224_at | 1429697_at |
| 164073_at | 1429699_at |
| 167273_at | 1429700_at |
| 167542_f_at | 1429704_at |
| 171093_r_at | 1429706_at |
| 96611_at | 1429708_at |
| 169389_at | 1429716_at |
| 162886_r_at | 1429730_at |
| 167171_at | 1429741_at |
| 165406_at | 1429744_at |
| 169961_at | 1429745_at |
| 166641_at | 1429747_at |
| 166872_r_at | 1429753_at |
| 166324_at | 1429754_a_at |
| 163856_at | 1429762_a_at |
| 163263_at | 1429781_s_at |
| 163704_at | 1429783_at |
| 163159_at | 1429793_at |
| 165921_r_at | 1429794_a_at |
| 166134_at | 1429796_at |
| 167034_at | 1429797_at |
| 165893_f_at | 1429803_at |
| 163836_at | 1429804_at |
| 163839_at | 1429808_at |
| 165880_f_at | 1429826_at |
| 171091_f_at | 1429827_at |
| 165834_r_at | 1429828_at |
| 96839_at | 1429834_a_at |
| 168004_r_at | 1429835_at |
| 166250_at | 1429838_at |
| 166789_f_at | 1429841_at |
| 164075_at | 1429851_at |

| | |
|---|---|
| 168801_at | 1429854_at |
| 166326_at | 1429855_at |
| 166821_r_at | 1429861_at |
| 165872_at | 1429869_at |
| 167652_i_at | 1429872_at |
| 167484_at | 1429874_at |
| 96516_at | 1429884_at |
| 163720_at | 1429889_at |
| 163834_at | 1429892_at |
| 166466_r_at | 1429902_at |
| 169713_r_at | 1429908_at |
| 165680_at | 1429920_at |
| 167781_at | 1429930_at |
| 167811_at | 1429936_at |
| 164454_i_at | 1429945_at |
| 109179_at | 1429958_x_at |
| 164127_at | 1429965_at |
| 170326_i_at | 1429974_at |
| 166899_at | 1429982_at |
| 167526_i_at | 1429985_at |
| 166590_at | 1429987_at |
| 168879_i_at | 1429989_at |
| 167517_r_at | 1429990_at |
| 170399_at | 1429998_at |
| 99827_at | 1429999_at |
| 170153_r_at | 1430001_at |
| 163515_r_at | 1430006_x_at |
| 168149_r_at | 1430008_x_at |
| 168576_i_at | 1430015_at |
| 167318_i_at | 1430016_at |
| 167137_at | 1430027_at |
| 164173_at | 1430030_at |
| 166117_i_at | 1430045_at |
| 166903_at | 1430047_at |
| 166359_at | 1430049_at |
| 167204_at | 1430060_at |
| 167601_at | 1430061_at |
| 166609_at | 1430067_at |
| 165829_r_at | 1430077_at |
| 165929_at | 1430078_a_at |
| 166779_at | 1430083_at |
| 171067_f_at | 1430105_at |
| 169095_i_at | 1430106_at |
| 164178_i_at | 1430136_at |
| 166184_r_at | 1430140_at |
| 168032_at | 1430151_at |
| 168047_at | 1430169_at |
| 166927_i_at | 1430170_at |
| 171119_at | 1430194_at |
| 166321_at | 1430205_a_at |
| 170595_at | 1430209_at |
| 168046_at | 1430227_at |
| 168425_at | 1430234_at |
| 167707_f_at | 1430237_at |
| 169371_at | 1430245_at |
| 167659_at | 1430254_at |

| | |
|---|---|
| 167214_at | 1430257_at |
| 170192_at | 1430269_at |
| 162983_at | 1430274_a_at |
| 166967_at | 1430279_at |
| 168886_at | 1430281_at |
| 167126_at | 1430284_at |
| 166738_at | 1430296_at |
| 166762_r_at | 1430300_at |
| 166270_at | 1430306_a_at |
| 166295_f_at | 1430315_at |
| 166944_r_at | 1430316_at |
| 167761_at | 1430321_at |
| 164238_at | 1430322_at |
| 167485_at | 1430325_at |
| 167622_at | 1430338_at |
| 168146_at | 1430351_at |
| 170421_at | 1430358_at |
| 168063_at | 1430391_a_at |
| 168213_at | 1430398_at |
| 168222_at | 1430405_at |
| 166733_at | 1430412_at |
| 167036_at | 1430419_at |
| 167546_r_at | 1430441_at |
| 167851_at | 1430444_at |
| 169748_r_at | 1430460_at |
| 161747_i_at | 1430461_at |
| 166709_at | 1430468_at |
| 165319_at | 1430480_at |
| 168892_i_at | 1430499_at |
| 167572_at | 1430507_at |
| 168565_at | 1430513_at |
| 167817_i_at | 1430518_at |
| 92496_at | 1430522_a_at |
| 167733_at | 1430524_at |
| 94555_at | 1430527_a_at |
| 170063_at | 1430531_at |
| 168237_at | 1430557_at |
| 164251_at | 1430558_at |
| 162046_at | 1430559_at |
| 163940_at | 1430566_at |
| 167305_at | 1430567_at |
| 167003_at | 1430579_at |
| 171186_at | 1430591_at |
| 166799_r_at | 1430598_at |
| 165824_at | 1430611_at |
| 167281_at | 1430624_at |
| 164959_r_at | 1430631_at |
| 166717_at | 1430638_at |
| 163665_at | 1430649_at |
| 97101_at | 1430661_at |
| 167939_s_at | 1430674_at |
| 169066_at | 1430677_at |
| 168217_at | 1430684_s_at |
| 129584_at | 1430698_a_at |
| 171440_at | 1430700_a_at |
| 164192_at | 1430702_at |

| | |
|---|---|
| 98358_at | 1430711_at |
| 93764_at | 1430713_s_at |
| 168310_r_at | 1430723_at |
| 163877_at | 1430754_at |
| 168005_at | 1430784_a_at |
| 171577_at | 1430806_at |
| 169922_at | 1430819_at |
| 167825_r_at | 1430827_a_at |
| 161886_at | 1430845_at |
| 171125_at | 1430850_x_at |
| 170871_f_at | 1430867_at |
| 168128_at | 1430872_at |
| 170417_at | 1430903_at |
| 171089_at | 1430908_at |
| 98301_at | 1430912_a_at |
| 168672_at | 1430916_at |
| 171375_at | 1430922_at |
| 170304_at | 1430923_at |
| 170690_at | 1430933_at |
| 169408_at | 1430946_at |
| 167264_at | 1430974_a_at |
| 99608_at | 1430979_a_at |
| 170954_at | 1430986_at |
| 94485_at | 1431012_a_at |
| 169435_at | 1431017_at |
| 163832_at | 1431021_at |
| 168395_at | 1431024_a_at |
| 166902_at | 1431029_at |
| 94556_at | 1431055_a_at |
| 166688_i_at | 1431073_at |
| 164117_at | 1431089_at |
| 170291_at | 1431091_at |
| 92238_at | 1431092_at |
| 93221_at | 1431099_at |
| 163872_i_at | 1431100_at |
| 167948_f_at | 1431102_at |
| 165857_at | 1431104_at |
| 167236_at | 1431130_at |
| 167842_r_at | 1431146_a_at |
| 167528_at | 1431152_at |
| 167386_at | 1431157_at |
| 167823_r_at | 1431172_at |
| 164209_at | 1431183_at |
| 171305_at | 1431196_at |
| 166661_at | 1431209_s_at |
| 163047_at | 1431211_s_at |
| 167258_r_at | 1431223_at |
| 168111_r_at | 1431226_a_at |
| 163612_at | 1431230_a_at |
| 163762_at | 1431244_s_at |
| 165566_i_at | 1431255_at |
| 168023_at | 1431263_at |
| 167008_i_at | 1431264_at |
| 165970_i_at | 1431281_at |
| 167127_f_at | 1431290_at |
| 165849_f_at | 1431306_at |

| | |
|---|---|
| 166407_at | 1431346_at |
| 167940_at | 1431348_at |
| 168006_at | 1431349_at |
| 170205_at | 1431356_at |
| 169589_at | 1431365_at |
| 165654_at | 1431376_at |
| 167046_at | 1431377_at |
| 168267_i_at | 1431382_a_at |
| 167060_i_at | 1431386_s_at |
| 93748_at | 1431390_a_at |
| 166783_r_at | 1431394_a_at |
| 164319_r_at | 1431409_at |
| 160077_at | 1431411_a_at |
| 97893_at | 1431415_a_at |
| 162922_at | 1431423_a_at |
| 171480_at | 1431431_a_at |
| 165906_at | 1431446_at |
| 167775_r_at | 1431454_at |
| 167661_i_at | 1431456_at |
| 167942_i_at | 1431460_at |
| 171362_r_at | 1431467_at |
| 92970_at | 1431475_a_at |
| 166475_r_at | 1431477_at |
| 170617_at | 1431483_at |
| 168370_at | 1431495_at |
| 168235_at | 1431508_at |
| 167450_at | 1431509_at |
| 92426_at | 1431530_a_at |
| 168027_at | 1431533_at |
| 163758_at | 1431554_a_at |
| 166952_i_at | 1431563_at |
| 164187_at | 1431567_at |
| 170911_at | 1431572_at |
| 170344_at | 1431588_at |
| 168774_at | 1431589_at |
| 97903_at | 1431593_a_at |
| 98859_at | 1431609_a_at |
| 170993_at | 1431624_a_at |
| 167961_r_at | 1431640_at |
| 171092_r_at | 1431643_at |
| 165879_at | 1431647_a_at |
| 164203_at | 1431666_at |
| 171562_at | 1431674_at |
| 169020_at | 1431720_at |
| 93853_at | 1431734_a_at |
| 163545_at | 1431737_at |
| 170887_at | 1431742_at |
| 97380_at | 1431745_a_at |
| 167064_r_at | 1431751_a_at |
| 169596_at | 1431758_at |
| 160744_r_at | 1431763_a_at |
| 163158_at | 1431772_a_at |
| 171447_at | 1431783_at |
| 97131_at | 1431792_a_at |
| 161801_r_at | 1431795_a_at |
| 98467_at | 1431808_a_at |

| | |
|---|---|
| 171085_at | 1431818_at |
| 166854_at | 1431826_a_at |
| 92590_at | 1431833_a_at |
| 98324_at | 1431900_a_at |
| 164242_r_at | 1431930_x_at |
| 165579_at | 1431931_a_at |
| 167085_at | 1431936_a_at |
| 163519_at | 1431946_a_at |
| 166096_f_at | 1431972_a_at |
| 162734_at | 1431980_a_at |
| 111116_at | 1431985_at |
| 169437_at | 1432002_at |
| 167112_at | 1432054_at |
| 170613_r_at | 1432076_at |
| 160566_at | 1432080_s_at |
| 169590_at | 1432082_at |
| 168655_r_at | 1432124_at |
| 162800_at | 1432136_s_at |
| 167180_at | 1432155_at |
| 166365_at | 1432156_a_at |
| 162867_r_at | 1432158_a_at |
| 98979_at | 1432177_a_at |
| 164104_at | 1432205_a_at |
| 169493_r_at | 1432214_at |
| 166970_at | 1432227_at |
| 111198_at | 1432228_at |
| 168388_at | 1432245_s_at |
| 114187_at | 1432250_at |
| 163953_at | 1432269_a_at |
| 93412_at | 1432273_a_at |
| 167770_at | 1432283_at |
| 163678_at | 1432289_a_at |
| 170886_at | 1432300_at |
| 170163_at | 1432308_at |
| 171253_at | 1432319_at |
| 93807_at | 1432322_at |
| 169319_r_at | 1432326_at |
| 165042_f_at | 1432332_a_at |
| 164171_at | 1432333_a_at |
| 167573_f_at | 1432348_at |
| 167480_at | 1432381_a_at |
| 169576_r_at | 1432386_a_at |
| 167640_at | 1432420_a_at |
| 170167_at | 1432422_at |
| 160186_at | 1432435_s_at |
| 163652_at | 1432443_at |
| 165695_at | 1432444_a_at |
| 163500_at | 1432447_a_at |
| 165931_i_at | 1432453_a_at |
| 169523_at | 1432454_at |
| 169885_at | 1432469_at |
| 168144_r_at | 1432473_a_at |
| 167738_f_at | 1432486_a_at |
| 168326_at | 1432489_a_at |
| 169680_at | 1432493_at |
| 169886_at | 1432504_at |

| | |
|---|---|
| 163176_at | 1432513_a_at |
| 170181_at | 1432536_at |
| 169972_at | 1432541_at |
| 163954_at | 1432556_a_at |
| 170903_at | 1432565_at |
| 164969_r_at | 1432571_at |
| 160664_at | 1432848_a_at |
| 135608_at | 1432852_at |
| 99168_at | 1433429_at |
| 162523_at | 1433431_at |
| 162559_at | 1433434_at |
| 168765_at | 1433435_at |
| 97282_at | 1433438_x_at |
| 96031_r_at | 1433441_at |
| 94325_at | 1433444_at |
| 160392_at | 1433448_at |
| 162962_at | 1433452_at |
| 162695_at | 1433456_at |
| 162565_r_at | 1433458_at |
| 163114_at | 1433462_a_at |
| 96079_at | 1433463_at |
| 160762_at | 1433477_at |
| 160155_at | 1433483_s_at |
| 94463_at | 1433487_at |
| 96925_at | 1433495_at |
| 162694_at | 1433498_at |
| 162952_at | 1433503_at |
| 168371_f_at | 1433505_a_at |
| 163390_r_at | 1433511_at |
| 166214_at | 1433518_at |
| 163308_at | 1433522_at |
| 163690_at | 1433525_at |
| 163897_i_at | 1433526_at |
| 160442_at | 1433534_a_at |
| 98036_at | 1433538_at |
| 162739_at | 1433542_at |
| 96784_at | 1433543_at |
| 162615_at | 1433547_s_at |
| 160825_at | 1433550_at |
| 163615_at | 1433553_at |
| 93983_at | 1433563_s_at |
| 163930_at | 1433566_at |
| 163084_at | 1433568_at |
| 163066_at | 1433577_at |
| 163714_at | 1433593_at |
| 94030_at | 1433594_at |
| 166068_at | 1433602_at |
| 96223_at | 1433606_at |
| 163226_at | 1433608_at |
| 96261_at | 1433616_a_at |
| 95604_at | 1433618_at |
| 165799_i_at | 1433619_at |
| 95587_at | 1433628_at |
| 93799_at | 1433629_s_at |
| 160460_at | 1433638_s_at |
| 163248_at | 1433643_at |

| | |
|---|---|
| 166917_at | 1433644_at |
| 112086_at | 1433650_at |
| 163224_at | 1433652_at |
| 94499_at | 1433654_at |
| 94355_at | 1433659_at |
| 96667_at | 1433666_s_at |
| 163637_at | 1433667_at |
| 93175_at | 1433671_at |
| 162872_at | 1433677_at |
| 96097_at | 1433684_at |
| 160682_at | 1433685_a_at |
| 162626_at | 1433691_at |
| 163599_at | 1433695_at |
| 169187_at | 1433696_at |
| 96113_at | 1433698_a_at |
| 160136_r_at | 1433704_s_at |
| 165688_r_at | 1433707_at |
| 95692_at | 1433708_at |
| 96829_at | 1433717_at |
| 96165_at | 1433737_at |
| 160994_at | 1433756_at |
| 163522_at | 1433762_at |
| 166594_at | 1433763_at |
| 163317_at | 1433778_at |
| 163037_at | 1433783_at |
| 163639_at | 1433787_at |
| 166763_at | 1433788_at |
| 166650_f_at | 1433790_at |
| 166894_r_at | 1433791_at |
| 94765_at | 1433799_at |
| 94026_at | 1433800_a_at |
| 163526_at | 1433815_at |
| 166318_at | 1433817_at |
| 140899_at | 1433818_at |
| 162933_at | 1433823_at |
| 163089_at | 1433841_at |
| 92411_at | 1433843_at |
| 167395_r_at | 1433847_at |
| 163378_at | 1433858_at |
| 163874_at | 1433860_at |
| 166832_f_at | 1433864_at |
| 162924_at | 1433870_at |
| 96864_at | 1433878_at |
| 162744_at | 1433879_a_at |
| 162923_at | 1433885_at |
| 96822_at | 1433886_at |
| 160644_at | 1433904_at |
| 161015_at | 1433906_at |
| 163075_at | 1433919_at |
| 94447_at | 1433923_at |
| 163598_at | 1433929_at |
| 164470_r_at | 1433930_at |
| 93832_at | 1433940_at |
| 162768_at | 1433943_at |
| 171318_at | 1433946_at |
| 165766_at | 1433950_at |

| | |
|---|---|
| 96751_at | 1433951_at |
| 94937_at | 1433953_at |
| 96230_at | 1433958_at |
| 165729_at | 1433962_at |
| 160815_at | 1433964_s_at |
| 171160_r_at | 1433966_x_at |
| 162570_at | 1433967_at |
| 163152_at | 1433969_at |
| 160178_r_at | 1433970_at |
| 164152_at | 1433989_at |
| 171051_at | 1433990_at |
| 162660_at | 1433993_at |
| 96130_at | 1433999_at |
| 169455_at | 1434010_at |
| 162722_at | 1434018_at |
| 163995_at | 1434024_at |
| 160995_at | 1434044_at |
| 163274_at | 1434051_s_at |
| 92615_at | 1434057_at |
| 97923_at | 1434060_at |
| 98017_at | 1434071_a_at |
| 163351_at | 1434076_at |
| 163697_at | 1434082_at |
| 162584_at | 1434083_a_at |
| 94991_at | 1434089_at |
| 163161_at | 1434090_at |
| 162792_at | 1434092_at |
| 163202_at | 1434094_at |
| 112498_at | 1434103_at |
| 166669_f_at | 1434125_at |
| 164378_f_at | 1434128_a_at |
| 92182_at | 1434131_at |
| 160776_at | 1434133_s_at |
| 166852_at | 1434139_at |
| 163859_f_at | 1434142_at |
| 162662_at | 1434144_s_at |
| 162630_at | 1434158_at |
| 94349_at | 1434164_s_at |
| 163919_at | 1434171_at |
| 163292_at | 1434189_at |
| 160630_at | 1434190_at |
| 163506_at | 1434196_at |
| 163939_at | 1434201_at |
| 164035_at | 1434211_at |
| 93184_at | 1434224_at |
| 99654_s_at | 1434227_at |
| 96217_at | 1434229_a_at |
| 93098_at | 1434231_x_at |
| 163392_at | 1434241_at |
| 163299_at | 1434242_at |
| 165429_at | 1434247_at |
| 164133_at | 1434249_s_at |
| 163606_at | 1434250_at |
| 99512_at | 1434251_at |
| 161081_at | 1434272_at |
| 97752_at | 1434292_at |

| | |
|---|---|
| 166711_at | 1434293_at |
| 169285_f_at | 1434298_at |
| 160432_at | 1434305_at |
| 171062_at | 1434321_at |
| 93144_at | 1434335_at |
| 162791_at | 1434338_at |
| 164029_at | 1434344_at |
| 160855_at | 1434345_at |
| 170770_r_at | 1434346_at |
| 164031_at | 1434363_x_at |
| 163728_at | 1434368_at |
| 163812_at | 1434388_at |
| 93621_at | 1434400_at |
| 164019_at | 1434407_at |
| 163244_at | 1434410_at |
| 171094_i_at | 1434420_x_at |
| 166622_at | 1434421_at |
| 165586_f_at | 1434422_at |
| 160586_r_at | 1434425_at |
| 168158_r_at | 1434427_a_at |
| 165770_at | 1434450_s_at |
| 164077_at | 1434451_at |
| 92654_at | 1434453_at |
| 165672_at | 1434455_at |
| 169370_at | 1434464_at |
| 166877_at | 1434474_at |
| 162799_at | 1434493_at |
| 166399_at | 1434495_at |
| 163595_at | 1434497_at |
| 163557_at | 1434501_at |
| 160732_at | 1434507_at |
| 161002_at | 1434514_at |
| 99190_at | 1434516_at |
| 165752_at | 1434526_at |
| 166817_i_at | 1434535_at |
| 161006_at | 1434537_at |
| 163573_at | 1434539_at |
| 97992_at | 1434546_at |
| 163323_at | 1434571_at |
| 163336_at | 1434574_at |
| 167634_i_at | 1434583_at |
| 160853_at | 1434586_a_at |
| 98142_at | 1434604_at |
| 161701_at | 1434611_at |
| 162988_at | 1434618_at |
| 163221_at | 1434619_at |
| 160725_at | 1434625_at |
| 165394_f_at | 1434626_at |
| 163469_at | 1434630_at |
| 163232_at | 1434634_at |
| 163957_at | 1434654_at |
| 98029_at | 1434658_at |
| 162987_at | 1434670_at |
| 164224_at | 1434685_at |
| 96731_at | 1434697_at |
| 95591_at | 1434703_at |

| | |
|---|---|
| 92293_at | 1434709_at |
| 171581_f_at | 1434711_at |
| 162884_at | 1434722_at |
| 165725_at | 1434724_at |
| 163150_at | 1434725_at |
| 162583_at | 1434726_at |
| 163396_at | 1434728_at |
| 163635_at | 1434730_at |
| 165745_at | 1434742_s_at |
| 97365_at | 1434755_at |
| 99864_at | 1434772_at |
| 160607_at | 1434775_at |
| 164023_at | 1434785_at |
| 95889_at | 1434787_at |
| 162868_at | 1434792_at |
| 163490_at | 1434795_at |
| 93191_at | 1434796_at |
| 92420_at | 1434802_s_at |
| 167984_r_at | 1434818_at |
| 162907_at | 1434827_at |
| 165529_r_at | 1434852_at |
| 99093_at | 1434854_a_at |
| 164154_at | 1434857_at |
| 166720_at | 1434870_at |
| 163911_at | 1434877_at |
| 94048_at | 1434879_at |
| 161024_at | 1434889_at |
| 161056_at | 1434901_at |
| 166599_at | 1434921_at |
| 93153_at | 1434932_at |
| 163277_at | 1434947_at |
| 166268_at | 1434957_at |
| 161111_f_at | 1434959_at |
| 165577_at | 1434961_at |
| 167479_at | 1434973_at |
| 115421_at | 1434974_at |
| 160997_at | 1434981_at |
| 164004_at | 1434983_at |
| 92816_r_at | 1434985_a_at |
| 163651_at | 1434990_at |
| 93158_at | 1434992_at |
| 160160_at | 1434994_at |
| 94440_at | 1435007_s_at |
| 93190_at | 1435013_at |
| 163679_at | 1435014_at |
| 161019_at | 1435015_at |
| 163418_at | 1435023_at |
| 93181_r_at | 1435030_at |
| 163417_at | 1435034_at |
| 163530_at | 1435047_at |
| 168387_i_at | 1435051_at |
| 163191_at | 1435052_at |
| 165753_at | 1435060_at |
| 165578_at | 1435089_at |
| 163577_at | 1435101_at |
| 96625_at | 1435114_at |

| | |
|---|---|
| 164124_at | 1435126_at |
| 99337_at | 1435128_at |
| 165749_r_at | 1435131_at |
| 163444_at | 1435132_at |
| 164016_at | 1435136_at |
| 163618_i_at | 1435138_at |
| 94380_at | 1435140_at |
| 160989_r_at | 1435184_at |
| 163586_at | 1435197_at |
| 92432_at | 1435202_at |
| 95153_at | 1435220_s_at |
| 92261_at | 1435231_at |
| 97936_at | 1435243_at |
| 166424_at | 1435252_at |
| 93232_at | 1435259_s_at |
| 163142_at | 1435263_at |
| 163413_at | 1435268_at |
| 165311_i_at | 1435274_at |
| 165836_at | 1435278_at |
| 167598_at | 1435286_at |
| 104799_at | 1435304_at |
| 165574_at | 1435312_at |
| 166204_at | 1435318_at |
| 97158_at | 1435360_at |
| 166651_i_at | 1435362_at |
| 166670_at | 1435366_at |
| 163421_at | 1435403_at |
| 166640_at | 1435410_at |
| 171524_at | 1435429_x_at |
| 163135_at | 1435435_at |
| 93994_at | 1435446_a_at |
| 166829_at | 1435456_at |
| 163385_at | 1435461_at |
| 93427_at | 1435463_s_at |
| 163653_at | 1435464_at |
| 163121_at | 1435472_at |
| 92374_at | 1435495_at |
| 165607_at | 1435502_a_at |
| 160975_at | 1435504_at |
| 164143_at | 1435510_at |
| 92241_at | 1435527_at |
| 93485_at | 1435537_at |
| 166830_at | 1435539_at |
| 96560_at | 1435559_at |
| 164080_at | 1435567_at |
| 94770_at | 1435573_at |
| 168159_at | 1435576_at |
| 163475_at | 1435580_at |
| 165562_r_at | 1435585_at |
| 166243_at | 1435596_at |
| 164196_at | 1435600_s_at |
| 171603_at | 1435607_at |
| 164141_at | 1435609_at |
| 166713_at | 1435617_at |
| 161103_at | 1435625_at |
| 92884_at | 1435638_at |

| | |
|---|---|
| 165645_at | 1435651_a_at |
| 164064_at | 1435662_at |
| 170175_at | 1435672_at |
| 162640_at | 1435677_at |
| 165744_at | 1435683_a_at |
| 165768_r_at | 1435688_at |
| 165657_f_at | 1435689_at |
| 163504_at | 1435693_at |
| 163424_at | 1435704_at |
| 167478_at | 1435706_at |
| 166686_at | 1435719_at |
| 166383_r_at | 1435724_at |
| 166693_at | 1435747_at |
| 165470_at | 1435761_at |
| 166913_at | 1435767_at |
| 164001_at | 1435786_at |
| 161057_at | 1435788_at |
| 167337_i_at | 1435794_at |
| 166638_at | 1435815_at |
| 98767_at | 1435824_at |
| 163190_at | 1435825_at |
| 163960_at | 1435839_at |
| 96050_at | 1435856_x_at |
| 94386_at | 1435862_at |
| 167597_at | 1435895_at |
| 135621_at | 1435901_at |
| 166719_at | 1435909_at |
| 166403_r_at | 1435922_at |
| 166809_i_at | 1435940_at |
| 96490_at | 1435941_at |
| 164679_at | 1435945_a_at |
| 97743_at | 1435949_at |
| 166880_i_at | 1435961_at |
| 164266_at | 1435970_at |
| 97687_at | 1435976_at |
| 165787_r_at | 1435977_at |
| 167096_f_at | 1435980_x_at |
| 168878_i_at | 1435983_at |
| 166692_at | 1435984_at |
| 168007_f_at | 1435987_x_at |
| 166698_at | 1436001_at |
| 167726_at | 1436003_at |
| 166233_at | 1436009_at |
| 95544_at | 1436014_a_at |
| 95629_at | 1436019_a_at |
| 163122_at | 1436022_at |
| 94439_at | 1436027_at |
| 163204_at | 1436041_at |
| 163597_at | 1436045_at |
| 170492_at | 1436063_at |
| 93121_at | 1436064_x_at |
| 99830_at | 1436066_at |
| 97118_at | 1436073_at |
| 165608_at | 1436086_at |
| 96391_at | 1436092_at |
| 163807_i_at | 1436104_a_at |

| | |
|---|---|
| 166178_at | 1436108_at |
| 95905_at | 1436112_at |
| 129436_at | 1436116_x_at |
| 167796_f_at | 1436126_at |
| 166844_i_at | 1436134_at |
| 168452_f_at | 1436140_at |
| 162880_at | 1436160_at |
| 164197_at | 1436174_at |
| 92941_at | 1436187_at |
| 93963_at | 1436212_at |
| 163171_at | 1436216_s_at |
| 93177_at | 1436228_at |
| 168451_at | 1436237_at |
| 93155_at | 1436243_at |
| 95302_at | 1436267_a_at |
| 167090_at | 1436270_at |
| 166608_at | 1436284_s_at |
| 165769_f_at | 1436285_at |
| 167607_at | 1436288_at |
| 169845_f_at | 1436293_x_at |
| 97097_at | 1436307_at |
| 99851_at | 1436308_at |
| 164262_at | 1436326_at |
| 93693_at | 1436341_at |
| 97241_at | 1436342_a_at |
| 93408_at | 1436348_at |
| 99365_at | 1436351_at |
| 163462_at | 1436369_at |
| 160418_at | 1436390_a_at |
| 163386_at | 1436401_at |
| 166750_at | 1436413_at |
| 99123_s_at | 1436416_x_at |
| 95034_f_at | 1436420_a_at |
| 165760_f_at | 1436421_s_at |
| 168626_r_at | 1436433_at |
| 163316_at | 1436437_at |
| 95597_at | 1436448_a_at |
| 164131_at | 1436455_at |
| 166824_at | 1436461_at |
| 94136_at | 1436491_at |
| 163401_at | 1436496_at |
| 160818_at | 1436510_a_at |
| 98621_at | 1436526_at |
| 160971_at | 1436528_at |
| 163211_at | 1436530_at |
| 166452_at | 1436554_at |
| 170556_at | 1436563_at |
| 163578_at | 1436578_at |
| 165842_at | 1436583_at |
| 165190_f_at | 1436586_x_at |
| 93636_at | 1436588_at |
| 166900_r_at | 1436611_at |
| 163481_at | 1436614_at |
| 94414_at | 1436615_a_at |
| 161063_r_at | 1436616_at |
| 166960_f_at | 1436625_at |

| | |
|---|---|
| 164205_at | 1436626_at |
| 166617_at | 1436653_at |
| 167632_at | 1436671_at |
| 167211_f_at | 1436683_at |
| 164130_at | 1436685_at |
| 96243_f_at | 1436689_a_at |
| 95910_f_at | 1436695_x_at |
| 160476_f_at | 1436699_x_at |
| 163311_at | 1436711_at |
| 163088_r_at | 1436712_at |
| 170841_at | 1436731_at |
| 166450_f_at | 1436744_x_at |
| 162823_at | 1436745_at |
| 164025_at | 1436753_at |
| 170816_at | 1436762_x_at |
| 164006_at | 1436776_x_at |
| 163148_at | 1436777_at |
| 163968_at | 1436781_at |
| 162542_r_at | 1436785_a_at |
| 163105_at | 1436795_at |
| 164207_at | 1436800_at |
| 162567_at | 1436802_at |
| 162803_at | 1436817_at |
| 165086_f_at | 1436820_at |
| 163879_r_at | 1436843_at |
| 93273_at | 1436853_a_at |
| 165492_at | 1436857_at |
| 165719_at | 1436873_at |
| 163946_at | 1436878_at |
| 161098_at | 1436882_at |
| 166815_at | 1436888_at |
| 162646_at | 1436899_at |
| 98057_at | 1436906_at |
| 166702_r_at | 1436916_at |
| 165485_r_at | 1436927_at |
| 167513_i_at | 1436930_x_at |
| 168207_f_at | 1436945_x_at |
| 160115_at | 1436947_a_at |
| 160587_at | 1436955_at |
| 165759_at | 1436956_at |
| 165832_at | 1436962_at |
| 167333_at | 1436966_at |
| 165509_at | 1436969_at |
| 169242_at | 1436972_at |
| 165508_i_at | 1436975_at |
| 114151_at | 1436976_a_at |
| 168414_at | 1436977_at |
| 169724_r_at | 1436996_x_at |
| 161242_f_at | 1437007_x_at |
| 163460_at | 1437017_at |
| 166834_i_at | 1437018_at |
| 160891_at | 1437028_at |
| 170626_i_at | 1437045_at |
| 99589_f_at | 1437054_x_at |
| 163471_at | 1437058_at |
| 163271_at | 1437060_at |

| | |
|---|---|
| 163253_at | 1437061_at |
| 138088_at | 1437079_at |
| 167828_r_at | 1437083_at |
| 163184_at | 1437085_at |
| 167714_at | 1437086_at |
| 165673_at | 1437087_at |
| 165518_f_at | 1437092_at |
| 166109_f_at | 1437096_at |
| 130540_at | 1437107_at |
| 97105_at | 1437111_at |
| 164051_at | 1437119_at |
| 93288_at | 1437148_at |
| 160956_r_at | 1437184_at |
| 163156_at | 1437190_at |
| 166261_f_at | 1437191_at |
| 171033_r_at | 1437192_x_at |
| 163402_at | 1437197_at |
| 96220_at | 1437198_at |
| 170825_i_at | 1437214_at |
| 166352_r_at | 1437219_at |
| 163857_at | 1437229_at |
| 133296_at | 1437240_at |
| 99966_at | 1437243_at |
| 168218_at | 1437248_at |
| 163888_at | 1437249_at |
| 165947_r_at | 1437258_at |
| 170129_r_at | 1437265_at |
| 164087_at | 1437271_at |
| 162796_at | 1437284_at |
| 93193_at | 1437302_at |
| 165552_i_at | 1437306_at |
| 95474_at | 1437308_s_at |
| 163201_at | 1437310_at |
| 166379_at | 1437321_at |
| 170082_i_at | 1437332_at |
| 164542_f_at | 1437344_x_at |
| 163124_s_at | 1437347_at |
| 167935_i_at | 1437352_at |
| 167063_f_at | 1437359_at |
| 166021_at | 1437371_at |
| 163944_at | 1437376_at |
| 163446_at | 1437384_at |
| 164932_f_at | 1437398_a_at |
| 165881_f_at | 1437412_at |
| 163120_at | 1437418_at |
| 93376_at | 1437419_at |
| 160323_at | 1437423_a_at |
| 163991_at | 1437429_at |
| 163272_at | 1437432_a_at |
| 93108_at | 1437465_a_at |
| 162551_at | 1437469_at |
| 133123_at | 1437499_at |
| 163153_at | 1437535_at |
| 162625_at | 1437541_at |
| 163234_at | 1437576_at |
| 165595_at | 1437596_at |

| | |
|---|---|
| 166397_at | 1437603_at |
| 163723_at | 1437631_at |
| 163703_r_at | 1437632_at |
| 163658_at | 1437640_at |
| 166036_f_at | 1437659_at |
| 93185_at | 1437661_at |
| 94664_at | 1437672_at |
| 167756_r_at | 1437694_at |
| 170538_i_at | 1437702_at |
| 165737_f_at | 1437709_x_at |
| 97355_at | 1437728_at |
| 162691_at | 1437731_at |
| 163961_at | 1437737_at |
| 163437_at | 1437740_at |
| 164265_at | 1437747_at |
| 93396_at | 1437758_a_at |
| 163374_at | 1437761_at |
| 163873_at | 1437764_at |
| 167465_at | 1437780_at |
| 165622_at | 1437788_at |
| 166632_at | 1437798_at |
| 168165_at | 1437813_at |
| 166123_at | 1437822_at |
| 166760_s_at | 1437880_at |
| 170743_at | 1437883_s_at |
| 170723_s_at | 1437884_at |
| 92653_at | 1437917_at |
| 161119_at | 1437920_at |
| 96355_at | 1437922_at |
| 160415_at | 1437932_a_at |
| 139160_at | 1437933_at |
| 163998_at | 1437954_at |
| 166272_f_at | 1437960_at |
| 166249_r_at | 1437962_at |
| 98363_at | 1437964_at |
| 164138_at | 1437966_at |
| 165900_f_at | 1437988_x_at |
| 164389_at | 1437994_x_at |
| 110183_at | 1438003_at |
| 167053_at | 1438009_at |
| 161074_at | 1438011_at |
| 165746_at | 1438012_at |
| 163008_at | 1438019_at |
| 164049_at | 1438035_at |
| 170083_r_at | 1438054_x_at |
| 168298_at | 1438086_at |
| 168552_i_at | 1438088_at |
| 170779_f_at | 1438102_at |
| 171592_at | 1438107_x_at |
| 166757_at | 1438109_at |
| 163273_at | 1438122_at |
| 136746_at | 1438135_at |
| 167721_at | 1438149_at |
| 166239_f_at | 1438162_x_at |
| 166066_r_at | 1438184_a_at |
| 162946_at | 1438191_a_at |

| | |
|---|---|
| 162658_at | 1438194_at |
| 163563_at | 1438197_at |
| 171422_at | 1438213_at |
| 164118_at | 1438232_at |
| 167093_at | 1438277_at |
| 167982_i_at | 1438296_at |
| 98357_at | 1438297_at |
| 163996_at | 1438298_a_at |
| 167257_i_at | 1438326_at |
| 166747_at | 1438330_at |
| 169999_at | 1438342_at |
| 165584_at | 1438356_x_at |
| 165935_at | 1438359_at |
| 165843_i_at | 1438375_at |
| 166182_f_at | 1438378_at |
| 166083_i_at | 1438384_at |
| 165538_r_at | 1438393_at |
| 162579_i_at | 1438396_at |
| 162661_at | 1438401_at |
| 166091_i_at | 1438427_at |
| 166120_at | 1438433_at |
| 166445_r_at | 1438444_at |
| 163854_at | 1438450_at |
| 96116_at | 1438456_at |
| 107494_at | 1438484_at |
| 163409_at | 1438496_a_at |
| 116490_at | 1438497_at |
| 163520_at | 1438498_at |
| 166362_at | 1438499_at |
| 170386_at | 1438540_at |
| 164011_at | 1438541_at |
| 163878_at | 1438544_at |
| 161069_at | 1438551_at |
| 166181_f_at | 1438555_x_at |
| 166027_f_at | 1438569_at |
| 97095_at | 1438571_at |
| 167771_r_at | 1438586_at |
| 166104_f_at | 1438587_at |
| 167731_r_at | 1438607_at |
| 162875_at | 1438618_at |
| 99653_at | 1438626_x_at |
| 168484_f_at | 1438639_x_at |
| 164545_f_at | 1438640_x_at |
| 162292_r_at | 1438646_x_at |
| 96732_at | 1438669_at |
| 164010_at | 1438681_at |
| 114192_at | 1438696_at |
| 167759_i_at | 1438697_at |
| 169223_at | 1438698_at |
| 166176_r_at | 1438702_at |
| 163672_at | 1438718_at |
| 170412_f_at | 1438756_at |
| 164303_at | 1438763_at |
| 166157_at | 1438766_at |
| 163405_at | 1438768_at |
| 167317_f_at | 1438791_at |

| | |
|---|---|
| 93027_r_at | 1438794_x_at |
| 162647_at | 1438797_at |
| 170818_i_at | 1438799_at |
| 166060_i_at | 1438813_at |
| 160262_at | 1438843_x_at |
| 163994_at | 1438869_at |
| 170575_i_at | 1438882_at |
| 168234_f_at | 1438885_at |
| 166164_at | 1438901_at |
| 164785_f_at | 1438909_at |
| 166734_at | 1438915_at |
| 169039_at | 1438935_at |
| 165883_r_at | 1438944_at |
| 166361_f_at | 1438979_s_at |
| 166074_i_at | 1438987_at |
| 166154_f_at | 1438996_at |
| 166378_at | 1439000_at |
| 96739_at | 1439028_at |
| 107788_at | 1439037_at |
| 93450_at | 1439040_at |
| 160741_at | 1439047_s_at |
| 168341_at | 1439056_at |
| 163169_at | 1439058_at |
| 171120_at | 1439066_at |
| 170883_i_at | 1439068_at |
| 92245_at | 1439082_at |
| 167004_r_at | 1439101_at |
| 166524_r_at | 1439109_at |
| 95931_at | 1439116_at |
| 163989_at | 1439149_s_at |
| 163466_at | 1439162_at |
| 165644_i_at | 1439181_at |
| 168200_at | 1439190_at |
| 167343_r_at | 1439213_at |
| 167132_r_at | 1439215_at |
| 166385_i_at | 1439230_at |
| 164272_at | 1439252_at |
| 164705_at | 1439262_x_at |
| 166737_at | 1439277_at |
| 99416_at | 1439283_at |
| 168340_at | 1439294_at |
| 168275_i_at | 1439309_at |
| 133484_at | 1439326_at |
| 166883_at | 1439331_at |
| 166390_at | 1439335_at |
| 166633_i_at | 1439353_x_at |
| 166146_i_at | 1439378_at |
| 164929_f_at | 1439381_x_at |
| 162061_f_at | 1439393_x_at |
| 166336_at | 1439402_at |
| 161279_f_at | 1439403_x_at |
| 165964_i_at | 1439408_a_at |
| 167154_f_at | 1439423_x_at |
| 93558_at | 1439433_a_at |
| 168140_at | 1439446_at |
| 93180_at | 1439483_at |

| | |
|---|---|
| 163488_at | 1439489_at |
| 167222_at | 1439512_at |
| 167604_f_at | 1439533_at |
| 166822_at | 1439536_at |
| 99874_at | 1439548_at |
| 167909_r_at | 1439554_at |
| 164277_at | 1439556_at |
| 168229_at | 1439559_at |
| 166296_r_at | 1439575_at |
| 161078_at | 1439625_at |
| 166858_at | 1439627_at |
| 167594_r_at | 1439640_at |
| 165925_at | 1439666_at |
| 167833_i_at | 1439689_at |
| 167901_at | 1439691_at |
| 163896_at | 1439700_at |
| 167908_r_at | 1439703_at |
| 167435_at | 1439713_at |
| 136213_at | 1439720_at |
| 168545_r_at | 1439726_at |
| 166731_at | 1439756_at |
| 169302_at | 1439794_at |
| 167807_i_at | 1439801_at |
| 166855_at | 1439807_at |
| 93245_at | 1439810_s_at |
| 167878_f_at | 1439861_at |
| 164559_at | 1439885_at |
| 168534_i_at | 1439888_at |
| 167115_i_at | 1439911_at |
| 167368_at | 1439917_at |
| 163958_at | 1439944_at |
| 167109_at | 1439952_at |
| 95377_at | 1439959_at |
| 161018_at | 1439962_at |
| 98849_at | 1439964_at |
| 166322_r_at | 1439966_x_at |
| 95353_at | 1439973_at |
| 171474_at | 1439994_at |
| 96481_at | 1439995_at |
| 133737_at | 1440024_at |
| 168446_at | 1440062_at |
| 92313_at | 1440101_at |
| 168136_at | 1440102_at |
| 165096_f_at | 1440120_at |
| 169318_r_at | 1440122_at |
| 98353_at | 1440134_at |
| 169456_at | 1440136_at |
| 164123_at | 1440150_at |
| 166843_at | 1440156_s_at |
| 166642_at | 1440159_at |
| 167993_at | 1440162_x_at |
| 166234_f_at | 1440172_s_at |
| 165903_r_at | 1440176_x_at |
| 166550_i_at | 1440181_at |
| 93462_at | 1440192_at |
| 93168_at | 1440201_at |

| | |
|---|---|
| 161105_at | 1440221_at |
| 98346_at | 1440230_at |
| 167911_at | 1440245_at |
| 94796_at | 1440253_at |
| 167323_r_at | 1440296_at |
| 165738_r_at | 1440303_at |
| 168017_at | 1440316_at |
| 93159_at | 1440323_at |
| 168122_i_at | 1440363_at |
| 167442_at | 1440460_at |
| 166610_r_at | 1440466_at |
| 168056_at | 1440469_at |
| 167838_at | 1440471_x_at |
| 99359_at | 1440499_at |
| 167846_r_at | 1440507_at |
| 167476_at | 1440513_at |
| 167424_at | 1440520_a_at |
| 171514_at | 1440523_at |
| 166969_at | 1440561_at |
| 161740_r_at | 1440574_at |
| 95999_at | 1440591_at |
| 170437_at | 1440617_at |
| 166576_at | 1440618_at |
| 131812_at | 1440627_at |
| 167804_r_at | 1440692_at |
| 168540_at | 1440716_at |
| 166847_at | 1440741_at |
| 166428_at | 1440766_at |
| 168423_f_at | 1440767_at |
| 164982_at | 1440784_at |
| 166062_f_at | 1440791_x_at |
| 166323_r_at | 1440810_x_at |
| 167730_r_at | 1440820_x_at |
| 168036_at | 1440835_at |
| 166230_at | 1440852_at |
| 164151_at | 1440855_at |
| 166842_r_at | 1440861_a_at |
| 95958_at | 1440862_at |
| 95303_at | 1440865_at |
| 164070_at | 1440887_at |
| 168041_at | 1440898_at |
| 166853_r_at | 1440902_at |
| 164105_at | 1440932_at |
| 99360_at | 1440936_at |
| 164234_at | 1440956_at |
| 95923_at | 1440959_s_at |
| 95902_at | 1440964_s_at |
| 93707_f_at | 1440971_x_at |
| 164227_at | 1440992_at |
| 94695_at | 1441013_at |
| 168897_i_at | 1441043_at |
| 166721_at | 1441048_at |
| 170784_at | 1441049_at |
| 134246_at | 1441057_at |
| 167436_i_at | 1441111_at |
| 169126_at | 1441124_at |

| | |
|---|---|
| 99351_at | 1441146_at |
| 97141_s_at | 1441147_at |
| 164084_at | 1441165_s_at |
| 95985_at | 1441182_at |
| 167378_i_at | 1441205_at |
| 166870_at | 1441228_at |
| 166545_r_at | 1441256_at |
| 164033_at | 1441259_s_at |
| 165909_at | 1441263_a_at |
| 166483_at | 1441280_at |
| 166306_at | 1441314_at |
| 92634_at | 1441342_at |
| 97146_g_at | 1441344_at |
| 92957_at | 1441350_at |
| 99354_s_at | 1441394_at |
| 95981_at | 1441401_at |
| 97147_at | 1441421_at |
| 166898_at | 1441430_at |
| 167656_at | 1441504_at |
| 96600_at | 1441618_at |
| 166963_at | 1441632_at |
| 170826_at | 1441633_at |
| 167928_at | 1441655_at |
| 95876_at | 1441659_at |
| 166584_at | 1441691_at |
| 162617_at | 1441757_at |
| 167888_at | 1441773_at |
| 166922_at | 1441849_at |
| 167623_r_at | 1441856_x_at |
| 165891_i_at | 1441861_at |
| 161864_f_at | 1441866_s_at |
| 168058_at | 1441875_at |
| 167555_i_at | 1441881_x_at |
| 165865_f_at | 1441883_at |
| 164885_f_at | 1441884_x_at |
| 167397_i_at | 1441886_at |
| 169199_at | 1441892_x_at |
| 167924_at | 1441893_at |
| 167895_f_at | 1441895_x_at |
| 167432_f_at | 1441909_s_at |
| 166128_r_at | 1441913_at |
| 167047_r_at | 1441927_at |
| 168154_i_at | 1441939_x_at |
| 167670_at | 1441943_x_at |
| 166341_r_at | 1441944_s_at |
| 167235_at | 1441950_s_at |
| 168157_at | 1441959_s_at |
| 163818_at | 1441965_at |
| 95360_at | 1441986_at |
| 163361_i_at | 1442002_at |
| 166543_r_at | 1442007_at |
| 167248_at | 1442021_at |
| 166774_r_at | 1442073_at |
| 167000_at | 1442138_at |
| 169339_r_at | 1442139_at |
| 171066_at | 1442141_at |

| | |
|---|---|
| 96534_at | 1442169_at |
| 166685_at | 1442173_at |
| 114214_at | 1442222_at |
| 167845_at | 1442247_at |
| 165353_f_at | 1442284_at |
| 96552_at | 1442296_at |
| 98362_s_at | 1442337_at |
| 168139_i_at | 1442345_at |
| 166046_at | 1442376_at |
| 168253_f_at | 1442391_at |
| 168124_at | 1442407_at |
| 161940_r_at | 1442414_at |
| 96367_at | 1442494_at |
| 137995_at | 1442518_at |
| 95967_at | 1442526_at |
| 94693_at | 1442529_at |
| 95953_at | 1442531_at |
| 166826_at | 1442536_at |
| 95966_at | 1442540_at |
| 95973_at | 1442566_at |
| 168666_i_at | 1442571_at |
| 92765_s_at | 1442581_at |
| 99818_at | 1442597_at |
| 171439_at | 1442659_at |
| 96483_at | 1442661_at |
| 95970_at | 1442665_at |
| 95997_at | 1442669_at |
| 168334_at | 1442676_at |
| 170169_at | 1442687_at |
| 95987_at | 1442694_at |
| 167110_r_at | 1442701_at |
| 164065_at | 1442743_at |
| 94689_at | 1442745_x_at |
| 97775_at | 1442778_at |
| 167190_r_at | 1442779_at |
| 161062_r_at | 1442786_s_at |
| 168286_i_at | 1442796_at |
| 167347_i_at | 1442797_x_at |
| 168661_r_at | 1442830_at |
| 99358_at | 1442833_at |
| 167394_at | 1442838_at |
| 168648_i_at | 1442896_at |
| 95945_at | 1442900_at |
| 166585_at | 1442902_at |
| 168441_at | 1442929_at |
| 99819_at | 1442932_at |
| 97137_at | 1442941_at |
| 167073_at | 1442945_at |
| 94783_at | 1442948_at |
| 96485_at | 1442975_at |
| 96487_at | 1442976_at |
| 163959_at | 1442982_at |
| 96484_at | 1442984_at |
| 97170_at | 1443001_at |
| 169269_i_at | 1443085_at |
| 166553_at | 1443093_at |

| | |
|---|---|
| 166954_at | 1443118_at |
| 134267_at | 1443236_at |
| 170997_at | 1443290_at |
| 97135_at | 1443312_at |
| 99363_at | 1443317_at |
| 95950_at | 1443326_at |
| 169588_r_at | 1443345_at |
| 97139_at | 1443352_at |
| 95975_at | 1443385_at |
| 161292_f_at | 1443408_at |
| 97693_at | 1443414_at |
| 95992_at | 1443416_at |
| 95959_at | 1443427_at |
| 95863_at | 1443428_at |
| 95947_at | 1443431_at |
| 95880_s_at | 1443461_at |
| 96486_at | 1443464_at |
| 95968_at | 1443472_at |
| 97080_at | 1443473_at |
| 95919_at | 1443482_at |
| 166764_at | 1443493_at |
| 138184_at | 1443529_at |
| 97142_at | 1443589_at |
| 166657_at | 1443594_at |
| 165946_r_at | 1443601_at |
| 165924_at | 1443671_x_at |
| 96517_at | 1443696_s_at |
| 96550_at | 1443706_at |
| 166887_at | 1443710_s_at |
| 99821_at | 1443711_at |
| 164904_i_at | 1443765_at |
| 169131_r_at | 1443778_at |
| 167072_r_at | 1443783_x_at |
| 171260_at | 1443790_x_at |
| 168339_i_at | 1443799_at |
| 168069_at | 1443813_x_at |
| 165823_s_at | 1443817_x_at |
| 165006_f_at | 1443820_x_at |
| 167868_f_at | 1443822_s_at |
| 168392_f_at | 1443830_x_at |
| 168900_i_at | 1443831_s_at |
| 167803_at | 1443839_at |
| 167327_at | 1443844_at |
| 168025_at | 1443875_at |
| 163301_at | 1443880_at |
| 169567_at | 1443899_at |
| 167062_at | 1443916_at |
| 166591_r_at | 1443929_at |
| 170846_at | 1443995_at |
| 95995_at | 1443996_at |
| 168881_at | 1444015_at |
| 94089_at | 1444160_at |
| 166939_at | 1444214_at |
| 136616_at | 1444254_at |
| 131080_at | 1444259_at |
| 96536_at | 1444274_at |

| | |
|---|---|
| 169962_i_at | 1444284_at |
| 164887_i_at | 1444335_at |
| 171434_at | 1444460_at |
| 170046_at | 1444564_at |
| 168474_r_at | 1444582_at |
| 168090_at | 1444584_at |
| 116500_at | 1444631_at |
| 170361_at | 1444680_at |
| 167268_at | 1444718_at |
| 165670_i_at | 1444732_at |
| 167920_at | 1444767_at |
| 160264_s_at | 1444791_at |
| 108890_at | 1444822_at |
| 95857_at | 1444866_at |
| 96431_at | 1444959_at |
| 164822_r_at | 1445003_at |
| 166912_at | 1445056_at |
| 97184_at | 1445079_at |
| 95858_at | 1445116_at |
| 167061_at | 1445121_at |
| 171064_r_at | 1445279_at |
| 164214_at | 1445303_at |
| 95852_at | 1445367_at |
| 169978_at | 1445424_at |
| 95934_at | 1445485_at |
| 169711_at | 1445503_at |
| 171333_at | 1445542_at |
| 168763_r_at | 1445593_at |
| 170240_f_at | 1445608_at |
| 166897_at | 1445642_at |
| 167280_f_at | 1445696_x_at |
| 165855_at | 1445708_x_at |
| 135390_at | 1445731_at |
| 166776_at | 1445813_at |
| 137324_at | 1445868_at |
| 139168_at | 1445928_at |
| 130726_at | 1446105_at |
| 169577_i_at | 1446106_at |
| 168692_i_at | 1446171_at |
| 166703_at | 1446238_at |
| 99779_at | 1446244_at |
| 169981_i_at | 1446307_at |
| 167902_i_at | 1446310_at |
| 166772_at | 1446368_at |
| 96394_at | 1446378_at |
| 167538_at | 1446413_at |
| 170442_at | 1446455_at |
| 169176_r_at | 1446485_at |
| 170359_at | 1446547_at |
| 96390_at | 1446572_at |
| 161746_i_at | 1446733_at |
| 95853_at | 1446828_at |
| 94668_at | 1446856_at |
| 92520_at | 1446914_at |
| 164037_at | 1446968_at |
| 169549_at | 1446977_at |

| | |
|---|---|
| 171364_at | 1446985_at |
| 94657_at | 1447130_at |
| 95864_at | 1447182_at |
| 166525_at | 1447241_at |
| 161737_at | 1447266_at |
| 166706_at | 1447361_at |
| 97187_at | 1447462_at |
| 166890_at | 1447487_at |
| 166971_at | 1447503_at |
| 162266_f_at | 1447530_at |
| 161730_f_at | 1447541_s_at |
| 168435_r_at | 1447548_at |
| 167100_s_at | 1447552_s_at |
| 168181_i_at | 1447563_at |
| 167504_at | 1447565_at |
| 169489_r_at | 1447572_at |
| 106268_at | 1447577_x_at |
| 167205_at | 1447594_at |
| 167936_at | 1447607_at |
| 92526_f_at | 1447608_x_at |
| 167481_at | 1447648_at |
| 167709_at | 1447650_at |
| 167580_f_at | 1447667_x_at |
| 167697_at | 1447673_x_at |
| 162274_f_at | 1447690_at |
| 167605_i_at | 1447710_at |
| 167813_f_at | 1447711_x_at |
| 168570_i_at | 1447714_x_at |
| 166187_f_at | 1447715_x_at |
| 166931_f_at | 1447729_s_at |
| 169392_at | 1447751_x_at |
| 168455_r_at | 1447768_at |
| 161120_r_at | 1447780_x_at |
| 166512_r_at | 1447782_x_at |
| 167834_f_at | 1447784_x_at |
| 168518_at | 1447787_x_at |
| 167411_i_at | 1447794_x_at |
| 167858_at | 1447795_at |
| 167348_at | 1447798_at |
| 167025_at | 1447802_x_at |
| 161969_f_at | 1447803_x_at |
| 164426_f_at | 1447806_s_at |
| 167913_r_at | 1447813_x_at |
| 167849_at | 1447821_at |
| 168332_f_at | 1447827_x_at |
| 165512_at | 1447831_s_at |
| 168488_i_at | 1447834_at |
| 167706_at | 1447844_at |
| 167486_i_at | 1447862_x_at |
| 164857_f_at | 1447873_x_at |
| 167460_at | 1447882_x_at |
| 167467_i_at | 1447886_at |
| 167751_f_at | 1447887_x_at |
| 170480_at | 1447899_x_at |
| 166980_at | 1447902_at |
| 167916_f_at | 1447914_x_at |

| | |
|---|---|
| 163600_at | 1447939_a_at |
| 92354_at | 1447945_at |
| 94787_at | 1447952_at |
| 167491_at | 1447955_at |
| 99357_at | 1447956_at |
| 94785_at | 1447957_at |
| 97688_at | 1447958_at |
| 95903_at | 1447959_at |
| 166770_at | 1447960_at |
| 96335_at | 1447961_s_at |
| 93166_at | 1447962_at |
| 95314_at | 1447964_at |
| 95994_at | 1447965_at |
| 95988_r_at | 1447968_at |
| 163335_at | 1447975_a_at |
| 97119_at | 1447976_at |
| 97144_at | 1447981_at |
| 95856_at | 1447983_at |
| 97140_at | 1447984_at |
| 95854_at | 1447990_at |
| 167591_at | 1448001_x_at |
| 161136_r_at | 1448002_x_at |
| 163906_at | 1448007_at |
| 138141_at | 1448015_at |
| 96579_at | 1448016_at |
| 92900_at | 1448018_at |
| 97711_at | 1448024_at |
| 96572_at | 1448032_at |
| 95378_at | 1448034_at |
| 160928_at | 1448040_at |
| 95895_at | 1448046_at |
| 96565_at | 1448047_at |
| 95920_at | 1448048_at |
| 95943_at | 1448051_at |
| 99346_at | 1448053_at |
| 97143_at | 1448054_at |
| 95938_at | 1448056_at |
| 167452_r_at | 1448057_at |
| 95855_at | 1448059_at |
| 95165_at | 1448060_at |
| 94792_at | 1448061_at |
| 161271_r_at | 1448065_at |
| 94131_at | 1448070_at |
| 96482_at | 1448071_at |
| 97088_at | 1448072_at |
| 97707_at | 1448073_at |
| 98837_at | 1448074_at |
| 97194_at | 1448075_at |
| 95962_at | 1448076_at |
| 94686_at | 1448078_at |
| 95871_at | 1448086_at |
| 97189_at | 1448097_at |
| 98325_at | 1448098_at |
| 165927_at | 1448099_at |
| 95445_at | 1448100_at |
| 160293_at | 1448102_a_at |

| | |
|---|---|
| 93830_at | 1448103_s_at |
| 160528_at | 1448105_at |
| 95101_at | 1448108_at |
| 94063_at | 1448110_at |
| 92616_at | 1448116_at |
| 160567_at | 1448120_at |
| 94218_at | 1448122_at |
| 92877_at | 1448123_s_at |
| 94419_at | 1448132_at |
| 162819_at | 1448139_at |
| 160382_at | 1448141_at |
| 93364_at | 1448149_at |
| 94001_at | 1448151_at |
| 96088_at | 1448154_at |
| 160300_at | 1448156_at |
| 99672_at | 1448161_a_at |
| 99049_at | 1448165_at |
| 98113_at | 1448166_a_at |
| 97906_at | 1448170_at |
| 95718_f_at | 1448179_at |
| 93276_at | 1448180_a_at |
| 95057_at | 1448185_at |
| 160070_at | 1448186_at |
| 96916_at | 1448190_at |
| 93581_at | 1448198_a_at |
| 95012_at | 1448209_a_at |
| 160421_r_at | 1448220_at |
| 98460_at | 1448223_at |
| 99172_at | 1448224_at |
| 99639_at | 1448230_at |
| 95754_at | 1448240_at |
| 98887_at | 1448243_at |
| 97750_at | 1448245_at |
| 92592_at | 1448249_at |
| 162974_at | 1448250_at |
| 96542_at | 1448255_a_at |
| 92950_at | 1448257_at |
| 98140_at | 1448261_at |
| 94219_at | 1448262_at |
| 160349_at | 1448263_a_at |
| 96076_at | 1448267_at |
| 96857_at | 1448274_at |
| 97473_at | 1448276_at |
| 160181_at | 1448280_at |
| 94037_at | 1448281_a_at |
| 94155_at | 1448285_at |
| 98082_at | 1448287_at |
| 96009_s_at | 1448290_at |
| 99618_at | 1448292_at |
| 94502_at | 1448295_at |
| 97222_at | 1448305_at |
| 97526_at | 1448308_at |
| 162996_at | 1448310_at |
| 98147_at | 1448311_at |
| 96712_at | 1448321_at |
| 93518_at | 1448324_at |

| | |
|---|---|
| 160463_at | 1448325_at |
| 160570_at | 1448327_at |
| 92975_at | 1448328_at |
| 98020_at | 1448336_at |
| 160179_at | 1448340_at |
| 98928_at | 1448351_at |
| 94237_at | 1448353_x_at |
| 163393_at | 1448355_at |
| 160520_at | 1448363_at |
| 99151_at | 1448365_at |
| 160390_at | 1448367_at |
| 92858_at | 1448377_at |
| 163529_at | 1448379_at |
| 168366_at | 1448381_at |
| 97316_at | 1448382_at |
| 160118_at | 1448383_at |
| 95516_at | 1448391_at |
| 93050_at | 1448394_at |
| 94391_at | 1448397_at |
| 93901_at | 1448408_at |
| 94229_at | 1448412_a_at |
| 99460_at | 1448415_a_at |
| 162650_at | 1448421_s_at |
| 96763_at | 1448426_at |
| 160748_at | 1448431_at |
| 96171_at | 1448446_at |
| 95148_at | 1448450_at |
| 160671_at | 1448455_at |
| 163225_at | 1448460_at |
| 162592_at | 1448467_a_at |
| 96918_at | 1448470_at |
| 97860_at | 1448476_at |
| 92769_at | 1448479_at |
| 99613_at | 1448486_at |
| 92563_at | 1448487_at |
| 94298_at | 1448495_at |
| 93602_at | 1448498_at |
| 96227_at | 1448506_at |
| 92786_at | 1448507_at |
| 96221_at | 1448508_at |
| 99478_at | 1448511_at |
| 160404_at | 1448512_at |
| 93348_at | 1448517_at |
| 163133_at | 1448520_at |
| 95630_at | 1448522_at |
| 162727_at | 1448528_at |
| 97276_at | 1448533_at |
| 94455_at | 1448536_at |
| 96921_at | 1448537_at |
| 92542_at | 1448538_a_at |
| 95725_at | 1448539_a_at |
| 160775_at | 1448548_at |
| 96123_at | 1448550_at |
| 98616_f_at | 1448553_at |
| 95030_at | 1448556_at |
| 94990_at | 1448567_at |

| | |
|---|---|
| 94000_at | 1448569_at |
| 92824_at | 1448574_at |
| 99030_at | 1448575_at |
| 93586_at | 1448577_x_at |
| 96131_at | 1448587_at |
| 95493_at | 1448590_at |
| 92461_at | 1448598_at |
| 160754_at | 1448602_at |
| 98529_at | 1448608_at |
| 98054_at | 1448613_at |
| 93008_at | 1448622_at |
| 163110_at | 1448625_at |
| 92386_at | 1448629_at |
| 94216_at | 1448630_a_at |
| 93255_at | 1448634_at |
| 94387_at | 1448639_a_at |
| 168019_r_at | 1448640_at |
| 94552_at | 1448642_at |
| 164014_at | 1448648_at |
| 98451_at | 1448657_a_at |
| 98449_at | 1448658_at |
| 93033_at | 1448671_at |
| 99644_at | 1448672_a_at |
| 163068_at | 1448673_at |
| 94540_at | 1448683_at |
| 97531_at | 1448700_at |
| 94416_at | 1448714_at |
| 160194_at | 1448717_at |
| 160294_at | 1448726_at |
| 96525_at | 1448731_at |
| 98333_at | 1448739_x_at |
| 93304_at | 1448741_at |
| 92624_r_at | 1448745_s_at |
| 99637_at | 1448755_at |
| 96616_at | 1448763_at |
| 93771_at | 1448767_s_at |
| 93984_at | 1448770_a_at |
| 96289_at | 1448774_at |
| 93321_at | 1448775_at |
| 92699_at | 1448783_at |
| 162669_at | 1448787_at |
| 99911_at | 1448790_at |
| 165456_s_at | 1448795_a_at |
| 96228_at | 1448804_at |
| 92625_at | 1448808_a_at |
| 97924_at | 1448810_at |
| 95067_at | 1448811_at |
| 160882_at | 1448815_at |
| 94336_at | 1448817_at |
| 92210_at | 1448831_at |
| 160831_at | 1448836_s_at |
| 99074_at | 1448837_at |
| 163680_at | 1448840_at |
| 94511_at | 1448843_at |
| 93766_at | 1448852_at |
| 99053_at | 1448862_at |

| | |
|---|---|
| 94866_at | 1448869_a_at |
| 96064_at | 1448872_at |
| 92332_at | 1448877_at |
| 96092_at | 1448881_at |
| 160267_at | 1448882_at |
| 98515_at | 1448884_at |
| 92228_at | 1448895_a_at |
| 95025_at | 1448900_at |
| 160360_at | 1448903_at |
| 93515_at | 1448906_at |
| 96147_at | 1448916_at |
| 160387_at | 1448919_at |
| 98502_at | 1448922_at |
| 160939_at | 1448925_at |
| 160277_at | 1448936_at |
| 92942_at | 1448940_at |
| 94377_at | 1448942_at |
| 93635_at | 1448946_at |
| 93914_at | 1448950_at |
| 97990_at | 1448962_at |
| 92943_at | 1448972_at |
| 98480_s_at | 1448975_s_at |
| 93178_at | 1448978_at |
| 99534_at | 1448980_at |
| 96694_at | 1448981_x_at |
| 92353_at | 1448982_at |
| 163032_at | 1448983_at |
| 164111_at | 1448984_at |
| 163756_at | 1448986_x_at |
| 93453_at | 1448996_at |
| 95325_at | 1448997_at |
| 94554_at | 1448999_at |
| 96757_at | 1449000_at |
| 163080_at | 1449001_at |
| 98056_at | 1449002_at |
| 165748_at | 1449010_at |
| 163206_at | 1449013_at |
| 162835_at | 1449015_at |
| 93429_at | 1449016_at |
| 96618_at | 1449021_at |
| 93956_at | 1449025_at |
| 163198_at | 1449032_at |
| 165519_f_at | 1449035_at |
| 167959_at | 1449037_at |
| 97867_at | 1449038_at |
| 95041_at | 1449046_a_at |
| 163450_at | 1449049_at |
| 96772_at | 1449061_a_at |
| 162666_at | 1449075_at |
| 162888_at | 1449085_at |
| 97379_at | 1449088_at |
| 163719_at | 1449096_at |
| 160437_at | 1449097_at |
| 92423_at | 1449100_at |
| 163192_at | 1449103_at |
| 165910_f_at | 1449104_at |

| | |
|---|---|
| 92587_at | 1449108_at |
| 92612_at | 1449112_at |
| 160909_at | 1449133_at |
| 94205_at | 1449136_at |
| 160569_at | 1449139_at |
| 94406_at | 1449148_a_at |
| 96128_at | 1449151_at |
| 93194_at | 1449156_at |
| 99238_at | 1449159_at |
| 96553_at | 1449175_at |
| 92911_at | 1449177_at |
| 94139_at | 1449179_at |
| 95071_at | 1449180_at |
| 92595_r_at | 1449181_at |
| 99900_at | 1449185_at |
| 93589_at | 1449190_a_at |
| 160914_at | 1449191_at |
| 93445_at | 1449193_at |
| 93030_at | 1449196_a_at |
| 92213_at | 1449201_at |
| 161033_at | 1449208_at |
| 94151_at | 1449212_at |
| 92486_at | 1449215_at |
| 93961_at | 1449216_at |
| 160851_r_at | 1449218_at |
| 93608_at | 1449222_at |
| 163752_at | 1449233_at |
| 163442_at | 1449234_at |
| 97113_at | 1449235_at |
| 97784_at | 1449237_at |
| 164126_at | 1449239_at |
| 165877_at | 1449240_at |
| 97726_at | 1449245_at |
| 163366_at | 1449247_at |
| 92976_at | 1449251_at |
| 163343_at | 1449257_at |
| 92189_at | 1449263_at |
| 166587_i_at | 1449270_at |
| 163420_at | 1449274_at |
| 99810_at | 1449279_at |
| 163553_at | 1449280_at |
| 93673_at | 1449281_at |
| 164125_at | 1449286_at |
| 98794_at | 1449287_at |
| 93072_at | 1449296_a_at |
| 92488_at | 1449297_at |
| 160861_s_at | 1449298_a_at |
| 163314_at | 1449307_at |
| 98768_at | 1449310_at |
| 99925_f_at | 1449318_at |
| 98312_at | 1449319_at |
| 94632_at | 1449320_at |
| 93827_at | 1449337_at |
| 169287_i_at | 1449339_at |
| 162986_at | 1449340_at |
| 94372_at | 1449349_at |

| | |
|---|---|
| 167256_at | 1449352_at |
| 92262_at | 1449353_at |
| 98497_at | 1449355_a_at |
| 163436_at | 1449356_at |
| 96595_at | 1449359_at |
| 94769_at | 1449366_at |
| 165582_f_at | 1449367_at |
| 93800_f_at | 1449378_at |
| 92949_at | 1449380_at |
| 166381_f_at | 1449384_at |
| 97973_at | 1449389_at |
| 92557_at | 1449392_at |
| 168225_i_at | 1449395_at |
| 168263_f_at | 1449400_at |
| 95358_at | 1449404_at |
| 163151_at | 1449409_at |
| 92480_f_at | 1449414_at |
| 93692_f_at | 1449417_at |
| 162698_at | 1449418_s_at |
| 93382_at | 1449420_at |
| 165816_at | 1449421_a_at |
| 95898_at | 1449422_at |
| 165693_r_at | 1449425_at |
| 92494_at | 1449426_a_at |
| 163687_at | 1449430_a_at |
| 99909_at | 1449431_at |
| 160915_at | 1449435_at |
| 92619_at | 1449441_a_at |
| 163128_at | 1449451_at |
| 165494_i_at | 1449452_a_at |
| 165610_at | 1449462_at |
| 99322_at | 1449464_at |
| 92224_at | 1449466_at |
| 161053_at | 1449467_at |
| 98394_at | 1449470_at |
| 98784_at | 1449472_at |
| 92962_at | 1449473_s_at |
| 168163_at | 1449475_at |
| 92917_at | 1449478_at |
| 98534_at | 1449480_at |
| 166097_at | 1449487_at |
| 92920_at | 1449488_at |
| 168080_i_at | 1449496_at |
| 95363_at | 1449501_a_at |
| 166149_at | 1449513_at |
| 163702_i_at | 1449517_at |
| 93393_at | 1449524_at |
| 92365_at | 1449528_at |
| 95639_at | 1449532_at |
| 165573_f_at | 1449533_at |
| 98310_at | 1449535_at |
| 97100_at | 1449536_at |
| 92655_at | 1449538_a_at |
| 94799_at | 1449558_at |
| 166338_i_at | 1449562_at |
| 92936_at | 1449563_at |

| | |
|---|---|
| 97777_at | 1449566_at |
| 97734_at | 1449569_at |
| 93380_at | 1449570_at |
| 97735_at | 1449571_at |
| 98035_g_at | 1449580_s_at |
| 93689_at | 1449581_at |
| 99996_at | 1449586_at |
| 97730_at | 1449588_at |
| 93409_at | 1449593_at |
| 94798_at | 1449594_at |
| 94690_at | 1449595_at |
| 94782_at | 1449596_at |
| 94684_at | 1449598_at |
| 97191_at | 1449599_at |
| 96492_at | 1449600_at |
| 169292_r_at | 1449601_x_at |
| 96499_at | 1449603_at |
| 95892_at | 1449609_at |
| 99585_at | 1449611_at |
| 163249_at | 1449616_s_at |
| 94043_at | 1449622_s_at |
| 95182_at | 1449624_at |
| 161665_at | 1449630_s_at |
| 95982_at | 1449638_at |
| 95937_at | 1449639_at |
| 95884_at | 1449640_at |
| 96576_at | 1449642_at |
| 93057_at | 1449643_s_at |
| 93223_at | 1449644_at |
| 95882_at | 1449647_at |
| 161688_r_at | 1449651_x_at |
| 160998_at | 1449677_s_at |
| 93278_at | 1449686_s_at |
| 96571_at | 1449692_at |
| 160973_at | 1449699_s_at |
| 96374_at | 1449704_at |
| 160661_at | 1449714_at |
| 163241_at | 1449734_s_at |
| 99555_at | 1449742_at |
| 166919_r_at | 1449766_at |
| 164754_at | 1449769_at |
| 161736_r_at | 1449771_at |
| 161543_at | 1449783_at |
| 95177_at | 1449785_at |
| 165957_f_at | 1449789_x_at |
| 164427_f_at | 1449794_x_at |
| 167987_r_at | 1449798_at |
| 169275_at | 1449802_x_at |
| 162278_r_at | 1449803_x_at |
| 161917_i_at | 1449804_at |
| 99708_at | 1449805_at |
| 92695_at | 1449814_at |
| 93434_at | 1449816_at |
| 94733_at | 1449818_at |
| 99831_at | 1449820_at |
| 167445_at | 1449825_at |

| | |
|---|---|
| 93216_at | 1449826_a_at |
| 94171_at | 1449829_at |
| 92274_at | 1449830_at |
| 94120_s_at | 1449833_at |
| 98836_at | 1449835_at |
| 94730_at | 1449838_at |
| 98437_at | 1449839_at |
| 160497_at | 1449842_at |
| 98446_s_at | 1449845_a_at |
| 93619_at | 1449851_at |
| 92930_at | 1449863_a_at |
| 98835_at | 1449865_at |
| 92891_f_at | 1449867_at |
| 93611_at | 1449868_at |
| 92982_at | 1449873_at |
| 163705_at | 1449876_at |
| 163026_at | 1449894_at |
| 93207_at | 1449895_at |
| 96540_at | 1449896_at |
| 92276_at | 1449901_a_at |
| 98334_at | 1449903_at |
| 98858_at | 1449908_at |
| 164719_at | 1449909_at |
| 98392_at | 1449911_at |
| 99843_at | 1449917_at |
| 92253_at | 1449919_at |
| 93151_at | 1449921_s_at |
| 95300_at | 1449924_at |
| 94173_at | 1449925_at |
| 98318_at | 1449926_at |
| 95301_at | 1449927_at |
| 93725_at | 1449949_a_at |
| 93709_at | 1449952_s_at |
| 94161_at | 1449956_at |
| 99424_at | 1449958_a_at |
| 92895_at | 1449967_at |
| 98343_s_at | 1449978_at |
| 99342_at | 1449980_a_at |
| 92746_at | 1449981_a_at |
| 92266_at | 1449982_at |
| 163042_at | 1449983_a_at |
| 168903_at | 1449985_at |
| 96506_at | 1449987_at |
| 92489_at | 1449988_at |
| 99958_at | 1449989_at |
| 95380_at | 1449991_at |
| 93266_at | 1449997_at |
| 97157_at | 1449998_at |
| 94276_at | 1450011_at |
| 166873_at | 1450013_at |
| 97546_at | 1450014_at |
| 95335_at | 1450019_at |
| 160214_at | 1450028_a_at |
| 163193_at | 1450029_s_at |
| 98806_s_at | 1450041_a_at |
| 93520_at | 1450045_at |

| | |
|---|---|
| 95447_at | 1450067_a_at |
| 161003_at | 1450073_at |
| 93919_at | 1450078_at |
| 99963_at | 1450090_at |
| 163215_at | 1450098_at |
| 93492_at | 1450103_a_at |
| 96154_at | 1450107_a_at |
| 95283_at | 1450109_s_at |
| 92764_at | 1450114_at |
| 99981_at | 1450115_at |
| 92885_at | 1450118_a_at |
| 162255_s_at | 1450121_at |
| 96500_at | 1450125_at |
| 92735_at | 1450128_at |
| 163948_at | 1450129_a_at |
| 94149_at | 1450132_at |
| 95329_at | 1450139_at |
| 98853_at | 1450144_at |
| 167316_f_at | 1450145_at |
| 93442_at | 1450151_at |
| 93855_at | 1450159_at |
| 94196_at | 1450161_at |
| 166459_at | 1450162_at |
| 168970_f_at | 1450168_at |
| 93129_at | 1450181_at |
| 93939_at | 1450183_a_at |
| 99973_s_at | 1450185_a_at |
| 95792_at | 1450190_at |
| 93698_at | 1450191_a_at |
| 99902_at | 1450193_at |
| 96886_at | 1450199_a_at |
| 92485_at | 1450210_at |
| 164787_at | 1450221_at |
| 94773_at | 1450222_x_at |
| 94119_at | 1450228_a_at |
| 97796_at | 1450229_at |
| 95392_at | 1450238_at |
| 97150_at | 1450245_at |
| 93920_at | 1450248_at |
| 96583_s_at | 1450249_s_at |
| 94104_at | 1450254_at |
| 138095_at | 1450255_at |
| 93452_at | 1450256_at |
| 92361_at | 1450258_a_at |
| 98302_at | 1450266_at |
| 98384_at | 1450271_at |
| 96585_at | 1450277_at |
| 99806_at | 1450286_at |
| 94621_at | 1450289_at |
| 95781_at | 1450293_at |
| 94772_at | 1450296_at |
| 95932_at | 1450306_at |
| 94631_at | 1450327_at |
| 95799_s_at | 1450331_s_at |
| 160088_at | 1450332_s_at |
| 93932_at | 1450335_at |

| | |
|---|---|
| 96414_at | 1450336_at |
| 96588_at | 1450344_a_at |
| 96979_at | 1450347_at |
| 99899_at | 1450357_a_at |
| 99381_at | 1450359_at |
| 98290_at | 1450360_at |
| 99237_at | 1450362_at |
| 94627_at | 1450369_at |
| 97741_at | 1450371_at |
| 132443_at | 1450384_at |
| 96010_at | 1450386_at |
| 97511_at | 1450391_a_at |
| 94504_at | 1450410_a_at |
| 160475_at | 1450418_a_at |
| 92689_at | 1450424_a_at |
| 96996_at | 1450426_at |
| 97943_at | 1450429_at |
| 93101_s_at | 1450431_a_at |
| 92493_at | 1450433_at |
| 93872_at | 1450440_at |
| 96502_at | 1450445_at |
| 92832_at | 1450446_a_at |
| 92713_at | 1450447_at |
| 95015_at | 1450455_s_at |
| 96508_at | 1450458_at |
| 94122_at | 1450468_at |
| 92455_at | 1450469_at |
| 93613_at | 1450471_at |
| 94169_at | 1450473_at |
| 99328_at | 1450475_at |
| 97692_at | 1450477_at |
| 92902_at | 1450481_at |
| 92264_at | 1450485_at |
| 94193_at | 1450490_at |
| 93678_s_at | 1450495_a_at |
| 98854_at | 1450499_at |
| 98834_at | 1450501_at |
| 98298_at | 1450502_at |
| 98823_at | 1450503_at |
| 92933_at | 1450507_at |
| 92674_at | 1450508_at |
| 94763_at | 1450511_at |
| 98787_at | 1450515_at |
| 92329_at | 1450516_a_at |
| 92501_s_at | 1450533_a_at |
| 97125_f_at | 1450534_x_at |
| 92215_at | 1450535_at |
| 94735_s_at | 1450538_s_at |
| 93710_at | 1450539_at |
| 92707_at | 1450541_at |
| 98374_at | 1450550_at |
| 99427_at | 1450553_at |
| 97782_at | 1450554_at |
| 94571_at | 1450558_at |
| 99437_at | 1450562_at |
| 96574_at | 1450565_at |

| | |
|---|---|
| 94086_at | 1450566_at |
| 95410_s_at | 1450567_a_at |
| 99837_at | 1450568_at |
| 99945_at | 1450570_a_at |
| 99556_s_at | 1450579_x_at |
| 95773_at | 1450582_at |
| 95770_s_at | 1450583_s_at |
| 92951_at | 1450584_at |
| 97003_at | 1450590_at |
| 96983_at | 1450600_at |
| 96986_at | 1450604_at |
| 94987_at | 1450612_a_at |
| 99702_at | 1450617_at |
| 94049_at | 1450624_at |
| 163350_at | 1450625_at |
| 93863_f_at | 1450631_x_at |
| 93744_at | 1450633_at |
| 93968_at | 1450638_at |
| 160406_at | 1450652_at |
| 163917_at | 1450658_at |
| 93645_at | 1450659_at |
| 93006_at | 1450661_x_at |
| 99666_at | 1450667_a_at |
| 92829_at | 1450668_s_at |
| 98027_at | 1450673_at |
| 96219_at | 1450675_at |
| 93683_at | 1450680_at |
| 99977_at | 1450682_at |
| 160185_at | 1450683_at |
| 93085_at | 1450696_at |
| 92758_at | 1450698_at |
| 162649_at | 1450700_at |
| 92981_at | 1450708_at |
| 99551_f_at | 1450709_at |
| 167299_at | 1450712_at |
| 160606_r_at | 1450716_at |
| 94392_f_at | 1450717_at |
| 160816_at | 1450728_at |
| 93306_at | 1450740_a_at |
| 93601_at | 1450746_at |
| 166886_at | 1450748_at |
| 93955_at | 1450751_at |
| 92929_at | 1450752_at |
| 160187_at | 1450755_at |
| 161028_at | 1450759_at |
| 163970_s_at | 1450770_at |
| 171058_i_at | 1450773_at |
| 98286_at | 1450774_at |
| 96521_at | 1450775_at |
| 99414_at | 1450789_at |
| 94154_at | 1450790_at |
| 93381_at | 1450794_at |
| 98361_at | 1450799_at |
| 92682_at | 1450800_at |
| 92433_at | 1450804_at |
| 98561_at | 1450813_a_at |

| | |
|---|---|
| 95035_at | 1450814_a_at |
| 93699_at | 1450816_at |
| 139754_f_at | 1450822_at |
| 93668_at | 1450823_at |
| 170966_r_at | 1450827_at |
| 99385_at | 1450831_at |
| 95312_at | 1450832_at |
| 99406_at | 1450834_at |
| 93230_at | 1450836_at |
| 94524_at | 1450848_at |
| 98971_at | 1450862_at |
| 99140_at | 1450880_at |
| 93332_at | 1450884_at |
| 99064_at | 1450892_a_at |
| 93362_at | 1450894_a_at |
| 160556_at | 1450895_a_at |
| 99097_at | 1450909_at |
| 160389_r_at | 1450925_a_at |
| 99944_at | 1450930_at |
| 96770_at | 1450936_a_at |
| 160774_at | 1450939_at |
| 163789_at | 1450943_at |
| 99542_at | 1450962_at |
| 160128_at | 1450969_at |
| 166863_f_at | 1450972_at |
| 98418_at | 1450978_at |
| 165403_at | 1450979_at |
| 98297_at | 1450988_at |
| 163101_r_at | 1450991_at |
| 98790_s_at | 1450992_a_at |
| 163383_at | 1450993_at |
| 163027_at | 1450999_a_at |
| 96870_at | 1451002_at |
| 97950_at | 1451006_at |
| 99044_at | 1451011_at |
| 97336_at | 1451019_at |
| 94188_at | 1451023_at |
| 160733_at | 1451030_at |
| 92469_at | 1451031_at |
| 94100_s_at | 1451033_a_at |
| 97318_at | 1451040_at |
| 164142_at | 1451045_at |
| 93505_at | 1451048_at |
| 93252_at | 1451049_at |
| 166038_r_at | 1451059_at |
| 165890_at | 1451061_at |
| 96872_at | 1451067_at |
| 98908_at | 1451073_at |
| 94918_at | 1451083_s_at |
| 97869_at | 1451084_at |
| 94514_s_at | 1451089_a_at |
| 163645_at | 1451094_at |
| 93972_at | 1451096_at |
| 160325_r_at | 1451100_a_at |
| 97391_at | 1451103_at |
| 162764_at | 1451106_at |

| | |
|---|---|
| 96737_at | 1451107_at |
| 160716_at | 1451118_a_at |
| 98081_at | 1451120_at |
| 96269_at | 1451122_at |
| 93490_at | 1451126_at |
| 160715_at | 1451127_at |
| 160225_at | 1451135_at |
| 94530_at | 1451136_a_at |
| 162939_at | 1451138_x_at |
| 97862_s_at | 1451145_s_at |
| 160865_at | 1451156_s_at |
| 98977_at | 1451163_at |
| 95737_at | 1451172_at |
| 165466_r_at | 1451176_at |
| 99153_at | 1451178_at |
| 96226_at | 1451180_a_at |
| 160677_at | 1451198_at |
| 163942_at | 1451204_at |
| 98557_f_at | 1451205_at |
| 163109_at | 1451209_at |
| 99469_at | 1451226_at |
| 95509_at | 1451228_a_at |
| 96232_at | 1451231_a_at |
| 98416_at | 1451233_at |
| 162670_at | 1451237_s_at |
| 160302_at | 1451238_at |
| 163823_at | 1451245_at |
| 98469_at | 1451246_s_at |
| 97325_at | 1451253_at |
| 163447_at | 1451257_at |
| 160508_at | 1451258_at |
| 160341_at | 1451262_a_at |
| 96879_at | 1451274_at |
| 170985_at | 1451278_a_at |
| 160947_at | 1451282_at |
| 94983_at | 1451283_at |
| 160211_at | 1451284_at |
| 163641_at | 1451288_s_at |
| 97200_f_at | 1451294_s_at |
| 163780_at | 1451315_at |
| 96122_at | 1451322_at |
| 164193_at | 1451325_at |
| 160061_at | 1451327_a_at |
| 99455_s_at | 1451333_a_at |
| 163486_at | 1451334_at |
| 160099_at | 1451336_at |
| 163056_at | 1451337_at |
| 163674_at | 1451355_at |
| 163454_at | 1451362_at |
| 98878_r_at | 1451377_a_at |
| 165675_at | 1451384_at |
| 95114_s_at | 1451387_s_at |
| 162817_at | 1451390_s_at |
| 163473_at | 1451393_at |
| 98287_at | 1451394_at |
| 92192_s_at | 1451406_a_at |

| | |
|---|---|
| 163753_at | 1451407_at |
| 95062_at | 1451413_at |
| 97228_at | 1451421_a_at |
| 163918_at | 1451424_at |
| 99641_at | 1451431_a_at |
| 98073_at | 1451435_at |
| 167311_i_at | 1451440_at |
| 94914_at | 1451441_at |
| 99506_at | 1451453_at |
| 167839_at | 1451468_s_at |
| 95697_at | 1451470_s_at |
| 97967_at | 1451475_at |
| 163816_at | 1451478_at |
| 162585_at | 1451479_a_at |
| 160978_at | 1451481_s_at |
| 168024_i_at | 1451482_at |
| 163508_at | 1451488_at |
| 163803_r_at | 1451490_at |
| 160079_i_at | 1451495_at |
| 99108_s_at | 1451501_a_at |
| 93815_at | 1451504_at |
| 97357_at | 1451507_at |
| 95449_at | 1451523_a_at |
| 164883_at | 1451524_at |
| 164240_at | 1451532_s_at |
| 99952_at | 1451537_at |
| 162874_at | 1451540_at |
| 160817_at | 1451546_s_at |
| 93469_at | 1451550_at |
| 96503_at | 1451551_at |
| 163182_at | 1451555_at |
| 96326_at | 1451557_at |
| 163233_at | 1451562_at |
| 95140_at | 1451572_a_at |
| 93476_at | 1451576_at |
| 98296_at | 1451582_at |
| 97700_at | 1451593_at |
| 99449_at | 1451595_a_at |
| 167157_f_at | 1451597_at |
| 99908_at | 1451598_at |
| 99941_at | 1451600_s_at |
| 168876_f_at | 1451607_at |
| 166991_at | 1451613_at |
| 94707_s_at | 1451614_a_at |
| 167852_r_at | 1451620_at |
| 93907_f_at | 1451626_x_at |
| 96202_at | 1451627_a_at |
| 164164_at | 1451634_at |
| 92195_at | 1451639_at |
| 99378_f_at | 1451644_a_at |
| 163608_at | 1451658_a_at |
| 165767_at | 1451674_at |
| 92768_s_at | 1451675_a_at |
| 96617_at | 1451676_at |
| 93654_at | 1451693_a_at |
| 93282_at | 1451703_s_at |

| | |
|---|---|
| 92940_s_at | 1451706_a_at |
| 94184_at | 1451708_at |
| 93315_at | 1451714_a_at |
| 94374_at | 1451728_at |
| 96592_at | 1451737_at |
| 160969_at | 1451752_at |
| 95368_at | 1451753_at |
| 98453_at | 1451756_at |
| 92255_at | 1451761_at |
| 95389_at | 1451763_at |
| 97756_s_at | 1451769_s_at |
| 92711_at | 1451802_at |
| 92522_at | 1451808_at |
| 161039_at | 1451812_at |
| 99886_at | 1451813_at |
| 163734_at | 1451822_a_at |
| 139020_at | 1451831_at |
| 98131_at | 1451848_a_at |
| 98030_at | 1451860_a_at |
| 93931_at | 1451862_a_at |
| 95306_at | 1451863_at |
| 92523_at | 1451868_at |
| 99107_at | 1451871_a_at |
| 97465_at | 1451879_a_at |
| 98417_at | 1451905_a_at |
| 92204_at | 1451910_a_at |
| 164487_at | 1451923_at |
| 97678_r_at | 1451933_a_at |
| 97540_f_at | 1451934_at |
| 93906_s_at | 1451950_a_at |
| 97073_at | 1451954_at |
| 99885_at | 1451958_at |
| 141073_at | 1451959_a_at |
| 93448_at | 1451970_at |
| 162676_at | 1451972_at |
| 160161_at | 1451981_at |
| 162566_at | 1451997_at |
| 98010_at | 1452001_at |
| 95546_g_at | 1452014_a_at |
| 99873_at | 1452018_at |
| 162814_at | 1452031_at |
| 93499_at | 1452038_at |
| 95538_at | 1452044_at |
| 99606_at | 1452060_a_at |
| 160750_at | 1452062_at |
| 95616_at | 1452064_at |
| 97370_at | 1452076_at |
| 163431_at | 1452081_a_at |
| 162723_at | 1452089_at |
| 165430_r_at | 1452091_a_at |
| 94245_at | 1452095_a_at |
| 160735_at | 1452096_s_at |
| 163589_at | 1452113_a_at |
| 98476_at | 1452124_at |
| 99115_at | 1452133_at |
| 163737_at | 1452135_at |

| | |
|---|---|
| 161059_at | 1452142_at |
| 93571_at | 1452143_at |
| 97296_at | 1452144_a_at |
| 99617_at | 1452147_at |
| 94236_at | 1452156_a_at |
| 160217_at | 1452159_at |
| 94321_at | 1452166_a_at |
| 97292_at | 1452167_at |
| 96619_at | 1452177_at |
| 162594_at | 1452194_at |
| 95508_at | 1452196_a_at |
| 160125_at | 1452200_at |
| 163793_at | 1452201_at |
| 96187_at | 1452209_at |
| 163738_at | 1452218_at |
| 160964_at | 1452234_s_at |
| 167557_i_at | 1452243_at |
| 166130_at | 1452244_at |
| 96825_at | 1452264_at |
| 94951_at | 1452266_at |
| 93618_at | 1452269_at |
| 165782_at | 1452284_at |
| 160541_at | 1452285_a_at |
| 163001_at | 1452307_at |
| 166564_f_at | 1452312_at |
| 163770_at | 1452313_at |
| 165918_at | 1452326_at |
| 162753_at | 1452330_a_at |
| 97962_at | 1452344_at |
| 163307_at | 1452345_at |
| 167352_r_at | 1452353_at |
| 93399_at | 1452358_at |
| 163603_at | 1452361_at |
| 96609_at | 1452371_at |
| 163740_at | 1452374_at |
| 92906_at | 1452380_at |
| 93873_s_at | 1452400_a_at |
| 92439_at | 1452403_a_at |
| 166484_at | 1452404_at |
| 98525_f_at | 1452406_x_at |
| 167850_r_at | 1452407_at |
| 94022_at | 1452409_at |
| 92188_s_at | 1452410_a_at |
| 92866_at | 1452431_s_at |
| 166892_at | 1452435_at |
| 93917_at | 1452440_at |
| 94417_at | 1452446_a_at |
| 98512_at | 1452462_a_at |
| 92457_at | 1452473_at |
| 98924_at | 1452474_a_at |
| 92194_at | 1452475_at |
| 167106_at | 1452477_at |
| 168206_r_at | 1452490_a_at |
| 168309_i_at | 1452506_a_at |
| 98866_at | 1452507_at |
| 97007_at | 1452516_at |

| | |
|---|---|
| 92239_at | 1452524_a_at |
| 98809_s_at | 1452525_a_at |
| 94291_at | 1452543_a_at |
| 95645_at | 1452579_at |
| 95076_at | 1452584_at |
| 98610_at | 1452585_at |
| 95641_at | 1452590_a_at |
| 94010_g_at | 1452593_a_at |
| 94980_at | 1452594_at |
| 95003_at | 1452596_at |
| 166228_at | 1452604_at |
| 96148_at | 1452627_at |
| 163357_at | 1452650_at |
| 160685_at | 1452660_s_at |
| 160348_at | 1452673_at |
| 95715_at | 1452674_a_at |
| 95049_at | 1452680_at |
| 96014_at | 1452686_s_at |
| 163360_at | 1452689_at |
| 94081_at | 1452691_at |
| 94244_at | 1452694_at |
| 162534_at | 1452695_at |
| 163842_at | 1452696_a_at |
| 160576_at | 1452697_at |
| 97403_at | 1452698_at |
| 95907_at | 1452701_x_at |
| 160703_at | 1452709_at |
| 95595_at | 1452714_at |
| 97371_at | 1452715_at |
| 163103_at | 1452724_at |
| 96621_at | 1452726_a_at |
| 95740_at | 1452732_at |
| 162994_at | 1452739_at |
| 160743_at | 1452743_at |
| 162809_at | 1452745_at |
| 93913_at | 1452751_at |
| 160119_at | 1452754_at |
| 170882_r_at | 1452763_at |
| 96104_at | 1452769_at |
| 95709_at | 1452770_at |
| 97916_at | 1452773_at |
| 98130_at | 1452782_a_at |
| 163062_at | 1452786_at |
| 164009_at | 1452793_at |
| 163023_at | 1452794_x_at |
| 165446_at | 1452802_at |
| 164686_f_at | 1452804_at |
| 165805_at | 1452817_at |
| 162802_at | 1452826_s_at |
| 160738_at | 1452835_a_at |
| 96806_at | 1452836_at |
| 163743_at | 1452840_at |
| 160793_at | 1452844_at |
| 163445_at | 1452861_at |
| 95070_at | 1452866_at |
| 94801_at | 1452882_at |

| | |
|---|---|
| 163924_at | 1452892_at |
| 93217_at | 1452895_at |
| 163293_at | 1452902_at |
| 163895_at | 1452904_at |
| 163610_at | 1452910_at |
| 97297_at | 1452913_at |
| 164184_at | 1452914_at |
| 98890_at | 1452919_a_at |
| 95110_at | 1452920_a_at |
| 165756_at | 1452922_at |
| 163239_r_at | 1452923_at |
| 166300_at | 1452924_at |
| 163847_at | 1452928_at |
| 162895_at | 1452931_at |
| 163363_at | 1452932_at |
| 163118_r_at | 1452934_at |
| 163725_at | 1452935_at |
| 160486_at | 1452937_s_at |
| 164528_at | 1452945_at |
| 166213_at | 1452951_at |
| 166839_at | 1452952_at |
| 97469_at | 1452957_at |
| 165772_at | 1452962_at |
| 162755_at | 1452975_at |
| 98582_at | 1452986_at |
| 168965_at | 1452995_at |
| 166871_at | 1453006_at |
| 165809_at | 1453011_at |
| 165950_r_at | 1453022_at |
| 167303_r_at | 1453030_at |
| 164599_at | 1453032_at |
| 165904_at | 1453043_at |
| 163031_at | 1453049_at |
| 164108_at | 1453050_at |
| 170297_at | 1453053_at |
| 164114_at | 1453058_at |
| 99553_f_at | 1453060_at |
| 166645_at | 1453062_at |
| 163181_at | 1453075_at |
| 93465_at | 1453076_at |
| 166780_at | 1453079_at |
| 166494_f_at | 1453080_at |
| 164057_at | 1453085_at |
| 161928_at | 1453089_at |
| 163550_at | 1453101_at |
| 163971_at | 1453109_at |
| 165479_at | 1453112_a_at |
| 92421_at | 1453113_at |
| 163028_at | 1453121_at |
| 163511_at | 1453128_at |
| 162743_at | 1453129_a_at |
| 162547_at | 1453132_a_at |
| 165992_f_at | 1453141_at |
| 166155_i_at | 1453142_at |
| 162789_at | 1453147_at |
| 163817_at | 1453152_at |

| | |
|---|---|
| 164008_at | 1453157_at |
| 163883_at | 1453166_at |
| 169485_at | 1453167_at |
| 161031_at | 1453175_at |
| 166251_f_at | 1453203_at |
| 166057_r_at | 1453205_at |
| 166331_at | 1453209_at |
| 163493_at | 1453210_at |
| 166235_at | 1453213_at |
| 163346_at | 1453221_at |
| 98932_at | 1453222_at |
| 163419_at | 1453231_at |
| 164570_r_at | 1453234_at |
| 165859_f_at | 1453235_at |
| 165592_r_at | 1453236_at |
| 166968_f_at | 1453248_at |
| 93640_s_at | 1453249_a_at |
| 166718_at | 1453252_at |
| 168244_at | 1453261_at |
| 160985_at | 1453265_at |
| 166147_f_at | 1453272_at |
| 167419_f_at | 1453275_at |
| 167980_r_at | 1453284_at |
| 165143_at | 1453294_at |
| 164113_at | 1453305_at |
| 166791_at | 1453310_at |
| 167241_at | 1453311_at |
| 164237_at | 1453317_a_at |
| 165845_at | 1453320_at |
| 93443_at | 1453321_at |
| 165952_r_at | 1453327_at |
| 164159_at | 1453330_at |
| 95952_at | 1453355_at |
| 168489_r_at | 1453363_at |
| 171347_at | 1453379_at |
| 164176_at | 1453383_at |
| 170734_at | 1453389_a_at |
| 167712_r_at | 1453394_at |
| 99341_r_at | 1453397_at |
| 166596_at | 1453403_at |
| 167800_at | 1453405_at |
| 165940_at | 1453411_at |
| 92672_at | 1453413_at |
| 163395_at | 1453415_at |
| 167917_at | 1453420_at |
| 166896_at | 1453432_at |
| 167150_f_at | 1453438_x_at |
| 167944_f_at | 1453447_at |
| 169332_at | 1453449_at |
| 167788_at | 1453453_at |
| 168582_f_at | 1453460_at |
| 164106_at | 1453461_at |
| 93730_at | 1453467_s_at |
| 167919_at | 1453471_at |
| 165589_at | 1453484_at |
| 135511_at | 1453489_at |

| | |
|---|---|
| 167780_i_at | 1453493_at |
| 93888_at | 1453501_at |
| 167854_at | 1453502_at |
| 170248_at | 1453511_at |
| 166643_at | 1453518_at |
| 171556_at | 1453519_at |
| 168420_r_at | 1453525_at |
| 167349_i_at | 1453536_at |
| 165463_at | 1453546_at |
| 98052_at | 1453570_x_at |
| 171555_at | 1453577_at |
| 164153_at | 1453583_at |
| 171201_at | 1453604_a_at |
| 164241_at | 1453609_s_at |
| 167867_at | 1453621_at |
| 98076_at | 1453634_a_at |
| 99377_at | 1453644_at |
| 169420_at | 1453645_at |
| 168109_at | 1453666_at |
| 170321_at | 1453672_at |
| 162870_at | 1453677_a_at |
| 169312_f_at | 1453708_a_at |
| 96028_at | 1453719_at |
| 92577_f_at | 1453729_a_at |
| 165603_i_at | 1453730_at |
| 163965_at | 1453735_at |
| 163394_at | 1453747_at |
| 163693_at | 1453755_at |
| 164204_at | 1453763_at |
| 164177_at | 1453769_at |
| 166675_at | 1453775_at |
| 96750_at | 1453784_at |
| 165308_f_at | 1453785_at |
| 171586_at | 1453791_at |
| 171576_at | 1453814_at |
| 165945_at | 1453818_a_at |
| 167140_at | 1453870_at |
| 167233_at | 1453875_at |
| 168785_at | 1453884_at |
| 167962_at | 1453888_at |
| 163136_at | 1453915_a_at |
| 169578_at | 1453933_at |
| 94172_at | 1453950_a_at |
| 170170_at | 1453959_at |
| 166690_i_at | 1453974_at |
| 167750_at | 1453977_at |
| 167059_at | 1453985_at |
| 167798_i_at | 1453992_at |
| 163021_at | 1453996_a_at |
| 170349_at | 1454004_at |
| 160803_at | 1454006_a_at |
| 163727_at | 1454010_a_at |
| 167671_at | 1454013_at |
| 97387_at | 1454023_a_at |
| 95027_at | 1454034_a_at |
| 170104_at | 1454051_at |

| | |
|---|---|
| 167089_i_at | 1454052_at |
| 94362_at | 1454060_a_at |
| 96810_at | 1454086_a_at |
| 169574_at | 1454111_at |
| 160425_at | 1454116_a_at |
| 96093_at | 1454161_s_at |
| 166671_at | 1454262_at |
| 171485_at | 1454271_at |
| 169671_at | 1454321_at |
| 94281_at | 1454602_s_at |
| 160307_at | 1454605_a_at |
| 164433_at | 1454613_at |
| 97265_at | 1454614_at |
| 160907_at | 1454622_at |
| 162539_at | 1454623_at |
| 160252_at | 1454626_at |
| 94240_i_at | 1454627_a_at |
| 95530_at | 1454631_at |
| 162684_at | 1454637_at |
| 95407_at | 1454638_a_at |
| 96676_at | 1454640_at |
| 160707_at | 1454648_s_at |
| 165728_at | 1454659_at |
| 165471_f_at | 1454660_at |
| 160598_at | 1454662_at |
| 94856_r_at | 1454673_at |
| 92348_at | 1454675_at |
| 163929_at | 1454685_at |
| 166861_at | 1454693_at |
| 99578_at | 1454694_a_at |
| 95731_at | 1454699_at |
| 163102_r_at | 1454701_at |
| 163467_at | 1454702_at |
| 98013_at | 1454705_at |
| 163024_at | 1454710_at |
| 97410_at | 1454712_at |
| 93328_at | 1454713_s_at |
| 165581_at | 1454715_at |
| 164000_at | 1454721_at |
| 162842_at | 1454723_at |
| 163347_at | 1454729_at |
| 163353_at | 1454743_at |
| 162631_at | 1454751_at |
| 163539_at | 1454752_at |
| 97339_at | 1454759_at |
| 160669_at | 1454760_at |
| 163549_at | 1454762_at |
| 165800_at | 1454770_at |
| 98085_f_at | 1454778_x_at |
| 163004_i_at | 1454800_at |
| 162940_at | 1454825_at |
| 162844_at | 1454836_at |
| 93978_at | 1454842_a_at |
| 163359_at | 1454843_at |
| 162891_at | 1454854_at |
| 162736_at | 1454861_at |

| | |
|---|---|
| 163657_at | 1454868_at |
| 162665_at | 1454871_at |
| 161011_at | 1454884_at |
| 97823_g_at | 1454887_at |
| 162745_at | 1454888_at |
| 163517_at | 1454895_at |
| 164273_at | 1454924_at |
| 162589_at | 1454926_at |
| 168188_at | 1454927_at |
| 94413_at | 1454929_s_at |
| 165721_f_at | 1454968_at |
| 161044_at | 1454985_at |
| 96176_at | 1455013_at |
| 162918_at | 1455020_at |
| 163675_r_at | 1455034_at |
| 161100_at | 1455040_s_at |
| 98578_at | 1455041_at |
| 163554_at | 1455055_at |
| 165798_s_at | 1455063_at |
| 164050_at | 1455074_at |
| 165621_at | 1455085_at |
| 99862_at | 1455093_a_at |
| 163827_at | 1455095_at |
| 163303_at | 1455097_at |
| 163916_i_at | 1455100_at |
| 167837_at | 1455101_at |
| 163117_at | 1455108_at |
| 166215_r_at | 1455117_at |
| 164122_at | 1455124_at |
| 96538_at | 1455125_at |
| 168115_f_at | 1455148_at |
| 94459_at | 1455152_at |
| 165399_i_at | 1455163_at |
| 99340_at | 1455168_a_at |
| 163214_at | 1455169_at |
| 163218_at | 1455183_at |
| 165596_at | 1455199_at |
| 93751_at | 1455205_a_at |
| 94210_at | 1455211_a_at |
| 165771_r_at | 1455212_at |
| 163227_at | 1455216_at |
| 165850_r_at | 1455224_at |
| 92744_at | 1455231_s_at |
| 163484_at | 1455232_at |
| 166710_at | 1455241_at |
| 168284_i_at | 1455251_at |
| 167667_i_at | 1455260_at |
| 166628_r_at | 1455267_at |
| 166914_i_at | 1455275_at |
| 92377_at | 1455279_at |
| 165551_at | 1455297_at |
| 165757_i_at | 1455301_at |
| 92272_at | 1455310_at |
| 161085_r_at | 1455338_at |
| 166615_at | 1455343_at |
| 93179_at | 1455350_at |

| | |
|---|---|
| 165758_at | 1455360_at |
| 92528_at | 1455363_at |
| 164061_at | 1455372_at |
| 165803_s_at | 1455379_at |
| 166305_r_at | 1455381_at |
| 114347_at | 1455391_at |
| 92851_at | 1455393_at |
| 161108_r_at | 1455408_at |
| 93410_at | 1455424_at |
| 166891_at | 1455445_at |
| 162725_at | 1455446_x_at |
| 95397_at | 1455447_at |
| 167376_at | 1455455_at |
| 115287_at | 1455460_at |
| 164231_at | 1455465_at |
| 98355_at | 1455466_at |
| 166722_at | 1455498_at |
| 93145_at | 1455505_at |
| 94793_at | 1455511_at |
| 166658_at | 1455521_at |
| 166605_at | 1455528_at |
| 98974_at | 1455530_at |
| 93479_at | 1455531_at |
| 93824_at | 1455540_at |
| 170305_at | 1455590_at |
| 96543_at | 1455601_at |
| 166823_i_at | 1455603_at |
| 163349_at | 1455608_at |
| 168127_at | 1455614_at |
| 168281_r_at | 1455625_at |
| 95904_at | 1455628_at |
| 166623_at | 1455631_at |
| 163567_at | 1455649_at |
| 163887_at | 1455668_at |
| 97107_at | 1455700_at |
| 167001_at | 1455712_at |
| 165614_i_at | 1455723_at |
| 98489_at | 1455730_at |
| 161083_at | 1455733_at |
| 95090_at | 1455752_a_at |
| 164083_at | 1455769_at |
| 167345_at | 1455775_at |
| 166551_at | 1455779_at |
| 167441_at | 1455807_at |
| 160505_at | 1455808_at |
| 96856_at | 1455821_x_at |
| 166226_f_at | 1455824_x_at |
| 163098_at | 1455837_at |
| 162777_at | 1455838_at |
| 163125_at | 1455851_at |
| 166694_at | 1455852_at |
| 165427_f_at | 1455859_at |
| 160799_at | 1455869_at |
| 160380_at | 1455880_s_at |
| 162609_at | 1455883_a_at |
| 164078_at | 1455885_at |

| | |
|---|---|
| 163388_at | 1455887_at |
| 93375_at | 1455893_at |
| 161016_at | 1455915_at |
| 170649_at | 1455919_at |
| 166443_f_at | 1455945_at |
| 99503_at | 1455958_s_at |
| 165783_at | 1455971_at |
| 167938_f_at | 1455973_at |
| 96168_at | 1455990_at |
| 165497_i_at | 1456001_at |
| 166652_at | 1456008_at |
| 160216_f_at | 1456017_x_at |
| 165027_at | 1456018_at |
| 163691_at | 1456022_at |
| 166291_at | 1456034_at |
| 167941_r_at | 1456035_at |
| 163069_i_at | 1456045_at |
| 163594_at | 1456052_at |
| 167617_r_at | 1456057_x_at |
| 92392_at | 1456064_at |
| 168472_at | 1456070_at |
| 166278_f_at | 1456076_at |
| 167388_f_at | 1456092_at |
| 162788_at | 1456101_at |
| 166220_r_at | 1456111_at |
| 163805_at | 1456114_at |
| 166289_at | 1456129_at |
| 168407_at | 1456160_at |
| 93523_at | 1456170_x_at |
| 166374_i_at | 1456171_at |
| 93341_r_at | 1456175_a_at |
| 163659_at | 1456181_at |
| 164048_at | 1456185_at |
| 96910_at | 1456190_a_at |
| 165882_r_at | 1456192_x_at |
| 164101_i_at | 1456203_at |
| 171296_f_at | 1456210_at |
| 94877_at | 1456222_at |
| 162586_at | 1456224_x_at |
| 163400_at | 1456230_at |
| 165956_f_at | 1456248_at |
| 164088_at | 1456259_at |
| 167592_at | 1456265_at |
| 165661_i_at | 1456298_at |
| 163886_at | 1456301_at |
| 166133_i_at | 1456334_s_at |
| 166619_f_at | 1456362_at |
| 165887_at | 1456366_at |
| 166240_r_at | 1456368_at |
| 166928_at | 1456371_a_at |
| 165914_r_at | 1456382_at |
| 160283_at | 1456383_at |
| 166559_at | 1456414_at |
| 98371_at | 1456416_at |
| 167879_at | 1456421_at |
| 164212_at | 1456422_at |

| | |
|---|---|
| 163927_at | 1456423_at |
| 165875_at | 1456428_at |
| 99828_at | 1456430_at |
| 167494_f_at | 1456436_x_at |
| 166193_at | 1456445_at |
| 167805_i_at | 1456452_at |
| 161075_at | 1456481_at |
| 168462_at | 1456512_at |
| 165674_f_at | 1456538_at |
| 165712_at | 1456539_at |
| 166793_r_at | 1456544_at |
| 169573_at | 1456549_at |
| 165662_at | 1456555_at |
| 167831_at | 1456559_at |
| 166729_at | 1456563_at |
| 161145_f_at | 1456580_s_at |
| 163085_i_at | 1456596_at |
| 97836_at | 1456600_a_at |
| 166404_r_at | 1456602_at |
| 96791_at | 1456603_at |
| 95512_at | 1456604_a_at |
| 164853_r_at | 1456605_at |
| 97328_at | 1456618_at |
| 163997_at | 1456631_at |
| 93779_at | 1456635_at |
| 162824_at | 1456653_a_at |
| 95385_at | 1456672_at |
| 166052_at | 1456693_at |
| 167967_at | 1456696_x_at |
| 94662_at | 1456706_at |
| 160433_at | 1456716_s_at |
| 166974_at | 1456723_at |
| 168813_r_at | 1456742_x_at |
| 97754_at | 1456757_at |
| 95315_at | 1456770_at |
| 163465_at | 1456779_a_at |
| 162651_at | 1456786_at |
| 166754_i_at | 1456792_at |
| 168183_f_at | 1456793_at |
| 167631_at | 1456801_at |
| 166073_at | 1456802_at |
| 167547_at | 1456823_at |
| 166761_f_at | 1456830_at |
| 93001_at | 1456862_at |
| 92206_at | 1456870_at |
| 164155_at | 1456879_at |
| 163830_at | 1456899_at |
| 167529_at | 1456906_at |
| 170032_at | 1456945_at |
| 95996_at | 1456972_at |
| 168683_f_at | 1457021_x_at |
| 92251_f_at | 1457035_at |
| 163950_r_at | 1457055_at |
| 165831_at | 1457057_at |
| 161099_at | 1457094_at |
| 167475_i_at | 1457111_at |

| | |
|---|---|
| 167887_at | 1457125_at |
| 164091_at | 1457127_at |
| 165869_at | 1457130_at |
| 97138_at | 1457147_at |
| 164767_at | 1457158_at |
| 167015_at | 1457210_at |
| 164003_at | 1457213_a_at |
| 169792_at | 1457216_at |
| 169091_f_at | 1457258_at |
| 94106_at | 1457272_at |
| 160966_at | 1457285_at |
| 96817_at | 1457303_at |
| 98327_at | 1457308_at |
| 166911_at | 1457337_at |
| 164235_at | 1457349_at |
| 97188_at | 1457372_at |
| 99438_at | 1457379_at |
| 97084_at | 1457475_at |
| 97085_at | 1457476_at |
| 95948_at | 1457520_at |
| 99824_at | 1457521_at |
| 167385_at | 1457541_at |
| 168990_r_at | 1457623_x_at |
| 169386_at | 1457631_at |
| 167039_at | 1457647_x_at |
| 170681_i_at | 1457651_x_at |
| 167642_f_at | 1457659_x_at |
| 167346_f_at | 1457663_at |
| 167672_f_at | 1457664_x_at |
| 161173_f_at | 1457666_s_at |
| 92281_at | 1457675_at |
| 96529_at | 1457695_at |
| 165271_at | 1457755_at |
| 170829_at | 1457763_at |
| 96520_at | 1457776_at |
| 167272_at | 1457779_at |
| 166320_r_at | 1457828_at |
| 99020_at | 1457834_at |
| 95979_at | 1457860_at |
| 95998_at | 1457872_at |
| 95986_at | 1457887_at |
| 166592_at | 1457889_at |
| 165916_at | 1457893_at |
| 96556_at | 1457899_at |
| 97120_at | 1457905_at |
| 167848_at | 1457928_at |
| 160983_at | 1457964_at |
| 97714_r_at | 1457970_at |
| 165202_r_at | 1457981_x_at |
| 167351_f_at | 1457982_at |
| 95960_at | 1458011_s_at |
| 166755_at | 1458118_at |
| 95946_at | 1458173_at |
| 95957_at | 1458215_at |
| 97701_at | 1458219_at |
| 168503_at | 1458282_at |

| | |
|---|---|
| 167720_i_at | 1458318_at |
| 168170_r_at | 1458319_at |
| 166486_at | 1458331_x_at |
| 168835_s_at | 1458345_s_at |
| 168416_at | 1458350_at |
| 164042_at | 1458367_at |
| 164085_at | 1458370_at |
| 167482_f_at | 1458375_at |
| 165726_at | 1458404_at |
| 169146_i_at | 1458408_at |
| 170221_at | 1458435_at |
| 167042_i_at | 1458444_at |
| 168620_at | 1458446_at |
| 92451_at | 1458452_at |
| 164040_at | 1458559_at |
| 167027_at | 1458591_at |
| 166788_at | 1458644_at |
| 167145_at | 1458652_at |
| 170848_at | 1458761_at |
| 168479_at | 1458842_at |
| 95983_at | 1458846_at |
| 98598_at | 1458854_at |
| 163073_at | 1458909_at |
| 171081_at | 1458937_at |
| 170823_at | 1458949_at |
| 167732_r_at | 1459013_at |
| 166841_at | 1459030_at |
| 96365_at | 1459092_at |
| 136081_at | 1459130_at |
| 167389_i_at | 1459142_at |
| 95875_at | 1459220_at |
| 95878_at | 1459410_at |
| 94667_at | 1459478_at |
| 169181_at | 1459481_at |
| 97112_at | 1459665_s_at |
| 168138_at | 1459759_s_at |
| 168627_at | 1459763_at |
| 164735_at | 1459791_at |
| 167054_at | 1459810_at |
| 167507_at | 1459813_at |
| 166618_at | 1459815_at |
| 161367_f_at | 1459827_x_at |
| 166938_at | 1459833_x_at |
| 168461_at | 1459840_s_at |
| 164321_at | 1459864_at |
| 167679_f_at | 1459877_x_at |
| 168173_at | 1459881_at |
| 96709_at | 1459890_s_at |
| 95972_at | 1459891_at |
| 160993_at | 1459894_at |
| 95984_at | 1459900_at |
| 93411_at | 1459903_at |
| 167289_at | 1459904_at |
| 96524_at | 1459911_at |
| 94759_at | 1459914_at |
| 96513_at | 1459917_at |

| | |
|---|---|
| 94113_at | 1459920_at |
| 95382_at | 1459922_at |
| 95891_at | 1459923_at |
| 96000_at | 1459924_at |
| 95971_at | 1459925_at |
| 95956_at | 1459926_at |
| 95906_at | 1459927_at |
| 94203_at | 1459928_at |
| 96373_at | 1459930_at |
| 97115_at | 1459931_a_at |
| 95352_at | 1459932_at |
| 97708_at | 1459933_at |
| 97190_f_at | 1459935_at |
| 92372_at | 1459947_at |
| 98420_at | 1459983_at |
| 99590_at | 1459986_a_at |
| 166730_at | 1460019_at |
| 98800_at | 1460042_at |
| 171574_r_at | 1460048_at |
| 164256_at | 1460056_at |
| 169327_r_at | 1460058_at |
| 169793_at | 1460069_at |
| 170824_i_at | 1460075_x_at |
| 169983_at | 1460106_at |
| 171258_r_at | 1460114_at |
| 167080_i_at | 1460120_at |
| 166728_r_at | 1460127_at |
| 99929_at | 1460170_at |
| 93793_at | 1460173_at |
| 162945_at | 1460174_at |
| 96358_at | 1460175_at |
| 94065_at | 1460176_at |
| 96890_at | 1460178_at |
| 97818_at | 1460182_at |
| 99569_at | 1460185_at |
| 97309_at | 1460193_at |
| 96608_at | 1460194_at |
| 163330_at | 1460200_s_at |
| 98995_at | 1460207_s_at |
| 96646_at | 1460209_at |
| 97375_at | 1460210_at |
| 99860_at | 1460212_at |
| 98023_r_at | 1460217_at |
| 92666_at | 1460222_at |
| 96331_at | 1460224_at |
| 160954_at | 1460230_at |
| 93425_at | 1460231_at |
| 95569_at | 1460233_at |
| 92963_at | 1460234_at |
| 165959_at | 1460236_at |
| 93677_at | 1460245_at |
| 99465_at | 1460246_at |
| 166202_at | 1460250_at |
| 165981_i_at | 1460258_at |
| 98786_at | 1460275_at |
| 99834_at | 1460289_at |

| | |
|---|---|
| 92416_at | 1460293_at |
| 94791_s_at | 1460300_a_at |
| 99150_at | 1460308_a_at |
| 92783_at | 1460310_a_at |
| 94170_at | 1460322_at |
| 98947_at | 1460328_at |
| 163928_at | 1460333_at |
| 95529_at | 1460334_at |
| 97459_at | 1460339_at |
| 97915_at | 1460341_at |
| 162972_at | 1460356_at |
| 163426_at | 1460364_at |
| 169831_at | 1460370_at |
| 92732_at | 1460374_at |
| 98319_at | 1460380_at |
| 96726_at | 1460389_at |
| 160166_r_at | 1460391_at |
| 98428_at | 1460400_at |
| 93657_at | 1460407_at |
| 98284_f_at | 1460418_x_at |
| 99510_at | 1460419_a_at |
| 97356_at | 1460424_at |
| 160338_at | 1460428_at |
| 160554_at | 1460432_a_at |
| 93165_at | 1460438_at |
| 163279_at | 1460439_at |
| 166678_at | 1460457_at |
| 163039_at | 1460458_at |
| 166061_f_at | 1460461_at |
| 166943_at | 1460478_at |
| 161097_at | 1460480_at |
| 169757_at | 1460484_at |
| 169081_at | 1460489_at |
| 167144_i_at | 1460502_at |
| 168089_at | 1460514_s_at |
| 97478_at | 1460544_at |
| 94261_at | 1460545_at |
| 163003_at | 1460553_at |
| 162953_at | 1460556_at |
| 165616_at | 1460572_a_at |
| 167753_f_at | 1460576_at |
| 163904_at | 1460577_at |
| 164116_at | 1460578_at |
| 163177_at | 1460580_at |
| 167342_r_at | 1460587_at |
| 164115_at | 1460604_at |
| 169788_r_at | 1460609_at |
| 163901_at | 1460615_at |
| 165527_f_at | 1460618_x_at |
| 98778_at | 1460626_at |
| 97800_at | 1460635_at |
| 92543_at | 1460636_at |
| 93564_at | 1460638_at |
| 94482_at | 1460646_at |
| 98862_at | 1460657_at |
| 160518_at | 1460660_x_at |

| | |
|---|---|
| 92352_at | 1460661_at |
| 98277_at | 1460664_at |
| 160301_at | 1460670_at |
| 99142_at | 1460674_at |
| 97517_at | 1460679_at |
| 96668_at | 1460685_at |
| 97933_at | 1460695_a_at |
| 160250_at | 1460698_a_at |
| 96301_at | 1460699_at |
| 97443_at | 1460701_a_at |
| 97277_at | 1460702_at |
| 92375_at | 1460703_at |
| 97179_at | 1460713_at |
| 95326_at | 1460717_at |
| 95602_at | 1460720_at |
| 99896_at | 1460723_at |
| 92384_at | 1460725_at |
| 92278_at | 1460727_at |
| 99635_at | 1460728_s_at |
| 96493_at | 1460744_at |
| 95305_at | 1460745_at |
| 96569_at | 1460746_at |
| 160122_at | 1460360_at |
| 160967_at | 1440786_x_at |
| 160986_r_at | 1452138_a_at |
| 161093_at | 1442476_at |
| 161123_i_at | 1453612_at |
| 161189_r_at | 1418604_at |
| 161197_r_at | 1439097_at |
| 161234_f_at | 1455422_x_at |
| 161239_r_at | 1443713_at |
| 161512_r_at | 1452900_at |
| 161554_r_at | 1443829_x_at |
| 161576_f_at | 1439507_at |
| 161648_at | 1438920_x_at |
| 161733_at | 1430998_at |
| 161760_s_at | 1425115_at |
| 161773_i_at | 1437851_x_at |
| 161795_r_at | 1452688_at |
| 161842_r_at | 1443381_at |
| 161903_f_at | 1448728_a_at |
| 161989_f_at | 1441919_x_at |
| 162050_at | 1447671_x_at |
| 162064_at | 1426580_at |
| 162088_r_at | 1431432_at |
| 162098_i_at | 1459134_at |
| 162123_f_at | 1449744_at |
| 162182_f_at | 1425885_a_at |
| 162205_f_at | 1454267_a_at |
| 162227_r_at | 1457922_at |
| 162273_at | 1457387_at |
| 162276_i_at | 1437726_x_at |
| 162350_at | 1459218_at |
| 162447_f_at | 1456586_x_at |
| 162504_at | 1439744_at |
| 162853_r_at | 1453185_at |

| | |
|---|---|
| 162882_at | 1439461_x_at |
| 163082_i_at | 1460359_at |
| 163165_at | 1420006_at |
| 163238_f_at | 1458223_at |
| 163329_i_at | 1437639_at |
| 163593_i_at | 1452936_at |
| 164013_i_at | 1426222_s_at |
| 164185_at | 1453854_at |
| 164202_at | 1428818_at |
| 164292_r_at | 1431634_at |
| 164310_f_at | 1447664_x_at |
| 164312_i_at | 1426021_a_at |
| 164329_f_at | 1444291_at |
| 164423_at | 1453984_at |
| 164440_f_at | 1451700_a_at |
| 164441_at | 1457804_at |
| 164456_f_at | 1458603_at |
| 164472_f_at | 1425170_a_at |
| 164531_f_at | 1439718_at |
| 164561_f_at | 1424401_at |
| 164644_r_at | 1451795_at |
| 164692_i_at | 1442572_at |
| 164711_r_at | 1444844_at |
| 164727_r_at | 1460362_at |
| 164768_f_at | 1447898_s_at |
| 164828_r_at | 1453450_at |
| 164830_r_at | 1455941_s_at |
| 164831_f_at | 1444849_at |
| 164842_i_at | 1449975_a_at |
| 164874_f_at | 1428606_at |
| 164967_f_at | 1440553_at |
| 164970_at | 1458657_at |
| 164989_i_at | 1445454_at |
| 164992_f_at | 1429760_at |
| 165025_f_at | 1447741_x_at |
| 165037_r_at | 1441846_x_at |
| 165088_r_at | 1446302_at |
| 165180_f_at | 1426129_at |
| 165193_i_at | 1438261_at |
| 165296_f_at | 1454003_at |
| 165334_s_at | 1445514_at |
| 165337_i_at | 1454137_s_at |
| 165376_f_at | 1449386_at |
| 165493_r_at | 1431727_at |
| 165532_r_at | 1447018_at |
| 165547_at | 1428757_at |
| 165716_at | 1429866_at |
| 165789_at | 1429217_at |
| 165791_f_at | 1451032_at |
| 165878_r_at | 1436889_at |
| 165943_f_at | 1427292_at |
| 166127_f_at | 1437147_at |
| 166174_r_at | 1443741_x_at |
| 166180_f_at | 1453881_x_at |
| 166263_at | 1448424_at |
| 166288_at | 1447817_at |

| | |
|---|---|
| 166333_at | 1417832_at |
| 166426_i_at | 1441501_at |
| 166444_f_at | 1451900_at |
| 166565_i_at | 1432262_at |
| 166572_f_at | 1453188_at |
| 166606_at | 1431706_at |
| 166674_r_at | 1431605_at |
| 166775_at | 1446296_at |
| 166792_at | 1437262_x_at |
| 166807_at | 1455417_at |
| 166827_at | 1432644_at |
| 166874_r_at | 1436894_at |
| 166941_f_at | 1418059_at |
| 166949_at | 1416314_at |
| 166953_r_at | 1444563_at |
| 167051_at | 1445626_at |
| 167208_f_at | 1443825_x_at |
| 167223_at | 1450459_at |
| 167251_r_at | 1434502_x_at |
| 167279_at | 1430572_at |
| 167312_i_at | 1431834_a_at |
| 167404_f_at | 1429221_at |
| 167405_r_at | 1445948_at |
| 167514_f_at | 1454649_at |
| 167548_i_at | 1440128_s_at |
| 167575_r_at | 1424808_at |
| 167576_at | 1429767_at |
| 167681_at | 1443396_at |
| 167684_f_at | 1460449_at |
| 167749_r_at | 1427395_a_at |
| 167830_at | 1443867_at |
| 167847_at | 1451533_at |
| 167921_r_at | 1458672_at |
| 167954_at | 1439950_at |
| 168008_at | 1459607_at |
| 168084_r_at | 1457624_at |
| 168121_i_at | 1438251_x_at |
| 168228_i_at | 1423458_at |
| 168342_r_at | 1453524_at |
| 168344_r_at | 1440525_at |
| 168391_f_at | 1450543_at |
| 168408_at | 1451873_a_at |
| 168541_i_at | 1459812_x_at |
| 168554_i_at | 1437348_at |
| 168849_at | 1417338_at |
| 169043_r_at | 1430231_a_at |
| 169094_at | 1439972_at |
| 169139_r_at | 1443110_at |
| 169182_f_at | 1451451_at |
| 169220_i_at | 1431926_a_at |
| 169346_at | 1439261_x_at |
| 169412_at | 1443562_at |
| 169601_i_at | 1436253_at |
| 169641_r_at | 1435628_x_at |
| 169762_at | 1438114_x_at |
| 169798_r_at | 1439425_x_at |

| | |
|---|---|
| 169814_at | 1420708_at |
| 169958_at | 1453812_at |
| 169993_r_at | 1446534_at |
| 170060_r_at | 1457646_at |
| 170086_at | 1453468_at |
| 170338_at | 1430294_at |
| 170490_i_at | 1444414_at |
| 170491_r_at | 1440291_at |
| 170498_at | 1458692_at |
| 170550_i_at | 1440580_at |
| 170552_at | 1458206_at |
| 170567_i_at | 1440243_at |
| 170586_r_at | 1445437_at |
| 170608_at | 1454803_a_at |
| 170662_at | 1458589_at |
| 170682_i_at | 1459251_at |
| 170696_at | 1459248_at |
| 170847_i_at | 1458608_at |
| 170866_at | 1446794_at |
| 170999_r_at | 1457459_at |
| 171028_r_at | 1446987_at |
| 171065_at | 1451966_at |
| 171079_at | 1459000_at |
| 171199_r_at | 1457838_at |
| 171211_at | 1416691_at |
| 171310_i_at | 1459296_at |
| 171324_at | 1443620_at |
| 171397_at | 1455881_at |
| 171448_at | 1444774_at |
| 171541_at | 1430955_at |
| 171559_i_at | 1443293_at |
| 92312_at | 1443655_s_at |
| 92687_at | 1426805_at |
| 92725_at | 1440788_at |
| 93432_at | 1418313_at |
| 93886_at | 1423048_a_at |
| 94411_at | 1447617_at |
| 94780_at | 1439278_at |
| 96214_at | 1452566_at |
| 96433_at | 1427678_at |
| 97136_at | 1446504_at |
| 97482_at | 1446539_at |
| 97694_at | 1459480_at |
| 97761_f_at | 1451664_x_at |
| 98922_at | 1450841_at |
| 99195_at | 1452735_at |
| 99418_at | 1459794_at |
| 99778_at | 1435725_x_at |
